# PacBio Single-Molecule Long-Read Sequencing Reveals Genes Tolerating Manganese Stress in *Schima superba* Saplings

**DOI:** 10.3389/fgene.2021.635043

**Published:** 2021-04-06

**Authors:** Fiza Liaquat, Muhammad Farooq Hussain Munis, Samiah Arif, Urooj Haroon, Jianxin Shi, Saddam Saqib, Wajid Zaman, Shengquan Che, Qunlu Liu

**Affiliations:** ^1^School of Agriculture and Biology, Shanghai Jiao Tong University, Shanghai, China; ^2^Department of Plant Sciences, Faculty of Biological Sciences, Quaid-i-Azam University, Islamabad, Pakistan; ^3^Joint International Research Laboratory of Metabolic and Developmental Sciences, School of Life Sciences and Biotechnology, Shanghai Jiao Tong University, Shanghai, China; ^4^State Key Laboratory of Systematic and Evolutionary Botany, Institute of Botany, Chinese Academy of Sciences, Beijing, China; ^5^University of Chinese Academy of Sciences, Beijing, China; ^6^Department of Landscape Architecture, School of Design, Shanghai Jiao Tong University, Shanghai, China

**Keywords:** *Schima superba*, manganese, transcriptome, full-length transcript, alternative splicing

## Abstract

*Schima superba* (Theaceae) is a subtropical evergreen tree and is used widely for forest firebreaks and gardening. It is a plant that tolerates salt and typically accumulates elevated amounts of manganese in the leaves. With large ecological amplitude, this tree species grows quickly. Due to its substantial biomass, it has a great potential for soil remediation. To evaluate the thorough framework of the mRNA, we employed PacBio sequencing technology for the first time to generate *S. Superba* transcriptome. In this analysis, overall, 511,759 full length non-chimeric reads were acquired, and 163,834 high-quality full-length reads were obtained. Overall, 93,362 open reading frames were obtained, of which 78,255 were complete. In gene annotation analyses, the Kyoto Encyclopedia of Genes and Genomes (KEGG), Clusters of Orthologous Genes (COG), Gene Ontology (GO), and Non-Redundant (Nr) databases were allocated 91,082, 71,839, 38,914, and 38,376 transcripts, respectively. To identify long non-coding RNAs (lncRNAs), we utilized four computational methods associated with protein families (Pfam), Cooperative Data Classification (CPC), Coding Assessing Potential Tool (CPAT), and Coding Non-Coding Index (CNCI) databases and observed 8,551, 9,174, 20,720, and 18,669 lncRNAs, respectively. Moreover, nine genes were randomly selected for the expression analysis, which showed the highest expression of Gene 6 (Na_Ca_ex gene), and CAX (CAX-interacting protein 4) was higher in manganese (Mn)-treated group. This work provided significant number of full-length transcripts and refined the annotation of the reference genome, which will ease advanced genetic analyses of *S. superba.*

## Introduction

*Schima superba* is naturally grown in mining areas and abundantly absorbs different metals, particularly manganese (Mn). Due to this absorbance ability, this tree is being used as phytoremediation agent in metal toxic soils (Yang et al., 2008). This tree belongs to the family Theaceae, and it is commonly used for gardening and forest firebreak. Characteristically, the leaves of this tree contain an unusual high concentration of Mn (Yang et al., 2021).

Hyper-accumulation of harmful metals in different plants helps in cleaning or decontaminating soils. Contamination of metals in the soil can be reduced through plants’ absorbance and transportation, which is a kind of green environmental remediation technology (Padmavathiamma and Li, 2007). Woody plants have high biomass and long-life cycle, and those are unique advantages in the phytoremediation of heavy metal. But the competency of woody trees to accumulate heavy metals is usually very low, and most of heavy metals are accumulated in the roots of woody trees (Capuana, 2020). As reported by Baker and Brooks (1989), more than 10,000 mg/kg concentrations of Mn in above ground tissues of plants are considered as the hyper-accumulation of Mn. Along with having a potential to accumulate Mn, *S. superba* has vast ecological amplitude, dominant canopy species (Tang et al., 2020) and has considerable biomass, which suggests its potential use in the soil remediation (Luo et al., 2006).

Although, researchers have reported the hyper-accumulation of Mn in leaves, roots, and shoots, no study has performed the transcriptomic analysis of *S. superba* till date. Since the establishment of large-scale sequencing technology, transcript sequences have become a significant source of studying gene expression and regulation (Levy and Myers, 2016). The restriction of sequencing read length in the second-generation, the full-length record acquired by splicing is not finished, and the third-generation sequencing technology signified by Pacific Biosciences (PacBio) has successfully overcome this issue (Jiao and Schneeberger, 2017; Slatko et al., 2018). Based on its long-read length, high-quality full-length record can be legitimately acquired to distinguish simple sequence repeat (SSR), fulfill functional annotation of transcripts, and lncRNA (Wang et al., 2015; Xie et al., 2020). Transcriptomics provides information of the complete set of RNA transcripts in a specific tissue or cell under different developmental or physiological stages, involving ribosomal RNA, transfer RNA, messenger RNA, and non-coding RNAs. Moreover, it concentrates on the gene expression, gene structure, and function at RNA level that help to reveal the molecular processes involved in various biological mechanisms (Dong and Chen, 2013).

In this research, a single molecule real-time sequencing (SMRT) has been completed to get the full-length transcriptome of *S. superba*. Utilizing the transcriptome data functional annotations of transcripts, transcriptional factors (TFs) analysis, prediction of lnRNA, and basic sequence repeat (SSR) analysis were performed. In addition, the expression pattern of various genes provides basic understanding of transcriptional regulation of *S. superba*. This study will not only assist future functional genomics research but also provide an avenue for further selective genetic engineering and breeding projects of *S. superba*.

## Materials and Methods

### Pot Experiment and Sample Collection

A pot test was carried out in the glasshouse of Shanghai Jiao Tong University, China, by using 1-year-old *S. superba* saplings. For this purpose, 18 saplings, with same growth potential (about 35 cm high), were taken and randomly allocated into two groups: control (CK) and Mn concentration 100 mM (WT), in three replicates. Plant tissues were collected after 1, 5, and 10 days of transplantation in quarter-strength of Hoagland solution. The tissues were collected in liquid nitrogen and processed through the isolation of RNA at −80°C.

### RNA Extraction

RNA extraction from the collected plant samples (0.2 g leaves) was performed with the RNeasy plus Mini Kit (Qiagen, Valencia, United States). Using the Nano drop ND-1000 (Nano drop Technologies, Rockland, DE, United States) spectrophotometer, its quality and quantity were calculated after observing RNA on agarose gel. By using Qubit^®^ RNA Assay Kit and RNA Nano 6000 Assay Kit, RNA quality has been identified clearly.

### Library Construction and SMRT Sequencing

For sequencing, C2 sequencing reagents were used in Pacific Biosciences (PacBio) real-time sequencer. Purified RNA was utilized for synthesizing cDNA by SMRT PCR cDNA Synthesis Kit (Clontech, CA, United States). Full-length cDNA of different sizes was selected and cDNA libraries were constructed using the BluePippin^®^ (SageScience, Beverly, MA, United States). After BluePippin screening, the fragments were pacified to large-scale PCR to get sufficient total cDNA and evaluate using Qubit fluorometer (Life Technologies, Carlsbad, CA, United States). The libraries’ uniqueness was maintained by using Agilent Bio analyzer 2100 system and SMRT sequencing was achieved.

### Error Correction and Quality Filtering

Sequence statistics were obtained by using the SMRT link 5.1 software. Relevant parameters such as no-polish TRUE, min-length 50, max-drop-fraction 0.8, min-predicted-accuracy 0.8, min-passes 2, min-z score -9999.0, and max-length 15000 were followed to produce CCS by subreading BAM files. The output was CCS.BAM files, which, using pbclassify.py, were categorized into full length and non-full length reads. Full and non-full length FASTA files were fed into the cluster stage, and additional nucleotide errors were corrected by LoRDEC software in the consensus readings. Unnecessary data in the corrected consensus reads were separated using CD-HIT (-c 0.95 -T 6 -G 0 -aL 0.00 -aS 0.99) to acquire final transcripts for the subsequent investigation.

### Functional Annotation of Transcripts

We recognized functional annotations matching by searching to the non-redundant nucleotide database (Nr), Swiss-Prot, Pfam, non-supervised orthologous groups egg (NOG), clusters of orthologous groups (COG), eukaryotic orthologous groups (KOG), Gene Ontology (GO), Kyoto Encyclopedia of Genes and Genomes (KEGG), and BLAST alignment databases, and all transcript sequences were examined for homology (Bairoch and Apweiler, 2000; Li et al., 2002; Tatusov et al., 2003; Kanehisa et al., 2004).

### Identification of TFs, lncRNAs, and SSR

Plant transcription factors were predicted using iTAK v1.7a (Zheng et al., 2016). Four tools, CNCIv2 (Sun et al., 2013), CPCvcpc-0.9-r2 with e-value “1e-10” (Kong et al., 2007), Pfam-scan (E 0.001 -domE 0.001) (Finn et al., 2016) and PLEKv1.2 with min length 200 (Li et al., 2002) were chosen to predict candidate lncRNAs. Transcripts predicted with coding capacity by all of the mentioned above tools were separated out, and those without coding prospective were considered as candidate set of lncRNAs.

### Development of SSR Markers

Simple sequence repeats were identified by MISA v1.0^1^ (Beier et al., 2017), with default parameters. MISA can recognize seven types of SSR, namely mono, di, tri, tetra, penta, and hexa nucleotide by analyzing transcript sequences. By using tool Batch primer 3, SSR primers were designed. DNA was extracted from *S. superba* for PCR amplification, and the product was extracted in 8% polyacrylamide gel.

### qRT-PCR Analysis

All 36 saplings of control group (CK) and Mn-treated group (WT) were utilized by RNA extraction via RNeasy plus Mini Kit (Qiagen, Valencia, United States). The cDNA was synthesized using SMRT PCR cDNA synthesis kit (Clontech, United States). The qRT PCR was performed to see the expression of nine different transporter genes (Supplementary Table S5). The reaction mixture consisted of 7.8 μl of cDNA and 10 μl of 2× qPCR. The process involve initial denaturation at 95°C for 10 min, which is followed by 45 cycles of denaturation at 95°C for 15 s, while annealing and extension at 60°C for 60 1 min.

## Results

### SMRT Sequencing Data of *S. superba*

**FIGURE 1:**
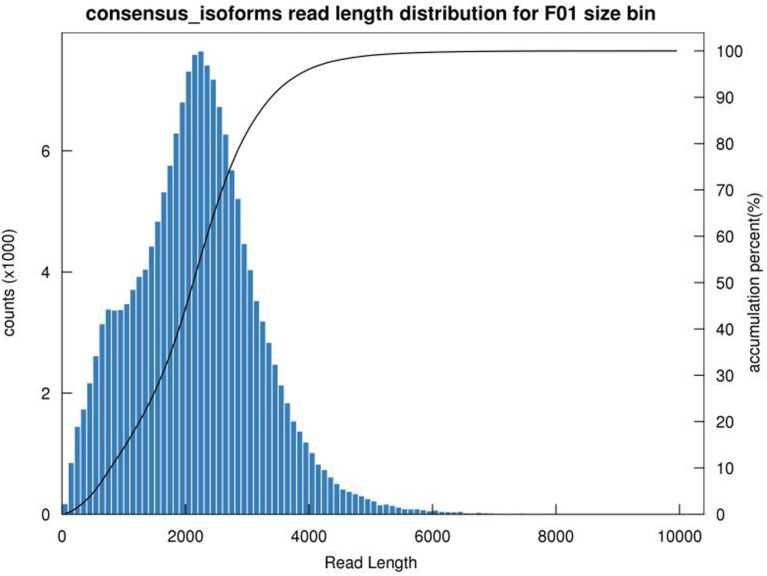
Length distribution of full-length non-chimeric sequence.

Plant tissues were used for RNA extraction and library construction of cDNA. After removing adaptor sequences, a total of 1,311,552,27 low-quality sequences were obtained. It comprised of 1–6 kb data. After self-correction of subread sequences (with minPasses = 39), overall 588,479 circular consensus sequence (CCS) were obtained. Moreover, 511,759 full-length non-chimeric (FLNC) picks were also identified. Iterative sequence clustering revealed 167,782 consensus isoforms, with a mean read length of 2187 bp. Together with non-FL sequence, the Quiver program corrected the consistent sequence in each cluster, yielding 163834 high-quality transcripts (high- quality isoforms) with more than 97.65% accuracy. In addition, the low-quality record was corrected with the corresponding sequencing data to enhance the accuracy of the isoforms. Finally, using CD HIT (3.3) tools, 97,520 bar non-redundant transcript sequences were obtained (Tables 1, 2 and Figure 1).

**TABLE 1:** Sequencing data statistics table.

Sample name	Sample ID	cDNA size	SMRT cells	Data size
*Schima superba*	F01	1–6 k	1	40 G

**TABLE 2:** Reads summary insert from single molecule long-read sequencing.

Samples	cDNA size	CCS no.	Read bases of CCS	Mean read length	Mean no. of passes
F01	1–6 k	588,479	1,311,552,271	2,228	39
F02	All	588,479	1,311,552,271	2,228	39

### Open Reading Frame and AS Event Prediction

**FIGURE 2:**
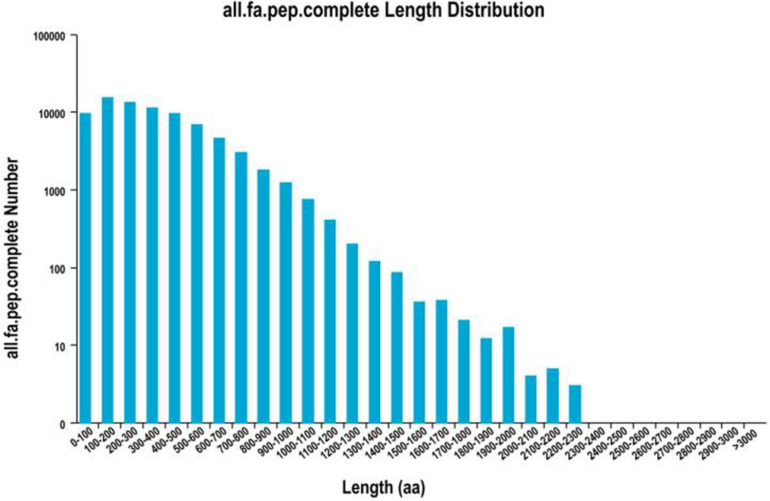
Division of predicted CDS-encoded protein length.

About 93,362 ORFs were predicted using Trans Decoder software. Overall, 78,255 complete ORFs were recognized, and the complete ORFs were successfully analyzed for length distribution (Figure 2); 7,489 AS events were revealed out of all transcripts obtained by SMRT sequencing (Supplementary Table S1). Owing to the lack of an unavailable *S. Superba* reference genome, the classification of the forms of AS events in future research would be justified (Figure 2).

### Long Non-coding RNA Identification

**FIGURE 3:**
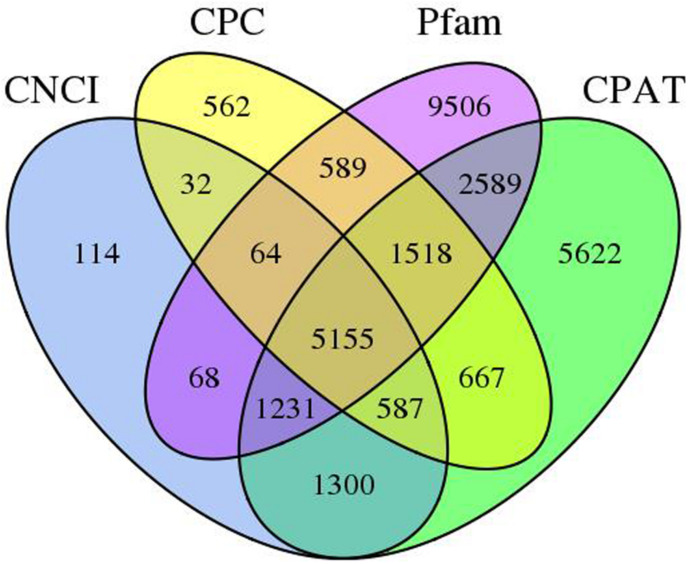
LncRNAs predicted by the CNCI, CPAT, Pfam, and CPC protein structure domain analysis.

These are a group of non-coding poly-A RNAs that are involved in growth and stress response of the plant. In this work, we utilize four computational methods to recognize lncRNAs, associated with Pfam, CPC, CPAT, and CNCI databases. Overall, 8,551, 9,174, 20,720, and 18,669 lncRNAs were recognized in the Pfam, CPC, CPAT, and CNCI, respectively (Supplementary Table S2). By filtering the record of less than 300 bp, 2,011 transcripts were examined as lncRNAs by all four mechanisms (Figure 3).

### Transcription Factor Prediction

**FIGURE 4:**
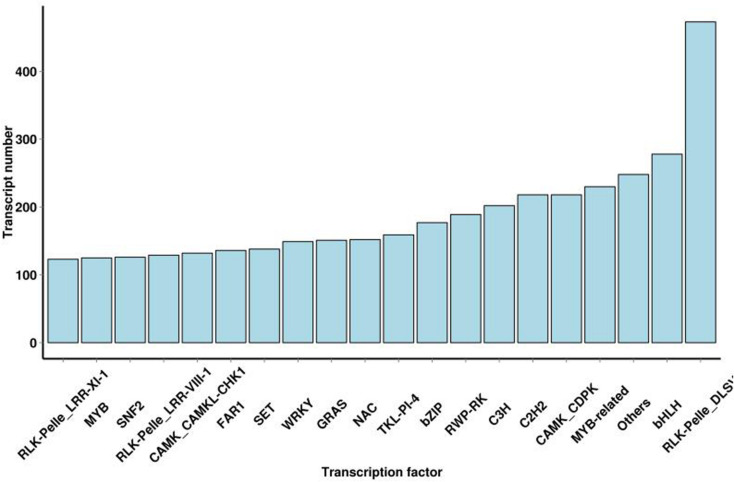
Distribution of transcription factor types.

These are the key regulators of gene expression and play a significant role in plant growth and development. In this study, TFs are divided into 64 families, and 9423 putative TFs were identified (Supplementary Table S3). TFs in *S. superba* transcriptome were mostly related to the RLK-Pelle-DLS (473,5.02%), bHLH (278,2.95%), MYB-related (230,2.44%), CAMK-CDPK (218,2.31%), C2H2 (218,2.31%), C3H (202,2.14%), RWP-RK (189,2.01%), bZIP (177,1.88%), TKL-PI-4 (159,1.69%), and NAC (152,1.61%) families (Figure 4).

### Functional Annotation of Transcripts

**FIGURE 5:**
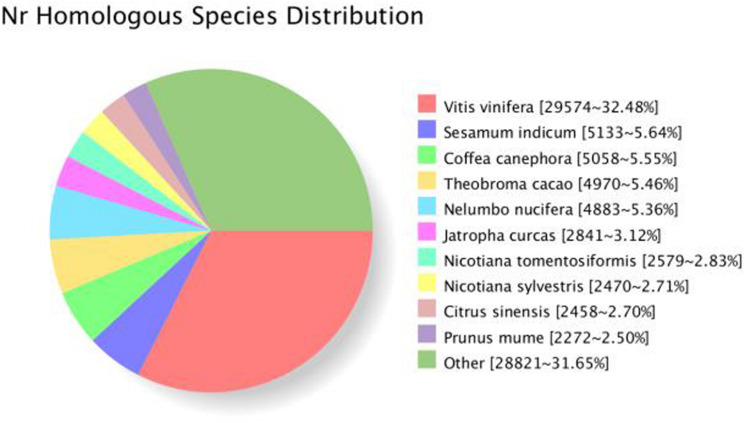
Species distribution of *S. superba* transcriptome annotated in the Nr database.

All 16,3834 unique SMRT transcripts were practically annotated by seven data storage, such as GO, KOG, Pfam, Swiss-Prot, COG, Nr, and KEGG using BLAST (7) software [version 2.2.26] (Table 3). By comparing the transcript sequence to NR with homologous species, 68,630 genes were annotated (Figure 5).

**TABLE 3:** Functional annotation of *S. superba* transcriptome.

Annotated databases	Isoform number
COG	38,914
GO	71,839
KEGG	38,376
KOG	58,193
PfAM	73,647
Eggnog	89,727
Swiss-Prot	66,790
Nr	91,082
All	91,617

### GO Classified Transcripts

**FIGURE 6:**
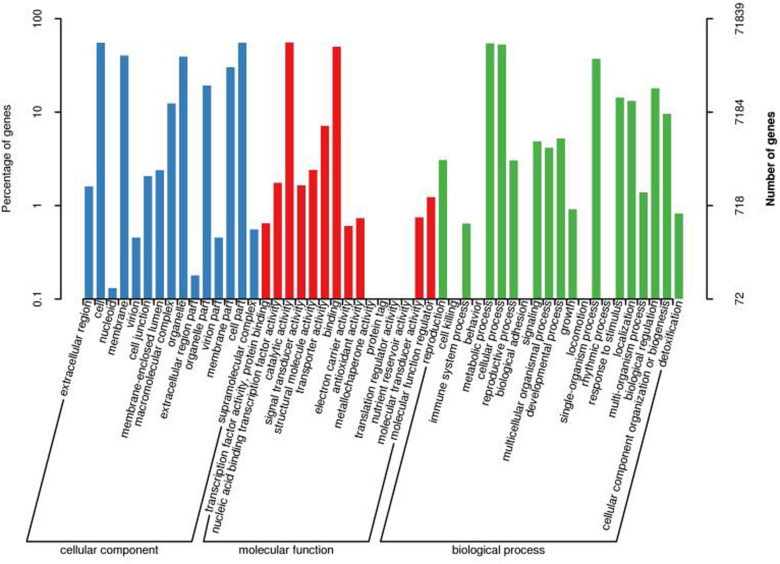
Transcript GO annotation classification statistics graph.

Transcripts GO classification statistics demonstrated 71,839 unique genes, which were enriched in major categories of molecular function, cellular component, biological component, and catalytic activity (Figures 3, 5). This analysis also helped us to get transcripts COG classification statistics (Figure 6).

### COG Classification

**FIGURE 7:**
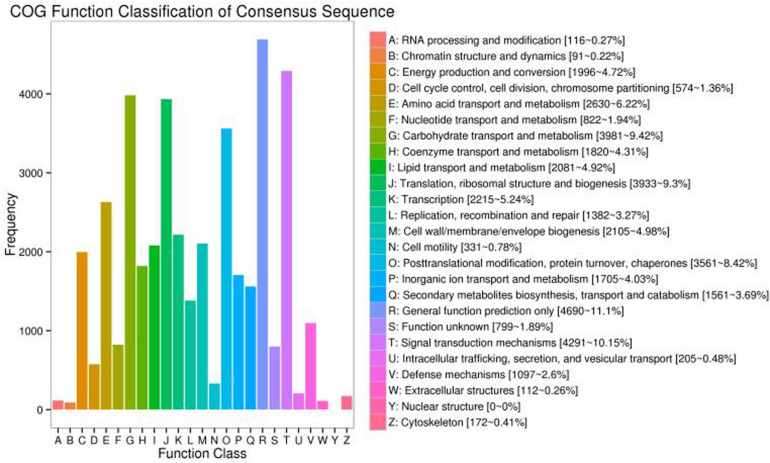
COG annotation classification statistics of transcripts.

To examine the functional annotation and classification of *S. Superba* for further study, all the transcripts were checked against the COG database clusters^2^. This investigation indicated that 38,914 transcripts were allocated into 24 categories (Figure 7). The highest group was general function prediction only (4,690, 11.1%), followed by signal transduction (4,291, 10.15%) whereas carbohydrate transport and metabolism was 3,981, 9.42%. Our results revealed six groups percentage were less than 1.00%, i.e., nuclear structure and modification, chromatin structure and dynamics including extracellular structure (Figure 7).

### KEGG Annotated Transcripts

KEGG data storage interpreted a total of 67,426 sequences and plotted 367 operative categories in *S. superba*. Among them, metabolism was the largest category. The functional annotations of all 78,559 unique transcripts were detected in this work (Supplementary Table S4). A significant number of genes, especially interrelated in salt-tolerance and fatty acid component of *S. superba* were annotated, such as oxidative phosphorylation (1073), plant hormone signal transduction (506), fatty acid biosynthesis (246), biosynthesis of unsaturated fatty acids (94), and α-linolenic acid metabolism (199). We also recognized matches to our unique transcripts in clusters of orthologous class of proteins (COG) (44,376, 56.49%), Pfam database (41,535, 52.87%), and Swiss-Prot (58,535, 74.51%) (Table 4).

**TABLE 4:** Topmost pathways annotated by the KEGG database.

No.	Name of pathways	Pathway ID	Transcripts (%)
1.	Carbon metabolism	ko01200	886(9.68%)
2.	Protein of processing in endoplasmic reticulum	Ko04141	462 (5.05%)
3.	Biosynthesis of amino acid	ko01230	621 (6.79%)
4.	Spliceosome	ko03040	520 (5.68%)
5.	Ribosome	ko03010	332 (3.63%)
6.	RNA transport	ko03013	356 (3.89%)
7.	Sucrose and starch metabolism	ko00500	384 (4.20%)
8.	Plant hormone signal transduction	ko04075	401 (4.38%)
9.	Oxidative phosphorylation	ko00190	240 (2.62%)
10.	Glycolysis/gluconeogenesis	ko00010	350 (3.82%)
11.	Plant pathogen interaction	ko04626	388 (4.24%)
12.	mRNA surveillance pathway	ko03015	339 (3.7%)
13.	Ubiquitin mediated proteolysis	ko04120	253 (2.76%)
14.	Amino sugar and nucleotide sugar metabolism	ko00520	245 (2.68%)
15.	Endocytosis	ko04144	270 (2.95%)

### Analysis of Gene Expression in qRT PCR

**FIGURE 8:**
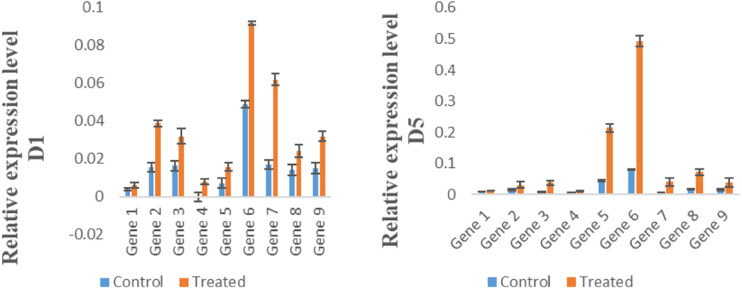
Expression of genes in control group (CK) and Mn-treated group (WT) at day 1, day 5, and day 10. Error bars represent the standard deviation.

For the expression analysis of potential high expression genes, nine transporter genes were selected randomly for qRT PCR. Results of qRT PCR analysis showed the highest expression by Gene 6 (Na_Ca_ex), in Mn-treated group (WT) on day 1, 5, and 10, while the expression of CAX (CAX-interacting protein 4) was higher in Mn-treated group (WT) at day 5 (Figure 8).

### SSR Identification

After the screening of 95,258 obtained transcripts, about 58,396 possible SSRs were recognized from 29,075 transcripts. Among them 26,312 composed one SSR, and 19,007 contained two loci or more. In addition, 29,075 and 18,324 transcripts contained one SSR and at least two SSRs, respectively. Whereas observed compound formations were 18,324 SSRs. As shown in Table 5, the number of mono-, di-, tri-, tetra-, penta-, and hexa-nucleotide repeats were 44,045, 51,716, 14,521, 1,675, 753, and 988, accordingly. SSRs with 10 repeat units (16949, 14.9%) were the most abundant, followed by those with 6 (12563, 11%), 11 (10564, 9.2%), and 7 (7995, 87.03%) (Table 6).

**TABLE 5:** Summary of SSR identified in *Schima superba.*

Searching item	Numbers
Total no. of sequence examined	95,258
Total size of examined sequences (bp)	225,274,809
No. of SSR containing sequences	58,396
No. of sequences containing more than one SSR	29,075
No. of SSRs present in compound formation	18,324
Mono-nucleotide	44,045
Di-nucleotide	51,716
Tri-nucleotide	14,521
Tetra-nucleotide	1,675
Penta-nucleotide	753
Hexa-nucleotide	988

**TABLE 6:** SSRs distribution based on the number of repeat units.

No. of repeat units	Mono	Di	Tri	Tetra	Penta	Hexa	Total	Percentage
5	0	0	6914	1146	573	763	9396	8.263997608
6	0	8785	3099	353	150	176	12563	11.04944678
7	0	6163	1661	113	26	32	7995	7.031785959
8	0	5669	977	44	3	15	6708	5.899839927
9	0	5588	734	17	0	0	6339	5.575295959
10	11953	4542	452	2	0	0	16949	14.90703442
11	7151	3099	314	0	0	0	10564	9.29128041
12	5263	2203	178	0	0	0	7644	6.723073405
13	3813	2115	82	0	0	0	6010	5.28593291
14	3294	2111	45	0	0	0	5450	4.79340006
≥15	12571	11441	65	0	1	2	24080	21.17891256

### Development of SSR Markers

By using Primer 3.0 software, 95,374 pair of primers were designed, and among them, 200 were randomly selected for PCR (Supplementary Table S6). The product of 180 PCR pairs was examined successfully, with an efficiency rate of 90%. However, the remaining 20 pairs of primer failed to amplify at various annealing temperatures.

## Discussion

Since the development of high-throughput sequencing technology, transcriptomic analysis has become a valuable technology to study gene expression and regulation (Wang et al., 2015; Dharshini et al., 2016). However, due to read length limitation of the second-generation sequencing in different organisms, the full-length transcript obtained by splicing is not complete (Rhoads and Au, 2015). SMRT sequencing technology effectively solves this problem. This study is the first transcriptome analysis of *S. superba* using SMRT sequencing technology. The sequencing peaks were gained from leaf samples using the Pacific Biosciences Iso-Seq platform. After sequencing, 511,759 full-length non-chimerics (FLNC) were identified. Previous studies also reported similar results that newly discovered transcripts by SMRT in red clover and wheat were 45 bp longer than the known transcripts (Dong et al., 2015; Chao et al., 2018). Subsequently, our results also showed that the SMRT technology is very efficient for full-length cDNA sequencing and provided a rich resource for further functional genomics analysis in *S. superba* (Workman et al., 2018; Zuo et al., 2018).

In past years, PacBio platform was used to sequence the full-length transcriptional group of tea (*Camellia sinensis*) (Qiao et al., 2019). Finally, 213,389 polished consensus sequences were obtained, 223,120 CDS sequences were predicted, 195,062 SSR loci were detected, and 5,785 transcription factors belonging to 60 transcription factor families were predicted. PacBio RS II platform was used to analyze the full-length transcription group of sunflowers, 10.43 Gb clean data were obtained, and 38,302 de-redundant sequences were obtained (Chen et al., 2018). Previous research on *S. superba* was mainly focused on genetic mapping, gene expression, and proteomic analysis (Chen et al., 2014; Zhang et al., 2019, Yang et al., 2021). However, these techniques could not provide and assembled full-length transcripts. On the other hand, PacBio SMRT single-molecule long-read sequencing revealed a better capacity in capturing transcript sequences, mainly long transcript sequences. The transcriptome sequencing of 18 samples was completed, and a total of 124.45 G of clean data were obtained, with Q30 reaching 85%. These data were corrected by Illumina clean reads, after the integration and quality control of data from the two platforms. After sequencing, 588,479 circular consensus (CCS) reads were obtained, including 511,759 full-length reads non-chimeric (FLNC) sequences. Previous studies showed that the length of the FLNC sequence emulates the length of the cDNA sequence in whole sequence library, whereas the library can be examined by the length of FLNC sequence (Chao et al., 2018). The full-length non-chimeric sequences were clustered to obtain 167,782 consensus sequences, and 163,834 high-quality consensus sequences were obtained by polishing the consensus sequences. The low-quality consensus sequences were corrected with the second-generation transcriptome data, and they were consistent with high-quality. The sequences were merged and subjected to de-redundancy analysis to obtain 41,112 transcript sequences. Each sample obtained 7,489 alternative splicing, and a total of 113,698 SSR and 78,255 complete CDS regions were obtained. Previous studies showed variations in the percentage of tetra, penta, and hexa nucleotide repeats observed in sorghum (5.4%), rice (2.54%), Populus (1.66%), and Medicago (0.94%) (Ref 4). Our findings of sequence library provide basic information for improving the *S. superba* draft genome annotation and complete characterization of this species transcriptome.

Results of qRT PCR analysis indicated the highest expression of Gene 6 (CAX-interacting protein 4), in Mn-treated group (WT) on day 1, 5, and 10. Our results are similar with the study of Pittman and Hirschi (2016). Previous studies indicated that CAX shows high expression under salt stress. Moreover, the higher expression of CAX (Na_Ca_ex) in Mn-treated group (WT) was also observed. The CAX proteins are one of the five transporter families that constitute Ca^2+^/cation antiporters (CaCA) superfamily (Shigaki et al., 2006; Emery et al., 2012). Although the CAX family members were at first identified as Ca^2+^ transporters, related work revealed their ability to transport a wide array of ions (Pittman and Hirschi, 2016). In plants, the CAXs mediate efflux of ions present into the vacuole. This study has provided enriched information about expression and regulation of genes in stress conditions of *S. superba*. In addition, this study provided for the first time a full-length transcriptome of the *S. superba* using the SMRT sequencing method. The transcriptome design in this research will assist future research on functional genomics and facilitate support for advance genetic engineering of *S. superba*.

## Data Availability Statement

Data are available at the SRA portal (https://www.ncbi.nlm.nih.gov/sra/PRJNA679388) of NCBI, accession number: PRJNA679388.

## Author Contributions

QL designed the experiments and supervised the research work. FL executed the experiments and wrote the manuscript. MM analyzed the results and formatted the manuscript. SA, UH, and JS did data compilation. WZ and SS did analyses and sample collection. SC supervised the research work. All authors read and approved the final manuscript.

## Conflict of Interest

The authors declare that the research was conducted in the absence of any commercial or financial relationships that could be construed as a potential conflict of interest.

## Footnotes

1 http://pgrc.ipk-gatersleben.de/misa/

2 http://www.ncbi.nlm.nih.gov/COG

## Supplementary Material

Supplementary Table 1 Summary of the predicted alternative splicing (AS) events.

Supplementary Table 2 Summary of lncRNA predicted by CPC, CNCI, CPAT, and Pfam protein structure domain analysis.

Supplementary Table 3 Summary of identified TFs.

Supplementary Table 4 Summary of the transcripts annotated to the reference canonical pathways in the KEGG database.

Supplementary Table 5 The primers used for qRT-PCR analysis.

Supplementary Table 6 The characterization of EST-SSR markers.
