# Supplementary material for: PacBio Single-Molecule Long-Read Sequencing Reveals Genes Tolerating Manganese Stress in Schima superba Saplings
Source: Front Genet. 2021 Apr 6;12:635043. doi: 10.3389/fgene.2021.635043 (PMC8057201; doi:10.3389/fgene.2021.635043)
Supplement: Supplementary Table 1 — Summary of the predicted alternative splicing (AS) events. [file Data_Sheet_1.PDF]

PacBio single-molecule long-read sequencing successfully explores the transcriptome of *Schima superba*

Fiza Liaquat[1], Muhammad Farooq Hussain Munis[2], Samiah Arif[1], Urooj Haroon[2], Muhammad Ashraf[3], Saddam Saqib[4,5], Wajid Zaman[4,5], Che Shengquan[6] and Liu Qunlu[6*]

1 School of Agriculture and Biology, Shanghai Jiao Tong University, Shanghai, 200240, China;

2 Department of Plant Sciences, Faculty of Biological Sciences, Quaid-i-Azam University, Islamabad, 45320, Pakistan;

3 Joint International Research Laboratory of Metabolic and Developmental Sciences, School of Life Science and Biotechnology, Shanghai Jiao Tong University, Shanghai, 200240, China;

4 State Key Laboratory of Systematic and Evolutionary Botany, Institute of Botany, Chinese Academy of Sciences, Beijing 100093, China;

5 University of Chinese Academy of Sciences, Beijing 100049, China;

6 Department of Landscape Architecture, School of Design, Shanghai Jiao Tong University, 200240, China;

*Correspondence: liuql@sjtu.edu.cn

Table S1 Summary of the predicted alternative splicing (AS) events

QueryName	SubjectName	QhspStart1	QhspEnd1	QhspStart2	QhspEnd2	ShspStart1	ShspEnd1	ShspStart2	ShspEnd2
transcript_100043	gnl|BL_ORD_ID|69933 transcript_130828	158	2495	1	158	787	3126	1	153
transcript_100063	gnl|BL_ORD_ID|72068 transcript_134290	1	1636	1635	2325	42	1695	1866	2556
transcript_100063	gnl|BL_ORD_ID|63354 transcript_121710	1022	2327	1	1019	1291	2642	101	1138
transcript_10008	gnl|BL_ORD_ID|97427 transcript_158243	16	2264	2262	2872	24	2272	3086	3713
transcript_100095	gnl|BL_ORD_ID|11750 transcript_2441	397	3495	5	396	543	3652	8	401
transcript_100144	gnl|BL_ORD_ID|21835 transcript_59064	1	2641	2640	3244	1505	4138	4289	4894
transcript_100148	gnl|BL_ORD_ID|58260 transcript_113524	438	1666	8	440	1708	2937	2	434
transcript_100163	gnl|BL_ORD_ID|24897 transcript_5269	2	2297	2293	2806	142	2437	2681	3194
transcript_100203	gnl|BL_ORD_ID|1045 transcript_1876	1189	2597	1	1191	2392	3800	4	1201
transcript_10021	gnl|BL_ORD_ID|28026 transcript_67470	1280	2837	1	1285	1556	3118	1	1301
transcript_10021	gnl|BL_ORD_ID|33085 transcript_75647	1	2405	2402	2758	1	2404	2520	2876
transcript_100238	gnl|BL_ORD_ID|18317 transcript_53305	328	2641	93	329	504	2816	140	376
transcript_100263	gnl|BL_ORD_ID|86245 transcript_156204	1	2373	2368	2637	2609	4976	5177	5446
transcript_100263	gnl|BL_ORD_ID|60962 transcript_117889	1	2373	2368	2637	477	2848	3049	3318
transcript_100302	gnl|BL_ORD_ID|85204 transcript_154491	1008	2089	1	1010	1173	2254	2	1011
transcript_1004	gnl|BL_ORD_ID|70043 transcript_131006	1857	4023	14	1859	2382	4548	1	1846
transcript_100469	gnl|BL_ORD_ID|25304 transcript_6140	1	2665	2664	2988	1	2657	2811	3135
transcript_100469	gnl|BL_ORD_ID|38025 transcript_6754	1	2665	2664	2890	1	2665	2914	3140
transcript_10047	gnl|BL_ORD_ID|88753 transcript_160312	19	2217	2218	2823	2	2202	2397	3002
transcript_10047	gnl|BL_ORD_ID|38787 transcript_8400	2	2217	2218	2791	1	2228	2423	2996
transcript_100495	gnl|BL_ORD_ID|19970 transcript_55978	1	1699	1696	3375	1	1699	2171	3850
transcript_100507	gnl|BL_ORD_ID|38325 transcript_7378	18	1886	1885	2447	1	1868	2479	3044
transcript_100511	gnl|BL_ORD_ID|42546 transcript_89268	1	2043	2042	2340	667	2689	3926	4224
transcript_100525	gnl|BL_ORD_ID|46585 transcript_95997	1343	2638	106	1346	1986	3280	301	1542
transcript_100530	gnl|BL_ORD_ID|93683 transcript_166639	269	2356	33	274	562	2667	2	243
transcript_100559	gnl|BL_ORD_ID|30140 transcript_70869	302	2994	4	306	464	3170	2	305
transcript_100586	gnl|BL_ORD_ID|11798 transcript_2545	1596	3449	1	1598	1785	3639	61	1658
transcript_100586	gnl|BL_ORD_ID|37437 transcript_82660	1596	3474	1	1598	1787	3663	58	1660
transcript_100609	gnl|BL_ORD_ID|51186 transcript_8562	170	2238	1	171	889	2958	16	184
transcript_100628	gnl|BL_ORD_ID|76045 transcript_140758	1	1287	1288	2241	24	1288	1390	2341
transcript_100647	gnl|BL_ORD_ID|2299 transcript_23916	1	1273	1273	1771	83	1353	1798	2298

transcript_100655	gnl|BL_ORD_ID|76151 transcript_140935	1	1609	1605	2379	766	2374	3075	3849
transcript_100663	gnl|BL_ORD_ID|50578 transcript_102317	1	1907	1903	2288	1	1905	2529	2913
transcript_100679	gnl|BL_ORD_ID|51454 transcript_9137	1	1497	1495	2649	2	1498	1701	2856
transcript_100679	gnl|BL_ORD_ID|38262 transcript_7234	1	1497	1495	2645	201	1696	1898	3048
transcript_100715	gnl|BL_ORD_ID|53459 transcript_105382	658	2338	7	661	767	2447	2	656
transcript_100769	gnl|BL_ORD_ID|49389 transcript_100409	1	1865	1864	2499	74	1939	2055	2692
transcript_100770	gnl|BL_ORD_ID|73436 transcript_136481	24	2768	2768	3096	2	2746	3367	3695
transcript_100832	gnl|BL_ORD_ID|48805 transcript_99498	169	3149	1	168	3569	6552	2136	2303
transcript_100872	gnl|BL_ORD_ID|27868 transcript_67202	1	1228	1227	2266	147	1385	1525	2565
transcript_100872	gnl|BL_ORD_ID|82004 transcript_148983	1	1228	1227	2266	202	1429	1569	2609
transcript_100872	gnl|BL_ORD_ID|38940 transcript_83460	1	1228	1227	2266	144	1354	1493	2531
transcript_100873	gnl|BL_ORD_ID|50669 transcript_102472	1	1361	1358	1591	1	1362	2200	2439
transcript_100880	gnl|BL_ORD_ID|95736 transcript_20192	188	2054	1	188	576	2372	2	181
transcript_100881	gnl|BL_ORD_ID|36710 transcript_81512	1	1303	1298	1997	3	1305	1441	2140
transcript_100881	gnl|BL_ORD_ID|1653 transcript_22517	1	1303	1298	1997	45	1345	1481	2180
transcript_100881	gnl|BL_ORD_ID|1835 transcript_22900	1	1303	1298	1997	123	1423	1559	2258
transcript_100881	gnl|BL_ORD_ID|44450 transcript_92444	1	1303	1298	1997	3	1279	1415	2114
transcript_100907	gnl|BL_ORD_ID|11748 transcript_2437	133	3336	1	137	452	3657	1	137
transcript_100907	gnl|BL_ORD_ID|898 transcript_1602	133	3336	1	137	656	3862	1	135
transcript_100931	gnl|BL_ORD_ID|53377 transcript_105251	13	1624	1621	2595	121	1732	2005	2979
transcript_100941	gnl|BL_ORD_ID|67430 transcript_126764	269	3854	2	274	433	4018	8	280
transcript_100941	gnl|BL_ORD_ID|590 transcript_1001	269	3854	2	274	443	4027	19	291
transcript_100941	gnl|BL_ORD_ID|89914 transcript_162150	269	3854	16	274	412	3997	2	260
transcript_100975	gnl|BL_ORD_ID|58954 transcript_114670	1	2029	2026	2373	236	2272	2410	2757
transcript_100975	gnl|BL_ORD_ID|88373 transcript_159694	1	2029	2026	2345	1782	3818	3956	4275
transcript_100976	gnl|BL_ORD_ID|36522 transcript_81233	199	1825	3	201	645	2272	199	397
transcript_1010	gnl|BL_ORD_ID|39836 transcript_84895	121	4097	1	121	351	4311	1	121
transcript_10104	gnl|BL_ORD_ID|60383 transcript_116960	277	2839	23	276	670	3232	2	260
transcript_10104	gnl|BL_ORD_ID|70441 transcript_131677	275	2853	2	277	686	3265	8	293
transcript_101041	gnl|BL_ORD_ID|23363 transcript_61624	1	2112	2110	2486	1027	3136	3595	3955
transcript_10107	gnl|BL_ORD_ID|38781 transcript_8393	2	2259	2259	2862	25	2278	2396	2999
transcript_101089	gnl|BL_ORD_ID|314 transcript_519	1337	2749	1	1337	3043	4455	1	1337
transcript_10114	gnl|BL_ORD_ID|37147 transcript_82196	658	2841	9	661	2954	5135	2	640
transcript_101149	gnl|BL_ORD_ID|82696 transcript_150209	1	1075	1075	1361	152	1226	2691	2977

transcript_101237	gnl|BL_ORD_ID|56951 transcript_111356	1	1965	1963	2672	155	2120	2274	2983
transcript_101268	gnl|BL_ORD_ID|51690 transcript_9641	1	2345	2345	2741	5	2348	2500	2895
transcript_101293	gnl|BL_ORD_ID|27172 transcript_66097	1	1334	1333	2558	1209	2538	2665	3886
transcript_101347	gnl|BL_ORD_ID|78273 transcript_14249	1	1753	1749	1972	1	1751	2240	2463
transcript_101360	gnl|BL_ORD_ID|49074 transcript_99907	1	2808	2808	3259	143	2952	3352	3804
transcript_101366	gnl|BL_ORD_ID|81554 transcript_148177	314	2280	4	314	453	2420	2	312
transcript_10137	gnl|BL_ORD_ID|29976 transcript_70601	1	1673	1672	2828	2	1650	2998	4148
transcript_101399	gnl|BL_ORD_ID|87354 transcript_158054	1	1464	1461	2074	2	1469	3098	3714
transcript_10143	gnl|BL_ORD_ID|39889 transcript_84981	1	1800	1801	2799	1	1802	2233	3231
transcript_101440	gnl|BL_ORD_ID|76524 transcript_141528	132	3164	1	133	258	3292	1	133
transcript_101477	gnl|BL_ORD_ID|78887 transcript_143848	1	1815	1812	3103	862	2680	2781	4069
transcript_101544	gnl|BL_ORD_ID|71478 transcript_133340	1	1815	1811	2103	1	1840	2078	2377
transcript_101555	gnl|BL_ORD_ID|33711 transcript_76656	202	2455	2	205	827	3106	6	208
transcript_101555	gnl|BL_ORD_ID|1068 transcript_1914	202	2457	2	205	829	3086	4	210
transcript_101555	gnl|BL_ORD_ID|59381 transcript_115367	202	2457	2	205	829	3089	4	210
transcript_101555	gnl|BL_ORD_ID|37945 transcript_6595	202	2457	2	205	827	3084	6	208
transcript_101561	gnl|BL_ORD_ID|78567 transcript_143334	2	2183	2181	2909	22	2203	2966	3693
transcript_10167	gnl|BL_ORD_ID|35332 transcript_79303	10	1654	1651	2780	2	1655	2718	3833
transcript_101689	gnl|BL_ORD_ID|89963 transcript_162228	259	3971	2	259	357	4057	1	257
transcript_101742	gnl|BL_ORD_ID|12649 transcript_4202	1	2202	2197	2525	665	2866	3038	3367
transcript_101742	gnl|BL_ORD_ID|11779 transcript_2503	1	2202	2197	2525	935	3136	3308	3638
transcript_101742	gnl|BL_ORD_ID|82737 transcript_150273	1	2202	2197	2497	966	3167	3339	3638
transcript_101742	gnl|BL_ORD_ID|24663 transcript_4785	1	2202	2197	2525	554	2755	2927	3255
transcript_101742	gnl|BL_ORD_ID|12197 transcript_3313	1	2202	2197	2525	781	2982	3154	3482
transcript_101808	gnl|BL_ORD_ID|35638 transcript_79775	1	1202	1200	1647	9	1194	2228	2675
transcript_101819	gnl|BL_ORD_ID|51662 transcript_9574	127	2608	1	132	495	2973	236	367
transcript_101870	gnl|BL_ORD_ID|22141 transcript_59584	1	1000	998	1431	31	1022	1320	1754
transcript_101870	gnl|BL_ORD_ID|6994 transcript_33915	1	1000	998	1430	30	1029	1351	1784
transcript_101895	gnl|BL_ORD_ID|24899 transcript_5274	2	2232	2229	3077	43	2273	2402	3250
transcript_101922	gnl|BL_ORD_ID|18223 transcript_53144	192	2572	1	197	576	2955	178	374
transcript_10193	gnl|BL_ORD_ID|68130 transcript_127900	248	2839	1	250	2006	4607	1	249
transcript_10193	gnl|BL_ORD_ID|38251 transcript_7216	248	2841	1	250	461	3055	1	250
transcript_10193	gnl|BL_ORD_ID|45728 transcript_94593	249	2841	1	250	495	3083	1	250
transcript_101951	gnl|BL_ORD_ID|61577 transcript_118871	1121	3521	1	1124	1613	4012	317	1451

transcript_102025	gnl|BL_ORD_ID|62099 transcript_119684	1	1754	1753	2242	2	1755	1864	2354
transcript_102028	gnl|BL_ORD_ID|11858 transcript_2648	1	2894	2894	3455	101	2989	3105	3665
transcript_102028	gnl|BL_ORD_ID|12139 transcript_3211	1	2894	2894	3349	45	2934	3050	3504
transcript_102028	gnl|BL_ORD_ID|11800 transcript_2548	1	2894	2894	3455	45	2933	3049	3609
transcript_102028	gnl|BL_ORD_ID|44590 transcript_92683	124	2894	2894	3452	2	2765	2881	3436
transcript_10208	gnl|BL_ORD_ID|42371 transcript_88998	1	1408	1407	2781	1	1406	2329	3703
transcript_102088	gnl|BL_ORD_ID|20705 transcript_57174	2	2685	2684	3665	94	2773	3173	4154
transcript_102091	gnl|BL_ORD_ID|81902 transcript_148793	1	1602	1598	2554	373	1955	2096	3037
transcript_102091	gnl|BL_ORD_ID|63498 transcript_121956	1	1486	1486	2534	369	1836	2115	3147
transcript_102091	gnl|BL_ORD_ID|39138 transcript_83767	1153	2555	1	1152	2079	3464	397	1532
transcript_102114	gnl|BL_ORD_ID|4622 transcript_28957	120	1158	4	120	936	1974	2	110
transcript_102131	gnl|BL_ORD_ID|51338 transcript_8910	361	2370	65	361	893	2919	205	503
transcript_102163	gnl|BL_ORD_ID|32792 transcript_75215	1	2280	2279	2713	655	2938	3052	3491
transcript_102192	gnl|BL_ORD_ID|283 transcript_469	1	3171	3167	3750	621	3778	3945	4524
transcript_102269	gnl|BL_ORD_ID|12328 transcript_3556	1297	3310	1	1298	1454	3467	19	1316
transcript_102360	gnl|BL_ORD_ID|6905 transcript_33737	295	1658	80	297	500	1863	151	368
transcript_102364	gnl|BL_ORD_ID|77765 transcript_13082	2	2444	2448	2582	1	2441	2555	2687
transcript_102375	gnl|BL_ORD_ID|52902 transcript_104447	18	2626	2625	2857	38	2646	2861	3092
transcript_10238	gnl|BL_ORD_ID|83228 transcript_151138	242	2826	103	242	420	3003	85	224
transcript_10238	gnl|BL_ORD_ID|17901 transcript_52626	241	2845	103	242	342	2945	85	224
transcript_102391	gnl|BL_ORD_ID|5497 transcript_30774	1	1488	1485	1798	1	1490	1615	1928
transcript_102397	gnl|BL_ORD_ID|3598 transcript_26763	620	1871	65	621	860	2111	164	720
transcript_102399	gnl|BL_ORD_ID|69797 transcript_130599	1	1914	1911	2541	875	2788	2893	3523
transcript_102427	gnl|BL_ORD_ID|21861 transcript_59115	1	1396	1393	2214	1	1400	2409	3230
transcript_102427	gnl|BL_ORD_ID|33014 transcript_75547	12	1394	1393	2211	2	1384	1651	2469
transcript_10244	gnl|BL_ORD_ID|49732 transcript_100959	235	2875	1	240	1471	4113	1	239
transcript_10244	gnl|BL_ORD_ID|69393 transcript_129934	235	2852	2	240	2545	5163	1	239
transcript_10244	gnl|BL_ORD_ID|525 transcript_892	235	2875	2	240	1488	4126	6	243
transcript_10247	gnl|BL_ORD_ID|51436 transcript_9107	1	1633	1633	2819	1	1633	1744	2928
transcript_10248	gnl|BL_ORD_ID|951 transcript_1690	266	2845	1	266	1265	3855	1	266
transcript_10248	gnl|BL_ORD_ID|30841 transcript_72022	266	2846	1	267	1240	3807	1	262
transcript_102483	gnl|BL_ORD_ID|11629 transcript_2208	2	2283	2282	3526	24	2314	2457	3715
transcript_102483	gnl|BL_ORD_ID|12153 transcript_3232	125	2283	2282	3528	5	2170	2313	3562
transcript_102483	gnl|BL_ORD_ID|63999 transcript_122753	2	2283	2282	3450	24	2308	2451	3621

transcript_102543	gnl|BL_ORD_ID|96066 transcript_20956	322	2164	61	326	466	2306	95	360
transcript_102543	gnl|BL_ORD_ID|81934 transcript_148858	322	2164	61	326	1120	2960	749	1014
transcript_102558	gnl|BL_ORD_ID|77724 transcript_13007	238	2453	2	239	386	2601	29	266
transcript_10258	gnl|BL_ORD_ID|25028 transcript_5563	1	2105	2104	2770	2	2102	2550	3216
transcript_10258	gnl|BL_ORD_ID|27098 transcript_65985	1	2105	2104	2798	2	2099	2547	3241
transcript_102599	gnl|BL_ORD_ID|57206 transcript_111781	11	1576	1576	1766	1	1563	2067	2255
transcript_102629	gnl|BL_ORD_ID|32333 transcript_74487	288	3137	1	290	2943	5783	1820	2107
transcript_102650	gnl|BL_ORD_ID|66031 transcript_124469	999	2012	5	1000	1111	2124	2	996
transcript_102651	gnl|BL_ORD_ID|47625 transcript_97604	1	1559	1558	2278	1	1558	1669	2389
transcript_102651	gnl|BL_ORD_ID|75934 transcript_140582	13	1393	1392	2292	2	1381	1530	2431
transcript_102651	gnl|BL_ORD_ID|62204 transcript_119858	11	1632	1628	2253	1	1617	1731	2356
transcript_102674	gnl|BL_ORD_ID|93672 transcript_166617	1	1503	1501	2000	6	1508	2426	2925
transcript_102691	gnl|BL_ORD_ID|66193 transcript_124762	1	1956	1956	2748	88	2043	3559	4352
transcript_102714	gnl|BL_ORD_ID|65199 transcript_12550	1	1733	1730	1924	65	1797	2495	2689
transcript_102717	gnl|BL_ORD_ID|67184 transcript_126373	1	1464	1460	2245	1	1464	3987	4772
transcript_102760	gnl|BL_ORD_ID|19973 transcript_55984	240	2408	107	240	619	2787	353	486
transcript_102762	gnl|BL_ORD_ID|61311 transcript_118464	10	1635	1632	1778	2	1626	2328	2474
transcript_102829	gnl|BL_ORD_ID|40458 transcript_85885	280	2830	1	281	1025	3571	6	285
transcript_102847	gnl|BL_ORD_ID|67256 transcript_126494	1	1812	1811	2468	1	1810	1956	2613
transcript_102890	gnl|BL_ORD_ID|96520 transcript_71122	1	2277	2277	2952	1	2279	2945	3620
transcript_102893	gnl|BL_ORD_ID|38991 transcript_83553	1	1516	1516	2543	1	1517	2050	3077
transcript_102931	gnl|BL_ORD_ID|20511 transcript_56852	1	1080	1076	1795	1	1080	1312	2031
transcript_102950	gnl|BL_ORD_ID|62884 transcript_120958	126	2484	2480	2671	1	2359	2983	3177
transcript_102974	gnl|BL_ORD_ID|21861 transcript_59115	1	1398	1396	2067	1	1398	2559	3230
transcript_102974	gnl|BL_ORD_ID|50640 transcript_102427	1	1398	1396	2067	1	1394	1543	2214
transcript_102990	gnl|BL_ORD_ID|65934 transcript_124316	1105	3352	108	1110	1109	3356	1	1004
transcript_103102	gnl|BL_ORD_ID|24949 transcript_5362	878	2281	9	879	1675	3079	3	850
transcript_103149	gnl|BL_ORD_ID|51964 transcript_10225	1	1854	1855	2062	683	2535	2651	2857
transcript_103149	gnl|BL_ORD_ID|64547 transcript_11080	1	1854	1855	1990	700	2552	2668	2803
transcript_103149	gnl|BL_ORD_ID|78583 transcript_143366	1	1854	1855	2062	566	2424	2540	2748
transcript_103194	gnl|BL_ORD_ID|71267 transcript_132995	13	1787	1783	1925	2	1775	1921	2063
transcript_103194	gnl|BL_ORD_ID|1280 transcript_21592	1	1785	1783	1925	51	1835	2020	2162
transcript_103194	gnl|BL_ORD_ID|1194 transcript_21408	1	1785	1783	1925	6	1790	1975	2117
transcript_103307	gnl|BL_ORD_ID|26291 transcript_64695	154	3181	1	155	811	3838	131	285

transcript_103340	gnl|BL_ORD_ID|62557 transcript_120455	1	1366	1366	2359	1	1367	1826	2821
transcript_103353	gnl|BL_ORD_ID|19054 transcript_54464	11	1777	1777	3098	1	1794	1912	3233
transcript_103387	gnl|BL_ORD_ID|77082 transcript_142426	26	3111	3109	3835	2	3060	3238	3962
transcript_103422	gnl|BL_ORD_ID|59821 transcript_116062	500	2070	96	501	529	2099	2	407
transcript_103519	gnl|BL_ORD_ID|32464 transcript_74682	2	2005	2006	2381	18	2017	2460	2835
transcript_103519	gnl|BL_ORD_ID|40487 transcript_85941	1	1725	1722	2385	1	1723	2790	3456
transcript_103542	gnl|BL_ORD_ID|12318 transcript_3540	216	3204	2	219	484	3469	142	357
transcript_103542	gnl|BL_ORD_ID|12252 transcript_3415	216	3286	2	219	427	3508	81	301
transcript_103558	gnl|BL_ORD_ID|39789 transcript_84825	289	1465	34	290	359	1531	2	258
transcript_103558	gnl|BL_ORD_ID|80893 transcript_147123	291	1465	38	290	374	1548	2	254
transcript_10358	gnl|BL_ORD_ID|23504 transcript_61870	1	2004	2001	2805	1	2022	2345	3137
transcript_10358	gnl|BL_ORD_ID|37866 transcript_6442	1	2004	2001	2808	1	1999	2323	3118
transcript_103622	gnl|BL_ORD_ID|27118 transcript_66013	686	2044	8	687	1218	2578	7	686
transcript_103658	gnl|BL_ORD_ID|27463 transcript_66546	1	1705	1704	2374	16	1721	2260	2930
transcript_103691	gnl|BL_ORD_ID|51288 transcript_8790	1	1724	1724	2710	1	1724	1859	2844
transcript_103721	gnl|BL_ORD_ID|21479 transcript_58481	1	1392	1392	2243	3	1411	3175	4024
transcript_103734	gnl|BL_ORD_ID|51861 transcript_10018	2	2259	2255	2458	17	2274	2646	2837
transcript_103734	gnl|BL_ORD_ID|17439 transcript_51913	2	2259	2255	2458	16	2273	2740	2943
transcript_103734	gnl|BL_ORD_ID|35296 transcript_79251	2	2259	2255	2458	17	2273	2621	2824
transcript_103734	gnl|BL_ORD_ID|52172 transcript_10673	2	2259	2255	2398	58	2315	2688	2831
transcript_103734	gnl|BL_ORD_ID|51654 transcript_9558	2	2259	2255	2430	16	2287	2664	2841
transcript_10375	gnl|BL_ORD_ID|26292 transcript_64696	505	2840	6	505	601	2937	2	501
transcript_10375	gnl|BL_ORD_ID|12571 transcript_4051	1	1950	1946	2840	1	1935	2391	3285
transcript_103772	gnl|BL_ORD_ID|56392 transcript_110451	1	1605	1602	1946	2313	3917	4100	4444
transcript_103772	gnl|BL_ORD_ID|62038 transcript_119585	1	1605	1602	1883	566	2222	2412	2710
transcript_103772	gnl|BL_ORD_ID|81627 transcript_148316	1	1605	1602	1965	2279	3883	4066	4430
transcript_103772	gnl|BL_ORD_ID|35747 transcript_79940	1	1605	1602	1914	2363	3967	4150	4462
transcript_103772	gnl|BL_ORD_ID|22255 transcript_59764	1	1605	1602	1909	2158	3762	3945	4253
transcript_103772	gnl|BL_ORD_ID|411 transcript_703	1	1605	1602	1965	2167	3771	3954	4318
transcript_103772	gnl|BL_ORD_ID|73389 transcript_136399	1	1605	1602	1927	2092	3696	3971	4297
transcript_103784	gnl|BL_ORD_ID|89086 transcript_160815	1	1204	1200	1541	1	1204	2275	2616
transcript_103793	gnl|BL_ORD_ID|18976 transcript_54326	217	2532	1	216	728	3034	21	232
transcript_103800	gnl|BL_ORD_ID|90803 transcript_15259	1	1168	1166	2184	54	1222	1500	2520
transcript_103826	gnl|BL_ORD_ID|31169 transcript_72529	1	1823	1822	2341	648	2446	2862	3382

transcript_103868	gnl|BL_ORD_ID|24484 transcript_4415	1	2021	2019	3205	1	2013	2149	3334
transcript_103898	gnl|BL_ORD_ID|30728 transcript_71824	491	2625	54	496	792	2927	3	444
transcript_103902	gnl|BL_ORD_ID|86102 transcript_155964	1	2578	2576	3267	1	2611	2820	3513
transcript_103925	gnl|BL_ORD_ID|83617 transcript_151832	10	1424	1423	1833	1	1429	4842	5252
transcript_103927	gnl|BL_ORD_ID|34390 transcript_77777	112	2694	1	113	635	3231	321	433
transcript_103944	gnl|BL_ORD_ID|44600 transcript_92696	147	1633	1	152	797	2286	1	152
transcript_103953	gnl|BL_ORD_ID|25656 transcript_63699	380	4910	186	381	488	5019	141	341
transcript_10396	gnl|BL_ORD_ID|12363 transcript_3627	269	2770	1	268	984	3485	1	268
transcript_103976	gnl|BL_ORD_ID|58573 transcript_114052	205	2729	1	209	1142	3673	95	303
transcript_103976	gnl|BL_ORD_ID|11777 transcript_2501	205	2729	1	209	1112	3640	95	303
transcript_103986	gnl|BL_ORD_ID|39927 transcript_85044	226	2087	23	229	319	2179	2	207
transcript_103994	gnl|BL_ORD_ID|12669 transcript_4236	1	2286	2285	3210	2	2287	2389	3330
transcript_103994	gnl|BL_ORD_ID|50924 transcript_102897	17	2286	2285	3190	36	2305	2407	3326
transcript_104002	gnl|BL_ORD_ID|87166 transcript_157727	1058	2415	1	1057	1380	2739	196	1253
transcript_104022	gnl|BL_ORD_ID|22967 transcript_60973	1	2518	2515	3127	987	3503	3611	4222
transcript_104066	gnl|BL_ORD_ID|56847 transcript_111187	1	2010	2010	3087	930	2927	3081	4160
transcript_104066	gnl|BL_ORD_ID|48580 transcript_99123	1	2010	2010	3085	906	2949	3103	4180
transcript_10414	gnl|BL_ORD_ID|35030 transcript_78834	2	2482	2481	2832	99	2575	2860	3211
transcript_104150	gnl|BL_ORD_ID|3091 transcript_25619	1	1177	1179	1832	2	1172	1506	2159
transcript_104164	gnl|BL_ORD_ID|20659 transcript_57087	10	2985	2985	3798	2	2978	3124	3936
transcript_104164	gnl|BL_ORD_ID|82531 transcript_149917	10	2985	2985	3795	2	2973	3117	3927
transcript_104164	gnl|BL_ORD_ID|715 transcript_1245	1	2985	2985	3798	4	2994	3138	3940
transcript_104189	gnl|BL_ORD_ID|25700 transcript_63764	1024	2322	1	1026	1152	2451	1	992
transcript_104242	gnl|BL_ORD_ID|11688 transcript_2321	2	3505	3505	3724	13	3498	3377	3597
transcript_104245	gnl|BL_ORD_ID|71127 transcript_132787	1	2388	2387	2973	1	2387	2512	3098
transcript_104256	gnl|BL_ORD_ID|4860 transcript_29429	383	1775	9	384	488	1900	2	372
transcript_104270	gnl|BL_ORD_ID|63771 transcript_122400	1	1205	1200	1917	155	1348	2493	3207
transcript_104272	gnl|BL_ORD_ID|8649 transcript_37209	426	1390	5	428	669	1625	50	473
transcript_104292	gnl|BL_ORD_ID|28822 transcript_68788	1	3535	3530	5038	1	3516	3792	5285
transcript_104314	gnl|BL_ORD_ID|56606 transcript_110795	519	2295	61	520	2542	4318	6	463
transcript_104320	gnl|BL_ORD_ID|49135 transcript_100005	1	1449	1448	1973	16	1474	1579	2101
transcript_104352	gnl|BL_ORD_ID|18976 transcript_54326	277	2492	41	276	728	2935	1	232
transcript_104354	gnl|BL_ORD_ID|60430 transcript_117031	210	3050	2	211	1155	3996	55	264
transcript_104381	gnl|BL_ORD_ID|41333 transcript_87335	1	1089	1085	1587	1	1080	2387	2889

transcript_10440	gnl|BL_ORD_ID|46532 transcript_95902	1134	2848	1	1134	1495	3209	1	1135
transcript_104443	gnl|BL_ORD_ID|50187 transcript_101679	1	3429	3426	3986	2	3428	5031	5590
transcript_104480	gnl|BL_ORD_ID|79106 transcript_144220	228	1121	6	230	4031	4924	2	225
transcript_104483	gnl|BL_ORD_ID|58779 transcript_114386	182	2547	1	184	896	3254	71	255
transcript_1045	gnl|BL_ORD_ID|62842 transcript_120894	16	3318	3319	4024	2	3310	3522	4227
transcript_104524	gnl|BL_ORD_ID|97474 transcript_163033	508	5115	134	509	615	5197	1	375
transcript_104575	gnl|BL_ORD_ID|87765 transcript_158704	1	1217	1217	1645	1	1217	1943	2371
transcript_104594	gnl|BL_ORD_ID|77222 transcript_142650	279	1119	3	279	1049	1889	1	276
transcript_104597	gnl|BL_ORD_ID|69766 transcript_130551	16	2808	2806	4458	1	2777	2984	4639
transcript_10471	gnl|BL_ORD_ID|38128 transcript_6963	226	2869	12	226	454	3094	97	312
transcript_104727	gnl|BL_ORD_ID|51473 transcript_9169	1	1272	1268	1620	90	1361	1513	1865
transcript_104727	gnl|BL_ORD_ID|24565 transcript_4569	1	1272	1268	1617	18	1283	1435	1784
transcript_104727	gnl|BL_ORD_ID|24875 transcript_5220	1	1272	1268	1620	28	1299	1451	1803
transcript_104734	gnl|BL_ORD_ID|30835 transcript_72013	1	2859	2856	3330	1	2858	3407	3881
transcript_104734	gnl|BL_ORD_ID|932 transcript_1661	1	2859	2856	3332	4	2861	3411	3871
transcript_10479	gnl|BL_ORD_ID|23106 transcript_61209	1187	2816	1	1189	1301	2934	1	1190
transcript_104888	gnl|BL_ORD_ID|87485 transcript_158252	176	1538	2	181	523	1883	239	406
transcript_104888	gnl|BL_ORD_ID|43254 transcript_90419	176	1570	9	181	393	1785	1	164
transcript_104888	gnl|BL_ORD_ID|64100 transcript_122916	176	1570	2	181	531	1923	244	414
transcript_104888	gnl|BL_ORD_ID|59673 transcript_115825	176	1538	9	181	285	1645	2	168
transcript_104926	gnl|BL_ORD_ID|92085 transcript_164033	1222	3772	1	1224	2456	5007	601	1824
transcript_104936	gnl|BL_ORD_ID|65167 transcript_12473	2	2223	2223	2386	1	2228	2354	2517
transcript_104951	gnl|BL_ORD_ID|39149 transcript_83786	129	1078	2	134	1700	2649	677	809
transcript_104951	gnl|BL_ORD_ID|59482 transcript_115525	129	1089	2	134	1518	2478	495	627
transcript_104951	gnl|BL_ORD_ID|86506 transcript_156627	129	1089	2	134	1564	2524	539	671
transcript_105010	gnl|BL_ORD_ID|66178 transcript_124736	1	2613	2611	3282	284	2894	3256	3927
transcript_105057	gnl|BL_ORD_ID|3820 transcript_27241	142	1985	1	141	244	2086	1	141
transcript_105103	gnl|BL_ORD_ID|37280 transcript_82401	1	1057	1056	1646	7	1063	2375	2965
transcript_10517	gnl|BL_ORD_ID|11938 transcript_2816	15	2111	2108	2841	1	2091	2913	3647
transcript_105227	gnl|BL_ORD_ID|24275 transcript_63087	1	1231	1229	1621	9	1218	3024	3411
transcript_105230	gnl|BL_ORD_ID|11281 transcript_42233	127	1149	13	129	223	1238	2	118
transcript_10524	gnl|BL_ORD_ID|27074 transcript_65949	1	2370	2369	2863	5	2362	4018	4512
transcript_105305	gnl|BL_ORD_ID|5812 transcript_31466	1	1563	1563	1801	52	1613	1718	1956
transcript_105306	gnl|BL_ORD_ID|73382 transcript_136391	306	2651	75	305	1966	4302	1620	1848

transcript_105343	gnl|BL_ORD_ID|81033 transcript_147332	1	1341	1340	1973	107	1447	3134	3767
transcript_105405	gnl|BL_ORD_ID|51512 transcript_9243	1	1662	1658	2656	2	1663	1788	2786
transcript_105424	gnl|BL_ORD_ID|12346 transcript_3585	1	2679	2675	3244	2	2680	2870	3439
transcript_105464	gnl|BL_ORD_ID|22932 transcript_60922	1	1021	1020	1609	23	1040	2768	3357
transcript_105482	gnl|BL_ORD_ID|91771 transcript_163527	1	1627	1629	2006	523	2149	2733	3096
transcript_10553	gnl|BL_ORD_ID|24484 transcript_4415	1	1609	1607	2792	405	2013	2149	3333
transcript_10553	gnl|BL_ORD_ID|90038 transcript_162337	1	1609	1607	2792	12	1617	1753	2938
transcript_10556	gnl|BL_ORD_ID|46084 transcript_95182	14	1416	1415	2817	261	1662	1780	3177
transcript_105566	gnl|BL_ORD_ID|90928 transcript_15535	1	1672	1671	2368	1	1672	1854	2549
transcript_105645	gnl|BL_ORD_ID|19559 transcript_55335	2	2029	2024	2465	20	2047	2469	2910
transcript_10570	gnl|BL_ORD_ID|45983 transcript_95026	2	2099	2096	2820	5	2104	2393	3116
transcript_105723	gnl|BL_ORD_ID|58816 transcript_114450	1	2688	2685	3243	503	3191	3405	3964
transcript_105724	gnl|BL_ORD_ID|68944 transcript_129218	1	1339	1338	1645	1	1326	2165	2473
transcript_105731	gnl|BL_ORD_ID|88527 transcript_159921	1	1593	1590	1874	1	1597	2571	2855
transcript_105744	gnl|BL_ORD_ID|56673 transcript_110911	1	2377	2374	3366	460	2834	3047	4038
transcript_105748	gnl|BL_ORD_ID|556 transcript_940	1	1633	1633	2788	1	1633	2965	4122
transcript_10576	gnl|BL_ORD_ID|67756 transcript_127293	1	1546	1546	2801	1	1543	2617	3875
transcript_105810	gnl|BL_ORD_ID|68199 transcript_128007	272	2621	33	271	422	2766	2	239
transcript_105833	gnl|BL_ORD_ID|422 transcript_726	618	3310	79	619	1440	4128	1	541
transcript_105835	gnl|BL_ORD_ID|11713 transcript_2368	2	3130	3130	3475	7	3133	3251	3596
transcript_105835	gnl|BL_ORD_ID|54986 transcript_108022	2	3130	3130	3349	49	3372	3498	3731
transcript_105835	gnl|BL_ORD_ID|47460 transcript_97357	2	3130	3130	3475	153	3278	3396	3741
transcript_105839	gnl|BL_ORD_ID|88373 transcript_159694	1	2364	2361	2681	1427	3818	3956	4276
transcript_105846	gnl|BL_ORD_ID|38502 transcript_7744	22	2547	2547	2952	2	2515	2617	3021
transcript_10588	gnl|BL_ORD_ID|88820 transcript_160418	417	2836	5	416	515	2928	2	413
transcript_105885	gnl|BL_ORD_ID|3226 transcript_25934	1	1068	1066	2018	3	1063	1182	2142
transcript_105885	gnl|BL_ORD_ID|4036 transcript_27719	1	1068	1066	2018	4	1071	1180	2133
transcript_105894	gnl|BL_ORD_ID|87097 transcript_157619	1	1444	1439	2734	67	1509	2299	3595
transcript_105906	gnl|BL_ORD_ID|67990 transcript_127668	1	1466	1466	1978	712	2156	2259	2772
transcript_10593	gnl|BL_ORD_ID|57672 transcript_112584	1157	2754	1	1158	1574	3170	1	1156
transcript_105944	gnl|BL_ORD_ID|76194 transcript_141017	1	2210	2208	2928	8	2221	4928	5648
transcript_1060	gnl|BL_ORD_ID|53931 transcript_106200	292	4144	136	295	336	4187	1	162
transcript_106026	gnl|BL_ORD_ID|62151 transcript_119768	366	1123	4	366	1055	1821	563	932
transcript_106073	gnl|BL_ORD_ID|64378 transcript_10729	103	1582	1582	2134	101	1580	2259	2813

transcript_1061	gnl|BL_ORD_ID|507 transcript_859	201	4071	2	204	308	4178	6	208
transcript_1061	gnl|BL_ORD_ID|63058 transcript_121239	205	4101	2	204	378	4274	11	213
transcript_106103	gnl|BL_ORD_ID|19348 transcript_54958	152	1060	8	154	275	1183	2	148
transcript_106133	gnl|BL_ORD_ID|74559 transcript_138345	1	2271	2268	2719	462	2730	2856	3307
transcript_106166	gnl|BL_ORD_ID|28569 transcript_68381	7	654	649	1180	1	648	1070	1601
transcript_10617	gnl|BL_ORD_ID|21842 transcript_59077	1	1711	1711	2827	1	1711	1837	2950
transcript_106186	gnl|BL_ORD_ID|1300 transcript_21629	1	1038	1036	1998	28	1063	1323	2284
transcript_106186	gnl|BL_ORD_ID|74946 transcript_138981	1	1038	1036	1998	20	1054	1345	2307
transcript_106217	gnl|BL_ORD_ID|19366 transcript_54990	1	1951	1952	2670	432	2372	2889	3607
transcript_106223	gnl|BL_ORD_ID|53322 transcript_105142	2	2442	2442	3829	1	2440	3086	4477
transcript_106230	gnl|BL_ORD_ID|85481 transcript_154926	1	1498	1496	2238	222	1723	3120	3865
transcript_106327	gnl|BL_ORD_ID|80201 transcript_145979	108	2197	2193	2422	921	3009	3264	3493
transcript_106327	gnl|BL_ORD_ID|51532 transcript_9279	2	2197	2193	2422	147	2342	2597	2826
transcript_106368	gnl|BL_ORD_ID|72761 transcript_135388	457	2253	53	457	1029	2826	32	437
transcript_106394	gnl|BL_ORD_ID|90891 transcript_15450	1	1023	1023	1864	75	1097	1725	2566
transcript_106394	gnl|BL_ORD_ID|12623 transcript_4143	1	1023	1023	1866	18	1040	2544	3387
transcript_106457	gnl|BL_ORD_ID|33329 transcript_76026	1	2089	2088	2761	55	2148	2355	3028
transcript_10650	gnl|BL_ORD_ID|38395 transcript_7539	2	2317	2318	2797	131	2431	2559	3037
transcript_10650	gnl|BL_ORD_ID|34704 transcript_78300	2	2317	2318	2779	86	2401	2529	2990
transcript_10650	gnl|BL_ORD_ID|91659 transcript_163338	2	2317	2318	2708	52	2368	2476	2866
transcript_106531	gnl|BL_ORD_ID|31863 transcript_73665	274	3833	2	276	429	3986	4	278
transcript_106582	gnl|BL_ORD_ID|76410 transcript_141338	2	2007	2008	2484	187	2192	3067	3541
transcript_10661	gnl|BL_ORD_ID|60383 transcript_116960	306	2825	43	306	713	3232	2	260
transcript_106625	gnl|BL_ORD_ID|85870 transcript_155565	384	1807	45	383	690	2111	237	575
transcript_106670	gnl|BL_ORD_ID|11677 transcript_2298	1	1648	1647	3174	2	1648	1889	3413
transcript_106679	gnl|BL_ORD_ID|65868 transcript_124206	1	2473	2473	2881	44	2516	3326	3734
transcript_106715	gnl|BL_ORD_ID|89356 transcript_161259	174	1407	2	178	574	1815	4	180
transcript_106715	gnl|BL_ORD_ID|56701 transcript_110961	174	1381	7	178	903	2118	331	503
transcript_10673	gnl|BL_ORD_ID|19665 transcript_55491	309	2831	75	308	428	2951	2	235
transcript_106741	gnl|BL_ORD_ID|68078 transcript_127813	1	1541	1537	2250	1	1559	3014	3736
transcript_106793	gnl|BL_ORD_ID|32880 transcript_75350	1	2096	2097	2522	137	2232	2897	3322
transcript_106849	gnl|BL_ORD_ID|96638 transcript_81324	11	1614	1614	3126	4	1607	1758	3269
transcript_106868	gnl|BL_ORD_ID|57450 transcript_112206	1	2493	2493	2884	279	2768	2870	3261
transcript_106868	gnl|BL_ORD_ID|37942 transcript_6590	1	2493	2493	2884	68	2563	2665	3056

transcript_106881	gnl|BL_ORD_ID|84870 transcript_153963	641	1907	7	644	770	2036	3	638
transcript_106881	gnl|BL_ORD_ID|49355 transcript_100348	1	1647	1646	1931	1	1646	1756	2041
transcript_1069	gnl|BL_ORD_ID|86871 transcript_157234	21	2218	2218	4001	162	2360	2463	4246
transcript_106943	gnl|BL_ORD_ID|2025 transcript_23283	143	985	1	143	1420	2263	1073	1215
transcript_107000	gnl|BL_ORD_ID|66665 transcript_125534	224	1088	50	225	1602	2466	158	333
transcript_107002	gnl|BL_ORD_ID|347 transcript_597	1378	3627	1	1381	2173	4417	9	1389
transcript_107003	gnl|BL_ORD_ID|729 transcript_1269	130	2724	1	134	1420	4011	487	621
transcript_107003	gnl|BL_ORD_ID|805 transcript_1404	130	2723	1	134	1184	3773	251	385
transcript_107003	gnl|BL_ORD_ID|1122 transcript_2014	130	2723	1	134	953	3542	20	154
transcript_107026	gnl|BL_ORD_ID|34793 transcript_78438	1	1839	1840	2551	18	1858	2087	2798
transcript_10707	gnl|BL_ORD_ID|44530 transcript_92577	354	2826	96	355	526	2998	2	261
transcript_107082	gnl|BL_ORD_ID|34107 transcript_77315	169	1572	2	174	1064	2461	19	179
transcript_107082	gnl|BL_ORD_ID|62154 transcript_119776	169	1572	2	174	1992	3399	15	173
transcript_107082	gnl|BL_ORD_ID|84453 transcript_153292	169	1572	2	174	1056	2452	25	185
transcript_107082	gnl|BL_ORD_ID|91047 transcript_15788	169	1572	2	174	1044	2448	1	172
transcript_107082	gnl|BL_ORD_ID|90700 transcript_15017	169	1572	2	174	1174	2577	30	202
transcript_107082	gnl|BL_ORD_ID|23975 transcript_62627	169	1572	4	174	1851	3253	3	172
transcript_107082	gnl|BL_ORD_ID|92231 transcript_164265	169	1572	2	174	1831	3230	1	161
transcript_10711	gnl|BL_ORD_ID|41434 transcript_87511	1086	2811	106	1090	1221	2946	2	986
transcript_10711	gnl|BL_ORD_ID|46585 transcript_95997	106	1646	1643	2811	2	1542	1986	3154
transcript_107116	gnl|BL_ORD_ID|3553 transcript_26662	1	1503	1504	2005	1	1515	1618	2119
transcript_107150	gnl|BL_ORD_ID|42013 transcript_88439	384	2206	42	387	490	2312	2	347
transcript_107177	gnl|BL_ORD_ID|27618 transcript_66789	190	2335	1	193	312	2458	20	212
transcript_107177	gnl|BL_ORD_ID|94901 transcript_18246	190	2310	1	193	339	2462	47	239
transcript_107231	gnl|BL_ORD_ID|10406 transcript_40626	276	1139	9	276	516	1379	1	265
transcript_107258	gnl|BL_ORD_ID|56969 transcript_111381	1	1955	1953	2273	102	2060	3099	3420
transcript_107267	gnl|BL_ORD_ID|23264 transcript_61472	501	1860	56	502	2531	3888	2	450
transcript_107270	gnl|BL_ORD_ID|73911 transcript_137285	272	2419	38	272	341	2483	3	237
transcript_107271	gnl|BL_ORD_ID|97307 transcript_146551	12	1402	1402	2663	115	1505	1667	2919
transcript_107288	gnl|BL_ORD_ID|51267 transcript_8735	252	2069	3	254	650	2468	7	258
transcript_107295	gnl|BL_ORD_ID|90767 transcript_15167	1	1127	1126	1529	838	1961	2116	2519
transcript_107379	gnl|BL_ORD_ID|2063 transcript_23361	1	1606	1601	2052	1	1611	1711	2167
transcript_107398	gnl|BL_ORD_ID|51977 transcript_10248	1	2376	2373	2708	1	2377	2512	2845
transcript_107398	gnl|BL_ORD_ID|51759 transcript_9760	2	2376	2373	2708	1	2374	2537	2873

transcript_107398	gnl|BL_ORD_ID|51325 transcript_8886	2	2376	2373	2708	69	2444	2591	2927
transcript_107462	gnl|BL_ORD_ID|43905 transcript_91507	1	1707	1704	2140	1	1684	1811	2247
transcript_107484	gnl|BL_ORD_ID|89997 transcript_162280	1	2043	2040	2470	8	2043	2196	2624
transcript_10750	gnl|BL_ORD_ID|20367 transcript_56603	1	2227	2227	2838	2	2229	2346	2957
transcript_107544	gnl|BL_ORD_ID|77610 transcript_143255	608	3049	7	610	732	3174	10	600
transcript_107548	gnl|BL_ORD_ID|18318 transcript_53308	1	1441	1438	2285	1	1441	1620	2467
transcript_10758	gnl|BL_ORD_ID|47877 transcript_97997	307	2790	6	309	431	2913	2	305
transcript_107596	gnl|BL_ORD_ID|11833 transcript_2606	1	3045	3041	3447	5	3049	3170	3576
transcript_107638	gnl|BL_ORD_ID|64853 transcript_11734	232	2458	2	236	407	2633	11	245
transcript_10764	gnl|BL_ORD_ID|39138 transcript_83767	1	1535	1536	2837	1	1532	2079	3379
transcript_10767	gnl|BL_ORD_ID|90555 transcript_163105	20	2576	2573	2815	2	2565	2708	2950
transcript_10775	gnl|BL_ORD_ID|35332 transcript_79303	1	1648	1645	2761	1	1655	2718	3833
transcript_107759	gnl|BL_ORD_ID|50295 transcript_101847	350	4187	162	352	1284	5120	2	193
transcript_107760	gnl|BL_ORD_ID|25213 transcript_5939	12	2752	2751	3002	2	2743	2944	3196
transcript_107780	gnl|BL_ORD_ID|9077 transcript_38061	244	1280	7	244	493	1529	2	239
transcript_107780	gnl|BL_ORD_ID|59460 transcript_115490	244	1245	7	244	472	1473	2	218
transcript_1078	gnl|BL_ORD_ID|518 transcript_877	135	3988	1	138	311	4164	1	138
transcript_10784	gnl|BL_ORD_ID|84655 transcript_153625	202	2704	2	202	526	3013	165	368
transcript_10784	gnl|BL_ORD_ID|47877 transcript_97997	354	2785	53	356	431	2875	2	305
transcript_107874	gnl|BL_ORD_ID|92835 transcript_165258	109	1969	1970	2853	2	1862	1983	2867
transcript_107885	gnl|BL_ORD_ID|5126 transcript_30008	383	1829	5	385	531	1974	2	380
transcript_107911	gnl|BL_ORD_ID|11774 transcript_2497	427	3433	7	428	641	3643	92	513
transcript_107911	gnl|BL_ORD_ID|11620 transcript_2187	427	3481	7	428	596	3618	47	468
transcript_107911	gnl|BL_ORD_ID|11691 transcript_2327	427	3481	7	428	614	3662	65	486
transcript_107948	gnl|BL_ORD_ID|52914 transcript_104463	17	2415	2414	2691	2	2399	2622	2899
transcript_107953	gnl|BL_ORD_ID|21506 transcript_58536	455	1573	8	455	717	1837	1	448
transcript_107960	gnl|BL_ORD_ID|28405 transcript_68130	121	2517	1	121	265	2660	1	121
transcript_10797	gnl|BL_ORD_ID|28914 transcript_68936	1	2116	2116	2728	1	2116	2268	2874
transcript_107990	gnl|BL_ORD_ID|51066 transcript_103127	1	1143	1141	1807	14	1156	1442	2108
transcript_107990	gnl|BL_ORD_ID|3550 transcript_26651	1	1040	1041	1807	149	1188	1307	2073
transcript_108049	gnl|BL_ORD_ID|76005 transcript_140698	1	2333	2333	2600	7	2338	3713	3980
transcript_108051	gnl|BL_ORD_ID|59578 transcript_115680	1	1184	1181	1673	1	1185	1394	1890
transcript_108051	gnl|BL_ORD_ID|23740 transcript_62259	255	1673	8	255	489	1906	6	254
transcript_108055	gnl|BL_ORD_ID|31311 transcript_72767	196	1059	2	198	415	1281	101	297

transcript_108059	gnl|BL_ORD_ID|41129 transcript_87002	223	2793	2	224	1074	3649	4	235
transcript_10807	gnl|BL_ORD_ID|51764 transcript_9774	219	2700	1	220	405	2885	1	220
transcript_108112	gnl|BL_ORD_ID|52602 transcript_103974	1	1650	1649	2077	1	1650	1791	2218
transcript_108112	gnl|BL_ORD_ID|12430 transcript_3755	1	1650	1649	2077	1	1650	1791	2219
transcript_108118	gnl|BL_ORD_ID|95974 transcript_20739	9	646	645	1198	55	680	1543	2090
transcript_108121	gnl|BL_ORD_ID|46362 transcript_95625	116	2010	2008	3280	51	1950	2057	3333
transcript_108128	gnl|BL_ORD_ID|38781 transcript_8393	2	2235	2234	2691	22	2279	2541	2999
transcript_108170	gnl|BL_ORD_ID|24263 transcript_63066	1	1975	1971	3352	1	2011	2611	3992
transcript_108190	gnl|BL_ORD_ID|22579 transcript_60304	1354	2935	1	1355	2185	3790	218	1612
transcript_108228	gnl|BL_ORD_ID|90882 transcript_15427	1	1355	1353	2219	1	1355	1701	2565
transcript_108228	gnl|BL_ORD_ID|64763 transcript_11562	1	1355	1353	2158	1	1355	1701	2504
transcript_108247	gnl|BL_ORD_ID|43548 transcript_90897	1	1075	1073	1521	1	1064	2336	2759
transcript_108296	gnl|BL_ORD_ID|92847 transcript_165278	134	1649	1	135	369	1885	83	217
transcript_108309	gnl|BL_ORD_ID|55371 transcript_108704	136	2007	2007	3610	1	1879	2031	3633
transcript_108382	gnl|BL_ORD_ID|43421 transcript_90677	1467	3471	1	1466	1579	3586	11	1473
transcript_108441	gnl|BL_ORD_ID|38799 transcript_8425	1	1595	1594	2799	29	1624	1780	2986
transcript_108504	gnl|BL_ORD_ID|84958 transcript_154100	1467	3423	1	1470	1575	3532	1	1439
transcript_108519	gnl|BL_ORD_ID|87822 transcript_158799	1	1871	1871	2732	743	2614	3324	4186
transcript_108538	gnl|BL_ORD_ID|6675 transcript_33279	1	1340	1341	1677	2	1341	1444	1780
transcript_10859	gnl|BL_ORD_ID|72502 transcript_134970	1	2469	2465	2787	1	2453	3103	3421
transcript_108641	gnl|BL_ORD_ID|17919 transcript_52657	1	1399	1398	2200	2	1398	2606	3408
transcript_108656	gnl|BL_ORD_ID|76369 transcript_141275	1	2002	1997	2773	225	2226	2914	3689
transcript_108656	gnl|BL_ORD_ID|86877 transcript_157242	1	2002	1997	2777	214	2215	3014	3793
transcript_108656	gnl|BL_ORD_ID|767 transcript_1338	1	2002	1997	2782	307	2308	3152	3936
transcript_108656	gnl|BL_ORD_ID|52899 transcript_104443	1	2002	1997	2792	346	2347	3191	3985
transcript_108656	gnl|BL_ORD_ID|33489 transcript_76290	1	2002	1997	2792	191	2192	4015	4809
transcript_108656	gnl|BL_ORD_ID|674 transcript_1166	1	2002	1997	2792	346	2348	3287	4082
transcript_108696	gnl|BL_ORD_ID|43624 transcript_91031	262	1569	34	261	1143	2454	287	515
transcript_108712	gnl|BL_ORD_ID|73053 transcript_135845	1	2165	2164	2897	1	2167	2750	3480
transcript_108719	gnl|BL_ORD_ID|88892 transcript_160517	15	2094	2090	2658	1	2076	2583	3164
transcript_10889	gnl|BL_ORD_ID|30403 transcript_71296	2	2217	2218	2789	6	2220	2856	3427
transcript_109015	gnl|BL_ORD_ID|4587 transcript_28867	160	1374	18	164	607	1821	2	148
transcript_109015	gnl|BL_ORD_ID|21591 transcript_58669	160	1374	18	164	529	1744	1	147
transcript_109018	gnl|BL_ORD_ID|30900 transcript_72109	1	1023	1022	1792	736	1758	2511	3271

transcript_109022	gnl|BL_ORD_ID|684 transcript_1180	2	3459	3458	3770	60	3517	3628	3940
transcript_10903	gnl|BL_ORD_ID|69498 transcript_130102	1221	2784	1	1220	1440	3004	1	1218
transcript_109038	gnl|BL_ORD_ID|66453 transcript_125189	451	1675	60	452	1257	2481	104	496
transcript_109054	gnl|BL_ORD_ID|65159 transcript_12456	340	1578	4	342	1500	2738	1	341
transcript_109077	gnl|BL_ORD_ID|67753 transcript_127286	311	1399	6	312	562	1650	1	320
transcript_109087	gnl|BL_ORD_ID|81056 transcript_147365	2	3134	3130	3284	63	3192	3330	3484
transcript_109122	gnl|BL_ORD_ID|25045 transcript_5600	148	1912	1	146	1143	2916	6	151
transcript_109162	gnl|BL_ORD_ID|45140 transcript_93587	1	1479	1478	2350	1	1466	1687	2560
transcript_109165	gnl|BL_ORD_ID|82438 transcript_149746	1	1588	1587	2908	1612	3183	3434	4750
transcript_109205	gnl|BL_ORD_ID|25287 transcript_6111	291	2690	4	295	775	3171	28	318
transcript_109205	gnl|BL_ORD_ID|37913 transcript_6541	291	2652	4	295	751	3115	2	292
transcript_109205	gnl|BL_ORD_ID|36525 transcript_81236	291	2690	4	295	749	3146	2	292
transcript_109205	gnl|BL_ORD_ID|53728 transcript_105876	291	2650	5	295	731	3089	1	290
transcript_109254	gnl|BL_ORD_ID|94093 transcript_167268	1	2159	2158	2371	1508	3665	4272	4485
transcript_109344	gnl|BL_ORD_ID|30928 transcript_72147	1	2136	2136	2641	1	2139	2695	3200
transcript_109396	gnl|BL_ORD_ID|44675 transcript_92810	1	2003	2001	2470	2	2034	2136	2608
transcript_109405	gnl|BL_ORD_ID|12261 transcript_3430	2	3009	3009	3202	142	3164	3280	3469
transcript_109405	gnl|BL_ORD_ID|12342 transcript_3580	2	3009	3009	3163	173	3181	3297	3444
transcript_109416	gnl|BL_ORD_ID|24770 transcript_5020	1488	3024	1	1490	1757	3300	152	1651
transcript_10942	gnl|BL_ORD_ID|92831 transcript_165252	309	2805	6	309	807	3303	2	305
transcript_109449	gnl|BL_ORD_ID|84789 transcript_153829	2	3256	3255	3682	6	3257	3710	4138
transcript_109490	gnl|BL_ORD_ID|12397 transcript_3694	1	2015	2012	2904	34	2040	2556	3448
transcript_109490	gnl|BL_ORD_ID|12330 transcript_3561	1	2015	2012	2899	6	2056	2572	3436
transcript_109490	gnl|BL_ORD_ID|12297 transcript_3505	1	2015	2012	2899	9	2024	2540	3429
transcript_10952	gnl|BL_ORD_ID|38156 transcript_7020	1	1572	1567	2795	1	1572	1873	3101
transcript_10959	gnl|BL_ORD_ID|38720 transcript_8250	1	1827	1825	2792	1	1823	2013	2971
transcript_109598	gnl|BL_ORD_ID|82679 transcript_150177	1	1976	1974	2484	1031	3004	4325	4854
transcript_109709	gnl|BL_ORD_ID|28929 transcript_68957	1	1637	1634	2177	1750	3377	4176	4713
transcript_109717	gnl|BL_ORD_ID|28054 transcript_67510	571	1618	65	572	831	1878	16	523
transcript_109770	gnl|BL_ORD_ID|920 transcript_1642	223	3696	1	224	345	3819	1	224
transcript_109805	gnl|BL_ORD_ID|56804 transcript_111117	235	2042	5	236	382	2209	23	252
transcript_109805	gnl|BL_ORD_ID|25608 transcript_63614	235	2042	5	236	434	2261	46	304
transcript_109805	gnl|BL_ORD_ID|75641 transcript_140090	235	2042	5	236	403	2230	15	273
transcript_109805	gnl|BL_ORD_ID|27111 transcript_66004	235	2042	7	236	945	2766	36	293

transcript_109805	gnl|BL_ORD_ID|76808 transcript_141974	235	2042	5	236	408	2233	49	278
transcript_10982	gnl|BL_ORD_ID|54403 transcript_106995	534	2792	6	536	1649	3907	1	531
transcript_10982	gnl|BL_ORD_ID|73591 transcript_136732	534	2792	6	536	2758	5009	2	532
transcript_10987	gnl|BL_ORD_ID|28077 transcript_67545	1	2387	2386	2791	1	2389	2845	3251
transcript_10987	gnl|BL_ORD_ID|29968 transcript_70589	1	1858	1856	2795	2	1853	1978	2917
transcript_10989	gnl|BL_ORD_ID|54485 transcript_107144	2	2579	2580	2792	37	2611	2731	2943
transcript_109897	gnl|BL_ORD_ID|25090 transcript_5704	998	2378	4	1000	1770	3163	2	995
transcript_109903	gnl|BL_ORD_ID|47513 transcript_97444	17	2185	2184	2666	3	2174	2383	2854
transcript_109903	gnl|BL_ORD_ID|27550 transcript_66674	1	2185	2184	2666	3	2197	2301	2774
transcript_109903	gnl|BL_ORD_ID|25031 transcript_5567	1	2185	2184	2665	26	2323	2429	2926
transcript_109903	gnl|BL_ORD_ID|88413 transcript_159752	20	2180	2184	2666	2	2165	3090	3574
transcript_109903	gnl|BL_ORD_ID|51991 transcript_10275	12	2185	2184	2666	28	2204	2308	2789
transcript_109903	gnl|BL_ORD_ID|38657 transcript_8102	17	2185	2184	2666	14	2185	2289	2759
transcript_109903	gnl|BL_ORD_ID|51894 transcript_10073	1	2185	2184	2666	29	2214	2318	2791
transcript_109918	gnl|BL_ORD_ID|90927 transcript_15533	137	2414	11	139	285	2563	2	130
transcript_10993	gnl|BL_ORD_ID|63618 transcript_122155	1416	2828	1	1417	2852	4278	1	1395
transcript_109972	gnl|BL_ORD_ID|1072 transcript_1919	2	2634	2633	3449	81	2713	2968	3784
transcript_110048	gnl|BL_ORD_ID|79896 transcript_145484	1	1272	1272	1843	1	1295	1459	2030
transcript_110048	gnl|BL_ORD_ID|25408 transcript_6355	1	1272	1271	1843	1	1295	2558	3129
transcript_11005	gnl|BL_ORD_ID|38688 transcript_8180	1406	2828	1	1406	1508	2927	1	1406
transcript_11005	gnl|BL_ORD_ID|38706 transcript_8217	1406	2820	1	1406	1567	2982	58	1465
transcript_11007	gnl|BL_ORD_ID|67270 transcript_126521	478	2685	8	480	1733	3941	2	474
transcript_110094	gnl|BL_ORD_ID|60196 transcript_116653	421	1581	6	423	1668	2824	2	418
transcript_110126	gnl|BL_ORD_ID|28968 transcript_69017	211	3669	1	213	1330	4788	33	247
transcript_110143	gnl|BL_ORD_ID|95155 transcript_18811	1	1146	1144	2096	36	1181	1385	2337
transcript_110144	gnl|BL_ORD_ID|64532 transcript_11052	258	1944	82	258	1112	2802	236	412
transcript_110251	gnl|BL_ORD_ID|24095 transcript_62805	1	2609	2607	3295	1381	3984	4095	4784
transcript_11027	gnl|BL_ORD_ID|49431 transcript_100484	1	1367	1365	2729	1	1367	1673	3036
transcript_110282	gnl|BL_ORD_ID|44050 transcript_91774	1	1606	1604	2564	1	1606	1800	2760
transcript_11033	gnl|BL_ORD_ID|38841 transcript_8527	803	2761	91	802	1020	2977	2	713
transcript_110330	gnl|BL_ORD_ID|84736 transcript_153751	280	3243	132	282	1333	4297	782	932
transcript_110344	gnl|BL_ORD_ID|24627 transcript_4704	1	1847	1845	2212	52	1901	2931	3298
transcript_110360	gnl|BL_ORD_ID|46665 transcript_96119	1	1316	1316	2513	300	1615	1746	2949
transcript_110433	gnl|BL_ORD_ID|18119 transcript_52972	1	1087	1085	1619	481	1581	2234	2767

transcript_110448	gnl|BL_ORD_ID|21620 transcript_58725	2	2405	2405	3891	164	2568	2670	4156
transcript_110448	gnl|BL_ORD_ID|49937 transcript_101287	2	2405	2405	4047	164	2569	2671	4313
transcript_110598	gnl|BL_ORD_ID|40047 transcript_85236	1	2458	2456	2939	10	2464	2612	3102
transcript_110599	gnl|BL_ORD_ID|97145 transcript_131498	124	1183	2	124	313	1372	36	158
transcript_110616	gnl|BL_ORD_ID|574 transcript_969	1	3092	3092	3840	90	3189	3325	4072
transcript_110616	gnl|BL_ORD_ID|840 transcript_1473	1	3092	3092	3840	35	3117	3253	4000
transcript_110676	gnl|BL_ORD_ID|56911 transcript_111292	2	3910	3909	4124	63	3987	4115	4330
transcript_110689	gnl|BL_ORD_ID|18745 transcript_53960	23	3568	3568	3994	1	3552	3658	4083
transcript_110689	gnl|BL_ORD_ID|28251 transcript_67854	2	2529	2528	3966	1	2528	2695	4133
transcript_110697	gnl|BL_ORD_ID|80824 transcript_147009	1	1161	1158	1757	2	1171	2144	2765
transcript_110699	gnl|BL_ORD_ID|77760 transcript_13074	485	2011	5	488	1136	2661	72	555
transcript_110702	gnl|BL_ORD_ID|22452 transcript_60083	2	2551	2552	3264	1	2553	2662	3375
transcript_110702	gnl|BL_ORD_ID|11741 transcript_2430	1	2551	2552	3264	1	2552	2661	3373
transcript_110702	gnl|BL_ORD_ID|12178 transcript_3280	2	2551	2552	3199	193	2745	2854	3501
transcript_110719	gnl|BL_ORD_ID|905 transcript_1618	1	1429	1428	2668	68	1498	2583	3823
transcript_11074	gnl|BL_ORD_ID|65811 transcript_124126	1	1867	1866	2803	1	1869	2046	2984
transcript_11075	gnl|BL_ORD_ID|48091 transcript_98332	1	2334	2334	2715	2459	4792	4893	5274
transcript_110759	gnl|BL_ORD_ID|88294 transcript_159567	113	1798	1	113	1374	3058	960	1072
transcript_110764	gnl|BL_ORD_ID|96394 transcript_59329	1	2755	2752	3544	329	3083	2834	3626
transcript_110871	gnl|BL_ORD_ID|64445 transcript_10880	1	2320	2318	2419	2	2368	2523	2626
transcript_110871	gnl|BL_ORD_ID|73981 transcript_137389	1	2320	2318	2458	27	2342	2494	2633
transcript_110871	gnl|BL_ORD_ID|38578 transcript_7937	1	2320	2318	2462	4	2331	2483	2624
transcript_110871	gnl|BL_ORD_ID|88132 transcript_159309	1	2320	2318	2462	2	2323	2476	2621
transcript_110905	gnl|BL_ORD_ID|4598 transcript_28903	593	1417	6	596	1200	2024	8	602
transcript_110924	gnl|BL_ORD_ID|75881 transcript_140489	1	3196	3195	3721	247	3444	3711	4237
transcript_110929	gnl|BL_ORD_ID|95650 transcript_19994	364	1472	4	367	1196	2304	444	807
transcript_110936	gnl|BL_ORD_ID|88526 transcript_159920	1	1385	1383	1611	166	1556	1684	1909
transcript_110970	gnl|BL_ORD_ID|9991 transcript_39866	223	1240	32	226	319	1338	1	195
transcript_111030	gnl|BL_ORD_ID|23914 transcript_62531	16	2386	2383	3120	8	2350	2535	3271
transcript_111032	gnl|BL_ORD_ID|17864 transcript_52573	1	2458	2456	3107	2	2451	3826	4477
transcript_111032	gnl|BL_ORD_ID|59339 transcript_115299	25	2530	2530	3107	2	2495	2597	3173
transcript_111033	gnl|BL_ORD_ID|55239 transcript_108474	1219	3094	1	1218	1943	3818	524	1741
transcript_111180	gnl|BL_ORD_ID|2438 transcript_24221	188	1701	2	183	710	2221	6	187
transcript_111230	gnl|BL_ORD_ID|78431 transcript_14635	1	2152	2149	2393	50	2222	2353	2600

transcript_111230	gnl|BL_ORD_ID|84202 transcript_152860	1	2152	2149	2453	8	2179	2310	2617
transcript_111230	gnl|BL_ORD_ID|78150 transcript_13989	1	2152	2149	2441	49	2221	2352	2647
transcript_111242	gnl|BL_ORD_ID|55926 transcript_109714	1	1540	1541	2012	1	1564	1719	2190
transcript_111296	gnl|BL_ORD_ID|18709 transcript_53903	1	2032	2030	2589	5	2155	2548	3151
transcript_111319	gnl|BL_ORD_ID|24701 transcript_4884	697	2708	98	699	1122	3134	2	603
transcript_111357	gnl|BL_ORD_ID|93736 transcript_166711	238	3348	129	239	1291	4401	729	839
transcript_111358	gnl|BL_ORD_ID|93462 transcript_166265	120	1880	1	119	350	2110	17	135
transcript_111407	gnl|BL_ORD_ID|42351 transcript_88965	1	1559	1559	1758	1	1558	2020	2219
transcript_111415	gnl|BL_ORD_ID|63498 transcript_121956	105	1918	1918	2809	26	1836	2115	3007
transcript_111478	gnl|BL_ORD_ID|420 transcript_723	1	3447	3447	4137	26	3472	3586	4276
transcript_111484	gnl|BL_ORD_ID|22711 transcript_60541	1	1247	1246	2233	4	1250	2075	3069
transcript_111484	gnl|BL_ORD_ID|24727 transcript_4929	1	1247	1246	2184	8	1260	2091	3047
transcript_111503	gnl|BL_ORD_ID|58500 transcript_113938	2	2589	2585	2919	1	2587	3631	3964
transcript_111509	gnl|BL_ORD_ID|11643 transcript_2232	125	2753	1	124	995	3624	2	132
transcript_111526	gnl|BL_ORD_ID|67634 transcript_127105	239	1989	74	240	1049	2798	2	168
transcript_111583	gnl|BL_ORD_ID|21817 transcript_59028	370	2246	5	375	843	2721	1	370
transcript_111615	gnl|BL_ORD_ID|24055 transcript_62741	1	1206	1204	1570	1	1205	1742	2108
transcript_111615	gnl|BL_ORD_ID|65142 transcript_12412	1	1206	1204	1570	2	1205	1935	2301
transcript_111615	gnl|BL_ORD_ID|44572 transcript_92658	1	1206	1204	1570	2	1205	2179	2545
transcript_11162	gnl|BL_ORD_ID|38588 transcript_7958	186	2824	1	189	377	3014	7	194
transcript_111708	gnl|BL_ORD_ID|28301 transcript_67955	1	2347	2346	2668	1	2346	2759	3081
transcript_111760	gnl|BL_ORD_ID|2319 transcript_23952	1	1271	1266	1764	1	1282	1537	2038
transcript_111760	gnl|BL_ORD_ID|5107 transcript_29967	1	1520	1517	1764	1	1534	1650	1900
transcript_111760	gnl|BL_ORD_ID|51864 transcript_10021	1	1358	1357	1764	1	1372	2242	2652
transcript_111763	gnl|BL_ORD_ID|95785 transcript_20313	1	1553	1552	2248	64	1617	1728	2429
transcript_111763	gnl|BL_ORD_ID|47625 transcript_97604	1	1553	1552	2249	7	1558	1669	2371
transcript_111763	gnl|BL_ORD_ID|26451 transcript_64940	1	1553	1552	2249	5	1565	1677	2380
transcript_111771	gnl|BL_ORD_ID|56445 transcript_110531	1	1141	1141	1895	124	1264	1370	2124
transcript_111788	gnl|BL_ORD_ID|92437 transcript_164609	360	2622	68	355	439	2701	2	289
transcript_111788	gnl|BL_ORD_ID|51327 transcript_8888	356	2622	68	355	443	2680	4	291
transcript_111804	gnl|BL_ORD_ID|42826 transcript_89720	1	2057	2058	2469	1	2050	2963	3373
transcript_111862	gnl|BL_ORD_ID|67306 transcript_126573	1	1467	1468	1943	30	1498	1625	2098
transcript_111862	gnl|BL_ORD_ID|86880 transcript_157246	1	1469	1465	1943	29	1499	1960	2436
transcript_1119	gnl|BL_ORD_ID|34345 transcript_77703	337	4061	1	338	551	4271	1	338

transcript_111920	gnl|BL_ORD_ID|17170 transcript_51482	149	2997	1	148	1331	4167	1071	1217
transcript_111920	gnl|BL_ORD_ID|31370 transcript_72860	1105	2934	1	1109	2348	4156	1135	2233
transcript_111920	gnl|BL_ORD_ID|58998 transcript_114740	1105	2933	1	1109	1576	3400	362	1461
transcript_11193	gnl|BL_ORD_ID|69036 transcript_129360	1371	2786	1	1370	1591	3005	97	1465
transcript_111973	gnl|BL_ORD_ID|41564 transcript_87728	1	1818	1818	2577	474	2297	2404	3163
transcript_111984	gnl|BL_ORD_ID|45932 transcript_94943	153	2989	1	152	1686	4504	1	152
transcript_111984	gnl|BL_ORD_ID|241 transcript_390	153	2998	1	152	1815	4654	1	152
transcript_111984	gnl|BL_ORD_ID|49396 transcript_100419	2	2007	2005	2903	6	2008	2941	3835
transcript_111984	gnl|BL_ORD_ID|41438 transcript_87522	153	2992	1	152	1919	4751	1	152
transcript_111990	gnl|BL_ORD_ID|84067 transcript_152621	1412	3078	1	1414	1974	3654	270	1669
transcript_111990	gnl|BL_ORD_ID|67816 transcript_127397	1387	3078	1	1391	1781	3496	283	1670
transcript_11200	gnl|BL_ORD_ID|84655 transcript_153625	228	2729	2	228	526	3013	140	368
transcript_11200	gnl|BL_ORD_ID|47877 transcript_97997	380	2765	79	382	431	2824	2	305
transcript_112005	gnl|BL_ORD_ID|28301 transcript_67955	1	2326	2325	2646	1	2346	2759	3081
transcript_112005	gnl|BL_ORD_ID|41144 transcript_87027	1	1994	1992	2697	1	2000	2102	2808
transcript_112041	gnl|BL_ORD_ID|25688 transcript_63748	1	2217	2215	2551	2	2219	2452	2789
transcript_112089	gnl|BL_ORD_ID|12570 transcript_4050	1	1585	1586	3025	147	1731	1879	3319
transcript_112090	gnl|BL_ORD_ID|447 transcript_760	181	3386	1	185	734	3937	321	505
transcript_112090	gnl|BL_ORD_ID|473 transcript_805	181	3386	1	185	766	3969	353	537
transcript_112091	gnl|BL_ORD_ID|33180 transcript_75801	442	1789	5	441	565	1909	18	452
transcript_112127	gnl|BL_ORD_ID|73209 transcript_136103	269	1022	3	269	1013	1761	421	688
transcript_112127	gnl|BL_ORD_ID|70834 transcript_132314	269	1022	3	269	1051	1802	458	725
transcript_112130	gnl|BL_ORD_ID|32894 transcript_75372	198	1540	50	200	2101	3463	1	151
transcript_112142	gnl|BL_ORD_ID|613 transcript_1049	25	3081	3076	3749	2	3059	3200	3873
transcript_112158	gnl|BL_ORD_ID|89685 transcript_161794	1132	2475	1	1132	1748	3091	465	1596
transcript_112204	gnl|BL_ORD_ID|52062 transcript_10436	1	2352	2351	2737	1	2352	2453	2839
transcript_112251	gnl|BL_ORD_ID|95097 transcript_18663	1	1644	1643	2238	1	1643	1812	2407
transcript_112259	gnl|BL_ORD_ID|24689 transcript_4852	1265	2471	1	1266	2079	3285	247	1473
transcript_1123	gnl|BL_ORD_ID|60114 transcript_116528	2	3317	3316	3907	69	3375	3516	4099
transcript_112316	gnl|BL_ORD_ID|27295 transcript_66286	1	1535	1534	2944	95	1640	2867	4277
transcript_112364	gnl|BL_ORD_ID|78074 transcript_13820	1045	2534	100	1044	1126	2613	1	944
transcript_112385	gnl|BL_ORD_ID|96626 transcript_80562	1221	2538	1	1222	1615	2931	97	1318
transcript_112389	gnl|BL_ORD_ID|64691 transcript_11399	159	2673	1	162	265	2785	1	162
transcript_112453	gnl|BL_ORD_ID|90439 transcript_162924	1	1390	1385	2383	114	1503	2025	3023

transcript_112494	gnl|BL_ORD_ID|48559 transcript_99086	1	2149	2147	3349	107	2280	2686	3905
transcript_112494	gnl|BL_ORD_ID|984 transcript_1751	1	2148	2147	3349	107	2279	2467	3673
transcript_112494	gnl|BL_ORD_ID|35179 transcript_79069	1	3089	3087	3349	2	3120	3310	3571
transcript_112513	gnl|BL_ORD_ID|92274 transcript_164334	343	1979	75	346	583	2219	2	277
transcript_11253	gnl|BL_ORD_ID|79506 transcript_144855	1339	2827	1	1342	1660	3146	68	1407
transcript_112536	gnl|BL_ORD_ID|22876 transcript_60840	173	1794	1	174	944	2565	4	177
transcript_112545	gnl|BL_ORD_ID|19306 transcript_54882	411	2266	48	412	727	2582	2	371
transcript_112561	gnl|BL_ORD_ID|60196 transcript_116653	127	1287	2	129	1669	2824	29	156
transcript_112562	gnl|BL_ORD_ID|73211 transcript_136105	1	1231	1230	2447	289	1513	1647	2867
transcript_112562	gnl|BL_ORD_ID|77649 transcript_143317	1	1231	1230	2447	345	1567	1700	2919
transcript_112562	gnl|BL_ORD_ID|38358 transcript_7449	1	1231	1230	2432	476	1700	1834	3039
transcript_112562	gnl|BL_ORD_ID|79369 transcript_144629	1	1231	1230	2429	355	1587	1721	2861
transcript_112562	gnl|BL_ORD_ID|38466 transcript_7683	1230	2447	1	1231	1818	3035	475	1684
transcript_112562	gnl|BL_ORD_ID|57630 transcript_112516	1	1231	1230	2448	1078	2302	2436	3657
transcript_112562	gnl|BL_ORD_ID|38115 transcript_6941	1	1231	1230	2447	474	1703	1837	3054
transcript_112562	gnl|BL_ORD_ID|25428 transcript_6390	1	1231	1230	2447	538	1762	1896	3116
transcript_112568	gnl|BL_ORD_ID|11896 transcript_2726	1	1750	1746	2995	50	1804	2329	3578
transcript_112572	gnl|BL_ORD_ID|41571 transcript_87743	1	2878	2873	3815	1	2882	3226	4188
transcript_112572	gnl|BL_ORD_ID|583 transcript_986	1	2875	2873	3954	2	2868	2988	4091
transcript_112572	gnl|BL_ORD_ID|586 transcript_995	1	2875	2873	3859	2	2875	2995	4004
transcript_112572	gnl|BL_ORD_ID|469 transcript_799	1	2878	2873	3858	16	2884	3228	4234
transcript_11260	gnl|BL_ORD_ID|29216 transcript_69395	1375	2780	107	1378	1461	2866	2	1275
transcript_112623	gnl|BL_ORD_ID|30712 transcript_71789	1	1609	1606	2759	556	2166	2413	3565
transcript_112633	gnl|BL_ORD_ID|42641 transcript_89423	1	1308	1303	2126	3	1306	2740	3562
transcript_112633	gnl|BL_ORD_ID|68069 transcript_127801	1	1308	1305	2127	3	1304	1441	2261
transcript_11266	gnl|BL_ORD_ID|19072 transcript_54494	1	1477	1477	2762	2	1482	2621	3907
transcript_11266	gnl|BL_ORD_ID|88206 transcript_159427	1	1477	1477	2720	1	1468	2616	3859
transcript_112747	gnl|BL_ORD_ID|70461 transcript_131705	3	1001	997	1889	1	998	1391	2283
transcript_112747	gnl|BL_ORD_ID|75549 transcript_139958	997	1901	5	1000	1473	2413	3	1012
transcript_112747	gnl|BL_ORD_ID|52741 transcript_104189	3	1001	997	1899	1	1027	1420	2322
transcript_112792	gnl|BL_ORD_ID|48483 transcript_98963	307	2659	9	310	903	3239	2	304
transcript_11281	gnl|BL_ORD_ID|42653 transcript_89449	230	2547	2	230	1087	3313	1	223
transcript_112812	gnl|BL_ORD_ID|74448 transcript_138157	1	1222	1218	2338	1	1221	1333	2474
transcript_112812	gnl|BL_ORD_ID|91549 transcript_16885	1	1222	1218	2338	1	1221	1333	2453

transcript_112846	gnl|BL_ORD_ID|10512 transcript_40805	197	1081	2	198	436	1320	16	211
transcript_112895	gnl|BL_ORD_ID|94 transcript_138	117	4983	1	116	223	5091	1	116
transcript_112912	gnl|BL_ORD_ID|19204 transcript_54727	1	2239	2239	3852	1	2240	3204	4816
transcript_112978	gnl|BL_ORD_ID|91992 transcript_163869	2	2423	2423	3468	7	2428	2824	3883
transcript_113031	gnl|BL_ORD_ID|17588 transcript_52137	19	3083	3084	3647	2	3065	3319	3882
transcript_113066	gnl|BL_ORD_ID|53762 transcript_105932	1	1343	1342	1872	6	1322	1427	1957
transcript_113084	gnl|BL_ORD_ID|81924 transcript_148838	1	1135	1133	1467	499	1623	1724	2057
transcript_113092	gnl|BL_ORD_ID|63069 transcript_121266	202	2473	1	202	556	2827	1	202
transcript_113093	gnl|BL_ORD_ID|61577 transcript_118871	1586	3969	151	1589	1613	4012	2	1451
transcript_113102	gnl|BL_ORD_ID|12396 transcript_3693	145	3222	1	146	378	3450	16	161
transcript_113118	gnl|BL_ORD_ID|12656 transcript_4213	2	2257	2253	2546	99	2354	3080	3375
transcript_113118	gnl|BL_ORD_ID|28684 transcript_68570	2	2257	2253	2546	151	2399	3125	3422
transcript_113145	gnl|BL_ORD_ID|29358 transcript_69597	193	1054	20	192	915	1782	12	184
transcript_113158	gnl|BL_ORD_ID|44419 transcript_92401	1225	5885	1	1228	1460	6119	1	1240
transcript_113158	gnl|BL_ORD_ID|60601 transcript_117297	1225	5884	1	1228	1542	6197	2	1244
transcript_113247	gnl|BL_ORD_ID|97302 transcript_146156	156	1053	4	155	1080	1976	2	152
transcript_113247	gnl|BL_ORD_ID|4622 transcript_28957	156	1053	4	155	937	1851	2	159
transcript_113248	gnl|BL_ORD_ID|67686 transcript_127194	15	2591	2589	3001	174	2750	2892	3299
transcript_113253	gnl|BL_ORD_ID|97060 transcript_122879	431	1726	71	433	881	2183	1	364
transcript_113274	gnl|BL_ORD_ID|49666 transcript_100854	1	1636	1634	2629	2	1645	1782	2777
transcript_113305	gnl|BL_ORD_ID|49881 transcript_101189	136	1062	2	137	1379	2300	212	346
transcript_113341	gnl|BL_ORD_ID|2614 transcript_24582	1	1254	1253	2025	16	1271	1420	2188
transcript_113350	gnl|BL_ORD_ID|38768 transcript_8369	1	1418	1421	2684	38	1456	1703	2966
transcript_113401	gnl|BL_ORD_ID|58987 transcript_114717	1	1665	1662	2608	28	1692	1993	2939
transcript_113435	gnl|BL_ORD_ID|54699 transcript_107506	13	2333	2332	2777	1	2339	2561	3007
transcript_113442	gnl|BL_ORD_ID|12385 transcript_3664	1	1378	1376	2367	1	1377	2356	3346
transcript_11361	gnl|BL_ORD_ID|46315 transcript_95550	696	2691	9	695	1217	3216	1	687
transcript_11365	gnl|BL_ORD_ID|63498 transcript_121956	17	1742	1742	2767	24	1741	2123	3147
transcript_11365	gnl|BL_ORD_ID|64582 transcript_11167	17	1742	1742	2676	52	1770	1873	2807
transcript_11365	gnl|BL_ORD_ID|51294 transcript_8804	17	1742	1742	2770	27	1749	1852	2880
transcript_11365	gnl|BL_ORD_ID|37722 transcript_83103	17	1742	1742	2770	26	1738	1841	2868
transcript_11365	gnl|BL_ORD_ID|64394 transcript_10764	1	1742	1742	2703	25	1772	1875	2837
transcript_113680	gnl|BL_ORD_ID|37559 transcript_82857	1183	2650	1	1183	2028	3510	707	1888
transcript_113680	gnl|BL_ORD_ID|22378 transcript_59968	1183	2663	1	1183	1340	2839	5	1199

transcript_113680	gnl|BL_ORD_ID|83129 transcript_150971	1183	2663	1	1183	1886	3365	565	1746
transcript_113680	gnl|BL_ORD_ID|58791 transcript_114405	1183	2663	1	1183	2246	3725	924	2106
transcript_113712	gnl|BL_ORD_ID|39761 transcript_84779	1	2557	2553	2904	39	2603	2731	3083
transcript_113712	gnl|BL_ORD_ID|25055 transcript_5621	11	2557	2553	2904	2	2551	2679	3031
transcript_113775	gnl|BL_ORD_ID|53236 transcript_104999	232	1467	7	233	549	1784	2	228
transcript_113783	gnl|BL_ORD_ID|779 transcript_1360	119	3368	1	122	700	3950	1	122
transcript_113783	gnl|BL_ORD_ID|865 transcript_1533	119	3360	1	118	673	3915	1	118
transcript_113792	gnl|BL_ORD_ID|71903 transcript_134028	2	2308	2307	3840	7	2311	2905	4437
transcript_113797	gnl|BL_ORD_ID|87048 transcript_157525	13	2298	2297	2706	1	2285	2407	2816
transcript_1138	gnl|BL_ORD_ID|31594 transcript_73210	318	4040	15	317	410	4139	2	304
transcript_113828	gnl|BL_ORD_ID|95497 transcript_19590	241	2145	5	240	419	2323	62	299
transcript_113831	gnl|BL_ORD_ID|68597 transcript_128635	1	1403	1402	2028	1	1403	2565	3191
transcript_113923	gnl|BL_ORD_ID|37897 transcript_6500	13	1439	1437	2615	13	1489	1652	2879
transcript_113923	gnl|BL_ORD_ID|51991 transcript_10275	11	1439	1437	2614	29	1457	1613	2798
transcript_113923	gnl|BL_ORD_ID|51894 transcript_10073	13	1439	1437	2614	41	1467	1623	2800
transcript_113980	gnl|BL_ORD_ID|2187 transcript_23645	284	2110	38	285	446	2272	72	319
transcript_113985	gnl|BL_ORD_ID|58422 transcript_113808	1	1531	1530	2222	27	1559	2634	3326
transcript_113986	gnl|BL_ORD_ID|90263 transcript_162679	1	1054	1054	1585	241	1270	2389	2904
transcript_113986	gnl|BL_ORD_ID|39292 transcript_84015	1	1054	1052	1600	423	1451	3079	3611
transcript_113986	gnl|BL_ORD_ID|62674 transcript_120633	1	1053	1054	1595	408	1424	1526	2050
transcript_114044	gnl|BL_ORD_ID|80391 transcript_146316	1	1872	1871	2308	1	1851	2044	2481
transcript_114044	gnl|BL_ORD_ID|17412 transcript_51870	1	1872	1871	2349	1	1872	2065	2543
transcript_114105	gnl|BL_ORD_ID|65441 transcript_123506	1	1476	1474	1890	1	1480	2644	3060
transcript_114134	gnl|BL_ORD_ID|25688 transcript_63748	2	2174	2175	2560	47	2219	2406	2791
transcript_114140	gnl|BL_ORD_ID|80798 transcript_146968	250	1994	5	249	2332	4080	2	246
transcript_114140	gnl|BL_ORD_ID|35543 transcript_79634	250	1996	5	249	768	2518	1	245
transcript_114142	gnl|BL_ORD_ID|53171 transcript_104896	7	589	588	1062	2	584	1369	1843
transcript_114143	gnl|BL_ORD_ID|33720 transcript_76666	1	3184	3183	3719	218	3394	5324	5859
transcript_11417	gnl|BL_ORD_ID|87684 transcript_158573	161	2733	1	162	1418	3985	1	169
transcript_114216	gnl|BL_ORD_ID|24634 transcript_4717	2	2825	2825	3058	6	2847	3020	3253
transcript_114216	gnl|BL_ORD_ID|24560 transcript_4557	1	2825	2825	3058	1	2826	2999	3232
transcript_114221	gnl|BL_ORD_ID|42806 transcript_89690	165	2803	2800	3284	111	2750	2875	3361
transcript_114234	gnl|BL_ORD_ID|83692 transcript_151967	1	1056	1051	2098	189	1245	1712	2756
transcript_114261	gnl|BL_ORD_ID|51299 transcript_8818	1279	2763	1	1280	1426	2911	3	1285

transcript_114318	gnl|BL_ORD_ID|38556 transcript_7874	1	1784	1784	2873	3	1794	1915	3004
transcript_114329	gnl|BL_ORD_ID|87831 transcript_158812	12	1603	1603	2459	1	1592	1888	2743
transcript_11434	gnl|BL_ORD_ID|76956 transcript_142219	1332	2748	1	1336	1493	2909	1	1340
transcript_114430	gnl|BL_ORD_ID|72277 transcript_134624	1	1370	1365	1833	1	1345	1678	2146
transcript_114458	gnl|BL_ORD_ID|95360 transcript_19302	1	1452	1448	2155	117	1567	1675	2381
transcript_114481	gnl|BL_ORD_ID|63344 transcript_121698	1113	3170	1	1112	1263	3320	45	1150
transcript_114481	gnl|BL_ORD_ID|12353 transcript_3596	1113	3170	1	1112	1346	3403	122	1233
transcript_114491	gnl|BL_ORD_ID|53341 transcript_105177	1	1815	1816	2303	2	1843	1974	2460
transcript_114534	gnl|BL_ORD_ID|29102 transcript_69213	114	1892	1891	2684	23	1798	1902	2695
transcript_114535	gnl|BL_ORD_ID|32515 transcript_74771	1143	3385	1	1145	1336	3588	12	1157
transcript_11456	gnl|BL_ORD_ID|73789 transcript_137075	1210	2749	1	1210	5063	6602	1	1208
transcript_114599	gnl|BL_ORD_ID|861 transcript_1524	102	2388	1	107	1603	3889	1310	1416
transcript_114609	gnl|BL_ORD_ID|23440 transcript_61769	1074	2244	1	1075	1202	2372	3	1081
transcript_114628	gnl|BL_ORD_ID|71 transcript_99	1	1323	1322	1899	2	1326	4786	5363
transcript_114628	gnl|BL_ORD_ID|49764 transcript_101008	1	1323	1322	1899	2	1352	4813	5390
transcript_11465	gnl|BL_ORD_ID|51308 transcript_8847	2	2350	2348	2771	70	2418	2560	2984
transcript_114667	gnl|BL_ORD_ID|60251 transcript_116740	1141	2254	1	1144	1585	2698	68	1242
transcript_114672	gnl|BL_ORD_ID|61162 transcript_118226	1	1534	1531	1721	1873	3423	4022	4212
transcript_114686	gnl|BL_ORD_ID|46783 transcript_96297	1	1562	1557	1928	2	1563	1956	2327
transcript_114686	gnl|BL_ORD_ID|46619 transcript_96048	1	1562	1557	1928	2	1562	1835	2206
transcript_114752	gnl|BL_ORD_ID|46747 transcript_96242	1	1512	1511	2088	681	2192	2614	3191
transcript_114808	gnl|BL_ORD_ID|32379 transcript_74564	1	1075	1076	2116	848	1922	2301	3341
transcript_114854	gnl|BL_ORD_ID|29666 transcript_70087	1	1777	1774	3455	1	1784	1969	3651
transcript_114857	gnl|BL_ORD_ID|2069 transcript_23372	216	1760	48	218	429	1973	146	316
transcript_114857	gnl|BL_ORD_ID|5598 transcript_30979	216	1760	48	218	352	1895	69	239
transcript_114860	gnl|BL_ORD_ID|59630 transcript_115759	1	1235	1235	1985	2517	3750	4476	5225
transcript_114860	gnl|BL_ORD_ID|46 transcript_64	1	1235	1235	1990	3031	4265	4991	5746
transcript_114860	gnl|BL_ORD_ID|69344 transcript_129860	1	1235	1235	1990	1352	2586	3312	4061
transcript_114860	gnl|BL_ORD_ID|88811 transcript_160407	1	1235	1235	1988	3178	4412	5138	5891
transcript_114860	gnl|BL_ORD_ID|62885 transcript_120959	1	1235	1235	1961	1457	2690	3415	4141
transcript_114860	gnl|BL_ORD_ID|33428 transcript_76183	1	1235	1235	1989	3234	4469	5193	5941
transcript_114860	gnl|BL_ORD_ID|61093 transcript_118121	1	1235	1235	1990	3169	4406	5134	5883
transcript_114860	gnl|BL_ORD_ID|69424 transcript_129984	1	1235	1235	1989	949	2183	2908	3663
transcript_114866	gnl|BL_ORD_ID|12587 transcript_4077	2	2573	2573	3062	11	2582	2884	3377

transcript_114866	gnl|BL_ORD_ID|71767 transcript_133813	2	2573	2576	3004	12	2583	3324	3756
transcript_114897	gnl|BL_ORD_ID|80165 transcript_145915	1	2943	2941	3781	3	2950	3133	3974
transcript_11490	gnl|BL_ORD_ID|25103 transcript_5735	1	1988	1989	2752	1	1981	2424	3187
transcript_114919	gnl|BL_ORD_ID|45983 transcript_95026	1	1639	1636	2359	466	2104	2393	3116
transcript_114925	gnl|BL_ORD_ID|51786 transcript_9827	2	2127	2125	2617	94	2220	2407	2898
transcript_114925	gnl|BL_ORD_ID|74475 transcript_138205	2	2127	2125	2617	63	2183	2370	2862
transcript_114960	gnl|BL_ORD_ID|21988 transcript_59337	2	2921	2919	3869	254	3149	3345	4296
transcript_114960	gnl|BL_ORD_ID|544 transcript_921	2	2921	2919	3883	105	3024	3220	4185
transcript_114960	gnl|BL_ORD_ID|29753 transcript_70215	2	2921	2919	3903	329	3369	3574	4623
transcript_11497	gnl|BL_ORD_ID|23722 transcript_62225	230	2724	1	234	398	2892	1	234
transcript_115008	gnl|BL_ORD_ID|57555 transcript_112390	573	2754	7	574	1245	3423	2	589
transcript_115027	gnl|BL_ORD_ID|25550 transcript_63509	2	2313	2313	3256	7	2314	2648	3590
transcript_115072	gnl|BL_ORD_ID|91179 transcript_16076	1	1385	1383	2316	97	1481	1614	2547
transcript_1151	gnl|BL_ORD_ID|61302 transcript_118451	2	3645	3646	4012	1	3641	3914	4279
transcript_115182	gnl|BL_ORD_ID|23343 transcript_61592	3	3276	3272	3857	26	3299	3581	4169
transcript_115183	gnl|BL_ORD_ID|27267 transcript_66243	1	2109	2106	2550	45	2161	2263	2708
transcript_115183	gnl|BL_ORD_ID|90646 transcript_163246	1	2109	2106	2592	68	2180	2282	2769
transcript_115185	gnl|BL_ORD_ID|79948 transcript_145566	1	2034	2033	3479	1	2037	2434	3877
transcript_115186	gnl|BL_ORD_ID|41105 transcript_86965	1	2454	2450	3534	36	2482	3484	4568
transcript_11521	gnl|BL_ORD_ID|50097 transcript_101539	272	2830	1	272	915	3477	193	464
transcript_115235	gnl|BL_ORD_ID|17188 transcript_51510	1	1565	1561	2081	8	1573	1691	2212
transcript_115237	gnl|BL_ORD_ID|22956 transcript_60962	1	2089	2087	3195	2	2091	2228	3342
transcript_115296	gnl|BL_ORD_ID|85224 transcript_154514	1	1271	1267	1762	7	1282	3400	3895
transcript_115330	gnl|BL_ORD_ID|73932 transcript_137316	101	3588	3588	3862	57	3520	3626	3900
transcript_115330	gnl|BL_ORD_ID|621 transcript_1063	1	3588	3588	3839	2	3599	3705	3956
transcript_115330	gnl|BL_ORD_ID|18745 transcript_53960	23	3588	3588	3766	1	3552	3658	3835
transcript_115330	gnl|BL_ORD_ID|28251 transcript_67854	2	2549	2548	3766	1	2528	2695	3912
transcript_115332	gnl|BL_ORD_ID|30161 transcript_70899	1	1217	1215	1754	1	1207	1477	2013
transcript_115427	gnl|BL_ORD_ID|11890 transcript_2711	1	1823	1819	2688	61	1881	2722	3590
transcript_115473	gnl|BL_ORD_ID|73428 transcript_136465	1	1060	1061	2028	8	1067	1213	2180
transcript_115473	gnl|BL_ORD_ID|91153 transcript_16017	1	1060	1061	2028	8	1067	1571	2538
transcript_115473	gnl|BL_ORD_ID|78278 transcript_14256	1061	2028	1	1060	1649	2612	8	1073
transcript_115473	gnl|BL_ORD_ID|78140 transcript_13970	1061	2028	1	1065	1679	2642	8	1077
transcript_115478	gnl|BL_ORD_ID|11922 transcript_2783	280	3137	2	281	472	3331	53	332

transcript_11548	gnl|BL_ORD_ID|34571 transcript_78076	1193	2770	11	1196	1573	3157	2	1175
transcript_115519	gnl|BL_ORD_ID|68582 transcript_128614	1	2195	2197	2405	613	2808	3313	3522
transcript_115519	gnl|BL_ORD_ID|11872 transcript_2677	1	2195	2191	2405	617	2811	3324	3538
transcript_115519	gnl|BL_ORD_ID|51110 transcript_103200	1	2195	2197	2405	607	2801	5894	6102
transcript_11556	gnl|BL_ORD_ID|25257 transcript_6048	1	1645	1644	2772	114	1779	1936	3064
transcript_11556	gnl|BL_ORD_ID|37692 transcript_83055	1146	2772	1	1146	1407	3033	113	1279
transcript_11556	gnl|BL_ORD_ID|38181 transcript_7072	1341	2772	1	1343	1621	3052	114	1477
transcript_11556	gnl|BL_ORD_ID|37920 transcript_6551	1	1645	1644	2772	111	1776	1948	3076
transcript_115586	gnl|BL_ORD_ID|74354 transcript_138020	2422	4924	2	2421	3120	5623	1	2412
transcript_115619	gnl|BL_ORD_ID|1436 transcript_21970	248	2008	3	249	529	2288	63	308
transcript_115619	gnl|BL_ORD_ID|1524 transcript_22205	248	2008	3	249	477	2235	20	256
transcript_11569	gnl|BL_ORD_ID|29442 transcript_69733	1353	2720	1	1353	1453	2818	1	1353
transcript_115743	gnl|BL_ORD_ID|90139 transcript_162496	265	2443	3	268	632	2800	1	264
transcript_115743	gnl|BL_ORD_ID|49992 transcript_101380	265	2451	71	268	565	2758	1	198
transcript_115759	gnl|BL_ORD_ID|46 transcript_64	1674	5225	1	1673	2188	5741	4	1676
transcript_115798	gnl|BL_ORD_ID|71489 transcript_133359	260	2379	3	262	442	2565	5	272
transcript_115798	gnl|BL_ORD_ID|24021 transcript_62692	260	2379	3	263	1268	3391	82	350
transcript_115848	gnl|BL_ORD_ID|20064 transcript_56121	208	1408	7	211	397	1633	2	206
transcript_115859	gnl|BL_ORD_ID|46738 transcript_96231	14	2112	2110	2451	1	2099	2293	2644
transcript_115862	gnl|BL_ORD_ID|92195 transcript_164210	1	1331	1329	1802	71	1404	1660	2130
transcript_115884	gnl|BL_ORD_ID|80892 transcript_147121	3	3097	3098	3753	1	3074	3401	4074
transcript_115917	gnl|BL_ORD_ID|1824 transcript_22876	1	1298	1293	1819	2	1301	1556	2083
transcript_115917	gnl|BL_ORD_ID|51613 transcript_9460	1	1385	1384	1819	2	1386	2260	2696
transcript_115933	gnl|BL_ORD_ID|88280 transcript_159538	185	2291	10	190	318	2414	1	181
transcript_115951	gnl|BL_ORD_ID|51418 transcript_9075	1	1345	1342	2344	161	1504	1851	2852
transcript_115970	gnl|BL_ORD_ID|88573 transcript_160004	2	2938	2936	3826	44	2985	3127	4013
transcript_1160	gnl|BL_ORD_ID|18745 transcript_53960	1	3557	3557	4042	6	3552	3658	4141
transcript_1160	gnl|BL_ORD_ID|28251 transcript_67854	1	2518	2517	3955	27	2528	2695	4133
transcript_116001	gnl|BL_ORD_ID|48187 transcript_98490	11	2016	2013	2634	1	2007	2107	2728
transcript_116025	gnl|BL_ORD_ID|5025 transcript_29799	168	1583	2	172	573	1987	18	188
transcript_116027	gnl|BL_ORD_ID|94120 transcript_167309	1	1488	1489	2719	717	2205	2320	3550
transcript_116030	gnl|BL_ORD_ID|93778 transcript_166771	478	1782	5	477	1562	2875	1	475
transcript_116049	gnl|BL_ORD_ID|36095 transcript_80515	235	3337	1	239	485	3592	2	242
transcript_11605	gnl|BL_ORD_ID|12191 transcript_3302	1407	2764	1	1410	2144	3503	1	1403

transcript_11606	gnl|BL_ORD_ID|24915 transcript_5300	1	1767	1766	2586	1	1767	2407	3227
transcript_116135	gnl|BL_ORD_ID|94185 transcript_167408	1	2069	2065	2649	84	2156	2259	2842
transcript_116152	gnl|BL_ORD_ID|38324 transcript_7377	204	2180	2	208	997	2974	39	243
transcript_116180	gnl|BL_ORD_ID|27210 transcript_66156	1160	3026	1	1161	1700	3563	1	1154
transcript_116186	gnl|BL_ORD_ID|62419 transcript_120215	1655	3354	1	1656	1820	3518	1	1655
transcript_116214	gnl|BL_ORD_ID|87654 transcript_158525	157	4772	14	160	1575	6184	2	148
transcript_11628	gnl|BL_ORD_ID|51743 transcript_9730	1	2062	2062	2717	1	2054	2213	2875
transcript_11632	gnl|BL_ORD_ID|52799 transcript_104289	419	2707	57	419	919	3207	1	363
transcript_116320	gnl|BL_ORD_ID|70597 transcript_131925	1	1538	1537	1856	5	1546	1868	2189
transcript_11634	gnl|BL_ORD_ID|41474 transcript_87589	100	1749	1750	2782	1	1650	2080	3112
transcript_11634	gnl|BL_ORD_ID|65543 transcript_123678	100	1749	1750	2777	1	1651	1856	2883
transcript_116366	gnl|BL_ORD_ID|63243 transcript_121536	1	2218	2217	3173	2	2191	2524	3476
transcript_116465	gnl|BL_ORD_ID|48454 transcript_98922	477	992	2	479	1273	1787	631	1108
transcript_116465	gnl|BL_ORD_ID|80385 transcript_146308	302	992	2	303	1554	2243	826	1127
transcript_116490	gnl|BL_ORD_ID|25984 transcript_64229	1119	2340	11	1122	1322	2541	2	1130
transcript_11657	gnl|BL_ORD_ID|49463 transcript_100534	2	2249	2247	2782	33	2255	2638	3173
transcript_116570	gnl|BL_ORD_ID|25897 transcript_64096	1	2005	2004	2821	1	2003	2781	3599
transcript_116579	gnl|BL_ORD_ID|30539 transcript_71520	1	1268	1267	2055	152	1405	1512	2299
transcript_116596	gnl|BL_ORD_ID|36906 transcript_81823	1097	2831	1	1102	1247	2965	1	1103
transcript_116629	gnl|BL_ORD_ID|58416 transcript_113800	1	2720	2716	3263	1	2720	2852	3399
transcript_116654	gnl|BL_ORD_ID|37866 transcript_6442	1	2013	2013	2804	2	2019	2327	3118
transcript_116654	gnl|BL_ORD_ID|23504 transcript_61870	1	2013	2013	2801	2	2042	2349	3137
transcript_116680	gnl|BL_ORD_ID|54724 transcript_107547	238	2159	5	240	839	2742	2	238
transcript_116706	gnl|BL_ORD_ID|92439 transcript_164611	174	1806	1	179	333	1966	1	179
transcript_11676	gnl|BL_ORD_ID|61912 transcript_119390	365	2781	71	365	426	2844	2	296
transcript_116761	gnl|BL_ORD_ID|87354 transcript_158054	1	1290	1287	1902	181	1469	3098	3703
transcript_116774	gnl|BL_ORD_ID|12365 transcript_3631	1	2992	2987	3107	1	2997	3261	3381
transcript_116779	gnl|BL_ORD_ID|95983 transcript_20769	1	1832	1833	2165	1	1837	1976	2320
transcript_11683	gnl|BL_ORD_ID|37767 transcript_83171	1059	2736	10	1059	1170	2847	2	1051
transcript_11687	gnl|BL_ORD_ID|51456 transcript_9139	1325	2812	1	1326	1435	2921	6	1331
transcript_116889	gnl|BL_ORD_ID|66136 transcript_124648	1	1975	1973	2767	2085	4079	4232	5026
transcript_116890	gnl|BL_ORD_ID|65016 transcript_12102	1	1648	1643	2123	1	1631	2208	2688
transcript_116909	gnl|BL_ORD_ID|45085 transcript_93503	1	1737	1735	2055	6	1742	1856	2176
transcript_117051	gnl|BL_ORD_ID|12344 transcript_3583	1424	3008	1	1425	1781	3365	156	1580

transcript_117084	gnl|BL_ORD_ID|18293 transcript_53252	1	2491	2490	2899	1	2490	2613	3021
transcript_117096	gnl|BL_ORD_ID|17500 transcript_52004	697	2049	74	698	1687	3066	41	662
transcript_11711	gnl|BL_ORD_ID|38755 transcript_8341	1	2327	2326	2753	1	2342	2540	2961
transcript_11711	gnl|BL_ORD_ID|38686 transcript_8176	2	2327	2326	2753	1	2320	2518	2947
transcript_117164	gnl|BL_ORD_ID|73724 transcript_136953	486	2156	7	486	715	2383	2	473
transcript_117202	gnl|BL_ORD_ID|73253 transcript_136175	164	2266	10	163	727	2825	1	156
transcript_117209	gnl|BL_ORD_ID|12339 transcript_3574	1	2147	2147	3122	2	2141	2471	3446
transcript_117209	gnl|BL_ORD_ID|30733 transcript_71831	2	2147	2145	3121	21	2166	2283	3259
transcript_117261	gnl|BL_ORD_ID|33150 transcript_75748	1	2397	2399	2721	1	2376	2492	2816
transcript_117261	gnl|BL_ORD_ID|29306 transcript_69530	1	2397	2399	2721	1	2409	2525	2849
transcript_117296	gnl|BL_ORD_ID|43088 transcript_90144	1162	2806	1	1165	1320	2959	1	1155
transcript_117329	gnl|BL_ORD_ID|5434 transcript_30655	543	1444	58	545	1073	1974	213	700
transcript_117329	gnl|BL_ORD_ID|35611 transcript_79732	543	1444	6	545	892	1791	1	521
transcript_11734	gnl|BL_ORD_ID|25131 transcript_5796	583	2662	6	585	1112	3193	2	584
transcript_117369	gnl|BL_ORD_ID|91895 transcript_163730	1	1546	1545	2427	2	1548	2450	3332
transcript_117369	gnl|BL_ORD_ID|84861 transcript_153952	1	1546	1546	2475	1	1549	1759	2684
transcript_1174	gnl|BL_ORD_ID|73047 transcript_135838	1706	4027	134	1707	1779	4100	2	1591
transcript_117422	gnl|BL_ORD_ID|79770 transcript_145271	260	4722	1	259	381	4856	2	261
transcript_117462	gnl|BL_ORD_ID|85551 transcript_155049	12	1705	1705	2459	3	1697	1819	2576
transcript_117489	gnl|BL_ORD_ID|20850 transcript_57410	313	2801	46	312	917	3404	1	267
transcript_117508	gnl|BL_ORD_ID|92495 transcript_164699	188	1608	2	188	2707	4127	408	594
transcript_117569	gnl|BL_ORD_ID|83193 transcript_151082	1	2076	2075	2798	17	2094	2573	3297
transcript_117569	gnl|BL_ORD_ID|49438 transcript_100495	1	2076	2075	2798	2	2079	2651	3375
transcript_117582	gnl|BL_ORD_ID|599 transcript_1013	3	3554	3552	3835	25	3574	3755	4044
transcript_117613	gnl|BL_ORD_ID|97117 transcript_128821	1	1366	1363	2147	46	1416	1517	2303
transcript_117624	gnl|BL_ORD_ID|53338 transcript_105171	1	2089	2090	2421	10	2096	2511	2842
transcript_117636	gnl|BL_ORD_ID|95064 transcript_18597	1	1134	1132	2030	1	1133	1541	2439
transcript_117668	gnl|BL_ORD_ID|59974 transcript_116312	331	2261	61	332	443	2373	2	273
transcript_11770	gnl|BL_ORD_ID|49619 transcript_100783	273	2752	1	275	523	3008	1	272
transcript_117729	gnl|BL_ORD_ID|70059 transcript_131033	1	1259	1258	1816	1	1232	1344	1883
transcript_117733	gnl|BL_ORD_ID|80527 transcript_146513	108	2258	2257	3266	1	2133	2828	3843
transcript_117733	gnl|BL_ORD_ID|64090 transcript_122895	2	2862	2860	3266	25	2859	2994	3402
transcript_117738	gnl|BL_ORD_ID|25049 transcript_5607	1113	2567	1	1113	1761	3201	250	1362
transcript_117738	gnl|BL_ORD_ID|25209 transcript_5929	1113	2558	1	1113	1735	3180	224	1336

transcript_11774	gnl|BL_ORD_ID|37091 transcript_82103	368	2659	72	367	440	2731	3	298
transcript_117793	gnl|BL_ORD_ID|81418 transcript_147949	319	3240	1	323	548	3469	1	323
transcript_117810	gnl|BL_ORD_ID|38539 transcript_7839	1356	2844	1	1357	1518	3007	57	1414
transcript_117844	gnl|BL_ORD_ID|5326 transcript_30420	137	1558	1	141	463	1886	198	338
transcript_117844	gnl|BL_ORD_ID|51008 transcript_103028	137	1558	1	141	402	1825	137	277
transcript_11787	gnl|BL_ORD_ID|38002 transcript_6714	200	2749	1	201	550	3101	43	243
transcript_117904	gnl|BL_ORD_ID|83424 transcript_151487	1	1061	1060	2015	4	1066	2088	3043
transcript_117918	gnl|BL_ORD_ID|75975 transcript_140653	2	2010	2010	3046	8	2016	3482	4518
transcript_117953	gnl|BL_ORD_ID|65900 transcript_124260	536	1882	6	539	1290	2625	2	531
transcript_118045	gnl|BL_ORD_ID|1117 transcript_2005	1	1079	1076	1513	2	1081	3245	3682
transcript_118068	gnl|BL_ORD_ID|36095 transcript_80515	1	1465	1463	1915	1	1465	3126	3578
transcript_118152	gnl|BL_ORD_ID|586 transcript_995	2	2481	2478	3024	4	2488	3458	4004
transcript_118152	gnl|BL_ORD_ID|766 transcript_1337	2	2481	2478	3022	1	2458	3428	3970
transcript_118152	gnl|BL_ORD_ID|469 transcript_799	2	2481	2478	3023	18	2494	3691	4234
transcript_118152	gnl|BL_ORD_ID|27012 transcript_65849	2	2481	2478	3118	4	2480	3771	4410
transcript_118152	gnl|BL_ORD_ID|57667 transcript_112572	2	2481	2478	3117	3	2488	3314	3952
transcript_118152	gnl|BL_ORD_ID|583 transcript_986	2	2481	2478	3117	4	2481	3451	4089
transcript_118152	gnl|BL_ORD_ID|41571 transcript_87743	2	2481	2478	2980	3	2492	3686	4188
transcript_118152	gnl|BL_ORD_ID|337 transcript_580	2	2481	2478	3026	59	2536	3827	4375
transcript_118279	gnl|BL_ORD_ID|88947 transcript_160603	1	1799	1794	2297	3178	4976	5080	5582
transcript_118279	gnl|BL_ORD_ID|36375 transcript_80983	1	1799	1794	2293	1571	3371	3475	3974
transcript_11828	gnl|BL_ORD_ID|43462 transcript_90757	2	2319	2317	2715	87	2405	2678	3076
transcript_11828	gnl|BL_ORD_ID|75138 transcript_139281	15	1834	1832	2736	1	1833	2000	2898
transcript_11828	gnl|BL_ORD_ID|79142 transcript_144287	1	2260	2258	2662	15	2270	3209	3612
transcript_118301	gnl|BL_ORD_ID|18196 transcript_53102	1	1318	1315	2022	2348	3654	4375	5082
transcript_118334	gnl|BL_ORD_ID|69242 transcript_129706	1272	3059	1	1271	1461	3248	26	1300
transcript_118341	gnl|BL_ORD_ID|60091 transcript_116493	2	2716	2715	3108	1	2723	2973	3366
transcript_118371	gnl|BL_ORD_ID|74290 transcript_137910	2	2715	2713	3271	13	2730	2835	3393
transcript_118371	gnl|BL_ORD_ID|1107 transcript_1990	2	2637	2636	3273	80	2718	3166	3804
transcript_118388	gnl|BL_ORD_ID|87394 transcript_158113	13	1817	1818	3076	1	1805	1929	3194
transcript_11840	gnl|BL_ORD_ID|79506 transcript_144855	1207	2764	1	1210	1660	3215	201	1407
transcript_118404	gnl|BL_ORD_ID|37748 transcript_83144	14	2625	2625	2952	31	2685	2819	3147
transcript_118452	gnl|BL_ORD_ID|64924 transcript_11891	521	2653	66	526	596	2728	1	461
transcript_118499	gnl|BL_ORD_ID|43421 transcript_90677	1335	3308	1	1334	1579	3551	143	1473

transcript_118536	gnl|BL_ORD_ID|766 transcript_1337	1	1669	1670	2951	915	2554	2694	3970
transcript_118536	gnl|BL_ORD_ID|583 transcript_986	1	1669	1670	2952	918	2577	2717	3994
transcript_118536	gnl|BL_ORD_ID|20969 transcript_57619	1	1669	1670	2952	1002	2660	2800	4078
transcript_118536	gnl|BL_ORD_ID|586 transcript_995	1	1669	1670	2952	926	2584	2724	4003
transcript_118559	gnl|BL_ORD_ID|28866 transcript_68858	1	3111	3110	4106	1	3123	3526	4522
transcript_118572	gnl|BL_ORD_ID|40233 transcript_85513	270	1994	35	270	383	2103	2	237
transcript_118643	gnl|BL_ORD_ID|23609 transcript_62039	1	1319	1318	2186	413	1732	1857	2726
transcript_118657	gnl|BL_ORD_ID|60615 transcript_117319	1	2649	2649	3779	31	2683	2802	3932
transcript_11866	gnl|BL_ORD_ID|45972 transcript_95007	1042	2720	1	1044	1521	3197	1	1044
transcript_11866	gnl|BL_ORD_ID|62840 transcript_120891	1	1958	1956	2692	1	1963	2139	2874
transcript_118687	gnl|BL_ORD_ID|91346 transcript_16432	1	1981	1980	2299	87	2067	2196	2517
transcript_118702	gnl|BL_ORD_ID|57075 transcript_111575	2	2644	2644	2833	217	2859	2969	3157
transcript_118709	gnl|BL_ORD_ID|3 transcript_5	1	1125	1120	1660	5237	6361	6893	7433
transcript_118709	gnl|BL_ORD_ID|82602 transcript_150048	1	1125	1120	1660	494	1618	2150	2690
transcript_118709	gnl|BL_ORD_ID|62945 transcript_121051	1	1125	1120	1658	1545	2667	3199	3738
transcript_118709	gnl|BL_ORD_ID|36892 transcript_81804	1	1125	1120	1660	937	2064	2596	3137
transcript_118709	gnl|BL_ORD_ID|35316 transcript_79277	1	1125	1120	1660	5219	6337	6869	7407
transcript_118716	gnl|BL_ORD_ID|21790 transcript_58987	1	1534	1533	2015	1	1535	1688	2170
transcript_118778	gnl|BL_ORD_ID|66178 transcript_124736	1	2850	2848	3457	46	2894	3256	3865
transcript_118804	gnl|BL_ORD_ID|3545 transcript_26643	1	1326	1323	1928	1	1326	1511	2116
transcript_118855	gnl|BL_ORD_ID|93701 transcript_166665	342	2070	43	342	718	2447	2	325
transcript_118869	gnl|BL_ORD_ID|80338 transcript_146226	649	2772	87	652	1093	3213	121	684
transcript_118882	gnl|BL_ORD_ID|53922 transcript_106187	1	2824	2823	3056	1	2825	2974	3207
transcript_11892	gnl|BL_ORD_ID|49463 transcript_100534	1	2224	2222	2757	34	2255	2638	3173
transcript_118927	gnl|BL_ORD_ID|74905 transcript_138914	1	1631	1630	2449	11	1639	1740	2556
transcript_118927	gnl|BL_ORD_ID|22764 transcript_60635	1	1631	1630	2449	11	1649	1751	2575
transcript_118927	gnl|BL_ORD_ID|78046 transcript_13740	1	1631	1630	2449	131	1762	1863	2682
transcript_118927	gnl|BL_ORD_ID|49806 transcript_101071	1	1631	1628	2449	1	1624	2980	3800
transcript_118930	gnl|BL_ORD_ID|95401 transcript_19387	1	1117	1120	2015	101	1216	1467	2360
transcript_118930	gnl|BL_ORD_ID|38012 transcript_6733	1	1323	1324	1961	101	1422	2500	3140
transcript_118930	gnl|BL_ORD_ID|58705 transcript_114281	1	1251	1250	1973	99	1349	1687	2411
transcript_118942	gnl|BL_ORD_ID|24310 transcript_63140	2	3348	3343	3790	39	3354	4293	4740
transcript_118949	gnl|BL_ORD_ID|72 transcript_100	194	3153	1	193	2500	5456	1	196
transcript_118949	gnl|BL_ORD_ID|361 transcript_620	192	3126	1	193	952	4048	2	211

transcript_118963	gnl|BL_ORD_ID|92324 transcript_164413	1471	3730	1	1476	3488	5747	1201	2677
transcript_118963	gnl|BL_ORD_ID|47941 transcript_98093	1472	3612	1	1472	2294	4435	462	1934
transcript_118975	gnl|BL_ORD_ID|30003 transcript_70641	206	1517	23	210	1372	2683	366	554
transcript_11898	gnl|BL_ORD_ID|33456 transcript_76227	1	1419	1420	2681	1	1419	2476	3737
transcript_11898	gnl|BL_ORD_ID|87413 transcript_158139	17	1990	1988	2681	2	1975	2994	3687
transcript_118992	gnl|BL_ORD_ID|70246 transcript_131340	1	1473	1473	2147	474	1946	2300	2972
transcript_118992	gnl|BL_ORD_ID|73443 transcript_136494	1	1473	1473	2147	485	1956	2310	2982
transcript_119019	gnl|BL_ORD_ID|73030 transcript_135810	1	1052	1049	2283	7	1064	1784	2988
transcript_11903	gnl|BL_ORD_ID|51381 transcript_8993	371	2755	96	374	542	2925	2	281
transcript_119053	gnl|BL_ORD_ID|71902 transcript_134027	361	3803	1	362	1786	5236	1	377
transcript_119093	gnl|BL_ORD_ID|42107 transcript_88573	109	2329	1	109	370	2590	153	261
transcript_119119	gnl|BL_ORD_ID|24707 transcript_4892	1	2001	2001	2472	1	1975	2819	3285
transcript_119119	gnl|BL_ORD_ID|18145 transcript_53013	1	1702	1698	2472	1	1692	3392	4152
transcript_119166	gnl|BL_ORD_ID|51308 transcript_8847	269	2735	2	269	501	2984	126	393
transcript_119166	gnl|BL_ORD_ID|25639 transcript_63666	269	2719	1	269	648	3116	1	269
transcript_119175	gnl|BL_ORD_ID|91729 transcript_163445	1185	2377	10	1190	2178	3369	1	1187
transcript_119175	gnl|BL_ORD_ID|53786 transcript_105982	13	1426	1427	2417	6	1421	1523	2513
transcript_119187	gnl|BL_ORD_ID|61848 transcript_119288	1378	2967	1	1380	2319	3908	1	1387
transcript_119187	gnl|BL_ORD_ID|626 transcript_1071	1378	2967	1	1380	2340	3934	1	1397
transcript_119215	gnl|BL_ORD_ID|85934 transcript_155675	1	2708	2708	2999	1	2722	2873	3164
transcript_119215	gnl|BL_ORD_ID|25370 transcript_6278	2	2707	2708	2999	1	2722	2891	3182
transcript_119215	gnl|BL_ORD_ID|37333 transcript_82489	15	2707	2708	2999	2	2680	2849	3140
transcript_119215	gnl|BL_ORD_ID|93211 transcript_165870	1	2707	2708	2998	1	2700	2869	3159
transcript_119230	gnl|BL_ORD_ID|27098 transcript_65985	119	2227	2226	2891	2	2099	2547	3212
transcript_119247	gnl|BL_ORD_ID|61414 transcript_118631	107	1998	1	112	4047	5939	3829	3940
transcript_119247	gnl|BL_ORD_ID|94959 transcript_18380	107	1998	1	112	415	2306	197	308
transcript_119247	gnl|BL_ORD_ID|77506 transcript_143100	107	1998	1	112	2721	4611	2503	2614
transcript_119247	gnl|BL_ORD_ID|36193 transcript_80682	107	1998	1	112	412	2275	194	305
transcript_119247	gnl|BL_ORD_ID|41936 transcript_88320	107	1998	1	112	2757	4649	2541	2652
transcript_119247	gnl|BL_ORD_ID|56428 transcript_110504	107	1998	1	112	915	2806	697	808
transcript_119281	gnl|BL_ORD_ID|64067 transcript_122857	126	1938	1	127	246	2058	1	127
transcript_119281	gnl|BL_ORD_ID|27691 transcript_66910	126	2038	1	131	270	2184	1	130
transcript_119289	gnl|BL_ORD_ID|42689 transcript_89511	1	1086	1083	1484	1	1081	1434	1833
transcript_11934	gnl|BL_ORD_ID|49619 transcript_100783	270	2738	1	272	523	3009	1	272

transcript_119358	gnl|BL_ORD_ID|88435 transcript_159784	1	1430	1425	2237	2	1411	1620	2421
transcript_119360	gnl|BL_ORD_ID|82523 transcript_149905	1	1333	1331	2347	13	1347	1713	2730
transcript_119370	gnl|BL_ORD_ID|76363 transcript_141266	1	2146	2143	2715	1232	3370	3485	4050
transcript_119396	gnl|BL_ORD_ID|62121 transcript_119720	314	1643	4	315	1004	2333	3	314
transcript_119408	gnl|BL_ORD_ID|4853 transcript_29418	198	1525	5	196	565	1859	13	198
transcript_119408	gnl|BL_ORD_ID|45866 transcript_94838	198	1525	2	196	415	1709	1	189
transcript_119408	gnl|BL_ORD_ID|38214 transcript_7135	198	1516	5	196	1785	3071	7	192
transcript_11941	gnl|BL_ORD_ID|12085 transcript_3112	201	2636	2	202	1106	3541	36	238
transcript_11941	gnl|BL_ORD_ID|74100 transcript_137596	1	2430	2429	2635	1	2430	3285	3491
transcript_119420	gnl|BL_ORD_ID|38440 transcript_7633	169	2437	1	168	706	2974	416	583
transcript_119420	gnl|BL_ORD_ID|51298 transcript_8815	169	2437	1	168	614	2883	324	491
transcript_119439	gnl|BL_ORD_ID|25488 transcript_63403	144	3455	13	148	427	3739	1	136
transcript_119455	gnl|BL_ORD_ID|79715 transcript_145187	1	1996	1996	3101	464	2459	2564	3674
transcript_119462	gnl|BL_ORD_ID|40214 transcript_85489	1	1584	1582	2674	1	1560	1761	2853
transcript_119462	gnl|BL_ORD_ID|89690 transcript_161801	1053	2671	1	1052	1162	2780	4	1032
transcript_119600	gnl|BL_ORD_ID|46083 transcript_95181	120	2731	2731	3663	1	2644	2796	3728
transcript_119614	gnl|BL_ORD_ID|51270 transcript_8754	194	2622	12	198	483	2913	3	189
transcript_119616	gnl|BL_ORD_ID|64768 transcript_11571	2	2194	2191	2577	124	2316	2425	2811
transcript_119620	gnl|BL_ORD_ID|66245 transcript_124852	2	2164	2162	2406	12	2151	2344	2589
transcript_119696	gnl|BL_ORD_ID|31438 transcript_72963	2	2728	2727	3062	86	2794	3056	3391
transcript_119858	gnl|BL_ORD_ID|95109 transcript_18691	1	1544	1543	2356	5	1553	1664	2473
transcript_119869	gnl|BL_ORD_ID|70065 transcript_131043	1	1148	1144	1752	973	2120	2221	2827
transcript_119891	gnl|BL_ORD_ID|42431 transcript_89089	224	1803	24	227	389	1968	2	205
transcript_119893	gnl|BL_ORD_ID|21451 transcript_58438	1	1077	1074	1389	904	1975	2519	2834
transcript_119895	gnl|BL_ORD_ID|71865 transcript_133969	10	1610	1608	2143	2	1602	1993	2528
transcript_119895	gnl|BL_ORD_ID|39667 transcript_84630	1	1610	1608	2143	1	1612	2452	2988
transcript_119895	gnl|BL_ORD_ID|38355 transcript_7438	1	1610	1608	2143	1	1612	2427	2963
transcript_119914	gnl|BL_ORD_ID|85319 transcript_154670	1	2857	2856	3145	59	2914	3308	3597
transcript_119914	gnl|BL_ORD_ID|84917 transcript_154042	1	2288	2290	3145	50	2335	2551	3392
transcript_119948	gnl|BL_ORD_ID|27437 transcript_66504	123	2741	12	125	275	2890	1	130
transcript_119948	gnl|BL_ORD_ID|65879 transcript_124224	123	2741	1	125	274	2892	5	129
transcript_119949	gnl|BL_ORD_ID|174 transcript_275	1	2521	2518	3924	4	2523	3413	4822
transcript_119949	gnl|BL_ORD_ID|170 transcript_270	1	2521	2518	3924	1	2513	3403	4812
transcript_11995	gnl|BL_ORD_ID|46510 transcript_95865	218	2775	10	217	378	2934	1	208

transcript_120025	gnl|BL_ORD_ID|12629 transcript_4158	1	1734	1733	1870	1314	3047	3151	3288
transcript_120025	gnl|BL_ORD_ID|83453 transcript_151532	1	1734	1733	1870	612	2345	2449	2583
transcript_120025	gnl|BL_ORD_ID|64229 transcript_123113	1	1734	1733	1870	995	2747	2854	2992
transcript_120025	gnl|BL_ORD_ID|84536 transcript_153424	1	1734	1733	1870	317	2050	2154	2288
transcript_120025	gnl|BL_ORD_ID|32487 transcript_74722	1	1734	1733	1870	1183	2916	3020	3155
transcript_12004	gnl|BL_ORD_ID|51743 transcript_9730	1	2064	2064	2727	1	2054	2213	2871
transcript_120068	gnl|BL_ORD_ID|18222 transcript_53141	250	1460	34	253	1135	2345	2	224
transcript_120082	gnl|BL_ORD_ID|81080 transcript_147404	1	1488	1485	2383	1867	3350	4469	5384
transcript_120091	gnl|BL_ORD_ID|55747 transcript_109397	1	2398	2398	2888	566	2963	3616	4106
transcript_12012	gnl|BL_ORD_ID|49316 transcript_100291	200	2654	1	202	500	2956	30	229
transcript_12012	gnl|BL_ORD_ID|51519 transcript_9251	200	2740	1	202	376	2917	38	237
transcript_12012	gnl|BL_ORD_ID|64957 transcript_11952	200	2661	1	202	347	2809	7	208
transcript_120142	gnl|BL_ORD_ID|41387 transcript_87421	292	3541	18	292	386	3631	1	274
transcript_120142	gnl|BL_ORD_ID|53512 transcript_105478	292	3448	2	292	423	3578	20	310
transcript_120146	gnl|BL_ORD_ID|1113 transcript_1997	1	3036	3033	3551	3	3040	3239	3756
transcript_120253	gnl|BL_ORD_ID|25816 transcript_63961	1	1414	1414	1842	261	1673	1942	2369
transcript_120277	gnl|BL_ORD_ID|65226 transcript_12607	1	1452	1449	2307	1	1460	1735	2604
transcript_120314	gnl|BL_ORD_ID|32298 transcript_74421	1	1478	1477	2852	59	1536	2195	3570
transcript_120338	gnl|BL_ORD_ID|66534 transcript_125312	2	2095	2095	2842	69	2167	2478	3225
transcript_120455	gnl|BL_ORD_ID|34319 transcript_77661	1	2092	2090	2825	1	2091	2216	2949
transcript_120501	gnl|BL_ORD_ID|37238 transcript_82336	2	2152	2150	2509	21	2167	2457	2816
transcript_120513	gnl|BL_ORD_ID|38488 transcript_7720	1	1087	1086	1554	5	1105	2564	3032
transcript_120524	gnl|BL_ORD_ID|71735 transcript_133753	1	1604	1605	2600	1	1588	1749	2744
transcript_120528	gnl|BL_ORD_ID|49409 transcript_100440	1	1453	1452	2415	1	1452	2725	3690
transcript_120530	gnl|BL_ORD_ID|95366 transcript_19310	251	2180	3	256	453	2381	2	251
transcript_120537	gnl|BL_ORD_ID|76230 transcript_141077	1	1318	1313	2084	2	1319	2253	3025
transcript_120544	gnl|BL_ORD_ID|79948 transcript_145566	1	1908	1907	3246	132	2037	2434	3772
transcript_120556	gnl|BL_ORD_ID|42977 transcript_89964	2	2139	2137	2789	108	2247	3092	3744
transcript_120559	gnl|BL_ORD_ID|57868 transcript_112912	263	2835	1	262	1284	3852	679	940
transcript_120559	gnl|BL_ORD_ID|83398 transcript_151439	263	2883	1	262	644	3250	39	300
transcript_120571	gnl|BL_ORD_ID|17979 transcript_52748	1	1726	1724	2383	425	2149	4634	5280
transcript_120583	gnl|BL_ORD_ID|79119 transcript_144250	1	1282	1281	2378	2	1307	1413	2517
transcript_120583	gnl|BL_ORD_ID|94616 transcript_17567	1	1282	1281	2335	8	1317	1423	2484
transcript_120638	gnl|BL_ORD_ID|31308 transcript_72759	1	2172	2170	2459	1511	3682	3809	4100

transcript_120667	gnl|BL_ORD_ID|57383 transcript_112085	275	3089	22	278	380	3182	2	258
transcript_120680	gnl|BL_ORD_ID|58921 transcript_114620	1318	3224	1	1319	2799	4705	1025	2343
transcript_120680	gnl|BL_ORD_ID|61749 transcript_119122	1	1672	1670	3228	18	1688	1791	3361
transcript_120707	gnl|BL_ORD_ID|75370 transcript_139656	1	1517	1518	1634	561	2079	2927	3042
transcript_120744	gnl|BL_ORD_ID|28323 transcript_67990	136	2535	1	140	823	3213	274	412
transcript_120744	gnl|BL_ORD_ID|72995 transcript_135765	136	2535	1	140	969	3372	419	557
transcript_120744	gnl|BL_ORD_ID|77387 transcript_142905	136	2533	1	140	825	3197	275	414
transcript_120757	gnl|BL_ORD_ID|36095 transcript_80515	163	3902	1	167	485	4224	76	242
transcript_120791	gnl|BL_ORD_ID|31235 transcript_72632	297	2346	55	297	444	2493	2	244
transcript_120829	gnl|BL_ORD_ID|61408 transcript_118619	17	2102	2100	2278	24	2106	2308	2485
transcript_120834	gnl|BL_ORD_ID|22323 transcript_59888	354	3305	8	353	489	3415	1	347
transcript_120875	gnl|BL_ORD_ID|95956 transcript_20702	179	1937	1	184	356	2113	1	184
transcript_120875	gnl|BL_ORD_ID|84532 transcript_153420	179	1902	1	184	356	2053	1	184
transcript_120875	gnl|BL_ORD_ID|94637 transcript_17614	179	1937	1	184	387	2264	1	202
transcript_120875	gnl|BL_ORD_ID|2474 transcript_24285	179	1937	1	184	356	2114	1	184
transcript_120875	gnl|BL_ORD_ID|44988 transcript_93337	179	1937	1	184	356	2114	1	184
transcript_120875	gnl|BL_ORD_ID|1404 transcript_21887	179	1928	1	184	356	2093	1	184
transcript_120890	gnl|BL_ORD_ID|83773 transcript_152118	1	1765	1765	2122	1464	3228	3375	3733
transcript_120919	gnl|BL_ORD_ID|58463 transcript_113881	1	1385	1382	1737	3	1401	1862	2218
transcript_120947	gnl|BL_ORD_ID|27295 transcript_66286	1	1541	1540	2969	95	1640	2867	4277
transcript_120950	gnl|BL_ORD_ID|47194 transcript_96932	1	1565	1564	2483	334	1821	1967	2862
transcript_120950	gnl|BL_ORD_ID|51462 transcript_9149	1	1565	1564	2483	332	1821	1967	2861
transcript_1210	gnl|BL_ORD_ID|617 transcript_1056	1994	4018	121	1993	2079	4102	9	1880
transcript_121008	gnl|BL_ORD_ID|93018 transcript_165553	1	1231	1229	1724	249	1479	2115	2610
transcript_121015	gnl|BL_ORD_ID|57785 transcript_112774	15	2170	2165	2693	5	2114	2217	2746
transcript_121015	gnl|BL_ORD_ID|96688 transcript_86751	11	2170	2165	2693	13	2174	2277	2795
transcript_121015	gnl|BL_ORD_ID|25358 transcript_6259	1	2170	2165	2693	2	2172	2275	2804
transcript_12109	gnl|BL_ORD_ID|54449 transcript_107081	112	1456	1456	2728	2	1347	1504	2776
transcript_12113	gnl|BL_ORD_ID|88702 transcript_160232	208	2719	1	209	1829	4355	1	209
transcript_121146	gnl|BL_ORD_ID|84302 transcript_153045	1	2646	2645	3125	2800	5444	5576	6056
transcript_121271	gnl|BL_ORD_ID|36401 transcript_81023	11	2148	2146	2615	1	2118	2871	3337
transcript_121271	gnl|BL_ORD_ID|29054 transcript_69145	1	2148	2146	2614	8	2155	2459	2927
transcript_121277	gnl|BL_ORD_ID|28962 transcript_69009	1	1205	1205	1839	1112	2316	2489	3124
transcript_121287	gnl|BL_ORD_ID|35888 transcript_80182	1	2833	2834	3039	115	2933	3040	3244

transcript_121287	gnl|BL_ORD_ID|65934 transcript_124316	1	2833	2834	3039	19	2852	2959	3164
transcript_121287	gnl|BL_ORD_ID|24570 transcript_4578	1	2833	2834	3039	28	2861	2968	3173
transcript_121296	gnl|BL_ORD_ID|64394 transcript_10764	1	1859	1856	2714	5	1872	1978	2837
transcript_121296	gnl|BL_ORD_ID|64582 transcript_11167	1	1859	1856	2687	6	1870	1976	2807
transcript_121319	gnl|BL_ORD_ID|74109 transcript_137609	1128	2639	1	1129	1244	2769	1	1129
transcript_121333	gnl|BL_ORD_ID|40160 transcript_85412	241	2253	3	242	367	2381	1	232
transcript_121357	gnl|BL_ORD_ID|904 transcript_1616	1119	2658	1	1119	2281	3818	1024	2141
transcript_121357	gnl|BL_ORD_ID|41921 transcript_88298	1118	2658	1	1119	2779	4317	1147	2265
transcript_121357	gnl|BL_ORD_ID|94032 transcript_167164	1119	2658	1	1119	2589	4127	1330	2449
transcript_121375	gnl|BL_ORD_ID|85718 transcript_155310	1	1181	1180	1823	15	1222	1387	2020
transcript_121377	gnl|BL_ORD_ID|705 transcript_1227	587	3922	66	587	642	3980	2	525
transcript_12140	gnl|BL_ORD_ID|51908 transcript_10107	1	2258	2258	2733	1	2259	2404	2879
transcript_12140	gnl|BL_ORD_ID|38781 transcript_8393	2	2259	2258	2716	25	2279	2541	2999
transcript_121406	gnl|BL_ORD_ID|39309 transcript_84036	2	2032	2030	2426	108	2137	2688	3084
transcript_121431	gnl|BL_ORD_ID|18604 transcript_53733	1	1867	1866	2602	33	1878	2089	2828
transcript_121431	gnl|BL_ORD_ID|231 transcript_373	1	1867	1866	2701	17	1886	3871	4709
transcript_121485	gnl|BL_ORD_ID|11634 transcript_2215	2	2922	2920	3120	449	3371	3485	3685
transcript_121485	gnl|BL_ORD_ID|24391 transcript_63274	2	2922	2920	3120	162	3080	3194	3394
transcript_121489	gnl|BL_ORD_ID|42855 transcript_89769	1	1973	1970	2461	1	1973	2352	2842
transcript_121522	gnl|BL_ORD_ID|87612 transcript_158460	257	2304	27	259	461	2512	42	274
transcript_12158	gnl|BL_ORD_ID|30516 transcript_71488	1	2139	2137	2723	2	2136	2282	2868
transcript_12158	gnl|BL_ORD_ID|17677 transcript_52288	1	2139	2137	2723	62	2190	2312	2898
transcript_12158	gnl|BL_ORD_ID|52022 transcript_10341	1	2139	2137	2723	6	2113	2235	2821
transcript_121590	gnl|BL_ORD_ID|28985 transcript_69041	1	1267	1265	2224	5	1272	1892	2850
transcript_1216	gnl|BL_ORD_ID|28251 transcript_67854	2	2569	2568	4006	1	2528	2695	4133
transcript_1216	gnl|BL_ORD_ID|18745 transcript_53960	24	3608	3608	4028	1	3552	3658	4077
transcript_121602	gnl|BL_ORD_ID|64729 transcript_11480	1	707	703	987	3	707	2497	2779
transcript_121653	gnl|BL_ORD_ID|50176 transcript_101660	1	1374	1371	1951	1	1373	1591	2171
transcript_121653	gnl|BL_ORD_ID|26975 transcript_65786	1	1374	1374	1951	1	1396	1498	2076
transcript_121659	gnl|BL_ORD_ID|87722 transcript_158625	1	1917	1915	2560	394	2311	3009	3655
transcript_121673	gnl|BL_ORD_ID|61867 transcript_119326	105	1285	1	109	212	1407	1	109
transcript_121673	gnl|BL_ORD_ID|84894 transcript_154000	105	1288	1	109	212	1394	1	109
transcript_121731	gnl|BL_ORD_ID|64798 transcript_11625	21	2426	2424	2570	33	2428	2631	2778
transcript_121735	gnl|BL_ORD_ID|94647 transcript_17639	1	1842	1838	2228	1	1841	2002	2392

transcript_121756	gnl|BL_ORD_ID|77761 transcript_13075	203	2365	2	205	513	2672	130	343
transcript_121766	gnl|BL_ORD_ID|76065 transcript_140786	1089	2427	1	1094	1271	2588	24	1124
transcript_121767	gnl|BL_ORD_ID|71461 transcript_133308	1	1531	1530	3027	2	1532	1667	3165
transcript_121786	gnl|BL_ORD_ID|42338 transcript_88943	1	1383	1384	2028	48	1429	1535	2161
transcript_121795	gnl|BL_ORD_ID|43907 transcript_91511	134	2454	2454	3848	1944	4253	4827	6221
transcript_12185	gnl|BL_ORD_ID|64430 transcript_10842	437	2742	86	440	505	2809	2	356
transcript_121850	gnl|BL_ORD_ID|34544 transcript_78036	332	1923	54	332	991	2583	152	430
transcript_121913	gnl|BL_ORD_ID|88433 transcript_159781	1	1552	1551	2711	1	1554	1803	2959
transcript_121913	gnl|BL_ORD_ID|38204 transcript_7116	1	1853	1852	2715	1	1852	2187	3050
transcript_121913	gnl|BL_ORD_ID|51333 transcript_8897	1	1853	1853	2714	1	1855	2066	2927
transcript_122018	gnl|BL_ORD_ID|36784 transcript_81634	336	2324	58	335	436	2426	2	277
transcript_122082	gnl|BL_ORD_ID|41891 transcript_88255	1	1246	1245	1588	38	1287	1472	1815
transcript_122082	gnl|BL_ORD_ID|89980 transcript_162253	1	1246	1245	1606	13	1260	1449	1810
transcript_122086	gnl|BL_ORD_ID|70723 transcript_132134	1	2088	2087	3679	106	2195	2343	3935
transcript_122133	gnl|BL_ORD_ID|32253 transcript_74345	201	2844	1	202	309	2953	1	204
transcript_122137	gnl|BL_ORD_ID|41726 transcript_88002	119	2868	2868	3455	165	2912	4200	4784
transcript_122162	gnl|BL_ORD_ID|42860 transcript_89775	14	2120	2120	4147	25	2124	2523	4532
transcript_122174	gnl|BL_ORD_ID|24766 transcript_5009	1	2238	2234	2645	1	2238	2520	2930
transcript_122174	gnl|BL_ORD_ID|97063 transcript_123436	2	2238	2234	2645	2	2238	2400	2808
transcript_122174	gnl|BL_ORD_ID|24784 transcript_5047	1	2238	2234	2645	1	2238	2520	2928
transcript_122201	gnl|BL_ORD_ID|63486 transcript_121937	1	1431	1428	2101	32	1462	2065	2748
transcript_12222	gnl|BL_ORD_ID|48432 transcript_98883	21	2157	2157	2684	10	2155	2517	3044
transcript_122228	gnl|BL_ORD_ID|18546 transcript_53639	1	1495	1490	2140	2	1495	2268	2918
transcript_122228	gnl|BL_ORD_ID|38138 transcript_6982	1	1495	1490	2114	121	1618	2475	3100
transcript_122228	gnl|BL_ORD_ID|20077 transcript_56138	1	1495	1490	2040	2	1499	2228	2778
transcript_122228	gnl|BL_ORD_ID|67672 transcript_127174	1	1495	1490	2092	2	1475	2332	2934
transcript_122228	gnl|BL_ORD_ID|17103 transcript_51375	1	1495	1490	2056	2	1503	2154	2719
transcript_122228	gnl|BL_ORD_ID|37920 transcript_6551	1	1495	1490	2078	2	1495	2525	3113
transcript_122228	gnl|BL_ORD_ID|65599 transcript_123770	11	1495	1490	2112	1	1489	2298	2919
transcript_122228	gnl|BL_ORD_ID|38359 transcript_7451	1	1495	1490	2140	2	1498	2355	3005
transcript_122228	gnl|BL_ORD_ID|12585 transcript_4074	1	1495	1490	2116	3	1542	2431	3077
transcript_122228	gnl|BL_ORD_ID|38310 transcript_7347	1	1495	1490	2050	127	1627	2484	3044
transcript_122228	gnl|BL_ORD_ID|28367 transcript_68061	1	1495	1490	2092	2	1498	2233	2834
transcript_122228	gnl|BL_ORD_ID|50242 transcript_101771	1	1495	1490	2099	2	1500	2410	3020

transcript_122228	gnl|BL_ORD_ID|44432 transcript_92421	11	1495	1490	2074	2	1489	2279	2863
transcript_122228	gnl|BL_ORD_ID|47846 transcript_97952	1	1495	1490	2112	2	1498	2050	2672
transcript_122228	gnl|BL_ORD_ID|17297 transcript_51683	1	1495	1490	2140	2	1502	2256	2906
transcript_122228	gnl|BL_ORD_ID|25257 transcript_6048	1	1495	1490	2140	2	1498	2513	3163
transcript_122254	gnl|BL_ORD_ID|88049 transcript_159163	10	1013	1012	1860	1	1024	1241	2089
transcript_122274	gnl|BL_ORD_ID|35423 transcript_79440	227	3036	2	232	2302	5111	57	295
transcript_122285	gnl|BL_ORD_ID|38729 transcript_8280	456	2757	7	457	608	2910	2	453
transcript_122285	gnl|BL_ORD_ID|84820 transcript_153888	457	2757	63	457	552	2853	2	396
transcript_122294	gnl|BL_ORD_ID|916 transcript_1638	1	1931	1929	3674	1	1949	2058	3798
transcript_122300	gnl|BL_ORD_ID|81923 transcript_148836	1	1280	1279	2123	2	1302	1470	2311
transcript_122300	gnl|BL_ORD_ID|96229 transcript_21275	1	1156	1157	2195	1	1177	1284	2322
transcript_122342	gnl|BL_ORD_ID|42553 transcript_89276	1	1375	1371	1720	10	1383	1524	1875
transcript_122342	gnl|BL_ORD_ID|4581 transcript_28861	1	1375	1371	1701	168	1541	1682	2014
transcript_122353	gnl|BL_ORD_ID|63321 transcript_121666	1	2149	2146	2692	50	2193	3026	3567
transcript_122367	gnl|BL_ORD_ID|80119 transcript_145833	1	1268	1267	2212	510	1777	2528	3473
transcript_122367	gnl|BL_ORD_ID|78898 transcript_143864	1	1268	1267	2255	216	1480	2232	3220
transcript_122368	gnl|BL_ORD_ID|75042 transcript_139123	1028	2132	1	1029	1547	2651	411	1440
transcript_122377	gnl|BL_ORD_ID|43667 transcript_91098	294	2593	75	298	886	3179	371	594
transcript_122413	gnl|BL_ORD_ID|23899 transcript_62508	1396	3187	1	1396	1558	3337	47	1452
transcript_122441	gnl|BL_ORD_ID|29639 transcript_70044	1	2962	2960	3366	2	2963	3888	4294
transcript_122444	gnl|BL_ORD_ID|42234 transcript_88780	611	1765	77	612	1115	2269	2	537
transcript_122497	gnl|BL_ORD_ID|37045 transcript_82035	322	2602	4	322	654	2947	211	532
transcript_122497	gnl|BL_ORD_ID|51303 transcript_8835	322	2627	4	322	526	2844	83	404
transcript_122505	gnl|BL_ORD_ID|84290 transcript_153021	158	2372	12	153	532	2748	35	176
transcript_122526	gnl|BL_ORD_ID|25883 transcript_64077	2	2285	2282	3694	2	2288	2648	4062
transcript_122587	gnl|BL_ORD_ID|70890 transcript_132399	1	1779	1778	2177	234	2004	2780	3188
transcript_122720	gnl|BL_ORD_ID|21420 transcript_58384	1255	2553	1	1255	3882	5180	2402	3655
transcript_122720	gnl|BL_ORD_ID|39140 transcript_83771	1255	2581	1	1255	4230	5556	2751	4003
transcript_122720	gnl|BL_ORD_ID|60060 transcript_116446	1255	2584	1	1255	3457	4783	1977	3230
transcript_122729	gnl|BL_ORD_ID|29970 transcript_70591	1	1644	1648	2265	247	1890	2051	2678
transcript_122758	gnl|BL_ORD_ID|72612 transcript_135138	1	1629	1626	2973	103	1739	1948	3280
transcript_122782	gnl|BL_ORD_ID|18317 transcript_53305	542	2543	6	547	835	2835	132	674
transcript_12279	gnl|BL_ORD_ID|25615 transcript_63625	1071	2647	1	1072	1367	2942	1	1072
transcript_122826	gnl|BL_ORD_ID|39475 transcript_84309	1320	3114	1	1325	3030	4825	1088	2412

transcript_122882	gnl|BL_ORD_ID|89750 transcript_161892	234	1867	52	234	294	1921	2	184
transcript_122941	gnl|BL_ORD_ID|46476 transcript_95815	1	1303	1304	1555	234	1537	1722	1972
transcript_123005	gnl|BL_ORD_ID|27119 transcript_66015	325	3550	2	324	442	3678	1	323
transcript_123015	gnl|BL_ORD_ID|18015 transcript_52800	1	1210	1209	1849	2	1214	1372	2012
transcript_123016	gnl|BL_ORD_ID|38134 transcript_6977	2	2220	2217	2870	10	2212	2363	3015
transcript_123016	gnl|BL_ORD_ID|38136 transcript_6979	1	2220	2217	2868	1	2204	2428	3078
transcript_123024	gnl|BL_ORD_ID|24951 transcript_5367	223	2629	2	227	588	2994	39	263
transcript_123024	gnl|BL_ORD_ID|38170 transcript_7050	223	2629	2	227	637	3043	89	312
transcript_123024	gnl|BL_ORD_ID|18789 transcript_54033	223	2629	11	227	565	2971	1	216
transcript_123054	gnl|BL_ORD_ID|95064 transcript_18597	1	1141	1139	1889	1	1133	1541	2293
transcript_123054	gnl|BL_ORD_ID|61896 transcript_119362	1	1141	1139	1887	1	1134	1463	2217
transcript_123094	gnl|BL_ORD_ID|39791 transcript_84827	638	1308	8	639	1992	2662	12	626
transcript_12310	gnl|BL_ORD_ID|52745 transcript_104201	17	1749	1747	2677	1	1732	1857	2785
transcript_123105	gnl|BL_ORD_ID|17971 transcript_52731	493	4334	93	493	657	4476	2	409
transcript_123113	gnl|BL_ORD_ID|81492 transcript_148062	1	1712	1711	2959	297	1984	2101	3337
transcript_123117	gnl|BL_ORD_ID|85494 transcript_154945	1121	2625	1	1120	1261	2765	2	1134
transcript_123118	gnl|BL_ORD_ID|18554 transcript_53649	10	1969	1967	2542	2	1969	2890	3465
transcript_123131	gnl|BL_ORD_ID|76851 transcript_142046	161	3136	1	166	310	3285	2	168
transcript_123131	gnl|BL_ORD_ID|82194 transcript_149332	1	2248	2245	3199	2	2250	2674	3635
transcript_123131	gnl|BL_ORD_ID|76057 transcript_140777	1	2437	2438	3199	2	2439	3677	4445
transcript_123141	gnl|BL_ORD_ID|90687 transcript_14988	348	2196	4	349	744	2592	123	468
transcript_123176	gnl|BL_ORD_ID|36197 transcript_80689	1108	3425	1	1109	2196	4513	101	1209
transcript_123246	gnl|BL_ORD_ID|37316 transcript_82457	107	1255	1	107	636	1798	1	106
transcript_12330	gnl|BL_ORD_ID|11938 transcript_2816	2	2024	2021	2754	60	2091	2913	3647
transcript_123326	gnl|BL_ORD_ID|85746 transcript_155361	1	1643	1641	2117	161	1804	2273	2749
transcript_123330	gnl|BL_ORD_ID|31453 transcript_72982	263	3369	2	268	544	3646	21	287
transcript_123342	gnl|BL_ORD_ID|20599 transcript_56994	250	3419	1	255	878	4051	1	256
transcript_123415	gnl|BL_ORD_ID|80089 transcript_145791	10	1023	1021	1669	2	992	1112	1761
transcript_123436	gnl|BL_ORD_ID|24784 transcript_5047	2	2244	2240	2896	2	2244	2360	3016
transcript_123436	gnl|BL_ORD_ID|24766 transcript_5009	2	2244	2240	2896	2	2244	2360	3018
transcript_123472	gnl|BL_ORD_ID|87512 transcript_158298	1	1873	1869	3616	2	1873	2001	3751
transcript_123472	gnl|BL_ORD_ID|661 transcript_1144	1	1873	1869	3616	24	1907	2035	3783
transcript_123481	gnl|BL_ORD_ID|89652 transcript_161743	9	788	787	1399	16	797	912	1525
transcript_123491	gnl|BL_ORD_ID|30065 transcript_70752	125	3461	10	126	260	3575	2	117

transcript_123498	gnl|BL_ORD_ID|24377 transcript_63257	275	3205	2	277	489	3419	32	308
transcript_123498	gnl|BL_ORD_ID|46742 transcript_96236	275	3205	2	277	523	3453	66	342
transcript_123509	gnl|BL_ORD_ID|35638 transcript_79775	1	1179	1177	1745	9	1194	2228	2796
transcript_123537	gnl|BL_ORD_ID|11717 transcript_2378	1	2609	2609	3398	45	2648	2825	3614
transcript_123537	gnl|BL_ORD_ID|1180 transcript_2127	1	2609	2609	3399	155	2765	2942	3732
transcript_123574	gnl|BL_ORD_ID|56811 transcript_111130	17	2357	2355	2719	22	2363	2615	2980
transcript_123606	gnl|BL_ORD_ID|41329 transcript_87328	1	1685	1684	2163	4	1688	1807	2287
transcript_123636	gnl|BL_ORD_ID|60841 transcript_117688	20	3577	3576	3827	1	3564	3677	3927
transcript_12367	gnl|BL_ORD_ID|32075 transcript_74048	264	2712	1	265	818	3267	1	266
transcript_123678	gnl|BL_ORD_ID|41474 transcript_87589	1	1855	1853	2883	1	1854	2077	3107
transcript_123678	gnl|BL_ORD_ID|39889 transcript_84981	1	1855	1853	2854	152	2006	2230	3231
transcript_123752	gnl|BL_ORD_ID|24230 transcript_63015	1165	2537	1	1167	1268	2621	2	1168
transcript_123752	gnl|BL_ORD_ID|64815 transcript_11650	1165	2537	1	1167	1268	2643	2	1168
transcript_123757	gnl|BL_ORD_ID|55429 transcript_108827	30	3618	3618	4033	7	3605	3731	4145
transcript_123770	gnl|BL_ORD_ID|25257 transcript_6048	1	1720	1718	2919	12	1729	1935	3135
transcript_123770	gnl|BL_ORD_ID|37920 transcript_6551	1	1720	1718	2885	12	1726	1947	3113
transcript_123776	gnl|BL_ORD_ID|47419 transcript_97292	1	2919	2918	4108	85	3003	4474	5664
transcript_123776	gnl|BL_ORD_ID|44678 transcript_92818	1	2640	2639	4108	114	2757	2882	4327
transcript_123798	gnl|BL_ORD_ID|71343 transcript_133123	12	1310	1310	2216	1	1319	1581	2488
transcript_12389	gnl|BL_ORD_ID|24113 transcript_62836	1	1344	1343	2666	1	1344	2264	3587
transcript_123895	gnl|BL_ORD_ID|21425 transcript_58391	1	1393	1392	2054	86	1497	1662	2325
transcript_123895	gnl|BL_ORD_ID|90112 transcript_162454	1	1393	1392	2029	103	1533	1698	2334
transcript_123895	gnl|BL_ORD_ID|66853 transcript_125839	1	1393	1392	2054	85	1475	1640	2303
transcript_123954	gnl|BL_ORD_ID|19424 transcript_55085	1	1538	1537	1940	239	1776	1956	2359
transcript_123954	gnl|BL_ORD_ID|39831 transcript_84889	1	1538	1537	1908	231	1773	1953	2324
transcript_123971	gnl|BL_ORD_ID|35198 transcript_79101	167	2725	1	169	308	2858	2	172
transcript_123986	gnl|BL_ORD_ID|78721 transcript_143593	1	1708	1711	3155	2	1709	1865	3293
transcript_123986	gnl|BL_ORD_ID|24631 transcript_4710	1	1711	1711	3149	1	1711	1864	3300
transcript_123989	gnl|BL_ORD_ID|80114 transcript_145828	1	1271	1272	2390	208	1478	1749	2867
transcript_124016	gnl|BL_ORD_ID|17136 transcript_51431	1	3358	3355	3845	40	3398	3532	4022
transcript_124032	gnl|BL_ORD_ID|733 transcript_1278	199	2060	1	200	2111	3977	36	234
transcript_124032	gnl|BL_ORD_ID|2045 transcript_23321	201	2038	1	200	406	2249	1	209
transcript_124036	gnl|BL_ORD_ID|31316 transcript_72779	1	1117	1115	1686	113	1230	1725	2296
transcript_124039	gnl|BL_ORD_ID|85109 transcript_154346	1	1628	1628	2396	1	1628	1849	2619

transcript_12412	gnl|BL_ORD_ID|44572 transcript_92658	1	1396	1393	2672	1	1396	1637	2916
transcript_124134	gnl|BL_ORD_ID|46078 transcript_95176	160	3002	1	159	335	3177	1	159
transcript_124153	gnl|BL_ORD_ID|2476 transcript_24298	177	2098	14	176	280	2202	1	163
transcript_124172	gnl|BL_ORD_ID|5 transcript_7	146	3542	1	147	3618	7014	2851	2998
transcript_124172	gnl|BL_ORD_ID|74055 transcript_137515	146	3542	1	146	838	4226	87	231
transcript_124173	gnl|BL_ORD_ID|66455 transcript_125193	1	1500	1501	2429	1	1497	1657	2585
transcript_12419	gnl|BL_ORD_ID|59734 transcript_115923	10	2084	2085	2625	1	2075	2943	3483
transcript_12419	gnl|BL_ORD_ID|38597 transcript_7980	2	2084	2085	2712	89	2171	2366	2993
transcript_124193	gnl|BL_ORD_ID|20251 transcript_56402	489	1676	74	488	561	1769	2	417
transcript_12420	gnl|BL_ORD_ID|49766 transcript_101012	14	2028	2028	2709	2	2015	2468	3149
transcript_12423	gnl|BL_ORD_ID|55507 transcript_108951	473	2704	60	474	1078	3309	1	427
transcript_124257	gnl|BL_ORD_ID|48407 transcript_98843	1502	3745	1	1507	1910	4151	1	1508
transcript_124316	gnl|BL_ORD_ID|73103 transcript_135917	1260	3305	1	1260	1373	3393	7	1264
transcript_12434	gnl|BL_ORD_ID|48775 transcript_99450	1	1461	1459	2720	2	1467	1634	2892
transcript_124383	gnl|BL_ORD_ID|35209 transcript_79115	114	1247	1	119	1439	2572	303	421
transcript_124383	gnl|BL_ORD_ID|63512 transcript_121978	114	1247	1	119	2235	3367	300	418
transcript_124383	gnl|BL_ORD_ID|63802 transcript_122447	114	1247	1	119	1367	2499	302	420
transcript_124383	gnl|BL_ORD_ID|40276 transcript_85578	114	1247	1	119	2360	3492	416	534
transcript_124397	gnl|BL_ORD_ID|85784 transcript_155418	1	2060	2058	3252	767	2748	2941	4103
transcript_124421	gnl|BL_ORD_ID|29959 transcript_70575	103	2215	1	106	405	2514	6	111
transcript_124421	gnl|BL_ORD_ID|64104 transcript_122922	109	2215	1	106	3125	5235	2	107
transcript_124421	gnl|BL_ORD_ID|95851 transcript_20468	103	2215	1	106	223	2335	6	111
transcript_124421	gnl|BL_ORD_ID|94995 transcript_18458	109	2215	1	106	290	2396	6	111
transcript_124421	gnl|BL_ORD_ID|19600 transcript_55392	103	2215	1	106	311	2423	3	108
transcript_124421	gnl|BL_ORD_ID|77588 transcript_143217	103	2215	1	106	401	2518	2	107
transcript_124429	gnl|BL_ORD_ID|87269 transcript_157907	328	2652	7	328	1522	3846	1036	1357
transcript_124476	gnl|BL_ORD_ID|1232 transcript_21491	587	2137	6	587	674	2227	2	553
transcript_124476	gnl|BL_ORD_ID|91332 transcript_16406	587	2137	6	587	769	2323	2	583
transcript_124476	gnl|BL_ORD_ID|75549 transcript_139958	587	2137	6	587	703	2325	3	578
transcript_124476	gnl|BL_ORD_ID|92909 transcript_165372	587	2140	6	587	669	2203	2	552
transcript_124476	gnl|BL_ORD_ID|36120 transcript_80550	587	2140	7	587	671	2225	4	554
transcript_1245	gnl|BL_ORD_ID|80675 transcript_146760	30	3218	3214	3991	1	3187	3438	4215
transcript_124508	gnl|BL_ORD_ID|20212 transcript_56352	1	2751	2750	3152	76	2826	3185	3587
transcript_124525	gnl|BL_ORD_ID|50356 transcript_101954	1	1784	1785	2571	1089	2869	2614	3397

transcript_124543	gnl|BL_ORD_ID|78512 transcript_14852	116	2312	1	116	311	2530	2	125
transcript_124552	gnl|BL_ORD_ID|45295 transcript_93845	15	1855	1854	2171	2	1842	1949	2267
transcript_124562	gnl|BL_ORD_ID|38637 transcript_8062	1122	2451	1	1123	1625	2953	2	1130
transcript_124570	gnl|BL_ORD_ID|4986 transcript_29715	617	1432	7	622	1016	1831	2	615
transcript_124576	gnl|BL_ORD_ID|38005 transcript_6725	1	1997	1997	2551	220	2216	2330	2884
transcript_124576	gnl|BL_ORD_ID|17762 transcript_52422	1	1997	1997	2551	303	2299	2413	2967
transcript_124586	gnl|BL_ORD_ID|84194 transcript_152849	1	1359	1354	2592	117	1474	1670	2910
transcript_124596	gnl|BL_ORD_ID|25311 transcript_6156	1	1245	1244	1560	36	1280	2831	3146
transcript_124596	gnl|BL_ORD_ID|17714 transcript_52341	1	1245	1244	1560	2	1246	2839	3155
transcript_12463	gnl|BL_ORD_ID|89093 transcript_160830	395	2689	41	394	1778	4070	2	359
transcript_124641	gnl|BL_ORD_ID|7449 transcript_34822	170	1337	4	170	584	1753	2	168
transcript_124664	gnl|BL_ORD_ID|91225 transcript_16167	1	1715	1716	2417	3	1717	1835	2518
transcript_124683	gnl|BL_ORD_ID|74325 transcript_137956	795	2020	80	796	984	2209	2	717
transcript_124691	gnl|BL_ORD_ID|17569 transcript_52111	113	2744	2743	3078	4	2636	2753	3088
transcript_124724	gnl|BL_ORD_ID|71616 transcript_133554	1	1627	1624	2997	1	1629	1794	3167
transcript_12476	gnl|BL_ORD_ID|30747 transcript_71858	14	2339	2336	2699	2	2350	2476	2839
transcript_124790	gnl|BL_ORD_ID|11787 transcript_2519	477	3480	56	478	632	3636	2	404
transcript_124816	gnl|BL_ORD_ID|37410 transcript_82616	1	1123	1124	1762	190	1312	1996	2632
transcript_124832	gnl|BL_ORD_ID|71118 transcript_132773	1007	2140	1	1008	1561	2680	1	1007
transcript_124881	gnl|BL_ORD_ID|34678 transcript_78255	1	2926	2926	5065	1	2928	3111	5247
transcript_124938	gnl|BL_ORD_ID|94325 transcript_167621	1	1598	1593	2422	507	2104	3436	4267
transcript_124938	gnl|BL_ORD_ID|73255 transcript_136177	1	1598	1593	2422	507	2104	3436	4267
transcript_124954	gnl|BL_ORD_ID|79990 transcript_145634	1	1900	1895	2314	2	1900	2280	2699
transcript_124954	gnl|BL_ORD_ID|64874 transcript_11774	1	1900	1895	2314	71	1969	2239	2658
transcript_124954	gnl|BL_ORD_ID|35381 transcript_79378	1	1900	1895	2314	36	1934	2763	3182
transcript_124954	gnl|BL_ORD_ID|48475 transcript_98950	1	1900	1895	2315	2	1899	2025	2445
transcript_124981	gnl|BL_ORD_ID|37333 transcript_82489	1	2900	2898	3341	46	2945	3220	3663
transcript_124996	gnl|BL_ORD_ID|36791 transcript_81644	1	1497	1500	2225	793	2289	3999	4722
transcript_125012	gnl|BL_ORD_ID|73769 transcript_137038	105	2868	2867	3701	2	2784	2893	3730
transcript_125012	gnl|BL_ORD_ID|59340 transcript_115301	1	2868	2867	3641	1	2884	2993	3765
transcript_125026	gnl|BL_ORD_ID|46080 transcript_95178	1197	2426	1	1197	2110	3342	73	1271
transcript_125026	gnl|BL_ORD_ID|96322 transcript_52676	1197	2426	1	1197	1915	3145	73	1271
transcript_125057	gnl|BL_ORD_ID|44780 transcript_92990	1	2165	2163	2837	1715	3876	4000	4670
transcript_125074	gnl|BL_ORD_ID|29760 transcript_70226	100	1522	1519	2298	84	1511	1835	2615

transcript_125103	gnl|BL_ORD_ID|95925 transcript_20628	1154	2242	1	1155	1273	2360	1	1160
transcript_125213	gnl|BL_ORD_ID|38582 transcript_7949	166	2791	1	171	336	2961	2	172
transcript_125232	gnl|BL_ORD_ID|70553 transcript_131853	1	1648	1646	1938	848	2495	2672	2960
transcript_125232	gnl|BL_ORD_ID|30260 transcript_71065	1	1648	1646	1998	930	2577	2723	3075
transcript_125234	gnl|BL_ORD_ID|11650 transcript_2252	2	3072	3071	3355	82	3159	3303	3587
transcript_125239	gnl|BL_ORD_ID|87383 transcript_158094	469	1929	60	468	3909	5373	1	409
transcript_125269	gnl|BL_ORD_ID|17192 transcript_51516	28	3081	3080	3338	5	3058	3795	4053
transcript_125280	gnl|BL_ORD_ID|48816 transcript_99511	1219	3112	1	1218	1437	3328	2	1219
transcript_125280	gnl|BL_ORD_ID|92413 transcript_164566	1219	3112	1	1218	1436	3344	2	1219
transcript_125280	gnl|BL_ORD_ID|31279 transcript_72709	1219	3112	1	1218	1442	3373	1	1223
transcript_12533	gnl|BL_ORD_ID|51813 transcript_9899	129	2705	1	128	301	2873	8	135
transcript_12534	gnl|BL_ORD_ID|51820 transcript_9922	168	2733	1	169	293	2860	1	165
transcript_12534	gnl|BL_ORD_ID|26046 transcript_64323	1	1766	1765	2721	80	1854	2772	3731
transcript_125358	gnl|BL_ORD_ID|53530 transcript_105502	131	2150	1	134	407	2414	2	135
transcript_125358	gnl|BL_ORD_ID|94733 transcript_17828	131	2176	1	134	453	2487	48	181
transcript_125358	gnl|BL_ORD_ID|64397 transcript_10771	131	2176	1	136	459	2507	57	192
transcript_125394	gnl|BL_ORD_ID|28121 transcript_67618	199	2985	1	201	603	3387	293	494
transcript_12540	gnl|BL_ORD_ID|83457 transcript_151541	555	2693	6	557	972	3110	2	553
transcript_12540	gnl|BL_ORD_ID|37981 transcript_6668	555	2693	6	557	973	3110	2	553
transcript_125402	gnl|BL_ORD_ID|58416 transcript_113800	1	2503	2499	3046	217	2720	2852	3399
transcript_12544	gnl|BL_ORD_ID|33709 transcript_76654	133	1989	1984	2709	1	1855	2600	3323
transcript_125464	gnl|BL_ORD_ID|70460 transcript_131704	1	1186	1182	1856	14	1199	1323	1997
transcript_125466	gnl|BL_ORD_ID|64397 transcript_10771	242	2192	72	241	556	2507	2	170
transcript_12547	gnl|BL_ORD_ID|41702 transcript_87966	583	2726	6	585	786	2935	2	582
transcript_125486	gnl|BL_ORD_ID|81029 transcript_147327	1	1684	1681	2222	1	1689	2745	3286
transcript_125512	gnl|BL_ORD_ID|80512 transcript_146491	1	2065	2066	2811	2	2072	2528	3276
transcript_125589	gnl|BL_ORD_ID|23406 transcript_61705	179	1476	2	179	1917	3213	151	328
transcript_125589	gnl|BL_ORD_ID|48587 transcript_99132	179	1461	2	179	1934	3226	145	325
transcript_125626	gnl|BL_ORD_ID|1030 transcript_1848	1	1577	1572	2326	1	1571	3078	3827
transcript_125626	gnl|BL_ORD_ID|79822 transcript_145353	1	1577	1572	2316	23	1593	3046	3787
transcript_125642	gnl|BL_ORD_ID|94033 transcript_167166	1	2320	2317	2958	2	2319	2670	3310
transcript_125688	gnl|BL_ORD_ID|20195 transcript_56329	1	1402	1400	2192	10	1433	1697	2489
transcript_125688	gnl|BL_ORD_ID|53023 transcript_104650	1	1402	1400	2192	226	1636	1900	2692
transcript_125688	gnl|BL_ORD_ID|53424 transcript_105327	1	1402	1400	2194	70	1492	1962	2756

transcript_125716	gnl|BL_ORD_ID|73170 transcript_136026	1	1871	1871	2486	902	2772	2982	3601
transcript_125750	gnl|BL_ORD_ID|71389 transcript_133191	1	2406	2403	3084	207	2613	2820	3501
transcript_125750	gnl|BL_ORD_ID|12567 transcript_4043	1	2406	2403	3165	15	2421	2628	3390
transcript_12577	gnl|BL_ORD_ID|44504 transcript_92527	1249	2640	1	1254	1385	2776	1	1257
transcript_125838	gnl|BL_ORD_ID|60857 transcript_117713	143	2310	1	145	664	2831	413	557
transcript_125838	gnl|BL_ORD_ID|97444 transcript_159642	143	2225	1	145	1554	3636	1304	1448
transcript_125849	gnl|BL_ORD_ID|55682 transcript_109281	1	1506	1505	1829	1077	2582	3196	3517
transcript_125986	gnl|BL_ORD_ID|89864 transcript_162062	217	2540	113	220	310	2642	2	109
transcript_125986	gnl|BL_ORD_ID|51764 transcript_9774	217	2540	1	220	423	2755	2	222
transcript_125986	gnl|BL_ORD_ID|64756 transcript_11551	217	2540	1	220	468	2796	50	269
transcript_126011	gnl|BL_ORD_ID|71865 transcript_133969	1	1533	1531	2122	72	1602	1993	2582
transcript_126011	gnl|BL_ORD_ID|23326 transcript_61569	1	1533	1531	2122	76	1615	2429	3016
transcript_126011	gnl|BL_ORD_ID|39667 transcript_84630	1	1533	1531	2122	80	1612	2452	3042
transcript_126011	gnl|BL_ORD_ID|38606 transcript_7996	1	1533	1531	2099	84	1614	2428	2996
transcript_126011	gnl|BL_ORD_ID|38355 transcript_7438	1	1533	1531	2170	80	1612	2427	3065
transcript_126011	gnl|BL_ORD_ID|30805 transcript_71967	1	1090	1090	2170	54	1143	2369	3449
transcript_126030	gnl|BL_ORD_ID|38157 transcript_7025	1	1409	1407	2486	2	1411	2012	3092
transcript_126072	gnl|BL_ORD_ID|90728 transcript_15079	147	1924	1	152	807	2583	217	368
transcript_12613	gnl|BL_ORD_ID|12037 transcript_3012	2	2138	2137	2645	1	2137	3041	3549
transcript_126175	gnl|BL_ORD_ID|38141 transcript_6989	183	2946	1	184	305	3069	15	197
transcript_126347	gnl|BL_ORD_ID|2220 transcript_23728	1	1719	1720	1918	4	1724	2004	2202
transcript_12637	gnl|BL_ORD_ID|37567 transcript_82869	138	2672	12	138	300	2832	2	128
transcript_12638	gnl|BL_ORD_ID|89805 transcript_161972	1	1336	1331	2643	1	1340	1776	3090
transcript_126425	gnl|BL_ORD_ID|12516 transcript_3924	1	1134	1131	1539	1486	2647	2976	3384
transcript_126447	gnl|BL_ORD_ID|25171 transcript_5858	1	1436	1431	2471	1	1436	2144	3181
transcript_126482	gnl|BL_ORD_ID|7073 transcript_34070	103	1668	1	103	233	1798	2	104
transcript_126489	gnl|BL_ORD_ID|20069 transcript_56126	126	2936	2934	3968	165	2979	3079	4121
transcript_126540	gnl|BL_ORD_ID|28792 transcript_68741	1	2268	2266	2871	477	2742	3086	3691
transcript_126547	gnl|BL_ORD_ID|89667 transcript_161768	1673	3319	148	1674	2010	3658	1	1527
transcript_126605	gnl|BL_ORD_ID|43594 transcript_90976	1228	2789	1	1230	1345	2912	2	1231
transcript_126613	gnl|BL_ORD_ID|40090 transcript_85307	2	2894	2890	3267	1	2910	4195	4580
transcript_126613	gnl|BL_ORD_ID|60514 transcript_117165	2	2894	2890	3280	93	3002	4228	4615
transcript_12662	gnl|BL_ORD_ID|84131 transcript_152740	640	2703	70	644	1105	3171	2	579
transcript_12665	gnl|BL_ORD_ID|51962 transcript_10218	289	2712	5	290	419	2840	2	287

transcript_126658	gnl|BL_ORD_ID|19923 transcript_55900	135	1357	3	138	2626	3842	22	160
transcript_126670	gnl|BL_ORD_ID|1047 transcript_1880	519	2916	87	519	1438	3837	671	1103
transcript_126706	gnl|BL_ORD_ID|42054 transcript_88498	1	1361	1362	1544	85	1448	1807	1989
transcript_126706	gnl|BL_ORD_ID|4055 transcript_27756	1	1361	1362	1562	84	1446	1805	2005
transcript_126706	gnl|BL_ORD_ID|26141 transcript_64469	1	1361	1364	1510	85	1459	1817	1964
transcript_126734	gnl|BL_ORD_ID|131 transcript_199	123	2233	1	122	2916	5026	2	123
transcript_126781	gnl|BL_ORD_ID|97500 transcript_166228	31	3453	3452	3988	1	3424	4843	5379
transcript_126789	gnl|BL_ORD_ID|12516 transcript_3924	1	2135	2132	2559	494	2647	2976	3403
transcript_126842	gnl|BL_ORD_ID|29538 transcript_69892	1	1583	1580	2605	4	1593	2221	3249
transcript_126857	gnl|BL_ORD_ID|18422 transcript_53466	1	1004	1003	1398	1	1019	2242	2635
transcript_126868	gnl|BL_ORD_ID|20337 transcript_56545	110	1451	1451	2411	2	1343	1847	2801
transcript_126916	gnl|BL_ORD_ID|101 transcript_149	1	1476	1473	2028	2712	4170	4421	4963
transcript_126916	gnl|BL_ORD_ID|54424 transcript_107034	1	1476	1473	2028	1991	3422	3672	4213
transcript_126919	gnl|BL_ORD_ID|46408 transcript_95715	189	1293	27	188	1962	3090	2	164
transcript_126928	gnl|BL_ORD_ID|51571 transcript_9374	1	2321	2316	2534	273	2592	2704	2920
transcript_12698	gnl|BL_ORD_ID|26505 transcript_65023	348	2699	84	348	1639	3990	2	266
transcript_12698	gnl|BL_ORD_ID|27781 transcript_67047	115	1410	1410	2699	2	1297	1460	2749
transcript_126992	gnl|BL_ORD_ID|12642 transcript_4187	1	2146	2144	2575	3	2144	2944	3375
transcript_127036	gnl|BL_ORD_ID|12516 transcript_3924	1	1423	1420	1777	1173	2647	2976	3333
transcript_127059	gnl|BL_ORD_ID|52567 transcript_103909	106	2037	2036	3158	133	2065	2453	3575
transcript_127112	gnl|BL_ORD_ID|2187 transcript_23645	258	2065	37	258	447	2256	72	293
transcript_127144	gnl|BL_ORD_ID|84302 transcript_153045	1	1425	1424	1892	4026	5444	5576	6043
transcript_127174	gnl|BL_ORD_ID|25257 transcript_6048	1	1756	1755	2934	1	1779	1936	3115
transcript_127174	gnl|BL_ORD_ID|80195 transcript_145968	1033	2865	1	1030	1172	3009	1	1067
transcript_127174	gnl|BL_ORD_ID|37920 transcript_6551	1	1756	1755	2920	1	1776	1948	3113
transcript_127174	gnl|BL_ORD_ID|38181 transcript_7072	1452	2907	1	1454	1621	3076	1	1477
transcript_127183	gnl|BL_ORD_ID|31250 transcript_72652	1379	3079	1	1378	2809	4512	382	1767
transcript_12725	gnl|BL_ORD_ID|93407 transcript_166192	1077	2711	1	1077	3461	5097	22	1098
transcript_12725	gnl|BL_ORD_ID|51302 transcript_8833	1077	2711	1	1077	1286	2922	1	1077
transcript_12725	gnl|BL_ORD_ID|92261 transcript_164308	1077	2711	1	1077	3256	4892	1	1077
transcript_127273	gnl|BL_ORD_ID|47906 transcript_98040	1	1524	1522	2709	1	1524	1942	3129
transcript_127305	gnl|BL_ORD_ID|38391 transcript_7534	173	2827	1	177	361	3017	1	177
transcript_127380	gnl|BL_ORD_ID|61691 transcript_119036	176	2254	17	175	2050	4128	2	160
transcript_127439	gnl|BL_ORD_ID|18292 transcript_53250	1	1320	1318	1850	404	1727	2314	2845

transcript_127475	gnl|BL_ORD_ID|76452 transcript_141400	795	1965	8	796	1476	2646	1	790
transcript_12749	gnl|BL_ORD_ID|56692 transcript_110948	609	2610	7	614	2446	4430	1	608
transcript_127490	gnl|BL_ORD_ID|51003 transcript_103021	1	1692	1690	2834	1	1673	1814	2959
transcript_127494	gnl|BL_ORD_ID|89088 transcript_160820	2	3026	3024	3418	53	3077	3221	3615
transcript_127496	gnl|BL_ORD_ID|44258 transcript_92111	1	1299	1299	2039	42	1338	2022	2762
transcript_127540	gnl|BL_ORD_ID|82874 transcript_150508	291	1863	32	292	688	2267	4	274
transcript_12759	gnl|BL_ORD_ID|24879 transcript_5228	2	2181	2179	2686	1	2180	2736	3243
transcript_127676	gnl|BL_ORD_ID|61848 transcript_119288	300	3561	2	302	1052	4317	10	311
transcript_127679	gnl|BL_ORD_ID|42546 transcript_89268	863	2688	9	866	2424	4248	1	859
transcript_127680	gnl|BL_ORD_ID|51556 transcript_9349	1	1841	1840	2298	1	1841	2423	2881
transcript_127794	gnl|BL_ORD_ID|72230 transcript_134544	283	3155	1	282	463	3335	1	283
transcript_127843	gnl|BL_ORD_ID|29926 transcript_70517	1	1999	1997	2946	1169	3190	3366	4317
transcript_127855	gnl|BL_ORD_ID|56289 transcript_110303	1119	2603	1	1120	1753	3240	35	1150
transcript_127885	gnl|BL_ORD_ID|65091 transcript_12279	1	2087	2085	2468	2	2110	2289	2672
transcript_12789	gnl|BL_ORD_ID|83720 transcript_152014	2	2069	2066	2661	1	2060	2170	2752
transcript_127908	gnl|BL_ORD_ID|96280 transcript_14794	1	1533	1533	2401	95	1627	1728	2596
transcript_127913	gnl|BL_ORD_ID|4145 transcript_27960	354	1930	6	353	491	2066	41	388
transcript_127975	gnl|BL_ORD_ID|28947 transcript_68988	109	1807	1	111	587	2285	1	113
transcript_127976	gnl|BL_ORD_ID|95981 transcript_20764	1	1851	1848	2097	2	1862	2033	2282
transcript_127976	gnl|BL_ORD_ID|1622 transcript_22455	1	1851	1848	2097	5	1853	2024	2273
transcript_127976	gnl|BL_ORD_ID|49193 transcript_100101	1	1851	1848	2097	1	1828	1999	2248
transcript_127981	gnl|BL_ORD_ID|61218 transcript_118306	27	2988	2988	3614	1	2937	3088	3713
transcript_128004	gnl|BL_ORD_ID|31164 transcript_72524	2	2679	2679	3322	259	2936	3086	3728
transcript_128004	gnl|BL_ORD_ID|21133 transcript_57915	2	2679	2679	3175	462	3142	3286	3782
transcript_128028	gnl|BL_ORD_ID|92953 transcript_165448	160	2276	1	161	1285	3421	2	162
transcript_128050	gnl|BL_ORD_ID|78092 transcript_13861	1	1827	1828	2543	1	1827	1940	2656
transcript_128074	gnl|BL_ORD_ID|81620 transcript_148302	124	2091	1	124	1461	3429	1094	1217
transcript_128090	gnl|BL_ORD_ID|33501 transcript_76309	2	2119	2119	3117	76	2193	2304	3302
transcript_128176	gnl|BL_ORD_ID|85368 transcript_154761	1	1343	1342	1606	2	1338	1552	1816
transcript_128186	gnl|BL_ORD_ID|73684 transcript_136888	528	1533	59	533	619	1623	2	476
transcript_12819	gnl|BL_ORD_ID|52142 transcript_10617	1	2220	2220	2701	1	2221	2346	2827
transcript_128212	gnl|BL_ORD_ID|88465 transcript_159833	1	1479	1478	2369	159	1637	1872	2758
transcript_128212	gnl|BL_ORD_ID|1002 transcript_1781	1	1480	1478	2418	159	1638	2069	3004
transcript_128212	gnl|BL_ORD_ID|788 transcript_1374	1	1480	1478	2418	128	1607	2037	2972

transcript_128274	gnl|BL_ORD_ID|66340 transcript_125005	112	1959	1954	2928	308	2155	2454	3429
transcript_128274	gnl|BL_ORD_ID|56814 transcript_111133	112	2120	2116	2928	132	2140	3245	4058
transcript_128396	gnl|BL_ORD_ID|37981 transcript_6668	1	1398	1395	2554	1	1393	1932	3105
transcript_128396	gnl|BL_ORD_ID|83457 transcript_151541	1	1398	1395	2554	1	1393	1932	3105
transcript_128415	gnl|BL_ORD_ID|61251 transcript_118351	878	3601	92	878	1037	3760	95	881
transcript_128419	gnl|BL_ORD_ID|54731 transcript_107562	1	2314	2314	2494	6	2340	2643	2823
transcript_128419	gnl|BL_ORD_ID|51234 transcript_8675	17	2314	2314	2494	58	2357	2660	2840
transcript_128423	gnl|BL_ORD_ID|93604 transcript_166496	283	2955	2	286	551	3224	154	438
transcript_128428	gnl|BL_ORD_ID|24784 transcript_5047	1	2751	2750	3002	1	2752	2990	3242
transcript_128436	gnl|BL_ORD_ID|29235 transcript_69423	1	1186	1185	1928	762	2017	2220	3020
transcript_128532	gnl|BL_ORD_ID|591 transcript_1003	288	3868	2	289	451	4030	61	348
transcript_128537	gnl|BL_ORD_ID|84448 transcript_153286	1	1373	1368	2172	2226	3597	3723	4526
transcript_128551	gnl|BL_ORD_ID|41523 transcript_87657	1	1296	1294	1891	516	1813	2700	3299
transcript_128575	gnl|BL_ORD_ID|55273 transcript_108540	1	1401	1400	1874	10	1415	1527	2001
transcript_128587	gnl|BL_ORD_ID|56351 transcript_110390	135	2569	1	137	482	2915	226	362
transcript_128589	gnl|BL_ORD_ID|30455 transcript_71382	16	3235	3235	4251	2	3230	3490	4494
transcript_128589	gnl|BL_ORD_ID|19021 transcript_54410	16	3235	3236	4252	2	3221	3388	4404
transcript_128589	gnl|BL_ORD_ID|32825 transcript_75266	16	3235	3236	4251	2	3232	3704	4705
transcript_128600	gnl|BL_ORD_ID|42550 transcript_89272	1	1725	1721	2100	83	1809	2541	2922
transcript_128600	gnl|BL_ORD_ID|92105 transcript_164069	1	1725	1721	2100	383	2104	2836	3211
transcript_128600	gnl|BL_ORD_ID|34257 transcript_77551	1	1425	1424	2050	126	1551	1770	2398
transcript_128606	gnl|BL_ORD_ID|26185 transcript_64536	426	2402	62	426	627	2603	2	363
transcript_128614	gnl|BL_ORD_ID|92793 transcript_165193	1	2055	2052	3602	1	2043	4941	6482
transcript_128635	gnl|BL_ORD_ID|39714 transcript_84705	2	2697	2696	3201	1	2707	3142	3647
transcript_128659	gnl|BL_ORD_ID|60046 transcript_116418	419	2672	73	421	482	2736	2	350
transcript_12868	gnl|BL_ORD_ID|90038 transcript_162337	101	1512	1510	2695	209	1617	1753	2939
transcript_128693	gnl|BL_ORD_ID|51570 transcript_9371	1	2116	2113	2709	1	2112	2224	2818
transcript_1287	gnl|BL_ORD_ID|62565 transcript_120466	30	3217	3216	3992	2	3190	3318	4092
transcript_128772	gnl|BL_ORD_ID|30645 transcript_71688	1	1785	1780	2211	163	1946	2072	2503
transcript_128772	gnl|BL_ORD_ID|22975 transcript_60985	1	1785	1780	2152	152	1932	2038	2410
transcript_128841	gnl|BL_ORD_ID|40624 transcript_86168	1	1831	1830	2165	214	2072	3089	3433
transcript_128884	gnl|BL_ORD_ID|18157 transcript_53031	229	1512	4	229	354	1637	2	227
transcript_128897	gnl|BL_ORD_ID|79121 transcript_144252	1	1337	1335	1565	38	1380	1977	2205
transcript_128910	gnl|BL_ORD_ID|34411 transcript_77808	146	1524	1	147	426	1815	1	147

transcript_128910	gnl|BL_ORD_ID|6416 transcript_32720	146	1525	1	147	441	1820	1	147
transcript_128910	gnl|BL_ORD_ID|6638 transcript_33202	146	1467	2	147	507	1835	84	229
transcript_128915	gnl|BL_ORD_ID|20016 transcript_56044	253	2762	1	254	1078	3589	4	257
transcript_128917	gnl|BL_ORD_ID|89101 transcript_160844	14	2666	2663	3254	2	2653	2892	3483
transcript_128933	gnl|BL_ORD_ID|69823 transcript_130640	1	2246	2245	2490	1	2246	2418	2663
transcript_128936	gnl|BL_ORD_ID|80004 transcript_145650	2	2270	2271	2979	114	2380	2502	3209
transcript_128936	gnl|BL_ORD_ID|38241 transcript_7195	2	2270	2271	2907	146	2414	2536	3172
transcript_128939	gnl|BL_ORD_ID|73840 transcript_137176	2	2186	2186	2743	29	2225	2347	2904
transcript_12899	gnl|BL_ORD_ID|59387 transcript_115379	1	2350	2351	2708	1	2349	2453	2811
transcript_128997	gnl|BL_ORD_ID|92937 transcript_165414	1	1024	1023	1684	48	1067	1338	1999
transcript_1290	gnl|BL_ORD_ID|401 transcript_685	2	3701	3700	3990	24	3720	4006	4291
transcript_129005	gnl|BL_ORD_ID|88228 transcript_159453	200	1757	3	200	613	2167	2	199
transcript_129005	gnl|BL_ORD_ID|70088 transcript_131079	200	1841	3	200	552	2189	2	198
transcript_129068	gnl|BL_ORD_ID|24577 transcript_4596	2	2529	2524	3225	1	2508	2610	3292
transcript_129073	gnl|BL_ORD_ID|90767 transcript_15167	1	1168	1167	1646	794	1961	2116	2595
transcript_129102	gnl|BL_ORD_ID|73321 transcript_136285	444	1302	6	447	569	1413	2	436
transcript_129122	gnl|BL_ORD_ID|20040 transcript_56075	2	2230	2230	2856	4	2234	2372	2997
transcript_129136	gnl|BL_ORD_ID|71462 transcript_133311	1	2137	2133	2406	260	2383	2500	2767
transcript_129136	gnl|BL_ORD_ID|52177 transcript_10687	1	2137	2132	2406	304	2440	2556	2829
transcript_129177	gnl|BL_ORD_ID|57471 transcript_112240	2	2055	2053	2310	27	2077	2780	3037
transcript_129184	gnl|BL_ORD_ID|49316 transcript_100291	248	2709	1	249	495	2956	116	364
transcript_129190	gnl|BL_ORD_ID|65091 transcript_12279	1	1284	1283	1991	3	1285	2009	2717
transcript_129198	gnl|BL_ORD_ID|85323 transcript_154682	23	2776	2776	3008	66	2818	3140	3374
transcript_129250	gnl|BL_ORD_ID|53301 transcript_105108	1	2044	2044	2989	30	2074	2248	3193
transcript_12929	gnl|BL_ORD_ID|11602 transcript_2156	240	2724	24	242	1240	3722	1	219
transcript_129322	gnl|BL_ORD_ID|94589 transcript_17493	489	1965	87	494	973	2449	2	409
transcript_129324	gnl|BL_ORD_ID|58313 transcript_113622	23	2370	2365	3212	4	2354	2464	3312
transcript_129335	gnl|BL_ORD_ID|88928 transcript_160570	1	2188	2184	2487	138	2318	2473	2778
transcript_129349	gnl|BL_ORD_ID|75705 transcript_140201	357	1096	5	360	1435	2176	2	355
transcript_12938	gnl|BL_ORD_ID|47195 transcript_96934	1	2115	2114	2690	19	2144	2303	2878
transcript_129391	gnl|BL_ORD_ID|76743 transcript_141869	1	1316	1313	2020	2	1317	3100	3807
transcript_129397	gnl|BL_ORD_ID|88006 transcript_159092	128	2587	2586	3725	160	2593	2834	3973
transcript_12943	gnl|BL_ORD_ID|51344 transcript_8923	1	1522	1520	2690	1	1522	1733	2903
transcript_129430	gnl|BL_ORD_ID|24915 transcript_5300	1	1469	1468	2291	319	1767	2407	3227

transcript_129460	gnl|BL_ORD_ID|94672 transcript_17696	1	1320	1317	2175	1	1320	1595	2464
transcript_129503	gnl|BL_ORD_ID|27148 transcript_66057	1	2056	2054	3236	1533	3588	3697	4888
transcript_129566	gnl|BL_ORD_ID|12026 transcript_2985	734	3208	93	733	949	3423	2	642
transcript_129592	gnl|BL_ORD_ID|67017 transcript_126098	258	2203	7	261	432	2377	1	270
transcript_129592	gnl|BL_ORD_ID|64775 transcript_11586	258	2203	7	261	420	2365	2	258
transcript_129612	gnl|BL_ORD_ID|67326 transcript_126605	1	1406	1407	2670	333	1737	1514	2782
transcript_129612	gnl|BL_ORD_ID|38203 transcript_7115	1	1406	1407	2670	661	2066	1842	3111
transcript_129612	gnl|BL_ORD_ID|51353 transcript_8938	1	1406	1407	2670	507	1912	1688	2957
transcript_129612	gnl|BL_ORD_ID|59927 transcript_116231	1	1406	1407	2585	828	2233	2009	3198
transcript_129612	gnl|BL_ORD_ID|38683 transcript_8171	1	1406	1407	2670	484	1889	1665	2936
transcript_129612	gnl|BL_ORD_ID|83290 transcript_151253	1	1406	1407	2670	507	1912	1688	2962
transcript_129612	gnl|BL_ORD_ID|66585 transcript_125394	1	1406	1407	2670	525	1929	1705	2980
transcript_129612	gnl|BL_ORD_ID|28121 transcript_67618	1	1406	1407	2670	929	2332	2108	3382
transcript_129612	gnl|BL_ORD_ID|302 transcript_497	1	1406	1407	2670	2012	3417	3193	4462
transcript_129612	gnl|BL_ORD_ID|25382 transcript_6298	1	1406	1407	2670	382	1787	1563	2837
transcript_129660	gnl|BL_ORD_ID|12584 transcript_4072	1	2111	2106	2355	7	2117	2558	2807
transcript_129794	gnl|BL_ORD_ID|86909 transcript_157291	469	4466	59	469	1911	5904	2	412
transcript_12983	gnl|BL_ORD_ID|41129 transcript_87002	222	2633	1	224	1273	3649	2	236
transcript_12983	gnl|BL_ORD_ID|26088 transcript_64384	222	2688	13	224	1386	3841	1	227
transcript_129844	gnl|BL_ORD_ID|81130 transcript_147488	1	1830	1826	3179	8	1837	1973	3326
transcript_129849	gnl|BL_ORD_ID|60890 transcript_117775	490	1933	6	492	1128	2610	4	496
transcript_129867	gnl|BL_ORD_ID|94423 transcript_17116	1	1382	1381	2126	209	1590	1738	2483
transcript_129867	gnl|BL_ORD_ID|78427 transcript_14622	1	1382	1381	2126	329	1710	1858	2603
transcript_12991	gnl|BL_ORD_ID|79447 transcript_144761	20	2293	2294	2623	2	2275	3134	3463
transcript_12991	gnl|BL_ORD_ID|12073 transcript_3085	20	2293	2294	2700	2	2272	3129	3535
transcript_129995	gnl|BL_ORD_ID|60941 transcript_117859	228	1307	29	228	1176	2255	1	202
transcript_130079	gnl|BL_ORD_ID|36197 transcript_80689	207	1462	3	211	3273	4531	3	211
transcript_130100	gnl|BL_ORD_ID|87364 transcript_158066	2	2006	2004	2607	1	1985	2098	2698
transcript_130150	gnl|BL_ORD_ID|47381 transcript_97234	1	2030	2026	2670	126	2168	2276	2922
transcript_130150	gnl|BL_ORD_ID|25061 transcript_5639	1	2030	2026	2670	44	2086	2195	2841
transcript_130150	gnl|BL_ORD_ID|36220 transcript_80722	14	2030	2026	2670	2	2018	2127	2773
transcript_130150	gnl|BL_ORD_ID|84781 transcript_153813	1	2030	2026	2670	42	2071	2180	2826
transcript_13019	gnl|BL_ORD_ID|41483 transcript_87601	262	2687	2	265	633	3057	21	284
transcript_130255	gnl|BL_ORD_ID|793 transcript_1383	348	3511	1	350	820	3982	4	349

transcript_130329	gnl|BL_ORD_ID|49694 transcript_100903	1	2050	2049	2654	1050	3098	3254	3858
transcript_13033	gnl|BL_ORD_ID|52062 transcript_10436	208	2686	1	209	365	2837	1	209
transcript_130335	gnl|BL_ORD_ID|304 transcript_499	1270	3617	125	1269	2154	4501	2	1146
transcript_130372	gnl|BL_ORD_ID|39374 transcript_84148	1	2594	2593	3374	605	3198	3303	4084
transcript_130372	gnl|BL_ORD_ID|507 transcript_859	1	2594	2593	3351	722	3315	3420	4178
transcript_130372	gnl|BL_ORD_ID|619 transcript_1061	1	2594	2593	3402	615	3208	3313	4124
transcript_130372	gnl|BL_ORD_ID|63058 transcript_121239	1	2594	2593	3381	788	3381	3486	4274
transcript_130402	gnl|BL_ORD_ID|45996 transcript_95046	2160	4373	15	2161	2271	4492	2	2151
transcript_130480	gnl|BL_ORD_ID|46659 transcript_96109	535	2139	8	539	1163	2772	499	1030
transcript_13049	gnl|BL_ORD_ID|71247 transcript_132963	1	2261	2260	2606	1	2261	2370	2716
transcript_13049	gnl|BL_ORD_ID|36401 transcript_81023	19	2189	2184	2684	1	2151	2868	3358
transcript_13049	gnl|BL_ORD_ID|67539 transcript_126954	1	1606	1606	2685	1	1606	1712	2798
transcript_13051	gnl|BL_ORD_ID|51199 transcript_8593	2	2455	2456	2682	22	2475	2719	2945
transcript_130526	gnl|BL_ORD_ID|10583 transcript_40943	1	1020	1018	1206	25	1044	1182	1370
transcript_130532	gnl|BL_ORD_ID|220 transcript_349	1512	3145	1	1516	3103	4739	1474	2990
transcript_130532	gnl|BL_ORD_ID|26556 transcript_65113	1512	3051	1	1516	2018	3560	389	1905
transcript_130532	gnl|BL_ORD_ID|35462 transcript_79504	1512	3145	1	1516	2076	3712	448	1963
transcript_130544	gnl|BL_ORD_ID|72079 transcript_134304	1	2171	2170	3634	1	2171	2686	4148
transcript_130544	gnl|BL_ORD_ID|74747 transcript_138660	2	2171	2171	3704	4	2174	2314	3848
transcript_130578	gnl|BL_ORD_ID|96733 transcript_90907	170	1020	2	171	849	1681	5	174
transcript_130578	gnl|BL_ORD_ID|8255 transcript_36404	170	1025	2	171	817	1664	7	176
transcript_130591	gnl|BL_ORD_ID|70377 transcript_131566	1	1298	1299	1717	468	1765	1988	2429
transcript_130633	gnl|BL_ORD_ID|3092 transcript_25620	1	1334	1333	1681	1	1330	1820	2169
transcript_13068	gnl|BL_ORD_ID|63498 transcript_121956	14	1825	1825	2859	15	1836	2115	3147
transcript_130684	gnl|BL_ORD_ID|56487 transcript_110605	188	1852	2	189	909	2564	40	216
transcript_130688	gnl|BL_ORD_ID|5142 transcript_30044	268	1788	5	270	498	2018	2	267
transcript_130768	gnl|BL_ORD_ID|96277 transcript_12087	1077	2324	1	1081	1207	2455	21	1101
transcript_130807	gnl|BL_ORD_ID|37592 transcript_82904	1	1625	1623	2638	322	1941	2215	3223
transcript_130807	gnl|BL_ORD_ID|24886 transcript_5245	1	1625	1623	2622	366	1987	2261	3253
transcript_130866	gnl|BL_ORD_ID|78351 transcript_14410	256	1444	3	260	1470	2654	39	308
transcript_130866	gnl|BL_ORD_ID|56385 transcript_110441	257	1447	3	260	1015	2255	23	299
transcript_130866	gnl|BL_ORD_ID|65098 transcript_12306	256	1446	3	260	1436	2626	24	281
transcript_130872	gnl|BL_ORD_ID|90981 transcript_15644	1	1464	1464	2255	1	1464	1772	2563
transcript_130887	gnl|BL_ORD_ID|1062 transcript_1906	2	2451	2446	3693	1	2450	2552	3796

transcript_130966	gnl|BL_ORD_ID|31836 transcript_73623	377	2921	40	377	560	3102	2	329
transcript_130966	gnl|BL_ORD_ID|70441 transcript_131677	315	2921	34	319	617	3231	2	292
transcript_130992	gnl|BL_ORD_ID|40160 transcript_85412	295	2325	64	296	367	2377	1	232
transcript_131013	gnl|BL_ORD_ID|74544 transcript_138325	1	1199	1198	1920	379	1575	1675	2400
transcript_131013	gnl|BL_ORD_ID|89783 transcript_161936	1	1200	1198	1920	321	1522	1654	2379
transcript_131021	gnl|BL_ORD_ID|4945 transcript_29612	1	1088	1090	1662	13	1089	1418	1991
transcript_131021	gnl|BL_ORD_ID|87001 transcript_157460	1	1088	1090	1594	63	1149	1478	1983
transcript_131021	gnl|BL_ORD_ID|3641 transcript_26855	1	1088	1090	1662	73	1171	1497	2070
transcript_131029	gnl|BL_ORD_ID|70402 transcript_131610	1	1507	1505	2264	26	1510	1666	2419
transcript_131101	gnl|BL_ORD_ID|7626 transcript_35194	295	1125	5	297	641	1471	2	294
transcript_131103	gnl|BL_ORD_ID|53238 transcript_105003	190	1526	36	190	307	1642	28	184
transcript_131122	gnl|BL_ORD_ID|35278 transcript_79222	1	2007	2004	3864	1	1992	2987	4848
transcript_131177	gnl|BL_ORD_ID|47322 transcript_97150	493	1580	6	494	2088	3151	2	487
transcript_131183	gnl|BL_ORD_ID|26687 transcript_65320	1	2831	2831	4843	108	2944	3127	5140
transcript_131183	gnl|BL_ORD_ID|66265 transcript_124881	1	4303	4301	4862	90	4399	4504	5064
transcript_131239	gnl|BL_ORD_ID|28566 transcript_68377	193	1008	2	194	3371	4186	1176	1368
transcript_131285	gnl|BL_ORD_ID|2474 transcript_24285	184	1966	1	187	332	2114	1	187
transcript_131285	gnl|BL_ORD_ID|84532 transcript_153420	185	1931	1	187	333	2053	1	187
transcript_131285	gnl|BL_ORD_ID|44988 transcript_93337	185	1966	1	187	333	2114	1	187
transcript_131285	gnl|BL_ORD_ID|1404 transcript_21887	184	1957	1	187	332	2093	1	187
transcript_131285	gnl|BL_ORD_ID|94637 transcript_17614	185	1966	1	187	361	2264	1	205
transcript_131285	gnl|BL_ORD_ID|95956 transcript_20702	184	1966	1	187	332	2113	1	187
transcript_131302	gnl|BL_ORD_ID|33061 transcript_75615	277	3116	14	276	1247	4086	2	264
transcript_131302	gnl|BL_ORD_ID|70338 transcript_131500	277	3115	2	276	702	3548	3	278
transcript_131302	gnl|BL_ORD_ID|27846 transcript_67166	277	3084	2	276	688	3498	3	277
transcript_131317	gnl|BL_ORD_ID|77243 transcript_142680	1	1026	1024	1387	1	1028	1228	1593
transcript_131430	gnl|BL_ORD_ID|26483 transcript_64993	451	2186	56	447	593	2328	2	403
transcript_131433	gnl|BL_ORD_ID|39925 transcript_85039	1	1943	1938	2223	1	1943	2081	2366
transcript_13146	gnl|BL_ORD_ID|81234 transcript_147651	14	2281	2276	2680	2	2272	2512	2910
transcript_131463	gnl|BL_ORD_ID|3274 transcript_26039	113	1786	1	113	441	2113	2	112
transcript_131599	gnl|BL_ORD_ID|42 transcript_60	286	3484	2	286	2642	5826	46	333
transcript_131599	gnl|BL_ORD_ID|62127 transcript_119729	286	3483	2	286	444	3641	46	330
transcript_131600	gnl|BL_ORD_ID|82634 transcript_150104	235	2703	2	237	1106	3576	418	653
transcript_131615	gnl|BL_ORD_ID|38127 transcript_6959	111	1921	1918	2626	7	1981	2156	2913

transcript_13174	gnl|BL_ORD_ID|89382 transcript_161295	2	2119	2120	2613	60	2175	2338	2828
transcript_131743	gnl|BL_ORD_ID|82358 transcript_149616	291	1550	62	291	1492	2751	2	231
transcript_131766	gnl|BL_ORD_ID|89060 transcript_160775	13	1341	1338	2158	92	1422	1542	2364
transcript_131766	gnl|BL_ORD_ID|26649 transcript_65259	1	1341	1338	2158	204	1544	1664	2484
transcript_131803	gnl|BL_ORD_ID|61794 transcript_119204	142	2425	1	143	306	2592	6	137
transcript_131803	gnl|BL_ORD_ID|77774 transcript_13098	142	2381	1	143	439	2682	147	284
transcript_131803	gnl|BL_ORD_ID|77856 transcript_13296	142	2425	1	143	411	2694	112	252
transcript_131803	gnl|BL_ORD_ID|70220 transcript_131298	142	2425	1	143	343	2628	51	188
transcript_131808	gnl|BL_ORD_ID|67756 transcript_127293	1104	2359	1	1104	2617	3875	441	1543
transcript_131811	gnl|BL_ORD_ID|79103 transcript_144217	99	2635	1	102	1405	3939	534	635
transcript_131811	gnl|BL_ORD_ID|65476 transcript_123562	101	2587	1	102	1545	4033	670	771
transcript_131811	gnl|BL_ORD_ID|17929 transcript_52674	101	2638	1	102	1503	4054	627	728
transcript_131823	gnl|BL_ORD_ID|24886 transcript_5245	114	2772	1	116	596	3253	327	442
transcript_131823	gnl|BL_ORD_ID|76291 transcript_141165	114	2671	1	116	664	3209	282	397
transcript_131897	gnl|BL_ORD_ID|24419 transcript_4281	1	1092	1089	2018	1	1096	2421	3349
transcript_131914	gnl|BL_ORD_ID|37608 transcript_82928	157	3028	1	161	668	3542	397	558
transcript_131914	gnl|BL_ORD_ID|27203 transcript_66147	157	3147	1	161	367	3361	96	257
transcript_131914	gnl|BL_ORD_ID|18848 transcript_54117	157	3028	1	161	493	3368	158	319
transcript_131914	gnl|BL_ORD_ID|232 transcript_375	157	3028	1	161	430	3304	159	320
transcript_131914	gnl|BL_ORD_ID|37782 transcript_83195	157	3028	1	161	429	3303	158	319
transcript_131970	gnl|BL_ORD_ID|230 transcript_372	1	1710	1709	3168	1	1712	3006	4464
transcript_131981	gnl|BL_ORD_ID|44102 transcript_91859	1007	2710	1	1010	1696	3398	575	1584
transcript_132020	gnl|BL_ORD_ID|83183 transcript_151062	223	2408	2	223	620	2786	63	284
transcript_132020	gnl|BL_ORD_ID|21435 transcript_58411	222	2385	12	223	683	2831	1	212
transcript_132024	gnl|BL_ORD_ID|10840 transcript_41421	446	1232	7	445	550	1337	2	444
transcript_132030	gnl|BL_ORD_ID|18899 transcript_54201	2	2235	2234	2649	20	2227	2353	2766
transcript_132058	gnl|BL_ORD_ID|22462 transcript_60101	2	2691	2689	3193	1	2683	2976	3482
transcript_132078	gnl|BL_ORD_ID|11734 transcript_2415	211	3361	2	212	526	3676	213	423
transcript_132078	gnl|BL_ORD_ID|11633 transcript_2214	211	3361	2	212	495	3643	182	392
transcript_132092	gnl|BL_ORD_ID|24782 transcript_5040	1	2627	2628	2918	1	2624	2913	3201
transcript_132095	gnl|BL_ORD_ID|78512 transcript_14852	124	2234	1	124	388	2504	3	126
transcript_132095	gnl|BL_ORD_ID|18879 transcript_54171	124	2246	1	124	388	2515	3	126
transcript_132099	gnl|BL_ORD_ID|90187 transcript_162567	122	2924	2921	3878	307	3085	3220	4187
transcript_132196	gnl|BL_ORD_ID|75579 transcript_140006	1	1083	1082	1289	383	1465	1610	1817

transcript_132225	gnl|BL_ORD_ID|61198 transcript_118280	1	2455	2456	3190	412	2849	3101	3829
transcript_132225	gnl|BL_ORD_ID|71067 transcript_132697	1	2455	2456	3216	416	2869	3121	3878
transcript_132244	gnl|BL_ORD_ID|67552 transcript_126971	192	1131	2	196	328	1263	17	211
transcript_132251	gnl|BL_ORD_ID|5479 transcript_30736	1	1040	1040	1719	3	1044	1253	1931
transcript_132251	gnl|BL_ORD_ID|36430 transcript_81078	1	1040	1040	1719	1	1052	2063	2741
transcript_132251	gnl|BL_ORD_ID|34140 transcript_77366	1	1040	1040	1676	5	1045	2060	2695
transcript_132251	gnl|BL_ORD_ID|6393 transcript_32671	1	1040	1041	1686	5	1044	1226	1871
transcript_132251	gnl|BL_ORD_ID|51769 transcript_9785	1	1040	1040	1719	5	1043	2187	2866
transcript_132267	gnl|BL_ORD_ID|48581 transcript_99124	1	1850	1848	2654	2	1860	2717	3523
transcript_132288	gnl|BL_ORD_ID|68571 transcript_128594	1	1013	1013	1524	333	1345	1446	1949
transcript_132368	gnl|BL_ORD_ID|56653 transcript_110881	1	2030	2028	2936	81	2109	2515	3424
transcript_132377	gnl|BL_ORD_ID|45015 transcript_93389	1	2134	2132	2614	861	3002	3614	4099
transcript_132393	gnl|BL_ORD_ID|49000 transcript_99796	1	1466	1464	2716	1	1466	1770	3022
transcript_132456	gnl|BL_ORD_ID|24879 transcript_5228	1	2169	2167	2653	1	2180	2736	3242
transcript_132461	gnl|BL_ORD_ID|65571 transcript_123725	2	2104	2104	2276	1	2091	2342	2514
transcript_13249	gnl|BL_ORD_ID|68797 transcript_128974	1	2287	2284	2678	147	2443	2703	3099
transcript_13249	gnl|BL_ORD_ID|73411 transcript_136437	1	2284	2284	2678	146	2442	2587	2982
transcript_132549	gnl|BL_ORD_ID|74491 transcript_138231	1	1300	1297	2282	2	1298	2238	3223
transcript_132575	gnl|BL_ORD_ID|38432 transcript_7620	1	1964	1964	2936	2	1963	2067	3038
transcript_132575	gnl|BL_ORD_ID|24601 transcript_4647	1	2458	2454	2985	2	2492	2678	3209
transcript_13260	gnl|BL_ORD_ID|93806 transcript_166817	1200	2578	1	1202	3872	5250	1	1202
transcript_13260	gnl|BL_ORD_ID|66815 transcript_125773	1	1703	1702	2566	1	1703	2213	3077
transcript_13261	gnl|BL_ORD_ID|81614 transcript_148293	1178	2716	1	1178	1331	2869	3	1178
transcript_132611	gnl|BL_ORD_ID|27253 transcript_66226	437	2062	5	436	577	2200	1	431
transcript_132619	gnl|BL_ORD_ID|38722 transcript_8262	424	2843	5	425	573	2992	1	417
transcript_132639	gnl|BL_ORD_ID|91003 transcript_15683	1	1642	1642	2286	4	1632	1917	2553
transcript_132642	gnl|BL_ORD_ID|89265 transcript_161111	1	1280	1279	2452	21	1310	1422	2598
transcript_132656	gnl|BL_ORD_ID|93318 transcript_166045	1	2532	2532	2897	1	2509	2658	3037
transcript_132656	gnl|BL_ORD_ID|38054 transcript_6815	2	2532	2532	2909	27	2557	2706	3083
transcript_132680	gnl|BL_ORD_ID|63829 transcript_122491	704	2528	9	706	984	2807	1	694
transcript_132683	gnl|BL_ORD_ID|53738 transcript_105894	650	2268	67	651	1117	2734	1	585
transcript_132799	gnl|BL_ORD_ID|60868 transcript_117735	1375	2963	1	1376	1656	3243	1	1343
transcript_13282	gnl|BL_ORD_ID|37930 transcript_6566	1015	2699	1	1020	1318	3002	22	1032
transcript_13282	gnl|BL_ORD_ID|51204 transcript_8607	1015	2611	1	1020	1374	2970	78	1088

transcript_132823	gnl|BL_ORD_ID|67444 transcript_126781	2	3232	3230	3850	29	3260	3366	3988
transcript_13284	gnl|BL_ORD_ID|38722 transcript_8262	424	2689	5	426	619	2884	1	417
transcript_132853	gnl|BL_ORD_ID|41931 transcript_88314	1	2673	2668	2906	10	2671	2871	3108
transcript_132898	gnl|BL_ORD_ID|23309 transcript_61542	275	1512	28	277	1342	2584	5	262
transcript_132898	gnl|BL_ORD_ID|43490 transcript_90798	277	1512	3	277	496	1731	12	301
transcript_132898	gnl|BL_ORD_ID|8729 transcript_37395	275	1512	32	276	362	1599	1	258
transcript_132898	gnl|BL_ORD_ID|23742 transcript_62261	277	1512	3	277	799	2033	2	287
transcript_132898	gnl|BL_ORD_ID|80147 transcript_145883	275	1512	29	276	348	1585	1	244
transcript_132898	gnl|BL_ORD_ID|86136 transcript_156018	275	1512	31	276	465	1702	2	250
transcript_132898	gnl|BL_ORD_ID|37721 transcript_83102	275	1512	46	277	873	2110	1	238
transcript_132898	gnl|BL_ORD_ID|80685 transcript_146777	277	1512	46	277	744	1979	2	245
transcript_132898	gnl|BL_ORD_ID|55106 transcript_108238	277	1490	3	277	974	2187	2	290
transcript_132898	gnl|BL_ORD_ID|75954 transcript_140614	1	1186	1184	1507	1	1199	2123	2446
transcript_132907	gnl|BL_ORD_ID|28507 transcript_68282	19	2544	2542	3349	1	2531	2697	3504
transcript_132914	gnl|BL_ORD_ID|66492 transcript_125248	367	2260	5	368	494	2387	1	364
transcript_132937	gnl|BL_ORD_ID|20425 transcript_56700	1	1476	1476	2234	238	1699	1945	2700
transcript_132937	gnl|BL_ORD_ID|48406 transcript_98842	1	1476	1476	2296	109	1584	1830	2650
transcript_132963	gnl|BL_ORD_ID|89586 transcript_161623	1	1607	1605	2704	1	1606	2036	3135
transcript_132977	gnl|BL_ORD_ID|18597 transcript_53725	2	2609	2609	2761	1	2608	2952	3105
transcript_133008	gnl|BL_ORD_ID|88800 transcript_160385	2	2077	2079	2305	62	2133	2248	2473
transcript_133022	gnl|BL_ORD_ID|26942 transcript_65730	1	1299	1298	1966	1	1299	1451	2109
transcript_133022	gnl|BL_ORD_ID|2924 transcript_25274	1	1299	1298	1966	1	1299	1451	2120
transcript_133086	gnl|BL_ORD_ID|60102 transcript_116509	10	1408	1406	2430	2	1398	2805	3820
transcript_133096	gnl|BL_ORD_ID|39520 transcript_84381	2	1998	2001	2350	21	1986	2830	3175
transcript_1331	gnl|BL_ORD_ID|67562 transcript_126988	366	3876	2	369	844	4367	6	375
transcript_133123	gnl|BL_ORD_ID|78358 transcript_14420	1319	2645	123	1321	1312	2638	1	1199
transcript_133158	gnl|BL_ORD_ID|24636 transcript_4719	1200	2586	1	1200	1934	3298	626	1817
transcript_133175	gnl|BL_ORD_ID|97210 transcript_137767	455	2043	5	455	851	2439	222	672
transcript_133185	gnl|BL_ORD_ID|19153 transcript_54626	1	1633	1631	2621	6	1656	1963	2946
transcript_133191	gnl|BL_ORD_ID|80963 transcript_147227	1091	3501	105	1093	1122	3508	1	990
transcript_133232	gnl|BL_ORD_ID|60416 transcript_117012	379	2939	8	382	589	3168	14	390
transcript_133269	gnl|BL_ORD_ID|877 transcript_1550	1682	3755	1	1684	1816	3889	3	1685
transcript_133273	gnl|BL_ORD_ID|75675 transcript_140152	1	1858	1854	2180	1147	3004	3171	3497
transcript_13331	gnl|BL_ORD_ID|31812 transcript_73588	18	2055	2052	2448	1	2032	5451	5846

transcript_13331	gnl|BL_ORD_ID|43181 transcript_90296	14	2055	2054	2457	1	2041	2155	2558
transcript_133364	gnl|BL_ORD_ID|33330 transcript_76027	1	1129	1128	1350	1	1142	1494	1716
transcript_133364	gnl|BL_ORD_ID|11635 transcript_2217	1	1129	1128	1350	60	1208	1560	1782
transcript_133364	gnl|BL_ORD_ID|26355 transcript_64791	1	1129	1128	1350	45	1191	1470	1692
transcript_13339	gnl|BL_ORD_ID|26227 transcript_64593	644	2677	83	644	686	2718	1	564
transcript_133472	gnl|BL_ORD_ID|52495 transcript_103810	142	1998	1	144	1356	3218	420	563
transcript_133476	gnl|BL_ORD_ID|66490 transcript_125246	2	3302	3299	4113	14	3326	4554	5361
transcript_133476	gnl|BL_ORD_ID|353 transcript_606	2	3302	3299	4113	18	3323	3521	4329
transcript_133476	gnl|BL_ORD_ID|42248 transcript_88801	140	3302	3299	4110	1	3168	3366	4177
transcript_133476	gnl|BL_ORD_ID|76842 transcript_142029	2	3302	3299	4042	61	3367	3565	4302
transcript_133517	gnl|BL_ORD_ID|85299 transcript_154638	1	2021	2024	3605	553	2578	2731	4311
transcript_133609	gnl|BL_ORD_ID|26793 transcript_65477	1	2094	2094	2618	118	2209	2684	3209
transcript_133690	gnl|BL_ORD_ID|1674 transcript_22554	1	1651	1647	2143	1	1645	1746	2242
transcript_133693	gnl|BL_ORD_ID|80043 transcript_145719	1163	2554	1	1164	1751	3142	41	1207
transcript_1337	gnl|BL_ORD_ID|41571 transcript_87743	1	2845	2842	3931	3	2879	3103	4188
transcript_1337	gnl|BL_ORD_ID|469 transcript_799	1	2845	2842	3970	18	2881	3105	4233
transcript_133713	gnl|BL_ORD_ID|65743 transcript_124004	1	1790	1789	2747	1235	3026	3317	4276
transcript_133718	gnl|BL_ORD_ID|94225 transcript_167466	2	3734	3734	4406	33	3764	4067	4739
transcript_133724	gnl|BL_ORD_ID|49316 transcript_100291	249	2674	2	250	495	2910	116	364
transcript_133730	gnl|BL_ORD_ID|46706 transcript_96182	345	3206	60	345	394	3256	1	286
transcript_133844	gnl|BL_ORD_ID|96148 transcript_21115	13	1691	1691	2187	1	1682	1796	2293
transcript_13385	gnl|BL_ORD_ID|49412 transcript_100447	531	2587	9	536	897	2955	9	542
transcript_133890	gnl|BL_ORD_ID|78140 transcript_13970	1	2359	2355	2535	2	2359	2466	2645
transcript_133925	gnl|BL_ORD_ID|65201 transcript_12558	126	1196	2	131	1546	2617	7	136
transcript_133933	gnl|BL_ORD_ID|74109 transcript_137609	1123	2638	1	1124	1244	2769	2	1129
transcript_133942	gnl|BL_ORD_ID|1158 transcript_2079	2	2226	2226	2515	58	2282	2397	2686
transcript_133942	gnl|BL_ORD_ID|1157 transcript_2077	2	2226	2226	2515	97	2320	2435	2724
transcript_133970	gnl|BL_ORD_ID|733 transcript_1278	1	2155	2155	3126	138	2293	2922	3893
transcript_133971	gnl|BL_ORD_ID|41778 transcript_88084	1009	2875	1	1011	1760	3626	5	1041
transcript_133975	gnl|BL_ORD_ID|65868 transcript_124206	1	2502	2502	3011	1	2516	3326	3836
transcript_134	gnl|BL_ORD_ID|48873 transcript_99599	45	4820	4816	5308	1	4787	4890	5382
transcript_134002	gnl|BL_ORD_ID|68397 transcript_128338	1	1253	1252	1644	3	1255	1519	1911
transcript_134078	gnl|BL_ORD_ID|12484 transcript_3866	224	3107	1	228	421	3303	13	238
transcript_13409	gnl|BL_ORD_ID|22323 transcript_59888	208	2679	1	207	489	2952	141	347

transcript_13409	gnl|BL_ORD_ID|27268 transcript_66244	1	1292	1291	2679	141	1429	1574	2957
transcript_13409	gnl|BL_ORD_ID|79835 transcript_145382	1	1292	1291	2679	273	1564	1709	3095
transcript_1341	gnl|BL_ORD_ID|60114 transcript_116528	23	3403	3398	3984	1	3380	3516	4099
transcript_134143	gnl|BL_ORD_ID|24496 transcript_4438	1075	2548	1	1078	1847	3322	648	1726
transcript_134143	gnl|BL_ORD_ID|97203 transcript_137091	1075	2537	1	1078	1828	3291	629	1707
transcript_13417	gnl|BL_ORD_ID|73215 transcript_136113	167	2632	1	168	284	2767	1	168
transcript_134185	gnl|BL_ORD_ID|39654 transcript_84608	285	3656	1	286	544	3915	2	287
transcript_134203	gnl|BL_ORD_ID|665 transcript_1153	2	3153	3152	3903	21	3175	3278	4031
transcript_134213	gnl|BL_ORD_ID|51618 transcript_9472	2	2103	2101	2521	97	2198	2385	2807
transcript_134213	gnl|BL_ORD_ID|22194 transcript_59662	2	2103	2100	2521	1	2102	2307	2729
transcript_134219	gnl|BL_ORD_ID|61900 transcript_119369	285	1128	7	287	392	1236	2	282
transcript_134279	gnl|BL_ORD_ID|92843 transcript_165271	1	1375	1374	1844	110	1484	2294	2764
transcript_134304	gnl|BL_ORD_ID|86554 transcript_156715	1	3240	3239	4146	1	3244	3378	4286
transcript_13436	gnl|BL_ORD_ID|69130 transcript_129525	18	2491	2491	2677	1	2487	2720	2906
transcript_134372	gnl|BL_ORD_ID|89122 transcript_160878	207	2827	2	209	608	3225	4	212
transcript_134372	gnl|BL_ORD_ID|72743 transcript_135356	207	2827	2	208	574	3188	8	215
transcript_134372	gnl|BL_ORD_ID|81289 transcript_147746	207	2827	2	209	826	3445	222	430
transcript_134378	gnl|BL_ORD_ID|97368 transcript_152583	12	1541	1540	2753	3	1551	1880	3096
transcript_134387	gnl|BL_ORD_ID|31898 transcript_73725	400	2700	44	401	519	2813	30	387
transcript_134412	gnl|BL_ORD_ID|73638 transcript_136810	517	1611	7	520	725	1819	1	514
transcript_134443	gnl|BL_ORD_ID|90865 transcript_15394	1	1419	1416	1804	2	1423	2140	2528
transcript_134450	gnl|BL_ORD_ID|51344 transcript_8923	1	1520	1518	2640	3	1522	1756	2878
transcript_134451	gnl|BL_ORD_ID|43462 transcript_90757	1	2390	2388	2785	2	2405	2678	3075
transcript_134461	gnl|BL_ORD_ID|95579 transcript_19803	1	1249	1245	1811	1	1251	1730	2296
transcript_134499	gnl|BL_ORD_ID|75449 transcript_139799	2	2850	2850	3544	25	2872	3197	3891
transcript_134503	gnl|BL_ORD_ID|52070 transcript_10458	11	1999	1995	2643	1	1992	2170	2818
transcript_134514	gnl|BL_ORD_ID|90810 transcript_15266	1112	2261	1	1113	1325	2476	107	1220
transcript_134514	gnl|BL_ORD_ID|26647 transcript_65256	1112	2261	1	1113	1305	2456	107	1200
transcript_13459	gnl|BL_ORD_ID|64667 transcript_11346	1	2269	2268	2637	2	2269	2371	2754
transcript_13465	gnl|BL_ORD_ID|12283 transcript_3464	241	2624	2	242	1091	3474	37	277
transcript_13466	gnl|BL_ORD_ID|12448 transcript_3790	1109	2627	1	1112	1929	3446	2	1113
transcript_134673	gnl|BL_ORD_ID|80308 transcript_146171	1	2211	2210	3257	73	2277	2516	3563
transcript_134693	gnl|BL_ORD_ID|25011 transcript_5511	2	2161	2160	3013	49	2211	2330	3180
transcript_134693	gnl|BL_ORD_ID|39873 transcript_84954	2	2161	2160	2967	145	2296	2415	3218

transcript_134739	gnl|BL_ORD_ID|23343 transcript_61592	1	2245	2241	2857	1055	3299	3581	4202
transcript_134739	gnl|BL_ORD_ID|76444 transcript_141389	1	2245	2241	2823	1652	3896	4178	4764
transcript_134799	gnl|BL_ORD_ID|93833 transcript_166857	1	2036	2034	3069	1	2035	2167	3203
transcript_134861	gnl|BL_ORD_ID|90859 transcript_15382	176	2276	1	176	353	2453	5	181
transcript_134861	gnl|BL_ORD_ID|46577 transcript_95986	176	2276	1	176	460	2563	105	289
transcript_134861	gnl|BL_ORD_ID|91137 transcript_15986	176	2276	1	176	362	2461	8	191
transcript_134861	gnl|BL_ORD_ID|91375 transcript_16492	176	2246	1	176	460	2530	114	288
transcript_134861	gnl|BL_ORD_ID|91381 transcript_16515	176	2276	1	176	424	2524	78	252
transcript_134863	gnl|BL_ORD_ID|80745 transcript_146882	395	2005	5	399	1062	2672	3	397
transcript_134869	gnl|BL_ORD_ID|84640 transcript_153598	108	1372	1	109	701	1965	242	353
transcript_134873	gnl|BL_ORD_ID|27148 transcript_66057	2	3541	3539	3999	47	3588	3697	4157
transcript_134943	gnl|BL_ORD_ID|77213 transcript_142638	1	1374	1375	2548	522	1895	2017	3191
transcript_135011	gnl|BL_ORD_ID|40428 transcript_85830	1007	3143	1	1008	1157	3291	1	1008
transcript_135085	gnl|BL_ORD_ID|47792 transcript_97875	1253	2761	1	1254	1367	2875	3	1257
transcript_13514	gnl|BL_ORD_ID|12207 transcript_3337	1	1412	1412	2654	1	1412	2251	3490
transcript_13516	gnl|BL_ORD_ID|27585 transcript_66726	222	2684	103	221	245	2708	2	120
transcript_135215	gnl|BL_ORD_ID|77937 transcript_13470	1	1874	1875	2432	2	1874	2112	2668
transcript_135215	gnl|BL_ORD_ID|65070 transcript_12237	1	1874	1875	2482	2	1877	2116	2724
transcript_135215	gnl|BL_ORD_ID|60465 transcript_117084	1	1874	1874	2432	1	1873	2341	2899
transcript_135215	gnl|BL_ORD_ID|68306 transcript_128183	1	1874	1874	2481	1	1874	2306	2912
transcript_135215	gnl|BL_ORD_ID|29740 transcript_70198	1	1874	1875	2457	2	1874	2016	2598
transcript_135216	gnl|BL_ORD_ID|65306 transcript_12780	1	2197	2192	2564	1	2197	2313	2686
transcript_135216	gnl|BL_ORD_ID|27173 transcript_66098	1	2197	2196	2544	1	2196	3377	3727
transcript_13529	gnl|BL_ORD_ID|71872 transcript_133977	1	2445	2444	2573	1	2444	2766	2895
transcript_135344	gnl|BL_ORD_ID|34806 transcript_78459	1102	4274	1	1101	1243	4419	1	1098
transcript_135346	gnl|BL_ORD_ID|64846 transcript_11718	163	2661	1	163	269	2752	2	164
transcript_13535	gnl|BL_ORD_ID|70954 transcript_132512	108	1552	1548	2655	2	1446	2672	3779
transcript_13538	gnl|BL_ORD_ID|54727 transcript_107556	294	2437	72	296	597	2741	2	218
transcript_13538	gnl|BL_ORD_ID|90854 transcript_15372	294	2461	36	294	375	2542	2	256
transcript_135431	gnl|BL_ORD_ID|38134 transcript_6977	1	2326	2323	2886	34	2362	2467	3030
transcript_135514	gnl|BL_ORD_ID|75013 transcript_139086	1	1698	1699	2121	264	1960	2077	2499
transcript_13554	gnl|BL_ORD_ID|63721 transcript_122321	555	2612	66	555	1291	3348	2	492
transcript_135545	gnl|BL_ORD_ID|86010 transcript_155802	1270	2524	1	1272	1418	2672	1	1298
transcript_135629	gnl|BL_ORD_ID|5489 transcript_30755	1	1516	1514	1691	6	1520	1667	1844

transcript_135642	gnl|BL_ORD_ID|45131 transcript_93571	1	1587	1586	2254	695	2282	2395	3063
transcript_135657	gnl|BL_ORD_ID|62523 transcript_120402	463	1561	9	464	564	1662	1	456
transcript_135661	gnl|BL_ORD_ID|65585 transcript_123745	202	2738	1	202	365	2910	25	220
transcript_135687	gnl|BL_ORD_ID|36341 transcript_80934	252	1811	40	253	900	2460	2	215
transcript_135691	gnl|BL_ORD_ID|89576 transcript_161609	12	2152	2150	2431	1	2137	2845	3126
transcript_13577	gnl|BL_ORD_ID|28681 transcript_68566	1112	2649	1	1112	1329	2862	1	1113
transcript_135775	gnl|BL_ORD_ID|38871 transcript_83337	1	1438	1437	2117	728	2165	2322	3002
transcript_135775	gnl|BL_ORD_ID|38126 transcript_6958	1	1438	1435	2034	729	2166	2483	3082
transcript_135775	gnl|BL_ORD_ID|42176 transcript_88677	1	1438	1435	2118	9	1444	1760	2443
transcript_135775	gnl|BL_ORD_ID|69292 transcript_129787	1	1438	1435	2118	239	1668	1984	2665
transcript_135775	gnl|BL_ORD_ID|38552 transcript_7866	1	1438	1437	2117	729	2166	2323	3003
transcript_135775	gnl|BL_ORD_ID|12247 transcript_3408	1	1438	1435	2118	731	2168	2485	3168
transcript_135775	gnl|BL_ORD_ID|25427 transcript_6388	1	1438	1435	2118	729	2160	2477	3159
transcript_135784	gnl|BL_ORD_ID|22745 transcript_60602	108	2048	1	104	356	2294	105	208
transcript_135787	gnl|BL_ORD_ID|86452 transcript_156541	1	1608	1605	2438	131	1734	3393	4225
transcript_135865	gnl|BL_ORD_ID|4549 transcript_28802	1	1509	1509	1616	2	1510	1712	1819
transcript_135865	gnl|BL_ORD_ID|35943 transcript_80279	1	1509	1509	1616	2	1524	1733	1840
transcript_135865	gnl|BL_ORD_ID|44034 transcript_91750	1	1509	1509	1616	2	1510	1709	1815
transcript_135870	gnl|BL_ORD_ID|80167 transcript_145918	167	2127	10	166	262	2220	2	158
transcript_135876	gnl|BL_ORD_ID|38560 transcript_7882	291	1890	6	293	1397	2995	2	289
transcript_135895	gnl|BL_ORD_ID|79406 transcript_144695	1	2299	2298	3411	1	2305	2671	3794
transcript_135913	gnl|BL_ORD_ID|17632 transcript_52199	1	1027	1028	1343	2	1029	1326	1641
transcript_13594	gnl|BL_ORD_ID|64565 transcript_11126	1164	2655	1	1167	1326	2817	2	1168
transcript_135946	gnl|BL_ORD_ID|2596 transcript_24546	312	1972	47	312	457	2115	2	267
transcript_135947	gnl|BL_ORD_ID|51474 transcript_9170	1	1886	1884	2225	1	1891	2559	2901
transcript_136105	gnl|BL_ORD_ID|38115 transcript_6941	231	2881	2	233	416	3068	37	266
transcript_136105	gnl|BL_ORD_ID|38466 transcript_7683	231	2868	2	233	417	3036	38	267
transcript_136105	gnl|BL_ORD_ID|38358 transcript_7449	231	2852	2	233	418	3039	38	268
transcript_136137	gnl|BL_ORD_ID|97417 transcript_157550	1033	2423	1	1038	1263	2650	76	1096
transcript_136137	gnl|BL_ORD_ID|18498 transcript_53575	1033	2434	1	1038	2786	4187	1581	2619
transcript_136137	gnl|BL_ORD_ID|81755 transcript_148541	1033	2470	1	1038	1997	3434	787	1830
transcript_136137	gnl|BL_ORD_ID|37366 transcript_82543	1033	2431	1	1038	2578	3976	1370	2411
transcript_136223	gnl|BL_ORD_ID|38170 transcript_7050	1	2341	2336	2860	62	2406	2554	3078
transcript_136223	gnl|BL_ORD_ID|24951 transcript_5367	1	2341	2336	2860	12	2357	2505	3029

transcript_136235	gnl|BL_ORD_ID|72985 transcript_135753	113	2211	1	112	318	2412	1	112
transcript_136235	gnl|BL_ORD_ID|78547 transcript_14938	113	2211	1	112	452	2550	1	112
transcript_136274	gnl|BL_ORD_ID|30789 transcript_71937	2	3156	3156	3257	13	3163	3274	3375
transcript_136308	gnl|BL_ORD_ID|48808 transcript_99501	1	1052	1048	2030	47	1078	1180	2159
transcript_136308	gnl|BL_ORD_ID|2555 transcript_24453	1	1052	1048	2030	47	1097	1199	2181
transcript_136308	gnl|BL_ORD_ID|20778 transcript_57299	1	1052	1048	2030	10	1060	1162	2144
transcript_136334	gnl|BL_ORD_ID|44637 transcript_92756	1	1911	1910	2579	1	1906	2371	3038
transcript_13637	gnl|BL_ORD_ID|89382 transcript_161295	2	2170	2171	2664	1	2175	2338	2825
transcript_136407	gnl|BL_ORD_ID|39700 transcript_84680	10	3530	3531	4582	12	3532	4415	5466
transcript_136407	gnl|BL_ORD_ID|81629 transcript_148320	10	3530	3531	4582	13	3532	4414	5466
transcript_136437	gnl|BL_ORD_ID|68797 transcript_128974	1	2442	2442	3131	2	2440	2558	3248
transcript_136465	gnl|BL_ORD_ID|91153 transcript_16017	1064	2182	1	1068	1422	2540	1	1068
transcript_136487	gnl|BL_ORD_ID|41943 transcript_88332	1	1298	1296	2069	3	1305	1501	2274
transcript_136531	gnl|BL_ORD_ID|50176 transcript_101660	1	1460	1458	2118	2	1477	1594	2254
transcript_136614	gnl|BL_ORD_ID|42052 transcript_88496	2	3241	3240	4023	16	3251	3486	4269
transcript_136614	gnl|BL_ORD_ID|459 transcript_778	2	3241	3240	4064	17	3255	3377	4200
transcript_136795	gnl|BL_ORD_ID|76854 transcript_142049	894	2503	90	895	930	2539	1	826
transcript_136822	gnl|BL_ORD_ID|93129 transcript_165726	1010	3103	1	1010	1351	3445	142	1175
transcript_136838	gnl|BL_ORD_ID|88047 transcript_159157	1131	2317	1	1136	1960	3144	6	1131
transcript_136838	gnl|BL_ORD_ID|40396 transcript_85772	1131	2338	1	1136	2243	3448	279	1414
transcript_136838	gnl|BL_ORD_ID|42363 transcript_88987	1131	2338	1	1136	2304	3511	342	1475
transcript_136838	gnl|BL_ORD_ID|12285 transcript_3468	1131	2338	1	1136	1969	3173	5	1140
transcript_136870	gnl|BL_ORD_ID|70745 transcript_132174	1	1558	1558	2967	28	1592	1726	3135
transcript_136870	gnl|BL_ORD_ID|12130 transcript_3200	1	1558	1558	2967	94	1648	1782	3191
transcript_136870	gnl|BL_ORD_ID|92733 transcript_165090	1	1558	1559	2967	17	1584	1717	3095
transcript_136878	gnl|BL_ORD_ID|32829 transcript_75270	107	2501	1	108	377	2770	1	106
transcript_136923	gnl|BL_ORD_ID|24424 transcript_4290	1047	2312	1	1049	2016	3280	1	1056
transcript_136940	gnl|BL_ORD_ID|68367 transcript_128287	1188	2510	1	1189	1368	2693	1	1189
transcript_136951	gnl|BL_ORD_ID|20854 transcript_57418	1	2380	2379	3142	3	2383	3901	4661
transcript_136952	gnl|BL_ORD_ID|38371 transcript_7488	137	1855	1853	2930	2	1719	1827	2904
transcript_136952	gnl|BL_ORD_ID|35941 transcript_80275	1	1852	1853	2930	120	1973	2084	3161
transcript_13698	gnl|BL_ORD_ID|67556 transcript_126976	1	1390	1389	2655	1	1390	1506	2771
transcript_136993	gnl|BL_ORD_ID|94797 transcript_17983	1	1142	1140	1754	569	1728	1847	2465
transcript_136993	gnl|BL_ORD_ID|24617 transcript_4684	1	1048	1044	1754	598	1652	2626	3342

transcript_137016	gnl|BL_ORD_ID|55966 transcript_109779	1	1235	1232	1564	3	1237	1343	1675
transcript_13706	gnl|BL_ORD_ID|86784 transcript_157106	2	2137	2134	2661	39	2179	2309	2825
transcript_13711	gnl|BL_ORD_ID|74359 transcript_138026	228	2653	1	230	1262	3688	6	227
transcript_13711	gnl|BL_ORD_ID|41129 transcript_87002	228	2631	1	230	1273	3649	9	236
transcript_13711	gnl|BL_ORD_ID|46721 transcript_96208	228	2650	1	230	1403	3834	6	226
transcript_13711	gnl|BL_ORD_ID|30290 transcript_71115	228	2653	1	229	430	2827	5	232
transcript_13711	gnl|BL_ORD_ID|19500 transcript_55227	228	2653	1	229	422	2856	5	224
transcript_137140	gnl|BL_ORD_ID|79978 transcript_145616	1	1699	1698	2144	2	1700	2137	2584
transcript_137164	gnl|BL_ORD_ID|64874 transcript_11774	1	1463	1464	2460	68	1530	1658	2654
transcript_137168	gnl|BL_ORD_ID|41623 transcript_87832	1	1694	1693	2220	1	1693	1828	2355
transcript_137186	gnl|BL_ORD_ID|62395 transcript_120177	1	2006	2005	3160	73	2079	2200	3357
transcript_137201	gnl|BL_ORD_ID|60060 transcript_116446	1	1426	1426	2748	1802	3230	3457	4776
transcript_137201	gnl|BL_ORD_ID|39140 transcript_83771	1	1426	1426	2748	2576	4003	4230	5552
transcript_137201	gnl|BL_ORD_ID|21420 transcript_58384	1	1426	1426	2724	2229	3655	3882	5180
transcript_137227	gnl|BL_ORD_ID|24952 transcript_5369	235	2393	1	239	1074	3236	1	238
transcript_137245	gnl|BL_ORD_ID|77228 transcript_142657	1	3804	3803	4219	77	3879	4052	4468
transcript_137245	gnl|BL_ORD_ID|299 transcript_492	1	3804	3803	4219	101	3905	4078	4493
transcript_137245	gnl|BL_ORD_ID|295 transcript_486	1	3804	3803	4176	149	3953	4126	4499
transcript_137272	gnl|BL_ORD_ID|88345 transcript_159657	266	1516	31	267	344	1597	1	238
transcript_137315	gnl|BL_ORD_ID|45972 transcript_95007	1042	2716	1	1044	1521	3197	1	1044
transcript_137319	gnl|BL_ORD_ID|92085 transcript_164033	1836	4405	1	1838	2456	5007	1	1824
transcript_137332	gnl|BL_ORD_ID|42842 transcript_89750	1	1455	1455	2308	174	1628	2075	2927
transcript_137332	gnl|BL_ORD_ID|38809 transcript_8446	1	1455	1455	2308	236	1690	2137	2990
transcript_137349	gnl|BL_ORD_ID|28962 transcript_69009	1	1246	1241	1880	1080	2327	2483	3124
transcript_137369	gnl|BL_ORD_ID|45982 transcript_95025	463	1068	6	465	768	1366	2	461
transcript_137391	gnl|BL_ORD_ID|26406 transcript_64871	243	1971	53	247	780	2508	1	195
transcript_137484	gnl|BL_ORD_ID|12379 transcript_3654	2	3051	3048	3169	117	3153	3284	3406
transcript_137484	gnl|BL_ORD_ID|47539 transcript_97483	2	3051	3048	3169	153	3198	3329	3451
transcript_137484	gnl|BL_ORD_ID|24545 transcript_4532	2	3051	3048	3169	5	3046	3177	3299
transcript_137493	gnl|BL_ORD_ID|63585 transcript_122106	1	2119	2117	2980	20	2139	2672	3533
transcript_137502	gnl|BL_ORD_ID|87464 transcript_158218	226	2603	15	225	598	2975	2	212
transcript_137544	gnl|BL_ORD_ID|12623 transcript_4143	1005	2476	1	1006	1916	3387	35	1041
transcript_137549	gnl|BL_ORD_ID|73411 transcript_136437	1	1642	1642	2186	803	2442	2587	3131
transcript_137549	gnl|BL_ORD_ID|68797 transcript_128974	1	1645	1642	2187	804	2443	2703	3249

transcript_137549	gnl|BL_ORD_ID|84466 transcript_153316	1	1642	1642	2184	871	2509	2654	3195
transcript_137553	gnl|BL_ORD_ID|11772 transcript_2480	2	3054	3050	3289	67	3108	3300	3539
transcript_137584	gnl|BL_ORD_ID|79022 transcript_144082	313	3870	1	314	1605	5163	4	323
transcript_137650	gnl|BL_ORD_ID|36289 transcript_80845	1	3098	3095	3771	1	3101	3238	3914
transcript_137655	gnl|BL_ORD_ID|67632 transcript_127103	165	1325	2	165	1520	2679	69	232
transcript_13770	gnl|BL_ORD_ID|55120 transcript_108270	1	1424	1422	2610	1	1425	1537	2720
transcript_137718	gnl|BL_ORD_ID|40446 transcript_85857	165	2777	1	168	387	3001	105	272
transcript_137718	gnl|BL_ORD_ID|69125 transcript_129517	165	2826	1	168	284	2948	2	169
transcript_137718	gnl|BL_ORD_ID|51420 transcript_9079	167	2780	1	169	308	2924	2	172
transcript_137730	gnl|BL_ORD_ID|93327 transcript_166062	576	1582	66	578	1323	2329	2	514
transcript_137767	gnl|BL_ORD_ID|92105 transcript_164069	10	2089	2085	2461	25	2104	2836	3213
transcript_137790	gnl|BL_ORD_ID|46706 transcript_96182	293	2677	8	293	394	2778	1	286
transcript_137846	gnl|BL_ORD_ID|11829 transcript_2602	1375	3430	1	1375	1485	3540	1	1374
transcript_137875	gnl|BL_ORD_ID|8953 transcript_37810	312	1341	38	313	598	1626	1	276
transcript_137877	gnl|BL_ORD_ID|80521 transcript_146506	16	1816	1816	2579	2	1811	1983	2746
transcript_137877	gnl|BL_ORD_ID|65218 transcript_12588	11	1816	1821	2579	66	1879	1994	2752
transcript_137877	gnl|BL_ORD_ID|28469 transcript_68221	1	1816	1816	2580	15	1858	2030	2794
transcript_137910	gnl|BL_ORD_ID|96663 transcript_84175	13	2652	2651	3393	2	2620	3069	3820
transcript_137919	gnl|BL_ORD_ID|50758 transcript_102613	201	2120	2	203	444	2364	98	299
transcript_137935	gnl|BL_ORD_ID|95414 transcript_19412	1	1619	1614	1839	448	2063	2209	2434
transcript_137935	gnl|BL_ORD_ID|4577 transcript_28856	1	1619	1614	1855	2	1618	1764	2007
transcript_137935	gnl|BL_ORD_ID|49588 transcript_100740	1	1619	1614	1855	429	2045	2191	2433
transcript_138000	gnl|BL_ORD_ID|50111 transcript_101561	2	2304	2305	2792	9	2310	2423	2909
transcript_138029	gnl|BL_ORD_ID|71343 transcript_133123	1	1397	1396	2458	1	1406	1580	2645
transcript_138047	gnl|BL_ORD_ID|60840 transcript_117687	1	1095	1094	1923	346	1432	1714	2530
transcript_138065	gnl|BL_ORD_ID|23702 transcript_62188	1065	3660	1	1068	1554	4156	70	1145
transcript_138088	gnl|BL_ORD_ID|25075 transcript_5669	1	1792	1792	2208	1	1804	2699	3115
transcript_13810	gnl|BL_ORD_ID|71776 transcript_133830	233	2608	13	234	417	2801	3	224
transcript_13810	gnl|BL_ORD_ID|25200 transcript_5914	1	2503	2506	2649	46	2550	3003	3147
transcript_138122	gnl|BL_ORD_ID|11714 transcript_2369	206	2951	1	206	409	3154	1	206
transcript_138135	gnl|BL_ORD_ID|52980 transcript_104574	1	1338	1338	1884	26	1363	1498	2030
transcript_138141	gnl|BL_ORD_ID|44217 transcript_92049	1	2744	2741	3726	1	2724	3970	4953
transcript_138157	gnl|BL_ORD_ID|21414 transcript_58377	114	2483	1	115	261	2609	1	115
transcript_13816	gnl|BL_ORD_ID|21453 transcript_58440	581	2646	6	583	2157	4217	2	576

transcript_138221	gnl|BL_ORD_ID|24926 transcript_5329	27	2727	2722	2953	1	2706	3031	3260
transcript_138221	gnl|BL_ORD_ID|88242 transcript_159477	1	2727	2722	2951	1	2732	3057	3285
transcript_138229	gnl|BL_ORD_ID|80150 transcript_145891	1	1024	1023	1748	1	1020	2828	3553
transcript_138230	gnl|BL_ORD_ID|38163 transcript_7034	1056	2653	1	1057	1506	3088	12	1043
transcript_138230	gnl|BL_ORD_ID|37954 transcript_6611	1056	2653	1	1057	1531	3113	12	1068
transcript_138249	gnl|BL_ORD_ID|42694 transcript_89518	129	2040	1	132	295	2199	23	157
transcript_138254	gnl|BL_ORD_ID|2104 transcript_23450	222	2134	24	226	329	2241	2	204
transcript_138254	gnl|BL_ORD_ID|77069 transcript_142403	226	2133	3	226	572	2479	2	225
transcript_138284	gnl|BL_ORD_ID|5025 transcript_29799	1	1053	1050	1741	132	1192	1295	1985
transcript_138284	gnl|BL_ORD_ID|20324 transcript_56524	1	1053	1052	1702	2	1056	1241	1891
transcript_138306	gnl|BL_ORD_ID|37897 transcript_6500	427	2118	5	431	1117	2878	3	442
transcript_138306	gnl|BL_ORD_ID|32124 transcript_74131	427	2141	8	431	1167	2880	2	425
transcript_138367	gnl|BL_ORD_ID|3406 transcript_26340	112	1202	1	116	1028	2118	381	496
transcript_138367	gnl|BL_ORD_ID|89397 transcript_161319	112	1202	1	116	906	1996	259	374
transcript_138372	gnl|BL_ORD_ID|29603 transcript_69997	1	1359	1360	2325	9	1354	1687	2625
transcript_138378	gnl|BL_ORD_ID|55239 transcript_108474	1753	3597	11	1752	1943	3785	4	1741
transcript_138478	gnl|BL_ORD_ID|37302 transcript_82436	133	2439	11	135	1093	3399	6	130
transcript_13848	gnl|BL_ORD_ID|38386 transcript_7523	1138	2690	1	1140	1286	2838	36	1174
transcript_138556	gnl|BL_ORD_ID|37992 transcript_6690	244	2570	1	246	753	3079	388	633
transcript_138556	gnl|BL_ORD_ID|65819 transcript_124140	244	2570	1	246	1009	3335	644	889
transcript_13857	gnl|BL_ORD_ID|19719 transcript_55577	21	2327	2324	2585	2	2309	2414	2676
transcript_13857	gnl|BL_ORD_ID|38019 transcript_6742	1	1380	1379	2572	1	1378	1946	3137
transcript_138590	gnl|BL_ORD_ID|26890 transcript_65641	123	2004	1	127	1590	3472	2	128
transcript_138641	gnl|BL_ORD_ID|24727 transcript_4929	1	1249	1248	2193	1	1260	2091	3047
transcript_138675	gnl|BL_ORD_ID|54955 transcript_107968	138	1247	2	141	273	1383	21	163
transcript_138704	gnl|BL_ORD_ID|41768 transcript_88067	2	3181	3180	3728	36	3268	3383	3931
transcript_13873	gnl|BL_ORD_ID|47041 transcript_96699	1	1680	1679	2654	46	1728	3146	4121
transcript_138769	gnl|BL_ORD_ID|65145 transcript_12422	2	761	756	998	2	760	2470	2712
transcript_138776	gnl|BL_ORD_ID|45002 transcript_93360	185	1792	2	188	566	2173	26	212
transcript_138776	gnl|BL_ORD_ID|1886 transcript_23009	185	1801	65	188	601	2217	124	247
transcript_138829	gnl|BL_ORD_ID|60811 transcript_117645	1	1091	1088	2207	586	1668	2399	3513
transcript_138829	gnl|BL_ORD_ID|48851 transcript_99571	1	1767	1766	2180	596	2359	2494	2906
transcript_138829	gnl|BL_ORD_ID|12074 transcript_3086	1	1091	1088	2203	596	1683	2417	3530
transcript_138857	gnl|BL_ORD_ID|24498 transcript_4440	1	2655	2656	3089	143	2801	2919	3352

transcript_138857	gnl|BL_ORD_ID|19237 transcript_54775	1	2656	2656	3089	1	2656	3043	3477
transcript_138857	gnl|BL_ORD_ID|52414 transcript_103683	20	2655	2655	3086	41	2676	2870	3301
transcript_138857	gnl|BL_ORD_ID|29294 transcript_69513	1	2655	2654	3087	24	2676	4211	4644
transcript_138921	gnl|BL_ORD_ID|1010 transcript_1799	1	2595	2591	3677	3	2595	2725	3799
transcript_138921	gnl|BL_ORD_ID|46736 transcript_96227	2	2595	2591	3646	1	2590	2722	3789
transcript_139020	gnl|BL_ORD_ID|9494 transcript_38891	1	1254	1250	1360	16	1269	1398	1510
transcript_139020	gnl|BL_ORD_ID|9051 transcript_38021	1	1254	1250	1419	13	1266	1395	1565
transcript_139078	gnl|BL_ORD_ID|24018 transcript_62688	799	2552	9	798	1136	2913	2	791
transcript_139086	gnl|BL_ORD_ID|51868 transcript_10026	1	2079	2076	2468	24	2104	2470	2862
transcript_139087	gnl|BL_ORD_ID|75314 transcript_139568	653	2203	87	652	715	2265	2	567
transcript_139114	gnl|BL_ORD_ID|52134 transcript_10604	1	1444	1442	1872	314	1755	2392	2822
transcript_139118	gnl|BL_ORD_ID|62070 transcript_119632	197	2395	1	198	352	2550	1	198
transcript_139172	gnl|BL_ORD_ID|58586 transcript_114079	151	1809	1	153	2466	4124	7	159
transcript_139172	gnl|BL_ORD_ID|20910 transcript_57525	152	1807	1	153	262	1917	8	160
transcript_139172	gnl|BL_ORD_ID|90368 transcript_162826	150	1809	1	151	1276	2933	9	160
transcript_139194	gnl|BL_ORD_ID|51517 transcript_9248	1	2036	2032	2214	7	2041	2541	2715
transcript_139194	gnl|BL_ORD_ID|85344 transcript_154724	1	2036	2032	2214	6	2042	2347	2521
transcript_139210	gnl|BL_ORD_ID|20850 transcript_57410	216	3237	2	215	377	3392	54	267
transcript_139214	gnl|BL_ORD_ID|59725 transcript_115910	1	2535	2532	3175	7	2540	2983	3625
transcript_139255	gnl|BL_ORD_ID|25223 transcript_5970	291	2627	3	295	623	2962	1	287
transcript_139291	gnl|BL_ORD_ID|29575 transcript_69949	1	1224	1227	2114	75	1271	1379	2262
transcript_139291	gnl|BL_ORD_ID|95585 transcript_19823	1	1224	1227	2114	206	1429	1537	2424
transcript_139292	gnl|BL_ORD_ID|51317 transcript_8872	130	1280	2	129	1786	2936	1	128
transcript_1393	gnl|BL_ORD_ID|76363 transcript_141266	20	3395	3392	3957	3	3370	3485	4043
transcript_139300	gnl|BL_ORD_ID|36118 transcript_80548	2	2116	2117	2358	28	2116	3498	3739
transcript_139300	gnl|BL_ORD_ID|80096 transcript_145805	10	2116	2117	2358	1	2109	3498	3736
transcript_139302	gnl|BL_ORD_ID|19561 transcript_55338	110	1579	11	111	239	1709	1	103
transcript_139379	gnl|BL_ORD_ID|86646 transcript_156871	473	1038	6	473	1854	2419	44	507
transcript_139405	gnl|BL_ORD_ID|38333 transcript_7392	1	2123	2118	2834	1	2123	2320	3036
transcript_139445	gnl|BL_ORD_ID|1532 transcript_22224	1	1172	1171	2109	48	1220	1351	2282
transcript_139450	gnl|BL_ORD_ID|31797 transcript_73567	1	1590	1587	2944	37	1623	1871	3230
transcript_139450	gnl|BL_ORD_ID|44709 transcript_92871	1	1588	1587	2949	126	1711	1835	3199
transcript_13946	gnl|BL_ORD_ID|88713 transcript_160247	2	2189	2187	2649	364	2551	2652	3113
transcript_139461	gnl|BL_ORD_ID|48645 transcript_99237	14	2401	2402	2618	21	2417	2535	2770

transcript_13950	gnl|BL_ORD_ID|25832 transcript_63989	112	2645	10	111	274	2804	1	104
transcript_139524	gnl|BL_ORD_ID|36261 transcript_80793	824	1742	9	824	969	1887	2	816
transcript_139557	gnl|BL_ORD_ID|72767 transcript_135397	163	1135	2	165	1917	2886	2	163
transcript_139578	gnl|BL_ORD_ID|66037 transcript_124480	1	1207	1204	1496	49	1272	2407	2698
transcript_139578	gnl|BL_ORD_ID|65122 transcript_12361	1	1207	1204	1513	70	1281	2413	2723
transcript_139578	gnl|BL_ORD_ID|49358 transcript_100353	1	1035	1035	1508	161	1199	1949	2422
transcript_139600	gnl|BL_ORD_ID|59141 transcript_114972	1	2496	2496	2798	72	2568	3236	3538
transcript_139618	gnl|BL_ORD_ID|86138 transcript_156020	573	2152	81	575	1607	3188	2	497
transcript_139635	gnl|BL_ORD_ID|96657 transcript_83749	376	1894	4	377	522	2041	17	390
transcript_139655	gnl|BL_ORD_ID|51252 transcript_8709	147	1725	1	152	1361	2939	1048	1199
transcript_139655	gnl|BL_ORD_ID|38802 transcript_8431	147	1774	1	152	1343	2973	1030	1181
transcript_139655	gnl|BL_ORD_ID|38131 transcript_6969	147	1774	1	152	1469	3100	1156	1307
transcript_139655	gnl|BL_ORD_ID|34019 transcript_77151	147	1723	1	152	1450	3021	1137	1288
transcript_139667	gnl|BL_ORD_ID|96861 transcript_104734	1	1705	1701	2884	3	1708	2023	3206
transcript_139668	gnl|BL_ORD_ID|86037 transcript_155855	11	1130	1129	1830	1	1119	2224	2927
transcript_139677	gnl|BL_ORD_ID|67017 transcript_126098	1	1402	1400	2253	1	1413	1524	2377
transcript_139677	gnl|BL_ORD_ID|64775 transcript_11586	1	1402	1400	2314	2	1401	1512	2426
transcript_139734	gnl|BL_ORD_ID|47994 transcript_98182	337	1766	4	342	1755	3171	294	632
transcript_139796	gnl|BL_ORD_ID|60628 transcript_117333	2	2108	2107	2655	13	2119	2221	2769
transcript_139812	gnl|BL_ORD_ID|23724 transcript_62228	1074	2634	1	1075	1665	3226	51	1125
transcript_139822	gnl|BL_ORD_ID|78301 transcript_14292	250	1694	6	254	1156	2596	1	249
transcript_139859	gnl|BL_ORD_ID|20891 transcript_57487	520	3253	53	522	711	3444	1	473
transcript_13987	gnl|BL_ORD_ID|135 transcript_204	1	1435	1434	2541	2	1438	3898	5008
transcript_13993	gnl|BL_ORD_ID|62884 transcript_120958	20	2377	2373	2586	1	2359	2983	3196
transcript_13999	gnl|BL_ORD_ID|64516 transcript_11010	1	2107	2106	2577	1	2111	2318	2789
transcript_140101	gnl|BL_ORD_ID|90555 transcript_163105	1	1414	1411	1813	1152	2565	2708	3118
transcript_140116	gnl|BL_ORD_ID|40072 transcript_85282	396	1676	8	394	1996	3272	2	382
transcript_140116	gnl|BL_ORD_ID|38281 transcript_7277	396	1676	8	394	1793	3061	3	379
transcript_140116	gnl|BL_ORD_ID|38812 transcript_8451	396	1678	8	394	1702	2972	2	378
transcript_140136	gnl|BL_ORD_ID|7702 transcript_35337	170	1485	2	174	370	1687	28	200
transcript_140162	gnl|BL_ORD_ID|63188 transcript_121454	1069	2251	1	1072	5146	6328	3729	4826
transcript_140162	gnl|BL_ORD_ID|31615 transcript_73242	1069	2245	1	1072	3169	4331	1751	2848
transcript_140162	gnl|BL_ORD_ID|796 transcript_1388	1069	2242	1	1072	2801	3977	1382	2480
transcript_140166	gnl|BL_ORD_ID|50176 transcript_101660	1	1472	1470	2307	1	1477	1594	2427

transcript_14017	gnl|BL_ORD_ID|756 transcript_1318	1105	2636	1	1108	2485	4016	17	1125
transcript_140205	gnl|BL_ORD_ID|39467 transcript_84294	1	1386	1381	1492	1	1368	1598	1709
transcript_140219	gnl|BL_ORD_ID|150 transcript_227	1	1700	1699	2432	9	1702	2331	3066
transcript_140248	gnl|BL_ORD_ID|69706 transcript_130467	1043	2711	1	1046	1786	3454	11	1055
transcript_14026	gnl|BL_ORD_ID|44540 transcript_92602	10	1498	1498	2642	1	1492	1827	2972
transcript_140296	gnl|BL_ORD_ID|38859 transcript_83319	208	4468	1	210	1029	5295	535	744
transcript_14033	gnl|BL_ORD_ID|64527 transcript_11044	1	1481	1478	2571	18	1495	1699	2795
transcript_140330	gnl|BL_ORD_ID|1682 transcript_22571	1	1770	1768	2110	7	1795	1925	2267
transcript_140330	gnl|BL_ORD_ID|69796 transcript_130598	1	1770	1768	2109	2	1768	1897	2237
transcript_140330	gnl|BL_ORD_ID|96103 transcript_21032	1	1770	1768	2110	2	1771	1901	2243
transcript_140389	gnl|BL_ORD_ID|62897 transcript_120974	2	2083	2083	3080	1	2084	3045	4042
transcript_1404	gnl|BL_ORD_ID|48559 transcript_99086	120	2398	2396	3922	2	2280	2686	4225
transcript_140436	gnl|BL_ORD_ID|62775 transcript_120789	1	2078	2077	2751	22	2084	3464	4138
transcript_140465	gnl|BL_ORD_ID|51940 transcript_10164	2	2222	2220	2573	2	2222	2500	2853
transcript_140525	gnl|BL_ORD_ID|52026 transcript_10347	1	1183	1179	2249	1	1183	1336	2407
transcript_140555	gnl|BL_ORD_ID|8615 transcript_37144	247	1559	37	248	315	1622	2	215
transcript_140612	gnl|BL_ORD_ID|97111 transcript_127431	1	2269	2270	3654	515	2802	3470	4854
transcript_140622	gnl|BL_ORD_ID|95890 transcript_20541	198	2199	1	199	342	2343	2	200
transcript_140664	gnl|BL_ORD_ID|59328 transcript_115280	151	3418	1	152	367	3618	5	156
transcript_140721	gnl|BL_ORD_ID|41096 transcript_86950	1	1866	1863	2711	3	1856	2131	2960
transcript_140721	gnl|BL_ORD_ID|38771 transcript_8372	1	1866	1863	2711	2	1862	2138	2980
transcript_140735	gnl|BL_ORD_ID|81902 transcript_148793	107	1916	1912	2854	146	1955	2096	3037
transcript_140737	gnl|BL_ORD_ID|18747 transcript_53965	1	1685	1681	2589	2	1690	1845	2753
transcript_140738	gnl|BL_ORD_ID|463 transcript_785	1	3192	3187	4034	1	3192	3380	4227
transcript_140773	gnl|BL_ORD_ID|12355 transcript_3604	197	3181	1	199	312	3297	2	203
transcript_140813	gnl|BL_ORD_ID|36436 transcript_81084	16	2632	2630	3029	1	2643	2981	3380
transcript_140813	gnl|BL_ORD_ID|12538 transcript_3971	16	2632	2630	3060	2	2618	2956	3386
transcript_140835	gnl|BL_ORD_ID|58979 transcript_114707	1	1348	1348	2218	82	1429	2116	2985
transcript_140849	gnl|BL_ORD_ID|78721 transcript_143593	250	3101	1	250	444	3279	3	251
transcript_140849	gnl|BL_ORD_ID|12533 transcript_3955	250	3101	1	250	554	3396	113	361
transcript_140849	gnl|BL_ORD_ID|24631 transcript_4710	250	3101	1	250	443	3292	2	250
transcript_140849	gnl|BL_ORD_ID|12146 transcript_3223	250	3077	1	250	654	3495	213	461
transcript_140849	gnl|BL_ORD_ID|36820 transcript_81688	250	3075	1	250	627	3453	210	458
transcript_140849	gnl|BL_ORD_ID|12460 transcript_3814	250	3101	1	250	509	3380	68	316

transcript_140849	gnl|BL_ORD_ID|12648 transcript_4201	250	3101	1	250	551	3376	110	358
transcript_14085	gnl|BL_ORD_ID|63561 transcript_122065	15	1600	1598	2608	1	1587	2079	3089
transcript_140884	gnl|BL_ORD_ID|4745 transcript_29241	1	1113	1114	1840	42	1160	1279	2008
transcript_140906	gnl|BL_ORD_ID|84161 transcript_152789	1	2400	2398	3911	1	2404	2527	4018
transcript_140961	gnl|BL_ORD_ID|53708 transcript_105844	1	2856	2851	3578	27	2885	3301	4028
transcript_140967	gnl|BL_ORD_ID|51864 transcript_10021	1	1156	1155	1764	217	1372	2242	2851
transcript_140967	gnl|BL_ORD_ID|51613 transcript_9460	1	1156	1155	1764	231	1386	2260	2870
transcript_140967	gnl|BL_ORD_ID|65114 transcript_12336	1	1156	1155	1764	213	1368	2118	2724
transcript_140967	gnl|BL_ORD_ID|2319 transcript_23952	1	1069	1064	1764	214	1282	1537	2237
transcript_140967	gnl|BL_ORD_ID|1824 transcript_22876	1	1069	1064	1764	233	1301	1556	2257
transcript_141	gnl|BL_ORD_ID|75489 transcript_139854	258	5247	2	257	373	5362	9	264
transcript_1410	gnl|BL_ORD_ID|88006 transcript_159092	162	2757	2756	3961	2	2593	2834	4039
transcript_141018	gnl|BL_ORD_ID|39710 transcript_84695	274	4650	1	273	677	5054	227	499
transcript_141034	gnl|BL_ORD_ID|38722 transcript_8262	503	2803	72	504	573	2873	2	417
transcript_141038	gnl|BL_ORD_ID|583 transcript_986	2	2804	2803	3806	6	2802	2990	3992
transcript_141038	gnl|BL_ORD_ID|469 transcript_799	2	2804	2803	3806	20	2815	3230	4232
transcript_141038	gnl|BL_ORD_ID|586 transcript_995	2	2804	2803	3806	6	2809	2997	4001
transcript_141038	gnl|BL_ORD_ID|27012 transcript_65849	2	2805	2803	3806	6	2802	3310	4312
transcript_141038	gnl|BL_ORD_ID|337 transcript_580	2	2805	2803	3806	61	2858	3366	4370
transcript_141038	gnl|BL_ORD_ID|766 transcript_1337	2	2804	2803	3806	3	2779	2967	3969
transcript_141038	gnl|BL_ORD_ID|41571 transcript_87743	2	2804	2803	3764	5	2813	3228	4188
transcript_141044	gnl|BL_ORD_ID|34272 transcript_77582	22	2502	2501	2815	15	2487	2612	2913
transcript_141044	gnl|BL_ORD_ID|38657 transcript_8102	16	2502	2501	2815	14	2492	2620	2927
transcript_141044	gnl|BL_ORD_ID|51991 transcript_10275	11	2502	2501	2710	28	2511	2641	2846
transcript_14107	gnl|BL_ORD_ID|70906 transcript_132426	11	1645	1645	2563	2	1632	1756	2674
transcript_14107	gnl|BL_ORD_ID|35492 transcript_79562	344	2633	4	347	855	3145	2	345
transcript_141080	gnl|BL_ORD_ID|26347 transcript_64778	1	1092	1090	1933	122	1213	1440	2285
transcript_141082	gnl|BL_ORD_ID|89178 transcript_160966	1445	3042	108	1446	1458	3034	1	1339
transcript_141082	gnl|BL_ORD_ID|38343 transcript_7412	1445	3042	108	1446	1459	3058	2	1340
transcript_141127	gnl|BL_ORD_ID|90774 transcript_15181	1	1767	1765	2451	38	1798	1909	2596
transcript_141127	gnl|BL_ORD_ID|78340 transcript_14393	1	1767	1765	2483	15	1775	1886	2606
transcript_141127	gnl|BL_ORD_ID|59246 transcript_115142	1	1767	1765	2456	9	1769	2032	2726
transcript_141127	gnl|BL_ORD_ID|24852 transcript_5174	1	1767	1765	2483	18	1778	2551	3271
transcript_141155	gnl|BL_ORD_ID|56077 transcript_109967	137	1352	6	139	244	1457	2	136

transcript_141178	gnl|BL_ORD_ID|52041 transcript_10390	1	1064	1063	1703	20	1083	2012	2652
transcript_141222	gnl|BL_ORD_ID|87937 transcript_158974	1	2416	2411	3181	1	2431	2838	3596
transcript_141228	gnl|BL_ORD_ID|25824 transcript_63978	160	3337	1	165	1460	4638	609	773
transcript_141249	gnl|BL_ORD_ID|92930 transcript_165403	141	1992	1	142	1779	3645	1	140
transcript_141275	gnl|BL_ORD_ID|52899 transcript_104443	2	2382	2381	3689	123	2503	2658	3966
transcript_141294	gnl|BL_ORD_ID|70632 transcript_131982	580	1891	7	582	2174	3489	2	577
transcript_141377	gnl|BL_ORD_ID|56692 transcript_110948	610	2585	7	615	2446	4430	1	608
transcript_141402	gnl|BL_ORD_ID|49307 transcript_100278	1	2275	2275	2795	66	2340	2446	2966
transcript_141408	gnl|BL_ORD_ID|31814 transcript_73591	2	2363	2360	3125	4	2351	2995	3760
transcript_141446	gnl|BL_ORD_ID|91099 transcript_15891	1	1777	1773	2297	1	1777	1918	2442
transcript_141446	gnl|BL_ORD_ID|59163 transcript_115016	1	1777	1773	2297	1	1777	1918	2442
transcript_141502	gnl|BL_ORD_ID|50191 transcript_101688	1	1881	1880	2157	60	1939	2094	2371
transcript_141522	gnl|BL_ORD_ID|20988 transcript_57650	173	1815	1	176	2607	4244	2147	2322
transcript_141522	gnl|BL_ORD_ID|66511 transcript_125282	173	1815	1	176	1144	2786	684	859
transcript_141522	gnl|BL_ORD_ID|69427 transcript_129987	173	1815	1	176	4890	6532	4430	4605
transcript_141522	gnl|BL_ORD_ID|79748 transcript_145243	173	1815	1	176	1892	3533	1432	1607
transcript_141522	gnl|BL_ORD_ID|31942 transcript_73806	173	1815	1	176	4992	6632	4533	4707
transcript_141552	gnl|BL_ORD_ID|51589 transcript_9405	1	2145	2146	2566	1	2144	2468	2895
transcript_141555	gnl|BL_ORD_ID|1937 transcript_23115	1	1099	1098	1947	16	1113	1251	2101
transcript_141567	gnl|BL_ORD_ID|96125 transcript_21079	198	1678	2	198	758	2227	136	332
transcript_141567	gnl|BL_ORD_ID|76334 transcript_141229	198	1678	2	198	563	2032	1	197
transcript_141567	gnl|BL_ORD_ID|57952 transcript_113038	198	1687	2	198	358	1847	1	196
transcript_141567	gnl|BL_ORD_ID|95861 transcript_20489	198	1651	2	198	915	2354	79	275
transcript_141567	gnl|BL_ORD_ID|63926 transcript_122643	198	1687	2	198	766	2255	146	342
transcript_141569	gnl|BL_ORD_ID|55010 transcript_108069	171	1598	2	173	1672	3100	6	177
transcript_14157	gnl|BL_ORD_ID|40546 transcript_86031	11	1427	1424	2636	3	1416	1902	3113
transcript_141574	gnl|BL_ORD_ID|42030 transcript_88463	1	1684	1682	3268	151	1814	1937	3524
transcript_141708	gnl|BL_ORD_ID|31687 transcript_73359	1	1064	1059	1275	1	1062	2702	2918
transcript_141711	gnl|BL_ORD_ID|57305 transcript_111969	15	3459	3457	3826	2	3434	3579	3948
transcript_141765	gnl|BL_ORD_ID|68321 transcript_128216	453	2134	9	456	596	2278	1	448
transcript_141779	gnl|BL_ORD_ID|42846 transcript_89758	148	3019	1	148	574	3459	2	150
transcript_1418	gnl|BL_ORD_ID|90550 transcript_163099	23	2900	2899	3915	2	2882	3447	4463
transcript_1418	gnl|BL_ORD_ID|96931 transcript_111138	1	3340	3337	3903	1	3341	3674	4240
transcript_1418	gnl|BL_ORD_ID|69346 transcript_129863	17	2955	2952	3972	2	2941	3179	4199

transcript_141804	gnl|BL_ORD_ID|22662 transcript_60451	206	2201	1	210	2019	24	2479	2270
transcript_141804	gnl|BL_ORD_ID|56826 transcript_111153	206	2201	1	210	470	2477	9	218
transcript_141804	gnl|BL_ORD_ID|903 transcript_1615	206	2201	1	210	2008	8	2468	2259
transcript_141815	gnl|BL_ORD_ID|12265 transcript_3436	2	3042	3040	3317	41	3081	3192	3469
transcript_141815	gnl|BL_ORD_ID|28819 transcript_68783	26	3042	3040	3373	1	3020	3131	3464
transcript_141815	gnl|BL_ORD_ID|60889 transcript_117773	29	3042	3040	3317	1	3031	3576	3853
transcript_141828	gnl|BL_ORD_ID|92346 transcript_164449	1	1531	1530	2049	117	1646	2065	2583
transcript_14187	gnl|BL_ORD_ID|38820 transcript_8473	241	2639	1	245	636	3010	2	244
transcript_141910	gnl|BL_ORD_ID|22132 transcript_59575	266	2044	6	267	962	2756	1	260
transcript_141937	gnl|BL_ORD_ID|47509 transcript_97435	184	2362	1	184	760	2940	248	431
transcript_141937	gnl|BL_ORD_ID|31379 transcript_72876	184	2362	1	184	509	2681	2	184
transcript_14194	gnl|BL_ORD_ID|26657 transcript_65271	1179	2622	10	1179	1311	2750	1	1179
transcript_14194	gnl|BL_ORD_ID|70920 transcript_132455	1179	2621	10	1179	2307	3745	1	1181
transcript_141996	gnl|BL_ORD_ID|2910 transcript_25245	112	1354	1	116	890	2132	1	116
transcript_14200	gnl|BL_ORD_ID|52006 transcript_10309	1	1719	1719	2631	1	1719	1921	2833
transcript_142003	gnl|BL_ORD_ID|17723 transcript_52357	2	2010	2009	2954	167	2176	2308	3254
transcript_142003	gnl|BL_ORD_ID|38059 transcript_6824	2	2151	2150	2954	1	2150	2276	3080
transcript_142003	gnl|BL_ORD_ID|50059 transcript_101481	2	2012	2009	2954	177	2188	2357	3300
transcript_142047	gnl|BL_ORD_ID|28709 transcript_68612	1152	2888	1	1151	1298	3037	36	1185
transcript_142050	gnl|BL_ORD_ID|53396 transcript_105281	1	2235	2233	2858	14	2248	2460	3085
transcript_142123	gnl|BL_ORD_ID|68961 transcript_129250	1101	2486	1	1100	1597	2989	206	1304
transcript_14218	gnl|BL_ORD_ID|29454 transcript_69747	1	2250	2248	2619	61	2319	3880	4251
transcript_14218	gnl|BL_ORD_ID|67651 transcript_127129	1	2250	2248	2619	2	2255	3817	4188
transcript_142181	gnl|BL_ORD_ID|65228 transcript_12610	1	1294	1294	2361	69	1361	1674	2742
transcript_142181	gnl|BL_ORD_ID|56773 transcript_111070	1	1895	1895	2290	36	1930	2035	2429
transcript_142279	gnl|BL_ORD_ID|8615 transcript_37144	218	1436	6	219	315	1510	2	215
transcript_142306	gnl|BL_ORD_ID|37662 transcript_83009	1	1980	1977	2273	723	2704	3095	3390
transcript_142350	gnl|BL_ORD_ID|74956 transcript_138995	1	1136	1135	1965	49	1184	2170	3015
transcript_142380	gnl|BL_ORD_ID|64367 transcript_10705	1	1716	1711	1946	68	1777	2395	2630
transcript_142380	gnl|BL_ORD_ID|51690 transcript_9641	1	1716	1711	1946	5	1714	2332	2567
transcript_142380	gnl|BL_ORD_ID|27533 transcript_66653	1	1716	1711	1946	3	1718	2336	2571
transcript_142380	gnl|BL_ORD_ID|64841 transcript_11704	1	1716	1711	1956	2	1717	2335	2581
transcript_142403	gnl|BL_ORD_ID|44307 transcript_92205	11	1581	1582	2408	2	1575	1727	2553
transcript_142408	gnl|BL_ORD_ID|619 transcript_1061	2	3212	3211	3922	2	3208	3313	4024

transcript_142420	gnl|BL_ORD_ID|40864 transcript_86579	1	2048	2046	2764	5	2077	2247	2965
transcript_142430	gnl|BL_ORD_ID|49593 transcript_100746	1	1043	1042	1614	2	1045	1158	1730
transcript_142430	gnl|BL_ORD_ID|7507 transcript_34948	1	1043	1042	1576	65	1108	1221	1755
transcript_14245	gnl|BL_ORD_ID|83757 transcript_152080	1	1334	1332	2534	1	1334	1475	2671
transcript_14245	gnl|BL_ORD_ID|47873 transcript_97993	526	2543	9	526	904	2921	5	505
transcript_142452	gnl|BL_ORD_ID|85304 transcript_154645	1	1153	1148	2167	252	1394	2135	3155
transcript_142452	gnl|BL_ORD_ID|60102 transcript_116509	1	1153	1151	2200	247	1398	2805	3854
transcript_142460	gnl|BL_ORD_ID|264 transcript_427	2	3445	3440	4295	7	3456	3711	4566
transcript_142483	gnl|BL_ORD_ID|95456 transcript_19500	391	1943	4	390	768	2296	4	391
transcript_142545	gnl|BL_ORD_ID|96120 transcript_21070	10	1427	1426	1991	3	1420	1796	2361
transcript_142558	gnl|BL_ORD_ID|66037 transcript_124480	1	1278	1275	1569	1	1272	2407	2701
transcript_142583	gnl|BL_ORD_ID|60677 transcript_117419	1	1559	1559	1744	249	1807	1936	2121
transcript_142583	gnl|BL_ORD_ID|4211 transcript_28102	1	1559	1559	1734	203	1761	1884	2059
transcript_142584	gnl|BL_ORD_ID|87700 transcript_158593	279	2792	2	278	927	3440	277	553
transcript_14259	gnl|BL_ORD_ID|85723 transcript_155322	1234	2651	1	1236	1326	2740	3	1214
transcript_142631	gnl|BL_ORD_ID|97145 transcript_131498	157	1250	2	157	313	1406	3	158
transcript_142651	gnl|BL_ORD_ID|578 transcript_979	1	2267	2264	2808	2	2284	2402	2948
transcript_142656	gnl|BL_ORD_ID|50348 transcript_101936	1	2696	2694	3383	3	2696	3017	3708
transcript_142707	gnl|BL_ORD_ID|63738 transcript_122349	1	1677	1676	2357	757	2433	2549	3231
transcript_142724	gnl|BL_ORD_ID|75079 transcript_139183	150	2162	1	152	915	2925	1	167
transcript_142736	gnl|BL_ORD_ID|64823 transcript_11665	157	2329	1	161	527	2700	154	314
transcript_142736	gnl|BL_ORD_ID|77733 transcript_13022	157	2329	1	161	462	2634	89	249
transcript_142736	gnl|BL_ORD_ID|64670 transcript_11354	157	2328	1	161	606	2777	233	393
transcript_142736	gnl|BL_ORD_ID|18839 transcript_54106	157	2329	1	161	408	2581	35	195
transcript_142736	gnl|BL_ORD_ID|18637 transcript_53790	157	2260	1	161	448	2569	72	231
transcript_1428	gnl|BL_ORD_ID|71902 transcript_134027	374	3861	1	375	1786	5277	1	377
transcript_14283	gnl|BL_ORD_ID|57293 transcript_111950	1	1625	1626	2635	1	1625	2366	3373
transcript_14284	gnl|BL_ORD_ID|64417 transcript_10807	240	2625	24	242	328	2714	2	221
transcript_14284	gnl|BL_ORD_ID|51764 transcript_9774	240	2611	24	241	514	2885	2	220
transcript_142850	gnl|BL_ORD_ID|11787 transcript_2519	455	3201	56	458	687	3433	2	405
transcript_142852	gnl|BL_ORD_ID|18014 transcript_52799	2	2224	2223	3128	4	2240	2346	3252
transcript_142870	gnl|BL_ORD_ID|17987 transcript_52760	1	1163	1160	1390	105	1311	2830	3063
transcript_142885	gnl|BL_ORD_ID|28335 transcript_68012	25	1000	999	1530	1	983	2447	2978
transcript_142897	gnl|BL_ORD_ID|68222 transcript_128039	1	2040	2038	3102	151	2190	2348	3410

transcript_142901	gnl|BL_ORD_ID|96650 transcript_82830	1	1436	1436	2806	127	1563	2054	3426
transcript_142905	gnl|BL_ORD_ID|23638 transcript_62083	2	2326	2324	3165	152	2469	2586	3433
transcript_142907	gnl|BL_ORD_ID|93564 transcript_166431	2	2831	2832	3231	16	2848	3040	3439
transcript_142922	gnl|BL_ORD_ID|74972 transcript_139026	484	1825	6	485	1337	2676	2	482
transcript_142939	gnl|BL_ORD_ID|27512 transcript_66618	2	2277	2276	3900	258	2534	3705	5359
transcript_142945	gnl|BL_ORD_ID|74820 transcript_138787	1	1923	1922	2534	2	1925	2284	2895
transcript_142956	gnl|BL_ORD_ID|615 transcript_1054	272	3688	1	273	603	3995	60	333
transcript_142958	gnl|BL_ORD_ID|86138 transcript_156020	500	2374	9	502	1607	3469	2	497
transcript_143075	gnl|BL_ORD_ID|58037 transcript_113176	769	1884	90	769	979	2093	1	680
transcript_143076	gnl|BL_ORD_ID|5405 transcript_30593	1	1497	1496	1766	1	1478	1584	1854
transcript_14309	gnl|BL_ORD_ID|57874 transcript_112920	112	1441	1441	2671	2	1344	1588	2820
transcript_143094	gnl|BL_ORD_ID|917 transcript_1639	2	2806	2801	2987	111	2913	3678	3864
transcript_143115	gnl|BL_ORD_ID|29443 transcript_69734	1617	3715	1	1619	2920	5009	1190	2804
transcript_143149	gnl|BL_ORD_ID|588 transcript_998	1	3669	3668	3992	1	3669	3776	4100
transcript_143157	gnl|BL_ORD_ID|64802 transcript_11632	195	2485	1	197	418	2709	12	200
transcript_14317	gnl|BL_ORD_ID|37897 transcript_6500	23	2501	2500	2648	15	2589	2723	2878
transcript_14317	gnl|BL_ORD_ID|38657 transcript_8102	10	2501	2500	2652	1	2492	2620	2772
transcript_14317	gnl|BL_ORD_ID|51894 transcript_10073	2	2501	2500	2651	20	2521	2652	2803
transcript_14317	gnl|BL_ORD_ID|27550 transcript_66674	23	2501	2500	2651	19	2505	2636	2786
transcript_14317	gnl|BL_ORD_ID|25031 transcript_5567	23	2502	2500	2646	44	2644	2778	2935
transcript_143178	gnl|BL_ORD_ID|93903 transcript_166958	1	2343	2339	2533	2	2365	2740	2934
transcript_143232	gnl|BL_ORD_ID|51883 transcript_10056	256	1987	5	259	1073	2803	2	256
transcript_143238	gnl|BL_ORD_ID|41192 transcript_87098	175	1087	7	177	801	1716	1	171
transcript_143238	gnl|BL_ORD_ID|8139 transcript_36185	175	1036	2	177	811	1673	1	179
transcript_143243	gnl|BL_ORD_ID|38474 transcript_7697	110	1366	1	112	1622	2879	18	129
transcript_143243	gnl|BL_ORD_ID|37924 transcript_6557	110	1348	1	110	1890	3129	2	113
transcript_143261	gnl|BL_ORD_ID|46317 transcript_95554	268	2220	5	268	831	2783	2	285
transcript_143301	gnl|BL_ORD_ID|17819 transcript_52506	1	1257	1257	1977	942	2198	2449	3170
transcript_143317	gnl|BL_ORD_ID|38466 transcript_7683	241	2920	1	243	371	3036	27	268
transcript_143317	gnl|BL_ORD_ID|38115 transcript_6941	241	2935	1	243	370	3070	26	267
transcript_143317	gnl|BL_ORD_ID|38358 transcript_7449	241	2904	1	243	372	3039	27	269
transcript_143384	gnl|BL_ORD_ID|37952 transcript_6609	2	2163	2163	2618	56	2206	2664	3119
transcript_143480	gnl|BL_ORD_ID|393 transcript_674	139	3399	1	142	1036	4319	1	141
transcript_143480	gnl|BL_ORD_ID|77347 transcript_142840	2	2487	2484	3399	3	2491	2635	3557

transcript_143516	gnl|BL_ORD_ID|90752 transcript_15139	1	1340	1339	2256	1	1341	1647	2564
transcript_143530	gnl|BL_ORD_ID|24428 transcript_4298	1	1861	1862	3184	1	1860	2016	3336
transcript_143530	gnl|BL_ORD_ID|54307 transcript_106831	1	1861	1862	3113	1	1857	2013	3245
transcript_143543	gnl|BL_ORD_ID|20706 transcript_57175	1	3365	3365	3692	937	4300	4422	4748
transcript_143543	gnl|BL_ORD_ID|69061 transcript_129401	1	3365	3365	3748	220	3583	3706	4087
transcript_143604	gnl|BL_ORD_ID|81163 transcript_147545	1	1568	1568	2565	1	1562	1704	2703
transcript_143637	gnl|BL_ORD_ID|23993 transcript_62650	2059	4081	11	2062	2837	4854	2	2023
transcript_14371	gnl|BL_ORD_ID|66968 transcript_126026	1	1643	1640	2623	1	1644	2411	3394
transcript_14371	gnl|BL_ORD_ID|35481 transcript_79542	12	1643	1643	2588	4	1645	2011	2964
transcript_143718	gnl|BL_ORD_ID|19626 transcript_55427	215	2365	2	215	349	2497	1	214
transcript_143743	gnl|BL_ORD_ID|59519 transcript_115586	545	4037	89	550	1433	4924	399	860
transcript_14378	gnl|BL_ORD_ID|32283 transcript_74399	1177	2612	1	1179	2693	4127	1	1179
transcript_143810	gnl|BL_ORD_ID|39667 transcript_84630	1	1593	1591	2268	29	1612	2452	3128
transcript_143810	gnl|BL_ORD_ID|23326 transcript_61569	1	1593	1591	2268	25	1615	2429	3104
transcript_143815	gnl|BL_ORD_ID|31590 transcript_73204	1	1070	1070	1488	2	1074	1498	1915
transcript_143823	gnl|BL_ORD_ID|74014 transcript_137441	2	2472	2470	3340	7	2476	4068	4938
transcript_143898	gnl|BL_ORD_ID|50502 transcript_102193	1	1979	1976	2984	424	2401	3792	4801
transcript_1439	gnl|BL_ORD_ID|597 transcript_1010	120	3948	1	123	243	4081	1	123
transcript_1439	gnl|BL_ORD_ID|39836 transcript_84895	120	3948	1	121	472	4295	1	121
transcript_1439	gnl|BL_ORD_ID|553 transcript_934	120	3948	1	125	324	4148	2	126
transcript_143914	gnl|BL_ORD_ID|30442 transcript_71353	1	1348	1345	2030	403	1749	1857	2561
transcript_143922	gnl|BL_ORD_ID|47012 transcript_96654	26	2692	2688	3072	1	2665	2769	3153
transcript_14393	gnl|BL_ORD_ID|24852 transcript_5174	1	1775	1773	2606	4	1778	2438	3271
transcript_143989	gnl|BL_ORD_ID|85041 transcript_154230	1	1592	1587	2620	1	1600	2250	3286
transcript_143996	gnl|BL_ORD_ID|17465 transcript_51951	483	3578	54	483	561	3683	30	461
transcript_144013	gnl|BL_ORD_ID|64735 transcript_11491	1	1444	1439	2017	589	2032	2189	2767
transcript_14407	gnl|BL_ORD_ID|84202 transcript_152860	1	2180	2177	2602	1	2179	2310	2735
transcript_144092	gnl|BL_ORD_ID|61001 transcript_117958	106	1079	1	105	1591	2565	1176	1280
transcript_144142	gnl|BL_ORD_ID|47529 transcript_97470	1	1598	1598	1791	199	1796	1906	2099
transcript_14419	gnl|BL_ORD_ID|52177 transcript_10687	1	2440	2435	2616	1	2440	2556	2736
transcript_144230	gnl|BL_ORD_ID|24060 transcript_62749	1268	3042	1	1273	1528	3302	1	1273
transcript_144230	gnl|BL_ORD_ID|34037 transcript_77186	1268	3023	1	1273	1552	3308	1	1297
transcript_144258	gnl|BL_ORD_ID|51752 transcript_9748	1	1781	1780	1947	771	2551	2689	2855
transcript_144258	gnl|BL_ORD_ID|17229 transcript_51577	1	1781	1780	1947	755	2535	2673	2840

transcript_144258	gnl|BL_ORD_ID|52869 transcript_104400	1	1781	1780	1947	35	1814	1951	2118
transcript_144258	gnl|BL_ORD_ID|56494 transcript_110615	1	1781	1780	1947	731	2511	2649	2816
transcript_144268	gnl|BL_ORD_ID|38097 transcript_6900	1	1609	1610	2263	133	1741	1980	2633
transcript_144268	gnl|BL_ORD_ID|56374 transcript_110428	1	1609	1610	2263	134	1757	1996	2653
transcript_144291	gnl|BL_ORD_ID|72656 transcript_135208	1	2510	2507	2922	13	2519	3431	3847
transcript_144291	gnl|BL_ORD_ID|79253 transcript_144456	1	2510	2507	2911	96	2624	3535	3941
transcript_144291	gnl|BL_ORD_ID|815 transcript_1422	1	2510	2507	2922	40	2549	3447	3863
transcript_144291	gnl|BL_ORD_ID|1185 transcript_2138	1	2509	2507	2922	50	2555	3216	3632
transcript_144291	gnl|BL_ORD_ID|39245 transcript_83946	1	2510	2507	2922	5	2514	3424	3840
transcript_144291	gnl|BL_ORD_ID|852 transcript_1504	1	2510	2507	2922	50	2559	3469	3884
transcript_144323	gnl|BL_ORD_ID|12223 transcript_3369	121	3354	1	120	246	3479	1	120
transcript_144379	gnl|BL_ORD_ID|489 transcript_829	1	3043	3042	3956	7	3041	3205	4119
transcript_144379	gnl|BL_ORD_ID|352 transcript_605	1	3043	3042	3956	216	3252	3416	4332
transcript_144379	gnl|BL_ORD_ID|85424 transcript_154840	1	3043	3044	3956	2	3060	3224	4138
transcript_144379	gnl|BL_ORD_ID|59286 transcript_115209	1	3043	3042	3956	6	3066	3230	4141
transcript_144389	gnl|BL_ORD_ID|25215 transcript_5947	1	1097	1097	1820	7	1105	2247	2969
transcript_144389	gnl|BL_ORD_ID|64046 transcript_122824	1	1098	1097	1820	84	1179	1409	2132
transcript_144389	gnl|BL_ORD_ID|58626 transcript_114140	1	1098	1097	1820	5	1098	1246	1969
transcript_144408	gnl|BL_ORD_ID|47822 transcript_97918	1622	3993	1	1623	1820	4214	95	1717
transcript_144425	gnl|BL_ORD_ID|65070 transcript_12237	116	1991	1992	2510	2	1877	2116	2635
transcript_144425	gnl|BL_ORD_ID|68306 transcript_128183	116	1991	1991	2510	1	1874	2306	2825
transcript_144456	gnl|BL_ORD_ID|34841 transcript_78516	548	3941	94	549	1299	4662	2	457
transcript_14447	gnl|BL_ORD_ID|94030 transcript_167162	441	2627	5	445	546	2734	2	442
transcript_144496	gnl|BL_ORD_ID|12644 transcript_4192	278	2948	1	280	678	3348	24	307
transcript_144496	gnl|BL_ORD_ID|49808 transcript_101074	278	2866	1	280	850	3438	208	479
transcript_144496	gnl|BL_ORD_ID|20940 transcript_57568	278	2866	27	280	439	3024	2	249
transcript_144497	gnl|BL_ORD_ID|25062 transcript_5640	1034	3085	1	1035	1230	3253	2	1039
transcript_144497	gnl|BL_ORD_ID|12456 transcript_3807	1034	3085	1	1035	1244	3296	15	1053
transcript_144508	gnl|BL_ORD_ID|68425 transcript_128386	1	1839	1838	2342	98	1940	2396	2900
transcript_144527	gnl|BL_ORD_ID|80633 transcript_146690	1	1234	1232	2256	1	1235	2500	3525
transcript_144529	gnl|BL_ORD_ID|85819 transcript_155470	2	2486	2484	3427	1	2485	3226	4191
transcript_144541	gnl|BL_ORD_ID|57641 transcript_112537	1	2015	2014	2329	62	2076	2231	2546
transcript_144546	gnl|BL_ORD_ID|4548 transcript_28800	1	1435	1432	1837	17	1454	1631	2036
transcript_14455	gnl|BL_ORD_ID|45972 transcript_95007	1120	2679	109	1122	1521	3083	31	1044

transcript_14455	gnl|BL_ORD_ID|62840 transcript_120891	109	2036	2034	2679	31	1963	2139	2788
transcript_144563	gnl|BL_ORD_ID|72174 transcript_134463	1	1967	1967	2644	1066	3031	3159	3836
transcript_144581	gnl|BL_ORD_ID|52699 transcript_104134	399	1306	6	402	662	1569	1	392
transcript_144618	gnl|BL_ORD_ID|26258 transcript_64647	1	1547	1546	1876	1	1546	2386	2723
transcript_144625	gnl|BL_ORD_ID|92396 transcript_164538	1	2789	2788	2998	48	2837	3129	3339
transcript_144675	gnl|BL_ORD_ID|57766 transcript_112744	1381	3034	104	1381	1508	3164	1	1278
transcript_144697	gnl|BL_ORD_ID|21545 transcript_58596	206	2559	1	206	479	2828	152	357
transcript_144697	gnl|BL_ORD_ID|71241 transcript_132955	206	2608	20	206	312	2722	2	188
transcript_1447	gnl|BL_ORD_ID|625 transcript_1070	223	3954	2	223	343	4074	18	237
transcript_144713	gnl|BL_ORD_ID|78389 transcript_14510	1	1432	1432	2284	102	1543	1748	2605
transcript_144713	gnl|BL_ORD_ID|77877 transcript_13338	1	1432	1432	2284	145	1590	1795	2652
transcript_144713	gnl|BL_ORD_ID|51555 transcript_9348	1	1432	1432	2191	169	1614	2130	2894
transcript_144713	gnl|BL_ORD_ID|40271 transcript_85569	1	1432	1432	2191	194	1634	1839	2602
transcript_144759	gnl|BL_ORD_ID|54425 transcript_107037	150	2485	1	154	513	2869	3	155
transcript_144759	gnl|BL_ORD_ID|50087 transcript_101519	150	2485	1	154	570	2905	59	212
transcript_144759	gnl|BL_ORD_ID|51241 transcript_8687	150	2485	1	154	520	2879	36	191
transcript_144759	gnl|BL_ORD_ID|37171 transcript_82236	150	2473	1	154	488	2835	4	159
transcript_144759	gnl|BL_ORD_ID|28316 transcript_67982	150	2485	1	154	584	2919	100	255
transcript_144759	gnl|BL_ORD_ID|51361 transcript_8960	150	2485	1	154	544	2903	60	215
transcript_144760	gnl|BL_ORD_ID|11795 transcript_2540	2	2674	2675	3427	84	2772	2916	3672
transcript_144760	gnl|BL_ORD_ID|11738 transcript_2420	2	2674	2675	3427	84	2756	2900	3651
transcript_144791	gnl|BL_ORD_ID|58922 transcript_114623	1	1995	1993	2220	1523	3513	3648	3872
transcript_144831	gnl|BL_ORD_ID|47939 transcript_98089	286	2499	64	289	1124	3284	419	644
transcript_144835	gnl|BL_ORD_ID|3798 transcript_27192	1	1086	1086	1839	2	1105	1245	2002
transcript_144837	gnl|BL_ORD_ID|75869 transcript_140472	246	1806	76	247	695	2255	2	173
transcript_144851	gnl|BL_ORD_ID|1299 transcript_21625	652	2241	68	656	721	2310	2	591
transcript_144858	gnl|BL_ORD_ID|3685 transcript_26951	113	1479	1	115	777	2145	1	114
transcript_144879	gnl|BL_ORD_ID|25332 transcript_6211	1	1495	1492	2368	463	1956	2295	3171
transcript_144879	gnl|BL_ORD_ID|23068 transcript_61146	1	1495	1492	2397	546	2040	2380	3286
transcript_14488	gnl|BL_ORD_ID|59141 transcript_114972	99	2542	2	103	1096	3538	157	258
transcript_14495	gnl|BL_ORD_ID|75691 transcript_140177	13	1671	1669	2565	1	1658	2216	3117
transcript_1450	gnl|BL_ORD_ID|52906 transcript_104451	15	2167	2165	3944	2	2153	2511	4290
transcript_145011	gnl|BL_ORD_ID|58170 transcript_113366	1578	4644	1	1577	2622	5676	2	1578
transcript_145014	gnl|BL_ORD_ID|21160 transcript_57959	17	2311	2309	3064	1	2298	2519	3275

transcript_145041	gnl|BL_ORD_ID|44995 transcript_93346	130	1234	2	131	418	1521	184	313
transcript_145101	gnl|BL_ORD_ID|5362 transcript_30498	1	1143	1143	1625	1	1144	1393	1875
transcript_145118	gnl|BL_ORD_ID|4 transcript_6	4	4642	4643	7042	269	4906	5013	7409
transcript_145122	gnl|BL_ORD_ID|73473 transcript_136548	257	2652	103	259	273	2668	17	172
transcript_145166	gnl|BL_ORD_ID|80448 transcript_146392	2	2297	2297	3044	43	2326	2433	3180
transcript_145218	gnl|BL_ORD_ID|523 transcript_886	160	3503	1	163	829	4172	4	162
transcript_145255	gnl|BL_ORD_ID|36449 transcript_81105	1	586	585	986	8	594	827	1228
transcript_145269	gnl|BL_ORD_ID|58580 transcript_114064	1	2464	2461	2749	1	2467	2791	3080
transcript_145312	gnl|BL_ORD_ID|86 transcript_127	289	3494	1	289	2107	5312	3	291
transcript_145321	gnl|BL_ORD_ID|74849 transcript_138838	1028	2158	1	1027	1255	2385	119	1145
transcript_145321	gnl|BL_ORD_ID|58948 transcript_114663	1028	2158	1	1027	1312	2448	173	1202
transcript_14538	gnl|BL_ORD_ID|64547 transcript_11080	2	2493	2494	2617	61	2552	2668	2793
transcript_14538	gnl|BL_ORD_ID|51964 transcript_10225	2	2493	2494	2617	44	2535	2651	2774
transcript_145435	gnl|BL_ORD_ID|57433 transcript_112176	345	1503	4	345	1264	2423	2	343
transcript_14547	gnl|BL_ORD_ID|92765 transcript_165139	261	2615	13	261	870	3221	2	250
transcript_145526	gnl|BL_ORD_ID|51574 transcript_9378	216	2722	1	215	327	2833	1	215
transcript_145531	gnl|BL_ORD_ID|95462 transcript_19509	1	1695	1694	2183	50	1741	1883	2372
transcript_145532	gnl|BL_ORD_ID|63003 transcript_121142	1	1491	1490	2398	25	1527	1632	2543
transcript_145532	gnl|BL_ORD_ID|25902 transcript_64103	763	2406	9	764	1086	2726	43	811
transcript_145552	gnl|BL_ORD_ID|69130 transcript_129525	1	2455	2455	2744	37	2487	2720	3009
transcript_145556	gnl|BL_ORD_ID|23691 transcript_62168	298	1576	65	301	966	2245	60	296
transcript_145597	gnl|BL_ORD_ID|64266 transcript_123172	1	1255	1255	2281	701	1956	2619	3645
transcript_145614	gnl|BL_ORD_ID|23767 transcript_62304	199	1348	2	201	468	1617	1	198
transcript_145635	gnl|BL_ORD_ID|92933 transcript_165409	1	1438	1436	2004	963	2400	4236	4808
transcript_14565	gnl|BL_ORD_ID|11707 transcript_2360	431	2623	58	432	1443	3635	943	1317
transcript_145674	gnl|BL_ORD_ID|186 transcript_296	1	2474	2469	2888	1903	4376	4501	4920
transcript_145692	gnl|BL_ORD_ID|39315 transcript_84046	1	2115	2117	2584	1035	3149	3992	4459
transcript_145705	gnl|BL_ORD_ID|82483 transcript_149833	105	3910	3909	4447	2	3807	3936	4474
transcript_145706	gnl|BL_ORD_ID|5373 transcript_30520	1	1249	1253	1751	14	1257	1465	1964
transcript_145720	gnl|BL_ORD_ID|46440 transcript_95765	2	2152	2151	2560	80	2246	2644	3052
transcript_145730	gnl|BL_ORD_ID|55926 transcript_109714	1	1560	1561	2032	1	1564	1719	2190
transcript_145731	gnl|BL_ORD_ID|19358 transcript_54972	2	3833	3833	4244	12	3835	4725	5136
transcript_145773	gnl|BL_ORD_ID|35421 transcript_79438	1	2787	2788	4263	45	2831	2940	4424
transcript_145812	gnl|BL_ORD_ID|58460 transcript_113877	1	1939	1938	2913	309	2244	2346	3321

transcript_145818	gnl|BL_ORD_ID|45495 transcript_94208	1076	3325	1	1075	1621	3836	421	1484
transcript_14584	gnl|BL_ORD_ID|59148 transcript_114985	12	1694	1694	2608	3	1684	1899	2813
transcript_14584	gnl|BL_ORD_ID|96712 transcript_89489	20	2177	2174	2571	3	2160	2980	3378
transcript_14586	gnl|BL_ORD_ID|67995 transcript_127676	641	2617	9	643	1566	3539	2	635
transcript_145881	gnl|BL_ORD_ID|46585 transcript_95997	1	1545	1542	2938	1	1542	1986	3380
transcript_145888	gnl|BL_ORD_ID|21930 transcript_59230	1	1698	1698	2106	82	1779	1961	2367
transcript_145960	gnl|BL_ORD_ID|66735 transcript_125649	1	1521	1520	2855	2	1522	1694	3029
transcript_146007	gnl|BL_ORD_ID|29943 transcript_70546	133	1080	2	133	1109	2068	4	137
transcript_146051	gnl|BL_ORD_ID|76554 transcript_141573	1	2395	2390	3393	1	2393	2672	3675
transcript_146059	gnl|BL_ORD_ID|73217 transcript_136118	1085	4715	1	1084	1308	4939	2	1087
transcript_146067	gnl|BL_ORD_ID|24897 transcript_5269	1	1968	1964	2943	1	1960	2163	3142
transcript_1461	gnl|BL_ORD_ID|56335 transcript_110362	791	3923	95	793	1376	4507	2	700
transcript_146130	gnl|BL_ORD_ID|80329 transcript_146212	1091	2546	1	1091	1213	2667	1	1093
transcript_146144	gnl|BL_ORD_ID|96634 transcript_81061	1	1370	1365	2303	9	1368	1555	2505
transcript_146189	gnl|BL_ORD_ID|29013 transcript_69087	338	2149	4	339	1812	3626	2	337
transcript_146237	gnl|BL_ORD_ID|52386 transcript_103638	164	1383	5	164	854	2062	3	162
transcript_146237	gnl|BL_ORD_ID|62954 transcript_121069	164	1380	5	164	838	2049	2	161
transcript_14624	gnl|BL_ORD_ID|78285 transcript_14266	1	1575	1574	2522	1	1569	1675	2624
transcript_146299	gnl|BL_ORD_ID|11978 transcript_2890	102	3227	1	104	425	3561	162	262
transcript_146299	gnl|BL_ORD_ID|1165 transcript_2091	102	3227	1	104	295	3428	29	132
transcript_146309	gnl|BL_ORD_ID|71123 transcript_132780	19	1954	1953	3437	2	1916	2612	4090
transcript_146356	gnl|BL_ORD_ID|28820 transcript_68784	712	3031	77	713	1002	3314	137	773
transcript_146428	gnl|BL_ORD_ID|90342 transcript_162787	756	2565	8	760	1132	2920	90	832
transcript_146501	gnl|BL_ORD_ID|11771 transcript_2479	108	3479	1	111	227	3605	1	116
transcript_146501	gnl|BL_ORD_ID|11752 transcript_2445	108	3479	1	111	227	3590	1	116
transcript_146505	gnl|BL_ORD_ID|25803 transcript_63934	16	2594	2595	3127	2	2581	2767	3299
transcript_146642	gnl|BL_ORD_ID|76151 transcript_140935	1	2174	2170	2892	202	2374	3075	3800
transcript_146663	gnl|BL_ORD_ID|79782 transcript_145290	1	2064	2065	2572	9	2054	2160	2677
transcript_146704	gnl|BL_ORD_ID|52980 transcript_104574	313	1829	9	315	407	1921	1	307
transcript_146706	gnl|BL_ORD_ID|32333 transcript_74487	2099	4940	1	2101	2943	5783	6	2107
transcript_146803	gnl|BL_ORD_ID|51810 transcript_9894	1	2452	2452	2690	1	2480	2587	2828
transcript_146803	gnl|BL_ORD_ID|51803 transcript_9882	2	2452	2452	2676	83	2534	2641	2868
transcript_146803	gnl|BL_ORD_ID|51510 transcript_9241	1	2452	2452	2690	1	2453	2560	2801
transcript_14689	gnl|BL_ORD_ID|64642 transcript_11293	18	2130	2131	2667	1	2113	2302	2838

transcript_146970	gnl|BL_ORD_ID|35286 transcript_79237	25	2904	2903	3507	24	2868	2983	3601
transcript_14702	gnl|BL_ORD_ID|87166 transcript_157727	1252	2609	1	1251	1380	2739	4	1253
transcript_147028	gnl|BL_ORD_ID|93442 transcript_166240	305	3411	1	306	2586	5691	2175	2480
transcript_147037	gnl|BL_ORD_ID|33186 transcript_75809	1	1645	1643	2621	716	2353	2687	3663
transcript_14709	gnl|BL_ORD_ID|48701 transcript_99322	1	2217	2216	2602	1	2222	2519	2907
transcript_147091	gnl|BL_ORD_ID|79631 transcript_145048	1	1965	1963	3167	1	1962	2066	3270
transcript_147128	gnl|BL_ORD_ID|20833 transcript_57383	305	2446	76	304	619	2760	272	501
transcript_147188	gnl|BL_ORD_ID|23 transcript_31	2	2125	2124	2947	2	2135	5449	6271
transcript_147236	gnl|BL_ORD_ID|55168 transcript_108345	1	1845	1843	2225	2	1846	2764	3146
transcript_147345	gnl|BL_ORD_ID|3557 transcript_26671	287	1707	7	284	706	2094	2	273
transcript_147363	gnl|BL_ORD_ID|77067 transcript_142400	1	1308	1308	2184	103	1411	1855	2731
transcript_147363	gnl|BL_ORD_ID|73143 transcript_135983	1	1307	1308	2184	53	1360	1711	2596
transcript_147363	gnl|BL_ORD_ID|78509 transcript_14848	1	1307	1308	2184	71	1377	1728	2604
transcript_147432	gnl|BL_ORD_ID|22248 transcript_59752	1	1363	1362	1873	6	1356	2230	2731
transcript_147432	gnl|BL_ORD_ID|65114 transcript_12336	1	1363	1362	1873	2	1368	2118	2621
transcript_147432	gnl|BL_ORD_ID|51864 transcript_10021	1	1363	1362	1873	2	1372	2242	2747
transcript_147432	gnl|BL_ORD_ID|26607 transcript_65194	492	1873	63	494	876	2251	88	518
transcript_147432	gnl|BL_ORD_ID|32615 transcript_74943	492	1873	7	494	844	2217	1	486
transcript_147432	gnl|BL_ORD_ID|5107 transcript_29967	1	1527	1524	1873	2	1534	1650	1995
transcript_147435	gnl|BL_ORD_ID|28543 transcript_68341	2	2988	2990	3508	64	3050	3159	3660
transcript_14747	gnl|BL_ORD_ID|60839 transcript_117685	231	2542	2	230	1053	3364	6	237
transcript_147483	gnl|BL_ORD_ID|93405 transcript_166189	1	1723	1723	1983	387	2109	2227	2487
transcript_147485	gnl|BL_ORD_ID|88281 transcript_159539	1	2276	2273	2799	2	2281	2407	2934
transcript_147503	gnl|BL_ORD_ID|84546 transcript_153437	1	1048	1046	2083	2	1044	1436	2432
transcript_147503	gnl|BL_ORD_ID|434 transcript_743	1046	2083	1	1048	1597	2637	2	1059
transcript_147525	gnl|BL_ORD_ID|64410 transcript_10792	1	1325	1324	1704	53	1377	2436	2817
transcript_147590	gnl|BL_ORD_ID|12183 transcript_3292	2	2112	2113	3072	1	2110	2534	3491
transcript_147609	gnl|BL_ORD_ID|77715 transcript_12983	1	1504	1508	2378	1	1492	1817	2686
transcript_147667	gnl|BL_ORD_ID|49712 transcript_100924	1	1815	1816	2709	1042	2860	3294	4187
transcript_147682	gnl|BL_ORD_ID|49101 transcript_99950	1	2306	2302	3630	1	2292	2412	3739
transcript_147727	gnl|BL_ORD_ID|12673 transcript_4247	1	1987	1986	2675	78	2064	2710	3399
transcript_147747	gnl|BL_ORD_ID|37895 transcript_6498	350	2563	5	354	925	3118	1	349
transcript_147762	gnl|BL_ORD_ID|35332 transcript_79303	1	1617	1614	2739	6	1655	2718	3833
transcript_147849	gnl|BL_ORD_ID|41610 transcript_87803	1	1264	1260	2114	10	1269	1380	2253

transcript_147849	gnl|BL_ORD_ID|26156 transcript_64491	1	1264	1260	2114	49	1309	1428	2286
transcript_147849	gnl|BL_ORD_ID|95488 transcript_19565	1	1264	1260	2114	50	1313	1438	2313
transcript_147875	gnl|BL_ORD_ID|61495 transcript_118748	110	1811	1	111	649	2350	2	112
transcript_147877	gnl|BL_ORD_ID|65584 transcript_123743	1	1663	1661	2351	49	1711	2993	3700
transcript_147878	gnl|BL_ORD_ID|3399 transcript_26330	1	1087	1087	1689	10	1096	1255	1857
transcript_147878	gnl|BL_ORD_ID|5187 transcript_30141	1	1087	1090	1741	76	1153	1315	1966
transcript_147878	gnl|BL_ORD_ID|90671 transcript_163290	1	1087	1090	1770	6	1090	1252	1932
transcript_147917	gnl|BL_ORD_ID|76006 transcript_140699	481	1777	6	484	1048	2342	2	476
transcript_147919	gnl|BL_ORD_ID|80390 transcript_146314	2230	4939	1	2230	2348	5056	12	2239
transcript_147925	gnl|BL_ORD_ID|56097 transcript_110003	634	4022	72	636	738	4131	1	566
transcript_14794	gnl|BL_ORD_ID|35699 transcript_79866	11	1573	1573	2596	3	1565	2156	3175
transcript_147941	gnl|BL_ORD_ID|3971 transcript_27571	187	1884	1	187	364	2064	1	184
transcript_147941	gnl|BL_ORD_ID|1676 transcript_22556	187	1884	1	187	564	2258	2	187
transcript_147946	gnl|BL_ORD_ID|20647 transcript_57071	2	2428	2427	2861	3	2423	2549	2983
transcript_148025	gnl|BL_ORD_ID|3739 transcript_27065	292	1852	6	293	396	1956	2	289
transcript_148025	gnl|BL_ORD_ID|24355 transcript_63222	292	1852	47	293	362	1919	23	255
transcript_148025	gnl|BL_ORD_ID|52298 transcript_103496	292	1852	62	293	619	2179	282	512
transcript_148040	gnl|BL_ORD_ID|12000 transcript_2937	11	3346	3346	3514	5	3339	3218	3386
transcript_148042	gnl|BL_ORD_ID|68039 transcript_127753	1	1491	1492	2879	1896	3388	3571	4954
transcript_148068	gnl|BL_ORD_ID|781 transcript_1362	322	3857	1	323	425	3962	2	325
transcript_14813	gnl|BL_ORD_ID|64531 transcript_11050	443	2609	67	445	628	2793	148	526
transcript_148309	gnl|BL_ORD_ID|63527 transcript_122001	1	1161	1162	2283	3	1163	1410	2531
transcript_14832	gnl|BL_ORD_ID|90646 transcript_163246	1	2153	2152	2602	20	2168	2282	2732
transcript_148381	gnl|BL_ORD_ID|94085 transcript_167255	134	1320	2	137	851	2032	1	137
transcript_148384	gnl|BL_ORD_ID|11821 transcript_2588	22	3054	3051	3526	2	3033	3159	3634
transcript_148389	gnl|BL_ORD_ID|57450 transcript_112206	1045	2831	1	1048	1492	3276	320	1357
transcript_148389	gnl|BL_ORD_ID|62876 transcript_120947	1045	2831	1	1048	1180	2983	1	1045
transcript_148389	gnl|BL_ORD_ID|37942 transcript_6590	1045	2831	1	1049	1287	3071	109	1153
transcript_148389	gnl|BL_ORD_ID|57512 transcript_112316	1045	2831	1	1049	1174	2960	1	1040
transcript_148405	gnl|BL_ORD_ID|24566 transcript_4572	97	1718	1	100	1693	3315	74	171
transcript_148405	gnl|BL_ORD_ID|55645 transcript_109201	97	1714	1	100	1608	3226	3	102
transcript_148405	gnl|BL_ORD_ID|96481 transcript_67586	97	1716	1	100	1692	3312	78	177
transcript_148438	gnl|BL_ORD_ID|79583 transcript_144976	362	1617	8	367	1381	2638	20	379
transcript_148438	gnl|BL_ORD_ID|60564 transcript_117238	362	1617	68	367	1048	2303	1	300

transcript_148438	gnl|BL_ORD_ID|78021 transcript_13678	362	1617	68	367	1304	2559	2	301
transcript_148438	gnl|BL_ORD_ID|45366 transcript_93979	362	1617	8	367	1366	2591	5	364
transcript_148454	gnl|BL_ORD_ID|36041 transcript_80419	1061	2244	1	1062	2379	3557	24	1082
transcript_148465	gnl|BL_ORD_ID|53865 transcript_106106	476	972	1	477	1321	1817	38	517
transcript_148472	gnl|BL_ORD_ID|91632 transcript_17076	146	1498	2	150	1108	2460	42	190
transcript_148472	gnl|BL_ORD_ID|72951 transcript_135691	146	1498	2	150	1078	2430	12	160
transcript_148484	gnl|BL_ORD_ID|67439 transcript_126775	245	3071	1	247	552	3377	2	248
transcript_148502	gnl|BL_ORD_ID|25745 transcript_63849	1798	3721	1	1799	3513	5443	1605	3402
transcript_148555	gnl|BL_ORD_ID|56554 transcript_110710	1	1251	1248	2147	125	1372	1484	2384
transcript_14858	gnl|BL_ORD_ID|64946 transcript_11936	1	2357	2356	2611	1	2357	2473	2727
transcript_148644	gnl|BL_ORD_ID|56806 transcript_111119	1	1203	1200	2106	2	1190	1306	2213
transcript_148658	gnl|BL_ORD_ID|5495 transcript_30767	121	1810	1	120	265	1943	1	120
transcript_148675	gnl|BL_ORD_ID|93710 transcript_166675	1	1414	1409	1791	676	2102	2271	2653
transcript_148675	gnl|BL_ORD_ID|21507 transcript_58538	1	1414	1409	1792	679	2105	2274	2656
transcript_148675	gnl|BL_ORD_ID|65213 transcript_12577	1	1414	1409	1805	677	2089	2258	2653
transcript_148698	gnl|BL_ORD_ID|56774 transcript_111071	2	3189	3188	3545	55	3247	3360	3717
transcript_148700	gnl|BL_ORD_ID|72352 transcript_134739	138	2449	1	139	515	2824	131	269
transcript_148700	gnl|BL_ORD_ID|86355 transcript_156373	138	2449	1	139	2370	4681	1986	2124
transcript_148700	gnl|BL_ORD_ID|59270 transcript_115182	138	2449	1	139	1546	3857	1162	1300
transcript_148700	gnl|BL_ORD_ID|961 transcript_1708	138	2449	1	139	1523	3836	1139	1277
transcript_148700	gnl|BL_ORD_ID|635 transcript_1085	138	2449	1	139	1772	4086	1388	1526
transcript_148826	gnl|BL_ORD_ID|63456 transcript_121899	1	1571	1570	2489	67	1635	1937	2857
transcript_148835	gnl|BL_ORD_ID|59616 transcript_115739	524	1717	64	526	1163	2352	330	792
transcript_148836	gnl|BL_ORD_ID|78019 transcript_13669	1	1552	1551	2311	1	1553	1818	2581
transcript_148866	gnl|BL_ORD_ID|38035 transcript_6781	13	1840	1839	2277	2	1827	1929	2368
transcript_148878	gnl|BL_ORD_ID|25850 transcript_64022	1	2361	2362	2832	1	2360	3834	4304
transcript_148879	gnl|BL_ORD_ID|87620 transcript_158473	1669	3901	124	1668	1914	4146	1	1544
transcript_149014	gnl|BL_ORD_ID|12027 transcript_2986	1	1339	1338	2381	2	1329	2513	3558
transcript_149014	gnl|BL_ORD_ID|92312 transcript_164396	1173	2406	1	1174	1281	2518	2	1164
transcript_149061	gnl|BL_ORD_ID|24021 transcript_62692	227	2400	1	229	1185	3363	116	349
transcript_149184	gnl|BL_ORD_ID|90351 transcript_162804	440	1396	46	443	921	1877	1	394
transcript_149261	gnl|BL_ORD_ID|8514 transcript_36924	7	508	505	1043	2	503	1082	1608
transcript_149261	gnl|BL_ORD_ID|8154 transcript_36206	505	1043	7	508	1082	1627	2	503
transcript_149261	gnl|BL_ORD_ID|89389 transcript_161309	7	508	505	1043	2	503	879	1406

transcript_149261	gnl|BL_ORD_ID|6572 transcript_33045	505	1030	9	508	1162	1714	9	550
transcript_149261	gnl|BL_ORD_ID|32720 transcript_75120	7	508	505	1043	1	501	1356	1881
transcript_149283	gnl|BL_ORD_ID|66340 transcript_125005	1	1305	1300	2334	869	2155	2454	3490
transcript_149283	gnl|BL_ORD_ID|56814 transcript_111133	1	1466	1462	2274	693	2140	3245	4059
transcript_149330	gnl|BL_ORD_ID|52065 transcript_10443	357	2382	5	359	801	2808	18	372
transcript_149351	gnl|BL_ORD_ID|38207 transcript_7124	394	2680	7	397	814	3100	46	433
transcript_149385	gnl|BL_ORD_ID|48914 transcript_99662	1	868	863	998	37	901	1093	1228
transcript_149417	gnl|BL_ORD_ID|53424 transcript_105327	1	1359	1357	2150	118	1492	1962	2754
transcript_149417	gnl|BL_ORD_ID|20195 transcript_56329	1	1359	1357	2191	58	1433	1697	2530
transcript_149417	gnl|BL_ORD_ID|53023 transcript_104650	1	1359	1357	2191	274	1636	1900	2733
transcript_149531	gnl|BL_ORD_ID|48984 transcript_99772	241	2356	31	244	1157	3273	1	216
transcript_14957	gnl|BL_ORD_ID|57448 transcript_112204	208	2580	1	209	365	2737	1	209
transcript_14957	gnl|BL_ORD_ID|77739 transcript_13033	1	2195	2194	2578	1	2196	2302	2686
transcript_149630	gnl|BL_ORD_ID|31720 transcript_73418	1	1734	1733	2338	27	1769	2776	3383
transcript_149770	gnl|BL_ORD_ID|38262 transcript_7234	2	2144	2143	2471	80	2220	2681	3009
transcript_149773	gnl|BL_ORD_ID|67363 transcript_126660	1030	2634	1	1033	2192	3796	919	1951
transcript_149773	gnl|BL_ORD_ID|86160 transcript_156058	1030	2591	1	1033	2461	4022	1190	2220
transcript_149777	gnl|BL_ORD_ID|35876 transcript_80164	1	1370	1370	2292	604	1974	2084	3005
transcript_14980	gnl|BL_ORD_ID|36464 transcript_81129	372	2598	43	374	782	3008	2	333
transcript_149803	gnl|BL_ORD_ID|44243 transcript_92089	138	1426	12	139	326	1612	99	226
transcript_149825	gnl|BL_ORD_ID|30687 transcript_71747	1	1820	1819	2460	3	1828	3542	4180
transcript_149836	gnl|BL_ORD_ID|9138 transcript_38188	1	1175	1173	1421	2	1178	1283	1544
transcript_1499	gnl|BL_ORD_ID|65 transcript_91	205	3906	1	206	1808	5509	1	206
transcript_149917	gnl|BL_ORD_ID|80675 transcript_146760	19	3197	3193	3927	1	3187	3438	4161
transcript_149972	gnl|BL_ORD_ID|77190 transcript_142610	247	1195	3	252	525	1473	3	252
transcript_150003	gnl|BL_ORD_ID|32706 transcript_75095	1	1179	1175	1748	3	1185	1832	2409
transcript_150003	gnl|BL_ORD_ID|91202 transcript_16125	1	1179	1175	1820	4	1188	1883	2530
transcript_150003	gnl|BL_ORD_ID|21479 transcript_58481	1	1179	1175	1820	3	1185	3632	4277
transcript_150003	gnl|BL_ORD_ID|78446 transcript_14685	1	1179	1175	1820	3	1185	1880	2527
transcript_150023	gnl|BL_ORD_ID|48549 transcript_99072	1	1266	1263	2043	674	1937	2097	2877
transcript_150023	gnl|BL_ORD_ID|92707 transcript_165052	1	1266	1263	2043	276	1541	1701	2488
transcript_150023	gnl|BL_ORD_ID|11892 transcript_2716	1	1266	1263	2043	1388	2653	2813	3600
transcript_150023	gnl|BL_ORD_ID|86590 transcript_156775	1	1266	1263	2043	943	2209	2369	3164
transcript_150031	gnl|BL_ORD_ID|67512 transcript_126911	338	1048	7	342	1003	1714	2	335

transcript_150038	gnl|BL_ORD_ID|25849 transcript_64020	134	1405	1	135	542	1813	201	335
transcript_150039	gnl|BL_ORD_ID|33771 transcript_76754	205	1602	4	210	1153	2554	2	208
transcript_150099	gnl|BL_ORD_ID|72381 transcript_134777	1	1286	1285	2230	19	1304	1412	2357
transcript_150124	gnl|BL_ORD_ID|1722 transcript_22666	144	2060	1	144	285	2201	2	148
transcript_150129	gnl|BL_ORD_ID|72638 transcript_135181	1	1914	1913	2647	1158	3074	3271	4005
transcript_150161	gnl|BL_ORD_ID|91929 transcript_163780	12	1301	1298	1936	2	1298	1947	2586
transcript_150180	gnl|BL_ORD_ID|101 transcript_149	1	3075	3072	3809	1073	4170	4421	5158
transcript_150238	gnl|BL_ORD_ID|61843 transcript_119279	299	1182	3	299	2345	3229	840	1136
transcript_15026	gnl|BL_ORD_ID|11677 transcript_2298	1	1992	1991	2550	2	1992	2872	3428
transcript_150365	gnl|BL_ORD_ID|78514 transcript_14854	1	1423	1420	1832	2	1425	2191	2603
transcript_150403	gnl|BL_ORD_ID|61266 transcript_118377	189	1053	29	189	264	1127	1	161
transcript_150416	gnl|BL_ORD_ID|12232 transcript_3385	2	2327	2325	3012	1	2326	2804	3490
transcript_15042	gnl|BL_ORD_ID|93857 transcript_166891	501	2593	6	506	768	2859	1	504
transcript_150467	gnl|BL_ORD_ID|95177 transcript_18881	1	1747	1744	2158	1	1747	1853	2268
transcript_150577	gnl|BL_ORD_ID|31528 transcript_73092	2	2057	2056	2444	4	2058	4512	4900
transcript_150627	gnl|BL_ORD_ID|24884 transcript_5243	204	2200	1	205	1264	3260	803	1007
transcript_150627	gnl|BL_ORD_ID|17762 transcript_52422	204	2200	1	205	1116	3112	655	859
transcript_150627	gnl|BL_ORD_ID|45193 transcript_93685	204	2194	1	205	999	2988	538	742
transcript_150627	gnl|BL_ORD_ID|25399 transcript_6331	204	2166	1	205	1181	3143	720	924
transcript_150627	gnl|BL_ORD_ID|38005 transcript_6725	204	2200	1	205	1033	3029	572	776
transcript_150655	gnl|BL_ORD_ID|59498 transcript_115554	249	1247	45	249	1999	3003	1	205
transcript_150702	gnl|BL_ORD_ID|35451 transcript_79485	1029	2321	1	1032	2020	3318	814	1857
transcript_150711	gnl|BL_ORD_ID|51201 transcript_8595	1	1509	1508	2064	10	1518	2403	2959
transcript_15072	gnl|BL_ORD_ID|19501 transcript_55230	595	2541	6	596	3025	4971	1	600
transcript_150726	gnl|BL_ORD_ID|51375 transcript_8981	1	1117	1115	2228	511	1627	1846	2959
transcript_150726	gnl|BL_ORD_ID|25421 transcript_6377	1	1117	1115	2227	716	1832	2051	3164
transcript_150726	gnl|BL_ORD_ID|38060 transcript_6825	1115	2227	1	1117	1977	3090	619	1758
transcript_150782	gnl|BL_ORD_ID|40787 transcript_86447	1	1510	1510	1884	1	1522	1795	2169
transcript_150788	gnl|BL_ORD_ID|38669 transcript_8131	1	1867	1867	2565	272	2138	2248	2946
transcript_150788	gnl|BL_ORD_ID|38674 transcript_8148	1	1867	1867	2567	310	2176	2286	2986
transcript_150788	gnl|BL_ORD_ID|38127 transcript_6959	1	1867	1872	2571	8	2040	2167	2913
transcript_150788	gnl|BL_ORD_ID|60595 transcript_117286	1	1867	1867	2570	625	2491	2601	3304
transcript_150788	gnl|BL_ORD_ID|38507 transcript_7757	1	1867	1867	2571	341	2207	2317	3024
transcript_150801	gnl|BL_ORD_ID|63520 transcript_121991	282	2946	1	281	471	3106	12	292

transcript_150801	gnl|BL_ORD_ID|96924 transcript_110976	1130	2933	1	1132	2096	3871	30	1161
transcript_150815	gnl|BL_ORD_ID|86871 transcript_157234	468	2251	74	468	2463	4246	1965	2360
transcript_15082	gnl|BL_ORD_ID|529 transcript_896	1261	2601	1	1264	2828	4169	1	1277
transcript_15083	gnl|BL_ORD_ID|65324 transcript_12819	1	1949	1950	2588	1	1948	2066	2704
transcript_150849	gnl|BL_ORD_ID|31717 transcript_73414	1	1262	1261	1789	364	1617	2002	2524
transcript_150880	gnl|BL_ORD_ID|41857 transcript_88202	1287	3266	127	1287	2647	4639	2	1166
transcript_15089	gnl|BL_ORD_ID|58416 transcript_113800	1	2048	2044	2584	686	2720	2852	3391
transcript_150893	gnl|BL_ORD_ID|43486 transcript_90794	410	2618	61	409	1086	3298	1	349
transcript_150910	gnl|BL_ORD_ID|51868 transcript_10026	1	1961	1957	2353	3	1984	2467	2862
transcript_15092	gnl|BL_ORD_ID|91966 transcript_163835	578	2617	67	579	617	2656	1	514
transcript_150924	gnl|BL_ORD_ID|96842 transcript_102945	1088	2243	10	1088	1236	2407	5	1100
transcript_150924	gnl|BL_ORD_ID|80454 transcript_146402	1088	2243	1	1088	1224	2379	1	1088
transcript_150971	gnl|BL_ORD_ID|74747 transcript_138660	1400	3424	1	1402	1829	3853	146	1549
transcript_150972	gnl|BL_ORD_ID|422 transcript_726	268	3092	1	269	1440	4259	273	541
transcript_150988	gnl|BL_ORD_ID|8902 transcript_37708	121	1469	1	125	233	1586	1	125
transcript_151013	gnl|BL_ORD_ID|17630 transcript_52197	1685	3844	1	1686	1966	4124	1	1696
transcript_151013	gnl|BL_ORD_ID|21190 transcript_58008	1685	3921	1	1686	1802	4038	1	1696
transcript_151025	gnl|BL_ORD_ID|75615 transcript_140053	152	1501	5	155	366	1718	1	152
transcript_151049	gnl|BL_ORD_ID|36968 transcript_81912	1	1957	1953	2266	1	1957	2098	2411
transcript_151062	gnl|BL_ORD_ID|21435 transcript_58411	620	2763	73	620	684	2831	1	550
transcript_151116	gnl|BL_ORD_ID|10281 transcript_40391	451	1251	9	453	596	1399	39	496
transcript_151231	gnl|BL_ORD_ID|150 transcript_227	1	1673	1668	2467	6	1702	4241	5040
transcript_151255	gnl|BL_ORD_ID|73844 transcript_137183	121	1494	1	122	232	1605	1	122
transcript_151264	gnl|BL_ORD_ID|70943 transcript_132494	1	1274	1269	1422	47	1326	1472	1625
transcript_151264	gnl|BL_ORD_ID|89801 transcript_161966	1	1274	1269	1422	100	1369	1515	1668
transcript_151289	gnl|BL_ORD_ID|428 transcript_732	1096	2658	1	1101	2629	4178	8	1108
transcript_151289	gnl|BL_ORD_ID|405 transcript_691	1096	2658	1	1101	2624	4185	2	1102
transcript_151289	gnl|BL_ORD_ID|20336 transcript_56544	1096	2658	1	1101	2405	3966	2	1103
transcript_151289	gnl|BL_ORD_ID|17613 transcript_52169	1096	2658	106	1101	2517	4082	1	995
transcript_151303	gnl|BL_ORD_ID|788 transcript_1374	1	1618	1616	2469	1	1607	2037	2890
transcript_151309	gnl|BL_ORD_ID|28488 transcript_68250	1	1683	1681	1960	1	1699	2147	2427
transcript_151322	gnl|BL_ORD_ID|47912 transcript_98052	1	1318	1318	2459	42	1359	1628	2770
transcript_151350	gnl|BL_ORD_ID|46545 transcript_95933	1073	2394	107	1076	1309	2630	28	1005
transcript_15139	gnl|BL_ORD_ID|34823 transcript_78486	221	2582	17	224	435	2796	1	208

transcript_15139	gnl|BL_ORD_ID|42177 transcript_88678	221	2532	2	221	472	2783	40	259
transcript_151422	gnl|BL_ORD_ID|29133 transcript_69263	1084	2976	1	1083	1240	3131	2	1096
transcript_151439	gnl|BL_ORD_ID|19204 transcript_54727	1606	3227	1	1605	3220	4841	642	2239
transcript_151458	gnl|BL_ORD_ID|18384 transcript_53399	1247	2661	1	1249	4612	6027	3227	4475
transcript_151469	gnl|BL_ORD_ID|39723 transcript_84722	1	1446	1445	1725	1	1432	1534	1814
transcript_151527	gnl|BL_ORD_ID|69123 transcript_129514	1211	2466	10	1210	1449	2704	2	1202
transcript_151585	gnl|BL_ORD_ID|97423 transcript_158105	10	1458	1458	2062	52	1500	1813	2417
transcript_151604	gnl|BL_ORD_ID|30403 transcript_71296	2	2131	2132	2702	79	2220	2856	3427
transcript_151623	gnl|BL_ORD_ID|45597 transcript_94367	244	2658	1	245	413	2830	4	248
transcript_151623	gnl|BL_ORD_ID|51723 transcript_9704	244	2757	1	245	356	2873	8	252
transcript_151623	gnl|BL_ORD_ID|63540 transcript_122022	244	2757	1	245	382	2899	6	250
transcript_151631	gnl|BL_ORD_ID|65536 transcript_123670	1	1048	1045	1401	1	1063	1627	1984
transcript_1517	gnl|BL_ORD_ID|617 transcript_1056	2004	3941	123	2003	2079	4016	2	1880
transcript_151707	gnl|BL_ORD_ID|38175 transcript_7058	1	2164	2159	2589	3	2176	2557	2987
transcript_151707	gnl|BL_ORD_ID|51314 transcript_8866	1	2164	2159	2493	49	2212	2593	2927
transcript_151707	gnl|BL_ORD_ID|38375 transcript_7496	1	2164	2159	2573	2	2165	2546	2960
transcript_151707	gnl|BL_ORD_ID|46256 transcript_95469	1	2164	2159	2560	5	2168	2297	2698
transcript_151797	gnl|BL_ORD_ID|90021 transcript_162311	1216	2508	1	1220	2295	3585	864	2081
transcript_151797	gnl|BL_ORD_ID|12060 transcript_3059	1216	2562	1	1220	2196	3541	761	1980
transcript_15181	gnl|BL_ORD_ID|40156 transcript_85403	10	2154	2153	2575	3	2150	2604	3027
transcript_151828	gnl|BL_ORD_ID|1003 transcript_1785	2	3573	3573	3736	49	3618	3718	3881
transcript_151851	gnl|BL_ORD_ID|46638 transcript_96075	1	1470	1468	1799	2	1471	1580	1911
transcript_151939	gnl|BL_ORD_ID|79502 transcript_144850	1	1529	1528	2164	1	1529	4946	5582
transcript_151939	gnl|BL_ORD_ID|90033 transcript_162331	1	1436	1437	2191	1	1448	1556	2310
transcript_151951	gnl|BL_ORD_ID|95981 transcript_20764	1	1832	1829	2133	1	1862	2033	2338
transcript_151986	gnl|BL_ORD_ID|36176 transcript_80650	170	1313	37	171	4158	5301	2	136
transcript_152014	gnl|BL_ORD_ID|38122 transcript_6951	1100	2751	1	1103	1429	3093	1	1111
transcript_152064	gnl|BL_ORD_ID|91424 transcript_16619	1	1919	1915	2211	104	2023	2190	2486
transcript_152068	gnl|BL_ORD_ID|42020 transcript_88447	1	1443	1443	2313	32	1489	1616	2483
transcript_152096	gnl|BL_ORD_ID|51743 transcript_9730	1	1519	1519	2187	536	2054	2213	2875
transcript_152252	gnl|BL_ORD_ID|94637 transcript_17614	1	1238	1236	1846	1	1333	1598	2254
transcript_152252	gnl|BL_ORD_ID|2474 transcript_24285	1	1238	1236	1846	1	1237	1484	2105
transcript_152252	gnl|BL_ORD_ID|44988 transcript_93337	1	1238	1236	1846	1	1234	1481	2105
transcript_152252	gnl|BL_ORD_ID|95956 transcript_20702	1	1238	1236	1846	1	1237	1484	2104

transcript_152252	gnl|BL_ORD_ID|84532 transcript_153420	1	1238	1236	1818	1	1237	1484	2053
transcript_152252	gnl|BL_ORD_ID|1404 transcript_21887	1	1238	1236	1847	1	1237	1484	2094
transcript_152258	gnl|BL_ORD_ID|89248 transcript_161083	1274	2569	1	1275	1412	2707	1	1273
transcript_152265	gnl|BL_ORD_ID|74738 transcript_138650	290	1066	40	293	913	1691	3	256
transcript_152314	gnl|BL_ORD_ID|35995 transcript_80349	10	1413	1411	2157	132	1533	2513	3248
transcript_15251	gnl|BL_ORD_ID|64732 transcript_11486	1	2263	2261	2581	33	2295	2469	2789
transcript_15251	gnl|BL_ORD_ID|17617 transcript_52175	1	2345	2342	2580	1	2318	2490	2728
transcript_152582	gnl|BL_ORD_ID|46934 transcript_96525	2	2242	2242	2516	44	2258	2375	2648
transcript_152638	gnl|BL_ORD_ID|28038 transcript_67485	169	2449	1	168	518	2810	113	280
transcript_15264	gnl|BL_ORD_ID|42653 transcript_89449	225	2393	1	225	1087	3207	1	223
transcript_152671	gnl|BL_ORD_ID|27512 transcript_66618	1	2532	2531	4158	2	2534	3705	5359
transcript_152683	gnl|BL_ORD_ID|57375 transcript_112075	1	1309	1306	1881	114	1423	1535	2108
transcript_152732	gnl|BL_ORD_ID|37333 transcript_82489	15	2982	2980	3456	2	2945	3220	3696
transcript_15276	gnl|BL_ORD_ID|47312 transcript_97133	203	2582	1	204	402	2786	82	284
transcript_152770	gnl|BL_ORD_ID|32817 transcript_75255	189	2585	14	191	1133	3530	783	960
transcript_152770	gnl|BL_ORD_ID|34272 transcript_77582	189	2585	14	191	365	2758	15	192
transcript_152779	gnl|BL_ORD_ID|4398 transcript_28493	1	1499	1496	1915	1	1501	1613	2032
transcript_152813	gnl|BL_ORD_ID|37991 transcript_6689	1	1591	1588	3031	1	1589	1754	3195
transcript_152821	gnl|BL_ORD_ID|89600 transcript_161647	1	1251	1248	1778	1	1250	1470	2000
transcript_152845	gnl|BL_ORD_ID|12130 transcript_3200	1	1808	1808	3210	90	1897	2004	3404
transcript_152845	gnl|BL_ORD_ID|27010 transcript_65844	1	1808	1808	3210	92	1876	1983	3383
transcript_152845	gnl|BL_ORD_ID|70745 transcript_132174	1	1808	1808	3210	24	1841	1948	3348
transcript_152845	gnl|BL_ORD_ID|92733 transcript_165090	1	1808	1808	3210	13	1831	1938	3301
transcript_152852	gnl|BL_ORD_ID|47369 transcript_97214	206	3184	1	206	1940	4922	1567	1772
transcript_15286	gnl|BL_ORD_ID|65091 transcript_12279	1	2084	2083	2546	1	2109	2256	2717
transcript_152883	gnl|BL_ORD_ID|46241 transcript_95448	151	4731	1	151	310	4896	1	152
transcript_152909	gnl|BL_ORD_ID|96232 transcript_21282	186	2229	10	187	282	2324	2	179
transcript_152958	gnl|BL_ORD_ID|30620 transcript_71646	1	1087	1087	1504	1	1157	1312	1755
transcript_152958	gnl|BL_ORD_ID|80235 transcript_146046	1	1087	1087	1504	201	1299	1449	1874
transcript_152980	gnl|BL_ORD_ID|534 transcript_904	2	2286	2283	2890	3	2289	3486	4093
transcript_152981	gnl|BL_ORD_ID|95277 transcript_19097	103	2068	1	104	371	2337	36	139
transcript_152981	gnl|BL_ORD_ID|90982 transcript_15645	103	2068	1	104	392	2358	58	161
transcript_15302	gnl|BL_ORD_ID|86615 transcript_156813	221	2572	2	221	634	2980	91	310
transcript_153076	gnl|BL_ORD_ID|31121 transcript_72472	208	2506	2	211	385	2682	14	223

transcript_153121	gnl|BL_ORD_ID|90590 transcript_163161	1075	2239	1	1074	1189	2353	4	1077
transcript_153131	gnl|BL_ORD_ID|63802 transcript_122447	322	2138	80	326	406	2222	2	246
transcript_153157	gnl|BL_ORD_ID|43978 transcript_91652	1	1520	1517	2417	1	1525	2035	2943
transcript_153185	gnl|BL_ORD_ID|36769 transcript_81604	108	3113	1	110	291	3298	2	112
transcript_153189	gnl|BL_ORD_ID|41943 transcript_88332	1	1310	1308	2036	1	1305	1501	2228
transcript_153257	gnl|BL_ORD_ID|37343 transcript_82508	1	2197	2198	2524	1220	3417	3535	3863
transcript_153257	gnl|BL_ORD_ID|68439 transcript_128415	1	2197	2198	2523	959	3156	3274	3601
transcript_153257	gnl|BL_ORD_ID|61251 transcript_118351	1	2197	2198	2523	1118	3315	3433	3760
transcript_153257	gnl|BL_ORD_ID|1088 transcript_1960	1	2197	2198	2524	1114	3311	3429	3757
transcript_153257	gnl|BL_ORD_ID|22016 transcript_59387	1	2197	2198	2513	1038	3235	3353	3668
transcript_153272	gnl|BL_ORD_ID|38321 transcript_7373	1	2013	2018	2217	609	2620	2831	3031
transcript_153272	gnl|BL_ORD_ID|46557 transcript_95956	1	2013	2018	2209	359	2367	2577	2769
transcript_153274	gnl|BL_ORD_ID|73158 transcript_136006	1	2429	2430	2663	1	2416	2534	2767
transcript_153274	gnl|BL_ORD_ID|51798 transcript_9861	1	2429	2430	2739	1	2430	2549	2859
transcript_153351	gnl|BL_ORD_ID|36340 transcript_80933	1	1275	1273	1912	1	1271	1385	2003
transcript_153420	gnl|BL_ORD_ID|84106 transcript_152694	1	1238	1235	2053	1	1238	1513	2356
transcript_153437	gnl|BL_ORD_ID|434 transcript_743	1167	2432	1	1169	1328	2637	1	1186
transcript_1535	gnl|BL_ORD_ID|35278 transcript_79222	17	2024	2021	3931	2	1992	2987	4897
transcript_153537	gnl|BL_ORD_ID|1086 transcript_1946	1	3193	3192	3474	2	3194	3389	3671
transcript_153537	gnl|BL_ORD_ID|28090 transcript_67560	131	3193	3192	3474	1	3062	3257	3543
transcript_153537	gnl|BL_ORD_ID|67621 transcript_127080	1	3193	3192	3391	1	3204	3399	3597
transcript_153610	gnl|BL_ORD_ID|38241 transcript_7195	2	2418	2419	2978	1	2414	2536	3095
transcript_153653	gnl|BL_ORD_ID|85256 transcript_154571	410	4092	67	410	553	4236	2	349
transcript_15367	gnl|BL_ORD_ID|58965 transcript_114684	101	2008	2007	2574	3	1918	2497	3065
transcript_153697	gnl|BL_ORD_ID|2961 transcript_25346	1	1771	1767	1940	1	1772	1876	2049
transcript_153698	gnl|BL_ORD_ID|89686 transcript_161795	1354	3020	1	1357	1463	3128	24	1358
transcript_153705	gnl|BL_ORD_ID|79657 transcript_145089	1	2252	2249	2878	1	2256	2366	2996
transcript_15372	gnl|BL_ORD_ID|54727 transcript_107556	255	2518	38	256	477	2741	2	216
transcript_153722	gnl|BL_ORD_ID|12236 transcript_3390	159	2212	2210	3421	75	2128	2269	3480
transcript_153768	gnl|BL_ORD_ID|59222 transcript_115104	145	1481	1	147	959	2296	27	179
transcript_153768	gnl|BL_ORD_ID|50868 transcript_102792	145	1481	1	145	434	1769	55	219
transcript_153820	gnl|BL_ORD_ID|55239 transcript_108474	1362	3173	1	1361	1943	3754	381	1741
transcript_153820	gnl|BL_ORD_ID|58153 transcript_113338	200	3173	1	200	595	3570	293	492
transcript_153826	gnl|BL_ORD_ID|95481 transcript_19551	1	1228	1228	2194	1	1230	1384	2351

transcript_153856	gnl|BL_ORD_ID|37948 transcript_6599	163	2345	1	166	922	3106	183	347
transcript_153856	gnl|BL_ORD_ID|38500 transcript_7741	163	2362	1	166	847	3048	108	272
transcript_153857	gnl|BL_ORD_ID|70808 transcript_132271	241	2397	64	243	637	2797	225	405
transcript_153857	gnl|BL_ORD_ID|64884 transcript_11796	241	2397	64	243	536	2695	124	304
transcript_153857	gnl|BL_ORD_ID|96365 transcript_56534	241	2397	64	243	678	2838	266	446
transcript_153857	gnl|BL_ORD_ID|64759 transcript_11557	241	2397	64	243	532	2693	121	301
transcript_153857	gnl|BL_ORD_ID|52169 transcript_10668	241	2397	64	243	525	2686	113	293
transcript_153858	gnl|BL_ORD_ID|71903 transcript_134028	1	2258	2256	4248	9	2267	2385	4377
transcript_153889	gnl|BL_ORD_ID|55371 transcript_108704	1186	3627	117	1191	1186	3629	1	1075
transcript_153896	gnl|BL_ORD_ID|77569 transcript_143190	253	1034	43	254	3129	3910	2	213
transcript_153908	gnl|BL_ORD_ID|47176 transcript_96907	1	1182	1183	2029	2601	1421	1319	482
transcript_153908	gnl|BL_ORD_ID|24752 transcript_4985	1	1182	1183	2030	2368	1187	1085	239
transcript_153908	gnl|BL_ORD_ID|21751 transcript_58918	1	1182	1183	2030	9	1190	1292	2128
transcript_15392	gnl|BL_ORD_ID|46934 transcript_96525	1	2257	2257	2657	2	2258	2375	2775
transcript_153952	gnl|BL_ORD_ID|91895 transcript_163730	1	1759	1759	2637	2	1759	2451	3332
transcript_15397	gnl|BL_ORD_ID|77832 transcript_13238	1	1971	1971	2560	1	1971	2093	2683
transcript_15401	gnl|BL_ORD_ID|94007 transcript_167128	296	2576	85	295	360	2638	1	213
transcript_15401	gnl|BL_ORD_ID|41348 transcript_87358	296	2543	6	295	438	2687	2	291
transcript_154030	gnl|BL_ORD_ID|91771 transcript_163527	2	2150	2149	2514	4	2152	2733	3096
transcript_154037	gnl|BL_ORD_ID|25017 transcript_5526	1	1887	1885	2874	225	2111	2218	3209
transcript_154043	gnl|BL_ORD_ID|36870 transcript_81772	355	1217	4	354	1401	2263	6	375
transcript_154075	gnl|BL_ORD_ID|59992 transcript_116333	1	1361	1364	1706	1667	3027	3144	3485
transcript_154079	gnl|BL_ORD_ID|93359 transcript_166115	1	2436	2431	2902	3210	5647	5768	6242
transcript_154079	gnl|BL_ORD_ID|75764 transcript_140296	1	2436	2431	3009	1248	3679	3800	4379
transcript_154079	gnl|BL_ORD_ID|38859 transcript_83319	1	2436	2431	3010	2070	4506	4627	5206
transcript_154079	gnl|BL_ORD_ID|18 transcript_24	1	2436	2431	3010	3146	5581	5702	6282
transcript_1541	gnl|BL_ORD_ID|65492 transcript_123590	1	2462	2462	3921	9	2458	3247	4724
transcript_154100	gnl|BL_ORD_ID|585 transcript_993	662	3557	8	663	1211	4107	2	657
transcript_154104	gnl|BL_ORD_ID|37942 transcript_6590	2	2559	2559	3004	10	2563	2665	3110
transcript_154104	gnl|BL_ORD_ID|57450 transcript_112206	2	2559	2559	2965	216	2768	2870	3276
transcript_154121	gnl|BL_ORD_ID|50619 transcript_102393	441	2319	64	441	481	2351	2	379
transcript_154121	gnl|BL_ORD_ID|34270 transcript_77577	441	2319	70	441	546	2415	5	373
transcript_15414	gnl|BL_ORD_ID|64914 transcript_11872	154	2581	11	156	285	2716	2	147
transcript_15416	gnl|BL_ORD_ID|87420 transcript_158147	1	2129	2128	2573	1	2131	2570	3017

transcript_154176	gnl|BL_ORD_ID|60506 transcript_117155	1	2945	2943	3659	4	2952	3135	3849
transcript_154176	gnl|BL_ORD_ID|59094 transcript_114897	1	3038	3038	3666	2	3034	3153	3781
transcript_154183	gnl|BL_ORD_ID|695 transcript_1203	2	2693	2692	2988	2	2718	3657	3953
transcript_154199	gnl|BL_ORD_ID|84230 transcript_152918	166	3550	1	166	680	4065	312	477
transcript_1542	gnl|BL_ORD_ID|27148 transcript_66057	30	3511	3509	3915	96	3588	3697	4104
transcript_154310	gnl|BL_ORD_ID|64584 transcript_11170	1	2161	2161	2438	1	2163	2449	2728
transcript_154340	gnl|BL_ORD_ID|17613 transcript_52169	107	1846	1842	3226	1	1740	2697	4080
transcript_154340	gnl|BL_ORD_ID|405 transcript_691	1	1846	1842	3226	1	1847	2804	4183
transcript_154362	gnl|BL_ORD_ID|96002 transcript_20812	155	2120	1	160	389	2354	14	173
transcript_154374	gnl|BL_ORD_ID|93665 transcript_166601	1	1613	1609	2720	1	1614	2289	3401
transcript_154436	gnl|BL_ORD_ID|60547 transcript_117214	1	2045	2046	2287	2	2046	2180	2421
transcript_15448	gnl|BL_ORD_ID|51318 transcript_8873	538	2543	8	538	927	2928	2	532
transcript_15448	gnl|BL_ORD_ID|11618 transcript_2185	536	2542	8	538	1650	3652	2	532
transcript_15452	gnl|BL_ORD_ID|780 transcript_1361	117	2576	1	118	1531	3988	1	118
transcript_154524	gnl|BL_ORD_ID|24060 transcript_62749	1	2757	2754	3109	1	2757	2872	3227
transcript_154524	gnl|BL_ORD_ID|34037 transcript_77186	1	2757	2754	3109	1	2781	2895	3252
transcript_154525	gnl|BL_ORD_ID|53738 transcript_105894	607	2210	87	608	1117	2719	1	522
transcript_154550	gnl|BL_ORD_ID|41647 transcript_87879	1	1667	1667	2285	1	1669	1845	2463
transcript_154563	gnl|BL_ORD_ID|27812 transcript_67105	1202	2591	10	1205	1976	3365	2	1202
transcript_154563	gnl|BL_ORD_ID|64667 transcript_11346	2	2223	2222	2589	49	2269	2371	2754
transcript_154573	gnl|BL_ORD_ID|30670 transcript_71724	1	2573	2568	2818	301	2869	2991	3241
transcript_154583	gnl|BL_ORD_ID|65567 transcript_123721	411	1292	49	413	3109	3990	2	360
transcript_154625	gnl|BL_ORD_ID|87603 transcript_158449	1	1492	1490	1737	166	1652	2079	2328
transcript_154634	gnl|BL_ORD_ID|90755 transcript_15144	1	1442	1441	2257	84	1525	1632	2447
transcript_154634	gnl|BL_ORD_ID|80683 transcript_146775	1	1442	1441	2257	214	1657	1764	2576
transcript_154650	gnl|BL_ORD_ID|72481 transcript_134941	1	2313	2311	2755	2	2303	3159	3604
transcript_154650	gnl|BL_ORD_ID|17771 transcript_52437	23	2313	2311	2822	1	2291	3148	3660
transcript_154653	gnl|BL_ORD_ID|61079 transcript_118088	194	1572	25	195	443	1817	2	178
transcript_154673	gnl|BL_ORD_ID|31991 transcript_73892	1	1230	1230	2156	3	1232	1414	2342
transcript_154701	gnl|BL_ORD_ID|39925 transcript_85039	1	1616	1615	2190	2	1617	1730	2305
transcript_154701	gnl|BL_ORD_ID|96995 transcript_117150	1	1616	1615	2190	4	1620	1733	2308
transcript_154701	gnl|BL_ORD_ID|90728 transcript_15079	1	1616	1615	2190	219	1834	1947	2522
transcript_154701	gnl|BL_ORD_ID|77726 transcript_13011	1	1615	1615	2190	6	1662	1781	2367
transcript_154709	gnl|BL_ORD_ID|1531 transcript_22223	1	1080	1080	1764	10	1143	1254	1965

transcript_154709	gnl|BL_ORD_ID|17259 transcript_51616	1	1080	1080	1764	1	1088	1195	1877
transcript_154709	gnl|BL_ORD_ID|5558 transcript_30891	1	1080	1080	1764	2	1091	1198	1882
transcript_154709	gnl|BL_ORD_ID|6338 transcript_32561	1	1080	1080	1764	1	1086	1193	1877
transcript_154709	gnl|BL_ORD_ID|6213 transcript_32306	1	1080	1080	1764	1	1086	1193	1847
transcript_154709	gnl|BL_ORD_ID|81099 transcript_147432	1	1080	1080	1756	1	1081	1189	1873
transcript_154709	gnl|BL_ORD_ID|4240 transcript_28169	1	1080	1080	1764	2	1092	1199	1907
transcript_154724	gnl|BL_ORD_ID|51517 transcript_9248	1	2106	2101	2594	2	2105	2294	2788
transcript_154749	gnl|BL_ORD_ID|67583 transcript_127017	1	1971	1969	2119	1127	3097	3218	3367
transcript_154770	gnl|BL_ORD_ID|95478 transcript_19544	687	2356	70	688	742	2413	2	624
transcript_154778	gnl|BL_ORD_ID|65724 transcript_123975	1	2494	2493	3876	47	2566	3330	4712
transcript_154822	gnl|BL_ORD_ID|96365 transcript_56534	1	2163	2158	2615	55	2217	2348	2805
transcript_154822	gnl|BL_ORD_ID|72648 transcript_135198	1	2163	2158	2605	87	2241	2372	2818
transcript_154831	gnl|BL_ORD_ID|25103 transcript_5735	1	1802	1803	2567	180	1981	2424	3188
transcript_154831	gnl|BL_ORD_ID|27304 transcript_66300	1	1802	1803	2605	121	1921	2364	3166
transcript_154831	gnl|BL_ORD_ID|47749 transcript_97800	1	2088	2090	2605	121	2208	2790	3305
transcript_15484	gnl|BL_ORD_ID|54361 transcript_106916	1003	2578	1	1005	1420	2995	21	1026
transcript_154840	gnl|BL_ORD_ID|23795 transcript_62344	2	2215	2216	4178	1	2200	2306	4269
transcript_154840	gnl|BL_ORD_ID|171 transcript_271	2	2512	2512	4176	7	2500	3236	4899
transcript_154904	gnl|BL_ORD_ID|25277 transcript_6091	12	1506	1504	2903	2	1491	1596	3002
transcript_154904	gnl|BL_ORD_ID|25112 transcript_5755	1	1506	1504	2903	61	1562	1667	3073
transcript_154910	gnl|BL_ORD_ID|56779 transcript_111078	471	1798	61	467	1729	3063	1131	1537
transcript_154964	gnl|BL_ORD_ID|51685 transcript_9630	1	1146	1144	2097	131	1276	1925	2879
transcript_155040	gnl|BL_ORD_ID|76045 transcript_140758	1	1240	1241	2189	24	1288	1390	2336
transcript_155060	gnl|BL_ORD_ID|50821 transcript_102715	1	1042	1040	1845	1	1042	2962	3767
transcript_155116	gnl|BL_ORD_ID|31440 transcript_72967	138	1905	1	139	600	2367	308	444
transcript_155171	gnl|BL_ORD_ID|81648 transcript_148355	19	2259	2256	2611	2	2240	2708	3036
transcript_155199	gnl|BL_ORD_ID|27240 transcript_66206	384	2385	73	385	435	2435	1	309
transcript_155301	gnl|BL_ORD_ID|79772 transcript_145276	2	3775	3773	4701	53	3813	5110	6040
transcript_155310	gnl|BL_ORD_ID|78996 transcript_144039	167	2020	1	169	517	2382	243	411
transcript_155331	gnl|BL_ORD_ID|24671 transcript_4805	1	1537	1532	2594	373	1917	2091	3153
transcript_155332	gnl|BL_ORD_ID|39593 transcript_84499	1439	4164	1	1444	6641	9366	106	1548
transcript_155358	gnl|BL_ORD_ID|90571 transcript_163128	433	1971	9	433	536	2050	1	425
transcript_155366	gnl|BL_ORD_ID|2106 transcript_23456	1	1327	1323	1660	1	1386	1694	2045
transcript_155366	gnl|BL_ORD_ID|3977 transcript_27585	1	1327	1323	1678	1	1325	1720	2072

transcript_15538	gnl|BL_ORD_ID|69040 transcript_129369	1	1663	1662	2542	84	1747	3044	3925
transcript_155380	gnl|BL_ORD_ID|96076 transcript_20971	268	1839	66	267	764	2335	79	280
transcript_155426	gnl|BL_ORD_ID|12234 transcript_3387	2	2807	2803	3080	109	2914	3254	3532
transcript_155426	gnl|BL_ORD_ID|11730 transcript_2403	2	2807	2803	3111	104	2909	3248	3555
transcript_155426	gnl|BL_ORD_ID|12314 transcript_3533	1	2807	2803	3111	1	2807	3147	3455
transcript_155456	gnl|BL_ORD_ID|3602 transcript_26769	1	1374	1370	1566	431	1804	1921	2118
transcript_155471	gnl|BL_ORD_ID|59141 transcript_114972	1	1752	1752	2054	814	2568	3236	3538
transcript_155492	gnl|BL_ORD_ID|96860 transcript_104654	576	1679	75	579	863	1967	1	505
transcript_155544	gnl|BL_ORD_ID|31316 transcript_72779	1	1230	1228	1868	1	1230	1725	2365
transcript_155596	gnl|BL_ORD_ID|1039 transcript_1865	1	2358	2357	2596	570	2918	3577	3817
transcript_15561	gnl|BL_ORD_ID|66712 transcript_125606	19	2151	2151	2514	1	2134	2663	3026
transcript_155610	gnl|BL_ORD_ID|47919 transcript_98062	1	1674	1672	2015	572	2244	2544	2887
transcript_155634	gnl|BL_ORD_ID|96636 transcript_81269	283	2934	140	285	892	3544	3	148
transcript_155675	gnl|BL_ORD_ID|23381 transcript_61663	1	1863	1861	3164	1	1852	2075	3395
transcript_155707	gnl|BL_ORD_ID|82035 transcript_149031	1	1704	1700	2217	12	1729	2240	2750
transcript_155734	gnl|BL_ORD_ID|81664 transcript_148384	2	2590	2587	3250	22	2611	2727	3390
transcript_15575	gnl|BL_ORD_ID|71855 transcript_133945	12	2130	2129	2564	17	2161	2271	2709
transcript_155769	gnl|BL_ORD_ID|67536 transcript_126949	693	1930	71	693	1070	2304	2	624
transcript_155782	gnl|BL_ORD_ID|25575 transcript_63554	209	2076	72	210	724	2603	1	139
transcript_155789	gnl|BL_ORD_ID|37721 transcript_83102	362	1860	45	363	611	2110	1	322
transcript_155796	gnl|BL_ORD_ID|68660 transcript_128747	533	2001	7	537	1208	2659	38	568
transcript_1558	gnl|BL_ORD_ID|85819 transcript_155470	21	2510	2508	3579	1	2485	3226	4296
transcript_155803	gnl|BL_ORD_ID|34492 transcript_77955	124	1448	1	126	649	1973	3	128
transcript_155870	gnl|BL_ORD_ID|61771 transcript_119165	12	1621	1617	2486	2	1611	2051	2920
transcript_155923	gnl|BL_ORD_ID|1078 transcript_1932	1709	3521	1	1711	1937	3749	66	1775
transcript_155928	gnl|BL_ORD_ID|5948 transcript_31741	215	1796	51	220	312	1885	2	179
transcript_155928	gnl|BL_ORD_ID|6355 transcript_32599	215	1799	77	218	327	1908	71	212
transcript_155945	gnl|BL_ORD_ID|69526 transcript_130157	11	1465	1467	2225	1	1466	1585	2342
transcript_15596	gnl|BL_ORD_ID|56212 transcript_110185	1	1788	1785	2563	1	1782	2352	3126
transcript_155973	gnl|BL_ORD_ID|92787 transcript_165184	461	1357	52	462	2963	3860	9	419
transcript_155976	gnl|BL_ORD_ID|38203 transcript_7115	1	1900	1896	2765	181	2079	2252	3116
transcript_155976	gnl|BL_ORD_ID|38683 transcript_8171	1	1900	1896	2765	2	1902	2075	2941
transcript_156037	gnl|BL_ORD_ID|32576 transcript_74881	2	2167	2162	2921	69	2228	2411	3175
transcript_156037	gnl|BL_ORD_ID|38129 transcript_6966	2	2167	2162	2921	47	2203	2311	3076

transcript_156037	gnl|BL_ORD_ID|19859 transcript_55801	2	2167	2162	2812	24	2180	2288	2940
transcript_15607	gnl|BL_ORD_ID|72279 transcript_134626	11	1757	1754	2574	3	1751	1881	2703
transcript_156077	gnl|BL_ORD_ID|33733 transcript_76686	1273	2604	1	1274	1402	2733	3	1256
transcript_156100	gnl|BL_ORD_ID|61094 transcript_118122	1	1484	1482	2381	13	1485	2669	3567
transcript_156137	gnl|BL_ORD_ID|47540 transcript_97484	1	1727	1725	2499	2	1728	1924	2682
transcript_15616	gnl|BL_ORD_ID|26760 transcript_65427	19	2146	2145	2554	58	2184	6139	6546
transcript_156160	gnl|BL_ORD_ID|26458 transcript_64949	1	1390	1387	1873	247	1610	1938	2415
transcript_156160	gnl|BL_ORD_ID|30613 transcript_71634	1	1390	1387	1873	247	1607	1935	2412
transcript_156160	gnl|BL_ORD_ID|38758 transcript_8349	1	1390	1387	1873	275	1638	1965	2468
transcript_15620	gnl|BL_ORD_ID|49814 transcript_101082	1198	2576	100	1198	1217	2596	13	1112
transcript_156290	gnl|BL_ORD_ID|53169 transcript_104892	1	2312	2312	2967	2	2317	2485	3140
transcript_156290	gnl|BL_ORD_ID|64155 transcript_123007	1	2312	2312	2938	24	2339	2507	3133
transcript_156292	gnl|BL_ORD_ID|69090 transcript_129454	12	2210	2211	3184	80	2264	2847	3820
transcript_156309	gnl|BL_ORD_ID|80610 transcript_146651	1	1455	1454	1876	1	1455	2325	2748
transcript_156311	gnl|BL_ORD_ID|70125 transcript_131134	298	1451	3	299	437	1593	4	294
transcript_156322	gnl|BL_ORD_ID|39533 transcript_84406	15	1552	1550	2415	1	1528	1646	2510
transcript_156322	gnl|BL_ORD_ID|46083 transcript_95181	317	2411	79	318	1607	3728	1	240
transcript_156367	gnl|BL_ORD_ID|96704 transcript_88779	392	2112	8	393	501	2215	4	388
transcript_156401	gnl|BL_ORD_ID|87831 transcript_158812	16	1625	1626	2294	12	1591	1848	2516
transcript_156417	gnl|BL_ORD_ID|50799 transcript_102680	27	3981	3979	5402	2	3966	4087	5511
transcript_15644	gnl|BL_ORD_ID|19038 transcript_54439	1095	2563	1	1100	1251	2719	1	1104
transcript_156450	gnl|BL_ORD_ID|71410 transcript_133227	1	1388	1383	2221	174	1561	2359	3186
transcript_15653	gnl|BL_ORD_ID|25187 transcript_5889	1	1819	1820	2552	1	1817	2439	3171
transcript_15660	gnl|BL_ORD_ID|31940 transcript_73800	1082	2571	103	1084	1623	3109	2	992
transcript_156696	gnl|BL_ORD_ID|49817 transcript_101085	1	1333	1333	2041	36	1387	2484	3214
transcript_156707	gnl|BL_ORD_ID|24549 transcript_4539	1239	2953	1	1244	1596	3315	131	1369
transcript_15672	gnl|BL_ORD_ID|89266 transcript_161113	339	2545	58	337	1141	3348	2	281
transcript_156733	gnl|BL_ORD_ID|33165 transcript_75780	13	1520	1517	1947	2	1509	2923	3353
transcript_156778	gnl|BL_ORD_ID|83914 transcript_152355	1	1119	1117	1432	12	1129	1281	1590
transcript_15678	gnl|BL_ORD_ID|24915 transcript_5300	1	1745	1744	2564	1	1767	2407	3227
transcript_156791	gnl|BL_ORD_ID|36413 transcript_81040	1	2510	2510	3313	2	2499	2602	3405
transcript_156807	gnl|BL_ORD_ID|29118 transcript_69239	1	1333	1328	2201	194	1525	1849	2722
transcript_156861	gnl|BL_ORD_ID|33443 transcript_76208	1	1144	1140	1918	2	1145	1542	2319
transcript_156896	gnl|BL_ORD_ID|26897 transcript_65651	165	2924	2921	4824	1	2750	3122	5025

transcript_156897	gnl|BL_ORD_ID|88259 transcript_159507	1020	2346	1	1019	896	2222	1	1019
transcript_156908	gnl|BL_ORD_ID|80901 transcript_147132	208	2624	1	207	909	3325	572	778
transcript_156927	gnl|BL_ORD_ID|38826 transcript_8484	116	2779	1	117	288	2963	1	117
transcript_156927	gnl|BL_ORD_ID|38002 transcript_6714	242	2779	1	243	562	3101	1	243
transcript_157031	gnl|BL_ORD_ID|31433 transcript_72955	1	1795	1793	2982	1005	2790	4149	5337
transcript_157101	gnl|BL_ORD_ID|6225 transcript_32336	1	1462	1463	1698	15	1474	1588	1824
transcript_157101	gnl|BL_ORD_ID|21830 transcript_59049	1	1463	1463	1692	15	1478	1590	1819
transcript_157207	gnl|BL_ORD_ID|60547 transcript_117214	1	2062	2063	2274	1	2046	2207	2419
transcript_157207	gnl|BL_ORD_ID|29286 transcript_69503	2	2062	2063	2285	24	2094	2255	2480
transcript_157216	gnl|BL_ORD_ID|81902 transcript_148793	1	1826	1825	2748	65	1899	2096	3018
transcript_157263	gnl|BL_ORD_ID|22957 transcript_60963	408	2632	73	412	531	2769	1	341
transcript_157357	gnl|BL_ORD_ID|45986 transcript_95033	1	1142	1143	2018	1	1144	1310	2185
transcript_157357	gnl|BL_ORD_ID|32932 transcript_75430	1	1142	1143	2018	1	1144	1271	2146
transcript_157361	gnl|BL_ORD_ID|77967 transcript_13533	1	1569	1566	2236	39	1613	2021	2691
transcript_157477	gnl|BL_ORD_ID|30097 transcript_70805	243	2606	2	244	581	2926	55	297
transcript_157477	gnl|BL_ORD_ID|38130 transcript_6967	243	2606	2	244	600	2944	161	403
transcript_157490	gnl|BL_ORD_ID|48332 transcript_98715	257	3127	2	258	543	3396	24	278
transcript_157507	gnl|BL_ORD_ID|91681 transcript_163377	1	2040	2037	2434	859	2900	3064	3451
transcript_157555	gnl|BL_ORD_ID|26621 transcript_65219	203	2288	1	204	883	2968	254	437
transcript_157560	gnl|BL_ORD_ID|294 transcript_485	2	2869	2869	3578	59	2932	3770	4480
transcript_157561	gnl|BL_ORD_ID|17723 transcript_52357	1	2115	2115	2990	26	2127	2450	3326
transcript_157561	gnl|BL_ORD_ID|50059 transcript_101481	1	2115	2115	2963	26	2137	2499	3345
transcript_157561	gnl|BL_ORD_ID|36956 transcript_81895	1	2115	2112	3010	23	2135	3905	4804
transcript_157628	gnl|BL_ORD_ID|202 transcript_323	1646	3610	1	1650	2783	4767	55	1696
transcript_157654	gnl|BL_ORD_ID|58402 transcript_113779	1	1625	1625	2566	51	1675	1784	2725
transcript_157654	gnl|BL_ORD_ID|86958 transcript_157382	1	1625	1625	2566	51	1674	1783	2702
transcript_157721	gnl|BL_ORD_ID|18477 transcript_53544	112	1885	1885	2852	1	1775	1882	2849
transcript_157736	gnl|BL_ORD_ID|50100 transcript_101544	1	1417	1416	1777	12	1431	1708	2069
transcript_157756	gnl|BL_ORD_ID|33165 transcript_75780	1	1287	1284	1771	223	1509	2923	3410
transcript_157775	gnl|BL_ORD_ID|40984 transcript_86763	251	1229	3	251	2085	3063	1	249
transcript_157832	gnl|BL_ORD_ID|64876 transcript_11776	1	1618	1617	2384	2	1618	1981	2744
transcript_157835	gnl|BL_ORD_ID|25821 transcript_63972	598	2033	83	599	1817	3252	770	1285
transcript_157838	gnl|BL_ORD_ID|31966 transcript_73851	200	2875	1	201	564	3233	1	197
transcript_157846	gnl|BL_ORD_ID|30496 transcript_71450	777	1969	9	778	1211	2379	3	772

transcript_157927	gnl|BL_ORD_ID|1350 transcript_21765	1	1153	1154	1789	394	1546	1682	2319
transcript_157927	gnl|BL_ORD_ID|93503 transcript_166332	1	1261	1259	1788	1007	2283	4493	5024
transcript_157927	gnl|BL_ORD_ID|65904 transcript_124265	1	1153	1154	1755	398	1550	1688	2291
transcript_157927	gnl|BL_ORD_ID|95835 transcript_20423	1	1153	1154	1790	438	1588	1723	2360
transcript_157927	gnl|BL_ORD_ID|42505 transcript_89201	1	1153	1154	1791	74	1226	1362	2002
transcript_157927	gnl|BL_ORD_ID|57152 transcript_111689	1	1261	1259	1772	426	1702	3913	4428
transcript_157927	gnl|BL_ORD_ID|41374 transcript_87404	1	1261	1259	1792	752	2028	4031	4566
transcript_157940	gnl|BL_ORD_ID|2340 transcript_24007	1	1530	1527	2029	4	1542	1651	2153
transcript_157940	gnl|BL_ORD_ID|2948 transcript_25321	1	1530	1527	2000	74	1619	1728	2201
transcript_15795	gnl|BL_ORD_ID|36374 transcript_80982	1055	2512	1	1056	1238	2695	20	1079
transcript_158020	gnl|BL_ORD_ID|68351 transcript_128261	309	1352	4	309	538	1583	1	284
transcript_158076	gnl|BL_ORD_ID|61093 transcript_118121	1	2410	2410	3157	1972	4406	5134	5883
transcript_158076	gnl|BL_ORD_ID|59630 transcript_115759	1	2410	2410	3136	1344	3750	4476	5201
transcript_158078	gnl|BL_ORD_ID|42994 transcript_89988	1	2593	2591	2898	86	2675	2799	3106
transcript_158084	gnl|BL_ORD_ID|11901 transcript_2735	1	2721	2718	3210	118	2838	3022	3512
transcript_158084	gnl|BL_ORD_ID|12088 transcript_3119	2	2721	2718	3217	95	2814	2997	3496
transcript_158084	gnl|BL_ORD_ID|88606 transcript_160072	1	2721	2718	3183	185	2905	3089	3553
transcript_158090	gnl|BL_ORD_ID|19178 transcript_54682	1	2454	2453	3599	697	3140	5694	6838
transcript_158103	gnl|BL_ORD_ID|73373 transcript_136371	160	2464	1	162	756	3044	487	648
transcript_158114	gnl|BL_ORD_ID|73502 transcript_136591	329	2469	48	330	1676	3819	2	284
transcript_158119	gnl|BL_ORD_ID|74299 transcript_137922	11	1185	1182	2168	2	1176	1298	2285
transcript_158123	gnl|BL_ORD_ID|18052 transcript_52862	218	3218	115	218	554	3553	2	105
transcript_158140	gnl|BL_ORD_ID|37732 transcript_83122	289	3100	1	294	2039	4839	996	1288
transcript_158153	gnl|BL_ORD_ID|23813 transcript_62366	131	2204	1	132	1399	3476	160	291
transcript_158257	gnl|BL_ORD_ID|45011 transcript_93376	1	1189	1186	1525	27	1216	1469	1808
transcript_158257	gnl|BL_ORD_ID|30025 transcript_70677	1	1189	1186	1578	28	1212	1531	1923
transcript_158257	gnl|BL_ORD_ID|6242 transcript_32376	1	1189	1186	1523	4	1192	1511	1848
transcript_158298	gnl|BL_ORD_ID|30736 transcript_71838	1078	3751	1	1079	1271	3942	1	1091
transcript_158298	gnl|BL_ORD_ID|32643 transcript_74991	1	2853	2851	3563	1	2851	3639	4351
transcript_158414	gnl|BL_ORD_ID|38713 transcript_8240	1	2005	2000	2413	68	2065	2276	2689
transcript_158414	gnl|BL_ORD_ID|52071 transcript_10461	1	2005	2000	2413	4	2005	2214	2626
transcript_158414	gnl|BL_ORD_ID|38347 transcript_7423	1	2005	2000	2413	4	2001	2212	2625
transcript_158422	gnl|BL_ORD_ID|50979 transcript_102982	1	3644	3641	4152	81	3724	3919	4430
transcript_158428	gnl|BL_ORD_ID|12532 transcript_3954	1	2011	2011	3286	19	2024	2152	3428

transcript_158458	gnl|BL_ORD_ID|17384 transcript_51827	1	1870	1870	2958	103	1978	2564	3652
transcript_15846	gnl|BL_ORD_ID|92443 transcript_164618	1107	2551	1	1108	1533	2980	301	1408
transcript_158588	gnl|BL_ORD_ID|39714 transcript_84705	1	1534	1532	2613	1	1535	2574	3652
transcript_158588	gnl|BL_ORD_ID|48340 transcript_98734	1	1600	1601	2613	2	1601	1796	2808
transcript_158613	gnl|BL_ORD_ID|77848 transcript_13277	1	1659	1659	2522	14	1681	1829	2690
transcript_158663	gnl|BL_ORD_ID|83282 transcript_151237	328	1845	9	332	482	1999	13	336
transcript_158667	gnl|BL_ORD_ID|22186 transcript_59651	1483	2937	136	1484	1502	2954	2	1350
transcript_158708	gnl|BL_ORD_ID|68249 transcript_128080	1	1888	1887	2359	4	1891	2474	2943
transcript_158737	gnl|BL_ORD_ID|64547 transcript_11080	1166	2649	1	1167	1322	2803	54	1220
transcript_158737	gnl|BL_ORD_ID|51964 transcript_10225	1166	2690	1	1167	1305	2825	37	1203
transcript_158737	gnl|BL_ORD_ID|77895 transcript_13382	1166	2598	1	1167	1269	2705	1	1167
transcript_158779	gnl|BL_ORD_ID|38370 transcript_7485	379	2899	6	383	503	3025	2	374
transcript_158780	gnl|BL_ORD_ID|35075 transcript_78913	1	1465	1461	1836	348	1836	2094	2469
transcript_1588	gnl|BL_ORD_ID|49483 transcript_100560	1	2897	2894	3883	1	2899	3018	4008
transcript_158815	gnl|BL_ORD_ID|41167 transcript_87062	228	3202	2	229	350	3349	22	249
transcript_158835	gnl|BL_ORD_ID|31868 transcript_73672	1	1858	1859	2598	21	1876	1983	2722
transcript_158868	gnl|BL_ORD_ID|57856 transcript_112895	1071	2769	1	1076	3286	4983	1279	2354
transcript_158868	gnl|BL_ORD_ID|74279 transcript_137891	1071	2841	1	1076	3414	5184	1407	2482
transcript_158868	gnl|BL_ORD_ID|94 transcript_138	1071	2841	1	1076	3393	5164	1386	2461
transcript_158978	gnl|BL_ORD_ID|67992 transcript_127670	1	1185	1186	1941	303	1487	2173	2923
transcript_158999	gnl|BL_ORD_ID|84255 transcript_152960	1	1071	1069	1473	136	1207	6137	6540
transcript_159015	gnl|BL_ORD_ID|52239 transcript_103399	228	1654	7	227	359	1785	37	257
transcript_159017	gnl|BL_ORD_ID|1969 transcript_23170	1	1156	1155	2082	10	1164	1264	2191
transcript_159049	gnl|BL_ORD_ID|64853 transcript_11734	2	2118	2113	2491	14	2130	2258	2637
transcript_15906	gnl|BL_ORD_ID|24748 transcript_4978	879	2548	95	882	1587	3277	3	790
transcript_159066	gnl|BL_ORD_ID|38776 transcript_8385	1	2075	2072	2726	1	2074	2315	2969
transcript_159079	gnl|BL_ORD_ID|22528 transcript_60214	1198	2429	1	1199	1323	2554	1	1199
transcript_159134	gnl|BL_ORD_ID|61799 transcript_119214	1	2433	2434	2768	280	2706	2988	3323
transcript_159139	gnl|BL_ORD_ID|38180 transcript_7070	194	1935	1	190	1296	3037	2	191
transcript_159157	gnl|BL_ORD_ID|25924 transcript_64138	2	2658	2656	3283	89	2760	3653	4280
transcript_159181	gnl|BL_ORD_ID|74461 transcript_138180	548	2281	86	549	637	2369	1	463
transcript_159217	gnl|BL_ORD_ID|75675 transcript_140152	2	2602	2598	2924	404	3004	3171	3497
transcript_15926	gnl|BL_ORD_ID|44127 transcript_91904	672	2547	7	672	933	2810	2	664
transcript_159272	gnl|BL_ORD_ID|33644 transcript_76540	15	2199	2198	2474	1	2186	2338	2614

transcript_159282	gnl|BL_ORD_ID|78024 transcript_13686	1	1927	1922	2376	45	1968	2163	2618
transcript_159295	gnl|BL_ORD_ID|19103 transcript_54551	172	2492	1	171	755	3066	345	515
transcript_159295	gnl|BL_ORD_ID|38044 transcript_6799	172	2492	1	171	759	3070	349	519
transcript_159323	gnl|BL_ORD_ID|25334 transcript_6214	1	1430	1428	2644	1	1430	1673	2885
transcript_159325	gnl|BL_ORD_ID|17886 transcript_52603	1	2270	2267	2566	1	2288	2922	3225
transcript_159365	gnl|BL_ORD_ID|51172 transcript_103299	1	1731	1728	2154	2400	4125	4475	4899
transcript_159428	gnl|BL_ORD_ID|56487 transcript_110605	255	1869	31	256	909	2526	2	216
transcript_159438	gnl|BL_ORD_ID|61799 transcript_119214	1	2666	2667	2973	57	2706	2988	3294
transcript_159517	gnl|BL_ORD_ID|22387 transcript_59981	227	1945	3	227	792	2511	34	258
transcript_159556	gnl|BL_ORD_ID|85796 transcript_155433	1	1703	1703	2244	1	1699	1807	2348
transcript_159583	gnl|BL_ORD_ID|58176 transcript_113372	1	1840	1839	1971	860	2701	3089	3221
transcript_159583	gnl|BL_ORD_ID|31955 transcript_73833	174	1971	1	173	1635	3432	855	1027
transcript_159599	gnl|BL_ORD_ID|90108 transcript_162446	1	1909	1910	3559	590	2485	2622	4271
transcript_159599	gnl|BL_ORD_ID|433 transcript_741	1	1909	1910	3560	455	2360	2497	4146
transcript_159599	gnl|BL_ORD_ID|62166 transcript_119794	1	1909	1909	3562	451	2347	2499	4152
transcript_159599	gnl|BL_ORD_ID|77167 transcript_142570	1	1909	1910	3562	56	1964	2101	3753
transcript_159599	gnl|BL_ORD_ID|600 transcript_1014	1	1909	1910	3559	292	2185	2322	3969
transcript_159599	gnl|BL_ORD_ID|417 transcript_718	1	1909	1910	3562	456	2376	2513	4165
transcript_159599	gnl|BL_ORD_ID|59072 transcript_114858	1	1909	1910	3514	615	2535	2672	4276
transcript_159611	gnl|BL_ORD_ID|48485 transcript_98968	479	2015	6	483	2659	4194	3	480
transcript_159656	gnl|BL_ORD_ID|82509 transcript_149884	2	2687	2686	2964	4	2689	3561	3839
transcript_15966	gnl|BL_ORD_ID|26008 transcript_64257	136	2485	1	135	306	2656	1	135
transcript_159709	gnl|BL_ORD_ID|24810 transcript_5107	406	3184	9	405	514	3291	6	402
transcript_159731	gnl|BL_ORD_ID|62040 transcript_119587	1	2065	2065	2494	627	2691	3023	3451
transcript_159752	gnl|BL_ORD_ID|11665 transcript_2276	2	3042	3040	3579	21	3060	3163	3700
transcript_159781	gnl|BL_ORD_ID|24640 transcript_4727	1	2102	2101	2959	1	2105	2442	3301
transcript_159802	gnl|BL_ORD_ID|77390 transcript_142909	726	2575	9	728	1184	3020	3	719
transcript_159832	gnl|BL_ORD_ID|66601 transcript_125424	1253	3539	1	1254	1370	3653	1	1250
transcript_159833	gnl|BL_ORD_ID|1002 transcript_1781	1	1637	1635	2758	1	1637	1831	2955
transcript_159839	gnl|BL_ORD_ID|12541 transcript_3976	292	3045	1	291	634	3387	135	425
transcript_159839	gnl|BL_ORD_ID|47218 transcript_96969	292	3043	1	291	514	3272	2	289
transcript_159839	gnl|BL_ORD_ID|17544 transcript_52073	292	3004	1	291	538	3224	41	329
transcript_159839	gnl|BL_ORD_ID|24531 transcript_4509	292	3045	1	291	589	3342	89	380
transcript_159839	gnl|BL_ORD_ID|12176 transcript_3277	292	3045	1	291	724	3477	58	349

transcript_159839	gnl|BL_ORD_ID|12310 transcript_3528	292	3044	1	291	779	3519	112	403
transcript_159918	gnl|BL_ORD_ID|59080 transcript_114869	1	1402	1398	1686	734	2136	2343	2631
transcript_159983	gnl|BL_ORD_ID|59178 transcript_115039	127	1979	1979	3322	1	1853	3353	4697
transcript_160003	gnl|BL_ORD_ID|50559 transcript_102279	1	2292	2287	2759	2	2291	3092	3564
transcript_160004	gnl|BL_ORD_ID|31433 transcript_72955	17	2823	2821	4013	2	2790	4149	5341
transcript_160028	gnl|BL_ORD_ID|90453 transcript_162944	117	1886	1886	2725	2	1770	2452	3292
transcript_160031	gnl|BL_ORD_ID|38652 transcript_8087	262	2696	1	262	458	2893	49	308
transcript_160047	gnl|BL_ORD_ID|26924 transcript_65701	536	2681	77	536	658	2794	1	458
transcript_160101	gnl|BL_ORD_ID|85831 transcript_155491	295	2973	79	298	335	3014	2	223
transcript_160106	gnl|BL_ORD_ID|58599 transcript_114100	215	2863	14	214	576	3355	3	216
transcript_160115	gnl|BL_ORD_ID|44740 transcript_92930	1	2692	2689	3322	1	2713	5272	5905
transcript_160138	gnl|BL_ORD_ID|89814 transcript_161989	1	1628	1627	2569	165	1812	1972	2914
transcript_160138	gnl|BL_ORD_ID|29966 transcript_70587	1	1628	1627	2569	164	1809	2887	3828
transcript_160138	gnl|BL_ORD_ID|730 transcript_1271	1	1628	1627	2569	221	1868	2952	3894
transcript_16017	gnl|BL_ORD_ID|78140 transcript_13970	1068	2541	1	1067	1173	2645	1	1073
transcript_160172	gnl|BL_ORD_ID|61479 transcript_118725	307	4085	133	309	1453	5225	494	670
transcript_160172	gnl|BL_ORD_ID|118 transcript_173	307	4083	2	309	1314	5090	225	532
transcript_160172	gnl|BL_ORD_ID|112 transcript_165	307	4083	2	309	1180	4950	91	398
transcript_160197	gnl|BL_ORD_ID|38376 transcript_7498	208	2096	2	208	1080	2971	181	387
transcript_160276	gnl|BL_ORD_ID|72244 transcript_134567	2	805	800	977	1	804	1257	1434
transcript_160292	gnl|BL_ORD_ID|54992 transcript_108035	2	2330	2325	4205	133	2454	3346	5225
transcript_16031	gnl|BL_ORD_ID|64404 transcript_10782	2	2126	2124	2516	6	2121	2419	2812
transcript_160410	gnl|BL_ORD_ID|64610 transcript_11216	99	2581	2	103	336	2816	2	102
transcript_160410	gnl|BL_ORD_ID|73215 transcript_136113	98	2581	1	103	283	2764	3	105
transcript_160410	gnl|BL_ORD_ID|64944 transcript_11934	99	2581	1	100	270	2738	3	99
transcript_160410	gnl|BL_ORD_ID|49619 transcript_100783	98	2581	1	100	522	3009	3	99
transcript_160410	gnl|BL_ORD_ID|64870 transcript_11770	99	2580	1	100	273	2752	3	102
transcript_160410	gnl|BL_ORD_ID|65104 transcript_12318	99	2581	1	100	246	2727	3	101
transcript_160416	gnl|BL_ORD_ID|17170 transcript_51482	355	3066	77	354	1331	4072	940	1217
transcript_160431	gnl|BL_ORD_ID|11738 transcript_2420	12	2807	2808	3265	1	2783	2900	3359
transcript_160431	gnl|BL_ORD_ID|11795 transcript_2540	11	2807	2808	3265	1	2799	2916	3374
transcript_160468	gnl|BL_ORD_ID|3674 transcript_26933	199	1988	56	199	332	2121	1	144
transcript_16050	gnl|BL_ORD_ID|75327 transcript_139591	271	2547	37	272	773	3056	2	237
transcript_160522	gnl|BL_ORD_ID|40022 transcript_85195	1	3627	3622	3933	485	4111	5306	5616

transcript_160579	gnl|BL_ORD_ID|51559 transcript_9352	2	2234	2233	2685	6	2255	2453	2901
transcript_160579	gnl|BL_ORD_ID|51427 transcript_9092	2	2234	2233	2713	6	2234	2432	2916
transcript_160703	gnl|BL_ORD_ID|76191 transcript_141014	1	1128	1125	1623	1330	2449	2568	3067
transcript_160710	gnl|BL_ORD_ID|326 transcript_556	252	3363	2	254	1276	4385	107	359
transcript_160710	gnl|BL_ORD_ID|23901 transcript_62510	252	3363	1	254	1293	4415	101	361
transcript_16074	gnl|BL_ORD_ID|64589 transcript_11180	224	2548	1	225	464	2787	1	224
transcript_16074	gnl|BL_ORD_ID|66804 transcript_125758	224	2548	1	225	464	2785	1	224
transcript_160753	gnl|BL_ORD_ID|32685 transcript_75064	1	1192	1187	1757	1	1191	1321	1891
transcript_160757	gnl|BL_ORD_ID|63738 transcript_122349	1	2406	2405	3079	1	2433	2549	3225
transcript_160773	gnl|BL_ORD_ID|11708 transcript_2361	1679	3494	1	1679	1867	3667	1	1678
transcript_160785	gnl|BL_ORD_ID|27699 transcript_66924	1	2377	2376	2827	1059	3435	3620	4070
transcript_160785	gnl|BL_ORD_ID|392 transcript_673	1	2377	2376	2824	1146	3522	3707	4154
transcript_160785	gnl|BL_ORD_ID|86438 transcript_156520	1	2377	2376	2824	1228	3604	3789	4236
transcript_160794	gnl|BL_ORD_ID|76310 transcript_141187	192	1607	54	192	273	1688	2	140
transcript_16081	gnl|BL_ORD_ID|86328 transcript_156334	309	2582	50	308	368	2641	2	260
transcript_160845	gnl|BL_ORD_ID|39392 transcript_84182	2	2519	2518	3073	18	2535	3262	3817
transcript_160850	gnl|BL_ORD_ID|22967 transcript_60973	16	3522	3519	3969	1	3503	3611	4061
transcript_160854	gnl|BL_ORD_ID|12047 transcript_3030	1094	2354	1	1095	2272	3532	1	1095
transcript_16090	gnl|BL_ORD_ID|68875 transcript_129103	524	2564	77	529	636	2676	2	454
transcript_160930	gnl|BL_ORD_ID|66275 transcript_124899	356	1913	5	355	477	2032	2	354
transcript_160930	gnl|BL_ORD_ID|88726 transcript_160269	356	1912	5	355	481	2057	2	358
transcript_160941	gnl|BL_ORD_ID|35357 transcript_79346	1	2454	2453	2765	53	2506	2726	3039
transcript_160943	gnl|BL_ORD_ID|83704 transcript_151993	195	3126	13	194	1600	4533	9	190
transcript_16096	gnl|BL_ORD_ID|65459 transcript_123539	1	2207	2205	2574	1	2211	4110	4479
transcript_160992	gnl|BL_ORD_ID|39988 transcript_85147	308	3081	1	305	1333	4103	136	440
transcript_160992	gnl|BL_ORD_ID|22967 transcript_60973	308	3127	1	305	1250	4065	116	420
transcript_161037	gnl|BL_ORD_ID|60811 transcript_117645	1	1701	1698	2816	1	1668	2399	3513
transcript_161037	gnl|BL_ORD_ID|48851 transcript_99571	2	2378	2377	2788	1	2359	2494	2906
transcript_161037	gnl|BL_ORD_ID|12074 transcript_3086	1	1701	1698	2812	2	1683	2417	3531
transcript_161041	gnl|BL_ORD_ID|73220 transcript_136123	1	1295	1293	1970	1	1298	1702	2384
transcript_161076	gnl|BL_ORD_ID|81809 transcript_148627	12	1205	1206	1538	2	1196	1391	1723
transcript_16110	gnl|BL_ORD_ID|997 transcript_1774	1	1655	1652	2425	1	1655	3073	3846
transcript_161116	gnl|BL_ORD_ID|86206 transcript_156134	422	2970	55	422	780	3327	1	369
transcript_161135	gnl|BL_ORD_ID|39480 transcript_84319	1	1362	1360	1750	1	1368	2622	3013

transcript_161139	gnl|BL_ORD_ID|69050 transcript_129385	1100	3102	1	1101	1212	3214	1	1101
transcript_161139	gnl|BL_ORD_ID|25728 transcript_63818	211	3104	1	211	2046	4934	1	212
transcript_161139	gnl|BL_ORD_ID|49912 transcript_101239	1100	3104	1	1101	1226	3226	1	1115
transcript_161171	gnl|BL_ORD_ID|66016 transcript_124434	1	1044	1045	2038	5	1049	1151	2144
transcript_161191	gnl|BL_ORD_ID|90673 transcript_163296	1	2585	2584	2908	1	2581	2885	3214
transcript_161191	gnl|BL_ORD_ID|59077 transcript_114866	11	2648	2648	2964	2	2639	2741	3062
transcript_161191	gnl|BL_ORD_ID|22413 transcript_60017	1	2648	2646	2964	1	2648	3581	3904
transcript_161221	gnl|BL_ORD_ID|69195 transcript_129627	24	3567	3563	3812	1	3544	4467	4718
transcript_161233	gnl|BL_ORD_ID|20019 transcript_56048	1	1911	1908	2152	393	2286	2395	2639
transcript_161233	gnl|BL_ORD_ID|71534 transcript_133432	1	1911	1906	2154	401	2310	2416	2663
transcript_161235	gnl|BL_ORD_ID|62395 transcript_120177	12	2088	2087	3204	2	2079	2200	3318
transcript_161240	gnl|BL_ORD_ID|52254 transcript_103428	426	1998	5	429	907	2479	68	487
transcript_161241	gnl|BL_ORD_ID|68220 transcript_128037	228	2811	2	233	411	3006	1	228
transcript_161251	gnl|BL_ORD_ID|53791 transcript_105990	235	3326	1	234	732	3826	151	385
transcript_161251	gnl|BL_ORD_ID|11705 transcript_2357	235	3351	1	234	584	3702	2	236
transcript_161260	gnl|BL_ORD_ID|52538 transcript_103873	2	2231	2228	2632	6	2234	2384	2787
transcript_16128	gnl|BL_ORD_ID|12470 transcript_3835	342	2575	41	344	1178	3412	2	305
transcript_161335	gnl|BL_ORD_ID|30851 transcript_72035	1	2953	2953	3415	1	2941	3868	4328
transcript_161345	gnl|BL_ORD_ID|93709 transcript_166674	1	2146	2146	3941	47	2193	2314	4110
transcript_161345	gnl|BL_ORD_ID|62702 transcript_120670	1	2146	2146	3941	28	2173	2294	4089
transcript_161368	gnl|BL_ORD_ID|24499 transcript_4441	1037	2795	1	1040	1555	3313	1	1028
transcript_161372	gnl|BL_ORD_ID|96130 transcript_21089	1	1380	1379	1967	5	1384	1756	2331
transcript_161547	gnl|BL_ORD_ID|31373 transcript_72864	18	4073	4068	4802	20	4088	5121	5855
transcript_161550	gnl|BL_ORD_ID|75343 transcript_139609	1	1750	1748	2282	1	1750	2055	2592
transcript_161560	gnl|BL_ORD_ID|19333 transcript_54927	1375	2764	1	1376	1466	2855	1	1362
transcript_161568	gnl|BL_ORD_ID|60867 transcript_117733	279	3061	24	281	482	3266	2	262
transcript_161568	gnl|BL_ORD_ID|24691 transcript_4856	279	3061	1	280	479	3263	1	282
transcript_1616	gnl|BL_ORD_ID|41921 transcript_88298	16	2283	2281	3899	138	2407	2780	4398
transcript_1616	gnl|BL_ORD_ID|96527 transcript_71933	16	2836	2835	3877	9	2833	2967	4010
transcript_161623	gnl|BL_ORD_ID|788 transcript_1374	1	2620	2615	3135	1	2621	3335	3855
transcript_161656	gnl|BL_ORD_ID|24947 transcript_5359	205	2899	2	206	541	3235	10	214
transcript_161656	gnl|BL_ORD_ID|38304 transcript_7329	205	2896	13	206	408	3089	1	194
transcript_161715	gnl|BL_ORD_ID|46554 transcript_95950	2	2648	2646	3223	1	2649	2749	3325
transcript_16172	gnl|BL_ORD_ID|24618 transcript_4689	1216	2530	1	1218	1985	3304	1	1216

transcript_161737	gnl|BL_ORD_ID|11647 transcript_2243	2	2375	2374	3427	55	2426	2635	3689
transcript_16179	gnl|BL_ORD_ID|28960 transcript_69006	1	2106	2104	2520	1	2099	3387	3803
transcript_161797	gnl|BL_ORD_ID|90594 transcript_163168	1	2346	2343	2769	1	2346	2981	3406
transcript_161797	gnl|BL_ORD_ID|46998 transcript_96635	1	2346	2343	2769	1	2361	2995	3420
transcript_161797	gnl|BL_ORD_ID|58139 transcript_113319	1	2346	2343	2769	1	2364	2998	3427
transcript_161799	gnl|BL_ORD_ID|64987 transcript_12026	135	2620	12	135	237	2722	2	125
transcript_161812	gnl|BL_ORD_ID|71929 transcript_134073	2	2338	2334	2744	25	2376	2487	2897
transcript_161816	gnl|BL_ORD_ID|68323 transcript_128218	11	1449	1447	2360	21	1460	1572	2484
transcript_161823	gnl|BL_ORD_ID|47625 transcript_97604	1	1558	1557	2232	2	1558	1714	2389
transcript_161823	gnl|BL_ORD_ID|95785 transcript_20313	1	1558	1557	2231	59	1617	1773	2447
transcript_161833	gnl|BL_ORD_ID|88702 transcript_160232	182	2598	1	182	1830	4248	5	186
transcript_161846	gnl|BL_ORD_ID|35849 transcript_80121	1	1805	1806	2878	1372	3174	3307	4378
transcript_161857	gnl|BL_ORD_ID|47194 transcript_96932	1	1703	1702	2605	1	1706	1967	2877
transcript_161857	gnl|BL_ORD_ID|51462 transcript_9149	1	1703	1702	2634	1	1706	1967	2905
transcript_161857	gnl|BL_ORD_ID|51621 transcript_9480	1	1703	1701	2636	1	1708	1821	2761
transcript_161914	gnl|BL_ORD_ID|46585 transcript_95997	1	1542	1539	2748	2	1542	1986	3175
transcript_16194	gnl|BL_ORD_ID|37873 transcript_6455	16	1805	1800	2521	3	1792	2407	3128
transcript_16196	gnl|BL_ORD_ID|25681 transcript_63740	673	2556	9	674	843	2721	1	668
transcript_161994	gnl|BL_ORD_ID|59398 transcript_115396	2	2538	2537	3076	1	2537	3080	3618
transcript_161994	gnl|BL_ORD_ID|11630 transcript_2210	1	2538	2537	3100	7	2544	2872	3435
transcript_161996	gnl|BL_ORD_ID|12182 transcript_3290	1	1648	1645	3100	273	1914	2034	3489
transcript_162000	gnl|BL_ORD_ID|32406 transcript_74604	131	1364	1	132	776	2009	417	548
transcript_162000	gnl|BL_ORD_ID|3607 transcript_26776	131	1364	1	132	877	2110	418	549
transcript_162036	gnl|BL_ORD_ID|33924 transcript_77000	317	3187	61	321	454	3319	5	265
transcript_162057	gnl|BL_ORD_ID|25642 transcript_63670	1	2539	2542	3061	1	2538	2847	3365
transcript_162153	gnl|BL_ORD_ID|53169 transcript_104892	1006	3068	1	1008	1127	3187	1	1008
transcript_162157	gnl|BL_ORD_ID|35022 transcript_78823	1	1832	1830	2564	972	2803	2979	3713
transcript_162173	gnl|BL_ORD_ID|42509 transcript_89205	1	1389	1389	1910	11	1418	2532	3052
transcript_162173	gnl|BL_ORD_ID|25766 transcript_63881	1	1389	1389	1910	30	1437	2550	3070
transcript_162230	gnl|BL_ORD_ID|22745 transcript_60602	2	2524	2522	2786	1	2525	2630	2894
transcript_16225	gnl|BL_ORD_ID|37238 transcript_82336	21	2155	2153	2515	46	2167	2457	2818
transcript_162255	gnl|BL_ORD_ID|65119 transcript_12350	1	1763	1764	2600	11	1775	1876	2706
transcript_162255	gnl|BL_ORD_ID|78316 transcript_14327	1	1763	1764	2555	36	1800	1901	2686
transcript_162256	gnl|BL_ORD_ID|65868 transcript_124206	1	2514	2514	2848	2	2516	3326	3660

transcript_162262	gnl|BL_ORD_ID|95203 transcript_18928	1	1091	1089	2161	18	1104	1224	2298
transcript_162272	gnl|BL_ORD_ID|32031 transcript_73963	320	1985	9	322	603	2290	1	314
transcript_162286	gnl|BL_ORD_ID|4968 transcript_29670	1	1213	1211	1809	2	1216	1351	1949
transcript_162287	gnl|BL_ORD_ID|97166 transcript_133297	697	2818	84	698	1860	3981	4	618
transcript_162298	gnl|BL_ORD_ID|71582 transcript_133497	1	2064	2061	2827	143	2208	2393	3159
transcript_162304	gnl|BL_ORD_ID|26547 transcript_65100	1	1153	1151	1962	1	1132	3046	3855
transcript_162429	gnl|BL_ORD_ID|36983 transcript_81937	1	1841	1838	2473	35	1873	2053	2674
transcript_16246	gnl|BL_ORD_ID|45375 transcript_93994	10	1952	1950	2539	1	1942	2219	2810
transcript_16246	gnl|BL_ORD_ID|58960 transcript_114677	1	2345	2345	2560	14	2360	2861	3078
transcript_162475	gnl|BL_ORD_ID|617 transcript_1056	1372	3873	1	1372	1602	4102	15	1384
transcript_162532	gnl|BL_ORD_ID|69799 transcript_130603	136	1793	1	134	675	2332	1	133
transcript_162592	gnl|BL_ORD_ID|95563 transcript_19765	113	2100	1	113	245	2232	1	117
transcript_16261	gnl|BL_ORD_ID|12556 transcript_4013	222	2533	1	222	1082	3394	1	222
transcript_162614	gnl|BL_ORD_ID|47151 transcript_96872	473	2434	90	474	807	2768	1	385
transcript_162615	gnl|BL_ORD_ID|65129 transcript_12388	223	2385	1	226	648	2810	284	509
transcript_162615	gnl|BL_ORD_ID|65066 transcript_12225	223	2284	1	226	666	2727	302	527
transcript_162650	gnl|BL_ORD_ID|61268 transcript_118380	1	1129	1128	1831	219	1340	2192	2897
transcript_162679	gnl|BL_ORD_ID|39292 transcript_84015	1	2389	2389	2904	183	2569	3081	3596
transcript_162684	gnl|BL_ORD_ID|18621 transcript_53757	1031	4464	1	1030	1256	4698	14	1047
transcript_162741	gnl|BL_ORD_ID|46440 transcript_95765	1	1557	1556	2127	682	2246	2644	3222
transcript_162758	gnl|BL_ORD_ID|38810 transcript_8448	253	2624	2	253	588	2958	45	296
transcript_162771	gnl|BL_ORD_ID|88474 transcript_159847	156	1692	1	157	494	2030	1	157
transcript_162800	gnl|BL_ORD_ID|87333 transcript_158025	2	2878	2876	3433	91	2967	3279	3836
transcript_162812	gnl|BL_ORD_ID|82829 transcript_150426	1	1363	1361	2148	69	1447	2984	3769
transcript_162857	gnl|BL_ORD_ID|38738 transcript_8302	191	1987	1	192	1182	2978	536	727
transcript_162862	gnl|BL_ORD_ID|77669 transcript_12880	1089	2681	1	1091	1236	2829	37	1125
transcript_162876	gnl|BL_ORD_ID|23437 transcript_61761	1	1174	1172	1308	22	1195	1830	1966
transcript_1629	gnl|BL_ORD_ID|74383 transcript_138062	283	3972	1	283	503	4189	1	278
transcript_162959	gnl|BL_ORD_ID|35511 transcript_79588	27	2702	2703	3051	1	2690	2824	3170
transcript_162977	gnl|BL_ORD_ID|51942 transcript_10167	1	1773	1774	2698	10	1778	1885	2824
transcript_162977	gnl|BL_ORD_ID|64400 transcript_10775	1	1773	1774	2698	2	1772	1879	2805
transcript_163018	gnl|BL_ORD_ID|27409 transcript_66461	1263	3075	10	1266	1507	3331	1	1257
transcript_163018	gnl|BL_ORD_ID|24748 transcript_4978	1263	3104	104	1266	1415	3277	3	1165
transcript_163058	gnl|BL_ORD_ID|70658 transcript_132025	27	2912	2913	4562	2	2880	3369	5017

transcript_163088	gnl|BL_ORD_ID|51632 transcript_9514	1	1317	1317	2620	36	1350	1659	2962
transcript_163128	gnl|BL_ORD_ID|68079 transcript_127815	1	1225	1223	2012	1	1225	1828	2635
transcript_163175	gnl|BL_ORD_ID|35719 transcript_79897	215	2847	1	216	495	3124	1	216
transcript_163175	gnl|BL_ORD_ID|59514 transcript_115577	215	2847	2	219	496	3128	4	221
transcript_163186	gnl|BL_ORD_ID|567 transcript_958	1	2570	2570	3965	60	2626	2737	4131
transcript_163186	gnl|BL_ORD_ID|26269 transcript_64662	1	2570	2570	3963	103	2674	2785	4152
transcript_163191	gnl|BL_ORD_ID|17588 transcript_52137	20	3066	3067	3395	19	3065	3319	3647
transcript_163194	gnl|BL_ORD_ID|78080 transcript_13835	284	2039	4	284	770	2525	11	293
transcript_163265	gnl|BL_ORD_ID|2099 transcript_23436	108	1993	1	108	313	2208	10	116
transcript_163296	gnl|BL_ORD_ID|12587 transcript_4077	1	2949	2949	3214	1	2950	3052	3317
transcript_163336	gnl|BL_ORD_ID|39839 transcript_84899	293	1740	8	294	1469	2916	2	289
transcript_163354	gnl|BL_ORD_ID|46408 transcript_95715	186	1271	42	185	1962	3047	21	164
transcript_163391	gnl|BL_ORD_ID|73143 transcript_135983	1	1282	1283	2292	78	1360	1711	2729
transcript_163507	gnl|BL_ORD_ID|69760 transcript_130541	1360	3285	1	1359	2064	3987	429	1789
transcript_16352	gnl|BL_ORD_ID|48483 transcript_98963	311	2498	9	310	1049	3239	2	304
transcript_163584	gnl|BL_ORD_ID|12461 transcript_3817	25	3118	3115	3316	15	3106	3223	3425
transcript_163608	gnl|BL_ORD_ID|67129 transcript_126284	1	1417	1417	1883	429	1845	2091	2557
transcript_163613	gnl|BL_ORD_ID|57092 transcript_111604	246	1746	65	248	1555	3054	157	340
transcript_163649	gnl|BL_ORD_ID|40047 transcript_85236	105	2452	2450	2940	116	2464	2612	3102
transcript_163721	gnl|BL_ORD_ID|51802 transcript_9877	1	1181	1181	1849	1	1181	2204	2867
transcript_163799	gnl|BL_ORD_ID|25176 transcript_5866	234	3005	2	237	389	3167	4	241
transcript_1638	gnl|BL_ORD_ID|436 transcript_745	107	3811	1	107	454	4163	1	107
transcript_163812	gnl|BL_ORD_ID|25009 transcript_5507	1	1277	1273	1447	1499	2775	3060	3234
transcript_163812	gnl|BL_ORD_ID|67495 transcript_126885	1	1277	1273	1442	302	1581	1866	2035
transcript_163812	gnl|BL_ORD_ID|49303 transcript_100270	1	1277	1273	1446	2761	4035	4318	4491
transcript_163812	gnl|BL_ORD_ID|18873 transcript_54163	1	1277	1273	1447	1453	2728	3011	3184
transcript_163812	gnl|BL_ORD_ID|90668 transcript_163285	1	1277	1273	1428	1603	2879	3164	3319
transcript_163863	gnl|BL_ORD_ID|599 transcript_1013	2	2769	2766	3410	23	2788	3335	3984
transcript_16389	gnl|BL_ORD_ID|64674 transcript_11361	1	1656	1652	2536	2	1657	1812	2696
transcript_163899	gnl|BL_ORD_ID|46546 transcript_95934	1	1350	1349	2081	1	1347	1460	2191
transcript_163899	gnl|BL_ORD_ID|85305 transcript_154647	1	1350	1349	2081	1	1350	1478	2210
transcript_163900	gnl|BL_ORD_ID|80435 transcript_146374	16	2287	2285	2553	1	2270	2812	3079
transcript_164010	gnl|BL_ORD_ID|45926 transcript_94935	1122	2957	1	1123	1526	3361	1	1123
transcript_16405	gnl|BL_ORD_ID|87480 transcript_158242	402	2493	9	401	891	2982	2	392

transcript_16406	gnl|BL_ORD_ID|34119 transcript_77336	1	1546	1544	2406	1	1538	2813	3674
transcript_164072	gnl|BL_ORD_ID|86067 transcript_155907	1	2001	1998	2272	12	2019	2236	2515
transcript_164072	gnl|BL_ORD_ID|60962 transcript_117889	1	2001	2000	2271	851	2850	3070	3341
transcript_164072	gnl|BL_ORD_ID|86245 transcript_156204	1	2001	1998	2278	2981	4978	5196	5475
transcript_164093	gnl|BL_ORD_ID|95549 transcript_19723	1	1502	1501	2241	42	1523	1636	2389
transcript_164093	gnl|BL_ORD_ID|48331 transcript_98713	1	1502	1501	2240	6	1507	2126	2878
transcript_164093	gnl|BL_ORD_ID|68107 transcript_127861	1	1502	1501	2241	44	1545	1658	2411
transcript_164115	gnl|BL_ORD_ID|88253 transcript_159499	1329	2651	1	1331	1457	2779	1	1333
transcript_164133	gnl|BL_ORD_ID|578 transcript_979	2	2284	2281	2671	1	2284	2402	2792
transcript_16419	gnl|BL_ORD_ID|88892 transcript_160517	1	2001	1997	2576	75	2076	2583	3164
transcript_164191	gnl|BL_ORD_ID|70156 transcript_131181	342	2157	90	343	431	2244	1	254
transcript_16424	gnl|BL_ORD_ID|64506 transcript_10987	430	2530	6	435	688	2787	1	428
transcript_164244	gnl|BL_ORD_ID|39982 transcript_85134	11	1880	1875	2775	67	1915	3151	4062
transcript_164264	gnl|BL_ORD_ID|64655 transcript_11319	1	1337	1332	2100	52	1392	1950	2718
transcript_164266	gnl|BL_ORD_ID|84697 transcript_153690	1	1153	1149	2193	169	1323	1454	2500
transcript_164266	gnl|BL_ORD_ID|51378 transcript_8987	1	1153	1149	2193	603	1754	1885	2929
transcript_164266	gnl|BL_ORD_ID|29040 transcript_69126	1	1153	1149	2193	129	1281	1412	2456
transcript_164270	gnl|BL_ORD_ID|44020 transcript_91725	315	1615	47	317	1028	2328	1	271
transcript_164276	gnl|BL_ORD_ID|77220 transcript_142647	1176	3603	1	1178	1695	4125	399	1577
transcript_164304	gnl|BL_ORD_ID|923 transcript_1647	1	2644	2642	2915	228	2876	2980	3255
transcript_164308	gnl|BL_ORD_ID|93407 transcript_166192	2045	4893	1	2047	2254	5098	22	2067
transcript_164319	gnl|BL_ORD_ID|24118 transcript_62846	1	1859	1859	3179	2	1833	2951	4271
transcript_164319	gnl|BL_ORD_ID|92354 transcript_164463	1	1859	1859	3144	1	1814	2926	4210
transcript_16436	gnl|BL_ORD_ID|66383 transcript_125076	304	2544	4	303	428	2667	3	302
transcript_164367	gnl|BL_ORD_ID|751 transcript_1312	1479	3680	1	1479	1774	3974	130	1608
transcript_164489	gnl|BL_ORD_ID|66501 transcript_125265	1	1555	1552	1953	1	1544	2426	2828
transcript_164489	gnl|BL_ORD_ID|80131 transcript_145853	12	1555	1552	1953	2	1546	2429	2830
transcript_164509	gnl|BL_ORD_ID|22506 transcript_60178	479	3468	9	484	1297	4285	2	477
transcript_164602	gnl|BL_ORD_ID|84202 transcript_152860	1	1073	1071	1991	339	1415	1634	2557
transcript_164602	gnl|BL_ORD_ID|78150 transcript_13989	1	1073	1069	1991	380	1456	1672	2599
transcript_164602	gnl|BL_ORD_ID|78431 transcript_14635	1	1073	1071	1991	381	1457	1676	2600
transcript_164639	gnl|BL_ORD_ID|757 transcript_1322	107	3700	1	112	379	3980	149	260
transcript_164639	gnl|BL_ORD_ID|640 transcript_1100	107	3751	1	112	285	3937	55	166
transcript_164656	gnl|BL_ORD_ID|30819 transcript_71986	1	2755	2757	3501	41	2795	3022	3772

transcript_164680	gnl|BL_ORD_ID|69933 transcript_130828	147	2301	1	147	787	2944	7	153
transcript_164680	gnl|BL_ORD_ID|38829 transcript_8494	147	2301	10	147	793	2964	19	156
transcript_164758	gnl|BL_ORD_ID|90532 transcript_163071	1	2397	2396	2800	2	2391	2545	2951
transcript_164779	gnl|BL_ORD_ID|67311 transcript_126580	1	1334	1335	2297	14	1362	2330	3293
transcript_1648	gnl|BL_ORD_ID|81223 transcript_147633	30	3047	3048	3897	1	3020	5794	6642
transcript_164802	gnl|BL_ORD_ID|57250 transcript_111866	262	4534	143	262	271	4541	1	120
transcript_164811	gnl|BL_ORD_ID|148 transcript_225	1	4330	4330	4579	1	4330	4559	4808
transcript_164813	gnl|BL_ORD_ID|52903 transcript_104448	166	1236	2	166	2483	3551	21	186
transcript_164820	gnl|BL_ORD_ID|94599 transcript_17518	1	1481	1481	2088	30	1510	1878	2489
transcript_164820	gnl|BL_ORD_ID|91326 transcript_16398	1	1481	1481	2133	13	1495	1866	2523
transcript_164826	gnl|BL_ORD_ID|50644 transcript_102433	200	1909	58	202	282	1990	1	145
transcript_164880	gnl|BL_ORD_ID|42898 transcript_89834	1058	3643	1	1058	1162	3748	4	1061
transcript_164906	gnl|BL_ORD_ID|34345 transcript_77703	1	1702	1698	2239	1076	2777	3071	3611
transcript_164906	gnl|BL_ORD_ID|650 transcript_1119	1	1702	1698	2239	862	2563	2858	3399
transcript_164936	gnl|BL_ORD_ID|4177 transcript_28022	763	1547	9	766	1284	2067	418	1176
transcript_164948	gnl|BL_ORD_ID|79393 transcript_144671	1	1068	1068	1409	301	1363	2062	2403
transcript_164948	gnl|BL_ORD_ID|30382 transcript_71259	1	1068	1068	1395	293	1362	2061	2388
transcript_16496	gnl|BL_ORD_ID|51991 transcript_10275	1021	2528	1	1021	1300	2821	12	1033
transcript_16496	gnl|BL_ORD_ID|51894 transcript_10073	1021	2528	1	1021	1310	2823	18	1043
transcript_16496	gnl|BL_ORD_ID|78688 transcript_143533	1022	2501	1	1021	1155	2637	3	1026
transcript_164993	gnl|BL_ORD_ID|93666 transcript_166603	783	1756	8	787	989	1964	2	782
transcript_165	gnl|BL_ORD_ID|71774 transcript_133827	3	4712	4709	5052	2	4715	4907	5250
transcript_165002	gnl|BL_ORD_ID|72260 transcript_134595	1	2327	2328	3219	1	2324	2544	3436
transcript_165092	gnl|BL_ORD_ID|56512 transcript_110642	385	3081	41	390	524	3223	1	350
transcript_165109	gnl|BL_ORD_ID|61421 transcript_118641	450	2913	88	455	505	2975	4	371
transcript_165165	gnl|BL_ORD_ID|18296 transcript_53259	1	2048	2048	2274	138	2207	2338	2564
transcript_165190	gnl|BL_ORD_ID|77984 transcript_13571	732	2061	92	733	1086	2433	4	658
transcript_165209	gnl|BL_ORD_ID|30673 transcript_71729	153	1814	15	158	1379	3040	1	143
transcript_16523	gnl|BL_ORD_ID|64691 transcript_11399	1127	2349	1	1130	1566	2788	1	1130
transcript_165231	gnl|BL_ORD_ID|78119 transcript_13926	1	1226	1225	2249	2	1235	1614	2646
transcript_165231	gnl|BL_ORD_ID|94966 transcript_18393	1	1226	1224	2250	82	1315	1432	2466
transcript_165239	gnl|BL_ORD_ID|38728 transcript_8278	2	2564	2564	2769	64	2626	2777	2981
transcript_165269	gnl|BL_ORD_ID|77327 transcript_142809	1	1098	1097	1761	4	1100	1233	1894
transcript_165286	gnl|BL_ORD_ID|26460 transcript_64951	104	1869	1867	2454	1	1770	3798	4382

transcript_165293	gnl|BL_ORD_ID|53504 transcript_105455	408	2218	6	410	559	2381	23	427
transcript_16535	gnl|BL_ORD_ID|82436 transcript_149743	487	2451	5	486	1660	3623	2	484
transcript_165364	gnl|BL_ORD_ID|18052 transcript_52862	105	3102	1	105	554	3553	1	105
transcript_165370	gnl|BL_ORD_ID|28282 transcript_67916	209	2703	2	209	2786	5279	20	226
transcript_165372	gnl|BL_ORD_ID|64104 transcript_122922	164	2283	1	165	3125	5268	1	166
transcript_165372	gnl|BL_ORD_ID|56548 transcript_110700	998	2285	1	1002	1150	2456	1	1039
transcript_165456	gnl|BL_ORD_ID|44475 transcript_92481	311	1471	5	314	443	1602	1	311
transcript_165466	gnl|BL_ORD_ID|57921 transcript_112995	1	1405	1404	2026	1	1402	1560	2182
transcript_165466	gnl|BL_ORD_ID|95628 transcript_19924	1	1560	1557	2026	1	1558	1871	2340
transcript_165563	gnl|BL_ORD_ID|23526 transcript_61899	348	2525	8	349	451	2629	1	342
transcript_16558	gnl|BL_ORD_ID|27550 transcript_66674	1017	2515	1	1017	1292	2780	1	1025
transcript_165582	gnl|BL_ORD_ID|88581 transcript_160020	1	1145	1144	1609	124	1265	1538	2002
transcript_165599	gnl|BL_ORD_ID|74010 transcript_137432	134	2876	2873	3258	596	3339	3236	3619
transcript_165604	gnl|BL_ORD_ID|18258 transcript_53199	1	1511	1511	1694	32	1543	2054	2237
transcript_165621	gnl|BL_ORD_ID|95540 transcript_19700	174	2243	1	170	323	2392	2	171
transcript_165642	gnl|BL_ORD_ID|83782 transcript_152134	1	1195	1195	2210	13	1206	1503	2518
transcript_165656	gnl|BL_ORD_ID|78594 transcript_143389	224	2515	2	228	2011	4299	81	308
transcript_165681	gnl|BL_ORD_ID|91327 transcript_16399	2	2188	2188	2378	39	2225	2356	2546
transcript_165806	gnl|BL_ORD_ID|52118 transcript_10570	1290	2634	1	1293	1395	2738	1	1287
transcript_165822	gnl|BL_ORD_ID|46415 transcript_95723	1	1453	1451	2141	74	1526	1635	2325
transcript_165843	gnl|BL_ORD_ID|3174 transcript_25811	1	1731	1730	2007	1	1741	1866	2143
transcript_165870	gnl|BL_ORD_ID|23381 transcript_61663	1	1842	1840	3159	1	1852	2075	3394
transcript_16596	gnl|BL_ORD_ID|77863 transcript_13315	1045	2534	1	1048	1176	2665	1	1053
transcript_165974	gnl|BL_ORD_ID|22510 transcript_60185	15	2298	2296	2760	2	2285	2547	3011
transcript_166000	gnl|BL_ORD_ID|28566 transcript_68377	1	3324	3322	3903	139	3492	3605	4185
transcript_166018	gnl|BL_ORD_ID|82270 transcript_149454	1	3210	3211	3738	2	3195	3304	3831
transcript_166020	gnl|BL_ORD_ID|51204 transcript_8607	333	2709	5	337	587	2970	103	426
transcript_166020	gnl|BL_ORD_ID|37930 transcript_6566	333	2799	5	337	531	3005	47	370
transcript_16603	gnl|BL_ORD_ID|73981 transcript_137389	1	2327	2323	2485	27	2344	2470	2633
transcript_16603	gnl|BL_ORD_ID|38578 transcript_7937	1	2327	2323	2544	4	2333	2460	2679
transcript_16603	gnl|BL_ORD_ID|64445 transcript_10880	1	2327	2323	2446	2	2370	2499	2626
transcript_16603	gnl|BL_ORD_ID|88132 transcript_159309	1	2327	2323	2544	2	2325	2452	2675
transcript_166052	gnl|BL_ORD_ID|24542 transcript_4528	291	2692	5	293	904	3306	52	337
transcript_166084	gnl|BL_ORD_ID|51526 transcript_9266	1018	2351	1	1021	1483	2815	2	1034

transcript_166125	gnl|BL_ORD_ID|64718 transcript_11456	118	2411	2408	2734	2	2295	2413	2739
transcript_166125	gnl|BL_ORD_ID|51360 transcript_8959	2	2411	2408	2734	52	2456	2574	2899
transcript_166125	gnl|BL_ORD_ID|534 transcript_904	118	2411	2408	2734	1	2288	2406	2732
transcript_166125	gnl|BL_ORD_ID|84248 transcript_152949	2	2411	2408	2734	1	2404	2522	2848
transcript_166187	gnl|BL_ORD_ID|51820 transcript_9922	276	2795	122	277	293	2811	1	165
transcript_166251	gnl|BL_ORD_ID|27694 transcript_66917	160	1744	1	159	390	1975	54	212
transcript_166251	gnl|BL_ORD_ID|1877 transcript_22989	160	1744	1	159	398	1988	48	220
transcript_166262	gnl|BL_ORD_ID|32105 transcript_74092	1	1338	1336	2488	57	1392	2455	3610
transcript_166315	gnl|BL_ORD_ID|72436 transcript_134868	1	1598	1596	2556	2	1599	2158	3119
transcript_166329	gnl|BL_ORD_ID|6102 transcript_32054	1	1340	1335	1671	1	1340	1498	1834
transcript_166330	gnl|BL_ORD_ID|57181 transcript_111737	1	1824	1824	2306	385	2206	2319	2795
transcript_166330	gnl|BL_ORD_ID|66694 transcript_125576	1	1824	1824	2378	377	2200	2313	2867
transcript_166366	gnl|BL_ORD_ID|58384 transcript_113744	1	1021	1022	1728	2	1017	1252	1987
transcript_166407	gnl|BL_ORD_ID|29778 transcript_70256	1	1652	1651	1969	1	1653	2182	2530
transcript_166421	gnl|BL_ORD_ID|47196 transcript_96935	303	2397	5	303	637	2729	4	302
transcript_166429	gnl|BL_ORD_ID|1717 transcript_22657	1	1884	1883	2046	89	1980	2115	2278
transcript_166429	gnl|BL_ORD_ID|2158 transcript_23587	1	1884	1883	2046	44	1916	2051	2214
transcript_166453	gnl|BL_ORD_ID|1059 transcript_1898	1	1513	1509	2505	1048	2560	2797	3793
transcript_166453	gnl|BL_ORD_ID|21704 transcript_58846	1	1513	1509	2505	2	1514	1751	2748
transcript_166503	gnl|BL_ORD_ID|64634 transcript_11271	216	2651	1	218	368	2788	4	221
transcript_166506	gnl|BL_ORD_ID|69061 transcript_129401	1	2479	2474	2823	1081	3565	3739	4087
transcript_166506	gnl|BL_ORD_ID|20706 transcript_57175	1	2479	2474	2767	1798	4282	4455	4748
transcript_166517	gnl|BL_ORD_ID|40160 transcript_85412	268	2167	33	269	367	2268	1	232
transcript_166546	gnl|BL_ORD_ID|25058 transcript_5627	2	2410	2411	2958	7	2414	2571	3118
transcript_166546	gnl|BL_ORD_ID|37904 transcript_6518	2	2410	2411	2909	66	2473	2630	3128
transcript_166547	gnl|BL_ORD_ID|93006 transcript_165535	331	2485	66	333	405	2562	2	269
transcript_166553	gnl|BL_ORD_ID|27097 transcript_65983	143	1435	2	148	416	1705	155	301
transcript_166553	gnl|BL_ORD_ID|30826 transcript_72000	143	1436	7	144	265	1558	8	145
transcript_166553	gnl|BL_ORD_ID|25592 transcript_63592	143	1435	2	148	389	1681	127	273
transcript_16658	gnl|BL_ORD_ID|52757 transcript_104223	680	2531	93	679	732	2583	2	587
transcript_166584	gnl|BL_ORD_ID|51868 transcript_10026	1	1584	1582	2678	1	1583	1766	2862
transcript_1666	gnl|BL_ORD_ID|42494 transcript_89181	23	2721	2719	3842	2	2699	3191	4311
transcript_166605	gnl|BL_ORD_ID|57498 transcript_112285	209	2740	1	209	557	3086	2	210
transcript_166605	gnl|BL_ORD_ID|88920 transcript_160560	1	1897	1896	2740	1	1897	2004	2848

transcript_166605	gnl|BL_ORD_ID|96309 transcript_51781	1019	2741	1	1020	1660	3383	2	1022
transcript_166631	gnl|BL_ORD_ID|29013 transcript_69087	1	2187	2184	2735	2	2186	2289	2842
transcript_166675	gnl|BL_ORD_ID|44504 transcript_92527	1262	2653	1	1267	1385	2775	1	1257
transcript_16669	gnl|BL_ORD_ID|64796 transcript_11621	201	2504	1	200	430	2733	2	201
transcript_166694	gnl|BL_ORD_ID|36389 transcript_81007	504	1100	51	504	655	1251	2	453
transcript_166706	gnl|BL_ORD_ID|46477 transcript_95816	2	2091	2091	2557	49	2138	2512	2978
transcript_166706	gnl|BL_ORD_ID|58914 transcript_114606	2	2091	2091	2536	4	2093	2379	2824
transcript_166789	gnl|BL_ORD_ID|42048 transcript_88489	329	3098	5	329	482	3238	1	325
transcript_166815	gnl|BL_ORD_ID|52768 transcript_104238	10	2431	2431	2812	3	2452	2808	3188
transcript_166815	gnl|BL_ORD_ID|25797 transcript_63925	17	2431	2431	2812	5	2448	2804	3160
transcript_166830	gnl|BL_ORD_ID|27741 transcript_66989	1	1533	1528	2750	2	1535	1745	2967
transcript_166854	gnl|BL_ORD_ID|53791 transcript_105990	392	3460	7	391	732	3803	2	385
transcript_166895	gnl|BL_ORD_ID|38558 transcript_7878	1	2757	2756	2858	11	2765	2895	2997
transcript_166957	gnl|BL_ORD_ID|46482 transcript_95827	1	1743	1743	2553	2	1744	2580	3390
transcript_166969	gnl|BL_ORD_ID|40285 transcript_85589	12	1225	1227	1772	1	1214	1481	2022
transcript_166969	gnl|BL_ORD_ID|52286 transcript_103478	1	1225	1227	1772	1966	3197	3467	4008
transcript_166972	gnl|BL_ORD_ID|87574 transcript_158401	1	1249	1248	2358	87	1335	1535	2649
transcript_166973	gnl|BL_ORD_ID|7131 transcript_34175	1	1059	1059	1702	1	1042	1142	1786
transcript_166997	gnl|BL_ORD_ID|79200 transcript_144372	1	1858	1859	2951	37	1894	2030	3137
transcript_167015	gnl|BL_ORD_ID|95289 transcript_19118	1027	2229	1	1027	1173	2375	5	1029
transcript_167078	gnl|BL_ORD_ID|87663 transcript_158542	1	2091	2091	2354	56	2158	2297	2560
transcript_16713	gnl|BL_ORD_ID|29008 transcript_69079	16	1664	1662	2505	2	1657	2063	2906
transcript_167174	gnl|BL_ORD_ID|84789 transcript_153829	2	3236	3235	3664	6	3257	3710	4138
transcript_16722	gnl|BL_ORD_ID|64724 transcript_11473	1	2063	2059	2503	1	2056	2320	2765
transcript_16723	gnl|BL_ORD_ID|90024 transcript_162314	1	1562	1560	2507	1	1567	2078	3031
transcript_16724	gnl|BL_ORD_ID|73689 transcript_136895	2	2051	2051	2250	1	2025	2150	2346
transcript_167263	gnl|BL_ORD_ID|27961 transcript_67346	1	3752	3749	3863	102	3852	4035	4149
transcript_167329	gnl|BL_ORD_ID|88484 transcript_159862	1	1564	1563	2272	260	1823	2407	3110
transcript_167334	gnl|BL_ORD_ID|74831 transcript_138807	472	1145	9	474	2061	2733	2	461
transcript_167347	gnl|BL_ORD_ID|50809 transcript_102695	211	2299	1	212	2356	4433	860	1071
transcript_167351	gnl|BL_ORD_ID|11998 transcript_2928	212	2344	1	215	1428	3561	10	224
transcript_167351	gnl|BL_ORD_ID|11759 transcript_2459	212	2344	1	215	1429	3582	11	225
transcript_167364	gnl|BL_ORD_ID|65167 transcript_12473	1	1536	1536	1794	691	2228	2354	2613
transcript_167454	gnl|BL_ORD_ID|85228 transcript_154524	1	1616	1615	2230	2	1620	2495	3109

transcript_167454	gnl|BL_ORD_ID|64321 transcript_123251	1	1616	1615	2309	1	1627	2379	3060
transcript_167501	gnl|BL_ORD_ID|61376 transcript_118569	339	2077	4	340	477	2213	2	338
transcript_167518	gnl|BL_ORD_ID|36425 transcript_81071	151	2544	11	151	984	3375	2	142
transcript_167518	gnl|BL_ORD_ID|74288 transcript_137905	11	1994	1990	2544	2	1969	2115	2669
transcript_167548	gnl|BL_ORD_ID|44258 transcript_92111	1	2674	2669	3257	1	2672	3166	3755
transcript_167550	gnl|BL_ORD_ID|6355 transcript_32599	226	1824	3	229	327	1932	6	212
transcript_167550	gnl|BL_ORD_ID|5405 transcript_30593	226	1826	88	229	258	1858	2	143
transcript_167656	gnl|BL_ORD_ID|82336 transcript_149568	1	1403	1400	1890	6	1403	1538	2029
transcript_167656	gnl|BL_ORD_ID|55273 transcript_108540	1	1403	1399	1847	16	1418	1552	2001
transcript_167678	gnl|BL_ORD_ID|28927 transcript_68952	1	1586	1585	2030	121	1705	1965	2412
transcript_167758	gnl|BL_ORD_ID|72865 transcript_135552	1	3396	3394	3530	685	4082	4286	4422
transcript_167758	gnl|BL_ORD_ID|86245 transcript_156204	1	3399	3394	3543	1579	4976	5177	5326
transcript_167769	gnl|BL_ORD_ID|63626 transcript_122167	1317	3012	1	1316	1709	3402	1	1314
transcript_167769	gnl|BL_ORD_ID|75361 transcript_139640	1317	3012	1	1316	1678	3369	1	1315
transcript_16783	gnl|BL_ORD_ID|38306 transcript_7334	1	1695	1693	2510	1	1670	2229	3046
transcript_16783	gnl|BL_ORD_ID|75691 transcript_140177	12	1695	1693	2511	1	1658	2216	3039
transcript_16791	gnl|BL_ORD_ID|78331 transcript_14372	10	2112	2112	2511	2	2108	2230	2629
transcript_16791	gnl|BL_ORD_ID|78396 transcript_14522	1	2112	2112	2496	1	2122	2244	2629
transcript_16847	gnl|BL_ORD_ID|86858 transcript_157215	1	2105	2106	2519	34	2136	2255	2669
transcript_16847	gnl|BL_ORD_ID|67852 transcript_127462	1	2105	2106	2519	96	2198	2323	2736
transcript_16882	gnl|BL_ORD_ID|37171 transcript_82236	142	2514	1	146	488	2864	14	159
transcript_16882	gnl|BL_ORD_ID|50087 transcript_101519	142	2511	1	146	570	2915	69	212
transcript_16882	gnl|BL_ORD_ID|51361 transcript_8960	142	2512	1	146	544	2914	70	215
transcript_16882	gnl|BL_ORD_ID|51241 transcript_8687	142	2517	1	146	520	2895	46	191
transcript_16882	gnl|BL_ORD_ID|28316 transcript_67982	142	2511	1	146	584	2927	110	255
transcript_16882	gnl|BL_ORD_ID|54425 transcript_107037	142	2514	1	146	513	2882	13	155
transcript_16885	gnl|BL_ORD_ID|21414 transcript_58377	114	2464	1	115	261	2611	1	115
transcript_16902	gnl|BL_ORD_ID|23476 transcript_61826	1	1605	1604	2593	5	1615	1756	2748
transcript_16939	gnl|BL_ORD_ID|66383 transcript_125076	299	2480	4	303	487	2667	3	302
transcript_16962	gnl|BL_ORD_ID|74088 transcript_137571	187	2509	12	182	560	2882	2	172
transcript_16991	gnl|BL_ORD_ID|90594 transcript_163168	1	2214	2211	2509	1	2215	2981	3279
transcript_16991	gnl|BL_ORD_ID|46998 transcript_96635	1	2214	2211	2509	1	2230	2995	3293
transcript_16991	gnl|BL_ORD_ID|17886 transcript_52603	1	2214	2211	2509	1	2233	2922	3224
transcript_16991	gnl|BL_ORD_ID|58139 transcript_113319	1	2214	2211	2509	1	2233	2998	3300

transcript_16991	gnl|BL_ORD_ID|89687 transcript_161797	1	2214	2211	2509	1	2215	2343	2642
transcript_16998	gnl|BL_ORD_ID|59688 transcript_115849	1	1713	1714	2498	82	1792	2336	3115
transcript_17013	gnl|BL_ORD_ID|70432 transcript_131658	1	1377	1377	2492	1	1378	1981	3097
transcript_17018	gnl|BL_ORD_ID|94706 transcript_17764	1	1118	1116	2265	2	1120	1289	2434
transcript_17018	gnl|BL_ORD_ID|57128 transcript_111654	1116	2260	1	1118	1438	2580	152	1268
transcript_17031	gnl|BL_ORD_ID|22879 transcript_60843	320	2507	33	320	1349	3537	904	1191
transcript_1704	gnl|BL_ORD_ID|28555 transcript_68359	290	3849	152	290	361	3920	1	139
transcript_17056	gnl|BL_ORD_ID|71953 transcript_134109	184	2365	12	184	325	2513	2	174
transcript_17067	gnl|BL_ORD_ID|65162 transcript_12463	201	2500	20	200	395	2696	2	194
transcript_17069	gnl|BL_ORD_ID|92836 transcript_165259	1	1400	1400	2445	1	1400	1553	2591
transcript_1708	gnl|BL_ORD_ID|23343 transcript_61592	3	3253	3249	3856	38	3299	3581	4189
transcript_17093	gnl|BL_ORD_ID|56904 transcript_111282	1	1249	1250	2453	1	1249	1398	2607
transcript_17106	gnl|BL_ORD_ID|20207 transcript_56344	1	1876	1872	2525	1	1865	2319	2973
transcript_17125	gnl|BL_ORD_ID|74109 transcript_137609	1	1770	1768	2514	1	1773	2026	2769
transcript_1713	gnl|BL_ORD_ID|61196 transcript_118276	10	2674	2671	3862	2	2672	2959	4150
transcript_17132	gnl|BL_ORD_ID|71512 transcript_133401	1	1920	1915	2424	1	1926	2712	3225
transcript_17177	gnl|BL_ORD_ID|63298 transcript_121619	297	2454	3	298	527	2679	2	296
transcript_172	gnl|BL_ORD_ID|84 transcript_124	2021	5177	1	2021	2136	5295	6	2025
transcript_1722	gnl|BL_ORD_ID|599 transcript_1013	24	3645	3640	3880	2	3623	3752	3992
transcript_17222	gnl|BL_ORD_ID|88294 transcript_159567	10	1625	1626	2618	2	1603	2088	3080
transcript_1728	gnl|BL_ORD_ID|69195 transcript_129627	23	3569	3566	3863	1	3546	4477	4770
transcript_173	gnl|BL_ORD_ID|71774 transcript_133827	151	4852	4849	5190	20	4715	4907	5248
transcript_17335	gnl|BL_ORD_ID|90594 transcript_163168	1	2232	2229	2500	1	2215	2981	3254
transcript_17335	gnl|BL_ORD_ID|17886 transcript_52603	1	2232	2229	2500	1	2233	2922	3199
transcript_17335	gnl|BL_ORD_ID|46998 transcript_96635	1	2232	2229	2500	1	2230	2995	3268
transcript_17335	gnl|BL_ORD_ID|58139 transcript_113319	1	2232	2229	2500	1	2233	2998	3275
transcript_17335	gnl|BL_ORD_ID|89687 transcript_161797	1	2232	2229	2500	1	2215	2343	2617
transcript_17427	gnl|BL_ORD_ID|64389 transcript_10754	1	1438	1436	2458	2	1439	1805	2827
transcript_17450	gnl|BL_ORD_ID|83113 transcript_150943	1	1271	1271	2493	6	1275	1548	2776
transcript_17505	gnl|BL_ORD_ID|38356 transcript_7444	1	1971	1969	2476	1	1971	2527	3034
transcript_1751	gnl|BL_ORD_ID|48559 transcript_99086	1	2279	2278	3846	1	2279	2497	4078
transcript_17556	gnl|BL_ORD_ID|69268 transcript_129740	115	1329	1328	2522	2	1216	1322	2517
transcript_17604	gnl|BL_ORD_ID|12682 transcript_4263	1	1549	1548	2472	1	1549	2459	3384
transcript_17606	gnl|BL_ORD_ID|78172 transcript_14040	2	2045	2044	2498	35	2075	2179	2633

transcript_17606	gnl|BL_ORD_ID|78133 transcript_13951	2	2045	2044	2496	29	2074	2178	2630
transcript_17606	gnl|BL_ORD_ID|77768 transcript_13085	2	2045	2044	2501	35	2071	2175	2632
transcript_17631	gnl|BL_ORD_ID|40125 transcript_85363	11	1330	1329	2494	2	1322	1848	3020
transcript_17644	gnl|BL_ORD_ID|31712 transcript_73402	1	1407	1404	2467	563	1969	2207	3265
transcript_17644	gnl|BL_ORD_ID|35106 transcript_78953	1	1818	1815	2463	565	2383	3325	3965
transcript_17661	gnl|BL_ORD_ID|55211 transcript_108420	1	1385	1384	2369	9	1393	1542	2527
transcript_17677	gnl|BL_ORD_ID|43553 transcript_90904	116	1848	1847	2487	2	1733	2315	2955
transcript_17679	gnl|BL_ORD_ID|77856 transcript_13296	242	2465	1	241	475	2694	1	250
transcript_17679	gnl|BL_ORD_ID|77774 transcript_13098	242	2421	2	241	503	2682	37	282
transcript_1768	gnl|BL_ORD_ID|27491 transcript_66581	1645	3855	1	1646	1791	3998	1	1635
transcript_17728	gnl|BL_ORD_ID|24852 transcript_5174	1	1671	1669	2468	114	1778	2438	3239
transcript_17728	gnl|BL_ORD_ID|40156 transcript_85403	1	2027	2026	2447	129	2150	2604	3027
transcript_17728	gnl|BL_ORD_ID|46738 transcript_96231	1	2028	2026	2382	79	2100	2750	3108
transcript_17739	gnl|BL_ORD_ID|64473 transcript_10934	358	2482	53	361	438	2562	2	310
transcript_17751	gnl|BL_ORD_ID|36716 transcript_81520	1	1432	1433	2480	1	1430	1603	2649
transcript_17770	gnl|BL_ORD_ID|58708 transcript_114284	17	2000	1998	2483	1	1984	2664	3149
transcript_1781	gnl|BL_ORD_ID|788 transcript_1374	33	3444	3443	3839	2	3412	3521	3917
transcript_17815	gnl|BL_ORD_ID|47721 transcript_97753	2	2158	2155	2401	66	2224	2369	2615
transcript_17846	gnl|BL_ORD_ID|64606 transcript_11210	1	1878	1875	2464	1	1878	2166	2755
transcript_1785	gnl|BL_ORD_ID|37169 transcript_82233	128	2809	2807	3881	2	2682	3932	5005
transcript_1786	gnl|BL_ORD_ID|566 transcript_956	229	3920	1	233	448	4139	39	272
transcript_1786	gnl|BL_ORD_ID|94292 transcript_167565	229	3920	1	233	409	4088	1	232
transcript_17892	gnl|BL_ORD_ID|77851 transcript_13281	210	2465	1	211	422	2677	1	210
transcript_17892	gnl|BL_ORD_ID|46562 transcript_95967	210	2477	2	211	514	2780	110	318
transcript_17896	gnl|BL_ORD_ID|91340 transcript_16420	770	2370	8	769	922	2522	1	762
transcript_17917	gnl|BL_ORD_ID|91324 transcript_16393	1110	2403	1	1112	1278	2577	9	1120
transcript_17917	gnl|BL_ORD_ID|44659 transcript_92788	1	1656	1652	2451	7	1662	1797	2590
transcript_1793	gnl|BL_ORD_ID|35277 transcript_79221	1	2832	2827	3820	1	2828	3154	4147
transcript_17961	gnl|BL_ORD_ID|92813 transcript_165224	278	2444	95	283	637	2804	107	295
transcript_1797	gnl|BL_ORD_ID|61369 transcript_118559	1074	3828	100	1075	1372	4126	2	967
transcript_1797	gnl|BL_ORD_ID|39041 transcript_83620	786	3824	97	786	850	3883	2	693
transcript_17979	gnl|BL_ORD_ID|54277 transcript_106775	1	1310	1311	2467	1	1310	2623	3758
transcript_17986	gnl|BL_ORD_ID|37432 transcript_82654	224	2459	1	223	464	2699	122	344
transcript_18037	gnl|BL_ORD_ID|801 transcript_1396	1	1983	1982	2456	1	1983	3442	3916

transcript_18040	gnl|BL_ORD_ID|65310 transcript_12789	1	1542	1539	2417	2	1543	1818	2696
transcript_18051	gnl|BL_ORD_ID|400 transcript_684	1	1500	1500	2451	1	1487	3227	4179
transcript_18090	gnl|BL_ORD_ID|65708 transcript_123948	268	2458	3	269	540	2729	8	274
transcript_18098	gnl|BL_ORD_ID|42691 transcript_89513	338	2454	4	339	1699	3816	4	339
transcript_1823	gnl|BL_ORD_ID|31664 transcript_73325	1	2834	2832	3796	1	2836	3283	4247
transcript_1824	gnl|BL_ORD_ID|27491 transcript_66581	1608	3817	12	1609	1791	3998	23	1635
transcript_1830	gnl|BL_ORD_ID|79022 transcript_144082	318	3811	1	320	1677	5164	1	323
transcript_18301	gnl|BL_ORD_ID|38604 transcript_7992	205	2445	1	206	759	2999	33	239
transcript_18341	gnl|BL_ORD_ID|78509 transcript_14848	1	2165	2161	2467	35	2199	2302	2609
transcript_18341	gnl|BL_ORD_ID|73143 transcript_135983	1	2165	2161	2479	17	2182	2285	2613
transcript_18372	gnl|BL_ORD_ID|90767 transcript_15167	1	1957	1956	2435	1	1961	2116	2595
transcript_18385	gnl|BL_ORD_ID|26365 transcript_64803	473	2338	9	475	649	2518	1	467
transcript_18417	gnl|BL_ORD_ID|46853 transcript_96401	149	2442	1	151	378	2671	1	151
transcript_18430	gnl|BL_ORD_ID|51692 transcript_9643	1	1533	1533	2349	1	1540	2079	2895
transcript_18458	gnl|BL_ORD_ID|64104 transcript_122922	290	2429	6	291	3125	5268	2	287
transcript_18464	gnl|BL_ORD_ID|91480 transcript_16723	1	1662	1663	2429	1	1657	1762	2528
transcript_18467	gnl|BL_ORD_ID|96514 transcript_70750	169	2328	1	167	528	2688	1	167
transcript_18485	gnl|BL_ORD_ID|37962 transcript_6623	621	2443	88	625	1312	3135	2	540
transcript_18490	gnl|BL_ORD_ID|77739 transcript_13033	14	2206	2205	2462	3	2196	2302	2559
transcript_18490	gnl|BL_ORD_ID|57448 transcript_112204	219	2462	14	220	365	2608	3	209
transcript_18521	gnl|BL_ORD_ID|48984 transcript_99772	246	2433	32	249	1085	3273	1	217
transcript_18534	gnl|BL_ORD_ID|92159 transcript_164150	541	2420	66	540	831	2709	51	524
transcript_18534	gnl|BL_ORD_ID|38841 transcript_8527	541	2420	66	540	1020	2897	239	713
transcript_18555	gnl|BL_ORD_ID|71179 transcript_132863	119	2451	1	123	281	2616	1	126
transcript_18560	gnl|BL_ORD_ID|52670 transcript_104086	1	1612	1611	2363	1	1646	1791	2543
transcript_18563	gnl|BL_ORD_ID|23389 transcript_61675	591	2437	63	593	669	2515	37	567
transcript_18572	gnl|BL_ORD_ID|81154 transcript_147530	610	2443	66	610	1171	3004	1	545
transcript_18597	gnl|BL_ORD_ID|91225 transcript_16167	1	1715	1716	2397	2	1717	1835	2518
transcript_18608	gnl|BL_ORD_ID|19381 transcript_55010	405	2434	60	406	477	2520	2	348
transcript_18647	gnl|BL_ORD_ID|23859 transcript_62438	453	2455	54	455	519	2524	2	404
transcript_18660	gnl|BL_ORD_ID|64093 transcript_122902	1184	2407	1	1184	2156	3381	773	1960
transcript_18664	gnl|BL_ORD_ID|38695 transcript_8194	308	2410	4	312	884	2985	1	308
transcript_18766	gnl|BL_ORD_ID|90951 transcript_15586	202	2427	2	201	335	2560	21	220
transcript_18850	gnl|BL_ORD_ID|90841 transcript_15333	445	2420	84	446	615	2589	2	364

transcript_18895	gnl|BL_ORD_ID|86137 transcript_156019	12	1818	1814	2467	1	1806	1908	2561
transcript_18901	gnl|BL_ORD_ID|12642 transcript_4187	1	2026	2024	2456	120	2144	2944	3375
transcript_18903	gnl|BL_ORD_ID|91447 transcript_16659	288	2421	37	292	392	2524	1	256
transcript_1893	gnl|BL_ORD_ID|39392 transcript_84182	22	2539	2538	3776	18	2535	3262	4501
transcript_18931	gnl|BL_ORD_ID|91186 transcript_16090	1	1749	1750	2347	1	1749	1967	2564
transcript_1899	gnl|BL_ORD_ID|86230 transcript_156178	726	3767	80	727	1166	4204	1	646
transcript_19005	gnl|BL_ORD_ID|38262 transcript_7234	2	2068	2067	2439	157	2220	2681	3053
transcript_19012	gnl|BL_ORD_ID|45160 transcript_93630	1	1206	1204	2395	1	1208	1764	2954
transcript_19027	gnl|BL_ORD_ID|24698 transcript_4873	1	1866	1865	2416	1	1866	2746	3300
transcript_19047	gnl|BL_ORD_ID|87937 transcript_158974	1	1747	1742	2399	682	2431	2838	3495
transcript_19054	gnl|BL_ORD_ID|24969 transcript_5410	253	2412	9	253	1025	3184	2	246
transcript_19058	gnl|BL_ORD_ID|38780 transcript_8392	377	2412	91	379	1000	3034	1	289
transcript_19063	gnl|BL_ORD_ID|71870 transcript_133975	1	1238	1234	2418	1	1232	1796	2991
transcript_19071	gnl|BL_ORD_ID|76400 transcript_141325	1067	2444	1	1067	1390	2771	224	1290
transcript_19089	gnl|BL_ORD_ID|55976 transcript_109795	1034	2360	1	1037	2185	3510	1	1037
transcript_19095	gnl|BL_ORD_ID|97063 transcript_123436	1	2117	2114	2409	3	2119	2575	2867
transcript_19095	gnl|BL_ORD_ID|63630 transcript_122174	1	2117	2114	2350	3	2119	2409	2645
transcript_19095	gnl|BL_ORD_ID|17764 transcript_52426	1	2117	2114	2353	3	2119	2239	2476
transcript_19095	gnl|BL_ORD_ID|24766 transcript_5009	1	2117	2114	2409	3	2119	2695	2989
transcript_19095	gnl|BL_ORD_ID|24784 transcript_5047	1	2117	2114	2409	3	2119	2695	2987
transcript_19124	gnl|BL_ORD_ID|69490 transcript_130093	153	2387	1	154	1157	3390	1	154
transcript_19125	gnl|BL_ORD_ID|801 transcript_1396	1	1878	1877	2366	1	1878	3442	3931
transcript_19172	gnl|BL_ORD_ID|25195 transcript_5902	299	2481	7	302	1019	3201	2	298
transcript_19191	gnl|BL_ORD_ID|51792 transcript_9844	1	1978	1978	2410	2	1978	2437	2868
transcript_19212	gnl|BL_ORD_ID|47721 transcript_97753	2	2206	2203	2401	17	2224	2369	2567
transcript_19217	gnl|BL_ORD_ID|51244 transcript_8697	173	2399	1	175	699	2923	1	175
transcript_19271	gnl|BL_ORD_ID|63850 transcript_122528	446	2409	62	447	797	2762	1	384
transcript_1930	gnl|BL_ORD_ID|54710 transcript_107527	137	3444	3444	3780	1	3298	3524	3859
transcript_19318	gnl|BL_ORD_ID|17517 transcript_52030	462	2423	78	462	489	2448	2	386
transcript_19337	gnl|BL_ORD_ID|69308 transcript_129809	1	1274	1271	2386	1	1274	1411	2534
transcript_19342	gnl|BL_ORD_ID|46539 transcript_95921	280	2401	56	281	334	2455	2	226
transcript_19342	gnl|BL_ORD_ID|11666 transcript_2277	14	1870	1870	2396	2	1857	3137	3662
transcript_19348	gnl|BL_ORD_ID|17956 transcript_52714	596	2379	60	598	726	2512	48	603
transcript_1937	gnl|BL_ORD_ID|74383 transcript_138062	301	3771	23	301	503	3970	1	278

transcript_1937	gnl|BL_ORD_ID|85572 transcript_155078	12	3006	3004	3769	1	2994	3432	4198
transcript_19385	gnl|BL_ORD_ID|74088 transcript_137571	145	2402	12	146	559	2816	2	136
transcript_1941	gnl|BL_ORD_ID|49769 transcript_101017	496	3776	63	495	548	3832	2	434
transcript_19424	gnl|BL_ORD_ID|46410 transcript_95717	177	2424	1	179	2020	4267	24	202
transcript_19427	gnl|BL_ORD_ID|20109 transcript_56194	1	1211	1207	2389	2	1212	2271	3454
transcript_1946	gnl|BL_ORD_ID|72492 transcript_134956	1051	3756	10	1050	1145	3849	5	1045
transcript_19465	gnl|BL_ORD_ID|91003 transcript_15683	1	1632	1633	2279	1	1630	1916	2556
transcript_19500	gnl|BL_ORD_ID|87194 transcript_157772	251	2378	4	252	382	2533	4	251
transcript_19514	gnl|BL_ORD_ID|21772 transcript_58953	1	1301	1297	2364	1	1294	1791	2860
transcript_19553	gnl|BL_ORD_ID|59274 transcript_115188	1110	2363	1	1114	1748	3002	2	1114
transcript_19556	gnl|BL_ORD_ID|74278 transcript_137890	1134	2420	1	1135	1319	2607	2	1133
transcript_19561	gnl|BL_ORD_ID|78516 transcript_14857	182	2328	1	181	431	2577	1	181
transcript_19572	gnl|BL_ORD_ID|91169 transcript_16054	1133	2382	1	1136	1262	2511	1	1133
transcript_19581	gnl|BL_ORD_ID|89093 transcript_160830	169	2390	16	169	1777	3998	2	159
transcript_19581	gnl|BL_ORD_ID|65162 transcript_12463	170	2390	1	169	395	2615	26	194
transcript_19645	gnl|BL_ORD_ID|95225 transcript_18984	378	2388	42	379	464	2474	1	338
transcript_19661	gnl|BL_ORD_ID|91471 transcript_16713	193	2397	14	194	314	2518	1	183
transcript_19712	gnl|BL_ORD_ID|68424 transcript_128382	1	1422	1423	2397	6	1422	1527	2503
transcript_19712	gnl|BL_ORD_ID|26343 transcript_64773	1	2169	2170	2396	2	2160	3262	3488
transcript_19724	gnl|BL_ORD_ID|29199 transcript_69369	112	1288	1287	2404	2	1186	1292	2409
transcript_1974	gnl|BL_ORD_ID|958 transcript_1705	1	2066	2062	3701	41	2107	2229	3868
transcript_19757	gnl|BL_ORD_ID|77325 transcript_142807	1169	2327	1	1172	1582	2740	1	1172
transcript_19772	gnl|BL_ORD_ID|19708 transcript_55561	19	1976	1972	2400	84	2037	2832	3259
transcript_19800	gnl|BL_ORD_ID|53338 transcript_105171	17	2107	2108	2380	2	2096	2511	2783
transcript_19809	gnl|BL_ORD_ID|62113 transcript_119705	1	1213	1209	2396	132	1337	2058	3244
transcript_19809	gnl|BL_ORD_ID|45160 transcript_93630	1	1211	1209	2396	2	1208	1764	2952
transcript_19815	gnl|BL_ORD_ID|69490 transcript_130093	1	1834	1830	2404	846	2671	2789	3362
transcript_19815	gnl|BL_ORD_ID|50923 transcript_102896	1	1967	1966	2404	876	2834	3051	3491
transcript_19826	gnl|BL_ORD_ID|63003 transcript_121142	1	1529	1528	2386	1	1527	1632	2490
transcript_19837	gnl|BL_ORD_ID|91076 transcript_15850	2	2149	2148	2415	7	2163	2288	2556
transcript_19837	gnl|BL_ORD_ID|18179 transcript_53070	1178	2370	1	1181	1588	2781	1	1190
transcript_19837	gnl|BL_ORD_ID|90972 transcript_15620	2	2149	2148	2386	69	2212	2337	2576
transcript_19861	gnl|BL_ORD_ID|24893 transcript_5259	11	1774	1774	2381	1	1764	2637	3244
transcript_19892	gnl|BL_ORD_ID|42459 transcript_89129	1	1983	1984	2374	1	1981	2095	2485

transcript_199	gnl|BL_ORD_ID|76307 transcript_141184	2	3128	3125	5026	8	3134	3254	5155
transcript_1996	gnl|BL_ORD_ID|397 transcript_680	17	2536	2531	3760	1	2520	3083	4312
transcript_20034	gnl|BL_ORD_ID|90181 transcript_162558	250	2373	4	251	386	2485	4	252
transcript_2014	gnl|BL_ORD_ID|48559 transcript_99086	2	2166	2164	3689	115	2280	2686	4223
transcript_20146	gnl|BL_ORD_ID|56475 transcript_110585	339	2372	60	344	936	2969	2	286
transcript_20158	gnl|BL_ORD_ID|12096 transcript_3133	1	1786	1785	2344	780	2565	2957	3517
transcript_2019	gnl|BL_ORD_ID|87627 transcript_158483	30	3575	3573	3719	14	3552	3709	3855
transcript_2019	gnl|BL_ORD_ID|52668 transcript_104082	3	3575	3573	3756	5	3598	3756	3940
transcript_2019	gnl|BL_ORD_ID|869 transcript_1537	3	3575	3573	3708	43	3614	3771	3906
transcript_2020	gnl|BL_ORD_ID|79022 transcript_144082	203	3696	2	205	1677	5164	120	323
transcript_2021	gnl|BL_ORD_ID|89103 transcript_160850	379	3749	7	381	526	3896	2	375
transcript_20271	gnl|BL_ORD_ID|17330 transcript_51743	1	1565	1562	2257	1	1565	1868	2563
transcript_20271	gnl|BL_ORD_ID|87663 transcript_158542	2	2104	2104	2367	56	2158	2297	2560
transcript_20292	gnl|BL_ORD_ID|19600 transcript_55392	196	2334	1	197	317	2456	1	197
transcript_20299	gnl|BL_ORD_ID|78736 transcript_143622	1	1513	1510	2393	1	1527	3239	4121
transcript_20313	gnl|BL_ORD_ID|55015 transcript_108080	419	2447	64	417	592	2619	2	365
transcript_2038	gnl|BL_ORD_ID|54981 transcript_108016	1250	3531	1	1253	2242	4493	287	1524
transcript_20418	gnl|BL_ORD_ID|91007 transcript_15691	306	2386	37	307	409	2490	2	276
transcript_20418	gnl|BL_ORD_ID|52986 transcript_104584	306	2386	60	307	415	2495	36	284
transcript_20419	gnl|BL_ORD_ID|91158 transcript_16025	210	2365	1	210	357	2512	1	210
transcript_2042	gnl|BL_ORD_ID|44217 transcript_92049	1	2720	2717	3700	2	2724	3970	4953
transcript_20459	gnl|BL_ORD_ID|61150 transcript_118209	1	1548	1546	2317	1	1557	1752	2523
transcript_2049	gnl|BL_ORD_ID|434 transcript_743	1045	3744	1	1049	1500	4265	1	1061
transcript_20503	gnl|BL_ORD_ID|91120 transcript_15940	1	1761	1762	2375	38	1789	1958	2579
transcript_20503	gnl|BL_ORD_ID|26414 transcript_64881	1	1761	1762	2375	126	1887	2058	2671
transcript_20509	gnl|BL_ORD_ID|29714 transcript_70157	375	2259	40	376	454	2340	2	339
transcript_20525	gnl|BL_ORD_ID|76712 transcript_141817	10	1224	1225	2351	5	1221	1369	2496
transcript_20541	gnl|BL_ORD_ID|41597 transcript_87776	1	1555	1555	2264	1	1555	2804	3513
transcript_20572	gnl|BL_ORD_ID|61734 transcript_119098	1	1551	1549	2301	2	1549	1670	2425
transcript_20646	gnl|BL_ORD_ID|25195 transcript_5902	1	1581	1576	2358	76	1657	2405	3187
transcript_20702	gnl|BL_ORD_ID|84106 transcript_152694	1	1238	1235	2255	1	1238	1513	2533
transcript_2072	gnl|BL_ORD_ID|89002 transcript_160681	10	3272	3271	3736	2	3266	3834	4299
transcript_20731	gnl|BL_ORD_ID|59314 transcript_115254	481	2355	87	480	864	2738	2	395
transcript_20743	gnl|BL_ORD_ID|49607 transcript_100766	566	2362	69	566	1011	2807	1	496

transcript_20748	gnl|BL_ORD_ID|28985 transcript_69041	1	1272	1270	2275	1	1272	1846	2850
transcript_20752	gnl|BL_ORD_ID|23724 transcript_62228	768	2328	87	769	1665	3226	443	1125
transcript_20764	gnl|BL_ORD_ID|60870 transcript_117737	1	1711	1710	2279	1	1724	3322	3891
transcript_20785	gnl|BL_ORD_ID|38604 transcript_7992	101	2341	1	102	759	2999	138	239
transcript_20802	gnl|BL_ORD_ID|94901 transcript_18246	230	2351	2	233	339	2461	8	239
transcript_20802	gnl|BL_ORD_ID|27618 transcript_66789	230	2349	23	233	312	2430	2	212
transcript_20809	gnl|BL_ORD_ID|60102 transcript_116509	1	1389	1387	2353	1	1398	2805	3771
transcript_20849	gnl|BL_ORD_ID|62049 transcript_119604	343	2345	73	344	449	2455	2	275
transcript_20869	gnl|BL_ORD_ID|44800 transcript_93013	2	2062	2063	2293	42	2131	2612	2842
transcript_20888	gnl|BL_ORD_ID|51631 transcript_9511	122	2339	1	123	675	2892	2	124
transcript_2089	gnl|BL_ORD_ID|82759 transcript_150310	359	3688	34	358	452	3785	2	326
transcript_20910	gnl|BL_ORD_ID|42459 transcript_89129	15	1984	1985	2340	2	1981	2102	2457
transcript_20924	gnl|BL_ORD_ID|17412 transcript_51870	1	1879	1878	2314	1	1872	2065	2500
transcript_20924	gnl|BL_ORD_ID|80391 transcript_146316	1	1879	1878	2314	1	1851	2044	2479
transcript_20956	gnl|BL_ORD_ID|95045 transcript_18548	1	2037	2035	2342	1	2036	2148	2456
transcript_20960	gnl|BL_ORD_ID|34715 transcript_78315	20	2014	2010	2406	1	2009	2494	2889
transcript_2098	gnl|BL_ORD_ID|36206 transcript_80701	160	2712	2710	3773	63	2615	2753	3816
transcript_20982	gnl|BL_ORD_ID|63150 transcript_121395	1	1530	1526	2212	1	1529	1773	2451
transcript_20987	gnl|BL_ORD_ID|24012 transcript_62681	1	1938	1937	2312	1	1938	2063	2450
transcript_20991	gnl|BL_ORD_ID|52829 transcript_104339	1016	2331	1	1017	1805	3120	1	1017
transcript_20993	gnl|BL_ORD_ID|84836 transcript_153912	1	1808	1804	2340	1	1807	2104	2639
transcript_21	gnl|BL_ORD_ID|92262 transcript_164309	40	6222	6218	6486	24	6197	7288	7556
transcript_2103	gnl|BL_ORD_ID|32286 transcript_74403	2	3398	3398	3739	55	3464	3580	3918
transcript_2103	gnl|BL_ORD_ID|62877 transcript_120948	1	3398	3398	3690	1	3389	3505	3797
transcript_21044	gnl|BL_ORD_ID|65226 transcript_12607	1	1460	1457	2334	1	1460	1735	2613
transcript_21090	gnl|BL_ORD_ID|43996 transcript_91681	270	2338	5	268	409	2477	2	279
transcript_21090	gnl|BL_ORD_ID|46048 transcript_95130	11	1796	1795	2338	2	1788	3607	4153
transcript_21135	gnl|BL_ORD_ID|77562 transcript_143182	1	1415	1415	2323	1	1415	1567	2475
transcript_21144	gnl|BL_ORD_ID|90734 transcript_15093	143	2336	1	142	294	2487	1	142
transcript_21183	gnl|BL_ORD_ID|66946 transcript_125991	459	2327	56	460	495	2396	1	378
transcript_21218	gnl|BL_ORD_ID|62356 transcript_120105	437	2337	81	441	615	2515	2	368
transcript_21237	gnl|BL_ORD_ID|12437 transcript_3769	15	1770	1769	2299	15	1770	2890	3420
transcript_21237	gnl|BL_ORD_ID|62874 transcript_120943	1	1769	1769	2298	8	1772	1928	2457
transcript_21238	gnl|BL_ORD_ID|56773 transcript_111070	1	1930	1930	2324	1	1930	2035	2429

transcript_21241	gnl|BL_ORD_ID|51556 transcript_9349	1	1808	1807	2268	1	1808	2423	2884
transcript_21241	gnl|BL_ORD_ID|77567 transcript_143188	1	1297	1297	2267	1	1297	2749	3701
transcript_21255	gnl|BL_ORD_ID|97032 transcript_120833	127	2292	10	128	233	2398	1	122
transcript_21274	gnl|BL_ORD_ID|38940 transcript_83460	700	2311	72	702	957	2562	193	817
transcript_21275	gnl|BL_ORD_ID|95183 transcript_18892	1	1407	1406	2258	2	1411	1579	2431
transcript_21283	gnl|BL_ORD_ID|66887 transcript_125889	1121	2333	1	1122	1433	2646	12	1133
transcript_21292	gnl|BL_ORD_ID|12073 transcript_3085	218	2359	2	217	1395	3534	30	245
transcript_21304	gnl|BL_ORD_ID|88799 transcript_160384	330	2266	8	331	442	2379	2	325
transcript_21318	gnl|BL_ORD_ID|26395 transcript_64857	1	1403	1401	2262	1	1430	1548	2409
transcript_21333	gnl|BL_ORD_ID|11944 transcript_2826	229	2328	1	231	1542	3646	64	302
transcript_21347	gnl|BL_ORD_ID|25115 transcript_5763	1	1713	1713	2323	2	1714	2587	3196
transcript_2138	gnl|BL_ORD_ID|79253 transcript_144456	1	2555	2556	3621	47	2623	2876	3941
transcript_2138	gnl|BL_ORD_ID|815 transcript_1422	12	2555	2556	3656	2	2548	2801	3887
transcript_2138	gnl|BL_ORD_ID|852 transcript_1504	1	2555	2556	3656	1	2558	2811	3908
transcript_21438	gnl|BL_ORD_ID|66396 transcript_125101	495	2324	8	497	928	2758	7	502
transcript_21440	gnl|BL_ORD_ID|18879 transcript_54171	126	2318	1	126	388	2584	1	126
transcript_21440	gnl|BL_ORD_ID|78512 transcript_14852	126	2318	1	126	388	2580	1	126
transcript_21491	gnl|BL_ORD_ID|56548 transcript_110700	1012	2312	1	1013	1155	2456	1	1042
transcript_21491	gnl|BL_ORD_ID|64104 transcript_122922	165	2310	1	166	3125	5268	1	166
transcript_21578	gnl|BL_ORD_ID|24889 transcript_5251	102	2020	2018	2310	2	1920	2335	2628
transcript_2159	gnl|BL_ORD_ID|18215 transcript_53129	1	3205	3205	3783	2	3213	4042	4621
transcript_21600	gnl|BL_ORD_ID|97292 transcript_145115	1	1170	1168	2322	1	1170	1285	2433
transcript_2162	gnl|BL_ORD_ID|615 transcript_1054	274	3748	2	275	603	4074	60	333
transcript_21641	gnl|BL_ORD_ID|39854 transcript_84922	13	1721	1716	2311	1	1711	2548	3143
transcript_21698	gnl|BL_ORD_ID|76235 transcript_141084	1	1406	1404	2241	2	1406	1683	2520
transcript_21698	gnl|BL_ORD_ID|18878 transcript_54170	1	1406	1404	2295	2	1406	1674	2561
transcript_21735	gnl|BL_ORD_ID|82199 transcript_149339	307	2303	8	307	636	2631	2	301
transcript_21735	gnl|BL_ORD_ID|47196 transcript_96935	307	2303	8	307	637	2619	1	302
transcript_21741	gnl|BL_ORD_ID|23326 transcript_61569	1	1580	1578	2311	27	1615	2429	3167
transcript_21741	gnl|BL_ORD_ID|39667 transcript_84630	1	1580	1578	2298	31	1612	2452	3173
transcript_2178	gnl|BL_ORD_ID|40497 transcript_85957	353	3716	2	355	525	3888	4	367
transcript_21795	gnl|BL_ORD_ID|11944 transcript_2826	217	2322	2	219	1542	3646	87	302
transcript_21823	gnl|BL_ORD_ID|42493 transcript_89180	1	1411	1412	2308	1	1411	1570	2467
transcript_2185	gnl|BL_ORD_ID|54403 transcript_106995	2	2596	2591	3652	1	2596	2848	3909

transcript_21852	gnl|BL_ORD_ID|33183 transcript_75804	1	2019	2017	2307	2	2022	2130	2420
transcript_21891	gnl|BL_ORD_ID|20842 transcript_57396	318	2297	54	320	1009	2990	624	890
transcript_21928	gnl|BL_ORD_ID|74802 transcript_138751	1072	2269	1	1073	1317	2514	120	1192
transcript_21959	gnl|BL_ORD_ID|52487 transcript_103793	238	2313	26	238	449	2532	1	217
transcript_21959	gnl|BL_ORD_ID|18976 transcript_54326	238	2313	6	237	960	3034	1	232
transcript_21972	gnl|BL_ORD_ID|40476 transcript_85921	254	2280	5	254	1272	3297	2	252
transcript_21994	gnl|BL_ORD_ID|27463 transcript_66546	15	1733	1732	2309	2	1721	2260	2837
transcript_21999	gnl|BL_ORD_ID|52330 transcript_103549	436	2287	81	439	472	2323	1	359
transcript_22062	gnl|BL_ORD_ID|18008 transcript_52791	1115	2296	1	1114	1222	2402	1	1114
transcript_22068	gnl|BL_ORD_ID|27386 transcript_66426	1	1284	1279	2266	1	1285	1737	2719
transcript_2210	gnl|BL_ORD_ID|11750 transcript_2441	263	3374	1	263	542	3652	1	263
transcript_22100	gnl|BL_ORD_ID|38129 transcript_6966	1	1164	1163	2279	23	1181	1985	3105
transcript_22100	gnl|BL_ORD_ID|18572 transcript_53683	1	1165	1163	2236	8	1170	1394	2467
transcript_2211	gnl|BL_ORD_ID|59328 transcript_115280	131	3698	1	132	367	3915	25	156
transcript_22170	gnl|BL_ORD_ID|59986 transcript_116325	287	2288	7	288	1669	3668	1	282
transcript_22170	gnl|BL_ORD_ID|80065 transcript_145753	287	2226	7	286	1065	3005	2	282
transcript_22253	gnl|BL_ORD_ID|80344 transcript_146242	522	2285	8	523	886	2649	107	603
transcript_22253	gnl|BL_ORD_ID|90687 transcript_14988	522	2285	57	523	744	2507	2	468
transcript_22301	gnl|BL_ORD_ID|72857 transcript_135536	1	1707	1704	2292	1	1704	2129	2715
transcript_22335	gnl|BL_ORD_ID|54475 transcript_107123	811	2281	83	813	882	2352	1	731
transcript_22373	gnl|BL_ORD_ID|77567 transcript_143188	1	1316	1318	2270	2	1317	2750	3701
transcript_22373	gnl|BL_ORD_ID|51556 transcript_9349	1	1810	1808	2271	2	1790	2421	2884
transcript_22408	gnl|BL_ORD_ID|49333 transcript_100315	1034	2205	1	1033	1188	2360	2	1037
transcript_22408	gnl|BL_ORD_ID|25437 transcript_6416	1	1677	1674	2204	1	1668	2587	3116
transcript_22443	gnl|BL_ORD_ID|73012 transcript_135786	686	2271	8	687	2330	3913	2	681
transcript_22449	gnl|BL_ORD_ID|91607 transcript_17023	1	1406	1406	2278	1	1406	1617	2489
transcript_225	gnl|BL_ORD_ID|101 transcript_149	1	4169	4166	4963	1	4170	4421	5219
transcript_22505	gnl|BL_ORD_ID|78037 transcript_13714	1	1711	1708	2278	1	1705	1996	2566
transcript_22514	gnl|BL_ORD_ID|97229 transcript_139253	1	1937	1935	2267	93	2030	3064	3394
transcript_22521	gnl|BL_ORD_ID|92953 transcript_165448	161	2284	1	162	1285	3406	1	162
transcript_22538	gnl|BL_ORD_ID|67980 transcript_127654	12	1439	1437	2267	2	1455	1643	2474
transcript_22538	gnl|BL_ORD_ID|49903 transcript_101224	12	1439	1437	2234	1	1420	1608	2405
transcript_22543	gnl|BL_ORD_ID|1265 transcript_21560	1	1487	1488	2202	2	1488	1609	2324
transcript_2257	gnl|BL_ORD_ID|41105 transcript_86965	2	2465	2461	3545	19	2482	3484	4568

transcript_22614	gnl|BL_ORD_ID|93243 transcript_165919	338	2278	42	339	609	2549	2	299
transcript_22680	gnl|BL_ORD_ID|77683 transcript_12915	11	1504	1501	2272	2	1495	1902	2646
transcript_22689	gnl|BL_ORD_ID|91879 transcript_163705	1	1724	1724	2206	1	1723	2174	2656
transcript_22745	gnl|BL_ORD_ID|78055 transcript_13771	1	1624	1623	2257	1	1611	2016	2645
transcript_22757	gnl|BL_ORD_ID|26494 transcript_65004	215	2219	1	217	886	2888	116	332
transcript_22771	gnl|BL_ORD_ID|66946 transcript_125991	384	2253	8	385	495	2397	1	378
transcript_22792	gnl|BL_ORD_ID|95717 transcript_20163	10	1647	1644	2266	1	1657	1769	2395
transcript_22803	gnl|BL_ORD_ID|46410 transcript_95717	201	2247	2	203	2020	4066	1	202
transcript_22835	gnl|BL_ORD_ID|70527 transcript_131816	1	1527	1524	2236	2	1527	1654	2365
transcript_22850	gnl|BL_ORD_ID|67199 transcript_126392	351	2263	9	351	485	2399	1	344
transcript_22864	gnl|BL_ORD_ID|60825 transcript_117667	1	1960	1961	2230	1	1975	2086	2355
transcript_22876	gnl|BL_ORD_ID|28026 transcript_67470	1	1648	1647	2243	1	1648	2522	3118
transcript_22891	gnl|BL_ORD_ID|34837 transcript_78511	1	1620	1619	2048	1	1621	1726	2155
transcript_22891	gnl|BL_ORD_ID|35788 transcript_80009	1	1410	1409	2032	1	1418	2318	2945
transcript_22923	gnl|BL_ORD_ID|65859 transcript_124195	1	1335	1333	2147	1	1336	1559	2378
transcript_22929	gnl|BL_ORD_ID|86783 transcript_157104	1	1522	1520	2274	10	1530	1630	2381
transcript_22943	gnl|BL_ORD_ID|27111 transcript_66004	327	2325	49	328	945	2938	16	293
transcript_22943	gnl|BL_ORD_ID|76808 transcript_141974	327	2325	42	328	408	2404	10	278
transcript_22943	gnl|BL_ORD_ID|75641 transcript_140090	327	2325	55	328	403	2402	1	273
transcript_22943	gnl|BL_ORD_ID|25608 transcript_63614	327	2293	38	328	434	2401	11	304
transcript_22955	gnl|BL_ORD_ID|52256 transcript_103430	127	2267	1	128	324	2462	2	129
transcript_2298	gnl|BL_ORD_ID|11801 transcript_2556	1	3061	3061	3424	1	3062	3257	3619
transcript_22983	gnl|BL_ORD_ID|63494 transcript_121950	1	1737	1735	2255	1	1737	1857	2376
transcript_23005	gnl|BL_ORD_ID|79185 transcript_144350	10	1592	1589	2202	5	1599	2348	2961
transcript_23005	gnl|BL_ORD_ID|24987 transcript_5454	631	2248	8	631	1513	3132	12	638
transcript_23023	gnl|BL_ORD_ID|29447 transcript_69739	606	2261	73	607	1596	3251	2	536
transcript_23043	gnl|BL_ORD_ID|32507 transcript_74752	248	2259	29	250	673	2691	2	227
transcript_23059	gnl|BL_ORD_ID|24039 transcript_62717	1115	2297	1	1114	1309	2485	94	1205
transcript_23105	gnl|BL_ORD_ID|36305 transcript_80870	1	1347	1348	2250	1	1350	1978	2874
transcript_23140	gnl|BL_ORD_ID|92178 transcript_164181	398	2235	55	398	839	2674	2	345
transcript_23140	gnl|BL_ORD_ID|75265 transcript_139497	398	2245	55	398	912	2758	2	346
transcript_23147	gnl|BL_ORD_ID|71343 transcript_133123	13	1385	1384	2290	33	1406	1580	2487
transcript_23150	gnl|BL_ORD_ID|38080 transcript_6860	1	1940	1940	2255	157	2093	2748	3062
transcript_23170	gnl|BL_ORD_ID|86669 transcript_156912	210	2203	11	210	1497	3488	3	202

transcript_23208	gnl|BL_ORD_ID|51578 transcript_9385	1	1623	1622	2256	2	1626	2272	2906
transcript_23220	gnl|BL_ORD_ID|66455 transcript_125193	1	1499	1497	2134	1	1499	1948	2585
transcript_23220	gnl|BL_ORD_ID|61538 transcript_118809	1	1176	1172	2132	1	1176	1526	2487
transcript_23220	gnl|BL_ORD_ID|65847 transcript_124173	1	1497	1497	2207	1	1500	1792	2502
transcript_23234	gnl|BL_ORD_ID|63827 transcript_122489	1	1443	1442	2228	20	1462	2335	3121
transcript_23246	gnl|BL_ORD_ID|38780 transcript_8392	203	2236	2	205	1000	3035	86	289
transcript_23264	gnl|BL_ORD_ID|27618 transcript_66789	238	2294	27	240	366	2420	2	213
transcript_23270	gnl|BL_ORD_ID|38355 transcript_7438	1	1617	1615	2203	1	1612	2427	3018
transcript_23270	gnl|BL_ORD_ID|71865 transcript_133969	10	1617	1615	2203	2	1602	1993	2582
transcript_23270	gnl|BL_ORD_ID|39667 transcript_84630	1	1617	1615	2237	1	1612	2452	3077
transcript_23302	gnl|BL_ORD_ID|62099 transcript_119684	1	1783	1782	2270	1	1755	1864	2352
transcript_2331	gnl|BL_ORD_ID|22506 transcript_60178	1571	3682	1	1570	2174	4285	1	1571
transcript_2332	gnl|BL_ORD_ID|1055 transcript_1893	2	2096	2094	3609	5	2097	2296	3811
transcript_23385	gnl|BL_ORD_ID|95661 transcript_20014	1	1969	1966	2254	4	1972	2096	2384
transcript_23395	gnl|BL_ORD_ID|48129 transcript_98397	1	1681	1679	2228	164	1846	1995	2545
transcript_23482	gnl|BL_ORD_ID|30687 transcript_71747	1	1823	1818	2211	2	1831	3562	3954
transcript_23484	gnl|BL_ORD_ID|34823 transcript_78486	258	2285	54	261	435	2462	1	208
transcript_23505	gnl|BL_ORD_ID|42864 transcript_89779	1096	2246	1	1096	1295	2518	1	1195
transcript_2355	gnl|BL_ORD_ID|72479 transcript_134937	1222	3675	1	1222	1483	3938	102	1351
transcript_2355	gnl|BL_ORD_ID|19373 transcript_54998	1	2779	2775	3667	114	2915	3130	4024
transcript_23599	gnl|BL_ORD_ID|58860 transcript_114523	860	2237	94	862	871	2248	2	770
transcript_23609	gnl|BL_ORD_ID|48324 transcript_98703	331	2228	38	332	428	2326	2	295
transcript_23613	gnl|BL_ORD_ID|49957 transcript_101320	208	2233	106	208	363	2388	1	103
transcript_23616	gnl|BL_ORD_ID|35377 transcript_79373	176	2235	1	178	769	2828	461	638
transcript_23631	gnl|BL_ORD_ID|90755 transcript_15144	1	1577	1576	2231	58	1634	1840	2494
transcript_23690	gnl|BL_ORD_ID|92343 transcript_164445	1	1566	1566	2227	1	1566	1672	2333
transcript_237	gnl|BL_ORD_ID|46821 transcript_96349	1	2879	2877	4922	26	2912	3070	5116
transcript_23704	gnl|BL_ORD_ID|60939 transcript_117852	1	1458	1457	2236	1	1458	1871	2650
transcript_23739	gnl|BL_ORD_ID|29603 transcript_69997	1	1356	1357	2226	2	1354	1687	2544
transcript_23761	gnl|BL_ORD_ID|36041 transcript_80419	1085	2230	1	1086	2379	3518	1	1082
transcript_23768	gnl|BL_ORD_ID|90927 transcript_15533	128	2239	1	130	421	2532	1	130
transcript_2378	gnl|BL_ORD_ID|31422 transcript_72939	2	2073	2070	3587	45	2123	2336	3852
transcript_2379	gnl|BL_ORD_ID|27390 transcript_66430	1	2453	2451	3587	1	2463	2576	3713
transcript_23808	gnl|BL_ORD_ID|57259 transcript_111883	1	1373	1373	2203	1	1375	1564	2394

transcript_23826	gnl|BL_ORD_ID|1775 transcript_22765	1	1515	1511	2191	1	1514	1618	2298
transcript_23826	gnl|BL_ORD_ID|25142 transcript_5809	1	1619	1616	2197	1	1619	2608	3189
transcript_23846	gnl|BL_ORD_ID|1685 transcript_22576	253	2227	68	254	300	2259	1	187
transcript_23857	gnl|BL_ORD_ID|64697 transcript_11414	693	2227	8	696	1264	2791	1	688
transcript_23857	gnl|BL_ORD_ID|96564 transcript_75360	170	2195	11	173	625	2645	4	166
transcript_23874	gnl|BL_ORD_ID|36983 transcript_81937	11	1746	1744	2223	1	1709	2054	2523
transcript_23893	gnl|BL_ORD_ID|92102 transcript_164064	12	1519	1514	2205	1	1508	1815	2506
transcript_2393	gnl|BL_ORD_ID|542 transcript_919	357	3599	2	358	923	4173	41	399
transcript_23946	gnl|BL_ORD_ID|42493 transcript_89180	1	1411	1412	2239	1	1411	1570	2398
transcript_23952	gnl|BL_ORD_ID|28026 transcript_67470	1	1629	1628	2222	1	1648	2522	3118
transcript_23966	gnl|BL_ORD_ID|42013 transcript_88439	401	2223	59	404	490	2312	2	347
transcript_23980	gnl|BL_ORD_ID|23926 transcript_62551	574	2228	67	577	1148	2797	519	1029
transcript_24007	gnl|BL_ORD_ID|79722 transcript_145197	1	1733	1730	2111	1	1734	1861	2242
transcript_24007	gnl|BL_ORD_ID|78538 transcript_14905	1	1063	1062	2166	4	1069	1532	2620
transcript_24020	gnl|BL_ORD_ID|95414 transcript_19412	1	1265	1266	2213	45	1309	1489	2434
transcript_24020	gnl|BL_ORD_ID|49588 transcript_100740	1	1265	1266	2213	33	1291	1471	2417
transcript_24029	gnl|BL_ORD_ID|24916 transcript_5301	1	2022	2022	2241	1	2021	3022	3241
transcript_24070	gnl|BL_ORD_ID|42679 transcript_89495	1065	2180	1	1068	1258	2372	1	1068
transcript_2412	gnl|BL_ORD_ID|401 transcript_685	1	3414	3413	3629	291	3720	4006	4224
transcript_2412	gnl|BL_ORD_ID|448 transcript_761	1	3414	3413	3629	228	3660	3946	4164
transcript_2412	gnl|BL_ORD_ID|49483 transcript_100560	1	2685	2682	3637	203	2899	3018	3991
transcript_24130	gnl|BL_ORD_ID|49838 transcript_101122	1	1258	1258	2233	1	1259	1669	2644
transcript_2415	gnl|BL_ORD_ID|31084 transcript_72413	115	2567	2565	3676	2	2454	2603	3715
transcript_24155	gnl|BL_ORD_ID|79359 transcript_144616	838	2242	89	839	1745	3149	3	753
transcript_24161	gnl|BL_ORD_ID|18643 transcript_53801	1	1554	1555	2187	18	1575	1704	2350
transcript_24161	gnl|BL_ORD_ID|17979 transcript_52748	1	1554	1555	2236	255	1806	4637	5319
transcript_24178	gnl|BL_ORD_ID|88928 transcript_160570	215	2204	2	216	551	2539	59	281
transcript_24178	gnl|BL_ORD_ID|64383 transcript_10741	215	2289	2	216	516	2590	24	246
transcript_24178	gnl|BL_ORD_ID|78173 transcript_14041	215	2289	2	216	579	2653	84	306
transcript_24188	gnl|BL_ORD_ID|95292 transcript_19124	102	1616	1612	2236	153	1667	1785	2410
transcript_24202	gnl|BL_ORD_ID|985 transcript_1752	1	1317	1316	2168	1	1317	2988	3843
transcript_24204	gnl|BL_ORD_ID|28877 transcript_68879	490	2208	78	491	1049	2767	2	415
transcript_24210	gnl|BL_ORD_ID|87705 transcript_158601	1	1289	1285	2215	1	1288	3218	4146
transcript_24210	gnl|BL_ORD_ID|64706 transcript_11437	526	2217	56	528	1106	2796	2	474

transcript_24221	gnl|BL_ORD_ID|12224 transcript_3370	1	1624	1622	2222	2	1606	2894	3496
transcript_24221	gnl|BL_ORD_ID|12152 transcript_3231	1	1624	1622	2214	2	1627	2915	3509
transcript_24233	gnl|BL_ORD_ID|1265 transcript_21560	1	1488	1489	2206	1	1488	1609	2326
transcript_24254	gnl|BL_ORD_ID|26119 transcript_64429	1	1741	1740	2181	1	1745	1869	2310
transcript_24285	gnl|BL_ORD_ID|84106 transcript_152694	1	1238	1235	2208	1	1238	1513	2489
transcript_24339	gnl|BL_ORD_ID|62934 transcript_121030	622	2223	85	623	838	2579	2	584
transcript_24342	gnl|BL_ORD_ID|20106 transcript_56187	1	1812	1811	2205	1	1823	1984	2379
transcript_24348	gnl|BL_ORD_ID|90996 transcript_15674	1101	2222	1	1101	1206	2327	6	1106
transcript_24360	gnl|BL_ORD_ID|77827 transcript_13225	1	1936	1936	2222	2	1926	2036	2322
transcript_24360	gnl|BL_ORD_ID|85722 transcript_155319	1	1936	1936	2222	2	1939	2049	2335
transcript_24360	gnl|BL_ORD_ID|90768 transcript_15168	1	1936	1936	2222	37	1973	2101	2387
transcript_2437	gnl|BL_ORD_ID|898 transcript_1602	137	3656	1	138	341	3863	1	136
transcript_24383	gnl|BL_ORD_ID|95711 transcript_20150	194	2204	1	193	360	2379	55	247
transcript_24383	gnl|BL_ORD_ID|41654 transcript_87890	191	2207	1	193	614	2640	2	193
transcript_24383	gnl|BL_ORD_ID|69539 transcript_130180	194	2207	1	193	307	2319	2	194
transcript_24383	gnl|BL_ORD_ID|42037 transcript_88472	191	2207	1	193	400	2426	2	194
transcript_24398	gnl|BL_ORD_ID|42864 transcript_89779	1097	2213	1	1097	1295	2481	1	1195
transcript_24401	gnl|BL_ORD_ID|70128 transcript_131139	111	1957	1956	2226	2	1848	2331	2601
transcript_24420	gnl|BL_ORD_ID|1853 transcript_22943	163	2162	1	164	327	2325	38	202
transcript_24420	gnl|BL_ORD_ID|25608 transcript_63614	162	2130	1	164	433	2401	13	178
transcript_24420	gnl|BL_ORD_ID|27111 transcript_66004	161	2219	10	164	943	2995	16	167
transcript_24422	gnl|BL_ORD_ID|90936 transcript_15555	12	1478	1479	2212	8	1464	1582	2316
transcript_24433	gnl|BL_ORD_ID|97229 transcript_139253	1	1892	1890	2219	138	2030	3064	3394
transcript_24446	gnl|BL_ORD_ID|35995 transcript_80349	1	1422	1420	2164	118	1533	2513	3248
transcript_24459	gnl|BL_ORD_ID|1289 transcript_21606	200	2201	11	201	326	2324	2	192
transcript_2446	gnl|BL_ORD_ID|685 transcript_1184	1500	3577	12	1501	1992	4069	46	1562
transcript_24463	gnl|BL_ORD_ID|20857 transcript_57422	1	1749	1749	2252	1	1751	1897	2415
transcript_24483	gnl|BL_ORD_ID|95092 transcript_18650	307	2281	84	307	326	2291	1	223
transcript_24483	gnl|BL_ORD_ID|89561 transcript_161588	307	2279	85	307	337	2301	1	222
transcript_24492	gnl|BL_ORD_ID|34120 transcript_77337	102	1617	1615	2205	2	1517	2331	2918
transcript_24492	gnl|BL_ORD_ID|86884 transcript_157251	389	2205	9	389	572	2389	2	384
transcript_24492	gnl|BL_ORD_ID|23326 transcript_61569	13	1617	1615	2205	2	1615	2429	3016
transcript_24538	gnl|BL_ORD_ID|90855 transcript_15375	1016	2120	1	1017	1483	2591	1	1020
transcript_24538	gnl|BL_ORD_ID|43905 transcript_91507	15	1687	1684	2150	9	1684	1811	2277

transcript_24544	gnl|BL_ORD_ID|71865 transcript_133969	1	1615	1613	2201	18	1602	1993	2582
transcript_24544	gnl|BL_ORD_ID|38606 transcript_7996	1	1615	1613	2179	30	1614	2428	2996
transcript_24544	gnl|BL_ORD_ID|23326 transcript_61569	1	1615	1613	2201	22	1615	2429	3016
transcript_24544	gnl|BL_ORD_ID|39667 transcript_84630	1	1615	1613	2201	26	1612	2452	3042
transcript_24544	gnl|BL_ORD_ID|38355 transcript_7438	1	1615	1613	2201	26	1612	2427	3018
transcript_24549	gnl|BL_ORD_ID|91845 transcript_163651	1	1453	1452	2161	1	1453	1669	2381
transcript_2459	gnl|BL_ORD_ID|29488 transcript_69810	11	2043	2040	3578	1	2048	2337	3855
transcript_24648	gnl|BL_ORD_ID|51551 transcript_9333	1	1743	1743	2192	1	1742	2436	2885
transcript_24657	gnl|BL_ORD_ID|25766 transcript_63881	1	1418	1418	2205	18	1437	2550	3332
transcript_2466	gnl|BL_ORD_ID|81851 transcript_148707	110	2679	2677	3659	1	2578	3143	4133
transcript_24663	gnl|BL_ORD_ID|95661 transcript_20014	1	1944	1941	2233	30	1972	2096	2384
transcript_24688	gnl|BL_ORD_ID|31887 transcript_73709	11	1757	1757	2204	1	1747	1921	2368
transcript_24688	gnl|BL_ORD_ID|49957 transcript_101320	113	2174	11	113	363	2424	1	103
transcript_24700	gnl|BL_ORD_ID|27386 transcript_66426	1	1181	1176	2163	103	1285	1737	2719
transcript_24718	gnl|BL_ORD_ID|44342 transcript_92262	1	1342	1342	2144	1	1342	1462	2267
transcript_24753	gnl|BL_ORD_ID|48142 transcript_98419	1	1370	1368	2157	1	1387	1747	2534
transcript_24754	gnl|BL_ORD_ID|94838 transcript_18095	554	2163	58	555	782	2398	2	499
transcript_24779	gnl|BL_ORD_ID|31822 transcript_73603	1	1652	1652	2162	3	1630	1775	2285
transcript_24779	gnl|BL_ORD_ID|28124 transcript_67623	1	1652	1652	2195	3	1672	1817	2361
transcript_24794	gnl|BL_ORD_ID|1203 transcript_21432	1	1366	1363	2199	1	1365	1485	2322
transcript_24832	gnl|BL_ORD_ID|65993 transcript_124404	1	1621	1620	2254	1	1620	2495	3129
transcript_24931	gnl|BL_ORD_ID|78834 transcript_143772	303	2193	8	303	632	2516	2	297
transcript_24961	gnl|BL_ORD_ID|52839 transcript_104352	206	2191	1	206	509	2492	72	277
transcript_24961	gnl|BL_ORD_ID|52487 transcript_103793	206	2256	1	206	449	2498	12	217
transcript_24961	gnl|BL_ORD_ID|18976 transcript_54326	206	2256	1	205	960	3000	32	232
transcript_24963	gnl|BL_ORD_ID|1685 transcript_22576	266	2209	81	267	300	2243	1	187
transcript_24995	gnl|BL_ORD_ID|60523 transcript_117178	471	2188	78	472	1542	3259	1	395
transcript_250	gnl|BL_ORD_ID|40616 transcript_86156	1985	4888	1	1986	2228	5131	2	1981
transcript_25032	gnl|BL_ORD_ID|60580 transcript_117266	317	2160	5	318	1200	3043	2	315
transcript_25109	gnl|BL_ORD_ID|67688 transcript_127196	1	1648	1646	2164	1	1646	3175	3694
transcript_2511	gnl|BL_ORD_ID|92158 transcript_164149	1	1855	1855	3603	1	1865	2019	3767
transcript_2512	gnl|BL_ORD_ID|34944 transcript_78690	10	2972	2972	3623	2	2960	3105	3757
transcript_25130	gnl|BL_ORD_ID|95142 transcript_18789	449	2164	7	449	682	2429	2	444
transcript_25168	gnl|BL_ORD_ID|61547 transcript_118823	1	1621	1618	2146	2	1624	1751	2279

transcript_25168	gnl|BL_ORD_ID|1188 transcript_21395	1	1621	1618	2198	11	1631	1758	2338
transcript_25198	gnl|BL_ORD_ID|48142 transcript_98419	1	1381	1379	2146	1	1387	1747	2513
transcript_25241	gnl|BL_ORD_ID|76567 transcript_141590	105	1867	1866	2195	2	1764	1866	2195
transcript_25242	gnl|BL_ORD_ID|1551 transcript_22284	1	1405	1403	2164	1	1406	1531	2292
transcript_25270	gnl|BL_ORD_ID|44555 transcript_92631	1	1854	1854	2168	1	1847	2190	2505
transcript_25283	gnl|BL_ORD_ID|36322 transcript_80899	1	1380	1378	2098	1	1398	2067	2791
transcript_25301	gnl|BL_ORD_ID|71090 transcript_132732	231	2134	4	233	760	2663	9	235
transcript_25329	gnl|BL_ORD_ID|62776 transcript_120791	125	2172	1	126	297	2346	3	128
transcript_25346	gnl|BL_ORD_ID|121 transcript_176	1	1498	1497	2049	1	1497	4559	5111
transcript_25349	gnl|BL_ORD_ID|88777 transcript_160347	1	1335	1335	2176	22	1356	1469	2310
transcript_25426	gnl|BL_ORD_ID|72184 transcript_134475	732	2005	78	734	966	2239	2	656
transcript_25451	gnl|BL_ORD_ID|36175 transcript_80649	1010	2105	1	1011	2260	3356	1	1011
transcript_25451	gnl|BL_ORD_ID|52564 transcript_103902	1010	2072	1	1011	2208	3267	1	1012
transcript_25462	gnl|BL_ORD_ID|95352 transcript_19290	15	1726	1725	2166	2	1713	1935	2376
transcript_25532	gnl|BL_ORD_ID|29578 transcript_69952	518	2120	6	520	800	2405	2	522
transcript_25536	gnl|BL_ORD_ID|61284 transcript_118412	1	1626	1624	2156	1	1606	2366	2913
transcript_25544	gnl|BL_ORD_ID|46437 transcript_95757	139	2148	1	141	540	2565	119	258
transcript_25544	gnl|BL_ORD_ID|998 transcript_1776	139	2129	1	141	1834	3837	1411	1551
transcript_25546	gnl|BL_ORD_ID|79978 transcript_145616	1	1671	1670	2164	2	1700	2137	2632
transcript_2556	gnl|BL_ORD_ID|19510 transcript_55243	406	3619	78	407	2179	5396	2	331
transcript_25580	gnl|BL_ORD_ID|52347 transcript_103575	1068	2162	103	1067	1090	2184	1	963
transcript_25586	gnl|BL_ORD_ID|38995 transcript_83559	1102	2235	1	1103	1331	2464	51	1155
transcript_25586	gnl|BL_ORD_ID|77235 transcript_142666	680	2235	90	683	1138	2691	2	597
transcript_2561	gnl|BL_ORD_ID|76419 transcript_141354	2	3148	3144	3611	1	3148	3875	4342
transcript_25623	gnl|BL_ORD_ID|51520 transcript_9252	1	1716	1715	2157	1	1728	2500	2942
transcript_25671	gnl|BL_ORD_ID|54189 transcript_106631	1	1124	1124	2149	1	1124	1350	2353
transcript_25705	gnl|BL_ORD_ID|65868 transcript_124206	1	1240	1236	2152	1	1246	1810	2711
transcript_25724	gnl|BL_ORD_ID|18906 transcript_54219	207	2153	2	208	324	2267	1	212
transcript_25736	gnl|BL_ORD_ID|19203 transcript_54726	102	2108	1	102	285	2291	25	126
transcript_25773	gnl|BL_ORD_ID|79943 transcript_145558	239	2103	7	241	628	2491	2	235
transcript_25775	gnl|BL_ORD_ID|67873 transcript_127491	443	2143	56	442	1681	3382	2	388
transcript_25795	gnl|BL_ORD_ID|94767 transcript_17913	679	2121	74	681	1027	2477	1	608
transcript_25806	gnl|BL_ORD_ID|56253 transcript_110252	2	2009	2006	2144	8	2015	2216	2354
transcript_25808	gnl|BL_ORD_ID|48598 transcript_99152	10	1874	1870	2147	2	1868	2492	2774

transcript_25842	gnl|BL_ORD_ID|30042 transcript_70705	1	1401	1399	2088	1	1399	2327	3016
transcript_25842	gnl|BL_ORD_ID|30014 transcript_70663	1	1401	1399	2066	1	1399	2225	2892
transcript_25892	gnl|BL_ORD_ID|64375 transcript_10726	563	2192	72	563	1214	2845	1	491
transcript_25892	gnl|BL_ORD_ID|18962 transcript_54301	747	2184	76	747	811	2245	2	673
transcript_25909	gnl|BL_ORD_ID|80787 transcript_146951	410	2142	70	411	510	2242	1	342
transcript_25913	gnl|BL_ORD_ID|80281 transcript_146123	757	2164	93	758	1335	2742	2	667
transcript_25919	gnl|BL_ORD_ID|2103 transcript_23448	1	1453	1454	2163	1	1444	1552	2257
transcript_2593	gnl|BL_ORD_ID|91736 transcript_163458	10	2301	2301	3563	11	2307	2427	3689
transcript_25943	gnl|BL_ORD_ID|71254 transcript_132974	433	2089	7	434	720	2377	3	428
transcript_25943	gnl|BL_ORD_ID|55078 transcript_108189	432	2079	8	436	565	2213	1	428
transcript_25943	gnl|BL_ORD_ID|38663 transcript_8112	432	2113	8	434	1318	3000	2	428
transcript_25971	gnl|BL_ORD_ID|42037 transcript_88472	193	2148	1	194	475	2422	1	194
transcript_25971	gnl|BL_ORD_ID|69539 transcript_130180	193	2148	1	194	379	2315	1	194
transcript_25971	gnl|BL_ORD_ID|41654 transcript_87890	193	2148	1	196	689	2636	1	195
transcript_25974	gnl|BL_ORD_ID|56509 transcript_110638	432	2177	9	435	1069	2816	7	424
transcript_25974	gnl|BL_ORD_ID|38663 transcript_8112	432	2159	7	434	1273	3000	9	427
transcript_25974	gnl|BL_ORD_ID|55078 transcript_108189	437	2125	9	435	525	2213	8	425
transcript_25996	gnl|BL_ORD_ID|63516 transcript_121986	382	2150	8	383	744	2500	10	388
transcript_26065	gnl|BL_ORD_ID|27421 transcript_66479	1009	2124	1	1010	4131	5251	2	1008
transcript_26080	gnl|BL_ORD_ID|36716 transcript_81520	322	2146	7	322	853	2649	2	318
transcript_26114	gnl|BL_ORD_ID|45620 transcript_94406	226	2145	28	227	477	2399	3	203
transcript_26195	gnl|BL_ORD_ID|32031 transcript_73963	319	2109	8	321	603	2395	1	314
transcript_26211	gnl|BL_ORD_ID|89618 transcript_161687	499	2083	5	504	785	2370	1	506
transcript_26211	gnl|BL_ORD_ID|64122 transcript_122952	251	2143	5	253	356	2250	1	255
transcript_26215	gnl|BL_ORD_ID|25766 transcript_63881	1	1420	1421	2137	19	1436	2526	3241
transcript_26215	gnl|BL_ORD_ID|42509 transcript_89205	1	1420	1421	2113	1	1417	2508	3200
transcript_2623	gnl|BL_ORD_ID|36251 transcript_80775	2	2183	2180	3550	24	2179	2735	4103
transcript_26258	gnl|BL_ORD_ID|2275 transcript_23858	1	1202	1201	2111	18	1217	1317	2226
transcript_26260	gnl|BL_ORD_ID|29578 transcript_69952	526	2131	8	528	800	2405	2	522
transcript_26283	gnl|BL_ORD_ID|1607 transcript_22415	181	2049	1	182	441	2309	19	217
transcript_26290	gnl|BL_ORD_ID|60942 transcript_117860	1	1327	1325	2117	1	1329	1839	2606
transcript_26293	gnl|BL_ORD_ID|34120 transcript_77337	1	1413	1411	2130	101	1517	2331	3048
transcript_26293	gnl|BL_ORD_ID|39667 transcript_84630	1	1413	1411	2122	194	1612	2452	3163
transcript_26329	gnl|BL_ORD_ID|32896 transcript_75377	342	2120	64	342	2252	4028	29	307

transcript_26335	gnl|BL_ORD_ID|49605 transcript_100762	1023	2125	1	1024	1157	2259	13	1031
transcript_26349	gnl|BL_ORD_ID|22132 transcript_59575	349	2205	95	350	891	2759	1	260
transcript_26431	gnl|BL_ORD_ID|31861 transcript_73662	696	2127	80	698	1029	2457	64	681
transcript_26441	gnl|BL_ORD_ID|26326 transcript_64745	10	1338	1334	2071	5	1332	2691	3426
transcript_2648	gnl|BL_ORD_ID|29989 transcript_70618	124	2989	2988	3629	2	2867	3573	4214
transcript_26483	gnl|BL_ORD_ID|56806 transcript_111119	1	1190	1187	2093	2	1190	1306	2213
transcript_26487	gnl|BL_ORD_ID|96002 transcript_20812	178	2130	12	183	389	2341	6	173
transcript_26493	gnl|BL_ORD_ID|2338 transcript_23999	1	1567	1568	2142	6	1556	1660	2234
transcript_26534	gnl|BL_ORD_ID|35036 transcript_78846	334	2140	4	339	842	2643	45	381
transcript_26537	gnl|BL_ORD_ID|95981 transcript_20764	1	1875	1872	2125	1	1862	2033	2286
transcript_26598	gnl|BL_ORD_ID|95984 transcript_20770	251	2127	29	251	364	2240	2	228
transcript_26598	gnl|BL_ORD_ID|82676 transcript_150172	251	2077	29	251	344	2170	2	208
transcript_26601	gnl|BL_ORD_ID|21721 transcript_58872	13	1691	1688	2127	4	1712	1827	2266
transcript_26609	gnl|BL_ORD_ID|38080 transcript_6860	1	1757	1757	2078	337	2093	2748	3068
transcript_26633	gnl|BL_ORD_ID|24727 transcript_4929	1	1256	1255	2127	1	1260	2091	2970
transcript_26643	gnl|BL_ORD_ID|32170 transcript_74212	1	1908	1907	2116	2	1909	2516	2725
transcript_26720	gnl|BL_ORD_ID|3211 transcript_25904	1	1528	1529	2017	1	1534	1639	2127
transcript_26726	gnl|BL_ORD_ID|64527 transcript_11044	1015	2111	1	1018	1699	2795	478	1495
transcript_26733	gnl|BL_ORD_ID|56622 transcript_110828	1	1590	1587	2048	56	1634	2411	2872
transcript_2677	gnl|BL_ORD_ID|51110 transcript_103200	11	3019	3018	3538	1	3009	5596	6102
transcript_2677	gnl|BL_ORD_ID|65897 transcript_124257	1172	3610	11	1174	1335	3747	1	1148
transcript_26804	gnl|BL_ORD_ID|45677 transcript_94497	595	2113	61	599	749	2267	2	540
transcript_26811	gnl|BL_ORD_ID|37527 transcript_82797	1	1176	1174	2079	1	1186	1333	2238
transcript_26811	gnl|BL_ORD_ID|78426 transcript_14617	1	1281	1279	2104	1	1281	1773	2598
transcript_26821	gnl|BL_ORD_ID|95984 transcript_20770	233	2108	9	233	471	2346	2	228
transcript_2683	gnl|BL_ORD_ID|76419 transcript_141354	27	3155	3151	3618	1	3148	3875	4342
transcript_26831	gnl|BL_ORD_ID|45853 transcript_94819	11	1739	1737	2136	1	1734	1846	2245
transcript_26834	gnl|BL_ORD_ID|46217 transcript_95416	1	1115	1112	2040	1	1114	1215	2139
transcript_26859	gnl|BL_ORD_ID|38617 transcript_8021	1	1353	1352	2095	1	1353	1453	2196
transcript_2689	gnl|BL_ORD_ID|11614 transcript_2179	2	3346	3343	3584	1	3338	3470	3713
transcript_26895	gnl|BL_ORD_ID|96248 transcript_21320	439	2114	49	441	609	2284	1	393
transcript_26920	gnl|BL_ORD_ID|64816 transcript_11653	1	1688	1687	2109	1	1698	2197	2619
transcript_26922	gnl|BL_ORD_ID|70472 transcript_131720	1	1862	1861	2106	56	1914	2249	2494
transcript_26933	gnl|BL_ORD_ID|1259 transcript_21548	1	1176	1174	2094	34	1209	1417	2337

transcript_26933	gnl|BL_ORD_ID|94943 transcript_18335	1	1176	1174	2112	136	1321	1529	2467
transcript_26933	gnl|BL_ORD_ID|48079 transcript_98317	1	1176	1174	2121	25	1244	1452	2399
transcript_26935	gnl|BL_ORD_ID|80278 transcript_146120	1	1474	1475	2100	2	1462	2556	3181
transcript_26954	gnl|BL_ORD_ID|2123 transcript_23502	1	1916	1917	2105	1	1913	2048	2236
transcript_26972	gnl|BL_ORD_ID|34544 transcript_78036	490	2081	62	490	991	2583	2	430
transcript_26982	gnl|BL_ORD_ID|39672 transcript_84637	1	1441	1440	2004	2	1442	1555	2119
transcript_26982	gnl|BL_ORD_ID|66895 transcript_125901	1	1178	1178	2058	2	1179	1289	2167
transcript_27015	gnl|BL_ORD_ID|54242 transcript_106720	121	2084	1	123	1826	3797	1	123
transcript_27027	gnl|BL_ORD_ID|39395 transcript_84187	1	1313	1311	1999	1	1313	1474	2162
transcript_27042	gnl|BL_ORD_ID|84762 transcript_153785	522	2111	89	523	3060	4646	364	798
transcript_27073	gnl|BL_ORD_ID|72000 transcript_134184	302	2109	31	302	424	2233	3	275
transcript_27090	gnl|BL_ORD_ID|39634 transcript_84575	1	1098	1098	2092	45	1142	1819	2805
transcript_27117	gnl|BL_ORD_ID|87393 transcript_158111	1	1585	1583	2082	1	1585	3220	3719
transcript_2713	gnl|BL_ORD_ID|41726 transcript_88002	1	2900	2900	3521	2	2912	4200	4823
transcript_27135	gnl|BL_ORD_ID|12481 transcript_3855	1	1218	1217	2092	14	1232	2550	3425
transcript_27139	gnl|BL_ORD_ID|77708 transcript_12969	420	2104	8	420	729	2417	2	414
transcript_27183	gnl|BL_ORD_ID|93431 transcript_166221	222	2079	46	223	357	2215	2	179
transcript_27222	gnl|BL_ORD_ID|78193 transcript_14076	1	1582	1581	2101	1	1582	2114	2635
transcript_27230	gnl|BL_ORD_ID|27686 transcript_66898	1007	2183	100	1006	1014	2189	1	914
transcript_27263	gnl|BL_ORD_ID|38780 transcript_8392	224	2125	4	226	1000	2898	68	289
transcript_27278	gnl|BL_ORD_ID|68160 transcript_127956	381	2057	4	384	774	2450	2	382
transcript_27281	gnl|BL_ORD_ID|82339 transcript_149571	415	2088	54	415	1000	2672	28	386
transcript_27281	gnl|BL_ORD_ID|38873 transcript_83341	239	2088	72	238	424	2273	2	165
transcript_27281	gnl|BL_ORD_ID|18906 transcript_54219	237	2088	50	238	324	2175	27	216
transcript_2733	gnl|BL_ORD_ID|80892 transcript_147121	3	3076	3077	3598	1	3074	3401	3922
transcript_27375	gnl|BL_ORD_ID|1534 transcript_22226	256	2037	32	257	504	2285	17	243
transcript_27404	gnl|BL_ORD_ID|2824 transcript_25039	10	1539	1538	2093	1	1527	1634	2185
transcript_27409	gnl|BL_ORD_ID|1932 transcript_23105	1	1115	1115	2086	1	1114	1252	2223
transcript_27411	gnl|BL_ORD_ID|37852 transcript_83307	1	1224	1223	2103	378	1601	2475	3355
transcript_27480	gnl|BL_ORD_ID|35518 transcript_79597	275	2071	3	276	427	2222	1	274
transcript_27497	gnl|BL_ORD_ID|53023 transcript_104650	1	1208	1206	2080	427	1636	1900	2774
transcript_27497	gnl|BL_ORD_ID|53424 transcript_105327	1	1208	1206	1998	267	1492	1962	2754
transcript_27497	gnl|BL_ORD_ID|20195 transcript_56329	1	1208	1206	2091	207	1433	1697	2582
transcript_27571	gnl|BL_ORD_ID|1676 transcript_22556	184	2077	1	184	384	2271	2	187

transcript_27579	gnl|BL_ORD_ID|2266 transcript_23838	11	1381	1382	2035	1	1371	1576	2227
transcript_2768	gnl|BL_ORD_ID|79529 transcript_144884	2	2708	2708	3598	16	2716	4084	4963
transcript_27691	gnl|BL_ORD_ID|77790 transcript_13138	267	2095	32	269	856	2684	1	238
transcript_27696	gnl|BL_ORD_ID|49843 transcript_101128	597	2103	75	600	1273	2777	2	527
transcript_27710	gnl|BL_ORD_ID|29333 transcript_69566	1	1419	1418	2111	1	1420	1988	2681
transcript_27741	gnl|BL_ORD_ID|3536 transcript_26615	1	1461	1466	1928	2	1445	1693	2138
transcript_27939	gnl|BL_ORD_ID|3077 transcript_25596	1	1583	1580	2024	2	1584	1722	2166
transcript_27958	gnl|BL_ORD_ID|92036 transcript_163949	1	1147	1148	2058	1	1146	1515	2424
transcript_27966	gnl|BL_ORD_ID|82739 transcript_150275	1	1140	1138	2082	1	1138	1819	2766
transcript_28034	gnl|BL_ORD_ID|22084 transcript_59495	481	2073	85	481	3083	4675	2	398
transcript_28047	gnl|BL_ORD_ID|51616 transcript_9468	1	1722	1720	2051	1	1722	2518	2849
transcript_2805	gnl|BL_ORD_ID|93235 transcript_165910	1258	3527	1	1263	1515	3780	1	1261
transcript_28060	gnl|BL_ORD_ID|53408 transcript_105302	192	2052	1	197	489	2343	21	217
transcript_28060	gnl|BL_ORD_ID|49609 transcript_100768	192	2053	1	197	492	2351	21	218
transcript_28073	gnl|BL_ORD_ID|23316 transcript_61554	609	2066	8	609	1045	2502	2	603
transcript_28133	gnl|BL_ORD_ID|2122 transcript_23500	1	1671	1671	2084	23	1693	1870	2283
transcript_28169	gnl|BL_ORD_ID|1824 transcript_22876	1	1284	1279	1989	1	1301	1556	2243
transcript_28169	gnl|BL_ORD_ID|51613 transcript_9460	1	1370	1369	1989	1	1386	2260	2856
transcript_28169	gnl|BL_ORD_ID|51864 transcript_10021	1	1370	1369	1989	1	1372	2242	2837
transcript_28169	gnl|BL_ORD_ID|65114 transcript_12336	1	1370	1369	1989	1	1368	2118	2710
transcript_28169	gnl|BL_ORD_ID|2319 transcript_23952	1	1284	1279	1989	1	1282	1537	2222
transcript_28169	gnl|BL_ORD_ID|32615 transcript_74943	502	1945	8	504	844	2263	1	486
transcript_28177	gnl|BL_ORD_ID|2060 transcript_23357	256	2053	4	257	436	2233	1	256
transcript_2818	gnl|BL_ORD_ID|20217 transcript_56358	136	3581	1	138	397	3846	109	246
transcript_2818	gnl|BL_ORD_ID|89103 transcript_160850	136	3579	1	138	526	3969	238	375
transcript_28211	gnl|BL_ORD_ID|22130 transcript_59573	469	2049	54	470	532	2111	1	418
transcript_28218	gnl|BL_ORD_ID|52137 transcript_10609	1	1543	1540	2045	1	1550	2306	2807
transcript_28258	gnl|BL_ORD_ID|92930 transcript_165403	139	2000	1	140	1779	3640	1	140
transcript_28299	gnl|BL_ORD_ID|3553 transcript_26662	1	1508	1509	2011	1	1515	1618	2119
transcript_2831	gnl|BL_ORD_ID|11601 transcript_2155	155	3431	1	158	460	3730	1	160
transcript_28313	gnl|BL_ORD_ID|84110 transcript_152702	333	2030	62	332	489	2186	1	273
transcript_28327	gnl|BL_ORD_ID|45745 transcript_94621	352	2006	46	355	639	2288	194	503
transcript_28328	gnl|BL_ORD_ID|20426 transcript_56702	528	2076	55	531	807	2355	1	477
transcript_28329	gnl|BL_ORD_ID|59775 transcript_115993	276	2042	69	279	387	2153	2	213

transcript_28329	gnl|BL_ORD_ID|31891 transcript_73715	680	2043	71	682	2448	3810	2	612
transcript_28409	gnl|BL_ORD_ID|96224 transcript_21268	673	2070	7	675	926	2323	2	670
transcript_28439	gnl|BL_ORD_ID|35748 transcript_79942	1	1335	1334	2173	6	1341	1564	2403
transcript_28483	gnl|BL_ORD_ID|95738 transcript_20197	1	1662	1661	1977	1	1665	2037	2366
transcript_28483	gnl|BL_ORD_ID|3285 transcript_26074	1	1662	1663	2006	1	1662	1791	2134
transcript_28489	gnl|BL_ORD_ID|39363 transcript_84132	1	1334	1334	2029	2	1338	2175	2864
transcript_28551	gnl|BL_ORD_ID|21201 transcript_58031	1	1520	1520	1990	1244	2763	3353	3823
transcript_28555	gnl|BL_ORD_ID|2320 transcript_23954	275	2016	4	277	473	2217	28	301
transcript_28555	gnl|BL_ORD_ID|18744 transcript_53957	275	2021	4	277	451	2200	6	279
transcript_2858	gnl|BL_ORD_ID|65860 transcript_124196	1	1789	1789	3523	1	1789	2169	3903
transcript_2859	gnl|BL_ORD_ID|83972 transcript_152455	1	2109	2109	3497	1	2125	2240	3635
transcript_2859	gnl|BL_ORD_ID|92793 transcript_165193	1	2040	2037	3570	1	2043	4941	6480
transcript_28594	gnl|BL_ORD_ID|4805 transcript_29341	121	1856	1	120	246	1981	1	120
transcript_2860	gnl|BL_ORD_ID|11843 transcript_2620	202	3252	1	202	556	3604	1	202
transcript_28604	gnl|BL_ORD_ID|58058 transcript_113207	1	1224	1224	2028	2	1189	1896	2701
transcript_28659	gnl|BL_ORD_ID|22130 transcript_59573	474	2035	59	475	532	2111	1	418
transcript_28682	gnl|BL_ORD_ID|22084 transcript_59495	401	2025	5	401	3083	4707	2	398
transcript_28741	gnl|BL_ORD_ID|92925 transcript_165396	200	2031	1	201	335	2163	1	201
transcript_28741	gnl|BL_ORD_ID|20460 transcript_56762	200	2031	18	201	747	2585	1	183
transcript_28741	gnl|BL_ORD_ID|42018 transcript_88445	200	2031	1	201	381	2211	1	201
transcript_2877	gnl|BL_ORD_ID|523 transcript_886	227	3564	1	232	825	4162	1	232
transcript_28792	gnl|BL_ORD_ID|38811 transcript_8450	1	1250	1248	2029	5	1260	2167	2946
transcript_28828	gnl|BL_ORD_ID|49899 transcript_101218	743	1904	79	747	2962	4120	1	669
transcript_28828	gnl|BL_ORD_ID|30712 transcript_71789	743	1892	79	748	2413	3561	1	670
transcript_28845	gnl|BL_ORD_ID|92993 transcript_165511	1	1192	1189	1993	1	1194	1497	2302
transcript_28845	gnl|BL_ORD_ID|69252 transcript_129716	1	1780	1776	2026	1	1782	1900	2150
transcript_28960	gnl|BL_ORD_ID|94798 transcript_17986	283	2023	7	284	720	2457	15	290
transcript_28964	gnl|BL_ORD_ID|26547 transcript_65100	1	1132	1130	1941	1	1132	3046	3855
transcript_28993	gnl|BL_ORD_ID|20369 transcript_56606	1	1460	1458	1996	188	1648	2713	3251
transcript_28997	gnl|BL_ORD_ID|19287 transcript_54852	434	2022	6	439	1736	3324	1	434
transcript_29016	gnl|BL_ORD_ID|74403 transcript_138091	456	1867	8	457	807	2216	1	450
transcript_2903	gnl|BL_ORD_ID|79715 transcript_145187	2	2457	2457	3566	4	2459	2564	3674
transcript_29068	gnl|BL_ORD_ID|63039 transcript_121203	1	1224	1223	1956	1	1224	1431	2163
transcript_29076	gnl|BL_ORD_ID|31669 transcript_73334	1	1184	1181	1983	1	1184	1376	2178

transcript_29121	gnl|BL_ORD_ID|38688 transcript_8180	226	2034	5	227	1119	2923	32	254
transcript_29121	gnl|BL_ORD_ID|38706 transcript_8217	226	2030	5	227	1178	2982	89	311
transcript_29164	gnl|BL_ORD_ID|33674 transcript_76595	326	2009	7	325	858	2541	1	319
transcript_29177	gnl|BL_ORD_ID|2596 transcript_24546	314	2019	49	314	457	2161	2	267
transcript_29186	gnl|BL_ORD_ID|94702 transcript_17751	323	1949	7	323	855	2481	2	318
transcript_29186	gnl|BL_ORD_ID|76187 transcript_141001	323	1952	4	323	1097	2726	1	320
transcript_29197	gnl|BL_ORD_ID|91093 transcript_15880	15	1654	1653	2010	24	1663	2195	2552
transcript_29241	gnl|BL_ORD_ID|67456 transcript_126806	365	1973	42	365	544	2152	2	327
transcript_29249	gnl|BL_ORD_ID|65230 transcript_12613	1	1267	1264	2017	1	1267	1957	2711
transcript_2928	gnl|BL_ORD_ID|29488 transcript_69810	10	2042	2039	3557	1	2048	2337	3855
transcript_29290	gnl|BL_ORD_ID|43905 transcript_91507	10	1693	1690	2022	2	1684	1811	2143
transcript_29296	gnl|BL_ORD_ID|58058 transcript_113207	1	1196	1196	2001	1	1189	1896	2702
transcript_29297	gnl|BL_ORD_ID|32291 transcript_74410	516	2006	78	516	1377	2867	471	909
transcript_29318	gnl|BL_ORD_ID|80481 transcript_146441	1	1076	1076	2001	2	1078	1409	2333
transcript_29320	gnl|BL_ORD_ID|3285 transcript_26074	1	1662	1663	2009	1	1662	1791	2122
transcript_29320	gnl|BL_ORD_ID|95738 transcript_20197	1	1662	1661	1992	1	1665	2037	2366
transcript_29341	gnl|BL_ORD_ID|69902 transcript_130773	1	1072	1068	1981	1	1072	1186	2099
transcript_29347	gnl|BL_ORD_ID|67184 transcript_126373	1	1466	1462	2005	1	1466	4382	4924
transcript_29367	gnl|BL_ORD_ID|3656 transcript_26895	1	1199	1198	1953	44	1242	1359	2114
transcript_29381	gnl|BL_ORD_ID|42561 transcript_89286	1	1719	1716	1961	22	1740	1939	2184
transcript_29401	gnl|BL_ORD_ID|95628 transcript_19924	1	1516	1513	2025	40	1558	1871	2383
transcript_29401	gnl|BL_ORD_ID|57921 transcript_112995	1	1364	1363	2027	40	1402	1563	2227
transcript_29408	gnl|BL_ORD_ID|4321 transcript_28331	284	1969	32	284	379	2064	1	253
transcript_29423	gnl|BL_ORD_ID|19094 transcript_54534	1	1280	1277	1918	1	1276	1521	2163
transcript_29433	gnl|BL_ORD_ID|2719 transcript_24805	1	1572	1570	1992	1	1574	1774	2195
transcript_29437	gnl|BL_ORD_ID|3767 transcript_27120	1	1340	1339	1990	2	1341	1446	2098
transcript_29483	gnl|BL_ORD_ID|82882 transcript_150520	1	1031	1030	2004	1	1031	2017	2991
transcript_29514	gnl|BL_ORD_ID|36340 transcript_80933	1	1275	1273	1431	1	1271	1385	1543
transcript_29530	gnl|BL_ORD_ID|82811 transcript_150398	1	1337	1335	1958	1	1326	2947	3570
transcript_29538	gnl|BL_ORD_ID|72895 transcript_135613	700	1986	85	700	1153	2427	3	619
transcript_29578	gnl|BL_ORD_ID|2320 transcript_23954	300	1500	9	302	504	1707	26	300
transcript_29578	gnl|BL_ORD_ID|18744 transcript_53957	300	1500	9	302	482	1685	4	278
transcript_29763	gnl|BL_ORD_ID|88679 transcript_160193	205	1984	40	206	445	2224	2	168
transcript_2984	gnl|BL_ORD_ID|47574 transcript_97535	10	3070	3067	3390	3	3063	3358	3680

transcript_2984	gnl|BL_ORD_ID|77347 transcript_142840	2	2503	2500	3424	1	2491	2635	3558
transcript_29914	gnl|BL_ORD_ID|65985 transcript_124392	738	1993	92	737	2097	3356	2	646
transcript_29942	gnl|BL_ORD_ID|96189 transcript_21190	161	2083	1	164	300	2223	1	164
transcript_29942	gnl|BL_ORD_ID|1441 transcript_21984	161	2083	15	164	298	2221	2	151
transcript_29954	gnl|BL_ORD_ID|40914 transcript_86647	12	1571	1570	1987	2	1561	2122	2539
transcript_29962	gnl|BL_ORD_ID|77680 transcript_12906	238	1964	58	239	990	2716	2	183
transcript_29967	gnl|BL_ORD_ID|33085 transcript_75647	1	1372	1371	2004	1	1372	2241	2874
transcript_29985	gnl|BL_ORD_ID|4321 transcript_28331	298	1969	32	298	379	2051	1	253
transcript_30005	gnl|BL_ORD_ID|97266 transcript_142788	814	1718	9	814	948	1852	7	812
transcript_30025	gnl|BL_ORD_ID|32511 transcript_74762	293	1982	6	293	388	2077	1	288
transcript_30034	gnl|BL_ORD_ID|46587 transcript_96000	558	1972	74	559	856	2272	1	486
transcript_30056	gnl|BL_ORD_ID|77983 transcript_13567	1	1470	1471	1965	1	1467	2174	2669
transcript_30068	gnl|BL_ORD_ID|64625 transcript_11252	1	1004	1002	1919	1	1004	1869	2786
transcript_3008	gnl|BL_ORD_ID|62911 transcript_120991	1066	3566	106	1066	2035	4535	2	1006
transcript_30088	gnl|BL_ORD_ID|25156 transcript_5833	229	1992	93	234	497	2260	1	142
transcript_30125	gnl|BL_ORD_ID|40508 transcript_85976	1	1238	1239	2015	174	1409	1679	2455
transcript_30179	gnl|BL_ORD_ID|77431 transcript_142967	125	1914	1	127	231	2020	1	128
transcript_30264	gnl|BL_ORD_ID|12251 transcript_3414	1	1286	1285	1885	400	1684	2887	3487
transcript_30268	gnl|BL_ORD_ID|76187 transcript_141001	323	1953	4	323	1097	2726	1	320
transcript_30268	gnl|BL_ORD_ID|94702 transcript_17751	323	1950	7	323	855	2481	2	318
transcript_30367	gnl|BL_ORD_ID|50644 transcript_102433	215	1948	74	216	259	1990	1	144
transcript_30374	gnl|BL_ORD_ID|32978 transcript_75499	461	1935	56	460	539	2013	1	404
transcript_30392	gnl|BL_ORD_ID|2593 transcript_24538	399	1996	7	398	522	2120	2	392
transcript_30429	gnl|BL_ORD_ID|91575 transcript_16947	1	1357	1356	1963	1	1357	1851	2457
transcript_30433	gnl|BL_ORD_ID|88469 transcript_159840	424	1984	8	427	1335	2897	427	854
transcript_30437	gnl|BL_ORD_ID|51450 transcript_9131	219	1970	32	220	1150	2900	2	190
transcript_30448	gnl|BL_ORD_ID|94643 transcript_17631	1	1464	1464	1907	11	1467	2056	2494
transcript_30478	gnl|BL_ORD_ID|733 transcript_1278	212	1953	3	213	2111	3852	25	234
transcript_30478	gnl|BL_ORD_ID|3228 transcript_25936	214	1953	3	213	426	2164	19	229
transcript_30524	gnl|BL_ORD_ID|87947 transcript_158996	242	1954	54	241	1863	3573	1	188
transcript_30565	gnl|BL_ORD_ID|64956 transcript_11951	10	1561	1560	1954	2	1553	2300	2692
transcript_30588	gnl|BL_ORD_ID|75856 transcript_140450	399	1951	61	398	505	2062	1	338
transcript_30632	gnl|BL_ORD_ID|46673 transcript_96128	1	1057	1053	1956	1	1063	1171	2075
transcript_30637	gnl|BL_ORD_ID|33443 transcript_76208	10	1153	1149	1928	2	1145	1542	2319

transcript_30639	gnl|BL_ORD_ID|44342 transcript_92262	1	1103	1103	1893	240	1342	1462	2266
transcript_30650	gnl|BL_ORD_ID|20108 transcript_56193	1	1558	1557	1951	1	1558	1659	2053
transcript_30660	gnl|BL_ORD_ID|60358 transcript_116909	481	1923	6	483	612	2055	1	478
transcript_30660	gnl|BL_ORD_ID|4409 transcript_28512	1	1605	1603	1926	1	1605	1719	2041
transcript_30689	gnl|BL_ORD_ID|90264 transcript_162680	564	1945	57	566	1533	2914	2	511
transcript_30697	gnl|BL_ORD_ID|84392 transcript_153199	411	1947	68	411	750	2286	2	345
transcript_30699	gnl|BL_ORD_ID|4070 transcript_27786	1	1121	1122	1933	1	1121	1248	2059
transcript_30712	gnl|BL_ORD_ID|34627 transcript_78174	271	1922	55	273	1818	3467	2	220
transcript_30732	gnl|BL_ORD_ID|62272 transcript_119973	1	1428	1425	1900	1	1428	2087	2562
transcript_30767	gnl|BL_ORD_ID|64109 transcript_122930	325	1943	40	324	390	2007	2	286
transcript_308	gnl|BL_ORD_ID|87654 transcript_158525	256	4876	112	259	1575	6194	2	148
transcript_3080	gnl|BL_ORD_ID|11610 transcript_2172	647	3526	81	647	924	3803	2	568
transcript_30808	gnl|BL_ORD_ID|90737 transcript_15100	583	1953	63	587	1168	2535	2	527
transcript_30857	gnl|BL_ORD_ID|91079 transcript_15853	1	1042	1038	1917	1	1043	1655	2546
transcript_30865	gnl|BL_ORD_ID|51324 transcript_8885	1	1054	1055	1936	4	1057	2007	2888
transcript_30878	gnl|BL_ORD_ID|79398 transcript_144682	1	1155	1156	1934	1	1155	1780	2558
transcript_30891	gnl|BL_ORD_ID|2319 transcript_23952	1	1283	1278	1979	1	1282	1537	2238
transcript_30891	gnl|BL_ORD_ID|65114 transcript_12336	1	1370	1369	1979	1	1368	2118	2725
transcript_30891	gnl|BL_ORD_ID|1824 transcript_22876	1	1283	1278	1991	1	1301	1556	2270
transcript_30891	gnl|BL_ORD_ID|51864 transcript_10021	1	1370	1369	1988	1	1372	2242	2861
transcript_30891	gnl|BL_ORD_ID|32615 transcript_74943	501	1920	8	503	844	2263	1	486
transcript_30891	gnl|BL_ORD_ID|51613 transcript_9460	1	1370	1369	1991	1	1386	2260	2883
transcript_309	gnl|BL_ORD_ID|44419 transcript_92401	1	3848	3846	4810	2	3859	5153	6117
transcript_30919	gnl|BL_ORD_ID|74553 transcript_138338	1	1601	1600	1914	1	1601	2572	2886
transcript_30974	gnl|BL_ORD_ID|38553 transcript_7867	1	1601	1597	1929	1	1601	2645	2986
transcript_30974	gnl|BL_ORD_ID|62018 transcript_119557	1	1601	1597	1924	1	1601	2632	2957
transcript_31002	gnl|BL_ORD_ID|1952 transcript_23143	249	1948	47	251	508	2208	2	206
transcript_31006	gnl|BL_ORD_ID|38156 transcript_7020	1	1317	1317	1955	1	1317	2352	2990
transcript_31006	gnl|BL_ORD_ID|40467 transcript_85901	1	1317	1317	1955	1	1317	1874	2512
transcript_31006	gnl|BL_ORD_ID|64485 transcript_10952	1	1317	1317	1955	1	1317	2046	2684
transcript_31011	gnl|BL_ORD_ID|3038 transcript_25502	1	1548	1549	1921	1	1549	1822	2195
transcript_31011	gnl|BL_ORD_ID|90327 transcript_162764	1	1548	1549	1881	1	1583	1862	2199
transcript_31073	gnl|BL_ORD_ID|92930 transcript_165403	135	1906	1	138	1875	3646	5	140
transcript_311	gnl|BL_ORD_ID|92324 transcript_164413	2	2664	2659	4803	6	2677	3488	5632

transcript_3112	gnl|BL_ORD_ID|85614 transcript_155144	130	3335	3334	3539	2	3197	4040	4243
transcript_31129	gnl|BL_ORD_ID|31373 transcript_72864	1	1185	1180	1929	2902	4088	5121	5870
transcript_31132	gnl|BL_ORD_ID|30326 transcript_71180	1	1034	1032	1843	1	1035	1237	2046
transcript_31142	gnl|BL_ORD_ID|91233 transcript_16179	1	1017	1014	1926	1	1016	1624	2536
transcript_31145	gnl|BL_ORD_ID|82339 transcript_149571	254	1925	78	254	1000	2670	210	386
transcript_31167	gnl|BL_ORD_ID|77680 transcript_12906	211	1938	3	213	989	2716	393	603
transcript_31245	gnl|BL_ORD_ID|24712 transcript_4899	1	1509	1510	1935	87	1595	1770	2195
transcript_31248	gnl|BL_ORD_ID|78631 transcript_143448	265	1891	36	264	782	2418	2	230
transcript_3132	gnl|BL_ORD_ID|850 transcript_1499	1	2337	2335	3513	1	2337	2728	3906
transcript_31320	gnl|BL_ORD_ID|50100 transcript_101544	1	1436	1435	1879	1	1431	1708	2166
transcript_31374	gnl|BL_ORD_ID|30274 transcript_71091	1	1116	1117	1918	4	1113	2131	2938
transcript_31391	gnl|BL_ORD_ID|23488 transcript_61848	528	1910	61	529	572	1949	1	467
transcript_31417	gnl|BL_ORD_ID|77916 transcript_13430	321	1916	60	321	1072	2682	2	263
transcript_31419	gnl|BL_ORD_ID|90019 transcript_162309	393	1915	69	394	929	2449	1	326
transcript_31426	gnl|BL_ORD_ID|24712 transcript_4899	10	1605	1606	1982	1	1595	1770	2147
transcript_31468	gnl|BL_ORD_ID|28691 transcript_68580	405	1912	73	407	489	1994	2	336
transcript_3149	gnl|BL_ORD_ID|61238 transcript_118332	1	2242	2237	3502	1	2247	2738	4003
transcript_31502	gnl|BL_ORD_ID|36869 transcript_81767	1	1261	1260	1907	1	1258	1375	2021
transcript_31530	gnl|BL_ORD_ID|20395 transcript_56649	11	1540	1541	1909	2	1558	1679	2055
transcript_31530	gnl|BL_ORD_ID|55463 transcript_108885	11	1540	1541	1907	1	1533	1653	2021
transcript_31591	gnl|BL_ORD_ID|91639 transcript_17091	600	1763	9	601	1322	2488	3	587
transcript_31633	gnl|BL_ORD_ID|56653 transcript_110881	1	1001	999	1926	1108	2109	2515	3444
transcript_31663	gnl|BL_ORD_ID|69155 transcript_129566	1	1139	1138	1855	720	1856	2498	3208
transcript_31663	gnl|BL_ORD_ID|24915 transcript_5300	1	1139	1138	1909	631	1767	2407	3179
transcript_31669	gnl|BL_ORD_ID|78051 transcript_13762	11	1108	1108	1894	2	1099	1862	2649
transcript_31759	gnl|BL_ORD_ID|57037 transcript_111508	252	1910	44	253	1320	2997	2	211
transcript_31784	gnl|BL_ORD_ID|54490 transcript_107151	246	1910	46	248	439	2101	1	202
transcript_31802	gnl|BL_ORD_ID|53660 transcript_105748	116	1836	1	119	1080	2800	1	119
transcript_31842	gnl|BL_ORD_ID|40868 transcript_86583	342	1915	6	345	974	2547	20	359
transcript_31864	gnl|BL_ORD_ID|49511 transcript_100605	1	1330	1328	1822	1	1306	1831	2325
transcript_31869	gnl|BL_ORD_ID|67665 transcript_127158	465	1917	7	466	1935	3388	1	450
transcript_31924	gnl|BL_ORD_ID|67665 transcript_127158	443	1897	7	444	1935	3388	1	450
transcript_31946	gnl|BL_ORD_ID|41452 transcript_87544	130	1872	1	129	355	2094	1	131
transcript_31946	gnl|BL_ORD_ID|84659 transcript_153629	10	1220	1217	1854	2	1213	2048	2696

transcript_31953	gnl|BL_ORD_ID|18180 transcript_53071	499	1863	7	502	3062	4427	1	496
transcript_32032	gnl|BL_ORD_ID|24909 transcript_5291	1	1519	1520	1859	1	1514	2892	3236
transcript_32032	gnl|BL_ORD_ID|87710 transcript_158608	1	1519	1520	1890	1	1522	2885	3253
transcript_32043	gnl|BL_ORD_ID|57832 transcript_112850	1	1164	1162	1782	1	1118	1261	1853
transcript_32075	gnl|BL_ORD_ID|3093 transcript_25623	1	1299	1296	1860	82	1380	1593	2157
transcript_32075	gnl|BL_ORD_ID|80971 transcript_147240	1	1299	1296	1858	83	1355	1560	2122
transcript_32129	gnl|BL_ORD_ID|27453 transcript_66530	1	1295	1294	1919	1	1295	1395	2020
transcript_32157	gnl|BL_ORD_ID|82128 transcript_149200	149	1838	10	149	251	1940	2	141
transcript_32167	gnl|BL_ORD_ID|3491 transcript_26527	361	1879	6	362	588	2106	2	364
transcript_32171	gnl|BL_ORD_ID|43254 transcript_90419	286	1846	9	287	387	1947	1	276
transcript_32171	gnl|BL_ORD_ID|4866 transcript_29437	493	1846	9	494	633	1984	1	484
transcript_32174	gnl|BL_ORD_ID|43583 transcript_90950	169	1868	14	172	2128	3841	1	158
transcript_3221	gnl|BL_ORD_ID|34173 transcript_77416	2	2762	2761	3420	1	2759	3046	3704
transcript_32227	gnl|BL_ORD_ID|78311 transcript_14310	1	1104	1105	1871	1	1108	1876	2643
transcript_32228	gnl|BL_ORD_ID|82761 transcript_150312	463	1824	9	463	1681	3042	1	455
transcript_32228	gnl|BL_ORD_ID|50291 transcript_101842	802	1837	9	805	2962	3993	1	797
transcript_3223	gnl|BL_ORD_ID|93129 transcript_165726	1180	3495	1	1180	1351	3657	1	1175
transcript_3224	gnl|BL_ORD_ID|921 transcript_1645	1	2954	2952	3448	1	2961	3436	3932
transcript_32242	gnl|BL_ORD_ID|69359 transcript_129886	201	1863	43	204	680	2340	1	162
transcript_32257	gnl|BL_ORD_ID|96266 transcript_21355	1	1566	1566	1853	9	1564	2043	2330
transcript_32269	gnl|BL_ORD_ID|59628 transcript_115757	1	1429	1428	1866	1	1436	1558	1996
transcript_32283	gnl|BL_ORD_ID|37732 transcript_83122	271	1886	46	272	83	1683	2	228
transcript_32306	gnl|BL_ORD_ID|1824 transcript_22876	1	1278	1273	1942	2	1301	1556	2257
transcript_32306	gnl|BL_ORD_ID|2319 transcript_23952	1	1278	1273	1942	2	1282	1537	2237
transcript_32306	gnl|BL_ORD_ID|32615 transcript_74943	497	1885	6	499	844	2263	1	486
transcript_32309	gnl|BL_ORD_ID|57037 transcript_111508	253	1863	45	254	1320	2938	2	211
transcript_3231	gnl|BL_ORD_ID|11851 transcript_2632	1227	3509	1	1231	1330	3610	1	1230
transcript_32394	gnl|BL_ORD_ID|83567 transcript_151747	352	1873	5	352	868	2388	1	343
transcript_324	gnl|BL_ORD_ID|87654 transcript_158525	162	4779	1	161	1578	6194	68	228
transcript_32431	gnl|BL_ORD_ID|33394 transcript_76133	290	1866	6	293	907	2498	1	287
transcript_32508	gnl|BL_ORD_ID|3820 transcript_27241	179	1868	1	182	397	2086	1	182
transcript_32531	gnl|BL_ORD_ID|19515 transcript_55250	361	1854	52	362	1042	2534	2	312
transcript_32536	gnl|BL_ORD_ID|37421 transcript_82637	362	1867	44	364	467	1972	1	319
transcript_32558	gnl|BL_ORD_ID|57025 transcript_111484	616	1845	8	617	995	2217	1	610

transcript_32561	gnl|BL_ORD_ID|32615 transcript_74943	496	1915	7	498	844	2263	1	486
transcript_32561	gnl|BL_ORD_ID|1824 transcript_22876	1	1278	1273	1962	2	1301	1556	2246
transcript_32561	gnl|BL_ORD_ID|51864 transcript_10021	1	1365	1364	1962	2	1372	2242	2840
transcript_32561	gnl|BL_ORD_ID|65114 transcript_12336	1	1365	1364	1962	2	1368	2118	2713
transcript_32561	gnl|BL_ORD_ID|2319 transcript_23952	1	1278	1273	1962	2	1282	1537	2225
transcript_32561	gnl|BL_ORD_ID|51613 transcript_9460	1	1365	1364	1962	2	1386	2260	2859
transcript_32576	gnl|BL_ORD_ID|2299 transcript_23916	11	1287	1286	1859	81	1354	1723	2298
transcript_32576	gnl|BL_ORD_ID|5196 transcript_30161	1	1187	1185	1859	7	1193	1297	1971
transcript_32608	gnl|BL_ORD_ID|96657 transcript_83749	394	1836	8	395	522	1965	3	390
transcript_32610	gnl|BL_ORD_ID|71365 transcript_133154	1	1063	1061	1859	1	1063	1196	1991
transcript_32614	gnl|BL_ORD_ID|25265 transcript_6063	1	1068	1069	1858	2	1065	2154	2941
transcript_32668	gnl|BL_ORD_ID|87348 transcript_158045	1	1485	1486	1854	1	1478	1627	1995
transcript_32671	gnl|BL_ORD_ID|34140 transcript_77366	1	1225	1226	1861	1	1226	2061	2695
transcript_32700	gnl|BL_ORD_ID|62261 transcript_119950	609	1852	82	611	1004	2244	2	531
transcript_32736	gnl|BL_ORD_ID|26815 transcript_65512	338	1857	42	339	666	2185	3	300
transcript_32843	gnl|BL_ORD_ID|42737 transcript_89581	1	1275	1273	1818	1	1275	2699	3223
transcript_32864	gnl|BL_ORD_ID|96428 transcript_61846	247	1840	45	249	862	2463	1	202
transcript_32868	gnl|BL_ORD_ID|88553 transcript_159971	1	1361	1356	1838	8	1369	3420	3902
transcript_32873	gnl|BL_ORD_ID|76408 transcript_141334	582	1870	69	585	684	1972	2	518
transcript_3292	gnl|BL_ORD_ID|61455 transcript_118691	1674	3479	1	1678	2106	3918	1	1678
transcript_32944	gnl|BL_ORD_ID|56599 transcript_110786	174	1808	1	178	308	1942	1	179
transcript_32947	gnl|BL_ORD_ID|48894 transcript_99629	1	1456	1456	1878	1	1456	1784	2205
transcript_32958	gnl|BL_ORD_ID|87765 transcript_158704	1	1250	1248	1782	1	1250	1941	2475
transcript_3297	gnl|BL_ORD_ID|685 transcript_1184	1459	3534	1	1460	1992	4067	104	1562
transcript_32989	gnl|BL_ORD_ID|50238 transcript_101765	1	1236	1236	1830	1	1236	1408	2002
transcript_3299	gnl|BL_ORD_ID|68910 transcript_129163	576	3522	75	579	637	3583	1	500
transcript_33000	gnl|BL_ORD_ID|72709 transcript_135301	715	1848	76	716	811	1944	1	641
transcript_33032	gnl|BL_ORD_ID|52810 transcript_104309	444	1846	5	443	900	2315	30	468
transcript_33041	gnl|BL_ORD_ID|29073 transcript_69176	1	1324	1322	1849	1	1307	2315	2842
transcript_33053	gnl|BL_ORD_ID|59409 transcript_115411	392	1832	45	394	1140	2580	1	350
transcript_33060	gnl|BL_ORD_ID|17478 transcript_51969	1	1215	1214	1830	82	1297	1514	2129
transcript_33093	gnl|BL_ORD_ID|1587 transcript_22366	532	1872	9	532	974	2312	2	524
transcript_33165	gnl|BL_ORD_ID|93778 transcript_166771	484	1812	5	483	1562	2875	1	475
transcript_33219	gnl|BL_ORD_ID|79794 transcript_145305	194	1676	2	195	327	1812	9	202

transcript_33231	gnl|BL_ORD_ID|3971 transcript_27571	186	1844	4	187	414	2078	4	185
transcript_33239	gnl|BL_ORD_ID|90731 transcript_15085	783	1806	8	785	1579	2602	3	780
transcript_3328	gnl|BL_ORD_ID|89002 transcript_160681	10	3265	3262	3448	2	3266	4186	4372
transcript_33295	gnl|BL_ORD_ID|72277 transcript_134624	1	1345	1340	1808	1	1345	1678	2146
transcript_333	gnl|BL_ORD_ID|28588 transcript_68411	241	4761	112	240	485	5000	2	130
transcript_33313	gnl|BL_ORD_ID|38300 transcript_7318	1	1210	1209	1762	1	1210	2434	2987
transcript_33325	gnl|BL_ORD_ID|46736 transcript_96227	399	1811	5	400	2381	3789	1	396
transcript_33347	gnl|BL_ORD_ID|93778 transcript_166771	546	1841	71	545	1562	2854	1	475
transcript_3335	gnl|BL_ORD_ID|69319 transcript_129824	1	2187	2187	3492	34	2221	2385	3690
transcript_33353	gnl|BL_ORD_ID|67665 transcript_127158	427	1820	5	428	1935	3328	1	450
transcript_33365	gnl|BL_ORD_ID|78803 transcript_143730	224	1836	62	225	2361	3973	2	165
transcript_33365	gnl|BL_ORD_ID|37363 transcript_82540	225	1849	59	225	680	2304	1	167
transcript_33367	gnl|BL_ORD_ID|61433 transcript_118654	1	1515	1513	1832	33	1547	2511	2831
transcript_33367	gnl|BL_ORD_ID|51712 transcript_9677	1	1515	1513	1832	34	1548	2523	2842
transcript_33370	gnl|BL_ORD_ID|35211 transcript_79118	162	1826	1	166	1484	3149	1	166
transcript_33474	gnl|BL_ORD_ID|48102 transcript_98352	1	1100	1101	1825	2	1101	1202	1926
transcript_3351	gnl|BL_ORD_ID|43853 transcript_91412	110	2688	2683	3489	2	2570	3611	4418
transcript_33520	gnl|BL_ORD_ID|83406 transcript_151449	145	1739	1	149	1529	3122	1	149
transcript_33523	gnl|BL_ORD_ID|73446 transcript_136499	750	1786	9	753	1373	2407	1	736
transcript_33526	gnl|BL_ORD_ID|5250 transcript_30276	1	1490	1490	1808	1	1492	1641	1959
transcript_33555	gnl|BL_ORD_ID|41373 transcript_87403	331	1884	39	334	2502	4055	604	899
transcript_33558	gnl|BL_ORD_ID|67328 transcript_126607	1	1471	1470	1804	1	1488	2926	3261
transcript_33558	gnl|BL_ORD_ID|53476 transcript_105409	1	1238	1236	1804	1	1238	1350	1919
transcript_33625	gnl|BL_ORD_ID|77916 transcript_13430	323	1833	66	323	1072	2582	6	263
transcript_33649	gnl|BL_ORD_ID|4243 transcript_28176	1	1106	1102	1822	119	1224	1331	2050
transcript_33649	gnl|BL_ORD_ID|5465 transcript_30709	1	1106	1102	1820	4	1109	1216	1933
transcript_33659	gnl|BL_ORD_ID|35638 transcript_79775	1	1194	1192	1760	1	1194	2228	2796
transcript_33659	gnl|BL_ORD_ID|63863 transcript_122548	313	1803	65	316	1482	2964	1	252
transcript_33674	gnl|BL_ORD_ID|5142 transcript_30044	326	1795	66	328	498	1967	5	267
transcript_33674	gnl|BL_ORD_ID|55082 transcript_108200	781	1795	82	781	897	1911	2	701
transcript_33689	gnl|BL_ORD_ID|75154 transcript_139306	267	1813	54	268	1520	3066	2	220
transcript_33696	gnl|BL_ORD_ID|35638 transcript_79775	1	1200	1198	1766	1	1194	2228	2796
transcript_3370	gnl|BL_ORD_ID|11851 transcript_2632	1206	3496	1	1210	1330	3618	1	1230
transcript_33779	gnl|BL_ORD_ID|53840 transcript_106065	242	1765	3	244	609	2132	1	242

transcript_33810	gnl|BL_ORD_ID|66990 transcript_126056	1	1111	1110	1798	2	1112	1787	2472
transcript_33827	gnl|BL_ORD_ID|79033 transcript_144108	114	1715	1	119	1172	2772	1	119
transcript_33835	gnl|BL_ORD_ID|32241 transcript_74322	343	1786	6	345	1138	2578	3	340
transcript_33883	gnl|BL_ORD_ID|51715 transcript_9689	359	1790	5	360	1454	2885	2	359
transcript_33942	gnl|BL_ORD_ID|57832 transcript_112850	1	1110	1108	1740	1	1118	1261	1893
transcript_33956	gnl|BL_ORD_ID|54341 transcript_106881	1	1118	1118	1696	1	1117	1364	1943
transcript_33977	gnl|BL_ORD_ID|96476 transcript_66912	12	1288	1286	1790	9	1285	2188	2690
transcript_33986	gnl|BL_ORD_ID|3042 transcript_25511	286	1769	36	286	698	2183	2	258
transcript_340	gnl|BL_ORD_ID|32703 transcript_75092	1	4221	4217	4753	1	4218	4325	4861
transcript_34013	gnl|BL_ORD_ID|39480 transcript_84319	1	1368	1366	1762	1	1368	2622	3018
transcript_34014	gnl|BL_ORD_ID|57486 transcript_112260	1	1275	1274	1769	1	1252	1686	2180
transcript_34014	gnl|BL_ORD_ID|45718 transcript_94572	1	1144	1142	1782	1	1144	2184	2826
transcript_34014	gnl|BL_ORD_ID|51876 transcript_10047	1	1144	1142	1782	1	1122	2149	2789
transcript_34014	gnl|BL_ORD_ID|60503 transcript_117148	1	1275	1274	1776	1	1252	1678	2176
transcript_34014	gnl|BL_ORD_ID|69716 transcript_130485	118	1764	1	120	735	2381	1	120
transcript_34019	gnl|BL_ORD_ID|62608 transcript_120536	607	1785	8	607	1519	2697	2	583
transcript_34021	gnl|BL_ORD_ID|77844 transcript_13267	13	1383	1382	1700	2	1372	2333	2649
transcript_3403	gnl|BL_ORD_ID|109 transcript_162	1	2299	2299	3479	2	2299	3948	5128
transcript_34040	gnl|BL_ORD_ID|26030 transcript_64294	1	1227	1224	1667	1	1226	1326	1770
transcript_34044	gnl|BL_ORD_ID|24909 transcript_5291	1	1513	1514	1787	1	1514	2892	3164
transcript_34044	gnl|BL_ORD_ID|87710 transcript_158608	1	1513	1514	1787	1	1522	2885	3151
transcript_34044	gnl|BL_ORD_ID|76276 transcript_141145	1	1513	1514	1787	1	1511	2894	3166
transcript_34164	gnl|BL_ORD_ID|60786 transcript_117600	1	1295	1294	1774	4	1298	4936	5417
transcript_34183	gnl|BL_ORD_ID|25957 transcript_64188	263	1746	4	263	1282	2765	3	262
transcript_34196	gnl|BL_ORD_ID|43156 transcript_90255	1	1176	1171	1778	1	1176	1431	2038
transcript_34199	gnl|BL_ORD_ID|23958 transcript_62602	435	1812	87	436	486	1863	1	350
transcript_3420	gnl|BL_ORD_ID|36089 transcript_80505	3	3288	3285	3488	126	3405	3613	3816
transcript_3420	gnl|BL_ORD_ID|58791 transcript_114405	3	3286	3285	3481	168	3444	3582	3778
transcript_34205	gnl|BL_ORD_ID|1019 transcript_1816	115	1774	1	115	2015	3673	1	115
transcript_34220	gnl|BL_ORD_ID|70451 transcript_131691	1	1439	1438	1755	1	1437	1612	1932
transcript_34225	gnl|BL_ORD_ID|33163 transcript_75777	197	1764	44	198	1452	3019	1	155
transcript_34237	gnl|BL_ORD_ID|25609 transcript_63617	1	1052	1049	1632	1	1051	2438	3022
transcript_34249	gnl|BL_ORD_ID|17105 transcript_51380	885	1785	9	888	987	1887	2	879
transcript_34260	gnl|BL_ORD_ID|58384 transcript_113744	1	1019	1016	1760	1	1017	1239	1987

transcript_34263	gnl|BL_ORD_ID|31532 transcript_73098	1	1248	1247	1766	1	1249	1741	2260
transcript_34276	gnl|BL_ORD_ID|71365 transcript_133154	1	1062	1060	1810	2	1063	1196	1946
transcript_34335	gnl|BL_ORD_ID|81119 transcript_147470	486	1763	84	486	550	1824	1	403
transcript_34439	gnl|BL_ORD_ID|24471 transcript_4391	346	1798	38	348	1889	3339	31	341
transcript_34439	gnl|BL_ORD_ID|425 transcript_729	346	1798	50	348	1848	3299	2	300
transcript_3444	gnl|BL_ORD_ID|11674 transcript_2289	1416	3471	1	1416	1571	3625	2	1417
transcript_3445	gnl|BL_ORD_ID|176 transcript_277	1	3058	3055	3461	1	3049	4412	4822
transcript_3446	gnl|BL_ORD_ID|30563 transcript_71563	12	2643	2638	3442	2	2633	2734	3540
transcript_3450	gnl|BL_ORD_ID|90176 transcript_162549	296	3487	101	296	307	3498	3	197
transcript_3450	gnl|BL_ORD_ID|50720 transcript_102559	102	2606	2603	3474	2	2506	2680	3551
transcript_3450	gnl|BL_ORD_ID|34447 transcript_77870	1332	3474	102	1333	1407	3549	2	1233
transcript_34534	gnl|BL_ORD_ID|32432 transcript_74637	840	1757	9	842	1388	2305	3	842
transcript_34548	gnl|BL_ORD_ID|47345 transcript_97183	173	1796	14	172	2282	3907	1	158
transcript_3456	gnl|BL_ORD_ID|1154 transcript_2072	1	3269	3265	3491	3	3274	3509	3736
transcript_3461	gnl|BL_ORD_ID|886 transcript_1575	311	3470	2	312	453	3614	42	352
transcript_34618	gnl|BL_ORD_ID|30890 transcript_72094	1	1320	1318	1728	112	1431	3017	3426
transcript_34618	gnl|BL_ORD_ID|81181 transcript_147571	1	1455	1454	1728	126	1586	1694	1967
transcript_34620	gnl|BL_ORD_ID|5558 transcript_30891	1	1404	1400	1757	1	1388	1621	1979
transcript_34620	gnl|BL_ORD_ID|3977 transcript_27585	1	1404	1400	1757	2	1387	1717	2074
transcript_34620	gnl|BL_ORD_ID|4240 transcript_28169	1	1404	1400	1742	1	1388	1646	1989
transcript_34620	gnl|BL_ORD_ID|1531 transcript_22223	1	1404	1400	1743	9	1448	1685	2053
transcript_34620	gnl|BL_ORD_ID|22153 transcript_59602	1	1404	1400	1740	1	1397	1630	1971
transcript_34626	gnl|BL_ORD_ID|30202 transcript_70966	1	1377	1372	1746	34	1413	2295	2669
transcript_3463	gnl|BL_ORD_ID|434 transcript_743	1	1797	1793	3462	2	1792	2572	4267
transcript_34649	gnl|BL_ORD_ID|53840 transcript_106065	269	1759	30	271	609	2099	1	242
transcript_34661	gnl|BL_ORD_ID|19740 transcript_55615	2	1000	999	1766	102	1100	1702	2469
transcript_34669	gnl|BL_ORD_ID|43639 transcript_91054	13	1403	1402	1784	2	1392	1497	1880
transcript_34677	gnl|BL_ORD_ID|27130 transcript_66028	259	1799	37	258	1447	2976	259	478
transcript_3468	gnl|BL_ORD_ID|25924 transcript_64138	2	2666	2664	3465	90	2760	3653	4450
transcript_34698	gnl|BL_ORD_ID|66176 transcript_124733	489	1705	56	491	1759	2974	2	438
transcript_34710	gnl|BL_ORD_ID|60033 transcript_116397	1	1462	1460	1777	73	1527	1730	2047
transcript_34711	gnl|BL_ORD_ID|5084 transcript_29914	404	1784	71	409	613	1993	2	340
transcript_34741	gnl|BL_ORD_ID|91639 transcript_17091	580	1746	9	581	1322	2488	3	587
transcript_34814	gnl|BL_ORD_ID|56770 transcript_111066	1	1164	1163	1747	3	1166	2108	2692

transcript_34824	gnl|BL_ORD_ID|3477 transcript_26498	553	1797	65	556	850	2093	1	492
transcript_34839	gnl|BL_ORD_ID|276 transcript_448	175	1721	41	174	3042	4588	41	174
transcript_34839	gnl|BL_ORD_ID|6152 transcript_32174	175	1727	6	175	325	1876	2	169
transcript_34839	gnl|BL_ORD_ID|42315 transcript_88912	175	1719	27	174	2336	3897	3	148
transcript_34839	gnl|BL_ORD_ID|43583 transcript_90950	175	1719	19	174	2285	3841	1	154
transcript_34839	gnl|BL_ORD_ID|934 transcript_1665	175	1721	2	174	2337	3882	40	210
transcript_3484	gnl|BL_ORD_ID|1095 transcript_1970	2	2484	2483	3466	81	2560	2687	3670
transcript_34843	gnl|BL_ORD_ID|53503 transcript_105452	203	1735	83	208	824	2356	212	337
transcript_34843	gnl|BL_ORD_ID|85278 transcript_154606	356	1735	60	357	1001	2380	2	299
transcript_3489	gnl|BL_ORD_ID|93014 transcript_165549	359	3472	95	358	794	3907	2	265
transcript_34912	gnl|BL_ORD_ID|73776 transcript_137050	577	1745	8	577	713	1876	3	552
transcript_34912	gnl|BL_ORD_ID|18946 transcript_54278	1	1260	1257	1740	1	1260	1757	2239
transcript_34969	gnl|BL_ORD_ID|19071 transcript_54492	583	1726	7	584	1938	3081	3	600
transcript_34970	gnl|BL_ORD_ID|44712 transcript_92876	400	1758	50	402	1326	2684	1	353
transcript_35040	gnl|BL_ORD_ID|28101 transcript_67579	1	1019	1019	1728	1	1019	1156	1865
transcript_35054	gnl|BL_ORD_ID|81124 transcript_147480	1	1046	1045	1714	1	1046	2826	3493
transcript_35057	gnl|BL_ORD_ID|56599 transcript_110786	198	1748	26	202	308	1858	2	179
transcript_35118	gnl|BL_ORD_ID|97079 transcript_124801	509	1732	53	511	860	2084	2	462
transcript_35185	gnl|BL_ORD_ID|83097 transcript_150916	384	1750	47	385	834	2202	1	339
transcript_35191	gnl|BL_ORD_ID|71016 transcript_132617	120	1729	1	120	572	2174	1	120
transcript_35209	gnl|BL_ORD_ID|5580 transcript_30931	1	1440	1440	1735	2	1459	1623	1919
transcript_3524	gnl|BL_ORD_ID|25881 transcript_64073	2	2296	2294	3471	1	2298	2689	3867
transcript_35286	gnl|BL_ORD_ID|88452 transcript_159814	466	1731	8	465	1127	2392	1	456
transcript_35293	gnl|BL_ORD_ID|42315 transcript_88912	162	1705	15	161	2336	3897	3	148
transcript_35293	gnl|BL_ORD_ID|385 transcript_658	162	1712	1	161	2765	4316	1	161
transcript_35296	gnl|BL_ORD_ID|80648 transcript_146715	343	1733	58	345	1491	2880	2	289
transcript_35300	gnl|BL_ORD_ID|31172 transcript_72536	210	1658	8	212	1449	2897	2	206
transcript_35317	gnl|BL_ORD_ID|35341 transcript_79318	591	1718	66	595	989	2114	84	613
transcript_35317	gnl|BL_ORD_ID|43728 transcript_91193	465	1718	66	465	836	2089	86	485
transcript_35327	gnl|BL_ORD_ID|80720 transcript_146826	419	1722	55	419	1232	2535	2	366
transcript_3536	gnl|BL_ORD_ID|66304 transcript_124946	458	3479	61	455	720	3742	1	390
transcript_35377	gnl|BL_ORD_ID|93485 transcript_166301	1	1281	1278	1713	448	1728	2014	2448
transcript_35393	gnl|BL_ORD_ID|19087 transcript_54515	263	1720	79	265	319	1780	2	188
transcript_35399	gnl|BL_ORD_ID|33235 transcript_75881	487	1726	60	486	2687	3926	2	429

transcript_35484	gnl|BL_ORD_ID|34147 transcript_77379	294	1658	3	295	1260	2623	4	298
transcript_35486	gnl|BL_ORD_ID|12568 transcript_4047	1	1026	1027	1628	99	1124	2780	3381
transcript_35494	gnl|BL_ORD_ID|95738 transcript_20197	1	1379	1378	1708	288	1665	2037	2366
transcript_35494	gnl|BL_ORD_ID|3285 transcript_26074	1	1379	1380	1729	285	1662	1791	2127
transcript_35494	gnl|BL_ORD_ID|33836 transcript_76858	1	1379	1380	1728	284	1661	1831	2166
transcript_35543	gnl|BL_ORD_ID|77009 transcript_142293	300	1701	51	299	1308	2709	4	257
transcript_35613	gnl|BL_ORD_ID|20496 transcript_56829	173	1638	18	172	759	2224	125	279
transcript_35619	gnl|BL_ORD_ID|93856 transcript_166890	545	1738	69	545	677	1873	45	521
transcript_35624	gnl|BL_ORD_ID|88821 transcript_160419	1	1055	1052	1706	2	1061	1180	1833
transcript_35630	gnl|BL_ORD_ID|73159 transcript_136009	1	1138	1136	1675	1	1154	1424	1963
transcript_35681	gnl|BL_ORD_ID|28432 transcript_68165	171	1639	2	174	561	2029	187	359
transcript_3569	gnl|BL_ORD_ID|199 transcript_313	2	2665	2665	3490	18	2681	3964	4789
transcript_35694	gnl|BL_ORD_ID|92934 transcript_165410	595	1702	8	596	1363	2464	1	590
transcript_3570	gnl|BL_ORD_ID|71249 transcript_132966	1605	3459	123	1605	1918	3773	321	1801
transcript_3570	gnl|BL_ORD_ID|1044 transcript_1875	1605	3459	102	1605	1944	3798	324	1827
transcript_3570	gnl|BL_ORD_ID|27119 transcript_66015	1605	3459	100	1605	1824	3678	202	1707
transcript_3570	gnl|BL_ORD_ID|55959 transcript_109770	1605	3459	102	1605	1843	3696	223	1726
transcript_3570	gnl|BL_ORD_ID|83151 transcript_151008	1605	3459	102	1605	2066	3918	447	1949
transcript_35708	gnl|BL_ORD_ID|77474 transcript_143039	10	1254	1252	1597	2	1252	2440	2811
transcript_35708	gnl|BL_ORD_ID|67395 transcript_126712	1	1052	1053	1611	1	1051	1948	2506
transcript_35732	gnl|BL_ORD_ID|78367 transcript_14446	1	1046	1044	1571	1	1045	2088	2615
transcript_35736	gnl|BL_ORD_ID|80648 transcript_146715	329	1712	42	331	1491	2889	2	289
transcript_35737	gnl|BL_ORD_ID|6427 transcript_32745	134	1671	1	134	241	1778	1	134
transcript_35772	gnl|BL_ORD_ID|95930 transcript_20646	280	1694	61	283	944	2358	1	223
transcript_3584	gnl|BL_ORD_ID|23368 transcript_61631	110	2025	2024	3418	1	1917	2397	3791
transcript_35852	gnl|BL_ORD_ID|48072 transcript_98304	208	1698	3	207	1160	2651	3	207
transcript_35883	gnl|BL_ORD_ID|72166 transcript_134450	185	1672	3	186	1182	2640	1	186
transcript_35883	gnl|BL_ORD_ID|91608 transcript_17025	185	1693	2	186	984	2486	1	187
transcript_35883	gnl|BL_ORD_ID|3971 transcript_27571	185	1693	2	185	587	2095	1	184
transcript_35883	gnl|BL_ORD_ID|81413 transcript_147941	185	1662	2	185	410	1884	1	187
transcript_3590	gnl|BL_ORD_ID|36251 transcript_80775	1	2175	2172	3486	9	2179	2735	4047
transcript_35925	gnl|BL_ORD_ID|72472 transcript_134926	494	1693	6	495	2192	3386	1	492
transcript_35932	gnl|BL_ORD_ID|6535 transcript_32964	180	1699	8	179	341	1858	1	172
transcript_35933	gnl|BL_ORD_ID|82168 transcript_149273	1	1294	1293	1702	31	1324	1488	1896

transcript_35946	gnl|BL_ORD_ID|74562 transcript_138348	839	1676	9	841	2392	3231	1	821
transcript_36010	gnl|BL_ORD_ID|56730 transcript_111001	139	1693	1	140	1406	2961	1	140
transcript_36056	gnl|BL_ORD_ID|34563 transcript_78063	1	1176	1173	1666	1	1176	2049	2542
transcript_36073	gnl|BL_ORD_ID|33163 transcript_75777	152	1621	1	151	1551	3019	1	155
transcript_36144	gnl|BL_ORD_ID|30533 transcript_71511	384	1674	39	386	753	2042	1	348
transcript_36152	gnl|BL_ORD_ID|4812 transcript_29351	1	1383	1384	1673	1	1383	1703	1992
transcript_36161	gnl|BL_ORD_ID|41160 transcript_87049	345	1650	39	349	887	2192	2	311
transcript_36167	gnl|BL_ORD_ID|93654 transcript_166584	402	1663	5	402	1417	2678	2	399
transcript_36178	gnl|BL_ORD_ID|49283 transcript_100238	116	1726	1	120	1031	2641	31	153
transcript_36242	gnl|BL_ORD_ID|76307 transcript_141184	129	1664	1	130	3603	5138	1	131
transcript_3625	gnl|BL_ORD_ID|30065 transcript_70752	125	3438	11	126	260	3575	2	117
transcript_36266	gnl|BL_ORD_ID|47322 transcript_97150	492	1671	8	493	2088	3268	2	487
transcript_36281	gnl|BL_ORD_ID|69096 transcript_129463	292	1652	3	293	939	2296	1	291
transcript_3629	gnl|BL_ORD_ID|11601 transcript_2155	190	3455	1	193	460	3719	1	193
transcript_36374	gnl|BL_ORD_ID|25214 transcript_5942	198	1749	73	199	1386	2938	3	128
transcript_36374	gnl|BL_ORD_ID|77431 transcript_142967	200	1749	73	199	306	1856	2	128
transcript_3640	gnl|BL_ORD_ID|97499 transcript_166183	1	2608	2603	3436	1	2612	2869	3699
transcript_36476	gnl|BL_ORD_ID|29603 transcript_69997	224	1656	48	225	1145	2568	2	179
transcript_36476	gnl|BL_ORD_ID|69096 transcript_129463	338	1656	49	339	939	2257	1	291
transcript_36586	gnl|BL_ORD_ID|45336 transcript_93923	1	1219	1218	1593	2	1206	1951	2326
transcript_36586	gnl|BL_ORD_ID|63787 transcript_122427	1	1219	1218	1600	2	1212	3675	4057
transcript_36595	gnl|BL_ORD_ID|61820 transcript_119245	1	1260	1259	1631	2	1261	1389	1769
transcript_36611	gnl|BL_ORD_ID|95958 transcript_20707	165	1607	4	165	895	2337	1	162
transcript_36611	gnl|BL_ORD_ID|2364 transcript_24055	163	1643	4	165	658	2137	41	194
transcript_36611	gnl|BL_ORD_ID|62256 transcript_119943	165	1643	6	165	885	2362	2	149
transcript_36611	gnl|BL_ORD_ID|46534 transcript_95908	166	1643	2	165	1341	2818	16	179
transcript_36611	gnl|BL_ORD_ID|17453 transcript_51928	165	1633	4	165	279	1746	19	168
transcript_3663	gnl|BL_ORD_ID|75031 transcript_139110	335	3450	3	337	618	3731	1	342
transcript_3663	gnl|BL_ORD_ID|56570 transcript_110737	335	3445	3	337	611	3725	1	335
transcript_3666	gnl|BL_ORD_ID|20341 transcript_56554	28	2879	2876	3453	2	2852	2958	3534
transcript_36770	gnl|BL_ORD_ID|87576 transcript_158406	470	1646	6	475	1672	2850	2	471
transcript_36798	gnl|BL_ORD_ID|56111 transcript_110022	198	1666	31	198	395	1864	2	175
transcript_36798	gnl|BL_ORD_ID|22364 transcript_59949	198	1660	49	200	923	2385	4	149
transcript_36798	gnl|BL_ORD_ID|94943 transcript_18335	198	1651	2	200	1014	2467	82	281

transcript_36798	gnl|BL_ORD_ID|75079 transcript_139183	198	1666	49	200	915	2383	7	167
transcript_36798	gnl|BL_ORD_ID|6038 transcript_31902	198	1666	2	198	433	1901	20	216
transcript_36798	gnl|BL_ORD_ID|48079 transcript_98317	198	1666	49	200	937	2405	31	191
transcript_36798	gnl|BL_ORD_ID|1963 transcript_23159	198	1666	50	200	780	2248	1	151
transcript_36804	gnl|BL_ORD_ID|79121 transcript_144252	1	1392	1390	1691	1	1380	1977	2278
transcript_36852	gnl|BL_ORD_ID|86242 transcript_156200	435	1651	5	434	875	2091	2	431
transcript_36932	gnl|BL_ORD_ID|32395 transcript_74590	210	1639	29	211	609	2038	10	192
transcript_36964	gnl|BL_ORD_ID|7010 transcript_33949	172	1632	2	172	410	1877	138	308
transcript_36971	gnl|BL_ORD_ID|46053 transcript_95141	1	1178	1177	1625	1	1176	2071	2519
transcript_36971	gnl|BL_ORD_ID|23145 transcript_61278	614	1616	7	615	783	1785	2	609
transcript_36987	gnl|BL_ORD_ID|82109 transcript_149164	156	1632	1	157	2181	3657	10	166
transcript_36988	gnl|BL_ORD_ID|96191 transcript_21195	1	1213	1210	1626	23	1235	1901	2317
transcript_37010	gnl|BL_ORD_ID|91096 transcript_15884	1	1153	1151	1628	1	1153	2079	2557
transcript_37033	gnl|BL_ORD_ID|61487 transcript_118738	185	1627	79	185	231	1673	1	107
transcript_37060	gnl|BL_ORD_ID|71271 transcript_133002	434	1617	53	435	1247	2428	13	395
transcript_37069	gnl|BL_ORD_ID|50012 transcript_101405	1	1016	1014	1626	2	1020	1993	2605
transcript_37084	gnl|BL_ORD_ID|48906 transcript_99648	193	1621	3	196	858	2287	1	194
transcript_37095	gnl|BL_ORD_ID|17744 transcript_52391	9	1002	998	1545	1	994	2131	2653
transcript_37116	gnl|BL_ORD_ID|44211 transcript_92034	1	1020	1017	1578	1	1017	1739	2292
transcript_37142	gnl|BL_ORD_ID|62585 transcript_120500	303	1625	4	304	550	1873	21	323
transcript_3715	gnl|BL_ORD_ID|41726 transcript_88002	2	2890	2890	3514	27	2912	4200	4823
transcript_372	gnl|BL_ORD_ID|76179 transcript_140987	1	3071	3071	4474	1	3074	4621	6024
transcript_3727	gnl|BL_ORD_ID|11771 transcript_2479	1	3110	3109	3468	1	3131	3250	3608
transcript_3727	gnl|BL_ORD_ID|12122 transcript_3187	2	3110	3109	3382	6	3120	3240	3516
transcript_3727	gnl|BL_ORD_ID|11752 transcript_2445	1	3110	3109	3468	1	3114	3235	3593
transcript_37288	gnl|BL_ORD_ID|96087 transcript_20991	1	1226	1223	1613	1	1226	1943	2333
transcript_3729	gnl|BL_ORD_ID|11593 transcript_2140	2	2723	2722	3436	3	2724	3022	3736
transcript_37297	gnl|BL_ORD_ID|86120 transcript_155996	1	1052	1049	1586	1	1052	2396	2933
transcript_37297	gnl|BL_ORD_ID|25609 transcript_63617	1	1052	1049	1608	1	1051	2438	2999
transcript_3732	gnl|BL_ORD_ID|12127 transcript_3193	106	3051	3050	3435	32	2975	3124	3510
transcript_3732	gnl|BL_ORD_ID|32348 transcript_74506	2	3053	3050	3459	3	3055	3203	3612
transcript_37372	gnl|BL_ORD_ID|7162 transcript_34225	145	1602	1	146	313	1770	49	198
transcript_37372	gnl|BL_ORD_ID|33163 transcript_75777	145	1596	1	147	1568	3019	6	156
transcript_37425	gnl|BL_ORD_ID|4280 transcript_28253	247	1594	9	249	625	1976	2	244

transcript_37425	gnl|BL_ORD_ID|92453 transcript_164638	247	1594	35	249	585	1936	2	216
transcript_37435	gnl|BL_ORD_ID|90427 transcript_162911	368	1599	5	370	503	1735	1	366
transcript_3745	gnl|BL_ORD_ID|89268 transcript_161118	2	2542	2542	3430	42	2582	2699	3587
transcript_37473	gnl|BL_ORD_ID|43639 transcript_91054	1	1214	1213	1596	176	1392	1497	1880
transcript_37486	gnl|BL_ORD_ID|3406 transcript_26340	495	1594	7	499	1028	2127	5	496
transcript_37495	gnl|BL_ORD_ID|1344 transcript_21750	1	1126	1125	1583	1	1126	1834	2288
transcript_37561	gnl|BL_ORD_ID|87037 transcript_157510	145	1594	1	148	675	2126	25	172
transcript_37603	gnl|BL_ORD_ID|96161 transcript_21140	164	1594	5	166	880	2310	3	164
transcript_37655	gnl|BL_ORD_ID|36082 transcript_80495	749	1548	8	753	1365	2165	1	736
transcript_37667	gnl|BL_ORD_ID|63006 transcript_121149	247	1599	51	249	345	1696	2	201
transcript_37667	gnl|BL_ORD_ID|7822 transcript_35550	247	1599	29	249	366	1718	1	221
transcript_37671	gnl|BL_ORD_ID|6039 transcript_31909	1	1033	1033	1476	1	1030	1412	1855
transcript_37689	gnl|BL_ORD_ID|75865 transcript_140465	1	1025	1024	1610	19	1031	1981	2567
transcript_3769	gnl|BL_ORD_ID|54184 transcript_106624	1623	3357	10	1622	2476	4205	16	1627
transcript_37692	gnl|BL_ORD_ID|71172 transcript_132853	431	1611	9	432	1728	2906	1	424
transcript_377	gnl|BL_ORD_ID|193 transcript_307	124	4663	1	125	243	4782	1	124
transcript_37701	gnl|BL_ORD_ID|59225 transcript_115109	548	1510	6	552	1114	2077	5	550
transcript_37752	gnl|BL_ORD_ID|21506 transcript_58536	216	1607	4	216	448	1839	1	213
transcript_37762	gnl|BL_ORD_ID|96031 transcript_20881	186	1582	2	183	918	2339	210	391
transcript_37822	gnl|BL_ORD_ID|47746 transcript_97793	146	1564	1	145	1659	3077	29	171
transcript_37829	gnl|BL_ORD_ID|6343 transcript_32576	535	1576	8	536	818	1859	13	544
transcript_37859	gnl|BL_ORD_ID|9069 transcript_38047	193	1440	5	192	315	1562	2	186
transcript_37869	gnl|BL_ORD_ID|30379 transcript_71253	165	1479	2	165	1853	3158	10	173
transcript_37876	gnl|BL_ORD_ID|86612 transcript_156810	1	1020	1017	1563	350	1369	2413	2956
transcript_37926	gnl|BL_ORD_ID|30620 transcript_71646	1	1305	1300	1527	1	1368	1522	1759
transcript_37926	gnl|BL_ORD_ID|80235 transcript_146046	1	1305	1300	1527	201	1504	1650	1877
transcript_37933	gnl|BL_ORD_ID|3139 transcript_25740	252	1796	4	252	573	2124	7	263
transcript_37954	gnl|BL_ORD_ID|19052 transcript_54462	104	1538	1	103	968	2402	1	103
transcript_3798	gnl|BL_ORD_ID|90257 transcript_162672	1	2466	2462	3427	2	2467	2786	3751
transcript_38026	gnl|BL_ORD_ID|23032 transcript_61083	427	1562	51	427	1463	2601	1	377
transcript_38048	gnl|BL_ORD_ID|18422 transcript_53466	1	1019	1018	1544	1	1019	2206	2732
transcript_38051	gnl|BL_ORD_ID|47396 transcript_97256	516	1563	7	516	679	1727	2	508
transcript_38173	gnl|BL_ORD_ID|40644 transcript_86199	254	1551	30	254	1350	2652	2	226
transcript_3819	gnl|BL_ORD_ID|36874 transcript_81777	300	3339	1	302	1070	4108	32	333

transcript_3819	gnl|BL_ORD_ID|59536 transcript_115622	300	3339	3	302	1031	4066	27	327
transcript_38211	gnl|BL_ORD_ID|53105 transcript_104781	1	1029	1027	1527	2	1020	1168	1672
transcript_38222	gnl|BL_ORD_ID|2210 transcript_23705	1	1306	1304	1571	45	1351	1879	2146
transcript_38228	gnl|BL_ORD_ID|5922 transcript_31687	1	1005	1005	1498	1	1005	1400	1894
transcript_38267	gnl|BL_ORD_ID|95342 transcript_19254	1	1061	1060	1563	25	1085	1845	2348
transcript_38286	gnl|BL_ORD_ID|44020 transcript_91725	323	1554	55	325	1028	2259	1	271
transcript_38407	gnl|BL_ORD_ID|7404 transcript_34707	420	1535	7	425	654	1766	7	426
transcript_38437	gnl|BL_ORD_ID|76750 transcript_141881	140	1468	1	141	1224	2552	103	243
transcript_38456	gnl|BL_ORD_ID|1683 transcript_22572	196	1527	9	196	1001	2334	64	251
transcript_38456	gnl|BL_ORD_ID|72297 transcript_134656	196	1540	2	196	1057	2403	110	304
transcript_38490	gnl|BL_ORD_ID|24493 transcript_4428	215	1534	51	214	2036	3356	2	165
transcript_3850	gnl|BL_ORD_ID|28792 transcript_68741	1	2744	2742	3347	1	2742	3086	3691
transcript_38536	gnl|BL_ORD_ID|67833 transcript_127427	1	1022	1020	1539	1	1014	2068	2587
transcript_38546	gnl|BL_ORD_ID|89121 transcript_160877	1	1192	1191	1534	1	1190	1444	1787
transcript_38580	gnl|BL_ORD_ID|7233 transcript_34365	555	1524	6	560	792	1765	29	578
transcript_38582	gnl|BL_ORD_ID|18422 transcript_53466	1	1010	1009	1527	1	1019	2206	2725
transcript_38585	gnl|BL_ORD_ID|38714 transcript_8242	356	1571	39	357	1787	3002	2	317
transcript_38654	gnl|BL_ORD_ID|66199 transcript_124774	199	1445	2	202	2024	3278	2	202
transcript_38663	gnl|BL_ORD_ID|76105 transcript_140856	156	1539	2	157	1292	2672	21	176
transcript_38703	gnl|BL_ORD_ID|93476 transcript_166284	1	1030	1028	1544	2	1031	3124	3640
transcript_38704	gnl|BL_ORD_ID|69692 transcript_130432	175	1549	2	174	448	1822	151	323
transcript_38704	gnl|BL_ORD_ID|53354 transcript_105203	174	1548	2	179	368	1741	48	224
transcript_38707	gnl|BL_ORD_ID|92453 transcript_164638	247	1508	35	249	585	1842	2	216
transcript_38707	gnl|BL_ORD_ID|4280 transcript_28253	247	1508	9	249	625	1881	2	244
transcript_38724	gnl|BL_ORD_ID|12598 transcript_4095	240	1619	27	244	2025	3402	21	238
transcript_38727	gnl|BL_ORD_ID|90925 transcript_15520	10	1144	1144	1528	1	1130	2201	2585
transcript_38732	gnl|BL_ORD_ID|78181 transcript_14057	113	1545	1	116	1204	2637	136	251
transcript_38738	gnl|BL_ORD_ID|58616 transcript_114127	206	1531	9	208	1023	2348	1	199
transcript_3877	gnl|BL_ORD_ID|84048 transcript_152588	1	1876	1874	3416	10	1885	2054	3596
transcript_38798	gnl|BL_ORD_ID|19978 transcript_55989	180	1489	2	182	464	1773	1	181
transcript_38809	gnl|BL_ORD_ID|7870 transcript_35640	209	1512	3	213	398	1698	54	264
transcript_38934	gnl|BL_ORD_ID|53265 transcript_105049	450	1457	60	450	1212	2219	1	390
transcript_38960	gnl|BL_ORD_ID|51009 transcript_103033	341	1518	4	341	1649	2832	2	335
transcript_3897	gnl|BL_ORD_ID|69319 transcript_129824	2	2209	2209	3424	6	2221	2385	3600

transcript_39022	gnl|BL_ORD_ID|76065 transcript_140786	268	1443	5	270	1422	2596	23	288
transcript_39022	gnl|BL_ORD_ID|63388 transcript_121766	268	1435	6	270	1240	2427	1	265
transcript_39022	gnl|BL_ORD_ID|88182 transcript_159385	486	1439	5	486	790	1742	1	482
transcript_39032	gnl|BL_ORD_ID|75601 transcript_140032	200	1490	29	201	282	1572	9	181
transcript_39045	gnl|BL_ORD_ID|53951 transcript_106231	392	1530	43	392	516	1657	2	351
transcript_39050	gnl|BL_ORD_ID|84323 transcript_153078	13	1396	1395	1502	23	1409	1577	1684
transcript_3906	gnl|BL_ORD_ID|71684 transcript_133671	2	2609	2609	3390	1	2622	3142	3923
transcript_39065	gnl|BL_ORD_ID|7931 transcript_35746	1	1245	1243	1524	1	1245	1376	1657
transcript_3913	gnl|BL_ORD_ID|73895 transcript_137261	352	3386	5	356	978	4016	1	362
transcript_39175	gnl|BL_ORD_ID|39483 transcript_84324	229	1490	8	232	503	1764	1	225
transcript_39222	gnl|BL_ORD_ID|6602 transcript_33113	1	1088	1087	1512	1	1088	1407	1831
transcript_39291	gnl|BL_ORD_ID|7231 transcript_34362	9	844	839	1496	2	837	946	1603
transcript_3931	gnl|BL_ORD_ID|52828 transcript_104338	1	3081	3080	3380	2	3082	4029	4329
transcript_39381	gnl|BL_ORD_ID|55966 transcript_109779	1	1235	1233	1491	3	1237	1344	1602
transcript_39381	gnl|BL_ORD_ID|8667 transcript_37248	1	1235	1233	1491	2	1239	1347	1605
transcript_39381	gnl|BL_ORD_ID|23925 transcript_62548	1	1235	1233	1491	965	2200	2308	2566
transcript_39401	gnl|BL_ORD_ID|27471 transcript_66556	407	1473	46	409	1214	2303	2	365
transcript_39432	gnl|BL_ORD_ID|55060 transcript_108156	245	1478	46	244	2132	3365	2	200
transcript_39481	gnl|BL_ORD_ID|50868 transcript_102792	271	1523	67	271	434	1686	1	219
transcript_39489	gnl|BL_ORD_ID|78081 transcript_13836	1	1060	1060	1469	1	1061	2233	2642
transcript_39489	gnl|BL_ORD_ID|78364 transcript_14437	1	1060	1060	1436	1	1055	2221	2597
transcript_39532	gnl|BL_ORD_ID|56739 transcript_111016	1	1163	1162	1391	1	1164	1320	1546
transcript_39540	gnl|BL_ORD_ID|63008 transcript_121152	1	1277	1276	1485	1	1273	2357	2565
transcript_39562	gnl|BL_ORD_ID|66199 transcript_124774	211	1456	3	214	2024	3278	3	202
transcript_39703	gnl|BL_ORD_ID|88857 transcript_160468	188	1455	3	188	736	2003	12	199
transcript_39703	gnl|BL_ORD_ID|50868 transcript_102792	188	1455	2	188	434	1701	13	219
transcript_39703	gnl|BL_ORD_ID|59222 transcript_115104	188	1455	39	190	959	2227	34	179
transcript_39703	gnl|BL_ORD_ID|8617 transcript_37149	188	1428	21	188	386	1626	2	169
transcript_3976	gnl|BL_ORD_ID|12176 transcript_3277	523	3409	79	523	613	3499	2	447
transcript_39760	gnl|BL_ORD_ID|9069 transcript_38047	198	1422	4	197	316	1536	6	187
transcript_3979	gnl|BL_ORD_ID|860 transcript_1523	714	3407	88	716	1206	3906	2	632
transcript_39791	gnl|BL_ORD_ID|77731 transcript_13020	595	1459	63	597	1815	2680	4	538
transcript_39830	gnl|BL_ORD_ID|43658 transcript_91084	137	1429	1	141	1957	3245	1	142
transcript_39967	gnl|BL_ORD_ID|17616 transcript_52173	183	1442	8	184	991	2250	2	178

transcript_39992	gnl|BL_ORD_ID|2106 transcript_23456	1	1082	1078	1415	3	1117	1691	2045
transcript_39992	gnl|BL_ORD_ID|1824 transcript_22876	1	1082	1078	1435	2	1085	1901	2258
transcript_39992	gnl|BL_ORD_ID|6338 transcript_32561	1	1082	1078	1423	1	1062	1616	1962
transcript_39992	gnl|BL_ORD_ID|28026 transcript_67470	1	1082	1078	1420	2	1085	2776	3118
transcript_39992	gnl|BL_ORD_ID|22153 transcript_59602	1	1082	1078	1418	2	1077	1630	1971
transcript_39992	gnl|BL_ORD_ID|5558 transcript_30891	1	1082	1078	1435	2	1067	1621	1979
transcript_39992	gnl|BL_ORD_ID|7360 transcript_34620	1	1082	1078	1435	2	1083	1400	1757
transcript_39992	gnl|BL_ORD_ID|51864 transcript_10021	1	1082	1078	1435	2	1069	2494	2852
transcript_39992	gnl|BL_ORD_ID|2910 transcript_25245	1	1082	1078	1435	2	1083	1639	1996
transcript_39992	gnl|BL_ORD_ID|85749 transcript_155366	1	1082	1081	1433	3	1068	1323	1678
transcript_39992	gnl|BL_ORD_ID|34037 transcript_77186	1	1082	1078	1397	2	1081	2989	3308
transcript_39992	gnl|BL_ORD_ID|3847 transcript_27298	1	1082	1078	1435	2	1083	1738	2097
transcript_39992	gnl|BL_ORD_ID|51613 transcript_9460	1	1082	1078	1435	2	1083	2514	2871
transcript_39992	gnl|BL_ORD_ID|2319 transcript_23952	1	1082	1078	1435	2	1066	1880	2238
transcript_39992	gnl|BL_ORD_ID|1531 transcript_22223	1	1082	1078	1421	10	1118	1685	2053
transcript_39992	gnl|BL_ORD_ID|4240 transcript_28169	1	1082	1078	1420	2	1068	1646	1989
transcript_39992	gnl|BL_ORD_ID|22871 transcript_60831	1	1082	1081	1435	2	1067	1364	1719
transcript_39992	gnl|BL_ORD_ID|55077 transcript_108186	1	1082	1078	1420	161	1242	1796	2138
transcript_40052	gnl|BL_ORD_ID|9685 transcript_39260	201	1439	41	203	309	1548	26	186
transcript_4011	gnl|BL_ORD_ID|12297 transcript_3505	573	3406	87	572	618	3454	4	487
transcript_4014	gnl|BL_ORD_ID|46442 transcript_95770	1	2222	2220	3123	9	2204	3353	4256
transcript_40146	gnl|BL_ORD_ID|39483 transcript_84324	229	1427	8	232	503	1702	1	225
transcript_40146	gnl|BL_ORD_ID|56471 transcript_110576	229	1425	8	232	520	1718	2	226
transcript_40165	gnl|BL_ORD_ID|75015 transcript_139089	605	1417	7	607	915	1729	199	799
transcript_40181	gnl|BL_ORD_ID|24448 transcript_4345	194	1370	2	194	2183	3361	7	199
transcript_40189	gnl|BL_ORD_ID|29628 transcript_70030	146	1367	3	147	3422	4641	2	146
transcript_40189	gnl|BL_ORD_ID|41508 transcript_87635	146	1396	23	147	3391	4641	2	126
transcript_40223	gnl|BL_ORD_ID|31590 transcript_73204	1	1071	1071	1423	2	1074	1558	1911
transcript_4023	gnl|BL_ORD_ID|66329 transcript_124993	130	2790	2790	3397	1	2661	2846	3453
transcript_4032	gnl|BL_ORD_ID|71684 transcript_133671	1	2617	2617	3383	14	2622	3142	3908
transcript_40343	gnl|BL_ORD_ID|57280 transcript_111918	496	1426	56	499	739	1667	2	445
transcript_40361	gnl|BL_ORD_ID|47574 transcript_97535	1	1081	1078	1400	1983	3063	3358	3680
transcript_4039	gnl|BL_ORD_ID|11863 transcript_2658	209	3384	1	209	428	3603	1	209
transcript_4039	gnl|BL_ORD_ID|12189 transcript_3299	209	3230	2	209	501	3522	75	282

transcript_40406	gnl|BL_ORD_ID|18463 transcript_53521	206	1444	46	207	849	2087	1	162
transcript_40424	gnl|BL_ORD_ID|3674 transcript_26933	166	1418	23	168	869	2121	1	146
transcript_40465	gnl|BL_ORD_ID|87723 transcript_158626	288	1383	3	287	2375	3471	111	395
transcript_4049	gnl|BL_ORD_ID|19693 transcript_55539	464	3297	8	463	642	3474	5	450
transcript_4049	gnl|BL_ORD_ID|73154 transcript_135998	1	2166	2164	3258	1	2178	2522	3616
transcript_40492	gnl|BL_ORD_ID|54692 transcript_107496	177	1424	8	176	1507	2743	356	525
transcript_4050	gnl|BL_ORD_ID|31833 transcript_73618	1	2656	2656	3365	1	2664	2776	3487
transcript_40521	gnl|BL_ORD_ID|90923 transcript_15515	494	1404	5	494	1684	2587	1	486
transcript_40545	gnl|BL_ORD_ID|25438 transcript_63320	163	1423	48	165	307	1567	1	118
transcript_40551	gnl|BL_ORD_ID|36988 transcript_81942	151	1406	2	151	1054	2316	1	149
transcript_40591	gnl|BL_ORD_ID|83910 transcript_152349	197	1395	57	197	294	1493	2	142
transcript_40627	gnl|BL_ORD_ID|32957 transcript_75469	423	1382	9	422	860	1819	3	416
transcript_40646	gnl|BL_ORD_ID|9419 transcript_38742	172	1366	2	175	319	1512	1	171
transcript_40689	gnl|BL_ORD_ID|51889 transcript_10066	308	1381	6	307	1770	2843	1	302
transcript_40691	gnl|BL_ORD_ID|23250 transcript_61455	238	1376	3	239	1926	3064	1195	1426
transcript_40699	gnl|BL_ORD_ID|78139 transcript_13969	487	1379	6	487	1686	2578	2	485
transcript_40755	gnl|BL_ORD_ID|43078 transcript_90130	174	1373	4	174	293	1491	2	172
transcript_40769	gnl|BL_ORD_ID|38474 transcript_7697	160	1394	2	159	1623	2862	18	174
transcript_40774	gnl|BL_ORD_ID|52227 transcript_103383	126	1310	1	130	750	1941	24	154
transcript_4080	gnl|BL_ORD_ID|78904 transcript_143873	1329	3370	107	1331	1921	3956	2	1225
transcript_41006	gnl|BL_ORD_ID|95612 transcript_19893	143	1371	2	147	1154	2380	7	164
transcript_41050	gnl|BL_ORD_ID|84615 transcript_153548	426	1351	7	425	1605	2531	2	420
transcript_4107	gnl|BL_ORD_ID|37436 transcript_82659	2	2049	2049	3422	86	2135	2246	3619
transcript_41090	gnl|BL_ORD_ID|43821 transcript_91350	243	1357	4	242	2642	3756	2	250
transcript_41117	gnl|BL_ORD_ID|71861 transcript_133961	480	1212	7	482	583	1315	2	477
transcript_4113	gnl|BL_ORD_ID|84722 transcript_153731	1228	3254	1	1229	2073	4098	1	1228
transcript_4113	gnl|BL_ORD_ID|23523 transcript_61896	2	2808	2807	3303	1	2820	2936	3432
transcript_41136	gnl|BL_ORD_ID|59222 transcript_115104	218	1382	34	220	959	2124	2	179
transcript_4114	gnl|BL_ORD_ID|12182 transcript_3290	1	1914	1911	3369	1	1914	2034	3492
transcript_41159	gnl|BL_ORD_ID|74577 transcript_138372	178	1338	9	179	1150	2325	1	171
transcript_41164	gnl|BL_ORD_ID|40361 transcript_85709	487	1256	6	486	1577	2344	807	1297
transcript_41198	gnl|BL_ORD_ID|43821 transcript_91350	215	1331	6	216	2640	3756	2	216
transcript_41284	gnl|BL_ORD_ID|73517 transcript_136619	198	1337	30	202	1851	2990	141	313
transcript_4133	gnl|BL_ORD_ID|1112 transcript_1996	193	3356	1	194	609	3756	2	195

transcript_4133	gnl|BL_ORD_ID|1142 transcript_2057	193	3360	1	194	612	3751	2	195
transcript_41351	gnl|BL_ORD_ID|30457 transcript_71387	401	1344	59	403	1159	2102	2	346
transcript_41437	gnl|BL_ORD_ID|9871 transcript_39609	137	1340	2	139	253	1456	14	151
transcript_41503	gnl|BL_ORD_ID|51949 transcript_10183	1	1016	1015	1330	1	1013	2357	2672
transcript_41510	gnl|BL_ORD_ID|4322 transcript_28333	416	1318	51	419	1144	2046	2	370
transcript_41539	gnl|BL_ORD_ID|70422 transcript_131644	10	1054	1057	1317	9	1054	2259	2519
transcript_41671	gnl|BL_ORD_ID|48134 transcript_98406	251	1298	9	251	1535	2583	99	342
transcript_417	gnl|BL_ORD_ID|46286 transcript_95514	2	3202	3202	4603	1	3193	3299	4700
transcript_41704	gnl|BL_ORD_ID|67658 transcript_127146	1	1135	1130	1329	84	1218	1364	1563
transcript_41704	gnl|BL_ORD_ID|20076 transcript_56137	432	1329	57	436	735	1632	226	605
transcript_41707	gnl|BL_ORD_ID|86429 transcript_156499	210	1312	3	212	1151	2253	5	214
transcript_4175	gnl|BL_ORD_ID|71684 transcript_133671	2	2600	2600	3381	15	2622	3142	3923
transcript_41793	gnl|BL_ORD_ID|56342 transcript_110372	10	1000	1001	1274	3	993	1276	1549
transcript_41809	gnl|BL_ORD_ID|73321 transcript_136285	449	1259	6	452	569	1366	2	436
transcript_41844	gnl|BL_ORD_ID|63147 transcript_121391	113	1287	1	114	1042	2216	1	114
transcript_41885	gnl|BL_ORD_ID|9561 transcript_39022	104	1281	1	109	265	1441	2	111
transcript_41895	gnl|BL_ORD_ID|88558 transcript_159977	8	727	728	1293	2	721	1164	1729
transcript_41998	gnl|BL_ORD_ID|84214 transcript_152884	420	1271	5	424	1293	2144	1	437
transcript_4201	gnl|BL_ORD_ID|93129 transcript_165726	1077	3375	1	1077	1351	3680	99	1175
transcript_4201	gnl|BL_ORD_ID|37668 transcript_83021	104	1820	1818	3375	2	1716	1827	3430
transcript_42066	gnl|BL_ORD_ID|58388 transcript_113751	306	1277	7	308	2305	3277	26	327
transcript_42128	gnl|BL_ORD_ID|85546 transcript_155039	242	1273	4	246	388	1417	1	239
transcript_42149	gnl|BL_ORD_ID|50933 transcript_102911	9	660	658	1229	1	652	1903	2474
transcript_42189	gnl|BL_ORD_ID|51317 transcript_8872	134	1257	7	134	1745	2868	2	129
transcript_42209	gnl|BL_ORD_ID|17616 transcript_52173	223	1266	7	223	991	2035	2	219
transcript_42209	gnl|BL_ORD_ID|36512 transcript_81213	223	1266	6	223	1019	2063	31	249
transcript_4226	gnl|BL_ORD_ID|68468 transcript_128452	1	2128	2126	3338	1	2128	2269	3494
transcript_4226	gnl|BL_ORD_ID|12236 transcript_3390	1	2128	2126	3337	1	2128	2269	3480
transcript_42265	gnl|BL_ORD_ID|34803 transcript_78455	129	1259	2	130	1959	3089	1	129
transcript_42281	gnl|BL_ORD_ID|74577 transcript_138372	177	1278	8	178	1150	2243	1	171
transcript_4229	gnl|BL_ORD_ID|27268 transcript_66244	1439	3356	13	1440	1574	3492	1	1429
transcript_4233	gnl|BL_ORD_ID|48809 transcript_99502	2	2698	2695	3263	40	2727	3058	3623
transcript_4233	gnl|BL_ORD_ID|814 transcript_1418	1	1815	1815	3330	1	1814	2457	3972
transcript_42371	gnl|BL_ORD_ID|52152 transcript_10635	387	1265	54	389	1950	2828	1	336

transcript_42424	gnl|BL_ORD_ID|10285 transcript_40397	117	1254	1	117	265	1403	1	118
transcript_4250	gnl|BL_ORD_ID|38025 transcript_6754	1	2661	2660	2955	1	2665	2845	3140
transcript_42589	gnl|BL_ORD_ID|20461 transcript_56764	520	1209	9	519	1429	2118	2	512
transcript_42598	gnl|BL_ORD_ID|44789 transcript_93000	410	1256	9	411	1383	2229	2	402
transcript_42598	gnl|BL_ORD_ID|35177 transcript_79067	409	1256	49	411	1859	2706	2	360
transcript_4261	gnl|BL_ORD_ID|47574 transcript_97535	28	3089	3086	3392	3	3063	3358	3665
transcript_4261	gnl|BL_ORD_ID|77347 transcript_142840	20	2522	2519	3396	1	2491	2635	3514
transcript_42647	gnl|BL_ORD_ID|11649 transcript_2246	418	1256	51	420	2806	3645	2	372
transcript_427	gnl|BL_ORD_ID|101 transcript_149	1	4173	4168	4581	2	4169	4808	5221
transcript_427	gnl|BL_ORD_ID|148 transcript_225	1	4173	4168	4582	2	4168	4553	4967
transcript_42735	gnl|BL_ORD_ID|76617 transcript_141670	211	1230	4	212	347	1366	2	210
transcript_42739	gnl|BL_ORD_ID|9954 transcript_39777	307	1229	5	310	467	1389	48	353
transcript_42739	gnl|BL_ORD_ID|71609 transcript_133542	307	1229	4	308	1096	2020	3	306
transcript_42767	gnl|BL_ORD_ID|31716 transcript_73412	206	1271	4	207	420	1485	54	248
transcript_42781	gnl|BL_ORD_ID|52176 transcript_10686	168	1216	2	169	1763	2810	1	168
transcript_42848	gnl|BL_ORD_ID|30457 transcript_71387	384	1212	42	386	1159	1987	2	346
transcript_42850	gnl|BL_ORD_ID|8571 transcript_37038	271	1277	49	271	517	1523	2	224
transcript_42862	gnl|BL_ORD_ID|73100 transcript_135913	270	1209	5	269	376	1315	1	266
transcript_42876	gnl|BL_ORD_ID|41268 transcript_87227	393	1209	5	394	667	1484	2	391
transcript_42900	gnl|BL_ORD_ID|25870 transcript_64059	299	1196	7	301	3104	4001	2	296
transcript_42903	gnl|BL_ORD_ID|23859 transcript_62438	184	1217	2	183	1497	2526	648	830
transcript_42903	gnl|BL_ORD_ID|95091 transcript_18647	184	1215	2	183	1428	2455	582	764
transcript_4298	gnl|BL_ORD_ID|23134 transcript_61261	11	2259	2256	3348	2	2249	4189	5280
transcript_42993	gnl|BL_ORD_ID|29897 transcript_70465	369	1212	7	371	1277	2120	2	366
transcript_43148	gnl|BL_ORD_ID|52031 transcript_10358	106	1196	1	108	1726	2808	1	108
transcript_43167	gnl|BL_ORD_ID|49718 transcript_100934	160	1159	2	159	949	1948	4	169
transcript_43211	gnl|BL_ORD_ID|64232 transcript_123118	302	1158	5	304	1703	2559	2	301
transcript_43293	gnl|BL_ORD_ID|75860 transcript_140458	285	1177	33	286	1137	2028	4	254
transcript_43308	gnl|BL_ORD_ID|56263 transcript_110265	558	1164	6	562	1940	2545	1	557
transcript_43373	gnl|BL_ORD_ID|1162 transcript_2086	315	1100	6	315	2965	3746	74	384
transcript_43441	gnl|BL_ORD_ID|7626 transcript_35194	288	1163	3	290	641	1515	7	294
transcript_43510	gnl|BL_ORD_ID|69099 transcript_129473	124	1165	25	125	1009	2050	1	101
transcript_43617	gnl|BL_ORD_ID|79241 transcript_144436	248	1153	4	252	2921	3827	4	252
transcript_4362	gnl|BL_ORD_ID|36095 transcript_80515	232	3349	2	236	485	3580	8	242

transcript_43632	gnl|BL_ORD_ID|22144 transcript_59590	232	1171	8	231	2425	3364	1	223
transcript_43637	gnl|BL_ORD_ID|58449 transcript_113858	145	1150	4	144	2274	3276	2	142
transcript_43659	gnl|BL_ORD_ID|58616 transcript_114127	228	1127	31	230	1023	1922	1	199
transcript_43670	gnl|BL_ORD_ID|10156 transcript_40181	411	1145	9	411	685	1425	2	404
transcript_43670	gnl|BL_ORD_ID|61522 transcript_118784	204	1145	9	202	324	1273	2	195
transcript_43670	gnl|BL_ORD_ID|91655 transcript_163333	411	1145	6	411	659	1393	2	407
transcript_43686	gnl|BL_ORD_ID|50675 transcript_102482	7	623	619	1123	1	617	2929	3433
transcript_43697	gnl|BL_ORD_ID|66450 transcript_125183	9	825	825	1140	3	818	1514	1824
transcript_43720	gnl|BL_ORD_ID|85887 transcript_155599	334	1131	35	339	881	1678	2	306
transcript_43812	gnl|BL_ORD_ID|82380 transcript_149651	500	1168	9	502	1386	2053	3	496
transcript_43838	gnl|BL_ORD_ID|74721 transcript_138617	412	1124	8	412	528	1240	1	405
transcript_43858	gnl|BL_ORD_ID|88805 transcript_160391	371	1142	40	371	1378	2151	24	355
transcript_43929	gnl|BL_ORD_ID|48623 transcript_99195	101	1087	1	103	2076	3061	1	103
transcript_4393	gnl|BL_ORD_ID|11879 transcript_2689	1258	3341	1	1263	1515	3600	1	1263
transcript_43963	gnl|BL_ORD_ID|17193 transcript_51517	476	1124	8	480	859	1507	1	474
transcript_43971	gnl|BL_ORD_ID|77911 transcript_13416	397	1114	4	398	1770	2488	1	395
transcript_44017	gnl|BL_ORD_ID|89638 transcript_161724	158	1114	35	157	277	1232	1	123
transcript_44070	gnl|BL_ORD_ID|33484 transcript_76274	456	1105	8	455	626	1275	2	448
transcript_44139	gnl|BL_ORD_ID|88805 transcript_160391	372	1103	41	372	1378	2110	24	355
transcript_44151	gnl|BL_ORD_ID|74738 transcript_138650	257	1108	7	260	913	1765	3	256
transcript_4424	gnl|BL_ORD_ID|1174 transcript_2109	1	1729	1726	3325	1	1729	2132	3733
transcript_4426	gnl|BL_ORD_ID|23544 transcript_61933	588	3194	7	588	708	3314	1	582
transcript_4426	gnl|BL_ORD_ID|84517 transcript_153394	588	3293	7	585	687	3383	2	576
transcript_4426	gnl|BL_ORD_ID|83016 transcript_150778	588	3292	67	588	1537	4235	1	519
transcript_4426	gnl|BL_ORD_ID|45111 transcript_93542	588	3181	7	588	1148	3740	2	583
transcript_44315	gnl|BL_ORD_ID|56616 transcript_110816	146	1090	2	149	1342	2286	237	384
transcript_44329	gnl|BL_ORD_ID|58616 transcript_114127	230	1087	27	232	1023	1881	1	199
transcript_44366	gnl|BL_ORD_ID|12502 transcript_3900	248	1117	6	247	2538	3407	1	244
transcript_44366	gnl|BL_ORD_ID|24524 transcript_4495	248	1117	6	247	2477	3346	1	226
transcript_44377	gnl|BL_ORD_ID|11281 transcript_42233	164	1086	50	166	223	1145	2	118
transcript_4440	gnl|BL_ORD_ID|29294 transcript_69513	120	2918	2919	3350	1	2793	4213	4644
transcript_44480	gnl|BL_ORD_ID|77448 transcript_142999	380	1070	4	381	811	1484	138	511
transcript_44484	gnl|BL_ORD_ID|87223 transcript_157822	404	1089	5	406	513	1182	1	402
transcript_44487	gnl|BL_ORD_ID|64057 transcript_122843	563	1099	57	567	637	1173	1	510

transcript_44567	gnl|BL_ORD_ID|96835 transcript_102422	335	1059	4	334	1848	2572	25	355
transcript_4464	gnl|BL_ORD_ID|61143 transcript_118200	2	2762	2759	3158	1	2765	2911	3313
transcript_4466	gnl|BL_ORD_ID|11787 transcript_2519	455	3320	55	457	783	3649	2	405
transcript_4468	gnl|BL_ORD_ID|68468 transcript_128452	1	2128	2126	3332	1	2128	2269	3474
transcript_4468	gnl|BL_ORD_ID|12236 transcript_3390	1	2128	2126	3332	1	2128	2269	3461
transcript_44778	gnl|BL_ORD_ID|74591 transcript_138397	8	715	714	1052	2	709	1202	1540
transcript_44855	gnl|BL_ORD_ID|67036 transcript_126130	182	1051	2	183	2582	3451	85	266
transcript_44910	gnl|BL_ORD_ID|40868 transcript_86583	358	1039	4	361	974	1655	2	359
transcript_44950	gnl|BL_ORD_ID|44184 transcript_91993	452	998	1	453	2097	2646	1	463
transcript_44964	gnl|BL_ORD_ID|38288 transcript_7297	479	1037	6	484	2489	3047	2	478
transcript_45024	gnl|BL_ORD_ID|95117 transcript_18713	9	860	856	1027	2	853	1301	1472
transcript_45024	gnl|BL_ORD_ID|90912 transcript_15486	9	860	856	1027	2	853	1328	1499
transcript_45028	gnl|BL_ORD_ID|33252 transcript_75905	464	1025	8	465	1564	2125	2	459
transcript_45035	gnl|BL_ORD_ID|79393 transcript_144671	9	692	693	1036	1	696	2063	2406
transcript_45058	gnl|BL_ORD_ID|79160 transcript_144316	1	715	713	994	2	717	1724	2005
transcript_45072	gnl|BL_ORD_ID|66487 transcript_125242	1	669	668	981	1	669	2405	2718
transcript_45127	gnl|BL_ORD_ID|56251 transcript_110248	139	1009	2	141	3536	4405	2194	2333
transcript_45149	gnl|BL_ORD_ID|55469 transcript_108893	2	497	495	985	1	496	2046	2534
transcript_45159	gnl|BL_ORD_ID|75052 transcript_139142	354	977	1	356	2342	2963	1	356
transcript_45181	gnl|BL_ORD_ID|79282 transcript_144505	457	986	1	462	1643	2172	2	464
transcript_45242	gnl|BL_ORD_ID|59350 transcript_115315	460	975	1	464	1332	1843	172	633
transcript_45253	gnl|BL_ORD_ID|62882 transcript_120956	2	626	623	976	13	638	773	1126
transcript_45291	gnl|BL_ORD_ID|91387 transcript_16526	264	1003	4	264	1791	2530	1	261
transcript_4532	gnl|BL_ORD_ID|45434 transcript_94089	364	3307	5	363	811	3745	2	368
transcript_45351	gnl|BL_ORD_ID|51923 transcript_10130	6	593	591	1001	9	597	2147	2558
transcript_45352	gnl|BL_ORD_ID|83095 transcript_150913	296	1016	37	296	418	1137	1	260
transcript_45611	gnl|BL_ORD_ID|71709 transcript_133712	1	505	501	976	1	504	1345	1820
transcript_4565	gnl|BL_ORD_ID|1112 transcript_1996	194	3329	1	195	609	3756	1	195
transcript_4565	gnl|BL_ORD_ID|1142 transcript_2057	194	3333	1	195	612	3751	1	195
transcript_4573	gnl|BL_ORD_ID|41166 transcript_87061	1603	3322	149	1602	1907	3626	145	1598
transcript_4591	gnl|BL_ORD_ID|74290 transcript_137910	10	2748	2746	3305	2	2730	2835	3393
transcript_4591	gnl|BL_ORD_ID|1107 transcript_1990	2	2670	2669	3307	61	2718	3166	3804
transcript_4605	gnl|BL_ORD_ID|12248 transcript_3410	1	1932	1929	3285	1	1931	2118	3474
transcript_4609	gnl|BL_ORD_ID|19693 transcript_55539	522	3330	54	521	642	3449	5	450

transcript_4635	gnl|BL_ORD_ID|92023 transcript_163927	1169	3318	1	1170	1455	3604	1	1175
transcript_4641	gnl|BL_ORD_ID|24676 transcript_4817	1	1765	1765	3211	1	1763	1887	3324
transcript_4661	gnl|BL_ORD_ID|11858 transcript_2648	2	2937	2937	3312	54	2989	3105	3480
transcript_4679	gnl|BL_ORD_ID|1121 transcript_2013	1	2901	2901	3312	1	2900	3409	3820
transcript_4684	gnl|BL_ORD_ID|80153 transcript_145895	1	2790	2789	3342	117	2916	3419	3972
transcript_4684	gnl|BL_ORD_ID|57987 transcript_113093	1565	3342	1	1565	2204	3981	118	1681
transcript_4684	gnl|BL_ORD_ID|50359 transcript_101960	1565	3330	1	1565	2249	4015	123	1710
transcript_4694	gnl|BL_ORD_ID|86792 transcript_157118	1	1717	1718	3248	1	1717	1823	3354
transcript_4694	gnl|BL_ORD_ID|36257 transcript_80785	111	3283	1	112	238	3411	1	112
transcript_4719	gnl|BL_ORD_ID|48796 transcript_99480	1400	3282	12	1399	2829	4712	1	1388
transcript_4727	gnl|BL_ORD_ID|12508 transcript_3911	2	2681	2682	3300	1	2674	2792	3410
transcript_4748	gnl|BL_ORD_ID|11787 transcript_2519	316	3278	2	319	687	3649	88	405
transcript_4751	gnl|BL_ORD_ID|45751 transcript_94629	1	1846	1844	3292	51	1896	2284	3732
transcript_4751	gnl|BL_ORD_ID|89371 transcript_161280	1367	3292	1	1372	2089	4012	492	1862
transcript_4751	gnl|BL_ORD_ID|942 transcript_1674	1	2042	2041	3292	474	2514	2620	3870
transcript_481	gnl|BL_ORD_ID|89226 transcript_161042	1	3341	3337	4482	1	3341	3514	4655
transcript_4812	gnl|BL_ORD_ID|89667 transcript_161768	1674	3306	149	1675	2029	3661	1	1527
transcript_4856	gnl|BL_ORD_ID|64090 transcript_122895	1	2858	2856	3263	1	2859	2994	3402
transcript_4856	gnl|BL_ORD_ID|80527 transcript_146513	129	2255	2254	3263	1	2133	2828	3843
transcript_4863	gnl|BL_ORD_ID|12278 transcript_3454	1	1894	1891	3270	1	1895	2081	3461
transcript_4873	gnl|BL_ORD_ID|11677 transcript_2298	279	3302	1	279	406	3426	1	279
transcript_4914	gnl|BL_ORD_ID|84917 transcript_154042	10	2343	2345	3262	2	2335	2551	3465
transcript_4914	gnl|BL_ORD_ID|85319 transcript_154670	1	2901	2900	3222	2	2914	3308	3630
transcript_4926	gnl|BL_ORD_ID|47491 transcript_97404	244	3193	2	243	736	3686	1	239
transcript_4942	gnl|BL_ORD_ID|85849 transcript_155524	2	2244	2245	3291	52	2303	2417	3480
transcript_4947	gnl|BL_ORD_ID|32983 transcript_75506	1136	3312	109	1141	1486	3655	1	1033
transcript_4953	gnl|BL_ORD_ID|30297 transcript_71134	1	2529	2526	3321	18	2550	3256	4052
transcript_5041	gnl|BL_ORD_ID|24174 transcript_62933	1	2661	2659	3255	1	2662	3148	3744
transcript_5058	gnl|BL_ORD_ID|26618 transcript_65213	291	3262	2	291	1712	4683	564	853
transcript_5071	gnl|BL_ORD_ID|40140 transcript_85385	555	3276	78	557	621	3340	2	480
transcript_5102	gnl|BL_ORD_ID|714 transcript_1243	103	1619	1618	3122	2	1518	2507	4011
transcript_5118	gnl|BL_ORD_ID|46362 transcript_95625	1	1945	1943	3215	1	1950	2057	3333
transcript_51348	gnl|BL_ORD_ID|1534 transcript_22226	233	1981	4	234	504	2252	11	243
transcript_51360	gnl|BL_ORD_ID|32628 transcript_74970	1	1023	1021	1828	86	1108	1888	2694

transcript_51372	gnl|BL_ORD_ID|91662 transcript_163342	260	2624	3	264	521	2886	31	293
transcript_51372	gnl|BL_ORD_ID|51521 transcript_9253	260	2624	3	264	536	2899	46	308
transcript_5139	gnl|BL_ORD_ID|26355 transcript_64791	1191	3272	1	1192	1470	3544	2	1191
transcript_51391	gnl|BL_ORD_ID|72860 transcript_135543	481	3009	62	483	1342	3868	1	422
transcript_51395	gnl|BL_ORD_ID|25224 transcript_5971	1	1579	1575	3061	2	1580	1685	3171
transcript_51418	gnl|BL_ORD_ID|65041 transcript_12168	221	2631	15	223	350	2758	1	208
transcript_51443	gnl|BL_ORD_ID|87846 transcript_158831	1	1902	1901	2575	2	1882	1985	2658
transcript_51443	gnl|BL_ORD_ID|73741 transcript_136983	1148	2555	1	1148	1329	2714	2	1149
transcript_51460	gnl|BL_ORD_ID|38407 transcript_7562	1	2695	2694	2898	1	2699	2817	3021
transcript_51559	gnl|BL_ORD_ID|95742 transcript_20206	1005	2067	1	1006	1303	2367	132	1163
transcript_51559	gnl|BL_ORD_ID|96246 transcript_21318	1005	2067	1	1006	1242	2306	98	1102
transcript_51574	gnl|BL_ORD_ID|86122 transcript_155999	1	1606	1604	1954	1349	2955	3065	3415
transcript_51575	gnl|BL_ORD_ID|46556 transcript_95955	494	1872	7	496	847	2225	3	507
transcript_51591	gnl|BL_ORD_ID|81047 transcript_147354	573	2337	66	574	686	2452	2	511
transcript_51592	gnl|BL_ORD_ID|29801 transcript_70303	2	2117	2112	3122	188	2302	2493	3507
transcript_51592	gnl|BL_ORD_ID|52335 transcript_103557	2	2113	2112	3132	13	2123	2531	3550
transcript_51606	gnl|BL_ORD_ID|91065 transcript_15826	1	1237	1238	2158	27	1267	1625	2546
transcript_51613	gnl|BL_ORD_ID|5687 transcript_31188	1	1348	1346	1498	137	1478	1632	1784
transcript_51613	gnl|BL_ORD_ID|7320 transcript_34534	1	1348	1346	1498	4	1345	1499	1651
transcript_51613	gnl|BL_ORD_ID|6556 transcript_33013	1	1348	1346	1498	4	1351	1505	1657
transcript_51613	gnl|BL_ORD_ID|6115 transcript_32090	1	1348	1346	1498	220	1567	1721	1873
transcript_51628	gnl|BL_ORD_ID|81299 transcript_147762	1	1909	1906	2311	324	2211	2332	2737
transcript_51628	gnl|BL_ORD_ID|51942 transcript_10167	1	1909	1906	2311	342	2250	2372	2778
transcript_51628	gnl|BL_ORD_ID|64400 transcript_10775	1	1909	1906	2311	336	2244	2366	2759
transcript_51629	gnl|BL_ORD_ID|33720 transcript_76666	14	3406	3405	3940	2	3394	5324	5859
transcript_51658	gnl|BL_ORD_ID|48985 transcript_99773	1	2013	2012	2489	25	2029	4286	4763
transcript_51658	gnl|BL_ORD_ID|94304 transcript_167591	1	2013	2012	2446	3	2014	4275	4709
transcript_51670	gnl|BL_ORD_ID|31146 transcript_72502	272	2161	29	276	934	2820	1	252
transcript_51722	gnl|BL_ORD_ID|71410 transcript_133227	1	1555	1550	2266	6	1561	2359	3076
transcript_51727	gnl|BL_ORD_ID|47184 transcript_96920	1114	2270	1	1117	1253	2409	44	1139
transcript_51771	gnl|BL_ORD_ID|6128 transcript_32118	1	1128	1126	1675	69	1196	1329	1878
transcript_51773	gnl|BL_ORD_ID|55390 transcript_108746	288	2443	4	288	609	2759	111	396
transcript_51787	gnl|BL_ORD_ID|78631 transcript_143448	116	1581	1	115	954	2418	116	230
transcript_51787	gnl|BL_ORD_ID|5714 transcript_31248	116	1581	1	115	437	1891	150	264

transcript_51797	gnl|BL_ORD_ID|42975 transcript_89962	2	2412	2409	2829	17	2428	2546	2966
transcript_51803	gnl|BL_ORD_ID|88947 transcript_160603	1	3606	3601	4102	1372	4976	5080	5581
transcript_51855	gnl|BL_ORD_ID|63517 transcript_121987	1	1014	1012	1604	181	1199	2959	3553
transcript_51961	gnl|BL_ORD_ID|18556 transcript_53652	265	2659	3	269	1330	3721	755	1021
transcript_52052	gnl|BL_ORD_ID|86329 transcript_156340	1	1008	1009	1525	57	1064	1457	1972
transcript_52062	gnl|BL_ORD_ID|97071 transcript_123965	19	2015	2015	2720	4	2000	2168	2873
transcript_5207	gnl|BL_ORD_ID|20706 transcript_57175	1	2873	2872	3198	1407	4278	4421	4748
transcript_5207	gnl|BL_ORD_ID|69061 transcript_129401	1	2873	2872	3255	690	3561	3705	4088
transcript_52167	gnl|BL_ORD_ID|71813 transcript_133887	1	1611	1608	2255	1	1611	1726	2375
transcript_52167	gnl|BL_ORD_ID|44098 transcript_91855	1	1611	1608	2153	1	1611	1726	2273
transcript_52221	gnl|BL_ORD_ID|12058 transcript_3056	2	2008	2006	2425	6	2013	3036	3457
transcript_52237	gnl|BL_ORD_ID|286 transcript_473	1	1806	1804	3302	1	1806	3021	4519
transcript_52299	gnl|BL_ORD_ID|70408 transcript_131619	2	3803	3802	4085	218	4018	4163	4446
transcript_52299	gnl|BL_ORD_ID|36578 transcript_81317	2	3803	3802	4085	164	3949	4094	4372
transcript_52299	gnl|BL_ORD_ID|309 transcript_511	2	3803	3802	4085	197	4033	4178	4461
transcript_52302	gnl|BL_ORD_ID|61727 transcript_119087	1316	2688	1	1316	2227	3600	1	1312
transcript_5236	gnl|BL_ORD_ID|34684 transcript_78268	132	1947	1945	3261	2	1818	2022	3338
transcript_52403	gnl|BL_ORD_ID|85658 transcript_155197	1001	2031	1	1006	3282	4312	25	1030
transcript_5244	gnl|BL_ORD_ID|58081 transcript_113235	2	2287	2286	3256	2	2287	2711	3682
transcript_5244	gnl|BL_ORD_ID|25784 transcript_63905	1	2166	2164	3225	1	2166	2295	3354
transcript_52451	gnl|BL_ORD_ID|80985 transcript_147260	1	1173	1171	1888	1	1173	3639	4356
transcript_52459	gnl|BL_ORD_ID|12275 transcript_3450	2	2660	2655	2851	251	2911	3268	3464
transcript_5250	gnl|BL_ORD_ID|12295 transcript_3502	2	2310	2308	3225	1	2309	2547	3464
transcript_52502	gnl|BL_ORD_ID|91292 transcript_16316	229	2326	1	232	363	2458	1	232
transcript_52507	gnl|BL_ORD_ID|30128 transcript_70854	2	2011	2010	2114	116	2126	2610	2712
transcript_52530	gnl|BL_ORD_ID|11708 transcript_2361	1582	3360	1	1582	1867	3619	177	1751
transcript_52530	gnl|BL_ORD_ID|11611 transcript_2173	1582	3360	1	1582	1900	3667	209	1784
transcript_52576	gnl|BL_ORD_ID|50484 transcript_102154	154	1515	4	154	1653	3018	1	151
transcript_52582	gnl|BL_ORD_ID|28184 transcript_67728	1072	3431	1	1076	1515	3886	2	1080
transcript_52588	gnl|BL_ORD_ID|23914 transcript_62531	1	2308	2306	3070	1	2307	2533	3298
transcript_52651	gnl|BL_ORD_ID|40337 transcript_85667	1	1056	1057	1656	2	1043	1182	1783
transcript_52651	gnl|BL_ORD_ID|73159 transcript_136009	1	1123	1121	1658	26	1154	1424	1963
transcript_52651	gnl|BL_ORD_ID|74113 transcript_137618	1	1123	1121	1658	10	1142	1243	1782
transcript_52665	gnl|BL_ORD_ID|85784 transcript_155418	138	2887	2885	4025	2	2748	2941	4081

transcript_52692	gnl|BL_ORD_ID|78654 transcript_143480	1	1779	1779	3051	234	2010	2111	3372
transcript_52692	gnl|BL_ORD_ID|393 transcript_674	1	1779	1779	3051	1131	2909	3020	4292
transcript_52692	gnl|BL_ORD_ID|12681 transcript_4261	1	1779	1779	3035	254	2032	2143	3396
transcript_52692	gnl|BL_ORD_ID|12025 transcript_2984	1	1779	1779	3051	234	2012	2123	3396
transcript_52692	gnl|BL_ORD_ID|1074 transcript_1927	1	1779	1779	3051	273	2052	2163	3435
transcript_52696	gnl|BL_ORD_ID|6945 transcript_33827	251	1474	3	254	493	1713	4	255
transcript_5271	gnl|BL_ORD_ID|12536 transcript_3968	2	2192	2191	3262	8	2198	2331	3399
transcript_52734	gnl|BL_ORD_ID|51375 transcript_8981	2	2468	2466	2792	66	2530	2631	2957
transcript_52734	gnl|BL_ORD_ID|25421 transcript_6377	2	2468	2466	2792	269	2735	2837	3163
transcript_52734	gnl|BL_ORD_ID|38060 transcript_6825	2	2468	2466	2792	172	2661	2763	3089
transcript_52741	gnl|BL_ORD_ID|90968 transcript_15616	1	1934	1934	2362	30	1963	2064	2493
transcript_5276	gnl|BL_ORD_ID|73614 transcript_136770	23	2364	2365	3126	1	2334	2923	3685
transcript_5277	gnl|BL_ORD_ID|96897 transcript_108170	19	2183	2182	3247	1	2154	2287	3352
transcript_5277	gnl|BL_ORD_ID|12536 transcript_3968	13	2183	2182	3250	2	2198	2331	3399
transcript_52778	gnl|BL_ORD_ID|33996 transcript_77120	166	2658	1	167	1406	3905	1	167
transcript_528	gnl|BL_ORD_ID|237 transcript_381	206	4454	1	208	425	4672	32	251
transcript_528	gnl|BL_ORD_ID|63934 transcript_122657	206	4454	1	208	410	4673	12	233
transcript_528	gnl|BL_ORD_ID|59262 transcript_115168	206	4439	1	208	411	4625	30	237
transcript_52830	gnl|BL_ORD_ID|54955 transcript_107968	179	1194	22	182	273	1291	1	163
transcript_52878	gnl|BL_ORD_ID|23744 transcript_62264	1	1821	1817	2552	556	2376	2826	3561
transcript_52896	gnl|BL_ORD_ID|74774 transcript_138701	269	2338	3	267	534	2606	4	268
transcript_52898	gnl|BL_ORD_ID|82339 transcript_149571	254	1812	78	254	1072	2624	210	386
transcript_52903	gnl|BL_ORD_ID|19204 transcript_54727	1	2316	2311	3949	1	2299	3204	4842
transcript_52914	gnl|BL_ORD_ID|4535 transcript_28766	1	1626	1625	1910	1	1628	1736	2023
transcript_52917	gnl|BL_ORD_ID|42192 transcript_88705	119	1861	1	120	244	1990	5	124
transcript_52944	gnl|BL_ORD_ID|59630 transcript_115759	1	2311	2307	3061	1073	3382	4473	5225
transcript_52960	gnl|BL_ORD_ID|57867 transcript_112911	1	1022	1017	1486	995	2016	2290	2757
transcript_52960	gnl|BL_ORD_ID|38652 transcript_8087	1	1022	1017	1486	1174	2194	2468	2937
transcript_52960	gnl|BL_ORD_ID|88587 transcript_160031	1	1022	1017	1441	978	1998	2272	2696
transcript_52960	gnl|BL_ORD_ID|48806 transcript_99499	1	1022	1017	1486	1139	2159	2433	2901
transcript_53021	gnl|BL_ORD_ID|34812 transcript_78469	18	2339	2339	3238	2	2336	2474	3383
transcript_53069	gnl|BL_ORD_ID|27295 transcript_66286	101	1802	1801	3211	3	1704	2867	4277
transcript_53298	gnl|BL_ORD_ID|69331 transcript_129841	1	2265	2266	3609	217	2481	2639	3988
transcript_53328	gnl|BL_ORD_ID|57869 transcript_112913	125	2866	1	128	3082	5829	1889	2016

transcript_53334	gnl|BL_ORD_ID|3760 transcript_27103	1	1049	1047	1675	7	1055	1445	2070
transcript_53390	gnl|BL_ORD_ID|66208 transcript_124788	1	1518	1516	1941	41	1557	2615	3038
transcript_5341	gnl|BL_ORD_ID|12039 transcript_3015	2	2674	2674	3240	45	2701	2861	3427
transcript_53415	gnl|BL_ORD_ID|86221 transcript_156159	129	1831	1831	2612	1	1727	1854	2634
transcript_53456	gnl|BL_ORD_ID|38023 transcript_6752	1143	2630	1	1145	1526	3014	2	1158
transcript_53456	gnl|BL_ORD_ID|31314 transcript_72775	1143	2630	1	1145	1407	2895	2	1154
transcript_53457	gnl|BL_ORD_ID|11977 transcript_2887	1	2891	2892	3385	1	2876	3067	3562
transcript_535	gnl|BL_ORD_ID|71880 transcript_133990	1	4164	4164	4443	1	4167	4350	4632
transcript_53540	gnl|BL_ORD_ID|24975 transcript_5425	133	3121	1	133	240	3229	2	134
transcript_53542	gnl|BL_ORD_ID|54956 transcript_107969	247	2565	1	247	587	2907	49	295
transcript_5355	gnl|BL_ORD_ID|24482 transcript_4410	1365	3226	1	1365	1478	3339	5	1369
transcript_53558	gnl|BL_ORD_ID|67145 transcript_126309	1	1350	1350	2339	12	1361	2581	3570
transcript_53595	gnl|BL_ORD_ID|40214 transcript_85489	1	1563	1561	2650	1	1560	1761	2853
transcript_53595	gnl|BL_ORD_ID|89690 transcript_161801	1034	2647	1	1033	1162	2780	4	1032
transcript_53597	gnl|BL_ORD_ID|64406 transcript_10784	1	1607	1603	2335	171	1771	1973	2700
transcript_53597	gnl|BL_ORD_ID|64391 transcript_10758	1	1607	1603	2335	124	1730	1932	2664
transcript_53597	gnl|BL_ORD_ID|64598 transcript_11200	1	1607	1603	2335	197	1797	1999	2725
transcript_53622	gnl|BL_ORD_ID|52488 transcript_103795	1	1848	1847	2703	2	1844	1982	2815
transcript_53685	gnl|BL_ORD_ID|46448 transcript_95778	303	3987	3	303	414	4098	5	305
transcript_53694	gnl|BL_ORD_ID|50082 transcript_101513	1	1738	1737	2598	292	2038	2149	3012
transcript_53694	gnl|BL_ORD_ID|25423 transcript_6384	1	1738	1737	2605	411	2155	2266	3131
transcript_53694	gnl|BL_ORD_ID|93787 transcript_166789	1	1738	1737	2605	476	2221	2332	3200
transcript_53694	gnl|BL_ORD_ID|37113 transcript_82137	1	1738	1737	2597	920	2672	2783	3635
transcript_53694	gnl|BL_ORD_ID|26698 transcript_65335	1	1738	1737	2605	646	2392	2503	3373
transcript_53694	gnl|BL_ORD_ID|12165 transcript_3253	1	1738	1737	2605	730	2476	2587	3445
transcript_53694	gnl|BL_ORD_ID|40396 transcript_85772	1	1738	1737	2605	826	2572	2683	3550
transcript_53694	gnl|BL_ORD_ID|42363 transcript_88987	1	1738	1737	2605	887	2633	2744	3613
transcript_53694	gnl|BL_ORD_ID|88047 transcript_159157	1	1738	1737	2597	545	2289	2400	3251
transcript_53694	gnl|BL_ORD_ID|42048 transcript_88489	1	1738	1737	2503	614	2360	2471	3238
transcript_53694	gnl|BL_ORD_ID|72687 transcript_135258	1	1738	1737	2597	790	2535	2646	3495
transcript_53694	gnl|BL_ORD_ID|12285 transcript_3468	1	1738	1737	2605	552	2297	2408	3275
transcript_53708	gnl|BL_ORD_ID|85136 transcript_154385	2	2653	2653	3092	6	2657	2805	3242
transcript_53730	gnl|BL_ORD_ID|145 transcript_219	1	2329	2326	2453	2141	4469	4655	4782
transcript_53730	gnl|BL_ORD_ID|74161 transcript_137703	1	2329	2326	2453	3397	5723	5909	6036

transcript_53730	gnl|BL_ORD_ID|42092 transcript_88551	1	2329	2326	2453	3532	5862	6048	6175
transcript_53730	gnl|BL_ORD_ID|27 transcript_35	1	2329	2326	2452	3442	5769	5955	6081
transcript_53730	gnl|BL_ORD_ID|81427 transcript_147961	1	2329	2326	2453	3531	5859	6045	6172
transcript_53742	gnl|BL_ORD_ID|77298 transcript_142767	1	1082	1080	2152	2	1083	1739	2813
transcript_53864	gnl|BL_ORD_ID|61710 transcript_119061	1	1762	1760	2303	1	1764	2795	3337
transcript_53879	gnl|BL_ORD_ID|51664 transcript_9581	273	2533	3	270	606	2877	40	308
transcript_53879	gnl|BL_ORD_ID|24775 transcript_5027	273	2531	6	270	1020	3289	111	376
transcript_53879	gnl|BL_ORD_ID|51564 transcript_9358	273	2533	3	270	617	2888	11	279
transcript_53879	gnl|BL_ORD_ID|24809 transcript_5105	273	2531	3	270	947	3216	38	306
transcript_53904	gnl|BL_ORD_ID|57555 transcript_112390	576	2749	99	577	1245	3420	111	589
transcript_5393	gnl|BL_ORD_ID|12536 transcript_3968	1	2180	2179	3247	2	2198	2331	3396
transcript_53936	gnl|BL_ORD_ID|24521 transcript_4488	1	1862	1863	2959	227	2094	2212	3305
transcript_53936	gnl|BL_ORD_ID|20111 transcript_56197	1	1917	1916	2959	227	2149	2282	3325
transcript_53945	gnl|BL_ORD_ID|85715 transcript_155301	1633	4549	144	1635	1745	4660	2	1486
transcript_53963	gnl|BL_ORD_ID|42076 transcript_88532	1	1820	1815	3137	2	1814	2076	3382
transcript_53963	gnl|BL_ORD_ID|24694 transcript_4866	1	1820	1815	3051	2	1824	2086	3324
transcript_53973	gnl|BL_ORD_ID|33359 transcript_76074	1	2959	2956	3190	457	3419	3525	3759
transcript_53976	gnl|BL_ORD_ID|32728 transcript_75130	119	2654	2650	3184	2	2542	2820	3356
transcript_54004	gnl|BL_ORD_ID|5499 transcript_30781	718	1792	8	719	853	1927	2	713
transcript_5403	gnl|BL_ORD_ID|12009 transcript_2951	1	2724	2724	3230	2	2727	3016	3521
transcript_54033	gnl|BL_ORD_ID|59846 transcript_116108	996	2992	85	1001	1116	3112	1	888
transcript_54039	gnl|BL_ORD_ID|43281 transcript_90452	1290	3942	1	1289	1561	4216	1	1296
transcript_54136	gnl|BL_ORD_ID|69953 transcript_130854	1	1694	1694	2420	1135	2826	2933	3658
transcript_54136	gnl|BL_ORD_ID|89136 transcript_160905	1	1694	1694	2479	1055	2749	2856	3632
transcript_54136	gnl|BL_ORD_ID|81836 transcript_148684	1000	2430	1	1003	2526	3949	1272	2275
transcript_54158	gnl|BL_ORD_ID|38194 transcript_7096	98	2136	1	103	1027	3065	29	131
transcript_54164	gnl|BL_ORD_ID|19950 transcript_55944	1	2562	2561	2975	81	2642	3415	3829
transcript_54254	gnl|BL_ORD_ID|88292 transcript_159562	1	1275	1273	2519	38	1312	1453	2699
transcript_54301	gnl|BL_ORD_ID|51320 transcript_8878	489	2245	61	489	1152	2922	2	429
transcript_54342	gnl|BL_ORD_ID|69760 transcript_130541	1232	2829	1	1236	2465	4062	428	1669
transcript_54342	gnl|BL_ORD_ID|82155 transcript_149258	1232	2823	1	1236	1821	3413	191	1426
transcript_5438	gnl|BL_ORD_ID|93297 transcript_166011	1301	3195	1	1301	1695	3589	1	1302
transcript_54422	gnl|BL_ORD_ID|61930 transcript_119418	150	2268	2268	3494	1	2119	2219	3445
transcript_54441	gnl|BL_ORD_ID|80704 transcript_146801	1101	2953	1	1105	1653	3491	185	1289

transcript_54441	gnl|BL_ORD_ID|19792 transcript_55692	1104	2824	1	1105	2571	4277	184	1288
transcript_54457	gnl|BL_ORD_ID|96477 transcript_67182	1468	4562	1	1470	2407	5499	11	1477
transcript_5446	gnl|BL_ORD_ID|79200 transcript_144372	101	2012	2013	3121	2	1894	2030	3137
transcript_5447	gnl|BL_ORD_ID|52567 transcript_103909	120	2185	2184	3226	1	2065	2453	3495
transcript_54491	gnl|BL_ORD_ID|25357 transcript_6258	2	2457	2457	2679	30	2460	2816	3038
transcript_54491	gnl|BL_ORD_ID|25797 transcript_63925	16	2457	2457	2679	5	2448	2804	3026
transcript_54506	gnl|BL_ORD_ID|53840 transcript_106065	311	1648	71	313	609	1946	1	242
transcript_54519	gnl|BL_ORD_ID|59 transcript_83	2136	5480	2	2137	2251	5618	1	2136
transcript_5454	gnl|BL_ORD_ID|35226 transcript_79141	1	2475	2472	3215	11	2461	3210	3954
transcript_54596	gnl|BL_ORD_ID|43078 transcript_90130	220	1382	50	220	308	1469	2	172
transcript_5460	gnl|BL_ORD_ID|30294 transcript_71128	1	2452	2449	3232	1	2449	2596	3378
transcript_54603	gnl|BL_ORD_ID|36089 transcript_80505	1	3107	3104	3306	299	3405	3613	3816
transcript_54603	gnl|BL_ORD_ID|37559 transcript_82857	1	3105	3104	3233	123	3226	3380	3510
transcript_54603	gnl|BL_ORD_ID|12173 transcript_3274	17	3107	3104	3328	2	3091	3299	3524
transcript_54603	gnl|BL_ORD_ID|58791 transcript_114405	1	3105	3104	3300	341	3444	3582	3779
transcript_54603	gnl|BL_ORD_ID|83129 transcript_150971	21	3105	3104	3369	1	3083	3222	3488
transcript_54607	gnl|BL_ORD_ID|64047 transcript_122826	1	1528	1527	2030	644	2171	2624	3125
transcript_54626	gnl|BL_ORD_ID|24780 transcript_5035	2	2447	2446	2946	6	2452	2787	3288
transcript_54636	gnl|BL_ORD_ID|36722 transcript_81527	130	2106	1	132	393	2371	1	128
transcript_54636	gnl|BL_ORD_ID|78342 transcript_14396	130	2106	1	132	483	2460	91	218
transcript_54730	gnl|BL_ORD_ID|69514 transcript_130131	291	1480	9	294	389	1576	2	289
transcript_54730	gnl|BL_ORD_ID|8932 transcript_37762	291	1480	7	294	417	1581	2	287
transcript_54744	gnl|BL_ORD_ID|27512 transcript_66618	1	2350	2349	4022	182	2534	3705	5359
transcript_5475	gnl|BL_ORD_ID|24499 transcript_4441	180	3163	1	182	325	3320	1	172
transcript_54760	gnl|BL_ORD_ID|1180 transcript_2127	1	2803	2803	3530	72	2874	2976	3703
transcript_5479	gnl|BL_ORD_ID|11963 transcript_2858	327	3307	3	328	542	3526	15	344
transcript_54829	gnl|BL_ORD_ID|29454 transcript_69747	1	2113	2111	2443	207	2319	3880	4212
transcript_54829	gnl|BL_ORD_ID|67651 transcript_127129	1	2113	2111	2443	162	2255	3817	4149
transcript_54835	gnl|BL_ORD_ID|28361 transcript_68053	2	2106	2101	2735	199	2304	3079	3713
transcript_54851	gnl|BL_ORD_ID|11630 transcript_2210	1	1669	1668	3273	2	1674	1813	3411
transcript_54899	gnl|BL_ORD_ID|27932 transcript_67297	412	2031	48	415	1196	2812	2	366
transcript_54899	gnl|BL_ORD_ID|71312 transcript_133073	412	2028	48	415	1629	3242	1	365
transcript_54899	gnl|BL_ORD_ID|19693 transcript_55539	412	2031	51	415	1818	3435	2	363
transcript_54899	gnl|BL_ORD_ID|12569 transcript_4049	412	2031	48	415	1640	3258	2	376

transcript_54951	gnl|BL_ORD_ID|53879 transcript_106127	3	3174	3174	4212	1	3168	3274	4310
transcript_54996	gnl|BL_ORD_ID|56653 transcript_110881	1	1675	1676	2601	322	1995	2518	3444
transcript_54996	gnl|BL_ORD_ID|70866 transcript_132368	1	1675	1676	2581	242	1916	2031	2936
transcript_54996	gnl|BL_ORD_ID|41360 transcript_87379	1	1675	1676	2518	297	1971	2086	2927
transcript_55001	gnl|BL_ORD_ID|63131 transcript_121355	1	3440	3438	4401	1	3436	3551	4513
transcript_55037	gnl|BL_ORD_ID|41287 transcript_87256	1	1933	1928	3048	344	2276	2485	3600
transcript_55054	gnl|BL_ORD_ID|89542 transcript_161556	2	3325	3324	3506	2	3323	3723	3913
transcript_55107	gnl|BL_ORD_ID|33034 transcript_75575	1188	3549	1	1188	1540	3901	25	1205
transcript_55136	gnl|BL_ORD_ID|86156 transcript_156053	2	2410	2408	2718	149	2556	2715	3025
transcript_5517	gnl|BL_ORD_ID|62231 transcript_119903	331	3226	4	330	432	3328	2	328
transcript_55227	gnl|BL_ORD_ID|74359 transcript_138026	223	2889	2	224	1063	3721	2	226
transcript_55227	gnl|BL_ORD_ID|26088 transcript_64384	222	2889	10	224	1187	3841	1	226
transcript_55227	gnl|BL_ORD_ID|46721 transcript_96208	222	2853	1	224	1203	3834	2	225
transcript_55239	gnl|BL_ORD_ID|77387 transcript_142905	192	2587	1	193	830	3197	1	194
transcript_55245	gnl|BL_ORD_ID|52234 transcript_103391	226	2626	18	227	672	3071	64	267
transcript_55245	gnl|BL_ORD_ID|84855 transcript_153941	228	2633	2	227	1160	3564	38	263
transcript_55253	gnl|BL_ORD_ID|54472 transcript_107120	1	1142	1141	2183	360	1501	1776	2818
transcript_55271	gnl|BL_ORD_ID|56673 transcript_110911	2	2723	2720	3665	113	2834	3047	3991
transcript_55303	gnl|BL_ORD_ID|80512 transcript_146491	1	2073	2074	2784	1	2072	2528	3238
transcript_55304	gnl|BL_ORD_ID|57750 transcript_112716	2	2478	2475	3017	5	2469	2952	3494
transcript_55313	gnl|BL_ORD_ID|74050 transcript_137508	190	3338	11	190	350	3498	1	180
transcript_55333	gnl|BL_ORD_ID|90776 transcript_15183	13	1478	1476	2152	29	1497	1725	2405
transcript_5534	gnl|BL_ORD_ID|19054 transcript_54464	11	1805	1805	3203	1	1794	1912	3310
transcript_55343	gnl|BL_ORD_ID|6479 transcript_32848	1	1154	1151	1642	1	1147	1256	1747
transcript_55362	gnl|BL_ORD_ID|48610 transcript_99173	322	1441	45	325	2397	3511	2	282
transcript_55364	gnl|BL_ORD_ID|93883 transcript_166931	1	2848	2848	3412	102	2956	3562	4127
transcript_55365	gnl|BL_ORD_ID|61691 transcript_119036	174	2892	16	173	1407	4130	2	159
transcript_55366	gnl|BL_ORD_ID|33307 transcript_75988	135	2325	1	137	777	2967	531	667
transcript_554	gnl|BL_ORD_ID|46241 transcript_95448	250	4419	100	250	318	4494	2	152
transcript_5540	gnl|BL_ORD_ID|19373 transcript_54998	2	2293	2289	3182	28	2345	3131	4024
transcript_55418	gnl|BL_ORD_ID|30968 transcript_72210	2	2037	2038	2523	56	2091	2243	2723
transcript_55490	gnl|BL_ORD_ID|87937 transcript_158974	128	2577	2576	3299	1	2449	2842	3565
transcript_55545	gnl|BL_ORD_ID|74820 transcript_138787	1	1636	1635	2247	291	1925	2284	2895
transcript_55598	gnl|BL_ORD_ID|39092 transcript_83694	1	2808	2807	3709	1	2807	2917	3818

transcript_55622	gnl|BL_ORD_ID|48004 transcript_98200	1	1557	1558	2220	20	1576	1879	2541
transcript_55682	gnl|BL_ORD_ID|60196 transcript_116653	104	1264	1	106	1668	2824	314	418
transcript_55686	gnl|BL_ORD_ID|60362 transcript_116914	162	1906	1	164	1006	2724	2	165
transcript_55686	gnl|BL_ORD_ID|27795 transcript_67077	162	1923	1	164	687	2437	182	357
transcript_55686	gnl|BL_ORD_ID|81719 transcript_148482	162	1923	1	164	532	2280	27	202
transcript_55708	gnl|BL_ORD_ID|90977 transcript_15636	396	2204	4	396	517	2332	1	393
transcript_55712	gnl|BL_ORD_ID|51873 transcript_10040	1	1798	1796	2455	1	1798	2195	2854
transcript_55756	gnl|BL_ORD_ID|51928 transcript_10140	101	2637	1	106	329	2851	81	186
transcript_55779	gnl|BL_ORD_ID|79606 transcript_145007	152	1043	5	156	1933	2824	691	842
transcript_55779	gnl|BL_ORD_ID|84678 transcript_153661	152	1071	5	156	1836	2755	618	769
transcript_55779	gnl|BL_ORD_ID|53860 transcript_106099	152	1071	5	156	1864	2783	624	775
transcript_55947	gnl|BL_ORD_ID|18173 transcript_53058	1232	2738	1	1235	1650	3160	29	1269
transcript_55990	gnl|BL_ORD_ID|30295 transcript_71132	1	1951	1952	2875	152	2119	2227	3151
transcript_56001	gnl|BL_ORD_ID|84420 transcript_153242	1	1302	1302	2514	36	1362	1846	3056
transcript_56006	gnl|BL_ORD_ID|82967 transcript_150688	1275	3270	1	1277	2516	4509	21	1298
transcript_56012	gnl|BL_ORD_ID|39745 transcript_84754	1	4181	4183	4490	1	4163	4534	4841
transcript_56014	gnl|BL_ORD_ID|44503 transcript_92526	272	1731	60	277	1004	2464	2	220
transcript_56017	gnl|BL_ORD_ID|151 transcript_229	1	2034	2030	2287	1450	3484	4681	4939
transcript_56045	gnl|BL_ORD_ID|304 transcript_499	1222	3542	1	1221	2154	4501	1	1221
transcript_56047	gnl|BL_ORD_ID|58553 transcript_114020	2	2941	2942	3798	143	3053	3327	4184
transcript_56052	gnl|BL_ORD_ID|65628 transcript_123809	11	1472	1473	2385	279	1740	1935	2847
transcript_56053	gnl|BL_ORD_ID|12592 transcript_4085	676	2833	73	678	1211	3368	479	1084
transcript_56066	gnl|BL_ORD_ID|65165 transcript_12467	2	2053	2052	2322	58	2102	2448	2717
transcript_56066	gnl|BL_ORD_ID|52657 transcript_104068	2	2053	2052	2316	56	2086	2447	2711
transcript_56126	gnl|BL_ORD_ID|64152 transcript_123003	290	4130	2	291	653	4478	184	470
transcript_56129	gnl|BL_ORD_ID|64667 transcript_11346	2	2233	2232	2589	39	2269	2371	2728
transcript_56137	gnl|BL_ORD_ID|2348 transcript_24020	1	1438	1433	1660	403	1840	1986	2213
transcript_56137	gnl|BL_ORD_ID|6187 transcript_32266	1	1438	1433	1676	2	1438	1584	1826
transcript_56162	gnl|BL_ORD_ID|84916 transcript_154041	1	2003	2000	3137	7	2006	2558	3695
transcript_56211	gnl|BL_ORD_ID|64518 transcript_11021	2	2043	2039	2251	216	2259	2400	2613
transcript_5623	gnl|BL_ORD_ID|79350 transcript_144605	1077	3141	1	1078	2092	4156	1	1082
transcript_5627	gnl|BL_ORD_ID|34844 transcript_78522	14	1943	1943	3154	2	1932	3636	4847
transcript_5627	gnl|BL_ORD_ID|24813 transcript_5113	287	3116	18	286	437	3267	3	271
transcript_56282	gnl|BL_ORD_ID|45728 transcript_94593	228	2862	1	227	481	3114	1	227

transcript_56282	gnl|BL_ORD_ID|38251 transcript_7216	228	2831	1	227	448	3055	1	227
transcript_56282	gnl|BL_ORD_ID|68130 transcript_127900	223	2829	1	227	1988	4607	1	226
transcript_563	gnl|BL_ORD_ID|74949 transcript_138985	1955	4399	101	1956	2096	4539	128	1983
transcript_563	gnl|BL_ORD_ID|284 transcript_470	1955	4401	1	1956	2070	4516	1	1957
transcript_56304	gnl|BL_ORD_ID|34645 transcript_78196	1	1775	1773	2136	1	1775	1905	2269
transcript_56346	gnl|BL_ORD_ID|4161 transcript_27987	8	794	792	1343	150	942	1345	1904
transcript_56346	gnl|BL_ORD_ID|17299 transcript_51686	8	794	792	1343	6	798	1899	2460
transcript_56346	gnl|BL_ORD_ID|91079 transcript_15853	8	794	792	1343	6	798	1817	2375
transcript_56358	gnl|BL_ORD_ID|22967 transcript_60973	1	3397	3394	4009	116	3503	3611	4222
transcript_56367	gnl|BL_ORD_ID|21479 transcript_58481	1	1077	1072	1958	345	1422	3170	4073
transcript_56389	gnl|BL_ORD_ID|77980 transcript_13559	190	2457	1	192	403	2674	15	208
transcript_56393	gnl|BL_ORD_ID|66329 transcript_124993	2	2461	2461	3068	201	2661	2846	3453
transcript_56433	gnl|BL_ORD_ID|25324 transcript_6195	226	2569	2	227	495	2838	30	255
transcript_56433	gnl|BL_ORD_ID|38784 transcript_8397	226	2569	2	227	609	2952	144	369
transcript_56433	gnl|BL_ORD_ID|38026 transcript_6755	226	2569	2	227	466	2809	1	226
transcript_56445	gnl|BL_ORD_ID|36405 transcript_81028	1	1272	1269	2064	1	1272	3232	4027
transcript_5645	gnl|BL_ORD_ID|23914 transcript_62531	103	2433	2430	3192	18	2350	2535	3298
transcript_56480	gnl|BL_ORD_ID|22296 transcript_59835	1	1491	1490	2505	109	1598	1883	2900
transcript_56515	gnl|BL_ORD_ID|25706 transcript_63775	603	2506	86	604	2231	4133	983	1501
transcript_56544	gnl|BL_ORD_ID|405 transcript_691	1607	4033	1	1612	1826	4252	1	1611
transcript_56544	gnl|BL_ORD_ID|17613 transcript_52169	1607	4033	107	1612	1719	4149	1	1504
transcript_56656	gnl|BL_ORD_ID|41923 transcript_88302	392	2816	43	395	2927	5365	2	349
transcript_56693	gnl|BL_ORD_ID|32348 transcript_74506	1	2121	2118	2547	934	3055	3203	3632
transcript_56723	gnl|BL_ORD_ID|25681 transcript_63740	1054	2600	1	1056	1179	2723	16	1072
transcript_56846	gnl|BL_ORD_ID|21494 transcript_58513	1	1473	1474	2145	114	1586	2199	2881
transcript_56869	gnl|BL_ORD_ID|47460 transcript_97357	1	2159	2154	2741	153	2316	3211	3801
transcript_56869	gnl|BL_ORD_ID|11713 transcript_2368	1	2159	2154	2741	7	2172	3066	3656
transcript_56871	gnl|BL_ORD_ID|77967 transcript_13533	152	2501	1	157	340	2691	18	174
transcript_56895	gnl|BL_ORD_ID|56538 transcript_110689	124	2643	1	127	1474	3994	1169	1295
transcript_56895	gnl|BL_ORD_ID|669 transcript_1160	124	2658	1	127	1463	3998	1158	1284
transcript_56895	gnl|BL_ORD_ID|700 transcript_1216	124	2637	1	127	1514	4028	1209	1335
transcript_56907	gnl|BL_ORD_ID|64383 transcript_10741	140	2259	1	141	516	2635	106	246
transcript_56907	gnl|BL_ORD_ID|78173 transcript_14041	140	2218	1	141	579	2657	166	306
transcript_56972	gnl|BL_ORD_ID|44814 transcript_93039	1	1778	1778	2119	1	1779	2026	2366

transcript_57055	gnl|BL_ORD_ID|82606 transcript_150055	450	995	2	450	554	1099	1	449
transcript_57066	gnl|BL_ORD_ID|31693 transcript_73369	415	1711	5	418	2756	4053	94	507
transcript_57072	gnl|BL_ORD_ID|74521 transcript_138290	271	2077	4	271	667	2469	2	269
transcript_57087	gnl|BL_ORD_ID|80675 transcript_146760	19	3204	3200	3987	1	3187	3438	4215
transcript_57172	gnl|BL_ORD_ID|4691 transcript_29129	294	1190	30	295	1110	2006	2	266
transcript_57176	gnl|BL_ORD_ID|52908 transcript_104455	220	1604	4	221	569	1961	2	219
transcript_57183	gnl|BL_ORD_ID|24680 transcript_4827	1	2039	2038	2652	4	2039	2721	3335
transcript_57187	gnl|BL_ORD_ID|60603 transcript_117299	1	1156	1155	1363	266	1422	1552	1760
transcript_572	gnl|BL_ORD_ID|232 transcript_375	315	4390	1	316	439	4515	1	316
transcript_57220	gnl|BL_ORD_ID|87863 transcript_158858	144	2825	2826	3550	80	2766	2915	3639
transcript_57277	gnl|BL_ORD_ID|83773 transcript_152118	1	2854	2852	3251	381	3234	3374	3768
transcript_57279	gnl|BL_ORD_ID|67990 transcript_127668	2	2176	2176	2676	1	2156	2259	2757
transcript_57361	gnl|BL_ORD_ID|78183 transcript_14059	747	1809	9	751	1282	2344	2	747
transcript_57361	gnl|BL_ORD_ID|81976 transcript_148921	747	1809	80	751	1126	2188	2	676
transcript_57378	gnl|BL_ORD_ID|38023 transcript_6752	1146	2714	1	1148	1553	3121	1	1158
transcript_57380	gnl|BL_ORD_ID|66300 transcript_124942	639	1743	9	641	1697	2801	2	634
transcript_57388	gnl|BL_ORD_ID|73143 transcript_135983	2	2127	2123	2520	56	2182	2285	2692
transcript_57388	gnl|BL_ORD_ID|78509 transcript_14848	2	2127	2123	2429	74	2199	2302	2609
transcript_57393	gnl|BL_ORD_ID|46355 transcript_95615	1012	3171	1	1012	1762	3923	552	1568
transcript_57404	gnl|BL_ORD_ID|41707 transcript_87977	312	1751	5	312	2876	4276	6	306
transcript_57413	gnl|BL_ORD_ID|26456 transcript_64946	108	2186	2185	3516	2	2072	2278	3599
transcript_57439	gnl|BL_ORD_ID|63344 transcript_121698	2	2791	2787	3136	75	2864	3114	3463
transcript_57439	gnl|BL_ORD_ID|12353 transcript_3596	2	2791	2787	3059	152	2947	3197	3469
transcript_57448	gnl|BL_ORD_ID|34741 transcript_78355	1	1605	1606	2229	1458	3062	3316	3942
transcript_57530	gnl|BL_ORD_ID|44814 transcript_93039	1	1772	1772	2020	1	1779	2026	2273
transcript_57535	gnl|BL_ORD_ID|43026 transcript_90037	2	2165	2163	2462	54	2212	2353	2652
transcript_57535	gnl|BL_ORD_ID|68468 transcript_128452	112	2165	2163	2462	75	2128	2269	2568
transcript_57535	gnl|BL_ORD_ID|12236 transcript_3390	112	2165	2163	2462	75	2128	2269	2567
transcript_57556	gnl|BL_ORD_ID|51675 transcript_9611	1	1929	1925	2511	107	2035	2257	2841
transcript_57564	gnl|BL_ORD_ID|54733 transcript_107564	182	2838	12	181	434	3095	2	172
transcript_57623	gnl|BL_ORD_ID|11960 transcript_2850	1	1955	1954	3257	46	2000	2270	3581
transcript_57640	gnl|BL_ORD_ID|4835 transcript_29386	1	1327	1327	1757	1	1326	1571	1996
transcript_57659	gnl|BL_ORD_ID|41597 transcript_87776	1	1411	1411	1722	145	1555	1807	2118
transcript_57659	gnl|BL_ORD_ID|25966 transcript_64199	1	1411	1411	1722	43	1453	1705	2016

transcript_57659	gnl|BL_ORD_ID|92531 transcript_164760	1	1411	1411	1722	167	1576	1935	2246
transcript_57670	gnl|BL_ORD_ID|43371 transcript_90591	1290	3423	1	1290	1669	3798	5	1276
transcript_57689	gnl|BL_ORD_ID|51904 transcript_10098	108	1439	1	108	1439	2780	1168	1275
transcript_57689	gnl|BL_ORD_ID|81423 transcript_147956	108	1439	1	108	1511	2852	1240	1347
transcript_57689	gnl|BL_ORD_ID|31734 transcript_73443	110	1432	1	108	1157	2490	886	993
transcript_57689	gnl|BL_ORD_ID|27776 transcript_67042	107	1437	1	108	1554	2883	1206	1313
transcript_57689	gnl|BL_ORD_ID|53553 transcript_105547	108	1431	1	108	1500	2821	1229	1336
transcript_57689	gnl|BL_ORD_ID|84962 transcript_154107	108	1433	1	108	1524	2846	1253	1360
transcript_57689	gnl|BL_ORD_ID|36366 transcript_80969	108	1436	1	108	1432	2771	1161	1268
transcript_57689	gnl|BL_ORD_ID|52171 transcript_10672	108	1439	1	108	1488	2817	1217	1324
transcript_57697	gnl|BL_ORD_ID|78057 transcript_13781	1	1239	1240	1938	1	1233	1946	2639
transcript_57714	gnl|BL_ORD_ID|36365 transcript_80967	7	603	602	1107	2	598	741	1246
transcript_5773	gnl|BL_ORD_ID|72442 transcript_134878	1	2978	2975	3192	1	2976	3575	3791
transcript_57739	gnl|BL_ORD_ID|31195 transcript_72570	1	1399	1400	1986	1340	2733	4120	4705
transcript_57787	gnl|BL_ORD_ID|86118 transcript_155993	534	2860	8	535	864	3181	1	525
transcript_5782	gnl|BL_ORD_ID|72729 transcript_135335	2	2208	2208	3209	1	2208	2314	3314
transcript_57847	gnl|BL_ORD_ID|59974 transcript_116312	208	2210	2	209	443	2447	66	273
transcript_57882	gnl|BL_ORD_ID|874 transcript_1545	2	2434	2434	3799	5	2434	2552	3915
transcript_57890	gnl|BL_ORD_ID|31373 transcript_72864	1	3077	3072	3871	1002	4088	5121	5921
transcript_57898	gnl|BL_ORD_ID|58464 transcript_113883	1	1720	1718	2134	1	1719	2869	3285
transcript_57910	gnl|BL_ORD_ID|30563 transcript_71563	1	2624	2623	3374	4	2629	2787	3540
transcript_57959	gnl|BL_ORD_ID|73436 transcript_136481	10	2755	2755	3321	2	2746	3367	3932
transcript_5796	gnl|BL_ORD_ID|52409 transcript_103675	10	1595	1592	3164	1	1583	1987	3557
transcript_57965	gnl|BL_ORD_ID|2170 transcript_23605	1	1393	1391	2052	68	1460	1606	2240
transcript_57987	gnl|BL_ORD_ID|91647 transcript_17108	1	1671	1666	2295	1	1674	1824	2451
transcript_58008	gnl|BL_ORD_ID|17630 transcript_52197	1703	3961	1	1704	1868	4124	1	1704
transcript_5808	gnl|BL_ORD_ID|12125 transcript_3190	1340	3200	1	1340	1650	3525	81	1420
transcript_5808	gnl|BL_ORD_ID|80238 transcript_146051	1340	3195	1	1340	1571	3400	2	1341
transcript_58142	gnl|BL_ORD_ID|27202 transcript_66146	1	2647	2647	2912	60	2704	2951	3216
transcript_58183	gnl|BL_ORD_ID|59236 transcript_115125	304	2612	75	304	447	2755	2	230
transcript_58200	gnl|BL_ORD_ID|89633 transcript_161715	1	2107	2107	2897	1	2124	2432	3223
transcript_5821	gnl|BL_ORD_ID|85327 transcript_154696	27	2735	2730	3183	2	2709	4048	4501
transcript_58214	gnl|BL_ORD_ID|65993 transcript_124404	1	2332	2329	2685	320	2658	2773	3129
transcript_58279	gnl|BL_ORD_ID|85277 transcript_154603	2	2096	2093	2451	62	2156	2307	2666

transcript_58285	gnl|BL_ORD_ID|12236 transcript_3390	1	2116	2115	3220	1	2127	2290	3375
transcript_58285	gnl|BL_ORD_ID|68468 transcript_128452	1	2116	2115	3220	1	2127	2290	3388
transcript_58316	gnl|BL_ORD_ID|74147 transcript_137683	1	1995	1998	2710	1	1977	2114	2825
transcript_58316	gnl|BL_ORD_ID|38076 transcript_6847	1	1995	1998	2710	1	1975	2112	2824
transcript_58338	gnl|BL_ORD_ID|41564 transcript_87728	1	1660	1660	2002	642	2297	2404	2746
transcript_58338	gnl|BL_ORD_ID|40149 transcript_85394	1	1660	1660	2002	125	1772	1879	2221
transcript_58339	gnl|BL_ORD_ID|47776 transcript_97846	121	1604	1	124	1690	3175	991	1114
transcript_58339	gnl|BL_ORD_ID|29604 transcript_69999	121	1662	1	124	1690	3231	991	1114
transcript_58339	gnl|BL_ORD_ID|64697 transcript_11414	121	1651	1	124	1264	2791	565	688
transcript_58339	gnl|BL_ORD_ID|27479 transcript_66565	121	1622	1	124	1701	3201	1001	1124
transcript_58373	gnl|BL_ORD_ID|27347 transcript_66365	791	2331	85	794	1496	3036	2	712
transcript_58373	gnl|BL_ORD_ID|68073 transcript_127806	792	2331	85	793	1057	2596	1	709
transcript_5838	gnl|BL_ORD_ID|37668 transcript_83021	1608	3206	149	1610	1827	3425	256	1716
transcript_58414	gnl|BL_ORD_ID|30350 transcript_71214	109	2976	2975	3747	2	2867	2976	3748
transcript_58424	gnl|BL_ORD_ID|91211 transcript_16144	1	1470	1465	2322	1	1471	1575	2433
transcript_58443	gnl|BL_ORD_ID|96817 transcript_99770	222	2813	1	222	1530	4123	11	221
transcript_58474	gnl|BL_ORD_ID|89137 transcript_160906	1	1149	1149	2112	48	1197	1845	2810
transcript_58492	gnl|BL_ORD_ID|7992 transcript_35877	1	1446	1442	1553	11	1455	1575	1686
transcript_58496	gnl|BL_ORD_ID|21652 transcript_58765	402	2203	85	402	1732	3534	593	912
transcript_58496	gnl|BL_ORD_ID|93766 transcript_166752	402	2203	85	402	1510	3316	367	686
transcript_58504	gnl|BL_ORD_ID|87097 transcript_157619	1	1526	1521	2728	2	1509	2299	3510
transcript_58536	gnl|BL_ORD_ID|39453 transcript_84274	137	1836	1	141	392	2093	8	148
transcript_58552	gnl|BL_ORD_ID|64868 transcript_11767	1	1045	1044	1777	2	1052	2018	2752
transcript_5856	gnl|BL_ORD_ID|77195 transcript_142619	227	3189	1	227	495	3457	1	228
transcript_58576	gnl|BL_ORD_ID|26585 transcript_65155	1273	2937	1	1272	1627	3291	206	1483
transcript_58611	gnl|BL_ORD_ID|71972 transcript_134144	1	1449	1444	2752	1292	2751	3061	4378
transcript_58622	gnl|BL_ORD_ID|93457 transcript_166256	1	1330	1329	2527	259	1586	2475	3673
transcript_58624	gnl|BL_ORD_ID|63336 transcript_121690	1	1768	1764	2194	778	2531	3606	4034
transcript_58624	gnl|BL_ORD_ID|89354 transcript_161255	1	1768	1764	2194	913	2666	3740	4169
transcript_58645	gnl|BL_ORD_ID|92847 transcript_165278	271	1786	57	272	369	1885	2	217
transcript_5865	gnl|BL_ORD_ID|68799 transcript_128976	1	2745	2741	3171	1	2746	3807	4239
transcript_5865	gnl|BL_ORD_ID|44960 transcript_93297	2	2826	2825	3171	1	2827	2945	3291
transcript_58653	gnl|BL_ORD_ID|26156 transcript_64491	1	1490	1489	1997	41	1523	1768	2290
transcript_58653	gnl|BL_ORD_ID|95488 transcript_19565	1	1490	1489	1997	42	1533	1793	2317

transcript_58653	gnl|BL_ORD_ID|41610 transcript_87803	1	1490	1489	1997	2	1475	1735	2257
transcript_58758	gnl|BL_ORD_ID|48559 transcript_99086	1155	2689	1	1157	2686	4223	1124	2280
transcript_58761	gnl|BL_ORD_ID|12111 transcript_3164	324	1098	5	325	2719	3493	2	322
transcript_58821	gnl|BL_ORD_ID|20013 transcript_56038	107	1825	1	109	1115	2829	750	858
transcript_58821	gnl|BL_ORD_ID|51996 transcript_10289	107	1847	1	109	1100	2837	735	843
transcript_58821	gnl|BL_ORD_ID|51780 transcript_9812	107	1869	1	109	1090	2852	725	833
transcript_58821	gnl|BL_ORD_ID|42403 transcript_89046	107	1838	1	109	1149	2877	784	892
transcript_58879	gnl|BL_ORD_ID|75667 transcript_140135	1	1933	1932	2900	77	2010	2432	3400
transcript_58897	gnl|BL_ORD_ID|1963 transcript_23159	171	1632	35	173	780	2242	1	151
transcript_58897	gnl|BL_ORD_ID|1259 transcript_21548	171	1605	2	173	902	2337	8	179
transcript_58897	gnl|BL_ORD_ID|94943 transcript_18335	171	1623	2	173	1014	2467	112	281
transcript_58897	gnl|BL_ORD_ID|48079 transcript_98317	171	1632	2	173	937	2399	1	191
transcript_58897	gnl|BL_ORD_ID|56111 transcript_110022	171	1632	30	171	395	1858	20	175
transcript_58897	gnl|BL_ORD_ID|22364 transcript_59949	171	1632	31	173	923	2385	1	149
transcript_58897	gnl|BL_ORD_ID|6038 transcript_31902	171	1632	34	171	433	1895	67	216
transcript_58897	gnl|BL_ORD_ID|75079 transcript_139183	171	1632	29	173	915	2377	2	167
transcript_58929	gnl|BL_ORD_ID|46757 transcript_96262	346	2765	76	351	1409	3828	1	276
transcript_58991	gnl|BL_ORD_ID|12344 transcript_3583	1008	2685	1	1009	1781	3458	572	1580
transcript_59003	gnl|BL_ORD_ID|7014 transcript_33957	361	1661	5	365	496	1795	4	363
transcript_59020	gnl|BL_ORD_ID|69243 transcript_129707	2	3183	3180	3416	29	3208	3472	3708
transcript_59126	gnl|BL_ORD_ID|72486 transcript_134948	138	2957	1	141	337	3156	33	171
transcript_59130	gnl|BL_ORD_ID|23283 transcript_61505	1141	2509	1	1144	1721	3068	404	1547
transcript_59138	gnl|BL_ORD_ID|54498 transcript_107163	373	1162	9	378	2248	3016	126	492
transcript_59138	gnl|BL_ORD_ID|32686 transcript_75065	373	1162	9	376	4036	4806	67	434
transcript_59189	gnl|BL_ORD_ID|63338 transcript_121692	7	689	689	1410	43	725	1412	2125
transcript_59285	gnl|BL_ORD_ID|38597 transcript_7980	1	2177	2178	2737	1	2171	2366	2924
transcript_5937	gnl|BL_ORD_ID|24499 transcript_4441	177	3166	1	179	325	3320	1	172
transcript_59390	gnl|BL_ORD_ID|39689 transcript_84665	1	2204	2203	3030	1238	3452	3570	4397
transcript_59439	gnl|BL_ORD_ID|38140 transcript_6988	1	1519	1516	2837	42	1560	1725	3049
transcript_59457	gnl|BL_ORD_ID|74383 transcript_138062	227	3943	1	227	503	4194	56	278
transcript_59471	gnl|BL_ORD_ID|36320 transcript_80895	1	4043	4043	4398	49	4082	4183	4539
transcript_5948	gnl|BL_ORD_ID|77533 transcript_143142	12	1864	1863	3115	2	1852	2581	3832
transcript_59491	gnl|BL_ORD_ID|25438 transcript_63320	111	1386	1	113	307	1566	6	118
transcript_59498	gnl|BL_ORD_ID|12282 transcript_3463	1054	2898	1	1058	1505	3348	1	1058

transcript_59498	gnl|BL_ORD_ID|37293 transcript_82422	1	1346	1342	2898	2	1351	2131	3724
transcript_59498	gnl|BL_ORD_ID|1140 transcript_2049	1	1346	1342	2898	2	1337	2073	3632
transcript_59503	gnl|BL_ORD_ID|3 transcript_5	1	2224	2224	2993	3790	6018	6328	7093
transcript_59503	gnl|BL_ORD_ID|35316 transcript_79277	1	2224	2224	2993	3776	5996	6304	7069
transcript_59515	gnl|BL_ORD_ID|45071 transcript_93477	268	1269	8	269	1383	2385	91	349
transcript_5952	gnl|BL_ORD_ID|87937 transcript_158974	12	2437	2437	3192	1	2426	2842	3596
transcript_59531	gnl|BL_ORD_ID|64172 transcript_123030	273	2509	3	274	379	2615	4	278
transcript_59531	gnl|BL_ORD_ID|38396 transcript_7541	273	2509	3	274	777	3013	120	393
transcript_59531	gnl|BL_ORD_ID|25433 transcript_6407	273	2509	3	274	665	2901	7	281
transcript_59531	gnl|BL_ORD_ID|54422 transcript_107031	273	2509	3	273	424	2660	6	279
transcript_59564	gnl|BL_ORD_ID|38759 transcript_8350	1154	2493	1	1154	1645	2980	6	1159
transcript_596	gnl|BL_ORD_ID|77284 transcript_142747	318	4383	2	318	969	5033	35	344
transcript_59723	gnl|BL_ORD_ID|45391 transcript_94021	1	1579	1579	2407	1726	3306	3912	4742
transcript_59725	gnl|BL_ORD_ID|32658 transcript_75014	1	1262	1257	2353	321	1580	1680	2774
transcript_59725	gnl|BL_ORD_ID|38117 transcript_6944	1	1262	1257	2353	286	1547	1647	2743
transcript_59725	gnl|BL_ORD_ID|94114 transcript_167297	1	1262	1257	2353	244	1503	1603	2699
transcript_59725	gnl|BL_ORD_ID|33058 transcript_75611	1	1262	1257	2353	367	1628	1728	2824
transcript_59747	gnl|BL_ORD_ID|41374 transcript_87404	1	1826	1824	2284	202	2028	4031	4491
transcript_59783	gnl|BL_ORD_ID|12146 transcript_3223	1	1955	1950	2397	3	1979	3036	3495
transcript_59783	gnl|BL_ORD_ID|36820 transcript_81688	1	1955	1950	2395	3	1952	3009	3453
transcript_59783	gnl|BL_ORD_ID|89607 transcript_161657	1	1955	1950	2421	3	1984	2856	3343
transcript_5980	gnl|BL_ORD_ID|35507 transcript_79583	133	3215	1	132	3252	6334	2839	2970
transcript_5981	gnl|BL_ORD_ID|17654 transcript_52237	1	1577	1575	3108	1	1577	1779	3312
transcript_59878	gnl|BL_ORD_ID|63199 transcript_121474	160	1429	3	163	377	1640	47	219
transcript_599	gnl|BL_ORD_ID|19358 transcript_54972	3	3783	3782	4374	71	3836	4728	5322
transcript_5995	gnl|BL_ORD_ID|12252 transcript_3415	386	3299	90	389	427	3340	1	301
transcript_5995	gnl|BL_ORD_ID|24494 transcript_4431	386	3292	48	389	470	3376	2	344
transcript_59972	gnl|BL_ORD_ID|17820 transcript_52507	433	1631	71	437	711	1907	2	368
transcript_60020	gnl|BL_ORD_ID|47159 transcript_96884	1	2729	2728	3048	1	2722	2897	3217
transcript_6003	gnl|BL_ORD_ID|79925 transcript_145534	2	2207	2202	3140	85	2279	2389	3326
transcript_60033	gnl|BL_ORD_ID|57740 transcript_112705	1	1301	1301	1890	1	1301	1561	2150
transcript_60045	gnl|BL_ORD_ID|52899 transcript_104443	144	2080	11	146	2042	3971	41	175
transcript_60080	gnl|BL_ORD_ID|59622 transcript_115746	1	1351	1350	2001	1	1350	1805	2456
transcript_6009	gnl|BL_ORD_ID|12605 transcript_4105	2	2131	2126	3171	1	2130	2365	3407

transcript_60096	gnl|BL_ORD_ID|97071 transcript_123965	1	1997	1997	2757	3	2000	2144	2908
transcript_60134	gnl|BL_ORD_ID|20675 transcript_57122	180	3623	17	180	286	3741	1	164
transcript_60278	gnl|BL_ORD_ID|95608 transcript_19880	1	1304	1300	2098	56	1360	1537	2335
transcript_60278	gnl|BL_ORD_ID|83126 transcript_150965	1	1304	1300	2098	41	1345	1522	2320
transcript_60331	gnl|BL_ORD_ID|43482 transcript_90790	419	2671	78	420	449	2710	1	343
transcript_60401	gnl|BL_ORD_ID|662 transcript_1147	157	2477	1	160	1250	3572	621	780
transcript_60401	gnl|BL_ORD_ID|12638 transcript_4178	157	2477	1	160	1043	3365	414	573
transcript_60417	gnl|BL_ORD_ID|29725 transcript_70173	704	2322	75	706	804	2437	2	627
transcript_60418	gnl|BL_ORD_ID|17819 transcript_52506	1	1542	1542	2261	637	2198	2449	3170
transcript_60438	gnl|BL_ORD_ID|92324 transcript_164413	1	2160	2157	2622	517	2675	2920	3385
transcript_60498	gnl|BL_ORD_ID|51515 transcript_9246	2	2300	2298	2502	5	2300	2698	2902
transcript_60578	gnl|BL_ORD_ID|53976 transcript_106266	1	1549	1546	2036	53	1608	1743	2233
transcript_6060	gnl|BL_ORD_ID|88258 transcript_159506	461	3154	5	464	566	3274	2	464
transcript_60686	gnl|BL_ORD_ID|5992 transcript_31818	1	1031	1031	1727	1	1031	1208	1903
transcript_60690	gnl|BL_ORD_ID|23363 transcript_61624	1	1629	1627	2936	3	1631	2659	3955
transcript_60702	gnl|BL_ORD_ID|93485 transcript_166301	1	1204	1201	1698	524	1728	2014	2510
transcript_60741	gnl|BL_ORD_ID|36936 transcript_81869	319	1156	7	320	1167	2003	138	451
transcript_60786	gnl|BL_ORD_ID|26974 transcript_65784	185	2537	1	188	807	3239	347	545
transcript_60786	gnl|BL_ORD_ID|32509 transcript_74755	185	2490	1	188	1323	3632	893	1080
transcript_60824	gnl|BL_ORD_ID|12422 transcript_3745	10	3101	3100	3317	2	3100	3219	3432
transcript_60831	gnl|BL_ORD_ID|51864 transcript_10021	1	1368	1364	1728	1	1370	2497	2861
transcript_60831	gnl|BL_ORD_ID|4240 transcript_28169	1	1368	1364	1704	1	1368	1649	1989
transcript_60831	gnl|BL_ORD_ID|3977 transcript_27585	1	1368	1364	1719	2	1367	1720	2074
transcript_60831	gnl|BL_ORD_ID|2106 transcript_23456	1	1368	1364	1699	2	1430	1694	2045
transcript_60831	gnl|BL_ORD_ID|65114 transcript_12336	1	1368	1364	1719	1	1366	2371	2725
transcript_60831	gnl|BL_ORD_ID|51613 transcript_9460	1	1368	1364	1728	1	1384	2517	2880
transcript_60831	gnl|BL_ORD_ID|2910 transcript_25245	1	1368	1364	1728	1	1384	1642	2005
transcript_60831	gnl|BL_ORD_ID|1531 transcript_22223	1	1368	1364	1705	9	1428	1688	2053
transcript_60831	gnl|BL_ORD_ID|22153 transcript_59602	1	1368	1364	1702	1	1378	1633	1971
transcript_60831	gnl|BL_ORD_ID|79112 transcript_144230	1	1368	1364	1700	1	1358	2707	3042
transcript_60831	gnl|BL_ORD_ID|5558 transcript_30891	1	1368	1364	1728	1	1368	1624	1988
transcript_60831	gnl|BL_ORD_ID|3847 transcript_27298	1	1368	1364	1719	1	1384	1741	2097
transcript_60929	gnl|BL_ORD_ID|44824 transcript_93063	1	1561	1560	2931	1804	3365	3576	4948
transcript_60935	gnl|BL_ORD_ID|74463 transcript_138183	1	1516	1512	1824	1799	3314	4374	4683

transcript_6094	gnl|BL_ORD_ID|69319 transcript_129824	1	1987	1987	3178	235	2221	2385	3576
transcript_60948	gnl|BL_ORD_ID|63270 transcript_121575	1	1087	1087	1974	1	1087	1219	2106
transcript_60954	gnl|BL_ORD_ID|65091 transcript_12279	1	2110	2109	2570	1	2109	2256	2717
transcript_60966	gnl|BL_ORD_ID|11677 transcript_2298	1	1906	1904	2462	378	2283	2871	3426
transcript_60985	gnl|BL_ORD_ID|92658 transcript_164967	1	1461	1458	2410	2	1463	2465	3435
transcript_61089	gnl|BL_ORD_ID|6657 transcript_33247	111	1598	1	110	365	1851	2	111
transcript_61096	gnl|BL_ORD_ID|20909 transcript_57523	1	1078	1078	1638	470	1516	3132	3691
transcript_61155	gnl|BL_ORD_ID|24007 transcript_62673	157	2805	1	160	435	3115	82	242
transcript_61171	gnl|BL_ORD_ID|64993 transcript_12045	10	1898	1898	2392	168	2056	2182	2676
transcript_61171	gnl|BL_ORD_ID|48068 transcript_98298	18	1898	1898	2392	136	2015	2141	2635
transcript_61190	gnl|BL_ORD_ID|82759 transcript_150310	340	3652	21	339	452	3785	2	326
transcript_61234	gnl|BL_ORD_ID|4906 transcript_29516	1	1508	1507	1826	2	1504	1679	1998
transcript_61234	gnl|BL_ORD_ID|77152 transcript_142545	1	1508	1507	1880	8	1497	1672	2043
transcript_61271	gnl|BL_ORD_ID|88217 transcript_159441	1025	2706	1	1028	1139	2819	1	1027
transcript_61347	gnl|BL_ORD_ID|46825 transcript_96354	269	1383	3	274	2162	3245	797	1061
transcript_61415	gnl|BL_ORD_ID|72436 transcript_134868	1	1598	1597	2555	1	1598	2094	3052
transcript_61450	gnl|BL_ORD_ID|11969 transcript_2868	1073	2899	1	1076	1743	3568	73	1134
transcript_6146	gnl|BL_ORD_ID|12595 transcript_4090	130	3148	1	129	285	3306	1	129
transcript_6146	gnl|BL_ORD_ID|12615 transcript_4127	130	3148	1	129	313	3331	30	158
transcript_61497	gnl|BL_ORD_ID|48766 transcript_99436	1	2222	2220	3362	1	2223	2332	3475
transcript_61553	gnl|BL_ORD_ID|43343 transcript_90552	401	2109	95	401	549	2252	120	425
transcript_61565	gnl|BL_ORD_ID|29659 transcript_70077	1	1982	1980	2198	214	2182	2341	2561
transcript_61565	gnl|BL_ORD_ID|18284 transcript_53236	1	1982	1980	2198	394	2376	2536	2756
transcript_61575	gnl|BL_ORD_ID|78484 transcript_14784	1225	2379	104	1225	1422	2576	2	1123
transcript_61591	gnl|BL_ORD_ID|91356 transcript_16450	145	1149	2	147	1526	2519	10	155
transcript_61635	gnl|BL_ORD_ID|51194 transcript_8581	224	2530	2	228	676	2981	114	340
transcript_61635	gnl|BL_ORD_ID|25223 transcript_5970	224	2607	2	228	623	3005	61	287
transcript_61635	gnl|BL_ORD_ID|38549 transcript_7857	224	2582	2	228	666	3023	98	330
transcript_61635	gnl|BL_ORD_ID|68162 transcript_127958	224	2509	2	226	1448	3731	183	413
transcript_61635	gnl|BL_ORD_ID|11644 transcript_2233	224	2562	2	226	1346	3678	85	309
transcript_6164	gnl|BL_ORD_ID|89904 transcript_162135	2	2441	2439	3101	45	2482	3401	4063
transcript_6164	gnl|BL_ORD_ID|17322 transcript_51733	2	2132	2132	3080	39	2165	2273	3227
transcript_61663	gnl|BL_ORD_ID|30851 transcript_72035	2	2935	2935	3395	1	2941	3868	4328
transcript_61671	gnl|BL_ORD_ID|80604 transcript_146639	199	1869	9	200	364	2033	14	207

transcript_61674	gnl|BL_ORD_ID|41313 transcript_87293	166	3469	3469	4207	2	3307	3513	4251
transcript_61683	gnl|BL_ORD_ID|51487 transcript_9197	1	1602	1602	2018	2	1604	2490	2906
transcript_61794	gnl|BL_ORD_ID|31881 transcript_73697	1	1800	1798	2038	7	1806	2001	2241
transcript_61794	gnl|BL_ORD_ID|2473 transcript_24284	1	1800	1798	2038	19	1818	1976	2217
transcript_61877	gnl|BL_ORD_ID|58081 transcript_113235	1	1304	1303	2410	983	2287	2711	3820
transcript_61894	gnl|BL_ORD_ID|39162 transcript_83802	1	1970	1966	2848	1637	3607	3724	4606
transcript_61911	gnl|BL_ORD_ID|76554 transcript_141573	2	2370	2370	3400	22	2389	2648	3677
transcript_61925	gnl|BL_ORD_ID|56259 transcript_110261	1	1376	1372	2148	8	1390	1506	2282
transcript_61949	gnl|BL_ORD_ID|58389 transcript_113753	1	1530	1527	2017	87	1617	2579	3069
transcript_61983	gnl|BL_ORD_ID|1132 transcript_2032	274	2961	1	274	998	3682	388	661
transcript_62001	gnl|BL_ORD_ID|72097 transcript_134339	2	2793	2792	2905	27	2816	2954	3066
transcript_62026	gnl|BL_ORD_ID|21402 transcript_58363	282	1055	3	278	3031	3798	376	650
transcript_62052	gnl|BL_ORD_ID|40154 transcript_85399	12	1606	1603	1905	2	1595	2360	2663
transcript_62053	gnl|BL_ORD_ID|12582 transcript_4069	573	3207	66	574	667	3304	2	514
transcript_62053	gnl|BL_ORD_ID|46892 transcript_96453	573	3206	66	574	656	3287	2	503
transcript_62112	gnl|BL_ORD_ID|52495 transcript_103810	1	1631	1630	2197	1	1592	2558	3125
transcript_62320	gnl|BL_ORD_ID|49227 transcript_100155	10	1175	1175	1980	2	1168	1269	2076
transcript_62325	gnl|BL_ORD_ID|94227 transcript_167471	1	1337	1332	1576	554	1891	2104	2348
transcript_62327	gnl|BL_ORD_ID|56317 transcript_110342	1	2602	2601	3017	120	2721	2825	3241
transcript_62411	gnl|BL_ORD_ID|18530 transcript_53620	204	2880	1	205	311	2968	1	205
transcript_62411	gnl|BL_ORD_ID|38155 transcript_7017	204	2880	2	205	327	3004	10	221
transcript_62413	gnl|BL_ORD_ID|75655 transcript_140117	18	1997	1995	2422	10	1983	2421	2845
transcript_62451	gnl|BL_ORD_ID|41878 transcript_88234	235	1138	5	236	367	1270	2	233
transcript_62451	gnl|BL_ORD_ID|20460 transcript_56762	235	1138	53	236	747	1649	1	183
transcript_62451	gnl|BL_ORD_ID|8078 transcript_36056	235	1138	37	236	765	1668	2	201
transcript_62451	gnl|BL_ORD_ID|92925 transcript_165396	235	1138	37	236	335	1238	2	201
transcript_62451	gnl|BL_ORD_ID|63268 transcript_121571	235	1138	37	236	334	1237	1	200
transcript_62451	gnl|BL_ORD_ID|42018 transcript_88445	235	1138	37	236	381	1284	2	201
transcript_62463	gnl|BL_ORD_ID|64004 transcript_122760	260	3766	1	261	408	3911	1	261
transcript_62498	gnl|BL_ORD_ID|451 transcript_768	1	2361	2361	4095	1	2359	2503	4224
transcript_62543	gnl|BL_ORD_ID|56271 transcript_110281	2	2168	2169	2920	27	2197	2305	3056
transcript_6255	gnl|BL_ORD_ID|32840 transcript_75290	126	1805	1802	3172	2	1685	3196	4570
transcript_62570	gnl|BL_ORD_ID|35401 transcript_79408	1123	2795	1	1125	1318	3011	1	1124
transcript_62629	gnl|BL_ORD_ID|69799 transcript_130603	200	1821	68	202	727	2348	2	135

transcript_6265	gnl|BL_ORD_ID|24636 transcript_4719	1	1788	1788	3149	2	1817	1934	3295
transcript_62661	gnl|BL_ORD_ID|72628 transcript_135165	1	1268	1266	1892	1	1267	5104	5728
transcript_62709	gnl|BL_ORD_ID|69797 transcript_130599	1	1720	1717	2337	1069	2788	2893	3513
transcript_62723	gnl|BL_ORD_ID|76158 transcript_140952	379	2288	47	382	4610	6520	2	337
transcript_62779	gnl|BL_ORD_ID|55101 transcript_108232	1	1593	1593	2117	1	1593	1711	2234
transcript_6278	gnl|BL_ORD_ID|89405 transcript_161335	1	1864	1862	3182	1	1870	2093	3415
transcript_6278	gnl|BL_ORD_ID|23381 transcript_61663	1	1864	1862	3182	2	1852	2075	3395
transcript_62782	gnl|BL_ORD_ID|89246 transcript_161080	1	1172	1167	1925	2	1177	1941	2699
transcript_62782	gnl|BL_ORD_ID|51863 transcript_10020	1	1172	1167	1925	2	1245	1846	2643
transcript_62790	gnl|BL_ORD_ID|25054 transcript_5618	1	2093	2093	3015	80	2184	2336	3260
transcript_62790	gnl|BL_ORD_ID|96664 transcript_84180	1	2094	2093	3015	38	2124	2275	3197
transcript_62797	gnl|BL_ORD_ID|82567 transcript_149986	612	1564	7	615	1122	2074	2	610
transcript_62799	gnl|BL_ORD_ID|33795 transcript_76792	10	1687	1691	2072	110	1777	2376	2758
transcript_62799	gnl|BL_ORD_ID|95013 transcript_18485	10	1696	1691	2072	115	1790	2020	2404
transcript_62881	gnl|BL_ORD_ID|80512 transcript_146491	1	2071	2069	2783	1	2072	2558	3276
transcript_62916	gnl|BL_ORD_ID|95269 transcript_19082	1	1070	1075	1310	998	2066	2189	2415
transcript_62916	gnl|BL_ORD_ID|74008 transcript_137430	1	1070	1069	1310	785	1854	1973	2214
transcript_62963	gnl|BL_ORD_ID|61099 transcript_118128	1246	2858	1	1249	2064	3671	29	1278
transcript_62968	gnl|BL_ORD_ID|26985 transcript_65798	10	1751	1750	2480	4	1746	2244	2974
transcript_63012	gnl|BL_ORD_ID|6351 transcript_32589	1	1183	1184	1716	3	1190	1298	1832
transcript_63014	gnl|BL_ORD_ID|51656 transcript_9561	322	2139	5	321	939	2756	2	342
transcript_63036	gnl|BL_ORD_ID|78247 transcript_14203	1	1425	1422	2403	36	1460	1570	2551
transcript_63036	gnl|BL_ORD_ID|89383 transcript_161299	1	1425	1422	2403	45	1497	1607	2586
transcript_63036	gnl|BL_ORD_ID|78152 transcript_13991	1	1425	1424	2403	37	1461	1573	2547
transcript_6309	gnl|BL_ORD_ID|22462 transcript_60101	1	2697	2695	3148	1	2683	2976	3431
transcript_6313	gnl|BL_ORD_ID|35886 transcript_80180	209	3129	1	211	318	3244	1	211
transcript_63133	gnl|BL_ORD_ID|49514 transcript_100609	177	1233	9	177	1179	2230	3	170
transcript_63133	gnl|BL_ORD_ID|51186 transcript_8562	177	1233	2	177	1898	2950	6	183
transcript_63182	gnl|BL_ORD_ID|11657 transcript_2265	287	2769	47	289	1239	3721	548	790
transcript_63205	gnl|BL_ORD_ID|85303 transcript_154644	2	2026	2026	2188	52	2080	2206	2367
transcript_63252	gnl|BL_ORD_ID|4452 transcript_28604	186	1894	1	186	308	2028	1	186
transcript_63252	gnl|BL_ORD_ID|4775 transcript_29296	186	1895	1	186	309	2001	2	187
transcript_63256	gnl|BL_ORD_ID|40373 transcript_85727	1207	3029	1	1208	2344	4166	1037	2244
transcript_63271	gnl|BL_ORD_ID|89012 transcript_160695	313	1430	34	315	406	1504	3	284

transcript_6329	gnl|BL_ORD_ID|52567 transcript_103909	158	2090	2089	3130	133	2065	2453	3495
transcript_63302	gnl|BL_ORD_ID|39873 transcript_84954	2	2429	2424	2855	72	2483	2789	3218
transcript_63306	gnl|BL_ORD_ID|4911 transcript_29531	123	1737	1	124	372	1995	49	173
transcript_63333	gnl|BL_ORD_ID|77661 transcript_12860	1	1628	1627	2413	1	1627	1911	2697
transcript_63345	gnl|BL_ORD_ID|95030 transcript_18517	1	1221	1221	2292	22	1247	1358	2429
transcript_63354	gnl|BL_ORD_ID|88862 transcript_160475	213	2665	1	214	314	2763	1	214
transcript_63355	gnl|BL_ORD_ID|48102 transcript_98352	1	1100	1101	1778	1	1101	1202	1870
transcript_63357	gnl|BL_ORD_ID|24429 transcript_4299	1	2088	2086	2557	470	2558	2702	3173
transcript_63387	gnl|BL_ORD_ID|92262 transcript_164309	1	4977	4973	5241	1255	6197	7288	7556
transcript_63396	gnl|BL_ORD_ID|38293 transcript_7305	1134	2849	1	1138	1325	3041	10	1145
transcript_63396	gnl|BL_ORD_ID|38029 transcript_6766	1134	2747	1	1138	1323	2965	8	1143
transcript_63396	gnl|BL_ORD_ID|71119 transcript_132775	1134	2849	1	1138	1307	3031	7	1127
transcript_63396	gnl|BL_ORD_ID|38414 transcript_7576	1134	2829	1	1138	1376	3072	61	1196
transcript_6341	gnl|BL_ORD_ID|53911 transcript_106173	2	2846	2846	3110	19	2863	2993	3257
transcript_6342	gnl|BL_ORD_ID|59337 transcript_115297	134	2495	2495	3190	2	2364	3280	3980
transcript_63424	gnl|BL_ORD_ID|75857 transcript_140453	886	1966	92	887	2526	3605	3	798
transcript_63454	gnl|BL_ORD_ID|18599 transcript_53727	1756	3524	135	1760	2004	3772	242	1867
transcript_63455	gnl|BL_ORD_ID|56814 transcript_111133	126	2133	2129	2941	133	2140	3245	4058
transcript_63455	gnl|BL_ORD_ID|66340 transcript_125005	126	1972	1967	2941	309	2155	2454	3429
transcript_63458	gnl|BL_ORD_ID|96380 transcript_57885	1	1376	1375	2508	1	1373	2182	3316
transcript_63463	gnl|BL_ORD_ID|63745 transcript_122362	383	2973	6	386	968	3553	3	382
transcript_63491	gnl|BL_ORD_ID|51288 transcript_8790	1	1755	1756	2832	2	1756	1860	2936
transcript_63587	gnl|BL_ORD_ID|78247 transcript_14203	2	2145	2141	2262	57	2200	2430	2551
transcript_63587	gnl|BL_ORD_ID|78152 transcript_13991	2	2145	2141	2262	58	2201	2426	2547
transcript_63587	gnl|BL_ORD_ID|89383 transcript_161299	2	2145	2141	2262	66	2235	2465	2586
transcript_63596	gnl|BL_ORD_ID|581 transcript_984	1	3629	3624	3936	69	3698	3804	4116
transcript_63613	gnl|BL_ORD_ID|71263 transcript_132989	134	3326	11	135	577	3745	3	128
transcript_63669	gnl|BL_ORD_ID|34175 transcript_77418	197	2135	10	198	299	2237	2	190
transcript_63695	gnl|BL_ORD_ID|42498 transcript_89190	10	1566	1562	1719	2	1559	1669	1826
transcript_63709	gnl|BL_ORD_ID|12668 transcript_4235	1544	3215	113	1547	1670	3341	2	1437
transcript_6376	gnl|BL_ORD_ID|45850 transcript_94813	551	3130	6	552	2132	4711	1	547
transcript_63764	gnl|BL_ORD_ID|88566 transcript_159990	1	1591	1589	2370	2	1590	3170	3951
transcript_63764	gnl|BL_ORD_ID|28676 transcript_68561	1	1591	1589	2453	2	1566	2698	3562
transcript_63784	gnl|BL_ORD_ID|82179 transcript_149293	1058	3269	1	1062	1223	3433	1	1064

transcript_63814	gnl|BL_ORD_ID|59286 transcript_115209	1237	3149	1	1240	2073	3985	1	1264
transcript_63814	gnl|BL_ORD_ID|489 transcript_829	1237	3149	1	1240	2048	3960	2	1239
transcript_63815	gnl|BL_ORD_ID|35226 transcript_79141	2	2467	2464	3116	41	2461	3210	3863
transcript_6390	gnl|BL_ORD_ID|57630 transcript_112516	297	3116	17	296	837	3656	1	280
transcript_63906	gnl|BL_ORD_ID|202 transcript_323	1688	3606	11	1692	2783	4723	15	1696
transcript_6393	gnl|BL_ORD_ID|63407 transcript_121800	1	2430	2431	3126	269	2706	2831	3527
transcript_63960	gnl|BL_ORD_ID|52394 transcript_103650	261	1781	3	263	1219	2714	4	264
transcript_6398	gnl|BL_ORD_ID|48002 transcript_98197	2	2636	2637	3149	30	2663	2763	3273
transcript_64	gnl|BL_ORD_ID|88811 transcript_160407	1507	5744	1	1506	1633	5891	1	1506
transcript_64032	gnl|BL_ORD_ID|58081 transcript_113235	2	2286	2285	3391	2	2287	2711	3820
transcript_64073	gnl|BL_ORD_ID|65 transcript_91	162	3867	1	164	1807	5508	2	165
transcript_64084	gnl|BL_ORD_ID|92238 transcript_164276	1	1553	1553	2801	451	2001	2356	3603
transcript_64097	gnl|BL_ORD_ID|918 transcript_1640	1	2791	2791	3059	60	2839	3342	3610
transcript_64118	gnl|BL_ORD_ID|25157 transcript_5837	262	2987	2	262	455	3180	22	282
transcript_64118	gnl|BL_ORD_ID|25422 transcript_6382	262	2987	12	262	422	3147	2	252
transcript_64133	gnl|BL_ORD_ID|73 transcript_103	1	2794	2794	5270	3	2795	2960	5438
transcript_64138	gnl|BL_ORD_ID|17971 transcript_52731	500	4450	93	500	738	4678	2	409
transcript_64139	gnl|BL_ORD_ID|69581 transcript_130245	322	1689	49	325	410	1781	2	278
transcript_6415	gnl|BL_ORD_ID|55682 transcript_109281	2	2572	2571	3059	8	2582	3196	3684
transcript_64162	gnl|BL_ORD_ID|30467 transcript_71403	1	3715	3710	3837	1	3729	3831	3959
transcript_6417	gnl|BL_ORD_ID|43980 transcript_91655	492	3172	79	492	945	3625	382	789
transcript_64206	gnl|BL_ORD_ID|48485 transcript_98968	1	2721	2719	3291	3	2717	3619	4194
transcript_643	gnl|BL_ORD_ID|92085 transcript_164033	1833	4334	1	1835	2456	4957	1	1824
transcript_64350	gnl|BL_ORD_ID|72758 transcript_135384	150	2763	2763	3536	2	2614	2897	3670
transcript_64364	gnl|BL_ORD_ID|56683 transcript_110930	296	2531	89	298	863	3097	546	755
transcript_64381	gnl|BL_ORD_ID|11786 transcript_2516	1152	3386	1	1157	1302	3536	1	1153
transcript_64426	gnl|BL_ORD_ID|47583 transcript_97547	102	3184	1	106	485	3568	1	106
transcript_64441	gnl|BL_ORD_ID|84970 transcript_154117	12	1930	1930	2671	2	1938	2083	2832
transcript_64454	gnl|BL_ORD_ID|33186 transcript_75809	178	2781	1	181	321	2925	1	181
transcript_6447	gnl|BL_ORD_ID|24599 transcript_4642	123	2736	2735	3192	23	2634	2838	3295
transcript_6447	gnl|BL_ORD_ID|38862 transcript_83326	405	3173	75	410	1261	4013	2	337
transcript_64507	gnl|BL_ORD_ID|41923 transcript_88302	1	3001	3001	3358	277	3295	4562	4920
transcript_64543	gnl|BL_ORD_ID|71620 transcript_133564	1000	2579	1	1003	1518	3097	112	1114
transcript_64555	gnl|BL_ORD_ID|11814 transcript_2576	209	3035	1	208	815	3642	161	378

transcript_64563	gnl|BL_ORD_ID|81885 transcript_148766	162	2098	1	162	267	2212	1	162
transcript_64616	gnl|BL_ORD_ID|71684 transcript_133671	1	2575	2575	3356	54	2622	3142	3923
transcript_64638	gnl|BL_ORD_ID|92852 transcript_165286	262	2232	46	261	484	2454	1	215
transcript_64638	gnl|BL_ORD_ID|65031 transcript_12130	262	2249	6	261	525	2547	1	264
transcript_6465	gnl|BL_ORD_ID|28380 transcript_68085	370	3014	5	371	795	3439	2	391
transcript_6465	gnl|BL_ORD_ID|11943 transcript_2825	119	3095	1	123	290	3272	1	123
transcript_64684	gnl|BL_ORD_ID|49918 transcript_101249	1	1238	1237	2022	284	1514	1625	2412
transcript_64684	gnl|BL_ORD_ID|21936 transcript_59239	1	1238	1237	2022	275	1503	1614	2399
transcript_64687	gnl|BL_ORD_ID|467 transcript_791	1	3710	3706	3994	104	3814	3939	4227
transcript_64767	gnl|BL_ORD_ID|42237 transcript_88784	352	1022	44	356	878	1540	2	314
transcript_64789	gnl|BL_ORD_ID|2978 transcript_25393	1	1241	1236	1883	1	1240	1382	2029
transcript_64813	gnl|BL_ORD_ID|96628 transcript_80568	123	2156	2154	3699	2	2048	2821	4369
transcript_64856	gnl|BL_ORD_ID|56599 transcript_110786	172	1798	1	176	308	1913	2	179
transcript_64877	gnl|BL_ORD_ID|39309 transcript_84036	1	2046	2044	2437	90	2137	2688	3081
transcript_64892	gnl|BL_ORD_ID|75480 transcript_139839	183	1798	2	188	1392	3005	2	183
transcript_64893	gnl|BL_ORD_ID|59473 transcript_115510	14	522	520	984	2	510	635	1099
transcript_64903	gnl|BL_ORD_ID|44482 transcript_92491	110	1813	1813	2348	2	1711	3137	3673
transcript_64922	gnl|BL_ORD_ID|19501 transcript_55230	597	2541	6	597	3006	4950	1	599
transcript_6493	gnl|BL_ORD_ID|57348 transcript_112036	1	2689	2685	3125	1	2689	3749	4189
transcript_64939	gnl|BL_ORD_ID|33468 transcript_76243	1294	2966	1	1295	1778	3448	2	1297
transcript_64940	gnl|BL_ORD_ID|55015 transcript_108080	365	2402	5	366	589	2624	2	365
transcript_64970	gnl|BL_ORD_ID|49316 transcript_100291	250	2666	25	252	494	2909	2	229
transcript_64970	gnl|BL_ORD_ID|51519 transcript_9251	251	2666	18	252	371	2785	2	237
transcript_64990	gnl|BL_ORD_ID|94032 transcript_167164	1649	3739	1	1651	2104	4194	162	1821
transcript_64995	gnl|BL_ORD_ID|52551 transcript_103887	207	2709	1	210	347	2859	8	215
transcript_64995	gnl|BL_ORD_ID|64957 transcript_11952	206	2648	1	210	368	2809	1	210
transcript_64995	gnl|BL_ORD_ID|51519 transcript_9251	206	2709	1	210	397	2899	32	239
transcript_64995	gnl|BL_ORD_ID|49316 transcript_100291	206	2641	1	210	521	2956	24	231
transcript_65	gnl|BL_ORD_ID|30 transcript_40	273	5809	1	274	510	6047	1	273
transcript_6500	gnl|BL_ORD_ID|88413 transcript_159752	18	2261	2259	2878	2	2165	2979	3583
transcript_65022	gnl|BL_ORD_ID|26393 transcript_64855	1	2307	2302	2591	994	3306	3424	3715
transcript_6506	gnl|BL_ORD_ID|41377 transcript_87408	253	3131	1	255	1513	4396	42	294
transcript_6507	gnl|BL_ORD_ID|55039 transcript_108121	2	3000	2998	3117	56	3053	3187	3306
transcript_65130	gnl|BL_ORD_ID|45286 transcript_93831	228	2208	6	230	353	2328	1	225

transcript_65143	gnl|BL_ORD_ID|71086 transcript_132727	113	3768	1	116	297	3952	1	116
transcript_65147	gnl|BL_ORD_ID|57537 transcript_112359	564	2790	90	565	581	2807	1	481
transcript_6518	gnl|BL_ORD_ID|24813 transcript_5113	346	3128	77	345	437	3220	3	271
transcript_6518	gnl|BL_ORD_ID|48492 transcript_98980	1525	3128	10	1527	1725	3328	2	1520
transcript_6521	gnl|BL_ORD_ID|45751 transcript_94629	1	1666	1664	3142	231	1896	2284	3762
transcript_6521	gnl|BL_ORD_ID|89371 transcript_161280	1187	3142	1	1192	2089	4042	672	1862
transcript_6521	gnl|BL_ORD_ID|942 transcript_1674	1	1862	1861	3142	654	2514	2620	3900
transcript_65228	gnl|BL_ORD_ID|11664 transcript_2273	1670	3546	1	1670	1783	3666	17	1682
transcript_65241	gnl|BL_ORD_ID|51701 transcript_9662	1104	2572	1	1107	1451	2917	43	1147
transcript_65277	gnl|BL_ORD_ID|46456 transcript_95789	513	3096	67	514	818	3399	121	562
transcript_6529	gnl|BL_ORD_ID|24730 transcript_4935	416	3151	89	415	554	3289	3	329
transcript_65295	gnl|BL_ORD_ID|21830 transcript_59049	1	1482	1480	1733	1	1480	1600	1853
transcript_65295	gnl|BL_ORD_ID|6225 transcript_32336	1	1485	1480	1735	4	1481	1598	1854
transcript_65305	gnl|BL_ORD_ID|73272 transcript_136203	1	2465	2465	2810	3	2467	2574	2919
transcript_65308	gnl|BL_ORD_ID|52987 transcript_104585	1	1738	1737	1960	264	1996	2147	2370
transcript_65320	gnl|BL_ORD_ID|34678 transcript_78255	20	4599	4597	5141	2	4582	4687	5230
transcript_65324	gnl|BL_ORD_ID|80481 transcript_146441	1	1078	1075	1925	2	1081	1478	2331
transcript_65335	gnl|BL_ORD_ID|64223 transcript_123105	2	2761	2759	3372	89	2836	3729	4340
transcript_65364	gnl|BL_ORD_ID|38659 transcript_8106	1	1832	1831	2083	1	1830	2423	2675
transcript_65371	gnl|BL_ORD_ID|92353 transcript_164460	1	2484	2479	2974	2	2481	3398	3894
transcript_65419	gnl|BL_ORD_ID|63850 transcript_122528	463	2375	87	464	797	2713	9	384
transcript_6549	gnl|BL_ORD_ID|29421 transcript_69700	132	2742	2741	3134	22	2633	2813	3206
transcript_65537	gnl|BL_ORD_ID|17592 transcript_52141	1	1808	1808	2467	908	2715	3707	4366
transcript_65545	gnl|BL_ORD_ID|80892 transcript_147121	1	1870	1871	2552	1205	3074	3401	4084
transcript_65546	gnl|BL_ORD_ID|50894 transcript_102839	1	1911	1910	2426	385	2296	2488	3004
transcript_65546	gnl|BL_ORD_ID|89771 transcript_161919	1	1911	1910	2367	469	2380	2572	3029
transcript_65572	gnl|BL_ORD_ID|19431 transcript_55096	1	1671	1668	2552	174	1844	2036	2920
transcript_65581	gnl|BL_ORD_ID|60160 transcript_116602	1	1267	1270	1497	1	1274	1496	1719
transcript_65611	gnl|BL_ORD_ID|20004 transcript_56025	1	2140	2138	2633	1	2129	2867	3361
transcript_65673	gnl|BL_ORD_ID|38229 transcript_7165	164	2484	1	168	748	3063	129	292
transcript_65713	gnl|BL_ORD_ID|35417 transcript_79431	597	2614	66	598	702	2722	1	534
transcript_65745	gnl|BL_ORD_ID|18196 transcript_53102	121	3777	3774	4482	2	3654	4375	5082
transcript_65752	gnl|BL_ORD_ID|95738 transcript_20197	1	1682	1681	1997	1	1665	2037	2366
transcript_65752	gnl|BL_ORD_ID|3285 transcript_26074	1	1682	1683	2006	1	1662	1791	2114

transcript_65786	gnl|BL_ORD_ID|50176 transcript_101660	1	1500	1498	2336	1	1477	1594	2427
transcript_65810	gnl|BL_ORD_ID|94093 transcript_167268	1	1081	1082	1831	34	1113	2454	3203
transcript_65837	gnl|BL_ORD_ID|77707 transcript_12967	1	1335	1336	2505	2	1336	1447	2607
transcript_65842	gnl|BL_ORD_ID|11984 transcript_2899	2	2327	2325	3009	111	2435	2914	3598
transcript_65899	gnl|BL_ORD_ID|66990 transcript_126056	1	1094	1093	1525	19	1112	1787	2220
transcript_6590	gnl|BL_ORD_ID|27295 transcript_66286	12	1648	1647	3056	3	1640	2867	4277
transcript_65916	gnl|BL_ORD_ID|76462 transcript_141413	14	2575	2575	3248	2	2565	3426	4081
transcript_65922	gnl|BL_ORD_ID|89432 transcript_161382	2	2016	2016	2703	33	2024	2171	2858
transcript_65922	gnl|BL_ORD_ID|51656 transcript_9561	2	2016	2016	2703	25	2040	2187	2874
transcript_65969	gnl|BL_ORD_ID|72442 transcript_134878	1	1878	1873	3515	1	1876	2129	3783
transcript_6598	gnl|BL_ORD_ID|46442 transcript_95770	2	2188	2186	3090	17	2204	3353	4257
transcript_65985	gnl|BL_ORD_ID|70716 transcript_132121	28	2815	2813	3241	1	2795	3539	3967
transcript_65989	gnl|BL_ORD_ID|78631 transcript_143448	257	1709	28	256	954	2418	2	230
transcript_6604	gnl|BL_ORD_ID|87976 transcript_159047	1097	3116	1	1097	1459	3475	2	1099
transcript_66044	gnl|BL_ORD_ID|47936 transcript_98085	366	4302	2	369	584	4521	103	470
transcript_66048	gnl|BL_ORD_ID|72167 transcript_134451	603	1773	8	604	1614	2785	1	595
transcript_66100	gnl|BL_ORD_ID|50395 transcript_102010	2	2830	2828	3662	41	2869	3431	4264
transcript_66119	gnl|BL_ORD_ID|12167 transcript_3260	1293	2930	1	1297	1846	3489	237	1544
transcript_66119	gnl|BL_ORD_ID|27966 transcript_67359	1293	2902	1	1297	1651	3270	42	1349
transcript_66142	gnl|BL_ORD_ID|76414 transcript_141344	1486	3888	1	1487	1625	4029	2	1478
transcript_66142	gnl|BL_ORD_ID|45787 transcript_94696	1	3197	3195	3888	4	3192	3422	4117
transcript_66194	gnl|BL_ORD_ID|28052 transcript_67508	337	2807	68	337	457	3032	1	284
transcript_66259	gnl|BL_ORD_ID|232 transcript_375	231	4435	2	235	430	4636	86	320
transcript_66314	gnl|BL_ORD_ID|46376 transcript_95660	1	1752	1753	2402	1	1748	1849	2498
transcript_66320	gnl|BL_ORD_ID|40373 transcript_85727	1416	3235	1	1417	2344	4166	827	2244
transcript_66357	gnl|BL_ORD_ID|57515 transcript_112319	1	2464	2461	2656	1490	3951	4091	4286
transcript_66357	gnl|BL_ORD_ID|66840 transcript_125817	1	2464	2461	2656	2876	5340	5480	5675
transcript_66421	gnl|BL_ORD_ID|74510 transcript_138271	544	2066	7	547	1116	2638	2	564
transcript_66432	gnl|BL_ORD_ID|545 transcript_922	2	3288	3287	3988	22	3303	3444	4146
transcript_66458	gnl|BL_ORD_ID|87087 transcript_157598	139	2667	1	141	621	3150	250	390
transcript_66461	gnl|BL_ORD_ID|72378 transcript_134774	1	2760	2760	3331	9	2756	2858	3429
transcript_66474	gnl|BL_ORD_ID|90579 transcript_163143	1	1342	1341	2064	5	1347	1554	2277
transcript_66510	gnl|BL_ORD_ID|48496 transcript_98987	1534	3108	101	1536	1613	3184	50	1491
transcript_66524	gnl|BL_ORD_ID|53922 transcript_106187	2	2073	2072	2988	24	2082	2296	3207

transcript_66543	gnl|BL_ORD_ID|68889 transcript_129127	1	2482	2481	3083	31	2509	2965	3565
transcript_6656	gnl|BL_ORD_ID|36364 transcript_80965	222	3110	16	223	306	3190	4	206
transcript_66574	gnl|BL_ORD_ID|78123 transcript_13936	187	2406	1	191	351	2569	5	196
transcript_66592	gnl|BL_ORD_ID|30410 transcript_71307	119	2924	1	120	225	3030	1	120
transcript_66598	gnl|BL_ORD_ID|38114 transcript_6940	214	2779	1	215	394	2953	62	277
transcript_66598	gnl|BL_ORD_ID|25312 transcript_6164	214	2779	1	215	347	2912	14	230
transcript_66639	gnl|BL_ORD_ID|84828 transcript_153899	13	1995	1992	2509	2	1991	2611	3134
transcript_6664	gnl|BL_ORD_ID|61127 transcript_118173	1	1971	1970	3027	61	2032	2165	3222
transcript_6668	gnl|BL_ORD_ID|79830 transcript_145368	2	2164	2161	2977	65	2227	3759	4575
transcript_66709	gnl|BL_ORD_ID|64019 transcript_122785	1	1447	1442	2626	2	1438	1594	2779
transcript_6672	gnl|BL_ORD_ID|90555 transcript_163105	1	2571	2568	2979	1	2565	2708	3118
transcript_66808	gnl|BL_ORD_ID|25200 transcript_5914	115	2367	1	116	278	2532	1	116
transcript_6681	gnl|BL_ORD_ID|87394 transcript_158113	1	1829	1830	3101	1	1829	1929	3207
transcript_6681	gnl|BL_ORD_ID|25005 transcript_5498	1	1829	1830	3101	30	1858	1958	3229
transcript_66817	gnl|BL_ORD_ID|38122 transcript_6951	1107	2771	1	1110	1429	3093	2	1111
transcript_66850	gnl|BL_ORD_ID|52567 transcript_103909	2	2033	2032	3154	34	2065	2453	3575
transcript_66856	gnl|BL_ORD_ID|48766 transcript_99436	1	1612	1610	2602	616	2223	2332	3339
transcript_6688	gnl|BL_ORD_ID|72086 transcript_134318	124	2025	2020	3097	1	1903	2744	3824
transcript_66910	gnl|BL_ORD_ID|33964 transcript_77067	1	1121	1121	2181	1	1121	1773	2833
transcript_66979	gnl|BL_ORD_ID|59429 transcript_115446	1	1184	1179	1512	264	1447	1586	1919
transcript_66979	gnl|BL_ORD_ID|75025 transcript_139101	1	1184	1179	1512	634	1818	1957	2293
transcript_66979	gnl|BL_ORD_ID|49981 transcript_101363	1	1184	1179	1512	608	1792	1931	2264
transcript_66979	gnl|BL_ORD_ID|92213 transcript_164241	1	1184	1179	1512	403	1586	1725	2061
transcript_66983	gnl|BL_ORD_ID|36512 transcript_81213	224	1319	7	224	1019	2115	31	249
transcript_66983	gnl|BL_ORD_ID|17616 transcript_52173	224	1319	8	224	991	2087	2	219
transcript_67	gnl|BL_ORD_ID|43907 transcript_91511	106	4356	4356	5750	2	4253	4827	6221
transcript_67025	gnl|BL_ORD_ID|88097 transcript_159248	1498	3544	1	1497	1641	3694	12	1508
transcript_67025	gnl|BL_ORD_ID|11696 transcript_2335	1498	3546	1	1497	1708	3743	80	1576
transcript_67028	gnl|BL_ORD_ID|80390 transcript_146314	1604	4337	1	1604	2348	5078	637	2239
transcript_67034	gnl|BL_ORD_ID|17849 transcript_52555	1	2421	2421	2725	596	3022	3926	4230
transcript_67036	gnl|BL_ORD_ID|69795 transcript_130597	339	2582	43	339	389	2632	2	284
transcript_67076	gnl|BL_ORD_ID|22682 transcript_60485	1	1452	1451	2219	1	1451	2312	3080
transcript_67081	gnl|BL_ORD_ID|1838 transcript_22911	1	1245	1240	1977	8	1256	1521	2259
transcript_67081	gnl|BL_ORD_ID|62075 transcript_119638	1	1245	1240	1977	4	1251	1615	2353

transcript_67081	gnl|BL_ORD_ID|95105 transcript_18682	1	1245	1240	1977	91	1337	1701	2440
transcript_67166	gnl|BL_ORD_ID|33061 transcript_75615	278	3498	15	277	820	4054	2	264
transcript_67181	gnl|BL_ORD_ID|41167 transcript_87062	226	3117	2	225	422	3337	25	247
transcript_67184	gnl|BL_ORD_ID|22742 transcript_60596	1	1270	1271	2427	9	1287	2054	3215
transcript_67191	gnl|BL_ORD_ID|88413 transcript_159752	19	1917	1912	2180	2	1900	3317	3585
transcript_67191	gnl|BL_ORD_ID|11665 transcript_2276	1	1917	1912	2174	1	1919	3438	3700
transcript_67191	gnl|BL_ORD_ID|51991 transcript_10275	11	1917	1912	2180	28	1934	2535	2800
transcript_67191	gnl|BL_ORD_ID|48645 transcript_99237	12	1917	1912	2141	9	1914	2542	2771
transcript_67191	gnl|BL_ORD_ID|37897 transcript_6500	1	1917	1913	2178	2	1987	2614	2878
transcript_67191	gnl|BL_ORD_ID|47513 transcript_97444	16	1917	1912	2170	3	1904	2608	2855
transcript_67191	gnl|BL_ORD_ID|38657 transcript_8102	16	1917	1912	2180	14	1915	2514	2770
transcript_6721	gnl|BL_ORD_ID|24952 transcript_5369	1	2884	2883	3104	1	2884	3011	3232
transcript_67235	gnl|BL_ORD_ID|2454 transcript_24254	1	1474	1471	1966	2	1474	1578	2074
transcript_67238	gnl|BL_ORD_ID|96129 transcript_21087	1	1561	1557	2021	1	1560	1843	2305
transcript_67238	gnl|BL_ORD_ID|1971 transcript_23176	1	1561	1557	1973	1	1560	1843	2259
transcript_67244	gnl|BL_ORD_ID|31401 transcript_72912	16	1712	1710	3312	2	1696	1830	3432
transcript_6725	gnl|BL_ORD_ID|25399 transcript_6331	261	2995	1	262	409	3143	1	262
transcript_67297	gnl|BL_ORD_ID|12569 transcript_4049	1022	2812	1	1021	1465	3258	1	1032
transcript_67330	gnl|BL_ORD_ID|51531 transcript_9278	140	1965	1	143	1103	2927	69	211
transcript_67332	gnl|BL_ORD_ID|49204 transcript_100118	125	2180	1	125	240	2291	1	125
transcript_67332	gnl|BL_ORD_ID|88050 transcript_159165	1	1430	1428	2174	1	1427	3188	3934
transcript_67337	gnl|BL_ORD_ID|68910 transcript_129163	454	3439	62	450	640	3627	113	496
transcript_6736	gnl|BL_ORD_ID|56166 transcript_110105	19	1913	1913	3101	1	1897	2875	4063
transcript_67414	gnl|BL_ORD_ID|39885 transcript_84976	1	2180	2179	2763	23	2215	2677	3260
transcript_67429	gnl|BL_ORD_ID|38181 transcript_7072	1132	2587	109	1134	1621	3076	452	1477
transcript_67429	gnl|BL_ORD_ID|37920 transcript_6551	109	1436	1435	2597	449	1776	1948	3110
transcript_67429	gnl|BL_ORD_ID|25257 transcript_6048	109	1436	1435	2597	452	1779	1936	3098
transcript_67458	gnl|BL_ORD_ID|38839 transcript_8522	1	2284	2279	2720	99	2381	2501	2942
transcript_67458	gnl|BL_ORD_ID|51243 transcript_8695	1	2284	2279	2720	95	2354	2473	2912
transcript_67458	gnl|BL_ORD_ID|40830 transcript_86522	1	2284	2279	2720	92	2333	2453	2891
transcript_67458	gnl|BL_ORD_ID|29188 transcript_69352	1	2284	2279	2694	98	2357	2477	2890
transcript_67458	gnl|BL_ORD_ID|52036 transcript_10375	1	2284	2279	2624	99	2376	2496	2840
transcript_67458	gnl|BL_ORD_ID|51274 transcript_8762	1	2284	2279	2720	97	2377	2497	2937
transcript_67569	gnl|BL_ORD_ID|62462 transcript_120290	416	2226	44	419	520	2330	1	383

transcript_67617	gnl|BL_ORD_ID|26743 transcript_65402	1018	2373	1	1017	1321	2676	159	1175
transcript_67649	gnl|BL_ORD_ID|71782 transcript_133838	1	1992	1988	2990	1	2009	3392	4394
transcript_6765	gnl|BL_ORD_ID|37709 transcript_83087	522	3131	64	525	1127	3737	1	455
transcript_67681	gnl|BL_ORD_ID|51551 transcript_9333	1	1740	1740	2156	4	1742	2436	2853
transcript_67681	gnl|BL_ORD_ID|87292 transcript_157956	1	1740	1740	2156	3	1742	2434	2851
transcript_67707	gnl|BL_ORD_ID|76428 transcript_141369	2	2059	2057	3109	2	2069	2176	3231
transcript_67710	gnl|BL_ORD_ID|85183 transcript_154462	10	2369	2365	2678	178	2546	2705	3018
transcript_67710	gnl|BL_ORD_ID|32277 transcript_74385	20	2369	2365	2686	6	2360	2519	2853
transcript_67710	gnl|BL_ORD_ID|93954 transcript_167041	18	2369	2365	2688	4	2341	2500	2836
transcript_67744	gnl|BL_ORD_ID|23809 transcript_62359	161	2335	1	164	906	3080	5	168
transcript_67759	gnl|BL_ORD_ID|32658 transcript_75014	1	2520	2519	2883	81	2597	2779	3143
transcript_67759	gnl|BL_ORD_ID|94114 transcript_167297	103	2520	2519	2901	107	2519	2704	3086
transcript_67759	gnl|BL_ORD_ID|38117 transcript_6944	103	2520	2519	2859	148	2565	2748	3088
transcript_67798	gnl|BL_ORD_ID|69935 transcript_130830	114	1678	1	115	268	1833	1	115
transcript_6788	gnl|BL_ORD_ID|81130 transcript_147488	13	1841	1841	3088	2	1833	2084	3326
transcript_67909	gnl|BL_ORD_ID|59337 transcript_115297	135	2475	2473	2936	2	2343	3278	3741
transcript_67929	gnl|BL_ORD_ID|33757 transcript_76725	11	2198	2196	2845	5	2170	2281	2930
transcript_67929	gnl|BL_ORD_ID|54508 transcript_107178	1	2198	2196	2845	86	2261	3638	4287
transcript_68016	gnl|BL_ORD_ID|47637 transcript_97618	300	3656	1	299	576	3932	1	299
transcript_68018	gnl|BL_ORD_ID|5233 transcript_30236	1	1211	1207	1794	65	1272	1415	2003
transcript_68018	gnl|BL_ORD_ID|3897 transcript_27404	1	1211	1207	1794	41	1248	1392	1979
transcript_68018	gnl|BL_ORD_ID|1836 transcript_22902	1	1207	1207	1794	115	1319	1463	2046
transcript_68034	gnl|BL_ORD_ID|24362 transcript_63233	1	1640	1641	3271	1	1632	1735	3363
transcript_68034	gnl|BL_ORD_ID|88961 transcript_160620	1315	3254	1	1311	1513	3448	1	1290
transcript_68045	gnl|BL_ORD_ID|29474 transcript_69780	168	2691	1	168	1540	4063	1237	1403
transcript_68045	gnl|BL_ORD_ID|61832 transcript_119259	168	2691	1	168	1408	3931	1104	1271
transcript_68050	gnl|BL_ORD_ID|89316 transcript_161195	2	2601	2600	3238	147	2745	3195	3831
transcript_68061	gnl|BL_ORD_ID|38359 transcript_7451	1	1567	1569	2834	1	1567	1691	2957
transcript_68061	gnl|BL_ORD_ID|12585 transcript_4074	1	1567	1569	2834	2	1615	1742	3053
transcript_68061	gnl|BL_ORD_ID|67672 transcript_127174	1	1567	1569	2834	1	1544	1668	2934
transcript_68085	gnl|BL_ORD_ID|71550 transcript_133458	1723	3436	119	1718	1920	3634	8	1607
transcript_68085	gnl|BL_ORD_ID|20483 transcript_56802	2	2684	2684	3426	5	2662	2769	3511
transcript_6810	gnl|BL_ORD_ID|87126 transcript_157667	1347	3101	1	1348	1537	3283	1	1348
transcript_68182	gnl|BL_ORD_ID|62655 transcript_120604	1	2064	2061	2233	2649	4720	4854	5026

transcript_68202	gnl|BL_ORD_ID|26409 transcript_64875	1470	5164	118	1475	2801	6493	59	1413
transcript_68202	gnl|BL_ORD_ID|53401 transcript_105290	1470	5090	1	1475	2867	6489	1	1476
transcript_6831	gnl|BL_ORD_ID|36478 transcript_81151	1	2421	2420	3051	1	2421	2583	3214
transcript_68336	gnl|BL_ORD_ID|12184 transcript_3293	134	2419	12	139	810	3091	21	148
transcript_68336	gnl|BL_ORD_ID|24791 transcript_5061	134	2419	1	139	939	3220	1	137
transcript_68352	gnl|BL_ORD_ID|61792 transcript_119201	1105	2301	1	1108	1411	2605	3	1097
transcript_68352	gnl|BL_ORD_ID|34999 transcript_78789	1	1601	1597	2390	3	1602	1757	2549
transcript_68366	gnl|BL_ORD_ID|82155 transcript_149258	250	3088	116	251	564	3402	123	258
transcript_68366	gnl|BL_ORD_ID|24621 transcript_4696	116	3088	1	116	340	3312	81	197
transcript_68387	gnl|BL_ORD_ID|40047 transcript_85236	1	2387	2385	2784	38	2464	2612	3014
transcript_684	gnl|BL_ORD_ID|305 transcript_501	3	3297	3293	4202	5	3310	3565	4474
transcript_68402	gnl|BL_ORD_ID|888 transcript_1577	326	3643	29	327	417	3733	2	298
transcript_6847	gnl|BL_ORD_ID|29976 transcript_70601	1	1653	1652	2808	1	1650	2998	4148
transcript_68503	gnl|BL_ORD_ID|91655 transcript_163333	410	1090	6	414	707	1380	2	410
transcript_68503	gnl|BL_ORD_ID|36098 transcript_80522	411	1094	6	414	903	1586	1	409
transcript_68503	gnl|BL_ORD_ID|10156 transcript_40181	410	1090	9	414	733	1412	2	407
transcript_68503	gnl|BL_ORD_ID|85859 transcript_155542	411	1049	9	410	630	1268	1	402
transcript_68504	gnl|BL_ORD_ID|38089 transcript_6884	16	2113	2113	2510	1	2105	2708	3105
transcript_68535	gnl|BL_ORD_ID|37979 transcript_6665	112	2808	1	113	403	3099	1	113
transcript_68558	gnl|BL_ORD_ID|17170 transcript_51482	1139	3926	1	1138	1331	4144	80	1217
transcript_68558	gnl|BL_ORD_ID|31370 transcript_72860	1	2089	2085	3887	146	2233	2348	4156
transcript_68567	gnl|BL_ORD_ID|89240 transcript_161070	167	2811	1	168	908	3548	116	280
transcript_68580	gnl|BL_ORD_ID|89599 transcript_161645	1	1131	1129	1994	1	1130	1465	2331
transcript_68596	gnl|BL_ORD_ID|89371 transcript_161280	1382	3179	1	1387	2089	3876	489	1862
transcript_68596	gnl|BL_ORD_ID|45751 transcript_94629	1	1864	1862	3179	48	1896	2284	3596
transcript_68596	gnl|BL_ORD_ID|942 transcript_1674	1	2062	2061	3179	471	2514	2620	3736
transcript_68597	gnl|BL_ORD_ID|73190 transcript_136072	1	1751	1747	2358	7	1757	2707	3318
transcript_68598	gnl|BL_ORD_ID|31415 transcript_72928	470	3236	7	472	711	3478	1	466
transcript_68626	gnl|BL_ORD_ID|91421 transcript_16611	1097	2142	1	1099	1357	2406	44	1123
transcript_68650	gnl|BL_ORD_ID|32552 transcript_74835	167	2507	1	172	336	2687	1	172
transcript_68656	gnl|BL_ORD_ID|25045 transcript_5600	149	2213	1	147	1143	3205	5	151
transcript_68716	gnl|BL_ORD_ID|60939 transcript_117852	1	1458	1457	2189	1	1458	1871	2604
transcript_68751	gnl|BL_ORD_ID|489 transcript_829	360	3923	2	359	559	4120	7	362
transcript_68751	gnl|BL_ORD_ID|85424 transcript_154840	360	3923	1	359	566	4139	1	366

transcript_68751	gnl|BL_ORD_ID|352 transcript_605	360	3923	2	359	770	4333	216	573
transcript_68751	gnl|BL_ORD_ID|59286 transcript_115209	360	3923	2	359	578	4142	6	381
transcript_68751	gnl|BL_ORD_ID|46418 transcript_95728	360	3761	2	360	764	4165	6	364
transcript_68757	gnl|BL_ORD_ID|72361 transcript_134750	999	2635	49	1004	1073	2703	2	957
transcript_68757	gnl|BL_ORD_ID|64425 transcript_10827	999	2635	2	1004	1158	2792	39	1042
transcript_68757	gnl|BL_ORD_ID|51231 transcript_8670	999	2635	2	1004	1154	2789	36	1038
transcript_68767	gnl|BL_ORD_ID|85828 transcript_155485	1	1468	1467	1807	7	1484	1834	2174
transcript_68796	gnl|BL_ORD_ID|27254 transcript_66227	1017	2207	1	1018	900	2101	1	1019
transcript_68797	gnl|BL_ORD_ID|76629 transcript_141687	1111	2943	1	1112	1434	3267	1	1110
transcript_68824	gnl|BL_ORD_ID|42041 transcript_88480	1	1213	1210	1847	10	1219	2080	2717
transcript_68829	gnl|BL_ORD_ID|38158 transcript_7026	2	2054	2052	2856	77	2113	2275	3072
transcript_68829	gnl|BL_ORD_ID|26041 transcript_64314	2	2054	2052	2931	2	2038	2200	3075
transcript_68851	gnl|BL_ORD_ID|90742 transcript_15113	1	1404	1399	1890	141	1545	2029	2518
transcript_68851	gnl|BL_ORD_ID|68851 transcript_129060	1	1404	1399	1890	77	1480	1964	2455
transcript_68851	gnl|BL_ORD_ID|78433 transcript_14642	1	1404	1399	1890	241	1645	2129	2617
transcript_68904	gnl|BL_ORD_ID|18390 transcript_53408	1	2100	2098	3399	78	2178	2280	3581
transcript_68942	gnl|BL_ORD_ID|73678 transcript_136879	1	1333	1330	2420	35	1365	1719	2812
transcript_68944	gnl|BL_ORD_ID|54740 transcript_107573	1	1175	1176	2018	1	1176	1537	2385
transcript_69013	gnl|BL_ORD_ID|19545 transcript_55304	1	1761	1761	2632	54	1814	2145	3016
transcript_69013	gnl|BL_ORD_ID|50070 transcript_101495	1	1761	1761	2632	99	1858	1987	2859
transcript_69083	gnl|BL_ORD_ID|92735 transcript_165093	1052	2457	1	1052	1824	3233	3	1055
transcript_69101	gnl|BL_ORD_ID|90820 transcript_15286	1	1289	1288	2432	1	1286	1430	2577
transcript_69101	gnl|BL_ORD_ID|22952 transcript_60954	1	1289	1288	2428	1	1286	1430	2597
transcript_69118	gnl|BL_ORD_ID|21300 transcript_58193	313	1948	70	315	3949	5584	2060	2304
transcript_69139	gnl|BL_ORD_ID|74931 transcript_138956	1533	3865	136	1534	4553	6881	2	1400
transcript_69141	gnl|BL_ORD_ID|86125 transcript_156003	1	1483	1482	2279	1	1483	2424	3246
transcript_69145	gnl|BL_ORD_ID|36401 transcript_81023	18	2187	2187	2927	1	2150	2599	3336
transcript_69146	gnl|BL_ORD_ID|61219 transcript_118307	725	2293	9	726	1752	3325	14	735
transcript_69171	gnl|BL_ORD_ID|27963 transcript_67349	1062	2247	1	1064	1246	2430	50	1115
transcript_69196	gnl|BL_ORD_ID|61133 transcript_118185	614	2743	8	615	751	2880	2	609
transcript_69209	gnl|BL_ORD_ID|56192 transcript_110150	1	1451	1447	2276	39	1504	1709	2539
transcript_69259	gnl|BL_ORD_ID|38782 transcript_8394	222	2259	2	223	928	2963	9	241
transcript_69275	gnl|BL_ORD_ID|24967 transcript_5408	1	2548	2549	3043	3	2553	2677	3171
transcript_69277	gnl|BL_ORD_ID|67631 transcript_127099	729	2171	83	734	990	2435	181	831

transcript_69345	gnl|BL_ORD_ID|87070 transcript_157568	1	1894	1891	2636	2	1893	2296	3040
transcript_69347	gnl|BL_ORD_ID|25216 transcript_5948	2	2247	2243	2400	55	2311	2905	3062
transcript_69352	gnl|BL_ORD_ID|12571 transcript_4051	1	1930	1926	2890	1	1935	2391	3354
transcript_69352	gnl|BL_ORD_ID|26292 transcript_64696	489	2890	6	489	601	3006	2	501
transcript_6940	gnl|BL_ORD_ID|17322 transcript_51733	14	2179	2179	3118	3	2165	2273	3224
transcript_6941	gnl|BL_ORD_ID|12516 transcript_3924	1	2649	2646	3077	2	2647	2976	3406
transcript_69411	gnl|BL_ORD_ID|91858 transcript_163671	1	1268	1265	1955	45	1310	1458	2149
transcript_69417	gnl|BL_ORD_ID|1528 transcript_22216	115	2092	1	116	325	2302	63	178
transcript_69425	gnl|BL_ORD_ID|78013 transcript_13645	1	1564	1560	2262	1	1564	1722	2422
transcript_69460	gnl|BL_ORD_ID|51589 transcript_9405	2	2333	2332	2752	8	2337	2466	2886
transcript_69468	gnl|BL_ORD_ID|46083 transcript_95181	269	2364	31	270	1607	3703	1	240
transcript_69468	gnl|BL_ORD_ID|39533 transcript_84406	1	1530	1528	2364	35	1528	1646	2481
transcript_69474	gnl|BL_ORD_ID|18180 transcript_53071	541	1906	67	544	3062	4427	19	496
transcript_69481	gnl|BL_ORD_ID|40384 transcript_85749	1	1081	1080	1542	43	1123	2157	2620
transcript_69481	gnl|BL_ORD_ID|90518 transcript_163047	1	1081	1080	1542	52	1140	2167	2629
transcript_69488	gnl|BL_ORD_ID|74630 transcript_138460	2	2298	2296	2516	42	2338	4812	5031
transcript_69603	gnl|BL_ORD_ID|38466 transcript_7683	1	2227	2225	2802	3	2208	2459	3036
transcript_69603	gnl|BL_ORD_ID|38115 transcript_6941	1	2227	2225	2809	2	2227	2478	3062
transcript_69603	gnl|BL_ORD_ID|38358 transcript_7449	1	2227	2225	2786	3	2224	2475	3039
transcript_69623	gnl|BL_ORD_ID|72735 transcript_135344	10	1395	1394	2798	21	1403	2838	4212
transcript_69632	gnl|BL_ORD_ID|63270 transcript_121575	220	1060	4	220	1219	2077	871	1087
transcript_69659	gnl|BL_ORD_ID|52221 transcript_103374	1	2864	2860	3350	453	3317	3452	3943
transcript_69665	gnl|BL_ORD_ID|64513 transcript_11005	1	1956	1955	2655	48	1983	2122	2821
transcript_69665	gnl|BL_ORD_ID|51972 transcript_10242	1	1956	1955	2655	50	2003	2142	2841
transcript_69677	gnl|BL_ORD_ID|24840 transcript_5154	2	2149	2149	2956	71	2219	2470	3277
transcript_69683	gnl|BL_ORD_ID|72260 transcript_134595	1	2682	2679	3191	3	2688	2879	3387
transcript_69702	gnl|BL_ORD_ID|34198 transcript_77462	137	2839	2838	3616	19	2721	3027	3805
transcript_69795	gnl|BL_ORD_ID|36727 transcript_81533	1326	3349	1	1326	1647	3670	1	1295
transcript_6982	gnl|BL_ORD_ID|25257 transcript_6048	121	1899	1898	3100	2	1779	1936	3137
transcript_69864	gnl|BL_ORD_ID|77707 transcript_12967	103	2230	1	106	399	2527	1	106
transcript_69869	gnl|BL_ORD_ID|53791 transcript_105990	304	3401	14	306	729	3826	2	292
transcript_69880	gnl|BL_ORD_ID|40886 transcript_86603	1	1021	1022	1492	1	1021	1286	1755
transcript_69923	gnl|BL_ORD_ID|77331 transcript_142814	1	2036	2032	2262	603	2636	2829	3060
transcript_69924	gnl|BL_ORD_ID|86122 transcript_155999	22	2974	2972	3354	2	2955	3065	3447

transcript_6996	gnl|BL_ORD_ID|57071 transcript_111561	1	2344	2342	3069	2	2345	2486	3205
transcript_69996	gnl|BL_ORD_ID|84067 transcript_152621	1663	3406	1	1665	1974	3714	1	1669
transcript_70004	gnl|BL_ORD_ID|12261 transcript_3430	120	3175	1	119	414	3469	1	119
transcript_70043	gnl|BL_ORD_ID|53581 transcript_105599	1245	3483	1	1245	1820	4058	429	1673
transcript_70043	gnl|BL_ORD_ID|57705 transcript_112637	1245	3483	1	1245	1719	3966	328	1572
transcript_70043	gnl|BL_ORD_ID|39354 transcript_84119	1245	3483	1	1245	1761	4000	370	1614
transcript_70053	gnl|BL_ORD_ID|96070 transcript_20962	1	1462	1466	2157	21	1483	1639	2331
transcript_70053	gnl|BL_ORD_ID|95726 transcript_20178	1	1462	1466	2157	15	1477	1633	2325
transcript_70058	gnl|BL_ORD_ID|38368 transcript_7483	1050	2578	1	1053	1456	2972	6	1054
transcript_70058	gnl|BL_ORD_ID|52066 transcript_10450	1052	2578	1	1053	1263	2777	9	1057
transcript_7009	gnl|BL_ORD_ID|49385 transcript_100404	1115	3092	1	1118	2008	3985	1	1116
transcript_70115	gnl|BL_ORD_ID|63006 transcript_121149	220	1687	25	222	345	1811	2	201
transcript_70117	gnl|BL_ORD_ID|68910 transcript_129163	367	3356	73	370	637	3627	207	500
transcript_70121	gnl|BL_ORD_ID|31583 transcript_73189	232	2197	6	235	361	2325	2	231
transcript_70154	gnl|BL_ORD_ID|41400 transcript_87452	667	1497	7	670	3056	3885	2	667
transcript_70166	gnl|BL_ORD_ID|70329 transcript_131474	1	1684	1683	2621	1	1701	1807	2740
transcript_70264	gnl|BL_ORD_ID|34248 transcript_77542	261	2581	86	266	669	2989	105	285
transcript_70284	gnl|BL_ORD_ID|56904 transcript_111282	1060	2276	1	1059	1398	2607	191	1249
transcript_70296	gnl|BL_ORD_ID|1446 transcript_21997	1	1543	1541	2108	104	1646	1758	2325
transcript_70296	gnl|BL_ORD_ID|2399 transcript_24135	1	1543	1541	2102	51	1593	1705	2249
transcript_70296	gnl|BL_ORD_ID|1302 transcript_21634	1	1543	1541	2126	11	1553	1665	2251
transcript_70336	gnl|BL_ORD_ID|64838 transcript_11698	319	2017	43	319	1095	2780	237	515
transcript_7039	gnl|BL_ORD_ID|50034 transcript_101440	1	2889	2892	3067	1	2888	2989	3164
transcript_70420	gnl|BL_ORD_ID|44543 transcript_92606	1113	2418	1	1113	1231	2540	1	1123
transcript_70518	gnl|BL_ORD_ID|56951 transcript_111356	1	2120	2118	2809	1	2120	2274	2964
transcript_70595	gnl|BL_ORD_ID|71774 transcript_133827	1	2103	2100	2441	2613	4715	4907	5248
transcript_70616	gnl|BL_ORD_ID|90257 transcript_162672	24	2480	2476	3439	2	2467	2786	3749
transcript_70741	gnl|BL_ORD_ID|361 transcript_620	1	3440	3438	3566	3	3628	3915	4048
transcript_70755	gnl|BL_ORD_ID|96167 transcript_21149	1	1537	1533	2134	2	1540	1661	2262
transcript_70755	gnl|BL_ORD_ID|2316 transcript_23949	1	1537	1533	2152	1	1533	1654	2247
transcript_70755	gnl|BL_ORD_ID|96705 transcript_88786	1	1537	1533	2131	1	1533	1654	2230
transcript_70786	gnl|BL_ORD_ID|285 transcript_471	1	2190	2189	3123	624	2815	3577	4511
transcript_70786	gnl|BL_ORD_ID|94319 transcript_167612	2	2190	2189	3123	790	2975	3737	4671
transcript_70828	gnl|BL_ORD_ID|26849 transcript_65563	254	1829	52	255	1091	2666	1	204

transcript_7083	gnl|BL_ORD_ID|36478 transcript_81151	2	2446	2445	3055	1	2421	2583	3191
transcript_7083	gnl|BL_ORD_ID|556 transcript_940	114	3055	1	119	1168	4108	1	119
transcript_70834	gnl|BL_ORD_ID|97281 transcript_144367	496	2455	80	500	794	2753	75	494
transcript_70834	gnl|BL_ORD_ID|39951 transcript_85079	496	2455	86	500	1642	3598	928	1342
transcript_70839	gnl|BL_ORD_ID|21672 transcript_58795	1	2310	2308	2944	1	2316	2477	3115
transcript_70848	gnl|BL_ORD_ID|27250 transcript_66223	1	1286	1283	2036	2	1289	2098	2851
transcript_70880	gnl|BL_ORD_ID|70712 transcript_132115	123	1480	1	124	1023	2380	489	612
transcript_70894	gnl|BL_ORD_ID|80791 transcript_146957	1	2777	2775	4003	2	2781	3001	4230
transcript_7092	gnl|BL_ORD_ID|56814 transcript_111133	2	2141	2137	2950	1	2140	3245	4059
transcript_70930	gnl|BL_ORD_ID|81462 transcript_148012	116	2540	1	121	588	3017	42	164
transcript_70933	gnl|BL_ORD_ID|73013 transcript_135787	319	2203	45	323	509	2394	1	279
transcript_70936	gnl|BL_ORD_ID|47602 transcript_97569	262	2938	1	262	2419	5110	1870	2128
transcript_70980	gnl|BL_ORD_ID|77694 transcript_12938	2	2222	2222	2502	39	2261	2378	2658
transcript_70980	gnl|BL_ORD_ID|47736 transcript_97774	2	2222	2222	2502	22	2244	2361	2641
transcript_70980	gnl|BL_ORD_ID|90718 transcript_15057	2	2222	2222	2408	96	2318	2435	2621
transcript_70993	gnl|BL_ORD_ID|82150 transcript_149247	1571	3184	141	1576	1698	3312	10	1444
transcript_71017	gnl|BL_ORD_ID|60939 transcript_117852	1	1458	1457	2217	1	1458	1871	2654
transcript_71034	gnl|BL_ORD_ID|80113 transcript_145827	1	1031	1029	1748	30	1058	1510	2219
transcript_71049	gnl|BL_ORD_ID|31164 transcript_72524	2	2801	2800	3434	130	2937	3104	3738
transcript_71076	gnl|BL_ORD_ID|48715 transcript_99346	244	4964	19	243	432	5149	58	281
transcript_71092	gnl|BL_ORD_ID|68452 transcript_128431	463	2993	68	463	1633	4163	35	440
transcript_7110	gnl|BL_ORD_ID|37507 transcript_82767	2	2407	2405	3016	17	2439	2590	3202
transcript_71115	gnl|BL_ORD_ID|26088 transcript_64384	230	2858	10	232	1187	3839	1	226
transcript_71115	gnl|BL_ORD_ID|46721 transcript_96208	230	2824	1	232	1203	3834	2	225
transcript_71115	gnl|BL_ORD_ID|41129 transcript_87002	231	2805	1	232	1074	3649	5	235
transcript_71115	gnl|BL_ORD_ID|74359 transcript_138026	231	2858	2	232	1063	3719	2	226
transcript_7116	gnl|BL_ORD_ID|24640 transcript_4727	1	1554	1553	3047	1	1554	1803	3302
transcript_71204	gnl|BL_ORD_ID|89895 transcript_162114	21	3420	3420	3762	1	3398	3923	4268
transcript_7121	gnl|BL_ORD_ID|75238 transcript_139454	373	3094	80	372	444	3178	2	292
transcript_7121	gnl|BL_ORD_ID|57399 transcript_112111	373	3094	64	372	461	3192	2	309
transcript_7121	gnl|BL_ORD_ID|42164 transcript_88656	371	3065	52	372	731	3427	4	324
transcript_71293	gnl|BL_ORD_ID|68127 transcript_127896	12	1880	1878	2245	29	1894	2005	2372
transcript_71303	gnl|BL_ORD_ID|76151 transcript_140935	1	2337	2333	2921	37	2374	3075	3663
transcript_71350	gnl|BL_ORD_ID|49025 transcript_99833	1077	3676	1	1076	1259	3857	11	1068

transcript_71369	gnl|BL_ORD_ID|48546 transcript_99069	1	1365	1362	1942	397	1759	2999	3579
transcript_7138	gnl|BL_ORD_ID|32370 transcript_74545	466	3064	77	467	498	3093	2	392
transcript_71503	gnl|BL_ORD_ID|96637 transcript_81300	1	1939	1938	2378	657	2598	4462	4902
transcript_71566	gnl|BL_ORD_ID|32225 transcript_74295	999	2344	5	1000	5983	7321	3	1000
transcript_71653	gnl|BL_ORD_ID|66340 transcript_125005	1	2154	2149	3042	2	2155	2454	3348
transcript_71656	gnl|BL_ORD_ID|37149 transcript_82198	16	2252	2252	3581	88	2324	2564	3893
transcript_71656	gnl|BL_ORD_ID|627 transcript_1073	2	2253	2252	3569	66	2315	2783	4099
transcript_71658	gnl|BL_ORD_ID|65348 transcript_123342	1	2527	2524	2926	1	2539	3004	3405
transcript_7168	gnl|BL_ORD_ID|76151 transcript_140935	2	2286	2282	3052	95	2374	3075	3849
transcript_71688	gnl|BL_ORD_ID|92658 transcript_164967	12	1476	1473	2497	2	1463	2465	3487
transcript_71760	gnl|BL_ORD_ID|2179 transcript_23624	1	1227	1228	1999	62	1283	1385	2160
transcript_71760	gnl|BL_ORD_ID|95092 transcript_18650	1	1227	1228	1997	2	1213	1315	2088
transcript_71760	gnl|BL_ORD_ID|17517 transcript_52030	1	1227	1228	1997	166	1376	1478	2254
transcript_71760	gnl|BL_ORD_ID|89561 transcript_161588	1	1227	1228	1999	1	1223	1325	2099
transcript_71760	gnl|BL_ORD_ID|79039 transcript_144119	1	1227	1228	1997	240	1451	1553	2326
transcript_7179	gnl|BL_ORD_ID|74718 transcript_138611	2	2493	2491	2997	5	2496	2809	3315
transcript_71831	gnl|BL_ORD_ID|12339 transcript_3574	21	2286	2285	3259	3	2261	2471	3445
transcript_71841	gnl|BL_ORD_ID|48842 transcript_99553	1	1446	1444	1882	1	1414	1587	2019
transcript_71841	gnl|BL_ORD_ID|66178 transcript_124736	1	1447	1444	1882	2	1416	3432	3863
transcript_71841	gnl|BL_ORD_ID|1246 transcript_21520	1	1446	1444	1882	26	1439	1612	2047
transcript_71841	gnl|BL_ORD_ID|71907 transcript_134034	1	1445	1444	1882	19	1431	1794	2226
transcript_71845	gnl|BL_ORD_ID|27512 transcript_66618	1	2531	2530	4182	2	2534	3705	5359
transcript_71858	gnl|BL_ORD_ID|84299 transcript_153041	2	2132	2131	3080	14	2138	2316	3264
transcript_7189	gnl|BL_ORD_ID|22259 transcript_59773	1457	3028	1	1456	1660	3232	1	1456
transcript_719	gnl|BL_ORD_ID|19021 transcript_54410	1	3232	3233	4265	1	3221	3388	4428
transcript_719	gnl|BL_ORD_ID|32825 transcript_75266	1	3232	3233	4256	1	3232	3704	4725
transcript_719	gnl|BL_ORD_ID|30455 transcript_71382	1	3232	3232	4266	1	3230	3490	4524
transcript_71907	gnl|BL_ORD_ID|866 transcript_1534	159	3601	1	161	487	3929	19	181
transcript_71942	gnl|BL_ORD_ID|50504 transcript_102195	1	1529	1525	2447	348	1886	2504	3423
transcript_72011	gnl|BL_ORD_ID|30510 transcript_71470	1	1971	1967	2984	1	1970	2725	3742
transcript_72016	gnl|BL_ORD_ID|28596 transcript_68424	174	2679	1	172	274	2803	1	172
transcript_72094	gnl|BL_ORD_ID|71592 transcript_133514	1	3154	3153	3426	48	3193	3303	3582
transcript_72101	gnl|BL_ORD_ID|2285 transcript_23883	1	1094	1094	2047	2	1096	1196	2149
transcript_72124	gnl|BL_ORD_ID|31110 transcript_72455	1	1142	1139	1436	1	1144	1389	1686

transcript_72148	gnl|BL_ORD_ID|97504 transcript_166466	1204	2503	116	1208	2087	3386	1	1093
transcript_7215	gnl|BL_ORD_ID|50324 transcript_101902	1187	3067	1	1189	3349	5228	1975	3163
transcript_72204	gnl|BL_ORD_ID|35600 transcript_79711	1	2135	2133	2645	1	2135	2619	3130
transcript_72236	gnl|BL_ORD_ID|63667 transcript_122240	1168	2367	1	1172	1282	2481	2	1175
transcript_72244	gnl|BL_ORD_ID|30588 transcript_71598	1	1361	1359	1728	11	1395	2109	2478
transcript_72244	gnl|BL_ORD_ID|48020 transcript_98226	694	1728	7	696	1053	2087	3	692
transcript_72278	gnl|BL_ORD_ID|31610 transcript_73235	245	2334	8	248	374	2464	2	256
transcript_72371	gnl|BL_ORD_ID|74609 transcript_138422	2	2232	2230	2601	125	2355	2459	2830
transcript_72440	gnl|BL_ORD_ID|17642 transcript_52213	1	4063	4059	4324	1	4063	4318	4585
transcript_72448	gnl|BL_ORD_ID|37908 transcript_6531	2	2608	2607	2981	31	2638	2751	3125
transcript_72456	gnl|BL_ORD_ID|70076 transcript_131064	2	2277	2275	3334	119	2401	2697	3756
transcript_72470	gnl|BL_ORD_ID|93883 transcript_166931	1	2550	2550	3114	401	2956	3562	4127
transcript_72475	gnl|BL_ORD_ID|78906 transcript_143877	125	1393	1	124	681	1949	21	144
transcript_72507	gnl|BL_ORD_ID|12027 transcript_2986	1	3055	3054	3387	2	3053	3226	3558
transcript_7251	gnl|BL_ORD_ID|25161 transcript_5844	18	2338	2337	3059	1	2334	2444	3157
transcript_72513	gnl|BL_ORD_ID|68665 transcript_128756	1	1258	1257	1808	5	1253	1383	1961
transcript_72580	gnl|BL_ORD_ID|36581 transcript_81323	1	1739	1737	2319	1	1733	1837	2425
transcript_72603	gnl|BL_ORD_ID|64787 transcript_11606	1120	2390	1	1123	1264	2542	1	1127
transcript_72603	gnl|BL_ORD_ID|91000 transcript_15678	1120	2390	1	1123	1242	2520	1	1105
transcript_72615	gnl|BL_ORD_ID|9987 transcript_39857	1	1033	1028	1195	1	1033	1154	1320
transcript_72615	gnl|BL_ORD_ID|39546 transcript_84424	1	1033	1028	1195	1	1033	1154	1321
transcript_72622	gnl|BL_ORD_ID|76410 transcript_141338	1	2190	2191	2664	1	2192	3067	3538
transcript_72638	gnl|BL_ORD_ID|91629 transcript_17067	188	2354	1	189	333	2499	44	232
transcript_72715	gnl|BL_ORD_ID|11571 transcript_42742	131	999	1	133	359	1227	9	141
transcript_72743	gnl|BL_ORD_ID|21177 transcript_57988	112	2654	1	111	1028	3578	592	702
transcript_72745	gnl|BL_ORD_ID|12321 transcript_3545	1	1665	1661	3266	91	1769	1871	3476
transcript_72745	gnl|BL_ORD_ID|29602 transcript_69996	1	1665	1661	3304	1	1666	1768	3404
transcript_72745	gnl|BL_ORD_ID|84067 transcript_152621	1	1664	1661	3304	1	1669	2079	3712
transcript_72745	gnl|BL_ORD_ID|12309 transcript_3525	1661	3304	1	1665	1803	3446	23	1701
transcript_72745	gnl|BL_ORD_ID|12369 transcript_3639	1661	3304	1	1665	1802	3445	2	1700
transcript_72777	gnl|BL_ORD_ID|12357 transcript_3618	1	2243	2241	2508	91	2322	2787	3054
transcript_72851	gnl|BL_ORD_ID|38767 transcript_8368	215	2875	21	217	306	2967	2	198
transcript_72854	gnl|BL_ORD_ID|79184 transcript_144349	367	2591	4	371	1063	3288	71	438
transcript_72877	gnl|BL_ORD_ID|2211 transcript_23706	1	1339	1337	2072	1	1326	1502	2237

transcript_72877	gnl|BL_ORD_ID|62740 transcript_120735	1	1339	1337	2066	1	1339	1515	2244
transcript_72889	gnl|BL_ORD_ID|45594 transcript_94361	14	1619	1617	2517	4	1609	1799	2699
transcript_7291	gnl|BL_ORD_ID|37507 transcript_82767	2	2424	2422	3034	17	2439	2590	3202
transcript_72911	gnl|BL_ORD_ID|37109 transcript_82132	185	2720	1	187	446	2985	1	183
transcript_72979	gnl|BL_ORD_ID|37501 transcript_82760	1	1205	1204	1942	11	1217	1350	2104
transcript_7298	gnl|BL_ORD_ID|35106 transcript_78953	1	2382	2379	3022	1	2383	3325	3965
transcript_7300	gnl|BL_ORD_ID|64305 transcript_123228	2	2365	2363	3024	1	2364	3132	3797
transcript_73066	gnl|BL_ORD_ID|50271 transcript_101811	1	1802	1802	2560	229	2028	2187	2942
transcript_731	gnl|BL_ORD_ID|50979 transcript_102982	3	3694	3691	4211	57	3724	3919	4430
transcript_73139	gnl|BL_ORD_ID|63479 transcript_121929	666	3123	7	670	861	3319	2	665
transcript_73146	gnl|BL_ORD_ID|12271 transcript_3445	322	3279	2	324	504	3461	55	378
transcript_73155	gnl|BL_ORD_ID|62753 transcript_120757	2	3202	3199	3762	10	3210	3337	3900
transcript_73167	gnl|BL_ORD_ID|61248 transcript_118348	16	1981	1977	2841	2	1947	2502	3360
transcript_7322	gnl|BL_ORD_ID|55682 transcript_109281	2	2521	2520	3030	63	2582	3196	3684
transcript_73249	gnl|BL_ORD_ID|31947 transcript_73815	1	1307	1303	2257	297	1603	2015	2965
transcript_73249	gnl|BL_ORD_ID|51831 transcript_9947	1	1307	1303	2257	184	1490	1902	2854
transcript_73249	gnl|BL_ORD_ID|38469 transcript_7687	1	1307	1303	2257	280	1586	1998	2952
transcript_73281	gnl|BL_ORD_ID|36413 transcript_81040	1	2497	2497	3318	1	2499	2602	3405
transcript_7329	gnl|BL_ORD_ID|24947 transcript_5359	296	3089	1	294	429	3232	21	314
transcript_73392	gnl|BL_ORD_ID|78134 transcript_13958	335	2300	65	337	691	2674	2	272
transcript_73424	gnl|BL_ORD_ID|78869 transcript_143823	1	2695	2692	3109	53	2747	2924	3341
transcript_73462	gnl|BL_ORD_ID|36280 transcript_80833	660	2222	92	664	1425	2982	268	849
transcript_7347	gnl|BL_ORD_ID|37920 transcript_6551	127	1908	1907	3045	2	1776	1948	3086
transcript_7347	gnl|BL_ORD_ID|25257 transcript_6048	127	1908	1907	3044	2	1779	1936	3073
transcript_7347	gnl|BL_ORD_ID|38181 transcript_7072	1604	3045	127	1606	1621	3062	2	1477
transcript_73471	gnl|BL_ORD_ID|42639 transcript_89421	2	2403	2401	3079	60	2445	2596	3274
transcript_73493	gnl|BL_ORD_ID|88566 transcript_159990	1	2017	2014	3649	4	1991	2313	3951
transcript_7356	gnl|BL_ORD_ID|90479 transcript_162992	117	1788	1789	3036	13	1712	1864	3113
transcript_73569	gnl|BL_ORD_ID|541 transcript_918	1	1985	1983	3927	3	1986	2211	4159
transcript_73569	gnl|BL_ORD_ID|25497 transcript_63417	1983	3927	142	1985	2568	4513	502	2343
transcript_73616	gnl|BL_ORD_ID|95239 transcript_19018	1	1414	1412	1906	154	1567	1738	2234
transcript_73623	gnl|BL_ORD_ID|60383 transcript_116960	270	3122	23	272	369	3232	2	261
transcript_7365	gnl|BL_ORD_ID|30240 transcript_71027	227	2932	1	228	628	3332	1	228
transcript_73688	gnl|BL_ORD_ID|95253 transcript_19049	1	1104	1100	1954	14	1088	1266	2121

transcript_73688	gnl|BL_ORD_ID|3206 transcript_25897	1	1104	1100	1954	34	1108	1286	2141
transcript_73697	gnl|BL_ORD_ID|90900 transcript_15461	1	1470	1468	2240	1	1470	1781	2554
transcript_73699	gnl|BL_ORD_ID|61569 transcript_118859	1	1983	1978	3435	2334	4295	4451	5892
transcript_73703	gnl|BL_ORD_ID|80457 transcript_146406	1	1367	1366	1907	561	1927	1718	2260
transcript_73703	gnl|BL_ORD_ID|1600 transcript_22401	1	1367	1366	1873	634	2000	1791	2299
transcript_73757	gnl|BL_ORD_ID|24935 transcript_5341	1	2088	2088	2910	2	2089	2418	3240
transcript_738	gnl|BL_ORD_ID|53137 transcript_104841	18	2406	2404	4238	1	2389	2544	4372
transcript_73821	gnl|BL_ORD_ID|24901 transcript_5276	1	2456	2453	2902	1	2460	2661	3111
transcript_73821	gnl|BL_ORD_ID|24896 transcript_5268	1	2456	2453	2902	32	2461	2662	3113
transcript_73841	gnl|BL_ORD_ID|64832 transcript_11681	1	1957	1953	2590	1	1957	2145	2782
transcript_73878	gnl|BL_ORD_ID|47095 transcript_96780	2	2334	2330	3089	41	2372	2486	3245
transcript_73885	gnl|BL_ORD_ID|64904 transcript_11852	1	2299	2298	2633	1	2294	2404	2736
transcript_73886	gnl|BL_ORD_ID|11696 transcript_2335	1	3162	3161	3552	60	3219	3321	3712
transcript_73886	gnl|BL_ORD_ID|88097 transcript_159248	11	3162	3161	3552	2	3172	3274	3665
transcript_73904	gnl|BL_ORD_ID|70432 transcript_131658	1	1377	1377	2492	1	1378	1981	3097
transcript_73934	gnl|BL_ORD_ID|27860 transcript_67192	1	1689	1687	2227	21	1709	2300	2840
transcript_7394	gnl|BL_ORD_ID|84116 transcript_152715	1	2799	2796	3043	1	2813	3352	3599
transcript_7394	gnl|BL_ORD_ID|17841 transcript_52537	22	2799	2796	3043	2	2779	3318	3565
transcript_73978	gnl|BL_ORD_ID|46093 transcript_95206	110	1664	1	109	214	1770	1	109
transcript_73978	gnl|BL_ORD_ID|93561 transcript_166428	110	1664	1	110	282	1836	1	110
transcript_73978	gnl|BL_ORD_ID|1037 transcript_1859	110	1664	1	109	226	1780	2	110
transcript_73978	gnl|BL_ORD_ID|56487 transcript_110605	108	1664	1	109	907	2463	2	110
transcript_73978	gnl|BL_ORD_ID|97441 transcript_159428	110	1664	1	109	255	1807	31	139
transcript_73983	gnl|BL_ORD_ID|12619 transcript_4133	1	1781	1780	3090	1	1782	2005	3315
transcript_73983	gnl|BL_ORD_ID|24555 transcript_4551	16	1781	1780	3090	2	1752	1975	3285
transcript_74086	gnl|BL_ORD_ID|72901 transcript_135620	1166	2748	11	1167	1386	2968	2	1158
transcript_74086	gnl|BL_ORD_ID|79185 transcript_144350	1166	2707	1	1167	1420	2961	18	1192
transcript_74086	gnl|BL_ORD_ID|38178 transcript_7065	1166	2748	1	1167	1396	2978	2	1168
transcript_74147	gnl|BL_ORD_ID|92432 transcript_164604	891	2478	9	893	2701	4287	3	886
transcript_74191	gnl|BL_ORD_ID|35499 transcript_79571	1	1242	1242	1736	345	1587	1732	2227
transcript_74197	gnl|BL_ORD_ID|48594 transcript_99144	1	1849	1845	2244	3593	5458	6811	7227
transcript_74217	gnl|BL_ORD_ID|56637 transcript_110852	570	1329	6	572	1516	2275	2	568
transcript_74236	gnl|BL_ORD_ID|1214 transcript_21449	190	2055	1	190	459	2324	61	250
transcript_74256	gnl|BL_ORD_ID|64916 transcript_11875	1	2014	2012	2464	1	1986	2289	2742

transcript_74295	gnl|BL_ORD_ID|63387 transcript_121765	1	6108	6104	7322	1	6096	6250	7469
transcript_74331	gnl|BL_ORD_ID|11814 transcript_2576	292	3111	1	291	815	3642	99	378
transcript_74341	gnl|BL_ORD_ID|12620 transcript_4135	1	2050	2046	2820	1	2052	2278	3052
transcript_74341	gnl|BL_ORD_ID|12247 transcript_3408	2	2050	2046	2820	4	2070	2265	3052
transcript_74341	gnl|BL_ORD_ID|38126 transcript_6958	1	2050	2046	2820	1	2068	2263	3050
transcript_74341	gnl|BL_ORD_ID|25427 transcript_6388	1	2050	2046	2820	1	2062	2257	3042
transcript_74341	gnl|BL_ORD_ID|38084 transcript_6869	2	2050	2046	2820	65	2127	2322	3109
transcript_74344	gnl|BL_ORD_ID|24766 transcript_5009	2	2031	2027	2784	3	2032	2151	2910
transcript_74344	gnl|BL_ORD_ID|24784 transcript_5047	2	2031	2027	2784	3	2032	2151	2908
transcript_7438	gnl|BL_ORD_ID|48603 transcript_99158	384	3018	43	384	527	3180	1	340
transcript_74394	gnl|BL_ORD_ID|61155 transcript_118219	18	1888	1885	2183	3	1875	1986	2286
transcript_74401	gnl|BL_ORD_ID|44447 transcript_92441	1	1762	1760	2053	1	1760	1881	2177
transcript_74401	gnl|BL_ORD_ID|43753 transcript_91232	1	1762	1760	2053	1	1761	1882	2177
transcript_74420	gnl|BL_ORD_ID|91296 transcript_16331	337	2152	54	339	769	2580	1	293
transcript_74434	gnl|BL_ORD_ID|31903 transcript_73737	1	1278	1276	1894	436	1718	2513	3136
transcript_74492	gnl|BL_ORD_ID|62403 transcript_120189	1106	2232	10	1106	1282	2407	2	1095
transcript_7451	gnl|BL_ORD_ID|38181 transcript_7072	1	1477	1475	2930	1	1477	1621	3076
transcript_7451	gnl|BL_ORD_ID|37920 transcript_6551	1	1779	1778	2943	1	1776	1948	3113
transcript_7451	gnl|BL_ORD_ID|25257 transcript_6048	1	1779	1778	3009	1	1779	1936	3167
transcript_74526	gnl|BL_ORD_ID|53045 transcript_104687	1169	2528	1	1173	2239	3599	2	1165
transcript_74528	gnl|BL_ORD_ID|1812 transcript_22856	1	1092	1090	1562	1	1091	1789	2261
transcript_74534	gnl|BL_ORD_ID|78487 transcript_14789	1006	2038	1	1007	1459	2492	31	1037
transcript_74535	gnl|BL_ORD_ID|91301 transcript_16344	1	1356	1351	2290	2	1357	1464	2403
transcript_74579	gnl|BL_ORD_ID|22793 transcript_60689	296	4068	1	295	1043	4825	513	808
transcript_74610	gnl|BL_ORD_ID|31836 transcript_73623	349	2924	9	349	560	3135	5	329
transcript_74618	gnl|BL_ORD_ID|2713 transcript_24796	98	1782	1	102	498	2181	24	125
transcript_74625	gnl|BL_ORD_ID|76924 transcript_142167	218	1662	52	219	1325	2775	4	173
transcript_74627	gnl|BL_ORD_ID|5618 transcript_31017	274	1669	5	274	499	1897	2	272
transcript_74645	gnl|BL_ORD_ID|45903 transcript_94893	1	1355	1353	2072	1	1355	1701	2425
transcript_74645	gnl|BL_ORD_ID|71790 transcript_133850	1	1357	1354	2140	1	1356	2262	3048
transcript_74645	gnl|BL_ORD_ID|64763 transcript_11562	1	1355	1353	2144	1	1355	1701	2493
transcript_74645	gnl|BL_ORD_ID|90882 transcript_15427	1	1355	1353	2144	1	1355	1701	2492
transcript_74664	gnl|BL_ORD_ID|81829 transcript_148672	123	1246	2	125	423	1546	71	193
transcript_7467	gnl|BL_ORD_ID|30260 transcript_71065	20	2604	2602	2962	1	2577	2723	3087

transcript_74723	gnl|BL_ORD_ID|93522 transcript_166369	1	1908	1904	2154	1	1908	2624	2873
transcript_74723	gnl|BL_ORD_ID|60825 transcript_117667	1	1908	1904	2154	1	1923	2091	2341
transcript_74750	gnl|BL_ORD_ID|27752 transcript_67004	367	2099	8	368	467	2225	1	357
transcript_74760	gnl|BL_ORD_ID|22807 transcript_60711	506	2063	7	508	633	2194	2	503
transcript_74782	gnl|BL_ORD_ID|44143 transcript_91928	1	1128	1127	1873	1	1128	1513	2259
transcript_74825	gnl|BL_ORD_ID|40373 transcript_85727	2	2256	2254	4006	1	2245	2414	4166
transcript_74835	gnl|BL_ORD_ID|18372 transcript_53382	1244	2737	1	1246	1358	2852	1	1246
transcript_74871	gnl|BL_ORD_ID|18002 transcript_52779	122	1342	1	124	394	1614	1	124
transcript_74879	gnl|BL_ORD_ID|72080 transcript_134306	2	3387	3385	4439	26	3411	3530	4585
transcript_75021	gnl|BL_ORD_ID|33374 transcript_76098	1	1362	1360	1855	22	1362	1551	2048
transcript_75021	gnl|BL_ORD_ID|2239 transcript_23771	1	1361	1360	1855	22	1355	1677	2173
transcript_75021	gnl|BL_ORD_ID|75516 transcript_139901	1	1154	1156	1855	6	1137	1258	1959
transcript_75036	gnl|BL_ORD_ID|40851 transcript_86558	189	1038	2	192	2124	2974	318	510
transcript_75052	gnl|BL_ORD_ID|25816 transcript_63961	1	1669	1666	2168	2	1675	1948	2450
transcript_75052	gnl|BL_ORD_ID|61866 transcript_119324	1	1198	1197	2106	2	1202	3655	4560
transcript_75067	gnl|BL_ORD_ID|51418 transcript_9075	197	1248	2	200	1851	2900	1307	1504
transcript_75095	gnl|BL_ORD_ID|21479 transcript_58481	1	1412	1412	2409	1	1411	3212	4205
transcript_75145	gnl|BL_ORD_ID|31419 transcript_72935	1	2318	2317	2796	1	2322	2797	3276
transcript_75161	gnl|BL_ORD_ID|11600 transcript_2152	1345	3466	1	1345	1549	3668	49	1395
transcript_75161	gnl|BL_ORD_ID|11674 transcript_2289	1345	3466	1	1345	1571	3691	73	1417
transcript_7522	gnl|BL_ORD_ID|79506 transcript_144855	1392	2989	1	1395	1660	3261	1	1407
transcript_75231	gnl|BL_ORD_ID|107 transcript_157	282	4827	2	281	450	5020	4	299
transcript_75231	gnl|BL_ORD_ID|48715 transcript_99346	282	4827	1	281	432	4979	1	281
transcript_75241	gnl|BL_ORD_ID|42052 transcript_88496	106	3907	1	106	455	4257	2	107
transcript_7526	gnl|BL_ORD_ID|63238 transcript_121529	1	1927	1928	3006	1	1927	2039	3116
transcript_7526	gnl|BL_ORD_ID|25341 transcript_6228	395	3029	4	400	509	3144	8	404
transcript_75284	gnl|BL_ORD_ID|91478 transcript_16721	235	1762	26	238	970	2497	2	214
transcript_75320	gnl|BL_ORD_ID|87480 transcript_158242	434	2349	71	434	890	2805	47	410
transcript_75430	gnl|BL_ORD_ID|64246 transcript_123139	1	1902	1900	2155	1	1902	2052	2307
transcript_75471	gnl|BL_ORD_ID|58597 transcript_114098	1	2323	2318	2806	455	2780	2915	3401
transcript_75540	gnl|BL_ORD_ID|72409 transcript_134817	1	1429	1428	2499	364	1792	2806	3877
transcript_75540	gnl|BL_ORD_ID|74685 transcript_138565	1	1431	1428	2499	198	1630	1957	3027
transcript_75583	gnl|BL_ORD_ID|96862 transcript_104735	1	2553	2554	2898	2	2553	2694	3040
transcript_7562	gnl|BL_ORD_ID|75207 transcript_139399	1	2424	2422	3021	1	2424	2773	3396

transcript_75642	gnl|BL_ORD_ID|49578 transcript_100719	1	1472	1470	2332	47	1518	1633	2479
transcript_75735	gnl|BL_ORD_ID|51079 transcript_103150	106	1872	1	108	1480	3249	2	109
transcript_75735	gnl|BL_ORD_ID|24553 transcript_4545	106	1876	1	108	1553	3326	4	111
transcript_75735	gnl|BL_ORD_ID|53694 transcript_105826	106	1874	1	108	1154	2926	4	111
transcript_75735	gnl|BL_ORD_ID|65449 transcript_123524	106	1853	1	108	1800	3552	2	109
transcript_7580	gnl|BL_ORD_ID|32564 transcript_74860	655	3050	90	656	1134	3528	2	567
transcript_75825	gnl|BL_ORD_ID|34996 transcript_78784	1	1277	1279	1648	2	1278	2588	2957
transcript_75825	gnl|BL_ORD_ID|45750 transcript_94628	1	1129	1125	1648	2	1130	1918	2441
transcript_75826	gnl|BL_ORD_ID|38104 transcript_6916	153	2916	1	149	318	3088	37	188
transcript_75902	gnl|BL_ORD_ID|71123 transcript_132780	1	1912	1911	3386	1	1916	2612	4090
transcript_75949	gnl|BL_ORD_ID|56616 transcript_110816	219	1061	3	222	1342	2184	165	384
transcript_75963	gnl|BL_ORD_ID|32573 transcript_74876	1	1641	1639	2299	625	2263	2396	3056
transcript_75977	gnl|BL_ORD_ID|3472 transcript_26487	1	1315	1313	1846	1	1317	1597	2130
transcript_7601	gnl|BL_ORD_ID|64093 transcript_122902	1	1949	1949	3107	6	1960	2156	3316
transcript_76013	gnl|BL_ORD_ID|41847 transcript_88188	1	2222	2221	3645	375	2595	2718	4141
transcript_76016	gnl|BL_ORD_ID|94644 transcript_17632	1	1788	1783	2146	12	1807	2068	2425
transcript_76060	gnl|BL_ORD_ID|45926 transcript_94935	1231	2863	110	1232	1526	3159	1	1123
transcript_76060	gnl|BL_ORD_ID|92267 transcript_164319	1231	2863	110	1232	1562	3196	1	1162
transcript_76060	gnl|BL_ORD_ID|39041 transcript_83620	107	1527	1527	2862	2	1433	2549	3883
transcript_76063	gnl|BL_ORD_ID|17279 transcript_51652	1	1725	1724	2212	1	1723	2812	3301
transcript_76077	gnl|BL_ORD_ID|62107 transcript_119697	1	1238	1235	2307	2	1237	1395	2467
transcript_7609	gnl|BL_ORD_ID|55206 transcript_108411	1	2303	2304	3025	28	2330	2536	3257
transcript_76098	gnl|BL_ORD_ID|2239 transcript_23771	1	1365	1360	2087	1	1359	1487	2212
transcript_7610	gnl|BL_ORD_ID|25125 transcript_5781	1179	3037	1	1179	1338	3184	24	1202
transcript_76162	gnl|BL_ORD_ID|25180 transcript_5874	1	1470	1470	2609	1	1476	1848	2987
transcript_76162	gnl|BL_ORD_ID|63028 transcript_121186	1	1470	1470	2609	1	1476	1800	2939
transcript_762	gnl|BL_ORD_ID|37782 transcript_83195	315	4269	2	316	438	4392	1	315
transcript_762	gnl|BL_ORD_ID|18848 transcript_54117	315	4267	2	316	502	4456	1	315
transcript_762	gnl|BL_ORD_ID|334 transcript_572	1	3248	3244	4092	1	3248	3542	4390
transcript_762	gnl|BL_ORD_ID|37608 transcript_82928	315	4090	2	316	677	4452	240	554
transcript_76232	gnl|BL_ORD_ID|77821 transcript_13203	1	1629	1625	2477	10	1638	1807	2658
transcript_76232	gnl|BL_ORD_ID|81348 transcript_147843	1	1629	1625	2477	2	1627	1795	2647
transcript_7625	gnl|BL_ORD_ID|27295 transcript_66286	1	1611	1610	3020	95	1704	2867	4277
transcript_76297	gnl|BL_ORD_ID|12536 transcript_3968	2	2163	2162	3151	3	2198	2331	3323

transcript_76312	gnl|BL_ORD_ID|923 transcript_1647	1	2497	2496	2891	379	2875	3485	3880
transcript_76363	gnl|BL_ORD_ID|25882 transcript_64076	1	2537	2534	3197	78	2612	2924	3583
transcript_7637	gnl|BL_ORD_ID|66431 transcript_125155	537	3052	6	537	694	3210	3	534
transcript_76424	gnl|BL_ORD_ID|70276 transcript_131391	295	1559	75	294	844	2131	2	222
transcript_76441	gnl|BL_ORD_ID|764 transcript_1335	1640	3572	1	1643	1958	3892	1	1644
transcript_76530	gnl|BL_ORD_ID|96448 transcript_64208	1141	2570	110	1144	1221	2650	88	1121
transcript_76536	gnl|BL_ORD_ID|77399 transcript_142920	1	1744	1744	2427	238	1978	4143	4826
transcript_76541	gnl|BL_ORD_ID|94892 transcript_18227	173	1655	2	176	1010	2490	10	184
transcript_76541	gnl|BL_ORD_ID|45657 transcript_94465	173	1655	2	176	613	2091	10	184
transcript_76554	gnl|BL_ORD_ID|65873 transcript_124215	139	3642	1	142	4489	7993	3792	3933
transcript_76554	gnl|BL_ORD_ID|86670 transcript_156913	139	3641	1	142	1670	5173	1028	1169
transcript_76554	gnl|BL_ORD_ID|85729 transcript_155332	139	3597	1	142	767	4226	124	265
transcript_7663	gnl|BL_ORD_ID|37507 transcript_82767	1	2450	2448	3060	4	2439	2590	3202
transcript_76671	gnl|BL_ORD_ID|1166 transcript_2092	29	3011	3010	3645	2	2984	3102	3737
transcript_76685	gnl|BL_ORD_ID|11978 transcript_2890	1	1776	1773	3222	198	1970	2100	3551
transcript_76686	gnl|BL_ORD_ID|32433 transcript_74639	329	2734	65	328	1068	3471	2	265
transcript_76706	gnl|BL_ORD_ID|748 transcript_1308	190	2858	1	193	1348	4018	44	236
transcript_76718	gnl|BL_ORD_ID|57682 transcript_112605	1	1792	1793	2733	443	2233	2375	3315
transcript_76718	gnl|BL_ORD_ID|80427 transcript_146361	1	1792	1793	2733	440	2231	2332	3272
transcript_76718	gnl|BL_ORD_ID|64085 transcript_122888	1	1792	1793	2733	508	2299	2441	3381
transcript_76718	gnl|BL_ORD_ID|12593 transcript_4087	1	1792	1793	2733	542	2333	2475	3415
transcript_76772	gnl|BL_ORD_ID|1176 transcript_2118	343	3475	1	345	574	3703	10	351
transcript_76854	gnl|BL_ORD_ID|12103 transcript_3149	1	1890	1891	3412	1	1863	1980	3502
transcript_76931	gnl|BL_ORD_ID|89536 transcript_161548	643	2277	69	638	2526	4161	1	571
transcript_77032	gnl|BL_ORD_ID|36425 transcript_81071	1	1144	1139	1763	1517	2662	2817	3444
transcript_77040	gnl|BL_ORD_ID|50324 transcript_101902	1	1871	1869	3680	1293	3163	3349	5160
transcript_77092	gnl|BL_ORD_ID|47543 transcript_97487	1	1817	1815	2611	978	2789	2905	3701
transcript_77092	gnl|BL_ORD_ID|67925 transcript_127569	1	1817	1815	2611	1370	3185	3301	4097
transcript_77092	gnl|BL_ORD_ID|74191 transcript_137760	1	1817	1815	2611	2531	4347	4463	5258
transcript_77092	gnl|BL_ORD_ID|51782 transcript_9815	1	1817	1815	2611	214	2006	2122	2918
transcript_77092	gnl|BL_ORD_ID|29495 transcript_69822	1	1817	1815	2611	70	1886	2002	2798
transcript_77092	gnl|BL_ORD_ID|37282 transcript_82403	1	1817	1815	2611	3316	5133	5249	6045
transcript_7712	gnl|BL_ORD_ID|11726 transcript_2397	1	1997	1996	3029	4	2001	2614	3647
transcript_77149	gnl|BL_ORD_ID|90620 transcript_163204	11	1281	1282	2209	2	1276	1598	2528

transcript_77158	gnl|BL_ORD_ID|38072 transcript_6837	1	2619	2619	2925	1	2610	2777	3082
transcript_77158	gnl|BL_ORD_ID|35267 transcript_79210	11	2619	2620	2925	2	2604	2794	3099
transcript_77158	gnl|BL_ORD_ID|62646 transcript_120590	2	2619	2620	2925	1	2609	2819	3123
transcript_7718	gnl|BL_ORD_ID|24601 transcript_4647	1	2489	2485	3017	4	2492	2678	3210
transcript_77195	gnl|BL_ORD_ID|84496 transcript_153364	2524	5383	2	2528	2758	5612	68	2595
transcript_77274	gnl|BL_ORD_ID|56475 transcript_110585	361	2301	85	366	936	2891	5	286
transcript_7728	gnl|BL_ORD_ID|38383 transcript_7518	142	2903	1	141	277	3038	1	141
transcript_77287	gnl|BL_ORD_ID|81086 transcript_147411	246	1106	27	245	387	1247	15	233
transcript_77324	gnl|BL_ORD_ID|80241 transcript_146057	1	1068	1069	2071	219	1285	1584	2586
transcript_77337	gnl|BL_ORD_ID|48603 transcript_99158	289	3040	2	289	527	3300	53	340
transcript_77366	gnl|BL_ORD_ID|51769 transcript_9785	1146	2695	1	1148	1272	2823	1	1147
transcript_77391	gnl|BL_ORD_ID|40864 transcript_86579	2	2031	2029	2724	48	2077	2253	2948
transcript_7744	gnl|BL_ORD_ID|61868 transcript_119327	105	1599	1598	3008	2	1496	2729	4139
transcript_7744	gnl|BL_ORD_ID|66271 transcript_124891	1121	3021	105	1123	1153	3053	2	1028
transcript_77477	gnl|BL_ORD_ID|89536 transcript_161548	659	3725	90	660	1091	4161	1	571
transcript_77492	gnl|BL_ORD_ID|67622 transcript_127083	1	2298	2295	3366	1192	3489	3758	4818
transcript_7750	gnl|BL_ORD_ID|29966 transcript_70587	133	1943	1942	2975	1	1809	2887	3919
transcript_77514	gnl|BL_ORD_ID|25766 transcript_63881	1	1413	1414	2220	23	1436	2526	3332
transcript_7752	gnl|BL_ORD_ID|25078 transcript_5672	1	2677	2674	3012	1	2677	2866	3203
transcript_77522	gnl|BL_ORD_ID|92912 transcript_165375	1	1249	1251	1734	5	1255	1522	2009
transcript_77551	gnl|BL_ORD_ID|40151 transcript_85396	12	2072	2068	2398	1	2061	2794	3124
transcript_77600	gnl|BL_ORD_ID|52776 transcript_104251	1	1137	1135	2064	55	1185	1285	2214
transcript_77645	gnl|BL_ORD_ID|79033 transcript_144108	1	1065	1065	1356	1274	2339	2482	2772
transcript_77645	gnl|BL_ORD_ID|6981 transcript_33891	1	1065	1065	1363	273	1338	1475	1769
transcript_77645	gnl|BL_ORD_ID|6945 transcript_33827	1	1065	1065	1371	217	1282	1425	1730
transcript_77676	gnl|BL_ORD_ID|78337 transcript_14385	1	1453	1454	2256	39	1491	1834	2639
transcript_77677	gnl|BL_ORD_ID|23097 transcript_61196	1	1604	1605	2017	1064	2658	2781	3193
transcript_77677	gnl|BL_ORD_ID|27427 transcript_66489	1	1531	1528	2017	389	1911	2142	2631
transcript_77677	gnl|BL_ORD_ID|70219 transcript_131297	1	1531	1528	2017	2392	3923	4154	4643
transcript_777	gnl|BL_ORD_ID|49937 transcript_101287	25	2593	2593	4235	1	2569	2671	4313
transcript_77700	gnl|BL_ORD_ID|77752 transcript_13056	1	1916	1916	2486	1	1921	2065	2638
transcript_77719	gnl|BL_ORD_ID|12247 transcript_3408	2	2166	2163	2747	4	2168	2485	3069
transcript_77719	gnl|BL_ORD_ID|38871 transcript_83337	2	2166	2165	2747	1	2165	2322	2904
transcript_77719	gnl|BL_ORD_ID|25427 transcript_6388	1	2166	2163	2747	1	2160	2477	3059

transcript_77719	gnl|BL_ORD_ID|38084 transcript_6869	2	2166	2163	2731	65	2225	2542	3110
transcript_77719	gnl|BL_ORD_ID|38552 transcript_7866	1	2166	2165	2747	1	2166	2323	2905
transcript_77719	gnl|BL_ORD_ID|38126 transcript_6958	1	2166	2163	2747	1	2166	2483	3067
transcript_77754	gnl|BL_ORD_ID|83773 transcript_152118	1	1808	1807	2182	1462	3251	3374	3736
transcript_77768	gnl|BL_ORD_ID|12351 transcript_3593	1	1851	1848	2622	582	2423	2653	3426
transcript_77774	gnl|BL_ORD_ID|46 transcript_64	1	2440	2436	3196	1455	3897	4988	5747
transcript_7778	gnl|BL_ORD_ID|24975 transcript_5425	1	1939	1937	3007	1	1942	2159	3229
transcript_7780	gnl|BL_ORD_ID|41271 transcript_87232	1	2624	2623	2949	57	2655	5201	5526
transcript_77806	gnl|BL_ORD_ID|38820 transcript_8473	179	2584	1	183	636	3015	62	244
transcript_77807	gnl|BL_ORD_ID|58183 transcript_113386	1	1782	1784	2090	1	1782	2023	2329
transcript_77807	gnl|BL_ORD_ID|2166 transcript_23600	1	1782	1784	2090	1	1767	1922	2228
transcript_77824	gnl|BL_ORD_ID|24954 transcript_5375	1	2049	2048	3070	56	2105	2205	3227
transcript_77908	gnl|BL_ORD_ID|55918 transcript_109701	310	994	1	313	1089	1766	1	313
transcript_77942	gnl|BL_ORD_ID|17188 transcript_51510	1	1465	1461	1981	106	1573	1691	2209
transcript_77993	gnl|BL_ORD_ID|25678 transcript_63736	2	2575	2571	3051	3	2582	2768	3250
transcript_78002	gnl|BL_ORD_ID|74294 transcript_137916	13	1357	1356	2109	2	1346	2882	3635
transcript_78029	gnl|BL_ORD_ID|32811 transcript_75247	1	1937	1937	2892	2759	4695	4919	5874
transcript_78029	gnl|BL_ORD_ID|31990 transcript_73891	1	1937	1937	2902	2746	4685	4907	5870
transcript_78106	gnl|BL_ORD_ID|45984 transcript_95028	1	1365	1363	2376	51	1416	1539	2552
transcript_78125	gnl|BL_ORD_ID|95353 transcript_19292	1	1242	1240	1984	5	1254	1695	2439
transcript_78145	gnl|BL_ORD_ID|64022 transcript_122788	224	2522	1	229	356	2660	2	230
transcript_78173	gnl|BL_ORD_ID|60949 transcript_117868	1	1104	1103	1440	67	1170	1284	1625
transcript_78214	gnl|BL_ORD_ID|57348 transcript_112036	1	2613	2609	3047	70	2689	3749	4189
transcript_78222	gnl|BL_ORD_ID|64867 transcript_11766	241	2309	9	240	524	2593	72	303
transcript_78285	gnl|BL_ORD_ID|69401 transcript_129948	1	1763	1760	2287	135	1897	2007	2535
transcript_78298	gnl|BL_ORD_ID|29793 transcript_70285	18	2106	2106	2287	2	2087	2221	2402
transcript_78330	gnl|BL_ORD_ID|40641 transcript_86195	2	2554	2551	2920	59	2609	2979	3348
transcript_78335	gnl|BL_ORD_ID|60942 transcript_117860	1	1062	1060	1852	270	1329	1839	2606
transcript_78413	gnl|BL_ORD_ID|12468 transcript_3826	157	2924	1	162	667	3423	1	162
transcript_78426	gnl|BL_ORD_ID|62127 transcript_119729	138	3434	1	139	330	3631	12	150
transcript_78433	gnl|BL_ORD_ID|43343 transcript_90552	457	2116	47	457	549	2207	1	425
transcript_78450	gnl|BL_ORD_ID|48511 transcript_99013	1304	4293	1	1303	1423	4415	2	1295
transcript_7848	gnl|BL_ORD_ID|25125 transcript_5781	1211	3035	12	1211	1338	3150	3	1202
transcript_78511	gnl|BL_ORD_ID|38157 transcript_7025	1	1411	1410	2155	1	1410	2309	3054

transcript_78511	gnl|BL_ORD_ID|37933 transcript_6571	1	1585	1580	2155	2	1584	2501	3077
transcript_78530	gnl|BL_ORD_ID|72502 transcript_134970	18	2376	2372	2554	88	2453	3103	3285
transcript_78532	gnl|BL_ORD_ID|94505 transcript_17320	175	2247	1	175	408	2477	131	305
transcript_78532	gnl|BL_ORD_ID|11666 transcript_2277	1	1716	1716	2242	119	1857	3137	3662
transcript_78560	gnl|BL_ORD_ID|479 transcript_813	1	3635	3635	4034	1	3640	3768	4167
transcript_78579	gnl|BL_ORD_ID|38367 transcript_7481	271	2929	1	271	374	3031	1	271
transcript_78597	gnl|BL_ORD_ID|74014 transcript_137441	1	2293	2291	3156	184	2476	4068	4938
transcript_7860	gnl|BL_ORD_ID|89496 transcript_161489	1	1993	1992	2995	1	1989	2170	3199
transcript_78624	gnl|BL_ORD_ID|95198 transcript_18918	1	1133	1134	2229	1	1132	1331	2427
transcript_7866	gnl|BL_ORD_ID|12247 transcript_3408	2	2324	2323	3003	4	2326	2487	3167
transcript_7866	gnl|BL_ORD_ID|38126 transcript_6958	1	2324	2323	2920	1	2324	2485	3082
transcript_7866	gnl|BL_ORD_ID|25427 transcript_6388	1	2324	2323	3003	1	2318	2479	3158
transcript_78724	gnl|BL_ORD_ID|37721 transcript_83102	278	1836	46	283	607	2165	1	239
transcript_78740	gnl|BL_ORD_ID|12248 transcript_3410	1	1971	1968	3283	2	1931	2118	3438
transcript_7877	gnl|BL_ORD_ID|38010 transcript_6730	1	1655	1654	2976	5	1659	1777	3099
transcript_7885	gnl|BL_ORD_ID|60868 transcript_117735	1349	2961	1	1350	1656	3267	1	1343
transcript_78862	gnl|BL_ORD_ID|65162 transcript_12463	156	2523	1	156	320	2688	41	194
transcript_78877	gnl|BL_ORD_ID|92458 transcript_164645	294	988	2	296	1111	1805	91	385
transcript_78891	gnl|BL_ORD_ID|78991 transcript_144033	240	1955	37	243	1883	3598	8	238
transcript_78907	gnl|BL_ORD_ID|24770 transcript_5020	1	1638	1636	3201	2	1651	1757	3316
transcript_7892	gnl|BL_ORD_ID|64305 transcript_123228	2	2372	2370	3002	1	2364	3132	3779
transcript_78937	gnl|BL_ORD_ID|57965 transcript_113061	1	1929	1925	2191	1	1931	1729	1997
transcript_78939	gnl|BL_ORD_ID|82168 transcript_149273	1	1196	1195	1633	128	1324	1488	1924
transcript_7897	gnl|BL_ORD_ID|18297 transcript_53260	23	2756	2754	2967	2	2730	4229	4442
transcript_79016	gnl|BL_ORD_ID|51235 transcript_8676	1	1874	1875	2756	5	1878	2064	2946
transcript_79102	gnl|BL_ORD_ID|62345 transcript_120088	1432	3682	1	1434	2546	4800	2	1431
transcript_7911	gnl|BL_ORD_ID|29966 transcript_70587	101	1911	1910	3027	1	1809	2887	4006
transcript_79182	gnl|BL_ORD_ID|21857 transcript_59108	127	4893	1	128	577	5341	1	128
transcript_79200	gnl|BL_ORD_ID|80231 transcript_146042	119	1529	1529	2868	1	1412	1522	2861
transcript_79216	gnl|BL_ORD_ID|23795 transcript_62344	1	1688	1683	2977	98	1784	2943	4236
transcript_79216	gnl|BL_ORD_ID|85424 transcript_154840	1	1688	1683	2977	99	1797	2855	4145
transcript_79216	gnl|BL_ORD_ID|171 transcript_271	1	1688	1683	2977	104	1788	3579	4868
transcript_79234	gnl|BL_ORD_ID|18393 transcript_53413	455	1786	55	457	2222	3551	775	1177
transcript_79251	gnl|BL_ORD_ID|17439 transcript_51913	2	2398	2396	2840	1	2398	2515	2959

transcript_79290	gnl|BL_ORD_ID|88007 transcript_159093	127	2644	1	126	819	3337	518	643
transcript_79318	gnl|BL_ORD_ID|57661 transcript_112564	1053	2114	1	1054	1482	2541	295	1348
transcript_79352	gnl|BL_ORD_ID|38491 transcript_7725	200	2235	1	203	980	3013	1	203
transcript_79389	gnl|BL_ORD_ID|61303 transcript_118452	294	2180	4	297	767	2653	90	383
transcript_79389	gnl|BL_ORD_ID|51959 transcript_10212	294	2194	4	297	951	2850	136	427
transcript_79389	gnl|BL_ORD_ID|64924 transcript_11891	294	2217	4	297	842	2765	25	318
transcript_79423	gnl|BL_ORD_ID|66625 transcript_125468	164	3380	1	165	409	3622	2	165
transcript_79442	gnl|BL_ORD_ID|51441 transcript_9119	1	1526	1523	2118	632	2157	2290	2885
transcript_79442	gnl|BL_ORD_ID|52589 transcript_103946	1	1526	1523	2118	434	1959	2092	2687
transcript_79535	gnl|BL_ORD_ID|17277 transcript_51648	1	1968	1967	2499	1469	3435	4836	5367
transcript_79550	gnl|BL_ORD_ID|38986 transcript_83547	290	1596	3	290	405	1709	2	297
transcript_79565	gnl|BL_ORD_ID|66990 transcript_126056	1	1109	1108	1794	3	1112	1787	2472
transcript_79570	gnl|BL_ORD_ID|46854 transcript_96402	1098	2245	1	1103	1440	2586	35	1161
transcript_79599	gnl|BL_ORD_ID|33364 transcript_76082	274	4398	1	275	379	4501	1	275
transcript_79605	gnl|BL_ORD_ID|29277 transcript_69486	1	2230	2227	2580	53	2282	2604	2957
transcript_79622	gnl|BL_ORD_ID|622 transcript_1064	2	2827	2825	3121	8	2830	3188	3482
transcript_79622	gnl|BL_ORD_ID|23891 transcript_62498	2	2827	2825	3121	1	2828	3207	3503
transcript_79634	gnl|BL_ORD_ID|80798 transcript_146968	246	2516	1	246	1811	4080	2	247
transcript_79637	gnl|BL_ORD_ID|77815 transcript_13185	229	2556	100	233	356	2690	2	136
transcript_79637	gnl|BL_ORD_ID|52702 transcript_104139	229	2488	2	231	503	2763	46	276
transcript_79637	gnl|BL_ORD_ID|64599 transcript_11202	229	2532	2	233	489	2799	37	269
transcript_79650	gnl|BL_ORD_ID|26726 transcript_65374	1	1189	1186	2283	110	1299	1575	2670
transcript_79674	gnl|BL_ORD_ID|81418 transcript_147949	1	3057	3054	3257	54	3116	3374	3577
transcript_79675	gnl|BL_ORD_ID|41374 transcript_87404	2	2028	2026	2561	3	2028	4031	4566
transcript_79695	gnl|BL_ORD_ID|77964 transcript_13529	1	1447	1447	2344	1	1443	1773	2668
transcript_79701	gnl|BL_ORD_ID|17891 transcript_52611	1	2635	2631	2863	2	2643	232	465
transcript_79722	gnl|BL_ORD_ID|64213 transcript_123089	1	2457	2452	2788	51	2506	3978	4315
transcript_79744	gnl|BL_ORD_ID|20811 transcript_57350	187	1147	2	189	908	1870	9	196
transcript_79745	gnl|BL_ORD_ID|1148 transcript_2063	153	3598	1	153	268	3714	8	160
transcript_79745	gnl|BL_ORD_ID|49211 transcript_100127	154	3598	1	153	290	3735	8	160
transcript_79756	gnl|BL_ORD_ID|39339 transcript_84093	2	2222	2219	2606	82	2288	3246	3632
transcript_79789	gnl|BL_ORD_ID|35577 transcript_79680	1	1505	1503	2191	18	1529	1978	2668
transcript_79805	gnl|BL_ORD_ID|25932 transcript_64146	1	1062	1061	1567	86	1147	1396	1902
transcript_79814	gnl|BL_ORD_ID|51436 transcript_9107	1	1565	1565	2751	83	1633	1744	2928

transcript_79815	gnl|BL_ORD_ID|60334 transcript_116871	2	2355	2354	2731	6	2347	5455	5830
transcript_79835	gnl|BL_ORD_ID|51991 transcript_10275	11	2492	2490	2694	28	2509	2641	2842
transcript_79842	gnl|BL_ORD_ID|21763 transcript_58940	1	1043	1043	1621	465	1505	1765	2343
transcript_79842	gnl|BL_ORD_ID|57887 transcript_112938	1	1043	1043	1621	1223	2263	2523	3101
transcript_79902	gnl|BL_ORD_ID|55443 transcript_108853	173	2089	1	173	863	2778	2	174
transcript_79916	gnl|BL_ORD_ID|39401 transcript_84200	332	3550	1	335	810	4031	360	694
transcript_79916	gnl|BL_ORD_ID|665 transcript_1153	1	2829	2828	3572	351	3175	3278	4031
transcript_7996	gnl|BL_ORD_ID|48603 transcript_99158	386	2996	47	386	527	3158	1	340
transcript_80009	gnl|BL_ORD_ID|38157 transcript_7025	1	2529	2528	2945	1	2520	2625	3038
transcript_80023	gnl|BL_ORD_ID|91207 transcript_16133	1056	2374	1	1058	1226	2541	1	1058
transcript_80047	gnl|BL_ORD_ID|37655 transcript_83000	2	2279	2279	3030	6	2257	2364	3115
transcript_80093	gnl|BL_ORD_ID|56314 transcript_110339	261	1751	31	261	346	1832	2	240
transcript_8011	gnl|BL_ORD_ID|40047 transcript_85236	16	2485	2483	2966	1	2464	2612	3102
transcript_8013	gnl|BL_ORD_ID|45942 transcript_94958	617	2988	81	618	1432	3804	1	538
transcript_80139	gnl|BL_ORD_ID|20705 transcript_57174	2	2120	2119	3102	671	2773	3173	4154
transcript_80181	gnl|BL_ORD_ID|26228 transcript_64594	1	1607	1607	2666	1	1616	2056	3115
transcript_80190	gnl|BL_ORD_ID|24288 transcript_63112	2	2106	2107	2846	212	2316	2417	3154
transcript_80216	gnl|BL_ORD_ID|52815 transcript_104320	135	1402	1	136	706	1973	1	136
transcript_80244	gnl|BL_ORD_ID|12437 transcript_3769	1	1475	1474	2003	323	1770	2890	3419
transcript_80244	gnl|BL_ORD_ID|87649 transcript_158515	1	1475	1474	1936	237	1684	2589	3051
transcript_80244	gnl|BL_ORD_ID|62874 transcript_120943	1	1474	1474	2003	326	1772	1928	2457
transcript_80336	gnl|BL_ORD_ID|3012 transcript_25451	1	1591	1589	2025	1	1590	1699	2135
transcript_80341	gnl|BL_ORD_ID|17627 transcript_52192	1	1947	1946	2502	1	1948	2050	2606
transcript_80359	gnl|BL_ORD_ID|77798 transcript_13153	576	2071	58	576	1138	2632	241	765
transcript_80389	gnl|BL_ORD_ID|25187 transcript_5889	1	1841	1842	2557	2	1817	2439	3154
transcript_80399	gnl|BL_ORD_ID|46965 transcript_96581	211	3350	2	212	438	3578	20	230
transcript_80449	gnl|BL_ORD_ID|87937 transcript_158974	1	1598	1593	2351	830	2431	2838	3596
transcript_80475	gnl|BL_ORD_ID|26261 transcript_64652	1	1227	1228	1790	1010	2238	2352	2916
transcript_80484	gnl|BL_ORD_ID|89362 transcript_161267	2	2113	2112	2583	110	2221	2982	3453
transcript_80563	gnl|BL_ORD_ID|38152 transcript_7012	2	2051	2052	2909	7	2060	2212	3067
transcript_8061	gnl|BL_ORD_ID|12224 transcript_3370	195	2987	1	190	715	3486	1	190
transcript_8061	gnl|BL_ORD_ID|12152 transcript_3231	195	2987	1	190	713	3507	1	190
transcript_80633	gnl|BL_ORD_ID|64418 transcript_10809	23	2412	2410	2572	72	2453	2651	2814
transcript_80633	gnl|BL_ORD_ID|51845 transcript_9987	1	2415	2410	2572	1	2411	2532	2695

transcript_80633	gnl|BL_ORD_ID|54057 transcript_106403	23	2414	2410	2572	88	2470	2691	2854
transcript_80645	gnl|BL_ORD_ID|17561 transcript_52099	1152	2407	1	1155	2820	4075	6	1160
transcript_80751	gnl|BL_ORD_ID|82879 transcript_150516	118	2000	1	116	2378	4259	1039	1154
transcript_80757	gnl|BL_ORD_ID|40283 transcript_85586	1137	4288	1	1138	1565	4704	51	1188
transcript_8077	gnl|BL_ORD_ID|20196 transcript_56330	1	1835	1834	2983	235	2069	2587	3736
transcript_8077	gnl|BL_ORD_ID|28109 transcript_67596	1	1835	1834	2983	2	1839	2464	3615
transcript_8077	gnl|BL_ORD_ID|11675 transcript_2294	1	1835	1834	2983	2	1836	2354	3503
transcript_80786	gnl|BL_ORD_ID|38441 transcript_7634	1	2436	2437	2870	31	2464	2600	3032
transcript_8081	gnl|BL_ORD_ID|17390 transcript_51835	2	2087	2087	2970	1	2088	3048	3912
transcript_8082	gnl|BL_ORD_ID|748 transcript_1308	211	2871	2	214	1348	4018	23	236
transcript_80854	gnl|BL_ORD_ID|41019 transcript_86826	200	2397	12	202	1480	3680	50	241
transcript_80897	gnl|BL_ORD_ID|72686 transcript_135257	1	2045	2045	3785	16	2058	2299	4040
transcript_80979	gnl|BL_ORD_ID|37169 transcript_82233	129	2801	2799	3858	2	2682	3932	5005
transcript_80986	gnl|BL_ORD_ID|38378 transcript_7505	271	1893	72	273	1304	2926	2	203
transcript_81020	gnl|BL_ORD_ID|42960 transcript_89937	1	1676	1676	2510	34	1706	1814	2650
transcript_81020	gnl|BL_ORD_ID|64891 transcript_11822	1116	2510	1	1116	1333	2729	36	1142
transcript_81023	gnl|BL_ORD_ID|35907 transcript_80209	1	2945	2944	3427	17	2983	3092	3575
transcript_81023	gnl|BL_ORD_ID|1002 transcript_1781	1585	3361	1	1587	2069	3862	50	1638
transcript_81031	gnl|BL_ORD_ID|92085 transcript_164033	1818	4326	1	1823	2501	5009	2	1824
transcript_81036	gnl|BL_ORD_ID|26837 transcript_65547	2	2225	2225	3163	41	2264	2445	3383
transcript_81070	gnl|BL_ORD_ID|5381 transcript_30543	1	1194	1195	1548	255	1448	1592	1945
transcript_81078	gnl|BL_ORD_ID|51769 transcript_9785	1153	2743	1	1155	1272	2868	5	1147
transcript_81081	gnl|BL_ORD_ID|24553 transcript_4545	2	2721	2716	3064	4	2723	2974	3322
transcript_81082	gnl|BL_ORD_ID|80868 transcript_147084	1	1810	1810	3335	1	1815	2228	3752
transcript_81090	gnl|BL_ORD_ID|33002 transcript_75531	1	1191	1188	1543	1	1188	1844	2197
transcript_81097	gnl|BL_ORD_ID|54724 transcript_107547	248	4695	14	250	839	5279	2	238
transcript_81210	gnl|BL_ORD_ID|37861 transcript_6431	207	2268	1	212	1076	3137	2	213
transcript_81233	gnl|BL_ORD_ID|74917 transcript_138933	260	2272	84	261	1083	3094	2	179
transcript_81243	gnl|BL_ORD_ID|23343 transcript_61592	1	1317	1313	1936	1983	3299	3581	4202
transcript_81243	gnl|BL_ORD_ID|76444 transcript_141389	1	1317	1313	1902	2580	3896	4178	4764
transcript_81258	gnl|BL_ORD_ID|70718 transcript_132124	129	1993	1991	2650	395	2260	3143	3802
transcript_81258	gnl|BL_ORD_ID|38708 transcript_8220	129	2596	1	133	502	2970	3	135
transcript_81261	gnl|BL_ORD_ID|62992 transcript_121127	469	2462	5	468	843	2840	90	545
transcript_81307	gnl|BL_ORD_ID|76342 transcript_141238	1	2056	2057	2411	851	2918	3023	3383

transcript_81348	gnl|BL_ORD_ID|38251 transcript_7216	209	2776	1	211	460	3055	1	211
transcript_81348	gnl|BL_ORD_ID|51415 transcript_9069	211	2785	1	211	327	2925	1	211
transcript_81348	gnl|BL_ORD_ID|68130 transcript_127900	209	2774	1	211	2005	4607	1	210
transcript_81348	gnl|BL_ORD_ID|45728 transcript_94593	211	2807	1	211	495	3114	1	211
transcript_81388	gnl|BL_ORD_ID|24711 transcript_4897	116	2761	1	118	684	3329	441	558
transcript_81463	gnl|BL_ORD_ID|88937 transcript_160586	2	3038	3036	3347	15	3059	3299	3609
transcript_81491	gnl|BL_ORD_ID|96394 transcript_59329	2	2752	2752	3506	329	3081	2836	3595
transcript_81514	gnl|BL_ORD_ID|90290 transcript_162714	1178	2705	1	1181	3509	5042	2197	3377
transcript_81514	gnl|BL_ORD_ID|90306 transcript_162735	1178	2706	1	1181	2255	3789	976	2124
transcript_81514	gnl|BL_ORD_ID|86984 transcript_157433	1178	2706	1	1181	4142	5675	2833	4011
transcript_81514	gnl|BL_ORD_ID|52512 transcript_103833	1178	2706	1	1181	1880	3409	574	1749
transcript_81523	gnl|BL_ORD_ID|348 transcript_598	1	2304	2301	2474	778	3081	3950	4122
transcript_81525	gnl|BL_ORD_ID|95109 transcript_18691	1	1648	1647	2274	65	1735	1848	2475
transcript_81525	gnl|BL_ORD_ID|55211 transcript_108420	1	1300	1299	2274	71	1393	1542	2517
transcript_81540	gnl|BL_ORD_ID|33720 transcript_76666	2	3367	3366	3901	41	3394	5324	5859
transcript_81591	gnl|BL_ORD_ID|31776 transcript_73518	636	2327	98	635	1749	3440	2	538
transcript_81609	gnl|BL_ORD_ID|44051 transcript_91776	1	1491	1489	2357	377	1868	2282	3153
transcript_8163	gnl|BL_ORD_ID|40639 transcript_86191	2	2662	2662	2981	28	2686	2915	3235
transcript_81635	gnl|BL_ORD_ID|44211 transcript_92034	1	1016	1013	1552	1	1017	1739	2292
transcript_81636	gnl|BL_ORD_ID|51137 transcript_103240	1	1683	1686	2001	1096	2775	3363	3678
transcript_81659	gnl|BL_ORD_ID|24561 transcript_4560	1	1588	1587	2559	2	1592	2342	3314
transcript_81659	gnl|BL_ORD_ID|24544 transcript_4530	10	1588	1587	2559	1	1578	2313	3284
transcript_81684	gnl|BL_ORD_ID|2380 transcript_24090	1	1012	1007	1353	13	1022	1871	2217
transcript_81684	gnl|BL_ORD_ID|91787 transcript_163559	1	1012	1007	1349	12	1021	1427	1769
transcript_81688	gnl|BL_ORD_ID|93129 transcript_165726	1153	3453	1	1153	1351	3655	1	1175
transcript_81730	gnl|BL_ORD_ID|35320 transcript_79288	151	3074	3075	4516	2	2927	3053	4492
transcript_81767	gnl|BL_ORD_ID|50054 transcript_101473	13	1376	1375	2021	2	1362	2164	2811
transcript_81791	gnl|BL_ORD_ID|41640 transcript_87867	1	1682	1680	1927	126	1799	1950	2198
transcript_8181	gnl|BL_ORD_ID|69125 transcript_129517	140	2951	14	143	284	3108	37	169
transcript_81826	gnl|BL_ORD_ID|62638 transcript_120579	1	1094	1094	1784	2	1094	1219	1911
transcript_81826	gnl|BL_ORD_ID|5092 transcript_29937	1	1094	1094	1784	2	1082	1207	1899
transcript_81826	gnl|BL_ORD_ID|4403 transcript_28503	1	1094	1094	1784	31	1123	1248	1940
transcript_81843	gnl|BL_ORD_ID|24380 transcript_63261	1117	2313	1	1116	1924	3125	316	1417
transcript_81889	gnl|BL_ORD_ID|84722 transcript_153731	1232	2563	1	1233	2073	3402	1	1228

transcript_81897	gnl|BL_ORD_ID|61401 transcript_118607	1	2285	2281	2458	2	2313	2937	3114
transcript_81910	gnl|BL_ORD_ID|70276 transcript_131391	286	1570	63	285	844	2131	2	222
transcript_81925	gnl|BL_ORD_ID|76745 transcript_141873	1108	4219	1	1109	1313	4421	47	1155
transcript_81925	gnl|BL_ORD_ID|80079 transcript_145773	1108	4219	1	1109	1255	4349	3	1097
transcript_81927	gnl|BL_ORD_ID|21930 transcript_59230	1	1755	1755	2140	24	1779	1961	2346
transcript_81976	gnl|BL_ORD_ID|19333 transcript_54927	1328	2756	1	1329	1466	2923	35	1362
transcript_82015	gnl|BL_ORD_ID|26008 transcript_64257	136	2434	1	135	356	2654	1	135
transcript_82029	gnl|BL_ORD_ID|37975 transcript_6656	1	2494	2495	2918	73	2566	2683	3106
transcript_82029	gnl|BL_ORD_ID|38261 transcript_7231	1	2494	2495	2918	50	2520	2637	3060
transcript_8205	gnl|BL_ORD_ID|25078 transcript_5672	1	2639	2637	2978	1	2639	2867	3206
transcript_82085	gnl|BL_ORD_ID|38940 transcript_83460	461	2292	73	460	736	2562	193	577
transcript_82092	gnl|BL_ORD_ID|88395 transcript_159730	377	1500	7	376	718	1841	1	370
transcript_82106	gnl|BL_ORD_ID|64276 transcript_123186	1070	3016	1	1071	3524	5470	2262	3327
transcript_82136	gnl|BL_ORD_ID|74931 transcript_138956	1	2137	2135	2958	2500	4640	6060	6881
transcript_82187	gnl|BL_ORD_ID|20440 transcript_56724	1	1095	1092	1646	120	1205	1353	1908
transcript_82187	gnl|BL_ORD_ID|71378 transcript_133172	1	1095	1092	1611	144	1238	1387	1906
transcript_82187	gnl|BL_ORD_ID|39305 transcript_84031	1	1236	1236	1647	127	1362	2654	3067
transcript_8220	gnl|BL_ORD_ID|70718 transcript_132124	116	2367	2365	2970	2	2260	3143	3748
transcript_82221	gnl|BL_ORD_ID|31428 transcript_72948	1	2837	2837	3116	1202	4035	4240	4519
transcript_82250	gnl|BL_ORD_ID|37091 transcript_82103	280	2565	30	279	440	2731	45	298
transcript_82316	gnl|BL_ORD_ID|58081 transcript_113235	1	1334	1333	2437	955	2287	2711	3820
transcript_82323	gnl|BL_ORD_ID|66178 transcript_124736	1	1242	1239	1662	175	1416	3432	3854
transcript_82323	gnl|BL_ORD_ID|71907 transcript_134034	1	1240	1239	1662	192	1431	1794	2217
transcript_82323	gnl|BL_ORD_ID|48842 transcript_99553	1	1241	1239	1662	174	1414	1587	2010
transcript_82323	gnl|BL_ORD_ID|1246 transcript_21520	1	1241	1239	1679	199	1439	1612	2051
transcript_82323	gnl|BL_ORD_ID|61517 transcript_118778	1	1242	1239	1662	130	1371	3023	3446
transcript_82354	gnl|BL_ORD_ID|59630 transcript_115759	1551	3378	1	1556	3377	5203	687	2244
transcript_82356	gnl|BL_ORD_ID|38191 transcript_7091	131	2010	2009	2871	161	2040	2176	3037
transcript_82356	gnl|BL_ORD_ID|24495 transcript_4432	2	2010	2009	2871	1	2011	2147	3008
transcript_82356	gnl|BL_ORD_ID|38252 transcript_7217	131	2010	2009	2871	138	2016	2152	3013
transcript_82356	gnl|BL_ORD_ID|52423 transcript_103697	131	2010	2009	2870	202	2081	2217	3077
transcript_82356	gnl|BL_ORD_ID|63687 transcript_122274	2	2010	2009	2800	100	2110	2246	3036
transcript_82356	gnl|BL_ORD_ID|46609 transcript_96035	2	2010	2009	2871	50	2058	2194	3055
transcript_82356	gnl|BL_ORD_ID|73757 transcript_137009	2	2010	2009	2871	1	2008	2144	3024

transcript_82367	gnl|BL_ORD_ID|47195 transcript_96934	1	1110	1109	2026	1034	2144	2303	3219
transcript_8242	gnl|BL_ORD_ID|12357 transcript_3618	2	2305	2303	2981	22	2322	2787	3464
transcript_82422	gnl|BL_ORD_ID|434 transcript_743	1059	3825	1	1063	1500	4267	1	1061
transcript_82451	gnl|BL_ORD_ID|42318 transcript_88915	156	2275	2274	3093	3	2122	2231	3050
transcript_82454	gnl|BL_ORD_ID|6865 transcript_33671	10	1030	1028	1661	2	1022	1167	1800
transcript_82481	gnl|BL_ORD_ID|41261 transcript_87216	1	1094	1091	1452	3	1096	1482	1844
transcript_82485	gnl|BL_ORD_ID|637 transcript_1096	201	3935	1	200	318	4054	2	201
transcript_8249	gnl|BL_ORD_ID|25422 transcript_6382	262	2937	12	262	474	3148	2	252
transcript_8249	gnl|BL_ORD_ID|25157 transcript_5837	262	2966	2	262	507	3210	22	282
transcript_8249	gnl|BL_ORD_ID|20452 transcript_56747	631	2967	9	636	792	3126	1	629
transcript_8249	gnl|BL_ORD_ID|33629 transcript_76521	570	2936	9	571	824	3189	1	563
transcript_82490	gnl|BL_ORD_ID|88923 transcript_160565	1	1309	1306	2047	1	1308	1712	2453
transcript_82578	gnl|BL_ORD_ID|12221 transcript_3366	315	3325	2	316	470	3474	16	330
transcript_82583	gnl|BL_ORD_ID|80516 transcript_146500	174	2055	1	177	898	2778	616	792
transcript_82583	gnl|BL_ORD_ID|58664 transcript_114212	174	2055	1	177	966	2846	684	860
transcript_82603	gnl|BL_ORD_ID|81612 transcript_148289	2	2141	2142	2584	22	2162	2268	2706
transcript_82606	gnl|BL_ORD_ID|51519 transcript_9251	214	2670	2	215	371	2827	23	237
transcript_82606	gnl|BL_ORD_ID|49316 transcript_100291	213	2670	2	215	494	2951	16	229
transcript_82606	gnl|BL_ORD_ID|64957 transcript_11952	214	2668	10	215	342	2795	1	208
transcript_8262	gnl|BL_ORD_ID|80659 transcript_146734	1	2484	2483	2875	72	2559	2672	3064
transcript_82684	gnl|BL_ORD_ID|72901 transcript_135620	12	1561	1558	2317	2	1565	2314	3073
transcript_82719	gnl|BL_ORD_ID|70527 transcript_131816	1	1495	1492	2172	21	1527	1654	2354
transcript_82769	gnl|BL_ORD_ID|75300 transcript_139544	1	1797	1796	2330	1	1797	2570	3104
transcript_82791	gnl|BL_ORD_ID|30712 transcript_71789	2	2046	2046	3195	5	2047	2416	3565
transcript_82791	gnl|BL_ORD_ID|26407 transcript_64872	2	2046	2046	3176	59	2112	2227	3362
transcript_82796	gnl|BL_ORD_ID|95390 transcript_19365	1	1209	1210	2183	2	1210	1367	2340
transcript_82814	gnl|BL_ORD_ID|78930 transcript_143916	1242	3428	1	1242	2571	4737	1007	2250
transcript_82814	gnl|BL_ORD_ID|45930 transcript_94940	1242	3436	1	1242	2664	4859	1103	2344
transcript_82814	gnl|BL_ORD_ID|87654 transcript_158525	1242	3437	1	1242	3987	6184	2422	3667
transcript_82814	gnl|BL_ORD_ID|38966 transcript_83512	1242	3436	1	1242	2653	4881	1089	2332
transcript_82814	gnl|BL_ORD_ID|59915 transcript_116214	1242	3504	1	1242	2570	4839	1007	2249
transcript_82814	gnl|BL_ORD_ID|203 transcript_324	1242	3436	1	1242	2573	4774	1009	2252
transcript_82814	gnl|BL_ORD_ID|194 transcript_308	1242	3461	1	1242	2670	4899	1106	2349
transcript_82841	gnl|BL_ORD_ID|28882 transcript_68890	225	1494	57	222	1287	2557	2	171

transcript_82846	gnl|BL_ORD_ID|37968 transcript_6640	1	2451	2451	2973	2	2452	2562	3085
transcript_82848	gnl|BL_ORD_ID|95280 transcript_19101	1076	2180	1	1080	1342	2443	20	1122
transcript_82848	gnl|BL_ORD_ID|29527 transcript_69873	1076	2264	1	1080	1310	2498	1	1090
transcript_82860	gnl|BL_ORD_ID|84291 transcript_153023	25	2616	2617	3260	1	2616	2766	3410
transcript_82860	gnl|BL_ORD_ID|841 transcript_1479	1	2616	2617	3260	5	2619	2770	3413
transcript_82865	gnl|BL_ORD_ID|35320 transcript_79288	2	2924	2925	4430	4	2927	3053	4557
transcript_82895	gnl|BL_ORD_ID|31237 transcript_72635	16	1680	1681	2794	121	1792	2035	3148
transcript_82895	gnl|BL_ORD_ID|86220 transcript_156158	15	1680	1681	2793	13	1686	1928	3040
transcript_82895	gnl|BL_ORD_ID|90479 transcript_162992	15	1680	1681	2794	12	1712	1864	2977
transcript_82902	gnl|BL_ORD_ID|89445 transcript_161404	138	1563	1	138	1021	2447	771	908
transcript_82902	gnl|BL_ORD_ID|20603 transcript_57002	138	1563	1	138	1097	2522	847	984
transcript_82902	gnl|BL_ORD_ID|30201 transcript_70965	138	1563	1	138	1153	2578	902	1039
transcript_82902	gnl|BL_ORD_ID|63296 transcript_121617	138	1563	1	138	1059	2484	808	945
transcript_82902	gnl|BL_ORD_ID|90979 transcript_15639	138	1563	1	138	963	2388	712	849
transcript_82902	gnl|BL_ORD_ID|35821 transcript_80071	138	1563	1	138	978	2402	727	864
transcript_82902	gnl|BL_ORD_ID|77655 transcript_12843	138	1563	1	138	1095	2520	844	981
transcript_82902	gnl|BL_ORD_ID|59086 transcript_114880	138	1563	1	138	1067	2491	816	953
transcript_82902	gnl|BL_ORD_ID|73865 transcript_137215	138	1563	1	138	734	2158	483	620
transcript_82918	gnl|BL_ORD_ID|26057 transcript_64337	1	3224	3222	3490	3264	6483	6595	6863
transcript_8292	gnl|BL_ORD_ID|46860 transcript_96412	521	3015	65	521	623	3117	1	457
transcript_82926	gnl|BL_ORD_ID|48341 transcript_98735	1	1411	1412	1814	1	1416	1777	2179
transcript_82949	gnl|BL_ORD_ID|31849 transcript_73642	1	2745	2744	3078	1934	4674	4776	5109
transcript_82972	gnl|BL_ORD_ID|82745 transcript_150288	260	2812	2	257	1761	4320	24	278
transcript_8299	gnl|BL_ORD_ID|35392 transcript_79394	2	2203	2202	2956	34	2248	2356	3117
transcript_83053	gnl|BL_ORD_ID|41660 transcript_87898	256	2283	73	256	805	2834	2	185
transcript_83055	gnl|BL_ORD_ID|46737 transcript_96230	1492	3067	13	1497	1629	3204	1	1484
transcript_83055	gnl|BL_ORD_ID|62534 transcript_120419	1	1906	1905	3041	1	1905	2062	3198
transcript_83127	gnl|BL_ORD_ID|53946 transcript_106223	268	3262	2	267	698	3691	5	270
transcript_83127	gnl|BL_ORD_ID|70338 transcript_131500	268	3262	2	267	702	3705	12	278
transcript_83146	gnl|BL_ORD_ID|51982 transcript_10256	302	1408	6	301	1754	2842	2	297
transcript_83146	gnl|BL_ORD_ID|51673 transcript_9606	302	1423	6	301	1760	2881	2	297
transcript_83147	gnl|BL_ORD_ID|27390 transcript_66430	1	2284	2282	3419	179	2463	2576	3713
transcript_83195	gnl|BL_ORD_ID|232 transcript_375	1	3371	3367	4413	2	3372	3667	4713
transcript_832	gnl|BL_ORD_ID|90665 transcript_163282	135	2434	2435	4211	2	2299	2444	4220

transcript_83202	gnl|BL_ORD_ID|85628 transcript_155160	309	2292	77	309	381	2369	3	235
transcript_83261	gnl|BL_ORD_ID|23843 transcript_62414	11	1768	1766	2145	1	1759	2174	2554
transcript_83305	gnl|BL_ORD_ID|61577 transcript_118871	1	1902	1898	2602	432	2334	3308	4012
transcript_83307	gnl|BL_ORD_ID|29884 transcript_70444	1	2560	2560	3353	184	2743	2845	3638
transcript_83331	gnl|BL_ORD_ID|24707 transcript_4892	1	1704	1704	2170	272	1975	2819	3285
transcript_83331	gnl|BL_ORD_ID|18145 transcript_53013	1	1414	1410	2170	280	1692	3392	4152
transcript_83337	gnl|BL_ORD_ID|38126 transcript_6958	1	2323	2322	2919	2	2324	2485	3082
transcript_83337	gnl|BL_ORD_ID|38084 transcript_6869	1	2323	2322	2888	65	2383	2544	3110
transcript_83337	gnl|BL_ORD_ID|12247 transcript_3408	1	2323	2322	3002	4	2326	2487	3167
transcript_83337	gnl|BL_ORD_ID|25427 transcript_6388	1	2323	2322	3002	2	2318	2479	3158
transcript_83367	gnl|BL_ORD_ID|78538 transcript_14905	1012	2098	1	1013	1532	2617	55	1069
transcript_83367	gnl|BL_ORD_ID|50798 transcript_102678	11	1144	1141	2098	20	1120	1660	2616
transcript_83367	gnl|BL_ORD_ID|43905 transcript_91507	1	1668	1665	2122	2	1684	1811	2268
transcript_83387	gnl|BL_ORD_ID|47239 transcript_97010	229	2425	1	229	373	2570	1	229
transcript_83405	gnl|BL_ORD_ID|44419 transcript_92401	1	3669	3667	4630	184	3859	5153	6119
transcript_83454	gnl|BL_ORD_ID|46792 transcript_96309	1	1818	1817	2435	2187	4021	4325	4943
transcript_83483	gnl|BL_ORD_ID|68249 transcript_128080	1	1654	1653	2890	2	1655	1763	3001
transcript_83503	gnl|BL_ORD_ID|19811 transcript_55723	1	1958	1957	2423	855	2806	2932	3404
transcript_83503	gnl|BL_ORD_ID|48834 transcript_99540	1	1829	1828	2346	273	2100	2777	3295
transcript_83542	gnl|BL_ORD_ID|12300 transcript_3511	1	1704	1699	2470	1	1702	2736	3509
transcript_83544	gnl|BL_ORD_ID|27421 transcript_66479	1139	3017	1	1143	2774	4652	1472	2614
transcript_83577	gnl|BL_ORD_ID|65719 transcript_123968	13	2010	2008	2416	2	1998	2438	2846
transcript_83577	gnl|BL_ORD_ID|38617 transcript_8021	2	2010	2008	2416	11	2024	2463	2871
transcript_83600	gnl|BL_ORD_ID|27390 transcript_66430	1	1206	1204	2341	1257	2463	2576	3713
transcript_8363	gnl|BL_ORD_ID|72735 transcript_135344	1515	2952	114	1516	2838	4274	2	1403
transcript_83712	gnl|BL_ORD_ID|50963 transcript_102956	19	3043	3039	3550	2	3024	5395	5906
transcript_8372	gnl|BL_ORD_ID|38122 transcript_6951	1	2389	2386	2980	2	2389	2499	3093
transcript_83744	gnl|BL_ORD_ID|75450 transcript_139800	1	1860	1858	2213	1	1854	1973	2329
transcript_83759	gnl|BL_ORD_ID|28242 transcript_67834	204	4235	2	205	349	4399	39	242
transcript_83766	gnl|BL_ORD_ID|33097 transcript_75664	2	2829	2828	5203	1	2827	3125	5502
transcript_83821	gnl|BL_ORD_ID|90768 transcript_15168	1	1953	1953	2153	40	1991	2101	2301
transcript_83821	gnl|BL_ORD_ID|77906 transcript_13407	1	1953	1953	2153	2	1953	2063	2263
transcript_83821	gnl|BL_ORD_ID|83037 transcript_150810	1	1953	1953	2153	2	1953	2063	2264
transcript_8384	gnl|BL_ORD_ID|73053 transcript_135845	16	2186	2186	2973	2	2166	2700	3484

transcript_83897	gnl|BL_ORD_ID|926 transcript_1652	230	3327	2	233	427	3514	1	228
transcript_83897	gnl|BL_ORD_ID|29591 transcript_69977	230	3327	2	233	447	3534	21	248
transcript_83926	gnl|BL_ORD_ID|64884 transcript_11796	316	2452	34	318	533	2666	2	304
transcript_8399	gnl|BL_ORD_ID|34111 transcript_77323	1	2445	2445	2985	1	2444	2603	3143
transcript_83998	gnl|BL_ORD_ID|40946 transcript_86699	1	2581	2578	2786	1254	3839	3949	4157
transcript_84045	gnl|BL_ORD_ID|6072 transcript_31982	399	1725	7	399	517	1844	2	393
transcript_8406	gnl|BL_ORD_ID|65868 transcript_124206	1	2490	2490	2964	2	2516	3326	3817
transcript_84071	gnl|BL_ORD_ID|84345 transcript_153121	281	1748	56	280	775	2242	71	295
transcript_84106	gnl|BL_ORD_ID|47184 transcript_96920	1245	2401	110	1248	1253	2409	1	1139
transcript_84115	gnl|BL_ORD_ID|42318 transcript_88915	111	2228	2227	3087	3	2122	2231	3094
transcript_84115	gnl|BL_ORD_ID|66345 transcript_125012	1	1883	1884	3056	1	1886	2540	3701
transcript_84129	gnl|BL_ORD_ID|25157 transcript_5837	255	2866	2	258	568	3179	29	286
transcript_84129	gnl|BL_ORD_ID|25909 transcript_64118	255	2866	2	255	375	2986	9	262
transcript_84129	gnl|BL_ORD_ID|38249 transcript_7213	255	2866	2	258	453	3064	4	261
transcript_84139	gnl|BL_ORD_ID|75207 transcript_139399	1	2424	2422	3041	1	2424	2773	3396
transcript_84140	gnl|BL_ORD_ID|17136 transcript_51431	1	2338	2335	2823	1057	3398	3532	4020
transcript_84151	gnl|BL_ORD_ID|53879 transcript_106127	2	2968	2968	4006	203	3166	3272	4310
transcript_84151	gnl|BL_ORD_ID|96607 transcript_78833	2	2767	2764	3974	61	2823	2940	4149
transcript_84155	gnl|BL_ORD_ID|12000 transcript_2937	2	3280	3280	3508	74	3339	3218	3446
transcript_84164	gnl|BL_ORD_ID|1399 transcript_21878	1	1939	1938	2132	75	2016	2123	2316
transcript_84164	gnl|BL_ORD_ID|1692 transcript_22596	1	1939	1938	2157	2	1942	2049	2267
transcript_84175	gnl|BL_ORD_ID|66265 transcript_124881	1004	3820	1	1004	2255	5063	1	1022
transcript_84206	gnl|BL_ORD_ID|36631 transcript_81399	1	1958	1958	2340	427	2384	2514	2902
transcript_84210	gnl|BL_ORD_ID|35266 transcript_79209	2	2992	2991	3208	1	2967	3524	3742
transcript_84210	gnl|BL_ORD_ID|11595 transcript_2142	25	2992	2991	3215	1	2947	3506	3730
transcript_84210	gnl|BL_ORD_ID|1063 transcript_1907	2	2992	2991	3215	6	2999	3556	3781
transcript_84294	gnl|BL_ORD_ID|24472 transcript_4393	1	1351	1348	1709	1	1351	2923	3284
transcript_84294	gnl|BL_ORD_ID|86641 transcript_156864	106	1709	1	107	894	2497	1	107
transcript_84294	gnl|BL_ORD_ID|4732 transcript_29210	1	1263	1258	1709	2	1263	1517	1971
transcript_84324	gnl|BL_ORD_ID|80525 transcript_146511	1	1241	1242	1733	1	1256	2600	3093
transcript_84327	gnl|BL_ORD_ID|45322 transcript_93902	2	2418	2421	2585	9	2431	1860	2024
transcript_8435	gnl|BL_ORD_ID|50059 transcript_101481	2	2011	2011	2861	177	2186	2499	3345
transcript_8435	gnl|BL_ORD_ID|38059 transcript_6824	2	2011	2011	2851	1	2009	2277	3114
transcript_8435	gnl|BL_ORD_ID|17723 transcript_52357	2	2011	2011	2888	167	2176	2450	3326

transcript_8435	gnl|BL_ORD_ID|36956 transcript_81895	2	2011	2010	2970	176	2184	3907	4866
transcript_84417	gnl|BL_ORD_ID|87452 transcript_158201	2	3238	3236	3629	155	3390	3714	4107
transcript_84417	gnl|BL_ORD_ID|624 transcript_1069	1	3238	3236	3675	1	3240	3564	4003
transcript_84467	gnl|BL_ORD_ID|42777 transcript_89649	212	2899	2	212	539	3227	187	397
transcript_84469	gnl|BL_ORD_ID|80193 transcript_145965	1	2667	2664	2960	815	3500	3628	3924
transcript_84469	gnl|BL_ORD_ID|50138 transcript_101608	1	2667	2664	2896	747	3432	3561	3796
transcript_8448	gnl|BL_ORD_ID|22817 transcript_60726	2	2787	2786	2958	6	2793	2897	3069
transcript_84481	gnl|BL_ORD_ID|24021 transcript_62692	325	2469	5	328	1268	3409	33	350
transcript_84481	gnl|BL_ORD_ID|71489 transcript_133359	325	2468	51	327	442	2582	2	272
transcript_84483	gnl|BL_ORD_ID|655 transcript_1129	358	3931	1	360	501	4071	2	372
transcript_84496	gnl|BL_ORD_ID|21479 transcript_58481	1	1375	1370	2487	6	1422	3170	4277
transcript_84546	gnl|BL_ORD_ID|62633 transcript_120571	1	1282	1277	2051	104	1384	1595	2383
transcript_84613	gnl|BL_ORD_ID|11794 transcript_2539	1249	3541	1	1250	1389	3682	2	1250
transcript_84630	gnl|BL_ORD_ID|48603 transcript_99158	383	3173	43	383	527	3310	1	340
transcript_84633	gnl|BL_ORD_ID|27828 transcript_67133	325	1225	6	324	1403	2302	1	319
transcript_84705	gnl|BL_ORD_ID|1012 transcript_1801	1	2642	2643	3644	2	2643	2838	3842
transcript_8473	gnl|BL_ORD_ID|81361 transcript_147863	839	3015	91	840	1015	3191	2	745
transcript_84742	gnl|BL_ORD_ID|41726 transcript_88002	1	2877	2877	3501	2	2912	4200	4823
transcript_84788	gnl|BL_ORD_ID|20036 transcript_56071	1	1240	1239	2020	2	1240	2139	2919
transcript_84788	gnl|BL_ORD_ID|52236 transcript_103394	1	1414	1409	2019	2	1415	2332	2944
transcript_84791	gnl|BL_ORD_ID|73052 transcript_135844	194	2119	18	195	611	2535	1	176
transcript_84849	gnl|BL_ORD_ID|32154 transcript_74187	1	1736	1736	2890	1130	2864	2994	4148
transcript_84849	gnl|BL_ORD_ID|92803 transcript_165208	1	1736	1736	2905	4274	6008	6138	7307
transcript_84895	gnl|BL_ORD_ID|64152 transcript_123003	472	4295	5	473	653	4478	2	470
transcript_85023	gnl|BL_ORD_ID|38776 transcript_8385	10	2013	2010	2530	2	2005	2445	2967
transcript_85023	gnl|BL_ORD_ID|87989 transcript_159066	10	2013	2010	2530	2	2006	2202	2724
transcript_85034	gnl|BL_ORD_ID|91224 transcript_16165	166	2214	1	167	275	2323	2	168
transcript_85074	gnl|BL_ORD_ID|19583 transcript_55365	188	2632	17	186	369	2809	4	173
transcript_85075	gnl|BL_ORD_ID|19826 transcript_55751	1	1993	1991	2584	55	2046	2176	2771
transcript_85083	gnl|BL_ORD_ID|90449 transcript_162935	1	1077	1072	1431	176	1252	1721	2081
transcript_85140	gnl|BL_ORD_ID|46892 transcript_96453	211	3079	2	210	420	3287	81	289
transcript_85140	gnl|BL_ORD_ID|12511 transcript_3914	211	3127	2	210	523	3439	173	381
transcript_85140	gnl|BL_ORD_ID|12582 transcript_4069	211	3154	2	210	431	3379	81	289
transcript_85216	gnl|BL_ORD_ID|51386 transcript_9001	408	1354	44	411	2053	1106	2759	2392

transcript_85216	gnl|BL_ORD_ID|76487 transcript_141459	408	1354	53	411	2048	1101	2743	2387
transcript_8522	gnl|BL_ORD_ID|12571 transcript_4051	1	1955	1951	2958	2	1935	2391	3396
transcript_85253	gnl|BL_ORD_ID|48851 transcript_99571	2	2354	2353	2764	5	2359	2494	2906
transcript_85329	gnl|BL_ORD_ID|19518 transcript_55254	1	1170	1171	2056	94	1262	1526	2410
transcript_8537	gnl|BL_ORD_ID|64305 transcript_123228	1	2365	2363	3024	1	2364	3132	3795
transcript_85425	gnl|BL_ORD_ID|67578 transcript_127011	2	2197	2196	2656	47	2237	2572	3029
transcript_85427	gnl|BL_ORD_ID|56739 transcript_111016	1	1158	1157	1386	7	1164	1320	1546
transcript_8543	gnl|BL_ORD_ID|66880 transcript_125876	1088	3039	107	1087	1204	3157	89	1073
transcript_85430	gnl|BL_ORD_ID|40768 transcript_86415	1	1664	1664	2271	129	1782	1907	2495
transcript_85463	gnl|BL_ORD_ID|79772 transcript_145276	1	3151	3149	4151	675	3813	5110	6112
transcript_85465	gnl|BL_ORD_ID|35571 transcript_79673	2	2351	2350	2832	31	2379	2488	2970
transcript_85527	gnl|BL_ORD_ID|71763 transcript_133806	1	2003	1999	2227	143	2133	2283	2511
transcript_85527	gnl|BL_ORD_ID|91147 transcript_16007	1	2003	1999	2227	68	2072	2222	2450
transcript_85527	gnl|BL_ORD_ID|91392 transcript_16533	1	2003	1999	2227	66	2060	2210	2438
transcript_85527	gnl|BL_ORD_ID|52392 transcript_103647	1	2003	1999	2227	1	2005	2155	2383
transcript_85577	gnl|BL_ORD_ID|29625 transcript_70026	2	2504	2501	2806	82	2584	2745	3050
transcript_85578	gnl|BL_ORD_ID|90269 transcript_162686	118	2904	2903	3498	1	2778	3182	3777
transcript_85584	gnl|BL_ORD_ID|76016 transcript_140716	282	4033	2	285	587	4349	28	311
transcript_85591	gnl|BL_ORD_ID|77697 transcript_12943	188	1357	2	189	1521	2690	1	188
transcript_85591	gnl|BL_ORD_ID|1676 transcript_22556	188	1335	2	189	1124	2271	1	188
transcript_85591	gnl|BL_ORD_ID|81413 transcript_147941	188	1322	3	188	747	1884	1	187
transcript_85591	gnl|BL_ORD_ID|3971 transcript_27571	188	1355	3	188	924	2097	1	184
transcript_85591	gnl|BL_ORD_ID|51344 transcript_8923	188	1372	2	189	1734	2918	1	188
transcript_85591	gnl|BL_ORD_ID|7994 transcript_35883	188	1353	2	191	522	1693	1	188
transcript_85591	gnl|BL_ORD_ID|91608 transcript_17025	188	1372	3	189	1321	2505	1	187
transcript_85591	gnl|BL_ORD_ID|6649 transcript_33231	188	1336	2	188	696	1844	1	186
transcript_85622	gnl|BL_ORD_ID|90895 transcript_15455	1	1752	1756	2015	542	2293	2133	2398
transcript_8564	gnl|BL_ORD_ID|25136 transcript_5803	1162	2935	10	1161	1410	3183	1	1152
transcript_85663	gnl|BL_ORD_ID|984 transcript_1751	1	2279	2278	3404	1	2279	2467	3593
transcript_85667	gnl|BL_ORD_ID|92062 transcript_163996	1	1248	1246	1783	8	1254	1524	2061
transcript_85684	gnl|BL_ORD_ID|62717 transcript_120698	1	1677	1673	2099	1	1677	2221	2648
transcript_85692	gnl|BL_ORD_ID|49798 transcript_101060	2	2864	2862	3284	44	2905	3710	4115
transcript_85692	gnl|BL_ORD_ID|28543 transcript_68341	2	2863	2860	3382	72	2933	3156	3660
transcript_85692	gnl|BL_ORD_ID|70395 transcript_131599	105	3393	1	105	286	3574	1	105

transcript_8576	gnl|BL_ORD_ID|33468 transcript_76243	1299	2966	1	1300	1778	3449	5	1297
transcript_85767	gnl|BL_ORD_ID|7923 transcript_35736	1	1097	1099	1478	2	1100	1271	1650
transcript_85767	gnl|BL_ORD_ID|7680 transcript_35296	1	1097	1096	1470	18	1114	1282	1656
transcript_85777	gnl|BL_ORD_ID|44170 transcript_91970	174	1156	2	177	2282	3262	29	200
transcript_85795	gnl|BL_ORD_ID|34507 transcript_77977	122	1907	1	124	459	2244	1	126
transcript_85795	gnl|BL_ORD_ID|92578 transcript_164838	122	1908	1	124	541	2326	1	124
transcript_85839	gnl|BL_ORD_ID|57407 transcript_112128	1	1290	1286	1972	6	1295	1551	2237
transcript_85839	gnl|BL_ORD_ID|94940 transcript_18332	1	1290	1286	1972	105	1394	1650	2336
transcript_85839	gnl|BL_ORD_ID|81123 transcript_147478	1	1290	1286	1972	2	1291	2182	2868
transcript_85839	gnl|BL_ORD_ID|90745 transcript_15120	1	1290	1286	1972	209	1498	1754	2440
transcript_8587	gnl|BL_ORD_ID|25365 transcript_6270	1031	2955	1	1034	1207	3131	1	1034
transcript_8587	gnl|BL_ORD_ID|87527 transcript_158325	1031	2953	1	1034	1195	3118	1	1022
transcript_85881	gnl|BL_ORD_ID|81950 transcript_148878	555	1151	7	554	2270	2863	2	548
transcript_85881	gnl|BL_ORD_ID|84589 transcript_153508	555	1132	8	554	2383	2961	123	663
transcript_85901	gnl|BL_ORD_ID|38156 transcript_7020	1	1496	1492	2512	1	1496	1970	2990
transcript_85901	gnl|BL_ORD_ID|64485 transcript_10952	1	1496	1492	2512	1	1497	1664	2684
transcript_85910	gnl|BL_ORD_ID|5848 transcript_31536	755	1727	8	755	861	1833	2	747
transcript_85915	gnl|BL_ORD_ID|38696 transcript_8195	1	1771	1770	2546	295	2079	2208	2988
transcript_85915	gnl|BL_ORD_ID|88747 transcript_160303	1	1771	1770	2577	144	1928	2057	2868
transcript_85915	gnl|BL_ORD_ID|38843 transcript_8529	1	1771	1770	2577	248	2017	2146	2952
transcript_85938	gnl|BL_ORD_ID|25222 transcript_5968	2	2295	2294	2948	109	2402	2529	3184
transcript_860	gnl|BL_ORD_ID|50979 transcript_102982	1	3720	3717	4234	2	3724	3919	4430
transcript_86030	gnl|BL_ORD_ID|46578 transcript_95988	124	2278	1	124	2639	4803	157	280
transcript_86030	gnl|BL_ORD_ID|55342 transcript_108658	124	2336	1	127	2617	4829	149	274
transcript_86030	gnl|BL_ORD_ID|98 transcript_144	124	2278	1	124	2593	4747	125	248
transcript_86030	gnl|BL_ORD_ID|77011 transcript_142295	124	2246	1	124	2577	4699	109	232
transcript_8608	gnl|BL_ORD_ID|85091 transcript_154320	194	2944	12	196	335	3084	2	185
transcript_86090	gnl|BL_ORD_ID|34844 transcript_78522	1	1931	1931	3110	2	1932	3636	4796
transcript_86090	gnl|BL_ORD_ID|48492 transcript_98980	1454	3110	1	1456	1725	3362	65	1520
transcript_86090	gnl|BL_ORD_ID|69551 transcript_130194	16	1930	1931	3110	2	1917	2048	3205
transcript_86090	gnl|BL_ORD_ID|88511 transcript_159902	1	1931	1931	3110	6	1929	3233	4392
transcript_86099	gnl|BL_ORD_ID|83415 transcript_151464	1429	3151	1	1431	2547	4269	2	1432
transcript_86123	gnl|BL_ORD_ID|63528 transcript_122003	162	1522	42	162	1331	2713	28	148
transcript_86156	gnl|BL_ORD_ID|64086 transcript_122889	2	3715	3716	5131	7	3725	3912	5327

transcript_86186	gnl|BL_ORD_ID|46577 transcript_95986	1	1222	1218	2394	92	1320	1453	2631
transcript_86186	gnl|BL_ORD_ID|90859 transcript_15382	11	1222	1218	2394	2	1210	1342	2520
transcript_86186	gnl|BL_ORD_ID|91381 transcript_16515	1	1222	1218	2346	65	1281	1413	2546
transcript_86214	gnl|BL_ORD_ID|38811 transcript_8450	1	1235	1233	1661	21	1260	2167	2596
transcript_86226	gnl|BL_ORD_ID|586 transcript_995	1295	3206	1	1296	2091	4000	560	1850
transcript_86226	gnl|BL_ORD_ID|20969 transcript_57619	1295	3206	1	1296	2167	4075	636	1926
transcript_86226	gnl|BL_ORD_ID|583 transcript_986	1295	3206	1	1296	2084	3991	552	1843
transcript_86226	gnl|BL_ORD_ID|766 transcript_1337	1295	3206	1	1296	2080	3968	549	1839
transcript_8625	gnl|BL_ORD_ID|57766 transcript_112744	1410	2972	133	1410	1508	3071	1	1278
transcript_86312	gnl|BL_ORD_ID|77726 transcript_13011	1	1445	1440	2095	6	1484	1764	2434
transcript_86312	gnl|BL_ORD_ID|90728 transcript_15079	1	1445	1440	2095	219	1663	1930	2585
transcript_86312	gnl|BL_ORD_ID|39925 transcript_85039	1	1445	1440	2095	2	1446	1713	2368
transcript_86318	gnl|BL_ORD_ID|64798 transcript_11625	19	2287	2285	2425	163	2428	2631	2776
transcript_86318	gnl|BL_ORD_ID|68725 transcript_128857	21	2287	2285	2425	11	2273	2475	2620
transcript_86318	gnl|BL_ORD_ID|64619 transcript_11238	2	2287	2282	2425	146	2426	2629	2777
transcript_86382	gnl|BL_ORD_ID|61626 transcript_118949	186	1763	27	188	1546	3125	33	193
transcript_86453	gnl|BL_ORD_ID|96083 transcript_20982	1	1086	1081	1880	3	1088	1324	2123
transcript_86453	gnl|BL_ORD_ID|89662 transcript_161758	1	1083	1081	1880	29	1111	1348	2147
transcript_86453	gnl|BL_ORD_ID|59322 transcript_115271	1	1086	1081	1880	1	1086	1322	2122
transcript_86472	gnl|BL_ORD_ID|65207 transcript_12567	17	2055	2051	2598	2	2023	2161	2708
transcript_86472	gnl|BL_ORD_ID|17341 transcript_51762	113	2053	2051	2621	1	1936	2075	2643
transcript_86477	gnl|BL_ORD_ID|21267 transcript_58130	1	1160	1156	2162	1	1171	2762	3769
transcript_86487	gnl|BL_ORD_ID|38817 transcript_8462	1	2006	2006	2274	1	2007	2696	2964
transcript_86504	gnl|BL_ORD_ID|63115 transcript_121329	157	1312	2	158	1134	2289	89	245
transcript_86522	gnl|BL_ORD_ID|12571 transcript_4051	1	1907	1903	2907	6	1935	2391	3396
transcript_86564	gnl|BL_ORD_ID|31187 transcript_72559	1	1867	1866	3620	47	1913	2093	3845
transcript_86619	gnl|BL_ORD_ID|4214 transcript_28110	214	1654	22	215	602	2042	2	195
transcript_86644	gnl|BL_ORD_ID|24535 transcript_4514	2	2588	2586	2686	142	2733	3221	3321
transcript_86644	gnl|BL_ORD_ID|50438 transcript_102078	1	2588	2586	2686	1	2590	3076	3176
transcript_86644	gnl|BL_ORD_ID|19271 transcript_54821	2	2588	2586	2686	1	2595	3082	3182
transcript_86647	gnl|BL_ORD_ID|46828 transcript_96362	252	2537	10	253	368	2576	1	244
transcript_86659	gnl|BL_ORD_ID|68546 transcript_128564	1	1895	1894	2531	1752	3646	3767	4404
transcript_86672	gnl|BL_ORD_ID|70081 transcript_131070	1	1036	1037	1500	2	1032	1171	1631
transcript_86674	gnl|BL_ORD_ID|68367 transcript_128287	1196	2707	10	1197	1306	2818	2	1189

transcript_86695	gnl|BL_ORD_ID|1082 transcript_1938	1	2360	2355	3029	56	2427	3097	3784
transcript_86745	gnl|BL_ORD_ID|18441 transcript_53486	1	1685	1686	2027	54	1736	1846	2188
transcript_86758	gnl|BL_ORD_ID|76224 transcript_141068	1	1320	1319	1973	1	1313	1428	2081
transcript_86766	gnl|BL_ORD_ID|79497 transcript_144842	15	2157	2154	2327	1	2143	2276	2449
transcript_86803	gnl|BL_ORD_ID|92636 transcript_164934	1	1222	1219	2110	133	1331	1798	2689
transcript_86824	gnl|BL_ORD_ID|62919 transcript_121005	1	2818	2818	4116	117	2928	3040	4344
transcript_86824	gnl|BL_ORD_ID|48032 transcript_98244	11	2818	2818	4134	2	2814	2926	4244
transcript_86827	gnl|BL_ORD_ID|4903 transcript_29511	1	1037	1033	1768	1	1044	1258	1994
transcript_86833	gnl|BL_ORD_ID|26456 transcript_64946	1	1425	1424	2724	648	2072	2278	3566
transcript_86916	gnl|BL_ORD_ID|3441 transcript_26414	1	1070	1066	1848	150	1225	1326	2116
transcript_86976	gnl|BL_ORD_ID|75701 transcript_140196	1109	2968	1	1109	1591	3438	1	1126
transcript_86976	gnl|BL_ORD_ID|46078 transcript_95176	1	1671	1672	3016	1	1667	1867	3211
transcript_86999	gnl|BL_ORD_ID|80675 transcript_146760	1	1377	1373	2161	1811	3187	3438	4215
transcript_87015	gnl|BL_ORD_ID|24641 transcript_4734	1	2178	2176	2761	1	2181	2737	3323
transcript_87027	gnl|BL_ORD_ID|87678 transcript_158564	1	2262	2261	2758	1	2253	3595	4091
transcript_87074	gnl|BL_ORD_ID|24919 transcript_5309	346	2819	4	349	573	3046	3	348
transcript_87076	gnl|BL_ORD_ID|30788 transcript_71932	1606	3367	1	1606	2359	4118	145	1741
transcript_87097	gnl|BL_ORD_ID|20574 transcript_56949	2	2148	2148	2996	56	2202	2451	3306
transcript_8711	gnl|BL_ORD_ID|40047 transcript_85236	1	2463	2461	2949	1	2464	2612	3102
transcript_87120	gnl|BL_ORD_ID|77325 transcript_142807	1097	2238	1	1098	1581	2722	2	1099
transcript_87169	gnl|BL_ORD_ID|40436 transcript_85841	1	1135	1133	1489	69	1204	2120	2476
transcript_87177	gnl|BL_ORD_ID|22886 transcript_60854	1	2803	2802	3126	546	3351	3992	4317
transcript_8720	gnl|BL_ORD_ID|67941 transcript_127593	2	2581	2582	2912	8	2589	2692	3024
transcript_87254	gnl|BL_ORD_ID|9964 transcript_39804	121	1040	6	123	478	1370	2	119
transcript_87254	gnl|BL_ORD_ID|23020 transcript_61066	121	1040	6	123	478	1398	2	119
transcript_87273	gnl|BL_ORD_ID|48747 transcript_99404	1	1309	1307	1810	2	1310	1566	2074
transcript_87303	gnl|BL_ORD_ID|25550 transcript_63509	1	2222	2222	3283	98	2314	2648	3708
transcript_87376	gnl|BL_ORD_ID|30924 transcript_72141	306	2866	34	309	505	3065	2	277
transcript_87379	gnl|BL_ORD_ID|56653 transcript_110881	2	2085	2083	2927	27	2109	2515	3361
transcript_874	gnl|BL_ORD_ID|248 transcript_402	1	3420	3418	4028	1	3417	4025	4635
transcript_87413	gnl|BL_ORD_ID|12266 transcript_3437	1	1896	1897	2778	452	2358	2479	3360
transcript_87413	gnl|BL_ORD_ID|11772 transcript_2480	1	1896	1897	2778	536	2427	2548	3430
transcript_87413	gnl|BL_ORD_ID|11669 transcript_2281	1	1896	1897	2777	519	2413	2534	3417
transcript_87429	gnl|BL_ORD_ID|91153 transcript_16017	1	2252	2248	2427	1	2251	2362	2540

transcript_87452	gnl|BL_ORD_ID|93763 transcript_166748	2	3377	3375	3884	55	3435	4795	5304
transcript_87539	gnl|BL_ORD_ID|53797 transcript_105996	143	2378	1	143	340	2574	90	233
transcript_8762	gnl|BL_ORD_ID|12571 transcript_4051	1	1951	1947	2948	3	1935	2391	3391
transcript_87632	gnl|BL_ORD_ID|22132 transcript_59575	192	1785	4	193	962	2567	71	260
transcript_87636	gnl|BL_ORD_ID|88017 transcript_159108	1	1446	1447	2106	1	1445	1945	2603
transcript_87637	gnl|BL_ORD_ID|29801 transcript_70303	2	2114	2111	3196	188	2299	2418	3507
transcript_87637	gnl|BL_ORD_ID|52335 transcript_103557	2	2115	2112	3206	13	2124	2457	3550
transcript_87649	gnl|BL_ORD_ID|30314 transcript_71159	1	2151	2152	2670	20	2170	2298	2816
transcript_8767	gnl|BL_ORD_ID|12026 transcript_2985	1	2091	2090	2878	1	2071	2713	3490
transcript_87746	gnl|BL_ORD_ID|1268 transcript_21565	1	1739	1739	2056	2	1740	1946	2263
transcript_87830	gnl|BL_ORD_ID|69417 transcript_129973	307	3165	2	310	610	3473	198	507
transcript_87936	gnl|BL_ORD_ID|11975 transcript_2883	1	1727	1724	2683	708	2437	2609	3566
transcript_87936	gnl|BL_ORD_ID|11885 transcript_2699	1	1727	1724	2683	731	2457	2629	3587
transcript_87985	gnl|BL_ORD_ID|11963 transcript_2858	1	2454	2453	3237	1	2458	2727	3513
transcript_87999	gnl|BL_ORD_ID|69931 transcript_130826	177	2288	1	177	285	2390	1	177
transcript_8812	gnl|BL_ORD_ID|38367 transcript_7481	219	2877	1	220	373	3031	1	220
transcript_88141	gnl|BL_ORD_ID|27471 transcript_66556	368	1457	6	370	1214	2303	2	365
transcript_88229	gnl|BL_ORD_ID|63324 transcript_121670	279	2654	5	281	894	3274	1	281
transcript_88260	gnl|BL_ORD_ID|48506 transcript_99003	1	2106	2102	2580	43	2150	2745	3223
transcript_88307	gnl|BL_ORD_ID|37165 transcript_82226	1	1394	1392	2190	293	1678	1961	2759
transcript_88317	gnl|BL_ORD_ID|49431 transcript_100484	1336	2673	1	1338	1673	3013	1	1367
transcript_8833	gnl|BL_ORD_ID|93407 transcript_166192	1077	2930	1	1077	3252	5105	22	1098
transcript_8833	gnl|BL_ORD_ID|92261 transcript_164308	1077	2923	1	1077	3047	4893	1	1077
transcript_88359	gnl|BL_ORD_ID|12142 transcript_3216	1	2710	2710	3379	8	2714	2821	3490
transcript_88442	gnl|BL_ORD_ID|29194 transcript_69360	712	2695	91	711	1465	3450	2	620
transcript_88489	gnl|BL_ORD_ID|64223 transcript_123105	1	2729	2727	3238	93	2836	3729	4240
transcript_88525	gnl|BL_ORD_ID|19374 transcript_54999	1	1751	1750	2310	2195	3943	4484	5047
transcript_88567	gnl|BL_ORD_ID|51559 transcript_9352	1	2328	2328	2748	1	2343	2454	2868
transcript_88567	gnl|BL_ORD_ID|51427 transcript_9092	1	2328	2328	2748	1	2322	2433	2855
transcript_88621	gnl|BL_ORD_ID|37908 transcript_6531	2	2620	2619	2965	20	2638	2751	3097
transcript_88626	gnl|BL_ORD_ID|17214 transcript_51556	512	2430	53	512	661	2582	2	461
transcript_88626	gnl|BL_ORD_ID|46416 transcript_95726	512	2437	93	512	621	2575	2	421
transcript_88629	gnl|BL_ORD_ID|91228 transcript_16172	1	1571	1570	2344	55	1626	1750	2520
transcript_88629	gnl|BL_ORD_ID|78464 transcript_14721	1	1571	1570	2349	50	1621	1821	2602

transcript_8863	gnl|BL_ORD_ID|27372 transcript_66406	106	1833	1834	2945	1	1728	2382	3493
transcript_8863	gnl|BL_ORD_ID|40864 transcript_86579	104	2178	2177	2945	2	2076	2185	2953
transcript_88687	gnl|BL_ORD_ID|30140 transcript_70869	269	2976	2	273	464	3171	34	305
transcript_88694	gnl|BL_ORD_ID|53879 transcript_106127	3	3175	3175	4182	1	3166	3272	4279
transcript_8872	gnl|BL_ORD_ID|31062 transcript_72378	1	2150	2150	2914	1	2154	2305	3069
transcript_8873	gnl|BL_ORD_ID|27086 transcript_65969	2	2714	2711	2919	7	2716	3313	3515
transcript_88743	gnl|BL_ORD_ID|58322 transcript_113641	326	2636	8	328	1326	3640	2	319
transcript_88766	gnl|BL_ORD_ID|73869 transcript_137222	1790	3758	1	1794	2041	4010	146	1940
transcript_88784	gnl|BL_ORD_ID|88686 transcript_160201	1	1334	1333	1622	1	1342	2166	2455
transcript_88801	gnl|BL_ORD_ID|66490 transcript_125246	1	3369	3365	4177	153	3527	4553	5358
transcript_88802	gnl|BL_ORD_ID|52481 transcript_103783	1	1474	1473	2165	531	2011	2205	2897
transcript_8886	gnl|BL_ORD_ID|951 transcript_1690	335	2927	69	334	1267	3855	2	267
transcript_88892	gnl|BL_ORD_ID|89933 transcript_162181	546	1809	91	547	884	2149	2	458
transcript_8890	gnl|BL_ORD_ID|34005 transcript_77133	1	1514	1515	2927	1	1513	1620	3032
transcript_88905	gnl|BL_ORD_ID|78376 transcript_14473	1	1321	1321	2198	1	1327	1738	2617
transcript_8891	gnl|BL_ORD_ID|38179 transcript_7066	270	2927	2	272	425	3082	53	324
transcript_88953	gnl|BL_ORD_ID|87705 transcript_158601	1713	3860	1	1714	1990	4146	168	1881
transcript_88962	gnl|BL_ORD_ID|84448 transcript_153286	1	2562	2557	3245	1037	3597	3723	4410
transcript_88963	gnl|BL_ORD_ID|24773 transcript_5025	110	2605	1	110	792	3290	90	199
transcript_8897	gnl|BL_ORD_ID|38204 transcript_7116	1	2066	2066	2927	1	2063	2188	3049
transcript_89030	gnl|BL_ORD_ID|52062 transcript_10436	2	2304	2303	2636	47	2352	2453	2786
transcript_89033	gnl|BL_ORD_ID|21450 transcript_58437	1	1862	1862	2786	196	2057	2180	3103
transcript_89060	gnl|BL_ORD_ID|12636 transcript_4176	229	3275	2	230	381	3431	1	232
transcript_89093	gnl|BL_ORD_ID|70034 transcript_130989	2	2471	2466	2721	25	2485	2702	2957
transcript_89093	gnl|BL_ORD_ID|93533 transcript_166385	2	2471	2466	2721	20	2504	2719	2974
transcript_8910	gnl|BL_ORD_ID|25109 transcript_5748	1	2635	2632	2920	1	2636	2912	3200
transcript_89102	gnl|BL_ORD_ID|30092 transcript_70791	1038	2622	1	1039	1667	3250	122	1172
transcript_89102	gnl|BL_ORD_ID|51798 transcript_9861	1	2311	2312	2622	78	2430	2549	2859
transcript_89102	gnl|BL_ORD_ID|73158 transcript_136006	1	2311	2312	2546	78	2416	2534	2767
transcript_89108	gnl|BL_ORD_ID|11889 transcript_2710	1	1663	1660	2073	65	1729	3188	3601
transcript_89140	gnl|BL_ORD_ID|84121 transcript_152726	1	1136	1136	1417	1	1137	1353	1634
transcript_8916	gnl|BL_ORD_ID|25425 transcript_6386	270	2805	12	272	593	3125	2	272
transcript_89194	gnl|BL_ORD_ID|45015 transcript_93389	1	1984	1983	2769	1	1981	2087	2872
transcript_89198	gnl|BL_ORD_ID|23257 transcript_61465	1	2724	2720	2923	325	3046	4061	4264

transcript_89198	gnl|BL_ORD_ID|74463 transcript_138183	1	2724	2720	3017	593	3314	4374	4671
transcript_8923	gnl|BL_ORD_ID|61584 transcript_118882	2	2083	2082	2903	1	2081	2294	3116
transcript_8923	gnl|BL_ORD_ID|27449 transcript_66524	25	2611	2610	2881	2	2604	2754	3026
transcript_89234	gnl|BL_ORD_ID|63344 transcript_121698	2	2865	2861	3171	1	2864	3114	3424
transcript_89234	gnl|BL_ORD_ID|12353 transcript_3596	1	2865	2861	3133	77	2947	3197	3469
transcript_89242	gnl|BL_ORD_ID|42885 transcript_89811	1153	2651	1	1155	1539	3033	1	1144
transcript_89285	gnl|BL_ORD_ID|78133 transcript_13951	1	2036	2035	2484	30	2074	2181	2630
transcript_89285	gnl|BL_ORD_ID|78172 transcript_14040	1	2036	2035	2490	36	2075	2182	2637
transcript_89285	gnl|BL_ORD_ID|77768 transcript_13085	1	2036	2035	2528	36	2071	2178	2671
transcript_8933	gnl|BL_ORD_ID|93079 transcript_165644	120	1955	1953	2933	2	1836	2397	3377
transcript_89384	gnl|BL_ORD_ID|815 transcript_1422	279	3717	1	278	438	3875	2	279
transcript_89384	gnl|BL_ORD_ID|852 transcript_1504	279	3713	1	278	448	3892	12	289
transcript_89384	gnl|BL_ORD_ID|79253 transcript_144456	279	3694	1	278	494	3941	58	335
transcript_89497	gnl|BL_ORD_ID|46408 transcript_95715	186	1314	23	185	1962	3090	2	164
transcript_89498	gnl|BL_ORD_ID|48834 transcript_99540	1	2066	2065	2582	38	2100	2777	3295
transcript_89498	gnl|BL_ORD_ID|19811 transcript_55723	1	2195	2194	2612	618	2806	2932	3357
transcript_89528	gnl|BL_ORD_ID|87988 transcript_159064	1	1117	1117	1860	23	1139	1624	2367
transcript_89569	gnl|BL_ORD_ID|76666 transcript_141745	11	1125	1124	1621	6	1115	1387	1887
transcript_89587	gnl|BL_ORD_ID|427 transcript_731	1	2290	2285	4024	51	2333	2496	4238
transcript_89596	gnl|BL_ORD_ID|93297 transcript_166011	1030	2888	1	1030	1695	3553	318	1347
transcript_89620	gnl|BL_ORD_ID|63850 transcript_122528	156	2041	1	157	797	2672	229	384
transcript_89627	gnl|BL_ORD_ID|18761 transcript_53989	1	2608	2606	3361	7	2614	3263	4018
transcript_89629	gnl|BL_ORD_ID|32170 transcript_74212	1	1237	1235	2434	1	1237	1513	2712
transcript_89660	gnl|BL_ORD_ID|94351 transcript_167665	1	1550	1546	2140	6	1555	1796	2390
transcript_89685	gnl|BL_ORD_ID|51560 transcript_9353	255	2410	78	256	556	2711	2	182
transcript_89704	gnl|BL_ORD_ID|68130 transcript_127900	126	2706	1	125	1993	4586	1569	1690
transcript_89731	gnl|BL_ORD_ID|43978 transcript_91652	1	1604	1602	2512	1	1608	2037	2946
transcript_89735	gnl|BL_ORD_ID|79220 transcript_144408	671	1910	94	675	2252	3491	1	582
transcript_89735	gnl|BL_ORD_ID|49398 transcript_100422	671	1910	74	675	2131	3368	2	603
transcript_89738	gnl|BL_ORD_ID|17956 transcript_52714	663	2448	80	665	726	2512	20	603
transcript_89769	gnl|BL_ORD_ID|66172 transcript_124724	118	2842	1	119	225	2948	2	120
transcript_89769	gnl|BL_ORD_ID|48616 transcript_99185	1	1522	1519	2842	2	1523	1688	3012
transcript_89792	gnl|BL_ORD_ID|55239 transcript_108474	1094	2949	1	1093	1943	3818	649	1741
transcript_8986	gnl|BL_ORD_ID|50476 transcript_102136	1	1984	1984	2916	1	1984	2260	3192

transcript_89870	gnl|BL_ORD_ID|27268 transcript_66244	1549	3333	125	1550	1574	3359	1	1429
transcript_89972	gnl|BL_ORD_ID|38781 transcript_8393	1095	2616	1	1097	1345	2866	25	1121
transcript_89989	gnl|BL_ORD_ID|89854 transcript_162048	152	1493	2	155	377	1718	29	183
transcript_89989	gnl|BL_ORD_ID|7018 transcript_33963	153	1545	2	155	261	1653	6	160
transcript_89989	gnl|BL_ORD_ID|7980 transcript_35848	152	1529	2	155	329	1706	75	229
transcript_90038	gnl|BL_ORD_ID|86507 transcript_156629	171	3134	1	170	283	3244	1	170
transcript_90055	gnl|BL_ORD_ID|46408 transcript_95715	170	1334	7	169	1962	3126	2	164
transcript_9007	gnl|BL_ORD_ID|25157 transcript_5837	255	2911	2	255	547	3203	29	282
transcript_9007	gnl|BL_ORD_ID|38249 transcript_7213	255	2887	2	255	432	3064	4	257
transcript_9008	gnl|BL_ORD_ID|62144 transcript_119758	105	1793	1790	2937	1	1688	1923	3069
transcript_90106	gnl|BL_ORD_ID|23406 transcript_61705	176	1452	2	176	1917	3218	151	328
transcript_90106	gnl|BL_ORD_ID|48587 transcript_99132	176	1432	2	176	1934	3226	145	325
transcript_90136	gnl|BL_ORD_ID|77960 transcript_13524	224	1003	6	226	1839	2631	17	239
transcript_90221	gnl|BL_ORD_ID|26550 transcript_65105	1	1852	1852	3488	1	1874	2839	4475
transcript_90240	gnl|BL_ORD_ID|91471 transcript_16713	180	2383	1	180	315	2518	20	197
transcript_90258	gnl|BL_ORD_ID|89136 transcript_160905	1	2382	2382	3109	368	2749	2856	3577
transcript_90267	gnl|BL_ORD_ID|45633 transcript_94422	1	1315	1311	1725	1476	2787	2960	3374
transcript_90267	gnl|BL_ORD_ID|12450 transcript_3794	1	1315	1311	1725	1417	2731	2903	3317
transcript_9031	gnl|BL_ORD_ID|42508 transcript_89204	228	2748	100	233	628	3147	1	134
transcript_90316	gnl|BL_ORD_ID|82717 transcript_150241	1	1201	1198	1935	54	1255	1889	2626
transcript_90347	gnl|BL_ORD_ID|41202 transcript_87123	14	2264	2265	2555	8	2270	2440	2730
transcript_9038	gnl|BL_ORD_ID|60536 transcript_117200	1215	2916	111	1215	1998	3699	1	1079
transcript_90397	gnl|BL_ORD_ID|76363 transcript_141266	1	2524	2521	3088	856	3370	3485	4050
transcript_90424	gnl|BL_ORD_ID|11758 transcript_2457	1	2024	2022	3574	1	2030	2159	3716
transcript_90430	gnl|BL_ORD_ID|42082 transcript_88539	296	3689	1	295	585	4002	151	445
transcript_90522	gnl|BL_ORD_ID|72337 transcript_134717	1	2097	2093	3457	104	2208	4660	6026
transcript_90538	gnl|BL_ORD_ID|40687 transcript_86273	366	1209	45	371	466	1304	2	328
transcript_90546	gnl|BL_ORD_ID|11795 transcript_2540	1	2186	2185	2949	84	2285	2914	3677
transcript_90546	gnl|BL_ORD_ID|79446 transcript_144760	1	2186	2187	2944	2	2187	2675	3427
transcript_90546	gnl|BL_ORD_ID|11738 transcript_2420	1	2186	2185	2948	84	2269	2898	3655
transcript_9055	gnl|BL_ORD_ID|38395 transcript_7539	2	2427	2428	2906	14	2431	2559	3037
transcript_90556	gnl|BL_ORD_ID|28737 transcript_68657	262	1508	4	264	1241	2489	4	264
transcript_90578	gnl|BL_ORD_ID|57448 transcript_112204	139	2455	1	143	397	2709	2	144
transcript_90606	gnl|BL_ORD_ID|1090 transcript_1962	334	1821	58	336	2298	3784	2	280

transcript_90646	gnl|BL_ORD_ID|61727 transcript_119087	1286	2657	1	1286	2227	3600	1	1312
transcript_90667	gnl|BL_ORD_ID|25849 transcript_64020	162	1434	2	163	542	1814	101	260
transcript_90717	gnl|BL_ORD_ID|92303 transcript_164383	1	1735	1733	1975	1	1738	2563	2805
transcript_90730	gnl|BL_ORD_ID|74186 transcript_137751	203	2281	1	203	523	2594	26	227
transcript_90807	gnl|BL_ORD_ID|58153 transcript_113338	573	3590	83	573	595	3634	2	492
transcript_90807	gnl|BL_ORD_ID|55239 transcript_108474	16	1734	1735	3590	23	1741	1943	3818
transcript_90900	gnl|BL_ORD_ID|46937 transcript_96530	1	2071	2066	2600	1	2068	3592	4126
transcript_9095	gnl|BL_ORD_ID|11692 transcript_2331	526	2914	58	531	1295	3685	2	475
transcript_90958	gnl|BL_ORD_ID|788 transcript_1374	1	1524	1521	2409	1	1524	2035	2923
transcript_90958	gnl|BL_ORD_ID|91108 transcript_15910	1	1524	1524	2395	1	1524	1706	2575
transcript_90958	gnl|BL_ORD_ID|31839 transcript_73628	1	1524	1524	2399	1	1524	1667	2541
transcript_90966	gnl|BL_ORD_ID|46477 transcript_95816	1	1866	1861	2140	652	2517	2678	2957
transcript_9097	gnl|BL_ORD_ID|26570 transcript_65137	386	2797	4	386	894	3304	7	388
transcript_91073	gnl|BL_ORD_ID|91699 transcript_163405	1	1292	1288	1886	13	1305	1417	2015
transcript_91089	gnl|BL_ORD_ID|94569 transcript_17446	1	1222	1223	1997	1	1228	1394	2168
transcript_9110	gnl|BL_ORD_ID|61174 transcript_118244	1054	2917	1	1057	1173	3036	1	1066
transcript_91159	gnl|BL_ORD_ID|60601 transcript_117297	2	3801	3799	4647	140	3939	5231	6080
transcript_91166	gnl|BL_ORD_ID|50396 transcript_102012	116	1605	1	117	851	2336	463	579
transcript_91166	gnl|BL_ORD_ID|94767 transcript_17913	115	1605	1	117	1027	2518	492	608
transcript_91199	gnl|BL_ORD_ID|671 transcript_1162	266	4008	18	265	361	4102	2	248
transcript_91210	gnl|BL_ORD_ID|58191 transcript_113402	207	1259	51	211	1287	2340	265	425
transcript_9122	gnl|BL_ORD_ID|93991 transcript_167102	258	2737	2	258	392	2871	24	282
transcript_91263	gnl|BL_ORD_ID|3015 transcript_25455	113	1841	1	116	427	2154	5	121
transcript_91283	gnl|BL_ORD_ID|91327 transcript_16399	1211	2409	1	1214	1342	2535	2	1234
transcript_91286	gnl|BL_ORD_ID|62939 transcript_121037	1	1715	1713	2415	201	1919	2079	2777
transcript_9132	gnl|BL_ORD_ID|71782 transcript_133838	1	1955	1951	2953	61	2009	3392	4394
transcript_9135	gnl|BL_ORD_ID|11967 transcript_2862	14	2255	2254	2921	22	2263	2890	3561
transcript_91354	gnl|BL_ORD_ID|64370 transcript_10708	1	1397	1396	2644	64	1469	1596	2844
transcript_91368	gnl|BL_ORD_ID|11601 transcript_2155	155	3353	1	156	526	3719	2	159
transcript_91381	gnl|BL_ORD_ID|43853 transcript_91412	159	2683	2678	3448	49	2570	3611	4383
transcript_91485	gnl|BL_ORD_ID|94225 transcript_167466	1	2113	2113	2865	1649	3764	4067	4819
transcript_915	gnl|BL_ORD_ID|31370 transcript_72860	1	2237	2233	4057	2	2233	2348	4156
transcript_91512	gnl|BL_ORD_ID|37981 transcript_6668	1	1392	1389	2579	2	1393	1932	3111
transcript_91512	gnl|BL_ORD_ID|83457 transcript_151541	1	1392	1389	2579	2	1393	1932	3111

transcript_91517	gnl|BL_ORD_ID|69799 transcript_130603	141	1700	1	141	756	2303	1	133
transcript_91591	gnl|BL_ORD_ID|96220 transcript_21261	1	1124	1124	2178	2	1127	1246	2297
transcript_916	gnl|BL_ORD_ID|45032 transcript_93414	2	3760	3760	4139	299	4057	4650	5029
transcript_9161	gnl|BL_ORD_ID|69774 transcript_130570	1	1611	1609	2839	9	1619	2425	3654
transcript_9168	gnl|BL_ORD_ID|38515 transcript_7778	134	2910	1	134	241	3013	1	134
transcript_91686	gnl|BL_ORD_ID|96150 transcript_21122	267	2109	3	268	443	2288	2	267
transcript_91705	gnl|BL_ORD_ID|73916 transcript_137291	1	1038	1039	2021	1571	2630	2748	3730
transcript_91705	gnl|BL_ORD_ID|44217 transcript_92049	1	1079	1076	2021	1622	2724	3970	4915
transcript_91707	gnl|BL_ORD_ID|78015 transcript_13660	1	1295	1295	2504	2	1294	1417	2630
transcript_91707	gnl|BL_ORD_ID|78344 transcript_14398	1	1295	1295	2494	2	1296	1419	2622
transcript_91769	gnl|BL_ORD_ID|36305 transcript_80870	176	2146	1	179	843	2813	2	180
transcript_91774	gnl|BL_ORD_ID|51471 transcript_9166	1	1909	1908	2760	1	1909	2038	2883
transcript_91774	gnl|BL_ORD_ID|51930 transcript_10146	1	1909	1908	2760	1	1907	2034	2862
transcript_91841	gnl|BL_ORD_ID|94841 transcript_18098	160	2281	1	160	339	2456	2	161
transcript_9187	gnl|BL_ORD_ID|19950 transcript_55944	1	2625	2624	2928	6	2642	3415	3719
transcript_919	gnl|BL_ORD_ID|42025 transcript_88455	347	4156	23	348	1393	5202	1	326
transcript_91904	gnl|BL_ORD_ID|83016 transcript_150778	581	4048	62	581	620	4085	1	520
transcript_91952	gnl|BL_ORD_ID|59529 transcript_115606	204	2894	1	203	417	3099	72	274
transcript_91973	gnl|BL_ORD_ID|97448 transcript_160340	11	2231	2230	3034	1	2201	2707	3511
transcript_92083	gnl|BL_ORD_ID|24601 transcript_4647	1	2487	2483	3016	6	2492	2678	3211
transcript_92083	gnl|BL_ORD_ID|33024 transcript_75561	2	2487	2484	3026	1	2486	3219	3761
transcript_92096	gnl|BL_ORD_ID|50041 transcript_101449	1	2174	2173	3050	4	2178	2483	3360
transcript_92100	gnl|BL_ORD_ID|83562 transcript_151735	1138	2751	1	1142	1419	3032	1	1142
transcript_92112	gnl|BL_ORD_ID|26464 transcript_64957	1002	2395	1	1005	1399	2798	1	1009
transcript_92131	gnl|BL_ORD_ID|2548 transcript_24440	1	1405	1404	2108	1	1405	1510	2214
transcript_92144	gnl|BL_ORD_ID|90777 transcript_15186	1	1385	1382	2080	51	1446	1875	2573
transcript_92172	gnl|BL_ORD_ID|42081 transcript_88537	998	2397	4	1001	1891	3288	687	1690
transcript_92182	gnl|BL_ORD_ID|170 transcript_270	1	2107	2107	2444	2305	4411	4511	4848
transcript_92182	gnl|BL_ORD_ID|174 transcript_275	1	2107	2107	2444	2314	4420	4521	4858
transcript_92182	gnl|BL_ORD_ID|148 transcript_225	1	2107	2107	2444	2353	4459	4559	4896
transcript_92182	gnl|BL_ORD_ID|82681 transcript_150180	1	2107	2107	2444	1260	3365	3465	3802
transcript_92182	gnl|BL_ORD_ID|32324 transcript_74470	1	2107	2107	2393	157	2263	2364	2650
transcript_92182	gnl|BL_ORD_ID|221 transcript_351	1	2107	2107	2357	2287	4393	4493	4743
transcript_92207	gnl|BL_ORD_ID|78147 transcript_13983	1035	2388	1	1034	1300	2650	116	1149

transcript_92215	gnl|BL_ORD_ID|3645 transcript_26869	370	1844	5	371	607	2081	99	465
transcript_92228	gnl|BL_ORD_ID|84302 transcript_153045	1	1811	1812	2157	3627	5443	5643	5988
transcript_92286	gnl|BL_ORD_ID|64533 transcript_11054	1	2103	2101	2644	2	2106	2227	2793
transcript_92294	gnl|BL_ORD_ID|33734 transcript_76687	1167	3060	1	1169	1836	3731	538	1706
transcript_92377	gnl|BL_ORD_ID|19665 transcript_55491	1	2265	2260	2787	1	2275	2443	2972
transcript_92381	gnl|BL_ORD_ID|87408 transcript_158134	341	3849	2	340	529	4037	36	374
transcript_92421	gnl|BL_ORD_ID|37920 transcript_6551	10	1712	1711	2863	14	1718	1957	3109
transcript_92426	gnl|BL_ORD_ID|39292 transcript_84015	1	1441	1439	2028	1	1451	3079	3671
transcript_92427	gnl|BL_ORD_ID|8293 transcript_36475	131	1526	1	132	270	1662	1	132
transcript_9243	gnl|BL_ORD_ID|88241 transcript_159476	1	2084	2085	2820	1	2084	2308	3043
transcript_9243	gnl|BL_ORD_ID|1151 transcript_2067	1	1945	1942	2820	1	1945	2868	3746
transcript_9249	gnl|BL_ORD_ID|92518 transcript_164742	1	1498	1498	2880	1	1497	1607	2988
transcript_92495	gnl|BL_ORD_ID|87063 transcript_157556	13	1521	1519	1831	1	1535	1677	1989
transcript_9251	gnl|BL_ORD_ID|49316 transcript_100291	371	2832	9	372	495	2956	2	364
transcript_9254	gnl|BL_ORD_ID|37431 transcript_82652	14	2588	2586	2900	2	2572	2836	3151
transcript_92562	gnl|BL_ORD_ID|95184 transcript_18893	13	1760	1758	1968	340	2090	2240	2450
transcript_92562	gnl|BL_ORD_ID|95325 transcript_19210	13	1760	1758	1968	266	2016	2166	2376
transcript_92562	gnl|BL_ORD_ID|91076 transcript_15850	1	1760	1758	1968	403	2165	2315	2525
transcript_92562	gnl|BL_ORD_ID|86967 transcript_157402	1	1760	1757	1968	459	2221	2370	2581
transcript_92562	gnl|BL_ORD_ID|90972 transcript_15620	1	1760	1757	1968	453	2214	2363	2574
transcript_92576	gnl|BL_ORD_ID|85154 transcript_154411	1586	3806	118	1586	1883	4104	1	1468
transcript_92576	gnl|BL_ORD_ID|82837 transcript_150442	1586	3806	1	1585	3899	6117	2010	3590
transcript_9266	gnl|BL_ORD_ID|38251 transcript_7216	208	2817	2	211	444	3056	1	210
transcript_9266	gnl|BL_ORD_ID|51415 transcript_9069	212	2816	2	211	313	2916	1	210
transcript_9266	gnl|BL_ORD_ID|45728 transcript_94593	208	2816	2	211	477	3083	1	210
transcript_9266	gnl|BL_ORD_ID|68130 transcript_127900	210	2814	2	211	1991	4607	1	209
transcript_92683	gnl|BL_ORD_ID|29989 transcript_70618	2	2765	2764	3403	101	2867	3573	4214
transcript_92714	gnl|BL_ORD_ID|80868 transcript_147084	1095	2620	1	1095	2228	3752	721	1815
transcript_92725	gnl|BL_ORD_ID|89812 transcript_161986	1	2023	2023	2874	184	2207	2383	3235
transcript_92743	gnl|BL_ORD_ID|12239 transcript_3393	1261	3218	11	1261	1525	3484	1	1272
transcript_92803	gnl|BL_ORD_ID|42506 transcript_89202	2	2793	2795	3043	130	2923	3150	3398
transcript_92803	gnl|BL_ORD_ID|30819 transcript_71986	2	2793	2795	3043	5	2795	3022	3270
transcript_92803	gnl|BL_ORD_ID|64287 transcript_123203	2	2793	2795	3043	5	2796	3420	3667
transcript_9281	gnl|BL_ORD_ID|35267 transcript_79210	14	2612	2613	2917	2	2604	2794	3099

transcript_92860	gnl|BL_ORD_ID|48141 transcript_98417	1281	2759	110	1282	2967	4443	140	1308
transcript_92860	gnl|BL_ORD_ID|39138 transcript_83767	110	1476	1477	2759	167	1532	2079	3360
transcript_92860	gnl|BL_ORD_ID|63498 transcript_121956	110	1808	1808	2759	138	1836	2115	3065
transcript_92860	gnl|BL_ORD_ID|81902 transcript_148793	110	1923	1919	2759	142	1955	2096	2934
transcript_92867	gnl|BL_ORD_ID|38396 transcript_7541	1	1697	1693	2081	52	1752	2646	3038
transcript_92878	gnl|BL_ORD_ID|22518 transcript_60197	150	1689	1	153	1836	3376	809	961
transcript_92918	gnl|BL_ORD_ID|68326 transcript_128222	307	1370	48	307	1914	2973	80	339
transcript_92919	gnl|BL_ORD_ID|32259 transcript_74352	1	1924	1920	2436	49	1970	2339	2854
transcript_92942	gnl|BL_ORD_ID|87627 transcript_158483	19	3543	3541	3687	14	3552	3709	3855
transcript_92942	gnl|BL_ORD_ID|869 transcript_1537	1	3543	3541	3676	52	3614	3771	3906
transcript_9295	gnl|BL_ORD_ID|12525 transcript_3941	1020	2914	1	1020	1574	3468	48	1066
transcript_93023	gnl|BL_ORD_ID|25077 transcript_5671	2	2670	2669	2807	69	2737	2987	3125
transcript_93023	gnl|BL_ORD_ID|21475 transcript_58475	2	2670	2669	2842	11	2679	2937	3110
transcript_9303	gnl|BL_ORD_ID|40446 transcript_85857	247	2854	1	250	387	2998	1	272
transcript_93086	gnl|BL_ORD_ID|54109 transcript_106489	1	1377	1375	1704	1	1367	1714	2045
transcript_93092	gnl|BL_ORD_ID|34039 transcript_77188	11	2097	2093	2796	98	2181	2308	3010
transcript_93099	gnl|BL_ORD_ID|47784 transcript_97859	11	2632	2632	2927	2	2624	2797	3091
transcript_93130	gnl|BL_ORD_ID|12026 transcript_2985	1	1615	1614	2391	457	2071	2713	3490
transcript_93130	gnl|BL_ORD_ID|61119 transcript_118156	1	1615	1614	2426	303	1914	2554	3373
transcript_9326	gnl|BL_ORD_ID|37147 transcript_82196	723	2905	88	726	2954	5135	2	640
transcript_93267	gnl|BL_ORD_ID|40373 transcript_85727	2	2255	2253	4030	2	2245	2414	4166
transcript_93313	gnl|BL_ORD_ID|321 transcript_540	355	4257	1	357	523	4443	8	365
transcript_93323	gnl|BL_ORD_ID|38166 transcript_7044	1231	2684	1	1232	1630	3083	2	1233
transcript_93432	gnl|BL_ORD_ID|21936 transcript_59239	10	1521	1516	3045	1	1508	3134	4660
transcript_93470	gnl|BL_ORD_ID|23422 transcript_61729	1	1332	1328	1987	503	1829	2254	2901
transcript_93470	gnl|BL_ORD_ID|71424 transcript_133248	1	1332	1328	2013	461	1788	2213	2897
transcript_93470	gnl|BL_ORD_ID|51331 transcript_8892	1	1332	1328	2011	506	1832	2257	2936
transcript_93473	gnl|BL_ORD_ID|80590 transcript_146611	177	1904	1	178	1443	3173	865	1042
transcript_93480	gnl|BL_ORD_ID|87717 transcript_158619	651	1696	7	655	2600	3647	2	643
transcript_93496	gnl|BL_ORD_ID|17078 transcript_51331	1	1638	1638	2441	443	2081	2192	2993
transcript_93509	gnl|BL_ORD_ID|76910 transcript_142137	114	2875	1	115	361	3107	1	115
transcript_93516	gnl|BL_ORD_ID|95977 transcript_20746	1	1554	1549	2217	1	1552	1670	2338
transcript_93516	gnl|BL_ORD_ID|95830 transcript_20412	11	1554	1549	2217	2	1563	1681	2347
transcript_93542	gnl|BL_ORD_ID|83016 transcript_150778	583	3740	62	583	972	4124	1	519

transcript_93544	gnl|BL_ORD_ID|26057 transcript_64337	1	1484	1483	2702	4047	5531	5644	6862
transcript_9355	gnl|BL_ORD_ID|28361 transcript_68053	1	2303	2298	2890	1	2304	3079	3671
transcript_93560	gnl|BL_ORD_ID|55266 transcript_108530	159	1236	3	158	919	1997	1	142
transcript_93572	gnl|BL_ORD_ID|89760 transcript_161906	1	1186	1181	2253	67	1255	1492	2565
transcript_9358	gnl|BL_ORD_ID|24775 transcript_5027	279	2886	1	279	682	3289	98	376
transcript_9358	gnl|BL_ORD_ID|24809 transcript_5105	279	2886	1	279	609	3216	28	306
transcript_93631	gnl|BL_ORD_ID|32584 transcript_74894	163	1621	1	162	819	2281	1	163
transcript_93631	gnl|BL_ORD_ID|59887 transcript_116170	163	1621	1	162	1633	3095	17	176
transcript_93643	gnl|BL_ORD_ID|12536 transcript_3968	1	2160	2159	3231	1	2198	2331	3399
transcript_93680	gnl|BL_ORD_ID|95304 transcript_19155	1	1875	1872	2245	1	1875	2043	2415
transcript_93682	gnl|BL_ORD_ID|11795 transcript_2540	21	2310	2309	3073	1	2285	2914	3672
transcript_93682	gnl|BL_ORD_ID|11738 transcript_2420	22	2310	2309	3073	1	2269	2898	3651
transcript_93685	gnl|BL_ORD_ID|25399 transcript_6331	261	2960	1	263	443	3143	1	263
transcript_93739	gnl|BL_ORD_ID|24800 transcript_5085	1	1664	1666	3070	3	1674	1846	3261
transcript_93752	gnl|BL_ORD_ID|69125 transcript_129517	113	2645	1	116	284	2826	54	169
transcript_93752	gnl|BL_ORD_ID|40446 transcript_85857	113	2645	1	116	387	2929	157	272
transcript_93752	gnl|BL_ORD_ID|51420 transcript_9079	115	2645	1	117	308	2848	56	172
transcript_93752	gnl|BL_ORD_ID|54374 transcript_106941	113	2645	1	115	334	2876	50	164
transcript_9376	gnl|BL_ORD_ID|31885 transcript_73705	16	1895	1892	2861	2	1864	2022	2988
transcript_9382	gnl|BL_ORD_ID|25332 transcript_6211	19	1978	1975	2851	2	1956	2295	3171
transcript_9385	gnl|BL_ORD_ID|96906 transcript_109067	1107	2906	1	1110	1599	3398	53	1161
transcript_93895	gnl|BL_ORD_ID|41307 transcript_87286	137	2284	1	138	1228	3376	6	145
transcript_93895	gnl|BL_ORD_ID|91173 transcript_16059	137	2284	1	138	401	2548	37	174
transcript_93895	gnl|BL_ORD_ID|74597 transcript_138407	137	2284	1	138	414	2550	26	165
transcript_93895	gnl|BL_ORD_ID|37223 transcript_82317	137	2284	1	138	1209	3360	6	144
transcript_93912	gnl|BL_ORD_ID|77677 transcript_12902	145	1051	2	147	1526	2426	7	152
transcript_93912	gnl|BL_ORD_ID|54308 transcript_106833	145	1051	2	147	1580	2487	5	150
transcript_93912	gnl|BL_ORD_ID|41310 transcript_87289	145	1051	2	147	1521	2428	6	151
transcript_93923	gnl|BL_ORD_ID|63787 transcript_122427	1	1210	1205	2326	1	1216	2929	4050
transcript_93926	gnl|BL_ORD_ID|1957 transcript_23149	140	2070	1	143	322	2254	19	161
transcript_9394	gnl|BL_ORD_ID|54485 transcript_107144	2	2583	2584	2891	30	2611	2731	3039
transcript_93963	gnl|BL_ORD_ID|32643 transcript_74991	1	2069	2067	2769	814	2851	3639	4351
transcript_93963	gnl|BL_ORD_ID|24137 transcript_62875	176	2854	1	175	1140	3812	806	980
transcript_93963	gnl|BL_ORD_ID|30736 transcript_71838	279	2854	1	280	1271	3840	812	1091

transcript_93966	gnl|BL_ORD_ID|68797 transcript_128974	2	2224	2221	2764	208	2443	2703	3248
transcript_93966	gnl|BL_ORD_ID|84466 transcript_153316	2	2221	2221	2762	287	2509	2654	3195
transcript_93966	gnl|BL_ORD_ID|73411 transcript_136437	2	2221	2221	2764	207	2442	2587	3131
transcript_9398	gnl|BL_ORD_ID|27397 transcript_66438	254	2896	2	255	1075	3715	1	254
transcript_93999	gnl|BL_ORD_ID|96125 transcript_21079	301	1783	39	303	756	2227	2	267
transcript_940	gnl|BL_ORD_ID|476 transcript_808	2	3499	3498	4107	1	3498	3660	4267
transcript_94043	gnl|BL_ORD_ID|926 transcript_1652	242	3723	20	245	427	3908	1	228
transcript_94058	gnl|BL_ORD_ID|93380 transcript_166147	300	3199	2	301	1215	4109	1	300
transcript_94058	gnl|BL_ORD_ID|50926 transcript_102901	302	3196	1	301	504	3417	1	301
transcript_94213	gnl|BL_ORD_ID|41714 transcript_87985	372	2897	40	373	542	3065	11	344
transcript_94277	gnl|BL_ORD_ID|54702 transcript_107511	1	1480	1481	2182	2	1475	1745	2446
transcript_94282	gnl|BL_ORD_ID|35253 transcript_79182	1	1738	1738	3173	1073	2808	3188	4625
transcript_94282	gnl|BL_ORD_ID|21857 transcript_59108	1	1738	1738	3173	1523	3258	3637	5073
transcript_94299	gnl|BL_ORD_ID|65283 transcript_12720	1	1862	1861	2068	1	1872	2491	2700
transcript_94321	gnl|BL_ORD_ID|76825 transcript_142008	1	1493	1490	2412	1	1470	1660	2582
transcript_94359	gnl|BL_ORD_ID|47686 transcript_97697	1	2086	2081	2310	48	2115	2233	2462
transcript_94376	gnl|BL_ORD_ID|714 transcript_1243	1	1515	1514	3014	3	1518	2507	4011
transcript_94431	gnl|BL_ORD_ID|93678 transcript_166631	165	1211	2	166	1665	2714	173	337
transcript_94432	gnl|BL_ORD_ID|54316 transcript_106846	1	1143	1140	1601	237	1383	1857	2318
transcript_94440	gnl|BL_ORD_ID|32395 transcript_74590	204	1586	26	205	609	1992	13	192
transcript_94446	gnl|BL_ORD_ID|72407 transcript_134814	3	3547	3545	4121	1	3546	4156	4732
transcript_945	gnl|BL_ORD_ID|407 transcript_693	193	4108	18	195	374	4289	21	198
transcript_94520	gnl|BL_ORD_ID|51539 transcript_9295	572	2475	99	575	946	2849	1	477
transcript_94543	gnl|BL_ORD_ID|78783 transcript_143694	1116	2915	1	1115	1230	3054	2	1117
transcript_94572	gnl|BL_ORD_ID|88753 transcript_160312	19	2252	2254	2828	2	2202	2397	2970
transcript_94572	gnl|BL_ORD_ID|38787 transcript_8400	2	2252	2254	2828	1	2228	2423	2996
transcript_94573	gnl|BL_ORD_ID|64657 transcript_11323	1	1802	1802	2631	1	1813	1947	2769
transcript_94596	gnl|BL_ORD_ID|82473 transcript_149815	10	1862	1862	3459	1	1825	2288	3873
transcript_9460	gnl|BL_ORD_ID|28026 transcript_67470	1294	2856	1	1299	1556	3118	1	1301
transcript_94650	gnl|BL_ORD_ID|25347 transcript_6240	10	1446	1444	2754	2	1436	1595	2905
transcript_94650	gnl|BL_ORD_ID|50070 transcript_101495	1	1446	1444	2754	34	1490	1649	2960
transcript_94650	gnl|BL_ORD_ID|60017 transcript_116369	1	1446	1444	2754	171	1614	1773	3083
transcript_94650	gnl|BL_ORD_ID|37909 transcript_6532	1	1446	1444	2754	38	1483	1642	2952
transcript_94672	gnl|BL_ORD_ID|85373 transcript_154766	1	2600	2598	3662	522	3125	3225	4289

transcript_94672	gnl|BL_ORD_ID|62709 transcript_120684	1	2600	2598	3644	565	3164	3264	4310
transcript_94708	gnl|BL_ORD_ID|1271 transcript_21571	14	1409	1408	1991	2	1397	1544	2127
transcript_9480	gnl|BL_ORD_ID|47194 transcript_96932	1	1823	1822	2730	1	1821	1967	2877
transcript_9480	gnl|BL_ORD_ID|51462 transcript_9149	1	1823	1822	2784	1	1821	1967	2930
transcript_94829	gnl|BL_ORD_ID|26414 transcript_64881	182	1246	2	181	1583	2649	130	310
transcript_94829	gnl|BL_ORD_ID|91120 transcript_15940	182	1224	2	181	1485	2526	42	222
transcript_94831	gnl|BL_ORD_ID|48443 transcript_98899	207	1847	4	210	401	2087	8	225
transcript_94838	gnl|BL_ORD_ID|4853 transcript_29418	188	1863	4	189	338	2014	13	198
transcript_9484	gnl|BL_ORD_ID|30260 transcript_71065	2	2575	2573	2878	4	2577	2723	3028
transcript_94879	gnl|BL_ORD_ID|46585 transcript_95997	104	1344	1341	2482	301	1542	1986	3154
transcript_94896	gnl|BL_ORD_ID|67601 transcript_127045	1	2263	2262	2614	4	2277	2386	2734
transcript_94906	gnl|BL_ORD_ID|39813 transcript_84862	109	1227	1	108	215	1331	1	108
transcript_94942	gnl|BL_ORD_ID|76493 transcript_141472	734	2602	80	736	820	2688	24	682
transcript_94990	gnl|BL_ORD_ID|66678 transcript_125553	1	1674	1672	2749	850	2527	2653	3733
transcript_95012	gnl|BL_ORD_ID|52062 transcript_10436	2	2135	2134	2520	220	2352	2453	2839
transcript_95039	gnl|BL_ORD_ID|29206 transcript_69382	198	2480	14	200	330	2595	2	180
transcript_95052	gnl|BL_ORD_ID|11815 transcript_2577	2	2496	2494	2811	118	2610	3319	3632
transcript_95060	gnl|BL_ORD_ID|91166 transcript_16049	249	2194	7	252	532	2477	2	247
transcript_95076	gnl|BL_ORD_ID|79228 transcript_144419	135	1604	1599	2898	1	1466	2012	3309
transcript_95082	gnl|BL_ORD_ID|926 transcript_1652	1	3095	3097	3406	396	3490	3604	3921
transcript_95115	gnl|BL_ORD_ID|86721 transcript_157000	672	2441	83	673	1243	3016	535	1125
transcript_95169	gnl|BL_ORD_ID|25981 transcript_64225	13	2908	2907	3179	100	2996	3299	3571
transcript_95169	gnl|BL_ORD_ID|12089 transcript_3121	25	2908	2907	3125	108	2993	3296	3514
transcript_95172	gnl|BL_ORD_ID|71737 transcript_133755	2	2343	2341	3157	9	2348	3690	4506
transcript_9532	gnl|BL_ORD_ID|49834 transcript_101115	1	2445	2444	2881	1	2445	2558	2995
transcript_95370	gnl|BL_ORD_ID|25360 transcript_6263	229	2742	1	228	607	3110	3	232
transcript_95391	gnl|BL_ORD_ID|72760 transcript_135387	1	1446	1446	2451	1	1413	1614	2619
transcript_95444	gnl|BL_ORD_ID|39162 transcript_83802	280	3015	1	282	1860	4595	5	285
transcript_95449	gnl|BL_ORD_ID|11770 transcript_2474	330	3447	105	333	562	3674	119	347
transcript_95449	gnl|BL_ORD_ID|11705 transcript_2357	330	3444	2	333	584	3702	38	369
transcript_95449	gnl|BL_ORD_ID|53791 transcript_105990	330	3418	1	333	732	3826	186	518
transcript_95449	gnl|BL_ORD_ID|11803 transcript_2559	330	3384	105	333	569	3623	127	355
transcript_95469	gnl|BL_ORD_ID|51314 transcript_8866	2	2252	2250	2631	46	2296	2546	2927
transcript_9549	gnl|BL_ORD_ID|40446 transcript_85857	264	2878	23	267	387	3001	2	272

transcript_95526	gnl|BL_ORD_ID|28280 transcript_67913	1182	5192	1	1182	1848	5858	1	1182
transcript_95541	gnl|BL_ORD_ID|28879 transcript_68882	221	2555	104	220	417	2752	1	117
transcript_9558	gnl|BL_ORD_ID|69706 transcript_130467	1059	2841	1	1060	1697	3464	1	1054
transcript_95587	gnl|BL_ORD_ID|19488 transcript_55206	351	2339	68	352	455	2438	1	285
transcript_95610	gnl|BL_ORD_ID|49200 transcript_100113	14	1715	1716	2766	2	1713	2108	3157
transcript_95637	gnl|BL_ORD_ID|94161 transcript_167368	269	1604	6	270	4255	5588	2	267
transcript_9565	gnl|BL_ORD_ID|38439 transcript_7631	1	1739	1737	2863	1	1740	1900	3026
transcript_95662	gnl|BL_ORD_ID|71805 transcript_133874	317	2051	38	318	2035	3749	1	281
transcript_9567	gnl|BL_ORD_ID|35106 transcript_78953	1	2381	2378	2897	2	2383	3325	3837
transcript_95684	gnl|BL_ORD_ID|56197 transcript_110157	346	1872	59	348	1053	2566	2	289
transcript_95704	gnl|BL_ORD_ID|50070 transcript_101495	262	2650	2	263	548	2936	45	318
transcript_95704	gnl|BL_ORD_ID|37909 transcript_6532	262	2650	2	263	540	2928	49	310
transcript_95704	gnl|BL_ORD_ID|60017 transcript_116369	262	2650	2	263	671	3059	182	441
transcript_95704	gnl|BL_ORD_ID|25347 transcript_6240	262	2650	2	263	493	2881	4	265
transcript_95744	gnl|BL_ORD_ID|164 transcript_258	1	3292	3291	4246	555	3846	3950	4905
transcript_95755	gnl|BL_ORD_ID|65459 transcript_123539	2	2173	2171	2554	36	2211	4110	4495
transcript_95775	gnl|BL_ORD_ID|58322 transcript_113641	326	2824	8	327	1141	3639	2	318
transcript_9578	gnl|BL_ORD_ID|45904 transcript_94894	2	2046	2043	2859	1	2045	2335	3151
transcript_95787	gnl|BL_ORD_ID|64674 transcript_11361	1	1812	1813	2628	3	1814	1980	2794
transcript_95804	gnl|BL_ORD_ID|21765 transcript_58944	235	2239	47	238	621	2625	1	191
transcript_9581	gnl|BL_ORD_ID|24809 transcript_5105	308	2875	6	308	649	3216	2	306
transcript_95847	gnl|BL_ORD_ID|11778 transcript_2502	1	2758	2757	3167	612	3368	3267	3679
transcript_95847	gnl|BL_ORD_ID|74010 transcript_137432	1	2758	2755	3206	583	3339	3236	3687
transcript_95879	gnl|BL_ORD_ID|31812 transcript_73588	16	1738	1733	1921	1	1713	5659	5846
transcript_95879	gnl|BL_ORD_ID|54751 transcript_107594	1	1738	1733	1921	2	1735	2125	2313
transcript_95879	gnl|BL_ORD_ID|77872 transcript_13331	1	1738	1733	1921	3	1735	2260	2448
transcript_95879	gnl|BL_ORD_ID|43181 transcript_90296	12	1738	1733	1921	1	1721	2361	2549
transcript_95933	gnl|BL_ORD_ID|58075 transcript_113228	1	1428	1424	2632	126	1552	1716	2918
transcript_95954	gnl|BL_ORD_ID|49725 transcript_100945	1	3599	3598	3893	2	3599	3792	4087
transcript_95974	gnl|BL_ORD_ID|1640 transcript_22498	184	1422	2	186	1033	2269	18	202
transcript_96006	gnl|BL_ORD_ID|18744 transcript_53957	182	1960	1	184	451	2232	96	279
transcript_96006	gnl|BL_ORD_ID|2564 transcript_24481	182	1922	1	184	463	2203	94	291
transcript_96006	gnl|BL_ORD_ID|2320 transcript_23954	182	1923	1	184	473	2217	118	301
transcript_96006	gnl|BL_ORD_ID|60885 transcript_117767	182	1890	1	184	484	2192	115	312

transcript_96025	gnl|BL_ORD_ID|51987 transcript_10266	1	1190	1188	1826	1	1191	2220	2858
transcript_96048	gnl|BL_ORD_ID|46783 transcript_96297	1	1619	1614	2260	1	1620	1735	2381
transcript_96057	gnl|BL_ORD_ID|24955 transcript_5378	1	2094	2091	2515	600	2698	2806	3231
transcript_96057	gnl|BL_ORD_ID|12326 transcript_3551	1	2094	2091	2515	826	2924	3032	3457
transcript_96057	gnl|BL_ORD_ID|52387 transcript_103639	1	2094	2091	2515	820	2918	3026	3452
transcript_96057	gnl|BL_ORD_ID|67372 transcript_126671	1	2094	2091	2460	647	2743	2851	3222
transcript_96057	gnl|BL_ORD_ID|12472 transcript_3838	1	2094	2091	2515	777	2875	2983	3408
transcript_96073	gnl|BL_ORD_ID|71902 transcript_134027	372	3827	1	373	1818	5277	2	377
transcript_96100	gnl|BL_ORD_ID|18372 transcript_53382	167	2401	1	169	618	2853	1	169
transcript_96113	gnl|BL_ORD_ID|26967 transcript_65775	776	2549	8	780	876	2632	2	770
transcript_96130	gnl|BL_ORD_ID|80646 transcript_146713	1	2393	2391	3443	81	2473	2589	3641
transcript_96227	gnl|BL_ORD_ID|708 transcript_1231	1	2878	2879	3787	2	2892	3043	3956
transcript_96227	gnl|BL_ORD_ID|763 transcript_1334	1	2878	2879	3789	4	2884	3035	3950
transcript_96230	gnl|BL_ORD_ID|29422 transcript_69701	1	2043	2042	3169	12	2061	2232	3359
transcript_96262	gnl|BL_ORD_ID|79061 transcript_144153	1565	3828	1	1565	2771	5048	876	2441
transcript_96274	gnl|BL_ORD_ID|60599 transcript_117295	289	1413	3	291	710	1834	208	496
transcript_96274	gnl|BL_ORD_ID|80851 transcript_147053	289	1413	3	294	707	1832	205	496
transcript_96280	gnl|BL_ORD_ID|87923 transcript_158955	198	1757	7	202	961	2521	2	197
transcript_96302	gnl|BL_ORD_ID|17781 transcript_52452	343	3664	1	345	496	3803	1	344
transcript_96326	gnl|BL_ORD_ID|95563 transcript_19765	115	2203	1	115	245	2361	1	117
transcript_96346	gnl|BL_ORD_ID|37950 transcript_6604	2	2194	2194	2980	1	2192	2331	3116
transcript_96386	gnl|BL_ORD_ID|79354 transcript_144611	505	1518	6	505	669	1682	1	500
transcript_96403	gnl|BL_ORD_ID|38041 transcript_6792	340	2940	4	341	458	3062	1	338
transcript_9643	gnl|BL_ORD_ID|57987 transcript_113093	2	2165	2161	2877	151	2291	3265	3981
transcript_9643	gnl|BL_ORD_ID|50359 transcript_101960	2	2165	2161	2865	156	2336	3311	4015
transcript_96451	gnl|BL_ORD_ID|85386 transcript_154780	1	1985	1983	2433	400	2384	2491	2944
transcript_96479	gnl|BL_ORD_ID|2103 transcript_23448	1	1443	1444	2182	2	1444	1552	2287
transcript_96481	gnl|BL_ORD_ID|56247 transcript_110239	603	2125	83	604	635	2158	2	523
transcript_96526	gnl|BL_ORD_ID|32850 transcript_75305	1	2388	2385	2723	94	2476	2650	2976
transcript_96578	gnl|BL_ORD_ID|73639 transcript_136811	1	3720	3715	4184	3396	7117	7245	7714
transcript_96624	gnl|BL_ORD_ID|662 transcript_1147	188	2272	1	189	938	3022	3023	3211
transcript_96624	gnl|BL_ORD_ID|12638 transcript_4178	188	2272	1	189	731	2815	2816	3004
transcript_96633	gnl|BL_ORD_ID|97214 transcript_138202	1	2887	2887	3422	1	2885	3328	3863
transcript_96642	gnl|BL_ORD_ID|23742 transcript_62261	317	1564	32	318	716	1962	2	286

transcript_96658	gnl|BL_ORD_ID|91318 transcript_16384	217	2329	1	218	409	2523	2	219
transcript_96659	gnl|BL_ORD_ID|55101 transcript_108232	1	1443	1442	1876	162	1594	1856	2287
transcript_96685	gnl|BL_ORD_ID|58774 transcript_114380	408	2693	7	409	1118	3374	1	398
transcript_9669	gnl|BL_ORD_ID|36364 transcript_80965	153	2890	15	155	306	3039	29	169
transcript_96695	gnl|BL_ORD_ID|91341 transcript_16421	1	1834	1832	2279	116	1948	2088	2535
transcript_96695	gnl|BL_ORD_ID|78016 transcript_13663	1	1834	1832	2357	49	1882	2022	2547
transcript_96695	gnl|BL_ORD_ID|78185 transcript_14062	1	1834	1832	2356	61	1894	2034	2558
transcript_96695	gnl|BL_ORD_ID|40865 transcript_86580	1	1834	1832	2356	37	1870	2010	2534
transcript_96704	gnl|BL_ORD_ID|12296 transcript_3504	2	2423	2418	3176	29	2449	2591	3348
transcript_96704	gnl|BL_ORD_ID|12603 transcript_4100	2	2423	2418	3176	55	2476	2618	3376
transcript_96704	gnl|BL_ORD_ID|33541 transcript_76375	2	2420	2418	3176	102	2518	2663	3420
transcript_96714	gnl|BL_ORD_ID|71903 transcript_134028	1	2273	2270	3805	1	2269	2903	4437
transcript_96714	gnl|BL_ORD_ID|41987 transcript_88399	2	2271	2271	3762	190	2460	2600	4091
transcript_96714	gnl|BL_ORD_ID|764 transcript_1335	1	2271	2271	3817	1	2270	2410	3956
transcript_9675	gnl|BL_ORD_ID|66734 transcript_125647	2	2473	2469	2868	17	2488	2652	3055
transcript_96825	gnl|BL_ORD_ID|24900 transcript_5275	198	2331	1	200	1098	3234	204	403
transcript_96854	gnl|BL_ORD_ID|50034 transcript_101440	114	1716	1716	2998	1	1602	1885	3164
transcript_96854	gnl|BL_ORD_ID|24435 transcript_4309	1	1716	1716	2998	59	1772	2055	3337
transcript_96872	gnl|BL_ORD_ID|80904 transcript_147136	1	2261	2261	2718	1	2261	2460	2917
transcript_96884	gnl|BL_ORD_ID|19923 transcript_55900	1	2959	2954	3217	1	2989	3437	3700
transcript_96901	gnl|BL_ORD_ID|61704 transcript_119051	13	1305	1306	2061	1	1293	1444	2201
transcript_96906	gnl|BL_ORD_ID|24423 transcript_4289	11	1645	1640	2235	22	1657	2501	3096
transcript_96954	gnl|BL_ORD_ID|82578 transcript_150002	1	1488	1483	2797	357	1845	2125	3441
transcript_96974	gnl|BL_ORD_ID|42731 transcript_89574	1	2399	2398	2590	465	2863	3241	3433
transcript_96984	gnl|BL_ORD_ID|82967 transcript_150688	361	4020	145	361	729	4387	37	253
transcript_96985	gnl|BL_ORD_ID|45730 transcript_94596	2	2663	2659	2904	123	2784	3260	3505
transcript_96985	gnl|BL_ORD_ID|41954 transcript_88350	2	2663	2659	2829	28	2688	3156	3326
transcript_97037	gnl|BL_ORD_ID|42032 transcript_88466	207	2281	2	206	562	2642	148	352
transcript_97081	gnl|BL_ORD_ID|95752 transcript_20232	330	2314	47	330	386	2370	2	286
transcript_97114	gnl|BL_ORD_ID|78332 transcript_14375	1	1439	1441	2482	46	1484	1585	2626
transcript_97120	gnl|BL_ORD_ID|37470 transcript_82714	180	1645	38	181	285	1769	1	144
transcript_97120	gnl|BL_ORD_ID|6394 transcript_32673	180	1645	23	181	301	1767	2	160
transcript_9714	gnl|BL_ORD_ID|87702 transcript_158595	1	1819	1818	2875	1	1821	2599	3655
transcript_97195	gnl|BL_ORD_ID|39839 transcript_84899	318	1748	42	319	1469	2897	2	289

transcript_9720	gnl|BL_ORD_ID|50412 transcript_102038	636	2652	71	634	755	2772	2	564
transcript_97223	gnl|BL_ORD_ID|26625 transcript_65223	1	1714	1711	2442	2	1710	2513	3244
transcript_9724	gnl|BL_ORD_ID|12152 transcript_3231	190	2961	1	187	713	3509	2	188
transcript_9724	gnl|BL_ORD_ID|12224 transcript_3370	190	2969	1	187	715	3496	2	188
transcript_9725	gnl|BL_ORD_ID|51592 transcript_9412	239	2877	110	238	264	2902	2	130
transcript_97278	gnl|BL_ORD_ID|41193 transcript_87102	246	2737	1	247	415	2905	1	247
transcript_97346	gnl|BL_ORD_ID|85950 transcript_155704	1	1142	1143	1668	3	1144	2123	2648
transcript_97349	gnl|BL_ORD_ID|97380 transcript_153863	1293	2911	1	1294	1420	3063	1	1296
transcript_97349	gnl|BL_ORD_ID|12670 transcript_4237	1293	2911	1	1294	1421	3039	4	1297
transcript_97349	gnl|BL_ORD_ID|38212 transcript_7133	1293	2911	1	1294	1450	3068	31	1326
transcript_97367	gnl|BL_ORD_ID|47380 transcript_97233	1	1210	1208	1835	1	1216	2245	2872
transcript_97387	gnl|BL_ORD_ID|637 transcript_1096	1	1809	1804	2265	1680	3488	3591	4052
transcript_97387	gnl|BL_ORD_ID|52667 transcript_104081	1	1809	1804	2265	1234	3042	3145	3606
transcript_97387	gnl|BL_ORD_ID|37330 transcript_82485	1	1809	1804	2265	1563	3370	3472	3933
transcript_97387	gnl|BL_ORD_ID|901 transcript_1611	1	1809	1804	2265	1513	3321	3424	3885
transcript_97387	gnl|BL_ORD_ID|32336 transcript_74491	1	1809	1804	2265	1156	2964	3067	3528
transcript_97387	gnl|BL_ORD_ID|25808 transcript_63946	1	1809	1804	2265	1210	3017	3120	3579
transcript_97387	gnl|BL_ORD_ID|68519 transcript_128532	1	1809	1804	2197	1564	3372	3475	3868
transcript_97387	gnl|BL_ORD_ID|591 transcript_1003	1	1809	1804	2265	1726	3534	3637	4098
transcript_97387	gnl|BL_ORD_ID|37880 transcript_6467	1	1809	1804	2265	575	2383	2486	2947
transcript_97396	gnl|BL_ORD_ID|24845 transcript_5161	1	1054	1051	1884	1	1060	1695	2528
transcript_97396	gnl|BL_ORD_ID|51863 transcript_10020	1	1054	1051	1870	1	1115	1778	2643
transcript_97396	gnl|BL_ORD_ID|89246 transcript_161080	1	1054	1051	1884	1	1054	1880	2713
transcript_97421	gnl|BL_ORD_ID|936 transcript_1668	298	3640	1	301	418	3786	2	302
transcript_97444	gnl|BL_ORD_ID|11665 transcript_2276	1	2169	2167	2855	16	2184	2997	3696
transcript_97447	gnl|BL_ORD_ID|25882 transcript_64076	1	2449	2448	3068	198	2611	2916	3534
transcript_97456	gnl|BL_ORD_ID|80512 transcript_146491	1	1097	1098	1808	976	2072	2528	3238
transcript_97540	gnl|BL_ORD_ID|41015 transcript_86820	1828	3693	1	1833	6365	8232	4198	6033
transcript_97557	gnl|BL_ORD_ID|33041 transcript_75587	1	1514	1510	2202	15	1528	1632	2324
transcript_97558	gnl|BL_ORD_ID|45604 transcript_94377	240	2994	2	243	374	3126	1	242
transcript_97558	gnl|BL_ORD_ID|25176 transcript_5866	241	2952	1	245	457	3167	1	244
transcript_97558	gnl|BL_ORD_ID|37939 transcript_6580	240	2952	2	245	405	3115	11	255
transcript_9756	gnl|BL_ORD_ID|83185 transcript_151064	347	2680	6	347	486	2819	1	344
transcript_97585	gnl|BL_ORD_ID|64990 transcript_12035	250	2503	2	249	392	2646	26	289

transcript_9760	gnl|BL_ORD_ID|951 transcript_1690	265	2873	1	265	1265	3855	2	266
transcript_9760	gnl|BL_ORD_ID|79256 transcript_144459	265	2807	1	266	1275	3817	1	266
transcript_97601	gnl|BL_ORD_ID|27137 transcript_66037	2	2487	2486	2993	55	2542	3134	3642
transcript_97625	gnl|BL_ORD_ID|92262 transcript_164309	1	3314	3312	3729	2884	6195	7139	7556
transcript_97660	gnl|BL_ORD_ID|43088 transcript_90144	1152	2778	1	1155	1320	2959	1	1155
transcript_97668	gnl|BL_ORD_ID|41387 transcript_87421	1	2959	2958	3539	69	3021	3139	3720
transcript_97701	gnl|BL_ORD_ID|91405 transcript_16577	463	2298	83	464	502	2339	2	382
transcript_97706	gnl|BL_ORD_ID|24611 transcript_4667	1	1967	1962	2864	64	2030	2175	3079
transcript_97711	gnl|BL_ORD_ID|17170 transcript_51482	447	3167	90	446	1331	4068	861	1217
transcript_97727	gnl|BL_ORD_ID|31860 transcript_73661	1	2018	2018	2837	12	2030	2657	3476
transcript_97754	gnl|BL_ORD_ID|58375 transcript_113730	1469	3139	138	1471	2180	3851	1	1336
transcript_97762	gnl|BL_ORD_ID|58393 transcript_113757	460	2760	83	459	571	2869	2	378
transcript_97800	gnl|BL_ORD_ID|40487 transcript_85941	1636	3305	1	1639	2790	4459	87	1723
transcript_97830	gnl|BL_ORD_ID|48531 transcript_99044	1	1663	1663	1983	65	1732	2020	2340
transcript_97830	gnl|BL_ORD_ID|24241 transcript_63030	1	1664	1663	1983	2	1665	2035	2356
transcript_97830	gnl|BL_ORD_ID|42897 transcript_89833	1	1663	1663	1983	8	1670	1958	2282
transcript_97830	gnl|BL_ORD_ID|95144 transcript_18794	1	1663	1663	1983	150	1812	2100	2421
transcript_97830	gnl|BL_ORD_ID|94586 transcript_17487	1	1663	1664	1983	66	1728	2164	2484
transcript_97860	gnl|BL_ORD_ID|1169 transcript_2098	2	2123	2120	2851	146	2269	2930	3661
transcript_97860	gnl|BL_ORD_ID|36206 transcript_80701	16	2123	2120	2851	63	2172	2973	3704
transcript_97860	gnl|BL_ORD_ID|66018 transcript_124444	2	2123	2120	2851	431	2554	3215	3946
transcript_97878	gnl|BL_ORD_ID|63808 transcript_122459	364	2790	7	365	507	2935	7	369
transcript_97922	gnl|BL_ORD_ID|3429 transcript_26390	726	2020	90	726	819	2114	2	639
transcript_97945	gnl|BL_ORD_ID|388 transcript_664	1422	3908	1	1421	1835	4322	301	1717
transcript_9797	gnl|BL_ORD_ID|32809 transcript_75241	1	2241	2239	2892	712	2944	3133	3788
transcript_9797	gnl|BL_ORD_ID|42052 transcript_88496	1	2241	2239	2892	1059	3297	3485	4137
transcript_98023	gnl|BL_ORD_ID|35024 transcript_78825	174	1197	2	171	1159	2183	157	326
transcript_98035	gnl|BL_ORD_ID|51189 transcript_8570	120	2628	11	123	438	2948	2	114
transcript_98060	gnl|BL_ORD_ID|44258 transcript_92111	2	2661	2656	3142	6	2672	3166	3658
transcript_98069	gnl|BL_ORD_ID|27268 transcript_66244	1092	3009	108	1093	1574	3492	444	1429
transcript_98078	gnl|BL_ORD_ID|79523 transcript_144875	2	2125	2122	2395	5	2126	2253	2527
transcript_98082	gnl|BL_ORD_ID|36364 transcript_80965	106	2879	1	107	307	3076	44	150
transcript_98082	gnl|BL_ORD_ID|22234 transcript_59729	106	2879	1	107	394	3167	28	134
transcript_98140	gnl|BL_ORD_ID|41605 transcript_87794	1	1372	1370	1978	21	1407	2091	2699

transcript_98159	gnl|BL_ORD_ID|44447 transcript_92441	1	1408	1406	2365	352	1760	1881	2845
transcript_98166	gnl|BL_ORD_ID|29991 transcript_70622	11	3208	3208	3791	2	3200	3355	3938
transcript_98186	gnl|BL_ORD_ID|4790 transcript_29318	1	1511	1507	1717	1	1510	1675	1885
transcript_98217	gnl|BL_ORD_ID|1864 transcript_22967	1	1479	1474	1879	258	1736	1849	2254
transcript_98252	gnl|BL_ORD_ID|51272 transcript_8759	1	1405	1404	2802	6	1410	1524	2922
transcript_98257	gnl|BL_ORD_ID|74014 transcript_137441	2	2427	2425	3060	49	2476	4068	4728
transcript_98362	gnl|BL_ORD_ID|51245 transcript_8698	235	2500	2	240	508	2776	5	243
transcript_98362	gnl|BL_ORD_ID|41934 transcript_88317	235	2432	1	240	477	2673	1	239
transcript_98362	gnl|BL_ORD_ID|64519 transcript_11027	235	2455	1	240	505	2729	1	240
transcript_98362	gnl|BL_ORD_ID|59126 transcript_114950	235	2500	13	240	496	2744	4	231
transcript_98362	gnl|BL_ORD_ID|66399 transcript_125105	236	2434	1	240	450	2651	1	240
transcript_98363	gnl|BL_ORD_ID|49061 transcript_99890	1	1835	1833	2035	1	1835	2263	2460
transcript_98394	gnl|BL_ORD_ID|24646 transcript_4744	1	2391	2391	2584	53	2447	3111	3303
transcript_98394	gnl|BL_ORD_ID|24999 transcript_5480	1	2391	2391	2566	53	2447	3087	3261
transcript_98445	gnl|BL_ORD_ID|20076 transcript_56137	1	1090	1086	1301	1	1072	1417	1630
transcript_98446	gnl|BL_ORD_ID|37861 transcript_6431	1	2016	2015	2925	1	2016	2227	3137
transcript_98448	gnl|BL_ORD_ID|23287 transcript_61509	1	3532	3527	3925	1	3545	3805	4205
transcript_98448	gnl|BL_ORD_ID|75535 transcript_139928	2	3532	3527	3925	1	3528	3788	4187
transcript_98493	gnl|BL_ORD_ID|71872 transcript_133977	430	2180	45	432	948	2699	2	381
transcript_98503	gnl|BL_ORD_ID|10469 transcript_40727	168	1145	3	167	400	1377	107	271
transcript_98506	gnl|BL_ORD_ID|43443 transcript_90718	1	2451	2448	4100	1214	3682	3940	5598
transcript_98506	gnl|BL_ORD_ID|42600 transcript_89353	1	2451	2448	4043	1519	3997	4255	5858
transcript_98506	gnl|BL_ORD_ID|69476 transcript_130066	1	2451	2448	4100	1312	3779	4037	5697
transcript_98524	gnl|BL_ORD_ID|61112 transcript_118146	2	2471	2472	3397	4	2474	2642	3567
transcript_98530	gnl|BL_ORD_ID|83888 transcript_152313	230	1925	84	233	472	2167	2	151
transcript_98546	gnl|BL_ORD_ID|26981 transcript_65793	231	3492	1	230	490	3751	46	275
transcript_98571	gnl|BL_ORD_ID|31659 transcript_73318	17	1845	1844	2374	2	1830	3331	3861
transcript_986	gnl|BL_ORD_ID|41571 transcript_87743	2	2868	2865	3954	1	2879	3103	4188
transcript_986	gnl|BL_ORD_ID|469 transcript_799	1	2868	2865	3994	15	2881	3105	4234
transcript_98734	gnl|BL_ORD_ID|1012 transcript_1801	1	1535	1533	2800	1	1535	2575	3842
transcript_98744	gnl|BL_ORD_ID|91290 transcript_16305	1	2090	2085	2376	39	2128	2235	2526
transcript_98744	gnl|BL_ORD_ID|46897 transcript_96464	1	2090	2085	2376	175	2287	2393	2687
transcript_98744	gnl|BL_ORD_ID|46308 transcript_95541	1	2090	2085	2344	101	2189	2296	2555
transcript_98795	gnl|BL_ORD_ID|43853 transcript_91412	107	2677	2672	3468	2	2570	3611	4383

transcript_98821	gnl|BL_ORD_ID|38352 transcript_7433	1	1572	1567	2755	90	1672	1821	3011
transcript_98881	gnl|BL_ORD_ID|44316 transcript_92223	1	1353	1354	2400	253	1618	1732	2786
transcript_98885	gnl|BL_ORD_ID|4106 transcript_27848	1	1288	1289	1875	10	1291	1445	2031
transcript_98896	gnl|BL_ORD_ID|55692 transcript_109297	16	1810	1810	2333	1	1796	2150	2673
transcript_98918	gnl|BL_ORD_ID|68597 transcript_128635	1	1534	1532	2168	2	1535	2564	3201
transcript_98918	gnl|BL_ORD_ID|87696 transcript_158588	1	1665	1664	2173	1	1665	2103	2613
transcript_98948	gnl|BL_ORD_ID|94851 transcript_18127	1	1508	1505	2370	1	1508	1624	2489
transcript_98967	gnl|BL_ORD_ID|41520 transcript_87652	1	1357	1355	1927	314	1665	2193	2768
transcript_98981	gnl|BL_ORD_ID|33996 transcript_77120	295	3043	23	299	1119	3875	2	278
transcript_98981	gnl|BL_ORD_ID|41527 transcript_87668	295	3043	2	299	1282	4031	144	441
transcript_98983	gnl|BL_ORD_ID|24998 transcript_5479	11	2267	2266	2764	3	2242	2511	3009
transcript_99044	gnl|BL_ORD_ID|94586 transcript_17487	1	2021	2021	2346	2	2017	2164	2490
transcript_9905	gnl|BL_ORD_ID|76910 transcript_142137	114	2868	1	115	361	3106	1	115
transcript_991	gnl|BL_ORD_ID|80791 transcript_146957	2	2738	2736	3965	45	2781	3001	4230
transcript_99126	gnl|BL_ORD_ID|73508 transcript_136601	1	1494	1491	2711	1796	3311	5176	6392
transcript_99133	gnl|BL_ORD_ID|55682 transcript_109281	1	1351	1350	1835	1232	2582	3196	3684
transcript_99148	gnl|BL_ORD_ID|46448 transcript_95778	305	3390	3	305	414	3498	3	305
transcript_99148	gnl|BL_ORD_ID|42167 transcript_88665	1575	3390	1	1577	1888	3702	1	1578
transcript_99152	gnl|BL_ORD_ID|61401 transcript_118607	1	1402	1399	2808	1	1401	1844	3253
transcript_99185	gnl|BL_ORD_ID|71905 transcript_134031	119	3061	1	120	401	3343	1	120
transcript_99185	gnl|BL_ORD_ID|71616 transcript_133554	119	3059	1	120	225	3165	1	120
transcript_99271	gnl|BL_ORD_ID|64480 transcript_10942	177	2674	1	176	309	2781	21	196
transcript_99271	gnl|BL_ORD_ID|92831 transcript_165252	174	2674	1	176	804	3279	17	192
transcript_99272	gnl|BL_ORD_ID|34999 transcript_78789	1	1486	1482	2321	118	1602	1757	2595
transcript_99272	gnl|BL_ORD_ID|63298 transcript_121619	177	2321	1	178	527	2669	120	296
transcript_99288	gnl|BL_ORD_ID|52022 transcript_10341	2	2127	2125	2712	8	2113	2235	2823
transcript_99288	gnl|BL_ORD_ID|30516 transcript_71488	2	2127	2125	2714	4	2136	2282	2872
transcript_99288	gnl|BL_ORD_ID|17677 transcript_52288	2	2127	2125	2714	64	2190	2312	2902
transcript_99296	gnl|BL_ORD_ID|72025 transcript_134223	244	2307	88	248	543	2607	2	162
transcript_99311	gnl|BL_ORD_ID|95176 transcript_18872	98	2093	1	100	373	2368	4	103
transcript_99311	gnl|BL_ORD_ID|91525 transcript_16825	98	2093	1	100	371	2366	5	104
transcript_99311	gnl|BL_ORD_ID|94978 transcript_18421	96	2093	1	100	451	2448	91	190
transcript_99319	gnl|BL_ORD_ID|82806 transcript_150391	1	1660	1659	2338	785	2447	3573	4251
transcript_99384	gnl|BL_ORD_ID|63706 transcript_122301	158	1423	17	158	261	1525	2	134

transcript_99399	gnl|BL_ORD_ID|52535 transcript_103868	288	2887	2	287	657	3256	68	353
transcript_99406	gnl|BL_ORD_ID|24807 transcript_5101	190	2714	1	192	593	3117	96	287
transcript_99406	gnl|BL_ORD_ID|33558 transcript_76403	190	2714	1	192	634	3158	137	328
transcript_99458	gnl|BL_ORD_ID|572 transcript_966	157	2095	1	156	2191	4120	1	154
transcript_9946	gnl|BL_ORD_ID|38251 transcript_7216	247	2847	1	247	447	3053	1	248
transcript_9946	gnl|BL_ORD_ID|68130 transcript_127900	247	2848	1	247	1992	4608	1	247
transcript_9946	gnl|BL_ORD_ID|45728 transcript_94593	247	2860	1	247	480	3094	1	248
transcript_99467	gnl|BL_ORD_ID|74774 transcript_138701	219	2125	4	220	531	2437	4	220
transcript_99469	gnl|BL_ORD_ID|41271 transcript_87232	2	2501	2497	2823	43	2526	5201	5526
transcript_99469	gnl|BL_ORD_ID|38516 transcript_7780	16	2498	2495	2923	1	2483	2621	3049
transcript_99489	gnl|BL_ORD_ID|61673 transcript_119013	1	4004	4004	4350	59	4060	4262	4611
transcript_99493	gnl|BL_ORD_ID|92407 transcript_164554	1142	3617	1	1143	2491	4966	1165	2306
transcript_99493	gnl|BL_ORD_ID|133 transcript_202	1142	3615	1	1143	2556	5032	1229	2371
transcript_99499	gnl|BL_ORD_ID|38652 transcript_8087	388	2974	81	388	423	3010	1	308
transcript_995	gnl|BL_ORD_ID|469 transcript_799	1	2875	2872	4003	15	2881	3105	4234
transcript_995	gnl|BL_ORD_ID|41571 transcript_87743	2	2875	2872	3960	1	2879	3103	4188
transcript_99524	gnl|BL_ORD_ID|19212 transcript_54738	133	1211	4	135	1195	2273	6	143
transcript_99553	gnl|BL_ORD_ID|66178 transcript_124736	1	1417	1412	2021	2	1419	3256	3865
transcript_99553	gnl|BL_ORD_ID|71907 transcript_134034	1	1413	1410	2021	19	1431	1617	2228
transcript_99558	gnl|BL_ORD_ID|42042 transcript_88481	1	2456	2453	3392	18	2471	2629	3572
transcript_99600	gnl|BL_ORD_ID|27881 transcript_67225	2	2406	2405	2777	101	2505	2616	2988
transcript_99601	gnl|BL_ORD_ID|4186 transcript_28042	1	1135	1132	1922	1	1135	1272	2062
transcript_99601	gnl|BL_ORD_ID|3807 transcript_27212	1	1135	1132	1924	1	1121	1258	2048
transcript_99646	gnl|BL_ORD_ID|38246 transcript_7208	1	1431	1431	2268	297	1727	2250	3087
transcript_99683	gnl|BL_ORD_ID|85041 transcript_154230	1	1527	1522	2593	65	1600	2250	3321
transcript_99683	gnl|BL_ORD_ID|70329 transcript_131474	1	1620	1619	2554	90	1701	1807	2740
transcript_9969	gnl|BL_ORD_ID|18297 transcript_53260	2	2652	2650	2863	93	2730	4229	4442
transcript_99696	gnl|BL_ORD_ID|67505 transcript_126898	1	1752	1747	2386	1	1752	1900	2539
transcript_99701	gnl|BL_ORD_ID|39492 transcript_84336	308	2486	5	308	447	2621	4	306
transcript_99703	gnl|BL_ORD_ID|719 transcript_1250	191	3738	1	191	355	3902	2	192
transcript_99710	gnl|BL_ORD_ID|34069 transcript_77243	215	7356	2	218	547	7707	18	234
transcript_99742	gnl|BL_ORD_ID|19628 transcript_55431	253	2027	56	258	1954	3726	1	203
transcript_99742	gnl|BL_ORD_ID|83016 transcript_150778	253	2045	56	258	2320	4105	1	200
transcript_99753	gnl|BL_ORD_ID|65240 transcript_12627	14	1626	1625	2168	2	1651	2170	2714

transcript_99759	gnl|BL_ORD_ID|37767 transcript_83171	1161	2662	109	1161	1170	2670	2	1051
transcript_99763	gnl|BL_ORD_ID|42005 transcript_88424	1009	2540	1	1010	1189	2720	1	1034
transcript_99815	gnl|BL_ORD_ID|94833 transcript_18085	153	2139	1	158	406	2392	2	159
transcript_99876	gnl|BL_ORD_ID|25803 transcript_63934	15	2589	2588	3203	2	2581	2683	3299
transcript_99899	gnl|BL_ORD_ID|70441 transcript_131677	273	2803	1	273	731	3265	14	292
transcript_99899	gnl|BL_ORD_ID|70022 transcript_130966	273	2769	1	273	421	2921	46	319
transcript_99899	gnl|BL_ORD_ID|32411 transcript_74610	273	2803	1	276	393	2925	12	282
transcript_99899	gnl|BL_ORD_ID|31836 transcript_73623	273	2799	1	273	604	3132	8	271
transcript_99899	gnl|BL_ORD_ID|60383 transcript_116960	273	2789	16	273	713	3232	2	260
transcript_99965	gnl|BL_ORD_ID|67392 transcript_126708	1096	3032	1	1097	3961	5922	1185	2278
transcript_99974	gnl|BL_ORD_ID|84546 transcript_153437	1	1047	1045	1716	1	1044	1436	2107
transcript_99974	gnl|BL_ORD_ID|434 transcript_743	1	1047	1045	1716	1	1059	1597	2314
