# Supplementary material for: PacBio Single-Molecule Long-Read Sequencing Reveals Genes Tolerating Manganese Stress in Schima superba Saplings
Source: Front Genet. 2021 Apr 6;12:635043. doi: 10.3389/fgene.2021.635043 (PMC8057201; doi:10.3389/fgene.2021.635043)
Supplement: Supplementary Table 3 — Summary of identified TFs. [file Data_Sheet_6.PDF]

PacBio single-molecule long-read sequencing successfully explores the transcriptome of *Schima superba*

Fiza Liaquat[1], Muhammad Farooq Hussain Munis[2], Samiah Arif[1], Urooj Haroon[2], Muhammad Ashraf[3], Saddam Saqib[4,5], Wajid Zaman[4,5], Che Shengquan[6] and Liu Qunlu[6*]

1 School of Agriculture and Biology, Shanghai Jiao Tong University, Shanghai, 200240, China;

2 Department of Plant Sciences, Faculty of Biological Sciences, Quaid-i-Azam University, Islamabad, 45320, Pakistan;

3 Joint International Research Laboratory of Metabolic and Developmental Sciences, School of Life Science and Biotechnology, Shanghai Jiao Tong University, Shanghai, 200240, China;

4 State Key Laboratory of Systematic and Evolutionary Botany, Institute of Botany, Chinese Academy of Sciences, Beijing 100093, China;

5 University of Chinese Academy of Sciences, Beijing 100049, China;

6 Department of Landscape Architecture, School of Design, Shanghai Jiao Tong University, 200240, China;

*Correspondence: liuql@sjtu.edu.cn

Table S3 Summary of identified TFs

#ID	Family	Type
F01_transcript_100023	C2C2-GATA	TF
F01_transcript_100055	SET	TR
F01_transcript_100104	HB-other	TF
F01_transcript_100113	TUB	TF
F01_transcript_100127	SNF2	TR
F01_transcript_100151	bZIP	TF
F01_transcript_100165	PHD	TR
F01_transcript_100170	WRKY	TF
F01_transcript_100196	PHD	TR
F01_transcript_100201	GRAS	TF
F01_transcript_100215	SNF2	TR
F01_transcript_100228	FAR1	TF
F01_transcript_100233	Others	TR
F01_transcript_100250	RWP-RK	TF
F01_transcript_100270	HB-BELL	TF
F01_transcript_100282	PHD	TR
F01_transcript_100434	RWP-RK	TF
F01_transcript_100438	MYB	TF
F01_transcript_100510	NAC	TF
F01_transcript_100513	GARP-ARR-B	TF
F01_transcript_100559	Trihelix	TF
F01_transcript_100599	Trihelix	TF
F01_transcript_100655	GARP-G2-like	TF
F01_transcript_100709	C2C2-GATA	TF
F01_transcript_100728	GARP-G2-like	TF
F01_transcript_100745	C2H2	TF
F01_transcript_100752	GARP-G2-like	TF
F01_transcript_100760	MYB-related	TF
F01_transcript_100799	SBP	TF
F01_transcript_100832	SET	TR
F01_transcript_100837	LUG	TR
F01_transcript_100860	HB-BELL	TF
F01_transcript_100883	mTERF	TR
F01_transcript_100888	bHLH	TF
F01_transcript_100903	GRAS	TF
F01_transcript_100907	SBP	TF
F01_transcript_100942	MYB-related	TF
F01_transcript_101019	AUX/IAA	TR
F01_transcript_101034	bHLH	TF
F01_transcript_101036	WRKY	TF
F01_transcript_101055	SNF2	TR
F01_transcript_101077	TUB	TF
F01_transcript_101085	GRF	TF
F01_transcript_101099	MYB-related	TF
F01_transcript_101108	BBR-BPC	TF
F01_transcript_101115	FAR1	TF
F01_transcript_10123	ARID	TR
F01_transcript_10134	HB-BELL	TF
F01_transcript_101359	Trihelix	TF
F01_transcript_101360	B3-ARF	TF
F01_transcript_1014	SBP	TF

F01_transcript_10140	Pseudo ARR-B	TR
F01_transcript_101454	MYB-related	TF
F01_transcript_101467	bZIP	TF
F01_transcript_10148	DDT	TR
F01_transcript_101552	SNF2	TR
F01_transcript_101598	SET	TR
F01_transcript_101626	HMG	TR
F01_transcript_10163	GARP-G2-like	TF
F01_transcript_101654	WRKY	TF
F01_transcript_101696	RWP-RK	TF
F01_transcript_1017	HB-HD-ZIP	TF
F01_transcript_101734	bHLH	TF
F01_transcript_10174	Trihelix	TF
F01_transcript_101746	HSF	TF
F01_transcript_101780	HB-BELL	TF
F01_transcript_101797	SET	TR
F01_transcript_101844	HB-BELL	TF
F01_transcript_101920	RWP-RK	TF
F01_transcript_101961	IWS1	TR
F01_transcript_101985	HB-KNOX	TF
F01_transcript_101988	WRKY	TF
F01_transcript_102006	MYB-related	TF
F01_transcript_102051	MYB-related	TF
F01_transcript_102073	IWS1	TR
F01_transcript_102130	C2H2	TF
F01_transcript_102132	GRAS	TF
F01_transcript_102230	C3H	TF
F01_transcript_10236	HB-BELL	TF
F01_transcript_102396	AP2/ERF-ERF	TF
F01_transcript_102466	Trihelix	TF
F01_transcript_102489	HB-HD-ZIP	TF
F01_transcript_102490	bHLH	TF
F01_transcript_10250	TRAF	TR
F01_transcript_102549	VOZ	TF
F01_transcript_102563	HB-BELL	TF
F01_transcript_102585	B3	TF
F01_transcript_102586	MYB	TF
F01_transcript_102597	PHD	TR
F01_transcript_102659	C3H	TF
F01_transcript_102666	Trihelix	TF
F01_transcript_10270	GARP-ARR-B	TF
F01_transcript_102705	RB	TR
F01_transcript_102710	HB-BELL	TF
F01_transcript_102781	SNF2	TR
F01_transcript_102813	MYB-related	TF
F01_transcript_102814	Others	TR
F01_transcript_102844	AP2/ERF-ERF	TF
F01_transcript_102852	B3-ARF	TF
F01_transcript_102893	Jumonji	TR
F01_transcript_102908	RWP-RK	TF
F01_transcript_102909	PHD	TR
F01_transcript_102924	zf-HD	TF
F01_transcript_10293	GRAS	TF
F01_transcript_102935	SNF2	TR
F01_transcript_102937	C3H	TF

F01_transcript_102955	GARP-G2-like	TF
F01_transcript_103007	PHD	TR
F01_transcript_103045	bHLH	TF
F01_transcript_103063	HB-other	TF
F01_transcript_103101	WRKY	TF
F01_transcript_103107	Coactivator p15	TR
F01_transcript_103150	C3H	TF
F01_transcript_103151	Others	TR
F01_transcript_103193	GRAS	TF
F01_transcript_1032	B3-ARF	TF
F01_transcript_103203	C3H	TF
F01_transcript_103229	HSF	TF
F01_transcript_103240	C3H	TF
F01_transcript_103242	HB-BELL	TF
F01_transcript_103257	bHLH	TF
F01_transcript_103275	SBP	TF
F01_transcript_103317	PHD	TR
F01_transcript_103358	SET	TR
F01_transcript_10336	WRKY	TF
F01_transcript_103469	bHLH	TF
F01_transcript_103546	MYB	TF
F01_transcript_103565	MYB-related	TF
F01_transcript_103603	SET	TR
F01_transcript_10364	SET	TR
F01_transcript_103640	B3	TF
F01_transcript_103649	GARP-G2-like	TF
F01_transcript_103683	FAR1	TF
F01_transcript_103724	B3-ARF	TF
F01_transcript_103727	Others	TR
F01_transcript_103753	HSF	TF
F01_transcript_103763	AP2/ERF-AP2	TF
F01_transcript_103841	bHLH	TF
F01_transcript_103849	MYB-related	TF
F01_transcript_103929	WRKY	TF
F01_transcript_103936	AUX/IAA	TR
F01_transcript_103965	bZIP	TF
F01_transcript_103969	NAC	TF
F01_transcript_103997	MYB	TF
F01_transcript_104018	ARID	TR
F01_transcript_10407	PHD	TR
F01_transcript_104132	Jumonji	TR
F01_transcript_104139	TCP	TF
F01_transcript_104181	HB-BELL	TF
F01_transcript_104183	mTERF	TR
F01_transcript_104184	NAC	TF
F01_transcript_104220	HB-other	TF
F01_transcript_104223	Others	TR
F01_transcript_10425	SWI/SNF-BAF60b	TR
F01_transcript_104253	LUG	TR
F01_transcript_104262	HSF	TF
F01_transcript_104263	PHD	TR
F01_transcript_104264	GRAS	TF
F01_transcript_104283	bZIP	TF
F01_transcript_104322	NAC	TF

F01_transcript_104333	BBR-BPC	TF
F01_transcript_104339	C2H2	TF
F01_transcript_104347	SET	TR
F01_transcript_10435	GRAS	TF
F01_transcript_104381	ARID	TR
F01_transcript_10439	RWP-RK	TF
F01_transcript_104401	C2C2-CO-like	TF
F01_transcript_104412	Others	TR
F01_transcript_104413	Alfin-like	TF
F01_transcript_104415	FAR1	TF
F01_transcript_104449	C2C2-GATA	TF
F01_transcript_104503	GARP-G2-like	TF
F01_transcript_104521	TCP	TF
F01_transcript_104525	SNF2	TR
F01_transcript_104543	TUB	TF
F01_transcript_104573	bHLH	TF
F01_transcript_104671	WRKY	TF
F01_transcript_104739	NAC	TF
F01_transcript_104753	AUX/IAA	TR
F01_transcript_104788	C3H	TF
F01_transcript_104793	RWP-RK	TF
F01_transcript_104795	Others	TR
F01_transcript_104807	MYB	TF
F01_transcript_104851	AUX/IAA	TR
F01_transcript_10487	C3H	TF
F01_transcript_104928	SET	TR
F01_transcript_104994	Others	TR
F01_transcript_105007	MYB	TF
F01_transcript_105020	HB-BELL	TF
F01_transcript_105040	MYB-related	TF
F01_transcript_105090	DBB	TF
F01_transcript_105142	RWP-RK	TF
F01_transcript_10518	CPP	TF
F01_transcript_10529	DBB	TF
F01_transcript_105307	HRT	TF
F01_transcript_105338	bHLH	TF
F01_transcript_105341	IWS1	TR
F01_transcript_1054	RWP-RK	TF
F01_transcript_10545	Jumonji	TR
F01_transcript_105468	mTERF	TR
F01_transcript_105499	FAR1	TF
F01_transcript_105552	HMG	TR
F01_transcript_105602	TRAF	TR
F01_transcript_105603	TUB	TF
F01_transcript_105823	Others	TR
F01_transcript_105826	C3H	TF
F01_transcript_105832	HB-BELL	TF
F01_transcript_105876	HB-BELL	TF
F01_transcript_10589	TRAF	TR
F01_transcript_105898	bHLH	TF
F01_transcript_105930	Others	TR
F01_transcript_105944	GNAT	TR
F01_transcript_106045	TRAF	TR
F01_transcript_106054	PHD	TR
F01_transcript_106066	HB-other	TF

F01_transcript_10609	mTERF	TR
F01_transcript_106109	HB-BELL	TF
F01_transcript_10612	GRAS	TF
F01_transcript_106125	bHLH	TF
F01_transcript_106147	GRAS	TF
F01_transcript_106163	GARP-G2-like	TF
F01_transcript_106173	FAR1	TF
F01_transcript_106216	HB-WOX	TF
F01_transcript_106236	C2C2-YABBY	TF
F01_transcript_106256	bZIP	TF
F01_transcript_106269	GRAS	TF
F01_transcript_106394	RWP-RK	TF
F01_transcript_106536	AP2/ERF-ERF	TF
F01_transcript_106545	SET	TR
F01_transcript_106568	HB-WOX	TF
F01_transcript_106581	bHLH	TF
F01_transcript_10662	B3-ARF	TF
F01_transcript_106637	RWP-RK	TF
F01_transcript_106654	GRAS	TF
F01_transcript_106689	Tify	TF
F01_transcript_106715	HB-other	TF
F01_transcript_106725	mTERF	TR
F01_transcript_106734	HB-other	TF
F01_transcript_106754	IWS1	TR
F01_transcript_106758	NF-YB	TF
F01_transcript_106810	AP2/ERF-ERF	TF
F01_transcript_106902	bHLH	TF
F01_transcript_106905	B3	TF
F01_transcript_106934	C2H2	TF
F01_transcript_106953	Rcd1-like	TR
F01_transcript_107	SBP	TF
F01_transcript_107045	SBP	TF
F01_transcript_10707	HB-BELL	TF
F01_transcript_107127	SWI/SNF-BAF60b	TR
F01_transcript_107159	FAR1	TF
F01_transcript_107208	AUX/IAA	TR
F01_transcript_107218	C2C2-Dof	TF
F01_transcript_107290	MYB-related	TF
F01_transcript_107295	RWP-RK	TF
F01_transcript_10731	SET	TR
F01_transcript_10734	TRAF	TR
F01_transcript_107382	SNF2	TR
F01_transcript_107405	TCP	TF
F01_transcript_107440	HB-other	TF
F01_transcript_107532	bZIP	TF
F01_transcript_107545	bHLH	TF
F01_transcript_10758	HB-BELL	TF
F01_transcript_107581	AP2/ERF-ERF	TF
F01_transcript_107650	C2C2-Dof	TF
F01_transcript_107655	GRAS	TF
F01_transcript_107696	AUX/IAA	TR
F01_transcript_107740	RWP-RK	TF
F01_transcript_107763	MADS-M-type	TF
F01_transcript_107781	FAR1	TF

F01_transcript_107789	HB-KNOX	TF
F01_transcript_107802	C2C2-YABBY	TF
F01_transcript_107837	GARP-G2-like	TF
F01_transcript_10784	HB-BELL	TF
F01_transcript_107847	TRAF	TR
F01_transcript_107859	Others	TR
F01_transcript_107877	Pseudo ARR-B	TR
F01_transcript_107929	C2H2	TF
F01_transcript_107932	TCP	TF
F01_transcript_107938	C3H	TF
F01_transcript_107944	SET	TR
F01_transcript_107989	FAR1	TF
F01_transcript_1080	DDT	TR
F01_transcript_108065	MADS-M-type	TF
F01_transcript_108076	AUX/IAA	TR
F01_transcript_108148	RWP-RK	TF
F01_transcript_108155	Others	TR
F01_transcript_108231	HB-BELL	TF
F01_transcript_108237	Trihelix	TF
F01_transcript_108270	TRAF	TR
F01_transcript_108305	MYB	TF
F01_transcript_108322	bZIP	TF
F01_transcript_108323	C3H	TF
F01_transcript_108415	C3H	TF
F01_transcript_108628	FAR1	TF
F01_transcript_108633	GNAT	TR
F01_transcript_108670	Others	TR
F01_transcript_108690	SWI/SNF-BAF60b	TR
F01_transcript_108695	Others	TR
F01_transcript_10873	AP2/ERF-AP2	TF
F01_transcript_108938	TRAF	TR
F01_transcript_108994	TUB	TF
F01_transcript_10902	MYB-related	TF
F01_transcript_109152	HB-HD-ZIP	TF
F01_transcript_109205	HB-BELL	TF
F01_transcript_109270	FAR1	TF
F01_transcript_109308	Alfin-like	TF
F01_transcript_109373	C2H2	TF
F01_transcript_10938	C3H	TF
F01_transcript_109397	Others	TR
F01_transcript_109403	C2C2-Dof	TF
F01_transcript_109429	Jumonji	TR
F01_transcript_109449	HB-HD-ZIP	TF
F01_transcript_10948	ARID	TR
F01_transcript_10954	bHLH	TF
F01_transcript_109548	MYB-related	TF
F01_transcript_10956	CPP	TF
F01_transcript_109570	IWS1	TR
F01_transcript_109639	bZIP	TF
F01_transcript_109702	HSF	TF
F01_transcript_109727	BES1	TF
F01_transcript_109732	bHLH	TF
F01_transcript_109759	MYB-related	TF
F01_transcript_109781	TRAF	TR
F01_transcript_109795	C2H2	TF

F01_transcript_109808	Trihelix	TF
F01_transcript_109852	AUX/IAA	TR
F01_transcript_109984	bHLH	TF
F01_transcript_110058	C3H	TF
F01_transcript_110065	C2H2	TF
F01_transcript_110087	bZIP	TF
F01_transcript_110095	zf-HD	TF
F01_transcript_110183	C2H2	TF
F01_transcript_110190	B3-ARF	TF
F01_transcript_110218	TCP	TF
F01_transcript_110221	FAR1	TF
F01_transcript_110244	RWP-RK	TF
F01_transcript_110299	WRKY	TF
F01_transcript_11034	Pseudo ARR-B	TR
F01_transcript_110341	BBR-BPC	TF
F01_transcript_110366	SET	TR
F01_transcript_110404	BES1	TF
F01_transcript_11057	C2H2	TF
F01_transcript_110576	C2C2-GATA	TF
F01_transcript_110687	C2H2	TF
F01_transcript_110715	E2F-DP	TF
F01_transcript_110741	HB-HD-ZIP	TF
F01_transcript_110748	PHD	TR
F01_transcript_110784	GRAS	TF
F01_transcript_11088	CPP	TF
F01_transcript_110937	SNF2	TR
F01_transcript_110956	NF-YA	TF
F01_transcript_110961	HB-KNOX	TF
F01_transcript_110971	AUX/IAA	TR
F01_transcript_111043	GARP-G2-like	TF
F01_transcript_111059	GARP-ARR-B	TF
F01_transcript_111069	NAC	TF
F01_transcript_111085	ARID	TR
F01_transcript_111094	Others	TR
F01_transcript_11111	Others	TR
F01_transcript_111162	FAR1	TF
F01_transcript_111197	mTERF	TR
F01_transcript_111206	TUB	TF
F01_transcript_11133	SET	TR
F01_transcript_111398	RWP-RK	TF
F01_transcript_111400	MYB	TF
F01_transcript_111407	MADS-MIKC	TF
F01_transcript_111423	RWP-RK	TF
F01_transcript_111464	MYB	TF
F01_transcript_111578	SET	TR
F01_transcript_111587	HB-KNOX	TF
F01_transcript_111612	B3-ARF	TF
F01_transcript_111622	MADS-M-type	TF
F01_transcript_111626	GNAT	TR
F01_transcript_111646	C3H	TF
F01_transcript_111654	C3H	TF
F01_transcript_111669	mTERF	TR
F01_transcript_111670	TCP	TF
F01_transcript_111788	HB-BELL	TF

F01_transcript_111800	LOB	TF
F01_transcript_111804	NF-YC	TF
F01_transcript_111817	NF-YC	TF
F01_transcript_111837	MYB-related	TF
F01_transcript_111862	C2H2	TF
F01_transcript_111896	C2C2-CO-like	TF
F01_transcript_111916	Tify	TF
F01_transcript_111943	bHLH	TF
F01_transcript_111944	MYB	TF
F01_transcript_111960	AP2/ERF-ERF	TF
F01_transcript_111969	DDT	TR
F01_transcript_111973	B3	TF
F01_transcript_111975	PHD	TR
F01_transcript_11200	HB-BELL	TF
F01_transcript_11202	TCP	TF
F01_transcript_112025	AP2/ERF-AP2	TF
F01_transcript_112030	RWP-RK	TF
F01_transcript_112042	bZIP	TF
F01_transcript_112053	bHLH	TF
F01_transcript_112074	B3-ARF	TF
F01_transcript_11209	HB-BELL	TF
F01_transcript_112187	C2H2	TF
F01_transcript_112188	bHLH	TF
F01_transcript_112258	Others	TR
F01_transcript_112308	GARP-G2-like	TF
F01_transcript_112446	AP2/ERF-ERF	TF
F01_transcript_112450	NAC	TF
F01_transcript_112481	HB-BELL	TF
F01_transcript_112516	Others	TR
F01_transcript_11253	B3-ARF	TF
F01_transcript_112537	SNF2	TR
F01_transcript_112549	RWP-RK	TF
F01_transcript_112562	Pseudo ARR-B	TR
F01_transcript_112651	MADS-MIKC	TF
F01_transcript_112653	AP2/ERF-ERF	TF
F01_transcript_112657	HB-other	TF
F01_transcript_112686	HB-BELL	TF
F01_transcript_11272	HB-BELL	TF
F01_transcript_112772	Others	TR
F01_transcript_112773	SBP	TF
F01_transcript_112792	FAR1	TF
F01_transcript_112821	C2H2	TF
F01_transcript_112830	mTERF	TR
F01_transcript_112850	AUX/IAA	TR
F01_transcript_112878	TUB	TF
F01_transcript_11294	RWP-RK	TF
F01_transcript_112960	C2C2-GATA	TF
F01_transcript_1131	C2H2	TF
F01_transcript_113106	MYB	TF
F01_transcript_113228	AP2/ERF-ERF	TF
F01_transcript_113250	WRKY	TF
F01_transcript_113251	C2H2	TF
F01_transcript_113254	MYB-related	TF
F01_transcript_113262	SET	TR
F01_transcript_113276	SNF2	TR

F01_transcript_113279	bZIP	TF
F01_transcript_113303	bHLH	TF
F01_transcript_113321	Others	TR
F01_transcript_113341	GARP-G2-like	TF
F01_transcript_113345	C2H2	TF
F01_transcript_113376	TRAF	TR
F01_transcript_113381	bZIP	TF
F01_transcript_113408	AUX/IAA	TR
F01_transcript_113446	RWP-RK	TF
F01_transcript_113454	MYB	TF
F01_transcript_113490	C3H	TF
F01_transcript_113503	MYB-related	TF
F01_transcript_113506	B3	TF
F01_transcript_113510	MYB-related	TF
F01_transcript_113548	Whirly	TF
F01_transcript_113613	bZIP	TF
F01_transcript_113694	C3H	TF
F01_transcript_11372	GRAS	TF
F01_transcript_113734	C2H2	TF
F01_transcript_113788	WRKY	TF
F01_transcript_113793	mTERF	TR
F01_transcript_113817	TUB	TF
F01_transcript_113869	SET	TR
F01_transcript_113907	C2H2	TF
F01_transcript_113922	ARID	TR
F01_transcript_113928	TUB	TF
F01_transcript_113989	RWP-RK	TF
F01_transcript_114029	MYB-related	TF
F01_transcript_114034	C2C2-GATA	TF
F01_transcript_114077	TCP	TF
F01_transcript_114089	MYB-related	TF
F01_transcript_114225	MYB-related	TF
F01_transcript_114247	C2C2-GATA	TF
F01_transcript_114281	bHLH	TF
F01_transcript_114282	Trihelix	TF
F01_transcript_114348	WRKY	TF
F01_transcript_114352	LOB	TF
F01_transcript_114414	FAR1	TF
F01_transcript_114447	C2H2	TF
F01_transcript_114465	mTERF	TR
F01_transcript_114509	SNF2	TR
F01_transcript_114514	EIL	TF
F01_transcript_114522	WRKY	TF
F01_transcript_114529	B3	TF
F01_transcript_114556	mTERF	TR
F01_transcript_11456	Pseudo ARR-B	TR
F01_transcript_114563	C2H2	TF
F01_transcript_114664	C2C2-YABBY	TF
F01_transcript_114666	Trihelix	TF
F01_transcript_114669	Others	TR
F01_transcript_114685	mTERF	TR
F01_transcript_114720	TRAF	TR
F01_transcript_114733	Others	TR
F01_transcript_1148	PHD	TR

F01_transcript_114808	RWP-RK	TF
F01_transcript_11481	HB-BELL	TF
F01_transcript_114829	C2H2	TF
F01_transcript_114850	SET	TR
F01_transcript_114858	SBP	TF
F01_transcript_114912	NAC	TF
F01_transcript_114973	mTERF	TR
F01_transcript_114976	MYB-related	TF
F01_transcript_114988	NF-YC	TF
F01_transcript_11499	Pseudo ARR-B	TR
F01_transcript_11500	bHLH	TF
F01_transcript_115083	MYB-related	TF
F01_transcript_115086	RWP-RK	TF
F01_transcript_115161	BBR-BPC	TF
F01_transcript_115183	FAR1	TF
F01_transcript_11523	Others	TR
F01_transcript_115238	HSF	TF
F01_transcript_115241	C2H2	TF
F01_transcript_115242	B3	TF
F01_transcript_115337	bHLH	TF
F01_transcript_115348	B3-ARF	TF
F01_transcript_115379	PHD	TR
F01_transcript_115510	MADS-MIKC	TF
F01_transcript_11554	GRAS	TF
F01_transcript_115542	SET	TR
F01_transcript_115547	bHLH	TF
F01_transcript_115550	HMG	TR
F01_transcript_115585	WRKY	TF
F01_transcript_115597	NAC	TF
F01_transcript_115658	IWS1	TR
F01_transcript_115686	WRKY	TF
F01_transcript_115691	bHLH	TF
F01_transcript_115699	TUB	TF
F01_transcript_1157	IWS1	TR
F01_transcript_115740	C2H2	TF
F01_transcript_115793	HB-KNOX	TF
F01_transcript_115805	FAR1	TF
F01_transcript_115816	B3	TF
F01_transcript_115826	NAC	TF
F01_transcript_115862	TRAF	TR
F01_transcript_115866	MYB-related	TF
F01_transcript_115868	SBP	TF
F01_transcript_11587	FAR1	TF
F01_transcript_115878	BBR-BPC	TF
F01_transcript_115889	RWP-RK	TF
F01_transcript_11590	HB-BELL	TF
F01_transcript_115900	NAC	TF
F01_transcript_115912	AP2/ERF-ERF	TF
F01_transcript_115922	AP2/ERF-ERF	TF
F01_transcript_115949	bHLH	TF
F01_transcript_115970	MYB-related	TF
F01_transcript_115986	MYB-related	TF
F01_transcript_115993	VOZ	TF
F01_transcript_116013	bHLH	TF
F01_transcript_116017	C2C2-GATA	TF

F01_transcript_116019	NAC	TF
F01_transcript_116022	FAR1	TF
F01_transcript_116041	AUX/IAA	TR
F01_transcript_116049	bZIP	TF
F01_transcript_116052	PHD	TR
F01_transcript_116098	NAC	TF
F01_transcript_116119	C3H	TF
F01_transcript_116134	Trihelix	TF
F01_transcript_116157	HSF	TF
F01_transcript_11616	SET	TR
F01_transcript_11620	bHLH	TF
F01_transcript_116210	MYB-related	TF
F01_transcript_116219	Others	TR
F01_transcript_116255	TUB	TF
F01_transcript_116270	Others	TR
F01_transcript_116297	RWP-RK	TF
F01_transcript_116314	FAR1	TF
F01_transcript_116349	ARID	TR
F01_transcript_116368	C2H2	TF
F01_transcript_11638	Others	TR
F01_transcript_116436	MYB-related	TF
F01_transcript_116502	mTERF	TR
F01_transcript_116505	GNAT	TR
F01_transcript_116566	PHD	TR
F01_transcript_116605	B3-ARF	TF
F01_transcript_116612	HB-KNOX	TF
F01_transcript_11662	bHLH	TF
F01_transcript_116623	B3	TF
F01_transcript_116650	MYB-related	TF
F01_transcript_116736	bHLH	TF
F01_transcript_116746	bHLH	TF
F01_transcript_116756	Others	TR
F01_transcript_116797	SET	TR
F01_transcript_116803	C2H2	TF
F01_transcript_116809	C2C2-YABBY	TF
F01_transcript_116826	C2C2-Dof	TF
F01_transcript_116840	GARP-G2-like	TF
F01_transcript_116852	MYB	TF
F01_transcript_116863	WRKY	TF
F01_transcript_116931	MYB	TF
F01_transcript_116952	bHLH	TF
F01_transcript_116961	MADS-M-type	TF
F01_transcript_1170	NF-X1	TF
F01_transcript_117024	GARP-ARR-B	TF
F01_transcript_117026	TRAF	TR
F01_transcript_117030	B3-ARF	TF
F01_transcript_117049	SWI/SNF-SWI3	TR
F01_transcript_117115	GNAT	TR
F01_transcript_117124	GARP-G2-like	TF
F01_transcript_117139	C3H	TF
F01_transcript_117158	RWP-RK	TF
F01_transcript_117197	HB-other	TF
F01_transcript_117200	RWP-RK	TF
F01_transcript_117246	AUX/IAA	TR

F01_transcript_117333	HB-HD-ZIP	TF
F01_transcript_117337	MADS-M-type	TF
F01_transcript_117338	C2C2-YABBY	TF
F01_transcript_117399	RWP-RK	TF
F01_transcript_117403	GRAS	TF
F01_transcript_117418	bHLH	TF
F01_transcript_117431	bHLH	TF
F01_transcript_117444	bHLH	TF
F01_transcript_117460	SBP	TF
F01_transcript_117461	B3	TF
F01_transcript_117489	HB-HD-ZIP	TF
F01_transcript_117565	MADS-MIKC	TF
F01_transcript_11758	Others	TR
F01_transcript_117583	PHD	TR
F01_transcript_117612	Jumonji	TR
F01_transcript_117630	SNF2	TR
F01_transcript_117637	SNF2	TR
F01_transcript_117655	FAR1	TF
F01_transcript_11769	MYB	TF
F01_transcript_11771	TRAF	TR
F01_transcript_117725	Tify	TF
F01_transcript_117761	B3-ARF	TF
F01_transcript_117767	C2H2	TF
F01_transcript_117800	C2H2	TF
F01_transcript_117905	MYB	TF
F01_transcript_117907	MYB-related	TF
F01_transcript_117912	NAC	TF
F01_transcript_117995	Others	TR
F01_transcript_118045	SBP	TF
F01_transcript_118068	bZIP	TF
F01_transcript_118075	MYB-related	TF
F01_transcript_118086	SWI/SNF-BAF60b	TR
F01_transcript_118095	TCP	TF
F01_transcript_118097	Jumonji	TR
F01_transcript_118116	NAC	TF
F01_transcript_118127	NAC	TF
F01_transcript_118171	C2C2-Dof	TF
F01_transcript_118187	B3-ARF	TF
F01_transcript_118195	RWP-RK	TF
F01_transcript_118198	Jumonji	TR
F01_transcript_118274	Trihelix	TF
F01_transcript_11828	Tify	TF
F01_transcript_118307	RWP-RK	TF
F01_transcript_118320	C3H	TF
F01_transcript_118332	PHD	TR
F01_transcript_11840	B3-ARF	TF
F01_transcript_118447	HB-BELL	TF
F01_transcript_118478	C2H2	TF
F01_transcript_118488	WRKY	TF
F01_transcript_118491	C2C2-GATA	TF
F01_transcript_118566	NAC	TF
F01_transcript_118572	mTERF	TR
F01_transcript_118654	bHLH	TF
F01_transcript_118696	TCP	TF
F01_transcript_118699	AP2/ERF-ERF	TF

F01_transcript_118706	GNAT	TR
F01_transcript_118757	FAR1	TF
F01_transcript_118808	B3-ARF	TF
F01_transcript_118809	mTERF	TR
F01_transcript_118823	bZIP	TF
F01_transcript_118828	bZIP	TF
F01_transcript_118845	BBR-BPC	TF
F01_transcript_118859	MYB	TF
F01_transcript_118902	RWP-RK	TF
F01_transcript_118906	Others	TR
F01_transcript_118930	bHLH	TF
F01_transcript_118943	Others	TR
F01_transcript_118996	TRAF	TR
F01_transcript_119013	SBP	TF
F01_transcript_119020	Others	TR
F01_transcript_119041	B3-ARF	TF
F01_transcript_119044	Others	TR
F01_transcript_119077	WRKY	TF
F01_transcript_119086	TRAF	TR
F01_transcript_119093	C2H2	TF
F01_transcript_119214	B3-ARF	TF
F01_transcript_119217	AP2/ERF-AP2	TF
F01_transcript_119231	SBP	TF
F01_transcript_119238	C2H2	TF
F01_transcript_119276	RWP-RK	TF
F01_transcript_119294	PHD	TR
F01_transcript_11935	SET	TR
F01_transcript_119406	NAC	TF
F01_transcript_11941	GRAS	TF
F01_transcript_119440	Tify	TF
F01_transcript_11948	RWP-RK	TF
F01_transcript_119494	FAR1	TF
F01_transcript_119513	GARP-ARR-B	TF
F01_transcript_11954	HB-BELL	TF
F01_transcript_11956	C3H	TF
F01_transcript_119574	bZIP	TF
F01_transcript_119595	Others	TR
F01_transcript_1196	DDT	TR
F01_transcript_119620	C3H	TF
F01_transcript_119626	C3H	TF
F01_transcript_119666	SNF2	TR
F01_transcript_1197	IWS1	TR
F01_transcript_119707	AP2/ERF-ERF	TF
F01_transcript_119716	TUB	TF
F01_transcript_119794	SBP	TF
F01_transcript_119821	IWS1	TR
F01_transcript_11986	C2H2	TF
F01_transcript_119895	ARID	TR
F01_transcript_119973	TRAF	TR
F01_transcript_119982	C2C2-CO-like	TF
F01_transcript_120091	Others	TR
F01_transcript_1201	Others	TR
F01_transcript_120100	C3H	TF
F01_transcript_120128	GARP-G2-like	TF

F01_transcript_120234	GNAT	TR
F01_transcript_120237	Jumonji	TR
F01_transcript_120278	C3H	TF
F01_transcript_120283	mTERF	TR
F01_transcript_120337	LUG	TR
F01_transcript_12034	GRAS	TF
F01_transcript_120500	SBP	TF
F01_transcript_12052	bZIP	TF
F01_transcript_120563	NAC	TF
F01_transcript_120571	Others	TR
F01_transcript_120576	bZIP	TF
F01_transcript_120584	GNAT	TR
F01_transcript_1207	RWP-RK	TF
F01_transcript_120711	RWP-RK	TF
F01_transcript_120738	TRAF	TR
F01_transcript_120744	WRKY	TF
F01_transcript_120757	bZIP	TF
F01_transcript_120784	WRKY	TF
F01_transcript_120797	NF-YA	TF
F01_transcript_120820	SNF2	TR
F01_transcript_120832	SET	TR
F01_transcript_120898	B3	TF
F01_transcript_120900	NAC	TF
F01_transcript_120927	B3-ARF	TF
F01_transcript_120928	Others	TR
F01_transcript_12094	NF-YA	TF
F01_transcript_120973	WRKY	TF
F01_transcript_120989	HSF	TF
F01_transcript_121022	C2H2	TF
F01_transcript_121032	bHLH	TF
F01_transcript_121088	MADS-MIKC	TF
F01_transcript_121092	Others	TR
F01_transcript_121108	C3H	TF
F01_transcript_121152	bHLH	TF
F01_transcript_121155	MYB	TF
F01_transcript_121167	PHD	TR
F01_transcript_121172	HSF	TF
F01_transcript_121192	HB-BELL	TF
F01_transcript_121194	bHLH	TF
F01_transcript_121219	LOB	TF
F01_transcript_121227	TCP	TF
F01_transcript_121230	NF-X1	TF
F01_transcript_12124	RWP-RK	TF
F01_transcript_121244	B3	TF
F01_transcript_121263	GNAT	TR
F01_transcript_12130	WRKY	TF
F01_transcript_121316	Jumonji	TR
F01_transcript_121331	WRKY	TF
F01_transcript_121379	WRKY	TF
F01_transcript_1214	B3-ARF	TF
F01_transcript_121412	Tify	TF
F01_transcript_121455	FAR1	TF
F01_transcript_121479	NAC	TF
F01_transcript_121509	SNF2	TR
F01_transcript_121531	TCP	TF

F01_transcript_121550	TUB	TF
F01_transcript_121579	HB-KNOX	TF
F01_transcript_121747	NF-YA	TF
F01_transcript_121789	Tify	TF
F01_transcript_121801	DBB	TF
F01_transcript_121854	PHD	TR
F01_transcript_121900	Trihelix	TF
F01_transcript_121983	FAR1	TF
F01_transcript_12200	RWP-RK	TF
F01_transcript_122020	Whirly	TF
F01_transcript_122055	TUB	TF
F01_transcript_122100	Others	TR
F01_transcript_122104	bZIP	TF
F01_transcript_122122	RWP-RK	TF
F01_transcript_122138	RWP-RK	TF
F01_transcript_122146	Trihelix	TF
F01_transcript_12222	MYB	TF
F01_transcript_122224	MADS-MIKC	TF
F01_transcript_122237	C3H	TF
F01_transcript_122243	RWP-RK	TF
F01_transcript_122265	PHD	TR
F01_transcript_122356	AUX/IAA	TR
F01_transcript_12236	Others	TR
F01_transcript_122383	SET	TR
F01_transcript_122395	B3	TF
F01_transcript_122397	C3H	TF
F01_transcript_122414	RWP-RK	TF
F01_transcript_122427	GARP-G2-like	TF
F01_transcript_122467	MYB-related	TF
F01_transcript_122469	MYB	TF
F01_transcript_122473	RWP-RK	TF
F01_transcript_122475	GNAT	TR
F01_transcript_122497	RWP-RK	TF
F01_transcript_122538	B3-ARF	TF
F01_transcript_122542	C2C2-GATA	TF
F01_transcript_122543	RWP-RK	TF
F01_transcript_122558	DDT	TR
F01_transcript_122567	SNF2	TR
F01_transcript_122572	bZIP	TF
F01_transcript_122574	MYB-related	TF
F01_transcript_12261	bZIP	TF
F01_transcript_122641	SNF2	TR
F01_transcript_122693	RWP-RK	TF
F01_transcript_122705	MADS-M-type	TF
F01_transcript_122711	PLATZ	TF
F01_transcript_122713	mTERF	TR
F01_transcript_122758	HB-HD-ZIP	TF
F01_transcript_122785	NAC	TF
F01_transcript_122792	TCP	TF
F01_transcript_1228	CAMTA	TF
F01_transcript_122800	NF-YC	TF
F01_transcript_122819	Trihelix	TF
F01_transcript_122861	B3	TF
F01_transcript_122966	NAC	TF

F01_transcript_123006	bHLH	TF
F01_transcript_123023	bZIP	TF
F01_transcript_12305	HB-BELL	TF
F01_transcript_123057	BES1	TF
F01_transcript_12309	HB-BELL	TF
F01_transcript_123131	B3-ARF	TF
F01_transcript_123195	GNAT	TR
F01_transcript_123211	HB-HD-ZIP	TF
F01_transcript_12323	GARP-ARR-B	TF
F01_transcript_123294	TRAF	TR
F01_transcript_123297	AUX/IAA	TR
F01_transcript_123333	B3	TF
F01_transcript_123368	B3-ARF	TF
F01_transcript_123369	C2C2-GATA	TF
F01_transcript_123376	GARP-G2-like	TF
F01_transcript_123516	Others	TR
F01_transcript_123524	C3H	TF
F01_transcript_123530	MADS-MIKC	TF
F01_transcript_123536	SNF2	TR
F01_transcript_123541	GNAT	TR
F01_transcript_123567	Others	TR
F01_transcript_123572	bZIP	TF
F01_transcript_123573	HSF	TF
F01_transcript_123578	MYB	TF
F01_transcript_123588	bHLH	TF
F01_transcript_123623	B3	TF
F01_transcript_123635	Rcd1-like	TR
F01_transcript_123640	SNF2	TR
F01_transcript_123698	bHLH	TF
F01_transcript_123709	bHLH	TF
F01_transcript_123734	Trihelix	TF
F01_transcript_123738	SWI/SNF-BAF60b	TR
F01_transcript_123746	C3H	TF
F01_transcript_123792	GARP-G2-like	TF
F01_transcript_123802	C3H	TF
F01_transcript_123842	MYB-related	TF
F01_transcript_123849	Jumonji	TR
F01_transcript_123856	MYB	TF
F01_transcript_123882	Others	TR
F01_transcript_1239	RWP-RK	TF
F01_transcript_12391	Others	TR
F01_transcript_123936	bHLH	TF
F01_transcript_123953	C3H	TF
F01_transcript_124065	ARID	TR
F01_transcript_124075	Trihelix	TF
F01_transcript_124100	Jumonji	TR
F01_transcript_124107	NAC	TF
F01_transcript_124173	mTERF	TR
F01_transcript_124218	WRKY	TF
F01_transcript_124241	bHLH	TF
F01_transcript_124243	TUB	TF
F01_transcript_124335	B3	TF
F01_transcript_124376	zf-HD	TF
F01_transcript_124380	C2C2-Dof	TF
F01_transcript_124511	TRAF	TR

F01_transcript_124558	GARP-G2-like	TF
F01_transcript_12458	FAR1	TF
F01_transcript_124635	C3H	TF
F01_transcript_124699	C2C2-YABBY	TF
F01_transcript_1247	HB-HD-ZIP	TF
F01_transcript_124720	FAR1	TF
F01_transcript_124738	GARP-G2-like	TF
F01_transcript_124768	SWI/SNF-BAF60b	TR
F01_transcript_124847	RWP-RK	TF
F01_transcript_124848	C2H2	TF
F01_transcript_124852	C3H	TF
F01_transcript_124974	Others	TR
F01_transcript_125006	SNF2	TR
F01_transcript_125010	PHD	TR
F01_transcript_125052	HB-BELL	TF
F01_transcript_125083	AP2/ERF-ERF	TF
F01_transcript_125132	SBP	TF
F01_transcript_125149	C2C2-Dof	TF
F01_transcript_125166	bZIP	TF
F01_transcript_125193	mTERF	TR
F01_transcript_125197	SWI/SNF-BAF60b	TR
F01_transcript_125228	C2C2-Dof	TF
F01_transcript_125238	NAC	TF
F01_transcript_125255	HB-HD-ZIP	TF
F01_transcript_125276	MYB-related	TF
F01_transcript_125280	RWP-RK	TF
F01_transcript_125290	GARP-G2-like	TF
F01_transcript_125388	SWI/SNF-BAF60b	TR
F01_transcript_125421	B3-ARF	TF
F01_transcript_125435	IWS1	TR
F01_transcript_125461	AUX/IAA	TR
F01_transcript_125551	RWP-RK	TF
F01_transcript_125591	AP2/ERF-ERF	TF
F01_transcript_125603	AP2/ERF-ERF	TF
F01_transcript_125628	RWP-RK	TF
F01_transcript_125642	Whirly	TF
F01_transcript_125710	HB-HD-ZIP	TF
F01_transcript_125713	B3	TF
F01_transcript_125734	C2C2-GATA	TF
F01_transcript_125735	bZIP	TF
F01_transcript_12576	SET	TR
F01_transcript_125763	SBP	TF
F01_transcript_125789	GRAS	TF
F01_transcript_125791	Others	TR
F01_transcript_125819	HB-HD-ZIP	TF
F01_transcript_125824	B3-ARF	TF
F01_transcript_125855	AUX/IAA	TR
F01_transcript_125946	mTERF	TR
F01_transcript_125978	TUB	TF
F01_transcript_125982	RWP-RK	TF
F01_transcript_125988	SWI/SNF-SWI3	TR
F01_transcript_126011	ARID	TR
F01_transcript_126012	WRKY	TF
F01_transcript_126054	MYB	TF

F01_transcript_126061	Others	TR
F01_transcript_126088	NAC	TF
F01_transcript_1261	NF-X1	TF
F01_transcript_126236	SNF2	TR
F01_transcript_126290	HB-HD-ZIP	TF
F01_transcript_12632	bHLH	TF
F01_transcript_126326	Jumonji	TR
F01_transcript_126353	GARP-G2-like	TF
F01_transcript_126421	RWP-RK	TF
F01_transcript_126425	Others	TR
F01_transcript_126449	NF-YA	TF
F01_transcript_126454	MYB-related	TF
F01_transcript_126466	PHD	TR
F01_transcript_126485	bZIP	TF
F01_transcript_126507	B3-ARF	TF
F01_transcript_126562	B3	TF
F01_transcript_126573	C2H2	TF
F01_transcript_126630	MYB	TF
F01_transcript_126653	TRAF	TR
F01_transcript_126665	SET	TR
F01_transcript_126713	GARP-G2-like	TF
F01_transcript_126716	HB-other	TF
F01_transcript_126748	GARP-G2-like	TF
F01_transcript_126789	Pseudo ARR-B	TR
F01_transcript_126827	BBR-BPC	TF
F01_transcript_126858	TRAF	TR
F01_transcript_126871	C3H	TF
F01_transcript_126883	RWP-RK	TF
F01_transcript_126885	HB-BELL	TF
F01_transcript_126913	zf-HD	TF
F01_transcript_12693	EIL	TF
F01_transcript_126941	Others	TR
F01_transcript_126948	GeBP	TF
F01_transcript_126953	bHLH	TF
F01_transcript_126989	C2C2-CO-like	TF
F01_transcript_12699	DDT	TR
F01_transcript_126992	TRAF	TR
F01_transcript_126993	SET	TR
F01_transcript_127036	Others	TR
F01_transcript_127037	AP2/ERF-AP2	TF
F01_transcript_127039	GARP-G2-like	TF
F01_transcript_127124	Pseudo ARR-B	TR
F01_transcript_127129	SBP	TF
F01_transcript_127158	C2C2-Dof	TF
F01_transcript_127160	HB-BELL	TF
F01_transcript_127185	HB-other	TF
F01_transcript_127192	TUB	TF
F01_transcript_127235	AP2/ERF-ERF	TF
F01_transcript_127258	C2H2	TF
F01_transcript_127267	C3H	TF
F01_transcript_12729	SBP	TF
F01_transcript_127309	GRAS	TF
F01_transcript_127385	Others	TR
F01_transcript_127406	mTERF	TR
F01_transcript_12742	DDT	TR

F01_transcript_127461	C2H2	TF
F01_transcript_127480	HB-HD-ZIP	TF
F01_transcript_127503	AP2/ERF-ERF	TF
F01_transcript_127519	C2C2-GATA	TF
F01_transcript_127520	Whirly	TF
F01_transcript_127593	mTERF	TR
F01_transcript_127649	GRAS	TF
F01_transcript_127661	bHLH	TF
F01_transcript_127707	C2H2	TF
F01_transcript_127728	Tify	TF
F01_transcript_127729	GARP-ARR-B	TF
F01_transcript_127734	SNF2	TR
F01_transcript_127756	HB-BELL	TF
F01_transcript_127776	WRKY	TF
F01_transcript_127806	C3H	TF
F01_transcript_127853	Others	TR
F01_transcript_127872	bHLH	TF
F01_transcript_12794	C2H2	TF
F01_transcript_128074	bHLH	TF
F01_transcript_128132	B3	TF
F01_transcript_128239	SET	TR
F01_transcript_128254	MYB-related	TF
F01_transcript_128275	C2H2	TF
F01_transcript_128291	SBP	TF
F01_transcript_128296	AP2/ERF-ERF	TF
F01_transcript_128297	AP2/ERF-ERF	TF
F01_transcript_128306	C3H	TF
F01_transcript_128337	RWP-RK	TF
F01_transcript_12834	HB-BELL	TF
F01_transcript_128598	C2H2	TF
F01_transcript_128636	AP2/ERF-AP2	TF
F01_transcript_128760	TCP	TF
F01_transcript_128762	SNF2	TR
F01_transcript_128801	HB-other	TF
F01_transcript_128915	TRAF	TR
F01_transcript_128933	MYB	TF
F01_transcript_128953	zf-HD	TF
F01_transcript_128987	HB-BELL	TF
F01_transcript_12899	PHD	TR
F01_transcript_129048	MYB-related	TF
F01_transcript_12906	Others	TR
F01_transcript_129073	RWP-RK	TF
F01_transcript_129096	bHLH	TF
F01_transcript_129101	bZIP	TF
F01_transcript_129138	Whirly	TF
F01_transcript_129269	SET	TR
F01_transcript_129288	B3-ARF	TF
F01_transcript_129321	MYB-related	TF
F01_transcript_129521	WRKY	TF
F01_transcript_129533	WRKY	TF
F01_transcript_129587	Others	TR
F01_transcript_129629	SBP	TF
F01_transcript_129637	BBR-BPC	TF
F01_transcript_129655	C2H2	TF

F01_transcript_129730	CPP	TF
F01_transcript_12980	GRAS	TF
F01_transcript_12982	mTERF	TR
F01_transcript_129832	MYB-related	TF
F01_transcript_12990	SET	TR
F01_transcript_129919	FAR1	TF
F01_transcript_129936	FAR1	TF
F01_transcript_129940	MYB	TF
F01_transcript_129980	WRKY	TF
F01_transcript_130006	LIM	TF
F01_transcript_130015	HSF	TF
F01_transcript_130038	ARID	TR
F01_transcript_130055	MYB	TF
F01_transcript_130092	HSF	TF
F01_transcript_130094	Others	TR
F01_transcript_13010	MYB	TF
F01_transcript_130171	Tify	TF
F01_transcript_130173	bZIP	TF
F01_transcript_130185	C2H2	TF
F01_transcript_130210	PHD	TR
F01_transcript_130214	Whirly	TF
F01_transcript_130329	GRAS	TF
F01_transcript_130349	NF-YC	TF
F01_transcript_13036	Jumonji	TR
F01_transcript_130389	Others	TR
F01_transcript_130428	GeBP	TF
F01_transcript_130429	MADS-MIKC	TF
F01_transcript_130453	NF-YB	TF
F01_transcript_130471	HB-PHD	TF
F01_transcript_130489	AP2/ERF-ERF	TF
F01_transcript_130492	AP2/ERF-ERF	TF
F01_transcript_130522	C2C2-GATA	TF
F01_transcript_130541	ARID	TR
F01_transcript_130584	B3	TF
F01_transcript_130633	GARP-G2-like	TF
F01_transcript_130640	MYB	TF
F01_transcript_130682	HB-BELL	TF
F01_transcript_13070	Pseudo ARR-B	TR
F01_transcript_130727	Others	TR
F01_transcript_130757	C2H2	TF
F01_transcript_130765	NAC	TF
F01_transcript_130826	Others	TR
F01_transcript_130847	C2C2-GATA	TF
F01_transcript_130857	mTERF	TR
F01_transcript_130878	Jumonji	TR
F01_transcript_130906	C3H	TF
F01_transcript_13091	FAR1	TF
F01_transcript_130973	WRKY	TF
F01_transcript_131041	bHLH	TF
F01_transcript_131056	bZIP	TF
F01_transcript_131122	SBP	TF
F01_transcript_131158	WRKY	TF
F01_transcript_131230	IWS1	TR
F01_transcript_131233	bZIP	TF
F01_transcript_13124	Pseudo ARR-B	TR

F01_transcript_131270	Jumonji	TR
F01_transcript_131293	bZIP	TF
F01_transcript_131302	RWP-RK	TF
F01_transcript_131317	bHLH	TF
F01_transcript_131319	S1Fa-like	TF
F01_transcript_131322	C3H	TF
F01_transcript_131405	C2H2	TF
F01_transcript_131424	HSF	TF
F01_transcript_131428	LIM	TF
F01_transcript_131462	SWI/SNF-BAF60b	TR
F01_transcript_131484	CPP	TF
F01_transcript_131549	FAR1	TF
F01_transcript_131550	Others	TR
F01_transcript_13161	TUB	TF
F01_transcript_131619	B3-ARF	TF
F01_transcript_131644	NF-YC	TF
F01_transcript_131696	RWP-RK	TF
F01_transcript_131738	GARP-G2-like	TF
F01_transcript_131784	FAR1	TF
F01_transcript_131816	bZIP	TF
F01_transcript_13185	TCP	TF
F01_transcript_131850	HMG	TR
F01_transcript_131853	FAR1	TF
F01_transcript_131901	HB-BELL	TF
F01_transcript_131924	WRKY	TF
F01_transcript_132006	C3H	TF
F01_transcript_132023	GNAT	TR
F01_transcript_132034	bHLH	TF
F01_transcript_132086	C2H2	TF
F01_transcript_132092	RWP-RK	TF
F01_transcript_132095	SWI/SNF-BAF60b	TR
F01_transcript_132108	CPP	TF
F01_transcript_132110	NF-YC	TF
F01_transcript_132181	Others	TR
F01_transcript_132187	RWP-RK	TF
F01_transcript_132213	HB-WOX	TF
F01_transcript_132221	C2C2-YABBY	TF
F01_transcript_132237	Others	TR
F01_transcript_132276	PHD	TR
F01_transcript_132358	GRAS	TF
F01_transcript_132371	bHLH	TF
F01_transcript_132386	GNAT	TR
F01_transcript_132398	B3-ARF	TF
F01_transcript_132509	E2F-DP	TF
F01_transcript_132516	AP2/ERF-ERF	TF
F01_transcript_132517	BBR-BPC	TF
F01_transcript_132564	B3	TF
F01_transcript_132587	bZIP	TF
F01_transcript_132629	SET	TR
F01_transcript_132631	Rcd1-like	TR
F01_transcript_132639	HB-KNOX	TF
F01_transcript_132711	Jumonji	TR
F01_transcript_132761	bZIP	TF
F01_transcript_132803	BBR-BPC	TF

F01_transcript_132827	bHLH	TF
F01_transcript_132948	HB-BELL	TF
F01_transcript_132986	TCP	TF
F01_transcript_133002	C2H2	TF
F01_transcript_133022	mTERF	TR
F01_transcript_133052	C2H2	TF
F01_transcript_133071	SNF2	TR
F01_transcript_133095	FAR1	TF
F01_transcript_133124	WRKY	TF
F01_transcript_133127	GARP-G2-like	TF
F01_transcript_13315	C3H	TF
F01_transcript_133151	HSF	TF
F01_transcript_133172	AUX/IAA	TR
F01_transcript_133192	MADS-M-type	TF
F01_transcript_133254	C3H	TF
F01_transcript_133274	MYB-related	TF
F01_transcript_133278	SBP	TF
F01_transcript_133286	C2H2	TF
F01_transcript_133323	MYB-related	TF
F01_transcript_133354	SBP	TF
F01_transcript_13340	TRAF	TR
F01_transcript_133403	BES1	TF
F01_transcript_133410	AUX/IAA	TR
F01_transcript_133423	GARP-G2-like	TF
F01_transcript_133516	C2H2	TF
F01_transcript_133548	LOB	TF
F01_transcript_13356	FAR1	TF
F01_transcript_133671	TRAF	TR
F01_transcript_133681	C2H2	TF
F01_transcript_133691	PHD	TR
F01_transcript_133743	GRAS	TF
F01_transcript_133772	C2H2	TF
F01_transcript_133949	bZIP	TF
F01_transcript_133958	AUX/IAA	TR
F01_transcript_133969	ARID	TR
F01_transcript_133972	C2H2	TF
F01_transcript_133976	MYB-related	TF
F01_transcript_133990	SET	TR
F01_transcript_133992	GNAT	TR
F01_transcript_134099	MYB-related	TF
F01_transcript_13414	GARP-G2-like	TF
F01_transcript_134144	Others	TR
F01_transcript_134154	CAMTA	TF
F01_transcript_134184	GNAT	TR
F01_transcript_134262	SET	TR
F01_transcript_134275	SBP	TF
F01_transcript_134279	mTERF	TR
F01_transcript_134314	PHD	TR
F01_transcript_134330	TCP	TF
F01_transcript_134372	B3-ARF	TF
F01_transcript_134384	bHLH	TF
F01_transcript_134435	Others	TR
F01_transcript_134451	Tify	TF
F01_transcript_134496	PHD	TR
F01_transcript_13452	GARP-ARR-B	TF

F01_transcript_134532	SET	TR
F01_transcript_134541	FAR1	TF
F01_transcript_134555	mTERF	TR
F01_transcript_134556	B3	TF
F01_transcript_134574	GARP-G2-like	TF
F01_transcript_134655	C2C2-GATA	TF
F01_transcript_134674	WRKY	TF
F01_transcript_134686	GARP-G2-like	TF
F01_transcript_134691	GRAS	TF
F01_transcript_134703	MADS-M-type	TF
F01_transcript_134779	SNF2	TR
F01_transcript_134817	Jumonji	TR
F01_transcript_134829	MYB-related	TF
F01_transcript_13483	GRAS	TF
F01_transcript_134843	SWI/SNF-BAF60b	TR
F01_transcript_134845	Whirly	TF
F01_transcript_13488	TRAF	TR
F01_transcript_134881	IWS1	TR
F01_transcript_134883	SNF2	TR
F01_transcript_134913	bZIP	TF
F01_transcript_134924	HB-other	TF
F01_transcript_134943	HB-BELL	TF
F01_transcript_134944	AUX/IAA	TR
F01_transcript_134966	SNF2	TR
F01_transcript_134985	mTERF	TR
F01_transcript_135055	Jumonji	TR
F01_transcript_135071	RWP-RK	TF
F01_transcript_135129	SNF2	TR
F01_transcript_135138	HB-HD-ZIP	TF
F01_transcript_13516	FAR1	TF
F01_transcript_135161	Trihelix	TF
F01_transcript_13522	C3H	TF
F01_transcript_13532	HB-HD-ZIP	TF
F01_transcript_135356	B3-ARF	TF
F01_transcript_135376	HB-HD-ZIP	TF
F01_transcript_135392	BBR-BPC	TF
F01_transcript_1354	CAMTA	TF
F01_transcript_135410	E2F-DP	TF
F01_transcript_135430	RWP-RK	TF
F01_transcript_135432	FAR1	TF
F01_transcript_135446	AP2/ERF-ERF	TF
F01_transcript_135484	MYB-related	TF
F01_transcript_135547	bHLH	TF
F01_transcript_135568	BES1	TF
F01_transcript_135584	bHLH	TF
F01_transcript_135596	ARID	TR
F01_transcript_135629	bZIP	TF
F01_transcript_135652	bZIP	TF
F01_transcript_135679	HSF	TF
F01_transcript_135695	C2C2-GATA	TF
F01_transcript_135765	WRKY	TF
F01_transcript_135823	MADS-M-type	TF
F01_transcript_135862	C2C2-GATA	TF
F01_transcript_135891	AUX/IAA	TR

F01_transcript_135940	FAR1	TF
F01_transcript_136018	HSF	TF
F01_transcript_136020	GARP-G2-like	TF
F01_transcript_136022	Trihelix	TF
F01_transcript_136105	Pseudo ARR-B	TR
F01_transcript_136167	PLATZ	TF
F01_transcript_1362	IWS1	TR
F01_transcript_136205	Trihelix	TF
F01_transcript_136280	HB-other	TF
F01_transcript_136317	NAC	TF
F01_transcript_136327	HB-HD-ZIP	TF
F01_transcript_136344	MYB-related	TF
F01_transcript_136368	WRKY	TF
F01_transcript_136400	CPP	TF
F01_transcript_136401	IWS1	TR
F01_transcript_136453	GNAT	TR
F01_transcript_136457	TRAF	TR
F01_transcript_136473	Others	TR
F01_transcript_136480	bHLH	TF
F01_transcript_136515	WRKY	TF
F01_transcript_13663	EIL	TF
F01_transcript_136630	Others	TR
F01_transcript_136676	bHLH	TF
F01_transcript_136689	SNF2	TR
F01_transcript_136698	WRKY	TF
F01_transcript_136736	C2H2	TF
F01_transcript_136762	Jumonji	TR
F01_transcript_136766	SET	TR
F01_transcript_136804	B3	TF
F01_transcript_13681	EIL	TF
F01_transcript_136811	SET	TR
F01_transcript_136841	SET	TR
F01_transcript_136856	C2C2-YABBY	TF
F01_transcript_136859	bHLH	TF
F01_transcript_136861	C3H	TF
F01_transcript_136923	AUX/IAA	TR
F01_transcript_136951	TUB	TF
F01_transcript_136996	AP2/ERF-ERF	TF
F01_transcript_137	C2H2	TF
F01_transcript_137049	MYB-related	TF
F01_transcript_137057	Others	TR
F01_transcript_137075	Others	TR
F01_transcript_137080	TRAF	TR
F01_transcript_137222	C3H	TF
F01_transcript_137277	CPP	TF
F01_transcript_137279	AP2/ERF-ERF	TF
F01_transcript_137293	FAR1	TF
F01_transcript_137304	PHD	TR
F01_transcript_137318	bHLH	TF
F01_transcript_137367	MYB	TF
F01_transcript_13738	HSF	TF
F01_transcript_137388	C2C2-GATA	TF
F01_transcript_137406	SWI/SNF-BAF60b	TR
F01_transcript_137444	GNAT	TR
F01_transcript_137484	HB-HD-ZIP	TF

F01_transcript_137491	GARP-G2-like	TF
F01_transcript_137544	RWP-RK	TF
F01_transcript_137596	GRAS	TF
F01_transcript_13761	TRAF	TR
F01_transcript_137663	Others	TR
F01_transcript_13770	TRAF	TR
F01_transcript_137700	MYB	TF
F01_transcript_137753	Others	TR
F01_transcript_137829	C2H2	TF
F01_transcript_137840	MYB-related	TF
F01_transcript_13785	FAR1	TF
F01_transcript_137870	HSF	TF
F01_transcript_137887	bZIP	TF
F01_transcript_13793	bHLH	TF
F01_transcript_137955	Jumonji	TR
F01_transcript_137975	TCP	TF
F01_transcript_137999	PHD	TR
F01_transcript_138052	mTERF	TR
F01_transcript_138083	Jumonji	TR
F01_transcript_138096	RWP-RK	TF
F01_transcript_138100	Jumonji	TR
F01_transcript_138124	C2C2-GATA	TF
F01_transcript_138214	FAR1	TF
F01_transcript_138222	ARID	TR
F01_transcript_138258	mTERF	TR
F01_transcript_138278	Others	TR
F01_transcript_138285	EIL	TF
F01_transcript_138296	bZIP	TF
F01_transcript_138346	AUX/IAA	TR
F01_transcript_138383	TCP	TF
F01_transcript_138432	GRAS	TF
F01_transcript_138455	WRKY	TF
F01_transcript_138460	FAR1	TF
F01_transcript_138474	GNAT	TR
F01_transcript_138478	EIL	TF
F01_transcript_138595	GRAS	TF
F01_transcript_138678	DDT	TR
F01_transcript_138691	TAZ	TR
F01_transcript_138694	WRKY	TF
F01_transcript_138698	SET	TR
F01_transcript_138718	MYB	TF
F01_transcript_13872	Pseudo ARR-B	TR
F01_transcript_138722	RWP-RK	TF
F01_transcript_138727	NAC	TF
F01_transcript_138747	MYB	TF
F01_transcript_138796	MYB	TF
F01_transcript_13885	GRAS	TF
F01_transcript_138857	FAR1	TF
F01_transcript_138945	C2C2-CO-like	TF
F01_transcript_138968	PHD	TR
F01_transcript_138993	Pseudo ARR-B	TR
F01_transcript_139	SWI/SNF-BAF60b	TR
F01_transcript_13900	AP2/ERF-AP2	TF
F01_transcript_139019	MYB-related	TF

F01_transcript_139042	MYB-related	TF
F01_transcript_139062	MYB-related	TF
F01_transcript_139067	LUG	TR
F01_transcript_139138	GARP-G2-like	TF
F01_transcript_139149	Others	TR
F01_transcript_139171	Others	TR
F01_transcript_139174	AP2/ERF-ERF	TF
F01_transcript_139210	HB-HD-ZIP	TF
F01_transcript_139233	Others	TR
F01_transcript_13926	IWS1	TR
F01_transcript_139262	SBP	TF
F01_transcript_139281	Tify	TF
F01_transcript_139372	MYB	TF
F01_transcript_139378	FAR1	TF
F01_transcript_139445	Rcd1-like	TR
F01_transcript_139475	HB-other	TF
F01_transcript_139487	GRAS	TF
F01_transcript_139617	FAR1	TF
F01_transcript_139652	MADS-MIKC	TF
F01_transcript_139655	HB-BELL	TF
F01_transcript_139682	C3H	TF
F01_transcript_139705	AP2/ERF-ERF	TF
F01_transcript_139752	Jumonji	TR
F01_transcript_139760	SET	TR
F01_transcript_139796	HB-HD-ZIP	TF
F01_transcript_139802	HB-other	TF
F01_transcript_139817	SBP	TF
F01_transcript_139829	AP2/ERF-ERF	TF
F01_transcript_139859	B3-ARF	TF
F01_transcript_139894	OFP	TF
F01_transcript_139909	Jumonji	TR
F01_transcript_139925	SET	TR
F01_transcript_139975	MYB-related	TF
F01_transcript_139978	MADS-M-type	TF
F01_transcript_139981	GRAS	TF
F01_transcript_139986	OFP	TF
F01_transcript_139987	bHLH	TF
F01_transcript_140010	AUX/IAA	TR
F01_transcript_14005	Others	TR
F01_transcript_140050	C2C2-Dof	TF
F01_transcript_140082	MYB	TF
F01_transcript_140084	NF-YA	TF
F01_transcript_14013	WRKY	TF
F01_transcript_140161	ARID	TR
F01_transcript_140164	PHD	TR
F01_transcript_140182	C2C2-GATA	TF
F01_transcript_140207	TUB	TF
F01_transcript_140224	GRAS	TF
F01_transcript_140252	bHLH	TF
F01_transcript_140274	bHLH	TF
F01_transcript_1403	Others	TR
F01_transcript_140319	SNF2	TR
F01_transcript_140342	SBP	TF
F01_transcript_140363	bZIP	TF
F01_transcript_14044	HB-HD-ZIP	TF

F01_transcript_14048	C2H2	TF
F01_transcript_140503	NAC	TF
F01_transcript_140536	SNF2	TR
F01_transcript_140539	C2H2	TF
F01_transcript_140545	BES1	TF
F01_transcript_140583	MYB-related	TF
F01_transcript_140611	DBB	TF
F01_transcript_140615	SNF2	TR
F01_transcript_14062	EIL	TF
F01_transcript_140652	MADS-MIKC	TF
F01_transcript_140656	bHLH	TF
F01_transcript_140679	C3H	TF
F01_transcript_140702	C2H2	TF
F01_transcript_140718	MYB-related	TF
F01_transcript_140724	C2C2-GATA	TF
F01_transcript_140755	TAZ	TR
F01_transcript_140757	RWP-RK	TF
F01_transcript_140762	NAC	TF
F01_transcript_140777	B3-ARF	TF
F01_transcript_140905	GRAS	TF
F01_transcript_140926	NAC	TF
F01_transcript_140935	GARP-ARR-B	TF
F01_transcript_140942	bHLH	TF
F01_transcript_14097	CPP	TF
F01_transcript_140983	mTERF	TR
F01_transcript_1410	CAMTA	TF
F01_transcript_141017	GNAT	TR
F01_transcript_141067	GRAS	TF
F01_transcript_141073	HB-KNOX	TF
F01_transcript_14112	C2H2	TF
F01_transcript_141125	Others	TR
F01_transcript_141164	SNF2	TR
F01_transcript_141170	HMG	TR
F01_transcript_141222	MYB-related	TF
F01_transcript_141251	bHLH	TF
F01_transcript_141261	GeBP	TF
F01_transcript_141273	SET	TR
F01_transcript_141304	GNAT	TR
F01_transcript_141335	SNF2	TR
F01_transcript_141344	SET	TR
F01_transcript_141406	ULT	TF
F01_transcript_141408	Trihelix	TF
F01_transcript_141412	bHLH	TF
F01_transcript_141515	MYB-related	TF
F01_transcript_14155	MYB-related	TF
F01_transcript_141553	GARP-G2-like	TF
F01_transcript_141577	C3H	TF
F01_transcript_141611	MYB-related	TF
F01_transcript_141709	C2C2-GATA	TF
F01_transcript_141711	DDT	TR
F01_transcript_141735	PHD	TR
F01_transcript_141748	RWP-RK	TF
F01_transcript_141750	GRAS	TF
F01_transcript_141771	Jumonji	TR

F01_transcript_141812	SNF2	TR
F01_transcript_141874	SET	TR
F01_transcript_141885	PHD	TR
F01_transcript_141891	C2H2	TF
F01_transcript_141898	Jumonji	TR
F01_transcript_141916	MYB	TF
F01_transcript_141936	GNAT	TR
F01_transcript_141959	Trihelix	TF
F01_transcript_141982	SNF2	TR
F01_transcript_141988	MYB	TF
F01_transcript_142	SNF2	TR
F01_transcript_142035	PHD	TR
F01_transcript_142046	B3-ARF	TF
F01_transcript_142066	C2C2-YABBY	TF
F01_transcript_142073	TCP	TF
F01_transcript_142084	C3H	TF
F01_transcript_142085	LUG	TR
F01_transcript_142100	MYB-related	TF
F01_transcript_142101	bZIP	TF
F01_transcript_142133	BBR-BPC	TF
F01_transcript_142137	FAR1	TF
F01_transcript_142163	bHLH	TF
F01_transcript_14218	SBP	TF
F01_transcript_14220	bHLH	TF
F01_transcript_142216	IWS1	TR
F01_transcript_142221	SET	TR
F01_transcript_142269	RWP-RK	TF
F01_transcript_142276	Others	TR
F01_transcript_14243	BSD	TF
F01_transcript_142455	WRKY	TF
F01_transcript_142469	C2C2-Dof	TF
F01_transcript_142497	DDT	TR
F01_transcript_14250	TUB	TF
F01_transcript_14252	C2H2	TF
F01_transcript_142570	SBP	TF
F01_transcript_142584	HB-BELL	TF
F01_transcript_14259	FAR1	TF
F01_transcript_142598	HB-BELL	TF
F01_transcript_142605	GRAS	TF
F01_transcript_142616	SBP	TF
F01_transcript_142651	Others	TR
F01_transcript_142658	NAC	TF
F01_transcript_142680	bHLH	TF
F01_transcript_142697	TUB	TF
F01_transcript_142727	C2C2-GATA	TF
F01_transcript_142752	SBP	TF
F01_transcript_142784	WRKY	TF
F01_transcript_142828	MYB-related	TF
F01_transcript_142848	Trihelix	TF
F01_transcript_142905	WRKY	TF
F01_transcript_142935	TCP	TF
F01_transcript_142956	RWP-RK	TF
F01_transcript_142991	ARID	TR
F01_transcript_143010	mTERF	TR
F01_transcript_143018	SOH1	TR

F01_transcript_143066	SET	TR
F01_transcript_143084	S1Fa-like	TF
F01_transcript_143089	DBP	TF
F01_transcript_143091	MYB	TF
F01_transcript_143163	GRF	TF
F01_transcript_143164	mTERF	TR
F01_transcript_143169	NAC	TF
F01_transcript_143174	RWP-RK	TF
F01_transcript_143200	bHLH	TF
F01_transcript_143236	GARP-G2-like	TF
F01_transcript_143284	C3H	TF
F01_transcript_143287	mTERF	TR
F01_transcript_143313	TCP	TF
F01_transcript_143317	Others	TR
F01_transcript_143350	GRAS	TF
F01_transcript_143351	TAZ	TR
F01_transcript_143455	EIL	TF
F01_transcript_143475	bHLH	TF
F01_transcript_14350	LUG	TR
F01_transcript_143502	C3H	TF
F01_transcript_143515	bHLH	TF
F01_transcript_143532	PHD	TR
F01_transcript_143537	bZIP	TF
F01_transcript_143570	RWP-RK	TF
F01_transcript_143580	FAR1	TF
F01_transcript_143595	NAC	TF
F01_transcript_143624	C3H	TF
F01_transcript_143625	HB-HD-ZIP	TF
F01_transcript_143635	SBP	TF
F01_transcript_143638	B3-ARF	TF
F01_transcript_143659	B3	TF
F01_transcript_14372	bHLH	TF
F01_transcript_143738	bHLH	TF
F01_transcript_143747	AUX/IAA	TR
F01_transcript_143760	Trihelix	TF
F01_transcript_143761	mTERF	TR
F01_transcript_143777	NAC	TF
F01_transcript_143791	HB-HD-ZIP	TF
F01_transcript_143810	ARID	TR
F01_transcript_143813	IWS1	TR
F01_transcript_143822	C2H2	TF
F01_transcript_143863	GRAS	TF
F01_transcript_143875	LUG	TR
F01_transcript_143900	Pseudo ARR-B	TR
F01_transcript_143923	GRAS	TF
F01_transcript_143928	HB-PHD	TF
F01_transcript_144045	WRKY	TF
F01_transcript_144051	C2C2-LSD	TF
F01_transcript_144092	B3	TF
F01_transcript_144106	SET	TR
F01_transcript_144155	MYB-related	TF
F01_transcript_144168	bHLH	TF
F01_transcript_144206	GRAS	TF
F01_transcript_14422	IWS1	TR

F01_transcript_144283	MYB	TF
F01_transcript_144300	MYB-related	TF
F01_transcript_14431	FAR1	TF
F01_transcript_144314	E2F-DP	TF
F01_transcript_144339	bHLH	TF
F01_transcript_144374	MYB-related	TF
F01_transcript_144384	DDT	TR
F01_transcript_144413	MADS-M-type	TF
F01_transcript_144498	C2C2-Dof	TF
F01_transcript_144541	SNF2	TR
F01_transcript_144579	HB-BELL	TF
F01_transcript_144614	NAC	TF
F01_transcript_144619	ULT	TF
F01_transcript_144629	Others	TR
F01_transcript_144683	B3-ARF	TF
F01_transcript_144684	FAR1	TF
F01_transcript_144741	IWS1	TR
F01_transcript_144813	MYB-related	TF
F01_transcript_144855	AUX/IAA	TR
F01_transcript_144883	bHLH	TF
F01_transcript_144956	NAC	TF
F01_transcript_144965	PHD	TR
F01_transcript_144975	GARP-G2-like	TF
F01_transcript_144997	RWP-RK	TF
F01_transcript_144999	RWP-RK	TF
F01_transcript_145031	GRAS	TF
F01_transcript_145056	B3	TF
F01_transcript_145080	SNF2	TR
F01_transcript_145088	C2H2	TF
F01_transcript_145118	SNF2	TR
F01_transcript_14518	Others	TR
F01_transcript_145181	SET	TR
F01_transcript_145190	bHLH	TF
F01_transcript_145196	MYB	TF
F01_transcript_14522	bHLH	TF
F01_transcript_145230	HSF	TF
F01_transcript_145308	AP2/ERF-ERF	TF
F01_transcript_145311	BES1	TF
F01_transcript_145313	GNAT	TR
F01_transcript_145343	BES1	TF
F01_transcript_145348	BBR-BPC	TF
F01_transcript_14539	GRAS	TF
F01_transcript_145438	C2C2-GATA	TF
F01_transcript_145534	MYB-related	TF
F01_transcript_145546	SNF2	TR
F01_transcript_145557	NAC	TF
F01_transcript_14556	Tify	TF
F01_transcript_145615	RWP-RK	TF
F01_transcript_145684	PHD	TR
F01_transcript_145692	SET	TR
F01_transcript_145702	FAR1	TF
F01_transcript_145703	Jumonji	TR
F01_transcript_145729	MYB	TF
F01_transcript_145750	C2H2	TF
F01_transcript_145817	LUG	TR

F01_transcript_14589	C2H2	TF
F01_transcript_145953	PHD	TR
F01_transcript_14603	SET	TR
F01_transcript_146040	AP2/ERF-AP2	TF
F01_transcript_146056	WRKY	TF
F01_transcript_146086	ARID	TR
F01_transcript_146087	C3H	TF
F01_transcript_146119	bHLH	TF
F01_transcript_146175	HB-BELL	TF
F01_transcript_146216	SBP	TF
F01_transcript_146221	Jumonji	TR
F01_transcript_146226	MYB-related	TF
F01_transcript_146296	C2C2-CO-like	TF
F01_transcript_146328	CAMTA	TF
F01_transcript_146332	AUX/IAA	TR
F01_transcript_146334	CPP	TF
F01_transcript_146403	AP2/ERF-ERF	TF
F01_transcript_146467	MADS-MIKC	TF
F01_transcript_146509	GNAT	TR
F01_transcript_146511	Tify	TF
F01_transcript_146554	SET	TR
F01_transcript_146567	C2H2	TF
F01_transcript_146627	AP2/ERF-AP2	TF
F01_transcript_146642	Others	TR
F01_transcript_14667	GARP-ARR-B	TF
F01_transcript_146725	bZIP	TF
F01_transcript_146834	TUB	TF
F01_transcript_14685	BES1	TF
F01_transcript_146878	HB-BELL	TF
F01_transcript_146910	Others	TR
F01_transcript_146922	AP2/ERF-ERF	TF
F01_transcript_146950	MYB-related	TF
F01_transcript_146956	HB-other	TF
F01_transcript_146983	TRAF	TR
F01_transcript_146986	TCP	TF
F01_transcript_146996	BBR-BPC	TF
F01_transcript_147012	bZIP	TF
F01_transcript_147018	BES1	TF
F01_transcript_147045	NAC	TF
F01_transcript_147087	HB-HD-ZIP	TF
F01_transcript_147130	FAR1	TF
F01_transcript_147179	MADS-M-type	TF
F01_transcript_147180	Trihelix	TF
F01_transcript_147199	bHLH	TF
F01_transcript_147200	BES1	TF
F01_transcript_147208	GNAT	TR
F01_transcript_147254	TRAF	TR
F01_transcript_147290	bHLH	TF
F01_transcript_147380	MYB	TF
F01_transcript_147390	SNF2	TR
F01_transcript_147457	MYB-related	TF
F01_transcript_147533	bHLH	TF
F01_transcript_147540	C2C2-LSD	TF
F01_transcript_147577	RWP-RK	TF

F01_transcript_147605	C2C2-Dof	TF
F01_transcript_147624	PHD	TR
F01_transcript_147733	GARP-G2-like	TF
F01_transcript_147746	B3-ARF	TF
F01_transcript_147764	TRAF	TR
F01_transcript_147782	C2C2-CO-like	TF
F01_transcript_147784	C2C2-Dof	TF
F01_transcript_147821	LOB	TF
F01_transcript_147822	RWP-RK	TF
F01_transcript_147867	NAC	TF
F01_transcript_147925	TUB	TF
F01_transcript_147938	GNAT	TR
F01_transcript_147957	bZIP	TF
F01_transcript_147969	bHLH	TF
F01_transcript_1480	HB-HD-ZIP	TF
F01_transcript_148017	HMG	TR
F01_transcript_148069	Jumonji	TR
F01_transcript_148145	WRKY	TF
F01_transcript_148167	NAC	TF
F01_transcript_148259	mTERF	TR
F01_transcript_148282	SWI/SNF-BAF60b	TR
F01_transcript_148305	Tify	TF
F01_transcript_14832	FAR1	TF
F01_transcript_148327	bZIP	TF
F01_transcript_148340	DDT	TR
F01_transcript_14836	NAC	TF
F01_transcript_148377	HB-HD-ZIP	TF
F01_transcript_148379	HSF	TF
F01_transcript_14838	TCP	TF
F01_transcript_148414	bHLH	TF
F01_transcript_148416	SBP	TF
F01_transcript_148436	WRKY	TF
F01_transcript_14846	GARP-ARR-B	TF
F01_transcript_148514	TAZ	TR
F01_transcript_14852	SWI/SNF-BAF60b	TR
F01_transcript_148540	GARP-G2-like	TF
F01_transcript_148648	PHD	TR
F01_transcript_148656	MYB-related	TF
F01_transcript_148791	HB-BELL	TF
F01_transcript_148845	Others	TR
F01_transcript_148852	bZIP	TF
F01_transcript_148859	C2H2	TF
F01_transcript_148864	C2H2	TF
F01_transcript_148912	bZIP	TF
F01_transcript_148991	SET	TR
F01_transcript_149028	HSF	TF
F01_transcript_149064	C3H	TF
F01_transcript_149087	GARP-ARR-B	TF
F01_transcript_149127	MYB-related	TF
F01_transcript_14913	C3H	TF
F01_transcript_149156	bHLH	TF
F01_transcript_14920	Others	TR
F01_transcript_149240	MYB-related	TF
F01_transcript_149258	ARID	TR
F01_transcript_149281	TUB	TF

F01_transcript_149293	Jumonji	TR
F01_transcript_149332	B3-ARF	TF
F01_transcript_149406	AP2/ERF-ERF	TF
F01_transcript_149485	Others	TR
F01_transcript_149525	C3H	TF
F01_transcript_149590	MYB	TF
F01_transcript_149593	Jumonji	TR
F01_transcript_14960	AP2/ERF-AP2	TF
F01_transcript_149628	bHLH	TF
F01_transcript_149641	C2H2	TF
F01_transcript_149663	MYB-related	TF
F01_transcript_149704	MYB-related	TF
F01_transcript_149706	bZIP	TF
F01_transcript_149746	PHD	TR
F01_transcript_149768	PHD	TR
F01_transcript_149787	bHLH	TF
F01_transcript_1498	RWP-RK	TF
F01_transcript_149809	GARP-G2-like	TF
F01_transcript_149828	NAC	TF
F01_transcript_149845	C2C2-YABBY	TF
F01_transcript_149862	mTERF	TR
F01_transcript_149874	NAC	TF
F01_transcript_149926	MYB-related	TF
F01_transcript_149968	mTERF	TR
F01_transcript_149977	Others	TR
F01_transcript_149999	NF-YB	TF
F01_transcript_150003	BES1	TF
F01_transcript_15004	C2C2-CO-like	TF
F01_transcript_150124	TRAF	TR
F01_transcript_150141	FAR1	TF
F01_transcript_150175	C2C2-GATA	TF
F01_transcript_150225	BBR-BPC	TF
F01_transcript_150317	C2C2-CO-like	TF
F01_transcript_150326	FAR1	TF
F01_transcript_150409	MYB-related	TF
F01_transcript_150483	OFP	TF
F01_transcript_150539	Trihelix	TF
F01_transcript_15054	RWP-RK	TF
F01_transcript_150575	Others	TR
F01_transcript_150717	SET	TR
F01_transcript_150726	SBP	TF
F01_transcript_150735	MYB-related	TF
F01_transcript_150782	C3H	TF
F01_transcript_150813	C2C2-Dof	TF
F01_transcript_150875	bHLH	TF
F01_transcript_150884	AUX/IAA	TR
F01_transcript_150904	GeBP	TF
F01_transcript_15093	SWI/SNF-BAF60b	TR
F01_transcript_150938	C2C2-GATA	TF
F01_transcript_151012	HB-HD-ZIP	TF
F01_transcript_151096	GNAT	TR
F01_transcript_151116	HB-other	TF
F01_transcript_151121	bZIP	TF
F01_transcript_151134	HB-HD-ZIP	TF

F01_transcript_151223	MYB-related	TF
F01_transcript_151285	AP2/ERF-AP2	TF
F01_transcript_151288	AUX/IAA	TR
F01_transcript_151338	WRKY	TF
F01_transcript_151350	AP2/ERF-ERF	TF
F01_transcript_151372	SET	TR
F01_transcript_151387	C2C2-GATA	TF
F01_transcript_151405	TRAF	TR
F01_transcript_151412	RWP-RK	TF
F01_transcript_151421	bHLH	TF
F01_transcript_151451	WRKY	TF
F01_transcript_151495	Jumonji	TR
F01_transcript_151509	C2C2-GATA	TF
F01_transcript_151530	bZIP	TF
F01_transcript_151536	TCP	TF
F01_transcript_151542	BBR-BPC	TF
F01_transcript_151601	AP2/ERF-AP2	TF
F01_transcript_151616	HSF	TF
F01_transcript_151620	MYB-related	TF
F01_transcript_151650	bHLH	TF
F01_transcript_151687	B3-ARF	TF
F01_transcript_151741	CPP	TF
F01_transcript_151781	WRKY	TF
F01_transcript_151790	OFP	TF
F01_transcript_1518	SBP	TF
F01_transcript_151840	LOB	TF
F01_transcript_151885	Tify	TF
F01_transcript_151936	BBR-BPC	TF
F01_transcript_151937	zf-HD	TF
F01_transcript_151950	GRAS	TF
F01_transcript_152003	MADS-MIKC	TF
F01_transcript_152013	Others	TR
F01_transcript_152032	SNF2	TR
F01_transcript_152038	MYB	TF
F01_transcript_15208	bHLH	TF
F01_transcript_152125	Trihelix	TF
F01_transcript_152181	ARID	TR
F01_transcript_152192	FAR1	TF
F01_transcript_152225	Others	TR
F01_transcript_152238	mTERF	TR
F01_transcript_152262	NF-YB	TF
F01_transcript_152290	DBB	TF
F01_transcript_152298	mTERF	TR
F01_transcript_152344	Others	TR
F01_transcript_152363	AUX/IAA	TR
F01_transcript_152370	SBP	TF
F01_transcript_152411	DBB	TF
F01_transcript_152418	GRAS	TF
F01_transcript_152483	Tify	TF
F01_transcript_152498	AUX/IAA	TR
F01_transcript_152515	RWP-RK	TF
F01_transcript_15260	C2H2	TF
F01_transcript_15265	CPP	TF
F01_transcript_152766	PHD	TR
F01_transcript_152802	bHLH	TF

F01_transcript_152881	MYB-related	TF
F01_transcript_152929	WRKY	TF
F01_transcript_152949	Pseudo ARR-B	TR
F01_transcript_152967	TRAF	TR
F01_transcript_152972	MYB-related	TF
F01_transcript_152978	AP2/ERF-AP2	TF
F01_transcript_152980	Pseudo ARR-B	TR
F01_transcript_153045	IWS1	TR
F01_transcript_153088	BBR-BPC	TF
F01_transcript_1531	MYB	TF
F01_transcript_153118	C2C2-GATA	TF
F01_transcript_153163	GeBP	TF
F01_transcript_153230	Others	TR
F01_transcript_153311	CAMTA	TF
F01_transcript_153319	DDT	TR
F01_transcript_153334	MYB	TF
F01_transcript_15334	EIL	TF
F01_transcript_153479	bZIP	TF
F01_transcript_153490	MYB	TF
F01_transcript_1535	SBP	TF
F01_transcript_153589	Rcd1-like	TR
F01_transcript_15359	GRAS	TF
F01_transcript_153604	NAC	TF
F01_transcript_153625	HB-BELL	TF
F01_transcript_153629	ARID	TR
F01_transcript_153656	Others	TR
F01_transcript_153665	TRAF	TR
F01_transcript_153672	Others	TR
F01_transcript_153770	GeBP	TF
F01_transcript_153771	MADS-M-type	TF
F01_transcript_153775	bHLH	TF
F01_transcript_153776	AP2/ERF-AP2	TF
F01_transcript_153786	C3H	TF
F01_transcript_153819	bHLH	TF
F01_transcript_153839	Jumonji	TR
F01_transcript_153850	PHD	TR
F01_transcript_153880	FAR1	TF
F01_transcript_153913	GARP-G2-like	TF
F01_transcript_153964	PHD	TR
F01_transcript_153999	AUX/IAA	TR
F01_transcript_154012	mTERF	TR
F01_transcript_154013	GARP-G2-like	TF
F01_transcript_154026	SWI/SNF-BAF60b	TR
F01_transcript_154028	MADS-M-type	TF
F01_transcript_154113	HMG	TR
F01_transcript_154117	NAC	TF
F01_transcript_15420	HB-BELL	TF
F01_transcript_154275	AP2/ERF-AP2	TF
F01_transcript_154277	TCP	TF
F01_transcript_154387	TRAF	TR
F01_transcript_154423	MYB-related	TF
F01_transcript_154466	VOZ	TF
F01_transcript_154484	PHD	TR
F01_transcript_15450	RWP-RK	TF

F01_transcript_154506	AP2/ERF-ERF	TF
F01_transcript_154507	HB-HD-ZIP	TF
F01_transcript_154510	C2H2	TF
F01_transcript_154513	bZIP	TF
F01_transcript_154569	PHD	TR
F01_transcript_154589	mTERF	TR
F01_transcript_154617	bZIP	TF
F01_transcript_154662	HB-BELL	TF
F01_transcript_15468	Others	TR
F01_transcript_154688	AP2/ERF-ERF	TF
F01_transcript_154732	C2H2	TF
F01_transcript_154745	HB-HD-ZIP	TF
F01_transcript_154798	Others	TR
F01_transcript_154816	NF-YC	TF
F01_transcript_154848	AUX/IAA	TR
F01_transcript_154858	NF-YC	TF
F01_transcript_15487	ARID	TR
F01_transcript_15495	bHLH	TF
F01_transcript_155001	Others	TR
F01_transcript_15501	Pseudo ARR-B	TR
F01_transcript_155032	SET	TR
F01_transcript_155051	FAR1	TF
F01_transcript_155063	MYB-related	TF
F01_transcript_15512	TCP	TF
F01_transcript_155144	GRAS	TF
F01_transcript_155153	HB-HD-ZIP	TF
F01_transcript_155282	FAR1	TF
F01_transcript_155322	FAR1	TF
F01_transcript_155380	RWP-RK	TF
F01_transcript_155413	AUX/IAA	TR
F01_transcript_155432	MYB	TF
F01_transcript_155612	bHLH	TF
F01_transcript_155634	GRAS	TF
F01_transcript_155637	AP2/ERF-ERF	TF
F01_transcript_155649	TRAF	TR
F01_transcript_155662	HSF	TF
F01_transcript_155704	bZIP	TF
F01_transcript_15572	C3H	TF
F01_transcript_15576	MYB-related	TF
F01_transcript_155784	C3H	TF
F01_transcript_155826	AP2/ERF-ERF	TF
F01_transcript_15583	Others	TR
F01_transcript_155922	MYB-related	TF
F01_transcript_156007	MYB-related	TF
F01_transcript_156011	HB-BELL	TF
F01_transcript_156115	C2C2-GATA	TF
F01_transcript_156171	bHLH	TF
F01_transcript_156268	MYB	TF
F01_transcript_156469	bHLH	TF
F01_transcript_15649	Pseudo ARR-B	TR
F01_transcript_156533	E2F-DP	TF
F01_transcript_156538	GNAT	TR
F01_transcript_156544	SBP	TF
F01_transcript_156637	bHLH	TF
F01_transcript_15664	WRKY	TF

F01_transcript_156652	FAR1	TF
F01_transcript_156716	SNF2	TR
F01_transcript_156790	NAC	TF
F01_transcript_156837	NF-YC	TF
F01_transcript_156846	BES1	TF
F01_transcript_156896	WRKY	TF
F01_transcript_156939	SBP	TF
F01_transcript_156957	IWS1	TR
F01_transcript_156998	B3-ARF	TF
F01_transcript_157	Others	TR
F01_transcript_157023	GRAS	TF
F01_transcript_157047	TUB	TF
F01_transcript_157185	SET	TR
F01_transcript_15721	FAR1	TF
F01_transcript_157225	GeBP	TF
F01_transcript_157246	C2H2	TF
F01_transcript_157251	ARID	TR
F01_transcript_15727	C2H2	TF
F01_transcript_157270	bZIP	TF
F01_transcript_157365	HB-PHD	TF
F01_transcript_157438	GNAT	TR
F01_transcript_157445	SNF2	TR
F01_transcript_157598	C3H	TF
F01_transcript_157620	B3	TF
F01_transcript_157626	RWP-RK	TF
F01_transcript_157633	AP2/ERF-ERF	TF
F01_transcript_157661	TRAF	TR
F01_transcript_157682	mTERF	TR
F01_transcript_157747	HB-HD-ZIP	TF
F01_transcript_157753	PHD	TR
F01_transcript_157774	bZIP	TF
F01_transcript_157782	CAMTA	TF
F01_transcript_157784	MYB-related	TF
F01_transcript_1578	SNF2	TR
F01_transcript_157802	MYB-related	TF
F01_transcript_157830	Coactivator p15	TR
F01_transcript_15795	AP2/ERF-ERF	TF
F01_transcript_157992	GNAT	TR
F01_transcript_158036	HB-BELL	TF
F01_transcript_158058	bHLH	TF
F01_transcript_15809	HSF	TF
F01_transcript_158124	Trihelix	TF
F01_transcript_158149	PHD	TR
F01_transcript_158174	GRAS	TF
F01_transcript_158180	AP2/ERF-AP2	TF
F01_transcript_158217	HB-other	TF
F01_transcript_158237	C2H2	TF
F01_transcript_158239	HMG	TR
F01_transcript_158286	MYB-related	TF
F01_transcript_15829	FAR1	TF
F01_transcript_158386	C3H	TF
F01_transcript_158392	MADS-M-type	TF
F01_transcript_158395	C3H	TF
F01_transcript_158453	HB-HD-ZIP	TF

F01_transcript_158464	RWP-RK	TF
F01_transcript_158476	PHD	TR
F01_transcript_158478	NF-YC	TF
F01_transcript_158485	MYB-related	TF
F01_transcript_158490	Others	TR
F01_transcript_158541	MYB	TF
F01_transcript_158557	Others	TR
F01_transcript_158582	BBR-BPC	TF
F01_transcript_158586	WRKY	TF
F01_transcript_158593	HB-BELL	TF
F01_transcript_158622	BBR-BPC	TF
F01_transcript_158635	HB-HD-ZIP	TF
F01_transcript_158790	MADS-MIKC	TF
F01_transcript_158829	RB	TR
F01_transcript_158843	HB-BELL	TF
F01_transcript_158892	NAC	TF
F01_transcript_158923	Others	TR
F01_transcript_158931	HB-BELL	TF
F01_transcript_158962	TUB	TF
F01_transcript_158998	BBR-BPC	TF
F01_transcript_15909	NAC	TF
F01_transcript_159136	FAR1	TF
F01_transcript_159140	B3-ARF	TF
F01_transcript_159172	SNF2	TR
F01_transcript_159178	PHD	TR
F01_transcript_159179	AUX/IAA	TR
F01_transcript_159209	PHD	TR
F01_transcript_159288	GARP-G2-like	TF
F01_transcript_159317	SBP	TF
F01_transcript_159323	TRAF	TR
F01_transcript_159324	GNAT	TR
F01_transcript_159346	B3-ARF	TF
F01_transcript_159347	bZIP	TF
F01_transcript_159402	FAR1	TF
F01_transcript_159438	B3-ARF	TF
F01_transcript_159455	mTERF	TR
F01_transcript_159478	LIM	TF
F01_transcript_159494	SBP	TF
F01_transcript_159503	C2H2	TF
F01_transcript_159524	bZIP	TF
F01_transcript_159599	SBP	TF
F01_transcript_15966	PHD	TR
F01_transcript_159687	AUX/IAA	TR
F01_transcript_159743	Others	TR
F01_transcript_159806	WRKY	TF
F01_transcript_159818	C3H	TF
F01_transcript_159852	GARP-G2-like	TF
F01_transcript_159864	DDT	TR
F01_transcript_159956	Trihelix	TF
F01_transcript_160004	MYB-related	TF
F01_transcript_160109	bHLH	TF
F01_transcript_160137	C2C2-GATA	TF
F01_transcript_1602	SBP	TF
F01_transcript_16020	TRAF	TR
F01_transcript_160236	GARP-G2-like	TF

F01_transcript_160242	bHLH	TF
F01_transcript_160297	bHLH	TF
F01_transcript_160318	Trihelix	TF
F01_transcript_160362	MYB-related	TF
F01_transcript_160374	MYB-related	TF
F01_transcript_160383	Coactivator p15	TR
F01_transcript_160388	MYB	TF
F01_transcript_160391	bHLH	TF
F01_transcript_160422	SET	TR
F01_transcript_16049	RWP-RK	TF
F01_transcript_160495	Others	TR
F01_transcript_160506	FAR1	TF
F01_transcript_160531	SNF2	TR
F01_transcript_160550	SBP	TF
F01_transcript_16057	RWP-RK	TF
F01_transcript_160617	TCP	TF
F01_transcript_160674	B3-ARF	TF
F01_transcript_1608	SBP	TF
F01_transcript_160805	SNF2	TR
F01_transcript_160814	Jumonji	TR
F01_transcript_160831	DBB	TF
F01_transcript_160856	Others	TR
F01_transcript_160860	bHLH	TF
F01_transcript_160861	C2C2-GATA	TF
F01_transcript_160878	B3-ARF	TF
F01_transcript_160907	EIL	TF
F01_transcript_160999	GRAS	TF
F01_transcript_161008	RWP-RK	TF
F01_transcript_161042	bZIP	TF
F01_transcript_161075	Others	TR
F01_transcript_161089	TRAF	TR
F01_transcript_161131	bHLH	TF
F01_transcript_161138	GNAT	TR
F01_transcript_161167	NF-YB	TF
F01_transcript_161202	SBP	TF
F01_transcript_161232	BBR-BPC	TF
F01_transcript_16125	BES1	TF
F01_transcript_161259	HB-KNOX	TF
F01_transcript_161310	DBB	TF
F01_transcript_161382	MADS-M-type	TF
F01_transcript_161396	GRAS	TF
F01_transcript_161413	TRAF	TR
F01_transcript_161432	bZIP	TF
F01_transcript_16144	C2H2	TF
F01_transcript_161445	MYB-related	TF
F01_transcript_161454	GNAT	TR
F01_transcript_161466	C3H	TF
F01_transcript_16148	ARID	TR
F01_transcript_16152	GARP-G2-like	TF
F01_transcript_161525	TAZ	TR
F01_transcript_16155	C3H	TF
F01_transcript_16157	bZIP	TF
F01_transcript_161591	bHLH	TF
F01_transcript_161772	C3H	TF

F01_transcript_161775	ARID	TR
F01_transcript_161782	bHLH	TF
F01_transcript_161787	C3H	TF
F01_transcript_161789	C3H	TF
F01_transcript_161826	C2C2-CO-like	TF
F01_transcript_161830	bHLH	TF
F01_transcript_161840	NF-YA	TF
F01_transcript_161850	GRAS	TF
F01_transcript_161908	bHLH	TF
F01_transcript_161925	C3H	TF
F01_transcript_161970	Jumonji	TR
F01_transcript_162131	SET	TR
F01_transcript_162169	NAC	TF
F01_transcript_162173	GARP-G2-like	TF
F01_transcript_162202	Jumonji	TR
F01_transcript_162259	C2C2-Dof	TF
F01_transcript_162272	NAC	TF
F01_transcript_162278	C3H	TF
F01_transcript_162281	bHLH	TF
F01_transcript_162285	Trihelix	TF
F01_transcript_162333	Others	TR
F01_transcript_162335	NAC	TF
F01_transcript_162402	GARP-G2-like	TF
F01_transcript_162411	HB-HD-ZIP	TF
F01_transcript_16242	C2H2	TF
F01_transcript_162428	GARP-G2-like	TF
F01_transcript_162438	Others	TR
F01_transcript_162440	C2H2	TF
F01_transcript_162446	SBP	TF
F01_transcript_162494	bZIP	TF
F01_transcript_162509	HSF	TF
F01_transcript_162515	B3	TF
F01_transcript_162533	NAC	TF
F01_transcript_162559	GRAS	TF
F01_transcript_162596	HB-BELL	TF
F01_transcript_162638	MYB	TF
F01_transcript_162649	NF-YC	TF
F01_transcript_162681	C2H2	TF
F01_transcript_162751	GNAT	TR
F01_transcript_162777	LUG	TR
F01_transcript_16279	C2H2	TF
F01_transcript_162803	Others	TR
F01_transcript_162820	C2H2	TF
F01_transcript_162823	PHD	TR
F01_transcript_162835	GARP-G2-like	TF
F01_transcript_162932	Trihelix	TF
F01_transcript_16298	Pseudo ARR-B	TR
F01_transcript_163009	TUB	TF
F01_transcript_163011	SNF2	TR
F01_transcript_163029	NF-YB	TF
F01_transcript_163057	GRAS	TF
F01_transcript_163153	MYB-related	TF
F01_transcript_163176	C2C2-Dof	TF
F01_transcript_163188	EIL	TF
F01_transcript_163203	C3H	TF

F01_transcript_163269	RWP-RK	TF
F01_transcript_163284	MADS-M-type	TF
F01_transcript_163285	HB-BELL	TF
F01_transcript_163314	AUX/IAA	TR
F01_transcript_163348	EIL	TF
F01_transcript_163361	HB-KNOX	TF
F01_transcript_163455	bHLH	TF
F01_transcript_163493	Trihelix	TF
F01_transcript_163507	ARID	TR
F01_transcript_163512	C2H2	TF
F01_transcript_16352	FAR1	TF
F01_transcript_163589	SET	TR
F01_transcript_163644	MYB-related	TF
F01_transcript_163657	ARID	TR
F01_transcript_16366	GRAS	TF
F01_transcript_16367	WRKY	TF
F01_transcript_16373	SWI/SNF-BAF60b	TR
F01_transcript_163746	RWP-RK	TF
F01_transcript_163765	AP2/ERF-AP2	TF
F01_transcript_163770	PHD	TR
F01_transcript_163808	bZIP	TF
F01_transcript_163812	HB-BELL	TF
F01_transcript_163813	MYB	TF
F01_transcript_163814	ARID	TR
F01_transcript_163843	C2H2	TF
F01_transcript_163882	SNF2	TR
F01_transcript_163888	Others	TR
F01_transcript_163937	Tify	TF
F01_transcript_163983	PHD	TR
F01_transcript_164002	Others	TR
F01_transcript_164005	SBP	TF
F01_transcript_16401	NAC	TF
F01_transcript_164017	C2C2-Dof	TF
F01_transcript_164059	TCP	TF
F01_transcript_164077	Whirly	TF
F01_transcript_164079	GARP-G2-like	TF
F01_transcript_1641	MADS-M-type	TF
F01_transcript_16411	HSF	TF
F01_transcript_164133	Others	TR
F01_transcript_164149	PHD	TR
F01_transcript_164162	MADS-MIKC	TF
F01_transcript_164168	Trihelix	TF
F01_transcript_164170	MYB-related	TF
F01_transcript_164173	NAC	TF
F01_transcript_164189	bHLH	TF
F01_transcript_16421	EIL	TF
F01_transcript_164210	TRAF	TR
F01_transcript_164244	bHLH	TF
F01_transcript_164245	C2H2	TF
F01_transcript_164248	TUB	TF
F01_transcript_164281	WRKY	TF
F01_transcript_164339	Others	TR
F01_transcript_164368	TRAF	TR
F01_transcript_164420	MADS-M-type	TF

F01_transcript_164478	PHD	TR
F01_transcript_164486	RWP-RK	TF
F01_transcript_164507	FAR1	TF
F01_transcript_164534	GARP-ARR-B	TF
F01_transcript_164566	RWP-RK	TF
F01_transcript_1646	MYB-related	TF
F01_transcript_164607	RWP-RK	TF
F01_transcript_164609	HB-BELL	TF
F01_transcript_164703	MYB	TF
F01_transcript_164720	ARID	TR
F01_transcript_164743	AP2/ERF-ERF	TF
F01_transcript_16475	AP2/ERF-AP2	TF
F01_transcript_164757	MYB-related	TF
F01_transcript_164768	WRKY	TF
F01_transcript_164790	SET	TR
F01_transcript_164849	WRKY	TF
F01_transcript_164936	C3H	TF
F01_transcript_164995	HB-BELL	TF
F01_transcript_165004	BBR-BPC	TF
F01_transcript_165056	NF-YA	TF
F01_transcript_165209	GARP-G2-like	TF
F01_transcript_165231	IWS1	TR
F01_transcript_165251	DBB	TF
F01_transcript_165271	mTERF	TR
F01_transcript_165280	bHLH	TF
F01_transcript_165286	WRKY	TF
F01_transcript_16531	bZIP	TF
F01_transcript_165311	SET	TR
F01_transcript_165318	HMG	TR
F01_transcript_165330	NAC	TF
F01_transcript_165395	HB-other	TF
F01_transcript_165472	HB-other	TF
F01_transcript_165477	MYB-related	TF
F01_transcript_165510	SNF2	TR
F01_transcript_165601	AP2/ERF-ERF	TF
F01_transcript_165614	mTERF	TR
F01_transcript_165624	MYB	TF
F01_transcript_165625	C3H	TF
F01_transcript_165692	B3-ARF	TF
F01_transcript_165705	B3	TF
F01_transcript_165731	MYB	TF
F01_transcript_165732	SET	TR
F01_transcript_165756	NAC	TF
F01_transcript_165783	C2H2	TF
F01_transcript_165789	SNF2	TR
F01_transcript_165836	AUX/IAA	TR
F01_transcript_165840	NAC	TF
F01_transcript_165861	AP2/ERF-RAV	TF
F01_transcript_165864	MYB-related	TF
F01_transcript_165892	bZIP	TF
F01_transcript_165909	Others	TR
F01_transcript_165952	AP2/ERF-AP2	TF
F01_transcript_16596	C3H	TF
F01_transcript_165963	C2H2	TF
F01_transcript_165979	WRKY	TF

F01_transcript_166013	C2H2	TF
F01_transcript_166031	SWI/SNF-SWI3	TR
F01_transcript_16604	HB-BELL	TF
F01_transcript_166125	Pseudo ARR-B	TR
F01_transcript_166142	MYB-related	TF
F01_transcript_166148	bHLH	TF
F01_transcript_166196	FAR1	TF
F01_transcript_16622	C3H	TF
F01_transcript_166222	mTERF	TR
F01_transcript_166250	GRAS	TF
F01_transcript_166272	LIM	TF
F01_transcript_166277	MADS-MIKC	TF
F01_transcript_166301	NAC	TF
F01_transcript_16639	HSF	TF
F01_transcript_16642	bHLH	TF
F01_transcript_166464	C3H	TF
F01_transcript_166504	SNF2	TR
F01_transcript_16658	Pseudo ARR-B	TR
F01_transcript_166685	NAC	TF
F01_transcript_166698	WRKY	TF
F01_transcript_166717	PHD	TR
F01_transcript_166790	C2H2	TF
F01_transcript_166846	RWP-RK	TF
F01_transcript_166870	Others	TR
F01_transcript_166890	NF-YA	TF
F01_transcript_166938	MYB-related	TF
F01_transcript_166950	ARID	TR
F01_transcript_167034	FAR1	TF
F01_transcript_167102	SET	TR
F01_transcript_167130	LUG	TR
F01_transcript_16715	bHLH	TF
F01_transcript_167156	HB-HD-ZIP	TF
F01_transcript_167166	Whirly	TF
F01_transcript_167174	HB-HD-ZIP	TF
F01_transcript_167182	C2H2	TF
F01_transcript_16725	BSD	TF
F01_transcript_167283	bHLH	TF
F01_transcript_167288	MADS-M-type	TF
F01_transcript_167301	B3	TF
F01_transcript_167303	MYB-related	TF
F01_transcript_167310	GARP-G2-like	TF
F01_transcript_167315	AP2/ERF-AP2	TF
F01_transcript_167421	Others	TR
F01_transcript_167437	Others	TR
F01_transcript_167443	C2H2	TF
F01_transcript_167559	B3	TF
F01_transcript_167568	SWI/SNF-BAF60b	TR
F01_transcript_167604	bHLH	TF
F01_transcript_16761	GRAS	TF
F01_transcript_167696	ARID	TR
F01_transcript_167717	AP2/ERF-ERF	TF
F01_transcript_167726	B3	TF
F01_transcript_16779	C3H	TF
F01_transcript_16791	bHLH	TF

F01_transcript_16799	C3H	TF
F01_transcript_168	SNF2	TR
F01_transcript_16801	bHLH	TF
F01_transcript_16811	GNAT	TR
F01_transcript_16838	STAT	TF
F01_transcript_16869	WRKY	TF
F01_transcript_16904	SWI/SNF-BAF60b	TR
F01_transcript_1701	NF-YC	TF
F01_transcript_17022	FAR1	TF
F01_transcript_17144	ARID	TR
F01_transcript_1717	HB-other	TF
F01_transcript_17227	WRKY	TF
F01_transcript_17228	AP2/ERF-AP2	TF
F01_transcript_1729	HB-HD-ZIP	TF
F01_transcript_17320	bZIP	TF
F01_transcript_17341	SWI/SNF-BAF60b	TR
F01_transcript_17390	TCP	TF
F01_transcript_1742	MYB	TF
F01_transcript_17491	C3H	TF
F01_transcript_17501	C3H	TF
F01_transcript_17608	B3	TF
F01_transcript_17636	GRAS	TF
F01_transcript_17654	NAC	TF
F01_transcript_17659	GARP-ARR-B	TF
F01_transcript_1767	SNF2	TR
F01_transcript_17686	GRAS	TF
F01_transcript_17735	Pseudo ARR-B	TR
F01_transcript_17814	C2C2-GATA	TF
F01_transcript_17820	GNAT	TR
F01_transcript_17823	EIL	TF
F01_transcript_17824	bHLH	TF
F01_transcript_17831	GRAS	TF
F01_transcript_17832	C2H2	TF
F01_transcript_17870	bZIP	TF
F01_transcript_17952	SNF2	TR
F01_transcript_18036	MYB-related	TF
F01_transcript_18098	GRAS	TF
F01_transcript_18148	WRKY	TF
F01_transcript_18150	Pseudo ARR-B	TR
F01_transcript_18170	C2C2-GATA	TF
F01_transcript_18177	bHLH	TF
F01_transcript_1819	TRAF	TR
F01_transcript_1823	SNF2	TR
F01_transcript_18259	RWP-RK	TF
F01_transcript_18288	bZIP	TF
F01_transcript_18294	HB-HD-ZIP	TF
F01_transcript_18297	RWP-RK	TF
F01_transcript_18301	EIL	TF
F01_transcript_18302	bHLH	TF
F01_transcript_18367	PHD	TR
F01_transcript_18372	RWP-RK	TF
F01_transcript_18393	IWS1	TR
F01_transcript_18396	TRAF	TR
F01_transcript_18413	HB-KNOX	TF
F01_transcript_18417	GRAS	TF

F01_transcript_18440	GRAS	TF
F01_transcript_18461	NAC	TF
F01_transcript_18506	TRAF	TR
F01_transcript_18509	C3H	TF
F01_transcript_18529	MYB	TF
F01_transcript_18554	HMG	TR
F01_transcript_18556	Trihelix	TF
F01_transcript_18570	bHLH	TF
F01_transcript_186	Others	TR
F01_transcript_18625	B3-ARF	TF
F01_transcript_18626	GRAS	TF
F01_transcript_18629	MADS-MIKC	TF
F01_transcript_1864	PHD	TR
F01_transcript_18693	HMG	TR
F01_transcript_18720	GRAS	TF
F01_transcript_18819	WRKY	TF
F01_transcript_18829	GRAS	TF
F01_transcript_1883	PHD	TR
F01_transcript_18858	MYB-related	TF
F01_transcript_1890	RB	TR
F01_transcript_18901	TRAF	TR
F01_transcript_18961	GeBP	TF
F01_transcript_19047	MYB-related	TF
F01_transcript_19064	bZIP	TF
F01_transcript_19089	C2H2	TF
F01_transcript_19113	RWP-RK	TF
F01_transcript_19165	mTERF	TR
F01_transcript_19256	TRAF	TR
F01_transcript_19276	Others	TR
F01_transcript_19290	MYB-related	TF
F01_transcript_19342	bZIP	TF
F01_transcript_19348	C2H2	TF
F01_transcript_19379	SWI/SNF-BAF60b	TR
F01_transcript_19392	RWP-RK	TF
F01_transcript_19406	B3	TF
F01_transcript_1941	SET	TR
F01_transcript_19411	ARID	TR
F01_transcript_19413	B3-ARF	TF
F01_transcript_19446	HB-KNOX	TF
F01_transcript_19461	GNAT	TR
F01_transcript_19465	HB-KNOX	TF
F01_transcript_19505	mTERF	TR
F01_transcript_19535	SWI/SNF-BAF60b	TR
F01_transcript_19559	AP2/ERF-ERF	TF
F01_transcript_1961	SBP	TF
F01_transcript_19635	NAC	TF
F01_transcript_19672	GARP-ARR-B	TF
F01_transcript_19676	AP2/ERF-ERF	TF
F01_transcript_19728	bZIP	TF
F01_transcript_1975	GRAS	TF
F01_transcript_19909	RWP-RK	TF
F01_transcript_19919	bZIP	TF
F01_transcript_19999	C3H	TF
F01_transcript_20003	NAC	TF

F01_transcript_20043	CPP	TF
F01_transcript_2005	SBP	TF
F01_transcript_20054	GRAS	TF
F01_transcript_20072	HB-KNOX	TF
F01_transcript_20090	FAR1	TF
F01_transcript_20104	ARID	TR
F01_transcript_20129	MYB-related	TF
F01_transcript_20188	MYB-related	TF
F01_transcript_20190	GARP-G2-like	TF
F01_transcript_2023	MYB	TF
F01_transcript_20230	MADS-M-type	TF
F01_transcript_20260	GARP-G2-like	TF
F01_transcript_20316	B3	TF
F01_transcript_20317	GRAS	TF
F01_transcript_20335	HRT	TF
F01_transcript_20371	C3H	TF
F01_transcript_20387	C2C2-CO-like	TF
F01_transcript_20407	NAC	TF
F01_transcript_20412	MYB-related	TF
F01_transcript_20460	bHLH	TF
F01_transcript_20467	C2H2	TF
F01_transcript_20471	NAC	TF
F01_transcript_20509	MYB-related	TF
F01_transcript_20512	HMG	TR
F01_transcript_20531	TRAF	TR
F01_transcript_2059	RWP-RK	TF
F01_transcript_2063	SNF2	TR
F01_transcript_20664	GARP-ARR-B	TF
F01_transcript_20704	TCP	TF
F01_transcript_20716	HMG	TR
F01_transcript_20719	GNAT	TR
F01_transcript_2073	SBP	TF
F01_transcript_20730	bHLH	TF
F01_transcript_20746	MYB-related	TF
F01_transcript_20785	EIL	TF
F01_transcript_20837	C3H	TF
F01_transcript_20918	GRAS	TF
F01_transcript_20971	RWP-RK	TF
F01_transcript_20991	C2H2	TF
F01_transcript_21000	FAR1	TF
F01_transcript_21012	bHLH	TF
F01_transcript_21041	Others	TR
F01_transcript_21056	HB-KNOX	TF
F01_transcript_21084	MADS-M-type	TF
F01_transcript_21094	GNAT	TR
F01_transcript_211	Others	TR
F01_transcript_21118	Trihelix	TF
F01_transcript_21143	WRKY	TF
F01_transcript_21144	SWI/SNF-BAF60b	TR
F01_transcript_21180	Trihelix	TF
F01_transcript_21217	Trihelix	TF
F01_transcript_21281	MYB-related	TF
F01_transcript_213	SNF2	TR
F01_transcript_2136	GRAS	TF
F01_transcript_21395	bZIP	TF

F01_transcript_21440	SWI/SNF-BAF60b	TR
F01_transcript_21441	GRAS	TF
F01_transcript_21457	HB-other	TF
F01_transcript_21472	C2H2	TF
F01_transcript_2149	B3	TF
F01_transcript_21536	TCP	TF
F01_transcript_21573	MYB	TF
F01_transcript_21578	bHLH	TF
F01_transcript_21599	RWP-RK	TF
F01_transcript_21603	C2C2-Dof	TF
F01_transcript_21609	ARID	TR
F01_transcript_2162	RWP-RK	TF
F01_transcript_2165	SNF2	TR
F01_transcript_21700	Others	TR
F01_transcript_21719	MADS-MIKC	TF
F01_transcript_21741	ARID	TR
F01_transcript_21801	Others	TR
F01_transcript_21863	B3-ARF	TF
F01_transcript_21900	NAC	TF
F01_transcript_21992	Others	TR
F01_transcript_22016	NAC	TF
F01_transcript_22022	GNAT	TR
F01_transcript_2206	NF-YA	TF
F01_transcript_22066	WRKY	TF
F01_transcript_22074	GRAS	TF
F01_transcript_22080	WRKY	TF
F01_transcript_22115	GARP-ARR-B	TF
F01_transcript_22118	GNAT	TR
F01_transcript_22193	TCP	TF
F01_transcript_22194	bZIP	TF
F01_transcript_22224	Rcd1-like	TR
F01_transcript_22238	B3	TF
F01_transcript_22243	GRAS	TF
F01_transcript_22254	WRKY	TF
F01_transcript_22307	GRAS	TF
F01_transcript_22323	Trihelix	TF
F01_transcript_22353	C3H	TF
F01_transcript_22366	bZIP	TF
F01_transcript_22380	mTERF	TR
F01_transcript_22399	AP2/ERF-ERF	TF
F01_transcript_22440	Others	TR
F01_transcript_2246	Rcd1-like	TR
F01_transcript_22466	NAC	TF
F01_transcript_22468	Others	TR
F01_transcript_22494	Others	TR
F01_transcript_22520	TUB	TF
F01_transcript_2253	SET	TR
F01_transcript_22559	MYB-related	TF
F01_transcript_22576	C2H2	TF
F01_transcript_22600	C3H	TF
F01_transcript_22604	mTERF	TR
F01_transcript_22624	WRKY	TF
F01_transcript_22644	TCP	TF
F01_transcript_22648	GARP-G2-like	TF

F01_transcript_22666	TRAF	TR
F01_transcript_22678	C2H2	TF
F01_transcript_2268	GRAS	TF
F01_transcript_2270	CAMTA	TF
F01_transcript_2277	bZIP	TF
F01_transcript_2278	B3	TF
F01_transcript_22788	HB-HD-ZIP	TF
F01_transcript_22814	WRKY	TF
F01_transcript_2283	RWP-RK	TF
F01_transcript_22835	bZIP	TF
F01_transcript_22853	mTERF	TR
F01_transcript_23044	RWP-RK	TF
F01_transcript_2311	Jumonji	TR
F01_transcript_23127	TUB	TF
F01_transcript_23163	TUB	TF
F01_transcript_23191	RWP-RK	TF
F01_transcript_23220	mTERF	TR
F01_transcript_23270	ARID	TR
F01_transcript_23285	RWP-RK	TF
F01_transcript_23289	RWP-RK	TF
F01_transcript_23342	GARP-G2-like	TF
F01_transcript_23369	RWP-RK	TF
F01_transcript_23384	C2H2	TF
F01_transcript_23390	Trihelix	TF
F01_transcript_23402	TUB	TF
F01_transcript_23415	Tify	TF
F01_transcript_23418	TCP	TF
F01_transcript_23448	Trihelix	TF
F01_transcript_23457	HRT	TF
F01_transcript_2347	RWP-RK	TF
F01_transcript_23494	AP2/ERF-ERF	TF
F01_transcript_23507	TCP	TF
F01_transcript_23518	GRAS	TF
F01_transcript_23553	HSF	TF
F01_transcript_23588	mTERF	TR
F01_transcript_23597	AP2/ERF-ERF	TF
F01_transcript_23604	RWP-RK	TF
F01_transcript_23609	WRKY	TF
F01_transcript_23641	bHLH	TF
F01_transcript_23674	C2H2	TF
F01_transcript_23776	C3H	TF
F01_transcript_23795	C2H2	TF
F01_transcript_23846	C2H2	TF
F01_transcript_23875	bHLH	TF
F01_transcript_23926	mTERF	TR
F01_transcript_23954	C2H2	TF
F01_transcript_2400	PHD	TR
F01_transcript_2402	C2C2-Dof	TF
F01_transcript_2403	CAMTA	TF
F01_transcript_24054	bHLH	TF
F01_transcript_24060	CPP	TF
F01_transcript_24069	GRAS	TF
F01_transcript_24081	HSF	TF
F01_transcript_24125	Others	TR
F01_transcript_24129	AP2/ERF-ERF	TF

F01_transcript_24161	Others	TR
F01_transcript_24169	Others	TR
F01_transcript_24186	Others	TR
F01_transcript_24257	bHLH	TF
F01_transcript_24277	B3	TF
F01_transcript_24338	C2C2-Dof	TF
F01_transcript_2437	SBP	TF
F01_transcript_24391	C2H2	TF
F01_transcript_24394	bHLH	TF
F01_transcript_24396	HRT	TF
F01_transcript_24418	PHD	TR
F01_transcript_24428	HB-HD-ZIP	TF
F01_transcript_24481	C2H2	TF
F01_transcript_24488	C2C2-CO-like	TF
F01_transcript_24492	ARID	TR
F01_transcript_24534	IWS1	TR
F01_transcript_24544	ARID	TR
F01_transcript_24547	TCP	TF
F01_transcript_24564	AP2/ERF-AP2	TF
F01_transcript_24573	WRKY	TF
F01_transcript_24582	GARP-G2-like	TF
F01_transcript_24587	GARP-G2-like	TF
F01_transcript_24603	mTERF	TR
F01_transcript_24629	Others	TR
F01_transcript_24657	GARP-G2-like	TF
F01_transcript_24680	GRAS	TF
F01_transcript_24718	NAC	TF
F01_transcript_24742	MYB-related	TF
F01_transcript_24780	WRKY	TF
F01_transcript_24784	bHLH	TF
F01_transcript_24799	SBP	TF
F01_transcript_24825	HB-HD-ZIP	TF
F01_transcript_24865	WRKY	TF
F01_transcript_24911	MYB-related	TF
F01_transcript_24921	ARID	TR
F01_transcript_24946	Others	TR
F01_transcript_24963	C2H2	TF
F01_transcript_24968	C2H2	TF
F01_transcript_24969	IWS1	TR
F01_transcript_25004	RWP-RK	TF
F01_transcript_25018	C3H	TF
F01_transcript_25020	C2H2	TF
F01_transcript_25022	HSF	TF
F01_transcript_25040	Others	TR
F01_transcript_25044	RWP-RK	TF
F01_transcript_2511	PHD	TR
F01_transcript_25121	mTERF	TR
F01_transcript_25131	C2H2	TF
F01_transcript_25162	C2H2	TF
F01_transcript_25168	bZIP	TF
F01_transcript_25238	AP2/ERF-ERF	TF
F01_transcript_2525	C3H	TF
F01_transcript_25274	mTERF	TR
F01_transcript_25306	AP2/ERF-AP2	TF

F01_transcript_25309	GeBP	TF
F01_transcript_25338	GARP-G2-like	TF
F01_transcript_25370	C2C2-CO-like	TF
F01_transcript_25389	VOZ	TF
F01_transcript_25393	GARP-G2-like	TF
F01_transcript_25397	WRKY	TF
F01_transcript_25455	Trihelix	TF
F01_transcript_25462	MYB-related	TF
F01_transcript_25476	WRKY	TF
F01_transcript_25495	bHLH	TF
F01_transcript_25527	MYB-related	TF
F01_transcript_25530	TRAF	TR
F01_transcript_25557	GARP-G2-like	TF
F01_transcript_25578	TUB	TF
F01_transcript_25597	PHD	TR
F01_transcript_25620	GARP-G2-like	TF
F01_transcript_25625	C2H2	TF
F01_transcript_25627	C3H	TF
F01_transcript_2567	MYB	TF
F01_transcript_25682	AP2/ERF-ERF	TF
F01_transcript_25686	NAC	TF
F01_transcript_25718	mTERF	TR
F01_transcript_258	SWI/SNF-BAF60b	TR
F01_transcript_25807	TRAF	TR
F01_transcript_25812	bZIP	TF
F01_transcript_25831	C2H2	TF
F01_transcript_25900	Others	TR
F01_transcript_25919	Trihelix	TF
F01_transcript_25953	TCP	TF
F01_transcript_26066	C2H2	TF
F01_transcript_2608	FAR1	TF
F01_transcript_26094	bHLH	TF
F01_transcript_26111	WRKY	TF
F01_transcript_26176	WRKY	TF
F01_transcript_26195	NAC	TF
F01_transcript_26215	GARP-G2-like	TF
F01_transcript_26293	ARID	TR
F01_transcript_26342	bZIP	TF
F01_transcript_26393	C3H	TF
F01_transcript_26395	GRAS	TF
F01_transcript_26408	bHLH	TF
F01_transcript_26414	HB-HD-ZIP	TF
F01_transcript_26474	SRS	TF
F01_transcript_26494	B3	TF
F01_transcript_26566	bHLH	TF
F01_transcript_2659	CAMTA	TF
F01_transcript_2662	RWP-RK	TF
F01_transcript_26645	TUB	TF
F01_transcript_26680	C2C2-CO-like	TF
F01_transcript_26682	C2H2	TF
F01_transcript_26685	bHLH	TF
F01_transcript_26692	HSF	TF
F01_transcript_26766	bHLH	TF
F01_transcript_26782	bZIP	TF
F01_transcript_26783	GARP-G2-like	TF

F01_transcript_26788	GARP-G2-like	TF
F01_transcript_26989	Trihelix	TF
F01_transcript_26991	WRKY	TF
F01_transcript_26997	TCP	TF
F01_transcript_27062	AP2/ERF-AP2	TF
F01_transcript_27063	GNAT	TR
F01_transcript_27073	GNAT	TR
F01_transcript_27083	mTERF	TR
F01_transcript_27105	Trihelix	TF
F01_transcript_27118	IWS1	TR
F01_transcript_27249	bHLH	TF
F01_transcript_27285	BBR-BPC	TF
F01_transcript_27357	SWI/SNF-BAF60b	TR
F01_transcript_27388	C3H	TF
F01_transcript_27456	C2C2-CO-like	TF
F01_transcript_27567	TRAF	TR
F01_transcript_2759	NF-YA	TF
F01_transcript_27662	bHLH	TF
F01_transcript_27723	B3	TF
F01_transcript_27726	RWP-RK	TF
F01_transcript_2774	RWP-RK	TF
F01_transcript_27746	mTERF	TR
F01_transcript_27752	mTERF	TR
F01_transcript_27828	Tify	TF
F01_transcript_27844	bZIP	TF
F01_transcript_27862	AUX/IAA	TR
F01_transcript_27875	GARP-G2-like	TF
F01_transcript_27947	C2H2	TF
F01_transcript_27983	AP2/ERF-ERF	TF
F01_transcript_27984	C2H2	TF
F01_transcript_27993	Tify	TF
F01_transcript_28022	C3H	TF
F01_transcript_28062	FAR1	TF
F01_transcript_28078	bHLH	TF
F01_transcript_28124	TCP	TF
F01_transcript_28161	GRAS	TF
F01_transcript_28182	LOB	TF
F01_transcript_28218	mTERF	TR
F01_transcript_28231	TCP	TF
F01_transcript_28251	WRKY	TF
F01_transcript_2830	RWP-RK	TF
F01_transcript_28324	Others	TR
F01_transcript_28329	VOZ	TF
F01_transcript_28384	SWI/SNF-BAF60b	TR
F01_transcript_28466	bHLH	TF
F01_transcript_28468	PHD	TR
F01_transcript_28488	bHLH	TF
F01_transcript_28489	GARP-G2-like	TF
F01_transcript_28506	C2C2-GATA	TF
F01_transcript_28508	bHLH	TF
F01_transcript_28547	WRKY	TF
F01_transcript_28555	C2H2	TF
F01_transcript_28556	bHLH	TF
F01_transcript_28585	GARP-G2-like	TF

F01_transcript_28614	C3H	TF
F01_transcript_28643	bHLH	TF
F01_transcript_28686	GRAS	TF
F01_transcript_28692	C3H	TF
F01_transcript_28701	C3H	TF
F01_transcript_28706	BBR-BPC	TF
F01_transcript_28790	C3H	TF
F01_transcript_28792	SWI/SNF-BAF60b	TR
F01_transcript_28860	GARP-G2-like	TF
F01_transcript_28898	C2H2	TF
F01_transcript_29055	SET	TR
F01_transcript_29067	BES1	TF
F01_transcript_291	GARP-G2-like	TF
F01_transcript_29132	C2C2-GATA	TF
F01_transcript_29208	HSF	TF
F01_transcript_29300	TUB	TF
F01_transcript_29313	TCP	TF
F01_transcript_29337	SET	TR
F01_transcript_29339	bHLH	TF
F01_transcript_29385	TUB	TF
F01_transcript_29400	SET	TR
F01_transcript_29402	bHLH	TF
F01_transcript_29418	TRAF	TR
F01_transcript_29441	AUX/IAA	TR
F01_transcript_29449	WRKY	TF
F01_transcript_29531	HB-KNOX	TF
F01_transcript_29546	Jumonji	TR
F01_transcript_29578	C2H2	TF
F01_transcript_29598	GARP-G2-like	TF
F01_transcript_29726	GNAT	TR
F01_transcript_29734	AP2/ERF-ERF	TF
F01_transcript_29785	bZIP	TF
F01_transcript_29810	bZIP	TF
F01_transcript_29838	GARP-G2-like	TF
F01_transcript_29847	GARP-ARR-B	TF
F01_transcript_29861	bHLH	TF
F01_transcript_29864	C3H	TF
F01_transcript_29871	MYB	TF
F01_transcript_29962	Others	TR
F01_transcript_29974	TUB	TF
F01_transcript_30064	WRKY	TF
F01_transcript_30200	TUB	TF
F01_transcript_30221	Others	TR
F01_transcript_30223	C2C2-Dof	TF
F01_transcript_30248	HB-other	TF
F01_transcript_30304	C3H	TF
F01_transcript_30305	C2C2-Dof	TF
F01_transcript_30337	TUB	TF
F01_transcript_30343	bHLH	TF
F01_transcript_30372	mTERF	TR
F01_transcript_30394	GRAS	TF
F01_transcript_30457	mTERF	TR
F01_transcript_30479	TRAF	TR
F01_transcript_30493	NAC	TF
F01_transcript_30544	WRKY	TF

F01_transcript_30556	GRAS	TF
F01_transcript_30598	bZIP	TF
F01_transcript_30639	NAC	TF
F01_transcript_30677	SBP	TF
F01_transcript_30679	SET	TR
F01_transcript_30714	SBP	TF
F01_transcript_30716	Trihelix	TF
F01_transcript_30732	TRAF	TR
F01_transcript_30740	MYB-related	TF
F01_transcript_30755	bZIP	TF
F01_transcript_30788	GARP-G2-like	TF
F01_transcript_30799	SET	TR
F01_transcript_30831	AP2/ERF-ERF	TF
F01_transcript_30860	GNAT	TR
F01_transcript_30916	NAC	TF
F01_transcript_31008	mTERF	TR
F01_transcript_31038	AP2/ERF-ERF	TF
F01_transcript_3105	RWP-RK	TF
F01_transcript_31118	GARP-G2-like	TF
F01_transcript_3112	GRAS	TF
F01_transcript_31149	SBP	TF
F01_transcript_31167	Others	TR
F01_transcript_31180	C2C2-Dof	TF
F01_transcript_31201	HSF	TF
F01_transcript_31220	AP2/ERF-ERF	TF
F01_transcript_31231	AP2/ERF-ERF	TF
F01_transcript_31257	GRAS	TF
F01_transcript_3128	FAR1	TF
F01_transcript_31286	SET	TR
F01_transcript_31368	MYB	TF
F01_transcript_31369	bHLH	TF
F01_transcript_31408	TCP	TF
F01_transcript_3142	Jumonji	TR
F01_transcript_31455	TUB	TF
F01_transcript_3149	PHD	TR
F01_transcript_31515	TCP	TF
F01_transcript_31522	GRAS	TF
F01_transcript_31631	WRKY	TF
F01_transcript_31654	SET	TR
F01_transcript_31726	GRAS	TF
F01_transcript_31748	SET	TR
F01_transcript_31757	SBP	TF
F01_transcript_31779	NAC	TF
F01_transcript_31786	SET	TR
F01_transcript_31790	MYB-related	TF
F01_transcript_31869	C2C2-Dof	TF
F01_transcript_31897	TUB	TF
F01_transcript_31901	TUB	TF
F01_transcript_31924	C2C2-Dof	TF
F01_transcript_31946	ARID	TR
F01_transcript_31970	SET	TR
F01_transcript_31976	TUB	TF
F01_transcript_32031	bZIP	TF
F01_transcript_32043	AUX/IAA	TR

F01_transcript_32073	mTERF	TR
F01_transcript_32128	NAC	TF
F01_transcript_32168	C3H	TF
F01_transcript_3220	SNF2	TR
F01_transcript_32205	bZIP	TF
F01_transcript_32270	TUB	TF
F01_transcript_32357	TRAF	TR
F01_transcript_32361	bZIP	TF
F01_transcript_32364	C2C2-Dof	TF
F01_transcript_32385	C2H2	TF
F01_transcript_32400	C3H	TF
F01_transcript_32425	IWS1	TR
F01_transcript_32438	TRAF	TR
F01_transcript_32486	Trihelix	TF
F01_transcript_32507	C2H2	TF
F01_transcript_32559	bHLH	TF
F01_transcript_3259	RWP-RK	TF
F01_transcript_32692	C2C2-CO-like	TF
F01_transcript_327	HB-PHD	TF
F01_transcript_3277	LUG	TR
F01_transcript_32790	B3	TF
F01_transcript_3289	RWP-RK	TF
F01_transcript_33031	C2C2-GATA	TF
F01_transcript_33093	bZIP	TF
F01_transcript_33131	AP2/ERF-ERF	TF
F01_transcript_33132	C2H2	TF
F01_transcript_33177	WRKY	TF
F01_transcript_33223	WRKY	TF
F01_transcript_33313	C2C2-CO-like	TF
F01_transcript_33353	C2C2-Dof	TF
F01_transcript_33367	bHLH	TF
F01_transcript_33430	C2H2	TF
F01_transcript_33474	SET	TR
F01_transcript_33637	AP2/ERF-ERF	TF
F01_transcript_33651	bZIP	TF
F01_transcript_33714	MYB	TF
F01_transcript_33757	TUB	TF
F01_transcript_3380	FAR1	TF
F01_transcript_3387	CAMTA	TF
F01_transcript_33872	NF-YC	TF
F01_transcript_33942	AUX/IAA	TR
F01_transcript_33945	HB-KNOX	TF
F01_transcript_33998	MYB-related	TF
F01_transcript_34033	TUB	TF
F01_transcript_34049	SWI/SNF-BAF60b	TR
F01_transcript_3406	Others	TR
F01_transcript_34101	TUB	TF
F01_transcript_3411	CAMTA	TF
F01_transcript_34117	MYB-related	TF
F01_transcript_34184	GRAS	TF
F01_transcript_34216	C3H	TF
F01_transcript_34225	bZIP	TF
F01_transcript_3434	MYB-related	TF
F01_transcript_34364	AP2/ERF-ERF	TF
F01_transcript_34387	HB-KNOX	TF

F01_transcript_34409	C2C2-Dof	TF
F01_transcript_3448	PHD	TR
F01_transcript_34512	bZIP	TF
F01_transcript_34520	AUX/IAA	TR
F01_transcript_3466	PHD	TR
F01_transcript_34679	MYB-related	TF
F01_transcript_34681	HB-KNOX	TF
F01_transcript_34725	bZIP	TF
F01_transcript_34789	AUX/IAA	TR
F01_transcript_34826	MYB	TF
F01_transcript_34829	bZIP	TF
F01_transcript_34835	HB-HD-ZIP	TF
F01_transcript_34852	MYB-related	TF
F01_transcript_34877	MYB	TF
F01_transcript_34881	Trihelix	TF
F01_transcript_34959	TRAF	TR
F01_transcript_34969	DBP	TF
F01_transcript_34981	TCP	TF
F01_transcript_34983	C2H2	TF
F01_transcript_34992	bZIP	TF
F01_transcript_35030	HMG	TR
F01_transcript_35034	MYB	TF
F01_transcript_35088	bZIP	TF
F01_transcript_35090	bZIP	TF
F01_transcript_35096	C3H	TF
F01_transcript_35142	C3H	TF
F01_transcript_3518	HB-HD-ZIP	TF
F01_transcript_35204	SWI/SNF-BAF60b	TR
F01_transcript_35205	bHLH	TF
F01_transcript_3528	LUG	TR
F01_transcript_35304	bZIP	TF
F01_transcript_35315	C2C2-GATA	TF
F01_transcript_35321	Trihelix	TF
F01_transcript_3533	CAMTA	TF
F01_transcript_35335	BES1	TF
F01_transcript_35340	AUX/IAA	TR
F01_transcript_35377	NAC	TF
F01_transcript_35387	HMG	TR
F01_transcript_35409	NAC	TF
F01_transcript_35509	AP2/ERF-ERF	TF
F01_transcript_35529	bHLH	TF
F01_transcript_35532	bHLH	TF
F01_transcript_35562	C2C2-Dof	TF
F01_transcript_35582	bZIP	TF
F01_transcript_35619	NF-YA	TF
F01_transcript_35627	TUB	TF
F01_transcript_35647	C2C2-GATA	TF
F01_transcript_35652	NAC	TF
F01_transcript_35713	AUX/IAA	TR
F01_transcript_35723	AUX/IAA	TR
F01_transcript_35733	HSF	TF
F01_transcript_35767	bZIP	TF
F01_transcript_3578	SBP	TF
F01_transcript_35784	WRKY	TF

F01_transcript_35795	C2C2-CO-like	TF
F01_transcript_35813	NAC	TF
F01_transcript_35846	bZIP	TF
F01_transcript_35863	MYB-related	TF
F01_transcript_36028	bHLH	TF
F01_transcript_36042	MYB-related	TF
F01_transcript_36058	SET	TR
F01_transcript_36061	SWI/SNF-BAF60b	TR
F01_transcript_36073	bZIP	TF
F01_transcript_36088	C3H	TF
F01_transcript_36097	MYB-related	TF
F01_transcript_36220	TCP	TF
F01_transcript_36367	bHLH	TF
F01_transcript_36397	mTERF	TR
F01_transcript_36410	AUX/IAA	TR
F01_transcript_36413	MYB-related	TF
F01_transcript_36445	C3H	TF
F01_transcript_36473	NF-YA	TF
F01_transcript_36515	MYB	TF
F01_transcript_3654	HB-HD-ZIP	TF
F01_transcript_36547	Jumonji	TR
F01_transcript_36548	ARID	TR
F01_transcript_36560	Trihelix	TF
F01_transcript_36562	GARP-G2-like	TF
F01_transcript_36586	GARP-G2-like	TF
F01_transcript_36596	Tify	TF
F01_transcript_36604	Tify	TF
F01_transcript_36621	MYB-related	TF
F01_transcript_36653	FAR1	TF
F01_transcript_36706	bZIP	TF
F01_transcript_36722	C2C2-Dof	TF
F01_transcript_36792	NAC	TF
F01_transcript_36819	Trihelix	TF
F01_transcript_36821	bHLH	TF
F01_transcript_36867	HB-HD-ZIP	TF
F01_transcript_36884	C2H2	TF
F01_transcript_36891	HB-HD-ZIP	TF
F01_transcript_37037	NF-YA	TF
F01_transcript_37044	HB-HD-ZIP	TF
F01_transcript_37050	DBB	TF
F01_transcript_37060	C2H2	TF
F01_transcript_3707	RWP-RK	TF
F01_transcript_37083	Tify	TF
F01_transcript_37111	bHLH	TF
F01_transcript_3712	FAR1	TF
F01_transcript_37142	SBP	TF
F01_transcript_37153	MYB-related	TF
F01_transcript_37179	SWI/SNF-BAF60b	TR
F01_transcript_3720	Jumonji	TR
F01_transcript_37209	DBP	TF
F01_transcript_37281	Trihelix	TF
F01_transcript_37288	C2H2	TF
F01_transcript_37290	C2H2	TF
F01_transcript_37317	bZIP	TF
F01_transcript_37371	Trihelix	TF

F01_transcript_37372	bZIP	TF
F01_transcript_37393	C3H	TF
F01_transcript_37414	HB-HD-ZIP	TF
F01_transcript_37441	TAZ	TR
F01_transcript_3746	SNF2	TR
F01_transcript_37472	C3H	TF
F01_transcript_37524	bHLH	TF
F01_transcript_37532	C2C2-GATA	TF
F01_transcript_37584	NAC	TF
F01_transcript_37591	GARP-G2-like	TF
F01_transcript_37622	C2H2	TF
F01_transcript_37639	C3H	TF
F01_transcript_37677	C2C2-CO-like	TF
F01_transcript_37682	MYB	TF
F01_transcript_37691	bHLH	TF
F01_transcript_37692	AP2/ERF-ERF	TF
F01_transcript_37800	HB-HD-ZIP	TF
F01_transcript_37938	C2C2-CO-like	TF
F01_transcript_37960	TUB	TF
F01_transcript_37990	SWI/SNF-BAF60b	TR
F01_transcript_38012	B3	TF
F01_transcript_38036	TAZ	TR
F01_transcript_38081	C2C2-CO-like	TF
F01_transcript_38091	C2H2	TF
F01_transcript_38167	C3H	TF
F01_transcript_3820	RWP-RK	TF
F01_transcript_38233	bZIP	TF
F01_transcript_38292	bZIP	TF
F01_transcript_38295	MYB-related	TF
F01_transcript_38298	MYB-related	TF
F01_transcript_38344	C2C2-GATA	TF
F01_transcript_38357	SWI/SNF-BAF60b	TR
F01_transcript_38375	HB-HD-ZIP	TF
F01_transcript_38401	AUX/IAA	TR
F01_transcript_38447	C2H2	TF
F01_transcript_38453	PLATZ	TF
F01_transcript_38493	MYB	TF
F01_transcript_38529	TCP	TF
F01_transcript_3853	RWP-RK	TF
F01_transcript_38567	bHLH	TF
F01_transcript_3858	Jumonji	TR
F01_transcript_38581	GARP-G2-like	TF
F01_transcript_38586	SET	TR
F01_transcript_38597	HB-HD-ZIP	TF
F01_transcript_38602	C2C2-GATA	TF
F01_transcript_38646	Trihelix	TF
F01_transcript_38656	GARP-G2-like	TF
F01_transcript_38659	Trihelix	TF
F01_transcript_38660	FAR1	TF
F01_transcript_38676	Alfin-like	TF
F01_transcript_38677	C3H	TF
F01_transcript_38711	HB-HD-ZIP	TF
F01_transcript_38742	BBR-BPC	TF
F01_transcript_38818	GeBP	TF

F01_transcript_38836	mTERF	TR
F01_transcript_38875	NF-YA	TF
F01_transcript_38896	NAC	TF
F01_transcript_38899	NAC	TF
F01_transcript_38916	BBR-BPC	TF
F01_transcript_38939	bZIP	TF
F01_transcript_39029	SWI/SNF-BAF60b	TR
F01_transcript_39049	bZIP	TF
F01_transcript_3906	TRAF	TR
F01_transcript_39066	C2H2	TF
F01_transcript_39084	E2F-DP	TF
F01_transcript_39089	GeBP	TF
F01_transcript_39171	TCP	TF
F01_transcript_39175	C2C2-GATA	TF
F01_transcript_39202	BBR-BPC	TF
F01_transcript_39220	MYB	TF
F01_transcript_39234	bZIP	TF
F01_transcript_3924	Others	TR
F01_transcript_39255	bHLH	TF
F01_transcript_39256	TCP	TF
F01_transcript_39258	GNAT	TR
F01_transcript_39261	C2C2-Dof	TF
F01_transcript_39263	NF-YB	TF
F01_transcript_39282	C2H2	TF
F01_transcript_39290	GNAT	TR
F01_transcript_39297	NF-YA	TF
F01_transcript_39298	C2C2-CO-like	TF
F01_transcript_39308	Trihelix	TF
F01_transcript_39342	Trihelix	TF
F01_transcript_39343	PLATZ	TF
F01_transcript_39485	C2H2	TF
F01_transcript_39540	bHLH	TF
F01_transcript_39544	SRS	TF
F01_transcript_39587	NAC	TF
F01_transcript_39610	MYB	TF
F01_transcript_39714	GeBP	TF
F01_transcript_39719	C2H2	TF
F01_transcript_39757	HSF	TF
F01_transcript_3976	LUG	TR
F01_transcript_39800	C3H	TF
F01_transcript_39843	AP2/ERF-ERF	TF
F01_transcript_39844	NF-YA	TF
F01_transcript_39894	MYB-related	TF
F01_transcript_39973	CSD	TF
F01_transcript_39979	C2C2-Dof	TF
F01_transcript_4003	RWP-RK	TF
F01_transcript_40076	C2C2-CO-like	TF
F01_transcript_40104	B3	TF
F01_transcript_40146	C2C2-GATA	TF
F01_transcript_4016	B3-ARF	TF
F01_transcript_40189	NF-YA	TF
F01_transcript_40207	HMG	TR
F01_transcript_40218	bHLH	TF
F01_transcript_40273	zf-HD	TF
F01_transcript_4032	TRAF	TR

F01_transcript_40382	PLATZ	TF
F01_transcript_40391	HB-WOX	TF
F01_transcript_40416	Tify	TF
F01_transcript_40420	C2H2	TF
F01_transcript_40423	Trihelix	TF
F01_transcript_40480	HB-HD-ZIP	TF
F01_transcript_4049	CAMTA	TF
F01_transcript_4051	MYB-related	TF
F01_transcript_40531	Trihelix	TF
F01_transcript_40535	bZIP	TF
F01_transcript_40603	NF-YC	TF
F01_transcript_40629	HSF	TF
F01_transcript_4063	SNF2	TR
F01_transcript_40646	BBR-BPC	TF
F01_transcript_40673	DBB	TF
F01_transcript_40694	C3H	TF
F01_transcript_40738	C2H2	TF
F01_transcript_40777	NAC	TF
F01_transcript_40785	C2C2-GATA	TF
F01_transcript_4080	B3-ARF	TF
F01_transcript_40862	Others	TR
F01_transcript_40865	MYB	TF
F01_transcript_40889	C3H	TF
F01_transcript_4090	LUG	TR
F01_transcript_40903	bHLH	TF
F01_transcript_40906	AP2/ERF-ERF	TF
F01_transcript_40913	MYB-related	TF
F01_transcript_40958	C2H2	TF
F01_transcript_40996	C2C2-GATA	TF
F01_transcript_41026	NF-YC	TF
F01_transcript_41071	C3H	TF
F01_transcript_41081	AP2/ERF-ERF	TF
F01_transcript_41085	NAC	TF
F01_transcript_41087	GARP-G2-like	TF
F01_transcript_41090	NF-YC	TF
F01_transcript_41094	TRAF	TR
F01_transcript_41127	bZIP	TF
F01_transcript_41146	MYB-related	TF
F01_transcript_41169	C2H2	TF
F01_transcript_41198	NF-YC	TF
F01_transcript_41201	NAC	TF
F01_transcript_4127	LUG	TR
F01_transcript_41314	BBR-BPC	TF
F01_transcript_41322	WRKY	TF
F01_transcript_41349	Tify	TF
F01_transcript_41368	bZIP	TF
F01_transcript_41389	DBB	TF
F01_transcript_41408	Alfin-like	TF
F01_transcript_41425	MYB	TF
F01_transcript_4143	RWP-RK	TF
F01_transcript_41441	HB-WOX	TF
F01_transcript_41453	GARP-G2-like	TF
F01_transcript_41492	MYB	TF
F01_transcript_41508	C2H2	TF

F01_transcript_41521	MYB	TF
F01_transcript_41539	NF-YC	TF
F01_transcript_41569	TRAF	TR
F01_transcript_41579	Alfin-like	TF
F01_transcript_41671	C2C2-GATA	TF
F01_transcript_41683	Alfin-like	TF
F01_transcript_41708	BBR-BPC	TF
F01_transcript_41740	HB-HD-ZIP	TF
F01_transcript_4175	TRAF	TR
F01_transcript_41779	NAC	TF
F01_transcript_41790	Others	TR
F01_transcript_41815	C3H	TF
F01_transcript_41853	Others	TR
F01_transcript_41868	WRKY	TF
F01_transcript_41892	AP2/ERF-ERF	TF
F01_transcript_41896	Others	TR
F01_transcript_41925	DBB	TF
F01_transcript_41947	Tify	TF
F01_transcript_41950	MYB	TF
F01_transcript_41951	Alfin-like	TF
F01_transcript_41992	PHD	TR
F01_transcript_42	IWS1	TR
F01_transcript_42010	MYB	TF
F01_transcript_42042	MYB	TF
F01_transcript_42085	Others	TR
F01_transcript_42086	WRKY	TF
F01_transcript_4210	GRAS	TF
F01_transcript_42112	MYB-related	TF
F01_transcript_42130	C2C2-GATA	TF
F01_transcript_42140	AUX/IAA	TR
F01_transcript_42142	GARP-G2-like	TF
F01_transcript_42185	MYB	TF
F01_transcript_42223	mTERF	TR
F01_transcript_42235	C2H2	TF
F01_transcript_42315	Rcd1-like	TR
F01_transcript_42375	bHLH	TF
F01_transcript_42407	Rcd1-like	TR
F01_transcript_42467	bZIP	TF
F01_transcript_42478	C2C2-GATA	TF
F01_transcript_42479	Alfin-like	TF
F01_transcript_4249	B3-ARF	TF
F01_transcript_42506	NF-YA	TF
F01_transcript_42526	Alfin-like	TF
F01_transcript_42543	C2H2	TF
F01_transcript_42557	AUX/IAA	TR
F01_transcript_42588	MYB	TF
F01_transcript_42637	NF-YC	TF
F01_transcript_42647	Rcd1-like	TR
F01_transcript_42677	AP2/ERF-ERF	TF
F01_transcript_42722	AUX/IAA	TR
F01_transcript_42767	NF-YC	TF
F01_transcript_42772	AUX/IAA	TR
F01_transcript_428	SNF2	TR
F01_transcript_42828	Others	TR
F01_transcript_4290	B3-ARF	TF

F01_transcript_42925	GARP-G2-like	TF
F01_transcript_42932	B3-ARF	TF
F01_transcript_42945	C2C2-GATA	TF
F01_transcript_42969	MADS-M-type	TF
F01_transcript_42970	MYB	TF
F01_transcript_4304	mTERF	TR
F01_transcript_43048	AP2/ERF-ERF	TF
F01_transcript_4308	B3	TF
F01_transcript_43123	C2H2	TF
F01_transcript_43220	mTERF	TR
F01_transcript_43279	PHD	TR
F01_transcript_43285	GNAT	TR
F01_transcript_43299	GNAT	TR
F01_transcript_43316	bHLH	TF
F01_transcript_43320	WRKY	TF
F01_transcript_43427	HB-HD-ZIP	TF
F01_transcript_43465	MADS-MIKC	TF
F01_transcript_43540	C3H	TF
F01_transcript_43596	Whirly	TF
F01_transcript_4362	bZIP	TF
F01_transcript_43658	MYB-related	TF
F01_transcript_43683	LOB	TF
F01_transcript_43705	mTERF	TR
F01_transcript_43743	mTERF	TR
F01_transcript_43825	AUX/IAA	TR
F01_transcript_43829	LIM	TF
F01_transcript_43840	AUX/IAA	TR
F01_transcript_43858	bHLH	TF
F01_transcript_43866	Others	TR
F01_transcript_43892	HB-other	TF
F01_transcript_43904	FAR1	TF
F01_transcript_43920	NAC	TF
F01_transcript_43931	LIM	TF
F01_transcript_43946	Whirly	TF
F01_transcript_43977	bZIP	TF
F01_transcript_43981	WRKY	TF
F01_transcript_43983	FAR1	TF
F01_transcript_43989	bZIP	TF
F01_transcript_44076	HB-other	TF
F01_transcript_44099	AUX/IAA	TR
F01_transcript_44139	bHLH	TF
F01_transcript_4415	TRAF	TR
F01_transcript_44194	C2C2-GATA	TF
F01_transcript_44221	AP2/ERF-ERF	TF
F01_transcript_44263	MYB	TF
F01_transcript_44291	NF-YC	TF
F01_transcript_44321	OFP	TF
F01_transcript_44332	FAR1	TF
F01_transcript_4439	B3-ARF	TF
F01_transcript_4440	FAR1	TF
F01_transcript_44400	LOB	TF
F01_transcript_44462	Whirly	TF
F01_transcript_44534	LIM	TF
F01_transcript_44561	HMG	TR

F01_transcript_44594	MYB-related	TF
F01_transcript_44626	C2H2	TF
F01_transcript_44638	NAC	TF
F01_transcript_44645	NF-YB	TF
F01_transcript_44649	MYB-related	TF
F01_transcript_44678	HB-HD-ZIP	TF
F01_transcript_44681	LIM	TF
F01_transcript_44705	bHLH	TF
F01_transcript_44751	NF-YC	TF
F01_transcript_44782	DBB	TF
F01_transcript_44801	MYB	TF
F01_transcript_4481	bZIP	TF
F01_transcript_44830	Alfin-like	TF
F01_transcript_44887	LIM	TF
F01_transcript_44942	BBR-BPC	TF
F01_transcript_44980	DBB	TF
F01_transcript_45061	bHLH	TF
F01_transcript_4509	LUG	TR
F01_transcript_45154	MADS-MIKC	TF
F01_transcript_45164	bHLH	TF
F01_transcript_4519	B3-ARF	TF
F01_transcript_45212	AP2/ERF-ERF	TF
F01_transcript_45262	MYB	TF
F01_transcript_45265	C2C2-GATA	TF
F01_transcript_4530	HB-HD-ZIP	TF
F01_transcript_4532	HB-HD-ZIP	TF
F01_transcript_45348	AP2/ERF-ERF	TF
F01_transcript_45364	AP2/ERF-ERF	TF
F01_transcript_45422	AP2/ERF-ERF	TF
F01_transcript_4545	C3H	TF
F01_transcript_45452	MED7	TR
F01_transcript_45521	C2H2	TF
F01_transcript_45560	LIM	TF
F01_transcript_45586	C2C2-YABBY	TF
F01_transcript_4560	HB-HD-ZIP	TF
F01_transcript_45608	bHLH	TF
F01_transcript_45631	C2C2-LSD	TF
F01_transcript_45892	HMG	TR
F01_transcript_45916	HMG	TR
F01_transcript_45939	HMG	TR
F01_transcript_45953	RWP-RK	TF
F01_transcript_46008	mTERF	TR
F01_transcript_4607	HB-HD-ZIP	TF
F01_transcript_4609	CAMTA	TF
F01_transcript_46124	MADS-MIKC	TF
F01_transcript_46150	AP2/ERF-ERF	TF
F01_transcript_46191	MADS-MIKC	TF
F01_transcript_46320	HMG	TR
F01_transcript_46444	GNAT	TR
F01_transcript_46446	HMG	TR
F01_transcript_46542	NAC	TF
F01_transcript_46609	HMG	TR
F01_transcript_46652	HMG	TR
F01_transcript_467	PHD	TR
F01_transcript_46711	HMG	TR

F01_transcript_46799	GNAT	TR
F01_transcript_46809	AP2/ERF-ERF	TF
F01_transcript_46871	bHLH	TF
F01_transcript_46878	bZIP	TF
F01_transcript_46885	SWI/SNF-BAF60b	TR
F01_transcript_46887	MYB-related	TF
F01_transcript_4696	ARID	TR
F01_transcript_46968	MYB	TF
F01_transcript_46988	AP2/ERF-ERF	TF
F01_transcript_47022	GNAT	TR
F01_transcript_47039	HB-other	TF
F01_transcript_47121	MYB	TF
F01_transcript_47241	GNAT	TR
F01_transcript_475	Others	TR
F01_transcript_47549	NAC	TF
F01_transcript_47593	GNAT	TR
F01_transcript_4761	SET	TR
F01_transcript_47651	SWI/SNF-BAF60b	TR
F01_transcript_47662	GNAT	TR
F01_transcript_47726	MBF1	TR
F01_transcript_47739	NF-YC	TF
F01_transcript_47828	SWI/SNF-BAF60b	TR
F01_transcript_47895	SWI/SNF-BAF60b	TR
F01_transcript_47999	bZIP	TF
F01_transcript_48	MYB-related	TF
F01_transcript_48053	SBP	TF
F01_transcript_481	bZIP	TF
F01_transcript_48107	MBF1	TR
F01_transcript_48111	C2H2	TF
F01_transcript_48149	SWI/SNF-BAF60b	TR
F01_transcript_48186	MYB-related	TF
F01_transcript_48395	MBF1	TR
F01_transcript_48403	Others	TR
F01_transcript_48455	MBF1	TR
F01_transcript_48476	AP2/ERF-ERF	TF
F01_transcript_48796	Coactivator p15	TR
F01_transcript_48827	Others	TR
F01_transcript_4896	mTERF	TR
F01_transcript_49039	MBF1	TR
F01_transcript_49062	MYB-related	TF
F01_transcript_49117	SWI/SNF-BAF60b	TR
F01_transcript_49264	MYB-related	TF
F01_transcript_4933	RWP-RK	TF
F01_transcript_49386	GNAT	TR
F01_transcript_4944	CAMTA	TF
F01_transcript_49628	MYB-related	TF
F01_transcript_4963	B3	TF
F01_transcript_49730	Coactivator p15	TR
F01_transcript_49951	Coactivator p15	TR
F01_transcript_49956	S1Fa-like	TF
F01_transcript_50093	WRKY	TF
F01_transcript_5012	B3-ARF	TF
F01_transcript_5020	B3	TF
F01_transcript_5030	B3-ARF	TF

F01_transcript_5081	mTERF	TR
F01_transcript_511	B3-ARF	TF
F01_transcript_51333	MYB	TF
F01_transcript_51334	GRAS	TF
F01_transcript_51400	B3-ARF	TF
F01_transcript_51415	MADS-M-type	TF
F01_transcript_51505	bHLH	TF
F01_transcript_51519	RWP-RK	TF
F01_transcript_51535	HB-HD-ZIP	TF
F01_transcript_51568	SNF2	TR
F01_transcript_51573	NF-YA	TF
F01_transcript_51653	PHD	TR
F01_transcript_51657	bHLH	TF
F01_transcript_51741	AP2/ERF-ERF	TF
F01_transcript_51783	SWI/SNF-SWI3	TR
F01_transcript_51790	bHLH	TF
F01_transcript_51795	Others	TR
F01_transcript_51806	WRKY	TF
F01_transcript_51811	MYB	TF
F01_transcript_51831	SET	TR
F01_transcript_51836	ARID	TR
F01_transcript_51843	NF-YA	TF
F01_transcript_5185	NAC	TF
F01_transcript_51856	SET	TR
F01_transcript_5190	B3-ARF	TF
F01_transcript_51901	MYB	TF
F01_transcript_51958	C2C2-Dof	TF
F01_transcript_51989	bHLH	TF
F01_transcript_52022	RB	TR
F01_transcript_52044	Trihelix	TF
F01_transcript_52047	TCP	TF
F01_transcript_52075	B3	TF
F01_transcript_5208	SBP	TF
F01_transcript_52089	HB-HD-ZIP	TF
F01_transcript_52108	TRAF	TR
F01_transcript_52138	NAC	TF
F01_transcript_52140	MBF1	TR
F01_transcript_52180	MBF1	TR
F01_transcript_52192	HB-HD-ZIP	TF
F01_transcript_52200	ARID	TR
F01_transcript_52203	B3-ARF	TF
F01_transcript_52211	ARID	TR
F01_transcript_52213	SET	TR
F01_transcript_5222	LUG	TR
F01_transcript_52243	MYB-related	TF
F01_transcript_52286	RWP-RK	TF
F01_transcript_5229	B3-ARF	TF
F01_transcript_52299	B3-ARF	TF
F01_transcript_52304	SET	TR
F01_transcript_52318	MYB	TF
F01_transcript_52320	SBP	TF
F01_transcript_52324	bHLH	TF
F01_transcript_52350	bZIP	TF
F01_transcript_52371	SNF2	TR
F01_transcript_52433	C2H2	TF

F01_transcript_52434	C2C2-Dof	TF
F01_transcript_52469	NF-YB	TF
F01_transcript_52489	TRAF	TR
F01_transcript_52501	MYB	TF
F01_transcript_5251	bHLH	TF
F01_transcript_52541	ARID	TR
F01_transcript_52546	B3-ARF	TF
F01_transcript_52574	AP2/ERF-AP2	TF
F01_transcript_52587	NAC	TF
F01_transcript_52622	AUX/IAA	TR
F01_transcript_52650	PHD	TR
F01_transcript_52662	PHD	TR
F01_transcript_52682	MYB-related	TF
F01_transcript_52716	RWP-RK	TF
F01_transcript_52734	SBP	TF
F01_transcript_52748	Others	TR
F01_transcript_5275	bHLH	TF
F01_transcript_52789	TCP	TF
F01_transcript_52834	MADS-M-type	TF
F01_transcript_52852	Others	TR
F01_transcript_52858	C3H	TF
F01_transcript_5286	NAC	TF
F01_transcript_52867	AUX/IAA	TR
F01_transcript_52935	HB-HD-ZIP	TF
F01_transcript_52958	HB-BELL	TF
F01_transcript_53079	TCP	TF
F01_transcript_53097	bHLH	TF
F01_transcript_53128	Jumonji	TR
F01_transcript_53133	HB-HD-ZIP	TF
F01_transcript_53195	WRKY	TF
F01_transcript_53208	WRKY	TF
F01_transcript_53234	C2H2	TF
F01_transcript_53255	GRAS	TF
F01_transcript_53350	C3H	TF
F01_transcript_53370	AUX/IAA	TR
F01_transcript_53375	HB-HD-ZIP	TF
F01_transcript_535	SET	TR
F01_transcript_53516	HB-HD-ZIP	TF
F01_transcript_53530	MYB	TF
F01_transcript_53539	MYB-related	TF
F01_transcript_53561	RWP-RK	TF
F01_transcript_53580	SNF2	TR
F01_transcript_53591	NAC	TF
F01_transcript_53593	MADS-M-type	TF
F01_transcript_53597	HB-BELL	TF
F01_transcript_53634	NF-YB	TF
F01_transcript_53644	SNF2	TR
F01_transcript_53666	zf-HD	TF
F01_transcript_53671	VOZ	TF
F01_transcript_53674	Tify	TF
F01_transcript_53714	LUG	TR
F01_transcript_5373	GRAS	TF
F01_transcript_53757	B3-ARF	TF
F01_transcript_53801	Others	TR

F01_transcript_53836	HB-HD-ZIP	TF
F01_transcript_53957	C2H2	TF
F01_transcript_53970	SET	TR
F01_transcript_53998	GARP-ARR-B	TF
F01_transcript_54045	Others	TR
F01_transcript_54052	HB-BELL	TF
F01_transcript_54078	HMG	TR
F01_transcript_54171	SWI/SNF-BAF60b	TR
F01_transcript_54187	MYB-related	TF
F01_transcript_54246	C2H2	TF
F01_transcript_5429	Trihelix	TF
F01_transcript_54297	mTERF	TR
F01_transcript_54299	Jumonji	TR
F01_transcript_54338	RWP-RK	TF
F01_transcript_54342	ARID	TR
F01_transcript_54382	HB-BELL	TF
F01_transcript_54449	C3H	TF
F01_transcript_545	SNF2	TR
F01_transcript_54509	HB-HD-ZIP	TF
F01_transcript_54546	MYB	TF
F01_transcript_54574	RWP-RK	TF
F01_transcript_54579	Trihelix	TF
F01_transcript_54580	PHD	TR
F01_transcript_54662	B3	TF
F01_transcript_54676	GARP-G2-like	TF
F01_transcript_54711	RWP-RK	TF
F01_transcript_54754	B3-ARF	TF
F01_transcript_54769	NAC	TF
F01_transcript_54775	FAR1	TF
F01_transcript_54779	bHLH	TF
F01_transcript_54811	C2H2	TF
F01_transcript_54829	SBP	TF
F01_transcript_54862	FAR1	TF
F01_transcript_54906	FAR1	TF
F01_transcript_54933	BBR-BPC	TF
F01_transcript_54969	SET	TR
F01_transcript_54972	C2H2	TF
F01_transcript_54994	SNF2	TR
F01_transcript_55023	C3H	TF
F01_transcript_5507	HB-BELL	TF
F01_transcript_55126	SBP	TF
F01_transcript_55177	mTERF	TR
F01_transcript_55239	WRKY	TF
F01_transcript_55248	SNF2	TR
F01_transcript_55300	FAR1	TF
F01_transcript_55306	B3	TF
F01_transcript_55328	GNAT	TR
F01_transcript_55344	DDT	TR
F01_transcript_5535	B3-ARF	TF
F01_transcript_55351	TRAF	TR
F01_transcript_55357	NAC	TF
F01_transcript_55371	MYB	TF
F01_transcript_55407	C3H	TF
F01_transcript_55417	TRAF	TR
F01_transcript_55422	SET	TR

F01_transcript_55490	MYB-related	TF
F01_transcript_55539	CAMTA	TF
F01_transcript_55546	Whirly	TF
F01_transcript_55592	C3H	TF
F01_transcript_55598	MYB-related	TF
F01_transcript_55604	SET	TR
F01_transcript_55626	TRAF	TR
F01_transcript_55671	GRAS	TF
F01_transcript_55756	Pseudo ARR-B	TR
F01_transcript_55758	NAC	TF
F01_transcript_55796	B3	TF
F01_transcript_55866	PHD	TR
F01_transcript_55934	SBP	TF
F01_transcript_55941	VOZ	TF
F01_transcript_55944	FAR1	TF
F01_transcript_55967	NAC	TF
F01_transcript_56009	C2C2-CO-like	TF
F01_transcript_56012	CSD	TF
F01_transcript_5604	BSD	TF
F01_transcript_56094	C2H2	TF
F01_transcript_56121	C3H	TF
F01_transcript_56144	Others	TR
F01_transcript_56266	S1Fa-like	TF
F01_transcript_56281	bHLH	TF
F01_transcript_56283	GRF	TF
F01_transcript_56290	C2C2-YABBY	TF
F01_transcript_5633	C3H	TF
F01_transcript_5635	PHD	TR
F01_transcript_56363	NF-YC	TF
F01_transcript_56365	HB-HD-ZIP	TF
F01_transcript_56395	LUG	TR
F01_transcript_56414	Alfin-like	TF
F01_transcript_56442	GNAT	TR
F01_transcript_56471	ARID	TR
F01_transcript_56473	PHD	TR
F01_transcript_56495	MYB-related	TF
F01_transcript_5653	B3	TF
F01_transcript_56543	NF-YA	TF
F01_transcript_56575	mTERF	TR
F01_transcript_56579	SWI/SNF-BAF60b	TR
F01_transcript_56586	bHLH	TF
F01_transcript_56631	PHD	TR
F01_transcript_56638	NF-YB	TF
F01_transcript_56714	TUB	TF
F01_transcript_56724	AUX/IAA	TR
F01_transcript_56735	Jumonji	TR
F01_transcript_56747	MYB-related	TF
F01_transcript_56756	Others	TR
F01_transcript_56760	BBR-BPC	TF
F01_transcript_56792	C2C2-LSD	TF
F01_transcript_56846	mTERF	TR
F01_transcript_56875	LOB	TF
F01_transcript_56884	C2H2	TF
F01_transcript_56993	RWP-RK	TF

F01_transcript_570	Others	TR
F01_transcript_57043	HMG	TR
F01_transcript_57059	RWP-RK	TF
F01_transcript_57061	NAC	TF
F01_transcript_571	SET	TR
F01_transcript_57120	EIL	TF
F01_transcript_57127	bHLH	TF
F01_transcript_57171	mTERF	TR
F01_transcript_57179	Trihelix	TF
F01_transcript_57253	SNF2	TR
F01_transcript_5727	FAR1	TF
F01_transcript_57273	Others	TR
F01_transcript_57323	ARID	TR
F01_transcript_57331	RWP-RK	TF
F01_transcript_57348	SET	TR
F01_transcript_57410	HB-HD-ZIP	TF
F01_transcript_57418	TUB	TF
F01_transcript_57419	C3H	TF
F01_transcript_57421	GNAT	TR
F01_transcript_57444	C2H2	TF
F01_transcript_5745	CAMTA	TF
F01_transcript_57487	B3-ARF	TF
F01_transcript_57501	C3H	TF
F01_transcript_57553	MYB-related	TF
F01_transcript_57601	C2C2-GATA	TF
F01_transcript_5764	TRAF	TR
F01_transcript_57651	SET	TR
F01_transcript_5769	GRAS	TF
F01_transcript_57725	SBP	TF
F01_transcript_57733	NF-YB	TF
F01_transcript_57824	mTERF	TR
F01_transcript_57835	Jumonji	TR
F01_transcript_57914	C3H	TF
F01_transcript_57920	MYB	TF
F01_transcript_57945	HMG	TR
F01_transcript_57969	C2C2-GATA	TF
F01_transcript_5797	TCP	TF
F01_transcript_58033	SWI/SNF-BAF60b	TR
F01_transcript_58064	IWS1	TR
F01_transcript_58071	MADS-MIKC	TF
F01_transcript_58086	RWP-RK	TF
F01_transcript_58125	C2C2-Dof	TF
F01_transcript_5821	Jumonji	TR
F01_transcript_58230	Tify	TF
F01_transcript_58299	PLATZ	TF
F01_transcript_58343	HMG	TR
F01_transcript_58354	bHLH	TF
F01_transcript_58362	NF-YA	TF
F01_transcript_5837	MYB-related	TF
F01_transcript_58373	C3H	TF
F01_transcript_58380	SNF2	TR
F01_transcript_58417	E2F-DP	TF
F01_transcript_58424	C2H2	TF
F01_transcript_58431	Others	TR
F01_transcript_58438	C2H2	TF

F01_transcript_5844	SWI/SNF-BAF60b	TR
F01_transcript_58443	C3H	TF
F01_transcript_58471	CPP	TF
F01_transcript_58513	mTERF	TR
F01_transcript_58600	Others	TR
F01_transcript_58603	WRKY	TF
F01_transcript_58611	Others	TR
F01_transcript_58623	bHLH	TF
F01_transcript_58659	HB-BELL	TF
F01_transcript_58689	PHD	TR
F01_transcript_58695	CPP	TF
F01_transcript_58698	HB-BELL	TF
F01_transcript_58712	SBP	TF
F01_transcript_58714	IWS1	TR
F01_transcript_58726	C2H2	TF
F01_transcript_58760	WRKY	TF
F01_transcript_5878	GRAS	TF
F01_transcript_58800	MYB	TF
F01_transcript_58804	Others	TR
F01_transcript_58824	AUX/IAA	TR
F01_transcript_58861	AUX/IAA	TR
F01_transcript_58883	NAC	TF
F01_transcript_589	C2H2	TF
F01_transcript_5891	FAR1	TF
F01_transcript_58917	GRAS	TF
F01_transcript_58973	HB-other	TF
F01_transcript_5900	RWP-RK	TF
F01_transcript_59022	bHLH	TF
F01_transcript_59037	bZIP	TF
F01_transcript_59044	HB-HD-ZIP	TF
F01_transcript_5908	Trihelix	TF
F01_transcript_59148	C2H2	TF
F01_transcript_59157	Others	TR
F01_transcript_59180	TCP	TF
F01_transcript_59184	GARP-ARR-B	TF
F01_transcript_59188	LOB	TF
F01_transcript_59239	HB-BELL	TF
F01_transcript_59305	AUX/IAA	TR
F01_transcript_59372	HRT	TF
F01_transcript_59390	PHD	TR
F01_transcript_59403	C2C2-CO-like	TF
F01_transcript_59463	WRKY	TF
F01_transcript_59591	TUB	TF
F01_transcript_59593	mTERF	TR
F01_transcript_5963	PHD	TR
F01_transcript_59637	C3H	TF
F01_transcript_59645	ARID	TR
F01_transcript_59682	mTERF	TR
F01_transcript_59754	C3H	TF
F01_transcript_5976	C3H	TF
F01_transcript_59768	WRKY	TF
F01_transcript_59808	B3	TF
F01_transcript_59815	bHLH	TF
F01_transcript_59833	DBP	TF

F01_transcript_59913	Trihelix	TF
F01_transcript_59948	SNF2	TR
F01_transcript_59975	Others	TR
F01_transcript_6	SNF2	TR
F01_transcript_60008	IWS1	TR
F01_transcript_60043	bHLH	TF
F01_transcript_60049	WRKY	TF
F01_transcript_60061	FAR1	TF
F01_transcript_60109	SET	TR
F01_transcript_60116	C2C2-GATA	TF
F01_transcript_60136	HB-HD-ZIP	TF
F01_transcript_60141	Jumonji	TR
F01_transcript_60152	MYB	TF
F01_transcript_60177	SNF2	TR
F01_transcript_60184	bHLH	TF
F01_transcript_60219	HB-KNOX	TF
F01_transcript_60236	NAC	TF
F01_transcript_60250	CAMTA	TF
F01_transcript_60297	HB-HD-ZIP	TF
F01_transcript_60310	AP2/ERF-ERF	TF
F01_transcript_60417	WRKY	TF
F01_transcript_60423	C3H	TF
F01_transcript_60434	GNAT	TR
F01_transcript_60484	Trihelix	TF
F01_transcript_60518	C2H2	TF
F01_transcript_6056	GRAS	TF
F01_transcript_60568	WRKY	TF
F01_transcript_60650	GARP-ARR-B	TF
F01_transcript_60679	bHLH	TF
F01_transcript_60689	SNF2	TR
F01_transcript_60702	NAC	TF
F01_transcript_60732	Coactivator p15	TR
F01_transcript_60742	Others	TR
F01_transcript_60852	NAC	TF
F01_transcript_60854	PHD	TR
F01_transcript_60865	GRAS	TF
F01_transcript_60900	Others	TR
F01_transcript_60905	MYB	TF
F01_transcript_60906	B3-ARF	TF
F01_transcript_60915	Others	TR
F01_transcript_60947	ARID	TR
F01_transcript_60991	TRAF	TR
F01_transcript_61011	NAC	TF
F01_transcript_61022	NF-X1	TF
F01_transcript_61063	bHLH	TF
F01_transcript_61076	NF-YB	TF
F01_transcript_61078	AUX/IAA	TR
F01_transcript_6111	HB-BELL	TF
F01_transcript_61170	bHLH	TF
F01_transcript_61186	MYB-related	TF
F01_transcript_61220	Others	TR
F01_transcript_61246	bZIP	TF
F01_transcript_6125	GRAS	TF
F01_transcript_61269	bZIP	TF
F01_transcript_61279	BES1	TF

F01_transcript_61318	AP2/ERF-ERF	TF
F01_transcript_61350	RWP-RK	TF
F01_transcript_61366	B3-ARF	TF
F01_transcript_6139	GRAS	TF
F01_transcript_6146	LUG	TR
F01_transcript_61490	TUB	TF
F01_transcript_61529	AUX/IAA	TR
F01_transcript_61569	ARID	TR
F01_transcript_61580	WRKY	TF
F01_transcript_61622	NAC	TF
F01_transcript_61626	AP2/ERF-AP2	TF
F01_transcript_61657	zf-HD	TF
F01_transcript_61660	GARP-ARR-B	TF
F01_transcript_61669	SWI/SNF-BAF60b	TR
F01_transcript_6167	Others	TR
F01_transcript_61680	SBP	TF
F01_transcript_61859	C2C2-GATA	TF
F01_transcript_61872	MYB-related	TF
F01_transcript_61923	Trihelix	TF
F01_transcript_62	IWS1	TR
F01_transcript_6205	GRAS	TF
F01_transcript_62083	WRKY	TF
F01_transcript_62103	MYB-related	TF
F01_transcript_62109	HB-HD-ZIP	TF
F01_transcript_6214	TRAF	TR
F01_transcript_62149	Trihelix	TF
F01_transcript_62156	C2H2	TF
F01_transcript_62246	PHD	TR
F01_transcript_62252	E2F-DP	TF
F01_transcript_62255	GRAS	TF
F01_transcript_62266	SWI/SNF-BAF60b	TR
F01_transcript_6231	B3-ARF	TF
F01_transcript_62322	bHLH	TF
F01_transcript_62355	bHLH	TF
F01_transcript_62373	SNF2	TR
F01_transcript_62414	bHLH	TF
F01_transcript_62497	C2H2	TF
F01_transcript_625	C2H2	TF
F01_transcript_62500	bHLH	TF
F01_transcript_62527	mTERF	TR
F01_transcript_62536	C2H2	TF
F01_transcript_62618	Others	TR
F01_transcript_62632	Others	TR
F01_transcript_62665	MYB-related	TF
F01_transcript_62673	SET	TR
F01_transcript_62716	AUX/IAA	TR
F01_transcript_62762	C2C2-GATA	TF
F01_transcript_62826	TRAF	TR
F01_transcript_62832	bHLH	TF
F01_transcript_62857	FAR1	TF
F01_transcript_62877	SNF2	TR
F01_transcript_62885	RWP-RK	TF
F01_transcript_62932	C2C2-CO-like	TF
F01_transcript_62962	NF-YC	TF

F01_transcript_62968	C2H2	TF
F01_transcript_63006	Trihelix	TF
F01_transcript_63014	MADS-M-type	TF
F01_transcript_63035	bHLH	TF
F01_transcript_63082	HB-HD-ZIP	TF
F01_transcript_63087	GARP-G2-like	TF
F01_transcript_63097	SET	TR
F01_transcript_63125	NAC	TF
F01_transcript_63142	C3H	TF
F01_transcript_63147	SWI/SNF-BAF60b	TR
F01_transcript_63254	NAC	TF
F01_transcript_63280	WRKY	TF
F01_transcript_63285	NAC	TF
F01_transcript_63293	bHLH	TF
F01_transcript_63306	HB-other	TF
F01_transcript_63308	TRAF	TR
F01_transcript_63317	HB-HD-ZIP	TF
F01_transcript_63330	SNF2	TR
F01_transcript_63333	HB-KNOX	TF
F01_transcript_63355	SET	TR
F01_transcript_63368	FAR1	TF
F01_transcript_63402	SET	TR
F01_transcript_63403	MYB	TF
F01_transcript_6341	FAR1	TF
F01_transcript_63429	bHLH	TF
F01_transcript_6343	C3H	TF
F01_transcript_63461	B3-ARF	TF
F01_transcript_63498	PHD	TR
F01_transcript_63504	C3H	TF
F01_transcript_63512	MYB-related	TF
F01_transcript_63533	NF-YA	TF
F01_transcript_63539	C2H2	TF
F01_transcript_63547	SNF2	TR
F01_transcript_63597	IWS1	TR
F01_transcript_63701	MED6	TR
F01_transcript_63706	Tify	TF
F01_transcript_63742	Others	TR
F01_transcript_6376	Jumonji	TR
F01_transcript_6377	SBP	TF
F01_transcript_63784	Jumonji	TR
F01_transcript_6382	MYB-related	TF
F01_transcript_63828	NAC	TF
F01_transcript_63847	C2C2-LSD	TF
F01_transcript_63863	PHD	TR
F01_transcript_63873	Pseudo ARR-B	TR
F01_transcript_63881	GARP-G2-like	TF
F01_transcript_63898	RWP-RK	TF
F01_transcript_6390	Pseudo ARR-B	TR
F01_transcript_63916	C3H	TF
F01_transcript_63927	MADS-M-type	TF
F01_transcript_63948	bZIP	TF
F01_transcript_63953	GARP-G2-like	TF
F01_transcript_64017	WRKY	TF
F01_transcript_64018	C2H2	TF
F01_transcript_64039	Others	TR

F01_transcript_64118	MYB-related	TF
F01_transcript_64125	bHLH	TF
F01_transcript_64153	C3H	TF
F01_transcript_64181	C2H2	TF
F01_transcript_64213	DDT	TR
F01_transcript_64215	Others	TR
F01_transcript_64224	Others	TR
F01_transcript_64226	C3H	TF
F01_transcript_64257	PHD	TR
F01_transcript_64260	GRAS	TF
F01_transcript_64268	PHD	TR
F01_transcript_64286	bHLH	TF
F01_transcript_6436	TRAF	TR
F01_transcript_64403	AP2/ERF-ERF	TF
F01_transcript_64436	B3-ARF	TF
F01_transcript_64437	TRAF	TR
F01_transcript_64442	C2H2	TF
F01_transcript_64461	B3-ARF	TF
F01_transcript_64510	CAMTA	TF
F01_transcript_64520	bZIP	TF
F01_transcript_64554	B3	TF
F01_transcript_6457	SET	TR
F01_transcript_64592	B3	TF
F01_transcript_64600	NAC	TF
F01_transcript_64601	FAR1	TF
F01_transcript_64616	TRAF	TR
F01_transcript_64625	IWS1	TR
F01_transcript_64638	WRKY	TF
F01_transcript_64669	HMG	TR
F01_transcript_64676	HB-BELL	TF
F01_transcript_64681	TRAF	TR
F01_transcript_64697	Tify	TF
F01_transcript_64771	MYB	TF
F01_transcript_64789	GARP-G2-like	TF
F01_transcript_64808	Jumonji	TR
F01_transcript_64821	Others	TR
F01_transcript_64851	MADS-M-type	TF
F01_transcript_64871	bZIP	TF
F01_transcript_64890	WRKY	TF
F01_transcript_64893	MADS-MIKC	TF
F01_transcript_64924	WRKY	TF
F01_transcript_64932	HB-other	TF
F01_transcript_64939	bHLH	TF
F01_transcript_64951	WRKY	TF
F01_transcript_64955	Others	TR
F01_transcript_65006	MYB-related	TF
F01_transcript_65010	TCP	TF
F01_transcript_65045	C2H2	TF
F01_transcript_65070	MYB-related	TF
F01_transcript_65087	WRKY	TF
F01_transcript_65098	EIL	TF
F01_transcript_65122	SNF2	TR
F01_transcript_65124	bZIP	TF
F01_transcript_65125	AP2/ERF-ERF	TF

F01_transcript_65169	C3H	TF
F01_transcript_6519	C3H	TF
F01_transcript_652	C2H2	TF
F01_transcript_65206	GARP-G2-like	TF
F01_transcript_65242	bHLH	TF
F01_transcript_65251	WRKY	TF
F01_transcript_65317	Jumonji	TR
F01_transcript_65318	CPP	TF
F01_transcript_65348	HB-BELL	TF
F01_transcript_65367	C2C2-Dof	TF
F01_transcript_6541	HB-BELL	TF
F01_transcript_65412	MYB	TF
F01_transcript_65434	TCP	TF
F01_transcript_6552	GRAS	TF
F01_transcript_65534	Trihelix	TF
F01_transcript_65544	MYB-related	TF
F01_transcript_65632	bZIP	TF
F01_transcript_65651	WRKY	TF
F01_transcript_65660	MYB-related	TF
F01_transcript_65695	HB-HD-ZIP	TF
F01_transcript_65696	RWP-RK	TF
F01_transcript_65730	mTERF	TR
F01_transcript_65741	MYB	TF
F01_transcript_65746	C2C2-Dof	TF
F01_transcript_65769	MYB-related	TF
F01_transcript_65831	RWP-RK	TF
F01_transcript_65851	MYB	TF
F01_transcript_65873	B3	TF
F01_transcript_65922	MADS-M-type	TF
F01_transcript_65932	SNF2	TR
F01_transcript_65938	VOZ	TF
F01_transcript_65946	SNF2	TR
F01_transcript_65970	C2H2	TF
F01_transcript_66006	GARP-ARR-B	TF
F01_transcript_66048	Tify	TF
F01_transcript_66075	C3H	TF
F01_transcript_66110	B3	TF
F01_transcript_66142	PHD	TR
F01_transcript_66161	NAC	TF
F01_transcript_66165	Others	TR
F01_transcript_6618	LUG	TR
F01_transcript_66182	Jumonji	TR
F01_transcript_66243	FAR1	TF
F01_transcript_66250	C2C2-GATA	TF
F01_transcript_66255	FAR1	TF
F01_transcript_66267	C3H	TF
F01_transcript_66285	SET	TR
F01_transcript_66318	RWP-RK	TF
F01_transcript_66323	IWS1	TR
F01_transcript_66355	GRAS	TF
F01_transcript_66377	MADS-M-type	TF
F01_transcript_66384	WRKY	TF
F01_transcript_66431	SET	TR
F01_transcript_66458	C3H	TF
F01_transcript_66483	C2H2	TF

F01_transcript_66494	Alfin-like	TF
F01_transcript_66497	MYB-related	TF
F01_transcript_66536	SWI/SNF-BAF60b	TR
F01_transcript_66538	B3-ARF	TF
F01_transcript_66582	IWS1	TR
F01_transcript_6660	FAR1	TF
F01_transcript_66661	AP2/ERF-AP2	TF
F01_transcript_66668	MYB-related	TF
F01_transcript_66709	NAC	TF
F01_transcript_6671	MADS-M-type	TF
F01_transcript_66718	B3-ARF	TF
F01_transcript_66726	FAR1	TF
F01_transcript_66729	NF-YC	TF
F01_transcript_66735	NF-YB	TF
F01_transcript_66752	bZIP	TF
F01_transcript_66759	HB-HD-ZIP	TF
F01_transcript_66771	SBP	TF
F01_transcript_66791	bZIP	TF
F01_transcript_66829	DDT	TR
F01_transcript_66844	NF-YA	TF
F01_transcript_66861	HMG	TR
F01_transcript_66877	HSF	TF
F01_transcript_66880	Trihelix	TF
F01_transcript_66891	C3H	TF
F01_transcript_66922	C2H2	TF
F01_transcript_66963	AP2/ERF-ERF	TF
F01_transcript_67056	bHLH	TF
F01_transcript_67067	AUX/IAA	TR
F01_transcript_67115	TCP	TF
F01_transcript_67125	SBP	TF
F01_transcript_67147	C2C2-CO-like	TF
F01_transcript_67166	RWP-RK	TF
F01_transcript_67196	GNAT	TR
F01_transcript_67205	C3H	TF
F01_transcript_67220	SRS	TF
F01_transcript_67226	MADS-M-type	TF
F01_transcript_6727	HB-BELL	TF
F01_transcript_67275	MYB-related	TF
F01_transcript_6728	GRAS	TF
F01_transcript_67301	SNF2	TR
F01_transcript_6733	bHLH	TF
F01_transcript_67368	B3	TF
F01_transcript_67408	LIM	TF
F01_transcript_6744	C3H	TF
F01_transcript_67455	C3H	TF
F01_transcript_67465	bHLH	TF
F01_transcript_67483	EIL	TF
F01_transcript_67486	C2H2	TF
F01_transcript_6753	FAR1	TF
F01_transcript_67556	RWP-RK	TF
F01_transcript_67558	S1Fa-like	TF
F01_transcript_67575	mTERF	TR
F01_transcript_67693	bZIP	TF
F01_transcript_67718	mTERF	TR

F01_transcript_67732	C2H2	TF
F01_transcript_67756	C3H	TF
F01_transcript_67771	FAR1	TF
F01_transcript_67783	SOH1	TR
F01_transcript_67792	MYB-related	TF
F01_transcript_678	C2H2	TF
F01_transcript_67800	Coactivator p15	TR
F01_transcript_67808	GARP-G2-like	TF
F01_transcript_67812	SNF2	TR
F01_transcript_67868	Trihelix	TF
F01_transcript_67876	MYB	TF
F01_transcript_67990	WRKY	TF
F01_transcript_67995	GARP-G2-like	TF
F01_transcript_68	SWI/SNF-BAF60b	TR
F01_transcript_68028	C3H	TF
F01_transcript_6803	B3-ARF	TF
F01_transcript_68039	WRKY	TF
F01_transcript_68059	MYB-related	TF
F01_transcript_68086	HB-HD-ZIP	TF
F01_transcript_68097	HB-BELL	TF
F01_transcript_68103	Whirly	TF
F01_transcript_68132	VOZ	TF
F01_transcript_68248	GRAS	TF
F01_transcript_6825	SBP	TF
F01_transcript_68318	FAR1	TF
F01_transcript_68334	Others	TR
F01_transcript_68339	Tify	TF
F01_transcript_6834	HB-BELL	TF
F01_transcript_68366	ARID	TR
F01_transcript_68375	MYB-related	TF
F01_transcript_6842	B3-ARF	TF
F01_transcript_6845	NF-X1	TF
F01_transcript_68469	Others	TR
F01_transcript_68485	C3H	TF
F01_transcript_68488	LIM	TF
F01_transcript_68588	WRKY	TF
F01_transcript_68619	MYB	TF
F01_transcript_6864	SET	TR
F01_transcript_68646	C2H2	TF
F01_transcript_68753	BES1	TF
F01_transcript_68782	HMG	TR
F01_transcript_68813	GARP-G2-like	TF
F01_transcript_68854	DBB	TF
F01_transcript_68919	B3	TF
F01_transcript_68937	CPP	TF
F01_transcript_68946	TRAF	TR
F01_transcript_68956	HB-BELL	TF
F01_transcript_690	C2H2	TF
F01_transcript_69015	Trihelix	TF
F01_transcript_69089	TCP	TF
F01_transcript_69124	HB-HD-ZIP	TF
F01_transcript_69136	B3-ARF	TF
F01_transcript_69146	RWP-RK	TF
F01_transcript_692	SNF2	TR
F01_transcript_69201	SET	TR

F01_transcript_69228	GNAT	TR
F01_transcript_69254	bHLH	TF
F01_transcript_69260	PHD	TR
F01_transcript_69309	OFP	TF
F01_transcript_6934	HB-BELL	TF
F01_transcript_69352	MYB-related	TF
F01_transcript_69365	Trihelix	TF
F01_transcript_69379	HSF	TF
F01_transcript_69383	SNF2	TR
F01_transcript_6941	Others	TR
F01_transcript_69422	bHLH	TF
F01_transcript_69424	Trihelix	TF
F01_transcript_69445	C3H	TF
F01_transcript_69471	bZIP	TF
F01_transcript_69488	FAR1	TF
F01_transcript_69497	bHLH	TF
F01_transcript_69513	FAR1	TF
F01_transcript_69518	HB-other	TF
F01_transcript_69535	Alfin-like	TF
F01_transcript_6954	Coactivator p15	TR
F01_transcript_69572	C2C2-Dof	TF
F01_transcript_69600	SNF2	TR
F01_transcript_69603	Others	TR
F01_transcript_69614	Tify	TF
F01_transcript_69628	TCP	TF
F01_transcript_69651	IWS1	TR
F01_transcript_6969	HB-BELL	TF
F01_transcript_69699	C2C2-GATA	TF
F01_transcript_69706	SNF2	TR
F01_transcript_69708	MADS-M-type	TF
F01_transcript_69747	SBP	TF
F01_transcript_6978	FAR1	TF
F01_transcript_69831	WRKY	TF
F01_transcript_69835	NAC	TF
F01_transcript_69852	HSF	TF
F01_transcript_69936	HB-HD-ZIP	TF
F01_transcript_69948	AP2/ERF-ERF	TF
F01_transcript_69975	Others	TR
F01_transcript_69985	Others	TR
F01_transcript_70013	C3H	TF
F01_transcript_70026	FAR1	TF
F01_transcript_70030	NF-YA	TF
F01_transcript_70037	SBP	TF
F01_transcript_70069	TAZ	TR
F01_transcript_70074	Tify	TF
F01_transcript_70079	HSF	TF
F01_transcript_70127	PHD	TR
F01_transcript_70153	SBP	TF
F01_transcript_70173	WRKY	TF
F01_transcript_70219	B3-ARF	TF
F01_transcript_70244	PHD	TR
F01_transcript_70249	MADS-M-type	TF
F01_transcript_70261	E2F-DP	TF
F01_transcript_7029	B3	TF

F01_transcript_7030	C3H	TF
F01_transcript_70445	C2C2-GATA	TF
F01_transcript_70474	HRT	TF
F01_transcript_70505	MYB-related	TF
F01_transcript_70528	HB-HD-ZIP	TF
F01_transcript_70529	C3H	TF
F01_transcript_70553	PHD	TR
F01_transcript_70560	bZIP	TF
F01_transcript_7060	SNF2	TR
F01_transcript_70689	Others	TR
F01_transcript_70698	Trihelix	TF
F01_transcript_70707	SBP	TF
F01_transcript_70710	TRAF	TR
F01_transcript_70742	PHD	TR
F01_transcript_70822	C3H	TF
F01_transcript_70835	MYB-related	TF
F01_transcript_70856	HB-KNOX	TF
F01_transcript_7086	HB-BELL	TF
F01_transcript_70869	Trihelix	TF
F01_transcript_70887	FAR1	TF
F01_transcript_70934	TRAF	TR
F01_transcript_70964	C2H2	TF
F01_transcript_71015	bZIP	TF
F01_transcript_7106	bHLH	TF
F01_transcript_71065	FAR1	TF
F01_transcript_71073	C2C2-Dof	TF
F01_transcript_71076	Others	TR
F01_transcript_71156	SNF2	TR
F01_transcript_71161	HB-BELL	TF
F01_transcript_71170	SWI/SNF-BAF60b	TR
F01_transcript_7125	RWP-RK	TF
F01_transcript_71256	AP2/ERF-AP2	TF
F01_transcript_71303	GARP-ARR-B	TF
F01_transcript_71309	C2C2-GATA	TF
F01_transcript_71321	C2C2-LSD	TF
F01_transcript_7134	FAR1	TF
F01_transcript_71349	mTERF	TR
F01_transcript_7135	TRAF	TR
F01_transcript_71424	C2H2	TF
F01_transcript_71455	HRT	TF
F01_transcript_7151	FAR1	TF
F01_transcript_71587	HB-HD-ZIP	TF
F01_transcript_7168	GARP-ARR-B	TF
F01_transcript_71721	NAC	TF
F01_transcript_71723	NAC	TF
F01_transcript_71729	GARP-G2-like	TF
F01_transcript_7178	Others	TR
F01_transcript_718	SBP	TF
F01_transcript_71899	MYB-related	TF
F01_transcript_71921	C2C2-Dof	TF
F01_transcript_71928	TCP	TF
F01_transcript_71944	HB-HD-ZIP	TF
F01_transcript_71967	ARID	TR
F01_transcript_72004	WRKY	TF
F01_transcript_72038	MYB-related	TF

F01_transcript_72058	SNF2	TR
F01_transcript_72066	C2C2-GATA	TF
F01_transcript_72084	HB-other	TF
F01_transcript_7209	Jumonji	TR
F01_transcript_72095	HB-other	TF
F01_transcript_72106	B3	TF
F01_transcript_7213	MYB-related	TF
F01_transcript_72138	C2C2-CO-like	TF
F01_transcript_72139	NAC	TF
F01_transcript_72153	SET	TR
F01_transcript_72157	AP2/ERF-ERF	TF
F01_transcript_72174	HSF	TF
F01_transcript_72211	GARP-G2-like	TF
F01_transcript_72278	WRKY	TF
F01_transcript_72303	C3H	TF
F01_transcript_72316	B3	TF
F01_transcript_72327	TCP	TF
F01_transcript_72386	SET	TR
F01_transcript_72410	RWP-RK	TF
F01_transcript_72440	SET	TR
F01_transcript_72453	C2C2-LSD	TF
F01_transcript_72479	MYB	TF
F01_transcript_72529	Others	TR
F01_transcript_72547	SET	TR
F01_transcript_72565	SNF2	TR
F01_transcript_72566	Tify	TF
F01_transcript_72606	HB-other	TF
F01_transcript_72626	mTERF	TR
F01_transcript_72642	TRAF	TR
F01_transcript_72671	C3H	TF
F01_transcript_72682	C3H	TF
F01_transcript_7269	HB-HD-ZIP	TF
F01_transcript_72709	Others	TR
F01_transcript_7271	NAC	TF
F01_transcript_72875	C2H2	TF
F01_transcript_7288	FAR1	TF
F01_transcript_72921	MYB-related	TF
F01_transcript_72924	mTERF	TR
F01_transcript_72981	B3	TF
F01_transcript_73026	RWP-RK	TF
F01_transcript_73030	C2C2-Dof	TF
F01_transcript_7307	FAR1	TF
F01_transcript_73102	MADS-MIKC	TF
F01_transcript_73140	SBP	TF
F01_transcript_73155	bZIP	TF
F01_transcript_7318	Others	TR
F01_transcript_73191	FAR1	TF
F01_transcript_73213	MYB-related	TF
F01_transcript_73235	WRKY	TF
F01_transcript_73238	MYB-related	TF
F01_transcript_73314	GNAT	TR
F01_transcript_73317	B3-ARF	TF
F01_transcript_73325	SNF2	TR
F01_transcript_73330	C3H	TF

F01_transcript_73332	bZIP	TF
F01_transcript_73337	MADS-M-type	TF
F01_transcript_73344	Others	TR
F01_transcript_73364	SNF2	TR
F01_transcript_73381	bZIP	TF
F01_transcript_73398	C2H2	TF
F01_transcript_73412	NF-YC	TF
F01_transcript_73452	ARID	TR
F01_transcript_73478	bZIP	TF
F01_transcript_73497	AUX/IAA	TR
F01_transcript_73523	C2H2	TF
F01_transcript_73557	bHLH	TF
F01_transcript_73573	GARP-G2-like	TF
F01_transcript_73591	Trihelix	TF
F01_transcript_73602	Others	TR
F01_transcript_73618	RWP-RK	TF
F01_transcript_73715	VOZ	TF
F01_transcript_73728	Trihelix	TF
F01_transcript_73740	C2H2	TF
F01_transcript_73742	HB-BELL	TF
F01_transcript_73750	TCP	TF
F01_transcript_73768	TCP	TF
F01_transcript_73774	CPP	TF
F01_transcript_73775	HSF	TF
F01_transcript_73781	Others	TR
F01_transcript_7379	FAR1	TF
F01_transcript_73809	Trihelix	TF
F01_transcript_73822	MYB-related	TF
F01_transcript_73828	NAC	TF
F01_transcript_73831	Trihelix	TF
F01_transcript_7385	Coactivator p15	TR
F01_transcript_73908	PHD	TR
F01_transcript_73950	C3H	TF
F01_transcript_73963	NAC	TF
F01_transcript_73968	MADS-MIKC	TF
F01_transcript_74022	SET	TR
F01_transcript_74042	RWP-RK	TF
F01_transcript_74047	GARP-G2-like	TF
F01_transcript_741	SBP	TF
F01_transcript_74114	GARP-G2-like	TF
F01_transcript_7414	RWP-RK	TF
F01_transcript_7415	FAR1	TF
F01_transcript_74186	AP2/ERF-ERF	TF
F01_transcript_74202	E2F-DP	TF
F01_transcript_74232	TUB	TF
F01_transcript_74248	bHLH	TF
F01_transcript_74271	SBP	TF
F01_transcript_74282	Jumonji	TR
F01_transcript_74296	BBR-BPC	TF
F01_transcript_74311	VOZ	TF
F01_transcript_74315	TCP	TF
F01_transcript_7438	ARID	TR
F01_transcript_7449	Pseudo ARR-B	TR
F01_transcript_74496	GARP-G2-like	TF
F01_transcript_74514	Others	TR

F01_transcript_74564	RWP-RK	TF
F01_transcript_74579	SNF2	TR
F01_transcript_7459	NF-X1	TF
F01_transcript_74617	TUB	TF
F01_transcript_74630	bZIP	TF
F01_transcript_7467	FAR1	TF
F01_transcript_74691	bHLH	TF
F01_transcript_74720	SBP	TF
F01_transcript_74735	Others	TR
F01_transcript_74742	ARID	TR
F01_transcript_74749	HB-BELL	TF
F01_transcript_7481	Others	TR
F01_transcript_74891	AP2/ERF-ERF	TF
F01_transcript_74931	GNAT	TR
F01_transcript_74942	SNF2	TR
F01_transcript_74995	MYB	TF
F01_transcript_75051	Others	TR
F01_transcript_7506	CAMTA	TF
F01_transcript_75073	SET	TR
F01_transcript_75076	mTERF	TR
F01_transcript_75095	BES1	TF
F01_transcript_75125	Pseudo ARR-B	TR
F01_transcript_75171	Pseudo ARR-B	TR
F01_transcript_7518	FAR1	TF
F01_transcript_7522	B3-ARF	TF
F01_transcript_75221	HB-HD-ZIP	TF
F01_transcript_75228	HMG	TR
F01_transcript_75231	Others	TR
F01_transcript_75256	MYB	TF
F01_transcript_75264	SNF2	TR
F01_transcript_7531	FAR1	TF
F01_transcript_75321	C3H	TF
F01_transcript_75400	bZIP	TF
F01_transcript_75506	C3H	TF
F01_transcript_75529	AP2/ERF-ERF	TF
F01_transcript_75530	SBP	TF
F01_transcript_75533	E2F-DP	TF
F01_transcript_75545	C3H	TF
F01_transcript_75556	mTERF	TR
F01_transcript_75560	MADS-M-type	TF
F01_transcript_75606	bZIP	TF
F01_transcript_75615	RWP-RK	TF
F01_transcript_75620	C2C2-GATA	TF
F01_transcript_75637	NAC	TF
F01_transcript_75640	C2C2-GATA	TF
F01_transcript_75661	SNF2	TR
F01_transcript_75662	MADS-M-type	TF
F01_transcript_75682	RWP-RK	TF
F01_transcript_75730	HSF	TF
F01_transcript_75777	bZIP	TF
F01_transcript_75802	TRAF	TR
F01_transcript_7581	PHD	TR
F01_transcript_7582	GARP-ARR-B	TF
F01_transcript_75906	PHD	TR

F01_transcript_75910	PLATZ	TF
F01_transcript_75945	C2H2	TF
F01_transcript_75954	AP2/ERF-ERF	TF
F01_transcript_75989	Tify	TF
F01_transcript_76024	MYB	TF
F01_transcript_76082	SNF2	TR
F01_transcript_76165	SWI/SNF-BAF60b	TR
F01_transcript_7618	GRAS	TF
F01_transcript_76206	GNAT	TR
F01_transcript_76243	bHLH	TF
F01_transcript_76245	B3	TF
F01_transcript_76355	bZIP	TF
F01_transcript_76384	GRAS	TF
F01_transcript_76405	bZIP	TF
F01_transcript_76413	bZIP	TF
F01_transcript_76422	FAR1	TF
F01_transcript_76464	MYB-related	TF
F01_transcript_76497	CAMTA	TF
F01_transcript_76508	bZIP	TF
F01_transcript_76521	MYB-related	TF
F01_transcript_76561	VOZ	TF
F01_transcript_76599	MYB-related	TF
F01_transcript_7660	PHD	TR
F01_transcript_76641	AP2/ERF-ERF	TF
F01_transcript_76655	B3	TF
F01_transcript_76676	AP2/ERF-ERF	TF
F01_transcript_76773	WRKY	TF
F01_transcript_76828	CAMTA	TF
F01_transcript_7683	Pseudo ARR-B	TR
F01_transcript_76836	NAC	TF
F01_transcript_76854	PHD	TR
F01_transcript_76888	HMG	TR
F01_transcript_76901	Others	TR
F01_transcript_77016	HB-other	TF
F01_transcript_77158	NAC	TF
F01_transcript_77206	HSF	TF
F01_transcript_77235	WRKY	TF
F01_transcript_7728	FAR1	TF
F01_transcript_77291	GRAS	TF
F01_transcript_77317	SWI/SNF-BAF60b	TR
F01_transcript_7732	FAR1	TF
F01_transcript_77337	ARID	TR
F01_transcript_77357	GARP-ARR-B	TF
F01_transcript_77368	bHLH	TF
F01_transcript_77377	AP2/ERF-ERF	TF
F01_transcript_77409	HB-HD-ZIP	TF
F01_transcript_77463	DBB	TF
F01_transcript_77482	MYB-related	TF
F01_transcript_77514	GARP-G2-like	TF
F01_transcript_77570	SBP	TF
F01_transcript_77612	BBR-BPC	TF
F01_transcript_77613	NF-YA	TF
F01_transcript_77619	NF-YA	TF
F01_transcript_77676	C2C2-CO-like	TF
F01_transcript_7768	GRAS	TF

F01_transcript_77680	GRAS	TF
F01_transcript_77709	bHLH	TF
F01_transcript_77722	TRAF	TR
F01_transcript_7774	B3-ARF	TF
F01_transcript_77792	bZIP	TF
F01_transcript_7785	NAC	TF
F01_transcript_77890	Others	TR
F01_transcript_77972	IWS1	TR
F01_transcript_77973	C3H	TF
F01_transcript_77985	C2C2-Dof	TF
F01_transcript_78062	PHD	TR
F01_transcript_78080	HSF	TF
F01_transcript_78092	Others	TR
F01_transcript_78102	mTERF	TR
F01_transcript_78141	AP2/ERF-ERF	TF
F01_transcript_78195	SBP	TF
F01_transcript_78213	ULT	TF
F01_transcript_78242	AUX/IAA	TR
F01_transcript_78269	CSD	TF
F01_transcript_78272	SET	TR
F01_transcript_78280	bHLH	TF
F01_transcript_7831	RWP-RK	TF
F01_transcript_78313	HMG	TR
F01_transcript_78336	C3H	TF
F01_transcript_78359	MYB	TF
F01_transcript_78416	Others	TR
F01_transcript_78422	SBP	TF
F01_transcript_78434	MYB	TF
F01_transcript_78532	bZIP	TF
F01_transcript_78563	SWI/SNF-BAF60b	TR
F01_transcript_78571	GRAS	TF
F01_transcript_78579	Others	TR
F01_transcript_78584	PHD	TR
F01_transcript_78606	B3	TF
F01_transcript_78645	AP2/ERF-ERF	TF
F01_transcript_78678	GNAT	TR
F01_transcript_7872	LUG	TR
F01_transcript_78777	bZIP	TF
F01_transcript_78783	NAC	TF
F01_transcript_78804	NF-YB	TF
F01_transcript_78812	MYB-related	TF
F01_transcript_78865	bZIP	TF
F01_transcript_78879	bHLH	TF
F01_transcript_78907	B3	TF
F01_transcript_78947	SBP	TF
F01_transcript_78954	ARID	TR
F01_transcript_79002	AP2/ERF-ERF	TF
F01_transcript_79057	HB-WOX	TF
F01_transcript_79120	GNAT	TR
F01_transcript_79142	NAC	TF
F01_transcript_79197	C3H	TF
F01_transcript_79210	NAC	TF
F01_transcript_79222	SBP	TF
F01_transcript_79260	C2C2-GATA	TF

F01_transcript_79267	MYB-related	TF
F01_transcript_79291	NF-YB	TF
F01_transcript_79355	Others	TR
F01_transcript_79362	Jumonji	TR
F01_transcript_79395	BBR-BPC	TF
F01_transcript_7940	WRKY	TF
F01_transcript_79432	Others	TR
F01_transcript_79455	zf-HD	TF
F01_transcript_7950	GARP-ARR-B	TF
F01_transcript_79510	STAT	TF
F01_transcript_79572	MADS-M-type	TF
F01_transcript_79580	Rcd1-like	TR
F01_transcript_79599	SNF2	TR
F01_transcript_7961	CPP	TF
F01_transcript_79637	TCP	TF
F01_transcript_79680	mTERF	TR
F01_transcript_79687	HB-HD-ZIP	TF
F01_transcript_79718	Others	TR
F01_transcript_79734	SNF2	TR
F01_transcript_79745	SNF2	TR
F01_transcript_79789	mTERF	TR
F01_transcript_79819	bHLH	TF
F01_transcript_79832	TCP	TF
F01_transcript_79843	NF-YA	TF
F01_transcript_79864	bHLH	TF
F01_transcript_7992	EIL	TF
F01_transcript_7996	ARID	TR
F01_transcript_79970	C2H2	TF
F01_transcript_79975	C2C2-Dof	TF
F01_transcript_80011	PHD	TR
F01_transcript_80024	SWI/SNF-SWI3	TR
F01_transcript_80053	C3H	TF
F01_transcript_80068	C3H	TF
F01_transcript_801	SNF2	TR
F01_transcript_80143	GRAS	TF
F01_transcript_80147	Others	TR
F01_transcript_80176	GRAS	TF
F01_transcript_80205	Others	TR
F01_transcript_80212	B3-ARF	TF
F01_transcript_80218	C2C2-Dof	TF
F01_transcript_80257	bZIP	TF
F01_transcript_8026	RWP-RK	TF
F01_transcript_80304	AP2/ERF-ERF	TF
F01_transcript_80341	HB-HD-ZIP	TF
F01_transcript_804	SBP	TF
F01_transcript_80449	MYB-related	TF
F01_transcript_80453	B3	TF
F01_transcript_80478	bHLH	TF
F01_transcript_80501	SBP	TF
F01_transcript_80513	HB-BELL	TF
F01_transcript_80515	bZIP	TF
F01_transcript_80519	GRAS	TF
F01_transcript_80544	GRAS	TF
F01_transcript_80552	WRKY	TF
F01_transcript_80554	GeBP	TF

F01_transcript_80612	mTERF	TR
F01_transcript_8068	HB-BELL	TF
F01_transcript_80707	C2H2	TF
F01_transcript_80728	bHLH	TF
F01_transcript_80740	GRAS	TF
F01_transcript_80744	Others	TR
F01_transcript_80771	C3H	TF
F01_transcript_80835	MADS-M-type	TF
F01_transcript_80842	B3	TF
F01_transcript_80899	MYB-related	TF
F01_transcript_8091	DDT	TR
F01_transcript_80920	Others	TR
F01_transcript_80930	SBP	TF
F01_transcript_80974	GRAS	TF
F01_transcript_80995	HSF	TF
F01_transcript_81015	WRKY	TF
F01_transcript_81018	SET	TR
F01_transcript_81021	bHLH	TF
F01_transcript_81041	MYB-related	TF
F01_transcript_81076	LOB	TF
F01_transcript_81081	C3H	TF
F01_transcript_8109	SBP	TF
F01_transcript_81100	SWI/SNF-BAF60b	TR
F01_transcript_81135	WRKY	TF
F01_transcript_81236	HB-BELL	TF
F01_transcript_81269	GRAS	TF
F01_transcript_81313	MYB-related	TF
F01_transcript_81317	B3-ARF	TF
F01_transcript_81343	AUX/IAA	TR
F01_transcript_81359	NAC	TF
F01_transcript_81363	GRAS	TF
F01_transcript_81425	DDT	TR
F01_transcript_81448	SET	TR
F01_transcript_81454	RWP-RK	TF
F01_transcript_8147	SNF2	TR
F01_transcript_81519	PHD	TR
F01_transcript_81550	TUB	TF
F01_transcript_81570	TUB	TF
F01_transcript_81601	PHD	TR
F01_transcript_81636	C3H	TF
F01_transcript_81637	B3-ARF	TF
F01_transcript_81642	PHD	TR
F01_transcript_81659	HB-other	TF
F01_transcript_81662	C2H2	TF
F01_transcript_81678	SWI/SNF-BAF60b	TR
F01_transcript_81696	mTERF	TR
F01_transcript_81722	RWP-RK	TF
F01_transcript_81806	Others	TR
F01_transcript_81899	HB-HD-ZIP	TF
F01_transcript_81907	bZIP	TF
F01_transcript_81920	bHLH	TF
F01_transcript_82015	PHD	TR
F01_transcript_82035	RWP-RK	TF
F01_transcript_82049	bZIP	TF

F01_transcript_82105	Trihelix	TF
F01_transcript_82114	bHLH	TF
F01_transcript_82174	AP2/ERF-ERF	TF
F01_transcript_82179	C3H	TF
F01_transcript_82187	AUX/IAA	TR
F01_transcript_82244	DDT	TR
F01_transcript_82273	SRS	TF
F01_transcript_82292	TUB	TF
F01_transcript_82325	B3-ARF	TF
F01_transcript_82331	RWP-RK	TF
F01_transcript_82332	C2H2	TF
F01_transcript_82400	bHLH	TF
F01_transcript_82424	Others	TR
F01_transcript_82436	EIL	TF
F01_transcript_82440	RWP-RK	TF
F01_transcript_82463	WRKY	TF
F01_transcript_82487	SNF2	TR
F01_transcript_8249	MYB-related	TF
F01_transcript_82506	HB-HD-ZIP	TF
F01_transcript_82544	Trihelix	TF
F01_transcript_82567	HB-other	TF
F01_transcript_82579	bHLH	TF
F01_transcript_82609	MYB-related	TF
F01_transcript_82719	bZIP	TF
F01_transcript_82750	PHD	TR
F01_transcript_82868	bHLH	TF
F01_transcript_82921	MYB	TF
F01_transcript_82937	PHD	TR
F01_transcript_82954	WRKY	TF
F01_transcript_82970	Others	TR
F01_transcript_82987	Others	TR
F01_transcript_83044	Trihelix	TF
F01_transcript_83096	IWS1	TR
F01_transcript_83127	RWP-RK	TF
F01_transcript_83134	C2C2-GATA	TF
F01_transcript_83163	Others	TR
F01_transcript_83217	Trihelix	TF
F01_transcript_83237	C3H	TF
F01_transcript_83261	bHLH	TF
F01_transcript_83332	NAC	TF
F01_transcript_8337	bHLH	TF
F01_transcript_83376	GRAS	TF
F01_transcript_83408	PHD	TR
F01_transcript_83440	DDT	TR
F01_transcript_83488	MADS-MIKC	TF
F01_transcript_83516	SNF2	TR
F01_transcript_83525	AP2/ERF-ERF	TF
F01_transcript_83538	SNF2	TR
F01_transcript_83553	Jumonji	TR
F01_transcript_83572	NF-YA	TF
F01_transcript_83633	Others	TR
F01_transcript_8364	SET	TR
F01_transcript_83642	Trihelix	TF
F01_transcript_83694	MYB-related	TF
F01_transcript_83724	C3H	TF

F01_transcript_83808	RWP-RK	TF
F01_transcript_83854	C3H	TF
F01_transcript_83867	bHLH	TF
F01_transcript_83931	C2H2	TF
F01_transcript_83954	C2C2-GATA	TF
F01_transcript_84030	E2F-DP	TF
F01_transcript_84031	AUX/IAA	TR
F01_transcript_84046	SET	TR
F01_transcript_84047	mTERF	TR
F01_transcript_84109	GARP-G2-like	TF
F01_transcript_84128	SET	TR
F01_transcript_84129	MYB-related	TF
F01_transcript_84134	WRKY	TF
F01_transcript_8420	GARP-ARR-B	TF
F01_transcript_84208	WRKY	TF
F01_transcript_84209	NF-YC	TF
F01_transcript_84233	AP2/ERF-ERF	TF
F01_transcript_84237	GRAS	TF
F01_transcript_84247	BBR-BPC	TF
F01_transcript_84252	MYB-related	TF
F01_transcript_84287	TRAF	TR
F01_transcript_84309	HB-HD-ZIP	TF
F01_transcript_8431	HB-BELL	TF
F01_transcript_84324	C2C2-GATA	TF
F01_transcript_84333	GARP-G2-like	TF
F01_transcript_84436	C3H	TF
F01_transcript_84438	C2C2-Dof	TF
F01_transcript_84449	SET	TR
F01_transcript_84467	HB-BELL	TF
F01_transcript_84471	C2H2	TF
F01_transcript_8450	SWI/SNF-BAF60b	TR
F01_transcript_84503	mTERF	TR
F01_transcript_8452	SET	TR
F01_transcript_84546	Others	TR
F01_transcript_8462	MYB-related	TF
F01_transcript_84630	ARID	TR
F01_transcript_84631	Jumonji	TR
F01_transcript_84665	PHD	TR
F01_transcript_84724	HMG	TR
F01_transcript_84735	GNAT	TR
F01_transcript_84752	Tify	TF
F01_transcript_84754	CSD	TF
F01_transcript_8476	B3-ARF	TF
F01_transcript_84761	GARP-G2-like	TF
F01_transcript_84766	NAC	TF
F01_transcript_8483	HB-BELL	TF
F01_transcript_84856	mTERF	TR
F01_transcript_84896	HB-PHD	TF
F01_transcript_84916	RWP-RK	TF
F01_transcript_84918	C3H	TF
F01_transcript_84939	MYB-related	TF
F01_transcript_84960	GARP-G2-like	TF
F01_transcript_84983	SBP	TF
F01_transcript_84997	B3-ARF	TF

F01_transcript_85015	SNF2	TR
F01_transcript_85092	C3H	TF
F01_transcript_85098	HB-HD-ZIP	TF
F01_transcript_851	SNF2	TR
F01_transcript_85145	SNF2	TR
F01_transcript_85179	GRAS	TF
F01_transcript_85191	RWP-RK	TF
F01_transcript_85203	bHLH	TF
F01_transcript_8521	bHLH	TF
F01_transcript_85216	MYB	TF
F01_transcript_85243	AP2/ERF-ERF	TF
F01_transcript_85294	HB-HD-ZIP	TF
F01_transcript_85305	C2H2	TF
F01_transcript_85314	Others	TR
F01_transcript_8533	Others	TR
F01_transcript_85331	bZIP	TF
F01_transcript_85353	GNAT	TR
F01_transcript_85361	GRAS	TF
F01_transcript_85376	Others	TR
F01_transcript_85392	AUX/IAA	TR
F01_transcript_85394	B3	TF
F01_transcript_8543	LUG	TR
F01_transcript_8548	bZIP	TF
F01_transcript_85491	C2H2	TF
F01_transcript_85492	C3H	TF
F01_transcript_85495	SBP	TF
F01_transcript_85513	mTERF	TR
F01_transcript_85516	WRKY	TF
F01_transcript_85570	NF-YB	TF
F01_transcript_85577	FAR1	TF
F01_transcript_85619	MADS-M-type	TF
F01_transcript_85644	MBF1	TR
F01_transcript_85662	bHLH	TF
F01_transcript_85671	C3H	TF
F01_transcript_85697	GeBP	TF
F01_transcript_85706	SWI/SNF-BAF60b	TR
F01_transcript_85732	Tify	TF
F01_transcript_85735	AP2/ERF-ERF	TF
F01_transcript_8575	mTERF	TR
F01_transcript_8576	bHLH	TF
F01_transcript_85842	GARP-G2-like	TF
F01_transcript_85878	C2C2-GATA	TF
F01_transcript_85894	GRAS	TF
F01_transcript_86002	Others	TR
F01_transcript_86023	RWP-RK	TF
F01_transcript_86114	GNAT	TR
F01_transcript_86141	SWI/SNF-BAF60b	TR
F01_transcript_8615	HB-BELL	TF
F01_transcript_86184	C2H2	TF
F01_transcript_8621	HB-HD-ZIP	TF
F01_transcript_86214	SWI/SNF-BAF60b	TR
F01_transcript_86304	MADS-MIKC	TF
F01_transcript_86348	MYB-related	TF
F01_transcript_86352	TCP	TF
F01_transcript_86369	NAC	TF

F01_transcript_86433	SNF2	TR
F01_transcript_86447	C3H	TF
F01_transcript_86473	SNF2	TR
F01_transcript_86487	MYB-related	TF
F01_transcript_86530	C2H2	TF
F01_transcript_86535	GARP-G2-like	TF
F01_transcript_86555	NAC	TF
F01_transcript_86563	C2H2	TF
F01_transcript_8657	GARP-ARR-B	TF
F01_transcript_86580	EIL	TF
F01_transcript_86620	bZIP	TF
F01_transcript_86623	SET	TR
F01_transcript_86663	STAT	TF
F01_transcript_86688	GRAS	TF
F01_transcript_86750	TCP	TF
F01_transcript_86754	MYB-related	TF
F01_transcript_86762	GNAT	TR
F01_transcript_86791	RWP-RK	TF
F01_transcript_86793	C2C2-GATA	TF
F01_transcript_86796	NAC	TF
F01_transcript_8681	HB-HD-ZIP	TF
F01_transcript_86812	Others	TR
F01_transcript_86822	HB-HD-ZIP	TF
F01_transcript_86873	RWP-RK	TF
F01_transcript_86877	C2C2-Dof	TF
F01_transcript_86916	HB-HD-ZIP	TF
F01_transcript_86946	Trihelix	TF
F01_transcript_86980	GRAS	TF
F01_transcript_86981	bHLH	TF
F01_transcript_87022	SNF2	TR
F01_transcript_87029	bHLH	TF
F01_transcript_87038	SWI/SNF-SWI3	TR
F01_transcript_87072	Jumonji	TR
F01_transcript_8709	HB-BELL	TF
F01_transcript_87121	C3H	TF
F01_transcript_87144	MYB-related	TF
F01_transcript_87183	RWP-RK	TF
F01_transcript_8720	mTERF	TR
F01_transcript_87224	Jumonji	TR
F01_transcript_87248	NAC	TF
F01_transcript_87250	mTERF	TR
F01_transcript_87255	LUG	TR
F01_transcript_87282	bHLH	TF
F01_transcript_873	NF-X1	TF
F01_transcript_87335	ARID	TR
F01_transcript_87341	C2H2	TF
F01_transcript_87351	bHLH	TF
F01_transcript_87370	TRAF	TR
F01_transcript_87412	C2H2	TF
F01_transcript_87415	WRKY	TF
F01_transcript_8743	SWI/SNF-SWI3	TR
F01_transcript_87477	SET	TR
F01_transcript_87495	C2H2	TF
F01_transcript_87502	GRAS	TF

F01_transcript_87537	Alfin-like	TF
F01_transcript_87544	ARID	TR
F01_transcript_87603	GARP-G2-like	TF
F01_transcript_87630	MYB	TF
F01_transcript_87635	NF-YA	TF
F01_transcript_87728	B3	TF
F01_transcript_87856	GARP-G2-like	TF
F01_transcript_87859	TCP	TF
F01_transcript_87922	Tify	TF
F01_transcript_87937	GARP-G2-like	TF
F01_transcript_87999	GARP-ARR-B	TF
F01_transcript_88060	GARP-ARR-B	TF
F01_transcript_88066	SET	TR
F01_transcript_88088	NF-YC	TF
F01_transcript_8812	Others	TR
F01_transcript_88233	LIM	TF
F01_transcript_88248	Others	TR
F01_transcript_88258	AP2/ERF-ERF	TF
F01_transcript_88312	NAC	TF
F01_transcript_88349	bHLH	TF
F01_transcript_8835	RWP-RK	TF
F01_transcript_88374	NF-YC	TF
F01_transcript_88375	WRKY	TF
F01_transcript_88427	MADS-MIKC	TF
F01_transcript_88453	bHLH	TF
F01_transcript_88482	SBP	TF
F01_transcript_88533	PHD	TR
F01_transcript_88555	B3-ARF	TF
F01_transcript_88557	GRAS	TF
F01_transcript_88565	MADS-M-type	TF
F01_transcript_88573	C2H2	TF
F01_transcript_8860	SET	TR
F01_transcript_88606	NF-YA	TF
F01_transcript_88666	MYB-related	TF
F01_transcript_88680	bZIP	TF
F01_transcript_88687	Trihelix	TF
F01_transcript_88733	RWP-RK	TF
F01_transcript_88741	MYB-related	TF
F01_transcript_88746	SET	TR
F01_transcript_88764	DDT	TR
F01_transcript_88795	Others	TR
F01_transcript_88796	C2H2	TF
F01_transcript_88857	MYB-related	TF
F01_transcript_8888	HB-BELL	TF
F01_transcript_88935	SET	TR
F01_transcript_88965	MADS-MIKC	TF
F01_transcript_89007	C3H	TF
F01_transcript_89061	BES1	TF
F01_transcript_89066	RWP-RK	TF
F01_transcript_89090	RWP-RK	TF
F01_transcript_89103	Trihelix	TF
F01_transcript_89105	C3H	TF
F01_transcript_89110	NF-YC	TF
F01_transcript_89169	MYB-related	TF
F01_transcript_89204	SET	TR

F01_transcript_89205	GARP-G2-like	TF
F01_transcript_89238	bZIP	TF
F01_transcript_8924	HB-BELL	TF
F01_transcript_8927	GRAS	TF
F01_transcript_89306	Jumonji	TR
F01_transcript_89367	NF-YA	TF
F01_transcript_89370	IWS1	TR
F01_transcript_89402	bHLH	TF
F01_transcript_89406	HB-BELL	TF
F01_transcript_89413	FAR1	TF
F01_transcript_89457	GARP-G2-like	TF
F01_transcript_89467	GRAS	TF
F01_transcript_89509	SNF2	TR
F01_transcript_89513	GRAS	TF
F01_transcript_89521	C2C2-GATA	TF
F01_transcript_89588	HB-KNOX	TF
F01_transcript_8959	Others	TR
F01_transcript_89608	SET	TR
F01_transcript_8961	SBP	TF
F01_transcript_89645	AUX/IAA	TR
F01_transcript_89649	HB-BELL	TF
F01_transcript_8965	AP2/ERF-ERF	TF
F01_transcript_89653	NAC	TF
F01_transcript_89684	SNF2	TR
F01_transcript_89694	C3H	TF
F01_transcript_89720	NF-YC	TF
F01_transcript_89729	NF-YC	TF
F01_transcript_89738	C2H2	TF
F01_transcript_89763	bHLH	TF
F01_transcript_89772	HB-WOX	TF
F01_transcript_8981	SBP	TF
F01_transcript_89810	NAC	TF
F01_transcript_89822	DDT	TR
F01_transcript_89902	TUB	TF
F01_transcript_89935	B3-ARF	TF
F01_transcript_8995	Others	TR
F01_transcript_89955	MYB-related	TF
F01_transcript_89970	bZIP	TF
F01_transcript_89971	AP2/ERF-ERF	TF
F01_transcript_89973	Others	TR
F01_transcript_89999	WRKY	TF
F01_transcript_90005	BBR-BPC	TF
F01_transcript_9002	GRAS	TF
F01_transcript_9007	MYB-related	TF
F01_transcript_90086	HB-HD-ZIP	TF
F01_transcript_90152	Jumonji	TR
F01_transcript_9019	B3-ARF	TF
F01_transcript_90213	PLATZ	TF
F01_transcript_90229	SBP	TF
F01_transcript_90250	MADS-M-type	TF
F01_transcript_9029	FAR1	TF
F01_transcript_9031	SET	TR
F01_transcript_90327	HB-HD-ZIP	TF
F01_transcript_9038	RWP-RK	TF

F01_transcript_90399	MADS-MIKC	TF
F01_transcript_904	Pseudo ARR-B	TR
F01_transcript_90453	bHLH	TF
F01_transcript_90485	B3-ARF	TF
F01_transcript_90510	MYB-related	TF
F01_transcript_90526	NAC	TF
F01_transcript_90530	GRAS	TF
F01_transcript_90533	RWP-RK	TF
F01_transcript_90536	bHLH	TF
F01_transcript_90553	B3-ARF	TF
F01_transcript_9057	GARP-ARR-B	TF
F01_transcript_9062	SWI/SNF-SWI3	TR
F01_transcript_90647	NAC	TF
F01_transcript_90675	WRKY	TF
F01_transcript_90691	GRAS	TF
F01_transcript_90694	SET	TR
F01_transcript_90725	AUX/IAA	TR
F01_transcript_90753	B3-ARF	TF
F01_transcript_90757	Tify	TF
F01_transcript_90767	GRAS	TF
F01_transcript_90792	ARID	TR
F01_transcript_90797	Others	TR
F01_transcript_90844	AUX/IAA	TR
F01_transcript_90943	bHLH	TF
F01_transcript_9096	B3-ARF	TF
F01_transcript_90980	MYB-related	TF
F01_transcript_90995	GNAT	TR
F01_transcript_91099	AP2/ERF-ERF	TF
F01_transcript_91113	bZIP	TF
F01_transcript_91122	GARP-G2-like	TF
F01_transcript_91147	MYB	TF
F01_transcript_91191	HMG	TR
F01_transcript_91201	HMG	TR
F01_transcript_9122	SET	TR
F01_transcript_91263	Trihelix	TF
F01_transcript_91272	C2H2	TF
F01_transcript_913	MYB-related	TF
F01_transcript_91309	bZIP	TF
F01_transcript_91322	HB-other	TF
F01_transcript_91342	Jumonji	TR
F01_transcript_91348	AUX/IAA	TR
F01_transcript_91350	NF-YC	TF
F01_transcript_91372	FAR1	TF
F01_transcript_91386	Others	TR
F01_transcript_91395	B3-ARF	TF
F01_transcript_91450	C3H	TF
F01_transcript_91540	SET	TR
F01_transcript_91604	GARP-G2-like	TF
F01_transcript_91614	GRAS	TF
F01_transcript_91675	BES1	TF
F01_transcript_91699	WRKY	TF
F01_transcript_91716	bHLH	TF
F01_transcript_9182	B3-ARF	TF
F01_transcript_91832	AUX/IAA	TR
F01_transcript_91840	BBR-BPC	TF

F01_transcript_91841	GRAS	TF
F01_transcript_9187	FAR1	TF
F01_transcript_91960	WRKY	TF
F01_transcript_91976	SET	TR
F01_transcript_91992	GRF	TF
F01_transcript_92002	IWS1	TR
F01_transcript_92029	GRAS	TF
F01_transcript_92048	BBR-BPC	TF
F01_transcript_92056	bHLH	TF
F01_transcript_9214	SET	TR
F01_transcript_92143	AUX/IAA	TR
F01_transcript_92194	TRAF	TR
F01_transcript_9223	MYB-related	TF
F01_transcript_92262	NAC	TF
F01_transcript_9227	SNF2	TR
F01_transcript_92288	NAC	TF
F01_transcript_92348	ARID	TR
F01_transcript_92379	C3H	TF
F01_transcript_92399	MYB	TF
F01_transcript_92432	C2H2	TF
F01_transcript_92435	MED7	TR
F01_transcript_92494	AP2/ERF-ERF	TF
F01_transcript_92546	bHLH	TF
F01_transcript_92565	GARP-G2-like	TF
F01_transcript_92577	HB-BELL	TF
F01_transcript_92591	LOB	TF
F01_transcript_92596	NF-YC	TF
F01_transcript_9260	MYB	TF
F01_transcript_92616	Trihelix	TF
F01_transcript_92627	GARP-G2-like	TF
F01_transcript_92661	SWI/SNF-SWI3	TR
F01_transcript_92706	mTERF	TR
F01_transcript_92761	bZIP	TF
F01_transcript_92771	Rcd1-like	TR
F01_transcript_92772	IWS1	TR
F01_transcript_9281	NAC	TF
F01_transcript_92853	ARID	TR
F01_transcript_92941	GNAT	TR
F01_transcript_92956	GRAS	TF
F01_transcript_92961	BBR-BPC	TF
F01_transcript_93	mTERF	TR
F01_transcript_93029	Rcd1-like	TR
F01_transcript_93032	HSF	TF
F01_transcript_93067	MYB	TF
F01_transcript_93093	C2H2	TF
F01_transcript_93107	C3H	TF
F01_transcript_93186	Trihelix	TF
F01_transcript_93187	MADS-MIKC	TF
F01_transcript_93208	NF-YA	TF
F01_transcript_93257	C2H2	TF
F01_transcript_93298	TCP	TF
F01_transcript_93311	HB-HD-ZIP	TF
F01_transcript_9334	LUG	TR
F01_transcript_93347	AP2/ERF-AP2	TF

F01_transcript_93387	SNF2	TR
F01_transcript_93396	zf-HD	TF
F01_transcript_93449	C2H2	TF
F01_transcript_93516	MYB-related	TF
F01_transcript_93592	C3H	TF
F01_transcript_93704	MYB-related	TF
F01_transcript_93707	AUX/IAA	TR
F01_transcript_93710	MADS-M-type	TF
F01_transcript_9376	HB-BELL	TF
F01_transcript_93781	TCP	TF
F01_transcript_93806	mTERF	TR
F01_transcript_93825	MYB	TF
F01_transcript_93837	HB-HD-ZIP	TF
F01_transcript_93844	MYB-related	TF
F01_transcript_93923	GARP-G2-like	TF
F01_transcript_9398	TRAF	TR
F01_transcript_93992	bHLH	TF
F01_transcript_93996	GARP-ARR-B	TF
F01_transcript_94190	MYB-related	TF
F01_transcript_94200	HB-HD-ZIP	TF
F01_transcript_94208	HB-other	TF
F01_transcript_94284	SWI/SNF-BAF60b	TR
F01_transcript_94306	HMG	TR
F01_transcript_94315	HB-BELL	TF
F01_transcript_9432	Others	TR
F01_transcript_94350	IWS1	TR
F01_transcript_94372	EIL	TF
F01_transcript_94413	MYB-related	TF
F01_transcript_94489	bHLH	TF
F01_transcript_94493	RWP-RK	TF
F01_transcript_94501	C3H	TF
F01_transcript_94531	PLATZ	TF
F01_transcript_94616	HB-KNOX	TF
F01_transcript_94635	C2C2-GATA	TF
F01_transcript_94642	C2H2	TF
F01_transcript_94696	PHD	TR
F01_transcript_94698	bZIP	TF
F01_transcript_94738	Others	TR
F01_transcript_94744	ARID	TR
F01_transcript_94792	bHLH	TF
F01_transcript_94803	HB-HD-ZIP	TF
F01_transcript_94813	Jumonji	TR
F01_transcript_94824	AP2/ERF-ERF	TF
F01_transcript_94838	TRAF	TR
F01_transcript_94839	PLATZ	TF
F01_transcript_9484	FAR1	TF
F01_transcript_94886	SNF2	TR
F01_transcript_94946	SET	TR
F01_transcript_94954	NAC	TF
F01_transcript_94999	SBP	TF
F01_transcript_95010	bZIP	TF
F01_transcript_95020	Jumonji	TR
F01_transcript_9505	MYB-related	TF
F01_transcript_95060	RWP-RK	TF
F01_transcript_95121	HB-other	TF

F01_transcript_95125	SET	TR
F01_transcript_95156	MYB	TF
F01_transcript_95188	Others	TR
F01_transcript_9519	NF-X1	TF
F01_transcript_95218	MYB-related	TF
F01_transcript_95248	Others	TR
F01_transcript_95252	NF-X1	TF
F01_transcript_9526	HB-BELL	TF
F01_transcript_9532	FAR1	TF
F01_transcript_95359	VOZ	TF
F01_transcript_9536	TRAF	TR
F01_transcript_95394	HB-other	TF
F01_transcript_95398	AP2/ERF-ERF	TF
F01_transcript_95434	FAR1	TF
F01_transcript_95439	SET	TR
F01_transcript_9546	SWI/SNF-SWI3	TR
F01_transcript_95461	MYB-related	TF
F01_transcript_95476	HSF	TF
F01_transcript_95529	C3H	TF
F01_transcript_95579	bHLH	TF
F01_transcript_95601	RWP-RK	TF
F01_transcript_9561	MADS-M-type	TF
F01_transcript_95610	TUB	TF
F01_transcript_95613	Trihelix	TF
F01_transcript_9571	GRAS	TF
F01_transcript_95743	SWI/SNF-BAF60b	TR
F01_transcript_95744	SWI/SNF-BAF60b	TR
F01_transcript_95762	B3-ARF	TF
F01_transcript_95821	BES1	TF
F01_transcript_9584	AUX/IAA	TR
F01_transcript_95860	FAR1	TF
F01_transcript_95896	TUB	TF
F01_transcript_95900	PHD	TR
F01_transcript_95909	Trihelix	TF
F01_transcript_95921	bZIP	TF
F01_transcript_95932	AUX/IAA	TR
F01_transcript_95933	AP2/ERF-ERF	TF
F01_transcript_95943	GRAS	TF
F01_transcript_95969	C2H2	TF
F01_transcript_95976	HB-PHD	TF
F01_transcript_95984	bZIP	TF
F01_transcript_96006	C2H2	TF
F01_transcript_96013	MADS-MIKC	TF
F01_transcript_96023	Whirly	TF
F01_transcript_96036	C2C2-YABBY	TF
F01_transcript_96123	MYB	TF
F01_transcript_96135	Others	TR
F01_transcript_96152	SBP	TF
F01_transcript_96166	MADS-M-type	TF
F01_transcript_96190	SET	TR
F01_transcript_96254	Jumonji	TR
F01_transcript_96276	Others	TR
F01_transcript_96324	TCP	TF
F01_transcript_96345	SNF2	TR

F01_transcript_96375	NAC	TF
F01_transcript_96401	GRAS	TF
F01_transcript_96461	HSF	TF
F01_transcript_96466	mTERF	TR
F01_transcript_96479	Trihelix	TF
F01_transcript_96519	C2C2-GATA	TF
F01_transcript_96563	RB	TR
F01_transcript_96578	SET	TR
F01_transcript_96644	RWP-RK	TF
F01_transcript_96653	DBB	TF
F01_transcript_96695	EIL	TF
F01_transcript_9677	bHLH	TF
F01_transcript_96771	Whirly	TF
F01_transcript_96797	bHLH	TF
F01_transcript_96808	MYB-related	TF
F01_transcript_96825	bHLH	TF
F01_transcript_96837	C2H2	TF
F01_transcript_96847	Others	TR
F01_transcript_9685	DDT	TR
F01_transcript_96877	LUG	TR
F01_transcript_96909	HB-other	TF
F01_transcript_97044	C3H	TF
F01_transcript_97047	Rcd1-like	TR
F01_transcript_97098	bHLH	TF
F01_transcript_97146	bHLH	TF
F01_transcript_97166	SNF2	TR
F01_transcript_9723	MYB-related	TF
F01_transcript_97299	Others	TR
F01_transcript_973	C2H2	TF
F01_transcript_97340	SBP	TF
F01_transcript_97346	bZIP	TF
F01_transcript_97361	B3	TF
F01_transcript_9738	TRAF	TR
F01_transcript_97381	RWP-RK	TF
F01_transcript_97401	B3	TF
F01_transcript_97462	SBP	TF
F01_transcript_97463	C3H	TF
F01_transcript_97476	B3-ARF	TF
F01_transcript_97514	B3	TF
F01_transcript_97581	AP2/ERF-ERF	TF
F01_transcript_97679	NAC	TF
F01_transcript_97707	GARP-G2-like	TF
F01_transcript_97722	GNAT	TR
F01_transcript_97729	NAC	TF
F01_transcript_97758	zf-HD	TF
F01_transcript_97850	GRAS	TF
F01_transcript_97882	bHLH	TF
F01_transcript_979	Pseudo ARR-B	TR
F01_transcript_97948	Others	TR
F01_transcript_97991	B3	TF
F01_transcript_97997	HB-BELL	TF
F01_transcript_9801	SET	TR
F01_transcript_98038	C2H2	TF
F01_transcript_98071	Jumonji	TR
F01_transcript_98117	DBB	TF

F01_transcript_98156	AP2/ERF-ERF	TF
F01_transcript_9818	RWP-RK	TF
F01_transcript_98215	C3H	TF
F01_transcript_98233	MADS-M-type	TF
F01_transcript_98240	PHD	TR
F01_transcript_98245	SWI/SNF-BAF60b	TR
F01_transcript_98286	WRKY	TF
F01_transcript_98307	C2C2-GATA	TF
F01_transcript_98331	PHD	TR
F01_transcript_98333	AUX/IAA	TR
F01_transcript_98357	C2C2-GATA	TF
F01_transcript_98368	SET	TR
F01_transcript_98406	C2C2-GATA	TF
F01_transcript_98498	BBR-BPC	TF
F01_transcript_98516	RWP-RK	TF
F01_transcript_98542	AP2/ERF-ERF	TF
F01_transcript_98547	WRKY	TF
F01_transcript_98555	MYB	TF
F01_transcript_98581	SET	TR
F01_transcript_98623	Others	TR
F01_transcript_98631	RWP-RK	TF
F01_transcript_98687	FAR1	TF
F01_transcript_98703	WRKY	TF
F01_transcript_98705	NF-YA	TF
F01_transcript_98796	C3H	TF
F01_transcript_98859	MYB-related	TF
F01_transcript_98861	GeBP	TF
F01_transcript_98865	MYB-related	TF
F01_transcript_98869	FAR1	TF
F01_transcript_98883	MYB	TF
F01_transcript_98897	Others	TR
F01_transcript_98907	RWP-RK	TF
F01_transcript_98972	B3-ARF	TF
F01_transcript_98992	mTERF	TR
F01_transcript_99033	HB-HD-ZIP	TF
F01_transcript_9905	FAR1	TF
F01_transcript_99054	SWI/SNF-BAF60b	TR
F01_transcript_99081	SNF2	TR
F01_transcript_99108	C2C2-GATA	TF
F01_transcript_99121	HB-BELL	TF
F01_transcript_99130	C2H2	TF
F01_transcript_99155	SET	TR
F01_transcript_99158	ARID	TR
F01_transcript_99162	Others	TR
F01_transcript_99217	Others	TR
F01_transcript_99289	B3	TF
F01_transcript_99344	RWP-RK	TF
F01_transcript_99378	C2C2-Dof	TF
F01_transcript_99385	Jumonji	TR
F01_transcript_99403	TRAF	TR
F01_transcript_9943	GRAS	TF
F01_transcript_99489	SBP	TF
F01_transcript_99497	PHD	TR
F01_transcript_99498	SET	TR

F01_transcript_99511	Others	TR
F01_transcript_99517	GRAS	TF
F01_transcript_99628	Jumonji	TR
F01_transcript_9964	GRAS	TF
F01_transcript_99655	NAC	TF
F01_transcript_99671	CPP	TF
F01_transcript_99696	RWP-RK	TF
F01_transcript_99712	TAZ	TR
F01_transcript_99769	TRAF	TR
F01_transcript_99770	C3H	TF
F01_transcript_99898	SWI/SNF-BAF60b	TR
F01_transcript_99907	B3-ARF	TF
F01_transcript_99914	FAR1	TF
F01_transcript_99956	NF-X1	TF
F01_transcript_99985	MYB-related	TF
F01_transcript_10002	CK1_CK1-Pl	PK
F01_transcript_100049	RLK-Pelle_DLSV	PK
F01_transcript_100063	AGC_PDK1	PK
F01_transcript_100130	CAMK_CDPK	PK
F01_transcript_100136	CAMK_CAMKL-CHK1	PK
F01_transcript_100171	RLK-Pelle_LRR-II	PK
F01_transcript_100172	TKL-Pl-4	PK
F01_transcript_100178	RLK-Pelle_Extensin	PK
F01_transcript_100235	STE_STE7	PK
F01_transcript_100278	RLK-Pelle_DLSV	PK
F01_transcript_100334	RLK-Pelle_CrRLK1L-1	PK
F01_transcript_100339	RLK-Pelle_LRR-VIII-1	PK
F01_transcript_100382	RLK-Pelle_LRR-VIII-1	PK
F01_transcript_100384	NEK	PK
F01_transcript_100397	CK1_CK1	PK
F01_transcript_100420	RLK-Pelle_LRR-VIII-1	PK
F01_transcript_100447	RLK-Pelle_RLCK-IXb	PK
F01_transcript_100464	RLK-Pelle_DLSV	PK
F01_transcript_100475	RLK-Pelle_LRR-VI-2	PK
F01_transcript_100502	CAMK_CDPK	PK
F01_transcript_100518	AGC_RSK-2	PK
F01_transcript_100551	CAMK_CAMKL-CHK1	PK
F01_transcript_100616	STE_STE11	PK
F01_transcript_100633	RLK-Pelle_LRR-IX	PK
F01_transcript_100635	RLK-Pelle_RLCK-VIIa-2	PK
F01_transcript_100693	TKL-Pl-4	PK
F01_transcript_100701	RLK-Pelle_RLCK-VI	PK
F01_transcript_100829	CMGC_MAPK	PK
F01_transcript_100835	RLK-Pelle_RLCK-IXb	PK
F01_transcript_100839	TKL-Pl-4	PK
F01_transcript_100843	RLK-Pelle_RLCK-VI	PK
F01_transcript_100881	CAMK_CDPK	PK
F01_transcript_101007	RLK-Pelle_LRR-XI-1	PK
F01_transcript_101009	RLK-Pelle_RLCK-VIIa-2	PK
F01_transcript_101061	RLK-Pelle_CR4L	PK
F01_transcript_101076	RLK-Pelle_RLCK-XII-1	PK
F01_transcript_10116	RLK-Pelle_LRR-VI-2	PK
F01_transcript_101161	CMGC_GSK	PK
F01_transcript_101171	STE_STE20-Fray	PK
F01_transcript_101178	RLK-Pelle_LRK10L-2	PK

F01_transcript_101247	RLK-Pelle_RLCK-VIIa-2	PK
F01_transcript_101334	CAMK_CDPK	PK
F01_transcript_101339	CMGC_CLK	PK
F01_transcript_101388	RLK-Pelle_RLCK-XII-1	PK
F01_transcript_101392	RLK-Pelle_LRK10L-2	PK
F01_transcript_101416	RLK-Pelle_DLSV	PK
F01_transcript_101513	RLK-Pelle_LRR-VI-1	PK
F01_transcript_101519	STE_STE20-Fray	PK
F01_transcript_101546	STE_STE11	PK
F01_transcript_101625	RLK-Pelle_RLCK-VIIb	PK
F01_transcript_101662	RLK-Pelle_LRR-III	PK
F01_transcript_101716	TKL-Pl-4	PK
F01_transcript_101729	CMGC_CDK-CRK7-CDK9	PK
F01_transcript_101769	TKL-Pl-1	PK
F01_transcript_10177	STE_STE20-Fray	PK
F01_transcript_10178	STE_STE20-YSK	PK
F01_transcript_101818	CAMK_AMPK	PK
F01_transcript_10184	RLK-Pelle_RLCK-VIIb	PK
F01_transcript_101858	RLK-Pelle_DLSV	PK
F01_transcript_101903	Group-Pl-4	PK
F01_transcript_101919	RLK-Pelle_LRK10L-2	PK
F01_transcript_101980	RLK-Pelle_DLSV	PK
F01_transcript_102079	RLK-Pelle_LRR-VIII-1	PK
F01_transcript_102081	TKL-Pl-4	PK
F01_transcript_102084	RLK-Pelle_LRR-VIII-1	PK
F01_transcript_102136	RLK-Pelle_DLSV	PK
F01_transcript_10214	WNK_NRBP	PK
F01_transcript_10221	CK1_CK1-Pl	PK
F01_transcript_102250	TKL-Pl-4	PK
F01_transcript_102263	CMGC_CDK-PITSLRE	PK
F01_transcript_102287	CMGC_CDK-CRK7-CDK9	PK
F01_transcript_102393	CAMK_AMPK	PK
F01_transcript_102441	RLK-Pelle_LRR-I-1	PK
F01_transcript_102465	RLK-Pelle_LRR-II	PK
F01_transcript_102486	AGC_RSK-2	PK
F01_transcript_102493	AGC-Pl	PK
F01_transcript_102503	WNK_NRBP	PK
F01_transcript_102537	CK1_CK1	PK
F01_transcript_102559	PEK_PEK	PK
F01_transcript_102566	CAMK_CDPK	PK
F01_transcript_102576	RLK-Pelle_LRR-XII-1	PK
F01_transcript_102598	TKL_CTR1-DRK-2	PK
F01_transcript_102621	CMGC_CDK-Pl	PK
F01_transcript_102626	CK1_CK1-Pl	PK
F01_transcript_102654	IRE1	PK
F01_transcript_102664	RLK-Pelle_CrRLK1L-1	PK
F01_transcript_10268	RLK-Pelle_RLCK-IXb	PK
F01_transcript_102720	RLK-Pelle_LRR-VII-1	PK
F01_transcript_102760	AGC_RSK-2	PK
F01_transcript_102762	STE_STE11	PK
F01_transcript_102809	RLK-Pelle_PERK-2	PK
F01_transcript_102828	RLK-Pelle_LysM	PK
F01_transcript_102857	RLK-Pelle_RLCK-XVI	PK
F01_transcript_102873	RLK-Pelle_RLCK-VIIb	PK

F01_transcript_102889	CAMK_CAMKL-CHK1	PK
F01_transcript_102890	CMGC_CDK-Pl	PK
F01_transcript_103010	RLK-Pelle_LRR-V	PK
F01_transcript_103027	RLK-Pelle_LRR-VIII-1	PK
F01_transcript_10305	RLK-Pelle_CR4L	PK
F01_transcript_103054	RLK-Pelle_RLCK-VIIa-2	PK
F01_transcript_103066	RLK-Pelle_DLSV	PK
F01_transcript_103146	CMGC_MAPK	PK
F01_transcript_103147	TKL-Pl-4	PK
F01_transcript_103239	RLK-Pelle_LRR-III	PK
F01_transcript_103320	RLK-Pelle_SD-2b	PK
F01_transcript_103369	NEK	PK
F01_transcript_103384	CAMK_CDPK	PK
F01_transcript_103395	RLK-Pelle_RLCK-IXb	PK
F01_transcript_103406	RLK-Pelle_DLSV	PK
F01_transcript_103426	CMGC_GSK	PK
F01_transcript_103429	TKL-Cr-3	PK
F01_transcript_103545	RLK-Pelle_RLCK-VIIa-2	PK
F01_transcript_103566	RLK-Pelle_DLSV	PK
F01_transcript_103635	STE_STE20-Fray	PK
F01_transcript_103726	RLK-Pelle_LRR-III	PK
F01_transcript_103800	CMGC_DYRK-PRP4	PK
F01_transcript_103808	STE_STE11	PK
F01_transcript_103813	RLK-Pelle_LRR-VIII-1	PK
F01_transcript_103832	TKL-Pl-5	PK
F01_transcript_103862	TKL-Pl-6	PK
F01_transcript_103864	RLK-Pelle_DLSV	PK
F01_transcript_103867	RLK-Pelle_DLSV	PK
F01_transcript_103870	CAMK_CAMKL-CHK1	PK
F01_transcript_103919	RLK-Pelle_RLCK-IV	PK
F01_transcript_10394	RLK-Pelle_LRR-V	PK
F01_transcript_103945	RLK-Pelle_LRR-VIII-1	PK
F01_transcript_103952	RLK-Pelle_LRK10L-2	PK
F01_transcript_103968	RLK-Pelle_LRK10L-2	PK
F01_transcript_104025	SCY1_SCYL2	PK
F01_transcript_104057	RLK-Pelle_DLSV	PK
F01_transcript_104059	STE_STE20-Fray	PK
F01_transcript_104068	CMGC_GSK	PK
F01_transcript_104098	CAMK_CAMKL-LKB	PK
F01_transcript_104138	Group-Pl-2	PK
F01_transcript_104152	RLK-Pelle_LRK10L-2	PK
F01_transcript_104161	RLK-Pelle_DLSV	PK
F01_transcript_104168	RLK-Pelle_RLCK-VIIa-1	PK
F01_transcript_104232	TKL-Pl-4	PK
F01_transcript_104259	WNK_NRBP	PK
F01_transcript_104307	CAMK_CDPK	PK
F01_transcript_104405	CMGC_CLK	PK
F01_transcript_104469	RLK-Pelle_LRR-II	PK
F01_transcript_104684	CAMK_CDPK	PK
F01_transcript_104789	RLK-Pelle_RLCK-VI	PK
F01_transcript_104819	RLK-Pelle_URK-1	PK
F01_transcript_104825	RLK-Pelle_LRR-V	PK
F01_transcript_104898	TKL-Pl-4	PK
F01_transcript_10502	STE_STE20-Fray	PK
F01_transcript_105055	RLK-Pelle_WAK	PK

F01_transcript_105064	RLK-Pelle_RLCK-VIIa-1	PK
F01_transcript_105119	RLK-Pelle_RLCK-VIIa-2	PK
F01_transcript_10515	RLK-Pelle_RLCK-VI	PK
F01_transcript_105158	CMGC_CK2	PK
F01_transcript_105162	RLK-Pelle_LRR-III	PK
F01_transcript_105198	RLK-Pelle_RLCK-VI	PK
F01_transcript_105392	RLK-Pelle_DLSV	PK
F01_transcript_105397	RLK-Pelle_RLCK-VIIb	PK
F01_transcript_105438	RLK-Pelle_RLCK-VIII	PK
F01_transcript_10554	RLK-Pelle_CrRLK1L-1	PK
F01_transcript_10558	RLK-Pelle_RLCK-IV	PK
F01_transcript_105723	TKL-Pl-6	PK
F01_transcript_10580	CAMK_CDPK	PK
F01_transcript_105800	RLK-Pelle_PERK-1	PK
F01_transcript_105807	CMGC_CDK-CRK7-CDK9	PK
F01_transcript_105998	RLK-Pelle_DLSV	PK
F01_transcript_106086	CMGC_MAPK	PK
F01_transcript_10617	RLK-Pelle_DLSV	PK
F01_transcript_106174	RLK-Pelle_LRR-VIII-1	PK
F01_transcript_106185	NEK	PK
F01_transcript_106211	RLK-Pelle_LRK10L-2	PK
F01_transcript_106239	RLK-Pelle_LRR-VII-2	PK
F01_transcript_106267	CMGC_MAPK	PK
F01_transcript_106281	RLK-Pelle_DLSV	PK
F01_transcript_1063	AGC_RSK-2	PK
F01_transcript_106327	CMGC_RCK	PK
F01_transcript_106337	RLK-Pelle_RLCK-V	PK
F01_transcript_106355	RLK-Pelle_RLCK-VI	PK
F01_transcript_106367	CK1_CK1	PK
F01_transcript_106373	RLK-Pelle_LRR-XI-1	PK
F01_transcript_106395	TKL-Pl-4	PK
F01_transcript_106508	CAMK_CAMKL-LKB	PK
F01_transcript_106557	RLK-Pelle_DLSV	PK
F01_transcript_106562	CAMK_CAMKL-CHK1	PK
F01_transcript_106573	CMGC_GSK	PK
F01_transcript_106585	RLK-Pelle_CrRLK1L-1	PK
F01_transcript_10659	Group-Pl-3	PK
F01_transcript_106598	CMGC_GSK	PK
F01_transcript_10660	RLK-Pelle_DLSV	PK
F01_transcript_106609	CMGC_DYRK-PRP4	PK
F01_transcript_106614	AGC_NDR	PK
F01_transcript_106617	TKL_CTR1-DRK-2	PK
F01_transcript_106762	RLK-Pelle_PERK-2	PK
F01_transcript_106771	RLK-Pelle_WAK_LRK10L-1	PK
F01_transcript_106788	Group-Pl-3	PK
F01_transcript_10680	RLK-Pelle_DLSV	PK
F01_transcript_106808	RLK-Pelle_LRR-II	PK
F01_transcript_106831	RLK-Pelle_LRR-I-1	PK
F01_transcript_106892	RLK-Pelle_RLCK-VIIa-2	PK
F01_transcript_106909	RLK-Pelle_LRR-IX	PK
F01_transcript_107029	WNK_NRBP	PK
F01_transcript_107037	STE_STE20-Fray	PK
F01_transcript_107041	RLK-Pelle_LRR-VIII-1	PK
F01_transcript_10706	RLK-Pelle_LRR-III	PK

F01_transcript_107081	RLK-Pelle_RLCK-XII-1	PK
F01_transcript_107156	STE_STE20-Fray	PK
F01_transcript_107167	RLK-Pelle_DLSV	PK
F01_transcript_107203	RLK-Pelle_LRR-I-2	PK
F01_transcript_107232	TKL-Pl-4	PK
F01_transcript_1073	STE_STE11	PK
F01_transcript_107304	RLK-Pelle_RLCK-VIII	PK
F01_transcript_10741	RLK-Pelle_LRR-Xa	PK
F01_transcript_107424	RLK-Pelle_DLSV	PK
F01_transcript_107429	TKL_CTR1-DRK-2	PK
F01_transcript_107443	NEK	PK
F01_transcript_10745	TKL-Pl-4	PK
F01_transcript_107450	CAMK_CDPK	PK
F01_transcript_107453	RLK-Pelle_LRR-V	PK
F01_transcript_107483	WNK_NRBP	PK
F01_transcript_107598	CAMK_CAMKL-LKB	PK
F01_transcript_10767	RLK-Pelle_RLCK-IXb	PK
F01_transcript_107686	CMGC_CLK	PK
F01_transcript_107745	RLK-Pelle_LRR-I-1	PK
F01_transcript_10777	RLK-Pelle_CR4L	PK
F01_transcript_107773	RLK-Pelle_LRR-III	PK
F01_transcript_107833	CAMK_OST1L	PK
F01_transcript_107857	TKL_CTR1-DRK-2	PK
F01_transcript_107875	CAMK_CDPK	PK
F01_transcript_10788	RLK-Pelle_DLSV	PK
F01_transcript_107930	RLK-Pelle_RLCK-VIIa-2	PK
F01_transcript_108044	RLK-Pelle_LRR-I-1	PK
F01_transcript_108084	CMGC_DYRK-PRP4	PK
F01_transcript_108102	RLK-Pelle_RLCK-IXb	PK
F01_transcript_108248	STE_STE20-Fray	PK
F01_transcript_10827	RLK-Pelle_RLCK-VI	PK
F01_transcript_108344	CAMK_CAMKL-CHK1	PK
F01_transcript_108382	RLK-Pelle_LRR-VIII-1	PK
F01_transcript_10842	WNK_NRBP	PK
F01_transcript_108490	AGC_NDR	PK
F01_transcript_108514	RLK-Pelle_RLCK-VIIa-2	PK
F01_transcript_108519	STE_STE11	PK
F01_transcript_108570	TKL_CTR1-DRK-2	PK
F01_transcript_10860	RLK-Pelle_RLCK-IV	PK
F01_transcript_108618	RLK-Pelle_LRR-II	PK
F01_transcript_108642	TKL-Pl-4	PK
F01_transcript_108662	CMGC_CK2	PK
F01_transcript_108719	CAMK_CDPK	PK
F01_transcript_108735	CMGC_CLK	PK
F01_transcript_108745	CMGC_MAPK	PK
F01_transcript_10881	RLK-Pelle_DLSV	PK
F01_transcript_10883	NEK	PK
F01_transcript_108876	RLK-Pelle_RLCK-XII-1	PK
F01_transcript_108883	TKL_CTR1-DRK-2	PK
F01_transcript_10889	STE_STE11	PK
F01_transcript_108895	CAMK_CAMKL-CHK1	PK
F01_transcript_108952	RLK-Pelle_DLSV	PK
F01_transcript_10897	STE_STE11	PK
F01_transcript_10904	CK1_CK1-Pl	PK
F01_transcript_109054	TKL-Pl-4	PK

F01_transcript_109090	TKL-Pl-4	PK
F01_transcript_109118	CAMK_CAMKL-CHK1	PK
F01_transcript_109157	CAMK_CAMKL-CHK1	PK
F01_transcript_109189	RLK-Pelle_DLSV	PK
F01_transcript_109292	CAMK_CAMKL-CHK1	PK
F01_transcript_10933	RLK-Pelle_SD-2b	PK
F01_transcript_10934	WNK_NRBP	PK
F01_transcript_109396	CAMK_CDPK	PK
F01_transcript_10953	RLK-Pelle_LRR-VI-2	PK
F01_transcript_109661	TKL-Pl-1	PK
F01_transcript_109737	RLK-Pelle_DLSV	PK
F01_transcript_109740	RLK-Pelle_DLSV	PK
F01_transcript_109770	RLK-Pelle_LRR-VIII-1	PK
F01_transcript_109822	RLK-Pelle_DLSV	PK
F01_transcript_109830	RLK-Pelle_RLCK-V	PK
F01_transcript_109845	RLK-Pelle_RLCK-VIIa-2	PK
F01_transcript_109850	RLK-Pelle_DLSV	PK
F01_transcript_109912	CK1_CK1	PK
F01_transcript_109921	RLK-Pelle_LRR-III	PK
F01_transcript_109926	RLK-Pelle_WAK	PK
F01_transcript_109939	STE_STE11	PK
F01_transcript_10994	RLK-Pelle_SD-2b	PK
F01_transcript_109955	CMGC_RCK	PK
F01_transcript_109980	WNK_NRBP	PK
F01_transcript_110025	STE_STE7	PK
F01_transcript_110026	CMGC_CLK	PK
F01_transcript_110080	RLK-Pelle_WAK	PK
F01_transcript_110102	RLK-Pelle_LRR-XI-1	PK
F01_transcript_11015	RLK-Pelle_SD-2b	PK
F01_transcript_110192	RLK-Pelle_DLSV	PK
F01_transcript_110200	CAMK_CAMKL-LKB	PK
F01_transcript_110237	CAMK_CDPK	PK
F01_transcript_1103	TKL-Pl-2	PK
F01_transcript_110326	CAMK_CAMKL-CHK1	PK
F01_transcript_110327	RLK-Pelle_DLSV	PK
F01_transcript_110342	TKL_CTR1-DRK-1	PK
F01_transcript_11037	RLK-Pelle_WAK	PK
F01_transcript_110400	RLK-Pelle_DLSV	PK
F01_transcript_110428	RLK-Pelle_DLSV	PK
F01_transcript_11046	STE_STE20-Fray	PK
F01_transcript_110474	RLK-Pelle_LRR-Xb-1	PK
F01_transcript_110524	RLK-Pelle_DLSV	PK
F01_transcript_110562	CAMK_CAMKL-CHK1	PK
F01_transcript_110646	AGC_RSK-2	PK
F01_transcript_110689	AGC_RSK-2	PK
F01_transcript_11069	RLK-Pelle_SD-2b	PK
F01_transcript_110709	RLK-Pelle_DLSV	PK
F01_transcript_110719	CMGC_RCK	PK
F01_transcript_110790	TKL-Pl-4	PK
F01_transcript_110833	CAMK_OST1L	PK
F01_transcript_110838	RLK-Pelle_LRR-XV	PK
F01_transcript_11084	RLK-Pelle_RLCK-IV	PK
F01_transcript_110886	RLK-Pelle_LRR-III	PK
F01_transcript_110894	RLK-Pelle_DLSV	PK

F01_transcript_110964	CMGC_CLK	PK
F01_transcript_110989	RLK-Pelle_DLSV	PK
F01_transcript_111004	TLK	PK
F01_transcript_111093	RLK-Pelle_RLCK-VIIa-2	PK
F01_transcript_111145	CK1_CK1-Pl	PK
F01_transcript_111151	RLK-Pelle_LRR-VI-2	PK
F01_transcript_111178	RLK-Pelle_DLSV	PK
F01_transcript_111190	CAMK_CDPK	PK
F01_transcript_111193	WNK_NRBP	PK
F01_transcript_111220	RLK-Pelle_RLCK-IXa	PK
F01_transcript_11126	RLK-Pelle_URK-1	PK
F01_transcript_11127	RLK-Pelle_DLSV	PK
F01_transcript_111282	STE_STE11	PK
F01_transcript_111311	RLK-Pelle_LRR-XI-1	PK
F01_transcript_111350	TKL-Pl-4	PK
F01_transcript_111418	RLK-Pelle_DLSV	PK
F01_transcript_11147	RLK-Pelle_SD-2b	PK
F01_transcript_111494	RLK-Pelle_DLSV	PK
F01_transcript_111509	RLK-Pelle_RLCK-IXb	PK
F01_transcript_111534	WNK_NRBP	PK
F01_transcript_111540	RLK-Pelle_RLCK-VIIa-2	PK
F01_transcript_111633	RLK-Pelle_LRR-XI-1	PK
F01_transcript_111640	NEK	PK
F01_transcript_11165	RLK-Pelle_PERK-2	PK
F01_transcript_11168	CMGC_GSK	PK
F01_transcript_111789	RLK-Pelle_LRR-IV	PK
F01_transcript_111796	CAMK_CDPK	PK
F01_transcript_111832	RLK-Pelle_LysM	PK
F01_transcript_111957	RLK-Pelle_LRR-VI-1	PK
F01_transcript_11196	CMGC_CDK-CRK7-CDK9	PK
F01_transcript_112008	CAMK_CAMKL-CHK1	PK
F01_transcript_112039	RLK-Pelle_LRR-XV	PK
F01_transcript_112072	AGC_RSK-2	PK
F01_transcript_112179	RLK-Pelle_LRR-III	PK
F01_transcript_112181	CAMK_CDPK	PK
F01_transcript_112211	CMGC_CDK-CRK7-CDK9	PK
F01_transcript_11231	RLK-Pelle_DLSV	PK
F01_transcript_112385	CMGC_DYRK-PRP4	PK
F01_transcript_112438	TKL_CTR1-DRK-2	PK
F01_transcript_11247	TKL-Pl-4	PK
F01_transcript_112541	TKL-Pl-4	PK
F01_transcript_112554	RLK-Pelle_RLCK-VIIa-1	PK
F01_transcript_112576	NAK	PK
F01_transcript_112582	RLK-Pelle_PERK-1	PK
F01_transcript_112606	STE_STE11	PK
F01_transcript_112668	RLK-Pelle_LRR-VI-2	PK
F01_transcript_112677	CK1_CK1-Pl	PK
F01_transcript_112695	RLK-Pelle_RLCK-XII-1	PK
F01_transcript_11270	WNK_NRBP	PK
F01_transcript_112730	CAMK_CDPK	PK
F01_transcript_112822	RLK-Pelle_LRR-XII-1	PK
F01_transcript_112857	CAMK_CAMKL-CHK1	PK
F01_transcript_11286	RLK-Pelle_SD-2b	PK
F01_transcript_112902	CMGC_GSK	PK
F01_transcript_11292	RLK-Pelle_LRR-VI-2	PK

F01_transcript_112925	NAK	PK
F01_transcript_11293	CK1_CK1	PK
F01_transcript_112937	RLK-Pelle_DLSV	PK
F01_transcript_112940	AGC_RSK-2	PK
F01_transcript_113027	AGC_NDR	PK
F01_transcript_113036	AGC_PDK1	PK
F01_transcript_113041	TKL-Pl-4	PK
F01_transcript_113124	STE_STE20-YSK	PK
F01_transcript_11317	RLK-Pelle_RLCK-IXb	PK
F01_transcript_113172	STE_STE11	PK
F01_transcript_1132	RLK-Pelle_LRR-XV	PK
F01_transcript_113236	STE_STE7	PK
F01_transcript_113328	TKL_CTR1-DRK-2	PK
F01_transcript_113340	AGC_RSK-2	PK
F01_transcript_11343	TKL-Pl-4	PK
F01_transcript_113432	CAMK_CDPK	PK
F01_transcript_113462	CAMK_CAMKL-CHK1	PK
F01_transcript_113513	RLK-Pelle_PERK-1	PK
F01_transcript_113525	TKL-Pl-6	PK
F01_transcript_113584	RLK-Pelle_LRR-II	PK
F01_transcript_113614	RLK-Pelle_DLSV	PK
F01_transcript_113730	RLK-Pelle_DLSV	PK
F01_transcript_113743	CMGC_CLK	PK
F01_transcript_113779	CMGC_RCK	PK
F01_transcript_113787	STE_STE11	PK
F01_transcript_11388	SCY1_SCYL1	PK
F01_transcript_113893	RLK-Pelle_DLSV	PK
F01_transcript_113900	RLK-Pelle_DLSV	PK
F01_transcript_113917	RLK-Pelle_DLSV	PK
F01_transcript_113943	WNK_NRBP	PK
F01_transcript_11396	RLK-Pelle_LRR-VI-2	PK
F01_transcript_114000	RLK-Pelle_CrRLK1L-1	PK
F01_transcript_114095	RLK-Pelle_DLSV	PK
F01_transcript_114099	RLK-Pelle_RLCK-VIIa-2	PK
F01_transcript_114116	RLK-Pelle_LRR-XI-1	PK
F01_transcript_114184	STE_STE11	PK
F01_transcript_114280	RLK-Pelle_DLSV	PK
F01_transcript_11434	RLK-Pelle_LRR-VI-2	PK
F01_transcript_114350	CAMK_CDPK	PK
F01_transcript_114398	CAMK_CAMKL-CHK1	PK
F01_transcript_114450	TKL-Pl-6	PK
F01_transcript_114645	CAMK_OST1L	PK
F01_transcript_114676	CMGC_SRPK	PK
F01_transcript_114708	RLK-Pelle_WAK_LRK10L-1	PK
F01_transcript_11475	RLK-Pelle_SD-2b	PK
F01_transcript_114754	TKL-Pl-4	PK
F01_transcript_114789	CAMK_CDPK	PK
F01_transcript_114807	CK1_CK1	PK
F01_transcript_114849	RLK-Pelle_SD-2b	PK
F01_transcript_114906	RLK-Pelle_DLSV	PK
F01_transcript_114914	CMGC_DYRK-PRP4	PK
F01_transcript_114918	RLK-Pelle_LRR-VIII-1	PK
F01_transcript_114946	TKL_CTR1-DRK-2	PK
F01_transcript_115038	RLK-Pelle_LRR-IV	PK

F01_transcript_115047	AGC_PDK1	PK
F01_transcript_115092	RLK-Pelle_DLSV	PK
F01_transcript_115095	RLK-Pelle_DLSV	PK
F01_transcript_115101	RLK-Pelle_DLSV	PK
F01_transcript_115114	IRE1	PK
F01_transcript_115125	RLK-Pelle_LRR-III	PK
F01_transcript_115144	CK1_CK1	PK
F01_transcript_115148	CAMK_CAMKL-CHK1	PK
F01_transcript_115168	TKL-Pl-6	PK
F01_transcript_115172	RLK-Pelle_CR4L	PK
F01_transcript_115203	CMGC_SRPK	PK
F01_transcript_115254	CMGC_SRPK	PK
F01_transcript_11529	RLK-Pelle_LRR-V	PK
F01_transcript_115303	RLK-Pelle_LRR-VIII-1	PK
F01_transcript_115309	RLK-Pelle_DLSV	PK
F01_transcript_115330	AGC_RSK-2	PK
F01_transcript_115351	WNK_NRBP	PK
F01_transcript_115364	STE_STE20-Fray	PK
F01_transcript_115371	RLK-Pelle_WAK_LRK10L-1	PK
F01_transcript_115375	RLK-Pelle_RLCK-V	PK
F01_transcript_115494	RLK-Pelle_RLCK-XII-1	PK
F01_transcript_115546	RLK-Pelle_DLSV	PK
F01_transcript_11558	RLK-Pelle_DLSV	PK
F01_transcript_115587	AGC_PDK1	PK
F01_transcript_115621	CAMK_CDPK	PK
F01_transcript_115626	RLK-Pelle_RLCK-VIIa-1	PK
F01_transcript_115627	RLK-Pelle_PERK-1	PK
F01_transcript_115731	RLK-Pelle_LRR-VIII-1	PK
F01_transcript_115739	RLK-Pelle_PERK-1	PK
F01_transcript_115810	RLK-Pelle_LRR-XI-1	PK
F01_transcript_11583	TKL-Pl-4	PK
F01_transcript_115838	STE_STE11	PK
F01_transcript_115849	CMGC_CDK-CRK7-CDK9	PK
F01_transcript_115899	RLK-Pelle_SD-2b	PK
F01_transcript_115905	RLK-Pelle_SD-2b	PK
F01_transcript_115972	CK1_CK1	PK
F01_transcript_1160	AGC_RSK-2	PK
F01_transcript_116025	CAMK_OST1L	PK
F01_transcript_116033	TKL-Pl-4	PK
F01_transcript_116036	RLK-Pelle_LRR-VIII-1	PK
F01_transcript_116039	RLK-Pelle_RLCK-VIIa-2	PK
F01_transcript_116125	RLK-Pelle_LRR-XI-1	PK
F01_transcript_116126	AGC_RSK-2	PK
F01_transcript_11615	RLK-Pelle_SD-2b	PK
F01_transcript_116162	RLK-Pelle_RLCK-VIIa-2	PK
F01_transcript_116213	CMGC_MAPK	PK
F01_transcript_116272	RLK-Pelle_LRR-VIII-1	PK
F01_transcript_116305	RLK-Pelle_RLCK-VIIa-2	PK
F01_transcript_116355	CMGC_CDK-CDK7	PK
F01_transcript_116358	RLK-Pelle_WAK	PK
F01_transcript_11636	CMGC_CDK-CRK7-CDK9	PK
F01_transcript_116366	RLK-Pelle_DLSV	PK
F01_transcript_116373	TKL_CTR1-DRK-2	PK
F01_transcript_116418	RLK-Pelle_RLCK-XII-1	PK
F01_transcript_116491	RLK-Pelle_CR4L	PK

F01_transcript_116506	RLK-Pelle_RLCK-VIII	PK
F01_transcript_116559	Group-Pl-4	PK
F01_transcript_116617	RLK-Pelle_DLSV	PK
F01_transcript_116701	AGC_PKA-PKG	PK
F01_transcript_116711	RLK-Pelle_CrRLK1L-1	PK
F01_transcript_11672	WNK_NRBP	PK
F01_transcript_116778	NEK	PK
F01_transcript_116812	RLK-Pelle_RLCK-VIIa-1	PK
F01_transcript_116814	RLK-Pelle_LRK10L-2	PK
F01_transcript_11683	RLK-Pelle_LRR-II	PK
F01_transcript_116847	RLK-Pelle_LRR-V	PK
F01_transcript_11697	RLK-Pelle_RLCK-XII-1	PK
F01_transcript_116976	CMGC_MAPK	PK
F01_transcript_117036	RLK-Pelle_URK-1	PK
F01_transcript_117073	RLK-Pelle_LRR-VIII-1	PK
F01_transcript_117084	RLK-Pelle_DLSV	PK
F01_transcript_117118	CMGC_CDK-CRK7-CDK9	PK
F01_transcript_117174	RLK-Pelle_LRR-II	PK
F01_transcript_117188	RLK-Pelle_RLCK-XII-2	PK
F01_transcript_117209	RLK-Pelle_DLSV	PK
F01_transcript_117235	RLK-Pelle_LRR-III	PK
F01_transcript_117253	RLK-Pelle_DLSV	PK
F01_transcript_117264	TKL-Pl-4	PK
F01_transcript_117276	RLK-Pelle_DLSV	PK
F01_transcript_117311	RLK-Pelle_DLSV	PK
F01_transcript_117387	WNK_NRBP	PK
F01_transcript_117389	RLK-Pelle_CrRLK1L-1	PK
F01_transcript_117439	CAMK_CDPK	PK
F01_transcript_117479	RLK-Pelle_DLSV	PK
F01_transcript_11751	RLK-Pelle_WAK	PK
F01_transcript_117534	CAMK_CDPK	PK
F01_transcript_117543	NEK	PK
F01_transcript_117571	RLK-Pelle_CR4L	PK
F01_transcript_11761	RLK-Pelle_SD-2b	PK
F01_transcript_117620	Group-Pl-4	PK
F01_transcript_117621	RLK-Pelle_LRR-III	PK
F01_transcript_117644	RLK-Pelle_DLSV	PK
F01_transcript_117652	RLK-Pelle_DLSV	PK
F01_transcript_117713	RLK-Pelle_RLCK-VI	PK
F01_transcript_117738	WNK_NRBP	PK
F01_transcript_117762	RLK-Pelle_DLSV	PK
F01_transcript_117777	CK1_CK1-Pl	PK
F01_transcript_117783	RLK-Pelle_RLCK-VIIa-1	PK
F01_transcript_11789	WNK_NRBP	PK
F01_transcript_117974	RLK-Pelle_LRR-XI-1	PK
F01_transcript_117984	IRE1	PK
F01_transcript_118019	RLK-Pelle_RLCK-VIIa-2	PK
F01_transcript_118036	CMGC_GSK	PK
F01_transcript_118042	RLK-Pelle_RLCK-IXb	PK
F01_transcript_118048	IRE1	PK
F01_transcript_118154	CK1_CK1-Pl	PK
F01_transcript_118183	TKL_CTR1-DRK-2	PK
F01_transcript_118193	RLK-Pelle_LRR-VIII-1	PK
F01_transcript_118215	RLK-Pelle_RLCK-XII-1	PK

F01_transcript_118225	AGC_RSK-2	PK
F01_transcript_118230	RLK-Pelle_RLCK-VIII	PK
F01_transcript_118247	CMGC_CDK-CRK7-CDK9	PK
F01_transcript_118261	PEK_GCN2	PK
F01_transcript_118313	CAMK_CAMKL-CHK1	PK
F01_transcript_118380	Group-Pl-4	PK
F01_transcript_118425	RLK-Pelle_LRK10L-2	PK
F01_transcript_118464	STE_STE11	PK
F01_transcript_118499	RLK-Pelle_LRR-VIII-1	PK
F01_transcript_11854	TKL-Pl-4	PK
F01_transcript_118565	RLK-Pelle_LRR-VII-1	PK
F01_transcript_118578	RLK-Pelle_CrRLK1L-1	PK
F01_transcript_118582	RLK-Pelle_LRR-VIII-1	PK
F01_transcript_118594	RLK-Pelle_WAK	PK
F01_transcript_118633	ULK_ULK4	PK
F01_transcript_118637	RLK-Pelle_LRR-IV	PK
F01_transcript_118655	RLK-Pelle_DLSV	PK
F01_transcript_118693	RLK-Pelle_LRR-III	PK
F01_transcript_118694	WNK_NRBP	PK
F01_transcript_118711	WNK_NRBP	PK
F01_transcript_11872	CK1_CK1-Pl	PK
F01_transcript_118730	RLK-Pelle_DLSV	PK
F01_transcript_118731	RLK-Pelle_DLSV	PK
F01_transcript_118732	RLK-Pelle_DLSV	PK
F01_transcript_11874	CMGC_MAPK	PK
F01_transcript_118749	RLK-Pelle_DLSV	PK
F01_transcript_118768	RLK-Pelle_DLSV	PK
F01_transcript_118785	TKL-Pl-4	PK
F01_transcript_118835	RLK-Pelle_LRR-XII-1	PK
F01_transcript_118867	RLK-Pelle_DLSV	PK
F01_transcript_11889	RLK-Pelle_DLSV	PK
F01_transcript_118898	RLK-Pelle_RLCK-VIIa-2	PK
F01_transcript_11893	RLK-Pelle_SD-2b	PK
F01_transcript_11895	RLK-Pelle_LRR-III	PK
F01_transcript_118951	TKL-Pl-4	PK
F01_transcript_119037	RLK-Pelle_RKF3	PK
F01_transcript_119042	RLK-Pelle_RLCK-VIIa-2	PK
F01_transcript_119043	RLK-Pelle_SD-2b	PK
F01_transcript_119056	RLK-Pelle_LRR-II	PK
F01_transcript_119204	RLK-Pelle_LRR-Xa	PK
F01_transcript_119211	CK1_CK1	PK
F01_transcript_119216	RLK-Pelle_DLSV	PK
F01_transcript_119273	RLK-Pelle_RLCK-IXb	PK
F01_transcript_119340	RLK-Pelle_LRR-XI-1	PK
F01_transcript_119375	CMGC_GSK	PK
F01_transcript_119381	RLK-Pelle_Extensin	PK
F01_transcript_119420	RLK-Pelle_RLCK-IXb	PK
F01_transcript_119437	RLK-Pelle_C-LEC	PK
F01_transcript_119444	RLK-Pelle_DLSV	PK
F01_transcript_119453	CAMK_CDPK	PK
F01_transcript_119460	STE_STE11	PK
F01_transcript_11953	RLK-Pelle_LRR-VI-2	PK
F01_transcript_119556	RLK-Pelle_L-LEC	PK
F01_transcript_119593	CAMK_CAMKL-CHK1	PK
F01_transcript_119699	RLK-Pelle_LRR-III	PK

F01_transcript_119737	TLK	PK
F01_transcript_119752	RLK-Pelle_LRR-I-1	PK
F01_transcript_119778	RLK-Pelle_DLSV	PK
F01_transcript_119853	CK1_CK1-Pl	PK
F01_transcript_119879	TKL-Pl-4	PK
F01_transcript_119901	STE_STE7	PK
F01_transcript_119977	CMGC_GSK	PK
F01_transcript_11999	AGC_RSK-2	PK
F01_transcript_119998	AGC_NDR	PK
F01_transcript_120006	CAMK_CDPK	PK
F01_transcript_120031	RLK-Pelle_DLSV	PK
F01_transcript_120065	RLK-Pelle_DLSV	PK
F01_transcript_120088	CMGC_RCK	PK
F01_transcript_120187	RLK-Pelle_LRR-XII-1	PK
F01_transcript_120204	RLK-Pelle_DLSV	PK
F01_transcript_120338	RLK-Pelle_DLSV	PK
F01_transcript_120340	RLK-Pelle_LRR-XI-1	PK
F01_transcript_120347	TKL-Pl-4	PK
F01_transcript_12035	CAMK_CDPK	PK
F01_transcript_120364	RLK-Pelle_LRR-XII-1	PK
F01_transcript_120382	RLK-Pelle_LRR-XI-1	PK
F01_transcript_120406	TLK	PK
F01_transcript_120426	RLK-Pelle_DLSV	PK
F01_transcript_120447	RLK-Pelle_RLCK-V	PK
F01_transcript_120463	RLK-Pelle_LRR-IX	PK
F01_transcript_120511	AGC_RSK-2	PK
F01_transcript_120513	RLK-Pelle_DLSV	PK
F01_transcript_120516	RLK-Pelle_LRR-I-1	PK
F01_transcript_120595	RLK-Pelle_RLCK-IXb	PK
F01_transcript_12062	RLK-Pelle_LRR-IV	PK
F01_transcript_120653	RLK-Pelle_LRR-XI-1	PK
F01_transcript_120658	RLK-Pelle_LRR-XI-1	PK
F01_transcript_120688	TKL_CTR1-DRK-1	PK
F01_transcript_120700	RLK-Pelle_LRK10L-2	PK
F01_transcript_120812	CMGC_GSK	PK
F01_transcript_120814	RLK-Pelle_CrRLK1L-1	PK
F01_transcript_120817	RLK-Pelle_CrRLK1L-1	PK
F01_transcript_120823	RLK-Pelle_LRK10L-2	PK
F01_transcript_120842	RLK-Pelle_DLSV	PK
F01_transcript_120847	TKL_CTR1-DRK-2	PK
F01_transcript_12086	RLK-Pelle_SD-2b	PK
F01_transcript_120864	RLK-Pelle_LRR-VIII-1	PK
F01_transcript_120868	CAMK_CAMKL-CHK1	PK
F01_transcript_120957	RLK-Pelle_LysM	PK
F01_transcript_120980	TKL-Pl-4	PK
F01_transcript_121005	TKL-Pl-4	PK
F01_transcript_12109	RLK-Pelle_RLCK-XII-1	PK
F01_transcript_121123	RLK-Pelle_LRR-III	PK
F01_transcript_121129	RLK-Pelle_RLCK-XII-1	PK
F01_transcript_121164	TKL-Pl-4	PK
F01_transcript_121181	TKL_CTR1-DRK-2	PK
F01_transcript_12123	CAMK_CDPK	PK
F01_transcript_121290	RLK-Pelle_LRR-VI-1	PK
F01_transcript_121411	RLK-Pelle_SD-2b	PK

F01_transcript_121413	TKL-Pl-6	PK
F01_transcript_121418	RLK-Pelle_SD-2b	PK
F01_transcript_121475	RLK-Pelle_LRR-II	PK
F01_transcript_121477	AGC_NDR	PK
F01_transcript_121584	TKL_Gdt	PK
F01_transcript_121598	TKL-Pl-4	PK
F01_transcript_1216	AGC_RSK-2	PK
F01_transcript_121621	STE_STE11	PK
F01_transcript_121623	CMGC_CDK-CCRK	PK
F01_transcript_121655	AGC_RSK-2	PK
F01_transcript_121684	RLK-Pelle_RLCK-IXb	PK
F01_transcript_121701	TKL_CTR1-DRK-2	PK
F01_transcript_121710	AGC_PDK1	PK
F01_transcript_121712	Group-Pl-4	PK
F01_transcript_121751	CAMK_CDPK	PK
F01_transcript_121838	RLK-Pelle_DLSV	PK
F01_transcript_12185	WNK_NRBP	PK
F01_transcript_121860	STE_STE20-Fray	PK
F01_transcript_12188	RLK-Pelle_RLCK-VI	PK
F01_transcript_121892	RLK-Pelle_LRR-XII-1	PK
F01_transcript_121929	RLK-Pelle_LRR-XII-1	PK
F01_transcript_122004	RLK-Pelle_LRK10L-2	PK
F01_transcript_122039	RLK-Pelle_DLSV	PK
F01_transcript_122073	RLK-Pelle_LRR-XII-1	PK
F01_transcript_122074	CAMK_CAMKL-CHK1	PK
F01_transcript_122093	RLK-Pelle_RLCK-XII-1	PK
F01_transcript_1221	CMGC_GSKL	PK
F01_transcript_122130	RLK-Pelle_RLCK-VI	PK
F01_transcript_122134	CK1_CK1	PK
F01_transcript_122137	RLK-Pelle_LRR-XI-1	PK
F01_transcript_122160	RLK-Pelle_Extensin	PK
F01_transcript_122234	STE_STE11	PK
F01_transcript_12225	AGC_RSK-2	PK
F01_transcript_122278	RLK-Pelle_CrRLK1L-1	PK
F01_transcript_122323	RLK-Pelle_RLCK-VIIa-2	PK
F01_transcript_12237	RLK-Pelle_DLSV	PK
F01_transcript_122424	RLK-Pelle_RLCK-VIIa-2	PK
F01_transcript_122426	TKL-Pl-4	PK
F01_transcript_122430	RLK-Pelle_DLSV	PK
F01_transcript_122461	RLK-Pelle_WAK_LRK10L-1	PK
F01_transcript_122505	AGC_RSK-2	PK
F01_transcript_122514	RLK-Pelle_DLSV	PK
F01_transcript_12254	CAMK_CDPK	PK
F01_transcript_122627	RLK-Pelle_RLCK-VIIa-2	PK
F01_transcript_12263	RLK-Pelle_DLSV	PK
F01_transcript_122657	TKL-Pl-6	PK
F01_transcript_122731	STE_STE11	PK
F01_transcript_122732	CAMK_CDPK	PK
F01_transcript_12275	RLK-Pelle_SD-2b	PK
F01_transcript_122772	TKL_CTR1-DRK-2	PK
F01_transcript_122814	TKL-Pl-5	PK
F01_transcript_122835	RLK-Pelle_CrRLK1L-1	PK
F01_transcript_122853	AGC_RSK-2	PK
F01_transcript_122859	RLK-Pelle_RLCK-VIIa-2	PK
F01_transcript_122940	PEK_PEK	PK

F01_transcript_122968	CAMK_CDPK	PK
F01_transcript_122995	RLK-Pelle_LysM	PK
F01_transcript_123005	RLK-Pelle_LRR-VIII-1	PK
F01_transcript_123009	CAMK_OST1L	PK
F01_transcript_123076	CAMK_CDPK	PK
F01_transcript_123105	RLK-Pelle_LRR-VI-1	PK
F01_transcript_123174	CMGC_CDK-CRK7-CDK9	PK
F01_transcript_123240	STE_STE20-Fray	PK
F01_transcript_123278	RLK-Pelle_LRR-III	PK
F01_transcript_123331	CAMK_AMPK	PK
F01_transcript_123343	RLK-Pelle_DLSV	PK
F01_transcript_123360	TKL_CTR1-DRK-2	PK
F01_transcript_123365	RLK-Pelle_PERK-2	PK
F01_transcript_1234	AGC_RSK-2	PK
F01_transcript_123493	RLK-Pelle_DLSV	PK
F01_transcript_123498	RLK-Pelle_DLSV	PK
F01_transcript_1235	RLK-Pelle_LRR-XV	PK
F01_transcript_123500	CAMK_CDPK	PK
F01_transcript_123523	STE_STE11	PK
F01_transcript_123543	CK1_CK1-Pl	PK
F01_transcript_123566	TKL-Pl-4	PK
F01_transcript_123571	RLK-Pelle_CR4L	PK
F01_transcript_123597	AGC_RSK-2	PK
F01_transcript_123648	CMGC_MAPK	PK
F01_transcript_123685	CMGC_CDK-CRK7-CDK9	PK
F01_transcript_123705	RLK-Pelle_RLCK-IV	PK
F01_transcript_123771	TKL-Pl-4	PK
F01_transcript_123794	AGC_RSK-2	PK
F01_transcript_123804	RLK-Pelle_DLSV	PK
F01_transcript_123835	CAMK_CDPK	PK
F01_transcript_12388	AGC_RSK-2	PK
F01_transcript_123884	RLK-Pelle_RLCK-VIIa-2	PK
F01_transcript_123910	RLK-Pelle_LRR-I-1	PK
F01_transcript_123937	CAMK_CAMKL-CHK1	PK
F01_transcript_123986	RLK-Pelle_LRR-VIII-1	PK
F01_transcript_124020	RLK-Pelle_LysM	PK
F01_transcript_124032	CMGC_CLK	PK
F01_transcript_124048	RLK-Pelle_RLCK-V	PK
F01_transcript_12406	CAMK_CDPK	PK
F01_transcript_12409	RLK-Pelle_DLSV	PK
F01_transcript_124134	RLK-Pelle_DLSV	PK
F01_transcript_124151	STE_STE20-YSK	PK
F01_transcript_124154	CMGC_CDK-CRK7-CDK9	PK
F01_transcript_124247	CMGC_GSK	PK
F01_transcript_124304	CK1_CK1-Pl	PK
F01_transcript_124349	AGC_RSK-2	PK
F01_transcript_124457	RLK-Pelle_LRR-Xb-1	PK
F01_transcript_124481	CMGC_CK2	PK
F01_transcript_12456	TKL-Pl-4	PK
F01_transcript_124565	RLK-Pelle_RLCK-VIIa-2	PK
F01_transcript_124609	RLK-Pelle_LRR-XII-1	PK
F01_transcript_124636	RLK-Pelle_SD-2b	PK
F01_transcript_12467	CMGC_GSK	PK
F01_transcript_124674	CAMK_CDPK	PK

F01_transcript_124712	RLK-Pelle_DLSV	PK
F01_transcript_124714	TKL-Pl-4	PK
F01_transcript_124731	CMGC_CDK-PITSLRE	PK
F01_transcript_124758	RLK-Pelle_LRR-VIII-1	PK
F01_transcript_124771	RLK-Pelle_LRR-XII-1	PK
F01_transcript_12479	RLK-Pelle_SD-2b	PK
F01_transcript_124878	CAMK_CDPK	PK
F01_transcript_124895	RLK-Pelle_DLSV	PK
F01_transcript_12491	RLK-Pelle_WAK	PK
F01_transcript_124931	RLK-Pelle_LRR-III	PK
F01_transcript_124988	RLK-Pelle_DLSV	PK
F01_transcript_124995	RLK-Pelle_LRR-IV	PK
F01_transcript_125001	RLK-Pelle_DLSV	PK
F01_transcript_125047	AGC_RSK-2	PK
F01_transcript_125053	RLK-Pelle_LysM	PK
F01_transcript_125060	CMGC_CLK	PK
F01_transcript_125139	TKL-Pl-4	PK
F01_transcript_125155	IRE1	PK
F01_transcript_125178	CMGC_GSK	PK
F01_transcript_125183	CAMK_CDPK	PK
F01_transcript_12523	RLK-Pelle_RLCK-IV	PK
F01_transcript_125253	RLK-Pelle_LRR-II	PK
F01_transcript_125261	RLK-Pelle_Extensin	PK
F01_transcript_125312	RLK-Pelle_DLSV	PK
F01_transcript_125353	RLK-Pelle_WAK	PK
F01_transcript_125364	RLK-Pelle_RLCK-XII-1	PK
F01_transcript_125418	RLK-Pelle_DLSV	PK
F01_transcript_125475	Group-Pl-4	PK
F01_transcript_125508	CAMK_CDPK	PK
F01_transcript_125518	RLK-Pelle_LRK10L-2	PK
F01_transcript_125579	RLK-Pelle_LRR-VIII-1	PK
F01_transcript_125606	CAMK_CDPK	PK
F01_transcript_125627	NEK	PK
F01_transcript_125699	RLK-Pelle_CrRLK1L-1	PK
F01_transcript_125733	STE_STE11	PK
F01_transcript_12580	RLK-Pelle_LRR-IV	PK
F01_transcript_125815	RLK-Pelle_WAK	PK
F01_transcript_12582	CMGC_CDK-CRK7-CDK9	PK
F01_transcript_125838	RLK-Pelle_RLCK-VI	PK
F01_transcript_125844	RLK-Pelle_DLSV	PK
F01_transcript_125889	STE_STE20-Fray	PK
F01_transcript_125950	STE_STE11	PK
F01_transcript_126001	CMGC_CK2	PK
F01_transcript_126026	STE_STE11	PK
F01_transcript_126038	RLK-Pelle_LRR-VI-2	PK
F01_transcript_126046	RLK-Pelle_RKF3	PK
F01_transcript_126092	CMGC_CLK	PK
F01_transcript_126093	RLK-Pelle_LRR-II	PK
F01_transcript_12610	RLK-Pelle_DLSV	PK
F01_transcript_126151	CAMK_CDPK	PK
F01_transcript_126157	TKL-Pl-4	PK
F01_transcript_126176	CK1_CK1-Pl	PK
F01_transcript_126214	STE_STE20-Fray	PK
F01_transcript_126219	RLK-Pelle_L-LEC	PK
F01_transcript_126248	TKL-Pl-6	PK

F01_transcript_126331	TKL_CTR1-DRK-2	PK
F01_transcript_126342	RLK-Pelle_LRR-IV	PK
F01_transcript_126387	RLK-Pelle_RLCK-V	PK
F01_transcript_126396	Group-Pl-4	PK
F01_transcript_126435	RLK-Pelle_DLSV	PK
F01_transcript_126518	TKL-Pl-4	PK
F01_transcript_126522	CMGC_CDK-CRK7-CDK9	PK
F01_transcript_126540	PEK_PEK	PK
F01_transcript_12656	RLK-Pelle_CR4L	PK
F01_transcript_126566	RLK-Pelle_DLSV	PK
F01_transcript_126606	CAMK_CAMKL-CHK1	PK
F01_transcript_126656	TKL_CTR1-DRK-2	PK
F01_transcript_126810	RLK-Pelle_LRR-XI-1	PK
F01_transcript_126834	RLK-Pelle_DLSV	PK
F01_transcript_126888	STE_STE7	PK
F01_transcript_126901	TKL-Pl-6	PK
F01_transcript_126908	TKL-Pl-6	PK
F01_transcript_126943	RLK-Pelle_LRR-III	PK
F01_transcript_12697	RLK-Pelle_SD-2b	PK
F01_transcript_127017	RLK-Pelle_LRR-XII-1	PK
F01_transcript_127018	CAMK_OST1L	PK
F01_transcript_127021	AGC_NDR	PK
F01_transcript_127038	RLK-Pelle_WAK	PK
F01_transcript_12706	Group-Pl-3	PK
F01_transcript_127088	RLK-Pelle_DLSV	PK
F01_transcript_127153	STE_STE11	PK
F01_transcript_127195	CAMK_OST1L	PK
F01_transcript_127261	CMGC_GSK	PK
F01_transcript_127269	CAMK_CAMKL-LKB	PK
F01_transcript_127305	RLK-Pelle_SD-2b	PK
F01_transcript_127362	CMGC_RCK	PK
F01_transcript_127378	TKL-Pl-5	PK
F01_transcript_127433	CMGC_CDK-PITSLRE	PK
F01_transcript_127445	TKL-Pl-4	PK
F01_transcript_127508	CAMK_CDPK	PK
F01_transcript_127526	RLK-Pelle_DLSV	PK
F01_transcript_127575	RLK-Pelle_SD-2b	PK
F01_transcript_127576	CAMK_CAMKL-CHK1	PK
F01_transcript_127610	RLK-Pelle_WAK_LRK10L-1	PK
F01_transcript_127619	CMGC_CK2	PK
F01_transcript_127627	RLK-Pelle_LRR-III	PK
F01_transcript_127636	TKL-Pl-4	PK
F01_transcript_127796	RLK-Pelle_PERK-1	PK
F01_transcript_1278	CMGC_CLK	PK
F01_transcript_127807	RLK-Pelle_RLCK-VI	PK
F01_transcript_12788	RLK-Pelle_DLSV	PK
F01_transcript_127890	RLK-Pelle_SD-2b	PK
F01_transcript_127898	RLK-Pelle_RLCK-VIIa-2	PK
F01_transcript_127928	RLK-Pelle_LRK10L-2	PK
F01_transcript_127940	CAMK_OST1L	PK
F01_transcript_127996	AGC_RSK-2	PK
F01_transcript_12801	RLK-Pelle_CrRLK1L-1	PK
F01_transcript_128018	RLK-Pelle_CrRLK1L-1	PK
F01_transcript_128022	RLK-Pelle_LRR-XI-1	PK

F01_transcript_128085	RLK-Pelle_RLCK-IXb	PK
F01_transcript_128104	RLK-Pelle_LRR-IX	PK
F01_transcript_12812	RLK-Pelle_LRR-VI-2	PK
F01_transcript_128122	RLK-Pelle_LRR-XI-1	PK
F01_transcript_128183	RLK-Pelle_DLSV	PK
F01_transcript_12819	RLK-Pelle_DLSV	PK
F01_transcript_128195	RLK-Pelle_CrRLK1L-1	PK
F01_transcript_12821	RLK-Pelle_LRR-III	PK
F01_transcript_128224	RLK-Pelle_CR4L	PK
F01_transcript_128290	STE_STE11	PK
F01_transcript_128363	STE_STE11	PK
F01_transcript_128386	CMGC_CLK	PK
F01_transcript_128409	CMGC_CDK-CRK7-CDK9	PK
F01_transcript_128483	CAMK_CDPK	PK
F01_transcript_128512	AGC_PDK1	PK
F01_transcript_128561	RLK-Pelle_DLSV	PK
F01_transcript_128596	CAMK_CAMKL-CHK1	PK
F01_transcript_128621	CK1_CK1-Pl	PK
F01_transcript_128659	RLK-Pelle_RLCK-XII-1	PK
F01_transcript_128670	RLK-Pelle_LRK10L-2	PK
F01_transcript_128853	CMGC_DYRK-PRP4	PK
F01_transcript_128903	RLK-Pelle_RLCK-VIIa-2	PK
F01_transcript_128954	CAMK_CDPK	PK
F01_transcript_128955	RLK-Pelle_RLCK-VIIa-2	PK
F01_transcript_129023	RLK-Pelle_LRR-III	PK
F01_transcript_129030	RLK-Pelle_DLSV	PK
F01_transcript_129063	RLK-Pelle_SD-2b	PK
F01_transcript_129131	CMGC_CK2	PK
F01_transcript_129132	CK1_CK1	PK
F01_transcript_129135	RLK-Pelle_SD-2b	PK
F01_transcript_129199	CMGC_MAPK	PK
F01_transcript_129267	AGC_NDR	PK
F01_transcript_129292	RLK-Pelle_LRR-II	PK
F01_transcript_129298	RLK-Pelle_DLSV	PK
F01_transcript_129335	RLK-Pelle_LRR-Xa	PK
F01_transcript_129369	RLK-Pelle_LRR-II	PK
F01_transcript_129461	RLK-Pelle_DLSV	PK
F01_transcript_129536	RLK-Pelle_LRR-VIII-1	PK
F01_transcript_12962	CAMK_CDPK	PK
F01_transcript_129696	STE_STE11	PK
F01_transcript_129698	CAMK_CDPK	PK
F01_transcript_129733	CK1_CK1-Pl	PK
F01_transcript_129749	CAMK_CAMKL-CHK1	PK
F01_transcript_129752	RLK-Pelle_RLCK-XII-1	PK
F01_transcript_129766	RLK-Pelle_DLSV	PK
F01_transcript_1298	RLK-Pelle_LRR-Xb-1	PK
F01_transcript_129808	CAMK_CAMKL-CHK1	PK
F01_transcript_129816	RLK-Pelle_LRK10L-2	PK
F01_transcript_129837	RLK-Pelle_LysM	PK
F01_transcript_129839	CMGC_GSK	PK
F01_transcript_130016	TKL-Pl-4	PK
F01_transcript_130021	CAMK_AMPK	PK
F01_transcript_130042	RLK-Pelle_LysM	PK
F01_transcript_130053	CAMK_CAMKL-CHK1	PK
F01_transcript_130158	WNK_NRBP	PK

F01_transcript_130174	CMGC_SRPK	PK
F01_transcript_130226	RLK-Pelle_LRR-XI-1	PK
F01_transcript_130237	RLK-Pelle_WAK_LRK10L-1	PK
F01_transcript_13024	RLK-Pelle_LRR-III	PK
F01_transcript_130335	RLK-Pelle_LRR-VIII-1	PK
F01_transcript_130338	AGC_RSK-2	PK
F01_transcript_130355	CMGC_CDK-Pl	PK
F01_transcript_130494	CAMK_OST1L	PK
F01_transcript_130525	CMGC_CDK-Pl	PK
F01_transcript_130582	CAMK_OST1L	PK
F01_transcript_130637	RLK-Pelle_LRK10L-2	PK
F01_transcript_130714	RLK-Pelle_LRR-VIII-1	PK
F01_transcript_130728	CMGC_GSK	PK
F01_transcript_130789	CMGC_CDK-CRK7-CDK9	PK
F01_transcript_130838	RLK-Pelle_LRR-VII-1	PK
F01_transcript_13084	RLK-Pelle_LRR-XI-1	PK
F01_transcript_130840	CK1_CK1	PK
F01_transcript_130855	RLK-Pelle_DLSV	PK
F01_transcript_130856	CK1_CK1	PK
F01_transcript_13086	CAMK_CDPK	PK
F01_transcript_130875	RLK-Pelle_LRR-I-1	PK
F01_transcript_130911	RLK-Pelle_DLSV	PK
F01_transcript_130930	CAMK_CDPK	PK
F01_transcript_130933	RLK-Pelle_LRK10L-2	PK
F01_transcript_130978	AGC_RSK-2	PK
F01_transcript_13098	RLK-Pelle_LRR-Xa	PK
F01_transcript_131062	RLK-Pelle_CrRLK1L-1	PK
F01_transcript_131104	CMGC_MAPK	PK
F01_transcript_13111	RLK-Pelle_RLCK-VI	PK
F01_transcript_131111	RLK-Pelle_LRR-XII-1	PK
F01_transcript_131113	CK1_CK1-Pl	PK
F01_transcript_131143	NEK	PK
F01_transcript_131149	RLK-Pelle_LRR-IX	PK
F01_transcript_131150	RLK-Pelle_LRR-XI-1	PK
F01_transcript_131195	RLK-Pelle_LysM	PK
F01_transcript_1312	STE_STE11	PK
F01_transcript_131240	CMGC_GSK	PK
F01_transcript_131245	WNK_NRBP	PK
F01_transcript_131251	RLK-Pelle_LRR-VIII-1	PK
F01_transcript_131265	RLK-Pelle_DLSV	PK
F01_transcript_131284	RLK-Pelle_DLSV	PK
F01_transcript_131298	RLK-Pelle_LRR-Xa	PK
F01_transcript_131319	RLK-Pelle_LRR-III	PK
F01_transcript_131358	RLK-Pelle_SD-2b	PK
F01_transcript_131385	RLK-Pelle_DLSV	PK
F01_transcript_131391	CMGC_CLK	PK
F01_transcript_131401	RLK-Pelle_DLSV	PK
F01_transcript_131407	AGC_RSK-2	PK
F01_transcript_131410	RLK-Pelle_DLSV	PK
F01_transcript_131411	RLK-Pelle_LRR-XII-1	PK
F01_transcript_131457	TKL-Pl-4	PK
F01_transcript_13153	RLK-Pelle_LRR-II	PK
F01_transcript_13156	CK1_CK1-Pl	PK
F01_transcript_131562	RLK-Pelle_DLSV	PK

F01_transcript_131647	RLK-Pelle_WAK_LRK10L-1	PK
F01_transcript_131742	RLK-Pelle_DLSV	PK
F01_transcript_131768	RLK-Pelle_LRR-VI-1	PK
F01_transcript_131776	RLK-Pelle_LRR-III	PK
F01_transcript_131803	RLK-Pelle_LRR-Xa	PK
F01_transcript_131842	AGC_NDR	PK
F01_transcript_131852	RLK-Pelle_LRK10L-2	PK
F01_transcript_13187	RLK-Pelle_RLCK-VI	PK
F01_transcript_131872	RLK-Pelle_LRR-XI-1	PK
F01_transcript_131873	RLK-Pelle_LysM	PK
F01_transcript_131897	STE_STE7	PK
F01_transcript_131904	RLK-Pelle_L-LEC	PK
F01_transcript_131906	RLK-Pelle_DLSV	PK
F01_transcript_131958	AGC_PDK1	PK
F01_transcript_131985	TKL-Pl-4	PK
F01_transcript_132020	AGC_RSK-2	PK
F01_transcript_132058	RLK-Pelle_LRR-XII-1	PK
F01_transcript_132125	RLK-Pelle_DLSV	PK
F01_transcript_132173	RLK-Pelle_RLCK-V	PK
F01_transcript_132186	CAMK_CAMKL-CHK1	PK
F01_transcript_132202	RLK-Pelle_LRR-II	PK
F01_transcript_132219	RLK-Pelle_RLCK-V	PK
F01_transcript_132226	RLK-Pelle_CrRLK1L-1	PK
F01_transcript_132265	CK1_CK1-Pl	PK
F01_transcript_132295	CMGC_CDK-Pl	PK
F01_transcript_13235	RLK-Pelle_SD-2b	PK
F01_transcript_132405	TKL-Pl-4	PK
F01_transcript_13241	RLK-Pelle_LRR-VI-2	PK
F01_transcript_132436	AGC_RSK-2	PK
F01_transcript_132437	RLK-Pelle_LRR-XII-1	PK
F01_transcript_132488	RLK-Pelle_PERK-1	PK
F01_transcript_132497	RLK-Pelle_LRR-VI-1	PK
F01_transcript_1325	RLK-Pelle_LRR-VII-1	PK
F01_transcript_132523	RLK-Pelle_LRR-VIII-1	PK
F01_transcript_132617	CMGC_GSK	PK
F01_transcript_132669	CMGC_MAPK	PK
F01_transcript_132778	RLK-Pelle_RLCK-VIIa-1	PK
F01_transcript_132790	RLK-Pelle_L-LEC	PK
F01_transcript_132808	RLK-Pelle_DLSV	PK
F01_transcript_132863	CAMK_CDPK	PK
F01_transcript_132875	RLK-Pelle_CrRLK1L-1	PK
F01_transcript_132876	CAMK_CAMKL-CHK1	PK
F01_transcript_13289	CMGC_RCK	PK
F01_transcript_132915	RLK-Pelle_RLCK-IV	PK
F01_transcript_13296	RLK-Pelle_LRR-Xa	PK
F01_transcript_132966	RLK-Pelle_LRR-VIII-1	PK
F01_transcript_133034	RLK-Pelle_RLCK-VIIa-2	PK
F01_transcript_13304	RLK-Pelle_SD-2b	PK
F01_transcript_133076	CMGC_RCK	PK
F01_transcript_1331	AGC_RSK-2	PK
F01_transcript_133178	TKL-Pl-4	PK
F01_transcript_13319	Group-Pl-3	PK
F01_transcript_133282	RLK-Pelle_RLCK-VIIa-2	PK
F01_transcript_133336	CAMK_CAMKL-CHK1	PK
F01_transcript_133347	TKL-Pl-6	PK

F01_transcript_133351	NAK	PK
F01_transcript_13336	RLK-Pelle_DLSV	PK
F01_transcript_133392	TKL-Pl-6	PK
F01_transcript_133401	RLK-Pelle_PERK-1	PK
F01_transcript_133409	CMGC_GSK	PK
F01_transcript_133419	CAMK_CDPK	PK
F01_transcript_133438	STE_STE11	PK
F01_transcript_133595	RLK-Pelle_LRR-XIIIa	PK
F01_transcript_133631	RLK-Pelle_LRR-XI-1	PK
F01_transcript_133660	AGC_NDR	PK
F01_transcript_1337	AGC_PKA-PKG	PK
F01_transcript_133821	CK1_CK1	PK
F01_transcript_13385	RLK-Pelle_RLCK-IXb	PK
F01_transcript_133878	CMGC_MAPK	PK
F01_transcript_133970	CMGC_CLK	PK
F01_transcript_134185	STE_STE11	PK
F01_transcript_134213	CK1_CK1-Pl	PK
F01_transcript_134261	RLK-Pelle_LRR-VIII-1	PK
F01_transcript_134266	RLK-Pelle_RLCK-VIIa-2	PK
F01_transcript_134280	IRE1	PK
F01_transcript_134290	AGC_PDK1	PK
F01_transcript_134308	CAMK_CAMKL-CHK1	PK
F01_transcript_134366	RLK-Pelle_DLSV	PK
F01_transcript_13439	NAK	PK
F01_transcript_134504	CMGC_CDK-CRK7-CDK9	PK
F01_transcript_134516	RLK-Pelle_RLCK-VI	PK
F01_transcript_134519	RLK-Pelle_SD-2b	PK
F01_transcript_134531	RLK-Pelle_RLCK-XII-1	PK
F01_transcript_134560	RLK-Pelle_LRR-XII-1	PK
F01_transcript_134597	RLK-Pelle_LRR-XI-1	PK
F01_transcript_134604	RLK-Pelle_RLCK-Os	PK
F01_transcript_13465	RLK-Pelle_DLSV	PK
F01_transcript_134662	NEK	PK
F01_transcript_134735	CAMK_OST1L	PK
F01_transcript_134749	CAMK_CDPK	PK
F01_transcript_134750	RLK-Pelle_RLCK-VI	PK
F01_transcript_134753	CK1_CK1	PK
F01_transcript_134767	CMGC_CLK	PK
F01_transcript_134775	RLK-Pelle_SD-2b	PK
F01_transcript_134783	RLK-Pelle_LRR-VIII-1	PK
F01_transcript_134916	RLK-Pelle_DLSV	PK
F01_transcript_134935	RLK-Pelle_RLCK-VIIa-2	PK
F01_transcript_135004	RLK-Pelle_L-LEC	PK
F01_transcript_135011	RLK-Pelle_LRR-XII-1	PK
F01_transcript_13502	RLK-Pelle_L-LEC	PK
F01_transcript_135036	RLK-Pelle_DLSV	PK
F01_transcript_135135	RLK-Pelle_LRR-XI-1	PK
F01_transcript_135215	RLK-Pelle_DLSV	PK
F01_transcript_135245	RLK-Pelle_RLCK-VI	PK
F01_transcript_135258	RLK-Pelle_LRR-VI-1	PK
F01_transcript_135270	RLK-Pelle_LRR-XII-1	PK
F01_transcript_135286	CMGC_CLK	PK
F01_transcript_135302	RLK-Pelle_RLCK-VIIa-2	PK
F01_transcript_135340	RLK-Pelle_LRR-VIII-1	PK

F01_transcript_135363	CAMK_OST1L	PK
F01_transcript_13537	RLK-Pelle_DLSV	PK
F01_transcript_135393	RLK-Pelle_WAK	PK
F01_transcript_135406	RLK-Pelle_RLCK-V	PK
F01_transcript_135424	RLK-Pelle_LRR-XI-1	PK
F01_transcript_135486	RLK-Pelle_RLCK-VIIa-2	PK
F01_transcript_135527	RLK-Pelle_LRR-Xa	PK
F01_transcript_135619	CAMK_AMPK	PK
F01_transcript_135690	CAMK_CAMKL-CHK1	PK
F01_transcript_135731	CAMK_OST1L	PK
F01_transcript_135773	CAMK_CAMKL-CHK1	PK
F01_transcript_13583	CMGC_CDK-CRK7-CDK9	PK
F01_transcript_135840	STE_STE11	PK
F01_transcript_1359	RLK-Pelle_LRR-Xb-1	PK
F01_transcript_13591	RLK-Pelle_DLSV	PK
F01_transcript_135927	RLK-Pelle_LRR-VIII-1	PK
F01_transcript_13594	RLK-Pelle_URK-1	PK
F01_transcript_135986	RLK-Pelle_LRR-VI-1	PK
F01_transcript_136089	RLK-Pelle_URK-1	PK
F01_transcript_13616	RLK-Pelle_RLCK-XVI	PK
F01_transcript_136163	TKL-Pl-6	PK
F01_transcript_136169	RLK-Pelle_SD-2b	PK
F01_transcript_136176	RLK-Pelle_LRR-VIII-1	PK
F01_transcript_13621	CAMK_CDPK	PK
F01_transcript_136250	CMGC_MAPK	PK
F01_transcript_136342	TKL-Pl-6	PK
F01_transcript_136398	RLK-Pelle_DLSV	PK
F01_transcript_136424	WNK_NRBP	PK
F01_transcript_136436	RLK-Pelle_CrRLK1L-1	PK
F01_transcript_136535	CAMK_CAMKL-CHK1	PK
F01_transcript_13660	RLK-Pelle_DLSV	PK
F01_transcript_136609	RLK-Pelle_LRR-III	PK
F01_transcript_136737	CAMK_CAMKL-LKB	PK
F01_transcript_136774	CMGC_GSK	PK
F01_transcript_136822	RLK-Pelle_LRR-VIII-1	PK
F01_transcript_136838	RLK-Pelle_LRR-VI-1	PK
F01_transcript_13686	CAMK_CDPK	PK
F01_transcript_136862	RLK-Pelle_LRR-V	PK
F01_transcript_13687	WNK_NRBP	PK
F01_transcript_136879	TKL_CTR1-DRK-2	PK
F01_transcript_13689	TKL-Pl-4	PK
F01_transcript_136903	TKL-Pl-6	PK
F01_transcript_13691	STE_STE20-Fray	PK
F01_transcript_136922	RLK-Pelle_DLSV	PK
F01_transcript_136932	Group-Pl-3	PK
F01_transcript_136939	RLK-Pelle_LRK10L-2	PK
F01_transcript_136959	RLK-Pelle_L-LEC	PK
F01_transcript_136981	CMGC_MAPK	PK
F01_transcript_137130	AGC-Pl	PK
F01_transcript_137135	CK1_CK1-Pl	PK
F01_transcript_137185	RLK-Pelle_RLCK-VIIa-2	PK
F01_transcript_137227	CMGC_CDK-PITSLRE	PK
F01_transcript_137264	CAMK_CAMKL-CHK1	PK
F01_transcript_137267	RLK-Pelle_LRR-II	PK
F01_transcript_137282	RLK-Pelle_RLCK-VIIa-2	PK

F01_transcript_137316	AGC_RSK-2	PK
F01_transcript_137323	RLK-Pelle_WAK_LRK10L-1	PK
F01_transcript_137384	WNK_NRBP	PK
F01_transcript_137426	STE_STE20-Fray	PK
F01_transcript_137430	RLK-Pelle_RLCK-VIIa-1	PK
F01_transcript_137465	CMGC_CK2	PK
F01_transcript_137488	RLK-Pelle_RLCK-VIIa-2	PK
F01_transcript_13757	AGC_RSK-2	PK
F01_transcript_137571	RLK-Pelle_LRR-III	PK
F01_transcript_137607	WNK_NRBP	PK
F01_transcript_137641	TKL-Pl-4	PK
F01_transcript_137656	TKL-Pl-4	PK
F01_transcript_137796	RLK-Pelle_DLSV	PK
F01_transcript_137798	RLK-Pelle_LRR-XII-1	PK
F01_transcript_137866	RLK-Pelle_LRR-Xb-1	PK
F01_transcript_13787	IRE1	PK
F01_transcript_137945	CK1_CK1-Pl	PK
F01_transcript_137994	TKL-Pl-5	PK
F01_transcript_13804	RLK-Pelle_CrRLK1L-1	PK
F01_transcript_138061	IRE1	PK
F01_transcript_138078	RLK-Pelle_LRR-IX	PK
F01_transcript_138081	RLK-Pelle_LRR-XII-1	PK
F01_transcript_138139	RLK-Pelle_LRK10L-2	PK
F01_transcript_13815	RLK-Pelle_CrRLK1L-1	PK
F01_transcript_138284	CAMK_OST1L	PK
F01_transcript_138288	RLK-Pelle_LRR-II	PK
F01_transcript_138292	STE_STE20-Fray	PK
F01_transcript_138293	CAMK_CAMKL-CHK1	PK
F01_transcript_138332	RLK-Pelle_RLCK-X	PK
F01_transcript_138348	CMGC_GSK	PK
F01_transcript_138379	TKL-Pl-4	PK
F01_transcript_13839	STE_STE11	PK
F01_transcript_13840	RLK-Pelle_L-LEC	PK
F01_transcript_138409	RLK-Pelle_DLSV	PK
F01_transcript_138417	CMGC_MAPK	PK
F01_transcript_138435	RLK-Pelle_DLSV	PK
F01_transcript_13845	RLK-Pelle_RLCK-VIIa-1	PK
F01_transcript_138472	CMGC_CDK-CRK7-CDK9	PK
F01_transcript_138484	RLK-Pelle_LRR-Xa	PK
F01_transcript_138526	RLK-Pelle_PERK-1	PK
F01_transcript_138609	RLK-Pelle_LRR-VIII-1	PK
F01_transcript_138611	CK1_CK1-Pl	PK
F01_transcript_138623	AGC_RSK-2	PK
F01_transcript_138634	TKL-Pl-4	PK
F01_transcript_138668	TKL-Pl-4	PK
F01_transcript_138687	RLK-Pelle_RLCK-VIIa-1	PK
F01_transcript_138744	CAMK_CAMKL-CHK1	PK
F01_transcript_138746	RLK-Pelle_RLCK-VIIa-2	PK
F01_transcript_138784	RLK-Pelle_DLSV	PK
F01_transcript_1389	TKL_CTR1-DRK-2	PK
F01_transcript_138923	RLK-Pelle_WAK_LRK10L-1	PK
F01_transcript_138950	RLK-Pelle_PERK-2	PK
F01_transcript_13897	RLK-Pelle_DLSV	PK
F01_transcript_139064	CAMK_OST1L	PK

F01_transcript_139065	CAMK_CDPK	PK
F01_transcript_139066	RLK-Pelle_DLSV	PK
F01_transcript_139076	CMGC_CDKL-Cr	PK
F01_transcript_139154	STE_STE11	PK
F01_transcript_139261	RLK-Pelle_LRR-XI-1	PK
F01_transcript_139288	CAMK_CDPK	PK
F01_transcript_139319	RLK-Pelle_LRK10L-2	PK
F01_transcript_13936	CAMK_CDPK	PK
F01_transcript_139396	CK1_CK1	PK
F01_transcript_13942	RLK-Pelle_SD-2b	PK
F01_transcript_139432	CK1_CK1	PK
F01_transcript_139434	CAMK_CAMKL-CHK1	PK
F01_transcript_139466	CAMK_CDPK	PK
F01_transcript_139471	RLK-Pelle_DLSV	PK
F01_transcript_139508	RLK-Pelle_DLSV	PK
F01_transcript_139524	CAMK_CDPK	PK
F01_transcript_139534	CK1_CK1	PK
F01_transcript_139581	RLK-Pelle_DLSV	PK
F01_transcript_139595	RLK-Pelle_LRR-VIII-1	PK
F01_transcript_139634	RLK-Pelle_WAK_LRK10L-1	PK
F01_transcript_139668	Group-Pl-4	PK
F01_transcript_139701	RLK-Pelle_LRR-VI-1	PK
F01_transcript_139718	RLK-Pelle_CrRLK1L-1	PK
F01_transcript_13974	RLK-Pelle_LRR-II	PK
F01_transcript_13977	RLK-Pelle_LRR-II	PK
F01_transcript_139787	CMGC_DYRK-PRP4	PK
F01_transcript_139811	CMGC_MAPK	PK
F01_transcript_139881	RLK-Pelle_LRR-VIII-1	PK
F01_transcript_139954	AGC_NDR	PK
F01_transcript_13998	RLK-Pelle_WAK_LRK10L-1	PK
F01_transcript_140000	RLK-Pelle_DLSV	PK
F01_transcript_140057	CAMK_CDPK	PK
F01_transcript_14008	RLK-Pelle_WAK_LRK10L-1	PK
F01_transcript_140101	RLK-Pelle_RLCK-IXb	PK
F01_transcript_140103	RLK-Pelle_LRR-VIII-1	PK
F01_transcript_140174	CMGC_GSK	PK
F01_transcript_140218	CMGC_Pl-Tthe	PK
F01_transcript_140272	RLK-Pelle_URK-2	PK
F01_transcript_140310	CAMK_OST1L	PK
F01_transcript_140326	WNK_NRBP	PK
F01_transcript_14041	RLK-Pelle_LRR-Xa	PK
F01_transcript_140457	CAMK_CAMKL-CHK1	PK
F01_transcript_140474	STE_STE7	PK
F01_transcript_140535	RLK-Pelle_DLSV	PK
F01_transcript_140566	RLK-Pelle_WAK	PK
F01_transcript_140580	RLK-Pelle_CrRLK1L-1	PK
F01_transcript_14061	CMGC_MAPK	PK
F01_transcript_140630	RLK-Pelle_RLCK-VIIa-2	PK
F01_transcript_140658	CMGC_CDK-Pl	PK
F01_transcript_140695	RLK-Pelle_WAK	PK
F01_transcript_140712	RLK-Pelle_LRR-VI-2	PK
F01_transcript_140761	RLK-Pelle_LRR-V	PK
F01_transcript_140766	RLK-Pelle_WAK_LRK10L-1	PK
F01_transcript_140784	RLK-Pelle_LRR-VI-2	PK
F01_transcript_140794	RLK-Pelle_DLSV	PK

F01_transcript_140805	RLK-Pelle_DLSV	PK
F01_transcript_14081	RLK-Pelle_L-LEC	PK
F01_transcript_140849	RLK-Pelle_LRR-VIII-1	PK
F01_transcript_140857	RLK-Pelle_LRR-VIII-1	PK
F01_transcript_140950	NEK	PK
F01_transcript_14098	CMGC_DYRK-PRP4	PK
F01_transcript_140994	CAMK_CDPK	PK
F01_transcript_141	AGC_RSK-2	PK
F01_transcript_141018	RLK-Pelle_LRR-VIII-1	PK
F01_transcript_141020	RLK-Pelle_LRR-XII-1	PK
F01_transcript_141038	AGC_PKA-PKG	PK
F01_transcript_141041	RLK-Pelle_WAK_LRK10L-1	PK
F01_transcript_141045	CMGC_CLK	PK
F01_transcript_141080	RLK-Pelle_RLCK-VIIa-2	PK
F01_transcript_141131	CAMK_CDPK	PK
F01_transcript_141135	CMGC_CDK-Pl	PK
F01_transcript_14117	RLK-Pelle_LRR-II	PK
F01_transcript_141202	RLK-Pelle_LysM	PK
F01_transcript_141220	STE_STE11	PK
F01_transcript_141401	CMGC_GSK	PK
F01_transcript_141402	RLK-Pelle_DLSV	PK
F01_transcript_141426	RLK-Pelle_CR4L	PK
F01_transcript_141436	NAK	PK
F01_transcript_141443	RLK-Pelle_DLSV	PK
F01_transcript_141525	RLK-Pelle_PERK-2	PK
F01_transcript_141581	CAMK_CDPK	PK
F01_transcript_141589	RLK-Pelle_CrRLK1L-1	PK
F01_transcript_141613	AGC_RSK-2	PK
F01_transcript_14162	CAMK_CDPK	PK
F01_transcript_141662	RLK-Pelle_DLSV	PK
F01_transcript_141783	RLK-Pelle_RLCK-VIIa-2	PK
F01_transcript_141824	TKL-Pl-4	PK
F01_transcript_141826	TKL-Pl-4	PK
F01_transcript_141832	TKL-Pl-4	PK
F01_transcript_14184	RLK-Pelle_SD-2b	PK
F01_transcript_141881	RLK-Pelle_RLCK-VIIa-2	PK
F01_transcript_141893	CMGC_MAPK	PK
F01_transcript_141915	RLK-Pelle_LRR-XI-1	PK
F01_transcript_141919	CMGC_SRPK	PK
F01_transcript_141937	Group-Pl-4	PK
F01_transcript_141961	RLK-Pelle_LRR-III	PK
F01_transcript_142021	CK1_CK1-Pl	PK
F01_transcript_142103	CMGC_MAPK	PK
F01_transcript_142118	RLK-Pelle_LRR-XII-1	PK
F01_transcript_142140	RLK-Pelle_LRR-II	PK
F01_transcript_142219	RLK-Pelle_LRR-VI-2	PK
F01_transcript_142236	RLK-Pelle_DLSV	PK
F01_transcript_14228	NEK	PK
F01_transcript_142282	TKL-Pl-4	PK
F01_transcript_14229	CAMK_CDPK	PK
F01_transcript_142326	CAMK_CDPK	PK
F01_transcript_142334	RLK-Pelle_RLCK-VI	PK
F01_transcript_14234	CAMK_CDPK	PK
F01_transcript_142356	WNK_NRBP	PK

F01_transcript_142399	RLK-Pelle_DLSV	PK
F01_transcript_142405	CAMK_CDPK	PK
F01_transcript_142409	RLK-Pelle_SD-2b	PK
F01_transcript_142445	CMGC_CDK-Pl	PK
F01_transcript_142472	TKL-Pl-6	PK
F01_transcript_142496	TKL-Pl-2	PK
F01_transcript_14268	RLK-Pelle_RLCK-V	PK
F01_transcript_142715	RLK-Pelle_LRK10L-2	PK
F01_transcript_14272	RLK-Pelle_LRR-V	PK
F01_transcript_14274	RLK-Pelle_LRR-III	PK
F01_transcript_142741	RLK-Pelle_CrRLK1L-1	PK
F01_transcript_142758	TKL-Pl-5	PK
F01_transcript_14278	RLK-Pelle_LRR-II	PK
F01_transcript_142783	TKL-Cr-3	PK
F01_transcript_142793	TKL-Pl-6	PK
F01_transcript_142823	RLK-Pelle_RLCK-VIIa-2	PK
F01_transcript_142826	CMGC_MAPK	PK
F01_transcript_142946	CAMK_CDPK	PK
F01_transcript_142953	RLK-Pelle_LRR-XI-1	PK
F01_transcript_142966	AGC_PKA-PKG	PK
F01_transcript_142969	Group-Pl-4	PK
F01_transcript_142970	AGC_RSK-2	PK
F01_transcript_143063	CAMK_CAMKL-CHK1	PK
F01_transcript_143087	CAMK_CAMKL-CHK1	PK
F01_transcript_143113	RLK-Pelle_LRR-XI-1	PK
F01_transcript_143182	RLK-Pelle_DLSV	PK
F01_transcript_143203	RLK-Pelle_DLSV	PK
F01_transcript_143279	TKL-Pl-4	PK
F01_transcript_143301	RLK-Pelle_DLSV	PK
F01_transcript_143318	RLK-Pelle_LRR-II	PK
F01_transcript_143434	CAMK_AMPK	PK
F01_transcript_143530	RLK-Pelle_LRR-I-1	PK
F01_transcript_14356	RLK-Pelle_LRR-III	PK
F01_transcript_143565	CMGC_RCK	PK
F01_transcript_143593	RLK-Pelle_LRR-VIII-1	PK
F01_transcript_143605	RLK-Pelle_LRR-XV	PK
F01_transcript_143642	TKL-Pl-4	PK
F01_transcript_14371	STE_STE11	PK
F01_transcript_143742	STE_STE11	PK
F01_transcript_143774	TKL-Pl-4	PK
F01_transcript_143776	RLK-Pelle_DLSV	PK
F01_transcript_143849	CAMK_CAMKL-CHK1	PK
F01_transcript_143885	RLK-Pelle_LRR-IV	PK
F01_transcript_143902	RLK-Pelle_SD-2b	PK
F01_transcript_14394	RLK-Pelle_SD-2b	PK
F01_transcript_14398	RLK-Pelle_DLSV	PK
F01_transcript_143996	CAMK_OST1L	PK
F01_transcript_14400	RLK-Pelle_RLCK-VIIa-1	PK
F01_transcript_144072	CAMK_CAMKL-LKB	PK
F01_transcript_144078	RLK-Pelle_PERK-1	PK
F01_transcript_144110	RLK-Pelle_DLSV	PK
F01_transcript_144111	TKL_CTR1-DRK-2	PK
F01_transcript_14413	RLK-Pelle_LRR-III	PK
F01_transcript_144167	RLK-Pelle_DLSV	PK
F01_transcript_144190	RLK-Pelle_DLSV	PK

F01_transcript_14426	TKL-Pl-4	PK
F01_transcript_144268	RLK-Pelle_DLSV	PK
F01_transcript_14434	RLK-Pelle_LRR-VI-2	PK
F01_transcript_144365	RLK-Pelle_CrRLK1L-1	PK
F01_transcript_144396	CAMK_OST1L	PK
F01_transcript_144425	RLK-Pelle_DLSV	PK
F01_transcript_144448	RLK-Pelle_RLCK-VIIa-2	PK
F01_transcript_14446	CMGC_MAPK	PK
F01_transcript_144488	STE_STE-Pl	PK
F01_transcript_144502	RLK-Pelle_LRR-II	PK
F01_transcript_144504	TKL-Pl-4	PK
F01_transcript_144508	CMGC_CLK	PK
F01_transcript_144515	RLK-Pelle_LRR-VI-1	PK
F01_transcript_144530	RLK-Pelle_RLCK-VIIa-2	PK
F01_transcript_144568	RLK-Pelle_RLCK-IXb	PK
F01_transcript_144627	RLK-Pelle_CrRLK1L-1	PK
F01_transcript_144637	RLK-Pelle_CrRLK1L-1	PK
F01_transcript_144681	RLK-Pelle_LRR-XIIIb	PK
F01_transcript_144710	AGC_NDR	PK
F01_transcript_144725	STE_STE11	PK
F01_transcript_14473	RLK-Pelle_DLSV	PK
F01_transcript_144759	STE_STE20-Fray	PK
F01_transcript_144775	RLK-Pelle_DLSV	PK
F01_transcript_144776	RLK-Pelle_WAK	PK
F01_transcript_144833	WNK_NRBP	PK
F01_transcript_144843	RLK-Pelle_RLCK-IV	PK
F01_transcript_144927	RLK-Pelle_LRR-IV	PK
F01_transcript_14493	RLK-Pelle_WAK	PK
F01_transcript_144951	RLK-Pelle_DLSV	PK
F01_transcript_144967	RLK-Pelle_LRR-VIII-1	PK
F01_transcript_144980	RLK-Pelle_LRR-XII-1	PK
F01_transcript_145012	RLK-Pelle_DLSV	PK
F01_transcript_145060	RLK-Pelle_LRR-VI-1	PK
F01_transcript_145107	WNK_NRBP	PK
F01_transcript_145120	CAMK_CAMKL-CHK1	PK
F01_transcript_145140	WNK_NRBP	PK
F01_transcript_145167	CAMK_OST1L	PK
F01_transcript_145178	RLK-Pelle_RLCK-XII-1	PK
F01_transcript_145206	WNK_NRBP	PK
F01_transcript_145218	RLK-Pelle_LRR-VII-1	PK
F01_transcript_145225	RLK-Pelle_LRR-XII-1	PK
F01_transcript_145247	CK1_CK1-Pl	PK
F01_transcript_145257	CAMK_AMPK	PK
F01_transcript_145267	TKL-Pl-4	PK
F01_transcript_14529	WNK_NRBP	PK
F01_transcript_145354	RLK-Pelle_RLCK-VI	PK
F01_transcript_145395	RLK-Pelle_CrRLK1L-1	PK
F01_transcript_145399	RLK-Pelle_RLCK-XV	PK
F01_transcript_145415	RLK-Pelle_Extensin	PK
F01_transcript_145447	RLK-Pelle_DLSV	PK
F01_transcript_145479	PEK_PEK	PK
F01_transcript_14566	RLK-Pelle_LysM	PK
F01_transcript_145689	STE_STE11	PK
F01_transcript_145697	AGC_RSK-2	PK

F01_transcript_145765	CK1_CK1	PK
F01_transcript_145777	CK1_CK1	PK
F01_transcript_145824	CK1_CK1	PK
F01_transcript_145835	RLK-Pelle_LRK10L-2	PK
F01_transcript_145840	CAMK_CAMKL-LKB	PK
F01_transcript_145896	RLK-Pelle_RLCK-VIII	PK
F01_transcript_145979	CMGC_RCK	PK
F01_transcript_146009	RLK-Pelle_L-LEC	PK
F01_transcript_146042	TKL-Pl-4	PK
F01_transcript_146049	RLK-Pelle_DLSV	PK
F01_transcript_14605	RLK-Pelle_LRR-Xa	PK
F01_transcript_146125	CMGC_CDK-Pl	PK
F01_transcript_146153	RLK-Pelle_DLSV	PK
F01_transcript_146168	RLK-Pelle_DLSV	PK
F01_transcript_146187	RLK-Pelle_LRR-XV	PK
F01_transcript_146236	RLK-Pelle_CrRLK1L-1	PK
F01_transcript_146265	RLK-Pelle_LysM	PK
F01_transcript_146281	CAMK_AMPK	PK
F01_transcript_146327	RLK-Pelle_LRR-VII-2	PK
F01_transcript_146333	CAMK_CAMKL-CHK1	PK
F01_transcript_146344	CAMK_CAMKL-CHK1	PK
F01_transcript_146404	RLK-Pelle_LRR-III	PK
F01_transcript_146437	RLK-Pelle_LRR-IX	PK
F01_transcript_146442	RLK-Pelle_LRR-IX	PK
F01_transcript_146485	CMGC_RCK	PK
F01_transcript_146693	RLK-Pelle_LRR-VI-2	PK
F01_transcript_146705	RLK-Pelle_L-LEC	PK
F01_transcript_146729	CMGC_MAPK	PK
F01_transcript_146737	CAMK_CDPK	PK
F01_transcript_146768	AGC_RSK-2	PK
F01_transcript_146783	RLK-Pelle_CrRLK1L-1	PK
F01_transcript_14686	Group-Pl-3	PK
F01_transcript_14689	CK1_CK1	PK
F01_transcript_146899	RLK-Pelle_LRR-XI-1	PK
F01_transcript_146916	RLK-Pelle_DLSV	PK
F01_transcript_146951	CK1_CK1	PK
F01_transcript_147016	RLK-Pelle_WAK	PK
F01_transcript_147050	STE_STE11	PK
F01_transcript_147077	RLK-Pelle_WAK_LRK10L-1	PK
F01_transcript_147144	RLK-Pelle_WAK	PK
F01_transcript_147161	TKL_CTR1-DRK-2	PK
F01_transcript_147233	RLK-Pelle_RLCK-IXa	PK
F01_transcript_147319	RLK-Pelle_LysM	PK
F01_transcript_147361	RLK-Pelle_DLSV	PK
F01_transcript_147422	TKL-Pl-5	PK
F01_transcript_147427	RLK-Pelle_CrRLK1L-1	PK
F01_transcript_147474	RLK-Pelle_LRR-XII-1	PK
F01_transcript_147532	CMGC_MAPK	PK
F01_transcript_147564	STE_STE11	PK
F01_transcript_147579	STE_STE20-Fray	PK
F01_transcript_14759	RLK-Pelle_SD-2b	PK
F01_transcript_147593	RLK-Pelle_LRR-VI-2	PK
F01_transcript_147597	RLK-Pelle_RLCK-VI	PK
F01_transcript_147648	RLK-Pelle_DLSV	PK
F01_transcript_147653	RLK-Pelle_LRR-XII-1	PK

F01_transcript_147669	CAMK_CDPK	PK
F01_transcript_147800	RLK-Pelle_DLSV	PK
F01_transcript_14782	TKL-Pl-4	PK
F01_transcript_147853	RLK-Pelle_DLSV	PK
F01_transcript_147884	TKL-Pl-5	PK
F01_transcript_147890	RLK-Pelle_DLSV	PK
F01_transcript_148001	RLK-Pelle_RLCK-Os	PK
F01_transcript_148048	RLK-Pelle_RLCK-VIIa-1	PK
F01_transcript_148086	RLK-Pelle_WAK_LRK10L-1	PK
F01_transcript_148182	CAMK_CDPK	PK
F01_transcript_14822	RLK-Pelle_CrRLK1L-1	PK
F01_transcript_148289	STE_STE20-Fray	PK
F01_transcript_148386	RLK-Pelle_DLSV	PK
F01_transcript_148433	RLK-Pelle_RLCK-IV	PK
F01_transcript_148454	TKL-Pl-4	PK
F01_transcript_148456	RLK-Pelle_DLSV	PK
F01_transcript_148480	RLK-Pelle_RLCK-VI	PK
F01_transcript_148519	RLK-Pelle_DLSV	PK
F01_transcript_148719	RLK-Pelle_DLSV	PK
F01_transcript_148835	RLK-Pelle_PERK-1	PK
F01_transcript_14888	WNK_NRBP	PK
F01_transcript_148880	RLK-Pelle_LRR-XII-1	PK
F01_transcript_149017	RLK-Pelle_DLSV	PK
F01_transcript_149047	RLK-Pelle_LRR-VII-2	PK
F01_transcript_149131	RLK-Pelle_LRR-VIII-1	PK
F01_transcript_149194	WNK_NRBP	PK
F01_transcript_149250	CAMK_CAMKL-CHK1	PK
F01_transcript_149270	RLK-Pelle_LRR-XII-1	PK
F01_transcript_149284	RLK-Pelle_DLSV	PK
F01_transcript_149290	WEE	PK
F01_transcript_149345	RLK-Pelle_WAK	PK
F01_transcript_149348	RLK-Pelle_DLSV	PK
F01_transcript_149472	TKL-Pl-4	PK
F01_transcript_149515	RLK-Pelle_DLSV	PK
F01_transcript_14966	CAMK_CDPK	PK
F01_transcript_1497	RLK-Pelle_LRR-XV	PK
F01_transcript_149724	STE_STE20-Fray	PK
F01_transcript_149938	WNK_NRBP	PK
F01_transcript_14996	STE_STE11	PK
F01_transcript_149988	RLK-Pelle_DLSV	PK
F01_transcript_14999	CMGC_MAPK	PK
F01_transcript_150059	RLK-Pelle_CrRLK1L-1	PK
F01_transcript_150154	RLK-Pelle_DLSV	PK
F01_transcript_150171	RLK-Pelle_DLSV	PK
F01_transcript_150172	CMGC_RCK	PK
F01_transcript_150186	RLK-Pelle_DLSV	PK
F01_transcript_150222	RLK-Pelle_DLSV	PK
F01_transcript_150259	RLK-Pelle_RLCK-VIIa-2	PK
F01_transcript_150274	WNK_NRBP	PK
F01_transcript_150335	CAMK_OST1L	PK
F01_transcript_15034	RLK-Pelle_LysM	PK
F01_transcript_150360	RLK-Pelle_DLSV	PK
F01_transcript_150369	RLK-Pelle_DLSV	PK
F01_transcript_15045	RLK-Pelle_LRR-III	PK

F01_transcript_150475	AGC_RSK-2	PK
F01_transcript_150498	CAMK_CDPK	PK
F01_transcript_150518	CMGC_CDK-Pl	PK
F01_transcript_150573	RLK-Pelle_SD-2b	PK
F01_transcript_150574	RLK-Pelle_LRR-III	PK
F01_transcript_150590	TKL-Pl-4	PK
F01_transcript_150693	CK1_CK1	PK
F01_transcript_150765	WNK_NRBP	PK
F01_transcript_150804	CK1_CK1	PK
F01_transcript_15083	RLK-Pelle_DLSV	PK
F01_transcript_150849	CAMK_CAMKL-CHK1	PK
F01_transcript_150857	WNK_NRBP	PK
F01_transcript_150903	CAMK_CDPK	PK
F01_transcript_150918	STE_STE-Pl	PK
F01_transcript_15095	RLK-Pelle_LRR-VI-2	PK
F01_transcript_151032	CMGC_MAPK	PK
F01_transcript_151062	AGC_RSK-2	PK
F01_transcript_151087	RLK-Pelle_DLSV	PK
F01_transcript_151090	RLK-Pelle_RLCK-X	PK
F01_transcript_151214	NAK	PK
F01_transcript_151233	TKL-Pl-6	PK
F01_transcript_151322	RLK-Pelle_DLSV	PK
F01_transcript_151364	RLK-Pelle_SD-2b	PK
F01_transcript_151378	AGC_PDK1	PK
F01_transcript_151392	RLK-Pelle_CrRLK1L-1	PK
F01_transcript_151420	WNK_NRBP	PK
F01_transcript_151464	CMGC_RCK	PK
F01_transcript_15153	RLK-Pelle_CR4L	PK
F01_transcript_151549	CMGC_GSK	PK
F01_transcript_151555	CAMK_AMPK	PK
F01_transcript_151604	STE_STE11	PK
F01_transcript_151607	RLK-Pelle_RLCK-IV	PK
F01_transcript_151608	RLK-Pelle_RKF3	PK
F01_transcript_151643	RLK-Pelle_DLSV	PK
F01_transcript_15166	AGC_RSK-2	PK
F01_transcript_15171	RLK-Pelle_RLCK-VIIa-1	PK
F01_transcript_151713	CAMK_CDPK	PK
F01_transcript_151729	RLK-Pelle_DLSV	PK
F01_transcript_151742	TKL_CTR1-DRK-2	PK
F01_transcript_15176	CAMK_CDPK	PK
F01_transcript_151777	TKL-Pl-6	PK
F01_transcript_151803	RLK-Pelle_RLCK-V	PK
F01_transcript_151819	RLK-Pelle_CrRLK1L-1	PK
F01_transcript_151886	RLK-Pelle_CrRLK1L-1	PK
F01_transcript_151996	CMGC_CLK	PK
F01_transcript_152004	RLK-Pelle_LRR-IV	PK
F01_transcript_152023	RLK-Pelle_DLSV	PK
F01_transcript_15212	RLK-Pelle_LRR-II	PK
F01_transcript_152378	RLK-Pelle_SD-2b	PK
F01_transcript_152545	RLK-Pelle_CrRLK1L-1	PK
F01_transcript_152548	CAMK_CDPK	PK
F01_transcript_15259	CMGC_DYRK-PRP4	PK
F01_transcript_152660	RLK-Pelle_DLSV	PK
F01_transcript_15272	TKL_CTR1-DRK-2	PK
F01_transcript_152833	CMGC_DYRK-PRP4	PK

F01_transcript_152906	RLK-Pelle_RLCK-IXb	PK
F01_transcript_15293	CMGC_MAPK	PK
F01_transcript_15299	RLK-Pelle_LRR-II	PK
F01_transcript_153021	AGC_RSK-2	PK
F01_transcript_153057	CMGC_CK2	PK
F01_transcript_153059	RLK-Pelle_DLSV	PK
F01_transcript_153071	CAMK_CDPK	PK
F01_transcript_153092	CAMK_CDPK	PK
F01_transcript_153096	RLK-Pelle_LRR-VIII-1	PK
F01_transcript_15310	CAMK_CDPK	PK
F01_transcript_153122	STE_STE11	PK
F01_transcript_153259	RLK-Pelle_DLSV	PK
F01_transcript_153262	RLK-Pelle_LRR-III	PK
F01_transcript_153278	RLK-Pelle_LRR-XII-1	PK
F01_transcript_15328	RLK-Pelle_LRR-IV	PK
F01_transcript_153432	RLK-Pelle_CR4L	PK
F01_transcript_153439	CAMK_OST1L	PK
F01_transcript_153462	WEE	PK
F01_transcript_15348	CK1_CK1	PK
F01_transcript_153543	TKL-Pl-6	PK
F01_transcript_153552	CK1_CK1	PK
F01_transcript_153808	CMGC_CLK	PK
F01_transcript_153814	RLK-Pelle_LRR-II	PK
F01_transcript_153832	STE_STE11	PK
F01_transcript_153848	WNK_NRBP	PK
F01_transcript_153910	RLK-Pelle_DLSV	PK
F01_transcript_153925	RLK-Pelle_WAK	PK
F01_transcript_153954	CMGC_SRPK	PK
F01_transcript_153985	RLK-Pelle_DLSV	PK
F01_transcript_154027	RLK-Pelle_LRR-XI-1	PK
F01_transcript_154078	STE_STE11	PK
F01_transcript_15411	CK1_CK1	PK
F01_transcript_15414	CK1_CK1-Pl	PK
F01_transcript_154258	RLK-Pelle_SD-2b	PK
F01_transcript_154303	RLK-Pelle_LRK10L-2	PK
F01_transcript_154338	RLK-Pelle_RLCK-IXb	PK
F01_transcript_15441	WNK_NRBP	PK
F01_transcript_154422	RLK-Pelle_LRR-VIII-1	PK
F01_transcript_15444	RLK-Pelle_LRR-II	PK
F01_transcript_15445	CK1_CK1	PK
F01_transcript_154550	STE_STE11	PK
F01_transcript_154639	RLK-Pelle_CrRLK1L-1	PK
F01_transcript_15466	RLK-Pelle_LRR-III	PK
F01_transcript_154673	RLK-Pelle_RLCK-V	PK
F01_transcript_154717	RLK-Pelle_DLSV	PK
F01_transcript_154749	RLK-Pelle_LRR-XII-1	PK
F01_transcript_154878	CMGC_CDK-CRK7-CDK9	PK
F01_transcript_154913	CMGC_CDK-Pl	PK
F01_transcript_154955	CAMK_AMPK	PK
F01_transcript_155000	RLK-Pelle_DLSV	PK
F01_transcript_155024	RLK-Pelle_RLCK-VIII	PK
F01_transcript_155156	RLK-Pelle_DLSV	PK
F01_transcript_155178	RLK-Pelle_RLCK-IXb	PK
F01_transcript_155180	CAMK_CAMKL-CHK1	PK

F01_transcript_155199	RLK-Pelle_WAK	PK
F01_transcript_155212	RLK-Pelle_RLCK-IXa	PK
F01_transcript_155223	TKL-Pl-4	PK
F01_transcript_155236	RLK-Pelle_SD-2b	PK
F01_transcript_155245	RLK-Pelle_RLCK-VIIa-2	PK
F01_transcript_155265	RLK-Pelle_LRR-XI-1	PK
F01_transcript_155298	RLK-Pelle_DLSV	PK
F01_transcript_155347	CMGC_CLK	PK
F01_transcript_15538	RLK-Pelle_LRR-II	PK
F01_transcript_155394	Group-Pl-3	PK
F01_transcript_15544	RLK-Pelle_RLCK-VIIa-1	PK
F01_transcript_15550	RLK-Pelle_LysM	PK
F01_transcript_155506	RLK-Pelle_DLSV	PK
F01_transcript_155514	RLK-Pelle_RLCK-VI	PK
F01_transcript_155525	CAMK_CDPK	PK
F01_transcript_155529	CAMK_AMPK	PK
F01_transcript_155589	RLK-Pelle_RLCK-IXa	PK
F01_transcript_15561	CAMK_CDPK	PK
F01_transcript_15566	RLK-Pelle_LysM	PK
F01_transcript_155703	RLK-Pelle_LRR-III	PK
F01_transcript_155786	RLK-Pelle_DLSV	PK
F01_transcript_155855	Group-Pl-4	PK
F01_transcript_15594	TKL-Pl-4	PK
F01_transcript_155946	RLK-Pelle_DLSV	PK
F01_transcript_15595	TKL-Pl-1	PK
F01_transcript_156022	CMGC_GSK	PK
F01_transcript_156040	CAMK_CDPK	PK
F01_transcript_156059	CMGC_CDK-PITSLRE	PK
F01_transcript_156139	RLK-Pelle_WAK_LRK10L-1	PK
F01_transcript_156275	TKL-Pl-4	PK
F01_transcript_156324	RLK-Pelle_LRR-Xa	PK
F01_transcript_156325	RLK-Pelle_LRR-VIII-1	PK
F01_transcript_156393	RLK-Pelle_DLSV	PK
F01_transcript_156492	AGC_RSK-2	PK
F01_transcript_156568	AGC_RSK-2	PK
F01_transcript_15660	RLK-Pelle_LRR-II	PK
F01_transcript_156626	CK1_CK1	PK
F01_transcript_156631	RLK-Pelle_LRK10L-2	PK
F01_transcript_156682	NEK	PK
F01_transcript_15670	AGC_RSK-2	PK
F01_transcript_156750	RLK-Pelle_RLCK-VI	PK
F01_transcript_156763	CMGC_CLK	PK
F01_transcript_156840	RLK-Pelle_LRR-IX	PK
F01_transcript_15688	RLK-Pelle_SD-2b	PK
F01_transcript_156918	RLK-Pelle_RLCK-VIIa-1	PK
F01_transcript_15692	STE_STE20-Fray	PK
F01_transcript_156954	RLK-Pelle_DLSV	PK
F01_transcript_156977	RLK-Pelle_RLCK-IXb	PK
F01_transcript_156997	RLK-Pelle_RLCK-XII-1	PK
F01_transcript_157000	STE_STE20-Fray	PK
F01_transcript_157048	STE_STE20-YSK	PK
F01_transcript_157075	CMGC_CK2	PK
F01_transcript_157129	RLK-Pelle_RLCK-VIIa-2	PK
F01_transcript_157150	TKL-Pl-4	PK
F01_transcript_157209	RLK-Pelle_RLCK-XII-1	PK

F01_transcript_157210	RLK-Pelle_LRR-XII-1	PK
F01_transcript_157254	WEE	PK
F01_transcript_157278	TKL-Pl-4	PK
F01_transcript_157292	RLK-Pelle_RLCK-Os	PK
F01_transcript_157299	CAMK_CAMKL-CHK1	PK
F01_transcript_157347	IRE1	PK
F01_transcript_157352	TKL-Pl-4	PK
F01_transcript_157355	CMGC_MAPK	PK
F01_transcript_157382	CMGC_RCK	PK
F01_transcript_157486	CAMK_CAMKL-CHK1	PK
F01_transcript_157519	CAMK_AMPK	PK
F01_transcript_157602	TKL-Pl-4	PK
F01_transcript_157652	RLK-Pelle_LRR-I-1	PK
F01_transcript_157654	CMGC_RCK	PK
F01_transcript_157662	RLK-Pelle_LRR-II	PK
F01_transcript_157689	TKL-Pl-5	PK
F01_transcript_157692	STE_STE11	PK
F01_transcript_157707	RLK-Pelle_RLCK-XII-1	PK
F01_transcript_157719	RLK-Pelle_WAK	PK
F01_transcript_157801	RLK-Pelle_DLSV	PK
F01_transcript_157944	NAK	PK
F01_transcript_158064	STE_STE11	PK
F01_transcript_158107	RLK-Pelle_WAK_LRK10L-1	PK
F01_transcript_158143	TKL-Pl-4	PK
F01_transcript_158154	RLK-Pelle_WAK_LRK10L-1	PK
F01_transcript_158165	RLK-Pelle_LRR-VI-1	PK
F01_transcript_158190	TKL-Pl-6	PK
F01_transcript_158198	RLK-Pelle_DLSV	PK
F01_transcript_158221	TKL_CTR1-DRK-1	PK
F01_transcript_15825	CK1_CK1	PK
F01_transcript_15827	RLK-Pelle_RLCK-VIIa-2	PK
F01_transcript_15832	CMGC_RCK	PK
F01_transcript_158322	RLK-Pelle_RLCK-V	PK
F01_transcript_158338	CAMK_CAMKL-CHK1	PK
F01_transcript_158408	CAMK_CDPK	PK
F01_transcript_158411	RLK-Pelle_DLSV	PK
F01_transcript_158416	CMGC_CDK-PITSLRE	PK
F01_transcript_158441	CAMK_CDPK	PK
F01_transcript_158495	RLK-Pelle_LRR-VIII-1	PK
F01_transcript_15858	RLK-Pelle_LysM	PK
F01_transcript_158683	CMGC_MAPK	PK
F01_transcript_158690	CAMK_OST1L	PK
F01_transcript_158712	CAMK_OST1L	PK
F01_transcript_158753	TKL_CTR1-DRK-2	PK
F01_transcript_158799	STE_STE11	PK
F01_transcript_158823	RLK-Pelle_LRR-II	PK
F01_transcript_158911	RLK-Pelle_LRR-VIII-1	PK
F01_transcript_158976	CK1_CK1-Pl	PK
F01_transcript_159012	RLK-Pelle_URK-1	PK
F01_transcript_15911	CK1_CK1	PK
F01_transcript_159129	RLK-Pelle_DLSV	PK
F01_transcript_159157	RLK-Pelle_LRR-VI-1	PK
F01_transcript_159230	RLK-Pelle_LRK10L-2	PK
F01_transcript_159238	RLK-Pelle_RLCK-IV	PK

F01_transcript_159243	CAMK_CDPK	PK
F01_transcript_159263	RLK-Pelle_LRK10L-2	PK
F01_transcript_159275	RLK-Pelle_L-LEC	PK
F01_transcript_15928	WNK_NRBP	PK
F01_transcript_159282	CAMK_CDPK	PK
F01_transcript_159284	CAMK_CDPK	PK
F01_transcript_159337	CAMK_OST1L	PK
F01_transcript_159349	CK1_CK1-Pl	PK
F01_transcript_15936	RLK-Pelle_LRR-XIIIa	PK
F01_transcript_159363	RLK-Pelle_RLCK-VIIa-1	PK
F01_transcript_159371	RLK-Pelle_RLCK-XII-2	PK
F01_transcript_15944	CMGC_MAPK	PK
F01_transcript_159490	CAMK_CDPK	PK
F01_transcript_159492	RLK-Pelle_DLSV	PK
F01_transcript_159546	RLK-Pelle_LRR-XII-1	PK
F01_transcript_159551	RLK-Pelle_LRR-XII-1	PK
F01_transcript_159571	CMGC_MAPK	PK
F01_transcript_159582	TKL-Pl-6	PK
F01_transcript_159609	RLK-Pelle_LRR-XI-1	PK
F01_transcript_159642	RLK-Pelle_RLCK-VI	PK
F01_transcript_159665	AGC_PDK1	PK
F01_transcript_159682	RLK-Pelle_RLCK-VIIa-2	PK
F01_transcript_159683	RLK-Pelle_LRR-VI-1	PK
F01_transcript_159699	WNK_NRBP	PK
F01_transcript_159704	RLK-Pelle_RLCK-VIIa-2	PK
F01_transcript_159768	CK1_CK1	PK
F01_transcript_159776	RLK-Pelle_DLSV	PK
F01_transcript_159888	CAMK_CAMKL-CHK1	PK
F01_transcript_159891	STE_STE7	PK
F01_transcript_15995	NEK	PK
F01_transcript_159952	AGC_RSK-2	PK
F01_transcript_159983	RLK-Pelle_LRR-VIII-1	PK
F01_transcript_160028	RLK-Pelle_DLSV	PK
F01_transcript_160080	RLK-Pelle_LRR-XI-1	PK
F01_transcript_160117	CMGC_RCK	PK
F01_transcript_160120	CAMK_CDPK	PK
F01_transcript_160194	RLK-Pelle_SD-2b	PK
F01_transcript_16023	NEK	PK
F01_transcript_160234	CK1_CK1-Pl	PK
F01_transcript_160268	RLK-Pelle_WAK	PK
F01_transcript_160303	RLK-Pelle_LRR-I-1	PK
F01_transcript_160322	AGC-Pl	PK
F01_transcript_160340	CMGC_GSKL	PK
F01_transcript_160375	CK1_CK1-Pl	PK
F01_transcript_160461	STE_STE20-Fray	PK
F01_transcript_160482	AGC_NDR	PK
F01_transcript_160508	RLK-Pelle_RLCK-VI	PK
F01_transcript_160517	CAMK_CDPK	PK
F01_transcript_160535	RLK-Pelle_LRR-XII-1	PK
F01_transcript_160570	RLK-Pelle_LRR-Xa	PK
F01_transcript_160576	RLK-Pelle_LRK10L-2	PK
F01_transcript_160624	RLK-Pelle_RLCK-VIIa-1	PK
F01_transcript_160654	TKL-Pl-4	PK
F01_transcript_160666	RLK-Pelle_RLCK-VIIa-1	PK
F01_transcript_160720	CMGC_RCK	PK

F01_transcript_160736	STE_STE11	PK
F01_transcript_160743	RLK-Pelle_LRR-VII-2	PK
F01_transcript_160786	TKL-Pl-6	PK
F01_transcript_16079	CMGC_DYRK-PRP4	PK
F01_transcript_160821	RLK-Pelle_RLCK-IXb	PK
F01_transcript_160828	TKL-Pl-5	PK
F01_transcript_16091	CAMK_CDPK	PK
F01_transcript_160957	CMGC_MAPK	PK
F01_transcript_160971	RLK-Pelle_LRR-XI-1	PK
F01_transcript_160990	RLK-Pelle_C-LEC	PK
F01_transcript_161006	RLK-Pelle_SD-2b	PK
F01_transcript_16103	RLK-Pelle_LysM	PK
F01_transcript_161109	RLK-Pelle_DLSV	PK
F01_transcript_161266	CMGC_SRPK	PK
F01_transcript_16127	TKL-Pl-1	PK
F01_transcript_161285	RLK-Pelle_LRR-VI-2	PK
F01_transcript_161312	RLK-Pelle_DLSV	PK
F01_transcript_161316	RLK-Pelle_CrRLK1L-1	PK
F01_transcript_161337	RLK-Pelle_RLCK-VIIa-1	PK
F01_transcript_161364	RLK-Pelle_RLCK-V	PK
F01_transcript_1614	STE_STE11	PK
F01_transcript_161403	RLK-Pelle_RLCK-VIIa-1	PK
F01_transcript_161438	STE_STE20-Pl	PK
F01_transcript_16147	RLK-Pelle_LysM	PK
F01_transcript_161499	CMGC_DYRK-PRP4	PK
F01_transcript_1615	RLK-Pelle_LRR-XI-1	PK
F01_transcript_161522	RLK-Pelle_LRR-VIII-1	PK
F01_transcript_161563	CAMK_CAMKL-CHK1	PK
F01_transcript_161589	CAMK_CDPK	PK
F01_transcript_161596	CAMK_CDPK	PK
F01_transcript_161648	RLK-Pelle_SD-2b	PK
F01_transcript_161657	RLK-Pelle_LRR-VIII-1	PK
F01_transcript_161696	RLK-Pelle_CrRLK1L-1	PK
F01_transcript_161722	RLK-Pelle_WAK_LRK10L-1	PK
F01_transcript_161728	RLK-Pelle_LRR-III	PK
F01_transcript_161747	TKL_CTR1-DRK-1	PK
F01_transcript_1618	CMGC_RCK	PK
F01_transcript_161808	STE_STE7	PK
F01_transcript_161845	CK1_CK1-Pl	PK
F01_transcript_161855	RLK-Pelle_WAK_LRK10L-1	PK
F01_transcript_161856	RLK-Pelle_PERK-1	PK
F01_transcript_16187	RLK-Pelle_LRR-I-1	PK
F01_transcript_161879	RLK-Pelle_RLCK-VIIa-2	PK
F01_transcript_161883	RLK-Pelle_CrRLK1L-1	PK
F01_transcript_161904	RLK-Pelle_RLCK-IXb	PK
F01_transcript_16195	CAMK_CDPK	PK
F01_transcript_161987	TKL-Pl-4	PK
F01_transcript_161991	RLK-Pelle_RLCK-VIIa-2	PK
F01_transcript_162033	RLK-Pelle_LRR-I-1	PK
F01_transcript_162041	CMGC_GSK	PK
F01_transcript_162060	TKL-Pl-4	PK
F01_transcript_162071	RLK-Pelle_DLSV	PK
F01_transcript_162110	TKL_CTR1-DRK-2	PK
F01_transcript_162117	RLK-Pelle_WAK	PK

F01_transcript_162128	RLK-Pelle_DLSV	PK
F01_transcript_162183	PEK_PEK	PK
F01_transcript_162189	RLK-Pelle_DLSV	PK
F01_transcript_162224	RLK-Pelle_LRR-XI-1	PK
F01_transcript_162288	CAMK_CAMKL-CHK1	PK
F01_transcript_162294	AGC_RSK-2	PK
F01_transcript_16230	CMGC_CDK-PITSLRE	PK
F01_transcript_162330	RLK-Pelle_RLCK-IV	PK
F01_transcript_16234	RLK-Pelle_LRR-III	PK
F01_transcript_162350	RLK-Pelle_RLCK-IXb	PK
F01_transcript_162392	RLK-Pelle_LRR-III	PK
F01_transcript_162393	CMGC_DYRK-YAK	PK
F01_transcript_16243	RLK-Pelle_LRR-VI-1	PK
F01_transcript_162488	RLK-Pelle_LRR-I-1	PK
F01_transcript_16251	CAMK_CDPK	PK
F01_transcript_162525	TTK	PK
F01_transcript_162549	PEK_PEK	PK
F01_transcript_162594	CAMK_CDPK	PK
F01_transcript_162615	AGC_RSK-2	PK
F01_transcript_162650	Group-Pl-4	PK
F01_transcript_162656	RLK-Pelle_DLSV	PK
F01_transcript_162678	RLK-Pelle_RLCK-VIIb	PK
F01_transcript_162739	STE_STE20-YSK	PK
F01_transcript_162765	RLK-Pelle_RKF3	PK
F01_transcript_16277	CAMK_CDPK	PK
F01_transcript_162834	RLK-Pelle_DLSV	PK
F01_transcript_162944	RLK-Pelle_DLSV	PK
F01_transcript_162955	RLK-Pelle_DLSV	PK
F01_transcript_163045	TKL_CTR1-DRK-2	PK
F01_transcript_163097	WNK_NRBP	PK
F01_transcript_163099	RLK-Pelle_LRR-VIII-1	PK
F01_transcript_163105	RLK-Pelle_RLCK-IXb	PK
F01_transcript_163202	RLK-Pelle_RLCK-IXb	PK
F01_transcript_163221	TKL-Pl-4	PK
F01_transcript_163239	RLK-Pelle_LRR-II	PK
F01_transcript_163251	RLK-Pelle_DLSV	PK
F01_transcript_163351	RLK-Pelle_DLSV	PK
F01_transcript_163383	RLK-Pelle_DLSV	PK
F01_transcript_163413	RLK-Pelle_SD-2b	PK
F01_transcript_163433	RLK-Pelle_LysM	PK
F01_transcript_163442	RLK-Pelle_RLCK-X	PK
F01_transcript_163510	CMGC_MAPK	PK
F01_transcript_163526	RLK-Pelle_LRR-Xb-1	PK
F01_transcript_163577	CMGC_MAPK	PK
F01_transcript_163579	RLK-Pelle_DLSV	PK
F01_transcript_16359	CAMK_CDPK	PK
F01_transcript_163591	STE_STE11	PK
F01_transcript_163692	RLK-Pelle_LRR-I-1	PK
F01_transcript_163718	RLK-Pelle_PERK-1	PK
F01_transcript_163730	RLK-Pelle_DLSV	PK
F01_transcript_163764	RLK-Pelle_CrRLK1L-1	PK
F01_transcript_163823	RLK-Pelle_LRR-V	PK
F01_transcript_163896	TKL-Pl-4	PK
F01_transcript_163936	CAMK_CDPK	PK
F01_transcript_163958	CMGC_MAPK	PK

F01_transcript_163971	RLK-Pelle_RLCK-V	PK
F01_transcript_164035	RLK-Pelle_PERK-1	PK
F01_transcript_164084	TKL-Pl-4	PK
F01_transcript_164089	RLK-Pelle_DLSV	PK
F01_transcript_164099	CAMK_CDPK	PK
F01_transcript_16410	RLK-Pelle_LRR-II	PK
F01_transcript_164104	RLK-Pelle_LRR-XII-1	PK
F01_transcript_164137	CAMK_AMPK	PK
F01_transcript_164141	RLK-Pelle_LRR-III	PK
F01_transcript_164152	RLK-Pelle_DLSV	PK
F01_transcript_164183	RLK-Pelle_LRR-II	PK
F01_transcript_16419	CAMK_CDPK	PK
F01_transcript_164197	CAMK_OST1L	PK
F01_transcript_1642	RLK-Pelle_LRR-VIII-1	PK
F01_transcript_16426	RLK-Pelle_RLCK-IXb	PK
F01_transcript_164263	CAMK_CAMKL-CHK1	PK
F01_transcript_164280	RLK-Pelle_RLCK-XII-1	PK
F01_transcript_164367	STE_STE11	PK
F01_transcript_164465	TKL-Pl-5	PK
F01_transcript_16466	RLK-Pelle_LRR-II	PK
F01_transcript_164660	RLK-Pelle_DLSV	PK
F01_transcript_164674	TKL_CTR1-DRK-2	PK
F01_transcript_164779	CAMK_CAMKL-CHK1	PK
F01_transcript_164816	RLK-Pelle_LRR-XIIIa	PK
F01_transcript_164843	RLK-Pelle_DLSV	PK
F01_transcript_164845	RLK-Pelle_RLCK-IV	PK
F01_transcript_164982	CAMK_CAMKL-CHK1	PK
F01_transcript_164984	CMGC_CDK-PITSLRE	PK
F01_transcript_164993	CAMK_OST1L	PK
F01_transcript_165000	CAMK_CDPK	PK
F01_transcript_165014	RLK-Pelle_SD-2b	PK
F01_transcript_165020	RLK-Pelle_LRK10L-2	PK
F01_transcript_165096	RLK-Pelle_DLSV	PK
F01_transcript_165198	RLK-Pelle_RLCK-IXb	PK
F01_transcript_165226	RLK-Pelle_RLCK-IV	PK
F01_transcript_165288	RLK-Pelle_WAK	PK
F01_transcript_165291	RLK-Pelle_DLSV	PK
F01_transcript_165316	RLK-Pelle_DLSV	PK
F01_transcript_165319	RLK-Pelle_DLSV	PK
F01_transcript_165329	RLK-Pelle_RLCK-X	PK
F01_transcript_165340	RLK-Pelle_DLSV	PK
F01_transcript_165349	CAMK_CAMKL-CHK1	PK
F01_transcript_165392	RLK-Pelle_LRR-III	PK
F01_transcript_165542	CMGC_CDK-PITSLRE	PK
F01_transcript_165554	RLK-Pelle_LysM	PK
F01_transcript_165590	CK1_CK1	PK
F01_transcript_165596	TTK	PK
F01_transcript_165637	RLK-Pelle_LRR-V	PK
F01_transcript_165653	RLK-Pelle_CrRLK1L-1	PK
F01_transcript_165660	RLK-Pelle_L-LEC	PK
F01_transcript_165716	RLK-Pelle_CrRLK1L-1	PK
F01_transcript_165726	RLK-Pelle_LRR-VIII-1	PK
F01_transcript_165822	STE_STE11	PK
F01_transcript_165891	STE_STE11	PK

F01_transcript_165945	CMGC_GSK	PK
F01_transcript_165968	RLK-Pelle_Extensin	PK
F01_transcript_165985	RLK-Pelle_RLCK-VIIa-1	PK
F01_transcript_166037	STE_STE11	PK
F01_transcript_166067	CMGC_DYRK-PRP4	PK
F01_transcript_166127	RLK-Pelle_DLSV	PK
F01_transcript_166137	CK1_CK1	PK
F01_transcript_16614	RLK-Pelle_L-LEC	PK
F01_transcript_16616	RLK-Pelle_DLSV	PK
F01_transcript_166188	CAMK_CDPK	PK
F01_transcript_166221	WNK_NRBP	PK
F01_transcript_166247	CMGC_RCK	PK
F01_transcript_166252	CMGC_CDK-CRK7-CDK9	PK
F01_transcript_166284	CMGC_CK2	PK
F01_transcript_166371	CMGC_GSK	PK
F01_transcript_166411	CMGC_CDK-CRK7-CDK9	PK
F01_transcript_16648	RLK-Pelle_LRR-II	PK
F01_transcript_16655	RLK-Pelle_L-LEC	PK
F01_transcript_166573	RLK-Pelle_LRR-III	PK
F01_transcript_166592	CAMK_AMPK	PK
F01_transcript_166603	CAMK_OST1L	PK
F01_transcript_166616	Group-Pl-3	PK
F01_transcript_166657	STE_STE11	PK
F01_transcript_166668	RLK-Pelle_PERK-1	PK
F01_transcript_16672	CAMK_CDPK	PK
F01_transcript_166725	RLK-Pelle_LRK10L-2	PK
F01_transcript_166726	RLK-Pelle_LRR-XI-1	PK
F01_transcript_166753	RLK-Pelle_LRR-XII-1	PK
F01_transcript_166789	RLK-Pelle_LRR-VI-1	PK
F01_transcript_16679	CAMK_CDPK	PK
F01_transcript_166873	CK1_CK1-Pl	PK
F01_transcript_1670	AGC_RSK-2	PK
F01_transcript_167010	RLK-Pelle_LRR-II	PK
F01_transcript_167062	RLK-Pelle_LRR-II	PK
F01_transcript_167083	CAMK_CAMKL-LKB	PK
F01_transcript_167105	CAMK_CDPK	PK
F01_transcript_167123	CK1_CK1-Pl	PK
F01_transcript_167124	CMGC_CDK-CRK7-CDK9	PK
F01_transcript_16714	RLK-Pelle_LRR-VI-2	PK
F01_transcript_167167	TKL_CTR1-DRK-2	PK
F01_transcript_167179	RLK-Pelle_RLCK-VIIa-2	PK
F01_transcript_167211	RLK-Pelle_LRR-XII-1	PK
F01_transcript_167237	RLK-Pelle_RLCK-VIIa-1	PK
F01_transcript_167279	RLK-Pelle_LRR-I-1	PK
F01_transcript_167346	RLK-Pelle_LRR-XI-1	PK
F01_transcript_167372	RLK-Pelle_PERK-1	PK
F01_transcript_167379	AGC_RSK-2	PK
F01_transcript_167399	RLK-Pelle_RLCK-VIIa-1	PK
F01_transcript_167446	RLK-Pelle_LRR-IX	PK
F01_transcript_167471	CAMK_CAMKL-CHK1	PK
F01_transcript_167506	RLK-Pelle_Extensin	PK
F01_transcript_167514	AGC_RSK-2	PK
F01_transcript_167557	RLK-Pelle_DLSV	PK
F01_transcript_167642	RLK-Pelle_LRR-II	PK
F01_transcript_167669	RLK-Pelle_L-LEC	PK

F01_transcript_167694	RLK-Pelle_RLCK-V	PK
F01_transcript_167707	TKL-Pl-5	PK
F01_transcript_167730	AGC_RSK-2	PK
F01_transcript_16777	RLK-Pelle_RLCK-VIIa-2	PK
F01_transcript_1678	RLK-Pelle_LRR-XII-1	PK
F01_transcript_16781	CAMK_OST1L	PK
F01_transcript_16812	CAMK_CDPK	PK
F01_transcript_16882	STE_STE20-Fray	PK
F01_transcript_16917	RLK-Pelle_LRR-III	PK
F01_transcript_16956	RLK-Pelle_RLCK-XI	PK
F01_transcript_16962	RLK-Pelle_LRR-III	PK
F01_transcript_16996	CMGC_MAPK	PK
F01_transcript_16997	RLK-Pelle_LRR-II	PK
F01_transcript_16998	CMGC_CDK-CRK7-CDK9	PK
F01_transcript_17023	RLK-Pelle_DLSV	PK
F01_transcript_17052	RLK-Pelle_LRR-Xb-1	PK
F01_transcript_17063	RLK-Pelle_DLSV	PK
F01_transcript_1707	CMGC_CLK	PK
F01_transcript_17093	STE_STE11	PK
F01_transcript_17102	RLK-Pelle_CrRLK1L-1	PK
F01_transcript_17132	RLK-Pelle_PERK-1	PK
F01_transcript_17155	CMGC_GSK	PK
F01_transcript_17209	RLK-Pelle_CrRLK1L-1	PK
F01_transcript_17240	CAMK_CDPK	PK
F01_transcript_17252	CAMK_CDPK	PK
F01_transcript_17310	RLK-Pelle_LRR-III	PK
F01_transcript_17318	RLK-Pelle_RLCK-VIIa-2	PK
F01_transcript_17336	RLK-Pelle_RLCK-VIIa-1	PK
F01_transcript_17359	TKL-Pl-1	PK
F01_transcript_17386	AGC_PDK1	PK
F01_transcript_17398	RLK-Pelle_RLCK-VIIa-1	PK
F01_transcript_17405	RLK-Pelle_C-LEC	PK
F01_transcript_17415	CAMK_CDPK	PK
F01_transcript_1743	CMGC_DYRK-YAK	PK
F01_transcript_17435	RLK-Pelle_LRR-VII-2	PK
F01_transcript_1746	RLK-Pelle_LRR-XII-1	PK
F01_transcript_1749	RLK-Pelle_LRR-XI-1	PK
F01_transcript_17551	CMGC_RCK	PK
F01_transcript_17559	RLK-Pelle_DLSV	PK
F01_transcript_17571	WNK_NRBP	PK
F01_transcript_17589	RLK-Pelle_RLCK-XI	PK
F01_transcript_17593	RLK-Pelle_DLSV	PK
F01_transcript_17625	TKL-Pl-3	PK
F01_transcript_17633	RLK-Pelle_RLCK-XI	PK
F01_transcript_17679	RLK-Pelle_LRR-Xa	PK
F01_transcript_17736	RLK-Pelle_RLCK-XII-1	PK
F01_transcript_17739	WNK_NRBP	PK
F01_transcript_17773	RLK-Pelle_PERK-1	PK
F01_transcript_17802	RLK-Pelle_LRR-III	PK
F01_transcript_17830	RLK-Pelle_WAK	PK
F01_transcript_17905	RLK-Pelle_WAK_LRK10L-1	PK
F01_transcript_18019	CAMK_CAMKL-LKB	PK
F01_transcript_18050	RLK-Pelle_DLSV	PK
F01_transcript_18070	RLK-Pelle_DLSV	PK

F01_transcript_18089	RLK-Pelle_RLCK-VIIa-2	PK
F01_transcript_18110	CK1_CK1	PK
F01_transcript_18111	RLK-Pelle_PERK-1	PK
F01_transcript_18124	CAMK_CDPK	PK
F01_transcript_1816	RLK-Pelle_RLCK-Os	PK
F01_transcript_18198	TKL-Pl-4	PK
F01_transcript_18212	NEK	PK
F01_transcript_18268	CAMK_CDPK	PK
F01_transcript_18269	STE_STE11	PK
F01_transcript_18278	AGC_PDK1	PK
F01_transcript_18333	RLK-Pelle_RLCK-VIIa-2	PK
F01_transcript_18354	RLK-Pelle_LRR-III	PK
F01_transcript_18360	RLK-Pelle_WAK	PK
F01_transcript_18377	CAMK_CDPK	PK
F01_transcript_18391	STE_STE11	PK
F01_transcript_18424	RLK-Pelle_LRR-III	PK
F01_transcript_18432	RLK-Pelle_LRR-II	PK
F01_transcript_1854	RLK-Pelle_LRR-XI-1	PK
F01_transcript_18552	RLK-Pelle_WAK	PK
F01_transcript_18555	CAMK_CDPK	PK
F01_transcript_18600	RLK-Pelle_WAK_LRK10L-1	PK
F01_transcript_18610	RLK-Pelle_LysM	PK
F01_transcript_18614	STE_STE11	PK
F01_transcript_18628	RLK-Pelle_L-LEC	PK
F01_transcript_18698	RLK-Pelle_LRR-II	PK
F01_transcript_18707	RLK-Pelle_LRR-XIIIa	PK
F01_transcript_18734	RLK-Pelle_WAK	PK
F01_transcript_1875	RLK-Pelle_LRR-VIII-1	PK
F01_transcript_18795	CMGC_SRPK	PK
F01_transcript_1880	RLK-Pelle_DLSV	PK
F01_transcript_18809	RLK-Pelle_DLSV	PK
F01_transcript_18867	RLK-Pelle_L-LEC	PK
F01_transcript_18922	STE_STE11	PK
F01_transcript_18924	RLK-Pelle_LRR-VI-2	PK
F01_transcript_1895	RLK-Pelle_LRR-XIIIb	PK
F01_transcript_18983	RLK-Pelle_RLCK-V	PK
F01_transcript_18984	RLK-Pelle_WAK	PK
F01_transcript_19010	RLK-Pelle_WAK	PK
F01_transcript_19041	RLK-Pelle_WAK_LRK10L-1	PK
F01_transcript_19055	RLK-Pelle_LRR-V	PK
F01_transcript_19082	RLK-Pelle_RLCK-VIIa-1	PK
F01_transcript_19168	RLK-Pelle_WAK	PK
F01_transcript_19171	RLK-Pelle_WAK	PK
F01_transcript_19273	CAMK_CDPK	PK
F01_transcript_19306	CK1_CK1	PK
F01_transcript_19314	RLK-Pelle_WAK_LRK10L-1	PK
F01_transcript_19385	RLK-Pelle_LRR-III	PK
F01_transcript_1939	RLK-Pelle_LRR-XII-1	PK
F01_transcript_19414	CAMK_CDPK	PK
F01_transcript_19415	RLK-Pelle_LRK10L-2	PK
F01_transcript_19417	CK1_CK1	PK
F01_transcript_19419	RLK-Pelle_LysM	PK
F01_transcript_19468	RLK-Pelle_RLCK-XII-1	PK
F01_transcript_19508	RLK-Pelle_RLCK-V	PK
F01_transcript_19529	NEK	PK

F01_transcript_19578	RLK-Pelle_RLCK-V	PK
F01_transcript_19603	RLK-Pelle_LysM	PK
F01_transcript_19645	RLK-Pelle_WAK	PK
F01_transcript_19665	CAMK_CDPK	PK
F01_transcript_19681	TKL-Pl-4	PK
F01_transcript_19691	RLK-Pelle_LRR-I-1	PK
F01_transcript_19787	RLK-Pelle_LRR-III	PK
F01_transcript_19839	RLK-Pelle_WAK_LRK10L-1	PK
F01_transcript_19852	TKL-Pl-4	PK
F01_transcript_19862	RLK-Pelle_WAK	PK
F01_transcript_19869	RLK-Pelle_WAK	PK
F01_transcript_19888	RLK-Pelle_LRR-Xb-1	PK
F01_transcript_19898	RLK-Pelle_RLCK-XI	PK
F01_transcript_19902	RLK-Pelle_RLCK-V	PK
F01_transcript_19942	CMGC_MAPK	PK
F01_transcript_19991	TKL-Pl-1	PK
F01_transcript_20026	RLK-Pelle_RLCK-V	PK
F01_transcript_20046	CMGC_MAPK	PK
F01_transcript_20075	RLK-Pelle_RLCK-VIIa-2	PK
F01_transcript_20081	RLK-Pelle_LRR-II	PK
F01_transcript_20099	RLK-Pelle_WAK_LRK10L-1	PK
F01_transcript_20113	RLK-Pelle_RLCK-XI	PK
F01_transcript_20154	CMGC_GSK	PK
F01_transcript_20174	CK1_CK1-Pl	PK
F01_transcript_20273	RLK-Pelle_LRR-III	PK
F01_transcript_20336	AGC_RSK-2	PK
F01_transcript_20343	CAMK_CAMKL-LKB	PK
F01_transcript_20370	RLK-Pelle_WAK_LRK10L-1	PK
F01_transcript_20384	RLK-Pelle_WAK	PK
F01_transcript_20422	RLK-Pelle_LRR-IV	PK
F01_transcript_20431	CMGC_MAPK	PK
F01_transcript_20480	RLK-Pelle_RLCK-V	PK
F01_transcript_20495	CAMK_CAMKL-CHK1	PK
F01_transcript_20508	RLK-Pelle_RLCK-V	PK
F01_transcript_20511	RLK-Pelle_RLCK-VIIa-2	PK
F01_transcript_20513	CMGC_MAPK	PK
F01_transcript_20527	RLK-Pelle_LRR-II	PK
F01_transcript_20602	RLK-Pelle_LRR-II	PK
F01_transcript_20634	RLK-Pelle_LRK10L-2	PK
F01_transcript_2064	RLK-Pelle_RLCK-VIIa-1	PK
F01_transcript_20648	STE_STE11	PK
F01_transcript_20713	CMGC_MAPK	PK
F01_transcript_20729	CAMK_CDPK	PK
F01_transcript_20731	CMGC_SRPK	PK
F01_transcript_20733	TKL-Pl-1	PK
F01_transcript_20770	CMGC_RCK	PK
F01_transcript_20782	RLK-Pelle_RLCK-XI	PK
F01_transcript_20793	TKL-Pl-4	PK
F01_transcript_20829	RLK-Pelle_LRK10L-2	PK
F01_transcript_20830	RLK-Pelle_LRR-VII-2	PK
F01_transcript_20834	CAMK_CAMKL-LKB	PK
F01_transcript_20838	CMGC_MAPK	PK
F01_transcript_20869	AGC_PDK1	PK
F01_transcript_20872	CMGC_CDK-CRK7-CDK9	PK

F01_transcript_20977	CAMK_CDPK	PK
F01_transcript_20987	STE_STE20-Fray	PK
F01_transcript_21086	TKL-Pl-1	PK
F01_transcript_21102	RLK-Pelle_LRK10L-2	PK
F01_transcript_21113	RLK-Pelle_DLSV	PK
F01_transcript_21130	RLK-Pelle_LRR-Xa	PK
F01_transcript_21135	RLK-Pelle_DLSV	PK
F01_transcript_21140	RLK-Pelle_CrRLK1L-1	PK
F01_transcript_21145	WNK_NRBP	PK
F01_transcript_21148	RLK-Pelle_DLSV	PK
F01_transcript_21154	CAMK_AMPK	PK
F01_transcript_21172	RLK-Pelle_L-LEC	PK
F01_transcript_21232	NEK	PK
F01_transcript_2124	AGC_MAST	PK
F01_transcript_21269	RLK-Pelle_LysM	PK
F01_transcript_21283	STE_STE20-Fray	PK
F01_transcript_21330	CAMK_CAMKL-CHK1	PK
F01_transcript_21496	TKL-Pl-1	PK
F01_transcript_21512	RLK-Pelle_LRR-Xa	PK
F01_transcript_21525	RLK-Pelle_DLSV	PK
F01_transcript_21558	TKL-Pl-4	PK
F01_transcript_21565	CMGC_CDK-CRK7-CDK9	PK
F01_transcript_21580	RLK-Pelle_RLCK-VIIa-1	PK
F01_transcript_21591	CMGC_GSK	PK
F01_transcript_21620	RLK-Pelle_LRR-Xa	PK
F01_transcript_21647	STE_STE11	PK
F01_transcript_21648	TKL-Pl-1	PK
F01_transcript_21650	RLK-Pelle_L-LEC	PK
F01_transcript_21689	RLK-Pelle_LRK10L-2	PK
F01_transcript_21805	RLK-Pelle_LRK10L-2	PK
F01_transcript_2182	CMGC_CDK-CRK7-CDK9	PK
F01_transcript_21829	RLK-Pelle_LRK10L-2	PK
F01_transcript_21840	CAMK_CDPK	PK
F01_transcript_21852	CAMK_CAMKL-CHK1	PK
F01_transcript_21880	RLK-Pelle_WAK	PK
F01_transcript_21931	RLK-Pelle_DLSV	PK
F01_transcript_21939	RLK-Pelle_LRK10L-2	PK
F01_transcript_21941	RLK-Pelle_LRR-IV	PK
F01_transcript_22088	RLK-Pelle_DLSV	PK
F01_transcript_22154	RLK-Pelle_LRR-II	PK
F01_transcript_22189	AGC_NDR	PK
F01_transcript_22216	TKL-Pl-4	PK
F01_transcript_22221	RLK-Pelle_RLCK-VIIa-2	PK
F01_transcript_22295	RLK-Pelle_LRR-XI-1	PK
F01_transcript_2232	RLK-Pelle_RLCK-IXb	PK
F01_transcript_22322	CAMK_CDPK	PK
F01_transcript_22333	RLK-Pelle_L-LEC	PK
F01_transcript_22341	RLK-Pelle_WAK_LRK10L-1	PK
F01_transcript_22363	RLK-Pelle_L-LEC	PK
F01_transcript_22449	RLK-Pelle_DLSV	PK
F01_transcript_22462	RLK-Pelle_LRR-Xa	PK
F01_transcript_22517	CAMK_CDPK	PK
F01_transcript_22535	CAMK_CDPK	PK
F01_transcript_2260	RLK-Pelle_LRR-Xb-1	PK
F01_transcript_22672	RLK-Pelle_DLSV	PK

F01_transcript_22743	CAMK_AMPK	PK
F01_transcript_22748	TKL-Pl-4	PK
F01_transcript_22750	RLK-Pelle_RLCK-VIIa-2	PK
F01_transcript_22762	RLK-Pelle_C-LEC	PK
F01_transcript_22774	RLK-Pelle_LysM	PK
F01_transcript_22795	RLK-Pelle_LRR-III	PK
F01_transcript_22847	CAMK_CDPK	PK
F01_transcript_22900	CAMK_CDPK	PK
F01_transcript_22926	CAMK_CDPK	PK
F01_transcript_22969	RLK-Pelle_DLSV	PK
F01_transcript_23013	CAMK_CAMKL-CHK1	PK
F01_transcript_23025	AGC_PDK1	PK
F01_transcript_23076	RLK-Pelle_LRK10L-2	PK
F01_transcript_23108	RLK-Pelle_RLCK-IXa	PK
F01_transcript_2317	AGC_RSK-2	PK
F01_transcript_23245	CAMK_CDPK	PK
F01_transcript_2325	RLK-Pelle_LRR-Xb-1	PK
F01_transcript_23308	RLK-Pelle_LRK10L-2	PK
F01_transcript_23321	CMGC_CLK	PK
F01_transcript_23383	RLK-Pelle_LRK10L-2	PK
F01_transcript_23395	RLK-Pelle_RLCK-V	PK
F01_transcript_23491	RLK-Pelle_C-LEC	PK
F01_transcript_23517	RLK-Pelle_RLCK-VIIa-1	PK
F01_transcript_23544	AGC_NDR	PK
F01_transcript_23601	RLK-Pelle_LRR-II	PK
F01_transcript_23644	RLK-Pelle_LRR-II	PK
F01_transcript_23659	CAMK_CDPK	PK
F01_transcript_23694	RLK-Pelle_LysM	PK
F01_transcript_23724	RLK-Pelle_LysM	PK
F01_transcript_23761	TKL-Pl-4	PK
F01_transcript_23797	RLK-Pelle_LRK10L-2	PK
F01_transcript_23803	RLK-Pelle_L-LEC	PK
F01_transcript_23849	RLK-Pelle_LRR-III	PK
F01_transcript_23852	RLK-Pelle_DLSV	PK
F01_transcript_23860	CAMK_CDPK	PK
F01_transcript_23889	AGC_PDK1	PK
F01_transcript_23908	AGC_NDR	PK
F01_transcript_2391	RLK-Pelle_LRR-VI-2	PK
F01_transcript_23924	RLK-Pelle_C-LEC	PK
F01_transcript_23951	RLK-Pelle_LRR-II	PK
F01_transcript_24089	CAMK_CDPK	PK
F01_transcript_24111	RLK-Pelle_LRK10L-2	PK
F01_transcript_24114	CK1_CK1	PK
F01_transcript_24119	RLK-Pelle_LRR-XII-1	PK
F01_transcript_24165	RLK-Pelle_LRK10L-2	PK
F01_transcript_24178	RLK-Pelle_LRR-Xa	PK
F01_transcript_24180	CMGC_RCK	PK
F01_transcript_24206	CAMK_CDPK	PK
F01_transcript_24209	RLK-Pelle_LRK10L-2	PK
F01_transcript_24249	RLK-Pelle_SD-2b	PK
F01_transcript_24255	AGC_NDR	PK
F01_transcript_24264	TKL-Pl-5	PK
F01_transcript_24273	RLK-Pelle_RKF3	PK
F01_transcript_2431	RLK-Pelle_Extensin	PK

F01_transcript_24310	RLK-Pelle_LRK10L-2	PK
F01_transcript_24332	RLK-Pelle_L-LEC	PK
F01_transcript_24361	RLK-Pelle_LRK10L-2	PK
F01_transcript_244	STE_STE11	PK
F01_transcript_24419	CAMK_CDPK	PK
F01_transcript_24434	RLK-Pelle_LRK10L-2	PK
F01_transcript_24440	CAMK_CDPK	PK
F01_transcript_24463	TKL-Pl-4	PK
F01_transcript_24476	RLK-Pelle_RLCK-XII-1	PK
F01_transcript_2448	RLK-Pelle_LRR-IX	PK
F01_transcript_2450	RLK-Pelle_RLCK-VIIa-2	PK
F01_transcript_24528	CMGC_CDK-CRK7-CDK9	PK
F01_transcript_24631	RLK-Pelle_LRR-II	PK
F01_transcript_24724	RLK-Pelle_LRR-II	PK
F01_transcript_24889	CAMK_CAMKL-CHK1	PK
F01_transcript_24937	TKL-Pl-1	PK
F01_transcript_25006	CMGC_CDK-CRK7-CDK9	PK
F01_transcript_25047	RLK-Pelle_LRR-VI-2	PK
F01_transcript_25093	CAMK_CAMKL-CHK1	PK
F01_transcript_25097	RLK-Pelle_LRK10L-2	PK
F01_transcript_25112	RLK-Pelle_DLSV	PK
F01_transcript_25119	RLK-Pelle_LRK10L-2	PK
F01_transcript_25122	CMGC_CDK-CRK7-CDK9	PK
F01_transcript_25261	RLK-Pelle_DLSV	PK
F01_transcript_25290	CAMK_CDPK	PK
F01_transcript_2535	RLK-Pelle_LRR-VII-2	PK
F01_transcript_25454	CK1_CK1	PK
F01_transcript_25505	RLK-Pelle_LRK10L-2	PK
F01_transcript_25567	CAMK_CDPK	PK
F01_transcript_25668	CMGC_CDK-CCRK	PK
F01_transcript_25692	CK1_CK1	PK
F01_transcript_25725	RLK-Pelle_SD-2b	PK
F01_transcript_2577	TKL_CTR1-DRK-2	PK
F01_transcript_25856	TKL-Pl-5	PK
F01_transcript_25909	CK1_CK1	PK
F01_transcript_2591	RLK-Pelle_LRR-Xb-1	PK
F01_transcript_25914	CAMK_CDPK	PK
F01_transcript_25925	CAMK_CAMKL-CHK1	PK
F01_transcript_25936	CMGC_CLK	PK
F01_transcript_25976	CK1_CK1	PK
F01_transcript_26021	RLK-Pelle_RLCK-VIIa-1	PK
F01_transcript_26036	RLK-Pelle_RLCK-VIIa-2	PK
F01_transcript_26037	AGC_NDR	PK
F01_transcript_26075	TKL-Pl-4	PK
F01_transcript_26105	AGC_NDR	PK
F01_transcript_26115	CMGC_MAPK	PK
F01_transcript_26219	RLK-Pelle_DLSV	PK
F01_transcript_2623	RLK-Pelle_LRR-VIII-1	PK
F01_transcript_26254	RLK-Pelle_PERK-1	PK
F01_transcript_26316	CMGC_CLK	PK
F01_transcript_26353	RLK-Pelle_LRK10L-2	PK
F01_transcript_26354	RLK-Pelle_RLCK-VI	PK
F01_transcript_26369	TKL-Pl-4	PK
F01_transcript_26399	RLK-Pelle_LRR-Xa	PK
F01_transcript_26400	RLK-Pelle_L-LEC	PK

F01_transcript_26485	RLK-Pelle_RLCK-V	PK
F01_transcript_26517	CAMK_CDPK	PK
F01_transcript_2652	CMGC_CDK-CRK7-CDK9	PK
F01_transcript_26552	RLK-Pelle_LRR-XII-1	PK
F01_transcript_26598	CMGC_RCK	PK
F01_transcript_26604	TKL-Pl-4	PK
F01_transcript_2663	RLK-Pelle_LRR-VII-2	PK
F01_transcript_26768	WNK_NRBP	PK
F01_transcript_2679	RLK-Pelle_RLCK-IXb	PK
F01_transcript_26815	RLK-Pelle_LRK10L-2	PK
F01_transcript_26821	CMGC_RCK	PK
F01_transcript_26901	RLK-Pelle_LRR-XI-1	PK
F01_transcript_27025	STE_STE7	PK
F01_transcript_2713	RLK-Pelle_LRR-XI-1	PK
F01_transcript_27183	WNK_NRBP	PK
F01_transcript_2732	RLK-Pelle_LRR-XI-1	PK
F01_transcript_27351	RLK-Pelle_CR4L	PK
F01_transcript_2744	AGC_RSK-2	PK
F01_transcript_27476	Group-Pl-4	PK
F01_transcript_27495	CMGC_GSK	PK
F01_transcript_27501	CAMK_CAMKL-CHK1	PK
F01_transcript_27518	RLK-Pelle_RKF3	PK
F01_transcript_27707	TKL-Pl-5	PK
F01_transcript_27744	RLK-Pelle_RLCK-VIIa-2	PK
F01_transcript_27749	RLK-Pelle_WAK_LRK10L-1	PK
F01_transcript_27753	RLK-Pelle_RLCK-XII-2	PK
F01_transcript_27791	RLK-Pelle_RLCK-XI	PK
F01_transcript_278	TKL-Pl-6	PK
F01_transcript_27833	RLK-Pelle_RLCK-XII-1	PK
F01_transcript_27837	RLK-Pelle_RLCK-VIIa-2	PK
F01_transcript_27891	CAMK_CAMKL-CHK1	PK
F01_transcript_2804	RLK-Pelle_LRR-XI-1	PK
F01_transcript_28065	AGC-Pl	PK
F01_transcript_28068	RLK-Pelle_DLSV	PK
F01_transcript_28069	CAMK_CAMKL-CHK1	PK
F01_transcript_28110	RLK-Pelle_RLCK-VIIa-2	PK
F01_transcript_28159	AGC-Pl	PK
F01_transcript_28206	RLK-Pelle_CR4L	PK
F01_transcript_2821	RLK-Pelle_CrRLK1L-1	PK
F01_transcript_28242	CMGC_RCK	PK
F01_transcript_28295	TKL-Pl-6	PK
F01_transcript_28331	CMGC_GSK	PK
F01_transcript_2836	TKL_CTR1-DRK-2	PK
F01_transcript_2839	STE_STE20-YSK	PK
F01_transcript_2842	RLK-Pelle_LRR-I-1	PK
F01_transcript_28427	Group-Pl-4	PK
F01_transcript_28433	CAMK_CAMKL-CHK1	PK
F01_transcript_28452	TKL-Pl-4	PK
F01_transcript_2848	RLK-Pelle_CrRLK1L-1	PK
F01_transcript_2850	RLK-Pelle_DLSV	PK
F01_transcript_28523	TKL-Pl-4	PK
F01_transcript_28617	RLK-Pelle_LRR-XII-1	PK
F01_transcript_28623	RLK-Pelle_RLCK-VI	PK
F01_transcript_28626	CAMK_CAMKL-CHK1	PK

F01_transcript_28631	CAMK_CAMKL-CHK1	PK
F01_transcript_2870	RLK-Pelle_CrRLK1L-1	PK
F01_transcript_28715	RLK-Pelle_RLCK-VIIa-1	PK
F01_transcript_28740	CAMK_CAMKL-CHK1	PK
F01_transcript_28753	RLK-Pelle_LRK10L-2	PK
F01_transcript_2877	RLK-Pelle_LRR-VII-1	PK
F01_transcript_28780	CAMK_CDPK	PK
F01_transcript_28938	TKL-Pl-4	PK
F01_transcript_28996	RLK-Pelle_LRR-VI-1	PK
F01_transcript_29050	CAMK_CDPK	PK
F01_transcript_29095	TKL-Pl-4	PK
F01_transcript_2913	RLK-Pelle_DLSV	PK
F01_transcript_29138	RLK-Pelle_LRK10L-2	PK
F01_transcript_2915	RLK-Pelle_LRR-XI-1	PK
F01_transcript_2921	RLK-Pelle_LRR-XIV	PK
F01_transcript_29231	STE_STE20-Fray	PK
F01_transcript_29307	RLK-Pelle_RLCK-VI	PK
F01_transcript_29308	TKL-Pl-4	PK
F01_transcript_29372	RLK-Pelle_RLCK-XII-1	PK
F01_transcript_29375	RLK-Pelle_RLCK-VIIa-2	PK
F01_transcript_29408	CMGC_GSK	PK
F01_transcript_29473	RLK-Pelle_RLCK-V	PK
F01_transcript_2950	RLK-Pelle_LRR-IX	PK
F01_transcript_29505	RLK-Pelle_SD-2b	PK
F01_transcript_29544	RLK-Pelle_RLCK-IV	PK
F01_transcript_29555	CMGC_CLK	PK
F01_transcript_2966	RLK-Pelle_LRR-VIII-1	PK
F01_transcript_29725	STE_STE7	PK
F01_transcript_29795	CAMK_OST1L	PK
F01_transcript_29799	CAMK_OST1L	PK
F01_transcript_29834	RLK-Pelle_RLCK-XII-1	PK
F01_transcript_29877	CMGC_GSK	PK
F01_transcript_29938	CAMK_CAMKL-CHK1	PK
F01_transcript_29975	CMGC_GSK	PK
F01_transcript_29985	CMGC_GSK	PK
F01_transcript_30042	TKL-Pl-5	PK
F01_transcript_30046	CMGC_MAPK	PK
F01_transcript_30168	CMGC_MAPK	PK
F01_transcript_30181	RLK-Pelle_LRR-XI-2	PK
F01_transcript_30198	CMGC_GSK	PK
F01_transcript_302	TKL-Pl-6	PK
F01_transcript_30206	RLK-Pelle_RLCK-XII-1	PK
F01_transcript_30229	CAMK_CAMKL-CHK1	PK
F01_transcript_3028	TKL_CTR1-DRK-2	PK
F01_transcript_30298	RLK-Pelle_RLCK-VIIa-2	PK
F01_transcript_30324	CAMK_OST1L	PK
F01_transcript_30330	CAMK_CDPK	PK
F01_transcript_30364	STE_STE20-Fray	PK
F01_transcript_3038	RLK-Pelle_Extensin	PK
F01_transcript_304	TKL-Pl-6	PK
F01_transcript_30404	CMGC_GSK	PK
F01_transcript_3043	RLK-Pelle_LRR-XI-1	PK
F01_transcript_3044	SCY1_SCYL2	PK
F01_transcript_30478	CMGC_CLK	PK
F01_transcript_30500	TKL-Pl-4	PK

F01_transcript_3051	RLK-Pelle_DLSV	PK
F01_transcript_30691	CAMK_CAMKL-CHK1	PK
F01_transcript_30702	CAMK_CDPK	PK
F01_transcript_30805	CAMK_CAMKL-CHK1	PK
F01_transcript_30847	CAMK_CAMKL-CHK1	PK
F01_transcript_30958	CAMK_CAMKL-CHK1	PK
F01_transcript_30967	RLK-Pelle_RLCK-XII-1	PK
F01_transcript_3100	RLK-Pelle_LRR-VIII-1	PK
F01_transcript_31019	CAMK_CAMKL-CHK1	PK
F01_transcript_31022	CMGC_MAPK	PK
F01_transcript_31127	RLK-Pelle_RLCK-VIIa-2	PK
F01_transcript_31134	CMGC_CDK-CRK7-CDK9	PK
F01_transcript_31210	RLK-Pelle_RLCK-V	PK
F01_transcript_31224	CAMK_CAMKL-CHK1	PK
F01_transcript_3127	RLK-Pelle_DLSV	PK
F01_transcript_31287	RLK-Pelle_RLCK-VIII	PK
F01_transcript_3130	RLK-Pelle_Singleton	PK
F01_transcript_31366	CAMK_AMPK	PK
F01_transcript_31372	CAMK_CAMKL-CHK1	PK
F01_transcript_3145	RLK-Pelle_LRR-VIII-1	PK
F01_transcript_31490	TKL-Pl-5	PK
F01_transcript_3152	RLK-Pelle_CrRLK1L-1	PK
F01_transcript_31541	RLK-Pelle_RLCK-VI	PK
F01_transcript_31592	TKL-Pl-4	PK
F01_transcript_31634	CMGC_MAPK	PK
F01_transcript_31716	CAMK_OST1L	PK
F01_transcript_3172	RLK-Pelle_LRR-XI-1	PK
F01_transcript_31800	RLK-Pelle_RLCK-VI	PK
F01_transcript_31805	CAMK_OST1L	PK
F01_transcript_31944	RLK-Pelle_RLCK-VI	PK
F01_transcript_3204	NAK	PK
F01_transcript_32079	TKL-Pl-4	PK
F01_transcript_3214	RLK-Pelle_LRR-XI-1	PK
F01_transcript_32156	CAMK_CDPK	PK
F01_transcript_32229	RLK-Pelle_RLCK-XII-1	PK
F01_transcript_3223	RLK-Pelle_LRR-VIII-1	PK
F01_transcript_32255	CAMK_CAMKL-CHK1	PK
F01_transcript_32269	CAMK_CAMKL-CHK1	PK
F01_transcript_3227	TKL_CTR1-DRK-2	PK
F01_transcript_32275	CMGC_MAPK	PK
F01_transcript_32281	RLK-Pelle_RLCK-VI	PK
F01_transcript_32354	CMGC_GSK	PK
F01_transcript_32443	CAMK_OST1L	PK
F01_transcript_32448	WEE	PK
F01_transcript_32477	CAMK_AMPK	PK
F01_transcript_32491	RLK-Pelle_RLCK-XII-1	PK
F01_transcript_32501	CAMK_OST1L	PK
F01_transcript_32504	RLK-Pelle_CR4L	PK
F01_transcript_3253	RLK-Pelle_LRR-VI-1	PK
F01_transcript_32536	CMGC_CLK	PK
F01_transcript_32584	CAMK_CAMKL-CHK1	PK
F01_transcript_32603	TKL-Pl-5	PK
F01_transcript_3270	RLK-Pelle_LRR-XI-1	PK
F01_transcript_32770	CAMK_CAMKL-CHK1	PK

F01_transcript_3278	RLK-Pelle_LRR-XI-1	PK
F01_transcript_3283	RLK-Pelle_LRR-XII-1	PK
F01_transcript_32879	CAMK_CAMKL-CHK1	PK
F01_transcript_32915	RLK-Pelle_RLCK-VIIa-2	PK
F01_transcript_32921	CMGC_MAPK	PK
F01_transcript_32925	RLK-Pelle_RLCK-XV	PK
F01_transcript_32930	CAMK_OST1L	PK
F01_transcript_3295	RLK-Pelle_LRR-VIII-1	PK
F01_transcript_32962	TKL-Pl-4	PK
F01_transcript_33003	RLK-Pelle_RLCK-VIII	PK
F01_transcript_3303	RLK-Pelle_CrRLK1L-1	PK
F01_transcript_33060	CAMK_CAMKL-CHK1	PK
F01_transcript_33063	TKL-Pl-4	PK
F01_transcript_331	TKL-Pl-6	PK
F01_transcript_33106	CMGC_GSK	PK
F01_transcript_33169	CAMK_CAMKL-CHK1	PK
F01_transcript_33332	RLK-Pelle_RLCK-VIIa-2	PK
F01_transcript_33339	RLK-Pelle_RLCK-V	PK
F01_transcript_3335	RLK-Pelle_LRR-VI-1	PK
F01_transcript_33441	CAMK_CAMKL-CHK1	PK
F01_transcript_33457	CMGC_SRPK	PK
F01_transcript_33494	CAMK_OST1L	PK
F01_transcript_33546	RLK-Pelle_RLCK-IV	PK
F01_transcript_33565	RLK-Pelle_RLCK-VIIa-2	PK
F01_transcript_33583	TKL-Pl-4	PK
F01_transcript_33617	RLK-Pelle_RLCK-VIIa-2	PK
F01_transcript_33639	CK1_CK1	PK
F01_transcript_3366	RLK-Pelle_DLSV	PK
F01_transcript_3372	RLK-Pelle_LRR-XI-1	PK
F01_transcript_33747	CK1_CK1	PK
F01_transcript_3376	RLK-Pelle_LRR-XI-1	PK
F01_transcript_3389	RLK-Pelle_DLSV	PK
F01_transcript_3398	RLK-Pelle_LRR-XI-1	PK
F01_transcript_340	TKL_CTR1-DRK-2	PK
F01_transcript_34004	Group-Pl-4	PK
F01_transcript_34027	RLK-Pelle_RLCK-VIIa-2	PK
F01_transcript_34048	RLK-Pelle_LRR-I-2	PK
F01_transcript_34058	RLK-Pelle_RLCK-VIIa-1	PK
F01_transcript_341	RLK-Pelle_DLSV	PK
F01_transcript_34121	CMGC_GSK	PK
F01_transcript_34168	CMGC_MAPK	PK
F01_transcript_34178	RLK-Pelle_LRR-I-2	PK
F01_transcript_34205	RLK-Pelle_RLCK-Os	PK
F01_transcript_34289	CAMK_OST1L	PK
F01_transcript_34359	RLK-Pelle_RLCK-VIII	PK
F01_transcript_34360	RLK-Pelle_RLCK-VIIa-2	PK
F01_transcript_34372	RLK-Pelle_RLCK-VI	PK
F01_transcript_34382	RLK-Pelle_RLCK-XII-1	PK
F01_transcript_3440	RLK-Pelle_LRR-IX	PK
F01_transcript_34460	Group-Pl-4	PK
F01_transcript_3450	PEK_PEK	PK
F01_transcript_3464	RLK-Pelle_DLSV	PK
F01_transcript_34641	STE_STE11	PK
F01_transcript_34661	RLK-Pelle_RLCK-XII-1	PK
F01_transcript_3473	RLK-Pelle_CrRLK1L-1	PK

F01_transcript_34744	CMGC_GSK	PK
F01_transcript_34787	RLK-Pelle_RLCK-VIIa-1	PK
F01_transcript_34885	RLK-Pelle_RLCK-VIIa-2	PK
F01_transcript_34937	CAMK_OST1L	PK
F01_transcript_34942	CMGC_GSK	PK
F01_transcript_34979	RLK-Pelle_LRR-I-2	PK
F01_transcript_35021	CMGC_CK2	PK
F01_transcript_35023	RLK-Pelle_RLCK-VIIa-2	PK
F01_transcript_35038	CAMK_CDPK	PK
F01_transcript_35128	CMGC_GSK	PK
F01_transcript_35180	CMGC_GSK	PK
F01_transcript_35191	CMGC_GSK	PK
F01_transcript_35193	RLK-Pelle_RLCK-XV	PK
F01_transcript_35196	TKL-Pl-4	PK
F01_transcript_35226	CMGC_MAPK	PK
F01_transcript_35294	RLK-Pelle_LRK10L-2	PK
F01_transcript_35300	CMGC_CLK	PK
F01_transcript_35324	RLK-Pelle_RLCK-VIIa-2	PK
F01_transcript_35331	RLK-Pelle_RLCK-VIII	PK
F01_transcript_35374	RLK-Pelle_RLCK-VIIa-2	PK
F01_transcript_35464	WNK_NRBP	PK
F01_transcript_35583	RLK-Pelle_LRR-VI-2	PK
F01_transcript_35658	CAMK_CAMKL-CHK1	PK
F01_transcript_3566	RLK-Pelle_PERK-1	PK
F01_transcript_35674	CMGC_GSK	PK
F01_transcript_3568	RLK-Pelle_DLSV	PK
F01_transcript_3570	RLK-Pelle_LRR-VIII-1	PK
F01_transcript_3571	RLK-Pelle_CrRLK1L-1	PK
F01_transcript_35716	RLK-Pelle_RLCK-VIIa-1	PK
F01_transcript_35732	CMGC_MAPK	PK
F01_transcript_3574	RLK-Pelle_DLSV	PK
F01_transcript_35741	RLK-Pelle_RLCK-VIIa-2	PK
F01_transcript_35790	RLK-Pelle_RLCK-VIIa-1	PK
F01_transcript_3590	RLK-Pelle_LRR-VIII-1	PK
F01_transcript_3593	RLK-Pelle_DLSV	PK
F01_transcript_35946	CMGC_GSK	PK
F01_transcript_35996	CMGC_MAPK	PK
F01_transcript_36012	RLK-Pelle_RLCK-VIIa-1	PK
F01_transcript_36039	RLK-Pelle_RLCK-VIIa-2	PK
F01_transcript_36047	RLK-Pelle_DLSV	PK
F01_transcript_36131	CMGC_MAPK	PK
F01_transcript_36260	RLK-Pelle_RLCK-VIIa-2	PK
F01_transcript_36301	RLK-Pelle_RLCK-XII-2	PK
F01_transcript_36316	RLK-Pelle_RLCK-VIIa-1	PK
F01_transcript_3635	SCY1_SCYL2	PK
F01_transcript_36373	RLK-Pelle_RLCK-VI	PK
F01_transcript_36450	CMGC_MAPK	PK
F01_transcript_3646	TKL_CTR1-DRK-2	PK
F01_transcript_3647	RLK-Pelle_LRR-XI-1	PK
F01_transcript_36532	CMGC_CK2	PK
F01_transcript_3683	TKL_CTR1-DRK-2	PK
F01_transcript_36834	CMGC_CK2	PK
F01_transcript_36910	CMGC_GSK	PK
F01_transcript_36941	RLK-Pelle_RLCK-Os	PK

F01_transcript_37028	RLK-Pelle_RLCK-VIIa-2	PK
F01_transcript_3715	RLK-Pelle_LRR-XI-1	PK
F01_transcript_3750	RLK-Pelle_DLSV	PK
F01_transcript_37603	RLK-Pelle_CrRLK1L-1	PK
F01_transcript_37654	CMGC_CLK	PK
F01_transcript_37782	CMGC_CLK	PK
F01_transcript_3781	RLK-Pelle_LRR-XI-1	PK
F01_transcript_37816	RLK-Pelle_Extensin	PK
F01_transcript_3784	RLK-Pelle_LRR-XI-1	PK
F01_transcript_37904	CMGC_CK2	PK
F01_transcript_381	TKL-Pl-6	PK
F01_transcript_3814	RLK-Pelle_LRR-VIII-1	PK
F01_transcript_38143	RLK-Pelle_RLCK-X	PK
F01_transcript_38223	RLK-Pelle_DLSV	PK
F01_transcript_3839	RLK-Pelle_LRR-VIII-1	PK
F01_transcript_3842	RLK-Pelle_LRR-VIII-1	PK
F01_transcript_38437	RLK-Pelle_RLCK-VIIa-2	PK
F01_transcript_38559	CMGC_MAPK	PK
F01_transcript_38626	RLK-Pelle_RLCK-IV	PK
F01_transcript_38641	AGC_RSK-2	PK
F01_transcript_38645	CAMK_CAMKL-CHK1	PK
F01_transcript_38703	CMGC_CK2	PK
F01_transcript_3884	RLK-Pelle_LRR-XI-1	PK
F01_transcript_3896	RLK-Pelle_LRR-VI-1	PK
F01_transcript_3897	RLK-Pelle_LRR-VI-1	PK
F01_transcript_39135	RLK-Pelle_RLCK-VIII	PK
F01_transcript_39167	RLK-Pelle_RLCK-VIIa-2	PK
F01_transcript_3919	RLK-Pelle_LRR-XI-1	PK
F01_transcript_39354	CMGC_CK2	PK
F01_transcript_39486	RLK-Pelle_DLSV	PK
F01_transcript_39547	AGC_RSK-2	PK
F01_transcript_3955	RLK-Pelle_LRR-VIII-1	PK
F01_transcript_39665	RLK-Pelle_RLCK-Os	PK
F01_transcript_39670	RLK-Pelle_RLCK-VIIb	PK
F01_transcript_39745	NAK	PK
F01_transcript_3986	RLK-Pelle_LRR-XII-1	PK
F01_transcript_40071	AGC_RSK-2	PK
F01_transcript_40246	CMGC_CDK-Pl	PK
F01_transcript_40294	WNK_NRBP	PK
F01_transcript_40310	RLK-Pelle_RLCK-VIIb	PK
F01_transcript_40368	RLK-Pelle_RLCK-VIIa-2	PK
F01_transcript_4040	RLK-Pelle_LRR-XI-1	PK
F01_transcript_40409	RLK-Pelle_URK-1	PK
F01_transcript_4055	RLK-Pelle_DLSV	PK
F01_transcript_40875	CMGC_CDK-Pl	PK
F01_transcript_4110	RLK-Pelle_LRR-VIII-1	PK
F01_transcript_41394	CMGC_CK2	PK
F01_transcript_41628	CAMK_OST1L	PK
F01_transcript_41649	STE_STE7	PK
F01_transcript_41675	CAMK_CDPK	PK
F01_transcript_4196	RLK-Pelle_CrRLK1L-1	PK
F01_transcript_41994	WNK_NRBP	PK
F01_transcript_4201	RLK-Pelle_LRR-VIII-1	PK
F01_transcript_4233	RLK-Pelle_LRR-VIII-1	PK
F01_transcript_42447	RLK-Pelle_L-LEC	PK

F01_transcript_42565	STE_STE7	PK
F01_transcript_4263	RLK-Pelle_DLSV	PK
F01_transcript_4281	STE_STE7	PK
F01_transcript_4298	RLK-Pelle_LRR-I-1	PK
F01_transcript_43176	CMGC_CDKL-Os	PK
F01_transcript_4348	RLK-Pelle_DLSV	PK
F01_transcript_436	TKL-Pl-6	PK
F01_transcript_43697	CAMK_CDPK	PK
F01_transcript_4459	CAMK_CAMKL-CHK1	PK
F01_transcript_44791	TKL-Pl-4	PK
F01_transcript_4503	RLK-Pelle_LRR-VII-1	PK
F01_transcript_452	STE_STE-Pl	PK
F01_transcript_4521	RLK-Pelle_LRR-XII-1	PK
F01_transcript_4527	RLK-Pelle_CrRLK1L-1	PK
F01_transcript_4542	RLK-Pelle_DLSV	PK
F01_transcript_4556	RLK-Pelle_LRR-XI-1	PK
F01_transcript_4682	RLK-Pelle_LRR-XI-1	PK
F01_transcript_4699	RLK-Pelle_LRR-XI-1	PK
F01_transcript_4710	RLK-Pelle_LRR-VIII-1	PK
F01_transcript_4753	AGC_RSK-2	PK
F01_transcript_4792	RLK-Pelle_LRR-I-1	PK
F01_transcript_480	RLK-Pelle_LRR-Xb-1	PK
F01_transcript_484	RLK-Pelle_CrRLK1L-1	PK
F01_transcript_4854	RLK-Pelle_LRR-XII-1	PK
F01_transcript_4886	RLK-Pelle_LRR-XI-1	PK
F01_transcript_4894	RLK-Pelle_LRR-XIV	PK
F01_transcript_4903	RLK-Pelle_PERK-1	PK
F01_transcript_4926	RLK-Pelle_LRR-VIII-1	PK
F01_transcript_495	TKL-Pl-6	PK
F01_transcript_49587	RLK-Pelle_DLSV	PK
F01_transcript_4981	RLK-Pelle_URK-1	PK
F01_transcript_4983	RLK-Pelle_LRR-XI-1	PK
F01_transcript_4989	RLK-Pelle_CrRLK1L-1	PK
F01_transcript_499	RLK-Pelle_LRR-VIII-1	PK
F01_transcript_5041	RLK-Pelle_LRR-XI-1	PK
F01_transcript_5058	RLK-Pelle_LRR-VIII-1	PK
F01_transcript_5076	TKL_CTR1-DRK-2	PK
F01_transcript_508	RLK-Pelle_LRR-III	PK
F01_transcript_5101	CMGC_CDK-CRK7-CDK9	PK
F01_transcript_5115	RLK-Pelle_LRR-XI-1	PK
F01_transcript_5124	RLK-Pelle_LRR-XI-1	PK
F01_transcript_51331	RLK-Pelle_RLCK-VI	PK
F01_transcript_51390	AGC-Pl	PK
F01_transcript_51399	RLK-Pelle_LRR-III	PK
F01_transcript_51456	CAMK_CDPK	PK
F01_transcript_51457	AGC_RSK-2	PK
F01_transcript_51465	RLK-Pelle_PERK-2	PK
F01_transcript_51479	RLK-Pelle_DLSV	PK
F01_transcript_5149	RLK-Pelle_CrRLK1L-1	PK
F01_transcript_51513	CMGC_CDK-PITSLRE	PK
F01_transcript_5154	RLK-Pelle_DLSV	PK
F01_transcript_51564	RLK-Pelle_SD-2b	PK
F01_transcript_51602	RLK-Pelle_LRR-Xb-1	PK
F01_transcript_51704	RLK-Pelle_DLSV	PK

F01_transcript_51763	RLK-Pelle_LRR-XI-1	PK
F01_transcript_51813	RLK-Pelle_RLCK-XII-1	PK
F01_transcript_51819	CAMK_CDPK	PK
F01_transcript_51821	RLK-Pelle_WAK_LRK10L-1	PK
F01_transcript_51824	CAMK_CAMKL-CHK1	PK
F01_transcript_51829	RLK-Pelle_LRR-II	PK
F01_transcript_51900	AGC_NDR	PK
F01_transcript_51951	CAMK_OST1L	PK
F01_transcript_51962	RLK-Pelle_L-LEC	PK
F01_transcript_51969	CAMK_CAMKL-CHK1	PK
F01_transcript_52029	CAMK_OST1L	PK
F01_transcript_52078	RLK-Pelle_RLCK-XII-2	PK
F01_transcript_52146	RLK-Pelle_RLCK-IXb	PK
F01_transcript_52151	CAMK_CAMKL-CHK1	PK
F01_transcript_52328	TKL-Pl-4	PK
F01_transcript_52376	RLK-Pelle_LRR-XI-1	PK
F01_transcript_5242	RLK-Pelle_SD-2b	PK
F01_transcript_52440	RLK-Pelle_LRR-II	PK
F01_transcript_52459	PEK_PEK	PK
F01_transcript_52490	CMGC_DYRK-PRP4	PK
F01_transcript_52506	RLK-Pelle_DLSV	PK
F01_transcript_52522	AGC_PDK1	PK
F01_transcript_52584	RLK-Pelle_DLSV	PK
F01_transcript_52604	CAMK_CDPK	PK
F01_transcript_52627	CMGC_CLK	PK
F01_transcript_52663	CAMK_CDPK	PK
F01_transcript_52733	RLK-Pelle_RLCK-VI	PK
F01_transcript_52752	RLK-Pelle_LysM	PK
F01_transcript_52776	TKL_CTR1-DRK-2	PK
F01_transcript_528	TKL-Pl-6	PK
F01_transcript_52818	CK1_CK1	PK
F01_transcript_52846	AGC_RSK-2	PK
F01_transcript_52865	RLK-Pelle_LRR-III	PK
F01_transcript_52875	TKL-Pl-4	PK
F01_transcript_52879	RLK-Pelle_LRR-II	PK
F01_transcript_5292	IRE1	PK
F01_transcript_52953	RLK-Pelle_LRR-XII-1	PK
F01_transcript_52989	WNK_NRBP	PK
F01_transcript_52995	CMGC_CDK-CRK7-CDK9	PK
F01_transcript_53014	RLK-Pelle_LRR-I-1	PK
F01_transcript_53106	RLK-Pelle_LRK10L-2	PK
F01_transcript_53108	RLK-Pelle_LRR-II	PK
F01_transcript_53119	AGC_PDK1	PK
F01_transcript_53206	CMGC_CLK	PK
F01_transcript_53213	RLK-Pelle_DLSV	PK
F01_transcript_53239	RLK-Pelle_RLCK-VIIa-1	PK
F01_transcript_53252	RLK-Pelle_DLSV	PK
F01_transcript_53321	CAMK_CDPK	PK
F01_transcript_53326	RLK-Pelle_RLCK-IV	PK
F01_transcript_53339	RLK-Pelle_DLSV	PK
F01_transcript_53404	RLK-Pelle_L-LEC	PK
F01_transcript_53423	CMGC_CLK	PK
F01_transcript_53470	RLK-Pelle_DLSV	PK
F01_transcript_53486	TKL-Pl-4	PK
F01_transcript_5357	RLK-Pelle_LRR-II	PK

F01_transcript_53607	RLK-Pelle_RLCK-IXa	PK
F01_transcript_53613	RLK-Pelle_CrRLK1L-1	PK
F01_transcript_53640	CMGC_CDK-CCRK	PK
F01_transcript_53651	RLK-Pelle_RLCK-XII-1	PK
F01_transcript_5369	CMGC_CDK-PITSLRE	PK
F01_transcript_53694	RLK-Pelle_LRR-VI-1	PK
F01_transcript_5375	TKL_CTR1-DRK-2	PK
F01_transcript_53822	RLK-Pelle_LysM	PK
F01_transcript_53844	RLK-Pelle_DLSV	PK
F01_transcript_53886	RLK-Pelle_LysM	PK
F01_transcript_53888	RLK-Pelle_RLCK-IV	PK
F01_transcript_53891	RLK-Pelle_LRR-Xb-1	PK
F01_transcript_53960	AGC_RSK-2	PK
F01_transcript_53989	STE_STE11	PK
F01_transcript_54050	RLK-Pelle_L-LEC	PK
F01_transcript_54125	RLK-Pelle_LRR-III	PK
F01_transcript_54200	RLK-Pelle_LRR-VIII-1	PK
F01_transcript_54202	CMGC_CLK	PK
F01_transcript_54207	WNK_NRBP	PK
F01_transcript_54268	CAMK_CDPK	PK
F01_transcript_54276	CMGC_CLK	PK
F01_transcript_54360	CMGC_MAPK	PK
F01_transcript_54363	AGC_NDR	PK
F01_transcript_5438	RLK-Pelle_LRR-VI-2	PK
F01_transcript_54413	RLK-Pelle_RLCK-VIII	PK
F01_transcript_5460	STE_STE11	PK
F01_transcript_54640	TKL_CTR1-DRK-2	PK
F01_transcript_5465	RLK-Pelle_LRR-IX	PK
F01_transcript_54653	RLK-Pelle_PERK-2	PK
F01_transcript_54681	CAMK_CAMKL-CHK1	PK
F01_transcript_54693	RLK-Pelle_DLSV	PK
F01_transcript_54709	STE_STE11	PK
F01_transcript_54718	RLK-Pelle_LRR-VIII-1	PK
F01_transcript_54761	RLK-Pelle_RLCK-VIIa-2	PK
F01_transcript_54783	RLK-Pelle_LRR-XI-1	PK
F01_transcript_54804	STE_STE20-Fray	PK
F01_transcript_54815	CAMK_CDPK	PK
F01_transcript_54855	RLK-Pelle_LRR-XIIIb	PK
F01_transcript_54873	RLK-Pelle_LRR-XII-1	PK
F01_transcript_54877	RLK-Pelle_LRR-VIII-1	PK
F01_transcript_54885	AGC_RSK-2	PK
F01_transcript_54910	RLK-Pelle_DLSV	PK
F01_transcript_54912	RLK-Pelle_RLCK-IV	PK
F01_transcript_54926	CMGC_CDK-CRK7-CDK9	PK
F01_transcript_54960	RLK-Pelle_CrRLK1L-1	PK
F01_transcript_55003	RLK-Pelle_LRK10L-2	PK
F01_transcript_55024	RLK-Pelle_DLSV	PK
F01_transcript_55107	RLK-Pelle_DLSV	PK
F01_transcript_5514	RLK-Pelle_CrRLK1L-1	PK
F01_transcript_55140	Group-Pl-4	PK
F01_transcript_55150	RLK-Pelle_LRR-VIII-1	PK
F01_transcript_55193	Group-Pl-4	PK
F01_transcript_5522	RLK-Pelle_LRR-XI-1	PK
F01_transcript_55256	CMGC_CDK-CCRK	PK

F01_transcript_55274	STE_STE11	PK
F01_transcript_55276	RLK-Pelle_WAK	PK
F01_transcript_55279	RLK-Pelle_LRR-I-2	PK
F01_transcript_55418	RLK-Pelle_DLSV	PK
F01_transcript_55445	RLK-Pelle_DLSV	PK
F01_transcript_55461	TKL-Pl-4	PK
F01_transcript_55465	CAMK_AMPK	PK
F01_transcript_55487	STE_STE11	PK
F01_transcript_55540	CK1_CK1	PK
F01_transcript_55542	STE_STE11	PK
F01_transcript_55576	RLK-Pelle_DLSV	PK
F01_transcript_55615	RLK-Pelle_RLCK-XII-1	PK
F01_transcript_55631	RLK-Pelle_LRK10L-2	PK
F01_transcript_55638	CAMK_CDPK	PK
F01_transcript_5570	CAMK_CAMKL-CHK1	PK
F01_transcript_55707	RLK-Pelle_RLCK-VIIa-1	PK
F01_transcript_55754	CK1_CK1	PK
F01_transcript_55783	RLK-Pelle_LRR-VIII-1	PK
F01_transcript_55795	RLK-Pelle_RLCK-V	PK
F01_transcript_55818	Group-Pl-4	PK
F01_transcript_55876	CAMK_OST1L	PK
F01_transcript_5590	RLK-Pelle_LRR-IX	PK
F01_transcript_55927	Group-Pl-3	PK
F01_transcript_55984	AGC_RSK-2	PK
F01_transcript_55995	RLK-Pelle_LRK10L-2	PK
F01_transcript_55997	CAMK_CAMKL-LKB	PK
F01_transcript_55999	RLK-Pelle_DLSV	PK
F01_transcript_56045	RLK-Pelle_LRR-VIII-1	PK
F01_transcript_56066	CMGC_GSK	PK
F01_transcript_5607	WNK_NRBP	PK
F01_transcript_56093	AGC_RSK-2	PK
F01_transcript_56140	TKL_CTR1-DRK-2	PK
F01_transcript_56163	RLK-Pelle_RLCK-V	PK
F01_transcript_56175	STE_STE11	PK
F01_transcript_56210	AGC_PDK1	PK
F01_transcript_56266	RLK-Pelle_LRR-III	PK
F01_transcript_56284	CMGC_CDK-CRK7-CDK9	PK
F01_transcript_56339	RLK-Pelle_LRR-II	PK
F01_transcript_56387	RLK-Pelle_LRK10L-2	PK
F01_transcript_56392	RLK-Pelle_DLSV	PK
F01_transcript_56396	RLK-Pelle_LRR-VII-2	PK
F01_transcript_56434	CMGC_CDK-PITSLRE	PK
F01_transcript_56465	CK1_CK1	PK
F01_transcript_56484	CK1_CK1	PK
F01_transcript_5651	RLK-Pelle_LRR-XI-1	PK
F01_transcript_5652	CMGC_CDK-PITSLRE	PK
F01_transcript_56524	CAMK_OST1L	PK
F01_transcript_56559	RLK-Pelle_LRK10L-2	PK
F01_transcript_56583	WNK_NRBP	PK
F01_transcript_56620	Group-Pl-4	PK
F01_transcript_56624	RLK-Pelle_WAK_LRK10L-1	PK
F01_transcript_56669	RLK-Pelle_LRR-II	PK
F01_transcript_56688	RLK-Pelle_PERK-2	PK
F01_transcript_56746	RLK-Pelle_RLCK-XII-1	PK
F01_transcript_56895	AGC_RSK-2	PK

F01_transcript_56896	RLK-Pelle_LRR-I-1	PK
F01_transcript_57021	RLK-Pelle_DLSV	PK
F01_transcript_57029	CAMK_CDPK	PK
F01_transcript_5707	RLK-Pelle_RLCK-V	PK
F01_transcript_57130	RLK-Pelle_WAK_LRK10L-1	PK
F01_transcript_57231	STE_STE11	PK
F01_transcript_57272	RLK-Pelle_RLCK-VIIa-2	PK
F01_transcript_5732	RLK-Pelle_LRR-XI-1	PK
F01_transcript_57356	RLK-Pelle_DLSV	PK
F01_transcript_57369	RLK-Pelle_LysM	PK
F01_transcript_57422	TKL-Pl-4	PK
F01_transcript_57426	RLK-Pelle_DLSV	PK
F01_transcript_57471	CMGC_MAPK	PK
F01_transcript_57555	RLK-Pelle_DLSV	PK
F01_transcript_57556	CK1_CK1-Pl	PK
F01_transcript_57574	RLK-Pelle_LRK10L-2	PK
F01_transcript_57619	AGC_PKA-PKG	PK
F01_transcript_57623	RLK-Pelle_DLSV	PK
F01_transcript_57787	Group-Pl-4	PK
F01_transcript_57797	TKL-Pl-5	PK
F01_transcript_5781	RLK-Pelle_LRR-I-1	PK
F01_transcript_57833	RLK-Pelle_LysM	PK
F01_transcript_5786	RLK-Pelle_DLSV	PK
F01_transcript_57870	RLK-Pelle_LRR-XII-1	PK
F01_transcript_57911	RLK-Pelle_LRR-XI-1	PK
F01_transcript_57913	CAMK_CDPK	PK
F01_transcript_57979	STE_STE11	PK
F01_transcript_58036	CAMK_CDPK	PK
F01_transcript_58073	CMGC_CDK-CRK7-CDK9	PK
F01_transcript_58114	RLK-Pelle_LRR-II	PK
F01_transcript_58124	RLK-Pelle_LRR-XI-1	PK
F01_transcript_58130	RLK-Pelle_RLCK-VIIa-2	PK
F01_transcript_5816	RLK-Pelle_LRR-VIII-1	PK
F01_transcript_58167	RLK-Pelle_LRR-VIII-1	PK
F01_transcript_58183	RLK-Pelle_LRR-III	PK
F01_transcript_58184	CMGC_MAPK	PK
F01_transcript_5820	CMGC_CDK-PITSLRE	PK
F01_transcript_58220	CMGC_CDK-CRK7-CDK9	PK
F01_transcript_58318	CK1_CK1	PK
F01_transcript_58371	RLK-Pelle_WAK_LRK10L-1	PK
F01_transcript_5838	RLK-Pelle_LRR-VIII-1	PK
F01_transcript_58411	AGC_RSK-2	PK
F01_transcript_58473	TKL_CTR1-DRK-1	PK
F01_transcript_5851	CMGC_CLK	PK
F01_transcript_58576	RLK-Pelle_RLCK-IXb	PK
F01_transcript_58652	CAMK_CAMKL-CHK1	PK
F01_transcript_58674	RLK-Pelle_DLSV	PK
F01_transcript_58768	RLK-Pelle_DLSV	PK
F01_transcript_58770	CMGC_GSKL	PK
F01_transcript_58826	RLK-Pelle_RLCK-VIIa-2	PK
F01_transcript_58998	CMGC_CLK	PK
F01_transcript_59019	RLK-Pelle_LRR-I-1	PK
F01_transcript_59140	TKL_CTR1-DRK-2	PK
F01_transcript_59228	TKL-Pl-4	PK

F01_transcript_59240	RLK-Pelle_WAK	PK
F01_transcript_5929	WNK_NRBP	PK
F01_transcript_59352	RLK-Pelle_LysM	PK
F01_transcript_59369	STE_STE11	PK
F01_transcript_59436	RLK-Pelle_LRR-III	PK
F01_transcript_59521	CAMK_CDPK	PK
F01_transcript_59541	TKL-Pl-4	PK
F01_transcript_59633	RLK-Pelle_LRR-II	PK
F01_transcript_59662	CK1_CK1-Pl	PK
F01_transcript_59738	CK1_CK1	PK
F01_transcript_59769	RLK-Pelle_RLCK-VIIa-2	PK
F01_transcript_59774	TKL-Pl-4	PK
F01_transcript_59780	AGC_RSK-2	PK
F01_transcript_59788	RLK-Pelle_WAK	PK
F01_transcript_59814	RLK-Pelle_L-LEC	PK
F01_transcript_59837	STE_STE11	PK
F01_transcript_59851	TKL-Pl-4	PK
F01_transcript_59884	RLK-Pelle_RLCK-IXb	PK
F01_transcript_5990	RLK-Pelle_LRR-XV	PK
F01_transcript_60006	RLK-Pelle_RLCK-VIIa-2	PK
F01_transcript_60078	RLK-Pelle_DLSV	PK
F01_transcript_60093	RLK-Pelle_LRR-VI-1	PK
F01_transcript_60101	RLK-Pelle_LRR-XII-1	PK
F01_transcript_60126	RLK-Pelle_LRR-Xb-1	PK
F01_transcript_60245	RLK-Pelle_RLCK-VIIa-2	PK
F01_transcript_60327	CAMK_CAMKL-CHK1	PK
F01_transcript_60418	RLK-Pelle_DLSV	PK
F01_transcript_60427	RLK-Pelle_LRR-Xb-1	PK
F01_transcript_60433	RLK-Pelle_LRK10L-2	PK
F01_transcript_60439	RLK-Pelle_LRR-III	PK
F01_transcript_60442	CMGC_MAPK	PK
F01_transcript_60451	RLK-Pelle_LRR-XI-1	PK
F01_transcript_60478	RLK-Pelle_LRR-VI-2	PK
F01_transcript_6051	RLK-Pelle_CrRLK1L-1	PK
F01_transcript_60554	RLK-Pelle_DLSV	PK
F01_transcript_60651	RLK-Pelle_DLSV	PK
F01_transcript_60725	RLK-Pelle_DLSV	PK
F01_transcript_60781	CAMK_CDPK	PK
F01_transcript_60815	RLK-Pelle_LRK10L-2	PK
F01_transcript_60837	STE_STE7	PK
F01_transcript_60874	STE_STE20-Fray	PK
F01_transcript_60924	CMGC_CLK	PK
F01_transcript_60928	TKL-Pl-4	PK
F01_transcript_6094	RLK-Pelle_LRR-VI-1	PK
F01_transcript_60974	RLK-Pelle_DLSV	PK
F01_transcript_61033	TKL-Pl-4	PK
F01_transcript_6106	TKL_CTR1-DRK-2	PK
F01_transcript_61085	CMGC_CK2	PK
F01_transcript_6109	TTK	PK
F01_transcript_61156	TKL-Pl-4	PK
F01_transcript_61203	RLK-Pelle_LRR-XI-1	PK
F01_transcript_61204	CMGC_CLK	PK
F01_transcript_61260	CMGC_MAPK	PK
F01_transcript_61277	RLK-Pelle_LRR-VI-1	PK
F01_transcript_61289	RLK-Pelle_DLSV	PK

F01_transcript_61312	RLK-Pelle_LRR-VIII-1	PK
F01_transcript_6135	RLK-Pelle_LRR-VIII-1	PK
F01_transcript_61434	TKL-Pl-6	PK
F01_transcript_61483	RLK-Pelle_DLSV	PK
F01_transcript_61571	RLK-Pelle_L-LEC	PK
F01_transcript_61627	RLK-Pelle_RLCK-IV	PK
F01_transcript_61696	RLK-Pelle_LRR-VI-2	PK
F01_transcript_61707	RLK-Pelle_RLCK-VIIa-1	PK
F01_transcript_61712	CAMK_CAMKL-CHK1	PK
F01_transcript_61772	WNK_NRBP	PK
F01_transcript_61792	CMGC_MAPK	PK
F01_transcript_61811	CAMK_CAMKL-CHK1	PK
F01_transcript_61813	RLK-Pelle_CrRLK1L-1	PK
F01_transcript_61838	RLK-Pelle_DLSV	PK
F01_transcript_6188	RLK-Pelle_LRR-V	PK
F01_transcript_619	TKL-Pl-4	PK
F01_transcript_61914	RLK-Pelle_RLCK-IV	PK
F01_transcript_61921	RLK-Pelle_LRR-XI-1	PK
F01_transcript_61960	RLK-Pelle_SD-2b	PK
F01_transcript_62012	RLK-Pelle_DLSV	PK
F01_transcript_62019	TKL-Pl-6	PK
F01_transcript_62090	RLK-Pelle_RLCK-VI	PK
F01_transcript_62091	RLK-Pelle_RLCK-VIII	PK
F01_transcript_62173	CAMK_CDPK	PK
F01_transcript_62205	TKL-Pl-4	PK
F01_transcript_62237	RLK-Pelle_LRR-VI-1	PK
F01_transcript_62263	CMGC_CLK	PK
F01_transcript_62281	RLK-Pelle_LRR-V	PK
F01_transcript_62327	TKL_CTR1-DRK-1	PK
F01_transcript_62346	TKL-Pl-6	PK
F01_transcript_62389	AGC_RSK-2	PK
F01_transcript_62405	RLK-Pelle_LRR-XII-1	PK
F01_transcript_6253	RLK-Pelle_DLSV	PK
F01_transcript_62541	WNK_NRBP	PK
F01_transcript_62564	RLK-Pelle_LysM	PK
F01_transcript_62570	STE_STE20-YSK	PK
F01_transcript_62681	STE_STE20-Fray	PK
F01_transcript_62746	RLK-Pelle_DLSV	PK
F01_transcript_6280	RLK-Pelle_CrRLK1L-1	PK
F01_transcript_62916	RLK-Pelle_RLCK-VIIa-1	PK
F01_transcript_62936	RLK-Pelle_LRR-II	PK
F01_transcript_62958	TKL-Pl-6	PK
F01_transcript_62991	RLK-Pelle_RLCK-VIIa-2	PK
F01_transcript_63025	CK1_CK1	PK
F01_transcript_6309	RLK-Pelle_LRR-XII-1	PK
F01_transcript_63106	RLK-Pelle_CrRLK1L-1	PK
F01_transcript_63156	AGC_NDR	PK
F01_transcript_63185	RLK-Pelle_DLSV	PK
F01_transcript_63257	RLK-Pelle_DLSV	PK
F01_transcript_63265	RLK-Pelle_RLCK-VI	PK
F01_transcript_63272	RLK-Pelle_DLSV	PK
F01_transcript_63275	RLK-Pelle_LysM	PK
F01_transcript_63299	RLK-Pelle_Extensin	PK
F01_transcript_63388	CMGC_CDK-PITSLRE	PK

F01_transcript_63451	RLK-Pelle_LRR-VII-2	PK
F01_transcript_63511	STE_STE11	PK
F01_transcript_63649	RLK-Pelle_LRR-VIII-1	PK
F01_transcript_63686	CK1_CK1-Pl	PK
F01_transcript_6374	RLK-Pelle_LRR-XI-1	PK
F01_transcript_63747	RLK-Pelle_LRR-XII-1	PK
F01_transcript_63754	RLK-Pelle_DLSV	PK
F01_transcript_63790	RLK-Pelle_LRR-XII-1	PK
F01_transcript_6384	RLK-Pelle_LRR-VI-1	PK
F01_transcript_63862	RLK-Pelle_LRR-VIII-1	PK
F01_transcript_63895	RLK-Pelle_LRR-II	PK
F01_transcript_63897	CAMK_CDPK	PK
F01_transcript_63923	RLK-Pelle_LRR-VI-1	PK
F01_transcript_64078	RLK-Pelle_DLSV	PK
F01_transcript_64138	RLK-Pelle_LRR-VI-1	PK
F01_transcript_64149	RLK-Pelle_LRK10L-2	PK
F01_transcript_64159	RLK-Pelle_LRR-V	PK
F01_transcript_64223	TKL-Pl-4	PK
F01_transcript_64256	RLK-Pelle_RLCK-IXb	PK
F01_transcript_64289	RLK-Pelle_RLCK-VIIa-1	PK
F01_transcript_6429	RLK-Pelle_CrRLK1L-1	PK
F01_transcript_64373	CMGC_CDK-Pl	PK
F01_transcript_64389	RLK-Pelle_LRR-III	PK
F01_transcript_64398	STE_STE20-YSK	PK
F01_transcript_64417	RLK-Pelle_DLSV	PK
F01_transcript_64418	TKL-Pl-6	PK
F01_transcript_64447	RLK-Pelle_LRR-Xa	PK
F01_transcript_64509	RLK-Pelle_LRR-VI-1	PK
F01_transcript_64512	RLK-Pelle_DLSV	PK
F01_transcript_64518	RLK-Pelle_DLSV	PK
F01_transcript_64532	RLK-Pelle_CrRLK1L-1	PK
F01_transcript_646	NEK	PK
F01_transcript_6462	STE_STE20-Fray	PK
F01_transcript_64621	RLK-Pelle_RLCK-XII-1	PK
F01_transcript_64629	RLK-Pelle_LRR-XI-1	PK
F01_transcript_64670	RLK-Pelle_CrRLK1L-1	PK
F01_transcript_64699	RLK-Pelle_RKF3	PK
F01_transcript_64721	CAMK_CDPK	PK
F01_transcript_64762	CAMK_CDPK	PK
F01_transcript_64778	RLK-Pelle_RLCK-VIIa-2	PK
F01_transcript_64848	TKL_CTR1-DRK-2	PK
F01_transcript_64874	CAMK_OST1L	PK
F01_transcript_64931	STE_STE11	PK
F01_transcript_65074	IRE1	PK
F01_transcript_65103	CMGC_CDK-CRK7-CDK9	PK
F01_transcript_65137	RLK-Pelle_LRR-VI-2	PK
F01_transcript_65141	STE_STE11	PK
F01_transcript_65155	RLK-Pelle_RLCK-IXb	PK
F01_transcript_65159	RLK-Pelle_DLSV	PK
F01_transcript_65193	RLK-Pelle_LRR-XI-1	PK
F01_transcript_65213	RLK-Pelle_LRR-VIII-1	PK
F01_transcript_65244	AGC-Pl	PK
F01_transcript_65254	RLK-Pelle_RLCK-IV	PK
F01_transcript_65263	RLK-Pelle_DLSV	PK
F01_transcript_65335	RLK-Pelle_LRR-VI-1	PK

F01_transcript_65336	STE_STE20-Fray	PK
F01_transcript_65349	TKL_CTR1-DRK-2	PK
F01_transcript_65409	RLK-Pelle_LRR-Xa	PK
F01_transcript_65447	RLK-Pelle_LRR-VIII-1	PK
F01_transcript_65478	RLK-Pelle_LRR-XI-1	PK
F01_transcript_65507	RLK-Pelle_WAK	PK
F01_transcript_65520	RLK-Pelle_LRK10L-2	PK
F01_transcript_6553	STE_STE11	PK
F01_transcript_65598	RLK-Pelle_LRR-Xa	PK
F01_transcript_65658	CAMK_CAMKL-CHK1	PK
F01_transcript_65755	CMGC_DYRK-PRP4	PK
F01_transcript_65828	AGC_MAST	PK
F01_transcript_65880	TKL-Pl-6	PK
F01_transcript_65964	RLK-Pelle_DLSV	PK
F01_transcript_65999	CMGC_GSK	PK
F01_transcript_66005	RLK-Pelle_RLCK-VI	PK
F01_transcript_66015	RLK-Pelle_LRR-VIII-1	PK
F01_transcript_66033	RLK-Pelle_RLCK-VIIa-2	PK
F01_transcript_66040	RLK-Pelle_LRR-I-1	PK
F01_transcript_66046	RLK-Pelle_LRR-XIIIa	PK
F01_transcript_66050	CMGC_GSK	PK
F01_transcript_66055	RLK-Pelle_WAK	PK
F01_transcript_66079	CAMK_OST1L	PK
F01_transcript_66080	RLK-Pelle_LRR-XI-1	PK
F01_transcript_66087	CMGC_CDK-CRK7-CDK9	PK
F01_transcript_6610	RLK-Pelle_CrRLK1L-1	PK
F01_transcript_66131	CMGC_RCK	PK
F01_transcript_66163	RLK-Pelle_DLSV	PK
F01_transcript_66172	RLK-Pelle_LRR-Xb-1	PK
F01_transcript_66192	RLK-Pelle_DLSV	PK
F01_transcript_66200	RLK-Pelle_RKF3	PK
F01_transcript_66206	RLK-Pelle_WAK	PK
F01_transcript_66222	CAMK_CAMKL-LKB	PK
F01_transcript_66228	TKL-Pl-5	PK
F01_transcript_66242	RLK-Pelle_LRR-XV	PK
F01_transcript_66246	RLK-Pelle_L-LEC	PK
F01_transcript_66351	TKL-Pl-4	PK
F01_transcript_66413	RLK-Pelle_CR4L	PK
F01_transcript_66439	RLK-Pelle_DLSV	PK
F01_transcript_66551	CAMK_CAMKL-CHK1	PK
F01_transcript_66574	CAMK_CDPK	PK
F01_transcript_6661	STE_STE20-Fray	PK
F01_transcript_66621	AGC_RSK-2	PK
F01_transcript_66640	RLK-Pelle_LRR-XI-1	PK
F01_transcript_6672	RLK-Pelle_RLCK-IXb	PK
F01_transcript_66806	NEK	PK
F01_transcript_66823	RLK-Pelle_DLSV	PK
F01_transcript_66967	RLK-Pelle_RLCK-V	PK
F01_transcript_67014	CAMK_CDPK	PK
F01_transcript_67029	RLK-Pelle_LRR-II	PK
F01_transcript_67074	STE_STE20-Pl	PK
F01_transcript_67100	CAMK_CDPK	PK
F01_transcript_67168	CAMK_CDPK	PK
F01_transcript_67178	CMGC_CDK-CDK8	PK

F01_transcript_6721	CMGC_CDK-PITSLRE	PK
F01_transcript_67222	RLK-Pelle_LRR-V	PK
F01_transcript_67237	Group-Pl-4	PK
F01_transcript_67299	TKL-Pl-4	PK
F01_transcript_67318	CAMK_CAMKL-CHK1	PK
F01_transcript_67322	AGC_RSK-2	PK
F01_transcript_6737	CMGC_MAPK	PK
F01_transcript_67454	RLK-Pelle_SD-2b	PK
F01_transcript_67523	CAMK_OST1L	PK
F01_transcript_67542	TKL-Pl-3	PK
F01_transcript_67589	WNK_NRBP	PK
F01_transcript_67606	RLK-Pelle_DLSV	PK
F01_transcript_67620	CMGC_RCK	PK
F01_transcript_67642	TKL-Pl-4	PK
F01_transcript_67664	CAMK_CDPK	PK
F01_transcript_67672	AGC_NDR	PK
F01_transcript_67761	CMGC_CDK-CRK7-CDK9	PK
F01_transcript_67855	CAMK_CDPK	PK
F01_transcript_6788	TKL_CTR1-DRK-2	PK
F01_transcript_6790	RLK-Pelle_LRR-VIII-1	PK
F01_transcript_67924	RLK-Pelle_LRR-XI-1	PK
F01_transcript_67941	RLK-Pelle_DLSV	PK
F01_transcript_67978	TKL-Pl-5	PK
F01_transcript_67982	STE_STE20-Fray	PK
F01_transcript_6808	RLK-Pelle_CrRLK1L-1	PK
F01_transcript_68153	RLK-Pelle_CrRLK1L-1	PK
F01_transcript_68180	RLK-Pelle_DLSV	PK
F01_transcript_68184	TKL-Pl-4	PK
F01_transcript_68203	CMGC_CDK-CDK8	PK
F01_transcript_68204	RLK-Pelle_LRR-XI-1	PK
F01_transcript_68243	RLK-Pelle_RLCK-IXb	PK
F01_transcript_6826	RLK-Pelle_LRR-VIII-1	PK
F01_transcript_68285	STE_STE20-Fray	PK
F01_transcript_68289	CAMK_CAMKL-CHK1	PK
F01_transcript_68343	RLK-Pelle_WAK	PK
F01_transcript_6839	RLK-Pelle_CrRLK1L-1	PK
F01_transcript_68472	TKL-Pl-4	PK
F01_transcript_6849	STE_STE11	PK
F01_transcript_68501	TKL-Pl-5	PK
F01_transcript_68516	TKL-Pl-1	PK
F01_transcript_68523	RLK-Pelle_LRR-XI-2	PK
F01_transcript_68529	TKL_CTR1-DRK-2	PK
F01_transcript_68543	RLK-Pelle_DLSV	PK
F01_transcript_68605	CMGC_CDK-PITSLRE	PK
F01_transcript_68621	CMGC_RCK	PK
F01_transcript_68721	RLK-Pelle_Extensin	PK
F01_transcript_68749	CMGC_CLK	PK
F01_transcript_68750	TKL-Pl-4	PK
F01_transcript_68757	RLK-Pelle_RLCK-VI	PK
F01_transcript_68800	RLK-Pelle_LRR-III	PK
F01_transcript_68826	RLK-Pelle_L-LEC	PK
F01_transcript_68836	RLK-Pelle_LRR-VIII-1	PK
F01_transcript_68860	RLK-Pelle_LRR-VIII-1	PK
F01_transcript_68874	RLK-Pelle_LRR-II	PK
F01_transcript_68888	RLK-Pelle_DLSV	PK

F01_transcript_68894	CMGC_CDK-CRK7-CDK9	PK
F01_transcript_68911	RLK-Pelle_LRR-VIII-1	PK
F01_transcript_68947	CK1_CK1-Pl	PK
F01_transcript_6900	RLK-Pelle_DLSV	PK
F01_transcript_69004	RLK-Pelle_DLSV	PK
F01_transcript_69114	TTK	PK
F01_transcript_69286	CMGC_GSK	PK
F01_transcript_69296	CAMK_CDPK	PK
F01_transcript_69350	CMGC_GSKL	PK
F01_transcript_69360	WNK_NRBP	PK
F01_transcript_69392	RLK-Pelle_LRR-II	PK
F01_transcript_69417	TKL-Pl-4	PK
F01_transcript_69457	STE_STE7	PK
F01_transcript_69512	RLK-Pelle_RLCK-VIIa-2	PK
F01_transcript_69538	CMGC_CDK-CRK7-CDK9	PK
F01_transcript_69558	CMGC_GSK	PK
F01_transcript_69599	CAMK_OST1L	PK
F01_transcript_69602	STE_STE20-Fray	PK
F01_transcript_69613	TKL_Gdt	PK
F01_transcript_69647	RLK-Pelle_SD-2b	PK
F01_transcript_69672	RLK-Pelle_DLSV	PK
F01_transcript_69677	RLK-Pelle_DLSV	PK
F01_transcript_69871	RLK-Pelle_L-LEC	PK
F01_transcript_69912	RLK-Pelle_SD-2b	PK
F01_transcript_69914	TKL_CTR1-DRK-2	PK
F01_transcript_70032	RLK-Pelle_LRR-VI-2	PK
F01_transcript_7012	RLK-Pelle_DLSV	PK
F01_transcript_70146	RLK-Pelle_DLSV	PK
F01_transcript_70198	RLK-Pelle_DLSV	PK
F01_transcript_70267	AGC_RSK-2	PK
F01_transcript_70284	STE_STE11	PK
F01_transcript_70293	RLK-Pelle_DLSV	PK
F01_transcript_70404	RLK-Pelle_CrRLK1L-1	PK
F01_transcript_70441	CMGC_GSK	PK
F01_transcript_70449	CMGC_CDK-PITSLRE	PK
F01_transcript_70519	RLK-Pelle_RLCK-VIIa-2	PK
F01_transcript_70544	AGC_NDR	PK
F01_transcript_70658	RLK-Pelle_RLCK-IXb	PK
F01_transcript_70712	RLK-Pelle_DLSV	PK
F01_transcript_70775	RLK-Pelle_CrRLK1L-1	PK
F01_transcript_70820	RLK-Pelle_LRR-VIII-1	PK
F01_transcript_70826	RLK-Pelle_LRR-XII-1	PK
F01_transcript_70836	RLK-Pelle_RLCK-IV	PK
F01_transcript_70851	RLK-Pelle_LRR-Xa	PK
F01_transcript_70864	RLK-Pelle_LRR-VIII-1	PK
F01_transcript_70901	CK1_CK1-Pl	PK
F01_transcript_70916	RLK-Pelle_DLSV	PK
F01_transcript_70961	RLK-Pelle_SD-2b	PK
F01_transcript_70987	CAMK_CAMKL-CHK1	PK
F01_transcript_71037	RLK-Pelle_CrRLK1L-1	PK
F01_transcript_71048	CK1_CK1	PK
F01_transcript_7109	RLK-Pelle_LRR-XI-1	PK
F01_transcript_71102	RLK-Pelle_DLSV	PK
F01_transcript_71128	STE_STE11	PK

F01_transcript_71148	RLK-Pelle_LRR-XI-1	PK
F01_transcript_7116	RLK-Pelle_DLSV	PK
F01_transcript_71187	RLK-Pelle_Extensin	PK
F01_transcript_71196	RLK-Pelle_DLSV	PK
F01_transcript_712	RLK-Pelle_LRR-Xb-1	PK
F01_transcript_7121	RLK-Pelle_LRR-VIII-1	PK
F01_transcript_71243	RLK-Pelle_Extensin	PK
F01_transcript_71296	STE_STE11	PK
F01_transcript_7139	RLK-Pelle_LRR-V	PK
F01_transcript_71402	RLK-Pelle_DLSV	PK
F01_transcript_71427	NAK	PK
F01_transcript_71504	RLK-Pelle_LRR-III	PK
F01_transcript_71508	WNK_NRBP	PK
F01_transcript_71524	RLK-Pelle_LRR-II	PK
F01_transcript_71656	STE_STE11	PK
F01_transcript_71667	RLK-Pelle_LRR-Xb-1	PK
F01_transcript_7179	CK1_CK1-Pl	PK
F01_transcript_71804	CK1_CK1-Pl	PK
F01_transcript_71898	TKL-Pl-6	PK
F01_transcript_71932	RLK-Pelle_LRR-VII-1	PK
F01_transcript_71953	CMGC_MAPK	PK
F01_transcript_72031	RLK-Pelle_Extensin	PK
F01_transcript_72041	RLK-Pelle_LRR-II	PK
F01_transcript_72093	RLK-Pelle_RLCK-VI	PK
F01_transcript_72100	TKL-Pl-4	PK
F01_transcript_72111	RLK-Pelle_RLCK-VIIa-1	PK
F01_transcript_72181	AGC_RSK-2	PK
F01_transcript_72210	RLK-Pelle_DLSV	PK
F01_transcript_72221	RLK-Pelle_RLCK-VIIa-2	PK
F01_transcript_72228	STE_STE11	PK
F01_transcript_72254	CK1_CK1	PK
F01_transcript_72328	RLK-Pelle_RLCK-IXb	PK
F01_transcript_72358	CAMK_CAMKL-CHK1	PK
F01_transcript_72408	RLK-Pelle_LRR-I-2	PK
F01_transcript_72441	CMGC_GSK	PK
F01_transcript_72458	TKL-Pl-2	PK
F01_transcript_72460	CMGC_MAPK	PK
F01_transcript_72486	RLK-Pelle_RLCK-VIIa-2	PK
F01_transcript_72498	RLK-Pelle_LRR-I-1	PK
F01_transcript_72508	NEK	PK
F01_transcript_72536	CMGC_CLK	PK
F01_transcript_72590	RLK-Pelle_CR4L	PK
F01_transcript_72596	STE_STE11	PK
F01_transcript_7261	RLK-Pelle_RLCK-IXb	PK
F01_transcript_72662	CMGC_CK2	PK
F01_transcript_72669	WNK_NRBP	PK
F01_transcript_72710	RLK-Pelle_RLCK-VI	PK
F01_transcript_72736	CMGC_DYRK-PRP4	PK
F01_transcript_72749	RLK-Pelle_SD-2b	PK
F01_transcript_72753	AGC_MAST	PK
F01_transcript_7282	CMGC_CK2	PK
F01_transcript_72907	RLK-Pelle_LRR-XI-1	PK
F01_transcript_72911	RLK-Pelle_RLCK-IXb	PK
F01_transcript_72982	RLK-Pelle_LRR-IX	PK
F01_transcript_73002	STE_STE11	PK

F01_transcript_73009	CMGC_DYRK-PRP4	PK
F01_transcript_73050	PEK_PEK	PK
F01_transcript_73122	RLK-Pelle_DLSV	PK
F01_transcript_7317	IRE1	PK
F01_transcript_73175	STE_STE20-Fray	PK
F01_transcript_73382	RLK-Pelle_RLCK-VIIa-2	PK
F01_transcript_7354	RLK-Pelle_LRK10L-2	PK
F01_transcript_73576	RLK-Pelle_WAK_LRK10L-1	PK
F01_transcript_73632	STE_STE11	PK
F01_transcript_73664	CMGC_SRPK	PK
F01_transcript_73708	TKL-Pl-4	PK
F01_transcript_73778	RLK-Pelle_LRR-I-1	PK
F01_transcript_73800	RLK-Pelle_LRR-II	PK
F01_transcript_73840	AGC_RSK-2	PK
F01_transcript_73873	CMGC_MAPK	PK
F01_transcript_73892	RLK-Pelle_RLCK-V	PK
F01_transcript_74027	CMGC_GSK	PK
F01_transcript_74058	TKL-Pl-4	PK
F01_transcript_74068	AGC_RSK-2	PK
F01_transcript_74070	CAMK_AMPK	PK
F01_transcript_74119	RLK-Pelle_RLCK-IV	PK
F01_transcript_74143	RLK-Pelle_RLCK-IXb	PK
F01_transcript_74155	RLK-Pelle_LRR-IX	PK
F01_transcript_74162	RLK-Pelle_LRR-XI-1	PK
F01_transcript_74279	RLK-Pelle_WAK	PK
F01_transcript_7428	RLK-Pelle_PERK-2	PK
F01_transcript_74298	RLK-Pelle_LRR-VI-2	PK
F01_transcript_74312	TKL_CTR1-DRK-2	PK
F01_transcript_74354	AGC_NDR	PK
F01_transcript_74413	RLK-Pelle_RLCK-VIIa-2	PK
F01_transcript_74419	RLK-Pelle_DLSV	PK
F01_transcript_74437	CMGC_CK2	PK
F01_transcript_74448	STE_STE20-Fray	PK
F01_transcript_74500	RLK-Pelle_RLCK-II	PK
F01_transcript_74501	RLK-Pelle_L-LEC	PK
F01_transcript_74513	RLK-Pelle_RLCK-XII-1	PK
F01_transcript_74515	CK1_CK1	PK
F01_transcript_74553	CK1_CK1-Pl	PK
F01_transcript_74563	AGC_RSK-2	PK
F01_transcript_74592	NEK	PK
F01_transcript_74640	RLK-Pelle_WAK_LRK10L-1	PK
F01_transcript_74642	CAMK_CDPK	PK
F01_transcript_74679	RLK-Pelle_DLSV	PK
F01_transcript_74699	RLK-Pelle_RLCK-IXb	PK
F01_transcript_74711	RLK-Pelle_LRR-XI-1	PK
F01_transcript_74746	RLK-Pelle_DLSV	PK
F01_transcript_7484	STE_STE11	PK
F01_transcript_74924	RLK-Pelle_RLCK-XI	PK
F01_transcript_74946	WNK_NRBP	PK
F01_transcript_74967	CMGC_RCK	PK
F01_transcript_74985	Group-Pl-4	PK
F01_transcript_74987	RLK-Pelle_LRR-Xb-1	PK
F01_transcript_75026	CAMK_CDPK	PK
F01_transcript_75035	CMGC_CLK	PK

F01_transcript_75092	TKL_CTR1-DRK-2	PK
F01_transcript_75105	RLK-Pelle_RLCK-VIIa-1	PK
F01_transcript_7511	RLK-Pelle_CrRLK1L-1	PK
F01_transcript_75110	CAMK_CDPK	PK
F01_transcript_75126	CAMK_CDPK	PK
F01_transcript_75137	RLK-Pelle_CrRLK1L-1	PK
F01_transcript_7514	RLK-Pelle_PERK-2	PK
F01_transcript_75141	RLK-Pelle_LRR-VI-1	PK
F01_transcript_75149	TKL-Pl-6	PK
F01_transcript_75181	RLK-Pelle_RLCK-IXb	PK
F01_transcript_75235	CMGC_CDK-PITSLRE	PK
F01_transcript_75236	RLK-Pelle_LRK10L-2	PK
F01_transcript_75275	WNK_NRBP	PK
F01_transcript_75280	CAMK_CDPK	PK
F01_transcript_75328	RLK-Pelle_LRR-VI-2	PK
F01_transcript_7534	RLK-Pelle_SD-2b	PK
F01_transcript_7536	TKL_CTR1-DRK-2	PK
F01_transcript_75507	STE_STE11	PK
F01_transcript_75575	RLK-Pelle_DLSV	PK
F01_transcript_75597	RLK-Pelle_LRR-VI-1	PK
F01_transcript_75628	RLK-Pelle_LRR-XII-1	PK
F01_transcript_75654	RLK-Pelle_DLSV	PK
F01_transcript_75728	RLK-Pelle_RLCK-VIIa-1	PK
F01_transcript_75729	AGC_PKA-PKG	PK
F01_transcript_75804	CAMK_CAMKL-CHK1	PK
F01_transcript_75830	WNK_NRBP	PK
F01_transcript_75866	CAMK_CDPK	PK
F01_transcript_75888	RLK-Pelle_LRR-V	PK
F01_transcript_76097	CAMK_CDPK	PK
F01_transcript_7610	RLK-Pelle_LRR-I-1	PK
F01_transcript_76152	CAMK_CDPK	PK
F01_transcript_76170	RLK-Pelle_RLCK-VIIa-2	PK
F01_transcript_76203	RLK-Pelle_SD-2b	PK
F01_transcript_76209	CMGC_MAPK	PK
F01_transcript_76294	CMGC_CDK-PITSLRE	PK
F01_transcript_76316	RLK-Pelle_RLCK-XII-1	PK
F01_transcript_7633	RLK-Pelle_RLCK-IXb	PK
F01_transcript_76340	RLK-Pelle_RLCK-VI	PK
F01_transcript_76351	RLK-Pelle_DLSV	PK
F01_transcript_76358	CK1_CK1	PK
F01_transcript_7637	IRE1	PK
F01_transcript_76377	CAMK_CDPK	PK
F01_transcript_76394	WNK_NRBP	PK
F01_transcript_76403	CMGC_CDK-CRK7-CDK9	PK
F01_transcript_7641	RLK-Pelle_LRR-IX	PK
F01_transcript_76424	CMGC_CLK	PK
F01_transcript_76438	RLK-Pelle_LRR-XI-1	PK
F01_transcript_76490	CMGC_MAPK	PK
F01_transcript_76534	RLK-Pelle_RLCK-VIIa-2	PK
F01_transcript_76575	RLK-Pelle_L-LEC	PK
F01_transcript_7664	RLK-Pelle_DLSV	PK
F01_transcript_76665	CAMK_CDPK	PK
F01_transcript_76679	CMGC_CDK-PITSLRE	PK
F01_transcript_76751	RLK-Pelle_RLCK-VIIa-2	PK
F01_transcript_76784	RLK-Pelle_LRR-III	PK

F01_transcript_76799	RLK-Pelle_DLSV	PK
F01_transcript_76808	AGC_NDR	PK
F01_transcript_76847	RLK-Pelle_LRR-XII-1	PK
F01_transcript_76886	RLK-Pelle_LRR-VIII-1	PK
F01_transcript_76909	RLK-Pelle_LysM	PK
F01_transcript_76916	RLK-Pelle_CrRLK1L-1	PK
F01_transcript_76974	RLK-Pelle_WAK	PK
F01_transcript_76983	CAMK_CDPK	PK
F01_transcript_77006	CAMK_CDPK	PK
F01_transcript_77142	CK1_CK1-Pl	PK
F01_transcript_7720	RLK-Pelle_DLSV	PK
F01_transcript_77203	CK1_CK1-Pl	PK
F01_transcript_77253	RLK-Pelle_LRK10L-2	PK
F01_transcript_77285	CMGC_GSK	PK
F01_transcript_77329	RLK-Pelle_DLSV	PK
F01_transcript_77413	CAMK_CAMKL-CHK1	PK
F01_transcript_77495	RLK-Pelle_LRR-I-1	PK
F01_transcript_77553	RLK-Pelle_LRR-XI-1	PK
F01_transcript_77577	CAMK_AMPK	PK
F01_transcript_77695	RLK-Pelle_SD-2b	PK
F01_transcript_77699	RLK-Pelle_LRK10L-2	PK
F01_transcript_77725	RLK-Pelle_CrRLK1L-1	PK
F01_transcript_77727	RLK-Pelle_CrRLK1L-1	PK
F01_transcript_77734	RLK-Pelle_LRR-XI-1	PK
F01_transcript_7776	RLK-Pelle_CrRLK1L-1	PK
F01_transcript_77761	RLK-Pelle_LRR-VII-1	PK
F01_transcript_7777	CMGC_CDK-CRK7-CDK9	PK
F01_transcript_77796	RLK-Pelle_RLCK-V	PK
F01_transcript_77846	TKL-Pl-4	PK
F01_transcript_77914	CAMK_AMPK	PK
F01_transcript_78020	CAMK_CAMKL-CHK1	PK
F01_transcript_78022	RLK-Pelle_Extensin	PK
F01_transcript_7803	RLK-Pelle_RLCK-IXb	PK
F01_transcript_78049	TKL-Pl-6	PK
F01_transcript_78050	TKL_CTR1-DRK-2	PK
F01_transcript_78081	CAMK_OST1L	PK
F01_transcript_78132	RLK-Pelle_LRR-XII-1	PK
F01_transcript_78149	RLK-Pelle_DLSV	PK
F01_transcript_78161	RLK-Pelle_LRK10L-2	PK
F01_transcript_78178	CMGC_CDK-CRK7-CDK9	PK
F01_transcript_78180	RLK-Pelle_DLSV	PK
F01_transcript_78183	RLK-Pelle_LRR-VIII-1	PK
F01_transcript_78201	RLK-Pelle_DLSV	PK
F01_transcript_7821	CAMK_OST1L	PK
F01_transcript_78248	CMGC_MAPK	PK
F01_transcript_78353	RLK-Pelle_PERK-2	PK
F01_transcript_78371	RLK-Pelle_LRR-VIII-1	PK
F01_transcript_7842	RLK-Pelle_LRR-XI-1	PK
F01_transcript_7844	CMGC_CDK-CRK7-CDK9	PK
F01_transcript_78440	RLK-Pelle_LRR-VIII-1	PK
F01_transcript_78451	CAMK_CAMKL-LKB	PK
F01_transcript_7847	RLK-Pelle_DLSV	PK
F01_transcript_78478	RLK-Pelle_DLSV	PK
F01_transcript_7848	RLK-Pelle_LRR-I-1	PK

F01_transcript_78632	CMGC_CDK-CDK8	PK
F01_transcript_78637	TKL-Pl-4	PK
F01_transcript_78654	CAMK_CAMKL-CHK1	PK
F01_transcript_78684	CMGC_CDK-CRK7-CDK9	PK
F01_transcript_78704	CMGC_GSK	PK
F01_transcript_78729	RLK-Pelle_CrRLK1L-1	PK
F01_transcript_788	PEK_GCN2	PK
F01_transcript_78802	RLK-Pelle_LysM	PK
F01_transcript_78922	TKL_CTR1-DRK-2	PK
F01_transcript_78924	RLK-Pelle_LRR-XIV	PK
F01_transcript_78983	RLK-Pelle_LRK10L-2	PK
F01_transcript_79007	RLK-Pelle_SD-2b	PK
F01_transcript_79021	RLK-Pelle_DLSV	PK
F01_transcript_79066	RLK-Pelle_DLSV	PK
F01_transcript_79102	CMGC_RCK	PK
F01_transcript_79154	TKL-Pl-4	PK
F01_transcript_79200	TKL-Pl-4	PK
F01_transcript_79233	CAMK_OST1L	PK
F01_transcript_79257	RLK-Pelle_RLCK-VI	PK
F01_transcript_79258	RLK-Pelle_PERK-2	PK
F01_transcript_79264	CK1_CK1	PK
F01_transcript_79273	RLK-Pelle_LRR-XIV	PK
F01_transcript_79295	WNK_NRBP	PK
F01_transcript_79332	CAMK_CAMKL-CHK1	PK
F01_transcript_79359	RLK-Pelle_CR4L	PK
F01_transcript_79360	TKL-Pl-4	PK
F01_transcript_79368	CAMK_CDPK	PK
F01_transcript_79408	STE_STE20-YSK	PK
F01_transcript_79443	CK1_CK1-Pl	PK
F01_transcript_79463	RLK-Pelle_LRR-IV	PK
F01_transcript_79478	AGC_RSK-2	PK
F01_transcript_79526	CAMK_CAMKL-CHK1	PK
F01_transcript_79542	STE_STE11	PK
F01_transcript_79573	RLK-Pelle_DLSV	PK
F01_transcript_79612	TKL-Pl-4	PK
F01_transcript_79627	RLK-Pelle_DLSV	PK
F01_transcript_79670	SCY1_SCYL2	PK
F01_transcript_7970	RLK-Pelle_RLCK-VI	PK
F01_transcript_79742	TKL-Pl-4	PK
F01_transcript_79804	RLK-Pelle_LRR-I-2	PK
F01_transcript_7981	RLK-Pelle_RLCK-IXb	PK
F01_transcript_79895	CMGC_MAPK	PK
F01_transcript_79920	RLK-Pelle_RLCK-VIIa-2	PK
F01_transcript_79951	CMGC_CDK-CCRK	PK
F01_transcript_80026	RLK-Pelle_SD-2b	PK
F01_transcript_80086	RLK-Pelle_LRR-VI-2	PK
F01_transcript_80130	RLK-Pelle_DLSV	PK
F01_transcript_80138	CK1_CK1	PK
F01_transcript_8020	TKL-Pl-4	PK
F01_transcript_80238	STE_STE11	PK
F01_transcript_8024	RLK-Pelle_Extensin	PK
F01_transcript_80260	STE_STE11	PK
F01_transcript_80277	RLK-Pelle_RLCK-V	PK
F01_transcript_80291	RLK-Pelle_LRR-VII-1	PK
F01_transcript_80313	RLK-Pelle_RLCK-IXb	PK

F01_transcript_80350	RLK-Pelle_DLSV	PK
F01_transcript_80359	RLK-Pelle_LRR-II	PK
F01_transcript_80411	RLK-Pelle_CrRLK1L-1	PK
F01_transcript_80419	TKL-Pl-4	PK
F01_transcript_80462	Group-Pl-4	PK
F01_transcript_80562	CMGC_DYRK-PRP4	PK
F01_transcript_80563	RLK-Pelle_DLSV	PK
F01_transcript_80587	RLK-Pelle_RLCK-IXb	PK
F01_transcript_80615	STE_STE11	PK
F01_transcript_80647	RLK-Pelle_LRK10L-2	PK
F01_transcript_80662	CMGC_CK2	PK
F01_transcript_80743	RLK-Pelle_CrRLK1L-1	PK
F01_transcript_80758	STE_STE7	PK
F01_transcript_80775	RLK-Pelle_LRR-VIII-1	PK
F01_transcript_80781	CAMK_CDPK	PK
F01_transcript_80793	CAMK_CDPK	PK
F01_transcript_80797	RLK-Pelle_LRR-XII-1	PK
F01_transcript_80822	RLK-Pelle_LRR-VIII-1	PK
F01_transcript_80895	PEK_GCN2	PK
F01_transcript_8090	CMGC_CDK-CRK7-CDK9	PK
F01_transcript_80954	RLK-Pelle_RLCK-VIIa-2	PK
F01_transcript_80957	CK1_CK1-Pl	PK
F01_transcript_8099	TKL_CTR1-DRK-1	PK
F01_transcript_81008	RLK-Pelle_DLSV	PK
F01_transcript_81134	RLK-Pelle_RLCK-VIIa-1	PK
F01_transcript_81136	PEK_GCN2	PK
F01_transcript_81207	CAMK_CDPK	PK
F01_transcript_81230	CMGC_CDK-CDK7	PK
F01_transcript_81242	AGC-Pl	PK
F01_transcript_81260	RLK-Pelle_WAK	PK
F01_transcript_81321	AGC_RSK-2	PK
F01_transcript_81360	RLK-Pelle_DLSV	PK
F01_transcript_81424	CK1_CK1-Pl	PK
F01_transcript_81468	WNK_NRBP	PK
F01_transcript_81472	RLK-Pelle_RLCK-V	PK
F01_transcript_81495	RLK-Pelle_LRR-XI-1	PK
F01_transcript_81508	TKL-Pl-6	PK
F01_transcript_81512	CAMK_CDPK	PK
F01_transcript_81535	RLK-Pelle_RLCK-VIIa-2	PK
F01_transcript_8154	TKL_CTR1-DRK-1	PK
F01_transcript_81575	TKL-Pl-5	PK
F01_transcript_81609	CK1_CK1	PK
F01_transcript_81626	Group-Pl-3	PK
F01_transcript_8163	RLK-Pelle_SD-2b	PK
F01_transcript_81664	RLK-Pelle_DLSV	PK
F01_transcript_81688	RLK-Pelle_LRR-VIII-1	PK
F01_transcript_8174	RLK-Pelle_RLCK-IXb	PK
F01_transcript_81783	TKL-Pl-4	PK
F01_transcript_81814	RLK-Pelle_LRK10L-2	PK
F01_transcript_81845	RLK-Pelle_DLSV	PK
F01_transcript_81864	TKL-Pl-4	PK
F01_transcript_81871	CAMK_CAMKL-CHK1	PK
F01_transcript_81900	CAMK_AMPK	PK
F01_transcript_81910	CMGC_CLK	PK

F01_transcript_8195	RLK-Pelle_LRR-I-1	PK
F01_transcript_81961	STE_STE11	PK
F01_transcript_82007	WNK_NRBP	PK
F01_transcript_82064	RLK-Pelle_DLSV	PK
F01_transcript_82131	CMGC_CK2	PK
F01_transcript_82132	RLK-Pelle_RLCK-IXb	PK
F01_transcript_82137	RLK-Pelle_LRR-VI-1	PK
F01_transcript_82140	RLK-Pelle_LRR-I-2	PK
F01_transcript_82171	RLK-Pelle_PERK-1	PK
F01_transcript_82198	STE_STE11	PK
F01_transcript_82199	RLK-Pelle_DLSV	PK
F01_transcript_82203	PEK_PEK	PK
F01_transcript_82220	RLK-Pelle_SD-2b	PK
F01_transcript_82236	STE_STE20-Fray	PK
F01_transcript_82272	RLK-Pelle_DLSV	PK
F01_transcript_82324	RLK-Pelle_DLSV	PK
F01_transcript_82347	TLK	PK
F01_transcript_82558	RLK-Pelle_DLSV	PK
F01_transcript_82578	RLK-Pelle_DLSV	PK
F01_transcript_82603	STE_STE20-Fray	PK
F01_transcript_82634	RLK-Pelle_WAK_LRK10L-1	PK
F01_transcript_82637	CMGC_CLK	PK
F01_transcript_82666	RLK-Pelle_LRR-I-1	PK
F01_transcript_82692	RLK-Pelle_LRR-II	PK
F01_transcript_82729	TKL_CTR1-DRK-2	PK
F01_transcript_82752	TKL-Pl-4	PK
F01_transcript_82877	RLK-Pelle_DLSV	PK
F01_transcript_82916	AGC_NDR	PK
F01_transcript_82920	CMGC_RCK	PK
F01_transcript_82922	CK1_CK1	PK
F01_transcript_83143	RLK-Pelle_CrRLK1L-1	PK
F01_transcript_83149	RLK-Pelle_LRR-XI-1	PK
F01_transcript_8316	RLK-Pelle_CrRLK1L-1	PK
F01_transcript_83168	CMGC_GSK	PK
F01_transcript_83171	RLK-Pelle_LRR-II	PK
F01_transcript_83216	RLK-Pelle_DLSV	PK
F01_transcript_83221	RLK-Pelle_LRR-VI-1	PK
F01_transcript_83264	CMGC_RCK	PK
F01_transcript_83286	RLK-Pelle_LRR-IX	PK
F01_transcript_83324	CAMK_CDPK	PK
F01_transcript_83352	CMGC_MAPK	PK
F01_transcript_83363	RLK-Pelle_RLCK-VI	PK
F01_transcript_8339	RLK-Pelle_SD-2b	PK
F01_transcript_83409	RLK-Pelle_DLSV	PK
F01_transcript_83435	RLK-Pelle_LysM	PK
F01_transcript_8346	TKL_CTR1-DRK-2	PK
F01_transcript_83508	TKL_CTR1-DRK-1	PK
F01_transcript_8352	TKL_CTR1-DRK-2	PK
F01_transcript_83524	RLK-Pelle_RLCK-VIIa-2	PK
F01_transcript_83582	RLK-Pelle_LRR-II	PK
F01_transcript_83588	RLK-Pelle_RLCK-XII-1	PK
F01_transcript_83602	RLK-Pelle_LRR-II	PK
F01_transcript_83653	RLK-Pelle_DLSV	PK
F01_transcript_83659	RLK-Pelle_LRR-II	PK
F01_transcript_83683	RLK-Pelle_RLCK-IXb	PK

F01_transcript_83720	RLK-Pelle_LRR-V	PK
F01_transcript_83732	RLK-Pelle_DLSV	PK
F01_transcript_83752	CAMK_CDPK	PK
F01_transcript_83765	RLK-Pelle_LRR-XI-1	PK
F01_transcript_83779	RLK-Pelle_LRK10L-2	PK
F01_transcript_83811	RLK-Pelle_LRR-XI-1	PK
F01_transcript_83815	CAMK_CDPK	PK
F01_transcript_83838	RLK-Pelle_RLCK-XII-1	PK
F01_transcript_83891	AGC_RSK-2	PK
F01_transcript_83924	RLK-Pelle_LRR-XI-1	PK
F01_transcript_83941	TKL_CTR1-DRK-2	PK
F01_transcript_8396	CAMK_CAMKL-CHK1	PK
F01_transcript_83965	RLK-Pelle_RLCK-VIIa-1	PK
F01_transcript_83991	CAMK_CDPK	PK
F01_transcript_8408	CAMK_CDPK	PK
F01_transcript_84113	TKL-Pl-4	PK
F01_transcript_8412	RLK-Pelle_CrRLK1L-1	PK
F01_transcript_84122	RLK-Pelle_PERK-2	PK
F01_transcript_84143	SCY1_SCYL2	PK
F01_transcript_84244	RLK-Pelle_LRR-VIII-1	PK
F01_transcript_84254	RLK-Pelle_DLSV	PK
F01_transcript_84295	STE_STE20-Fray	PK
F01_transcript_84346	RLK-Pelle_LRR-XI-1	PK
F01_transcript_84364	RLK-Pelle_CrRLK1L-1	PK
F01_transcript_84418	RLK-Pelle_DLSV	PK
F01_transcript_84440	AGC_MAST	PK
F01_transcript_84469	RLK-Pelle_DLSV	PK
F01_transcript_84490	RLK-Pelle_RLCK-IXb	PK
F01_transcript_84493	CAMK_CDPK	PK
F01_transcript_84506	RLK-Pelle_SD-2b	PK
F01_transcript_8456	RLK-Pelle_CrRLK1L-1	PK
F01_transcript_8458	STE_STE20-Pl	PK
F01_transcript_84596	RLK-Pelle_LRR-II	PK
F01_transcript_84597	RLK-Pelle_LRR-XII-1	PK
F01_transcript_84606	CAMK_CDPK	PK
F01_transcript_84608	STE_STE11	PK
F01_transcript_84639	RLK-Pelle_DLSV	PK
F01_transcript_84669	RLK-Pelle_DLSV	PK
F01_transcript_84700	RLK-Pelle_LRR-VIII-1	PK
F01_transcript_84742	RLK-Pelle_LRR-XI-1	PK
F01_transcript_84743	CMGC_DYRK-YAK	PK
F01_transcript_84800	RLK-Pelle_LRK10L-2	PK
F01_transcript_84824	STE_STE20-Fray	PK
F01_transcript_8486	CMGC_CDK-CRK7-CDK9	PK
F01_transcript_84869	RLK-Pelle_LRR-VII-2	PK
F01_transcript_84885	RLK-Pelle_LRK10L-2	PK
F01_transcript_84924	RLK-Pelle_LRR-I-1	PK
F01_transcript_8497	RLK-Pelle_SD-2b	PK
F01_transcript_84973	RLK-Pelle_L-LEC	PK
F01_transcript_84975	AGC-Pl	PK
F01_transcript_85009	RLK-Pelle_LRR-XII-1	PK
F01_transcript_85024	RLK-Pelle_RLCK-XI	PK
F01_transcript_85027	NEK	PK
F01_transcript_85067	RLK-Pelle_RLCK-VI	PK

F01_transcript_85080	TKL-Pl-3	PK
F01_transcript_85135	TKL-Pl-5	PK
F01_transcript_85250	CAMK_CAMKL-CHK1	PK
F01_transcript_8529	RLK-Pelle_LRR-I-1	PK
F01_transcript_8534	CMGC_CDK-PITSLRE	PK
F01_transcript_85343	RLK-Pelle_SD-2b	PK
F01_transcript_85346	RLK-Pelle_RLCK-V	PK
F01_transcript_85354	RLK-Pelle_WAK_LRK10L-1	PK
F01_transcript_85386	CK1_CK1-Pl	PK
F01_transcript_85421	RLK-Pelle_LRR-XI-1	PK
F01_transcript_85451	RLK-Pelle_DLSV	PK
F01_transcript_85477	RLK-Pelle_RLCK-Os	PK
F01_transcript_85483	STE_STE11	PK
F01_transcript_85496	STE_STE7	PK
F01_transcript_85517	WNK_NRBP	PK
F01_transcript_85537	RLK-Pelle_LRR-II	PK
F01_transcript_85546	RLK-Pelle_L-LEC	PK
F01_transcript_85612	CMGC_GSK	PK
F01_transcript_85634	RLK-Pelle_DLSV	PK
F01_transcript_85665	CAMK_CAMKL-CHK1	PK
F01_transcript_8567	WNK_NRBP	PK
F01_transcript_85772	RLK-Pelle_LRR-VI-1	PK
F01_transcript_85773	STE_STE11	PK
F01_transcript_85819	STE_STE11	PK
F01_transcript_85830	RLK-Pelle_LRR-XII-1	PK
F01_transcript_8588	CK1_CK1-Pl	PK
F01_transcript_86022	CAMK_OST1L	PK
F01_transcript_86028	CMGC_GSK	PK
F01_transcript_86069	RLK-Pelle_LRR-V	PK
F01_transcript_86099	CMGC_RCK	PK
F01_transcript_86191	RLK-Pelle_SD-2b	PK
F01_transcript_86226	AGC_PKA-PKG	PK
F01_transcript_86234	STE_STE11	PK
F01_transcript_86235	RLK-Pelle_DLSV	PK
F01_transcript_86241	RLK-Pelle_L-LEC	PK
F01_transcript_86274	RLK-Pelle_RLCK-XII-1	PK
F01_transcript_86292	RLK-Pelle_RLCK-V	PK
F01_transcript_8632	RLK-Pelle_DLSV	PK
F01_transcript_86398	CAMK_CAMKL-CHK1	PK
F01_transcript_86465	CAMK_CAMKL-CHK1	PK
F01_transcript_86477	RLK-Pelle_RLCK-VIIa-2	PK
F01_transcript_86478	RLK-Pelle_LRR-XII-1	PK
F01_transcript_86497	CMGC_MAPK	PK
F01_transcript_86543	SCY1_SCYL2	PK
F01_transcript_86578	CMGC_CDK-PITSLRE	PK
F01_transcript_86585	AGC_RSK-2	PK
F01_transcript_86598	RLK-Pelle_DLSV	PK
F01_transcript_86600	TKL-Pl-5	PK
F01_transcript_86619	RLK-Pelle_RLCK-VIIa-2	PK
F01_transcript_86682	RLK-Pelle_LRK10L-2	PK
F01_transcript_86696	RLK-Pelle_DLSV	PK
F01_transcript_8670	RLK-Pelle_RLCK-VI	PK
F01_transcript_86710	RLK-Pelle_RLCK-V	PK
F01_transcript_86718	RLK-Pelle_DLSV	PK
F01_transcript_8672	TKL_CTR1-DRK-1	PK

F01_transcript_86745	TKL-Pl-4	PK
F01_transcript_86824	TKL-Pl-4	PK
F01_transcript_8687	STE_STE20-Fray	PK
F01_transcript_86904	WNK_NRBP	PK
F01_transcript_87039	AGC_MAST	PK
F01_transcript_87045	CMGC_GSK	PK
F01_transcript_87066	RLK-Pelle_LRR-XI-1	PK
F01_transcript_87067	RLK-Pelle_DLSV	PK
F01_transcript_87076	RLK-Pelle_LRR-VII-1	PK
F01_transcript_8713	TLK	PK
F01_transcript_87148	RLK-Pelle_LysM	PK
F01_transcript_87179	RLK-Pelle_SD-2b	PK
F01_transcript_87228	RLK-Pelle_LRR-VIII-1	PK
F01_transcript_87243	STE_STE11	PK
F01_transcript_87246	RLK-Pelle_DLSV	PK
F01_transcript_87292	CAMK_CDPK	PK
F01_transcript_87346	RLK-Pelle_RLCK-VIIa-2	PK
F01_transcript_87355	RLK-Pelle_DLSV	PK
F01_transcript_87359	CAMK_CAMKL-CHK1	PK
F01_transcript_874	RLK-Pelle_LRR-XI-1	PK
F01_transcript_87432	RLK-Pelle_LRR-XI-1	PK
F01_transcript_87486	RLK-Pelle_LRR-XI-1	PK
F01_transcript_87508	CMGC_GSK	PK
F01_transcript_87535	RLK-Pelle_SD-2b	PK
F01_transcript_87549	RLK-Pelle_RLCK-IXb	PK
F01_transcript_87555	RLK-Pelle_URK-1	PK
F01_transcript_87573	CAMK_CDPK	PK
F01_transcript_87593	CK1_CK1	PK
F01_transcript_87624	CAMK_CDPK	PK
F01_transcript_87648	RLK-Pelle_LRR-III	PK
F01_transcript_87653	RLK-Pelle_LRR-XI-1	PK
F01_transcript_87669	CAMK_CAMKL-CHK1	PK
F01_transcript_87693	RLK-Pelle_LRR-IX	PK
F01_transcript_87746	CMGC_CDK-CRK7-CDK9	PK
F01_transcript_87747	RLK-Pelle_DLSV	PK
F01_transcript_87800	RLK-Pelle_LRR-III	PK
F01_transcript_87879	STE_STE11	PK
F01_transcript_87884	RLK-Pelle_DLSV	PK
F01_transcript_8791	RLK-Pelle_RLCK-VI	PK
F01_transcript_87910	CMGC_GSK	PK
F01_transcript_87931	AGC_NDR	PK
F01_transcript_88006	AGC_MAST	PK
F01_transcript_88033	RLK-Pelle_LRR-IX	PK
F01_transcript_88061	CMGC_GSK	PK
F01_transcript_88062	RLK-Pelle_DLSV	PK
F01_transcript_88105	RLK-Pelle_WAK	PK
F01_transcript_8813	RLK-Pelle_DLSV	PK
F01_transcript_8815	RLK-Pelle_RLCK-IXb	PK
F01_transcript_88160	CMGC_CDK-PITSLRE	PK
F01_transcript_88178	RLK-Pelle_LRR-VIII-1	PK
F01_transcript_88216	RLK-Pelle_CR4L	PK
F01_transcript_88285	RLK-Pelle_LRR-I-1	PK
F01_transcript_88294	WNK_NRBP	PK
F01_transcript_88304	RLK-Pelle_DLSV	PK

F01_transcript_88355	RLK-Pelle_RLCK-IXb	PK
F01_transcript_88395	RLK-Pelle_LRR-XI-1	PK
F01_transcript_88442	WNK_NRBP	PK
F01_transcript_88489	RLK-Pelle_LRR-VI-1	PK
F01_transcript_88522	CAMK_CDPK	PK
F01_transcript_88548	TKL-Pl-4	PK
F01_transcript_8855	RLK-Pelle_LRR-V	PK
F01_transcript_88571	CAMK_CDPK	PK
F01_transcript_88585	TKL-Pl-4	PK
F01_transcript_886	RLK-Pelle_LRR-VII-1	PK
F01_transcript_88644	RLK-Pelle_LRR-XI-1	PK
F01_transcript_88656	RLK-Pelle_LRR-VIII-1	PK
F01_transcript_88661	RLK-Pelle_LRR-V	PK
F01_transcript_88683	RLK-Pelle_LRR-XI-1	PK
F01_transcript_88771	RLK-Pelle_LRR-Xb-1	PK
F01_transcript_88905	RLK-Pelle_DLSV	PK
F01_transcript_88956	RLK-Pelle_RLCK-IXb	PK
F01_transcript_8897	RLK-Pelle_DLSV	PK
F01_transcript_88987	RLK-Pelle_LRR-VI-1	PK
F01_transcript_89002	CK1_CK1-Pl	PK
F01_transcript_89015	CAMK_CDPK	PK
F01_transcript_89116	CMGC_MAPK	PK
F01_transcript_89122	CAMK_CDPK	PK
F01_transcript_89186	AGC_RSK-2	PK
F01_transcript_89241	RLK-Pelle_WAK_LRK10L-1	PK
F01_transcript_89323	RLK-Pelle_DLSV	PK
F01_transcript_89335	RLK-Pelle_DLSV	PK
F01_transcript_89348	RLK-Pelle_RLCK-XI	PK
F01_transcript_89369	CAMK_CAMKL-CHK1	PK
F01_transcript_89376	CMGC_CDK-CRK7-CDK9	PK
F01_transcript_89405	RLK-Pelle_LRR-Xb-1	PK
F01_transcript_89416	CAMK_CDPK	PK
F01_transcript_89484	RLK-Pelle_RLCK-IV	PK
F01_transcript_89492	RLK-Pelle_RLCK-VIIa-2	PK
F01_transcript_89502	CAMK_CAMKL-CHK1	PK
F01_transcript_89520	CAMK_OST1L	PK
F01_transcript_89576	CAMK_CDPK	PK
F01_transcript_8960	STE_STE20-Fray	PK
F01_transcript_89627	STE_STE11	PK
F01_transcript_89633	RLK-Pelle_DLSV	PK
F01_transcript_89761	RLK-Pelle_DLSV	PK
F01_transcript_89765	TKL_CTR1-DRK-2	PK
F01_transcript_89785	RLK-Pelle_LRR-XIV	PK
F01_transcript_89847	CMGC_RCK	PK
F01_transcript_89851	RLK-Pelle_RLCK-XII-1	PK
F01_transcript_89859	RLK-Pelle_RLCK-VIIa-2	PK
F01_transcript_8986	RLK-Pelle_DLSV	PK
F01_transcript_89899	RLK-Pelle_DLSV	PK
F01_transcript_89918	RLK-Pelle_DLSV	PK
F01_transcript_89919	RLK-Pelle_RLCK-VIIa-2	PK
F01_transcript_90077	RLK-Pelle_LRR-III	PK
F01_transcript_90125	RLK-Pelle_LRR-XII-1	PK
F01_transcript_90159	RLK-Pelle_LRR-II	PK
F01_transcript_9022	RLK-Pelle_CrRLK1L-1	PK
F01_transcript_90272	RLK-Pelle_DLSV	PK

F01_transcript_90298	RLK-Pelle_LRR-XII-1	PK
F01_transcript_90315	STE_STE11	PK
F01_transcript_90377	RLK-Pelle_L-LEC	PK
F01_transcript_90477	RLK-Pelle_RLCK-IV	PK
F01_transcript_90676	CAMK_CDPK	PK
F01_transcript_90677	RLK-Pelle_LRR-VIII-1	PK
F01_transcript_90690	RLK-Pelle_RLCK-VIIa-1	PK
F01_transcript_90744	RLK-Pelle_RLCK-IV	PK
F01_transcript_90751	CAMK_CAMK1-Tthe	PK
F01_transcript_90856	RLK-Pelle_DLSV	PK
F01_transcript_9090	RLK-Pelle_L-LEC	PK
F01_transcript_90924	TKL_CTR1-DRK-2	PK
F01_transcript_90938	RLK-Pelle_RLCK-VIIa-1	PK
F01_transcript_9094	RLK-Pelle_DLSV	PK
F01_transcript_90955	RLK-Pelle_RLCK-VIIa-2	PK
F01_transcript_90961	CMGC_CLK	PK
F01_transcript_9097	RLK-Pelle_LRR-VI-2	PK
F01_transcript_91018	RLK-Pelle_RLCK-VIIa-2	PK
F01_transcript_91029	RLK-Pelle_RLCK-VIII	PK
F01_transcript_91046	STE_STE20-Fray	PK
F01_transcript_91067	STE_STE11	PK
F01_transcript_91087	RLK-Pelle_RLCK-VI	PK
F01_transcript_91090	CAMK_CAMKL-CHK1	PK
F01_transcript_91149	RLK-Pelle_DLSV	PK
F01_transcript_91184	RLK-Pelle_DLSV	PK
F01_transcript_91216	STE_STE11	PK
F01_transcript_9134	RLK-Pelle_SD-2b	PK
F01_transcript_91378	NAK	PK
F01_transcript_91418	RLK-Pelle_RLCK-IV	PK
F01_transcript_91449	TKL-Pl-4	PK
F01_transcript_9145	RLK-Pelle_RLCK-IV	PK
F01_transcript_91509	CAMK_CAMKL-CHK1	PK
F01_transcript_91520	RLK-Pelle_LRR-VIII-1	PK
F01_transcript_9155	CAMK_CDPK	PK
F01_transcript_91598	Group-Pl-4	PK
F01_transcript_91680	AGC-Pl	PK
F01_transcript_91737	CAMK_OST1L	PK
F01_transcript_91741	RLK-Pelle_LRK10L-2	PK
F01_transcript_91761	RLK-Pelle_LRR-XIIIa	PK
F01_transcript_91771	RLK-Pelle_DLSV	PK
F01_transcript_91776	CK1_CK1	PK
F01_transcript_91802	RLK-Pelle_SD-2b	PK
F01_transcript_91839	RLK-Pelle_RLCK-XII-1	PK
F01_transcript_9189	RLK-Pelle_DLSV	PK
F01_transcript_91921	RLK-Pelle_RLCK-VIIa-2	PK
F01_transcript_91958	RLK-Pelle_DLSV	PK
F01_transcript_91973	CMGC_GSKL	PK
F01_transcript_92035	RLK-Pelle_LRR-VIII-1	PK
F01_transcript_92037	RLK-Pelle_LRR-XII-1	PK
F01_transcript_92057	STE_STE7	PK
F01_transcript_92131	CAMK_CDPK	PK
F01_transcript_92150	TKL-Pl-4	PK
F01_transcript_92186	RLK-Pelle_RLCK-VIIa-2	PK
F01_transcript_92254	STE_STE11	PK

F01_transcript_92303	CMGC_CK2	PK
F01_transcript_92396	CMGC_CDK-PITSLRE	PK
F01_transcript_92397	RLK-Pelle_RLCK-IV	PK
F01_transcript_9240	RLK-Pelle_Extensin	PK
F01_transcript_92425	CMGC_MAPK	PK
F01_transcript_92436	CMGC_CLK	PK
F01_transcript_92456	RLK-Pelle_DLSV	PK
F01_transcript_92473	RLK-Pelle_PERK-1	PK
F01_transcript_92500	AGC_NDR	PK
F01_transcript_92534	CAMK_CDPK	PK
F01_transcript_92622	RLK-Pelle_DLSV	PK
F01_transcript_92667	RLK-Pelle_DLSV	PK
F01_transcript_92672	CK1_CK1	PK
F01_transcript_9279	CMGC_RCK	PK
F01_transcript_92791	RLK-Pelle_LRR-VIII-1	PK
F01_transcript_92810	CAMK_CDPK	PK
F01_transcript_92910	RLK-Pelle_DLSV	PK
F01_transcript_92999	AGC-Pl	PK
F01_transcript_93013	AGC_PDK1	PK
F01_transcript_93118	RLK-Pelle_LRR-VIII-1	PK
F01_transcript_93132	RLK-Pelle_LRR-VIII-1	PK
F01_transcript_93264	RLK-Pelle_RKF3	PK
F01_transcript_93282	RLK-Pelle_LRR-I-1	PK
F01_transcript_93317	RLK-Pelle_RLCK-IXb	PK
F01_transcript_93341	RLK-Pelle_WAK_LRK10L-1	PK
F01_transcript_93368	RLK-Pelle_DLSV	PK
F01_transcript_93428	CAMK_OST1L	PK
F01_transcript_93496	RLK-Pelle_RLCK-VI	PK
F01_transcript_93527	WNK_NRBP	PK
F01_transcript_93581	CAMK_CAMKL-CHK1	PK
F01_transcript_93678	RLK-Pelle_RLCK-IXb	PK
F01_transcript_93741	CAMK_CAMKL-CHK1	PK
F01_transcript_93819	CMGC_GSK	PK
F01_transcript_93820	RLK-Pelle_LRR-VI-1	PK
F01_transcript_9392	TLK	PK
F01_transcript_93937	NAK	PK
F01_transcript_93989	RLK-Pelle_LysM	PK
F01_transcript_94041	CMGC_CDK-PITSLRE	PK
F01_transcript_9406	RLK-Pelle_LRR-III	PK
F01_transcript_94063	RLK-Pelle_RLCK-VIIa-2	PK
F01_transcript_94105	AGC_RSK-2	PK
F01_transcript_94185	WNK_NRBP	PK
F01_transcript_94191	WNK_NRBP	PK
F01_transcript_94212	STE_STE7	PK
F01_transcript_94224	RLK-Pelle_LRR-IX	PK
F01_transcript_9423	RLK-Pelle_Extensin	PK
F01_transcript_94237	AGC_RSK-2	PK
F01_transcript_94253	TKL-Pl-4	PK
F01_transcript_94283	RLK-Pelle_RLCK-VIIa-2	PK
F01_transcript_94293	CMGC_GSK	PK
F01_transcript_94303	RLK-Pelle_DLSV	PK
F01_transcript_94342	RLK-Pelle_LRR-XII-1	PK
F01_transcript_94354	CAMK_CDPK	PK
F01_transcript_94389	CMGC_RCK	PK
F01_transcript_94442	RLK-Pelle_DLSV	PK

F01_transcript_94535	RLK-Pelle_LRK10L-2	PK
F01_transcript_94538	WNK_NRBP	PK
F01_transcript_94562	CAMK_CDPK	PK
F01_transcript_9458	STE_STE20-YSK	PK
F01_transcript_94694	RLK-Pelle_LRR-VIII-1	PK
F01_transcript_9472	CK1_CK1-Pl	PK
F01_transcript_94781	RLK-Pelle_RLCK-VIIb	PK
F01_transcript_94843	STE_STE11	PK
F01_transcript_9501	RLK-Pelle_LRR-VI-2	PK
F01_transcript_95014	RLK-Pelle_DLSV	PK
F01_transcript_95052	TKL_CTR1-DRK-2	PK
F01_transcript_95115	STE_STE20-Fray	PK
F01_transcript_9515	RLK-Pelle_CR4L	PK
F01_transcript_95176	RLK-Pelle_DLSV	PK
F01_transcript_95214	CMGC_GSK	PK
F01_transcript_95368	RLK-Pelle_LRR-XII-1	PK
F01_transcript_95487	RLK-Pelle_LRR-VI-2	PK
F01_transcript_95513	TKL-Pl-4	PK
F01_transcript_95520	WNK_NRBP	PK
F01_transcript_9554	RLK-Pelle_LRR-IV	PK
F01_transcript_95558	RLK-Pelle_DLSV	PK
F01_transcript_95586	RLK-Pelle_LRR-VI-1	PK
F01_transcript_95602	RLK-Pelle_LRR-I-1	PK
F01_transcript_95608	RLK-Pelle_WAK	PK
F01_transcript_95611	CK1_CK1-Pl	PK
F01_transcript_95657	CMGC_CLK	PK
F01_transcript_95671	TKL-Pl-4	PK
F01_transcript_95680	CMGC_CDK-PITSLRE	PK
F01_transcript_95706	ULK_ULK4	PK
F01_transcript_95719	RLK-Pelle_L-LEC	PK
F01_transcript_95723	STE_STE11	PK
F01_transcript_95808	RLK-Pelle_DLSV	PK
F01_transcript_95858	RLK-Pelle_RLCK-XII-1	PK
F01_transcript_95863	RLK-Pelle_Singleton	PK
F01_transcript_95864	RLK-Pelle_LRR-IX	PK
F01_transcript_9596	STE_STE20-Fray	PK
F01_transcript_95972	RLK-Pelle_DLSV	PK
F01_transcript_9604	RLK-Pelle_RLCK-VI	PK
F01_transcript_96043	RLK-Pelle_LysM	PK
F01_transcript_96047	CMGC_GSK	PK
F01_transcript_96052	CK1_CK1-Pl	PK
F01_transcript_96059	RLK-Pelle_RLCK-IXb	PK
F01_transcript_96077	RLK-Pelle_LRR-VIII-1	PK
F01_transcript_9611	CK1_CK1-Pl	PK
F01_transcript_96133	CK1_CK1	PK
F01_transcript_96236	RLK-Pelle_DLSV	PK
F01_transcript_96285	AGC_NDR	PK
F01_transcript_96290	CAMK_CDPK	PK
F01_transcript_96291	RLK-Pelle_RLCK-V	PK
F01_transcript_96354	CK1_CK1	PK
F01_transcript_96417	RLK-Pelle_LRR-VI-1	PK
F01_transcript_96419	RLK-Pelle_RLCK-VI	PK
F01_transcript_96423	WEE	PK
F01_transcript_96435	RLK-Pelle_LRR-XI-1	PK

F01_transcript_96458	RLK-Pelle_RLCK-XII-1	PK
F01_transcript_96498	WNK_NRBP	PK
F01_transcript_96530	TKL-Pl-2	PK
F01_transcript_96542	RLK-Pelle_LRR-XI-1	PK
F01_transcript_96611	CAMK_CAMKL-CHK1	PK
F01_transcript_96645	RLK-Pelle_LRR-XIV	PK
F01_transcript_96648	CMGC_CDK-CRK7-CDK9	PK
F01_transcript_9672	WNK_NRBP	PK
F01_transcript_96732	CMGC_GSK	PK
F01_transcript_9676	RLK-Pelle_LRR-V	PK
F01_transcript_96827	Group-Pl-3	PK
F01_transcript_96896	RLK-Pelle_DLSV	PK
F01_transcript_969	AGC_RSK-2	PK
F01_transcript_9692	RLK-Pelle_LRR-VI-2	PK
F01_transcript_97027	TKL_CTR1-DRK-2	PK
F01_transcript_9706	RLK-Pelle_LRR-III	PK
F01_transcript_97089	RLK-Pelle_DLSV	PK
F01_transcript_97092	RLK-Pelle_DLSV	PK
F01_transcript_97109	RLK-Pelle_SD-2b	PK
F01_transcript_97143	RLK-Pelle_PERK-1	PK
F01_transcript_97197	AGC_RSK-2	PK
F01_transcript_97243	CAMK_CDPK	PK
F01_transcript_97300	CAMK_CDPK	PK
F01_transcript_97328	RLK-Pelle_LRR-VI-1	PK
F01_transcript_97334	CAMK_CAMKL-CHK1	PK
F01_transcript_97382	RLK-Pelle_RLCK-IV	PK
F01_transcript_97395	RLK-Pelle_LRR-XIIIa	PK
F01_transcript_97404	RLK-Pelle_LRR-VIII-1	PK
F01_transcript_97435	Group-Pl-4	PK
F01_transcript_97475	WNK_NRBP	PK
F01_transcript_97499	RLK-Pelle_LRR-II	PK
F01_transcript_97512	RLK-Pelle_LRR-XI-1	PK
F01_transcript_9754	RLK-Pelle_Extensin	PK
F01_transcript_97556	TKL-Pl-4	PK
F01_transcript_97563	Group-Pl-4	PK
F01_transcript_97572	CAMK_CDPK	PK
F01_transcript_97585	CAMK_CDPK	PK
F01_transcript_97590	CMGC_MAPK	PK
F01_transcript_97600	SCY1_SCYL2	PK
F01_transcript_97619	RLK-Pelle_LRR-XI-1	PK
F01_transcript_9762	STE_STE20-Fray	PK
F01_transcript_97629	WNK_NRBP	PK
F01_transcript_97648	RLK-Pelle_LRR-XI-1	PK
F01_transcript_97685	TKL_CTR1-DRK-1	PK
F01_transcript_97723	Group-Pl-4	PK
F01_transcript_97746	RLK-Pelle_LRR-XI-1	PK
F01_transcript_97749	RLK-Pelle_LRR-IX	PK
F01_transcript_97754	RLK-Pelle_DLSV	PK
F01_transcript_97863	RLK-Pelle_DLSV	PK
F01_transcript_97871	RLK-Pelle_LRR-XI-1	PK
F01_transcript_97901	TKL-Pl-4	PK
F01_transcript_97944	RLK-Pelle_DLSV	PK
F01_transcript_98018	RLK-Pelle_LRK10L-2	PK
F01_transcript_98028	CAMK_CDPK	PK
F01_transcript_98052	RLK-Pelle_DLSV	PK

F01_transcript_98059	RLK-Pelle_LRR-VIII-1	PK
F01_transcript_98206	RLK-Pelle_LRR-VIII-1	PK
F01_transcript_98244	TKL-Pl-4	PK
F01_transcript_98335	WEE	PK
F01_transcript_98349	RLK-Pelle_LRR-III	PK
F01_transcript_98397	RLK-Pelle_RLCK-V	PK
F01_transcript_98521	WNK_NRBP	PK
F01_transcript_98528	STE_STE11	PK
F01_transcript_98594	CMGC_GSK	PK
F01_transcript_986	AGC_PKA-PKG	PK
F01_transcript_98602	CK1_CK1	PK
F01_transcript_98670	RLK-Pelle_CrRLK1L-1	PK
F01_transcript_98709	RLK-Pelle_LRR-I-1	PK
F01_transcript_98715	RLK-Pelle_LRR-VIII-1	PK
F01_transcript_98727	RLK-Pelle_LRR-V	PK
F01_transcript_98929	RLK-Pelle_LRR-XI-1	PK
F01_transcript_98937	CMGC_CLK	PK
F01_transcript_98942	STE_STE-Pl	PK
F01_transcript_99009	RLK-Pelle_RLCK-VIIa-2	PK
F01_transcript_99068	CMGC_DYRK-PRP4	PK
F01_transcript_9912	RLK-Pelle_Extensin	PK
F01_transcript_9923	RLK-Pelle_DLSV	PK
F01_transcript_99245	NAK	PK
F01_transcript_99258	RLK-Pelle_DLSV	PK
F01_transcript_99274	CMGC_CDK-CRK7-CDK9	PK
F01_transcript_99281	STE_STE20-Fray	PK
F01_transcript_99292	RLK-Pelle_DLSV	PK
F01_transcript_99298	RLK-Pelle_DLSV	PK
F01_transcript_99318	NEK	PK
F01_transcript_99337	RLK-Pelle_LRR-VIII-1	PK
F01_transcript_99355	RLK-Pelle_LRR-VII-1	PK
F01_transcript_99357	RLK-Pelle_DLSV	PK
F01_transcript_99406	CMGC_CDK-CRK7-CDK9	PK
F01_transcript_99429	AGC_NDR	PK
F01_transcript_99473	RLK-Pelle_LRR-III	PK
F01_transcript_99495	RLK-Pelle_RLCK-XI	PK
F01_transcript_995	AGC_PKA-PKG	PK
F01_transcript_99515	RLK-Pelle_DLSV	PK
F01_transcript_99525	CK1_CK1-Pl	PK
F01_transcript_99535	RLK-Pelle_WAK_LRK10L-1	PK
F01_transcript_99556	CK1_CK1	PK
F01_transcript_99582	CAMK_CDPK	PK
F01_transcript_99598	CMGC_MAPK	PK
F01_transcript_99611	RLK-Pelle_DLSV	PK
F01_transcript_99638	RLK-Pelle_LRR-VIII-1	PK
F01_transcript_99676	CAMK_CAMKL-CHK1	PK
F01_transcript_99688	RLK-Pelle_LRR-III	PK
F01_transcript_99719	STE_STE11	PK
F01_transcript_99725	RLK-Pelle_DLSV	PK
F01_transcript_99759	RLK-Pelle_LRR-II	PK
F01_transcript_99869	RLK-Pelle_RLCK-VIII	PK
F01_transcript_99955	CAMK_OST1L	PK
