# Supplementary material for: PacBio Single-Molecule Long-Read Sequencing Reveals Genes Tolerating Manganese Stress in Schima superba Saplings
Source: Front Genet. 2021 Apr 6;12:635043. doi: 10.3389/fgene.2021.635043 (PMC8057201; doi:10.3389/fgene.2021.635043)
Supplement: Supplementary Table 4 — Summary of the transcripts annotated to the reference canonical pathways in the KEGG database. [file Data_Sheet_2.PDF]

PacBio single-molecule long-read sequencing successfully explores the transcriptome of *Schima superba*

Fiza Liaquat[1], Muhammad Farooq Hussain Munis[2], Samiah Arif[1], Urooj Haroon[2], Muhammad Ashraf[3], Saddam Saqib[4,5], Wajid Zaman[4,5], Che Shengquan[6] and Liu Qunlu[6*]

1 School of Agriculture and Biology, Shanghai Jiao Tong University, Shanghai, 200240, China;

2 Department of Plant Sciences, Faculty of Biological Sciences, Quaid-i-Azam University, Islamabad, 45320, Pakistan;

3 Joint International Research Laboratory of Metabolic and Developmental Sciences, School of Life Science and Biotechnology, Shanghai Jiao Tong University, Shanghai, 200240, China;

4 State Key Laboratory of Systematic and Evolutionary Botany, Institute of Botany, Chinese Academy of Sciences, Beijing 100093, China;

5 University of Chinese Academy of Sciences, Beijing 100049, China;

6 Department of Landscape Architecture, School of Design, Shanghai Jiao Tong University, 200240, China;

*Correspondence: liuql@sjtu.edu.cn

Table S4 Summary of the transcripts annotated to the reference canonical pathways in the KEGG database

pathway	pathway_id	Gene_number	Gene_id	KOs
Glycolysis / Gluconeogenesis	ko00010	752	F01_transcript_100214;F01_transcript_100740;F01_transcript_100783;F01_transcript_100809;F01_transcript_101187;F01_transcript_101574;F01_transcript_10158;F01_transcript_101609;F01_transcript_101871;F01_transcript_102262;F01_transcript_102370;F01_transcript_102373;F01_transcript_102523;F01_transcript_102731;F01_transcript_102834;F01_transcript_102845;F01_transcript_102902;F01_transcript_103451;F01_transcript_103571;F01_transcript_103634;F01_transcript_103691;F01_transcript_103925;F01_transcript_104096;F01_transcript_104489;F01_transcript_104691;F01_transcript_104746;F01_transcript_104756;F01_transcript_104986;F01_transcript_105213;F01_transcript_105365;F01_transcript_105726;F01_transcript_105738;F01_transcript_105972;F01_transcript_106765;F01_transcript_108019;F01_transcript_108216;F01_transcript_108221;F01_transcript_108502;F01_transcript_108868;F01_transcript_108947;F01_transcript_109032;F01_transcript_109312;F01_transcript_109676;F01_transcript_109885;F01_transcript_109968;F01_transcript_110541;F01_transcript_110559;F01_transcript_110879;F01_transcript_111322;F01_transcript_111485;F01_transcript_111496;F01_transcript_112117;F01_transcript_11216;F01_transcript_11219;F01_transcript_112269;F01_transcript_112669;F01_transcript_112685;F01_transcript_112884;F01_transcript_113973;F01_transcript_114303;F01_transcript_114384;F01_transcript_114570;F01_transcript_114898;F01_transcript_114952;F01_transcript_115044;F01_transcript_115059;F01_transcript_115624;F01_transcript_115636;F01_transcript_116260;F01_transcript_116289;F01_transcript_116347;F01_transcript_116379;F01_transcript_116451;F	K01803+K00128+K00873+K15634+K00873+K00850+K01895+K01792+K00121+K00895+K00162+K01803+K18857+K00162+K00162+K01623+K01689+K01568+K01810+K00134+K14085+K00128+K00873+K00121+K00627+K00382+K03841+K00850+K00128+K01623+K00162+K00873+K00161+K03841+K00873+K00382+K00128+K03841+K00850+K01803+K03841+K00128+K01623+K00161+K01792+K03841+K00850+K00161+K00895+K00134+K03103+K00382+K00873+K15634+K01803+K03841+K00128+K00128+K00895+K00895+K01792+K01689+K00627+K03103+K01810+K00895+K00382+K14085+K01810+K01810+K01810+K00873+K00382+K01810+K00161+K01623+K01792+K01810+K00162+K01610+K00873+K00161+K01689+K01835+K00161+K00128+K00627+K00128+K03841+K00895+K00873+K00128+K00895+K00382+K01623+K03841+K01792+K00873+K00121+K01792+K01810+K01623+K00128+K15633+K00627+K01792+K00134+K18857+K00128+K00873+K00927+K00873+K01623+K15633+K03841+K00844+K01792+K00627+K00895+K00001+K00128+K01568+K00128+K01792+K01810+K01834+K01803+K00128+K00627+K00134+K01792+K00128+K01895+K03841+K01792+K03103+K00627+K01623+K00873+K15633+K01792+K01835+K00128+K01835+K01810+K00128+K00128+K00927+K01803+K01803+K00927+K00873+K00128+K00128+K00134+K01835+K00895+K01689+K00162+K01810+K01792+K01803+K00844+K00927+K00873+K00121+K00850+K00121+K03841+K15633+K01610+K00128+K01835+K01792+K00128+K00128+K01803+K01810+K01568+K00128+K01792+K00128+K00121+K00128+K00131+K

	01_transcript_116518;F01_transcript_116556;F01_transcript_117151;F01_transcript_117237;F01_transcript_117555;F01_transcript_117574;F01_transcript_117586;F01_transcript_11770;F01_transcript_117732;F01_transcript_117798;F01_transcript_117826;F01_transcript_118433;F01_transcript_118736;F01_transcript_11882;F01_transcript_118848;F01_transcript_118900;F01_transcript_119152;F01_transcript_11934;F01_transcript_119343;F01_transcript_119475;F01_transcript_11951;F01_transcript_119854;F01_transcript_119939;F01_transcript_120402;F01_transcript_120942;F01_transcript_121145;F01_transcript_121238;F01_transcript_121401;F01_transcript_121571;F01_transcript_121819;F01_transcript_122012;F01_transcript_122215;F01_transcript_122638;F01_transcript_122893;F01_transcript_122972;F01_transcript_1231;F01_transcript_12318;F01_transcript_123349;F01_transcript_124464;F01_transcript_124658;F01_transcript_125563;F01_transcript_126086;F01_transcript_126146;F01_transcript_126284;F01_transcript_126405;F01_transcript_126465;F01_transcript_126836;F01_transcript_127146;F01_transcript_127282;F01_transcript_127326;F01_transcript_127660;F01_transcript_127751;F01_transcript_127834;F01_transcript_128187;F01_transcript_128218;F01_transcript_128243;F01_transcript_128590;F01_transcript_128662;F01_transcript_128872;F01_transcript_129084;F01_transcript_129206;F01_transcript_129712;F01_transcript_129886;F01_transcript_129897;F01_transcript_129946;F01_transcript_130000;F01_transcript_131057;F01_transcript_13143;F01_transcript_132330;F01_transcript_132494;F01_transcript_132804;F01_transcript_132921;F01_transcript_132930;F01_transcript_1334;F01_transcript_133545;F01_transcript_133587;F01_transcript_13368;F01_transcript_134102;F01_transcript_13417;F01_transcript_134251;F01_transcript_134507;F01_transcript_135113;F01_transcript_135131;F01_transcript_135142;F01_transcript_135187;F01_transcript_135612;F01_transcript_135613;F01_transcript_135657;F01_transcript_135767;F01_transcript_135864;F01_transcript_135995;F01_transcript_136113;F01_transcript_136325;F01_transcript_136831;F01_transcript_136852;F01_transcript_136868;F01_transcript_137134;F01_transcript_137514;F01_transcript_137525;F01_transcript_137623;F01_transcript_13771;F01_transcript_137935;F01_transcript_138093;F01_tran	01623+K00134+K01792+K00927+K01895+K01785+K00131+K15633+K03103+K01792+K00873+K15634+K01792+K00121+K15633+K00382+K01835+K00873+K01568+K00895+K00627+K00128+K01792+K00128+K00134+K01610+K01623+K00128+K00128+K01623+K01568+K00128+K00162+K00121+K03841+K00850+K00128+K00927+K15633+K03841+K01792+K00128+K01623+K00844+K01610+K03103+K00382+K00128+K00895+K00382+K01803+K00128+K01623+K01895+K00134+K00128+K00128+K00128+K00161+K01689+K01623+K00128+K01792+K01623+K00131+K00873+K01785+K01623+K01895+K00927+K00161+K01689+K00131+K01803+K00128+K00927+K01835+K00895+K00131+K01835+K00927+K01623+K00131+K01835+K00850+K00850+K00161+K00121+K00873+K00850+K00873+K00850+K00128+K01623+K01803+K00850+K14085+K00873+K00128+K00873+K01792+K01568+K00873+K03841+K00134+K03103+K18857+K00128+K00844+K00128+K00001+K00128+K00128+K00895+K00162+K00161+K00134+K00382+K00162+K01792+K00873+K00895+K00121+K01810+K01792+K03841+K01610+K18857+K00131+K01834+K00850+K01810+K01623+K03841+K01835+K00131+K01835+K00873+K00162+K01803+K03841+K01792+K00895+K01689+K01623+K01610+K00121+K00131+K01568+K01689+K01610+K00128+K01610+K01810+K00128+K01689+K01623+K01568+K01835+K00873+K00895+K01810+K00927+K00895+K00895+K01803+K00895+K00895+K01792+K00627+K01835+K00382+K00627+K01568+K01792+K00873+K00927+K00873+K00873+K00131+K00128+K01568+K00128+K00873+K00382+K00895+K00873+K01803+K00850+K00128+K01792+K01792+K01568+K01835+K00873+K01835+K00382+K00128+K00850+K01835+K00873+K00128+K00895+K15633+K00873+K00844+K01610+K00627+K00382+K00627+K00128+K01610+K00382+K00844+K00850+K15634+K00873+K00873+K00873+K00128+K01623+K00873+K00128+K00382+K00873+K01689+K00873+K00873+K01792+K01810+K00627+K00873+K00128+K00873+K00627+K14085+K14085+K00128+K03103+K01689+K15634+K00128+K15634+K00627+K00131+K00927+K00627+K01623+K00128+K00627+K00128+K00131+K00927+K03103+K00128+K00873+K00001+K03103+K00927+K00873+K0012

	script_138326;F01_transcript_138795;F01_transcript_138828;F01_transcript_138897;F01_transcript_138906;F01_transcript_138921;F01_transcript_139169;F01_transcript_139567;F01_transcript_139573;F01_transcript_140250;F01_transcript_140783;F01_transcript_140840;F01_transcript_140868;F01_transcript_141140;F01_transcript_141154;F01_transcript_141376;F01_transcript_141418;F01_transcript_141635;F01_transcript_141754;F01_transcript_141836;F01_transcript_141901;F01_transcript_141980;F01_transcript_142036;F01_transcript_142290;F01_transcript_143349;F01_transcript_143548;F01_transcript_143572;F01_transcript_143951;F01_transcript_14447;F01_transcript_144572;F01_transcript_144636;F01_transcript_144822;F01_transcript_144850;F01_transcript_144853;F01_transcript_145043;F01_transcript_145324;F01_transcript_145352;F01_transcript_145474;F01_transcript_145851;F01_transcript_145932;F01_transcript_146046;F01_transcript_146217;F01_transcript_146237;F01_transcript_146244;F01_transcript_146888;F01_transcript_147257;F01_transcript_147408;F01_transcript_147492;F01_transcript_147603;F01_transcript_147704;F01_transcript_147783;F01_transcript_147987;F01_transcript_148041;F01_transcript_148049;F01_transcript_148772;F01_transcript_149234;F01_transcript_149292;F01_transcript_149792;F01_transcript_150228;F01_transcript_150940;F01_transcript_151264;F01_transcript_151300;F01_transcript_151518;F01_transcript_151553;F01_transcript_151832;F01_transcript_151939;F01_transcript_152756;F01_transcript_152795;F01_transcript_152798;F01_transcript_152812;F01_transcript_152958;F01_transcript_153108;F01_transcript_153307;F01_transcript_153422;F01_transcript_153523;F01_transcript_153664;F01_transcript_153773;F01_transcript_153897;F01_transcript_153933;F01_transcript_154172;F01_transcript_154485;F01_transcript_154540;F01_transcript_154897;F01_transcript_154982;F01_transcript_155306;F01_transcript_155598;F01_transcript_155659;F01_transcript_156057;F01_transcript_156116;F01_transcript_156346;F01_transcript_156435;F01_transcript_156464;F01_transcript_156780;F01_transcript_157081;F01_transcript_157674;F01_transcript_157893;F01_transcript_158132;F01_transcript_158451;F01_transcript_158480;F01_transcript_158716;F01_tra	8+K00134+K00873+K00128+K00162+K00128+K00131+K00001+K01623+K01689+K00382+K00162+K00927+K18857+K01834+K00161+K00128+K00134+K00927+K00131+K01610+K00161+K00162+K01623+K00162+K00121+K01792+K01623+K00128+K00128+K00134+K00161+K00162+K01623+K00162+K00927+K00161+K00121+K00128+K03841+K01623+K00128+K00128+K01623+K00927+K00927+K00128+K00121+K01792+K00134+K01792+K00162+K01803+K00134+K01803+K00134+K00927+K00627+K00121+K01792+K03841+K01792+K18857+K00134+K03841+K00134+K01785+K00134+K00134+K00134+K00121+K01803+K01623+K03841+K01785+K00134+K00128+K01792+K01803+K01623+K00382+K01803+K03841+K01803+K01623+K01803+K00134+K00121+K00895+K00128+K00134+K01623+K01623+K01623+K01623+K00927+K01792+K15634+K03841+K00162+K15633+K00873+K01792+K00895+K00927+K00134+K00873+K00927+K01792+K01803+K01803+K00121+K00128+K01623+K01785+K00128+K01835+K14085+K00895+K00850+K15634+K01623+K00850+K00927+K01810+K01568+K01792+K00382+K00627+K03841+K00162+K00895+K00895+K00121+K15633+K00162+K01792+K00121+K00850+K00850+K00382+K03103+K01792+K01568+K00844+K00895+K00873+K00873+K01610+K00382+K01623+K01792+K00873+K01623+K01810+K14085+K00873+K15633+K03841+K00873+K01785+K00128+K00128+K00844+K00128+K00128+K15633+K01610+K01835+K01810+K00134+K00162+K00128+K01792+K03841+K01568+K03103+K00134+K03841+K00895+K01792+K00627+K01792+K00128+K01689+K00844+K00128+K03841+K00128+K01792+K01792+K00927+K03841+K00627+K00128+K01623+K00382+K01568+K15633+K00128+K00627+K00850+K01810+K01568+K00382+K00895+K00927+K01810+K00161+K03841+K00128+K01623+K00850+K00134+K00016+K00927+K03841+K00161+K01792+K03841+K00844+K00873+K15634+K00161+K15633+K15633+K14085+K01623+K01568+K00162+K01689+K01810+K01835+K00382+K00134+K00128+K00128+K00121+K00134+K03103+K00128+K15633+K01792+K01895+K01810+K00873+K00382+K01792+K01792+K00161+K01792+K00873+K01623+K01803+K00

	nscript_158736;F01_transcript_158912;F01_transcript_159245;F01_transcript_159358;F01_transcript_159443;F01_transcript_159514;F01_transcript_159748;F01_transcript_159814;F01_transcript_160044;F01_transcript_160064;F01_transcript_160090;F01_transcript_160410;F01_transcript_160426;F01_transcript_160634;F01_transcript_161132;F01_transcript_161429;F01_transcript_161816;F01_transcript_161918;F01_transcript_161966;F01_transcript_162066;F01_transcript_162134;F01_transcript_162331;F01_transcript_162372;F01_transcript_162648;F01_transcript_162736;F01_transcript_162882;F01_transcript_162906;F01_transcript_16293;F01_transcript_163608;F01_transcript_163795;F01_transcript_164007;F01_transcript_164021;F01_transcript_164032;F01_transcript_164164;F01_transcript_164751;F01_transcript_164754;F01_transcript_164766;F01_transcript_164870;F01_transcript_165106;F01_transcript_165153;F01_transcript_165159;F01_transcript_165396;F01_transcript_165440;F01_transcript_165871;F01_transcript_165904;F01_transcript_166075;F01_transcript_166121;F01_transcript_166454;F01_transcript_166475;F01_transcript_166935;F01_transcript_166974;F01_transcript_167162;F01_transcript_167640;F01_transcript_167733;F01_transcript_16829;F01_transcript_17183;F01_transcript_17385;F01_transcript_17878;F01_transcript_17879;F01_transcript_17942;F01_transcript_1799;F01_transcript_18092;F01_transcript_19381;F01_transcript_19412;F01_transcript_19656;F01_transcript_1968;F01_transcript_19714;F01_transcript_19835;F01_transcript_20518;F01_transcript_20997;F01_transcript_21106;F01_transcript_21167;F01_transcript_21327;F01_transcript_21988;F01_transcript_22107;F01_transcript_22140;F01_transcript_22373;F01_transcript_22745;F01_transcript_22766;F01_transcript_22972;F01_transcript_23074;F01_transcript_23078;F01_transcript_23097;F01_transcript_23224;F01_transcript_23258;F01_transcript_23299;F01_transcript_23327;F01_transcript_23407;F01_transcript_23477;F01_transcript_23785;F01_transcript_23979;F01_transcript_24020;F01_transcript_24502;F01_transcript_24513;F01_transcript_24531;F01_transcript_24533;F01_transcript_24566;F01_transcript_24624;F01_transcript_24677;F01_transcript_24751;F01_transcript_25167;F01_transcript_25379;F01_transcript_25381;F01_transcript_25467;F01_transcript_25470;F01_transcript_	927+K18857+K00927+K01610+K00873+K00844+K01835+K00873+K01785+K01785+K00895+K01895+K00895+K14085+K00873+K01623+K01623+K15634+K00121+K00873+K00134+K00873+K01803+K01792+K00121+K00382+K00001+K00121+K00382+K01792+K00128+K00873+K03103+K00927+K01610+K01792+K01835+K01810+K00162+K00895+K00128+K00128+K00873+K00382+K00128+K00128+K01689+K00844+K00895+K00162+K00128+K00895+K01610

25482;F01_transcript_25659;F01_transcript_25920;F01_transcript_25979;F01_transcript_26085;F01_transcript_26120;F01_transcript_26163;F01_transcript_26406;F01_transcript_26508;F01_transcript_26688;F01_transcript_27039;F01_transcript_27096;F01_transcript_27300;F01_transcript_27373;F01_transcript_27376;F01_transcript_27479;F01_transcript_27827;F01_transcript_27931;F01_transcript_27932;F01_transcript_28043;F01_transcript_28090;F01_transcript_28152;F01_transcript_28168;F01_transcript_28572;F01_transcript_28741;F01_transcript_28763;F01_transcript_28856;F01_transcript_28894;F01_transcript_28992;F01_transcript_29149;F01_transcript_29161;F01_transcript_29309;F01_transcript_29525;F01_transcript_29538;F01_transcript_29760;F01_transcript_29765;F01_transcript_29858;F01_transcript_29908;F01_transcript_30176;F01_transcript_30292;F01_transcript_30362;F01_transcript_30379;F01_transcript_30543;F01_transcript_30852;F01_transcript_30880;F01_transcript_30935;F01_transcript_31066;F01_transcript_31148;F01_transcript_31162;F01_transcript_31188;F01_transcript_31562;F01_transcript_31687;F01_transcript_31894;F01_transcript_31945;F01_transcript_32017;F01_transcript_32044;F01_transcript_32090;F01_transcript_32242;F01_transcript_32266;F01_transcript_32422;F01_transcript_32640;F01_transcript_32765;F01_transcript_33013;F01_transcript_33286;F01_transcript_33325;F01_transcript_33400;F01_transcript_33449;F01_transcript_33600;F01_transcript_34179;F01_transcript_34271;F01_transcript_34275;F01_transcript_34354;F01_transcript_34413;F01_transcript_34418;F01_transcript_34439;F01_transcript_34513;F01_transcript_34534;F01_transcript_34541;F01_transcript_34832;F01_transcript_35239;F01_transcript_35286;F01_transcript_35302;F01_transcript_35313;F01_transcript_35332;F01_transcript_35540;F01_transcript_35667;F01_transcript_36043;F01_transcript_36056;F01_transcript_36124;F01_transcript_36465;F01_transcript_36468;F01_transcript_36584;F01_transcript_36697;F01_transcript_36801;F01_transcript_36808;F01_transcript_37154;F01_transcript_37233;F01_transcript_37274;F01_transcript_37456;F01_transcript_37523;F01_transcript_37614;F01_transcript_37617;F01_transcript_37696;F01_transcript_37740;F01_transcript_37835;F01_transcript_37926;

F01_transcript_37987;F01_transcript_38228;F01_transcript_382
74;F01_transcript_38315;F01_transcript_38323;F01_transcript_
38373;F01_transcript_38610;F01_transcript_38937;F01_transcri
pt_39009;F01_transcript_39026;F01_transcript_39223;F01_tran
script_39305;F01_transcript_39520;F01_transcript_39525;F01_t
ranscript_39557;F01_transcript_39600;F01_transcript_39701;F0
1_transcript_39763;F01_transcript_39895;F01_transcript_39949
;F01_transcript_39959;F01_transcript_40033;F01_transcript_40
114;F01_transcript_40209;F01_transcript_40233;F01_transcript
_40354;F01_transcript_40448;F01_transcript_40536;F01_transc
ript_40578;F01_transcript_40883;F01_transcript_41015;F01_tra
nscript_41061;F01_transcript_41388;F01_transcript_41482;F01
_transcript_41704;F01_transcript_41742;F01_transcript_43050;
F01_transcript_43061;F01_transcript_4391;F01_transcript_4427
4;F01_transcript_44495;F01_transcript_44524;F01_transcript_4
5361;F01_transcript_47618;F01_transcript_47886;F01_transcrip
t_48006;F01_transcript_48211;F01_transcript_48424;F01_trans
cript_48625;F01_transcript_49278;F01_transcript_49281;F01_tr
anscript_49964;F01_transcript_50604;F01_transcript_51613;F0
1_transcript_51738;F01_transcript_51810;F01_transcript_51889
;F01_transcript_51976;F01_transcript_52139;F01_transcript_52
465;F01_transcript_52590;F01_transcript_52817;F01_transcript
_53223;F01_transcript_53385;F01_transcript_53589;F01_transc
ript_54119;F01_transcript_54273;F01_transcript_54487;F01_tra
nscript_55263;F01_transcript_55528;F01_transcript_55541;F01
_transcript_55560;F01_transcript_55798;F01_transcript_56137;
F01_transcript_56580;F01_transcript_56690;F01_transcript_566
95;F01_transcript_56717;F01_transcript_56754;F01_transcript_
56762;F01_transcript_56794;F01_transcript_56950;F01_transcri
pt_56958;F01_transcript_57537;F01_transcript_57602;F01_tran
script_57636;F01_transcript_57807;F01_transcript_57906;F01_t
ranscript_58405;F01_transcript_59220;F01_transcript_59277;F0
1_transcript_59371;F01_transcript_59406;F01_transcript_59734
;F01_transcript_59798;F01_transcript_59821;F01_transcript_59
961;F01_transcript_60235;F01_transcript_60314;F01_transcript
_60325;F01_transcript_6047;F01_transcript_60677;F01_transcri
pt_60736;F01_transcript_61026;F01_transcript_61112;F01_tran
script_61414;F01_transcript_61908;F01_transcript_62135;F01_t

ranscript_62451;F01_transcript_62781;F01_transcript_63160;F01_transcript_63171;F01_transcript_63241;F01_transcript_63491;F01_transcript_63521;F01_transcript_63669;F01_transcript_63911;F01_transcript_64577;F01_transcript_64674;F01_transcript_65034;F01_transcript_65403;F01_transcript_65494;F01_transcript_65979;F01_transcript_66261;F01_transcript_66352;F01_transcript_66550;F01_transcript_66664;F01_transcript_66746;F01_transcript_66793;F01_transcript_67026;F01_transcript_67211;F01_transcript_67303;F01_transcript_67319;F01_transcript_67473;F01_transcript_67492;F01_transcript_68495;F01_transcript_68522;F01_transcript_68679;F01_transcript_69105;F01_transcript_69884;F01_transcript_69945;F01_transcript_70471;F01_transcript_70950;F01_transcript_70970;F01_transcript_71260;F01_transcript_71315;F01_transcript_71646;F01_transcript_71752;F01_transcript_71827;F01_transcript_72018;F01_transcript_72250;F01_transcript_72311;F01_transcript_72349;F01_transcript_72421;F01_transcript_729;F01_transcript_72990;F01_transcript_73266;F01_transcript_73295;F01_transcript_73477;F01_transcript_73635;F01_transcript_73888;F01_transcript_74268;F01_transcript_74401;F01_transcript_74585;F01_transcript_74637;F01_transcript_74751;F01_transcript_74764;F01_transcript_74788;F01_transcript_75083;F01_transcript_75206;F01_transcript_75573;F01_transcript_76121;F01_transcript_76175;F01_transcript_76178;F01_transcript_76279;F01_transcript_76335;F01_transcript_76574;F01_transcript_76721;F01_transcript_76768;F01_transcript_76856;F01_transcript_77136;F01_transcript_77252;F01_transcript_77418;F01_transcript_77873;F01_transcript_77976;F01_transcript_78063;F01_transcript_78185;F01_transcript_78525;F01_transcript_78831;F01_transcript_7908;F01_transcript_79249;F01_transcript_79704;F01_transcript_79736;F01_transcript_79852;F01_transcript_80510;F01_transcript_80594;F01_transcript_80994;F01_transcript_81070;F01_transcript_81584;F01_transcript_81686;F01_transcript_81931;F01_transcript_8208;F01_transcript_8235;F01_transcript_82389;F01_transcript_82589;F01_transcript_82652;F01_transcript_82739;F01_transcript_82889;F01_transcript_83165;F01_transcript_83284;F01_transcript_83682;F01_transcript_83748;F01_transcript_83916;F01_transcript_84

			296;F01_transcript_84920;F01_transcript_85267;F01_transcript_85796;F01_transcript_86116;F01_transcript_86207;F01_transcript_8631;F01_transcript_86630;F01_transcript_86802;F01_transcript_87312;F01_transcript_8733;F01_transcript_87825;F01_transcript_8790;F01_transcript_87964;F01_transcript_88234;F01_transcript_88445;F01_transcript_8856;F01_transcript_88860;F01_transcript_89459;F01_transcript_89719;F01_transcript_89922;F01_transcript_90169;F01_transcript_90499;F01_transcript_91037;F01_transcript_91232;F01_transcript_91452;F01_transcript_91795;F01_transcript_92441;F01_transcript_9254;F01_transcript_92804;F01_transcript_92968;F01_transcript_93794;F01_transcript_93824;F01_transcript_93976;F01_transcript_94052;F01_transcript_94317;F01_transcript_95055;F01_transcript_95452;F01_transcript_96218;F01_transcript_96227;F01_transcript_97230;F01_transcript_97966;F01_transcript_98159;F01_transcript_98282;F01_transcript_98445;F01_transcript_98475;F01_transcript_98699;F01_transcript_99038;F01_transcript_99341;F01_transcript_99528;F01_transcript_99768;F01_transcript_99977;	
Citrate cycle (TCA cycle)	ko00020	324	F01_transcript_100005;F01_transcript_100254;F01_transcript_101218;F01_transcript_102294;F01_transcript_102370;F01_transcript_102731;F01_transcript_102834;F01_transcript_103394;F01_transcript_103488;F01_transcript_104320;F01_transcript_104691;F01_transcript_104746;F01_transcript_105291;F01_transcript_105634;F01_transcript_105726;F01_transcript_105972;F01_transcript_107153;F01_transcript_107970;F01_transcript_108216;F01_transcript_108617;F01_transcript_109283;F01_transcript_109885;F01_transcript_110879;F01_transcript_111778;F01_transcript_112117;F01_transcript_112623;F01_transcript_113031;F01_transcript_114667;F01_transcript_114898;F01_transcript_114966;F01_transcript_115077;F01_transcript_115594;F01_transcript_115624;F01_transcript_116381;F01_transcript_116451;F01_transcript_116556;F01_transcript_116740;F01_transcript_116945;F01_transcript_117319;F01_transcript_117415;F01_transcript_117574;F01_transcript_117586;F01_transcript_11767;F01_transcript_117732;F01_transcript_117804;F01_transcript_118433;F01_transcript_118657;F01_transcript_118710;F01_transcript_11882;F01_transcript_119034;F01_transcript_119411;F01_transcript_11951;F01_transcript_119534;F01_transcript_120543;F01	K00026+K01679+K01647+K01899+K00162+K00162+K00162+K00026+K00026+K00026+K00627+K00382+K01647+K01681+K00162+K00161+K01679+K00658+K00382+K00026+K00026+K00161+K00161+K00164+K00382+K01647+K00164+K00234+K00627+K00031+K00030+K00026+K00382+K00026+K00382+K00161+K00234+K01647+K01647+K00026+K00162+K01610+K00030+K00161+K00234+K00161+K01647+K00026+K00627+K00031+K00026+K00382+K00031+K01899+K01681+K00627+K00235+K01647+K00234+K00026+K00627+K00026+K01647+K00627+K00026+K00235+K01899+K00627+K00025+K00026+K01648+K01648+K00658+K01647+K01648+K00658+K00026+K00162+K00026+K00025+K00030+K01610+K00234+K01681+K00026+K00026+K01899+K00030+K00382+K00030+K00627+K00026+K01610+K00030+K00162+K00031+K00026+K00026+K00026+K01610+K01647+K00026+K00658+K00382+K01681+K01648+K01648+K00026+K00030+K00382+K00161+K01681+K00161+K01648+K00234+K00164+K00161+K00026+K00026+K01647+K00026+K01647+K00234+K01681+K01648+K00026+K00162+K00161+K00026+K00382+K00162+K00026+K00164+K01647+K01899+K00026+K01610+K

	_transcript_121896;F01_transcript_122215;F01_transcript_123542;F01_transcript_123773;F01_transcript_124944;F01_transcript_126030;F01_transcript_126405;F01_transcript_126482;F01_transcript_127049;F01_transcript_128243;F01_transcript_128256;F01_transcript_129207;F01_transcript_129651;F01_transcript_129897;F01_transcript_130017;F01_transcript_130127;F01_transcript_131000;F01_transcript_131367;F01_transcript_131432;F01_transcript_131587;F01_transcript_132349;F01_transcript_132504;F01_transcript_134955;F01_transcript_135612;F01_transcript_135698;F01_transcript_136111;F01_transcript_136221;F01_transcript_137514;F01_transcript_137621;F01_transcript_138295;F01_transcript_139464;F01_transcript_140879;F01_transcript_141489;F01_transcript_142960;F01_transcript_143349;F01_transcript_144117;F01_transcript_144572;F01_transcript_144706;F01_transcript_145043;F01_transcript_145058;F01_transcript_146217;F01_transcript_147008;F01_transcript_147154;F01_transcript_147445;F01_transcript_147898;F01_transcript_148049;F01_transcript_148941;F01_transcript_149184;F01_transcript_149233;F01_transcript_149234;F01_transcript_149398;F01_transcript_149454;F01_transcript_149801;F01_transcript_149909;F01_transcript_149933;F01_transcript_150228;F01_transcript_152795;F01_transcript_153015;F01_transcript_154172;F01_transcript_154201;F01_transcript_155034;F01_transcript_1562;F01_transcript_157893;F01_transcript_158374;F01_transcript_160122;F01_transcript_160446;F01_transcript_161406;F01_transcript_161671;F01_transcript_161943;F01_transcript_162211;F01_transcript_162251;F01_transcript_162570;F01_transcript_162648;F01_transcript_162736;F01_transcript_162804;F01_transcript_162906;F01_transcript_16293;F01_transcript_163028;F01_transcript_163191;F01_transcript_163468;F01_transcript_163897;F01_transcript_164460;F01_transcript_164754;F01_transcript_166018;F01_transcript_166454;F01_transcript_167137;F01_transcript_167536;F01_transcript_167751;F01_transcript_16829;F01_transcript_17239;F01_transcript_17942;F01_transcript_18092;F01_transcript_18156;F01_transcript_19052;F01_transcript_19652;F01_transcript_20561;F01_transcript_20578;F01_transcript_22454;F01_transcript_22766;F01_transcript_22891;F01_transcript_230	01648+K00162+K00026+K01648+K01647+K01610+K01648+K01610+K01610+K01648+K00234+K01648+K01648+K01899+K01648+K00627+K00026+K00382+K00627+K00382+K01900+K00382+K00030+K01681+K00164+K01610+K00627+K00382+K00627+K01610+K01679+K00382+K01647+K00382+K00627+K00627+K01647+K00030+K00026+K00627+K00658+K00234+K00658+K00627+K00627+K01648+K00658+K00026+K00162+K00030+K01900+K00026+K00382+K00162+K00031+K00161+K01610+K01647+K00161+K01648+K01648+K01648+K00162+K00162+K00026+K01648+K00026+K00161+K00162+K00162+K01648+K00161+K00026+K00026+K00025+K00162+K00026+K00627+K00026+K00025+K00026+K00025+K00025+K01899+K00235+K00382+K00026+K00162+K00164+K01648+K01647+K00031+K00031+K00658+K00235+K00234+K00164+K00382+K00030+K00627+K00162+K00030+K00026+K00162+K01679+K00026+K00235+K00382+K00658+K01681+K00235+K01610+K00658+K00382+K00030+K01648+K01647+K00026+K01681+K00026+K01610+K00234+K00162+K00026+K00026+K00026+K00627+K00030+K00026+K01648+K00030+K01647+K00627+K00025+K00382+K00627+K00026+K00658+K00382+K00161+K00031+K00161+K01900+K00161+K01679+K00164+K01681+K00026+K00162+K00026+K01681+K00026+K01648+K00025+K00382+K00026+K00658+K00026+K00382+K01647+K00161+K01648+K00026+K00026+K01610+K01647+K00030+K00026+K00030+K00382+K00026+K00382+K01610+K01647+K00026+K00162+K00026+K00026+K00026+K00234+K00382+K00026+K00031+K00162+K01647+K00026+K01610

74;F01_transcript_23078;F01_transcript_24513;F01_transcript_
24919;F01_transcript_25482;F01_transcript_26583;F01_transcri
pt_2680;F01_transcript_2693;F01_transcript_27039;F01_transcr
ipt_27096;F01_transcript_27300;F01_transcript_27373;F01_tra
nscript_27479;F01_transcript_27528;F01_transcript_27827;F01
_transcript_28828;F01_transcript_28894;F01_transcript_29760;
F01_transcript_30176;F01_transcript_30906;F01_transcript_309
97;F01_transcript_31005;F01_transcript_31148;F01_transcript_
31205;F01_transcript_31236;F01_transcript_31297;F01_transcri
pt_31562;F01_transcript_31945;F01_transcript_33372;F01_tran
script_33503;F01_transcript_34070;F01_transcript_34179;F01_t
ranscript_34218;F01_transcript_34332;F01_transcript_34420;F0
1_transcript_34439;F01_transcript_34513;F01_transcript_34767
;F01_transcript_35239;F01_transcript_35540;F01_transcript_35
664;F01_transcript_35667;F01_transcript_35729;F01_transcript
_35809;F01_transcript_35844;F01_transcript_36043;F01_transc
ript_36124;F01_transcript_36309;F01_transcript_36483;F01_tra
nscript_36873;F01_transcript_37154;F01_transcript_37233;F01
_transcript_37456;F01_transcript_37600;F01_transcript_37614;
F01_transcript_37900;F01_transcript_38243;F01_transcript_382
73;F01_transcript_39026;F01_transcript_39388;F01_transcript_
39600;F01_transcript_39641;F01_transcript_40133;F01_transcri
pt_40284;F01_transcript_40490;F01_transcript_40600;F01_tran
script_41010;F01_transcript_43846;F01_transcript_4391;F01_tr
anscript_49083;F01_transcript_51976;F01_transcript_52137;F0
1_transcript_52230;F01_transcript_52381;F01_transcript_52686
;F01_transcript_52750;F01_transcript_53752;F01_transcript_56
233;F01_transcript_56927;F01_transcript_57448;F01_transcript
_57636;F01_transcript_57701;F01_transcript_57807;F01_transc
ript_58405;F01_transcript_58552;F01_transcript_59025;F01_tra
nscript_59734;F01_transcript_59767;F01_transcript_60128;F01
_transcript_60229;F01_transcript_60314;F01_transcript_61072;
F01_transcript_61449;F01_transcript_61538;F01_transcript_619
08;F01_transcript_61978;F01_transcript_62135;F01_transcript_
63028;F01_transcript_64173;F01_transcript_64872;F01_transcri
pt_65371;F01_transcript_65464;F01_transcript_6571;F01_trans
cript_66550;F01_transcript_66560;F01_transcript_67026;F01_tr
anscript_68075;F01_transcript_69158;F01_transcript_69697;F0

			1_transcript_69884;F01_transcript_70154;F01_transcript_7025;F01_transcript_70771;F01_transcript_70881;F01_transcript_71789;F01_transcript_72311;F01_transcript_72893;F01_transcript_729;F01_transcript_73477;F01_transcript_73713;F01_transcript_74262;F01_transcript_74401;F01_transcript_74764;F01_transcript_75938;F01_transcript_76335;F01_transcript_76476;F01_transcript_77252;F01_transcript_77968;F01_transcript_78355;F01_transcript_78472;F01_transcript_78511;F01_transcript_78525;F01_transcript_78775;F01_transcript_78791;F01_transcript_79019;F01_transcript_79119;F01_transcript_79482;F01_transcript_79704;F01_transcript_80009;F01_transcript_80046;F01_transcript_80216;F01_transcript_82589;F01_transcript_82791;F01_transcript_82889;F01_transcript_83987;F01_transcript_84462;F01_transcript_84788;F01_transcript_85267;F01_transcript_86113;F01_transcript_87452;F01_transcript_89898;F01_transcript_90207;F01_transcript_91232;F01_transcript_91546;F01_transcript_92441;F01_transcript_93976;F01_transcript_94796;F01_transcript_95043;F01_transcript_95452;F01_transcript_95502;F01_transcript_959;F01_transcript_97342;F01_transcript_97960;F01_transcript_98159;F01_transcript_98458;F01_transcript_98527;F01_transcript_99341;F01_transcript_99615;F01_transcript_99915;F01_transcript_99977;	
Pentose phosphate pathway	ko00030	374	F01_transcript_100238;F01_transcript_100430;F01_transcript_100797;F01_transcript_100825;F01_transcript_101574;F01_transcript_102189;F01_transcript_102262;F01_transcript_102681;F01_transcript_102826;F01_transcript_102845;F01_transcript_103331;F01_transcript_103366;F01_transcript_103571;F01_transcript_103675;F01_transcript_10450;F01_transcript_104756;F01_transcript_104986;F01_transcript_105258;F01_transcript_105365;F01_transcript_105445;F01_transcript_105645;F01_transcript_10578;F01_transcript_105919;F01_transcript_106166;F01_transcript_106765;F01_transcript_107113;F01_transcript_107638;F01_transcript_107770;F01_transcript_108371;F01_transcript_108502;F01_transcript_108868;F01_transcript_109032;F01_transcript_109271;F01_transcript_109676;F01_transcript_110541;F01_transcript_110549;F01_transcript_110559;F01_transcript_110771;F01_transcript_111322;F01_transcript_112669;F01_transcript	K00615+K01783+K00036+K00615+K00850+K01783+K00895+K01807+K01783+K01623+K00615+K00033+K01810+K00615+K00615+K03841+K00850+K00948+K01623+K00036+K01807+K00615+K01807+K01807+K03841+K01783+K00615+K00852+K00615+K03841+K00850+K03841+K00036+K01623+K03841+K00615+K00850+K00615+K00895+K03841+K00033+K00895+K00895+K01807+K01810+K00895+K00948+K01810+K01810+K01810+K00036+K01810+K01623+K00033+K00615+K01783+K01810+K00616+K01057+K01835+K03841+K00895+K01807+K01807+K00895+K01623+K03841+K01807+K00615+K01783+K01810+K01623+K00615+K01783+K01623+K00615+K03841+K00895+K01810+K01783+K03841+K01783+K01623+K00851+K01835+K01835+K01810+K00615+K00615+K00948+K00948+K01835+K00895+K01057+K01810+K00615+K00615+K01783+K00850+K03841+K00036+K01835+K00615

	_113356;F01_transcript_113973;F01_transcript_114303;F01_transcript_114958;F01_transcript_115044;F01_transcript_115059;F01_transcript_115763;F01_transcript_116260;F01_transcript_116289;F01_transcript_116347;F01_transcript_116357;F01_transcript_116518;F01_transcript_117151;F01_transcript_117248;F01_transcript_11734;F01_transcript_117388;F01_transcript_117555;F01_transcript_117662;F01_transcript_117820;F01_transcript_117826;F01_transcript_118900;F01_transcript_119152;F01_transcript_119183;F01_transcript_119312;F01_transcript_119475;F01_transcript_119854;F01_transcript_119939;F01_transcript_120193;F01_transcript_120459;F01_transcript_121130;F01_transcript_121401;F01_transcript_121571;F01_transcript_122782;F01_transcript_124491;F01_transcript_124658;F01_transcript_12547;F01_transcript_126086;F01_transcript_126465;F01_transcript_127751;F01_transcript_128717;F01_transcript_129206;F01_transcript_129302;F01_transcript_129946;F01_transcript_131837;F01_transcript_132330;F01_transcript_132804;F01_transcript_132921;F01_transcript_13355;F01_transcript_133564;F01_transcript_133891;F01_transcript_134405;F01_transcript_135131;F01_transcript_135142;F01_transcript_135405;F01_transcript_135613;F01_transcript_135726;F01_transcript_135831;F01_transcript_136826;F01_transcript_136831;F01_transcript_136868;F01_transcript_136877;F01_transcript_137623;F01_transcript_138105;F01_transcript_138795;F01_transcript_138810;F01_transcript_139092;F01_transcript_139300;F01_transcript_139573;F01_transcript_139738;F01_transcript_140038;F01_transcript_140250;F01_transcript_140526;F01_transcript_141350;F01_transcript_141376;F01_transcript_143511;F01_transcript_143548;F01_transcript_144469;F01_transcript_14447;F01_transcript_145324;F01_transcript_145805;F01_transcript_145851;F01_transcript_146244;F01_transcript_146888;F01_transcript_147603;F01_transcript_147987;F01_transcript_149792;F01_transcript_149827;F01_transcript_151079;F01_transcript_151300;F01_transcript_151431;F01_transcript_151839;F01_transcript_152000;F01_transcript_152812;F01_transcript_152838;F01_transcript_153307;F01_transcript_153422;F01_transcript_153773;F01_transcript_154540;F01_transcript_154770;F01_transcript_155598;F01_transcript_155659;F01_transcript_156057;F01_transcript_156116;F01_trans	+K01810+K00036+K01807+K01807+K00131+K00616+K00948+K01623+K01057+K00036+K00131+K01807+K01835+K01807+K00895+K01623+K01807+K01623+K03841+K00850+K03841+K01623+K00895+K01807+K00615+K01623+K00948+K01057+K00036+K01623+K01807+K01623+K00131+K01623+K00131+K00036+K01835+K00895+K00131+K01835+K01623+K00131+K01835+K00850+K00850+K00850+K00850+K00036+K00615+K01623+K00850+K01807+K03841+K00948+K00036+K00895+K00615+K00948+K00895+K01810+K01783+K00036+K00615+K03841+K00131+K00036+K00850+K01810+K01783+K01623+K03841+K01835+K00131+K01835+K03841+K00895+K01623+K00131+K00036+K00036+K01810+K01623+K01835+K00895+K00615+K01810+K00895+K00895+K00895+K00895+K01835+K00131+K00895+K00850+K01835+K01835+K00850+K01835+K00895+K00036+K00850+K00033+K00036+K00948+K00036+K01623+K00036+K01783+K01810+K00036+K00033+K00036+K00033+K00131+K00033+K01623+K00033+K00131+K00033+K00948+K00948+K00131+K01623+K00948+K00131+K00852+K00616+K01623+K00615+K00616+K01623+K00852+K01623+K00616+K03841+K01623+K01623+K01807+K01783+K01807+K01807+K00616+K03841+K03841+K01807+K01623+K03841+K01783+K01807+K01807+K01057+K01057+K01783+K01783+K01057+K01623+K01807+K00615+K03841+K01807+K01807+K00851+K01623+K00851+K00895+K01623+K01623+K01623+K01623+K03841+K00036+K00895+K00615+K00615+K01623+K00852+K01835+K00895+K00850+K01623+K01783+K00850+K01057+K01810+K03841+K00615+K00895+K00895+K00850+K01783+K00850+K00895+K01807+K01623+K01807+K01623+K01810+K03841+K00615+K00852+K00615+K01835+K01810+K00033+K03841+K00615+K01807+K03841+K01783+K00895+K00033+K00033+K01057+K00851+K00615+K00615+K03841+K00851+K03841+K01623+K00036+K00615+K00033+K00850+K01810+K00895+K00615+K01810+K03841+K00615+K01623+K00850+K03841+K03841+K00036+K01623+K01057+K00615+K01810+K01835+K00615+K01807+K00948+K01057+K01807+K00033+K01810+K01623+K00615+K01835+K00895+K00895+K01807+K01623+K01623+K00615+K00033+K01807+K01783+K00036+K00

	cript_156435;F01_transcript_156464;F01_transcript_156780;F01_transcript_157081;F01_transcript_157674;F01_transcript_158480;F01_transcript_158736;F01_transcript_159010;F01_transcript_159049;F01_transcript_159245;F01_transcript_159443;F01_transcript_160206;F01_transcript_160426;F01_transcript_161004;F01_transcript_161713;F01_transcript_162372;F01_transcript_162722;F01_transcript_163703;F01_transcript_164007;F01_transcript_164032;F01_transcript_164521;F01_transcript_164702;F01_transcript_164718;F01_transcript_164751;F01_transcript_164870;F01_transcript_165059;F01_transcript_165153;F01_transcript_165159;F01_transcript_165336;F01_transcript_165396;F01_transcript_165440;F01_transcript_165871;F01_transcript_165904;F01_transcript_166075;F01_transcript_166935;F01_transcript_167162;F01_transcript_167733;F01_transcript_17385;F01_transcript_18744;F01_transcript_19222;F01_transcript_19381;F01_transcript_1968;F01_transcript_19835;F01_transcript_20997;F01_transcript_2109;F01_transcript_21106;F01_transcript_21327;F01_transcript_21988;F01_transcript_22140;F01_transcript_22373;F01_transcript_22972;F01_transcript_23477;F01_transcript_24531;F01_transcript_24624;F01_transcript_25381;F01_transcript_25470;F01_transcript_25920;F01_transcript_25979;F01_transcript_26163;F01_transcript_27689;F01_transcript_27932;F01_transcript_28236;F01_transcript_28474;F01_transcript_28633;F01_transcript_28678;F01_transcript_28741;F01_transcript_29080;F01_transcript_29301;F01_transcript_29538;F01_transcript_29825;F01_transcript_29902;F01_transcript_30407;F01_transcript_30823;F01_transcript_31162;F01_transcript_31274;F01_transcript_31687;F01_transcript_31959;F01_transcript_32044;F01_transcript_32428;F01_transcript_32449;F01_transcript_33728;F01_transcript_34275;F01_transcript_34413;F01_transcript_34996;F01_transcript_35332;F01_transcript_35884;F01_transcript_35990;F01_transcript_36056;F01_transcript_36178;F01_transcript_36447;F01_transcript_36584;F01_transcript_36789;F01_transcript_37274;F01_transcript_37402;F01_transcript_37740;F01_transcript_37835;F01_transcript_38228;F01_transcript_38229;F01_transcript_38522;F01_transcript_39110;F01_transcript_39348;F01_transcript_39842;F01_transcript_39895;F01_transcript_40	616+K00616+K01835+K01810+K00615+K00895+K00036+K00895+K00851+K00895+K00036

114;F01_transcript_40501;F01_transcript_41015;F01_transcript_41061;F01_transcript_41074;F01_transcript_41430;F01_transcript_41738;F01_transcript_42148;F01_transcript_42312;F01_transcript_42505;F01_transcript_42589;F01_transcript_42962;F01_transcript_43061;F01_transcript_44079;F01_transcript_4424;F01_transcript_44495;F01_transcript_44824;F01_transcript_45041;F01_transcript_45203;F01_transcript_45361;F01_transcript_45700;F01_transcript_48211;F01_transcript_49278;F01_transcript_49281;F01_transcript_49964;F01_transcript_50604;F01_transcript_51889;F01_transcript_51926;F01_transcript_52817;F01_transcript_53212;F01_transcript_53305;F01_transcript_55560;F01_transcript_56504;F01_transcript_56580;F01_transcript_56695;F01_transcript_56717;F01_transcript_56762;F01_transcript_56764;F01_transcript_56794;F01_transcript_56939;F01_transcript_56958;F01_transcript_57906;F01_transcript_5796;F01_transcript_59220;F01_transcript_59277;F01_transcript_59961;F01_transcript_60194;F01_transcript_60235;F01_transcript_61026;F01_transcript_61535;F01_transcript_62451;F01_transcript_62775;F01_transcript_63171;F01_transcript_63241;F01_transcript_63911;F01_transcript_64543;F01_transcript_65654;F01_transcript_65775;F01_transcript_66664;F01_transcript_66746;F01_transcript_6697;F01_transcript_67319;F01_transcript_67829;F01_transcript_68381;F01_transcript_68522;F01_transcript_68670;F01_transcript_68679;F01_transcript_68820;F01_transcript_69051;F01_transcript_69084;F01_transcript_70015;F01_transcript_70058;F01_transcript_71194;F01_transcript_71315;F01_transcript_72063;F01_transcript_72250;F01_transcript_72421;F01_transcript_72676;F01_transcript_73118;F01_transcript_73620;F01_transcript_73635;F01_transcript_73888;F01_transcript_74585;F01_transcript_74740;F01_transcript_74751;F01_transcript_74788;F01_transcript_7483;F01_transcript_75206;F01_transcript_75573;F01_transcript_76279;F01_transcript_76721;F01_transcript_76887;F01_transcript_78063;F01_transcript_78617;F01_transcript_78912;F01_transcript_7908;F01_transcript_79249;F01_transcript_79375;F01_transcript_80548;F01_transcript_80918;F01_transcript_81146;F01_transcript_81466;F01_transcript_82090;F01_transcript_8235;F01_transcript_83682;F01_transcript_85721;F01_transcript_86207;F01_transcript_87312;F01_transcript_87825;F01_tr

			anscript_88131;F01_transcript_88234;F01_transcript_88445;F01_transcript_88662;F01_transcript_89371;F01_transcript_8980;F01_transcript_90503;F01_transcript_90574;F01_transcript_91161;F01_transcript_92003;F01_transcript_94317;F01_transcript_95055;F01_transcript_96113;F01_transcript_96218;F01_transcript_97564;F01_transcript_99038;F01_transcript_99744;F01_transcript_99768;F01_transcript_99818;	
Pentose and glucuronate interconversions	ko00040	258	F01_transcript_100430;F01_transcript_100740;F01_transcript_101619;F01_transcript_102016;F01_transcript_102189;F01_transcript_102519;F01_transcript_102826;F01_transcript_103180;F01_transcript_103186;F01_transcript_103713;F01_transcript_103925;F01_transcript_105085;F01_transcript_105213;F01_transcript_105490;F01_transcript_106862;F01_transcript_107113;F01_transcript_107350;F01_transcript_108221;F01_transcript_108698;F01_transcript_109312;F01_transcript_10957;F01_transcript_110022;F01_transcript_110613;F01_transcript_111765;F01_transcript_112054;F01_transcript_112685;F01_transcript_112884;F01_transcript_114336;F01_transcript_11718;F01_transcript_117388;F01_transcript_118006;F01_transcript_118736;F01_transcript_118848;F01_transcript_119343;F01_transcript_120622;F01_transcript_121070;F01_transcript_121098;F01_transcript_121130;F01_transcript_121575;F01_transcript_121819;F01_transcript_122038;F01_transcript_122236;F01_transcript_1231;F01_transcript_124323;F01_transcript_124491;F01_transcript_124783;F01_transcript_125344;F01_transcript_125586;F01_transcript_126186;F01_transcript_127146;F01_transcript_127326;F01_transcript_127895;F01_transcript_128218;F01_transcript_128717;F01_transcript_128872;F01_transcript_129302;F01_transcript_130293;F01_transcript_131014;F01_transcript_131486;F01_transcript_132401;F01_transcript_132494;F01_transcript_132918;F01_transcript_132930;F01_transcript_1334;F01_transcript_134251;F01_transcript_134507;F01_transcript_134893;F01_transcript_135333;F01_transcript_135346;F01_transcript_136826;F01_transcript_137482;F01_transcript_137525;F01_transcript_137900;F01_transcript_137935;F01_transcript_138093;F01_transcript_138897;F01_transcript_138921;F01_transcript_139183;F01_transcript_139567;F01_transcript_140966;F01_transcript_142724;F01_tra	K01783+K00128+K00854+K01728+K01783+K00963+K01783+K01728+K01805+K00008+K00128+K01051+K00128+K01051+K16190+K01783+K01805+K00128+K00008+K00128+K01051+K00008+K01051+K00963+K01728+K00128+K00128+K01805+K01051+K01783+K01051+K00128+K00128+K00128+K01805+K00012+K00008+K01783+K01051+K00128+K00008+K00008+K00128+K01728+K01783+K01728+K00008+K00008+K01051+K00128+K00128+K00008+K00128+K01783+K00128+K01783+K00008+K01051+K01728+K01051+K00128+K00012+K00128+K00128+K00128+K00128+K01805+K00012+K01051+K01783+K01805+K00128+K00008+K00128+K00128+K00128+K00128+K00008+K00128+K00854+K00008+K01728+K01051+K00008+K00963+K00128+K00128+K01728+K00128+K00128+K00128+K00128+K00128+K00963+K01728+K01805+K00128+K00128+K00128+K00128+K01051+K00128+K00128+K01728+K01051+K00008+K00128+K01805+K00963+K12447+K01051+K00012+K00128+K00128+K00128+K00128+K00128+K00128+K00008+K01783+K01783+K00008+K00963+K00963+K00128+K00008+K12447+K00963+K00128+K00008+K00008+K00128+K00128+K00128+K00128+K01051+K00128+K00854+K00128+K00854+K01805+K00128+K00128+K01051+K01783+K00128+K01051+K00008+K00963+K00128+K00963+K01051+K00128+K00963+K00012+K00128+K00008+K00128+K00128+K01051+K00963+K01051+K00012+K00128+K00128+K00128+K00128+K00128+K00008+K00128+K00008+K01805+K00128+K00128+K00128+K01728+K00128+K01783+K01051+K01728+K00008+K00008+K01728+K00008+K00008+K01783+K00008+K00854+K00128+K01783+K01783+K00128+K01051+K00854+K00963+K00128+K00128+K01051+K01783+K01051+K00008+K00008+K01783+K00854+K00012+K0105

	nscript_143152;F01_transcript_143707;F01_transcript_143967;F01_transcript_144131;F01_transcript_144636;F01_transcript_144850;F01_transcript_144898;F01_transcript_145352;F01_transcript_145474;F01_transcript_146046;F01_transcript_147257;F01_transcript_147783;F01_transcript_147923;F01_transcript_148232;F01_transcript_148258;F01_transcript_149292;F01_transcript_151264;F01_transcript_151832;F01_transcript_151939;F01_transcript_152598;F01_transcript_152756;F01_transcript_152958;F01_transcript_153243;F01_transcript_153400;F01_transcript_153768;F01_transcript_154982;F01_transcript_155013;F01_transcript_155592;F01_transcript_156063;F01_transcript_156540;F01_transcript_157611;F01_transcript_158912;F01_transcript_159814;F01_transcript_161816;F01_transcript_161966;F01_transcript_162134;F01_transcript_162331;F01_transcript_163884;F01_transcript_164521;F01_transcript_165336;F01_transcript_166925;F01_transcript_167654;F01_transcript_17487;F01_transcript_1799;F01_transcript_18335;F01_transcript_18784;F01_transcript_18794;F01_transcript_19412;F01_transcript_21548;F01_transcript_23159;F01_transcript_23785;F01_transcript_24020;F01_transcript_24677;F01_transcript_25659;F01_transcript_25848;F01_transcript_26120;F01_transcript_27293;F01_transcript_27376;F01_transcript_27383;F01_transcript_27763;F01_transcript_28572;F01_transcript_28856;F01_transcript_28995;F01_transcript_29301;F01_transcript_29858;F01_transcript_30166;F01_transcript_30272;F01_transcript_30280;F01_transcript_30379;F01_transcript_30433;F01_transcript_30911;F01_transcript_30935;F01_transcript_31186;F01_transcript_31810;F01_transcript_31894;F01_transcript_31902;F01_transcript_32017;F01_transcript_32266;F01_transcript_32468;F01_transcript_32619;F01_transcript_32847;F01_transcript_33161;F01_transcript_33325;F01_transcript_33600;F01_transcript_34271;F01_transcript_35286;F01_transcript_36697;F01_transcript_36798;F01_transcript_36801;F01_transcript_36850;F01_transcript_37085;F01_transcript_37696;F01_transcript_37926;F01_transcript_37987;F01_transcript_38068;F01_transcript_38323;F01_transcript_38522;F01_transcript_38532;F01_transcript_38629;F01_transcript_39166;F01_transcript_39481;F01_transcript_39594;F01_transcript_39703;F01_transcript_40424;F01_transcript_41074;F01_transcript_41136;F01_t	1+K00128+K00128+K00008+K00128+K00128+K01051+K01051+K00128+K01783+K01051+K00128+K00128+K00128+K00128+K00128+K00128+K01051+K00854+K00128+K01051+K00128+K00128+K01805+K01051+K01051+K00854+K00854+K01728+K01805+K00963+K01051+K01783+K01805+K10268+K00128+K01728+K01051+K00008+K00128+K01051+K00854+K00128+K01051+K00128+K00008+K00128+K00008+K00963+K00128+K01184+K01728+K01728

			ranscript_4137;F01_transcript_41704;F01_transcript_42505;F01_transcript_42589;F01_transcript_48424;F01_transcript_5060;F01_transcript_54426;F01_transcript_54712;F01_transcript_55541;F01_transcript_56137;F01_transcript_56576;F01_transcript_56764;F01_transcript_57557;F01_transcript_58897;F01_transcript_59949;F01_transcript_60194;F01_transcript_60300;F01_transcript_60764;F01_transcript_60948;F01_transcript_65034;F01_transcript_65403;F01_transcript_65675;F01_transcript_65979;F01_transcript_66261;F01_transcript_66723;F01_transcript_67044;F01_transcript_67211;F01_transcript_68670;F01_transcript_69632;F01_transcript_70471;F01_transcript_71260;F01_transcript_71646;F01_transcript_72349;F01_transcript_73295;F01_transcript_75083;F01_transcript_75657;F01_transcript_76902;F01_transcript_79852;F01_transcript_80245;F01_transcript_80510;F01_transcript_81584;F01_transcript_81830;F01_transcript_84044;F01_transcript_86371;F01_transcript_86665;F01_transcript_86835;F01_transcript_87943;F01_transcript_88529;F01_transcript_89833;F01_transcript_90237;F01_transcript_90503;F01_transcript_90813;F01_transcript_91198;F01_transcript_92804;F01_transcript_93547;F01_transcript_93594;F01_transcript_93946;F01_transcript_96227;F01_transcript_96468;F01_transcript_97038;F01_transcript_97230;F01_transcript_97412;F01_transcript_98282;F01_transcript_98317;F01_transcript_98445;F01_transcript_98487;F01_transcript_99044;F01_transcript_99528;F01_transcript_99670;F01_transcript_99672;F01_transcript_99842;	
Fructose and mannose metabolism	ko00051	368	F01_transcript_100214;F01_transcript_101574;F01_transcript_102262;F01_transcript_102373;F01_transcript_102845;F01_transcript_103174;F01_transcript_103186;F01_transcript_103713;F01_transcript_104021;F01_transcript_104756;F01_transcript_10497;F01_transcript_104986;F01_transcript_105365;F01_transcript_106765;F01_transcript_107350;F01_transcript_108502;F01_transcript_108641;F01_transcript_108698;F01_transcript_108868;F01_transcript_108947;F01_transcript_109032;F01_transcript_109676;F01_transcript_110022;F01_transcript_110195;F01_transcript_110294;F01_transcript_110373;F01_transcript_110541;F01_transcript_110559;F01_transcript_111322;F01_transcript_112202;F01_transcript_112269;F01_transcript_112669;F01_transc	K01803+K00850+K00895+K01803+K01623+K01711+K01805+K00008+K00966+K03841+K01103+K00850+K01623+K03841+K01805+K03841+K01809+K00008+K00850+K01803+K03841+K01623+K00008+K17497+K01103+K00966+K03841+K00850+K00895+K00966+K01803+K03841+K17497+K01103+K00895+K01809+K00895+K01805+K00895+K19355+K01103+K01623+K03841+K00895+K00895+K19355+K01623+K03841+K01805+K17497+K01103+K00008+K01103+K01623+K00008+K00008+K19355+K01103+K01103+K01103+K01623+K00008+K00008+K03841+K00844+K00895+K00008+K01803+K17497+K03841+K01623+K00008+K01103+K01809+K01803+K01803+K19355+K00847+K01103+K01103+K01103+K01805+K0

	ript_113335;F01_transcript_113847;F01_transcript_113973;F01_transcript_114021;F01_transcript_114303;F01_transcript_114336;F01_transcript_115059;F01_transcript_116253;F01_transcript_116647;F01_transcript_117151;F01_transcript_118900;F01_transcript_119152;F01_transcript_119475;F01_transcript_119723;F01_transcript_119854;F01_transcript_119939;F01_transcript_120622;F01_transcript_120692;F01_transcript_121067;F01_transcript_121098;F01_transcript_121484;F01_transcript_121571;F01_transcript_122038;F01_transcript_122236;F01_transcript_122763;F01_transcript_123776;F01_transcript_124606;F01_transcript_124617;F01_transcript_124658;F01_transcript_125344;F01_transcript_125586;F01_transcript_126086;F01_transcript_126146;F01_transcript_126465;F01_transcript_127895;F01_transcript_128187;F01_transcript_128321;F01_transcript_129206;F01_transcript_129946;F01_transcript_130293;F01_transcript_13099;F01_transcript_131928;F01_transcript_133587;F01_transcript_133368;F01_transcript_134344;F01_transcript_134473;F01_transcript_134598;F01_transcript_134603;F01_transcript_134792;F01_transcript_134893;F01_transcript_135142;F01_transcript_135767;F01_transcript_135864;F01_transcript_136831;F01_transcript_136868;F01_transcript_137276;F01_transcript_137482;F01_transcript_137900;F01_transcript_138073;F01_transcript_138326;F01_transcript_138467;F01_transcript_139183;F01_transcript_140172;F01_transcript_140250;F01_transcript_141500;F01_transcript_142724;F01_transcript_143368;F01_transcript_143967;F01_transcript_14447;F01_transcript_145324;F01_transcript_145851;F01_transcript_146244;F01_transcript_146888;F01_transcript_147320;F01_transcript_147603;F01_transcript_147987;F01_transcript_148041;F01_transcript_148258;F01_transcript_148281;F01_transcript_149478;F01_transcript_149729;F01_transcript_149792;F01_transcript_150940;F01_transcript_151300;F01_transcript_152812;F01_transcript_153214;F01_transcript_153307;F01_transcript_153768;F01_transcript_153773;F01_transcript_153774;F01_transcript_154897;F01_transcript_155013;F01_transcript_155151;F01_transcript_155204;F01_transcript_155659;F01_transcript_155996;F01_transcript_156179;F01_transcript_156354;F01_transcript_156435;F01_transcript_156916;F01_transcript_157081;F01_transcript_157672;F01_transcript_157674;F01_t	0895+K01803+K00844+K00850+K03841+K00966+K01805+K00008+K00847+K01803+K17497+K00008+K01103+K01623+K18447+K00008+K01103+K00008+K00895+K01623+K01623+K03841+K00850+K01103+K03841+K01623+K00844+K01805+K00847+K05305+K00966+K00895+K01803+K01623+K01623+K17497+K01623+K00008+K01623+K01103+K01803+K01805+K01809+K01809+K00895+K01809+K01103+K17497+K01623+K00966+K00850+K00966+K00850+K00847+K00850+K00850+K01103+K01623+K01803+K19355+K00850+K19355+K03841+K19355+K00844+K00895+K00008+K19355+K00895+K03841+K00850+K01809+K01623+K03841+K01103+K01803+K00008+K03841+K00895+K17497+K01103+K01623+K00008+K01623+K00966+K00966+K00895+K00895+K00008+K00895+K01803+K00895+K00895+K00008+K05305+K05305+K00895+K01803+K00850+K00850+K00895+K00844+K19355+K19355+K01805+K00844+K00850+K01623+K19355+K19355+K19355+K00008+K19355+K01623+K00966+K00008+K00966+K00847+K01809+K01623+K00966+K00847+K00847+K01809+K01623+K00966+K01623+K00008+K00008+K00966+K01805+K01623+K01809+K03841+K01623+K19355+K01623+K19355+K19355+K19355+K00008+K01803+K00008+K01803+K00008+K00847+K03841+K03841+K19355+K00008+K18447+K01803+K01623+K03841+K00008+K00966+K19355+K01803+K01623+K01803+K03841+K01803+K01623+K00966+K00966+K01803+K00895+K01623+K01623+K01623+K00847+K01623+K03841+K00895+K17497+K05305+K01803+K01803+K01623+K01809+K00895+K00850+K01623+K01103+K00850+K03841+K17497+K00008+K00895+K00895+K00966+K00008+K00850+K00850+K01809+K00844+K00895+K19355+K00966+K19355+K01623+K01623+K01103+K01809+K03841+K00844+K00008+K01103+K03841+K00966+K03841+K00895+K17497+K00844+K03841+K00847+K03841+K01623+K02377+K00850+K00966+K00895+K03841+K01623+K01103+K00850+K03841+K00847+K03841+K00844+K00966+K19355+K01809+K01623+K00847+K17497+K01103+K01103+K01103+K01805+K19355+K00863+K01623+K00847+K01803+K00847+K19355+K05305+K19355+K00844+K00966+K01103+K00847+K00895+K00895+K01103+K01623+K01623+K05305+K01805+K19355

	ranscript_157846;F01_transcript_158480;F01_transcript_158736;F01_transcript_158780;F01_transcript_159245;F01_transcript_159358;F01_transcript_159374;F01_transcript_159443;F01_transcript_160325;F01_transcript_160426;F01_transcript_161538;F01_transcript_161918;F01_transcript_162372;F01_transcript_163884;F01_transcript_163963;F01_transcript_164007;F01_transcript_164751;F01_transcript_165153;F01_transcript_165243;F01_transcript_165396;F01_transcript_165440;F01_transcript_166383;F01_transcript_166475;F01_transcript_166925;F01_transcript_166935;F01_transcript_167162;F01_transcript_167432;F01_transcript_167598;F01_transcript_167733;F01_transcript_18335;F01_transcript_1968;F01_transcript_1996;F01_transcript_2057;F01_transcript_20997;F01_transcript_21327;F01_transcript_21548;F01_transcript_21988;F01_transcript_22107;F01_transcript_22140;F01_transcript_22373;F01_transcript_23159;F01_transcript_2318;F01_transcript_2451;F01_transcript_24531;F01_transcript_24566;F01_transcript_24624;F01_transcript_25920;F01_transcript_26163;F01_transcript_26688;F01_transcript_26869;F01_transcript_27090;F01_transcript_27763;F01_transcript_27931;F01_transcript_27932;F01_transcript_28741;F01_transcript_28815;F01_transcript_29583;F01_transcript_29948;F01_transcript_30272;F01_transcript_31494;F01_transcript_31687;F01_transcript_31713;F01_transcript_31902;F01_transcript_33874;F01_transcript_34086;F01_transcript_34237;F01_transcript_34413;F01_transcript_34419;F01_transcript_34671;F01_transcript_34966;F01_transcript_35258;F01_transcript_36056;F01_transcript_36365;F01_transcript_36584;F01_transcript_36798;F01_transcript_36850;F01_transcript_36879;F01_transcript_37085;F01_transcript_37274;F01_transcript_37297;F01_transcript_37740;F01_transcript_37835;F01_transcript_37981;F01_transcript_38228;F01_transcript_38366;F01_transcript_38561;F01_transcript_39090;F01_transcript_39166;F01_transcript_39223;F01_transcript_39481;F01_transcript_39520;F01_transcript_39703;F01_transcript_39890;F01_transcript_39895;F01_transcript_40114;F01_transcript_40318;F01_transcript_40424;F01_transcript_40717;F01_transcript_40883;F01_transcript_41015;F01_transcript_41061;F01_transcript_41136;F01_transcript_4133;F01_transcript_42889;F01_tr	+K17497+K17497+K01803+K01805+K19355+K01103+K01103+K00966+K19355+K00008+K00966+K01103+K00847+K00895+K00966+K01103+K01103+K01103+K00008+K00008+K01103+K00844+K19355+K00895+K00895

	anscript_43050;F01_transcript_43061;F01_transcript_44274;F01_transcript_44495;F01_transcript_44524;F01_transcript_45361;F01_transcript_4551;F01_transcript_4565;F01_transcript_47618;F01_transcript_48211;F01_transcript_49278;F01_transcript_49281;F01_transcript_49964;F01_transcript_50425;F01_transcript_50604;F01_transcript_51889;F01_transcript_52817;F01_transcript_52997;F01_transcript_54102;F01_transcript_54487;F01_transcript_55263;F01_transcript_55560;F01_transcript_56539;F01_transcript_56695;F01_transcript_56717;F01_transcript_56762;F01_transcript_56765;F01_transcript_56794;F01_transcript_57906;F01_transcript_58217;F01_transcript_58897;F01_transcript_59220;F01_transcript_59277;F01_transcript_59297;F01_transcript_59949;F01_transcript_59961;F01_transcript_60235;F01_transcript_60364;F01_transcript_60736;F01_transcript_61026;F01_transcript_61370;F01_transcript_61428;F01_transcript_62323;F01_transcript_62451;F01_transcript_63171;F01_transcript_63350;F01_transcript_63617;F01_transcript_63911;F01_transcript_65494;F01_transcript_65675;F01_transcript_66032;F01_transcript_67319;F01_transcript_680;F01_transcript_68522;F01_transcript_68679;F01_transcript_70537;F01_transcript_70970;F01_transcript_71315;F01_transcript_71450;F01_transcript_72250;F01_transcript_72421;F01_transcript_72450;F01_transcript_73635;F01_transcript_73983;F01_transcript_74585;F01_transcript_74788;F01_transcript_75206;F01_transcript_75285;F01_transcript_75573;F01_transcript_76279;F01_transcript_76648;F01_transcript_76721;F01_transcript_76768;F01_transcript_76938;F01_transcript_77347;F01_transcript_78016;F01_transcript_78063;F01_transcript_78310;F01_transcript_78510;F01_transcript_78913;F01_transcript_78979;F01_transcript_79936;F01_transcript_81830;F01_transcript_82749;F01_transcript_83385;F01_transcript_83682;F01_transcript_8370;F01_transcript_83748;F01_transcript_83764;F01_transcript_83921;F01_transcript_84250;F01_transcript_84575;F01_transcript_86116;F01_transcript_86361;F01_transcript_86638;F01_transcript_86749;F01_transcript_87312;F01_transcript_87825;F01_transcript_87839;F01_transcript_88234;F01_transcript_88445;F01_transcript_88491;F01_transcript_88529;F01_transcript_88921;F01_transcript_89165;F01_transcript_89529;F01_transcript_90169;F01_transcript_90813;F01_transcript	

			_92215;F01_transcript_9228;F01_transcript_92818;F01_transcript_93239;F01_transcript_93284;F01_transcript_93946;F01_transcript_94111;F01_transcript_94998;F01_transcript_95936;F01_transcript_96218;F01_transcript_96838;F01_transcript_96958;F01_transcript_97292;F01_transcript_97778;F01_transcript_98317;F01_transcript_98487;F01_transcript_9864;F01_transcript_98699;F01_transcript_98768;F01_transcript_99038;F01_transcript_99768;	
Galactose metabolism	ko00052	239	F01_transcript_100741;F01_transcript_101239;F01_transcript_101574;F01_transcript_101603;F01_transcript_10193;F01_transcript_102376;F01_transcript_102519;F01_transcript_104654;F01_transcript_104986;F01_transcript_105550;F01_transcript_105731;F01_transcript_108122;F01_transcript_108387;F01_transcript_108868;F01_transcript_109592;F01_transcript_110029;F01_transcript_110559;F01_transcript_111765;F01_transcript_112177;F01_transcript_112285;F01_transcript_112922;F01_transcript_113188;F01_transcript_113345;F01_transcript_113710;F01_transcript_114697;F01_transcript_115630;F01_transcript_117826;F01_transcript_118531;F01_transcript_121543;F01_transcript_121673;F01_transcript_122065;F01_transcript_123915;F01_transcript_125167;F01_transcript_12544;F01_transcript_126146;F01_transcript_126182;F01_transcript_12739;F01_transcript_127900;F01_transcript_129385;F01_transcript_131624;F01_transcript_132330;F01_transcript_132804;F01_transcript_132878;F01_transcript_132978;F01_transcript_133699;F01_transcript_134092;F01_transcript_135131;F01_transcript_135864;F01_transcript_136831;F01_transcript_137006;F01_transcript_137623;F01_transcript_138414;F01_transcript_139405;F01_transcript_13991;F01_transcript_140613;F01_transcript_14085;F01_transcript_140989;F01_transcript_141154;F01_transcript_14203;F01_transcript_142687;F01_transcript_142834;F01_transcript_143548;F01_transcript_143764;F01_transcript_143975;F01_transcript_144131;F01_transcript_144857;F01_transcript_145526;F01_transcript_145772;F01_transcript_145966;F01_transcript_146888;F01_transcript_147923;F01_transcript_148041;F01_transcript_148896;F01_transcript_14964;F01_transcript_150629;F01_transcript_153205;F01_transcript_153664;F01_transcript_153936;F01_transcript_1	K01190+K06617+K00850+K06617+K06617+K00965+K00963+K00965+K00850+K18819+K18819+K18819+K12309+K00850+K07407+K01784+K00850+K00963+K12309+K06617+K12309+K12309+K12309+K01193+K01784+K18819+K01835+K06617+K06617+K18819+K01187+K01190+K07407+K06617+K00844+K12309+K06617+K06617+K06617+K07407+K01835+K01835+K07407+K00965+K06617+K06617+K01835+K00844+K00850+K01193+K01835+K01784+K06617+K12309+K07407+K01187+K06617+K01785+K12309+K06617+K00965+K01835+K18819+K18819+K00963+K12309+K06617+K01190+K01187+K00850+K00963+K00844+K06617+K06617+K07407+K00965+K01785+K07407+K18819+K00965+K00963+K01835+K06617+K12447+K01835+K01835+K00850+K00850+K18819+K06617+K00850+K00850+K00850+K18819+K01190+K06617+K06617+K12309+K07407+K00965+K00844+K01190+K12309+K00850+K01835+K01784+K01835+K06617+K06617+K01190+K00963+K00963+K01193+K12447+K00963+K18819+K01193+K01193+K01835+K12309+K01835+K00850+K18819+K01835+K01835+K00850+K01835+K00965+K00844+K18819+K06611+K00844+K00850+K00963+K00963+K01193+K00963+K00963+K01193+K07407+K00965+K00965+K00965+K07407+K01784+K01784+K01784+K01784+K01784+K01785+K18819+K01785+K18819+K06617+K00965+K00963+K01785+K06617+K01193+K01835+K00850+K00850+K06617+K00965+K12309+K01190+K07407+K00850+K00850+K00844+K06617+K12309+K12309+K01785+K00844+K06617+K01190+K01835+K18819+K01187+K18819+K07407+K00844+K01190+K06617+K06617+K06617+K06617+K07407+K00850+K06617+K06617+K06617+K18819+K18819+K00850+K01784+K06617+K01784+K00844

	54000;F01_transcript_155492;F01_transcript_155592;F01_transcript_155598;F01_transcript_156048;F01_transcript_156063;F01_transcript_156116;F01_transcript_156780;F01_transcript_157081;F01_transcript_157674;F01_transcript_157956;F01_transcript_158039;F01_transcript_158480;F01_transcript_158736;F01_transcript_159443;F01_transcript_159921;F01_transcript_1603;F01_transcript_160560;F01_transcript_161139;F01_transcript_161299;F01_transcript_161354;F01_transcript_161488;F01_transcript_161918;F01_transcript_162418;F01_transcript_163495;F01_transcript_165153;F01_transcript_165871;F01_transcript_165880;F01_transcript_166075;F01_transcript_166084;F01_transcript_166605;F01_transcript_1668;F01_transcript_167654;F01_transcript_17487;F01_transcript_17520;F01_transcript_18784;F01_transcript_18794;F01_transcript_19191;F01_transcript_19300;F01_transcript_19639;F01_transcript_19835;F01_transcript_2181;F01_transcript_22972;F01_transcript_24624;F01_transcript_24648;F01_transcript_25381;F01_transcript_25470;F01_transcript_25920;F01_transcript_25979;F01_transcript_26498;F01_transcript_26688;F01_transcript_27101;F01_transcript_27522;F01_transcript_27931;F01_transcript_27932;F01_transcript_30280;F01_transcript_30433;F01_transcript_30850;F01_transcript_31186;F01_transcript_32619;F01_transcript_32632;F01_transcript_32988;F01_transcript_34824;F01_transcript_35769;F01_transcript_35857;F01_transcript_36267;F01_transcript_37601;F01_transcript_38768;F01_transcript_39043;F01_transcript_39177;F01_transcript_39411;F01_transcript_40233;F01_transcript_40644;F01_transcript_41388;F01_transcript_50206;F01_transcript_51781;F01_transcript_53956;F01_transcript_54712;F01_transcript_55798;F01_transcript_56282;F01_transcript_56372;F01_transcript_56580;F01_transcript_56717;F01_transcript_56794;F01_transcript_5705;F01_transcript_57114;F01_transcript_57444;F01_transcript_58059;F01_transcript_58571;F01_transcript_59961;F01_transcript_60235;F01_transcript_60736;F01_transcript_62809;F01_transcript_63036;F01_transcript_63587;F01_transcript_64674;F01_transcript_65494;F01_transcript_65779;F01_transcript_66447;F01_transcript_66664;F01_transcript_67681;F01_transcript_67795;F01_transcript_68731;F01_transcript_69846;F01_transcript_70970;F01_transcript_71581;F01_transcript_71808;F01_transc	+K07407+K01835+K00965+K06617+K00965+K06617+K06617+K01193+K06617+K01187+K00844+K01835+K01785+K01785+K12309+K01187+K06617+K01187+K01784+K06617+K00963+K07407+K06617+K06617+K18819+K06617+K18819+K18819+K18819+K06617+K01835+K06617+K07407+K01190+K01187+K18819+K00844+K00963+K06617+K00965

			ript_71982;F01_transcript_7216;F01_transcript_72977;F01_transcript_73635;F01_transcript_7392;F01_transcript_74231;F01_transcript_74925;F01_transcript_75200;F01_transcript_75451;F01_transcript_75573;F01_transcript_76212;F01_transcript_76654;F01_transcript_76689;F01_transcript_76768;F01_transcript_78851;F01_transcript_79249;F01_transcript_79518;F01_transcript_80203;F01_transcript_81122;F01_transcript_81348;F01_transcript_81811;F01_transcript_82183;F01_transcript_84758;F01_transcript_8481;F01_transcript_86116;F01_transcript_86207;F01_transcript_86630;F01_transcript_86802;F01_transcript_87412;F01_transcript_8759;F01_transcript_87654;F01_transcript_89020;F01_transcript_89411;F01_transcript_89704;F01_transcript_89833;F01_transcript_89903;F01_transcript_9069;F01_transcript_9193;F01_transcript_92472;F01_transcript_9266;F01_transcript_92838;F01_transcript_9333;F01_transcript_93738;F01_transcript_9378;F01_transcript_94317;F01_transcript_94593;F01_transcript_96959;F01_transcript_97421;F01_transcript_98252;F01_transcript_9844;F01_transcript_98699;F01_transcript_99044;F01_transcript_9946;F01_transcript_99970;	
Ascorbate and aldarate metabolism	ko00053	296	F01_transcript_100740;F01_transcript_103099;F01_transcript_103691;F01_transcript_103733;F01_transcript_103925;F01_transcript_105213;F01_transcript_106663;F01_transcript_106862;F01_transcript_107021;F01_transcript_107572;F01_transcript_108221;F01_transcript_108771;F01_transcript_109253;F01_transcript_109312;F01_transcript_112685;F01_transcript_112884;F01_transcript_113038;F01_transcript_114016;F01_transcript_115554;F01_transcript_115636;F01_transcript_116615;F01_transcript_11714;F01_transcript_117909;F01_transcript_118736;F01_transcript_118848;F01_transcript_118899;F01_transcript_119023;F01_transcript_119343;F01_transcript_120277;F01_transcript_120875;F01_transcript_121070;F01_transcript_121153;F01_transcript_121508;F01_transcript_121819;F01_transcript_122054;F01_transcript_122643;F01_transcript_122700;F01_transcript_1231;F01_transcript_123521;F01_transcript_124756;F01_transcript_125301;F01_transcript_126659;F01_transcript_127146;F01_transcript_127326;F01_transcript_12789;F01_transcript_128218;F01_transcript_128872;F01_transcript_129460;F01_transcript_131	K00128+K00434+K14085+K14190+K00128+K00128+K00434+K16190+K08232+K10046+K00128+K14190+K14190+K00128+K00128+K00128+K14190+K00434+K00434+K14085+K00434+K14190+K14190+K00128+K00128+K08232+K00434+K00128+K14190+K14190+K00012+K14190+K14190+K00128+K00434+K14190+K00434+K00128+K14190+K00469+K00434+K10047+K00128+K00128+K14190+K00128+K00128+K14190+K14190+K14190+K18649+K00128+K00012+K00128+K00434+K00469+K00434+K00128+K00128+K00128+K14190+K00434+K08232+K00012+K00434+K10047+K10046+K14190+K00434+K17744+K00128+K00128+K00128+K00128+K00128+K00434+K00434+K00128+K00434+K14190+K14190+K14190+K14190+K10046+K14190+K00128+K00128+K00225+K14190+K14190+K00128+K00128+K14190+K00128+K00128+K00128+K00469+K17744+K00128+K00434+K00434+K10046+K00128+K00128+K00128+K14190+K00128+K00128+K14190+K00434+K00434+K14190+K00434+K00128+K12447+K00434+K00012+K14190+K00128+K14085+K00128+K00434+K19642+K00

	179;F01_transcript_131285;F01_transcript_132042;F01_transcript_132494;F01_transcript_132918;F01_transcript_132930;F01_transcript_133029;F01_transcript_133289;F01_transcript_133331;F01_transcript_1334;F01_transcript_134251;F01_transcript_134507;F01_transcript_134680;F01_transcript_134810;F01_transcript_135227;F01_transcript_135333;F01_transcript_135567;F01_transcript_135803;F01_transcript_135890;F01_transcript_136154;F01_transcript_136249;F01_transcript_137379;F01_transcript_137525;F01_transcript_137935;F01_transcript_138093;F01_transcript_138897;F01_transcript_138921;F01_transcript_139000;F01_transcript_139223;F01_transcript_139567;F01_transcript_140055;F01_transcript_140642;F01_transcript_140721;F01_transcript_141229;F01_transcript_141567;F01_transcript_14271;F01_transcript_143373;F01_transcript_144636;F01_transcript_144850;F01_transcript_145061;F01_transcript_145089;F01_transcript_145275;F01_transcript_145352;F01_transcript_145474;F01_transcript_145514;F01_transcript_146046;F01_transcript_147257;F01_transcript_147783;F01_transcript_148129;F01_transcript_149029;F01_transcript_149292;F01_transcript_149651;F01_transcript_150655;F01_transcript_150921;F01_transcript_151264;F01_transcript_151832;F01_transcript_151939;F01_transcript_152577;F01_transcript_152756;F01_transcript_152958;F01_transcript_153420;F01_transcript_153586;F01_transcript_153624;F01_transcript_153705;F01_transcript_153946;F01_transcript_154982;F01_transcript_156063;F01_transcript_156980;F01_transcript_157611;F01_transcript_158321;F01_transcript_158912;F01_transcript_159514;F01_transcript_159814;F01_transcript_159904;F01_transcript_160293;F01_transcript_160400;F01_transcript_161020;F01_transcript_161816;F01_transcript_161832;F01_transcript_161966;F01_transcript_162134;F01_transcript_162331;F01_transcript_162345;F01_transcript_165142;F01_transcript_166901;F01_transcript_17614;F01_transcript_1799;F01_transcript_18040;F01_transcript_18480;F01_transcript_18784;F01_transcript_19412;F01_transcript_19640;F01_transcript_20702;F01_transcript_21044;F01_transcript_21079;F01_transcript_21093;F01_transcript_21887;F01_transcript_22936;F01_transcript_23785;F01_transcript_24020;F01_transcript_24285;F01_transcript_24677;F01_transcript_24785;F01_transcript_24827;F01_transcript	434+K00434+K00128+K00434+K00128+K00128+K00128+K00434+K00434+K14190+K14190+K00128+K14190+K14190+K12447+K00128+K14190+K14190+K14190+K14190+K14190+K14190+K14190+K00128+K00128+K14190+K00128+K08232+K00225+K14190+K00225+K00128+K00128+K00423+K08232+K08232+K08232+K00128+K00128+K14190+K00128+K00423+K08232+K14190+K14190+K00128+K14085+K14085+K00128+K00128+K00012+K00128+K00128+K00128+K00012+K00128+K00128+K00128+K08232+K08232+K00128+K08232+K00434+K08232+K00434+K08232+K00128+K00128+K08232+K10046+K00434+K00128+K00128+K00128+K00128+K10046+K00434+K17744+K00434+K00128+K00434+K14190+K00434+K10047+K00434+K00434+K00434+K00434+K00128+K00434+K08232+K00434+K14190+K00128+K00128+K17744+K14085+K00434+K00434+K00434+K00434+K00012+K14190+K14085+K00434+K00128+K10047+K00128+K00128+K10047+K14190+K00434+K00128+K00434+K00434+K14190+K00128+K00434+K00434+K14190+K00434+K00128+K00128+K00128+K00128+K00128+K00434+K10046+K00128+K14190+K00434+K14085+K10046+K00128+K00128+K08232+K00434+K08232+K19642+K00434+K00434+K00434+K00128+K10046+K14190+K14190+K08232+K14190+K14190+K00434+K08232+K14190+K14085+K08232+K08232+K00434+K00434+K00128+K10046+K14190+K14190+K10047+K00128+K08232+K00128+K10047+K00128+K10047+K14190+K00128+K00434+K00128+K14190

_25045;F01_transcript_25222;F01_transcript_25659;F01_transc
ript_26120;F01_transcript_26597;F01_transcript_26786;F01_tra
nscript_26795;F01_transcript_26875;F01_transcript_27376;F01
_transcript_28572;F01_transcript_28854;F01_transcript_28856;
F01_transcript_28857;F01_transcript_28868;F01_transcript_290
03;F01_transcript_29093;F01_transcript_29858;F01_transcript_
30292;F01_transcript_30362;F01_transcript_30379;F01_transcri
pt_30935;F01_transcript_31810;F01_transcript_31894;F01_tran
script_32017;F01_transcript_32266;F01_transcript_33161;F01_t
ranscript_33325;F01_transcript_33600;F01_transcript_34271;F0
1_transcript_34374;F01_transcript_34570;F01_transcript_35286
;F01_transcript_35528;F01_transcript_36017;F01_transcript_36
190;F01_transcript_36246;F01_transcript_36378;F01_transcript
_36697;F01_transcript_36801;F01_transcript_37129;F01_transc
ript_37199;F01_transcript_37604;F01_transcript_37696;F01_tra
nscript_37926;F01_transcript_37987;F01_transcript_38323;F01
_transcript_38847;F01_transcript_39355;F01_transcript_40557;
F01_transcript_40663;F01_transcript_41704;F01_transcript_420
59;F01_transcript_4343;F01_transcript_43812;F01_transcript_4
4007;F01_transcript_44061;F01_transcript_44350;F01_transcrip
t_44753;F01_transcript_47535;F01_transcript_48424;F01_trans
cript_52419;F01_transcript_53709;F01_transcript_54110;F01_tr
anscript_55118;F01_transcript_55541;F01_transcript_56137;F0
1_transcript_56548;F01_transcript_56690;F01_transcript_57076
;F01_transcript_57714;F01_transcript_59141;F01_transcript_59
243;F01_transcript_60764;F01_transcript_61014;F01_transcript
_63491;F01_transcript_64343;F01_transcript_65034;F01_transc
ript_65191;F01_transcript_65403;F01_transcript_65979;F01_tra
nscript_66026;F01_transcript_66074;F01_transcript_66090;F01
_transcript_66261;F01_transcript_66794;F01_transcript_66881;
F01_transcript_67003;F01_transcript_67211;F01_transcript_680
47;F01_transcript_68526;F01_transcript_6951;F01_transcript_7
0328;F01_transcript_70471;F01_transcript_71260;F01_transcrip
t_71646;F01_transcript_72349;F01_transcript_73295;F01_trans
cript_73951;F01_transcript_74436;F01_transcript_75083;F01_tr
anscript_76682;F01_transcript_77591;F01_transcript_77976;F0
1_transcript_79642;F01_transcript_79852;F01_transcript_80510

			;F01_transcript_80956;F01_transcript_80967;F01_transcript_81167;F01_transcript_81244;F01_transcript_81257;F01_transcript_81378;F01_transcript_81549;F01_transcript_81584;F01_transcript_83475;F01_transcript_8372;F01_transcript_83970;F01_transcript_85016;F01_transcript_85021;F01_transcript_85608;F01_transcript_85750;F01_transcript_86238;F01_transcript_86950;F01_transcript_8790;F01_transcript_89697;F01_transcript_89706;F01_transcript_91493;F01_transcript_92430;F01_transcript_92804;F01_transcript_93078;F01_transcript_93337;F01_transcript_93999;F01_transcript_95171;F01_transcript_96227;F01_transcript_96521;F01_transcript_97230;F01_transcript_97327;F01_transcript_98282;F01_transcript_98320;F01_transcript_98363;F01_transcript_98445;F01_transcript_98488;F01_transcript_99528;F01_transcript_99890;	
Fatty acid biosynthesis	ko00061	209	F01_transcript_10004;F01_transcript_100288;F01_transcript_101409;F01_transcript_101823;F01_transcript_103130;F01_transcript_104073;F01_transcript_10466;F01_transcript_104730;F01_transcript_104782;F01_transcript_104998;F01_transcript_105202;F01_transcript_105292;F01_transcript_105720;F01_transcript_106220;F01_transcript_106677;F01_transcript_108901;F01_transcript_10985;F01_transcript_113382;F01_transcript_113401;F01_transcript_114458;F01_transcript_114684;F01_transcript_114717;F01_transcript_115230;F01_transcript_115996;F01_transcript_117227;F01_transcript_117636;F01_transcript_117649;F01_transcript_118673;F01_transcript_119362;F01_transcript_119532;F01_transcript_121035;F01_transcript_121478;F01_transcript_123054;F01_transcript_124664;F01_transcript_125612;F01_transcript_12599;F01_transcript_127333;F01_transcript_128013;F01_transcript_12879;F01_transcript_12985;F01_transcript_129865;F01_transcript_129901;F01_transcript_130423;F01_transcript_130464;F01_transcript_13109;F01_transcript_13154;F01_transcript_131603;F01_transcript_131659;F01_transcript_131715;F01_transcript_132668;F01_transcript_133721;F01_transcript_134920;F01_transcript_135733;F01_transcript_135955;F01_transcript_139502;F01_transcript_139851;F01_transcript_140123;F01_transcript_140225;F01_transcript_141554;F01_transcript_141747;F01_transcript_143258;F01_transcript_143805;F01_transcript_144542;F01_transcript_144717;F01_transcript_144779;F01_t	K09458+K01962+K01962+K09458+K00059+K10782+K01897+K09458+K01897+K01897+K01962+K11262+K11262+K01962+K01962+K11262+K01962+K09458+K09458+K01897+K01897+K09458+K01897+K01897+K01897+K10781+K09458+K00208+K10781+K01897+K11262+K01897+K10781+K10781+K00648+K01897+K10781+K01897+K01897+K01897+K03921+K10781+K03921+K09458+K01897+K01962+K00059+K00208+K01897+K01897+K10781+K01897+K01897+K01962+K01897+K09458+K01897+K03921+K11262+K10781+K01897+K09458+K10782+K01897+K11262+K00208+K09458+K00059+K01897+K01897+K01897+K09458+K10781+K01897+K01897+K01897+K01897+K02372+K01897+K01897+K01897+K01963+K09458+K09458+K01897+K10781+K01897+K01897+K01897+K01897+K09458+K01962+K10781+K09458+K00208+K01897+K01897+K01897+K10781+K01897+K01897+K01897+K01897+K01897+K10781+K01897+K10781+K01897+K11262+K00208+K01961+K00208+K09458+K03921+K00648+K00648+K03921+K03921+K00059+K00059+K03921+K00059+K00645+K02160+K02160+K02160+K00059+K02372+K01962+K01897+K00648+K01961+K02372+K01897+K10781+K01897+K01962+K01962+K00059+K01962+K09458+K02372+K01897+K01897+K09458+K10781+K01897+K00208+K10781+K01897+K09458+K01897+K11262+K10781+K01961+K09458+K00208+K01963+K01897+K01897+K00059+K01897+K09458+K09458+K039

	ranscript_144932;F01_transcript_147293;F01_transcript_147705;F01_transcript_147795;F01_transcript_14899;F01_transcript_149352;F01_transcript_15003;F01_transcript_153478;F01_transcript_153488;F01_transcript_15349;F01_transcript_15367;F01_transcript_154600;F01_transcript_156365;F01_transcript_15661;F01_transcript_15780;F01_transcript_158278;F01_transcript_158628;F01_transcript_15890;F01_transcript_158942;F01_transcript_159135;F01_transcript_16167;F01_transcript_161773;F01_transcript_162421;F01_transcript_16301;F01_transcript_16433;F01_transcript_164916;F01_transcript_164977;F01_transcript_165897;F01_transcript_165923;F01_transcript_166283;F01_transcript_167396;F01_transcript_17966;F01_transcript_18447;F01_transcript_18597;F01_transcript_19074;F01_transcript_19302;F01_transcript_19520;F01_transcript_19705;F01_transcript_20309;F01_transcript_21972;F01_transcript_23121;F01_transcript_23503;F01_transcript_28357;F01_transcript_29158;F01_transcript_29866;F01_transcript_30491;F01_transcript_31883;F01_transcript_32112;F01_transcript_32634;F01_transcript_32829;F01_transcript_33263;F01_transcript_34915;F01_transcript_36214;F01_transcript_36847;F01_transcript_36931;F01_transcript_37206;F01_transcript_37378;F01_transcript_38219;F01_transcript_40383;F01_transcript_41552;F01_transcript_41596;F01_transcript_44928;F01_transcript_46128;F01_transcript_4651;F01_transcript_4986;F01_transcript_53543;F01_transcript_53796;F01_transcript_54259;F01_transcript_54622;F01_transcript_55556;F01_transcript_55736;F01_transcript_56614;F01_transcript_5825;F01_transcript_60150;F01_transcript_6084;F01_transcript_61151;F01_transcript_61462;F01_transcript_61928;F01_transcript_62811;F01_transcript_62859;F01_transcript_65211;F01_transcript_65891;F01_transcript_65988;F01_transcript_664;F01_transcript_67276;F01_transcript_67593;F01_transcript_68887;F01_transcript_68924;F01_transcript_69192;F01_transcript_69815;F01_transcript_69883;F01_transcript_70315;F01_transcript_70327;F01_transcript_71725;F01_transcript_72007;F01_transcript_72026;F01_transcript_72096;F01_transcript_72848;F01_transcript_73024;F01_transcript_73288;F01_transcript_74275;F01_transcript_76033;F01_transcript_76982;F01_transcript_77290;F01_transcript	21+K10781+K09458+K01897+K02372+K09458+K01897+K01962+K11262+K09458+K01897+K11262+K01962+K01961+K09458+K00648+K01897+K01962+K10781+K01897+K01897+K00059+K01897+K09458+K01897+K00648+K01962+K10781+K01962+K00059+K02160+K01897+K00648+K09458+K00208+K01897+K11262+K00648+K00059+K01897+K10781+K01897+K01897+K01897+K01897

			_77795;F01_transcript_78246;F01_transcript_78484;F01_transcript_80148;F01_transcript_80702;F01_transcript_82184;F01_transcript_82337;F01_transcript_82396;F01_transcript_82656;F01_transcript_83638;F01_transcript_84001;F01_transcript_84935;F01_transcript_85424;F01_transcript_85921;F01_transcript_86684;F01_transcript_88028;F01_transcript_88933;F01_transcript_89994;F01_transcript_90527;F01_transcript_90896;F01_transcript_90940;F01_transcript_91076;F01_transcript_91258;F01_transcript_91910;F01_transcript_92715;F01_transcript_92735;F01_transcript_92831;F01_transcript_93026;F01_transcript_93122;F01_transcript_94689;F01_transcript_95989;F01_transcript_96058;F01_transcript_96369;F01_transcript_96815;F01_transcript_97377;F01_transcript_97945;F01_transcript_98172;F01_transcript_98478;F01_transcript_99276;F01_transcript_99279;	
Fatty acid elongation	ko00062	96	F01_transcript_105432;F01_transcript_106311;F01_transcript_110694;F01_transcript_111506;F01_transcript_112515;F01_transcript_113046;F01_transcript_113706;F01_transcript_114795;F01_transcript_119757;F01_transcript_120483;F01_transcript_121926;F01_transcript_122671;F01_transcript_122692;F01_transcript_126252;F01_transcript_128927;F01_transcript_129744;F01_transcript_130395;F01_transcript_131263;F01_transcript_131404;F01_transcript_133828;F01_transcript_135787;F01_transcript_136158;F01_transcript_138847;F01_transcript_140469;F01_transcript_141759;F01_transcript_14337;F01_transcript_145685;F01_transcript_146347;F01_transcript_148949;F01_transcript_151304;F01_transcript_152691;F01_transcript_153426;F01_transcript_154923;F01_transcript_156541;F01_transcript_16110;F01_transcript_163409;F01_transcript_165303;F01_transcript_165616;F01_transcript_165801;F01_transcript_166122;F01_transcript_167185;F01_transcript_1774;F01_transcript_18009;F01_transcript_18477;F01_transcript_20745;F01_transcript_22348;F01_transcript_26117;F01_transcript_26159;F01_transcript_27292;F01_transcript_28116;F01_transcript_28634;F01_transcript_28677;F01_transcript_28904;F01_transcript_29590;F01_transcript_30681;F01_transcript_31627;F01_transcript_32122;F01_transcript_32331;F01_transcript_32482;F01_transcript_33717;F01_transcript_38790;F01_transcript_38861;F01_transcript_40457;F01_transcript_40823;F01_transcript_41243;F01_transcript_41358;F01_t	K15397+K01074+K01068+K10703+K10258+K10703+K10703+K15397+K01074+K01074+K10251+K01074+K10251+K10258+K01074+K10703+K01074+K01074+K15397+K10703+K15397+K15397+K15397+K01074+K10703+K15397+K01074+K10703+K10703+K15397+K15397+K01074+K01074+K15397+K15397+K15397+K10251+K10251+K10251+K10251+K01068+K15397+K15397+K15397+K01074+K15397+K15397+K10251+K15397+K15397+K15397+K15397+K15397+K15397+K15397+K15397+K15397+K15397+K15397+K01068+K01074+K01074+K07512+K10251+K10251+K01074+K01074+K15397+K10703+K01074+K10703+K15397+K15397+K10703+K01074+K01068+K07512+K01068+K10251+K15397+K07512+K15397+K15397+K10251+K01074+K01074+K01074+K01074+K15397+K07512+K10703+K01074+K01074+K10251+K15397+K01068

			ranscript_53233;F01_transcript_55295;F01_transcript_56417;F01_transcript_57503;F01_transcript_59428;F01_transcript_59509;F01_transcript_59822;F01_transcript_60200;F01_transcript_66972;F01_transcript_67882;F01_transcript_68598;F01_transcript_70434;F01_transcript_70525;F01_transcript_70933;F01_transcript_72928;F01_transcript_73619;F01_transcript_73785;F01_transcript_77538;F01_transcript_86897;F01_transcript_91879;F01_transcript_92575;F01_transcript_93476;F01_transcript_94219;F01_transcript_96104;F01_transcript_96460;F01_transcript_96469;F01_transcript_97231;F01_transcript_97408;F01_transcript_97674;F01_transcript_98034;	
Fatty acid degradation	ko00071	335	F01_transcript_100740;F01_transcript_10098;F01_transcript_101871;F01_transcript_102523;F01_transcript_103691;F01_transcript_103925;F01_transcript_104489;F01_transcript_10466;F01_transcript_104782;F01_transcript_104998;F01_transcript_105213;F01_transcript_105403;F01_transcript_105547;F01_transcript_106095;F01_transcript_106217;F01_transcript_10672;F01_transcript_108221;F01_transcript_109312;F01_transcript_112685;F01_transcript_112884;F01_transcript_114458;F01_transcript_114684;F01_transcript_115230;F01_transcript_115636;F01_transcript_115996;F01_transcript_11601;F01_transcript_117227;F01_transcript_117687;F01_transcript_118434;F01_transcript_118736;F01_transcript_118848;F01_transcript_119343;F01_transcript_119532;F01_transcript_120801;F01_transcript_121145;F01_transcript_121478;F01_transcript_121819;F01_transcript_122368;F01_transcript_122721;F01_transcript_122818;F01_transcript_122972;F01_transcript_1231;F01_transcript_124769;F01_transcript_124788;F01_transcript_12599;F01_transcript_126836;F01_transcript_127146;F01_transcript_127326;F01_transcript_128013;F01_transcript_128218;F01_transcript_12879;F01_transcript_128872;F01_transcript_12985;F01_transcript_130717;F01_transcript_13109;F01_transcript_131715;F01_transcript_132494;F01_transcript_132668;F01_transcript_132695;F01_transcript_132930;F01_transcript_1334;F01_transcript_134251;F01_transcript_134507;F01_transcript_134920;F01_transcript_135733;F01_transcript_13624;F01_transcript_136325;F01_transcript_136852;F01_transcript_137525;F01_transcript_137935;F01_transcript_1380	K00128+K10527+K00121+K18857+K14085+K00128+K00121+K01897+K01897+K01897+K00128+K00626+K10527+K10527+K00232+K10527+K00128+K00128+K00128+K00128+K01897+K01897+K01897+K14085+K01897+K00232+K01897+K00232+K00232+K00128+K00128+K00128+K01897+K00232+K00121+K01897+K00128+K00232+K10527+K00232+K18857+K00128+K00232+K00232+K01897+K00001+K00128+K00128+K01897+K00128+K01897+K00128+K01897+K07513+K01897+K01897+K00128+K01897+K10527+K00128+K00128+K00128+K00128+K01897+K01897+K10527+K00121+K00121+K00128+K00128+K00232+K00128+K00626+K10527+K00128+K00128+K00232+K00121+K01897+K00128+K10527+K01897+K00121+K00232+K01897+K00232+K10527+K00128+K01897+K00128+K00128+K00128+K00128+K00121+K00232+K00128+K00232+K07513+K00128+K01897+K10527+K01897+K00128+K01897+K00232+K00128+K00128+K00128+K00128+K00232+K00128+K01897+K01897+K01897+K10527+K01897+K00128+K00232+K00626+K10527+K01897+K00232+K01897+K00121+K00232+K01897+K00128+K01897+K00232+K14085+K10527+K00128+K07513+K00232+K10527+K18857+K01897+K00128+K00128+K10527+K00001+K00128+K00128+K01897+K00232+K01897+K00121+K01897+K18857+K10527+K10527+K00626+K01897+K07513+K00121+K10527+K10527+K01897+K00232+K00128+K01897+K01897+K01897+K00128+K01897+K01897+K01897+K00232+K01897+K00128+K00128+K00128+K00128+K00128+K00232+K00232+K00128+K01897+K0

	47;F01_transcript_138093;F01_transcript_138114;F01_transcript_13822;F01_transcript_138897;F01_transcript_138921;F01_transcript_139123;F01_transcript_139169;F01_transcript_139502;F01_transcript_139567;F01_transcript_139625;F01_transcript_140123;F01_transcript_142036;F01_transcript_142214;F01_transcript_143258;F01_transcript_143781;F01_transcript_144395;F01_transcript_144636;F01_transcript_144717;F01_transcript_144850;F01_transcript_145352;F01_transcript_145474;F01_transcript_146046;F01_transcript_146237;F01_transcript_147167;F01_transcript_147257;F01_transcript_147259;F01_transcript_147398;F01_transcript_147783;F01_transcript_147795;F01_transcript_147956;F01_transcript_14899;F01_transcript_149292;F01_transcript_149352;F01_transcript_149791;F01_transcript_151264;F01_transcript_151832;F01_transcript_151939;F01_transcript_152756;F01_transcript_15284;F01_transcript_152958;F01_transcript_153488;F01_transcript_15349;F01_transcript_15367;F01_transcript_154107;F01_transcript_154600;F01_transcript_154982;F01_transcript_155474;F01_transcript_155685;F01_transcript_156381;F01_transcript_15661;F01_transcript_157406;F01_transcript_15780;F01_transcript_158132;F01_transcript_15817;F01_transcript_158278;F01_transcript_158912;F01_transcript_159135;F01_transcript_15946;F01_transcript_159514;F01_transcript_159785;F01_transcript_159814;F01_transcript_159968;F01_transcript_160683;F01_transcript_161110;F01_transcript_161429;F01_transcript_161773;F01_transcript_161816;F01_transcript_161966;F01_transcript_161981;F01_transcript_162066;F01_transcript_162134;F01_transcript_162331;F01_transcript_162421;F01_transcript_16286;F01_transcript_16301;F01_transcript_164021;F01_transcript_16433;F01_transcript_164766;F01_transcript_164901;F01_transcript_165924;F01_transcript_166964;F01_transcript_167396;F01_transcript_167595;F01_transcript_17183;F01_transcript_17281;F01_transcript_17857;F01_transcript_17966;F01_transcript_17987;F01_transcript_1799;F01_transcript_18447;F01_transcript_19074;F01_transcript_19302;F01_transcript_19412;F01_transcript_19520;F01_transcript_19705;F01_transcript_20309;F01_transcript_22669;F01_transcript_23121;F01_transcript_23785;F01_transcript_24020;F01_transcript_24677;F01_transcript_25659;F01_transcript_26120;F01_transcript_27104;F01_t	0128+K00128+K00232+K00128+K14085+K14085+K00128+K00232+K00128+K00128+K00128+K00128+K00001+K07513+K00128+K07513+K00128+K00128+K00001+K07513+K18857+K00128+K07513+K00232+K00121+K00232+K00128+K00128+K00626+K00121+K00626+K00128+K00128+K00128+K00626+K00128+K00121+K00626+K00121+K18857+K00121+K00128+K00121+K00128+K01897+K00249+K00232+K00626+K00232+K00232+K01897+K10527+K00232+K00232+K00232+K00121+K00128+K01897+K00232+K00128+K14085+K10527+K00232+K00232+K00626+K00121+K00121+K10527+K07513+K01897+K01897+K14085+K00128+K00128+K01897+K00128+K00232+K00128+K10527+K00128+K01897+K00249+K00232+K10527+K01897+K00626+K00128+K00249+K00128+K00128+K10527+K01897+K01897+K10527+K01897+K00128+K00232+K10527+K00128+K10527+K00128+K00249+K01897+K00626+K14085+K01897+K00626+K10527+K00128+K00232+K00128+K00121+K00232+K00626+K10527+K00128+K01897+K00232+K00232+K10527+K00232+K18857+K00626+K01897+K00232+K01897+K14085+K01897+K07513+K00121+K01897+K01897+K00232+K00121+K00001+K00121+K00249+K00232+K00232+K00128+K01897+K00232+K01897+K00128+K00232+K00128+K01897+K10527+K00626+K01897+K00128+K00128+K07513+K01897+K01897+K01897+K00128

ranscript_27232;F01_transcript_27376;F01_transcript_28357;F01_transcript_28572;F01_transcript_28856;F01_transcript_29191;F01_transcript_29858;F01_transcript_30292;F01_transcript_30362;F01_transcript_30379;F01_transcript_30801;F01_transcript_30935;F01_transcript_31894;F01_transcript_32017;F01_transcript_32266;F01_transcript_32640;F01_transcript_32889;F01_transcript_33325;F01_transcript_33549;F01_transcript_33600;F01_transcript_34271;F01_transcript_34354;F01_transcript_34454;F01_transcript_34541;F01_transcript_35286;F01_transcript_35442;F01_transcript_35988;F01_transcript_36465;F01_transcript_36482;F01_transcript_36697;F01_transcript_36801;F01_transcript_37377;F01_transcript_37617;F01_transcript_37686;F01_transcript_37696;F01_transcript_37926;F01_transcript_37987;F01_transcript_38302;F01_transcript_38323;F01_transcript_38373;F01_transcript_38657;F01_transcript_39701;F01_transcript_39959;F01_transcript_40578;F01_transcript_41704;F01_transcript_48006;F01_transcript_48424;F01_transcript_4986;F01_transcript_51625;F01_transcript_52099;F01_transcript_52332;F01_transcript_52548;F01_transcript_53390;F01_transcript_54622;F01_transcript_54950;F01_transcript_54990;F01_transcript_55240;F01_transcript_55281;F01_transcript_55528;F01_transcript_55541;F01_transcript_55736;F01_transcript_56128;F01_transcript_56137;F01_transcript_56690;F01_transcript_57689;F01_transcript_57777;F01_transcript_58171;F01_transcript_58368;F01_transcript_59371;F01_transcript_59821;F01_transcript_60135;F01_transcript_61866;F01_transcript_61928;F01_transcript_62811;F01_transcript_63491;F01_transcript_65034;F01_transcript_65403;F01_transcript_65891;F01_transcript_65979;F01_transcript_66022;F01_transcript_66261;F01_transcript_67042;F01_transcript_67211;F01_transcript_67276;F01_transcript_6767;F01_transcript_67738;F01_transcript_68865;F01_transcript_68887;F01_transcript_69739;F01_transcript_70471;F01_transcript_7124;F01_transcript_71260;F01_transcript_71646;F01_transcript_71676;F01_transcript_71725;F01_transcript_72007;F01_transcript_72010;F01_transcript_72096;F01_transcript_72349;F01_transcript_72826;F01_transcript_7309;F01_transcript_73295;F01_transcript_73443;F01_transcript_75083;F01_transcript_7550;F01_transcript_76

			982;F01_transcript_77451;F01_transcript_77976;F01_transcript_78246;F01_transcript_79052;F01_transcript_79452;F01_transcript_79852;F01_transcript_80183;F01_transcript_80510;F01_transcript_80594;F01_transcript_80645;F01_transcript_80843;F01_transcript_80969;F01_transcript_81584;F01_transcript_82184;F01_transcript_82368;F01_transcript_82742;F01_transcript_83118;F01_transcript_83317;F01_transcript_84296;F01_transcript_84463;F01_transcript_84935;F01_transcript_85292;F01_transcript_86684;F01_transcript_8790;F01_transcript_88028;F01_transcript_88720;F01_transcript_88860;F01_transcript_89994;F01_transcript_90896;F01_transcript_90931;F01_transcript_91037;F01_transcript_91452;F01_transcript_91795;F01_transcript_9209;F01_transcript_92632;F01_transcript_92699;F01_transcript_92804;F01_transcript_92831;F01_transcript_93534;F01_transcript_95989;F01_transcript_96227;F01_transcript_97024;F01_transcript_97230;F01_transcript_97377;F01_transcript_97546;F01_transcript_97808;F01_transcript_98172;F01_transcript_98282;F01_transcript_98445;F01_transcript_98462;F01_transcript_98478;F01_transcript_99276;F01_transcript_99279;F01_transcript_99528;	
Synthesis and degradation of ketone bodies	ko00072	28	F01_transcript_100488;F01_transcript_105403;F01_transcript_123569;F01_transcript_138114;F01_transcript_154880;F01_transcript_155685;F01_transcript_166964;F01_transcript_28787;F01_transcript_30530;F01_transcript_31485;F01_transcript_32485;F01_transcript_33312;F01_transcript_37377;F01_transcript_37686;F01_transcript_37841;F01_transcript_38302;F01_transcript_38657;F01_transcript_52332;F01_transcript_58368;F01_transcript_61404;F01_transcript_69739;F01_transcript_72756;F01_transcript_77451;F01_transcript_79052;F01_transcript_80843;F01_transcript_81816;F01_transcript_84463;F01_transcript_97808;	K01640+K00626+K01640+K00626+K01641+K00626+K00626+K01641+K01641+K01640+K01641+K01640+K00626+K00626+K01640+K00626+K00626+K00626+K00626+K01640+K00626+K01640+K00626+K00626+K00626+K01640+K00626+K00626
Cutin, suberine and wax biosynthesis	ko00073	42	F01_transcript_111390;F01_transcript_112221;F01_transcript_123081;F01_transcript_125042;F01_transcript_128433;F01_transcript_130689;F01_transcript_131023;F01_transcript_13880;F01_transcript_145047;F01_transcript_152744;F01_transcript_158436;F01_transcript_162352;F01_transcript_164224;F01_transcript_166266;F01_transcript_18319;F01_transcript_24637;F01_transcript_26026;F01_transcript_26288;F01_transcript_33783;F01_transcript_33972;F01_transcript_35086;F01_transcript_36201;F0	K15400+K15403+K15400+K15398+K17991+K15400+K15400+K15402+K15398+K15400+K15403+K15404+K15400+K15400+K15398+K15404+K15403+K15404+K13407+K15404+K13407+K15400+K15400+K15400+K15400+K15400+K15400+K13356+K15403+K13407+K15398+K15400+K15404+K15398+K15400+K15400+K13356+K15403+K17991+K17991+K15400+K15401

			1_transcript_36774;F01_transcript_37944;F01_transcript_3933;F01_transcript_51982;F01_transcript_58198;F01_transcript_61935;F01_transcript_62553;F01_transcript_64488;F01_transcript_66072;F01_transcript_6929;F01_transcript_72220;F01_transcript_73700;F01_transcript_84399;F01_transcript_87181;F01_transcript_89458;F01_transcript_89536;F01_transcript_90835;F01_transcript_97368;F01_transcript_97994;F01_transcript_99098;	
Steroid biosynthesis	ko00100	123	F01_transcript_100010;F01_transcript_10026;F01_transcript_100264;F01_transcript_101455;F01_transcript_101494;F01_transcript_101530;F01_transcript_102483;F01_transcript_103873;F01_transcript_104969;F01_transcript_106083;F01_transcript_106484;F01_transcript_110972;F01_transcript_111725;F01_transcript_112777;F01_transcript_112791;F01_transcript_113367;F01_transcript_113984;F01_transcript_116037;F01_transcript_117487;F01_transcript_119852;F01_transcript_120411;F01_transcript_121993;F01_transcript_122157;F01_transcript_122561;F01_transcript_122753;F01_transcript_123034;F01_transcript_123459;F01_transcript_126068;F01_transcript_126610;F01_transcript_126947;F01_transcript_129354;F01_transcript_129640;F01_transcript_130870;F01_transcript_135049;F01_transcript_135086;F01_transcript_135514;F01_transcript_137286;F01_transcript_137598;F01_transcript_139086;F01_transcript_139100;F01_transcript_139989;F01_transcript_141816;F01_transcript_142302;F01_transcript_142791;F01_transcript_143690;F01_transcript_144176;F01_transcript_145472;F01_transcript_145482;F01_transcript_147251;F01_transcript_147757;F01_transcript_148513;F01_transcript_149842;F01_transcript_150082;F01_transcript_150397;F01_transcript_150910;F01_transcript_152112;F01_transcript_156823;F01_transcript_159698;F01_transcript_161260;F01_transcript_163393;F01_transcript_163876;F01_transcript_164947;F01_transcript_16528;F01_transcript_165941;F01_transcript_166472;F01_transcript_166584;F01_transcript_16784;F01_transcript_18241;F01_transcript_1856;F01_transcript_19129;F01_transcript_20978;F01_transcript_2208;F01_transcript_22713;F01_transcript_23017;F01_transcript_28293;F01_transcript_29159;F01_transcript_29649;F01_transcript_3071;F01_transcript_31806;F01_transcript_3191;F01_transcript_32272;F01_transcript_3232;F01_	K08242+K01052+K01052+K08246+K00511+K01052+K14674+K01853+K01853+K01052+K08246+K00213+K08246+K00511+K01052+K08242+K01052+K00213+K01853+K00511+K01052+K07748+K07748+K00511+K14674+K01853+K00213+K09828+K01052+K07748+K05917+K00222+K00511+K00222+K00511+K01052+K14423+K01052+K01052+K07748+K01052+K07748+K00511+K14424+K07748+K00213+K00213+K00511+K01853+K00222+K00559+K07748+K00222+K01052+K01052+K08242+K01052+K00801+K01853+K00222+K14674+K01052+K01052+K08242+K01052+K01052+K09828+K01052+K14674+K07748+K05917+K14674+K00511+K00511+K00511+K00511+K05917+K14674+K05917+K00801+K00511+K14674+K05917+K00801+K00213+K00801+K00559+K01052+K14423+K08242+K00222+K08246+K07748+K01824+K00222+K01824+K01052+K14674+K00801+K01052+K00801+K00213+K01853+K01052+K05917+K01052+K01052+K07748+K01052+K00801+K00559+K01824+K01052+K07748+K09828+K00801+K00511+K14424+K14423+K00801+K14674+K00801+K00801

			transcript_33260;F01_transcript_33759;F01_transcript_35739;F01_transcript_36198;F01_transcript_36895;F01_transcript_38747;F01_transcript_40188;F01_transcript_40538;F01_transcript_40836;F01_transcript_41182;F01_transcript_458;F01_transcript_45877;F01_transcript_51837;F01_transcript_54101;F01_transcript_58534;F01_transcript_59089;F01_transcript_59628;F01_transcript_61158;F01_transcript_61655;F01_transcript_63636;F01_transcript_67298;F01_transcript_67316;F01_transcript_67494;F01_transcript_67583;F01_transcript_68560;F01_transcript_69882;F01_transcript_70122;F01_transcript_72821;F01_transcript_73209;F01_transcript_77828;F01_transcript_79995;F01_transcript_82290;F01_transcript_82691;F01_transcript_85783;F01_transcript_87614;F01_transcript_90025;F01_transcript_92201;F01_transcript_9222;F01_transcript_93920;F01_transcript_96071;F01_transcript_98094;	
Ubiquinone and other terpenoid-quinone biosynthesis	ko00130	201	F01_transcript_100618;F01_transcript_100818;F01_transcript_102029;F01_transcript_105511;F01_transcript_105544;F01_transcript_106628;F01_transcript_106666;F01_transcript_107947;F01_transcript_109102;F01_transcript_109370;F01_transcript_109637;F01_transcript_110607;F01_transcript_111129;F01_transcript_112411;F01_transcript_113062;F01_transcript_114433;F01_transcript_115169;F01_transcript_115332;F01_transcript_117065;F01_transcript_117133;F01_transcript_118602;F01_transcript_118832;F01_transcript_119046;F01_transcript_120210;F01_transcript_122119;F01_transcript_122634;F01_transcript_123831;F01_transcript_124326;F01_transcript_126044;F01_transcript_126362;F01_transcript_126805;F01_transcript_127284;F01_transcript_127371;F01_transcript_128948;F01_transcript_130539;F01_transcript_131515;F01_transcript_132157;F01_transcript_133078;F01_transcript_133094;F01_transcript_133186;F01_transcript_133648;F01_transcript_134247;F01_transcript_134424;F01_transcript_135806;F01_transcript_136009;F01_transcript_136534;F01_transcript_137618;F01_transcript_137702;F01_transcript_140176;F01_transcript_141833;F01_transcript_141972;F01_transcript_142218;F01_transcript_142629;F01_transcript_143278;F01_transcript_14328;F01_transcript_143895;F01_transcript_144347;F01_transcript_144444;F01_transcript_144749;F01_transcript_146359;F01_transcript_146924;F01_transcript_14720;F01_	K00815+K00487+K14759+K03809+K02548+K06125+K00815+K03809+K03809+K05928+K12502+K09834+K01661+K00457+K00591+K00487+K02548+K02548+K09834+K06125+K09833+K12502+K14759+K05928+K00815+K02548+K06127+K00457+K14759+K12501+K05928+K09833+K09833+K12502+K01661+K01904+K00815+K12502+K00487+K14759+K00815+K01661+K02548+K00815+K02548+K00815+K02548+K01904+K02548+K03809+K00591+K05928+K06125+K01904+K00815+K14759+K02548+K05928+K00815+K09834+K00815+K05928+K01904+K00487+K02548+K02548+K00815+K09834+K09834+K00457+K06125+K06127+K00487+K09833+K02548+K09833+K12502+K00457+K12502+K00815+K00815+K12502+K09833+K05928+K02548+K03809+K05928+K02548+K14760+K14760+K00815+K09834+K02548+K06126+K02552+K14760+K02548+K01904+K01904+K14760+K02548+K00457+K01904+K00487+K02548+K00815+K02548+K00815+K12502+K00815+K00487+K00457+K02548+K02548+K12502+K00815+K09833+K12502+K18606+K18606+K05928+K12501+K03809+K18606+K12502+K01661+K00815+K05928+K05928+K03809+K03183+K03809+K03809+K05928+K02548+K09834+K03183+K02548+K09834+K09833+K06126+K02548+K03183+K09834+K06127+K12502+K06127+K18606+K05928+K01904+K00815+K06125+K02548+K01904+K09833+K00487+K06125+K0318

	transcript_147651;F01_transcript_148330;F01_transcript_149022;F01_transcript_149448;F01_transcript_150132;F01_transcript_152025;F01_transcript_153517;F01_transcript_154276;F01_transcript_154398;F01_transcript_154823;F01_transcript_155813;F01_transcript_156053;F01_transcript_157281;F01_transcript_158135;F01_transcript_158349;F01_transcript_158999;F01_transcript_160030;F01_transcript_161264;F01_transcript_161557;F01_transcript_162035;F01_transcript_162556;F01_transcript_162705;F01_transcript_163389;F01_transcript_163469;F01_transcript_163672;F01_transcript_163996;F01_transcript_165325;F01_transcript_165673;F01_transcript_19390;F01_transcript_19799;F01_transcript_21271;F01_transcript_25369;F01_transcript_26290;F01_transcript_27361;F01_transcript_27474;F01_transcript_28040;F01_transcript_29461;F01_transcript_29759;F01_transcript_29833;F01_transcript_29880;F01_transcript_29890;F01_transcript_30745;F01_transcript_30806;F01_transcript_31061;F01_transcript_31652;F01_transcript_31700;F01_transcript_32004;F01_transcript_32147;F01_transcript_32473;F01_transcript_35520;F01_transcript_35574;F01_transcript_35630;F01_transcript_35919;F01_transcript_37457;F01_transcript_37833;F01_transcript_37888;F01_transcript_38995;F01_transcript_39596;F01_transcript_39674;F01_transcript_39860;F01_transcript_40445;F01_transcript_40724;F01_transcript_41031;F01_transcript_41196;F01_transcript_42165;F01_transcript_42168;F01_transcript_42485;F01_transcript_43742;F01_transcript_45319;F01_transcript_46062;F01_transcript_46179;F01_transcript_51615;F01_transcript_51833;F01_transcript_52261;F01_transcript_52296;F01_transcript_52651;F01_transcript_54830;F01_transcript_55136;F01_transcript_58215;F01_transcript_58922;F01_transcript_59111;F01_transcript_59902;F01_transcript_60842;F01_transcript_60972;F01_transcript_62574;F01_transcript_63163;F01_transcript_63991;F01_transcript_64346;F01_transcript_65744;F01_transcript_65855;F01_transcript_66125;F01_transcript_66441;F01_transcript_66966;F01_transcript_68082;F01_transcript_68880;F01_transcript_69616;F01_transcript_69918;F01_transcript_70885;F01_transcript_70899;F01_transcript_71133;F01_transcript_71598;F01_transcript_72244;F01_transcript_72452;F01_transcript_73063;	3+K14759+K00457+K02548+K02548+K02548+K02548+K03809+K14759+K05928+K09834+K06126+K18606+K00815+K01661+K14760+K02548+K02552+K06127+K02548+K05928+K14759+K05928+K01661+K01904+K12502+K02548+K01904+K03809+K02548+K09833+K12502+K01904+K00815+K05928+K09833+K12501+K00591+K09834+K14759+K01661+K02548+K03809+K02548

			F01_transcript_73642;F01_transcript_73802;F01_transcript_74216;F01_transcript_75254;F01_transcript_76908;F01_transcript_77099;F01_transcript_77578;F01_transcript_77887;F01_transcript_78335;F01_transcript_79703;F01_transcript_80427;F01_transcript_80489;F01_transcript_81941;F01_transcript_82949;F01_transcript_83780;F01_transcript_84574;F01_transcript_84657;F01_transcript_85667;F01_transcript_86407;F01_transcript_86854;F01_transcript_88064;F01_transcript_88878;F01_transcript_89279;F01_transcript_90345;F01_transcript_90364;F01_transcript_91451;F01_transcript_92235;F01_transcript_92251;F01_transcript_94247;F01_transcript_95334;F01_transcript_95850;F01_transcript_98150;F01_transcript_98226;F01_transcript_99453;F01_transcript_99850;	
Oxidative phosphorylation	ko00190	678	F01_transcript_100496;F01_transcript_10077;F01_transcript_100856;F01_transcript_101172;F01_transcript_101332;F01_transcript_101495;F01_transcript_101659;F01_transcript_101808;F01_transcript_102624;F01_transcript_102877;F01_transcript_103249;F01_transcript_103299;F01_transcript_103303;F01_transcript_103533;F01_transcript_103664;F01_transcript_103783;F01_transcript_103833;F01_transcript_104066;F01_transcript_104787;F01_transcript_105351;F01_transcript_105452;F01_transcript_105669;F01_transcript_105956;F01_transcript_106439;F01_transcript_106619;F01_transcript_106622;F01_transcript_106875;F01_transcript_106911;F01_transcript_106959;F01_transcript_107476;F01_transcript_107664;F01_transcript_107933;F01_transcript_108068;F01_transcript_108380;F01_transcript_108396;F01_transcript_108842;F01_transcript_108893;F01_transcript_109437;F01_transcript_109710;F01_transcript_109903;F01_transcript_110259;F01_transcript_110347;F01_transcript_110884;F01_transcript_111050;F01_transcript_111187;F01_transcript_111446;F01_transcript_111455;F01_transcript_111546;F01_transcript_111558;F01_transcript_111651;F01_transcript_11207;F01_transcript_112319;F01_transcript_112429;F01_transcript_11246;F01_transcript_11256;F01_transcript_112621;F01_transcript_112716;F01_transcript_112913;F01_transcript_112977;F01_transcript_113492;F01_transcript_11373;F01_transcript_113958;F01_transcript_113970;F01_transcript_113977;F01_transcript_114667;F01_transcript_115030;F01_transcript_115407;F01_transcript_11	K02147+K03934+K03952+K03942+K03939+K02154+K03881+K02259+K02267+K02150+K01507+K03881+K02147+K03953+K03879+K02147+K05575+K01535+K01507+K03937+K01507+K00411+K03943+K02154+K02134+K02262+K02133+K01507+K02152+K02140+K02144+K01535+K01507+K03965+K02133+K02133+K02155+K02140+K01535+K01507+K01507+K03940+K03935+K00413+K01535+K02128+K01507+K02136+K02257+K02136+K02140+K02111+K01507+K01507+K03965+K01507+K02154+K01535+K11353+K03881+K03934+K02147+K01507+K02266+K00234+K02150+K02133+K02149+K02154+K02154+K02155+K01507+K02154+K02112+K00234+K01535+K00417+K01535+K01535+K01507+K00234+K02147+K01535+K01507+K02155+K02150+K02148+K11352+K02146+K02154+K01507+K02145+K02154+K02152+K02111+K00413+K01507+K02259+K02154+K02112+K02259+K00235+K02153+K02154+K05577+K02147+K03661+K00234+K01507+K02155+K02112+K02140+K01507+K02111+K03883+K00416+K00413+K00413+K03941+K01535+K01535+K01507+K01535+K02262+K00235+K02134+K00411+K01507+K03949+K01535+K02155+K01535+K02265+K03953+K02112+K00417+K01507+K03943+K02111+K01535+K02147+K02134+K01507+K00417+K01507+K01535+K00417+K02147+K02154+K03934+K01507+K05575+K02265+K02109+K01507+K01507+K01535+K00234+K02147+K01507+K01507+K02147+K05575+K03943+K03965+K02261+K03942+K02133+K02154+K01507+K02137+K03

	5891;F01_transcript_115923;F01_transcript_116015;F01_transcript_116212;F01_transcript_116224;F01_transcript_116369;F01_transcript_116568;F01_transcript_116740;F01_transcript_116774;F01_transcript_116776;F01_transcript_117240;F01_transcript_117320;F01_transcript_11774;F01_transcript_117804;F01_transcript_118118;F01_transcript_119455;F01_transcript_119570;F01_transcript_11974;F01_transcript_119898;F01_transcript_120079;F01_transcript_120414;F01_transcript_120442;F01_transcript_120999;F01_transcript_121117;F01_transcript_121128;F01_transcript_121150;F01_transcript_121600;F01_transcript_122159;F01_transcript_122229;F01_transcript_122387;F01_transcript_122548;F01_transcript_122562;F01_transcript_123322;F01_transcript_123509;F01_transcript_123542;F01_transcript_124147;F01_transcript_12419;F01_transcript_124482;F01_transcript_124805;F01_transcript_124828;F01_transcript_124944;F01_transcript_124954;F01_transcript_125359;F01_transcript_125530;F01_transcript_125584;F01_transcript_12565;F01_transcript_125817;F01_transcript_125854;F01_transcript_125975;F01_transcript_126194;F01_transcript_126441;F01_transcript_126571;F01_transcript_126776;F01_transcript_127275;F01_transcript_127581;F01_transcript_127843;F01_transcript_127877;F01_transcript_129207;F01_transcript_129674;F01_transcript_129680;F01_transcript_129761;F01_transcript_129891;F01_transcript_130036;F01_transcript_130192;F01_transcript_130222;F01_transcript_130414;F01_transcript_130472;F01_transcript_130626;F01_transcript_130794;F01_transcript_130810;F01_transcript_131101;F01_transcript_131777;F01_transcript_13258;F01_transcript_133020;F01_transcript_133241;F01_transcript_134474;F01_transcript_134625;F01_transcript_134971;F01_transcript_134977;F01_transcript_134982;F01_transcript_135068;F01_transcript_135298;F01_transcript_135464;F01_transcript_135913;F01_transcript_135987;F01_transcript_136429;F01_transcript_136568;F01_transcript_136958;F01_transcript_137164;F01_transcript_137172;F01_transcript_137621;F01_transcript_138220;F01_transcript_138524;F01_transcript_138659;F01_transcript_138846;F01_transcript_138911;F01_transcript_139317;F01_transcript_139423;F01_transcript_139715;F01_transcript_140537;F01_transcript_14113	938+K01535+K02134+K02113+K03965+K02108+K01507+K05579+K02146+K02109+K01535+K02148+K02258+K01535+K01507+K02260+K01507+K01535+K01507+K02147+K02148+K02148+K02147+K02137+K02149+K11352+K02133+K02261+K02147+K03939+K01535+K03938+K02136+K01507+K03965+K02133+K01535+K01507+K00416+K01507+K02256+K01535+K02147+K03661+K02155+K02147+K01535+K02112+K03934+K02147+K01507+K01507+K02154+K00234+K02154+K02147+K03943+K02147+K05572+K11353+K03939+K03937+K01507+K01507+K02140+K01507+K02138+K03940+K01535+K02150+K01507+K01507+K02155+K01535+K03952+K02108+K02150+K01507+K03941+K02147+K00234+K03941+K05582+K02138+K05575+K02265+K02258+K02259+K02112+K05577+K02153+K01535+K03940+K02112+K01535+K05575+K05577+K01507+K02267+K02258+K03965+K01507+K00419+K01535+K02260+K01535+K03965+K02154+K02265+K02133+K02145+K02138+K02145+K02155+K05575+K00234+K01535+K01535+K02256+K01507+K03879+K02112+K02133+K01507+K02133+K02133+K02147+K03963+K02133+K02138+K01535+K01535+K02148+K02138+K01507+K02155+K01535+K02147+K05579+K02133+K05575+K03942+K01507+K00234+K11353+K11353+K02132+K03942+K01507+K02144+K02133+K02259+K02259+K05575+K01535+K01507+K03881+K03945+K03943+K03881+K02115+K03953+K03953+K03965+K02115+K01535+K02148+K02136+K02144+K02136+K02256+K02148+K03953+K02146+K02115+K02148+K02136+K02148+K02115+K02146+K02146+K03943+K01507+K00411+K00413+K01507+K02136+K00411+K00413+K01507+K00411+K03943+K01507+K02150+K03943+K02149+K01507+K00235+K02137+K01507+K03940+K02134+K02150+K03941+K02134+K02149+K03941+K03661+K02113+K02109+K02155+K02155+K03661+K03941+K02155+K02155+K02265+K02155+K02155+K02267+K02155+K02134+K01507+K02267+K01535+K01507+K02155+K02267+K02138+K02140+K01507+K02155+K00417+K02265+K02138+K03949+K11353+K02138+K02153+K00417+K11353+K02155+K03952+K02152+K11352+K02155+K03965+K03950+K00417+K03937+K00417+K11353+K02151+K03966+K

	4;F01_transcript_141509;F01_transcript_141889;F01_transcript_142063;F01_transcript_142393;F01_transcript_142647;F01_transcript_142728;F01_transcript_142990;F01_transcript_143162;F01_transcript_144301;F01_transcript_144311;F01_transcript_144367;F01_transcript_144387;F01_transcript_145129;F01_transcript_145187;F01_transcript_145300;F01_transcript_145537;F01_transcript_145607;F01_transcript_145634;F01_transcript_145961;F01_transcript_146204;F01_transcript_146207;F01_transcript_146579;F01_transcript_146671;F01_transcript_146945;F01_transcript_147090;F01_transcript_147572;F01_transcript_147806;F01_transcript_147882;F01_transcript_148033;F01_transcript_148310;F01_transcript_148578;F01_transcript_148778;F01_transcript_148842;F01_transcript_148999;F01_transcript_149280;F01_transcript_149982;F01_transcript_150078;F01_transcript_15023;F01_transcript_15038;F01_transcript_151075;F01_transcript_151954;F01_transcript_152179;F01_transcript_152397;F01_transcript_152624;F01_transcript_152835;F01_transcript_153084;F01_transcript_153405;F01_transcript_153407;F01_transcript_153673;F01_transcript_153686;F01_transcript_153690;F01_transcript_153859;F01_transcript_154015;F01_transcript_154259;F01_transcript_154606;F01_transcript_154669;F01_transcript_155034;F01_transcript_155072;F01_transcript_155620;F01_transcript_155773;F01_transcript_155890;F01_transcript_156096;F01_transcript_156350;F01_transcript_156455;F01_transcript_15676;F01_transcript_157018;F01_transcript_15710;F01_transcript_157161;F01_transcript_157307;F01_transcript_157583;F01_transcript_158069;F01_transcript_158343;F01_transcript_158869;F01_transcript_159057;F01_transcript_159119;F01_transcript_159285;F01_transcript_160258;F01_transcript_160361;F01_transcript_160534;F01_transcript_160710;F01_transcript_160937;F01_transcript_161274;F01_transcript_161923;F01_transcript_161943;F01_transcript_162015;F01_transcript_162616;F01_transcript_162690;F01_transcript_162714;F01_transcript_162866;F01_transcript_162956;F01_transcript_163522;F01_transcript_163681;F01_transcript_163772;F01_transcript_163783;F01_transcript_163865;F01_transcript_164217;F01_transcript_164266;F01_transcript_164276;F01_transcript_164822;F01_transcript_164975;F01_transcript_165655;F01_transcript_166114;F01_transcript_1	02261+K03950+K02267+K03937+K01535+K03965+K02266+K02140+K02266+K02152+K03965+K02153+K03952+K02267+K03966+K00419+K02135+K03938+K02265+K03945+K00416+K03946+K03963+K00416+K03955+K02267+K02135+K00419+K00416+K02135+K03945+K01507+K02113+K01535+K02154+K01535+K01507+K02112+K01507+K01535+K01535+K02155+K01535+K02150+K01535+K03937+K02154+K02154+K05573+K01535+K02150+K01535+K00235+K01535+K00234+K00411+K02147+K03965+K03955+K02147+K03963+K02112+K03881+K01535+K01535+K02154+K02111+K02113+K02151+K02112+K02154+K02152+K00235+K02109+K02147+K01535+K02109+K02133+K02147+K02109+K03940+K00235+K02256+K01535+K02154+K02150+K02115+K02154+K02154+K01507+K01535+K03938+K02115+K01507+K03943+K02151+K02154+K03881+K01507+K02111+K03943+K01507+K02140+K00234+K02145+K02144+K02147+K02154+K01535+K01535+K02148+K02155+K00411+K02150+K02154+K03953+K02112+K02115+K02133+K01507+K02265+K01535+K03945+K05579+K02145+K01535+K02146+K02154+K02155+K01507+K02154+K01507+K02154+K02138+K03883+K02112+K02137+K03966+K02154+K02147+K02147+K02155+K02155+K02138+K01507+K03937+K01507+K01507+K02115+K03965+K02257+K00416+K01535+K03963+K02147+K01507+K00412+K02259+K02154+K00413+K01507+K02266+K01507+K02147+K02147+K02112+K00417+K05575+K03953+K01507+K01507+K01507+K01535+K03942+K03955+K02153+K01507+K01535+K05579+K02154+K02147+K01507+K02154+K01535+K01535+K02109+K05575+K02148+K02112+K02145+K01535+K01535+K01507+K02147+K01535+K03883+K01507+K02154+K00412+K02112+K02154+K02112+K01507+K02150+K03965+K01507+K05577+K01535+K02111+K03661+K02266+K03937+K03953+K02147+K01507+K03939+K02261+K03965+K03953+K02109+K03937+K05582+K00413+K02154+K01507+K02154+K02115+K02154+K03935+K03963+K05579+K05575+K02145+K02153+K02115+K02154+K03955+K05577+K02145+K01507+K02140+K01535+K02148+K01507+K02147+K00234+K05582+K03941+K02140+K01507+K05579+K01535+K01535

	66145;F01_transcript_166193;F01_transcript_166223;F01_transcript_166663;F01_transcript_166853;F01_transcript_167059;F01_transcript_167425;F01_transcript_167492;F01_transcript_167677;F01_transcript_167712;F01_transcript_16824;F01_transcript_17061;F01_transcript_17432;F01_transcript_18162;F01_transcript_1857;F01_transcript_18972;F01_transcript_19052;F01_transcript_19546;F01_transcript_20405;F01_transcript_20592;F01_transcript_22605;F01_transcript_22945;F01_transcript_23252;F01_transcript_23485;F01_transcript_23611;F01_transcript_24240;F01_transcript_24328;F01_transcript_24750;F01_transcript_25318;F01_transcript_25456;F01_transcript_25647;F01_transcript_2572;F01_transcript_2605;F01_transcript_26207;F01_transcript_26329;F01_transcript_26539;F01_transcript_28359;F01_transcript_2903;F01_transcript_29571;F01_transcript_2959;F01_transcript_29807;F01_transcript_30515;F01_transcript_30760;F01_transcript_30921;F01_transcript_31236;F01_transcript_31363;F01_transcript_31418;F01_transcript_31473;F01_transcript_31811;F01_transcript_31848;F01_transcript_33092;F01_transcript_33626;F01_transcript_33659;F01_transcript_33696;F01_transcript_33996;F01_transcript_3453;F01_transcript_34843;F01_transcript_34993;F01_transcript_3515;F01_transcript_35194;F01_transcript_35474;F01_transcript_35489;F01_transcript_35573;F01_transcript_35585;F01_transcript_3592;F01_transcript_35982;F01_transcript_3631;F01_transcript_36313;F01_transcript_36359;F01_transcript_36825;F01_transcript_37576;F01_transcript_37704;F01_transcript_38360;F01_transcript_38395;F01_transcript_38485;F01_transcript_38548;F01_transcript_38873;F01_transcript_38967;F01_transcript_39078;F01_transcript_39149;F01_transcript_39463;F01_transcript_39977;F01_transcript_40876;F01_transcript_41271;F01_transcript_41355;F01_transcript_41497;F01_transcript_41519;F01_transcript_41916;F01_transcript_41999;F01_transcript_42615;F01_transcript_42862;F01_transcript_43039;F01_transcript_43146;F01_transcript_43170;F01_transcript_43205;F01_transcript_43441;F01_transcript_43486;F01_transcript_43732;F01_transcript_43846;F01_transcript_43901;F01_transcript_44522;F01_transcript_44544;F01_transcript_44583;F01_transcript_44613;F01_transcript_44671;F01_transcript_446

94;F01_transcript_44703;F01_transcript_44708;F01_transcript_
44714;F01_transcript_44732;F01_transcript_44951;F01_transcri
pt_45149;F01_transcript_45252;F01_transcript_45403;F01_tran
script_45476;F01_transcript_45589;F01_transcript_45603;F01_t
ranscript_45647;F01_transcript_45652;F01_transcript_45772;F0
1_transcript_45781;F01_transcript_45814;F01_transcript_45841
;F01_transcript_45856;F01_transcript_45948;F01_transcript_45
97;F01_transcript_46074;F01_transcript_46116;F01_transcript_
46176;F01_transcript_46190;F01_transcript_46228;F01_transcri
pt_46280;F01_transcript_46466;F01_transcript_46480;F01_tran
script_46615;F01_transcript_46718;F01_transcript_46782;F01_t
ranscript_46804;F01_transcript_47060;F01_transcript_47128;F0
1_transcript_47138;F01_transcript_47261;F01_transcript_47323
;F01_transcript_47505;F01_transcript_47528;F01_transcript_47
554;F01_transcript_47640;F01_transcript_47641;F01_transcript
_47672;F01_transcript_47826;F01_transcript_47897;F01_transc
ript_48141;F01_transcript_48222;F01_transcript_48268;F01_tra
nscript_48388;F01_transcript_48527;F01_transcript_48541;F01
_transcript_48614;F01_transcript_48634;F01_transcript_4866;F
01_transcript_48700;F01_transcript_48780;F01_transcript_4879
4;F01_transcript_48848;F01_transcript_49010;F01_transcript_4
9012;F01_transcript_49040;F01_transcript_49105;F01_transcrip
t_49156;F01_transcript_49251;F01_transcript_49404;F01_trans
cript_49431;F01_transcript_49450;F01_transcript_49463;F01_tr
anscript_49629;F01_transcript_49665;F01_transcript_49748;F0
1_transcript_49757;F01_transcript_49869;F01_transcript_49939
;F01_transcript_49955;F01_transcript_50007;F01_transcript_50
063;F01_transcript_50120;F01_transcript_50185;F01_transcript
_50210;F01_transcript_50420;F01_transcript_50802;F01_transc
ript_5133;F01_transcript_51417;F01_transcript_51523;F01_tran
script_52199;F01_transcript_52831;F01_transcript_53238;F01_t
ranscript_53328;F01_transcript_53361;F01_transcript_53586;F0
1_transcript_53839;F01_transcript_53857;F01_transcript_53963
;F01_transcript_54014;F01_transcript_5415;F01_transcript_553
04;F01_transcript_55391;F01_transcript_55394;F01_transcript_
556;F01_transcript_55925;F01_transcript_56233;F01_transcript
_56851;F01_transcript_56927;F01_transcript_57106;F01_transc
ript_57129;F01_transcript_57504;F01_transcript_57767;F01_tra

nscript_57803;F01_transcript_58057;F01_transcript_58452;F01
_transcript_5867;F01_transcript_58931;F01_transcript_59212;F
01_transcript_5931;F01_transcript_59415;F01_transcript_59494
;F01_transcript_59500;F01_transcript_59676;F01_transcript_59
773;F01_transcript_60016;F01_transcript_60229;F01_transcript
_60343;F01_transcript_60346;F01_transcript_60440;F01_transc
ript_60572;F01_transcript_60752;F01_transcript_60834;F01_tra
nscript_61040;F01_transcript_61049;F01_transcript_61538;F01
_transcript_61837;F01_transcript_62198;F01_transcript_6240;F
01_transcript_62510;F01_transcript_6290;F01_transcript_6295;
F01_transcript_6365;F01_transcript_63752;F01_transcript_6408
4;F01_transcript_64089;F01_transcript_64143;F01_transcript_6
4503;F01_transcript_64977;F01_transcript_65163;F01_transcrip
t_6532;F01_transcript_65680;F01_transcript_66195;F01_transcr
ipt_66357;F01_transcript_66417;F01_transcript_66544;F01_tra
nscript_66558;F01_transcript_66560;F01_transcript_66625;F01
_transcript_67046;F01_transcript_67078;F01_transcript_67400;
F01_transcript_67646;F01_transcript_68004;F01_transcript_680
62;F01_transcript_68163;F01_transcript_68687;F01_transcript_
68917;F01_transcript_69013;F01_transcript_69040;F01_transcri
pt_69126;F01_transcript_69674;F01_transcript_69719;F01_tran
script_69788;F01_transcript_70054;F01_transcript_70517;F01_t
ranscript_70592;F01_transcript_70834;F01_transcript_709;F01_
transcript_70907;F01_transcript_71095;F01_transcript_71108;F
01_transcript_71299;F01_transcript_71522;F01_transcript_7157
2;F01_transcript_71715;F01_transcript_7189;F01_transcript_72
08;F01_transcript_72191;F01_transcript_72265;F01_transcript_
72742;F01_transcript_72968;F01_transcript_7313;F01_transcrip
t_73334;F01_transcript_74347;F01_transcript_74431;F01_trans
cript_74657;F01_transcript_75377;F01_transcript_75463;F01_tr
anscript_75480;F01_transcript_76061;F01_transcript_76246;F0
1_transcript_77152;F01_transcript_7783;F01_transcript_78105;
F01_transcript_78356;F01_transcript_78652;F01_transcript_787
63;F01_transcript_78998;F01_transcript_79378;F01_transcript_
79563;F01_transcript_79775;F01_transcript_7980;F01_transcrip
t_79833;F01_transcript_80063;F01_transcript_80090;F01_trans
cript_8018;F01_transcript_80267;F01_transcript_80436;F01_tra

			nscript_80631;F01_transcript_81350;F01_transcript_81514;F01_transcript_81577;F01_transcript_82103;F01_transcript_82250;F01_transcript_82269;F01_transcript_82647;F01_transcript_82780;F01_transcript_8308;F01_transcript_8355;F01_transcript_84146;F01_transcript_84443;F01_transcript_85079;F01_transcript_85090;F01_transcript_85222;F01_transcript_86197;F01_transcript_86400;F01_transcript_86648;F01_transcript_86774;F01_transcript_87112;F01_transcript_87222;F01_transcript_87445;F01_transcript_88065;F01_transcript_88104;F01_transcript_88111;F01_transcript_88532;F01_transcript_88641;F01_transcript_88802;F01_transcript_88851;F01_transcript_89352;F01_transcript_89609;F01_transcript_89771;F01_transcript_89782;F01_transcript_89798;F01_transcript_89806;F01_transcript_8987;F01_transcript_90121;F01_transcript_90171;F01_transcript_90388;F01_transcript_9054;F01_transcript_90689;F01_transcript_91038;F01_transcript_91226;F01_transcript_91237;F01_transcript_91239;F01_transcript_91549;F01_transcript_91649;F01_transcript_91857;F01_transcript_91907;F01_transcript_92076;F01_transcript_92489;F01_transcript_92688;F01_transcript_93293;F01_transcript_93659;F01_transcript_93762;F01_transcript_93980;F01_transcript_94248;F01_transcript_94249;F01_transcript_94362;F01_transcript_94387;F01_transcript_94529;F01_transcript_94650;F01_transcript_94692;F01_transcript_94914;F01_transcript_95251;F01_transcript_95351;F01_transcript_95466;F01_transcript_95669;F01_transcript_95697;F01_transcript_95704;F01_transcript_95959;F01_transcript_96258;F01_transcript_96368;F01_transcript_96500;F01_transcript_96968;F01_transcript_97147;F01_transcript_9728;F01_transcript_9740;F01_transcript_97661;F01_transcript_97960;F01_transcript_98290;F01_transcript_98619;F01_transcript_9895;F01_transcript_98950;F01_transcript_98998;F01_transcript_99123;F01_transcript_99605;	
Photosynthesis	ko00195	369	F01_transcript_100092;F01_transcript_101590;F01_transcript_101959;F01_transcript_102040;F01_transcript_103160;F01_transcript_103461;F01_transcript_103750;F01_transcript_103751;F01_transcript_105052;F01_transcript_105238;F01_transcript_105467;F01_transcript_105834;F01_transcript_106122;F01_transcript_106661;F01_transcript_107179;F01_transcript_107887;F01_transcript_108982;F01_transcript_109108;F01_transcript_10926	K08901+K02641+K02639+K03541+K08901+K02639+K02694+K03541+K03542+K02639+K02721+K03541+K02639+K08901+K02692+K02717+K02641+K02641+K02717+K02706+K02717+K02717+K08905+K02717+K03541+K02694+K02721+K08903+K02111+K03541+K03541+K02638+K08903+K02705+K02636+K02717+K03541+K02698+K02112+K03541+K02717+K02705+K02635+K03541+K02717+K02717+K02717+K02701+

	8;F01_transcript_109893;F01_transcript_110051;F01_transcript_110247;F01_transcript_110273;F01_transcript_110297;F01_transcript_110405;F01_transcript_110666;F01_transcript_110907;F01_transcript_111430;F01_transcript_112319;F01_transcript_112338;F01_transcript_112701;F01_transcript_112923;F01_transcript_112997;F01_transcript_113043;F01_transcript_113728;F01_transcript_113744;F01_transcript_114340;F01_transcript_115096;F01_transcript_116568;F01_transcript_116843;F01_transcript_116875;F01_transcript_117692;F01_transcript_119519;F01_transcript_119584;F01_transcript_119825;F01_transcript_120538;F01_transcript_120800;F01_transcript_121321;F01_transcript_121366;F01_transcript_121523;F01_transcript_122159;F01_transcript_12267;F01_transcript_122925;F01_transcript_123322;F01_transcript_124251;F01_transcript_124268;F01_transcript_124370;F01_transcript_124389;F01_transcript_124533;F01_transcript_125530;F01_transcript_125659;F01_transcript_125798;F01_transcript_125817;F01_transcript_126309;F01_transcript_126506;F01_transcript_127249;F01_transcript_128995;F01_transcript_129252;F01_transcript_129256;F01_transcript_129965;F01_transcript_130328;F01_transcript_130444;F01_transcript_130626;F01_transcript_131777;F01_transcript_13285;F01_transcript_133163;F01_transcript_133488;F01_transcript_133896;F01_transcript_13455;F01_transcript_135110;F01_transcript_136568;F01_transcript_136638;F01_transcript_137106;F01_transcript_137292;F01_transcript_137698;F01_transcript_139102;F01_transcript_142381;F01_transcript_142714;F01_transcript_142990;F01_transcript_143148;F01_transcript_143493;F01_transcript_143796;F01_transcript_144109;F01_transcript_144301;F01_transcript_144382;F01_transcript_145129;F01_transcript_146962;F01_transcript_147321;F01_transcript_148768;F01_transcript_148920;F01_transcript_149766;F01_transcript_150151;F01_transcript_150255;F01_transcript_150285;F01_transcript_151696;F01_transcript_152233;F01_transcript_152653;F01_transcript_152836;F01_transcript_152925;F01_transcript_153239;F01_transcript_153258;F01_transcript_153690;F01_transcript_154196;F01_transcript_154260;F01_transcript_154704;F01_transcript_154896;F01_transcript_155248;F01_transcript_155743;F01_transcript_155775	K02641+K03541+K02111+K02695+K03541+K02112+K03542+K02639+K02698+K03542+K02694+K02112+K02704+K02690+K02111+K02706+K02709+K03541+K03542+K02699+K03541+K02690+K02636+K02717+K02112+K02111+K02635+K14332+K02639+K02717+K03541+K02698+K02109+K02641+K02723+K02721+K02639+K08901+K02636+K02634+K02113+K02717+K02717+K03542+K02721+K02108+K02689+K02109+K03541+K03541+K02693+K03542+K02693+K08905+K03541+K02641+K03541+K02636+K02641+K02717+K02701+K02694+K03542+K02112+K03542+K02695+K02692+K02706+K02692+K03541+K02692+K02634+K02717+K02695+K02723+K14332+K02641+K02634+K03541+K02717+K14332+K14332+K08901+K08901+K02108+K03541+K02634+K02692+K14332+K02112+K02706+K02112+K02716+K03541+K02689+K14332+K03541+K02639+K02637+K02717+K02641+K02694+K02640+K02636+K02695+K08901+K02695+K03542+K03542+K03542+K02112+K08901+K03542+K08901+K02717+K03541+K08901+K03541+K02689+K02695+K02717+K02698+K02115+K03541+K02115+K02641+K02641+K02115+K02641+K02641+K02641+K02115+K02641+K02705+K03542+K03542+K02717+K02717+K03542+K03541+K03541+K03542+K03541+K03542+K03542+K02717+K03541+K02113+K02717+K02717+K03541+K02109+K02636+K02694+K02636+K02717+K02723+K02694+K02717+K08901+K08901+K08901+K08901+K08901+K03541+K02723+K03542+K08901+K08901+K08901+K02717+K02639+K08901+K02639+K02723+K03541+K02723+K03541+K02698+K03541+K02701+K02639+K02694+K03541+K14332+K02701+K08906+K08902+K02723+K08905+K03541+K14332+K02721+K03541+K02695+K08902+K02639+K03541+K08901+K02721+K02698+K02639+K02721+K14332+K03541+K02721+K02701+K02639+K02699+K08905+K02721+K03541+K02639+K02721+K03541+K02693+K02693+K02721+K02698+K03541+K02693+K02693+K02721+K02716+K02692+K02638+K02694+K02113+K02639+K02635+K02694+K02638+K02692+K02716+K02639+K02638+K02639+K02701+K03542+K02112+K08903+K02706+K02716+K02723+K02690+K02717+K02695+K02704+K03542+K02112+K03542+K02111+K02113+K0211

	;F01_transcript_156136;F01_transcript_156452;F01_transcript_156545;F01_transcript_157009;F01_transcript_157053;F01_transcript_157343;F01_transcript_157618;F01_transcript_157891;F01_transcript_158240;F01_transcript_158437;F01_transcript_159828;F01_transcript_160350;F01_transcript_160496;F01_transcript_160534;F01_transcript_160866;F01_transcript_161701;F01_transcript_162724;F01_transcript_163618;F01_transcript_163681;F01_transcript_164185;F01_transcript_164266;F01_transcript_164614;F01_transcript_164905;F01_transcript_165128;F01_transcript_165167;F01_transcript_165444;F01_transcript_165693;F01_transcript_166100;F01_transcript_166366;F01_transcript_166734;F01_transcript_167114;F01_transcript_167193;F01_transcript_167600;F01_transcript_1962;F01_transcript_19816;F01_transcript_20799;F01_transcript_21759;F01_transcript_2186;F01_transcript_22056;F01_transcript_23252;F01_transcript_23502;F01_transcript_24681;F01_transcript_25778;F01_transcript_26691;F01_transcript_26934;F01_transcript_26954;F01_transcript_27757;F01_transcript_300;F01_transcript_30214;F01_transcript_34260;F01_transcript_35216;F01_transcript_35489;F01_transcript_35726;F01_transcript_35982;F01_transcript_38136;F01_transcript_38278;F01_transcript_38548;F01_transcript_38784;F01_transcript_38801;F01_transcript_38975;F01_transcript_39149;F01_transcript_40411;F01_transcript_410;F01_transcript_42635;F01_transcript_42723;F01_transcript_42844;F01_transcript_42860;F01_transcript_4327;F01_transcript_43421;F01_transcript_43461;F01_transcript_43552;F01_transcript_43770;F01_transcript_44204;F01_transcript_44211;F01_transcript_44226;F01_transcript_44538;F01_transcript_44732;F01_transcript_44741;F01_transcript_44745;F01_transcript_44862;F01_transcript_44951;F01_transcript_44978;F01_transcript_45106;F01_transcript_45205;F01_transcript_45264;F01_transcript_45331;F01_transcript_45563;F01_transcript_45680;F01_transcript_45706;F01_transcript_45802;F01_transcript_45868;F01_transcript_46138;F01_transcript_46163;F01_transcript_46235;F01_transcript_46287;F01_transcript_46351;F01_transcript_46375;F01_transcript_46455;F01_transcript_46500;F01_transcript_46639;F01_transcript_46659;F01_transcript_46695;F01_transcript_46710;F01_transcript_46740;F01_transcript_46786;F01_transcript_46908;F01_transcript_	2+K02695+K02109+K02109+K02109+K02115+K02115+K02717+K02693+K02111+K02112+K08901+K08906+K02115+K03542+K02692+K03541+K02641+K03541+K02112+K03541+K02705+K02639+K02639+K02701+K02636+K03542+K02717+K02115+K02689+K02634+K08903+K02636+K02704+K02112+K02706+K02706+K02636+K02706+K02723+K02641+K02695+K02693+K02109+K08905+K02112+K02717+K02695+K03542+K02112+K02112+K02695+K02638+K02111+K02695+K02639+K02689+K02721+K02109+K03541+K02695+K02639+K02717+K02115+K08901+K02115+K02636+K02717

47059;F01_transcript_47149;F01_transcript_47221;F01_transcript_47291;F01_transcript_47297;F01_transcript_47444;F01_transcript_47499;F01_transcript_47510;F01_transcript_47565;F01_transcript_47656;F01_transcript_47747;F01_transcript_47804;F01_transcript_47841;F01_transcript_47850;F01_transcript_47911;F01_transcript_48084;F01_transcript_48093;F01_transcript_48133;F01_transcript_48272;F01_transcript_48291;F01_transcript_48323;F01_transcript_48358;F01_transcript_48496;F01_transcript_48497;F01_transcript_48499;F01_transcript_48572;F01_transcript_48577;F01_transcript_48662;F01_transcript_48703;F01_transcript_48711;F01_transcript_48716;F01_transcript_48722;F01_transcript_48759;F01_transcript_48776;F01_transcript_48781;F01_transcript_48793;F01_transcript_48957;F01_transcript_49006;F01_transcript_49077;F01_transcript_49115;F01_transcript_49360;F01_transcript_49593;F01_transcript_49599;F01_transcript_49751;F01_transcript_49781;F01_transcript_49854;F01_transcript_50177;F01_transcript_50500;F01_transcript_50571;F01_transcript_50742;F01_transcript_50802;F01_transcript_50834;F01_transcript_5095;F01_transcript_50950;F01_transcript_50979;F01_transcript_51102;F01_transcript_51112;F01_transcript_51128;F01_transcript_51167;F01_transcript_51229;F01_transcript_51387;F01_transcript_5159;F01_transcript_52831;F01_transcript_53421;F01_transcript_53558;F01_transcript_53664;F01_transcript_55816;F01_transcript_56195;F01_transcript_56264;F01_transcript_56474;F01_transcript_57208;F01_transcript_58054;F01_transcript_58452;F01_transcript_58843;F01_transcript_59415;F01_transcript_59494;F01_transcript_59676;F01_transcript_59977;F01_transcript_60343;F01_transcript_60572;F01_transcript_61040;F01_transcript_6290;F01_transcript_64143;F01_transcript_64767;F01_transcript_65548;F01_transcript_66357;F01_transcript_69126;F01_transcript_69138;F01_transcript_69561;F01_transcript_69674;F01_transcript_70877;F01_transcript_71167;F01_transcript_71441;F01_transcript_71487;F01_transcript_72124;F01_transcript_72265;F01_transcript_72455;F01_transcript_727;F01_transcript_73005;F01_transcript_73257;F01_transcript_73388;F01_transcript_75068;F01_transcript_75884;F01_transcript_76894;F01_transcript_77152;F01_transcript_77227;F01_tr

			anscript_77855;F01_transcript_78658;F01_transcript_78906;F01_transcript_79017;F01_transcript_80631;F01_transcript_81947;F01_transcript_82011;F01_transcript_82074;F01_transcript_82576;F01_transcript_83218;F01_transcript_84982;F01_transcript_86302;F01_transcript_87046;F01_transcript_87112;F01_transcript_87799;F01_transcript_88065;F01_transcript_88784;F01_transcript_8963;F01_transcript_89670;F01_transcript_89798;F01_transcript_8987;F01_transcript_90606;F01_transcript_90920;F01_transcript_91226;F01_transcript_92050;F01_transcript_92245;F01_transcript_92440;F01_transcript_93425;F01_transcript_93659;F01_transcript_93984;F01_transcript_94116;F01_transcript_94290;F01_transcript_94454;F01_transcript_94529;F01_transcript_95677;F01_transcript_95697;F01_transcript_97333;F01_transcript_97963;	
Photosynthesis - antenna proteins	ko00196	201	F01_transcript_101361;F01_transcript_101617;F01_transcript_101862;F01_transcript_104455;F01_transcript_105309;F01_transcript_105581;F01_transcript_105583;F01_transcript_106088;F01_transcript_106247;F01_transcript_106463;F01_transcript_106828;F01_transcript_108571;F01_transcript_108822;F01_transcript_109128;F01_transcript_109410;F01_transcript_110047;F01_transcript_110248;F01_transcript_110710;F01_transcript_110880;F01_transcript_111879;F01_transcript_111998;F01_transcript_114032;F01_transcript_114367;F01_transcript_116462;F01_transcript_116550;F01_transcript_117353;F01_transcript_117741;F01_transcript_118387;F01_transcript_118887;F01_transcript_118958;F01_transcript_119575;F01_transcript_121003;F01_transcript_121090;F01_transcript_121844;F01_transcript_122742;F01_transcript_124299;F01_transcript_125123;F01_transcript_125792;F01_transcript_126731;F01_transcript_128410;F01_transcript_129145;F01_transcript_129351;F01_transcript_129899;F01_transcript_130578;F01_transcript_131868;F01_transcript_134755;F01_transcript_135226;F01_transcript_135622;F01_transcript_136267;F01_transcript_136270;F01_transcript_136314;F01_transcript_137007;F01_transcript_138677;F01_transcript_139032;F01_transcript_139328;F01_transcript_139726;F01_transcript_141107;F01_transcript_141954;F01_transcript_142023;F01_transcript_143116;F01_transcript_143268;F01_transcript_145305;F01_transcript_145748;F01_transcript_148555;F01_transcript_148	K08908+K08914+K08910+K08914+K08908+K14172+K08912+K08908+K08910+K08908+K08909+K08909+K08916+K08911+K08912+K08915+K08909+K08908+K08908+K08908+K08908+K08907+K08908+K08909+K08908+K08914+K08911+K08916+K08912+K08916+K08907+K08917+K08907+K08907+K08914+K08914+K08908+K14172+K08917+K08908+K08914+K08912+K08914+K08909+K08908+K08910+K08916+K08914+K08911+K08914+K08916+K08908+K08908+K08916+K08916+K14172+K08909+K08908+K08915+K08911+K08911+K08914+K08912+K08908+K08916+K08911+K08908+K08908+K08907+K08912+K08915+K08914+K08908+K08908+K08909+K08910+K08916+K08916+K08912+K08915+K08914+K08908+K08917+K08908+K08916+K08916+K08916+K08910+K14172+K08908+K08917+K08917+K08911+K08909+K08914+K08914+K08909+K08907+K08909+K08916+K08916+K14172+K08912+K08916+K08917+K08917+K08916+K08910+K08909+K08917+K08915+K08912+K08917+K08908+K08916+K08912+K08910+K08908+K08912+K08908+K08909+K08912+K08908+K08913+K08912+K08912+K08912+K08909+K08917+K08914+K08910+K08912+K08917+K08908+K08914+K08908+K08917+K08908+K08908+K08910+K08917+K08909+K08914+K08908+K08910+K08917+K08909+K08914+K08912+K08916+K08917+K08914+K08912+K08912+K08916+K08908+K08914+K08917+K08908+K08908+K08916+K08908+K08916+K08909+K089

	564;F01_transcript_149051;F01_transcript_150907;F01_transcript_151018;F01_transcript_151497;F01_transcript_153011;F01_transcript_153303;F01_transcript_153699;F01_transcript_153769;F01_transcript_154022;F01_transcript_154543;F01_transcript_154744;F01_transcript_155857;F01_transcript_155912;F01_transcript_157923;F01_transcript_158200;F01_transcript_160287;F01_transcript_161125;F01_transcript_161612;F01_transcript_163008;F01_transcript_163695;F01_transcript_167032;F01_transcript_17708;F01_transcript_22920;F01_transcript_23707;F01_transcript_24029;F01_transcript_28966;F01_transcript_30396;F01_transcript_31896;F01_transcript_33185;F01_transcript_33205;F01_transcript_33219;F01_transcript_33533;F01_transcript_33849;F01_transcript_36404;F01_transcript_40715;F01_transcript_42036;F01_transcript_42119;F01_transcript_42133;F01_transcript_42219;F01_transcript_42492;F01_transcript_42494;F01_transcript_42628;F01_transcript_42875;F01_transcript_43175;F01_transcript_43177;F01_transcript_43300;F01_transcript_43344;F01_transcript_43447;F01_transcript_43506;F01_transcript_43508;F01_transcript_43513;F01_transcript_43569;F01_transcript_43639;F01_transcript_43681;F01_transcript_43684;F01_transcript_43787;F01_transcript_43819;F01_transcript_43929;F01_transcript_43937;F01_transcript_44029;F01_transcript_44138;F01_transcript_44158;F01_transcript_44165;F01_transcript_44243;F01_transcript_44419;F01_transcript_44539;F01_transcript_44695;F01_transcript_44747;F01_transcript_44814;F01_transcript_44864;F01_transcript_45054;F01_transcript_45121;F01_transcript_45273;F01_transcript_45471;F01_transcript_45499;F01_transcript_45511;F01_transcript_45516;F01_transcript_45637;F01_transcript_45867;F01_transcript_45936;F01_transcript_46305;F01_transcript_46380;F01_transcript_46987;F01_transcript_47612;F01_transcript_47780;F01_transcript_48333;F01_transcript_48702;F01_transcript_48730;F01_transcript_49076;F01_transcript_50322;F01_transcript_50373;F01_transcript_50391;F01_transcript_50522;F01_transcript_50744;F01_transcript_5301;F01_transcript_53049;F01_transcript_5381;F01_transcript_53946;F01_transcript_55267;F01_transcript_55649;F01_transcript_56564;F01_transcript_57176;F01_transcript_58143;F01_transcript_58308;	09+K08913+K08914+K08914+K08909+K08912+K08908+K08908+K08907+K08908+K08907+K08912+K08911+K08916+K08917+K08916+K08916+K08907+K08916+K08908+K14172+K08910+K08916+K08910+K08916+K08917+K08911+K08914+K08916+K08914+K08914+K08909+K08907+K14172+K08908+K08908+K08908

			F01_transcript_58703;F01_transcript_58994;F01_transcript_59796;F01_transcript_60386;F01_transcript_61515;F01_transcript_61945;F01_transcript_62850;F01_transcript_64365;F01_transcript_6891;F01_transcript_69327;F01_transcript_70949;F01_transcript_72195;F01_transcript_72617;F01_transcript_74998;F01_transcript_7553;F01_transcript_76622;F01_transcript_77737;F01_transcript_78291;F01_transcript_81852;F01_transcript_83717;F01_transcript_85170;F01_transcript_85582;F01_transcript_86869;F01_transcript_8769;F01_transcript_90389;F01_transcript_90636;F01_transcript_90907;F01_transcript_94266;F01_transcript_97041;F01_transcript_99026;F01_transcript_99195;F01_transcript_99398;	
Arginine biosynthesis	ko00220	228	F01_transcript_10047;F01_transcript_101062;F01_transcript_101386;F01_transcript_101473;F01_transcript_102048;F01_transcript_10266;F01_transcript_102942;F01_transcript_103318;F01_transcript_103356;F01_transcript_103532;F01_transcript_104113;F01_transcript_105845;F01_transcript_106024;F01_transcript_106156;F01_transcript_107151;F01_transcript_107931;F01_transcript_108303;F01_transcript_108633;F01_transcript_109463;F01_transcript_110137;F01_transcript_110169;F01_transcript_110317;F01_transcript_11182;F01_transcript_112260;F01_transcript_113831;F01_transcript_114452;F01_transcript_114576;F01_transcript_116166;F01_transcript_116580;F01_transcript_116648;F01_transcript_116793;F01_transcript_117148;F01_transcript_117385;F01_transcript_119869;F01_transcript_120116;F01_transcript_120234;F01_transcript_122101;F01_transcript_122816;F01_transcript_123393;F01_transcript_123427;F01_transcript_123753;F01_transcript_123819;F01_transcript_12434;F01_transcript_124982;F01_transcript_125144;F01_transcript_125768;F01_transcript_126185;F01_transcript_126272;F01_transcript_127209;F01_transcript_127620;F01_transcript_128203;F01_transcript_129374;F01_transcript_130485;F01_transcript_130674;F01_transcript_131043;F01_transcript_132266;F01_transcript_132527;F01_transcript_132815;F01_transcript_133532;F01_transcript_134077;F01_transcript_135578;F01_transcript_136085;F01_transcript_137444;F01_transcript_137593;F01_transcript_137850;F01_transcript_138956;F01_transcript_14157;F01_transcript_141936;F01_transcript_142458;F01_transcript_142888;F01_transc	K14272+K01915+K00261+K14454+K14272+K14272+K14677+K01915+K01755+K14272+K01940+K01476+K01915+K14454+K00811+K14272+K01915+K14682+K01915+K14272+K00261+K01915+K14272+K14272+K14272+K14455+K01915+K01915+K14677+K14272+K00814+K14272+K14677+K14455+K01915+K14682+K14272+K01940+K00145+K01915+K14272+K00262+K00262+K00262+K01940+K14272+K14455+K00930+K00818+K01755+K00620+K01915+K14272+K01915+K14455+K01915+K14272+K01915+K01940+K00261+K14454+K00262+K14682+K01915+K00262+K00262+K14677+K14682+K01915+K00620+K00262+K00611+K14272+K00262+K14272+K14272+K00611+K00262+K01915+K01915+K14454+K01427+K14454+K14272+K14455+K01755+K01755+K14682+K01755+K14272+K14272+K00811+K00262+K00261+K14272+K00262+K00262+K14682+K01915+K00611+K14682+K00814+K14682+K01755+K14272+K14272+K14454+K01755+K14272+K00145+K14454+K13427+K14677+K00811+K14272+K14272+K14455+K01940+K14272+K01915+K14272+K01940+K01940+K00818+K14677+K01915+K00818+K01915+K01915+K14677+K14272+K01438+K14455+K01755+K14455+K00145+K01915+K01915+K01915+K00611+K01915+K01915+K01915+K01915+K00930+K00930+K14272+K01427+K00262+K00814+K01755+K14682+K14677+K00814+K14272+K00145+K14272+K00818+K00611+K00930+K01940+K01915+K00814+K14272+K14454+K01427+K00145+K01915+K14272+K00262+K01427+K00261+K00814+K01915+K14677+K00262+K00930+K00620+K0

	ript_143068;F01_transcript_144293;F01_transcript_144458;F01_transcript_144745;F01_transcript_145616;F01_transcript_146963;F01_transcript_147721;F01_transcript_148005;F01_transcript_149327;F01_transcript_149467;F01_transcript_150639;F01_transcript_150688;F01_transcript_152726;F01_transcript_154837;F01_transcript_156344;F01_transcript_157104;F01_transcript_157378;F01_transcript_159324;F01_transcript_159730;F01_transcript_160034;F01_transcript_160312;F01_transcript_160500;F01_transcript_162049;F01_transcript_166551;F01_transcript_167098;F01_transcript_16898;F01_transcript_18796;F01_transcript_19461;F01_transcript_19893;F01_transcript_20546;F01_transcript_20719;F01_transcript_21833;F01_transcript_22118;F01_transcript_22929;F01_transcript_25546;F01_transcript_27248;F01_transcript_28405;F01_transcript_28486;F01_transcript_28733;F01_transcript_29855;F01_transcript_30736;F01_transcript_31330;F01_transcript_31456;F01_transcript_31784;F01_transcript_31881;F01_transcript_32376;F01_transcript_32622;F01_transcript_33472;F01_transcript_33668;F01_transcript_33768;F01_transcript_34014;F01_transcript_34197;F01_transcript_34402;F01_transcript_34701;F01_transcript_34756;F01_transcript_34873;F01_transcript_35241;F01_transcript_35262;F01_transcript_35441;F01_transcript_35621;F01_transcript_35906;F01_transcript_36194;F01_transcript_36375;F01_transcript_3653;F01_transcript_36639;F01_transcript_36937;F01_transcript_37859;F01_transcript_38047;F01_transcript_39760;F01_transcript_40196;F01_transcript_40430;F01_transcript_40444;F01_transcript_41006;F01_transcript_41423;F01_transcript_42030;F01_transcript_42523;F01_transcript_52222;F01_transcript_56006;F01_transcript_56883;F01_transcript_56914;F01_transcript_57385;F01_transcript_57421;F01_transcript_5774;F01_transcript_58763;F01_transcript_58783;F01_transcript_60833;F01_transcript_61178;F01_transcript_63041;F01_transcript_63456;F01_transcript_63480;F01_transcript_63860;F01_transcript_65509;F01_transcript_65515;F01_transcript_65567;F01_transcript_65858;F01_transcript_66164;F01_transcript_66578;F01_transcript_66852;F01_transcript_67228;F01_transcript_69139;F01_transcript_7104;F01_transcript_71219;F01_transcript_72598;F01_transcript_72651;F01_transcri	0262+K14272+K14272+K14272+K01915+K14272+K01940+K14682+K14455+K00811+K14682+K00811+K14454+K00145+K14454+K01755+K00262+K01427+K00145+K00145+K14272+K14677+K01427+K00814+K00811+K14455+K14272+K00145+K14454+K00620+K00620+K00145+K00814+K14272+K14272+K14455+K14272+K14272+K14272+K14272+K00262+K01915+K01427+K14272+K14454+K00262+K14272+K14677+K00262+K00262

			pt_72718;F01_transcript_73112;F01_transcript_73717;F01_transcript_73953;F01_transcript_73969;F01_transcript_75477;F01_transcript_76748;F01_transcript_77213;F01_transcript_77389;F01_transcript_77476;F01_transcript_78437;F01_transcript_78678;F01_transcript_78720;F01_transcript_78919;F01_transcript_79120;F01_transcript_80769;F01_transcript_81078;F01_transcript_81489;F01_transcript_81767;F01_transcript_82092;F01_transcript_82136;F01_transcript_8325;F01_transcript_83737;F01_transcript_84746;F01_transcript_85168;F01_transcript_86031;F01_transcript_8678;F01_transcript_86847;F01_transcript_86970;F01_transcript_87504;F01_transcript_87957;F01_transcript_88765;F01_transcript_89140;F01_transcript_90013;F01_transcript_90688;F01_transcript_91391;F01_transcript_91443;F01_transcript_92797;F01_transcript_92895;F01_transcript_93052;F01_transcript_93248;F01_transcript_93376;F01_transcript_94572;F01_transcript_96025;F01_transcript_96820;F01_transcript_96851;F01_transcript_96984;F01_transcript_97367;F01_transcript_9785;F01_transcript_98863;F01_transcript_98918;F01_transcript_99014;F01_transcript_99450;F01_transcript_99860;	
Purine metabolism	ko00230	686	F01_transcript_100336;F01_transcript_100703;F01_transcript_100783;F01_transcript_101187;F01_transcript_101484;F01_transcript_101864;F01_transcript_101926;F01_transcript_102080;F01_transcript_102357;F01_transcript_102678;F01_transcript_103096;F01_transcript_103677;F01_transcript_103869;F01_transcript_104096;F01_transcript_104227;F01_transcript_104313;F01_transcript_104394;F01_transcript_104791;F01_transcript_105182;F01_transcript_105214;F01_transcript_105258;F01_transcript_105368;F01_transcript_105409;F01_transcript_105727;F01_transcript_105738;F01_transcript_106390;F01_transcript_106604;F01_transcript_107412;F01_transcript_107438;F01_transcript_107462;F01_transcript_107564;F01_transcript_107589;F01_transcript_107776;F01_transcript_108019;F01_transcript_108543;F01_transcript_108621;F01_transcript_108778;F01_transcript_108782;F01_transcript_110032;F01_transcript_1101;F01_transcript_110555;F01_transcript_110847;F01_transcript_111060;F01_transcript_111158;F01_transcript_111241;F01_transcript_111486;F01_transcript_111586;F01_transcript_111679;F01_transcript_112056;F01_transcript_11216;F01_transcript_112497;F01_transc	K03026+K01488+K00873+K00873+K14721+K01490+K00939+K03787+K00365+K00939+K18151+K03027+K00939+K00873+K02999+K14977+K00759+K01939+K03023+K01923+K00948+K03018+K03023+K00940+K00873+K18532+K14977+K03022+K00962+K00939+K03023+K03787+K00940+K00873+K03014+K00942+K01510+K01490+K03023+K02335+K03025+K03023+K03787+K01933+K01514+K00940+K03023+K00365+K02325+K00873+K03023+K00939+K00939+K01490+K00962+K00962+K02326+K03017+K01514+K00948+K01519+K00962+K01756+K00873+K03023+K01466+K01522+K00939+K00873+K01835+K13484+K03012+K00760+K00602+K01756+K02083+K00873+K00942+K00602+K01490+K00942+K11808+K01490+K01488+K00106+K00962+K03018+K03006+K00873+K01514+K00939+K11808+K01514+K01514+K03018+K03015+K10807+K03018+K01514+K01081+K00856+K11808+K03787+K00873+K06928+K01514+K01952+K00764+K03019+K00873+K00939+K03006+K03015+K14977+K13484+K03023+K00939+K00939+K03016+K11808+K10807+K03012+K03007+K03002+K03012+K00764+K02327+K00856+K11808+K00873+K0

	ript_113212;F01_transcript_113393;F01_transcript_113416;F01	3023+K01522+K01514+K03012+K03023+K00088+K00939+K
	_transcript_113808;F01_transcript_113985;F01_transcript_1149	03021+K01835+K13484+K10807+K03016+K14977+K01835+
	68;F01_transcript_115240;F01_transcript_115715;F01_transcrip	K00860+K01514+K11808+K00948+K00873+K03046+K00942
	t_115763;F01_transcript_115902;F01_transcript_115918;F01_tr	+K03002+K00948+K02083+K01835+K01514+K01952+K0151
	anscript_116189;F01_transcript_116379;F01_transcript_116651	4+K01490+K03018+K03006+K00873+K03005+K03787+K023
	;F01_transcript_116973;F01_transcript_117475;F01_transcript_	35+K01514+K00962+K11808+K03012+K01835+K03006+K03
	117532;F01_transcript_11770;F01_transcript_117826;F01_trans	014+K11808+K00860+K00365+K00939+K01933+K02083+K0
	cript_117841;F01_transcript_117913;F01_transcript_118569;F0	2328+K03023+K03006+K03006+K03787+K02083+K01490+K
	1_transcript_118714;F01_transcript_118806;F01_transcript_118	00948+K18532+K01513+K14977+K00939+K18447+K03023+
	826;F01_transcript_11934;F01_transcript_119451;F01_transcrip	K01522+K00939+K00873+K03012+K00602+K02335+K00088
	t_119520;F01_transcript_119940;F01_transcript_120045;F01_tr	+K14977+K03021+K00106+K01835+K00873+K00962+K0093
	anscript_120086;F01_transcript_120180;F01_transcript_120361	9+K03007+K13811+K03018+K00942+K01490+K00602+K009
	;F01_transcript_120521;F01_transcript_120539;F01_transcript_	39+K00939+K00939+K13811+K03007+K00856+K00962+K01
	120701;F01_transcript_120865;F01_transcript_120942;F01_tran	490+K00939+K02328+K00759+K03023+K00939+K13811+K0
	script_121274;F01_transcript_121313;F01_transcript_121522;F	2335+K00962+K00601+K01756+K03025+K03006+K03018+K
	01_transcript_1220;F01_transcript_122107;F01_transcript_1222	02083+K03018+K00939+K00939+K02335+K00940+K03007+
	44;F01_transcript_12253;F01_transcript_122649;F01_transcript	K00939+K00939+K03504+K00760+K01427+K00948+K01513
	_122706;F01_transcript_122734;F01_transcript_122779;F01_tra	+K03016+K03046+K02328+K03021+K03023+K03026+K0087
	nscript_122784;F01_transcript_122883;F01_transcript_123026;	3+K03787+K00939+K03006+K01510+K03024+K02083+K118
	F01_transcript_12318;F01_transcript_123657;F01_transcript_12	08+K14977+K01835+K03026+K00939+K03787+K01835+K00
	3679;F01_transcript_123745;F01_transcript_123875;F01_transc	942+K01835+K13811+K14721+K10807+K03787+K00939+K0
	ript_123958;F01_transcript_124464;F01_transcript_124871;F01	1514+K02328+K03023+K00873+K11808+K00873+K00939+K
	_transcript_125165;F01_transcript_126015;F01_transcript_1263	02083+K00106+K03026+K01756+K02328+K00873+K00873+
	11;F01_transcript_126433;F01_transcript_126607;F01_transcrip	K03787+K01933+K00873+K00939+K00940+K00948+K00939
	t_126646;F01_transcript_126697;F01_transcript_127244;F01_tr	+K02083+K03787+K13484+K14641+K10808+K01490+K0301
	anscript_127551;F01_transcript_127934;F01_transcript_128043	3+K02326+K00948+K00873+K03022+K01514+K11808+K118
	;F01_transcript_128722;F01_transcript_1292;F01_transcript_12	08+K03023+K01514+K01835+K01835+K00873+K03040+K03
	9204;F01_transcript_12927;F01_transcript_129404;F01_transcri	025+K03013+K03040+K11808+K00601+K00962+K03046+K0
	pt_129720;F01_transcript_129809;F01_transcript_130000;F01_t	0760+K00602+K01756+K01510+K11808+K00602+K01510+K
	ranscript_131083;F01_transcript_13113;F01_transcript_131136;	00602+K11808+K10808+K01510+K00602+K01835+K00602+
	F01_transcript_131349;F01_transcript_131700;F01_transcript_1	K02083+K00873+K01510+K03006+K01756+K01835+K00873
	32191;F01_transcript_132192;F01_transcript_132216;F01_trans	+K00873+K00873+K14641+K00939+K01756+K00873+K0087
	cript_132330;F01_transcript_132467;F01_transcript_132505;F0	3+K00939+K00962+K01945+K00764+K01490+K00939+K018
	1_transcript_132637;F01_transcript_132770;F01_transcript_132	35+K00873+K01835+K01945+K01835+K00962+K00873+K03
	804;F01_transcript_133802;F01_transcript_133833;F01_transcri	018+K00873+K00764+K01513+K01939+K01939+K01756+K0
	pt_133879;F01_transcript_133891;F01_transcript_13417;F01_tr	0873+K00873+K00873+K03504+K00948+K00873+K01466+K
	anscript_134237;F01_transcript_134300;F01_transcript_134341	00873+K00873+K03046+K01466+K00962+K00939+K00873+

	;F01_transcript_134405;F01_transcript_134930;F01_transcript_135131;F01_transcript_135347;F01_transcript_135661;F01_transcript_135717;F01_transcript_135895;F01_transcript_135929;F01_transcript_136028;F01_transcript_136113;F01_transcript_136150;F01_transcript_136197;F01_transcript_136652;F01_transcript_136998;F01_transcript_137065;F01_transcript_137309;F01_transcript_137575;F01_transcript_137623;F01_transcript_137703;F01_transcript_137852;F01_transcript_137868;F01_transcript_137976;F01_transcript_138099;F01_transcript_138230;F01_transcript_138895;F01_transcript_138916;F01_transcript_139121;F01_transcript_139189;F01_transcript_139341;F01_transcript_139382;F01_transcript_139692;F01_transcript_139888;F01_transcript_139941;F01_transcript_140038;F01_transcript_140054;F01_transcript_140192;F01_transcript_140740;F01_transcript_140925;F01_transcript_141500;F01_transcript_141582;F01_transcript_14172;F01_transcript_141744;F01_transcript_141836;F01_transcript_141997;F01_transcript_142008;F01_transcript_142708;F01_transcript_142767;F01_transcript_142877;F01_transcript_142939;F01_transcript_143330;F01_transcript_143548;F01_transcript_143572;F01_transcript_143703;F01_transcript_143897;F01_transcript_144532;F01_transcript_144538;F01_transcript_144621;F01_transcript_144643;F01_transcript_144695;F01_transcript_144874;F01_transcript_144875;F01_transcript_145197;F01_transcript_145411;F01_transcript_145873;F01_transcript_145905;F01_transcript_146286;F01_transcript_146362;F01_transcript_146481;F01_transcript_146546;F01_transcript_146556;F01_transcript_146684;F01_transcript_146748;F01_transcript_146889;F01_transcript_146938;F01_transcript_146957;F01_transcript_147245;F01_transcript_147294;F01_transcript_147298;F01_transcript_147483;F01_transcript_147961;F01_transcript_148285;F01_transcript_148535;F01_transcript_148890;F01_transcript_14905;F01_transcript_149057;F01_transcript_14906;F01_transcript_149323;F01_transcript_149386;F01_transcript_149387;F01_transcript_149752;F01_transcript_150196;F01_transcript_150489;F01_transcript_150688;F01_transcript_151431;F01_transcript_151441;F01_transcript_151626;F01_transcript_151710;F01_transcript_152398;F01_transcript_152671;F01_transcript_153188;F01_transcript_15324;F01_transcript_153523;F01_transcript_15	K00088+K00873+K01951+K01466+K00873+K00939+K13811+K01490+K02328+K02083+K01466+K02083+K00873+K00948+K02328+K00939+K00873+K00873+K03023+K00948+K02685+K02328+K01510+K13811+K00948+K03006+K18151+K00942+K01923+K01490+K00860+K00860+K01923+K03026+K00106+K00856+K03018+K10808+K03787+K00939+K01517+K03011+K03787+K01952+K18447+K03011+K00939+K00939+K03787+K18453+K03024+K00759+K00365+K00940+K13484+K01519+K00939+K03012+K03013+K00940+K00939+K00760+K03016+K03013+K03013+K03013+K00759+K01522+K18532+K03015+K03015+K01522+K00940+K00365+K03015+K03015+K03019+K00940+K03017+K00940+K03000+K03012+K00940+K03012+K03012+K01490+K03505+K03017+K03008+K03007+K03009+K10807+K00873+K01490+K00939+K00873+K03006+K00088+K03022+K01514+K03505+K03011+K00088+K03021+K18151+K01466+K11808+K01427+K01514+K01490+K03023+K01835+K03015+K02999+K00939+K03023+K01490+K14977+K01490+K02083+K00942+K01514+K03012+K02083+K01519+K01514+K01514+K01490+K00873+K03026+K02328+K00873+K01945+K00759+K02083+K00088+K10808+K00942+K00873+K03011+K00873+K01952+K03024+K00106+K01923+K01514+K18151+K02083+K00873+K01939+K02083+K00759+K00939+K00939+K00940+K00088+K02335+K03787+K11808+K00939+K01427+K03006+K03021+K01835+K02325+K03012+K03023+K00939+K01756+K10807+K01514+K03006+K00962+K00940+K01510+K00962+K00939+K03023+K01466+K02335+K01427+K00759+K00939+K03006+K00856+K03021+K14977+K03787+K00939+K02335+K11808+K03018+K03021+K03787+K00940+K11808+K13484+K00939+K02083+K00106+K02335+K03018+K03787+K11808+K13811+K00942+K00939+K00939+K00873+K03015+K03022+K03018+K10808+K03006+K01490+K02326+K03043+K03023+K11808+K03026+K01835+K00759+K00942+K02324+K01510+K00764+K00939+K00948+K00939+K01756+K00939+K01510+K03012+K03019+K00873+K00940+K00940+K01427+K00873+K00939+K00942+K10807+K00939+K00962+K00873+K13484+K02335+K01835+K00873+K03018+K13484+K00939+K01427+K11808+K00873+K01514+K02335+K03006+K02335+K00873+K008

	3582;F01_transcript_15375;F01_transcript_153763;F01_transcript_153789;F01_transcript_154052;F01_transcript_154566;F01_transcript_155143;F01_transcript_155586;F01_transcript_155598;F01_transcript_155788;F01_transcript_155841;F01_transcript_155930;F01_transcript_156116;F01_transcript_156418;F01_transcript_156780;F01_transcript_156861;F01_transcript_15700;F01_transcript_157170;F01_transcript_157467;F01_transcript_157940;F01_transcript_158056;F01_transcript_158296;F01_transcript_158336;F01_transcript_158451;F01_transcript_158460;F01_transcript_158716;F01_transcript_158939;F01_transcript_158963;F01_transcript_159122;F01_transcript_159126;F01_transcript_159456;F01_transcript_159626;F01_transcript_159748;F01_transcript_160044;F01_transcript_160163;F01_transcript_160229;F01_transcript_160410;F01_transcript_160427;F01_transcript_160752;F01_transcript_161004;F01_transcript_161183;F01_transcript_161361;F01_transcript_161932;F01_transcript_162076;F01_transcript_162245;F01_transcript_162389;F01_transcript_162717;F01_transcript_162916;F01_transcript_163029;F01_transcript_163703;F01_transcript_163795;F01_transcript_164219;F01_transcript_164286;F01_transcript_16449;F01_transcript_164909;F01_transcript_165423;F01_transcript_165819;F01_transcript_165871;F01_transcript_166075;F01_transcript_166121;F01_transcript_166129;F01_transcript_166189;F01_transcript_166241;F01_transcript_167163;F01_transcript_167228;F01_transcript_167275;F01_transcript_167351;F01_transcript_167486;F01_transcript_167501;F01_transcript_16782;F01_transcript_17035;F01_transcript_17186;F01_transcript_17725;F01_transcript_18129;F01_transcript_18262;F01_transcript_18683;F01_transcript_19337;F01_transcript_19398;F01_transcript_19420;F01_transcript_19643;F01_transcript_19835;F01_transcript_20123;F01_transcript_20325;F01_transcript_20518;F01_transcript_21043;F01_transcript_219;F01_transcript_22645;F01_transcript_22972;F01_transcript_23258;F01_transcript_23327;F01_transcript_23407;F01_transcript_23941;F01_transcript_24007;F01_transcript_24204;F01_transcript_24502;F01_transcript_24533;F01_transcript_24538;F01_transcript_2459;F01_transcript_25098;F01_transcript_25173;F01_transcript_25320;F01_transcript_25321;F01_transcript_	73+K03046+K01490+K03018+K01514+K00939+K01756+K01514+K03022+K00873+K03012+K14977+K00962+K01835+K00602+K13811+K00962+K02335+K03002+K02083+K03012+K03023+K02325+K03006+K03020+K01522+K03027+K14977+K01427+K03016+K01488+K11808+K02325+K00873+K02327+K00939+K03023+K03026+K01488+K02335+K00601+K00939

	25381;F01_transcript_25467;F01_transcript_25470;F01_transcript_25868;F01_transcript_25979;F01_transcript_2604;F01_transcript_26085;F01_transcript_265;F01_transcript_26508;F01_transcript_26892;F01_transcript_27218;F01_transcript_27371;F01_transcript_27672;F01_transcript_27949;F01_transcript_28090;F01_transcript_28152;F01_transcript_28168;F01_transcript_28257;F01_transcript_28633;F01_transcript_28763;F01_transcript_28935;F01_transcript_28992;F01_transcript_29161;F01_transcript_2923;F01_transcript_29279;F01_transcript_2928;F01_transcript_29290;F01_transcript_29309;F01_transcript_29423;F01_transcript_29765;F01_transcript_29816;F01_transcript_29857;F01_transcript_29908;F01_transcript_30392;F01_transcript_30637;F01_transcript_30739;F01_transcript_30875;F01_transcript_31391;F01_transcript_31658;F01_transcript_31839;F01_transcript_32422;F01_transcript_32449;F01_transcript_32534;F01_transcript_32767;F01_transcript_33286;F01_transcript_33449;F01_transcript_33558;F01_transcript_33728;F01_transcript_33994;F01_transcript_34563;F01_transcript_34597;F01_transcript_34975;F01_transcript_34996;F01_transcript_35;F01_transcript_35201;F01_transcript_35276;F01_transcript_36151;F01_transcript_3637;F01_transcript_36857;F01_transcript_37554;F01_transcript_37788;F01_transcript_37905;F01_transcript_37979;F01_transcript_38803;F01_transcript_3912;F01_transcript_39835;F01_transcript_40039;F01_transcript_40055;F01_transcript_40169;F01_transcript_40205;F01_transcript_40373;F01_transcript_404;F01_transcript_40717;F01_transcript_41047;F01_transcript_41576;F01_transcript_41669;F01_transcript_41800;F01_transcript_41814;F01_transcript_42046;F01_transcript_42382;F01_transcript_42747;F01_transcript_42766;F01_transcript_43126;F01_transcript_43958;F01_transcript_44417;F01_transcript_44486;F01_transcript_44662;F01_transcript_44713;F01_transcript_44730;F01_transcript_44866;F01_transcript_45093;F01_transcript_45318;F01_transcript_45505;F01_transcript_45583;F01_transcript_45718;F01_transcript_46205;F01_transcript_46213;F01_transcript_46776;F01_transcript_46905;F01_transcript_47147;F01_transcript_47162;F01_transcript_47259;F01_transcript_47363;F01_transcript_47443;F01_transcript_47453;F01_transcript_47517;F01_transcript_47943;F01_transcript_48041;F01_transcript_48111;F01_tran	

script_48117;F01_transcript_48182;F01_transcript_48332;F01_t
ranscript_48335;F01_transcript_48359;F01_transcript_48686;F0
1_transcript_49116;F01_transcript_49309;F01_transcript_49943
;F01_transcript_50112;F01_transcript_5119;F01_transcript_524
65;F01_transcript_53170;F01_transcript_5332;F01_transcript_5
3589;F01_transcript_53730;F01_transcript_53742;F01_transcrip
t_53743;F01_transcript_54133;F01_transcript_54343;F01_trans
cript_54444;F01_transcript_54534;F01_transcript_54744;F01_tr
anscript_55036;F01_transcript_55056;F01_transcript_55890;F0
1_transcript_56006;F01_transcript_56202;F01_transcript_56235
;F01_transcript_56240;F01_transcript_56580;F01_transcript_56
634;F01_transcript_56841;F01_transcript_57496;F01_transcript
_57564;F01_transcript_5780;F01_transcript_58039;F01_transcri
pt_5876;F01_transcript_59014;F01_transcript_59121;F01_trans
cript_59399;F01_transcript_59667;F01_transcript_59830;F01_tr
anscript_59901;F01_transcript_59997;F01_transcript_60493;F0
1_transcript_60637;F01_transcript_61112;F01_transcript_61242
;F01_transcript_61368;F01_transcript_61414;F01_transcript_61
639;F01_transcript_61759;F01_transcript_61848;F01_transcript
_61851;F01_transcript_61954;F01_transcript_62041;F01_transc
ript_63160;F01_transcript_63353;F01_transcript_63521;F01_tra
nscript_63680;F01_transcript_63945;F01_transcript_64077;F01
_transcript_64310;F01_transcript_64452;F01_transcript_64556;
F01_transcript_64564;F01_transcript_64577;F01_transcript_647
18;F01_transcript_64891;F01_transcript_64936;F01_transcript_
65020;F01_transcript_65387;F01_transcript_65702;F01_transcri
pt_65760;F01_transcript_65789;F01_transcript_65868;F01_tran
script_66091;F01_transcript_6611;F01_transcript_66164;F01_tr
anscript_66564;F01_transcript_66618;F01_transcript_66664;F0
1_transcript_67697;F01_transcript_68330;F01_transcript_68587
;F01_transcript_68681;F01_transcript_68879;F01_transcript_68
925;F01_transcript_69027;F01_transcript_69789;F01_transcript
_69810;F01_transcript_69823;F01_transcript_69987;F01_transc
ript_70063;F01_transcript_7034;F01_transcript_70696;F01_tran
script_70859;F01_transcript_70894;F01_transcript_7104;F01_tr
anscript_71164;F01_transcript_71263;F01_transcript_71458;F0
1_transcript_71737;F01_transcript_71845;F01_transcript_72131

;F01_transcript_72394;F01_transcript_72545;F01_transcript_728;F01_transcript_73289;F01_transcript_73296;F01_transcript_73704;F01_transcript_74371;F01_transcript_74461;F01_transcript_74581;F01_transcript_7460;F01_transcript_74732;F01_transcript_75012;F01_transcript_75032;F01_transcript_75496;F01_transcript_75974;F01_transcript_75987;F01_transcript_76064;F01_transcript_76208;F01_transcript_76220;F01_transcript_76392;F01_transcript_76416;F01_transcript_76856;F01_transcript_76961;F01_transcript_77171;F01_transcript_77269;F01_transcript_77936;F01_transcript_78239;F01_transcript_78730;F01_transcript_78804;F01_transcript_78867;F01_transcript_78988;F01_transcript_79048;F01_transcript_79059;F01_transcript_79249;F01_transcript_79363;F01_transcript_79550;F01_transcript_79636;F01_transcript_80006;F01_transcript_80300;F01_transcript_80344;F01_transcript_80918;F01_transcript_80927;F01_transcript_81590;F01_transcript_81622;F01_transcript_81979;F01_transcript_82288;F01_transcript_82302;F01_transcript_82389;F01_transcript_83105;F01_transcript_83170;F01_transcript_8325;F01_transcript_83284;F01_transcript_83367;F01_transcript_83547;F01_transcript_84620;F01_transcript_84866;F01_transcript_84893;F01_transcript_85796;F01_transcript_86105;F01_transcript_862;F01_transcript_86207;F01_transcript_8631;F01_transcript_86322;F01_transcript_86424;F01_transcript_86697;F01_transcript_8678;F01_transcript_87264;F01_transcript_87964;F01_transcript_88242;F01_transcript_88462;F01_transcript_88551;F01_transcript_89051;F01_transcript_89459;F01_transcript_89922;F01_transcript_90069;F01_transcript_90198;F01_transcript_91330;F01_transcript_91440;F01_transcript_91507;F01_transcript_92282;F01_transcript_92532;F01_transcript_92965;F01_transcript_92968;F01_transcript_93012;F01_transcript_93724;F01_transcript_93798;F01_transcript_94317;F01_transcript_94321;F01_transcript_94345;F01_transcript_94392;F01_transcript_944;F01_transcript_95338;F01_transcript_95364;F01_transcript_95488;F01_transcript_95575;F01_transcript_95940;F01_transcript_96015;F01_transcript_96269;F01_transcript_96554;F01_transcript_96668;F01_transcript_96786;F01_transcript_96984;F01_transcript_97410;F01_transcript_97561;F01_transcript_97870;F01_transcript_97909;F01_transcript_97966;F01_transcript_98012;F01_transcript

			_98078;F01_transcript_98513;F01_transcript_98674;F01_transcript_99079;F01_transcript_991;F01_transcript_99941;F01_transcript_99953;	
Caffeine metabolism	ko00232	11	F01_transcript_102357;F01_transcript_111679;F01_transcript_120521;F01_transcript_138099;F01_transcript_143330;F01_transcript_159122;F01_transcript_37979;F01_transcript_42747;F01_transcript_47259;F01_transcript_64077;F01_transcript_75032;	K00365+K00365+K00106+K00365+K00106+K00106+K00106+K00365+K00365+K00106+K00106
Pyrimidine metabolism	ko00240	448	F01_transcript_100110;F01_transcript_100336;F01_transcript_101484;F01_transcript_101870;F01_transcript_102080;F01_transcript_102734;F01_transcript_10306;F01_transcript_103677;F01_transcript_104227;F01_transcript_105182;F01_transcript_105368;F01_transcript_105409;F01_transcript_105727;F01_transcript_105803;F01_transcript_1060;F01_transcript_106200;F01_transcript_106282;F01_transcript_106441;F01_transcript_107060;F01_transcript_107412;F01_transcript_107438;F01_transcript_107564;F01_transcript_107589;F01_transcript_107776;F01_transcript_1078;F01_transcript_108238;F01_transcript_108543;F01_transcript_108650;F01_transcript_108778;F01_transcript_109354;F01_transcript_109625;F01_transcript_110032;F01_transcript_1101;F01_transcript_110555;F01_transcript_110602;F01_transcript_110836;F01_transcript_110847;F01_transcript_111060;F01_transcript_111486;F01_transcript_111586;F01_transcript_112056;F01_transcript_112497;F01_transcript_113808;F01_transcript_113985;F01_transcript_114306;F01_transcript_114968;F01_transcript_115240;F01_transcript_115902;F01_transcript_115918;F01_transcript_116429;F01_transcript_116651;F01_transcript_117067;F01_transcript_117281;F01_transcript_117913;F01_transcript_119910;F01_transcript_120539;F01_transcript_120701;F01_transcript_120865;F01_transcript_122244;F01_transcript_122417;F01_transcript_12253;F01_transcript_122649;F01_transcript_122706;F01_transcript_122779;F01_transcript_123026;F01_transcript_123958;F01_transcript_124166;F01_transcript_124478;F01_transcript_125165;F01_transcript_125448;F01_transcript_126015;F01_transcript_126601;F01_transcript_126607;F01_tra	K00609+K03026+K14721+K00761+K03787+K01937+K01937+K03027+K02999+K03023+K03018+K03023+K00940+K00609+K01955+K01955+K00384+K00876+K13998+K03022+K00962+K03023+K03787+K00940+K01955+K01465+K03014+K01431+K01510+K00876+K01489+K03023+K02335+K03025+K00876+K00876+K03023+K03787+K00940+K03023+K02325+K03023+K00962+K00962+K00609+K02326+K03017+K01519+K00962+K16329+K03023+K00609+K13998+K03012+K01955+K00962+K03018+K03006+K03018+K01465+K03015+K10807+K03018+K01081+K03787+K03019+K13800+K01955+K03006+K00384+K03015+K01240+K03023+K13800+K03016+K13998+K10807+K01465+K03012+K01956+K16329+K01937+K03007+K01464+K00876+K03002+K03012+K02327+K01465+K03023+K03012+K03023+K03021+K00384+K01937+K10807+K03016+K01465+K00857+K13800+K16329+K03046+K00254+K03002+K13800+K03018+K03006+K03005+K03787+K13800+K02335+K00962+K03012+K03006+K03014+K13998+K02328+K03023+K03006+K03006+K03787+K01465+K00609+K13998+K03023+K03012+K02335+K03021+K00962+K03007+K03018+K01465+K03007+K00962+K02328+K03023+K01465+K02335+K00962+K03025+K03006+K03018+K03018+K02335+K00940+K03007+K01520+K03504+K13998+K13998+K03016+K03046+K02328+K03021+K13998+K03023+K03026+K03787+K01240+K03006+K01510+K03024+K01431+K00761+K13421+K03026+K01465+K03787+K01465+K01955+K01240+K00384+K14721+K10807+K03787+K13998+K02328+K03023+K03026+K02328+K00876+K03787+K00940+K00207+K03787+K

	nscript_127220;F01_transcript_127244;F01_transcript_127626;F01_transcript_127934;F01_transcript_128025;F01_transcript_128043;F01_transcript_128466;F01_transcript_128629;F01_transcript_128694;F01_transcript_128722;F01_transcript_128756;F01_transcript_129122;F01_transcript_1292;F01_transcript_129204;F01_transcript_129404;F01_transcript_129851;F01_transcript_131083;F01_transcript_131349;F01_transcript_131700;F01_transcript_132216;F01_transcript_132217;F01_transcript_132379;F01_transcript_132505;F01_transcript_132637;F01_transcript_132898;F01_transcript_132961;F01_transcript_132997;F01_transcript_133906;F01_transcript_134237;F01_transcript_134331;F01_transcript_134341;F01_transcript_134702;F01_transcript_135929;F01_transcript_136028;F01_transcript_136150;F01_transcript_136197;F01_transcript_136583;F01_transcript_136652;F01_transcript_137065;F01_transcript_137575;F01_transcript_137703;F01_transcript_137852;F01_transcript_137890;F01_transcript_139121;F01_transcript_139189;F01_transcript_139341;F01_transcript_139382;F01_transcript_139692;F01_transcript_140614;F01_transcript_140856;F01_transcript_141179;F01_transcript_141582;F01_transcript_141997;F01_transcript_142708;F01_transcript_142939;F01_transcript_143703;F01_transcript_144532;F01_transcript_144621;F01_transcript_145883;F01_transcript_145905;F01_transcript_146362;F01_transcript_146556;F01_transcript_146748;F01_transcript_146777;F01_transcript_146957;F01_transcript_147245;F01_transcript_147483;F01_transcript_147961;F01_transcript_148285;F01_transcript_148890;F01_transcript_14906;F01_transcript_149323;F01_transcript_149386;F01_transcript_150061;F01_transcript_150196;F01_transcript_150666;F01_transcript_150827;F01_transcript_151626;F01_transcript_151710;F01_transcript_152398;F01_transcript_152671;F01_transcript_153160;F01_transcript_153188;F01_transcript_15324;F01_transcript_153582;F01_transcript_153643;F01_transcript_153763;F01_transcript_153789;F01_transcript_154052;F01_transcript_154093;F01_transcript_154341;F01_transcript_155235;F01_transcript_155788;F01_transcript_155789;F01_transcript_155930;F01_transcript_156018;F01_transcript_156073;F01_transcript_156552;F01_transcript_156595;F01_transcript_15700;F01_transcript_157170;F01_transcript_157467;F01_transcript_158206;F01	14641+K10808+K00384+K13800+K01955+K03013+K02326+K01464+K03022+K13800+K01937+K03023+K03040+K03025+K03013+K13998+K03040+K00962+K03046+K01510+K01955+K13998+K01510+K01937+K01464+K10808+K01510+K13998+K01510+K01431+K01937+K03006+K01937+K13998+K14641+K13998+K00962+K01464+K13998+K00962+K03018+K00876+K00254+K13998+K03504+K01464+K03046+K00962+K01431+K01464+K00254+K02328+K01464+K00384+K00384+K00384+K02328+K00876+K03023+K00876+K00761+K02685+K00207+K02328+K01510+K00876+K03006+K01465+K03026+K01240+K00609+K03018+K10808+K03787+K03011+K03787+K00384+K03011+K00384+K03787+K03024+K00940+K13800+K01519+K00761+K03012+K03013+K00940+K03016+K13800+K03013+K03013+K03013+K01520+K03015+K03015+K00940+K03015+K03015+K03019+K00940+K03017+K00940+K03000+K03012+K00940+K16904+K03012+K03012+K03505+K03017+K03008+K03007+K03009+K10807+K01431+K00384+K01937+K03006+K03022+K03505+K03011+K03021+K13998+K00876+K03023+K00384+K03015+K02999+K01956+K01464+K03023+K00207+K01465+K00761+K03012+K01519+K13800+K01240+K01465+K03026+K02328+K01955+K01465+K00876+K10808+K01465+K00876+K03011+K00609+K03024+K00609+K00384+K13998+K00940+K02335+K03787+K03006+K03021+K13998+K02325+K03012+K03023+K10807+K03006+K00962+K00940+K01510+K00962+K01955+K00609+K03023+K02335+K03006+K00384+K03021+K03787+K01464+K00761+K02335+K03018+K01493+K03021+K01937+K03787+K00940+K00207+K01956+K02335+K03018+K03787+K01937+K03015+K03022+K03018+K16329+K10808+K03006+K01431+K01465+K02326+K03043+K03023+K03026+K01465+K02324+K01510+K00384+K01510+K03012+K03019+K01465+K00940+K00940+K01937+K01937+K10807+K00962+K01465+K02335+K03018+K01465+K01464+K01955+K02335+K03006+K02335+K00876+K03046+K01465+K01465+K03018+K01956+K01955+K03022+K03012+K00962+K13998+K00962+K02335+K00384+K00384+K03002+K03012+K03023+K01956+K02325+K03006+K13998+K03020+K00207+K01465+K03027+K00761

	_transcript_158296;F01_transcript_158336;F01_transcript_159126;F01_transcript_159626;F01_transcript_159848;F01_transcript_160163;F01_transcript_160752;F01_transcript_160755;F01_transcript_161932;F01_transcript_162245;F01_transcript_162389;F01_transcript_162587;F01_transcript_162692;F01_transcript_162815;F01_transcript_162916;F01_transcript_163029;F01_transcript_164154;F01_transcript_164219;F01_transcript_164330;F01_transcript_165023;F01_transcript_165423;F01_transcript_166129;F01_transcript_166189;F01_transcript_166241;F01_transcript_167049;F01_transcript_167163;F01_transcript_167351;F01_transcript_167486;F01_transcript_17186;F01_transcript_1772;F01_transcript_17722;F01_transcript_18262;F01_transcript_18293;F01_transcript_19217;F01_transcript_19398;F01_transcript_19420;F01_transcript_19556;F01_transcript_21043;F01_transcript_21265;F01_transcript_21878;F01_transcript_219;F01_transcript_22596;F01_transcript_22881;F01_transcript_23941;F01_transcript_24211;F01_transcript_2459;F01_transcript_24871;F01_transcript_25078;F01_transcript_2604;F01_transcript_265;F01_transcript_26656;F01_transcript_27195;F01_transcript_27420;F01_transcript_28257;F01_transcript_29103;F01_transcript_2923;F01_transcript_2928;F01_transcript_29933;F01_transcript_30416;F01_transcript_30438;F01_transcript_30875;F01_transcript_31229;F01_transcript_31774;F01_transcript_31988;F01_transcript_32454;F01_transcript_32534;F01_transcript_33271;F01_transcript_33558;F01_transcript_33908;F01_transcript_33915;F01_transcript_33994;F01_transcript_34128;F01_transcript_34563;F01_transcript_34597;F01_transcript_34740;F01_transcript_35;F01_transcript_37395;F01_transcript_37905;F01_transcript_38137;F01_transcript_38663;F01_transcript_3912;F01_transcript_39835;F01_transcript_40039;F01_transcript_40205;F01_transcript_40373;F01_transcript_40484;F01_transcript_41047;F01_transcript_41499;F01_transcript_41800;F01_transcript_42046;F01_transcript_42766;F01_transcript_43595;F01_transcript_43958;F01_transcript_44120;F01_transcript_44486;F01_transcript_44662;F01_transcript_44713;F01_transcript_45093;F01_transcript_45206;F01_transcript_45318;F01_transcript_45505;F01_transcript_45583;F01_transcript_46729;F01_transcript_46776;F01_transcript	+K03016+K02325+K01955+K02327+K03023+K03026+K00207+K02335+K01464

_46905;F01_transcript_47162;F01_transcript_47363;F01_transc
ript_47443;F01_transcript_47453;F01_transcript_47517;F01_tra
nscript_47943;F01_transcript_48041;F01_transcript_48111;F01
_transcript_48117;F01_transcript_48182;F01_transcript_48318;
F01_transcript_48332;F01_transcript_48335;F01_transcript_486
86;F01_transcript_49116;F01_transcript_49309;F01_transcript_
49943;F01_transcript_50112;F01_transcript_5119;F01_transcrip
t_52477;F01_transcript_52832;F01_transcript_53219;F01_trans
cript_53730;F01_transcript_53743;F01_transcript_54343;F01_tr
anscript_54444;F01_transcript_54744;F01_transcript_55158;F0
1_transcript_56075;F01_transcript_56240;F01_transcript_56598
;F01_transcript_56634;F01_transcript_56841;F01_transcript_57
089;F01_transcript_57483;F01_transcript_57564;F01_transcript
_57595;F01_transcript_57700;F01_transcript_59584;F01_transc
ript_59667;F01_transcript_59901;F01_transcript_60041;F01_tra
nscript_60230;F01_transcript_61095;F01_transcript_61242;F01
_transcript_61368;F01_transcript_61511;F01_transcript_61542;
F01_transcript_61812;F01_transcript_61954;F01_transcript_622
61;F01_transcript_62706;F01_transcript_63353;F01_transcript_
63892;F01_transcript_63945;F01_transcript_64296;F01_transcri
pt_64528;F01_transcript_65118;F01_transcript_65702;F01_tran
script_65789;F01_transcript_65868;F01_transcript_66564;F01_t
ranscript_66618;F01_transcript_67403;F01_transcript_67697;F0
1_transcript_68330;F01_transcript_68587;F01_transcript_68925
;F01_transcript_69789;F01_transcript_69810;F01_transcript_69
823;F01_transcript_69987;F01_transcript_70063;F01_transcript
_70147;F01_transcript_70427;F01_transcript_70696;F01_transc
ript_70894;F01_transcript_71458;F01_transcript_71551;F01_tra
nscript_71845;F01_transcript_72394;F01_transcript_72513;F01
_transcript_72798;F01_transcript_728;F01_transcript_73296;F0
1_transcript_73537;F01_transcript_73704;F01_transcript_74040
;F01_transcript_74371;F01_transcript_74461;F01_transcript_74
548;F01_transcript_74626;F01_transcript_75496;F01_transcript
_75974;F01_transcript_75987;F01_transcript_76469;F01_transc
ript_76961;F01_transcript_77171;F01_transcript_77269;F01_tra
nscript_77604;F01_transcript_77936;F01_transcript_78239;F01
_transcript_78714;F01_transcript_78724;F01_transcript_78804;
F01_transcript_78867;F01_transcript_78988;F01_transcript_790

			59;F01_transcript_79313;F01_transcript_79636;F01_transcript_80006;F01_transcript_81743;F01_transcript_81979;F01_transcript_82288;F01_transcript_82302;F01_transcript_83102;F01_transcript_83105;F01_transcript_83170;F01_transcript_84164;F01_transcript_84521;F01_transcript_84620;F01_transcript_84893;F01_transcript_85833;F01_transcript_862;F01_transcript_86322;F01_transcript_86727;F01_transcript_8697;F01_transcript_877;F01_transcript_88462;F01_transcript_88551;F01_transcript_89051;F01_transcript_89897;F01_transcript_90069;F01_transcript_90798;F01_transcript_90941;F01_transcript_91330;F01_transcript_91551;F01_transcript_92400;F01_transcript_92965;F01_transcript_93012;F01_transcript_93798;F01_transcript_93974;F01_transcript_94392;F01_transcript_944;F01_transcript_95099;F01_transcript_95175;F01_transcript_95338;F01_transcript_95488;F01_transcript_95575;F01_transcript_95605;F01_transcript_95940;F01_transcript_96015;F01_transcript_96086;F01_transcript_96269;F01_transcript_96558;F01_transcript_96642;F01_transcript_96668;F01_transcript_97101;F01_transcript_97410;F01_transcript_97909;F01_transcript_97999;F01_transcript_98012;F01_transcript_98513;F01_transcript_98674;F01_transcript_98781;F01_transcript_991;F01_transcript_99795;	
Alanine, aspartate and glutamate metabolism	ko00250	313	F01_transcript_100110;F01_transcript_10047;F01_transcript_100692;F01_transcript_101062;F01_transcript_101386;F01_transcript_101473;F01_transcript_102048;F01_transcript_10266;F01_transcript_103318;F01_transcript_103356;F01_transcript_103532;F01_transcript_104113;F01_transcript_10428;F01_transcript_104791;F01_transcript_105803;F01_transcript_1060;F01_transcript_106024;F01_transcript_106156;F01_transcript_106200;F01_transcript_107151;F01_transcript_1078;F01_transcript_107931;F01_transcript_108303;F01_transcript_109418;F01_transcript_109463;F01_transcript_110137;F01_transcript_110169;F01_transcript_110317;F01_transcript_11182;F01_transcript_111872;F01_transcript_112260;F01_transcript_112458;F01_transcript_113831;F01_transcript_113986;F01_transcript_114306;F01_transcript_114452;F01_transcript_114576;F01_transcript_115316;F01_transcript_116166;F01_transcript_116189;F01_transcript_116648;F01_transcript_116793;F01_transcript_117067;F01_transcript	K00609+K14272+K01580+K01915+K00261+K14454+K14272+K14272+K01915+K01755+K14272+K01940+K17761+K01939+K00609+K01955+K01915+K14454+K01955+K00811+K01955+K14272+K01915+K00830+K01915+K14272+K00261+K01915+K14272+K13566+K14272+K16871+K14272+K17761+K00609+K14455+K01915+K00278+K01915+K01756+K14272+K00814+K00609+K14272+K00830+K01756+K00830+K14455+K01955+K01915+K00294+K17761+K16871+K00264+K14272+K00830+K00264+K01940+K01915+K14272+K00262+K00764+K00262+K01955+K00262+K01940+K14272+K14455+K01755+K00830+K01956+K00764+K01915+K00278+K14272+K01915+K14455+K01915+K14272+K01915+K01940+K00261+K01953+K00264+K14454+K00262+K01915+K00262+K00264+K00262+K00609+K01915+K00262+K14272+K01953+K00262+K00820+K01953+K00820+K14272+K00830+K14272+K01953+K01756+K00262+K01915+K01915+K00830+K00264+K1445

	_117148;F01_transcript_118373;F01_transcript_118806;F01_transcript_119569;F01_transcript_119869;F01_transcript_119910;F01_transcript_120116;F01_transcript_120261;F01_transcript_120633;F01_transcript_121867;F01_transcript_121985;F01_transcript_122101;F01_transcript_122499;F01_transcript_122790;F01_transcript_122816;F01_transcript_123427;F01_transcript_123753;F01_transcript_123819;F01_transcript_123875;F01_transcript_12434;F01_transcript_124478;F01_transcript_124982;F01_transcript_125144;F01_transcript_125768;F01_transcript_126185;F01_transcript_127620;F01_transcript_127687;F01_transcript_128466;F01_transcript_12927;F01_transcript_129374;F01_transcript_12979;F01_transcript_130485;F01_transcript_130674;F01_transcript_131043;F01_transcript_132266;F01_transcript_132527;F01_transcript_132815;F01_transcript_133532;F01_transcript_134077;F01_transcript_134652;F01_transcript_135156;F01_transcript_135578;F01_transcript_136085;F01_transcript_137593;F01_transcript_137850;F01_transcript_138648;F01_transcript_138956;F01_transcript_140856;F01_transcript_142458;F01_transcript_143068;F01_transcript_144458;F01_transcript_144723;F01_transcript_144745;F01_transcript_144876;F01_transcript_145172;F01_transcript_14523;F01_transcript_145616;F01_transcript_146447;F01_transcript_146963;F01_transcript_147198;F01_transcript_147298;F01_transcript_148005;F01_transcript_149327;F01_transcript_149467;F01_transcript_150046;F01_transcript_150354;F01_transcript_150639;F01_transcript_152726;F01_transcript_154837;F01_transcript_156073;F01_transcript_156134;F01_transcript_156344;F01_transcript_156809;F01_transcript_157104;F01_transcript_157378;F01_transcript_15819;F01_transcript_158968;F01_transcript_159360;F01_transcript_159456;F01_transcript_159651;F01_transcript_159730;F01_transcript_160021;F01_transcript_160034;F01_transcript_160312;F01_transcript_160500;F01_transcript_160783;F01_transcript_161116;F01_transcript_162049;F01_transcript_162679;F01_transcript_162815;F01_transcript_16355;F01_transcript_163651;F01_transcript_165317;F01_transcript_16570;F01_transcript_166087;F01_transcript_166551;F01_transcript_166993;F01_transcript_167098;F01_transcript_16810;F01_transcript_16898;F01_transcript_17035;F01_transcript_17474;F01_transcript_1772;F01_transcript_1879	4+K14454+K14272+K01955+K00278+K14455+K00827+K01755+K01755+K00820+K16871+K00827+K01756+K00830+K01755+K00827+K14272+K14272+K00811+K00294+K00278+K00262+K17761+K01955+K00264+K01953+K00830+K00278+K17761+K00261+K00830+K14272+K00830+K00262+K01756+K00278+K01955+K00262+K01915+K00278+K00814+K01953+K01756+K01755+K01953+K01756+K01953+K00764+K14272+K13566+K00764+K14272+K01939+K01939+K01756+K00294+K14454+K01755+K14272+K14454+K16871+K00811+K14272+K14272+K00827+K17761+K14455+K01940+K01580+K14272+K01915+K14272+K01940+K01940+K01915+K01915+K01915+K01580+K14272+K14455+K01755+K14455+K01915+K00830+K01580+K01915+K01580+K00609+K00830+K01915+K01915+K01915+K13566+K01915+K01915+K01580+K13566+K14272+K16871+K16871+K00262+K00814+K01956+K01755+K16871+K00814+K14272+K17761+K00827+K14272+K01955+K00278+K00264+K01580+K01940+K00609+K01953+K00609+K01939+K00830+K01915+K00814+K14272+K00827+K14454+K00830+K01915+K14272+K00278+K01756+K00262+K13566+K01955+K00609+K00820+K00261+K00278+K00814+K01915+K00262+K00262+K01956+K00827+K14272+K00830+K14272+K16871+K14272+K00264+K01915+K01580+K14272+K01940+K14455+K00811+K16871+K00820+K00764+K00811+K14454+K13566+K01756+K14454+K01755+K00262+K01580+K00278+K17761+K14272+K00830+K00814+K00811+K14455+K01955+K14272+K14454+K16871+K00830+K00814+K01956+K01756+K01955+K17761+K14272+K14272+K14455+K14272+K14272+K17761+K14272+K13566+K00294+K01956+K14272+K00262+K01915+K14272+K13566+K14454+K01955+K00262+K14272+K00262+K00262

6;F01_transcript_19893;F01_transcript_20575;F01_transcript_2
1833;F01_transcript_22038;F01_transcript_22645;F01_transcrip
t_22929;F01_transcript_24116;F01_transcript_24204;F01_trans
cript_24549;F01_transcript_25173;F01_transcript_25546;F01_tr
anscript_26560;F01_transcript_26892;F01_transcript_27248;F0
1_transcript_27371;F01_transcript_27672;F01_transcript_27949
;F01_transcript_28205;F01_transcript_28405;F01_transcript_28
486;F01_transcript_28733;F01_transcript_30736;F01_transcript
_31067;F01_transcript_31784;F01_transcript_31881;F01_transc
ript_32376;F01_transcript_32442;F01_transcript_32554;F01_tra
nscript_32622;F01_transcript_33472;F01_transcript_33656;F01
_transcript_33668;F01_transcript_33768;F01_transcript_34014;
F01_transcript_34197;F01_transcript_34402;F01_transcript_348
73;F01_transcript_35262;F01_transcript_35441;F01_transcript_
35762;F01_transcript_35906;F01_transcript_36375;F01_transcri
pt_3653;F01_transcript_36639;F01_transcript_37859;F01_trans
cript_37978;F01_transcript_37988;F01_transcript_38047;F01_tr
anscript_38235;F01_transcript_38663;F01_transcript_38799;F0
1_transcript_39760;F01_transcript_40430;F01_transcript_40444
;F01_transcript_40989;F01_transcript_41006;F01_transcript_41
423;F01_transcript_49750;F01_transcript_51794;F01_transcript
_52222;F01_transcript_52463;F01_transcript_55549;F01_transc
ript_56883;F01_transcript_56914;F01_transcript_57089;F01_tra
nscript_57385;F01_transcript_58341;F01_transcript_58763;F01
_transcript_58783;F01_transcript_58881;F01_transcript_60971;
F01_transcript_61178;F01_transcript_61511;F01_transcript_620
06;F01_transcript_62329;F01_transcript_62577;F01_transcript_
63860;F01_transcript_63892;F01_transcript_64069;F01_transcri
pt_64296;F01_transcript_64718;F01_transcript_65073;F01_tran
script_65509;F01_transcript_65515;F01_transcript_65567;F01_t
ranscript_65841;F01_transcript_65858;F01_transcript_65973;F0
1_transcript_66852;F01_transcript_67228;F01_transcript_68428
;F01_transcript_68879;F01_transcript_69139;F01_transcript_69
220;F01_transcript_70147;F01_transcript_70427;F01_transcript
_71019;F01_transcript_71219;F01_transcript_72257;F01_transc
ript_72598;F01_transcript_72651;F01_transcript_73112;F01_tra
nscript_73969;F01_transcript_74626;F01_transcript_75129;F01

			_transcript_75477;F01_transcript_75487;F01_transcript_76748;F01_transcript_77012;F01_transcript_77213;F01_transcript_77264;F01_transcript_77389;F01_transcript_77468;F01_transcript_77476;F01_transcript_78437;F01_transcript_78720;F01_transcript_78919;F01_transcript_79307;F01_transcript_80252;F01_transcript_80300;F01_transcript_80769;F01_transcript_81078;F01_transcript_81217;F01_transcript_81590;F01_transcript_81767;F01_transcript_82092;F01_transcript_82136;F01_transcript_83562;F01_transcript_83908;F01_transcript_84015;F01_transcript_85168;F01_transcript_86483;F01_transcript_86847;F01_transcript_86970;F01_transcript_87504;F01_transcript_877;F01_transcript_87957;F01_transcript_89140;F01_transcript_90459;F01_transcript_90815;F01_transcript_91443;F01_transcript_91551;F01_transcript_92282;F01_transcript_92400;F01_transcript_92426;F01_transcript_92797;F01_transcript_92895;F01_transcript_93052;F01_transcript_93248;F01_transcript_93376;F01_transcript_93536;F01_transcript_94572;F01_transcript_94860;F01_transcript_95090;F01_transcript_95605;F01_transcript_96025;F01_transcript_96820;F01_transcript_96851;F01_transcript_97367;F01_transcript_97628;F01_transcript_9785;F01_transcript_97999;F01_transcript_98863;F01_transcript_98918;F01_transcript_99450;F01_transcript_99860;	
Glycine, serine and threonine metabolism	ko00260	590	F01_transcript_100118;F01_transcript_100230;F01_transcript_100330;F01_transcript_100363;F01_transcript_10047;F01_transcript_100647;F01_transcript_100809;F01_transcript_100871;F01_transcript_101071;F01_transcript_101371;F01_transcript_101567;F01_transcript_102048;F01_transcript_102135;F01_transcript_102306;F01_transcript_10266;F01_transcript_102714;F01_transcript_103227;F01_transcript_103345;F01_transcript_103532;F01_transcript_103691;F01_transcript_104167;F01_transcript_104745;F01_transcript_104746;F01_transcript_105405;F01_transcript_105644;F01_transcript_106158;F01_transcript_106462;F01_transcript_106623;F01_transcript_106679;F01_transcript_106967;F01_transcript_107003;F01_transcript_107677;F01_transcript_107764;F01_transcript_107931;F01_transcript_10799;F01_transcript_108216;F01_transcript_108345;F01_transcript_108584;F01_transcript_108602;F01_transcript_109068;F01_transcript_109254;F01_transcript_109418;F01_transcript_109519;F01_tran	K00600+K00058+K00600+K00928+K14272+K15918+K15634+K00281+K00600+K00281+K00276+K14272+K00600+K00281+K14272+K00600+K00600+K15918+K14272+K14085+K00600+K12235+K00382+K00276+K00928+K00133+K15893+K12524+K00928+K00600+K00281+K00058+K00276+K14272+K00600+K00382+K00281+K00600+K00600+K00281+K00600+K00830+K00130+K15919+K14272+K15893+K00600+K14272+K00281+K00382+K15634+K14272+K00281+K00600+K01079+K00928+K14272+K00281+K00058+K00600+K00281+K00281+K00058+K00382+K14085+K00382+K15893+K00281+K14272+K00281+K14272+K00928+K00600+K00600+K00281+K00281+K00830+K00600+K01733+K00600+K00382+K00830+K00600+K00605+K00276+K00600+K00130+K15633+K14272+K00600+K00600+K00830+K15919+K01696+K00605+K15893+K14272+K00600+K00600+K00281+K00928+K00276+K15893+K15918+K00600+K15633+K14272+K00281+K12524+K1591

	script_109993;F01_transcript_110137;F01_transcript_110477;F01_transcript_111549;F01_transcript_11182;F01_transcript_112020;F01_transcript_112117;F01_transcript_11219;F01_transcript_112260;F01_transcript_112494;F01_transcript_112563;F01_transcript_113436;F01_transcript_113828;F01_transcript_113831;F01_transcript_113837;F01_transcript_114614;F01_transcript_114677;F01_transcript_114689;F01_transcript_115186;F01_transcript_115188;F01_transcript_115624;F01_transcript_115636;F01_transcript_116451;F01_transcript_116525;F01_transcript_116591;F01_transcript_116648;F01_transcript_116855;F01_transcript_117148;F01_transcript_117214;F01_transcript_117422;F01_transcript_117699;F01_transcript_117863;F01_transcript_118084;F01_transcript_118373;F01_transcript_118804;F01_transcript_118908;F01_transcript_118927;F01_transcript_11951;F01_transcript_119569;F01_transcript_119952;F01_transcript_120212;F01_transcript_12072;F01_transcript_121306;F01_transcript_121491;F01_transcript_122012;F01_transcript_122101;F01_transcript_122115;F01_transcript_122331;F01_transcript_122499;F01_transcript_122872;F01_transcript_122875;F01_transcript_12302;F01_transcript_123124;F01_transcript_123753;F01_transcript_123848;F01_transcript_124153;F01_transcript_124200;F01_transcript_124206;F01_transcript_124604;F01_transcript_125370;F01_transcript_125371;F01_transcript_12550;F01_transcript_125563;F01_transcript_125768;F01_transcript_125984;F01_transcript_126096;F01_transcript_126285;F01_transcript_1269;F01_transcript_126994;F01_transcript_127297;F01_transcript_127687;F01_transcript_127834;F01_transcript_128000;F01_transcript_129051;F01_transcript_130313;F01_transcript_130382;F01_transcript_130447;F01_transcript_130485;F01_transcript_130612;F01_transcript_130708;F01_transcript_130742;F01_transcript_130895;F01_transcript_130923;F01_transcript_131057;F01_transcript_131196;F01_transcript_131459;F01_transcript_131892;F01_transcript_132156;F01_transcript_132238;F01_transcript_132264;F01_transcript_132310;F01_transcript_132483;F01_transcript_132512;F01_transcript_132527;F01_transcript_133019;F01_transcript_133265;F01_transcript_133975;F01_transcript_133996;F01_transcript_134020;F01_transcript_134653;F01_transcript_	8+K00281+K00281+K00600+K00830+K01834+K15918+K00600+K00276+K00600+K15893+K14272+K00600+K00058+K00605+K00600+K00281+K15633+K00605+K15893+K00058+K00600+K00600+K02437+K00058+K00600+K00276+K14272+K00600+K00600+K00928+K15918+K00281+K00130+K00276+K00281+K12524+K00928+K15918+K15633+K00600+K00600+K00600+K00928+K00600+K00600+K00281+K00281+K00276+K00276+K15919+K00928+K15633+K00600+K00281+K00928+K15893+K15634+K15633+K15893+K00600+K00605+K00382+K02437+K14272+K00133+K00600+K14272+K15919+K15918+K15893+K00281+K00281+K00830+K00600+K14272+K00600+K00281+K15633+K01733+K00276+K00928+K00276+K00058+K15893+K00382+K00830+K00382+K00600+K15893+K00281+K00281+K15893+K00306+K00928+K15919+K00928+K15918+K01079+K00928+K00276+K14272+K00600+K00600+K00928+K15919+K00058+K00133+K00827+K00281+K00928+K15919+K00281+K00600+K00605+K00600+K00276+K00600+K15893+K00827+K00276+K14085+K00830+K00827+K14272+K15893+K15918+K14272+K00600+K00928+K00281+K00600+K15918+K02437+K00281+K00281+K00928+K00928+K00600+K00382+K00281+K00600+K00276+K00276+K00600+K15893+K01834+K00600+K00306+K00830+K01696+K00928+K00872+K00058+K00276+K15919+K00830+K14272+K00600+K00830+K00600+K00928+K00281+K00600+K15918+K00605+K00276+K00928+K15918+K15893+K00058+K00928+K00058+K00600+K00281+K00276+K00928+K15918+K00928+K00276+K00058+K00281+K01754+K00382+K00605+K00058+K15919+K15918+K00600+K00600+K00276+K00382+K01758+K00600+K00382+K14272+K00130+K01754+K00928+K00600+K00058+K00058+K15633+K00600+K00600+K00600+K00600+K14272+K00382+K00049+K00382+K15634+K00600+K06001+K00600+K01733+K14272+K00600+K00600+K00382+K00281+K00600+K00600+K06001+K00600+K06001+K00281+K00600+K00600+K00600+K15918+K00600+K00600+K14085+K14085+K00600+K00600+K15634+K00600+K15634+K00130+K14272+K14272+K00827+K01696+K15918+K00600+K00281+K00600+K14272+K14272+K00605+K00382+K00605+K01

	13535;F01_transcript_135610;F01_transcript_136273;F01_transcript_136367;F01_transcript_136997;F01_transcript_137134;F01_transcript_13740;F01_transcript_138453;F01_transcript_138914;F01_transcript_139074;F01_transcript_139819;F01_transcript_139822;F01_transcript_140347;F01_transcript_1404;F01_transcript_140406;F01_transcript_140632;F01_transcript_140744;F01_transcript_140944;F01_transcript_141418;F01_transcript_141510;F01_transcript_141575;F01_transcript_141642;F01_transcript_141657;F01_transcript_141901;F01_transcript_142290;F01_transcript_142720;F01_transcript_14292;F01_transcript_142920;F01_transcript_143349;F01_transcript_144002;F01_transcript_144458;F01_transcript_144716;F01_transcript_145271;F01_transcript_145616;F01_transcript_145718;F01_transcript_145724;F01_transcript_145854;F01_transcript_145862;F01_transcript_146329;F01_transcript_146447;F01_transcript_146840;F01_transcript_146963;F01_transcript_147232;F01_transcript_147236;F01_transcript_147492;F01_transcript_147534;F01_transcript_147552;F01_transcript_148355;F01_transcript_148409;F01_transcript_148710;F01_transcript_148713;F01_transcript_149234;F01_transcript_150046;F01_transcript_150228;F01_transcript_150241;F01_transcript_150492;F01_transcript_150811;F01_transcript_151605;F01_transcript_152060;F01_transcript_153078;F01_transcript_153395;F01_transcript_153435;F01_transcript_153912;F01_transcript_154298;F01_transcript_154314;F01_transcript_154436;F01_transcript_154647;F01_transcript_154837;F01_transcript_154869;F01_transcript_155116;F01_transcript_155171;F01_transcript_155558;F01_transcript_15571;F01_transcript_156046;F01_transcript_156809;F01_transcript_157013;F01_transcript_157207;F01_transcript_158189;F01_transcript_158216;F01_transcript_158283;F01_transcript_158425;F01_transcript_158617;F01_transcript_158722;F01_transcript_159165;F01_transcript_159272;F01_transcript_159360;F01_transcript_159476;F01_transcript_159514;F01_transcript_159651;F01_transcript_160021;F01_transcript_160034;F01_transcript_16005;F01_transcript_16018;F01_transcript_160312;F01_transcript_160408;F01_transcript_160462;F01_transcript_160612;F01_transcript_160895;F01_transcript_161372;F01_transcript_161395;F01_transcript_161508;F01_transcript_161905;F01_transcript_16207;F01_transcript_1	834+K15918+K00600+K14272+K00058+K00831+K00133+K00600+K00600+K00605+K00600+K01620+K12524+K01620+K00830+K01620+K01696+K00830+K01620+K00605+K00306+K15893+K00605+K01620+K12235+K01695+K12235+K00049+K00600+K01079+K01079+K15919+K01079+K01079+K15919+K00382+K12524+K00605+K00281+K02437+K02437+K02437+K02437+K02437+K02437+K02437+K02437+K00600+K15634+K00605+K01733+K15633+K14272+K00133+K00276+K12524+K00600+K02437+K00600+K15893+K00281+K00281+K00276+K14085+K15634+K00600+K00130+K00382+K00831+K00281+K00281+K14272+K15633+K01620+K00600+K00600+K00281+K00276+K15918+K00382+K00600+K00600+K00049+K00827+K14272+K00058+K00600+K00276+K00276+K15893+K00058+K00382+K00600+K00281+K12524+K06001+K14085+K15633+K00276+K00600+K00600+K00830+K14272+K00600+K00827+K01696+K00830+K15633+K00276+K14272+K00600+K12235+K00928+K00600+K00382+K00281+K00600+K00058+K15633+K00600+K00281+K02437+K00600+K00276+K15893+K00382+K00281+K00827+K00130+K14272+K00830+K00600+K00600+K15918+K00281+K00600+K15919+K00600+K00605+K15893+K15918+K14272+K15918+K00600+K00600+K15634+K14272+K15633+K14272+K00928+K15633+K14085+K01696+K00605+K00281+K15893+K00600+K15893+K15893+K00382+K12524+K01696+K00600+K15633+K00600+K00600+K00382+K00276+K00281+K00928+K02437+K00600+K14272+K00281+K00928+K00281+K00830+K00600+K00281+K00281+K14085+K14272+K15634+K00281+K00281+K00600+K00600+K00276+K00600+K00830+K15893+K00382+K00276+K00133+K15918+K00276+K00382+K14272+K14272+K00281+K14272+K14272+K00600+K00281+K00600+K00600+K00281+K14272+K00276+K14272+K00600+K00600+K00605+K00928+K00281+K00600+K00281+K14272+K00600+K01733+K00600+K00382+K00281+K00600+K00276+K14272+K00281

62256;F01_transcript_16246;F01_transcript_162906;F01_transcript_1635;F01_transcript_163735;F01_transcript_163899;F01_transcript_16418;F01_transcript_164525;F01_transcript_164548;F01_transcript_165106;F01_transcript_165169;F01_transcript_165256;F01_transcript_165317;F01_transcript_165464;F01_transcript_165529;F01_transcript_165571;F01_transcript_16580;F01_transcript_16644;F01_transcript_166703;F01_transcript_166993;F01_transcript_167098;F01_transcript_167268;F01_transcript_16810;F01_transcript_17308;F01_transcript_17449;F01_transcript_1751;F01_transcript_17809;F01_transcript_18138;F01_transcript_18500;F01_transcript_18563;F01_transcript_19063;F01_transcript_19340;F01_transcript_19355;F01_transcript_19553;F01_transcript_19590;F01_transcript_19876;F01_transcript_20069;F01_transcript_2014;F01_transcript_2067;F01_transcript_20993;F01_transcript_21089;F01_transcript_21550;F01_transcript_22000;F01_transcript_22500;F01_transcript_2257;F01_transcript_22758;F01_transcript_23074;F01_transcript_23148;F01_transcript_23168;F01_transcript_23231;F01_transcript_23916;F01_transcript_24050;F01_transcript_24298;F01_transcript_24430;F01_transcript_24513;F01_transcript_24651;F01_transcript_25171;F01_transcript_25482;F01_transcript_25546;F01_transcript_25609;F01_transcript_25633;F01_transcript_25705;F01_transcript_25780;F01_transcript_25903;F01_transcript_26171;F01_transcript_26406;F01_transcript_26643;F01_transcript_26845;F01_transcript_2695;F01_transcript_27140;F01_transcript_27248;F01_transcript_27300;F01_transcript_27683;F01_transcript_27827;F01_transcript_28043;F01_transcript_28121;F01_transcript_28267;F01_transcript_28268;F01_transcript_28502;F01_transcript_28733;F01_transcript_28812;F01_transcript_28865;F01_transcript_28894;F01_transcript_2900;F01_transcript_29058;F01_transcript_29214;F01_transcript_29399;F01_transcript_29482;F01_transcript_29494;F01_transcript_2952;F01_transcript_29697;F01_transcript_29873;F01_transcript_30092;F01_transcript_30161;F01_transcript_30170;F01_transcript_30222;F01_transcript_30292;F01_transcript_30362;F01_transcript_30482;F01_transcript_30802;F01_transcript_30880;F01_transcript_31015;F01_transcript_31066;F01_transcript_31710;F01_transcript_31881;F01_transc

ript_32376;F01_transcript_32442;F01_transcript_32492;F01_tra
nscript_32576;F01_transcript_32834;F01_transcript_3321;F01_t
ranscript_33418;F01_transcript_33668;F01_transcript_34014;F0
1_transcript_34189;F01_transcript_34439;F01_transcript_34659
;F01_transcript_34832;F01_transcript_34949;F01_transcript_35
430;F01_transcript_35906;F01_transcript_35927;F01_transcript
_35945;F01_transcript_36269;F01_transcript_36398;F01_transc
ript_366;F01_transcript_36649;F01_transcript_36997;F01_trans
cript_37409;F01_transcript_3744;F01_transcript_37558;F01_tra
nscript_37978;F01_transcript_38394;F01_transcript_38511;F01
_transcript_38799;F01_transcript_38877;F01_transcript_39000;
F01_transcript_39050;F01_transcript_39467;F01_transcript_395
79;F01_transcript_40206;F01_transcript_40693;F01_transcript_
40811;F01_transcript_40908;F01_transcript_4197;F01_transcrip
t_42368;F01_transcript_42378;F01_transcript_42658;F01_trans
cript_42808;F01_transcript_43237;F01_transcript_43764;F01_tr
anscript_43886;F01_transcript_4391;F01_transcript_4410;F01_t
ranscript_45006;F01_transcript_4584;F01_transcript_46515;F01
_transcript_46967;F01_transcript_47239;F01_transcript_47558;
F01_transcript_47625;F01_transcript_47881;F01_transcript_481
19;F01_transcript_48690;F01_transcript_50115;F01_transcript_
51810;F01_transcript_51868;F01_transcript_51986;F01_transcri
pt_52139;F01_transcript_52222;F01_transcript_52630;F01_tran
script_52920;F01_transcript_5355;F01_transcript_53824;F01_tr
anscript_54286;F01_transcript_55946;F01_transcript_55951;F0
1_transcript_55975;F01_transcript_56493;F01_transcript_56629
;F01_transcript_56690;F01_transcript_56754;F01_transcript_56
98;F01_transcript_57582;F01_transcript_57636;F01_transcript_
57970;F01_transcript_58208;F01_transcript_58758;F01_transcri
pt_58783;F01_transcript_59406;F01_transcript_59608;F01_tran
script_59629;F01_transcript_59737;F01_transcript_60064;F01_t
ranscript_60104;F01_transcript_60113;F01_transcript_60314;F0
1_transcript_60381;F01_transcript_60635;F01_transcript_60687
;F01_transcript_60971;F01_transcript_61178;F01_transcript_61
243;F01_transcript_61445;F01_transcript_61675;F01_transcript
_61722;F01_transcript_62010;F01_transcript_62037;F01_transc
ript_62135;F01_transcript_62260;F01_transcript_62390;F01_tra
nscript_6274;F01_transcript_63045;F01_transcript_63491;F01_t

ranscript_63669;F01_transcript_63755;F01_transcript_64095;F01_transcript_64897;F01_transcript_65073;F01_transcript_65567;F01_transcript_65810;F01_transcript_65841;F01_transcript_65935;F01_transcript_65973;F01_transcript_66352;F01_transcript_66411;F01_transcript_67228;F01_transcript_67332;F01_transcript_67395;F01_transcript_69503;F01_transcript_70704;F01_transcript_729;F01_transcript_72917;F01_transcript_72967;F01_transcript_73233;F01_transcript_73266;F01_transcript_73464;F01_transcript_73498;F01_transcript_73584;F01_transcript_74212;F01_transcript_74237;F01_transcript_74379;F01_transcript_74401;F01_transcript_75040;F01_transcript_75129;F01_transcript_75267;F01_transcript_75477;F01_transcript_75487;F01_transcript_75531;F01_transcript_75678;F01_transcript_75819;F01_transcript_75876;F01_transcript_75942;F01_transcript_76142;F01_transcript_76460;F01_transcript_76536;F01_transcript_76540;F01_transcript_76633;F01_transcript_76748;F01_transcript_76878;F01_transcript_76950;F01_transcript_77082;F01_transcript_77136;F01_transcript_77213;F01_transcript_77418;F01_transcript_77476;F01_transcript_77531;F01_transcript_77873;F01_transcript_77976;F01_transcript_78254;F01_transcript_78456;F01_transcript_79069;F01_transcript_79175;F01_transcript_79353;F01_transcript_79364;F01_transcript_79630;F01_transcript_79704;F01_transcript_80154;F01_transcript_80889;F01_transcript_81090;F01_transcript_81686;F01_transcript_82110;F01_transcript_82373;F01_transcript_82589;F01_transcript_8293;F01_transcript_83616;F01_transcript_8406;F01_transcript_84138;F01_transcript_84894;F01_transcript_85168;F01_transcript_85447;F01_transcript_85599;F01_transcript_85663;F01_transcript_86483;F01_transcript_86731;F01_transcript_86965;F01_transcript_87507;F01_transcript_8790;F01_transcript_87957;F01_transcript_8856;F01_transcript_88614;F01_transcript_89475;F01_transcript_89629;F01_transcript_89641;F01_transcript_90314;F01_transcript_90316;F01_transcript_90815;F01_transcript_90853;F01_transcript_91232;F01_transcript_91560;F01_transcript_91694;F01_transcript_91900;F01_transcript_9243;F01_transcript_92441;F01_transcript_92797;F01_transcript_92895;F01_transcript_92931;F01_transcript_93248;F01_transcript_93376;F01_transcript_935

			9;F01_transcript_93656;F01_transcript_93722;F01_transcript_93994;F01_transcript_94039;F01_transcript_94572;F01_transcript_95934;F01_transcript_96025;F01_transcript_96028;F01_transcript_96153;F01_transcript_96758;F01_transcript_97035;F01_transcript_97196;F01_transcript_97239;F01_transcript_97343;F01_transcript_97367;F01_transcript_97597;F01_transcript_98005;F01_transcript_98080;F01_transcript_98159;F01_transcript_98369;F01_transcript_98413;F01_transcript_98894;F01_transcript_98918;F01_transcript_99086;	
Monobactam biosynthesis	ko00261	73	F01_transcript_100363;F01_transcript_105644;F01_transcript_106158;F01_transcript_106623;F01_transcript_106679;F01_transcript_109944;F01_transcript_113828;F01_transcript_116785;F01_transcript_117214;F01_transcript_124206;F01_transcript_125014;F01_transcript_125956;F01_transcript_126096;F01_transcript_131899;F01_transcript_133975;F01_transcript_134494;F01_transcript_135345;F01_transcript_136273;F01_transcript_136367;F01_transcript_138692;F01_transcript_139074;F01_transcript_140944;F01_transcript_141642;F01_transcript_144538;F01_transcript_144716;F01_transcript_145873;F01_transcript_146938;F01_transcript_148355;F01_transcript_153395;F01_transcript_153912;F01_transcript_154155;F01_transcript_154436;F01_transcript_154557;F01_transcript_155171;F01_transcript_156046;F01_transcript_156861;F01_transcript_157207;F01_transcript_160462;F01_transcript_16207;F01_transcript_162256;F01_transcript_163833;F01_transcript_165529;F01_transcript_17449;F01_transcript_19063;F01_transcript_19590;F01_transcript_20993;F01_transcript_21550;F01_transcript_25705;F01_transcript_26308;F01_transcript_30637;F01_transcript_34975;F01_transcript_36269;F01_transcript_3744;F01_transcript_39170;F01_transcript_4410;F01_transcript_51569;F01_transcript_52630;F01_transcript_5355;F01_transcript_6274;F01_transcript_69503;F01_transcript_76208;F01_transcript_77531;F01_transcript_80154;F01_transcript_83649;F01_transcript_8406;F01_transcript_85599;F01_transcript_88527;F01_transcript_88550;F01_transcript_91694;F01_transcript_93694;F01_transcript_94345;F01_transcript_97035;F01_transcript_98454;	K00928+K00928+K00133+K12524+K00928+K00215+K00928+K00215+K00928+K00928+K00215+K00215+K12524+K00215+K00928+K00215+K00215+K12524+K00928+K00215+K00928+K00928+K00928+K13811+K00133+K13811+K13811+K00928+K00928+K00928+K00215+K00928+K00215+K00928+K00133+K13811+K00928+K00928+K00928+K00928+K00215+K00928+K00928+K00928+K00928+K00928+K00928+K00928+K00215+K13811+K13811+K00133+K12524+K01714+K12524+K00215+K00133+K12524+K12524+K00928+K13811+K00928+K12524+K00215+K00928+K00928+K00215+K00215+K00133+K00215+K13811+K00928+K00215
Cysteine	ko	488	F01_transcript_100005;F01_transcript_100363;F01_transcript_100487;F01_transcript_100516;F01_transcript_100618;F01_trans	K00026+K00928+K00826+K00826+K00815+K00640+K14454+K00826+K00549+K00547+K17398+K01739+K00026+K0101

and methi onine metab olism	0 0 2 7 0	cript_101296;F01_transcript_101473;F01_transcript_101584;F01_transcript_101758;F01_transcript_102018;F01_transcript_102613;F01_transcript_103028;F01_transcript_103394;F01_transcript_103399;F01_transcript_103488;F01_transcript_10354;F01_transcript_103700;F01_transcript_103884;F01_transcript_104142;F01_transcript_104320;F01_transcript_104378;F01_transcript_104794;F01_transcript_105281;F01_transcript_105644;F01_transcript_105710;F01_transcript_106156;F01_transcript_106158;F01_transcript_106222;F01_transcript_106243;F01_transcript_10658;F01_transcript_106623;F01_transcript_106666;F01_transcript_106679;F01_transcript_107012;F01_transcript_10704;F01_transcript_107151;F01_transcript_107168;F01_transcript_107578;F01_transcript_108617;F01_transcript_109267;F01_transcript_109283;F01_transcript_109749;F01_transcript_110674;F01_transcript_110728;F01_transcript_110921;F01_transcript_111001;F01_transcript_111470;F01_transcript_111520;F01_transcript_111627;F01_transcript_11185;F01_transcript_112262;F01_transcript_113621;F01_transcript_113659;F01_transcript_113828;F01_transcript_114007;F01_transcript_114030;F01_transcript_114327;F01_transcript_114452;F01_transcript_114551;F01_transcript_114713;F01_transcript_114920;F01_transcript_115361;F01_transcript_115397;F01_transcript_115594;F01_transcript_116381;F01_transcript_116956;F01_transcript_117214;F01_transcript_117415;F01_transcript_117844;F01_transcript_117943;F01_transcript_117992;F01_transcript_118012;F01_transcript_118376;F01_transcript_118538;F01_transcript_118580;F01_transcript_118710;F01_transcript_118935;F01_transcript_119076;F01_transcript_119306;F01_transcript_119411;F01_transcript_119690;F01_transcript_119869;F01_transcript_120073;F01_transcript_120145;F01_transcript_120425;F01_transcript_120613;F01_transcript_120997;F01_transcript_121171;F01_transcript_12118;F01_transcript_121295;F01_transcript_121502;F01_transcript_121908;F01_transcript_122119;F01_transcript_122379;F01_transcript_123859;F01_transcript_124206;F01_transcript_124408;F01_transcript_124416;F01_transcript_12464;F01_transcript_125334;F01_transcript_125649;F01_transcript_125818;F01_transcript_126030;F01_transcript_126096;F01_transcript_126139;F01_transcript	1+K00026+K00549+K01738+K16054+K01611+K00026+K01251+K08963+K01738+K00928+K17398+K14454+K00133+K00797+K01611+K00549+K12524+K00815+K00928+K00797+K00549+K00811+K01919+K00899+K00026+K00640+K00026+K05933+K01251+K00826+K00789+K00789+K05933+K00558+K16054+K00549+K00797+K00547+K01761+K00928+K00549+K01738+K00640+K14455+K01738+K20772+K01738+K01738+K01611+K00026+K00026+K00826+K00928+K00026+K01739+K01611+K00558+K00899+K01738+K00899+K00797+K00026+K01920+K01251+K13034+K00026+K00826+K14455+K00640+K01738+K17398+K01251+K13034+K01251+K00549+K01920+K01739+K00797+K00815+K01251+K01739+K00928+K08963+K00549+K01251+K01738+K00558+K01738+K00026+K12524+K00549+K14455+K00026+K00549+K01611+K01738+K00549+K01739+K01919+K01738+K00558+K00797+K01244+K00026+K20772+K01739+K00025+K00026+K00899+K16054+K14455+K01251+K00815+K01251+K17398+K00797+K00549+K00815+K00928+K13034+K16054+K00547+K00549+K00026+K14454+K00026+K00815+K00547+K00025+K12524+K00928+K00815+K17398+K01738+K01611+K00928+K00026+K01611+K00026+K00928+K00789+K00549+K00928+K01011+K01738+K00797+K00549+K01739+K00789+K00815+K05933+K16054+K00026+K00133+K00815+K01244+K00558+K17398+K01738+K01251+K00815+K00026+K00026+K00797+K00026+K00928+K00549+K00826+K00026+K00789+K01738+K00026+K00815+K08967+K14454+K01738+K00797+K01738+K16054+K01920+K01738+K00558+K13034+K14454+K00558+K00547+K00928+K00928+K16054+K00928+K01738+K00558+K00826+K00928+K01738+K01739+K01611+K01738+K00133+K14455+K00827+K00797+K00928+K01920+K00026+K01011+K00789+K00827+K00549+K00827+K00026+K01920+K00928+K00811+K00547+K00815+K00026+K01738+K00815+K00928+K01244+K00928+K00026+K00026+K00026+K00026+K01738+K00928+K00026+K00928+K01920+K00928+K00549+K00815+K00928+K00928+K00928+K00549+K01919+K00026+K00549+K01738+K01251+K01758+K00797+K00928+K01739+K01611+K01611+K01919+K14454+K01611+K01739+K01

	_126185;F01_transcript_126482;F01_transcript_12663;F01_transcript_126818;F01_transcript_127193;F01_transcript_127328;F01_transcript_127418;F01_transcript_127422;F01_transcript_127989;F01_transcript_128098;F01_transcript_128143;F01_transcript_128198;F01_transcript_128256;F01_transcript_129083;F01_transcript_129435;F01_transcript_130017;F01_transcript_130127;F01_transcript_130829;F01_transcript_13091;F01_transcript_131043;F01_transcript_131353;F01_transcript_132157;F01_transcript_13240;F01_transcript_132632;F01_transcript_132867;F01_transcript_133224;F01_transcript_133648;F01_transcript_133975;F01_transcript_134216;F01_transcript_134338;F01_transcript_134683;F01_transcript_134823;F01_transcript_134955;F01_transcript_135578;F01_transcript_135698;F01_transcript_135806;F01_transcript_135829;F01_transcript_136111;F01_transcript_136273;F01_transcript_136367;F01_transcript_136534;F01_transcript_137919;F01_transcript_138596;F01_transcript_138913;F01_transcript_139074;F01_transcript_139464;F01_transcript_140464;F01_transcript_140879;F01_transcript_140944;F01_transcript_140974;F01_transcript_141325;F01_transcript_141642;F01_transcript_142028;F01_transcript_142050;F01_transcript_142320;F01_transcript_142448;F01_transcript_142449;F01_transcript_142963;F01_transcript_14328;F01_transcript_143626;F01_transcript_143753;F01_transcript_144706;F01_transcript_144716;F01_transcript_144749;F01_transcript_14542;F01_transcript_145726;F01_transcript_145866;F01_transcript_146107;F01_transcript_146109;F01_transcript_146924;F01_transcript_147154;F01_transcript_147445;F01_transcript_147521;F01_transcript_147898;F01_transcript_148355;F01_transcript_148697;F01_transcript_148792;F01_transcript_149184;F01_transcript_149261;F01_transcript_149508;F01_transcript_149909;F01_transcript_150132;F01_transcript_150213;F01_transcript_150639;F01_transcript_151028;F01_transcript_151102;F01_transcript_151234;F01_transcript_152192;F01_transcript_152255;F01_transcript_152349;F01_transcript_152382;F01_transcript_152454;F01_transcript_152726;F01_transcript_152908;F01_transcript_153020;F01_transcript_153395;F01_transcript_153912;F01_transcript_154327;F01_transcript_154436;F01_transcript_154618;F01_transcript_154763;F01_transcript_154917;F01_transcript_155171;F01_transc	739+K14454+K01251+K01611+K00026+K00815+K01611+K00815+K00811+K20772+K00815+K00827+K14455+K01738+K01251+K01761+K01611+K00026+K00640+K00826+K00026+K01011+K01611+K00797+K00640+K00826+K00133+K00026+K14455+K08963+K14455+K00026+K01611+K13034+K12524+K00815+K00826+K13034+K16054+K00797+K00026+K00789+K00026+K00025+K00547+K01738+K01738+K01738+K00899+K00026+K00026+K00797+K00640+K00025+K05933+K01738+K00026+K00025+K01738+K00025+K05933+K01738+K01738+K01738+K05933+K00640+K00815+K05933+K00640+K00640+K12524+K08967+K08967+K08967+K08967+K00026+K05933+K13034+K00797+K00826+K00133+K00899+K12524+K01611+K00826+K00558+K01738+K01919+K17398+K01611+K01739+K01251+K16054+K00789+K00826+K01738+K00026+K01244+K00026+K00827+K00549+K01251+K00797+K00558+K01244+K12524+K01251+K17398+K00797+K00026+K00026+K00815+K00827+K14454+K00549+K17398+K00026+K00640+K01251+K13034+K13034+K00026+K16054+K00928+K00026+K01738+K00026+K01611+K01251+K01738+K00826+K00558+K00549+K16054+K00025+K08963+K00826+K00026+K05933+K01920+K00789+K00827+K01738+K01611+K01919+K00016+K01738+K00815+K01611+K01920+K00928+K13034+K01611+K00026+K01739+K14455+K00547+K00026+K00811+K00558+K00026+K00025+K00026+K12524+K00026+K00811+K14454+K14454+K01738+K01920+K00826+K00549+K00928+K00797+K00026+K01738+K00026+K00826+K01738+K16054+K00928+K01611+K00811+K01738+K00826+K14455+K17398+K14454+K00026+K01611+K00815+K01738+K00640+K16054+K00826+K00026+K00133+K00547+K14455+K00826+K13034+K00026+K00549+K00026+K01920+K00026+K01739+K01920+K01738+K00899+K16054+K00928+K00026+K14454+K01251+K00558+K00026+K01919+K01244+K00026+K01738

ript_155318;F01_transcript_155339;F01_transcript_155521;F01_transcript_155840;F01_transcript_156046;F01_transcript_156344;F01_transcript_156809;F01_transcript_157007;F01_transcript_157207;F01_transcript_157906;F01_transcript_158374;F01_transcript_159015;F01_transcript_159027;F01_transcript_159360;F01_transcript_159723;F01_transcript_160021;F01_transcript_160122;F01_transcript_160271;F01_transcript_160462;F01_transcript_160500;F01_transcript_160991;F01_transcript_161264;F01_transcript_161406;F01_transcript_161478;F01_transcript_161557;F01_transcript_16207;F01_transcript_162141;F01_transcript_162256;F01_transcript_162570;F01_transcript_162804;F01_transcript_163028;F01_transcript_164460;F01_transcript_165501;F01_transcript_165529;F01_transcript_167137;F01_transcript_17449;F01_transcript_18940;F01_transcript_19063;F01_transcript_19071;F01_transcript_19390;F01_transcript_19590;F01_transcript_20993;F01_transcript_21550;F01_transcript_21773;F01_transcript_22691;F01_transcript_22891;F01_transcript_2341;F01_transcript_23969;F01_transcript_24647;F01_transcript_24651;F01_transcript_24957;F01_transcript_25705;F01_transcript_26005;F01_transcript_26011;F01_transcript_27043;F01_transcript_27457;F01_transcript_28405;F01_transcript_28426;F01_transcript_29328;F01_transcript_30420;F01_transcript_30736;F01_transcript_30825;F01_transcript_30842;F01_transcript_31005;F01_transcript_31061;F01_transcript_31534;F01_transcript_31700;F01_transcript_31784;F01_transcript_31841;F01_transcript_32147;F01_transcript_32442;F01_transcript_32622;F01_transcript_32913;F01_transcript_33473;F01_transcript_33564;F01_transcript_33947;F01_transcript_34070;F01_transcript_34211;F01_transcript_34357;F01_transcript_34420;F01_transcript_34493;F01_transcript_34634;F01_transcript_35071;F01_transcript_35081;F01_transcript_35824;F01_transcript_36269;F01_transcript_36309;F01_transcript_36375;F01_transcript_36503;F01_transcript_36639;F01_transcript_36873;F01_transcript_37030;F01_transcript_37334;F01_transcript_3744;F01_transcript_37457;F01_transcript_37725;F01_transcript_37739;F01_transcript_37744;F01_transcript_37802;F01_transcript_37900;F01_transcript_38117;F01_transcript_38243;F01_transcript_38273;F01_transcript_38512

;F01_transcript_38763;F01_transcript_38874;F01_transcript_39013;F01_transcript_39061;F01_transcript_39388;F01_transcript_39641;F01_transcript_39898;F01_transcript_39940;F01_transcript_40133;F01_transcript_40208;F01_transcript_40243;F01_transcript_40284;F01_transcript_40490;F01_transcript_40591;F01_transcript_40600;F01_transcript_40945;F01_transcript_41338;F01_transcript_41547;F01_transcript_41905;F01_transcript_41968;F01_transcript_42029;F01_transcript_42165;F01_transcript_43288;F01_transcript_43371;F01_transcript_43614;F01_transcript_4410;F01_transcript_46393;F01_transcript_46451;F01_transcript_46733;F01_transcript_47078;F01_transcript_49083;F01_transcript_50367;F01_transcript_51442;F01_transcript_51841;F01_transcript_52367;F01_transcript_52630;F01_transcript_53363;F01_transcript_5355;F01_transcript_54080;F01_transcript_54318;F01_transcript_55419;F01_transcript_55774;F01_transcript_56280;F01_transcript_56491;F01_transcript_56672;F01_transcript_56992;F01_transcript_58080;F01_transcript_58311;F01_transcript_58463;F01_transcript_58468;F01_transcript_5896;F01_transcript_59025;F01_transcript_59951;F01_transcript_60128;F01_transcript_60971;F01_transcript_61029;F01_transcript_61994;F01_transcript_62377;F01_transcript_62558;F01_transcript_62684;F01_transcript_6274;F01_transcript_62787;F01_transcript_63378;F01_transcript_63834;F01_transcript_65371;F01_transcript_6571;F01_transcript_65744;F01_transcript_65841;F01_transcript_65858;F01_transcript_66754;F01_transcript_67098;F01_transcript_68075;F01_transcript_68093;F01_transcript_68144;F01_transcript_68454;F01_transcript_68627;F01_transcript_69158;F01_transcript_69177;F01_transcript_69503;F01_transcript_69697;F01_transcript_70160;F01_transcript_7025;F01_transcript_71519;F01_transcript_71662;F01_transcript_71954;F01_transcript_72229;F01_transcript_72237;F01_transcript_72578;F01_transcript_72825;F01_transcript_72893;F01_transcript_73362;F01_transcript_73375;F01_transcript_73713;F01_transcript_74254;F01_transcript_74849;F01_transcript_75120;F01_transcript_75129;F01_transcript_75379;F01_transcript_75621;F01_transcript_75840;F01_transcript_76175;F01_transcript_76723;F01_transcript_76908;F01_transcript_77184;F01_transcript_77193;F01_transcript_77531;F01_transcript_78302;F01_transcript_78418;F01_tra

			nscript_78511;F01_transcript_78612;F01_transcript_78720;F01_transcript_78764;F01_transcript_78775;F01_transcript_78919;F01_transcript_78970;F01_transcript_79019;F01_transcript_79482;F01_transcript_80009;F01_transcript_80154;F01_transcript_80216;F01_transcript_80769;F01_transcript_81078;F01_transcript_81767;F01_transcript_82746;F01_transcript_83097;F01_transcript_83526;F01_transcript_83801;F01_transcript_8406;F01_transcript_84285;F01_transcript_84462;F01_transcript_84568;F01_transcript_84788;F01_transcript_84934;F01_transcript_85473;F01_transcript_85480;F01_transcript_85599;F01_transcript_86863;F01_transcript_86970;F01_transcript_87136;F01_transcript_87267;F01_transcript_87504;F01_transcript_883;F01_transcript_89140;F01_transcript_89898;F01_transcript_90343;F01_transcript_90364;F01_transcript_90435;F01_transcript_90820;F01_transcript_90827;F01_transcript_91214;F01_transcript_91546;F01_transcript_91694;F01_transcript_92428;F01_transcript_93052;F01_transcript_93866;F01_transcript_94201;F01_transcript_95043;F01_transcript_95332;F01_transcript_95502;F01_transcript_95560;F01_transcript_959;F01_transcript_96054;F01_transcript_96138;F01_transcript_96511;F01_transcript_96686;F01_transcript_96880;F01_transcript_97035;F01_transcript_97342;F01_transcript_9785;F01_transcript_97938;F01_transcript_98321;F01_transcript_98458;F01_transcript_99309;F01_transcript_99423;F01_transcript_99915;F01_transcript_99924;	
Valine, leucine and isoleucine degradation	ko00280	350	F01_transcript_100487;F01_transcript_100488;F01_transcript_100516;F01_transcript_100740;F01_transcript_101584;F01_transcript_102741;F01_transcript_103175;F01_transcript_103691;F01_transcript_103925;F01_transcript_104346;F01_transcript_104696;F01_transcript_104746;F01_transcript_105213;F01_transcript_105377;F01_transcript_105403;F01_transcript_108216;F01_transcript_108221;F01_transcript_109141;F01_transcript_109312;F01_transcript_110728;F01_transcript_110861;F01_transcript_112117;F01_transcript_112210;F01_transcript_112685;F01_transcript_112884;F01_transcript_113444;F01_transcript_115085;F01_transcript_115624;F01_transcript_115636;F01_transcript_116451;F01_transcript_116665;F01_transcript_116861;F01_transcript_116956;F01_transcript_118736;F01_transcript_118763;F0	K00826+K01640+K00826+K00128+K00826+K00166+K05605+K14085+K00128+K05605+K05605+K00382+K00128+K05605+K00626+K00382+K00128+K01968+K00128+K00826+K09699+K00382+K05605+K00128+K00128+K18660+K05605+K00382+K14085+K00382+K05605+K00166+K00826+K00128+K05607+K00128+K00128+K00382+K01968+K00826+K01968+K00140+K00128+K00140+K18660+K00128+K05605+K05605+K01640+K05605+K01968+K05605+K05605+K00166+K00128+K00128+K05605+K00128+K00128+K01968+K00166+K07513+K00140+K05605+K00128+K00128+K00128+K01968+K00128+K00128+K18660+K00128+K00128+K00128+K00626+K01968+K00128+K00128+K00128+K05605+K00140+K01969+K05605+K18660+K01968+K01968+K00382+K00128+K05605+K

	1_transcript_118848;F01_transcript_119343;F01_transcript_11951;F01_transcript_119516;F01_transcript_119690;F01_transcript_121062;F01_transcript_121237;F01_transcript_121819;F01_transcript_122090;F01_transcript_122129;F01_transcript_1231;F01_transcript_123185;F01_transcript_123463;F01_transcript_123569;F01_transcript_124506;F01_transcript_126097;F01_transcript_126314;F01_transcript_126346;F01_transcript_126875;F01_transcript_127146;F01_transcript_127326;F01_transcript_127971;F01_transcript_128218;F01_transcript_128872;F01_transcript_130159;F01_transcript_130417;F01_transcript_130717;F01_transcript_130773;F01_transcript_132279;F01_transcript_132494;F01_transcript_132930;F01_transcript_1334;F01_transcript_133945;F01_transcript_134251;F01_transcript_134507;F01_transcript_136418;F01_transcript_137525;F01_transcript_137935;F01_transcript_138093;F01_transcript_138114;F01_transcript_138420;F01_transcript_138897;F01_transcript_138921;F01_transcript_139567;F01_transcript_139604;F01_transcript_140307;F01_transcript_140479;F01_transcript_140486;F01_transcript_141084;F01_transcript_14231;F01_transcript_143139;F01_transcript_143349;F01_transcript_144636;F01_transcript_144740;F01_transcript_144850;F01_transcript_145352;F01_transcript_145474;F01_transcript_145665;F01_transcript_146046;F01_transcript_14631;F01_transcript_147002;F01_transcript_147257;F01_transcript_147398;F01_transcript_147783;F01_transcript_148792;F01_transcript_149234;F01_transcript_149292;F01_transcript_150228;F01_transcript_150299;F01_transcript_151264;F01_transcript_151674;F01_transcript_151832;F01_transcript_151939;F01_transcript_152756;F01_transcript_152958;F01_transcript_15331;F01_transcript_15347;F01_transcript_154730;F01_transcript_154880;F01_transcript_154917;F01_transcript_154982;F01_transcript_155120;F01_transcript_155685;F01_transcript_15575;F01_transcript_156809;F01_transcript_157489;F01_transcript_157909;F01_transcript_158912;F01_transcript_159360;F01_transcript_159514;F01_transcript_159814;F01_transcript_159968;F01_transcript_160021;F01_transcript_160676;F01_transcript_16133;F01_transcript_161816;F01_transcript_161966;F01_transcript_162134;F01_transcript_162331;F01_transcript_162763;F01_transcript_162906;F01_transcript_163870;F01_transcript_164546;F01_tr	00128+K00128+K00128+K00253+K00128+K18660+K00166+K00128+K07513+K00128+K00826+K00382+K00128+K00382+K05605+K00128+K05605+K00128+K00128+K00128+K00128+K00140+K00140+K05605+K01641+K00826+K00128+K00020+K00626+K01968+K00827+K01969+K18660+K00128+K00827+K14085+K00128+K07513+K00827+K01968+K18660+K00128+K00128+K00128+K00128+K05605+K00382+K01969+K05605+K18660+K00626+K18660+K07513+K00020+K09699+K00140+K00128+K00128+K00140+K01969+K18660+K18660+K18660+K05605+K00382+K00128+K00128+K00382+K00128+K00166+K00382+K01969+K00128+K00128+K00382+K00128+K00382+K00128+K00140+K01641+K00128+K00382+K00140+K00140+K00128+K14085+K14085+K00128+K01641+K00166+K00128+K00140+K01640+K00128+K00128+K00128+K05605+K00827+K01641+K00166+K07513+K01640+K00128+K07513+K01968+K00128+K00166+K00128+K00826+K00382+K07513+K00128+K07513+K00166+K00826+K00253+K05605+K00128+K00128+K05605+K00626+K00626+K00128+K00826+K01640+K00128+K00128+K00626+K00128+K00626+K05605+K05605+K00020+K00128+K00382+K00128+K00249+K05605+K00253+K00626+K00826+K05605+K00167+K18660+K00826+K05605+K00166+K00128+K00128+K05605+K14085+K00140+K00382+K00166+K00626+K00826+K09699+K00382+K00827+K01640+K07513+K00382+K14085+K00140+K00128+K00128+K00827+K00128+K00128+K00128+K00249+K00140+K01968+K00626+K18660+K00128+K01969+K01968+K00249+K00128+K00128+K00167+K00826+K00128+K00166+K01640+K00382+K00128+K00826+K05605+K00382+K05605+K09699+K00128+K00827+K00020+K00249+K00140+K00626+K01969+K00140+K14085+K00166+K01969+K00626+K00140+K00382+K00128+K09699+K00140+K18660+K05605+K00128+K00626+K05605+K05605+K01969+K00128+K01640+K01968+K00382+K00253+K00826+K00167+K00626+K00826+K00166+K00166+K00826+K01968+K14085+K07513+K05605+K00826+K00382+K05605+K09699+K00249+K00382+K05605+K00128+K00826+K00166+K18660+K00128+K05605+K00128+K18660+K00626+K00253+K05605+K00382+K00128+K00128

	anscript_166407;F01_transcript_166964;F01_transcript_167075;F01_transcript_167595;F01_transcript_16760;F01_transcript_167649;F01_transcript_1704;F01_transcript_1799;F01_transcript_19412;F01_transcript_19633;F01_transcript_21027;F01_transcript_21612;F01_transcript_21698;F01_transcript_22849;F01_transcript_22856;F01_transcript_23074;F01_transcript_23785;F01_transcript_24020;F01_transcript_24513;F01_transcript_24677;F01_transcript_24810;F01_transcript_25482;F01_transcript_25646;F01_transcript_25659;F01_transcript_26120;F01_transcript_27300;F01_transcript_27376;F01_transcript_27827;F01_transcript_28572;F01_transcript_28594;F01_transcript_28787;F01_transcript_28856;F01_transcript_28894;F01_transcript_28947;F01_transcript_29341;F01_transcript_29858;F01_transcript_30292;F01_transcript_30362;F01_transcript_30379;F01_transcript_30530;F01_transcript_30868;F01_transcript_30935;F01_transcript_31414;F01_transcript_31485;F01_transcript_31894;F01_transcript_32017;F01_transcript_32266;F01_transcript_32386;F01_transcript_32442;F01_transcript_32485;F01_transcript_32562;F01_transcript_32889;F01_transcript_33312;F01_transcript_33325;F01_transcript_33549;F01_transcript_33555;F01_transcript_33600;F01_transcript_33822;F01_transcript_34271;F01_transcript_34357;F01_transcript_34439;F01_transcript_34454;F01_transcript_35286;F01_transcript_35442;F01_transcript_35669;F01_transcript_35824;F01_transcript_35874;F01_transcript_3669;F01_transcript_36697;F01_transcript_36801;F01_transcript_36901;F01_transcript_37377;F01_transcript_37686;F01_transcript_37696;F01_transcript_37725;F01_transcript_37841;F01_transcript_37926;F01_transcript_37987;F01_transcript_38302;F01_transcript_38323;F01_transcript_38657;F01_transcript_38936;F01_transcript_39168;F01_transcript_39281;F01_transcript_41704;F01_transcript_4391;F01_transcript_48424;F01_transcript_51625;F01_transcript_52158;F01_transcript_52314;F01_transcript_52332;F01_transcript_52367;F01_transcript_52494;F01_transcript_52864;F01_transcript_54170;F01_transcript_54318;F01_transcript_54938;F01_transcript_55336;F01_transcript_55541;F01_transcript_56137;F01_transcript_56268;F01_transcript_56690;F01_transcript_57145;F01_transcript_57636;F01_transcript_58172;F01_transc	+K07513+K00166+K01969+K18660+K00128+K01968+K18660

	ript_58368;F01_transcript_58468;F01_transcript_58882;F01_transcript_60314;F01_transcript_60971;F01_transcript_61404;F01_transcript_61866;F01_transcript_62135;F01_transcript_63491;F01_transcript_64572;F01_transcript_65034;F01_transcript_65403;F01_transcript_65841;F01_transcript_65979;F01_transcript_66261;F01_transcript_67211;F01_transcript_6767;F01_transcript_68359;F01_transcript_68981;F01_transcript_69739;F01_transcript_70256;F01_transcript_70471;F01_transcript_70876;F01_transcript_71233;F01_transcript_7124;F01_transcript_71260;F01_transcript_71646;F01_transcript_72143;F01_transcript_72229;F01_transcript_72349;F01_transcript_72537;F01_transcript_72756;F01_transcript_729;F01_transcript_73295;F01_transcript_73375;F01_transcript_73519;F01_transcript_74401;F01_transcript_74528;F01_transcript_75055;F01_transcript_75083;F01_transcript_75129;F01_transcript_75180;F01_transcript_7550;F01_transcript_76336;F01_transcript_77451;F01_transcript_77535;F01_transcript_77784;F01_transcript_77976;F01_transcript_78651;F01_transcript_78973;F01_transcript_79052;F01_transcript_79569;F01_transcript_79704;F01_transcript_79852;F01_transcript_79878;F01_transcript_79982;F01_transcript_80023;F01_transcript_80229;F01_transcript_80510;F01_transcript_80843;F01_transcript_80964;F01_transcript_81280;F01_transcript_81504;F01_transcript_81584;F01_transcript_81816;F01_transcript_82522;F01_transcript_82589;F01_transcript_82984;F01_transcript_83526;F01_transcript_83597;F01_transcript_84463;F01_transcript_84934;F01_transcript_85703;F01_transcript_86353;F01_transcript_87267;F01_transcript_87403;F01_transcript_8790;F01_transcript_88720;F01_transcript_89647;F01_transcript_91214;F01_transcript_91232;F01_transcript_91526;F01_transcript_91641;F01_transcript_9209;F01_transcript_92441;F01_transcript_92567;F01_transcript_92804;F01_transcript_93866;F01_transcript_94471;F01_transcript_95540;F01_transcript_96227;F01_transcript_97153;F01_transcript_97230;F01_transcript_97389;F01_transcript_97808;F01_transcript_97969;F01_transcript_98121;F01_transcript_98159;F01_transcript_98282;F01_transcript_98445;F01_transcript_98462;F01_transcript_98561;F01_transcript_98751;F01_transcript_99078;F01_transcript_99528;F01_transcript_99811;F01_transcript_99997;	

Valine, leucine and isoleucine biosynthesis	ko00290	100	F01_transcript_100487;F01_transcript_100516;F01_transcript_101584;F01_transcript_105548;F01_transcript_106479;F01_transcript_107447;F01_transcript_110728;F01_transcript_111759;F01_transcript_115383;F01_transcript_116956;F01_transcript_118645;F01_transcript_119338;F01_transcript_119690;F01_transcript_120201;F01_transcript_125864;F01_transcript_127540;F01_transcript_127921;F01_transcript_129549;F01_transcript_133194;F01_transcript_133321;F01_transcript_135402;F01_transcript_140089;F01_transcript_140510;F01_transcript_145367;F01_transcript_146194;F01_transcript_146322;F01_transcript_148792;F01_transcript_1503;F01_transcript_150508;F01_transcript_151854;F01_transcript_152848;F01_transcript_153213;F01_transcript_153480;F01_transcript_15454;F01_transcript_154917;F01_transcript_160665;F01_transcript_160691;F01_transcript_161767;F01_transcript_167653;F01_transcript_18842;F01_transcript_19555;F01_transcript_19790;F01_transcript_19945;F01_transcript_20059;F01_transcript_20342;F01_transcript_20537;F01_transcript_20928;F01_transcript_22601;F01_transcript_22646;F01_transcript_22758;F01_transcript_25633;F01_transcript_28993;F01_transcript_29072;F01_transcript_29737;F01_transcript_30778;F01_transcript_31526;F01_transcript_32304;F01_transcript_33011;F01_transcript_34357;F01_transcript_35824;F01_transcript_36862;F01_transcript_37086;F01_transcript_37725;F01_transcript_51931;F01_transcript_52367;F01_transcript_52882;F01_transcript_54318;F01_transcript_54539;F01_transcript_56606;F01_transcript_58468;F01_transcript_58777;F01_transcript_60186;F01_transcript_60455;F01_transcript_61708;F01_transcript_61852;F01_transcript_62228;F01_transcript_64900;F01_transcript_66275;F01_transcript_67213;F01_transcript_70967;F01_transcript_71528;F01_transcript_72229;F01_transcript_73375;F01_transcript_74222;F01_transcript_74843;F01_transcript_77518;F01_transcript_78308;F01_transcript_80747;F01_transcript_83526;F01_transcript_84934;F01_transcript_85279;F01_transcript_85411;F01_transcript_85624;F01_transcript_85969;F01_transcript_87267;F01_transcript_90977;F01_transcript_91214;F01_transcript_93119;F01_transcript_93866;F01_transcript_97229;	K00826+K00826+K00826+K00053+K01649+K01649+K00826+K01703+K00053+K00826+K01649+K01649+K00826+K01653+K00053+K00053+K01649+K00053+K00053+K01653+K00053+K01703+K01649+K01649+K01653+K00053+K00826+K00053+K00053+K00053+K00053+K01653+K01703+K01649+K00826+K01649+K01652+K01703+K00053+K01652+K01653+K00053+K01652+K01687+K00053+K01652+K00053+K01649+K00053+K01754+K01754+K00053+K01703+K01703+K01653+K01703+K00053+K01653+K00826+K00826+K00052+K00052+K00826+K01652+K00826+K01649+K00826+K00052+K00053+K00826+K00052+K00053+K00053+K01649+K01652+K01652+K00053+K01703+K00053+K00053+K01703+K00826+K00826+K00053+K01652+K01702+K01687+K01703+K00826+K00826+K01649+K01649+K01703+K00053+K00826+K01653+K00826+K01703+K00826+K00052

Lysine biosynthesis	ko00300	92	F01_transcript_100363;F01_transcript_103691;F01_transcript_104908;F01_transcript_105644;F01_transcript_106158;F01_transcript_106623;F01_transcript_106679;F01_transcript_107162;F01_transcript_109944;F01_transcript_113249;F01_transcript_113828;F01_transcript_115636;F01_transcript_116785;F01_transcript_117214;F01_transcript_123162;F01_transcript_124206;F01_transcript_125014;F01_transcript_125956;F01_transcript_126096;F01_transcript_131899;F01_transcript_133975;F01_transcript_134494;F01_transcript_135345;F01_transcript_136273;F01_transcript_136367;F01_transcript_138314;F01_transcript_138605;F01_transcript_138692;F01_transcript_139074;F01_transcript_140944;F01_transcript_141642;F01_transcript_142287;F01_transcript_143862;F01_transcript_144716;F01_transcript_148016;F01_transcript_148355;F01_transcript_153395;F01_transcript_153912;F01_transcript_154155;F01_transcript_154436;F01_transcript_154557;F01_transcript_155171;F01_transcript_156046;F01_transcript_157207;F01_transcript_159514;F01_transcript_160462;F01_transcript_16207;F01_transcript_162256;F01_transcript_163833;F01_transcript_165529;F01_transcript_17449;F01_transcript_18459;F01_transcript_19063;F01_transcript_19590;F01_transcript_20993;F01_transcript_21550;F01_transcript_25705;F01_transcript_26308;F01_transcript_30292;F01_transcript_30362;F01_transcript_31511;F01_transcript_31983;F01_transcript_33912;F01_transcript_36269;F01_transcript_3744;F01_transcript_39170;F01_transcript_41181;F01_transcript_4410;F01_transcript_51241;F01_transcript_51569;F01_transcript_52630;F01_transcript_53240;F01_transcript_5355;F01_transcript_56690;F01_transcript_6089;F01_transcript_6274;F01_transcript_63491;F01_transcript_69503;F01_transcript_77531;F01_transcript_77976;F01_transcript_80154;F01_transcript_80698;F01_transcript_83649;F01_transcript_8406;F01_transcript_85599;F01_transcript_8790;F01_transcript_88527;F01_transcript_88550;F01_transcript_91694;F01_transcript_93694;F01_transcript_97035;F01_transcript_98454;	K00928+K14085+K10206+K00928+K00133+K12524+K00928+K01586+K00215+K10206+K00928+K14085+K00215+K00928+K10206+K00928+K00215+K00215+K12524+K00215+K00928+K00215+K00215+K12524+K00928+K10206+K10206+K00215+K00928+K00928+K00928+K01778+K10206+K00133+K10206+K00928+K00928+K00928+K00215+K00928+K00215+K00928+K00133+K00928+K14085+K00928+K00928+K00928+K00215+K00928+K00928+K01586+K00928+K00928+K00928+K00928+K00928+K00215+K14085+K14085+K01586+K10206+K01586+K00133+K12524+K01714+K01778+K12524+K01586+K00215+K00133+K01586+K12524+K14085+K10206+K12524+K14085+K00928+K00928+K14085+K12524+K10206+K00215+K00928+K00928+K14085+K00215+K00215+K00133+K00215+K00928+K00215
Lysine degradation	ko00	212	F01_transcript_100740;F01_transcript_101797;F01_transcript_102683;F01_transcript_103358;F01_transcript_10364;F01_transcript_103691;F01_transcript_103925;F01_transcript_104293;F01_transcript_104347;F01_transcript_104928;F01_transcript_1052	K00128+K11420+K14157+K11420+K11420+K14085+K00128+K14157+K11420+K11420+K00128+K00626+K14157+K00658+K00128+K00128+K11420+K00164+K00128+K00128+K00164+K14085+K14157+K11420+K14157+K00128+K00128+K00

3<br>1<br>0	13;F01_transcript_105403;F01_transcript_105636;F01_transcript_107970;F01_transcript_108221;F01_transcript_109312;F01_transcript_11133;F01_transcript_111778;F01_transcript_112685;F01_transcript_112884;F01_transcript_113031;F01_transcript_115636;F01_transcript_115939;F01_transcript_116797;F01_transcript_11680;F01_transcript_118736;F01_transcript_118848;F01_transcript_119343;F01_transcript_121819;F01_transcript_122383;F01_transcript_1231;F01_transcript_12576;F01_transcript_12620;F01_transcript_126937;F01_transcript_127146;F01_transcript_127326;F01_transcript_128218;F01_transcript_128872;F01_transcript_129269;F01_transcript_12990;F01_transcript_130234;F01_transcript_13059;F01_transcript_131432;F01_transcript_132494;F01_transcript_132504;F01_transcript_132629;F01_transcript_132930;F01_transcript_1334;F01_transcript_134251;F01_transcript_134507;F01_transcript_135959;F01_transcript_136337;F01_transcript_136766;F01_transcript_136841;F01_transcript_137525;F01_transcript_137935;F01_transcript_138093;F01_transcript_138114;F01_transcript_138698;F01_transcript_138897;F01_transcript_138921;F01_transcript_139567;F01_transcript_139760;F01_transcript_142221;F01_transcript_144636;F01_transcript_144850;F01_transcript_145352;F01_transcript_145474;F01_transcript_146046;F01_transcript_147257;F01_transcript_147783;F01_transcript_148990;F01_transcript_149233;F01_transcript_149292;F01_transcript_151264;F01_transcript_151832;F01_transcript_151939;F01_transcript_152756;F01_transcript_152958;F01_transcript_153078;F01_transcript_154982;F01_transcript_155017;F01_transcript_155685;F01_transcript_1562;F01_transcript_157185;F01_transcript_158224;F01_transcript_158912;F01_transcript_159514;F01_transcript_159814;F01_transcript_161590;F01_transcript_161595;F01_transcript_161816;F01_transcript_161966;F01_transcript_162134;F01_transcript_162331;F01_transcript_163191;F01_transcript_165256;F01_transcript_16537;F01_transcript_166964;F01_transcript_167102;F01_transcript_167272;F01_transcript_1799;F01_transcript_19412;F01_transcript_22272;F01_transcript_2253;F01_transcript_23111;F01_transcript_23471;F01_transcript_23785;F01_transcript_24020;F01_transcript_24677;F01_transcript_25659;F01_transcript_26120;F0	128+K00128+K11420+K00128+K11420+K11420+K14157+K00128+K00128+K00128+K00128+K11420+K11420+K11420+K14157+K00658+K00128+K00658+K11420+K00128+K00128+K00128+K00128+K14157+K14157+K11420+K11420+K00128+K00128+K00128+K00626+K11420+K00128+K00128+K00128+K11420+K11420+K00128+K00128+K00128+K00128+K00128+K00128+K00128+K11420+K00658+K00128+K00128+K00128+K00128+K00128+K00128+K00306+K00128+K14157+K00626+K00164+K11420+K14157+K00128+K14085+K00128+K14157+K14157+K00128+K00128+K00128+K00128+K00164+K00306+K11423+K00626+K11420+K11420+K00128+K00128+K14157+K11420+K14157+K14157+K00128+K00128+K00128+K00128+K00128+K00164+K00128+K00128+K00128+K00128+K14085+K14085+K00128+K00128+K00658+K00658+K00128+K00128+K00128+K00128+K00658+K00128+K00128+K00128+K11423+K00128+K00128+K00626+K00626+K00128+K00128+K00128+K00626+K00128+K00626+K00306+K00128+K11420+K00128+K11420+K00164+K11422+K11420+K00626+K00658+K11420+K00128+K00128+K14085+K00164+K00626+K00658+K00658+K11423+K14085+K00128+K00128+K00128+K11422+K00128+K00128+K11420+K00626+K00128+K14157+K00128+K00128+K00128+K11423+K00128+K11420+K00658+K00128+K00626+K14085+K11420+K00164+K00626+K11420+K00128+K00658+K00128+K00626+K00128+K00626+K11420+K11420+K14085+K11420+K11420+K11420+K11420+K11420+K11420+K00128+K11420+K00128+K00128+K00626+K00128+K11420+K00128+K11420+K14157+K00128

			1_transcript_2693;F01_transcript_27376;F01_transcript_28572;F01_transcript_28856;F01_transcript_29858;F01_transcript_30292;F01_transcript_30362;F01_transcript_30379;F01_transcript_30935;F01_transcript_31205;F01_transcript_31297;F01_transcript_31894;F01_transcript_32017;F01_transcript_32266;F01_transcript_33325;F01_transcript_33503;F01_transcript_33600;F01_transcript_34271;F01_transcript_35286;F01_transcript_36058;F01_transcript_36697;F01_transcript_36801;F01_transcript_37377;F01_transcript_37686;F01_transcript_37696;F01_transcript_37926;F01_transcript_37987;F01_transcript_38302;F01_transcript_38323;F01_transcript_38657;F01_transcript_39050;F01_transcript_41704;F01_transcript_4349;F01_transcript_48424;F01_transcript_51831;F01_transcript_52137;F01_transcript_52213;F01_transcript_52304;F01_transcript_52332;F01_transcript_53752;F01_transcript_53970;F01_transcript_55541;F01_transcript_56137;F01_transcript_56690;F01_transcript_57448;F01_transcript_58368;F01_transcript_61072;F01_transcript_61978;F01_transcript_63402;F01_transcript_63491;F01_transcript_65034;F01_transcript_65403;F01_transcript_65979;F01_transcript_66044;F01_transcript_66261;F01_transcript_67211;F01_transcript_6864;F01_transcript_69739;F01_transcript_70471;F01_transcript_70637;F01_transcript_71260;F01_transcript_71646;F01_transcript_72349;F01_transcript_72547;F01_transcript_73295;F01_transcript_73486;F01_transcript_74262;F01_transcript_75083;F01_transcript_77451;F01_transcript_77976;F01_transcript_78272;F01_transcript_78355;F01_transcript_79052;F01_transcript_79499;F01_transcript_79852;F01_transcript_80046;F01_transcript_80510;F01_transcript_80843;F01_transcript_81584;F01_transcript_84463;F01_transcript_85418;F01_transcript_87477;F01_transcript_8790;F01_transcript_88746;F01_transcript_89204;F01_transcript_89608;F01_transcript_9031;F01_transcript_9122;F01_transcript_9214;F01_transcript_92804;F01_transcript_95439;F01_transcript_96227;F01_transcript_97230;F01_transcript_97808;F01_transcript_98282;F01_transcript_98368;F01_transcript_98445;F01_transcript_99155;F01_transcript_99364;F01_transcript_99528;	
Arginine and	ko0	298	F01_transcript_100740;F01_transcript_101473;F01_transcript_103590;F01_transcript_103691;F01_transcript_103925;F01_transcript_103963;F01_transcript_104142;F01_transcript_105213;F0	K00128+K14454+K01426+K14085+K00128+K10536+K01611+K00128+K01426+K01476+K14454+K00797+K01611+K00318+K00797+K00811+K00318+K00128+K00128+K00797+K126

proline metabolism	0330	1_transcript_105220;F01_transcript_105845;F01_transcript_106156;F01_transcript_106222;F01_transcript_106243;F01_transcript_106816;F01_transcript_107012;F01_transcript_107151;F01_transcript_107226;F01_transcript_108221;F01_transcript_109312;F01_transcript_112262;F01_transcript_112405;F01_transcript_112685;F01_transcript_112884;F01_transcript_114452;F01_transcript_114572;F01_transcript_115397;F01_transcript_115636;F01_transcript_116470;F01_transcript_116989;F01_transcript_117360;F01_transcript_117943;F01_transcript_118580;F01_transcript_118736;F01_transcript_118810;F01_transcript_118848;F01_transcript_119343;F01_transcript_119869;F01_transcript_120261;F01_transcript_121186;F01_transcript_121819;F01_transcript_121908;F01_transcript_122275;F01_transcript_1231;F01_transcript_126185;F01_transcript_126818;F01_transcript_127146;F01_transcript_127326;F01_transcript_128143;F01_transcript_128218;F01_transcript_128872;F01_transcript_129050;F01_transcript_130287;F01_transcript_131043;F01_transcript_131162;F01_transcript_131181;F01_transcript_131204;F01_transcript_132426;F01_transcript_132494;F01_transcript_132867;F01_transcript_132930;F01_transcript_1334;F01_transcript_134044;F01_transcript_134132;F01_transcript_134251;F01_transcript_134507;F01_transcript_135578;F01_transcript_136180;F01_transcript_136206;F01_transcript_137525;F01_transcript_137935;F01_transcript_138093;F01_transcript_138897;F01_transcript_138913;F01_transcript_138921;F01_transcript_139567;F01_transcript_140464;F01_transcript_14107;F01_transcript_141828;F01_transcript_142320;F01_transcript_144636;F01_transcript_144685;F01_transcript_144850;F01_transcript_145352;F01_transcript_145474;F01_transcript_146046;F01_transcript_146192;F01_transcript_147257;F01_transcript_147521;F01_transcript_147783;F01_transcript_148402;F01_transcript_149292;F01_transcript_149388;F01_transcript_150639;F01_transcript_151102;F01_transcript_151264;F01_transcript_151832;F01_transcript_151939;F01_transcript_15205;F01_transcript_152726;F01_transcript_152756;F01_transcript_152958;F01_transcript_154937;F01_transcript_154982;F01_transcript_155215;F01_transcript_155521;F01_transcript_156344;F01_transcript_157007;F01_transcript_158912;F01_tra	57+K00128+K00128+K14455+K01583+K01611+K14085+K12657+K00472+K17839+K01611+K00797+K00128+K00819+K00128+K00128+K14455+K00294+K01583+K00128+K00797+K01583+K00128+K14455+K01611+K00128+K00128+K00797+K00128+K00128+K00472+K01426+K14455+K01583+K00819+K01259+K12657+K00128+K00797+K00128+K00128+K01426+K00819+K00128+K00128+K14454+K01426+K00318+K00128+K00128+K00128+K00128+K01611+K00128+K00128+K01611+K12657+K01426+K00797+K00128+K01426+K00128+K00128+K00128+K00128+K00472+K00128+K00797+K00128+K12259+K00128+K00318+K14454+K00797+K00128+K00128+K00128+K12657+K14454+K00128+K00128+K01426+K00128+K00472+K01611+K14455+K00797+K00128+K14085+K00128+K00811+K00294+K00128+K00128+K10536+K01426+K00128+K00128+K01426+K00819+K01583+K10536+K01426+K00128+K17839+K01426+K00318+K00318+K00128+K00128+K00128+K00128+K00797+K00128+K01611+K00128+K00472+K01611+K17839+K01426+K00128+K12259+K01426+K00294+K14454+K01611+K00128+K00128+K12251+K00128+K14085+K14085+K00128+K14454+K01611+K00128+K01426+K01426+K13427+K01611+K00811+K00128+K00819+K00128+K00128+K00819+K14455+K00128+K00318+K00128+K01611+K00128+K01611+K00318+K00797+K01426+K00128+K01426+K01426+K14455+K14455+K00128+K00128+K01611+K00128+K10536+K00797+K00472+K00128+K01259+K00128+K10536+K00472+K00128+K00797+K12251+K00472+K00128+K10536+K00286+K12251+K10536+K00128+K01426+K00797+K00472+K00472+K01426+K01583+K01611+K01583+K00128+K00819+K00128+K01426+K01611+K14085+K00286+K01583+K12251+K00472+K12251+K00797+K00472+K14085+K00472+K00797+K00128+K00128+K01583+K14454+K00128+K00128+K00128+K01426+K00128+K01426+K01426+K00128+K01611+K00128+K00128+K00128+K00128+K01611+K01426+K01583+K10536+K01611+K14085+K01611+K14455+K00811+K00472+K00286+K12657+K00472+K00128+K00128+K00811+K14454+K00128+K14454+K00472+K00819+K12657+K12251+K00472+K00797+K01426+K00472+K00472+K01426+K01611+K0

	nscript_159514;F01_transcript_159814;F01_transcript_160500;F01_transcript_160783;F01_transcript_161816;F01_transcript_161966;F01_transcript_162031;F01_transcript_162077;F01_transcript_162134;F01_transcript_162331;F01_transcript_164449;F01_transcript_165762;F01_transcript_165942;F01_transcript_166782;F01_transcript_17106;F01_transcript_1799;F01_transcript_18524;F01_transcript_18644;F01_transcript_18708;F01_transcript_19336;F01_transcript_19412;F01_transcript_23785;F01_transcript_24020;F01_transcript_24677;F01_transcript_24957;F01_transcript_25659;F01_transcript_26011;F01_transcript_26120;F01_transcript_2624;F01_transcript_27043;F01_transcript_27158;F01_transcript_27335;F01_transcript_27376;F01_transcript_27498;F01_transcript_27851;F01_transcript_28205;F01_transcript_28405;F01_transcript_28426;F01_transcript_28572;F01_transcript_28856;F01_transcript_29122;F01_transcript_29858;F01_transcript_30292;F01_transcript_30362;F01_transcript_30379;F01_transcript_30736;F01_transcript_30842;F01_transcript_30935;F01_transcript_30994;F01_transcript_31171;F01_transcript_31330;F01_transcript_31534;F01_transcript_31784;F01_transcript_31894;F01_transcript_31930;F01_transcript_32017;F01_transcript_32266;F01_transcript_32612;F01_transcript_32622;F01_transcript_33325;F01_transcript_33326;F01_transcript_33600;F01_transcript_33947;F01_transcript_34271;F01_transcript_34634;F01_transcript_34924;F01_transcript_35071;F01_transcript_35213;F01_transcript_35286;F01_transcript_35356;F01_transcript_35724;F01_transcript_36375;F01_transcript_36639;F01_transcript_36697;F01_transcript_36801;F01_transcript_37030;F01_transcript_37696;F01_transcript_37712;F01_transcript_37802;F01_transcript_37918;F01_transcript_37926;F01_transcript_37961;F01_transcript_37987;F01_transcript_38230;F01_transcript_38279;F01_transcript_38323;F01_transcript_39898;F01_transcript_40044;F01_transcript_41253;F01_transcript_41704;F01_transcript_42413;F01_transcript_43837;F01_transcript_44325;F01_transcript_44391;F01_transcript_48424;F01_transcript_51652;F01_transcript_51841;F01_transcript_52556;F01_transcript_52593;F01_transcript_52764;F01_transcript_53243;F01_transcript_54080;F01_transcript_55223;F01_transcript_55541;F01_transcript_56065;F01_transcript_56137;F01_transcript_56344;F01_transcript_566	0811+K14455+K14085+K14454+K01583+K01426+K01611+K00472+K01426+K00128+K14455+K00472+K00318+K00819+K00472+K00294+K00128+K17839+K00128+K14454+K00128+K00128+K00318+K00128

			72;F01_transcript_56690;F01_transcript_58355;F01_transcript_5874;F01_transcript_59317;F01_transcript_60505;F01_transcript_61726;F01_transcript_62377;F01_transcript_62641;F01_transcript_63491;F01_transcript_63698;F01_transcript_63834;F01_transcript_65034;F01_transcript_65403;F01_transcript_6561;F01_transcript_65858;F01_transcript_65979;F01_transcript_66261;F01_transcript_67211;F01_transcript_69560;F01_transcript_70471;F01_transcript_70746;F01_transcript_71207;F01_transcript_71260;F01_transcript_71519;F01_transcript_71646;F01_transcript_72349;F01_transcript_73295;F01_transcript_75083;F01_transcript_75621;F01_transcript_76063;F01_transcript_76162;F01_transcript_76620;F01_transcript_77184;F01_transcript_77976;F01_transcript_78418;F01_transcript_78720;F01_transcript_78919;F01_transcript_78930;F01_transcript_79045;F01_transcript_79562;F01_transcript_79656;F01_transcript_79852;F01_transcript_80510;F01_transcript_80769;F01_transcript_81078;F01_transcript_81584;F01_transcript_81767;F01_transcript_82549;F01_transcript_82689;F01_transcript_83381;F01_transcript_83609;F01_transcript_84005;F01_transcript_84285;F01_transcript_84583;F01_transcript_84607;F01_transcript_84862;F01_transcript_85460;F01_transcript_86863;F01_transcript_86970;F01_transcript_87504;F01_transcript_8790;F01_transcript_89140;F01_transcript_89512;F01_transcript_89614;F01_transcript_90343;F01_transcript_91503;F01_transcript_92454;F01_transcript_92804;F01_transcript_93052;F01_transcript_93657;F01_transcript_93763;F01_transcript_94109;F01_transcript_94906;F01_transcript_95090;F01_transcript_96227;F01_transcript_97151;F01_transcript_97230;F01_transcript_9785;F01_transcript_98282;F01_transcript_98445;F01_transcript_99285;F01_transcript_99528;	
Histidine metabolism	ko00340	162	F01_transcript_100740;F01_transcript_103691;F01_transcript_103925;F01_transcript_105011;F01_transcript_105213;F01_transcript_107871;F01_transcript_108221;F01_transcript_109116;F01_transcript_109312;F01_transcript_110772;F01_transcript_112685;F01_transcript_112884;F01_transcript_114312;F01_transcript_115636;F01_transcript_117037;F01_transcript_117335;F01_transcript_118736;F01_transcript_118848;F01_transcript_119343;F01_transcript_121819;F01_transcript_122487;F01_transcript	K00128+K14085+K00128+K01590+K00128+K01693+K00128+K01814+K00128+K00013+K00128+K00128+K11755+K14085+K00013+K01693+K00128+K00128+K00128+K00128+K01814+K00128+K01590+K01814+K00128+K11755+K00128+K00128+K00128+K01693+K18649+K00817+K00128+K00128+K00128+K00128+K00128+K00013+K00817+K01814+K00128+K00128+K00128+K00128+K00128+K00128+K00128+K01590+K00128+K00128+K00128+K00013+K00128+K00128+K01663

	_1231;F01_transcript_125724;F01_transcript_126762;F01_transcript_127146;F01_transcript_127322;F01_transcript_127326;F01_transcript_128218;F01_transcript_128872;F01_transcript_132024;F01_transcript_132042;F01_transcript_132374;F01_transcript_132494;F01_transcript_132930;F01_transcript_1334;F01_transcript_134251;F01_transcript_134507;F01_transcript_135870;F01_transcript_136643;F01_transcript_136970;F01_transcript_137525;F01_transcript_137935;F01_transcript_138093;F01_transcript_138897;F01_transcript_138921;F01_transcript_139567;F01_transcript_144636;F01_transcript_144807;F01_transcript_144850;F01_transcript_145352;F01_transcript_145474;F01_transcript_145918;F01_transcript_146046;F01_transcript_147257;F01_transcript_147523;F01_transcript_147783;F01_transcript_149292;F01_transcript_151264;F01_transcript_151832;F01_transcript_151939;F01_transcript_152756;F01_transcript_152958;F01_transcript_154982;F01_transcript_158172;F01_transcript_158912;F01_transcript_159514;F01_transcript_159814;F01_transcript_160884;F01_transcript_161816;F01_transcript_161966;F01_transcript_162134;F01_transcript_162331;F01_transcript_162654;F01_transcript_163671;F01_transcript_163792;F01_transcript_165377;F01_transcript_165543;F01_transcript_17452;F01_transcript_1799;F01_transcript_19412;F01_transcript_23785;F01_transcript_24020;F01_transcript_24062;F01_transcript_24677;F01_transcript_25334;F01_transcript_25659;F01_transcript_26120;F01_transcript_26805;F01_transcript_27376;F01_transcript_28572;F01_transcript_28856;F01_transcript_29858;F01_transcript_30292;F01_transcript_30362;F01_transcript_30379;F01_transcript_30935;F01_transcript_31894;F01_transcript_32017;F01_transcript_32266;F01_transcript_32881;F01_transcript_33325;F01_transcript_33600;F01_transcript_33879;F01_transcript_34271;F01_transcript_35210;F01_transcript_35286;F01_transcript_35646;F01_transcript_36103;F01_transcript_36697;F01_transcript_36801;F01_transcript_37696;F01_transcript_37926;F01_transcript_37987;F01_transcript_38323;F01_transcript_41421;F01_transcript_41704;F01_transcript_41924;F01_transcript_4472;F01_transcript_48424;F01_transcript_55541;F01_transcript_56137;F01_transcript_56690;F01_transcript_63328;F01_transcript_63491;F01_transcript_65034;F01_transcript_65403;F01_transcript_65979;F0	+K00128+K00128+K00128+K00128+K00128+K00128+K00128+K00128+K00765+K00128+K14085+K00128+K11755+K00128+K00128+K00128+K00128+K11755+K11755+K11755+K00765+K00765+K01693+K00128+K00128+K00128+K00128+K01663+K00128+K00013+K00128+K00128+K01663+K00128+K00128+K00128+K00128+K14085+K14085+K00128+K00128+K00128+K00128+K00128+K00013+K00128+K00128+K00817+K00128+K00013+K00128+K00765+K00765+K00128+K00128+K00128+K00128+K00128+K00128+K01693+K00128+K01693+K00765+K00128+K00128+K00128+K14085+K01663+K14085+K00128+K00128+K00128+K00128+K00128+K00765+K11755+K00128+K00128+K11755+K00128+K11755+K00128+K00128+K00128+K11755+K01590+K01590+K14085+K11755+K00128+K00765+K00128+K00128+K01693+K01663+K01590+K14085+K00817+K01590+K00128+K00128+K01693+K11755+K00128+K00128+K00128+K00128

			1_transcript_66261;F01_transcript_67211;F01_transcript_67540;F01_transcript_69411;F01_transcript_70471;F01_transcript_71260;F01_transcript_71361;F01_transcript_71646;F01_transcript_72176;F01_transcript_72349;F01_transcript_73295;F01_transcript_75083;F01_transcript_75870;F01_transcript_75903;F01_transcript_76531;F01_transcript_77976;F01_transcript_79320;F01_transcript_79852;F01_transcript_80225;F01_transcript_80510;F01_transcript_81584;F01_transcript_83984;F01_transcript_85872;F01_transcript_86294;F01_transcript_8790;F01_transcript_87962;F01_transcript_91897;F01_transcript_92804;F01_transcript_96227;F01_transcript_96296;F01_transcript_96799;F01_transcript_97230;F01_transcript_98282;F01_transcript_98445;F01_transcript_99528;	
Tyrosine metabolism	ko00350	195	F01_transcript_100618;F01_transcript_101473;F01_transcript_101567;F01_transcript_101871;F01_transcript_102388;F01_transcript_102523;F01_transcript_104489;F01_transcript_105405;F01_transcript_106156;F01_transcript_106666;F01_transcript_107151;F01_transcript_107764;F01_transcript_109059;F01_transcript_10939;F01_transcript_112411;F01_transcript_113517;F01_transcript_114452;F01_transcript_119806;F01_transcript_119869;F01_transcript_12072;F01_transcript_121145;F01_transcript_122119;F01_transcript_122972;F01_transcript_123492;F01_transcript_123595;F01_transcript_12429;F01_transcript_124326;F01_transcript_124604;F01_transcript_125225;F01_transcript_126185;F01_transcript_126836;F01_transcript_127780;F01_transcript_130313;F01_transcript_130548;F01_transcript_131043;F01_transcript_132157;F01_transcript_132374;F01_transcript_132512;F01_transcript_133648;F01_transcript_13535;F01_transcript_135578;F01_transcript_135802;F01_transcript_135806;F01_transcript_136316;F01_transcript_136325;F01_transcript_136534;F01_transcript_136643;F01_transcript_136852;F01_transcript_137874;F01_transcript_138249;F01_transcript_139169;F01_transcript_139669;F01_transcript_140406;F01_transcript_140632;F01_transcript_142036;F01_transcript_142762;F01_transcript_14328;F01_transcript_143473;F01_transcript_144749;F01_transcript_146237;F01_transcript_146924;F01_transcript_147552;F01_transcript_148409;F01_transcript_150132;F01_transcript_150639;F0	K00815+K14454+K00276+K00121+K01592+K18857+K00121+K00276+K14454+K00815+K00811+K00276+K00451+K07253+K00457+K01592+K14455+K07253+K14455+K00276+K00121+K00815+K18857+K00422+K07253+K07253+K00457+K00276+K01592+K14455+K00001+K01592+K00276+K01592+K14455+K00815+K00817+K00276+K00815+K00276+K14454+K01800+K00815+K15849+K00121+K00815+K00817+K00121+K01800+K07253+K00121+K00422+K00276+K00276+K00121+K00422+K00815+K07253+K00815+K00121+K00815+K00276+K00276+K00815+K14454+K14454+K00457+K01800+K00276+K14455+K00121+K00276+K00457+K00276+K00811+K00815+K18857+K00815+K01592+K00001+K00422+K00276+K00121+K00276+K18857+K00276+K15849+K00121+K00276+K00815+K00276+K00276+K00276+K01592+K00422+K14454+K00422+K00422+K00457+K00422+K14454+K00815+K00451+K15849+K01592+K00815+K00811+K01592+K00451+K15849+K00815+K14455+K00001+K00817+K00001+K18857+K07253+K00457+K14455+K00121+K14455+K00815+K00121+K00121+K18606+K18606+K00121+K18857+K00121+K18606+K00451+K00815+K01800+K00451+K01800+K01800+K07253+K00121+K00451+K00276+K00121+K00276+K07253+K00121+K00121+K00276+K07253+K00276+K00276+K18606+K00276+K01557+K00815+K14454+K00276+K01592+K00457+K01592+K00422+K00276+K18606+K00815+K01800+K14455+K008

	1_transcript_152726;F01_transcript_154276;F01_transcript_154299;F01_transcript_154647;F01_transcript_156344;F01_transcript_158132;F01_transcript_158722;F01_transcript_158999;F01_transcript_159476;F01_transcript_160500;F01_transcript_161264;F01_transcript_161429;F01_transcript_161557;F01_transcript_161741;F01_transcript_162066;F01_transcript_163139;F01_transcript_163899;F01_transcript_164021;F01_transcript_16418;F01_transcript_164766;F01_transcript_16644;F01_transcript_166505;F01_transcript_17183;F01_transcript_18563;F01_transcript_19390;F01_transcript_2067;F01_transcript_22000;F01_transcript_24430;F01_transcript_24431;F01_transcript_28048;F01_transcript_28405;F01_transcript_28975;F01_transcript_29563;F01_transcript_29880;F01_transcript_30484;F01_transcript_30736;F01_transcript_31061;F01_transcript_31193;F01_transcript_31276;F01_transcript_31346;F01_transcript_31700;F01_transcript_31784;F01_transcript_31814;F01_transcript_31833;F01_transcript_31834;F01_transcript_32147;F01_transcript_32622;F01_transcript_32640;F01_transcript_33879;F01_transcript_34354;F01_transcript_34541;F01_transcript_34850;F01_transcript_35520;F01_transcript_36375;F01_transcript_36465;F01_transcript_36639;F01_transcript_37457;F01_transcript_37617;F01_transcript_38373;F01_transcript_38995;F01_transcript_39596;F01_transcript_39701;F01_transcript_39959;F01_transcript_40578;F01_transcript_40724;F01_transcript_41689;F01_transcript_42165;F01_transcript_43911;F01_transcript_44798;F01_transcript_45075;F01_transcript_46787;F01_transcript_47177;F01_transcript_48006;F01_transcript_50365;F01_transcript_52920;F01_transcript_55528;F01_transcript_56629;F01_transcript_58841;F01_transcript_59371;F01_transcript_59821;F01_transcript_60104;F01_transcript_61042;F01_transcript_61675;F01_transcript_61722;F01_transcript_63163;F01_transcript_63755;F01_transcript_64362;F01_transcript_65744;F01_transcript_65858;F01_transcript_66411;F01_transcript_67201;F01_transcript_70885;F01_transcript_72857;F01_transcript_73675;F01_transcript_74237;F01_transcript_75254;F01_transcript_76908;F01_transcript_77071;F01_transcript_78720;F01_transcript_78919;F01_transcript_80594;F01_transcript_80637;F01_transcript_80769;F01_transcript_80944;F01_transcript_81078;F01_transcript_81767;F01_transcript_8293;F01_tr	11+K00121+K00451+K00811+K01592+K14454+K14454+K00276+K18857+K01800+K00811+K00422+K14455+K00817+K15849+K01592+K00121+K14454+K07253+K00276+K00815+K00121+K00001+K00276+K00121+K00451+K00276+K14455+K00276+K14454+K00276

			anscript_84296;F01_transcript_85991;F01_transcript_86970;F01_transcript_87463;F01_transcript_87504;F01_transcript_87962;F01_transcript_88383;F01_transcript_88838;F01_transcript_88860;F01_transcript_89140;F01_transcript_89518;F01_transcript_90314;F01_transcript_90364;F01_transcript_91037;F01_transcript_91452;F01_transcript_91560;F01_transcript_91795;F01_transcript_92290;F01_transcript_9243;F01_transcript_93052;F01_transcript_95934;F01_transcript_9785;F01_transcript_98894;	
Phenylalanine metabolism	ko00360	204	F01_transcript_100509;F01_transcript_100618;F01_transcript_100818;F01_transcript_101473;F01_transcript_101567;F01_transcript_103590;F01_transcript_105220;F01_transcript_105405;F01_transcript_106156;F01_transcript_106666;F01_transcript_107151;F01_transcript_107764;F01_transcript_10939;F01_transcript_109502;F01_transcript_110418;F01_transcript_112190;F01_transcript_112411;F01_transcript_114433;F01_transcript_114452;F01_transcript_116533;F01_transcript_119806;F01_transcript_119869;F01_transcript_120452;F01_transcript_120561;F01_transcript_12072;F01_transcript_122119;F01_transcript_123595;F01_transcript_12429;F01_transcript_124326;F01_transcript_124604;F01_transcript_125968;F01_transcript_126185;F01_transcript_12967;F01_transcript_129687;F01_transcript_129894;F01_transcript_130287;F01_transcript_130313;F01_transcript_131043;F01_transcript_131515;F01_transcript_132157;F01_transcript_132374;F01_transcript_132512;F01_transcript_133094;F01_transcript_133648;F01_transcript_134044;F01_transcript_13535;F01_transcript_135578;F01_transcript_135806;F01_transcript_136180;F01_transcript_136316;F01_transcript_13645;F01_transcript_136534;F01_transcript_136643;F01_transcript_137702;F01_transcript_138249;F01_transcript_140406;F01_transcript_140632;F01_transcript_141828;F01_transcript_143278;F01_transcript_14328;F01_transcript_143473;F01_transcript_144685;F01_transcript_144749;F01_transcript_146924;F01_transcript_147552;F01_transcript_147651;F01_transcript_148330;F01_transcript_148409;F01_transcript_150132;F01_transcript_150639;F01_transcript_15146;F01_transcript_152726;F01_transcript_153198;F01_transcript_15388;F01_transcript_154276;F01_transcript_154647;F01_transcript_154937;F01_transcript_155813;F01_transcript_15	K00588+K00815+K00487+K14454+K00276+K01426+K01426+K00276+K14454+K00815+K00811+K00276+K07253+K00588+K10775+K10775+K00457+K00487+K14455+K10775+K07253+K14455+K00588+K00588+K00276+K00815+K07253+K07253+K00457+K00276+K10775+K14455+K10775+K10775+K00074+K01426+K00276+K14455+K01904+K00815+K00817+K00276+K00487+K00815+K01426+K00276+K14454+K00815+K01426+K15849+K10775+K00815+K00817+K01904+K07253+K00276+K00276+K01426+K01904+K00815+K07253+K01426+K00815+K00815+K00276+K01904+K00487+K00276+K00815+K14454+K10775+K14454+K10775+K10775+K00457+K00276+K01426+K00487+K14455+K00276+K00457+K00276+K00811+K00815+K00815+K01426+K00276+K00276+K10775+K01426+K00276+K15849+K01426+K00276+K01426+K10775+K00815+K00276+K00276+K00276+K10775+K01426+K01426+K01904+K14454+K01904+K00457+K01904+K14454+K00487+K01426+K00815+K01426+K15849+K00815+K00811+K15849+K00815+K00487+K14455+K00817+K07253+K01426+K01426+K00457+K01426+K14455+K14455+K00588+K00815+K18606+K18606+K00074+K18606+K00815+K00074+K00588+K07253+K01426+K01426+K00276+K10775+K01426+K00276+K07253+K00588+K00276+K10775+K07253+K00276+K00276+K18606+K00276+K01904+K10775+K00815+K00588+K14454+K00276+K01904+K10775+K00487+K10775+K10775+K01426+K01426+K00588+K00457+K01426+K10775+K00276+K18606+K00074+K01426+K00815+K00588+K14455+K00811+K00074+K00811+K14454+K14454+K00276+K01904+K01426+K01426+K01904+K00811+K14455+K00817+K15849+K14454+

	6344;F01_transcript_158722;F01_transcript_158999;F01_transcript_159476;F01_transcript_160500;F01_transcript_161264;F01_transcript_161557;F01_transcript_162077;F01_transcript_163899;F01_transcript_16418;F01_transcript_164372;F01_transcript_164449;F01_transcript_16644;F01_transcript_166505;F01_transcript_17106;F01_transcript_18563;F01_transcript_18644;F01_transcript_18993;F01_transcript_19390;F01_transcript_2067;F01_transcript_22000;F01_transcript_24430;F01_transcript_26246;F01_transcript_27335;F01_transcript_27851;F01_transcript_28040;F01_transcript_28405;F01_transcript_29461;F01_transcript_29880;F01_transcript_29890;F01_transcript_30736;F01_transcript_30745;F01_transcript_30994;F01_transcript_31061;F01_transcript_31171;F01_transcript_31276;F01_transcript_31700;F01_transcript_31784;F01_transcript_31834;F01_transcript_32147;F01_transcript_32473;F01_transcript_32622;F01_transcript_33879;F01_transcript_34850;F01_transcript_35213;F01_transcript_35356;F01_transcript_35520;F01_transcript_35724;F01_transcript_36375;F01_transcript_36639;F01_transcript_36939;F01_transcript_37457;F01_transcript_38995;F01_transcript_39596;F01_transcript_40530;F01_transcript_40724;F01_transcript_42165;F01_transcript_42881;F01_transcript_43609;F01_transcript_47177;F01_transcript_51652;F01_transcript_52764;F01_transcript_52920;F01_transcript_55819;F01_transcript_56344;F01_transcript_56629;F01_transcript_58841;F01_transcript_59357;F01_transcript_60104;F01_transcript_60998;F01_transcript_61042;F01_transcript_61675;F01_transcript_61722;F01_transcript_63163;F01_transcript_63755;F01_transcript_64346;F01_transcript_64918;F01_transcript_65744;F01_transcript_65829;F01_transcript_65858;F01_transcript_66411;F01_transcript_66441;F01_transcript_66825;F01_transcript_68082;F01_transcript_68591;F01_transcript_69425;F01_transcript_69560;F01_transcript_70746;F01_transcript_70761;F01_transcript_70885;F01_transcript_71207;F01_transcript_72984;F01_transcript_74237;F01_transcript_75254;F01_transcript_75779;F01_transcript_76063;F01_transcript_76908;F01_transcript_78475;F01_transcript_78720;F01_transcript_78919;F01_transcript_79587;F01_transcript_80769;F01_transcript_81078;F01_transcript_81767;F01_transcript_8293;F01_transcript_84574;F01_transcript_84583;F01_transcript_85460;F01_trans	K07253+K01426+K00276+K01904+K00815+K00276+K00276+K01426+K14455+K00276+K14454+K00276

			cript_86407;F01_transcript_86970;F01_transcript_87504;F01_transcript_87962;F01_transcript_88383;F01_transcript_89140;F01_transcript_89518;F01_transcript_89614;F01_transcript_90314;F01_transcript_90345;F01_transcript_90364;F01_transcript_91560;F01_transcript_9243;F01_transcript_92454;F01_transcript_93052;F01_transcript_95934;F01_transcript_9785;F01_transcript_98894;	
Tryptophan metabolism	ko00380	293	F01_transcript_10021;F01_transcript_100740;F01_transcript_100780;F01_transcript_103590;F01_transcript_103691;F01_transcript_103925;F01_transcript_105213;F01_transcript_105220;F01_transcript_105403;F01_transcript_107228;F01_transcript_107966;F01_transcript_108186;F01_transcript_108221;F01_transcript_109312;F01_transcript_110588;F01_transcript_111760;F01_transcript_111778;F01_transcript_112685;F01_transcript_112884;F01_transcript_113031;F01_transcript_11473;F01_transcript_114737;F01_transcript_115636;F01_transcript_115917;F01_transcript_116320;F01_transcript_118736;F01_transcript_118848;F01_transcript_119285;F01_transcript_119343;F01_transcript_121105;F01_transcript_121247;F01_transcript_121819;F01_transcript_121960;F01_transcript_1231;F01_transcript_123251;F01_transcript_12336;F01_transcript_124404;F01_transcript_124803;F01_transcript_127146;F01_transcript_127239;F01_transcript_127326;F01_transcript_127607;F01_transcript_127884;F01_transcript_128218;F01_transcript_128872;F01_transcript_130287;F01_transcript_131015;F01_transcript_131343;F01_transcript_131925;F01_transcript_132298;F01_transcript_132494;F01_transcript_132822;F01_transcript_132930;F01_transcript_1334;F01_transcript_134044;F01_transcript_134251;F01_transcript_134507;F01_transcript_136180;F01_transcript_136746;F01_transcript_137525;F01_transcript_137935;F01_transcript_138093;F01_transcript_138114;F01_transcript_138897;F01_transcript_138921;F01_transcript_139567;F01_transcript_140205;F01_transcript_14076;F01_transcript_140967;F01_transcript_141828;F01_transcript_141996;F01_transcript_144230;F01_transcript_144636;F01_transcript_144685;F01_transcript_144850;F01_transcript_145352;F01_transcript_145474;F01_transcript_146046;F01_transcript_147257;F01_transcript_147432;F01_transcript_147783;F01_trans	K03781+K00128+K11816+K01426+K14085+K00128+K00128+K01426+K00626+K03781+K03781+K03781+K00128+K00128+K11816+K03781+K00164+K00128+K00128+K00164+K03781+K03781+K14085+K03781+K03781+K00128+K00128+K03781+K00128+K03781+K16903+K00128+K03781+K00128+K03781+K03781+K03781+K11816+K00128+K03781+K00128+K03781+K03781+K00128+K00128+K01426+K03781+K03781+K03781+K03781+K00128+K11816+K00128+K00128+K01426+K00128+K00128+K01426+K03781+K00128+K00128+K00128+K00626+K00128+K00128+K00128+K03781+K03781+K03781+K01426+K03781+K03781+K00128+K01426+K00128+K00128+K00128+K00128+K00128+K03781+K00128+K00128+K11816+K00128+K00128+K00128+K03781+K03781+K00128+K00128+K03781+K03781+K03781+K03781+K01426+K00128+K03781+K03781+K00626+K00164+K03781+K03781+K11816+K03781+K00128+K14085+K11816+K00128+K11817+K03781+K00128+K00128+K01426+K00128+K00128+K00164+K03781+K03781+K01426+K03781+K03781+K00626+K03781+K03781+K01426+K00128+K01426+K00128+K03781+K03781+K03781+K03781+K03781+K00128+K03781+K00128+K03781+K00128+K03781+K03781+K00128+K00128+K03781+K00164+K03781+K03781+K01426+K00128+K03781+K01426+K03781+K03781+K00128+K00128+K03781+K03781+K00128+K03781+K14085+K14085+K00128+K03781+K03781+K03781+K00128+K01426+K01426+K03781+K03781+K00128+K00128+K00128+K03781+K03781+K00128+K00128+K03781+K00128+K03781+K03781+K03781+K01426+K00128+K03781+K01426+K01426+K00128+K00128+K11820+K00626+K00626+K00128+K00128+K00128+K03781+K03781+K00626+K00128+K00626+K11820+K03781+K00128+K03781+K03781+K00128+K0378

	cript_149292;F01_transcript_149654;F01_transcript_151264;F01_transcript_151832;F01_transcript_151939;F01_transcript_15195;F01_transcript_152474;F01_transcript_152756;F01_transcript_152958;F01_transcript_153615;F01_transcript_154347;F01_transcript_154524;F01_transcript_154709;F01_transcript_154937;F01_transcript_154982;F01_transcript_15505;F01_transcript_155366;F01_transcript_155685;F01_transcript_1562;F01_transcript_156564;F01_transcript_156864;F01_transcript_157287;F01_transcript_15880;F01_transcript_158912;F01_transcript_159514;F01_transcript_159542;F01_transcript_159814;F01_transcript_160549;F01_transcript_161709;F01_transcript_161816;F01_transcript_161966;F01_transcript_162077;F01_transcript_162134;F01_transcript_162331;F01_transcript_163191;F01_transcript_163559;F01_transcript_163584;F01_transcript_164449;F01_transcript_165910;F01_transcript_166597;F01_transcript_166964;F01_transcript_16722;F01_transcript_167454;F01_transcript_17106;F01_transcript_1799;F01_transcript_18644;F01_transcript_19412;F01_transcript_2179;F01_transcript_22223;F01_transcript_22876;F01_transcript_23456;F01_transcript_23618;F01_transcript_23785;F01_transcript_23952;F01_transcript_24020;F01_transcript_24090;F01_transcript_24677;F01_transcript_24832;F01_transcript_25245;F01_transcript_25659;F01_transcript_26120;F01_transcript_2689;F01_transcript_2693;F01_transcript_27222;F01_transcript_27298;F01_transcript_27335;F01_transcript_27376;F01_transcript_27585;F01_transcript_27851;F01_transcript_2805;F01_transcript_28169;F01_transcript_28572;F01_transcript_28856;F01_transcript_29197;F01_transcript_29210;F01_transcript_29858;F01_transcript_29967;F01_transcript_30292;F01_transcript_30362;F01_transcript_30379;F01_transcript_30553;F01_transcript_30696;F01_transcript_30891;F01_transcript_30935;F01_transcript_30994;F01_transcript_31171;F01_transcript_31325;F01_transcript_31749;F01_transcript_31894;F01_transcript_32017;F01_transcript_32266;F01_transcript_32306;F01_transcript_32561;F01_transcript_33325;F01_transcript_33600;F01_transcript_34196;F01_transcript_34271;F01_transcript_34440;F01_transcript_34620;F01_transcript_35037;F01_transcript_35213;F01_transcript_35286;F01_transcript_35349;F01_transcript_35356;F01_transcript_35724;F01_transcript_36697;F01_transcript_36801	1+K03781+K01426+K03781+K00164+K00626+K01426+K00128+K00128+K01426+K14085+K00164+K03781+K00626+K03781+K03781+K03781+K03781+K03781+K03781+K03781+K14085+K03781+K00128+K03781+K16903+K00128+K00128+K00128+K00128+K03781+K03781+K01426+K00626+K00128+K01426+K01426+K00128+K03781+K00128+K00128+K11816+K00128+K16903+K03781+K00128+K03781+K01426+K03781+K00626+K14085+K00164+K11816+K00626+K00128+K00128+K00626+K00128+K03781+K11816+K03781+K00626+K01426+K03781+K01426+K03781+K03781+K14085+K16903+K01426+K03781+K03781+K03781+K01426+K00128+K11816+K03781+K11816+K03781+K00128+K00128+K03781+K00626+K03781+K11820+K00128+K00128+K00128

;F01_transcript_37001;F01_transcript_37377;F01_transcript_37686;F01_transcript_37696;F01_transcript_37926;F01_transcript_37987;F01_transcript_38062;F01_transcript_3817;F01_transcript_38302;F01_transcript_38323;F01_transcript_38657;F01_transcript_39196;F01_transcript_39992;F01_transcript_41704;F01_transcript_4393;F01_transcript_47321;F01_transcript_48424;F01_transcript_50401;F01_transcript_51616;F01_transcript_51652;F01_transcript_5211;F01_transcript_52137;F01_transcript_52332;F01_transcript_52764;F01_transcript_55541;F01_transcript_56137;F01_transcript_56344;F01_transcript_56690;F01_transcript_57448;F01_transcript_58214;F01_transcript_58368;F01_transcript_59602;F01_transcript_59752;F01_transcript_59771;F01_transcript_60004;F01_transcript_60831;F01_transcript_61725;F01_transcript_62749;F01_transcript_63491;F01_transcript_63540;F01_transcript_65034;F01_transcript_65194;F01_transcript_65245;F01_transcript_65403;F01_transcript_65979;F01_transcript_66261;F01_transcript_67211;F01_transcript_67470;F01_transcript_68283;F01_transcript_69560;F01_transcript_69739;F01_transcript_70471;F01_transcript_70746;F01_transcript_71207;F01_transcript_71260;F01_transcript_71512;F01_transcript_71646;F01_transcript_72349;F01_transcript_72832;F01_transcript_73295;F01_transcript_73898;F01_transcript_74943;F01_transcript_75083;F01_transcript_75647;F01_transcript_76063;F01_transcript_77186;F01_transcript_77451;F01_transcript_77976;F01_transcript_78355;F01_transcript_78887;F01_transcript_79052;F01_transcript_79852;F01_transcript_80510;F01_transcript_80843;F01_transcript_81584;F01_transcript_81684;F01_transcript_82993;F01_transcript_84294;F01_transcript_84463;F01_transcript_84583;F01_transcript_84919;F01_transcript_85460;F01_transcript_8635;F01_transcript_87291;F01_transcript_8790;F01_transcript_88193;F01_transcript_89614;F01_transcript_90255;F01_transcript_91939;F01_transcript_92190;F01_transcript_92454;F01_transcript_92804;F01_transcript_93139;F01_transcript_93559;F01_transcript_93668;F01_transcript_9460;F01_transcript_96227;F01_transcript_97230;F01_transcript_97417;F01_transcript_97808;F01_transcript_97828;F01_transcript_98138;F01_transcript_98282;F01_transcript_98445;F01_transcript_99528;

Phenylalanine, tyrosine and tryptophan biosynthesis	ko00400	236	F01_transcript_100228;F01_transcript_100287;F01_transcript_100408;F01_transcript_100412;F01_transcript_100618;F01_transcript_101473;F01_transcript_102803;F01_transcript_102962;F01_transcript_105879;F01_transcript_106156;F01_transcript_106666;F01_transcript_106845;F01_transcript_107151;F01_transcript_107736;F01_transcript_109009;F01_transcript_110305;F01_transcript_111377;F01_transcript_113901;F01_transcript_114297;F01_transcript_114452;F01_transcript_118803;F01_transcript_119838;F01_transcript_119869;F01_transcript_121116;F01_transcript_122119;F01_transcript_122875;F01_transcript_124009;F01_transcript_124133;F01_transcript_124596;F01_transcript_125391;F01_transcript_126185;F01_transcript_131043;F01_transcript_131784;F01_transcript_131919;F01_transcript_132157;F01_transcript_132374;F01_transcript_132514;F01_transcript_133648;F01_transcript_134096;F01_transcript_134635;F01_transcript_135438;F01_transcript_135578;F01_transcript_135806;F01_transcript_135900;F01_transcript_136316;F01_transcript_136534;F01_transcript_136574;F01_transcript_136643;F01_transcript_138287;F01_transcript_138639;F01_transcript_139912;F01_transcript_140216;F01_transcript_140305;F01_transcript_141326;F01_transcript_141398;F01_transcript_141694;F01_transcript_142847;F01_transcript_14328;F01_transcript_144749;F01_transcript_144901;F01_transcript_145241;F01_transcript_146307;F01_transcript_146336;F01_transcript_146649;F01_transcript_146924;F01_transcript_147260;F01_transcript_147740;F01_transcript_148580;F01_transcript_150132;F01_transcript_150511;F01_transcript_150639;F01_transcript_150809;F01_transcript_152726;F01_transcript_153754;F01_transcript_154468;F01_transcript_154598;F01_transcript_154711;F01_transcript_155169;F01_transcript_155404;F01_transcript_156344;F01_transcript_156638;F01_transcript_157138;F01_transcript_157390;F01_transcript_157461;F01_transcript_158415;F01_transcript_159156;F01_transcript_160500;F01_transcript_160849;F01_transcript_161264;F01_transcript_161557;F01_transcript_161949;F01_transcript_161971;F01_transcript_165464;F01_transcript_166505;F01_transcript_167269;F01_transcript_17768;F01_transcript_19390;F01_transcript_20197;F01_transcript_20769;F01_transcript_25348;F01_transcript_26074;F01_transcript_26177;F01_transcript_26222;F0	K05359+K01626+K05359+K00891+K00815+K14454+K01657+K00766+K01736+K14454+K00815+K01850+K00811+K13832+K15227+K01850+K13832+K00800+K01850+K14455+K01609+K01609+K14455+K01609+K00815+K01696+K15227+K01626+K05359+K01850+K14455+K14455+K05359+K01626+K00815+K00817+K01609+K00815+K15227+K01657+K00891+K14454+K00815+K05359+K15849+K00815+K13832+K00817+K00800+K01850+K13832+K00800+K05359+K05359+K01850+K13832+K00891+K00815+K00815+K01735+K01609+K01850+K00766+K01850+K00815+K13832+K00766+K01850+K00815+K01817+K14454+K00891+K14454+K13832+K00891+K01850+K13832+K05359+K01609+K14455+K01850+K01609+K05359+K01735+K00891+K00891+K00811+K05359+K00815+K00815+K13832+K01626+K01696+K15849+K13832+K05359+K00815+K13832+K13832+K00800+K13832+K15227+K01657+K00800+K01657+K06001+K14454+K13832+K01626+K01657+K15227+K13832+K06001+K06001+K15227+K01626+K05359+K14454+K00815+K15849+K01736+K00815+K00811+K15849+K05359+K00815+K13832+K01696+K14455+K13832+K15227+K01735+K00817+K00766+K01609+K05359+K15227+K14455+K14455+K15227+K00815+K00766+K05359+K00766+K01696+K01695+K01850+K00815+K01850+K00891+K00891+K01850+K15227+K15227+K00891+K05359+K13832+K01609+K05359+K13832+K13832+K00891+K00800+K01626+K15227+K15227+K13832+K05359+K05359+K00891+K15227+K00891+K06001+K00800+K05359+K01736+K00815+K14454+K01696+K15227+K01609+K01626+K00800+K01626+K01658+K13832+K00800+K01850+K00891+K01609+K13832+K00815+K13832+K13832+K00800+K01696+K14455+K00811+K00891+K01736+K05359+K15227+K13832+K13832+K00811+K01696+K01657+K15227+K14454+K14454+K00800+K13832+K05359+K00811+K01735+K14455+K00817+K00800+K15849+K01658+K00800+K14454+K01735+K00815+K00800+K01609+K01609+K05359+K14455+K01626+K13832+K14454+K05359+K00891+K01735+K01658

1_transcript_26226;F01_transcript_27425;F01_transcript_28267
;F01_transcript_28405;F01_transcript_28483;F01_transcript_28
852;F01_transcript_28916;F01_transcript_29257;F01_transcript
_29320;F01_transcript_29399;F01_transcript_29494;F01_transc
ript_30075;F01_transcript_30413;F01_transcript_30625;F01_tra
nscript_30736;F01_transcript_31061;F01_transcript_31276;F01
_transcript_31382;F01_transcript_31700;F01_transcript_31784;
F01_transcript_31834;F01_transcript_32005;F01_transcript_321
47;F01_transcript_32382;F01_transcript_32492;F01_transcript_
32622;F01_transcript_32642;F01_transcript_33713;F01_transcri
pt_33776;F01_transcript_33879;F01_transcript_34385;F01_tran
script_35368;F01_transcript_35768;F01_transcript_35775;F01_t
ranscript_36375;F01_transcript_36639;F01_transcript_37385;F0
1_transcript_37457;F01_transcript_37512;F01_transcript_37687
;F01_transcript_38382;F01_transcript_38511;F01_transcript_40
811;F01_transcript_41216;F01_transcript_42165;F01_transcript
_42350;F01_transcript_42671;F01_transcript_43042;F01_transc
ript_43089;F01_transcript_45390;F01_transcript_46630;F01_tra
nscript_52054;F01_transcript_52341;F01_transcript_52451;F01
_transcript_52540;F01_transcript_53081;F01_transcript_54568;
F01_transcript_54799;F01_transcript_55149;F01_transcript_561
59;F01_transcript_57782;F01_transcript_58806;F01_transcript_
58847;F01_transcript_59248;F01_transcript_59641;F01_transcri
pt_6156;F01_transcript_61628;F01_transcript_61840;F01_trans
cript_62575;F01_transcript_63045;F01_transcript_63781;F01_tr
anscript_64505;F01_transcript_65614;F01_transcript_65744;F0
1_transcript_65858;F01_transcript_65935;F01_transcript_66391
;F01_transcript_66486;F01_transcript_69688;F01_transcript_70
971;F01_transcript_71324;F01_transcript_71564;F01_transcript
_72187;F01_transcript_74064;F01_transcript_74308;F01_transc
ript_75740;F01_transcript_76532;F01_transcript_76858;F01_tra
nscript_76908;F01_transcript_76978;F01_transcript_77068;F01
_transcript_77574;F01_transcript_78254;F01_transcript_78720;
F01_transcript_78919;F01_transcript_79280;F01_transcript_794
81;F01_transcript_79966;F01_transcript_8028;F01_transcript_8
0310;F01_transcript_80555;F01_transcript_80769;F01_transcrip
t_80889;F01_transcript_80923;F01_transcript_80943;F01_trans

			cript_81078;F01_transcript_81767;F01_transcript_82718;F01_transcript_83229;F01_transcript_84352;F01_transcript_86970;F01_transcript_87223;F01_transcript_87504;F01_transcript_87962;F01_transcript_88003;F01_transcript_88383;F01_transcript_88898;F01_transcript_88942;F01_transcript_89140;F01_transcript_90147;F01_transcript_90364;F01_transcript_91410;F01_transcript_92372;F01_transcript_92825;F01_transcript_92835;F01_transcript_93052;F01_transcript_93624;F01_transcript_94953;F01_transcript_9785;F01_transcript_98067;F01_transcript_98682;F01_transcript_98971;F01_transcript_99996;	
beta-Alanine metabolism	ko00410	268	F01_transcript_100692;F01_transcript_100740;F01_transcript_101567;F01_transcript_103175;F01_transcript_103691;F01_transcript_103925;F01_transcript_104346;F01_transcript_104696;F01_transcript_105213;F01_transcript_105377;F01_transcript_105405;F01_transcript_106222;F01_transcript_107012;F01_transcript_107764;F01_transcript_108221;F01_transcript_108650;F01_transcript_109312;F01_transcript_112210;F01_transcript_112262;F01_transcript_112685;F01_transcript_112884;F01_transcript_115085;F01_transcript_115636;F01_transcript_116665;F01_transcript_117360;F01_transcript_118580;F01_transcript_118736;F01_transcript_118848;F01_transcript_119343;F01_transcript_12072;F01_transcript_121237;F01_transcript_121819;F01_transcript_121908;F01_transcript_122090;F01_transcript_1231;F01_transcript_123185;F01_transcript_123463;F01_transcript_124506;F01_transcript_124604;F01_transcript_126314;F01_transcript_126346;F01_transcript_127146;F01_transcript_127326;F01_transcript_127971;F01_transcript_128143;F01_transcript_128218;F01_transcript_128756;F01_transcript_128872;F01_transcript_130313;F01_transcript_130773;F01_transcript_132279;F01_transcript_132494;F01_transcript_132512;F01_transcript_132867;F01_transcript_132930;F01_transcript_1334;F01_transcript_134251;F01_transcript_134507;F01_transcript_13535;F01_transcript_137525;F01_transcript_137935;F01_transcript_138093;F01_transcript_138897;F01_transcript_138921;F01_transcript_139567;F01_transcript_139604;F01_transcript_140307;F01_transcript_140406;F01_transcript_140486;F01_transcript_140632;F01_transcript_142320;F01_transcript_144636;F01_transcript_144740;F01_transcript_144850;F01_transcript_145352;F01_transcript_14547	K01580+K00128+K00276+K05605+K14085+K00128+K05605+K05605+K00128+K05605+K00276+K00797+K00797+K00276+K00128+K01431+K00128+K05605+K00797+K00128+K00128+K05605+K14085+K05605+K17839+K00797+K00128+K00128+K00128+K00276+K00140+K00128+K00797+K00140+K00128+K05605+K05605+K05605+K00276+K05605+K05605+K00128+K00128+K05605+K00797+K00128+K01464+K00128+K00276+K00140+K05605+K00128+K00276+K00797+K00128+K00128+K00128+K00128+K00276+K00128+K00128+K00128+K00128+K00128+K00128+K05605+K00140+K00276+K05605+K00276+K00797+K00128+K05605+K00128+K00128+K00128+K00128+K00128+K00797+K00276+K00128+K12259+K00276+K00128+K05605+K01918+K00797+K00128+K05605+K00128+K00128+K00128+K00128+K00140+K00140+K01431+K00276+K05605+K00128+K00797+K00276+K00128+K00276+K14085+K00128+K00207+K00128+K00128+K00128+K00128+K05605+K00276+K01464+K00276+K05605+K00276+K00140+K00128+K17839+K00276+K01464+K00128+K00140+K00276+K01431+K00276+K05605+K00128+K00128+K00276+K00128+K01464+K00797+K00128+K00128+K17839+K00128+K12259+K00128+K00140+K00128+K00140+K01464+K00140+K00128+K01431+K14085+K14085+K00128+K01464+K00128+K01464+K00140+K00128+K00128+K00128+K05605+K00128+K00128+K01580+K01578+K00207+K00128+K00797+K00128+K01580+K05605+K00128+K00128+K05605+K00128+K00797+K00128+K00128+K01580+K01580+K00128+K05605+K05605+K00797+K00128+K00128+K01580+K00249+K00797+K05605+K01431+K05605+K00276+K05605+K00128+K00128+

	4;F01_transcript_146046;F01_transcript_147257;F01_transcript_147521;F01_transcript_147552;F01_transcript_147783;F01_transcript_148402;F01_transcript_148409;F01_transcript_149292;F01_transcript_150299;F01_transcript_150546;F01_transcript_151102;F01_transcript_151264;F01_transcript_151674;F01_transcript_151832;F01_transcript_151939;F01_transcript_152756;F01_transcript_152958;F01_transcript_15331;F01_transcript_15347;F01_transcript_154093;F01_transcript_154647;F01_transcript_154730;F01_transcript_154982;F01_transcript_157007;F01_transcript_158722;F01_transcript_158912;F01_transcript_159476;F01_transcript_159514;F01_transcript_159814;F01_transcript_160755;F01_transcript_161816;F01_transcript_161966;F01_transcript_162134;F01_transcript_162331;F01_transcript_162763;F01_transcript_163899;F01_transcript_164154;F01_transcript_16418;F01_transcript_164546;F01_transcript_16644;F01_transcript_1704;F01_transcript_1799;F01_transcript_18524;F01_transcript_18563;F01_transcript_19217;F01_transcript_19412;F01_transcript_19633;F01_transcript_2067;F01_transcript_21265;F01_transcript_22000;F01_transcript_22856;F01_transcript_23785;F01_transcript_24020;F01_transcript_24430;F01_transcript_24677;F01_transcript_24871;F01_transcript_24957;F01_transcript_25659;F01_transcript_26120;F01_transcript_27158;F01_transcript_27376;F01_transcript_27498;F01_transcript_28572;F01_transcript_28594;F01_transcript_28856;F01_transcript_28947;F01_transcript_29103;F01_transcript_29341;F01_transcript_29858;F01_transcript_29933;F01_transcript_30292;F01_transcript_30362;F01_transcript_30379;F01_transcript_30416;F01_transcript_30935;F01_transcript_31229;F01_transcript_31414;F01_transcript_31894;F01_transcript_32017;F01_transcript_32266;F01_transcript_32386;F01_transcript_33325;F01_transcript_33600;F01_transcript_33656;F01_transcript_33685;F01_transcript_34128;F01_transcript_34271;F01_transcript_35071;F01_transcript_35286;F01_transcript_35762;F01_transcript_3669;F01_transcript_36697;F01_transcript_36801;F01_transcript_36901;F01_transcript_37696;F01_transcript_37802;F01_transcript_37926;F01_transcript_37987;F01_transcript_37988;F01_transcript_38235;F01_transcript_38323;F01_transcript_38936;F01_transcript_39168;F01_trans	K05605+K00276+K14085+K00140+K01464+K00207+K00276+K00276+K00276+K00797+K01580+K14085+K00276+K00797+K00140+K00128+K00128+K00128+K00128+K00276+K00128+K00249+K00140+K00128+K00249+K00128+K00128+K00128+K01464+K00128+K05605+K00276+K05605+K00207+K00128+K00249+K00140+K01580+K00140+K14085+K01431+K00140+K00128+K00140+K05605+K00128+K05605+K05605+K00128+K00276+K01580+K00797+K01464+K14085+K01578+K05605+K00276+K05605+K00276+K00249+K00276+K05605+K00128+K00276+K00128+K00207+K17839+K05605+K00128+K05605+K00128+K00128+K00207+K00276+K00128+K01464

			cript_39898;F01_transcript_41704;F01_transcript_48424;F01_transcript_49750;F01_transcript_51625;F01_transcript_51841;F01_transcript_52158;F01_transcript_52477;F01_transcript_52494;F01_transcript_52920;F01_transcript_54938;F01_transcript_55541;F01_transcript_56137;F01_transcript_56268;F01_transcript_56629;F01_transcript_56690;F01_transcript_57145;F01_transcript_57483;F01_transcript_57595;F01_transcript_60104;F01_transcript_61675;F01_transcript_61722;F01_transcript_62377;F01_transcript_62577;F01_transcript_63491;F01_transcript_63755;F01_transcript_63834;F01_transcript_64572;F01_transcript_65034;F01_transcript_65403;F01_transcript_65979;F01_transcript_66261;F01_transcript_66411;F01_transcript_67211;F01_transcript_6767;F01_transcript_68359;F01_transcript_70471;F01_transcript_7124;F01_transcript_71260;F01_transcript_71646;F01_transcript_72349;F01_transcript_72513;F01_transcript_73295;F01_transcript_73519;F01_transcript_74237;F01_transcript_74528;F01_transcript_74548;F01_transcript_75083;F01_transcript_7550;F01_transcript_76336;F01_transcript_77468;F01_transcript_77784;F01_transcript_77976;F01_transcript_78714;F01_transcript_79569;F01_transcript_79852;F01_transcript_79982;F01_transcript_80229;F01_transcript_80510;F01_transcript_80964;F01_transcript_81280;F01_transcript_81584;F01_transcript_8293;F01_transcript_83562;F01_transcript_84285;F01_transcript_8697;F01_transcript_8790;F01_transcript_88199;F01_transcript_89647;F01_transcript_90314;F01_transcript_91526;F01_transcript_91560;F01_transcript_9209;F01_transcript_9243;F01_transcript_92567;F01_transcript_92804;F01_transcript_95934;F01_transcript_96227;F01_transcript_96558;F01_transcript_97151;F01_transcript_97153;F01_transcript_97230;F01_transcript_98121;F01_transcript_98282;F01_transcript_98445;F01_transcript_98781;F01_transcript_98894;F01_transcript_99528;F01_transcript_99795;	
Taurine and hypotaurine	ko004	43	F01_transcript_100692;F01_transcript_107788;F01_transcript_107892;F01_transcript_115052;F01_transcript_127998;F01_transcript_130647;F01_transcript_13324;F01_transcript_146015;F01_transcript_149300;F01_transcript_155997;F01_transcript_157631;F01_transcript_165383;F01_transcript_16611;F01_transcript_18485;F01_transcript_20900;F01_transcript_21864;F01_transc	K01580+K10712+K10712+K10712+K10712+K10712+K10712+K10712+K18592+K18592+K18592+K18592+K18592+K18592+K18592+K18592+K18592+K10712+K01580+K01580+K10712+K01580+K01580+K10712+K10712+K01580+K10712+K10712+K01580+K18592+K18592+K18592+K18592+K18592+K1

metab olism	3 0		ript_21913;F01_transcript_27280;F01_transcript_33656;F01_transcript_35762;F01_transcript_36015;F01_transcript_37988;F01_transcript_38235;F01_transcript_40697;F01_transcript_43828;F01_transcript_49750;F01_transcript_51484;F01_transcript_55688;F01_transcript_62577;F01_transcript_62799;F01_transcript_64276;F01_transcript_6623;F01_transcript_68626;F01_transcript_72920;F01_transcript_75845;F01_transcript_76792;F01_transcript_77351;F01_transcript_77468;F01_transcript_78466;F01_transcript_78880;F01_transcript_78958;F01_transcript_80683;F01_transcript_83562;	0712+K18592+K10712+K01580+K10712+K10712+K10712+K18592+K01580
Selen ocom pound metab olism	k o 0 0 4 5 0	10 9	F01_transcript_100400;F01_transcript_101627;F01_transcript_101758;F01_transcript_103028;F01_transcript_10354;F01_transcript_104967;F01_transcript_106282;F01_transcript_10658;F01_transcript_10704;F01_transcript_111000;F01_transcript_11185;F01_transcript_113659;F01_transcript_114007;F01_transcript_117844;F01_transcript_12118;F01_transcript_121344;F01_transcript_121502;F01_transcript_123859;F01_transcript_124416;F01_transcript_124552;F01_transcript_125424;F01_transcript_125448;F01_transcript_125548;F01_transcript_126139;F01_transcript_126432;F01_transcript_12663;F01_transcript_127328;F01_transcript_127418;F01_transcript_129435;F01_transcript_131941;F01_transcript_132217;F01_transcript_133224;F01_transcript_134633;F01_transcript_134823;F01_transcript_138008;F01_transcript_141325;F01_transcript_142448;F01_transcript_142449;F01_transcript_144538;F01_transcript_144983;F01_transcript_145306;F01_transcript_145873;F01_transcript_146745;F01_transcript_146938;F01_transcript_148697;F01_transcript_155163;F01_transcript_155339;F01_transcript_156015;F01_transcript_156595;F01_transcript_156861;F01_transcript_158496;F01_transcript_159723;F01_transcript_159832;F01_transcript_162587;F01_transcript_163553;F01_transcript_19071;F01_transcript_19153;F01_transcript_2016;F01_transcript_21773;F01_transcript_2341;F01_transcript_24651;F01_transcript_26005;F01_transcript_26048;F01_transcript_26446;F01_transcript_26648;F01_transcript_27011;F01_transcript_27447;F01_transcript_29328;F01_transcript_30420;F01_transcript_30637;F01_transcript_31774;F01_transcript_31988;F01_transcript_32454;F01_transcript_33564;F01_tr	K01874+K01874+K00549+K01739+K00549+K01874+K00384+K00549+K00549+K01874+K00549+K01761+K00549+K01739+K00549+K11717+K01739+K01739+K00549+K01874+K08247+K00384+K01874+K00549+K01874+K00549+K00549+K01739+K01739+K01874+K00384+K00549+K01008+K00549+K01874+K00549+K00549+K01739+K13811+K01874+K01874+K13811+K01874+K13811+K00549+K01874+K01739+K01874+K00384+K13811+K01874+K00549+K08247+K00384+K01874+K00549+K01874+K08247+K00549+K00549+K01758+K01739+K01874+K01874+K01874+K01874+K01874+K01739+K01739+K13811+K00384+K00384+K00384+K01761+K11717+K13811+K11717+K00384+K00384+K00384+K11717+K00384+K01739+K00549+K01874+K01874+K00384+K01874+K01874+K00549+K00384+K00549+K13811+K01739+K01874+K01874+K08247+K00384+K00549+K11717+K01874+K01874+K01874+K13811+K00384+K00384+K00549+K01739+K11717

			anscript_34423;F01_transcript_34975;F01_transcript_35908;F01_transcript_40484;F01_transcript_41499;F01_transcript_52832;F01_transcript_5452;F01_transcript_56598;F01_transcript_56992;F01_transcript_61029;F01_transcript_61806;F01_transcript_62202;F01_transcript_64528;F01_transcript_6454;F01_transcript_66588;F01_transcript_66754;F01_transcript_71551;F01_transcript_72578;F01_transcript_76208;F01_transcript_78612;F01_transcript_79092;F01_transcript_8073;F01_transcript_81155;F01_transcript_81743;F01_transcript_83801;F01_transcript_86641;F01_transcript_8978;F01_transcript_93629;F01_transcript_93845;F01_transcript_94345;F01_transcript_95099;F01_transcript_95175;F01_transcript_95332;F01_transcript_96054;F01_transcript_98887;	
Cyano amino acid metabolism	ko00460	320	F01_transcript_100118;F01_transcript_100330;F01_transcript_100848;F01_transcript_101071;F01_transcript_101092;F01_transcript_101377;F01_transcript_102135;F01_transcript_102714;F01_transcript_103227;F01_transcript_104167;F01_transcript_104888;F01_transcript_105138;F01_transcript_105197;F01_transcript_105374;F01_transcript_105932;F01_transcript_106618;F01_transcript_106967;F01_transcript_10799;F01_transcript_108584;F01_transcript_108602;F01_transcript_109254;F01_transcript_109794;F01_transcript_110100;F01_transcript_110932;F01_transcript_111549;F01_transcript_111966;F01_transcript_112563;F01_transcript_113066;F01_transcript_114677;F01_transcript_115746;F01_transcript_115825;F01_transcript_117422;F01_transcript_117699;F01_transcript_117950;F01_transcript_118053;F01_transcript_118804;F01_transcript_118927;F01_transcript_119306;F01_transcript_119952;F01_transcript_120858;F01_transcript_120997;F01_transcript_121306;F01_transcript_122115;F01_transcript_122331;F01_transcript_122844;F01_transcript_122916;F01_transcript_123750;F01_transcript_123848;F01_transcript_124153;F01_transcript_12550;F01_transcript_127221;F01_transcript_127297;F01_transcript_128370;F01_transcript_128562;F01_transcript_128875;F01_transcript_129051;F01_transcript_129319;F01_transcript_130382;F01_transcript_130383;F01_transcript_130612;F01_transcript_130895;F01_transcript_131304;F01_transcript_131327;F01_transcript_132067;F01_transcript_132156;F01_transcript_132238;F01_transcript_132483;F01_transcript_	K00600+K00600+K05350+K00600+K01188+K01188+K00600+K00600+K00600+K00600+K05350+K05350+K01188+K05349+K05350+K05349+K00600+K00600+K00600+K00600+K00600+K01188+K05350+K05349+K00600+K05350+K00600+K05350+K00600+K01188+K05350+K00600+K00600+K05349+K01455+K00600+K00600+K13034+K00600+K05350+K13034+K00600+K00600+K00600+K05350+K05350+K01188+K00600+K00600+K00600+K05349+K00600+K05350+K05350+K01188+K00600+K01188+K00600+K01188+K00600+K00600+K05350+K05349+K01188+K00600+K00600+K00600+K00600+K05349+K00600+K05349+K13034+K01188+K01455+K01455+K01188+K00600+K05349+K00600+K05350+K00600+K01188+K01188+K05350+K01188+K00600+K00600+K01188+K08248+K00600+K05350+K01455+K00600+K01188+K00600+K05349+K00600+K00600+K01188+K05350+K18592+K01188+K00600+K05349+K01188+K01188+K13034+K01188+K00600+K00600+K18592+K01188+K05350+K18592+K01188+K01188+K05350+K00600+K00600+K00600+K13035+K01188+K00600+K01188+K05350+K05350+K00600+K05349+K01188+K00600+K00600+K05349+K05350+K00600+K05349+K05350+K00600+K05350+K18592+K18592+K01188+K01188+K01188+K05349+K01188+K00600+K00600+K00600+K18592+K05349+K00600+K05349+K18592+K01188+K18592+K18592+K05349+K01188+K00600+K00600+K05349+K00600+K05349+K00600+K05349+K05349+K00600+K00600+K00600+K05350+K05350+K00

	133019;F01_transcript_133256;F01_transcript_133265;F01_transcript_133921;F01_transcript_134216;F01_transcript_134748;F01_transcript_135878;F01_transcript_136192;F01_transcript_136619;F01_transcript_13740;F01_transcript_137403;F01_transcript_138453;F01_transcript_138640;F01_transcript_138914;F01_transcript_138970;F01_transcript_139196;F01_transcript_139446;F01_transcript_139496;F01_transcript_139819;F01_transcript_139822;F01_transcript_140007;F01_transcript_140490;F01_transcript_141510;F01_transcript_142532;F01_transcript_142863;F01_transcript_14292;F01_transcript_144580;F01_transcript_145271;F01_transcript_145380;F01_transcript_146840;F01_transcript_147232;F01_transcript_147992;F01_transcript_148902;F01_transcript_149300;F01_transcript_149911;F01_transcript_150241;F01_transcript_150421;F01_transcript_150926;F01_transcript_152198;F01_transcript_152454;F01_transcript_153240;F01_transcript_154869;F01_transcript_155116;F01_transcript_155997;F01_transcript_156051;F01_transcript_156083;F01_transcript_157631;F01_transcript_157986;F01_transcript_158249;F01_transcript_158252;F01_transcript_158283;F01_transcript_158617;F01_transcript_159165;F01_transcript_159208;F01_transcript_159376;F01_transcript_160408;F01_transcript_160671;F01_transcript_160753;F01_transcript_160784;F01_transcript_160895;F01_transcript_160988;F01_transcript_161192;F01_transcript_16246;F01_transcript_163735;F01_transcript_164292;F01_transcript_164390;F01_transcript_164525;F01_transcript_165033;F01_transcript_165071;F01_transcript_165169;F01_transcript_165335;F01_transcript_165383;F01_transcript_16611;F01_transcript_166184;F01_transcript_16620;F01_transcript_166833;F01_transcript_166888;F01_transcript_167210;F01_transcript_167268;F01_transcript_17308;F01_transcript_17809;F01_transcript_18485;F01_transcript_19594;F01_transcript_20069;F01_transcript_20653;F01_transcript_20900;F01_transcript_21793;F01_transcript_21864;F01_transcript_21913;F01_transcript_22769;F01_transcript_23513;F01_transcript_24050;F01_transcript_24298;F01_transcript_24985;F01_transcript_25171;F01_transcript_25483;F01_transcript_25780;F01_transcript_25948;F01_transcript_26197;F01_transcript_26643;F01_transcript_26845;F01_transcript_2695;F	600+K01188+K00600+K00600+K00600+K00600+K05350+K00600+K00600+K01188+K01188+K05350+K00600+K00600+K05350+K00600+K00600+K00600+K00600+K00600+K01455+K05350+K00600+K00600+K01455+K01188+K05350+K05350+K00600+K01188+K00600+K01188+K01188+K05350+K01188+K01188+K01455+K00600+K01455+K00600+K00600+K00600+K13034+K13034+K13035+K01188+K00600+K00600+K01188+K13034+K01188+K00600+K05349+K05350+K00600+K01188+K05350+K00600+K01188+K05349+K00600+K00600+K01188+K00600+K01455+K00600+K00600+K00600+K01188+K05349+K18592+K00600+K18592+K00600+K01455+K00600+K05349+K18592+K00600+K13034+K18592+K13034+K01188+K05350+K00600+K05349+K13035+K18592+K00600+K05349+K00600+K00600+K05350+K05350+K00600+K00600+K00600+K00600+K18592+K00600+K00600+K05349+K13034+K00600+K01188+K18592+K00600+K01188+K08248+K01188+K00600+K00600+K01188+K05350+K05350+K05350+K05350+K01188+K00600+K05350+K00600+K05349+K05349+K00600+K00600+K01188+K00600+K05350+K01188+K05349+K00600+K00600+K00600+K13034+K05350+K05350+K05349+K00600+K00600+K01188+K01188+K00600+K00600+K01188+K00600+K00600+K05350+K05350+K05349

01_transcript_26984;F01_transcript_27120;F01_transcript_2714
0;F01_transcript_28050;F01_transcript_28121;F01_transcript_2
8268;F01_transcript_28812;F01_transcript_28865;F01_transcrip
t_29052;F01_transcript_29058;F01_transcript_29214;F01_trans
cript_29294;F01_transcript_29312;F01_transcript_29437;F01_tr
anscript_29482;F01_transcript_29697;F01_transcript_29784;F0
1_transcript_29873;F01_transcript_30092;F01_transcript_30170
;F01_transcript_30222;F01_transcript_30482;F01_transcript_30
503;F01_transcript_30650;F01_transcript_30802;F01_transcript
_31015;F01_transcript_31271;F01_transcript_31528;F01_transc
ript_32171;F01_transcript_32819;F01_transcript_32834;F01_tra
nscript_33214;F01_transcript_33418;F01_transcript_33678;F01
_transcript_34009;F01_transcript_34522;F01_transcript_35049;
F01_transcript_35183;F01_transcript_35426;F01_transcript_354
30;F01_transcript_36320;F01_transcript_36398;F01_transcript_
366;F01_transcript_36997;F01_transcript_37334;F01_transcript
_37739;F01_transcript_38686;F01_transcript_41284;F01_transc
ript_42368;F01_transcript_50115;F01_transcript_50524;F01_tra
nscript_51442;F01_transcript_52855;F01_transcript_53824;F01
_transcript_53935;F01_transcript_53994;F01_transcript_55946;
F01_transcript_55994;F01_transcript_56193;F01_transcript_569
8;F01_transcript_57117;F01_transcript_58663;F01_transcript_5
9629;F01_transcript_59737;F01_transcript_60080;F01_transcrip
t_60381;F01_transcript_60431;F01_transcript_60635;F01_trans
cript_61445;F01_transcript_62260;F01_transcript_62639;F01_tr
anscript_62701;F01_transcript_62799;F01_transcript_64095;F0
1_transcript_64276;F01_transcript_64897;F01_transcript_65162
;F01_transcript_65810;F01_transcript_65838;F01_transcript_66
23;F01_transcript_67332;F01_transcript_68454;F01_transcript_
68626;F01_transcript_68627;F01_transcript_68840;F01_transcri
pt_69406;F01_transcript_70704;F01_transcript_71241;F01_tran
script_71753;F01_transcript_72920;F01_transcript_72967;F01_t
ranscript_73186;F01_transcript_73464;F01_transcript_74212;F0
1_transcript_74577;F01_transcript_75064;F01_transcript_75531
;F01_transcript_75678;F01_transcript_75942;F01_transcript_76
460;F01_transcript_76792;F01_transcript_76950;F01_transcript
_77082;F01_transcript_78182;F01_transcript_78302;F01_transc
ript_79353;F01_transcript_79800;F01_transcript_80683;F01_tra

			nscript_81090;F01_transcript_81126;F01_transcript_81288;F01_transcript_81833;F01_transcript_82110;F01_transcript_82373;F01_transcript_82466;F01_transcript_83222;F01_transcript_83296;F01_transcript_83393;F01_transcript_83418;F01_transcript_84796;F01_transcript_84894;F01_transcript_86419;F01_transcript_86731;F01_transcript_88605;F01_transcript_89585;F01_transcript_89629;F01_transcript_89641;F01_transcript_90214;F01_transcript_90316;F01_transcript_90419;F01_transcript_91915;F01_transcript_92681;F01_transcript_9359;F01_transcript_93722;F01_transcript_93994;F01_transcript_94201;F01_transcript_94343;F01_transcript_94626;F01_transcript_95784;F01_transcript_96028;F01_transcript_96153;F01_transcript_96241;F01_transcript_96323;F01_transcript_97239;F01_transcript_97597;F01_transcript_97889;F01_transcript_98080;F01_transcript_98413;F01_transcript_98615;F01_transcript_98978;F01_transcript_99106;	
Glutathione metabolism	ko00480	396	F01_transcript_100277;F01_transcript_100797;F01_transcript_101015;F01_transcript_101456;F01_transcript_101488;F01_transcript_102954;F01_transcript_103099;F01_transcript_103366;F01_transcript_103486;F01_transcript_104060;F01_transcript_104395;F01_transcript_105445;F01_transcript_106222;F01_transcript_106663;F01_transcript_107012;F01_transcript_107168;F01_transcript_108039;F01_transcript_10823;F01_transcript_108546;F01_transcript_108684;F01_transcript_109271;F01_transcript_109520;F01_transcript_109701;F01_transcript_110626;F01_transcript_112145;F01_transcript_112262;F01_transcript_113356;F01_transcript_113507;F01_transcript_113845;F01_transcript_114016;F01_transcript_114037;F01_transcript_114966;F01_transcript_115428;F01_transcript_115554;F01_transcript_116357;F01_transcript_116397;F01_transcript_116615;F01_transcript_116815;F01_transcript_117189;F01_transcript_117248;F01_transcript_118580;F01_transcript_118681;F01_transcript_118935;F01_transcript_119023;F01_transcript_119034;F01_transcript_119534;F01_transcript_120416;F01_transcript_120644;F01_transcript_121295;F01_transcript_121908;F01_transcript_122054;F01_transcript_122649;F01_transcript_122700;F01_transcript_125194;F01_transcript_125223;F01_transcript_125301;F01_transcript_125941;F01_transcript_126578;F01_transcript_127422;F01_transcri	K01256+K00036+K01256+K00799+K00799+K00799+K00434+K00033+K00799+K00799+K00799+K00036+K00797+K00434+K00797+K01919+K00799+K01256+K00799+K00799+K00036+K00799+K00799+K00799+K00799+K00797+K00033+K00799+K00799+K00434+K01255+K00031+K00799+K00434+K00036+K00383+K00434+K00799+K00799+K00033+K00797+K00383+K01920+K00434+K00031+K00031+K00383+K00432+K01920+K00797+K00434+K10807+K00434+K01256+K00383+K00434+K01256+K00799+K01919+K10807+K00797+K01256+K01256+K00799+K00383+K01256+K00383+K10807+K00797+K00434+K00434+K01256+K00799+K00799+K00799+K00383+K00434+K00434+K00434+K01256+K00383+K00432+K00036+K00432+K01255+K00036+K00434+K00383+K00434+K01256+K00434+K01256+K00799+K00799+K00799+K00036+K00797+K00432+K00799+K01255+K00383+K00799+K00383+K00031+K00799+K00797+K00799+K00383+K18592+K00434+K00799+K00434+K00799+K00797+K00799+K01255+K00036+K01920+K00434+K00434+K00434+K00799+K00036+K00799+K00799+K18592+K00799+K01256+K00434+K00797+K10807+K01256+K18592+K01920+K00799+K00036+K01256+K00434+K01920+K00434+K00799+K00434+K00036+K00434+K00799+K00434+K10808+K00036+K00036+K00434+K18592

	pt_127934;F01_transcript_128143;F01_transcript_129539;F01_transcript_129605;F01_transcript_129636;F01_transcript_130486;F01_transcript_130787;F01_transcript_131469;F01_transcript_132505;F01_transcript_132867;F01_transcript_133029;F01_transcript_133331;F01_transcript_133458;F01_transcript_133764;F01_transcript_134065;F01_transcript_13416;F01_transcript_134317;F01_transcript_134810;F01_transcript_135567;F01_transcript_136249;F01_transcript_136383;F01_transcript_136549;F01_transcript_136710;F01_transcript_136877;F01_transcript_137083;F01_transcript_138787;F01_transcript_138810;F01_transcript_139000;F01_transcript_139166;F01_transcript_139223;F01_transcript_139438;F01_transcript_140055;F01_transcript_140311;F01_transcript_140361;F01_transcript_140429;F01_transcript_141236;F01_transcript_141350;F01_transcript_142320;F01_transcript_142413;F01_transcript_142434;F01_transcript_142945;F01_transcript_144653;F01_transcript_144793;F01_transcript_145319;F01_transcript_147008;F01_transcript_147268;F01_transcript_147521;F01_transcript_147652;F01_transcript_148422;F01_transcript_149300;F01_transcript_149651;F01_transcript_150537;F01_transcript_150655;F01_transcript_150680;F01_transcript_151102;F01_transcript_151182;F01_transcript_151816;F01_transcript_152000;F01_transcript_152255;F01_transcript_153586;F01_transcript_153624;F01_transcript_153946;F01_transcript_154349;F01_transcript_154770;F01_transcript_154933;F01_transcript_155068;F01_transcript_155997;F01_transcript_156472;F01_transcript_156709;F01_transcript_156980;F01_transcript_157007;F01_transcript_157170;F01_transcript_157235;F01_transcript_157631;F01_transcript_157906;F01_transcript_158941;F01_transcript_159010;F01_transcript_159560;F01_transcript_159904;F01_transcript_160271;F01_transcript_160400;F01_transcript_160877;F01_transcript_161020;F01_transcript_161713;F01_transcript_161832;F01_transcript_16233;F01_transcript_162345;F01_transcript_162389;F01_transcript_164702;F01_transcript_165059;F01_transcript_165142;F01_transcript_165383;F01_transcript_16611;F01_transcript_166214;F01_transcript_166342;F01_transcript_167047;F01_transcript_167406;F01_transcript_167660;F01_transcript_18485;F01_transcript_18744;F01_transcript_18940;F01_transcript_19222;F01_transcript_19398;F01_transcri	+K18592+K00799+K00799+K00799+K00799+K00799+K18592+K00036+K01920+K00036+K10808+K18592+K18592+K18592+K00799+K00799+K01919+K00799+K00797+K00383+K00799+K01919+K00036+K00033+K01256+K00036+K00036+K00799+K00036+K00799+K01255+K00036+K01255+K00033+K00036+K00033+K01255+K00799+K00033+K00383+K00033+K00033+K00383+K00383+K00799+K00383+K00031+K00797+K00434+K00434+K00434+K00797+K00799+K00434+K10808+K00797+K00799+K00434+K00799+K00434+K00432+K00799+K00432+K00434+K00799+K00799+K00799+K00799+K00434+K00799+K01256+K00799+K00434+K00799+K00799+K00432+K00799+K00434+K00799+K00799+K00799+K00799+K00799+K00432+K01256+K00799+K00799+K00799+K00799+K00432+K00432+K00799+K00799+K00799+K00799+K00799+K00432+K00799+K00432+K00799+K00434+K00799+K01255+K10807+K01256+K00797+K00036+K00434+K00031+K00031+K00799+K01256+K01256+K00434+K00799+K01255+K00799+K01256+K01919+K00799+K00799+K01255+K01256+K00799+K00799+K00799+K00434+K00799+K00434+K01255+K00799+K00432+K00434+K00434+K00799+K00799+K10808+K00799+K00797+K18592+K00797+K00799+K00432+K18592+K00434+K01256+K00799+K00434+K00799+K18592+K00799+K01256+K00434+K00799+K00434+K00033+K00434+K01256+K01256+K01256+K00799+K00434+K18592+K00033+K10807+K00033+K00434+K01256+K00383+K00383+K00036+K00383+K18592+K00383+K00033+K00434+K00383+K01920+K01256+K01919+K00799+K00031+K18592+K00799+K00036+K01920+K00799+K00434+K01256+K00799+K00799+K10808+K01469+K00799+K01256+K00799+K00799+K00799+K18592+K00434+K00434+K00434+K00434+K00033+K01256+K01920+K01256+K00432+K00797+K10807+K00799+K01256+K00434+K01256+K00799+K01256+K01256+K01469+K00383+K00432+K00799+K01256+K00033+K00383+K00799+K00799+K00036+K01256+K00434+K00799+K00799+K00799+K00434+K00799+K00799+K01920+K01920+K00383+K00799+K00036+K00434+K00031+K01919+K00799+K00036

	pt_20900;F01_transcript_21864;F01_transcript_21913;F01_transcript_22083;F01_transcript_22117;F01_transcript_22691;F01_transcript_23143;F01_transcript_24957;F01_transcript_25291;F01_transcript_27362;F01_transcript_27457;F01_transcript_27689;F01_transcript_28236;F01_transcript_2825;F01_transcript_28474;F01_transcript_28678;F01_transcript_28841;F01_transcript_29080;F01_transcript_29176;F01_transcript_29805;F01_transcript_29825;F01_transcript_29845;F01_transcript_29902;F01_transcript_30407;F01_transcript_30823;F01_transcript_30900;F01_transcript_31002;F01_transcript_31274;F01_transcript_31499;F01_transcript_31959;F01_transcript_32428;F01_transcript_32651;F01_transcript_32973;F01_transcript_33748;F01_transcript_34710;F01_transcript_34767;F01_transcript_35071;F01_transcript_36017;F01_transcript_36246;F01_transcript_37604;F01_transcript_37802;F01_transcript_38546;F01_transcript_39355;F01_transcript_39835;F01_transcript_39898;F01_transcript_40494;F01_transcript_40663;F01_transcript_4076;F01_transcript_42059;F01_transcript_42340;F01_transcript_43354;F01_transcript_43737;F01_transcript_43812;F01_transcript_43960;F01_transcript_43966;F01_transcript_43971;F01_transcript_44012;F01_transcript_44061;F01_transcript_44150;F01_transcript_4425;F01_transcript_44258;F01_transcript_44350;F01_transcript_44371;F01_transcript_44412;F01_transcript_44570;F01_transcript_44595;F01_transcript_44753;F01_transcript_45047;F01_transcript_45066;F01_transcript_45261;F01_transcript_45550;F01_transcript_45576;F01_transcript_45616;F01_transcript_4573;F01_transcript_45742;F01_transcript_45794;F01_transcript_45901;F01_transcript_46039;F01_transcript_46093;F01_transcript_46117;F01_transcript_46169;F01_transcript_46303;F01_transcript_46307;F01_transcript_46546;F01_transcript_46605;F01_transcript_46758;F01_transcript_46915;F01_transcript_46934;F01_transcript_47099;F01_transcript_47535;F01_transcript_49023;F01_transcript_50807;F01_transcript_5119;F01_transcript_51449;F01_transcript_51841;F01_transcript_51926;F01_transcript_52419;F01_transcript_52686;F01_transcript_52750;F01_transcript_52876;F01_transcript_53317;F01_transcript_53534;F01_transcript_54110;F01_transcript_54409;F01_transcript_55545;F01_transcript_55773;F0	

1_transcript_56257;F01_transcript_56280;F01_transcript_56308
;F01_transcript_56694;F01_transcript_56766;F01_transcript_56
802;F01_transcript_56805;F01_transcript_56842;F01_transcript
_57035;F01_transcript_57076;F01_transcript_57665;F01_transc
ript_57714;F01_transcript_58594;F01_transcript_58735;F01_tra
nscript_58773;F01_transcript_59141;F01_transcript_59243;F01
_transcript_60858;F01_transcript_61354;F01_transcript_61954;
F01_transcript_62197;F01_transcript_62377;F01_transcript_627
99;F01_transcript_63834;F01_transcript_63994;F01_transcript_
64255;F01_transcript_64276;F01_transcript_64343;F01_transcri
pt_6465;F01_transcript_65382;F01_transcript_66090;F01_trans
cript_66210;F01_transcript_6623;F01_transcript_66380;F01_tra
nscript_66567;F01_transcript_66794;F01_transcript_66816;F01
_transcript_66881;F01_transcript_6697;F01_transcript_68047;F
01_transcript_68085;F01_transcript_6817;F01_transcript_6836;
F01_transcript_68431;F01_transcript_68526;F01_transcript_686
26;F01_transcript_68820;F01_transcript_68925;F01_transcript_
69051;F01_transcript_70328;F01_transcript_7099;F01_transcrip
t_72388;F01_transcript_72504;F01_transcript_72676;F01_trans
cript_72707;F01_transcript_72920;F01_transcript_73433;F01_tr
anscript_73620;F01_transcript_73951;F01_transcript_74502;F0
1_transcript_74849;F01_transcript_75542;F01_transcript_75840
;F01_transcript_75899;F01_transcript_75938;F01_transcript_76
792;F01_transcript_76841;F01_transcript_76887;F01_transcript
_77193;F01_transcript_77249;F01_transcript_77591;F01_transc
ript_77603;F01_transcript_77643;F01_transcript_77908;F01_tra
nscript_77936;F01_transcript_79296;F01_transcript_79792;F01
_transcript_80047;F01_transcript_80112;F01_transcript_80236;
F01_transcript_8064;F01_transcript_80683;F01_transcript_8096
7;F01_transcript_81257;F01_transcript_81378;F01_transcript_8
1549;F01_transcript_82090;F01_transcript_83000;F01_transcrip
t_83097;F01_transcript_83501;F01_transcript_83884;F01_trans
cript_84285;F01_transcript_84620;F01_transcript_85235;F01_tr
anscript_85598;F01_transcript_85750;F01_transcript_86188;F0
1_transcript_86591;F01_transcript_86592;F01_transcript_87061
;F01_transcript_884;F01_transcript_88655;F01_transcript_8890
8;F01_transcript_89001;F01_transcript_89130;F01_transcript_8
9371;F01_transcript_89702;F01_transcript_90095;F01_transcrip

			t_90312;F01_transcript_90574;F01_transcript_90579;F01_transcript_91493;F01_transcript_91494;F01_transcript_91676;F01_transcript_91888;F01_transcript_92430;F01_transcript_94334;F01_transcript_95474;F01_transcript_95560;F01_transcript_96138;F01_transcript_96189;F01_transcript_96332;F01_transcript_97564;F01_transcript_98488;F01_transcript_98527;F01_transcript_99309;F01_transcript_99750;F01_transcript_99818;	
Starch and sucrose metabolism	ko00500	910	F01_transcript_10016;F01_transcript_10019;F01_transcript_100525;F01_transcript_100753;F01_transcript_100848;F01_transcript_101092;F01_transcript_101139;F01_transcript_101290;F01_transcript_101377;F01_transcript_101449;F01_transcript_101499;F01_transcript_101600;F01_transcript_101689;F01_transcript_101742;F01_transcript_101811;F01_transcript_102175;F01_transcript_102251;F01_transcript_102519;F01_transcript_10258;F01_transcript_102912;F01_transcript_103409;F01_transcript_103547;F01_transcript_103571;F01_transcript_103655;F01_transcript_104698;F01_transcript_104888;F01_transcript_104904;F01_transcript_105085;F01_transcript_105138;F01_transcript_105197;F01_transcript_105374;F01_transcript_105490;F01_transcript_105612;F01_transcript_105808;F01_transcript_105932;F01_transcript_106102;F01_transcript_106472;F01_transcript_106618;F01_transcript_107108;F01_transcript_10711;F01_transcript_107234;F01_transcript_108190;F01_transcript_108504;F01_transcript_108702;F01_transcript_10912;F01_transcript_109156;F01_transcript_10957;F01_transcript_109794;F01_transcript_109878;F01_transcript_109951;F01_transcript_110100;F01_transcript_110301;F01_transcript_110613;F01_transcript_110932;F01_transcript_111133;F01_transcript_111325;F01_transcript_111533;F01_transcript_111765;F01_transcript_111869;F01_transcript_111966;F01_transcript_112228;F01_transcript_112251;F01_transcript_112919;F01_transcript_113009;F01_transcript_113066;F01_transcript_11319;F01_transcript_113616;F01_transcript_113710;F01_transcript_113834;F01_transcript_113898;F01_transcript_114010;F01_transcript_114056;F01_transcript_114270;F01_transcript_11440;F01_transcript_114526;F01_transcript_114743;F01_transcript_114931;F01_transcript_114945;F01_transcript_11504;F01_transcript_115044;F01_transcript_115261;F01_transc	K00703+K00695+K00695+K13648+K05350+K01188+K00700+K00703+K01188+K01177+K01177+K15920+K00700+K00705+K13648+K00700+K13648+K00963+K00700+K00696+K00703+K00975+K01810+K00700+K01177+K05350+K00975+K01051+K05350+K01188+K05349+K01051+K00688+K16055+K05350+K00703+K00696+K05349+K01177+K00695+K16055+K00696+K00696+K00695+K13648+K13648+K01051+K01188+K01176+K00695+K05350+K00705+K01051+K05349+K00688+K13648+K00703+K00963+K08679+K05350+K00688+K13648+K00688+K00696+K05350+K00695+K13648+K01193+K13648+K20867+K01176+K00696+K00695+K00695+K00703+K16055+K13648+K00703+K13648+K01810+K13648+K01177+K00700+K00695+K01177+K01188+K16055+K05350+K16055+K00695+K01810+K13648+K01810+K01810+K01810+K01177+K01051+K00703+K01810+K00703+K13648+K01835+K13648+K05349+K01177+K01051+K00975+K16055+K00695+K16055+K00703+K00700+K01177+K13648+K13648+K16055+K00975+K05350+K13648+K00012+K01810+K13648+K00975+K00705+K01051+K00975+K00696+K01187+K00975+K00688+K05350+K05350+K00705+K00695+K01188+K08678+K16055+K00695+K16055+K00688+K00695+K01179+K00696+K16055+K00844+K01051+K01179+K00695+K00695+K16055+K00700+K00695+K16055+K05349+K00975+K15920+K13648+K01810+K00975+K00696+K00688+K05350+K05350+K00975+K16055+K01188+K00770+K13648+K13648+K13648+K00696+K00695+K00975+K01188+K00700+K01087+K00703+K01087+K00695+K01087+K00696+K01177+K00700+K00700+K01188+K00695+K00703+K13648+K01051+K05350+K05349+K00695+K00975+K01087+K00703+K01188+K00700+K15920+K13648+K01835+K13648+K01051+K13648+K16055+K01087+K0183

	ript_115338;F01_transcript_115568;F01_transcript_115602;F01_transcript_11561;F01_transcript_115746;F01_transcript_115783;F01_transcript_115825;F01_transcript_116135;F01_transcript_116231;F01_transcript_116260;F01_transcript_11628;F01_transcript_116289;F01_transcript_116347;F01_transcript_116518;F01_transcript_116752;F01_transcript_11718;F01_transcript_117181;F01_transcript_117555;F01_transcript_11778;F01_transcript_11781;F01_transcript_117826;F01_transcript_117829;F01_transcript_117950;F01_transcript_117969;F01_transcript_118006;F01_transcript_118040;F01_transcript_118051;F01_transcript_118131;F01_transcript_118282;F01_transcript_119096;F01_transcript_119230;F01_transcript_119458;F01_transcript_12004;F01_transcript_120369;F01_transcript_120374;F01_transcript_120405;F01_transcript_120858;F01_transcript_121042;F01_transcript_121070;F01_transcript_121401;F01_transcript_121410;F01_transcript_121518;F01_transcript_121545;F01_transcript_121575;F01_transcript_121687;F01_transcript_121945;F01_transcript_122065;F01_transcript_12279;F01_transcript_122807;F01_transcript_122844;F01_transcript_122916;F01_transcript_123438;F01_transcript_123733;F01_transcript_123750;F01_transcript_124036;F01_transcript_124056;F01_transcript_124163;F01_transcript_124608;F01_transcript_125005;F01_transcript_125394;F01_transcript_125495;F01_transcript_125806;F01_transcript_126035;F01_transcript_126146;F01_transcript_126186;F01_transcript_126250;F01_transcript_12633;F01_transcript_126605;F01_transcript_126746;F01_transcript_12698;F01_transcript_127019;F01_transcript_127138;F01_transcript_127221;F01_transcript_127250;F01_transcript_12737;F01_transcript_127568;F01_transcript_127751;F01_transcript_127885;F01_transcript_128233;F01_transcript_128274;F01_transcript_128370;F01_transcript_128562;F01_transcript_128844;F01_transcript_128862;F01_transcript_128875;F01_transcript_128893;F01_transcript_128959;F01_transcript_128960;F01_transcript_129072;F01_transcript_129093;F01_transcript_129113;F01_transcript_129190;F01_transcript_129319;F01_transcript_129392;F01_transcript_129477;F01_transcript_129602;F01_transcript_129606;F01_transcript_129612;F01_transcript_129845;F01_transcript_129917;F01_transcript_130179;F01_transcript_130194;F01_transcript_130378;F01_transcrip	5+K00012+K01810+K00695+K05349+K01177+K16055+K16055+K05349+K16055+K00703+K00975+K00975+K00847+K19893+K01188+K01835+K00012+K01051+K01176+K01194+K01810+K13648+K13648+K00844+K00703+K20867+K01087+K01188+K00703+K16055+K01193+K00703+K01177+K13648+K05349+K01835+K00847+K05350+K00703+K01810+K00703+K16055+K01188+K00975+K01188+K00700+K05350+K01188+K16055+K16055+K00700+K00696+K01188+K01513+K00703+K01187+K16055+K00688+K18447+K13648+K16055+K00695+K05350+K01176+K01177+K19892+K08678+K00688+K01835+K00696+K01051+K00963+K01177+K00700+K01188+K00696+K00695+K00695+K05349+K00695+K01187+K16055+K00700+K00703+K00695+K01087+K13648+K16055+K15920+K00696+K19891+K00963+K01188+K00844+K00847+K08679+K08678+K16055+K01194+K13648+K05350+K13648+K00688+K00696+K00695+K00688+K01188+K00705+K05349+K01087+K00688+K00975+K01188+K00700+K13648+K00700+K00695+K01087+K01513+K13648+K00688+K13648+K01188+K00703+K00703+K01177+K01051+K00975+K16055+K00695+K01188+K01051+K01087+K00696+K13648+K08678+K00963+K01835+K20867+K00695+K01188+K05350+K01835+K00696+K08678+K01051+K08678+K16055+K01835+K13648+K00012+K00847+K01188+K16055+K00688+K01188+K05350+K08678+K20867+K00703+K01188+K00975+K08678+K00688+K00695+K01179+K00700+K16055+K15920+K01177+K01188+K00695+K05350+K05350+K05349+K16055+K01188+K00696+K13648+K00695+K00695+K00844+K00700+K00975+K19891+K08678+K00703+K13648+K00700+K00696+K19893+K00975+K08679+K01810+K00695+K00695+K05349+K05350+K16055+K13648+K05349+K05350+K16055+K01810+K00696+K05350+K16055+K01177+K00975+K00703+K01835+K01835+K19893+K01188+K01188+K00700+K00700+K00975+K00705+K01188+K05349+K13648+K00695+K01188+K16055+K00963+K00975+K00963+K01193+K00696+K00696+K13648+K00963+K15920+K00696+K20867+K01193+K20867+K01810+K13648+K05349+K01193+K01835+K00703+K00696+K13648+K05349+K01176+K01810+K00703+K00703+K00703+K01087+K20867+K01188+K20867+K00696+K00696+K05349+K01835+K0

	t_130383;F01_transcript_130480;F01_transcript_130570;F01_tr	1177+K01177+K01177+K01188+K00975+K20867+K01194+K
	anscript_130777;F01_transcript_131014;F01_transcript_131304	13648+K01177+K00700+K00700+K05349+K00705+K13648+
	;F01_transcript_131327;F01_transcript_131338;F01_transcript_	K01835+K01835+K05349+K01051+K13648+K05349+K01835
	131553;F01_transcript_131846;F01_transcript_131849;F01_tran	+K00975+K01177+K05349+K00844+K01177+K13648+K0535
	script_132067;F01_transcript_132121;F01_transcript_132136;F	0+K08679+K05350+K13648+K01177+K01513+K01177+K011
	01_transcript_132273;F01_transcript_132330;F01_transcript_13	77+K19891+K01177+K00844+K08678+K01188+K08678+K01
	237;F01_transcript_132401;F01_transcript_132414;F01_transcri	051+K00975+K00703+K05350+K01177+K01188+K01188+K1
	pt_132585;F01_transcript_132740;F01_transcript_132804;F01_t	9892+K05350+K00705+K01810+K00705+K08678+K05350+K
	ranscript_132918;F01_transcript_132921;F01_transcript_13295	00975+K01177+K08679+K08678+K01051+K00963+K00975+
	5;F01_transcript_133256;F01_transcript_133403;F01_transcript	K08678+K00963+K05350+K01193+K01051+K00696+K00963
	_133762;F01_transcript_133779;F01_transcript_133921;F01_tra	+K00975+K01188+K16055+K08678+K00012+K00700+K1989
	nscript_134078;F01_transcript_134167;F01_transcript_134380;	2+K05350+K08678+K13648+K01177+K01051+K20867+K009
	F01_transcript_134387;F01_transcript_134473;F01_transcript_1	63+K01193+K05350+K01051+K08678+K00705+K08679+K00
	34505;F01_transcript_134748;F01_transcript_135131;F01_trans	012+K01188+K01188+K01188+K00847+K05350+K00847+K0
	cript_135333;F01_transcript_135346;F01_transcript_135427;F0	0847+K01188+K01188+K16055+K00688+K08678+K16055+K
	1_transcript_135608;F01_transcript_135613;F01_transcript_135	00700+K16055+K13648+K00705+K01051+K01087+K16055+
	678;F01_transcript_135756;F01_transcript_135864;F01_transcri	K01176+K08678+K08678+K00847+K16055+K08678+K01176
	pt_135889;F01_transcript_13631;F01_transcript_136576;F01_tr	+K18447+K16055+K01188+K00705+K16055+K00688+K0068
	anscript_136619;F01_transcript_13674;F01_transcript_136949;	8+K00705+K16055+K00695+K00700+K00847+K01188+K010
	F01_transcript_137006;F01_transcript_137051;F01_transcript_1	51+K00688+K00688+K00700+K13648+K00703+K16055+K20
	37289;F01_transcript_13739;F01_transcript_137403;F01_transc	867+K01177+K00695+K00696+K16055+K00975+K00703+K0
	ript_137623;F01_transcript_138073;F01_transcript_138640;F01	0688+K00688+K01188+K16055+K16055+K13648+K00703+K
	_transcript_138739;F01_transcript_138795;F01_transcript_1388	05349+K05350+K01087+K16055+K00963+K00696+K08679+
	77;F01_transcript_138933;F01_transcript_138970;F01_transcrip	K00700+K00695+K01176+K01188+K05350+K00700+K01193
	t_139107;F01_transcript_139196;F01_transcript_139337;F01_tr	+K15920+K01051+K01835+K01177+K01810+K01188+K0070
	anscript_139446;F01_transcript_139496;F01_transcript_139597	3+K16055+K13648+K00975+K13648+K01051+K01177+K011
	;F01_transcript_139751;F01_transcript_139883;F01_transcript_	76+K16055+K01194+K16055+K00695+K05349+K13648+K00
	139915;F01_transcript_140007;F01_transcript_140192;F01_tran	703+K00703+K01188+K00696+K16055+K00975+K16055+K0
	script_140638;F01_transcript_14085;F01_transcript_141047;F0	0700+K00844+K00012+K16055+K16055+K16055+K01051+K
	1_transcript_141384;F01_transcript_141500;F01_transcript_141	00975+K00688+K00695+K16055+K13648+K01188+K00695+
	572;F01_transcript_141737;F01_transcript_141920;F01_transcri	K05349+K00700+K00695+K16055+K01810+K00688+K01087
	pt_142532;F01_transcript_142913;F01_transcript_143101;F01_t	+K00688+K00975+K16055+K13648+K00703+K00688+K0069
	ranscript_143171;F01_transcript_143463;F01_transcript_14352	6+K00705+K01177+K16055+K00703+K01177+K00700+K006
	4;F01_transcript_143548;F01_transcript_143688;F01_transcript	95+K01177+K00700+K00700+K00696+K08678+K00844+K00
	_143707;F01_transcript_144131;F01_transcript_144286;F01_tra	696+K08679+K05349+K20867+K00696+K00700+K00695+K0
	nscript_144510;F01_transcript_144580;F01_transcript_144655;	0696+K01835+K01051+K01810+K16055+K01051+K00700+K
	F01_transcript_144697;F01_transcript_145128;F01_transcript_1	13648+K00695+K01187+K20867+K01177+K13648+K01188+

	45380;F01_transcript_145881;F01_transcript_145966;F01_transcript_146395;F01_transcript_146501;F01_transcript_146512;F01_transcript_146609;F01_transcript_146657;F01_transcript_146687;F01_transcript_146837;F01_transcript_147498;F01_transcript_147688;F01_transcript_147788;F01_transcript_147923;F01_transcript_147992;F01_transcript_148041;F01_transcript_148281;F01_transcript_148470;F01_transcript_148567;F01_transcript_148717;F01_transcript_148771;F01_transcript_148899;F01_transcript_148902;F01_transcript_148971;F01_transcript_149283;F01_transcript_149414;F01_transcript_149772;F01_transcript_149823;F01_transcript_149911;F01_transcript_150273;F01_transcript_150421;F01_transcript_150535;F01_transcript_150552;F01_transcript_150556;F01_transcript_150926;F01_transcript_151093;F01_transcript_151133;F01_transcript_151155;F01_transcript_151253;F01_transcript_151433;F01_transcript_151441;F01_transcript_15179;F01_transcript_152009;F01_transcript_152096;F01_transcript_152198;F01_transcript_152295;F01_transcript_152365;F01_transcript_152559;F01_transcript_152598;F01_transcript_15286;F01_transcript_152983;F01_transcript_153136;F01_transcript_153240;F01_transcript_153400;F01_transcript_153903;F01_transcript_154100;F01_transcript_154191;F01_transcript_155544;F01_transcript_155592;F01_transcript_155598;F01_transcript_155968;F01_transcript_155976;F01_transcript_156051;F01_transcript_156083;F01_transcript_156116;F01_transcript_156178;F01_transcript_156470;F01_transcript_156540;F01_transcript_156579;F01_transcript_156765;F01_transcript_156780;F01_transcript_156789;F01_transcript_157611;F01_transcript_157846;F01_transcript_157986;F01_transcript_158123;F01_transcript_158173;F01_transcript_158249;F01_transcript_158252;F01_transcript_15905;F01_transcript_159145;F01_transcript_159225;F01_transcript_159376;F01_transcript_159430;F01_transcript_159513;F01_transcript_159596;F01_transcript_159800;F01_transcript_159850;F01_transcript_159902;F01_transcript_159905;F01_transcript_160085;F01_transcript_160423;F01_transcript_160671;F01_transcript_160731;F01_transcript_160753;F01_transcript_160784;F01_transcript_160988;F01_transcript_161118;F01_transcript_161192;F01_transcript_161548;F01_transcript_16165;F01_transcript_161814;F01_transcript_161914;F01_transcrip	K16055+K00975+K16055+K05350+K01051+K13648+K00696+K16055+K00696+K20867+K16055+K00695+K16055+K19893+K00688+K00844+K00695+K05349+K01177+K00847+K00696+K00688+K01177+K00700+K08678+K13648+K05349+K13648+K00700+K13648+K00688+K00975+K16055+K00695+K01810+K00695+K00700+K05350+K01810+K00705+K05350+K16055+K01051+K00975+K00688+K00703+K00703+K00847+K00688+K00844+K00696+K00700+K00696+K01087+K00700+K16055+K05349+K00847+K00700+K01810+K01177+K01835+K16055+K16055+K01188+K08678+K01051+K13648+K00700+K00696+K01188+K16055+K13648+K00695+K00703+K00695+K01188+K16055+K01193+K01810+K00703+K01188+K00705+K00688+K05350+K05350+K05350+K05350+K00847+K00847+K01051+K01177+K01177+K19891+K13648+K00703+K16055+K01188+K01187+K13648+K00700+K00844+K01835+K01051+K05350+K20867+K00847+K00695+K01187+K00703+K01176+K00975+K00703+K01177+K05349+K00703+K01187+K13648+K00695+K00975+K00695+K05349+K01177+K00963+K00696+K01188+K01051+K05350+K00695+K16055+K00703+K00695+K10268+K00703+K00696+K00703+K01188+K13648+K01177+K13648+K00703+K05349+K00695+K00975+K01177+K00703+K01051+K00696+K00695+K00695+K01835+K05350+K19891+K05350+K19893+K13648+K08678+K00695+K01810+K00703+K00696+K16055+K05349+K00695+K00847+K01177+K00695+K00695+K01188+K01188+K01051+K16055+K01087+K13648+K00695+K01051+K01176+K01188+K00975+K01187+K05350+K00844+K05350+K00700+K00963+K05349+K00695+K00696+K00696+K01184

t_161918;F01_transcript_162228;F01_transcript_162246;F01_transcript_162413;F01_transcript_162516;F01_transcript_162574;F01_transcript_162778;F01_transcript_163066;F01_transcript_163438;F01_transcript_163697;F01_transcript_163919;F01_transcript_164028;F01_transcript_164032;F01_transcript_164096;F01_transcript_164264;F01_transcript_164292;F01_transcript_164390;F01_transcript_164470;F01_transcript_164741;F01_transcript_165033;F01_transcript_165071;F01_transcript_165116;F01_transcript_165159;F01_transcript_165247;F01_transcript_165335;F01_transcript_165364;F01_transcript_165486;F01_transcript_165502;F01_transcript_165690;F01_transcript_165871;F01_transcript_166075;F01_transcript_166119;F01_transcript_166184;F01_transcript_16620;F01_transcript_166546;F01_transcript_166552;F01_transcript_166761;F01_transcript_166813;F01_transcript_166833;F01_transcript_166888;F01_transcript_16700;F01_transcript_167157;F01_transcript_167210;F01_transcript_167408;F01_transcript_167654;F01_transcript_16844;F01_transcript_17487;F01_transcript_17520;F01_transcript_1768;F01_transcript_1824;F01_transcript_18663;F01_transcript_18794;F01_transcript_18799;F01_transcript_1899;F01_transcript_19039;F01_transcript_19300;F01_transcript_19344;F01_transcript_19381;F01_transcript_19477;F01_transcript_19594;F01_transcript_19639;F01_transcript_19835;F01_transcript_19957;F01_transcript_2041;F01_transcript_20473;F01_transcript_20653;F01_transcript_20797;F01_transcript_21106;F01_transcript_21161;F01_transcript_21223;F01_transcript_21326;F01_transcript_21377;F01_transcript_21459;F01_transcript_21793;F01_transcript_22146;F01_transcript_2242;F01_transcript_2273;F01_transcript_22769;F01_transcript_22972;F01_transcript_23222;F01_transcript_23295;F01_transcript_23505;F01_transcript_23513;F01_transcript_23808;F01_transcript_23934;F01_transcript_23992;F01_transcript_23996;F01_transcript_24398;F01_transcript_2445;F01_transcript_2479;F01_transcript_24985;F01_transcript_2503;F01_transcript_25375;F01_transcript_25381;F01_transcript_25470;F01_transcript_25483;F01_transcript_25848;F01_transcript_25878;F01_transcript_25948;F01_transcript_25979;F01_transcript_26024;F01_transcript_26156;F01_transcript_26197;F01_transcript_26688;

	F01_transcript_26696;F01_transcript_26853;F01_transcript_26984;F01_transcript_27087;F01_transcript_27120;F01_transcript_27125;F01_transcript_27166;F01_transcript_27218;F01_transcript_27467;F01_transcript_27604;F01_transcript_27630;F01_transcript_27680;F01_transcript_27931;F01_transcript_28011;F01_transcript_28050;F01_transcript_28813;F01_transcript_28995;F01_transcript_29026;F01_transcript_29030;F01_transcript_29052;F01_transcript_29268;F01_transcript_29294;F01_transcript_29312;F01_transcript_29331;F01_transcript_29437;F01_transcript_29528;F01_transcript_29538;F01_transcript_29773;F01_transcript_29783;F01_transcript_29784;F01_transcript_29910;F01_transcript_29960;F01_transcript_30065;F01_transcript_30159;F01_transcript_30166;F01_transcript_30280;F01_transcript_30303;F01_transcript_30341;F01_transcript_30433;F01_transcript_30650;F01_transcript_30850;F01_transcript_30911;F01_transcript_3109;F01_transcript_31186;F01_transcript_31524;F01_transcript_31528;F01_transcript_3153;F01_transcript_31677;F01_transcript_31810;F01_transcript_3187;F01_transcript_31891;F01_transcript_32171;F01_transcript_32240;F01_transcript_32267;F01_transcript_32355;F01_transcript_32468;F01_transcript_32617;F01_transcript_32619;F01_transcript_32632;F01_transcript_32819;F01_transcript_32847;F01_transcript_33069;F01_transcript_3313;F01_transcript_33130;F01_transcript_33161;F01_transcript_33214;F01_transcript_33678;F01_transcript_34009;F01_transcript_34086;F01_transcript_34522;F01_transcript_34671;F01_transcript_34966;F01_transcript_35049;F01_transcript_35183;F01_transcript_3539;F01_transcript_3543;F01_transcript_36518;F01_transcript_3652;F01_transcript_3727;F01_transcript_3745;F01_transcript_38255;F01_transcript_3849;F01_transcript_38532;F01_transcript_38572;F01_transcript_3866;F01_transcript_38798;F01_transcript_39309;F01_transcript_39815;F01_transcript_39890;F01_transcript_4010;F01_transcript_40185;F01_transcript_40443;F01_transcript_40717;F01_transcript_4096;F01_transcript_41284;F01_transcript_4202;F01_transcript_4280;F01_transcript_4346;F01_transcript_4644;F01_transcript_4785;F01_transcript_4809;F01_transcript_497;F01_transcript_5022;F01_transcript_50425;F01_transcript_50524;F01_transcript_5060;F01_transcript_5074;F01_transcript_5100;F01_transcript_5113;F01_transcript	

_51506;F01_transcript_51719;F01_transcript_5176;F01_transcript_518;F01_transcript_52031;F01_transcript_52218;F01_transcript_52234;F01_transcript_52335;F01_transcript_52513;F01_transcript_52514;F01_transcript_5256;F01_transcript_5281;F01_transcript_52855;F01_transcript_52862;F01_transcript_5294;F01_transcript_53289;F01_transcript_53771;F01_transcript_53935;F01_transcript_53994;F01_transcript_54041;F01_transcript_5469;F01_transcript_54712;F01_transcript_55236;F01_transcript_55265;F01_transcript_5563;F01_transcript_55799;F01_transcript_55989;F01_transcript_55994;F01_transcript_56193;F01_transcript_5627;F01_transcript_56372;F01_transcript_56571;F01_transcript_56576;F01_transcript_56580;F01_transcript_56592;F01_transcript_56958;F01_transcript_57117;F01_transcript_57252;F01_transcript_5728;F01_transcript_57284;F01_transcript_57304;F01_transcript_57399;F01_transcript_57557;F01_transcript_57844;F01_transcript_57856;F01_transcript_58151;F01_transcript_58262;F01_transcript_58317;F01_transcript_58596;F01_transcript_58663;F01_transcript_59072;F01_transcript_59257;F01_transcript_59356;F01_transcript_60080;F01_transcript_60304;F01_transcript_60358;F01_transcript_60443;F01_transcript_60483;F01_transcript_60704;F01_transcript_60736;F01_transcript_60764;F01_transcript_60802;F01_transcript_60824;F01_transcript_6088;F01_transcript_60948;F01_transcript_60954;F01_transcript_61876;F01_transcript_62427;F01_transcript_62457;F01_transcript_62612;F01_transcript_62639;F01_transcript_62656;F01_transcript_62701;F01_transcript_62865;F01_transcript_6298;F01_transcript_63203;F01_transcript_63241;F01_transcript_63455;F01_transcript_63468;F01_transcript_635;F01_transcript_63625;F01_transcript_63688;F01_transcript_63974;F01_transcript_64050;F01_transcript_64243;F01_transcript_64329;F01_transcript_6445;F01_transcript_64450;F01_transcript_64769;F01_transcript_64796;F01_transcript_64810;F01_transcript_64859;F01_transcript_64907;F01_transcript_64921;F01_transcript_65023;F01_transcript_6518;F01_transcript_65228;F01_transcript_65469;F01_transcript_65494;F01_transcript_65529;F01_transcript_65836;F01_transcript_65838;F01_transcript_65854;F01_transcript_65874;F01_transcript_65985;F01_transcript_66007;F01_transcript_66

581;F01_transcript_66664;F01_transcript_66723;F01_transcript_66746;F01_transcript_6685;F01_transcript_67044;F01_transcript_67047;F01_transcript_67357;F01_transcript_67618;F01_transcript_67795;F01_transcript_68723;F01_transcript_68753;F01_transcript_68819;F01_transcript_68840;F01_transcript_69023;F01_transcript_69101;F01_transcript_69180;F01_transcript_69406;F01_transcript_69632;F01_transcript_70121;F01_transcript_70209;F01_transcript_70230;F01_transcript_70274;F01_transcript_70354;F01_transcript_70381;F01_transcript_70383;F01_transcript_70526;F01_transcript_70577;F01_transcript_7092;F01_transcript_70970;F01_transcript_7115;F01_transcript_71241;F01_transcript_71250;F01_transcript_71450;F01_transcript_71570;F01_transcript_71653;F01_transcript_71924;F01_transcript_72235;F01_transcript_72779;F01_transcript_73066;F01_transcript_73186;F01_transcript_73189;F01_transcript_73411;F01_transcript_73460;F01_transcript_7358;F01_transcript_73725;F01_transcript_7386;F01_transcript_73876;F01_transcript_73888;F01_transcript_74032;F01_transcript_74285;F01_transcript_74577;F01_transcript_74751;F01_transcript_74919;F01_transcript_75064;F01_transcript_75335;F01_transcript_75657;F01_transcript_76141;F01_transcript_76547;F01_transcript_76551;F01_transcript_76586;F01_transcript_76648;F01_transcript_76669;F01_transcript_76768;F01_transcript_76931;F01_transcript_77465;F01_transcript_77477;F01_transcript_78025;F01_transcript_7805;F01_transcript_78148;F01_transcript_78182;F01_transcript_78310;F01_transcript_78522;F01_transcript_7908;F01_transcript_79193;F01_transcript_79249;F01_transcript_7944;F01_transcript_79538;F01_transcript_79800;F01_transcript_80240;F01_transcript_80245;F01_transcript_80661;F01_transcript_80818;F01_transcript_81108;F01_transcript_81126;F01_transcript_81233;F01_transcript_81435;F01_transcript_81477;F01_transcript_8161;F01_transcript_8171;F01_transcript_81833;F01_transcript_81950;F01_transcript_82183;F01_transcript_8235;F01_transcript_82390;F01_transcript_82466;F01_transcript_82721;F01_transcript_82990;F01_transcript_83222;F01_transcript_83296;F01_transcript_83393;F01_transcript_83418;F01_transcript_8370;F01_transcript_83764;F01_transcript_84044;F01_transcript_84158;F01_transcript_84322;F01_transcript_84347;F01_transcript_84420;F01_transcrip

			t_84572;F01_transcript_84738;F01_transcript_84796;F01_transcript_8481;F01_transcript_85034;F01_transcript_86090;F01_transcript_86116;F01_transcript_86207;F01_transcript_86371;F01_transcript_86419;F01_transcript_86553;F01_transcript_86749;F01_transcript_87511;F01_transcript_8759;F01_transcript_87754;F01_transcript_87775;F01_transcript_87929;F01_transcript_87952;F01_transcript_88127;F01_transcript_88605;F01_transcript_8868;F01_transcript_89020;F01_transcript_891;F01_transcript_8938;F01_transcript_89486;F01_transcript_89571;F01_transcript_89585;F01_transcript_89779;F01_transcript_89833;F01_transcript_90143;F01_transcript_90214;F01_transcript_90237;F01_transcript_90419;F01_transcript_90474;F01_transcript_90634;F01_transcript_9091;F01_transcript_90976;F01_transcript_91198;F01_transcript_9161;F01_transcript_91826;F01_transcript_9190;F01_transcript_91915;F01_transcript_92001;F01_transcript_92096;F01_transcript_92149;F01_transcript_92310;F01_transcript_92681;F01_transcript_92745;F01_transcript_93416;F01_transcript_93485;F01_transcript_93563;F01_transcript_93594;F01_transcript_93843;F01_transcript_93906;F01_transcript_93990;F01_transcript_94317;F01_transcript_94343;F01_transcript_94378;F01_transcript_94626;F01_transcript_9486;F01_transcript_94861;F01_transcript_94872;F01_transcript_94879;F01_transcript_95055;F01_transcript_95336;F01_transcript_95572;F01_transcript_95749;F01_transcript_95784;F01_transcript_9588;F01_transcript_95936;F01_transcript_95981;F01_transcript_95997;F01_transcript_96109;F01_transcript_96241;F01_transcript_96323;F01_transcript_96468;F01_transcript_96528;F01_transcript_96826;F01_transcript_9730;F01_transcript_97306;F01_transcript_97412;F01_transcript_97517;F01_transcript_97889;F01_transcript_98212;F01_transcript_98252;F01_transcript_98615;F01_transcript_98699;F01_transcript_98978;F01_transcript_98980;F01_transcript_99044;F01_transcript_99106;F01_transcript_99214;F01_transcript_993;F01_transcript_99439;F01_transcript_99670;	
N-Glycan biosy	ko00	244	F01_transcript_100026;F01_transcript_100573;F01_transcript_101391;F01_transcript_101445;F01_transcript_102463;F01_transcript_103656;F01_transcript_105656;F01_transcript_106876;F01_transcript_107104;F01_transcript_10728;F01_transcript_1076	K09659+K03847+K03842+K03844+K07252+K05546+K12669+K07441+K07441+K12666+K09659+K03842+K01230+K07441+K12667+K03850+K12667+K00721+K01230+K07432+K09659+K12666+K01228+K01231+K03842+K03850+K03846+K00

nthesis	510	14;F01_transcript_109345;F01_transcript_110472;F01_transcript_110758;F01_transcript_110957;F01_transcript_111808;F01_transcript_112978;F01_transcript_114239;F01_transcript_114682;F01_transcript_115672;F01_transcript_116311;F01_transcript_116659;F01_transcript_117645;F01_transcript_117955;F01_transcript_118293;F01_transcript_119358;F01_transcript_119499;F01_transcript_119886;F01_transcript_121923;F01_transcript_123328;F01_transcript_123452;F01_transcript_123879;F01_transcript_124942;F01_transcript_125688;F01_transcript_126007;F01_transcript_126192;F01_transcript_126462;F01_transcript_12657;F01_transcript_127867;F01_transcript_128405;F01_transcript_128462;F01_transcript_129848;F01_transcript_13043;F01_transcript_130579;F01_transcript_130653;F01_transcript_131325;F01_transcript_131456;F01_transcript_132229;F01_transcript_132380;F01_transcript_13245;F01_transcript_132719;F01_transcript_132932;F01_transcript_134100;F01_transcript_13412;F01_transcript_134175;F01_transcript_134361;F01_transcript_13437;F01_transcript_13466;F01_transcript_136521;F01_transcript_138009;F01_transcript_138829;F01_transcript_138863;F01_transcript_139321;F01_transcript_139514;F01_transcript_139729;F01_transcript_141830;F01_transcript_145265;F01_transcript_145647;F01_transcript_145712;F01_transcript_147309;F01_transcript_148356;F01_transcript_148610;F01_transcript_149268;F01_transcript_149417;F01_transcript_149434;F01_transcript_150466;F01_transcript_150471;F01_transcript_15048;F01_transcript_151097;F01_transcript_151449;F01_transcript_151966;F01_transcript_152678;F01_transcript_152976;F01_transcript_153687;F01_transcript_153971;F01_transcript_154365;F01_transcript_154406;F01_transcript_155919;F01_transcript_156131;F01_transcript_156608;F01_transcript_157046;F01_transcript_158004;F01_transcript_158267;F01_transcript_159784;F01_transcript_161007;F01_transcript_161037;F01_transcript_16248;F01_transcript_163015;F01_transcript_163230;F01_transcript_163255;F01_transcript_163684;F01_transcript_163851;F01_transcript_163869;F01_transcript_163911;F01_transcript_164425;F01_transcript_16470;F01_transcript_165168;F01_transcript_166356;F01_transcript_166948;F01_transcript_19928;F01_transcript_20289;F01_transcript_23167;F01_transcript_23777;F01_transcript_23894;F01	737+K07432+K03850+K03843+K12669+K03845+K03846+K07432+K03850+K00721+K07151+K03847+K01228+K03848+K12669+K07151+K03849+K01228+K01231+K00737+K00721+K03845+K01230+K03845+K03850+K07151+K07151+K12670+K07441+K07151+K12667+K00737+K01228+K01228+K01230+K00737+K00729+K01230+K00726+K00902+K05546+K01230+K00729+K00737+K01230+K07151+K03846+K01001+K03846+K07252+K12667+K03848+K12666+K03842+K12666+K07441+K12666+K12666+K00737+K12666+K09658+K01230+K00737+K03850+K03847+K03842+K03850+K00729+K01228+K07151+K01230+K00726+K03842+K12666+K00737+K12667+K12667+K05546+K01230+K00737+K07151+K07441+K07441+K01230+K01230+K03850+K03846+K00737+K07441+K07252+K03844+K00737+K01230+K03844+K12666+K01001+K03845+K00737+K03848+K00726+K00737+K00726+K12670+K01228+K03848+K12666+K03842+K12670+K01001+K01001+K12667+K12670+K12666+K03848+K07252+K12669+K07252+K07252+K12668+K09658+K12668+K01231+K03845+K07151+K00736+K03850+K00729+K01001+K03846+K03844+K07151+K01230+K01230+K00729+K05546+K03846+K05546+K00726+K03843+K03845+K07151+K01001+K01001+K09659+K03845+K05546+K03843+K07151+K01230+K03844+K12666+K03843+K01001+K03849+K00729+K03842+K01230+K03850+K12667+K03843+K07252+K01231+K03849+K12667+K03843+K12666+K07151+K03842+K03847+K03842+K01001+K07441+K00726+K03848+K07151+K03843+K01228+K01228+K03847+K01231+K01231+K03848+K12668+K12667+K03846+K05546+K03848+K01228+K03845+K00902+K01230+K03850+K01231+K01001+K12667+K12666+K03843+K03846+K01231+K05546+K03848+K03845+K03848+K00721+K01231+K00726+K03842+K05546+K03848+K00726+K00729+K01231+K07151+K03848+K03850+K05546+K01228

_transcript_24623;F01_transcript_25385;F01_transcript_26178;F01_transcript_26826;F01_transcript_26963;F01_transcript_27416;F01_transcript_27568;F01_transcript_28621;F01_transcript_29178;F01_transcript_29304;F01_transcript_29748;F01_transcript_30124;F01_transcript_30300;F01_transcript_30386;F01_transcript_30536;F01_transcript_30672;F01_transcript_3086;F01_transcript_30938;F01_transcript_33520;F01_transcript_34416;F01_transcript_35046;F01_transcript_35565;F01_transcript_35682;F01_transcript_3790;F01_transcript_38884;F01_transcript_40228;F01_transcript_41426;F01_transcript_42134;F01_transcript_42331;F01_transcript_42550;F01_transcript_45929;F01_transcript_49086;F01_transcript_49148;F01_transcript_49326;F01_transcript_51416;F01_transcript_51732;F01_transcript_51785;F01_transcript_51793;F01_transcript_51957;F01_transcript_51974;F01_transcript_53373;F01_transcript_53426;F01_transcript_53779;F01_transcript_54047;F01_transcript_54082;F01_transcript_54567;F01_transcript_56131;F01_transcript_5614;F01_transcript_56329;F01_transcript_5647;F01_transcript_56622;F01_transcript_56719;F01_transcript_57380;F01_transcript_57585;F01_transcript_5830;F01_transcript_5845;F01_transcript_61903;F01_transcript_63110;F01_transcript_6334;F01_transcript_63502;F01_transcript_63812;F01_transcript_64004;F01_transcript_64837;F01_transcript_64863;F01_transcript_65064;F01_transcript_65172;F01_transcript_65761;F01_transcript_66069;F01_transcript_66472;F01_transcript_68433;F01_transcript_68676;F01_transcript_68846;F01_transcript_68907;F01_transcript_69735;F01_transcript_69784;F01_transcript_70078;F01_transcript_70807;F01_transcript_71755;F01_transcript_72387;F01_transcript_73614;F01_transcript_74624;F01_transcript_74917;F01_transcript_74977;F01_transcript_75009;F01_transcript_75424;F01_transcript_75672;F01_transcript_75782;F01_transcript_79109;F01_transcript_79424;F01_transcript_79632;F01_transcript_79713;F01_transcript_79783;F01_transcript_811;F01_transcript_81224;F01_transcript_82449;F01_transcript_82658;F01_transcript_82852;F01_transcript_83050;F01_transcript_8310;F01_transcript_83348;F01_transcript_85253;F01_transcript_85875;F01_transcript_86111;F01_transcript_86542;F01_transcript_8674;F01_transcript_870;F0

			1_transcript_88212;F01_transcript_88310;F01_transcript_89652;F01_transcript_89809;F01_transcript_90094;F01_transcript_90317;F01_transcript_91421;F01_transcript_92027;F01_transcript_92243;F01_transcript_92993;F01_transcript_93646;F01_transcript_939;F01_transcript_95564;F01_transcript_95731;F01_transcript_96158;F01_transcript_96791;F01_transcript_96795;F01_transcript_96845;F01_transcript_96928;F01_transcript_9741;F01_transcript_98766;F01_transcript_98826;F01_transcript_99477;F01_transcript_99571;	
Other glycan degradation	ko00511	220	F01_transcript_100724;F01_transcript_100741;F01_transcript_102327;F01_transcript_104288;F01_transcript_108175;F01_transcript_108387;F01_transcript_110359;F01_transcript_110365;F01_transcript_110407;F01_transcript_1109;F01_transcript_112177;F01_transcript_112922;F01_transcript_113188;F01_transcript_113345;F01_transcript_113680;F01_transcript_113792;F01_transcript_114405;F01_transcript_114730;F01_transcript_115234;F01_transcript_115346;F01_transcript_116161;F01_transcript_116511;F01_transcript_116673;F01_transcript_118827;F01_transcript_121357;F01_transcript_122091;F01_transcript_122366;F01_transcript_122378;F01_transcript_123915;F01_transcript_125881;F01_transcript_126182;F01_transcript_126483;F01_transcript_126872;F01_transcript_128440;F01_transcript_129841;F01_transcript_130544;F01_transcript_131516;F01_transcript_1335;F01_transcript_134028;F01_transcript_134304;F01_transcript_134517;F01_transcript_135688;F01_transcript_136287;F01_transcript_137546;F01_transcript_137553;F01_transcript_138035;F01_transcript_138660;F01_transcript_138772;F01_transcript_139036;F01_transcript_13991;F01_transcript_140383;F01_transcript_140684;F01_transcript_14203;F01_transcript_142092;F01_transcript_142941;F01_transcript_143645;F01_transcript_143719;F01_transcript_143884;F01_transcript_143925;F01_transcript_144342;F01_transcript_144857;F01_transcript_145772;F01_transcript_146835;F01_transcript_147021;F01_transcript_148565;F01_transcript_148631;F01_transcript_149436;F01_transcript_149690;F01_transcript_150720;F01_transcript_150760;F01_transcript_150823;F01_transcript_150856;F01_transcript_150971;F01_transcript_151375;F01_transcript_151522;F01_transcript_152172;F01_transcript_153747;F01_transcript_153858;F01_transcript_	K12373+K01190+K01227+K17108+K12373+K12309+K01191+K01191+K01191+K17108+K12309+K12309+K12309+K12309+K01191+K01191+K01191+K15923+K01191+K01191+K01444+K17108+K01191+K17108+K01191+K01191+K15923+K01191+K01190+K12373+K12309+K17108+K12373+K12373+K01191+K01191+K01191+K01191+K01191+K01191+K01191+K15923+K01444+K15923+K01191+K17108+K01191+K01191+K01191+K12309+K17108+K17108+K12309+K01191+K01191+K01444+K01191+K12373+K01444+K12373+K12309+K01190+K12373+K17108+K12373+K17108+K01191+K17108+K01191+K01444+K12373+K01191+K01191+K01191+K17108+K01444+K17108+K01191+K17108+K01191+K01206+K01191+K12373+K01191+K12373+K12373+K01191+K12373+K01190+K01444+K12309+K01191+K01190+K01191+K12309+K15923+K01191+K01191+K01191+K17108+K12373+K17108+K01190+K01191+K01206+K01191+K01191+K01191+K01227+K12309+K01191+K12373+K01191+K12373+K01191+K12373+K12373+K01191+K12373+K12373+K01191+K01191+K01206+K01206+K01206+K01191+K01191+K12373+K17108+K17108+K17108+K17108+K12373+K01191+K01191+K01191+K17108+K17108+K12373+K12309+K01191+K01190+K01227+K17108+K17108+K01191+K12373+K15923+K01191+K01191+K12373+K15923+K12309+K12373+K12309+K12373+K17108+K01444+K17108+K01191+K12373+K12373+K12373+K01191+K01190+K12373+K12373+K17108+K01191+K17108+K17108+K12373+K01190+K01191+K01191+K01206+K01191+K01191+K12373+K12373+K01191+K12373+K12373+K01191+K12373+K17108+K17108+K17108+K17108+K12373+K15923+K01191+K17108+K01191+K12373+K01191+K12373+K12373+K12373

	154208;F01_transcript_155435;F01_transcript_156396;F01_transcript_156715;F01_transcript_156812;F01_transcript_157064;F01_transcript_157698;F01_transcript_158489;F01_transcript_159866;F01_transcript_160207;F01_transcript_1603;F01_transcript_160512;F01_transcript_161299;F01_transcript_1616;F01_transcript_162418;F01_transcript_162430;F01_transcript_163495;F01_transcript_163534;F01_transcript_163895;F01_transcript_165134;F01_transcript_165299;F01_transcript_165484;F01_transcript_165965;F01_transcript_166200;F01_transcript_1668;F01_transcript_167164;F01_transcript_17486;F01_transcript_1771;F01_transcript_1776;F01_transcript_1867;F01_transcript_19266;F01_transcript_2181;F01_transcript_2281;F01_transcript_24661;F01_transcript_2480;F01_transcript_25080;F01_transcript_25544;F01_transcript_26723;F01_transcript_27130;F01_transcript_2894;F01_transcript_30356;F01_transcript_31504;F01_transcript_3161;F01_transcript_3166;F01_transcript_32570;F01_transcript_33273;F01_transcript_34092;F01_transcript_3420;F01_transcript_3437;F01_transcript_388;F01_transcript_4235;F01_transcript_4718;F01_transcript_4878;F01_transcript_52171;F01_transcript_52616;F01_transcript_53179;F01_transcript_53248;F01_transcript_53298;F01_transcript_5538;F01_transcript_55450;F01_transcript_57260;F01_transcript_57444;F01_transcript_57476;F01_transcript_58059;F01_transcript_58520;F01_transcript_5966;F01_transcript_59871;F01_transcript_59968;F01_transcript_60062;F01_transcript_60340;F01_transcript_60765;F01_transcript_61684;F01_transcript_61860;F01_transcript_62636;F01_transcript_63036;F01_transcript_63270;F01_transcript_63587;F01_transcript_6361;F01_transcript_63709;F01_transcript_64144;F01_transcript_64239;F01_transcript_64990;F01_transcript_65180;F01_transcript_65894;F01_transcript_6630;F01_transcript_66429;F01_transcript_66447;F01_transcript_6655;F01_transcript_6741;F01_transcript_68989;F01_transcript_69428;F01_transcript_69795;F01_transcript_7045;F01_transcript_7118;F01_transcript_71581;F01_transcript_71933;F01_transcript_72305;F01_transcript_72840;F01_transcript_73595;F01_transcript_74129;F01_transcript_75592;F01_transcript_75702;F01_transcript_76441;F01_transcript_76707;F01_transcript_77895;F01_transcript_79534;F0	+K01191+K12309+K01191+K15923+K17108+K01191+K01191+K01191+K01191+K01191+K01206+K12373+K01191+K17108+K01191+K15923+K01191+K12373+K01190+K01191+K12373

			1_transcript_79756;F01_transcript_8017;F01_transcript_81172;F01_transcript_8132;F01_transcript_81533;F01_transcript_82388;F01_transcript_82420;F01_transcript_82857;F01_transcript_82913;F01_transcript_82925;F01_transcript_83155;F01_transcript_83593;F01_transcript_84093;F01_transcript_84220;F01_transcript_84272;F01_transcript_84805;F01_transcript_87412;F01_transcript_87413;F01_transcript_87722;F01_transcript_87749;F01_transcript_88298;F01_transcript_88315;F01_transcript_88399;F01_transcript_89280;F01_transcript_90418;F01_transcript_90868;F01_transcript_93660;F01_transcript_94695;F01_transcript_94962;F01_transcript_95757;F01_transcript_9620;F01_transcript_96714;F01_transcript_96767;F01_transcript_97421;F01_transcript_97914;F01_transcript_98049;	
Other types of O-glycan biosynthesis	ko00514	42	F01_transcript_101190;F01_transcript_113508;F01_transcript_130096;F01_transcript_133853;F01_transcript_14565;F01_transcript_147621;F01_transcript_147889;F01_transcript_150002;F01_transcript_159951;F01_transcript_162386;F01_transcript_21854;F01_transcript_2294;F01_transcript_2360;F01_transcript_2512;F01_transcript_2751;F01_transcript_28577;F01_transcript_29545;F01_transcript_3244;F01_transcript_33485;F01_transcript_4107;F01_transcript_516;F01_transcript_54992;F01_transcript_56330;F01_transcript_57308;F01_transcript_58782;F01_transcript_61878;F01_transcript_67596;F01_transcript_68700;F01_transcript_70622;F01_transcript_74614;F01_transcript_78690;F01_transcript_80175;F01_transcript_8077;F01_transcript_82659;F01_transcript_85196;F01_transcript_85683;F01_transcript_87556;F01_transcript_88672;F01_transcript_89786;F01_transcript_96954;F01_transcript_98166;F01_transcript_99551;	K09667+K09667+K09667+K03691+K09667+K03691+K09667+K09667+K09667+K09667+K03691+K09667+K09667+K09667+K09667+K13667+K13667+K09667+K13667+K09667+K09667+K09667+K09667+K09667+K13667+K09667+K09667+K13667+K09667+K09667+K09667+K18134+K09667+K09667+K03691+K09667+K09667+K09667+K09667+K09667+K09667+K03691
Amino sugar and nucleotide sugar metabolism	ko00520	528	F01_transcript_100470;F01_transcript_100622;F01_transcript_100724;F01_transcript_100753;F01_transcript_101030;F01_transcript_101600;F01_transcript_101811;F01_transcript_102251;F01_transcript_102376;F01_transcript_102519;F01_transcript_103174;F01_transcript_103547;F01_transcript_103571;F01_transcript_104021;F01_transcript_104118;F01_transcript_104303;F01_transcript_104654;F01_transcript_104904;F01_transcript_104965;F01_transcript_105944;F01_transcript_106862;F01_transcript_107461;F01_transcript_107572;F01_transcript_108117;F01_transcript_108175;F01_transcript_108641;F01_transcript_10912;F	K01183+K01209+K12373+K13648+K00972+K15920+K13648+K13648+K00965+K00963+K01711+K00975+K01810+K00966+K18677+K00326+K00965+K00975+K13379+K00621+K16190+K01183+K10046+K01183+K12373+K01809+K13648+K13648+K01183+K01784+K17497+K00966+K13648+K00621+K00963+K08679+K00326+K00966+K13648+K17497+K12451+K13648+K13648+K01836+K20867+K01809+K01784+K13648+K13648+K01810+K13648+K01810+K13648+K01810+K01810+K01810+K12446+K01810+K12450+K13648+K01835+K13648+K00972+K00975+K12448+K20547+K13648+K13648+K009

	01_transcript_109156;F01_transcript_109163;F01_transcript_110029;F01_transcript_110195;F01_transcript_110373;F01_transcript_111325;F01_transcript_111626;F01_transcript_111765;F01_transcript_111869;F01_transcript_111996;F01_transcript_112202;F01_transcript_112251;F01_transcript_113335;F01_transcript_113549;F01_transcript_113616;F01_transcript_113834;F01_transcript_113853;F01_transcript_113898;F01_transcript_114021;F01_transcript_114697;F01_transcript_114931;F01_transcript_11504;F01_transcript_115044;F01_transcript_115261;F01_transcript_116260;F01_transcript_11628;F01_transcript_116289;F01_transcript_116347;F01_transcript_116518;F01_transcript_116561;F01_transcript_117555;F01_transcript_117685;F01_transcript_11781;F01_transcript_117826;F01_transcript_117829;F01_transcript_117881;F01_transcript_118040;F01_transcript_118360;F01_transcript_119495;F01_transcript_12004;F01_transcript_120369;F01_transcript_120405;F01_transcript_120692;F01_transcript_121042;F01_transcript_121070;F01_transcript_121401;F01_transcript_121410;F01_transcript_121518;F01_transcript_121687;F01_transcript_122468;F01_transcript_12279;F01_transcript_124036;F01_transcript_125031;F01_transcript_125881;F01_transcript_125915;F01_transcript_126146;F01_transcript_126872;F01_transcript_127250;F01_transcript_12737;F01_transcript_127568;F01_transcript_127751;F01_transcript_127885;F01_transcript_128321;F01_transcript_128440;F01_transcript_128844;F01_transcript_128893;F01_transcript_128959;F01_transcript_128960;F01_transcript_129072;F01_transcript_129190;F01_transcript_129726;F01_transcript_130777;F01_transcript_131553;F01_transcript_131832;F01_transcript_131928;F01_transcript_132136;F01_transcript_132273;F01_transcript_132330;F01_transcript_13237;F01_transcript_132386;F01_transcript_132414;F01_transcript_132804;F01_transcript_132918;F01_transcript_132921;F01_transcript_132978;F01_transcript_13373;F01_transcript_134380;F01_transcript_134387;F01_transcript_134473;F01_transcript_135131;F01_transcript_135333;F01_transcript_135613;F01_transcript_135678;F01_transcript_135756;F01_transcript_135864;F01_transcript_135890;F01_transcript_136055;F01_transcript_13631;F01_transcript_136408;F01_transcript_137276;F01_trans	75+K17497+K13648+K00012+K01810+K13648+K00975+K00975+K00972+K00975+K08678+K00326+K12373+K13379+K00844+K12373+K00975+K15920+K13648+K01810+K00975+K17497+K12373+K00975+K00770+K13648+K13648+K13648+K00975+K06118+K13648+K00975+K00972+K01809+K15920+K13648+K01835+K13648+K00621+K13648+K01835+K00012+K01810+K00965+K12450+K00975+K00975+K00847+K01835+K00012+K01810+K13648+K13648+K00844+K10046+K12448+K20867+K13379+K00966+K13648+K01835+K12450+K18677+K00847+K01784+K17497+K00621+K01810+K00975+K18677+K12446+K00621+K13648+K12450+K10046+K00965+K08678+K01835+K12373+K00963+K12373+K00820+K00820+K13648+K12373+K12450+K15920+K00963+K00844+K00847+K08679+K12373+K08678+K13648+K13648+K05305+K00966+K00972+K00975+K12373+K10046+K13648+K20547+K13648+K13648+K00972+K00975+K00965+K17497+K13648+K18677+K20547+K01809+K01809+K00965+K06118+K08678+K00963+K01835+K20867+K01809+K12447+K01835+K17497+K08678+K08678+K01835+K13648+K12373+K00966+K18677+K00326+K00012+K00966+K12373+K01209+K00847+K00820+K12373+K01209+K00326+K08678+K20867+K00975+K08678+K15920+K12451+K12373+K00965+K13648+K00844+K00975+K08678+K13648+K00975+K08679+K01810+K13648+K12450+K01810+K01809+K00975+K12450+K01835+K01784+K12373+K01835+K00975+K13648+K17497+K00963+K12450+K00975+K00963+K12450+K12450+K13648+K12447+K00963+K15920+K20867+K01209+K20867+K01810+K13648+K01835+K00966+K13648+K00966+K01810+K20867+K20867+K01209+K01835+K05305+K00975+K20867+K13648+K05305+K12373+K12373+K13648+K01835+K01835+K13648+K01209+K01835+K00975+K00965+K00844+K12373+K13648+K06118+K08679+K13648+K12373+K12448+K00844+K08678+K08678+K00975+K01810+K08678+K00975+K08679+K08678+K00963+K00975+K08678+K12373+K00963+K00963+K12373+K00975+K08678+K00966+K00972+K00012+K08678+K13648+K13379+K20867+K00963+K08678+K08679+K00012+K13379+K00972+K00966+K00847+K01809+K00966+K12449+K00847+K0

	cript_13739;F01_transcript_137623;F01_transcript_13777;F01_transcript_137926;F01_transcript_138073;F01_transcript_138414;F01_transcript_138467;F01_transcript_138474;F01_transcript_138795;F01_transcript_139107;F01_transcript_139332;F01_transcript_140703;F01_transcript_141017;F01_transcript_141572;F01_transcript_142701;F01_transcript_14271;F01_transcript_142834;F01_transcript_143463;F01_transcript_143548;F01_transcript_143884;F01_transcript_144131;F01_transcript_144342;F01_transcript_144876;F01_transcript_14523;F01_transcript_146687;F01_transcript_146835;F01_transcript_14747;F01_transcript_147498;F01_transcript_147923;F01_transcript_148041;F01_transcript_148281;F01_transcript_148470;F01_transcript_148565;F01_transcript_148567;F01_transcript_148899;F01_transcript_148971;F01_transcript_149478;F01_transcript_149729;F01_transcript_150181;F01_transcript_150556;F01_transcript_150823;F01_transcript_150921;F01_transcript_151133;F01_transcript_151281;F01_transcript_15179;F01_transcript_152096;F01_transcript_152436;F01_transcript_15286;F01_transcript_153205;F01_transcript_153214;F01_transcript_154191;F01_transcript_154335;F01_transcript_154760;F01_transcript_155151;F01_transcript_155204;F01_transcript_155492;F01_transcript_155532;F01_transcript_155544;F01_transcript_155592;F01_transcript_155598;F01_transcript_155968;F01_transcript_155996;F01_transcript_156063;F01_transcript_156116;F01_transcript_156354;F01_transcript_156470;F01_transcript_156579;F01_transcript_156780;F01_transcript_156789;F01_transcript_156812;F01_transcript_156916;F01_transcript_157019;F01_transcript_157179;F01_transcript_157611;F01_transcript_157672;F01_transcript_157698;F01_transcript_157711;F01_transcript_157846;F01_transcript_15819;F01_transcript_158489;F01_transcript_158570;F01_transcript_158707;F01_transcript_15905;F01_transcript_159145;F01_transcript_159430;F01_transcript_159513;F01_transcript_160085;F01_transcript_160141;F01_transcript_160207;F01_transcript_161488;F01_transcript_16165;F01_transcript_161918;F01_transcript_162246;F01_transcript_162516;F01_transcript_162778;F01_transcript_163919;F01_transcript_164028;F01_transcript_164032;F01_transcript_164741;F01_transcript_165046;F01_transcript_165159;F01_transcript_165243;F01_transcript_165502;F01_transcri	0965+K00847+K12449+K01809+K00965+K12449+K00965+K00966+K08678+K00966+K10046+K01809+K01784+K12449+K13648+K20547+K01784+K12373+K10046+K01784+K20547+K01784+K08678+K01784+K13379+K20547+K08678+K00847+K08678+K12451+K00326+K13379+K00326+K00966+K12451+K20547+K00326+K01183+K00966+K00966+K12446+K06118+K00847+K13648+K20867+K06118+K00975+K12373+K17497+K13648+K00972+K00965+K05305+K20547+K00963+K08679+K01183+K20547+K01809+K15920+K01835+K01810+K00965+K12373+K13648+K00975+K00972+K13648+K17497+K13648+K00966+K13379+K12373+K01809+K00621+K00975+K00844+K00012+K00975+K01183+K13379+K00966+K12373+K13648+K01810+K12373+K12373+K01809+K00975+K13648+K12373+K08678+K00844+K08679+K20867+K12373+K12373+K12373+K01835+K01810+K13648+K12373+K00966+K20867+K13648+K01183+K00975+K12450+K13648+K20867+K17497+K00844+K00820+K12450+K12373+K00847+K02377+K08678+K13648+K13648+K13648+K00975+K01810+K00966+K10046+K00326+K01810+K12373+K12373+K00975+K01784+K00847+K01784+K12373+K00844+K20547+K00966+K12373+K01809+K00847+K17497+K01810+K01835+K00965+K10046+K12373+K08678+K00820+K13648+K00965+K13648+K01183+K01810+K12373+K12450+K12373+K12449+K10046+K00847+K00847+K12373+K12373+K05305+K12373+K13648+K13648+K00844+K01835+K00966+K20867+K00847+K00975+K12450+K05305+K13648+K17497+K01784+K00975+K17497+K00963+K13648+K13648+K10046+K18677+K00966+K00975+K12373+K00966+K01835+K13648+K08678+K01810+K01209+K00847+K12373+K00966+K12449+K13648+K00621+K12373+K00975+K00844+K00963+K12449+K00972+K00965

pt_16566;F01_transcript_165871;F01_transcript_165880;F01_tr
anscript_165965;F01_transcript_166075;F01_transcript_166761
;F01_transcript_16700;F01_transcript_167432;F01_transcript_1
67654;F01_transcript_167673;F01_transcript_16844;F01_transc
ript_17487;F01_transcript_18314;F01_transcript_18623;F01_tra
nscript_18663;F01_transcript_18784;F01_transcript_18794;F01
_transcript_18799;F01_transcript_19039;F01_transcript_19239;
F01_transcript_19344;F01_transcript_19381;F01_transcript_194
77;F01_transcript_19835;F01_transcript_1996;F01_transcript_2
0473;F01_transcript_2057;F01_transcript_21106;F01_transcript
_21459;F01_transcript_22146;F01_transcript_22202;F01_transc
ript_22972;F01_transcript_2318;F01_transcript_23808;F01_tran
script_23934;F01_transcript_23996;F01_transcript_2451;F01_tr
anscript_24661;F01_transcript_25080;F01_transcript_25375;F0
1_transcript_25381;F01_transcript_25470;F01_transcript_25878
;F01_transcript_25899;F01_transcript_25979;F01_transcript_26
024;F01_transcript_26498;F01_transcript_26688;F01_transcript
_26723;F01_transcript_26853;F01_transcript_26974;F01_transc
ript_27087;F01_transcript_27125;F01_transcript_27130;F01_tra
nscript_27690;F01_transcript_27931;F01_transcript_28011;F01
_transcript_28813;F01_transcript_29026;F01_transcript_29538;
F01_transcript_29783;F01_transcript_29910;F01_transcript_300
65;F01_transcript_30159;F01_transcript_30280;F01_transcript_
30303;F01_transcript_30341;F01_transcript_30356;F01_transcri
pt_30433;F01_transcript_31186;F01_transcript_31504;F01_tran
script_31524;F01_transcript_31677;F01_transcript_31713;F01_t
ranscript_31732;F01_transcript_31810;F01_transcript_32240;F0
1_transcript_32267;F01_transcript_32527;F01_transcript_32617
;F01_transcript_32619;F01_transcript_33069;F01_transcript_33
130;F01_transcript_33161;F01_transcript_33247;F01_transcript
_33248;F01_transcript_33874;F01_transcript_34086;F01_transc
ript_34237;F01_transcript_34419;F01_transcript_34600;F01_tra
nscript_34671;F01_transcript_34824;F01_transcript_34966;F01
_transcript_35045;F01_transcript_35258;F01_transcript_35769;
F01_transcript_35805;F01_transcript_35857;F01_transcript_363
65;F01_transcript_36518;F01_transcript_36879;F01_transcript_
37199;F01_transcript_37297;F01_transcript_37601;F01_transcri

pt_37784;F01_transcript_38255;F01_transcript_38716;F01_tran
script_38768;F01_transcript_388;F01_transcript_38847;F01_tra
nscript_39043;F01_transcript_39164;F01_transcript_39177;F01
_transcript_39309;F01_transcript_39411;F01_transcript_39529;
F01_transcript_39601;F01_transcript_39815;F01_transcript_398
90;F01_transcript_40185;F01_transcript_40201;F01_transcript_
40308;F01_transcript_40827;F01_transcript_40920;F01_transcri
pt_4133;F01_transcript_42816;F01_transcript_43259;F01_trans
cript_43519;F01_transcript_44015;F01_transcript_4551;F01_tra
nscript_4565;F01_transcript_4640;F01_transcript_47229;F01_tr
anscript_50425;F01_transcript_51506;F01_transcript_518;F01_t
ranscript_52271;F01_transcript_52513;F01_transcript_52616;F0
1_transcript_52997;F01_transcript_53289;F01_transcript_53776
;F01_transcript_53956;F01_transcript_54102;F01_transcript_54
460;F01_transcript_54712;F01_transcript_55265;F01_transcript
_55320;F01_transcript_55727;F01_transcript_56539;F01_transc
ript_56571;F01_transcript_56580;F01_transcript_56958;F01_tra
nscript_57114;F01_transcript_57260;F01_transcript_57284;F01
_transcript_57304;F01_transcript_57316;F01_transcript_57399;
F01_transcript_58217;F01_transcript_59072;F01_transcript_592
97;F01_transcript_59648;F01_transcript_60062;F01_transcript_
60364;F01_transcript_60434;F01_transcript_60443;F01_transcri
pt_60736;F01_transcript_60764;F01_transcript_60954;F01_tran
script_60956;F01_transcript_61089;F01_transcript_61428;F01_t
ranscript_61860;F01_transcript_62612;F01_transcript_63241;F0
1_transcript_63270;F01_transcript_6361;F01_transcript_63617;
F01_transcript_63625;F01_transcript_63974;F01_transcript_651
80;F01_transcript_65469;F01_transcript_65494;F01_transcript_
65836;F01_transcript_65854;F01_transcript_65894;F01_transcri
pt_6630;F01_transcript_6655;F01_transcript_66664;F01_transcr
ipt_66746;F01_transcript_67357;F01_transcript_6741;F01_trans
cript_680;F01_transcript_68723;F01_transcript_68819;F01_tran
script_69018;F01_transcript_69101;F01_transcript_69465;F01_t
ranscript_70121;F01_transcript_70354;F01_transcript_70537;F0
1_transcript_70970;F01_transcript_71019;F01_transcript_71153
;F01_transcript_7118;F01_transcript_71450;F01_transcript_724
50;F01_transcript_72779;F01_transcript_73066;F01_transcript_
73189;F01_transcript_73460;F01_transcript_73725;F01_transcri

			pt_73888;F01_transcript_73983;F01_transcript_74436;F01_transcript_74516;F01_transcript_74751;F01_transcript_75592;F01_transcript_75702;F01_transcript_76141;F01_transcript_76212;F01_transcript_76648;F01_transcript_76689;F01_transcript_76707;F01_transcript_76768;F01_transcript_76814;F01_transcript_76938;F01_transcript_77895;F01_transcript_78016;F01_transcript_78310;F01_transcript_78510;F01_transcript_7908;F01_transcript_79249;F01_transcript_79518;F01_transcript_79642;F01_transcript_79756;F01_transcript_80240;F01_transcript_80252;F01_transcript_80661;F01_transcript_81122;F01_transcript_81435;F01_transcript_82030;F01_transcript_8235;F01_transcript_82388;F01_transcript_83121;F01_transcript_83155;F01_transcript_83364;F01_transcript_83475;F01_transcript_8370;F01_transcript_83764;F01_transcript_84093;F01_transcript_84220;F01_transcript_84250;F01_transcript_84272;F01_transcript_84420;F01_transcript_85034;F01_transcript_86116;F01_transcript_86207;F01_transcript_86361;F01_transcript_86553;F01_transcript_86749;F01_transcript_87929;F01_transcript_88211;F01_transcript_88491;F01_transcript_891;F01_transcript_89165;F01_transcript_89411;F01_transcript_89486;F01_transcript_89529;F01_transcript_89833;F01_transcript_92001;F01_transcript_92149;F01_transcript_93078;F01_transcript_93204;F01_transcript_93239;F01_transcript_93416;F01_transcript_93660;F01_transcript_94111;F01_transcript_94317;F01_transcript_94861;F01_transcript_94872;F01_transcript_95055;F01_transcript_95803;F01_transcript_95936;F01_transcript_96767;F01_transcript_96838;F01_transcript_97160;F01_transcript_9730;F01_transcript_97722;F01_transcript_98049;F01_transcript_98212;F01_transcript_98699;F01_transcript_99044;F01_transcript_99176;F01_transcript_99607;F01_transcript_99970;	
Glycosaminoglycan degradation	ko00531	121	F01_transcript_100022;F01_transcript_100670;F01_transcript_100724;F01_transcript_101774;F01_transcript_103169;F01_transcript_108175;F01_transcript_108387;F01_transcript_112177;F01_transcript_112922;F01_transcript_113188;F01_transcript_113345;F01_transcript_114140;F01_transcript_115112;F01_transcript_116282;F01_transcript_118623;F01_transcript_119810;F01_transcript_119856;F01_transcript_121557;F01_transcript_12282	K10532+K07964+K12373+K07964+K07964+K12373+K12309+K12309+K12309+K12309+K12309+K10532+K10532+K10532+K10532+K01205+K07964+K07964+K10532+K12373+K12309+K12373+K12373+K10532+K01205+K12309+K07964+K12309+K01205+K01205+K12373+K12373+K10532+K12309+K12373+K10532+K12373+K07964+K12373+K10532+K12373+K12373+K12373+K10532+K12373+K12309+K12309+K07964+

	4;F01_transcript_125881;F01_transcript_126182;F01_transcript_126872;F01_transcript_128440;F01_transcript_131692;F01_transcript_13272;F01_transcript_13991;F01_transcript_141309;F01_transcript_14203;F01_transcript_143292;F01_transcript_143797;F01_transcript_143884;F01_transcript_144342;F01_transcript_144389;F01_transcript_144857;F01_transcript_146835;F01_transcript_146968;F01_transcript_148565;F01_transcript_150118;F01_transcript_150823;F01_transcript_156668;F01_transcript_156812;F01_transcript_157698;F01_transcript_158489;F01_transcript_159946;F01_transcript_160207;F01_transcript_161299;F01_transcript_163495;F01_transcript_164654;F01_transcript_164732;F01_transcript_164959;F01_transcript_165041;F01_transcript_165334;F01_transcript_165965;F01_transcript_17742;F01_transcript_21375;F01_transcript_21800;F01_transcript_2181;F01_transcript_2212;F01_transcript_24661;F01_transcript_25080;F01_transcript_25788;F01_transcript_25935;F01_transcript_26723;F01_transcript_27130;F01_transcript_29405;F01_transcript_30356;F01_transcript_31189;F01_transcript_31307;F01_transcript_31504;F01_transcript_32538;F01_transcript_388;F01_transcript_52616;F01_transcript_57260;F01_transcript_57444;F01_transcript_5947;F01_transcript_60062;F01_transcript_61860;F01_transcript_61946;F01_transcript_63036;F01_transcript_63137;F01_transcript_63270;F01_transcript_63587;F01_transcript_6361;F01_transcript_65180;F01_transcript_65894;F01_transcript_6630;F01_transcript_6655;F01_transcript_6741;F01_transcript_7118;F01_transcript_71578;F01_transcript_72698;F01_transcript_73361;F01_transcript_74089;F01_transcript_74958;F01_transcript_75592;F01_transcript_75702;F01_transcript_76115;F01_transcript_76707;F01_transcript_77895;F01_transcript_78715;F01_transcript_78732;F01_transcript_79634;F01_transcript_79756;F01_transcript_82388;F01_transcript_82866;F01_transcript_83155;F01_transcript_84093;F01_transcript_84220;F01_transcript_84272;F01_transcript_87412;F01_transcript_90446;F01_transcript_91291;F01_transcript_93458;F01_transcript_93660;F01_transcript_94001;F01_transcript_96156;F01_transcript_96767;F01_transcript_96810;F01_transcript_98049;F01_transcript_98636;F01_transcript_98932;	K10532+K07964+K10532+K10532+K12373+K07964+K10532+K07964+K12309+K07964+K12373+K12373+K07964+K10532+K12373+K12373+K10532+K12373+K10532+K10532+K12373+K07964+K12373+K12373+K12373+K12309+K10532+K12373+K12373+K01205+K12309+K07964+K12373+K12309+K12373+K12373+K12373+K12373+K12373+K12373+K12373+K10532+K10532+K10532+K01205+K10532+K12373+K12373+K01205+K12373+K12373+K07964+K01205+K10532+K12373+K12373+K07964+K12373+K12373+K12373+K12373+K12309+K10532+K07964+K10532+K12373+K07964+K01205+K12373+K10532+K12373+K10532+K07964

Glycerolipid metabolism	ko00561	395	F01_transcript_100463;F01_transcript_100647;F01_transcript_100678;F01_transcript_100740;F01_transcript_102169;F01_transcript_102483;F01_transcript_103345;F01_transcript_103691;F01_transcript_103925;F01_transcript_104517;F01_transcript_104539;F01_transcript_104554;F01_transcript_104608;F01_transcript_104892;F01_transcript_10505;F01_transcript_105213;F01_transcript_106090;F01_transcript_106912;F01_transcript_107385;F01_transcript_10759;F01_transcript_107955;F01_transcript_108221;F01_transcript_108229;F01_transcript_109312;F01_transcript_109592;F01_transcript_110447;F01_transcript_110865;F01_transcript_11109;F01_transcript_111460;F01_transcript_112305;F01_transcript_11252;F01_transcript_112685;F01_transcript_112884;F01_transcript_11301;F01_transcript_113123;F01_transcript_11320;F01_transcript_113363;F01_transcript_11377;F01_transcript_115558;F01_transcript_115636;F01_transcript_116757;F01_transcript_117096;F01_transcript_11728;F01_transcript_117870;F01_transcript_117894;F01_transcript_118736;F01_transcript_118787;F01_transcript_118848;F01_transcript_119343;F01_transcript_121707;F01_transcript_121800;F01_transcript_121819;F01_transcript_122753;F01_transcript_123007;F01_transcript_1231;F01_transcript_123318;F01_transcript_123865;F01_transcript_123965;F01_transcript_124067;F01_transcript_124128;F01_transcript_125167;F01_transcript_125371;F01_transcript_125531;F01_transcript_125755;F01_transcript_126233;F01_transcript_126285;F01_transcript_126618;F01_transcript_127146;F01_transcript_127326;F01_transcript_127355;F01_transcript_127588;F01_transcript_128000;F01_transcript_128218;F01_transcript_128872;F01_transcript_129384;F01_transcript_129726;F01_transcript_129760;F01_transcript_12984;F01_transcript_13151;F01_transcript_131624;F01_transcript_13167;F01_transcript_132089;F01_transcript_132245;F01_transcript_132254;F01_transcript_132428;F01_transcript_132494;F01_transcript_132878;F01_transcript_132930;F01_transcript_1334;F01_transcript_133496;F01_transcript_133565;F01_transcript_133996;F01_transcript_134168;F01_transcript_134251;F01_transcript_134404;F01_transcript_134507;F01_transcript_134693;F01_transcript_13541;F01_transcript_135743;F01_transcript_136134;F01_transcript_1	K13523+K15918+K00901+K00128+K00630+K14674+K15918+K14085+K00128+K01054+K13523+K11155+K00901+K09480+K09480+K00128+K01054+K00679+K09480+K00679+K09480+K00128+K13506+K00128+K07407+K00679+K13523+K01054+K00679+K01054+K00901+K00128+K00128+K00901+K13523+K09480+K11155+K09480+K03715+K14085+K00901+K09480+K13513+K13523+K00901+K00128+K03715+K00128+K00128+K15728+K01054+K00128+K14674+K09480+K00128+K00679+K00901+K09480+K13513+K00901+K07407+K15918+K00679+K13519+K00901+K15918+K06119+K00128+K00128+K00901+K00679+K15918+K00128+K00128+K14457+K06118+K00655+K00679+K00679+K07407+K00679+K13523+K00901+K00679+K03715+K00128+K07407+K00128+K00128+K00901+K13523+K15918+K00630+K00128+K15728+K00128+K00901+K00864+K00901+K14457+K03715+K15918+K13513+K00128+K00679+K00128+K00128+K00128+K00128+K03715+K00128+K13506+K07407+K01054+K01054+K01054+K00630+K00128+K00901+K00128+K03715+K13523+K00128+K00128+K15918+K00679+K00128+K13508+K00128+K13513+K03715+K00128+K01054+K03715+K00128+K09480+K07407+K00630+K13523+K00128+K00901+K00128+K01054+K00128+K01054+K00128+K03715+K00128+K00864+K13508+K07407+K15918+K01054+K13506+K00128+K06118+K09480+K09480+K13523+K00901+K00128+K13508+K14085+K13513+K00128+K00901+K15918+K01054+K00901+K00901+K07407+K15918+K13508+K00128+K00128+K00128+K09480+K00128+K03715+K13513+K01054+K00901+K01054+K13508+K14674+K01054+K13523+K13519+K00901+K00128+K15918+K14674+K00679+K15918+K00128+K15918+K14674+K01054+K00901+K15728+K00128+K15918+K00128+K00128+K00128+K09480+K00128+K11155+K01054+K06119+K06118+K13508+K00128+K06119+K00128+K00128+K00630+K06119+K00655+K00128+K06119+K00630+K00901+K01054+K15918+K01054+K14085+K14085+K00128+K13519+K03715+K14674+K00901+K00128+K00864+K13519+K03715+K13506+K01054+K00128+K00630+K00128+K03715+K00128+K00655+K14674+K15918+K13508+K01054+K01054+K07407+K00128+K06119+K0012

	36776;F01_transcript_136997;F01_transcript_137272;F01_transcript_137525;F01_transcript_137671;F01_transcript_137935;F01_transcript_138093;F01_transcript_138897;F01_transcript_138921;F01_transcript_139359;F01_transcript_139567;F01_transcript_13985;F01_transcript_140613;F01_transcript_141550;F01_transcript_142438;F01_transcript_142604;F01_transcript_142818;F01_transcript_144636;F01_transcript_144803;F01_transcript_144850;F01_transcript_145037;F01_transcript_145053;F01_transcript_145352;F01_transcript_145474;F01_transcript_145724;F01_transcript_145950;F01_transcript_146046;F01_transcript_146621;F01_transcript_147257;F01_transcript_147437;F01_transcript_147517;F01_transcript_147783;F01_transcript_147878;F01_transcript_148611;F01_transcript_149292;F01_transcript_149971;F01_transcript_150629;F01_transcript_150915;F01_transcript_151058;F01_transcript_151264;F01_transcript_151754;F01_transcript_151832;F01_transcript_151847;F01_transcript_151939;F01_transcript_152130;F01_transcript_152756;F01_transcript_152957;F01_transcript_152958;F01_transcript_153095;F01_transcript_153448;F01_transcript_153936;F01_transcript_154298;F01_transcript_154572;F01_transcript_154804;F01_transcript_154982;F01_transcript_155532;F01_transcript_156290;F01_transcript_15696;F01_transcript_157203;F01_transcript_158880;F01_transcript_158912;F01_transcript_158981;F01_transcript_159514;F01_transcript_159657;F01_transcript_159814;F01_transcript_160115;F01_transcript_16018;F01_transcript_160486;F01_transcript_160561;F01_transcript_160655;F01_transcript_161354;F01_transcript_161372;F01_transcript_161640;F01_transcript_161816;F01_transcript_161966;F01_transcript_162134;F01_transcript_162153;F01_transcript_162331;F01_transcript_162680;F01_transcript_162876;F01_transcript_162925;F01_transcript_162994;F01_transcript_163290;F01_transcript_163859;F01_transcript_163876;F01_transcript_165652;F01_transcript_166414;F01_transcript_166610;F01_transcript_167641;F01_transcript_1799;F01_transcript_18138;F01_transcript_1856;F01_transcript_19294;F01_transcript_19340;F01_transcript_19412;F01_transcript_21089;F01_transcript_2208;F01_transcript_22338;F01_transcript_22916;F01_transcript_2334;F01_transcript_23785;F01_transcript_23916;F01_transcript_24020;F01_transcript_24677;F01_trans	8+K00901+K00128+K00901+K13508+K15918+K00128+K13508+K13508+K13508+K13523+K07407+K00128+K00128+K00128+K13513+K00128+K00128+K00128+K01054+K13523+K13523+K00128+K14457+K01054+K13508+K01054+K06118+K00128+K13519+K00630+K03715+K03715+K09480+K09480+K06118+K00864+K09480+K00901+K00128+K00901+K13523+K00128+K01054+K14085+K00901+K09480+K03715+K07407+K14674+K09480+K15918+K00901+K13513+K00679+K13513+K00901+K14085+K09480+K01054+K09480+K00128+K13506+K00128+K00128+K00128+K09480+K00128+K13506+K03715+K07407+K00679+K00128+K13519+K00901+K00128+K00128+K13508+K01054+K01054+K00630+K00128+K07407+K00679+K00128+K13519+K13508+K00128+K15918+K15918+K15918+K15728+K14085+K01054+K07407+K00901+K00128+K06119+K00128+K00679+K00679+K00901+K15728+K00128+K00863+K00679+K03715+K00901+K00679+K13506+K13506+K01054+K14085+K14457+K00655+K13513+K07407+K06119+K00679+K01054+K00901+K15918+K03715+K00128+K00901+K14674+K00901+K00128+K00901+K00901+K15728+K07407+K00128+K00901+K13523+K00901+K00128+K00128+K15728+K00128+K00655+K13506

	cript_25659;F01_transcript_25794;F01_transcript_26120;F01_transcript_26134;F01_transcript_26330;F01_transcript_26373;F01_transcript_26974;F01_transcript_27004;F01_transcript_27376;F01_transcript_27843;F01_transcript_28572;F01_transcript_28856;F01_transcript_29153;F01_transcript_29326;F01_transcript_29430;F01_transcript_29858;F01_transcript_29940;F01_transcript_29947;F01_transcript_30068;F01_transcript_30141;F01_transcript_30161;F01_transcript_30195;F01_transcript_30292;F01_transcript_30362;F01_transcript_30379;F01_transcript_30631;F01_transcript_30689;F01_transcript_3071;F01_transcript_30863;F01_transcript_30935;F01_transcript_31177;F01_transcript_31374;F01_transcript_31409;F01_transcript_31681;F01_transcript_31872;F01_transcript_31894;F01_transcript_31922;F01_transcript_32017;F01_transcript_32177;F01_transcript_32266;F01_transcript_32298;F01_transcript_3232;F01_transcript_32576;F01_transcript_32611;F01_transcript_32679;F01_transcript_32897;F01_transcript_32988;F01_transcript_33325;F01_transcript_33455;F01_transcript_33600;F01_transcript_34137;F01_transcript_34271;F01_transcript_34466;F01_transcript_34504;F01_transcript_34949;F01_transcript_35286;F01_transcript_35419;F01_transcript_35889;F01_transcript_36211;F01_transcript_36216;F01_transcript_36267;F01_transcript_36697;F01_transcript_36801;F01_transcript_37696;F01_transcript_37773;F01_transcript_37926;F01_transcript_37987;F01_transcript_38323;F01_transcript_38624;F01_transcript_39370;F01_transcript_39913;F01_transcript_41704;F01_transcript_41839;F01_transcript_42351;F01_transcript_42466;F01_transcript_44180;F01_transcript_47229;F01_transcript_48424;F01_transcript_50079;F01_transcript_51402;F01_transcript_51782;F01_transcript_51820;F01_transcript_52004;F01_transcript_52062;F01_transcript_52271;F01_transcript_53523;F01_transcript_54767;F01_transcript_5511;F01_transcript_55541;F01_transcript_55547;F01_transcript_55804;F01_transcript_56137;F01_transcript_56665;F01_transcript_56690;F01_transcript_56945;F01_transcript_58312;F01_transcript_58542;F01_transcript_58571;F01_transcript_59089;F01_transcript_60096;F01_transcript_60113;F01_transcript_60567;F01_transcript_61761;F01_transcript_61938;F01_transcript_631;F01_transcript_6330	

			2;F01_transcript_63491;F01_transcript_63856;F01_transcript_6393;F01_transcript_63935;F01_transcript_65034;F01_transcript_65042;F01_transcript_65403;F01_transcript_65979;F01_transcript_66261;F01_transcript_66366;F01_transcript_67211;F01_transcript_69301;F01_transcript_69766;F01_transcript_69846;F01_transcript_69885;F01_transcript_70471;F01_transcript_71091;F01_transcript_71252;F01_transcript_71260;F01_transcript_71646;F01_transcript_71732;F01_transcript_71998;F01_transcript_72024;F01_transcript_72057;F01_transcript_72349;F01_transcript_72977;F01_transcript_7324;F01_transcript_73295;F01_transcript_73874;F01_transcript_74668;F01_transcript_75083;F01_transcript_75819;F01_transcript_76633;F01_transcript_76878;F01_transcript_779;F01_transcript_77976;F01_transcript_7868;F01_transcript_78851;F01_transcript_79124;F01_transcript_79852;F01_transcript_80428;F01_transcript_80510;F01_transcript_8111;F01_transcript_81312;F01_transcript_81385;F01_transcript_81426;F01_transcript_81584;F01_transcript_83385;F01_transcript_8385;F01_transcript_84344;F01_transcript_84954;F01_transcript_85023;F01_transcript_86198;F01_transcript_86588;F01_transcript_87814;F01_transcript_8790;F01_transcript_87930;F01_transcript_88424;F01_transcript_89230;F01_transcript_89903;F01_transcript_90087;F01_transcript_90781;F01_transcript_91004;F01_transcript_91252;F01_transcript_91900;F01_transcript_92078;F01_transcript_92804;F01_transcript_92930;F01_transcript_93920;F01_transcript_95059;F01_transcript_96227;F01_transcript_96475;F01_transcript_96737;F01_transcript_96945;F01_transcript_96959;F01_transcript_97230;F01_transcript_9743;F01_transcript_9755;F01_transcript_97607;F01_transcript_98282;F01_transcript_98445;F01_transcript_99421;F01_transcript_99528;F01_transcript_99763;F01_transcript_99797;	
Inositol phosphate metabolism	ko00562	319	F01_transcript_100214;F01_transcript_100291;F01_transcript_100774;F01_transcript_101123;F01_transcript_101744;F01_transcript_102373;F01_transcript_103125;F01_transcript_103304;F01_transcript_10363;F01_transcript_103887;F01_transcript_104015;F01_transcript_104277;F01_transcript_104353;F01_transcript_105010;F01_transcript_105886;F01_transcript_106165;F01_transcript_106454;F01_transcript_10798;F01_transcript_108947;F01_transcript_109177;F01_transcript_109590;F01_transcript_1	K01803+K00889+K19801+K00921+K01110+K01803+K00921+K00999+K00921+K00889+K00921+K05857+K00914+K00889+K00999+K01114+K05857+K00914+K01803+K01092+K19801+K00889+K00913+K00914+K01858+K03103+K19801+K01803+K01114+K00889+K01114+K00888+K00889+K03103+K05857+K00914+K00914+K00888+K00889+K00889+K00921+K00889+K05857+K00915+K01110+K00140+K00915+K00889+K00914+K00140+K05857+K01858+K00889+K00889+K00889

	09860;F01_transcript_109925;F01_transcript_110751;F01_transcript_111119;F01_transcript_111496;F01_transcript_111866;F01_transcript_112269;F01_transcript_112935;F01_transcript_112941;F01_transcript_113679;F01_transcript_114431;F01_transcript_114629;F01_transcript_114952;F01_transcript_115711;F01_transcript_117270;F01_transcript_117794;F01_transcript_117975;F01_transcript_118778;F01_transcript_11952;F01_transcript_119968;F01_transcript_12012;F01_transcript_120207;F01_transcript_120640;F01_transcript_120833;F01_transcript_121237;F01_transcript_121391;F01_transcript_121489;F01_transcript_121786;F01_transcript_122090;F01_transcript_122263;F01_transcript_122342;F01_transcript_123225;F01_transcript_124691;F01_transcript_124724;F01_transcript_124736;F01_transcript_124756;F01_transcript_125162;F01_transcript_125516;F01_transcript_125705;F01_transcript_126659;F01_transcript_127179;F01_transcript_127233;F01_transcript_127812;F01_transcript_128187;F01_transcript_128721;F01_transcript_129146;F01_transcript_129184;F01_transcript_129886;F01_transcript_130320;F01_transcript_130443;F01_transcript_130773;F01_transcript_131437;F01_transcript_131625;F01_transcript_132042;F01_transcript_133072;F01_transcript_133289;F01_transcript_133384;F01_transcript_133554;F01_transcript_133587;F01_transcript_13368;F01_transcript_133724;F01_transcript_133730;F01_transcript_134031;F01_transcript_134034;F01_transcript_134616;F01_transcript_134796;F01_transcript_135701;F01_transcript_135724;F01_transcript_135767;F01_transcript_135803;F01_transcript_136099;F01_transcript_13665;F01_transcript_137504;F01_transcript_137790;F01_transcript_137791;F01_transcript_137832;F01_transcript_138185;F01_transcript_138326;F01_transcript_138842;F01_transcript_140237;F01_transcript_140307;F01_transcript_140625;F01_transcript_141635;F01_transcript_143489;F01_transcript_14366;F01_transcript_143704;F01_transcript_143711;F01_transcript_144552;F01_transcript_14513;F01_transcript_145512;F01_transcript_146185;F01_transcript_146478;F01_transcript_146545;F01_transcript_147722;F01_transcript_148129;F01_transcript_148252;F01_transcript_148772;F01_transcript_149745;F01_transcript_149881;F01_transcript_149901;F01_transcript_15006	+K00889+K00469+K19801+K00914+K00913+K10047+K00889+K00914+K00889+K01803+K01114+K00914+K00889+K03103+K00889+K05857+K00140+K01110+K01114+K18649+K01110+K00469+K00914+K00889+K01803+K01803+K00889+K18081+K00889+K00889+K01858+K19801+K01110+K00889+K01803+K10047+K00913+K00921+K15422+K18081+K01110+K05857+K00889+K01803+K00914+K05857+K00140+K01110+K03103+K01858+K10572+K00889+K01106+K00921+K10572+K10572+K00921+K00914+K00913+K05857+K00469+K01858+K03103+K18081+K18081+K00914+K01114+K18081+K10572+K00914+K01803+K01858+K00915+K19517+K05857+K00889+K00140+K00140+K00914+K00889+K00921+K01803+K00921+K05857+K00921+K01110+K05857+K01114+K00889+K00914+K01803+K01110+K03103+K00889+K01114+K00999+K00889+K19801+K01858+K00999+K01114+K01803+K00914+K05857+K05857+K01114+K00140+K01110+K10572+K00913+K00140+K01110+K01110+K10572+K01110+K01858+K01110+K00889+K05857+K01803+K00889+K00921+K05857+K01110+K01803+K01858+K01114+K00921+K01858+K05857+K15422+K00921+K00140+K00889+K00140+K10572+K00140+K03103+K00140+K03103+K03103+K01858+K00913+K00913+K00913+K00913+K00913+K01803+K01803+K01092+K15422+K01803+K00915+K01803+K10047+K01803+K01803+K01803+K01114+K19517+K00913+K00921+K00889+K00921+K05857+K01803+K01803+K05857+K00140+K05857+K10572+K00889+K01110+K03103+K10572+K01858+K00889+K00914+K00913+K00889+K00140+K00889+K00889+K00914+K10047+K00914+K10047+K00889+K05857+K00889+K03103+K00921+K01114+K00140+K00921+K00913+K00889+K00889+K18081+K05857+K00889+K00889+K01858+K00914+K10572+K05857+K01110+K10572+K00914+K00140+K10572+K01110+K00914+K00140+K00889+K19517+K00914+K19801+K00140+K00140+K00921+K03103+K00889+K19801+K00889+K00889+K19801+K00889+K01803+K01858+K01858+K10572+K10572+K19801+K00914+K00914+K00889+K00888+K01803+K19801+K00913+K00889+K00889+K15422+K15422+K00921+K03103+K00889+K00889+K00888+K00889+K10047+K10572+K009

	8;F01_transcript_150218;F01_transcript_15030;F01_transcript_150423;F01_transcript_150940;F01_transcript_151040;F01_transcript_151119;F01_transcript_152029;F01_transcript_152601;F01_transcript_152813;F01_transcript_15331;F01_transcript_15347;F01_transcript_153720;F01_transcript_153811;F01_transcript_154350;F01_transcript_154897;F01_transcript_156217;F01_transcript_156315;F01_transcript_156666;F01_transcript_157038;F01_transcript_157286;F01_transcript_157545;F01_transcript_157718;F01_transcript_158433;F01_transcript_159358;F01_transcript_160089;F01_transcript_161132;F01_transcript_161895;F01_transcript_164158;F01_transcript_164415;F01_transcript_164709;F01_transcript_164802;F01_transcript_165228;F01_transcript_165856;F01_transcript_166441;F01_transcript_166475;F01_transcript_166806;F01_transcript_166884;F01_transcript_167180;F01_transcript_167256;F01_transcript_1704;F01_transcript_17337;F01_transcript_17957;F01_transcript_18566;F01_transcript_19633;F01_transcript_20178;F01_transcript_20962;F01_transcript_21125;F01_transcript_21255;F01_transcript_21268;F01_transcript_21279;F01_transcript_21520;F01_transcript_21865;F01_transcript_22107;F01_transcript_22882;F01_transcript_23;F01_transcript_23080;F01_transcript_23214;F01_transcript_24566;F01_transcript_25304;F01_transcript_25399;F01_transcript_25412;F01_transcript_25516;F01_transcript_26104;F01_transcript_27724;F01_transcript_28;F01_transcript_28594;F01_transcript_2861;F01_transcript_28947;F01_transcript_29014;F01_transcript_29341;F01_transcript_30543;F01_transcript_31414;F01_transcript_32242;F01_transcript_32765;F01_transcript_34140;F01_transcript_34615;F01_transcript_34791;F01_transcript_34807;F01_transcript_36200;F01_transcript_36222;F01_transcript_39223;F01_transcript_39520;F01_transcript_40331;F01_transcript_40512;F01_transcript_40883;F01_transcript_41844;F01_transcript_43050;F01_transcript_44007;F01_transcript_44274;F01_transcript_44524;F01_transcript_47618;F01_transcript_4840;F01_transcript_51461;F01_transcript_51537;F01_transcript_51990;F01_transcript_52111;F01_transcript_52496;F01_transcript_54432;F01_transcript_54487;F01_transcript_55263;F01_transcript_55919;F01_transcript_57145;F01_transcript_57879;F01_transcript_58506;F01_transcript_59439;F01_transcript_60254;F01_transcript	21+K18081+K00915+K01114+K00921+K10047+K10047+K05857+K00889+K10572+K00889

			_60325;F01_transcript_61325;F01_transcript_62349;F01_transcript_6257;F01_transcript_63398;F01_transcript_63470;F01_transcript_64409;F01_transcript_64572;F01_transcript_64970;F01_transcript_64995;F01_transcript_65003;F01_transcript_65191;F01_transcript_6591;F01_transcript_66026;F01_transcript_66592;F01_transcript_66677;F01_transcript_6689;F01_transcript_67492;F01_transcript_67527;F01_transcript_67983;F01_transcript_68359;F01_transcript_68408;F01_transcript_68866;F01_transcript_6988;F01_transcript_70192;F01_transcript_70203;F01_transcript_70648;F01_transcript_71307;F01_transcript_71841;F01_transcript_72511;F01_transcript_72837;F01_transcript_73080;F01_transcript_73787;F01_transcript_7424;F01_transcript_75023;F01_transcript_75395;F01_transcript_76336;F01_transcript_76406;F01_transcript_76899;F01_transcript_77133;F01_transcript_77784;F01_transcript_78675;F01_transcript_79088;F01_transcript_79219;F01_transcript_79288;F01_transcript_79569;F01_transcript_79982;F01_transcript_80155;F01_transcript_81070;F01_transcript_81263;F01_transcript_81730;F01_transcript_82323;F01_transcript_82606;F01_transcript_82865;F01_transcript_83591;F01_transcript_83748;F01_transcript_87105;F01_transcript_88037;F01_transcript_88245;F01_transcript_88365;F01_transcript_88603;F01_transcript_8890;F01_transcript_88943;F01_transcript_89769;F01_transcript_9;F01_transcript_90169;F01_transcript_90463;F01_transcript_91968;F01_transcript_9251;F01_transcript_93174;F01_transcript_93426;F01_transcript_93589;F01_transcript_93671;F01_transcript_93794;F01_transcript_9461;F01_transcript_94770;F01_transcript_94951;F01_transcript_95120;F01_transcript_95171;F01_transcript_95193;F01_transcript_96011;F01_transcript_96182;F01_transcript_9636;F01_transcript_96439;F01_transcript_97203;F01_transcript_97327;F01_transcript_98320;F01_transcript_98548;F01_transcript_99185;F01_transcript_99324;F01_transcript_99553;	
Glycosylphosphatidylinositol(	ko005	105	F01_transcript_100302;F01_transcript_100928;F01_transcript_100931;F01_transcript_102990;F01_transcript_103857;F01_transcript_105251;F01_transcript_108309;F01_transcript_108704;F01_transcript_11346;F01_transcript_114370;F01_transcript_114880;F01_transcript_115402;F01_transcript_116038;F01_transcrip	K03857+K03858+K05288+K05285+K05288+K05288+K05288+K05288+K03860+K05287+K05284+K05288+K05285+K03858+K05287+K05288+K05294+K05285+K05293+K05285+K05284+K05285+K05284+K05286+K03858+K05310+K05289+K05293+K03860+K05285+K05285+K05284+K05287+K03861+K0

GPI)-anchor biosynthesis	63		t_116087;F01_transcript_118158;F01_transcript_118713;F01_transcript_119521;F01_transcript_119606;F01_transcript_120536;F01_transcript_121287;F01_transcript_121617;F01_transcript_124316;F01_transcript_12843;F01_transcript_128661;F01_transcript_133847;F01_transcript_134258;F01_transcript_13453;F01_transcript_134582;F01_transcript_13459;F01_transcript_135364;F01_transcript_135917;F01_transcript_137215;F01_transcript_137265;F01_transcript_137454;F01_transcript_139638;F01_transcript_140975;F01_transcript_147308;F01_transcript_147696;F01_transcript_150496;F01_transcript_153613;F01_transcript_153889;F01_transcript_154491;F01_transcript_154563;F01_transcript_155371;F01_transcript_155919;F01_transcript_15639;F01_transcript_156920;F01_transcript_160116;F01_transcript_161404;F01_transcript_161460;F01_transcript_16351;F01_transcript_163909;F01_transcript_165232;F01_transcript_166682;F01_transcript_167008;F01_transcript_18798;F01_transcript_19283;F01_transcript_19455;F01_transcript_29664;F01_transcript_34019;F01_transcript_4163;F01_transcript_45073;F01_transcript_4578;F01_transcript_49148;F01_transcript_5197;F01_transcript_52122;F01_transcript_53901;F01_transcript_56129;F01_transcript_57002;F01_transcript_57895;F01_transcript_65031;F01_transcript_65584;F01_transcript_65756;F01_transcript_66215;F01_transcript_66480;F01_transcript_66584;F01_transcript_67105;F01_transcript_67823;F01_transcript_68449;F01_transcript_69427;F01_transcript_70965;F01_transcript_71916;F01_transcript_7447;F01_transcript_76512;F01_transcript_7792;F01_transcript_78045;F01_transcript_78610;F01_transcript_79122;F01_transcript_79685;F01_transcript_80071;F01_transcript_80182;F01_transcript_80305;F01_transcript_82902;F01_transcript_83975;F01_transcript_84782;F01_transcript_85638;F01_transcript_87161;F01_transcript_87186;F01_transcript_92098;F01_transcript_92781;F01_transcript_94569;F01_transcript_96388;F01_transcript_97167;F01_transcript_98466;F01_transcript_99733;	3857+K03857+K05288+K03857+K03858+K07542+K05288+K03857+K03860+K05310+K09658+K05284+K03858+K05287+K05284+K05293+K05291+K05285+K05310+K03857+K05291+K05292+K05291+K05291+K07542+K05293+K05310+K03434+K05285+K09658+K05287+K03857+K05286+K03860+K05284+K03861+K03860+K05292+K05283+K05285+K05291+K03857+K03860+K05288+K05310+K05288+K05284+K05285+K05310+K05291+K05287+K05290+K07542+K05290+K05290+K05284+K05285+K05310+K05284+K05283+K05310+K05286+K05290+K05290+K05310+K03857+K05283+K05310+K05285+K05284+K05289
Glycerophospholipid	ko005	400	F01_transcript_100463;F01_transcript_100678;F01_transcript_101258;F01_transcript_101718;F01_transcript_101957;F01_transcript_102169;F01_transcript_102321;F01_transcript_102483;F01_transcript_102573;F01_transcript_102716;F01_transcript_103277;F01_transcript_103304;F01_transcript_104539;F01_transcri	K13523+K00901+K00967+K01613+K00993+K00630+K01613+K14674+K01613+K13510+K13510+K00999+K13523+K00901+K13510+K06130+K00968+K01126+K00999+K01114+K01613+K01115+K00550+K01115+K01115+K01115+K06130+K13506+K01115+K08730+K01115+K06130+K13510+K13523+K0

metabolism	64	pt_104608;F01_transcript_104860;F01_transcript_105079;F01_transcript_105239;F01_transcript_105421;F01_transcript_105886;F01_transcript_106165;F01_transcript_106445;F01_transcript_106580;F01_transcript_106667;F01_transcript_107465;F01_transcript_107508;F01_transcript_10774;F01_transcript_108184;F01_transcript_108229;F01_transcript_10833;F01_transcript_108465;F01_transcript_108515;F01_transcript_110534;F01_transcript_110567;F01_transcript_110865;F01_transcript_112146;F01_transcript_11229;F01_transcript_11252;F01_transcript_112935;F01_transcript_11301;F01_transcript_113123;F01_transcript_113199;F01_transcript_113242;F01_transcript_113286;F01_transcript_113467;F01_transcript_113679;F01_transcript_114962;F01_transcript_115561;F01_transcript_11607;F01_transcript_116201;F01_transcript_116466;F01_transcript_116757;F01_transcript_11728;F01_transcript_117729;F01_transcript_117870;F01_transcript_117894;F01_transcript_1198;F01_transcript_120590;F01_transcript_120899;F01_transcript_121569;F01_transcript_121695;F01_transcript_121707;F01_transcript_122067;F01_transcript_122341;F01_transcript_122753;F01_transcript_123307;F01_transcript_123687;F01_transcript_123865;F01_transcript_124067;F01_transcript_124105;F01_transcript_124128;F01_transcript_124305;F01_transcript_125077;F01_transcript_125755;F01_transcript_126209;F01_transcript_126233;F01_transcript_126313;F01_transcript_126654;F01_transcript_127355;F01_transcript_127481;F01_transcript_127690;F01_transcript_127740;F01_transcript_128492;F01_transcript_128721;F01_transcript_128990;F01_transcript_129463;F01_transcript_129760;F01_transcript_131033;F01_transcript_131625;F01_transcript_132089;F01_transcript_132174;F01_transcript_132245;F01_transcript_132372;F01_transcript_132887;F01_transcript_133496;F01_transcript_133565;F01_transcript_133905;F01_transcript_134168;F01_transcript_134243;F01_transcript_134404;F01_transcript_134693;F01_transcript_134997;F01_transcript_135743;F01_transcript_135988;F01_transcript_136123;F01_transcript_136127;F01_transcript_136633;F01_transcript_136870;F01_transcript_136984;F01_transcript_137225;F01_transcript_13723;F01_transcript_137272;F01_transcript_137650;F01_transcript_138130;F01_transcript_138142;	0894+K13510+K00901+K01114+K00901+K13523+K00894+K13510+K01115+K13510+K01114+K01115+K00993+K01613+K01613+K00981+K00901+K13513+K00968+K13523+K00901+K01115+K01115+K08744+K13510+K00993+K15728+K00981+K00968+K14674+K00993+K00993+K00901+K13513+K01126+K00901+K01115+K08730+K13519+K01613+K00901+K13510+K00968+K00901+K01613+K01613+K00550+K01115+K01114+K13510+K00968+K00655+K00968+K01114+K13523+K01115+K00901+K00967+K06129+K00901+K13523+K06130+K00630+K06130+K15728+K00901+K00967+K00901+K16860+K13510+K16860+K01115+K01115+K00993+K01115+K13510+K13513+K01126+K00968+K00995+K00968+K01047+K01115+K06130+K13506+K01115+K01126+K01115+K00630+K13510+K08730+K01115+K13510+K00901+K13523+K01613+K00968+K01115+K13508+K18696+K00993+K13513+K00968+K00967+K01613+K00967+K00968+K13510+K01114+K00630+K00995+K13523+K00901+K00006+K13510+K01115+K08730+K13508+K00981+K06130+K14156+K13506+K08730+K13511+K13510+K13523+K01114+K01115+K13510+K00993+K01115+K08730+K01115+K00901+K13508+K13513+K00901+K00894+K00901+K00901+K06130+K13510+K13510+K01613+K13508+K13513+K00901+K14156+K13510+K13508+K14674+K01114+K00999+K01115+K00995+K01115+K00995+K00999+K01047+K01115+K13523+K01114+K13519+K01114+K13510+K06130+K00901+K01613+K00111+K14674+K00006+K14674+K00901+K15728+K00968+K01115+K01114+K13508+K05929+K13510+K00630+K00655+K00630+K00901+K13519+K14674+K00901+K00967+K08730+K13519+K13506+K00630+K01115+K00981+K00655+K14674+K00967+K13508+K16860+K13511+K00967+K13510+K00901+K00995+K00901+K13508+K13508+K00995+K13508+K13508+K13523+K00968+K00968+K13510+K06129+K13513+K08744+K01126+K06129+K01126+K00006+K13523+K00995+K01126+K13523+K01517+K06130+K01126+K00968+K00968+K00968+K00968+K13508+K01094+K01094+K06130+K06130+K06130+K01114+K13519+K00630+K00968+K00981+K06130+K00901+K01613+K00901+K13523+K00550+K06130+K00995+K00901+K01094+K01115+

	F01_transcript_138372;F01_transcript_138707;F01_transcript_139307;F01_transcript_139365;F01_transcript_13985;F01_transcript_141318;F01_transcript_142280;F01_transcript_1427;F01_transcript_142818;F01_transcript_143106;F01_transcript_143130;F01_transcript_143193;F01_transcript_143485;F01_transcript_144803;F01_transcript_145053;F01_transcript_145116;F01_transcript_145170;F01_transcript_145450;F01_transcript_146621;F01_transcript_146913;F01_transcript_147386;F01_transcript_147437;F01_transcript_148218;F01_transcript_148667;F01_transcript_148737;F01_transcript_149157;F01_transcript_149431;F01_transcript_149585;F01_transcript_150068;F01_transcript_150915;F01_transcript_150973;F01_transcript_151058;F01_transcript_151754;F01_transcript_15180;F01_transcript_152571;F01_transcript_152845;F01_transcript_153103;F01_transcript_153448;F01_transcript_153592;F01_transcript_153739;F01_transcript_154620;F01_transcript_154804;F01_transcript_155881;F01_transcript_156081;F01_transcript_156532;F01_transcript_157203;F01_transcript_157545;F01_transcript_157739;F01_transcript_158010;F01_transcript_158019;F01_transcript_158289;F01_transcript_15841;F01_transcript_158517;F01_transcript_158880;F01_transcript_158981;F01_transcript_159657;F01_transcript_160115;F01_transcript_160346;F01_transcript_160561;F01_transcript_160655;F01_transcript_160727;F01_transcript_160749;F01_transcript_161041;F01_transcript_161529;F01_transcript_161640;F01_transcript_162876;F01_transcript_162994;F01_transcript_163023;F01_transcript_163300;F01_transcript_163859;F01_transcript_163876;F01_transcript_164158;F01_transcript_164415;F01_transcript_164522;F01_transcript_164902;F01_transcript_165090;F01_transcript_165298;F01_transcript_165856;F01_transcript_165949;F01_transcript_1662;F01_transcript_166414;F01_transcript_166441;F01_transcript_166610;F01_transcript_167256;F01_transcript_167453;F01_transcript_167459;F01_transcript_167641;F01_transcript_17682;F01_transcript_18308;F01_transcript_1856;F01_transcript_21912;F01_transcript_2208;F01_transcript_22916;F01_transcript_2334;F01_transcript_23739;F01_transcript_2470;F01_transcript_25399;F01_transcript_27004;F01_transcript_28326;F01_transcript_29069;F01_transcript_29153;F01_transcript_29430;F01_transcript_29947;F01_transcript_30068;	K00968+K14674+K01115+K00995+K01047+K00981+K00901+K00006+K13513+K13513+K01115+K00901+K01126+K08730+K00006+K13506+K06130+K01126+K01115+K00967+K00993+K00993+K01114+K01115+K00993+K01115+K01115+K06130+K00981+K00993+K13506+K01115+K00968+K01115+K01115+K00993+K08730+K13519+K00901+K13508+K00630+K13519+K13508+K01126+K00993+K00981+K00967+K00967+K00006+K15728+K00006+K00901+K08730+K01115+K00006+K01126+K06130+K06129+K00901+K15728+K00967+K01094+K00901+K01094+K01613+K00968+K13506+K00981+K00993+K13506+K01115+K00655+K13513+K01613+K01115+K08744+K00006+K00006+K06130+K00995+K00901+K18696+K06130+K00968+K01115+K01126+K00901+K01115+K00968+K00006+K14674+K01126+K01115+K01613+K00901+K00968+K01114+K00901+K00901+K15728+K01115+K00901+K00993+K13523+K06129+K00901+K01115+K13510+K13510+K15728+K00655+K13506

F01_transcript_30631;F01_transcript_3071;F01_transcript_3086
3;F01_transcript_30991;F01_transcript_31182;F01_transcript_3
1374;F01_transcript_31681;F01_transcript_31922;F01_transcrip
t_3200;F01_transcript_32241;F01_transcript_32298;F01_transcr
ipt_3232;F01_transcript_32553;F01_transcript_32611;F01_trans
cript_32939;F01_transcript_33493;F01_transcript_34039;F01_tr
anscript_34093;F01_transcript_34137;F01_transcript_34434;F0
1_transcript_34466;F01_transcript_34504;F01_transcript_35419
;F01_transcript_35533;F01_transcript_35889;F01_transcript_36
211;F01_transcript_36216;F01_transcript_36281;F01_transcript
_36476;F01_transcript_3665;F01_transcript_37720;F01_transcri
pt_37773;F01_transcript_38340;F01_transcript_38403;F01_tran
script_38731;F01_transcript_38764;F01_transcript_38779;F01_t
ranscript_39370;F01_transcript_39551;F01_transcript_39740;F0
1_transcript_39913;F01_transcript_40169;F01_transcript_40346
;F01_transcript_40387;F01_transcript_41159;F01_transcript_41
703;F01_transcript_42064;F01_transcript_42281;F01_transcript
_42466;F01_transcript_43150;F01_transcript_43289;F01_transc
ript_44494;F01_transcript_44889;F01_transcript_45782;F01_tra
nscript_4840;F01_transcript_50079;F01_transcript_51402;F01_t
ranscript_51778;F01_transcript_52771;F01_transcript_53648;F0
1_transcript_5511;F01_transcript_55182;F01_transcript_55547;
F01_transcript_55804;F01_transcript_56222;F01_transcript_563
50;F01_transcript_56480;F01_transcript_56945;F01_transcript_
57042;F01_transcript_5800;F01_transcript_58649;F01_transcrip
t_59089;F01_transcript_59477;F01_transcript_59835;F01_trans
cript_60237;F01_transcript_60409;F01_transcript_60567;F01_tr
anscript_61109;F01_transcript_61761;F01_transcript_631;F01_t
ranscript_63238;F01_transcript_63302;F01_transcript_63777;F0
1_transcript_64413;F01_transcript_64798;F01_transcript_65042
;F01_transcript_65427;F01_transcript_65780;F01_transcript_65
844;F01_transcript_66238;F01_transcript_66648;F01_transcript
_67417;F01_transcript_67983;F01_transcript_6837;F01_transcri
pt_68425;F01_transcript_6867;F01_transcript_68724;F01_trans
cript_69111;F01_transcript_69123;F01_transcript_69232;F01_tr
anscript_69301;F01_transcript_6972;F01_transcript_69997;F01
_transcript_70159;F01_transcript_70343;F01_transcript_70376;

			F01_transcript_70422;F01_transcript_71091;F01_transcript_71252;F01_transcript_71732;F01_transcript_72057;F01_transcript_73874;F01_transcript_74668;F01_transcript_74889;F01_transcript_75503;F01_transcript_76017;F01_transcript_77445;F01_transcript_77530;F01_transcript_77543;F01_transcript_779;F01_transcript_77928;F01_transcript_79124;F01_transcript_79155;F01_transcript_79210;F01_transcript_79855;F01_transcript_80845;F01_transcript_80872;F01_transcript_8115;F01_transcript_81385;F01_transcript_81426;F01_transcript_81496;F01_transcript_81939;F01_transcript_84954;F01_transcript_85794;F01_transcript_85871;F01_transcript_85988;F01_transcript_86198;F01_transcript_86245;F01_transcript_86513;F01_transcript_86588;F01_transcript_8760;F01_transcript_88424;F01_transcript_89230;F01_transcript_89580;F01_transcript_89778;F01_transcript_89946;F01_transcript_90405;F01_transcript_90427;F01_transcript_90438;F01_transcript_90563;F01_transcript_91252;F01_transcript_91827;F01_transcript_91929;F01_transcript_92155;F01_transcript_92236;F01_transcript_92823;F01_transcript_92930;F01_transcript_9329;F01_transcript_93294;F01_transcript_93551;F01_transcript_93920;F01_transcript_94488;F01_transcript_9471;F01_transcript_94856;F01_transcript_95059;F01_transcript_96184;F01_transcript_96439;F01_transcript_96475;F01_transcript_96737;F01_transcript_96945;F01_transcript_97266;F01_transcript_9743;F01_transcript_97478;F01_transcript_9755;F01_transcript_97553;F01_transcript_97607;F01_transcript_98476;F01_transcript_98696;F01_transcript_98742;F01_transcript_99421;F01_transcript_99763;F01_transcript_99797;	
Ether lipid metabolism	ko00565	127	F01_transcript_101957;F01_transcript_102483;F01_transcript_102716;F01_transcript_103277;F01_transcript_104860;F01_transcript_106165;F01_transcript_106580;F01_transcript_107465;F01_transcript_107508;F01_transcript_10774;F01_transcript_10833;F01_transcript_108515;F01_transcript_110567;F01_transcript_11229;F01_transcript_112935;F01_transcript_113242;F01_transcript_113286;F01_transcript_113467;F01_transcript_113679;F01_transcript_114962;F01_transcript_115561;F01_transcript_1198;F01_transcript_120590;F01_transcript_121569;F01_transcript_121695;F01_transcript_122753;F01_transcript_123307;F01_transcript_123687;F01_transcript_124305;F01_transcript_125755	K00993+K14674+K13510+K13510+K13510+K01114+K01115+K01115+K01115+K01115+K01115+K01115+K13510+K13510+K01114+K13510+K01115+K13510+K01114+K01115+K00993+K01115+K01115+K13510+K00993+K14674+K00993+K00993+K01115+K13519+K13510+K01115+K01114+K13510+K01114+K01115+K16860+K13510+K16860+K01115+K01115+K00993+K01115+K13510+K01047+K01115+K01115+K01115+K13510+K01115+K13510+K01115+K00993+K13510+K01114+K13510+K01115+K13510+K01114+K01115+K13510+K00993+K01115+K01115+K13510+K13510+K13510+K14674+K01114+K01115+K01115+K01047+K01115+K01114+K13519+K01

			;F01_transcript_126313;F01_transcript_128492;F01_transcript_128721;F01_transcript_128990;F01_transcript_131625;F01_transcript_132174;F01_transcript_135988;F01_transcript_136123;F01_transcript_136127;F01_transcript_136633;F01_transcript_136870;F01_transcript_136984;F01_transcript_137225;F01_transcript_13723;F01_transcript_138707;F01_transcript_139307;F01_transcript_141318;F01_transcript_1427;F01_transcript_143106;F01_transcript_143193;F01_transcript_143485;F01_transcript_145450;F01_transcript_147386;F01_transcript_149585;F01_transcript_150068;F01_transcript_152571;F01_transcript_152845;F01_transcript_156532;F01_transcript_157545;F01_transcript_157739;F01_transcript_158010;F01_transcript_158019;F01_transcript_158289;F01_transcript_158517;F01_transcript_160749;F01_transcript_161041;F01_transcript_163300;F01_transcript_163876;F01_transcript_164158;F01_transcript_164522;F01_transcript_165090;F01_transcript_165949;F01_transcript_1662;F01_transcript_166441;F01_transcript_166610;F01_transcript_167256;F01_transcript_167453;F01_transcript_1856;F01_transcript_2208;F01_transcript_2470;F01_transcript_25399;F01_transcript_29069;F01_transcript_30631;F01_transcript_3071;F01_transcript_31374;F01_transcript_3200;F01_transcript_3232;F01_transcript_32939;F01_transcript_34093;F01_transcript_3665;F01_transcript_4840;F01_transcript_50079;F01_transcript_5800;F01_transcript_59089;F01_transcript_59477;F01_transcript_60237;F01_transcript_63238;F01_transcript_65844;F01_transcript_66648;F01_transcript_67417;F01_transcript_67983;F01_transcript_6837;F01_transcript_68425;F01_transcript_6867;F01_transcript_68724;F01_transcript_69232;F01_transcript_6972;F01_transcript_70159;F01_transcript_70343;F01_transcript_70376;F01_transcript_71091;F01_transcript_73874;F01_transcript_75503;F01_transcript_79210;F01_transcript_86513;F01_transcript_8760;F01_transcript_89778;F01_transcript_92236;F01_transcript_9329;F01_transcript_93920;F01_transcript_9471;F01_transcript_96439;F01_transcript_97266;F01_transcript_97478;F01_transcript_98476;F01_transcript_98696;F01_transcript_98742;	114+K13510+K14674+K14674+K01115+K01114+K13510+K13519+K14674+K13519+K01115+K14674+K16860+K13510+K13510+K01114+K13519+K01115+K14674+K01115+K01047+K01115+K01115+K00993+K00993+K01114+K01115+K00993+K01115+K01115+K00993+K01115+K01115+K01115+K00993+K13519+K13519+K00993+K01115+K00993+K01115+K01115+K01115+K01115+K14674+K01115+K01114+K01115+K00993+K01115+K13510+K13510
Arachidonic	ko	57	F01_transcript_102483;F01_transcript_110721;F01_transcript_120644;F01_transcript_122753;F01_transcript_136710;F01_trans	K14674+K01254+K00432+K14674+K00432+K00432+K01254+K01047+K05309+K00432+K18592+K01254+K18592+K0007

acid metabolism	00590		cript_137083;F01_transcript_138699;F01_transcript_138707;F01_transcript_139527;F01_transcript_142413;F01_transcript_149300;F01_transcript_153843;F01_transcript_155997;F01_transcript_157373;F01_transcript_157631;F01_transcript_163876;F01_transcript_165383;F01_transcript_165949;F01_transcript_16611;F01_transcript_18160;F01_transcript_18468;F01_transcript_18485;F01_transcript_1856;F01_transcript_20900;F01_transcript_21864;F01_transcript_21913;F01_transcript_2208;F01_transcript_22152;F01_transcript_3071;F01_transcript_3232;F01_transcript_40069;F01_transcript_41826;F01_transcript_42340;F01_transcript_42730;F01_transcript_43546;F01_transcript_43737;F01_transcript_44570;F01_transcript_45616;F01_transcript_46093;F01_transcript_46117;F01_transcript_46758;F01_transcript_46934;F01_transcript_58773;F01_transcript_59089;F01_transcript_60237;F01_transcript_62799;F01_transcript_64255;F01_transcript_64276;F01_transcript_6623;F01_transcript_68626;F01_transcript_72920;F01_transcript_76792;F01_transcript_80683;F01_transcript_83884;F01_transcript_88908;F01_transcript_93920;F01_transcript_97491;	9+K18592+K14674+K18592+K01047+K18592+K01254+K01254+K18592+K14674+K18592+K18592+K18592+K14674+K01254+K14674+K14674+K05309+K05309+K00432+K05309+K15717+K00432+K00432+K00432+K00432+K00432+K00432+K00432+K00432+K14674+K01047+K18592+K00432+K18592+K18592+K18592+K18592+K18592+K18592+K00432+K00432+K14674+K00079
Linoleic acid metabolism	ko00591	111	F01_transcript_101771;F01_transcript_102483;F01_transcript_105333;F01_transcript_105853;F01_transcript_109866;F01_transcript_114513;F01_transcript_114674;F01_transcript_114744;F01_transcript_11556;F01_transcript_116596;F01_transcript_117716;F01_transcript_120419;F01_transcript_122228;F01_transcript_122753;F01_transcript_123559;F01_transcript_123770;F01_transcript_125715;F01_transcript_127174;F01_transcript_132489;F01_transcript_132775;F01_transcript_133105;F01_transcript_133521;F01_transcript_135542;F01_transcript_136234;F01_transcript_13653;F01_transcript_138095;F01_transcript_138305;F01_transcript_14004;F01_transcript_140687;F01_transcript_142621;F01_transcript_144582;F01_transcript_145968;F01_transcript_151241;F01_transcript_152941;F01_transcript_153249;F01_transcript_157588;F01_transcript_158325;F01_transcript_158484;F01_transcript_158778;F01_transcript_159098;F01_transcript_160057;F01_transcript_161774;F01_transcript_163876;F01_transcript_165949;F01_transcript_166102;F01_transcript_1856;F01_transcript_2208;F01_transcript_3071;F01_transcript_3232;F01_transcript_36109;F01_transcript_39855;F01_transcript_40510;	K00454+K14674+K15718+K00454+K00454+K00454+K00454+K15718+K00454+K00454+K00454+K00454+K00454+K14674+K00454+K00454+K00454+K00454+K00454+K00454+K00454+K00454+K00454+K15718+K00454+K15718+K00454+K00454+K00454+K15718+K15718+K00454+K00454+K00454+K00454+K00454+K15718+K15718+K00454+K00454+K15718+K15718+K14674+K01047+K00454+K14674+K14674+K14674+K14674+K00454+K00454+K00454+K00454+K00454+K00454+K00454+K00454+K00454+K00454+K00454+K00454+K14674+K00454+K01047+K00454+K00454+K15718+K00454+K00454+K00454+K00454+K00454+K15718+K00454+K00454+K00454+K00454+K00454+K00454+K00454+K00454+K15718+K00454+K00454+K00454+K00454+K00454+K00454+K00454+K00454+K00454+K15718+K00454+K00454+K00454+K00454+K15718+K00454+K15718+K15718+K00454+K00454+K00454+K00454+K00454+K14674+K00454+K15718+K00454+K15718+K00454

			F01_transcript_4074;F01_transcript_50121;F01_transcript_51375;F01_transcript_5163;F01_transcript_51683;F01_transcript_53639;F01_transcript_54044;F01_transcript_56138;F01_transcript_57717;F01_transcript_59089;F01_transcript_5915;F01_transcript_60237;F01_transcript_6048;F01_transcript_61241;F01_transcript_6270;F01_transcript_63305;F01_transcript_63396;F01_transcript_64901;F01_transcript_65283;F01_transcript_6551;F01_transcript_67242;F01_transcript_67429;F01_transcript_6766;F01_transcript_68061;F01_transcript_68094;F01_transcript_6883;F01_transcript_69619;F01_transcript_69701;F01_transcript_6982;F01_transcript_70007;F01_transcript_7072;F01_transcript_7305;F01_transcript_73347;F01_transcript_7347;F01_transcript_7451;F01_transcript_75154;F01_transcript_75210;F01_transcript_7576;F01_transcript_7607;F01_transcript_76670;F01_transcript_81823;F01_transcript_8246;F01_transcript_83055;F01_transcript_84985;F01_transcript_8587;F01_transcript_86779;F01_transcript_89273;F01_transcript_8939;F01_transcript_8947;F01_transcript_89963;F01_transcript_9220;F01_transcript_92421;F01_transcript_93784;F01_transcript_93920;F01_transcript_95442;F01_transcript_9621;F01_transcript_96230;F01_transcript_9711;F01_transcript_97952;	
alpha-Linolenic acid metabolism	ko00592	239	F01_transcript_10098;F01_transcript_101771;F01_transcript_102483;F01_transcript_102523;F01_transcript_105547;F01_transcript_105853;F01_transcript_106095;F01_transcript_106217;F01_transcript_10672;F01_transcript_107251;F01_transcript_109866;F01_transcript_114513;F01_transcript_114674;F01_transcript_11556;F01_transcript_11601;F01_transcript_116306;F01_transcript_116596;F01_transcript_117687;F01_transcript_117716;F01_transcript_118434;F01_transcript_120419;F01_transcript_120801;F01_transcript_122228;F01_transcript_122368;F01_transcript_122721;F01_transcript_122753;F01_transcript_122818;F01_transcript_122972;F01_transcript_123559;F01_transcript_123770;F01_transcript_124769;F01_transcript_124788;F01_transcript_125715;F01_transcript_127174;F01_transcript_130717;F01_transcript_130899;F01_transcript_132489;F01_transcript_132695;F01_transcript_132775;F01_transcript_133105;F01_transcript_133521;F01_transcript_135542;F01_transcript_136239;F01_trans	K10527+K00454+K14674+K18857+K10527+K00454+K10527+K00232+K10527+K05894+K00454+K00454+K00454+K00454+K00232+K10525+K00454+K00232+K00454+K00232+K00454+K00232+K00454+K00232+K10527+K14674+K00232+K18857+K00454+K00454+K00232+K00232+K00454+K00454+K07513+K05894+K00454+K10527+K00454+K00454+K00454+K00454+K08241+K10527+K00454+K00232+K10527+K00454+K01047+K10526+K00232+K10527+K00454+K10528+K00454+K00232+K00232+K10526+K10527+K00454+K00232+K00232+K01723+K07513+K10527+K00232+K00454+K00232+K00454+K00454+K10527+K00232+K05894+K10527+K08241+K00232+K00454+K00232+K00454+K08241+K00454+K00232+K10527+K07513+K00232+K10529+K10527+K18857+K10527+K00232+K14674+K18857+K10527+K10527+K01047+K00454+K01723+K05894+K07513+K10526+K10527+K10527+K00232+K14674+K14674+K00232+K10529+K00232+K00232+K0172

	cript_13624;F01_transcript_13653;F01_transcript_138047;F01_transcript_13822;F01_transcript_138305;F01_transcript_138707;F01_transcript_138876;F01_transcript_139123;F01_transcript_139625;F01_transcript_14004;F01_transcript_140136;F01_transcript_140687;F01_transcript_142214;F01_transcript_143781;F01_transcript_143785;F01_transcript_144395;F01_transcript_145968;F01_transcript_147167;F01_transcript_147259;F01_transcript_147278;F01_transcript_147398;F01_transcript_147956;F01_transcript_149791;F01_transcript_151241;F01_transcript_15284;F01_transcript_152941;F01_transcript_153249;F01_transcript_154107;F01_transcript_155474;F01_transcript_155571;F01_transcript_156381;F01_transcript_156945;F01_transcript_157406;F01_transcript_157588;F01_transcript_15817;F01_transcript_158778;F01_transcript_158794;F01_transcript_159098;F01_transcript_15946;F01_transcript_159785;F01_transcript_159968;F01_transcript_160683;F01_transcript_160964;F01_transcript_161110;F01_transcript_161429;F01_transcript_161981;F01_transcript_16286;F01_transcript_163876;F01_transcript_164766;F01_transcript_164901;F01_transcript_165924;F01_transcript_165949;F01_transcript_166102;F01_transcript_166300;F01_transcript_167286;F01_transcript_167595;F01_transcript_16803;F01_transcript_17281;F01_transcript_17857;F01_transcript_17987;F01_transcript_1856;F01_transcript_2208;F01_transcript_22669;F01_transcript_24692;F01_transcript_27104;F01_transcript_27232;F01_transcript_27346;F01_transcript_28985;F01_transcript_29191;F01_transcript_29229;F01_transcript_3071;F01_transcript_30801;F01_transcript_31003;F01_transcript_31880;F01_transcript_32105;F01_transcript_3232;F01_transcript_32889;F01_transcript_33549;F01_transcript_34223;F01_transcript_34454;F01_transcript_34541;F01_transcript_35337;F01_transcript_35442;F01_transcript_35988;F01_transcript_36109;F01_transcript_36482;F01_transcript_37657;F01_transcript_39855;F01_transcript_39872;F01_transcript_39959;F01_transcript_40510;F01_transcript_4074;F01_transcript_40984;F01_transcript_44070;F01_transcript_45663;F01_transcript_50121;F01_transcript_51375;F01_transcript_5163;F01_transcript_51683;F01_transcript_51945;F01_transcript_52099;F01_transcript_52548;F01_transcript_53390;F01_transcript_53639;F01_transcript_54044;F01_transcript_54738;F01_tr	3+K01723+K00232+K05894+K14674+K00232+K01723+K10526+K10526+K14674+K07513+K07513+K10528+K07513+K18857+K10528+K07513+K00232+K00454+K00232+K05894+K00454+K05894+K18857+K00454+K00454+K08241+K10525+K05894+K00454+K00454+K00454+K00454+K05894+K00232+K00232+K00232+K00454+K00454+K08241+K10527+K00232+K00232+K00232+K05894+K00232+K00454+K10527+K00454+K00232+K00232+K14674+K00454+K10527+K01047+K00454+K00454+K10529+K07513+K00454+K00454+K00454+K00454+K00454+K00232+K10527+K00454+K00454+K00232+K00454+K00454+K00454+K10527+K00454+K00454+K00454+K00454+K10527+K10525+K10527+K00232+K00454+K10527+K00454+K10527+K00454+K00454+K00454+K00454+K00454+K00454+K10525+K05894+K10527+K00232+K00232+K10527+K00454+K00232+K00454+K00232+K00454+K10527+K00232+K18857+K00454+K00232+K00454+K07513+K00454+K10528+K00454+K10525+K00232+K00454+K00454+K05894+K00232+K00232+K00232+K00454+K14674+K00454+K00454+K00232+K10527+K00454+K07513+K05894+K08241

			anscript_54950;F01_transcript_54990;F01_transcript_55240;F01_transcript_55281;F01_transcript_55341;F01_transcript_56128;F01_transcript_56138;F01_transcript_57689;F01_transcript_57717;F01_transcript_57777;F01_transcript_58171;F01_transcript_59089;F01_transcript_5915;F01_transcript_60135;F01_transcript_60237;F01_transcript_6048;F01_transcript_61241;F01_transcript_61643;F01_transcript_61866;F01_transcript_63305;F01_transcript_63396;F01_transcript_64901;F01_transcript_65283;F01_transcript_6551;F01_transcript_66022;F01_transcript_67042;F01_transcript_67429;F01_transcript_6766;F01_transcript_67738;F01_transcript_68061;F01_transcript_68094;F01_transcript_6883;F01_transcript_68865;F01_transcript_69619;F01_transcript_69701;F01_transcript_6982;F01_transcript_7072;F01_transcript_71676;F01_transcript_71739;F01_transcript_72010;F01_transcript_72826;F01_transcript_7305;F01_transcript_7309;F01_transcript_73347;F01_transcript_73443;F01_transcript_7347;F01_transcript_7451;F01_transcript_75154;F01_transcript_75210;F01_transcript_7576;F01_transcript_7607;F01_transcript_76274;F01_transcript_77123;F01_transcript_79452;F01_transcript_80183;F01_transcript_80645;F01_transcript_80969;F01_transcript_81823;F01_transcript_82368;F01_transcript_8246;F01_transcript_82742;F01_transcript_83055;F01_transcript_83118;F01_transcript_83317;F01_transcript_84296;F01_transcript_84985;F01_transcript_85292;F01_transcript_86779;F01_transcript_88720;F01_transcript_8947;F01_transcript_89618;F01_transcript_89963;F01_transcript_90640;F01_transcript_90931;F01_transcript_9220;F01_transcript_92421;F01_transcript_92561;F01_transcript_92632;F01_transcript_92699;F01_transcript_93534;F01_transcript_93784;F01_transcript_93920;F01_transcript_95442;F01_transcript_96230;F01_transcript_97024;F01_transcript_97546;F01_transcript_97952;F01_transcript_98462;F01_transcript_98647;F01_transcript_99524;	
Sphingolipid metabolism	ko00006	178	F01_transcript_100741;F01_transcript_103094;F01_transcript_10316;F01_transcript_104288;F01_transcript_107336;F01_transcript_107601;F01_transcript_107707;F01_transcript_108283;F01_transcript_108387;F01_transcript_10942;F01_transcript_109592;F01_transcript_109686;F01_transcript_1109;F01_transcript_1	K01190+K00654+K00654+K17108+K04710+K12349+K00654+K04710+K12309+K12349+K07407+K04711+K17108+K12309+K12309+K12309+K12309+K00654+K04715+K04713+K17108+K04718+K12349+K17108+K01634+K00654+K04711+K04715+K00654+K04715+K04708+K01190+K04715+K07407+K1

0<br>0	12177;F01_transcript_112922;F01_transcript_113188;F01_transcript_113345;F01_transcript_11466;F01_transcript_114762;F01_transcript_116316;F01_transcript_116511;F01_transcript_117141;F01_transcript_117274;F01_transcript_118827;F01_transcript_120501;F01_transcript_120691;F01_transcript_121113;F01_transcript_121273;F01_transcript_122786;F01_transcript_122936;F01_transcript_123466;F01_transcript_123915;F01_transcript_124853;F01_transcript_125167;F01_transcript_126182;F01_transcript_126483;F01_transcript_126976;F01_transcript_130086;F01_transcript_13080;F01_transcript_131624;F01_transcript_132878;F01_transcript_133190;F01_transcript_133470;F01_transcript_134235;F01_transcript_13698;F01_transcript_138035;F01_transcript_139467;F01_transcript_13991;F01_transcript_140383;F01_transcript_140613;F01_transcript_140684;F01_transcript_141511;F01_transcript_14203;F01_transcript_143242;F01_transcript_143842;F01_transcript_144569;F01_transcript_144857;F01_transcript_145772;F01_transcript_145940;F01_transcript_147021;F01_transcript_148631;F01_transcript_149690;F01_transcript_150029;F01_transcript_150629;F01_transcript_151349;F01_transcript_151522;F01_transcript_153560;F01_transcript_153747;F01_transcript_153936;F01_transcript_154208;F01_transcript_157477;F01_transcript_157735;F01_transcript_159616;F01_transcript_1603;F01_transcript_161299;F01_transcript_161354;F01_transcript_161831;F01_transcript_162231;F01_transcript_16225;F01_transcript_162418;F01_transcript_162651;F01_transcript_163495;F01_transcript_164622;F01_transcript_165252;F01_transcript_165484;F01_transcript_166200;F01_transcript_1668;F01_transcript_167314;F01_transcript_18494;F01_transcript_2181;F01_transcript_26532;F01_transcript_26773;F01_transcript_27027;F01_transcript_27732;F01_transcript_30597;F01_transcript_32786;F01_transcript_32988;F01_transcript_36267;F01_transcript_37006;F01_transcript_38263;F01_transcript_40615;F01_transcript_4235;F01_transcript_42566;F01_transcript_4718;F01_transcript_4878;F01_transcript_51466;F01_transcript_52171;F01_transcript_53000;F01_transcript_53424;F01_transcript_5538;F01_transcript_55450;F01_transcript_56256;F01_transcript_57444;F01_transcript_57755;F01_transcript_58059;F01_transcript_58571;F01_transcript_5966;F01_transcript_59871;F01_transcript	2309+K17108+K12349+K04710+K04710+K07407+K07407+K04710+K12349+K00654+K12349+K17108+K00654+K12309+K17108+K07407+K17108+K00654+K12309+K00654+K04715+K04710+K12309+K01190+K00654+K17108+K17108+K17108+K04711+K07407+K12349+K17108+K04710+K17108+K07407+K17108+K12349+K01634+K04716+K01190+K12309+K07407+K04713+K12349+K01634+K01190+K01634+K12309+K04713+K12349+K17108+K17108+K01190+K04712+K04712+K12309+K00654+K00654+K04715+K00654+K01634+K00654+K07407+K07407+K04710+K04716+K04708+K17108+K04711+K17108+K17108+K04718+K17108+K04712+K01634+K17108+K17108+K04718+K12309+K04712+K01190+K07407+K17108+K17108+K00654+K12349+K04710+K00654+K12309+K04718+K12309+K17108+K17108+K12349+K00654+K01190+K12349+K04718+K17108+K17108+K07407+K17108+K04710+K00654+K01190+K04718+K12349+K12349+K12349+K07407+K04715+K01634+K12349+K00654+K00654+K07407+K17108+K17108+K17108+K17108+K01634+K17108+K04710+K04715+K01634+K04718+K04716+K12309+K04710+K17108+K12349+K04712+K07407+K00654+K12349+K04713+K12349+K17108+K04710+K04715+K07407+K01190+K12349+K12349

			_60234;F01_transcript_60914;F01_transcript_62744;F01_transcript_62886;F01_transcript_63036;F01_transcript_63349;F01_transcript_63587;F01_transcript_63709;F01_transcript_64239;F01_transcript_64908;F01_transcript_6565;F01_transcript_66447;F01_transcript_6694;F01_transcript_68781;F01_transcript_68989;F01_transcript_69795;F01_transcript_69846;F01_transcript_7045;F01_transcript_71113;F01_transcript_71460;F01_transcript_71581;F01_transcript_71833;F01_transcript_72580;F01_transcript_72673;F01_transcript_72813;F01_transcript_72977;F01_transcript_74386;F01_transcript_75843;F01_transcript_76374;F01_transcript_76651;F01_transcript_76853;F01_transcript_78851;F01_transcript_8017;F01_transcript_81172;F01_transcript_8132;F01_transcript_81533;F01_transcript_82336;F01_transcript_82913;F01_transcript_83802;F01_transcript_84187;F01_transcript_85061;F01_transcript_8594;F01_transcript_86027;F01_transcript_87412;F01_transcript_87431;F01_transcript_87749;F01_transcript_88235;F01_transcript_88772;F01_transcript_89903;F01_transcript_92030;F01_transcript_9284;F01_transcript_93545;F01_transcript_94023;F01_transcript_94962;F01_transcript_95444;F01_transcript_95538;F01_transcript_96959;F01_transcript_97421;F01_transcript_9802;F01_transcript_99271;	
Glycosphingolipid biosynthesis - globo series	ko00603	65	F01_transcript_100724;F01_transcript_103858;F01_transcript_108175;F01_transcript_109592;F01_transcript_114631;F01_transcript_125167;F01_transcript_125881;F01_transcript_126872;F01_transcript_128440;F01_transcript_131624;F01_transcript_132878;F01_transcript_140613;F01_transcript_143884;F01_transcript_144342;F01_transcript_146835;F01_transcript_148565;F01_transcript_150629;F01_transcript_150823;F01_transcript_153936;F01_transcript_156812;F01_transcript_157698;F01_transcript_158489;F01_transcript_160207;F01_transcript_161354;F01_transcript_165965;F01_transcript_24661;F01_transcript_25080;F01_transcript_26723;F01_transcript_27130;F01_transcript_30356;F01_transcript_31504;F01_transcript_32988;F01_transcript_36267;F01_transcript_388;F01_transcript_52616;F01_transcript_57260;F01_transcript_58571;F01_transcript_60062;F01_transcript_61860;F01_transcript_63270;F01_transcript_6361;F01_transcript_65180;F01_transcript_65894;F01_transcript_6630;F01_trans	K12373+K01988+K12373+K07407+K01988+K07407+K12373+K12373+K12373+K07407+K07407+K07407+K12373+K12373+K12373+K12373+K07407+K12373+K07407+K12373+K12373+K12373+K12373+K07407+K12373+K12373+K12373+K12373+K12373+K12373+K12373+K07407+K07407+K12373+K12373+K12373+K07407+K12373+K12373+K12373+K12373+K12373+K12373+K12373+K12373+K12373+K07407+K12373+K07407+K12373+K12373+K12373+K12373+K07407+K12373+K12373+K12373+K12373+K12373+K12373+K07407+K12373+K12373+K07407+K12373

			cript_6655;F01_transcript_6741;F01_transcript_69846;F01_transcript_7118;F01_transcript_72977;F01_transcript_75592;F01_transcript_75702;F01_transcript_76707;F01_transcript_77895;F01_transcript_78851;F01_transcript_79756;F01_transcript_82388;F01_transcript_83155;F01_transcript_84093;F01_transcript_84220;F01_transcript_84272;F01_transcript_89903;F01_transcript_93660;F01_transcript_96767;F01_transcript_96959;F01_transcript_98049;	
Glycosphingolipid biosynthesis - ganglio series	ko00604	63	F01_transcript_100724;F01_transcript_108175;F01_transcript_108387;F01_transcript_112177;F01_transcript_112922;F01_transcript_113188;F01_transcript_113345;F01_transcript_125881;F01_transcript_126182;F01_transcript_126872;F01_transcript_128440;F01_transcript_13991;F01_transcript_14203;F01_transcript_143884;F01_transcript_144342;F01_transcript_144857;F01_transcript_146835;F01_transcript_148565;F01_transcript_150823;F01_transcript_156812;F01_transcript_157698;F01_transcript_158489;F01_transcript_160207;F01_transcript_161299;F01_transcript_163495;F01_transcript_165965;F01_transcript_2181;F01_transcript_24661;F01_transcript_25080;F01_transcript_26723;F01_transcript_27130;F01_transcript_30356;F01_transcript_31504;F01_transcript_388;F01_transcript_52616;F01_transcript_57260;F01_transcript_57444;F01_transcript_60062;F01_transcript_61860;F01_transcript_63036;F01_transcript_63270;F01_transcript_63587;F01_transcript_6361;F01_transcript_65180;F01_transcript_65894;F01_transcript_6630;F01_transcript_6655;F01_transcript_6741;F01_transcript_7118;F01_transcript_75592;F01_transcript_75702;F01_transcript_76707;F01_transcript_77895;F01_transcript_79756;F01_transcript_82388;F01_transcript_83155;F01_transcript_84093;F01_transcript_84220;F01_transcript_84272;F01_transcript_87412;F01_transcript_93660;F01_transcript_96767;F01_transcript_98049;	K12373+K12373+K12309+K12309+K12309+K12309+K12309+K12373+K12309+K12373+K12373+K12309+K12309+K12373+K12373+K12309+K12373+K12373+K12373+K12373+K12373+K12373+K12373+K12309+K12309+K12373+K12309+K12373+K12373+K12373+K12373+K12373+K12373+K12373+K12373+K12373+K12309+K12373+K12373+K12309+K12373+K12309+K12373+K12373+K12373+K12373+K12373+K12373+K12373+K12373+K12373+K12373+K12373+K12373+K12373+K12373+K12373+K12373+K12373+K12309+K12373+K12373+K12373
Pyruvate metabolism	ko00620	620	F01_transcript_100005;F01_transcript_100254;F01_transcript_100288;F01_transcript_100740;F01_transcript_100783;F01_transcript_100917;F01_transcript_101187;F01_transcript_101409;F01_transcript_10158;F01_transcript_101685;F01_transcript_102370;F01_transcript_102731;F01_transcript_102834;F01_transcript_103394;F01_transcript_10347;F01_transcript_103488;F01_transcript_103691;F01_transcript_103925;F01_transcript_103974;	K00026+K01679+K01962+K00128+K00873+K01595+K00873+K01962+K01895+K01006+K00162+K00162+K00162+K00026+K00029+K00026+K14085+K00128+K01759+K00873+K00026+K00627+K00382+K01962+K00128+K11262+K00626+K11262+K00162+K00873+K00161+K01962+K01649+K00028+K01962+K00028+K01679+K00028+K01649+K00051+K00873+K01759+K00382+K00128+K00026+K11262+K00028+K00026+

	F01_transcript_104096;F01_transcript_104320;F01_transcript_104691;F01_transcript_104746;F01_transcript_105202;F01_transcript_105213;F01_transcript_105292;F01_transcript_105403;F01_transcript_105720;F01_transcript_105726;F01_transcript_105738;F01_transcript_105972;F01_transcript_106220;F01_transcript_106479;F01_transcript_106631;F01_transcript_106677;F01_transcript_107013;F01_transcript_107153;F01_transcript_107406;F01_transcript_107447;F01_transcript_107604;F01_transcript_108019;F01_transcript_108112;F01_transcript_108216;F01_transcript_108221;F01_transcript_108617;F01_transcript_108901;F01_transcript_109050;F01_transcript_109283;F01_transcript_109312;F01_transcript_109495;F01_transcript_10985;F01_transcript_109885;F01_transcript_110433;F01_transcript_110879;F01_transcript_111089;F01_transcript_111222;F01_transcript_111451;F01_transcript_111739;F01_transcript_112117;F01_transcript_11216;F01_transcript_112685;F01_transcript_112884;F01_transcript_114540;F01_transcript_114712;F01_transcript_114898;F01_transcript_115322;F01_transcript_115427;F01_transcript_115594;F01_transcript_115624;F01_transcript_115636;F01_transcript_115768;F01_transcript_116027;F01_transcript_116379;F01_transcript_116381;F01_transcript_116451;F01_transcript_116556;F01_transcript_116782;F01_transcript_117298;F01_transcript_117415;F01_transcript_117574;F01_transcript_117586;F01_transcript_11770;F01_transcript_117732;F01_transcript_118146;F01_transcript_118433;F01_transcript_118584;F01_transcript_118645;F01_transcript_118710;F01_transcript_118736;F01_transcript_118744;F01_transcript_11882;F01_transcript_118848;F01_transcript_119338;F01_transcript_11934;F01_transcript_119343;F01_transcript_119411;F01_transcript_11951;F01_transcript_119638;F01_transcript_120942;F01_transcript_121035;F01_transcript_121138;F01_transcript_121529;F01_transcript_121819;F01_transcript_122007;F01_transcript_122152;F01_transcript_122215;F01_transcript_1231;F01_transcript_12318;F01_transcript_123478;F01_transcript_124195;F01_transcript_124464;F01_transcript_126030;F01_transcript_126084;F01_transcript_126405;F01_transcript_126482;F01_transcript_127146;F01_transcript_127326;F01_transcript_127390;F01_transcript_127494;F01_trans	K00128+K00029+K01962+K00161+K00102+K00161+K01759+K00029+K00029+K00029+K00382+K00873+K00128+K00128+K00029+K01595+K00627+K00029+K01595+K00026+K00382+K14085+K01759+K01759+K00873+K00026+K00382+K00161+K01759+K01595+K00026+K00162+K01610+K00873+K00161+K01759+K00161+K00028+K01649+K00026+K00128+K01069+K00627+K00128+K01649+K00873+K00128+K00026+K00382+K00029+K00873+K11262+K01759+K01759+K00128+K18881+K00029+K00627+K00128+K00873+K00029+K00028+K00873+K00026+K01069+K00627+K00026+K00128+K00128+K01759+K01759+K01649+K01759+K00128+K00627+K00026+K00028+K01006+K00128+K01895+K00627+K00873+K00025+K00029+K00026+K00029+K00029+K01759+K01962+K00128+K00128+K00028+K00128+K00873+K00128+K00128+K00026+K00029+K00162+K00026+K01962+K00025+K00873+K01610+K00128+K18881+K01759+K00128+K00128+K00626+K00029+K01069+K01069+K00029+K00128+K00128+K01759+K00026+K00128+K01649+K00029+K01595+K00026+K01895+K11262+K00873+K00382+K00029+K01006+K00873+K01595+K00627+K00128+K00026+K11262+K00128+K01610+K00128+K01649+K00128+K01595+K00128+K00162+K00028+K00026+K00128+K00026+K00128+K00026+K01610+K00026+K00382+K00128+K00028+K00026+K00382+K18881+K01759+K00029+K00128+K01895+K00128+K00128+K00128+K00161+K00128+K01512+K00873+K01895+K00161+K01649+K00128+K01759+K00626+K01595+K00161+K00026+K00873+K00028+K01963+K00873+K00128+K00028+K14085+K01759+K00873+K00128+K00873+K00026+K00873+K01649+K01759+K00026+K01069+K00128+K00029+K00128+K00128+K00128+K00029+K00029+K00026+K00162+K00161+K00026+K00382+K00162+K00026+K00028+K00873+K00029+K00026+K01610+K00029+K01962+K00029+K00028+K00873+K00162+K01512+K00102+K01006+K00626+K00026+K01759+K01759+K01610+K00029+K00029+K00029+K00029+K01610+K00128+K01610+K00029+K01006+K00128+K00028+K00028+K00029+K00873+K00029+K00029+K01649+K00627+K00026+K00029+K00382+K00627+K00873+K00873+K00873+K00029+K0012

	cript_127921;F01_transcript_127968;F01_transcript_128218;F01_transcript_128243;F01_transcript_128256;F01_transcript_12827;F01_transcript_128611;F01_transcript_128872;F01_transcript_129084;F01_transcript_129897;F01_transcript_130000;F01_transcript_130017;F01_transcript_130078;F01_transcript_130127;F01_transcript_130673;F01_transcript_13108;F01_transcript_131255;F01_transcript_13154;F01_transcript_132494;F01_transcript_132930;F01_transcript_133379;F01_transcript_1334;F01_transcript_13417;F01_transcript_134251;F01_transcript_134507;F01_transcript_134955;F01_transcript_135366;F01_transcript_135612;F01_transcript_135698;F01_transcript_135955;F01_transcript_136111;F01_transcript_136113;F01_transcript_137514;F01_transcript_137525;F01_transcript_137679;F01_transcript_137715;F01_transcript_137935;F01_transcript_138093;F01_transcript_138114;F01_transcript_138133;F01_transcript_138279;F01_transcript_138493;F01_transcript_138594;F01_transcript_138897;F01_transcript_138921;F01_transcript_139159;F01_transcript_139464;F01_transcript_139567;F01_transcript_140510;F01_transcript_140525;F01_transcript_140859;F01_transcript_140879;F01_transcript_141140;F01_transcript_141554;F01_transcript_141836;F01_transcript_143349;F01_transcript_143362;F01_transcript_143480;F01_transcript_143572;F01_transcript_144497;F01_transcript_144572;F01_transcript_144636;F01_transcript_144706;F01_transcript_144779;F01_transcript_144850;F01_transcript_145043;F01_transcript_145352;F01_transcript_145367;F01_transcript_145474;F01_transcript_145524;F01_transcript_146046;F01_transcript_146217;F01_transcript_146770;F01_transcript_147154;F01_transcript_147257;F01_transcript_147445;F01_transcript_147783;F01_transcript_147898;F01_transcript_148049;F01_transcript_149184;F01_transcript_149234;F01_transcript_149292;F01_transcript_149403;F01_transcript_149909;F01_transcript_150228;F01_transcript_15029;F01_transcript_150950;F01_transcript_151069;F01_transcript_151264;F01_transcript_151518;F01_transcript_151832;F01_transcript_151939;F01_transcript_152756;F01_transcript_152795;F01_transcript_152958;F01_transcript_153431;F01_transcript_153523;F01_transcript_153897;F01_transcript_154172;F01_transcript_15454;F01_transcript_154982;F01_transcript_155342;F01_transcript_155685;F01_trans	8+K00128+K00029+K00873+K00382+K00873+K00128+K00028+K00873+K00382+K00128+K00873+K00128+K00029+K00873+K01610+K00102+K00627+K01595+K00382+K00627+K00128+K01610+K01679+K00049+K00382+K01595+K00873+K00873+K00873+K00128+K00873+K00128+K00382+K00873+K11262+K00873+K00873+K00102+K00627+K00873+K01006+K00128+K00873+K00627+K14085+K14085+K00128+K01961+K01595+K00128+K00026+K00627+K00627+K00128+K00627+K01595+K00128+K00128+K00873+K00051+K01595+K01759+K00873+K00128+K00873+K00128+K00051+K00026+K00162+K00128+K00026+K00382+K00162+K00161+K00128+K01595+K01610+K00161+K00162+K00162+K00026+K00128+K00051+K00128+K00026+K00161+K00051+K00162+K00626+K00162+K01759+K00161+K00626+K00128+K00026+K00128+K00128+K01595+K00026+K00025+K00626+K00128+K00626+K01069+K00162+K00026+K00627+K00026+K18881+K00025+K00026+K01006+K02160+K00025+K00025+K01006+K01069+K02160+K02160+K00128+K00049+K01006+K00382+K01069+K01759+K01512+K01759+K01962+K01006+K00128+K00026+K01759+K01595+K00162+K01759+K01069+K00626+K00873+K01006+K01649+K00102+K00029+K00873+K01961+K00028+K00028+K00128+K00128+K01595+K01962+K14085+K01595+K00382+K00627+K01962+K00626+K00162+K00026+K01006+K00162+K01679+K00026+K00382+K00029+K00049+K01962+K00873+K01759+K00873+K01595+K01649+K01610+K00029+K00382+K01759+K00873+K14085+K00873+K00873+K00128+K00026+K00128+K00026+K00128+K00128+K01069+K01610+K00162+K00029+K00128+K18881+K01006+K01759+K00026+K11262+K00026+K00026+K00626+K01961+K00627+K00026+K01963+K00128+K00128+K00128+K01069+K00627+K00128+K00025+K00382+K00128+K00627+K00029+K00026+K00029+K00029+K00051+K00382+K00161+K00029+K00128+K01759+K01759+K00029+K00029+K00016+K00161+K00873+K00028+K00161+K01759+K00626+K01759+K01679+K14085+K01759+K01962+K00026+K00162+K00026+K00026+K00626+K00025+K00382+K00128+K00026+K01759+K11262+K00026+K00128+K00626+K00128+K01895+K11262+K00873+K01962+K00382+K01961+K00161+K00873+K0

	cript_157503;F01_transcript_157893;F01_transcript_158374;F01_transcript_158451;F01_transcript_158513;F01_transcript_158628;F01_transcript_158716;F01_transcript_158912;F01_transcript_159070;F01_transcript_159514;F01_transcript_159709;F01_transcript_159748;F01_transcript_159814;F01_transcript_160044;F01_transcript_160122;F01_transcript_160410;F01_transcript_160665;F01_transcript_160820;F01_transcript_161406;F01_transcript_161518;F01_transcript_161816;F01_transcript_161915;F01_transcript_161966;F01_transcript_162134;F01_transcript_162331;F01_transcript_162362;F01_transcript_162554;F01_transcript_162570;F01_transcript_162648;F01_transcript_162736;F01_transcript_162804;F01_transcript_162906;F01_transcript_16293;F01_transcript_163028;F01_transcript_163184;F01_transcript_163795;F01_transcript_163836;F01_transcript_164460;F01_transcript_164754;F01_transcript_16479;F01_transcript_164977;F01_transcript_165558;F01_transcript_165757;F01_transcript_166121;F01_transcript_166454;F01_transcript_166638;F01_transcript_166733;F01_transcript_166915;F01_transcript_166964;F01_transcript_167137;F01_transcript_167309;F01_transcript_167464;F01_transcript_16829;F01_transcript_16930;F01_transcript_17447;F01_transcript_17632;F01_transcript_17931;F01_transcript_17942;F01_transcript_1799;F01_transcript_18092;F01_transcript_18682;F01_transcript_1927;F01_transcript_19412;F01_transcript_19821;F01_transcript_19895;F01_transcript_20036;F01_transcript_20518;F01_transcript_21586;F01_transcript_22470;F01_transcript_22601;F01_transcript_22766;F01_transcript_22891;F01_transcript_22911;F01_transcript_23074;F01_transcript_23078;F01_transcript_23258;F01_transcript_23327;F01_transcript_23407;F01_transcript_23534;F01_transcript_23785;F01_transcript_24020;F01_transcript_24261;F01_transcript_24502;F01_transcript_24513;F01_transcript_24533;F01_transcript_24677;F01_transcript_25019;F01_transcript_25467;F01_transcript_25482;F01_transcript_25659;F01_transcript_26085;F01_transcript_26120;F01_transcript_26328;F01_transcript_26508;F01_transcript_27039;F01_transcript_27064;F01_transcript_27096;F01_transcript_2711;F01_transcript_27300;F01_transcript_27373;F01_transcript_27376;F01_transcript_27479;F01_transcript_27528;F0	0026+K00626+K00026+K01610+K01649+K01649+K00028+K01962+K01759+K00873+K00873+K00029+K01895+K01512+K14085+K00873+K01759+K00873+K00026+K00873+K00029+K00029+K01962+K00382+K00026+K01069+K01962+K00382+K02160+K00128+K00873+K01610+K00026+K01595+K00162+K00026+K00051+K00026+K11262+K00128+K00029+K00128+K00026+K00029+K01006+K00626+K00873+K00382+K00128+K00128+K00026+K01759+K00028+K01595+K00162+K00128+K00026+K01610

1_transcript_27683;F01_transcript_27827;F01_transcript_2788;
F01_transcript_28090;F01_transcript_28152;F01_transcript_281
68;F01_transcript_28572;F01_transcript_28763;F01_transcript_
28856;F01_transcript_28894;F01_transcript_28992;F01_transcri
pt_29158;F01_transcript_29161;F01_transcript_29309;F01_tran
script_29689;F01_transcript_29760;F01_transcript_29765;F01_t
ranscript_2984;F01_transcript_29858;F01_transcript_29908;F01
_transcript_30176;F01_transcript_30292;F01_transcript_30362;
F01_transcript_30379;F01_transcript_30491;F01_transcript_306
4;F01_transcript_30935;F01_transcript_31005;F01_transcript_3
1148;F01_transcript_31562;F01_transcript_31894;F01_transcrip
t_31945;F01_transcript_3201;F01_transcript_32017;F01_transcr
ipt_32266;F01_transcript_32422;F01_transcript_32658;F01_tra
nscript_3314;F01_transcript_33152;F01_transcript_33286;F01_t
ranscript_33325;F01_transcript_33449;F01_transcript_33600;F0
1_transcript_34035;F01_transcript_34070;F01_transcript_34179
;F01_transcript_34271;F01_transcript_34420;F01_transcript_34
439;F01_transcript_34513;F01_transcript_35239;F01_transcript
_35286;F01_transcript_3546;F01_transcript_35540;F01_transcri
pt_35667;F01_transcript_36043;F01_transcript_36124;F01_tran
script_36309;F01_transcript_36697;F01_transcript_36718;F01_t
ranscript_36801;F01_transcript_36873;F01_transcript_37154;F0
1_transcript_37169;F01_transcript_37233;F01_transcript_37377
;F01_transcript_37456;F01_transcript_3755;F01_transcript_376
14;F01_transcript_37686;F01_transcript_37696;F01_transcript_
37900;F01_transcript_37926;F01_transcript_37987;F01_transcri
pt_3807;F01_transcript_38243;F01_transcript_38273;F01_trans
cript_38302;F01_transcript_38323;F01_transcript_38657;F01_tr
anscript_38998;F01_transcript_39026;F01_transcript_39388;F0
1_transcript_39600;F01_transcript_39641;F01_transcript_39951
;F01_transcript_40133;F01_transcript_40284;F01_transcript_40
361;F01_transcript_40383;F01_transcript_40490;F01_transcript
_40600;F01_transcript_4130;F01_transcript_41527;F01_transcri
pt_41552;F01_transcript_41596;F01_transcript_41704;F01_tran
script_4197;F01_transcript_4261;F01_transcript_4391;F01_tran
script_44758;F01_transcript_45676;F01_transcript_46456;F01_t
ranscript_46469;F01_transcript_4651;F01_transcript_4702;F01_
transcript_48424;F01_transcript_49083;F01_transcript_5107;F0

	1_transcript_51453;F01_transcript_51976;F01_transcript_5213;F01_transcript_52166;F01_transcript_52332;F01_transcript_52465;F01_transcript_52692;F01_transcript_52882;F01_transcript_52972;F01_transcript_53401;F01_transcript_53589;F01_transcript_53796;F01_transcript_54269;F01_transcript_55061;F01_transcript_55541;F01_transcript_56137;F01_transcript_5640;F01_transcript_56614;F01_transcript_56690;F01_transcript_56847;F01_transcript_57636;F01_transcript_57807;F01_transcript_5825;F01_transcript_58368;F01_transcript_58405;F01_transcript_59025;F01_transcript_5928;F01_transcript_59734;F01_transcript_59767;F01_transcript_60128;F01_transcript_60314;F01_transcript_60685;F01_transcript_60687;F01_transcript_6084;F01_transcript_61112;F01_transcript_61358;F01_transcript_61414;F01_transcript_61453;F01_transcript_61708;F01_transcript_61908;F01_transcript_62031;F01_transcript_62135;F01_transcript_6228;F01_transcript_63160;F01_transcript_63491;F01_transcript_63521;F01_transcript_64577;F01_transcript_65034;F01_transcript_65371;F01_transcript_65403;F01_transcript_6571;F01_transcript_65979;F01_transcript_66261;F01_transcript_66374;F01_transcript_66550;F01_transcript_67026;F01_transcript_67081;F01_transcript_67211;F01_transcript_67399;F01_transcript_674;F01_transcript_67850;F01_transcript_68075;F01_transcript_68924;F01_transcript_69158;F01_transcript_69697;F01_transcript_69739;F01_transcript_69815;F01_transcript_69884;F01_transcript_7025;F01_transcript_70327;F01_transcript_70471;F01_transcript_71260;F01_transcript_71646;F01_transcript_7219;F01_transcript_72311;F01_transcript_72349;F01_transcript_72893;F01_transcript_729;F01_transcript_73295;F01_transcript_73477;F01_transcript_73598;F01_transcript_73713;F01_transcript_73912;F01_transcript_73944;F01_transcript_74095;F01_transcript_74401;F01_transcript_74764;F01_transcript_74775;F01_transcript_75083;F01_transcript_7526;F01_transcript_75436;F01_transcript_75514;F01_transcript_76016;F01_transcript_76175;F01_transcript_76335;F01_transcript_76856;F01_transcript_77212;F01_transcript_77252;F01_transcript_77399;F01_transcript_77451;F01_transcript_77658;F01_transcript_77968;F01_transcript_77976;F01_transcript_78270;F01_transcript_78484;F01_transcript_78511;F0	

			1_transcript_78525;F01_transcript_78775;F01_transcript_79019;F01_transcript_79052;F01_transcript_79482;F01_transcript_79704;F01_transcript_79852;F01_transcript_80009;F01_transcript_80056;F01_transcript_80148;F01_transcript_80216;F01_transcript_80510;F01_transcript_80843;F01_transcript_81584;F01_transcript_8208;F01_transcript_82337;F01_transcript_82389;F01_transcript_82396;F01_transcript_82589;F01_transcript_82656;F01_transcript_82889;F01_transcript_83284;F01_transcript_84462;F01_transcript_84463;F01_transcript_84788;F01_transcript_85267;F01_transcript_85279;F01_transcript_85411;F01_transcript_85420;F01_transcript_85424;F01_transcript_8550;F01_transcript_85796;F01_transcript_8631;F01_transcript_8639;F01_transcript_8733;F01_transcript_87505;F01_transcript_8790;F01_transcript_87964;F01_transcript_88988;F01_transcript_89459;F01_transcript_89898;F01_transcript_89922;F01_transcript_8997;F01_transcript_90728;F01_transcript_91076;F01_transcript_91232;F01_transcript_91546;F01_transcript_91759;F01_transcript_91910;F01_transcript_92441;F01_transcript_92735;F01_transcript_92804;F01_transcript_92968;F01_transcript_93976;F01_transcript_95043;F01_transcript_95232;F01_transcript_95452;F01_transcript_95502;F01_transcript_95843;F01_transcript_959;F01_transcript_96058;F01_transcript_96227;F01_transcript_96917;F01_transcript_97230;F01_transcript_97342;F01_transcript_97371;F01_transcript_97535;F01_transcript_97808;F01_transcript_97966;F01_transcript_98159;F01_transcript_98282;F01_transcript_98445;F01_transcript_98458;F01_transcript_98524;F01_transcript_98525;F01_transcript_98771;F01_transcript_99341;F01_transcript_99528;F01_transcript_99915;F01_transcript_99977;	
Glyoxylate and dicarboxylate metabolism	ko00630	856	F01_transcript_100005;F01_transcript_100118;F01_transcript_10021;F01_transcript_100330;F01_transcript_10047;F01_transcript_100647;F01_transcript_100871;F01_transcript_101062;F01_transcript_101071;F01_transcript_101218;F01_transcript_101368;F01_transcript_101371;F01_transcript_102048;F01_transcript_102135;F01_transcript_102306;F01_transcript_102477;F01_transcript_10266;F01_transcript_102714;F01_transcript_103227;F01_transcript_103318;F01_transcript_103345;F01_transcript_103394;F01_transcript_103488;F01_transcript_103532;F01_transcript_103774;F01_transcript_103802;F01_transcript_104167;F01	K00026+K00600+K03781+K00600+K14272+K15918+K00281+K01915+K00600+K01647+K01601+K00281+K14272+K00600+K00281+K11517+K14272+K00600+K00600+K01915+K15918+K00026+K00026+K14272+K11517+K11517+K00600+K01602+K00026+K00382+K01433+K01647+K01602+K00626+K01681+K01915+K01601+K15893+K00600+K00281+K03781+K14272+K01602+K03781+K00600+K11517+K01601+K03781+K00382+K01915+K00281+K18121+K00600+K00600+K00026+K01602+K00281+K00600+K00026+K00830+K01915+K15919+K14272+K01915+K15893+K00284+K00600+K03781+K142

	_transcript_104170;F01_transcript_104320;F01_transcript_104746;F01_transcript_105092;F01_transcript_105291;F01_transcript_105385;F01_transcript_105403;F01_transcript_105634;F01_transcript_106024;F01_transcript_106422;F01_transcript_106462;F01_transcript_106967;F01_transcript_107003;F01_transcript_107228;F01_transcript_107931;F01_transcript_107952;F01_transcript_107966;F01_transcript_10799;F01_transcript_108062;F01_transcript_1081;F01_transcript_108186;F01_transcript_108216;F01_transcript_108303;F01_transcript_108345;F01_transcript_108512;F01_transcript_108584;F01_transcript_108602;F01_transcript_108617;F01_transcript_109006;F01_transcript_109068;F01_transcript_109254;F01_transcript_109283;F01_transcript_109418;F01_transcript_109463;F01_transcript_109993;F01_transcript_110137;F01_transcript_110317;F01_transcript_110477;F01_transcript_110642;F01_transcript_111549;F01_transcript_111760;F01_transcript_11182;F01_transcript_112020;F01_transcript_112117;F01_transcript_112260;F01_transcript_112494;F01_transcript_112563;F01_transcript_112623;F01_transcript_112663;F01_transcript_113095;F01_transcript_113831;F01_transcript_113837;F01_transcript_114576;F01_transcript_114677;F01_transcript_114689;F01_transcript_11473;F01_transcript_114737;F01_transcript_115186;F01_transcript_115594;F01_transcript_115624;F01_transcript_115789;F01_transcript_115870;F01_transcript_115917;F01_transcript_116166;F01_transcript_116320;F01_transcript_116381;F01_transcript_116451;F01_transcript_116525;F01_transcript_116591;F01_transcript_116648;F01_transcript_116855;F01_transcript_116945;F01_transcript_117148;F01_transcript_117319;F01_transcript_117415;F01_transcript_117422;F01_transcript_117699;F01_transcript_117863;F01_transcript_118053;F01_transcript_118084;F01_transcript_118373;F01_transcript_118657;F01_transcript_118710;F01_transcript_118804;F01_transcript_118820;F01_transcript_118927;F01_transcript_119014;F01_transcript_119285;F01_transcript_119411;F01_transcript_11951;F01_transcript_119569;F01_transcript_119894;F01_transcript_119952;F01_transcript_120050;F01_transcript_120116;F01_transcript_120212;F01_transcript_121105;F01_transcript_121306;F01_transcript_121672;F01_transcript_121896;F01_tran	72+K00281+K00382+K14272+K00281+K00600+K01647+K00284+K11517+K14272+K00281+K01915+K00600+K00281+K03781+K03781+K00281+K00026+K00382+K00284+K01602+K03781+K01915+K03781+K00026+K00382+K15893+K00281+K14272+K00281+K01647+K14272+K01647+K00026+K00600+K00600+K00281+K01455+K00281+K00830+K01647+K00026+K00600+K11517+K00600+K01601+K03781+K00026+K00382+K00830+K11517+K00600+K01602+K01915+K00605+K03781+K00600+K00284+K01681+K18121+K03781+K14272+K00600+K01601+K00600+K00830+K01602+K11517+K15919+K00605+K15893+K18121+K03781+K03781+K01915+K14272+K01647+K00600+K00600+K00281+K03781+K01433+K11517+K01433+K15893+K15918+K00600+K14272+K00281+K00026+K15918+K00026+K01602+K01602+K01602+K01433+K00281+K00281+K01647+K03781+K00600+K03781+K00830+K03781+K15918+K00026+K00122+K00122+K00600+K01915+K00025+K00026+K00600+K15893+K14272+K19269+K00600+K01915+K00605+K00600+K00281+K19269+K03781+K00605+K03781+K15893+K01647+K03781+K00600+K00600+K11517+K02437+K01915+K03781+K00600+K14272+K01915+K01433+K00600+K00600+K01602+K01602+K15918+K00281+K00284+K11517+K00026+K11517+K01602+K00281+K00026+K01433+K01455+K00025+K01455+K03781+K15918+K00600+K01602+K01915+K00626+K01681+K00600+K00600+K00026+K00600+K00600+K03781+K18121+K00281+K00281+K11517+K15919+K03781+K00026+K03781+K00600+K00281+K15893+K03781+K01915+K19269+K15893+K01455+K00600+K00605+K11517+K11517+K00382+K01433+K02437+K03781+K14272+K11517+K00284+K00026+K00600+K11517+K14272+K15919+K15918+K15893+K00281+K00281+K00284+K00830+K19269+K00600+K14272+K00026+K00600+K00281+K03781+K00026+K00026+K01602+K15893+K01647+K00026+K00382+K01915+K01681+K11517+K01915+K00026+K00830+K01602+K00382+K00600+K15893+K00281+K00281+K03781+K15893+K03781+K01681+K15919+K03781+K01602+K01602+K18121+K15918+K03781+K03781+K03781+K14272+K00600+K03781+K00600+K03781+K01602+K15919+K00626+K00284+K0

	script_121955;F01_transcript_121960;F01_transcript_122101;F01_transcript_122115;F01_transcript_122132;F01_transcript_122331;F01_transcript_122499;F01_transcript_122578;F01_transcript_122863;F01_transcript_122872;F01_transcript_12302;F01_transcript_123124;F01_transcript_123183;F01_transcript_123251;F01_transcript_12336;F01_transcript_123427;F01_transcript_123753;F01_transcript_123773;F01_transcript_123848;F01_transcript_124153;F01_transcript_124200;F01_transcript_124404;F01_transcript_124625;F01_transcript_125285;F01_transcript_125296;F01_transcript_125370;F01_transcript_125371;F01_transcript_12550;F01_transcript_125768;F01_transcript_125984;F01_transcript_126030;F01_transcript_126285;F01_transcript_126482;F01_transcript_126534;F01_transcript_126567;F01_transcript_126769;F01_transcript_126781;F01_transcript_1269;F01_transcript_126994;F01_transcript_127049;F01_transcript_127239;F01_transcript_127297;F01_transcript_127607;F01_transcript_127687;F01_transcript_127884;F01_transcript_128000;F01_transcript_128256;F01_transcript_128312;F01_transcript_128648;F01_transcript_129051;F01_transcript_129374;F01_transcript_130017;F01_transcript_130127;F01_transcript_130382;F01_transcript_130447;F01_transcript_130485;F01_transcript_130572;F01_transcript_130612;F01_transcript_130674;F01_transcript_130742;F01_transcript_130895;F01_transcript_130923;F01_transcript_130965;F01_transcript_131015;F01_transcript_131196;F01_transcript_131343;F01_transcript_131459;F01_transcript_131587;F01_transcript_131925;F01_transcript_132156;F01_transcript_132238;F01_transcript_132241;F01_transcript_132264;F01_transcript_132266;F01_transcript_132298;F01_transcript_132483;F01_transcript_132527;F01_transcript_132815;F01_transcript_132823;F01_transcript_133019;F01_transcript_133265;F01_transcript_133272;F01_transcript_133486;F01_transcript_133996;F01_transcript_134020;F01_transcript_134370;F01_transcript_134656;F01_transcript_134955;F01_transcript_135338;F01_transcript_135362;F01_transcript_135610;F01_transcript_135698;F01_transcript_135764;F01_transcript_135878;F01_transcript_136111;F01_transcript_136192;F01_transcript_136746;F01_transcript_136997;F01_transcript_13740;F01_transcript_137500;F01_transcript_137593;F01_transcript_138114;F01_transcript_138295;F01_tr	3781+K01602+K11517+K03781+K00281+K15919+K00281+K00600+K00026+K00605+K00600+K03781+K00600+K15893+K00830+K14272+K15893+K00026+K15918+K18121+K14272+K00600+K01647+K00281+K11517+K00600+K11517+K11517+K15918+K02437+K00026+K00284+K00281+K01647+K03781+K00281+K00284+K01681+K00600+K11517+K00026+K00026+K00382+K00026+K19269+K01647+K00281+K03781+K03781+K00600+K00026+K00600+K15893+K00284+K11517+K00600+K00830+K11517+K03781+K01433+K03781+K15919+K00626+K00830+K14272+K00026+K03781+K00600+K03781+K01647+K00830+K11517+K00600+K00281+K11517+K00600+K15918+K00605+K15918+K15893+K01915+K00600+K00281+K11517+K15918+K03781+K03781+K00281+K11517+K03781+K00026+K00382+K00605+K15919+K03781+K03781+K15918+K03781+K00600+K03781+K00600+K00382+K03781+K00600+K03781+K01433+K00382+K14272+K00600+K01602+K00600+K01681+K00600+K03781+K00600+K01602+K00600+K03781+K14272+K03781+K00382+K03781+K00049+K00382+K01602+K03781+K00600+K03781+K00600+K14272+K00600+K01647+K00600+K00382+K00281+K00600+K03781+K03781+K00600+K00600+K00281+K00600+K00600+K03781+K00600+K15918+K00600+K00600+K00600+K01455+K03781+K03781+K00600+K03781+K01647+K00026+K00600+K01455+K03781+K03781+K14272+K03781+K14272+K03781+K15918+K01433+K00600+K00281+K00600+K11517+K14272+K01433+K01915+K14272+K00026+K00605+K03781+K00026+K00382+K03781+K03781+K00605+K01915+K15918+K03781+K01915+K03781+K01455+K00600+K01915+K01602+K01647+K14272+K00026+K01455+K00600+K00600+K00605+K00026+K00600+K19269+K11517+K00626+K11517+K01433+K00626+K11517+K00122+K01915+K00026+K00830+K11517+K01915+K11517+K03781+K18121+K03781+K00026+K00025+K00626+K11517+K11517+K00626+K18121+K00830+K01602+K11517+K11517+K00605+K11517+K11517+K00026+K15893+K00605+K00026+K01915+K11517+K11517+K03781+K11517+K00025+K00026+K01915+K01915+K19269+K00025+K00025+K19269+K18121+K01915+K01915+K19269+K00049+K18121+K00600+K15919+K15919+K01602+K00382+K03781+K00605

	anscript_138453;F01_transcript_138914;F01_transcript_139464;F01_transcript_139819;F01_transcript_139822;F01_transcript_140205;F01_transcript_140217;F01_transcript_140347;F01_transcript_1404;F01_transcript_140463;F01_transcript_140744;F01_transcript_14076;F01_transcript_140879;F01_transcript_140967;F01_transcript_141510;F01_transcript_141575;F01_transcript_141657;F01_transcript_141996;F01_transcript_142458;F01_transcript_142711;F01_transcript_142720;F01_transcript_142863;F01_transcript_14292;F01_transcript_142920;F01_transcript_143118;F01_transcript_143180;F01_transcript_143349;F01_transcript_143976;F01_transcript_144002;F01_transcript_144230;F01_transcript_144458;F01_transcript_144482;F01_transcript_144514;F01_transcript_144706;F01_transcript_145271;F01_transcript_145345;F01_transcript_145616;F01_transcript_145718;F01_transcript_145724;F01_transcript_145854;F01_transcript_145862;F01_transcript_146329;F01_transcript_146434;F01_transcript_146447;F01_transcript_146785;F01_transcript_146840;F01_transcript_146963;F01_transcript_147154;F01_transcript_147232;F01_transcript_147236;F01_transcript_147432;F01_transcript_147445;F01_transcript_147898;F01_transcript_148274;F01_transcript_148713;F01_transcript_148941;F01_transcript_149184;F01_transcript_149234;F01_transcript_149327;F01_transcript_149398;F01_transcript_149413;F01_transcript_149467;F01_transcript_149909;F01_transcript_150046;F01_transcript_150123;F01_transcript_150228;F01_transcript_150241;F01_transcript_150492;F01_transcript_150811;F01_transcript_151605;F01_transcript_15195;F01_transcript_152060;F01_transcript_152474;F01_transcript_153015;F01_transcript_153435;F01_transcript_153615;F01_transcript_153780;F01_transcript_153807;F01_transcript_153837;F01_transcript_154298;F01_transcript_154347;F01_transcript_154524;F01_transcript_154709;F01_transcript_154837;F01_transcript_154869;F01_transcript_15505;F01_transcript_155116;F01_transcript_155366;F01_transcript_155367;F01_transcript_155558;F01_transcript_155685;F01_transcript_155884;F01_transcript_156564;F01_transcript_156653;F01_transcript_15677;F01_transcript_156864;F01_transcript_157013;F01_transcript_158189;F01_transcript_158216;F01_transcript_158283;F01_transcrip	+K00281+K01602+K00284+K02437+K02437+K02437+K01602+K03781+K02437+K02437+K02437+K01602+K02437+K02437+K00026+K01602+K01602+K01602+K00600+K01602+K03781+K01602+K01602+K01602+K03781+K00605+K03781+K14272+K00626+K01647+K18121+K00600+K01602+K02437+K00284+K19269+K00600+K15893+K00122+K00281+K00281+K00600+K01433+K11517+K00382+K00281+K03781+K00626+K00281+K14272+K00026+K01602+K03781+K00600+K00600+K03781+K03781+K11517+K03781+K00281+K15918+K00026+K00382+K00600+K01455+K00600+K00049+K03781+K14272+K00600+K01681+K03781+K15893+K00382+K00600+K11517+K00281+K03781+K01433+K03781+K00600+K19269+K01647+K00600+K00830+K01455+K03781+K00026+K01681+K01915+K14272+K00026+K00600+K00830+K11517+K01915+K14272+K00600+K03781+K11517+K00026+K03781+K11517+K00026+K00026+K00626+K00026+K00600+K11517+K03781+K01647+K01915+K00025+K00382+K00281+K00600+K11517+K11517+K00600+K00281+K02437+K00026+K00600+K15893+K00382+K00284+K03781+K00281+K14272+K00830+K00600+K11517+K03781+K00600+K15918+K00281+K00600+K01433+K15919+K01433+K00600+K00605+K15893+K15918+K14272+K19269+K15918+K00600+K00600+K03781+K14272+K11517+K01915+K00626+K14272+K00605+K01681+K00026+K00026+K01681+K00026+K00626+K00281+K15893+K00600+K15893+K00025+K11517+K15893+K00382+K00026+K00026+K11517+K00626+K00600+K03781+K00600+K00600+K00382+K01647+K00284+K00281+K00284+K02437+K03781+K00026+K00626+K01433+K00026+K00600+K03781+K11517+K14272+K00281+K00281+K00284+K01647+K03781+K00830+K01433+K00600+K00281+K03781+K00281+K14272+K01433+K00281+K11517+K00281+K00600+K01602+K00600+K00026+K11517+K11517+K03781+K00600+K00830+K15893+K00382+K00026+K15918+K03781+K11517+K03781+K00382+K14272+K14272+K00281+K14272+K14272+K03781+K00600+K00281+K00600+K00600+K00281+K14272+K03781+K01647+K00026+K00284+K00026+K00026+K14272+K00600+K00600+K11517+K00605+K01915+K00281+K00600+K11517+K000

	t_158374;F01_transcript_158425;F01_transcript_158617;F01_transcript_15880;F01_transcript_159165;F01_transcript_159272;F01_transcript_159651;F01_transcript_160034;F01_transcript_16005;F01_transcript_160122;F01_transcript_16018;F01_transcript_160238;F01_transcript_160312;F01_transcript_160408;F01_transcript_160446;F01_transcript_160612;F01_transcript_160623;F01_transcript_160895;F01_transcript_160955;F01_transcript_161267;F01_transcript_161372;F01_transcript_161395;F01_transcript_161406;F01_transcript_161505;F01_transcript_161508;F01_transcript_161671;F01_transcript_161709;F01_transcript_161905;F01_transcript_162163;F01_transcript_162211;F01_transcript_16246;F01_transcript_162492;F01_transcript_162570;F01_transcript_162804;F01_transcript_162906;F01_transcript_163028;F01_transcript_163170;F01_transcript_163468;F01_transcript_1635;F01_transcript_163559;F01_transcript_163584;F01_transcript_163735;F01_transcript_164460;F01_transcript_164525;F01_transcript_164548;F01_transcript_165092;F01_transcript_165154;F01_transcript_165169;F01_transcript_165317;F01_transcript_165385;F01_transcript_165910;F01_transcript_166228;F01_transcript_166597;F01_transcript_166703;F01_transcript_166964;F01_transcript_166993;F01_transcript_167098;F01_transcript_167137;F01_transcript_16722;F01_transcript_167268;F01_transcript_167454;F01_transcript_167751;F01_transcript_16810;F01_transcript_1702;F01_transcript_17308;F01_transcript_1751;F01_transcript_17645;F01_transcript_17809;F01_transcript_18138;F01_transcript_18500;F01_transcript_19340;F01_transcript_19355;F01_transcript_19893;F01_transcript_20069;F01_transcript_2014;F01_transcript_20901;F01_transcript_21089;F01_transcript_2179;F01_transcript_22223;F01_transcript_2257;F01_transcript_22572;F01_transcript_22876;F01_transcript_22891;F01_transcript_23074;F01_transcript_23148;F01_transcript_23231;F01_transcript_23456;F01_transcript_23618;F01_transcript_23916;F01_transcript_23952;F01_transcript_24050;F01_transcript_24090;F01_transcript_24298;F01_transcript_24513;F01_transcript_24832;F01_transcript_25171;F01_transcript_25245;F01_transcript_25296;F01_transcript_25482;F01_transcript_25546;F01_transcript_25780;F01_transcript_2583;F01_transcript_26643;F01_transcript_2680;F01_transcript_26845;F01_transcript_2689;F01_	26+K00281+K14272+K03781+K00600+K00284+K00626+K03781+K00600+K00382+K00281+K01601+K00600+K00026+K19269+K14272+K00281+K00284+K01647+K00026

transcript_2695;F01_transcript_26964;F01_transcript_27140;F0
1_transcript_27222;F01_transcript_27248;F01_transcript_27298
;F01_transcript_27300;F01_transcript_27585;F01_transcript_27
683;F01_transcript_27827;F01_transcript_28020;F01_transcript
_2805;F01_transcript_28121;F01_transcript_28169;F01_transcri
pt_28268;F01_transcript_28733;F01_transcript_28812;F01_tran
script_28828;F01_transcript_28865;F01_transcript_28894;F01_t
ranscript_2900;F01_transcript_29058;F01_transcript_29197;F01
_transcript_29210;F01_transcript_29214;F01_transcript_29482;
F01_transcript_2952;F01_transcript_29697;F01_transcript_2987
3;F01_transcript_29967;F01_transcript_30092;F01_transcript_3
0161;F01_transcript_30170;F01_transcript_30222;F01_transcrip
t_30482;F01_transcript_30503;F01_transcript_30553;F01_trans
cript_30696;F01_transcript_30802;F01_transcript_30891;F01_tr
anscript_30906;F01_transcript_31005;F01_transcript_31015;F0
1_transcript_31271;F01_transcript_31325;F01_transcript_31749
;F01_transcript_31881;F01_transcript_32306;F01_transcript_32
376;F01_transcript_32561;F01_transcript_32576;F01_transcript
_32737;F01_transcript_32834;F01_transcript_3321;F01_transcri
pt_33418;F01_transcript_33540;F01_transcript_33668;F01_tran
script_33743;F01_transcript_33768;F01_transcript_34014;F01_t
ranscript_34070;F01_transcript_34189;F01_transcript_34196;F0
1_transcript_34420;F01_transcript_34439;F01_transcript_34440
;F01_transcript_34620;F01_transcript_34659;F01_transcript_34
873;F01_transcript_34949;F01_transcript_35037;F01_transcript
_35262;F01_transcript_35349;F01_transcript_35426;F01_transc
ript_35430;F01_transcript_35441;F01_transcript_35640;F01_tra
nscript_35664;F01_transcript_35906;F01_transcript_36309;F01
_transcript_36320;F01_transcript_36398;F01_transcript_366;F0
1_transcript_36649;F01_transcript_36873;F01_transcript_36997
;F01_transcript_37253;F01_transcript_37345;F01_transcript_37
377;F01_transcript_37531;F01_transcript_37633;F01_transcript
_37686;F01_transcript_37690;F01_transcript_37806;F01_transc
ript_37859;F01_transcript_37900;F01_transcript_37978;F01_tra
nscript_38026;F01_transcript_38047;F01_transcript_38051;F01
_transcript_38062;F01_transcript_38083;F01_transcript_3817;F
01_transcript_38243;F01_transcript_38273;F01_transcript_3830

2;F01_transcript_38456;F01_transcript_38578;F01_transcript_38657;F01_transcript_38687;F01_transcript_38799;F01_transcript_38809;F01_transcript_38826;F01_transcript_38934;F01_transcript_39000;F01_transcript_39119;F01_transcript_39124;F01_transcript_39388;F01_transcript_39467;F01_transcript_39579;F01_transcript_39641;F01_transcript_39760;F01_transcript_39827;F01_transcript_39952;F01_transcript_39992;F01_transcript_40003;F01_transcript_40133;F01_transcript_40284;F01_transcript_40430;F01_transcript_40444;F01_transcript_40485;F01_transcript_40490;F01_transcript_40600;F01_transcript_40894;F01_transcript_40982;F01_transcript_41006;F01_transcript_41423;F01_transcript_41941;F01_transcript_4197;F01_transcript_42058;F01_transcript_42368;F01_transcript_42808;F01_transcript_43886;F01_transcript_43891;F01_transcript_4391;F01_transcript_4393;F01_transcript_45006;F01_transcript_4584;F01_transcript_46185;F01_transcript_4627;F01_transcript_46515;F01_transcript_46967;F01_transcript_47239;F01_transcript_47266;F01_transcript_47321;F01_transcript_47558;F01_transcript_47625;F01_transcript_47881;F01_transcript_47883;F01_transcript_48119;F01_transcript_48690;F01_transcript_49083;F01_transcript_49088;F01_transcript_49392;F01_transcript_50110;F01_transcript_50115;F01_transcript_50144;F01_transcript_50401;F01_transcript_50712;F01_transcript_50911;F01_transcript_51014;F01_transcript_51616;F01_transcript_51868;F01_transcript_5211;F01_transcript_52222;F01_transcript_52332;F01_transcript_52381;F01_transcript_53226;F01_transcript_53824;F01_transcript_54142;F01_transcript_54286;F01_transcript_55634;F01_transcript_55889;F01_transcript_55946;F01_transcript_55951;F01_transcript_55958;F01_transcript_55975;F01_transcript_56493;F01_transcript_5698;F01_transcript_57190;F01_transcript_57206;F01_transcript_57636;F01_transcript_58208;F01_transcript_58214;F01_transcript_58368;F01_transcript_58758;F01_transcript_58783;F01_transcript_59025;F01_transcript_59548;F01_transcript_59602;F01_transcript_59629;F01_transcript_59737;F01_transcript_59752;F01_transcript_59771;F01_transcript_59956;F01_transcript_60004;F01_transcript_60064;F01_transcript_60113;F01_transcript_60128;F01_transcript_60314;F01_transcript_60381;F01_transcript_60431;F01_transcript_60635;F01_transcript_60687;F01_tra

nscript_60831;F01_transcript_61178;F01_transcript_61445;F01_transcript_61449;F01_transcript_61725;F01_transcript_62010;F01_transcript_62135;F01_transcript_62260;F01_transcript_62337;F01_transcript_62390;F01_transcript_62749;F01_transcript_63248;F01_transcript_63540;F01_transcript_64095;F01_transcript_64420;F01_transcript_64872;F01_transcript_64897;F01_transcript_65073;F01_transcript_65162;F01_transcript_65194;F01_transcript_65371;F01_transcript_65464;F01_transcript_65509;F01_transcript_65567;F01_transcript_6571;F01_transcript_65810;F01_transcript_65973;F01_transcript_66719;F01_transcript_66852;F01_transcript_67228;F01_transcript_67332;F01_transcript_67470;F01_transcript_67774;F01_transcript_68075;F01_transcript_68283;F01_transcript_69129;F01_transcript_69158;F01_transcript_69697;F01_transcript_69739;F01_transcript_7025;F01_transcript_70704;F01_transcript_71223;F01_transcript_71512;F01_transcript_71789;F01_transcript_72651;F01_transcript_72893;F01_transcript_729;F01_transcript_72917;F01_transcript_72967;F01_transcript_73015;F01_transcript_73145;F01_transcript_73464;F01_transcript_73498;F01_transcript_73584;F01_transcript_73713;F01_transcript_74212;F01_transcript_74379;F01_transcript_74401;F01_transcript_74497;F01_transcript_74943;F01_transcript_75040;F01_transcript_75477;F01_transcript_75487;F01_transcript_75531;F01_transcript_75535;F01_transcript_75647;F01_transcript_75678;F01_transcript_75819;F01_transcript_75876;F01_transcript_75942;F01_transcript_76014;F01_transcript_76142;F01_transcript_76385;F01_transcript_76460;F01_transcript_76536;F01_transcript_76540;F01_transcript_76633;F01_transcript_76748;F01_transcript_76830;F01_transcript_76878;F01_transcript_76950;F01_transcript_77082;F01_transcript_77186;F01_transcript_77213;F01_transcript_77339;F01_transcript_77389;F01_transcript_77451;F01_transcript_77476;F01_transcript_78456;F01_transcript_78472;F01_transcript_78511;F01_transcript_78775;F01_transcript_78791;F01_transcript_79019;F01_transcript_79052;F01_transcript_79069;F01_transcript_79175;F01_transcript_79353;F01_transcript_79364;F01_transcript_79482;F01_transcript_79544;F01_transcript_79630;F01_transcript_79704;F01_transcript_80009;F01_transcript_80216;F01_transcript_80

			484;F01_transcript_80843;F01_transcript_81090;F01_transcript_81684;F01_transcript_82110;F01_transcript_82373;F01_transcript_82589;F01_transcript_82791;F01_transcript_83083;F01_transcript_83616;F01_transcript_84;F01_transcript_84138;F01_transcript_84294;F01_transcript_84462;F01_transcript_84463;F01_transcript_84585;F01_transcript_84788;F01_transcript_84894;F01_transcript_84919;F01_transcript_85164;F01_transcript_85168;F01_transcript_85447;F01_transcript_85663;F01_transcript_85759;F01_transcript_86113;F01_transcript_8635;F01_transcript_86483;F01_transcript_86512;F01_transcript_86731;F01_transcript_86965;F01_transcript_87291;F01_transcript_87507;F01_transcript_87957;F01_transcript_88253;F01_transcript_88614;F01_transcript_89027;F01_transcript_89475;F01_transcript_89629;F01_transcript_89640;F01_transcript_89641;F01_transcript_89898;F01_transcript_90051;F01_transcript_90156;F01_transcript_90255;F01_transcript_90316;F01_transcript_90815;F01_transcript_90853;F01_transcript_91232;F01_transcript_91546;F01_transcript_91900;F01_transcript_91939;F01_transcript_92070;F01_transcript_92190;F01_transcript_92441;F01_transcript_92797;F01_transcript_92895;F01_transcript_92931;F01_transcript_93248;F01_transcript_93376;F01_transcript_93559;F01_transcript_9359;F01_transcript_93656;F01_transcript_93722;F01_transcript_93994;F01_transcript_94039;F01_transcript_94572;F01_transcript_9460;F01_transcript_94796;F01_transcript_95043;F01_transcript_95330;F01_transcript_95502;F01_transcript_959;F01_transcript_96025;F01_transcript_96028;F01_transcript_96153;F01_transcript_96299;F01_transcript_96758;F01_transcript_96851;F01_transcript_97196;F01_transcript_97239;F01_transcript_97256;F01_transcript_97342;F01_transcript_97343;F01_transcript_97367;F01_transcript_97417;F01_transcript_97597;F01_transcript_97608;F01_transcript_97808;F01_transcript_97828;F01_transcript_98080;F01_transcript_98159;F01_transcript_98369;F01_transcript_98382;F01_transcript_98413;F01_transcript_98458;F01_transcript_98700;F01_transcript_98918;F01_transcript_99086;F01_transcript_99350;F01_transcript_99615;F01_transcript_99915;	
Propanoate	ko	196	F01_transcript_100288;F01_transcript_101409;F01_transcript_10158;F01_transcript_102294;F01_transcript_102741;F01_transc	K01962+K01962+K01895+K01899+K00166+K05605+K05605+K05605+K00382+K01962+K11262+K05605+K00626+K1126

metab olism	0 0 6 4 0	ript_103175;F01_transcript_104346;F01_transcript_104696;F01_transcript_104746;F01_transcript_105202;F01_transcript_105292;F01_transcript_105377;F01_transcript_105403;F01_transcript_105720;F01_transcript_106220;F01_transcript_106677;F01_transcript_108216;F01_transcript_108901;F01_transcript_10985;F01_transcript_110861;F01_transcript_112117;F01_transcript_112210;F01_transcript_115085;F01_transcript_115624;F01_transcript_116451;F01_transcript_116665;F01_transcript_116861;F01_transcript_11951;F01_transcript_120543;F01_transcript_121035;F01_transcript_121237;F01_transcript_122090;F01_transcript_123185;F01_transcript_123463;F01_transcript_124506;F01_transcript_126314;F01_transcript_126346;F01_transcript_126875;F01_transcript_127971;F01_transcript_129084;F01_transcript_129651;F01_transcript_130417;F01_transcript_130773;F01_transcript_13154;F01_transcript_132279;F01_transcript_135955;F01_transcript_138114;F01_transcript_139604;F01_transcript_140307;F01_transcript_140486;F01_transcript_141140;F01_transcript_141489;F01_transcript_141554;F01_transcript_143349;F01_transcript_144740;F01_transcript_144779;F01_transcript_147002;F01_transcript_149234;F01_transcript_150228;F01_transcript_150299;F01_transcript_151518;F01_transcript_151674;F01_transcript_15331;F01_transcript_15347;F01_transcript_153897;F01_transcript_154730;F01_transcript_155685;F01_transcript_158628;F01_transcript_162763;F01_transcript_162906;F01_transcript_163897;F01_transcript_164546;F01_transcript_164977;F01_transcript_166964;F01_transcript_167649;F01_transcript_1704;F01_transcript_19633;F01_transcript_20578;F01_transcript_22856;F01_transcript_23074;F01_transcript_24513;F01_transcript_24810;F01_transcript_24919;F01_transcript_25482;F01_transcript_27300;F01_transcript_27827;F01_transcript_28594;F01_transcript_28894;F01_transcript_28947;F01_transcript_29158;F01_transcript_29341;F01_transcript_30491;F01_transcript_30868;F01_transcript_31414;F01_transcript_32386;F01_transcript_32562;F01_transcript_33685;F01_transcript_33822;F01_transcript_34332;F01_transcript_34439;F01_transcript_35669;F01_transcript_3669;F01_transcript_36901;F01_transcript_37377;F01_transcript_37686;F01_transcript_38302;F01_transcript_38657;F01_tran	2+K01962+K01962+K00382+K11262+K01962+K09699+K00382+K05605+K05605+K00382+K00382+K05605+K00166+K00382+K01899+K11262+K00140+K00140+K05605+K05605+K05605+K05605+K05605+K00166+K05605+K01895+K01899+K00166+K00140+K01962+K05605+K01962+K00626+K05605+K00140+K05605+K01895+K01899+K11262+K00382+K05605+K11262+K00166+K00382+K00382+K05605+K01895+K05605+K00140+K00140+K01895+K05605+K00626+K01963+K05605+K00382+K01899+K05605+K01962+K00626+K09699+K00140+K00140+K01899+K05605+K00382+K00382+K00166+K01900+K00382+K00382+K00382+K00140+K00382+K00140+K11262+K00140+K01961+K00166+K00140+K05605+K00166+K01578+K00166+K01900+K00382+K00166+K05605+K05605+K00626+K00626+K00626+K00626+K05605+K05605+K02160+K01899+K02160+K02160+K00382+K01962+K00249+K05605+K00626+K05605+K00167+K01961+K05605+K00166+K05605+K01962+K00140+K00382+K00166+K01962+K00626+K09699+K00382+K01962+K00382+K00140+K00249+K00140+K11262+K00626+K01961+K01963+K00249+K00167+K00166+K00382+K05605+K00382+K05605+K09699+K00249+K00016+K00140+K01900+K00626+K00140+K01962+K00166+K00626+K00140+K00382+K09699+K00140+K11262+K05605+K00626+K05605+K05605+K01895+K11262+K01962+K00382+K01961+K00167+K00626+K01962+K00166+K00166+K01895+K01578+K05605+K01962+K00382+K05605+K09699+K01962+K00249+K00382+K05605+K02160+K00166+K11262+K05605+K00626+K05605+K00382+K00166

			script_38936;F01_transcript_39168;F01_transcript_40383;F01_transcript_41010;F01_transcript_41552;F01_transcript_41596;F01_transcript_4391;F01_transcript_4651;F01_transcript_51625;F01_transcript_52158;F01_transcript_52332;F01_transcript_52494;F01_transcript_52864;F01_transcript_53796;F01_transcript_54938;F01_transcript_55336;F01_transcript_56268;F01_transcript_56614;F01_transcript_57145;F01_transcript_57636;F01_transcript_58172;F01_transcript_5825;F01_transcript_58368;F01_transcript_58882;F01_transcript_60314;F01_transcript_6084;F01_transcript_62135;F01_transcript_64572;F01_transcript_6767;F01_transcript_68359;F01_transcript_68924;F01_transcript_69739;F01_transcript_69815;F01_transcript_70327;F01_transcript_7124;F01_transcript_72143;F01_transcript_72537;F01_transcript_729;F01_transcript_73519;F01_transcript_74401;F01_transcript_74528;F01_transcript_75055;F01_transcript_7550;F01_transcript_76175;F01_transcript_76336;F01_transcript_76476;F01_transcript_77451;F01_transcript_77784;F01_transcript_78484;F01_transcript_78651;F01_transcript_79052;F01_transcript_79569;F01_transcript_79704;F01_transcript_79878;F01_transcript_79982;F01_transcript_80148;F01_transcript_80229;F01_transcript_80843;F01_transcript_80964;F01_transcript_81280;F01_transcript_8208;F01_transcript_82337;F01_transcript_82396;F01_transcript_82589;F01_transcript_82656;F01_transcript_83597;F01_transcript_84463;F01_transcript_85424;F01_transcript_85703;F01_transcript_86353;F01_transcript_8733;F01_transcript_88199;F01_transcript_89647;F01_transcript_91076;F01_transcript_91232;F01_transcript_91526;F01_transcript_91641;F01_transcript_91910;F01_transcript_9209;F01_transcript_92441;F01_transcript_92567;F01_transcript_92735;F01_transcript_94471;F01_transcript_96058;F01_transcript_97153;F01_transcript_97808;F01_transcript_98121;F01_transcript_98159;F01_transcript_98561;	
Butanoate metabolism	ko00650	89	F01_transcript_100488;F01_transcript_100692;F01_transcript_10428;F01_transcript_105403;F01_transcript_108512;F01_transcript_112458;F01_transcript_113986;F01_transcript_120201;F01_transcript_120633;F01_transcript_121867;F01_transcript_121955;F01_transcript_123183;F01_transcript_123569;F01_transcript_129894;F01_transcript_133321;F01_transcript_138114;F01_transcript_140217;F01_transcript_146194;F01_transcript_153213	K01640+K01580+K17761+K00626+K18121+K16871+K17761+K01653+K17761+K16871+K18121+K18121+K01640+K00074+K01653+K00626+K18121+K01653+K01653+K18121+K01641+K00626+K16871+K18121+K01652+K17761+K17761+K00626+K01652+K01653+K01652+K01652+K01641+K01641+K01653+K16871+K01640+K01641+K17761+K01653+K01640+K01580+K01580+K00626+K00626+K01640+K01580+K18121+

			;F01_transcript_153837;F01_transcript_154880;F01_transcript_155685;F01_transcript_158968;F01_transcript_160238;F01_transcript_160691;F01_transcript_162679;F01_transcript_166087;F01_transcript_166964;F01_transcript_18842;F01_transcript_19555;F01_transcript_19945;F01_transcript_20537;F01_transcript_28787;F01_transcript_30530;F01_transcript_30778;F01_transcript_31067;F01_transcript_31485;F01_transcript_32485;F01_transcript_32554;F01_transcript_33011;F01_transcript_33312;F01_transcript_33656;F01_transcript_35762;F01_transcript_37377;F01_transcript_37686;F01_transcript_37841;F01_transcript_37988;F01_transcript_38083;F01_transcript_38235;F01_transcript_38302;F01_transcript_38657;F01_transcript_38687;F01_transcript_40530;F01_transcript_40982;F01_transcript_42058;F01_transcript_42881;F01_transcript_49750;F01_transcript_51931;F01_transcript_52332;F01_transcript_52463;F01_transcript_53226;F01_transcript_55549;F01_transcript_58341;F01_transcript_58368;F01_transcript_58881;F01_transcript_61404;F01_transcript_61852;F01_transcript_62228;F01_transcript_62577;F01_transcript_69739;F01_transcript_72756;F01_transcript_74843;F01_transcript_75779;F01_transcript_77012;F01_transcript_77451;F01_transcript_77468;F01_transcript_79052;F01_transcript_79307;F01_transcript_79587;F01_transcript_80843;F01_transcript_81816;F01_transcript_83562;F01_transcript_84015;F01_transcript_84463;F01_transcript_90459;F01_transcript_90977;F01_transcript_92426;F01_transcript_93536;F01_transcript_97808;	K01580+K00626+K00626+K18121+K00074+K18121+K18121+K00074+K01580+K01652+K00626+K16871+K18121+K16871+K16871+K00626+K17761+K01640+K01652+K01652+K01580+K00626+K01640+K01652+K00074+K16871+K00626+K01580+K00626+K16871+K00074+K00626+K01640+K01580+K17761+K00626+K16871+K01653+K17761+K17761+K00626
C5-Branched dibasic acid metabolism	ko00660	33	F01_transcript_111759;F01_transcript_120201;F01_transcript_133321;F01_transcript_140089;F01_transcript_146194;F01_transcript_153213;F01_transcript_153480;F01_transcript_160691;F01_transcript_161767;F01_transcript_18842;F01_transcript_19555;F01_transcript_19945;F01_transcript_20537;F01_transcript_29072;F01_transcript_29737;F01_transcript_30778;F01_transcript_31526;F01_transcript_33011;F01_transcript_36862;F01_transcript_37086;F01_transcript_51931;F01_transcript_54539;F01_transcript_58777;F01_transcript_61852;F01_transcript_62228;F01_transcript_66275;F01_transcript_71528;F01_transcript_74843;F01_transcript_80747;F01_transcript_85624;F01_transcript_90977;F01_transcript_93119;F01_transcript_97229;	K01703+K01653+K01653+K01703+K01653+K01653+K01703+K01652+K01703+K01652+K01653+K01652+K01652+K01703+K01703+K01653+K01703+K01653+K00052+K00052+K01652+K00052+K00052+K01652+K01652+K01703+K01703+K01652+K01703+K01703+K01653+K01703+K00052

One carbon pool by folate	ko00670	236	F01_transcript_100118;F01_transcript_100330;F01_transcript_101071;F01_transcript_101795;F01_transcript_102135;F01_transcript_102714;F01_transcript_103227;F01_transcript_104167;F01_transcript_105092;F01_transcript_106967;F01_transcript_107060;F01_transcript_10799;F01_transcript_108584;F01_transcript_108602;F01_transcript_109254;F01_transcript_111549;F01_transcript_112563;F01_transcript_114677;F01_transcript_117281;F01_transcript_117422;F01_transcript_117699;F01_transcript_118714;F01_transcript_118804;F01_transcript_118927;F01_transcript_119520;F01_transcript_119952;F01_transcript_120212;F01_transcript_121270;F01_transcript_121306;F01_transcript_122115;F01_transcript_122331;F01_transcript_12302;F01_transcript_123481;F01_transcript_123647;F01_transcript_123848;F01_transcript_124153;F01_transcript_124625;F01_transcript_125296;F01_transcript_12550;F01_transcript_126781;F01_transcript_127297;F01_transcript_127626;F01_transcript_129051;F01_transcript_12963;F01_transcript_130382;F01_transcript_130612;F01_transcript_130742;F01_transcript_130895;F01_transcript_131196;F01_transcript_131478;F01_transcript_132156;F01_transcript_132238;F01_transcript_132483;F01_transcript_132823;F01_transcript_133019;F01_transcript_133265;F01_transcript_135764;F01_transcript_13740;F01_transcript_137890;F01_transcript_137923;F01_transcript_138453;F01_transcript_138914;F01_transcript_139819;F01_transcript_139822;F01_transcript_140903;F01_transcript_141179;F01_transcript_141510;F01_transcript_142008;F01_transcript_14292;F01_transcript_142920;F01_transcript_143976;F01_transcript_144053;F01_transcript_144874;F01_transcript_145271;F01_transcript_14669;F01_transcript_146840;F01_transcript_147232;F01_transcript_147294;F01_transcript_148374;F01_transcript_150241;F01_transcript_150666;F01_transcript_150827;F01_transcript_151893;F01_transcript_153160;F01_transcript_154869;F01_transcript_154872;F01_transcript_155116;F01_transcript_15726;F01_transcript_158206;F01_transcript_158283;F01_transcript_158425;F01_transcript_158617;F01_transcript_159165;F01_transcript_160408;F01_transcript_160895;F01_transcript_161743;F01_transcript_16246;F01_transcript_163735;F01_transcript_164525;F01_transcript_165169;F01_transcript_166228;F01_transcript_167049;F01_transcript_167268;F0	K00600+K00600+K00600+K00297+K00600+K00600+K00600+K00600+K01433+K00600+K13998+K00600+K00600+K00600+K00600+K00600+K00600+K00600+K13998+K00600+K00600+K00602+K00600+K00600+K00602+K00600+K00605+K01938+K00600+K00600+K00600+K00605+K00604+K00297+K00600+K00600+K01433+K01433+K00600+K01433+K00600+K13998+K00600+K01934+K00600+K00600+K00605+K00600+K00605+K01934+K00600+K00600+K00600+K01433+K00600+K00600+K01433+K00600+K13998+K01938+K00600+K00600+K00600+K00600+K01934+K13998+K00600+K00602+K00600+K00605+K01433+K01934+K00602+K00600+K01938+K00600+K00600+K00601+K01934+K00600+K13998+K13998+K00297+K13998+K00600+K00297+K00600+K01938+K13998+K00600+K00605+K00600+K00600+K00600+K00600+K00604+K00600+K00600+K00600+K00600+K01433+K13998+K00600+K00601+K00602+K00600+K13998+K00600+K00602+K01938+K00605+K00602+K13998+K00602+K00600+K00602+K00297+K13998+K00605+K00600+K13998+K00600+K13998+K00600+K01433+K00600+K00600+K00600+K00600+K00600+K13998+K00600+K00600+K00600+K00600+K00600+K00600+K00600+K00600+K00600+K00600+K00600+K00600+K00600+K00600+K00600+K00604+K01433+K00600+K00600+K01433+K00605+K00605+K00600+K00600+K00600+K00605+K00600+K01433+K00605+K00605+K00604+K01934+K00600+K01934+K00605+K00600+K00605+K00604+K00600+K13998+K00604+K00600+K00600+K01433+K00600+K00600+K00600+K00600+K00600+K00600+K01433+K00600+K00600+K13998+K00600+K00600+K13998+K01938+K00600+K00600+K00600+K00600+K00297+K00600+K00600+K00600+K01433+K01433+K00600+K00605+K00600+K00600+K00605+K00600+K00600+K00600+K00600+K00604+K01433+K00600+K01938+K01433+K00600+K01433+K00600+K00600+K00600+K00600+K00600+K00604+K13998+K00600+K00602+K00600+K13998+K00600+K00605+K00297+K00600+K00600+K01934+K01938+K00600+K00600+K00601

1_transcript_167275;F01_transcript_16782;F01_transcript_17308;F01_transcript_17722;F01_transcript_17809;F01_transcript_18129;F01_transcript_18192;F01_transcript_18500;F01_transcript_18683;F01_transcript_19556;F01_transcript_19643;F01_transcript_20069;F01_transcript_20123;F01_transcript_22012;F01_transcript_22881;F01_transcript_23148;F01_transcript_24050;F01_transcript_24211;F01_transcript_24298;F01_transcript_25078;F01_transcript_25171;F01_transcript_25296;F01_transcript_25780;F01_transcript_26643;F01_transcript_26845;F01_transcript_2695;F01_transcript_27140;F01_transcript_27420;F01_transcript_28121;F01_transcript_28268;F01_transcript_28812;F01_transcript_28865;F01_transcript_29058;F01_transcript_29214;F01_transcript_29482;F01_transcript_29697;F01_transcript_29873;F01_transcript_30092;F01_transcript_30170;F01_transcript_30222;F01_transcript_30482;F01_transcript_30802;F01_transcript_31015;F01_transcript_31275;F01_transcript_32737;F01_transcript_32834;F01_transcript_33418;F01_transcript_33743;F01_transcript_34189;F01_transcript_34659;F01_transcript_35430;F01_transcript_36398;F01_transcript_366;F01_transcript_36649;F01_transcript_36997;F01_transcript_37633;F01_transcript_39000;F01_transcript_39579;F01_transcript_39616;F01_transcript_42044;F01_transcript_42368;F01_transcript_42975;F01_transcript_45006;F01_transcript_50115;F01_transcript_51868;F01_transcript_52566;F01_transcript_53824;F01_transcript_55158;F01_transcript_55563;F01_transcript_55946;F01_transcript_5698;F01_transcript_57190;F01_transcript_59629;F01_transcript_59737;F01_transcript_60381;F01_transcript_60635;F01_transcript_61445;F01_transcript_62260;F01_transcript_63248;F01_transcript_64095;F01_transcript_64897;F01_transcript_65118;F01_transcript_65810;F01_transcript_67332;F01_transcript_67403;F01_transcript_68031;F01_transcript_70704;F01_transcript_72967;F01_transcript_73464;F01_transcript_74212;F01_transcript_74996;F01_transcript_75531;F01_transcript_75678;F01_transcript_75942;F01_transcript_76014;F01_transcript_76385;F01_transcript_76460;F01_transcript_76536;F01_transcript_76950;F01_transcript_77082;F01_transcript_78456;F01_transcript_79353;F01_transcript_81090;F01_transcript_82110;F01_transcript_82373;F01_transcri

			pt_83673;F01_transcript_84585;F01_transcript_84894;F01_transcript_85526;F01_transcript_86512;F01_transcript_86731;F01_transcript_88253;F01_transcript_89629;F01_transcript_89641;F01_transcript_90316;F01_transcript_9359;F01_transcript_93722;F01_transcript_93736;F01_transcript_93974;F01_transcript_93994;F01_transcript_94321;F01_transcript_96028;F01_transcript_96086;F01_transcript_96153;F01_transcript_96758;F01_transcript_97015;F01_transcript_97239;F01_transcript_97597;F01_transcript_97755;F01_transcript_97894;F01_transcript_98080;F01_transcript_98413;F01_transcript_99941;	
Carbon fixation in photosynthetic organisms	ko00710	696	F01_transcript_100005;F01_transcript_100214;F01_transcript_100238;F01_transcript_100430;F01_transcript_10047;F01_transcript_100825;F01_transcript_100917;F01_transcript_101368;F01_transcript_101473;F01_transcript_101685;F01_transcript_102048;F01_transcript_102189;F01_transcript_102373;F01_transcript_10266;F01_transcript_102681;F01_transcript_102826;F01_transcript_102845;F01_transcript_103331;F01_transcript_103394;F01_transcript_10347;F01_transcript_103488;F01_transcript_103532;F01_transcript_103634;F01_transcript_103675;F01_transcript_103795;F01_transcript_104170;F01_transcript_104320;F01_transcript_10450;F01_transcript_104756;F01_transcript_105365;F01_transcript_105385;F01_transcript_105645;F01_transcript_10578;F01_transcript_105919;F01_transcript_106156;F01_transcript_106166;F01_transcript_106422;F01_transcript_106631;F01_transcript_106765;F01_transcript_106970;F01_transcript_107013;F01_transcript_107113;F01_transcript_107406;F01_transcript_107604;F01_transcript_107638;F01_transcript_107906;F01_transcript_107931;F01_transcript_107952;F01_transcript_1081;F01_transcript_108200;F01_transcript_108371;F01_transcript_108502;F01_transcript_108617;F01_transcript_108659;F01_transcript_108947;F01_transcript_109006;F01_transcript_109032;F01_transcript_109050;F01_transcript_109150;F01_transcript_109283;F01_transcript_109495;F01_transcript_109676;F01_transcript_110137;F01_transcript_110541;F01_transcript_110549;F01_transcript_110771;F01_transcript_111222;F01_transcript_111451;F01_transcript_111485;F01_transcript_111739;F01_transcript_11180;F01_transcript_11182;F01_transcript_112260;F01_transcript_112269;F01_transcript_112669;F01_transcript_113831;F	K00026+K01803+K00615+K01783+K14272+K00615+K01595+K01601+K14454+K01006+K14272+K01783+K01803+K14272+K01807+K01783+K01623+K00615+K00026+K00029+K00026+K14272+K00134+K00615+K01100+K01602+K00026+K00615+K03841+K01623+K01602+K01807+K00615+K01807+K14454+K01807+K01601+K00028+K03841+K01100+K00028+K01783+K00028+K00051+K00615+K01100+K14272+K01602+K01601+K05298+K00615+K03841+K00026+K05298+K01803+K01602+K03841+K00028+K05298+K00026+K00029+K01623+K14272+K03841+K00615+K00615+K00029+K00029+K00134+K00029+K01100+K14272+K14272+K01803+K03841+K14272+K14455+K00029+K01595+K01807+K00029+K01595+K00026+K01602+K00026+K14272+K00814+K14272+K01623+K01595+K00615+K01783+K00026+K01610+K00855+K00028+K00026+K03841+K01601+K01807+K01807+K05298+K00026+K00029+K01623+K14455+K03841+K01100+K01602+K01807+K00615+K01783+K00855+K01623+K14272+K01601+K00029+K01602+K00615+K00134+K00927+K00029+K14272+K00028+K01100+K05298+K01783+K01623+K00615+K01100+K14272+K00026+K03841+K14455+K00026+K01602+K01602+K05298+K01602+K01100+K01803+K00026+K00028+K00134+K01006+K01783+K03841+K01783+K01623+K00025+K00029+K00026+K14272+K00029+K05298+K14455+K00029+K01100+K14272+K05298+K01602+K00028+K01602+K00927+K00615+K00615+K01803+K01803+K00927+K00026+K00134+K01602+K00029+K14454+K00026+K00615+K01803+K00615+K00927+K00025+K01783+K03841+K01602+K01610+K00615+K00029+K01100+K01803+K00855+K00029+K01807+K01807+

	01_transcript_114452;F01_transcript_114540;F01_transcript_114712;F01_transcript_114958;F01_transcript_115322;F01_transcript_115427;F01_transcript_115594;F01_transcript_115870;F01_transcript_116381;F01_transcript_116648;F01_transcript_116793;F01_transcript_117148;F01_transcript_117151;F01_transcript_117298;F01_transcript_11734;F01_transcript_117388;F01_transcript_117415;F01_transcript_117586;F01_transcript_117834;F01_transcript_118584;F01_transcript_118710;F01_transcript_118900;F01_transcript_119014;F01_transcript_119183;F01_transcript_119312;F01_transcript_119351;F01_transcript_119411;F01_transcript_119638;F01_transcript_119854;F01_transcript_119869;F01_transcript_119939;F01_transcript_120034;F01_transcript_120050;F01_transcript_120193;F01_transcript_120459;F01_transcript_121130;F01_transcript_121500;F01_transcript_121571;F01_transcript_122101;F01_transcript_122132;F01_transcript_122152;F01_transcript_122578;F01_transcript_122782;F01_transcript_122893;F01_transcript_123349;F01_transcript_123478;F01_transcript_123753;F01_transcript_124195;F01_transcript_124288;F01_transcript_124392;F01_transcript_124491;F01_transcript_124658;F01_transcript_12547;F01_transcript_125758;F01_transcript_125768;F01_transcript_126030;F01_transcript_126086;F01_transcript_126185;F01_transcript_126482;F01_transcript_126534;F01_transcript_126567;F01_transcript_126766;F01_transcript_126769;F01_transcript_127785;F01_transcript_128187;F01_transcript_128256;F01_transcript_12827;F01_transcript_128590;F01_transcript_128611;F01_transcript_128717;F01_transcript_129206;F01_transcript_129302;F01_transcript_129946;F01_transcript_130017;F01_transcript_130078;F01_transcript_130127;F01_transcript_130485;F01_transcript_130673;F01_transcript_130688;F01_transcript_131043;F01_transcript_13108;F01_transcript_131895;F01_transcript_132527;F01_transcript_133162;F01_transcript_133272;F01_transcript_133379;F01_transcript_133486;F01_transcript_133545;F01_transcript_13355;F01_transcript_133564;F01_transcript_133587;F01_transcript_13368;F01_transcript_134102;F01_transcript_134955;F01_transcript_135113;F01_transcript_135362;F01_transcript_135366;F01_transcript_135578;F01_transcript_135698;F01_transcript_135726;F01_tr	K00026+K01623+K00029+K00134+K01595+K00927+K00026+K01100+K05298+K05298+K00029+K01006+K01807+K14272+K01807+K01595+K05298+K00026+K00134+K01610+K05298+K01623+K01595+K14272+K01807+K01623+K03841+K00028+K14272+K00026+K00927+K00026+K03841+K00026+K01623+K01610+K01602+K00026+K00028+K01807+K00026+K05298+K01602+K14454+K01803+K00029+K00615+K01623+K00134+K01100+K14454+K01623+K01807+K01623+K01623+K01602+K01602+K00927+K05298+K14272+K01803+K00927+K01602+K05298+K14455+K00927+K01623+K01602+K01595+K05298+K00026+K00028+K00615+K00028+K01623+K01803+K14272+K05298+K00026+K01100+K01807+K14272+K03841+K00134+K01100+K05298+K00026+K00029+K05298+K00029+K00029+K00026+K00615+K00026+K00134+K00026+K01100+K00028+K00029+K01100+K00026+K01783+K00615+K03841+K01610+K00029+K01783+K01623+K03841+K00029+K00028+K01803+K01006+K03841+K14272+K00026+K01100+K01623+K01610+K00029+K00029+K00029+K00029+K01610+K01100+K01610+K00029+K01006+K01623+K00028+K00028+K00029+K01100+K05298+K00615+K00927+K00029+K00814+K01100+K01803+K05298+K00029+K00026+K00029+K01100+K00927+K00029+K00029+K01803+K00028+K14272+K01602+K00029+K01602+K01610+K01595+K14272+K01610+K01595+K01602+K14454+K14272+K01623+K01783+K01100+K01006+K05298+K05298+K01595+K14454+K00026+K00927+K05298+K01623+K14272+K01595+K00927+K14272+K14455+K00051+K00927+K01595+K00134+K14272+K05298+K05298+K05298+K14272+K00051+K00026+K01100+K05298+K01100+K01623+K00026+K00927+K05298+K05298+K00134+K00927+K05298+K01595+K01610+K05298+K01602+K05298+K14272+K00855+K01623+K05298+K00615+K00855+K00026+K14455+K01100+K01100+K01623+K14455+K00051+K00134+K00026+K00051+K01623+K00927+K03841+K01623+K00026+K01595+K01623+K01807+K00026+K00025+K00927+K00927+K01783+K01602+K00134+K01807+K01100+K01803+K01100+K00134+K01807+K00026+K01803+K00134+K00927+K00026+K03841+K00134+K03841+K00025+K00134+K0002

	anscript_135767;F01_transcript_135831;F01_transcript_135995;F01_transcript_136111;F01_transcript_136826;F01_transcript_136868;F01_transcript_137500;F01_transcript_137514;F01_transcript_138105;F01_transcript_138133;F01_transcript_138307;F01_transcript_138326;F01_transcript_138396;F01_transcript_138594;F01_transcript_139092;F01_transcript_139300;F01_transcript_139464;F01_transcript_140250;F01_transcript_140525;F01_transcript_140783;F01_transcript_140859;F01_transcript_140868;F01_transcript_140879;F01_transcript_141555;F01_transcript_141782;F01_transcript_142610;F01_transcript_143362;F01_transcript_143480;F01_transcript_143511;F01_transcript_144458;F01_transcript_144469;F01_transcript_144497;F01_transcript_144688;F01_transcript_144706;F01_transcript_144853;F01_transcript_145043;F01_transcript_145193;F01_transcript_145324;F01_transcript_145524;F01_transcript_145616;F01_transcript_145805;F01_transcript_145851;F01_transcript_146244;F01_transcript_146770;F01_transcript_146963;F01_transcript_147154;F01_transcript_147408;F01_transcript_147445;F01_transcript_147603;F01_transcript_147898;F01_transcript_147987;F01_transcript_148049;F01_transcript_148274;F01_transcript_149184;F01_transcript_149403;F01_transcript_149827;F01_transcript_149909;F01_transcript_149972;F01_transcript_150123;F01_transcript_150639;F01_transcript_150940;F01_transcript_151069;F01_transcript_151079;F01_transcript_151300;F01_transcript_151553;F01_transcript_151732;F01_transcript_152726;F01_transcript_152812;F01_transcript_152838;F01_transcript_153307;F01_transcript_153773;F01_transcript_153780;F01_transcript_153807;F01_transcript_153933;F01_transcript_154457;F01_transcript_154837;F01_transcript_154897;F01_transcript_155306;F01_transcript_155367;F01_transcript_155594;F01_transcript_156344;F01_transcript_156346;F01_transcript_156435;F01_transcript_156653;F01_transcript_157503;F01_transcript_158337;F01_transcript_158374;F01_transcript_158513;F01_transcript_159049;F01_transcript_159070;F01_transcript_159245;F01_transcript_159358;F01_transcript_160034;F01_transcript_160119;F01_transcript_160122;F01_transcript_160193;F01_transcript_160206;F01_transcript_160312;F01_transcript_160426;F01_transcript_160634;F01_transcript_16074;F01_transcript_161388;F01_transcript_16140	6+K00134+K01006+K00134+K00025+K01807+K00134+K00025+K01803+K01623+K05298+K03841+K01783+K01006+K01807+K00134+K01807+K01783+K01783+K01006+K01803+K01623+K01602+K01807+K00615+K01803+K03841+K01803+K01807+K01807+K01623+K01602+K01006+K01602+K01803+K01602+K00134+K00134+K00026+K01602+K01623+K01623+K01602+K01623+K01602+K01602+K01623+K01602+K01602+K01602+K01595+K00927+K03841+K01100+K14272+K05298+K01006+K01100+K00615+K00927+K00615+K00134+K00029+K01100+K00927+K01602+K00028+K01803+K00028+K01803+K01623+K01595+K01623+K01783+K01595+K00814+K00927+K05298+K03841+K00615+K05298+K00814+K14272+K01100+K00026+K01006+K01602+K00026+K01783+K00029+K14272+K01595+K01807+K01100+K01610+K00029+K01623+K01807+K01623+K05298+K03841+K00615+K00026+K00814+K14272+K00026+K00615+K14454+K01610+K00134+K00029+K14272+K03841+K01006+K00615+K00026+K01807+K00134+K03841+K01783+K00026+K00026+K00615+K00026+K01100+K05298+K00615+K03841+K05298+K00927+K03841+K01623+K00814+K05298+K05298+K00025+K05298+K00615+K00029+K00026+K00029+K00029+K00051+K01100+K00927+K00615+K00029+K03841+K00615+K01100+K01623+K14272+K00029+K00029+K00134+K00927+K05298+K03841+K05298+K03841+K14272+K01100+K00028+K14272+K14272+K01623+K00026+K14455+K00026+K00615+K00026+K05298+K00615+K00025+K00134+K00026+K00026+K01807+K00134+K14454+K01807+K14454+K05298+K01100+K01623+K01803+K00927+K00026+K00026+K00927+K14272+K00855+K01610+K00028+K00615+K01100+K00029+K00814+K14455+K14272+K01807+K01623+K01623+K00615+K14454+K01602+K00134+K01807+K00026+K00029+K05298+K01803+K01783+K05298+K00029+K00814+K00026+K14272+K14272+K14455+K14272+K14272+K00927+K01610+K05298+K05298+K14272+K00026+K01595+K00026+K00855+K00051+K00026+K14272+K00615+K00855+K00029+K00026+K14272+K00029+K01006+K14454+K01100+K01601+K00026+K00028+K01100+K01595+K14272+K00026+K01610

	6;F01_transcript_161915;F01_transcript_162048;F01_transcript_162362;F01_transcript_162554;F01_transcript_162570;F01_transcript_162722;F01_transcript_162804;F01_transcript_162882;F01_transcript_163028;F01_transcript_163092;F01_transcript_163184;F01_transcript_163836;F01_transcript_16385;F01_transcript_164460;F01_transcript_164521;F01_transcript_164718;F01_transcript_164751;F01_transcript_164754;F01_transcript_16479;F01_transcript_165336;F01_transcript_165396;F01_transcript_165440;F01_transcript_165558;F01_transcript_165757;F01_transcript_166475;F01_transcript_166915;F01_transcript_166935;F01_transcript_167098;F01_transcript_167137;F01_transcript_167672;F01_transcript_167733;F01_transcript_16829;F01_transcript_16930;F01_transcript_17447;F01_transcript_17632;F01_transcript_17931;F01_transcript_17942;F01_transcript_17949;F01_transcript_18092;F01_transcript_18682;F01_transcript_1927;F01_transcript_1968;F01_transcript_19821;F01_transcript_19895;F01_transcript_20036;F01_transcript_20911;F01_transcript_20947;F01_transcript_2109;F01_transcript_21167;F01_transcript_21586;F01_transcript_21833;F01_transcript_21897;F01_transcript_22107;F01_transcript_22285;F01_transcript_22470;F01_transcript_22891;F01_transcript_22911;F01_transcript_23115;F01_transcript_23299;F01_transcript_23534;F01_transcript_24261;F01_transcript_24566;F01_transcript_25019;F01_transcript_25546;F01_transcript_2583;F01_transcript_26328;F01_transcript_26964;F01_transcript_27039;F01_transcript_2711;F01_transcript_27248;F01_transcript_27479;F01_transcript_2788;F01_transcript_28020;F01_transcript_28405;F01_transcript_28733;F01_transcript_28741;F01_transcript_29301;F01_transcript_29763;F01_transcript_2984;F01_transcript_29914;F01_transcript_30044;F01_transcript_3064;F01_transcript_30736;F01_transcript_31005;F01_transcript_31188;F01_transcript_31568;F01_transcript_31687;F01_transcript_31881;F01_transcript_3201;F01_transcript_32090;F01_transcript_32376;F01_transcript_32622;F01_transcript_32658;F01_transcript_33013;F01_transcript_3314;F01_transcript_33400;F01_transcript_33668;F01_transcript_33674;F01_transcript_33963;F01_transcript_33995;F01_transcript_34014;F01_transcript_34035;F01_transcript_34070;F01_transcript_34082;F01

_transcript_34351;F01_transcript_34362;F01_transcript_34413;F01_transcript_34420;F01_transcript_34534;F01_transcript_34711;F01_transcript_34844;F01_transcript_35302;F01_transcript_35313;F01_transcript_35385;F01_transcript_3546;F01_transcript_35540;F01_transcript_35628;F01_transcript_35640;F01_transcript_35848;F01_transcript_35906;F01_transcript_35994;F01_transcript_36056;F01_transcript_36126;F01_transcript_36178;F01_transcript_36268;F01_transcript_36309;F01_transcript_36375;F01_transcript_36494;F01_transcript_36576;F01_transcript_36584;F01_transcript_36639;F01_transcript_36718;F01_transcript_36808;F01_transcript_36873;F01_transcript_37169;F01_transcript_37274;F01_transcript_37523;F01_transcript_37740;F01_transcript_37835;F01_transcript_37900;F01_transcript_3807;F01_transcript_38228;F01_transcript_38229;F01_transcript_38243;F01_transcript_38273;F01_transcript_38274;F01_transcript_38315;F01_transcript_38522;F01_transcript_38809;F01_transcript_38937;F01_transcript_39110;F01_transcript_39160;F01_transcript_39223;F01_transcript_39291;F01_transcript_39305;F01_transcript_39348;F01_transcript_39388;F01_transcript_39520;F01_transcript_39525;F01_transcript_39557;F01_transcript_39641;F01_transcript_39895;F01_transcript_40033;F01_transcript_40114;F01_transcript_40133;F01_transcript_40209;F01_transcript_40284;F01_transcript_40354;F01_transcript_40361;F01_transcript_40448;F01_transcript_40490;F01_transcript_40501;F01_transcript_40536;F01_transcript_40600;F01_transcript_40883;F01_transcript_41015;F01_transcript_41035;F01_transcript_41061;F01_transcript_41074;F01_transcript_4130;F01_transcript_41430;F01_transcript_41482;F01_transcript_41738;F01_transcript_42505;F01_transcript_42589;F01_transcript_4261;F01_transcript_43050;F01_transcript_43061;F01_transcript_43891;F01_transcript_44079;F01_transcript_4424;F01_transcript_44274;F01_transcript_44495;F01_transcript_44524;F01_transcript_44824;F01_transcript_45041;F01_transcript_45361;F01_transcript_46185;F01_transcript_4702;F01_transcript_47266;F01_transcript_47618;F01_transcript_47883;F01_transcript_47886;F01_transcript_48625;F01_transcript_49083;F01_transcript_49088;F01_transcript_49278;F01_transcript_49281;F01_transcript_49392;F01_transcript_49964;F01_transcript_50110;F01_transcript_50144;F01_transcript

	_50604;F01_transcript_50712;F01_transcript_50911;F01_transcript_51014;F01_transcript_51453;F01_transcript_51613;F01_transcript_51889;F01_transcript_51891;F01_transcript_52222;F01_transcript_52265;F01_transcript_52692;F01_transcript_52699;F01_transcript_53212;F01_transcript_53223;F01_transcript_53305;F01_transcript_53385;F01_transcript_53401;F01_transcript_53622;F01_transcript_54119;F01_transcript_54142;F01_transcript_54269;F01_transcript_54487;F01_transcript_55061;F01_transcript_55263;F01_transcript_55560;F01_transcript_5640;F01_transcript_56762;F01_transcript_56764;F01_transcript_56847;F01_transcript_56914;F01_transcript_56950;F01_transcript_57473;F01_transcript_57906;F01_transcript_5796;F01_transcript_58667;F01_transcript_58763;F01_transcript_58783;F01_transcript_58795;F01_transcript_59025;F01_transcript_5928;F01_transcript_59548;F01_transcript_60128;F01_transcript_60194;F01_transcript_60685;F01_transcript_61178;F01_transcript_61453;F01_transcript_61535;F01_transcript_61865;F01_transcript_61908;F01_transcript_62031;F01_transcript_62451;F01_transcript_62775;F01_transcript_63171;F01_transcript_63482;F01_transcript_63911;F01_transcript_64543;F01_transcript_65371;F01_transcript_65515;F01_transcript_65567;F01_transcript_6571;F01_transcript_65775;F01_transcript_65858;F01_transcript_66550;F01_transcript_66793;F01_transcript_67081;F01_transcript_67228;F01_transcript_67319;F01_transcript_674;F01_transcript_67829;F01_transcript_68075;F01_transcript_68381;F01_transcript_68495;F01_transcript_68522;F01_transcript_68670;F01_transcript_69158;F01_transcript_69697;F01_transcript_70058;F01_transcript_7025;F01_transcript_70839;F01_transcript_71185;F01_transcript_71194;F01_transcript_71315;F01_transcript_71623;F01_transcript_72018;F01_transcript_72250;F01_transcript_72421;F01_transcript_72598;F01_transcript_72732;F01_transcript_72889;F01_transcript_72893;F01_transcript_73033;F01_transcript_73118;F01_transcript_73598;F01_transcript_73713;F01_transcript_73912;F01_transcript_73944;F01_transcript_74095;F01_transcript_74422;F01_transcript_74637;F01_transcript_74740;F01_transcript_74775;F01_transcript_74788;F01_transcript_7483;F01_transcript_75159;F01_transcript_75206;F01_transcript_75477;F01_tr	

			anscript_75514;F01_transcript_76016;F01_transcript_76121;F01_transcript_76178;F01_transcript_76231;F01_transcript_76279;F01_transcript_76449;F01_transcript_76721;F01_transcript_76748;F01_transcript_77028;F01_transcript_77212;F01_transcript_77213;F01_transcript_77476;F01_transcript_78063;F01_transcript_78511;F01_transcript_78720;F01_transcript_78775;F01_transcript_78912;F01_transcript_79019;F01_transcript_79202;F01_transcript_79375;F01_transcript_79482;F01_transcript_79736;F01_transcript_80009;F01_transcript_80216;F01_transcript_80548;F01_transcript_80994;F01_transcript_81078;F01_transcript_81466;F01_transcript_81767;F01_transcript_81807;F01_transcript_82545;F01_transcript_83682;F01_transcript_83748;F01_transcript_83916;F01_transcript_84462;F01_transcript_84788;F01_transcript_84920;F01_transcript_85168;F01_transcript_85230;F01_transcript_85267;F01_transcript_85420;F01_transcript_85721;F01_transcript_85922;F01_transcript_8639;F01_transcript_86847;F01_transcript_87504;F01_transcript_87957;F01_transcript_88131;F01_transcript_88234;F01_transcript_88445;F01_transcript_88662;F01_transcript_89140;F01_transcript_89640;F01_transcript_89719;F01_transcript_8980;F01_transcript_89898;F01_transcript_8997;F01_transcript_89989;F01_transcript_90169;F01_transcript_90503;F01_transcript_90517;F01_transcript_90728;F01_transcript_91443;F01_transcript_91546;F01_transcript_92797;F01_transcript_92895;F01_transcript_93052;F01_transcript_93248;F01_transcript_93376;F01_transcript_93824;F01_transcript_93976;F01_transcript_94045;F01_transcript_94361;F01_transcript_94572;F01_transcript_95043;F01_transcript_95232;F01_transcript_95502;F01_transcript_95544;F01_transcript_95843;F01_transcript_959;F01_transcript_96025;F01_transcript_96113;F01_transcript_96425;F01_transcript_96917;F01_transcript_97342;F01_transcript_97367;F01_transcript_97371;F01_transcript_97535;F01_transcript_9785;F01_transcript_97857;F01_transcript_98382;F01_transcript_98458;F01_transcript_98525;F01_transcript_98659;F01_transcript_98771;F01_transcript_98918;F01_transcript_99915;F01_transcript_99977;	
Thiamine	ko0	103	F01_transcript_100995;F01_transcript_10129;F01_transcript_101776;F01_transcript_103689;F01_transcript_104905;F01_transcript_10691;F01_transcript_110223;F01_transcript_111319;F01_	K01662+K01662+K01662+K14153+K03146+K01662+K03147+K01662+K01662+K03147+K01662+K03146+K01662+K14153+K01662+K01662+K01662+K14153+K01662+K03146+K031

metabolism	0730		transcript_112259;F01_transcript_112948;F01_transcript_11393;F01_transcript_114074;F01_transcript_11415;F01_transcript_114787;F01_transcript_114842;F01_transcript_11575;F01_transcript_117989;F01_transcript_119128;F01_transcript_12045;F01_transcript_12099;F01_transcript_121968;F01_transcript_12212;F01_transcript_123657;F01_transcript_127478;F01_transcript_128071;F01_transcript_128452;F01_transcript_128766;F01_transcript_130989;F01_transcript_132346;F01_transcript_136391;F01_transcript_136895;F01_transcript_138792;F01_transcript_139694;F01_transcript_145651;F01_transcript_146507;F01_transcript_146508;F01_transcript_147128;F01_transcript_153329;F01_transcript_153722;F01_transcript_155652;F01_transcript_15624;F01_transcript_157993;F01_transcript_160516;F01_transcript_160566;F01_transcript_165011;F01_transcript_166003;F01_transcript_166385;F01_transcript_166752;F01_transcript_16724;F01_transcript_19853;F01_transcript_20159;F01_transcript_21861;F01_transcript_22258;F01_transcript_23913;F01_transcript_31264;F01_transcript_3160;F01_transcript_3390;F01_transcript_35710;F01_transcript_36187;F01_transcript_36922;F01_transcript_38481;F01_transcript_39104;F01_transcript_40921;F01_transcript_41079;F01_transcript_41210;F01_transcript_4226;F01_transcript_43226;F01_transcript_4468;F01_transcript_44896;F01_transcript_45296;F01_transcript_4852;F01_transcript_4857;F01_transcript_4884;F01_transcript_53287;F01_transcript_54350;F01_transcript_55513;F01_transcript_57157;F01_transcript_57383;F01_transcript_57535;F01_transcript_58285;F01_transcript_58496;F01_transcript_58765;F01_transcript_59150;F01_transcript_61171;F01_transcript_65493;F01_transcript_68991;F01_transcript_77100;F01_transcript_78042;F01_transcript_82450;F01_transcript_82833;F01_transcript_82847;F01_transcript_85472;F01_transcript_85562;F01_transcript_85912;F01_transcript_88748;F01_transcript_89093;F01_transcript_89368;F01_transcript_90037;F01_transcript_92275;F01_transcript_92378;F01_transcript_94429;F01_transcript_9634;F01_transcript_98298;	46+K01662+K06928+K01662+K01662+K03147+K01662+K01662+K01662+K03147+K03147+K01662+K00878+K01662+K01662+K00949+K03147+K03146+K03147+K03146+K01662+K14153+K03146+K01662+K14153+K01662+K01662+K01662+K03147+K03146+K03147+K03147+K03147+K01662+K03147+K14153+K03147+K03146+K03147+K03146+K03146+K03146+K03146+K03146+K00949+K03147+K03146+K03147+K03146+K00878+K01662+K03147+K01662+K03146+K01662+K01662+K03146+K03147+K03147+K03147+K01662+K01662+K03146+K01662+K01662+K03147+K03146+K00949+K01662+K01662+K01662+K01662+K01662+K14153+K00949+K01662+K03147+K03147+K01662+K03146+K03146+K01662+K01662
Riboflavin	ko0	43	F01_transcript_101778;F01_transcript_103452;F01_transcript_105497;F01_transcript_109754;F01_transcript_110150;F01_transcript_111020;F01_transcript_114971;F01_transcript_118851;F0	K14652+K14652+K14652+K14652+K14652+K11752+K14652+K14652+K00794+K14652+K14652+K14652+K11752+K14652+K14652+K14652+K14652+K14652+K14652+K14652+K117

metab olism	0 7 4 0		1_transcript_129778;F01_transcript_135487;F01_transcript_14059;F01_transcript_148921;F01_transcript_151030;F01_transcript_155530;F01_transcript_16046;F01_transcript_160902;F01_transcript_161068;F01_transcript_162917;F01_transcript_16749;F01_transcript_28089;F01_transcript_28885;F01_transcript_30551;F01_transcript_33899;F01_transcript_41739;F01_transcript_41814;F01_transcript_43341;F01_transcript_43433;F01_transcript_44804;F01_transcript_53228;F01_transcript_53526;F01_transcript_57361;F01_transcript_57728;F01_transcript_62055;F01_transcript_68824;F01_transcript_69209;F01_transcript_70865;F01_transcript_71053;F01_transcript_81446;F01_transcript_83485;F01_transcript_85439;F01_transcript_88480;F01_transcript_89211;F01_transcript_96952;	52+K14652+K00794+K00793+K18453+K00794+K00794+K00793+K14652+K00794+K14652+K11752+K00793+K11752+K14652+K11752+K11752+K11752+K14652+K00794+K11752+K14652+K14652
Vitamin B6 metabolism	ko00750	52	F01_transcript_102433;F01_transcript_105435;F01_transcript_106692;F01_transcript_109307;F01_transcript_109886;F01_transcript_110485;F01_transcript_114138;F01_transcript_114956;F01_transcript_118908;F01_transcript_122898;F01_transcript_129850;F01_transcript_132549;F01_transcript_133475;F01_transcript_134902;F01_transcript_136518;F01_transcript_138231;F01_transcript_140500;F01_transcript_144096;F01_transcript_146656;F01_transcript_146746;F01_transcript_147534;F01_transcript_151115;F01_transcript_152086;F01_transcript_162619;F01_transcript_164756;F01_transcript_164826;F01_transcript_166538;F01_transcript_28502;F01_transcript_30367;F01_transcript_31138;F01_transcript_33950;F01_transcript_35945;F01_transcript_36455;F01_transcript_38669;F01_transcript_39718;F01_transcript_39769;F01_transcript_40260;F01_transcript_41598;F01_transcript_42515;F01_transcript_43372;F01_transcript_43523;F01_transcript_43810;F01_transcript_51986;F01_transcript_53068;F01_transcript_56566;F01_transcript_57236;F01_transcript_57970;F01_transcript_59991;F01_transcript_60095;F01_transcript_77620;F01_transcript_98005;F01_transcript_98379;	K00275+K00275+K05275+K06215+K13248+K00275+K00275+K06215+K01733+K00275+K06215+K00275+K05275+K00868+K06215+K00275+K13248+K06215+K00275+K08681+K01733+K13248+K13248+K00275+K06215+K00275+K08681+K01733+K00275+K00275+K00868+K00831+K05275+K05275+K06215+K06215+K06215+K06215+K08681+K08681+K08681+K13248+K01733+K00275+K06215+K13248+K00831+K08681+K05275+K00275+K01733+K08681
Nicotinate and nicotinamide	ko007	110	F01_transcript_100612;F01_transcript_102080;F01_transcript_107470;F01_transcript_107589;F01_transcript_108794;F01_transcript_111060;F01_transcript_112364;F01_transcript_112432;F01_transcript_115316;F01_transcript_115618;F01_transcript_115641;F01_transcript_116733;F01_transcript_117641;F01_transcript_118033;F01_transcript_11815;F01_transcript_118288;F01_tr	K00858+K03787+K00858+K03787+K00858+K03787+K01950+K00858+K00278+K03517+K06210+K00858+K00858+K00858+K01950+K00858+K03517+K00858+K01081+K03787+K03517+K18551+K01240+K03517+K00278+K00858+K00858+K00763+K03787+K03426+K00858+K01950+K03787+K01513+K03426+K00858+K00763+K00858+K03517+K03517+K03517+K

metab olism	6 0		anscript_12059;F01_transcript_122239;F01_transcript_122779;F01_transcript_123026;F01_transcript_12350;F01_transcript_124890;F01_transcript_126601;F01_transcript_127075;F01_transcript_12979;F01_transcript_133462;F01_transcript_133899;F01_transcript_134950;F01_transcript_136197;F01_transcript_136229;F01_transcript_137287;F01_transcript_13820;F01_transcript_139692;F01_transcript_140192;F01_transcript_140365;F01_transcript_140591;F01_transcript_140650;F01_transcript_141784;F01_transcript_14327;F01_transcript_14777;F01_transcript_149031;F01_transcript_151441;F01_transcript_153582;F01_transcript_153643;F01_transcript_153805;F01_transcript_153929;F01_transcript_154031;F01_transcript_154160;F01_transcript_15422;F01_transcript_155707;F01_transcript_155930;F01_transcript_156134;F01_transcript_156552;F01_transcript_157467;F01_transcript_160163;F01_transcript_160369;F01_transcript_161116;F01_transcript_161932;F01_transcript_162255;F01_transcript_16570;F01_transcript_167497;F01_transcript_17474;F01_transcript_20575;F01_transcript_20898;F01_transcript_24371;F01_transcript_25012;F01_transcript_25142;F01_transcript_25440;F01_transcript_27218;F01_transcript_2728;F01_transcript_3263;F01_transcript_32673;F01_transcript_34468;F01_transcript_35379;F01_transcript_38137;F01_transcript_40039;F01_transcript_40373;F01_transcript_41800;F01_transcript_43107;F01_transcript_43927;F01_transcript_44132;F01_transcript_52018;F01_transcript_54249;F01_transcript_54484;F01_transcript_54897;F01_transcript_56844;F01_transcript_58733;F01_transcript_60230;F01_transcript_61239;F01_transcript_62006;F01_transcript_65868;F01_transcript_68428;F01_transcript_69479;F01_transcript_70301;F01_transcript_72202;F01_transcript_72257;F01_transcript_72394;F01_transcript_74371;F01_transcript_75987;F01_transcript_76519;F01_transcript_79664;F01_transcript_80042;F01_transcript_82714;F01_transcript_83908;F01_transcript_85997;F01_transcript_89055;F01_transcript_96155;F01_transcript_97120;F01_transcript_98074;F01_transcript_98651;	01513+K03787+K01240+K06210+K00858+K01950+K03517+K03426+K03517+K03787+K00278+K01240+K03787+K03787+K00858+K00278+K03787+K03517+K00278+K03517+K00278+K00278+K00763+K00763+K00767+K00763+K00763+K01513+K00858+K00858+K00767+K00767+K03426+K01240+K03787+K03787+K03787+K00858+K18551+K06210+K18551+K03517+K00763+K00858+K00763+K06210+K01240+K00858+K00278+K03787+K00278+K00858+K00858+K00858+K00278+K03787+K03787+K03787+K00858+K18551+K00858+K00767+K00278+K06210+K00763+K06210+K00767+K03517+K00858
Pantot henate and	k o 0	15 5	F01_transcript_100070;F01_transcript_100487;F01_transcript_100516;F01_transcript_101584;F01_transcript_101694;F01_transcript_102061;F01_transcript_102210;F01_transcript_104244;F0	K01922+K00826+K00826+K00826+K09680+K00859+K02201+K02201+K00053+K06133+K00859+K01431+K00826+K09680+K09680+K01922+K00053+K06133+K06133+K00826+K096

CoA biosynthesis	0770	1_transcript_105548;F01_transcript_107441;F01_transcript_108503;F01_transcript_108650;F01_transcript_110728;F01_transcript_111718;F01_transcript_113740;F01_transcript_113925;F01_transcript_115383;F01_transcript_116365;F01_transcript_116578;F01_transcript_116956;F01_transcript_117063;F01_transcript_119690;F01_transcript_120201;F01_transcript_124927;F01_transcript_125864;F01_transcript_127540;F01_transcript_128756;F01_transcript_129549;F01_transcript_130510;F01_transcript_131371;F01_transcript_131664;F01_transcript_133194;F01_transcript_133321;F01_transcript_135402;F01_transcript_139672;F01_transcript_140192;F01_transcript_141532;F01_transcript_146194;F01_transcript_146322;F01_transcript_146892;F01_transcript_148792;F01_transcript_1503;F01_transcript_150508;F01_transcript_150546;F01_transcript_151441;F01_transcript_151854;F01_transcript_152421;F01_transcript_152555;F01_transcript_152848;F01_transcript_153213;F01_transcript_154093;F01_transcript_154917;F01_transcript_157416;F01_transcript_160691;F01_transcript_160755;F01_transcript_161184;F01_transcript_162480;F01_transcript_163491;F01_transcript_164154;F01_transcript_164487;F01_transcript_167520;F01_transcript_167653;F01_transcript_17406;F01_transcript_18842;F01_transcript_19217;F01_transcript_19555;F01_transcript_19790;F01_transcript_19945;F01_transcript_20059;F01_transcript_20342;F01_transcript_20537;F01_transcript_20928;F01_transcript_21265;F01_transcript_22646;F01_transcript_24871;F01_transcript_27218;F01_transcript_28993;F01_transcript_29103;F01_transcript_29933;F01_transcript_30416;F01_transcript_30778;F01_transcript_31229;F01_transcript_32304;F01_transcript_3260;F01_transcript_33011;F01_transcript_34128;F01_transcript_34357;F01_transcript_35824;F01_transcript_36001;F01_transcript_36481;F01_transcript_36577;F01_transcript_37725;F01_transcript_38052;F01_transcript_38232;F01_transcript_42528;F01_transcript_46295;F01_transcript_47335;F01_transcript_51931;F01_transcript_52367;F01_transcript_52477;F01_transcript_52510;F01_transcript_53384;F01_transcript_53605;F01_transcript_54318;F01_transcript_56606;F01_transcript_57483;F01_transcript_57595;F01_transcript_58468;F01_transcript_60186;F01_transcript_60455;F01_transcript_60812;F01_transcript_61852;F01_transcript_62228;F01_transc	80+K00826+K01653+K02201+K00053+K00053+K01464+K00053+K06133+K00859+K06133+K00053+K01653+K00053+K09680+K01513+K09680+K01653+K00053+K06133+K00826+K00053+K00053+K01918+K01513+K00053+K00859+K09680+K00053+K01653+K01431+K00826+K00606+K01652+K00207+K00859+K00606+K00859+K01464+K09680+K09680+K00053+K00859+K01652+K01464+K01653+K00053+K01652+K01687+K00053+K01652+K00053+K01431+K00053+K01464+K01513+K00053+K01464+K01431+K01464+K01653+K01464+K00053+K09680+K01653+K00207+K00826+K00826+K09680+K09680+K00859+K00826+K00606+K09680+K01922+K02201+K02201+K01652+K00826+K01431+K00859+K00606+K00859+K00826+K00053+K01464+K00207+K00826+K00053+K00053+K09680+K01652+K01652+K06133+K06133+K00053+K09680+K00053+K09680+K06133+K06133+K06133+K00859+K09680+K00859+K09680+K00053+K00826+K01464+K00826+K00053+K00207+K01652+K01687+K02201+K01431+K09680+K09680+K09680+K06133+K09680+K00826+K00826+K00053+K06133+K01464+K00826+K01653+K00826+K00859+K00826+K06133+K00207+K00207+K01464

			ript_62245;F01_transcript_62359;F01_transcript_64900;F01_transcript_66119;F01_transcript_67213;F01_transcript_67359;F01_transcript_67744;F01_transcript_69880;F01_transcript_70110;F01_transcript_70118;F01_transcript_70723;F01_transcript_70767;F01_transcript_70944;F01_transcript_70967;F01_transcript_72229;F01_transcript_72513;F01_transcript_73375;F01_transcript_74222;F01_transcript_74548;F01_transcript_74843;F01_transcript_78308;F01_transcript_78446;F01_transcript_78714;F01_transcript_8030;F01_transcript_80614;F01_transcript_81113;F01_transcript_82299;F01_transcript_82468;F01_transcript_83526;F01_transcript_84934;F01_transcript_85969;F01_transcript_86603;F01_transcript_8697;F01_transcript_87267;F01_transcript_90977;F01_transcript_91214;F01_transcript_92061;F01_transcript_93866;F01_transcript_94595;F01_transcript_96558;F01_transcript_98781;F01_transcript_99795;	
Biotin metabolism	ko00780	72	F01_transcript_10004;F01_transcript_101823;F01_transcript_103130;F01_transcript_104730;F01_transcript_110547;F01_transcript_113382;F01_transcript_113401;F01_transcript_114717;F01_transcript_117649;F01_transcript_118673;F01_transcript_121350;F01_transcript_123649;F01_transcript_124818;F01_transcript_130464;F01_transcript_131603;F01_transcript_131659;F01_transcript_135092;F01_transcript_138481;F01_transcript_139851;F01_transcript_141300;F01_transcript_143805;F01_transcript_143911;F01_transcript_144932;F01_transcript_147293;F01_transcript_147705;F01_transcript_15003;F01_transcript_151519;F01_transcript_156365;F01_transcript_15890;F01_transcript_158942;F01_transcript_162824;F01_transcript_164916;F01_transcript_165923;F01_transcript_166283;F01_transcript_29866;F01_transcript_31883;F01_transcript_32112;F01_transcript_33118;F01_transcript_36847;F01_transcript_36931;F01_transcript_37378;F01_transcript_44928;F01_transcript_46128;F01_transcript_54259;F01_transcript_57707;F01_transcript_60150;F01_transcript_61151;F01_transcript_61462;F01_transcript_62859;F01_transcript_65988;F01_transcript_67593;F01_transcript_69883;F01_transcript_70297;F01_transcript_70315;F01_transcript_72026;F01_transcript_72790;F01_transcript_72848;F01_transcript_73024;F01_transcript_76033;F01_transcript_77290;F01_transcript_77333	K09458+K09458+K00059+K09458+K01942+K09458+K09458+K09458+K09458+K00208+K19562+K00652+K01942+K09458+K00059+K00208+K01942+K01942+K09458+K01942+K09458+K01012+K00208+K09458+K00059+K09458+K19562+K02372+K09458+K09458+K01942+K09458+K09458+K00208+K00208+K00208+K09458+K01012+K00059+K00059+K00059+K00059+K02372+K02372+K01942+K00059+K09458+K02372+K09458+K00208+K09458+K09458+K00652+K00208+K00059+K00652+K09458+K09458+K09458+K02372+K00652+K00652+K09458+K09458+K09458+K01012+K00059+K09458+K00059+K09458+K00208+K00059

			;F01_transcript_77705;F01_transcript_77795;F01_transcript_80702;F01_transcript_83638;F01_transcript_87698;F01_transcript_88933;F01_transcript_90527;F01_transcript_92715;F01_transcript_93122;F01_transcript_94689;F01_transcript_96815;	
Lipoic acid metabolism	ko00785	17	F01_transcript_105895;F01_transcript_113208;F01_transcript_118716;F01_transcript_134743;F01_transcript_136721;F01_transcript_138577;F01_transcript_141216;F01_transcript_5054;F01_transcript_53087;F01_transcript_58987;F01_transcript_62138;F01_transcript_65685;F01_transcript_66879;F01_transcript_68871;F01_transcript_81883;F01_transcript_89446;F01_transcript_99262;	K03801+K03644+K03644+K03644+K03644+K03644+K03644+K03644+K03644+K03644+K03644+K03644+K03644+K03644+K03801+K03644+K03644
Folate biosynthesis	ko00790	89	F01_transcript_102208;F01_transcript_103906;F01_transcript_103941;F01_transcript_107060;F01_transcript_112001;F01_transcript_117281;F01_transcript_117340;F01_transcript_118978;F01_transcript_121649;F01_transcript_122672;F01_transcript_122684;F01_transcript_123673;F01_transcript_125179;F01_transcript_126472;F01_transcript_127626;F01_transcript_134472;F01_transcript_135687;F01_transcript_137890;F01_transcript_138271;F01_transcript_141179;F01_transcript_143129;F01_transcript_143142;F01_transcript_148417;F01_transcript_150656;F01_transcript_150666;F01_transcript_150827;F01_transcript_15301;F01_transcript_153160;F01_transcript_153791;F01_transcript_158206;F01_transcript_163423;F01_transcript_165236;F01_transcript_165810;F01_transcript_167049;F01_transcript_167360;F01_transcript_17612;F01_transcript_17722;F01_transcript_19357;F01_transcript_19556;F01_transcript_20272;F01_transcript_22178;F01_transcript_22881;F01_transcript_23045;F01_transcript_24211;F01_transcript_25078;F01_transcript_27236;F01_transcript_27420;F01_transcript_33831;F01_transcript_38745;F01_transcript_39710;F01_transcript_53355;F01_transcript_54011;F01_transcript_55158;F01_transcript_56102;F01_transcript_57139;F01_transcript_5818;F01_transcript_58367;F01_transcript_5948;F01_transcript_59506;F01_transcript_59562;F01_transcript_59699;F01_transcript_60683;F01_transcript_65118;F01_transcript_66421;F01_transcript_67403;F01_transcript_68055;F01_transcript_69347;F01_transcript_69748;F01_transcript_71756;F01_transcript_72487;F01_transcript_73924;F01_transcript_75365;F01_transcript_76915;F01_transcript_79347;F01_transcript_79629;F0	K01930+K01495+K01930+K13998+K01930+K13998+K01930+K20457+K01495+K01495+K01930+K01930+K13950+K00357+K13998+K01307+K13941+K13998+K01930+K13998+K21232+K01930+K01930+K03637+K13998+K13998+K20457+K13998+K01930+K13998+K20457+K01930+K01930+K13998+K13950+K20457+K13998+K20457+K13998+K01495+K01495+K13998+K01930+K13998+K13998+K01930+K13998+K20457+K01307+K18482+K01930+K01930+K13998+K01307+K01307+K01930+K01495+K01930+K21232+K01930+K01930+K13950+K13998+K01930+K13998+K01495+K01930+K01307+K18482+K01930+K13950+K01930+K01113+K01930+K01495+K13941+K01930+K01930+K01307+K01633+K01930+K01930+K13998+K01930+K18482+K13998+K13950+K01930+K01930

			1_transcript_80934;F01_transcript_84053;F01_transcript_84078;F01_transcript_88032;F01_transcript_89435;F01_transcript_90158;F01_transcript_910;F01_transcript_93974;F01_transcript_95432;F01_transcript_95598;F01_transcript_96086;F01_transcript_96627;F01_transcript_97423;F01_transcript_99764;	
Porphyrin and chlorophyll metabolism	ko00860	240	F01_transcript_101415;F01_transcript_101808;F01_transcript_102012;F01_transcript_103779;F01_transcript_104960;F01_transcript_105267;F01_transcript_105716;F01_transcript_108026;F01_transcript_109197;F01_transcript_111558;F01_transcript_111674;F01_transcript_112075;F01_transcript_112492;F01_transcript_113084;F01_transcript_114156;F01_transcript_115451;F01_transcript_117436;F01_transcript_117648;F01_transcript_118088;F01_transcript_119162;F01_transcript_121515;F01_transcript_122548;F01_transcript_123222;F01_transcript_123509;F01_transcript_123663;F01_transcript_123837;F01_transcript_124949;F01_transcript_125268;F01_transcript_12540;F01_transcript_126043;F01_transcript_128396;F01_transcript_128837;F01_transcript_130353;F01_transcript_130834;F01_transcript_130836;F01_transcript_131577;F01_transcript_131809;F01_transcript_132456;F01_transcript_132649;F01_transcript_133626;F01_transcript_133690;F01_transcript_134426;F01_transcript_134509;F01_transcript_135465;F01_transcript_135863;F01_transcript_136081;F01_transcript_136985;F01_transcript_137833;F01_transcript_139078;F01_transcript_139411;F01_transcript_139630;F01_transcript_140515;F01_transcript_142114;F01_transcript_143683;F01_transcript_144714;F01_transcript_144913;F01_transcript_145261;F01_transcript_145368;F01_transcript_14617;F01_transcript_146585;F01_transcript_147034;F01_transcript_147480;F01_transcript_147827;F01_transcript_147989;F01_transcript_148497;F01_transcript_148838;F01_transcript_149519;F01_transcript_149618;F01_transcript_149721;F01_transcript_150661;F01_transcript_151541;F01_transcript_151667;F01_transcript_15186;F01_transcript_152329;F01_transcript_152683;F01_transcript_153675;F01_transcript_154653;F01_transcript_156397;F01_transcript_158125;F01_transcript_158455;F01_transcript_158607;F01_transcript_159017;F01_transcript_159276;F01_transcript_159357;F01_transcript_160019;F01_transcript_161405;F01_transcrip	K00218+K02259+K00231+K01599+K10960+K13600+K13606+K10960+K00510+K02257+K01885+K03403+K03404+K01885+K01885+K03428+K00228+K00231+K00218+K13071+K13600+K02259+K03405+K02259+K00231+K01599+K01772+K00231+K00231+K03404+K00231+K03403+K02492+K00218+K01885+K01772+K01719+K04035+K00218+K01885+K02492+K01599+K13071+K00231+K01599+K03403+K13545+K18010+K00231+K13545+K00231+K19073+K00231+K00231+K01599+K03403+K03404+K00231+K01885+K13071+K01885+K00228+K00231+K01885+K00231+K01885+K00231+K18010+K01698+K13606+K00231+K00231+K03404+K19054+K03403+K19073+K00218+K00218+K01845+K03403+K02492+K01772+K01772+K03403+K01885+K01772+K08099+K01772+K00231+K13606+K02259+K13071+K01772+K01885+K00231+K03403+K01845+K03404+K00510+K01885+K02492+K02492+K01772+K01885+K02492+K01772+K01772+K01772+K13600+K13606+K00231+K00231+K01772+K01885+K00231+K00231+K02492+K13071+K00231+K01772+K13606+K01772+K00231+K00231+K01885+K01885+K03405+K02259+K02259+K03405+K18010+K03405+K00228+K01599+K00218+K01599+K01599+K03405+K00228+K10960+K04040+K01599+K00218+K19073+K08099+K19073+K08099+K00218+K03428+K03403+K13606+K00510+K13606+K08099+K08099+K13545+K03428+K00218+K04035+K04035+K03405+K00231+K18010+K03404+K01772+K01885+K01599+K18010+K01772+K03404+K00218+K03403+K18010+K03404+K00231+K01772+K00231+K03405+K01599+K18010+K00231+K00231+K01845+K00231+K01698+K00231+K00510+K01698+K04040+K01698+K13606+K08101+K00510+K01599+K00231+K00231+K01698+K04035+K08099+K19073+K04040+K01599+K03404+K00218+K02257+K01772+K02259+K08099+K00218+K03403+K00231+K02492+K00231+K01885+K02492+K01885+K00218+K00231+K08101+K00

	t_161780;F01_transcript_161926;F01_transcript_162102;F01_transcript_162450;F01_transcript_163522;F01_transcript_163949;F01_transcript_167233;F01_transcript_16743;F01_transcript_17913;F01_transcript_1817;F01_transcript_18225;F01_transcript_18325;F01_transcript_20115;F01_transcript_20923;F01_transcript_22529;F01_transcript_22554;F01_transcript_23170;F01_transcript_23223;F01_transcript_23338;F01_transcript_23565;F01_transcript_23858;F01_transcript_24372;F01_transcript_24991;F01_transcript_25509;F01_transcript_25795;F01_transcript_25855;F01_transcript_26258;F01_transcript_26811;F01_transcript_27001;F01_transcript_27294;F01_transcript_27699;F01_transcript_27958;F01_transcript_28214;F01_transcript_28361;F01_transcript_28385;F01_transcript_29593;F01_transcript_30570;F01_transcript_31034;F01_transcript_31250;F01_transcript_32410;F01_transcript_32780;F01_transcript_33659;F01_transcript_33696;F01_transcript_34037;F01_transcript_34346;F01_transcript_34976;F01_transcript_35054;F01_transcript_35495;F01_transcript_35632;F01_transcript_35849;F01_transcript_36620;F01_transcript_36630;F01_transcript_36953;F01_transcript_37282;F01_transcript_37431;F01_transcript_37446;F01_transcript_37487;F01_transcript_37552;F01_transcript_38163;F01_transcript_38285;F01_transcript_38378;F01_transcript_39253;F01_transcript_39798;F01_transcript_398;F01_transcript_41172;F01_transcript_41337;F01_transcript_41747;F01_transcript_41986;F01_transcript_42417;F01_transcript_43577;F01_transcript_44851;F01_transcript_46829;F01_transcript_4734;F01_transcript_5228;F01_transcript_52709;F01_transcript_53806;F01_transcript_54021;F01_transcript_5409;F01_transcript_54234;F01_transcript_54372;F01_transcript_54606;F01_transcript_54649;F01_transcript_54810;F01_transcript_5680;F01_transcript_56852;F01_transcript_58702;F01_transcript_59689;F01_transcript_60015;F01_transcript_61409;F01_transcript_62350;F01_transcript_62688;F01_transcript_63786;F01_transcript_64575;F01_transcript_64784;F01_transcript_65232;F01_transcript_65940;F01_transcript_66501;F01_transcript_6668;F01_transcript_66824;F01_transcript_67053;F01_transcript_67199;F01_transcript_67365;F01_transcript_68806;F01_transcript_70631;F01_transcript_70661;F01_transcript_70940;F01_transcript_72398;F01_transcript_72836;F01_transcript_	231+K03403+K04035+K03404+K03404+K03403+K00510+K00231+K00231+K00231+K01772+K02492+K00231+K00228+K00231+K01885+K01772+K13606+K18010+K02492+K13071

			72962;F01_transcript_73234;F01_transcript_74357;F01_transcript_7444;F01_transcript_74718;F01_transcript_75010;F01_transcript_75033;F01_transcript_75383;F01_transcript_75811;F01_transcript_77694;F01_transcript_78105;F01_transcript_78697;F01_transcript_79775;F01_transcript_80127;F01_transcript_80186;F01_transcript_80269;F01_transcript_80609;F01_transcript_80684;F01_transcript_80699;F01_transcript_81695;F01_transcript_82298;F01_transcript_82797;F01_transcript_83072;F01_transcript_8336;F01_transcript_83610;F01_transcript_84224;F01_transcript_86086;F01_transcript_86795;F01_transcript_8741;F01_transcript_89753;F01_transcript_90581;F01_transcript_90603;F01_transcript_91166;F01_transcript_91331;F01_transcript_91512;F01_transcript_92088;F01_transcript_92663;F01_transcript_93514;F01_transcript_94134;F01_transcript_94415;F01_transcript_95240;F01_transcript_95805;F01_transcript_96108;F01_transcript_96126;F01_transcript_96836;F01_transcript_97277;	
Terpenoid backbone biosynthesis	ko00900	276	F01_transcript_100148;F01_transcript_100265;F01_transcript_100995;F01_transcript_10129;F01_transcript_101776;F01_transcript_103120;F01_transcript_103159;F01_transcript_104960;F01_transcript_105403;F01_transcript_106241;F01_transcript_10691;F01_transcript_107207;F01_transcript_107939;F01_transcript_108026;F01_transcript_108243;F01_transcript_108455;F01_transcript_10877;F01_transcript_110216;F01_transcript_110265;F01_transcript_111207;F01_transcript_111303;F01_transcript_111319;F01_transcript_111859;F01_transcript_111915;F01_transcript_112259;F01_transcript_112794;F01_transcript_113524;F01_transcript_11393;F01_transcript_11415;F01_transcript_114438;F01_transcript_114642;F01_transcript_114842;F01_transcript_115644;F01_transcript_11575;F01_transcript_116600;F01_transcript_117429;F01_transcript_117966;F01_transcript_117989;F01_transcript_118905;F01_transcript_119530;F01_transcript_12045;F01_transcript_121368;F01_transcript_12212;F01_transcript_122207;F01_transcript_124639;F01_transcript_125004;F01_transcript_126486;F01_transcript_126739;F01_transcript_127478;F01_transcript_127869;F01_transcript_128071;F01_transcript_128766;F01_transcript_129139;F01_transcript_129261;F01_transcript_130989;F01_transcript_131427;F01_transcript_131876;F0	K00787+K05906+K01662+K01662+K01662+K11778+K00099+K10960+K00626+K05906+K01662+K11778+K13789+K10960+K00787+K03527+K00938+K01823+K01770+K00919+K08658+K01662+K00938+K00938+K01662+K11778+K00787+K01662+K01662+K15891+K15889+K01662+K00021+K01662+K00919+K12742+K05954+K01662+K05906+K15889+K01662+K11778+K01662+K13789+K03527+K00021+K00021+K00938+K01662+K00099+K01662+K01662+K05906+K03527+K01662+K11778+K00021+K01662+K06013+K05954+K05906+K00919+K15889+K11778+K00021+K08658+K08658+K13789+K05954+K00626+K08658+K01662+K00938+K00919+K05356+K00991+K11778+K14066+K00938+K05356+K01662+K00587+K01662+K03526+K00099+K15889+K08658+K00869+K00787+K00919+K00938+K13789+K00021+K11778+K11778+K00787+K01770+K01641+K13789+K00626+K01662+K06013+K00919+K14066+K06013+K00099+K11778+K01662+K00938+K00938+K01823+K05954+K03526+K01662+K01662+K05356+K01662+K00626+K00021+K14066+K01662+K00021+K00938+K01641+K05906+K00938+K00938+K01641+K05356+K00938+K14066+K06013+K15889+K05356+K15889+K05954+K05954+K01641+K06013+K05906+K01597+K00919+K06013+K00099+

	1_transcript_132346;F01_transcript_132519;F01_transcript_134360;F01_transcript_134709;F01_transcript_134747;F01_transcript_135048;F01_transcript_135616;F01_transcript_136231;F01_transcript_136510;F01_transcript_136513;F01_transcript_136546;F01_transcript_137177;F01_transcript_138114;F01_transcript_138365;F01_transcript_138792;F01_transcript_140370;F01_transcript_141253;F01_transcript_141287;F01_transcript_142108;F01_transcript_143818;F01_transcript_144696;F01_transcript_144734;F01_transcript_145203;F01_transcript_145651;F01_transcript_146469;F01_transcript_146507;F01_transcript_146803;F01_transcript_147391;F01_transcript_148617;F01_transcript_149804;F01_transcript_150083;F01_transcript_151418;F01_transcript_151613;F01_transcript_152821;F01_transcript_153588;F01_transcript_153761;F01_transcript_153970;F01_transcript_154333;F01_transcript_154414;F01_transcript_154465;F01_transcript_154880;F01_transcript_155296;F01_transcript_155685;F01_transcript_15624;F01_transcript_157022;F01_transcript_157136;F01_transcript_157848;F01_transcript_157849;F01_transcript_158081;F01_transcript_159031;F01_transcript_160566;F01_transcript_161511;F01_transcript_161647;F01_transcript_162568;F01_transcript_164081;F01_transcript_165576;F01_transcript_166003;F01_transcript_166385;F01_transcript_166529;F01_transcript_166752;F01_transcript_166964;F01_transcript_18265;F01_transcript_18685;F01_transcript_23913;F01_transcript_25962;F01_transcript_26778;F01_transcript_28787;F01_transcript_29424;F01_transcript_29911;F01_transcript_30352;F01_transcript_30530;F01_transcript_30662;F01_transcript_31185;F01_transcript_31230;F01_transcript_31665;F01_transcript_31706;F01_transcript_31723;F01_transcript_31793;F01_transcript_32163;F01_transcript_32360;F01_transcript_32485;F01_transcript_32630;F01_transcript_33088;F01_transcript_33514;F01_transcript_33801;F01_transcript_33895;F01_transcript_34285;F01_transcript_34365;F01_transcript_35362;F01_transcript_35463;F01_transcript_35502;F01_transcript_35950;F01_transcript_36071;F01_transcript_36778;F01_transcript_36969;F01_transcript_37282;F01_transcript_37377;F01_transcript_37686;F01_transcript_37737;F01_transcript_38302;F01_transcript_38313;F01_transcript_38580;F01_transcript_38657;F01_transcript_39328;F01_transcript_40008;	K01597+K11778+K00919+K15889+K11778+K00919+K13789+K13789+K10960+K00626+K00626+K03527+K00626+K00869+K01597+K00626+K13789+K08658+K12742+K00787+K01823+K00787+K00787+K12742+K01823+K13789+K01823+K13789+K13789+K13789+K05955+K11778+K01770+K11778+K01770+K01770+K05356+K01662+K01662+K15889+K15889+K00626+K13789+K01770+K03526+K01662+K00787+K01662+K00938+K01770+K13789+K05954+K00626+K00787+K01662+K01662+K11778+K00787+K13789+K11778+K13789+K01662+K00787+K06013+K01662+K11778+K15889+K08658+K13789+K05356+K00919+K15889+K05906+K00626+K13789+K00099+K13789+K15889+K12742+K13789+K15889+K15889+K00626+K00626+K15892+K00919+K03526+K00626+K05356+K01662+K00938+K13789+K00787+K01662+K01662+K00938+K00021+K00626+K01662+K01662+K15892+K00787+K15891+K00021+K13789+K11778+K14066+K00021+K01662+K00787+K15889+K00938+K00021+K15891+K11778+K05954+K00919+K01662+K03526+K15892+K14066+K14066+K00099+K01662+K05906+K00099+K05954+K00626+K01662+K03526+K03526+K14066

	F01_transcript_40036;F01_transcript_40844;F01_transcript_41280;F01_transcript_41626;F01_transcript_41794;F01_transcript_41906;F01_transcript_41963;F01_transcript_42132;F01_transcript_42210;F01_transcript_42324;F01_transcript_42345;F01_transcript_42872;F01_transcript_43030;F01_transcript_43072;F01_transcript_43308;F01_transcript_43733;F01_transcript_43940;F01_transcript_44064;F01_transcript_45691;F01_transcript_4852;F01_transcript_4884;F01_transcript_51584;F01_transcript_51740;F01_transcript_52332;F01_transcript_52847;F01_transcript_52902;F01_transcript_53947;F01_transcript_54350;F01_transcript_55346;F01_transcript_55513;F01_transcript_56106;F01_transcript_56139;F01_transcript_56587;F01_transcript_57336;F01_transcript_58368;F01_transcript_58397;F01_transcript_58496;F01_transcript_58765;F01_transcript_59353;F01_transcript_60503;F01_transcript_60913;F01_transcript_61021;F01_transcript_61140;F01_transcript_61171;F01_transcript_62935;F01_transcript_63394;F01_transcript_65493;F01_transcript_65936;F01_transcript_66468;F01_transcript_66765;F01_transcript_67462;F01_transcript_67857;F01_transcript_68960;F01_transcript_69442;F01_transcript_69576;F01_transcript_69739;F01_transcript_70703;F01_transcript_70858;F01_transcript_71338;F01_transcript_7154;F01_transcript_71560;F01_transcript_71784;F01_transcript_72788;F01_transcript_74939;F01_transcript_77451;F01_transcript_79052;F01_transcript_79067;F01_transcript_79584;F01_transcript_7989;F01_transcript_80843;F01_transcript_82043;F01_transcript_82450;F01_transcript_82496;F01_transcript_82516;F01_transcript_82831;F01_transcript_82833;F01_transcript_82847;F01_transcript_83020;F01_transcript_84010;F01_transcript_84463;F01_transcript_85472;F01_transcript_85562;F01_transcript_85630;F01_transcript_85955;F01_transcript_86279;F01_transcript_86560;F01_transcript_87524;F01_transcript_88306;F01_transcript_88554;F01_transcript_88675;F01_transcript_89093;F01_transcript_89324;F01_transcript_89355;F01_transcript_90167;F01_transcript_90478;F01_transcript_91168;F01_transcript_91197;F01_transcript_91267;F01_transcript_91753;F01_transcript_92275;F01_transcript_9241;F01_transcript_93000;F01_transcript_94680;F01_transcript_95790;F01_transcript_96082;F01_transcri

			pt_9634;F01_transcript_96445;F01_transcript_96693;F01_transcript_96757;F01_transcript_97808;F01_transcript_98298;F01_transcript_9882;F01_transcript_9894;F01_transcript_99438;	
Monoterpenoid biosynthesis	ko00902	41	F01_transcript_107886;F01_transcript_113255;F01_transcript_118837;F01_transcript_12613;F01_transcript_131120;F01_transcript_132015;F01_transcript_133570;F01_transcript_147841;F01_transcript_152986;F01_transcript_155893;F01_transcript_158492;F01_transcript_161365;F01_transcript_163623;F01_transcript_166001;F01_transcript_166138;F01_transcript_24212;F01_transcript_28925;F01_transcript_29249;F01_transcript_3012;F01_transcript_30498;F01_transcript_32948;F01_transcript_39877;F01_transcript_41351;F01_transcript_42316;F01_transcript_42848;F01_transcript_42884;F01_transcript_43122;F01_transcript_43528;F01_transcript_43533;F01_transcript_44112;F01_transcript_51960;F01_transcript_55690;F01_transcript_57508;F01_transcript_58839;F01_transcript_62088;F01_transcript_63366;F01_transcript_70426;F01_transcript_71387;F01_transcript_81253;F01_transcript_82673;F01_transcript_99633;	K15095+K15095+K15095+K15095+K15095+K15095+K15095+K15095+K15095+K15095+K15095+K18108+K15095+K15095+K15095+K15095+K15095+K15095+K15095+K18108+K15095+K15095+K15095+K18108+K15095+K15095+K15095+K15095+K15095+K15095+K15095+K15095+K15095+K15095+K15095+K15095+K15095+K15095+K15095+K15095+K15095
Limonene and pinene degradation	ko00903	18	F01_transcript_119343;F01_transcript_121819;F01_transcript_132494;F01_transcript_134507;F01_transcript_137935;F01_transcript_138921;F01_transcript_145352;F01_transcript_151264;F01_transcript_152958;F01_transcript_158912;F01_transcript_20002;F01_transcript_25031;F01_transcript_31371;F01_transcript_41704;F01_transcript_55231;F01_transcript_56137;F01_transcript_98445;F01_transcript_99528;	K00128+K00128+K00128+K00128+K00128+K00128+K00128+K00128+K00128+K00128+K00517+K00517+K00517+K00128+K00517+K00128+K00128+K00128
Diterpenoid biosynthesis	ko00904	18	F01_transcript_100098;F01_transcript_108937;F01_transcript_113784;F01_transcript_131686;F01_transcript_133453;F01_transcript_142502;F01_transcript_148150;F01_transcript_149306;F01_transcript_154219;F01_transcript_28136;F01_transcript_29995;F01_transcript_56410;F01_transcript_65040;F01_transcript_72369;F01_transcript_74381;F01_transcript_78734;F01_transcript_78931;F01_transcript_89846;	K04125+K04125+K17982+K04123+K05282+K04121+K04125+K17961+K04124+K04122+K17961+K17961+K04124+K17961+K17961+K04121+K04121+K04124
Brassinosteroid biosynthesis	ko009	24	F01_transcript_100429;F01_transcript_10290;F01_transcript_104891;F01_transcript_114467;F01_transcript_131526;F01_transcript_134209;F01_transcript_137858;F01_transcript_142048;F01_transcript_142808;F01_transcript_26916;F01_transcript_27171;F01_transcript_27394;F01_transcript_27509;F01_transcript_32070;F01_transcript_32301;F01_transcript_32663;F01_transcript	K09587+K12637+K09588+K12637+K09587+K15639+K09588+K09587+K12637+K09587+K12637+K15639+K09587+K12637+K12637+K09588+K09588+K12640+K09588+K09587+K12640+K12637+K12637+K12640

	05		_34668;F01_transcript_36971;F01_transcript_43364;F01_transcript_54499;F01_transcript_61278;F01_transcript_76432;F01_transcript_81202;F01_transcript_95141;	
Carotenoid biosynthesis	ko00906	153	F01_transcript_101672;F01_transcript_101719;F01_transcript_101752;F01_transcript_103383;F01_transcript_106186;F01_transcript_106198;F01_transcript_110252;F01_transcript_110354;F01_transcript_112256;F01_transcript_113883;F01_transcript_114063;F01_transcript_115545;F01_transcript_116463;F01_transcript_117057;F01_transcript_118301;F01_transcript_119769;F01_transcript_121772;F01_transcript_122324;F01_transcript_122604;F01_transcript_123535;F01_transcript_124486;F01_transcript_125269;F01_transcript_126413;F01_transcript_128117;F01_transcript_128489;F01_transcript_129027;F01_transcript_130088;F01_transcript_131532;F01_transcript_133233;F01_transcript_134581;F01_transcript_136012;F01_transcript_136045;F01_transcript_13612;F01_transcript_136596;F01_transcript_136789;F01_transcript_137552;F01_transcript_137761;F01_transcript_138981;F01_transcript_140820;F01_transcript_143347;F01_transcript_145454;F01_transcript_147959;F01_transcript_149200;F01_transcript_150562;F01_transcript_151511;F01_transcript_152036;F01_transcript_152788;F01_transcript_15453;F01_transcript_156010;F01_transcript_157455;F01_transcript_158292;F01_transcript_159545;F01_transcript_160338;F01_transcript_160798;F01_transcript_160864;F01_transcript_161055;F01_transcript_161764;F01_transcript_161990;F01_transcript_165753;F01_transcript_166424;F01_transcript_166995;F01_transcript_17541;F01_transcript_18517;F01_transcript_19170;F01_transcript_19309;F01_transcript_19345;F01_transcript_19401;F01_transcript_20517;F01_transcript_20792;F01_transcript_21629;F01_transcript_21926;F01_transcript_22072;F01_transcript_22346;F01_transcript_22944;F01_transcript_23137;F01_transcript_23204;F01_transcript_23510;F01_transcript_23696;F01_transcript_23799;F01_transcript_23816;F01_transcript_23899;F01_transcript_24830;F01_transcript_25806;F01_transcript_26148;F01_transcript_27103;F01_transcript_27223;F01_transcript_28944;F01_transcript_29280;F01_transcript_29610;F01_transcript_30984;F01_transcript_32157;F01_transcript_32505;F01_transcript_32589;F01_transcri	K14595+K02291+K06444+K09841+K00514+K09837+K02293+K09843+K02291+K09838+K09838+K09838+K15744+K09840+K09842+K09838+K06443+K09841+K09838+K02291+K09841+K09842+K02291+K02293+K09840+K06444+K09843+K06444+K02293+K06444+K09841+K09843+K09835+K09843+K09840+K06444+K09839+K00514+K09839+K09843+K09838+K09838+K06443+K09838+K09838+K15744+K06444+K00514+K15746+K15746+K02293+K06444+K09835+K09838+K06443+K09839+K15747+K09841+K09839+K02293+K09843+K09838+K09839+K00514+K09839+K09838+K09840+K15747+K09838+K00514+K02291+K02291+K02291+K09838+K02291+K02293+K06443+K02291+K02293+K09840+K09838+K09840+K02293+K09840+K09840+K06443+K06444+K06444+K09837+K09843+K06443+K09840+K09843+K14595+K09839+K14595+K09839+K02293+K09839+K09839+K09840+K09841+K15746+K09842+K14595+K09842+K09840+K02293+K15747+K15747+K09840+K02291+K09838+K02293+K02291+K06444+K09838+K02291+K09843+K09839+K09842+K09838+K15747+K09843+K09839+K02293+K09843+K02291+K09840+K00514+K09838+K15746+K06444+K02291+K09843+K09835+K09838+K02291+K02293+K02291+K09840+K09839+K15746+K09838+K15747+K09838+K09842+K09838+K09838+K09840+K09838+K02291+K09838

			pt_32931;F01_transcript_33383;F01_transcript_33709;F01_transcript_35593;F01_transcript_3564;F01_transcript_36031;F01_transcript_36078;F01_transcript_37032;F01_transcript_40774;F01_transcript_42502;F01_transcript_51516;F01_transcript_51907;F01_transcript_53102;F01_transcript_53334;F01_transcript_54059;F01_transcript_54254;F01_transcript_55131;F01_transcript_55815;F01_transcript_56057;F01_transcript_57898;F01_transcript_60208;F01_transcript_60621;F01_transcript_60785;F01_transcript_62844;F01_transcript_63002;F01_transcript_63012;F01_transcript_63345;F01_transcript_65745;F01_transcript_65878;F01_transcript_67532;F01_transcript_68076;F01_transcript_69092;F01_transcript_69375;F01_transcript_74428;F01_transcript_76046;F01_transcript_76963;F01_transcript_77225;F01_transcript_77361;F01_transcript_77408;F01_transcript_77423;F01_transcript_78531;F01_transcript_79561;F01_transcript_80496;F01_transcript_81034;F01_transcript_81576;F01_transcript_82437;F01_transcript_83624;F01_transcript_91138;F01_transcript_91218;F01_transcript_93167;F01_transcript_93865;F01_transcript_94775;F01_transcript_95164;F01_transcript_95569;F01_transcript_96144;F01_transcript_96800;F01_transcript_97554;F01_transcript_98009;F01_transcript_99082;F01_transcript_99579;	
Zeatin biosynthesis	ko00908	47	F01_transcript_100823;F01_transcript_104072;F01_transcript_107416;F01_transcript_112471;F01_transcript_114558;F01_transcript_121020;F01_transcript_124249;F01_transcript_125741;F01_transcript_129926;F01_transcript_132281;F01_transcript_137917;F01_transcript_138091;F01_transcript_140770;F01_transcript_141587;F01_transcript_144202;F01_transcript_144979;F01_transcript_152245;F01_transcript_157250;F01_transcript_158977;F01_transcript_24299;F01_transcript_24344;F01_transcript_26185;F01_transcript_26521;F01_transcript_28119;F01_transcript_29016;F01_transcript_29451;F01_transcript_30104;F01_transcript_33101;F01_transcript_33648;F01_transcript_35173;F01_transcript_35681;F01_transcript_3595;F01_transcript_36456;F01_transcript_36889;F01_transcript_4850;F01_transcript_53432;F01_transcript_54094;F01_transcript_57690;F01_transcript_68165;F01_transcript_68662;F01_transcript_75486;F01_transcript_76105;F01_transcript_79328;F01_transcript_79662;F01_transcript_85070;F01_transcript_85636;F01_transcript_97657;	K13495+K10760+K00791+K13495+K13495+K00791+K00791+K13495+K13495+K13495+K00279+K00791+K13495+K13495+K13495+K13495+K00791+K13495+K13495+K13495+K13495+K13495+K13495+K13495+K00791+K13495+K13495+K00279+K13495+K10760+K10760+K00279+K13495+K13495+K13495+K13495+K10760+K13495+K10760+K13495+K00279+K13495+K10760+K13495+K13495+K00279+K10760

Sesquiterpenoid and triterpenoid biosynthesis	ko00909	62	F01_transcript_101494;F01_transcript_103668;F01_transcript_106896;F01_transcript_107325;F01_transcript_111232;F01_transcript_112777;F01_transcript_114079;F01_transcript_114438;F01_transcript_117095;F01_transcript_119852;F01_transcript_122224;F01_transcript_122561;F01_transcript_124961;F01_transcript_130870;F01_transcript_13179;F01_transcript_134910;F01_transcript_135086;F01_transcript_136753;F01_transcript_139172;F01_transcript_13979;F01_transcript_142302;F01_transcript_145482;F01_transcript_148606;F01_transcript_15287;F01_transcript_158211;F01_transcript_159698;F01_transcript_159713;F01_transcript_16048;F01_transcript_163797;F01_transcript_164924;F01_transcript_165574;F01_transcript_22713;F01_transcript_23017;F01_transcript_28293;F01_transcript_29159;F01_transcript_3191;F01_transcript_32272;F01_transcript_32704;F01_transcript_33759;F01_transcript_36198;F01_transcript_41795;F01_transcript_57525;F01_transcript_59628;F01_transcript_61655;F01_transcript_70923;F01_transcript_72821;F01_transcript_75563;F01_transcript_78106;F01_transcript_83402;F01_transcript_85783;F01_transcript_86279;F01_transcript_87214;F01_transcript_87614;F01_transcript_91168;F01_transcript_9222;F01_transcript_93593;F01_transcript_95028;F01_transcript_95412;F01_transcript_96071;F01_transcript_96308;F01_transcript_97907;F01_transcript_98094;	K00511+K15803+K15813+K15813+K15813+K00511+K15803+K15891+K15813+K00511+K15813+K00511+K14173+K00511+K15813+K15813+K00511+K15472+K15803+K15813+K00511+K00511+K15813+K15813+K15472+K00801+K15813+K15813+K15472+K15813+K14173+K00511+K00511+K00511+K00511+K00801+K00511+K15472+K00801+K00801+K15813+K15803+K00801+K00801+K15803+K00801+K14173+K15803+K15803+K00801+K15891+K15813+K00511+K15891+K00801+K15472+K15803+K15803+K00801+K15803+K15813+K00801
Nitrogen metabolism	ko00910	216	F01_transcript_100683;F01_transcript_101062;F01_transcript_101386;F01_transcript_101671;F01_transcript_102131;F01_transcript_102482;F01_transcript_102819;F01_transcript_103318;F01_transcript_106024;F01_transcript_107619;F01_transcript_10787;F01_transcript_108303;F01_transcript_108706;F01_transcript_109463;F01_transcript_110169;F01_transcript_110317;F01_transcript_110535;F01_transcript_110642;F01_transcript_112476;F01_transcript_112663;F01_transcript_114576;F01_transcript_115789;F01_transcript_116166;F01_transcript_118053;F01_transcript_118784;F01_transcript_119228;F01_transcript_120116;F01_transcript_121672;F01_transcript_121985;F01_transcript_122171;F01_transcript_122790;F01_transcript_123427;F01_transcript_123819;F01_transcript_12434;F01_transcript_124982;F01_transcript_125501;F01_transcript_127070;F01_transcript_12840	K01674+K01915+K00261+K01673+K01673+K01673+K01673+K01915+K01915+K01673+K01673+K01915+K01673+K01915+K00261+K01915+K01673+K00284+K01673+K00284+K01915+K00284+K01915+K01455+K01673+K01673+K01915+K00284+K00264+K01673+K00264+K01915+K00262+K00262+K00262+K01673+K02575+K01674+K01673+K01915+K01915+K01915+K01915+K01673+K00261+K01673+K00284+K00264+K01455+K00262+K01455+K01673+K02575+K01673+K01915+K00262+K10534+K00264+K01674+K00262+K01673+K01673+K01915+K01455+K00262+K01673+K01725+K00284+K00262+K00284+K00262+K01915+K01673+K01915+K00264+K01673+K02575+K01673+K01673+K00284+K01673+K01673+K01673+K00284+K01673+K00262+K00284+K01673+K10534+K00264+K01673+K00284+K01673+K00261+K00262+K01673+

	0;F01_transcript_128487;F01_transcript_129374;F01_transcript_130674;F01_transcript_132266;F01_transcript_132815;F01_transcript_133154;F01_transcript_134077;F01_transcript_134160;F01_transcript_134370;F01_transcript_135156;F01_transcript_135878;F01_transcript_136085;F01_transcript_136192;F01_transcript_136215;F01_transcript_136977;F01_transcript_137527;F01_transcript_137593;F01_transcript_137850;F01_transcript_137930;F01_transcript_138648;F01_transcript_138702;F01_transcript_138956;F01_transcript_139823;F01_transcript_141953;F01_transcript_142458;F01_transcript_142863;F01_transcript_143068;F01_transcript_143153;F01_transcript_144112;F01_transcript_144514;F01_transcript_144745;F01_transcript_146434;F01_transcript_148005;F01_transcript_149327;F01_transcript_149439;F01_transcript_149467;F01_transcript_150354;F01_transcript_150523;F01_transcript_154356;F01_transcript_155110;F01_transcript_155542;F01_transcript_155884;F01_transcript_155955;F01_transcript_156629;F01_transcript_157775;F01_transcript_161505;F01_transcript_161903;F01_transcript_162049;F01_transcript_162163;F01_transcript_163333;F01_transcript_163368;F01_transcript_16355;F01_transcript_164553;F01_transcript_165092;F01_transcript_165327;F01_transcript_166551;F01_transcript_16898;F01_transcript_18365;F01_transcript_18796;F01_transcript_19893;F01_transcript_22555;F01_transcript_24939;F01_transcript_30503;F01_transcript_31271;F01_transcript_32610;F01_transcript_33768;F01_transcript_34276;F01_transcript_34873;F01_transcript_35262;F01_transcript_35426;F01_transcript_35441;F01_transcript_36320;F01_transcript_37859;F01_transcript_38047;F01_transcript_39760;F01_transcript_40097;F01_transcript_40364;F01_transcript_40430;F01_transcript_40444;F01_transcript_4067;F01_transcript_40951;F01_transcript_41006;F01_transcript_41130;F01_transcript_41164;F01_transcript_41423;F01_transcript_41592;F01_transcript_42208;F01_transcript_42511;F01_transcript_42571;F01_transcript_42754;F01_transcript_43147;F01_transcript_43383;F01_transcript_43415;F01_transcript_4345;F01_transcript_43541;F01_transcript_43589;F01_transcript_43670;F01_transcript_43686;F01_transcript_44129;F01_transcript_44922;F01_transcript_4627;F01_transcript_47377;F01_transcript_47394;F01_transcript_50305;F01_transcript_50490;F01_tr	K00262+K01915+K00366+K01673+K01455+K01455+K01673+K01915+K01673+K01915+K01915+K01455+K01915+K01455+K01915+K01915+K01915+K01673+K01673+K01915+K01915+K01673+K01673+K01915+K01673+K01673+K01915+K01673+K01673+K01673+K01673+K01673+K01673+K01673+K01674+K01673+K01674+K01673+K01673+K01673+K01673+K01673+K00284+K01725+K01725+K01673+K01673+K01673+K00284+K01673+K01673+K00262+K02575+K10534+K10534+K01673+K01673+K01673+K01673+K01455+K01673+K01673+K00264+K00366+K01455+K01915+K01673+K01915+K01673+K01673+K01673+K00262+K01673+K00261+K01673+K01673+K01673+K01673+K01915+K00262+K01673+K01673+K01673+K00262+K00284+K01673+K00264+K01915+K01674+K01673+K01673+K01673+K00262+K00284+K00284+K01673+K01673+K00284+K01673+K01673+K01673+K01673+K02575+K01673+K02575+K10534+K01673+K00284+K01673+K00262+K01915+K00284+K01673+K01673+K00262+K01673+K00284+K00262+K01673+K00262

			anscript_55109;F01_transcript_55634;F01_transcript_56446;F01_transcript_5664;F01_transcript_56883;F01_transcript_57173;F01_transcript_57192;F01_transcript_5739;F01_transcript_5748;F01_transcript_58112;F01_transcript_58939;F01_transcript_59178;F01_transcript_60431;F01_transcript_60975;F01_transcript_62297;F01_transcript_62329;F01_transcript_64725;F01_transcript_65162;F01_transcript_65509;F01_transcript_66422;F01_transcript_66852;F01_transcript_67225;F01_transcript_67794;F01_transcript_68503;F01_transcript_69139;F01_transcript_69154;F01_transcript_71219;F01_transcript_71679;F01_transcript_71720;F01_transcript_72186;F01_transcript_72214;F01_transcript_72651;F01_transcript_73112;F01_transcript_73245;F01_transcript_73542;F01_transcript_73638;F01_transcript_73969;F01_transcript_74497;F01_transcript_74606;F01_transcript_77264;F01_transcript_77389;F01_transcript_78801;F01_transcript_80522;F01_transcript_81515;F01_transcript_81683;F01_transcript_82136;F01_transcript_83083;F01_transcript_84;F01_transcript_84378;F01_transcript_85709;F01_transcript_85759;F01_transcript_86763;F01_transcript_8910;F01_transcript_89766;F01_transcript_90038;F01_transcript_90050;F01_transcript_92305;F01_transcript_93746;F01_transcript_9416;F01_transcript_95216;F01_transcript_95330;F01_transcript_95431;F01_transcript_96820;F01_transcript_96851;F01_transcript_97608;F01_transcript_97809;F01_transcript_98271;F01_transcript_98863;F01_transcript_99186;F01_transcript_99350;F01_transcript_99450;F01_transcript_99600;F01_transcript_99860;	
Sulfur metabolism	ko00920	154	F01_transcript_101296;F01_transcript_101986;F01_transcript_103028;F01_transcript_103399;F01_transcript_103700;F01_transcript_105281;F01_transcript_109267;F01_transcript_110570;F01_transcript_114030;F01_transcript_114327;F01_transcript_114415;F01_transcript_114551;F01_transcript_114920;F01_transcript_115361;F01_transcript_117844;F01_transcript_118376;F01_transcript_119306;F01_transcript_119615;F01_transcript_120073;F01_transcript_120145;F01_transcript_120997;F01_transcript_121502;F01_transcript_123859;F01_transcript_123935;F01_transcript_125334;F01_transcript_125818;F01_transcript_127193;F01_transcript_127418;F01_transcript_127989;F01_transcript_1	K00640+K00392+K01739+K01011+K01738+K01738+K00640+K05907+K01738+K00640+K01082+K01738+K01738+K01738+K01739+K01738+K13034+K01082+K00640+K01738+K13034+K01739+K01739+K17725+K01738+K01738+K01738+K01739+K01738+K01739+K00392+K00860+K13034+K15422+K00860+K01738+K00387+K00387+K05907+K01011+K01738+K00392+K00392+K01739+K13811+K00387+K13811+K01738+K00387+K13811+K00392+K08738+K01738+K01738+K01738+K01738+K13034+K05907+K01738+K01738+K01739+K01738+K13811+K08738+K01011+K05907+K00392+K01738+K01738+K00392+K01738+K01739+K15422+K05907+K01739+K05

	29435;F01_transcript_12953;F01_transcript_133802;F01_transcript_134216;F01_transcript_137504;F01_transcript_137976;F01_transcript_138596;F01_transcript_138868;F01_transcript_141749;F01_transcript_141990;F01_transcript_142028;F01_transcript_142050;F01_transcript_142264;F01_transcript_142300;F01_transcript_142449;F01_transcript_144538;F01_transcript_144770;F01_transcript_145873;F01_transcript_146107;F01_transcript_146253;F01_transcript_146938;F01_transcript_14772;F01_transcript_148380;F01_transcript_149508;F01_transcript_151028;F01_transcript_151234;F01_transcript_152349;F01_transcript_152454;F01_transcript_153191;F01_transcript_154618;F01_transcript_155318;F01_transcript_155339;F01_transcript_155840;F01_transcript_156861;F01_transcript_157543;F01_transcript_159015;F01_transcript_159059;F01_transcript_160165;F01_transcript_161478;F01_transcript_165501;F01_transcript_17893;F01_transcript_23969;F01_transcript_26005;F01_transcript_27724;F01_transcript_27822;F01_transcript_29328;F01_transcript_29702;F01_transcript_29815;F01_transcript_30420;F01_transcript_30637;F01_transcript_31318;F01_transcript_31458;F01_transcript_31937;F01_transcript_32913;F01_transcript_34211;F01_transcript_34493;F01_transcript_34975;F01_transcript_35081;F01_transcript_36857;F01_transcript_37334;F01_transcript_37554;F01_transcript_37739;F01_transcript_38414;F01_transcript_38763;F01_transcript_38874;F01_transcript_39013;F01_transcript_39940;F01_transcript_40243;F01_transcript_40512;F01_transcript_40591;F01_transcript_41338;F01_transcript_4149;F01_transcript_41547;F01_transcript_41769;F01_transcript_41905;F01_transcript_42029;F01_transcript_42729;F01_transcript_43371;F01_transcript_43614;F01_transcript_50910;F01_transcript_51442;F01_transcript_53695;F01_transcript_53734;F01_transcript_55553;F01_transcript_55774;F01_transcript_56778;F01_transcript_56992;F01_transcript_58267;F01_transcript_58630;F01_transcript_5896;F01_transcript_59040;F01_transcript_65708;F01_transcript_68093;F01_transcript_68454;F01_transcript_68627;F01_transcript_68830;F01_transcript_70160;F01_transcript_71697;F01_transcript_71954;F01_transcript_75379;F01_transcript_76208;F01_transcript_76431;F01_transcript_76723;F01_transcript_78302;F01_transcript_78612;F01_transcript_81824;F01_transcript_82746	907+K05907+K01739+K13811+K05907+K01082+K01082+K01738+K00640+K01011+K13811+K00640+K00860+K13034+K00860+K13034+K00387+K01738+K01738+K01738+K00640+K01738+K15422+K01738+K01738+K05907+K01738+K17725+K01738+K00640+K17725+K00640+K00640+K00387+K13034+K00387+K00387+K00392+K01738+K01082+K01739+K05907+K01082+K01738+K08738+K05907+K00640+K13034+K13034+K00392+K01738+K05907+K01738+K01738+K13811+K00392+K01738+K13034+K01739+K00387+K01738+K01738+K01738+K01738+K05907+K01738+K00640+K00387+K00387+K00387+K00392+K15422+K15422+K00387+K13034+K13811+K01739+K01738+K01738

			;F01_transcript_84568;F01_transcript_85473;F01_transcript_87136;F01_transcript_88648;F01_transcript_90435;F01_transcript_90820;F01_transcript_90989;F01_transcript_91240;F01_transcript_91299;F01_transcript_92349;F01_transcript_93426;F01_transcript_93589;F01_transcript_93851;F01_transcript_94201;F01_transcript_94345;F01_transcript_96054;F01_transcript_96511;F01_transcript_99924;	
Phenylpropanoid biosynthesis	ko00940	369	F01_transcript_100268;F01_transcript_100509;F01_transcript_100818;F01_transcript_100848;F01_transcript_101092;F01_transcript_101229;F01_transcript_101377;F01_transcript_102649;F01_transcript_103447;F01_transcript_103560;F01_transcript_104041;F01_transcript_104888;F01_transcript_105091;F01_transcript_105138;F01_transcript_105197;F01_transcript_105374;F01_transcript_105932;F01_transcript_106048;F01_transcript_106190;F01_transcript_106618;F01_transcript_107305;F01_transcript_107315;F01_transcript_108178;F01_transcript_108680;F01_transcript_109502;F01_transcript_109794;F01_transcript_110100;F01_transcript_110418;F01_transcript_110932;F01_transcript_111238;F01_transcript_111344;F01_transcript_111608;F01_transcript_111966;F01_transcript_112190;F01_transcript_112756;F01_transcript_113066;F01_transcript_114433;F01_transcript_114852;F01_transcript_115190;F01_transcript_115341;F01_transcript_115746;F01_transcript_115825;F01_transcript_116533;F01_transcript_116891;F01_transcript_117950;F01_transcript_118485;F01_transcript_119278;F01_transcript_120452;F01_transcript_120561;F01_transcript_120858;F01_transcript_121201;F01_transcript_122844;F01_transcript_122916;F01_transcript_123584;F01_transcript_123750;F01_transcript_125096;F01_transcript_125307;F01_transcript_125314;F01_transcript_125968;F01_transcript_126563;F01_transcript_126634;F01_transcript_126650;F01_transcript_127221;F01_transcript_127889;F01_transcript_128370;F01_transcript_128562;F01_transcript_128592;F01_transcript_128875;F01_transcript_128934;F01_transcript_129319;F01_transcript_129657;F01_transcript_12967;F01_transcript_129687;F01_transcript_130383;F01_transcript_130972;F01_transcript_131001;F01_transcript_131304;F01_transcript_131327;F01_transcript_131383;F01_transcript_131515;F01_transcript_132059;F0	K00430+K00588+K00487+K05350+K01188+K13065+K01188+K09754+K00430+K09755+K12355+K05350+K00083+K05350+K01188+K05349+K05350+K00430+K00083+K05349+K13066+K00430+K09753+K18368+K00588+K01188+K05350+K10775+K05349+K00430+K13065+K09755+K05350+K10775+K00430+K05350+K00487+K13066+K09755+K00430+K01188+K05350+K10775+K00430+K05349+K00430+K12356+K00588+K00588+K05350+K00430+K05350+K05350+K00430+K01188+K13065+K00430+K00430+K10775+K13065+K00430+K00083+K05349+K00430+K05350+K05350+K00083+K01188+K00430+K01188+K00430+K10775+K10775+K01188+K12356+K00430+K05350+K05349+K00430+K01904+K00083+K01188+K09754+K00430+K00430+K00487+K05349+K00083+K05349+K13065+K01188+K00083+K10775+K01188+K13065+K05349+K01904+K05350+K01188+K01188+K05350+K01188+K00430+K01188+K00430+K09755+K00083+K00083+K05350+K00430+K00430+K13065+K01904+K00430+K00083+K00430+K01188+K00430+K05349+K18368+K01904+K01188+K00487+K05350+K01188+K05349+K00083+K00083+K01188+K10775+K00083+K01188+K09753+K00083+K10775+K01188+K10775+K00430+K09753+K00487+K01188+K05350+K09755+K09754+K00430+K00083+K00083+K01188+K01188+K05350+K01188+K00430+K01188+K05350+K05350+K05349+K01188+K13066+K13066+K05349+K10775+K05350+K05349+K05350+K05350+K01188+K01188+K00430+K01188+K05349+K01188+K10775+K05349+K12355+K05349+K01188+K05349+K01188+K00430+K00430+K05349+K05349+K05349+K05349+K10775+K00430+K05350+K00083+K05350+K09755+K01904+K01188+K12356+K09755+K05350+K01188+K01188+K05350+K01904+K13065+K05350+K01904+K09755+K09754+K05350+K0048

	1_transcript_132067;F01_transcript_132236;F01_transcript_132256;F01_transcript_132313;F01_transcript_133094;F01_transcript_133256;F01_transcript_133412;F01_transcript_133921;F01_transcript_134183;F01_transcript_134748;F01_transcript_135957;F01_transcript_13645;F01_transcript_136619;F01_transcript_136934;F01_transcript_137403;F01_transcript_137702;F01_transcript_138640;F01_transcript_138970;F01_transcript_139196;F01_transcript_139446;F01_transcript_139496;F01_transcript_139539;F01_transcript_140007;F01_transcript_140399;F01_transcript_140779;F01_transcript_141172;F01_transcript_142299;F01_transcript_142532;F01_transcript_142617;F01_transcript_142921;F01_transcript_142998;F01_transcript_143278;F01_transcript_143674;F01_transcript_144107;F01_transcript_144392;F01_transcript_144580;F01_transcript_145363;F01_transcript_145380;F01_transcript_145774;F01_transcript_147651;F01_transcript_147992;F01_transcript_148330;F01_transcript_148902;F01_transcript_149911;F01_transcript_150421;F01_transcript_150650;F01_transcript_150887;F01_transcript_150926;F01_transcript_15146;F01_transcript_151502;F01_transcript_152198;F01_transcript_152855;F01_transcript_153019;F01_transcript_153198;F01_transcript_153240;F01_transcript_15388;F01_transcript_154421;F01_transcript_155253;F01_transcript_155813;F01_transcript_156051;F01_transcript_156083;F01_transcript_156089;F01_transcript_156326;F01_transcript_156431;F01_transcript_156465;F01_transcript_156507;F01_transcript_157986;F01_transcript_158249;F01_transcript_158252;F01_transcript_159376;F01_transcript_160010;F01_transcript_160671;F01_transcript_160753;F01_transcript_160784;F01_transcript_160988;F01_transcript_161192;F01_transcript_161294;F01_transcript_162539;F01_transcript_164292;F01_transcript_164372;F01_transcript_164390;F01_transcript_165033;F01_transcript_165071;F01_transcript_165335;F01_transcript_166184;F01_transcript_16620;F01_transcript_166232;F01_transcript_166833;F01_transcript_166888;F01_transcript_167210;F01_transcript_18993;F01_transcript_19594;F01_transcript_20126;F01_transcript_20653;F01_transcript_21793;F01_transcript_22769;F01_transcript_23513;F01_transcript_24047;F01_transcript_24702;F01_transcript_24985;F01_transcript_25483;F01_transcript_25948;F01_transcript_26197;F01_transcript	7+K01188+K13065+K13065+K05350+K09755+K00487+K05350+K01188+K01188+K01188+K00430+K05350+K01188+K01188+K12355+K13065+K00588+K00083+K13065+K00083+K09753+K13066+K00430+K00083+K09753+K18368+K13066+K00430+K00083+K00430+K00083+K01188+K00430+K00430+K00083+K00430+K09753+K00430+K00430+K00083+K00430+K00430+K00430+K00430+K00430+K00083+K00430+K00430+K00588+K00083+K09753+K00430+K01188+K12355+K01188+K09754+K05349+K05350+K10775+K01188+K05350+K13066+K13066+K00430+K01188+K00430+K00430+K05349+K00588+K01188+K10775+K01188+K05349+K00430+K01904+K10775+K00588+K05349+K01904+K10775+K00487+K10775+K01188+K00430+K05350+K10775+K00588+K00083+K05349+K00430+K12355+K09755+K10775+K09753+K00430+K05349+K00430+K05350+K12355+K05350+K09755+K00083+K00430+K00430+K12355+K00430+K00430+K05349+K00588+K00430+K01188+K00430+K00430+K01188+K01188+K01188+K05350+K05350+K05350+K05350+K00083+K01904+K01188+K00430+K01904+K05350+K00430+K09754+K00430+K00430+K05349+K12355+K00083+K05349+K01188+K01904+K05350+K00430+K00430+K13065+K09755+K00083+K01188+K09754+K13066+K05349+K00430+K09754+K05350+K05350+K05349+K09755+K00430+K01188+K01188+K00430+K13065+K01188+K00430+K05350+K05350+K05349+K09754

	_26246;F01_transcript_26647;F01_transcript_26984;F01_transcript_27084;F01_transcript_27120;F01_transcript_27340;F01_transcript_28040;F01_transcript_28050;F01_transcript_28454;F01_transcript_28752;F01_transcript_29052;F01_transcript_29294;F01_transcript_29312;F01_transcript_29437;F01_transcript_29461;F01_transcript_29708;F01_transcript_29784;F01_transcript_29890;F01_transcript_30234;F01_transcript_30480;F01_transcript_30650;F01_transcript_30745;F01_transcript_31528;F01_transcript_31916;F01_transcript_31925;F01_transcript_32171;F01_transcript_32249;F01_transcript_32473;F01_transcript_32819;F01_transcript_33214;F01_transcript_33678;F01_transcript_34009;F01_transcript_34183;F01_transcript_34522;F01_transcript_35049;F01_transcript_35183;F01_transcript_36253;F01_transcript_36668;F01_transcript_36939;F01_transcript_37817;F01_transcript_37939;F01_transcript_38949;F01_transcript_39519;F01_transcript_39573;F01_transcript_40000;F01_transcript_40105;F01_transcript_40301;F01_transcript_40540;F01_transcript_40712;F01_transcript_40835;F01_transcript_40868;F01_transcript_41002;F01_transcript_41008;F01_transcript_41284;F01_transcript_41371;F01_transcript_41381;F01_transcript_41433;F01_transcript_41528;F01_transcript_41687;F01_transcript_41820;F01_transcript_41846;F01_transcript_41920;F01_transcript_42258;F01_transcript_42302;F01_transcript_42468;F01_transcript_42610;F01_transcript_42650;F01_transcript_42851;F01_transcript_42982;F01_transcript_43401;F01_transcript_43609;F01_transcript_44478;F01_transcript_46564;F01_transcript_47842;F01_transcript_50524;F01_transcript_52725;F01_transcript_52855;F01_transcript_53224;F01_transcript_53935;F01_transcript_53994;F01_transcript_55819;F01_transcript_55994;F01_transcript_56193;F01_transcript_56288;F01_transcript_56397;F01_transcript_56743;F01_transcript_57117;F01_transcript_57851;F01_transcript_58138;F01_transcript_58663;F01_transcript_59357;F01_transcript_60080;F01_transcript_60998;F01_transcript_62639;F01_transcript_62701;F01_transcript_64188;F01_transcript_64346;F01_transcript_64918;F01_transcript_65829;F01_transcript_65838;F01_transcript_66441;F01_transcript_66825;F01_transcript_68082;F01_transcript_68591;F01_transcript_68840;F01_transcript_69237

			;F01_transcript_69406;F01_transcript_69425;F01_transcript_70761;F01_transcript_70982;F01_transcript_71241;F01_transcript_71335;F01_transcript_72582;F01_transcript_72870;F01_transcript_72984;F01_transcript_73055;F01_transcript_73150;F01_transcript_73186;F01_transcript_73939;F01_transcript_74577;F01_transcript_74876;F01_transcript_75064;F01_transcript_7525;F01_transcript_75317;F01_transcript_75795;F01_transcript_75955;F01_transcript_75963;F01_transcript_76055;F01_transcript_76356;F01_transcript_78182;F01_transcript_78475;F01_transcript_78543;F01_transcript_79800;F01_transcript_80632;F01_transcript_81123;F01_transcript_81126;F01_transcript_81833;F01_transcript_82466;F01_transcript_83222;F01_transcript_83296;F01_transcript_83393;F01_transcript_83418;F01_transcript_83631;F01_transcript_84574;F01_transcript_84796;F01_transcript_86334;F01_transcript_86407;F01_transcript_86419;F01_transcript_86634;F01_transcript_86929;F01_transcript_87916;F01_transcript_87919;F01_transcript_88605;F01_transcript_88814;F01_transcript_89025;F01_transcript_89585;F01_transcript_90214;F01_transcript_90345;F01_transcript_90419;F01_transcript_90834;F01_transcript_90951;F01_transcript_91140;F01_transcript_9125;F01_transcript_91297;F01_transcript_91915;F01_transcript_92300;F01_transcript_92650;F01_transcript_92681;F01_transcript_93411;F01_transcript_94124;F01_transcript_94343;F01_transcript_94626;F01_transcript_95784;F01_transcript_95871;F01_transcript_96008;F01_transcript_96241;F01_transcript_96323;F01_transcript_96386;F01_transcript_96547;F01_transcript_97889;F01_transcript_98189;F01_transcript_98615;F01_transcript_98978;F01_transcript_99106;F01_transcript_99241;	
Flavonoid biosynthesis	ko00941	101	F01_transcript_100509;F01_transcript_100818;F01_transcript_100833;F01_transcript_101229;F01_transcript_102649;F01_transcript_108442;F01_transcript_109502;F01_transcript_110372;F01_transcript_111344;F01_transcript_113330;F01_transcript_113417;F01_transcript_113654;F01_transcript_113795;F01_transcript_114433;F01_transcript_120014;F01_transcript_120452;F01_transcript_120561;F01_transcript_120984;F01_transcript_121339;F01_transcript_122661;F01_transcript_124029;F01_transcript_124353;F01_transcript_125096;F01_transcript_126563;F01_transcript_128823;F01_transcript_132236;F01_transcript_133094;	K00588+K00487+K05277+K13065+K09754+K13082+K00588+K21102+K13065+K00660+K05278+K00660+K13082+K00487+K00660+K00588+K00588+K05278+K21102+K05278+K13081+K00475+K13065+K13065+K00660+K09754+K00487+K13065+K13065+K05280+K13065+K05278+K00487+K05278+K05278+K08695+K00487+K09754+K21102+K21102+K13083+K05280+K05280+K13065+K09754+K00487+K05280+K13065+K13065+K05280+K00487+K13065+K00588+K13065+K00660+K05280+K00660+K05278+K05277+K05277+K21102+K21102+K00475+K00475+K00475+K21102+K13081+K00588+K018

			F01_transcript_134183;F01_transcript_136934;F01_transcript_137351;F01_transcript_142998;F01_transcript_147017;F01_transcript_148330;F01_transcript_149429;F01_transcript_152098;F01_transcript_154415;F01_transcript_155813;F01_transcript_156326;F01_transcript_157513;F01_transcript_160567;F01_transcript_163104;F01_transcript_26543;F01_transcript_27925;F01_transcript_29708;F01_transcript_30480;F01_transcript_30745;F01_transcript_31836;F01_transcript_31916;F01_transcript_31925;F01_transcript_32014;F01_transcript_32473;F01_transcript_36668;F01_transcript_36939;F01_transcript_37939;F01_transcript_39092;F01_transcript_39415;F01_transcript_39477;F01_transcript_40198;F01_transcript_40437;F01_transcript_40676;F01_transcript_40763;F01_transcript_41398;F01_transcript_41454;F01_transcript_41509;F01_transcript_41670;F01_transcript_41793;F01_transcript_42060;F01_transcript_43609;F01_transcript_44283;F01_transcript_45303;F01_transcript_4631;F01_transcript_46484;F01_transcript_47167;F01_transcript_50588;F01_transcript_51873;F01_transcript_53224;F01_transcript_59357;F01_transcript_65829;F01_transcript_66151;F01_transcript_68082;F01_transcript_68160;F01_transcript_69390;F01_transcript_70761;F01_transcript_7290;F01_transcript_76185;F01_transcript_78475;F01_transcript_80642;F01_transcript_83668;F01_transcript_86929;F01_transcript_88593;F01_transcript_89041;F01_transcript_89189;F01_transcript_91140;F01_transcript_91911;F01_transcript_92300;F01_transcript_93533;F01_transcript_93805;F01_transcript_94083;F01_transcript_94124;F01_transcript_96547;F01_transcript_99241;	59+K01859+K05278+K05278+K05278+K05278+K21102+K09754+K00588+K00588+K05278+K00487+K01859+K21102+K00588+K05280+K21102+K00588+K13081+K13081+K09754+K05277+K13082+K05278+K13065+K13081+K09754+K05278+K13082+K13081+K09754+K13065+K09754
Anthocyanin biosynthesis	ko00942	2	F01_transcript_138327;F01_transcript_36470;	K17193+K12930
Flavone and	ko0	10	F01_transcript_106254;F01_transcript_137351;F01_transcript_163104;F01_transcript_26543;F01_transcript_27925;F01_transcri	K13269+K05280+K13083+K05280+K05280+K05280+K05280+K05280+K13272+K05280

flavonol biosynthesis	0944		pt_31836;F01_transcript_32014;F01_transcript_39415;F01_transcript_66826;F01_transcript_7290;	
Stilbenoid, diarylheptanoid and gingerol biosynthesis	ko00945	45	F01_transcript_100509;F01_transcript_100818;F01_transcript_101229;F01_transcript_102649;F01_transcript_109502;F01_transcript_111344;F01_transcript_114433;F01_transcript_120452;F01_transcript_120561;F01_transcript_125096;F01_transcript_126563;F01_transcript_132236;F01_transcript_133094;F01_transcript_134183;F01_transcript_136934;F01_transcript_142998;F01_transcript_148330;F01_transcript_155813;F01_transcript_156326;F01_transcript_20002;F01_transcript_25031;F01_transcript_29708;F01_transcript_30480;F01_transcript_30745;F01_transcript_31371;F01_transcript_31916;F01_transcript_31925;F01_transcript_32473;F01_transcript_36668;F01_transcript_36939;F01_transcript_37939;F01_transcript_43609;F01_transcript_53224;F01_transcript_55231;F01_transcript_59357;F01_transcript_65829;F01_transcript_68082;F01_transcript_70761;F01_transcript_78475;F01_transcript_86929;F01_transcript_91140;F01_transcript_92300;F01_transcript_94124;F01_transcript_96547;F01_transcript_99241;	K00588+K00487+K13065+K09754+K00588+K13065+K00487+K00588+K00588+K13065+K13065+K09754+K00487+K13065+K13065+K13065+K00487+K00487+K09754+K00517+K00517+K13065+K09754+K00487+K00517+K13065+K13065+K00487+K13065+K00588+K13065+K00588+K09754+K00517+K00588+K00588+K00487+K00588+K00588+K09754+K13065+K09754+K09754+K13065+K09754
Isoquinoline alkaloid biosynthesis	ko00950	117	F01_transcript_100618;F01_transcript_101473;F01_transcript_101567;F01_transcript_102388;F01_transcript_105405;F01_transcript_106156;F01_transcript_106666;F01_transcript_107151;F01_transcript_107764;F01_transcript_113517;F01_transcript_114452;F01_transcript_119869;F01_transcript_12072;F01_transcript_122119;F01_transcript_123492;F01_transcript_124604;F01_transcript_125225;F01_transcript_126185;F01_transcript_127780;F01_transcript_130313;F01_transcript_130548;F01_transcript_131043;F01_transcript_132157;F01_transcript_132512;F01_transcript_133648;F01_transcript_13535;F01_transcript_135578;F01_transcript_135806;F01_transcript_136316;F01_transcript_136534;F01_transcript_139669;F01_transcript_140406;F01_transcript_140632;F01_transcript_142762;F01_transcript_14328;F01_transcript_144749;F01_transcript_146924;F01_transcript_147552;F01_transcript_148409;F01_transcript_150132;F01_transcript_150639;F01_transcript_152726;F01_transcript_154647;F01_tran	K00815+K14454+K00276+K01592+K00276+K14454+K00815+K00811+K00276+K01592+K14455+K14455+K00276+K00815+K00422+K00276+K01592+K14455+K01592+K00276+K01592+K14455+K00815+K00276+K00815+K00276+K14454+K00815+K15849+K00815+K00422+K00276+K00276+K00422+K00815+K00815+K00815+K00276+K00276+K00815+K14454+K14454+K00276+K14455+K00276+K00276+K00811+K00815+K00815+K01592+K00422+K13384+K00276+K00276+K00276+K15849+K00276+K00815+K00276+K00276+K00276+K01592+K00422+K14454+K00422+K00422+K00422+K14454+K00815+K15849+K01592+K00815+K00811+K01592+K15849+K00815+K14455+K14455+K14455+K13384+K00815+K00815+K00276+K00276+K00276+K00276+K00276+K00276+K00815+K14454+K00276+K01592+K01592+K00422+K00276+K00815+K14455+K00811+K00811+K01592+K14454+K14454+K00276+K00811+K00422+K14455+K15849+K01592+K14454+K0027

			script_156344;F01_transcript_158722;F01_transcript_159476;F01_transcript_160500;F01_transcript_161264;F01_transcript_161557;F01_transcript_161741;F01_transcript_163139;F01_transcript_163691;F01_transcript_163899;F01_transcript_16418;F01_transcript_16644;F01_transcript_166505;F01_transcript_18563;F01_transcript_19390;F01_transcript_2067;F01_transcript_22000;F01_transcript_24430;F01_transcript_24431;F01_transcript_28048;F01_transcript_28405;F01_transcript_28975;F01_transcript_29563;F01_transcript_30484;F01_transcript_30736;F01_transcript_31061;F01_transcript_31276;F01_transcript_31346;F01_transcript_31700;F01_transcript_31784;F01_transcript_31814;F01_transcript_31834;F01_transcript_32147;F01_transcript_32622;F01_transcript_36375;F01_transcript_36639;F01_transcript_36988;F01_transcript_37457;F01_transcript_42165;F01_transcript_52920;F01_transcript_56629;F01_transcript_60104;F01_transcript_61675;F01_transcript_61722;F01_transcript_63755;F01_transcript_65744;F01_transcript_65858;F01_transcript_66411;F01_transcript_67201;F01_transcript_72857;F01_transcript_73675;F01_transcript_74237;F01_transcript_76908;F01_transcript_78720;F01_transcript_78919;F01_transcript_80769;F01_transcript_80944;F01_transcript_81078;F01_transcript_81767;F01_transcript_8293;F01_transcript_86970;F01_transcript_87463;F01_transcript_87504;F01_transcript_88383;F01_transcript_88838;F01_transcript_89140;F01_transcript_90314;F01_transcript_90364;F01_transcript_91560;F01_transcript_9243;F01_transcript_93052;F01_transcript_95934;F01_transcript_9785;F01_transcript_98894;	6+K00815+K00276+K00276+K14455+K00276+K14454+K00276
Tropane, piperidine and pyridine alkaloid biosy	ko00960	140	F01_transcript_100618;F01_transcript_101473;F01_transcript_101567;F01_transcript_103473;F01_transcript_105405;F01_transcript_106156;F01_transcript_106666;F01_transcript_107151;F01_transcript_107764;F01_transcript_114452;F01_transcript_114987;F01_transcript_117887;F01_transcript_119761;F01_transcript_119869;F01_transcript_119950;F01_transcript_12072;F01_transcript_122119;F01_transcript_124604;F01_transcript_125242;F01_transcript_126185;F01_transcript_127519;F01_transcript_128535;F01_transcript_130313;F01_transcript_131043;F01_transcript_132157;F01_transcript_132374;F01_transcript_132512;F	K00815+K14454+K00276+K08081+K00276+K14454+K00815+K00811+K00276+K14455+K08081+K08081+K08081+K14455+K08081+K00276+K00815+K00276+K08081+K14455+K08081+K08081+K00276+K14455+K00815+K00817+K00276+K00815+K08081+K00276+K14454+K00815+K08081+K15849+K00815+K00817+K08081+K08081+K00276+K00276+K08081+K00815+K00815+K08081+K00815+K00276+K00276+K08081+K08081+K00815+K14454+K08081+K14454+K00276+K14455+K08081+K00276+K00276+K00811+K08081+K00815+K00815+K00276+K00276+K00276+K15849+K00276+K00815+K002

nthesis	01_transcript_133648;F01_transcript_134128;F01_transcript_13535;F01_transcript_135578;F01_transcript_135806;F01_transcript_136302;F01_transcript_136316;F01_transcript_136534;F01_transcript_136643;F01_transcript_140086;F01_transcript_140372;F01_transcript_140406;F01_transcript_140632;F01_transcript_143002;F01_transcript_14328;F01_transcript_144749;F01_transcript_146524;F01_transcript_146924;F01_transcript_147552;F01_transcript_148409;F01_transcript_149489;F01_transcript_149627;F01_transcript_150132;F01_transcript_150639;F01_transcript_152618;F01_transcript_152726;F01_transcript_154647;F01_transcript_156344;F01_transcript_157788;F01_transcript_158722;F01_transcript_159476;F01_transcript_160500;F01_transcript_160881;F01_transcript_161264;F01_transcript_161557;F01_transcript_163899;F01_transcript_16418;F01_transcript_16644;F01_transcript_166505;F01_transcript_18563;F01_transcript_19390;F01_transcript_2067;F01_transcript_20778;F01_transcript_22000;F01_transcript_24430;F01_transcript_27289;F01_transcript_28405;F01_transcript_30736;F01_transcript_31061;F01_transcript_31276;F01_transcript_31700;F01_transcript_31784;F01_transcript_31834;F01_transcript_32147;F01_transcript_32622;F01_transcript_32700;F01_transcript_33879;F01_transcript_35387;F01_transcript_36375;F01_transcript_36639;F01_transcript_37191;F01_transcript_37210;F01_transcript_37457;F01_transcript_38955;F01_transcript_38995;F01_transcript_39596;F01_transcript_40724;F01_transcript_42057;F01_transcript_42165;F01_transcript_44410;F01_transcript_44421;F01_transcript_44543;F01_transcript_45072;F01_transcript_52920;F01_transcript_55052;F01_transcript_56164;F01_transcript_56629;F01_transcript_58126;F01_transcript_60104;F01_transcript_61675;F01_transcript_61722;F01_transcript_63163;F01_transcript_63755;F01_transcript_65744;F01_transcript_65858;F01_transcript_66225;F01_transcript_66411;F01_transcript_67721;F01_transcript_74237;F01_transcript_75254;F01_transcript_76908;F01_transcript_78720;F01_transcript_78919;F01_transcript_80769;F01_transcript_81078;F01_transcript_81767;F01_transcript_8293;F01_transcript_84689;F01_transcript_86970;F01_transcript_87504;F01_transcript_87877;F01_transcript_87962;F01_transcript_88383;F01_transcript_89140;F01_transcript_90314;F01_transcript_90364;F01_tran	76+K08081+K00276+K00276+K08081+K14454+K14454+K00815+K15849+K00815+K00811+K15849+K00815+K14455+K08081+K00817+K08081+K14455+K14455+K08081+K08081+K00815+K08081+K18606+K18606+K18606+K08081+K00815+K08081+K08081+K08081+K08081+K00276+K08081+K08081+K00276+K08081+K00276+K00276+K00276+K18606+K00276+K00815+K14454+K08081+K00276+K08081+K00276+K18606+K00815+K14455+K00811+K00811+K14454+K14454+K00276+K08081+K00811+K14455+K08081+K00817+K15849+K14454+K00276+K00815+K00276+K08081+K00276+K14455+K00276+K14454+K00276

			script_91560;F01_transcript_92110;F01_transcript_9243;F01_transcript_93052;F01_transcript_95934;F01_transcript_9785;F01_transcript_98894;	
Glucosinolate biosynthesis	ko00966	3	F01_transcript_37001;F01_transcript_39196;F01_transcript_98138;	K11820+K11820+K11820
Aminoacyl-tRNA biosynthesis	ko00970	503	F01_transcript_10037;F01_transcript_100400;F01_transcript_101215;F01_transcript_101627;F01_transcript_101633;F01_transcript_101660;F01_transcript_101663;F01_transcript_101775;F01_transcript_102151;F01_transcript_102313;F01_transcript_102719;F01_transcript_103194;F01_transcript_103220;F01_transcript_104384;F01_transcript_104387;F01_transcript_104487;F01_transcript_104607;F01_transcript_104967;F01_transcript_106020;F01_transcript_106457;F01_transcript_106757;F01_transcript_107728;F01_transcript_108067;F01_transcript_108170;F01_transcript_108311;F01_transcript_108591;F01_transcript_109630;F01_transcript_109871;F01_transcript_110082;F01_transcript_110791;F01_transcript_111000;F01_transcript_111007;F01_transcript_111255;F01_transcript_111576;F01_transcript_111674;F01_transcript_112723;F01_transcript_113084;F01_transcript_113122;F01_transcript_113291;F01_transcript_113324;F01_transcript_113362;F01_transcript_114156;F01_transcript_114213;F01_transcript_114430;F01_transcript_114535;F01_transcript_114564;F01_transcript_114580;F01_transcript_114869;F01_transcript_115549;F01_transcript_116044;F01_transcript_116254;F01_transcript_116256;F01_transcript_116370;F01_transcript_11650;F01_transcript_116543;F01_transcript_116599;F01_transcript_116693;F01_transcript_116926;F01_transcript_117135;F01_transcript_117689;F01_transcript_117773;F01_transcript_117866;F01_transcript_117906;F01_transcript_118587;F01_transcript_118652;F01_transcript_119903;F01_transcript_119911;F01_transcript_119987;F01_transcript_120320;F01_transcript_120486;F01_transcript_120583;F01_transcript_120765;F01_transcript_120843;	K01892+K01874+K01872+K01874+K04567+K02433+K01868+K01890+K01873+K01869+K01872+K01880+K14164+K01872+K01872+K01892+K01881+K01874+K04567+K01886+K01880+K01875+K01873+K01869+K01870+K01893+K01866+K01872+K01883+K01872+K01874+K01873+K01881+K01872+K01885+K01892+K01885+K01892+K14164+K01889+K01889+K01885+K04567+K01875+K01872+K01873+K01870+K01870+K01887+K01881+K01875+K01881+K01889+K01886+K01881+K01869+K01875+K01872+K02433+K01866+K01873+K01889+K01870+K01873+K01868+K01886+K01889+K01893+K01876+K01872+K02434+K01868+K01870+K01872+K01870+K02433+K01873+K01872+K00604+K01886+K01890+K01868+K01869+K01874+K01868+K01873+K01874+K14164+K01874+K01872+K01870+K01869+K04567+K01893+K01870+K01889+K01887+K01893+K01883+K01875+K01885+K01870+K01867+K01870+K01872+K01874+K01868+K01870+K01875+K01883+K01880+K01885+K01887+K01873+K04567+K14164+K01875+K02434+K01867+K01868+K02433+K01875+K01883+K01872+K01874+K01889+K01889+K01881+K01887+K01890+K04567+K01893+K01870+K01889+K02433+K01892+K01893+K01873+K01889+K01887+K01875+K01876+K01870+K01872+K01869+K01872+K02434+K01874+K01874+K01869+K01881+K01887+K01889+K01868+K01883+K01885+K01874+K01885+K01883+K01873+K01869+K01885+K01873+K01870+K01885+K01870+K14164+K01880+K01889+K01876+K01870+K04567+K01872+K01893+K01889+K01868+K02433+K01874+K01876+K01874+K14164+K01872+K01889+K01872+K01869+K

	F01_transcript_120990;F01_transcript_121180;F01_transcript_121653;F01_transcript_122524;F01_transcript_122832;F01_transcript_123481;F01_transcript_123752;F01_transcript_124338;F01_transcript_124339;F01_transcript_124405;F01_transcript_124552;F01_transcript_12501;F01_transcript_125527;F01_transcript_125548;F01_transcript_126057;F01_transcript_126432;F01_transcript_126719;F01_transcript_127154;F01_transcript_127238;F01_transcript_127294;F01_transcript_127512;F01_transcript_1280;F01_transcript_128247;F01_transcript_129556;F01_transcript_130197;F01_transcript_130265;F01_transcript_130595;F01_transcript_130836;F01_transcript_130854;F01_transcript_131458;F01_transcript_131684;F01_transcript_131767;F01_transcript_131941;F01_transcript_132101;F01_transcript_132210;F01_transcript_132407;F01_transcript_132562;F01_transcript_132995;F01_transcript_133626;F01_transcript_133861;F01_transcript_133894;F01_transcript_134197;F01_transcript_134358;F01_transcript_134624;F01_transcript_135659;F01_transcript_135908;F01_transcript_136141;F01_transcript_136531;F01_transcript_136908;F01_transcript_137375;F01_transcript_137441;F01_transcript_138008;F01_transcript_138210;F01_transcript_13830;F01_transcript_138781;F01_transcript_138830;F01_transcript_139047;F01_transcript_139439;F01_transcript_13960;F01_transcript_139713;F01_transcript_140018;F01_transcript_140166;F01_transcript_140430;F01_transcript_141288;F01_transcript_141815;F01_transcript_142646;F01_transcript_142789;F01_transcript_142832;F01_transcript_143125;F01_transcript_143483;F01_transcript_143508;F01_transcript_143740;F01_transcript_143823;F01_transcript_144250;F01_transcript_144983;F01_transcript_145306;F01_transcript_145390;F01_transcript_145424;F01_transcript_145505;F01_transcript_145578;F01_transcript_145666;F01_transcript_145823;F01_transcript_14617;F01_transcript_146745;F01_transcript_147034;F01_transcript_147423;F01_transcript_147860;F01_transcript_147870;F01_transcript_147989;F01_transcript_148142;F01_transcript_148684;F01_transcript_148838;F01_transcript_14927;F01_transcript_150239;F01_transcript_150719;F01_transcript_150833;F01_transcript_151861;F01_transcript_151945;F01_transcript_152236;F01_transcript_152381;F01_transcript_152556;F01_transcript_154326;F01_transcript_15458;	04567+K01869+K02434+K01876+K01883+K01874+K01892+K04567+K01886+K01883+K01870+K01885+K01868+K01880+K01889+K01893+K01893+K01870+K01889+K01883+K00604+K01867+K01893+K01883+K01881+K01893+K01892+K01873+K01886+K01874+K14164+K01868+K01868+K01876+K01883+K01870+K02433+K01872+K01873+K01870+K01868+K01890+K01866+K01885+K01887+K02434+K02434+K01880+K01868+K04567+K01893+K01874+K01876+K01873+K01875+K04567+K04567+K04567+K01869+K01887+K01885+K01887+K04567+K01890+K01890+K01880+K01880+K04567+K04567+K04567+K02434+K01869+K01885+K01893+K01870+K04567+K01890+K01889+K04567+K01883+K01887+K01883+K01880+K01875+K01869+K01893+K01874+K01893+K01874+K01874+K01885+K01883+K01874+K01883+K01881+K01874+K01876+K01883+K01887+K01875+K01881+K01881+K01881+K02435+K01881+K01892+K01889+K01881+K01885+K00604+K01892+K01889+K01885+K01881+K01889+K01875+K01866+K01892+K01867+K01889+K01873+K01869+K01889+K01873+K01872+K01889+K01873+K01872+K01872+K01867+K01872+K01866+K01873+K00604+K01869+K01872+K01881+K01872+K01869+K02435+K01869+K01886+K00604+K01869+K01869+K01873+K01869+K01869+K01890+K01869+K01869+K01893+K01870+K01885+K04567+K01890+K01886+K01869+K01873+K00604+K01889+K04567+K01893+K01869+K01883+K01872+K01872+K01889+K01873+K01868+K01883+K01876+K01892+K01892+K01873+K01867+K01880+K01866+K01872+K01866+K01874+K01889+K01868+K01892+K01869+K01881+K01874+K01872+K01886+K01869+K01875+K01883+K01874+K01883+K01875+K14164+K04567+K02433+K01876+K04567+K01874+K01869+K04567+K01889+K01883+K01886+K01870+K04567+K01867+K01873+K01872+K04567+K02435+K01867+K04567+K01887+K01869+K01866+K01881+K01872+K01872+K01892+K01881+K01883+K01872+K01870+K01889+K01886+K01870+K01869+K01892+K01872+K01886+K01873+K01881+K01880+K01876+K01872+K01873+K01868+K01892+K01874+K01886+K01869+K01873+K04567+K01870+K01889+K01874+K01873+K01892+K01870+K01870+K01885+K01873+K14164+K01873+K01885+K02435+K01889+K0188

	F01_transcript_155125;F01_transcript_155163;F01_transcript_155824;F01_transcript_156015;F01_transcript_156135;F01_transcript_156234;F01_transcript_156299;F01_transcript_156569;F01_transcript_156587;F01_transcript_157842;F01_transcript_157989;F01_transcript_158185;F01_transcript_158261;F01_transcript_158334;F01_transcript_158496;F01_transcript_158933;F01_transcript_159220;F01_transcript_159737;F01_transcript_159879;F01_transcript_159918;F01_transcript_160019;F01_transcript_160098;F01_transcript_160200;F01_transcript_160311;F01_transcript_160470;F01_transcript_160726;F01_transcript_160905;F01_transcript_161545;F01_transcript_161704;F01_transcript_161743;F01_transcript_162284;F01_transcript_162353;F01_transcript_162397;F01_transcript_162731;F01_transcript_162797;F01_transcript_162922;F01_transcript_163108;F01_transcript_163193;F01_transcript_163553;F01_transcript_164349;F01_transcript_164750;F01_transcript_164893;F01_transcript_165473;F01_transcript_165517;F01_transcript_165535;F01_transcript_165993;F01_transcript_166183;F01_transcript_166299;F01_transcript_166547;F01_transcript_16676;F01_transcript_166762;F01_transcript_167276;F01_transcript_16743;F01_transcript_16763;F01_transcript_17567;F01_transcript_17810;F01_transcript_18101;F01_transcript_18498;F01_transcript_1876;F01_transcript_18904;F01_transcript_19153;F01_transcript_19472;F01_transcript_1970;F01_transcript_19752;F01_transcript_19836;F01_transcript_19989;F01_transcript_20168;F01_transcript_2017;F01_transcript_20323;F01_transcript_20923;F01_transcript_20959;F01_transcript_21149;F01_transcript_21314;F01_transcript_21329;F01_transcript_21408;F01_transcript_21592;F01_transcript_21810;F01_transcript_21895;F01_transcript_22095;F01_transcript_22407;F01_transcript_2302;F01_transcript_23223;F01_transcript_23668;F01_transcript_23805;F01_transcript_23949;F01_transcript_24198;F01_transcript_24442;F01_transcript_24460;F01_transcript_25174;F01_transcript_25380;F01_transcript_25383;F01_transcript_25424;F01_transcript_25576;F01_transcript_2575;F01_transcript_25896;F01_transcript_26048;F01_transcript_26224;F01_transcript_26446;F01_transcript_26648;F01_transcript_26811;F01_transcript_26829;F01_transcript_27011;F01_transcript_27	0+K01886+K04567+K02434+K01889+K00604+K01881+K04567+K01873+K01866+K01873+K01872+K01881+K02433+K01889+K02435+K01881+K01892+K04567+K01881+K01874+K01889+K01870+K01892+K01886+K01868+K01873+K01892+K01887+K02434+K01886+K01872+K01874+K01869+K04567+K00604+K01874+K01883+K01892+K01885+K01881+K01868+K01889+K01889+K01872+K02435+K01872+K04567+K02433+K01893+K01870+K01867+K01886+K01880+K01872+K01893+K14164+K01883

315;F01_transcript_27402;F01_transcript_27447;F01_transcript_28173;F01_transcript_28363;F01_transcript_28730;F01_transcript_28942;F01_transcript_28943;F01_transcript_29381;F01_transcript_29656;F01_transcript_29954;F01_transcript_30036;F01_transcript_30083;F01_transcript_30418;F01_transcript_30722;F01_transcript_31250;F01_transcript_31275;F01_transcript_31660;F01_transcript_32353;F01_transcript_32410;F01_transcript_32696;F01_transcript_32938;F01_transcript_33295;F01_transcript_33377;F01_transcript_33654;F01_transcript_33818;F01_transcript_33890;F01_transcript_3436;F01_transcript_3439;F01_transcript_34533;F01_transcript_3484;F01_transcript_3640;F01_transcript_36843;F01_transcript_3713;F01_transcript_3716;F01_transcript_3738;F01_transcript_38767;F01_transcript_3879;F01_transcript_39254;F01_transcript_3928;F01_transcript_39616;F01_transcript_3968;F01_transcript_4254;F01_transcript_42748;F01_transcript_4398;F01_transcript_4488;F01_transcript_47789;F01_transcript_5130;F01_transcript_52034;F01_transcript_52566;F01_transcript_5271;F01_transcript_5277;F01_transcript_52992;F01_transcript_53171;F01_transcript_53183;F01_transcript_53266;F01_transcript_5393;F01_transcript_53936;F01_transcript_54104;F01_transcript_54136;F01_transcript_54372;F01_transcript_54512;F01_transcript_54581;F01_transcript_5517;F01_transcript_55377;F01_transcript_55537;F01_transcript_55563;F01_transcript_5575;F01_transcript_55790;F01_transcript_56158;F01_transcript_56197;F01_transcript_56732;F01_transcript_57254;F01_transcript_57604;F01_transcript_57681;F01_transcript_58427;F01_transcript_58448;F01_transcript_58494;F01_transcript_58558;F01_transcript_59274;F01_transcript_60321;F01_transcript_60699;F01_transcript_60861;F01_transcript_61162;F01_transcript_61297;F01_transcript_61474;F01_transcript_61723;F01_transcript_61806;F01_transcript_61807;F01_transcript_62029;F01_transcript_62078;F01_transcript_62141;F01_transcript_62187;F01_transcript_62202;F01_transcript_62428;F01_transcript_63015;F01_transcript_63066;F01_transcript_63230;F01_transcript_63510;F01_transcript_6454;F01_transcript_64576;F01_transcript_64835;F01_transcript_6486;F01_transcript_65129;F01_transcript_65786;F01_transcript_6582;F01_transcript_66194;F01_transcript_66588;F01_transcript_66785;F01_transcript_66956;

F01_transcript_67093;F01_transcript_67288;F01_transcript_673
30;F01_transcript_67390;F01_transcript_67508;F01_transcript_
67925;F01_transcript_68783;F01_transcript_69247;F01_transcri
pt_69893;F01_transcript_70684;F01_transcript_70717;F01_tran
script_70755;F01_transcript_71106;F01_transcript_71593;F01_t
ranscript_72082;F01_transcript_72263;F01_transcript_72294;F0
1_transcript_73424;F01_transcript_73528;F01_transcript_74453
;F01_transcript_74671;F01_transcript_74771;F01_transcript_74
961;F01_transcript_75425;F01_transcript_76026;F01_transcript
_76219;F01_transcript_76297;F01_transcript_7647;F01_transcri
pt_77242;F01_transcript_77384;F01_transcript_77874;F01_tran
script_78089;F01_transcript_78534;F01_transcript_78585;F01_t
ranscript_78597;F01_transcript_78780;F01_transcript_79010;F0
1_transcript_79039;F01_transcript_79092;F01_transcript_79379
;F01_transcript_79390;F01_transcript_79559;F01_transcript_79
791;F01_transcript_79925;F01_transcript_80088;F01_transcript
_8073;F01_transcript_80961;F01_transcript_8127;F01_transcrip
t_81488;F01_transcript_81658;F01_transcript_81695;F01_trans
cript_81940;F01_transcript_82081;F01_transcript_82725;F01_tr
anscript_82797;F01_transcript_82888;F01_transcript_82891;F0
1_transcript_82999;F01_transcript_83076;F01_transcript_83243
;F01_transcript_83377;F01_transcript_83442;F01_transcript_83
673;F01_transcript_84228;F01_transcript_84555;F01_transcript
_84777;F01_transcript_84814;F01_transcript_84847;F01_transc
ript_85711;F01_transcript_86106;F01_transcript_86110;F01_tra
nscript_86554;F01_transcript_86647;F01_transcript_87948;F01
_transcript_8839;F01_transcript_88786;F01_transcript_89286;F
01_transcript_8978;F01_transcript_90047;F01_transcript_90258
;F01_transcript_9056;F01_transcript_9087;F01_transcript_9145
3;F01_transcript_91873;F01_transcript_92126;F01_transcript_9
25;F01_transcript_92711;F01_transcript_9278;F01_transcript_9
2952;F01_transcript_93629;F01_transcript_93643;F01_transcrip
t_93709;F01_transcript_93736;F01_transcript_93845;F01_trans
cript_93933;F01_transcript_94164;F01_transcript_95240;F01_tr
anscript_95322;F01_transcript_9564;F01_transcript_95663;F01
_transcript_95730;F01_transcript_96325;F01_transcript_96362;
F01_transcript_96398;F01_transcript_96716;F01_transcript_967

			53;F01_transcript_96776;F01_transcript_977;F01_transcript_97728;F01_transcript_97769;F01_transcript_97795;F01_transcript_98257;F01_transcript_98372;F01_transcript_98729;F01_transcript_99140;	
Biosynthesis of unsaturated fatty acids	ko01040	124	F01_transcript_103130;F01_transcript_106217;F01_transcript_110694;F01_transcript_111506;F01_transcript_112515;F01_transcript_113046;F01_transcript_113706;F01_transcript_11601;F01_transcript_116845;F01_transcript_117687;F01_transcript_118434;F01_transcript_120801;F01_transcript_121926;F01_transcript_122368;F01_transcript_122692;F01_transcript_122818;F01_transcript_124368;F01_transcript_124769;F01_transcript_124788;F01_transcript_126252;F01_transcript_129744;F01_transcript_129865;F01_transcript_130423;F01_transcript_130717;F01_transcript_131603;F01_transcript_133828;F01_transcript_138047;F01_transcript_139123;F01_transcript_140225;F01_transcript_141759;F01_transcript_142214;F01_transcript_143781;F01_transcript_146347;F01_transcript_147167;F01_transcript_147259;F01_transcript_147398;F01_transcript_147705;F01_transcript_148949;F01_transcript_149791;F01_transcript_15284;F01_transcript_155474;F01_transcript_157406;F01_transcript_15817;F01_transcript_15946;F01_transcript_159968;F01_transcript_160683;F01_transcript_16286;F01_transcript_165303;F01_transcript_165616;F01_transcript_165801;F01_transcript_166122;F01_transcript_167185;F01_transcript_167595;F01_transcript_17987;F01_transcript_22669;F01_transcript_26159;F01_transcript_27104;F01_transcript_27232;F01_transcript_29191;F01_transcript_30801;F01_transcript_32634;F01_transcript_32889;F01_transcript_33549;F01_transcript_33717;F01_transcript_34454;F01_transcript_34472;F01_transcript_34915;F01_transcript_35442;F01_transcript_35988;F01_transcript_36146;F01_transcript_36214;F01_transcript_36482;F01_transcript_36847;F01_transcript_36931;F01_transcript_37206;F01_transcript_37378;F01_transcript_38069;F01_transcript_40823;F01_transcript_41243;F01_transcript_44928;F01_transcript_52099;F01_transcript_52548;F01_transcript_53390;F01_transcript_54990;F01_transcript_55240;F01_transcript_55281;F01_transcript_56128;F01_transcript_56417;F01_transcript_57777;F01_transcript_58171;F01_transcript_59428;F01_transcript_60150;F01_transcript_60200;F01_transcript_61866;F01	K00059+K00232+K01068+K10703+K10258+K10703+K10703+K00232+K10256+K00232+K00232+K00232+K10251+K00232+K10251+K00232+K10256+K00232+K00232+K10258+K10703+K03921+K03921+K07513+K00059+K10703+K00232+K00232+K03921+K10703+K00232+K00232+K10703+K00232+K00232+K07513+K00059+K10703+K00232+K00232+K00232+K00232+K00232+K00232+K07513+K00232+K00232+K10251+K10251+K10251+K10251+K01068+K07513+K00232+K00232+K10251+K00232+K00232+K00232+K00232+K03921+K07513+K07513+K01068+K07513+K10256+K03921+K07513+K00232+K10256+K03921+K00232+K00059+K00059+K03921+K00059+K10256+K10251+K10251+K00059+K00232+K00232+K00232+K00232+K00232+K00232+K00232+K10703+K00232+K00232+K10703+K00059+K10703+K07513+K00232+K00232+K01068+K01068+K10251+K00059+K00232+K03921+K10251+K00232+K00232+K00232+K00232+K00232+K00232+K07513+K00059+K00232+K00232+K00232+K00059+K00232+K10703+K00059+K00232+K10251+K10256+K01068+K07513+K10256

			_transcript_66022;F01_transcript_67738;F01_transcript_67882;F01_transcript_70434;F01_transcript_70525;F01_transcript_72026;F01_transcript_72826;F01_transcript_73288;F01_transcript_77538;F01_transcript_80183;F01_transcript_80645;F01_transcript_82368;F01_transcript_82742;F01_transcript_83317;F01_transcript_85292;F01_transcript_88720;F01_transcript_88933;F01_transcript_90931;F01_transcript_92632;F01_transcript_92699;F01_transcript_92715;F01_transcript_93534;F01_transcript_96460;F01_transcript_96815;F01_transcript_97024;F01_transcript_97408;F01_transcript_97838;F01_transcript_98034;F01_transcript_98462;F01_transcript_99157;	
Carbon metabolism	ko01200	1946	F01_transcript_100005;F01_transcript_100118;F01_transcript_10021;F01_transcript_100214;F01_transcript_100230;F01_transcript_100238;F01_transcript_100254;F01_transcript_100288;F01_transcript_100330;F01_transcript_100430;F01_transcript_10047;F01_transcript_100647;F01_transcript_100783;F01_transcript_100797;F01_transcript_100809;F01_transcript_100825;F01_transcript_100871;F01_transcript_100917;F01_transcript_101071;F01_transcript_101187;F01_transcript_101218;F01_transcript_101296;F01_transcript_101368;F01_transcript_101371;F01_transcript_101386;F01_transcript_101409;F01_transcript_101473;F01_transcript_101574;F01_transcript_10158;F01_transcript_101685;F01_transcript_101795;F01_transcript_101871;F01_transcript_102048;F01_transcript_102135;F01_transcript_102189;F01_transcript_102294;F01_transcript_102306;F01_transcript_102370;F01_transcript_102373;F01_transcript_102477;F01_transcript_10266;F01_transcript_102681;F01_transcript_102714;F01_transcript_102731;F01_transcript_102826;F01_transcript_102834;F01_transcript_102845;F01_transcript_102902;F01_transcript_103175;F01_transcript_103227;F01_transcript_103331;F01_transcript_103345;F01_transcript_103366;F01_transcript_103394;F01_transcript_10347;F01_transcript_103488;F01_transcript_103532;F01_transcript_103571;F01_transcript_103634;F01_transcript_103675;F01_transcript_103700;F01_transcript_103774;F01_transcript_103795;F01_transcript_103802;F01_transcript_104096;F01_transcript_104167;F01_transcript_104170;F01_transcript_104300;F01_transcript_104320;F01_transcript_104346;F01_transcri	K00026+K00600+K03781+K01803+K00058+K00615+K01679+K01962+K00600+K01783+K14272+K15918+K00873+K00036+K15634+K00615+K00281+K01595+K00600+K00873+K01647+K00640+K01601+K00281+K00261+K01962+K14454+K00850+K01895+K01006+K00297+K00121+K14272+K00600+K01783+K01899+K00281+K00162+K01803+K11517+K14272+K01807+K00600+K00162+K01783+K00162+K01623+K01689+K05605+K00600+K00615+K15918+K00033+K00026+K00029+K00026+K14272+K01810+K00134+K00615+K01738+K11517+K01100+K11517+K00873+K00600+K01602+K01070+K00026+K05605+K00121+K00615+K00627+K05605+K00382+K03841+K00850+K01962+K00948+K01738+K01647+K01623+K05605+K01602+K00626+K00036+K01681+K01807+K00162+K00873+K00615+K01807+K00161+K14454+K01807+K01962+K01601+K15893+K00028+K01962+K03841+K00600+K01100+K00281+K00028+K01783+K01679+K03781+K00028+K00051+K00615+K00058+K01100+K14272+K01602+K03781+K00658+K00600+K00873+K11517+K01601+K03781+K05298+K00382+K00281+K00615+K03841+K18121+K00600+K00600+K00026+K05298+K00850+K01803+K01602+K03841+K00028+K00281+K05298+K00600+K00640+K00036+K00026+K00830+K00029+K01623+K01962+K00161+K15919+K14272+K00261+K15893+K03841+K00615+K00850+K00615+K00161+K00029+K00029+K00134+K00600+K00029+K03781+K00164+K01100+K14272+K00281+K00382+K00873+K15634+K05605+K14272+K01803+K00281+K00600+K01647+K03841+K00164+K1

	pt_104489;F01_transcript_10450;F01_transcript_104691;F01_transcript_104696;F01_transcript_104746;F01_transcript_104756;F01_transcript_104986;F01_transcript_105202;F01_transcript_105258;F01_transcript_105281;F01_transcript_105291;F01_transcript_105365;F01_transcript_105377;F01_transcript_105385;F01_transcript_105403;F01_transcript_105445;F01_transcript_105634;F01_transcript_105645;F01_transcript_105726;F01_transcript_105738;F01_transcript_10578;F01_transcript_105919;F01_transcript_105972;F01_transcript_106156;F01_transcript_106166;F01_transcript_106220;F01_transcript_106422;F01_transcript_106462;F01_transcript_106631;F01_transcript_106677;F01_transcript_106765;F01_transcript_106967;F01_transcript_106970;F01_transcript_107003;F01_transcript_107013;F01_transcript_107113;F01_transcript_107153;F01_transcript_107228;F01_transcript_107406;F01_transcript_107604;F01_transcript_107638;F01_transcript_107677;F01_transcript_107906;F01_transcript_107931;F01_transcript_107952;F01_transcript_107966;F01_transcript_107970;F01_transcript_10799;F01_transcript_108019;F01_transcript_108062;F01_transcript_1081;F01_transcript_108186;F01_transcript_108200;F01_transcript_108216;F01_transcript_108345;F01_transcript_108371;F01_transcript_108502;F01_transcript_108512;F01_transcript_108584;F01_transcript_108602;F01_transcript_108617;F01_transcript_108659;F01_transcript_108868;F01_transcript_108947;F01_transcript_109006;F01_transcript_109032;F01_transcript_109050;F01_transcript_109068;F01_transcript_109150;F01_transcript_109254;F01_transcript_109267;F01_transcript_109271;F01_transcript_109283;F01_transcript_109418;F01_transcript_109495;F01_transcript_109676;F01_transcript_10985;F01_transcript_109885;F01_transcript_109993;F01_transcript_110137;F01_transcript_110169;F01_transcript_110477;F01_transcript_110541;F01_transcript_110549;F01_transcript_110559;F01_transcript_110771;F01_transcript_110879;F01_transcript_111222;F01_transcript_111451;F01_transcript_111485;F01_transcript_111549;F01_transcript_111739;F01_transcript_111760;F01_transcript_111778;F01_transcript_11180;F01_transcript_11182;F01_transcript_112020;F01_transcript_112117;F01_transcript_11216;F01_transcript_11219;F01_transcript_112210;F01_transcript_112260;F01_transcript_112269;F01_transcript	1517+K00033+K01079+K14272+K00281+K01738+K01070+K00640+K14455+K00029+K01738+K01689+K00058+K00234+K00600+K00281+K01595+K03781+K03781+K00627+K01738+K01807+K00031+K01810+K00030+K05605+K00281+K00058+K00029+K01738+K01595+K00026+K00382+K00948+K01602+K03781+K01810+K01810+K03781+K01810+K00036+K00873+K00026+K00382+K01810+K15893+K00161+K00281+K14272+K05605+K00234+K00814+K00281+K01647+K14272+K01623+K00033+K01595+K01647+K00615+K01783+K00026+K00600+K01810+K00162+K01610+K00616+K00030+K00600+K00873+K00161+K01689+K00234+K01057+K00855+K00281+K01455+K00281+K00830+K01738+K00161+K00028+K01647+K00026+K00600+K00627+K11517+K03841+K00600+K01601+K00031+K01807+K03781+K13034+K01807+K00873+K05298+K00026+K00382+K00031+K00830+K00029+K01623+K14455+K11517+K03841+K00600+K01100+K01602+K00640+K01738+K01807+K00605+K00615+K01899+K00873+K13034+K03781+K01783+K00121+K00140+K01938+K00600+K01810+K00855+K01623+K01681+K18121+K03781+K15633+K00140+K14272+K00600+K01601+K00029+K00627+K00600+K00830+K01602+K00615+K11517+K15919+K00134+K00605+K15893+K00873+K18121+K05605+K03781+K00927+K03781+K05605+K00029+K00235+K00297+K14272+K01647+K00600+K00600+K00028+K00281+K01100+K05298+K03781+K00873+K01783+K05605+K01623+K00234+K11517+K01738+K15893+K15918+K00615+K00600+K15633+K01100+K14272+K01738+K00281+K00026+K03841+K00844+K14455+K15918+K05605+K05605+K00627+K00026+K01602+K01602+K05298+K01602+K00281+K00281+K01647+K01738+K03781+K00600+K03781+K00830+K01810+K01100+K01834+K03781+K05605+K01738+K15918+K01803+K00627+K00026+K00028+K00122+K00134+K01006+K00122+K01783+K00600+K01895+K03841+K00235+K01783+K01899+K00627+K01623+K00873+K00025+K00029+K00026+K00600+K15893+K14272+K19269+K00600+K00029+K05298+K00058+K00605+K00140+K00600+K00281+K19269+K03781+K14455+K15633+K00029+K00605+K03781+K00658+K15893+K01962+K01647+K00851+K00058+K01100+K03781+K00600+K00600+K11517+K05605+K03781

	_112494;F01_transcript_112563;F01_transcript_112623;F01_transcript_112669;F01_transcript_113031;F01_transcript_113095;F01_transcript_113356;F01_transcript_113436;F01_transcript_113831;F01_transcript_113837;F01_transcript_114030;F01_transcript_114127;F01_transcript_114327;F01_transcript_114452;F01_transcript_114540;F01_transcript_114551;F01_transcript_114570;F01_transcript_114614;F01_transcript_114667;F01_transcript_114677;F01_transcript_114689;F01_transcript_114712;F01_transcript_11473;F01_transcript_114737;F01_transcript_114898;F01_transcript_114920;F01_transcript_114958;F01_transcript_114966;F01_transcript_115044;F01_transcript_115077;F01_transcript_115085;F01_transcript_115186;F01_transcript_115188;F01_transcript_115322;F01_transcript_115361;F01_transcript_115427;F01_transcript_115594;F01_transcript_115624;F01_transcript_115763;F01_transcript_115870;F01_transcript_115917;F01_transcript_116260;F01_transcript_116289;F01_transcript_116320;F01_transcript_116347;F01_transcript_116357;F01_transcript_116379;F01_transcript_116381;F01_transcript_116451;F01_transcript_116518;F01_transcript_116525;F01_transcript_116556;F01_transcript_116591;F01_transcript_116648;F01_transcript_116665;F01_transcript_116740;F01_transcript_116793;F01_transcript_116855;F01_transcript_116945;F01_transcript_117148;F01_transcript_117151;F01_transcript_117248;F01_transcript_117298;F01_transcript_117319;F01_transcript_11734;F01_transcript_117388;F01_transcript_117415;F01_transcript_117422;F01_transcript_117555;F01_transcript_117574;F01_transcript_117586;F01_transcript_117662;F01_transcript_11767;F01_transcript_117699;F01_transcript_11770;F01_transcript_117732;F01_transcript_117798;F01_transcript_117804;F01_transcript_117820;F01_transcript_117834;F01_transcript_117863;F01_transcript_118053;F01_transcript_118084;F01_transcript_118373;F01_transcript_118376;F01_transcript_118433;F01_transcript_118584;F01_transcript_118657;F01_transcript_118710;F01_transcript_118804;F01_transcript_11882;F01_transcript_118820;F01_transcript_118900;F01_transcript_118927;F01_transcript_119014;F01_transcript_119034;F01_transcript_119183;F01_transcript_119285;F01_transcript_119306;F01_transcript_119312;F01_transcript_119	+K00058+K00600+K00658+K14272+K01810+K00600+K05298+K00600+K01602+K00028+K01602+K00927+K00615+K00615+K01803+K01803+K00948+K15918+K00281+K00261+K00927+K00873+K13034+K00948+K11517+K00026+K00134+K01689+K11517+K01602+K00029+K01057+K14454+K00281+K00162+K01810+K00026+K00615+K01803+K00615+K00844+K01455+K01962+K00927+K00025+K00873+K01455+K00030+K00121+K03781+K01783+K00850+K00121+K03841+K00036+K15918+K15633+K00600+K01602+K01610+K00234+K01938+K00615+K00626+K00029+K01681+K01100+K01803+K00855+K00600+K00029+K01738+K01810+K00036+K00600+K01807+K00121+K01807+K00026+K00131+K05605+K00616+K00600+K00600+K00948+K03781+K18121+K01623+K00140+K00281+K00281+K11517+K05605+K00029+K01057+K15919+K03781+K00134+K01595+K00927+K00026+K03781+K01895+K00036+K00131+K15633+K01899+K00600+K01100+K00281+K15893+K05298+K00873+K15634+K03781+K00121+K01738+K15633+K05298+K19269+K15893+K01455+K00600+K00605+K00030+K11517+K11517+K00382+K00029+K01006+K01807+K00873+K00030+K03781+K14272+K01807+K11517+K01595+K00627+K05298+K00026+K05605+K00134+K01610+K00030+K05298+K00600+K01623+K11517+K01595+K14272+K15919+K15918+K01807+K01623+K15893+K00281+K01738+K00162+K00121+K03841+K00281+K00830+K01938+K00028+K19269+K00600+K00850+K14272+K00031+K00026+K00600+K00281+K00927+K03781+K00026+K15633+K03841+K00026+K01623+K00844+K01610+K01602+K00058+K15893+K01647+K00026+K00658+K00382+K01681+K00028+K11517+K01738+K01807+K00026+K00030+K05298+K00830+K01602+K00382+K00600+K05605+K15893+K14454+K00281+K01803+K01738+K00029+K00615+K01738+K01623+K00948+K01895+K00134+K00281+K05605+K01100+K01057+K00297+K03781+K00036+K15893+K01738+K13034+K03781+K14454+K00161+K01689+K01623+K01807+K01681+K01623+K00140+K00131+K15919+K00140+K00873+K03781+K01623+K01602+K01602+K18121+K01895+K00927+K00161+K15918+K01079+K03781+K05298+K01689+K03781+K00131+K01738+K037

	34;F01_transcript_119351;F01_transcript_119411;F01_transcript_11951;F01_transcript_119534;F01_transcript_119569;F01_transcript_119638;F01_transcript_119854;F01_transcript_119869;F01_transcript_119894;F01_transcript_119939;F01_transcript_119952;F01_transcript_120034;F01_transcript_120050;F01_transcript_120073;F01_transcript_120145;F01_transcript_120193;F01_transcript_120212;F01_transcript_120459;F01_transcript_120543;F01_transcript_120942;F01_transcript_120997;F01_transcript_121105;F01_transcript_121130;F01_transcript_121145;F01_transcript_121237;F01_transcript_121270;F01_transcript_121306;F01_transcript_121401;F01_transcript_121500;F01_transcript_121571;F01_transcript_121896;F01_transcript_121955;F01_transcript_121960;F01_transcript_122012;F01_transcript_122090;F01_transcript_122101;F01_transcript_122115;F01_transcript_122132;F01_transcript_122152;F01_transcript_122215;F01_transcript_122331;F01_transcript_122499;F01_transcript_122578;F01_transcript_122782;F01_transcript_122863;F01_transcript_122872;F01_transcript_122893;F01_transcript_12302;F01_transcript_123124;F01_transcript_12318;F01_transcript_123183;F01_transcript_123185;F01_transcript_123251;F01_transcript_123349;F01_transcript_12336;F01_transcript_123463;F01_transcript_123478;F01_transcript_123542;F01_transcript_123647;F01_transcript_123753;F01_transcript_123773;F01_transcript_123848;F01_transcript_124153;F01_transcript_124195;F01_transcript_124200;F01_transcript_124288;F01_transcript_124392;F01_transcript_124404;F01_transcript_124464;F01_transcript_124491;F01_transcript_124506;F01_transcript_124658;F01_transcript_124944;F01_transcript_125285;F01_transcript_125334;F01_transcript_125370;F01_transcript_125371;F01_transcript_12547;F01_transcript_12550;F01_transcript_125563;F01_transcript_125758;F01_transcript_125768;F01_transcript_125818;F01_transcript_125984;F01_transcript_126030;F01_transcript_126086;F01_transcript_126146;F01_transcript_126185;F01_transcript_126285;F01_transcript_126314;F01_transcript_126346;F01_transcript_126405;F01_transcript_126482;F01_transcript_126534;F01_transcript_126567;F01_transcript_126766;F01_transcript_126769;F01_transcript_1269;F01_transcript_126994;F01_transcript_127049;F01_transcript_127193;F01_transcript_127239;F01_transcript_1272	81+K05605+K00036+K14272+K00600+K00297+K01803+K00234+K03781+K00600+K00927+K01738+K03781+K01602+K15919+K05298+K00626+K00058+K01738+K00131+K00164+K14455+K00927+K01623+K00131+K03781+K01602+K11517+K03781+K00281+K00850+K01938+K01595+K00850+K00161+K00121+K15919+K00281+K00600+K05298+K00026+K00605+K00873+K00850+K00028+K00600+K01963+K00873+K00850+K03781+K00036+K00615+K00028+K00600+K01623+K15893+K01803+K00850+K00830+K00873+K14272+K00873+K15893+K05298+K00026+K15918+K01100+K01807+K18121+K14272+K00600+K00873+K03841+K01647+K00281+K11517+K00134+K01100+K00600+K11517+K00948+K11517+K15918+K05298+K00026+K01738+K00281+K01647+K03781+K00036+K00281+K00029+K00844+K00234+K05298+K01681+K00029+K00600+K11517+K00029+K00026+K00162+K00615+K00161+K05605+K00026+K00134+K00382+K00162+K00026+K01100+K19269+K00028+K00164+K01647+K00281+K03781+K03781+K00948+K00600+K00873+K00029+K01100+K01899+K00121+K01810+K00026+K01783+K00600+K05605+K15893+K00036+K00615+K03841+K01610+K00029+K00131+K01962+K00036+K01834+K00850+K11517+K01810+K00600+K00830+K01783+K11517+K01623+K03841+K01738+K00029+K00028+K00058+K00131+K03781+K00873+K00162+K01803+K00261+K03781+K15919+K01006+K03841+K00626+K00830+K14272+K00026+K03781+K00600+K03781+K01689+K01100+K01623+K01647+K00830+K01610+K00029+K11517+K00140+K00121+K00600+K00131+K00029+K00281+K00029+K11517+K00600+K01689+K00029+K01610+K01100+K01610+K15918+K01938+K00605+K00029+K00036+K00234+K00036+K01006+K15918+K15893+K01810+K00058+K00140+K01689+K01623+K00028+K00058+K00028+K00029+K00600+K00281+K00873+K01899+K11517+K01100+K05298+K15918+K00615+K01810+K00927+K00029+K03781+K00814+K01100+K00297+K01803+K03781+K05298+K00029+K00058+K00281+K11517+K01754+K00627+K05605+K03781+K00026+K00029+K00382+K00627+K01100+K00605+K00058+K15919+K00873+K00927+K00873+K00873+K03781+K00131+K00029+K03781+K15918+K03781+K01738+K00600+K03781+K00029+K00600+K

	97;F01_transcript_127607;F01_transcript_127687;F01_transcript_127751;F01_transcript_127785;F01_transcript_127834;F01_transcript_127884;F01_transcript_127971;F01_transcript_127989;F01_transcript_128000;F01_transcript_128187;F01_transcript_128243;F01_transcript_128256;F01_transcript_12827;F01_transcript_128312;F01_transcript_128590;F01_transcript_128611;F01_transcript_128648;F01_transcript_128717;F01_transcript_129051;F01_transcript_129084;F01_transcript_129206;F01_transcript_129207;F01_transcript_129302;F01_transcript_129651;F01_transcript_129897;F01_transcript_129946;F01_transcript_130000;F01_transcript_130017;F01_transcript_130078;F01_transcript_130127;F01_transcript_130382;F01_transcript_130447;F01_transcript_130485;F01_transcript_130572;F01_transcript_130612;F01_transcript_130673;F01_transcript_130688;F01_transcript_130708;F01_transcript_130742;F01_transcript_130773;F01_transcript_130895;F01_transcript_130923;F01_transcript_130965;F01_transcript_131015;F01_transcript_131043;F01_transcript_131057;F01_transcript_13108;F01_transcript_131196;F01_transcript_131343;F01_transcript_131432;F01_transcript_131459;F01_transcript_13154;F01_transcript_131587;F01_transcript_131837;F01_transcript_131892;F01_transcript_131895;F01_transcript_131925;F01_transcript_132156;F01_transcript_132238;F01_transcript_132241;F01_transcript_132279;F01_transcript_132298;F01_transcript_132310;F01_transcript_132483;F01_transcript_132504;F01_transcript_132527;F01_transcript_132921;F01_transcript_133019;F01_transcript_133162;F01_transcript_133265;F01_transcript_133272;F01_transcript_133379;F01_transcript_133486;F01_transcript_133545;F01_transcript_13355;F01_transcript_133564;F01_transcript_133587;F01_transcript_13368;F01_transcript_133891;F01_transcript_133996;F01_transcript_134020;F01_transcript_134077;F01_transcript_134102;F01_transcript_13417;F01_transcript_134216;F01_transcript_134405;F01_transcript_134656;F01_transcript_134955;F01_transcript_135113;F01_transcript_135187;F01_transcript_135338;F01_transcript_135362;F01_transcript_135366;F01_transcript_135405;F01_transcript_135578;F01_transcript_135610;F01_transcript_135612;F01_transcript_135613;F01_transcript_135698;F01_transcript_135726;F	00873+K00382+K00873+K01803+K00850+K03781+K01900+K00028+K00600+K03781+K00873+K00382+K14272+K01754+K00600+K01602+K00058+K00850+K00873+K00058+K00029+K15633+K00873+K00030+K00600+K00844+K01681+K00600+K03781+K00164+K00600+K01602+K01610+K00627+K01595+K00600+K03781+K14272+K03781+K00382+K00627+K01610+K01679+K03781+K00036+K00382+K01595+K00844+K00850+K01602+K15634+K03781+K00873+K00600+K00873+K00873+K03781+K00033+K00600+K14454+K00036+K00140+K00948+K00036+K14272+K01623+K00873+K00600+K01647+K00600+K00382+K00140+K00873+K00281+K00600+K00036+K01689+K00873+K03781+K03781+K00600+K01783+K00873+K00140+K00600+K00281+K01810+K00600+K00627+K01100+K00873+K00036+K01006+K00600+K00033+K00873+K05298+K03781+K05298+K00600+K15918+K00600+K00627+K00600+K00036+K00600+K01961+K01455+K03781+K01595+K03781+K14454+K00600+K00033+K01689+K15634+K03781+K01647+K00030+K00026+K00600+K15634+K00627+K00131+K00927+K00658+K00234+K01455+K00033+K00658+K03781+K00140+K00627+K05298+K01623+K03781+K14272+K00627+K00033+K01595+K00131+K00927+K03781+K14272+K05605+K00873+K00033+K00948+K03781+K15918+K14455+K00051+K00600+K01738+K00927+K01595+K00281+K00873+K00134+K00600+K00873+K00658+K11517+K14272+K05298+K00948+K05298+K05298+K14272+K00051+K00026+K01100+K00162+K00605+K03781+K00640+K00030+K00131+K01900+K05298+K01100+K01623+K01689+K00026+K00382+K03781+K00162+K00927+K03781+K00605+K05298+K00031+K01834+K05298+K15918+K00948+K03781+K00640+K00161+K00134+K00927+K00131+K03781+K05298+K01455+K00600+K01595+K01610+K05298+K01602+K01647+K00161+K05298+K14272+K00058+K00831+K00616+K00855+K00162+K01623+K00162+K05298+K00615+K00855+K00026+K01455+K14455+K00600+K00616+K00121+K01100+K01100+K01623+K00600+K14455+K00605+K05605+K00051+K00134+K00026+K05605+K00600+K00161+K00051+K00162+K19269+K01623+K13034+K11517+K00626+K00616+K00162+K00927+K11517

	01_transcript_135767;F01_transcript_135831;F01_transcript_135864;F01_transcript_135878;F01_transcript_135955;F01_transcript_135995;F01_transcript_136111;F01_transcript_136113;F01_transcript_136192;F01_transcript_136221;F01_transcript_136325;F01_transcript_136746;F01_transcript_136826;F01_transcript_136831;F01_transcript_136852;F01_transcript_136868;F01_transcript_136877;F01_transcript_136997;F01_transcript_137134;F01_transcript_13740;F01_transcript_137500;F01_transcript_137514;F01_transcript_137621;F01_transcript_137923;F01_transcript_138105;F01_transcript_138114;F01_transcript_138133;F01_transcript_138295;F01_transcript_138307;F01_transcript_138326;F01_transcript_138396;F01_transcript_138453;F01_transcript_138594;F01_transcript_138596;F01_transcript_138795;F01_transcript_138810;F01_transcript_138914;F01_transcript_139092;F01_transcript_139169;F01_transcript_139300;F01_transcript_139464;F01_transcript_139573;F01_transcript_139604;F01_transcript_139738;F01_transcript_139819;F01_transcript_139822;F01_transcript_140038;F01_transcript_140205;F01_transcript_140217;F01_transcript_140250;F01_transcript_140307;F01_transcript_140347;F01_transcript_1404;F01_transcript_140463;F01_transcript_140486;F01_transcript_140525;F01_transcript_140526;F01_transcript_140744;F01_transcript_14076;F01_transcript_140783;F01_transcript_140859;F01_transcript_140868;F01_transcript_140879;F01_transcript_140967;F01_transcript_141140;F01_transcript_141350;F01_transcript_141376;F01_transcript_141418;F01_transcript_141489;F01_transcript_141510;F01_transcript_141555;F01_transcript_141575;F01_transcript_141657;F01_transcript_141782;F01_transcript_141836;F01_transcript_141901;F01_transcript_141996;F01_transcript_142036;F01_transcript_142050;F01_transcript_142290;F01_transcript_142610;F01_transcript_142711;F01_transcript_142720;F01_transcript_142863;F01_transcript_14292;F01_transcript_142920;F01_transcript_142960;F01_transcript_143118;F01_transcript_143180;F01_transcript_143349;F01_transcript_143362;F01_transcript_143480;F01_transcript_143511;F01_transcript_143572;F01_transcript_144117;F01_transcript_144230;F01_transcript_144458;F01_transcript_144469;F01_transcript_144482;F01_transcript_144497;F01_transcript_144572;F01_transcript_144688;F01_transcript_14470	+K00161+K00121+K00626+K11517+K13034+K03841+K00122+K01623+K00026+K00830+K11517+K11517+K03781+K01595+K18121+K03781+K01623+K01807+K00026+K00025+K00927+K00626+K00927+K00121+K11517+K01783+K11517+K00626+K18121+K01070+K01738+K00830+K01602+K11517+K01738+K11517+K05605+K00134+K00605+K01738+K00162+K01807+K11517+K11517+K01100+K05605+K01803+K01100+K00134+K01807+K00026+K15893+K01803+K00134+K00927+K00605+K00627+K00026+K00121+K11517+K00616+K03841+K00640+K11517+K03781+K11517+K00134+K03841+K00025+K00134+K01738+K00026+K00134+K01006+K02160+K00134+K19269+K00025+K01807+K00134+K00121+K01738+K00025+K01803+K19269+K18121+K01899+K01623+K05298+K03841+K01783+K01006+K01738+K01807+K00134+K01738+K02160+K02160+K01807+K01738+K19269+K00640+K18121+K01057+K01057+K00600+K01079+K01783+K01783+K01006+K01079+K01070+K15919+K01057+K01803+K01623+K01079+K00640+K00640+K01070+K01079+K00235+K15919+K01602+K00382+K03781+K01807+K00615+K01803+K01070+K03841+K01803+K01807+K00605+K01807+K00851+K01623+K00851+K00281+K01602+K01962+K01006+K01602+K03781+K01803+K01602+K00134+K00121+K00134+K00026+K01602+K01623+K01623+K01602+K01623+K01602+K00600+K01602+K03781+K01623+K01602+K01602+K01602+K13034+K01595+K00927+K03781+K00249+K15634+K00605+K03841+K01100+K00036+K00162+K03781+K00164+K15633+K05605+K14272+K05298+K00626+K01647+K00873+K05605+K00031+K01006+K01100+K00031+K00615+K00927+K18121+K00615+K00134+K00029+K00873+K01100+K00658+K01961+K00600+K00927+K01602+K00028+K01803+K05605+K00028+K01803+K00121+K01623+K01738+K19269+K00600+K15893+K00122+K00281+K00235+K05605+K01595+K00281+K01962+K01070+K00850+K15634+K01623+K01783+K00850+K01595+K00814+K00234+K01057+K00927+K01810+K00600+K00140+K11517+K00164+K05298+K00382+K00030+K00627+K03841+K00615+K00831+K00281+K03781+K01962+K00626+K00162+K00030+K05298+K00281+K00814+K14272+K01100+K01738+K00026+K01006+K00121+K15633+K01602+K03781+K00

	6;F01_transcript_144740;F01_transcript_144853;F01_transcript_145043;F01_transcript_145058;F01_transcript_145193;F01_transcript_145271;F01_transcript_145324;F01_transcript_145345;F01_transcript_145524;F01_transcript_145616;F01_transcript_145718;F01_transcript_145724;F01_transcript_145805;F01_transcript_145851;F01_transcript_145854;F01_transcript_145862;F01_transcript_146107;F01_transcript_146217;F01_transcript_146237;F01_transcript_146244;F01_transcript_146329;F01_transcript_146447;F01_transcript_14669;F01_transcript_146770;F01_transcript_146785;F01_transcript_146840;F01_transcript_146888;F01_transcript_146963;F01_transcript_147008;F01_transcript_147154;F01_transcript_147232;F01_transcript_147236;F01_transcript_147408;F01_transcript_147432;F01_transcript_147445;F01_transcript_147492;F01_transcript_147603;F01_transcript_147898;F01_transcript_147987;F01_transcript_148041;F01_transcript_148049;F01_transcript_148274;F01_transcript_148710;F01_transcript_148713;F01_transcript_148941;F01_transcript_149184;F01_transcript_149233;F01_transcript_149234;F01_transcript_149398;F01_transcript_149403;F01_transcript_149413;F01_transcript_149508;F01_transcript_149827;F01_transcript_149909;F01_transcript_149933;F01_transcript_149972;F01_transcript_150046;F01_transcript_150123;F01_transcript_150228;F01_transcript_150241;F01_transcript_150299;F01_transcript_150492;F01_transcript_150639;F01_transcript_150811;F01_transcript_150940;F01_transcript_151028;F01_transcript_151069;F01_transcript_151079;F01_transcript_151234;F01_transcript_151300;F01_transcript_151431;F01_transcript_151518;F01_transcript_151553;F01_transcript_151605;F01_transcript_151674;F01_transcript_151732;F01_transcript_151839;F01_transcript_151893;F01_transcript_15195;F01_transcript_152000;F01_transcript_152060;F01_transcript_152349;F01_transcript_152454;F01_transcript_152474;F01_transcript_152726;F01_transcript_152795;F01_transcript_152798;F01_transcript_152812;F01_transcript_152838;F01_transcript_153015;F01_transcript_153307;F01_transcript_153331;F01_transcript_153422;F01_transcript_153435;F01_transcript_15347;F01_transcript_153523;F01_transcript_153615;F01_transcript_153773;F01_transcript_153780;F01_transcript_153807;	600+K00162+K00600+K03781+K01679+K03781+K00121+K11517+K00850+K03781+K00281+K15918+K00026+K01783+K00235+K00850+K00382+K00600+K01455+K00600+K00029+K00844+K03781+K01962+K00658+K00873+K14272+K00058+K00873+K00600+K01681+K01595+K01807+K00235+K03781+K01100+K01610+K00658+K15893+K00029+K00058+K00382+K00600+K11517+K00281+K01623+K03781+K01807+K00030+K00873+K01623+K01810+K05298+K00873+K03781+K15633+K03841+K00600+K19269+K01070+K00615+K00140+K00873+K01647+K00600+K00830+K01455+K03781+K00026+K01681+K00844+K00814+K14272+K00026+K00615+K00600+K14454+K00830+K15633+K01610+K00234+K11517+K01810+K00134+K00033+K00162+K00029+K14272+K03841+K00600+K01006+K03781+K00249+K11517+K00615+K01938+K00026+K00640+K03781+K00140+K01807+K13034+K00134+K03841+K13034+K01783+K00033+K00033+K01057+K11517+K00026+K00026+K00626+K01961+K00627+K00851+K00615+K00030+K01738+K00026+K01963+K00600+K01100+K00030+K01689+K00844+K05298+K00615+K00261+K11517+K00249+K03841+K03781+K05298+K01647+K01738+K00927+K00851+K03841+K00627+K01623+K00814+K00036+K05298+K05298+K00025+K00382+K00281+K00600+K11517+K05298+K00615+K11517+K00058+K15633+K00600+K00627+K00281+K05605+K00029+K00033+K00850+K00026+K01810+K00029+K00029+K00051+K00600+K00658+K15893+K00382+K01100+K05605+K00927+K00615+K01810+K00161+K00029+K03841+K00615+K03781+K00297+K00281+K01100+K01623+K01738+K14272+K00830+K00249+K00029+K00600+K11517+K00850+K03781+K00600+K15918+K00281+K00031+K00600+K00029+K00134+K15919+K00927+K05298+K03841+K00161+K00140+K05298+K00600+K01900+K00605+K15893+K15918+K03841+K01738+K14272+K00844+K19269+K00873+K15918+K00036+K00600+K01100+K00600+K15634+K03781+K00028+K14272+K00161+K11517+K15633+K00626+K14272+K00140+K15633+K01679+K01623+K13034+K00164+K00605+K01681+K01962+K00026+K00162+K01057+K14455+K00026+K01681+K01689+K00615+K00026+K00626+K00281+K01810+K

	F01_transcript_153837;F01_transcript_153897;F01_transcript_153933;F01_transcript_154172;F01_transcript_154298;F01_transcript_154314;F01_transcript_154347;F01_transcript_154457;F01_transcript_154485;F01_transcript_154524;F01_transcript_154540;F01_transcript_154618;F01_transcript_154709;F01_transcript_154730;F01_transcript_154770;F01_transcript_154837;F01_transcript_154869;F01_transcript_154872;F01_transcript_154897;F01_transcript_155034;F01_transcript_15505;F01_transcript_155116;F01_transcript_155306;F01_transcript_155318;F01_transcript_155366;F01_transcript_155367;F01_transcript_155558;F01_transcript_155594;F01_transcript_155685;F01_transcript_15571;F01_transcript_155840;F01_transcript_156057;F01_transcript_1562;F01_transcript_156344;F01_transcript_156346;F01_transcript_156435;F01_transcript_156464;F01_transcript_156564;F01_transcript_156653;F01_transcript_15677;F01_transcript_156864;F01_transcript_157013;F01_transcript_157081;F01_transcript_15726;F01_transcript_157503;F01_transcript_157674;F01_transcript_157893;F01_transcript_158132;F01_transcript_158189;F01_transcript_158216;F01_transcript_158283;F01_transcript_158337;F01_transcript_158374;F01_transcript_158425;F01_transcript_158451;F01_transcript_158480;F01_transcript_158513;F01_transcript_158617;F01_transcript_158628;F01_transcript_158716;F01_transcript_158736;F01_transcript_15880;F01_transcript_159010;F01_transcript_159049;F01_transcript_159070;F01_transcript_159165;F01_transcript_159245;F01_transcript_159272;F01_transcript_159358;F01_transcript_159443;F01_transcript_159651;F01_transcript_159748;F01_transcript_160034;F01_transcript_160044;F01_transcript_16005;F01_transcript_160119;F01_transcript_160122;F01_transcript_16018;F01_transcript_160193;F01_transcript_160206;F01_transcript_160238;F01_transcript_160312;F01_transcript_160408;F01_transcript_160410;F01_transcript_160426;F01_transcript_160446;F01_transcript_160612;F01_transcript_160623;F01_transcript_160634;F01_transcript_16074;F01_transcript_160895;F01_transcript_160955;F01_transcript_161004;F01_transcript_161267;F01_transcript_161372;F01_transcript_161388;F01_transcript_161406;F01_transcript_161478;F01_transcript_161508;F01_transcript_161671;F01_transcript_161709;F01_transcript_161713;F01_transcript_161905;F0	15893+K05298+K00600+K15893+K00615+K00025+K11517+K00140+K15893+K00382+K00134+K00140+K00026+K00658+K00026+K05605+K11517+K01807+K00121+K00626+K00948+K05605+K00134+K14454+K00600+K01057+K05605+K01807+K03781+K15633+K14454+K05298+K01895+K00033+K00600+K01810+K00600+K00873+K01962+K01100+K00382+K01961+K01738+K01647+K00161+K00873+K00863+K00281+K01623+K01803+K00927+K03781+K00026+K00626+K01738+K00026+K00600+K03781+K00927+K11517+K14272+K00855+K01610+K00028+K01962+K00281+K01738+K01938+K00281+K00615+K00873+K01100+K01647+K00844+K00873+K03781+K00029+K00830+K00600+K00814+K00281+K01738+K03781+K01895+K00030+K14455+K00281+K14272+K00873+K01807+K01623+K01070+K01623+K15634+K00281+K00615+K00121+K11517+K14454+K00033+K00873+K00281+K00600+K01602+K00600+K05605+K00134+K01807+K00026+K00873+K00029+K05298+K11517+K11517+K01803+K00030+K03781+K00600+K01738+K01783+K05298+K00036+K00029+K00830+K00640+K15893+K00121+K01962+K00616+K00382+K00814+K05605+K00026+K00121+K15918+K01962+K03781+K00616+K11517+K00249+K03781+K00382+K05605+K02160+K14272+K14272+K00281+K00873+K14455+K14272+K14272+K03781+K00600+K00281+K00600+K00927+K01610+K00600+K00281+K05298+K13034+K05298+K14272+K03781+K01647+K00026+K01810+K01595+K00162+K00026+K00855+K00051+K00026+K14272+K00600+K00615+K00600+K11517+K00855+K01738+K00605+K00029+K00297+K05605+K00281+K00600+K11517+K00026+K00281+K14272+K00029+K03781+K01006+K00036+K00600+K00626+K03781+K14454+K01100+K01938+K00234+K00873+K00600+K05605+K00382+K00281+K01601+K00600+K00026+K01689+K00028+K00031+K01100+K00844+K19269+K01595+K14272+K00281+K00162+K01647+K00851+K00036+K00026+K01738+K01610

	1_transcript_161915;F01_transcript_161918;F01_transcript_161943;F01_transcript_162048;F01_transcript_162211;F01_transcript_162362;F01_transcript_16246;F01_transcript_162492;F01_transcript_162554;F01_transcript_162570;F01_transcript_162648;F01_transcript_162722;F01_transcript_162736;F01_transcript_162763;F01_transcript_162804;F01_transcript_162882;F01_transcript_162906;F01_transcript_16293;F01_transcript_163028;F01_transcript_163092;F01_transcript_163170;F01_transcript_163184;F01_transcript_163191;F01_transcript_163468;F01_transcript_1635;F01_transcript_163559;F01_transcript_163584;F01_transcript_163703;F01_transcript_163735;F01_transcript_163795;F01_transcript_163836;F01_transcript_16385;F01_transcript_163897;F01_transcript_164021;F01_transcript_164032;F01_transcript_164460;F01_transcript_164521;F01_transcript_164525;F01_transcript_164546;F01_transcript_164548;F01_transcript_164702;F01_transcript_164718;F01_transcript_164751;F01_transcript_164754;F01_transcript_16479;F01_transcript_164870;F01_transcript_164977;F01_transcript_165059;F01_transcript_165106;F01_transcript_165153;F01_transcript_165154;F01_transcript_165159;F01_transcript_165169;F01_transcript_165317;F01_transcript_165336;F01_transcript_165385;F01_transcript_165396;F01_transcript_165440;F01_transcript_165501;F01_transcript_165558;F01_transcript_165757;F01_transcript_16580;F01_transcript_165904;F01_transcript_165910;F01_transcript_166121;F01_transcript_166454;F01_transcript_166475;F01_transcript_166551;F01_transcript_166597;F01_transcript_166703;F01_transcript_166915;F01_transcript_166935;F01_transcript_166964;F01_transcript_166993;F01_transcript_167098;F01_transcript_167137;F01_transcript_16722;F01_transcript_167268;F01_transcript_167454;F01_transcript_167640;F01_transcript_167672;F01_transcript_167733;F01_transcript_167751;F01_transcript_16810;F01_transcript_16829;F01_transcript_16930;F01_transcript_1702;F01_transcript_1704;F01_transcript_17183;F01_transcript_17308;F01_transcript_17385;F01_transcript_17447;F01_transcript_1751;F01_transcript_17632;F01_transcript_17645;F01_transcript_17809;F01_transcript_17879;F01_transcript_17931;F01_transcript_17942;F01_transcript_17949;F01_transcript_18092;F01_transc

ript_18138;F01_transcript_18192;F01_transcript_18500;F01_tra
nscript_18682;F01_transcript_18744;F01_transcript_19052;F01
_transcript_19222;F01_transcript_1927;F01_transcript_19340;F
01_transcript_19355;F01_transcript_19381;F01_transcript_1955
3;F01_transcript_19633;F01_transcript_19656;F01_transcript_1
968;F01_transcript_19821;F01_transcript_19876;F01_transcript
_19895;F01_transcript_20036;F01_transcript_20069;F01_transc
ript_2014;F01_transcript_20518;F01_transcript_20578;F01_tran
script_20901;F01_transcript_20911;F01_transcript_20947;F01_t
ranscript_21089;F01_transcript_2109;F01_transcript_21106;F01
_transcript_21167;F01_transcript_21586;F01_transcript_2179;F
01_transcript_21833;F01_transcript_21897;F01_transcript_2201
2;F01_transcript_22107;F01_transcript_22223;F01_transcript_2
2285;F01_transcript_22470;F01_transcript_22500;F01_transcrip
t_2257;F01_transcript_22572;F01_transcript_22758;F01_transcr
ipt_22766;F01_transcript_22856;F01_transcript_22876;F01_tra
nscript_22891;F01_transcript_22911;F01_transcript_23074;F01
_transcript_23078;F01_transcript_23115;F01_transcript_23148;
F01_transcript_23168;F01_transcript_23231;F01_transcript_232
58;F01_transcript_23299;F01_transcript_23327;F01_transcript_
23407;F01_transcript_23456;F01_transcript_23477;F01_transcri
pt_23534;F01_transcript_23618;F01_transcript_23916;F01_tran
script_23952;F01_transcript_23969;F01_transcript_24050;F01_t
ranscript_24090;F01_transcript_24261;F01_transcript_24298;F0
1_transcript_24502;F01_transcript_24513;F01_transcript_24533
;F01_transcript_24566;F01_transcript_24624;F01_transcript_24
832;F01_transcript_24919;F01_transcript_25019;F01_transcript
_25171;F01_transcript_25245;F01_transcript_25467;F01_transc
ript_25482;F01_transcript_25546;F01_transcript_25633;F01_tra
nscript_25780;F01_transcript_2583;F01_transcript_25903;F01_t
ranscript_25920;F01_transcript_26085;F01_transcript_26171;F0
1_transcript_26328;F01_transcript_26406;F01_transcript_26508
;F01_transcript_26583;F01_transcript_26643;F01_transcript_26
688;F01_transcript_2680;F01_transcript_26845;F01_transcript_
2689;F01_transcript_2693;F01_transcript_2695;F01_transcript_
26964;F01_transcript_27039;F01_transcript_27096;F01_transcri
pt_2711;F01_transcript_27140;F01_transcript_27222;F01_trans
cript_27248;F01_transcript_27298;F01_transcript_27300;F01_tr

anscript_27373;F01_transcript_27479;F01_transcript_27528;F0
1_transcript_27585;F01_transcript_27689;F01_transcript_27827
;F01_transcript_2788;F01_transcript_27931;F01_transcript_279
32;F01_transcript_28020;F01_transcript_28043;F01_transcript_
2805;F01_transcript_28090;F01_transcript_28121;F01_transcrip
t_28152;F01_transcript_28168;F01_transcript_28169;F01_trans
cript_28236;F01_transcript_28268;F01_transcript_28405;F01_tr
anscript_28474;F01_transcript_28594;F01_transcript_28633;F0
1_transcript_28678;F01_transcript_28733;F01_transcript_28741
;F01_transcript_28763;F01_transcript_28812;F01_transcript_28
828;F01_transcript_28865;F01_transcript_28894;F01_transcript
_28947;F01_transcript_28992;F01_transcript_2900;F01_transcri
pt_29058;F01_transcript_29080;F01_transcript_29149;F01_tran
script_29161;F01_transcript_29197;F01_transcript_29210;F01_t
ranscript_29214;F01_transcript_29301;F01_transcript_29309;F0
1_transcript_29341;F01_transcript_29482;F01_transcript_2952;
F01_transcript_29538;F01_transcript_29697;F01_transcript_297
60;F01_transcript_29763;F01_transcript_29765;F01_transcript_
29825;F01_transcript_2984;F01_transcript_29873;F01_transcrip
t_29902;F01_transcript_29908;F01_transcript_29914;F01_trans
cript_29967;F01_transcript_30044;F01_transcript_30092;F01_tr
anscript_30161;F01_transcript_30170;F01_transcript_30176;F0
1_transcript_30222;F01_transcript_30407;F01_transcript_30482
;F01_transcript_30491;F01_transcript_30503;F01_transcript_30
553;F01_transcript_3064;F01_transcript_30696;F01_transcript_
30736;F01_transcript_30802;F01_transcript_30823;F01_transcri
pt_30852;F01_transcript_30880;F01_transcript_30891;F01_tran
script_30906;F01_transcript_30997;F01_transcript_31005;F01_t
ranscript_31015;F01_transcript_31066;F01_transcript_31148;F0
1_transcript_31162;F01_transcript_31188;F01_transcript_31205
;F01_transcript_31236;F01_transcript_31271;F01_transcript_31
274;F01_transcript_31297;F01_transcript_31325;F01_transcript
_31414;F01_transcript_31562;F01_transcript_31568;F01_transc
ript_31687;F01_transcript_31749;F01_transcript_31881;F01_tra
nscript_31945;F01_transcript_31959;F01_transcript_3201;F01_t
ranscript_32044;F01_transcript_32090;F01_transcript_32306;F0
1_transcript_32376;F01_transcript_32386;F01_transcript_32422

;F01_transcript_32428;F01_transcript_32449;F01_transcript_32561;F01_transcript_32576;F01_transcript_32622;F01_transcript_32658;F01_transcript_32834;F01_transcript_32913;F01_transcript_33013;F01_transcript_3314;F01_transcript_3321;F01_transcript_33286;F01_transcript_33400;F01_transcript_33418;F01_transcript_33449;F01_transcript_33503;F01_transcript_33540;F01_transcript_33668;F01_transcript_33674;F01_transcript_33728;F01_transcript_33963;F01_transcript_33995;F01_transcript_34014;F01_transcript_34035;F01_transcript_34070;F01_transcript_34082;F01_transcript_34179;F01_transcript_34189;F01_transcript_34196;F01_transcript_34211;F01_transcript_34218;F01_transcript_34275;F01_transcript_34332;F01_transcript_34351;F01_transcript_34362;F01_transcript_34413;F01_transcript_34418;F01_transcript_34420;F01_transcript_34439;F01_transcript_34440;F01_transcript_34513;F01_transcript_34534;F01_transcript_34620;F01_transcript_34659;F01_transcript_34711;F01_transcript_34767;F01_transcript_34832;F01_transcript_34844;F01_transcript_34949;F01_transcript_34996;F01_transcript_35037;F01_transcript_35081;F01_transcript_35239;F01_transcript_35302;F01_transcript_35313;F01_transcript_35332;F01_transcript_35349;F01_transcript_35385;F01_transcript_35426;F01_transcript_35430;F01_transcript_3546;F01_transcript_35540;F01_transcript_35628;F01_transcript_35640;F01_transcript_35664;F01_transcript_35667;F01_transcript_35848;F01_transcript_35906;F01_transcript_35927;F01_transcript_35945;F01_transcript_35990;F01_transcript_35994;F01_transcript_36043;F01_transcript_36056;F01_transcript_36124;F01_transcript_36126;F01_transcript_36178;F01_transcript_36268;F01_transcript_36309;F01_transcript_36320;F01_transcript_36375;F01_transcript_36398;F01_transcript_36447;F01_transcript_36465;F01_transcript_36494;F01_transcript_36576;F01_transcript_36584;F01_transcript_366;F01_transcript_36639;F01_transcript_36649;F01_transcript_3669;F01_transcript_36718;F01_transcript_36808;F01_transcript_36873;F01_transcript_36901;F01_transcript_36997;F01_transcript_37154;F01_transcript_37169;F01_transcript_37233;F01_transcript_37253;F01_transcript_37274;F01_transcript_37334;F01_transcript_37345;F01_transcript_37377;F01_transcript_37402;F01_transcript_37456;F01_transcript_37523;F01_transcript_37531;F01_tran

	script_37614;F01_transcript_37617;F01_transcript_37686;F01_transcript_37690;F01_transcript_37739;F01_transcript_37740;F01_transcript_37806;F01_transcript_37835;F01_transcript_37900;F01_transcript_37978;F01_transcript_38026;F01_transcript_38051;F01_transcript_38062;F01_transcript_3807;F01_transcript_38083;F01_transcript_3817;F01_transcript_38228;F01_transcript_38229;F01_transcript_38243;F01_transcript_38273;F01_transcript_38274;F01_transcript_38302;F01_transcript_38315;F01_transcript_38373;F01_transcript_38456;F01_transcript_38522;F01_transcript_38578;F01_transcript_38657;F01_transcript_38687;F01_transcript_38738;F01_transcript_38763;F01_transcript_38799;F01_transcript_38809;F01_transcript_38826;F01_transcript_38874;F01_transcript_38934;F01_transcript_38936;F01_transcript_38937;F01_transcript_39000;F01_transcript_39013;F01_transcript_39026;F01_transcript_39110;F01_transcript_39119;F01_transcript_39124;F01_transcript_39160;F01_transcript_39168;F01_transcript_39223;F01_transcript_39291;F01_transcript_39305;F01_transcript_39348;F01_transcript_39388;F01_transcript_39467;F01_transcript_39520;F01_transcript_39525;F01_transcript_39557;F01_transcript_39579;F01_transcript_39600;F01_transcript_39641;F01_transcript_39701;F01_transcript_39827;F01_transcript_39842;F01_transcript_39895;F01_transcript_39940;F01_transcript_39952;F01_transcript_39992;F01_transcript_40003;F01_transcript_40033;F01_transcript_40114;F01_transcript_40133;F01_transcript_40209;F01_transcript_40243;F01_transcript_40284;F01_transcript_40354;F01_transcript_40361;F01_transcript_40383;F01_transcript_40448;F01_transcript_40485;F01_transcript_40490;F01_transcript_40501;F01_transcript_40536;F01_transcript_40578;F01_transcript_40591;F01_transcript_40600;F01_transcript_40883;F01_transcript_40894;F01_transcript_40982;F01_transcript_41010;F01_transcript_41015;F01_transcript_41035;F01_transcript_41061;F01_transcript_41074;F01_transcript_4130;F01_transcript_41338;F01_transcript_41430;F01_transcript_41482;F01_transcript_41547;F01_transcript_41552;F01_transcript_41596;F01_transcript_41738;F01_transcript_41905;F01_transcript_41941;F01_transcript_42029;F01_transcript_42058;F01_transcript_42148;F01_transcript_42312;F01_transcript_42

368;F01_transcript_42378;F01_transcript_42505;F01_transcript_42589;F01_transcript_4261;F01_transcript_42658;F01_transcript_42807;F01_transcript_42808;F01_transcript_42962;F01_transcript_43050;F01_transcript_43061;F01_transcript_43237;F01_transcript_43371;F01_transcript_43614;F01_transcript_43659;F01_transcript_43764;F01_transcript_43846;F01_transcript_43886;F01_transcript_43891;F01_transcript_4391;F01_transcript_4393;F01_transcript_44079;F01_transcript_4424;F01_transcript_44274;F01_transcript_44329;F01_transcript_44495;F01_transcript_44524;F01_transcript_44824;F01_transcript_45006;F01_transcript_45041;F01_transcript_45203;F01_transcript_45361;F01_transcript_45700;F01_transcript_4584;F01_transcript_46185;F01_transcript_4651;F01_transcript_4702;F01_transcript_47266;F01_transcript_47321;F01_transcript_47618;F01_transcript_47883;F01_transcript_47886;F01_transcript_48006;F01_transcript_48625;F01_transcript_49083;F01_transcript_49088;F01_transcript_49278;F01_transcript_49281;F01_transcript_49392;F01_transcript_49964;F01_transcript_50110;F01_transcript_50115;F01_transcript_50144;F01_transcript_50401;F01_transcript_50604;F01_transcript_50712;F01_transcript_50911;F01_transcript_51014;F01_transcript_51442;F01_transcript_51453;F01_transcript_51613;F01_transcript_51616;F01_transcript_51625;F01_transcript_51810;F01_transcript_51868;F01_transcript_51889;F01_transcript_51891;F01_transcript_51926;F01_transcript_51976;F01_transcript_5211;F01_transcript_52137;F01_transcript_52139;F01_transcript_52158;F01_transcript_52222;F01_transcript_52265;F01_transcript_52332;F01_transcript_52381;F01_transcript_52465;F01_transcript_52494;F01_transcript_52686;F01_transcript_52692;F01_transcript_52699;F01_transcript_52750;F01_transcript_53212;F01_transcript_53223;F01_transcript_53226;F01_transcript_53305;F01_transcript_53385;F01_transcript_53401;F01_transcript_53589;F01_transcript_53622;F01_transcript_53752;F01_transcript_53796;F01_transcript_53824;F01_transcript_54119;F01_transcript_54142;F01_transcript_54269;F01_transcript_54487;F01_transcript_54938;F01_transcript_55061;F01_transcript_55263;F01_transcript_55528;F01_transcript_55560;F01_transcript_55774;F01_transcript_55889;F01_transcript_55946;F01_transcript_55951;F01_transcript_55958;F01_transcript_55975;F01_tran

script_56233;F01_transcript_56268;F01_transcript_5640;F01_transcript_56493;F01_transcript_56614;F01_transcript_56656;F01_transcript_56717;F01_transcript_56754;F01_transcript_56762;F01_transcript_56764;F01_transcript_56794;F01_transcript_56847;F01_transcript_56914;F01_transcript_56927;F01_transcript_56939;F01_transcript_56950;F01_transcript_56958;F01_transcript_5698;F01_transcript_57145;F01_transcript_57206;F01_transcript_57448;F01_transcript_57473;F01_transcript_57636;F01_transcript_57701;F01_transcript_57807;F01_transcript_57906;F01_transcript_5796;F01_transcript_57970;F01_transcript_58208;F01_transcript_58214;F01_transcript_5825;F01_transcript_58368;F01_transcript_58405;F01_transcript_58552;F01_transcript_58667;F01_transcript_58758;F01_transcript_58763;F01_transcript_58783;F01_transcript_58795;F01_transcript_5896;F01_transcript_59025;F01_transcript_5928;F01_transcript_59371;F01_transcript_59406;F01_transcript_59548;F01_transcript_59602;F01_transcript_59629;F01_transcript_59734;F01_transcript_59737;F01_transcript_59752;F01_transcript_59767;F01_transcript_59771;F01_transcript_59821;F01_transcript_59956;F01_transcript_59961;F01_transcript_60004;F01_transcript_60064;F01_transcript_60113;F01_transcript_60128;F01_transcript_60194;F01_transcript_60229;F01_transcript_60235;F01_transcript_60314;F01_transcript_60381;F01_transcript_60431;F01_transcript_60635;F01_transcript_60685;F01_transcript_60736;F01_transcript_60831;F01_transcript_6084;F01_transcript_61072;F01_transcript_61112;F01_transcript_61178;F01_transcript_61243;F01_transcript_61414;F01_transcript_61445;F01_transcript_61449;F01_transcript_61453;F01_transcript_61535;F01_transcript_61538;F01_transcript_61725;F01_transcript_61865;F01_transcript_61908;F01_transcript_61978;F01_transcript_62010;F01_transcript_62031;F01_transcript_62037;F01_transcript_62135;F01_transcript_62260;F01_transcript_62337;F01_transcript_62390;F01_transcript_62451;F01_transcript_62749;F01_transcript_62775;F01_transcript_63028;F01_transcript_63160;F01_transcript_63171;F01_transcript_63241;F01_transcript_63482;F01_transcript_63521;F01_transcript_63540;F01_transcript_63669;F01_transcript_63911;F01_t

2-Oxocarboxylic acid metabolism	ko01210	334	F01_transcript_100363;F01_transcript_10047;F01_transcript_100487;F01_transcript_100516;F01_transcript_101218;F01_transcript_101473;F01_transcript_101584;F01_transcript_102048;F01_transcript_10266;F01_transcript_102942;F01_transcript_103532;F01_transcript_105291;F01_transcript_105548;F01_transcript_105634;F01_transcript_105644;F01_transcript_106156;F01_transcript_106158;F01_transcript_106479;F01_transcript_106679;F01_transcript_107151;F01_transcript_107447;F01_transcript_107931;F01_transcript_108633;F01_transcript_110137;F01_transcript_110728;F01_transcript_111759;F01_transcript_11182;F01_transcript_112260;F01_transcript_112623;F01_transcript_113828;F01_transcript_113831;F01_transcript_114452;F01_transcript_114966;F01_transcript_115077;F01_transcript_115383;F01_transcript_116580;F01_transcript_116648;F01_transcript_116793;F01_transcript_116945;F01_transcript_116956;F01_transcript_117148;F01_transcript_117214;F01_transcript_117319;F01_transcript_117385;F01_transcript_11767;F01_transcript_118645;F01_transcript_118657;F01_transcript_119034;F01_transcript_119338;F01_transcript_119534;F01_transcript_119690;F01_transcript_119869;F01_transcript_120201;F01_transcript_120234;F01_transcript_121896;F01_transcript_122101;F01_transcript_123393;F01_transcript_123753;F01_transcript_123773;F01_transcript_124206;F01_transcript_125768;F01_transcript_125864;F01_transcript_126185;F01_transcript_126272;F01_transcript_127049;F01_transcript_127209;F01_transcript_127540;F01_transcript_127921;F01_transcript_128203;F01_transcript_129549;F01_transcript_130485;F01_transcript_131043;F01_transcript_131587;F01_transcript_132527;F01_transcript_133194;F01_transcript_133321;F01_transcript_133975;F01_transcript_135402;F01_transcript_135578;F01_transcript_136221;F01_transcript_136367;F01_transcript_137444;F01_transcript_138295;F01_transcript_139074;F01_transcript_140089;F01_transcript_140510;F01_transcript_140944;F01_transcript_14157;F01_transcript_141642;F01_transcript_141936;F01_transcript_142888;F01_transcript_142960;F01_transcript_144117;F01_transcript_144458;F01_transcript_144716;F01_transcript_145058;F01_transcript_145367;F01_transcript_145616;F01_transcript_146194;F01_transcript_146322;F01_transcript_146963;F01_transcript_147008;F01_transcript_1483	K00928+K14272+K00826+K00826+K01647+K14454+K00826+K14272+K14272+K14677+K14272+K01647+K00053+K01681+K00928+K14454+K00133+K01649+K00928+K00811+K01649+K14272+K14682+K14272+K00826+K01703+K14272+K14272+K01647+K00928+K14272+K14455+K00031+K00030+K00053+K14677+K14272+K00814+K01647+K00826+K14272+K00928+K01647+K14677+K00030+K01649+K01647+K00031+K01649+K00031+K00826+K14455+K01653+K14682+K01681+K14272+K00145+K14272+K01647+K00928+K14272+K00053+K14455+K00930+K01647+K00818+K00053+K01649+K00620+K00053+K14272+K14455+K01647+K14272+K00053+K01653+K00928+K00053+K14454+K00030+K00928+K14682+K01681+K00928+K01703+K01649+K00928+K14677+K00928+K14682+K00620+K00030+K00030+K14272+K00133+K00030+K01649+K14272+K01653+K00053+K14272+K00031+K00928+K00826+K01647+K01681+K00030+K00053+K00053+K14454+K00053+K14454+K00053+K01681+K01653+K00928+K01703+K00928+K00928+K01649+K14272+K00826+K00928+K00133+K14455+K00928+K14682+K14272+K14272+K01647+K00928+K00811+K01649+K01652+K01647+K01703+K00928+K01681+K00928+K01647+K00928+K14272+K00053+K01647+K00928+K01652+K00928+K14682+K01653+K00928+K00053+K01652+K01687+K00053+K01652+K14682+K00053+K00928+K00928+K00814+K14682+K01649+K00053+K14272+K00928+K00030+K01681+K14272+K14454+K14272+K01647+K00053+K01703+K01703+K00145+K14454+K01653+K01647+K00030+K14677+K01703+K00811+K14272+K00053+K14272+K14455+K01653+K14272+K14272+K00030+K00826+K00818+K14677+K00031+K00818+K14677+K01647+K00826+K14272+K01438+K00133+K14455+K14455+K00052+K00145+K11820+K00052+K00826+K11820+K00930+K00930+K01652+K14272+K00826+K01647+K00133+K00031+K00031+K01649+K00826+K00052+K00053+K00814+K14682+K00030+K14677+K00826+K00030+K00814+K00052+K14272+K00053+K00053+K00145+K14272+K01681+K01649+K01652+K01652+K00030+K00818+K00930+K01647+K00053+K01681+K00814+K14272+K14454+K01703+K00145+K00053+K14272+K00928+K00030+K00030+K00053+K01703+K01647+K00826+K00814+K146

	55;F01_transcript_148792;F01_transcript_148941;F01_transcript_149398;F01_transcript_149933;F01_transcript_1503;F01_transcript_150508;F01_transcript_150639;F01_transcript_151854;F01_transcript_152726;F01_transcript_152848;F01_transcript_153015;F01_transcript_153213;F01_transcript_153395;F01_transcript_153480;F01_transcript_153912;F01_transcript_154436;F01_transcript_15454;F01_transcript_154837;F01_transcript_154917;F01_transcript_155171;F01_transcript_156046;F01_transcript_156344;F01_transcript_157207;F01_transcript_159324;F01_transcript_160034;F01_transcript_160312;F01_transcript_160446;F01_transcript_160462;F01_transcript_160500;F01_transcript_160665;F01_transcript_160691;F01_transcript_161671;F01_transcript_161767;F01_transcript_16207;F01_transcript_162211;F01_transcript_162256;F01_transcript_163468;F01_transcript_165529;F01_transcript_167098;F01_transcript_167653;F01_transcript_167751;F01_transcript_17449;F01_transcript_18842;F01_transcript_19063;F01_transcript_19461;F01_transcript_19555;F01_transcript_19590;F01_transcript_19790;F01_transcript_19945;F01_transcript_20059;F01_transcript_20342;F01_transcript_20537;F01_transcript_20719;F01_transcript_20928;F01_transcript_20993;F01_transcript_21550;F01_transcript_21833;F01_transcript_22118;F01_transcript_22601;F01_transcript_22646;F01_transcript_25546;F01_transcript_25705;F01_transcript_26583;F01_transcript_2680;F01_transcript_27248;F01_transcript_28405;F01_transcript_28733;F01_transcript_28828;F01_transcript_28993;F01_transcript_29072;F01_transcript_29737;F01_transcript_29855;F01_transcript_30736;F01_transcript_30778;F01_transcript_30906;F01_transcript_30997;F01_transcript_31456;F01_transcript_31526;F01_transcript_31784;F01_transcript_31881;F01_transcript_32304;F01_transcript_32376;F01_transcript_32622;F01_transcript_33011;F01_transcript_33668;F01_transcript_34014;F01_transcript_34218;F01_transcript_34357;F01_transcript_34701;F01_transcript_34756;F01_transcript_34767;F01_transcript_35241;F01_transcript_35621;F01_transcript_35664;F01_transcript_35824;F01_transcript_35906;F01_transcript_36194;F01_transcript_36269;F01_transcript_36375;F01_transcript_36639;F01_transcript_36862;F01_transcript_36937;F01_transcript_37001;F01_t	77+K00826+K00930+K00620+K00053+K01652+K14272+K00031+K14272+K14272+K14272+K01702+K00928+K01687+K01681+K14682+K14455+K01681+K00811+K14682+K01703+K00811+K14454+K00145+K14454+K01647+K00826+K00145+K00928+K00145+K00826+K14272+K01649+K01649+K00928+K01703+K00053+K14677+K01647+K00814+K00811+K00826+K00030+K14455+K14272+K00145+K14454+K00620+K00030+K00620+K01653+K00826+K00145+K00814+K00133+K14272+K14272+K14455+K01703+K14272+K14272+K00826+K14272+K01647+K14272+K00928+K00052+K14272+K14454+K11820+K00031+K14272+K14677+K01647

ranscript_37086;F01_transcript_37725;F01_transcript_39196;F0
1_transcript_42030;F01_transcript_42523;F01_transcript_51931
;F01_transcript_52222;F01_transcript_52367;F01_transcript_52
381;F01_transcript_52630;F01_transcript_52686;F01_transcript
_52750;F01_transcript_52882;F01_transcript_54318;F01_transc
ript_54539;F01_transcript_56606;F01_transcript_56914;F01_tra
nscript_57421;F01_transcript_57701;F01_transcript_5774;F01_t
ranscript_58468;F01_transcript_58552;F01_transcript_58763;F0
1_transcript_58777;F01_transcript_58783;F01_transcript_60186
;F01_transcript_60455;F01_transcript_60833;F01_transcript_61
178;F01_transcript_61449;F01_transcript_61708;F01_transcript
_61852;F01_transcript_62228;F01_transcript_63028;F01_transc
ript_63041;F01_transcript_63480;F01_transcript_64872;F01_tra
nscript_64900;F01_transcript_65464;F01_transcript_65515;F01
_transcript_65567;F01_transcript_65858;F01_transcript_66275;
F01_transcript_66578;F01_transcript_67213;F01_transcript_672
28;F01_transcript_69503;F01_transcript_70154;F01_transcript_
70881;F01_transcript_70967;F01_transcript_71528;F01_transcri
pt_71789;F01_transcript_72229;F01_transcript_72598;F01_tran
script_72718;F01_transcript_73375;F01_transcript_73717;F01_t
ranscript_73953;F01_transcript_74222;F01_transcript_74843;F0
1_transcript_75477;F01_transcript_75938;F01_transcript_76748
;F01_transcript_77213;F01_transcript_77476;F01_transcript_77
518;F01_transcript_77531;F01_transcript_78308;F01_transcript
_78472;F01_transcript_78678;F01_transcript_78720;F01_transc
ript_78791;F01_transcript_78919;F01_transcript_79120;F01_tra
nscript_80747;F01_transcript_80769;F01_transcript_81078;F01
_transcript_81489;F01_transcript_81767;F01_transcript_82791;
F01_transcript_83526;F01_transcript_83737;F01_transcript_840
6;F01_transcript_84746;F01_transcript_84934;F01_transcript_8
5168;F01_transcript_85279;F01_transcript_85411;F01_transcrip
t_85599;F01_transcript_85624;F01_transcript_85969;F01_trans
cript_86031;F01_transcript_86113;F01_transcript_86847;F01_tr
anscript_86970;F01_transcript_87267;F01_transcript_87452;F0
1_transcript_87504;F01_transcript_87957;F01_transcript_88765
;F01_transcript_89140;F01_transcript_90013;F01_transcript_90
207;F01_transcript_90688;F01_transcript_90977;F01_transcript
_91214;F01_transcript_91391;F01_transcript_91443;F01_transc

			ript_91694;F01_transcript_92797;F01_transcript_92895;F01_transcript_93052;F01_transcript_93119;F01_transcript_93248;F01_transcript_93376;F01_transcript_93866;F01_transcript_94572;F01_transcript_94796;F01_transcript_96025;F01_transcript_97035;F01_transcript_97229;F01_transcript_97367;F01_transcript_9785;F01_transcript_98138;F01_transcript_98527;F01_transcript_98918;F01_transcript_99014;F01_transcript_99615;	
Fatty acid metabolism	ko01212	387	F01_transcript_10004;F01_transcript_100288;F01_transcript_10098;F01_transcript_101409;F01_transcript_101823;F01_transcript_103130;F01_transcript_10466;F01_transcript_104730;F01_transcript_104782;F01_transcript_104998;F01_transcript_105202;F01_transcript_105292;F01_transcript_105403;F01_transcript_105547;F01_transcript_105720;F01_transcript_106095;F01_transcript_106217;F01_transcript_106220;F01_transcript_106311;F01_transcript_106677;F01_transcript_10672;F01_transcript_108901;F01_transcript_10985;F01_transcript_111506;F01_transcript_112515;F01_transcript_113046;F01_transcript_113382;F01_transcript_113401;F01_transcript_113706;F01_transcript_114458;F01_transcript_114684;F01_transcript_114717;F01_transcript_115230;F01_transcript_115996;F01_transcript_11601;F01_transcript_116845;F01_transcript_117227;F01_transcript_117636;F01_transcript_117649;F01_transcript_117687;F01_transcript_118434;F01_transcript_118673;F01_transcript_119362;F01_transcript_119532;F01_transcript_119757;F01_transcript_120483;F01_transcript_120801;F01_transcript_121035;F01_transcript_121478;F01_transcript_121926;F01_transcript_122368;F01_transcript_122671;F01_transcript_122692;F01_transcript_122721;F01_transcript_122818;F01_transcript_123054;F01_transcript_124368;F01_transcript_124664;F01_transcript_124769;F01_transcript_124788;F01_transcript_125612;F01_transcript_12599;F01_transcript_126252;F01_transcript_127333;F01_transcript_128013;F01_transcript_12879;F01_transcript_128927;F01_transcript_129744;F01_transcript_12985;F01_transcript_129865;F01_transcript_129901;F01_transcript_130395;F01_transcript_130423;F01_transcript_130464;F01_transcript_130717;F01_transcript_13109;F01_transcript_131263;F01_transcript_13154;F01_transcript_131603;F01_transcript_131659;F01_transcript_131715;F01_transcrip	K09458+K01962+K10527+K01962+K09458+K00059+K01897+K09458+K01897+K01897+K01962+K11262+K00626+K10527+K11262+K10527+K00232+K01962+K01074+K01962+K10527+K11262+K01962+K10703+K10258+K10703+K09458+K09458+K10703+K01897+K01897+K09458+K01897+K01897+K00232+K10256+K01897+K10781+K09458+K00232+K00232+K00208+K10781+K01897+K01074+K01074+K00232+K11262+K01897+K10251+K00232+K01074+K10251+K10527+K00232+K10781+K10256+K10781+K00232+K00232+K00648+K01897+K10258+K10781+K01897+K01897+K01074+K10703+K01897+K03921+K10781+K01074+K03921+K09458+K07513+K01897+K01074+K01962+K00059+K00208+K01897+K01897+K10527+K10781+K10703+K01897+K01897+K01962+K10527+K00232+K00626+K10527+K00232+K01897+K10527+K09458+K01897+K03921+K01074+K11262+K10781+K10703+K00232+K01897+K00232+K09458+K10527+K01897+K11262+K00208+K01074+K10703+K00232+K00232+K09458+K07513+K00059+K01897+K10527+K10703+K01897+K01897+K00232+K09458+K00232+K01074+K10781+K01897+K01897+K01897+K10527+K01897+K01074+K00232+K00626+K02372+K10527+K01897+K00232+K01897+K00232+K01897+K01963+K09458+K09458+K01897+K00232+K10527+K07513+K00232+K10527+K10781+K01897+K10527+K01897+K00232+K01897+K01897+K10527+K09458+K01962+K10251+K10251+K10251+K10781+K09458+K10527+K10251+K00208+K00626+K01897+K07513+K10527+K10527+K01897+K00232+K01897+K10781+K01897+K01897+K01897+K01897+K01897+K01074+K10781+K00232+K01897+K10781+K10251+K00232+K00232+K01897+K11262+K00232+K00208+K01961+K00232+K00208+K09458+K03921+K00648+K07513+K00648+K07513+K07513+K1025

	t_132668;F01_transcript_132695;F01_transcript_133721;F01_transcript_133828;F01_transcript_134920;F01_transcript_135733;F01_transcript_135955;F01_transcript_13624;F01_transcript_138047;F01_transcript_138114;F01_transcript_13822;F01_transcript_139123;F01_transcript_139502;F01_transcript_139625;F01_transcript_139851;F01_transcript_140123;F01_transcript_140225;F01_transcript_140469;F01_transcript_141554;F01_transcript_141747;F01_transcript_141759;F01_transcript_142214;F01_transcript_143258;F01_transcript_143781;F01_transcript_143805;F01_transcript_144395;F01_transcript_144717;F01_transcript_144779;F01_transcript_144932;F01_transcript_145685;F01_transcript_146347;F01_transcript_147167;F01_transcript_147259;F01_transcript_147293;F01_transcript_147398;F01_transcript_147705;F01_transcript_147795;F01_transcript_147956;F01_transcript_148949;F01_transcript_14899;F01_transcript_149352;F01_transcript_149791;F01_transcript_15003;F01_transcript_15284;F01_transcript_153426;F01_transcript_153478;F01_transcript_153488;F01_transcript_15349;F01_transcript_15367;F01_transcript_154107;F01_transcript_154600;F01_transcript_154923;F01_transcript_155474;F01_transcript_155685;F01_transcript_156365;F01_transcript_156381;F01_transcript_15661;F01_transcript_157406;F01_transcript_15780;F01_transcript_15817;F01_transcript_158278;F01_transcript_158628;F01_transcript_15890;F01_transcript_158942;F01_transcript_159135;F01_transcript_15946;F01_transcript_159785;F01_transcript_159968;F01_transcript_160683;F01_transcript_161110;F01_transcript_16167;F01_transcript_161773;F01_transcript_161981;F01_transcript_162421;F01_transcript_16286;F01_transcript_16301;F01_transcript_16433;F01_transcript_164901;F01_transcript_164916;F01_transcript_164977;F01_transcript_165303;F01_transcript_165616;F01_transcript_165801;F01_transcript_165897;F01_transcript_165923;F01_transcript_165924;F01_transcript_166122;F01_transcript_166283;F01_transcript_166964;F01_transcript_167396;F01_transcript_167595;F01_transcript_17281;F01_transcript_17857;F01_transcript_17966;F01_transcript_17987;F01_transcript_18447;F01_transcript_18597;F01_transcript_19074;F01_transcript_19302;F01_transcript_19520;F01_transcript_19705;F01_transcript_20309;F01_transcript_20745;F01_transcript_21972;F01_transcript	6+K03921+K07513+K00232+K10256+K03921+K00232+K00059+K00059+K03921+K00626+K00059+K00626+K10256+K00645+K00626+K00626+K01074+K01074+K02160+K07512+K10251+K10251+K01074+K02160+K02160+K00059+K02372+K01962+K01897+K00249+K00232+K00626+K00232+K01074+K00232+K00648+K01961+K02372+K01897+K10527+K00232+K00232+K00232+K10781+K01897+K00232+K10703+K01962+K01074+K10527+K00232+K00232+K01962+K00626+K10703+K10527+K00059+K10703+K01962+K09458+K02372+K07513+K01897+K01897+K09458+K10781+K01897+K00208+K00232+K10781+K01074+K10527+K01897+K09458+K00249+K00232+K07512+K10527+K01897+K11262+K10781+K00626+K01961+K09458+K00208+K01963+K10251+K00249+K10527+K01897+K01897+K10527+K00059+K01897+K00232+K09458+K07512+K09458+K10527+K03921+K10527+K10781+K00249+K09458+K01897+K02372+K00626+K10251+K09458+K01897+K01962+K00626+K10527+K11262+K00232+K00232+K09458+K00626+K10527+K01897+K11262+K00232+K01962+K01961+K00232+K10527+K00232+K09458+K00648+K00626+K01897+K00232+K01962+K10781+K01897+K01074+K01897+K07513+K00059+K01897+K09458+K01897+K00232+K00648+K01962+K10781+K01074+K01962+K00249+K01074+K00232+K00232+K00059+K02160+K01897+K00648+K09458+K01074+K00232+K00208+K01897+K11262+K07512+K00648+K10703+K01074+K00059+K00232+K01074+K01897+K10251+K10527+K00626+K10256+K10781+K01897+K07513+K01897+K10256+K01897+K01897

	_22669;F01_transcript_23121;F01_transcript_23503;F01_transcript_26159;F01_transcript_27104;F01_transcript_27232;F01_transcript_28357;F01_transcript_29158;F01_transcript_29191;F01_transcript_29866;F01_transcript_30491;F01_transcript_30801;F01_transcript_31883;F01_transcript_32112;F01_transcript_32634;F01_transcript_32829;F01_transcript_32889;F01_transcript_33263;F01_transcript_33549;F01_transcript_34454;F01_transcript_34472;F01_transcript_34915;F01_transcript_35442;F01_transcript_35988;F01_transcript_36146;F01_transcript_36214;F01_transcript_36482;F01_transcript_36847;F01_transcript_36931;F01_transcript_37206;F01_transcript_37377;F01_transcript_37378;F01_transcript_37686;F01_transcript_38069;F01_transcript_38219;F01_transcript_38302;F01_transcript_38657;F01_transcript_38790;F01_transcript_38861;F01_transcript_40383;F01_transcript_40457;F01_transcript_40823;F01_transcript_41243;F01_transcript_41358;F01_transcript_41552;F01_transcript_41596;F01_transcript_44928;F01_transcript_46128;F01_transcript_4651;F01_transcript_4986;F01_transcript_51625;F01_transcript_52099;F01_transcript_52332;F01_transcript_52548;F01_transcript_53233;F01_transcript_53390;F01_transcript_53543;F01_transcript_53796;F01_transcript_54259;F01_transcript_54622;F01_transcript_54950;F01_transcript_54990;F01_transcript_55240;F01_transcript_55281;F01_transcript_55556;F01_transcript_55736;F01_transcript_56128;F01_transcript_56417;F01_transcript_56614;F01_transcript_57503;F01_transcript_57689;F01_transcript_57777;F01_transcript_58171;F01_transcript_5825;F01_transcript_58368;F01_transcript_59428;F01_transcript_60135;F01_transcript_60150;F01_transcript_60200;F01_transcript_6084;F01_transcript_61151;F01_transcript_61462;F01_transcript_61866;F01_transcript_61928;F01_transcript_62811;F01_transcript_62859;F01_transcript_65211;F01_transcript_65891;F01_transcript_65988;F01_transcript_66022;F01_transcript_664;F01_transcript_66972;F01_transcript_67042;F01_transcript_67276;F01_transcript_67593;F01_transcript_6767;F01_transcript_67738;F01_transcript_68598;F01_transcript_68865;F01_transcript_68887;F01_transcript_68924;F01_transcript_69192;F01_transcript_69739;F01_transcript_69815;F01_transcript_69883;F01_transcript_70315;F01_t

			ranscript_70327;F01_transcript_70525;F01_transcript_7124;F01_transcript_71676;F01_transcript_71725;F01_transcript_72007;F01_transcript_72010;F01_transcript_72026;F01_transcript_72096;F01_transcript_72826;F01_transcript_72848;F01_transcript_72928;F01_transcript_73024;F01_transcript_7309;F01_transcript_73288;F01_transcript_73443;F01_transcript_74275;F01_transcript_7550;F01_transcript_76033;F01_transcript_76982;F01_transcript_77290;F01_transcript_77451;F01_transcript_77538;F01_transcript_77795;F01_transcript_78246;F01_transcript_78484;F01_transcript_79052;F01_transcript_79452;F01_transcript_80148;F01_transcript_80183;F01_transcript_80645;F01_transcript_80702;F01_transcript_80843;F01_transcript_80969;F01_transcript_82184;F01_transcript_82337;F01_transcript_82368;F01_transcript_82396;F01_transcript_82656;F01_transcript_82742;F01_transcript_83118;F01_transcript_83317;F01_transcript_83638;F01_transcript_84001;F01_transcript_84463;F01_transcript_84935;F01_transcript_85292;F01_transcript_85424;F01_transcript_85921;F01_transcript_86684;F01_transcript_86897;F01_transcript_88028;F01_transcript_88720;F01_transcript_88933;F01_transcript_89994;F01_transcript_90527;F01_transcript_90896;F01_transcript_90931;F01_transcript_90940;F01_transcript_91076;F01_transcript_91258;F01_transcript_91879;F01_transcript_91910;F01_transcript_9209;F01_transcript_92575;F01_transcript_92632;F01_transcript_92699;F01_transcript_92715;F01_transcript_92735;F01_transcript_92831;F01_transcript_93026;F01_transcript_93122;F01_transcript_93476;F01_transcript_93534;F01_transcript_94689;F01_transcript_95989;F01_transcript_96058;F01_transcript_96104;F01_transcript_96369;F01_transcript_96460;F01_transcript_96469;F01_transcript_96815;F01_transcript_97024;F01_transcript_97231;F01_transcript_97377;F01_transcript_97408;F01_transcript_97546;F01_transcript_97808;F01_transcript_97838;F01_transcript_97945;F01_transcript_98172;F01_transcript_98462;F01_transcript_98478;F01_transcript_99157;F01_transcript_99276;F01_transcript_99279;	
Biosynthesis of	ko01	1387	F01_transcript_100118;F01_transcript_100214;F01_transcript_100228;F01_transcript_100230;F01_transcript_100238;F01_transcript_100287;F01_transcript_100330;F01_transcript_100363;F01_transcript_100408;F01_transcript_100412;F01_transcript_100	K00600+K01803+K05359+K00058+K00615+K01626+K00600+K00928+K05359+K00891+K01783+K14272+K00826+K00826+K00815+K00873+K15634+K00615+K01915+K00600+K00873+K01647+K00640+K14454+K00850+K00826+K00549+K14

amino acids	230	430;F01_transcript_10047;F01_transcript_100487;F01_transcript_100516;F01_transcript_100618;F01_transcript_100783;F01_transcript_100809;F01_transcript_100825;F01_transcript_101062;F01_transcript_101071;F01_transcript_101187;F01_transcript_101218;F01_transcript_101296;F01_transcript_101473;F01_transcript_101574;F01_transcript_101584;F01_transcript_101758;F01_transcript_102048;F01_transcript_102135;F01_transcript_102189;F01_transcript_102373;F01_transcript_10266;F01_transcript_102681;F01_transcript_102714;F01_transcript_102803;F01_transcript_102826;F01_transcript_102845;F01_transcript_102902;F01_transcript_102942;F01_transcript_102962;F01_transcript_103028;F01_transcript_103227;F01_transcript_103318;F01_transcript_103331;F01_transcript_103356;F01_transcript_103532;F01_transcript_10354;F01_transcript_103634;F01_transcript_103675;F01_transcript_103691;F01_transcript_103700;F01_transcript_104096;F01_transcript_104113;F01_transcript_104167;F01_transcript_10450;F01_transcript_104908;F01_transcript_104986;F01_transcript_105258;F01_transcript_105281;F01_transcript_105291;F01_transcript_105365;F01_transcript_105548;F01_transcript_105634;F01_transcript_105644;F01_transcript_105645;F01_transcript_105738;F01_transcript_10578;F01_transcript_105845;F01_transcript_105879;F01_transcript_105919;F01_transcript_106024;F01_transcript_106156;F01_transcript_106158;F01_transcript_106166;F01_transcript_106479;F01_transcript_10658;F01_transcript_106623;F01_transcript_106666;F01_transcript_106679;F01_transcript_106845;F01_transcript_106967;F01_transcript_10704;F01_transcript_107113;F01_transcript_107151;F01_transcript_107162;F01_transcript_107447;F01_transcript_107638;F01_transcript_107677;F01_transcript_107736;F01_transcript_107871;F01_transcript_107931;F01_transcript_10799;F01_transcript_108019;F01_transcript_108303;F01_transcript_108371;F01_transcript_108584;F01_transcript_108602;F01_transcript_108633;F01_transcript_108868;F01_transcript_108947;F01_transcript_109009;F01_transcript_109116;F01_transcript_109254;F01_transcript_109267;F01_transcript_109463;F01_transcript_109676;F01_transcript_109944;F01_transcript_110137;F01_transcript_110305;F01_transcript_110317;F01_transcript_110549;F0	272+K00600+K01783+K01803+K14272+K01807+K00600+K01657+K01783+K01623+K01689+K14677+K00766+K01739+K00600+K01915+K00615+K01755+K14272+K00549+K00134+K00615+K14085+K01738+K00873+K01940+K00600+K00615+K10206+K00850+K00948+K01738+K01647+K01623+K00053+K01681+K00928+K01807+K00873+K00615+K01476+K01736+K01807+K01915+K14454+K00133+K01807+K01649+K00549+K12524+K00815+K00928+K01850+K00600+K00549+K01783+K00811+K01586+K01649+K00615+K00058+K13832+K01693+K14272+K00600+K00873+K01915+K00615+K00600+K00600+K14682+K00850+K01803+K15227+K01814+K00600+K00640+K01915+K01623+K00215+K14272+K01850+K01915+K00615+K00850+K00826+K00615+K00013+K00789+K00789+K13832+K00134+K00600+K01703+K14272+K00549+K00873+K15634+K14272+K01803+K12657+K00600+K01647+K10206+K01079+K00928+K14272+K00800+K00549+K01738+K01850+K11755+K00640+K14455+K01738+K01689+K01915+K00058+K00600+K01738+K01807+K00031+K00030+K00058+K01738+K00053+K14085+K00948+K01915+K00873+K12657+K14677+K14272+K00215+K00814+K01647+K00826+K00013+K14272+K01623+K00928+K01647+K01693+K00615+K14677+K01783+K00600+K00616+K00030+K00600+K00873+K01689+K01739+K01738+K01649+K01647+K01609+K00600+K01733+K00600+K00031+K01807+K13034+K01807+K01649+K00873+K00031+K00826+K01609+K01623+K14455+K00600+K00640+K01915+K01738+K01807+K01653+K14682+K00615+K00873+K13034+K01609+K01783+K00549+K00600+K01739+K01623+K01681+K00264+K15633+K14272+K00600+K00815+K00600+K01814+K00615+K00264+K01940+K01696+K00134+K10206+K00873+K00927+K00145+K01915+K14272+K01647+K00600+K01739+K15227+K01626+K00600+K00928+K00549+K00873+K01783+K05359+K01623+K00215+K01940+K01738+K01850+K00615+K00600+K15633+K14272+K01738+K00053+K00215+K12524+K00549+K14455+K00930+K00549+K01814+K01647+K01738+K00818+K00600+K11755+K00549+K01739+K00053+K01755+K01834+K01649+K01738+K01803+K00620+K00134+K01783+K00600+K01783+K01915+K

	1_transcript_110559;F01_transcript_110728;F01_transcript_110771;F01_transcript_110772;F01_transcript_110921;F01_transcript_111001;F01_transcript_111377;F01_transcript_111485;F01_transcript_111549;F01_transcript_111759;F01_transcript_11182;F01_transcript_11185;F01_transcript_11216;F01_transcript_11219;F01_transcript_112260;F01_transcript_112269;F01_transcript_112405;F01_transcript_112563;F01_transcript_112623;F01_transcript_113249;F01_transcript_113436;F01_transcript_113828;F01_transcript_113831;F01_transcript_113901;F01_transcript_114007;F01_transcript_114030;F01_transcript_114297;F01_transcript_114312;F01_transcript_114327;F01_transcript_114452;F01_transcript_114551;F01_transcript_114570;F01_transcript_114576;F01_transcript_114614;F01_transcript_114677;F01_transcript_114920;F01_transcript_114958;F01_transcript_114966;F01_transcript_115077;F01_transcript_115188;F01_transcript_115361;F01_transcript_115383;F01_transcript_115636;F01_transcript_115763;F01_transcript_116166;F01_transcript_116379;F01_transcript_116470;F01_transcript_116580;F01_transcript_116648;F01_transcript_116785;F01_transcript_116793;F01_transcript_116945;F01_transcript_116956;F01_transcript_117037;F01_transcript_117148;F01_transcript_117151;F01_transcript_117214;F01_transcript_117319;F01_transcript_117335;F01_transcript_11734;F01_transcript_117385;F01_transcript_117388;F01_transcript_117422;F01_transcript_117662;F01_transcript_11767;F01_transcript_117699;F01_transcript_11770;F01_transcript_117798;F01_transcript_117844;F01_transcript_118376;F01_transcript_118645;F01_transcript_118657;F01_transcript_118803;F01_transcript_118804;F01_transcript_118908;F01_transcript_118927;F01_transcript_119034;F01_transcript_119183;F01_transcript_119306;F01_transcript_119312;F01_transcript_119338;F01_transcript_11934;F01_transcript_119534;F01_transcript_119690;F01_transcript_119838;F01_transcript_119854;F01_transcript_119869;F01_transcript_119952;F01_transcript_120073;F01_transcript_120116;F01_transcript_120145;F01_transcript_120193;F01_transcript_120201;F01_transcript_120234;F01_transcript_120459;F01_transcript_120942;F01_transcript_120997;F01_transcript_121116;F01_transcript_121130;F01_transcript_12118;F01_transcript_121306;F01_transcript_121502;F01_transcript_121571;F01_t	01739+K00053+K01623+K00873+K00600+K14272+K00600+K01915+K00058+K00600+K14455+K15633+K01647+K05359+K00058+K00215+K01626+K01693+K18649+K00600+K00815+K00600+K01915+K00058+K00817+K12657+K00600+K01609+K14272+K01915+K00600+K00053+K00549+K00600+K01653+K01940+K00927+K00615+K00615+K01803+K00815+K01803+K00948+K00928+K15227+K00927+K00873+K13034+K00948+K00215+K01657+K00549+K00134+K00264+K01689+K00215+K00053+K00891+K14454+K00615+K01803+K00815+K00615+K00013+K05359+K00927+K00873+K00030+K12524+K15849+K00928+K00815+K13832+K00817+K01783+K00850+K01814+K15633+K00600+K14682+K01915+K00615+K00800+K01681+K10206+K01803+K00600+K01738+K10206+K01850+K00264+K00215+K00600+K00928+K01807+K01807+K00616+K00600+K00600+K13832+K00948+K01703+K00800+K01623+K05359+K01649+K00134+K00927+K00928+K00789+K12657+K00549+K05359+K01850+K15633+K00600+K14677+K00928+K13832+K00873+K15634+K14682+K01738+K01778+K15633+K00549+K01739+K01915+K00891+K00620+K00600+K00030+K00789+K00815+K01807+K00873+K10206+K00030+K00611+K14272+K01807+K00133+K00815+K00134+K01735+K00030+K01609+K00600+K01623+K01649+K14272+K01807+K01623+K00013+K01738+K01653+K01850+K00053+K00766+K01850+K00600+K00850+K00815+K14272+K00031+K00600+K13832+K00927+K15633+K01663+K01733+K00611+K00766+K01623+K10206+K00928+K01850+K00549+K00058+K00826+K01647+K00789+K01915+K01681+K01915+K01738+K01807+K00030+K00815+K00600+K00053+K00264+K00053+K01817+K14454+K00891+K01803+K01738+K00615+K01738+K01623+K00948+K00134+K00053+K12657+K01738+K13034+K14454+K01689+K01623+K01807+K00053+K01681+K01653+K01623+K00928+K01703+K00873+K13832+K01623+K00928+K00927+K00215+K01079+K00928+K00891+K01689+K01649+K00215+K01850+K01738+K13832+K14272+K00600+K01803+K00826+K00600+K05359+K00928+K00927+K01738+K01739+K01609+K00058+K01738+K00133+K14455+K00927+K01623+K01850+K00850+K01755+K01609+K00928+K01755+K05359+K01735+K00850+K00765+K00600+K0089

	ranscript_121896;F01_transcript_121985;F01_transcript_12201	1+K00873+K00850+K00600+K00873+K00850+K00789+K006
	2;F01_transcript_122101;F01_transcript_122115;F01_transcript	15+K00891+K00600+K01623+K14682+K01803+K00850+K14
	_122119;F01_transcript_122331;F01_transcript_122487;F01_tra	085+K00549+K01755+K00873+K14272+K00873+K01807+K1
	nscript_122782;F01_transcript_122790;F01_transcript_122816;	4272+K00600+K00873+K01647+K00928+K00811+K00134+K
	F01_transcript_122875;F01_transcript_122893;F01_transcript_1	01649+K01652+K05359+K11755+K00600+K00948+K00815+
	23162;F01_transcript_12318;F01_transcript_123349;F01_transc	K01738+K00815+K01647+K01703+K13832+K01626+K00928
	ript_123393;F01_transcript_123427;F01_transcript_123753;F01	+K01681+K00928+K00600+K11755+K00615+K00134+K0164
	_transcript_123773;F01_transcript_123848;F01_transcript_1238	7+K00264+K11755+K00948+K00600+K11755+K00873+K002
	59;F01_transcript_124009;F01_transcript_124133;F01_transcrip	15+K01783+K00600+K00615+K01834+K00850+K00600+K01
	t_124153;F01_transcript_124206;F01_transcript_124416;F01_tr	783+K00765+K01623+K01696+K01738+K00928+K00765+K0
	anscript_124464;F01_transcript_124491;F01_transcript_124596	0872+K00058+K00873+K01803+K15849+K14272+K00600+K
	;F01_transcript_124658;F01_transcript_125014;F01_transcript_	13832+K01689+K00053+K01623+K01647+K00600+K00928+
	125144;F01_transcript_125334;F01_transcript_125391;F01_tran	K01693+K05359+K00600+K01689+K01586+K01652+K00928
	script_12547;F01_transcript_12550;F01_transcript_125563;F01	+K00549+K00815+K14682+K00058+K01653+K00928+K0168
	_transcript_125768;F01_transcript_125818;F01_transcript_1258	9+K01623+K00053+K00058+K01915+K01652+K01687+K006
	64;F01_transcript_125956;F01_transcript_126096;F01_transcrip	00+K13832+K00053+K00873+K01652+K00611+K14682+K13
	t_126139;F01_transcript_126185;F01_transcript_126272;F01_tr	832+K00053+K00928+K00615+K00927+K00928+K00549+K0
	anscript_12663;F01_transcript_126762;F01_transcript_127049;	0814+K01803+K14682+K00058+K01649+K00053+K01754+K
	F01_transcript_127193;F01_transcript_127209;F01_transcript_1	01755+K00058+K00873+K00927+K00873+K00873+K00549+
	27297;F01_transcript_127322;F01_transcript_127328;F01_trans	K01738+K00600+K01663+K00600+K00873+K00873+K01803
	cript_127418;F01_transcript_127540;F01_transcript_127620;F0	+K00850+K01758+K00600+K00013+K00800+K00873+K1427
	1_transcript_127834;F01_transcript_127921;F01_transcript_127	2+K01754+K00928+K00600+K00058+K00850+K01739+K138
	989;F01_transcript_128187;F01_transcript_128203;F01_transcri	32+K00873+K00058+K15227+K01657+K00800+K00215+K15
	pt_128590;F01_transcript_128717;F01_transcript_129051;F01_t	633+K00873+K00030+K00600+K01681+K01663+K00600+K0
	ranscript_129302;F01_transcript_129374;F01_transcript_12943	0600+K00600+K14272+K01657+K00850+K15634+K00873+K
	5;F01_transcript_129549;F01_transcript_129946;F01_transcript	00600+K00873+K00873+K06001+K00600+K14454+K13832+
	_130000;F01_transcript_130382;F01_transcript_130485;F01_tra	K01755+K01733+K00948+K14272+K01623+K00873+K00600
	nscript_130612;F01_transcript_130674;F01_transcript_130708;	+K01647+K01626+K00600+K01657+K00873+K00053+K0060
	F01_transcript_130895;F01_transcript_131043;F01_transcript_1	0+K01703+K01689+K00873+K00600+K15227+K01783+K008
	31057;F01_transcript_131587;F01_transcript_131784;F01_trans	73+K13832+K01739+K06001+K00600+K06001+K00600+K01
	cript_131892;F01_transcript_131899;F01_transcript_131919;F0	703+K00873+K00145+K00600+K00873+K15227+K00600+K0
	1_transcript_132024;F01_transcript_132042;F01_transcript_132	0600+K00600+K14085+K14085+K01626+K01739+K00600+K
	156;F01_transcript_132157;F01_transcript_132238;F01_transcri	05359+K14454+K01653+K00600+K01689+K15634+K01647+
	pt_132266;F01_transcript_132310;F01_transcript_132374;F01_t	K00030+K00600+K00815+K15634+K00927+K15849+K01736
	ranscript_132426;F01_transcript_132483;F01_transcript_13251	+K14677+K01586+K01703+K01623+K00815+K00811+K1584
	4;F01_transcript_132527;F01_transcript_132815;F01_transcript	9+K14272+K10206+K05359+K00927+K00815+K00053+K142
	_133019;F01_transcript_133194;F01_transcript_133224;F01_tra	72+K13832+K00873+K00948+K01696+K14455+K13832+K00

	nscript_133265;F01_transcript_133321;F01_transcript_133532;F01_transcript_133545;F01_transcript_13355;F01_transcript_133564;F01_transcript_133587;F01_transcript_133648;F01_transcript_13368;F01_transcript_133891;F01_transcript_133975;F01_transcript_134096;F01_transcript_134102;F01_transcript_13417;F01_transcript_134216;F01_transcript_134405;F01_transcript_134494;F01_transcript_134635;F01_transcript_134823;F01_transcript_135113;F01_transcript_135156;F01_transcript_135187;F01_transcript_135345;F01_transcript_135402;F01_transcript_135438;F01_transcript_135578;F01_transcript_135726;F01_transcript_135767;F01_transcript_135806;F01_transcript_135831;F01_transcript_135870;F01_transcript_135900;F01_transcript_135995;F01_transcript_136113;F01_transcript_136221;F01_transcript_136273;F01_transcript_136316;F01_transcript_136367;F01_transcript_136534;F01_transcript_136574;F01_transcript_136643;F01_transcript_136826;F01_transcript_136831;F01_transcript_136970;F01_transcript_137134;F01_transcript_13740;F01_transcript_137444;F01_transcript_137593;F01_transcript_138105;F01_transcript_138287;F01_transcript_138295;F01_transcript_138314;F01_transcript_138326;F01_transcript_138453;F01_transcript_138596;F01_transcript_138605;F01_transcript_138639;F01_transcript_138648;F01_transcript_138692;F01_transcript_138914;F01_transcript_139074;F01_transcript_139092;F01_transcript_139300;F01_transcript_139738;F01_transcript_139819;F01_transcript_139822;F01_transcript_139912;F01_transcript_140038;F01_transcript_140089;F01_transcript_140216;F01_transcript_140250;F01_transcript_140305;F01_transcript_140510;F01_transcript_140783;F01_transcript_140868;F01_transcript_140944;F01_transcript_140974;F01_transcript_14107;F01_transcript_141325;F01_transcript_141326;F01_transcript_141398;F01_transcript_141418;F01_transcript_141510;F01_transcript_14157;F01_transcript_141642;F01_transcript_141694;F01_transcript_141836;F01_transcript_141901;F01_transcript_141936;F01_transcript_142050;F01_transcript_142287;F01_transcript_142290;F01_transcript_142448;F01_transcript_142449;F01_transcript_142458;F01_transcript_142847;F01_transcript_142888;F01_transcript_14292;F01_transcript_142960;F01_transcript_142963;F01_transcript_14328;F01_transcript_143511;F01_transcript_143572;F01_tra	600+K00013+K01738+K01653+K00927+K00873+K00134+K00600+K00873+K01940+K14272+K15227+K00948+K01915+K01735+K00817+K01586+K14272+K01940+K00640+K00030+K00826+K00766+K01940+K01623+K01689+K00927+K00818+K14677+K00031+K01834+K01915+K00948+K00640+K00013+K00818+K01915+K00134+K00927+K01609+K00600+K01915+K14677+K00765+K01647+K05359+K15227+K00826+K14272+K00058+K00831+K00616+K01623+K00765+K00615+K01438+K00133+K14455+K00600+K00616+K01755+K01623+K00600+K14455+K00134+K00052+K00145+K00600+K00052+K01623+K13034+K15227+K00616+K01620+K12524+K00815+K00766+K00927+K01620+K05359+K00826+K13034+K01623+K01915+K01915+K00789+K01623+K01807+K00927+K00927+K00766+K01620+K01696+K01783+K01738+K01738+K01620+K00134+K01738+K01807+K01714+K01803+K00134+K01807+K01803+K00134+K00927+K01915+K00616+K00640+K00134+K00611+K01620+K00134+K01738+K00134+K01915+K01915+K00134+K01807+K00134+K01738+K01695+K01803+K01915+K01623+K01783+K01778+K01850+K01738+K01693+K01915+K01807+K00134+K01738+K01807+K01738+K01693+K00640+K00930+K00815+K01850+K00600+K01079+K01783+K00930+K01783+K01079+K00891+K00891+K01803+K01623+K01850+K01079+K00640+K00640+K01079+K00286+K01807+K12524+K00615+K01803+K01803+K00765+K01807+K01807+K01623+K15227+K15227+K01803+K00134+K00134+K01623+K01623+K01623+K00600+K01623+K01586+K13034+K00215+K00927+K15634+K01652+K01733+K00891+K15633+K14272+K05359+K00826+K01647+K13832+K00873+K01609+K00133+K00031+K00031+K01649+K05359+K00615+K00927+K01586+K00615+K00134+K12524+K00873+K00600+K00927+K00826+K01803+K00052+K13832+K13832+K00891+K01803+K01623+K01738+K00600+K00800+K00053+K14085+K00850+K15634+K01623+K01783+K00850+K00814+K00927+K00600+K01739+K01755+K14682+K00030+K14677+K01626+K00615+K00831+K00286+K00789+K00826+K00030+K00814+K00052+K14272+K15227+K15227+K01738+K13832+K15633+K01620+K00600+K05359+K00600+K00850+K00053+K01783+K00850+K00600+K00053+K00600+K00145+K10206+

	nscript_143862;F01_transcript_144117;F01_transcript_144293;F01_transcript_144458;F01_transcript_144469;F01_transcript_144716;F01_transcript_144749;F01_transcript_144853;F01_transcript_144901;F01_transcript_145058;F01_transcript_145241;F01_transcript_145271;F01_transcript_145324;F01_transcript_145367;F01_transcript_145616;F01_transcript_145805;F01_transcript_145851;F01_transcript_145918;F01_transcript_146107;F01_transcript_146194;F01_transcript_146307;F01_transcript_146322;F01_transcript_146336;F01_transcript_146649;F01_transcript_146840;F01_transcript_146888;F01_transcript_146924;F01_transcript_146963;F01_transcript_147008;F01_transcript_147232;F01_transcript_147260;F01_transcript_147408;F01_transcript_147492;F01_transcript_147523;F01_transcript_147534;F01_transcript_147721;F01_transcript_147740;F01_transcript_147987;F01_transcript_148016;F01_transcript_148355;F01_transcript_148580;F01_transcript_148697;F01_transcript_148710;F01_transcript_148792;F01_transcript_148941;F01_transcript_149261;F01_transcript_149327;F01_transcript_149398;F01_transcript_149467;F01_transcript_149508;F01_transcript_149827;F01_transcript_149933;F01_transcript_150132;F01_transcript_150241;F01_transcript_1503;F01_transcript_150354;F01_transcript_150508;F01_transcript_150511;F01_transcript_150639;F01_transcript_150809;F01_transcript_150940;F01_transcript_151028;F01_transcript_151079;F01_transcript_151234;F01_transcript_151300;F01_transcript_151431;F01_transcript_151553;F01_transcript_151854;F01_transcript_15205;F01_transcript_152349;F01_transcript_152454;F01_transcript_152726;F01_transcript_152798;F01_transcript_152812;F01_transcript_152838;F01_transcript_152848;F01_transcript_153015;F01_transcript_153213;F01_transcript_153307;F01_transcript_153395;F01_transcript_153480;F01_transcript_153523;F01_transcript_153754;F01_transcript_153773;F01_transcript_153912;F01_transcript_153933;F01_transcript_154155;F01_transcript_154314;F01_transcript_154436;F01_transcript_154468;F01_transcript_154485;F01_transcript_15454;F01_transcript_154557;F01_transcript_154598;F01_transcript_154618;F01_transcript_154711;F01_transcript_154837;F01_transcript_154869;F01_transcript_154897;F01_transcript_154917;F01_trans	K00549+K00873+K14272+K00058+K00873+K00600+K01681+K01807+K05359+K00891+K01649+K15227+K01652+K00058+K01652+K00600+K00264+K01623+K00891+K12524+K01807+K00030+K00818+K06001+K00873+K01623+K01663+K00611+K00930+K14085+K00873+K15633+K00800+K01940+K00600+K05359+K00615+K00873+K01647+K00600+K00053+K01681+K01915+K00814+K14272+K01736+K00815+K00615+K00600+K14454+K01696+K01703+K15633+K15227+K01609+K00145+K00549+K00134+K01915+K00053+K14272+K00600+K00765+K00615+K00640+K01807+K13034+K00134+K13034+K01783+K11755+K00928+K01626+K00615+K00030+K01738+K00600+K00030+K01689+K00053+K00800+K00615+K01626+K11755+K01703+K01658+K01647+K01738+K00927+K11755+K13832+K00826+K01623+K00549+K00814+K01915+K14677+K00600+K00615+K00058+K15633+K00826+K00600+K00850+K00930+K00620+K00800+K00600+K00053+K01850+K00927+K00615+K00615+K01652+K00789+K01623+K01738+K14272+K00600+K00850+K00600+K00891+K11755+K00031+K00600+K00134+K00927+K00600+K01609+K01738+K14272+K00873+K13832+K00815+K00600+K13832+K13832+K00600+K15634+K14272+K00264+K01915+K15633+K14272+K01702+K00928+K00800+K15633+K14085+K01623+K01696+K13034+K01687+K01940+K01681+K01739+K14682+K14455+K01681+K01689+K00615+K00811+K00286+K14682+K00891+K11755+K00600+K00615+K01736+K12657+K00134+K05359+K12524+K00765+K15227+K13832+K01807+K13832+K10206+K01703+K00811+K01696+K00948+K01657+K15227+K00134+K14454+K00600+K01807+K00145+K15633+K14454+K01755+K00600+K00600+K00873+K00800+K01738+K01647+K13832+K00873+K12657+K00826+K00215+K01623+K00145+K01803+K00549+K00927+K01693+K00928+K05359+K01738+K00145+K00600+K00927+K00826+K14272+K01649+K01649+K01738+K00928+K01703+K00615+K00873+K01663+K00053+K14677+K01647+K00873+K00600+K00814+K00811+K01738+K01735+K00826+K00030+K14455+K14085+K14272+K00817+K00873+K00800+K01807+K01623+K15849+K01623+K00215+K00215+K15634+K00615+K00145+K01658+K0080

	cript_155116;F01_transcript_155169;F01_transcript_155171;F01_transcript_155306;F01_transcript_155318;F01_transcript_155339;F01_transcript_155404;F01_transcript_15571;F01_transcript_155840;F01_transcript_156046;F01_transcript_156344;F01_transcript_156346;F01_transcript_156435;F01_transcript_156638;F01_transcript_157081;F01_transcript_157104;F01_transcript_157138;F01_transcript_157207;F01_transcript_157378;F01_transcript_157390;F01_transcript_157461;F01_transcript_157674;F01_transcript_158172;F01_transcript_158283;F01_transcript_158415;F01_transcript_158451;F01_transcript_158480;F01_transcript_158617;F01_transcript_158716;F01_transcript_158736;F01_transcript_159027;F01_transcript_159049;F01_transcript_159156;F01_transcript_159165;F01_transcript_159245;F01_transcript_159324;F01_transcript_159358;F01_transcript_159443;F01_transcript_159514;F01_transcript_159723;F01_transcript_159730;F01_transcript_159748;F01_transcript_160034;F01_transcript_160044;F01_transcript_160206;F01_transcript_160312;F01_transcript_160408;F01_transcript_160410;F01_transcript_160446;F01_transcript_160462;F01_transcript_160500;F01_transcript_160634;F01_transcript_160665;F01_transcript_160691;F01_transcript_160849;F01_transcript_160884;F01_transcript_160895;F01_transcript_161004;F01_transcript_161264;F01_transcript_161478;F01_transcript_161557;F01_transcript_161671;F01_transcript_161767;F01_transcript_161949;F01_transcript_161971;F01_transcript_16207;F01_transcript_162211;F01_transcript_162256;F01_transcript_16246;F01_transcript_162654;F01_transcript_162722;F01_transcript_162882;F01_transcript_163468;F01_transcript_16355;F01_transcript_163671;F01_transcript_163703;F01_transcript_163735;F01_transcript_163792;F01_transcript_163795;F01_transcript_163833;F01_transcript_164521;F01_transcript_164525;F01_transcript_164718;F01_transcript_165106;F01_transcript_165153;F01_transcript_165169;F01_transcript_165336;F01_transcript_165377;F01_transcript_165396;F01_transcript_165464;F01_transcript_165501;F01_transcript_165529;F01_transcript_165543;F01_transcript_165571;F01_transcript_16580;F01_transcript_166121;F01_transcript_166475;F01_transcript_166505;F01_transcript_167098;F01_transcript_167268;F01_transcript_167269;F01_transcript_167640;F01_transcript_167653;F01_tr	0+K14454+K00873+K00600+K00600+K00134+K01807+K00873+K00620+K01735+K01803+K00030+K00600+K00815+K01738+K01783+K00620+K00640+K01653+K00616+K00826+K00145+K00800+K00814+K00133+K00616+K01609+K14272+K01609+K05359+K14272+K00873+K14455+K01703+K14272+K14272+K00600+K01626+K00215+K00600+K00927+K00826+K00600+K13034+K14272+K01647+K13832+K00549+K14272+K00600+K01739+K00615+K00600+K01693+K01738+K11755+K01915+K00928+K00052+K00600+K14272+K00600+K14454+K00873+K01733+K05359+K00600+K00600+K00215+K01689+K00031+K00891+K14272+K01735+K14677+K01647+K01738+K01658

anscript_167733;F01_transcript_167751;F01_transcript_17308;
F01_transcript_17449;F01_transcript_17452;F01_transcript_177
68;F01_transcript_17809;F01_transcript_17879;F01_transcript_
18459;F01_transcript_18842;F01_transcript_19063;F01_transcri
pt_19071;F01_transcript_19390;F01_transcript_19461;F01_tran
script_19553;F01_transcript_19555;F01_transcript_19590;F01_t
ranscript_19656;F01_transcript_1968;F01_transcript_19790;F01
_transcript_19876;F01_transcript_19893;F01_transcript_19945;
F01_transcript_20059;F01_transcript_20069;F01_transcript_201
97;F01_transcript_20342;F01_transcript_20518;F01_transcript_
20537;F01_transcript_20546;F01_transcript_20719;F01_transcri
pt_20769;F01_transcript_20928;F01_transcript_20993;F01_tran
script_2109;F01_transcript_21167;F01_transcript_21550;F01_tr
anscript_21773;F01_transcript_21833;F01_transcript_22107;F0
1_transcript_22118;F01_transcript_22500;F01_transcript_22601
;F01_transcript_22646;F01_transcript_22758;F01_transcript_22
929;F01_transcript_23168;F01_transcript_23258;F01_transcript
_23299;F01_transcript_23327;F01_transcript_23407;F01_transc
ript_2341;F01_transcript_23969;F01_transcript_24050;F01_tran
script_24062;F01_transcript_24298;F01_transcript_24502;F01_t
ranscript_24533;F01_transcript_24566;F01_transcript_24624;F0
1_transcript_24651;F01_transcript_25171;F01_transcript_25334
;F01_transcript_25348;F01_transcript_25467;F01_transcript_25
546;F01_transcript_25633;F01_transcript_25705;F01_transcript
_25780;F01_transcript_25903;F01_transcript_25920;F01_transc
ript_26005;F01_transcript_26074;F01_transcript_26085;F01_tra
nscript_26171;F01_transcript_26177;F01_transcript_26222;F01
_transcript_26226;F01_transcript_26308;F01_transcript_26406;
F01_transcript_26508;F01_transcript_26583;F01_transcript_266
43;F01_transcript_2680;F01_transcript_26805;F01_transcript_2
6845;F01_transcript_2695;F01_transcript_27140;F01_transcript
_27248;F01_transcript_27425;F01_transcript_27932;F01_transc
ript_28043;F01_transcript_28090;F01_transcript_28121;F01_tra
nscript_28152;F01_transcript_28168;F01_transcript_28267;F01
_transcript_28268;F01_transcript_28405;F01_transcript_28483;
F01_transcript_28486;F01_transcript_28502;F01_transcript_286
33;F01_transcript_28733;F01_transcript_28741;F01_transcript_

28763;F01_transcript_28812;F01_transcript_28828;F01_transcri
pt_28852;F01_transcript_28865;F01_transcript_28916;F01_tran
script_28992;F01_transcript_28993;F01_transcript_29058;F01_t
ranscript_29072;F01_transcript_29149;F01_transcript_29161;F0
1_transcript_29214;F01_transcript_29257;F01_transcript_29301
;F01_transcript_29309;F01_transcript_29320;F01_transcript_29
328;F01_transcript_29399;F01_transcript_29482;F01_transcript
_29494;F01_transcript_29697;F01_transcript_29737;F01_transc
ript_29765;F01_transcript_29855;F01_transcript_29873;F01_tra
nscript_29908;F01_transcript_30075;F01_transcript_30092;F01
_transcript_30170;F01_transcript_30222;F01_transcript_30292;
F01_transcript_30362;F01_transcript_30413;F01_transcript_304
20;F01_transcript_30482;F01_transcript_30625;F01_transcript_
30736;F01_transcript_30778;F01_transcript_30802;F01_transcri
pt_30852;F01_transcript_30880;F01_transcript_30906;F01_tran
script_30997;F01_transcript_31015;F01_transcript_31061;F01_t
ranscript_31066;F01_transcript_31188;F01_transcript_31276;F0
1_transcript_31382;F01_transcript_31456;F01_transcript_31511
;F01_transcript_31526;F01_transcript_31687;F01_transcript_31
700;F01_transcript_31784;F01_transcript_31834;F01_transcript
_31881;F01_transcript_31983;F01_transcript_32005;F01_transc
ript_32090;F01_transcript_32147;F01_transcript_32304;F01_tra
nscript_32376;F01_transcript_32382;F01_transcript_32422;F01
_transcript_32449;F01_transcript_32492;F01_transcript_32622;
F01_transcript_32642;F01_transcript_32834;F01_transcript_328
81;F01_transcript_32913;F01_transcript_33011;F01_transcript_
33013;F01_transcript_33286;F01_transcript_33400;F01_transcri
pt_33418;F01_transcript_33449;F01_transcript_33472;F01_tran
script_33668;F01_transcript_33713;F01_transcript_33728;F01_t
ranscript_33768;F01_transcript_33776;F01_transcript_33879;F0
1_transcript_33912;F01_transcript_34014;F01_transcript_34197
;F01_transcript_34211;F01_transcript_34218;F01_transcript_34
357;F01_transcript_34385;F01_transcript_34402;F01_transcript
_34413;F01_transcript_34418;F01_transcript_34534;F01_transc
ript_34701;F01_transcript_34756;F01_transcript_34767;F01_tra
nscript_34832;F01_transcript_34873;F01_transcript_34996;F01
_transcript_35081;F01_transcript_35210;F01_transcript_35241;
F01_transcript_35262;F01_transcript_35302;F01_transcript_353

13;F01_transcript_35368;F01_transcript_35430;F01_transcript_35441;F01_transcript_35621;F01_transcript_35646;F01_transcript_35664;F01_transcript_35768;F01_transcript_35775;F01_transcript_35824;F01_transcript_35906;F01_transcript_35927;F01_transcript_35945;F01_transcript_35990;F01_transcript_36056;F01_transcript_36103;F01_transcript_36178;F01_transcript_36194;F01_transcript_36269;F01_transcript_36375;F01_transcript_36398;F01_transcript_36447;F01_transcript_3653;F01_transcript_36584;F01_transcript_366;F01_transcript_36639;F01_transcript_36808;F01_transcript_36862;F01_transcript_36937;F01_transcript_36997;F01_transcript_37086;F01_transcript_37274;F01_transcript_37334;F01_transcript_37385;F01_transcript_37402;F01_transcript_37409;F01_transcript_3744;F01_transcript_37457;F01_transcript_37512;F01_transcript_37523;F01_transcript_37558;F01_transcript_37687;F01_transcript_37725;F01_transcript_37739;F01_transcript_37835;F01_transcript_37859;F01_transcript_38047;F01_transcript_38117;F01_transcript_38228;F01_transcript_38229;F01_transcript_38274;F01_transcript_38315;F01_transcript_38382;F01_transcript_38394;F01_transcript_38511;F01_transcript_38522;F01_transcript_38763;F01_transcript_38874;F01_transcript_38877;F01_transcript_38937;F01_transcript_39013;F01_transcript_39110;F01_transcript_39170;F01_transcript_39223;F01_transcript_39305;F01_transcript_39348;F01_transcript_39520;F01_transcript_39525;F01_transcript_39557;F01_transcript_39760;F01_transcript_39842;F01_transcript_39940;F01_transcript_40033;F01_transcript_40196;F01_transcript_40206;F01_transcript_40209;F01_transcript_40243;F01_transcript_40354;F01_transcript_40430;F01_transcript_40444;F01_transcript_40448;F01_transcript_40501;F01_transcript_40536;F01_transcript_40591;F01_transcript_40811;F01_transcript_40883;F01_transcript_41006;F01_transcript_41015;F01_transcript_41074;F01_transcript_41181;F01_transcript_41216;F01_transcript_41338;F01_transcript_41421;F01_transcript_41423;F01_transcript_41430;F01_transcript_41482;F01_transcript_41547;F01_transcript_41738;F01_transcript_41905;F01_transcript_41924;F01_transcript_42029;F01_transcript_42030;F01_transcript_42165;F01_transcript_42350;F01_transcript_42368;F01_transcript_42378;F01_tra

nscript_42505;F01_transcript_42523;F01_transcript_42589;F01_transcript_42658;F01_transcript_42671;F01_transcript_43042;F01_transcript_43050;F01_transcript_43061;F01_transcript_43089;F01_transcript_43237;F01_transcript_43371;F01_transcript_43614;F01_transcript_43764;F01_transcript_43837;F01_transcript_44079;F01_transcript_4410;F01_transcript_4424;F01_transcript_44274;F01_transcript_44524;F01_transcript_4472;F01_transcript_44824;F01_transcript_45041;F01_transcript_45361;F01_transcript_45390;F01_transcript_46630;F01_transcript_47618;F01_transcript_47886;F01_transcript_48625;F01_transcript_49278;F01_transcript_49281;F01_transcript_49964;F01_transcript_50115;F01_transcript_50604;F01_transcript_51241;F01_transcript_51442;F01_transcript_51569;F01_transcript_51613;F01_transcript_51810;F01_transcript_51931;F01_transcript_51986;F01_transcript_52054;F01_transcript_52139;F01_transcript_52222;F01_transcript_52341;F01_transcript_52367;F01_transcript_52381;F01_transcript_52451;F01_transcript_52465;F01_transcript_52540;F01_transcript_52630;F01_transcript_52686;F01_transcript_52750;F01_transcript_52882;F01_transcript_53081;F01_transcript_53212;F01_transcript_53223;F01_transcript_53240;F01_transcript_53305;F01_transcript_53385;F01_transcript_5355;F01_transcript_53589;F01_transcript_53824;F01_transcript_54119;F01_transcript_54318;F01_transcript_54487;F01_transcript_54539;F01_transcript_54568;F01_transcript_54799;F01_transcript_55149;F01_transcript_55263;F01_transcript_55560;F01_transcript_55774;F01_transcript_55946;F01_transcript_56159;F01_transcript_56606;F01_transcript_56690;F01_transcript_56717;F01_transcript_56754;F01_transcript_56762;F01_transcript_56764;F01_transcript_56794;F01_transcript_56914;F01_transcript_56950;F01_transcript_5698;F01_transcript_56992;F01_transcript_57385;F01_transcript_57421;F01_transcript_57701;F01_transcript_5774;F01_transcript_57782;F01_transcript_5796;F01_transcript_57970;F01_transcript_58355;F01_transcript_58463;F01_transcript_58468;F01_transcript_58552;F01_transcript_58763;F01_transcript_58777;F01_transcript_58783;F01_transcript_58806;F01_transcript_58847;F01_transcript_5896;F01_transcript_59248;F01_transcript_59406;F01_transcript_59608;F01_transcript_59629;F01_transcript_59641;F01_transcript_59737;F01_transcript_599

61;F01_transcript_60186;F01_transcript_60194;F01_transcript_60235;F01_transcript_60381;F01_transcript_60455;F01_transcript_60635;F01_transcript_60833;F01_transcript_6089;F01_transcript_61029;F01_transcript_61112;F01_transcript_61178;F01_transcript_61243;F01_transcript_61414;F01_transcript_61445;F01_transcript_61449;F01_transcript_61535;F01_transcript_6156;F01_transcript_61628;F01_transcript_61708;F01_transcript_61840;F01_transcript_61852;F01_transcript_62037;F01_transcript_62228;F01_transcript_62260;F01_transcript_62329;F01_transcript_62451;F01_transcript_62575;F01_transcript_6274;F01_transcript_62775;F01_transcript_63028;F01_transcript_63041;F01_transcript_63045;F01_transcript_63160;F01_transcript_63171;F01_transcript_63328;F01_transcript_63456;F01_transcript_63480;F01_transcript_63491;F01_transcript_63521;F01_transcript_63669;F01_transcript_63781;F01_transcript_63860;F01_transcript_64095;F01_transcript_64505;F01_transcript_64543;F01_transcript_64577;F01_transcript_64872;F01_transcript_64897;F01_transcript_64900;F01_transcript_65464;F01_transcript_65509;F01_transcript_65515;F01_transcript_65567;F01_transcript_65614;F01_transcript_65744;F01_transcript_65775;F01_transcript_65810;F01_transcript_65858;F01_transcript_65935;F01_transcript_66275;F01_transcript_66352;F01_transcript_66391;F01_transcript_66486;F01_transcript_66578;F01_transcript_66754;F01_transcript_66793;F01_transcript_66852;F01_transcript_67213;F01_transcript_67228;F01_transcript_67332;F01_transcript_67540;F01_transcript_67829;F01_transcript_68093;F01_transcript_68381;F01_transcript_68454;F01_transcript_68495;F01_transcript_68627;F01_transcript_68670;F01_transcript_69411;F01_transcript_69503;F01_transcript_69688;F01_transcript_70058;F01_transcript_70154;F01_transcript_70160;F01_transcript_70704;F01_transcript_70881;F01_transcript_70950;F01_transcript_70967;F01_transcript_70971;F01_transcript_71194;F01_transcript_71324;F01_transcript_71361;F01_transcript_71528;F01_transcript_71564;F01_transcript_71789;F01_transcript_71954;F01_transcript_72018;F01_transcript_72176;F01_transcript_72187;F01_transcript_72229;F01_transcript_72421;F01_transcript_72578;F01_transcript_72598;F01_transcript_72651;F01_transcript_72718;F01_t

ranscript_72967;F01_transcript_73118;F01_transcript_73233;F01_transcript_73266;F01_transcript_73375;F01_transcript_73464;F01_transcript_73635;F01_transcript_73717;F01_transcript_73953;F01_transcript_74064;F01_transcript_74212;F01_transcript_74222;F01_transcript_74308;F01_transcript_74637;F01_transcript_74740;F01_transcript_7483;F01_transcript_74843;F01_transcript_75120;F01_transcript_75206;F01_transcript_75379;F01_transcript_75477;F01_transcript_75531;F01_transcript_75573;F01_transcript_75678;F01_transcript_75740;F01_transcript_75870;F01_transcript_75938;F01_transcript_75942;F01_transcript_76121;F01_transcript_76178;F01_transcript_76460;F01_transcript_76532;F01_transcript_76723;F01_transcript_76748;F01_transcript_76856;F01_transcript_76858;F01_transcript_76908;F01_transcript_76950;F01_transcript_76978;F01_transcript_77068;F01_transcript_77082;F01_transcript_77136;F01_transcript_77213;F01_transcript_77264;F01_transcript_77389;F01_transcript_77418;F01_transcript_77476;F01_transcript_77518;F01_transcript_77531;F01_transcript_77574;F01_transcript_77873;F01_transcript_77976;F01_transcript_78063;F01_transcript_78254;F01_transcript_78302;F01_transcript_78308;F01_transcript_78437;F01_transcript_78472;F01_transcript_78612;F01_transcript_78678;F01_transcript_78720;F01_transcript_78791;F01_transcript_78831;F01_transcript_78912;F01_transcript_78919;F01_transcript_79045;F01_transcript_79120;F01_transcript_79280;F01_transcript_79320;F01_transcript_79353;F01_transcript_79375;F01_transcript_79481;F01_transcript_79562;F01_transcript_79736;F01_transcript_79966;F01_transcript_80154;F01_transcript_80225;F01_transcript_8028;F01_transcript_80310;F01_transcript_80548;F01_transcript_80555;F01_transcript_80698;F01_transcript_80747;F01_transcript_80769;F01_transcript_80889;F01_transcript_80918;F01_transcript_80923;F01_transcript_80943;F01_transcript_80994;F01_transcript_81078;F01_transcript_81090;F01_transcript_81466;F01_transcript_81489;F01_transcript_81686;F01_transcript_81767;F01_transcript_82092;F01_transcript_82110;F01_transcript_82373;F01_transcript_82389;F01_transcript_82718;F01_transcript_82746;F01_transcript_82791;F01_transcript_83229;F01_transcript_83284;F01_transcript_83381;F01_transcript_83526;F01_transcript_83649;F01_transcript_83682;F01_transc

	ript_83737;F01_transcript_83748;F01_transcript_83801;F01_transcript_83916;F01_transcript_83984;F01_transcript_8406;F01_transcript_84352;F01_transcript_84568;F01_transcript_84746;F01_transcript_84894;F01_transcript_84920;F01_transcript_84934;F01_transcript_85168;F01_transcript_85279;F01_transcript_85411;F01_transcript_85473;F01_transcript_85599;F01_transcript_85624;F01_transcript_85721;F01_transcript_85796;F01_transcript_85872;F01_transcript_85969;F01_transcript_86031;F01_transcript_86113;F01_transcript_8631;F01_transcript_86731;F01_transcript_86847;F01_transcript_86970;F01_transcript_87136;F01_transcript_87223;F01_transcript_87267;F01_transcript_87452;F01_transcript_87504;F01_transcript_8790;F01_transcript_87957;F01_transcript_87962;F01_transcript_87964;F01_transcript_88003;F01_transcript_88131;F01_transcript_88234;F01_transcript_88383;F01_transcript_88445;F01_transcript_88527;F01_transcript_88550;F01_transcript_8856;F01_transcript_88662;F01_transcript_88765;F01_transcript_88898;F01_transcript_88942;F01_transcript_89140;F01_transcript_89459;F01_transcript_89629;F01_transcript_89641;F01_transcript_89719;F01_transcript_8980;F01_transcript_89922;F01_transcript_90013;F01_transcript_90147;F01_transcript_90169;F01_transcript_90207;F01_transcript_90316;F01_transcript_90364;F01_transcript_90435;F01_transcript_90503;F01_transcript_90688;F01_transcript_90820;F01_transcript_90977;F01_transcript_91161;F01_transcript_91214;F01_transcript_91391;F01_transcript_91410;F01_transcript_91443;F01_transcript_91694;F01_transcript_92003;F01_transcript_92372;F01_transcript_92797;F01_transcript_92825;F01_transcript_92835;F01_transcript_92895;F01_transcript_92968;F01_transcript_93052;F01_transcript_93119;F01_transcript_93248;F01_transcript_93376;F01_transcript_9359;F01_transcript_93624;F01_transcript_93694;F01_transcript_93722;F01_transcript_93824;F01_transcript_93866;F01_transcript_93994;F01_transcript_94201;F01_transcript_94572;F01_transcript_94796;F01_transcript_94953;F01_transcript_95332;F01_transcript_96025;F01_transcript_96028;F01_transcript_96054;F01_transcript_96113;F01_transcript_96153;F01_transcript_96296;F01_transcript_96511;F01_transcript_96799;F01_transcript_96851;F01_transcript_97035;F01

			_transcript_97229;F01_transcript_97239;F01_transcript_97367;F01_transcript_97597;F01_transcript_9785;F01_transcript_97966;F01_transcript_98005;F01_transcript_98067;F01_transcript_98080;F01_transcript_98413;F01_transcript_98454;F01_transcript_98475;F01_transcript_98527;F01_transcript_98682;F01_transcript_98918;F01_transcript_98971;F01_transcript_99014;F01_transcript_99615;F01_transcript_99924;F01_transcript_99996;	
Vancomycin resistance	ko01502	4	F01_transcript_110802;F01_transcript_166929;F01_transcript_55650;F01_transcript_84103;	K02563+K01000+K01000+K01000
ABC transporters	ko02010	83	F01_transcript_100258;F01_transcript_102089;F01_transcript_1022;F01_transcript_103083;F01_transcript_103133;F01_transcript_10500;F01_transcript_107278;F01_transcript_108692;F01_transcript_108781;F01_transcript_109378;F01_transcript_110413;F01_transcript_110781;F01_transcript_117981;F01_transcript_120333;F01_transcript_122008;F01_transcript_122624;F01_transcript_124764;F01_transcript_127314;F01_transcript_127562;F01_transcript_128242;F01_transcript_130779;F01_transcript_131027;F01_transcript_131365;F01_transcript_131838;F01_transcript_135561;F01_transcript_136320;F01_transcript_137199;F01_transcript_138644;F01_transcript_138990;F01_transcript_140185;F01_transcript_142526;F01_transcript_144481;F01_transcript_144998;F01_transcript_145154;F01_transcript_148743;F01_transcript_148786;F01_transcript_151363;F01_transcript_151877;F01_transcript_153356;F01_transcript_153540;F01_transcript_154355;F01_transcript_154809;F01_transcript_155903;F01_transcript_155970;F01_transcript_157043;F01_transcript_157449;F01_transcript_159318;F01_transcript_159913;F01_transcript_160915;F01_transcript_161314;F01_transcript_162279;F01_transcript_1624;F01_transcript_163222;F01_transcript_165322;F01_transcript_167420;F01_transcript_19518;F01_transcript_19523;F01_transcript_23883;F01_transcript_2436;F01_transcript_342;F01_transcript_424;F01_transcript_53367;F01_transcript_55748;F01_transcript_56443;F01_transcript_57374;F01_transcript_585	K05674+K05658+K05658+K05658+K05674+K05663+K05663+K05658+K05643+K05658+K05674+K05658+K05658+K05658+K05674+K05658+K05674+K05658+K05663+K05643+K05658+K05663+K05658+K05674+K05658+K05656+K05674+K05658+K05658+K05674+K05658+K05674+K05658+K05674+K05674+K05674+K05674+K05658+K05658+K05674+K05658+K05663+K05657+K05658+K05643+K05658+K05643+K05657+K05658+K05674+K05674+K05658+K05674+K05674+K05677+K05674+K05658+K05681+K05658+K05674+K05658+K05657+K05658+K05658+K05658+K05658+K05658+K05658+K05658+K05658+K05658+K05681+K05658+K05658+K05663+K05674+K05658+K05643+K05643+K05674+K05658+K05663+K05674

			;F01_transcript_60277;F01_transcript_64748;F01_transcript_682;F01_transcript_70814;F01_transcript_71806;F01_transcript_72101;F01_transcript_74049;F01_transcript_76193;F01_transcript_81843;F01_transcript_82123;F01_transcript_84736;F01_transcript_86839;F01_transcript_94769;F01_transcript_94963;F01_transcript_95300;F01_transcript_97735;F01_transcript_99686;	
Ribosome biogenesis in eukaryotes	ko03008	363	F01_transcript_100921;F01_transcript_100978;F01_transcript_101093;F01_transcript_101418;F01_transcript_101629;F01_transcript_101787;F01_transcript_101911;F01_transcript_102399;F01_transcript_102864;F01_transcript_102963;F01_transcript_103124;F01_transcript_103456;F01_transcript_103645;F01_transcript_10416;F01_transcript_104514;F01_transcript_105158;F01_transcript_105860;F01_transcript_105913;F01_transcript_106365;F01_transcript_106390;F01_transcript_106460;F01_transcript_107317;F01_transcript_108301;F01_transcript_108655;F01_transcript_108662;F01_transcript_109281;F01_transcript_110726;F01_transcript_110734;F01_transcript_110821;F01_transcript_110843;F01_transcript_111371;F01_transcript_111607;F01_transcript_112735;F01_transcript_112927;F01_transcript_113368;F01_transcript_113536;F01_transcript_114230;F01_transcript_114874;F01_transcript_115016;F01_transcript_115271;F01_transcript_1158;F01_transcript_116508;F01_transcript_117002;F01_transcript_117106;F01_transcript_118385;F01_transcript_11876;F01_transcript_118913;F01_transcript_119200;F01_transcript_120118;F01_transcript_121395;F01_transcript_121676;F01_transcript_123063;F01_transcript_123354;F01_transcript_124185;F01_transcript_124481;F01_transcript_124513;F01_transcript_125284;F01_transcript_125326;F01_transcript_125702;F01_transcript_125849;F01_transcript_126001;F01_transcript_126461;F01_transcript_127155;F01_transcript_127619;F01_transcript_128311;F01_transcript_128334;F01_transcript_129004;F01_transcript_129131;F01_transcript_129155;F01_transcript_129355;F01_transcript_129869;F01_transcript_129889;F01_transcript_130260;F01_transcript_130282;F01_transcript_130599;F01_transcript_130601;F01_transcript_131341;F01_transcript_131418;F01_transcript_133710;F01_transcript_133720;F01_transcript_133799;F01_transcript_133838;F01_transcript_133931;F01_transcript_13433	K14550+K03115+K14537+K07936+K14563+K14568+K14537+K14570+K14573+K13288+K14569+K14570+K07936+K14548+K03537+K03097+K07936+K12619+K14563+K18532+K12845+K14548+K03097+K03539+K03097+K01164+K07936+K07936+K14552+K14521+K03264+K14564+K07178+K14521+K14564+K03115+K13288+K14570+K06943+K11883+K14290+K03115+K14554+K11108+K07562+K14556+K03115+K11131+K14556+K11883+K07178+K06943+K11108+K07936+K03097+K14571+K14525+K03115+K14552+K01164+K03097+K14566+K11131+K03097+K03538+K14537+K14544+K03097+K14537+K14544+K03115+K12619+K14290+K03115+K14570+K14563+K03115+K14557+K14544+K14570+K14556+K14555+K03115+K14555+K12619+K14290+K11129+K07936+K03538+K14548+K03115+K14574+K03097+K07562+K03264+K14567+K12619+K14571+K18532+K03115+K14546+K14568+K06943+K14546+K14545+K14556+K14570+K14525+K07936+K14538+K14555+K14290+K14572+K01164+K14573+K14568+K11131+K07936+K03097+K12619+K14561+K14290+K12619+K07179+K07179+K14573+K07936+K14561+K11108+K03097+K14548+K14568+K06943+K03264+K03264+K14525+K14546+K14566+K11883+K14571+K03115+K12619+K03539+K14566+K14550+K14570+K14569+K03115+K03097+K14571+K14537+K07936+K14290+K14290+K14570+K03539+K14546+K03115+K11883+K14539+K03115+K14539+K14557+K14553+K14565+K14561+K14557+K14570+K14570+K14544+K11131+K14570+K14570+K14559+K14570+K07178+K07562+K07562+K14538+K14564+K14570+K07562+K06943+K14521+K14564+K14564+K14565+K14564+K06943+K14549+K03115+K14558+K03097+K03115+K03115+K03097+K03115+K03097+K14521+K03115+K03097+K03097+K03115+K03115+K03097+K14566+K11108+K14545+K03115+K03538+K03115+K14568+K079

	9;F01_transcript_134536;F01_transcript_1347;F01_transcript_135828;F01_transcript_135846;F01_transcript_136237;F01_transcript_136305;F01_transcript_136969;F01_transcript_137456;F01_transcript_137465;F01_transcript_137689;F01_transcript_138012;F01_transcript_138088;F01_transcript_1387;F01_transcript_139342;F01_transcript_140054;F01_transcript_140244;F01_transcript_14051;F01_transcript_141255;F01_transcript_141446;F01_transcript_14287;F01_transcript_142899;F01_transcript_144600;F01_transcript_144828;F01_transcript_145030;F01_transcript_145427;F01_transcript_145937;F01_transcript_146576;F01_transcript_146582;F01_transcript_147182;F01_transcript_147215;F01_transcript_147681;F01_transcript_149422;F01_transcript_149692;F01_transcript_151633;F01_transcript_153057;F01_transcript_154231;F01_transcript_154233;F01_transcript_1544;F01_transcript_154404;F01_transcript_154812;F01_transcript_154875;F01_transcript_154909;F01_transcript_155365;F01_transcript_155383;F01_transcript_156296;F01_transcript_157075;F01_transcript_157405;F01_transcript_15815;F01_transcript_15891;F01_transcript_158965;F01_transcript_159598;F01_transcript_159842;F01_transcript_16112;F01_transcript_161227;F01_transcript_161758;F01_transcript_162026;F01_transcript_16205;F01_transcript_16239;F01_transcript_162998;F01_transcript_163205;F01_transcript_163662;F01_transcript_163721;F01_transcript_164012;F01_transcript_165853;F01_transcript_166284;F01_transcript_166399;F01_transcript_16742;F01_transcript_16907;F01_transcript_1703;F01_transcript_1929;F01_transcript_20021;F01_transcript_20428;F01_transcript_20492;F01_transcript_20535;F01_transcript_20982;F01_transcript_22472;F01_transcript_22509;F01_transcript_23166;F01_transcript_23388;F01_transcript_23744;F01_transcript_24318;F01_transcript_24326;F01_transcript_24436;F01_transcript_24753;F01_transcript_25198;F01_transcript_2536;F01_transcript_25439;F01_transcript_25846;F01_transcript_26116;F01_transcript_26451;F01_transcript_26579;F01_transcript_26699;F01_transcript_27482;F01_transcript_27530;F01_transcript_27545;F01_transcript_28323;F01_transcript_28699;F01_transcript_29230;F01_transcript_29446;F01_transcript_3022;F01_transcript_30370;F01_transcript_30382;F01_transcript_30567;F01_transcript_30622;F01_transcript_31309;F01_tran	36+K03097+K14539+K14556+K14566+K03264+K07936+K03264+K03264+K14568+K07936+K07936+K18532+K07936+K14560+K12845+K12845+K14539+K03097+K14290+K12619+K12619+K14548+K14558+K03097+K06943+K03264+K14561+K03264+K14521+K14568+K14567+K14552+K14561+K14290+K14563+K13288+K14571+K03115+K14560+K07562+K11883+K12619+K14557+K14571+K14536+K14546+K03097+K03115+K14563+K14555+K12619+K14570+K14552+K03537+K01164+K11129+K14569+K14557+K03264+K13288+K14536+K14574+K14567+K14554+K12619+K06943+K11131+K14555+K14563+K14546+K14546+K14552+K14290+K14544+K11108+K14536+K14537+K03097+K03115+K03097+K14568+K01164+K12619+K14570+K14555+K14564+K03097+K03538+K14571+K12619+K14570+K14571+K07936+K14552+K14290+K14570+K01164+K14552+K14555+K03097+K03097+K12619+K07178+K03115+K14570+K14555+K11129+K03115+K14537+K03115+K11130+K14290+K14539+K11883+K03115+K06943+K07179+K07936+K14570+K03097+K03115+K14569+K14290+K14553+K07936+K03537+K14570+K14555+K12619+K11129+K03538+K14568+K03264+K03115+K03097+K03264+K14556+K12619+K14559+K14544+K06943+K07178+K14290+K14290+K14290+K14570+K14570+K07179+K01164+K14521

script_31742;F01_transcript_34726;F01_transcript_3502;F01_tr
anscript_35021;F01_transcript_35486;F01_transcript_35909;F0
1_transcript_36532;F01_transcript_36669;F01_transcript_36834
;F01_transcript_3702;F01_transcript_37791;F01_transcript_379
04;F01_transcript_38703;F01_transcript_38800;F01_transcript_
38917;F01_transcript_39354;F01_transcript_39755;F01_transcri
pt_39981;F01_transcript_40028;F01_transcript_40149;F01_tran
script_40395;F01_transcript_4047;F01_transcript_40523;F01_tr
anscript_41141;F01_transcript_41394;F01_transcript_41524;F0
1_transcript_4330;F01_transcript_43612;F01_transcript_43766;
F01_transcript_44013;F01_transcript_44335;F01_transcript_444
03;F01_transcript_45301;F01_transcript_45507;F01_transcript_
45591;F01_transcript_46213;F01_transcript_46226;F01_transcri
pt_47989;F01_transcript_48322;F01_transcript_48349;F01_tran
script_51721;F01_transcript_51921;F01_transcript_52040;F01_t
ranscript_52105;F01_transcript_52249;F01_transcript_52345;F0
1_transcript_5250;F01_transcript_52669;F01_transcript_54083;
F01_transcript_54258;F01_transcript_55439;F01_transcript_560
25;F01_transcript_56326;F01_transcript_56370;F01_transcript_
5669;F01_transcript_56825;F01_transcript_57251;F01_transcrip
t_57268;F01_transcript_57625;F01_transcript_58528;F01_trans
cript_58585;F01_transcript_58625;F01_transcript_58852;F01_tr
anscript_58868;F01_transcript_58977;F01_transcript_59431;F0
1_transcript_59866;F01_transcript_60408;F01_transcript_60697
;F01_transcript_61012;F01_transcript_61085;F01_transcript_61
341;F01_transcript_6175;F01_transcript_62001;F01_transcript_
62686;F01_transcript_62709;F01_transcript_64101;F01_transcri
pt_64117;F01_transcript_6415;F01_transcript_64246;F01_trans
cript_64999;F01_transcript_65538;F01_transcript_65611;F01_tr
anscript_65749;F01_transcript_65958;F01_transcript_66076;F0
1_transcript_6650;F01_transcript_6667;F01_transcript_66698;F
01_transcript_66971;F01_transcript_67481;F01_transcript_6764
9;F01_transcript_68452;F01_transcript_68954;F01_transcript_6
9982;F01_transcript_7049;F01_transcript_70511;F01_transcript
_70963;F01_transcript_71606;F01_transcript_71876;F01_transc
ript_71983;F01_transcript_72662;F01_transcript_72687;F01_tra
nscript_7282;F01_transcript_7294;F01_transcript_7322;F01_tra

			nscript_73264;F01_transcript_73367;F01_transcript_73792;F01_transcript_74340;F01_transcript_74437;F01_transcript_74782;F01_transcript_74968;F01_transcript_75889;F01_transcript_75960;F01_transcript_77081;F01_transcript_77515;F01_transcript_77844;F01_transcript_78112;F01_transcript_78349;F01_transcript_7888;F01_transcript_78900;F01_transcript_8053;F01_transcript_80662;F01_transcript_82131;F01_transcript_82957;F01_transcript_83010;F01_transcript_83225;F01_transcript_83242;F01_transcript_83285;F01_transcript_83497;F01_transcript_84201;F01_transcript_84661;F01_transcript_84685;F01_transcript_84838;F01_transcript_85998;F01_transcript_86330;F01_transcript_86453;F01_transcript_87043;F01_transcript_87135;F01_transcript_87166;F01_transcript_87880;F01_transcript_88419;F01_transcript_88600;F01_transcript_88732;F01_transcript_895;F01_transcript_89564;F01_transcript_90142;F01_transcript_90183;F01_transcript_90627;F01_transcript_91277;F01_transcript_9132;F01_transcript_91558;F01_transcript_91638;F01_transcript_91928;F01_transcript_91990;F01_transcript_92222;F01_transcript_92298;F01_transcript_92303;F01_transcript_92501;F01_transcript_92633;F01_transcript_936;F01_transcript_93921;F01_transcript_94874;F01_transcript_95681;F01_transcript_96245;F01_transcript_96394;F01_transcript_96449;F01_transcript_97467;F01_transcript_98419;F01_transcript_9877;F01_transcript_98845;F01_transcript_99133;F01_transcript_99569;	
Ribosome	ko03010	879	F01_transcript_100200;F01_transcript_100255;F01_transcript_100394;F01_transcript_100398;F01_transcript_100654;F01_transcript_100747;F01_transcript_100902;F01_transcript_100934;F01_transcript_101032;F01_transcript_101389;F01_transcript_101657;F01_transcript_102114;F01_transcript_102404;F01_transcript_102620;F01_transcript_102745;F01_transcript_102867;F01_transcript_102967;F01_transcript_103226;F01_transcript_103291;F01_transcript_103339;F01_transcript_103457;F01_transcript_103592;F01_transcript_10430;F01_transcript_104463;F01_transcript_104802;F01_transcript_104820;F01_transcript_104924;F01_transcript_105074;F01_transcript_105134;F01_transcript_105164;F01_transcript_105186;F01_transcript_105293;F01_transcript_105849;F01_transcript_106004;F01_transcript_106025;F01_transcript_106391;F01_transcript_106493;F01_transcript_1065	K02870+K02921+K02881+K02863+K02914+K02931+K02963+K02935+K02976+K02886+K02890+K02935+K02897+K02966+K02966+K02981+K02985+K02982+K02918+K02998+K02888+K02903+K02953+K02874+K02953+K02887+K02953+K02888+K02913+K02991+K02977+K02879+K02881+K02985+K02917+K02970+K02883+K02984+K02939+K02888+K02870+K02896+K02898+K02976+K02922+K02876+K02930+K02874+K02870+K02929+K02922+K02866+K02870+K02914+K02997+K02876+K02993+K02890+K02941+K02971+K02926+K02912+K02870+K02893+K02976+K02921+K02979+K02961+K02906+K02907+K02977+K02870+K02988+K02968+K02939+K02932+K02935+K02907+K02947+K02898+K02935+K02992+K02985+K02925+K02976+K02902+K02936+K02979+K02871+K02886+K02866+K02871+K02988+K02937+K02931+K02895+

	49;F01_transcript_106657;F01_transcript_106700;F01_transcript_106935;F01_transcript_107092;F01_transcript_107381;F01_transcript_107444;F01_transcript_107561;F01_transcript_107752;F01_transcript_107757;F01_transcript_107948;F01_transcript_108172;F01_transcript_108191;F01_transcript_108476;F01_transcript_108790;F01_transcript_108814;F01_transcript_109136;F01_transcript_109194;F01_transcript_109313;F01_transcript_109331;F01_transcript_109483;F01_transcript_109629;F01_transcript_109780;F01_transcript_110288;F01_transcript_110345;F01_transcript_110869;F01_transcript_111103;F01_transcript_111143;F01_transcript_111267;F01_transcript_111345;F01_transcript_112031;F01_transcript_112138;F01_transcript_112340;F01_transcript_112403;F01_transcript_112525;F01_transcript_112552;F01_transcript_112626;F01_transcript_112793;F01_transcript_113145;F01_transcript_113247;F01_transcript_113571;F01_transcript_113695;F01_transcript_113947;F01_transcript_113976;F01_transcript_114302;F01_transcript_114317;F01_transcript_114715;F01_transcript_115675;F01_transcript_115702;F01_transcript_116001;F01_transcript_116063;F01_transcript_116193;F01_transcript_116281;F01_transcript_116460;F01_transcript_117090;F01_transcript_117212;F01_transcript_117576;F01_transcript_118057;F01_transcript_118066;F01_transcript_118886;F01_transcript_119540;F01_transcript_119753;F01_transcript_119897;F01_transcript_120328;F01_transcript_120408;F01_transcript_120760;F01_transcript_121268;F01_transcript_121566;F01_transcript_121608;F01_transcript_122351;F01_transcript_122438;F01_transcript_122958;F01_transcript_122985;F01_transcript_123022;F01_transcript_123078;F01_transcript_123154;F01_transcript_123191;F01_transcript_123325;F01_transcript_123345;F01_transcript_124051;F01_transcript_124101;F01_transcript_124156;F01_transcript_124379;F01_transcript_124415;F01_transcript_124580;F01_transcript_124643;F01_transcript_124759;F01_transcript_124839;F01_transcript_124854;F01_transcript_124902;F01_transcript_124986;F01_transcript_125152;F01_transcript_125217;F01_transcript_125286;F01_transcript_125295;F01_transcript_125361;F01_transcript_125706;F01_transcript_125811;F01_transcript_126166;F01_transcript_126234;F01_transcript_12	K02904+K02990+K02920+K02917+K02939+K02924+K02901+K02888+K02989+K02939+K02964+K02887+K02950+K02945+K02931+K02974+K02865+K02891+K02918+K02875+K02876+K02941+K02915+K02930+K02865+K02969+K02933+K02945+K02911+K02889+K02863+K02981+K02974+K02890+K02959+K02933+K02895+K02998+K02960+K02912+K02899+K02933+K02915+K02896+K02980+K02951+K02887+K02980+K02981+K02915+K02946+K02945+K02863+K02918+K02985+K02967+K02891+K02932+K02863+K02906+K02935+K02970+K02886+K02906+K02932+K02935+K02968+K02893+K02955+K02997+K02926+K02864+K02975+K02977+K02865+K02940+K02977+K02887+K02945+K02977+K02937+K02953+K02942+K02866+K02966+K02932+K02871+K02877+K02998+K02917+K02982+K02932+K02930+K02918+K02988+K02918+K02946+K02901+K02955+K02982+K02985+K02982+K02979+K02900+K02935+K02945+K02895+K02882+K02925+K02906+K02874+K02863+K02927+K02890+K02912+K02881+K02894+K02879+K02995+K02871+K02975+K02998+K02895+K02951+K02939+K02899+K02940+K02884+K02945+K02888+K02888+K02964+K02953+K02941+K02995+K02974+K02935+K02920+K02961+K02975+K02890+K02990+K02935+K02875+K02961+K02877+K02911+K02946+K02899+K02918+K02995+K02987+K02995+K02872+K02898+K02872+K02945+K02910+K02982+K02990+K02921+K02932+K02906+K02977+K02906+K02899+K02915+K02993+K02896+K02951+K02871+K02914+K02864+K02921+K02929+K02966+K02918+K02879+K02902+K02863+K02942+K02949+K02995+K02966+K02914+K02894+K02917+K02864+K02939+K02991+K02995+K02876+K02933+K02893+K02879+K02976+K02943+K02937+K02889+K02873+K02962+K02886+K02945+K02983+K02883+K02974+K02966+K02883+K02988+K02866+K02863+K02990+K02908+K02984+K02867+K02982+K02903+K02933+K02932+K02933+K02952+K02931+K02923+K02982+K02916+K02949+K02865+K02988+K02877+K02887+K02871+K02936+K02913+K02933+K02942+K02880+K02983+K02899+K02933+K02900+K02886+K02946+K02962+K02888+K02988+K02881+K02902+K02988+K02901+K02945+K02935+K02910+K02969+K0296

	625;F01_transcript_126446;F01_transcript_126581;F01_transcript_12670;F01_transcript_126735;F01_transcript_127283;F01_transcript_127521;F01_transcript_127534;F01_transcript_127722;F01_transcript_128177;F01_transcript_128225;F01_transcript_128335;F01_transcript_128364;F01_transcript_128723;F01_transcript_128792;F01_transcript_128925;F01_transcript_129210;F01_transcript_129272;F01_transcript_129344;F01_transcript_129447;F01_transcript_129486;F01_transcript_129797;F01_transcript_129967;F01_transcript_130187;F01_transcript_130346;F01_transcript_130841;F01_transcript_130876;F01_transcript_130886;F01_transcript_130915;F01_transcript_131366;F01_transcript_131558;F01_transcript_131875;F01_transcript_131894;F01_transcript_132148;F01_transcript_132366;F01_transcript_132588;F01_transcript_132613;F01_transcript_132848;F01_transcript_132869;F01_transcript_132906;F01_transcript_132912;F01_transcript_133174;F01_transcript_133808;F01_transcript_134130;F01_transcript_134591;F01_transcript_13463;F01_transcript_134727;F01_transcript_134740;F01_transcript_135148;F01_transcript_135318;F01_transcript_135444;F01_transcript_135648;F01_transcript_135714;F01_transcript_135729;F01_transcript_135834;F01_transcript_135901;F01_transcript_136189;F01_transcript_136397;F01_transcript_1364;F01_transcript_137061;F01_transcript_137996;F01_transcript_138174;F01_transcript_138405;F01_transcript_138470;F01_transcript_139457;F01_transcript_139629;F01_transcript_140413;F01_transcript_140521;F01_transcript_140626;F01_transcript_141479;F01_transcript_141995;F01_transcript_142328;F01_transcript_142914;F01_transcript_143025;F01_transcript_143083;F01_transcript_143086;F01_transcript_143190;F01_transcript_143428;F01_transcript_143754;F01_transcript_144139;F01_transcript_144172;F01_transcript_144198;F01_transcript_144237;F01_transcript_144316;F01_transcript_144592;F01_transcript_144609;F01_transcript_144856;F01_transcript_144936;F01_transcript_145180;F01_transcript_145350;F01_transcript_145499;F01_transcript_145576;F01_transcript_145786;F01_transcript_146105;F01_transcript_146146;F01_transcript_146156;F01_transcript_146338;F01_transcript_146382;F01_transcript_146431;F01_transcript_146563;F01_transcript_146831;F01_transcript_147070;F01_transcript_147202;F01_transcript	1+K02953+K02886+K02945+K02881+K02996+K02872+K02930+K02886+K02946+K02888+K02966+K02925+K02863+K02945+K02863+K02926+K02925+K02890+K02926+K02995+K02990+K02966+K02906+K02863+K02926+K02949+K02935+K02926+K02890+K02948+K02998+K02884+K02888+K02935+K02932+K02932+K02932+K02876+K02998+K02876+K02981+K02881+K02888+K02876+K02988+K02902+K02936+K02888+K02987+K02935+K02939+K02879+K02933+K02939+K02876+K02987+K02987+K02985+K02931+K02936+K02948+K02936+K02871+K02989+K02937+K02897+K02936+K02936+K02931+K02933+K02938+K02871+K02906+K02888+K02995+K02947+K02990+K02885+K02938+K02884+K02991+K02990+K02879+K02939+K02884+K02937+K02939+K02933+K02865+K02885+K02867+K02883+K02865+K02877+K02937+K02867+K02985+K02865+K02885+K02881+K02946+K02865+K02912+K02997+K02866+K02989+K02885+K02946+K02959+K02997+K02946+K02934+K02997+K02899+K02880+K02883+K02864+K02879+K02885+K02968+K02865+K02865+K02990+K02993+K02895+K02872+K02877+K02989+K02879+K02946+K02935+K02935+K02904+K02881+K02991+K02899+K02990+K02935+K02968+K02940+K02877+K02907+K02964+K02880+K02996+K02947+K02903+K02870+K02880+K02993+K02953+K02883+K02894+K02896+K02966+K02990+K02949+K02866+K02955+K02889+K02882+K02868+K02870+K02896+K02974+K02899+K02870+K02868+K02997+K02946+K02966+K02883+K02916+K02894+K02962+K02943+K02895+K02894+K02955+K02977+K02889+K02949+K02898+K02882+K02870+K02952+K02955+K02943+K02979+K02887+K02966+K02911+K02917+K02974+K02964+K02895+K02974+K02953+K02925+K02973+K02880+K02960+K02904+K02973+K02973+K02966+K02961+K02955+K02973+K02953+K02942+K02953+K02899+K02977+K02977+K02962+K02899+K02877+K02957+K02912+K02917+K02931+K02975+K02915+K02976+K02994+K02973+K02898+K02957+K02960+K02903+K02960+K02974+K02903+K02966+K02957+K02927+K02920+K02918+K02908+K02915+K02927+K02927+K02917+K02867+K02943+K02914+K02921+K02997+K02898+K02977+K02891+K02927+K02901+K02922+K02900+K02980+K02929+K02973+K02900+K0

	_147284;F01_transcript_147336;F01_transcript_147407;F01_transcript_147473;F01_transcript_148031;F01_transcript_148083;F01_transcript_148432;F01_transcript_148545;F01_transcript_148758;F01_transcript_148871;F01_transcript_149235;F01_transcript_149239;F01_transcript_149354;F01_transcript_149460;F01_transcript_149502;F01_transcript_149582;F01_transcript_149820;F01_transcript_150136;F01_transcript_150231;F01_transcript_150785;F01_transcript_151002;F01_transcript_151035;F01_transcript_151106;F01_transcript_151235;F01_transcript_151874;F01_transcript_152053;F01_transcript_152113;F01_transcript_15224;F01_transcript_152322;F01_transcript_152535;F01_transcript_152561;F01_transcript_153506;F01_transcript_153878;F01_transcript_153896;F01_transcript_154018;F01_transcript_154043;F01_transcript_154166;F01_transcript_154963;F01_transcript_155328;F01_transcript_155463;F01_transcript_155838;F01_transcript_155854;F01_transcript_156318;F01_transcript_156405;F01_transcript_156419;F01_transcript_156448;F01_transcript_156535;F01_transcript_156537;F01_transcript_156669;F01_transcript_156725;F01_transcript_156784;F01_transcript_156792;F01_transcript_157168;F01_transcript_157639;F01_transcript_157873;F01_transcript_157926;F01_transcript_158241;F01_transcript_158549;F01_transcript_158945;F01_transcript_159040;F01_transcript_159060;F01_transcript_159196;F01_transcript_159236;F01_transcript_159797;F01_transcript_160020;F01_transcript_160210;F01_transcript_160313;F01_transcript_161001;F01_transcript_161245;F01_transcript_161306;F01_transcript_161433;F01_transcript_161610;F01_transcript_161809;F01_transcript_161874;F01_transcript_162088;F01_transcript_162223;F01_transcript_162757;F01_transcript_163622;F01_transcript_163633;F01_transcript_164412;F01_transcript_164442;F01_transcript_164933;F01_transcript_164955;F01_transcript_165582;F01_transcript_165679;F01_transcript_165704;F01_transcript_165794;F01_transcript_166117;F01_transcript_166128;F01_transcript_166374;F01_transcript_166460;F01_transcript_166899;F01_transcript_167249;F01_transcript_167322;F01_transcript_167725;F01_transcript_18673;F01_transcript_19164;F01_transcript_20170;F01_transcript_22340;F01_transcript_24019;F01_transcript_269	2927+K02875+K02919+K02915+K02969+K02891+K02918+K02975+K02927+K02901+K02883+K02943+K02894+K02918+K02977+K02929+K02908+K02929+K02875+K02882+K02978+K02894+K02921+K02908+K02920+K02921+K02971+K02917+K02924+K02922+K02971+K02975+K02891+K02979+K02889+K02971+K02979+K02955+K02979+K02923+K02923+K02983+K02922+K02980+K02923+K02894+K02983+K02921+K02913+K02980+K02962+K02980+K02905+K02924+K02905+K02924+K02987+K02971+K02952+K02927+K02996+K02864+K02863+K02946+K02916+K02863+K02992+K02955+K02896+K02945+K02918+K02875+K02912+K02895+K02995+K02957+K02976+K02939+K02985+K02866+K02871+K02872+K02955+K02863+K02892+K02996+K02937+K02895+K02984+K02902+K02872+K02882+K02882+K02975+K02876+K02905+K02971+K02918+K02993+K02868+K02966+K02863+K02893+K02894+K02995+K02895+K02942+K02863+K02912+K02877+K02975+K02912+K02924+K02993+K02948+K02881+K02962+K02955+K02876+K02991+K02903+K02922+K02877+K02896+K02984+K02997+K02912+K02886+K02918+K02997+K02939+K02871+K02957+K02945+K02964+K02971+K02961+K02947+K02876+K02876+K02988+K02927+K02915+K02969+K02865+K02900+K02888+K02991+K02932+K02925+K02974+K02957+K02863+K02938+K02961+K02921+K02955+K02937+K02865+K02920+K02931+K02990+K02941+K02985+K02969+K02912+K02941+K02912+K02959+K02960+K02899+K02885+K02957+K02967+K02868+K02946+K02876+K02888+K02883+K02886+K02955+K02918+K02943+K02986+K02872+K02979+K02963+K02968+K02879+K02942+K02863+K02896+K02900+K02899+K02931+K02863+K02970+K02957+K02940+K02980+K02949+K02915+K02990+K02942+K02903+K02922+K02943+K02888+K02939+K02885+K02915+K02990+K02930+K02997+K02971+K02885+K02925+K02886+K02912+K02975+K02875+K02978+K02939+K02893+K02876+K02885+K02914+K02971+K02969+K02946+K02893+K02990+K02977+K02983+K02888+K02910+K02881+K02989+K02991+K02969+K02964+K02880+K02996+K02875+K02932+K02933+K02969+K02957+K02939+K02932+K02961+K02909+K02899+K02971+

	53;F01_transcript_26967;F01_transcript_27244;F01_transcript_27313;F01_transcript_27853;F01_transcript_28254;F01_transcript_28957;F01_transcript_30002;F01_transcript_30437;F01_transcript_31399;F01_transcript_31699;F01_transcript_3212;F01_transcript_32153;F01_transcript_32526;F01_transcript_34126;F01_transcript_35041;F01_transcript_35873;F01_transcript_35951;F01_transcript_36370;F01_transcript_36841;F01_transcript_37596;F01_transcript_38494;F01_transcript_38903;F01_transcript_39131;F01_transcript_39152;F01_transcript_39224;F01_transcript_39335;F01_transcript_40156;F01_transcript_40259;F01_transcript_40293;F01_transcript_40612;F01_transcript_40764;F01_transcript_40792;F01_transcript_40826;F01_transcript_41044;F01_transcript_4129;F01_transcript_41525;F01_transcript_41537;F01_transcript_41561;F01_transcript_41563;F01_transcript_41873;F01_transcript_41934;F01_transcript_41973;F01_transcript_42049;F01_transcript_42188;F01_transcript_42326;F01_transcript_42500;F01_transcript_42572;F01_transcript_42679;F01_transcript_42687;F01_transcript_42724;F01_transcript_42762;F01_transcript_42802;F01_transcript_42821;F01_transcript_42836;F01_transcript_42854;F01_transcript_42995;F01_transcript_43069;F01_transcript_43071;F01_transcript_43167;F01_transcript_43181;F01_transcript_43339;F01_transcript_43438;F01_transcript_43557;F01_transcript_43765;F01_transcript_43803;F01_transcript_43900;F01_transcript_43944;F01_transcript_43978;F01_transcript_43993;F01_transcript_44060;F01_transcript_44066;F01_transcript_44141;F01_transcript_44205;F01_transcript_44305;F01_transcript_44382;F01_transcript_44442;F01_transcript_44446;F01_transcript_44465;F01_transcript_44507;F01_transcript_44521;F01_transcript_44589;F01_transcript_44599;F01_transcript_44619;F01_transcript_44777;F01_transcript_44901;F01_transcript_44961;F01_transcript_44966;F01_transcript_44976;F01_transcript_44988;F01_transcript_45016;F01_transcript_45044;F01_transcript_45055;F01_transcript_45058;F01_transcript_45068;F01_transcript_45138;F01_transcript_45158;F01_transcript_45200;F01_transcript_45228;F01_transcript_45278;F01_transcript_45359;F01_transcript_45410;F01_transcript_45416;F01_transcript_45487;F01_transcript_45517;F01_transcript_45525;F01_transcript_45527;F01_transcript_45567;F01_transcript_45580;	K02866+K02941+K02915+K02918+K02946+K02980+K02996+K02936+K02930+K02990+K02910+K02991+K02888+K02872+K02905

F01_transcript_45590;F01_transcript_45639;F01_transcript_456
88;F01_transcript_45716;F01_transcript_45763;F01_transcript_
45771;F01_transcript_45785;F01_transcript_45815;F01_transcri
pt_45822;F01_transcript_45945;F01_transcript_45979;F01_tran
script_46025;F01_transcript_46036;F01_transcript_46053;F01_t
ranscript_46056;F01_transcript_46082;F01_transcript_46085;F0
1_transcript_46130;F01_transcript_46149;F01_transcript_46172
;F01_transcript_46219;F01_transcript_46239;F01_transcript_46
255;F01_transcript_46273;F01_transcript_46374;F01_transcript
_46376;F01_transcript_46389;F01_transcript_46434;F01_transc
ript_46460;F01_transcript_46476;F01_transcript_46563;F01_tra
nscript_46566;F01_transcript_46573;F01_transcript_46603;F01
_transcript_46608;F01_transcript_46627;F01_transcript_46679;
F01_transcript_46680;F01_transcript_46744;F01_transcript_467
69;F01_transcript_46773;F01_transcript_46790;F01_transcript_
46811;F01_transcript_46831;F01_transcript_46851;F01_transcri
pt_46891;F01_transcript_46902;F01_transcript_46953;F01_tran
script_46970;F01_transcript_46971;F01_transcript_47012;F01_t
ranscript_47043;F01_transcript_47066;F01_transcript_47071;F0
1_transcript_47076;F01_transcript_47098;F01_transcript_47114
;F01_transcript_47140;F01_transcript_47150;F01_transcript_47
152;F01_transcript_47223;F01_transcript_47233;F01_transcript
_47255;F01_transcript_47270;F01_transcript_47293;F01_transc
ript_47312;F01_transcript_47324;F01_transcript_47334;F01_tra
nscript_47345;F01_transcript_47359;F01_transcript_47370;F01
_transcript_47403;F01_transcript_47417;F01_transcript_47449;
F01_transcript_47485;F01_transcript_47538;F01_transcript_475
57;F01_transcript_47573;F01_transcript_47657;F01_transcript_
47664;F01_transcript_47688;F01_transcript_47699;F01_transcri
pt_47706;F01_transcript_47718;F01_transcript_47740;F01_tran
script_47743;F01_transcript_47753;F01_transcript_47756;F01_t
ranscript_47766;F01_transcript_47773;F01_transcript_47783;F0
1_transcript_47786;F01_transcript_47795;F01_transcript_47796
;F01_transcript_47827;F01_transcript_47829;F01_transcript_47
833;F01_transcript_47849;F01_transcript_47857;F01_transcript
_4786;F01_transcript_47872;F01_transcript_47882;F01_transcri
pt_47890;F01_transcript_47912;F01_transcript_47915;F01_tran

script_47983;F01_transcript_48039;F01_transcript_48048;F01_t
ranscript_48049;F01_transcript_48094;F01_transcript_48124;F0
1_transcript_48132;F01_transcript_48173;F01_transcript_48285
;F01_transcript_48294;F01_transcript_48295;F01_transcript_48
302;F01_transcript_48303;F01_transcript_48312;F01_transcript
_48325;F01_transcript_48338;F01_transcript_48387;F01_transc
ript_48405;F01_transcript_48419;F01_transcript_48438;F01_tra
nscript_48459;F01_transcript_48469;F01_transcript_48498;F01
_transcript_48518;F01_transcript_48542;F01_transcript_48567;
F01_transcript_48592;F01_transcript_48617;F01_transcript_486
33;F01_transcript_48636;F01_transcript_48637;F01_transcript_
48642;F01_transcript_48645;F01_transcript_48679;F01_transcri
pt_48683;F01_transcript_48687;F01_transcript_48689;F01_tran
script_48723;F01_transcript_48724;F01_transcript_48731;F01_t
ranscript_48737;F01_transcript_48744;F01_transcript_48767;F0
1_transcript_48771;F01_transcript_48869;F01_transcript_48875
;F01_transcript_48877;F01_transcript_48881;F01_transcript_48
907;F01_transcript_48931;F01_transcript_48939;F01_transcript
_48961;F01_transcript_48975;F01_transcript_48988;F01_transc
ript_48997;F01_transcript_49024;F01_transcript_49080;F01_tra
nscript_49098;F01_transcript_49111;F01_transcript_49112;F01
_transcript_49134;F01_transcript_49136;F01_transcript_49180;
F01_transcript_49193;F01_transcript_49243;F01_transcript_492
65;F01_transcript_49311;F01_transcript_49349;F01_transcript_
49384;F01_transcript_49393;F01_transcript_49394;F01_transcri
pt_49465;F01_transcript_49473;F01_transcript_49516;F01_tran
script_49519;F01_transcript_49542;F01_transcript_49574;F01_t
ranscript_49578;F01_transcript_49609;F01_transcript_49663;F0
1_transcript_49738;F01_transcript_49768;F01_transcript_49770
;F01_transcript_49777;F01_transcript_49813;F01_transcript_49
822;F01_transcript_49841;F01_transcript_49874;F01_transcript
_49987;F01_transcript_50011;F01_transcript_50018;F01_transc
ript_50027;F01_transcript_50030;F01_transcript_50058;F01_tra
nscript_50127;F01_transcript_50138;F01_transcript_50148;F01
_transcript_50173;F01_transcript_50195;F01_transcript_50223;
F01_transcript_50249;F01_transcript_50340;F01_transcript_503
64;F01_transcript_50385;F01_transcript_50436;F01_transcript_
50448;F01_transcript_50451;F01_transcript_50476;F01_transcri

pt_50486;F01_transcript_50580;F01_transcript_50601;F01_transcript_50610;F01_transcript_50798;F01_transcript_50799;F01_transcript_50822;F01_transcript_50846;F01_transcript_50971;F01_transcript_51108;F01_transcript_51395;F01_transcript_51706;F01_transcript_51863;F01_transcript_51865;F01_transcript_52132;F01_transcript_52207;F01_transcript_52835;F01_transcript_52939;F01_transcript_53161;F01_transcript_53574;F01_transcript_53948;F01_transcript_54131;F01_transcript_54199;F01_transcript_54438;F01_transcript_54480;F01_transcript_54498;F01_transcript_54613;F01_transcript_54808;F01_transcript_55370;F01_transcript_55495;F01_transcript_55590;F01_transcript_56074;F01_transcript_56312;F01_transcript_56360;F01_transcript_56391;F01_transcript_56416;F01_transcript_56525;F01_transcript_56537;F01_transcript_56542;F01_transcript_56681;F01_transcript_56785;F01_transcript_57164;F01_transcript_57646;F01_transcript_57753;F01_transcript_57827;F01_transcript_58000;F01_transcript_58168;F01_transcript_58461;F01_transcript_58574;F01_transcript_58588;F01_transcript_59018;F01_transcript_59502;F01_transcript_59543;F01_transcript_59595;F01_transcript_59640;F01_transcript_5971;F01_transcript_5975;F01_transcript_60048;F01_transcript_60106;F01_transcript_6015;F01_transcript_60182;F01_transcript_60284;F01_transcript_61469;F01_transcript_61710;F01_transcript_61817;F01_transcript_62290;F01_transcript_62348;F01_transcript_62909;F01_transcript_62919;F01_transcript_63020;F01_transcript_63139;F01_transcript_63260;F01_transcript_63891;F01_transcript_63902;F01_transcript_6470;F01_transcript_64886;F01_transcript_65018;F01_transcript_65151;F01_transcript_65344;F01_transcript_65757;F01_transcript_65781;F01_transcript_65981;F01_transcript_66180;F01_transcript_66395;F01_transcript_67265;F01_transcript_67563;F01_transcript_67988;F01_transcript_68413;F01_transcript_68636;F01_transcript_68672;F01_transcript_68837;F01_transcript_68859;F01_transcript_69110;F01_transcript_6912;F01_transcript_69185;F01_transcript_69506;F01_transcript_69597;F01_transcript_69743;F01_transcript_69840;F01_transcript_70029;F01_transcript_7013;F01_transcript_70205;F01_transcript_70365;F01_transcript_70667;F01_transcript_71875;F01_transcript_71939;F0

1_transcript_72193;F01_transcript_72611;F01_transcript_72738
;F01_transcript_73020;F01_transcript_74024;F01_transcript_74
4;F01_transcript_74489;F01_transcript_74594;F01_transcript_7
4814;F01_transcript_74983;F01_transcript_75156;F01_transcrip
t_75260;F01_transcript_75483;F01_transcript_75760;F01_trans
cript_75786;F01_transcript_76320;F01_transcript_76932;F01_tr
anscript_76965;F01_transcript_77478;F01_transcript_77559;F0
1_transcript_77787;F01_transcript_78013;F01_transcript_78305
;F01_transcript_78680;F01_transcript_78693;F01_transcript_79
107;F01_transcript_79954;F01_transcript_80311;F01_transcript
_80493;F01_transcript_80688;F01_transcript_80782;F01_transc
ript_80849;F01_transcript_81772;F01_transcript_82705;F01_tra
nscript_82817;F01_transcript_83065;F01_transcript_83576;F01
_transcript_83839;F01_transcript_83893;F01_transcript_83979;
F01_transcript_84147;F01_transcript_84524;F01_transcript_847
08;F01_transcript_85031;F01_transcript_85503;F01_transcript_
86034;F01_transcript_86557;F01_transcript_86606;F01_transcri
pt_86726;F01_transcript_87094;F01_transcript_87134;F01_tran
script_87195;F01_transcript_87277;F01_transcript_87753;F01_t
ranscript_87853;F01_transcript_87881;F01_transcript_88232;F0
1_transcript_88257;F01_transcript_88996;F01_transcript_89008
;F01_transcript_89247;F01_transcript_89552;F01_transcript_89
637;F01_transcript_89714;F01_transcript_90068;F01_transcript
_90180;F01_transcript_90329;F01_transcript_90988;F01_transc
ript_91052;F01_transcript_91183;F01_transcript_9131;F01_tran
script_91480;F01_transcript_91664;F01_transcript_91683;F01_t
ranscript_91995;F01_transcript_92119;F01_transcript_92153;F0
1_transcript_92318;F01_transcript_92506;F01_transcript_92980
;F01_transcript_93177;F01_transcript_93207;F01_transcript_93
51;F01_transcript_93510;F01_transcript_93616;F01_transcript_
93864;F01_transcript_93951;F01_transcript_94029;F01_transcri
pt_94209;F01_transcript_94791;F01_transcript_94820;F01_tran
script_94883;F01_transcript_94904;F01_transcript_95925;F01_t
ranscript_95971;F01_transcript_95994;F01_transcript_96110;F0
1_transcript_96385;F01_transcript_96400;F01_transcript_97204
;F01_transcript_97461;F01_transcript_97998;F01_transcript_98
210;F01_transcript_98490;F01_transcript_99012;F01_transcript

			_99030;F01_transcript_99071;F01_transcript_99200;F01_transcript_99238;F01_transcript_99443;F01_transcript_99577;	
RNA transport	ko03013	824	F01_transcript_10020;F01_transcript_100317;F01_transcript_100393;F01_transcript_100581;F01_transcript_100918;F01_transcript_101347;F01_transcript_101418;F01_transcript_101492;F01_transcript_101691;F01_transcript_101897;F01_transcript_101971;F01_transcript_101979;F01_transcript_102120;F01_transcript_102229;F01_transcript_102567;F01_transcript_103000;F01_transcript_103287;F01_transcript_103301;F01_transcript_103314;F01_transcript_103445;F01_transcript_103570;F01_transcript_103645;F01_transcript_103987;F01_transcript_104179;F01_transcript_10418;F01_transcript_104257;F01_transcript_104376;F01_transcript_104450;F01_transcript_104460;F01_transcript_104514;F01_transcript_104946;F01_transcript_105235;F01_transcript_105242;F01_transcript_105551;F01_transcript_105860;F01_transcript_106512;F01_transcript_106798;F01_transcript_106850;F01_transcript_106984;F01_transcript_107247;F01_transcript_107258;F01_transcript_107377;F01_transcript_107484;F01_transcript_107924;F01_transcript_108224;F01_transcript_108277;F01_transcript_108370;F01_transcript_108433;F01_transcript_108460;F01_transcript_108592;F01_transcript_108648;F01_transcript_108655;F01_transcript_108801;F01_transcript_108905;F01_transcript_108939;F01_transcript_109281;F01_transcript_10943;F01_transcript_109729;F01_transcript_110004;F01_transcript_110374;F01_transcript_110726;F01_transcript_110734;F01_transcript_11094;F01_transcript_110969;F01_transcript_111146;F01_transcript_111278;F01_transcript_111279;F01_transcript_111328;F01_transcript_111381;F01_transcript_111912;F01_transcript_112033;F01_transcript_112222;F01_transcript_112491;F01_transcript_112542;F01_transcript_113079;F01_transcript_113105;F01_transcript_113186;F01_transcript_113235;F01_transcript_113433;F01_transcript_113652;F01_transcript_113664;F01_transcript_114020;F01_transcript_114240;F01_transcript_114255;F01_transcript_114472;F01_transcript_114504;F01_transcript_114574;F01_transcript_114655;F01_transcript_114741;F01_transcript_114905;F01_transcript_115094;F01_transcript_115750;F01_transcript_1158;F01_transcript_115802;F01_transcript_116	K13126+K03260+K03238+K12878+K03262+K13126+K07936+K14297+K03260+K13126+K03680+K12880+K03680+K14328+K03259+K14297+K14292+K14328+K12878+K14298+K14295+K07936+K14292+K14297+K14292+K12879+K03254+K12875+K14301+K03537+K12878+K14312+K14004+K03680+K07936+K03253+K14304+K03260+K14301+K14318+K03680+K18213+K03251+K03238+K12878+K13025+K18213+K03240+K14309+K12160+K03260+K03539+K03242+K00784+K03257+K01164+K03260+K13176+K14305+K12877+K07936+K07936+K13126+K13130+K12875+K14301+K13126+K03262+K03680+K03259+K14297+K13126+K14304+K03253+K12879+K03251+K14292+K18213+K03231+K12160+K14328+K14289+K03259+K14297+K12878+K03250+K14327+K12878+K14325+K18213+K14306+K12881+K14290+K14326+K12880+K12876+K12812+K03253+K12881+K12881+K14326+K03257+K07562+K14306+K14297+K14309+K14297+K03259+K14325+K03243+K03254+K14328+K14328+K03259+K03243+K14004+K14304+K03254+K03242+K14297+K13126+K14288+K14306+K03754+K03257+K03113+K03237+K14325+K12880+K14318+K14308+K13126+K07936+K13171+K03240+K14315+K14292+K03253+K14327+K02516+K09291+K14525+K03242+K01164+K12881+K14301+K13174+K14293+K03240+K12881+K12878+K13171+K14289+K03231+K13171+K03538+K03236+K14328+K13174+K03240+K09291+K14293+K14290+K03259+K14293+K05749+K13126+K14295+K14291+K14310+K03236+K14297+K14309+K03238+K03113+K14294+K14318+K13171+K03260+K03246+K14298+K14295+K14290+K03236+K14327+K14310+K03240+K12875+K03240+K03259+K14328+K07936+K03231+K12879+K12875+K03238+K03754+K03538+K14314+K03242+K03254+K03238+K14328+K03240+K07562+K03243+K03236+K14327+K03257+K14301+K03253+K12812+K03260+K12812+K03113+K03257+K03260+K12880+K14295+K14327+K13126+K14293+K03246+K14319+K09291+K14295+K12875+K13126+K14313+K14326+K14004+K12812+K13171+K03231+K13126+K03257+K14308+K03254+K12882+K14301+

	564;F01_transcript_116577;F01_transcript_116677;F01_transcript_116981;F01_transcript_117177;F01_transcript_117205;F01_transcript_118027;F01_transcript_118265;F01_transcript_118385;F01_transcript_11841;F01_transcript_118530;F01_transcript_118801;F01_transcript_118830;F01_transcript_119012;F01_transcript_119146;F01_transcript_119255;F01_transcript_119331;F01_transcript_11945;F01_transcript_119462;F01_transcript_119548;F01_transcript_119891;F01_transcript_119915;F01_transcript_119983;F01_transcript_120265;F01_transcript_120295;F01_transcript_120480;F01_transcript_12087;F01_transcript_120890;F01_transcript_121307;F01_transcript_121430;F01_transcript_121822;F01_transcript_122592;F01_transcript_122676;F01_transcript_123205;F01_transcript_123234;F01_transcript_123315;F01_transcript_123527;F01_transcript_123951;F01_transcript_124185;F01_transcript_124209;F01_transcript_124359;F01_transcript_124644;F01_transcript_12469;F01_transcript_124997;F01_transcript_125088;F01_transcript_125274;F01_transcript_125282;F01_transcript_125284;F01_transcript_125698;F01_transcript_125849;F01_transcript_126045;F01_transcript_1262;F01_transcript_126200;F01_transcript_1265;F01_transcript_126583;F01_transcript_126986;F01_transcript_127041;F01_transcript_127424;F01_transcript_127618;F01_transcript_127684;F01_transcript_128109;F01_transcript_128311;F01_transcript_128488;F01_transcript_12849;F01_transcript_129522;F01_transcript_12969;F01_transcript_129987;F01_transcript_130090;F01_transcript_130260;F01_transcript_130557;F01_transcript_130629;F01_transcript_130655;F01_transcript_130768;F01_transcript_130894;F01_transcript_130926;F01_transcript_132016;F01_transcript_132141;F01_transcript_132350;F01_transcript_132434;F01_transcript_132884;F01_transcript_132959;F01_transcript_133021;F01_transcript_133117;F01_transcript_133247;F01_transcript_133376;F01_transcript_134070;F01_transcript_134111;F01_transcript_134396;F01_transcript_1347;F01_transcript_134706;F01_transcript_134708;F01_transcript_135445;F01_transcript_13552;F01_transcript_135662;F01_transcript_135670;F01_transcript_135718;F01_transcript_13577;F01_transcript_135846;F01_transcript_135865;F01_transcript_135961;F01_transcript_135983;F01_transcript_136140;F01_transcript_136200;F01_transcript_136237;F01	K14297+K03231+K12875+K14311+K14304+K14525+K14305+K03260+K09291+K07936+K03262+K03257+K03241+K13126+K12880+K14290+K03249+K01164+K12875+K14294+K14288+K14295+K12882+K14292+K10577+K12875+K03240+K03259+K13151+K12876+K03257+K14325+K03246+K03240+K12812+K03245+K14313+K12878+K07936+K14293+K14288+K12160+K03260+K02516+K09291+K12883+K14290+K03113+K12880+K13126+K03245+K12881+K07936+K14319+K14327+K03237+K12160+K03252+K02516+K14318+K14315+K14325+K03247+K14295+K03239+K14318+K14313+K14297+K03236+K03245+K03243+K12882+K12881+K14327+K14293+K12878+K14310+K14328+K14525+K14306+K14315+K12878+K03238+K09291+K13126+K14293+K14306+K03754+K14328+K14328+K13126+K03251+K09291+K14312+K03260+K03539+K14004+K12875+K03113+K14297+K14325+K12812+K14309+K09291+K13126+K14306+K03680+K13126+K14319+K03259+K14295+K14291+K12881+K03231+K14328+K03239+K13126+K03254+K03253+K14328+K14295+K03239+K03754+K07936+K03680+K14290+K14293+K03680+K03247+K02516+K03240+K02516+K12875+K14290+K03262+K03240+K12879+K14319+K03539+K12879+K03262+K03251+K14319+K14297+K03250+K12881+K03262+K03262+K14309+K14319+K14297+K03262+K12882+K14319+K03262+K14319+K14315+K03262+K14319+K03262+K14319+K03231+K18213+K14315+K03240+K07562+K07562+K03251+K03231+K14315+K14297+K03231+K03241+K12877+K03260+K03251+K07562+K12812+K03260+K12812+K03231+K13126+K03754+K14293+K14318+K03257+K12812+K03257+K13171+K03245+K14326+K14326+K13137+K03257+K13114+K03243+K14320+K12812+K03231+K13151+K13114+K03231+K03231+K03231+K03257+K10577+K03257+K03260+K03257+K13174+K13025+K13114+K14326+K13025+K03254+K14321+K03231+K00784+K14325+K13114+K13137+K13114+K13114+K03231+K03257+K14293+K03231+K14325+K03250+K03252+K14293+K14313+K03754+K13114+K14308+K14325+K14295+K03239+K03247+K00784+K13175+K03247+K14298+K03237+K03246+K03538+K14004+K03238+K03246+K03246+K03254+K14004+K07936+K03238+K13126+K13126+K03237+K12881+K03248+K13126+K143

	_transcript_136248;F01_transcript_136730;F01_transcript_137030;F01_transcript_137421;F01_transcript_137425;F01_transcript_137537;F01_transcript_137689;F01_transcript_137709;F01_transcript_137781;F01_transcript_138207;F01_transcript_138972;F01_transcript_139022;F01_transcript_139175;F01_transcript_139577;F01_transcript_139613;F01_transcript_139635;F01_transcript_139680;F01_transcript_139839;F01_transcript_139913;F01_transcript_140049;F01_transcript_140697;F01_transcript_140738;F01_transcript_140741;F01_transcript_140790;F01_transcript_141007;F01_transcript_141088;F01_transcript_141522;F01_transcript_141588;F01_transcript_142400;F01_transcript_14249;F01_transcript_142524;F01_transcript_142626;F01_transcript_142636;F01_transcript_142788;F01_transcript_142907;F01_transcript_142967;F01_transcript_143241;F01_transcript_143304;F01_transcript_143355;F01_transcript_143557;F01_transcript_143725;F01_transcript_144037;F01_transcript_144185;F01_transcript_144319;F01_transcript_144624;F01_transcript_144806;F01_transcript_144859;F01_transcript_145030;F01_transcript_145054;F01_transcript_145171;F01_transcript_145243;F01_transcript_145427;F01_transcript_145652;F01_transcript_145958;F01_transcript_146048;F01_transcript_146077;F01_transcript_146354;F01_transcript_146582;F01_transcript_146698;F01_transcript_147215;F01_transcript_147363;F01_transcript_147430;F01_transcript_147580;F01_transcript_147748;F01_transcript_148008;F01_transcript_148290;F01_transcript_148375;F01_transcript_14848;F01_transcript_148943;F01_transcript_149042;F01_transcript_149111;F01_transcript_149527;F01_transcript_149570;F01_transcript_150125;F01_transcript_150242;F01_transcript_150304;F01_transcript_150338;F01_transcript_150792;F01_transcript_150988;F01_transcript_151022;F01_transcript_151633;F01_transcript_151890;F01_transcript_152118;F01_transcript_152161;F01_transcript_153179;F01_transcript_153256;F01_transcript_153684;F01_transcript_154359;F01_transcript_1544;F01_transcript_154593;F01_transcript_154665;F01_transcript_15498;F01_transcript_155039;F01_transcript_155189;F01_transcript_155365;F01_transcript_155456;F01_transcript_155497;F01_transcript_155635;F01_transcript_155779;F01_transcript_155865;F01_tra	10+K12881+K03248+K03238+K05019+K03259+K05019+K07936+K12883+K14318+K05019+K14295+K03257+K07936+K07936+K03259+K10577+K03113+K03259+K12876+K14327+K07936+K10577+K03113+K03113+K03236+K14324+K03113+K12877+K03113+K03236+K10577+K03236+K14324+K03113+K12160+K03236+K03236+K12160+K12160+K03236+K03236+K03236+K12160+K12160+K03231+K13114+K03231+K12878+K13126+K03253+K14290+K18213+K03680+K12875+K14319+K03231+K14295+K12881+K18213+K03243+K12883+K03231+K12878+K14289+K12881+K13126+K03239+K14327+K14318+K12878+K03237+K14295+K13175+K14326+K03113+K03237+K14290+K14288+K12875+K12875+K14326+K14319+K09291+K03248+K13137+K03238+K03239+K13114+K12160+K07562+K14304+K03231+K03259+K14291+K14309+K03231+K03113+K09291+K14300+K13151+K14291+K03239+K18213+K14318+K05749+K05749+K14312+K12881+K03260+K18213+K12812+K03260+K03257+K14312+K13137+K13126+K14297+K03260+K14289+K14319+K13126+K03238+K14289+K18213+K03242+K18213+K03537+K01164+K12881+K03257+K14295+K14297+K14320+K14288+K13176+K05749+K12881+K12875+K03231+K14328+K14326+K14297+K12881+K13114+K14288+K14326+K00784+K14292+K12875+K03260+K13126+K14297+K14304+K14304+K14328+K14326+K12881+K03680+K18213+K12879+K14315+K14319+K14290+K12879+K14325+K03231+K14325+K03257+K13025+K14326+K14314+K14318+K12875+K14315+K01164+K03262+K03260+K03680+K00784+K09291+K14291+K14318+K03231+K03538+K03754+K03754+K14327+K03254+K13130+K14297+K03113+K03242+K00784+K03680+K03754+K12875+K12882+K03680+K14298+K07936+K03246+K14315+K14288+K14290+K03231+K02516+K14327+K01164+K14327+K03240+K03243+K14328+K03231+K14292+K13174+K02516+K12878+K03243+K14320+K14308+K03240+K03249+K18213+K13171+K03113+K03260+K03243+K03236+K14325+K13025+K03260+K03260+K14304+K03680+K14004+K03239+K12879+K12881+K03260+K14325+K13174+K03239+K03249+K13171+K03249+K03254+K13114+K03260+K14290+K03754+K14295+K14297+K03260+K1

	nscript_15615;F01_transcript_156321;F01_transcript_156550;F01_transcript_156551;F01_transcript_15707;F01_transcript_157282;F01_transcript_157295;F01_transcript_157441;F01_transcript_157585;F01_transcript_1577;F01_transcript_157749;F01_transcript_157961;F01_transcript_158140;F01_transcript_158256;F01_transcript_158294;F01_transcript_158530;F01_transcript_158672;F01_transcript_158840;F01_transcript_159440;F01_transcript_15963;F01_transcript_159842;F01_transcript_160162;F01_transcript_160282;F01_transcript_160336;F01_transcript_160695;F01_transcript_161029;F01_transcript_161080;F01_transcript_161142;F01_transcript_161149;F01_transcript_161172;F01_transcript_161801;F01_transcript_161852;F01_transcript_162165;F01_transcript_162280;F01_transcript_162416;F01_transcript_162581;F01_transcript_162982;F01_transcript_162998;F01_transcript_163113;F01_transcript_163391;F01_transcript_163403;F01_transcript_163940;F01_transcript_163956;F01_transcript_164123;F01_transcript_164509;F01_transcript_164570;F01_transcript_164620;F01_transcript_164749;F01_transcript_16478;F01_transcript_164830;F01_transcript_164951;F01_transcript_165118;F01_transcript_165408;F01_transcript_165451;F01_transcript_165682;F01_transcript_165838;F01_transcript_166047;F01_transcript_166066;F01_transcript_166716;F01_transcript_166905;F01_transcript_16718;F01_transcript_167251;F01_transcript_167418;F01_transcript_167710;F01_transcript_1687;F01_transcript_16907;F01_transcript_1700;F01_transcript_1703;F01_transcript_1723;F01_transcript_17256;F01_transcript_17468;F01_transcript_17737;F01_transcript_18063;F01_transcript_18303;F01_transcript_18341;F01_transcript_1929;F01_transcript_19424;F01_transcript_19619;F01_transcript_19910;F01_transcript_20014;F01_transcript_20428;F01_transcript_20504;F01_transcript_21661;F01_transcript_21677;F01_transcript_21686;F01_transcript_2170;F01_transcript_21750;F01_transcript_21777;F01_transcript_21811;F01_transcript_22803;F01_transcript_2331;F01_transcript_23385;F01_transcript_2345;F01_transcript_23558;F01_transcript_24229;F01_transcript_24663;F01_transcript_24816;F01_transcript_24878;F01_transcript_25395;F01_transcript_25421;F01_transcript_25864;F01_transcript_26757;F01_transcript_26769;F01_transcript_26776;F01_transcript_26851;F01_transcript_26	4326+K14304+K14309+K03242+K03239+K14324+K14327+K13174+K14328+K03680+K12879+K07936+K18213+K05749+K03262+K03243+K12875+K14303+K14311+K14326+K14290+K14309+K14321+K12875+K14324+K07936+K03537+K03260+K14309+K14297+K03113+K14307+K03231+K03538+K02516+K03250+K14327+K13174+K12883+K12875+K03246+K03260+K03240+K10577+K18213+K03262+K14310+K12160+K14290+K03754+K14290+K12879+K03254+K14289+K13126+K14297+K13126+K14290+K12160+K14310+K14289+K12881+K14324+K03240+K14324+K01164+K05749+K03754+K14288+K03262

994;F01_transcript_27139;F01_transcript_27482;F01_transcript_27530;F01_transcript_27602;F01_transcript_27819;F01_transcript_27873;F01_transcript_2867;F01_transcript_28802;F01_transcript_28919;F01_transcript_28952;F01_transcript_29;F01_transcript_29120;F01_transcript_29230;F01_transcript_29672;F01_transcript_30;F01_transcript_30005;F01_transcript_30179;F01_transcript_3024;F01_transcript_30483;F01_transcript_3063;F01_transcript_30652;F01_transcript_30725;F01_transcript_31413;F01_transcript_31436;F01_transcript_31465;F01_transcript_31893;F01_transcript_319;F01_transcript_321;F01_transcript_32178;F01_transcript_32189;F01_transcript_32219;F01_transcript_32283;F01_transcript_32463;F01_transcript_32608;F01_transcript_32862;F01_transcript_32946;F01_transcript_33198;F01_transcript_33431;F01_transcript_33529;F01_transcript_33672;F01_transcript_33703;F01_transcript_338;F01_transcript_33850;F01_transcript_34;F01_transcript_34173;F01_transcript_3418;F01_transcript_34182;F01_transcript_34249;F01_transcript_345;F01_transcript_34727;F01_transcript_3504;F01_transcript_35096;F01_transcript_35479;F01_transcript_3565;F01_transcript_35680;F01_transcript_35719;F01_transcript_35830;F01_transcript_35877;F01_transcript_35895;F01_transcript_35961;F01_transcript_36144;F01_transcript_3636;F01_transcript_36374;F01_transcript_36650;F01_transcript_37495;F01_transcript_3759;F01_transcript_3767;F01_transcript_37708;F01_transcript_37768;F01_transcript_38032;F01_transcript_38064;F01_transcript_38406;F01_transcript_38466;F01_transcript_38613;F01_transcript_38876;F01_transcript_38901;F01_transcript_39011;F01_transcript_39528;F01_transcript_39753;F01_transcript_40342;F01_transcript_40371;F01_transcript_40395;F01_transcript_40471;F01_transcript_40837;F01_transcript_40880;F01_transcript_40966;F01_transcript_4100;F01_transcript_41113;F01_transcript_41141;F01_transcript_41195;F01_transcript_41559;F01_transcript_41861;F01_transcript_41939;F01_transcript_42236;F01_transcript_42240;F01_transcript_42832;F01_transcript_43;F01_transcript_43140;F01_transcript_43282;F01_transcript_43581;F01_transcript_43736;F01_transcript_43780;F01_transcript_43905;F01_transcript_44013;F01_transcript_44475;F01_transcript_4469;F01_transcript_449

83;F01_transcript_4522;F01_transcript_45496;F01_transcript_4
5507;F01_transcript_45591;F01_transcript_45600;F01_transcrip
t_45643;F01_transcript_45891;F01_transcript_45956;F01_trans
cript_46017;F01_transcript_46118;F01_transcript_46226;F01_tr
anscript_47038;F01_transcript_47048;F01_transcript_47116;F0
1_transcript_47352;F01_transcript_47878;F01_transcript_47880
;F01_transcript_48050;F01_transcript_48193;F01_transcript_48
242;F01_transcript_48300;F01_transcript_48566;F01_transcript
_48613;F01_transcript_48631;F01_transcript_48760;F01_transc
ript_48807;F01_transcript_48856;F01_transcript_48934;F01_tra
nscript_48960;F01_transcript_49227;F01_transcript_49427;F01
_transcript_49524;F01_transcript_49826;F01_transcript_49853;
F01_transcript_50241;F01_transcript_51380;F01_transcript_514
78;F01_transcript_51502;F01_transcript_5161;F01_transcript_5
1980;F01_transcript_52040;F01_transcript_5244;F01_transcript
_52575;F01_transcript_53456;F01_transcript_54018;F01_transc
ript_54158;F01_transcript_54547;F01_transcript_55564;F01_tra
nscript_55639;F01_transcript_55676;F01_transcript_55685;F01
_transcript_55687;F01_transcript_55747;F01_transcript_56047;
F01_transcript_56167;F01_transcript_56260;F01_transcript_564
89;F01_transcript_56547;F01_transcript_5661;F01_transcript_5
6648;F01_transcript_56728;F01_transcript_56816;F01_transcrip
t_56887;F01_transcript_56898;F01_transcript_57100;F01_trans
cript_57228;F01_transcript_57268;F01_transcript_57277;F01_tr
anscript_57378;F01_transcript_57388;F01_transcript_57554;F0
1_transcript_57609;F01_transcript_57650;F01_transcript_57858
;F01_transcript_58161;F01_transcript_58179;F01_transcript_58
194;F01_transcript_58492;F01_transcript_58731;F01_transcript
_58868;F01_transcript_59269;F01_transcript_5942;F01_transcri
pt_59583;F01_transcript_59678;F01_transcript_60178;F01_tran
script_60196;F01_transcript_60211;F01_transcript_60260;F01_t
ranscript_6059;F01_transcript_60783;F01_transcript_60877;F01
_transcript_60944;F01_transcript_60949;F01_transcript_60979;
F01_transcript_61017;F01_transcript_612;F01_transcript_6150;
F01_transcript_61512;F01_transcript_61808;F01_transcript_618
77;F01_transcript_61899;F01_transcript_6220;F01_transcript_6
2520;F01_transcript_62525;F01_transcript_62634;F01_transcrip
t_62782;F01_transcript_62913;F01_transcript_62926;F01_trans

cript_62966;F01_transcript_63026;F01_transcript_63101;F01_tr
anscript_63271;F01_transcript_63859;F01_transcript_63905;F0
1_transcript_63908;F01_transcript_64032;F01_transcript_64117
;F01_transcript_6415;F01_transcript_64378;F01_transcript_648
92;F01_transcript_64991;F01_transcript_65154;F01_transcript_
65234;F01_transcript_65363;F01_transcript_65399;F01_transcri
pt_655;F01_transcript_65618;F01_transcript_65947;F01_transcr
ipt_65972;F01_transcript_66105;F01_transcript_66196;F01_tra
nscript_66239;F01_transcript_66370;F01_transcript_66378;F01
_transcript_66660;F01_transcript_67092;F01_transcript_6745;F
01_transcript_67512;F01_transcript_6752;F01_transcript_6765;
F01_transcript_68276;F01_transcript_68402;F01_transcript_684
22;F01_transcript_68477;F01_transcript_68566;F01_transcript_
68987;F01_transcript_69080;F01_transcript_69108;F01_transcri
pt_70031;F01_transcript_70051;F01_transcript_70491;F01_tran
script_70509;F01_transcript_70511;F01_transcript_70584;F01_t
ranscript_70903;F01_transcript_7096;F01_transcript_71245;F01
_transcript_71415;F01_transcript_71511;F01_transcript_7162;F
01_transcript_71852;F01_transcript_72175;F01_transcript_7277
5;F01_transcript_72974;F01_transcript_7322;F01_transcript_73
321;F01_transcript_73641;F01_transcript_73714;F01_transcript
_73765;F01_transcript_73806;F01_transcript_73887;F01_transc
ript_74103;F01_transcript_74604;F01_transcript_74782;F01_tra
nscript_75229;F01_transcript_75279;F01_transcript_76138;F01
_transcript_76375;F01_transcript_76397;F01_transcript_76447;
F01_transcript_76661;F01_transcript_76664;F01_transcript_768
22;F01_transcript_76829;F01_transcript_76882;F01_transcript_
77017;F01_transcript_77043;F01_transcript_77407;F01_transcri
pt_77428;F01_transcript_77515;F01_transcript_77550;F01_tran
script_77648;F01_transcript_77754;F01_transcript_78112;F01_t
ranscript_78261;F01_transcript_78306;F01_transcript_785;F01_
transcript_7888;F01_transcript_79135;F01_transcript_79215;F0
1_transcript_7956;F01_transcript_79998;F01_transcript_80279;
F01_transcript_80309;F01_transcript_80537;F01_transcript_805
60;F01_transcript_80620;F01_transcript_80724;F01_transcript_
81411;F01_transcript_81714;F01_transcript_81800;F01_transcri
pt_82181;F01_transcript_82316;F01_transcript_82538;F01_tran

			script_82674;F01_transcript_83087;F01_transcript_83122;F01_transcript_83139;F01_transcript_84163;F01_transcript_84256;F01_transcript_8432;F01_transcript_84373;F01_transcript_84393;F01_transcript_84439;F01_transcript_84498;F01_transcript_84655;F01_transcript_84729;F01_transcript_84811;F01_transcript_84823;F01_transcript_84941;F01_transcript_8496;F01_transcript_85181;F01_transcript_85242;F01_transcript_85510;F01_transcript_85514;F01_transcript_85929;F01_transcript_85943;F01_transcript_85956;F01_transcript_85998;F01_transcript_8602;F01_transcript_86256;F01_transcript_86423;F01_transcript_86599;F01_transcript_86611;F01_transcript_8664;F01_transcript_86702;F01_transcript_86737;F01_transcript_86858;F01_transcript_87107;F01_transcript_87193;F01_transcript_87332;F01_transcript_87481;F01_transcript_87674;F01_transcript_87755;F01_transcript_87880;F01_transcript_88179;F01_transcript_88438;F01_transcript_88488;F01_transcript_89089;F01_transcript_89242;F01_transcript_89297;F01_transcript_89341;F01_transcript_89516;F01_transcript_89564;F01_transcript_8967;F01_transcript_89694;F01_transcript_89811;F01_transcript_90102;F01_transcript_90183;F01_transcript_90627;F01_transcript_9078;F01_transcript_9095;F01_transcript_91078;F01_transcript_91577;F01_transcript_91722;F01_transcript_91750;F01_transcript_91928;F01_transcript_92374;F01_transcript_93689;F01_transcript_93759;F01_transcript_93767;F01_transcript_93814;F01_transcript_94580;F01_transcript_94858;F01_transcript_95160;F01_transcript_95519;F01_transcript_95546;F01_transcript_95633;F01_transcript_95717;F01_transcript_95975;F01_transcript_96079;F01_transcript_96394;F01_transcript_96424;F01_transcript_96449;F01_transcript_96545;F01_transcript_96704;F01_transcript_96889;F01_transcript_96979;F01_transcript_97358;F01_transcript_97396;F01_transcript_97467;F01_transcript_97772;F01_transcript_98293;F01_transcript_98403;F01_transcript_98629;F01_transcript_98689;F01_transcript_98776;F01_transcript_99097;F01_transcript_99133;F01_transcript_99360;F01_transcript_99425;F01_transcript_99592;F01_transcript_99730;	
mRNA survei	ko0	726	F01_transcript_100143;F01_transcript_100176;F01_transcript_10020;F01_transcript_100236;F01_transcript_10024;F01_transcript_100716;F01_transcript_101158;F01_transcript_101347;F01_t	K14398+K14376+K13126+K04382+K15542+K13917+K14962+K13126+K14376+K13126+K14328+K14401+K13917+K14376+K14401+K03265+K14405+K14328+K14407+K14416+K061

llance pathway	3015	ranscript_101612;F01_transcript_101897;F01_transcript_102229;F01_transcript_102334;F01_transcript_10247;F01_transcript_102473;F01_transcript_102629;F01_transcript_102938;F01_transcript_103234;F01_transcript_103301;F01_transcript_103578;F01_transcript_10384;F01_transcript_104029;F01_transcript_104450;F01_transcript_10486;F01_transcript_1053;F01_transcript_105630;F01_transcript_10588;F01_transcript_106015;F01_transcript_106042;F01_transcript_106084;F01_transcript_106393;F01_transcript_106880;F01_transcript_107787;F01_transcript_108021;F01_transcript_108185;F01_transcript_108277;F01_transcript_108712;F01_transcript_109014;F01_transcript_10920;F01_transcript_110134;F01_transcript_110210;F01_transcript_110296;F01_transcript_110374;F01_transcript_11094;F01_transcript_111118;F01_transcript_111146;F01_transcript_111211;F01_transcript_111279;F01_transcript_111598;F01_transcript_11170;F01_transcript_11191;F01_transcript_112222;F01_transcript_112229;F01_transcript_112266;F01_transcript_112368;F01_transcript_112464;F01_transcript_112853;F01_transcript_113048;F01_transcript_1131;F01_transcript_113209;F01_transcript_113248;F01_transcript_113364;F01_transcript_113372;F01_transcript_113664;F01_transcript_113899;F01_transcript_114143;F01_transcript_114574;F01_transcript_11465;F01_transcript_114741;F01_transcript_11477;F01_transcript_114890;F01_transcript_115054;F01_transcript_115290;F01_transcript_115291;F01_transcript_115750;F01_transcript_115802;F01_transcript_116004;F01_transcript_116084;F01_transcript_116577;F01_transcript_116677;F01_transcript_117177;F01_transcript_117205;F01_transcript_117683;F01_transcript_117799;F01_transcript_117970;F01_transcript_117988;F01_transcript_118027;F01_transcript_118222;F01_transcript_118371;F01_transcript_118992;F01_transcript_119049;F01_transcript_119146;F01_transcript_11915;F01_transcript_119166;F01_transcript_119334;F01_transcript_11945;F01_transcript_119462;F01_transcript_119467;F01_transcript_119792;F01_transcript_120052;F01_transcript_120186;F01_transcript_120747;F01_transcript_120773;F01_transcript_12087;F01_transcript_121037;F01_transcript_12116;F01_transcript_121736;F01_transcript_121802;F01_transcript_122111;F01_transcript_122690;	00+K12875+K14376+K14409+K11583+K14416+K14404+K11583+K13917+K14376+K11584+K14411+K14407+K14411+K13025+K11583+K14416+K11583+K11584+K04354+K14416+K12877+K13126+K14376+K12875+K14411+K13126+K14403+K14411+K11584+K13126+K06269+K06965+K04354+K06269+K14416+K04354+K14400+K15542+K14376+K14376+K14376+K14328+K11584+K14409+K14327+K14376+K14325+K11584+K14397+K04382+K14962+K14396+K12881+K14326+K04354+K06965+K12876+K12812+K12881+K12881+K14376+K06269+K14409+K06100+K14326+K00565+K14376+K11583+K14408+K14325+K14402+K14376+K14376+K14328+K14328+K14399+K11584+K14411+K04382+K14411+K14406+K13126+K14409+K14403+K14411+K03265+K14411+K14409+K14376+K14397+K06965+K14325+K14399+K14408+K14396+K13126+K04354+K14399+K13171+K14407+K04382+K14376+K14327+K14376+K14402+K14411+K03267+K14376+K12881+K14411+K14397+K11584+K11584+K12881+K14376+K11583+K13171+K11584+K13171+K06965+K14400+K14328+K14399+K14398+K11583+K06100+K11583+K13917+K14411+K03267+K13126+K03456+K14376+K04354+K11583+K11583+K04354+K04382+K15542+K11583+K04382+K14409+K04354+K14294+K06965+K14409+K13171+K03265+K06269+K14376+K14327+K03456+K03456+K04354+K12875+K06269+K14328+K11583+K12875+K14376+K11583+K14376+K11583+K14411+K14411+K14376+K14328+K14400+K14376+K11583+K14411+K14376+K14376+K06100+K14327+K11584+K14411+K14411+K11583+K14411+K12812+K14396+K12812+K14416+K14416+K04354+K14411+K14327+K13126+K11583+K14411+K14403+K04354+K14376+K06965+K14409+K14411+K14376+K14376+K12875+K13126+K14326+K14409+K14407+K12812+K13171+K14396+K13126+K13917+K14409+K06100+K12882+K14400+K14400+K12875+K14409+K14962+K14376+K14400+K14399+K13126+K04354+K14409+K14400+K14401+K14411+K13917+K08873+K14396+K14407+K12875+K04382+K14294+K03456+K15542+K12882+K14411+K14398+K14411+K12875+K14376+K14403+K14411+K14411+K06269+K06269+K12876+K11583+K14408+K11584+K14411+K06269+K14325+K1

	F01_transcript_122722;F01_transcript_122736;F01_transcript_123099;F01_transcript_123205;F01_transcript_123415;F01_transcript_12368;F01_transcript_123830;F01_transcript_123951;F01_transcript_124068;F01_transcript_124104;F01_transcript_124209;F01_transcript_124677;F01_transcript_124844;F01_transcript_124881;F01_transcript_125088;F01_transcript_125137;F01_transcript_12533;F01_transcript_1254;F01_transcript_125782;F01_transcript_125888;F01_transcript_126045;F01_transcript_12627;F01_transcript_126459;F01_transcript_126622;F01_transcript_126804;F01_transcript_126986;F01_transcript_127194;F01_transcript_127308;F01_transcript_127424;F01_transcript_12760;F01_transcript_128109;F01_transcript_128176;F01_transcript_128275;F01_transcript_12849;F01_transcript_128774;F01_transcript_129276;F01_transcript_129394;F01_transcript_129401;F01_transcript_129727;F01_transcript_129795;F01_transcript_129827;F01_transcript_130070;F01_transcript_130768;F01_transcript_131139;F01_transcript_131183;F01_transcript_131252;F01_transcript_131340;F01_transcript_13174;F01_transcript_131839;F01_transcript_131920;F01_transcript_13203;F01_transcript_132343;F01_transcript_132510;F01_transcript_132816;F01_transcript_133017;F01_transcript_133021;F01_transcript_133044;F01_transcript_133221;F01_transcript_133247;F01_transcript_133737;F01_transcript_134240;F01_transcript_134514;F01_transcript_134708;F01_transcript_134978;F01_transcript_135105;F01_transcript_135440;F01_transcript_135662;F01_transcript_135700;F01_transcript_13577;F01_transcript_135845;F01_transcript_135983;F01_transcript_136034;F01_transcript_13637;F01_transcript_136472;F01_transcript_136494;F01_transcript_136894;F01_transcript_136907;F01_transcript_137321;F01_transcript_137425;F01_transcript_137451;F01_transcript_137594;F01_transcript_13762;F01_transcript_137759;F01_transcript_137769;F01_transcript_137910;F01_transcript_138104;F01_transcript_138207;F01_transcript_13849;F01_transcript_138529;F01_transcript_138579;F01_transcript_13909;F01_transcript_13943;F01_transcript_139577;F01_transcript_139608;F01_transcript_139635;F01_transcript_13967;F01_transcript_140209;F01_transcript_140320;F01_transcript_140716;F01_transcript_140738;F01_transcript_140741;F01_transcript_140953;F01_transcript_141212;F01_trans	2812+K15542+K00565+K11584+K11583+K14399+K06269+K14376+K11584+K04354+K13917+K14408+K14376+K06269+K08873+K14411+K12883+K11584+K13126+K06269+K12881+K00565+K04382+K14327+K14411+K04354+K04382+K14411+K14325+K13917+K14396+K14411+K11584+K12882+K12881+K14327+K14376+K04354+K14376+K11583+K13917+K14411+K14376+K14328+K14398+K14416+K14376+K08873+K13917+K04354+K11584+K13126+K14376+K11583+K04382+K04354+K11583+K14328+K14328+K03265+K14400+K13126+K04354+K14397+K14411+K04354+K06269+K03456+K14376+K12875+K06269+K11583+K14325+K12812+K13126+K14411+K14397+K13126+K14400+K13917+K14376+K14409+K14407+K06269+K12881+K14400+K14376+K14411+K14328+K06100+K13126+K15542+K04354+K06269+K14328+K14411+K14411+K14409+K14396+K12875+K14404+K04354+K11584+K06269+K14376+K11584+K04354+K11583+K14407+K14400+K12881+K11583+K14376+K06269+K00565+K14411+K06269+K14407+K04354+K14376+K11584+K14400+K12882+K03456+K11584+K14411+K03456+K14411+K14404+K14400+K11584+K03265+K14400+K14407+K14411+K14407+K03267+K14411+K03265+K14376+K11583+K12877+K11583+K03265+K12812+K12812+K03265+K03265+K13126+K03267+K14411+K14411+K12812+K13171+K11583+K11583+K14326+K14326+K13114+K14399+K12812+K06269+K06269+K13114+K06269+K14399+K14401+K14399+K13025+K14411+K13114+K00565+K14326+K13025+K04382+K14411+K06269+K14411+K03265+K14325+K13114+K06269+K06269+K13114+K13114+K04382+K06269+K14411+K04382+K06269+K14325+K04354+K04382+K06269+K13114+K04382+K14409+K06269+K06269+K14376+K14325+K06269+K04382+K04382+K13126+K06100+K13126+K12881+K14407+K13126+K12881+K12883+K14396+K14376+K14396+K14397+K14397+K14397+K14376+K12876+K14327+K14376+K14324+K12877+K14324+K14396+K14400+K13114+K13126+K14409+K14376+K14408+K14405+K06100+K14411+K13917+K12875+K14411+K14409+K08873+K14400+K12881+K12883+K04354+K14404+K12881+K13126+K14327+K14326+K11584+K06100+K12875+K12875+K14326+K14404+K13114+K04382+K08873+K14402+K03456+K14400

	cript_141468;F01_transcript_141653;F01_transcript_14180;F01_transcript_141800;F01_transcript_141958;F01_transcript_142195;F01_transcript_142231;F01_transcript_142375;F01_transcript_142400;F01_transcript_14249;F01_transcript_142626;F01_transcript_142721;F01_transcript_142777;F01_transcript_142788;F01_transcript_142907;F01_transcript_142912;F01_transcript_143241;F01_transcript_14343;F01_transcript_143498;F01_transcript_143543;F01_transcript_143725;F01_transcript_143800;F01_transcript_143822;F01_transcript_144624;F01_transcript_145337;F01_transcript_145518;F01_transcript_145541;F01_transcript_145731;F01_transcript_145791;F01_transcript_146077;F01_transcript_146166;F01_transcript_146343;F01_transcript_146520;F01_transcript_146706;F01_transcript_146802;F01_transcript_146939;F01_transcript_146988;F01_transcript_147134;F01_transcript_147139;F01_transcript_147363;F01_transcript_147400;F01_transcript_147430;F01_transcript_147599;F01_transcript_147843;F01_transcript_148008;F01_transcript_148175;F01_transcript_148403;F01_transcript_148464;F01_transcript_14848;F01_transcript_148486;F01_transcript_14862;F01_transcript_148897;F01_transcript_148986;F01_transcript_149000;F01_transcript_149425;F01_transcript_149527;F01_transcript_149553;F01_transcript_14962;F01_transcript_149625;F01_transcript_149897;F01_transcript_150009;F01_transcript_150125;F01_transcript_150338;F01_transcript_15042;F01_transcript_150694;F01_transcript_15138;F01_transcript_151524;F01_transcript_151963;F01_transcript_152347;F01_transcript_15266;F01_transcript_15276;F01_transcript_15290;F01_transcript_153447;F01_transcript_153520;F01_transcript_153828;F01_transcript_153868;F01_transcript_154297;F01_transcript_154310;F01_transcript_154359;F01_transcript_15475;F01_transcript_15498;F01_transcript_155173;F01_transcript_155189;F01_transcript_155334;F01_transcript_155406;F01_transcript_155497;F01_transcript_156031;F01_transcript_15609;F01_transcript_15612;F01_transcript_156273;F01_transcript_156551;F01_transcript_156694;F01_transcript_156699;F01_transcript_157501;F01_transcript_15751;F01_transcript_158256;F01_transcript_158294;F01_transcript_158530;F01_transcript_158706;F01_transcript_158815;F01_transcript_158950;F0	+K03456+K04382+K14399+K14405+K14411+K14376+K04382+K12881+K00565+K14411+K03456+K12812+K14397+K13126+K14399+K04354+K13126+K06269+K14376+K08873+K14402+K06269+K14400+K06269+K12881+K13917+K11583+K14400+K14376+K14376+K14409+K12881+K03456+K12875+K06269+K14328+K11584+K04354+K14326+K14376+K12881+K13114+K11583+K04354+K14326+K14376+K04354+K11583+K00565+K12875+K14376+K13126+K14402+K14328+K11584+K14326+K12881+K11583+K13917+K06100+K14325+K14325+K13025+K14326+K11584+K11583+K14402+K14409+K12875+K11584+K04354+K14400+K14397+K14376+K13917+K14401+K14416+K11584+K14400+K14411+K14376+K14327+K15542+K14399+K06100+K04354+K00565+K13917+K14409+K03456+K14406+K14376+K12875+K12882+K03265+K14400+K14327+K14400+K14327+K11584+K06269+K13917+K14328+K14402+K14411+K14409+K06269+K14376+K03267+K14411+K14402+K14409+K14407+K14401+K13171+K14396+K14411+K11583+K14400+K14325+K14376+K13025+K14407+K12881+K14400+K14325+K00565+K13171+K14411+K14398+K13114+K14326+K13917+K14376+K04354+K14324+K14327+K14376+K14416+K14411+K14328+K14411+K00565+K06269+K14376+K08873+K12875+K14376+K14326+K12875+K14324+K14376+K13917+K14411+K14409+K00565+K00565+K14411+K14411+K14376+K14400+K14327+K12883+K12875+K14408+K13917+K11584+K14407+K14962+K14400+K11584+K14403+K13126+K11584+K14376+K14402+K14416+K13126+K03456+K08873+K14411+K11584+K12881+K14324+K14411+K14402+K14324+K04354+K11583+K14411+K14416

1_transcript_15898;F01_transcript_159311;F01_transcript_1593
52;F01_transcript_159583;F01_transcript_15963;F01_transcript
_159679;F01_transcript_160418;F01_transcript_160431;F01_tra
nscript_160438;F01_transcript_160920;F01_transcript_160975;
F01_transcript_161045;F01_transcript_161080;F01_transcript_1
61195;F01_transcript_161295;F01_transcript_161521;F01_trans
cript_161719;F01_transcript_161763;F01_transcript_161801;F0
1_transcript_161852;F01_transcript_161889;F01_transcript_162
157;F01_transcript_162165;F01_transcript_162197;F01_transcri
pt_162236;F01_transcript_162524;F01_transcript_16271;F01_tr
anscript_162968;F01_transcript_162983;F01_transcript_163195
;F01_transcript_163391;F01_transcript_163420;F01_transcript_
16348;F01_transcript_163956;F01_transcript_164123;F01_trans
cript_164620;F01_transcript_164668;F01_transcript_164782;F0
1_transcript_164830;F01_transcript_164837;F01_transcript_164
919;F01_transcript_165306;F01_transcript_165342;F01_transcri
pt_165388;F01_transcript_165588;F01_transcript_165682;F01_t
ranscript_165712;F01_transcript_165865;F01_transcript_16591
9;F01_transcript_166047;F01_transcript_166506;F01_transcript
_166716;F01_transcript_166891;F01_transcript_167007;F01_tra
nscript_16716;F01_transcript_167251;F01_transcript_17006;F0
1_transcript_17845;F01_transcript_1808;F01_transcript_1812;F
01_transcript_18341;F01_transcript_18509;F01_transcript_1857
5;F01_transcript_19099;F01_transcript_19376;F01_transcript_1
990;F01_transcript_19904;F01_transcript_20285;F01_transcript
_20668;F01_transcript_20953;F01_transcript_2122;F01_transcri
pt_21777;F01_transcript_21806;F01_transcript_2211;F01_trans
cript_22256;F01_transcript_22436;F01_transcript_22614;F01_tr
anscript_23705;F01_transcript_23754;F01_transcript_23762;F0
1_transcript_23841;F01_transcript_23920;F01_transcript_2417;
F01_transcript_24229;F01_transcript_24401;F01_transcript_244
27;F01_transcript_24520;F01_transcript_24673;F01_transcript_
25068;F01_transcript_25107;F01_transcript_2514;F01_transcrip
t_25496;F01_transcript_25596;F01_transcript_2586;F01_transcr
ipt_26362;F01_transcript_26615;F01_transcript_27363;F01_tra
nscript_27537;F01_transcript_27741;F01_transcript_27939;F01
_transcript_2862;F01_transcript_28900;F01_transcript_28952;F
01_transcript_29362;F01_transcript_29642;F01_transcript_2967

2;F01_transcript_30005;F01_transcript_30061;F01_transcript_30174;F01_transcript_3024;F01_transcript_30411;F01_transcript_30542;F01_transcript_31411;F01_transcript_31413;F01_transcript_31465;F01_transcript_31492;F01_transcript_31669;F01_transcript_319;F01_transcript_321;F01_transcript_32219;F01_transcript_32294;F01_transcript_32608;F01_transcript_33009;F01_transcript_33010;F01_transcript_33198;F01_transcript_33490;F01_transcript_33547;F01_transcript_33630;F01_transcript_34132;F01_transcript_34182;F01_transcript_34226;F01_transcript_34249;F01_transcript_34316;F01_transcript_345;F01_transcript_34727;F01_transcript_34851;F01_transcript_34891;F01_transcript_35212;F01_transcript_35214;F01_transcript_35472;F01_transcript_35680;F01_transcript_35719;F01_transcript_35734;F01_transcript_35746;F01_transcript_35877;F01_transcript_35895;F01_transcript_36013;F01_transcript_36069;F01_transcript_36387;F01_transcript_36452;F01_transcript_36527;F01_transcript_36650;F01_transcript_36962;F01_transcript_37542;F01_transcript_37928;F01_transcript_38032;F01_transcript_38038;F01_transcript_3808;F01_transcript_38197;F01_transcript_38222;F01_transcript_3836;F01_transcript_38406;F01_transcript_39065;F01_transcript_39203;F01_transcript_39558;F01_transcript_41559;F01_transcript_416;F01_transcript_41861;F01_transcript_42236;F01_transcript_42617;F01_transcript_42832;F01_transcript_43140;F01_transcript_44475;F01_transcript_44608;F01_transcript_4475;F01_transcript_44760;F01_transcript_44885;F01_transcript_44897;F01_transcript_45699;F01_transcript_4591;F01_transcript_46017;F01_transcript_46118;F01_transcript_4775;F01_transcript_47878;F01_transcript_48050;F01_transcript_48613;F01_transcript_50484;F01_transcript_51340;F01_transcript_51380;F01_transcript_5161;F01_transcript_51629;F01_transcript_5175;F01_transcript_51779;F01_transcript_51887;F01_transcript_5207;F01_transcript_52292;F01_transcript_52417;F01_transcript_53456;F01_transcript_53627;F01_transcript_54328;F01_transcript_54488;F01_transcript_54972;F01_transcript_55564;F01_transcript_55685;F01_transcript_55791;F01_transcript_55838;F01_transcript_56167;F01_transcript_56260;F01_transcript_56547;F01_transcript_56898;F01_transcript_57057;F01_transcript_57175;

F01_transcript_57378;F01_transcript_57388;F01_transcript_575
54;F01_transcript_57704;F01_transcript_58492;F01_transcript_
58510;F01_transcript_58643;F01_transcript_58812;F01_transcri
pt_59672;F01_transcript_599;F01_transcript_59922;F01_transcr
ipt_60221;F01_transcript_60270;F01_transcript_60444;F01_tra
nscript_60510;F01_transcript_61338;F01_transcript_61458;F01
_transcript_61512;F01_transcript_61551;F01_transcript_61611;
F01_transcript_61720;F01_transcript_61899;F01_transcript_623
79;F01_transcript_62782;F01_transcript_62801;F01_transcript_
6296;F01_transcript_63101;F01_transcript_6337;F01_transcript
_63666;F01_transcript_63803;F01_transcript_64016;F01_transc
ript_64146;F01_transcript_64181;F01_transcript_64242;F01_tra
nscript_64378;F01_transcript_6441;F01_transcript_64504;F01_t
ranscript_65181;F01_transcript_65256;F01_transcript_65320;F0
1_transcript_65405;F01_transcript_65618;F01_transcript_65897
;F01_transcript_65947;F01_transcript_66099;F01_transcript_66
105;F01_transcript_66109;F01_transcript_66150;F01_transcript
_66196;F01_transcript_66204;F01_transcript_66370;F01_transc
ript_66378;F01_transcript_66678;F01_transcript_66787;F01_tra
nscript_67092;F01_transcript_67154;F01_transcript_67181;F01
_transcript_67285;F01_transcript_67414;F01_transcript_6752;F
01_transcript_68050;F01_transcript_68276;F01_transcript_6842
4;F01_transcript_68566;F01_transcript_68816;F01_transcript_6
8987;F01_transcript_69080;F01_transcript_69802;F01_transcrip
t_69868;F01_transcript_70307;F01_transcript_70903;F01_trans
cript_71245;F01_transcript_71511;F01_transcript_7162;F01_tra
nscript_71620;F01_transcript_71975;F01_transcript_72016;F01
_transcript_72375;F01_transcript_72775;F01_transcript_72987;
F01_transcript_73292;F01_transcript_73651;F01_transcript_737
71;F01_transcript_73833;F01_transcript_7389;F01_transcript_7
4487;F01_transcript_74520;F01_transcript_75417;F01_transcrip
t_75773;F01_transcript_75912;F01_transcript_75936;F01_trans
cript_76138;F01_transcript_76232;F01_transcript_76242;F01_tr
anscript_76378;F01_transcript_76393;F01_transcript_76419;F0
1_transcript_76538;F01_transcript_76666;F01_transcript_76952
;F01_transcript_76953;F01_transcript_7696;F01_transcript_770
17;F01_transcript_77043;F01_transcript_77214;F01_transcript_
78457;F01_transcript_785;F01_transcript_78823;F01_transcript

			_79135;F01_transcript_79556;F01_transcript_79805;F01_transcript_79814;F01_transcript_79998;F01_transcript_80272;F01_transcript_80302;F01_transcript_80598;F01_transcript_80681;F01_transcript_81373;F01_transcript_81392;F01_transcript_81427;F01_transcript_81506;F01_transcript_81540;F01_transcript_81639;F01_transcript_81711;F01_transcript_82538;F01_transcript_82845;F01_transcript_83681;F01_transcript_8384;F01_transcript_839;F01_transcript_84163;F01_transcript_84175;F01_transcript_84256;F01_transcript_84409;F01_transcript_84811;F01_transcript_84888;F01_transcript_84941;F01_transcript_84976;F01_transcript_85510;F01_transcript_85584;F01_transcript_85882;F01_transcript_85943;F01_transcript_86611;F01_transcript_86908;F01_transcript_87016;F01_transcript_87062;F01_transcript_87107;F01_transcript_87193;F01_transcript_8728;F01_transcript_87454;F01_transcript_87468;F01_transcript_87481;F01_transcript_87515;F01_transcript_88029;F01_transcript_88154;F01_transcript_8847;F01_transcript_88847;F01_transcript_89242;F01_transcript_89291;F01_transcript_89516;F01_transcript_89811;F01_transcript_90102;F01_transcript_90422;F01_transcript_9107;F01_transcript_91112;F01_transcript_91286;F01_transcript_91613;F01_transcript_91943;F01_transcript_93192;F01_transcript_93304;F01_transcript_93682;F01_transcript_93727;F01_transcript_93759;F01_transcript_93814;F01_transcript_94580;F01_transcript_9465;F01_transcript_94731;F01_transcript_95111;F01_transcript_95424;F01_transcript_95672;F01_transcript_95969;F01_transcript_96003;F01_transcript_96363;F01_transcript_96979;F01_transcript_97133;F01_transcript_97189;F01_transcript_97325;F01_transcript_9735;F01_transcript_97396;F01_transcript_98079;F01_transcript_98090;F01_transcript_98135;F01_transcript_98167;F01_transcript_98629;F01_transcript_98689;F01_transcript_98848;F01_transcript_9899;F01_transcript_99097;F01_transcript_99396;F01_transcript_99494;F01_transcript_99753;F01_transcript_99873;	
RNA degradation	ko03	578	F01_transcript_10020;F01_transcript_100876;F01_transcript_100920;F01_transcript_101153;F01_transcript_101347;F01_transcript_101574;F01_transcript_101649;F01_transcript_101897;F01_transcript_102237;F01_transcript_102330;F01_transcript_1027	K13126+K12593+K10643+K04077+K13126+K00850+K12598+K13126+K12608+K12616+K12581+K01689+K12614+K12617+K04077+K12620+K12617+K03678+K14442+K12614+K00850+K12614+K12619+K12616+K03654+K12606+K00962+K12

018	71;F01_transcript_102902;F01_transcript_103102;F01_transcript_103898;F01_transcript_104064;F01_transcript_104249;F01_transcript_104270;F01_transcript_104410;F01_transcript_104841;F01_transcript_104968;F01_transcript_104986;F01_transcript_10508;F01_transcript_105913;F01_transcript_106021;F01_transcript_106295;F01_transcript_106953;F01_transcript_107438;F01_transcript_107726;F01_transcript_108144;F01_transcript_108686;F01_transcript_108807;F01_transcript_108868;F01_transcript_109064;F01_transcript_109807;F01_transcript_109922;F01_transcript_110380;F01_transcript_110461;F01_transcript_110463;F01_transcript_110559;F01_transcript_11094;F01_transcript_111008;F01_transcript_111279;F01_transcript_111595;F01_transcript_111716;F01_transcript_111768;F01_transcript_111990;F01_transcript_112222;F01_transcript_112630;F01_transcript_112633;F01_transcript_112771;F01_transcript_112867;F01_transcript_113498;F01_transcript_113808;F01_transcript_113985;F01_transcript_114014;F01_transcript_114151;F01_transcript_114227;F01_transcript_114475;F01_transcript_114498;F01_transcript_114570;F01_transcript_114893;F01_transcript_115365;F01_transcript_115399;F01_transcript_115918;F01_transcript_116319;F01_transcript_116988;F01_transcript_11706;F01_transcript_117164;F01_transcript_117798;F01_transcript_11812;F01_transcript_118252;F01_transcript_119165;F01_transcript_119196;F01_transcript_119473;F01_transcript_120038;F01_transcript_120539;F01_transcript_12087;F01_transcript_121058;F01_transcript_121392;F01_transcript_121426;F01_transcript_121690;F01_transcript_122400;F01_transcript_123000;F01_transcript_123204;F01_transcript_123442;F01_transcript_123539;F01_transcript_123557;F01_transcript_123558;F01_transcript_123635;F01_transcript_123951;F01_transcript_124777;F01_transcript_125244;F01_transcript_125260;F01_transcript_125669;F01_transcript_125709;F01_transcript_125787;F01_transcript_126377;F01_transcript_126674;F01_transcript_126693;F01_transcript_126738;F01_transcript_126751;F01_transcript_127397;F01_transcript_127548;F01_transcript_127801;F01_transcript_128491;F01_transcript_128564;F01_transcript_128623;F01_transcript_128804;F01_transcript_129230;F01_transcript_129889;F01_transcript_129991;F01_transcript_130259;F01_transcript_130312;F01_transcript_1	617+K01148+K10643+K12613+K00850+K12580+K12607+K12593+K03681+K10643+K12580+K00850+K13126+K12626+K13126+K12620+K12620+K12602+K12598+K13126+K12600+K03514+K14442+K12592+K03514+K00962+K00962+K12608+K12617+K12608+K03514+K12608+K01689+K10643+K12608+K14442+K00962+K04077+K12591+K01148+K03678+K01689+K14442+K14442+K03514+K12581+K12600+K12604+K00962+K13126+K12585+K12585+K03654+K12616+K12617+K03514+K12591+K01148+K12608+K04077+K10643+K12606+K13126+K12604+K03514+K12581+K12580+K12585+K04077+K01148+K03678+K12620+K12620+K12585+K12598+K01148+K03514+K03678+K12616+K12623+K03681+K07573+K12619+K12585+K10643+K12604+K12598+K13126+K12607+K14442+K12580+K12611+K12608+K12606+K12607+K12590+K12600+K03678+K12619+K01689+K12585+K12616+K04077+K00850+K03678+K12608+K00962+K12580+K12580+K04077+K12580+K12619+K04077+K12611+K12606+K13126+K12585+K12616+K12604+K12581+K12592+K04077+K12617+K04077+K12585+K12603+K14442+K13126+K12599+K12608+K13126+K12591+K12614+K14442+K12587+K12627+K00962+K12585+K12600+K12585+K12585+K10643+K03681+K03678+K12616+K13126+K12608+K00962+K14442+K12608+K10643+K00850+K12600+K00962+K04077+K12591+K03514+K12585+K12608+K12581+K12586+K12617+K03514+K12608+K12581+K12611+K12598+K01689+K12608+K12581+K12605+K03681+K12606+K12611+K12589+K12619+K12619+K01689+K13126+K12626+K12604+K12598+K12611+K03514+K12585+K04077+K00850+K00850+K12621+K12624+K00850+K12581+K03514+K14442+K00850+K12590+K01148+K04077+K12614+K07573+K00850+K04077+K12617+K12608+K12598+K12614+K10643+K12608+K13126+K12598+K12616+K14442+K12581+K04077+K12624+K01148+K12585+K13126+K12585+K12619+K01148+K04077+K03514+K14442+K13126+K13126+K12604+K10643+K12613+K00850+K12591+K04077+K12605+K03678+K12608+K13126+K00962+K01689+K12614+K01148+K01148+K12624+K12605+K10643+K01689+K03678+K12580+K03514+K01689+K04077+K12623+K04077+K03678+K12587+K12585+K12580+K12614+K12606+K12606+K12614+K04077+

	307;F01_transcript_130768;F01_transcript_130793;F01_transcript_131417;F01_transcript_131752;F01_transcript_131879;F01_transcript_132300;F01_transcript_132631;F01_transcript_132905;F01_transcript_133761;F01_transcript_133766;F01_transcript_133901;F01_transcript_134536;F01_transcript_135187;F01_transcript_135208;F01_transcript_136118;F01_transcript_136635;F01_transcript_136831;F01_transcript_136953;F01_transcript_137056;F01_transcript_137065;F01_transcript_137360;F01_transcript_138248;F01_transcript_138483;F01_transcript_138603;F01_transcript_1387;F01_transcript_138808;F01_transcript_139001;F01_transcript_139445;F01_transcript_140741;F01_transcript_1408;F01_transcript_140847;F01_transcript_140993;F01_transcript_141170;F01_transcript_141411;F01_transcript_141428;F01_transcript_141499;F01_transcript_141542;F01_transcript_1422;F01_transcript_142201;F01_transcript_142488;F01_transcript_14249;F01_transcript_142527;F01_transcript_142628;F01_transcript_143241;F01_transcript_143289;F01_transcript_143306;F01_transcript_143384;F01_transcript_143484;F01_transcript_143562;F01_transcript_143703;F01_transcript_143914;F01_transcript_143942;F01_transcript_144291;F01_transcript_144456;F01_transcript_1446;F01_transcript_144835;F01_transcript_145520;F01_transcript_146059;F01_transcript_146077;F01_transcript_146189;F01_transcript_146362;F01_transcript_146419;F01_transcript_146441;F01_transcript_146816;F01_transcript_146888;F01_transcript_147097;F01_transcript_147245;F01_transcript_147701;F01_transcript_148828;F01_transcript_150102;F01_transcript_1504;F01_transcript_150432;F01_transcript_150515;F01_transcript_151014;F01_transcript_151161;F01_transcript_151567;F01_transcript_151774;F01_transcript_152279;F01_transcript_152599;F01_transcript_152621;F01_transcript_152798;F01_transcript_153029;F01_transcript_153100;F01_transcript_15339;F01_transcript_153528;F01_transcript_153589;F01_transcript_153750;F01_transcript_153928;F01_transcript_154231;F01_transcript_154404;F01_transcript_154485;F01_transcript_15498;F01_transcript_155107;F01_transcript_155113;F01_transcript_155326;F01_transcript_155862;F01_transcript_155870;F01_transcript_156143;F01_transcript_156931;F01_transcript_157081;F01_tran	K12614+K04077+K04077+K00962+K00850+K12587+K10643+K04077+K04077+K00850+K03514+K00962+K04077+K03681+K12623+K03514+K10643+K04077+K00850+K01689+K00962+K12608+K12620+K13126+K12611+K10643+K01689+K01689+K12581+K12598+K12598+K12598+K01148+K12616+K12611+K12613+K12620+K12608+K12603+K12581+K12585+K12617+K12608+K13126+K12589+K13126+K10643+K12606+K12606+K03679+K12606+K12613+K13126+K12602+K11600+K12581+K12593+K07573+K12623+K12623+K12617+K12591+K12626+K10643+K12625+K12624+K12622+K12627+K12625+K12625+K12624+K12621+K14442+K14442+K12622+K12617+K10643+K13126+K12608+K12607+K12585+K12619+K12619+K04077+K14442+K12608+K12617+K12617+K12614+K10643+K12585+K12599+K12617+K14442+K12591+K01148+K12586+K14442+K12604+K12617+K12614+K12580+K13126+K12591+K12604+K12585+K00850+K00850+K12607+K12600+K12586+K12580+K04077+K12580+K03514+K11600+K12580+K12616+K12623+K01148+K12619+K12586+K00850+K12586+K12600+K00850+K03514+K12590+K12617+K12604+K12619+K13126+K12580+K13126+K14442+K10643+K11600+K12600+K12586+K12604+K12604+K12587+K03678+K03678+K03514+K14442+K12580+K12619+K12598+K12580+K10643+K12591+K13126+K12603+K12608+K12614+K00962+K03678+K12598+K00962+K01689+K12585+K14442+K12617+K03514+K12593+K12598+K03514+K12619+K12604+K12614+K00850+K14442+K12585+K03681+K12585+K00850+K04077+K03678+K01148+K12619+K12600+K12600+K03654+K12617+K12608+K12587+K12585+K12613+K12613+K10643+K12617+K03678+K12585+K01689+K12616+K12580+K04077+K12606+K04077+K12617+K04077+K03514+K10643+K12608+K12586+K11600+K03654+K03654+K14442+K14442+K12586+K12619+K04077+K12611+K10643+K12585+K12580+K12581+K12604+K00962+K03514+K10643+K04077+K12624+K12611+K12624+K12587+K14442+K12598+K12616+K03678+K12598+K14442+K01148+K12585+K12620+K12581+K12600+K12585+K03514+K12599+K12623+K12608+K12580+K12613+K12619+K12587+K12586+K03654+K12606+K10643+K12606+K1261

	script_157674;F01_transcript_158071;F01_transcript_158166;F01_transcript_158480;F01_transcript_158516;F01_transcript_15870;F01_transcript_158719;F01_transcript_158736;F01_transcript_158940;F01_transcript_158986;F01_transcript_159218;F01_transcript_159308;F01_transcript_159380;F01_transcript_159443;F01_transcript_159887;F01_transcript_160081;F01_transcript_160450;F01_transcript_160463;F01_transcript_160741;F01_transcript_160778;F01_transcript_16096;F01_transcript_161080;F01_transcript_161219;F01_transcript_161255;F01_transcript_161434;F01_transcript_161641;F01_transcript_161779;F01_transcript_161818;F01_transcript_161849;F01_transcript_161937;F01_transcript_162165;F01_transcript_162192;F01_transcript_16239;F01_transcript_162921;F01_transcript_163111;F01_transcript_164110;F01_transcript_164118;F01_transcript_164620;F01_transcript_164830;F01_transcript_164889;F01_transcript_164962;F01_transcript_165097;F01_transcript_165153;F01_transcript_165332;F01_transcript_165380;F01_transcript_165493;F01_transcript_16653;F01_transcript_166631;F01_transcript_166716;F01_transcript_167351;F01_transcript_167640;F01_transcript_167689;F01_transcript_17113;F01_transcript_17118;F01_transcript_17119;F01_transcript_17167;F01_transcript_1735;F01_transcript_17879;F01_transcript_18385;F01_transcript_1907;F01_transcript_19463;F01_transcript_19656;F01_transcript_20262;F01_transcript_20306;F01_transcript_20490;F01_transcript_20935;F01_transcript_21122;F01_transcript_2138;F01_transcript_2142;F01_transcript_21761;F01_transcript_22224;F01_transcript_2246;F01_transcript_22643;F01_transcript_22765;F01_transcript_23032;F01_transcript_23826;F01_transcript_24239;F01_transcript_2459;F01_transcript_24624;F01_transcript_25228;F01_transcript_25297;F01_transcript_25551;F01_transcript_25672;F01_transcript_25920;F01_transcript_25989;F01_transcript_2604;F01_transcript_26727;F01_transcript_27192;F01_transcript_27259;F01_transcript_27506;F01_transcript_2762;F01_transcript_27818;F01_transcript_27932;F01_transcript_29149;F01_transcript_2928;F01_transcript_29318;F01_transcript_30175;F01_transcript_3024;F01_transcript_30441;F01_transcript_3078;F01_transcript_30852;F01_transcript_34418;F01_transcript_35030;F01_transcript_3525;F01_transcript_3545;F01_transcript_3639;F01_transcript	9+K00962+K12593+K00962+K12608+K12580+K12604+K12626+K12580+K10643+K12604+K12600+K12616+K12608+K12585+K12605+K03514+K03654+K13126+K12606+K13126+K04077+K14442+K12600+K03514+K12608+K12611+K12617+K12600+K01689+K10643+K12620+K01148+K12620+K14442+K12591+K12589

_36570;F01_transcript_389;F01_transcript_39151;F01_transcrip
t_40048;F01_transcript_40542;F01_transcript_40773;F01_trans
cript_408;F01_transcript_40893;F01_transcript_4132;F01_trans
cript_4145;F01_transcript_41493;F01_transcript_41559;F01_tra
nscript_41788;F01_transcript_41861;F01_transcript_4222;F01_t
ranscript_42315;F01_transcript_42407;F01_transcript_42541;F0
1_transcript_42647;F01_transcript_42654;F01_transcript_42832
;F01_transcript_43641;F01_transcript_43682;F01_transcript_44
148;F01_transcript_44985;F01_transcript_45080;F01_transcript
_45789;F01_transcript_46160;F01_transcript_4744;F01_transcri
pt_4799;F01_transcript_48052;F01_transcript_4851;F01_transcr
ipt_48852;F01_transcript_49043;F01_transcript_49059;F01_tra
nscript_49064;F01_transcript_49291;F01_transcript_49331;F01
_transcript_49540;F01_transcript_49910;F01_transcript_502;F0
1_transcript_5032;F01_transcript_50758;F01_transcript_5122;F
01_transcript_5131;F01_transcript_5161;F01_transcript_51877;
F01_transcript_5191;F01_transcript_52094;F01_transcript_5210
5;F01_transcript_52249;F01_transcript_52375;F01_transcript_5
2926;F01_transcript_53022;F01_transcript_53408;F01_transcrip
t_53467;F01_transcript_5362;F01_transcript_53727;F01_transcr
ipt_53834;F01_transcript_541;F01_transcript_5480;F01_transcri
pt_55413;F01_transcript_5550;F01_transcript_55502;F01_trans
cript_55529;F01_transcript_55681;F01_transcript_55853;F01_tr
anscript_5592;F01_transcript_56018;F01_transcript_5610;F01_t
ranscript_56260;F01_transcript_56332;F01_transcript_56515;F0
1_transcript_56588;F01_transcript_56717;F01_transcript_56794
;F01_transcript_5686;F01_transcript_57122;F01_transcript_576
88;F01_transcript_57783;F01_transcript_5809;F01_transcript_5
813;F01_transcript_58174;F01_transcript_58522;F01_transcript
_5855;F01_transcript_58624;F01_transcript_58814;F01_transcri
pt_59199;F01_transcript_59431;F01_transcript_59441;F01_tran
script_59961;F01_transcript_59969;F01_transcript_60134;F01_t
ranscript_60235;F01_transcript_60290;F01_transcript_60982;F0
1_transcript_6133;F01_transcript_61890;F01_transcript_62686;
F01_transcript_62782;F01_transcript_6281;F01_transcript_6310
1;F01_transcript_63277;F01_transcript_63454;F01_transcript_6
3549;F01_transcript_63596;F01_transcript_63618;F01_transcrip

t_63727;F01_transcript_63775;F01_transcript_64559;F01_transcript_64803;F01_transcript_65340;F01_transcript_65462;F01_transcript_6609;F01_transcript_6619;F01_transcript_66698;F01_transcript_67249;F01_transcript_67281;F01_transcript_67484;F01_transcript_68011;F01_transcript_68276;F01_transcript_68564;F01_transcript_69087;F01_transcript_69526;F01_transcript_69810;F01_transcript_69886;F01_transcript_69996;F01_transcript_70063;F01_transcript_70950;F01_transcript_71353;F01_transcript_716;F01_transcript_71824;F01_transcript_72484;F01_transcript_72607;F01_transcript_72745;F01_transcript_73133;F01_transcript_73264;F01_transcript_73468;F01_transcript_73499;F01_transcript_73635;F01_transcript_738;F01_transcript_73844;F01_transcript_74407;F01_transcript_75493;F01_transcript_75573;F01_transcript_75587;F01_transcript_75817;F01_transcript_75877;F01_transcript_75889;F01_transcript_76181;F01_transcript_76423;F01_transcript_7648;F01_transcript_7662;F01_transcript_76787;F01_transcript_76870;F01_transcript_76933;F01_transcript_77064;F01_transcript_77224;F01_transcript_77782;F01_transcript_78096;F01_transcript_78266;F01_transcript_78516;F01_transcript_78831;F01_transcript_78996;F01_transcript_79209;F01_transcript_79358;F01_transcript_79580;F01_transcript_79673;F01_transcript_80013;F01_transcript_80438;F01_transcript_80639;F01_transcript_80861;F01_transcript_81001;F01_transcript_81012;F01_transcript_81201;F01_transcript_82197;F01_transcript_8247;F01_transcript_826;F01_transcript_827;F01_transcript_82755;F01_transcript_82957;F01_transcript_83057;F01_transcript_83545;F01_transcript_83836;F01_transcript_83946;F01_transcript_84210;F01_transcript_84430;F01_transcript_84621;F01_transcript_84893;F01_transcript_84940;F01_transcript_85180;F01_transcript_85465;F01_transcript_85494;F01_transcript_85543;F01_transcript_85580;F01_transcript_85716;F01_transcript_86218;F01_transcript_86297;F01_transcript_86659;F01_transcript_86951;F01_transcript_86953;F01_transcript_87252;F01_transcript_87925;F01_transcript_88210;F01_transcript_88225;F01_transcript_88897;F01_transcript_89096;F01_transcript_89384;F01_transcript_89423;F01_transcript_89605;F01_transcript_9024;F01_transcript_90338;F01_transcript_90525;F01_transcript_90902;F01_transcript_91558;F01_transcript_91686;F01_trans

			cript_91923;F01_transcript_91935;F01_transcript_92771;F01_transcript_92974;F01_transcript_93029;F01_transcript_936;F01_transcript_93798;F01_transcript_94244;F01_transcript_94392;F01_transcript_94431;F01_transcript_94477;F01_transcript_94640;F01_transcript_94833;F01_transcript_95009;F01_transcript_95360;F01_transcript_95534;F01_transcript_95542;F01_transcript_95747;F01_transcript_95755;F01_transcript_96164;F01_transcript_96655;F01_transcript_96760;F01_transcript_96770;F01_transcript_96979;F01_transcript_97047;F01_transcript_97396;F01_transcript_97557;F01_transcript_97773;F01_transcript_97796;F01_transcript_97881;F01_transcript_98186;F01_transcript_98353;F01_transcript_98394;F01_transcript_984;F01_transcript_98475;F01_transcript_99104;F01_transcript_99167;F01_transcript_99174;F01_transcript_99348;F01_transcript_99509;F01_transcript_99510;F01_transcript_99954;	
RNA polymerase	ko03020	156	F01_transcript_100336;F01_transcript_101484;F01_transcript_103677;F01_transcript_104227;F01_transcript_105182;F01_transcript_105368;F01_transcript_105409;F01_transcript_107412;F01_transcript_107564;F01_transcript_108543;F01_transcript_110032;F01_transcript_110555;F01_transcript_110847;F01_transcript_111586;F01_transcript_112497;F01_transcript_115240;F01_transcript_116651;F01_transcript_117913;F01_transcript_120701;F01_transcript_120865;F01_transcript_122244;F01_transcript_12253;F01_transcript_122706;F01_transcript_123958;F01_transcript_125165;F01_transcript_126015;F01_transcript_126607;F01_transcript_127244;F01_transcript_128043;F01_transcript_128722;F01_transcript_1292;F01_transcript_129204;F01_transcript_131083;F01_transcript_131349;F01_transcript_131700;F01_transcript_132216;F01_transcript_132637;F01_transcript_134237;F01_transcript_134341;F01_transcript_135929;F01_transcript_136028;F01_transcript_136150;F01_transcript_137575;F01_transcript_137703;F01_transcript_137852;F01_transcript_139189;F01_transcript_139341;F01_transcript_139382;F01_transcript_141582;F01_transcript_141997;F01_transcript_142939;F01_transcript_144532;F01_transcript_144621;F01_transcript_145905;F01_transcript_146748;F01_transcript_147483;F01_transcript_147961;F01_transcript_148285;F01_transcript_148890;F01_transcrip	K03026+K14721+K03027+K02999+K03023+K03018+K03023+K03022+K03023+K03014+K03023+K03025+K03023+K03023+K03023+K03017+K03023+K03012+K03018+K03006+K03018+K03015+K03018+K03019+K03006+K03015+K03023+K03016+K03012+K03007+K03002+K03012+K03023+K03012+K03023+K03021+K03016+K03046+K03002+K03018+K03006+K03005+K03012+K03006+K03014+K03023+K03006+K03006+K03023+K03012+K03021+K03007+K03018+K03007+K03023+K03025+K03006+K03018+K03018+K03007+K03016+K03046+K03021+K03023+K03026+K03006+K03024+K03026+K14721+K03023+K03026+K03013+K03022+K03023+K03040+K03025+K03013+K03040+K03046+K03006+K03018+K03046+K03023+K03006+K03026+K03018+K03011+K03011+K03024+K03012+K03013+K03016+K03013+K03013+K03013+K03015+K03015+K03015+K03015+K03019+K03017+K03000+K03012+K03012+K03012+K03017+K03008+K03007+K03009+K03006+K03022+K03011+K03021+K03023+K03015+K02999+K03023+K03012+K03026+K03011+K03024+K03006+K03021+K03012+K03023+K03006+K03023+K03006+K03021+K03018+K03021+K03018+K03015+K03022+K03018+K03006+K03043+K03023+K03026+K03012+K03019+K03018+K03006+K03046+

			t_149386;F01_transcript_151626;F01_transcript_151710;F01_transcript_152671;F01_transcript_153188;F01_transcript_15324;F01_transcript_153763;F01_transcript_154052;F01_transcript_155788;F01_transcript_15700;F01_transcript_158336;F01_transcript_159126;F01_transcript_162916;F01_transcript_164219;F01_transcript_165423;F01_transcript_166129;F01_transcript_166189;F01_transcript_166241;F01_transcript_167163;F01_transcript_167486;F01_transcript_219;F01_transcript_265;F01_transcript_2923;F01_transcript_33558;F01_transcript_35;F01_transcript_37905;F01_transcript_3912;F01_transcript_40205;F01_transcript_41047;F01_transcript_42046;F01_transcript_44486;F01_transcript_44662;F01_transcript_45093;F01_transcript_45318;F01_transcript_45505;F01_transcript_45583;F01_transcript_46776;F01_transcript_46905;F01_transcript_47363;F01_transcript_47443;F01_transcript_47453;F01_transcript_47943;F01_transcript_48111;F01_transcript_48117;F01_transcript_48332;F01_transcript_48335;F01_transcript_49116;F01_transcript_49309;F01_transcript_49943;F01_transcript_50112;F01_transcript_53730;F01_transcript_53743;F01_transcript_54444;F01_transcript_54744;F01_transcript_56240;F01_transcript_56634;F01_transcript_56841;F01_transcript_57564;F01_transcript_59667;F01_transcript_61242;F01_transcript_63353;F01_transcript_63945;F01_transcript_66564;F01_transcript_66618;F01_transcript_68330;F01_transcript_68587;F01_transcript_69789;F01_transcript_70696;F01_transcript_71458;F01_transcript_71845;F01_transcript_73296;F01_transcript_73704;F01_transcript_75974;F01_transcript_76961;F01_transcript_77171;F01_transcript_77269;F01_transcript_78239;F01_transcript_78867;F01_transcript_78988;F01_transcript_79059;F01_transcript_82288;F01_transcript_82302;F01_transcript_86322;F01_transcript_88551;F01_transcript_90069;F01_transcript_91330;F01_transcript_92965;F01_transcript_93012;F01_transcript_95338;F01_transcript_95488;F01_transcript_95575;F01_transcript_96015;F01_transcript_96269;F01_transcript_96668;F01_transcript_97410;F01_transcript_98513;F01_transcript_98674;	K03018+K03022+K03012+K03002+K03012+K03023+K03006+K03020+K03027+K03016+K03023+K03026
Basal transcriptio	ko0	197	F01_transcript_100379;F01_transcript_101938;F01_transcript_102056;F01_transcript_10324;F01_transcript_103889;F01_transcript_104069;F01_transcript_104310;F01_transcript_104424;F01	K03137+K03144+K03120+K10844+K03139+K03122+K03137+K03143+K03128+K14649+K03127+K03141+K02202+K03124+K03134+K03122+K03129+K03124+K03128+K03129+K031

n factors	3022	_transcript_104597;F01_transcript_104962;F01_transcript_106489;F01_transcript_107316;F01_transcript_108013;F01_transcript_109352;F01_transcript_109820;F01_transcript_110779;F01_transcript_112022;F01_transcript_112151;F01_transcript_1122;F01_transcript_11240;F01_transcript_113716;F01_transcript_114050;F01_transcript_114051;F01_transcript_115051;F01_transcript_115252;F01_transcript_115637;F01_transcript_116350;F01_transcript_116355;F01_transcript_118629;F01_transcript_119393;F01_transcript_120305;F01_transcript_120514;F01_transcript_120735;F01_transcript_121474;F01_transcript_122412;F01_transcript_125315;F01_transcript_126560;F01_transcript_127149;F01_transcript_128140;F01_transcript_130551;F01_transcript_133290;F01_transcript_133492;F01_transcript_133584;F01_transcript_134075;F01_transcript_134363;F01_transcript_134673;F01_transcript_135682;F01_transcript_136005;F01_transcript_137476;F01_transcript_137638;F01_transcript_137814;F01_transcript_137828;F01_transcript_139333;F01_transcript_139952;F01_transcript_140608;F01_transcript_14243;F01_transcript_143385;F01_transcript_144179;F01_transcript_144659;F01_transcript_145183;F01_transcript_145506;F01_transcript_145834;F01_transcript_146171;F01_transcript_148688;F01_transcript_149758;F01_transcript_150303;F01_transcript_151600;F01_transcript_152165;F01_transcript_15225;F01_transcript_152303;F01_transcript_153002;F01_transcript_153200;F01_transcript_153591;F01_transcript_153602;F01_transcript_153758;F01_transcript_153957;F01_transcript_154611;F01_transcript_155411;F01_transcript_156232;F01_transcript_156674;F01_transcript_159279;F01_transcript_159572;F01_transcript_15974;F01_transcript_160041;F01_transcript_163121;F01_transcript_163339;F01_transcript_164083;F01_transcript_164545;F01_transcript_164896;F01_transcript_165971;F01_transcript_166692;F01_transcript_16725;F01_transcript_17780;F01_transcript_18791;F01_transcript_21213;F01_transcript_23145;F01_transcript_23272;F01_transcript_23329;F01_transcript_23330;F01_transcript_23392;F01_transcript_23706;F01_transcript_24715;F01_transcript_25319;F01_transcript_26919;F01_transcript_29272;F01_transcript_29716;F01_transcript_31871;F01_transcript_32479;F01_transcript_33342;F01_tra	27+K03139+K03127+K10843+K03137+K03123+K03120+K02202+K03138+K03139+K03128+K03134+K03137+K03137+K03137+K03127+K03127+K10844+K03137+K03128+K03122+K03122+K03125+K03120+K03124+K03129+K03129+K03124+K03139+K03124+K03125+K14649+K03141+K03122+K03128+K03141+K10844+K03144+K03136+K03131+K03135+K03131+K03129+K03120+K03136+K03127+K03141+K03139+K03130+K03136+K03126+K03129+K03125+K03137+K03144+K03124+K03124+K03138+K03127+K03120+K03126+K03130+K03126+K03143+K14649+K10844+K03127+K03139+K03135+K03123+K03128+K03141+K03126+K03126+K03131+K03137+K03142+K03126+K03143+K03139+K03137+K03144+K03144+K03136+K03142+K03136+K03124+K03122+K14649+K14649+K03120+K03129+K03124+K03120+K03124+K03132+K03139+K10842+K03133+K03129+K03127+K03134+K03128+K03137+K03144+K03130+K10845+K03143+K03141+K03124+K02202+K03141+K03124+K03137+K03137+K03126+K03133+K03120+K03120+K03127+K03124+K03126+K03123+K03133+K03130+K03130+K10844+K03125+K03130+K03137+K03143+K03126+K03124+K03137+K03135+K10843+K03124+K10845+K03126+K03129+K03129+K03131+K02202+K03123+K03137+K03120+K03125+K03130+K03122+K03132+K03133+K03129+K03126+K03133+K03124+K03124+K03141+K03127+K03143+K03120+K03143+K03127+K03120+K03127+K10843+K03127+K03139+K03136+K03131+K03127+K03127+K03129+K03129+K10843+K03123+K03130+K03132

			nscript_34095;F01_transcript_36117;F01_transcript_3649;F01_transcript_40312;F01_transcript_40650;F01_transcript_42643;F01_transcript_43029;F01_transcript_45022;F01_transcript_45110;F01_transcript_46582;F01_transcript_4752;F01_transcript_47793;F01_transcript_47932;F01_transcript_53150;F01_transcript_53443;F01_transcript_53642;F01_transcript_53712;F01_transcript_54553;F01_transcript_54782;F01_transcript_5604;F01_transcript_56375;F01_transcript_56550;F01_transcript_58058;F01_transcript_59849;F01_transcript_59878;F01_transcript_6121;F01_transcript_62924;F01_transcript_64270;F01_transcript_65176;F01_transcript_65475;F01_transcript_65930;F01_transcript_6617;F01_transcript_67192;F01_transcript_67280;F01_transcript_6811;F01_transcript_68948;F01_transcript_71276;F01_transcript_71889;F01_transcript_72158;F01_transcript_7275;F01_transcript_72877;F01_transcript_73445;F01_transcript_73934;F01_transcript_74148;F01_transcript_74705;F01_transcript_75677;F01_transcript_7631;F01_transcript_76442;F01_transcript_7661;F01_transcript_77140;F01_transcript_78402;F01_transcript_79269;F01_transcript_80248;F01_transcript_81230;F01_transcript_81283;F01_transcript_82170;F01_transcript_82301;F01_transcript_82484;F01_transcript_8428;F01_transcript_84780;F01_transcript_85631;F01_transcript_86213;F01_transcript_8700;F01_transcript_87464;F01_transcript_88722;F01_transcript_89363;F01_transcript_89986;F01_transcript_90216;F01_transcript_91033;F01_transcript_91203;F01_transcript_92391;F01_transcript_92760;F01_transcript_92827;F01_transcript_92878;F01_transcript_93086;F01_transcript_9565;F01_transcript_96016;F01_transcript_97072;F01_transcript_97379;F01_transcript_97690;F01_transcript_97864;F01_transcript_97971;F01_transcript_98440;F01_transcript_98570;F01_transcript_98841;F01_transcript_99639;F01_transcript_99856;F01_transcript_99875;	
DNA replication	ko03030	147	F01_transcript_102104;F01_transcript_106285;F01_transcript_106305;F01_transcript_107999;F01_transcript_1101;F01_transcript_110896;F01_transcript_112056;F01_transcript_113185;F01_transcript_114411;F01_transcript_114864;F01_transcript_11492;F01_transcript_114968;F01_transcript_115417;F01_transcript_115738;F01_transcript_116687;F01_transcript_119625;F01_transcript_121040;F01_transcript_121601;F01_transcript_121657;F0	K10755+K10754+K10755+K07466+K02335+K10742+K02325+K10747+K02210+K07466+K07466+K02326+K04799+K10754+K10744+K10756+K10747+K10755+K10744+K07466+K10747+K10755+K10744+K10755+K02327+K10744+K10755+K10754+K07466+K10756+K07466+K10755+K10755+K02335+K10754+K10754+K02328+K02541+K02212+K02335+K07466+K10754+K02328+K10747+K02335+K02335+K04799+K03504+

	1_transcript_121703;F01_transcript_124264;F01_transcript_124661;F01_transcript_124733;F01_transcript_125272;F01_transcript_129404;F01_transcript_130026;F01_transcript_130442;F01_transcript_131237;F01_transcript_131999;F01_transcript_132323;F01_transcript_133243;F01_transcript_133706;F01_transcript_133770;F01_transcript_136652;F01_transcript_137291;F01_transcript_138141;F01_transcript_139121;F01_transcript_139902;F01_transcript_142440;F01_transcript_142708;F01_transcript_143196;F01_transcript_146387;F01_transcript_146556;F01_transcript_146806;F01_transcript_146957;F01_transcript_14906;F01_transcript_149855;F01_transcript_150196;F01_transcript_151343;F01_transcript_151783;F01_transcript_152398;F01_transcript_155052;F01_transcript_155603;F01_transcript_155687;F01_transcript_156736;F01_transcript_157558;F01_transcript_158044;F01_transcript_158296;F01_transcript_159626;F01_transcript_160006;F01_transcript_161577;F01_transcript_162212;F01_transcript_163029;F01_transcript_167070;F01_transcript_17688;F01_transcript_18802;F01_transcript_2042;F01_transcript_20485;F01_transcript_28257;F01_transcript_30875;F01_transcript_32534;F01_transcript_33994;F01_transcript_34563;F01_transcript_34698;F01_transcript_38014;F01_transcript_38198;F01_transcript_39293;F01_transcript_39420;F01_transcript_40950;F01_transcript_4207;F01_transcript_42381;F01_transcript_42392;F01_transcript_45535;F01_transcript_45921;F01_transcript_46906;F01_transcript_48686;F01_transcript_52470;F01_transcript_52773;F01_transcript_53178;F01_transcript_533;F01_transcript_54343;F01_transcript_55038;F01_transcript_55499;F01_transcript_57153;F01_transcript_5854;F01_transcript_5879;F01_transcript_59979;F01_transcript_61368;F01_transcript_6499;F01_transcript_65789;F01_transcript_66251;F01_transcript_66392;F01_transcript_66776;F01_transcript_67697;F01_transcript_68828;F01_transcript_70894;F01_transcript_70999;F01_transcript_71262;F01_transcript_7220;F01_transcript_728;F01_transcript_73069;F01_transcript_74164;F01_transcript_74712;F01_transcript_75496;F01_transcript_77639;F01_transcript_77701;F01_transcript_78804;F01_transcript_79595;F01_transcript_79636;F01_transcript_80132;F01_transcript_80286;F01_transcript_81168;F01_transc	K10755+K04799+K02328+K04799+K10747+K04802+K10743+K02210+K10755+K02328+K02328+K04799+K04799+K10754+K02326+K04799+K07466+K07466+K10754+K10756+K03504+K02328+K02328+K02685+K02328+K10744+K04799+K10756+K10756+K10756+K10755+K10756+K10755+K10755+K07466+K07466+K07466+K03505+K04799+K10755+K10747+K10747+K03505+K10756+K07466+K03111+K02540+K02540+K02210+K02328+K07466+K02335+K10754+K10747+K10743+K02325+K02212+K02335+K04799+K10747+K10747+K02335+K07466+K10747+K10755+K02335+K10756+K10747+K02326+K10747+K02324+K10743+K10739+K10747+K10754+K07466+K10747+K07466+K04799+K04799+K10747+K02335+K04799+K10754+K02335+K07466+K02335+K10747+K10747+K07466+K10754+K10745+K10754+K02335+K07466+K02325+K02325+K02327+K02335

			ript_82319;F01_transcript_82696;F01_transcript_83309;F01_transcript_83773;F01_transcript_85351;F01_transcript_85430;F01_transcript_85797;F01_transcript_862;F01_transcript_86415;F01_transcript_88147;F01_transcript_88462;F01_transcript_89037;F01_transcript_89051;F01_transcript_90918;F01_transcript_91162;F01_transcript_91407;F01_transcript_91705;F01_transcript_91981;F01_transcript_92049;F01_transcript_944;F01_transcript_95868;F01_transcript_95940;F01_transcript_97909;F01_transcript_98012;F01_transcript_991;	
Spliceosome	ko03040	1106	F01_transcript_100115;F01_transcript_100436;F01_transcript_100458;F01_transcript_100466;F01_transcript_100581;F01_transcript_100639;F01_transcript_100718;F01_transcript_100811;F01_transcript_10107;F01_transcript_101094;F01_transcript_101238;F01_transcript_101285;F01_transcript_101501;F01_transcript_101806;F01_transcript_101825;F01_transcript_10191;F01_transcript_101979;F01_transcript_102185;F01_transcript_102761;F01_transcript_10301;F01_transcript_103081;F01_transcript_103155;F01_transcript_103314;F01_transcript_103539;F01_transcript_103607;F01_transcript_103620;F01_transcript_103624;F01_transcript_103705;F01_transcript_103896;F01_transcript_104257;F01_transcript_10431;F01_transcript_104450;F01_transcript_104773;F01_transcript_104946;F01_transcript_105006;F01_transcript_105108;F01_transcript_105324;F01_transcript_10559;F01_transcript_105638;F01_transcript_106152;F01_transcript_10616;F01_transcript_106187;F01_transcript_106456;F01_transcript_106460;F01_transcript_106717;F01_transcript_107076;F01_transcript_107194;F01_transcript_107238;F01_transcript_107311;F01_transcript_10753;F01_transcript_107739;F01_transcript_107938;F01_transcript_107995;F01_transcript_108;F01_transcript_108118;F01_transcript_108128;F01_transcript_108224;F01_transcript_108232;F01_transcript_108277;F01_transcript_108341;F01_transcript_108565;F01_transcript_108579;F01_transcript_108694;F01_transcript_109158;F01_transcript_109348;F01_transcript_109373;F01_transcript_109538;F01_transcript_109717;F01_transcript_109803;F01_transcript_109844;F01_transcript_110112;F01_transcript_110281;F01_transcript_110374;F01_transcript_110480;F01_transcript_110606;F01_transcript_110675;F01_transcript_110798;F01_transcript_110845;F01_transcript_110	K12885+K12890+K12839+K12865+K12878+K03283+K12816+K12849+K12837+K12850+K03283+K11093+K12829+K12868+K12662+K12836+K12880+K12733+K11097+K12820+K12837+K12862+K12878+K12837+K12891+K11984+K12741+K12860+K10599+K12879+K12820+K12875+K12885+K12878+K12856+K12865+K12827+K12741+K03283+K12833+K12858+K12893+K12822+K12845+K12837+K12824+K12832+K12827+K03283+K12890+K12823+K12836+K12861+K12818+K12849+K12837+K12878+K12837+K13025+K12821+K12837+K12741+K10599+K12741+K12823+K12848+K12836+K12900+K12818+K12849+K12897+K12896+K12877+K12741+K12827+K12858+K12823+K12896+K12830+K12626+K12818+K12662+K12835+K12875+K12741+K12897+K12871+K12896+K12836+K12900+K12813+K12813+K12890+K12900+K12839+K12818+K10599+K11093+K12900+K12818+K12843+K12879+K12827+K11094+K12891+K12824+K12829+K12829+K12811+K12829+K12890+K12818+K12825+K12878+K12872+K12878+K12854+K12891+K12837+K12893+K12890+K12865+K12826+K12867+K12837+K12881+K12822+K12823+K12893+K12836+K12900+K12825+K12880+K12876+K12812+K03283+K12821+K12818+K12881+K12881+K12821+K12741+K12829+K12837+K12858+K12893+K12825+K12890+K12890+K12893+K12826+K12891+K12893+K03283+K12823+K12860+K12836+K12837+K11091+K12741+K12836+K12842+K12836+K12837+K12823+K11984+K12823+K12897+K12837+K12842+K12891+K12741+K12893+K12885+K12823+K12896+K12741+K06063+K12825+K12836+K12837+K12854+K12829+K06063+K12821+K12890+K12900+K12824+K12880+K12829+K12849+K12822+K12865+K12741+K12891+K12835+K12897+K12890+K12893

	900;F01_transcript_111008;F01_transcript_11104;F01_transcript_111044;F01_transcript_111110;F01_transcript_111146;F01_transcript_111273;F01_transcript_111307;F01_transcript_111351;F01_transcript_111531;F01_transcript_111654;F01_transcript_111664;F01_transcript_111851;F01_transcript_1119;F01_transcript_112004;F01_transcript_11210;F01_transcript_112469;F01_transcript_11266;F01_transcript_112734;F01_transcript_112841;F01_transcript_112995;F01_transcript_1130;F01_transcript_113011;F01_transcript_113079;F01_transcript_113300;F01_transcript_113530;F01_transcript_113693;F01_transcript_113782;F01_transcript_113911;F01_transcript_113944;F01_transcript_113963;F01_transcript_114041;F01_transcript_114174;F01_transcript_114180;F01_transcript_114216;F01_transcript_114472;F01_transcript_114489;F01_transcript_114655;F01_transcript_114859;F01_transcript_114921;F01_transcript_114985;F01_transcript_115015;F01_transcript_1151;F01_transcript_115111;F01_transcript_11524;F01_transcript_115459;F01_transcript_115611;F01_transcript_115750;F01_transcript_115861;F01_transcript_115903;F01_transcript_116009;F01_transcript_116197;F01_transcript_11631;F01_transcript_116493;F01_transcript_116564;F01_transcript_116577;F01_transcript_116677;F01_transcript_116810;F01_transcript_1169;F01_transcript_117064;F01_transcript_117177;F01_transcript_117205;F01_transcript_117433;F01_transcript_117499;F01_transcript_117708;F01_transcript_117775;F01_transcript_118111;F01_transcript_118232;F01_transcript_118341;F01_transcript_118451;F01_transcript_118497;F01_transcript_118576;F01_transcript_118689;F01_transcript_118822;F01_transcript_118882;F01_transcript_119258;F01_transcript_119324;F01_transcript_119439;F01_transcript_119510;F01_transcript_119696;F01_transcript_119698;F01_transcript_11991;F01_transcript_12017;F01_transcript_120731;F01_transcript_120808;F01_transcript_120964;F01_transcript_121126;F01_transcript_121234;F01_transcript_121255;F01_transcript_121299;F01_transcript_12140;F01_transcript_121535;F01_transcript_121544;F01_transcript_121614;F01_transcript_121658;F01_transcript_121810;F01_transcript_121999;F01_transcript_122031;F01_transcript_12208;F01_transcript_122346;F01_transcript_122377;F01_transcrip	+K12837+K12890+K03283+K12890+K12821+K12825+K12881+K12849+K12890+K12881+K12842+K12878+K12900+K11087+K12867+K03283+K12836+K12820+K12865+K12623+K12823+K12837+K12836+K12862+K12741+K12891+K12837+K12850+K12818+K12865+K12824+K03283+K12893+K12821+K12820+K12837+K12862+K12892+K12839+K11088+K12662+K12865+K12816+K12662+K12896+K12885+K12893+K12856+K03283+K11093+K12867+K03283+K12837+K12813+K12818+K12874+K12874+K12823+K03283+K12837+K03283+K12842+K12891+K12891+K12862+K12818+K12818+K06063+K03283+K11095+K12900+K12896+K12813+K12825+K12844+K12813+K12818+K12875+K12741+K12879+K12875+K12831+K12823+K12856+K12843+K12837+K12900+K12823+K12854+K12824+K11099+K12837+K12872+K12900+K12818+K12813+K12837+K12825+K12811+K12741+K12823+K12869+K12867+K12824+K12812+K12812+K12856+K12868+K11095+K12824+K12815+K12880+K12890+K12741+K12897+K12846+K12869+K12825+K12849+K12890+K03283+K12865+K12818+K12854+K12875+K12843+K12818+K12812+K12900+K12627+K12850+K12882+K12890+K12823+K12875+K12815+K12896+K12818+K12836+K12900+K12890+K12837+K12823+K12891+K12880+K12822+K12818+K12741+K12885+K12875+K12815+K12890+K12850+K12882+K12849+K12896+K12900+K12829+K12875+K12900+K12858+K12823+K12741+K12811+K12876+K09564+K12896+K12842+K12891+K12862+K12813+K11092+K12812+K12821+K12837+K12823+K12818+K12829+K12822+K12823+K12834+K03283+K12837+K12878+K12869+K12850+K12893+K12865+K12870+K12823+K12741+K12741+K12891+K12900+K12824+K12860+K12741+K12822+K12890+K12741+K12893+K12869+K12868+K12854+K12883+K03283+K12849+K12880+K12626+K12829+K12881+K12811+K12837+K12839+K12890+K12837+K12874+K12825+K12848+K12873+K12896+K12818+K11093+K12897+K12825+K12837+K11093+K12891+K12822+K12896+K12852+K12741+K12621+K12624+K12882+K12881+K12829+K12823+K03283+K12811+K12854+K12733+K12878+K11096+K12822+K12825+K03283+K12820+K12813+K12830+K12878+K12822+K12826+K128

	t_122397;F01_transcript_122423;F01_transcript_122534;F01_transcript_122646;F01_transcript_122717;F01_transcript_122773;F01_transcript_122813;F01_transcript_123015;F01_transcript_123107;F01_transcript_123234;F01_transcript_123426;F01_transcript_123474;F01_transcript_123514;F01_transcript_123878;F01_transcript_123927;F01_transcript_123968;F01_transcript_124510;F01_transcript_124520;F01_transcript_124641;F01_transcript_124665;F01_transcript_124748;F01_transcript_124876;F01_transcript_125130;F01_transcript_125348;F01_transcript_125672;F01_transcript_125814;F01_transcript_126045;F01_transcript_126354;F01_transcript_126556;F01_transcript_126986;F01_transcript_127035;F01_transcript_127041;F01_transcript_127186;F01_transcript_127541;F01_transcript_127583;F01_transcript_128228;F01_transcript_128249;F01_transcript_128369;F01_transcript_128490;F01_transcript_128623;F01_transcript_128731;F01_transcript_128747;F01_transcript_128752;F01_transcript_128757;F01_transcript_128850;F01_transcript_128902;F01_transcript_128974;F01_transcript_129038;F01_transcript_129225;F01_transcript_129250;F01_transcript_129279;F01_transcript_129423;F01_transcript_12943;F01_transcript_129453;F01_transcript_129544;F01_transcript_129849;F01_transcript_130191;F01_transcript_130239;F01_transcript_130419;F01_transcript_130702;F01_transcript_131029;F01_transcript_131369;F01_transcript_131372;F01_transcript_131610;F01_transcript_131623;F01_transcript_131733;F01_transcript_13188;F01_transcript_131884;F01_transcript_131910;F01_transcript_132106;F01_transcript_132263;F01_transcript_132381;F01_transcript_13249;F01_transcript_1326;F01_transcript_132672;F01_transcript_132984;F01_transcript_133;F01_transcript_133248;F01_transcript_133251;F01_transcript_133263;F01_transcript_133415;F01_transcript_133544;F01_transcript_133844;F01_transcript_133903;F01_transcript_133908;F01_transcript_134196;F01_transcript_134238;F01_transcript_134381;F01_transcript_134461;F01_transcript_134759;F01_transcript_134818;F01_transcript_134826;F01_transcript_135181;F01_transcript_135206;F01_transcript_135321;F01_transcript_1355;F01_transcript_135500;F01_transcript_135662;F01_transcript_135789;F01_transcript_135961;F01_transcript_135983;F01_transcript_136017;F01_transcript_136542;F01_transcript_	25+K12849+K12821+K11093+K12836+K03283+K11094+K12624+K10599+K12842+K12823+K12869+K12890+K12893+K12858+K12890+K12818+K12837+K03283+K12875+K12890+K12893+K12741+K12812+K12825+K12865+K12830+K12741+K03283+K12821+K12869+K12813+K12828+K12829+K12816+K12836+K12900+K12837+K12881+K12900+K12741+K10599+K03283+K12893+K12874+K12813+K12862+K12830+K11098+K12815+K12896+K11095+K12839+K12847+K12836+K12893+K12624+K12836+K12816+K12837+K12860+K12820+K12836+K12900+K12837+K12875+K12869+K03283+K12813+K03283+K03283+K12662+K12885+K12893+K03283+K12858+K12879+K12900+K12856+K12741+K03283+K03283+K03283+K12830+K12860+K12830+K12623+K10599+K12879+K12829+K12823+K03283+K12849+K12891+K12662+K12837+K11095+K12813+K12881+K03283+K12829+K12900+K12893+K12849+K12896+K12822+K12837+K12824+K12824+K12837+K03283+K12882+K12836+K12662+K12817+K12842+K12837+K12837+K12837+K12842+K12890+K12825+K12824+K12827+K10599+K12890+K12819+K12819+K12891+K12896+K12623+K12896+K12837+K10599+K12823+K12891+K12864+K10599+K12877+K12823+K11093+K12741+K12900+K10599+K12885+K03283+K12741+K12812+K12812+K12823+K12844+K12741+K12890+K12837+K12855+K12839+K12837+K12741+K12890+K12862+K11984+K12812+K12862+K12844+K12821+K12872+K12812+K12815+K12885+K12855+K12844+K12893+K12824+K12900+K12850+K12872+K11093+K13025+K12890+K12844+K12824+K11093+K12741+K12872+K12741+K13025+K12741+K12850+K12741+K12896+K12741+K12842+K12821+K12741+K12823+K12855+K12837+K12741+K12822+K12835+K12891+K12817+K12852+K12852+K12741+K12741+K12741+K12817+K12844+K12859+K11093+K12836+K12896+K12741+K12741+K12741+K12741+K12896+K12821+K12896+K12821+K12849+K11086+K12836+K12741+K12836+K11086+K12868+K12826+K12741+K12900+K12868+K12831+K12900+K12881+K12896+K12891+K12741+K12900+K12900+K11086+K12900+K12881+K12900+K11095+K12844+K11091+K11091+K12837+K12825+K12893+K12822+K12885+K12883+K12848+K11091+K12843+K12897+K12825+K12846+K12623+K

	136673;F01_transcript_136756;F01_transcript_137111;F01_transcript_13714;F01_transcript_137238;F01_transcript_137364;F01_transcript_137373;F01_transcript_137490;F01_transcript_137549;F01_transcript_137565;F01_transcript_137688;F01_transcript_137820;F01_transcript_137943;F01_transcript_138059;F01_transcript_138177;F01_transcript_138183;F01_transcript_138338;F01_transcript_138589;F01_transcript_138658;F01_transcript_139122;F01_transcript_139483;F01_transcript_139577;F01_transcript_139635;F01_transcript_139716;F01_transcript_139766;F01_transcript_139845;F01_transcript_139875;F01_transcript_139928;F01_transcript_140049;F01_transcript_140051;F01_transcript_140129;F01_transcript_140710;F01_transcript_140839;F01_transcript_14110;F01_transcript_141116;F01_transcript_141387;F01_transcript_141852;F01_transcript_141858;F01_transcript_142123;F01_transcript_142304;F01_transcript_142332;F01_transcript_142400;F01_transcript_14248;F01_transcript_142484;F01_transcript_142788;F01_transcript_143416;F01_transcript_143562;F01_transcript_143701;F01_transcript_143725;F01_transcript_144079;F01_transcript_144408;F01_transcript_144624;F01_transcript_144718;F01_transcript_144924;F01_transcript_144991;F01_transcript_145090;F01_transcript_145335;F01_transcript_145481;F01_transcript_14584;F01_transcript_146271;F01_transcript_146272;F01_transcript_146354;F01_transcript_146466;F01_transcript_146794;F01_transcript_14706;F01_transcript_147276;F01_transcript_147363;F01_transcript_147654;F01_transcript_147895;F01_transcript_147951;F01_transcript_148008;F01_transcript_148023;F01_transcript_148082;F01_transcript_148151;F01_transcript_148410;F01_transcript_14848;F01_transcript_148501;F01_transcript_14860;F01_transcript_148879;F01_transcript_149119;F01_transcript_149361;F01_transcript_149527;F01_transcript_149533;F01_transcript_149822;F01_transcript_149965;F01_transcript_150097;F01_transcript_150111;F01_transcript_150129;F01_transcript_150165;F01_transcript_150338;F01_transcript_150395;F01_transcript_15040;F01_transcript_150464;F01_transcript_150480;F01_transcript_150679;F01_transcript_150778;F01_transcript_150797;F01_transcript_150874;F01_transcript_150982;F01_transcript_15100;F01_transcript_151022;	11094+K12831+K12876+K11093+K12623+K11086+K09567+K12837+K12821+K12873+K12825+K12825+K12871+K12859+K11088+K12877+K12626+K11096+K12733+K12845+K12845+K12859+K11093+K12625+K12859+K12867+K11098+K11096+K12624+K12622+K12627+K11097+K12625+K12625+K11098+K12624+K11097+K11097+K12621+K12832+K12823+K11099+K11099+K11099+K12622+K12878+K12900+K12864+K12896+K12825+K12825+K12837+K12900+K12818+K12867+K12823+K12843+K12875+K03283+K12900+K12811+K12835+K12741+K06063+K12818+K12825+K12854+K03283+K12870+K12825+K12822+K12854+K12881+K12883+K12878+K12890+K10599+K12847+K12897+K12823+K10599+K12881+K12867+K12864+K12878+K12823+K12875+K12875+K03283+K12820+K12820+K12890+K12623+K12839+K12825+K12854+K12836+K12848+K12850+K12741+K12893+K12811+K12860+K12811+K12829+K12811+K12854+K12891+K11093+K12741+K12836+K12811+K12815+K12881+K12837+K12823+K12823+K12848+K12812+K12822+K12818+K12823+K12825+K12890+K12896+K12890+K12741+K12825+K12741+K12860+K12867+K03283+K12825+K12823+K12881+K12741+K12836+K11086+K12836+K11984+K12823+K12818+K12825+K12900+K12881+K12825+K12837+K12893+K12856+K12896+K12875+K12813+K12836+K12830+K12881+K12824+K12893+K12741+K12872+K12900+K12875+K12822+K12821+K12830+K12885+K12867+K12825+K12823+K12881+K12842+K12813+K12818+K12890+K12813+K12830+K12879+K12831+K12879+K12830+K12830+K12822+K13025+K12896+K12820+K12741+K12825+K12820+K12820+K12875+K12837+K12835+K12890+K12824+K12837+K12835+K12821+K12837+K12818+K12823+K12900+K12828+K12870+K12821+K12825+K12839+K03283+K12900+K12824+K12741+K12830+K12842+K12823+K12875+K12882+K11984+K12868+K12818+K12813+K12856+K12860+K12896+K12862+K11984+K12820+K11093+K12811+K12823+K12891+K12878+K12837+K12896+K12825+K12872+K12890+K12874+K12837+K12849+K11098+K12830+K03283+K12825+K03283+K12818+K12821+K12813+K12830+K12896+K12858+K12741+K12824+K12828+K12823+K03283+K12891

	F01_transcript_151271;F01_transcript_151287;F01_transcript_151295;F01_transcript_151889;F01_transcript_152050;F01_transcript_152510;F01_transcript_152523;F01_transcript_152735;F01_transcript_152777;F01_transcript_1530;F01_transcript_153066;F01_transcript_1531;F01_transcript_153151;F01_transcript_153394;F01_transcript_153558;F01_transcript_153577;F01_transcript_154070;F01_transcript_154164;F01_transcript_15424;F01_transcript_154267;F01_transcript_154359;F01_transcript_154605;F01_transcript_154637;F01_transcript_154665;F01_transcript_155107;F01_transcript_155127;F01_transcript_155189;F01_transcript_155523;F01_transcript_155796;F01_transcript_155814;F01_transcript_155882;F01_transcript_156165;F01_transcript_156170;F01_transcript_156196;F01_transcript_156305;F01_transcript_156356;F01_transcript_156505;F01_transcript_156603;F01_transcript_156689;F01_transcript_156704;F01_transcript_156924;F01_transcript_157118;F01_transcript_157155;F01_transcript_157188;F01_transcript_157408;F01_transcript_157443;F01_transcript_157705;F01_transcript_157779;F01_transcript_158071;F01_transcript_158166;F01_transcript_158256;F01_transcript_158294;F01_transcript_158454;F01_transcript_158473;F01_transcript_158686;F01_transcript_158717;F01_transcript_158744;F01_transcript_158833;F01_transcript_158840;F01_transcript_159214;F01_transcript_15926;F01_transcript_159269;F01_transcript_159283;F01_transcript_159415;F01_transcript_159760;F01_transcript_159937;F01_transcript_160336;F01_transcript_160392;F01_transcript_160544;F01_transcript_160554;F01_transcript_160591;F01_transcript_161230;F01_transcript_161356;F01_transcript_161466;F01_transcript_161611;F01_transcript_161660;F01_transcript_161818;F01_transcript_161973;F01_transcript_162036;F01_transcript_162171;F01_transcript_16275;F01_transcript_162764;F01_transcript_162852;F01_transcript_16291;F01_transcript_162935;F01_transcript_163229;F01_transcript_163289;F01_transcript_16338;F01_transcript_163391;F01_transcript_163479;F01_transcript_163632;F01_transcript_163669;F01_transcript_164123;F01_transcript_164139;F01_transcript_164529;F01_transcript_164554;F01_transcript_164640;F01_transcript_164652;F01_transcript_164743;F01_transcript_16490;F01_transcript_164906;F01_transcript_164911;F01_transcript_165060;F0	+K12824+K11984+K12843+K12837+K12837+K12825+K13025+K12839+K03283+K12823+K12858+K12842+K12830+K12879+K12881+K12890+K12823+K12854+K12837+K12624+K12847+K12624+K12893+K12836+K12890+K12837+K12896+K06063+K12891+K12890+K12900+K12741+K12890+K12858+K12867+K12835+K12824+K12818+K12879+K12741+K12858+K12741+K12741+K12818+K12842+K12823+K12811+K12893+K12875+K12842+K12837+K10599+K12823+K12858+K12854+K12875+K12821+K11984+K12837+K12623+K12890+K12873+K12890+K12892+K03283+K12825+K12890+K12864+K12896+K12856+K12822+K12835+K11093+K12741+K10599+K12823+K11097+K12818+K12890+K12891+K12811+K12823+K12822+K11091+K12883+K12837+K12829+K12900+K06063+K11093+K12896+K12858+K12875+K12626+K12874+K12858+K12893+K12821+K12896+K12874+K11097+K12879+K12837+K06063+K11093+K12837+K12837+K12860+K12890+K12836+K12823+K12836+K12836+K12896+K12842+K12896+K12837+K12823+K12823+K12830+K12865+K12815+K03283+K12881+K12862+K12890+K12852+K12839+K12896+K12815+K12830+K12900+K12837+K12842+K12822+K12825+K12896+K12860+K12823

1_transcript_165158;F01_transcript_165374;F01_transcript_165466;F01_transcript_165557;F01_transcript_165682;F01_transcript_165915;F01_transcript_165916;F01_transcript_165938;F01_transcript_166023;F01_transcript_166165;F01_transcript_166239;F01_transcript_166382;F01_transcript_166534;F01_transcript_166548;F01_transcript_166676;F01_transcript_166842;F01_transcript_167003;F01_transcript_167597;F01_transcript_167690;F01_transcript_16861;F01_transcript_17018;F01_transcript_17025;F01_transcript_17119;F01_transcript_17143;F01_transcript_17153;F01_transcript_17206;F01_transcript_1742;F01_transcript_17729;F01_transcript_17764;F01_transcript_17846;F01_transcript_18266;F01_transcript_18341;F01_transcript_18531;F01_transcript_18596;F01_transcript_1881;F01_transcript_19108;F01_transcript_19240;F01_transcript_19397;F01_transcript_19626;F01_transcript_19729;F01_transcript_19803;F01_transcript_19879;F01_transcript_19910;F01_transcript_19924;F01_transcript_2;F01_transcript_20005;F01_transcript_20030;F01_transcript_20138;F01_transcript_20149;F01_transcript_202;F01_transcript_2023;F01_transcript_2024;F01_transcript_20306;F01_transcript_20324;F01_transcript_20504;F01_transcript_20593;F01_transcript_20667;F01_transcript_20725;F01_transcript_20739;F01_transcript_21115;F01_transcript_21156;F01_transcript_21510;F01_transcript_21569;F01_transcript_2171;F01_transcript_21777;F01_transcript_22008;F01_transcript_22374;F01_transcript_22505;F01_transcript_22556;F01_transcript_22733;F01_transcript_23105;F01_transcript_2354;F01_transcript_23627;F01_transcript_2370;F01_transcript_2379;F01_transcript_23917;F01_transcript_24061;F01_transcript_24229;F01_transcript_2434;F01_transcript_24357;F01_transcript_24646;F01_transcript_2469;F01_transcript_24779;F01_transcript_24866;F01_transcript_24958;F01_transcript_2539;F01_transcript_25502;F01_transcript_2582;F01_transcript_2601;F01_transcript_26033;F01_transcript_26055;F01_transcript_26198;F01_transcript_26374;F01_transcript_26603;F01_transcript_26859;F01_transcript_2718;F01_transcript_27259;F01_transcript_27409;F01_transcript_27465;F01_transcript_27566;F01_transcript_28277;F01_transcript_28492;F01_transcript_28693;F01_transcript_28895;F01_transcript_28952;F01_trans

cript_29085;F01_transcript_2920;F01_transcript_29219;F01_tra
nscript_29401;F01_transcript_29434;F01_transcript_2948;F01_t
ranscript_29500;F01_transcript_29533;F01_transcript_29672;F0
1_transcript_30005;F01_transcript_30022;F01_transcript_30328
;F01_transcript_30508;F01_transcript_30555;F01_transcript_30
671;F01_transcript_3072;F01_transcript_30789;F01_transcript_
30808;F01_transcript_30919;F01_transcript_31011;F01_transcri
pt_31158;F01_transcript_3129;F01_transcript_31413;F01_trans
cript_31453;F01_transcript_31629;F01_transcript_3219;F01_tra
nscript_32234;F01_transcript_32608;F01_transcript_3266;F01_t
ranscript_32783;F01_transcript_3308;F01_transcript_33212;F01
_transcript_33231;F01_transcript_3327;F01_transcript_33767;F
01_transcript_33806;F01_transcript_34025;F01_transcript_3410
;F01_transcript_34182;F01_transcript_34188;F01_transcript_34
238;F01_transcript_3447;F01_transcript_3454;F01_transcript_3
4540;F01_transcript_34639;F01_transcript_34682;F01_transcrip
t_34727;F01_transcript_34797;F01_transcript_34868;F01_trans
cript_34884;F01_transcript_34968;F01_transcript_35160;F01_tr
anscript_3522;F01_transcript_3541;F01_transcript_35497;F01_t
ranscript_35517;F01_transcript_3557;F01_transcript_363;F01_t
ranscript_36315;F01_transcript_364;F01_transcript_3643;F01_t
ranscript_36529;F01_transcript_36948;F01_transcript_3717;F01
_transcript_3718;F01_transcript_37245;F01_transcript_37287;F
01_transcript_37373;F01_transcript_37625;F01_transcript_3780
9;F01_transcript_3786;F01_transcript_3791;F01_transcript_379
96;F01_transcript_3826;F01_transcript_38420;F01_transcript_3
8766;F01_transcript_38771;F01_transcript_38828;F01_transcrip
t_38988;F01_transcript_3918;F01_transcript_39267;F01_transcr
ipt_3944;F01_transcript_39725;F01_transcript_39965;F01_trans
cript_40163;F01_transcript_40566;F01_transcript_40751;F01_tr
anscript_40791;F01_transcript_40845;F01_transcript_40975;F0
1_transcript_41016;F01_transcript_41145;F01_transcript_41331
;F01_transcript_41622;F01_transcript_42111;F01_transcript_42
236;F01_transcript_42354;F01_transcript_42357;F01_transcript
_42559;F01_transcript_42596;F01_transcript_42711;F01_transc
ript_42763;F01_transcript_43081;F01_transcript_43140;F01_tra
nscript_43216;F01_transcript_43262;F01_transcript_4353;F01_t
ranscript_43941;F01_transcript_43951;F01_transcript_4399;F01

	_transcript_4400;F01_transcript_4422;F01_transcript_4426;F01_transcript_44374;F01_transcript_44475;F01_transcript_44626;F01_transcript_44990;F01_transcript_4546;F01_transcript_45466;F01_transcript_4557;F01_transcript_45719;F01_transcript_45789;F01_transcript_45873;F01_transcript_45972;F01_transcript_46017;F01_transcript_4605;F01_transcript_46160;F01_transcript_46394;F01_transcript_46592;F01_transcript_4694;F01_transcript_4697;F01_transcript_46973;F01_transcript_4717;F01_transcript_4722;F01_transcript_47536;F01_transcript_47727;F01_transcript_47803;F01_transcript_48050;F01_transcript_48052;F01_transcript_48218;F01_transcript_48257;F01_transcript_48322;F01_transcript_48349;F01_transcript_48392;F01_transcript_4863;F01_transcript_48852;F01_transcript_48872;F01_transcript_4890;F01_transcript_48978;F01_transcript_49001;F01_transcript_49043;F01_transcript_49059;F01_transcript_49064;F01_transcript_49089;F01_transcript_49291;F01_transcript_49331;F01_transcript_49518;F01_transcript_49540;F01_transcript_49652;F01_transcript_49773;F01_transcript_49910;F01_transcript_49927;F01_transcript_5016;F01_transcript_50207;F01_transcript_50471;F01_transcript_50532;F01_transcript_50758;F01_transcript_51502;F01_transcript_51714;F01_transcript_51830;F01_transcript_51893;F01_transcript_52162;F01_transcript_52455;F01_transcript_52586;F01_transcript_52800;F01_transcript_52912;F01_transcript_5309;F01_transcript_53131;F01_transcript_5319;F01_transcript_53456;F01_transcript_53641;F01_transcript_53827;F01_transcript_5384;F01_transcript_53880;F01_transcript_54022;F01_transcript_54451;F01_transcript_54494;F01_transcript_54566;F01_transcript_54697;F01_transcript_54768;F01_transcript_55113;F01_transcript_55372;F01_transcript_55431;F01_transcript_55525;F01_transcript_55564;F01_transcript_55685;F01_transcript_55747;F01_transcript_55767;F01_transcript_55813;F01_transcript_55834;F01_transcript_55886;F01_transcript_55963;F01_transcript_56026;F01_transcript_56167;F01_transcript_5625;F01_transcript_56334;F01_transcript_56648;F01_transcript_57017;F01_transcript_57378;F01_transcript_57388;F01_transcript_57404;F01_transcript_57988;F01_transcript_5858;F01_transcript_58708;F01_transcript_58814;F01_transcript_59088;F01_tra

nscript_5916;F01_transcript_59671;F01_transcript_59756;F01_transcript_59932;F01_transcript_60331;F01_transcript_60482;F01_transcript_60576;F01_transcript_60904;F01_transcript_60905;F01_transcript_60935;F01_transcript_61114;F01_transcript_61193;F01_transcript_61207;F01_transcript_61214;F01_transcript_61259;F01_transcript_61388;F01_transcript_61405;F01_transcript_61465;F01_transcript_61509;F01_transcript_61512;F01_transcript_61516;F01_transcript_61685;F01_transcript_61729;F01_transcript_61777;F01_transcript_61899;F01_transcript_61933;F01_transcript_62234;F01_transcript_62275;F01_transcript_62301;F01_transcript_62540;F01_transcript_62543;F01_transcript_62547;F01_transcript_62631;F01_transcript_6308;F01_transcript_63290;F01_transcript_63403;F01_transcript_6359;F01_transcript_63634;F01_transcript_63903;F01_transcript_63961;F01_transcript_64378;F01_transcript_64458;F01_transcript_64515;F01_transcript_64979;F01_transcript_65057;F01_transcript_65197;F01_transcript_65329;F01_transcript_65383;F01_transcript_6544;F01_transcript_65506;F01_transcript_65618;F01_transcript_65714;F01_transcript_65720;F01_transcript_65865;F01_transcript_65886;F01_transcript_65914;F01_transcript_65947;F01_transcript_66021;F01_transcript_6608;F01_transcript_66308;F01_transcript_66370;F01_transcript_66430;F01_transcript_66524;F01_transcript_66936;F01_transcript_67198;F01_transcript_67510;F01_transcript_6752;F01_transcript_67627;F01_transcript_67801;F01_transcript_68287;F01_transcript_68489;F01_transcript_68613;F01_transcript_68844;F01_transcript_68914;F01_transcript_69080;F01_transcript_69302;F01_transcript_69737;F01_transcript_69754;F01_transcript_69888;F01_transcript_69917;F01_transcript_70040;F01_transcript_70051;F01_transcript_70357;F01_transcript_70584;F01_transcript_70594;F01_transcript_70656;F01_transcript_71039;F01_transcript_71511;F01_transcript_71733;F01_transcript_71868;F01_transcript_7192;F01_transcript_7196;F01_transcript_72126;F01_transcript_72743;F01_transcript_72775;F01_transcript_72963;F01_transcript_72973;F01_transcript_73008;F01_transcript_73044;F01_transcript_73462;F01_transcript_73919;F01_transcript_74009;F01_transcript_74191;F01_transcript_74193;F01_transcript_74348;F01_transcript_74507;F01_transcript_746;F01_transcript_74739;F01_transcript_7490

6;F01_transcript_7538;F01_transcript_75398;F01_transcript_76090;F01_transcript_76157;F01_transcript_76315;F01_transcript_76590;F01_transcript_770;F01_transcript_77000;F01_transcript_77005;F01_transcript_77017;F01_transcript_77043;F01_transcript_77175;F01_transcript_77605;F01_transcript_77689;F01_transcript_77703;F01_transcript_77962;F01_transcript_78359;F01_transcript_78413;F01_transcript_78496;F01_transcript_78699;F01_transcript_78717;F01_transcript_78740;F01_transcript_79032;F01_transcript_79965;F01_transcript_8021;F01_transcript_80620;F01_transcript_80785;F01_transcript_80870;F01_transcript_81399;F01_transcript_81499;F01_transcript_815;F01_transcript_81712;F01_transcript_82008;F01_transcript_82109;F01_transcript_82141;F01_transcript_82182;F01_transcript_82517;F01_transcript_82520;F01_transcript_82523;F01_transcript_82584;F01_transcript_82734;F01_transcript_82862;F01_transcript_830;F01_transcript_83067;F01_transcript_83104;F01_transcript_83141;F01_transcript_83147;F01_transcript_833;F01_transcript_835;F01_transcript_83518;F01_transcript_83577;F01_transcript_83600;F01_transcript_83625;F01_transcript_83661;F01_transcript_8393;F01_transcript_83955;F01_transcript_84206;F01_transcript_84256;F01_transcript_84314;F01_transcript_84335;F01_transcript_84550;F01_transcript_84610;F01_transcript_84613;F01_transcript_84619;F01_transcript_84729;F01_transcript_84811;F01_transcript_85083;F01_transcript_85225;F01_transcript_85375;F01_transcript_85388;F01_transcript_85494;F01_transcript_85554;F01_transcript_85580;F01_transcript_85591;F01_transcript_85729;F01_transcript_86001;F01_transcript_861;F01_transcript_86163;F01_transcript_86299;F01_transcript_86442;F01_transcript_86594;F01_transcript_86686;F01_transcript_86764;F01_transcript_86789;F01_transcript_86807;F01_transcript_87074;F01_transcript_8721;F01_transcript_87326;F01_transcript_87748;F01_transcript_87755;F01_transcript_87990;F01_transcript_88102;F01_transcript_88203;F01_transcript_88291;F01_transcript_88714;F01_transcript_88844;F01_transcript_8892;F01_transcript_89198;F01_transcript_8923;F01_transcript_89242;F01_transcript_89427;F01_transcript_89489;F01_transcript_89687;F01_transcript_89735;F01_transcript_89762;F01_transcript_89800;

			F01_transcript_89811;F01_transcript_89841;F01_transcript_89909;F01_transcript_89972;F01_transcript_9024;F01_transcript_90310;F01_transcript_90456;F01_transcript_90551;F01_transcript_90609;F01_transcript_90998;F01_transcript_91098;F01_transcript_91186;F01_transcript_91357;F01_transcript_91769;F01_transcript_91789;F01_transcript_91904;F01_transcript_9208;F01_transcript_92267;F01_transcript_92277;F01_transcript_92343;F01_transcript_9242;F01_transcript_92662;F01_transcript_92726;F01_transcript_92983;F01_transcript_93005;F01_transcript_93230;F01_transcript_93470;F01_transcript_93542;F01_transcript_93663;F01_transcript_93814;F01_transcript_93966;F01_transcript_94061;F01_transcript_94122;F01_transcript_94142;F01_transcript_94189;F01_transcript_94465;F01_transcript_94524;F01_transcript_94580;F01_transcript_94833;F01_transcript_94976;F01_transcript_95154;F01_transcript_95473;F01_transcript_95788;F01_transcript_96220;F01_transcript_9631;F01_transcript_96384;F01_transcript_96545;F01_transcript_96659;F01_transcript_96848;F01_transcript_96868;F01_transcript_96875;F01_transcript_96946;F01_transcript_96988;F01_transcript_97042;F01_transcript_97044;F01_transcript_9744;F01_transcript_97463;F01_transcript_9749;F01_transcript_97519;F01_transcript_97536;F01_transcript_97787;F01_transcript_97905;F01_transcript_97918;F01_transcript_97947;F01_transcript_981;F01_transcript_9831;F01_transcript_98448;F01_transcript_98591;F01_transcript_98629;F01_transcript_98681;F01_transcript_98868;F01_transcript_98871;F01_transcript_98875;F01_transcript_98935;F01_transcript_99265;F01_transcript_99493;F01_transcript_99596;F01_transcript_99624;F01_transcript_99702;F01_transcript_99742;F01_transcript_99840;F01_transcript_99909;F01_transcript_99934;F01_transcript_9995;	
Proteasome	ko03050	237	F01_transcript_10040;F01_transcript_100608;F01_transcript_101751;F01_transcript_102026;F01_transcript_103106;F01_transcript_103195;F01_transcript_103402;F01_transcript_103874;F01_transcript_105788;F01_transcript_106246;F01_transcript_107281;F01_transcript_107433;F01_transcript_107785;F01_transcript_109777;F01_transcript_110870;F01_transcript_111383;F01_transcript_113074;F01_transcript_115342;F01_transcript_115445;F01_transcript_11585;F01_transcript_117312;F01_transcript_1	K03063+K02737+K03033+K03033+K03065+K03064+K03035+K06700+K03065+K03061+K03030+K02732+K02730+K03035+K02736+K03039+K02736+K03063+K03066+K03063+K02737+K02727+K02726+K03063+K03036+K03031+K11599+K03065+K03032+K02732+K03062+K03030+K02734+K03029+K02739+K02736+K03033+K02732+K03033+K02730+K03062+K02731+K03062+K02726+K03036+K06699+K03032+K03032+K02726+K03029+K06700+K03032+K03061+K02738+K06700

	17330;F01_transcript_119428;F01_transcript_119719;F01_transcript_119943;F01_transcript_120359;F01_transcript_120685;F01_transcript_121768;F01_transcript_122036;F01_transcript_122609;F01_transcript_127829;F01_transcript_129569;F01_transcript_129747;F01_transcript_130374;F01_transcript_130514;F01_transcript_131308;F01_transcript_131535;F01_transcript_131621;F01_transcript_132062;F01_transcript_132084;F01_transcript_132872;F01_transcript_133666;F01_transcript_134763;F01_transcript_135953;F01_transcript_136675;F01_transcript_13717;F01_transcript_138025;F01_transcript_138454;F01_transcript_138604;F01_transcript_138785;F01_transcript_140001;F01_transcript_140060;F01_transcript_141702;F01_transcript_141760;F01_transcript_145575;F01_transcript_147828;F01_transcript_148026;F01_transcript_15012;F01_transcript_150128;F01_transcript_150310;F01_transcript_151114;F01_transcript_151489;F01_transcript_152188;F01_transcript_152787;F01_transcript_153128;F01_transcript_154539;F01_transcript_154725;F01_transcript_157499;F01_transcript_159146;F01_transcript_159595;F01_transcript_161074;F01_transcript_161622;F01_transcript_162534;F01_transcript_163482;F01_transcript_164761;F01_transcript_165081;F01_transcript_166236;F01_transcript_166581;F01_transcript_166814;F01_transcript_166816;F01_transcript_166886;F01_transcript_167335;F01_transcript_20707;F01_transcript_2089;F01_transcript_24055;F01_transcript_26138;F01_transcript_28264;F01_transcript_28504;F01_transcript_28562;F01_transcript_29351;F01_transcript_29353;F01_transcript_30306;F01_transcript_32763;F01_transcript_34145;F01_transcript_34622;F01_transcript_35100;F01_transcript_35130;F01_transcript_35702;F01_transcript_35787;F01_transcript_36152;F01_transcript_36393;F01_transcript_36530;F01_transcript_36554;F01_transcript_36581;F01_transcript_36611;F01_transcript_36662;F01_transcript_36684;F01_transcript_36760;F01_transcript_37070;F01_transcript_37092;F01_transcript_37307;F01_transcript_37579;F01_transcript_37765;F01_transcript_37870;F01_transcript_37977;F01_transcript_38207;F01_transcript_39956;F01_transcript_39962;F01_transcript_40226;F01_transcript_40595;F01_transcript_4078;F01_transcript_40939;F01_transcript_40998;F01_transcript_41466;	+K02731+K03036+K03065+K03063+K03032+K03031+K03036+K03038+K03036+K02737+K03032+K02734+K03029+K03032+K02737+K02731+K03063+K02732+K03065+K03064+K03028+K03033+K03031+K03037+K02728+K06699+K03066+K03036+K03032+K03036+K03033+K02726+K03035+K03033+K03039+K03033+K03035+K03035+K03061+K03029+K03063+K03065+K03061+K03062+K03039+K03066+K03062+K03039+K03064+K03036+K03063+K03062+K03064+K03039+K03066+K03063+K03066+K03063+K03063+K02736+K03029+K03037+K03038+K03064+K03038+K03031+K03038+K06700+K03063+K03038+K03030+K02725+K03031+K02739+K02739+K02737+K02730+K02739+K02727+K02729+K03031+K03031+K02735+K02734+K02728+K02736+K02736+K02737+K02737+K02726+K02732+K02735+K02726+K02735+K02731+K02735+K02734+K02732+K11599+K11599+K03039+K11599+K11599+K10881+K10881+K02732+K03036+K03063+K03028+K03031+K03031+K03032+K11599+K11599+K03061+K02726+K03032+K11599+K06699+K03063+K03065+K02726+K03029+K03063+K03028+K03033+K03039+K02736+K03029+K03065+K03065+K03032+K02739+K02727+K02731+K03038+K03036+K03032+K03063+K02731+K03038+K10881+K03066+K03063+K02737+K06700+K02739+K03033+K02727+K03033+K02737+K03032+K03028+K03064+K02730+K02731+K03032+K03064+K02731+K03029+K03031+K03028+K03063+K03033+K03031+K03029+K06700+K02725+K02738+K03061+K02731+K03065+K03038+K06700+K03065+K03036+K02729+K02727+K03038+K03033+K02729+K03066

F01_transcript_41548;F01_transcript_41725;F01_transcript_41933;F01_transcript_42006;F01_transcript_42440;F01_transcript_42674;F01_transcript_42882;F01_transcript_42952;F01_transcript_42980;F01_transcript_43654;F01_transcript_44197;F01_transcript_44244;F01_transcript_44248;F01_transcript_44328;F01_transcript_44409;F01_transcript_44468;F01_transcript_44469;F01_transcript_44512;F01_transcript_44535;F01_transcript_44779;F01_transcript_44860;F01_transcript_45107;F01_transcript_45175;F01_transcript_45470;F01_transcript_45472;F01_transcript_45682;F01_transcript_45708;F01_transcript_46597;F01_transcript_46863;F01_transcript_47440;F01_transcript_48087;F01_transcript_48215;F01_transcript_48764;F01_transcript_49438;F01_transcript_49940;F01_transcript_50101;F01_transcript_51777;F01_transcript_51928;F01_transcript_52485;F01_transcript_53582;F01_transcript_56505;F01_transcript_56715;F01_transcript_57084;F01_transcript_57371;F01_transcript_57939;F01_transcript_58921;F01_transcript_59966;F01_transcript_61190;F01_transcript_62046;F01_transcript_62652;F01_transcript_63734;F01_transcript_64400;F01_transcript_64438;F01_transcript_66052;F01_transcript_66311;F01_transcript_6640;F01_transcript_66753;F01_transcript_68754;F01_transcript_69218;F01_transcript_69386;F01_transcript_69989;F01_transcript_70092;F01_transcript_70650;F01_transcript_70867;F01_transcript_7167;F01_transcript_72137;F01_transcript_72820;F01_transcript_73040;F01_transcript_73525;F01_transcript_7390;F01_transcript_74373;F01_transcript_76806;F01_transcript_77062;F01_transcript_77167;F01_transcript_78024;F01_transcript_79026;F01_transcript_7983;F01_transcript_79921;F01_transcript_79953;F01_transcript_80762;F01_transcript_82082;F01_transcript_82427;F01_transcript_82773;F01_transcript_82846;F01_transcript_82903;F01_transcript_84717;F01_transcript_86061;F01_transcript_86157;F01_transcript_86703;F01_transcript_87602;F01_transcript_87615;F01_transcript_89086;F01_transcript_89318;F01_transcript_8956;F01_transcript_89721;F01_transcript_90185;F01_transcript_90779;F01_transcript_91121;F01_transcript_91850;F01_transcript_91917;F01_transcript_92299;F01_transcript_92342;F01_transcript_92939;F01_transcript_93098;F01_transcript_9342;F01_transcript_95312;F01_transcript_95908;F01_transcript_95970;F01_trans

			cript_96038;F01_transcript_96422;F01_transcript_97759;F01_transcript_98350;F01_transcript_99046;	
Protein export	ko03060	242	F01_transcript_100414;F01_transcript_100528;F01_transcript_100537;F01_transcript_100593;F01_transcript_100604;F01_transcript_101112;F01_transcript_101607;F01_transcript_101917;F01_transcript_101967;F01_transcript_102342;F01_transcript_102822;F01_transcript_104480;F01_transcript_104781;F01_transcript_105670;F01_transcript_105767;F01_transcript_106075;F01_transcript_107845;F01_transcript_109873;F01_transcript_110625;F01_transcript_110650;F01_transcript_11072;F01_transcript_110853;F01_transcript_111148;F01_transcript_111501;F01_transcript_111521;F01_transcript_112828;F01_transcript_114001;F01_transcript_114181;F01_transcript_1144;F01_transcript_115055;F01_transcript_115212;F01_transcript_115764;F01_transcript_117305;F01_transcript_11733;F01_transcript_117951;F01_transcript_118364;F01_transcript_119538;F01_transcript_119543;F01_transcript_119673;F01_transcript_120601;F01_transcript_120724;F01_transcript_121735;F01_transcript_122532;F01_transcript_122755;F01_transcript_123127;F01_transcript_123472;F01_transcript_123898;F01_transcript_123972;F01_transcript_124324;F01_transcript_12453;F01_transcript_125106;F01_transcript_125719;F01_transcript_125820;F01_transcript_127274;F01_transcript_127703;F01_transcript_127826;F01_transcript_128287;F01_transcript_129182;F01_transcript_129713;F01_transcript_130426;F01_transcript_132359;F01_transcript_133212;F01_transcript_133887;F01_transcript_134825;F01_transcript_135413;F01_transcript_135632;F01_transcript_135858;F01_transcript_135919;F01_transcript_136940;F01_transcript_137003;F01_transcript_138401;F01_transcript_140106;F01_transcript_140902;F01_transcript_142014;F01_transcript_143826;F01_transcript_143887;F01_transcript_144074;F01_transcript_144170;F01_transcript_144220;F01_transcript_144251;F01_transcript_144928;F01_transcript_145747;F01_transcript_146376;F01_transcript_146408;F01_transcript_147080;F01_transcript_148351;F01_transcript_148393;F01_transcript_150161;F01_transcript_150378;F01_transcript_150433;F01_transcript_150891;F01_transcript_151516;F01_transcript_151688;F01_transcript_152451;F01_transcript_15	K03106+K03106+K03107+K03217+K09481+K09490+K03110+K09490+K12947+K03106+K03217+K10956+K03110+K03106+K13431+K03107+K03070+K03118+K03104+K03106+K10956+K03100+K12946+K03070+K03217+K03109+K03109+K03100+K03070+K10956+K09540+K03070+K10956+K12948+K03106+K03100+K03100+K03100+K09481+K03106+K10956+K09490+K03104+K03070+K09540+K03070+K03110+K03106+K13280+K10956+K03070+K03070+K12272+K03106+K03106+K12946+K10956+K12275+K03217+K09647+K03100+K03217+K09540+K03217+K03110+K03106+K03100+K12948+K10956+K03070+K03110+K03107+K03070+K12948+K10956+K03070+K13431+K09540+K10956+K07342+K12947+K12948+K09481+K10956+K03217+K03106+K10956+K10956+K10956+K12275+K12272+K09648+K10956+K03100+K12272+K09540+K09490+K03070+K12272+K03217+K10956+K03217+K03070+K09481+K09481+K03109+K03070+K03070+K03108+K10956+K09490+K10956+K12948+K12948+K10956+K09540+K03106+K09490+K03106+K09490+K09490+K09540+K03106+K03217+K03107+K03108+K03108+K03217+K13431+K03108+K03217+K03217+K03217+K03106+K03217+K03106+K10956+K10956+K03106+K10956+K10956+K03106+K03100+K10956+K03217+K03217+K03217+K03217+K10956+K03217+K03100+K12275+K03106+K03110+K03110+K03110+K12272+K12272+K12272+K12272+K03100+K12947+K12947+K13280+K12947+K12948+K13280+K13280+K07342+K03104+K03109+K07342+K03109+K09540+K03107+K03100+K12275+K03217+K09648+K12947+K09481+K03070+K09648+K03106+K03106+K13431+K12272+K03070+K12946+K03104+K09490+K03070+K03100+K10956+K10956+K09481+K03070+K03070+K03217+K10956+K03116+K09490+K07342+K03106+K03106+K10956+K10956+K03106+K09490+K03070+K09490+K03070+K09540+K03104+K03100+K03106+K03070+K03217+K03106+K03070+K03070+K03106+K03070+K03108+K03106+K09540+K09540+K09481+K10956+K03070+K07342+K09540+K03070+K

	2462;F01_transcript_15402;F01_transcript_154925;F01_transcript_155957;F01_transcript_156458;F01_transcript_157096;F01_transcript_157649;F01_transcript_158291;F01_transcript_158298;F01_transcript_158664;F01_transcript_159474;F01_transcript_160702;F01_transcript_160754;F01_transcript_162837;F01_transcript_163661;F01_transcript_163780;F01_transcript_164863;F01_transcript_165283;F01_transcript_165507;F01_transcript_167110;F01_transcript_167170;F01_transcript_16821;F01_transcript_16975;F01_transcript_17639;F01_transcript_17950;F01_transcript_18206;F01_transcript_18368;F01_transcript_19017;F01_transcript_20980;F01_transcript_21492;F01_transcript_21542;F01_transcript_22464;F01_transcript_23312;F01_transcript_23399;F01_transcript_23836;F01_transcript_24323;F01_transcript_24402;F01_transcript_25212;F01_transcript_25591;F01_transcript_27324;F01_transcript_27919;F01_transcript_28006;F01_transcript_29113;F01_transcript_29659;F01_transcript_29752;F01_transcript_29978;F01_transcript_30147;F01_transcript_31291;F01_transcript_32278;F01_transcript_32349;F01_transcript_32627;F01_transcript_32975;F01_transcript_33305;F01_transcript_33561;F01_transcript_33869;F01_transcript_33959;F01_transcript_34116;F01_transcript_35971;F01_transcript_36991;F01_transcript_38211;F01_transcript_38504;F01_transcript_39010;F01_transcript_39609;F01_transcript_40015;F01_transcript_41109;F01_transcript_41437;F01_transcript_41890;F01_transcript_45783;F01_transcript_46022;F01_transcript_46430;F01_transcript_46435;F01_transcript_47994;F01_transcript_48055;F01_transcript_48144;F01_transcript_48783;F01_transcript_48926;F01_transcript_49515;F01_transcript_50238;F01_transcript_50838;F01_transcript_52167;F01_transcript_52273;F01_transcript_52947;F01_transcript_53818;F01_transcript_54314;F01_transcript_54604;F01_transcript_54813;F01_transcript_55536;F01_transcript_55744;F01_transcript_55769;F01_transcript_56096;F01_transcript_56707;F01_transcript_57007;F01_transcript_57412;F01_transcript_57711;F01_transcript_59891;F01_transcript_60763;F01_transcript_61346;F01_transcript_62875;F01_transcript_63763;F01_transcript_64393;F01_transcript_66568;F01_transcript_66846;F01_transcript_67456;F01_transcript_68978;F01_transcript_69728;F01_transcript_69895;F01_transcript_70454;F01_transcript_719	03107+K03070+K03109+K12947+K09647+K03217+K13431+K03070+K03217

			93;F01_transcript_72052;F01_transcript_72079;F01_transcript_72281;F01_transcript_72495;F01_transcript_73247;F01_transcript_73946;F01_transcript_74006;F01_transcript_74398;F01_transcript_74425;F01_transcript_74991;F01_transcript_75304;F01_transcript_77434;F01_transcript_77647;F01_transcript_77798;F01_transcript_7963;F01_transcript_79764;F01_transcript_80316;F01_transcript_80357;F01_transcript_80406;F01_transcript_80846;F01_transcript_82892;F01_transcript_83587;F01_transcript_83951;F01_transcript_84312;F01_transcript_86227;F01_transcript_86265;F01_transcript_86674;F01_transcript_87190;F01_transcript_87534;F01_transcript_91855;F01_transcript_92031;F01_transcript_92090;F01_transcript_93963;F01_transcript_94250;F01_transcript_94660;F01_transcript_94681;F01_transcript_95870;F01_transcript_96710;F01_transcript_97074;F01_transcript_99801;	
Base excision repair	ko03410	182	F01_transcript_100194;F01_transcript_100685;F01_transcript_101146;F01_transcript_101626;F01_transcript_102876;F01_transcript_105169;F01_transcript_105318;F01_transcript_106039;F01_transcript_106511;F01_transcript_1101;F01_transcript_110683;F01_transcript_111881;F01_transcript_112056;F01_transcript_112417;F01_transcript_113185;F01_transcript_114825;F01_transcript_114839;F01_transcript_114968;F01_transcript_115417;F01_transcript_115550;F01_transcript_116353;F01_transcript_118984;F01_transcript_119005;F01_transcript_121040;F01_transcript_121872;F01_transcript_123334;F01_transcript_123860;F01_transcript_123892;F01_transcript_124264;F01_transcript_126956;F01_transcript_129404;F01_transcript_131373;F01_transcript_131874;F01_transcript_132403;F01_transcript_135072;F01_transcript_136652;F01_transcript_136872;F01_transcript_137249;F01_transcript_137937;F01_transcript_139121;F01_transcript_139722;F01_transcript_141109;F01_transcript_141574;F01_transcript_142708;F01_transcript_142813;F01_transcript_143128;F01_transcript_143718;F01_transcript_14521;F01_transcript_146556;F01_transcript_146806;F01_transcript_146957;F01_transcript_148549;F01_transcript_14906;F01_transcript_149855;F01_transcript_150196;F01_transcript_151783;F01_transcript_152398;F01_transcript_154800;F01_transcript_154874;F01_transcript	K10803+K03652+K10798+K10802+K03512+K10798+K01246+K01246+K01142+K02335+K01246+K03660+K02325+K10803+K10747+K03512+K01246+K02326+K04799+K10802+K01142+K10798+K10772+K10747+K10798+K01246+K10802+K01246+K10747+K01247+K02327+K10563+K01142+K03512+K10563+K02335+K10802+K10803+K03648+K02328+K10802+K10772+K10798+K02335+K10803+K10801+K10802+K10772+K02328+K10747+K02335+K01247+K02335+K04799+K03504+K04799+K02328+K01142+K10798+K04799+K10747+K04802+K03652+K02328+K10803+K02328+K04799+K10772+K04799+K10772+K02326+K10798+K10801+K10802+K10801+K04799+K01246+K01246+K01246+K01142+K03504+K10803+K10801+K01246+K02328+K10798+K02328+K01246+K10563+K10803+K02328+K03660+K01247+K10798+K04799+K01246+K03652+K01247+K03648+K01246+K01246+K10802+K10802+K10802+K10802+K10802+K10802+K03505+K04799+K10747+K10747+K03505+K10802+K01247+K10802+K10802+K10798+K10798+K01246+K10772+K02328+K01246+K01247+K10798+K01246+K02335+K03512+K03512+K10747+K01246+K10798+K02325+K01247+K10798+K10802+K01246+K10772+K03512+K02335+K04799+K10747+K10747+K02335+K10747+K02335+K10802+K10801+K01246+K10798+K10747+K01247

	_155052;F01_transcript_155603;F01_transcript_155687;F01_transcript_157439;F01_transcript_158296;F01_transcript_159185;F01_transcript_159626;F01_transcript_160006;F01_transcript_16130;F01_transcript_161577;F01_transcript_163021;F01_transcript_163029;F01_transcript_163186;F01_transcript_163975;F01_transcript_165318;F01_transcript_165685;F01_transcript_167070;F01_transcript_18527;F01_transcript_20643;F01_transcript_20895;F01_transcript_24594;F01_transcript_28257;F01_transcript_28696;F01_transcript_30028;F01_transcript_30866;F01_transcript_30875;F01_transcript_3125;F01_transcript_32534;F01_transcript_32825;F01_transcript_33144;F01_transcript_33166;F01_transcript_34563;F01_transcript_36989;F01_transcript_37026;F01_transcript_3719;F01_transcript_38014;F01_transcript_39421;F01_transcript_39560;F01_transcript_40899;F01_transcript_42075;F01_transcript_42311;F01_transcript_42461;F01_transcript_45892;F01_transcript_46320;F01_transcript_46446;F01_transcript_46609;F01_transcript_46652;F01_transcript_48135;F01_transcript_48686;F01_transcript_52470;F01_transcript_53178;F01_transcript_533;F01_transcript_54343;F01_transcript_55427;F01_transcript_56111;F01_transcript_56905;F01_transcript_57945;F01_transcript_58025;F01_transcript_59915;F01_transcript_60350;F01_transcript_61344;F01_transcript_61368;F01_transcript_63034;F01_transcript_63169;F01_transcript_64662;F01_transcript_65622;F01_transcript_65789;F01_transcript_66223;F01_transcript_66348;F01_transcript_66392;F01_transcript_67380;F01_transcript_67657;F01_transcript_67697;F01_transcript_68046;F01_transcript_68219;F01_transcript_69598;F01_transcript_69782;F01_transcript_70378;F01_transcript_70848;F01_transcript_70894;F01_transcript_70999;F01_transcript_71262;F01_transcript_7220;F01_transcript_728;F01_transcript_74164;F01_transcript_75496;F01_transcript_76888;F01_transcript_76934;F01_transcript_77148;F01_transcript_77536;F01_transcript_77701;F01_transcript_78124;F01_transcript_78396;F01_transcript_78656;F01_transcript_78804;F01_transcript_79595;F01_transcript_79636;F01_transcript_81168;F01_transcript_81872;F01_transcript_83309;F01_transcript_84198;F01_transcript_85351;F01_transcript_85430;F01_transcript_85797;F01_transcript_85990;F01_transcript_862;F01_transcript_86415;F01_transcript_88462;F01_tra	+K01247+K10772+K02326+K10747+K02324+K10747+K10803+K10747+K10801+K04799+K04799+K10747+K10798+K02335+K04799+K02335+K10798+K02335+K10772+K10747+K10747+K10802+K10803+K10802+K02335+K10798+K02325+K10563+K02325+K02327+K02335

			nscript_88463;F01_transcript_89051;F01_transcript_90205;F01_transcript_90918;F01_transcript_91162;F01_transcript_91191;F01_transcript_93401;F01_transcript_94306;F01_transcript_944;F01_transcript_958;F01_transcript_95940;F01_transcript_97520;F01_transcript_97909;F01_transcript_98012;F01_transcript_991;	
Nucleotide excision repair	ko03420	225	F01_transcript_101938;F01_transcript_102104;F01_transcript_10324;F01_transcript_104424;F01_transcript_105255;F01_transcript_106235;F01_transcript_106285;F01_transcript_106305;F01_transcript_107316;F01_transcript_107719;F01_transcript_107994;F01_transcript_107999;F01_transcript_108013;F01_transcript_109792;F01_transcript_1101;F01_transcript_110692;F01_transcript_112056;F01_transcript_113185;F01_transcript_114360;F01_transcript_114864;F01_transcript_11492;F01_transcript_114968;F01_transcript_115051;F01_transcript_115738;F01_transcript_116355;F01_transcript_119614;F01_transcript_119625;F01_transcript_120598;F01_transcript_121040;F01_transcript_121601;F01_transcript_121703;F01_transcript_122963;F01_transcript_124055;F01_transcript_124264;F01_transcript_124661;F01_transcript_125006;F01_transcript_125272;F01_transcript_127149;F01_transcript_129404;F01_transcript_129431;F01_transcript_130442;F01_transcript_131237;F01_transcript_131607;F01_transcript_131999;F01_transcript_132323;F01_transcript_133243;F01_transcript_133364;F01_transcript_133441;F01_transcript_133706;F01_transcript_133770;F01_transcript_133785;F01_transcript_134905;F01_transcript_136652;F01_transcript_137138;F01_transcript_137291;F01_transcript_138141;F01_transcript_138344;F01_transcript_139121;F01_transcript_139333;F01_transcript_14243;F01_transcript_142708;F01_transcript_143098;F01_transcript_143196;F01_transcript_143385;F01_transcript_144179;F01_transcript_144788;F01_transcript_146387;F01_transcript_146556;F01_transcript_146806;F01_transcript_146957;F01_transcript_147460;F01_transcript_14906;F01_transcript_149891;F01_transcript_150196;F01_transcript_151343;F01_transcript_151600;F01_transcript_152069;F01_transcript_152398;F01_transcript_152998;F01_transcript_153758;F01_transcript_154192;F01_transcript_155603;F01_transcript_155687;F01_transcript_155880;	K03144+K10755+K10844+K03143+K10609+K10848+K10754+K10755+K03141+K10846+K10839+K07466+K02202+K10838+K02335+K10839+K02325+K10747+K10609+K07466+K07466+K02326+K10843+K10754+K02202+K10609+K10756+K10140+K10747+K10755+K07466+K10570+K10839+K10747+K10755+K10841+K10755+K10844+K02327+K10839+K10755+K10754+K10570+K07466+K10756+K07466+K10846+K10841+K10755+K10755+K10838+K03868+K02335+K03868+K10754+K10754+K10838+K02328+K03141+K03141+K02335+K10839+K07466+K10844+K03144+K10610+K10754+K02328+K10747+K02335+K10846+K02335+K10841+K03504+K10755+K03141+K10839+K02328+K10609+K03144+K10838+K10747+K04802+K10838+K10755+K02328+K10839+K02328+K03143+K10754+K02326+K10844+K10839+K10609+K03141+K07466+K07466+K10846+K10754+K10756+K10610+K10140+K03142+K03143+K03144+K10140+K03144+K10838+K10570+K03504+K03142+K02328+K10838+K02328+K02328+K10849+K10839+K10839+K10839+K10756+K10839+K10839+K10756+K10756+K10755+K10756+K10755+K10755+K10842+K07466+K07466+K07466+K03868+K03868+K03505+K10839+K10755+K10747+K10747+K03144+K03505+K10845+K03143+K10756+K07466+K03141+K02202+K10838+K10838+K03141+K02328+K10839+K10849+K10609+K10841+K07466+K02335+K10754+K10747+K02325+K10846+K10849+K02335+K10747+K10844+K10747+K10839+K02335+K07466+K03143+K10747+K10755+K02335+K03868+K10843+K10845+K10756+K10747+K10609+K10846+K10609+K02326+K10848+K10747+K02324+K10841+K10739+K10747+K02202+K10754+K10841+K07466+K10747+K07466+K10839+K10841+K10839+K10747+K02335+K10609+K10609+K10140+K10754+K02335+K07466+K02335+K10609+K03141+K10747+K10747+K03143+K07466+K107

	F01_transcript_158044;F01_transcript_158296;F01_transcript_159096;F01_transcript_159626;F01_transcript_160041;F01_transcript_162212;F01_transcript_163029;F01_transcript_163339;F01_transcript_164437;F01_transcript_166895;F01_transcript_16725;F01_transcript_17688;F01_transcript_18802;F01_transcript_190;F01_transcript_2042;F01_transcript_20485;F01_transcript_2135;F01_transcript_23244;F01_transcript_23272;F01_transcript_23330;F01_transcript_24715;F01_transcript_25126;F01_transcript_25319;F01_transcript_2568;F01_transcript_28186;F01_transcript_28257;F01_transcript_29272;F01_transcript_30875;F01_transcript_3194;F01_transcript_32534;F01_transcript_34563;F01_transcript_35797;F01_transcript_36386;F01_transcript_37190;F01_transcript_37661;F01_transcript_38198;F01_transcript_38459;F01_transcript_39214;F01_transcript_39293;F01_transcript_39420;F01_transcript_40950;F01_transcript_4207;F01_transcript_42381;F01_transcript_42392;F01_transcript_45110;F01_transcript_45535;F01_transcript_45921;F01_transcript_46906;F01_transcript_47513;F01_transcript_47908;F01_transcript_48686;F01_transcript_51796;F01_transcript_52773;F01_transcript_53178;F01_transcript_533;F01_transcript_53642;F01_transcript_54343;F01_transcript_54553;F01_transcript_54782;F01_transcript_55038;F01_transcript_55499;F01_transcript_5604;F01_transcript_56550;F01_transcript_56913;F01_transcript_57485;F01_transcript_58058;F01_transcript_61368;F01_transcript_62076;F01_transcript_62151;F01_transcript_62689;F01_transcript_63547;F01_transcript_6499;F01_transcript_65789;F01_transcript_66251;F01_transcript_66392;F01_transcript_67697;F01_transcript_6777;F01_transcript_68654;F01_transcript_70894;F01_transcript_71262;F01_transcript_71889;F01_transcript_7220;F01_transcript_7277;F01_transcript_728;F01_transcript_73069;F01_transcript_73445;F01_transcript_74164;F01_transcript_74712;F01_transcript_75496;F01_transcript_75965;F01_transcript_7631;F01_transcript_7661;F01_transcript_77639;F01_transcript_77701;F01_transcript_77979;F01_transcript_78463;F01_transcript_7878;F01_transcript_78804;F01_transcript_79086;F01_transcript_79595;F01_transcript_79636;F01_transcript_801;F01_transcript_80286;F01_transcript_81168;F01_transcript_81230;F01_transcript_82319;F01_transcript_82487;F01_transcript_82696;F01_transcript	54+K10754+K03143+K03868+K02335+K10841+K10843+K07466+K02325+K02325+K02327+K10843+K02335

			_83309;F01_transcript_83773;F01_transcript_8451;F01_transcript_851;F01_transcript_85282;F01_transcript_85797;F01_transcript_862;F01_transcript_8628;F01_transcript_8754;F01_transcript_88096;F01_transcript_88147;F01_transcript_88462;F01_transcript_89037;F01_transcript_89051;F01_transcript_89537;F01_transcript_90216;F01_transcript_90918;F01_transcript_91162;F01_transcript_91203;F01_transcript_91407;F01_transcript_91705;F01_transcript_92049;F01_transcript_92760;F01_transcript_93472;F01_transcript_944;F01_transcript_94886;F01_transcript_9565;F01_transcript_95868;F01_transcript_95940;F01_transcript_97909;F01_transcript_98012;F01_transcript_98841;F01_transcript_991;	
Mismatch repair	ko03430	140	F01_transcript_100679;F01_transcript_102104;F01_transcript_106285;F01_transcript_106305;F01_transcript_107999;F01_transcript_113185;F01_transcript_11344;F01_transcript_114864;F01_transcript_11492;F01_transcript_115738;F01_transcript_116634;F01_transcript_116898;F01_transcript_118429;F01_transcript_119625;F01_transcript_121040;F01_transcript_121601;F01_transcript_121703;F01_transcript_122238;F01_transcript_124024;F01_transcript_124264;F01_transcript_124330;F01_transcript_124661;F01_transcript_125272;F01_transcript_127167;F01_transcript_128024;F01_transcript_129404;F01_transcript_130442;F01_transcript_131237;F01_transcript_131999;F01_transcript_132323;F01_transcript_133243;F01_transcript_133706;F01_transcript_133770;F01_transcript_135556;F01_transcript_136794;F01_transcript_137291;F01_transcript_137929;F01_transcript_138141;F01_transcript_138243;F01_transcript_139121;F01_transcript_139598;F01_transcript_143196;F01_transcript_145943;F01_transcript_146387;F01_transcript_146556;F01_transcript_146806;F01_transcript_149364;F01_transcript_149770;F01_transcript_150196;F01_transcript_15082;F01_transcript_151343;F01_transcript_152398;F01_transcript_155603;F01_transcript_155687;F01_transcript_158044;F01_transcript_158296;F01_transcript_159626;F01_transcript_162212;F01_transcript_162437;F01_transcript_165803;F01_transcript_17688;F01_transcript_18802;F01_transcript_19005;F01_transcript_2042;F01_transcript_20485;F01_transcript_20828;F01_transcript_28257;F01_transcript_30875;F0	K07456+K10755+K10754+K10755+K07466+K10747+K08734+K07466+K07466+K10754+K08737+K08736+K07456+K10756+K10747+K10755+K07466+K08739+K08737+K10747+K10746+K10755+K10755+K08739+K10858+K02327+K10755+K10754+K07466+K10756+K07466+K10755+K10755+K07456+K08734+K10754+K08734+K10754+K08736+K02328+K08734+K07466+K10858+K10754+K02328+K10747+K08737+K07456+K03504+K08739+K10755+K02328+K10747+K04802+K10755+K02328+K02328+K10754+K07456+K08734+K07466+K07466+K07456+K10754+K10756+K08736+K03504+K02328+K02328+K08737+K02328+K10746+K08737+K10756+K10756+K10756+K10858+K10755+K10756+K10755+K10755+K07466+K07466+K07466+K10858+K03505+K08736+K10755+K10747+K10747+K03505+K10756+K07466+K03111+K10858+K08739+K02328+K08737+K07466+K08739+K10746+K10754+K10747+K08739+K10747+K08734+K10747+K07456+K07466+K08734+K10747+K10755+K08739+K10756+K10747+K10747+K10739+K10747+K10754+K07466+K08736+K10746+K10747+K07466+K10747+K10754+K07466+K10858+K08739+K10747+K10747+K07456+K07466+K10754+K10754+K07466+K08739+K08737+K08739+K02327

			1_transcript_32534;F01_transcript_334;F01_transcript_34563;F01_transcript_35760;F01_transcript_370;F01_transcript_38198;F01_transcript_39293;F01_transcript_39420;F01_transcript_4056;F01_transcript_40950;F01_transcript_4207;F01_transcript_42381;F01_transcript_42392;F01_transcript_45535;F01_transcript_45921;F01_transcript_46906;F01_transcript_4779;F01_transcript_48686;F01_transcript_52087;F01_transcript_52773;F01_transcript_53178;F01_transcript_533;F01_transcript_54343;F01_transcript_55038;F01_transcript_55499;F01_transcript_57153;F01_transcript_58736;F01_transcript_593;F01_transcript_61368;F01_transcript_64062;F01_transcript_6499;F01_transcript_65805;F01_transcript_65910;F01_transcript_66251;F01_transcript_66392;F01_transcript_66892;F01_transcript_71262;F01_transcript_71365;F01_transcript_7220;F01_transcript_7234;F01_transcript_73069;F01_transcript_74157;F01_transcript_74164;F01_transcript_74712;F01_transcript_77089;F01_transcript_77639;F01_transcript_77701;F01_transcript_79595;F01_transcript_80286;F01_transcript_81168;F01_transcript_82319;F01_transcript_82696;F01_transcript_82835;F01_transcript_83279;F01_transcript_83309;F01_transcript_83773;F01_transcript_85797;F01_transcript_88147;F01_transcript_89037;F01_transcript_89048;F01_transcript_896;F01_transcript_90918;F01_transcript_91162;F01_transcript_9137;F01_transcript_91407;F01_transcript_91705;F01_transcript_92049;F01_transcript_95868;F01_transcript_96543;F01_transcript_97385;F01_transcript_97433;F01_transcript_98012;	
Homologous recombination	ko03440	159	F01_transcript_100577;F01_transcript_100821;F01_transcript_103735;F01_transcript_104093;F01_transcript_107999;F01_transcript_108049;F01_transcript_109342;F01_transcript_109986;F01_transcript_1101;F01_transcript_114864;F01_transcript_11492;F01_transcript_115208;F01_transcript_12128;F01_transcript_121349;F01_transcript_121509;F01_transcript_121703;F01_transcript_122563;F01_transcript_124711;F01_transcript_125801;F01_transcript_125929;F01_transcript_1267;F01_transcript_127856;F01_transcript_128172;F01_transcript_129163;F01_transcript_129404;F01_transcript_129921;F01_transcript_131757;F01_transcript_131972;F01_transcript_131999;F01_transcript_133243;F01_transcript_134883;F01_transcript_135045;F01_transcript_135313;F01_transcript_136652;F01_transcript_137806;F01_trans	K10879+K10879+K08991+K03165+K07466+K10865+K10882+K10875+K02335+K07466+K07466+K10870+K03655+K10901+K10875+K07466+K03655+K10901+K03165+K10867+K10879+K10867+K10866+K03655+K02327+K10870+K10875+K10901+K07466+K07466+K10875+K10901+K10867+K02335+K03655+K10865+K03553+K02328+K10875+K10879+K08991+K10879+K10865+K10875+K02335+K07466+K10901+K03165+K10867+K10866+K10879+K02328+K02335+K02335+K03504+K02328+K03165+K10865+K10875+K10901+K10866+K08991+K02328+K03165+K03165+K10865+K02328+K10870+K03553+K10901+K10879+K10875+K10901+K07466+K07466+K03165+K03655+K03504+K02328+K02328+K03655+K02328+K03553+K10875+K03655+K10879+K07466+K07466+K07466+K

	cript_137924;F01_transcript_138349;F01_transcript_139121;F01_transcript_140319;F01_transcript_140888;F01_transcript_141317;F01_transcript_141452;F01_transcript_141487;F01_transcript_141812;F01_transcript_142708;F01_transcript_143196;F01_transcript_14367;F01_transcript_144370;F01_transcript_144409;F01_transcript_145590;F01_transcript_146319;F01_transcript_146556;F01_transcript_146957;F01_transcript_14906;F01_transcript_150196;F01_transcript_152398;F01_transcript_153467;F01_transcript_15442;F01_transcript_155210;F01_transcript_15554;F01_transcript_156776;F01_transcript_157058;F01_transcript_158296;F01_transcript_158562;F01_transcript_158851;F01_transcript_15901;F01_transcript_159626;F01_transcript_159740;F01_transcript_160129;F01_transcript_16026;F01_transcript_165487;F01_transcript_165789;F01_transcript_167004;F01_transcript_17688;F01_transcript_18802;F01_transcript_20891;F01_transcript_2658;F01_transcript_28257;F01_transcript_30875;F01_transcript_32534;F01_transcript_3299;F01_transcript_34563;F01_transcript_34610;F01_transcript_3746;F01_transcript_4039;F01_transcript_41595;F01_transcript_45535;F01_transcript_45921;F01_transcript_46906;F01_transcript_48686;F01_transcript_49940;F01_transcript_50101;F01_transcript_51892;F01_transcript_53914;F01_transcript_54035;F01_transcript_54343;F01_transcript_55499;F01_transcript_56850;F01_transcript_57153;F01_transcript_60360;F01_transcript_61368;F01_transcript_61893;F01_transcript_62812;F01_transcript_6499;F01_transcript_65789;F01_transcript_67177;F01_transcript_67337;F01_transcript_67834;F01_transcript_68365;F01_transcript_6871;F01_transcript_692;F01_transcript_69753;F01_transcript_70016;F01_transcript_70117;F01_transcript_70344;F01_transcript_70894;F01_transcript_71156;F01_transcript_71266;F01_transcript_72565;F01_transcript_72616;F01_transcript_728;F01_transcript_73069;F01_transcript_74149;F01_transcript_75184;F01_transcript_75496;F01_transcript_75661;F01_transcript_76082;F01_transcript_77062;F01_transcript_77116;F01_transcript_77642;F01_transcript_79245;F01_transcript_79308;F01_transcript_79599;F01_transcript_79607;F01_transcript_80286;F01_transcript_82696;F01_transcript_8286;F01_transcript_83759;F01_transcript_83773;F01_transcrip	03505+K10881+K10881+K03165+K08991+K10865+K03505+K07466+K10879+K03111+K10870+K02328+K10866+K03165+K07466+K02335+K10901+K03655+K10901+K03165+K10879+K10875+K03165+K03165+K03655+K03165+K02335+K10875+K03553+K10875+K10869+K02335+K07466+K03553+K10866+K02335+K10875+K10875+K10881+K10875+K10901+K10875+K03165+K10875+K10866+K10739+K07466+K10875+K10901+K07466+K02335+K10865+K02335+K07466+K02335+K03165+K03165+K10882+K03553+K07466+K10901+K02335+K03553+K07466+K03165+K10901+K02327+K10882+K02335+K03165

			t_862;F01_transcript_86740;F01_transcript_88462;F01_transcript_89037;F01_transcript_89051;F01_transcript_89910;F01_transcript_90524;F01_transcript_91016;F01_transcript_91021;F01_transcript_91407;F01_transcript_92762;F01_transcript_944;F01_transcript_94448;F01_transcript_95868;F01_transcript_97088;F01_transcript_97267;F01_transcript_98012;F01_transcript_98924;F01_transcript_991;F01_transcript_9915;	
Non-homologous end-joining	ko03450	49	F01_transcript_102876;F01_transcript_108049;F01_transcript_114825;F01_transcript_115417;F01_transcript_121346;F01_transcript_126702;F01_transcript_128172;F01_transcript_132403;F01_transcript_137924;F01_transcript_139243;F01_transcript_141487;F01_transcript_145590;F01_transcript_149855;F01_transcript_151783;F01_transcript_15442;F01_transcript_155052;F01_transcript_156776;F01_transcript_15901;F01_transcript_160006;F01_transcript_161577;F01_transcript_167070;F01_transcript_24265;F01_transcript_29887;F01_transcript_30156;F01_transcript_33733;F01_transcript_38014;F01_transcript_47945;F01_transcript_52470;F01_transcript_53015;F01_transcript_54035;F01_transcript_54417;F01_transcript_61893;F01_transcript_65253;F01_transcript_66223;F01_transcript_66348;F01_transcript_68440;F01_transcript_70848;F01_transcript_70999;F01_transcript_71350;F01_transcript_75184;F01_transcript_75831;F01_transcript_79607;F01_transcript_85351;F01_transcript_85430;F01_transcript_85924;F01_transcript_86415;F01_transcript_86740;F01_transcript_94540;F01_transcript_99833;	K03512+K10865+K03512+K04799+K10885+K10885+K10866+K03512+K10865+K10777+K10865+K10866+K04799+K04799+K10865+K04799+K10866+K10865+K04799+K04799+K04799+K10884+K10886+K10884+K10884+K04799+K10885+K04799+K10884+K10865+K10885+K10866+K10885+K03512+K03512+K10777+K03512+K04799+K10777+K10866+K10885+K10866+K04799+K04799+K10885+K04799+K10865+K10886+K10777
Phosphatidylinositol signaling system	ko04070	310	F01_transcript_100291;F01_transcript_100678;F01_transcript_100774;F01_transcript_101123;F01_transcript_101744;F01_transcript_103125;F01_transcript_103304;F01_transcript_10363;F01_transcript_103887;F01_transcript_104015;F01_transcript_104277;F01_transcript_104353;F01_transcript_104608;F01_transcript_104981;F01_transcript_105010;F01_transcript_105886;F01_transcript_106454;F01_transcript_10798;F01_transcript_108461;F01_transcript_109177;F01_transcript_109590;F01_transcript_109860;F01_transcript_109925;F01_transcript_110584;F01_transcript_110751;F01_transcript_111866;F01_transcript_11252;F01_transcript_112941;F01_transcript_11301;F01_transcript_113539;F01_transcript_114431;F01_transcript_114629;F01_transcript_115711;F01_transcript_115766;F01_transcript_116466;F01_tra	K00889+K00901+K19801+K00921+K01110+K00921+K00999+K00921+K00889+K00921+K05857+K00914+K00901+K02183+K00889+K00999+K05857+K00914+K02183+K01092+K19801+K00889+K00913+K13024+K00914+K19801+K00901+K00889+K00901+K13024+K00888+K00889+K05857+K13024+K00981+K00901+K00914+K00914+K00901+K00888+K00889+K00889+K00921+K00889+K05857+K00915+K01110+K00915+K00889+K00914+K00981+K05857+K00889+K00901+K00901+K00889+K00889+K00889+K19801+K00914+K00913+K00901+K10047+K00889+K00914+K00901+K02183+K00889+K00914+K00889+K00889+K05857+K02183+K01110+K18649+K00901+K01110+K00914+K00901+K00889+K00889+K18081+K00889+K00889+K00901+K19801+K01110+K00889+K00901+K

	nscript_116757;F01_transcript_117270;F01_transcript_117794;F01_transcript_117894;F01_transcript_117975;F01_transcript_118778;F01_transcript_11952;F01_transcript_119968;F01_transcript_12012;F01_transcript_120207;F01_transcript_120640;F01_transcript_120833;F01_transcript_121391;F01_transcript_121489;F01_transcript_121786;F01_transcript_122067;F01_transcript_122263;F01_transcript_123225;F01_transcript_123865;F01_transcript_124128;F01_transcript_124691;F01_transcript_124724;F01_transcript_124736;F01_transcript_125162;F01_transcript_125516;F01_transcript_125705;F01_transcript_126233;F01_transcript_126659;F01_transcript_127179;F01_transcript_127233;F01_transcript_127355;F01_transcript_127598;F01_transcript_127812;F01_transcript_129146;F01_transcript_129184;F01_transcript_130320;F01_transcript_130443;F01_transcript_131361;F01_transcript_131437;F01_transcript_132042;F01_transcript_132245;F01_transcript_133072;F01_transcript_133384;F01_transcript_133496;F01_transcript_133554;F01_transcript_133724;F01_transcript_133730;F01_transcript_134031;F01_transcript_134034;F01_transcript_134693;F01_transcript_134796;F01_transcript_135701;F01_transcript_135724;F01_transcript_135743;F01_transcript_135803;F01_transcript_136099;F01_transcript_13665;F01_transcript_137504;F01_transcript_137790;F01_transcript_137791;F01_transcript_137832;F01_transcript_138185;F01_transcript_138842;F01_transcript_139028;F01_transcript_140237;F01_transcript_140625;F01_transcript_141569;F01_transcript_14366;F01_transcript_143704;F01_transcript_143711;F01_transcript_144552;F01_transcript_144803;F01_transcript_14513;F01_transcript_145512;F01_transcript_146185;F01_transcript_146478;F01_transcript_146545;F01_transcript_147722;F01_transcript_149745;F01_transcript_149881;F01_transcript_149901;F01_transcript_150218;F01_transcript_15030;F01_transcript_150423;F01_transcript_151119;F01_transcript_151754;F01_transcript_152601;F01_transcript_152813;F01_transcript_153592;F01_transcript_153720;F01_transcript_153811;F01_transcript_154350;F01_transcript_156217;F01_transcript_156315;F01_transcript_156666;F01_transcript_157038;F01_transcript_157286;F01_transcript_157718;F01_transcript_158433;F01_transcript_158880;F01_transcri	10047+K00913+K00921+K15422+K18081+K01110+K05857+K00889+K00914+K02183+K05857+K01110+K02183+K10572+K00889+K01106+K00921+K00901+K10572+K10572+K00921+K00914+K00913+K05857+K18081+K18081+K00914+K18081+K10572+K00914+K00915+K00901+K05857+K00889+K00981+K00914+K00889+K00921+K00921+K05857+K00921+K01110+K05857+K00889+K00914+K00901+K01110+K00901+K00901+K00901+K00889+K00901+K00999+K00889+K19801+K00999+K00914+K05857+K05857+K02183+K00901+K01110+K10572+K00913+K01110+K01110+K13024+K10572+K01110+K01110+K00889+K05857+K00889+K00901+K00921+K05857+K01110+K00921+K05857+K15422+K00921+K00889+K10572+K00901+K00901+K00981+K00901+K00901+K00913+K00913+K00913+K00913+K00913+K02183+K01092+K15422+K00915+K10047+K02183+K02183+K02183+K02183+K02183+K02183+K02183+K02183+K00913+K00921+K00889+K00921+K00981+K02183+K05857+K00901+K00901+K05857+K00901+K05857+K10572+K00889+K02183+K01110+K00981+K02183+K00901+K10572+K00889+K00901+K00914+K00913+K00889+K13024+K02183+K00889+K00889+K00914+K10047+K00914+K10047+K00889+K05857+K00889+K00921+K00921+K00913+K02183+K00981+K00889+K00889+K18081+K05857+K00901+K00889+K00889+K00914+K10572+K05857+K01110+K10572+K00914+K00981+K13024+K10572+K02183+K01110+K00914+K13024+K00889+K00901+K02183+K00914+K19801+K00921+K00889+K00901+K19801+K02183+K00889+K00889+K19801+K00889+K00901+K00981+K02183+K10572+K10572+K19801+K00914+K00914+K00889+K00888+K19801+K00901+K00913+K00889+K00901+K00889+K15422+K15422+K00921+K00889+K00889+K00888+K00901+K00889+K10047+K10572+K00921+K18081+K00915+K00901+K00901+K00921+K10047+K00901+K00901+K10047+K05857+K00889+K10572+K00889

pt_160089;F01_transcript_160115;F01_transcript_160561;F01_transcript_160655;F01_transcript_161895;F01_transcript_162994;F01_transcript_164415;F01_transcript_164709;F01_transcript_164802;F01_transcript_165856;F01_transcript_166806;F01_transcript_166884;F01_transcript_167180;F01_transcript_167405;F01_transcript_167641;F01_transcript_17337;F01_transcript_17957;F01_transcript_18566;F01_transcript_20178;F01_transcript_20962;F01_transcript_2100;F01_transcript_21125;F01_transcript_21255;F01_transcript_21279;F01_transcript_21520;F01_transcript_21865;F01_transcript_22882;F01_transcript_22916;F01_transcript_23;F01_transcript_23080;F01_transcript_23214;F01_transcript_25412;F01_transcript_26104;F01_transcript_27724;F01_transcript_28;F01_transcript_2861;F01_transcript_29014;F01_transcript_30068;F01_transcript_30863;F01_transcript_32241;F01_transcript_34137;F01_transcript_34466;F01_transcript_34615;F01_transcript_34791;F01_transcript_34807;F01_transcript_36200;F01_transcript_36222;F01_transcript_40244;F01_transcript_40331;F01_transcript_40512;F01_transcript_41844;F01_transcript_44007;F01_transcript_45009;F01_transcript_46272;F01_transcript_46373;F01_transcript_46402;F01_transcript_46836;F01_transcript_47112;F01_transcript_49102;F01_transcript_50493;F01_transcript_51537;F01_transcript_51990;F01_transcript_52111;F01_transcript_52496;F01_transcript_52771;F01_transcript_53835;F01_transcript_54432;F01_transcript_5511;F01_transcript_55547;F01_transcript_55919;F01_transcript_56945;F01_transcript_57879;F01_transcript_58506;F01_transcript_59439;F01_transcript_59535;F01_transcript_60254;F01_transcript_60409;F01_transcript_60545;F01_transcript_60567;F01_transcript_61325;F01_transcript_6257;F01_transcript_63302;F01_transcript_63398;F01_transcript_63470;F01_transcript_64409;F01_transcript_64590;F01_transcript_64888;F01_transcript_64970;F01_transcript_64995;F01_transcript_65003;F01_transcript_65191;F01_transcript_6591;F01_transcript_66026;F01_transcript_66592;F01_transcript_66677;F01_transcript_6689;F01_transcript_67527;F01_transcript_68408;F01_transcript_68866;F01_transcript_69054;F01_transcript_69123;F01_transcript_6988;F01_transcript_70192;F01_transcript_70203;F01_transcript_70648;F01_transcript_71252;F01_transcript_71307;F01_transcript_71841;F01_transcrip

			t_72837;F01_transcript_73080;F01_transcript_73787;F01_transcript_7424;F01_transcript_75023;F01_transcript_75395;F01_transcript_76017;F01_transcript_76146;F01_transcript_76406;F01_transcript_76546;F01_transcript_76899;F01_transcript_77133;F01_transcript_78607;F01_transcript_78675;F01_transcript_79124;F01_transcript_79214;F01_transcript_79219;F01_transcript_79288;F01_transcript_80155;F01_transcript_81263;F01_transcript_81385;F01_transcript_81730;F01_transcript_82062;F01_transcript_82323;F01_transcript_82606;F01_transcript_82865;F01_transcript_83591;F01_transcript_84954;F01_transcript_86245;F01_transcript_86575;F01_transcript_88245;F01_transcript_88365;F01_transcript_88603;F01_transcript_8890;F01_transcript_88943;F01_transcript_89769;F01_transcript_9;F01_transcript_90463;F01_transcript_91252;F01_transcript_91968;F01_transcript_9251;F01_transcript_92930;F01_transcript_93174;F01_transcript_93426;F01_transcript_93589;F01_transcript_93671;F01_transcript_9461;F01_transcript_94770;F01_transcript_94951;F01_transcript_95059;F01_transcript_95120;F01_transcript_95171;F01_transcript_95193;F01_transcript_96011;F01_transcript_96182;F01_transcript_9636;F01_transcript_96475;F01_transcript_96737;F01_transcript_97203;F01_transcript_97327;F01_transcript_9743;F01_transcript_97607;F01_transcript_98320;F01_transcript_98548;F01_transcript_99185;F01_transcript_99324;F01_transcript_99553;	
Plant hormone signal transduction	ko04075	836	F01_transcript_100151;F01_transcript_100308;F01_transcript_100655;F01_transcript_100752;F01_transcript_100796;F01_transcript_101019;F01_transcript_101076;F01_transcript_101388;F01_transcript_101467;F01_transcript_101555;F01_transcript_10163;F01_transcript_101759;F01_transcript_101992;F01_transcript_10270;F01_transcript_103024;F01_transcript_103600;F01_transcript_103615;F01_transcript_103678;F01_transcript_103936;F01_transcript_103965;F01_transcript_104145;F01_transcript_104319;F01_transcript_104717;F01_transcript_104753;F01_transcript_104851;F01_transcript_104937;F01_transcript_105315;F01_transcript_105908;F01_transcript_106267;F01_transcript_106902;F01_transcript_107081;F01_transcript_107208;F01_transcript_10734;F01_transcript_107429;F01_transcript_107629;F01_tr	K14432+K14431+K14491+K14491+K13463+K14486+K14500+K14500+K14431+K14515+K14491+K13946+K14489+K14491+K14487+K14486+K13946+K14505+K14484+K14432+K14486+K14510+K13946+K14484+K14484+K14488+K14497+K14488+K14512+K12126+K14500+K14484+K14508+K14510+K14496+K14484+K13946+K14498+K14510+K14497+K14432+K14500+K13422+K14489+K14432+K14484+K14431+K14500+K14503+K14496+K14498+K14484+K14491+K14497+K14489+K14485+K14485+K14497+K14432+K14488+K14486+K14509+K14497+K14484+K13463+K14490+K14506+K14506+K14509+K14514+K14498+K13946+K14508+K13464+K14506+K14496+K14486+K14500+K14506+K14513+K14498+K14484+K14489+K14489+K14500+K14509+K12126+K14500+K14500+K

	anscript_107696;F01_transcript_107796;F01_transcript_107833;F01_transcript_107857;F01_transcript_107912;F01_transcript_108322;F01_transcript_108876;F01_transcript_109302;F01_transcript_109397;F01_transcript_109639;F01_transcript_109852;F01_transcript_110087;F01_transcript_110117;F01_transcript_110404;F01_transcript_110410;F01_transcript_110833;F01_transcript_110971;F01_transcript_111059;F01_transcript_111084;F01_transcript_11113;F01_transcript_111514;F01_transcript_111589;F01_transcript_111689;F01_transcript_112042;F01_transcript_112247;F01_transcript_11253;F01_transcript_112741;F01_transcript_112751;F01_transcript_112850;F01_transcript_113331;F01_transcript_113697;F01_transcript_113798;F01_transcript_113827;F01_transcript_114261;F01_transcript_114514;F01_transcript_114645;F01_transcript_114719;F01_transcript_114720;F01_transcript_114733;F01_transcript_114803;F01_transcript_114969;F01_transcript_115348;F01_transcript_115494;F01_transcript_115518;F01_transcript_115827;F01_transcript_116025;F01_transcript_116041;F01_transcript_116219;F01_transcript_116270;F01_transcript_116418;F01_transcript_116500;F01_transcript_11662;F01_transcript_11697;F01_transcript_117018;F01_transcript_117024;F01_transcript_117246;F01_transcript_117669;F01_transcript_117761;F01_transcript_117840;F01_transcript_118215;F01_transcript_11828;F01_transcript_118321;F01_transcript_11840;F01_transcript_119044;F01_transcript_119113;F01_transcript_119214;F01_transcript_119513;F01_transcript_119574;F01_transcript_120091;F01_transcript_1201;F01_transcript_120141;F01_transcript_120393;F01_transcript_120436;F01_transcript_120636;F01_transcript_120837;F01_transcript_120847;F01_transcript_12109;F01_transcript_121129;F01_transcript_121412;F01_transcript_121635;F01_transcript_121701;F01_transcript_121709;F01_transcript_121789;F01_transcript_122093;F01_transcript_122104;F01_transcript_122122;F01_transcript_122169;F01_transcript_12236;F01_transcript_122449;F01_transcript_122572;F01_transcript_123009;F01_transcript_123131;F01_transcript_12323;F01_transcript_123244;F01_transcript_123297;F01_transcript_124265;F01_transcript_124310;F01_transcript_124345;F01_transcript_125166;F01_transcript_125364;F01_transcript_125397;F01_transcript_125445;F01_transcript_125461;F01_trans	14491+K14484+K14431+K14486+K13463+K14500+K13464+K14497+K14486+K14489+K14489+K14486+K14491+K14432+K14489+K14489+K14490+K14486+K14506+K14498+K14489+K14510+K14500+K14500+K13464+K14490+K14510+K14486+K13464+K14500+K14431+K14505+K14485+K14489+K13946+K14432+K14498+K14486+K14491+K14513+K14484+K14497+K14489+K14486+K14432+K14500+K14487+K14485+K14484+K14505+K13946+K14432+K14486+K13422+K14510+K14497+K14431+K14514+K14508+K14498+K14498+K14502+K14488+K13422+K13464+K14498+K13463+K14500+K14506+K14431+K14506+K14486+K14488+K14500+K14510+K14431+K14498+K14500+K14498+K14505+K13946+K14510+K14491+K14486+K14486+K14431+K14432+K14484+K14500+K14508+K14484+K14506+K14431+K14484+K14506+K14486+K13464+K14491+K14500+K14498+K14431+K14508+K14484+K14486+K14486+K14498+K14484+K14498+K14484+K13415+K14488+K13946+K14492+K14431+K14514+K14514+K14508+K14491+K14489+K14488+K14509+K14431+K14494+K14498+K14514+K14484+K14514+K14498+K13464+K14484+K14489+K14498+K14486+K14431+K14513+K14496+K14514+K14487+K14497+K14486+K13946+K14491+K14489+K14509+K14432+K13416+K13464+K12126+K14485+K14486+K12126+K14485+K14488+K14514+K12126+K14484+K14498+K13946+K14492+K14494+K13464+K14498+K14506+K14486+K14486+K14498+K14500+K14503+K13464+K14515+K14485+K14491+K14432+K13946+K13946+K14488+K14510+K14509+K14486+K14491+K14431+K14486+K14509+K14491+K14497+K14508+K14509+K14508+K14498+K14488+K14484+K14491+K14502+K14486+K13464+K14491+K14484+K14515+K14499+K14484+K14489+K14509+K14514+K14508+K14498+K14494+K14496+K14489+K14515+K14489+K13416+K14485+K14431+K14485+K14497+K14484+K13463+K14515+K14497+K14484+K14497+K14432+K13463+K14497+K14486+K13416+K14505+K13463+K14500+K14488+K13946+K14500+K14500+K14497+K13946+K14494+K14498+K14498+K13463+K14510+K14486+K14486+K14484+K14498+K14486+K14486+K14513+K14512+K14484+K14506+K13422+K14489+K14497+K14486+K14514+K14491+K14508+K13463+K14510+K13946+K1449

	cript_125628;F01_transcript_125720;F01_transcript_125735;F0	4+K13946+K14489+K14514+K14489+K14488+K14510+K145
	1_transcript_125824;F01_transcript_12632;F01_transcript_1263	09+K14514+K14486+K14484+K14514+K14509+K13416+K14
	31;F01_transcript_126444;F01_transcript_126485;F01_transcrip	498+K14500+K14491+K14510+K14498+K14485+K14509+K1
	t_12693;F01_transcript_126992;F01_transcript_127018;F01_tra	4497+K14484+K14497+K14498+K14486+K14494+K14498+K
	nscript_127195;F01_transcript_127261;F01_transcript_127429;	14509+K13416+K13415+K14494+K14500+K14514+K13946+
	F01_transcript_127661;F01_transcript_127728;F01_transcript_1	K13946+K14513+K14514+K14497+K13422+K14489+K13946
	27940;F01_transcript_12839;F01_transcript_128659;F01_transc	+K14508+K13946+K14431+K14500+K13946+K14500+K1448
	ript_128667;F01_transcript_129101;F01_transcript_129236;F01	5+K14432+K14494+K13946+K14497+K14509+K14514+K145
	_transcript_129288;F01_transcript_129735;F01_transcript_1297	13+K14497+K14491+K14432+K14485+K13946+K14508+K14
	52;F01_transcript_129844;F01_transcript_130173;F01_transcrip	506+K14485+K14506+K14506+K13415+K13946+K14497+K1
	t_130494;F01_transcript_130571;F01_transcript_130582;F01_tr	4497+K13422+K13946+K14500+K13946+K13946+K13422+K
	anscript_130827;F01_transcript_131416;F01_transcript_131529	14487+K14506+K14506+K14487+K14485+K13946+K14497+
	;F01_transcript_131550;F01_transcript_131619;F01_transcript_	K13422+K14432+K14491+K14486+K14506+K14500+K14484
	132398;F01_transcript_132587;F01_transcript_132761;F01_tran	+K13464+K13422+K14503+K14493+K14490+K14500+K1342
	script_133172;F01_transcript_133386;F01_transcript_13340;F0	2+K14484+K14431+K14498+K14498+K13422+K14502+K134
	1_transcript_133410;F01_transcript_133530;F01_transcript_133	22+K14498+K14500+K14505+K14498+K14498+K14432+K14
	949;F01_transcript_133958;F01_transcript_134232;F01_transcri	484+K14500+K14498+K14500+K14498+K14498+K14432+K1
	pt_134372;F01_transcript_134451;F01_transcript_13452;F01_tr	4502+K14498+K14497+K14431+K14484+K14510+K14509+K
	anscript_134531;F01_transcript_134735;F01_transcript_134913	14496+K14498+K14500+K14484+K14500+K14484+K14431+
	;F01_transcript_134914;F01_transcript_134944;F01_transcript_	K14498+K14432+K14431+K14503+K14484+K14497+K13946
	135115;F01_transcript_135356;F01_transcript_135363;F01_tran	+K14484+K14484+K14431+K14493+K14512+K13463+K1448
	script_135617;F01_transcript_135731;F01_transcript_135891;F	4+K14497+K13464+K13464+K13464+K14432+K14484+K144
	01_transcript_1359;F01_transcript_136065;F01_transcript_1363	89+K14512+K14497+K14488+K13464+K14496+K14488+K14
	71;F01_transcript_136473;F01_transcript_136490;F01_transcrip	498+K14508+K13464+K14484+K14497+K14484+K14484+K1
	t_13663;F01_transcript_13681;F01_transcript_137080;F01_tran	4484+K14496+K14486+K14496+K14504+K14484+K14484+K
	script_137491;F01_transcript_137673;F01_transcript_137737;F	14484+K14496+K14490+K14488+K14496+K14496+K14496+
	01_transcript_137753;F01_transcript_137887;F01_transcript_13	K14496+K14488+K14490+K14485+K14496+K14490+K14488
	8043;F01_transcript_138284;F01_transcript_138285;F01_transc	+K14488+K14497+K14489+K14490+K14490+K13449+K1448
	ript_138346;F01_transcript_138478;F01_transcript_139064;F01	8+K14488+K14486+K14486+K13946+K14500+K14498+K144
	_transcript_139281;F01_transcript_140010;F01_transcript_1403	98+K13946+K14486+K14486+K14432+K14506+K14490+K14
	;F01_transcript_140310;F01_transcript_140335;F01_transcript_	486+K14484+K14510+K14484+K14486+K14490+K14500+K1
	140363;F01_transcript_140414;F01_transcript_14053;F01_trans	3464+K14510+K14506+K14489+K14491+K14496+K14509+K
	cript_14062;F01_transcript_140709;F01_transcript_140752;F01	14509+K14486+K14486+K14500+K14508+K14498+K14432+
	_transcript_140777;F01_transcript_140830;F01_transcript_1409	K14509+K14506+K14498+K13946+K14484+K14500+K13415
	35;F01_transcript_141104;F01_transcript_141125;F01_transcrip	+K14489+K14514+K14496+K14515+K14490+K14487+K1449
	t_141167;F01_transcript_14117;F01_transcript_141365;F01_tra	1+K14486+K14484+K14431+K14491+K14499+K14496+K144
	nscript_141412;F01_transcript_141911;F01_transcript_142046;	84+K14488+K14497+K14500+K13946+K14485+K14505+K14

	F01_transcript_14220;F01_transcript_142807;F01_transcript_143291;F01_transcript_143455;F01_transcript_143515;F01_transcript_143747;F01_transcript_143996;F01_transcript_144119;F01_transcript_144130;F01_transcript_144206;F01_transcript_144287;F01_transcript_144396;F01_transcript_144432;F01_transcript_144676;F01_transcript_144855;F01_transcript_145167;F01_transcript_145178;F01_transcript_145343;F01_transcript_14556;F01_transcript_145636;F01_transcript_145728;F01_transcript_146642;F01_transcript_146725;F01_transcript_147219;F01_transcript_147354;F01_transcript_147439;F01_transcript_147488;F01_transcript_147637;F01_transcript_147746;F01_transcript_14846;F01_transcript_148852;F01_transcript_149332;F01_transcript_149485;F01_transcript_149809;F01_transcript_14987;F01_transcript_149912;F01_transcript_149977;F01_transcript_150124;F01_transcript_150335;F01_transcript_150528;F01_transcript_150884;F01_transcript_151412;F01_transcript_151549;F01_transcript_151687;F01_transcript_151885;F01_transcript_152225;F01_transcript_152363;F01_transcript_152446;F01_transcript_152480;F01_transcript_152498;F01_transcript_153;F01_transcript_153230;F01_transcript_15334;F01_transcript_153413;F01_transcript_153439;F01_transcript_15359;F01_transcript_153627;F01_transcript_153656;F01_transcript_153959;F01_transcript_154234;F01_transcript_15444;F01_transcript_154500;F01_transcript_154513;F01_transcript_154527;F01_transcript_15457;F01_transcript_154848;F01_transcript_154860;F01_transcript_155372;F01_transcript_155391;F01_transcript_155413;F01_transcript_15563;F01_transcript_155704;F01_transcript_155778;F01_transcript_156001;F01_transcript_156378;F01_transcript_15660;F01_transcript_156839;F01_transcript_156872;F01_transcript_156997;F01_transcript_157003;F01_transcript_157113;F01_transcript_157209;F01_transcript_157707;F01_transcript_157927;F01_transcript_158103;F01_transcript_158174;F01_transcript_158690;F01_transcript_158712;F01_transcript_15875;F01_transcript_158753;F01_transcript_159134;F01_transcript_159140;F01_transcript_159179;F01_transcript_159337;F01_transcript_159346;F01_transcript_159438;F01_transcript_159532;F01_transcript_159571;F01_transcript_159687;F01_transcript_159823;F01_transcript_160242;F01_transcript_160495;F01_transcript_160874;F01_tra	491+K14491+K14509+K14510+K14484+K14431+K14509+K14510+K14486+K14484+K14497+K14431+K14508+K14493+K14488+K13946+K13416+K14491+K14496+K14498+K14514+K14513+K14513+K13464+K14498+K14508+K13415+K14505+K14496+K14486+K14432+K14431+K14489+K14486+K14486+K14514+K14498+K14506+K14485+K13416+K14510+K14497+K14489+K13464+K14498+K14489+K13946+K14510+K14488+K14485+K14488+K12126+K14488+K13415+K14491+K14505+K14491+K13946+K13946+K14497+K14506+K14502+K14491+K14488+K13415+K14486+K14489+K14484+K14506+K14513+K13416+K14491+K14491+K14431+K14488+K12126+K14431+K14509+K14510+K14486+K14497+K14431+K14488+K14431+K14503+K13422+K14489+K14491+K13464+K14506+K14494+K14497+K14500+K14489+K14494+K14491+K12126+K14432+K14510+K14490+K14515+K14498+K14498+K14484+K14509+K14497+K14432+K14488+K13946+K14498+K13464+K14491+K14497+K14509+K14497+K14514+K14485+K14509+K14487+K14509+K14485+K14489+K14506+K14486+K14484+K14497+K14508+K13946+K14431+K14491+K14484+K14492+K12126+K14506+K14491+K14487+K14510+K14510+K14489+K14513+K14510+K14484+K14491+K14496+K14486+K14515+K14494+K14491+K14509+K14491+K14484+K14510+K14505+K14431+K14432+K13463+K13464+K13463+K14498+K14500+K14512+K14491+K14485+K13946+K14497+K14491+K14509+K14506+K14509+K14497+K13415+K14486+K14510+K14500+K14509+K14486+K14489+K14484+K14486+K13464+K14484+K14486+K14432+K13946+K14486+K14497+K14503+K14498+K14484+K14500+K14496+K14484+K14488+K14431+K14499+K14498+K13946+K14509+K14484+K14491+K12126+K14509+K13946+K14509+K14514+K14488+K14496+K13415+K14432+K14487+K14486+K14500+K14484+K14489+K14489+K14489+K14500+K12126+K14509+K14432+K14500+K14488+K14484+K14488+K13946+K14491+K13946+K14496+K14486+K14489+K13946+K14494+K14498

nscript_160878;F01_transcript_160907;F01_transcript_161008;
F01_transcript_161089;F01_transcript_161173;F01_transcript_1
61561;F01_transcript_161588;F01_transcript_161850;F01_trans
cript_16196;F01_transcript_162009;F01_transcript_162184;F01
_transcript_162621;F01_transcript_162929;F01_transcript_1630
45;F01_transcript_163055;F01_transcript_163188;F01_transcrip
t_163248;F01_transcript_163314;F01_transcript_163348;F01_tr
anscript_163775;F01_transcript_16410;F01_transcript_164197;
F01_transcript_164280;F01_transcript_164534;F01_transcript_1
64674;F01_transcript_164993;F01_transcript_16523;F01_transc
ript_165234;F01_transcript_165294;F01_transcript_165836;F01
_transcript_166332;F01_transcript_166603;F01_transcript_1672
36;F01_transcript_16761;F01_transcript_16781;F01_transcript_
16850;F01_transcript_16997;F01_transcript_17052;F01_transcri
pt_17686;F01_transcript_17736;F01_transcript_17823;F01_tran
script_17990;F01_transcript_18095;F01_transcript_183;F01_tra
nscript_18301;F01_transcript_18455;F01_transcript_18570;F01
_transcript_186;F01_transcript_18650;F01_transcript_18901;F0
1_transcript_18946;F01_transcript_19064;F01_transcript_19175
;F01_transcript_19318;F01_transcript_19468;F01_transcript_19
757;F01_transcript_19919;F01_transcript_20317;F01_transcript
_20379;F01_transcript_20423;F01_transcript_20698;F01_transc
ript_20785;F01_transcript_215;F01_transcript_21765;F01_trans
cript_22115;F01_transcript_22366;F01_transcript_22450;F01_tr
anscript_22525;F01_transcript_22666;F01_transcript_22724;F0
1_transcript_22775;F01_transcript_22977;F01_transcript_23198
;F01_transcript_2325;F01_transcript_23624;F01_transcript_239
82;F01_transcript_24013;F01_transcript_24054;F01_transcript_
24208;F01_transcript_24476;F01_transcript_24483;F01_transcri
pt_24754;F01_transcript_24784;F01_transcript_24802;F01_tran
script_24819;F01_transcript_25116;F01_transcript_25288;F01_t
ranscript_25404;F01_transcript_25471;F01_transcript_25538;F0
1_transcript_26408;F01_transcript_26782;F01_transcript_26788
;F01_transcript_26817;F01_transcript_27358;F01_transcript_27
833;F01_transcript_27862;F01_transcript_27993;F01_transcript
_28508;F01_transcript_29067;F01_transcript_29130;F01_transc
ript_29364;F01_transcript_29372;F01_transcript_29402;F01_tra

nscript_29441;F01_transcript_29785;F01_transcript_29795;F01
_transcript_29799;F01_transcript_29861;F01_transcript_29877;
F01_transcript_30189;F01_transcript_30324;F01_transcript_309
67;F01_transcript_31588;F01_transcript_31716;F01_transcript_
31805;F01_transcript_32031;F01_transcript_32043;F01_transcri
pt_32229;F01_transcript_32443;F01_transcript_32491;F01_tran
script_32501;F01_transcript_32930;F01_transcript_33093;F01_t
ranscript_33106;F01_transcript_33494;F01_transcript_33787;F0
1_transcript_33920;F01_transcript_33942;F01_transcript_340;F
01_transcript_3406;F01_transcript_34278;F01_transcript_34289
;F01_transcript_34382;F01_transcript_34520;F01_transcript_34
661;F01_transcript_34789;F01_transcript_34829;F01_transcript
_34937;F01_transcript_34992;F01_transcript_35088;F01_transc
ript_35335;F01_transcript_35340;F01_transcript_35551;F01_tra
nscript_35704;F01_transcript_35713;F01_transcript_35723;F01
_transcript_35846;F01_transcript_35962;F01_transcript_35996;
F01_transcript_36271;F01_transcript_36410;F01_transcript_365
38;F01_transcript_36596;F01_transcript_36604;F01_transcript_
37083;F01_transcript_38292;F01_transcript_38401;F01_transcri
pt_385;F01_transcript_38559;F01_transcript_38825;F01_transcr
ipt_39820;F01_transcript_40416;F01_transcript_40806;F01_tra
nscript_41091;F01_transcript_41628;F01_transcript_4187;F01_t
ranscript_41947;F01_transcript_42140;F01_transcript_42489;F0
1_transcript_42557;F01_transcript_42722;F01_transcript_42772
;F01_transcript_42947;F01_transcript_4308;F01_transcript_433
50;F01_transcript_43381;F01_transcript_43825;F01_transcript_
43840;F01_transcript_44099;F01_transcript_44818;F01_transcri
pt_44839;F01_transcript_45186;F01_transcript_45211;F01_tran
script_45432;F01_transcript_45720;F01_transcript_46072;F01_t
ranscript_46164;F01_transcript_46206;F01_transcript_46218;F0
1_transcript_46441;F01_transcript_46532;F01_transcript_46534
;F01_transcript_46596;F01_transcript_4672;F01_transcript_475;
F01_transcript_47819;F01_transcript_48612;F01_transcript_488
90;F01_transcript_49204;F01_transcript_49762;F01_transcript_
5012;F01_transcript_511;F01_transcript_51591;F01_transcript_
51813;F01_transcript_51951;F01_transcript_52029;F01_transcri
pt_52030;F01_transcript_5229;F01_transcript_52299;F01_trans
cript_52350;F01_transcript_52402;F01_transcript_52446;F01_tr

anscript_52546;F01_transcript_52867;F01_transcript_53078;F0
1_transcript_53370;F01_transcript_53455;F01_transcript_53629
;F01_transcript_53651;F01_transcript_53674;F01_transcript_53
75;F01_transcript_53842;F01_transcript_54045;F01_transcript_
54338;F01_transcript_54684;F01_transcript_55020;F01_transcri
pt_55030;F01_transcript_5509;F01_transcript_5535;F01_transcr
ipt_55615;F01_transcript_55626;F01_transcript_55876;F01_tra
nscript_56079;F01_transcript_56144;F01_transcript_56405;F01
_transcript_56524;F01_transcript_56723;F01_transcript_56724;
F01_transcript_56746;F01_transcript_56879;F01_transcript_570
;F01_transcript_57120;F01_transcript_57754;F01_transcript_57
999;F01_transcript_58333;F01_transcript_58497;F01_transcript
_58804;F01_transcript_58824;F01_transcript_58861;F01_transc
ript_59037;F01_transcript_59184;F01_transcript_59203;F01_tra
nscript_5926;F01_transcript_59305;F01_transcript_59559;F01_t
ranscript_59747;F01_transcript_59864;F01_transcript_59929;F0
1_transcript_5998;F01_transcript_60171;F01_transcript_60650;
F01_transcript_60742;F01_transcript_60915;F01_transcript_610
6;F01_transcript_61078;F01_transcript_61269;F01_transcript_6
1374;F01_transcript_61745;F01_transcript_6231;F01_transcript
_62716;F01_transcript_63120;F01_transcript_63311;F01_transc
ript_63464;F01_transcript_63476;F01_transcript_63515;F01_tra
nscript_63740;F01_transcript_63895;F01_transcript_64215;F01
_transcript_64291;F01_transcript_64874;F01_transcript_65098;
F01_transcript_65872;F01_transcript_659;F01_transcript_66048
;F01_transcript_66079;F01_transcript_66135;F01_transcript_66
172;F01_transcript_66318;F01_transcript_66340;F01_transcript
_66718;F01_transcript_66752;F01_transcript_66791;F01_transc
ript_66813;F01_transcript_67067;F01_transcript_67368;F01_tra
nscript_67483;F01_transcript_67523;F01_transcript_67529;F01
_transcript_67548;F01_transcript_67861;F01_transcript_6788;F
01_transcript_68010;F01_transcript_68334;F01_transcript_6833
9;F01_transcript_69599;F01_transcript_69975;F01_transcript_7
0012;F01_transcript_70100;F01_transcript_70514;F01_transcrip
t_70675;F01_transcript_70737;F01_transcript_7106;F01_transcr
ipt_71069;F01_transcript_712;F01_transcript_71303;F01_transc
ript_71597;F01_transcript_7168;F01_transcript_71760;F01_tran

script_72118;F01_transcript_72223;F01_transcript_72418;F01_transcript_72441;F01_transcript_72709;F01_transcript_72785;F01_transcript_72855;F01_transcript_73317;F01_transcript_73344;F01_transcript_73497;F01_transcript_73596;F01_transcript_73677;F01_transcript_73800;F01_transcript_74047;F01_transcript_74114;F01_transcript_74139;F01_transcript_74165;F01_transcript_74248;F01_transcript_74678;F01_transcript_7481;F01_transcript_75092;F01_transcript_7522;F01_transcript_75230;F01_transcript_75312;F01_transcript_75329;F01_transcript_75606;F01_transcript_75612;F01_transcript_75656;F01_transcript_75685;F01_transcript_7582;F01_transcript_75989;F01_transcript_76119;F01_transcript_76384;F01_transcript_76776;F01_transcript_76781;F01_transcript_76901;F01_transcript_77291;F01_transcript_77357;F01_transcript_77368;F01_transcript_77792;F01_transcript_77824;F01_transcript_77920;F01_transcript_77948;F01_transcript_78081;F01_transcript_7821;F01_transcript_78242;F01_transcript_78579;F01_transcript_78646;F01_transcript_78777;F01_transcript_78938;F01_transcript_79179;F01_transcript_79233;F01_transcript_79432;F01_transcript_7950;F01_transcript_79675;F01_transcript_79718;F01_transcript_79836;F01_transcript_7992;F01_transcript_80434;F01_transcript_80738;F01_transcript_80876;F01_transcript_80920;F01_transcript_80958;F01_transcript_80959;F01_transcript_81004;F01_transcript_81317;F01_transcript_81343;F01_transcript_81537;F01_transcript_8164;F01_transcript_81938;F01_transcript_82049;F01_transcript_82063;F01_transcript_82187;F01_transcript_82424;F01_transcript_82579;F01_transcript_82881;F01_transcript_82987;F01_transcript_83099;F01_transcript_8346;F01_transcript_8352;F01_transcript_83633;F01_transcript_83847;F01_transcript_84016;F01_transcript_84031;F01_transcript_8420;F01_transcript_84321;F01_transcript_8476;F01_transcript_8517;F01_transcript_85179;F01_transcript_85314;F01_transcript_8533;F01_transcript_85365;F01_transcript_85392;F01_transcript_85400;F01_transcript_85449;F01_transcript_8548;F01_transcript_85652;F01_transcript_85694;F01_transcript_85732;F01_transcript_85946;F01_transcript_86022;F01_transcript_86274;F01_transcript_86497;F01_transcript_8657;F01_transcript_87120;F01_transcript_87273;F01_transcript_87404;F01_transcript_88060;F01_transcript_8812;F01_trans

			cript_88138;F01_transcript_8818;F01_transcript_89201;F01_transcript_89405;F01_transcript_89645;F01_transcript_89765;F01_transcript_89851;F01_transcript_8995;F01_transcript_9019;F01_transcript_90367;F01_transcript_90725;F01_transcript_90753;F01_transcript_90757;F01_transcript_90844;F01_transcript_9096;F01_transcript_91113;F01_transcript_91202;F01_transcript_91395;F01_transcript_91489;F01_transcript_91675;F01_transcript_91737;F01_transcript_91832;F01_transcript_91839;F01_transcript_92011;F01_transcript_92143;F01_transcript_92367;F01_transcript_92761;F01_transcript_93227;F01_transcript_93428;F01_transcript_93435;F01_transcript_93634;F01_transcript_93707;F01_transcript_93996;F01_transcript_94019;F01_transcript_9412;F01_transcript_94255;F01_transcript_9432;F01_transcript_94372;F01_transcript_94764;F01_transcript_94780;F01_transcript_9482;F01_transcript_95010;F01_transcript_95259;F01_transcript_95762;F01_transcript_95858;F01_transcript_95932;F01_transcript_96135;F01_transcript_96276;F01_transcript_96416;F01_transcript_96458;F01_transcript_96797;F01_transcript_9725;F01_transcript_97346;F01_transcript_97892;F01_transcript_98108;F01_transcript_98333;F01_transcript_98348;F01_transcript_98535;F01_transcript_98623;F01_transcript_98758;F01_transcript_98834;F01_transcript_98972;F01_transcript_99217;F01_transcript_99404;F01_transcript_9943;F01_transcript_99955;	
Ubiquitin mediated proteolysis	ko04120	597	F01_transcript_100068;F01_transcript_100080;F01_transcript_100263;F01_transcript_10060;F01_transcript_100963;F01_transcript_101117;F01_transcript_101241;F01_transcript_101358;F01_transcript_101365;F01_transcript_101438;F01_transcript_101613;F01_transcript_101970;F01_transcript_102608;F01_transcript_102846;F01_transcript_102943;F01_transcript_103519;F01_transcript_103896;F01_transcript_103939;F01_transcript_104201;F01_transcript_104256;F01_transcript_104351;F01_transcript_104524;F01_transcript_105255;F01_transcript_106690;F01_transcript_106882;F01_transcript_107294;F01_transcript_107536;F01_transcript_10755;F01_transcript_107560;F01_transcript_1076;F01_transcript_107800;F01_transcript_108278;F01_transcript_108290;F01_transcript_108369;F01_transcript_108426;F01_transcript_108612;F01_transcript_108694;F01_transcript_108739;	K03352+K03347+K10590+K10143+K10143+K10143+K10588+K03178+K10580+K03347+K03354+K04554+K03364+K03869+K10143+K10143+K10599+K03347+K03347+K10581+K10590+K10590+K10609+K04506+K03178+K10688+K10604+K03350+K03355+K10588+K10684+K10686+K10588+K03094+K10573+K06689+K10599+K10606+K10576+K03347+K03347+K03094+K10592+K04506+K10590+K10580+K03347+K10579+K10592+K03354+K10575+K10599+K10591+K10575+K10143+K10688+K03351+K10573+K10609+K03094+K10589+K10143+K10684+K03872+K10592+K10143+K10589+K03347+K04506+K10143+K10581+K10581+K06689+K10590+K10588+K10590+K10589+K03347+K10609+K03347+K10140+K03347+K03351+K10589+K03347+K03348+K10590+K10591+K09561+K03347+K10570+K03354+K10144+K03347+K10591+K10143+

	F01_transcript_109153;F01_transcript_109778;F01_transcript_10987;F01_transcript_110104;F01_transcript_110436;F01_transcript_110487;F01_transcript_11075;F01_transcript_110763;F01_transcript_110976;F01_transcript_111122;F01_transcript_111186;F01_transcript_111244;F01_transcript_112197;F01_transcript_112734;F01_transcript_112837;F01_transcript_113211;F01_transcript_113309;F01_transcript_113512;F01_transcript_11391;F01_transcript_114191;F01_transcript_114360;F01_transcript_114461;F01_transcript_114520;F01_transcript_11490;F01_transcript_115214;F01_transcript_115469;F01_transcript_115479;F01_transcript_115800;F01_transcript_115858;F01_transcript_11609;F01_transcript_116602;F01_transcript_116877;F01_transcript_117688;F01_transcript_117769;F01_transcript_117805;F01_transcript_117889;F01_transcript_118302;F01_transcript_118638;F01_transcript_118983;F01_transcript_11958;F01_transcript_119614;F01_transcript_119713;F01_transcript_120598;F01_transcript_12092;F01_transcript_121122;F01_transcript_121753;F01_transcript_121991;F01_transcript_122339;F01_transcript_122398;F01_transcript_122441;F01_transcript_122591;F01_transcript_122829;F01_transcript_122963;F01_transcript_123050;F01_transcript_123095;F01_transcript_12310;F01_transcript_123157;F01_transcript_123395;F01_transcript_123417;F01_transcript_123636;F01_transcript_123904;F01_transcript_124474;F01_transcript_1246;F01_transcript_124919;F01_transcript_125021;F01_transcript_126539;F01_transcript_126595;F01_transcript_126734;F01_transcript_126842;F01_transcript_127363;F01_transcript_127980;F01_transcript_128459;F01_transcript_128591;F01_transcript_128685;F01_transcript_128742;F01_transcript_129187;F01_transcript_129271;F01_transcript_129924;F01_transcript_130019;F01_transcript_130347;F01_transcript_130376;F01_transcript_130991;F01_transcript_131126;F01_transcript_131474;F01_transcript_131607;F01_transcript_131747;F01_transcript_132301;F01_transcript_1324;F01_transcript_132586;F01_transcript_133300;F01_transcript_133407;F01_transcript_133892;F01_transcript_133911;F01_transcript_134;F01_transcript_134124;F01_transcript_134173;F01_transcript_134905;F01_transcript_135133;F01_transcript_135488;F01_transcript_135552;F01_transcript_135554;F01_transcript_135742;F01_transcript_135842;F01_trans	K10260+K10581+K10571+K03347+K10589+K10581+K03363+K10589+K03094+K10144+K10685+K10588+K10575+K03358+K03355+K10581+K04506+K10579+K03353+K04506+K04506+K10592+K10685+K04554+K03178+K10685+K10570+K03872+K10143+K10144+K03352+K03178+K10686+K10143+K10590+K10590+K03353+K04706+K03868+K10592+K03347+K10590+K06689+K10583+K10685+K10688+K10143+K10590+K03363+K10592+K10144+K03094+K03868+K03347+K03351+K04506+K03178+K04554+K03347+K03349+K03350+K10685+K10598+K04649+K10685+K12456+K10573+K03347+K03354+K10597+K10144+K10144+K03347+K10576+K03347+K03347+K10143+K10685+K10685+K03347+K03869+K10685+K10580+K03352+K04706+K10610+K10575+K10576+K10685+K10598+K10581+K03363+K10686+K10686+K10577+K10581+K10576+K03094+K04506+K10573+K03869+K10573+K10583+K09561+K10573+K10575+K03347+K10144+K10575+K03347+K04706+K10688+K10579+K10573+K10609+K10576+K10684+K10581+K10685+K10575+K10143+K10579+K03178+K03353+K04506+K03352+K10590+K10606+K10590+K10143+K10688+K10573+K04506+K10143+K10590+K10592+K03363+K10606+K03354+K10592+K03178+K04506+K10144+K10597+K03349+K10143+K10589+K04649+K03354+K03364+K03178+K04554+K10599+K10144+K10588+K10590+K03354+K03178+K10590+K03347+K03178+K04506+K10604+K03094+K10590+K03347+K10575+K03875+K03352+K10579+K10601+K10686+K10590+K10599+K10688+K10581+K03351+K03347+K10589+K10684+K10609+K03178+K10590+K10685+K10686+K10590+K10686+K03178+K10597+K10144+K03178+K10581+K10599+K10590+K10610+K03364+K03869+K10601+K03178+K10598+K10140+K03355+K10143+K10598+K10140+K10599+K10599+K10581+K10570+K10601+K10571+K10599+K10581+K03364+K10581+K10599+K03353+K10590+K10571+K03875+K10592+K10684+K10573+K10144+K10577+K10686+K10686+K06689+K10604+K10686+K10144+K09561+K04706+K10685+K09561+K10597+K06689+K03178+K04506+K04506+K03875+K04506+K10144+K03875+K10144+K04506+K03354+K10144+K04506+K10144+K03347+K03354+K10144+K04506+K10684+K09561+K04554+K10144+K09561+K10144+K105

	cript_135850;F01_transcript_135960;F01_transcript_136;F01_transcript_136315;F01_transcript_136435;F01_transcript_136653;F01_transcript_136778;F01_transcript_137138;F01_transcript_137156;F01_transcript_137342;F01_transcript_137667;F01_transcript_138062;F01_transcript_138723;F01_transcript_139007;F01_transcript_139265;F01_transcript_139877;F01_transcript_14015;F01_transcript_140169;F01_transcript_140263;F01_transcript_140374;F01_transcript_140417;F01_transcript_140836;F01_transcript_140864;F01_transcript_141002;F01_transcript_141158;F01_transcript_141184;F01_transcript_141192;F01_transcript_141297;F01_transcript_141448;F01_transcript_141623;F01_transcript_142009;F01_transcript_142632;F01_transcript_14266;F01_transcript_142852;F01_transcript_143591;F01_transcript_14390;F01_transcript_143989;F01_transcript_144274;F01_transcript_144507;F01_transcript_144605;F01_transcript_144788;F01_transcript_144960;F01_transcript_145613;F01_transcript_14624;F01_transcript_146477;F01_transcript_14713;F01_transcript_147242;F01_transcript_147626;F01_transcript_147747;F01_transcript_148375;F01_transcript_14900;F01_transcript_149106;F01_transcript_149187;F01_transcript_149420;F01_transcript_149562;F01_transcript_149736;F01_transcript_149853;F01_transcript_149923;F01_transcript_149986;F01_transcript_150245;F01_transcript_15069;F01_transcript_150801;F01_transcript_150846;F01_transcript_150893;F01_transcript_151366;F01_transcript_151506;F01_transcript_151697;F01_transcript_152614;F01_transcript_152709;F01_transcript_152998;F01_transcript_153300;F01_transcript_153391;F01_transcript_15409;F01_transcript_154230;F01_transcript_154363;F01_transcript_154831;F01_transcript_154836;F01_transcript_155078;F01_transcript_155247;F01_transcript_155384;F01_transcript_155680;F01_transcript_155907;F01_transcript_156156;F01_transcript_156204;F01_transcript_156759;F01_transcript_157484;F01_transcript_157717;F01_transcript_157750;F01_transcript_15803;F01_transcript_158554;F01_transcript_158590;F01_transcript_158959;F01_transcript_159153;F01_transcript_159397;F01_transcript_159502;F01_transcript_1600;F01_transcript_160257;F01_transcript_160323;F01_transcript_160730;F01_transcript_160923;F01_transcript_16097;F0	73+K10573+K10579+K04649+K10579+K04649+K10577+K10573+K10573+K10576+K10575+K06689+K10576+K10580+K10688+K10575+K10573+K03357+K10573+K10581+K10573+K10688+K06689+K10577+K03094+K10575+K03352+K10573+K03094+K20217+K03094+K03094+K10573+K03868+K10688+K03868+K03094+K10577+K10580+K10591+K03094+K06689+K10573+K12456+K03352+K10581+K10592+K10686+K10144+K03348+K03358+K03351+K04506+K10685+K03869+K03869+K10581+K10685+K09561+K10588+K10581+K10599+K03349+K10599+K10576+K04706+K03351+K10686+K03357+K10143+K10591+K10581+K10143+K03349+K10578+K10589+K10575+K03178+K10581+K10144+K03347+K10260+K03869+K06688+K03178+K03354+K10598+K03094+K10143+K10609+K09561+K03347+K10579+K10592+K03178+K10576+K03094+K03349+K10688+K10581+K10686+K10591+K03364+K04506+K10143+K10143+K03354+K03178+K03347+K10144+K10592+K03347+K10575+K10592+K03347+K03363+K04706+K10581+K10591+K03347+K10591+K03869+K03363+K03355+K10576+K03347+K10581+K10573+K10571+K03178+K03178+K10143+K03354+K10581+K10589+K10588+K03347+K10579+K03178+K10589+K10143+K10592+K10685+K03355+K03868+K03347+K10592+K10143+K03354+K10143+K03354+K10609+K03094+K10589+K03178+K10609+K10589+K10592+K04706+K03347+K10581+K10688+K10573+K10686+K03347+K10686+K10686+K04506+K10579+K10143+K03178+K10686+K10598+K10144+K09561+K03347+K10589+K10573+K10579+K10581+K10590+K10143+K10581+K10609+K10581+K03349+K10686+K10581+K10609+K03350+K10580+K10140+K10685+K10590+K03353+K10589+K10609+K10599+K10581+K10592+K10573+K10575+K03875+K10573+K03869+K10575+K03357+K10599+K03349+K10575+K03355+K10581+K03868+K10573+K03347+K10591+K10581+K03347+K10577+K10592+K03347+K03872+K04506+K10688+K03872+K03094+K03178+K10143+K03349+K10686+K10590+K10581+K10581+K10688+K10143+K10688+K10685+K10590+K10685+K10578+K03347

1_transcript_161327;F01_transcript_161471;F01_transcript_161
495;F01_transcript_161551;F01_transcript_161593;F01_transcri
pt_161781;F01_transcript_161973;F01_transcript_161996;F01_t
ranscript_162108;F01_transcript_162291;F01_transcript_16280
7;F01_transcript_1629;F01_transcript_163033;F01_transcript_1
63095;F01_transcript_163198;F01_transcript_163751;F01_trans
cript_163855;F01_transcript_163970;F01_transcript_164072;F0
1_transcript_16424;F01_transcript_164344;F01_transcript_1645
89;F01_transcript_164828;F01_transcript_164859;F01_transcrip
t_164915;F01_transcript_165021;F01_transcript_165391;F01_tr
anscript_165938;F01_transcript_166043;F01_transcript_166074
;F01_transcript_166346;F01_transcript_166493;F01_transcript_
1666;F01_transcript_166837;F01_transcript_166895;F01_transc
ript_167510;F01_transcript_167758;F01_transcript_16969;F01_
transcript_18664;F01_transcript_19;F01_transcript_19042;F01_t
ranscript_1937;F01_transcript_1988;F01_transcript_199;F01_tra
nscript_1992;F01_transcript_2013;F01_transcript_20324;F01_tr
anscript_2085;F01_transcript_2135;F01_transcript_21637;F01_t
ranscript_21838;F01_transcript_22097;F01_transcript_2219;F01
_transcript_23016;F01_transcript_23244;F01_transcript_23939;
F01_transcript_24220;F01_transcript_24281;F01_transcript_251
26;F01_transcript_26055;F01_transcript_27566;F01_transcript_
27772;F01_transcript_28186;F01_transcript_28344;F01_transcri
pt_28360;F01_transcript_28895;F01_transcript_29073;F01_tran
script_29387;F01_transcript_29429;F01_transcript_29434;F01_t
ranscript_29510;F01_transcript_30317;F01_transcript_30373;F0
1_transcript_30645;F01_transcript_3144;F01_transcript_32000;
F01_transcript_32645;F01_transcript_3290;F01_transcript_338;
F01_transcript_35270;F01_transcript_35343;F01_transcript_353
90;F01_transcript_35476;F01_transcript_36161;F01_transcript_
36242;F01_transcript_36265;F01_transcript_3671;F01_transcrip
t_37436;F01_transcript_37798;F01_transcript_3823;F01_transcr
ipt_38334;F01_transcript_38423;F01_transcript_38554;F01_tra
nscript_38631;F01_transcript_39538;F01_transcript_39584;F01
_transcript_39998;F01_transcript_40021;F01_transcript_40027;
F01_transcript_40934;F01_transcript_40936;F01_transcript_410
55;F01_transcript_41123;F01_transcript_4114;F01_transcript_4
123;F01_transcript_41239;F01_transcript_41311;F01_transcript

_41333;F01_transcript_41614;F01_transcript_42159;F01_transc
ript_42483;F01_transcript_42520;F01_transcript_43137;F01_tra
nscript_43549;F01_transcript_44552;F01_transcript_45153;F01
_transcript_45347;F01_transcript_45365;F01_transcript_45602;
F01_transcript_45609;F01_transcript_45643;F01_transcript_457
75;F01_transcript_45931;F01_transcript_45982;F01_transcript_
46051;F01_transcript_46076;F01_transcript_46158;F01_transcri
pt_46184;F01_transcript_46324;F01_transcript_46547;F01_tran
script_46587;F01_transcript_46694;F01_transcript_46715;F01_t
ranscript_4679;F01_transcript_46797;F01_transcript_46817;F01
_transcript_46917;F01_transcript_47038;F01_transcript_47086;
F01_transcript_47130;F01_transcript_4723;F01_transcript_4724
3;F01_transcript_47409;F01_transcript_47423;F01_transcript_4
7429;F01_transcript_47434;F01_transcript_47503;F01_transcrip
t_47513;F01_transcript_47617;F01_transcript_47908;F01_trans
cript_48118;F01_transcript_48300;F01_transcript_48526;F01_tr
anscript_4920;F01_transcript_49258;F01_transcript_49267;F01
_transcript_49674;F01_transcript_50039;F01_transcript_5021;F
01_transcript_504;F01_transcript_51800;F01_transcript_51911;
F01_transcript_51978;F01_transcript_52005;F01_transcript_521
14;F01_transcript_52129;F01_transcript_52641;F01_transcript_
52799;F01_transcript_53236;F01_transcript_53324;F01_transcri
pt_53621;F01_transcript_53623;F01_transcript_54177;F01_tran
script_55174;F01_transcript_55356;F01_transcript_55813;F01_t
ranscript_56013;F01_transcript_56026;F01_transcript_56073;F0
1_transcript_5623;F01_transcript_56337;F01_transcript_56630;
F01_transcript_56808;F01_transcript_57075;F01_transcript_571
1;F01_transcript_57289;F01_transcript_5735;F01_transcript_57
413;F01_transcript_58509;F01_transcript_59362;F01_transcript
_59395;F01_transcript_59457;F01_transcript_59654;F01_transc
ript_59724;F01_transcript_60757;F01_transcript_61013;F01_tra
nscript_61565;F01_transcript_61704;F01_transcript_61891;F01
_transcript_62059;F01_transcript_62454;F01_transcript_62475;
F01_transcript_62647;F01_transcript_62689;F01_transcript_627
97;F01_transcript_64090;F01_transcript_64167;F01_transcript_
64411;F01_transcript_64426;F01_transcript_64573;F01_transcri
pt_64899;F01_transcript_64946;F01_transcript_64953;F01_tran

script_64966;F01_transcript_6498;F01_transcript_65099;F01_transcript_65386;F01_transcript_65581;F01_transcript_66300;F01_transcript_66626;F01_transcript_67070;F01_transcript_67167;F01_transcript_67545;F01_transcript_67677;F01_transcript_67692;F01_transcript_67742;F01_transcript_67824;F01_transcript_67846;F01_transcript_6797;F01_transcript_68108;F01_transcript_6832;F01_transcript_69349;F01_transcript_69435;F01_transcript_69550;F01_transcript_70044;F01_transcript_70077;F01_transcript_70080;F01_transcript_70162;F01_transcript_70243;F01_transcript_70589;F01_transcript_70652;F01_transcript_7075;F01_transcript_71493;F01_transcript_71631;F01_transcript_71678;F01_transcript_72115;F01_transcript_72313;F01_transcript_72402;F01_transcript_72879;F01_transcript_72943;F01_transcript_7375;F01_transcript_74110;F01_transcript_74215;F01_transcript_74523;F01_transcript_74682;F01_transcript_7480;F01_transcript_75785;F01_transcript_75961;F01_transcript_75965;F01_transcript_7675;F01_transcript_77170;F01_transcript_77352;F01_transcript_77397;F01_transcript_7760;F01_transcript_77717;F01_transcript_77979;F01_transcript_78200;F01_transcript_78299;F01_transcript_78716;F01_transcript_7878;F01_transcript_79030;F01_transcript_79292;F01_transcript_79400;F01_transcript_79721;F01_transcript_79798;F01_transcript_80525;F01_transcript_80742;F01_transcript_81366;F01_transcript_8185;F01_transcript_8194;F01_transcript_82283;F01_transcript_82458;F01_transcript_82934;F01_transcript_83335;F01_transcript_836;F01_transcript_84022;F01_transcript_84145;F01_transcript_8459;F01_transcript_84668;F01_transcript_84996;F01_transcript_85224;F01_transcript_85241;F01_transcript_85359;F01_transcript_85592;F01_transcript_85869;F01_transcript_85941;F01_transcript_86072;F01_transcript_8628;F01_transcript_86747;F01_transcript_86833;F01_transcript_87049;F01_transcript_87225;F01_transcript_8754;F01_transcript_8787;F01_transcript_88012;F01_transcript_88096;F01_transcript_88367;F01_transcript_88436;F01_transcript_88770;F01_transcript_89181;F01_transcript_89537;F01_transcript_89687;F01_transcript_89700;F01_transcript_89965;F01_transcript_90295;F01_transcript_90359;F01_transcript_90428;F01_transcript_90571;F01_transcript_90769;F01_transcript_90794;F01_transcript_91230;F01_transcript_92343;F01_tra

			nscript_92917;F01_transcript_93046;F01_transcript_931;F01_transcript_93190;F01_transcript_93472;F01_transcript_94155;F01_transcript_9449;F01_transcript_9502;F01_transcript_95210;F01_transcript_9535;F01_transcript_95546;F01_transcript_95854;F01_transcript_96259;F01_transcript_96351;F01_transcript_96591;F01_transcript_96678;F01_transcript_96887;F01_transcript_97472;F01_transcript_97547;F01_transcript_97800;F01_transcript_98070;F01_transcript_98164;F01_transcript_98332;F01_transcript_98612;F01_transcript_98677;F01_transcript_98807;F01_transcript_98891;F01_transcript_99451;F01_transcript_99516;F01_transcript_99599;F01_transcript_99683;F01_transcript_99784;F01_transcript_9984;	
Sulfur relay system	ko04122	33	F01_transcript_103399;F01_transcript_106537;F01_transcript_116490;F01_transcript_119664;F01_transcript_126632;F01_transcript_136499;F01_transcript_136742;F01_transcript_142028;F01_transcript_143129;F01_transcript_146381;F01_transcript_147659;F01_transcript_149203;F01_transcript_150656;F01_transcript_158486;F01_transcript_159015;F01_transcript_160168;F01_transcript_160464;F01_transcript_161597;F01_transcript_161887;F01_transcript_31337;F01_transcript_33523;F01_transcript_34493;F01_transcript_36816;F01_transcript_37301;F01_transcript_37509;F01_transcript_56124;F01_transcript_59325;F01_transcript_59506;F01_transcript_64229;F01_transcript_72216;F01_transcript_73043;F01_transcript_75195;F01_transcript_94934;	K01011+K21027+K21027+K14169+K14169+K21027+K14168+K01011+K21232+K11996+K14168+K11996+K03637+K21027+K01011+K11996+K14169+K14169+K14168+K14169+K21027+K01011+K11996+K14168+K11996+K21027+K14168+K21232+K21027+K11996+K21027+K11996+K14168
SNARE interactions in vesicular transport	ko04130	99	F01_transcript_103775;F01_transcript_10458;F01_transcript_107846;F01_transcript_107956;F01_transcript_111261;F01_transcript_111452;F01_transcript_112750;F01_transcript_113091;F01_transcript_114163;F01_transcript_114432;F01_transcript_115971;F01_transcript_116731;F01_transcript_118614;F01_transcript_119609;F01_transcript_121106;F01_transcript_121725;F01_transcript_123223;F01_transcript_123258;F01_transcript_123906;F01_transcript_12422;F01_transcript_124225;F01_transcript_126339;F01_transcript_126772;F01_transcript_127470;F01_transcript_128682;F01_transcript_133500;F01_transcript_134291;F01_transcript_134503;F01_transcript_134515;F01_transcript_135437;F01_transcript_136240;F01_transcript_138769;F01_transcript_139151;F01_transcript_141601;F01_transcript_144133;F01_t	K08506+K08505+K08505+K08489+K08516+K08496+K08493+K08506+K08516+K08486+K08498+K08498+K08515+K08507+K08495+K08498+K08493+K08498+K08507+K08495+K08498+K08516+K08506+K08489+K08495+K08493+K08495+K08505+K08503+K08507+K08486+K08495+K08493+K08506+K08504+K08490+K08497+K08493+K08489+K08486+K08490+K08515+K08495+K08490+K08489+K08490+K08517+K08490+K08493+K08495+K08497+K08492+K08495+K08517+K08490+K08490+K08490+K08497+K08486+K08497+K08506+K08492+K08517+K08515+K08506+K08493+K08503+K08498+K08503+K08516+K08498+K08493+K08495+K08516+K08505+K08504+K08489+K08506+K08495+K08507+K08486+K08492+K08493+K08496+K08495+K08505+K08495+K08489+K08495+K

			ranscript_145194;F01_transcript_147979;F01_transcript_150526;F01_transcript_151882;F01_transcript_15191;F01_transcript_152191;F01_transcript_152501;F01_transcript_153756;F01_transcript_153788;F01_transcript_155628;F01_transcript_158814;F01_transcript_159854;F01_transcript_160949;F01_transcript_161156;F01_transcript_161324;F01_transcript_162639;F01_transcript_164142;F01_transcript_164285;F01_transcript_25227;F01_transcript_33007;F01_transcript_33399;F01_transcript_33461;F01_transcript_39399;F01_transcript_39580;F01_transcript_40492;F01_transcript_41374;F01_transcript_41427;F01_transcript_41487;F01_transcript_42469;F01_transcript_43203;F01_transcript_44001;F01_transcript_44009;F01_transcript_44228;F01_transcript_44635;F01_transcript_44739;F01_transcript_45071;F01_transcript_45661;F01_transcript_46151;F01_transcript_46291;F01_transcript_46736;F01_transcript_48987;F01_transcript_50303;F01_transcript_53855;F01_transcript_5427;F01_transcript_56464;F01_transcript_57846;F01_transcript_58106;F01_transcript_60500;F01_transcript_61736;F01_transcript_66926;F01_transcript_71826;F01_transcript_74044;F01_transcript_75885;F01_transcript_78792;F01_transcript_79621;F01_transcript_81267;F01_transcript_81873;F01_transcript_82417;F01_transcript_86314;F01_transcript_89948;F01_transcript_91682;F01_transcript_92694;F01_transcript_95794;F01_transcript_97425;	08507+K08490+K08517+K08497+K08517+K08505+K08517+K08515+K08486+K08498
Regulation of autophagy	ko04140	208	F01_transcript_100497;F01_transcript_101818;F01_transcript_102393;F01_transcript_102412;F01_transcript_103404;F01_transcript_104321;F01_transcript_104353;F01_transcript_10659;F01_transcript_106788;F01_transcript_106841;F01_transcript_10798;F01_transcript_110116;F01_transcript_110562;F01_transcript_110751;F01_transcript_112860;F01_transcript_11296;F01_transcript_113462;F01_transcript_113892;F01_transcript_114139;F01_transcript_114346;F01_transcript_116138;F01_transcript_117270;F01_transcript_117272;F01_transcript_117794;F01_transcript_120156;F01_transcript_121786;F01_transcript_122997;F01_transcript_123331;F01_transcript_123937;F01_transcript_125444;F01_transcript_125516;F01_transcript_125666;F01_transcript_126145;F01_transcript_126787;F01_transcript_12706;F01_transcript_127233;F01_transcript_129146;F01_transcript_12991;F01_transcript_130021;F01_transcript_13030;F01_transcript_132	K08331+K07198+K07198+K08337+K08342+K08337+K00914+K08269+K08269+K08341+K00914+K08341+K07198+K00914+K08342+K08331+K07198+K07198+K08332+K08342+K08342+K00914+K07198+K00914+K08343+K00914+K08341+K07198+K07198+K08342+K00914+K08333+K08337+K08341+K08269+K00914+K00914+K08341+K07198+K08337+K07198+K08337+K08336+K08269+K00914+K08337+K07198+K08341+K08331+K08269+K08334+K00914+K08333+K08342+K07198+K07198+K08341+K07198+K08342+K07198+K07198+K07198+K00914+K08269+K07198+K00914+K00914+K08341+K07198+K08337+K08337+K00914+K08337+K07198+K07198+K08269+K07198+K07198+K08341+K00914+K08331+K07198+K07198+K07198+K07198+K08341+K08337+K08341+K07198+K07198+K07198+K08269+K00914+K08337+K08341+K08333+K08337+K08331+K08343+K07198+K08342+K08341+K08331

	290;F01_transcript_132935;F01_transcript_133018;F01_transcript_13319;F01_transcript_133384;F01_transcript_13388;F01_transcript_135619;F01_transcript_136207;F01_transcript_136878;F01_transcript_136932;F01_transcript_138822;F01_transcript_138842;F01_transcript_140218;F01_transcript_142296;F01_transcript_143087;F01_transcript_143434;F01_transcript_144761;F01_transcript_145257;F01_transcript_145558;F01_transcript_146281;F01_transcript_146333;F01_transcript_146344;F01_transcript_146478;F01_transcript_14686;F01_transcript_148693;F01_transcript_149901;F01_transcript_150423;F01_transcript_150686;F01_transcript_151555;F01_transcript_152360;F01_transcript_152582;F01_transcript_153720;F01_transcript_15392;F01_transcript_154121;F01_transcript_154955;F01_transcript_155394;F01_transcript_155529;F01_transcript_157519;F01_transcript_157607;F01_transcript_158433;F01_transcript_158545;F01_transcript_159322;F01_transcript_159888;F01_transcript_161563;F01_transcript_162288;F01_transcript_162767;F01_transcript_163042;F01_transcript_163125;F01_transcript_164137;F01_transcript_165349;F01_transcript_166592;F01_transcript_166616;F01_transcript_166806;F01_transcript_167152;F01_transcript_17342;F01_transcript_174;F01_transcript_18371;F01_transcript_19149;F01_transcript_19308;F01_transcript_21154;F01_transcript_21201;F01_transcript_21292;F01_transcript_2222;F01_transcript_22743;F01_transcript_22776;F01_transcript_25773;F01_transcript_2616;F01_transcript_26584;F01_transcript_2691;F01_transcript_26946;F01_transcript_27793;F01_transcript_27891;F01_transcript_28626;F01_transcript_28631;F01_transcript_28740;F01_transcript_29938;F01_transcript_30217;F01_transcript_30423;F01_transcript_3054;F01_transcript_30691;F01_transcript_30847;F01_transcript_3085;F01_transcript_31224;F01_transcript_31366;F01_transcript_32477;F01_transcript_38634;F01_transcript_39834;F01_transcript_41923;F01_transcript_47588;F01_transcript_47720;F01_transcript_48404;F01_transcript_48501;F01_transcript_48714;F01_transcript_49095;F01_transcript_49099;F01_transcript_49355;F01_transcript_49725;F01_transcript_51975;F01_transcript_52765;F01_transcript_53033;F01_transcript_54540;F01_transcript_55435;F01_transcript_55465;F01_transcript_55	+K07198+K08334+K08342+K08331+K08339+K08332+K17890+K08342+K07198+K07198+K07198+K07198+K07198+K08334+K08334+K08331+K07198+K07198+K08341+K07198+K07198+K07198+K08341+K08343+K08339+K08341+K08341+K08341+K08341+K08341+K08341+K08341+K08341+K08336+K17888+K17888+K08341+K17888+K08337+K07198+K08336+K08269+K08341+K08341+K08341+K08341+K17888+K17888+K00914+K08342+K08341+K00914+K08331+K00914+K08341+K17888+K08333+K08342+K07198+K08334+K08337+K00914+K08339+K08339+K08343+K07198+K17890+K08331+K00914+K00914+K07198+K07198+K08337+K00914+K17890+K08341+K08269+K07198+K07198+K08341+K08341+K08337+K08331+K07198+K08343+K08342+K08334+K00914+K00914+K17888+K17888+K08343+K08337+K08341+K08342+K08337+K08343+K08343+K08337+K07198+K08269+K08342+K07198+K08337+K07198

			603;F01_transcript_55927;F01_transcript_59536;F01_transcript_60144;F01_transcript_61168;F01_transcript_61329;F01_transcript_61821;F01_transcript_62122;F01_transcript_63398;F01_transcript_63826;F01_transcript_64707;F01_transcript_65003;F01_transcript_65573;F01_transcript_6591;F01_transcript_66107;F01_transcript_66960;F01_transcript_68211;F01_transcript_71046;F01_transcript_72358;F01_transcript_72438;F01_transcript_72489;F01_transcript_72837;F01_transcript_73673;F01_transcript_73856;F01_transcript_73959;F01_transcript_74070;F01_transcript_74199;F01_transcript_75270;F01_transcript_75395;F01_transcript_77133;F01_transcript_77577;F01_transcript_77914;F01_transcript_78961;F01_transcript_79219;F01_transcript_80101;F01_transcript_80780;F01_transcript_81626;F01_transcript_81871;F01_transcript_81900;F01_transcript_82329;F01_transcript_84279;F01_transcript_84604;F01_transcript_85232;F01_transcript_87669;F01_transcript_88305;F01_transcript_88517;F01_transcript_88742;F01_transcript_8890;F01_transcript_88943;F01_transcript_89003;F01_transcript_91685;F01_transcript_91805;F01_transcript_93506;F01_transcript_94630;F01_transcript_94985;F01_transcript_95501;F01_transcript_96266;F01_transcript_96503;F01_transcript_96525;F01_transcript_96744;F01_transcript_96827;F01_transcript_97176;F01_transcript_97334;F01_transcript_97458;F01_transcript_99676;	
Protein processing in endoplasmic reticulum	ko04141	1023	F01_transcript_100080;F01_transcript_100604;F01_transcript_100637;F01_transcript_100639;F01_transcript_100664;F01_transcript_100672;F01_transcript_100793;F01_transcript_100894;F01_transcript_100958;F01_transcript_1010;F01_transcript_10100;F01_transcript_101098;F01_transcript_101112;F01_transcript_101238;F01_transcript_101253;F01_transcript_101330;F01_transcript_101438;F01_transcript_101553;F01_transcript_101917;F01_transcript_101970;F01_transcript_102362;F01_transcript_102577;F01_transcript_102654;F01_transcript_102660;F01_transcript_102764;F01_transcript_103656;F01_transcript_10371;F01_transcript_103792;F01_transcript_103939;F01_transcript_104056;F01_transcript_104201;F01_transcript_104448;F01_transcript_104480;F01_transcript_10457;F01_transcript_104865;F01_transcript_104996;F01_transcript_105033;F01_transcript_105242;F01_transcript_105631;F01_transcript_105638;F01_transcript_10	K03347+K09481+K14018+K03283+K14001+K09562+K14007+K16196+K04523+K14007+K13525+K10636+K09490+K03283+K10084+K09580+K03347+K07765+K09490+K04554+K13525+K14009+K08852+K04523+K10084+K05546+K13525+K14006+K03347+K10085+K03347+K10666+K10956+K13525+K14012+K08057+K13525+K14004+K10084+K03283+K12669+K14018+K13249+K14012+K09562+K09503+K08057+K14018+K13249+K13249+K13719+K13984+K12666+K03283+K10085+K07765+K10839+K08653+K14018+K03094+K06689+K09487+K04079+K13993+K13993+K09487+K03347+K14005+K03347+K09580+K09580+K03094+K14016+K07765+K14005+K14005+K14001+K14009+K01230+K09503+K14005+K10839+K10956+K08288+K09503+K12667+K03347+K13525+K10950+K08054+K07953+K09562+K09487+K10085+K04523+K10575+K13719+K10666+K04079+K14001+K12667+K09503+K10575

	5656;F01_transcript_10571;F01_transcript_105794;F01_transcript_105801;F01_transcript_105841;F01_transcript_105843;F01_transcript_106040;F01_transcript_106403;F01_transcript_106500;F01_transcript_106539;F01_transcript_106837;F01_transcript_106940;F01_transcript_10728;F01_transcript_107311;F01_transcript_107642;F01_transcript_107965;F01_transcript_107994;F01_transcript_108008;F01_transcript_10809;F01_transcript_108369;F01_transcript_108612;F01_transcript_108840;F01_transcript_109181;F01_transcript_109315;F01_transcript_109444;F01_transcript_10970;F01_transcript_109778;F01_transcript_109828;F01_transcript_10987;F01_transcript_109952;F01_transcript_110035;F01_transcript_110104;F01_transcript_110274;F01_transcript_110306;F01_transcript_110362;F01_transcript_1104;F01_transcript_110452;F01_transcript_110459;F01_transcript_110472;F01_transcript_110523;F01_transcript_1106;F01_transcript_110692;F01_transcript_11072;F01_transcript_110842;F01_transcript_110890;F01_transcript_110957;F01_transcript_110976;F01_transcript_11128;F01_transcript_111360;F01_transcript_111397;F01_transcript_111623;F01_transcript_111706;F01_transcript_111794;F01_transcript_11184;F01_transcript_112185;F01_transcript_112197;F01_transcript_112531;F01_transcript_112596;F01_transcript_11281;F01_transcript_112810;F01_transcript_112978;F01_transcript_112998;F01_transcript_113211;F01_transcript_113216;F01_transcript_113230;F01_transcript_113339;F01_transcript_113642;F01_transcript_113644;F01_transcript_113683;F01_transcript_114113;F01_transcript_114461;F01_transcript_114661;F01_transcript_114682;F01_transcript_114750;F01_transcript_114784;F01_transcript_114799;F01_transcript_11484;F01_transcript_115019;F01_transcript_115055;F01_transcript_115056;F01_transcript_115114;F01_transcript_115212;F01_transcript_11530;F01_transcript_115400;F01_transcript_115457;F01_transcript_11609;F01_transcript_116192;F01_transcript_116228;F01_transcript_116308;F01_transcript_116539;F01_transcript_116659;F01_transcript_116692;F01_transcript_116705;F01_transcript_116810;F01_transcript_116829;F01_transcript_117305;F01_transcript_1174;F01_transcript_117645;F01_transcript_117659;F01_transcript_117805;F01_transcript_117984;F01_transcript_	+K13993+K10950+K10636+K08057+K10085+K10084+K04523+K03094+K09487+K01230+K08288+K04079+K11718+K09487+K10088+K10956+K11863+K08852+K09540+K13993+K08653+K16196+K03347+K09523+K09487+K10088+K14006+K12666+K09580+K09562+K03283+K10661+K10956+K08653+K01228+K09487+K06689+K08852+K08852+K16196+K04079+K03283+K03347+K09481+K03347+K14004+K14007+K14007+K10956+K13993+K03347+K09487+K09487+K13993+K09490+K03347+K10661+K14001+K13984+K09561+K03237+K14012+K03347+K14003+K14007+K14007+K03347+K09540+K10661+K14012+K13525+K12669+K10839+K10085+K14006+K03347+K10956+K09580+K03283+K08852+K08054+K14003+K14001+K09584+K04079+K16196+K14007+K07151+K03094+K09487+K14012+K04079+K10575+K03283+K10956+K01456+K01228+K13525+K04079+K01456+K10088+K08057+K12275+K03283+K10839+K04079+K11718+K01456+K12669+K10084+K14007+K07151+K13719+K01228+K14012+K08653+K14005+K04554+K13989+K09487+K09487+K09584+K10636+K10666+K14009+K04523+K03283+K07765+K10085+K03283+K01230+K03283+K03283+K09584+K09540+K07151+K07151+K12670+K08852+K07151+K03283+K13525+K12667+K08653+K03868+K09487+K13993+K03347+K06689+K09503+K08653+K14003+K03094+K10956+K14006+K03868+K03347+K14005+K07953+K08852+K14026+K01228+K09517+K08852+K04554+K01228+K09580+K01230+K08653+K03347+K10084+K14001+K13993+K13525+K10666+K01456+K13993+K14016+K10084+K01230+K04523+K10950+K13249+K09487+K14007+K13993+K03347+K13249+K10597+K10084+K03347+K03347+K09503+K14003+K09487+K14001+K03283+K03347+K09580+K09487+K14007+K09580+K14004+K04079+K10666+K14018+K10839+K03347+K14018+K14018+K10956+K14007+K04523+K14006+K09540+K10956+K07342+K09562+K09584+K09584+K10575+K09486+K09503+K10084+K13525+K14026+K13993+K05546+K01230+K14018+K10084+K09584+K13250+K14005+K10084+K10085+K09481+K10956+K13250+K10636+K13719+K13525+K10956+K14001+K01230+K07953+K04079+K09518+K13525+K03094+K07151+K13984+K10666+K095

	118048;F01_transcript_118261;F01_transcript_119256;F01_transcript_119258;F01_transcript_11958;F01_transcript_119673;F01_transcript_119713;F01_transcript_119915;F01_transcript_120194;F01_transcript_120587;F01_transcript_120724;F01_transcript_120827;F01_transcript_12092;F01_transcript_120950;F01_transcript_121015;F01_transcript_121677;F01_transcript_121735;F01_transcript_121991;F01_transcript_122049;F01_transcript_122337;F01_transcript_122444;F01_transcript_122591;F01_transcript_122676;F01_transcript_122698;F01_transcript_122829;F01_transcript_122886;F01_transcript_123003;F01_transcript_123098;F01_transcript_12310;F01_transcript_123127;F01_transcript_123537;F01_transcript_123593;F01_transcript_123672;F01_transcript_123879;F01_transcript_124055;F01_transcript_124266;F01_transcript_124291;F01_transcript_124474;F01_transcript_12453;F01_transcript_124836;F01_transcript_125130;F01_transcript_125155;F01_transcript_125441;F01_transcript_125453;F01_transcript_125629;F01_transcript_125908;F01_transcript_126269;F01_transcript_126366;F01_transcript_126489;F01_transcript_12657;F01_transcript_126595;F01_transcript_126814;F01_transcript_12721;F01_transcript_127903;F01_transcript_127980;F01_transcript_128228;F01_transcript_128287;F01_transcript_12831;F01_transcript_128405;F01_transcript_128475;F01_transcript_128714;F01_transcript_12888;F01_transcript_129025;F01_transcript_12914;F01_transcript_129182;F01_transcript_129423;F01_transcript_129431;F01_transcript_12956;F01_transcript_129597;F01_transcript_12960;F01_transcript_129848;F01_transcript_130050;F01_transcript_1302;F01_transcript_13043;F01_transcript_130533;F01_transcript_130653;F01_transcript_130667;F01_transcript_130729;F01_transcript_130746;F01_transcript_130991;F01_transcript_131207;F01_transcript_131225;F01_transcript_131420;F01_transcript_131455;F01_transcript_131698;F01_transcript_131727;F01_transcript_131818;F01_transcript_131831;F01_transcript_131910;F01_transcript_131944;F01_transcript_132294;F01_transcript_132381;F01_transcript_13245;F01_transcript_133251;F01_transcript_133415;F01_transcript_133622;F01_transcript_133887;F01_transcript_134100;F01_transcript_13412;F01_transcript_134175;F01_transcript_134280;F01_transcript_13437;F01_transcript_134461;F01_transcript_134623;F0	61+K13249+K10956+K09584+K13525+K07953+K10956+K12275+K12667+K13984+K10575+K03347+K01456+K10575+K03283+K04079+K13525+K03347+K09487+K07953+K12666+K10666+K10956+K04523+K10839+K09517+K04079+K13989+K08288+K04079+K12666+K14016+K14007+K13993+K14016+K09487+K14012+K12666+K12666+K09540+K09523+K10666+K13993+K10575+K12666+K03283+K13525+K09490+K13989+K10084+K03237+K09580+K04523+K10636+K01230+K14003+K13249+K09487+K14026+K09487+K10956+K14015+K09503+K09481+K03283+K14007+K10839+K08288+K03283+K14003+K14005+K09481+K09518+K14005+K09580+K14006+K09518+K08288+K04523+K10597+K01228+K09487+K14006+K09503+K11863+K14003+K10636+K04079+K13525+K10084+K03283+K08653+K04554+K09487+K14018+K09580+K09503+K07151+K13525+K04079+K09584+K09584+K09487+K01230+K03347+K14004+K03283+K08057+K10084+K13993+K14011+K12666+K10956+K12667+K12667+K03094+K03347+K10575+K05546+K10839+K03283+K01230+K07953+K10666+K09490+K13993+K09580+K10601+K14003+K10956+K09580+K10661+K03283+K14006+K13993+K14005+K07151+K09487+K03347+K14011+K14009+K08057+K04079+K10636+K10956+K09487+K09540+K04079+K14007+K10661+K09490+K04079+K09490+K10666+K09490+K03283+K09540+K03283+K03283+K10636+K03283+K10597+K04523+K03283+K03283+K03283+K01230+K10666+K14026+K13249+K03283+K10661+K09580+K03283+K10601+K04523+K04523+K04523+K04523+K04523+K09580+K01230+K10661+K03283+K09518+K10666+K10636+K09580+K07953+K10084+K10666+K09580+K04523+K01230+K10666+K09580+K14012+K10601+K12666+K13719+K10956+K03283+K10956+K10956+K10956+K10950+K12670+K01228+K14009+K04079+K07765+K09518+K10956+K09518+K14011+K14015+K14001+K12666+K14001+K09518+K09518+K09518+K10956+K09584+K09503+K14003+K09503+K09503+K14015+K13525+K12670+K06689+K14001+K09503+K08057+K12275+K14003+K09561+K09584+K09518+K10839+K13250+K09584+K14015+K14003+K14003+K09517+K10839+K04079+K09562+K09584+K10839+K08057+K09561+K12667+K10597+K09503+K06689+K09517+K10839+K09503+K

	1_transcript_13466;F01_transcript_134766;F01_transcript_134905;F01_transcript_135179;F01_transcript_135229;F01_transcript_135488;F01_transcript_135554;F01_transcript_135769;F01_transcript_135838;F01_transcript_136060;F01_transcript_136778;F01_transcript_136940;F01_transcript_137078;F01_transcript_137138;F01_transcript_137156;F01_transcript_1372;F01_transcript_137784;F01_transcript_13787;F01_transcript_137886;F01_transcript_138009;F01_transcript_138033;F01_transcript_138061;F01_transcript_138723;F01_transcript_138829;F01_transcript_138862;F01_transcript_138863;F01_transcript_138946;F01_transcript_139007;F01_transcript_139061;F01_transcript_139139;F01_transcript_139142;F01_transcript_139194;F01_transcript_139335;F01_transcript_139596;F01_transcript_139610;F01_transcript_139684;F01_transcript_139706;F01_transcript_139729;F01_transcript_139806;F01_transcript_139947;F01_transcript_139953;F01_transcript_140178;F01_transcript_14026;F01_transcript_140478;F01_transcript_140864;F01_transcript_141029;F01_transcript_141158;F01_transcript_141245;F01_transcript_141297;F01_transcript_141623;F01_transcript_141681;F01_transcript_141697;F01_transcript_141708;F01_transcript_141786;F01_transcript_141858;F01_transcript_142009;F01_transcript_142251;F01_transcript_142258;F01_transcript_142314;F01_transcript_142581;F01_transcript_142636;F01_transcript_142803;F01_transcript_142811;F01_transcript_14301;F01_transcript_143098;F01_transcript_143591;F01_transcript_143609;F01_transcript_143799;F01_transcript_143826;F01_transcript_1439;F01_transcript_14391;F01_transcript_144068;F01_transcript_144170;F01_transcript_144220;F01_transcript_144251;F01_transcript_144610;F01_transcript_144661;F01_transcript_144918;F01_transcript_144960;F01_transcript_145028;F01_transcript_145042;F01_transcript_145121;F01_transcript_145202;F01_transcript_145418;F01_transcript_145441;F01_transcript_145647;F01_transcript_145712;F01_transcript_145741;F01_transcript_145863;F01_transcript_145949;F01_transcript_145954;F01_transcript_1461;F01_transcript_146123;F01_transcript_146302;F01_transcript_146376;F01_transcript_146408;F01_transcript_147185;F01_transcript_147291;F01_transcript_147364;F01_transcript_147613;F01_transcrip	12670+K09486+K10839+K04079+K14012+K12666+K03237+K14004+K14012+K13250+K13249+K14016+K14004+K03347+K14016+K08653+K03237+K10666+K07953+K09561+K13989+K12669+K04554+K07953+K13249+K10666+K09561+K10088+K13249+K14005+K13993+K10666+K14009+K07953+K07953+K07953+K13719+K07953+K07953+K13993+K13719+K13250+K13250+K13719+K13719+K13993+K13993+K07953+K13250+K13993+K10575+K07953+K06689+K13993+K10575+K13993+K13993+K13719+K13993+K06689+K03094+K13993+K10575+K13984+K08288+K04079+K03094+K13993+K03094+K03094+K03868+K08288+K09503+K03868+K04079+K03094+K13993+K13993+K07342+K07953+K13525+K12668+K03094+K06689+K12668+K13993+K07342+K13993+K09518+K07151+K10839+K08288+K14006+K13984+K09540+K08054+K09580+K09580+K09487+K10666+K13719+K08852+K13525+K10666+K13984+K14006+K01456+K07765+K03283+K04523+K12275+K13984+K07151+K01230+K09561+K07953+K10661+K01230+K10661+K03283+K14006+K13525+K10085+K13249+K09481+K04523+K14007+K13249+K05546+K14007+K14016+K05546+K03237+K13249+K03237+K03283+K07151+K08653+K07953+K14012+K14007+K04079+K10578+K10661+K14018+K10575+K16196+K09584+K08057+K09580+K13993+K14011+K04079+K10084+K03347+K14006+K09490+K13525+K13249+K14016+K10636+K09503+K10839+K09487+K13525+K03094+K07953+K09487+K09561+K14006+K09503+K10085+K05546+K09580+K03283+K13249+K07151+K01230+K09503+K03347+K13993+K10956+K10084+K09580+K10666+K13993+K12666+K03094+K09580+K14003+K13525+K10636+K10956+K10661+K09481+K14005+K13250+K13989+K14006+K03347+K03347+K10084+K10575+K03347+K14003+K01230+K14005+K04079+K13525+K12667+K10661+K14016+K10636+K14009+K03347+K04523+K14007+K08057+K10956+K13719+K03347+K12667+K10666+K09580+K14006+K09487+K09490+K07342+K12666+K10956+K08653+K04079+K10839+K10661+K04523+K07953+K07765+K08852+K10956+K09487+K09487+K10084+K08057+K07151+K03347+K13250+K14018+K09490+K14003+K09490+K10084+K04523+K01456+K09540

	t_148393;F01_transcript_148499;F01_transcript_148610;F01_transcript_148936;F01_transcript_14898;F01_transcript_149078;F01_transcript_149099;F01_transcript_149187;F01_transcript_149268;F01_transcript_149423;F01_transcript_149548;F01_transcript_149986;F01_transcript_150011;F01_transcript_150161;F01_transcript_150190;F01_transcript_150207;F01_transcript_150351;F01_transcript_150378;F01_transcript_150433;F01_transcript_15048;F01_transcript_150664;F01_transcript_15069;F01_transcript_150801;F01_transcript_150885;F01_transcript_150893;F01_transcript_150982;F01_transcript_15101;F01_transcript_151258;F01_transcript_151366;F01_transcript_151394;F01_transcript_151445;F01_transcript_151449;F01_transcript_151572;F01_transcript_151688;F01_transcript_151994;F01_transcript_152069;F01_transcript_152158;F01_transcript_152213;F01_transcript_152373;F01_transcript_152570;F01_transcript_15264;F01_transcript_152678;F01_transcript_152690;F01_transcript_152746;F01_transcript_152760;F01_transcript_152856;F01_transcript_152874;F01_transcript_153102;F01_transcript_153687;F01_transcript_153971;F01_transcript_15402;F01_transcript_154050;F01_transcript_154210;F01_transcript_154238;F01_transcript_154363;F01_transcript_154406;F01_transcript_154605;F01_transcript_154724;F01_transcript_154925;F01_transcript_155373;F01_transcript_155511;F01_transcript_155635;F01_transcript_155717;F01_transcript_156037;F01_transcript_156074;F01_transcript_156131;F01_transcript_15666;F01_transcript_15693;F01_transcript_15717;F01_transcript_15735;F01_transcript_157487;F01_transcript_157649;F01_transcript_157742;F01_transcript_157818;F01_transcript_158664;F01_transcript_158686;F01_transcript_159041;F01_transcript_159096;F01_transcript_159149;F01_transcript_159283;F01_transcript_159342;F01_transcript_159464;F01_transcript_159474;F01_transcript_159627;F01_transcript_1597;F01_transcript_160063;F01_transcript_16012;F01_transcript_16013;F01_transcript_160472;F01_transcript_160637;F01_transcript_160730;F01_transcript_161037;F01_transcript_161108;F01_transcript_161119;F01_transcript_161162;F01_transcript_161290;F01_transcript_161428;F01_transcript_16146;F01_transcript_161535;F01_transcript_161560;F01_transcript_161598;F01_transcript_161611;F01_transcript_161738;F01_transcript_16178	+K08288+K08057+K03868+K09503+K03283+K10084+K08852+K07953+K09518+K04079+K03347+K14006+K14009+K14018+K14007+K10084+K14005+K03094+K07765+K16196+K08057+K16196+K08288+K07151+K14015+K09487+K14003+K01228+K01228+K09584+K03347+K14018+K10084+K14018+K09487+K16196+K16196+K08057+K09503+K14007+K03347+K13525+K03283+K03283+K14005+K12668+K12667+K10084+K05546+K09580+K04079+K03283+K13993+K14007+K08057+K04079+K09487+K09540+K03283+K14004+K10839+K09561+K14007+K09503+K03347+K01228+K10839+K09580+K11718+K10666+K09540+K09481+K01230+K14018+K09523+K10956+K09487+K13249+K07342+K16196+K12667+K14016+K13984+K10661+K04079+K14005+K12666+K09487+K10636+K10575+K10636+K13989+K08653+K09503+K14001+K10575+K09518+K14016+K03283+K14006+K09487+K05546+K09487+K09540+K09580+K04523+K14006+K13993+K13525+K14007+K14007+K10575+K13249+K14007+K09487+K03868+K09517+K10088+K03347+K09487+K10085+K14007+K03347+K13989+K09580+K14007+K14007+K08288+K05546+K03347+K09487+K09487+K14015+K13525+K09487+K09487+K07151+K03094+K14007+K10666+K10084+K14006+K03283+K09487+K08054+K14003+K05546+K01228+K10578+K03347+K14018+K08288

1;F01_transcript_161857;F01_transcript_162371;F01_transcript_162410;F01_transcript_162474;F01_transcript_16248;F01_transcript_162511;F01_transcript_16261;F01_transcript_16262;F01_transcript_162658;F01_transcript_162886;F01_transcript_163015;F01_transcript_163095;F01_transcript_163113;F01_transcript_16338;F01_transcript_163434;F01_transcript_163642;F01_transcript_163664;F01_transcript_163665;F01_transcript_163684;F01_transcript_163780;F01_transcript_163869;F01_transcript_163911;F01_transcript_163970;F01_transcript_16424;F01_transcript_164344;F01_transcript_164425;F01_transcript_164437;F01_transcript_164652;F01_transcript_16470;F01_transcript_164711;F01_transcript_164813;F01_transcript_164863;F01_transcript_164897;F01_transcript_164914;F01_transcript_164915;F01_transcript_165227;F01_transcript_165283;F01_transcript_165869;F01_transcript_1659;F01_transcript_166023;F01_transcript_166057;F01_transcript_166282;F01_transcript_166294;F01_transcript_166356;F01_transcript_166423;F01_transcript_166493;F01_transcript_166743;F01_transcript_166831;F01_transcript_167025;F01_transcript_167055;F01_transcript_16713;F01_transcript_167170;F01_transcript_167441;F01_transcript_16821;F01_transcript_16871;F01_transcript_1744;F01_transcript_1757;F01_transcript_17639;F01_transcript_17855;F01_transcript_18206;F01_transcript_18330;F01_transcript_18368;F01_transcript_18596;F01_transcript_19017;F01_transcript_19108;F01_transcript_19240;F01_transcript_19661;F01_transcript_19803;F01_transcript_1988;F01_transcript_19935;F01_transcript_20030;F01_transcript_20138;F01_transcript_20149;F01_transcript_20289;F01_transcript_20330;F01_transcript_20466;F01_transcript_20685;F01_transcript_20725;F01_transcript_2127;F01_transcript_21477;F01_transcript_22008;F01_transcript_22097;F01_transcript_22100;F01_transcript_22228;F01_transcript_22475;F01_transcript_22664;F01_transcript_22693;F01_transcript_22946;F01_transcript_23167;F01_transcript_2378;F01_transcript_24061;F01_transcript_24405;F01_transcript_24455;F01_transcript_2457;F01_transcript_25016;F01_transcript_25442;F01_transcript_25913;F01_transcript_26139;F01_transcript_26443;F01_transcript_26488;F01_transcript_27416;F01_transcript_27460;F01_transcript_28232;F0

1_transcript_28301;F01_transcript_28344;F01_transcript_28621
;F01_transcript_28766;F01_transcript_29113;F01_transcript_29
500;F01_transcript_29659;F01_transcript_29978;F01_transcript
_30147;F01_transcript_30180;F01_transcript_30672;F01_transc
ript_3086;F01_transcript_3094;F01_transcript_30951;F01_trans
cript_31667;F01_transcript_31675;F01_transcript_32349;F01_tr
anscript_32687;F01_transcript_32953;F01_transcript_33094;F0
1_transcript_33478;F01_transcript_33520;F01_transcript_33532
;F01_transcript_33551;F01_transcript_33590;F01_transcript_33
845;F01_transcript_33869;F01_transcript_33882;F01_transcript
_34106;F01_transcript_34198;F01_transcript_34384;F01_transc
ript_34478;F01_transcript_34602;F01_transcript_34672;F01_tra
nscript_35046;F01_transcript_35390;F01_transcript_35498;F01
_transcript_35547;F01_transcript_35749;F01_transcript_35971;
F01_transcript_36258;F01_transcript_36265;F01_transcript_362
75;F01_transcript_36362;F01_transcript_36386;F01_transcript_
3656;F01_transcript_36599;F01_transcript_36640;F01_transcrip
t_36811;F01_transcript_37057;F01_transcript_37106;F01_trans
cript_37190;F01_transcript_37400;F01_transcript_37415;F01_tr
anscript_37501;F01_transcript_37661;F01_transcript_37723;F0
1_transcript_37798;F01_transcript_3790;F01_transcript_3823;F
01_transcript_38234;F01_transcript_38334;F01_transcript_3844
3;F01_transcript_38459;F01_transcript_38866;F01_transcript_3
8884;F01_transcript_3898;F01_transcript_39214;F01_transcript
_4013;F01_transcript_40138;F01_transcript_40228;F01_transcri
pt_40342;F01_transcript_40471;F01_transcript_40550;F01_tran
script_40604;F01_transcript_40691;F01_transcript_40733;F01_t
ranscript_41113;F01_transcript_4123;F01_transcript_41238;F01
_transcript_4168;F01_transcript_41939;F01_transcript_41997;F
01_transcript_42063;F01_transcript_42159;F01_transcript_4216
7;F01_transcript_42331;F01_transcript_42483;F01_transcript_4
2644;F01_transcript_42876;F01_transcript_42988;F01_transcrip
t_43137;F01_transcript_43193;F01_transcript_43613;F01_trans
cript_43716;F01_transcript_44425;F01_transcript_44467;F01_tr
anscript_44508;F01_transcript_44688;F01_transcript_44947;F0
1_transcript_44964;F01_transcript_45170;F01_transcript_45293
;F01_transcript_45339;F01_transcript_45377;F01_transcript_45
392;F01_transcript_45479;F01_transcript_45483;F01_transcript

_45552;F01_transcript_45555;F01_transcript_45565;F01_transcript_45644;F01_transcript_45819;F01_transcript_45825;F01_transcript_45834;F01_transcript_46051;F01_transcript_46058;F01_transcript_46076;F01_transcript_46498;F01_transcript_46547;F01_transcript_46625;F01_transcript_46631;F01_transcript_46819;F01_transcript_46883;F01_transcript_46917;F01_transcript_47086;F01_transcript_47118;F01_transcript_47130;F01_transcript_47176;F01_transcript_47232;F01_transcript_47281;F01_transcript_47409;F01_transcript_47418;F01_transcript_47429;F01_transcript_47434;F01_transcript_47513;F01_transcript_47665;F01_transcript_47741;F01_transcript_47908;F01_transcript_47990;F01_transcript_48118;F01_transcript_48138;F01_transcript_48452;F01_transcript_48783;F01_transcript_48905;F01_transcript_49055;F01_transcript_49086;F01_transcript_49258;F01_transcript_49267;F01_transcript_49326;F01_transcript_49810;F01_transcript_50238;F01_transcript_50450;F01_transcript_51570;F01_transcript_51785;F01_transcript_51796;F01_transcript_51883;F01_transcript_51955;F01_transcript_52003;F01_transcript_52167;F01_transcript_52270;F01_transcript_52300;F01_transcript_52507;F01_transcript_52660;F01_transcript_52843;F01_transcript_52914;F01_transcript_5292;F01_transcript_52969;F01_transcript_52970;F01_transcript_53032;F01_transcript_53454;F01_transcript_53508;F01_transcript_53538;F01_transcript_53641;F01_transcript_53683;F01_transcript_53818;F01_transcript_53882;F01_transcript_54047;F01_transcript_54082;F01_transcript_54177;F01_transcript_54356;F01_transcript_54377;F01_transcript_54567;F01_transcript_54760;F01_transcript_54768;F01_transcript_54849;F01_transcript_54927;F01_transcript_55402;F01_transcript_55441;F01_transcript_55536;F01_transcript_55801;F01_transcript_56126;F01_transcript_56136;F01_transcript_5614;F01_transcript_56311;F01_transcript_56444;F01_transcript_5647;F01_transcript_56728;F01_transcript_57109;F01_transcript_57228;F01_transcript_57404;F01_transcript_57585;F01_transcript_57670;F01_transcript_57682;F01_transcript_58254;F01_transcript_58330;F01_transcript_58445;F01_transcript_58509;F01_transcript_59262;F01_transcript_59268;F01_transcript_59395;F01_transcript_59471;F01_transcript_59606;F01_transcript_59867;

F01_transcript_59972;F01_transcript_6025;F01_transcript_6066
4;F01_transcript_60698;F01_transcript_60711;F01_transcript_6
0757;F01_transcript_6108;F01_transcript_61346;F01_transcript
_61446;F01_transcript_61455;F01_transcript_61464;F01_transc
ript_61585;F01_transcript_61932;F01_transcript_62076;F01_tra
nscript_62440;F01_transcript_62450;F01_transcript_62475;F01
_transcript_62571;F01_transcript_6259;F01_transcript_62797;F
01_transcript_6325;F01_transcript_63282;F01_transcript_63295
;F01_transcript_6334;F01_transcript_63362;F01_transcript_636
34;F01_transcript_63689;F01_transcript_63812;F01_transcript_
64004;F01_transcript_64036;F01_transcript_64090;F01_transcri
pt_64348;F01_transcript_64393;F01_transcript_64569;F01_tran
script_64586;F01_transcript_64646;F01_transcript_64723;F01_t
ranscript_64863;F01_transcript_64899;F01_transcript_65294;F0
1_transcript_65458;F01_transcript_66301;F01_transcript_66542
;F01_transcript_66568;F01_transcript_66797;F01_transcript_66
846;F01_transcript_66944;F01_transcript_67002;F01_transcript
_67022;F01_transcript_6738;F01_transcript_67545;F01_transcri
pt_67742;F01_transcript_67791;F01_transcript_67824;F01_tran
script_6797;F01_transcript_68150;F01_transcript_68433;F01_tr
anscript_68451;F01_transcript_68577;F01_transcript_68773;F0
1_transcript_68846;F01_transcript_68927;F01_transcript_69057
;F01_transcript_69079;F01_transcript_69545;F01_transcript_69
550;F01_transcript_6966;F01_transcript_69759;F01_transcript_
69797;F01_transcript_69895;F01_transcript_69920;F01_transcri
pt_70589;F01_transcript_70807;F01_transcript_70812;F01_tran
script_70854;F01_transcript_71235;F01_transcript_71750;F01_t
ranscript_71993;F01_transcript_72052;F01_transcript_72387;F0
1_transcript_72495;F01_transcript_72574;F01_transcript_72633
;F01_transcript_7277;F01_transcript_72939;F01_transcript_729
40;F01_transcript_7297;F01_transcript_73123;F01_transcript_7
317;F01_transcript_73247;F01_transcript_73359;F01_transcript
_73469;F01_transcript_73548;F01_transcript_73551;F01_transc
ript_73614;F01_transcript_7375;F01_transcript_73936;F01_tran
script_73999;F01_transcript_74006;F01_transcript_74250;F01_t
ranscript_74425;F01_transcript_74760;F01_transcript_74881;F0
1_transcript_75288;F01_transcript_75304;F01_transcript_75370
;F01_transcript_75718;F01_transcript_75965;F01_transcript_76

	037;F01_transcript_76090;F01_transcript_76233;F01_transcript_7637;F01_transcript_76445;F01_transcript_76448;F01_transcript_76606;F01_transcript_7675;F01_transcript_7704;F01_transcript_77046;F01_transcript_77076;F01_transcript_77127;F01_transcript_77189;F01_transcript_77334;F01_transcript_78200;F01_transcript_78573;F01_transcript_788;F01_transcript_78933;F01_transcript_78975;F01_transcript_79014;F01_transcript_79109;F01_transcript_79126;F01_transcript_79451;F01_transcript_79483;F01_transcript_79632;F01_transcript_79713;F01_transcript_79719;F01_transcript_79721;F01_transcript_79796;F01_transcript_80299;F01_transcript_80633;F01_transcript_80820;F01_transcript_80895;F01_transcript_81136;F01_transcript_81246;F01_transcript_81247;F01_transcript_81803;F01_transcript_8185;F01_transcript_81976;F01_transcript_82517;F01_transcript_82523;F01_transcript_82602;F01_transcript_82658;F01_transcript_82852;F01_transcript_82933;F01_transcript_8310;F01_transcript_83353;F01_transcript_83446;F01_transcript_83518;F01_transcript_83581;F01_transcript_83586;F01_transcript_83874;F01_transcript_83942;F01_transcript_84008;F01_transcript_84312;F01_transcript_84335;F01_transcript_84498;F01_transcript_8451;F01_transcript_84668;F01_transcript_84895;F01_transcript_84944;F01_transcript_84996;F01_transcript_85253;F01_transcript_85282;F01_transcript_85642;F01_transcript_86;F01_transcript_86175;F01_transcript_86227;F01_transcript_86265;F01_transcript_86542;F01_transcript_86590;F01_transcript_86646;F01_transcript_86674;F01_transcript_86751;F01_transcript_87227;F01_transcript_87534;F01_transcript_88268;F01_transcript_88310;F01_transcript_88639;F01_transcript_88780;F01_transcript_88861;F01_transcript_89449;F01_transcript_89481;F01_transcript_89652;F01_transcript_8969;F01_transcript_90240;F01_transcript_90359;F01_transcript_90424;F01_transcript_90436;F01_transcript_90591;F01_transcript_90626;F01_transcript_90709;F01_transcript_90794;F01_transcript_90843;F01_transcript_90947;F01_transcript_90998;F01_transcript_91041;F01_transcript_91268;F01_transcript_91421;F01_transcript_9149;F01_transcript_91855;F01_transcript_91919;F01_transcript_92304;F01_transcript_92358;F01_transcript_92459;F01_transcript_9248;F01_transcript_9	

			2485;F01_transcript_92602;F01_transcript_93046;F01_transcript_93106;F01_transcript_934;F01_transcript_93452;F01_transcript_93472;F01_transcript_93566;F01_transcript_94404;F01_transcript_9449;F01_transcript_9480;F01_transcript_95000;F01_transcript_953;F01_transcript_9535;F01_transcript_95389;F01_transcript_95425;F01_transcript_95521;F01_transcript_95857;F01_transcript_96150;F01_transcript_96158;F01_transcript_96259;F01_transcript_9663;F01_transcript_96932;F01_transcript_96971;F01_transcript_96990;F01_transcript_9709;F01_transcript_97280;F01_transcript_9741;F01_transcript_97472;F01_transcript_975;F01_transcript_98302;F01_transcript_98344;F01_transcript_9839;F01_transcript_98591;F01_transcript_9928;F01_transcript_99294;F01_transcript_99466;F01_transcript_99477;F01_transcript_99571;F01_transcript_99784;F01_transcript_9984;F01_transcript_9987;F01_transcript_99916;	
Endocytosis	ko04144	698	F01_transcript_100291;F01_transcript_100313;F01_transcript_100639;F01_transcript_101214;F01_transcript_101238;F01_transcript_101481;F01_transcript_101913;F01_transcript_102286;F01_transcript_102303;F01_transcript_102515;F01_transcript_102665;F01_transcript_103136;F01_transcript_103480;F01_transcript_103601;F01_transcript_103749;F01_transcript_103880;F01_transcript_103887;F01_transcript_104433;F01_transcript_104748;F01_transcript_105010;F01_transcript_105535;F01_transcript_105638;F01_transcript_10593;F01_transcript_106580;F01_transcript_107067;F01_transcript_107311;F01_transcript_107428;F01_transcript_107454;F01_transcript_107465;F01_transcript_107508;F01_transcript_10774;F01_transcript_107853;F01_transcript_108156;F01_transcript_108255;F01_transcript_10833;F01_transcript_108340;F01_transcript_108515;F01_transcript_108812;F01_transcript_109013;F01_transcript_10914;F01_transcript_10949;F01_transcript_109860;F01_transcript_111011;F01_transcript_111471;F01_transcript_111645;F01_transcript_111946;F01_transcript_112012;F01_transcript_112353;F01_transcript_112584;F01_transcript_112611;F01_transcript_112632;F01_transcript_112837;F01_transcript_112941;F01_transcript_113156;F01_transcript_113191;F01_transcript_113286;F01_transcript_113603;F01_transcript_113623;F01_transcript_113794;F01_transcript_114123;F01_transcript_114629;F01_transcript_114833;F01_tran	K00889+K18467+K03283+K12200+K03283+K12200+K12190+K12493+K12199+K07889+K12196+K07937+K07937+K12489+K11866+K01528+K00889+K12489+K18468+K00889+K17917+K03283+K18468+K01115+K07901+K03283+K12193+K05757+K01115+K01115+K01115+K12194+K12199+K07889+K01115+K12197+K01115+K18466+K19366+K12489+K18468+K00889+K10365+K11824+K12486+K12185+K12190+K07897+K18468+K07904+K07904+K10591+K00889+K12471+K07897+K01115+K11824+K12193+K12486+K12489+K00889+K12192+K01115+K12489+K11824+K12196+K12471+K12196+K05756+K12489+K03283+K18468+K12192+K18468+K19367+K07889+K12492+K12200+K12189+K00889+K04646+K05758+K03283+K04646+K00889+K01115+K07904+K11826+K07904+K00889+K12200+K18468+K01115+K07904+K19476+K18468+K04646+K00889+K12471+K10591+K05755+K12200+K10591+K00889+K12493+K01115+K07901+K00889+K00889+K00889+K11866+K12486+K03283+K11824+K11866+K18443+K04646+K18442+K00889+K19476+K01528+K00889+K03283+K01115+K12194+K00889+K01528+K03283+K12486+K12486+K07904+K00889+K03283+K12183+K01115+K03283+K12183+K03283+K03283+K00889+K00889+K00889+K00889+K12489+K03283+K12199+K12197+K00889+K07937+K10365+K01115+K01528+K07904+K01115+K12489+K01115+K17917+K0793

	script_114962;F01_transcript_115815;F01_transcript_115820;F01_transcript_115979;F01_transcript_116006;F01_transcript_116131;F01_transcript_116143;F01_transcript_116323;F01_transcript_116810;F01_transcript_116851;F01_transcript_117776;F01_transcript_117924;F01_transcript_117926;F01_transcript_118000;F01_transcript_118599;F01_transcript_118720;F01_transcript_11875;F01_transcript_118778;F01_transcript_119022;F01_transcript_119194;F01_transcript_119258;F01_transcript_119314;F01_transcript_11952;F01_transcript_1198;F01_transcript_119828;F01_transcript_119836;F01_transcript_120005;F01_transcript_12012;F01_transcript_120399;F01_transcript_120428;F01_transcript_120590;F01_transcript_120709;F01_transcript_121260;F01_transcript_121277;F01_transcript_121433;F01_transcript_121489;F01_transcript_121590;F01_transcript_122441;F01_transcript_1229;F01_transcript_123025;F01_transcript_123157;F01_transcript_123225;F01_transcript_123262;F01_transcript_124305;F01_transcript_124426;F01_transcript_124691;F01_transcript_124724;F01_transcript_124736;F01_transcript_125029;F01_transcript_125108;F01_transcript_125130;F01_transcript_125532;F01_transcript_125630;F01_transcript_125863;F01_transcript_126009;F01_transcript_126340;F01_transcript_127179;F01_transcript_127705;F01_transcript_127783;F01_transcript_127812;F01_transcript_128228;F01_transcript_128492;F01_transcript_129154;F01_transcript_129184;F01_transcript_129289;F01_transcript_129423;F01_transcript_129688;F01_transcript_129792;F01_transcript_130041;F01_transcript_130320;F01_transcript_131910;F01_transcript_132109;F01_transcript_132174;F01_transcript_132381;F01_transcript_132507;F01_transcript_133251;F01_transcript_133415;F01_transcript_133554;F01_transcript_133724;F01_transcript_134031;F01_transcript_134034;F01_transcript_134041;F01_transcript_134461;F01_transcript_135315;F01_transcript_135709;F01_transcript_135724;F01_transcript_135771;F01_transcript_135907;F01_transcript_136633;F01_transcript_136786;F01_transcript_136825;F01_transcript_136870;F01_transcript_136944;F01_transcript_137225;F01_transcript_137252;F01_transcript_137263;F01_transcript_137349;F01_transcript_137380;F01_transcript_137601;F01_transcript_137774;F01_transcript	7+K18468+K19476+K18468+K07901+K07937+K00889+K12191+K19367+K19366+K07904+K12196+K01115+K07889+K19367+K12185+K07904+K07889+K18468+K01115+K18468+K11824+K03283+K12200+K12194+K12196+K01115+K12483+K11824+K01115+K07904+K07901+K00889+K12486+K11824+K01528+K11866+K18465+K12196+K11866+K12194+K01115+K12193+K01528+K18468+K19366+K12196+K12489+K11824+K11824+K07904+K12489+K12486+K12195+K07897+K12196+K19476+K07889+K12197+K12471+K12483+K12194+K03283+K19476+K19367+K19476+K18468+K12471+K00889+K01115+K19366+K07904+K00889+K12197+K07937+K07889+K10365+K18466+K12486+K18442+K03283+K12486+K19476+K11866+K12471+K12489+K12194+K07901+K12196+K19476+K12200+K18468+K00889+K01115+K12492+K19367+K01115+K12471+K11866+K01115+K12471+K03283+K07889+K19476+K05755+K07937+K03283+K07889+K12471+K07937+K07937+K18468+K19366+K19367+K11866+K03283+K00889+K04646+K12199+K19367+K12479+K12486+K07937+K03283+K18468+K19476+K12489+K11826+K17917+K07904+K01115+K03283+K00889+K01115+K07904+K12471+K12185+K12486+K03283+K12471+K01115+K12489+K12199+K04646+K07904+K12194+K19367+K11824+K04646+K19476+K03283+K12199+K03283+K03283+K12471+K12471+K11824+K03283+K11824+K03283+K03283+K03283+K18442+K03283+K12471+K00889+K12479+K19476+K03283+K01528+K00889+K10364+K12489+K03283+K01115+K19476+K19476+K12489+K11866+K18467+K12471+K00889+K11866+K11826+K12190+K12486+K03283+K12483+K19476+K11866+K12199+K11866+K12492+K11866+K11826+K12193+K11866+K12471+K01115+K12471+K12196+K19476+K12196+K07889+K12486+K12489+K01528+K12493+K19366+K17917+K05757+K19366+K19366+K19367+K18466+K17917+K19366+K12199+K19476+K12183+K12183+K07897+K18466+K19476+K01528+K07904+K19366+K12199+K07937+K07937+K10364+K18466+K18466+K07904+K12185+K07901+K07904+K12185+K12193+K12194+K07904+K12193+K12198+K19476+K07901+K12193+K12194+K07897+K18442+K07904+K07897+K07937+K07904+K10365+K12

	_137884;F01_transcript_138185;F01_transcript_138335;F01_transcript_138551;F01_transcript_138928;F01_transcript_139021;F01_transcript_139208;F01_transcript_139307;F01_transcript_139606;F01_transcript_139808;F01_transcript_139832;F01_transcript_140873;F01_transcript_141197;F01_transcript_141210;F01_transcript_141318;F01_transcript_141472;F01_transcript_1416;F01_transcript_141858;F01_transcript_142003;F01_transcript_142209;F01_transcript_142311;F01_transcript_1427;F01_transcript_142858;F01_transcript_143189;F01_transcript_143193;F01_transcript_143262;F01_transcript_143277;F01_transcript_143704;F01_transcript_143730;F01_transcript_143941;F01_transcript_144025;F01_transcript_144054;F01_transcript_144183;F01_transcript_144872;F01_transcript_144990;F01_transcript_145131;F01_transcript_145450;F01_transcript_145714;F01_transcript_145892;F01_transcript_146917;F01_transcript_147013;F01_transcript_147297;F01_transcript_147615;F01_transcript_147636;F01_transcript_147731;F01_transcript_147883;F01_transcript_147952;F01_transcript_147997;F01_transcript_148159;F01_transcript_148395;F01_transcript_148904;F01_transcript_149572;F01_transcript_149872;F01_transcript_150013;F01_transcript_150236;F01_transcript_150262;F01_transcript_150853;F01_transcript_150982;F01_transcript_151192;F01_transcript_151857;F01_transcript_151946;F01_transcript_151962;F01_transcript_152247;F01_transcript_152813;F01_transcript_152845;F01_transcript_153368;F01_transcript_153727;F01_transcript_153811;F01_transcript_153953;F01_transcript_153996;F01_transcript_154389;F01_transcript_154397;F01_transcript_154522;F01_transcript_154545;F01_transcript_1546;F01_transcript_154605;F01_transcript_154729;F01_transcript_154957;F01_transcript_155216;F01_transcript_155960;F01_transcript_156254;F01_transcript_156368;F01_transcript_156616;F01_transcript_157084;F01_transcript_157323;F01_transcript_157561;F01_transcript_157629;F01_transcript_157718;F01_transcript_157739;F01_transcript_157910;F01_transcript_158025;F01_transcript_158289;F01_transcript_158417;F01_transcript_158423;F01_transcript_158517;F01_transcript_158566;F01_transcript_158686;F01_transcript_158738;F01_transcript_158809;F01_transcript_158882;F01_transcript_159188;F01_transcript_159283;F01_transcript_159294;F01_transcri	194+K12184+K07897+K07937+K07937+K10365+K07937+K07937+K07901+K18467+K07904+K07937+K12192+K07937+K07904+K12197+K07904+K12191+K07937+K12191+K07937+K07889+K07937+K12197+K05754+K05754+K10591+K05754+K07889+K18468+K18468+K12200+K18468+K00889+K12192+K12200+K12489+K01528+K12489+K12471+K12200+K18468+K12197+K03283+K11866+K12483+K03283+K12199+K07937+K18467+K12471+K18466+K11866+K12479+K18442+K07904+K10591+K03283+K12479+K01115+K07897+K19367+K00889+K01115+K17917+K11824+K18466+K01528+K12471+K12196+K07937+K07904+K07904+K18442+K18468+K12471+K12200+K12194+K12188+K00889+K18468+K01115+K10396+K03283+K18468+K07904+K00889+K11824+K11826+K00889+K00889+K04646+K10591+K19476+K04646+K01115+K12483+K00889+K12194+K00889+K07937+K17917+K12200+K01115+K18466+K01115+K01115+K18464+K12194+K18468+K18468+K12471+K12492+K11866+K05755+K19476+K12189+K10591+K18468+K12200+K12489+K01115+K00889+K10365+K10591+K01115+K00889+K01115+K01528+K18442+K12483+K11866+K18442+K12493+K00889+K19476+K00889+K12192+K10365+K12195+K11824+K05754+K07937+K07904+K12489+K18468+K12200+K05757+K12185+K01528+K12194+K07937+K07904+K03283+K01528+K11866+K12489+K12194+K19476+K18466+K19367+K05755+K00889+K18468+K11866+K12489+K12486+K01115+K12197+K07937+K18468+K07889+K12489+K12489+K04646+K00889+K12489+K12188+K12471+K18468+K18442+K12200+K00889+K19476+K03283+K03283+K12486+K00889+K12190+K12486+K12184+K12183+K12194+K12198+K03283+K12483+K00889+K11826+K19367+K12486+K07897+K03283+K12200+K18443+K18468+K12196+K12193+K12199+K07904+K19367+K12471+K19476+K01115+K11824+K12196+K18442+K07937+K07937+K12493+K07889+K19367+K12188+K11866+K00889+K01115+K07904+K07904+K11866+K07889+K05758+K18468+K05758+K01528+K12196+K07889+K12489+K04646+K03283+K12479+K01115+K18468+K18468+K00889+K10365+K18468+K12197+K12193+K00889+K11866+K01115+K15053+K07897+K01528+K07897+K00889+K01115+K00889+K07937+K07901+K18468+K18442+

	pt_159601;F01_transcript_159706;F01_transcript_160212;F01_transcript_160715;F01_transcript_160761;F01_transcript_161092;F01_transcript_161164;F01_transcript_161611;F01_transcript_161895;F01_transcript_162636;F01_transcript_162640;F01_transcript_162800;F01_transcript_163040;F01_transcript_163067;F01_transcript_163130;F01_transcript_16338;F01_transcript_163472;F01_transcript_163524;F01_transcript_163964;F01_transcript_164146;F01_transcript_164176;F01_transcript_164404;F01_transcript_164522;F01_transcript_164652;F01_transcript_164709;F01_transcript_165090;F01_transcript_16525;F01_transcript_165516;F01_transcript_165689;F01_transcript_165902;F01_transcript_166023;F01_transcript_166029;F01_transcript_1662;F01_transcript_166242;F01_transcript_166352;F01_transcript_166462;F01_transcript_166643;F01_transcript_166765;F01_transcript_167341;F01_transcript_1761;F01_transcript_18057;F01_transcript_18541;F01_transcript_18596;F01_transcript_18828;F01_transcript_19108;F01_transcript_19240;F01_transcript_1938;F01_transcript_19485;F01_transcript_1965;F01_transcript_19803;F01_transcript_1981;F01_transcript_20030;F01_transcript_20138;F01_transcript_20149;F01_transcript_20340;F01_transcript_20725;F01_transcript_20748;F01_transcript_21520;F01_transcript_21528;F01_transcript_21776;F01_transcript_22008;F01_transcript_22316;F01_transcript_22882;F01_transcript_23678;F01_transcript_2399;F01_transcript_24061;F01_transcript_2470;F01_transcript_24949;F01_transcript_26183;F01_transcript_2666;F01_transcript_26772;F01_transcript_27180;F01_transcript_2845;F01_transcript_2861;F01_transcript_28735;F01_transcript_28924;F01_transcript_29189;F01_transcript_29489;F01_transcript_29500;F01_transcript_29637;F01_transcript_29827;F01_transcript_30066;F01_transcript_30157;F01_transcript_30250;F01_transcript_30410;F01_transcript_30605;F01_transcript_30982;F01_transcript_31536;F01_transcript_31620;F01_transcript_3188;F01_transcript_3200;F01_transcript_3209;F01_transcript_32243;F01_transcript_32458;F01_transcript_32635;F01_transcript_3298;F01_transcript_33365;F01_transcript_3348;F01_transcript_3353;F01_transcript_33725;F01_transcript_34251;F01_transcript_34473;F01_transcript_34899;F01_transcript_35253;F01_transcript_3	K10591+K00889+K18466+K07904+K12489+K12189+K04646+K18466+K01115+K19476+K12189+K19367+K12479+K11866+K12489+K01115+K12486+K03283+K12486+K11826+K12471+K10364+K00889+K18468+K00889+K01528

5703;F01_transcript_36296;F01_transcript_36383;F01_transcrip
t_36517;F01_transcript_36637;F01_transcript_36657;F01_trans
cript_36696;F01_transcript_36983;F01_transcript_37295;F01_tr
anscript_37310;F01_transcript_37581;F01_transcript_37846;F0
1_transcript_3794;F01_transcript_38133;F01_transcript_38525;
F01_transcript_39432;F01_transcript_40079;F01_transcript_401
01;F01_transcript_40175;F01_transcript_40191;F01_transcript_
40398;F01_transcript_41222;F01_transcript_41404;F01_transcri
pt_42388;F01_transcript_42460;F01_transcript_42957;F01_tran
script_43099;F01_transcript_43328;F01_transcript_43338;F01_t
ranscript_43389;F01_transcript_43482;F01_transcript_43498;F0
1_transcript_43529;F01_transcript_43556;F01_transcript_43747
;F01_transcript_43928;F01_transcript_44;F01_transcript_44050;
F01_transcript_44166;F01_transcript_44318;F01_transcript_443
27;F01_transcript_44360;F01_transcript_44497;F01_transcript_
44513;F01_transcript_44738;F01_transcript_44821;F01_transcri
pt_44882;F01_transcript_44918;F01_transcript_45185;F01_tran
script_45230;F01_transcript_45260;F01_transcript_45311;F01_t
ranscript_45353;F01_transcript_45420;F01_transcript_45429;F0
1_transcript_45874;F01_transcript_45960;F01_transcript_46142
;F01_transcript_46254;F01_transcript_46262;F01_transcript_46
398;F01_transcript_46815;F01_transcript_46981;F01_transcript
_47131;F01_transcript_47166;F01_transcript_47581;F01_transc
ript_48650;F01_transcript_49157;F01_transcript_4920;F01_tran
script_50631;F01_transcript_5134;F01_transcript_5145;F01_tra
nscript_51469;F01_transcript_5157;F01_transcript_52042;F01_t
ranscript_52111;F01_transcript_52163;F01_transcript_52357;F0
1_transcript_52518;F01_transcript_52687;F01_transcript_52836
;F01_transcript_52871;F01_transcript_53050;F01_transcript_53
207;F01_transcript_53554;F01_transcript_53641;F01_transcript
_53691;F01_transcript_54287;F01_transcript_54768;F01_transc
ript_55292;F01_transcript_55339;F01_transcript_55574;F01_tra
nscript_55632;F01_transcript_55977;F01_transcript_56022;F01
_transcript_56259;F01_transcript_56647;F01_transcript_56872;
F01_transcript_5711;F01_transcript_57404;F01_transcript_5745
7;F01_transcript_5800;F01_transcript_58195;F01_transcript_58
231;F01_transcript_59439;F01_transcript_59477;F01_transcript
_5949;F01_transcript_59552;F01_transcript_59681;F01_transcri

pt_59893;F01_transcript_59926;F01_transcript_60421;F01_tran
script_60422;F01_transcript_60849;F01_transcript_60919;F01_t
ranscript_61282;F01_transcript_61390;F01_transcript_61630;F0
1_transcript_6229;F01_transcript_62326;F01_transcript_62358;
F01_transcript_6257;F01_transcript_62719;F01_transcript_6323
8;F01_transcript_63361;F01_transcript_63634;F01_transcript_6
3920;F01_transcript_64374;F01_transcript_64409;F01_transcrip
t_64799;F01_transcript_64819;F01_transcript_64970;F01_trans
cript_64995;F01_transcript_65001;F01_transcript_65099;F01_tr
anscript_65358;F01_transcript_65552;F01_transcript_65844;F0
1_transcript_65885;F01_transcript_66592;F01_transcript_66778
;F01_transcript_6689;F01_transcript_67099;F01_transcript_674
88;F01_transcript_6824;F01_transcript_6837;F01_transcript_68
648;F01_transcript_6867;F01_transcript_68724;F01_transcript_
68726;F01_transcript_68801;F01_transcript_68835;F01_transcri
pt_69009;F01_transcript_69041;F01_transcript_69072;F01_tran
script_69094;F01_transcript_69157;F01_transcript_69273;F01_t
ranscript_69322;F01_transcript_69435;F01_transcript_69455;F0
1_transcript_69562;F01_transcript_69690;F01_transcript_6972;
F01_transcript_6988;F01_transcript_69910;F01_transcript_7004
4;F01_transcript_70159;F01_transcript_70192;F01_transcript_7
0343;F01_transcript_70753;F01_transcript_70770;F01_transcrip
t_70833;F01_transcript_70898;F01_transcript_71;F01_transcript
_71232;F01_transcript_71307;F01_transcript_71643;F01_transc
ript_71841;F01_transcript_72352;F01_transcript_72420;F01_tra
nscript_72512;F01_transcript_72569;F01_transcript_72588;F01
_transcript_73271;F01_transcript_73273;F01_transcript_7382;F
01_transcript_73824;F01_transcript_74359;F01_transcript_7454
4;F01_transcript_74556;F01_transcript_74688;F01_transcript_7
5848;F01_transcript_75917;F01_transcript_76031;F01_transcrip
t_76090;F01_transcript_76306;F01_transcript_76593;F01_trans
cript_7695;F01_transcript_77048;F01_transcript_77915;F01_tra
nscript_78064;F01_transcript_78344;F01_transcript_78634;F01
_transcript_78675;F01_transcript_78705;F01_transcript_78798;
F01_transcript_78945;F01_transcript_79023;F01_transcript_792
10;F01_transcript_79338;F01_transcript_79979;F01_transcript_
8012;F01_transcript_80156;F01_transcript_80690;F01_transcrip

t_80749;F01_transcript_81;F01_transcript_81263;F01_transcript_81344;F01_transcript_81528;F01_transcript_81552;F01_transcript_81592;F01_transcript_81887;F01_transcript_81895;F01_transcript_82323;F01_transcript_82402;F01_transcript_82517;F01_transcript_82523;F01_transcript_82540;F01_transcript_82606;F01_transcript_82715;F01_transcript_82813;F01_transcript_82985;F01_transcript_83283;F01_transcript_83349;F01_transcript_83406;F01_transcript_83518;F01_transcript_83540;F01_transcript_83591;F01_transcript_83986;F01_transcript_83990;F01_transcript_84018;F01_transcript_84086;F01_transcript_84335;F01_transcript_8435;F01_transcript_84644;F01_transcript_8467;F01_transcript_85263;F01_transcript_85910;F01_transcript_86051;F01_transcript_86176;F01_transcript_86249;F01_transcript_86695;F01_transcript_87219;F01_transcript_8760;F01_transcript_87726;F01_transcript_88149;F01_transcript_88287;F01_transcript_88337;F01_transcript_88887;F01_transcript_88923;F01_transcript_88957;F01_transcript_89023;F01_transcript_89717;F01_transcript_89741;F01_transcript_89769;F01_transcript_89778;F01_transcript_89945;F01_transcript_89950;F01_transcript_90016;F01_transcript_90018;F01_transcript_90083;F01_transcript_9014;F01_transcript_90236;F01_transcript_90267;F01_transcript_90408;F01_transcript_90425;F01_transcript_90582;F01_transcript_90942;F01_transcript_90998;F01_transcript_92092;F01_transcript_92236;F01_transcript_9229;F01_transcript_9238;F01_transcript_9251;F01_transcript_92749;F01_transcript_92755;F01_transcript_92861;F01_transcript_93117;F01_transcript_93174;F01_transcript_93224;F01_transcript_9329;F01_transcript_93569;F01_transcript_94408;F01_transcript_94422;F01_transcript_94528;F01_transcript_9461;F01_transcript_9471;F01_transcript_94770;F01_transcript_94788;F01_transcript_94882;F01_transcript_94942;F01_transcript_94979;F01_transcript_9502;F01_transcript_95120;F01_transcript_95179;F01_transcript_95331;F01_transcript_95403;F01_transcript_95773;F01_transcript_96042;F01_transcript_97184;F01_transcript_97266;F01_transcript_97370;F01_transcript_9758;F01_transcript_97639;F01_transcript_97946;F01_transcript_98058;F01_transcript_98389;F01_transcript_98476;F01_transcript_98507;F01_transcript_98591;F01_transcript_98595;F01_transcript_98737;F01_transcript_99000;F01_transcri

			pt_99034;F01_transcript_99185;F01_transcript_99500;F01_transcript_99553;F01_transcript_99650;	
Phagosome	ko04145	424	F01_transcript_100496;F01_transcript_100604;F01_transcript_101123;F01_transcript_101495;F01_transcript_101950;F01_transcript_102282;F01_transcript_102328;F01_transcript_102515;F01_transcript_102877;F01_transcript_103125;F01_transcript_103303;F01_transcript_10363;F01_transcript_103783;F01_transcript_104015;F01_transcript_104353;F01_transcript_104480;F01_transcript_104594;F01_transcript_104996;F01_transcript_105116;F01_transcript_105729;F01_transcript_106017;F01_transcript_106019;F01_transcript_106040;F01_transcript_106439;F01_transcript_106594;F01_transcript_106959;F01_transcript_107347;F01_transcript_107664;F01_transcript_10798;F01_transcript_108255;F01_transcript_108893;F01_transcript_109384;F01_transcript_10945;F01_transcript_11072;F01_transcript_110751;F01_transcript_110828;F01_transcript_111397;F01_transcript_111857;F01_transcript_112353;F01_transcript_112716;F01_transcript_113191;F01_transcript_113642;F01_transcript_113645;F01_transcript_113958;F01_transcript_115030;F01_transcript_115055;F01_transcript_115411;F01_transcript_115682;F01_transcript_115891;F01_transcript_115923;F01_transcript_11593;F01_transcript_116015;F01_transcript_116212;F01_transcript_116369;F01_transcript_117270;F01_transcript_117305;F01_transcript_117794;F01_transcript_118000;F01_transcript_118008;F01_transcript_118118;F01_transcript_119673;F01_transcript_11974;F01_transcript_119898;F01_transcript_119968;F01_transcript_120079;F01_transcript_120434;F01_transcript_120442;F01_transcript_120724;F01_transcript_120999;F01_transcript_121061;F01_transcript_121128;F01_transcript_121150;F01_transcript_121351;F01_transcript_121498;F01_transcript_121600;F01_transcript_121786;F01_transcript_122562;F01_transcript_123713;F01_transcript_124147;F01_transcript_12419;F01_transcript_124354;F01_transcript_12453;F01_transcript_124805;F01_transcript_124828;F01_transcript_125359;F01_transcript_125441;F01_transcript_125505;F01_transcript_125516;F01_transcript_125621;F01_transcript_125964;F01_transcript_126698;F01_transcript_126962;F01_transcript_127233;F01_transcript_128287;F01_transcript_12858	K02147+K09481+K00921+K02154+K07374+K07375+K04392+K07889+K02150+K00921+K02147+K00921+K02147+K00921+K00914+K10956+K04392+K08057+K04392+K04392+K04392+K04392+K08057+K02154+K07375+K02152+K07375+K02144+K00914+K07889+K02155+K07374+K04392+K10956+K00914+K07374+K08054+K07375+K07897+K02154+K07897+K08057+K07375+K02147+K02150+K10956+K07374+K07374+K02149+K02154+K04392+K02154+K02155+K02154+K00914+K10956+K00914+K07889+K07374+K02147+K09481+K02155+K02150+K00921+K02148+K04392+K02146+K10956+K02154+K07375+K02145+K02154+K07374+K04392+K02152+K00914+K02154+K07375+K02153+K02154+K04392+K10956+K02147+K03661+K02155+K08054+K07374+K00914+K07375+K07375+K04392+K07375+K00914+K10956+K04392+K08057+K00914+K04392+K02155+K04392+K02147+K00914+K07375+K02147+K02154+K00921+K10956+K07375+K02147+K04392+K00914+K02147+K07889+K07374+K07374+K07889+K02154+K04392+K10956+K10956+K07342+K02146+K07374+K00921+K02148+K00921+K09481+K10956+K00914+K02147+K02148+K02148+K02147+K04392+K02149+K10956+K07897+K02147+K07889+K00914+K10956+K10956+K00914+K07375+K10956+K07374+K02147+K03661+K02155+K04392+K02147+K00914+K07375+K02147+K00921+K07889+K02154+K02154+K02147+K02147+K00921+K00921+K10956+K00914+K09481+K07889+K02150+K02155+K07889+K09481+K07375+K08517+K07375+K02150+K07375+K02147+K08057+K10956+K02153+K08492+K10956+K00914+K08057+K10956+K02154+K02145+K02145+K02155+K00921+K02147+K08517+K00921+K02148+K07374+K00921+K02155+K10956+K02147+K10956+K10956+K10956+K04392+K07375+K07375+K07374+K10956+K04392+K07375+K07889+K07374+K02144+K07374+K10956+K07374+K07374+K07374+K07374+K07374+K07375+K07374+K07374+K07374+K07374+K07374+K07375+K08057+K07375+K07374+K02148+K02144+K07374+K07374+K07897+K07374+K07375+K08057+K07374+K02148+K02146+K02148+

	2;F01_transcript_12914;F01_transcript_129146;F01_transcript_130063;F01_transcript_130192;F01_transcript_131602;F01_transcript_133020;F01_transcript_133384;F01_transcript_133408;F01_transcript_135068;F01_transcript_135298;F01_transcript_13665;F01_transcript_136940;F01_transcript_137262;F01_transcript_138220;F01_transcript_138341;F01_transcript_138842;F01_transcript_138846;F01_transcript_139606;F01_transcript_140286;F01_transcript_140914;F01_transcript_141197;F01_transcript_141509;F01_transcript_142650;F01_transcript_143826;F01_transcript_144220;F01_transcript_144251;F01_transcript_144387;F01_transcript_144410;F01_transcript_144552;F01_transcript_145300;F01_transcript_146185;F01_transcript_146376;F01_transcript_146408;F01_transcript_146478;F01_transcript_146671;F01_transcript_146945;F01_transcript_147090;F01_transcript_147572;F01_transcript_147743;F01_transcript_147882;F01_transcript_148393;F01_transcript_148395;F01_transcript_148778;F01_transcript_149872;F01_transcript_149901;F01_transcript_150161;F01_transcript_150378;F01_transcript_150423;F01_transcript_151682;F01_transcript_151688;F01_transcript_151749;F01_transcript_153084;F01_transcript_153405;F01_transcript_153407;F01_transcript_153666;F01_transcript_153673;F01_transcript_153720;F01_transcript_153759;F01_transcript_154015;F01_transcript_154350;F01_transcript_154389;F01_transcript_154669;F01_transcript_155072;F01_transcript_155620;F01_transcript_155890;F01_transcript_156217;F01_transcript_156666;F01_transcript_157649;F01_transcript_158433;F01_transcript_158664;F01_transcript_158738;F01_transcript_158869;F01_transcript_159285;F01_transcript_159294;F01_transcript_159474;F01_transcript_159708;F01_transcript_159854;F01_transcript_160071;F01_transcript_160710;F01_transcript_160911;F01_transcript_161923;F01_transcript_163434;F01_transcript_163780;F01_transcript_163783;F01_transcript_164142;F01_transcript_165283;F01_transcript_166806;F01_transcript_167025;F01_transcript_167170;F01_transcript_167677;F01_transcript_17061;F01_transcript_18162;F01_transcript_1857;F01_transcript_23;F01_transcript_24750;F01_transcript_25227;F01_transcript_25412;F01_transcript_26207;F01_transcript_26733;F01_transcript_28;F01_transcript_28359;F01_transcript_29113;F01_transcript_29571;F01_transcript_	K02148+K02146+K02146+K08492+K08517+K04392+K04392+K04392+K04392+K02150+K02149+K04392+K07374+K07897+K04392+K07897+K04392+K02150+K02149+K03661+K07897+K02155+K02155+K03661+K02155+K02155+K02155+K02155+K02155+K02155+K02155+K04392+K02153+K07889+K02155+K02152+K02155+K02151+K07342+K02152+K02153+K07342+K07889+K02154+K00921+K08054+K00921+K02155+K02150+K02154+K07374+K07375+K02154+K09481+K02150+K04392+K07375+K02147+K02147+K08492+K07897+K04392+K02154+K02151+K02154+K08057+K02152+K02147+K02147+K07374+K02154+K02150+K02154+K00914+K02154+K07375+K10956+K07374+K00914+K02151+K02154+K00914+K10956+K02145+K09481+K07374+K02144+K02147+K02154+K00921+K02148+K02155+K07375+K00921+K02150+K02154+K08057+K10956+K07375+K07375+K02145+K02146+K02154+K02155+K02154+K02154+K07342+K10956+K00914+K02154+K10956+K02147+K07375+K08057+K07374+K02147+K02155+K02155+K00914+K08057+K00914+K08057+K02147+K00914+K02154+K00921+K07889+K02147+K02147+K08057+K07374+K08517+K07375+K04392+K02153+K08057+K07897+K07375+K02154+K02147+K09481+K08517+K02154+K10956+K05692+K02148+K07342+K02145+K02147+K00914+K00914+K07889+K02154+K02154+K07889+K02150+K07889+K04392+K03661+K08517+K02147+K07375+K07374+K00921+K07375+K02154+K02154+K07897+K07375+K07897+K02154+K02145+K07375+K02153+K02154+K00921+K02145+K00921+K02148+K07374+K02147+K07375+K04392+K08054

29659;F01_transcript_29978;F01_transcript_30147;F01_transcri
pt_30602;F01_transcript_31278;F01_transcript_31354;F01_tran
script_32221;F01_transcript_32349;F01_transcript_32585;F01_t
ranscript_32971;F01_transcript_3298;F01_transcript_33053;F01
_transcript_33092;F01_transcript_33404;F01_transcript_33869;
F01_transcript_33977;F01_transcript_34042;F01_transcript_340
78;F01_transcript_34108;F01_transcript_34122;F01_transcript_
34674;F01_transcript_35179;F01_transcript_35257;F01_transcri
pt_35410;F01_transcript_35523;F01_transcript_35609;F01_tran
script_35745;F01_transcript_35749;F01_transcript_36236;F01_t
ranscript_36294;F01_transcript_36313;F01_transcript_36825;F0
1_transcript_36994;F01_transcript_37126;F01_transcript_37310
;F01_transcript_37488;F01_transcript_37573;F01_transcript_37
723;F01_transcript_38044;F01_transcript_38360;F01_transcript
_38485;F01_transcript_38873;F01_transcript_39078;F01_transc
ript_39463;F01_transcript_39977;F01_transcript_41427;F01_tra
nscript_41487;F01_transcript_41536;F01_transcript_42493;F01
_transcript_42584;F01_transcript_42839;F01_transcript_43205;
F01_transcript_43486;F01_transcript_43785;F01_transcript_438
96;F01_transcript_43928;F01_transcript_44098;F01_transcript_
44166;F01_transcript_44362;F01_transcript_44613;F01_transcri
pt_44703;F01_transcript_44714;F01_transcript_44738;F01_tran
script_45149;F01_transcript_45252;F01_transcript_45403;F01_t
ranscript_45589;F01_transcript_45603;F01_transcript_45652;F0
1_transcript_45772;F01_transcript_45814;F01_transcript_46116
;F01_transcript_46466;F01_transcript_4698;F01_transcript_471
28;F01_transcript_47131;F01_transcript_47323;F01_transcript_
47528;F01_transcript_47640;F01_transcript_48268;F01_transcri
pt_48783;F01_transcript_49010;F01_transcript_49040;F01_tran
script_50238;F01_transcript_5134;F01_transcript_51417;F01_tr
anscript_51990;F01_transcript_52270;F01_transcript_52496;F0
1_transcript_53586;F01_transcript_53857;F01_transcript_5415;
F01_transcript_54785;F01_transcript_54838;F01_transcript_553
04;F01_transcript_55536;F01_transcript_556;F01_transcript_55
752;F01_transcript_57066;F01_transcript_57129;F01_transcript
_57803;F01_transcript_58106;F01_transcript_58195;F01_transc
ript_58487;F01_transcript_5931;F01_transcript_59500;F01_tran

script_59773;F01_transcript_59867;F01_transcript_60016;F01_transcript_60346;F01_transcript_60834;F01_transcript_62084;F01_transcript_6240;F01_transcript_62510;F01_transcript_6295;F01_transcript_63398;F01_transcript_6365;F01_transcript_64232;F01_transcript_64393;F01_transcript_64539;F01_transcript_65003;F01_transcript_65163;F01_transcript_6532;F01_transcript_6591;F01_transcript_66568;F01_transcript_66625;F01_transcript_66846;F01_transcript_66912;F01_transcript_67046;F01_transcript_67078;F01_transcript_67400;F01_transcript_67527;F01_transcript_68062;F01_transcript_68163;F01_transcript_68216;F01_transcript_68408;F01_transcript_68917;F01_transcript_69013;F01_transcript_69797;F01_transcript_69895;F01_transcript_70410;F01_transcript_70739;F01_transcript_709;F01_transcript_71095;F01_transcript_71108;F01_transcript_71299;F01_transcript_71572;F01_transcript_7189;F01_transcript_72052;F01_transcript_72495;F01_transcript_72837;F01_transcript_7313;F01_transcript_73247;F01_transcript_73334;F01_transcript_73369;F01_transcript_73551;F01_transcript_74208;F01_transcript_74347;F01_transcript_74431;F01_transcript_74657;F01_transcript_75395;F01_transcript_75718;F01_transcript_77133;F01_transcript_78933;F01_transcript_78998;F01_transcript_79219;F01_transcript_7980;F01_transcript_80155;F01_transcript_80156;F01_transcript_80267;F01_transcript_80436;F01_transcript_81246;F01_transcript_81647;F01_transcript_81873;F01_transcript_81954;F01_transcript_83534;F01_transcript_8355;F01_transcript_83874;F01_transcript_84086;F01_transcript_84338;F01_transcript_85090;F01_transcript_85222;F01_transcript_86265;F01_transcript_86314;F01_transcript_86400;F01_transcript_86674;F01_transcript_87419;F01_transcript_87445;F01_transcript_87534;F01_transcript_88104;F01_transcript_88802;F01_transcript_8890;F01_transcript_88943;F01_transcript_88957;F01_transcript_89771;F01_transcript_89806;F01_transcript_90018;F01_transcript_90171;F01_transcript_90425;F01_transcript_90465;F01_transcript_91237;F01_transcript_91682;F01_transcript_91857;F01_transcript_91962;F01_transcript_92966;F01_transcript_93671;F01_transcript_93983;F01_transcript_94249;F01_transcript_94387;F01_transcript_94408;F01_transcript_94508;F01_transcript_94528;F01_transcript_94650;F01_transcript_95466;F01_transcript_95493;F01

			_transcript_95669;F01_transcript_95704;F01_transcript_96011;F01_transcript_96368;F01_transcript_97203;F01_transcript_97288;F01_transcript_97486;F01_transcript_97661;F01_transcript_98120;F01_transcript_99194;F01_transcript_99294;	
Peroxisome	ko04146	616	F01_transcript_10021;F01_transcript_100488;F01_transcript_100624;F01_transcript_101080;F01_transcript_102091;F01_transcript_102423;F01_transcript_102477;F01_transcript_102606;F01_transcript_103774;F01_transcript_103802;F01_transcript_103934;F01_transcript_103986;F01_transcript_10466;F01_transcript_104782;F01_transcript_104846;F01_transcript_104998;F01_transcript_105491;F01_transcript_105494;F01_transcript_106057;F01_transcript_106217;F01_transcript_106800;F01_transcript_107016;F01_transcript_107228;F01_transcript_10764;F01_transcript_107966;F01_transcript_108062;F01_transcript_108186;F01_transcript_108699;F01_transcript_109183;F01_transcript_109418;F01_transcript_110455;F01_transcript_111415;F01_transcript_11167;F01_transcript_111760;F01_transcript_113095;F01_transcript_11365;F01_transcript_114458;F01_transcript_114684;F01_transcript_11473;F01_transcript_114737;F01_transcript_114966;F01_transcript_115230;F01_transcript_115378;F01_transcript_115917;F01_transcript_115996;F01_transcript_11601;F01_transcript_116320;F01_transcript_116449;F01_transcript_117227;F01_transcript_117687;F01_transcript_118373;F01_transcript_118434;F01_transcript_118448;F01_transcript_118613;F01_transcript_118620;F01_transcript_118820;F01_transcript_119034;F01_transcript_119285;F01_transcript_119532;F01_transcript_119534;F01_transcript_119569;F01_transcript_119763;F01_transcript_119894;F01_transcript_12016;F01_transcript_120521;F01_transcript_120641;F01_transcript_120801;F01_transcript_121105;F01_transcript_121203;F01_transcript_121296;F01_transcript_121422;F01_transcript_121478;F01_transcript_121724;F01_transcript_121956;F01_transcript_121960;F01_transcript_122368;F01_transcript_122442;F01_transcript_122499;F01_transcript_122818;F01_transcript_122839;F01_transcript_122863;F01_transcript_123251;F01_transcript_12336;F01_transcript_123569;F01_transcript_123616;F01_transcript_124192;F01_transcript_124404;F01_transcript_124429;F01_transcript_124453;F01_transcrip	K03781+K01640+K13339+K13342+K13342+K13343+K11517+K13348+K11517+K11517+K13348+K13343+K01897+K01897+K13348+K01897+K13354+K13337+K13237+K00232+K04564+K12261+K03781+K13342+K03781+K11517+K03781+K13348+K13348+K00830+K13336+K13342+K13342+K03781+K11517+K13342+K01897+K01897+K03781+K03781+K00031+K01897+K13335+K03781+K01897+K00232+K03781+K13348+K01897+K00232+K00830+K00232+K13348+K13237+K04564+K11517+K00031+K03781+K01897+K00031+K00830+K13348+K11517+K13354+K00106+K13346+K00232+K03781+K13343+K13342+K13348+K01897+K13343+K13342+K03781+K00232+K13354+K00830+K00232+K13338+K11517+K03781+K03781+K01640+K13343+K13347+K03781+K13338+K13354+K00232+K00232+K13342+K11517+K13345+K01897+K03781+K03781+K00830+K03781+K01897+K12261+K13354+K01897+K00477+K11147+K13338+K01897+K00477+K13354+K13342+K07513+K03781+K12261+K01897+K03781+K13338+K01897+K13354+K03781+K11517+K03781+K01897+K04565+K13346+K13336+K11517+K13338+K01897+K11517+K01897+K13354+K03426+K03781+K06664+K13337+K00232+K13354+K00232+K04564+K01897+K13348+K01897+K03781+K13346+K03426+K11517+K13342+K12261+K03781+K03781+K12663+K03781+K00232+K11517+K11517+K01897+K00106+K00232+K03781+K11517+K01897+K06664+K13347+K11517+K13348+K12261+K13343+K13237+K00830+K04564+K00031+K00232+K13342+K00232+K07513+K03781+K13354+K01897+K13335+K13342+K01897+K01897+K11517+K00232+K00830+K00869+K04564+K13347+K03781+K03781+K04564+K00232+K00306+K01897+K01897+K03781+K01897+K12261+K04564+K13338+K03426+K03781+K03781+K01897+K03781+K13347+K03781+K03781+K00232+K13348+K03781+K01897+K11517+K03781+K13342+K13346+K13354+K00232+K01897+K13338+K13345+K00232+K01897+K03781+K00106+K01897+K0

	t_124769;F01_transcript_124788;F01_transcript_124923;F01_transcript_125285;F01_transcript_125345;F01_transcript_12599;F01_transcript_127239;F01_transcript_127607;F01_transcript_127687;F01_transcript_127884;F01_transcript_128013;F01_transcript_128036;F01_transcript_128702;F01_transcript_12879;F01_transcript_128817;F01_transcript_129286;F01_transcript_129503;F01_transcript_12985;F01_transcript_130142;F01_transcript_130339;F01_transcript_13068;F01_transcript_130717;F01_transcript_131015;F01_transcript_131055;F01_transcript_13109;F01_transcript_131343;F01_transcript_1317;F01_transcript_131715;F01_transcript_131758;F01_transcript_131925;F01_transcript_132241;F01_transcript_132298;F01_transcript_132668;F01_transcript_134005;F01_transcript_134543;F01_transcript_134575;F01_transcript_134656;F01_transcript_134873;F01_transcript_134920;F01_transcript_135338;F01_transcript_135733;F01_transcript_135783;F01_transcript_136229;F01_transcript_136746;F01_transcript_136833;F01_transcript_136880;F01_transcript_138047;F01_transcript_138732;F01_transcript_139123;F01_transcript_139452;F01_transcript_139502;F01_transcript_140118;F01_transcript_140123;F01_transcript_140205;F01_transcript_140269;F01_transcript_140365;F01_transcript_140463;F01_transcript_140735;F01_transcript_140743;F01_transcript_14076;F01_transcript_140967;F01_transcript_141058;F01_transcript_141996;F01_transcript_142214;F01_transcript_143118;F01_transcript_143180;F01_transcript_143258;F01_transcript_143330;F01_transcript_143781;F01_transcript_144230;F01_transcript_144482;F01_transcript_144717;F01_transcript_144958;F01_transcript_145099;F01_transcript_145345;F01_transcript_145620;F01_transcript_145644;F01_transcript_146251;F01_transcript_146268;F01_transcript_146447;F01_transcript_146799;F01_transcript_147008;F01_transcript_147167;F01_transcript_147222;F01_transcript_147259;F01_transcript_147398;F01_transcript_147432;F01_transcript_147448;F01_transcript_147795;F01_transcript_147885;F01_transcript_148793;F01_transcript_14899;F01_transcript_149352;F01_transcript_149413;F01_transcript_149791;F01_transcript_150046;F01_transcript_150083;F01_transcript_151112;F01_transcript_151592;F01_transcript_15195;F01_transcript_152474;F01_transcript_152542;F01_transcript_15284;F01_transcript_15	0232+K00830+K13335+K07513+K04565+K13345+K11517+K00232+K11517+K11517+K12663+K03781+K01897+K01897+K11517+K00232+K01897+K13342+K13343+K03781+K03781+K13354+K13342+K01897+K13354+K11517+K00306+K13338+K00830+K13345+K11517+K13342+K13348+K03781+K13348+K03781+K13347+K00830+K03781+K01897+K05677+K03781+K07513+K13346+K13342+K00830+K11517+K12261+K11517+K01897+K00232+K01897+K01897+K01897+K01897+K01897+K01897+K11517+K03781+K03781+K11517+K00232+K03781+K01897+K03781+K03781+K03781+K03781+K13345+K03781+K03781+K12261+K13343+K03781+K13343+K00232+K03781+K00232+K03781+K03781+K03781+K03781+K01897+K06664+K13346+K13343+K12261+K00232+K03781+K03781+K03781+K03781+K03781+K00232+K03781+K03781+K01640+K03781+K13354+K03781+K03781+K07513+K01640+K11517+K07513+K01578+K03781+K03781+K07513+K03781+K00031+K13354+K03781+K03781+K03426+K07513+K13354+K13345+K00232+K00232+K13345+K13335+K13348+K11517+K13348+K11517+K11517+K13348+K01640+K13354+K00830+K00106+K11517+K11517+K03781+K13336+K03781+K00869+K11517+K11517+K00830+K11517+K11517+K13348+K00306+K11517+K11517+K13346+K11517+K04564+K11517+K03781+K11517+K13354+K13341+K13348+K13354+K00477+K04564+K06664+K13341+K13237+K00477+K13348+K13347+K04564+K13237+K04564+K04565+K13237+K12663+K03781+K04564+K04564+K04564+K04565+K13348+K04565+K04565+K04565+K04565+K11147+K13347+K11147+K13347+K04565+K03781+K04565+K04565+K04565+K04565+K04565+K13348+K04565+K04565+K04565+K01897+K03781+K13336+K13237+K03781+K00232+K03781+K13342+K13347+K00232+K00031+K00031+K00232+K04565+K01897+K13348+K00232+K00232+K00232+K01897+K00232+K13348+K13348+K11517+K13346+K00232+K13348+K13348+K00232+K03781+K13237+K04564+K03781+K03781+K03781+K11517+K03781+K13348+K13354+K03781+K01640+K13354+K03781+K07513+K01897+K13356+K00477+K11517+K13339+K03781+K01897+K12261+K13348+K13348+K03781+K00106+K13348+K04564+K00830+K03781+K04565+K01897+K00830+K00232+K13338

	3078;F01_transcript_153488;F01_transcript_15349;F01_transcript_153615;F01_transcript_15367;F01_transcript_153697;F01_transcript_153704;F01_transcript_1542;F01_transcript_15422;F01_transcript_154347;F01_transcript_154524;F01_transcript_154600;F01_transcript_154709;F01_transcript_155047;F01_transcript_15505;F01_transcript_155366;F01_transcript_155474;F01_transcript_156224;F01_transcript_156564;F01_transcript_15661;F01_transcript_15677;F01_transcript_156864;F01_transcript_157216;F01_transcript_157239;F01_transcript_157335;F01_transcript_157406;F01_transcript_15780;F01_transcript_157907;F01_transcript_157974;F01_transcript_15817;F01_transcript_158278;F01_transcript_15880;F01_transcript_159122;F01_transcript_159135;F01_transcript_15946;F01_transcript_159651;F01_transcript_159892;F01_transcript_159968;F01_transcript_160377;F01_transcript_160511;F01_transcript_160623;F01_transcript_160683;F01_transcript_160955;F01_transcript_161267;F01_transcript_161279;F01_transcript_161709;F01_transcript_161773;F01_transcript_162421;F01_transcript_162492;F01_transcript_16286;F01_transcript_16301;F01_transcript_163076;F01_transcript_163143;F01_transcript_163559;F01_transcript_163584;F01_transcript_163645;F01_transcript_164048;F01_transcript_16433;F01_transcript_164407;F01_transcript_165154;F01_transcript_165256;F01_transcript_165272;F01_transcript_165317;F01_transcript_165378;F01_transcript_165385;F01_transcript_165496;F01_transcript_16581;F01_transcript_165910;F01_transcript_166307;F01_transcript_166597;F01_transcript_166618;F01_transcript_166993;F01_transcript_16722;F01_transcript_167396;F01_transcript_167420;F01_transcript_167454;F01_transcript_167595;F01_transcript_167723;F01_transcript_16774;F01_transcript_16810;F01_transcript_1702;F01_transcript_176;F01_transcript_17645;F01_transcript_17966;F01_transcript_17987;F01_transcript_18447;F01_transcript_19074;F01_transcript_19302;F01_transcript_19520;F01_transcript_19705;F01_transcript_20309;F01_transcript_20901;F01_transcript_2179;F01_transcript_22223;F01_transcript_22572;F01_transcript_22669;F01_transcript_22876;F01_transcript_23121;F01_transcript_23456;F01_transcript_23618;F01_transcript_23952;F01_transcript_24090;F01_transcript_24654;	+K13336+K13348+K13343+K11517+K01897+K03781+K00232+K11517+K03781+K01897+K11517+K13339+K04564+K13339+K13336+K13335+K11517+K03781+K01897+K01897+K01897+K13348+K01640+K00232+K13348+K11517+K11517+K03781+K12663+K00106+K00830+K11517+K03781+K00031+K13336+K13338+K13341+K01897+K03781+K11517+K13346+K01897+K11517+K00232+K11517+K00232+K04565+K13348+K03781+K01640+K13346+K13346+K01897+K00232+K13354+K13348+K00232+K13354+K13342+K13354+K00232+K13342+K03781+K03781+K01897+K13343+K11517+K00232+K03781+K00830+K13354+K01897+K03781+K11187+K13354+K01897+K13342+K13346+K01578+K07513+K11517+K13356+K13348+K01897+K11517+K11517+K03781+K13348+K00830+K01897+K00232+K13339+K13354+K04565+K13339+K03781+K11517+K03781+K00232+K00232+K01897+K13342+K13339+K00232+K03781+K04564+K03781+K13346+K01897+K13339+K11517+K13336+K13342+K00232+K11517+K11147+K01897+K03781+K13348+K03781+K01897+K13342+K07513+K01897+K00031+K13342+K01897+K01897+K13346

F01_transcript_24832;F01_transcript_25245;F01_transcript_253
46;F01_transcript_26371;F01_transcript_2689;F01_transcript_2
6938;F01_transcript_27104;F01_transcript_27222;F01_transcrip
t_27232;F01_transcript_27298;F01_transcript_27585;F01_trans
cript_2805;F01_transcript_28169;F01_transcript_28357;F01_tra
nscript_28477;F01_transcript_28994;F01_transcript_29068;F01
_transcript_29119;F01_transcript_29191;F01_transcript_29197;
F01_transcript_29210;F01_transcript_29967;F01_transcript_305
53;F01_transcript_30696;F01_transcript_30801;F01_transcript_
30891;F01_transcript_31325;F01_transcript_31485;F01_transcri
pt_31749;F01_transcript_31818;F01_transcript_32306;F01_tran
script_32561;F01_transcript_32889;F01_transcript_33312;F01_t
ranscript_33540;F01_transcript_33549;F01_transcript_33685;F0
1_transcript_34196;F01_transcript_34440;F01_transcript_34454
;F01_transcript_34620;F01_transcript_34767;F01_transcript_34
892;F01_transcript_35037;F01_transcript_35349;F01_transcript
_35379;F01_transcript_35442;F01_transcript_35833;F01_transc
ript_35968;F01_transcript_35988;F01_transcript_36482;F01_tra
nscript_36768;F01_transcript_36839;F01_transcript_37231;F01
_transcript_37345;F01_transcript_37438;F01_transcript_37531;
F01_transcript_37690;F01_transcript_37731;F01_transcript_378
41;F01_transcript_37893;F01_transcript_37978;F01_transcript_
37979;F01_transcript_38026;F01_transcript_38051;F01_transcri
pt_38062;F01_transcript_38141;F01_transcript_3817;F01_trans
cript_38313;F01_transcript_38456;F01_transcript_38578;F01_tr
anscript_38799;F01_transcript_38826;F01_transcript_38934;F0
1_transcript_38952;F01_transcript_39050;F01_transcript_39119
;F01_transcript_39124;F01_transcript_39311;F01_transcript_39
827;F01_transcript_39947;F01_transcript_39952;F01_transcript
_39992;F01_transcript_40003;F01_transcript_40238;F01_transc
ript_40843;F01_transcript_40933;F01_transcript_41617;F01_tra
nscript_41789;F01_transcript_41852;F01_transcript_41995;F01
_transcript_42004;F01_transcript_42137;F01_transcript_42205;
F01_transcript_42284;F01_transcript_42477;F01_transcript_427
15;F01_transcript_42998;F01_transcript_43353;F01_transcript_
43483;F01_transcript_43648;F01_transcript_43777;F01_transcri
pt_4393;F01_transcript_44030;F01_transcript_44356;F01_trans
cript_44397;F01_transcript_44472;F01_transcript_44481;F01_tr

anscript_44509;F01_transcript_44590;F01_transcript_45157;F01_transcript_45522;F01_transcript_45974;F01_transcript_46156;F01_transcript_46194;F01_transcript_47052;F01_transcript_47272;F01_transcript_47321;F01_transcript_47892;F01_transcript_48005;F01_transcript_48082;F01_transcript_48266;F01_transcript_48534;F01_transcript_48574;F01_transcript_48916;F01_transcript_49184;F01_transcript_49852;F01_transcript_4986;F01_transcript_50401;F01_transcript_51423;F01_transcript_51601;F01_transcript_51616;F01_transcript_52099;F01_transcript_5211;F01_transcript_52160;F01_transcript_52334;F01_transcript_52548;F01_transcript_52686;F01_transcript_52750;F01_transcript_53390;F01_transcript_53916;F01_transcript_54622;F01_transcript_54798;F01_transcript_54990;F01_transcript_55240;F01_transcript_55281;F01_transcript_55736;F01_transcript_56128;F01_transcript_56521;F01_transcript_57154;F01_transcript_57206;F01_transcript_57270;F01_transcript_57777;F01_transcript_57798;F01_transcript_58046;F01_transcript_58171;F01_transcript_58214;F01_transcript_59015;F01_transcript_59577;F01_transcript_59602;F01_transcript_59752;F01_transcript_59771;F01_transcript_59956;F01_transcript_60004;F01_transcript_60292;F01_transcript_60686;F01_transcript_60831;F01_transcript_61404;F01_transcript_61420;F01_transcript_61725;F01_transcript_61866;F01_transcript_61928;F01_transcript_61935;F01_transcript_61986;F01_transcript_62337;F01_transcript_6248;F01_transcript_62749;F01_transcript_62811;F01_transcript_62852;F01_transcript_63162;F01_transcript_63312;F01_transcript_63540;F01_transcript_64077;F01_transcript_64306;F01_transcript_64906;F01_transcript_65073;F01_transcript_65194;F01_transcript_65326;F01_transcript_65891;F01_transcript_65973;F01_transcript_66022;F01_transcript_66057;F01_transcript_66230;F01_transcript_66322;F01_transcript_66474;F01_transcript_66719;F01_transcript_67276;F01_transcript_67470;F01_transcript_67738;F01_transcript_67774;F01_transcript_68283;F01_transcript_68887;F01_transcript_69129;F01_transcript_69199;F01_transcript_69469;F01_transcript_7003;F01_transcript_70676;F01_transcript_71145;F01_transcript_71223;F01_transcript_71512;F01_transcript_71725;F01_transcript_72007;F01_transcript_72096;F01_transcript_7

2550;F01_transcript_72756;F01_transcript_72826;F01_transcrip
t_72969;F01_transcript_73015;F01_transcript_73145;F01_trans
cript_74943;F01_transcript_74952;F01_transcript_75032;F01_tr
anscript_75487;F01_transcript_75535;F01_transcript_75647;F0
1_transcript_75938;F01_transcript_76249;F01_transcript_76479
;F01_transcript_76975;F01_transcript_76982;F01_transcript_77
186;F01_transcript_77339;F01_transcript_78123;F01_transcript
_78246;F01_transcript_79544;F01_transcript_80183;F01_transc
ript_80484;F01_transcript_80645;F01_transcript_80850;F01_tra
nscript_81208;F01_transcript_81684;F01_transcript_81816;F01
_transcript_81977;F01_transcript_82120;F01_transcript_82184;
F01_transcript_82368;F01_transcript_82552;F01_transcript_827
09;F01_transcript_82742;F01_transcript_82756;F01_transcript_
83103;F01_transcript_83183;F01_transcript_83317;F01_transcri
pt_83767;F01_transcript_84294;F01_transcript_84919;F01_tran
script_84935;F01_transcript_85044;F01_transcript_85164;F01_t
ranscript_85292;F01_transcript_8635;F01_transcript_86483;F01
_transcript_86645;F01_transcript_86684;F01_transcript_87291;
F01_transcript_87738;F01_transcript_87798;F01_transcript_880
28;F01_transcript_8804;F01_transcript_88103;F01_transcript_8
8199;F01_transcript_88720;F01_transcript_89027;F01_transcrip
t_89458;F01_transcript_89867;F01_transcript_89994;F01_trans
cript_90051;F01_transcript_90156;F01_transcript_90255;F01_tr
anscript_90722;F01_transcript_90815;F01_transcript_90896;F0
1_transcript_90931;F01_transcript_90994;F01_transcript_91134
;F01_transcript_91144;F01_transcript_91894;F01_transcript_91
939;F01_transcript_92070;F01_transcript_92190;F01_transcript
_92632;F01_transcript_92699;F01_transcript_92831;F01_transc
ript_92860;F01_transcript_93422;F01_transcript_93534;F01_tra
nscript_93559;F01_transcript_94051;F01_transcript_9460;F01_t
ranscript_94970;F01_transcript_95989;F01_transcript_96196;F0
1_transcript_96299;F01_transcript_96588;F01_transcript_96649
;F01_transcript_97024;F01_transcript_97256;F01_transcript_97
369;F01_transcript_97377;F01_transcript_97417;F01_transcript
_97480;F01_transcript_97828;F01_transcript_98172;F01_transc
ript_98417;F01_transcript_98462;F01_transcript_98478;F01_tra
nscript_98527;F01_transcript_99261;F01_transcript_99276;F01
_transcript_99279;F01_transcript_99380;

Plant-pathogen interaction	ko04626	868	F01_transcript_100033;F01_transcript_100191;F01_transcript_100232;F01_transcript_100457;F01_transcript_10062;F01_transcript_100881;F01_transcript_101546;F01_transcript_101593;F01_transcript_102102;F01_transcript_102149;F01_transcript_102492;F01_transcript_102566;F01_transcript_102762;F01_transcript_102783;F01_transcript_102808;F01_transcript_102828;F01_transcript_102941;F01_transcript_103310;F01_transcript_10336;F01_transcript_103374;F01_transcript_103458;F01_transcript_103687;F01_transcript_103929;F01_transcript_103984;F01_transcript_104102;F01_transcript_104204;F01_transcript_104307;F01_transcript_104338;F01_transcript_104406;F01_transcript_104591;F01_transcript_104684;F01_transcript_104734;F01_transcript_104850;F01_transcript_104964;F01_transcript_104981;F01_transcript_105034;F01_transcript_105438;F01_transcript_10580;F01_transcript_106536;F01_transcript_106720;F01_transcript_106879;F01_transcript_1069;F01_transcript_107304;F01_transcript_107450;F01_transcript_107662;F01_transcript_107745;F01_transcript_107875;F01_transcript_108384;F01_transcript_108461;F01_transcript_108719;F01_transcript_108840;F01_transcript_108974;F01_transcript_10909;F01_transcript_109181;F01_transcript_10922;F01_transcript_10970;F01_transcript_10993;F01_transcript_110269;F01_transcript_110987;F01_transcript_111022;F01_transcript_111200;F01_transcript_111794;F01_transcript_111999;F01_transcript_11232;F01_transcript_112554;F01_transcript_112591;F01_transcript_112606;F01_transcript_112730;F01_transcript_11281;F01_transcript_11300;F01_transcript_113045;F01_transcript_113118;F01_transcript_113295;F01_transcript_113432;F01_transcript_114184;F01_transcript_114510;F01_transcript_114657;F01_transcript_114661;F01_transcript_114784;F01_transcript_114786;F01_transcript_114789;F01_transcript_11484;F01_transcript_115257;F01_transcript_115585;F01_transcript_115621;F01_transcript_115686;F01_transcript_115960;F01_transcript_116149;F01_transcript_116228;F01_transcript_116496;F01_transcript_116506;F01_transcript_116638;F01_transcript_116834;F01_transcript_117178;F01_transcript_117439;F01_transcript_117659;F01_transcript_118230;F01_transcript_118464;F01_transcript_118488;F01_transcript_118861;F01_transcript	K13457+K13459+K13457+K13457+K13459+K13412+K13414+K05391+K13457+K05391+K05391+K13412+K13414+K13459+K13457+K13429+K05391+K13457+K18835+K13459+K05391+K05391+K18834+K13448+K13459+K13447+K13412+K13459+K13436+K13448+K13412+K13457+K13412+K05391+K02183+K12795+K13436+K13412+K13434+K13456+K13457+K13447+K13436+K13412+K13458+K13429+K13412+K13447+K02183+K13412+K09487+K13448+K05391+K04079+K05391+K09487+K05391+K13457+K13457+K05391+K13457+K09487+K13448+K05391+K13430+K05391+K13414+K13412+K04079+K05391+K13414+K13457+K13456+K13412+K13414+K13448+K05391+K09487+K04079+K05391+K13412+K09487+K13457+K18835+K13412+K13424+K13429+K13448+K09487+K05391+K13436+K13457+K13457+K18875+K13412+K09487+K13436+K13414+K13424+K13457+K04079+K13412+K13414+K13457+K13457+K13447+K13429+K13448+K18835+K09487+K13429+K09487+K18835+K13447+K05391+K13414+K13412+K13457+K13457+K05391+K13447+K05391+K13412+K13414+K13412+K13448+K13414+K05391+K13412+K05391+K13412+K13448+K12795+K13412+K13429+K05391+K13412+K13414+K18834+K13414+K13412+K04079+K13447+K09487+K13420+K02183+K04079+K13457+K13414+K13412+K04079+K13457+K18835+K04079+K05391+K13412+K13457+K13414+K13459+K05391+K13434+K13457+K05391+K12795+K13429+K13414+K09487+K02183+K09487+K13429+K18835+K13459+K13412+K13412+K13434+K12795+K13430+K13412+K13412+K05391+K05391+K05391+K05391+K09487+K05391+K00864+K18835+K13414+K13448+K13412+K13448+K05391+K18834+K13459+K05391+K13448+K13412+K13457+K13458+K13459+K13420+K13457+K13459+K13459+K13456+K18834+K13430+K18835+K05391+K13412+K13414+K13458+K13412+K13412+K13457+K13457+K13412+K18835+K09487+K13459+K05391+K05391+K13414+K13416+K13429+K13414+K05391+K02183+K13412+K09487+K05391+K13447+K09487+K13412+K13412+K04079+K18835+K13412+K13459+K13459+K05391+K13414+K13414+K13430+K05391+K13412+K13459+K05391+K20536+K05391+K13448+K13412+K13457+K1341

	_119256;F01_transcript_119453;F01_transcript_119460;F01_transcript_119472;F01_transcript_119573;F01_transcript_119964;F01_transcript_120516;F01_transcript_120523;F01_transcript_120744;F01_transcript_120950;F01_transcript_120957;F01_transcript_121015;F01_transcript_121331;F01_transcript_121421;F01_transcript_121526;F01_transcript_121621;F01_transcript_121815;F01_transcript_121874;F01_transcript_121894;F01_transcript_122155;F01_transcript_122219;F01_transcript_122317;F01_transcript_12254;F01_transcript_122731;F01_transcript_123076;F01_transcript_123308;F01_transcript_123523;F01_transcript_123809;F01_transcript_123835;F01_transcript_124052;F01_transcript_12406;F01_transcript_124084;F01_transcript_124164;F01_transcript_124878;F01_transcript_125053;F01_transcript_125246;F01_transcript_125508;F01_transcript_125950;F01_transcript_126012;F01_transcript_126026;F01_transcript_126151;F01_transcript_126269;F01_transcript_126744;F01_transcript_126814;F01_transcript_126917;F01_transcript_127598;F01_transcript_127903;F01_transcript_128209;F01_transcript_128290;F01_transcript_128483;F01_transcript_128714;F01_transcript_129500;F01_transcript_129521;F01_transcript_12956;F01_transcript_12958;F01_transcript_12962;F01_transcript_129647;F01_transcript_129696;F01_transcript_129706;F01_transcript_129758;F01_transcript_130492;F01_transcript_130530;F01_transcript_130984;F01_transcript_131068;F01_transcript_131195;F01_transcript_1312;F01_transcript_131225;F01_transcript_131361;F01_transcript_131420;F01_transcript_131873;F01_transcript_131924;F01_transcript_132010;F01_transcript_132025;F01_transcript_132080;F01_transcript_132516;F01_transcript_132596;F01_transcript_132778;F01_transcript_132863;F01_transcript_133419;F01_transcript_133476;F01_transcript_134260;F01_transcript_13456;F01_transcript_134813;F01_transcript_135179;F01_transcript_135344;F01_transcript_13541;F01_transcript_135765;F01_transcript_135827;F01_transcript_136159;F01_transcript_13621;F01_transcript_136311;F01_transcript_13640;F01_transcript_136515;F01_transcript_136695;F01_transcript_136818;F01_transcript_136835;F01_transcript_13686;F01_transcript_136936;F01_transcript_137179;F01_transcript_137508;F01_transcript_138081;F01_transcript_13810;F01_transcript_138136;F01_transcript_13831	4+K13456+K13412+K13436+K13459+K13429+K13459+K13414+K13448+K05391+K13429+K13414+K13448+K13456+K13448+K05391+K13448+K13412+K13448+K04079+K13414+K13414+K13456+K13447+K13412+K05391+K04079+K09487+K18834+K05391+K05391+K13412+K18834+K13447+K04079+K04079+K13448+K09487+K13412+K00864+K13412+K13447+K13414+K13447+K13420+K13457+K13414+K12795+K13459+K13448+K13457+K13448+K13416+K05391+K13414+K13457+K13459+K13448+K13436+K05391+K05391+K05391+K05391+K05391+K13412+K13448+K05391+K13416+K18835+K09487+K13447+K09487+K13414+K13414+K13414+K13447+K13414+K13447+K13459+K13459+K13412+K13412+K13412+K13430+K05391+K05391+K05391+K13412+K13430+K13414+K13457+K05391+K05391+K09487+K04079+K13412+K13447+K05391+K09487+K13412+K18875+K04079+K13457+K13412+K09487+K13447+K13412+K13459+K13447+K13429+K13457+K18835+K13412+K13412+K13416+K05391+K13412+K05391+K13457+K13414+K05391+K13412+K05391+K05391+K13447+K13414+K13448+K13414+K13459+K13414+K13456+K18835+K13414+K13457+K09487+K13459+K13424+K13412+K05391+K04079+K13412+K13420+K13430+K02183+K09487+K13447+K13420+K05391+K04079+K13416+K13459+K18835+K13412+K13412+K05391+K04079+K13448+K13412+K13412+K13414+K13412+K13459+K13414+K13412+K13414+K18835+K13457+K13414+K05391+K13412+K13420+K13412+K13429+K05391+K13429+K13412+K13429+K13459+K05391+K13414+K18875+K13412+K13457+K13424+K13414+K13412+K05391+K05391+K13412+K13457+K13412+K13412+K13430+K13429+K13429+K13412+K13412+K13424+K13457+K18834+K18875+K18875+K13424+K13412+K13412+K18834+K13412+K13436+K13456+K13447+K13430+K13412+K13413+K20536+K13412+K13412+K04079+K05391+K20600+K00864+K13436+K13427+K13412+K20600+K20600+K13447+K13436+K13424+K13459+K13436+K13447+K13414+K13457+K13457+K20600+K13436+K13448+K20536+K13457+K13459+K13459+K20600+K04079+K12795+K13459+K12795+K13436+K13459+K12795+K13456+K04079+K13412+K12795+K05391+K13457+K13459+K13448+K13457+K13459+K13459+K05391+K1

	9;F01_transcript_138376;F01_transcript_138455;F01_transcript_138687;F01_transcript_138694;F01_transcript_138809;F01_transcript_139065;F01_transcript_139154;F01_transcript_139352;F01_transcript_13936;F01_transcript_139524;F01_transcript_139528;F01_transcript_139667;F01_transcript_140057;F01_transcript_14013;F01_transcript_140178;F01_transcript_140647;F01_transcript_14071;F01_transcript_140863;F01_transcript_141041;F01_transcript_14117;F01_transcript_141202;F01_transcript_141220;F01_transcript_141549;F01_transcript_141569;F01_transcript_14162;F01_transcript_141708;F01_transcript_142029;F01_transcript_142159;F01_transcript_142258;F01_transcript_14229;F01_transcript_14234;F01_transcript_142803;F01_transcript_142905;F01_transcript_142946;F01_transcript_143121;F01_transcript_143281;F01_transcript_143517;F01_transcript_14371;F01_transcript_143742;F01_transcript_14400;F01_transcript_144019;F01_transcript_144102;F01_transcript_144157;F01_transcript_144184;F01_transcript_14446;F01_transcript_144847;F01_transcript_144978;F01_transcript_145498;F01_transcript_145504;F01_transcript_145689;F01_transcript_145754;F01_transcript_145876;F01_transcript_145896;F01_transcript_146246;F01_transcript_146265;F01_transcript_146548;F01_transcript_147050;F01_transcript_147084;F01_transcript_147188;F01_transcript_147319;F01_transcript_147564;F01_transcript_147566;F01_transcript_147598;F01_transcript_147718;F01_transcript_147794;F01_transcript_148157;F01_transcript_148182;F01_transcript_148867;F01_transcript_14898;F01_transcript_149024;F01_transcript_149492;F01_transcript_149703;F01_transcript_150815;F01_transcript_150903;F01_transcript_150962;F01_transcript_15101;F01_transcript_151394;F01_transcript_151451;F01_transcript_151505;F01_transcript_151701;F01_transcript_15176;F01_transcript_151781;F01_transcript_152021;F01_transcript_152213;F01_transcript_15264;F01_transcript_152764;F01_transcript_152874;F01_transcript_153092;F01_transcript_153095;F01_transcript_15310;F01_transcript_15311;F01_transcript_153122;F01_transcript_153211;F01_transcript_153278;F01_transcript_153587;F01_transcript_153832;F01_transcript_153997;F01_transcript_154014;F01_transcript_154207;F01_transcript_154301;F01_transcript	3458+K13448+K13457+K13448+K13448+K02183+K13457+K13448+K13448+K02183+K02183+K02183+K02183+K02183+K13448+K13448+K04079+K13448+K13448+K13448+K13457+K13448+K13448+K04079+K13448+K13449+K13457+K02183+K13412+K02183+K13447+K13457+K05391+K13412+K13447+K13457+K13412+K13457+K13436+K09487+K13429+K13459+K13420+K13420+K13412+K00864+K13448+K13429+K02183+K13429+K13457+K13447+K05391+K13412+K13436+K13456+K13457+K13412+K18835+K13459+K13459+K13459+K13412+K13430+K05391+K05391+K12795+K05391+K13459+K13457+K13457+K13412+K13457+K20600+K13456+K13457+K13412+K13414+K13412+K13457+K05391+K04079+K13414+K13448+K13457+K13429+K13414+K18835+K02183+K13457+K18834+K13414+K05391+K02183+K05391+K04079+K13448+K13447+K13459+K05391+K13424+K13430+K13457+K18835+K13436+K13420+K05391+K12795+K09487+K09487+K18835+K13414+K13416+K13412+K13412+K05391+K13457+K13412+K02183+K13414+K12795+K05391+K13459+K13447+K05391+K13457+K13457+K13459+K13448+K13424+K13412+K13459+K13457+K13414+K13412+K13459+K13459+K13412+K18875+K05391+K13412+K13416+K05391+K05391+K18835+K13425+K13447+K13457+K04079+K13447+K13459+K05391+K02183+K13457+K13457+K13412+K13447+K05391+K13459+K13457+K13456+K05391+K13459+K13447+K05391+K13466+K13412+K09487+K13457+K13430+K13457+K13414+K13414+K13414+K04079+K13447+K13420+K13448+K09487+K09487+K13414+K13416+K13459+K13457+K05391+K05391+K13457+K13412+K13459+K13458+K13459+K05391+K13412+K13412+K13414+K05391+K05391+K13447+K13412+K13412+K13448+K13412+K05391+K13420+K02183+K04079+K13412+K13457+K13412+K13429+K13412+K13457+K12795+K20600+K05391+K13459+K05391+K13448+K02183+K13447+K13412+K18835+K09487+K13459+K13457+K05391+K13447+K13412+K13412+K09487+K13448+K13459+K13412+K13457+K13457+K02183+K13457+K05391+K13448+K13448+K13457+K13412+K13457+K13412+K05391+K04079+K13429+K05391+K05391+K13412+K13412+K04079+K13430+K09487+

	_154308;F01_transcript_15444;F01_transcript_154462;F01_transcript_154550;F01_transcript_154658;F01_transcript_154905;F01_transcript_154940;F01_transcript_155024;F01_transcript_155122;F01_transcript_155123;F01_transcript_155147;F01_transcript_155176;F01_transcript_155246;F01_transcript_156040;F01_transcript_156433;F01_transcript_156574;F01_transcript_15660;F01_transcript_15664;F01_transcript_15717;F01_transcript_157234;F01_transcript_157487;F01_transcript_157692;F01_transcript_157814;F01_transcript_158064;F01_transcript_158201;F01_transcript_158307;F01_transcript_158330;F01_transcript_158543;F01_transcript_158973;F01_transcript_159243;F01_transcript_159282;F01_transcript_159284;F01_transcript_159363;F01_transcript_15953;F01_transcript_160367;F01_transcript_160386;F01_transcript_160517;F01_transcript_160666;F01_transcript_160736;F01_transcript_160842;F01_transcript_160854;F01_transcript_160922;F01_transcript_161108;F01_transcript_161535;F01_transcript_161596;F01_transcript_161619;F01_transcript_161692;F01_transcript_161857;F01_transcript_16195;F01_transcript_162543;F01_transcript_16261;F01_transcript_162685;F01_transcript_16277;F01_transcript_162886;F01_transcript_163052;F01_transcript_163058;F01_transcript_163100;F01_transcript_163190;F01_transcript_163433;F01_transcript_163450;F01_transcript_16367;F01_transcript_163936;F01_transcript_164099;F01_transcript_16410;F01_transcript_164167;F01_transcript_16419;F01_transcript_164258;F01_transcript_164340;F01_transcript_164367;F01_transcript_164552;F01_transcript_165000;F01_transcript_165202;F01_transcript_165281;F01_transcript_165441;F01_transcript_165460;F01_transcript_165790;F01_transcript_165822;F01_transcript_165855;F01_transcript_165891;F01_transcript_165970;F01_transcript_165979;F01_transcript_166037;F01_transcript_1661;F01_transcript_166423;F01_transcript_166545;F01_transcript_166698;F01_transcript_16679;F01_transcript_167041;F01_transcript_167055;F01_transcript_167105;F01_transcript_167211;F01_transcript_167399;F01_transcript_167405;F01_transcript_167441;F01_transcript_167513;F01_transcript_1678;F01_transcript_16870;F01_transcript_16871;F01_transcript_16997;F01_transcript_1706;F01_transcript_17227;F01_transcript_17240;F01_transcript_17252;F01_transcript_17278;F01_transcript	K13412+K18835+K13457+K13447+K13447+K13412+K13414+K13413+K13457+K13414+K13414+K13457+K13412+K02183+K09487+K05391+K13414+K05391+K18835+K13412+K13414+K13447+K13457+K13457+K13412+K13412+K13459+K13457+K05391+K13412+K13412+K13412+K13457+K04079+K13456+K13412+K09487+K13457+K05391+K18835+K13414+K13459+K13457+K18835+K13430+K13412+K13436+K13414+K09487+K13457+K09487+K05391+K13457+K13414+K13457+K13457+K13457+K13448+K13448+K09487+K13429+K05391+K13459+K05391+K13412+K13448+K09487+K18875+K13447+K05391+K13414+K13436+K13429+K13412+K09487+K13448+K12795+K13448+K05391+K09487+K13457+K09487+K09487+K05391+K13457+K13457+K13412+K13448+K13459+K13413+K13459+K13457+K09487+K13448+K13412+K13414+K05391+K13436+K05391+K13448

	_17855;F01_transcript_17882;F01_transcript_18124;F01_transcript_18268;F01_transcript_18269;F01_transcript_18377;F01_transcript_1838;F01_transcript_18391;F01_transcript_18555;F01_transcript_18614;F01_transcript_18819;F01_transcript_18849;F01_transcript_18922;F01_transcript_19003;F01_transcript_19273;F01_transcript_1939;F01_transcript_19414;F01_transcript_19419;F01_transcript_19524;F01_transcript_19603;F01_transcript_19715;F01_transcript_19888;F01_transcript_1995;F01_transcript_20228;F01_transcript_20648;F01_transcript_20657;F01_transcript_20977;F01_transcript_21045;F01_transcript_21143;F01_transcript_21647;F01_transcript_21840;F01_transcript_21890;F01_transcript_22099;F01_transcript_22517;F01_transcript_22611;F01_transcript_22847;F01_transcript_22900;F01_transcript_23517;F01_transcript_23694;F01_transcript_23724;F01_transcript_23860;F01_transcript_24419;F01_transcript_24780;F01_transcript_24833;F01_transcript_24865;F01_transcript_24995;F01_transcript_25349;F01_transcript_25397;F01_transcript_25567;F01_transcript_25914;F01_transcript_26176;F01_transcript_26517;F01_transcript_26932;F01_transcript_27015;F01_transcript_2723;F01_transcript_28715;F01_transcript_28780;F01_transcript_29725;F01_transcript_30046;F01_transcript_30330;F01_transcript_30702;F01_transcript_30951;F01_transcript_31;F01_transcript_31022;F01_transcript_31177;F01_transcript_31287;F01_transcript_31330;F01_transcript_32156;F01_transcript_32275;F01_transcript_32921;F01_transcript_3293;F01_transcript_33003;F01_transcript_33223;F01_transcript_3386;F01_transcript_34359;F01_transcript_3442;F01_transcript_34641;F01_transcript_3471;F01_transcript_3512;F01_transcript_35226;F01_transcript_35331;F01_transcript_35508;F01_transcript_35732;F01_transcript_3580;F01_transcript_3581;F01_transcript_3625;F01_transcript_36450;F01_transcript_37400;F01_transcript_38824;F01_transcript_3888;F01_transcript_38974;F01_transcript_39135;F01_transcript_3931;F01_transcript_39385;F01_transcript_39893;F01_transcript_4013;F01_transcript_40279;F01_transcript_41041;F01_transcript_4151;F01_transcript_4213;F01_transcript_4230;F01_transcript_42852;F01_transcript_4358;F01_transcript_4385;F01_transcript_4395;F01_transcript_4419;F01_transcript_44268;F01_tra

nscript_44407;F01_transcript_4444;F01_transcript_44571;F01_transcript_44686;F01_transcript_45009;F01_transcript_4513;F01_transcript_45360;F01_transcript_45723;F01_transcript_46272;F01_transcript_46373;F01_transcript_46402;F01_transcript_46836;F01_transcript_47112;F01_transcript_47119;F01_transcript_47137;F01_transcript_47281;F01_transcript_47305;F01_transcript_47320;F01_transcript_47546;F01_transcript_4795;F01_transcript_47966;F01_transcript_47967;F01_transcript_47990;F01_transcript_48139;F01_transcript_48890;F01_transcript_4893;F01_transcript_49102;F01_transcript_50174;F01_transcript_50493;F01_transcript_5061;F01_transcript_51412;F01_transcript_51440;F01_transcript_51456;F01_transcript_5151;F01_transcript_51875;F01_transcript_51897;F01_transcript_5198;F01_transcript_52259;F01_transcript_52660;F01_transcript_52752;F01_transcript_52829;F01_transcript_52953;F01_transcript_53085;F01_transcript_53321;F01_transcript_53523;F01_transcript_53603;F01_transcript_53822;F01_transcript_53835;F01_transcript_53886;F01_transcript_5394;F01_transcript_54006;F01_transcript_54093;F01_transcript_54268;F01_transcript_54413;F01_transcript_54721;F01_transcript_54771;F01_transcript_54815;F01_transcript_55239;F01_transcript_55313;F01_transcript_55329;F01_transcript_55423;F01_transcript_55638;F01_transcript_55707;F01_transcript_55743;F01_transcript_56052;F01_transcript_56062;F01_transcript_56394;F01_transcript_56678;F01_transcript_56968;F01_transcript_57039;F01_transcript_57048;F01_transcript_57445;F01_transcript_57471;F01_transcript_57806;F01_transcript_57885;F01_transcript_57913;F01_transcript_57979;F01_transcript_58036;F01_transcript_58050;F01_transcript_58423;F01_transcript_58445;F01_transcript_58592;F01_transcript_58831;F01_transcript_5914;F01_transcript_59352;F01_transcript_59369;F01_transcript_59463;F01_transcript_59535;F01_transcript_5968;F01_transcript_59768;F01_transcript_59837;F01_transcript_59908;F01_transcript_60545;F01_transcript_606;F01_transcript_60698;F01_transcript_60823;F01_transcript_6087;F01_transcript_61149;F01_transcript_61533;F01_transcript_61580;F01_transcript_61707;F01_transcript_61979;F01_transcript_62083;F01_transcript_62091;F01_transcript_62106;F01_transcript_62312;F01_transcript_62419;F01_transcript_62440;F01_transcript_6259;F01_tr

anscript_63280;F01_transcript_63511;F01_transcript_63895;F0
1_transcript_63897;F01_transcript_64275;F01_transcript_6456;
F01_transcript_64679;F01_transcript_64762;F01_transcript_648
88;F01_transcript_65141;F01_transcript_65372;F01_transcript_
6548;F01_transcript_65556;F01_transcript_65560;F01_transcrip
t_65561;F01_transcript_6569;F01_transcript_65870;F01_transcr
ipt_65915;F01_transcript_6603;F01_transcript_66384;F01_trans
cript_66574;F01_transcript_66585;F01_transcript_66808;F01_tr
anscript_66899;F01_transcript_67168;F01_transcript_67450;F0
1_transcript_67468;F01_transcript_67664;F01_transcript_67674
;F01_transcript_67710;F01_transcript_67855;F01_transcript_67
861;F01_transcript_67927;F01_transcript_6796;F01_transcript_
67990;F01_transcript_68039;F01_transcript_68336;F01_transcri
pt_68570;F01_transcript_68577;F01_transcript_68799;F01_tran
script_68898;F01_transcript_69005;F01_transcript_69054;F01_t
ranscript_69128;F01_transcript_69262;F01_transcript_69317;F0
1_transcript_69517;F01_transcript_69623;F01_transcript_69659
;F01_transcript_69745;F01_transcript_69931;F01_transcript_70
653;F01_transcript_70752;F01_transcript_71063;F01_transcript
_71082;F01_transcript_71184;F01_transcript_71538;F01_transc
ript_71750;F01_transcript_72013;F01_transcript_72111;F01_tra
nscript_72148;F01_transcript_72228;F01_transcript_72380;F01
_transcript_72596;F01_transcript_72633;F01_transcript_72980;
F01_transcript_73192;F01_transcript_73306;F01_transcript_733
59;F01_transcript_73469;F01_transcript_73632;F01_transcript_
73800;F01_transcript_73835;F01_transcript_74098;F01_transcri
pt_74385;F01_transcript_74529;F01_transcript_74611;F01_tran
script_74642;F01_transcript_74730;F01_transcript_74833;F01_t
ranscript_74892;F01_transcript_74901;F01_transcript_75110;F0
1_transcript_75126;F01_transcript_75507;F01_transcript_7566;
F01_transcript_75789;F01_transcript_75992;F01_transcript_760
97;F01_transcript_76152;F01_transcript_76217;F01_transcript_
76377;F01_transcript_76486;F01_transcript_76527;F01_transcri
pt_76546;F01_transcript_76606;F01_transcript_76625;F01_tran
script_76635;F01_transcript_76665;F01_transcript_76909;F01_t
ranscript_77006;F01_transcript_77419;F01_transcript_77880;F0
1_transcript_78248;F01_transcript_78459;F01_transcript_78549

;F01_transcript_78898;F01_transcript_78971;F01_transcript_79214;F01_transcript_79243;F01_transcript_79368;F01_transcript_7940;F01_transcript_79451;F01_transcript_79701;F01_transcript_79893;F01_transcript_80004;F01_transcript_80362;F01_transcript_80781;F01_transcript_80793;F01_transcript_80820;F01_transcript_81082;F01_transcript_81159;F01_transcript_81512;F01_transcript_81729;F01_transcript_81808;F01_transcript_82062;F01_transcript_82088;F01_transcript_82683;F01_transcript_82774;F01_transcript_82844;F01_transcript_82931;F01_transcript_82986;F01_transcript_83304;F01_transcript_83324;F01_transcript_83423;F01_transcript_83446;F01_transcript_83499;F01_transcript_8363;F01_transcript_83656;F01_transcript_83752;F01_transcript_83815;F01_transcript_83942;F01_transcript_83965;F01_transcript_84008;F01_transcript_8408;F01_transcript_84208;F01_transcript_84371;F01_transcript_84417;F01_transcript_84476;F01_transcript_85130;F01_transcript_85483;F01_transcript_85496;F01_transcript_8570;F01_transcript_85773;F01_transcript_85819;F01_transcript_85938;F01_transcript_86344;F01_transcript_86575;F01_transcript_86751;F01_transcript_8719;F01_transcript_87243;F01_transcript_87409;F01_transcript_87415;F01_transcript_87624;F01_transcript_87879;F01_transcript_88319;F01_transcript_88394;F01_transcript_88503;F01_transcript_88522;F01_transcript_88571;F01_transcript_88587;F01_transcript_8870;F01_transcript_88801;F01_transcript_88983;F01_transcript_89122;F01_transcript_89349;F01_transcript_8937;F01_transcript_89449;F01_transcript_89524;F01_transcript_89576;F01_transcript_8969;F01_transcript_89730;F01_transcript_89978;F01_transcript_89999;F01_transcript_90315;F01_transcript_90588;F01_transcript_90629;F01_transcript_90675;F01_transcript_90690;F01_transcript_90751;F01_transcript_91029;F01_transcript_91216;F01_transcript_91268;F01_transcript_9128;F01_transcript_9149;F01_transcript_91678;F01_transcript_91781;F01_transcript_92254;F01_transcript_92402;F01_transcript_92437;F01_transcript_92710;F01_transcript_92714;F01_transcript_93237;F01_transcript_93452;F01_transcript_93989;F01_transcript_94035;F01_transcript_94320;F01_transcript_94351;F01_transcript_94354;F01_transcript_94553;F01_transcript_9480;F01_transcript_95032;F01_transcript_95289;F01_transcript_95297;F01_transcript_

			95723;F01_transcript_95742;F01_transcript_96043;F01_transcript_96290;F01_transcript_9663;F01_transcript_96634;F01_transcript_96670;F01_transcript_9683;F01_transcript_96898;F01_transcript_96932;F01_transcript_97084;F01_transcript_9709;F01_transcript_97280;F01_transcript_97419;F01_transcript_98035;F01_transcript_98408;F01_transcript_98499;F01_transcript_98515;F01_transcript_98957;F01_transcript_98958;F01_transcript_99048;F01_transcript_99120;F01_transcript_9928;F01_transcript_99581;F01_transcript_99582;F01_transcript_99719;F01_transcript_99741;F01_transcript_99869;F01_transcript_99894;F01_transcript_99923;	
Circadian rhythm - plant	ko04712	351	F01_transcript_100007;F01_transcript_10046;F01_transcript_10060;F01_transcript_100963;F01_transcript_100978;F01_transcript_101117;F01_transcript_10140;F01_transcript_101876;F01_transcript_102038;F01_transcript_102704;F01_transcript_102943;F01_transcript_103519;F01_transcript_103727;F01_transcript_104223;F01_transcript_104323;F01_transcript_104420;F01_transcript_104994;F01_transcript_105158;F01_transcript_106430;F01_transcript_106902;F01_transcript_107859;F01_transcript_107877;F01_transcript_108155;F01_transcript_108301;F01_transcript_108662;F01_transcript_110091;F01_transcript_11034;F01_transcript_11044;F01_transcript_111094;F01_transcript_11181;F01_transcript_112258;F01_transcript_11244;F01_transcript_112516;F01_transcript_112562;F01_transcript_113309;F01_transcript_113330;F01_transcript_113536;F01_transcript_11354;F01_transcript_113654;F01_transcript_11456;F01_transcript_114669;F01_transcript_11490;F01_transcript_114976;F01_transcript_11499;F01_transcript_115800;F01_transcript_116508;F01_transcript_11662;F01_transcript_11665;F01_transcript_116877;F01_transcript_117323;F01_transcript_11758;F01_transcript_118333;F01_transcript_11842;F01_transcript_118906;F01_transcript_118913;F01_transcript_119090;F01_transcript_120014;F01_transcript_121383;F01_transcript_122100;F01_transcript_122112;F01_transcript_123395;F01_transcript_123567;F01_transcript_12391;F01_transcript_124481;F01_transcript_125276;F01_transcript_125326;F01_transcript_125470;F01_transcript_126001;F01_transcript_126425;F01_transcript_126631;F01_transcript_126789;	K12118+K12118+K10143+K10143+K03115+K10143+K12130+K12118+K12118+K12125+K10143+K10143+K12127+K12130+K12130+K12124+K12129+K03097+K12124+K12126+K12129+K12129+K12127+K03097+K03097+K12118+K12130+K12125+K12129+K12121+K12130+K12125+K12129+K12129+K10143+K00660+K03115+K12118+K00660+K12127+K12127+K10143+K12133+K12130+K10143+K03115+K12126+K12118+K10143+K12129+K12130+K12115+K12118+K12129+K03115+K12125+K00660+K12124+K12130+K12119+K10143+K12129+K12127+K03097+K12133+K03115+K12118+K03097+K12129+K16240+K12129+K12125+K12129+K12129+K12133+K03097+K12127+K12115+K16240+K00660+K03097+K12120+K03115+K12118+K03115+K12127+K12130+K16240+K03115+K12124+K10143+K12125+K10143+K03115+K12129+K12127+K12118+K10143+K12129+K03115+K12120+K12127+K12125+K03097+K12119+K12130+K12124+K12130+K16240+K12127+K03115+K12125+K12126+K12133+K12126+K10143+K12127+K12118+K12120+K12129+K12126+K12119+K12130+K12118+K12129+K12118+K12125+K12124+K16240+K12118+K12127+K12130+K12125+K12127+K12127+K03097+K12130+K12130+K12130+K10143+K12127+K12130+K12129+K12130+K10143+K12120+K03097+K16240+K10143+K12129+K12127+K16240+K12133+K12130+K10143+K12121+K12129+K12119+K03115+K12115+K12130+K12118+K12127+K12124+K12133+K03115+K12127+K03097+K12125+K12130+K12125+K12130+K12124+K12130+K12118+K12127+K12116+K03115+K1

	F01_transcript_127032;F01_transcript_127036;F01_transcript_127124;F01_transcript_127230;F01_transcript_127619;F01_transcript_127853;F01_transcript_127991;F01_transcript_1283;F01_transcript_128823;F01_transcript_129131;F01_transcript_129814;F01_transcript_129869;F01_transcript_13022;F01_transcript_130282;F01_transcript_13070;F01_transcript_13124;F01_transcript_131301;F01_transcript_131341;F01_transcript_131914;F01_transcript_132301;F01_transcript_133045;F01_transcript_133892;F01_transcript_133931;F01_transcript_134144;F01_transcript_134435;F01_transcript_135758;F01_transcript_135960;F01_transcript_136105;F01_transcript_136969;F01_transcript_137071;F01_transcript_137075;F01_transcript_137253;F01_transcript_137465;F01_transcript_138436;F01_transcript_13872;F01_transcript_138975;F01_transcript_138993;F01_transcript_139025;F01_transcript_14005;F01_transcript_140244;F01_transcript_14033;F01_transcript_141412;F01_transcript_141611;F01_transcript_14220;F01_transcript_142632;F01_transcript_142651;F01_transcript_142736;F01_transcript_142747;F01_transcript_143317;F01_transcript_143515;F01_transcript_143694;F01_transcript_143900;F01_transcript_144361;F01_transcript_144629;F01_transcript_144925;F01_transcript_146612;F01_transcript_146870;F01_transcript_147808;F01_transcript_148797;F01_transcript_148845;F01_transcript_14920;F01_transcript_15074;F01_transcript_152949;F01_transcript_152980;F01_transcript_153057;F01_transcript_153645;F01_transcript_153672;F01_transcript_15468;F01_transcript_154831;F01_transcript_155001;F01_transcript_15501;F01_transcript_155484;F01_transcript_15649;F01_transcript_156759;F01_transcript_156795;F01_transcript_157075;F01_transcript_157658;F01_transcript_15803;F01_transcript_159613;F01_transcript_159743;F01_transcript_160180;F01_transcript_160362;F01_transcript_160856;F01_transcript_16097;F01_transcript_161206;F01_transcript_161359;F01_transcript_161953;F01_transcript_16205;F01_transcript_162741;F01_transcript_16298;F01_transcript_163200;F01_transcript_164133;F01_transcript_164701;F01_transcript_165477;F01_transcript_165853;F01_transcript_166125;F01_transcript_166284;F01_transcript_166306;F01_transcript_16658;F01_transcript_166874;F01_transcript_17735;F01_transcript_18051;F01_transcript_18150;F01_transcript_19	2130+K03115+K10143+K12125+K12129+K12125+K16241+K12133+K03115+K03097+K03115+K03115+K16240+K03097+K03115+K03097+K12124+K03115+K03097+K03097+K03115+K03115+K00660+K12129+K03097+K00660+K03115+K03115+K03097+K16241+K12124+K12119+K12129+K03097+K12124+K03097+K12118+K12118+K12118+K12124+K16241+K12130+K12133+K12124+K12124+K12115+K10143+K12124+K12129+K10143+K12125+K12124+K12133+K12129+K03115+K12120+K12124+K12119+K12125+K12124+K03097+K12130+K03115+K12129+K12125+K10143+K12124+K12125+K12120+K12133+K12130+K12129+K12124+K12133+K12129+K12125+K12124+K12124+K10143+K12118+K10143+K16241+K16240+K12125+K12124+K12124+K12129+K12121+K12129+K12130+K12124+K12127+K12126+K16223+K12129+K10143+K12133+K03097+K03115+K03097+K12116+K12126+K03097+K12129+K10143+K12127+K12129+K12120+K12125+K12118+K16240+K12124+K12124+K12133+K12129+K10143+K12126+K10143+K12125+K12124+K16240+K12124+K12115+K12130+K03097+K12121+K03097+K12133+K12126+K12118+K12124+K12124+K12124+K03115+K10143+K12133+K03115+K03115+K12127+K12125+K10143+K03115+K12130+K12124+K12118+K03097+K16241+K03115+K12127+K12133+K12127+K03115+K03097+K12126+K12125+K16240+K12124+K12119+K12118+K12130+K12133+K12115+K12120+K12124+K12126+K12133+K12118+K12124+K12124+K12125+K10143+K12127+K10143

182;F01_transcript_19276;F01_transcript_20376;F01_transcript
_20535;F01_transcript_21992;F01_transcript_22509;F01_transc
ript_24220;F01_transcript_26726;F01_transcript_31321;F01_tra
nscript_32347;F01_transcript_33308;F01_transcript_3434;F01_t
ranscript_34726;F01_transcript_35021;F01_transcript_35486;F0
1_transcript_35909;F01_transcript_36044;F01_transcript_36532
;F01_transcript_36669;F01_transcript_36834;F01_transcript_37
5;F01_transcript_37791;F01_transcript_37904;F01_transcript_3
8703;F01_transcript_38800;F01_transcript_38917;F01_transcrip
t_39092;F01_transcript_3924;F01_transcript_39354;F01_transcr
ipt_39477;F01_transcript_40149;F01_transcript_4047;F01_trans
cript_41394;F01_transcript_47999;F01_transcript_501;F01_tran
script_5028;F01_transcript_51795;F01_transcript_51921;F01_tr
anscript_52224;F01_transcript_52669;F01_transcript_52994;F0
1_transcript_53790;F01_transcript_54106;F01_transcript_54117
;F01_transcript_55211;F01_transcript_55756;F01_transcript_56
747;F01_transcript_569;F01_transcript_56984;F01_transcript_5
6999;F01_transcript_57075;F01_transcript_572;F01_transcript_
57273;F01_transcript_5735;F01_transcript_57765;F01_transcrip
t_57891;F01_transcript_5837;F01_transcript_58611;F01_transcr
ipt_58625;F01_transcript_596;F01_transcript_5980;F01_transcri
pt_60307;F01_transcript_60354;F01_transcript_60648;F01_tran
script_61085;F01_transcript_61220;F01_transcript_61341;F01_t
ranscript_6167;F01_transcript_6255;F01_transcript_62647;F01_
transcript_628;F01_transcript_62997;F01_transcript_63593;F01
_transcript_6382;F01_transcript_63873;F01_transcript_6390;F0
1_transcript_63983;F01_transcript_64118;F01_transcript_64821
;F01_transcript_65960;F01_transcript_66147;F01_transcript_66
259;F01_transcript_66300;F01_transcript_66393;F01_transcript
_66626;F01_transcript_66828;F01_transcript_67207;F01_transc
ript_68064;F01_transcript_684;F01_transcript_693;F01_transcri
pt_6941;F01_transcript_696;F01_transcript_69603;F01_transcri
pt_69985;F01_transcript_702;F01_transcript_70689;F01_transcr
ipt_7106;F01_transcript_71660;F01_transcript_7178;F01_transc
ript_72115;F01_transcript_7213;F01_transcript_72662;F01_tran
script_72687;F01_transcript_7282;F01_transcript_73159;F01_tr
anscript_74248;F01_transcript_74437;F01_transcript_7449;F01

			_transcript_74682;F01_transcript_75125;F01_transcript_75171;F01_transcript_75268;F01_transcript_75290;F01_transcript_75489;F01_transcript_759;F01_transcript_762;F01_transcript_76304;F01_transcript_76521;F01_transcript_7683;F01_transcript_77352;F01_transcript_77368;F01_transcript_7760;F01_transcript_78819;F01_transcript_79583;F01_transcript_797;F01_transcript_79839;F01_transcript_80197;F01_transcript_80205;F01_transcript_80662;F01_transcript_807;F01_transcript_82131;F01_transcript_8249;F01_transcript_82579;F01_transcript_82617;F01_transcript_82928;F01_transcript_82936;F01_transcript_83195;F01_transcript_83225;F01_transcript_83335;F01_transcript_84129;F01_transcript_84201;F01_transcript_84685;F01_transcript_85376;F01_transcript_85509;F01_transcript_85941;F01_transcript_87043;F01_transcript_88248;F01_transcript_88393;F01_transcript_88457;F01_transcript_88600;F01_transcript_88680;F01_transcript_88732;F01_transcript_8959;F01_transcript_9007;F01_transcript_904;F01_transcript_92298;F01_transcript_92303;F01_transcript_94019;F01_transcript_94123;F01_transcript_943;F01_transcript_945;F01_transcript_94543;F01_transcript_94789;F01_transcript_95188;F01_transcript_95285;F01_transcript_9574;F01_transcript_960;F01_transcript_96599;F01_transcript_96797;F01_transcript_96808;F01_transcript_9720;F01_transcript_97258;F01_transcript_97503;F01_transcript_97538;F01_transcript_97800;F01_transcript_979;F01_transcript_98891;	
AGE-RAGE signaling pathway in diabetic complications	ko04933	56	F01_transcript_102328;F01_transcript_104277;F01_transcript_104594;F01_transcript_105116;F01_transcript_106017;F01_transcript_106019;F01_transcript_106454;F01_transcript_10945;F01_transcript_115711;F01_transcript_11593;F01_transcript_120207;F01_transcript_120434;F01_transcript_121498;F01_transcript_122263;F01_transcript_124354;F01_transcript_126698;F01_transcript_128582;F01_transcript_130063;F01_transcript_130443;F01_transcript_131602;F01_transcript_137832;F01_transcript_138341;F01_transcript_140237;F01_transcript_142650;F01_transcript_147722;F01_transcript_147743;F01_transcript_152601;F01_transcript_153666;F01_transcript_156315;F01_transcript_157286;F01_transcript_166884;F01_transcript_167180;F01_transcript_21865;F01_transcript_23080;F01_transcript_26104;F01_transcript_30602;F01_transcript_32585;F01_transcript_36131;F01_t	K04392+K05857+K04392+K04392+K04392+K04392+K05857+K04392+K05857+K04392+K05857+K04392+K04392+K05857+K04392+K04392+K04392+K04392+K05857+K04392+K05857+K04392+K05857+K04392+K05857+K04392+K05857+K04392+K05857+K05857+K05857+K05857+K05857+K05857+K05857+K04392+K04392+K04371+K04392+K04392+K04392+K04392+K04392+K04392+K05857+K04392+K05857+K05857+K04392+K05857+K05857+K05857+K04392+K04392+K05857+K04392

	ranscript_42493;F01_transcript_42584;F01_transcript_42839;F01_transcript_43785;F01_transcript_44098;F01_transcript_44362;F01_transcript_54432;F01_transcript_55752;F01_transcript_55919;F01_transcript_57879;F01_transcript_58487;F01_transcript_66677;F01_transcript_70648;F01_transcript_73787;F01_transcript_83534;F01_transcript_90465;F01_transcript_98548;F01_transcript_99194;
