# Supplementary material for: PacBio Single-Molecule Long-Read Sequencing Reveals Genes Tolerating Manganese Stress in Schima superba Saplings
Source: Front Genet. 2021 Apr 6;12:635043. doi: 10.3389/fgene.2021.635043 (PMC8057201; doi:10.3389/fgene.2021.635043)
Supplement: Supplementary Table 5 — The primers used for qRT-PCR analysis. [file Data_Sheet_4.PDF]

PacBio single-molecule long-read sequencing successfully explores the transcriptome of *Schima superba*

Fiza Liaquat[1], Muhammad Farooq Hussain Munis[2], Samiah Arif[1], Urooj Haroon[2], Muhammad Ashraf[3], Saddam Saqib[4,5], Wajid Zaman[4,5], Che Shengquan[6] and Liu Qunlu[6*]

1 School of Agriculture and Biology, Shanghai Jiao Tong University, Shanghai, 200240, China;

2 Department of Plant Sciences, Faculty of Biological Sciences, Quaid-i-Azam University, Islamabad, 45320, Pakistan;

3 Joint International Research Laboratory of Metabolic and Developmental Sciences, School of Life Science and Biotechnology, Shanghai Jiao Tong University, Shanghai, 200240, China;

4 State Key Laboratory of Systematic and Evolutionary Botany, Institute of Botany, Chinese Academy of Sciences, Beijing 100093, China;

5 University of Chinese Academy of Sciences, Beijing 100049, China;

6 Department of Landscape Architecture, School of Design, Shanghai Jiao Tong University, 200240, China;

*Correspondence: liuql@sjtu.edu.cn

Table S5 Primer sequences of selected genes for q-PCR

Sr.	Gene ID	Gene Name	Function	Primers
1	88069	Nramp3	Natural resistance-associated macrophage protein	F: 5′ - AGCAGGCTGTGGGTGTTGTG -3′
				R: 5′ - GCTTCTTGAACCCGGCCAGT -3′
2	20654	Na_Ca_ex	Proton exchanger 2-like	F: 5′ - TGCTTCCTTTTGGGCCCCTG -3′
				R: 5′ - AAGGCGCATCCAAGCACCAA -3′
3	153047	YSL	yellow stripe-like protein	F: 5′ - GAATGTCTCTGCCGCCCTCC -3′
				R: 5′ - AAACCCGCCTCCAACAGCAA -3′
4	135198	Zip	Inorganic ion transport and	F: 5′ - GGGCTTTGCAGCTGGGTGTA-3′
				R: 5′ - AGCAACGGCCCAAGACCAAA -3′
5	33644	Na_Ca_ex	Proton exchanger 3 -like protein	F: 5 ′ -GCCGGACAGCGCAGAATCTA-3 ′
				R: 5′ - TCAGCAAGTGGAACGAGCCC -3′
6	151736	Na_Ca_ex	hypothetical protein POPTR_	F: 5′ - TGGGGGTTGCTTTGGGTTCA -3′
				R: 5′ - TCCTGCAGAGTGAAGGCTGTG -3′
7	99880	CAX	CAX-interacting protein 4	F: 5′ - ATTGGCCCTGTCCTCAAGGT -3′
				R: 5′ - GGCCGGGCCAATCCAGTTTC -3′
8	93796	Lzipper-MIP1	electron transporter, putative	F: 5′ - CGGCAACAGCAGGTAGGGTT -3′
				R: 5′ - CCTGGAAGCTGGCGTATGGG -3′
9	13424	Lzipper-ATHB	Electron transporter	F: 5′ - TCGCTTGCCAACCCATGGAA -3′
				R: 5′ - CGGGTACCAAGATGCTCTGCC -3′
