# Supplementary material for: PacBio Single-Molecule Long-Read Sequencing Reveals Genes Tolerating Manganese Stress in Schima superba Saplings
Source: Front Genet. 2021 Apr 6;12:635043. doi: 10.3389/fgene.2021.635043 (PMC8057201; doi:10.3389/fgene.2021.635043)
Supplement: Supplementary Table 6 — The characterization of EST-SSR markers. [file Data_Sheet_5.PDF]

PacBio single-molecule long-read sequencing successfully explores the transcriptome of *Schima superba*

Fiza Liaquat[1], Muhammad Farooq Hussain Munis[2], Samiah Arif[1], Urooj Haroon[2], Muhammad Ashraf[3], Saddam Saqib[4,5], Wajid Zaman[4,5], Che Shengquan[6] and Liu Qunlu[6*]

1 School of Agriculture and Biology, Shanghai Jiao Tong University, Shanghai, 200240, China;

2 Department of Plant Sciences, Faculty of Biological Sciences, Quaid-i-Azam University, Islamabad, 45320, Pakistan;

3 Joint International Research Laboratory of Metabolic and Developmental Sciences, School of Life Science and Biotechnology, Shanghai Jiao Tong University, Shanghai, 200240, China;

4 State Key Laboratory of Systematic and Evolutionary Botany, Institute of Botany, Chinese Academy of Sciences, Beijing 100093, China;

5 University of Chinese Academy of Sciences, Beijing 100049, China;

6 Department of Landscape Architecture, School of Design, Shanghai Jiao Tong University, 200240, China;

*Correspondence: liuql@sjtu.edu.cn

Table S6 The characterizations of EST-SSR markers

Primer -ID	#Gene_ID	Forward	Reverse	Product Size	Repeat motif	SSR length	Amplifi-cation Result
F01_1	F01_transcript_782	GTTTGAACCCTAGGAAGCCC	GCCCCTTGTGATGACATTTT	275	(T)12	12	S
F01_2	F01_transcript_6	CGTGAATTTTGGAGGGATTG	TGTTGTTGCAATGACTGCCT	275	(GCA)6	18	S
F01_3	F01_transcript_7	TTCACTTTGTGAACACCCCA	GGGGTTTTGCCTTACCTGTT	207	(A)12	12	S
F01_4	F01_transcript_8	GTCTTGCAAAAAGGCACTTG	TGAAAAGCACACGAATCTCAA	108	(TATTTT)6	36	S
F01_5	F01_transcript_11	ATCTGGGATGTCAGCCAAAG	GCCAAAAAGGCCACTAGGTT	246	(T)10	10	S
F01_6	F01_transcript_21	GAATGGTGAACAAGTTCTCTTCC	TTCTTATCCCCTTTCGGCTT	258	(CT)6	34	S
F01_7	F01_transcript_23	TCACGTAGATCCGAAAACCC	GGGGTCGTGGTTACAAATGA	241	(CA)17	34	S
F01_8	F01_transcript_28	GGTCGCAAGAAATCCAAAAC	GGTAGGTGGAGAAAGTTGCG	260	(TA)9	18	S
F01_9	F01_transcript_375	TCAGCAACACCAACAAAAGC	ACCTCTGCCAAAATCAGGAA	241	(T)10	10	S
F01_10	F01_transcript_379	CCACTCGTCAAGGATTTCG	CATTGATTTGAGCCCTCGTT	214	(CT)14	28	F
F01_11	F01_transcript_380	CCCTTCACAGGTTTGTGCTT	CGAGCGTGCTCACATAATTT	235	(AT)10	20	S

F01_12	F01_transcript_388	TGCTGAGTTCCTCTGTCCCT	GATTTGCAAGTGAAAACCTCC	227	(TA)8	16	S
F01_13	F01_transcript_389	GTCTACAAAGCTCTTCGCCG	CAGCTGGTTGGTTTGGATTT	200	(CT)15	48	S
F01_14	F01_transcript_392	ATAACCCCCTTATGCCTTGG	TGGGTGGACTTTTTGTCCTC	237	(A)13	13	S
F01_15	F01_transcript_394	AAAAAGCCGATTCACTCGAA	GCGAATCGAAGTAACGCTTC	271	(CAC)5	15	S
F01_16	F01_transcript_397	GCTTCCATTCACATGGATCA	TGATGGGAGAACAGAGCTACAA	233	(T)10	10	S
F01_17	F01_transcript_402	TGTTAATTTTCACCCCTCGG	AGGCACGCATGTCTAGAACC	101	(T)10	10	F
F01_18	F01_transcript_403	GATTGCAAATGGGTATTGGG	CCAGAAGGCTTTTTCCACAA	247	(TA)9	36	S
F01_19	F01_transcript_406	TCCATGCTGATGGGTGTAGA	AGGGTCGAAGATTGTTGACG	244	(TCA)6	18	S
F01_20	F01_transcript_407	ACCAGCCAGAAGAGGTGAGA	CCCTTTCCTTGAGTTTTCCC	145	(T)20	20	S
F01_21	F01_transcript_407	GTTTTTGTTTGGGACATGGG	AGTGCAAAGGAACGCAGATT	152	(T)10	10	S
F01_22	F01_transcript_408	TAAAACCCTAACGCCATTCG	AATTCCACCCCCACAATACA	225	(TC)6	12	S
F01_23	F01_transcript_410	CTTCGACCCGTGGAAAGATA	CCTATCCCCTTGTTCCATCA	262	(A)13	13	S
F01_24	F01_transcript_411	TGTGTGTCCAAGCATCAAATC	GAAGAAGATGCTTACATTTGCTAGT	280	(AC)11	22	S
F01_25	F01_transcript_416	ACTCCCTTCCACATTCGTTG	GAAACTCGGAGAGCAAAACG	277	(TC)10	20	S

F01_26	F01_transcript_164984	GCCGATTAGGGGATTCTCTC	CAGGCGAAATAAGATTGGGA	120	(CT)6	12	S
F01_27	F01_transcript_165783	TCGCACTTTCTCTTTAACCGA	ATGGCTCGAGAGAGTGGAGA	175	(CT)8	16	S
F01_28	F01_transcript_165875	TCGTTTTGGGGTTTTGATTC	TGAAAAATGGATTACACCAAACA	254	(T)17	17	S
F01_29	F01_transcript_165908	TCTCTTTTCCATCCACCACC	CCTTTTCTACCCCAAGAGGC	263	(GGC)6	18	S
F01_30	F01_transcript_166148	CTCCACCAACCCTAGGATCTC	TGCTTTTGGTTGGGTGTATTC	277	(CA)6	194	S
F01_31	F01_transcript_160521	TCAGCCACTTCTCTCTCCGT	TAGGCCAAAGTTCTGCTCGT	133	(CT)19	38	S
F01_32	F01_transcript_160521	GGAAGATTGGAAGTGTGGGA	TCATACCCCAAGACCACCAT	258	(T)23	71	S
F01_33	F01_transcript_160521	ATGGTGGTCTTGGGGTATGA	AGGGCCTTCAAGACCATTG	214	(T)17	17	S
F01_34	F01_transcript_166814	AAAGGAAGGAAAGGTCACCC	AGGAGGGGGAGTGAAGAGAA	223	(T)14	14	S
F01_35	F01_transcript_167228	CATGCTGGCAGGTTATGTCA	ATTGATTGGCCTGCATGACT	252	(T)10	23	F
F01_36	F01_transcript_163579	TCACCCTCCTGAGAAATTGG	GGTTGCTGAAGAAGTCCGAG	249	(GGT)7	21	S
F01_37	F01_transcript_163595	ACGTTGGTCAACCTAATCGC	ACAAACAAAGCCAGAAGCGT	182	(CT)10	20	S
F01_38	F01_transcript_163595	ACACCATATGCACGCTTTGA	CCAGATTCTCCTCGACCTGA	256	(TG)11	72	F

F01_39	F01_transcript_163595	ACAGCCTTGTACCAAATGGC	ACTCATACAATGCCCCCAAA	180	(TA)6	12	S
F01_40	F01_transcript_164237	ATTATCCAACAGCGGTGAGC	CCAGGGTTGACCACAGACTT	184	(TC)14	28	S
F01_41	F01_transcript_164237	AACATGCAAACCCTCTCCAC	ATGCGACTGTGAGGTGTCAG	249	(CT)7	14	S
F01_42	F01_transcript_158212	CGTGATTGCTTATGCTATTGTGA	GAGGCATGCCCTATTTCTGA	279	(T)10	10	S
F01_43	F01_transcript_158243	CGGCCAACCTAATTTACCCT	TGAGCCTTTCCGCTAAAGAA	201	(T)12	12	S
F01_44	F01_transcript_158265	ACTATGCTGATTGCAAGGGG	ATAGTGGGATGCTTCCGGTT	191	(CT)15	62	F
F01_45	F01_transcript_158582	AATGGATGGAGGATCCATGA	TACGGAATGGAAGCAAAAGG	227	(TC)20	40	S
F01_46	F01_transcript_158777	CCGAATCCATCTCTCCCTTT	CCGGAGAGAAGATCCTGGTA	226	(CT)15	106	S
F01_47	F01_transcript_158986	GGCTTTGTGAACTTGACTCCA	AATCAAGGGAATTTGGGGAC	256	(TG)6	12	S
F01_48	F01_transcript_159426	GGCAGAACTAATGCGAGAGG	GCTTGCAGCAGCATGTAAAA	268	(A)11	49	S
F01_49	F01_transcript_73197	GGCAGATCGACCTTCATTTT	CTTAAAAATCGGGCCACCTT	180	(CT)34	103	S
F01_50	F01_transcript_159642	TTTCGTTTCCTGCATTTTCC	CGGCTGAACTTGAGTGGAGT	251	(T)10	10	F
F01_51	F01_transcript_87526	TGTTTTTGGAGGGAGGCATA	TGTAGTCACCAAAGGAGGGG	250	(TTTG)5	50	S
F01_52	F01_transcript_88919	TTTTATTTGGGGCTTCATGC	CGAGCACAATTGCTGAAAGA	263	(CTAT)5	20	S

F01_53	F01_transcript_89333	CAGACTGTAGCTCTGAGTTGGG	TGAGCAGGCCTCCAGTTATT	141	(T)12	12	S
F01_54	F01_transcript_89670	GGGCTCCACAACAAGGAGTA	TGTTTTGGCAATTGCTCTTTT	202	(T)15	15	S
F01_55	F01_transcript_17474	GAGTTCGCGACAACTTCACA	TGGAAAACAGAAAATGAGAGTTCA	224	(A)13	13	S
F01_56	F01_transcript_17483	TGGTTGAATCGACTAAAAAGCA	AACCAATATTCTTGCAGTCTTGT	145	(T)21	21	S
F01_57	F01_transcript_17486	TGTCCTCAGTCCTAGCCTCC	TCACAATGGGTAGTGCCAAA	231	(TA)10	20	S
F01_58	F01_transcript_17486	GGCCCTATTGTGCTAACATGA	CAGTGGATGCACAAATCCTG	173	(TA)8	16	S
F01_59	F01_transcript_17491	GCGGGTACACAAACAAACG	ATCAGAAGAGCGAAGCGAAG	243	(AG)16	120	S
F01_60	F01_transcript_164100	TGGACCTTGGTTTTTCCTCA	GGAGCACAAAGAGTTCTCCG	172	(ATTT)7	100	S
F01_61	F01_transcript_164109	CCCAACCTCCTCTCCTCTCT	GATGTAGCTCTGCTGCAACG	192	(CT)28	56	S
F01_62	F01_transcript_164115	GGTTATCGATCAGCTCTCGC	GGAGAGATCGAAAGGGAAGG	155	(TCC)5	15	S
F01_63	F01_transcript_164118	ATGGACCTTGAGCCAAAATG	TGGACACATTGCCTACCAAC	268	(TTTA)5	20	S
F01_64	F01_transcript_164133	GAACAGAAGAAGACGACGGC	CGGCCCTAATTAGACGATGA	101	(TC)16	32	S
F01_65	F01_transcript_163034	CGCCTAATTGTTGAGTAGAATTTG	GGACGATGGGGAACACTAGA	204	(A)10	27	F

F01_66	F01_transcript_163035	AACATTTCCCTGACACAGGC	GCTTCCGACTTTGAGATTGC	260	(T)12	12	S
F01_67	F01_transcript_163037	ACCAAGGGACCTTCGACTCT	TCAGATTTCGCCATCTACCC	243	(T)10	10	S
F01_68	F01_transcript_163040	TGTTTGCAGAACACAGAAGGA	ACAAGAACAACCCAAACCCA	168	(AGA)5	15	S
F01_69	F01_transcript_163046	CTCGCCAACACTTCACTCAA	AAGGCGAGGATAAGGAGGAG	171	(T)10	10	S
F01_70	F01_transcript_163066	TCAAGATTAGGGCAAGTCGC	GGTTGGATAGCAAGCAAACAA	259	(CT)8	61	S
F01_71	F01_transcript_160465	GTATTGCCACCAAAGGGATG	GAAAGAAAGCAACGGGAAAA	271	(T)12	33	S
F01_72	F01_transcript_160465	GAGGGGAGTGCTAGACATCG	TTTCAATTCCCTGCCTACAGA	231	(T)18	18	S
F01_73	F01_transcript_160468	TTACCCAATCAAAGCCCATC	TGACATCAAGAGGAGGGAGG	178	(AG)14	82	S
F01_74	F01_transcript_160472	CACTCCACAGTGGCAGCTAA	TCGGGAGCAATTCCAAGTAG	258	(GATTTG)5	30	S
F01_75	F01_transcript_160479	ATGCCTTCGGAGGTTTTCTT	TTCCACAACAATCTACCCCAG	262	(A)18	18	S
F01_76	F01_transcript_160485	GCATACAGAGTTTCGTTATCGG	CCTAGGGCAAACAACGGTAA	243	(AG)17	34	S
F01_77	F01_transcript_158688	TCACACAAAGTGGCTTCTCG	CGCGCTACTTTTCCATACAA	218	(AT)6	12	S
F01_78	F01_transcript_158690	GGCTGCAACATCCAGAGATA	GTCAATGTTCAAAGGCGGTT	157	(TC)18	36	S
F01_79	F01_transcript_158691	GCACCTCCTTTGACTCCTGA	TGGAGGAAACACAATGGTTG	234	(AT)9	18	S

F01_80	F01_transcript_158692	GCTGCCAGCTTAATCCTCAA	AAATTCATGTCTTGCGTCCC	219	(T)10	10	S
F01_81	F01_transcript_158695	TTGCAGTTCAGGTTCCAGTG	TTTCTCCATCCACAAGGAGG	242	(CT)7	14	S
F01_82	F01_transcript_158707	GGTCTACACCGAAACCCTCA	AGCTTTGGCCAGTCTTTTGA	120	(CT)22	44	S
F01_83	F01_transcript_147623	TGCTCGTGGGATGTACCATA	GCCCAAAACTATGCCAGAAG	132	(T)10	55	S
F01_84	F01_transcript_147624	GGAGGTGAGGATGGTTGGTA	GCCTCCCCACCTTCTTATTC	245	(GAT)9	27	F
F01_85	F01_transcript_147626	TTCTGGGAGCTTAATGTGGTG	TGCTGTCTTGATGTGGTGGT	276	(T)12	12	S
F01_86	F01_transcript_147632	CCTCTTCGCCTTCTTCCTCT	CTCGTATTTGCTGTTTGCCA	262	(GA)6	12	S
F01_87	F01_transcript_147633	CCCTCTTGGATCCATTGTTG	CGCCTATGGGTTTAGCAAGA	174	(TA)7	14	S
F01_88	F01_transcript_147636	CAACCTATGAAGCACGGACA	TGTGAAGACAATTGTTGGATGA	275	(GACACT)6	103	F
F01_89	F01_transcript_147639	CGTGCTTTTTAGGTTCTTTTAGC	TTGTAACTCACGTTGCACCAA	137	(A)14	14	S
F01_90	F01_transcript_147639	ACTAGACTAGGCAAATCTAAGCAAA	CACTTTGGATACCTGCACCA	268	(GT)7	24	F
F01_91	F01_transcript_147644	AAAAAGCTGAGAGAGAAAATAGAGTG	GTGGACAGCAGCTGGTCTTT	257	(GA)6	107	S
F01_92	F01_transcript_147646	GCAACATGTGGTGGTATTGC	GGGGTTAACCAAGGTACAGGA	212	(TC)13	26	S

F01_93	F01_transcript_144687	TTGTTTCAAATTCCCTGCCT	CTGTGTGCCATAATTGGGTG	253	(GC)7	44	F
F01_94	F01_transcript_144695	TTCAAGCAGGACATCAGTCG	AGGATATCGCAGAACCAAACA	242	(TA)8	16	S
F01_95	F01_transcript_144698	ACACTCAGCCAGTGCCTTCT	GGAGTGGAAGAGAGTTCTGATGA	274	(CT)6	12	S
F01_96	F01_transcript_144714	GAAATGTGGACCCTGCTTGT	AGCTAGTGATCATGTGCCCC	237	(T)12	12	S
F01_97	F01_transcript_144715	TAGCACCAAAGGCCAAACTC	TGCGAGAAGCAATAGCAATG	275	(TGT)5	15	S
F01_98	F01_transcript_144716	GGACAACGCACCCTCATACT	AGACATCCGGACAGTGAAGG	250	(CT)12	24	S
F01_99	F01_transcript_129563	GGCTTTACAAGAAAATACCCAGA	TGCCATAGTTGCTTCCACAG	247	(TTA)5	15	S
F01_100	F01_transcript_129566	GGTTGACTGCCAAGTTTGCT	TGCACAAGAAGAAACCCAAA	275	(T)14	14	S
F01_101	F01_transcript_129583	GGAGTGCATAGGGGATCAAA	GGGGAATTAATGCCAACAAA	228	(CT)21	129	S
F01_102	F01_transcript_129592	GCTTCTACACACGATTTCCACA	AGGGTTTTGGTTTTGGCTCT	170	(CT)14	28	S
F01_103	F01_transcript_129595	AAAAATGAAAATTCAGTACATTTCG	CAGTTTTTGAGTTTTGAATTCGT	194	(T)12	81	S
F01_104	F01_transcript_129597	TTTGGTTGGCTCGTTCATTT	TGCAAACGAAACTAATTGGG	101	(T)10	10	S
F01_105	F01_transcript_125978	ATCTCAACCAGGCCAAACTG	GGCACTTGCTGCATACAAAA	248	(T)15	15	S
F01_106	F01_transcript_125979	TCCCCTCGTTTTGCTTATTG	TGGCATGATGGTCATATCGT	153	(T)12	12	S

F01_107	F01_transcript_125988	CAAAGCATGGGTTGATGTTG	GCCCCTCTAGGTTGATTTCC	258	(GCT)5	15	S
F01_108	F01_transcript_125991	ATTATTAGGGCGAGGGATGG	CAACCAAAAAGAAAATACAAATGG	214	(T)10	10	S
F01_109	F01_transcript_126001	TGCAACTGCTGTTCTGTACTCA	CAGCAAATTGCAGAAACGAA	262	(AT)9	18	S
F01_110	F01_transcript_126002	GAAGGCCAATCCATTTTGTG	TGGCTCACTACGGTTTGGTT	201	(T)14	14	S
F01_111	F01_transcript_116904	AGCACTGAGCCATCACCTTT	GCAAGTTTTGGAGCCTCTGA	176	(CT)17	47	F
F01_112	F01_transcript_116906	GGTGATCCAAAAGTGGAAGC	GAATTGGCATAAGAGCCAGG	220	(TG)10	20	S
F01_113	F01_transcript_116908	CGGCGTCGATACTTCTTCTC	ATTCACAGTGGACCGACCTC	238	(CT)26	52	S
F01_114	F01_transcript_116911	GAAGAGAAGAGGAAACCACCAG	CTGTTTCTGGGTTCCGTGAT	150	(GAC)5	73	S
F01_115	F01_transcript_116912	CATGTAGCTGACCCCATTTG	GGGTGGGTAATTGCAAGAGA	187	(TG)6	12	S
F01_116	F01_transcript_116914	TGGAAAAGAAATCAGGGGAA	CAACATCCATCCAAATGAACC	278	(T)10	10	S
F01_117	F01_transcript_116916	CCTTAGGGCAAGCTTTGTTG	ACTTGGTCGGCCTATGACAC	279	(T)12	12	S
F01_118	F01_transcript_116917	GTCGCGATACCATTTGGAGT	TTTTTGGGAATCCCTCACAG	251	(AG)7	14	S
F01_119	F01_transcript_116920	TTGGCCAAGGTTTGTCTTGT	TTATCCACTGCAGTTTCCCA	258	(T)14	14	S

F01_120	F01_transcript_116146	TTCCGAAAGGGTTTTGACAG	CAGCACCATTAATCATTGCG	196	(A)11	11	S
F01_121	F01_transcript_116156	TGATTCTGACGAACCCACAA	GGGGGATTCCTTCAAACATT	154	(TGA)7	21	S
F01_122	F01_transcript_116158	TCATTCCCCTCAAGATTTCG	CAATTTCAAATGGCCGTCTA	174	(A)11	11	S
F01_123	F01_transcript_116158	TCATTTTGTCCTGCTAACACTCTC	GCAAACAAAAGCAATACCCAA	125	(TA)9	18	S
F01_124	F01_transcript_116170	GATGAAACGCGGAAAGAGAG	AGAGGGAGAGAAACCCCAAA	270	(TGA)6	18	S
F01_125	F01_transcript_116170	CGACATAAATTGGTGCGAGA	ATATTTGGAGTGCGGTGGAG	263	(T)12	12	S
F01_126	F01_transcript_116176	GGTGCCCTGCAGAGAGAG	AACGTGATGATTACCCCCAA	277	(AG)9	18	S
F01_127	F01_transcript_116176	GCTGGTATCCCCTCTCAATG	TGGAAGTAAGGCAGGAATGG	212	(A)13	13	S
F01_128	F01_transcript_116177	TTCCAGCAGGTAGCTCAGGT	GGAGTCAGAGGCAGAAATCG	201	(TA)6	12	F
F01_129	F01_transcript_110299	GCACATCAAAGGGTTGTTCA	TGAAGGGTTGTTCTTGTTGG	252	(CCA)5	117	S
F01_130	F01_transcript_110301	GTCTCTCTCTGCTCCCGATG	AATGAAATTGGCCTCGTTTG	186	(CCT)8	24	S
F01_131	F01_transcript_110302	GACCAGTGCGTGGGACTAAT	CACTCCCCCTCTCCATAACA	217	(T)11	11	S
F01_132	F01_transcript_110303	CTACCCGCGTGATTTTGAAT	CAAATTGGGGACTAAGCCAA	264	(CT)26	52	S
F01_133	F01_transcript_110304	TCCCAGCAAGTTCTACTTTTTC	AAAGCCACACAAATTCAGGG	271	(AT)9	18	S

F01_134	F01_transcript_110307	CCTCTCCATCTCTTCTCCCC	TTTTGCAGAGATGATCGACG	200	(CGC)5	15	S
F01_135	F01_transcript_110311	CACAGGTTGAGCCAACAAGA	CGAACATGGACGAAGAGGAT	246	(A)12	12	S
F01_136	F01_transcript_110311	GGCGGGGACACACTAACTA	GACACGACATGGACATGGTAA	273	(T)15	15	S
F01_137	F01_transcript_110317	CACCCACACAAACACACACA	CTTTGGGGTCACTGACAGGT	219	(AG)10	20	S
F01_138	F01_transcript_9574	GGCTGTGGTTGGTTGGTACT	TCCAATGTGTCCAAGAGCAG	153	(GCT)6	18	S
F01_139	F01_transcript_9582	GCCAAAAGACTCTCCATTGC	ATGCAAAACAACAACACCGA	277	(GA)16	32	S
F01_140	F01_transcript_9584	TCTGCAGTCACAGGCTCTTG	CAGCTGGGATGGCATTAAGT	187	(GCA)6	43	F
F01_141	F01_transcript_9606	GACGTCGGAGGAGGAAGATT	GGTGAGATTGAGTGAGGGGA	274	(TC)7	14	S
F01_142	F01_transcript_9607	ACAAAAACACGTTTCGGAGG	GAAGAAAGAAGAGGGGCAAAA	207	(T)13	13	S
F01_143	F01_transcript_9613	ATATTCGATCCGAAAACCCG	TTGTCTCTTTCTAAGCCTCTTCG	219	(ATT)6	18	S
F01_144	F01_transcript_9613	GATCACATTTTATGGCTAAATTCAA	CGAATCCTACCCTGACCTGA	270	(A)10	10	S
F01_145	F01_transcript_9640	AGTAGCCGGAGCTGTGGTTT	ACTTCGCTTCGTTCCTTCAA	277	(TC)13	26	S
F01_146	F01_transcript_9628	TCCTCACACTCGCTCTCTGA	ATTTTGTCCCCCTCAACTCC	210	(GA)7	14	S

F01_147	F01_transcript_9641	TGCAATTGTTACTCCTCCCA	AGTTTCAGCGACAGCGATTT	188	(CT)15	53	S
F01_148	F01_transcript_91520	GGAATTTCTGGGATTGGGTT	TAACGGCAGCACTCAATGAC	257	(TC)17	34	S
F01_149	F01_transcript_91520	GTTTGTGGCAGTTGGAAGGT	CGACACAATCCTCCCGTACT	209	(TC)19	38	S
F01_150	F01_transcript_91522	TGCCATTAGATGTAGGGGAAA	TCCCATGCAATGTCGTAAAA	266	(TGTGCG)6	36	F
F01_151	F01_transcript_91524	ATTATTAGGGCGAGGGATGG	CAACCAAAAAGAAAATACAAATGG	214	(T)10	10	S
F01_152	F01_transcript_91527	CTGTGTGTGCCACTCATTCC	TAACGTCGGCCTATTTCCAG	139	(TA)6	12	S
F01_153	F01_transcript_91527	CAAACAGTTTAAAATTGCTGCC	TGTTTTGGCTTAGGCCATTC	272	(A)13	127	S
F01_154	F01_transcript_91533	TGGGGTTCCATTTGTGAAGT	ATGCGCACCTTATCTCCATC	278	(ATT)5	15	S
F01_155	F01_transcript_91543	CTTCTCCACCTTGGAGGTCA	GGGTCACATTTAGCCTGTGG	155	(TA)11	72	S
F01_156	F01_transcript_91543	ACAAACACACAAGGGCACAA	TGGCAAATCTGTTCAAACCA	214	(TC)26	52	S
F01_157	F01_transcript_91546	TTGATTTTGGGAGCTTGGAG	TCTGCATTGAACATTTCGTCA	195	(T)20	20	S
F01_158	F01_transcript_6457	TCTTGGTGGGATGCTTTTGT	AGGGACACCCTCCTGAAAAC	212	(T)10	10	S
F01_159	F01_transcript_6464	TGTTGGAGGAAATCCTTTCG	TGTTCAGTTCATGCCACAGG	263	(T)10	10	S
F01_160	F01_transcript_6481	TGATCTGGTCTCTGGAAGCC	GAAGAAGTCCTTCCGATCCC	254	(GT)9	18	S

F01_161	F01_transcript_6485	CCCAGCAGAAGTTGGTTTTC	GGAGCTTTAATCAGGCTGCTA	238	(TA)7	14	S
F01_162	F01_transcript_6489	CCCTTCATGTCTGACCTGTG	GCATGGAATCCTATGGCACT	127	(A)10	10	F
F01_163	F01_transcript_6496	TTTCTTTAATTTTCACTCTCTCTCTCG	AAACGTAAACGCGATGAAGG	256	(CT)8	16	S
F01_164	F01_transcript_6499	TCCCTCTTAAATTCATGCCG	TCGCCATTTTTCCACCTAAC	164	(TC)20	40	S
F01_165	F01_transcript_6506	TATAAAATGCCAAGGACCGC	TCCGAACTGTCCAAACACAA	145	(A)10	10	S
F01_166	F01_transcript_73617	GAGAGAGAAAGAGAGAGTGGTGG	AGAGGGAAAAGAGATGGGGA	251	(AG)8	16	S
F01_167	F01_transcript_73617	TTCATGTAGGTAAAGCCCGC	CTCGTCTAAAAGCCAGCCAG	162	(AG)9	18	S
F01_168	F01_transcript_73619	GGTGGGACCCAACACTCATA	AGCAAACAGATTGCCATGAA	265	(CT)14	28	S
F01_169	F01_transcript_73631	TAACGCAAGGCGTTTTACCT	AACACACAAAGCTATGGCTCAA	273	(A)10	10	S
F01_170	F01_transcript_73636	CACATAAGCGCGATTTCTCA	CCTGCAACAGAAAACAGAAACA	271	(T)16	102	S
F01_171	F01_transcript_73640	AGAACTTCTACGGCGACCAA	CAAACCCATAAAACCATGTCAA	227	(A)19	19	S
F01_172	F01_transcript_73642	AGCTGCACCTGTCAAAGTGA	CTTGACACGCATTCAGCCTA	133	(A)12	12	S
F01_173	F01_transcript_73642	CATAGGGAAAAGGCTGGTGA	AACTGCCTCCAATTCCACAC	279	(CT)9	47	S

F01_174	F01_transcript_73652	GGAAGGTTTTGGGGATCAAT	GAAGCACCATATCCGCTCTC	248	(T)15	15	S
F01_175	F01_transcript_65927	GGACAAAGGAATGGAGCAAA	ATCCACGCACTTTGGAAAAC	216	(C)12	12	S
F01_176	F01_transcript_65931	TGTAGTTCCATACCATCCGACA	ACCCGAGAAGAACTGGGTTT	114	(TC)8	16	S
F01_177	F01_transcript_65931	TTGATTCTTGGAAAATGGGC	AGCCTTCACTTAGGCAATGG	157	(CT)11	22	S
F01_178	F01_transcript_65938	CGTTGCTTTTCTGGTAGACG	TCGAAACCGGAGAGTCTGAT	204	(TC)6	12	S
F01_179	F01_transcript_65941	TCTCCATCCTTGTGGCAAAT	TCAAATTGCCTCCGTTTCTT	265	(GTTT)5	36	S
F01_180	F01_transcript_65946	GGAGAGAGAGGGAGAGAGCG	CATGGCGGGAAACGTATATC	161	(TA)8	16	S
F01_181	F01_transcript_65954	GTTCTGCGCAGTCACAGAGA	CTAGCTGAAGCCTGACCCAC	204	(GA)7	14	S
F01_182	F01_transcript_65955	TGGTTTCGATTCCTCTTTGG	ATTCACAAAGCACCCCTACG	265	(GA)43	86	S
F01_183	F01_transcript_65959	ATTGAGGCAGCTACACGGAT	TGTGATCAACTCAAAGACAACAAA	117	(T)10	10	F
F01_184	F01_transcript_65963	CAAAGGTCTCCTAACGCCTG	TGATTAATGTTTGATGGTTCTTGA	152	(T)12	12	S
F01_185	F01_transcript_73617	GAGAGAGAAAGAGAGAGTGGTGG	AGAGGGAAAAGAGATGGGGA	251	(AG)8	16	S
F01_186	F01_transcript_73617	TTCATGTAGGTAAAGCCCGC	CTCGTCTAAAAGCCAGCCAG	162	(AG)9	18	S
F01_187	F01_transcript_73619	GGTGGGACCCAACACTCATA	AGCAAACAGATTGCCATGAA	265	(CT)14	28	F

F01_188	F01_transcript_73631	TAACGCAAGGCGTTTTACCT	AACACACAAAGCTATGGCTCAA	273	(A)10	10	S
F01_189	F01_transcript_73636	CACATAAGCGCGATTTCTCA	CCTGCAACAGAAAACAGAAACA	271	(T)16	102	S
F01_190	F01_transcript_73640	AGAACTTCTACGGCGACCAA	CAAACCCATAAAACCATGTCAA	227	(A)19	19	S
F01_191	F01_transcript_73642	AGCTGCACCTGTCAAAGTGA	CTTGACACGCATTCAGCCTA	133	(A)12	12	S
F01_192	F01_transcript_73642	CATAGGGAAAAGGCTGGTGA	AACTGCCTCCAATTCCACAC	279	(CT)9	47	F
F01_193	F01_transcript_73652	GGAAGGTTTTGGGGATCAAT	GAAGCACCATATCCGCTCTC	248	(T)15	15	S
F01_194	F01_transcript_38183	TTGATCTGAATGAGCACCCA	CAAGGTTTTAGTGCACTGGTGA	168	(AT)11	22	S
F01_195	F01_transcript_38185	TGGCCAGAGACAGAGACAGA	TAGCCCTAGCCCTAGCCTTC	164	(GA)7	14	S
F01_196	F01_transcript_38201	AAGCAAATCATCGGTACCTCA	GAGCAACAAGAGGGGTCTCA	168	(T)10	10	S
F01_197	F01_transcript_38209	GACTTCGTCAGTTTCCGCTC	CTATTCCAGCTCACACGCAA	180	(TGT)5	60	S
F01_198	F01_transcript_38222	CGTCGATATAGAGAGAGAAAGTGG	GCGACGAAACAGTGATGTTG	192	(GA)6	12	S
F01_199	F01_transcript_38229	GTGAGAAAGAGGGGGAGAGG	CAAGGCCAGATCCTAATCCA	270	(GCA)5	15	S
F01_200	F01_transcript_38235	AGGCAAGCAAGCAACACTCT	ATTTATGTTGCCGGTCATCC	280	(AT)10	20	F
