# Supplementary material for: PacBio Single-Molecule Long-Read Sequencing Reveals Genes Tolerating Manganese Stress in Schima superba Saplings
Source: Front Genet. 2021 Apr 6;12:635043. doi: 10.3389/fgene.2021.635043 (PMC8057201; doi:10.3389/fgene.2021.635043)
Supplement: Supplementary Table 2 — Summary of lncRNA predicted by CPC, CNCI, CPAT, and Pfam protein structure domain analysis. [file Data_Sheet_3.PDF]

PacBio single-molecule long-read sequencing successfully explores the transcriptome of *Schima superba*

Fiza Liaquat[1], Muhammad Farooq Hussain Munis[2], Samiah Arif[1], Urooj Haroon[2], Muhammad Ashraf[3], Saddam Saqib[4,5], Wajid Zaman[4,5], Che Shengquan[6] and Liu Qunlu[6*]

1 School of Agriculture and Biology, Shanghai Jiao Tong University, Shanghai, 200240, China;

2 Department of Plant Sciences, Faculty of Biological Sciences, Quaid-i-Azam University, Islamabad, 45320, Pakistan;

3 Joint International Research Laboratory of Metabolic and Developmental Sciences, School of Life Science and Biotechnology, Shanghai Jiao Tong University, Shanghai, 200240, China;

4 State Key Laboratory of Systematic and Evolutionary Botany, Institute of Botany, Chinese Academy of Sciences, Beijing 100093, China;

5 University of Chinese Academy of Sciences, Beijing 100049, China;

6 Department of Landscape Architecture, School of Design, Shanghai Jiao Tong University, 200240, China;

*Correspondence: liuql@sjtu.edu.cn

Table S2 Summary of lncRNA predicted by CPC, CNCI, CPAT, and Pfam protein structure domain analysis

#ID	CNCI	CPAT	CPC	Pfam
F01_transcript_23711	NA	NA	NA	F01_transcript_23711
F01_transcript_60727	NA	NA	NA	F01_transcript_60727
F01_transcript_106582	NA	NA	NA	F01_transcript_106582
F01_transcript_96554	NA	F01_transcript_96554	NA	NA
F01_transcript_124337	NA	NA	F01_transcript_124337	NA
F01_transcript_42251	NA	NA	NA	F01_transcript_42251
F01_transcript_119660	NA	F01_transcript_119660	NA	NA
F01_transcript_114294	NA	F01_transcript_114294	NA	F01_transcript_114294
F01_transcript_69038	NA	NA	NA	F01_transcript_69038
F01_transcript_56889	F01_transcript_56889	F01_transcript_56889	F01_transcript_56889	NA
F01_transcript_101731	NA	F01_transcript_101731	NA	F01_transcript_101731
F01_transcript_147491	NA	NA	NA	F01_transcript_147491

F01_transcript_72155	F01_transcript_72155	F01_transcript_72155	F01_transcript_72155	F01_transcript_72155
F01_transcript_151933	NA	NA	NA	F01_transcript_151933
F01_transcript_107387	NA	NA	NA	F01_transcript_107387
F01_transcript_94320	NA	F01_transcript_94320	F01_transcript_94320	NA
F01_transcript_152006	F01_transcript_152006	F01_transcript_152006	NA	NA
F01_transcript_80744	NA	F01_transcript_80744	NA	NA
F01_transcript_98061	F01_transcript_98061	F01_transcript_98061	F01_transcript_98061	F01_transcript_98061
F01_transcript_93947	F01_transcript_93947	F01_transcript_93947	NA	F01_transcript_93947
F01_transcript_111870	F01_transcript_111870	F01_transcript_111870	F01_transcript_111870	F01_transcript_111870
F01_transcript_49853	NA	F01_transcript_49853	NA	NA
F01_transcript_155869	NA	NA	NA	F01_transcript_155869
F01_transcript_156201	F01_transcript_156201	F01_transcript_156201	F01_transcript_156201	F01_transcript_156201
F01_transcript_133670	F01_transcript_133670	F01_transcript_133670	F01_transcript_133670	F01_transcript_133670
F01_transcript_104870	NA	NA	NA	F01_transcript_104870

F01_transcript_76445	F01_transcript_76445	F01_transcript_76445	NA	NA
F01_transcript_82720	F01_transcript_82720	F01_transcript_82720	F01_transcript_82720	NA
F01_transcript_163571	F01_transcript_163571	F01_transcript_163571	F01_transcript_163571	F01_transcript_163571
F01_transcript_79259	NA	F01_transcript_79259	NA	NA
F01_transcript_147655	NA	NA	NA	F01_transcript_147655
F01_transcript_121402	NA	NA	NA	F01_transcript_121402
F01_transcript_153525	F01_transcript_153525	F01_transcript_153525	F01_transcript_153525	F01_transcript_153525
F01_transcript_87730	NA	F01_transcript_87730	F01_transcript_87730	F01_transcript_87730
F01_transcript_82581	F01_transcript_82581	F01_transcript_82581	NA	NA
F01_transcript_128459	F01_transcript_128459	F01_transcript_128459	NA	NA
F01_transcript_3444	NA	NA	NA	F01_transcript_3444
F01_transcript_132555	NA	F01_transcript_132555	NA	NA
F01_transcript_112750	NA	F01_transcript_112750	NA	NA
F01_transcript_150883	NA	NA	NA	F01_transcript_150883

F01_transcript_106554	NA	NA	NA	F01_transcript_106554
F01_transcript_121748	F01_transcript_121748	F01_transcript_121748	F01_transcript_121748	F01_transcript_121748
F01_transcript_124350	NA	NA	NA	F01_transcript_124350
F01_transcript_78189	NA	NA	NA	F01_transcript_78189
F01_transcript_108013	NA	F01_transcript_108013	NA	F01_transcript_108013
F01_transcript_128516	F01_transcript_128516	F01_transcript_128516	F01_transcript_128516	F01_transcript_128516
F01_transcript_73309	NA	NA	NA	F01_transcript_73309
F01_transcript_167760	NA	NA	NA	F01_transcript_167760
F01_transcript_74045	NA	NA	NA	F01_transcript_74045
F01_transcript_35761	NA	NA	NA	F01_transcript_35761
F01_transcript_83994	NA	F01_transcript_83994	NA	F01_transcript_83994
F01_transcript_149425	F01_transcript_149425	F01_transcript_149425	NA	NA
F01_transcript_1930	NA	NA	NA	F01_transcript_1930
F01_transcript_109215	F01_transcript_109215	F01_transcript_109215	NA	F01_transcript_109215

F01_transcript_143562	F01_transcript_143562	F01_transcript_143562	NA	NA
F01_transcript_81522	NA	NA	NA	F01_transcript_81522
F01_transcript_137934	NA	F01_transcript_137934	NA	NA
F01_transcript_128467	F01_transcript_128467	F01_transcript_128467	F01_transcript_128467	F01_transcript_128467
F01_transcript_139305	F01_transcript_139305	F01_transcript_139305	F01_transcript_139305	F01_transcript_139305
F01_transcript_79555	F01_transcript_79555	F01_transcript_79555	F01_transcript_79555	F01_transcript_79555
F01_transcript_125311	NA	F01_transcript_125311	F01_transcript_125311	F01_transcript_125311
F01_transcript_164260	F01_transcript_164260	F01_transcript_164260	NA	F01_transcript_164260
F01_transcript_130410	NA	NA	NA	F01_transcript_130410
F01_transcript_97057	NA	F01_transcript_97057	F01_transcript_97057	F01_transcript_97057
F01_transcript_147713	F01_transcript_147713	F01_transcript_147713	F01_transcript_147713	F01_transcript_147713
F01_transcript_98764	NA	F01_transcript_98764	NA	NA
F01_transcript_54538	NA	NA	NA	F01_transcript_54538
F01_transcript_5293	NA	NA	NA	F01_transcript_5293

F01_transcript_132248	NA	F01_transcript_132248	NA	NA
F01_transcript_89174	NA	NA	NA	F01_transcript_89174
F01_transcript_43380	NA	NA	NA	F01_transcript_43380
F01_transcript_35853	NA	F01_transcript_35853	NA	NA
F01_transcript_53612	NA	F01_transcript_53612	NA	NA
F01_transcript_96832	NA	NA	NA	F01_transcript_96832
F01_transcript_99052	NA	F01_transcript_99052	F01_transcript_99052	F01_transcript_99052
F01_transcript_137914	F01_transcript_137914	F01_transcript_137914	F01_transcript_137914	F01_transcript_137914
F01_transcript_108235	NA	F01_transcript_108235	NA	NA
F01_transcript_74864	NA	NA	NA	F01_transcript_74864
F01_transcript_114124	NA	F01_transcript_114124	NA	F01_transcript_114124
F01_transcript_139706	NA	F01_transcript_139706	NA	NA
F01_transcript_150722	F01_transcript_150722	F01_transcript_150722	F01_transcript_150722	F01_transcript_150722
F01_transcript_65484	NA	F01_transcript_65484	F01_transcript_65484	NA

F01_transcript_160169	NA	NA	F01_transcript_160169	NA
F01_transcript_56356	NA	NA	F01_transcript_56356	F01_transcript_56356
F01_transcript_1802	NA	NA	NA	F01_transcript_1802
F01_transcript_161540	NA	F01_transcript_161540	NA	F01_transcript_161540
F01_transcript_1512	NA	NA	NA	F01_transcript_1512
F01_transcript_110869	NA	F01_transcript_110869	NA	NA
F01_transcript_76634	NA	F01_transcript_76634	NA	NA
F01_transcript_93841	NA	NA	NA	F01_transcript_93841
F01_transcript_119096	NA	NA	NA	F01_transcript_119096
F01_transcript_145647	NA	F01_transcript_145647	NA	NA
F01_transcript_5387	NA	NA	NA	F01_transcript_5387
F01_transcript_61084	NA	F01_transcript_61084	NA	NA
F01_transcript_148106	F01_transcript_148106	F01_transcript_148106	F01_transcript_148106	F01_transcript_148106
F01_transcript_142007	NA	F01_transcript_142007	NA	NA

F01_transcript_70817	NA	NA	NA	F01_transcript_70817
F01_transcript_125400	NA	NA	NA	F01_transcript_125400
F01_transcript_163249	NA	F01_transcript_163249	NA	F01_transcript_163249
F01_transcript_87587	NA	F01_transcript_87587	NA	NA
F01_transcript_115306	F01_transcript_115306	F01_transcript_115306	NA	NA
F01_transcript_100483	NA	F01_transcript_100483	NA	F01_transcript_100483
F01_transcript_12840	NA	NA	NA	F01_transcript_12840
F01_transcript_7158	NA	NA	NA	F01_transcript_7158
F01_transcript_147881	F01_transcript_147881	F01_transcript_147881	NA	NA
F01_transcript_126300	NA	F01_transcript_126300	NA	F01_transcript_126300
F01_transcript_163782	NA	NA	NA	F01_transcript_163782
F01_transcript_37921	NA	NA	NA	F01_transcript_37921
F01_transcript_145216	NA	NA	NA	F01_transcript_145216
F01_transcript_194	NA	NA	NA	F01_transcript_194

F01_transcript_58053	F01_transcript_58053	F01_transcript_58053	F01_transcript_58053	F01_transcript_58053
F01_transcript_91812	F01_transcript_91812	NA	NA	NA
F01_transcript_156317	F01_transcript_156317	F01_transcript_156317	F01_transcript_156317	F01_transcript_156317
F01_transcript_107032	F01_transcript_107032	F01_transcript_107032	F01_transcript_107032	F01_transcript_107032
F01_transcript_156296	NA	F01_transcript_156296	NA	NA
F01_transcript_83936	NA	NA	NA	F01_transcript_83936
F01_transcript_33883	NA	NA	NA	F01_transcript_33883
F01_transcript_106150	F01_transcript_106150	F01_transcript_106150	F01_transcript_106150	F01_transcript_106150
F01_transcript_79088	NA	F01_transcript_79088	NA	F01_transcript_79088
F01_transcript_137908	F01_transcript_137908	F01_transcript_137908	F01_transcript_137908	F01_transcript_137908
F01_transcript_106647	NA	F01_transcript_106647	F01_transcript_106647	NA
F01_transcript_159491	F01_transcript_159491	F01_transcript_159491	F01_transcript_159491	NA
F01_transcript_118336	NA	F01_transcript_118336	NA	NA
F01_transcript_40223	NA	F01_transcript_40223	NA	NA

F01_transcript_127702	NA	NA	NA	F01_transcript_127702
F01_transcript_115052	F01_transcript_115052	F01_transcript_115052	NA	NA
F01_transcript_84955	F01_transcript_84955	F01_transcript_84955	F01_transcript_84955	F01_transcript_84955
F01_transcript_27807	NA	NA	NA	F01_transcript_27807
F01_transcript_49938	NA	NA	NA	F01_transcript_49938
F01_transcript_56899	NA	NA	F01_transcript_56899	NA
F01_transcript_64636	NA	F01_transcript_64636	NA	NA
F01_transcript_112547	F01_transcript_112547	F01_transcript_112547	NA	F01_transcript_112547
F01_transcript_127265	NA	F01_transcript_127265	NA	NA
F01_transcript_155339	F01_transcript_155339	F01_transcript_155339	NA	NA
F01_transcript_100796	NA	NA	NA	F01_transcript_100796
F01_transcript_166597	NA	F01_transcript_166597	NA	NA
F01_transcript_73972	F01_transcript_73972	F01_transcript_73972	F01_transcript_73972	NA
F01_transcript_85025	NA	F01_transcript_85025	NA	NA

F01_transcript_56562	F01_transcript_56562	F01_transcript_56562	F01_transcript_56562	F01_transcript_56562
F01_transcript_46024	NA	NA	NA	F01_transcript_46024
F01_transcript_132079	F01_transcript_132079	F01_transcript_132079	F01_transcript_132079	F01_transcript_132079
F01_transcript_115020	NA	NA	NA	F01_transcript_115020
F01_transcript_47052	NA	F01_transcript_47052	NA	NA
F01_transcript_59208	NA	NA	NA	F01_transcript_59208
F01_transcript_95460	F01_transcript_95460	F01_transcript_95460	NA	F01_transcript_95460
F01_transcript_115015	NA	F01_transcript_115015	NA	NA
F01_transcript_107976	NA	F01_transcript_107976	NA	F01_transcript_107976
F01_transcript_149660	NA	NA	NA	F01_transcript_149660
F01_transcript_65060	NA	NA	NA	F01_transcript_65060
F01_transcript_88117	NA	F01_transcript_88117	NA	NA
F01_transcript_156191	NA	NA	F01_transcript_156191	F01_transcript_156191
F01_transcript_80454	F01_transcript_80454	F01_transcript_80454	NA	NA

F01_transcript_97636	NA	NA	NA	F01_transcript_97636
F01_transcript_84838	F01_transcript_84838	F01_transcript_84838	F01_transcript_84838	NA
F01_transcript_148556	NA	F01_transcript_148556	NA	NA
F01_transcript_105896	F01_transcript_105896	F01_transcript_105896	NA	NA
F01_transcript_160063	NA	F01_transcript_160063	NA	F01_transcript_160063
F01_transcript_23896	NA	NA	F01_transcript_23896	F01_transcript_23896
F01_transcript_143718	NA	F01_transcript_143718	NA	F01_transcript_143718
F01_transcript_36772	NA	NA	F01_transcript_36772	F01_transcript_36772
F01_transcript_8516	NA	NA	NA	F01_transcript_8516
F01_transcript_63037	NA	NA	NA	F01_transcript_63037
F01_transcript_57045	NA	NA	NA	F01_transcript_57045
F01_transcript_161963	F01_transcript_161963	F01_transcript_161963	F01_transcript_161963	F01_transcript_161963
F01_transcript_58610	F01_transcript_58610	F01_transcript_58610	F01_transcript_58610	F01_transcript_58610
F01_transcript_79430	NA	F01_transcript_79430	NA	NA

F01_transcript_23425	NA	NA	NA	F01_transcript_23425
F01_transcript_72143	NA	F01_transcript_72143	NA	NA
F01_transcript_137755	F01_transcript_137755	F01_transcript_137755	F01_transcript_137755	F01_transcript_137755
F01_transcript_117332	F01_transcript_117332	F01_transcript_117332	NA	F01_transcript_117332
F01_transcript_65223	NA	NA	F01_transcript_65223	NA
F01_transcript_52577	NA	NA	NA	F01_transcript_52577
F01_transcript_66840	NA	F01_transcript_66840	NA	F01_transcript_66840
F01_transcript_134357	NA	NA	NA	F01_transcript_134357
F01_transcript_162646	F01_transcript_162646	F01_transcript_162646	F01_transcript_162646	NA
F01_transcript_111004	NA	F01_transcript_111004	NA	NA
F01_transcript_139308	F01_transcript_139308	F01_transcript_139308	F01_transcript_139308	F01_transcript_139308
F01_transcript_83745	NA	NA	NA	F01_transcript_83745
F01_transcript_58270	NA	F01_transcript_58270	NA	NA
F01_transcript_111288	F01_transcript_111288	F01_transcript_111288	F01_transcript_111288	NA

F01_transcript_104740	F01_transcript_104740	F01_transcript_104740	NA	NA
F01_transcript_54013	NA	NA	NA	F01_transcript_54013
F01_transcript_58387	F01_transcript_58387	F01_transcript_58387	NA	NA
F01_transcript_2574	NA	NA	NA	F01_transcript_2574
F01_transcript_99988	NA	NA	NA	F01_transcript_99988
F01_transcript_52858	NA	NA	NA	F01_transcript_52858
F01_transcript_86422	F01_transcript_86422	F01_transcript_86422	F01_transcript_86422	F01_transcript_86422
F01_transcript_116636	NA	NA	NA	F01_transcript_116636
F01_transcript_131652	NA	F01_transcript_131652	NA	F01_transcript_131652
F01_transcript_26616	NA	NA	F01_transcript_26616	F01_transcript_26616
F01_transcript_52103	NA	F01_transcript_52103	NA	NA
F01_transcript_129151	NA	NA	F01_transcript_129151	F01_transcript_129151
F01_transcript_65445	NA	NA	NA	F01_transcript_65445
F01_transcript_116743	F01_transcript_116743	F01_transcript_116743	F01_transcript_116743	F01_transcript_116743

F01_transcript_35359	NA	NA	F01_transcript_35359	F01_transcript_35359
F01_transcript_143234	NA	F01_transcript_143234	NA	NA
F01_transcript_149177	NA	F01_transcript_149177	F01_transcript_149177	NA
F01_transcript_118381	NA	NA	NA	F01_transcript_118381
F01_transcript_110231	F01_transcript_110231	F01_transcript_110231	NA	F01_transcript_110231
F01_transcript_124395	F01_transcript_124395	F01_transcript_124395	F01_transcript_124395	F01_transcript_124395
F01_transcript_85045	NA	F01_transcript_85045	F01_transcript_85045	F01_transcript_85045
F01_transcript_68638	F01_transcript_68638	F01_transcript_68638	F01_transcript_68638	NA
F01_transcript_106353	F01_transcript_106353	F01_transcript_106353	F01_transcript_106353	F01_transcript_106353
F01_transcript_163623	NA	F01_transcript_163623	NA	NA
F01_transcript_114285	NA	NA	F01_transcript_114285	F01_transcript_114285
F01_transcript_21613	NA	NA	NA	F01_transcript_21613
F01_transcript_65942	NA	NA	NA	F01_transcript_65942
F01_transcript_99134	F01_transcript_99134	F01_transcript_99134	F01_transcript_99134	F01_transcript_99134

F01_transcript_53186	NA	NA	NA	F01_transcript_53186
F01_transcript_50548	NA	F01_transcript_50548	F01_transcript_50548	F01_transcript_50548
F01_transcript_32036	F01_transcript_32036	F01_transcript_32036	F01_transcript_32036	F01_transcript_32036
F01_transcript_160078	NA	NA	NA	F01_transcript_160078
F01_transcript_137876	NA	NA	F01_transcript_137876	NA
F01_transcript_162856	NA	NA	NA	F01_transcript_162856
F01_transcript_44182	NA	NA	NA	F01_transcript_44182
F01_transcript_148886	NA	F01_transcript_148886	NA	NA
F01_transcript_66409	F01_transcript_66409	F01_transcript_66409	F01_transcript_66409	F01_transcript_66409
F01_transcript_80823	F01_transcript_80823	F01_transcript_80823	F01_transcript_80823	F01_transcript_80823
F01_transcript_158052	NA	NA	NA	F01_transcript_158052
F01_transcript_133266	NA	NA	NA	F01_transcript_133266
F01_transcript_161662	NA	NA	NA	F01_transcript_161662
F01_transcript_105032	F01_transcript_105032	F01_transcript_105032	NA	F01_transcript_105032

F01_transcript_135455	NA	F01_transcript_135455	F01_transcript_135455	NA
F01_transcript_158597	NA	NA	NA	F01_transcript_158597
F01_transcript_107194	F01_transcript_107194	F01_transcript_107194	NA	NA
F01_transcript_140908	NA	NA	NA	F01_transcript_140908
F01_transcript_159082	F01_transcript_159082	F01_transcript_159082	F01_transcript_159082	F01_transcript_159082
F01_transcript_151302	F01_transcript_151302	NA	NA	F01_transcript_151302
F01_transcript_52617	NA	F01_transcript_52617	NA	NA
F01_transcript_134228	NA	F01_transcript_134228	NA	NA
F01_transcript_132535	F01_transcript_132535	F01_transcript_132535	F01_transcript_132535	F01_transcript_132535
F01_transcript_126773	F01_transcript_126773	F01_transcript_126773	F01_transcript_126773	F01_transcript_126773
F01_transcript_152709	NA	F01_transcript_152709	NA	NA
F01_transcript_82352	NA	F01_transcript_82352	F01_transcript_82352	F01_transcript_82352
F01_transcript_139520	F01_transcript_139520	F01_transcript_139520	NA	NA
F01_transcript_137402	NA	NA	NA	F01_transcript_137402

F01_transcript_121790	NA	NA	NA	F01_transcript_121790
F01_transcript_87732	NA	NA	NA	F01_transcript_87732
F01_transcript_146689	F01_transcript_146689	F01_transcript_146689	F01_transcript_146689	F01_transcript_146689
F01_transcript_138881	F01_transcript_138881	F01_transcript_138881	F01_transcript_138881	F01_transcript_138881
F01_transcript_142389	F01_transcript_142389	F01_transcript_142389	F01_transcript_142389	F01_transcript_142389
F01_transcript_98893	NA	NA	NA	F01_transcript_98893
F01_transcript_13328	NA	NA	NA	F01_transcript_13328
F01_transcript_2940	NA	NA	NA	F01_transcript_2940
F01_transcript_51068	F01_transcript_51068	F01_transcript_51068	F01_transcript_51068	F01_transcript_51068
F01_transcript_71314	NA	F01_transcript_71314	F01_transcript_71314	F01_transcript_71314
F01_transcript_26924	NA	NA	NA	F01_transcript_26924
F01_transcript_100062	NA	F01_transcript_100062	NA	F01_transcript_100062
F01_transcript_37623	NA	NA	NA	F01_transcript_37623
F01_transcript_12658	NA	NA	NA	F01_transcript_12658

F01_transcript_90343	NA	F01_transcript_90343	NA	NA
F01_transcript_100235	NA	F01_transcript_100235	NA	NA
F01_transcript_36165	NA	NA	NA	F01_transcript_36165
F01_transcript_98744	NA	NA	NA	F01_transcript_98744
F01_transcript_136060	NA	F01_transcript_136060	NA	F01_transcript_136060
F01_transcript_166550	NA	F01_transcript_166550	NA	NA
F01_transcript_109086	F01_transcript_109086	F01_transcript_109086	F01_transcript_109086	F01_transcript_109086
F01_transcript_107922	F01_transcript_107922	F01_transcript_107922	F01_transcript_107922	F01_transcript_107922
F01_transcript_25799	NA	NA	NA	F01_transcript_25799
F01_transcript_154807	NA	F01_transcript_154807	F01_transcript_154807	F01_transcript_154807
F01_transcript_60182	F01_transcript_60182	F01_transcript_60182	NA	NA
F01_transcript_51974	NA	F01_transcript_51974	NA	NA
F01_transcript_55227	NA	NA	NA	F01_transcript_55227
F01_transcript_59498	NA	F01_transcript_59498	F01_transcript_59498	F01_transcript_59498

F01_transcript_57190	F01_transcript_57190	F01_transcript_57190	F01_transcript_57190	F01_transcript_57190
F01_transcript_69258	NA	NA	NA	F01_transcript_69258
F01_transcript_24922	NA	NA	NA	F01_transcript_24922
F01_transcript_88780	NA	F01_transcript_88780	NA	NA
F01_transcript_27542	F01_transcript_27542	F01_transcript_27542	F01_transcript_27542	F01_transcript_27542
F01_transcript_138086	F01_transcript_138086	F01_transcript_138086	F01_transcript_138086	F01_transcript_138086
F01_transcript_139986	NA	F01_transcript_139986	NA	NA
F01_transcript_67781	NA	F01_transcript_67781	NA	NA
F01_transcript_167647	NA	NA	F01_transcript_167647	F01_transcript_167647
F01_transcript_86984	NA	F01_transcript_86984	NA	F01_transcript_86984
F01_transcript_55025	F01_transcript_55025	F01_transcript_55025	F01_transcript_55025	F01_transcript_55025
F01_transcript_154896	NA	F01_transcript_154896	NA	NA
F01_transcript_104626	NA	F01_transcript_104626	F01_transcript_104626	NA
F01_transcript_126655	NA	F01_transcript_126655	NA	F01_transcript_126655

F01_transcript_75057	NA	F01_transcript_75057	NA	F01_transcript_75057
F01_transcript_67666	NA	F01_transcript_67666	NA	F01_transcript_67666
F01_transcript_116288	F01_transcript_116288	F01_transcript_116288	F01_transcript_116288	NA
F01_transcript_121179	NA	F01_transcript_121179	NA	NA
F01_transcript_133269	NA	NA	F01_transcript_133269	NA
F01_transcript_67725	NA	F01_transcript_67725	F01_transcript_67725	F01_transcript_67725
F01_transcript_114814	NA	NA	NA	F01_transcript_114814
F01_transcript_156304	NA	NA	NA	F01_transcript_156304
F01_transcript_120058	NA	F01_transcript_120058	NA	F01_transcript_120058
F01_transcript_119991	NA	NA	NA	F01_transcript_119991
F01_transcript_53112	F01_transcript_53112	F01_transcript_53112	F01_transcript_53112	F01_transcript_53112
F01_transcript_148201	NA	F01_transcript_148201	NA	NA
F01_transcript_105752	NA	F01_transcript_105752	NA	F01_transcript_105752
F01_transcript_56228	NA	NA	F01_transcript_56228	NA

F01_transcript_43108	NA	NA	NA	F01_transcript_43108
F01_transcript_114129	F01_transcript_114129	F01_transcript_114129	F01_transcript_114129	F01_transcript_114129
F01_transcript_141790	NA	NA	NA	F01_transcript_141790
F01_transcript_33675	NA	NA	NA	F01_transcript_33675
F01_transcript_132599	NA	F01_transcript_132599	NA	NA
F01_transcript_29323	NA	NA	NA	F01_transcript_29323
F01_transcript_52309	NA	NA	NA	F01_transcript_52309
F01_transcript_154779	NA	F01_transcript_154779	F01_transcript_154779	F01_transcript_154779
F01_transcript_47996	NA	F01_transcript_47996	NA	NA
F01_transcript_149997	NA	NA	NA	F01_transcript_149997
F01_transcript_155385	NA	F01_transcript_155385	NA	F01_transcript_155385
F01_transcript_155978	NA	NA	NA	F01_transcript_155978
F01_transcript_66863	NA	NA	NA	F01_transcript_66863
F01_transcript_58635	F01_transcript_58635	F01_transcript_58635	F01_transcript_58635	F01_transcript_58635

F01_transcript_137561	NA	F01_transcript_137561	F01_transcript_137561	NA
F01_transcript_116471	F01_transcript_116471	NA	NA	NA
F01_transcript_50846	NA	F01_transcript_50846	NA	NA
F01_transcript_47088	NA	F01_transcript_47088	NA	NA
F01_transcript_46823	NA	F01_transcript_46823	NA	F01_transcript_46823
F01_transcript_143677	NA	NA	NA	F01_transcript_143677
F01_transcript_51505	NA	F01_transcript_51505	NA	NA
F01_transcript_111901	NA	NA	NA	F01_transcript_111901
F01_transcript_72809	NA	F01_transcript_72809	NA	NA
F01_transcript_81464	NA	F01_transcript_81464	NA	NA
F01_transcript_79722	NA	F01_transcript_79722	NA	F01_transcript_79722
F01_transcript_105776	NA	F01_transcript_105776	NA	NA
F01_transcript_42430	NA	NA	NA	F01_transcript_42430
F01_transcript_106691	F01_transcript_106691	F01_transcript_106691	F01_transcript_106691	F01_transcript_106691

F01_transcript_77348	F01_transcript_77348	F01_transcript_77348	F01_transcript_77348	F01_transcript_77348
F01_transcript_84884	F01_transcript_84884	F01_transcript_84884	F01_transcript_84884	F01_transcript_84884
F01_transcript_117599	NA	F01_transcript_117599	NA	F01_transcript_117599
F01_transcript_101220	NA	NA	NA	F01_transcript_101220
F01_transcript_7219	NA	F01_transcript_7219	NA	NA
F01_transcript_43083	F01_transcript_43083	F01_transcript_43083	NA	NA
F01_transcript_137069	NA	NA	NA	F01_transcript_137069
F01_transcript_131838	NA	F01_transcript_131838	NA	F01_transcript_131838
F01_transcript_52126	NA	F01_transcript_52126	F01_transcript_52126	F01_transcript_52126
F01_transcript_68113	NA	F01_transcript_68113	F01_transcript_68113	F01_transcript_68113
F01_transcript_116858	NA	F01_transcript_116858	NA	NA
F01_transcript_86405	NA	F01_transcript_86405	NA	NA
F01_transcript_17989	F01_transcript_17989	F01_transcript_17989	F01_transcript_17989	F01_transcript_17989
F01_transcript_125583	NA	F01_transcript_125583	NA	NA

F01_transcript_108903	F01_transcript_108903	F01_transcript_108903	NA	F01_transcript_108903
F01_transcript_132418	NA	NA	NA	F01_transcript_132418
F01_transcript_151696	NA	F01_transcript_151696	NA	NA
F01_transcript_67289	F01_transcript_67289	F01_transcript_67289	F01_transcript_67289	NA
F01_transcript_121873	F01_transcript_121873	F01_transcript_121873	NA	NA
F01_transcript_120914	NA	F01_transcript_120914	NA	F01_transcript_120914
F01_transcript_113526	NA	NA	NA	F01_transcript_113526
F01_transcript_161702	NA	F01_transcript_161702	NA	F01_transcript_161702
F01_transcript_161939	NA	F01_transcript_161939	NA	NA
F01_transcript_163281	NA	NA	NA	F01_transcript_163281
F01_transcript_127782	NA	NA	F01_transcript_127782	F01_transcript_127782
F01_transcript_78625	F01_transcript_78625	F01_transcript_78625	F01_transcript_78625	F01_transcript_78625
F01_transcript_74598	F01_transcript_74598	F01_transcript_74598	F01_transcript_74598	F01_transcript_74598
F01_transcript_52333	NA	F01_transcript_52333	NA	NA

F01_transcript_132841	NA	NA	NA	F01_transcript_132841
F01_transcript_29178	NA	NA	NA	F01_transcript_29178
F01_transcript_40866	NA	F01_transcript_40866	NA	NA
F01_transcript_44491	NA	NA	NA	F01_transcript_44491
F01_transcript_123909	F01_transcript_123909	F01_transcript_123909	F01_transcript_123909	NA
F01_transcript_58697	NA	F01_transcript_58697	F01_transcript_58697	NA
F01_transcript_100993	NA	NA	NA	F01_transcript_100993
F01_transcript_119124	NA	NA	NA	F01_transcript_119124
F01_transcript_47669	NA	F01_transcript_47669	NA	NA
F01_transcript_62432	NA	F01_transcript_62432	NA	NA
F01_transcript_49993	F01_transcript_49993	F01_transcript_49993	F01_transcript_49993	F01_transcript_49993
F01_transcript_95112	NA	NA	NA	F01_transcript_95112
F01_transcript_88017	NA	NA	NA	F01_transcript_88017
F01_transcript_133466	NA	F01_transcript_133466	NA	NA

F01_transcript_97048	NA	NA	NA	F01_transcript_97048
F01_transcript_123944	F01_transcript_123944	F01_transcript_123944	F01_transcript_123944	F01_transcript_123944
F01_transcript_19340	NA	F01_transcript_19340	NA	F01_transcript_19340
F01_transcript_157418	NA	NA	NA	F01_transcript_157418
F01_transcript_108569	NA	NA	NA	F01_transcript_108569
F01_transcript_124896	NA	F01_transcript_124896	F01_transcript_124896	NA
F01_transcript_48769	NA	NA	NA	F01_transcript_48769
F01_transcript_143027	NA	NA	NA	F01_transcript_143027
F01_transcript_158882	NA	NA	F01_transcript_158882	NA
F01_transcript_131297	NA	F01_transcript_131297	NA	NA
F01_transcript_104649	NA	NA	F01_transcript_104649	F01_transcript_104649
F01_transcript_52527	NA	F01_transcript_52527	NA	NA
F01_transcript_50202	NA	F01_transcript_50202	NA	NA
F01_transcript_150192	F01_transcript_150192	F01_transcript_150192	F01_transcript_150192	F01_transcript_150192

F01_transcript_128479	F01_transcript_128479	F01_transcript_128479	F01_transcript_128479	F01_transcript_128479
F01_transcript_11964	NA	NA	NA	F01_transcript_11964
F01_transcript_142863	NA	F01_transcript_142863	F01_transcript_142863	NA
F01_transcript_50470	NA	F01_transcript_50470	NA	NA
F01_transcript_141117	F01_transcript_141117	F01_transcript_141117	F01_transcript_141117	F01_transcript_141117
F01_transcript_115466	NA	F01_transcript_115466	NA	NA
F01_transcript_83655	NA	NA	NA	F01_transcript_83655
F01_transcript_78031	NA	NA	NA	F01_transcript_78031
F01_transcript_62553	NA	F01_transcript_62553	NA	NA
F01_transcript_14740	NA	NA	NA	F01_transcript_14740
F01_transcript_41764	NA	NA	NA	F01_transcript_41764
F01_transcript_56822	NA	F01_transcript_56822	F01_transcript_56822	NA
F01_transcript_84352	NA	F01_transcript_84352	NA	NA
F01_transcript_82771	NA	F01_transcript_82771	NA	F01_transcript_82771

F01_transcript_117251	NA	NA	NA	F01_transcript_117251
F01_transcript_21979	NA	NA	NA	F01_transcript_21979
F01_transcript_70867	F01_transcript_70867	F01_transcript_70867	F01_transcript_70867	F01_transcript_70867
F01_transcript_104420	NA	NA	NA	F01_transcript_104420
F01_transcript_151904	F01_transcript_151904	F01_transcript_151904	F01_transcript_151904	F01_transcript_151904
F01_transcript_91686	NA	F01_transcript_91686	F01_transcript_91686	F01_transcript_91686
F01_transcript_161104	NA	NA	NA	F01_transcript_161104
F01_transcript_64266	NA	F01_transcript_64266	NA	F01_transcript_64266
F01_transcript_133657	F01_transcript_133657	F01_transcript_133657	F01_transcript_133657	F01_transcript_133657
F01_transcript_116680	NA	NA	NA	F01_transcript_116680
F01_transcript_127255	NA	NA	NA	F01_transcript_127255
F01_transcript_8029	F01_transcript_8029	F01_transcript_8029	F01_transcript_8029	F01_transcript_8029
F01_transcript_49100	NA	F01_transcript_49100	NA	NA
F01_transcript_10753	F01_transcript_10753	F01_transcript_10753	F01_transcript_10753	F01_transcript_10753

F01_transcript_157764	F01_transcript_157764	F01_transcript_157764	NA	NA
F01_transcript_73818	NA	NA	NA	F01_transcript_73818
F01_transcript_164726	NA	F01_transcript_164726	NA	F01_transcript_164726
F01_transcript_106649	NA	F01_transcript_106649	NA	F01_transcript_106649
F01_transcript_23771	NA	NA	NA	F01_transcript_23771
F01_transcript_121071	F01_transcript_121071	F01_transcript_121071	NA	NA
F01_transcript_50953	F01_transcript_50953	F01_transcript_50953	F01_transcript_50953	F01_transcript_50953
F01_transcript_137746	NA	F01_transcript_137746	F01_transcript_137746	NA
F01_transcript_121486	F01_transcript_121486	F01_transcript_121486	NA	F01_transcript_121486
F01_transcript_116536	NA	NA	F01_transcript_116536	NA
F01_transcript_157954	NA	F01_transcript_157954	F01_transcript_157954	F01_transcript_157954
F01_transcript_81260	NA	F01_transcript_81260	NA	NA
F01_transcript_23358	NA	NA	NA	F01_transcript_23358
F01_transcript_134929	F01_transcript_134929	F01_transcript_134929	F01_transcript_134929	F01_transcript_134929

F01_transcript_50206	NA	F01_transcript_50206	NA	F01_transcript_50206
F01_transcript_66433	F01_transcript_66433	F01_transcript_66433	F01_transcript_66433	F01_transcript_66433
F01_transcript_111567	NA	NA	NA	F01_transcript_111567
F01_transcript_154742	NA	NA	NA	F01_transcript_154742
F01_transcript_152414	NA	NA	NA	F01_transcript_152414
F01_transcript_151216	NA	NA	NA	F01_transcript_151216
F01_transcript_142178	NA	NA	NA	F01_transcript_142178
F01_transcript_143444	NA	NA	NA	F01_transcript_143444
F01_transcript_14199	NA	F01_transcript_14199	F01_transcript_14199	F01_transcript_14199
F01_transcript_161921	NA	NA	NA	F01_transcript_161921
F01_transcript_52669	F01_transcript_52669	F01_transcript_52669	NA	NA
F01_transcript_99812	NA	NA	NA	F01_transcript_99812
F01_transcript_64254	NA	F01_transcript_64254	F01_transcript_64254	NA
F01_transcript_162424	NA	NA	NA	F01_transcript_162424

F01_transcript_30080	NA	NA	NA	F01_transcript_30080
F01_transcript_97254	NA	NA	NA	F01_transcript_97254
F01_transcript_68322	F01_transcript_68322	F01_transcript_68322	F01_transcript_68322	F01_transcript_68322
F01_transcript_59255	NA	NA	F01_transcript_59255	F01_transcript_59255
F01_transcript_61567	F01_transcript_61567	F01_transcript_61567	F01_transcript_61567	F01_transcript_61567
F01_transcript_113319	F01_transcript_113319	F01_transcript_113319	F01_transcript_113319	F01_transcript_113319
F01_transcript_56838	F01_transcript_56838	NA	F01_transcript_56838	F01_transcript_56838
F01_transcript_111095	NA	F01_transcript_111095	NA	NA
F01_transcript_94970	NA	F01_transcript_94970	NA	F01_transcript_94970
F01_transcript_80395	NA	NA	NA	F01_transcript_80395
F01_transcript_108353	NA	F01_transcript_108353	NA	NA
F01_transcript_149471	NA	F01_transcript_149471	F01_transcript_149471	F01_transcript_149471
F01_transcript_127374	NA	F01_transcript_127374	NA	F01_transcript_127374
F01_transcript_117611	NA	F01_transcript_117611	NA	NA

F01_transcript_44737	NA	F01_transcript_44737	NA	F01_transcript_44737
F01_transcript_46116	NA	F01_transcript_46116	NA	NA
F01_transcript_123922	NA	NA	NA	F01_transcript_123922
F01_transcript_70099	F01_transcript_70099	F01_transcript_70099	F01_transcript_70099	F01_transcript_70099
F01_transcript_114054	F01_transcript_114054	F01_transcript_114054	F01_transcript_114054	NA
F01_transcript_108268	F01_transcript_108268	NA	NA	F01_transcript_108268
F01_transcript_73795	F01_transcript_73795	F01_transcript_73795	F01_transcript_73795	F01_transcript_73795
F01_transcript_65865	NA	F01_transcript_65865	NA	NA
F01_transcript_132391	NA	NA	NA	F01_transcript_132391
F01_transcript_155772	F01_transcript_155772	F01_transcript_155772	F01_transcript_155772	F01_transcript_155772
F01_transcript_157905	NA	NA	NA	F01_transcript_157905
F01_transcript_151531	NA	NA	NA	F01_transcript_151531
F01_transcript_106334	F01_transcript_106334	F01_transcript_106334	NA	F01_transcript_106334
F01_transcript_150564	F01_transcript_150564	F01_transcript_150564	F01_transcript_150564	F01_transcript_150564

F01_transcript_160528	NA	NA	NA	F01_transcript_160528
F01_transcript_157050	NA	NA	NA	F01_transcript_157050
F01_transcript_116569	F01_transcript_116569	F01_transcript_116569	F01_transcript_116569	F01_transcript_116569
F01_transcript_50865	F01_transcript_50865	F01_transcript_50865	F01_transcript_50865	F01_transcript_50865
F01_transcript_48073	NA	NA	NA	F01_transcript_48073
F01_transcript_47760	NA	NA	NA	F01_transcript_47760
F01_transcript_130342	NA	NA	NA	F01_transcript_130342
F01_transcript_139603	NA	F01_transcript_139603	F01_transcript_139603	F01_transcript_139603
F01_transcript_162241	F01_transcript_162241	F01_transcript_162241	F01_transcript_162241	F01_transcript_162241
F01_transcript_59610	F01_transcript_59610	F01_transcript_59610	F01_transcript_59610	F01_transcript_59610
F01_transcript_132798	F01_transcript_132798	F01_transcript_132798	F01_transcript_132798	F01_transcript_132798
F01_transcript_63135	NA	NA	NA	F01_transcript_63135
F01_transcript_89612	NA	F01_transcript_89612	F01_transcript_89612	F01_transcript_89612
F01_transcript_145304	NA	NA	NA	F01_transcript_145304

F01_transcript_26951	NA	F01_transcript_26951	NA	F01_transcript_26951
F01_transcript_165771	NA	F01_transcript_165771	NA	F01_transcript_165771
F01_transcript_131025	NA	F01_transcript_131025	NA	NA
F01_transcript_160512	NA	F01_transcript_160512	NA	NA
F01_transcript_139964	NA	F01_transcript_139964	NA	NA
F01_transcript_55246	NA	F01_transcript_55246	F01_transcript_55246	F01_transcript_55246
F01_transcript_68640	F01_transcript_68640	F01_transcript_68640	NA	F01_transcript_68640
F01_transcript_70767	NA	F01_transcript_70767	NA	NA
F01_transcript_68616	NA	F01_transcript_68616	NA	F01_transcript_68616
F01_transcript_124084	NA	F01_transcript_124084	NA	NA
F01_transcript_128944	F01_transcript_128944	F01_transcript_128944	F01_transcript_128944	F01_transcript_128944
F01_transcript_90438	NA	F01_transcript_90438	NA	NA
F01_transcript_160746	NA	NA	NA	F01_transcript_160746
F01_transcript_16720	NA	NA	NA	F01_transcript_16720

F01_transcript_90239	F01_transcript_90239	F01_transcript_90239	F01_transcript_90239	F01_transcript_90239
F01_transcript_139421	F01_transcript_139421	F01_transcript_139421	F01_transcript_139421	F01_transcript_139421
F01_transcript_137511	NA	F01_transcript_137511	NA	NA
F01_transcript_56531	NA	NA	NA	F01_transcript_56531
F01_transcript_160285	NA	F01_transcript_160285	F01_transcript_160285	F01_transcript_160285
F01_transcript_103122	NA	F01_transcript_103122	NA	NA
F01_transcript_28602	NA	NA	NA	F01_transcript_28602
F01_transcript_2279	NA	NA	NA	F01_transcript_2279
F01_transcript_136477	NA	F01_transcript_136477	NA	F01_transcript_136477
F01_transcript_68775	F01_transcript_68775	F01_transcript_68775	F01_transcript_68775	F01_transcript_68775
F01_transcript_63795	NA	NA	NA	F01_transcript_63795
F01_transcript_36122	NA	NA	NA	F01_transcript_36122
F01_transcript_65506	NA	NA	F01_transcript_65506	NA
F01_transcript_154612	NA	F01_transcript_154612	NA	NA

F01_transcript_123232	NA	F01_transcript_123232	F01_transcript_123232	NA
F01_transcript_88756	F01_transcript_88756	F01_transcript_88756	NA	F01_transcript_88756
F01_transcript_81804	NA	F01_transcript_81804	NA	F01_transcript_81804
F01_transcript_90312	F01_transcript_90312	F01_transcript_90312	F01_transcript_90312	F01_transcript_90312
F01_transcript_129712	F01_transcript_129712	F01_transcript_129712	NA	NA
F01_transcript_75440	NA	NA	NA	F01_transcript_75440
F01_transcript_106362	NA	F01_transcript_106362	NA	F01_transcript_106362
F01_transcript_132511	NA	NA	NA	F01_transcript_132511
F01_transcript_145004	NA	F01_transcript_145004	F01_transcript_145004	F01_transcript_145004
F01_transcript_147155	NA	F01_transcript_147155	F01_transcript_147155	NA
F01_transcript_136052	NA	NA	NA	F01_transcript_136052
F01_transcript_123748	NA	F01_transcript_123748	F01_transcript_123748	F01_transcript_123748
F01_transcript_117576	NA	F01_transcript_117576	NA	NA
F01_transcript_58021	F01_transcript_58021	F01_transcript_58021	NA	NA

F01_transcript_71237	NA	F01_transcript_71237	F01_transcript_71237	NA
F01_transcript_128541	NA	NA	NA	F01_transcript_128541
F01_transcript_124569	NA	NA	NA	F01_transcript_124569
F01_transcript_18423	NA	NA	NA	F01_transcript_18423
F01_transcript_116006	F01_transcript_116006	F01_transcript_116006	F01_transcript_116006	F01_transcript_116006
F01_transcript_150921	NA	F01_transcript_150921	NA	F01_transcript_150921
F01_transcript_122246	NA	F01_transcript_122246	F01_transcript_122246	F01_transcript_122246
F01_transcript_121843	NA	F01_transcript_121843	NA	F01_transcript_121843
F01_transcript_45705	NA	NA	NA	F01_transcript_45705
F01_transcript_6682	F01_transcript_6682	F01_transcript_6682	F01_transcript_6682	F01_transcript_6682
F01_transcript_38856	NA	NA	NA	F01_transcript_38856
F01_transcript_153743	NA	F01_transcript_153743	NA	NA
F01_transcript_55519	NA	NA	F01_transcript_55519	F01_transcript_55519
F01_transcript_77939	F01_transcript_77939	F01_transcript_77939	NA	NA

F01_transcript_19592	F01_transcript_19592	F01_transcript_19592	F01_transcript_19592	F01_transcript_19592
F01_transcript_164504	F01_transcript_164504	F01_transcript_164504	NA	NA
F01_transcript_28472	NA	F01_transcript_28472	NA	F01_transcript_28472
F01_transcript_67371	NA	F01_transcript_67371	NA	F01_transcript_67371
F01_transcript_165780	NA	F01_transcript_165780	F01_transcript_165780	NA
F01_transcript_69184	NA	F01_transcript_69184	NA	NA
F01_transcript_59270	NA	F01_transcript_59270	NA	F01_transcript_59270
F01_transcript_146374	NA	F01_transcript_146374	NA	NA
F01_transcript_163247	NA	NA	NA	F01_transcript_163247
F01_transcript_125552	NA	NA	NA	F01_transcript_125552
F01_transcript_59453	F01_transcript_59453	F01_transcript_59453	F01_transcript_59453	F01_transcript_59453
F01_transcript_125113	F01_transcript_125113	F01_transcript_125113	F01_transcript_125113	F01_transcript_125113
F01_transcript_65211	NA	NA	F01_transcript_65211	NA
F01_transcript_153637	F01_transcript_153637	F01_transcript_153637	F01_transcript_153637	F01_transcript_153637

F01_transcript_160689	NA	F01_transcript_160689	NA	F01_transcript_160689
F01_transcript_127832	F01_transcript_127832	F01_transcript_127832	F01_transcript_127832	F01_transcript_127832
F01_transcript_20570	NA	NA	NA	F01_transcript_20570
F01_transcript_85168	NA	F01_transcript_85168	NA	NA
F01_transcript_150108	F01_transcript_150108	F01_transcript_150108	NA	F01_transcript_150108
F01_transcript_104567	F01_transcript_104567	F01_transcript_104567	NA	NA
F01_transcript_134329	NA	NA	F01_transcript_134329	F01_transcript_134329
F01_transcript_97378	F01_transcript_97378	F01_transcript_97378	NA	NA
F01_transcript_68861	F01_transcript_68861	F01_transcript_68861	NA	NA
F01_transcript_135536	NA	NA	NA	F01_transcript_135536
F01_transcript_101981	NA	F01_transcript_101981	NA	NA
F01_transcript_49808	NA	F01_transcript_49808	NA	F01_transcript_49808
F01_transcript_153577	NA	NA	NA	F01_transcript_153577
F01_transcript_152524	F01_transcript_152524	F01_transcript_152524	F01_transcript_152524	F01_transcript_152524

F01_transcript_159939	NA	F01_transcript_159939	NA	F01_transcript_159939
F01_transcript_39986	NA	NA	NA	F01_transcript_39986
F01_transcript_6359	NA	NA	NA	F01_transcript_6359
F01_transcript_68547	NA	NA	NA	F01_transcript_68547
F01_transcript_50330	F01_transcript_50330	F01_transcript_50330	F01_transcript_50330	F01_transcript_50330
F01_transcript_113892	F01_transcript_113892	F01_transcript_113892	F01_transcript_113892	F01_transcript_113892
F01_transcript_10126	NA	NA	F01_transcript_10126	F01_transcript_10126
F01_transcript_48928	F01_transcript_48928	NA	NA	NA
F01_transcript_142294	NA	NA	NA	F01_transcript_142294
F01_transcript_58298	NA	F01_transcript_58298	NA	NA
F01_transcript_84775	F01_transcript_84775	F01_transcript_84775	F01_transcript_84775	F01_transcript_84775
F01_transcript_44040	NA	NA	NA	F01_transcript_44040
F01_transcript_59059	F01_transcript_59059	F01_transcript_59059	F01_transcript_59059	F01_transcript_59059
F01_transcript_150465	NA	F01_transcript_150465	F01_transcript_150465	F01_transcript_150465

F01_transcript_95094	F01_transcript_95094	F01_transcript_95094	F01_transcript_95094	F01_transcript_95094
F01_transcript_111667	NA	NA	NA	F01_transcript_111667
F01_transcript_126865	F01_transcript_126865	F01_transcript_126865	F01_transcript_126865	F01_transcript_126865
F01_transcript_72472	NA	F01_transcript_72472	NA	NA
F01_transcript_106265	F01_transcript_106265	F01_transcript_106265	F01_transcript_106265	F01_transcript_106265
F01_transcript_88341	F01_transcript_88341	F01_transcript_88341	F01_transcript_88341	F01_transcript_88341
F01_transcript_67236	NA	NA	NA	F01_transcript_67236
F01_transcript_88336	F01_transcript_88336	F01_transcript_88336	NA	F01_transcript_88336
F01_transcript_138711	NA	F01_transcript_138711	F01_transcript_138711	NA
F01_transcript_94638	NA	F01_transcript_94638	NA	NA
F01_transcript_153970	NA	F01_transcript_153970	NA	F01_transcript_153970
F01_transcript_100874	NA	NA	NA	F01_transcript_100874
F01_transcript_77156	NA	NA	NA	F01_transcript_77156
F01_transcript_140977	F01_transcript_140977	F01_transcript_140977	NA	F01_transcript_140977

F01_transcript_117962	NA	NA	NA	F01_transcript_117962
F01_transcript_155726	NA	NA	NA	F01_transcript_155726
F01_transcript_116888	NA	F01_transcript_116888	NA	NA
F01_transcript_151911	NA	NA	NA	F01_transcript_151911
F01_transcript_68056	F01_transcript_68056	F01_transcript_68056	F01_transcript_68056	F01_transcript_68056
F01_transcript_47868	NA	NA	NA	F01_transcript_47868
F01_transcript_135358	F01_transcript_135358	F01_transcript_135358	NA	F01_transcript_135358
F01_transcript_136663	NA	NA	NA	F01_transcript_136663
F01_transcript_50504	NA	F01_transcript_50504	F01_transcript_50504	F01_transcript_50504
F01_transcript_128064	NA	NA	NA	F01_transcript_128064
F01_transcript_146601	NA	F01_transcript_146601	NA	NA
F01_transcript_81975	NA	NA	NA	F01_transcript_81975
F01_transcript_122423	NA	F01_transcript_122423	NA	F01_transcript_122423
F01_transcript_74661	NA	NA	NA	F01_transcript_74661

F01_transcript_166076	F01_transcript_166076	F01_transcript_166076	NA	NA
F01_transcript_145987	F01_transcript_145987	F01_transcript_145987	NA	F01_transcript_145987
F01_transcript_61343	F01_transcript_61343	F01_transcript_61343	F01_transcript_61343	F01_transcript_61343
F01_transcript_95657	NA	F01_transcript_95657	NA	NA
F01_transcript_45265	NA	F01_transcript_45265	NA	NA
F01_transcript_91578	NA	F01_transcript_91578	F01_transcript_91578	F01_transcript_91578
F01_transcript_65387	NA	NA	F01_transcript_65387	NA
F01_transcript_76299	NA	NA	NA	F01_transcript_76299
F01_transcript_93692	F01_transcript_93692	F01_transcript_93692	F01_transcript_93692	F01_transcript_93692
F01_transcript_88823	NA	NA	NA	F01_transcript_88823
F01_transcript_104741	F01_transcript_104741	F01_transcript_104741	F01_transcript_104741	NA
F01_transcript_73570	NA	F01_transcript_73570	F01_transcript_73570	NA
F01_transcript_147958	NA	F01_transcript_147958	F01_transcript_147958	F01_transcript_147958
F01_transcript_138163	F01_transcript_138163	F01_transcript_138163	NA	F01_transcript_138163

F01_transcript_59967	NA	NA	NA	F01_transcript_59967
F01_transcript_165469	NA	NA	NA	F01_transcript_165469
F01_transcript_134442	NA	NA	NA	F01_transcript_134442
F01_transcript_148513	NA	NA	NA	F01_transcript_148513
F01_transcript_117834	F01_transcript_117834	F01_transcript_117834	NA	F01_transcript_117834
F01_transcript_90064	NA	NA	NA	F01_transcript_90064
F01_transcript_158494	NA	NA	NA	F01_transcript_158494
F01_transcript_83426	NA	F01_transcript_83426	F01_transcript_83426	F01_transcript_83426
F01_transcript_99846	NA	F01_transcript_99846	NA	NA
F01_transcript_63206	F01_transcript_63206	F01_transcript_63206	F01_transcript_63206	F01_transcript_63206
F01_transcript_164419	F01_transcript_164419	F01_transcript_164419	F01_transcript_164419	F01_transcript_164419
F01_transcript_51517	NA	F01_transcript_51517	NA	NA
F01_transcript_88560	NA	NA	NA	F01_transcript_88560
F01_transcript_88030	F01_transcript_88030	F01_transcript_88030	F01_transcript_88030	F01_transcript_88030

F01_transcript_155889	NA	F01_transcript_155889	NA	F01_transcript_155889
F01_transcript_106324	F01_transcript_106324	F01_transcript_106324	F01_transcript_106324	F01_transcript_106324
F01_transcript_94988	NA	F01_transcript_94988	F01_transcript_94988	F01_transcript_94988
F01_transcript_110863	NA	NA	NA	F01_transcript_110863
F01_transcript_118266	NA	NA	NA	F01_transcript_118266
F01_transcript_81284	NA	F01_transcript_81284	F01_transcript_81284	F01_transcript_81284
F01_transcript_136824	NA	F01_transcript_136824	NA	NA
F01_transcript_68770	F01_transcript_68770	F01_transcript_68770	F01_transcript_68770	F01_transcript_68770
F01_transcript_119070	F01_transcript_119070	F01_transcript_119070	F01_transcript_119070	F01_transcript_119070
F01_transcript_151167	F01_transcript_151167	F01_transcript_151167	NA	F01_transcript_151167
F01_transcript_134901	NA	F01_transcript_134901	F01_transcript_134901	NA
F01_transcript_51123	F01_transcript_51123	F01_transcript_51123	F01_transcript_51123	F01_transcript_51123
F01_transcript_22933	NA	NA	NA	F01_transcript_22933
F01_transcript_115268	F01_transcript_115268	F01_transcript_115268	NA	F01_transcript_115268

F01_transcript_92014	NA	F01_transcript_92014	F01_transcript_92014	NA
F01_transcript_112082	F01_transcript_112082	F01_transcript_112082	NA	NA
F01_transcript_31217	NA	NA	NA	F01_transcript_31217
F01_transcript_103693	F01_transcript_103693	F01_transcript_103693	F01_transcript_103693	F01_transcript_103693
F01_transcript_150385	NA	F01_transcript_150385	NA	F01_transcript_150385
F01_transcript_122911	NA	NA	NA	F01_transcript_122911
F01_transcript_134003	NA	NA	NA	F01_transcript_134003
F01_transcript_7460	NA	F01_transcript_7460	NA	NA
F01_transcript_137606	NA	F01_transcript_137606	NA	NA
F01_transcript_114109	NA	F01_transcript_114109	NA	NA
F01_transcript_88094	F01_transcript_88094	F01_transcript_88094	F01_transcript_88094	F01_transcript_88094
F01_transcript_66878	NA	F01_transcript_66878	F01_transcript_66878	F01_transcript_66878
F01_transcript_104036	NA	NA	NA	F01_transcript_104036
F01_transcript_104980	NA	F01_transcript_104980	NA	F01_transcript_104980

F01_transcript_99105	NA	NA	NA	F01_transcript_99105
F01_transcript_16534	NA	NA	NA	F01_transcript_16534
F01_transcript_128544	NA	NA	NA	F01_transcript_128544
F01_transcript_81715	NA	NA	NA	F01_transcript_81715
F01_transcript_104326	NA	F01_transcript_104326	NA	F01_transcript_104326
F01_transcript_59322	NA	NA	F01_transcript_59322	NA
F01_transcript_152394	NA	F01_transcript_152394	NA	F01_transcript_152394
F01_transcript_167176	NA	F01_transcript_167176	NA	F01_transcript_167176
F01_transcript_57164	NA	NA	F01_transcript_57164	NA
F01_transcript_28308	NA	NA	NA	F01_transcript_28308
F01_transcript_130408	NA	F01_transcript_130408	NA	NA
F01_transcript_145635	F01_transcript_145635	F01_transcript_145635	F01_transcript_145635	NA
F01_transcript_147274	F01_transcript_147274	F01_transcript_147274	F01_transcript_147274	F01_transcript_147274
F01_transcript_151577	NA	NA	NA	F01_transcript_151577

F01_transcript_93084	NA	NA	NA	F01_transcript_93084
F01_transcript_99179	F01_transcript_99179	F01_transcript_99179	F01_transcript_99179	NA
F01_transcript_6899	NA	NA	NA	F01_transcript_6899
F01_transcript_50129	F01_transcript_50129	F01_transcript_50129	NA	NA
F01_transcript_153484	NA	NA	NA	F01_transcript_153484
F01_transcript_73147	NA	F01_transcript_73147	NA	NA
F01_transcript_89060	NA	NA	NA	F01_transcript_89060
F01_transcript_158955	NA	F01_transcript_158955	F01_transcript_158955	NA
F01_transcript_157091	NA	NA	NA	F01_transcript_157091
F01_transcript_143723	NA	NA	NA	F01_transcript_143723
F01_transcript_65388	NA	NA	NA	F01_transcript_65388
F01_transcript_96406	NA	F01_transcript_96406	NA	NA
F01_transcript_71574	NA	NA	NA	F01_transcript_71574
F01_transcript_128357	F01_transcript_128357	F01_transcript_128357	NA	F01_transcript_128357

F01_transcript_85022	F01_transcript_85022	NA	NA	NA
F01_transcript_70787	NA	F01_transcript_70787	NA	NA
F01_transcript_106443	NA	F01_transcript_106443	NA	F01_transcript_106443
F01_transcript_6049	NA	NA	NA	F01_transcript_6049
F01_transcript_131165	F01_transcript_131165	F01_transcript_131165	F01_transcript_131165	F01_transcript_131165
F01_transcript_129231	F01_transcript_129231	F01_transcript_129231	F01_transcript_129231	F01_transcript_129231
F01_transcript_102123	NA	NA	NA	F01_transcript_102123
F01_transcript_4135	NA	F01_transcript_4135	NA	NA
F01_transcript_130769	F01_transcript_130769	F01_transcript_130769	F01_transcript_130769	F01_transcript_130769
F01_transcript_57112	F01_transcript_57112	F01_transcript_57112	F01_transcript_57112	F01_transcript_57112
F01_transcript_55950	NA	F01_transcript_55950	F01_transcript_55950	F01_transcript_55950
F01_transcript_97639	NA	F01_transcript_97639	NA	F01_transcript_97639
F01_transcript_48810	NA	NA	NA	F01_transcript_48810
F01_transcript_93816	NA	F01_transcript_93816	NA	NA

F01_transcript_129505	NA	NA	NA	F01_transcript_129505
F01_transcript_50425	NA	F01_transcript_50425	NA	NA
F01_transcript_144139	F01_transcript_144139	F01_transcript_144139	NA	NA
F01_transcript_67579	NA	NA	NA	F01_transcript_67579
F01_transcript_98459	NA	F01_transcript_98459	NA	NA
F01_transcript_163197	NA	NA	NA	F01_transcript_163197
F01_transcript_63583	NA	NA	NA	F01_transcript_63583
F01_transcript_74818	F01_transcript_74818	F01_transcript_74818	NA	NA
F01_transcript_129243	F01_transcript_129243	F01_transcript_129243	F01_transcript_129243	F01_transcript_129243
F01_transcript_14269	NA	NA	NA	F01_transcript_14269
F01_transcript_81160	F01_transcript_81160	F01_transcript_81160	F01_transcript_81160	F01_transcript_81160
F01_transcript_39792	NA	NA	NA	F01_transcript_39792
F01_transcript_97521	F01_transcript_97521	F01_transcript_97521	NA	F01_transcript_97521
F01_transcript_129056	NA	NA	NA	F01_transcript_129056

F01_transcript_128356	NA	NA	NA	F01_transcript_128356
F01_transcript_17193	NA	NA	NA	F01_transcript_17193
F01_transcript_13062	NA	NA	NA	F01_transcript_13062
F01_transcript_48669	NA	F01_transcript_48669	NA	NA
F01_transcript_142574	NA	NA	NA	F01_transcript_142574
F01_transcript_69574	F01_transcript_69574	F01_transcript_69574	F01_transcript_69574	F01_transcript_69574
F01_transcript_125454	F01_transcript_125454	F01_transcript_125454	F01_transcript_125454	F01_transcript_125454
F01_transcript_102185	F01_transcript_102185	F01_transcript_102185	NA	NA
F01_transcript_22509	F01_transcript_22509	NA	NA	F01_transcript_22509
F01_transcript_54108	NA	NA	NA	F01_transcript_54108
F01_transcript_49256	NA	F01_transcript_49256	NA	F01_transcript_49256
F01_transcript_105871	NA	NA	NA	F01_transcript_105871
F01_transcript_62333	NA	NA	NA	F01_transcript_62333
F01_transcript_119059	F01_transcript_119059	F01_transcript_119059	F01_transcript_119059	F01_transcript_119059

F01_transcript_159087	F01_transcript_159087	F01_transcript_159087	F01_transcript_159087	F01_transcript_159087
F01_transcript_139550	NA	F01_transcript_139550	F01_transcript_139550	F01_transcript_139550
F01_transcript_128076	NA	NA	NA	F01_transcript_128076
F01_transcript_113416	NA	F01_transcript_113416	NA	NA
F01_transcript_152784	NA	NA	NA	F01_transcript_152784
F01_transcript_62650	NA	F01_transcript_62650	NA	NA
F01_transcript_82975	NA	F01_transcript_82975	F01_transcript_82975	F01_transcript_82975
F01_transcript_64688	NA	F01_transcript_64688	NA	NA
F01_transcript_1392	NA	NA	NA	F01_transcript_1392
F01_transcript_89964	NA	F01_transcript_89964	NA	NA
F01_transcript_71405	F01_transcript_71405	F01_transcript_71405	F01_transcript_71405	F01_transcript_71405
F01_transcript_114928	F01_transcript_114928	F01_transcript_114928	F01_transcript_114928	F01_transcript_114928
F01_transcript_94187	F01_transcript_94187	F01_transcript_94187	F01_transcript_94187	F01_transcript_94187
F01_transcript_119465	NA	F01_transcript_119465	NA	NA

F01_transcript_95384	NA	NA	NA	F01_transcript_95384
F01_transcript_141940	NA	F01_transcript_141940	NA	F01_transcript_141940
F01_transcript_157773	NA	NA	NA	F01_transcript_157773
F01_transcript_135196	NA	NA	NA	F01_transcript_135196
F01_transcript_158951	NA	NA	F01_transcript_158951	F01_transcript_158951
F01_transcript_22986	NA	NA	NA	F01_transcript_22986
F01_transcript_60817	F01_transcript_60817	F01_transcript_60817	F01_transcript_60817	F01_transcript_60817
F01_transcript_144495	NA	NA	NA	F01_transcript_144495
F01_transcript_152774	NA	NA	NA	F01_transcript_152774
F01_transcript_74981	NA	NA	NA	F01_transcript_74981
F01_transcript_6833	F01_transcript_6833	F01_transcript_6833	NA	NA
F01_transcript_81326	NA	F01_transcript_81326	NA	NA
F01_transcript_137769	F01_transcript_137769	F01_transcript_137769	NA	F01_transcript_137769
F01_transcript_136950	NA	F01_transcript_136950	NA	F01_transcript_136950

F01_transcript_61201	F01_transcript_61201	F01_transcript_61201	F01_transcript_61201	F01_transcript_61201
F01_transcript_10183	NA	F01_transcript_10183	NA	NA
F01_transcript_80141	NA	NA	F01_transcript_80141	F01_transcript_80141
F01_transcript_23002	NA	F01_transcript_23002	NA	F01_transcript_23002
F01_transcript_161025	NA	NA	F01_transcript_161025	NA
F01_transcript_51924	NA	NA	NA	F01_transcript_51924
F01_transcript_31004	NA	NA	NA	F01_transcript_31004
F01_transcript_116747	NA	F01_transcript_116747	NA	NA
F01_transcript_139387	NA	F01_transcript_139387	NA	NA
F01_transcript_83882	NA	F01_transcript_83882	NA	NA
F01_transcript_155165	F01_transcript_155165	F01_transcript_155165	F01_transcript_155165	F01_transcript_155165
F01_transcript_115535	NA	F01_transcript_115535	NA	NA
F01_transcript_121867	NA	F01_transcript_121867	NA	NA
F01_transcript_154777	NA	NA	NA	F01_transcript_154777

F01_transcript_149847	F01_transcript_149847	F01_transcript_149847	F01_transcript_149847	F01_transcript_149847
F01_transcript_81309	NA	NA	F01_transcript_81309	NA
F01_transcript_160812	F01_transcript_160812	F01_transcript_160812	F01_transcript_160812	F01_transcript_160812
F01_transcript_64969	NA	F01_transcript_64969	NA	NA
F01_transcript_74539	F01_transcript_74539	F01_transcript_74539	F01_transcript_74539	F01_transcript_74539
F01_transcript_71322	NA	F01_transcript_71322	NA	NA
F01_transcript_81832	F01_transcript_81832	F01_transcript_81832	NA	F01_transcript_81832
F01_transcript_12047	NA	NA	NA	F01_transcript_12047
F01_transcript_111345	NA	F01_transcript_111345	NA	NA
F01_transcript_148962	F01_transcript_148962	F01_transcript_148962	F01_transcript_148962	F01_transcript_148962
F01_transcript_125575	NA	F01_transcript_125575	NA	NA
F01_transcript_63376	NA	NA	NA	F01_transcript_63376
F01_transcript_139044	NA	NA	NA	F01_transcript_139044
F01_transcript_59696	NA	NA	NA	F01_transcript_59696

F01_transcript_110046	NA	F01_transcript_110046	NA	NA
F01_transcript_125821	F01_transcript_125821	F01_transcript_125821	F01_transcript_125821	F01_transcript_125821
F01_transcript_120698	NA	F01_transcript_120698	NA	NA
F01_transcript_132012	NA	NA	NA	F01_transcript_132012
F01_transcript_125430	F01_transcript_125430	F01_transcript_125430	F01_transcript_125430	F01_transcript_125430
F01_transcript_63435	NA	F01_transcript_63435	NA	NA
F01_transcript_108499	F01_transcript_108499	F01_transcript_108499	F01_transcript_108499	F01_transcript_108499
F01_transcript_99035	NA	NA	NA	F01_transcript_99035
F01_transcript_150155	F01_transcript_150155	F01_transcript_150155	F01_transcript_150155	F01_transcript_150155
F01_transcript_119754	NA	F01_transcript_119754	NA	F01_transcript_119754
F01_transcript_147287	NA	NA	NA	F01_transcript_147287
F01_transcript_26211	NA	NA	NA	F01_transcript_26211
F01_transcript_99646	F01_transcript_99646	F01_transcript_99646	NA	F01_transcript_99646
F01_transcript_128613	NA	NA	NA	F01_transcript_128613

F01_transcript_47189	NA	F01_transcript_47189	NA	NA
F01_transcript_93801	F01_transcript_93801	NA	F01_transcript_93801	F01_transcript_93801
F01_transcript_161552	NA	F01_transcript_161552	NA	NA
F01_transcript_158565	NA	F01_transcript_158565	NA	NA
F01_transcript_99921	F01_transcript_99921	F01_transcript_99921	F01_transcript_99921	F01_transcript_99921
F01_transcript_53341	NA	F01_transcript_53341	F01_transcript_53341	F01_transcript_53341
F01_transcript_82752	NA	F01_transcript_82752	NA	NA
F01_transcript_112235	NA	NA	NA	F01_transcript_112235
F01_transcript_75704	NA	NA	NA	F01_transcript_75704
F01_transcript_115028	NA	F01_transcript_115028	F01_transcript_115028	F01_transcript_115028
F01_transcript_17428	NA	NA	NA	F01_transcript_17428
F01_transcript_121966	NA	NA	F01_transcript_121966	F01_transcript_121966
F01_transcript_62244	F01_transcript_62244	F01_transcript_62244	F01_transcript_62244	F01_transcript_62244
F01_transcript_117457	F01_transcript_117457	F01_transcript_117457	NA	F01_transcript_117457

F01_transcript_50617	F01_transcript_50617	NA	F01_transcript_50617	F01_transcript_50617
F01_transcript_44302	NA	NA	NA	F01_transcript_44302
F01_transcript_155046	NA	F01_transcript_155046	NA	F01_transcript_155046
F01_transcript_95423	NA	NA	NA	F01_transcript_95423
F01_transcript_74702	F01_transcript_74702	F01_transcript_74702	F01_transcript_74702	F01_transcript_74702
F01_transcript_100880	NA	F01_transcript_100880	NA	F01_transcript_100880
F01_transcript_84935	NA	F01_transcript_84935	NA	F01_transcript_84935
F01_transcript_432	F01_transcript_432	F01_transcript_432	F01_transcript_432	F01_transcript_432
F01_transcript_159495	NA	NA	F01_transcript_159495	NA
F01_transcript_30593	NA	NA	NA	F01_transcript_30593
F01_transcript_152046	F01_transcript_152046	F01_transcript_152046	F01_transcript_152046	F01_transcript_152046
F01_transcript_109428	F01_transcript_109428	F01_transcript_109428	F01_transcript_109428	F01_transcript_109428
F01_transcript_49790	NA	F01_transcript_49790	NA	F01_transcript_49790
F01_transcript_6307	F01_transcript_6307	F01_transcript_6307	F01_transcript_6307	NA

F01_transcript_141086	F01_transcript_141086	F01_transcript_141086	NA	F01_transcript_141086
F01_transcript_80669	NA	NA	NA	F01_transcript_80669
F01_transcript_96973	NA	F01_transcript_96973	F01_transcript_96973	F01_transcript_96973
F01_transcript_62341	NA	NA	NA	F01_transcript_62341
F01_transcript_100837	F01_transcript_100837	F01_transcript_100837	NA	NA
F01_transcript_30002	NA	F01_transcript_30002	NA	NA
F01_transcript_133816	F01_transcript_133816	F01_transcript_133816	F01_transcript_133816	F01_transcript_133816
F01_transcript_65234	NA	NA	F01_transcript_65234	F01_transcript_65234
F01_transcript_137309	NA	F01_transcript_137309	NA	NA
F01_transcript_148893	NA	F01_transcript_148893	NA	NA
F01_transcript_64759	NA	NA	NA	F01_transcript_64759
F01_transcript_48431	NA	NA	NA	F01_transcript_48431
F01_transcript_4421	F01_transcript_4421	F01_transcript_4421	F01_transcript_4421	F01_transcript_4421
F01_transcript_146716	F01_transcript_146716	F01_transcript_146716	NA	F01_transcript_146716

F01_transcript_166162	NA	F01_transcript_166162	NA	F01_transcript_166162
F01_transcript_27400	NA	NA	NA	F01_transcript_27400
F01_transcript_69593	NA	NA	NA	F01_transcript_69593
F01_transcript_28848	NA	NA	NA	F01_transcript_28848
F01_transcript_166932	F01_transcript_166932	F01_transcript_166932	F01_transcript_166932	F01_transcript_166932
F01_transcript_128787	NA	NA	NA	F01_transcript_128787
F01_transcript_138856	F01_transcript_138856	F01_transcript_138856	NA	NA
F01_transcript_32507	NA	NA	NA	F01_transcript_32507
F01_transcript_135809	F01_transcript_135809	F01_transcript_135809	F01_transcript_135809	F01_transcript_135809
F01_transcript_122994	NA	NA	NA	F01_transcript_122994
F01_transcript_62785	NA	F01_transcript_62785	NA	NA
F01_transcript_130324	NA	F01_transcript_130324	NA	F01_transcript_130324
F01_transcript_155639	F01_transcript_155639	F01_transcript_155639	NA	F01_transcript_155639
F01_transcript_112415	NA	F01_transcript_112415	NA	NA

F01_transcript_90103	NA	NA	NA	F01_transcript_90103
F01_transcript_108337	NA	NA	NA	F01_transcript_108337
F01_transcript_128100	NA	F01_transcript_128100	NA	F01_transcript_128100
F01_transcript_67652	F01_transcript_67652	F01_transcript_67652	F01_transcript_67652	F01_transcript_67652
F01_transcript_90479	NA	F01_transcript_90479	NA	F01_transcript_90479
F01_transcript_51941	NA	F01_transcript_51941	NA	NA
F01_transcript_138047	NA	F01_transcript_138047	NA	NA
F01_transcript_167771	NA	F01_transcript_167771	NA	F01_transcript_167771
F01_transcript_120440	F01_transcript_120440	F01_transcript_120440	F01_transcript_120440	F01_transcript_120440
F01_transcript_25516	NA	F01_transcript_25516	NA	NA
F01_transcript_127473	F01_transcript_127473	F01_transcript_127473	F01_transcript_127473	F01_transcript_127473
F01_transcript_62422	NA	NA	NA	F01_transcript_62422
F01_transcript_160370	F01_transcript_160370	F01_transcript_160370	NA	F01_transcript_160370
F01_transcript_5564	NA	F01_transcript_5564	NA	NA

F01_transcript_6152	NA	NA	NA	F01_transcript_6152
F01_transcript_59307	NA	NA	F01_transcript_59307	F01_transcript_59307
F01_transcript_50070	F01_transcript_50070	F01_transcript_50070	F01_transcript_50070	F01_transcript_50070
F01_transcript_13962	NA	NA	NA	F01_transcript_13962
F01_transcript_30793	NA	NA	NA	F01_transcript_30793
F01_transcript_142244	NA	NA	NA	F01_transcript_142244
F01_transcript_92660	F01_transcript_92660	F01_transcript_92660	F01_transcript_92660	F01_transcript_92660
F01_transcript_116158	F01_transcript_116158	F01_transcript_116158	F01_transcript_116158	F01_transcript_116158
F01_transcript_59607	F01_transcript_59607	F01_transcript_59607	F01_transcript_59607	F01_transcript_59607
F01_transcript_156313	F01_transcript_156313	F01_transcript_156313	F01_transcript_156313	F01_transcript_156313
F01_transcript_21494	NA	NA	NA	F01_transcript_21494
F01_transcript_132295	NA	F01_transcript_132295	NA	NA
F01_transcript_148269	NA	F01_transcript_148269	NA	F01_transcript_148269
F01_transcript_128463	NA	NA	NA	F01_transcript_128463

F01_transcript_116229	F01_transcript_116229	F01_transcript_116229	NA	NA
F01_transcript_78788	NA	NA	NA	F01_transcript_78788
F01_transcript_139202	NA	F01_transcript_139202	NA	NA
F01_transcript_128330	F01_transcript_128330	F01_transcript_128330	NA	NA
F01_transcript_119388	F01_transcript_119388	F01_transcript_119388	NA	NA
F01_transcript_53812	F01_transcript_53812	F01_transcript_53812	NA	NA
F01_transcript_106288	NA	F01_transcript_106288	NA	F01_transcript_106288
F01_transcript_133629	NA	F01_transcript_133629	NA	F01_transcript_133629
F01_transcript_87871	NA	F01_transcript_87871	NA	F01_transcript_87871
F01_transcript_89503	F01_transcript_89503	F01_transcript_89503	F01_transcript_89503	F01_transcript_89503
F01_transcript_48802	NA	NA	NA	F01_transcript_48802
F01_transcript_142839	NA	F01_transcript_142839	NA	F01_transcript_142839
F01_transcript_141621	NA	F01_transcript_141621	NA	NA
F01_transcript_67458	NA	NA	NA	F01_transcript_67458

F01_transcript_148298	F01_transcript_148298	F01_transcript_148298	F01_transcript_148298	F01_transcript_148298
F01_transcript_150927	F01_transcript_150927	F01_transcript_150927	NA	NA
F01_transcript_114382	NA	NA	NA	F01_transcript_114382
F01_transcript_126551	F01_transcript_126551	F01_transcript_126551	F01_transcript_126551	NA
F01_transcript_85550	F01_transcript_85550	F01_transcript_85550	F01_transcript_85550	F01_transcript_85550
F01_transcript_69477	NA	F01_transcript_69477	NA	NA
F01_transcript_102110	NA	F01_transcript_102110	NA	NA
F01_transcript_90348	NA	F01_transcript_90348	F01_transcript_90348	NA
F01_transcript_154476	F01_transcript_154476	F01_transcript_154476	F01_transcript_154476	F01_transcript_154476
F01_transcript_2272	F01_transcript_2272	F01_transcript_2272	F01_transcript_2272	NA
F01_transcript_86978	NA	NA	NA	F01_transcript_86978
F01_transcript_61906	NA	F01_transcript_61906	NA	NA
F01_transcript_59489	NA	NA	F01_transcript_59489	NA
F01_transcript_93749	F01_transcript_93749	F01_transcript_93749	F01_transcript_93749	F01_transcript_93749

F01_transcript_46575	NA	NA	NA	F01_transcript_46575
F01_transcript_113056	F01_transcript_113056	F01_transcript_113056	F01_transcript_113056	F01_transcript_113056
F01_transcript_40738	NA	NA	NA	F01_transcript_40738
F01_transcript_148432	NA	F01_transcript_148432	NA	NA
F01_transcript_117806	NA	NA	NA	F01_transcript_117806
F01_transcript_66768	F01_transcript_66768	F01_transcript_66768	NA	NA
F01_transcript_40272	NA	F01_transcript_40272	NA	NA
F01_transcript_50228	NA	F01_transcript_50228	NA	NA
F01_transcript_54798	NA	F01_transcript_54798	NA	NA
F01_transcript_52046	NA	F01_transcript_52046	NA	NA
F01_transcript_132543	NA	NA	NA	F01_transcript_132543
F01_transcript_47845	NA	NA	NA	F01_transcript_47845
F01_transcript_135701	NA	NA	NA	F01_transcript_135701
F01_transcript_73537	F01_transcript_73537	F01_transcript_73537	NA	NA

F01_transcript_58477	F01_transcript_58477	F01_transcript_58477	NA	NA
F01_transcript_144883	NA	F01_transcript_144883	NA	NA
F01_transcript_99647	NA	F01_transcript_99647	NA	NA
F01_transcript_147712	NA	NA	NA	F01_transcript_147712
F01_transcript_53467	NA	NA	NA	F01_transcript_53467
F01_transcript_87785	F01_transcript_87785	F01_transcript_87785	F01_transcript_87785	F01_transcript_87785
F01_transcript_151833	NA	NA	NA	F01_transcript_151833
F01_transcript_166437	NA	NA	NA	F01_transcript_166437
F01_transcript_5782	NA	NA	NA	F01_transcript_5782
F01_transcript_57722	F01_transcript_57722	F01_transcript_57722	F01_transcript_57722	NA
F01_transcript_159726	NA	NA	NA	F01_transcript_159726
F01_transcript_100221	NA	NA	NA	F01_transcript_100221
F01_transcript_167136	F01_transcript_167136	F01_transcript_167136	F01_transcript_167136	NA
F01_transcript_103047	NA	NA	NA	F01_transcript_103047

F01_transcript_66795	NA	F01_transcript_66795	F01_transcript_66795	NA
F01_transcript_91178	NA	NA	NA	F01_transcript_91178
F01_transcript_108833	NA	NA	NA	F01_transcript_108833
F01_transcript_148676	NA	F01_transcript_148676	NA	NA
F01_transcript_120656	NA	NA	NA	F01_transcript_120656
F01_transcript_133191	NA	NA	NA	F01_transcript_133191
F01_transcript_124007	NA	NA	NA	F01_transcript_124007
F01_transcript_116227	F01_transcript_116227	NA	NA	F01_transcript_116227
F01_transcript_126890	NA	F01_transcript_126890	NA	F01_transcript_126890
F01_transcript_51237	F01_transcript_51237	F01_transcript_51237	F01_transcript_51237	F01_transcript_51237
F01_transcript_165550	NA	NA	NA	F01_transcript_165550
F01_transcript_62732	F01_transcript_62732	F01_transcript_62732	NA	F01_transcript_62732
F01_transcript_59458	NA	NA	F01_transcript_59458	NA
F01_transcript_18096	NA	NA	NA	F01_transcript_18096

F01_transcript_48319	NA	NA	NA	F01_transcript_48319
F01_transcript_95159	NA	F01_transcript_95159	NA	F01_transcript_95159
F01_transcript_15244	F01_transcript_15244	F01_transcript_15244	F01_transcript_15244	F01_transcript_15244
F01_transcript_38381	F01_transcript_38381	F01_transcript_38381	F01_transcript_38381	F01_transcript_38381
F01_transcript_124554	NA	NA	NA	F01_transcript_124554
F01_transcript_82930	F01_transcript_82930	F01_transcript_82930	F01_transcript_82930	NA
F01_transcript_58453	F01_transcript_58453	F01_transcript_58453	F01_transcript_58453	F01_transcript_58453
F01_transcript_106519	F01_transcript_106519	F01_transcript_106519	F01_transcript_106519	F01_transcript_106519
F01_transcript_44238	NA	NA	NA	F01_transcript_44238
F01_transcript_105855	NA	F01_transcript_105855	NA	F01_transcript_105855
F01_transcript_114592	NA	F01_transcript_114592	F01_transcript_114592	F01_transcript_114592
F01_transcript_2995	F01_transcript_2995	F01_transcript_2995	F01_transcript_2995	F01_transcript_2995
F01_transcript_82822	NA	NA	NA	F01_transcript_82822
F01_transcript_156315	NA	F01_transcript_156315	NA	F01_transcript_156315

F01_transcript_56921	NA	F01_transcript_56921	F01_transcript_56921	F01_transcript_56921
F01_transcript_63548	F01_transcript_63548	F01_transcript_63548	F01_transcript_63548	F01_transcript_63548
F01_transcript_50639	F01_transcript_50639	F01_transcript_50639	F01_transcript_50639	NA
F01_transcript_49872	NA	F01_transcript_49872	NA	NA
F01_transcript_139721	NA	F01_transcript_139721	NA	NA
F01_transcript_4684	NA	NA	NA	F01_transcript_4684
F01_transcript_73685	NA	F01_transcript_73685	NA	F01_transcript_73685
F01_transcript_164081	NA	F01_transcript_164081	NA	NA
F01_transcript_86073	F01_transcript_86073	F01_transcript_86073	F01_transcript_86073	F01_transcript_86073
F01_transcript_13047	NA	F01_transcript_13047	F01_transcript_13047	F01_transcript_13047
F01_transcript_142909	NA	F01_transcript_142909	NA	NA
F01_transcript_156980	NA	F01_transcript_156980	NA	NA
F01_transcript_79875	NA	F01_transcript_79875	F01_transcript_79875	F01_transcript_79875
F01_transcript_84934	NA	F01_transcript_84934	NA	NA

F01_transcript_133756	NA	F01_transcript_133756	NA	NA
F01_transcript_131350	F01_transcript_131350	F01_transcript_131350	NA	NA
F01_transcript_107503	NA	F01_transcript_107503	NA	F01_transcript_107503
F01_transcript_52056	NA	NA	NA	F01_transcript_52056
F01_transcript_69271	NA	F01_transcript_69271	NA	F01_transcript_69271
F01_transcript_164257	NA	NA	F01_transcript_164257	NA
F01_transcript_49040	F01_transcript_49040	F01_transcript_49040	NA	NA
F01_transcript_156500	NA	NA	NA	F01_transcript_156500
F01_transcript_102637	NA	F01_transcript_102637	NA	F01_transcript_102637
F01_transcript_119329	NA	NA	NA	F01_transcript_119329
F01_transcript_96635	NA	F01_transcript_96635	F01_transcript_96635	F01_transcript_96635
F01_transcript_123965	NA	NA	NA	F01_transcript_123965
F01_transcript_20305	NA	NA	NA	F01_transcript_20305
F01_transcript_109986	F01_transcript_109986	F01_transcript_109986	NA	F01_transcript_109986

F01_transcript_107713	F01_transcript_107713	F01_transcript_107713	F01_transcript_107713	F01_transcript_107713
F01_transcript_152974	NA	F01_transcript_152974	NA	NA
F01_transcript_56719	NA	F01_transcript_56719	NA	F01_transcript_56719
F01_transcript_147379	NA	NA	NA	F01_transcript_147379
F01_transcript_103570	NA	F01_transcript_103570	NA	NA
F01_transcript_128360	NA	NA	NA	F01_transcript_128360
F01_transcript_149751	NA	NA	NA	F01_transcript_149751
F01_transcript_146264	F01_transcript_146264	F01_transcript_146264	F01_transcript_146264	F01_transcript_146264
F01_transcript_3477	F01_transcript_3477	F01_transcript_3477	F01_transcript_3477	NA
F01_transcript_161761	NA	NA	NA	F01_transcript_161761
F01_transcript_103742	F01_transcript_103742	F01_transcript_103742	NA	NA
F01_transcript_104992	NA	NA	NA	F01_transcript_104992
F01_transcript_147327	NA	NA	NA	F01_transcript_147327
F01_transcript_141728	NA	F01_transcript_141728	NA	F01_transcript_141728

F01_transcript_118006	F01_transcript_118006	F01_transcript_118006	F01_transcript_118006	NA
F01_transcript_10880	NA	F01_transcript_10880	NA	NA
F01_transcript_129227	NA	F01_transcript_129227	NA	F01_transcript_129227
F01_transcript_69005	NA	NA	NA	F01_transcript_69005
F01_transcript_116630	F01_transcript_116630	F01_transcript_116630	F01_transcript_116630	F01_transcript_116630
F01_transcript_60244	F01_transcript_60244	F01_transcript_60244	F01_transcript_60244	F01_transcript_60244
F01_transcript_162260	F01_transcript_162260	F01_transcript_162260	F01_transcript_162260	F01_transcript_162260
F01_transcript_15558	NA	NA	NA	F01_transcript_15558
F01_transcript_75112	NA	NA	NA	F01_transcript_75112
F01_transcript_65262	NA	NA	F01_transcript_65262	NA
F01_transcript_147986	NA	F01_transcript_147986	F01_transcript_147986	F01_transcript_147986
F01_transcript_57674	F01_transcript_57674	F01_transcript_57674	F01_transcript_57674	F01_transcript_57674
F01_transcript_154820	F01_transcript_154820	F01_transcript_154820	F01_transcript_154820	F01_transcript_154820
F01_transcript_93858	NA	F01_transcript_93858	NA	F01_transcript_93858

F01_transcript_16669	NA	F01_transcript_16669	NA	F01_transcript_16669
F01_transcript_92183	NA	NA	NA	F01_transcript_92183
F01_transcript_77388	NA	F01_transcript_77388	F01_transcript_77388	NA
F01_transcript_124345	NA	F01_transcript_124345	NA	F01_transcript_124345
F01_transcript_101562	NA	NA	NA	F01_transcript_101562
F01_transcript_51256	F01_transcript_51256	F01_transcript_51256	F01_transcript_51256	F01_transcript_51256
F01_transcript_164945	F01_transcript_164945	F01_transcript_164945	F01_transcript_164945	F01_transcript_164945
F01_transcript_112905	F01_transcript_112905	F01_transcript_112905	NA	NA
F01_transcript_126166	F01_transcript_126166	F01_transcript_126166	NA	NA
F01_transcript_158272	NA	F01_transcript_158272	F01_transcript_158272	NA
F01_transcript_149372	F01_transcript_149372	F01_transcript_149372	F01_transcript_149372	F01_transcript_149372
F01_transcript_126089	F01_transcript_126089	F01_transcript_126089	F01_transcript_126089	F01_transcript_126089
F01_transcript_160734	NA	F01_transcript_160734	NA	F01_transcript_160734
F01_transcript_156434	F01_transcript_156434	F01_transcript_156434	F01_transcript_156434	F01_transcript_156434

F01_transcript_159044	NA	NA	F01_transcript_159044	NA
F01_transcript_45118	NA	NA	NA	F01_transcript_45118
F01_transcript_77397	NA	F01_transcript_77397	NA	NA
F01_transcript_91497	F01_transcript_91497	F01_transcript_91497	F01_transcript_91497	F01_transcript_91497
F01_transcript_9219	NA	NA	NA	F01_transcript_9219
F01_transcript_129578	F01_transcript_129578	F01_transcript_129578	F01_transcript_129578	F01_transcript_129578
F01_transcript_158323	F01_transcript_158323	F01_transcript_158323	F01_transcript_158323	F01_transcript_158323
F01_transcript_85031	F01_transcript_85031	F01_transcript_85031	NA	NA
F01_transcript_81708	NA	NA	NA	F01_transcript_81708
F01_transcript_143394	F01_transcript_143394	F01_transcript_143394	F01_transcript_143394	F01_transcript_143394
F01_transcript_68240	NA	NA	NA	F01_transcript_68240
F01_transcript_57151	F01_transcript_57151	F01_transcript_57151	F01_transcript_57151	F01_transcript_57151
F01_transcript_127790	NA	NA	NA	F01_transcript_127790
F01_transcript_5865	NA	NA	NA	F01_transcript_5865

F01_transcript_45761	NA	F01_transcript_45761	NA	NA
F01_transcript_109614	F01_transcript_109614	F01_transcript_109614	NA	F01_transcript_109614
F01_transcript_133542	NA	F01_transcript_133542	NA	NA
F01_transcript_129500	NA	F01_transcript_129500	F01_transcript_129500	NA
F01_transcript_121016	NA	F01_transcript_121016	NA	F01_transcript_121016
F01_transcript_57997	NA	NA	NA	F01_transcript_57997
F01_transcript_144499	F01_transcript_144499	F01_transcript_144499	NA	F01_transcript_144499
F01_transcript_38809	NA	F01_transcript_38809	NA	NA
F01_transcript_76911	F01_transcript_76911	F01_transcript_76911	F01_transcript_76911	F01_transcript_76911
F01_transcript_65156	NA	NA	F01_transcript_65156	F01_transcript_65156
F01_transcript_123774	F01_transcript_123774	F01_transcript_123774	F01_transcript_123774	F01_transcript_123774
F01_transcript_166794	F01_transcript_166794	F01_transcript_166794	F01_transcript_166794	F01_transcript_166794
F01_transcript_128074	F01_transcript_128074	F01_transcript_128074	NA	NA
F01_transcript_85597	F01_transcript_85597	F01_transcript_85597	F01_transcript_85597	F01_transcript_85597

F01_transcript_129595	NA	F01_transcript_129595	F01_transcript_129595	F01_transcript_129595
F01_transcript_139916	NA	NA	NA	F01_transcript_139916
F01_transcript_11986	NA	NA	NA	F01_transcript_11986
F01_transcript_81788	NA	NA	NA	F01_transcript_81788
F01_transcript_65735	F01_transcript_65735	F01_transcript_65735	NA	F01_transcript_65735
F01_transcript_130611	NA	F01_transcript_130611	F01_transcript_130611	F01_transcript_130611
F01_transcript_65326	NA	F01_transcript_65326	F01_transcript_65326	NA
F01_transcript_62286	NA	F01_transcript_62286	F01_transcript_62286	NA
F01_transcript_71848	NA	NA	NA	F01_transcript_71848
F01_transcript_118070	F01_transcript_118070	NA	F01_transcript_118070	F01_transcript_118070
F01_transcript_86316	NA	NA	NA	F01_transcript_86316
F01_transcript_135714	NA	F01_transcript_135714	NA	F01_transcript_135714
F01_transcript_157261	NA	F01_transcript_157261	NA	F01_transcript_157261
F01_transcript_27663	NA	NA	NA	F01_transcript_27663

F01_transcript_28289	NA	NA	NA	F01_transcript_28289
F01_transcript_131046	NA	NA	NA	F01_transcript_131046
F01_transcript_7726	NA	F01_transcript_7726	F01_transcript_7726	F01_transcript_7726
F01_transcript_123083	F01_transcript_123083	F01_transcript_123083	F01_transcript_123083	F01_transcript_123083
F01_transcript_153222	F01_transcript_153222	F01_transcript_153222	F01_transcript_153222	F01_transcript_153222
F01_transcript_122897	NA	NA	NA	F01_transcript_122897
F01_transcript_109968	NA	F01_transcript_109968	NA	NA
F01_transcript_70610	NA	NA	NA	F01_transcript_70610
F01_transcript_26945	NA	NA	NA	F01_transcript_26945
F01_transcript_164196	F01_transcript_164196	F01_transcript_164196	NA	F01_transcript_164196
F01_transcript_103735	NA	NA	NA	F01_transcript_103735
F01_transcript_140955	NA	NA	NA	F01_transcript_140955
F01_transcript_82327	F01_transcript_82327	F01_transcript_82327	F01_transcript_82327	F01_transcript_82327
F01_transcript_130636	NA	F01_transcript_130636	NA	NA

F01_transcript_84567	NA	F01_transcript_84567	NA	F01_transcript_84567
F01_transcript_75827	NA	NA	NA	F01_transcript_75827
F01_transcript_105293	NA	F01_transcript_105293	NA	NA
F01_transcript_81078	NA	F01_transcript_81078	NA	F01_transcript_81078
F01_transcript_56971	F01_transcript_56971	F01_transcript_56971	F01_transcript_56971	F01_transcript_56971
F01_transcript_19640	NA	NA	NA	F01_transcript_19640
F01_transcript_154732	NA	NA	NA	F01_transcript_154732
F01_transcript_57012	NA	NA	F01_transcript_57012	F01_transcript_57012
F01_transcript_77512	F01_transcript_77512	F01_transcript_77512	F01_transcript_77512	F01_transcript_77512
F01_transcript_85415	NA	F01_transcript_85415	NA	F01_transcript_85415
F01_transcript_87002	NA	NA	NA	F01_transcript_87002
F01_transcript_140674	NA	NA	NA	F01_transcript_140674
F01_transcript_67450	NA	NA	F01_transcript_67450	NA
F01_transcript_109081	NA	NA	NA	F01_transcript_109081

F01_transcript_93158	NA	F01_transcript_93158	NA	NA
F01_transcript_96016	F01_transcript_96016	F01_transcript_96016	NA	F01_transcript_96016
F01_transcript_108651	F01_transcript_108651	F01_transcript_108651	F01_transcript_108651	F01_transcript_108651
F01_transcript_120872	F01_transcript_120872	F01_transcript_120872	F01_transcript_120872	NA
F01_transcript_153853	NA	NA	NA	F01_transcript_153853
F01_transcript_51618	F01_transcript_51618	F01_transcript_51618	F01_transcript_51618	F01_transcript_51618
F01_transcript_94836	F01_transcript_94836	F01_transcript_94836	NA	NA
F01_transcript_13184	NA	F01_transcript_13184	NA	NA
F01_transcript_56492	NA	F01_transcript_56492	F01_transcript_56492	F01_transcript_56492
F01_transcript_143473	NA	F01_transcript_143473	NA	NA
F01_transcript_134649	NA	NA	NA	F01_transcript_134649
F01_transcript_44501	NA	NA	NA	F01_transcript_44501
F01_transcript_143959	F01_transcript_143959	F01_transcript_143959	F01_transcript_143959	F01_transcript_143959
F01_transcript_146690	NA	F01_transcript_146690	NA	NA

F01_transcript_92050	NA	F01_transcript_92050	NA	NA
F01_transcript_74476	NA	F01_transcript_74476	NA	F01_transcript_74476
F01_transcript_120600	NA	NA	NA	F01_transcript_120600
F01_transcript_2049	F01_transcript_2049	F01_transcript_2049	NA	F01_transcript_2049
F01_transcript_93425	NA	F01_transcript_93425	NA	NA
F01_transcript_152017	NA	NA	NA	F01_transcript_152017
F01_transcript_84576	NA	F01_transcript_84576	F01_transcript_84576	NA
F01_transcript_124425	F01_transcript_124425	F01_transcript_124425	F01_transcript_124425	F01_transcript_124425
F01_transcript_148108	NA	NA	NA	F01_transcript_148108
F01_transcript_49681	NA	F01_transcript_49681	NA	NA
F01_transcript_66821	F01_transcript_66821	F01_transcript_66821	F01_transcript_66821	F01_transcript_66821
F01_transcript_81349	NA	F01_transcript_81349	NA	F01_transcript_81349
F01_transcript_159533	NA	NA	NA	F01_transcript_159533
F01_transcript_100971	NA	NA	NA	F01_transcript_100971

F01_transcript_112120	F01_transcript_112120	F01_transcript_112120	F01_transcript_112120	F01_transcript_112120
F01_transcript_57069	NA	NA	F01_transcript_57069	F01_transcript_57069
F01_transcript_136370	NA	NA	NA	F01_transcript_136370
F01_transcript_127652	F01_transcript_127652	F01_transcript_127652	F01_transcript_127652	NA
F01_transcript_107399	F01_transcript_107399	F01_transcript_107399	F01_transcript_107399	F01_transcript_107399
F01_transcript_137942	NA	F01_transcript_137942	NA	F01_transcript_137942
F01_transcript_156073	NA	F01_transcript_156073	NA	NA
F01_transcript_163752	F01_transcript_163752	F01_transcript_163752	F01_transcript_163752	F01_transcript_163752
F01_transcript_91187	NA	F01_transcript_91187	F01_transcript_91187	F01_transcript_91187
F01_transcript_135350	NA	NA	NA	F01_transcript_135350
F01_transcript_145321	F01_transcript_145321	F01_transcript_145321	F01_transcript_145321	F01_transcript_145321
F01_transcript_142576	NA	F01_transcript_142576	NA	NA
F01_transcript_41341	NA	NA	NA	F01_transcript_41341
F01_transcript_43513	NA	F01_transcript_43513	NA	F01_transcript_43513

F01_transcript_56290	NA	NA	F01_transcript_56290	NA
F01_transcript_71323	F01_transcript_71323	F01_transcript_71323	F01_transcript_71323	NA
F01_transcript_82094	F01_transcript_82094	F01_transcript_82094	NA	F01_transcript_82094
F01_transcript_61055	NA	F01_transcript_61055	F01_transcript_61055	F01_transcript_61055
F01_transcript_117011	NA	F01_transcript_117011	NA	NA
F01_transcript_70901	NA	NA	F01_transcript_70901	NA
F01_transcript_56845	NA	NA	NA	F01_transcript_56845
F01_transcript_96451	NA	F01_transcript_96451	NA	F01_transcript_96451
F01_transcript_145275	NA	NA	NA	F01_transcript_145275
F01_transcript_43967	NA	NA	NA	F01_transcript_43967
F01_transcript_91126	NA	F01_transcript_91126	NA	F01_transcript_91126
F01_transcript_130914	NA	NA	NA	F01_transcript_130914
F01_transcript_137037	NA	F01_transcript_137037	NA	NA
F01_transcript_96897	F01_transcript_96897	F01_transcript_96897	NA	NA

F01_transcript_7376	NA	NA	NA	F01_transcript_7376
F01_transcript_75198	F01_transcript_75198	F01_transcript_75198	F01_transcript_75198	NA
F01_transcript_132466	NA	NA	NA	F01_transcript_132466
F01_transcript_159220	NA	F01_transcript_159220	NA	NA
F01_transcript_143940	NA	F01_transcript_143940	F01_transcript_143940	F01_transcript_143940
F01_transcript_151612	F01_transcript_151612	F01_transcript_151612	NA	F01_transcript_151612
F01_transcript_74836	F01_transcript_74836	F01_transcript_74836	F01_transcript_74836	F01_transcript_74836
F01_transcript_81175	NA	NA	NA	F01_transcript_81175
F01_transcript_144860	NA	NA	NA	F01_transcript_144860
F01_transcript_69586	NA	NA	NA	F01_transcript_69586
F01_transcript_58591	NA	F01_transcript_58591	NA	NA
F01_transcript_122301	NA	NA	NA	F01_transcript_122301
F01_transcript_155114	F01_transcript_155114	F01_transcript_155114	F01_transcript_155114	F01_transcript_155114
F01_transcript_117273	NA	NA	NA	F01_transcript_117273

F01_transcript_158366	NA	F01_transcript_158366	NA	F01_transcript_158366
F01_transcript_81548	F01_transcript_81548	F01_transcript_81548	F01_transcript_81548	F01_transcript_81548
F01_transcript_141732	NA	F01_transcript_141732	NA	NA
F01_transcript_152776	NA	NA	NA	F01_transcript_152776
F01_transcript_70416	NA	NA	NA	F01_transcript_70416
F01_transcript_3345	F01_transcript_3345	F01_transcript_3345	F01_transcript_3345	F01_transcript_3345
F01_transcript_88125	F01_transcript_88125	F01_transcript_88125	F01_transcript_88125	F01_transcript_88125
F01_transcript_116983	NA	F01_transcript_116983	NA	NA
F01_transcript_96246	NA	F01_transcript_96246	NA	F01_transcript_96246
F01_transcript_78469	NA	F01_transcript_78469	NA	NA
F01_transcript_70585	NA	NA	NA	F01_transcript_70585
F01_transcript_142020	NA	F01_transcript_142020	NA	NA
F01_transcript_2140	NA	NA	NA	F01_transcript_2140
F01_transcript_129342	NA	F01_transcript_129342	NA	NA

F01_transcript_118415	F01_transcript_118415	F01_transcript_118415	F01_transcript_118415	F01_transcript_118415
F01_transcript_140608	NA	F01_transcript_140608	NA	F01_transcript_140608
F01_transcript_120008	F01_transcript_120008	F01_transcript_120008	F01_transcript_120008	F01_transcript_120008
F01_transcript_126119	F01_transcript_126119	F01_transcript_126119	F01_transcript_126119	F01_transcript_126119
F01_transcript_76802	NA	NA	NA	F01_transcript_76802
F01_transcript_73631	F01_transcript_73631	F01_transcript_73631	F01_transcript_73631	F01_transcript_73631
F01_transcript_114960	NA	NA	NA	F01_transcript_114960
F01_transcript_62812	NA	F01_transcript_62812	NA	NA
F01_transcript_49800	NA	NA	NA	F01_transcript_49800
F01_transcript_10743	NA	NA	NA	F01_transcript_10743
F01_transcript_48663	NA	NA	F01_transcript_48663	F01_transcript_48663
F01_transcript_100720	NA	F01_transcript_100720	NA	NA
F01_transcript_145861	F01_transcript_145861	F01_transcript_145861	F01_transcript_145861	F01_transcript_145861
F01_transcript_66424	NA	F01_transcript_66424	NA	F01_transcript_66424

F01_transcript_8272	NA	F01_transcript_8272	F01_transcript_8272	NA
F01_transcript_82079	NA	NA	NA	F01_transcript_82079
F01_transcript_65767	NA	NA	F01_transcript_65767	F01_transcript_65767
F01_transcript_167239	NA	NA	NA	F01_transcript_167239
F01_transcript_7743	NA	NA	NA	F01_transcript_7743
F01_transcript_69890	NA	NA	NA	F01_transcript_69890
F01_transcript_130155	F01_transcript_130155	F01_transcript_130155	F01_transcript_130155	F01_transcript_130155
F01_transcript_125532	NA	F01_transcript_125532	NA	F01_transcript_125532
F01_transcript_159465	F01_transcript_159465	F01_transcript_159465	F01_transcript_159465	F01_transcript_159465
F01_transcript_60237	NA	F01_transcript_60237	NA	F01_transcript_60237
F01_transcript_127392	NA	F01_transcript_127392	NA	NA
F01_transcript_50712	F01_transcript_50712	NA	NA	NA
F01_transcript_84684	NA	F01_transcript_84684	NA	F01_transcript_84684
F01_transcript_159809	NA	F01_transcript_159809	NA	NA

F01_transcript_47523	F01_transcript_47523	F01_transcript_47523	F01_transcript_47523	F01_transcript_47523
F01_transcript_39619	NA	NA	NA	F01_transcript_39619
F01_transcript_77895	NA	F01_transcript_77895	NA	NA
F01_transcript_81956	NA	F01_transcript_81956	F01_transcript_81956	F01_transcript_81956
F01_transcript_153015	NA	F01_transcript_153015	NA	NA
F01_transcript_94419	NA	F01_transcript_94419	F01_transcript_94419	F01_transcript_94419
F01_transcript_139458	F01_transcript_139458	F01_transcript_139458	F01_transcript_139458	F01_transcript_139458
F01_transcript_101608	NA	F01_transcript_101608	NA	NA
F01_transcript_56325	NA	NA	F01_transcript_56325	NA
F01_transcript_67265	F01_transcript_67265	NA	NA	NA
F01_transcript_48397	F01_transcript_48397	F01_transcript_48397	F01_transcript_48397	F01_transcript_48397
F01_transcript_96746	NA	NA	NA	F01_transcript_96746
F01_transcript_85996	NA	NA	NA	F01_transcript_85996
F01_transcript_87974	F01_transcript_87974	F01_transcript_87974	NA	F01_transcript_87974

F01_transcript_115563	NA	NA	NA	F01_transcript_115563
F01_transcript_123307	F01_transcript_123307	F01_transcript_123307	NA	F01_transcript_123307
F01_transcript_110025	NA	F01_transcript_110025	NA	NA
F01_transcript_12956	NA	F01_transcript_12956	NA	NA
F01_transcript_99173	NA	F01_transcript_99173	NA	NA
F01_transcript_19115	NA	F01_transcript_19115	NA	NA
F01_transcript_80200	F01_transcript_80200	NA	F01_transcript_80200	F01_transcript_80200
F01_transcript_44602	NA	F01_transcript_44602	NA	NA
F01_transcript_124651	F01_transcript_124651	F01_transcript_124651	NA	NA
F01_transcript_69840	F01_transcript_69840	F01_transcript_69840	NA	NA
F01_transcript_131905	F01_transcript_131905	F01_transcript_131905	F01_transcript_131905	F01_transcript_131905
F01_transcript_43210	NA	NA	NA	F01_transcript_43210
F01_transcript_167059	NA	F01_transcript_167059	NA	NA
F01_transcript_82730	F01_transcript_82730	F01_transcript_82730	F01_transcript_82730	NA

F01_transcript_96656	F01_transcript_96656	F01_transcript_96656	F01_transcript_96656	F01_transcript_96656
F01_transcript_82621	F01_transcript_82621	F01_transcript_82621	NA	NA
F01_transcript_124916	NA	NA	NA	F01_transcript_124916
F01_transcript_144194	NA	F01_transcript_144194	NA	NA
F01_transcript_143008	NA	NA	NA	F01_transcript_143008
F01_transcript_135431	NA	NA	NA	F01_transcript_135431
F01_transcript_105059	NA	F01_transcript_105059	F01_transcript_105059	NA
F01_transcript_154004	NA	F01_transcript_154004	NA	F01_transcript_154004
F01_transcript_119025	NA	F01_transcript_119025	NA	NA
F01_transcript_96719	NA	F01_transcript_96719	NA	NA
F01_transcript_82983	F01_transcript_82983	F01_transcript_82983	F01_transcript_82983	F01_transcript_82983
F01_transcript_79483	NA	F01_transcript_79483	NA	F01_transcript_79483
F01_transcript_159988	NA	NA	NA	F01_transcript_159988
F01_transcript_16054	NA	NA	NA	F01_transcript_16054

F01_transcript_109716	NA	NA	NA	F01_transcript_109716
F01_transcript_107518	NA	NA	NA	F01_transcript_107518
F01_transcript_136035	F01_transcript_136035	F01_transcript_136035	F01_transcript_136035	F01_transcript_136035
F01_transcript_86837	NA	F01_transcript_86837	NA	NA
F01_transcript_125182	NA	NA	NA	F01_transcript_125182
F01_transcript_129710	F01_transcript_129710	F01_transcript_129710	F01_transcript_129710	F01_transcript_129710
F01_transcript_70097	F01_transcript_70097	F01_transcript_70097	NA	F01_transcript_70097
F01_transcript_107294	NA	F01_transcript_107294	NA	NA
F01_transcript_81331	NA	NA	NA	F01_transcript_81331
F01_transcript_81077	NA	NA	NA	F01_transcript_81077
F01_transcript_12612	NA	NA	NA	F01_transcript_12612
F01_transcript_132959	NA	F01_transcript_132959	NA	NA
F01_transcript_140075	NA	F01_transcript_140075	NA	F01_transcript_140075
F01_transcript_124300	F01_transcript_124300	F01_transcript_124300	F01_transcript_124300	F01_transcript_124300

F01_transcript_91066	NA	NA	NA	F01_transcript_91066
F01_transcript_100493	NA	NA	NA	F01_transcript_100493
F01_transcript_161035	NA	F01_transcript_161035	F01_transcript_161035	F01_transcript_161035
F01_transcript_56487	F01_transcript_56487	F01_transcript_56487	F01_transcript_56487	NA
F01_transcript_157566	NA	F01_transcript_157566	NA	F01_transcript_157566
F01_transcript_147500	NA	NA	NA	F01_transcript_147500
F01_transcript_124993	NA	NA	NA	F01_transcript_124993
F01_transcript_98786	NA	NA	NA	F01_transcript_98786
F01_transcript_47672	NA	NA	NA	F01_transcript_47672
F01_transcript_98046	NA	F01_transcript_98046	F01_transcript_98046	F01_transcript_98046
F01_transcript_97467	F01_transcript_97467	F01_transcript_97467	NA	NA
F01_transcript_32292	F01_transcript_32292	F01_transcript_32292	F01_transcript_32292	F01_transcript_32292
F01_transcript_70858	NA	F01_transcript_70858	F01_transcript_70858	NA
F01_transcript_53648	F01_transcript_53648	F01_transcript_53648	F01_transcript_53648	F01_transcript_53648

F01_transcript_94028	NA	NA	NA	F01_transcript_94028
F01_transcript_162711	NA	F01_transcript_162711	NA	NA
F01_transcript_127867	NA	F01_transcript_127867	NA	NA
F01_transcript_61042	NA	F01_transcript_61042	NA	NA
F01_transcript_53369	F01_transcript_53369	F01_transcript_53369	F01_transcript_53369	F01_transcript_53369
F01_transcript_83490	NA	F01_transcript_83490	NA	F01_transcript_83490
F01_transcript_142999	NA	F01_transcript_142999	NA	NA
F01_transcript_94733	F01_transcript_94733	F01_transcript_94733	F01_transcript_94733	F01_transcript_94733
F01_transcript_91588	F01_transcript_91588	F01_transcript_91588	F01_transcript_91588	F01_transcript_91588
F01_transcript_42268	NA	NA	NA	F01_transcript_42268
F01_transcript_86631	NA	NA	NA	F01_transcript_86631
F01_transcript_156891	F01_transcript_156891	F01_transcript_156891	F01_transcript_156891	NA
F01_transcript_115460	F01_transcript_115460	F01_transcript_115460	F01_transcript_115460	F01_transcript_115460
F01_transcript_154248	NA	NA	NA	F01_transcript_154248

F01_transcript_99928	NA	NA	NA	F01_transcript_99928
F01_transcript_150901	F01_transcript_150901	NA	NA	NA
F01_transcript_101710	F01_transcript_101710	F01_transcript_101710	F01_transcript_101710	F01_transcript_101710
F01_transcript_72701	NA	F01_transcript_72701	NA	F01_transcript_72701
F01_transcript_115084	NA	F01_transcript_115084	NA	NA
F01_transcript_165794	NA	F01_transcript_165794	NA	NA
F01_transcript_79833	NA	F01_transcript_79833	NA	NA
F01_transcript_132637	NA	F01_transcript_132637	NA	NA
F01_transcript_85834	NA	F01_transcript_85834	NA	F01_transcript_85834
F01_transcript_7920	NA	NA	NA	F01_transcript_7920
F01_transcript_93748	F01_transcript_93748	F01_transcript_93748	F01_transcript_93748	F01_transcript_93748
F01_transcript_73607	NA	NA	NA	F01_transcript_73607
F01_transcript_155277	F01_transcript_155277	F01_transcript_155277	F01_transcript_155277	F01_transcript_155277
F01_transcript_106379	NA	NA	NA	F01_transcript_106379

F01_transcript_82936	NA	NA	NA	F01_transcript_82936
F01_transcript_112545	NA	F01_transcript_112545	NA	F01_transcript_112545
F01_transcript_30750	NA	F01_transcript_30750	NA	NA
F01_transcript_126647	NA	F01_transcript_126647	NA	NA
F01_transcript_87701	NA	F01_transcript_87701	F01_transcript_87701	F01_transcript_87701
F01_transcript_162027	NA	NA	NA	F01_transcript_162027
F01_transcript_88654	NA	F01_transcript_88654	F01_transcript_88654	F01_transcript_88654
F01_transcript_99424	NA	F01_transcript_99424	NA	NA
F01_transcript_50520	NA	F01_transcript_50520	F01_transcript_50520	F01_transcript_50520
F01_transcript_78990	F01_transcript_78990	F01_transcript_78990	F01_transcript_78990	F01_transcript_78990
F01_transcript_93710	F01_transcript_93710	F01_transcript_93710	NA	NA
F01_transcript_65169	NA	NA	NA	F01_transcript_65169
F01_transcript_58468	NA	F01_transcript_58468	NA	NA
F01_transcript_159985	F01_transcript_159985	F01_transcript_159985	F01_transcript_159985	F01_transcript_159985

F01_transcript_165608	NA	NA	NA	F01_transcript_165608
F01_transcript_58311	NA	F01_transcript_58311	NA	F01_transcript_58311
F01_transcript_144287	NA	F01_transcript_144287	NA	NA
F01_transcript_27945	NA	NA	NA	F01_transcript_27945
F01_transcript_56426	F01_transcript_56426	F01_transcript_56426	F01_transcript_56426	NA
F01_transcript_56351	NA	NA	F01_transcript_56351	F01_transcript_56351
F01_transcript_477	NA	NA	NA	F01_transcript_477
F01_transcript_71700	NA	NA	NA	F01_transcript_71700
F01_transcript_163613	NA	F01_transcript_163613	NA	NA
F01_transcript_55440	F01_transcript_55440	F01_transcript_55440	F01_transcript_55440	F01_transcript_55440
F01_transcript_65540	NA	F01_transcript_65540	NA	F01_transcript_65540
F01_transcript_150131	F01_transcript_150131	F01_transcript_150131	F01_transcript_150131	NA
F01_transcript_65489	NA	F01_transcript_65489	F01_transcript_65489	NA
F01_transcript_36783	NA	F01_transcript_36783	F01_transcript_36783	F01_transcript_36783

F01_transcript_80696	F01_transcript_80696	F01_transcript_80696	F01_transcript_80696	F01_transcript_80696
F01_transcript_8519	NA	F01_transcript_8519	NA	NA
F01_transcript_125101	NA	F01_transcript_125101	NA	NA
F01_transcript_121060	NA	F01_transcript_121060	NA	NA
F01_transcript_133783	NA	NA	F01_transcript_133783	F01_transcript_133783
F01_transcript_56543	NA	NA	F01_transcript_56543	NA
F01_transcript_49435	NA	NA	F01_transcript_49435	F01_transcript_49435
F01_transcript_147913	F01_transcript_147913	F01_transcript_147913	F01_transcript_147913	F01_transcript_147913
F01_transcript_59395	NA	NA	F01_transcript_59395	NA
F01_transcript_140340	F01_transcript_140340	F01_transcript_140340	NA	NA
F01_transcript_122850	F01_transcript_122850	F01_transcript_122850	NA	NA
F01_transcript_81237	NA	NA	NA	F01_transcript_81237
F01_transcript_89814	NA	F01_transcript_89814	NA	NA
F01_transcript_57641	NA	F01_transcript_57641	F01_transcript_57641	F01_transcript_57641

F01_transcript_112769	F01_transcript_112769	F01_transcript_112769	F01_transcript_112769	F01_transcript_112769
F01_transcript_150553	NA	F01_transcript_150553	NA	F01_transcript_150553
F01_transcript_136082	NA	NA	NA	F01_transcript_136082
F01_transcript_59189	NA	F01_transcript_59189	NA	NA
F01_transcript_97905	NA	F01_transcript_97905	NA	NA
F01_transcript_147925	NA	F01_transcript_147925	NA	NA
F01_transcript_100902	F01_transcript_100902	F01_transcript_100902	NA	NA
F01_transcript_97844	NA	F01_transcript_97844	NA	F01_transcript_97844
F01_transcript_3915	NA	F01_transcript_3915	NA	F01_transcript_3915
F01_transcript_153390	NA	F01_transcript_153390	NA	NA
F01_transcript_53017	F01_transcript_53017	F01_transcript_53017	NA	NA
F01_transcript_133697	NA	F01_transcript_133697	NA	NA
F01_transcript_86268	NA	F01_transcript_86268	F01_transcript_86268	F01_transcript_86268
F01_transcript_159352	NA	F01_transcript_159352	NA	NA

F01_transcript_118192	F01_transcript_118192	F01_transcript_118192	F01_transcript_118192	NA
F01_transcript_114904	F01_transcript_114904	F01_transcript_114904	F01_transcript_114904	F01_transcript_114904
F01_transcript_24558	NA	NA	NA	F01_transcript_24558
F01_transcript_141583	NA	F01_transcript_141583	NA	NA
F01_transcript_164288	NA	NA	NA	F01_transcript_164288
F01_transcript_49444	NA	F01_transcript_49444	NA	NA
F01_transcript_147522	F01_transcript_147522	NA	F01_transcript_147522	NA
F01_transcript_105560	NA	F01_transcript_105560	F01_transcript_105560	F01_transcript_105560
F01_transcript_94612	NA	NA	NA	F01_transcript_94612
F01_transcript_92440	F01_transcript_92440	F01_transcript_92440	NA	NA
F01_transcript_165670	F01_transcript_165670	F01_transcript_165670	F01_transcript_165670	F01_transcript_165670
F01_transcript_143865	NA	NA	NA	F01_transcript_143865
F01_transcript_146749	NA	NA	NA	F01_transcript_146749
F01_transcript_137222	NA	NA	NA	F01_transcript_137222

F01_transcript_128688	NA	NA	NA	F01_transcript_128688
F01_transcript_100675	F01_transcript_100675	F01_transcript_100675	NA	F01_transcript_100675
F01_transcript_126303	NA	NA	NA	F01_transcript_126303
F01_transcript_43646	NA	NA	F01_transcript_43646	F01_transcript_43646
F01_transcript_96836	F01_transcript_96836	F01_transcript_96836	NA	NA
F01_transcript_148320	NA	NA	NA	F01_transcript_148320
F01_transcript_163514	NA	NA	NA	F01_transcript_163514
F01_transcript_48715	NA	NA	NA	F01_transcript_48715
F01_transcript_6691	NA	NA	NA	F01_transcript_6691
F01_transcript_109749	F01_transcript_109749	F01_transcript_109749	F01_transcript_109749	NA
F01_transcript_153495	NA	F01_transcript_153495	F01_transcript_153495	NA
F01_transcript_70954	NA	F01_transcript_70954	F01_transcript_70954	F01_transcript_70954
F01_transcript_145583	F01_transcript_145583	F01_transcript_145583	F01_transcript_145583	F01_transcript_145583
F01_transcript_119468	NA	F01_transcript_119468	F01_transcript_119468	NA

F01_transcript_78061	NA	NA	NA	F01_transcript_78061
F01_transcript_133197	NA	NA	NA	F01_transcript_133197
F01_transcript_120270	NA	NA	NA	F01_transcript_120270
F01_transcript_166312	NA	F01_transcript_166312	NA	NA
F01_transcript_100844	NA	NA	NA	F01_transcript_100844
F01_transcript_154504	NA	F01_transcript_154504	F01_transcript_154504	NA
F01_transcript_111708	NA	NA	NA	F01_transcript_111708
F01_transcript_123803	NA	F01_transcript_123803	F01_transcript_123803	F01_transcript_123803
F01_transcript_104241	NA	F01_transcript_104241	F01_transcript_104241	F01_transcript_104241
F01_transcript_152598	F01_transcript_152598	F01_transcript_152598	F01_transcript_152598	NA
F01_transcript_114777	F01_transcript_114777	F01_transcript_114777	F01_transcript_114777	NA
F01_transcript_144322	F01_transcript_144322	F01_transcript_144322	F01_transcript_144322	F01_transcript_144322
F01_transcript_114159	NA	NA	NA	F01_transcript_114159
F01_transcript_119578	NA	F01_transcript_119578	NA	NA

F01_transcript_158637	NA	NA	NA	F01_transcript_158637
F01_transcript_34510	NA	NA	NA	F01_transcript_34510
F01_transcript_132170	F01_transcript_132170	F01_transcript_132170	NA	NA
F01_transcript_50848	NA	F01_transcript_50848	F01_transcript_50848	F01_transcript_50848
F01_transcript_40517	NA	NA	NA	F01_transcript_40517
F01_transcript_51477	NA	NA	NA	F01_transcript_51477
F01_transcript_64500	F01_transcript_64500	F01_transcript_64500	NA	F01_transcript_64500
F01_transcript_94856	NA	F01_transcript_94856	NA	NA
F01_transcript_155959	F01_transcript_155959	F01_transcript_155959	NA	F01_transcript_155959
F01_transcript_137964	F01_transcript_137964	F01_transcript_137964	F01_transcript_137964	F01_transcript_137964
F01_transcript_85381	F01_transcript_85381	F01_transcript_85381	NA	F01_transcript_85381
F01_transcript_117642	F01_transcript_117642	F01_transcript_117642	F01_transcript_117642	F01_transcript_117642
F01_transcript_79879	F01_transcript_79879	F01_transcript_79879	F01_transcript_79879	F01_transcript_79879
F01_transcript_76320	F01_transcript_76320	F01_transcript_76320	F01_transcript_76320	F01_transcript_76320

F01_transcript_59423	NA	NA	F01_transcript_59423	NA
F01_transcript_123164	NA	F01_transcript_123164	NA	NA
F01_transcript_87803	NA	F01_transcript_87803	NA	NA
F01_transcript_133674	NA	F01_transcript_133674	NA	NA
F01_transcript_46935	NA	NA	NA	F01_transcript_46935
F01_transcript_110395	NA	NA	NA	F01_transcript_110395
F01_transcript_157666	NA	NA	NA	F01_transcript_157666
F01_transcript_148317	NA	NA	NA	F01_transcript_148317
F01_transcript_126327	NA	NA	NA	F01_transcript_126327
F01_transcript_89403	NA	NA	NA	F01_transcript_89403
F01_transcript_141947	NA	NA	NA	F01_transcript_141947
F01_transcript_63629	NA	NA	NA	F01_transcript_63629
F01_transcript_55495	F01_transcript_55495	F01_transcript_55495	NA	F01_transcript_55495
F01_transcript_64827	NA	NA	F01_transcript_64827	F01_transcript_64827

F01_transcript_148916	NA	NA	NA	F01_transcript_148916
F01_transcript_111556	NA	NA	NA	F01_transcript_111556
F01_transcript_163745	F01_transcript_163745	F01_transcript_163745	F01_transcript_163745	F01_transcript_163745
F01_transcript_116865	NA	NA	NA	F01_transcript_116865
F01_transcript_83601	NA	F01_transcript_83601	NA	F01_transcript_83601
F01_transcript_79700	F01_transcript_79700	F01_transcript_79700	F01_transcript_79700	F01_transcript_79700
F01_transcript_156792	NA	F01_transcript_156792	NA	NA
F01_transcript_40084	NA	F01_transcript_40084	F01_transcript_40084	NA
F01_transcript_58515	NA	NA	NA	F01_transcript_58515
F01_transcript_122196	NA	NA	NA	F01_transcript_122196
F01_transcript_94578	NA	F01_transcript_94578	NA	NA
F01_transcript_86885	NA	F01_transcript_86885	NA	F01_transcript_86885
F01_transcript_101338	NA	F01_transcript_101338	NA	NA
F01_transcript_164206	F01_transcript_164206	F01_transcript_164206	NA	F01_transcript_164206

F01_transcript_20390	NA	NA	NA	F01_transcript_20390
F01_transcript_166059	F01_transcript_166059	F01_transcript_166059	NA	NA
F01_transcript_78311	NA	NA	F01_transcript_78311	NA
F01_transcript_80922	NA	NA	NA	F01_transcript_80922
F01_transcript_72941	NA	F01_transcript_72941	F01_transcript_72941	NA
F01_transcript_155716	NA	NA	NA	F01_transcript_155716
F01_transcript_120960	NA	NA	NA	F01_transcript_120960
F01_transcript_64793	NA	NA	NA	F01_transcript_64793
F01_transcript_49971	NA	F01_transcript_49971	NA	NA
F01_transcript_90422	NA	F01_transcript_90422	NA	F01_transcript_90422
F01_transcript_114263	NA	NA	NA	F01_transcript_114263
F01_transcript_59271	NA	NA	NA	F01_transcript_59271
F01_transcript_122258	NA	F01_transcript_122258	NA	F01_transcript_122258
F01_transcript_150649	F01_transcript_150649	F01_transcript_150649	F01_transcript_150649	F01_transcript_150649

F01_transcript_130653	F01_transcript_130653	NA	NA	NA
F01_transcript_71027	NA	F01_transcript_71027	F01_transcript_71027	F01_transcript_71027
F01_transcript_130027	NA	NA	NA	F01_transcript_130027
F01_transcript_152836	NA	NA	NA	F01_transcript_152836
F01_transcript_85533	NA	F01_transcript_85533	NA	F01_transcript_85533
F01_transcript_75767	NA	NA	NA	F01_transcript_75767
F01_transcript_147412	NA	NA	NA	F01_transcript_147412
F01_transcript_105779	F01_transcript_105779	F01_transcript_105779	NA	NA
F01_transcript_67555	NA	NA	NA	F01_transcript_67555
F01_transcript_159375	NA	F01_transcript_159375	NA	NA
F01_transcript_56108	NA	NA	NA	F01_transcript_56108
F01_transcript_78505	NA	F01_transcript_78505	NA	NA
F01_transcript_109579	NA	F01_transcript_109579	F01_transcript_109579	F01_transcript_109579
F01_transcript_72382	NA	NA	NA	F01_transcript_72382

F01_transcript_115134	NA	NA	NA	F01_transcript_115134
F01_transcript_142492	NA	F01_transcript_142492	F01_transcript_142492	NA
F01_transcript_56929	NA	F01_transcript_56929	F01_transcript_56929	NA
F01_transcript_47319	NA	NA	NA	F01_transcript_47319
F01_transcript_73967	F01_transcript_73967	F01_transcript_73967	F01_transcript_73967	F01_transcript_73967
F01_transcript_69313	F01_transcript_69313	F01_transcript_69313	F01_transcript_69313	NA
F01_transcript_54439	NA	NA	NA	F01_transcript_54439
F01_transcript_156850	F01_transcript_156850	F01_transcript_156850	F01_transcript_156850	F01_transcript_156850
F01_transcript_121506	F01_transcript_121506	F01_transcript_121506	NA	F01_transcript_121506
F01_transcript_163093	NA	NA	NA	F01_transcript_163093
F01_transcript_18365	NA	F01_transcript_18365	NA	F01_transcript_18365
F01_transcript_152561	F01_transcript_152561	F01_transcript_152561	NA	NA
F01_transcript_144922	NA	F01_transcript_144922	F01_transcript_144922	F01_transcript_144922
F01_transcript_163266	NA	NA	NA	F01_transcript_163266

F01_transcript_49160	NA	F01_transcript_49160	NA	F01_transcript_49160
F01_transcript_82601	NA	F01_transcript_82601	F01_transcript_82601	F01_transcript_82601
F01_transcript_156295	NA	NA	NA	F01_transcript_156295
F01_transcript_66657	NA	F01_transcript_66657	NA	F01_transcript_66657
F01_transcript_41316	NA	NA	NA	F01_transcript_41316
F01_transcript_85698	F01_transcript_85698	F01_transcript_85698	F01_transcript_85698	F01_transcript_85698
F01_transcript_142899	F01_transcript_142899	F01_transcript_142899	NA	NA
F01_transcript_48546	NA	F01_transcript_48546	NA	NA
F01_transcript_70458	F01_transcript_70458	F01_transcript_70458	NA	F01_transcript_70458
F01_transcript_154526	F01_transcript_154526	F01_transcript_154526	F01_transcript_154526	F01_transcript_154526
F01_transcript_113014	F01_transcript_113014	F01_transcript_113014	NA	NA
F01_transcript_693	NA	NA	NA	F01_transcript_693
F01_transcript_109991	NA	F01_transcript_109991	NA	NA
F01_transcript_35168	NA	NA	NA	F01_transcript_35168

F01_transcript_126087	NA	F01_transcript_126087	NA	F01_transcript_126087
F01_transcript_165932	NA	F01_transcript_165932	NA	NA
F01_transcript_153821	NA	NA	NA	F01_transcript_153821
F01_transcript_107667	NA	NA	F01_transcript_107667	F01_transcript_107667
F01_transcript_115103	F01_transcript_115103	F01_transcript_115103	NA	F01_transcript_115103
F01_transcript_45749	NA	NA	NA	F01_transcript_45749
F01_transcript_150639	F01_transcript_150639	F01_transcript_150639	NA	F01_transcript_150639
F01_transcript_124259	NA	NA	NA	F01_transcript_124259
F01_transcript_75007	F01_transcript_75007	F01_transcript_75007	NA	NA
F01_transcript_109585	NA	F01_transcript_109585	NA	NA
F01_transcript_70875	NA	F01_transcript_70875	F01_transcript_70875	NA
F01_transcript_65355	NA	F01_transcript_65355	F01_transcript_65355	NA
F01_transcript_143894	NA	F01_transcript_143894	NA	F01_transcript_143894
F01_transcript_73808	F01_transcript_73808	F01_transcript_73808	NA	F01_transcript_73808

F01_transcript_70191	NA	NA	NA	F01_transcript_70191
F01_transcript_130028	NA	NA	F01_transcript_130028	NA
F01_transcript_65864	NA	F01_transcript_65864	NA	F01_transcript_65864
F01_transcript_160420	NA	NA	NA	F01_transcript_160420
F01_transcript_98990	NA	NA	NA	F01_transcript_98990
F01_transcript_82444	NA	NA	NA	F01_transcript_82444
F01_transcript_135861	NA	NA	NA	F01_transcript_135861
F01_transcript_26726	NA	NA	NA	F01_transcript_26726
F01_transcript_111513	NA	NA	NA	F01_transcript_111513
F01_transcript_106985	NA	F01_transcript_106985	F01_transcript_106985	F01_transcript_106985
F01_transcript_104976	NA	F01_transcript_104976	NA	F01_transcript_104976
F01_transcript_80677	NA	F01_transcript_80677	NA	NA
F01_transcript_118456	F01_transcript_118456	F01_transcript_118456	F01_transcript_118456	F01_transcript_118456
F01_transcript_71417	NA	F01_transcript_71417	F01_transcript_71417	F01_transcript_71417

F01_transcript_3370	NA	F01_transcript_3370	NA	NA
F01_transcript_69288	NA	NA	NA	F01_transcript_69288
F01_transcript_99438	NA	F01_transcript_99438	NA	NA
F01_transcript_108632	NA	F01_transcript_108632	F01_transcript_108632	F01_transcript_108632
F01_transcript_32547	NA	NA	NA	F01_transcript_32547
F01_transcript_58633	F01_transcript_58633	F01_transcript_58633	F01_transcript_58633	F01_transcript_58633
F01_transcript_59870	NA	F01_transcript_59870	NA	F01_transcript_59870
F01_transcript_75209	F01_transcript_75209	F01_transcript_75209	F01_transcript_75209	F01_transcript_75209
F01_transcript_82154	F01_transcript_82154	F01_transcript_82154	F01_transcript_82154	F01_transcript_82154
F01_transcript_42368	NA	F01_transcript_42368	NA	NA
F01_transcript_70653	NA	F01_transcript_70653	NA	F01_transcript_70653
F01_transcript_50121	NA	NA	NA	F01_transcript_50121
F01_transcript_113355	F01_transcript_113355	F01_transcript_113355	F01_transcript_113355	F01_transcript_113355
F01_transcript_81001	NA	NA	NA	F01_transcript_81001

F01_transcript_132318	NA	F01_transcript_132318	NA	F01_transcript_132318
F01_transcript_148503	NA	NA	NA	F01_transcript_148503
F01_transcript_131280	NA	F01_transcript_131280	NA	NA
F01_transcript_59078	F01_transcript_59078	F01_transcript_59078	F01_transcript_59078	F01_transcript_59078
F01_transcript_119395	NA	F01_transcript_119395	NA	NA
F01_transcript_11376	NA	NA	NA	F01_transcript_11376
F01_transcript_22440	NA	F01_transcript_22440	NA	NA
F01_transcript_61938	F01_transcript_61938	F01_transcript_61938	NA	NA
F01_transcript_59538	F01_transcript_59538	F01_transcript_59538	F01_transcript_59538	F01_transcript_59538
F01_transcript_166153	F01_transcript_166153	F01_transcript_166153	F01_transcript_166153	F01_transcript_166153
F01_transcript_99906	NA	NA	NA	F01_transcript_99906
F01_transcript_121726	F01_transcript_121726	F01_transcript_121726	F01_transcript_121726	F01_transcript_121726
F01_transcript_21255	NA	NA	NA	F01_transcript_21255
F01_transcript_86032	NA	F01_transcript_86032	NA	NA

F01_transcript_19901	F01_transcript_19901	F01_transcript_19901	F01_transcript_19901	F01_transcript_19901
F01_transcript_42990	NA	NA	F01_transcript_42990	F01_transcript_42990
F01_transcript_147169	NA	F01_transcript_147169	NA	NA
F01_transcript_102865	NA	F01_transcript_102865	F01_transcript_102865	F01_transcript_102865
F01_transcript_127011	NA	F01_transcript_127011	NA	F01_transcript_127011
F01_transcript_62987	NA	NA	NA	F01_transcript_62987
F01_transcript_116148	NA	F01_transcript_116148	NA	NA
F01_transcript_117450	NA	F01_transcript_117450	NA	NA
F01_transcript_145054	NA	NA	NA	F01_transcript_145054
F01_transcript_104936	NA	F01_transcript_104936	NA	NA
F01_transcript_2759	NA	F01_transcript_2759	NA	NA
F01_transcript_65811	NA	F01_transcript_65811	NA	NA
F01_transcript_127896	NA	F01_transcript_127896	NA	NA
F01_transcript_79711	NA	F01_transcript_79711	NA	NA

F01_transcript_152480	NA	NA	NA	F01_transcript_152480
F01_transcript_101859	NA	NA	NA	F01_transcript_101859
F01_transcript_72722	F01_transcript_72722	F01_transcript_72722	F01_transcript_72722	F01_transcript_72722
F01_transcript_58805	NA	NA	NA	F01_transcript_58805
F01_transcript_116892	NA	NA	NA	F01_transcript_116892
F01_transcript_55002	F01_transcript_55002	F01_transcript_55002	F01_transcript_55002	F01_transcript_55002
F01_transcript_72527	F01_transcript_72527	F01_transcript_72527	F01_transcript_72527	F01_transcript_72527
F01_transcript_130402	F01_transcript_130402	F01_transcript_130402	NA	F01_transcript_130402
F01_transcript_57095	F01_transcript_57095	F01_transcript_57095	F01_transcript_57095	NA
F01_transcript_133215	NA	NA	NA	F01_transcript_133215
F01_transcript_162501	NA	NA	NA	F01_transcript_162501
F01_transcript_147413	NA	F01_transcript_147413	NA	F01_transcript_147413
F01_transcript_60929	NA	F01_transcript_60929	NA	NA
F01_transcript_111285	NA	NA	NA	F01_transcript_111285

F01_transcript_116273	NA	F01_transcript_116273	NA	F01_transcript_116273
F01_transcript_154898	NA	NA	NA	F01_transcript_154898
F01_transcript_119698	F01_transcript_119698	F01_transcript_119698	F01_transcript_119698	NA
F01_transcript_119209	NA	NA	NA	F01_transcript_119209
F01_transcript_153431	NA	F01_transcript_153431	NA	NA
F01_transcript_86736	NA	NA	NA	F01_transcript_86736
F01_transcript_61032	F01_transcript_61032	F01_transcript_61032	F01_transcript_61032	F01_transcript_61032
F01_transcript_166300	NA	NA	F01_transcript_166300	NA
F01_transcript_96023	NA	F01_transcript_96023	NA	NA
F01_transcript_94778	F01_transcript_94778	F01_transcript_94778	F01_transcript_94778	F01_transcript_94778
F01_transcript_16590	F01_transcript_16590	NA	NA	NA
F01_transcript_88454	F01_transcript_88454	F01_transcript_88454	NA	F01_transcript_88454
F01_transcript_13383	F01_transcript_13383	F01_transcript_13383	NA	F01_transcript_13383
F01_transcript_107592	NA	F01_transcript_107592	NA	NA

F01_transcript_102968	F01_transcript_102968	F01_transcript_102968	F01_transcript_102968	F01_transcript_102968
F01_transcript_112496	NA	F01_transcript_112496	NA	NA
F01_transcript_76491	F01_transcript_76491	F01_transcript_76491	F01_transcript_76491	F01_transcript_76491
F01_transcript_152311	F01_transcript_152311	F01_transcript_152311	F01_transcript_152311	NA
F01_transcript_120371	F01_transcript_120371	F01_transcript_120371	F01_transcript_120371	F01_transcript_120371
F01_transcript_120800	NA	F01_transcript_120800	NA	NA
F01_transcript_57076	NA	NA	F01_transcript_57076	NA
F01_transcript_135967	NA	NA	NA	F01_transcript_135967
F01_transcript_144170	NA	NA	F01_transcript_144170	F01_transcript_144170
F01_transcript_42874	NA	NA	NA	F01_transcript_42874
F01_transcript_125123	NA	F01_transcript_125123	NA	NA
F01_transcript_49499	NA	NA	NA	F01_transcript_49499
F01_transcript_162707	NA	NA	NA	F01_transcript_162707
F01_transcript_63404	NA	F01_transcript_63404	NA	NA

F01_transcript_90571	NA	F01_transcript_90571	NA	NA
F01_transcript_94882	NA	F01_transcript_94882	NA	NA
F01_transcript_126227	NA	NA	NA	F01_transcript_126227
F01_transcript_50461	NA	F01_transcript_50461	NA	NA
F01_transcript_65897	NA	NA	NA	F01_transcript_65897
F01_transcript_91027	NA	NA	NA	F01_transcript_91027
F01_transcript_70592	F01_transcript_70592	F01_transcript_70592	F01_transcript_70592	NA
F01_transcript_71455	NA	NA	NA	F01_transcript_71455
F01_transcript_61581	NA	NA	NA	F01_transcript_61581
F01_transcript_113951	NA	NA	F01_transcript_113951	NA
F01_transcript_55427	F01_transcript_55427	F01_transcript_55427	NA	F01_transcript_55427
F01_transcript_78405	F01_transcript_78405	F01_transcript_78405	F01_transcript_78405	F01_transcript_78405
F01_transcript_46211	NA	NA	F01_transcript_46211	F01_transcript_46211
F01_transcript_143858	NA	NA	NA	F01_transcript_143858

F01_transcript_91182	F01_transcript_91182	F01_transcript_91182	NA	F01_transcript_91182
F01_transcript_40023	NA	NA	NA	F01_transcript_40023
F01_transcript_40	NA	NA	NA	F01_transcript_40
F01_transcript_143504	NA	F01_transcript_143504	NA	NA
F01_transcript_53226	NA	F01_transcript_53226	NA	NA
F01_transcript_140774	NA	F01_transcript_140774	NA	NA
F01_transcript_16215	NA	NA	NA	F01_transcript_16215
F01_transcript_78411	NA	F01_transcript_78411	NA	NA
F01_transcript_133119	NA	F01_transcript_133119	F01_transcript_133119	F01_transcript_133119
F01_transcript_92232	F01_transcript_92232	F01_transcript_92232	NA	NA
F01_transcript_127943	NA	NA	NA	F01_transcript_127943
F01_transcript_63605	NA	F01_transcript_63605	F01_transcript_63605	NA
F01_transcript_110011	F01_transcript_110011	F01_transcript_110011	F01_transcript_110011	F01_transcript_110011
F01_transcript_52270	NA	NA	NA	F01_transcript_52270

F01_transcript_150127	NA	F01_transcript_150127	NA	F01_transcript_150127
F01_transcript_158013	NA	F01_transcript_158013	NA	NA
F01_transcript_66899	F01_transcript_66899	F01_transcript_66899	NA	NA
F01_transcript_18895	NA	NA	NA	F01_transcript_18895
F01_transcript_65163	NA	NA	F01_transcript_65163	NA
F01_transcript_46025	NA	F01_transcript_46025	NA	NA
F01_transcript_144391	NA	NA	NA	F01_transcript_144391
F01_transcript_3532	NA	NA	NA	F01_transcript_3532
F01_transcript_68630	NA	NA	NA	F01_transcript_68630
F01_transcript_42543	NA	NA	NA	F01_transcript_42543
F01_transcript_99670	NA	F01_transcript_99670	F01_transcript_99670	NA
F01_transcript_48090	NA	F01_transcript_48090	NA	F01_transcript_48090
F01_transcript_109169	NA	NA	NA	F01_transcript_109169
F01_transcript_133930	NA	F01_transcript_133930	NA	NA

F01_transcript_75717	NA	NA	NA	F01_transcript_75717
F01_transcript_103208	NA	F01_transcript_103208	NA	F01_transcript_103208
F01_transcript_62618	NA	F01_transcript_62618	NA	NA
F01_transcript_74468	NA	NA	NA	F01_transcript_74468
F01_transcript_91615	F01_transcript_91615	F01_transcript_91615	F01_transcript_91615	F01_transcript_91615
F01_transcript_104111	NA	NA	NA	F01_transcript_104111
F01_transcript_115857	F01_transcript_115857	F01_transcript_115857	F01_transcript_115857	F01_transcript_115857
F01_transcript_139908	F01_transcript_139908	F01_transcript_139908	F01_transcript_139908	F01_transcript_139908
F01_transcript_123860	NA	NA	F01_transcript_123860	F01_transcript_123860
F01_transcript_83736	NA	NA	NA	F01_transcript_83736
F01_transcript_37251	NA	NA	NA	F01_transcript_37251
F01_transcript_90352	NA	NA	NA	F01_transcript_90352
F01_transcript_61859	F01_transcript_61859	F01_transcript_61859	NA	NA
F01_transcript_148885	NA	F01_transcript_148885	F01_transcript_148885	F01_transcript_148885

F01_transcript_147592	F01_transcript_147592	F01_transcript_147592	F01_transcript_147592	F01_transcript_147592
F01_transcript_138888	NA	NA	NA	F01_transcript_138888
F01_transcript_45297	NA	F01_transcript_45297	NA	F01_transcript_45297
F01_transcript_75771	F01_transcript_75771	F01_transcript_75771	F01_transcript_75771	F01_transcript_75771
F01_transcript_44031	NA	NA	NA	F01_transcript_44031
F01_transcript_75030	NA	F01_transcript_75030	NA	NA
F01_transcript_101110	NA	F01_transcript_101110	NA	F01_transcript_101110
F01_transcript_68274	NA	F01_transcript_68274	F01_transcript_68274	F01_transcript_68274
F01_transcript_126319	NA	F01_transcript_126319	NA	NA
F01_transcript_73435	NA	F01_transcript_73435	F01_transcript_73435	F01_transcript_73435
F01_transcript_84039	NA	F01_transcript_84039	NA	NA
F01_transcript_151260	NA	NA	NA	F01_transcript_151260
F01_transcript_108310	F01_transcript_108310	F01_transcript_108310	F01_transcript_108310	F01_transcript_108310
F01_transcript_112676	NA	F01_transcript_112676	NA	F01_transcript_112676

F01_transcript_83051	NA	NA	F01_transcript_83051	F01_transcript_83051
F01_transcript_49655	NA	F01_transcript_49655	NA	F01_transcript_49655
F01_transcript_124004	NA	F01_transcript_124004	NA	F01_transcript_124004
F01_transcript_165535	NA	F01_transcript_165535	NA	NA
F01_transcript_116666	NA	NA	NA	F01_transcript_116666
F01_transcript_119384	NA	F01_transcript_119384	NA	NA
F01_transcript_27190	F01_transcript_27190	F01_transcript_27190	F01_transcript_27190	F01_transcript_27190
F01_transcript_134076	NA	NA	NA	F01_transcript_134076
F01_transcript_50633	F01_transcript_50633	F01_transcript_50633	NA	F01_transcript_50633
F01_transcript_137068	NA	F01_transcript_137068	NA	NA
F01_transcript_142172	NA	NA	NA	F01_transcript_142172
F01_transcript_155058	NA	F01_transcript_155058	NA	NA
F01_transcript_46889	NA	NA	NA	F01_transcript_46889
F01_transcript_77724	F01_transcript_77724	F01_transcript_77724	F01_transcript_77724	F01_transcript_77724

F01_transcript_99874	NA	NA	NA	F01_transcript_99874
F01_transcript_143308	F01_transcript_143308	F01_transcript_143308	F01_transcript_143308	F01_transcript_143308
F01_transcript_65299	NA	F01_transcript_65299	F01_transcript_65299	F01_transcript_65299
F01_transcript_70827	NA	NA	F01_transcript_70827	NA
F01_transcript_160558	F01_transcript_160558	F01_transcript_160558	F01_transcript_160558	F01_transcript_160558
F01_transcript_9944	NA	F01_transcript_9944	F01_transcript_9944	F01_transcript_9944
F01_transcript_19664	NA	NA	F01_transcript_19664	F01_transcript_19664
F01_transcript_82880	NA	NA	NA	F01_transcript_82880
F01_transcript_83902	NA	F01_transcript_83902	NA	F01_transcript_83902
F01_transcript_93823	NA	F01_transcript_93823	NA	NA
F01_transcript_7110	NA	NA	NA	F01_transcript_7110
F01_transcript_82632	NA	NA	NA	F01_transcript_82632
F01_transcript_116171	NA	NA	F01_transcript_116171	F01_transcript_116171
F01_transcript_83201	NA	F01_transcript_83201	NA	NA

F01_transcript_134596	NA	F01_transcript_134596	NA	F01_transcript_134596
F01_transcript_136721	NA	F01_transcript_136721	NA	F01_transcript_136721
F01_transcript_166625	NA	F01_transcript_166625	NA	F01_transcript_166625
F01_transcript_142413	NA	F01_transcript_142413	NA	NA
F01_transcript_123616	NA	F01_transcript_123616	F01_transcript_123616	F01_transcript_123616
F01_transcript_49969	F01_transcript_49969	NA	NA	NA
F01_transcript_43347	NA	NA	NA	F01_transcript_43347
F01_transcript_73636	NA	F01_transcript_73636	NA	NA
F01_transcript_127388	NA	NA	NA	F01_transcript_127388
F01_transcript_65709	NA	F01_transcript_65709	NA	NA
F01_transcript_67362	NA	F01_transcript_67362	F01_transcript_67362	F01_transcript_67362
F01_transcript_136428	NA	F01_transcript_136428	NA	NA
F01_transcript_139229	NA	NA	NA	F01_transcript_139229
F01_transcript_47256	NA	F01_transcript_47256	NA	F01_transcript_47256

F01_transcript_54851	NA	NA	NA	F01_transcript_54851
F01_transcript_126249	NA	F01_transcript_126249	NA	NA
F01_transcript_104862	F01_transcript_104862	F01_transcript_104862	NA	F01_transcript_104862
F01_transcript_35211	NA	NA	NA	F01_transcript_35211
F01_transcript_82888	NA	F01_transcript_82888	NA	F01_transcript_82888
F01_transcript_96118	NA	F01_transcript_96118	NA	NA
F01_transcript_150556	NA	F01_transcript_150556	F01_transcript_150556	F01_transcript_150556
F01_transcript_90712	F01_transcript_90712	F01_transcript_90712	F01_transcript_90712	F01_transcript_90712
F01_transcript_53198	F01_transcript_53198	F01_transcript_53198	F01_transcript_53198	F01_transcript_53198
F01_transcript_104966	NA	NA	NA	F01_transcript_104966
F01_transcript_39186	NA	NA	NA	F01_transcript_39186
F01_transcript_126380	F01_transcript_126380	F01_transcript_126380	F01_transcript_126380	NA
F01_transcript_41707	NA	NA	NA	F01_transcript_41707
F01_transcript_87283	NA	NA	NA	F01_transcript_87283

F01_transcript_95111	NA	F01_transcript_95111	NA	NA
F01_transcript_90384	NA	NA	NA	F01_transcript_90384
F01_transcript_166608	NA	F01_transcript_166608	F01_transcript_166608	F01_transcript_166608
F01_transcript_61099	NA	NA	NA	F01_transcript_61099
F01_transcript_20489	NA	NA	NA	F01_transcript_20489
F01_transcript_89270	NA	F01_transcript_89270	NA	NA
F01_transcript_126546	NA	F01_transcript_126546	NA	NA
F01_transcript_106584	NA	F01_transcript_106584	F01_transcript_106584	F01_transcript_106584
F01_transcript_130677	F01_transcript_130677	F01_transcript_130677	NA	F01_transcript_130677
F01_transcript_149295	F01_transcript_149295	F01_transcript_149295	F01_transcript_149295	F01_transcript_149295
F01_transcript_2583	NA	F01_transcript_2583	NA	NA
F01_transcript_131843	NA	NA	F01_transcript_131843	F01_transcript_131843
F01_transcript_71245	NA	NA	F01_transcript_71245	NA
F01_transcript_161016	NA	NA	NA	F01_transcript_161016

F01_transcript_158938	F01_transcript_158938	F01_transcript_158938	F01_transcript_158938	F01_transcript_158938
F01_transcript_113080	NA	F01_transcript_113080	F01_transcript_113080	F01_transcript_113080
F01_transcript_51913	NA	NA	NA	F01_transcript_51913
F01_transcript_161061	NA	F01_transcript_161061	NA	NA
F01_transcript_8209	NA	F01_transcript_8209	NA	F01_transcript_8209
F01_transcript_110731	NA	F01_transcript_110731	NA	NA
F01_transcript_133385	NA	NA	NA	F01_transcript_133385
F01_transcript_131916	F01_transcript_131916	F01_transcript_131916	NA	F01_transcript_131916
F01_transcript_161818	NA	F01_transcript_161818	NA	NA
F01_transcript_71697	NA	F01_transcript_71697	NA	F01_transcript_71697
F01_transcript_60498	NA	F01_transcript_60498	NA	NA
F01_transcript_78190	F01_transcript_78190	F01_transcript_78190	NA	NA
F01_transcript_55425	NA	NA	NA	F01_transcript_55425
F01_transcript_166551	NA	F01_transcript_166551	NA	NA

F01_transcript_60021	F01_transcript_60021	F01_transcript_60021	F01_transcript_60021	F01_transcript_60021
F01_transcript_154102	F01_transcript_154102	F01_transcript_154102	F01_transcript_154102	NA
F01_transcript_40619	NA	NA	F01_transcript_40619	F01_transcript_40619
F01_transcript_70600	NA	F01_transcript_70600	F01_transcript_70600	F01_transcript_70600
F01_transcript_69405	NA	NA	NA	F01_transcript_69405
F01_transcript_39684	NA	NA	NA	F01_transcript_39684
F01_transcript_75166	F01_transcript_75166	F01_transcript_75166	NA	F01_transcript_75166
F01_transcript_108182	NA	NA	F01_transcript_108182	NA
F01_transcript_111127	NA	NA	F01_transcript_111127	F01_transcript_111127
F01_transcript_151534	NA	NA	NA	F01_transcript_151534
F01_transcript_163878	NA	NA	NA	F01_transcript_163878
F01_transcript_121394	NA	F01_transcript_121394	NA	NA
F01_transcript_80538	F01_transcript_80538	F01_transcript_80538	NA	NA
F01_transcript_91155	NA	F01_transcript_91155	NA	NA

F01_transcript_154552	F01_transcript_154552	F01_transcript_154552	F01_transcript_154552	F01_transcript_154552
F01_transcript_132102	NA	F01_transcript_132102	F01_transcript_132102	F01_transcript_132102
F01_transcript_152361	NA	F01_transcript_152361	F01_transcript_152361	F01_transcript_152361
F01_transcript_126544	F01_transcript_126544	F01_transcript_126544	F01_transcript_126544	F01_transcript_126544
F01_transcript_57865	F01_transcript_57865	F01_transcript_57865	NA	NA
F01_transcript_137760	NA	NA	NA	F01_transcript_137760
F01_transcript_144424	F01_transcript_144424	F01_transcript_144424	F01_transcript_144424	NA
F01_transcript_129015	F01_transcript_129015	F01_transcript_129015	F01_transcript_129015	F01_transcript_129015
F01_transcript_135308	F01_transcript_135308	F01_transcript_135308	F01_transcript_135308	F01_transcript_135308
F01_transcript_86575	F01_transcript_86575	NA	F01_transcript_86575	F01_transcript_86575
F01_transcript_125528	NA	F01_transcript_125528	NA	NA
F01_transcript_55920	F01_transcript_55920	F01_transcript_55920	F01_transcript_55920	F01_transcript_55920
F01_transcript_115870	NA	F01_transcript_115870	NA	NA
F01_transcript_35760	NA	F01_transcript_35760	NA	NA

F01_transcript_155139	NA	NA	NA	F01_transcript_155139
F01_transcript_82474	NA	NA	NA	F01_transcript_82474
F01_transcript_82977	F01_transcript_82977	F01_transcript_82977	F01_transcript_82977	F01_transcript_82977
F01_transcript_138034	NA	NA	NA	F01_transcript_138034
F01_transcript_108808	NA	NA	NA	F01_transcript_108808
F01_transcript_77819	F01_transcript_77819	F01_transcript_77819	NA	NA
F01_transcript_118405	F01_transcript_118405	F01_transcript_118405	F01_transcript_118405	F01_transcript_118405
F01_transcript_126587	F01_transcript_126587	F01_transcript_126587	F01_transcript_126587	F01_transcript_126587
F01_transcript_152334	NA	NA	F01_transcript_152334	F01_transcript_152334
F01_transcript_163214	NA	F01_transcript_163214	NA	F01_transcript_163214
F01_transcript_75039	NA	NA	NA	F01_transcript_75039
F01_transcript_140405	NA	NA	NA	F01_transcript_140405
F01_transcript_152995	NA	F01_transcript_152995	NA	NA
F01_transcript_85786	NA	NA	NA	F01_transcript_85786

F01_transcript_145536	NA	NA	NA	F01_transcript_145536
F01_transcript_120026	F01_transcript_120026	F01_transcript_120026	F01_transcript_120026	F01_transcript_120026
F01_transcript_4207	NA	NA	NA	F01_transcript_4207
F01_transcript_88277	F01_transcript_88277	F01_transcript_88277	NA	NA
F01_transcript_159659	NA	F01_transcript_159659	NA	F01_transcript_159659
F01_transcript_129016	NA	F01_transcript_129016	F01_transcript_129016	F01_transcript_129016
F01_transcript_135554	F01_transcript_135554	NA	NA	NA
F01_transcript_46261	NA	NA	NA	F01_transcript_46261
F01_transcript_150838	F01_transcript_150838	F01_transcript_150838	F01_transcript_150838	F01_transcript_150838
F01_transcript_76965	F01_transcript_76965	F01_transcript_76965	NA	NA
F01_transcript_57905	NA	NA	NA	F01_transcript_57905
F01_transcript_126157	F01_transcript_126157	F01_transcript_126157	NA	NA
F01_transcript_39098	NA	NA	NA	F01_transcript_39098
F01_transcript_68255	F01_transcript_68255	F01_transcript_68255	NA	NA

F01_transcript_147781	F01_transcript_147781	F01_transcript_147781	F01_transcript_147781	F01_transcript_147781
F01_transcript_130454	NA	F01_transcript_130454	F01_transcript_130454	NA
F01_transcript_143839	F01_transcript_143839	F01_transcript_143839	F01_transcript_143839	F01_transcript_143839
F01_transcript_56128	NA	NA	NA	F01_transcript_56128
F01_transcript_100137	NA	NA	NA	F01_transcript_100137
F01_transcript_53344	NA	NA	NA	F01_transcript_53344
F01_transcript_72380	NA	F01_transcript_72380	NA	NA
F01_transcript_65551	NA	NA	NA	F01_transcript_65551
F01_transcript_141736	NA	F01_transcript_141736	NA	NA
F01_transcript_45680	F01_transcript_45680	NA	NA	F01_transcript_45680
F01_transcript_78129	F01_transcript_78129	F01_transcript_78129	NA	NA
F01_transcript_69579	F01_transcript_69579	F01_transcript_69579	F01_transcript_69579	F01_transcript_69579
F01_transcript_162145	NA	NA	NA	F01_transcript_162145
F01_transcript_164449	NA	F01_transcript_164449	NA	NA

F01_transcript_37008	NA	NA	NA	F01_transcript_37008
F01_transcript_112914	NA	NA	NA	F01_transcript_112914
F01_transcript_83267	F01_transcript_83267	F01_transcript_83267	F01_transcript_83267	F01_transcript_83267
F01_transcript_93247	F01_transcript_93247	F01_transcript_93247	F01_transcript_93247	F01_transcript_93247
F01_transcript_105979	NA	NA	NA	F01_transcript_105979
F01_transcript_80195	NA	NA	NA	F01_transcript_80195
F01_transcript_99433	F01_transcript_99433	F01_transcript_99433	F01_transcript_99433	F01_transcript_99433
F01_transcript_91533	NA	F01_transcript_91533	NA	NA
F01_transcript_114471	F01_transcript_114471	F01_transcript_114471	F01_transcript_114471	F01_transcript_114471
F01_transcript_123654	F01_transcript_123654	F01_transcript_123654	NA	F01_transcript_123654
F01_transcript_88791	NA	NA	NA	F01_transcript_88791
F01_transcript_108329	NA	F01_transcript_108329	NA	NA
F01_transcript_81755	NA	NA	NA	F01_transcript_81755
F01_transcript_155656	NA	F01_transcript_155656	NA	NA

F01_transcript_75207	NA	F01_transcript_75207	NA	F01_transcript_75207
F01_transcript_155499	F01_transcript_155499	F01_transcript_155499	F01_transcript_155499	F01_transcript_155499
F01_transcript_128485	NA	NA	NA	F01_transcript_128485
F01_transcript_87972	NA	NA	NA	F01_transcript_87972
F01_transcript_62071	NA	NA	NA	F01_transcript_62071
F01_transcript_28914	NA	NA	F01_transcript_28914	F01_transcript_28914
F01_transcript_124622	F01_transcript_124622	NA	F01_transcript_124622	F01_transcript_124622
F01_transcript_96351	NA	F01_transcript_96351	F01_transcript_96351	F01_transcript_96351
F01_transcript_54463	F01_transcript_54463	F01_transcript_54463	NA	NA
F01_transcript_50485	F01_transcript_50485	F01_transcript_50485	F01_transcript_50485	F01_transcript_50485
F01_transcript_159083	NA	F01_transcript_159083	F01_transcript_159083	NA
F01_transcript_155992	NA	NA	NA	F01_transcript_155992
F01_transcript_155221	NA	NA	NA	F01_transcript_155221
F01_transcript_48832	NA	F01_transcript_48832	NA	F01_transcript_48832

F01_transcript_284	F01_transcript_284	F01_transcript_284	F01_transcript_284	F01_transcript_284
F01_transcript_157935	NA	NA	NA	F01_transcript_157935
F01_transcript_154240	NA	F01_transcript_154240	NA	F01_transcript_154240
F01_transcript_160186	NA	NA	NA	F01_transcript_160186
F01_transcript_49175	NA	F01_transcript_49175	NA	F01_transcript_49175
F01_transcript_87521	F01_transcript_87521	F01_transcript_87521	F01_transcript_87521	F01_transcript_87521
F01_transcript_54058	NA	NA	NA	F01_transcript_54058
F01_transcript_134174	F01_transcript_134174	F01_transcript_134174	F01_transcript_134174	F01_transcript_134174
F01_transcript_80952	NA	F01_transcript_80952	NA	NA
F01_transcript_161372	NA	F01_transcript_161372	NA	F01_transcript_161372
F01_transcript_56442	NA	NA	F01_transcript_56442	NA
F01_transcript_56864	NA	F01_transcript_56864	F01_transcript_56864	F01_transcript_56864
F01_transcript_114989	NA	NA	NA	F01_transcript_114989
F01_transcript_43421	NA	F01_transcript_43421	NA	NA

F01_transcript_40547	NA	NA	NA	F01_transcript_40547
F01_transcript_166799	NA	NA	NA	F01_transcript_166799
F01_transcript_153053	NA	F01_transcript_153053	NA	NA
F01_transcript_125632	NA	F01_transcript_125632	NA	NA
F01_transcript_51366	F01_transcript_51366	F01_transcript_51366	F01_transcript_51366	F01_transcript_51366
F01_transcript_62589	F01_transcript_62589	F01_transcript_62589	F01_transcript_62589	NA
F01_transcript_49408	NA	NA	NA	F01_transcript_49408
F01_transcript_49470	NA	NA	F01_transcript_49470	F01_transcript_49470
F01_transcript_66250	NA	F01_transcript_66250	NA	NA
F01_transcript_53395	NA	NA	NA	F01_transcript_53395
F01_transcript_107237	NA	NA	NA	F01_transcript_107237
F01_transcript_87756	NA	NA	NA	F01_transcript_87756
F01_transcript_99230	NA	F01_transcript_99230	F01_transcript_99230	NA
F01_transcript_45713	NA	NA	NA	F01_transcript_45713

F01_transcript_66965	NA	F01_transcript_66965	NA	F01_transcript_66965
F01_transcript_155184	F01_transcript_155184	F01_transcript_155184	F01_transcript_155184	NA
F01_transcript_131089	NA	F01_transcript_131089	NA	NA
F01_transcript_62900	NA	F01_transcript_62900	NA	NA
F01_transcript_20444	NA	F01_transcript_20444	NA	NA
F01_transcript_2267	NA	NA	NA	F01_transcript_2267
F01_transcript_123293	NA	F01_transcript_123293	F01_transcript_123293	NA
F01_transcript_43064	NA	NA	NA	F01_transcript_43064
F01_transcript_136578	NA	NA	NA	F01_transcript_136578
F01_transcript_16884	NA	NA	NA	F01_transcript_16884
F01_transcript_48303	F01_transcript_48303	F01_transcript_48303	NA	NA
F01_transcript_159758	NA	NA	NA	F01_transcript_159758
F01_transcript_17469	NA	NA	NA	F01_transcript_17469
F01_transcript_64188	NA	F01_transcript_64188	NA	NA

F01_transcript_42165	NA	F01_transcript_42165	NA	NA
F01_transcript_166635	NA	NA	NA	F01_transcript_166635
F01_transcript_127023	NA	NA	NA	F01_transcript_127023
F01_transcript_166899	NA	F01_transcript_166899	NA	NA
F01_transcript_81914	F01_transcript_81914	F01_transcript_81914	F01_transcript_81914	F01_transcript_81914
F01_transcript_48852	NA	F01_transcript_48852	NA	NA
F01_transcript_158505	NA	F01_transcript_158505	F01_transcript_158505	F01_transcript_158505
F01_transcript_70233	F01_transcript_70233	F01_transcript_70233	F01_transcript_70233	F01_transcript_70233
F01_transcript_101700	NA	NA	NA	F01_transcript_101700
F01_transcript_22965	NA	NA	F01_transcript_22965	F01_transcript_22965
F01_transcript_103728	NA	F01_transcript_103728	NA	NA
F01_transcript_111380	NA	NA	NA	F01_transcript_111380
F01_transcript_162346	NA	NA	NA	F01_transcript_162346
F01_transcript_5329	NA	NA	NA	F01_transcript_5329

F01_transcript_93957	F01_transcript_93957	F01_transcript_93957	NA	F01_transcript_93957
F01_transcript_55189	NA	F01_transcript_55189	NA	F01_transcript_55189
F01_transcript_115480	F01_transcript_115480	F01_transcript_115480	F01_transcript_115480	F01_transcript_115480
F01_transcript_111695	F01_transcript_111695	F01_transcript_111695	F01_transcript_111695	F01_transcript_111695
F01_transcript_10375	NA	NA	NA	F01_transcript_10375
F01_transcript_107471	F01_transcript_107471	F01_transcript_107471	F01_transcript_107471	F01_transcript_107471
F01_transcript_108159	F01_transcript_108159	F01_transcript_108159	F01_transcript_108159	F01_transcript_108159
F01_transcript_157152	F01_transcript_157152	F01_transcript_157152	F01_transcript_157152	F01_transcript_157152
F01_transcript_118299	NA	F01_transcript_118299	NA	NA
F01_transcript_49605	NA	F01_transcript_49605	NA	NA
F01_transcript_58127	NA	NA	F01_transcript_58127	F01_transcript_58127
F01_transcript_150443	NA	F01_transcript_150443	NA	F01_transcript_150443
F01_transcript_54817	NA	F01_transcript_54817	NA	NA
F01_transcript_104471	F01_transcript_104471	F01_transcript_104471	F01_transcript_104471	F01_transcript_104471

F01_transcript_58364	NA	NA	NA	F01_transcript_58364
F01_transcript_53297	NA	NA	NA	F01_transcript_53297
F01_transcript_6057	NA	NA	NA	F01_transcript_6057
F01_transcript_6694	NA	F01_transcript_6694	NA	F01_transcript_6694
F01_transcript_140001	F01_transcript_140001	F01_transcript_140001	NA	NA
F01_transcript_34986	F01_transcript_34986	F01_transcript_34986	F01_transcript_34986	F01_transcript_34986
F01_transcript_131999	NA	NA	NA	F01_transcript_131999
F01_transcript_148170	NA	F01_transcript_148170	NA	NA
F01_transcript_26524	NA	F01_transcript_26524	NA	F01_transcript_26524
F01_transcript_75899	NA	F01_transcript_75899	NA	NA
F01_transcript_11812	NA	NA	NA	F01_transcript_11812
F01_transcript_89948	NA	F01_transcript_89948	NA	NA
F01_transcript_85694	NA	NA	NA	F01_transcript_85694
F01_transcript_71954	NA	F01_transcript_71954	NA	NA

F01_transcript_86968	NA	F01_transcript_86968	NA	NA
F01_transcript_155090	NA	F01_transcript_155090	F01_transcript_155090	NA
F01_transcript_114317	NA	F01_transcript_114317	NA	NA
F01_transcript_75140	NA	NA	NA	F01_transcript_75140
F01_transcript_109135	F01_transcript_109135	F01_transcript_109135	F01_transcript_109135	F01_transcript_109135
F01_transcript_103231	NA	NA	NA	F01_transcript_103231
F01_transcript_60977	NA	F01_transcript_60977	NA	NA
F01_transcript_60548	NA	F01_transcript_60548	NA	NA
F01_transcript_144334	NA	F01_transcript_144334	NA	NA
F01_transcript_124192	NA	F01_transcript_124192	NA	F01_transcript_124192
F01_transcript_162691	F01_transcript_162691	F01_transcript_162691	F01_transcript_162691	F01_transcript_162691
F01_transcript_132350	NA	NA	NA	F01_transcript_132350
F01_transcript_158513	F01_transcript_158513	F01_transcript_158513	NA	NA
F01_transcript_87579	NA	F01_transcript_87579	NA	F01_transcript_87579

F01_transcript_119466	NA	NA	NA	F01_transcript_119466
F01_transcript_14033	NA	NA	NA	F01_transcript_14033
F01_transcript_107371	F01_transcript_107371	F01_transcript_107371	F01_transcript_107371	F01_transcript_107371
F01_transcript_59699	NA	NA	NA	F01_transcript_59699
F01_transcript_91183	NA	F01_transcript_91183	NA	NA
F01_transcript_21253	NA	NA	NA	F01_transcript_21253
F01_transcript_35118	NA	NA	NA	F01_transcript_35118
F01_transcript_74366	NA	F01_transcript_74366	NA	NA
F01_transcript_119526	F01_transcript_119526	F01_transcript_119526	F01_transcript_119526	NA
F01_transcript_104720	F01_transcript_104720	F01_transcript_104720	F01_transcript_104720	F01_transcript_104720
F01_transcript_132823	NA	F01_transcript_132823	NA	F01_transcript_132823
F01_transcript_56881	NA	F01_transcript_56881	F01_transcript_56881	NA
F01_transcript_111302	NA	F01_transcript_111302	F01_transcript_111302	F01_transcript_111302
F01_transcript_92142	NA	F01_transcript_92142	F01_transcript_92142	F01_transcript_92142

F01_transcript_167409	F01_transcript_167409	F01_transcript_167409	NA	F01_transcript_167409
F01_transcript_28372	NA	NA	NA	F01_transcript_28372
F01_transcript_51019	F01_transcript_51019	F01_transcript_51019	F01_transcript_51019	F01_transcript_51019
F01_transcript_101814	NA	F01_transcript_101814	NA	F01_transcript_101814
F01_transcript_111143	NA	F01_transcript_111143	NA	NA
F01_transcript_140432	NA	NA	NA	F01_transcript_140432
F01_transcript_48530	NA	NA	NA	F01_transcript_48530
F01_transcript_150479	F01_transcript_150479	F01_transcript_150479	F01_transcript_150479	NA
F01_transcript_124962	NA	NA	NA	F01_transcript_124962
F01_transcript_149077	F01_transcript_149077	F01_transcript_149077	F01_transcript_149077	F01_transcript_149077
F01_transcript_61802	NA	F01_transcript_61802	NA	F01_transcript_61802
F01_transcript_51033	F01_transcript_51033	F01_transcript_51033	F01_transcript_51033	F01_transcript_51033
F01_transcript_115981	F01_transcript_115981	F01_transcript_115981	F01_transcript_115981	F01_transcript_115981
F01_transcript_117991	F01_transcript_117991	F01_transcript_117991	NA	NA

F01_transcript_77790	NA	NA	NA	F01_transcript_77790
F01_transcript_75673	NA	F01_transcript_75673	NA	NA
F01_transcript_139544	NA	F01_transcript_139544	NA	NA
F01_transcript_40626	NA	NA	NA	F01_transcript_40626
F01_transcript_119820	F01_transcript_119820	F01_transcript_119820	NA	F01_transcript_119820
F01_transcript_24679	NA	NA	NA	F01_transcript_24679
F01_transcript_77516	NA	NA	NA	F01_transcript_77516
F01_transcript_153496	NA	NA	NA	F01_transcript_153496
F01_transcript_113172	NA	F01_transcript_113172	NA	NA
F01_transcript_49575	F01_transcript_49575	F01_transcript_49575	F01_transcript_49575	F01_transcript_49575
F01_transcript_88269	F01_transcript_88269	F01_transcript_88269	F01_transcript_88269	F01_transcript_88269
F01_transcript_16504	NA	NA	NA	F01_transcript_16504
F01_transcript_144029	F01_transcript_144029	F01_transcript_144029	F01_transcript_144029	F01_transcript_144029
F01_transcript_148639	NA	F01_transcript_148639	NA	F01_transcript_148639

F01_transcript_59574	NA	NA	NA	F01_transcript_59574
F01_transcript_44545	NA	NA	NA	F01_transcript_44545
F01_transcript_86591	NA	F01_transcript_86591	NA	NA
F01_transcript_128129	F01_transcript_128129	NA	NA	NA
F01_transcript_136186	F01_transcript_136186	F01_transcript_136186	NA	F01_transcript_136186
F01_transcript_112742	NA	F01_transcript_112742	NA	NA
F01_transcript_72173	NA	NA	F01_transcript_72173	F01_transcript_72173
F01_transcript_75047	F01_transcript_75047	F01_transcript_75047	F01_transcript_75047	F01_transcript_75047
F01_transcript_126299	F01_transcript_126299	F01_transcript_126299	F01_transcript_126299	F01_transcript_126299
F01_transcript_105343	NA	F01_transcript_105343	NA	NA
F01_transcript_54486	F01_transcript_54486	F01_transcript_54486	NA	F01_transcript_54486
F01_transcript_167139	F01_transcript_167139	F01_transcript_167139	F01_transcript_167139	F01_transcript_167139
F01_transcript_98645	NA	F01_transcript_98645	NA	NA
F01_transcript_94102	NA	NA	NA	F01_transcript_94102

F01_transcript_166157	F01_transcript_166157	F01_transcript_166157	F01_transcript_166157	NA
F01_transcript_66788	NA	NA	F01_transcript_66788	F01_transcript_66788
F01_transcript_42144	NA	NA	NA	F01_transcript_42144
F01_transcript_162953	NA	NA	NA	F01_transcript_162953
F01_transcript_82654	NA	NA	NA	F01_transcript_82654
F01_transcript_98190	NA	NA	NA	F01_transcript_98190
F01_transcript_163787	NA	F01_transcript_163787	NA	NA
F01_transcript_125304	NA	NA	NA	F01_transcript_125304
F01_transcript_93677	F01_transcript_93677	F01_transcript_93677	F01_transcript_93677	F01_transcript_93677
F01_transcript_65220	NA	NA	F01_transcript_65220	F01_transcript_65220
F01_transcript_87606	NA	F01_transcript_87606	NA	F01_transcript_87606
F01_transcript_108997	F01_transcript_108997	F01_transcript_108997	F01_transcript_108997	NA
F01_transcript_94773	NA	NA	NA	F01_transcript_94773
F01_transcript_147410	NA	NA	NA	F01_transcript_147410

F01_transcript_97703	NA	F01_transcript_97703	NA	NA
F01_transcript_140466	F01_transcript_140466	F01_transcript_140466	NA	NA
F01_transcript_118143	NA	F01_transcript_118143	NA	F01_transcript_118143
F01_transcript_164555	NA	NA	NA	F01_transcript_164555
F01_transcript_162667	F01_transcript_162667	F01_transcript_162667	NA	NA
F01_transcript_152243	F01_transcript_152243	NA	F01_transcript_152243	F01_transcript_152243
F01_transcript_79902	NA	NA	NA	F01_transcript_79902
F01_transcript_96670	NA	F01_transcript_96670	NA	NA
F01_transcript_94050	F01_transcript_94050	F01_transcript_94050	F01_transcript_94050	F01_transcript_94050
F01_transcript_54125	NA	F01_transcript_54125	NA	NA
F01_transcript_117749	NA	F01_transcript_117749	NA	NA
F01_transcript_104198	NA	F01_transcript_104198	NA	NA
F01_transcript_163069	NA	NA	NA	F01_transcript_163069
F01_transcript_5257	NA	NA	NA	F01_transcript_5257

F01_transcript_130985	NA	NA	NA	F01_transcript_130985
F01_transcript_65798	NA	NA	NA	F01_transcript_65798
F01_transcript_61524	F01_transcript_61524	F01_transcript_61524	F01_transcript_61524	F01_transcript_61524
F01_transcript_16598	NA	NA	NA	F01_transcript_16598
F01_transcript_138590	NA	F01_transcript_138590	F01_transcript_138590	F01_transcript_138590
F01_transcript_167061	NA	NA	NA	F01_transcript_167061
F01_transcript_118213	F01_transcript_118213	F01_transcript_118213	F01_transcript_118213	F01_transcript_118213
F01_transcript_163104	NA	F01_transcript_163104	NA	NA
F01_transcript_57754	NA	F01_transcript_57754	NA	NA
F01_transcript_44775	NA	NA	NA	F01_transcript_44775
F01_transcript_124491	F01_transcript_124491	F01_transcript_124491	NA	NA
F01_transcript_110909	NA	F01_transcript_110909	NA	NA
F01_transcript_160488	F01_transcript_160488	F01_transcript_160488	F01_transcript_160488	F01_transcript_160488
F01_transcript_37931	NA	NA	NA	F01_transcript_37931

F01_transcript_85766	F01_transcript_85766	F01_transcript_85766	F01_transcript_85766	F01_transcript_85766
F01_transcript_164286	NA	F01_transcript_164286	NA	NA
F01_transcript_36848	NA	NA	NA	F01_transcript_36848
F01_transcript_28604	NA	NA	NA	F01_transcript_28604
F01_transcript_72367	NA	NA	NA	F01_transcript_72367
F01_transcript_125701	NA	F01_transcript_125701	NA	F01_transcript_125701
F01_transcript_76966	NA	NA	F01_transcript_76966	F01_transcript_76966
F01_transcript_166746	F01_transcript_166746	F01_transcript_166746	NA	F01_transcript_166746
F01_transcript_108452	F01_transcript_108452	F01_transcript_108452	F01_transcript_108452	F01_transcript_108452
F01_transcript_94260	F01_transcript_94260	F01_transcript_94260	NA	NA
F01_transcript_14410	NA	NA	NA	F01_transcript_14410
F01_transcript_36945	NA	NA	NA	F01_transcript_36945
F01_transcript_92451	NA	F01_transcript_92451	F01_transcript_92451	F01_transcript_92451
F01_transcript_57927	NA	NA	NA	F01_transcript_57927

F01_transcript_2156	NA	F01_transcript_2156	NA	NA
F01_transcript_90669	NA	NA	NA	F01_transcript_90669
F01_transcript_142538	F01_transcript_142538	F01_transcript_142538	NA	F01_transcript_142538
F01_transcript_96124	F01_transcript_96124	F01_transcript_96124	NA	F01_transcript_96124
F01_transcript_87214	NA	F01_transcript_87214	NA	NA
F01_transcript_135571	NA	F01_transcript_135571	NA	NA
F01_transcript_118287	NA	NA	NA	F01_transcript_118287
F01_transcript_64423	NA	NA	NA	F01_transcript_64423
F01_transcript_143250	F01_transcript_143250	F01_transcript_143250	NA	F01_transcript_143250
F01_transcript_113842	NA	F01_transcript_113842	NA	NA
F01_transcript_104531	NA	NA	NA	F01_transcript_104531
F01_transcript_89488	NA	F01_transcript_89488	NA	F01_transcript_89488
F01_transcript_149365	F01_transcript_149365	F01_transcript_149365	F01_transcript_149365	F01_transcript_149365
F01_transcript_128934	NA	F01_transcript_128934	NA	NA

F01_transcript_148330	F01_transcript_148330	F01_transcript_148330	NA	F01_transcript_148330
F01_transcript_78830	NA	F01_transcript_78830	F01_transcript_78830	F01_transcript_78830
F01_transcript_138799	NA	NA	NA	F01_transcript_138799
F01_transcript_87873	F01_transcript_87873	F01_transcript_87873	F01_transcript_87873	NA
F01_transcript_88115	F01_transcript_88115	F01_transcript_88115	F01_transcript_88115	F01_transcript_88115
F01_transcript_117390	F01_transcript_117390	F01_transcript_117390	F01_transcript_117390	F01_transcript_117390
F01_transcript_158077	NA	NA	NA	F01_transcript_158077
F01_transcript_14376	F01_transcript_14376	F01_transcript_14376	F01_transcript_14376	F01_transcript_14376
F01_transcript_88223	NA	NA	NA	F01_transcript_88223
F01_transcript_80127	NA	F01_transcript_80127	NA	NA
F01_transcript_102730	F01_transcript_102730	F01_transcript_102730	F01_transcript_102730	F01_transcript_102730
F01_transcript_72149	NA	F01_transcript_72149	NA	F01_transcript_72149
F01_transcript_77018	F01_transcript_77018	F01_transcript_77018	F01_transcript_77018	F01_transcript_77018
F01_transcript_101589	F01_transcript_101589	F01_transcript_101589	NA	F01_transcript_101589

F01_transcript_127685	F01_transcript_127685	F01_transcript_127685	NA	NA
F01_transcript_113090	NA	F01_transcript_113090	NA	F01_transcript_113090
F01_transcript_116318	F01_transcript_116318	F01_transcript_116318	NA	F01_transcript_116318
F01_transcript_136661	NA	NA	NA	F01_transcript_136661
F01_transcript_164229	NA	NA	NA	F01_transcript_164229
F01_transcript_152167	F01_transcript_152167	F01_transcript_152167	F01_transcript_152167	NA
F01_transcript_51945	NA	F01_transcript_51945	NA	NA
F01_transcript_98945	NA	NA	NA	F01_transcript_98945
F01_transcript_106619	NA	NA	NA	F01_transcript_106619
F01_transcript_126738	NA	F01_transcript_126738	NA	NA
F01_transcript_162433	NA	F01_transcript_162433	NA	NA
F01_transcript_78465	NA	NA	NA	F01_transcript_78465
F01_transcript_84723	NA	F01_transcript_84723	NA	NA
F01_transcript_27935	NA	F01_transcript_27935	F01_transcript_27935	NA

F01_transcript_142248	NA	NA	NA	F01_transcript_142248
F01_transcript_143294	NA	NA	F01_transcript_143294	NA
F01_transcript_158522	NA	NA	NA	F01_transcript_158522
F01_transcript_158677	NA	NA	NA	F01_transcript_158677
F01_transcript_163237	F01_transcript_163237	F01_transcript_163237	F01_transcript_163237	F01_transcript_163237
F01_transcript_47910	NA	NA	NA	F01_transcript_47910
F01_transcript_160133	F01_transcript_160133	NA	F01_transcript_160133	NA
F01_transcript_60034	NA	F01_transcript_60034	NA	NA
F01_transcript_77777	F01_transcript_77777	F01_transcript_77777	NA	NA
F01_transcript_141844	NA	F01_transcript_141844	F01_transcript_141844	F01_transcript_141844
F01_transcript_135968	F01_transcript_135968	F01_transcript_135968	F01_transcript_135968	F01_transcript_135968
F01_transcript_49264	NA	F01_transcript_49264	NA	NA
F01_transcript_155501	NA	F01_transcript_155501	NA	NA
F01_transcript_14764	F01_transcript_14764	F01_transcript_14764	F01_transcript_14764	F01_transcript_14764

F01_transcript_50611	NA	F01_transcript_50611	F01_transcript_50611	NA
F01_transcript_79467	F01_transcript_79467	F01_transcript_79467	F01_transcript_79467	F01_transcript_79467
F01_transcript_162184	NA	F01_transcript_162184	NA	F01_transcript_162184
F01_transcript_147117	F01_transcript_147117	F01_transcript_147117	F01_transcript_147117	F01_transcript_147117
F01_transcript_6731	NA	NA	NA	F01_transcript_6731
F01_transcript_108431	F01_transcript_108431	F01_transcript_108431	F01_transcript_108431	NA
F01_transcript_121087	F01_transcript_121087	F01_transcript_121087	F01_transcript_121087	F01_transcript_121087
F01_transcript_167158	F01_transcript_167158	F01_transcript_167158	NA	F01_transcript_167158
F01_transcript_52025	NA	F01_transcript_52025	NA	NA
F01_transcript_58195	F01_transcript_58195	F01_transcript_58195	F01_transcript_58195	NA
F01_transcript_28661	NA	NA	NA	F01_transcript_28661
F01_transcript_75081	NA	F01_transcript_75081	NA	NA
F01_transcript_50156	F01_transcript_50156	NA	NA	F01_transcript_50156
F01_transcript_103391	NA	NA	NA	F01_transcript_103391

F01_transcript_74345	NA	NA	NA	F01_transcript_74345
F01_transcript_66342	NA	F01_transcript_66342	NA	NA
F01_transcript_157174	NA	NA	NA	F01_transcript_157174
F01_transcript_54261	NA	F01_transcript_54261	NA	NA
F01_transcript_107464	F01_transcript_107464	F01_transcript_107464	F01_transcript_107464	F01_transcript_107464
F01_transcript_10307	NA	F01_transcript_10307	NA	F01_transcript_10307
F01_transcript_71031	NA	F01_transcript_71031	F01_transcript_71031	F01_transcript_71031
F01_transcript_101652	NA	NA	NA	F01_transcript_101652
F01_transcript_115453	NA	F01_transcript_115453	NA	NA
F01_transcript_102061	NA	F01_transcript_102061	NA	NA
F01_transcript_129345	F01_transcript_129345	F01_transcript_129345	F01_transcript_129345	F01_transcript_129345
F01_transcript_99942	NA	F01_transcript_99942	F01_transcript_99942	F01_transcript_99942
F01_transcript_52756	NA	NA	NA	F01_transcript_52756
F01_transcript_89635	NA	F01_transcript_89635	NA	NA

F01_transcript_66846	F01_transcript_66846	NA	NA	NA
F01_transcript_93760	NA	F01_transcript_93760	F01_transcript_93760	F01_transcript_93760
F01_transcript_132221	F01_transcript_132221	F01_transcript_132221	NA	NA
F01_transcript_38709	NA	NA	NA	F01_transcript_38709
F01_transcript_148257	F01_transcript_148257	F01_transcript_148257	F01_transcript_148257	F01_transcript_148257
F01_transcript_128792	F01_transcript_128792	F01_transcript_128792	NA	NA
F01_transcript_112817	NA	F01_transcript_112817	NA	NA
F01_transcript_121074	NA	NA	NA	F01_transcript_121074
F01_transcript_115584	F01_transcript_115584	F01_transcript_115584	NA	NA
F01_transcript_45558	NA	NA	NA	F01_transcript_45558
F01_transcript_47742	NA	F01_transcript_47742	NA	F01_transcript_47742
F01_transcript_38227	NA	NA	NA	F01_transcript_38227
F01_transcript_159265	NA	NA	F01_transcript_159265	NA
F01_transcript_90640	NA	F01_transcript_90640	NA	NA

F01_transcript_59670	NA	F01_transcript_59670	NA	F01_transcript_59670
F01_transcript_159444	NA	NA	NA	F01_transcript_159444
F01_transcript_104771	F01_transcript_104771	F01_transcript_104771	NA	F01_transcript_104771
F01_transcript_124802	NA	NA	NA	F01_transcript_124802
F01_transcript_105406	F01_transcript_105406	F01_transcript_105406	F01_transcript_105406	F01_transcript_105406
F01_transcript_93189	NA	F01_transcript_93189	NA	NA
F01_transcript_162513	F01_transcript_162513	F01_transcript_162513	F01_transcript_162513	F01_transcript_162513
F01_transcript_70164	F01_transcript_70164	F01_transcript_70164	F01_transcript_70164	F01_transcript_70164
F01_transcript_18641	NA	NA	NA	F01_transcript_18641
F01_transcript_124327	NA	F01_transcript_124327	NA	NA
F01_transcript_118205	F01_transcript_118205	F01_transcript_118205	NA	NA
F01_transcript_124236	NA	F01_transcript_124236	NA	F01_transcript_124236
F01_transcript_69510	NA	NA	F01_transcript_69510	F01_transcript_69510
F01_transcript_133723	NA	NA	F01_transcript_133723	F01_transcript_133723

F01_transcript_115152	NA	F01_transcript_115152	F01_transcript_115152	NA
F01_transcript_147516	F01_transcript_147516	F01_transcript_147516	F01_transcript_147516	F01_transcript_147516
F01_transcript_73865	NA	F01_transcript_73865	NA	F01_transcript_73865
F01_transcript_155160	NA	F01_transcript_155160	NA	NA
F01_transcript_111294	F01_transcript_111294	F01_transcript_111294	F01_transcript_111294	F01_transcript_111294
F01_transcript_129792	F01_transcript_129792	F01_transcript_129792	NA	F01_transcript_129792
F01_transcript_94553	NA	F01_transcript_94553	NA	NA
F01_transcript_90076	NA	NA	NA	F01_transcript_90076
F01_transcript_150586	F01_transcript_150586	F01_transcript_150586	F01_transcript_150586	F01_transcript_150586
F01_transcript_109723	F01_transcript_109723	F01_transcript_109723	F01_transcript_109723	F01_transcript_109723
F01_transcript_26207	NA	F01_transcript_26207	NA	NA
F01_transcript_133444	NA	NA	NA	F01_transcript_133444
F01_transcript_38834	NA	NA	NA	F01_transcript_38834
F01_transcript_59584	NA	NA	F01_transcript_59584	NA

F01_transcript_114495	NA	NA	NA	F01_transcript_114495
F01_transcript_129443	NA	F01_transcript_129443	NA	NA
F01_transcript_81850	F01_transcript_81850	F01_transcript_81850	F01_transcript_81850	F01_transcript_81850
F01_transcript_130645	F01_transcript_130645	F01_transcript_130645	F01_transcript_130645	F01_transcript_130645
F01_transcript_122033	NA	F01_transcript_122033	NA	NA
F01_transcript_137253	NA	NA	NA	F01_transcript_137253
F01_transcript_131322	NA	NA	NA	F01_transcript_131322
F01_transcript_29156	NA	NA	NA	F01_transcript_29156
F01_transcript_71814	F01_transcript_71814	F01_transcript_71814	F01_transcript_71814	F01_transcript_71814
F01_transcript_10925	F01_transcript_10925	F01_transcript_10925	NA	F01_transcript_10925
F01_transcript_151003	NA	NA	NA	F01_transcript_151003
F01_transcript_82762	NA	NA	NA	F01_transcript_82762
F01_transcript_68064	NA	NA	NA	F01_transcript_68064
F01_transcript_138132	NA	F01_transcript_138132	NA	F01_transcript_138132

F01_transcript_64013	NA	NA	NA	F01_transcript_64013
F01_transcript_128626	NA	NA	NA	F01_transcript_128626
F01_transcript_84767	F01_transcript_84767	F01_transcript_84767	F01_transcript_84767	F01_transcript_84767
F01_transcript_46960	NA	NA	NA	F01_transcript_46960
F01_transcript_26619	F01_transcript_26619	F01_transcript_26619	F01_transcript_26619	F01_transcript_26619
F01_transcript_134418	F01_transcript_134418	F01_transcript_134418	F01_transcript_134418	F01_transcript_134418
F01_transcript_164661	F01_transcript_164661	F01_transcript_164661	F01_transcript_164661	F01_transcript_164661
F01_transcript_97746	F01_transcript_97746	F01_transcript_97746	NA	NA
F01_transcript_41664	NA	NA	NA	F01_transcript_41664
F01_transcript_98813	NA	F01_transcript_98813	NA	F01_transcript_98813
F01_transcript_130724	F01_transcript_130724	F01_transcript_130724	F01_transcript_130724	NA
F01_transcript_111286	NA	NA	NA	F01_transcript_111286
F01_transcript_112247	NA	F01_transcript_112247	NA	NA
F01_transcript_65872	NA	F01_transcript_65872	NA	F01_transcript_65872

F01_transcript_10517	NA	NA	NA	F01_transcript_10517
F01_transcript_23053	F01_transcript_23053	F01_transcript_23053	F01_transcript_23053	NA
F01_transcript_113622	NA	NA	NA	F01_transcript_113622
F01_transcript_156923	F01_transcript_156923	F01_transcript_156923	F01_transcript_156923	F01_transcript_156923
F01_transcript_81566	NA	F01_transcript_81566	F01_transcript_81566	F01_transcript_81566
F01_transcript_75443	F01_transcript_75443	F01_transcript_75443	NA	F01_transcript_75443
F01_transcript_999	F01_transcript_999	F01_transcript_999	F01_transcript_999	F01_transcript_999
F01_transcript_7400	F01_transcript_7400	F01_transcript_7400	F01_transcript_7400	F01_transcript_7400
F01_transcript_135289	NA	F01_transcript_135289	NA	NA
F01_transcript_96072	NA	NA	NA	F01_transcript_96072
F01_transcript_122520	NA	F01_transcript_122520	NA	NA
F01_transcript_32809	NA	NA	NA	F01_transcript_32809
F01_transcript_90333	F01_transcript_90333	F01_transcript_90333	F01_transcript_90333	F01_transcript_90333
F01_transcript_118914	F01_transcript_118914	F01_transcript_118914	F01_transcript_118914	NA

F01_transcript_114384	NA	F01_transcript_114384	NA	NA
F01_transcript_53452	F01_transcript_53452	F01_transcript_53452	F01_transcript_53452	F01_transcript_53452
F01_transcript_96301	NA	F01_transcript_96301	NA	NA
F01_transcript_108124	NA	NA	NA	F01_transcript_108124
F01_transcript_165300	F01_transcript_165300	F01_transcript_165300	F01_transcript_165300	NA
F01_transcript_126055	NA	NA	NA	F01_transcript_126055
F01_transcript_134048	NA	NA	NA	F01_transcript_134048
F01_transcript_52124	NA	NA	NA	F01_transcript_52124
F01_transcript_18176	NA	F01_transcript_18176	NA	NA
F01_transcript_156008	NA	NA	NA	F01_transcript_156008
F01_transcript_154721	NA	NA	NA	F01_transcript_154721
F01_transcript_126280	NA	F01_transcript_126280	NA	NA
F01_transcript_56040	NA	NA	NA	F01_transcript_56040
F01_transcript_92230	NA	NA	NA	F01_transcript_92230

F01_transcript_122044	F01_transcript_122044	F01_transcript_122044	F01_transcript_122044	F01_transcript_122044
F01_transcript_78468	NA	NA	NA	F01_transcript_78468
F01_transcript_55013	NA	NA	NA	F01_transcript_55013
F01_transcript_2196	NA	F01_transcript_2196	NA	F01_transcript_2196
F01_transcript_144970	F01_transcript_144970	F01_transcript_144970	F01_transcript_144970	F01_transcript_144970
F01_transcript_121236	NA	F01_transcript_121236	NA	F01_transcript_121236
F01_transcript_48849	NA	NA	NA	F01_transcript_48849
F01_transcript_78051	F01_transcript_78051	F01_transcript_78051	NA	F01_transcript_78051
F01_transcript_59419	F01_transcript_59419	F01_transcript_59419	F01_transcript_59419	F01_transcript_59419
F01_transcript_53914	NA	NA	NA	F01_transcript_53914
F01_transcript_89994	NA	F01_transcript_89994	NA	NA
F01_transcript_126616	NA	F01_transcript_126616	NA	F01_transcript_126616
F01_transcript_164024	NA	F01_transcript_164024	NA	F01_transcript_164024
F01_transcript_153338	NA	NA	NA	F01_transcript_153338

F01_transcript_86276	NA	F01_transcript_86276	NA	NA
F01_transcript_59247	NA	F01_transcript_59247	NA	NA
F01_transcript_51267	F01_transcript_51267	F01_transcript_51267	F01_transcript_51267	F01_transcript_51267
F01_transcript_118971	F01_transcript_118971	F01_transcript_118971	NA	F01_transcript_118971
F01_transcript_61945	F01_transcript_61945	F01_transcript_61945	NA	F01_transcript_61945
F01_transcript_71503	NA	F01_transcript_71503	NA	F01_transcript_71503
F01_transcript_77466	NA	F01_transcript_77466	NA	NA
F01_transcript_131299	NA	NA	NA	F01_transcript_131299
F01_transcript_99650	NA	F01_transcript_99650	NA	F01_transcript_99650
F01_transcript_11176	NA	NA	NA	F01_transcript_11176
F01_transcript_89279	NA	F01_transcript_89279	NA	NA
F01_transcript_130813	NA	F01_transcript_130813	NA	NA
F01_transcript_113463	F01_transcript_113463	F01_transcript_113463	F01_transcript_113463	NA
F01_transcript_47010	NA	NA	NA	F01_transcript_47010

F01_transcript_94674	F01_transcript_94674	F01_transcript_94674	NA	F01_transcript_94674
F01_transcript_95806	F01_transcript_95806	F01_transcript_95806	NA	NA
F01_transcript_154558	NA	NA	NA	F01_transcript_154558
F01_transcript_159505	NA	F01_transcript_159505	NA	NA
F01_transcript_152135	F01_transcript_152135	F01_transcript_152135	NA	F01_transcript_152135
F01_transcript_80664	F01_transcript_80664	F01_transcript_80664	F01_transcript_80664	F01_transcript_80664
F01_transcript_142639	NA	NA	NA	F01_transcript_142639
F01_transcript_165336	NA	F01_transcript_165336	NA	NA
F01_transcript_158062	NA	NA	NA	F01_transcript_158062
F01_transcript_47586	NA	F01_transcript_47586	F01_transcript_47586	NA
F01_transcript_71968	NA	F01_transcript_71968	NA	NA
F01_transcript_74660	F01_transcript_74660	F01_transcript_74660	NA	F01_transcript_74660
F01_transcript_114003	NA	F01_transcript_114003	NA	NA
F01_transcript_32444	F01_transcript_32444	F01_transcript_32444	F01_transcript_32444	F01_transcript_32444

F01_transcript_25464	NA	F01_transcript_25464	F01_transcript_25464	NA
F01_transcript_116014	NA	NA	NA	F01_transcript_116014
F01_transcript_103138	NA	NA	NA	F01_transcript_103138
F01_transcript_80328	NA	NA	NA	F01_transcript_80328
F01_transcript_166980	F01_transcript_166980	F01_transcript_166980	F01_transcript_166980	F01_transcript_166980
F01_transcript_21110	NA	NA	NA	F01_transcript_21110
F01_transcript_54273	F01_transcript_54273	F01_transcript_54273	F01_transcript_54273	F01_transcript_54273
F01_transcript_101530	NA	F01_transcript_101530	NA	NA
F01_transcript_118330	NA	F01_transcript_118330	NA	NA
F01_transcript_96584	NA	NA	F01_transcript_96584	F01_transcript_96584
F01_transcript_73877	NA	F01_transcript_73877	NA	NA
F01_transcript_128978	NA	F01_transcript_128978	NA	F01_transcript_128978
F01_transcript_25932	NA	NA	NA	F01_transcript_25932
F01_transcript_53101	NA	NA	NA	F01_transcript_53101

F01_transcript_58166	NA	F01_transcript_58166	NA	F01_transcript_58166
F01_transcript_56358	F01_transcript_56358	F01_transcript_56358	F01_transcript_56358	F01_transcript_56358
F01_transcript_148942	NA	F01_transcript_148942	NA	NA
F01_transcript_55633	NA	NA	NA	F01_transcript_55633
F01_transcript_106744	NA	F01_transcript_106744	NA	NA
F01_transcript_157010	F01_transcript_157010	F01_transcript_157010	F01_transcript_157010	F01_transcript_157010
F01_transcript_145112	NA	F01_transcript_145112	NA	NA
F01_transcript_80202	F01_transcript_80202	F01_transcript_80202	F01_transcript_80202	F01_transcript_80202
F01_transcript_59771	NA	F01_transcript_59771	NA	NA
F01_transcript_118833	NA	F01_transcript_118833	NA	NA
F01_transcript_131042	F01_transcript_131042	F01_transcript_131042	F01_transcript_131042	F01_transcript_131042
F01_transcript_2026	F01_transcript_2026	NA	NA	F01_transcript_2026
F01_transcript_78399	F01_transcript_78399	F01_transcript_78399	F01_transcript_78399	F01_transcript_78399
F01_transcript_157670	NA	F01_transcript_157670	NA	NA

F01_transcript_46563	NA	F01_transcript_46563	NA	NA
F01_transcript_77847	NA	NA	NA	F01_transcript_77847
F01_transcript_79838	NA	F01_transcript_79838	F01_transcript_79838	F01_transcript_79838
F01_transcript_139303	NA	NA	NA	F01_transcript_139303
F01_transcript_75401	F01_transcript_75401	F01_transcript_75401	F01_transcript_75401	F01_transcript_75401
F01_transcript_97089	NA	F01_transcript_97089	NA	NA
F01_transcript_41857	NA	NA	NA	F01_transcript_41857
F01_transcript_33790	F01_transcript_33790	F01_transcript_33790	F01_transcript_33790	F01_transcript_33790
F01_transcript_95510	NA	NA	NA	F01_transcript_95510
F01_transcript_149900	F01_transcript_149900	F01_transcript_149900	F01_transcript_149900	F01_transcript_149900
F01_transcript_99652	NA	NA	NA	F01_transcript_99652
F01_transcript_94609	F01_transcript_94609	F01_transcript_94609	F01_transcript_94609	F01_transcript_94609
F01_transcript_167623	F01_transcript_167623	F01_transcript_167623	F01_transcript_167623	F01_transcript_167623
F01_transcript_102953	F01_transcript_102953	F01_transcript_102953	NA	NA

F01_transcript_151507	NA	NA	NA	F01_transcript_151507
F01_transcript_9081	NA	NA	NA	F01_transcript_9081
F01_transcript_1648	NA	NA	NA	F01_transcript_1648
F01_transcript_102335	F01_transcript_102335	F01_transcript_102335	F01_transcript_102335	F01_transcript_102335
F01_transcript_116652	NA	NA	NA	F01_transcript_116652
F01_transcript_15969	NA	NA	NA	F01_transcript_15969
F01_transcript_111953	NA	F01_transcript_111953	NA	NA
F01_transcript_133416	NA	F01_transcript_133416	NA	F01_transcript_133416
F01_transcript_87166	NA	F01_transcript_87166	NA	F01_transcript_87166
F01_transcript_27575	NA	NA	NA	F01_transcript_27575
F01_transcript_162899	NA	F01_transcript_162899	F01_transcript_162899	F01_transcript_162899
F01_transcript_117440	NA	F01_transcript_117440	NA	NA
F01_transcript_83545	F01_transcript_83545	F01_transcript_83545	NA	NA
F01_transcript_155868	NA	F01_transcript_155868	F01_transcript_155868	F01_transcript_155868

F01_transcript_153475	NA	NA	NA	F01_transcript_153475
F01_transcript_95129	NA	NA	NA	F01_transcript_95129
F01_transcript_154699	NA	NA	F01_transcript_154699	F01_transcript_154699
F01_transcript_121051	NA	F01_transcript_121051	NA	F01_transcript_121051
F01_transcript_106223	NA	F01_transcript_106223	NA	NA
F01_transcript_49521	F01_transcript_49521	NA	NA	F01_transcript_49521
F01_transcript_142548	F01_transcript_142548	F01_transcript_142548	F01_transcript_142548	F01_transcript_142548
F01_transcript_143284	NA	NA	NA	F01_transcript_143284
F01_transcript_140827	NA	F01_transcript_140827	NA	NA
F01_transcript_157795	NA	NA	NA	F01_transcript_157795
F01_transcript_16630	NA	F01_transcript_16630	F01_transcript_16630	NA
F01_transcript_163807	F01_transcript_163807	F01_transcript_163807	F01_transcript_163807	NA
F01_transcript_69152	NA	NA	NA	F01_transcript_69152
F01_transcript_86159	F01_transcript_86159	F01_transcript_86159	F01_transcript_86159	F01_transcript_86159

F01_transcript_139423	NA	F01_transcript_139423	NA	F01_transcript_139423
F01_transcript_118595	F01_transcript_118595	F01_transcript_118595	NA	F01_transcript_118595
F01_transcript_104802	NA	F01_transcript_104802	NA	NA
F01_transcript_53322	F01_transcript_53322	F01_transcript_53322	F01_transcript_53322	F01_transcript_53322
F01_transcript_65384	NA	NA	NA	F01_transcript_65384
F01_transcript_135793	F01_transcript_135793	F01_transcript_135793	NA	F01_transcript_135793
F01_transcript_48568	NA	NA	NA	F01_transcript_48568
F01_transcript_84281	NA	F01_transcript_84281	NA	NA
F01_transcript_162673	NA	NA	NA	F01_transcript_162673
F01_transcript_132899	F01_transcript_132899	F01_transcript_132899	F01_transcript_132899	F01_transcript_132899
F01_transcript_49543	NA	NA	F01_transcript_49543	F01_transcript_49543
F01_transcript_133926	NA	F01_transcript_133926	NA	F01_transcript_133926
F01_transcript_88475	F01_transcript_88475	F01_transcript_88475	F01_transcript_88475	F01_transcript_88475
F01_transcript_20354	NA	NA	NA	F01_transcript_20354

F01_transcript_167068	F01_transcript_167068	F01_transcript_167068	NA	NA
F01_transcript_145566	NA	NA	NA	F01_transcript_145566
F01_transcript_118212	NA	NA	NA	F01_transcript_118212
F01_transcript_29296	NA	NA	NA	F01_transcript_29296
F01_transcript_139698	F01_transcript_139698	F01_transcript_139698	F01_transcript_139698	F01_transcript_139698
F01_transcript_125714	NA	F01_transcript_125714	NA	F01_transcript_125714
F01_transcript_19880	NA	NA	NA	F01_transcript_19880
F01_transcript_108294	NA	F01_transcript_108294	NA	NA
F01_transcript_4384	NA	F01_transcript_4384	F01_transcript_4384	F01_transcript_4384
F01_transcript_143538	NA	F01_transcript_143538	NA	NA
F01_transcript_88948	NA	F01_transcript_88948	F01_transcript_88948	F01_transcript_88948
F01_transcript_137566	NA	NA	NA	F01_transcript_137566
F01_transcript_150294	F01_transcript_150294	F01_transcript_150294	NA	F01_transcript_150294
F01_transcript_134295	NA	F01_transcript_134295	NA	NA

F01_transcript_82548	NA	F01_transcript_82548	NA	NA
F01_transcript_123769	F01_transcript_123769	F01_transcript_123769	F01_transcript_123769	F01_transcript_123769
F01_transcript_76005	NA	NA	NA	F01_transcript_76005
F01_transcript_143768	NA	F01_transcript_143768	NA	NA
F01_transcript_72609	F01_transcript_72609	F01_transcript_72609	F01_transcript_72609	F01_transcript_72609
F01_transcript_70728	F01_transcript_70728	F01_transcript_70728	F01_transcript_70728	F01_transcript_70728
F01_transcript_135338	F01_transcript_135338	F01_transcript_135338	NA	NA
F01_transcript_75475	NA	NA	NA	F01_transcript_75475
F01_transcript_96256	NA	F01_transcript_96256	NA	NA
F01_transcript_53670	NA	F01_transcript_53670	NA	F01_transcript_53670
F01_transcript_45283	F01_transcript_45283	F01_transcript_45283	F01_transcript_45283	NA
F01_transcript_79907	NA	F01_transcript_79907	NA	F01_transcript_79907
F01_transcript_55743	NA	F01_transcript_55743	F01_transcript_55743	NA
F01_transcript_43640	NA	NA	NA	F01_transcript_43640

F01_transcript_60587	F01_transcript_60587	F01_transcript_60587	F01_transcript_60587	F01_transcript_60587
F01_transcript_151591	NA	F01_transcript_151591	NA	NA
F01_transcript_78539	F01_transcript_78539	F01_transcript_78539	F01_transcript_78539	F01_transcript_78539
F01_transcript_58382	F01_transcript_58382	F01_transcript_58382	F01_transcript_58382	F01_transcript_58382
F01_transcript_95320	F01_transcript_95320	F01_transcript_95320	NA	NA
F01_transcript_99973	NA	F01_transcript_99973	NA	F01_transcript_99973
F01_transcript_127131	F01_transcript_127131	F01_transcript_127131	F01_transcript_127131	F01_transcript_127131
F01_transcript_141830	NA	F01_transcript_141830	NA	NA
F01_transcript_8932	NA	NA	NA	F01_transcript_8932
F01_transcript_81111	F01_transcript_81111	F01_transcript_81111	F01_transcript_81111	NA
F01_transcript_55823	F01_transcript_55823	F01_transcript_55823	F01_transcript_55823	F01_transcript_55823
F01_transcript_79050	NA	NA	NA	F01_transcript_79050
F01_transcript_33985	NA	NA	NA	F01_transcript_33985
F01_transcript_124196	NA	NA	NA	F01_transcript_124196

F01_transcript_19763	NA	F01_transcript_19763	NA	NA
F01_transcript_157568	NA	F01_transcript_157568	NA	NA
F01_transcript_55491	NA	NA	NA	F01_transcript_55491
F01_transcript_16970	NA	F01_transcript_16970	F01_transcript_16970	F01_transcript_16970
F01_transcript_163360	NA	NA	NA	F01_transcript_163360
F01_transcript_75369	F01_transcript_75369	F01_transcript_75369	F01_transcript_75369	F01_transcript_75369
F01_transcript_133325	NA	NA	NA	F01_transcript_133325
F01_transcript_108503	NA	F01_transcript_108503	NA	NA
F01_transcript_108475	F01_transcript_108475	F01_transcript_108475	F01_transcript_108475	F01_transcript_108475
F01_transcript_115819	F01_transcript_115819	F01_transcript_115819	F01_transcript_115819	F01_transcript_115819
F01_transcript_153793	F01_transcript_153793	F01_transcript_153793	F01_transcript_153793	F01_transcript_153793
F01_transcript_130202	F01_transcript_130202	F01_transcript_130202	NA	NA
F01_transcript_156990	NA	NA	NA	F01_transcript_156990
F01_transcript_140060	NA	F01_transcript_140060	NA	F01_transcript_140060

F01_transcript_150748	NA	NA	NA	F01_transcript_150748
F01_transcript_77008	F01_transcript_77008	F01_transcript_77008	F01_transcript_77008	F01_transcript_77008
F01_transcript_113287	NA	NA	NA	F01_transcript_113287
F01_transcript_159038	NA	NA	NA	F01_transcript_159038
F01_transcript_88403	F01_transcript_88403	F01_transcript_88403	F01_transcript_88403	F01_transcript_88403
F01_transcript_63915	NA	NA	NA	F01_transcript_63915
F01_transcript_92892	NA	F01_transcript_92892	NA	NA
F01_transcript_86042	NA	F01_transcript_86042	NA	NA
F01_transcript_98180	NA	F01_transcript_98180	F01_transcript_98180	F01_transcript_98180
F01_transcript_104693	F01_transcript_104693	F01_transcript_104693	F01_transcript_104693	F01_transcript_104693
F01_transcript_163975	NA	NA	NA	F01_transcript_163975
F01_transcript_64472	NA	NA	NA	F01_transcript_64472
F01_transcript_161120	NA	F01_transcript_161120	NA	NA
F01_transcript_86685	NA	F01_transcript_86685	NA	NA

F01_transcript_74053	NA	F01_transcript_74053	NA	F01_transcript_74053
F01_transcript_37374	NA	NA	NA	F01_transcript_37374
F01_transcript_132742	NA	F01_transcript_132742	NA	F01_transcript_132742
F01_transcript_3938	NA	F01_transcript_3938	NA	F01_transcript_3938
F01_transcript_84284	NA	F01_transcript_84284	NA	F01_transcript_84284
F01_transcript_164975	F01_transcript_164975	F01_transcript_164975	NA	NA
F01_transcript_129376	F01_transcript_129376	F01_transcript_129376	F01_transcript_129376	F01_transcript_129376
F01_transcript_6789	NA	NA	NA	F01_transcript_6789
F01_transcript_87012	NA	NA	NA	F01_transcript_87012
F01_transcript_45131	NA	NA	NA	F01_transcript_45131
F01_transcript_120895	F01_transcript_120895	F01_transcript_120895	F01_transcript_120895	F01_transcript_120895
F01_transcript_122415	NA	F01_transcript_122415	NA	F01_transcript_122415
F01_transcript_143662	NA	F01_transcript_143662	NA	NA
F01_transcript_93461	F01_transcript_93461	F01_transcript_93461	F01_transcript_93461	F01_transcript_93461

F01_transcript_126234	NA	F01_transcript_126234	NA	NA
F01_transcript_81212	NA	NA	NA	F01_transcript_81212
F01_transcript_94056	NA	F01_transcript_94056	NA	F01_transcript_94056
F01_transcript_128719	NA	F01_transcript_128719	NA	F01_transcript_128719
F01_transcript_76562	NA	NA	NA	F01_transcript_76562
F01_transcript_116202	F01_transcript_116202	F01_transcript_116202	F01_transcript_116202	F01_transcript_116202
F01_transcript_145739	NA	NA	NA	F01_transcript_145739
F01_transcript_5479	NA	NA	NA	F01_transcript_5479
F01_transcript_109172	NA	F01_transcript_109172	NA	NA
F01_transcript_55111	NA	NA	NA	F01_transcript_55111
F01_transcript_136999	F01_transcript_136999	F01_transcript_136999	F01_transcript_136999	F01_transcript_136999
F01_transcript_59261	NA	NA	NA	F01_transcript_59261
F01_transcript_152471	F01_transcript_152471	F01_transcript_152471	F01_transcript_152471	F01_transcript_152471
F01_transcript_143494	NA	F01_transcript_143494	NA	F01_transcript_143494

F01_transcript_13882	NA	NA	NA	F01_transcript_13882
F01_transcript_120059	F01_transcript_120059	F01_transcript_120059	F01_transcript_120059	F01_transcript_120059
F01_transcript_20419	NA	NA	NA	F01_transcript_20419
F01_transcript_95644	NA	F01_transcript_95644	F01_transcript_95644	F01_transcript_95644
F01_transcript_70246	NA	NA	NA	F01_transcript_70246
F01_transcript_73311	NA	NA	NA	F01_transcript_73311
F01_transcript_114534	NA	NA	NA	F01_transcript_114534
F01_transcript_72718	NA	F01_transcript_72718	NA	F01_transcript_72718
F01_transcript_165665	NA	NA	NA	F01_transcript_165665
F01_transcript_155795	NA	F01_transcript_155795	F01_transcript_155795	NA
F01_transcript_100040	NA	NA	NA	F01_transcript_100040
F01_transcript_44372	NA	NA	NA	F01_transcript_44372
F01_transcript_164991	NA	NA	NA	F01_transcript_164991
F01_transcript_147547	NA	F01_transcript_147547	NA	NA

F01_transcript_121146	NA	NA	NA	F01_transcript_121146
F01_transcript_98107	NA	F01_transcript_98107	F01_transcript_98107	F01_transcript_98107
F01_transcript_146695	NA	F01_transcript_146695	NA	NA
F01_transcript_142773	NA	F01_transcript_142773	NA	F01_transcript_142773
F01_transcript_62169	NA	NA	NA	F01_transcript_62169
F01_transcript_92811	F01_transcript_92811	F01_transcript_92811	NA	F01_transcript_92811
F01_transcript_109699	F01_transcript_109699	F01_transcript_109699	F01_transcript_109699	F01_transcript_109699
F01_transcript_50819	F01_transcript_50819	F01_transcript_50819	F01_transcript_50819	F01_transcript_50819
F01_transcript_35192	NA	F01_transcript_35192	F01_transcript_35192	F01_transcript_35192
F01_transcript_84703	NA	NA	NA	F01_transcript_84703
F01_transcript_113343	F01_transcript_113343	F01_transcript_113343	F01_transcript_113343	NA
F01_transcript_107522	NA	F01_transcript_107522	NA	NA
F01_transcript_8769	NA	F01_transcript_8769	NA	NA
F01_transcript_136607	NA	NA	NA	F01_transcript_136607

F01_transcript_133686	NA	NA	NA	F01_transcript_133686
F01_transcript_116817	F01_transcript_116817	F01_transcript_116817	F01_transcript_116817	F01_transcript_116817
F01_transcript_82728	F01_transcript_82728	F01_transcript_82728	NA	F01_transcript_82728
F01_transcript_36717	NA	F01_transcript_36717	NA	F01_transcript_36717
F01_transcript_2236	F01_transcript_2236	F01_transcript_2236	NA	F01_transcript_2236
F01_transcript_80985	NA	NA	F01_transcript_80985	F01_transcript_80985
F01_transcript_69849	NA	NA	NA	F01_transcript_69849
F01_transcript_93313	NA	F01_transcript_93313	NA	F01_transcript_93313
F01_transcript_101929	F01_transcript_101929	F01_transcript_101929	F01_transcript_101929	F01_transcript_101929
F01_transcript_129635	NA	NA	F01_transcript_129635	F01_transcript_129635
F01_transcript_103235	NA	NA	NA	F01_transcript_103235
F01_transcript_158897	NA	NA	F01_transcript_158897	NA
F01_transcript_112106	F01_transcript_112106	F01_transcript_112106	F01_transcript_112106	F01_transcript_112106
F01_transcript_160614	NA	NA	NA	F01_transcript_160614

F01_transcript_167073	NA	NA	NA	F01_transcript_167073
F01_transcript_44615	NA	NA	NA	F01_transcript_44615
F01_transcript_101474	F01_transcript_101474	F01_transcript_101474	NA	NA
F01_transcript_67052	NA	F01_transcript_67052	NA	NA
F01_transcript_148382	NA	F01_transcript_148382	NA	NA
F01_transcript_139449	NA	F01_transcript_139449	NA	F01_transcript_139449
F01_transcript_77010	F01_transcript_77010	F01_transcript_77010	F01_transcript_77010	NA
F01_transcript_58762	F01_transcript_58762	F01_transcript_58762	F01_transcript_58762	F01_transcript_58762
F01_transcript_76777	NA	NA	NA	F01_transcript_76777
F01_transcript_128866	NA	F01_transcript_128866	NA	NA
F01_transcript_62995	NA	F01_transcript_62995	NA	NA
F01_transcript_63809	NA	F01_transcript_63809	NA	NA
F01_transcript_53548	F01_transcript_53548	F01_transcript_53548	F01_transcript_53548	F01_transcript_53548
F01_transcript_68179	NA	NA	NA	F01_transcript_68179

F01_transcript_46739	NA	F01_transcript_46739	NA	F01_transcript_46739
F01_transcript_108439	NA	NA	NA	F01_transcript_108439
F01_transcript_119966	NA	NA	NA	F01_transcript_119966
F01_transcript_166441	F01_transcript_166441	F01_transcript_166441	F01_transcript_166441	NA
F01_transcript_102547	NA	NA	NA	F01_transcript_102547
F01_transcript_17054	NA	NA	NA	F01_transcript_17054
F01_transcript_130413	NA	NA	NA	F01_transcript_130413
F01_transcript_121589	F01_transcript_121589	F01_transcript_121589	F01_transcript_121589	F01_transcript_121589
F01_transcript_132812	NA	NA	NA	F01_transcript_132812
F01_transcript_146799	NA	F01_transcript_146799	NA	NA
F01_transcript_67841	NA	NA	F01_transcript_67841	F01_transcript_67841
F01_transcript_162174	F01_transcript_162174	F01_transcript_162174	F01_transcript_162174	F01_transcript_162174
F01_transcript_76633	NA	F01_transcript_76633	NA	F01_transcript_76633
F01_transcript_48562	NA	NA	NA	F01_transcript_48562

F01_transcript_75480	NA	F01_transcript_75480	NA	NA
F01_transcript_79070	NA	F01_transcript_79070	NA	F01_transcript_79070
F01_transcript_161797	NA	F01_transcript_161797	F01_transcript_161797	F01_transcript_161797
F01_transcript_121961	F01_transcript_121961	F01_transcript_121961	F01_transcript_121961	F01_transcript_121961
F01_transcript_128256	NA	F01_transcript_128256	NA	NA
F01_transcript_48614	NA	F01_transcript_48614	NA	NA
F01_transcript_59460	NA	NA	F01_transcript_59460	F01_transcript_59460
F01_transcript_50753	NA	F01_transcript_50753	NA	NA
F01_transcript_20001	NA	F01_transcript_20001	NA	NA
F01_transcript_101130	NA	NA	NA	F01_transcript_101130
F01_transcript_92496	F01_transcript_92496	F01_transcript_92496	F01_transcript_92496	F01_transcript_92496
F01_transcript_82266	NA	NA	NA	F01_transcript_82266
F01_transcript_44832	NA	NA	NA	F01_transcript_44832
F01_transcript_69589	NA	F01_transcript_69589	NA	NA

F01_transcript_159912	NA	F01_transcript_159912	NA	F01_transcript_159912
F01_transcript_129266	F01_transcript_129266	F01_transcript_129266	F01_transcript_129266	F01_transcript_129266
F01_transcript_21023	NA	NA	NA	F01_transcript_21023
F01_transcript_47004	NA	NA	NA	F01_transcript_47004
F01_transcript_100754	NA	F01_transcript_100754	F01_transcript_100754	NA
F01_transcript_149633	F01_transcript_149633	F01_transcript_149633	F01_transcript_149633	F01_transcript_149633
F01_transcript_80693	F01_transcript_80693	F01_transcript_80693	F01_transcript_80693	F01_transcript_80693
F01_transcript_59001	NA	NA	NA	F01_transcript_59001
F01_transcript_88561	NA	NA	NA	F01_transcript_88561
F01_transcript_5122	NA	NA	NA	F01_transcript_5122
F01_transcript_59873	NA	NA	NA	F01_transcript_59873
F01_transcript_90442	NA	F01_transcript_90442	NA	F01_transcript_90442
F01_transcript_118840	F01_transcript_118840	F01_transcript_118840	NA	F01_transcript_118840
F01_transcript_94662	F01_transcript_94662	F01_transcript_94662	F01_transcript_94662	F01_transcript_94662

F01_transcript_98807	F01_transcript_98807	F01_transcript_98807	NA	NA
F01_transcript_136094	NA	F01_transcript_136094	NA	NA
F01_transcript_146795	F01_transcript_146795	F01_transcript_146795	F01_transcript_146795	F01_transcript_146795
F01_transcript_141820	NA	NA	NA	F01_transcript_141820
F01_transcript_152567	NA	F01_transcript_152567	NA	NA
F01_transcript_80753	F01_transcript_80753	F01_transcript_80753	NA	F01_transcript_80753
F01_transcript_48215	NA	F01_transcript_48215	NA	F01_transcript_48215
F01_transcript_88758	NA	F01_transcript_88758	NA	F01_transcript_88758
F01_transcript_104969	NA	F01_transcript_104969	F01_transcript_104969	F01_transcript_104969
F01_transcript_25087	NA	NA	NA	F01_transcript_25087
F01_transcript_48125	NA	NA	NA	F01_transcript_48125
F01_transcript_73381	NA	F01_transcript_73381	NA	NA
F01_transcript_49996	NA	F01_transcript_49996	F01_transcript_49996	F01_transcript_49996
F01_transcript_146783	NA	F01_transcript_146783	NA	NA

F01_transcript_14474	NA	NA	NA	F01_transcript_14474
F01_transcript_75839	NA	F01_transcript_75839	F01_transcript_75839	F01_transcript_75839
F01_transcript_79300	F01_transcript_79300	F01_transcript_79300	F01_transcript_79300	F01_transcript_79300
F01_transcript_7769	NA	NA	NA	F01_transcript_7769
F01_transcript_149653	F01_transcript_149653	F01_transcript_149653	F01_transcript_149653	NA
F01_transcript_100931	NA	NA	NA	F01_transcript_100931
F01_transcript_66499	F01_transcript_66499	F01_transcript_66499	F01_transcript_66499	F01_transcript_66499
F01_transcript_146448	F01_transcript_146448	F01_transcript_146448	NA	NA
F01_transcript_78621	NA	F01_transcript_78621	F01_transcript_78621	F01_transcript_78621
F01_transcript_60368	F01_transcript_60368	F01_transcript_60368	NA	F01_transcript_60368
F01_transcript_76941	NA	NA	NA	F01_transcript_76941
F01_transcript_85371	F01_transcript_85371	F01_transcript_85371	F01_transcript_85371	F01_transcript_85371
F01_transcript_42583	NA	F01_transcript_42583	NA	NA
F01_transcript_19518	NA	NA	NA	F01_transcript_19518

F01_transcript_63093	NA	NA	NA	F01_transcript_63093
F01_transcript_84612	NA	NA	NA	F01_transcript_84612
F01_transcript_86437	F01_transcript_86437	F01_transcript_86437	F01_transcript_86437	F01_transcript_86437
F01_transcript_51315	NA	F01_transcript_51315	F01_transcript_51315	F01_transcript_51315
F01_transcript_121915	NA	NA	NA	F01_transcript_121915
F01_transcript_56062	F01_transcript_56062	F01_transcript_56062	NA	NA
F01_transcript_163384	NA	F01_transcript_163384	NA	NA
F01_transcript_147267	NA	F01_transcript_147267	NA	NA
F01_transcript_56586	NA	NA	F01_transcript_56586	NA
F01_transcript_131541	NA	NA	NA	F01_transcript_131541
F01_transcript_139753	NA	F01_transcript_139753	F01_transcript_139753	NA
F01_transcript_50365	NA	F01_transcript_50365	NA	NA
F01_transcript_121555	NA	F01_transcript_121555	NA	F01_transcript_121555
F01_transcript_151114	NA	F01_transcript_151114	NA	NA

F01_transcript_100308	NA	F01_transcript_100308	NA	F01_transcript_100308
F01_transcript_119407	NA	NA	F01_transcript_119407	NA
F01_transcript_159860	NA	NA	NA	F01_transcript_159860
F01_transcript_142379	F01_transcript_142379	F01_transcript_142379	NA	F01_transcript_142379
F01_transcript_152865	F01_transcript_152865	F01_transcript_152865	NA	F01_transcript_152865
F01_transcript_104901	F01_transcript_104901	F01_transcript_104901	F01_transcript_104901	F01_transcript_104901
F01_transcript_94482	NA	F01_transcript_94482	NA	NA
F01_transcript_71358	NA	F01_transcript_71358	NA	NA
F01_transcript_83631	F01_transcript_83631	F01_transcript_83631	NA	NA
F01_transcript_110033	F01_transcript_110033	F01_transcript_110033	NA	F01_transcript_110033
F01_transcript_74715	F01_transcript_74715	F01_transcript_74715	F01_transcript_74715	F01_transcript_74715
F01_transcript_58952	F01_transcript_58952	F01_transcript_58952	NA	F01_transcript_58952
F01_transcript_165679	NA	F01_transcript_165679	NA	NA
F01_transcript_58353	NA	F01_transcript_58353	F01_transcript_58353	F01_transcript_58353

F01_transcript_148032	F01_transcript_148032	NA	NA	NA
F01_transcript_53062	NA	F01_transcript_53062	NA	F01_transcript_53062
F01_transcript_107220	NA	F01_transcript_107220	F01_transcript_107220	NA
F01_transcript_6869	NA	F01_transcript_6869	NA	NA
F01_transcript_59025	NA	F01_transcript_59025	NA	NA
F01_transcript_64011	NA	F01_transcript_64011	NA	NA
F01_transcript_71631	NA	F01_transcript_71631	NA	NA
F01_transcript_52838	F01_transcript_52838	F01_transcript_52838	F01_transcript_52838	F01_transcript_52838
F01_transcript_120662	NA	NA	NA	F01_transcript_120662
F01_transcript_102502	NA	F01_transcript_102502	NA	NA
F01_transcript_136974	NA	NA	NA	F01_transcript_136974
F01_transcript_59108	NA	NA	NA	F01_transcript_59108
F01_transcript_95548	NA	F01_transcript_95548	F01_transcript_95548	NA
F01_transcript_124665	NA	F01_transcript_124665	NA	NA

F01_transcript_49665	NA	F01_transcript_49665	NA	NA
F01_transcript_159571	NA	F01_transcript_159571	NA	NA
F01_transcript_129138	NA	F01_transcript_129138	NA	NA
F01_transcript_123606	NA	NA	NA	F01_transcript_123606
F01_transcript_105654	NA	NA	NA	F01_transcript_105654
F01_transcript_132613	F01_transcript_132613	F01_transcript_132613	NA	NA
F01_transcript_23354	F01_transcript_23354	F01_transcript_23354	F01_transcript_23354	F01_transcript_23354
F01_transcript_96592	F01_transcript_96592	F01_transcript_96592	F01_transcript_96592	F01_transcript_96592
F01_transcript_83153	NA	NA	NA	F01_transcript_83153
F01_transcript_134319	NA	F01_transcript_134319	NA	F01_transcript_134319
F01_transcript_57723	F01_transcript_57723	F01_transcript_57723	F01_transcript_57723	F01_transcript_57723
F01_transcript_159991	NA	NA	NA	F01_transcript_159991
F01_transcript_86589	NA	F01_transcript_86589	NA	NA
F01_transcript_22498	F01_transcript_22498	F01_transcript_22498	NA	F01_transcript_22498

F01_transcript_102419	NA	NA	F01_transcript_102419	F01_transcript_102419
F01_transcript_70024	NA	NA	NA	F01_transcript_70024
F01_transcript_149671	NA	NA	NA	F01_transcript_149671
F01_transcript_121422	NA	F01_transcript_121422	NA	NA
F01_transcript_50530	F01_transcript_50530	F01_transcript_50530	NA	NA
F01_transcript_64217	NA	NA	NA	F01_transcript_64217
F01_transcript_110082	NA	F01_transcript_110082	NA	NA
F01_transcript_137162	NA	F01_transcript_137162	NA	NA
F01_transcript_98557	NA	NA	NA	F01_transcript_98557
F01_transcript_33829	NA	NA	NA	F01_transcript_33829
F01_transcript_79251	NA	NA	NA	F01_transcript_79251
F01_transcript_82100	NA	F01_transcript_82100	NA	F01_transcript_82100
F01_transcript_33437	NA	NA	NA	F01_transcript_33437
F01_transcript_165121	F01_transcript_165121	F01_transcript_165121	F01_transcript_165121	F01_transcript_165121

F01_transcript_57960	F01_transcript_57960	F01_transcript_57960	F01_transcript_57960	F01_transcript_57960
F01_transcript_61879	NA	NA	NA	F01_transcript_61879
F01_transcript_154066	NA	NA	NA	F01_transcript_154066
F01_transcript_157437	NA	F01_transcript_157437	NA	NA
F01_transcript_125630	F01_transcript_125630	NA	F01_transcript_125630	NA
F01_transcript_52939	NA	F01_transcript_52939	NA	F01_transcript_52939
F01_transcript_164977	NA	NA	NA	F01_transcript_164977
F01_transcript_110449	F01_transcript_110449	F01_transcript_110449	F01_transcript_110449	F01_transcript_110449
F01_transcript_142969	NA	F01_transcript_142969	NA	NA
F01_transcript_45506	NA	NA	NA	F01_transcript_45506
F01_transcript_167619	NA	NA	NA	F01_transcript_167619
F01_transcript_52300	NA	F01_transcript_52300	F01_transcript_52300	F01_transcript_52300
F01_transcript_131486	NA	F01_transcript_131486	NA	F01_transcript_131486
F01_transcript_56291	NA	F01_transcript_56291	F01_transcript_56291	F01_transcript_56291

F01_transcript_147193	F01_transcript_147193	F01_transcript_147193	F01_transcript_147193	F01_transcript_147193
F01_transcript_155172	F01_transcript_155172	F01_transcript_155172	F01_transcript_155172	F01_transcript_155172
F01_transcript_104575	NA	NA	NA	F01_transcript_104575
F01_transcript_69514	NA	NA	NA	F01_transcript_69514
F01_transcript_133896	NA	F01_transcript_133896	NA	NA
F01_transcript_85479	F01_transcript_85479	F01_transcript_85479	NA	F01_transcript_85479
F01_transcript_77966	NA	F01_transcript_77966	NA	NA
F01_transcript_114364	NA	F01_transcript_114364	NA	NA
F01_transcript_47792	NA	F01_transcript_47792	F01_transcript_47792	F01_transcript_47792
F01_transcript_133133	F01_transcript_133133	F01_transcript_133133	F01_transcript_133133	F01_transcript_133133
F01_transcript_146901	NA	NA	NA	F01_transcript_146901
F01_transcript_14486	NA	NA	NA	F01_transcript_14486
F01_transcript_129255	NA	F01_transcript_129255	NA	NA
F01_transcript_26738	NA	NA	NA	F01_transcript_26738

F01_transcript_54328	NA	NA	NA	F01_transcript_54328
F01_transcript_152470	NA	F01_transcript_152470	NA	F01_transcript_152470
F01_transcript_121341	F01_transcript_121341	F01_transcript_121341	NA	NA
F01_transcript_119252	NA	NA	NA	F01_transcript_119252
F01_transcript_117244	F01_transcript_117244	F01_transcript_117244	F01_transcript_117244	F01_transcript_117244
F01_transcript_83880	F01_transcript_83880	F01_transcript_83880	F01_transcript_83880	F01_transcript_83880
F01_transcript_110966	NA	F01_transcript_110966	NA	NA
F01_transcript_53063	NA	NA	NA	F01_transcript_53063
F01_transcript_102670	NA	F01_transcript_102670	NA	NA
F01_transcript_120272	NA	NA	NA	F01_transcript_120272
F01_transcript_135027	NA	NA	NA	F01_transcript_135027
F01_transcript_119018	NA	NA	NA	F01_transcript_119018
F01_transcript_9230	F01_transcript_9230	F01_transcript_9230	F01_transcript_9230	F01_transcript_9230
F01_transcript_156623	F01_transcript_156623	F01_transcript_156623	F01_transcript_156623	F01_transcript_156623

F01_transcript_136042	NA	NA	NA	F01_transcript_136042
F01_transcript_33251	NA	NA	NA	F01_transcript_33251
F01_transcript_37306	NA	NA	NA	F01_transcript_37306
F01_transcript_160704	F01_transcript_160704	F01_transcript_160704	NA	NA
F01_transcript_149198	NA	F01_transcript_149198	F01_transcript_149198	NA
F01_transcript_152772	F01_transcript_152772	F01_transcript_152772	F01_transcript_152772	F01_transcript_152772
F01_transcript_24494	NA	NA	NA	F01_transcript_24494
F01_transcript_12637	NA	F01_transcript_12637	NA	F01_transcript_12637
F01_transcript_26381	NA	NA	NA	F01_transcript_26381
F01_transcript_125088	NA	F01_transcript_125088	NA	F01_transcript_125088
F01_transcript_162499	NA	NA	NA	F01_transcript_162499
F01_transcript_102148	NA	NA	NA	F01_transcript_102148
F01_transcript_158988	NA	F01_transcript_158988	F01_transcript_158988	NA
F01_transcript_128258	F01_transcript_128258	F01_transcript_128258	F01_transcript_128258	F01_transcript_128258

F01_transcript_111237	F01_transcript_111237	F01_transcript_111237	NA	NA
F01_transcript_50098	F01_transcript_50098	NA	F01_transcript_50098	F01_transcript_50098
F01_transcript_109389	NA	NA	NA	F01_transcript_109389
F01_transcript_88653	NA	NA	NA	F01_transcript_88653
F01_transcript_74838	NA	NA	NA	F01_transcript_74838
F01_transcript_110953	F01_transcript_110953	F01_transcript_110953	NA	NA
F01_transcript_5341	NA	F01_transcript_5341	F01_transcript_5341	F01_transcript_5341
F01_transcript_98962	F01_transcript_98962	F01_transcript_98962	F01_transcript_98962	F01_transcript_98962
F01_transcript_62342	NA	F01_transcript_62342	NA	NA
F01_transcript_31268	NA	NA	NA	F01_transcript_31268
F01_transcript_142446	NA	NA	NA	F01_transcript_142446
F01_transcript_28854	NA	NA	NA	F01_transcript_28854
F01_transcript_113013	NA	F01_transcript_113013	NA	NA
F01_transcript_50605	F01_transcript_50605	NA	F01_transcript_50605	F01_transcript_50605

F01_transcript_54694	NA	F01_transcript_54694	NA	F01_transcript_54694
F01_transcript_68346	NA	F01_transcript_68346	NA	F01_transcript_68346
F01_transcript_140934	NA	F01_transcript_140934	F01_transcript_140934	NA
F01_transcript_44659	NA	NA	NA	F01_transcript_44659
F01_transcript_119859	NA	F01_transcript_119859	NA	F01_transcript_119859
F01_transcript_73661	NA	NA	NA	F01_transcript_73661
F01_transcript_149799	F01_transcript_149799	F01_transcript_149799	F01_transcript_149799	F01_transcript_149799
F01_transcript_162175	NA	NA	NA	F01_transcript_162175
F01_transcript_130420	NA	F01_transcript_130420	NA	NA
F01_transcript_90361	NA	F01_transcript_90361	NA	NA
F01_transcript_147470	NA	NA	NA	F01_transcript_147470
F01_transcript_42420	NA	NA	NA	F01_transcript_42420
F01_transcript_97862	NA	F01_transcript_97862	NA	NA
F01_transcript_103161	NA	F01_transcript_103161	NA	F01_transcript_103161

F01_transcript_59990	F01_transcript_59990	F01_transcript_59990	F01_transcript_59990	F01_transcript_59990
F01_transcript_58007	NA	NA	NA	F01_transcript_58007
F01_transcript_88854	NA	F01_transcript_88854	NA	NA
F01_transcript_52062	NA	NA	NA	F01_transcript_52062
F01_transcript_71148	NA	NA	F01_transcript_71148	NA
F01_transcript_149265	NA	NA	NA	F01_transcript_149265
F01_transcript_132838	F01_transcript_132838	F01_transcript_132838	F01_transcript_132838	F01_transcript_132838
F01_transcript_68060	NA	F01_transcript_68060	F01_transcript_68060	F01_transcript_68060
F01_transcript_47627	NA	NA	NA	F01_transcript_47627
F01_transcript_121200	NA	F01_transcript_121200	NA	NA
F01_transcript_42235	NA	NA	NA	F01_transcript_42235
F01_transcript_145271	NA	F01_transcript_145271	NA	NA
F01_transcript_104056	NA	F01_transcript_104056	NA	NA
F01_transcript_71346	F01_transcript_71346	F01_transcript_71346	F01_transcript_71346	F01_transcript_71346

F01_transcript_72301	F01_transcript_72301	F01_transcript_72301	F01_transcript_72301	F01_transcript_72301
F01_transcript_45457	NA	NA	NA	F01_transcript_45457
F01_transcript_84797	NA	NA	NA	F01_transcript_84797
F01_transcript_157044	NA	F01_transcript_157044	NA	NA
F01_transcript_47168	NA	NA	NA	F01_transcript_47168
F01_transcript_49913	NA	NA	F01_transcript_49913	F01_transcript_49913
F01_transcript_95283	NA	F01_transcript_95283	NA	F01_transcript_95283
F01_transcript_45864	NA	NA	NA	F01_transcript_45864
F01_transcript_53117	NA	F01_transcript_53117	NA	NA
F01_transcript_122856	NA	F01_transcript_122856	NA	F01_transcript_122856
F01_transcript_92258	NA	F01_transcript_92258	NA	NA
F01_transcript_158854	NA	NA	NA	F01_transcript_158854
F01_transcript_155296	NA	NA	NA	F01_transcript_155296
F01_transcript_64719	NA	NA	NA	F01_transcript_64719

F01_transcript_92585	F01_transcript_92585	F01_transcript_92585	F01_transcript_92585	F01_transcript_92585
F01_transcript_3068	NA	NA	NA	F01_transcript_3068
F01_transcript_5243	NA	F01_transcript_5243	F01_transcript_5243	F01_transcript_5243
F01_transcript_39035	NA	NA	NA	F01_transcript_39035
F01_transcript_99543	F01_transcript_99543	F01_transcript_99543	F01_transcript_99543	F01_transcript_99543
F01_transcript_129594	F01_transcript_129594	F01_transcript_129594	F01_transcript_129594	F01_transcript_129594
F01_transcript_148965	F01_transcript_148965	F01_transcript_148965	F01_transcript_148965	F01_transcript_148965
F01_transcript_151516	NA	F01_transcript_151516	NA	NA
F01_transcript_102810	F01_transcript_102810	F01_transcript_102810	F01_transcript_102810	F01_transcript_102810
F01_transcript_94587	F01_transcript_94587	F01_transcript_94587	F01_transcript_94587	NA
F01_transcript_105260	NA	F01_transcript_105260	NA	F01_transcript_105260
F01_transcript_137195	NA	NA	NA	F01_transcript_137195
F01_transcript_123459	NA	F01_transcript_123459	F01_transcript_123459	F01_transcript_123459
F01_transcript_56930	F01_transcript_56930	F01_transcript_56930	F01_transcript_56930	F01_transcript_56930

F01_transcript_146720	NA	F01_transcript_146720	NA	NA
F01_transcript_55205	F01_transcript_55205	F01_transcript_55205	F01_transcript_55205	F01_transcript_55205
F01_transcript_120191	NA	F01_transcript_120191	NA	NA
F01_transcript_98809	NA	F01_transcript_98809	NA	NA
F01_transcript_135380	F01_transcript_135380	F01_transcript_135380	F01_transcript_135380	F01_transcript_135380
F01_transcript_52982	F01_transcript_52982	F01_transcript_52982	F01_transcript_52982	F01_transcript_52982
F01_transcript_116071	NA	NA	NA	F01_transcript_116071
F01_transcript_56096	NA	F01_transcript_56096	NA	NA
F01_transcript_81255	F01_transcript_81255	F01_transcript_81255	F01_transcript_81255	F01_transcript_81255
F01_transcript_96403	NA	NA	NA	F01_transcript_96403
F01_transcript_90850	NA	NA	NA	F01_transcript_90850
F01_transcript_106964	NA	NA	F01_transcript_106964	F01_transcript_106964
F01_transcript_40355	NA	F01_transcript_40355	NA	NA
F01_transcript_71543	F01_transcript_71543	NA	F01_transcript_71543	F01_transcript_71543

F01_transcript_48541	NA	NA	NA	F01_transcript_48541
F01_transcript_107899	NA	F01_transcript_107899	NA	NA
F01_transcript_117175	NA	F01_transcript_117175	NA	F01_transcript_117175
F01_transcript_149527	NA	F01_transcript_149527	NA	NA
F01_transcript_52600	NA	NA	NA	F01_transcript_52600
F01_transcript_164467	NA	NA	NA	F01_transcript_164467
F01_transcript_113998	NA	F01_transcript_113998	NA	NA
F01_transcript_16205	NA	F01_transcript_16205	NA	F01_transcript_16205
F01_transcript_51281	F01_transcript_51281	F01_transcript_51281	F01_transcript_51281	F01_transcript_51281
F01_transcript_56063	NA	F01_transcript_56063	NA	NA
F01_transcript_150370	NA	NA	NA	F01_transcript_150370
F01_transcript_44983	NA	F01_transcript_44983	NA	NA
F01_transcript_154366	F01_transcript_154366	F01_transcript_154366	F01_transcript_154366	F01_transcript_154366
F01_transcript_102607	F01_transcript_102607	F01_transcript_102607	F01_transcript_102607	F01_transcript_102607

F01_transcript_127910	NA	NA	NA	F01_transcript_127910
F01_transcript_57113	NA	F01_transcript_57113	F01_transcript_57113	F01_transcript_57113
F01_transcript_120954	F01_transcript_120954	F01_transcript_120954	F01_transcript_120954	F01_transcript_120954
F01_transcript_69467	F01_transcript_69467	F01_transcript_69467	F01_transcript_69467	F01_transcript_69467
F01_transcript_132994	NA	F01_transcript_132994	NA	F01_transcript_132994
F01_transcript_7142	NA	NA	NA	F01_transcript_7142
F01_transcript_145575	NA	NA	NA	F01_transcript_145575
F01_transcript_46351	NA	F01_transcript_46351	NA	F01_transcript_46351
F01_transcript_133238	F01_transcript_133238	F01_transcript_133238	F01_transcript_133238	F01_transcript_133238
F01_transcript_91872	NA	F01_transcript_91872	NA	NA
F01_transcript_137912	NA	NA	NA	F01_transcript_137912
F01_transcript_122646	NA	NA	NA	F01_transcript_122646
F01_transcript_71582	F01_transcript_71582	F01_transcript_71582	F01_transcript_71582	F01_transcript_71582
F01_transcript_99595	NA	F01_transcript_99595	F01_transcript_99595	F01_transcript_99595

F01_transcript_125199	NA	F01_transcript_125199	NA	F01_transcript_125199
F01_transcript_12771	F01_transcript_12771	F01_transcript_12771	F01_transcript_12771	F01_transcript_12771
F01_transcript_156427	NA	NA	NA	F01_transcript_156427
F01_transcript_29441	NA	F01_transcript_29441	NA	NA
F01_transcript_82367	NA	NA	NA	F01_transcript_82367
F01_transcript_100763	NA	F01_transcript_100763	NA	NA
F01_transcript_54280	F01_transcript_54280	F01_transcript_54280	F01_transcript_54280	F01_transcript_54280
F01_transcript_118010	NA	F01_transcript_118010	F01_transcript_118010	F01_transcript_118010
F01_transcript_83919	NA	F01_transcript_83919	NA	NA
F01_transcript_61172	F01_transcript_61172	F01_transcript_61172	NA	NA
F01_transcript_152163	F01_transcript_152163	F01_transcript_152163	NA	F01_transcript_152163
F01_transcript_106927	NA	NA	NA	F01_transcript_106927
F01_transcript_63869	NA	NA	NA	F01_transcript_63869
F01_transcript_62038	NA	F01_transcript_62038	NA	NA

F01_transcript_95832	F01_transcript_95832	F01_transcript_95832	NA	NA
F01_transcript_8125	NA	F01_transcript_8125	NA	NA
F01_transcript_114204	F01_transcript_114204	F01_transcript_114204	NA	F01_transcript_114204
F01_transcript_120528	NA	F01_transcript_120528	NA	NA
F01_transcript_165528	NA	NA	NA	F01_transcript_165528
F01_transcript_11278	NA	F01_transcript_11278	NA	F01_transcript_11278
F01_transcript_48561	NA	NA	NA	F01_transcript_48561
F01_transcript_141493	NA	F01_transcript_141493	NA	NA
F01_transcript_70239	NA	NA	NA	F01_transcript_70239
F01_transcript_130739	F01_transcript_130739	F01_transcript_130739	NA	NA
F01_transcript_3032	NA	F01_transcript_3032	F01_transcript_3032	F01_transcript_3032
F01_transcript_123359	NA	NA	NA	F01_transcript_123359
F01_transcript_78611	NA	F01_transcript_78611	NA	NA
F01_transcript_113083	NA	NA	NA	F01_transcript_113083

F01_transcript_51276	F01_transcript_51276	F01_transcript_51276	F01_transcript_51276	F01_transcript_51276
F01_transcript_21044	NA	NA	NA	F01_transcript_21044
F01_transcript_156478	NA	F01_transcript_156478	NA	F01_transcript_156478
F01_transcript_71783	NA	F01_transcript_71783	F01_transcript_71783	F01_transcript_71783
F01_transcript_162165	NA	F01_transcript_162165	NA	NA
F01_transcript_126348	NA	NA	F01_transcript_126348	F01_transcript_126348
F01_transcript_156804	NA	F01_transcript_156804	NA	F01_transcript_156804
F01_transcript_45121	NA	F01_transcript_45121	NA	NA
F01_transcript_129333	NA	F01_transcript_129333	NA	F01_transcript_129333
F01_transcript_83682	NA	F01_transcript_83682	NA	NA
F01_transcript_65035	F01_transcript_65035	F01_transcript_65035	F01_transcript_65035	F01_transcript_65035
F01_transcript_49737	NA	F01_transcript_49737	F01_transcript_49737	NA
F01_transcript_85331	F01_transcript_85331	F01_transcript_85331	F01_transcript_85331	NA
F01_transcript_69470	NA	F01_transcript_69470	F01_transcript_69470	F01_transcript_69470

F01_transcript_28182	F01_transcript_28182	F01_transcript_28182	NA	NA
F01_transcript_49862	NA	F01_transcript_49862	NA	NA
F01_transcript_145297	NA	NA	NA	F01_transcript_145297
F01_transcript_104887	F01_transcript_104887	F01_transcript_104887	F01_transcript_104887	F01_transcript_104887
F01_transcript_104921	NA	NA	NA	F01_transcript_104921
F01_transcript_149874	F01_transcript_149874	F01_transcript_149874	NA	NA
F01_transcript_116203	NA	F01_transcript_116203	NA	NA
F01_transcript_133331	NA	F01_transcript_133331	NA	NA
F01_transcript_122088	F01_transcript_122088	F01_transcript_122088	F01_transcript_122088	F01_transcript_122088
F01_transcript_74631	F01_transcript_74631	F01_transcript_74631	F01_transcript_74631	F01_transcript_74631
F01_transcript_55416	F01_transcript_55416	F01_transcript_55416	F01_transcript_55416	F01_transcript_55416
F01_transcript_54813	NA	F01_transcript_54813	NA	NA
F01_transcript_68391	NA	F01_transcript_68391	F01_transcript_68391	F01_transcript_68391
F01_transcript_63636	NA	F01_transcript_63636	NA	NA

F01_transcript_67838	NA	F01_transcript_67838	F01_transcript_67838	F01_transcript_67838
F01_transcript_120225	F01_transcript_120225	F01_transcript_120225	F01_transcript_120225	F01_transcript_120225
F01_transcript_106712	NA	NA	NA	F01_transcript_106712
F01_transcript_69580	NA	F01_transcript_69580	NA	NA
F01_transcript_165949	NA	NA	NA	F01_transcript_165949
F01_transcript_84279	NA	F01_transcript_84279	NA	NA
F01_transcript_34629	NA	NA	NA	F01_transcript_34629
F01_transcript_163900	NA	F01_transcript_163900	NA	NA
F01_transcript_98087	F01_transcript_98087	F01_transcript_98087	F01_transcript_98087	F01_transcript_98087
F01_transcript_96689	NA	F01_transcript_96689	NA	NA
F01_transcript_137622	NA	F01_transcript_137622	NA	F01_transcript_137622
F01_transcript_49389	NA	F01_transcript_49389	NA	F01_transcript_49389
F01_transcript_97084	NA	F01_transcript_97084	NA	NA
F01_transcript_70397	NA	F01_transcript_70397	NA	NA

F01_transcript_39245	NA	NA	NA	F01_transcript_39245
F01_transcript_76694	NA	NA	NA	F01_transcript_76694
F01_transcript_131081	NA	NA	NA	F01_transcript_131081
F01_transcript_162083	NA	F01_transcript_162083	F01_transcript_162083	F01_transcript_162083
F01_transcript_86855	NA	NA	NA	F01_transcript_86855
F01_transcript_4715	NA	NA	F01_transcript_4715	F01_transcript_4715
F01_transcript_123737	F01_transcript_123737	F01_transcript_123737	NA	F01_transcript_123737
F01_transcript_97487	NA	NA	NA	F01_transcript_97487
F01_transcript_93237	NA	F01_transcript_93237	NA	NA
F01_transcript_53544	NA	NA	NA	F01_transcript_53544
F01_transcript_140968	NA	F01_transcript_140968	NA	NA
F01_transcript_100381	F01_transcript_100381	F01_transcript_100381	F01_transcript_100381	F01_transcript_100381
F01_transcript_99315	F01_transcript_99315	F01_transcript_99315	F01_transcript_99315	F01_transcript_99315
F01_transcript_146152	NA	NA	NA	F01_transcript_146152

F01_transcript_47361	NA	NA	NA	F01_transcript_47361
F01_transcript_48607	F01_transcript_48607	F01_transcript_48607	F01_transcript_48607	F01_transcript_48607
F01_transcript_128568	F01_transcript_128568	F01_transcript_128568	F01_transcript_128568	F01_transcript_128568
F01_transcript_56399	NA	F01_transcript_56399	F01_transcript_56399	F01_transcript_56399
F01_transcript_162214	F01_transcript_162214	F01_transcript_162214	NA	NA
F01_transcript_144652	F01_transcript_144652	F01_transcript_144652	F01_transcript_144652	F01_transcript_144652
F01_transcript_167304	F01_transcript_167304	F01_transcript_167304	F01_transcript_167304	F01_transcript_167304
F01_transcript_86176	F01_transcript_86176	F01_transcript_86176	NA	NA
F01_transcript_107247	NA	F01_transcript_107247	NA	F01_transcript_107247
F01_transcript_52305	NA	NA	NA	F01_transcript_52305
F01_transcript_88887	NA	F01_transcript_88887	NA	NA
F01_transcript_109439	F01_transcript_109439	F01_transcript_109439	F01_transcript_109439	F01_transcript_109439
F01_transcript_165559	NA	F01_transcript_165559	NA	F01_transcript_165559
F01_transcript_118197	NA	NA	NA	F01_transcript_118197

F01_transcript_88749	NA	NA	NA	F01_transcript_88749
F01_transcript_128603	NA	NA	NA	F01_transcript_128603
F01_transcript_121553	NA	NA	NA	F01_transcript_121553
F01_transcript_167247	NA	NA	NA	F01_transcript_167247
F01_transcript_18562	NA	NA	NA	F01_transcript_18562
F01_transcript_128397	NA	NA	NA	F01_transcript_128397
F01_transcript_14835	NA	NA	NA	F01_transcript_14835
F01_transcript_92769	F01_transcript_92769	F01_transcript_92769	F01_transcript_92769	F01_transcript_92769
F01_transcript_98858	NA	F01_transcript_98858	F01_transcript_98858	F01_transcript_98858
F01_transcript_99830	NA	F01_transcript_99830	NA	NA
F01_transcript_114638	F01_transcript_114638	F01_transcript_114638	F01_transcript_114638	F01_transcript_114638
F01_transcript_90713	NA	NA	NA	F01_transcript_90713
F01_transcript_149323	NA	F01_transcript_149323	NA	NA
F01_transcript_50558	F01_transcript_50558	F01_transcript_50558	F01_transcript_50558	F01_transcript_50558

F01_transcript_100333	F01_transcript_100333	F01_transcript_100333	NA	F01_transcript_100333
F01_transcript_76425	NA	F01_transcript_76425	NA	NA
F01_transcript_125260	NA	F01_transcript_125260	NA	NA
F01_transcript_51762	NA	F01_transcript_51762	NA	NA
F01_transcript_42221	NA	NA	NA	F01_transcript_42221
F01_transcript_31818	NA	F01_transcript_31818	NA	NA
F01_transcript_149051	NA	F01_transcript_149051	NA	NA
F01_transcript_106258	F01_transcript_106258	F01_transcript_106258	F01_transcript_106258	NA
F01_transcript_164458	F01_transcript_164458	F01_transcript_164458	NA	F01_transcript_164458
F01_transcript_162891	NA	NA	NA	F01_transcript_162891
F01_transcript_112598	F01_transcript_112598	F01_transcript_112598	NA	F01_transcript_112598
F01_transcript_161863	F01_transcript_161863	F01_transcript_161863	F01_transcript_161863	F01_transcript_161863
F01_transcript_156648	NA	NA	NA	F01_transcript_156648
F01_transcript_147743	NA	F01_transcript_147743	NA	NA

F01_transcript_93022	F01_transcript_93022	F01_transcript_93022	F01_transcript_93022	F01_transcript_93022
F01_transcript_56220	NA	NA	F01_transcript_56220	F01_transcript_56220
F01_transcript_1640	NA	F01_transcript_1640	NA	NA
F01_transcript_68704	F01_transcript_68704	F01_transcript_68704	NA	NA
F01_transcript_92360	F01_transcript_92360	F01_transcript_92360	NA	F01_transcript_92360
F01_transcript_101602	NA	NA	NA	F01_transcript_101602
F01_transcript_76907	F01_transcript_76907	F01_transcript_76907	F01_transcript_76907	F01_transcript_76907
F01_transcript_119637	F01_transcript_119637	F01_transcript_119637	F01_transcript_119637	F01_transcript_119637
F01_transcript_21279	NA	NA	NA	F01_transcript_21279
F01_transcript_68907	NA	F01_transcript_68907	NA	NA
F01_transcript_107262	NA	F01_transcript_107262	NA	NA
F01_transcript_110065	NA	NA	NA	F01_transcript_110065
F01_transcript_7042	NA	NA	F01_transcript_7042	F01_transcript_7042
F01_transcript_35534	NA	NA	NA	F01_transcript_35534

F01_transcript_135570	NA	F01_transcript_135570	NA	NA
F01_transcript_116562	NA	F01_transcript_116562	NA	F01_transcript_116562
F01_transcript_106436	NA	NA	NA	F01_transcript_106436
F01_transcript_165177	NA	NA	F01_transcript_165177	F01_transcript_165177
F01_transcript_143466	F01_transcript_143466	F01_transcript_143466	F01_transcript_143466	F01_transcript_143466
F01_transcript_46906	NA	NA	NA	F01_transcript_46906
F01_transcript_156769	NA	NA	NA	F01_transcript_156769
F01_transcript_158471	F01_transcript_158471	F01_transcript_158471	NA	F01_transcript_158471
F01_transcript_163666	F01_transcript_163666	F01_transcript_163666	NA	NA
F01_transcript_139576	NA	NA	NA	F01_transcript_139576
F01_transcript_12794	NA	NA	NA	F01_transcript_12794
F01_transcript_134002	NA	NA	NA	F01_transcript_134002
F01_transcript_125977	F01_transcript_125977	F01_transcript_125977	NA	NA
F01_transcript_526	NA	NA	NA	F01_transcript_526

F01_transcript_134900	NA	F01_transcript_134900	NA	NA
F01_transcript_166193	F01_transcript_166193	F01_transcript_166193	NA	NA
F01_transcript_134379	NA	NA	NA	F01_transcript_134379
F01_transcript_29144	NA	NA	NA	F01_transcript_29144
F01_transcript_89026	F01_transcript_89026	F01_transcript_89026	F01_transcript_89026	F01_transcript_89026
F01_transcript_73928	NA	F01_transcript_73928	NA	NA
F01_transcript_129870	F01_transcript_129870	F01_transcript_129870	F01_transcript_129870	F01_transcript_129870
F01_transcript_65597	F01_transcript_65597	F01_transcript_65597	F01_transcript_65597	F01_transcript_65597
F01_transcript_150759	NA	F01_transcript_150759	F01_transcript_150759	F01_transcript_150759
F01_transcript_139549	F01_transcript_139549	F01_transcript_139549	NA	NA
F01_transcript_110467	F01_transcript_110467	F01_transcript_110467	F01_transcript_110467	F01_transcript_110467
F01_transcript_134534	NA	NA	NA	F01_transcript_134534
F01_transcript_47192	NA	NA	NA	F01_transcript_47192
F01_transcript_161135	NA	F01_transcript_161135	NA	NA

F01_transcript_147212	NA	NA	NA	F01_transcript_147212
F01_transcript_88797	NA	NA	NA	F01_transcript_88797
F01_transcript_76002	NA	NA	NA	F01_transcript_76002
F01_transcript_165687	NA	NA	NA	F01_transcript_165687
F01_transcript_117108	NA	F01_transcript_117108	NA	F01_transcript_117108
F01_transcript_394	F01_transcript_394	NA	NA	NA
F01_transcript_59657	F01_transcript_59657	F01_transcript_59657	F01_transcript_59657	F01_transcript_59657
F01_transcript_100424	F01_transcript_100424	F01_transcript_100424	F01_transcript_100424	F01_transcript_100424
F01_transcript_93445	F01_transcript_93445	F01_transcript_93445	F01_transcript_93445	F01_transcript_93445
F01_transcript_149530	NA	F01_transcript_149530	NA	NA
F01_transcript_94937	F01_transcript_94937	F01_transcript_94937	F01_transcript_94937	F01_transcript_94937
F01_transcript_100535	F01_transcript_100535	F01_transcript_100535	F01_transcript_100535	F01_transcript_100535
F01_transcript_129640	NA	F01_transcript_129640	NA	NA
F01_transcript_28757	NA	NA	NA	F01_transcript_28757

F01_transcript_127010	NA	NA	NA	F01_transcript_127010
F01_transcript_26155	NA	NA	NA	F01_transcript_26155
F01_transcript_160949	NA	F01_transcript_160949	NA	NA
F01_transcript_119425	NA	NA	NA	F01_transcript_119425
F01_transcript_149734	NA	F01_transcript_149734	NA	F01_transcript_149734
F01_transcript_155402	F01_transcript_155402	F01_transcript_155402	F01_transcript_155402	NA
F01_transcript_38611	NA	NA	NA	F01_transcript_38611
F01_transcript_87961	F01_transcript_87961	F01_transcript_87961	F01_transcript_87961	F01_transcript_87961
F01_transcript_59246	F01_transcript_59246	F01_transcript_59246	F01_transcript_59246	F01_transcript_59246
F01_transcript_132914	NA	NA	NA	F01_transcript_132914
F01_transcript_163374	NA	NA	NA	F01_transcript_163374
F01_transcript_52924	F01_transcript_52924	F01_transcript_52924	F01_transcript_52924	F01_transcript_52924
F01_transcript_81307	NA	NA	NA	F01_transcript_81307
F01_transcript_88001	NA	F01_transcript_88001	NA	F01_transcript_88001

F01_transcript_108543	F01_transcript_108543	F01_transcript_108543	NA	F01_transcript_108543
F01_transcript_78096	NA	NA	NA	F01_transcript_78096
F01_transcript_115088	NA	F01_transcript_115088	NA	NA
F01_transcript_141682	F01_transcript_141682	F01_transcript_141682	NA	F01_transcript_141682
F01_transcript_157308	F01_transcript_157308	F01_transcript_157308	F01_transcript_157308	F01_transcript_157308
F01_transcript_29158	NA	F01_transcript_29158	NA	NA
F01_transcript_56446	F01_transcript_56446	F01_transcript_56446	F01_transcript_56446	NA
F01_transcript_154147	NA	F01_transcript_154147	F01_transcript_154147	NA
F01_transcript_157201	NA	F01_transcript_157201	NA	NA
F01_transcript_55709	NA	NA	NA	F01_transcript_55709
F01_transcript_106412	NA	F01_transcript_106412	NA	NA
F01_transcript_129715	F01_transcript_129715	F01_transcript_129715	F01_transcript_129715	F01_transcript_129715
F01_transcript_94172	F01_transcript_94172	F01_transcript_94172	F01_transcript_94172	F01_transcript_94172
F01_transcript_49611	F01_transcript_49611	F01_transcript_49611	NA	F01_transcript_49611

F01_transcript_163072	F01_transcript_163072	F01_transcript_163072	F01_transcript_163072	F01_transcript_163072
F01_transcript_154768	F01_transcript_154768	F01_transcript_154768	F01_transcript_154768	NA
F01_transcript_68536	F01_transcript_68536	F01_transcript_68536	F01_transcript_68536	F01_transcript_68536
F01_transcript_98850	F01_transcript_98850	F01_transcript_98850	F01_transcript_98850	F01_transcript_98850
F01_transcript_105430	NA	NA	NA	F01_transcript_105430
F01_transcript_116467	NA	NA	NA	F01_transcript_116467
F01_transcript_79943	NA	F01_transcript_79943	F01_transcript_79943	F01_transcript_79943
F01_transcript_50240	NA	F01_transcript_50240	NA	F01_transcript_50240
F01_transcript_150865	F01_transcript_150865	F01_transcript_150865	F01_transcript_150865	F01_transcript_150865
F01_transcript_136997	NA	F01_transcript_136997	NA	F01_transcript_136997
F01_transcript_118191	F01_transcript_118191	F01_transcript_118191	NA	NA
F01_transcript_138557	NA	F01_transcript_138557	NA	NA
F01_transcript_148925	NA	F01_transcript_148925	F01_transcript_148925	F01_transcript_148925
F01_transcript_131623	NA	F01_transcript_131623	NA	NA

F01_transcript_53740	NA	F01_transcript_53740	NA	NA
F01_transcript_102700	F01_transcript_102700	F01_transcript_102700	F01_transcript_102700	NA
F01_transcript_87474	NA	NA	NA	F01_transcript_87474
F01_transcript_86469	NA	NA	NA	F01_transcript_86469
F01_transcript_92974	NA	NA	NA	F01_transcript_92974
F01_transcript_117538	NA	NA	NA	F01_transcript_117538
F01_transcript_54077	F01_transcript_54077	F01_transcript_54077	NA	NA
F01_transcript_33665	NA	NA	NA	F01_transcript_33665
F01_transcript_4744	NA	NA	NA	F01_transcript_4744
F01_transcript_114206	NA	F01_transcript_114206	NA	NA
F01_transcript_56337	NA	NA	F01_transcript_56337	NA
F01_transcript_100039	F01_transcript_100039	F01_transcript_100039	F01_transcript_100039	F01_transcript_100039
F01_transcript_147849	NA	F01_transcript_147849	NA	NA
F01_transcript_120552	NA	NA	NA	F01_transcript_120552

F01_transcript_118113	NA	F01_transcript_118113	F01_transcript_118113	F01_transcript_118113
F01_transcript_9441	F01_transcript_9441	F01_transcript_9441	F01_transcript_9441	F01_transcript_9441
F01_transcript_10773	F01_transcript_10773	F01_transcript_10773	F01_transcript_10773	F01_transcript_10773
F01_transcript_119423	NA	NA	NA	F01_transcript_119423
F01_transcript_101069	NA	F01_transcript_101069	F01_transcript_101069	F01_transcript_101069
F01_transcript_87622	NA	NA	NA	F01_transcript_87622
F01_transcript_156621	NA	NA	F01_transcript_156621	NA
F01_transcript_54308	NA	NA	NA	F01_transcript_54308
F01_transcript_64078	NA	F01_transcript_64078	NA	NA
F01_transcript_38323	NA	F01_transcript_38323	NA	F01_transcript_38323
F01_transcript_164243	NA	NA	NA	F01_transcript_164243
F01_transcript_156863	F01_transcript_156863	F01_transcript_156863	F01_transcript_156863	F01_transcript_156863
F01_transcript_73127	NA	F01_transcript_73127	NA	F01_transcript_73127
F01_transcript_48223	NA	F01_transcript_48223	F01_transcript_48223	NA

F01_transcript_57918	NA	F01_transcript_57918	NA	NA
F01_transcript_69532	NA	F01_transcript_69532	NA	F01_transcript_69532
F01_transcript_41288	NA	NA	F01_transcript_41288	NA
F01_transcript_144810	F01_transcript_144810	F01_transcript_144810	F01_transcript_144810	F01_transcript_144810
F01_transcript_112277	F01_transcript_112277	F01_transcript_112277	NA	F01_transcript_112277
F01_transcript_93096	NA	NA	F01_transcript_93096	NA
F01_transcript_123961	NA	NA	F01_transcript_123961	F01_transcript_123961
F01_transcript_105434	F01_transcript_105434	F01_transcript_105434	F01_transcript_105434	NA
F01_transcript_49464	NA	F01_transcript_49464	F01_transcript_49464	F01_transcript_49464
F01_transcript_112253	NA	NA	NA	F01_transcript_112253
F01_transcript_118265	NA	F01_transcript_118265	NA	F01_transcript_118265
F01_transcript_166156	NA	NA	NA	F01_transcript_166156
F01_transcript_51388	NA	F01_transcript_51388	NA	NA
F01_transcript_59086	NA	NA	NA	F01_transcript_59086

F01_transcript_75947	NA	NA	NA	F01_transcript_75947
F01_transcript_161687	NA	NA	NA	F01_transcript_161687
F01_transcript_152555	NA	F01_transcript_152555	NA	NA
F01_transcript_67814	F01_transcript_67814	F01_transcript_67814	F01_transcript_67814	F01_transcript_67814
F01_transcript_114005	F01_transcript_114005	F01_transcript_114005	NA	NA
F01_transcript_164452	NA	NA	NA	F01_transcript_164452
F01_transcript_78902	NA	F01_transcript_78902	NA	F01_transcript_78902
F01_transcript_109424	NA	NA	NA	F01_transcript_109424
F01_transcript_67113	NA	NA	F01_transcript_67113	NA
F01_transcript_162986	NA	F01_transcript_162986	NA	F01_transcript_162986
F01_transcript_165203	NA	NA	NA	F01_transcript_165203
F01_transcript_79710	NA	F01_transcript_79710	F01_transcript_79710	F01_transcript_79710
F01_transcript_143658	NA	F01_transcript_143658	NA	NA
F01_transcript_148716	F01_transcript_148716	F01_transcript_148716	F01_transcript_148716	F01_transcript_148716

F01_transcript_164710	NA	F01_transcript_164710	F01_transcript_164710	NA
F01_transcript_131201	F01_transcript_131201	F01_transcript_131201	NA	F01_transcript_131201
F01_transcript_94504	F01_transcript_94504	F01_transcript_94504	F01_transcript_94504	F01_transcript_94504
F01_transcript_94528	NA	F01_transcript_94528	NA	NA
F01_transcript_104013	F01_transcript_104013	F01_transcript_104013	F01_transcript_104013	F01_transcript_104013
F01_transcript_122703	NA	F01_transcript_122703	NA	NA
F01_transcript_89889	NA	F01_transcript_89889	NA	NA
F01_transcript_164258	NA	F01_transcript_164258	NA	F01_transcript_164258
F01_transcript_135794	F01_transcript_135794	F01_transcript_135794	F01_transcript_135794	F01_transcript_135794
F01_transcript_93355	NA	F01_transcript_93355	NA	NA
F01_transcript_45489	NA	NA	NA	F01_transcript_45489
F01_transcript_55942	NA	F01_transcript_55942	F01_transcript_55942	F01_transcript_55942
F01_transcript_81793	NA	NA	NA	F01_transcript_81793
F01_transcript_104823	F01_transcript_104823	F01_transcript_104823	F01_transcript_104823	F01_transcript_104823

F01_transcript_115594	NA	F01_transcript_115594	NA	NA
F01_transcript_129488	NA	NA	NA	F01_transcript_129488
F01_transcript_156934	NA	NA	NA	F01_transcript_156934
F01_transcript_144371	F01_transcript_144371	F01_transcript_144371	F01_transcript_144371	F01_transcript_144371
F01_transcript_57117	NA	F01_transcript_57117	F01_transcript_57117	NA
F01_transcript_69167	NA	F01_transcript_69167	NA	NA
F01_transcript_72021	NA	F01_transcript_72021	NA	F01_transcript_72021
F01_transcript_56527	NA	NA	F01_transcript_56527	NA
F01_transcript_111623	F01_transcript_111623	F01_transcript_111623	F01_transcript_111623	NA
F01_transcript_64383	NA	F01_transcript_64383	NA	F01_transcript_64383
F01_transcript_107652	F01_transcript_107652	F01_transcript_107652	F01_transcript_107652	F01_transcript_107652
F01_transcript_34018	F01_transcript_34018	F01_transcript_34018	F01_transcript_34018	F01_transcript_34018
F01_transcript_50471	F01_transcript_50471	F01_transcript_50471	NA	NA
F01_transcript_23688	NA	F01_transcript_23688	F01_transcript_23688	F01_transcript_23688

F01_transcript_128393	F01_transcript_128393	F01_transcript_128393	F01_transcript_128393	F01_transcript_128393
F01_transcript_26637	NA	F01_transcript_26637	NA	NA
F01_transcript_58906	NA	NA	NA	F01_transcript_58906
F01_transcript_160850	F01_transcript_160850	F01_transcript_160850	F01_transcript_160850	F01_transcript_160850
F01_transcript_3754	NA	NA	NA	F01_transcript_3754
F01_transcript_57162	NA	F01_transcript_57162	F01_transcript_57162	NA
F01_transcript_122609	NA	F01_transcript_122609	NA	NA
F01_transcript_1267	F01_transcript_1267	F01_transcript_1267	NA	F01_transcript_1267
F01_transcript_44198	NA	NA	NA	F01_transcript_44198
F01_transcript_158049	NA	F01_transcript_158049	F01_transcript_158049	F01_transcript_158049
F01_transcript_130014	NA	F01_transcript_130014	F01_transcript_130014	F01_transcript_130014
F01_transcript_159404	NA	F01_transcript_159404	NA	F01_transcript_159404
F01_transcript_166544	NA	NA	NA	F01_transcript_166544
F01_transcript_54963	NA	F01_transcript_54963	NA	F01_transcript_54963

F01_transcript_140256	NA	NA	NA	F01_transcript_140256
F01_transcript_63390	NA	F01_transcript_63390	NA	NA
F01_transcript_144991	F01_transcript_144991	F01_transcript_144991	NA	F01_transcript_144991
F01_transcript_160981	F01_transcript_160981	F01_transcript_160981	F01_transcript_160981	F01_transcript_160981
F01_transcript_67736	F01_transcript_67736	F01_transcript_67736	F01_transcript_67736	NA
F01_transcript_146722	NA	NA	NA	F01_transcript_146722
F01_transcript_79017	F01_transcript_79017	F01_transcript_79017	F01_transcript_79017	NA
F01_transcript_68928	NA	F01_transcript_68928	F01_transcript_68928	F01_transcript_68928
F01_transcript_78357	NA	NA	NA	F01_transcript_78357
F01_transcript_46607	NA	F01_transcript_46607	NA	F01_transcript_46607
F01_transcript_130219	NA	F01_transcript_130219	NA	NA
F01_transcript_132653	F01_transcript_132653	F01_transcript_132653	F01_transcript_132653	F01_transcript_132653
F01_transcript_131954	NA	NA	NA	F01_transcript_131954
F01_transcript_88908	NA	F01_transcript_88908	NA	NA

F01_transcript_115937	NA	F01_transcript_115937	F01_transcript_115937	F01_transcript_115937
F01_transcript_96235	NA	F01_transcript_96235	NA	NA
F01_transcript_140855	F01_transcript_140855	F01_transcript_140855	F01_transcript_140855	F01_transcript_140855
F01_transcript_109863	NA	NA	NA	F01_transcript_109863
F01_transcript_84832	NA	F01_transcript_84832	NA	F01_transcript_84832
F01_transcript_87921	F01_transcript_87921	F01_transcript_87921	F01_transcript_87921	F01_transcript_87921
F01_transcript_162853	F01_transcript_162853	F01_transcript_162853	F01_transcript_162853	NA
F01_transcript_74227	NA	F01_transcript_74227	NA	F01_transcript_74227
F01_transcript_155140	NA	F01_transcript_155140	NA	NA
F01_transcript_165551	F01_transcript_165551	F01_transcript_165551	F01_transcript_165551	NA
F01_transcript_43770	NA	F01_transcript_43770	NA	NA
F01_transcript_108053	NA	NA	NA	F01_transcript_108053
F01_transcript_111879	NA	F01_transcript_111879	NA	NA
F01_transcript_81539	NA	NA	NA	F01_transcript_81539

F01_transcript_167323	NA	F01_transcript_167323	NA	F01_transcript_167323
F01_transcript_40643	NA	NA	NA	F01_transcript_40643
F01_transcript_166164	NA	F01_transcript_166164	NA	F01_transcript_166164
F01_transcript_106406	F01_transcript_106406	F01_transcript_106406	F01_transcript_106406	F01_transcript_106406
F01_transcript_27121	NA	F01_transcript_27121	NA	NA
F01_transcript_154990	NA	F01_transcript_154990	NA	NA
F01_transcript_76298	NA	NA	NA	F01_transcript_76298
F01_transcript_109835	F01_transcript_109835	F01_transcript_109835	NA	NA
F01_transcript_102904	NA	F01_transcript_102904	NA	NA
F01_transcript_125493	F01_transcript_125493	F01_transcript_125493	F01_transcript_125493	F01_transcript_125493
F01_transcript_119063	NA	F01_transcript_119063	NA	NA
F01_transcript_113407	NA	NA	NA	F01_transcript_113407
F01_transcript_104788	NA	NA	NA	F01_transcript_104788
F01_transcript_123040	NA	NA	NA	F01_transcript_123040

F01_transcript_34590	NA	NA	NA	F01_transcript_34590
F01_transcript_127139	NA	NA	NA	F01_transcript_127139
F01_transcript_106415	F01_transcript_106415	F01_transcript_106415	F01_transcript_106415	F01_transcript_106415
F01_transcript_153467	NA	F01_transcript_153467	NA	NA
F01_transcript_145477	F01_transcript_145477	F01_transcript_145477	F01_transcript_145477	F01_transcript_145477
F01_transcript_122761	NA	NA	F01_transcript_122761	NA
F01_transcript_112531	NA	F01_transcript_112531	NA	F01_transcript_112531
F01_transcript_67600	NA	NA	NA	F01_transcript_67600
F01_transcript_74650	F01_transcript_74650	F01_transcript_74650	F01_transcript_74650	F01_transcript_74650
F01_transcript_138831	NA	F01_transcript_138831	F01_transcript_138831	F01_transcript_138831
F01_transcript_63577	NA	NA	NA	F01_transcript_63577
F01_transcript_77473	F01_transcript_77473	F01_transcript_77473	F01_transcript_77473	F01_transcript_77473
F01_transcript_26188	NA	NA	NA	F01_transcript_26188
F01_transcript_74805	NA	NA	NA	F01_transcript_74805

F01_transcript_78327	NA	F01_transcript_78327	NA	NA
F01_transcript_167021	NA	NA	NA	F01_transcript_167021
F01_transcript_70859	NA	NA	F01_transcript_70859	NA
F01_transcript_22997	NA	NA	NA	F01_transcript_22997
F01_transcript_148487	NA	F01_transcript_148487	NA	F01_transcript_148487
F01_transcript_161352	NA	NA	NA	F01_transcript_161352
F01_transcript_88222	NA	F01_transcript_88222	NA	F01_transcript_88222
F01_transcript_166210	F01_transcript_166210	F01_transcript_166210	F01_transcript_166210	F01_transcript_166210
F01_transcript_56628	NA	F01_transcript_56628	NA	NA
F01_transcript_75986	F01_transcript_75986	F01_transcript_75986	F01_transcript_75986	F01_transcript_75986
F01_transcript_66619	NA	F01_transcript_66619	NA	NA
F01_transcript_158789	NA	F01_transcript_158789	NA	NA
F01_transcript_113045	NA	NA	NA	F01_transcript_113045
F01_transcript_52585	F01_transcript_52585	F01_transcript_52585	F01_transcript_52585	F01_transcript_52585

F01_transcript_153009	NA	NA	NA	F01_transcript_153009
F01_transcript_140831	NA	F01_transcript_140831	NA	NA
F01_transcript_89912	F01_transcript_89912	F01_transcript_89912	F01_transcript_89912	F01_transcript_89912
F01_transcript_117847	F01_transcript_117847	F01_transcript_117847	F01_transcript_117847	F01_transcript_117847
F01_transcript_124514	F01_transcript_124514	F01_transcript_124514	NA	NA
F01_transcript_98328	NA	F01_transcript_98328	NA	NA
F01_transcript_86233	NA	NA	NA	F01_transcript_86233
F01_transcript_48960	NA	F01_transcript_48960	NA	NA
F01_transcript_67487	NA	F01_transcript_67487	F01_transcript_67487	F01_transcript_67487
F01_transcript_145322	NA	F01_transcript_145322	NA	NA
F01_transcript_65664	F01_transcript_65664	NA	F01_transcript_65664	F01_transcript_65664
F01_transcript_94397	F01_transcript_94397	F01_transcript_94397	F01_transcript_94397	F01_transcript_94397
F01_transcript_146099	F01_transcript_146099	F01_transcript_146099	F01_transcript_146099	NA
F01_transcript_166764	NA	F01_transcript_166764	NA	NA

F01_transcript_90625	F01_transcript_90625	F01_transcript_90625	NA	NA
F01_transcript_76955	NA	NA	NA	F01_transcript_76955
F01_transcript_81762	NA	NA	NA	F01_transcript_81762
F01_transcript_158729	NA	NA	NA	F01_transcript_158729
F01_transcript_94160	NA	NA	NA	F01_transcript_94160
F01_transcript_146701	F01_transcript_146701	F01_transcript_146701	F01_transcript_146701	F01_transcript_146701
F01_transcript_140803	NA	NA	NA	F01_transcript_140803
F01_transcript_118524	NA	NA	NA	F01_transcript_118524
F01_transcript_106471	NA	F01_transcript_106471	NA	NA
F01_transcript_163032	NA	NA	NA	F01_transcript_163032
F01_transcript_97998	F01_transcript_97998	F01_transcript_97998	NA	NA
F01_transcript_133750	NA	NA	NA	F01_transcript_133750
F01_transcript_86075	F01_transcript_86075	F01_transcript_86075	F01_transcript_86075	F01_transcript_86075
F01_transcript_40752	F01_transcript_40752	F01_transcript_40752	F01_transcript_40752	F01_transcript_40752

F01_transcript_68103	NA	F01_transcript_68103	NA	NA
F01_transcript_120707	NA	F01_transcript_120707	NA	F01_transcript_120707
F01_transcript_153266	F01_transcript_153266	F01_transcript_153266	NA	NA
F01_transcript_103920	F01_transcript_103920	F01_transcript_103920	F01_transcript_103920	F01_transcript_103920
F01_transcript_75651	NA	F01_transcript_75651	NA	NA
F01_transcript_147529	NA	NA	NA	F01_transcript_147529
F01_transcript_97303	NA	NA	NA	F01_transcript_97303
F01_transcript_84211	NA	F01_transcript_84211	NA	NA
F01_transcript_116039	NA	F01_transcript_116039	NA	NA
F01_transcript_160799	F01_transcript_160799	F01_transcript_160799	F01_transcript_160799	F01_transcript_160799
F01_transcript_63626	NA	NA	NA	F01_transcript_63626
F01_transcript_38336	F01_transcript_38336	F01_transcript_38336	NA	NA
F01_transcript_129507	NA	F01_transcript_129507	NA	NA
F01_transcript_73316	NA	F01_transcript_73316	NA	F01_transcript_73316

F01_transcript_153438	NA	F01_transcript_153438	NA	NA
F01_transcript_92119	NA	F01_transcript_92119	NA	NA
F01_transcript_137764	F01_transcript_137764	F01_transcript_137764	F01_transcript_137764	F01_transcript_137764
F01_transcript_83373	F01_transcript_83373	NA	NA	NA
F01_transcript_113986	F01_transcript_113986	F01_transcript_113986	NA	NA
F01_transcript_57578	F01_transcript_57578	F01_transcript_57578	F01_transcript_57578	F01_transcript_57578
F01_transcript_66060	NA	NA	NA	F01_transcript_66060
F01_transcript_38041	NA	NA	NA	F01_transcript_38041
F01_transcript_70551	F01_transcript_70551	F01_transcript_70551	NA	NA
F01_transcript_125882	NA	NA	NA	F01_transcript_125882
F01_transcript_88158	NA	NA	NA	F01_transcript_88158
F01_transcript_72677	NA	NA	NA	F01_transcript_72677
F01_transcript_103023	F01_transcript_103023	F01_transcript_103023	F01_transcript_103023	F01_transcript_103023
F01_transcript_59238	NA	NA	NA	F01_transcript_59238

F01_transcript_148737	F01_transcript_148737	NA	NA	F01_transcript_148737
F01_transcript_165352	NA	F01_transcript_165352	NA	NA
F01_transcript_107275	F01_transcript_107275	F01_transcript_107275	F01_transcript_107275	F01_transcript_107275
F01_transcript_148258	NA	F01_transcript_148258	NA	F01_transcript_148258
F01_transcript_118837	NA	F01_transcript_118837	NA	NA
F01_transcript_111126	NA	F01_transcript_111126	NA	NA
F01_transcript_64026	NA	NA	NA	F01_transcript_64026
F01_transcript_90839	F01_transcript_90839	F01_transcript_90839	F01_transcript_90839	F01_transcript_90839
F01_transcript_80782	F01_transcript_80782	F01_transcript_80782	NA	NA
F01_transcript_151130	F01_transcript_151130	F01_transcript_151130	F01_transcript_151130	F01_transcript_151130
F01_transcript_57579	NA	NA	NA	F01_transcript_57579
F01_transcript_147404	NA	F01_transcript_147404	NA	NA
F01_transcript_3598	NA	NA	NA	F01_transcript_3598
F01_transcript_2060	NA	NA	F01_transcript_2060	F01_transcript_2060

F01_transcript_84472	NA	F01_transcript_84472	NA	NA
F01_transcript_101803	NA	NA	NA	F01_transcript_101803
F01_transcript_167249	F01_transcript_167249	F01_transcript_167249	NA	NA
F01_transcript_134368	NA	F01_transcript_134368	F01_transcript_134368	F01_transcript_134368
F01_transcript_156844	NA	F01_transcript_156844	F01_transcript_156844	F01_transcript_156844
F01_transcript_154759	NA	F01_transcript_154759	NA	NA
F01_transcript_163654	NA	F01_transcript_163654	NA	NA
F01_transcript_97920	NA	F01_transcript_97920	NA	F01_transcript_97920
F01_transcript_104023	F01_transcript_104023	F01_transcript_104023	F01_transcript_104023	F01_transcript_104023
F01_transcript_164695	NA	F01_transcript_164695	NA	NA
F01_transcript_13501	F01_transcript_13501	F01_transcript_13501	NA	F01_transcript_13501
F01_transcript_147439	NA	F01_transcript_147439	NA	NA
F01_transcript_157265	F01_transcript_157265	F01_transcript_157265	F01_transcript_157265	F01_transcript_157265
F01_transcript_54214	NA	F01_transcript_54214	NA	NA

F01_transcript_30515	F01_transcript_30515	F01_transcript_30515	NA	F01_transcript_30515
F01_transcript_87616	NA	F01_transcript_87616	F01_transcript_87616	F01_transcript_87616
F01_transcript_85947	NA	NA	F01_transcript_85947	F01_transcript_85947
F01_transcript_68440	NA	NA	NA	F01_transcript_68440
F01_transcript_88213	F01_transcript_88213	F01_transcript_88213	F01_transcript_88213	NA
F01_transcript_138868	F01_transcript_138868	F01_transcript_138868	NA	NA
F01_transcript_6951	NA	NA	NA	F01_transcript_6951
F01_transcript_66154	NA	NA	NA	F01_transcript_66154
F01_transcript_87162	NA	NA	NA	F01_transcript_87162
F01_transcript_47507	NA	NA	NA	F01_transcript_47507
F01_transcript_120507	F01_transcript_120507	F01_transcript_120507	NA	NA
F01_transcript_79681	NA	F01_transcript_79681	NA	NA
F01_transcript_79141	NA	NA	NA	F01_transcript_79141
F01_transcript_97469	NA	NA	NA	F01_transcript_97469

F01_transcript_101448	NA	NA	NA	F01_transcript_101448
F01_transcript_77191	NA	F01_transcript_77191	F01_transcript_77191	NA
F01_transcript_164742	NA	NA	NA	F01_transcript_164742
F01_transcript_13763	NA	NA	NA	F01_transcript_13763
F01_transcript_65115	NA	NA	F01_transcript_65115	F01_transcript_65115
F01_transcript_59798	F01_transcript_59798	F01_transcript_59798	NA	F01_transcript_59798
F01_transcript_57921	NA	F01_transcript_57921	NA	F01_transcript_57921
F01_transcript_74230	NA	F01_transcript_74230	NA	NA
F01_transcript_20455	NA	NA	NA	F01_transcript_20455
F01_transcript_142795	NA	F01_transcript_142795	NA	NA
F01_transcript_115689	NA	NA	NA	F01_transcript_115689
F01_transcript_108972	NA	NA	NA	F01_transcript_108972
F01_transcript_90772	NA	F01_transcript_90772	NA	NA
F01_transcript_105827	F01_transcript_105827	F01_transcript_105827	NA	F01_transcript_105827

F01_transcript_5381	F01_transcript_5381	F01_transcript_5381	NA	F01_transcript_5381
F01_transcript_110821	NA	NA	NA	F01_transcript_110821
F01_transcript_92532	NA	F01_transcript_92532	F01_transcript_92532	NA
F01_transcript_79182	NA	NA	NA	F01_transcript_79182
F01_transcript_113585	NA	NA	NA	F01_transcript_113585
F01_transcript_127571	NA	NA	NA	F01_transcript_127571
F01_transcript_130387	F01_transcript_130387	F01_transcript_130387	F01_transcript_130387	F01_transcript_130387
F01_transcript_128167	F01_transcript_128167	F01_transcript_128167	NA	F01_transcript_128167
F01_transcript_37085	NA	NA	NA	F01_transcript_37085
F01_transcript_97075	NA	NA	NA	F01_transcript_97075
F01_transcript_37748	NA	F01_transcript_37748	NA	F01_transcript_37748
F01_transcript_33631	NA	NA	NA	F01_transcript_33631
F01_transcript_54126	F01_transcript_54126	F01_transcript_54126	F01_transcript_54126	NA
F01_transcript_90158	NA	F01_transcript_90158	NA	NA

F01_transcript_53415	NA	F01_transcript_53415	NA	NA
F01_transcript_139649	NA	F01_transcript_139649	NA	NA
F01_transcript_80736	NA	F01_transcript_80736	F01_transcript_80736	F01_transcript_80736
F01_transcript_82861	NA	F01_transcript_82861	NA	F01_transcript_82861
F01_transcript_122053	F01_transcript_122053	NA	F01_transcript_122053	F01_transcript_122053
F01_transcript_43766	F01_transcript_43766	F01_transcript_43766	NA	F01_transcript_43766
F01_transcript_135454	F01_transcript_135454	F01_transcript_135454	F01_transcript_135454	F01_transcript_135454
F01_transcript_118615	NA	F01_transcript_118615	NA	F01_transcript_118615
F01_transcript_149249	F01_transcript_149249	F01_transcript_149249	F01_transcript_149249	F01_transcript_149249
F01_transcript_122239	NA	F01_transcript_122239	NA	NA
F01_transcript_153715	NA	NA	NA	F01_transcript_153715
F01_transcript_148009	NA	NA	NA	F01_transcript_148009
F01_transcript_106703	NA	F01_transcript_106703	NA	F01_transcript_106703
F01_transcript_88514	NA	F01_transcript_88514	F01_transcript_88514	F01_transcript_88514

F01_transcript_55959	NA	F01_transcript_55959	NA	F01_transcript_55959
F01_transcript_43398	NA	F01_transcript_43398	NA	NA
F01_transcript_90039	NA	NA	NA	F01_transcript_90039
F01_transcript_92018	NA	F01_transcript_92018	NA	NA
F01_transcript_50181	NA	F01_transcript_50181	NA	NA
F01_transcript_140093	NA	NA	NA	F01_transcript_140093
F01_transcript_124079	NA	F01_transcript_124079	NA	NA
F01_transcript_56632	NA	F01_transcript_56632	NA	NA
F01_transcript_43661	NA	NA	NA	F01_transcript_43661
F01_transcript_147921	NA	F01_transcript_147921	F01_transcript_147921	F01_transcript_147921
F01_transcript_50668	F01_transcript_50668	F01_transcript_50668	F01_transcript_50668	NA
F01_transcript_139171	F01_transcript_139171	F01_transcript_139171	NA	NA
F01_transcript_149334	NA	NA	NA	F01_transcript_149334
F01_transcript_64799	NA	F01_transcript_64799	NA	F01_transcript_64799

F01_transcript_119347	F01_transcript_119347	F01_transcript_119347	F01_transcript_119347	F01_transcript_119347
F01_transcript_93457	NA	NA	NA	F01_transcript_93457
F01_transcript_67257	NA	NA	NA	F01_transcript_67257
F01_transcript_143255	NA	NA	NA	F01_transcript_143255
F01_transcript_95217	NA	F01_transcript_95217	NA	NA
F01_transcript_155452	F01_transcript_155452	F01_transcript_155452	F01_transcript_155452	F01_transcript_155452
F01_transcript_47646	NA	F01_transcript_47646	F01_transcript_47646	F01_transcript_47646
F01_transcript_56289	F01_transcript_56289	F01_transcript_56289	F01_transcript_56289	F01_transcript_56289
F01_transcript_142475	NA	F01_transcript_142475	F01_transcript_142475	F01_transcript_142475
F01_transcript_93437	NA	NA	NA	F01_transcript_93437
F01_transcript_47452	NA	NA	NA	F01_transcript_47452
F01_transcript_122936	NA	NA	NA	F01_transcript_122936
F01_transcript_8066	NA	F01_transcript_8066	NA	NA
F01_transcript_88862	NA	F01_transcript_88862	NA	NA

F01_transcript_69481	NA	F01_transcript_69481	NA	NA
F01_transcript_110861	NA	F01_transcript_110861	NA	NA
F01_transcript_41518	NA	NA	NA	F01_transcript_41518
F01_transcript_104359	NA	F01_transcript_104359	NA	NA
F01_transcript_112906	NA	NA	NA	F01_transcript_112906
F01_transcript_104533	F01_transcript_104533	F01_transcript_104533	NA	NA
F01_transcript_68374	NA	NA	NA	F01_transcript_68374
F01_transcript_95509	NA	F01_transcript_95509	NA	NA
F01_transcript_93663	NA	F01_transcript_93663	NA	NA
F01_transcript_132754	NA	F01_transcript_132754	NA	F01_transcript_132754
F01_transcript_45770	NA	NA	NA	F01_transcript_45770
F01_transcript_49722	NA	F01_transcript_49722	NA	NA
F01_transcript_19596	F01_transcript_19596	F01_transcript_19596	NA	NA
F01_transcript_103345	NA	NA	NA	F01_transcript_103345

F01_transcript_120575	F01_transcript_120575	F01_transcript_120575	F01_transcript_120575	F01_transcript_120575
F01_transcript_164657	NA	NA	NA	F01_transcript_164657
F01_transcript_167120	NA	F01_transcript_167120	NA	NA
F01_transcript_24285	NA	NA	NA	F01_transcript_24285
F01_transcript_44228	NA	F01_transcript_44228	NA	NA
F01_transcript_149270	NA	F01_transcript_149270	NA	NA
F01_transcript_79108	NA	F01_transcript_79108	NA	NA
F01_transcript_24633	NA	F01_transcript_24633	F01_transcript_24633	F01_transcript_24633
F01_transcript_102919	F01_transcript_102919	F01_transcript_102919	F01_transcript_102919	F01_transcript_102919
F01_transcript_53411	F01_transcript_53411	F01_transcript_53411	F01_transcript_53411	F01_transcript_53411
F01_transcript_132199	F01_transcript_132199	F01_transcript_132199	NA	NA
F01_transcript_88186	NA	F01_transcript_88186	NA	NA
F01_transcript_105701	F01_transcript_105701	F01_transcript_105701	F01_transcript_105701	F01_transcript_105701
F01_transcript_93124	F01_transcript_93124	F01_transcript_93124	F01_transcript_93124	F01_transcript_93124

F01_transcript_92087	F01_transcript_92087	F01_transcript_92087	F01_transcript_92087	F01_transcript_92087
F01_transcript_157543	F01_transcript_157543	F01_transcript_157543	F01_transcript_157543	F01_transcript_157543
F01_transcript_73949	NA	F01_transcript_73949	NA	F01_transcript_73949
F01_transcript_74789	F01_transcript_74789	F01_transcript_74789	F01_transcript_74789	NA
F01_transcript_92998	NA	F01_transcript_92998	NA	NA
F01_transcript_109771	NA	F01_transcript_109771	NA	F01_transcript_109771
F01_transcript_115174	NA	F01_transcript_115174	NA	NA
F01_transcript_109285	NA	F01_transcript_109285	NA	F01_transcript_109285
F01_transcript_109682	F01_transcript_109682	F01_transcript_109682	F01_transcript_109682	F01_transcript_109682
F01_transcript_65427	NA	F01_transcript_65427	F01_transcript_65427	NA
F01_transcript_147330	NA	F01_transcript_147330	F01_transcript_147330	F01_transcript_147330
F01_transcript_154927	NA	F01_transcript_154927	F01_transcript_154927	F01_transcript_154927
F01_transcript_110498	NA	F01_transcript_110498	F01_transcript_110498	F01_transcript_110498
F01_transcript_146516	F01_transcript_146516	F01_transcript_146516	F01_transcript_146516	F01_transcript_146516

F01_transcript_61306	NA	F01_transcript_61306	NA	F01_transcript_61306
F01_transcript_136241	F01_transcript_136241	F01_transcript_136241	F01_transcript_136241	F01_transcript_136241
F01_transcript_91684	NA	F01_transcript_91684	F01_transcript_91684	F01_transcript_91684
F01_transcript_157003	NA	F01_transcript_157003	NA	NA
F01_transcript_130684	NA	F01_transcript_130684	NA	NA
F01_transcript_140851	NA	NA	F01_transcript_140851	F01_transcript_140851
F01_transcript_57267	NA	NA	NA	F01_transcript_57267
F01_transcript_66723	F01_transcript_66723	F01_transcript_66723	F01_transcript_66723	NA
F01_transcript_29580	NA	NA	NA	F01_transcript_29580
F01_transcript_92960	NA	F01_transcript_92960	NA	F01_transcript_92960
F01_transcript_162044	NA	F01_transcript_162044	NA	NA
F01_transcript_89512	NA	NA	NA	F01_transcript_89512
F01_transcript_148113	F01_transcript_148113	F01_transcript_148113	NA	NA
F01_transcript_133576	NA	F01_transcript_133576	NA	NA

F01_transcript_125489	NA	NA	F01_transcript_125489	NA
F01_transcript_167410	F01_transcript_167410	F01_transcript_167410	F01_transcript_167410	F01_transcript_167410
F01_transcript_90471	NA	F01_transcript_90471	NA	NA
F01_transcript_107692	NA	F01_transcript_107692	NA	F01_transcript_107692
F01_transcript_54272	NA	F01_transcript_54272	NA	F01_transcript_54272
F01_transcript_106497	F01_transcript_106497	F01_transcript_106497	F01_transcript_106497	F01_transcript_106497
F01_transcript_55166	F01_transcript_55166	F01_transcript_55166	F01_transcript_55166	F01_transcript_55166
F01_transcript_306	NA	F01_transcript_306	NA	F01_transcript_306
F01_transcript_117906	F01_transcript_117906	F01_transcript_117906	NA	F01_transcript_117906
F01_transcript_166358	NA	F01_transcript_166358	NA	NA
F01_transcript_83269	NA	NA	NA	F01_transcript_83269
F01_transcript_65566	NA	F01_transcript_65566	NA	NA
F01_transcript_130691	F01_transcript_130691	F01_transcript_130691	F01_transcript_130691	F01_transcript_130691
F01_transcript_74885	NA	F01_transcript_74885	NA	F01_transcript_74885

F01_transcript_115207	NA	NA	NA	F01_transcript_115207
F01_transcript_73107	NA	NA	F01_transcript_73107	NA
F01_transcript_110312	NA	F01_transcript_110312	NA	F01_transcript_110312
F01_transcript_124745	NA	NA	NA	F01_transcript_124745
F01_transcript_93270	NA	NA	NA	F01_transcript_93270
F01_transcript_91755	NA	F01_transcript_91755	F01_transcript_91755	F01_transcript_91755
F01_transcript_128624	F01_transcript_128624	NA	F01_transcript_128624	F01_transcript_128624
F01_transcript_122680	NA	F01_transcript_122680	NA	NA
F01_transcript_118817	NA	F01_transcript_118817	F01_transcript_118817	NA
F01_transcript_63745	F01_transcript_63745	F01_transcript_63745	F01_transcript_63745	F01_transcript_63745
F01_transcript_79678	NA	NA	NA	F01_transcript_79678
F01_transcript_53772	NA	F01_transcript_53772	NA	F01_transcript_53772
F01_transcript_147534	NA	F01_transcript_147534	NA	NA
F01_transcript_112696	NA	F01_transcript_112696	NA	F01_transcript_112696

F01_transcript_129988	NA	F01_transcript_129988	F01_transcript_129988	F01_transcript_129988
F01_transcript_96513	NA	F01_transcript_96513	NA	NA
F01_transcript_65059	F01_transcript_65059	F01_transcript_65059	F01_transcript_65059	F01_transcript_65059
F01_transcript_16258	F01_transcript_16258	F01_transcript_16258	F01_transcript_16258	F01_transcript_16258
F01_transcript_51563	NA	F01_transcript_51563	NA	F01_transcript_51563
F01_transcript_82274	NA	F01_transcript_82274	NA	NA
F01_transcript_86315	NA	F01_transcript_86315	F01_transcript_86315	NA
F01_transcript_70039	NA	NA	NA	F01_transcript_70039
F01_transcript_68286	F01_transcript_68286	F01_transcript_68286	F01_transcript_68286	F01_transcript_68286
F01_transcript_122149	NA	NA	NA	F01_transcript_122149
F01_transcript_124459	F01_transcript_124459	F01_transcript_124459	F01_transcript_124459	F01_transcript_124459
F01_transcript_51263	F01_transcript_51263	F01_transcript_51263	F01_transcript_51263	F01_transcript_51263
F01_transcript_52754	NA	NA	NA	F01_transcript_52754
F01_transcript_101841	F01_transcript_101841	F01_transcript_101841	F01_transcript_101841	F01_transcript_101841

F01_transcript_84240	NA	NA	NA	F01_transcript_84240
F01_transcript_54219	NA	NA	NA	F01_transcript_54219
F01_transcript_32828	NA	F01_transcript_32828	NA	F01_transcript_32828
F01_transcript_93706	NA	F01_transcript_93706	NA	F01_transcript_93706
F01_transcript_126514	NA	NA	NA	F01_transcript_126514
F01_transcript_68998	NA	F01_transcript_68998	NA	F01_transcript_68998
F01_transcript_49482	NA	F01_transcript_49482	F01_transcript_49482	F01_transcript_49482
F01_transcript_112086	NA	NA	NA	F01_transcript_112086
F01_transcript_111643	NA	F01_transcript_111643	NA	NA
F01_transcript_46992	F01_transcript_46992	F01_transcript_46992	F01_transcript_46992	F01_transcript_46992
F01_transcript_99257	NA	NA	F01_transcript_99257	F01_transcript_99257
F01_transcript_156857	NA	NA	NA	F01_transcript_156857
F01_transcript_88453	NA	F01_transcript_88453	NA	NA
F01_transcript_47527	NA	NA	NA	F01_transcript_47527

F01_transcript_82524	F01_transcript_82524	F01_transcript_82524	F01_transcript_82524	F01_transcript_82524
F01_transcript_167474	NA	F01_transcript_167474	NA	NA
F01_transcript_61600	F01_transcript_61600	F01_transcript_61600	F01_transcript_61600	F01_transcript_61600
F01_transcript_146666	F01_transcript_146666	F01_transcript_146666	NA	NA
F01_transcript_87147	NA	F01_transcript_87147	NA	NA
F01_transcript_125117	NA	F01_transcript_125117	NA	NA
F01_transcript_82354	NA	NA	NA	F01_transcript_82354
F01_transcript_110936	NA	F01_transcript_110936	NA	NA
F01_transcript_92957	NA	F01_transcript_92957	NA	F01_transcript_92957
F01_transcript_107279	F01_transcript_107279	F01_transcript_107279	F01_transcript_107279	F01_transcript_107279
F01_transcript_156882	F01_transcript_156882	F01_transcript_156882	F01_transcript_156882	F01_transcript_156882
F01_transcript_71939	F01_transcript_71939	F01_transcript_71939	NA	NA
F01_transcript_151188	F01_transcript_151188	F01_transcript_151188	NA	NA
F01_transcript_126385	NA	F01_transcript_126385	NA	NA

F01_transcript_113507	NA	F01_transcript_113507	NA	NA
F01_transcript_160496	NA	F01_transcript_160496	NA	NA
F01_transcript_85359	F01_transcript_85359	F01_transcript_85359	NA	F01_transcript_85359
F01_transcript_148532	NA	F01_transcript_148532	NA	NA
F01_transcript_102920	NA	F01_transcript_102920	F01_transcript_102920	NA
F01_transcript_109364	NA	NA	NA	F01_transcript_109364
F01_transcript_56857	NA	F01_transcript_56857	F01_transcript_56857	NA
F01_transcript_71250	NA	NA	F01_transcript_71250	NA
F01_transcript_60710	NA	NA	NA	F01_transcript_60710
F01_transcript_78882	NA	NA	NA	F01_transcript_78882
F01_transcript_148231	NA	NA	NA	F01_transcript_148231
F01_transcript_79743	F01_transcript_79743	F01_transcript_79743	NA	NA
F01_transcript_64976	NA	F01_transcript_64976	F01_transcript_64976	F01_transcript_64976
F01_transcript_64584	F01_transcript_64584	F01_transcript_64584	NA	F01_transcript_64584

F01_transcript_81736	NA	NA	NA	F01_transcript_81736
F01_transcript_150341	NA	NA	NA	F01_transcript_150341
F01_transcript_48022	NA	NA	NA	F01_transcript_48022
F01_transcript_45542	NA	NA	NA	F01_transcript_45542
F01_transcript_97432	NA	F01_transcript_97432	F01_transcript_97432	F01_transcript_97432
F01_transcript_149788	NA	F01_transcript_149788	NA	F01_transcript_149788
F01_transcript_35022	NA	NA	NA	F01_transcript_35022
F01_transcript_85586	NA	NA	NA	F01_transcript_85586
F01_transcript_150042	NA	F01_transcript_150042	F01_transcript_150042	F01_transcript_150042
F01_transcript_77778	NA	F01_transcript_77778	F01_transcript_77778	F01_transcript_77778
F01_transcript_88416	NA	F01_transcript_88416	NA	NA
F01_transcript_53303	NA	F01_transcript_53303	F01_transcript_53303	F01_transcript_53303
F01_transcript_98669	NA	F01_transcript_98669	NA	NA
F01_transcript_51532	F01_transcript_51532	F01_transcript_51532	F01_transcript_51532	F01_transcript_51532

F01_transcript_140382	F01_transcript_140382	F01_transcript_140382	F01_transcript_140382	F01_transcript_140382
F01_transcript_133395	F01_transcript_133395	F01_transcript_133395	NA	F01_transcript_133395
F01_transcript_59818	NA	F01_transcript_59818	NA	NA
F01_transcript_86922	NA	F01_transcript_86922	NA	NA
F01_transcript_138666	F01_transcript_138666	NA	F01_transcript_138666	NA
F01_transcript_89392	F01_transcript_89392	F01_transcript_89392	F01_transcript_89392	F01_transcript_89392
F01_transcript_146167	NA	F01_transcript_146167	F01_transcript_146167	NA
F01_transcript_117047	NA	NA	NA	F01_transcript_117047
F01_transcript_117684	NA	NA	NA	F01_transcript_117684
F01_transcript_101318	F01_transcript_101318	F01_transcript_101318	F01_transcript_101318	F01_transcript_101318
F01_transcript_138388	NA	NA	NA	F01_transcript_138388
F01_transcript_63710	NA	NA	NA	F01_transcript_63710
F01_transcript_102186	NA	F01_transcript_102186	NA	F01_transcript_102186
F01_transcript_129708	F01_transcript_129708	F01_transcript_129708	NA	F01_transcript_129708

F01_transcript_80250	F01_transcript_80250	F01_transcript_80250	F01_transcript_80250	F01_transcript_80250
F01_transcript_13108	NA	F01_transcript_13108	NA	NA
F01_transcript_132951	NA	NA	F01_transcript_132951	NA
F01_transcript_145844	NA	F01_transcript_145844	NA	NA
F01_transcript_147778	F01_transcript_147778	F01_transcript_147778	F01_transcript_147778	F01_transcript_147778
F01_transcript_118651	NA	NA	NA	F01_transcript_118651
F01_transcript_9131	NA	F01_transcript_9131	NA	NA
F01_transcript_41200	NA	NA	NA	F01_transcript_41200
F01_transcript_109580	F01_transcript_109580	F01_transcript_109580	F01_transcript_109580	F01_transcript_109580
F01_transcript_62209	NA	F01_transcript_62209	F01_transcript_62209	F01_transcript_62209
F01_transcript_139409	F01_transcript_139409	F01_transcript_139409	F01_transcript_139409	F01_transcript_139409
F01_transcript_69966	NA	NA	F01_transcript_69966	F01_transcript_69966
F01_transcript_802	NA	NA	NA	F01_transcript_802
F01_transcript_145016	F01_transcript_145016	F01_transcript_145016	NA	F01_transcript_145016

F01_transcript_50538	NA	F01_transcript_50538	NA	F01_transcript_50538
F01_transcript_59310	NA	NA	F01_transcript_59310	NA
F01_transcript_162879	NA	NA	NA	F01_transcript_162879
F01_transcript_67975	F01_transcript_67975	NA	NA	F01_transcript_67975
F01_transcript_103033	NA	NA	NA	F01_transcript_103033
F01_transcript_154393	F01_transcript_154393	F01_transcript_154393	F01_transcript_154393	F01_transcript_154393
F01_transcript_149586	F01_transcript_149586	F01_transcript_149586	F01_transcript_149586	NA
F01_transcript_83766	NA	NA	NA	F01_transcript_83766
F01_transcript_102373	NA	F01_transcript_102373	NA	NA
F01_transcript_13458	NA	NA	NA	F01_transcript_13458
F01_transcript_165981	NA	NA	NA	F01_transcript_165981
F01_transcript_27150	NA	NA	NA	F01_transcript_27150
F01_transcript_114734	F01_transcript_114734	F01_transcript_114734	NA	F01_transcript_114734
F01_transcript_48882	NA	NA	NA	F01_transcript_48882

F01_transcript_115601	NA	NA	NA	F01_transcript_115601
F01_transcript_79831	F01_transcript_79831	F01_transcript_79831	F01_transcript_79831	F01_transcript_79831
F01_transcript_166525	NA	NA	NA	F01_transcript_166525
F01_transcript_76041	NA	NA	NA	F01_transcript_76041
F01_transcript_95218	NA	F01_transcript_95218	NA	NA
F01_transcript_71690	NA	F01_transcript_71690	F01_transcript_71690	F01_transcript_71690
F01_transcript_167081	NA	F01_transcript_167081	NA	NA
F01_transcript_139823	NA	NA	NA	F01_transcript_139823
F01_transcript_50382	NA	F01_transcript_50382	F01_transcript_50382	F01_transcript_50382
F01_transcript_101927	NA	F01_transcript_101927	NA	NA
F01_transcript_135178	NA	NA	NA	F01_transcript_135178
F01_transcript_93335	F01_transcript_93335	F01_transcript_93335	F01_transcript_93335	F01_transcript_93335
F01_transcript_49553	NA	F01_transcript_49553	NA	F01_transcript_49553
F01_transcript_84000	F01_transcript_84000	F01_transcript_84000	F01_transcript_84000	F01_transcript_84000

F01_transcript_23998	NA	NA	NA	F01_transcript_23998
F01_transcript_49492	F01_transcript_49492	F01_transcript_49492	F01_transcript_49492	F01_transcript_49492
F01_transcript_161126	NA	F01_transcript_161126	NA	NA
F01_transcript_62520	NA	NA	NA	F01_transcript_62520
F01_transcript_65261	NA	F01_transcript_65261	F01_transcript_65261	F01_transcript_65261
F01_transcript_52629	NA	F01_transcript_52629	NA	F01_transcript_52629
F01_transcript_52582	NA	NA	NA	F01_transcript_52582
F01_transcript_77745	NA	NA	NA	F01_transcript_77745
F01_transcript_93529	NA	F01_transcript_93529	F01_transcript_93529	F01_transcript_93529
F01_transcript_106594	NA	NA	F01_transcript_106594	F01_transcript_106594
F01_transcript_67412	NA	F01_transcript_67412	NA	NA
F01_transcript_102222	NA	F01_transcript_102222	NA	NA
F01_transcript_155143	NA	F01_transcript_155143	NA	NA
F01_transcript_46547	NA	F01_transcript_46547	NA	NA

F01_transcript_110780	F01_transcript_110780	F01_transcript_110780	F01_transcript_110780	F01_transcript_110780
F01_transcript_82488	NA	F01_transcript_82488	NA	NA
F01_transcript_116384	F01_transcript_116384	F01_transcript_116384	F01_transcript_116384	F01_transcript_116384
F01_transcript_50567	F01_transcript_50567	F01_transcript_50567	F01_transcript_50567	NA
F01_transcript_57298	NA	NA	NA	F01_transcript_57298
F01_transcript_3877	NA	F01_transcript_3877	NA	NA
F01_transcript_87983	F01_transcript_87983	F01_transcript_87983	F01_transcript_87983	NA
F01_transcript_20821	NA	F01_transcript_20821	NA	F01_transcript_20821
F01_transcript_47141	NA	F01_transcript_47141	F01_transcript_47141	F01_transcript_47141
F01_transcript_101868	F01_transcript_101868	F01_transcript_101868	F01_transcript_101868	F01_transcript_101868
F01_transcript_82170	NA	F01_transcript_82170	NA	F01_transcript_82170
F01_transcript_147359	NA	NA	NA	F01_transcript_147359
F01_transcript_120369	NA	F01_transcript_120369	NA	NA
F01_transcript_149895	F01_transcript_149895	F01_transcript_149895	F01_transcript_149895	F01_transcript_149895

F01_transcript_19552	NA	NA	F01_transcript_19552	F01_transcript_19552
F01_transcript_164301	NA	NA	NA	F01_transcript_164301
F01_transcript_106510	NA	F01_transcript_106510	F01_transcript_106510	NA
F01_transcript_45292	NA	NA	NA	F01_transcript_45292
F01_transcript_91618	F01_transcript_91618	F01_transcript_91618	F01_transcript_91618	NA
F01_transcript_27647	NA	NA	NA	F01_transcript_27647
F01_transcript_127281	NA	NA	NA	F01_transcript_127281
F01_transcript_137179	NA	F01_transcript_137179	NA	NA
F01_transcript_92356	NA	F01_transcript_92356	NA	NA
F01_transcript_118013	NA	NA	NA	F01_transcript_118013
F01_transcript_132946	NA	F01_transcript_132946	NA	NA
F01_transcript_123398	NA	NA	NA	F01_transcript_123398
F01_transcript_75290	NA	NA	NA	F01_transcript_75290
F01_transcript_29669	NA	NA	NA	F01_transcript_29669

F01_transcript_94414	F01_transcript_94414	F01_transcript_94414	NA	NA
F01_transcript_56416	NA	NA	F01_transcript_56416	NA
F01_transcript_72972	F01_transcript_72972	F01_transcript_72972	F01_transcript_72972	F01_transcript_72972
F01_transcript_66334	F01_transcript_66334	F01_transcript_66334	F01_transcript_66334	F01_transcript_66334
F01_transcript_92689	NA	NA	NA	F01_transcript_92689
F01_transcript_73919	NA	NA	NA	F01_transcript_73919
F01_transcript_149440	F01_transcript_149440	F01_transcript_149440	F01_transcript_149440	F01_transcript_149440
F01_transcript_41276	NA	NA	NA	F01_transcript_41276
F01_transcript_115753	NA	NA	NA	F01_transcript_115753
F01_transcript_102848	NA	NA	NA	F01_transcript_102848
F01_transcript_73878	NA	F01_transcript_73878	NA	NA
F01_transcript_148722	NA	NA	F01_transcript_148722	F01_transcript_148722
F01_transcript_55041	NA	F01_transcript_55041	NA	F01_transcript_55041
F01_transcript_167110	F01_transcript_167110	F01_transcript_167110	NA	NA

F01_transcript_115312	F01_transcript_115312	F01_transcript_115312	NA	NA
F01_transcript_137696	NA	NA	NA	F01_transcript_137696
F01_transcript_163355	NA	F01_transcript_163355	F01_transcript_163355	F01_transcript_163355
F01_transcript_78820	NA	F01_transcript_78820	NA	F01_transcript_78820
F01_transcript_53113	NA	NA	NA	F01_transcript_53113
F01_transcript_121517	F01_transcript_121517	F01_transcript_121517	F01_transcript_121517	F01_transcript_121517
F01_transcript_131632	NA	NA	NA	F01_transcript_131632
F01_transcript_123171	NA	NA	NA	F01_transcript_123171
F01_transcript_152607	NA	NA	NA	F01_transcript_152607
F01_transcript_72546	NA	F01_transcript_72546	NA	F01_transcript_72546
F01_transcript_132003	NA	F01_transcript_132003	NA	NA
F01_transcript_134287	NA	NA	NA	F01_transcript_134287
F01_transcript_2327	NA	NA	NA	F01_transcript_2327
F01_transcript_162758	F01_transcript_162758	F01_transcript_162758	NA	NA

F01_transcript_85946	NA	NA	NA	F01_transcript_85946
F01_transcript_79212	NA	F01_transcript_79212	NA	NA
F01_transcript_118629	F01_transcript_118629	F01_transcript_118629	NA	NA
F01_transcript_131058	NA	NA	NA	F01_transcript_131058
F01_transcript_149668	F01_transcript_149668	F01_transcript_149668	F01_transcript_149668	F01_transcript_149668
F01_transcript_125731	F01_transcript_125731	F01_transcript_125731	F01_transcript_125731	F01_transcript_125731
F01_transcript_30640	NA	NA	NA	F01_transcript_30640
F01_transcript_56273	NA	NA	F01_transcript_56273	F01_transcript_56273
F01_transcript_155556	NA	NA	NA	F01_transcript_155556
F01_transcript_113629	NA	F01_transcript_113629	F01_transcript_113629	F01_transcript_113629
F01_transcript_159862	NA	NA	NA	F01_transcript_159862
F01_transcript_149669	NA	F01_transcript_149669	NA	NA
F01_transcript_143575	NA	F01_transcript_143575	NA	NA
F01_transcript_147573	NA	F01_transcript_147573	NA	NA

F01_transcript_26934	NA	F01_transcript_26934	NA	NA
F01_transcript_78547	NA	NA	NA	F01_transcript_78547
F01_transcript_157816	NA	NA	NA	F01_transcript_157816
F01_transcript_151886	NA	F01_transcript_151886	NA	NA
F01_transcript_76486	NA	NA	NA	F01_transcript_76486
F01_transcript_16320	NA	NA	NA	F01_transcript_16320
F01_transcript_110907	NA	F01_transcript_110907	NA	NA
F01_transcript_143796	NA	F01_transcript_143796	NA	NA
F01_transcript_107620	F01_transcript_107620	F01_transcript_107620	F01_transcript_107620	F01_transcript_107620
F01_transcript_11000	NA	F01_transcript_11000	NA	NA
F01_transcript_100016	F01_transcript_100016	F01_transcript_100016	F01_transcript_100016	NA
F01_transcript_68474	F01_transcript_68474	F01_transcript_68474	F01_transcript_68474	NA
F01_transcript_159915	NA	F01_transcript_159915	F01_transcript_159915	F01_transcript_159915
F01_transcript_84184	NA	NA	NA	F01_transcript_84184

F01_transcript_61467	F01_transcript_61467	F01_transcript_61467	F01_transcript_61467	NA
F01_transcript_46704	NA	F01_transcript_46704	NA	NA
F01_transcript_102735	NA	NA	NA	F01_transcript_102735
F01_transcript_11901	NA	F01_transcript_11901	NA	NA
F01_transcript_112036	NA	NA	NA	F01_transcript_112036
F01_transcript_53157	F01_transcript_53157	F01_transcript_53157	F01_transcript_53157	F01_transcript_53157
F01_transcript_68149	NA	F01_transcript_68149	NA	NA
F01_transcript_113651	NA	NA	NA	F01_transcript_113651
F01_transcript_126710	F01_transcript_126710	F01_transcript_126710	F01_transcript_126710	F01_transcript_126710
F01_transcript_113725	NA	F01_transcript_113725	NA	F01_transcript_113725
F01_transcript_73318	NA	NA	NA	F01_transcript_73318
F01_transcript_157814	F01_transcript_157814	F01_transcript_157814	NA	NA
F01_transcript_166936	NA	NA	NA	F01_transcript_166936
F01_transcript_62073	NA	NA	NA	F01_transcript_62073

F01_transcript_138029	NA	F01_transcript_138029	NA	NA
F01_transcript_55660	NA	F01_transcript_55660	NA	NA
F01_transcript_123689	NA	F01_transcript_123689	NA	NA
F01_transcript_148504	NA	F01_transcript_148504	NA	NA
F01_transcript_162635	NA	F01_transcript_162635	NA	NA
F01_transcript_105719	NA	NA	F01_transcript_105719	F01_transcript_105719
F01_transcript_71614	NA	F01_transcript_71614	NA	NA
F01_transcript_119116	NA	F01_transcript_119116	NA	NA
F01_transcript_124505	NA	NA	NA	F01_transcript_124505
F01_transcript_16025	NA	NA	NA	F01_transcript_16025
F01_transcript_144380	NA	NA	NA	F01_transcript_144380
F01_transcript_116641	F01_transcript_116641	F01_transcript_116641	F01_transcript_116641	F01_transcript_116641
F01_transcript_8668	NA	NA	NA	F01_transcript_8668
F01_transcript_52680	F01_transcript_52680	F01_transcript_52680	F01_transcript_52680	F01_transcript_52680

F01_transcript_80839	NA	NA	NA	F01_transcript_80839
F01_transcript_71931	NA	F01_transcript_71931	NA	NA
F01_transcript_75620	NA	F01_transcript_75620	NA	NA
F01_transcript_159395	F01_transcript_159395	F01_transcript_159395	F01_transcript_159395	F01_transcript_159395
F01_transcript_128975	F01_transcript_128975	F01_transcript_128975	NA	F01_transcript_128975
F01_transcript_70811	NA	F01_transcript_70811	NA	NA
F01_transcript_27902	NA	NA	NA	F01_transcript_27902
F01_transcript_121404	NA	NA	NA	F01_transcript_121404
F01_transcript_50435	NA	NA	NA	F01_transcript_50435
F01_transcript_21503	NA	NA	NA	F01_transcript_21503
F01_transcript_86167	F01_transcript_86167	F01_transcript_86167	F01_transcript_86167	F01_transcript_86167
F01_transcript_124979	F01_transcript_124979	F01_transcript_124979	F01_transcript_124979	F01_transcript_124979
F01_transcript_150588	NA	NA	NA	F01_transcript_150588
F01_transcript_163533	F01_transcript_163533	F01_transcript_163533	F01_transcript_163533	F01_transcript_163533

F01_transcript_86374	F01_transcript_86374	F01_transcript_86374	NA	F01_transcript_86374
F01_transcript_1435	NA	NA	NA	F01_transcript_1435
F01_transcript_83137	NA	NA	NA	F01_transcript_83137
F01_transcript_79583	NA	NA	NA	F01_transcript_79583
F01_transcript_79674	NA	NA	NA	F01_transcript_79674
F01_transcript_107307	NA	NA	NA	F01_transcript_107307
F01_transcript_74136	F01_transcript_74136	F01_transcript_74136	F01_transcript_74136	F01_transcript_74136
F01_transcript_135661	NA	F01_transcript_135661	NA	NA
F01_transcript_39091	NA	NA	NA	F01_transcript_39091
F01_transcript_146570	F01_transcript_146570	F01_transcript_146570	F01_transcript_146570	F01_transcript_146570
F01_transcript_68325	F01_transcript_68325	F01_transcript_68325	F01_transcript_68325	NA
F01_transcript_69994	NA	F01_transcript_69994	NA	NA
F01_transcript_139561	F01_transcript_139561	F01_transcript_139561	F01_transcript_139561	F01_transcript_139561
F01_transcript_104313	NA	NA	NA	F01_transcript_104313

F01_transcript_89835	F01_transcript_89835	F01_transcript_89835	F01_transcript_89835	F01_transcript_89835
F01_transcript_103825	NA	NA	NA	F01_transcript_103825
F01_transcript_113977	NA	NA	NA	F01_transcript_113977
F01_transcript_93729	F01_transcript_93729	F01_transcript_93729	F01_transcript_93729	F01_transcript_93729
F01_transcript_99709	NA	F01_transcript_99709	NA	NA
F01_transcript_96243	F01_transcript_96243	F01_transcript_96243	F01_transcript_96243	F01_transcript_96243
F01_transcript_105978	NA	F01_transcript_105978	NA	NA
F01_transcript_131628	NA	F01_transcript_131628	F01_transcript_131628	NA
F01_transcript_70831	NA	NA	F01_transcript_70831	NA
F01_transcript_26988	NA	NA	NA	F01_transcript_26988
F01_transcript_134536	NA	NA	NA	F01_transcript_134536
F01_transcript_122124	NA	F01_transcript_122124	F01_transcript_122124	NA
F01_transcript_113333	NA	F01_transcript_113333	NA	F01_transcript_113333
F01_transcript_141485	NA	NA	NA	F01_transcript_141485

F01_transcript_157404	NA	NA	NA	F01_transcript_157404
F01_transcript_70429	F01_transcript_70429	F01_transcript_70429	NA	NA
F01_transcript_117321	F01_transcript_117321	F01_transcript_117321	F01_transcript_117321	F01_transcript_117321
F01_transcript_111097	NA	NA	NA	F01_transcript_111097
F01_transcript_55164	F01_transcript_55164	F01_transcript_55164	F01_transcript_55164	F01_transcript_55164
F01_transcript_125319	NA	F01_transcript_125319	F01_transcript_125319	F01_transcript_125319
F01_transcript_129496	NA	F01_transcript_129496	NA	NA
F01_transcript_73396	NA	F01_transcript_73396	NA	F01_transcript_73396
F01_transcript_162678	NA	F01_transcript_162678	NA	NA
F01_transcript_117127	NA	NA	NA	F01_transcript_117127
F01_transcript_118549	NA	NA	NA	F01_transcript_118549
F01_transcript_59372	NA	NA	NA	F01_transcript_59372
F01_transcript_139953	NA	F01_transcript_139953	NA	NA
F01_transcript_55387	NA	F01_transcript_55387	NA	NA

F01_transcript_139535	NA	F01_transcript_139535	NA	F01_transcript_139535
F01_transcript_156704	NA	F01_transcript_156704	NA	NA
F01_transcript_138225	NA	F01_transcript_138225	NA	NA
F01_transcript_119905	NA	NA	NA	F01_transcript_119905
F01_transcript_135994	NA	F01_transcript_135994	NA	NA
F01_transcript_106345	F01_transcript_106345	F01_transcript_106345	F01_transcript_106345	F01_transcript_106345
F01_transcript_119367	F01_transcript_119367	F01_transcript_119367	F01_transcript_119367	F01_transcript_119367
F01_transcript_55879	F01_transcript_55879	F01_transcript_55879	NA	F01_transcript_55879
F01_transcript_45318	NA	F01_transcript_45318	NA	NA
F01_transcript_109831	NA	F01_transcript_109831	NA	NA
F01_transcript_151810	NA	NA	NA	F01_transcript_151810
F01_transcript_58608	NA	F01_transcript_58608	NA	F01_transcript_58608
F01_transcript_53751	NA	NA	NA	F01_transcript_53751
F01_transcript_121471	NA	F01_transcript_121471	NA	F01_transcript_121471

F01_transcript_155903	NA	F01_transcript_155903	NA	NA
F01_transcript_78591	NA	NA	NA	F01_transcript_78591
F01_transcript_60968	F01_transcript_60968	F01_transcript_60968	F01_transcript_60968	F01_transcript_60968
F01_transcript_123197	F01_transcript_123197	F01_transcript_123197	F01_transcript_123197	F01_transcript_123197
F01_transcript_165178	NA	F01_transcript_165178	F01_transcript_165178	NA
F01_transcript_51664	F01_transcript_51664	F01_transcript_51664	NA	F01_transcript_51664
F01_transcript_87376	NA	F01_transcript_87376	NA	F01_transcript_87376
F01_transcript_83100	NA	F01_transcript_83100	NA	F01_transcript_83100
F01_transcript_87042	NA	NA	NA	F01_transcript_87042
F01_transcript_64566	NA	NA	NA	F01_transcript_64566
F01_transcript_99332	NA	NA	NA	F01_transcript_99332
F01_transcript_116687	NA	F01_transcript_116687	NA	NA
F01_transcript_116585	F01_transcript_116585	F01_transcript_116585	NA	F01_transcript_116585
F01_transcript_127067	NA	F01_transcript_127067	NA	NA

F01_transcript_134225	F01_transcript_134225	F01_transcript_134225	F01_transcript_134225	F01_transcript_134225
F01_transcript_156918	NA	F01_transcript_156918	NA	NA
F01_transcript_161861	F01_transcript_161861	F01_transcript_161861	F01_transcript_161861	F01_transcript_161861
F01_transcript_116437	NA	NA	NA	F01_transcript_116437
F01_transcript_569	NA	NA	NA	F01_transcript_569
F01_transcript_56155	NA	NA	NA	F01_transcript_56155
F01_transcript_134680	NA	NA	NA	F01_transcript_134680
F01_transcript_70474	NA	NA	NA	F01_transcript_70474
F01_transcript_162926	NA	F01_transcript_162926	F01_transcript_162926	F01_transcript_162926
F01_transcript_163003	NA	NA	NA	F01_transcript_163003
F01_transcript_78986	NA	F01_transcript_78986	F01_transcript_78986	F01_transcript_78986
F01_transcript_137424	NA	F01_transcript_137424	NA	F01_transcript_137424
F01_transcript_55921	NA	NA	NA	F01_transcript_55921
F01_transcript_148865	NA	F01_transcript_148865	NA	F01_transcript_148865

F01_transcript_51861	F01_transcript_51861	F01_transcript_51861	F01_transcript_51861	F01_transcript_51861
F01_transcript_20439	NA	NA	NA	F01_transcript_20439
F01_transcript_114846	NA	NA	NA	F01_transcript_114846
F01_transcript_19214	NA	NA	NA	F01_transcript_19214
F01_transcript_116593	F01_transcript_116593	F01_transcript_116593	NA	NA
F01_transcript_30728	NA	NA	NA	F01_transcript_30728
F01_transcript_156290	NA	NA	NA	F01_transcript_156290
F01_transcript_77033	F01_transcript_77033	F01_transcript_77033	F01_transcript_77033	F01_transcript_77033
F01_transcript_166784	NA	F01_transcript_166784	NA	F01_transcript_166784
F01_transcript_123680	NA	NA	NA	F01_transcript_123680
F01_transcript_142353	NA	F01_transcript_142353	NA	NA
F01_transcript_102524	NA	NA	F01_transcript_102524	F01_transcript_102524
F01_transcript_111174	F01_transcript_111174	F01_transcript_111174	F01_transcript_111174	NA
F01_transcript_113846	NA	NA	NA	F01_transcript_113846

F01_transcript_122346	NA	NA	NA	F01_transcript_122346
F01_transcript_128799	NA	F01_transcript_128799	NA	NA
F01_transcript_101616	NA	NA	NA	F01_transcript_101616
F01_transcript_44348	NA	F01_transcript_44348	NA	NA
F01_transcript_143184	F01_transcript_143184	F01_transcript_143184	F01_transcript_143184	NA
F01_transcript_137534	F01_transcript_137534	F01_transcript_137534	F01_transcript_137534	F01_transcript_137534
F01_transcript_87605	F01_transcript_87605	F01_transcript_87605	F01_transcript_87605	NA
F01_transcript_58740	F01_transcript_58740	F01_transcript_58740	F01_transcript_58740	NA
F01_transcript_6493	NA	NA	NA	F01_transcript_6493
F01_transcript_34424	NA	NA	NA	F01_transcript_34424
F01_transcript_116254	NA	F01_transcript_116254	NA	F01_transcript_116254
F01_transcript_9153	NA	NA	NA	F01_transcript_9153
F01_transcript_166254	NA	F01_transcript_166254	NA	NA
F01_transcript_135708	NA	F01_transcript_135708	NA	NA

F01_transcript_152992	F01_transcript_152992	F01_transcript_152992	F01_transcript_152992	NA
F01_transcript_96613	F01_transcript_96613	F01_transcript_96613	F01_transcript_96613	NA
F01_transcript_19994	NA	NA	NA	F01_transcript_19994
F01_transcript_142951	NA	NA	NA	F01_transcript_142951
F01_transcript_48004	NA	NA	NA	F01_transcript_48004
F01_transcript_18903	NA	F01_transcript_18903	NA	NA
F01_transcript_63293	F01_transcript_63293	F01_transcript_63293	NA	NA
F01_transcript_80391	F01_transcript_80391	F01_transcript_80391	F01_transcript_80391	F01_transcript_80391
F01_transcript_56293	NA	NA	F01_transcript_56293	NA
F01_transcript_127056	NA	NA	NA	F01_transcript_127056
F01_transcript_85925	NA	NA	NA	F01_transcript_85925
F01_transcript_113461	NA	F01_transcript_113461	NA	NA
F01_transcript_50354	F01_transcript_50354	F01_transcript_50354	F01_transcript_50354	F01_transcript_50354
F01_transcript_133977	NA	NA	NA	F01_transcript_133977

F01_transcript_120908	NA	F01_transcript_120908	NA	NA
F01_transcript_73688	NA	NA	NA	F01_transcript_73688
F01_transcript_27675	NA	NA	NA	F01_transcript_27675
F01_transcript_75522	NA	F01_transcript_75522	F01_transcript_75522	F01_transcript_75522
F01_transcript_120380	F01_transcript_120380	F01_transcript_120380	NA	F01_transcript_120380
F01_transcript_107185	NA	NA	F01_transcript_107185	NA
F01_transcript_140613	NA	F01_transcript_140613	NA	NA
F01_transcript_105053	NA	NA	NA	F01_transcript_105053
F01_transcript_52145	NA	F01_transcript_52145	NA	NA
F01_transcript_100164	NA	NA	NA	F01_transcript_100164
F01_transcript_149070	NA	NA	NA	F01_transcript_149070
F01_transcript_52065	NA	F01_transcript_52065	NA	F01_transcript_52065
F01_transcript_66679	NA	F01_transcript_66679	F01_transcript_66679	F01_transcript_66679
F01_transcript_101194	F01_transcript_101194	F01_transcript_101194	F01_transcript_101194	F01_transcript_101194

F01_transcript_65352	F01_transcript_65352	F01_transcript_65352	F01_transcript_65352	F01_transcript_65352
F01_transcript_64545	F01_transcript_64545	F01_transcript_64545	F01_transcript_64545	F01_transcript_64545
F01_transcript_137535	NA	NA	NA	F01_transcript_137535
F01_transcript_33384	NA	NA	NA	F01_transcript_33384
F01_transcript_140444	NA	F01_transcript_140444	NA	F01_transcript_140444
F01_transcript_25822	NA	F01_transcript_25822	NA	F01_transcript_25822
F01_transcript_80399	NA	F01_transcript_80399	NA	NA
F01_transcript_58235	NA	F01_transcript_58235	NA	F01_transcript_58235
F01_transcript_159215	F01_transcript_159215	F01_transcript_159215	F01_transcript_159215	F01_transcript_159215
F01_transcript_72980	NA	F01_transcript_72980	NA	NA
F01_transcript_158647	NA	F01_transcript_158647	NA	F01_transcript_158647
F01_transcript_150321	F01_transcript_150321	F01_transcript_150321	NA	F01_transcript_150321
F01_transcript_124492	F01_transcript_124492	F01_transcript_124492	F01_transcript_124492	F01_transcript_124492
F01_transcript_110891	NA	F01_transcript_110891	NA	NA

F01_transcript_79171	NA	F01_transcript_79171	NA	NA
F01_transcript_163187	NA	NA	NA	F01_transcript_163187
F01_transcript_138940	NA	NA	NA	F01_transcript_138940
F01_transcript_133195	NA	F01_transcript_133195	NA	NA
F01_transcript_59568	NA	F01_transcript_59568	NA	F01_transcript_59568
F01_transcript_157635	F01_transcript_157635	F01_transcript_157635	F01_transcript_157635	F01_transcript_157635
F01_transcript_141629	NA	NA	NA	F01_transcript_141629
F01_transcript_152588	NA	F01_transcript_152588	NA	NA
F01_transcript_56170	NA	NA	NA	F01_transcript_56170
F01_transcript_160325	NA	NA	NA	F01_transcript_160325
F01_transcript_65041	NA	F01_transcript_65041	NA	NA
F01_transcript_87168	NA	F01_transcript_87168	NA	NA
F01_transcript_142918	NA	F01_transcript_142918	F01_transcript_142918	F01_transcript_142918
F01_transcript_116504	NA	NA	NA	F01_transcript_116504

F01_transcript_23214	NA	NA	NA	F01_transcript_23214
F01_transcript_156741	NA	F01_transcript_156741	NA	NA
F01_transcript_48263	NA	NA	NA	F01_transcript_48263
F01_transcript_29439	NA	F01_transcript_29439	NA	NA
F01_transcript_89805	NA	NA	NA	F01_transcript_89805
F01_transcript_47825	F01_transcript_47825	F01_transcript_47825	F01_transcript_47825	F01_transcript_47825
F01_transcript_157809	NA	F01_transcript_157809	NA	NA
F01_transcript_152216	NA	F01_transcript_152216	NA	NA
F01_transcript_97433	F01_transcript_97433	F01_transcript_97433	NA	F01_transcript_97433
F01_transcript_133722	NA	F01_transcript_133722	F01_transcript_133722	F01_transcript_133722
F01_transcript_50262	F01_transcript_50262	F01_transcript_50262	F01_transcript_50262	F01_transcript_50262
F01_transcript_147283	NA	F01_transcript_147283	NA	NA
F01_transcript_16055	NA	NA	NA	F01_transcript_16055
F01_transcript_106801	NA	F01_transcript_106801	NA	NA

F01_transcript_160276	F01_transcript_160276	F01_transcript_160276	F01_transcript_160276	F01_transcript_160276
F01_transcript_104268	NA	F01_transcript_104268	NA	NA
F01_transcript_38209	NA	NA	NA	F01_transcript_38209
F01_transcript_39021	NA	NA	NA	F01_transcript_39021
F01_transcript_133287	NA	NA	NA	F01_transcript_133287
F01_transcript_45308	NA	NA	F01_transcript_45308	F01_transcript_45308
F01_transcript_58733	NA	F01_transcript_58733	NA	NA
F01_transcript_45465	NA	F01_transcript_45465	NA	NA
F01_transcript_82479	NA	NA	NA	F01_transcript_82479
F01_transcript_145134	NA	F01_transcript_145134	NA	NA
F01_transcript_165367	NA	NA	NA	F01_transcript_165367
F01_transcript_57172	NA	NA	NA	F01_transcript_57172
F01_transcript_110393	NA	NA	NA	F01_transcript_110393
F01_transcript_161873	NA	NA	NA	F01_transcript_161873

F01_transcript_124157	NA	NA	NA	F01_transcript_124157
F01_transcript_147123	NA	NA	NA	F01_transcript_147123
F01_transcript_65072	NA	NA	NA	F01_transcript_65072
F01_transcript_83684	NA	NA	NA	F01_transcript_83684
F01_transcript_146149	NA	F01_transcript_146149	NA	F01_transcript_146149
F01_transcript_55744	F01_transcript_55744	F01_transcript_55744	NA	NA
F01_transcript_62793	F01_transcript_62793	F01_transcript_62793	F01_transcript_62793	F01_transcript_62793
F01_transcript_100082	F01_transcript_100082	F01_transcript_100082	F01_transcript_100082	F01_transcript_100082
F01_transcript_44134	NA	NA	NA	F01_transcript_44134
F01_transcript_75297	NA	F01_transcript_75297	F01_transcript_75297	F01_transcript_75297
F01_transcript_45519	F01_transcript_45519	F01_transcript_45519	F01_transcript_45519	F01_transcript_45519
F01_transcript_35913	NA	F01_transcript_35913	NA	NA
F01_transcript_134237	NA	F01_transcript_134237	NA	NA
F01_transcript_71945	F01_transcript_71945	F01_transcript_71945	F01_transcript_71945	F01_transcript_71945

F01_transcript_120818	NA	F01_transcript_120818	F01_transcript_120818	F01_transcript_120818
F01_transcript_2210	NA	NA	NA	F01_transcript_2210
F01_transcript_134830	NA	F01_transcript_134830	F01_transcript_134830	F01_transcript_134830
F01_transcript_144635	NA	F01_transcript_144635	F01_transcript_144635	F01_transcript_144635
F01_transcript_96685	NA	F01_transcript_96685	NA	F01_transcript_96685
F01_transcript_90957	F01_transcript_90957	F01_transcript_90957	F01_transcript_90957	F01_transcript_90957
F01_transcript_108755	NA	F01_transcript_108755	NA	NA
F01_transcript_145347	NA	F01_transcript_145347	NA	NA
F01_transcript_160852	NA	NA	NA	F01_transcript_160852
F01_transcript_87778	NA	NA	NA	F01_transcript_87778
F01_transcript_396	NA	NA	NA	F01_transcript_396
F01_transcript_147993	NA	F01_transcript_147993	NA	NA
F01_transcript_92233	NA	NA	NA	F01_transcript_92233
F01_transcript_75885	NA	NA	NA	F01_transcript_75885

F01_transcript_70987	NA	F01_transcript_70987	F01_transcript_70987	NA
F01_transcript_70481	NA	F01_transcript_70481	NA	NA
F01_transcript_46348	NA	NA	NA	F01_transcript_46348
F01_transcript_136091	F01_transcript_136091	F01_transcript_136091	F01_transcript_136091	F01_transcript_136091
F01_transcript_157946	NA	F01_transcript_157946	F01_transcript_157946	F01_transcript_157946
F01_transcript_95775	NA	NA	NA	F01_transcript_95775
F01_transcript_55857	NA	F01_transcript_55857	F01_transcript_55857	F01_transcript_55857
F01_transcript_146893	F01_transcript_146893	F01_transcript_146893	F01_transcript_146893	F01_transcript_146893
F01_transcript_96341	NA	NA	NA	F01_transcript_96341
F01_transcript_77604	F01_transcript_77604	F01_transcript_77604	F01_transcript_77604	NA
F01_transcript_135084	F01_transcript_135084	F01_transcript_135084	NA	NA
F01_transcript_80878	NA	NA	NA	F01_transcript_80878
F01_transcript_127855	F01_transcript_127855	F01_transcript_127855	F01_transcript_127855	F01_transcript_127855
F01_transcript_74121	NA	F01_transcript_74121	NA	NA

F01_transcript_2718	NA	F01_transcript_2718	NA	NA
F01_transcript_77460	NA	NA	NA	F01_transcript_77460
F01_transcript_79044	F01_transcript_79044	F01_transcript_79044	F01_transcript_79044	F01_transcript_79044
F01_transcript_33988	NA	NA	NA	F01_transcript_33988
F01_transcript_61558	F01_transcript_61558	F01_transcript_61558	F01_transcript_61558	F01_transcript_61558
F01_transcript_59845	NA	F01_transcript_59845	NA	NA
F01_transcript_57046	F01_transcript_57046	F01_transcript_57046	F01_transcript_57046	NA
F01_transcript_50652	F01_transcript_50652	F01_transcript_50652	F01_transcript_50652	F01_transcript_50652
F01_transcript_126163	F01_transcript_126163	F01_transcript_126163	NA	NA
F01_transcript_72052	F01_transcript_72052	NA	NA	NA
F01_transcript_103141	F01_transcript_103141	F01_transcript_103141	F01_transcript_103141	NA
F01_transcript_84518	NA	F01_transcript_84518	NA	F01_transcript_84518
F01_transcript_86707	NA	NA	NA	F01_transcript_86707
F01_transcript_144603	NA	F01_transcript_144603	F01_transcript_144603	NA

F01_transcript_145089	NA	NA	NA	F01_transcript_145089
F01_transcript_165074	NA	F01_transcript_165074	F01_transcript_165074	F01_transcript_165074
F01_transcript_81945	NA	NA	NA	F01_transcript_81945
F01_transcript_97531	NA	NA	NA	F01_transcript_97531
F01_transcript_99329	NA	NA	NA	F01_transcript_99329
F01_transcript_17708	NA	F01_transcript_17708	NA	NA
F01_transcript_52741	NA	F01_transcript_52741	NA	NA
F01_transcript_154274	NA	NA	NA	F01_transcript_154274
F01_transcript_102180	NA	NA	NA	F01_transcript_102180
F01_transcript_69880	F01_transcript_69880	F01_transcript_69880	NA	F01_transcript_69880
F01_transcript_82454	NA	NA	F01_transcript_82454	NA
F01_transcript_98164	NA	F01_transcript_98164	NA	NA
F01_transcript_80123	NA	F01_transcript_80123	NA	NA
F01_transcript_59444	NA	NA	F01_transcript_59444	NA

F01_transcript_23971	F01_transcript_23971	F01_transcript_23971	F01_transcript_23971	F01_transcript_23971
F01_transcript_139522	NA	NA	NA	F01_transcript_139522
F01_transcript_50163	F01_transcript_50163	NA	NA	F01_transcript_50163
F01_transcript_30931	NA	NA	NA	F01_transcript_30931
F01_transcript_161547	NA	NA	NA	F01_transcript_161547
F01_transcript_39213	NA	NA	NA	F01_transcript_39213
F01_transcript_68807	NA	F01_transcript_68807	F01_transcript_68807	NA
F01_transcript_116787	F01_transcript_116787	F01_transcript_116787	F01_transcript_116787	F01_transcript_116787
F01_transcript_75969	F01_transcript_75969	F01_transcript_75969	F01_transcript_75969	F01_transcript_75969
F01_transcript_107329	NA	NA	NA	F01_transcript_107329
F01_transcript_142914	F01_transcript_142914	F01_transcript_142914	NA	NA
F01_transcript_35087	NA	F01_transcript_35087	NA	F01_transcript_35087
F01_transcript_27821	NA	NA	NA	F01_transcript_27821
F01_transcript_165998	NA	NA	NA	F01_transcript_165998

F01_transcript_77738	NA	NA	NA	F01_transcript_77738
F01_transcript_134893	NA	NA	NA	F01_transcript_134893
F01_transcript_78300	NA	NA	NA	F01_transcript_78300
F01_transcript_27696	NA	NA	NA	F01_transcript_27696
F01_transcript_160761	NA	NA	NA	F01_transcript_160761
F01_transcript_56972	NA	NA	F01_transcript_56972	F01_transcript_56972
F01_transcript_96338	F01_transcript_96338	F01_transcript_96338	NA	NA
F01_transcript_141253	NA	F01_transcript_141253	NA	NA
F01_transcript_87058	NA	NA	NA	F01_transcript_87058
F01_transcript_132086	NA	NA	NA	F01_transcript_132086
F01_transcript_71090	F01_transcript_71090	F01_transcript_71090	F01_transcript_71090	F01_transcript_71090
F01_transcript_129245	F01_transcript_129245	F01_transcript_129245	F01_transcript_129245	F01_transcript_129245
F01_transcript_127018	NA	F01_transcript_127018	NA	NA
F01_transcript_144772	F01_transcript_144772	F01_transcript_144772	F01_transcript_144772	F01_transcript_144772

F01_transcript_122293	NA	F01_transcript_122293	NA	NA
F01_transcript_64452	NA	F01_transcript_64452	NA	NA
F01_transcript_69284	F01_transcript_69284	F01_transcript_69284	F01_transcript_69284	F01_transcript_69284
F01_transcript_166413	NA	NA	NA	F01_transcript_166413
F01_transcript_138241	F01_transcript_138241	F01_transcript_138241	F01_transcript_138241	F01_transcript_138241
F01_transcript_11838	NA	F01_transcript_11838	NA	NA
F01_transcript_162368	F01_transcript_162368	F01_transcript_162368	F01_transcript_162368	F01_transcript_162368
F01_transcript_99224	NA	NA	NA	F01_transcript_99224
F01_transcript_159980	NA	NA	NA	F01_transcript_159980
F01_transcript_115025	NA	NA	NA	F01_transcript_115025
F01_transcript_151700	NA	NA	NA	F01_transcript_151700
F01_transcript_91480	NA	F01_transcript_91480	NA	NA
F01_transcript_93265	F01_transcript_93265	F01_transcript_93265	NA	NA
F01_transcript_95617	F01_transcript_95617	F01_transcript_95617	NA	NA

F01_transcript_94067	NA	F01_transcript_94067	NA	F01_transcript_94067
F01_transcript_67567	NA	NA	NA	F01_transcript_67567
F01_transcript_49054	NA	F01_transcript_49054	NA	NA
F01_transcript_163481	NA	NA	NA	F01_transcript_163481
F01_transcript_162066	F01_transcript_162066	NA	NA	F01_transcript_162066
F01_transcript_166649	NA	F01_transcript_166649	NA	NA
F01_transcript_78680	NA	F01_transcript_78680	NA	NA
F01_transcript_160128	F01_transcript_160128	F01_transcript_160128	F01_transcript_160128	F01_transcript_160128
F01_transcript_149966	NA	NA	NA	F01_transcript_149966
F01_transcript_75599	NA	F01_transcript_75599	NA	NA
F01_transcript_158145	NA	NA	NA	F01_transcript_158145
F01_transcript_92678	NA	NA	NA	F01_transcript_92678
F01_transcript_73769	F01_transcript_73769	F01_transcript_73769	F01_transcript_73769	F01_transcript_73769
F01_transcript_48047	F01_transcript_48047	F01_transcript_48047	F01_transcript_48047	F01_transcript_48047

F01_transcript_79127	NA	NA	NA	F01_transcript_79127
F01_transcript_141279	NA	F01_transcript_141279	F01_transcript_141279	F01_transcript_141279
F01_transcript_65136	F01_transcript_65136	F01_transcript_65136	F01_transcript_65136	F01_transcript_65136
F01_transcript_52271	NA	NA	NA	F01_transcript_52271
F01_transcript_103224	NA	F01_transcript_103224	NA	NA
F01_transcript_103861	NA	NA	NA	F01_transcript_103861
F01_transcript_108795	F01_transcript_108795	F01_transcript_108795	F01_transcript_108795	F01_transcript_108795
F01_transcript_83485	NA	F01_transcript_83485	NA	NA
F01_transcript_155383	F01_transcript_155383	F01_transcript_155383	NA	NA
F01_transcript_45492	NA	NA	NA	F01_transcript_45492
F01_transcript_101611	NA	NA	NA	F01_transcript_101611
F01_transcript_97101	NA	F01_transcript_97101	NA	NA
F01_transcript_75473	F01_transcript_75473	F01_transcript_75473	NA	F01_transcript_75473
F01_transcript_92117	NA	NA	F01_transcript_92117	F01_transcript_92117

F01_transcript_125939	F01_transcript_125939	F01_transcript_125939	F01_transcript_125939	F01_transcript_125939
F01_transcript_67040	NA	NA	NA	F01_transcript_67040
F01_transcript_132776	NA	NA	NA	F01_transcript_132776
F01_transcript_61179	F01_transcript_61179	F01_transcript_61179	F01_transcript_61179	F01_transcript_61179
F01_transcript_14644	NA	NA	NA	F01_transcript_14644
F01_transcript_103576	F01_transcript_103576	F01_transcript_103576	F01_transcript_103576	F01_transcript_103576
F01_transcript_89377	NA	F01_transcript_89377	F01_transcript_89377	F01_transcript_89377
F01_transcript_108482	NA	F01_transcript_108482	F01_transcript_108482	F01_transcript_108482
F01_transcript_101591	NA	NA	NA	F01_transcript_101591
F01_transcript_91219	NA	F01_transcript_91219	NA	NA
F01_transcript_110660	NA	F01_transcript_110660	NA	NA
F01_transcript_43275	NA	NA	NA	F01_transcript_43275
F01_transcript_82655	NA	F01_transcript_82655	NA	NA
F01_transcript_107122	F01_transcript_107122	F01_transcript_107122	F01_transcript_107122	F01_transcript_107122

F01_transcript_66319	NA	F01_transcript_66319	F01_transcript_66319	F01_transcript_66319
F01_transcript_160695	NA	F01_transcript_160695	NA	NA
F01_transcript_91462	F01_transcript_91462	F01_transcript_91462	NA	NA
F01_transcript_127272	F01_transcript_127272	F01_transcript_127272	NA	NA
F01_transcript_55798	NA	F01_transcript_55798	NA	NA
F01_transcript_54075	F01_transcript_54075	F01_transcript_54075	NA	NA
F01_transcript_110683	NA	NA	NA	F01_transcript_110683
F01_transcript_154216	F01_transcript_154216	F01_transcript_154216	F01_transcript_154216	F01_transcript_154216
F01_transcript_88077	F01_transcript_88077	F01_transcript_88077	F01_transcript_88077	NA
F01_transcript_68817	NA	NA	NA	F01_transcript_68817
F01_transcript_146983	NA	F01_transcript_146983	NA	NA
F01_transcript_70240	NA	NA	NA	F01_transcript_70240
F01_transcript_14072	NA	NA	NA	F01_transcript_14072
F01_transcript_48570	NA	NA	F01_transcript_48570	F01_transcript_48570

F01_transcript_77479	NA	F01_transcript_77479	NA	NA
F01_transcript_148179	F01_transcript_148179	F01_transcript_148179	F01_transcript_148179	F01_transcript_148179
F01_transcript_97216	NA	NA	NA	F01_transcript_97216
F01_transcript_58572	F01_transcript_58572	F01_transcript_58572	F01_transcript_58572	F01_transcript_58572
F01_transcript_7130	NA	NA	F01_transcript_7130	NA
F01_transcript_24864	NA	NA	NA	F01_transcript_24864
F01_transcript_74741	NA	F01_transcript_74741	NA	F01_transcript_74741
F01_transcript_65093	NA	NA	F01_transcript_65093	NA
F01_transcript_113191	F01_transcript_113191	F01_transcript_113191	NA	F01_transcript_113191
F01_transcript_131086	F01_transcript_131086	F01_transcript_131086	NA	NA
F01_transcript_92416	F01_transcript_92416	F01_transcript_92416	F01_transcript_92416	F01_transcript_92416
F01_transcript_124400	NA	F01_transcript_124400	F01_transcript_124400	F01_transcript_124400
F01_transcript_88306	NA	NA	NA	F01_transcript_88306
F01_transcript_79106	NA	F01_transcript_79106	NA	NA

F01_transcript_35224	NA	NA	NA	F01_transcript_35224
F01_transcript_55909	F01_transcript_55909	F01_transcript_55909	F01_transcript_55909	F01_transcript_55909
F01_transcript_48632	NA	NA	NA	F01_transcript_48632
F01_transcript_151189	F01_transcript_151189	F01_transcript_151189	F01_transcript_151189	F01_transcript_151189
F01_transcript_135507	NA	F01_transcript_135507	NA	NA
F01_transcript_122314	NA	NA	NA	F01_transcript_122314
F01_transcript_111834	NA	NA	NA	F01_transcript_111834
F01_transcript_46031	NA	NA	NA	F01_transcript_46031
F01_transcript_92076	F01_transcript_92076	F01_transcript_92076	NA	NA
F01_transcript_109487	F01_transcript_109487	F01_transcript_109487	F01_transcript_109487	F01_transcript_109487
F01_transcript_2045	NA	NA	NA	F01_transcript_2045
F01_transcript_113883	NA	F01_transcript_113883	NA	F01_transcript_113883
F01_transcript_138995	NA	NA	NA	F01_transcript_138995
F01_transcript_77158	F01_transcript_77158	NA	NA	NA

F01_transcript_134877	F01_transcript_134877	F01_transcript_134877	NA	F01_transcript_134877
F01_transcript_50327	F01_transcript_50327	NA	F01_transcript_50327	F01_transcript_50327
F01_transcript_80110	F01_transcript_80110	F01_transcript_80110	F01_transcript_80110	F01_transcript_80110
F01_transcript_93140	NA	NA	NA	F01_transcript_93140
F01_transcript_136951	NA	F01_transcript_136951	NA	NA
F01_transcript_72720	F01_transcript_72720	F01_transcript_72720	NA	NA
F01_transcript_96852	NA	NA	NA	F01_transcript_96852
F01_transcript_56875	NA	F01_transcript_56875	F01_transcript_56875	NA
F01_transcript_155253	F01_transcript_155253	F01_transcript_155253	NA	F01_transcript_155253
F01_transcript_85845	NA	F01_transcript_85845	NA	NA
F01_transcript_126375	NA	F01_transcript_126375	NA	NA
F01_transcript_155464	F01_transcript_155464	F01_transcript_155464	F01_transcript_155464	F01_transcript_155464
F01_transcript_115418	F01_transcript_115418	F01_transcript_115418	F01_transcript_115418	F01_transcript_115418
F01_transcript_89496	F01_transcript_89496	F01_transcript_89496	F01_transcript_89496	F01_transcript_89496

F01_transcript_129444	NA	F01_transcript_129444	F01_transcript_129444	F01_transcript_129444
F01_transcript_52485	NA	F01_transcript_52485	NA	NA
F01_transcript_155022	NA	NA	NA	F01_transcript_155022
F01_transcript_155154	NA	F01_transcript_155154	NA	F01_transcript_155154
F01_transcript_89079	NA	NA	NA	F01_transcript_89079
F01_transcript_19048	NA	NA	NA	F01_transcript_19048
F01_transcript_76218	F01_transcript_76218	F01_transcript_76218	F01_transcript_76218	F01_transcript_76218
F01_transcript_69094	F01_transcript_69094	F01_transcript_69094	NA	F01_transcript_69094
F01_transcript_69866	F01_transcript_69866	F01_transcript_69866	F01_transcript_69866	F01_transcript_69866
F01_transcript_133694	NA	F01_transcript_133694	NA	NA
F01_transcript_87361	NA	F01_transcript_87361	NA	F01_transcript_87361
F01_transcript_64599	NA	F01_transcript_64599	NA	NA
F01_transcript_130674	NA	NA	NA	F01_transcript_130674
F01_transcript_74977	NA	NA	NA	F01_transcript_74977

F01_transcript_54376	NA	F01_transcript_54376	NA	NA
F01_transcript_62176	NA	NA	NA	F01_transcript_62176
F01_transcript_134792	F01_transcript_134792	F01_transcript_134792	NA	F01_transcript_134792
F01_transcript_79881	F01_transcript_79881	F01_transcript_79881	NA	NA
F01_transcript_107963	F01_transcript_107963	F01_transcript_107963	NA	F01_transcript_107963
F01_transcript_100808	NA	NA	NA	F01_transcript_100808
F01_transcript_130654	NA	NA	NA	F01_transcript_130654
F01_transcript_57340	NA	NA	F01_transcript_57340	F01_transcript_57340
F01_transcript_68659	NA	NA	NA	F01_transcript_68659
F01_transcript_115165	NA	NA	NA	F01_transcript_115165
F01_transcript_210	NA	F01_transcript_210	NA	F01_transcript_210
F01_transcript_53483	NA	NA	NA	F01_transcript_53483
F01_transcript_125451	NA	NA	F01_transcript_125451	NA
F01_transcript_142627	F01_transcript_142627	F01_transcript_142627	F01_transcript_142627	F01_transcript_142627

F01_transcript_92606	NA	F01_transcript_92606	NA	NA
F01_transcript_116979	NA	NA	NA	F01_transcript_116979
F01_transcript_49398	NA	F01_transcript_49398	NA	NA
F01_transcript_37494	F01_transcript_37494	F01_transcript_37494	NA	F01_transcript_37494
F01_transcript_8277	NA	NA	NA	F01_transcript_8277
F01_transcript_98857	NA	F01_transcript_98857	NA	NA
F01_transcript_58872	NA	F01_transcript_58872	NA	NA
F01_transcript_56401	F01_transcript_56401	F01_transcript_56401	F01_transcript_56401	F01_transcript_56401
F01_transcript_23916	NA	F01_transcript_23916	NA	F01_transcript_23916
F01_transcript_140114	NA	F01_transcript_140114	NA	NA
F01_transcript_137554	F01_transcript_137554	F01_transcript_137554	F01_transcript_137554	F01_transcript_137554
F01_transcript_160651	F01_transcript_160651	F01_transcript_160651	F01_transcript_160651	F01_transcript_160651
F01_transcript_15135	NA	NA	NA	F01_transcript_15135
F01_transcript_113481	NA	NA	NA	F01_transcript_113481

F01_transcript_68472	NA	F01_transcript_68472	NA	NA
F01_transcript_94029	NA	F01_transcript_94029	NA	NA
F01_transcript_160838	F01_transcript_160838	F01_transcript_160838	F01_transcript_160838	F01_transcript_160838
F01_transcript_125944	NA	NA	F01_transcript_125944	F01_transcript_125944
F01_transcript_114313	NA	NA	NA	F01_transcript_114313
F01_transcript_106057	NA	F01_transcript_106057	NA	NA
F01_transcript_106576	F01_transcript_106576	F01_transcript_106576	F01_transcript_106576	F01_transcript_106576
F01_transcript_91252	NA	F01_transcript_91252	NA	NA
F01_transcript_107812	F01_transcript_107812	F01_transcript_107812	F01_transcript_107812	F01_transcript_107812
F01_transcript_51832	NA	NA	NA	F01_transcript_51832
F01_transcript_63064	NA	NA	NA	F01_transcript_63064
F01_transcript_64675	NA	F01_transcript_64675	NA	F01_transcript_64675
F01_transcript_131775	NA	F01_transcript_131775	F01_transcript_131775	F01_transcript_131775
F01_transcript_47101	F01_transcript_47101	F01_transcript_47101	F01_transcript_47101	F01_transcript_47101

F01_transcript_71320	NA	NA	NA	F01_transcript_71320
F01_transcript_8210	NA	NA	NA	F01_transcript_8210
F01_transcript_138025	NA	NA	NA	F01_transcript_138025
F01_transcript_161558	NA	F01_transcript_161558	F01_transcript_161558	F01_transcript_161558
F01_transcript_121570	NA	NA	NA	F01_transcript_121570
F01_transcript_57844	NA	F01_transcript_57844	NA	NA
F01_transcript_153878	NA	F01_transcript_153878	NA	NA
F01_transcript_66804	NA	F01_transcript_66804	F01_transcript_66804	F01_transcript_66804
F01_transcript_98415	NA	F01_transcript_98415	F01_transcript_98415	NA
F01_transcript_150419	F01_transcript_150419	F01_transcript_150419	NA	F01_transcript_150419
F01_transcript_73132	NA	NA	NA	F01_transcript_73132
F01_transcript_111289	NA	F01_transcript_111289	F01_transcript_111289	NA
F01_transcript_125202	NA	F01_transcript_125202	NA	NA
F01_transcript_90117	F01_transcript_90117	F01_transcript_90117	NA	NA

F01_transcript_95294	NA	F01_transcript_95294	NA	F01_transcript_95294
F01_transcript_103591	NA	F01_transcript_103591	F01_transcript_103591	F01_transcript_103591
F01_transcript_3577	NA	NA	NA	F01_transcript_3577
F01_transcript_82018	F01_transcript_82018	F01_transcript_82018	F01_transcript_82018	F01_transcript_82018
F01_transcript_50636	F01_transcript_50636	F01_transcript_50636	F01_transcript_50636	F01_transcript_50636
F01_transcript_57121	NA	F01_transcript_57121	F01_transcript_57121	F01_transcript_57121
F01_transcript_62472	NA	F01_transcript_62472	NA	F01_transcript_62472
F01_transcript_42424	NA	NA	NA	F01_transcript_42424
F01_transcript_68016	NA	F01_transcript_68016	NA	NA
F01_transcript_82588	NA	NA	NA	F01_transcript_82588
F01_transcript_86846	NA	F01_transcript_86846	NA	NA
F01_transcript_95443	F01_transcript_95443	F01_transcript_95443	F01_transcript_95443	F01_transcript_95443
F01_transcript_147189	NA	NA	NA	F01_transcript_147189
F01_transcript_41796	NA	NA	NA	F01_transcript_41796

F01_transcript_23738	NA	NA	NA	F01_transcript_23738
F01_transcript_75146	NA	F01_transcript_75146	NA	F01_transcript_75146
F01_transcript_86643	NA	NA	NA	F01_transcript_86643
F01_transcript_55076	NA	NA	NA	F01_transcript_55076
F01_transcript_87288	NA	F01_transcript_87288	NA	F01_transcript_87288
F01_transcript_161136	NA	NA	F01_transcript_161136	NA
F01_transcript_73513	NA	NA	NA	F01_transcript_73513
F01_transcript_57809	F01_transcript_57809	F01_transcript_57809	F01_transcript_57809	F01_transcript_57809
F01_transcript_167560	NA	NA	NA	F01_transcript_167560
F01_transcript_149886	NA	F01_transcript_149886	F01_transcript_149886	NA
F01_transcript_113268	NA	F01_transcript_113268	F01_transcript_113268	F01_transcript_113268
F01_transcript_117997	NA	NA	NA	F01_transcript_117997
F01_transcript_57320	NA	NA	NA	F01_transcript_57320
F01_transcript_5478	NA	NA	NA	F01_transcript_5478

F01_transcript_130693	NA	F01_transcript_130693	NA	NA
F01_transcript_149980	F01_transcript_149980	F01_transcript_149980	F01_transcript_149980	F01_transcript_149980
F01_transcript_166809	NA	NA	NA	F01_transcript_166809
F01_transcript_117099	NA	F01_transcript_117099	F01_transcript_117099	F01_transcript_117099
F01_transcript_116877	NA	F01_transcript_116877	NA	F01_transcript_116877
F01_transcript_128537	NA	NA	NA	F01_transcript_128537
F01_transcript_123310	NA	F01_transcript_123310	NA	NA
F01_transcript_61860	NA	F01_transcript_61860	NA	NA
F01_transcript_36432	NA	NA	NA	F01_transcript_36432
F01_transcript_153901	NA	NA	NA	F01_transcript_153901
F01_transcript_19089	NA	NA	NA	F01_transcript_19089
F01_transcript_121897	F01_transcript_121897	F01_transcript_121897	F01_transcript_121897	F01_transcript_121897
F01_transcript_133075	NA	F01_transcript_133075	NA	F01_transcript_133075
F01_transcript_144444	NA	F01_transcript_144444	NA	NA

F01_transcript_135242	NA	F01_transcript_135242	NA	F01_transcript_135242
F01_transcript_51988	NA	F01_transcript_51988	F01_transcript_51988	NA
F01_transcript_93012	F01_transcript_93012	F01_transcript_93012	NA	NA
F01_transcript_53437	F01_transcript_53437	F01_transcript_53437	F01_transcript_53437	F01_transcript_53437
F01_transcript_123657	NA	F01_transcript_123657	NA	NA
F01_transcript_167396	F01_transcript_167396	F01_transcript_167396	NA	F01_transcript_167396
F01_transcript_52229	NA	F01_transcript_52229	NA	F01_transcript_52229
F01_transcript_91056	F01_transcript_91056	F01_transcript_91056	F01_transcript_91056	NA
F01_transcript_118228	NA	NA	NA	F01_transcript_118228
F01_transcript_117470	F01_transcript_117470	F01_transcript_117470	F01_transcript_117470	F01_transcript_117470
F01_transcript_64060	NA	F01_transcript_64060	NA	NA
F01_transcript_118989	F01_transcript_118989	F01_transcript_118989	F01_transcript_118989	F01_transcript_118989
F01_transcript_116472	NA	F01_transcript_116472	NA	NA
F01_transcript_138664	F01_transcript_138664	F01_transcript_138664	NA	NA

F01_transcript_85153	NA	F01_transcript_85153	NA	NA
F01_transcript_146419	NA	NA	NA	F01_transcript_146419
F01_transcript_161400	NA	F01_transcript_161400	F01_transcript_161400	F01_transcript_161400
F01_transcript_47636	F01_transcript_47636	F01_transcript_47636	F01_transcript_47636	F01_transcript_47636
F01_transcript_131741	NA	F01_transcript_131741	NA	NA
F01_transcript_105739	F01_transcript_105739	F01_transcript_105739	NA	F01_transcript_105739
F01_transcript_5336	NA	NA	NA	F01_transcript_5336
F01_transcript_78733	F01_transcript_78733	F01_transcript_78733	F01_transcript_78733	F01_transcript_78733
F01_transcript_123955	F01_transcript_123955	F01_transcript_123955	F01_transcript_123955	F01_transcript_123955
F01_transcript_113551	F01_transcript_113551	F01_transcript_113551	F01_transcript_113551	F01_transcript_113551
F01_transcript_104757	NA	NA	NA	F01_transcript_104757
F01_transcript_60633	NA	F01_transcript_60633	NA	F01_transcript_60633
F01_transcript_116835	NA	NA	NA	F01_transcript_116835
F01_transcript_124834	F01_transcript_124834	F01_transcript_124834	F01_transcript_124834	F01_transcript_124834

F01_transcript_96411	F01_transcript_96411	F01_transcript_96411	F01_transcript_96411	F01_transcript_96411
F01_transcript_101954	NA	F01_transcript_101954	F01_transcript_101954	F01_transcript_101954
F01_transcript_167043	F01_transcript_167043	F01_transcript_167043	F01_transcript_167043	F01_transcript_167043
F01_transcript_144030	F01_transcript_144030	F01_transcript_144030	F01_transcript_144030	NA
F01_transcript_47190	NA	NA	NA	F01_transcript_47190
F01_transcript_24076	NA	F01_transcript_24076	F01_transcript_24076	F01_transcript_24076
F01_transcript_166800	F01_transcript_166800	F01_transcript_166800	F01_transcript_166800	F01_transcript_166800
F01_transcript_90045	NA	F01_transcript_90045	NA	F01_transcript_90045
F01_transcript_113905	NA	NA	NA	F01_transcript_113905
F01_transcript_124428	NA	NA	NA	F01_transcript_124428
F01_transcript_14118	F01_transcript_14118	F01_transcript_14118	F01_transcript_14118	F01_transcript_14118
F01_transcript_50987	F01_transcript_50987	F01_transcript_50987	F01_transcript_50987	F01_transcript_50987
F01_transcript_69186	NA	NA	NA	F01_transcript_69186
F01_transcript_86227	F01_transcript_86227	F01_transcript_86227	NA	F01_transcript_86227

F01_transcript_51126	F01_transcript_51126	F01_transcript_51126	F01_transcript_51126	F01_transcript_51126
F01_transcript_119408	F01_transcript_119408	F01_transcript_119408	NA	F01_transcript_119408
F01_transcript_123274	NA	F01_transcript_123274	NA	NA
F01_transcript_25385	NA	F01_transcript_25385	NA	NA
F01_transcript_103290	NA	NA	NA	F01_transcript_103290
F01_transcript_15379	NA	NA	NA	F01_transcript_15379
F01_transcript_138145	NA	F01_transcript_138145	NA	F01_transcript_138145
F01_transcript_74233	NA	F01_transcript_74233	NA	NA
F01_transcript_3972	NA	F01_transcript_3972	NA	F01_transcript_3972
F01_transcript_117226	F01_transcript_117226	F01_transcript_117226	F01_transcript_117226	F01_transcript_117226
F01_transcript_120641	F01_transcript_120641	F01_transcript_120641	NA	NA
F01_transcript_52529	NA	F01_transcript_52529	NA	NA
F01_transcript_51159	F01_transcript_51159	F01_transcript_51159	F01_transcript_51159	F01_transcript_51159
F01_transcript_59994	NA	F01_transcript_59994	NA	NA

F01_transcript_45842	NA	NA	NA	F01_transcript_45842
F01_transcript_76935	NA	F01_transcript_76935	F01_transcript_76935	F01_transcript_76935
F01_transcript_85860	NA	NA	F01_transcript_85860	NA
F01_transcript_2226	NA	NA	NA	F01_transcript_2226
F01_transcript_91009	F01_transcript_91009	F01_transcript_91009	NA	NA
F01_transcript_166587	F01_transcript_166587	F01_transcript_166587	NA	NA
F01_transcript_123588	F01_transcript_123588	F01_transcript_123588	NA	NA
F01_transcript_64855	NA	F01_transcript_64855	NA	F01_transcript_64855
F01_transcript_133406	NA	F01_transcript_133406	F01_transcript_133406	NA
F01_transcript_149620	F01_transcript_149620	F01_transcript_149620	NA	NA
F01_transcript_141315	NA	F01_transcript_141315	F01_transcript_141315	F01_transcript_141315
F01_transcript_5568	F01_transcript_5568	F01_transcript_5568	F01_transcript_5568	F01_transcript_5568
F01_transcript_76098	NA	F01_transcript_76098	NA	F01_transcript_76098
F01_transcript_165655	NA	F01_transcript_165655	NA	NA

F01_transcript_146274	NA	NA	NA	F01_transcript_146274
F01_transcript_63569	F01_transcript_63569	F01_transcript_63569	F01_transcript_63569	F01_transcript_63569
F01_transcript_21674	NA	NA	NA	F01_transcript_21674
F01_transcript_148126	F01_transcript_148126	F01_transcript_148126	F01_transcript_148126	F01_transcript_148126
F01_transcript_166387	F01_transcript_166387	F01_transcript_166387	F01_transcript_166387	F01_transcript_166387
F01_transcript_70279	NA	NA	NA	F01_transcript_70279
F01_transcript_47416	NA	NA	NA	F01_transcript_47416
F01_transcript_61678	NA	NA	F01_transcript_61678	F01_transcript_61678
F01_transcript_36287	NA	NA	NA	F01_transcript_36287
F01_transcript_131022	NA	F01_transcript_131022	NA	F01_transcript_131022
F01_transcript_129044	NA	NA	NA	F01_transcript_129044
F01_transcript_159380	F01_transcript_159380	NA	NA	NA
F01_transcript_67065	NA	NA	NA	F01_transcript_67065
F01_transcript_89041	F01_transcript_89041	F01_transcript_89041	NA	F01_transcript_89041

F01_transcript_136771	F01_transcript_136771	F01_transcript_136771	NA	F01_transcript_136771
F01_transcript_86224	F01_transcript_86224	F01_transcript_86224	F01_transcript_86224	NA
F01_transcript_135962	NA	F01_transcript_135962	F01_transcript_135962	F01_transcript_135962
F01_transcript_97715	NA	NA	NA	F01_transcript_97715
F01_transcript_108771	NA	NA	NA	F01_transcript_108771
F01_transcript_79454	F01_transcript_79454	F01_transcript_79454	F01_transcript_79454	F01_transcript_79454
F01_transcript_141103	NA	F01_transcript_141103	F01_transcript_141103	F01_transcript_141103
F01_transcript_140936	F01_transcript_140936	F01_transcript_140936	F01_transcript_140936	F01_transcript_140936
F01_transcript_87377	NA	NA	NA	F01_transcript_87377
F01_transcript_157190	F01_transcript_157190	F01_transcript_157190	F01_transcript_157190	F01_transcript_157190
F01_transcript_88932	NA	F01_transcript_88932	NA	NA
F01_transcript_84853	F01_transcript_84853	F01_transcript_84853	F01_transcript_84853	F01_transcript_84853
F01_transcript_70070	NA	NA	NA	F01_transcript_70070
F01_transcript_106430	NA	NA	NA	F01_transcript_106430

F01_transcript_72705	NA	F01_transcript_72705	NA	NA
F01_transcript_75545	NA	NA	NA	F01_transcript_75545
F01_transcript_23909	NA	NA	NA	F01_transcript_23909
F01_transcript_161371	NA	NA	NA	F01_transcript_161371
F01_transcript_95432	NA	NA	NA	F01_transcript_95432
F01_transcript_164401	NA	F01_transcript_164401	NA	NA
F01_transcript_148784	F01_transcript_148784	F01_transcript_148784	F01_transcript_148784	F01_transcript_148784
F01_transcript_13225	F01_transcript_13225	F01_transcript_13225	F01_transcript_13225	F01_transcript_13225
F01_transcript_41216	NA	NA	NA	F01_transcript_41216
F01_transcript_128395	NA	NA	NA	F01_transcript_128395
F01_transcript_111116	NA	NA	NA	F01_transcript_111116
F01_transcript_67680	NA	NA	NA	F01_transcript_67680
F01_transcript_16438	NA	NA	NA	F01_transcript_16438
F01_transcript_117877	F01_transcript_117877	F01_transcript_117877	F01_transcript_117877	F01_transcript_117877

F01_transcript_22823	NA	NA	NA	F01_transcript_22823
F01_transcript_54547	NA	F01_transcript_54547	NA	F01_transcript_54547
F01_transcript_109982	NA	F01_transcript_109982	NA	NA
F01_transcript_42180	NA	NA	NA	F01_transcript_42180
F01_transcript_121922	NA	NA	NA	F01_transcript_121922
F01_transcript_89947	F01_transcript_89947	F01_transcript_89947	F01_transcript_89947	F01_transcript_89947
F01_transcript_162023	NA	F01_transcript_162023	NA	F01_transcript_162023
F01_transcript_111176	F01_transcript_111176	F01_transcript_111176	NA	NA
F01_transcript_57137	NA	NA	F01_transcript_57137	NA
F01_transcript_36189	NA	NA	NA	F01_transcript_36189
F01_transcript_31078	NA	NA	NA	F01_transcript_31078
F01_transcript_47710	NA	F01_transcript_47710	NA	NA
F01_transcript_79548	F01_transcript_79548	F01_transcript_79548	F01_transcript_79548	F01_transcript_79548
F01_transcript_135155	NA	F01_transcript_135155	F01_transcript_135155	NA

F01_transcript_41561	NA	NA	F01_transcript_41561	NA
F01_transcript_58509	NA	NA	NA	F01_transcript_58509
F01_transcript_94049	NA	NA	NA	F01_transcript_94049
F01_transcript_145544	F01_transcript_145544	F01_transcript_145544	F01_transcript_145544	F01_transcript_145544
F01_transcript_131316	NA	F01_transcript_131316	NA	F01_transcript_131316
F01_transcript_132009	F01_transcript_132009	F01_transcript_132009	F01_transcript_132009	NA
F01_transcript_75662	NA	F01_transcript_75662	NA	NA
F01_transcript_83233	F01_transcript_83233	F01_transcript_83233	NA	F01_transcript_83233
F01_transcript_5662	NA	F01_transcript_5662	NA	NA
F01_transcript_90416	NA	NA	NA	F01_transcript_90416
F01_transcript_125355	F01_transcript_125355	F01_transcript_125355	NA	NA
F01_transcript_153707	F01_transcript_153707	F01_transcript_153707	F01_transcript_153707	F01_transcript_153707
F01_transcript_159749	F01_transcript_159749	F01_transcript_159749	NA	F01_transcript_159749
F01_transcript_116101	NA	NA	NA	F01_transcript_116101

F01_transcript_5680	F01_transcript_5680	F01_transcript_5680	NA	F01_transcript_5680
F01_transcript_15929	NA	NA	NA	F01_transcript_15929
F01_transcript_98650	NA	F01_transcript_98650	F01_transcript_98650	NA
F01_transcript_24751	NA	F01_transcript_24751	NA	NA
F01_transcript_132201	NA	NA	NA	F01_transcript_132201
F01_transcript_80163	NA	NA	NA	F01_transcript_80163
F01_transcript_125963	NA	NA	NA	F01_transcript_125963
F01_transcript_161620	NA	F01_transcript_161620	NA	NA
F01_transcript_105510	F01_transcript_105510	F01_transcript_105510	F01_transcript_105510	F01_transcript_105510
F01_transcript_61458	NA	F01_transcript_61458	NA	NA
F01_transcript_116633	NA	NA	NA	F01_transcript_116633
F01_transcript_96838	NA	F01_transcript_96838	NA	NA
F01_transcript_142185	NA	F01_transcript_142185	NA	F01_transcript_142185
F01_transcript_106402	NA	F01_transcript_106402	NA	F01_transcript_106402

F01_transcript_165029	NA	F01_transcript_165029	NA	NA
F01_transcript_49715	NA	F01_transcript_49715	F01_transcript_49715	NA
F01_transcript_88763	F01_transcript_88763	F01_transcript_88763	F01_transcript_88763	F01_transcript_88763
F01_transcript_149532	NA	NA	NA	F01_transcript_149532
F01_transcript_56035	NA	NA	NA	F01_transcript_56035
F01_transcript_79087	F01_transcript_79087	F01_transcript_79087	NA	F01_transcript_79087
F01_transcript_107407	F01_transcript_107407	F01_transcript_107407	NA	NA
F01_transcript_83315	F01_transcript_83315	F01_transcript_83315	F01_transcript_83315	F01_transcript_83315
F01_transcript_130389	NA	F01_transcript_130389	NA	NA
F01_transcript_153938	NA	NA	F01_transcript_153938	NA
F01_transcript_68973	NA	NA	F01_transcript_68973	F01_transcript_68973
F01_transcript_83798	F01_transcript_83798	F01_transcript_83798	F01_transcript_83798	F01_transcript_83798
F01_transcript_137333	F01_transcript_137333	F01_transcript_137333	F01_transcript_137333	F01_transcript_137333
F01_transcript_117705	F01_transcript_117705	F01_transcript_117705	F01_transcript_117705	NA

F01_transcript_68796	NA	NA	NA	F01_transcript_68796
F01_transcript_50155	NA	NA	NA	F01_transcript_50155
F01_transcript_88606	NA	F01_transcript_88606	NA	NA
F01_transcript_115138	F01_transcript_115138	F01_transcript_115138	NA	NA
F01_transcript_53150	NA	NA	NA	F01_transcript_53150
F01_transcript_26271	F01_transcript_26271	F01_transcript_26271	F01_transcript_26271	F01_transcript_26271
F01_transcript_60610	NA	F01_transcript_60610	NA	NA
F01_transcript_26956	NA	F01_transcript_26956	F01_transcript_26956	NA
F01_transcript_35690	NA	NA	F01_transcript_35690	NA
F01_transcript_140292	NA	F01_transcript_140292	NA	F01_transcript_140292
F01_transcript_65671	NA	F01_transcript_65671	NA	NA
F01_transcript_5480	NA	NA	NA	F01_transcript_5480
F01_transcript_133204	NA	F01_transcript_133204	NA	F01_transcript_133204
F01_transcript_118907	NA	NA	NA	F01_transcript_118907

F01_transcript_53138	F01_transcript_53138	F01_transcript_53138	F01_transcript_53138	F01_transcript_53138
F01_transcript_69839	NA	F01_transcript_69839	NA	NA
F01_transcript_144577	NA	NA	NA	F01_transcript_144577
F01_transcript_49963	NA	NA	F01_transcript_49963	F01_transcript_49963
F01_transcript_142660	NA	NA	NA	F01_transcript_142660
F01_transcript_69953	NA	NA	NA	F01_transcript_69953
F01_transcript_92688	F01_transcript_92688	F01_transcript_92688	NA	F01_transcript_92688
F01_transcript_95506	NA	NA	NA	F01_transcript_95506
F01_transcript_126759	NA	NA	NA	F01_transcript_126759
F01_transcript_36427	NA	NA	NA	F01_transcript_36427
F01_transcript_66153	NA	NA	NA	F01_transcript_66153
F01_transcript_64145	NA	NA	NA	F01_transcript_64145
F01_transcript_161793	NA	NA	F01_transcript_161793	F01_transcript_161793
F01_transcript_81547	NA	F01_transcript_81547	F01_transcript_81547	F01_transcript_81547

F01_transcript_137347	NA	F01_transcript_137347	NA	NA
F01_transcript_50802	NA	F01_transcript_50802	NA	NA
F01_transcript_90710	NA	NA	NA	F01_transcript_90710
F01_transcript_115711	F01_transcript_115711	F01_transcript_115711	NA	F01_transcript_115711
F01_transcript_70909	F01_transcript_70909	F01_transcript_70909	F01_transcript_70909	F01_transcript_70909
F01_transcript_50403	NA	F01_transcript_50403	NA	NA
F01_transcript_90112	NA	F01_transcript_90112	NA	NA
F01_transcript_127939	NA	F01_transcript_127939	NA	NA
F01_transcript_110265	F01_transcript_110265	F01_transcript_110265	NA	NA
F01_transcript_42632	NA	NA	NA	F01_transcript_42632
F01_transcript_87353	NA	NA	NA	F01_transcript_87353
F01_transcript_130864	NA	F01_transcript_130864	F01_transcript_130864	F01_transcript_130864
F01_transcript_152284	NA	F01_transcript_152284	F01_transcript_152284	F01_transcript_152284
F01_transcript_135907	F01_transcript_135907	NA	NA	NA

F01_transcript_161234	F01_transcript_161234	F01_transcript_161234	F01_transcript_161234	F01_transcript_161234
F01_transcript_167629	NA	NA	NA	F01_transcript_167629
F01_transcript_98899	NA	F01_transcript_98899	NA	F01_transcript_98899
F01_transcript_101028	NA	F01_transcript_101028	NA	NA
F01_transcript_68253	F01_transcript_68253	F01_transcript_68253	F01_transcript_68253	F01_transcript_68253
F01_transcript_44389	NA	NA	NA	F01_transcript_44389
F01_transcript_93815	F01_transcript_93815	F01_transcript_93815	F01_transcript_93815	F01_transcript_93815
F01_transcript_127615	NA	F01_transcript_127615	F01_transcript_127615	F01_transcript_127615
F01_transcript_50465	NA	NA	F01_transcript_50465	F01_transcript_50465
F01_transcript_111685	NA	F01_transcript_111685	NA	NA
F01_transcript_157911	NA	NA	NA	F01_transcript_157911
F01_transcript_148531	NA	NA	NA	F01_transcript_148531
F01_transcript_135514	NA	NA	NA	F01_transcript_135514
F01_transcript_143260	NA	F01_transcript_143260	NA	NA

F01_transcript_138630	F01_transcript_138630	F01_transcript_138630	F01_transcript_138630	F01_transcript_138630
F01_transcript_42202	NA	NA	NA	F01_transcript_42202
F01_transcript_107563	NA	F01_transcript_107563	NA	NA
F01_transcript_148527	NA	NA	NA	F01_transcript_148527
F01_transcript_26146	NA	NA	NA	F01_transcript_26146
F01_transcript_67312	F01_transcript_67312	F01_transcript_67312	F01_transcript_67312	F01_transcript_67312
F01_transcript_48643	F01_transcript_48643	F01_transcript_48643	F01_transcript_48643	F01_transcript_48643
F01_transcript_7185	NA	NA	NA	F01_transcript_7185
F01_transcript_69179	NA	NA	NA	F01_transcript_69179
F01_transcript_151237	NA	NA	NA	F01_transcript_151237
F01_transcript_111634	F01_transcript_111634	F01_transcript_111634	F01_transcript_111634	F01_transcript_111634
F01_transcript_106938	F01_transcript_106938	F01_transcript_106938	F01_transcript_106938	F01_transcript_106938
F01_transcript_77472	F01_transcript_77472	F01_transcript_77472	F01_transcript_77472	F01_transcript_77472
F01_transcript_97163	NA	NA	NA	F01_transcript_97163

F01_transcript_83573	F01_transcript_83573	F01_transcript_83573	F01_transcript_83573	F01_transcript_83573
F01_transcript_78206	NA	F01_transcript_78206	NA	NA
F01_transcript_139137	F01_transcript_139137	F01_transcript_139137	F01_transcript_139137	F01_transcript_139137
F01_transcript_105128	F01_transcript_105128	F01_transcript_105128	F01_transcript_105128	F01_transcript_105128
F01_transcript_32858	NA	NA	NA	F01_transcript_32858
F01_transcript_110309	NA	NA	NA	F01_transcript_110309
F01_transcript_61508	F01_transcript_61508	F01_transcript_61508	F01_transcript_61508	F01_transcript_61508
F01_transcript_72290	F01_transcript_72290	F01_transcript_72290	F01_transcript_72290	F01_transcript_72290
F01_transcript_78658	F01_transcript_78658	F01_transcript_78658	NA	NA
F01_transcript_21570	NA	F01_transcript_21570	NA	NA
F01_transcript_116208	NA	F01_transcript_116208	NA	NA
F01_transcript_28649	NA	NA	NA	F01_transcript_28649
F01_transcript_58997	NA	F01_transcript_58997	NA	NA
F01_transcript_132242	NA	F01_transcript_132242	NA	NA

F01_transcript_70713	F01_transcript_70713	F01_transcript_70713	NA	F01_transcript_70713
F01_transcript_140680	NA	NA	NA	F01_transcript_140680
F01_transcript_152499	NA	NA	NA	F01_transcript_152499
F01_transcript_104458	F01_transcript_104458	F01_transcript_104458	F01_transcript_104458	F01_transcript_104458
F01_transcript_106671	NA	NA	NA	F01_transcript_106671
F01_transcript_161055	NA	F01_transcript_161055	NA	NA
F01_transcript_164685	NA	F01_transcript_164685	NA	NA
F01_transcript_57950	NA	F01_transcript_57950	F01_transcript_57950	F01_transcript_57950
F01_transcript_161624	NA	F01_transcript_161624	NA	F01_transcript_161624
F01_transcript_118083	NA	F01_transcript_118083	NA	F01_transcript_118083
F01_transcript_67568	NA	F01_transcript_67568	F01_transcript_67568	F01_transcript_67568
F01_transcript_144269	F01_transcript_144269	F01_transcript_144269	NA	F01_transcript_144269
F01_transcript_100584	NA	F01_transcript_100584	F01_transcript_100584	F01_transcript_100584
F01_transcript_64303	NA	NA	NA	F01_transcript_64303

F01_transcript_65154	NA	NA	NA	F01_transcript_65154
F01_transcript_120920	F01_transcript_120920	F01_transcript_120920	F01_transcript_120920	F01_transcript_120920
F01_transcript_5026	NA	NA	NA	F01_transcript_5026
F01_transcript_65974	NA	NA	NA	F01_transcript_65974
F01_transcript_103754	NA	F01_transcript_103754	NA	NA
F01_transcript_139830	NA	NA	NA	F01_transcript_139830
F01_transcript_68700	F01_transcript_68700	F01_transcript_68700	NA	NA
F01_transcript_161681	NA	F01_transcript_161681	F01_transcript_161681	F01_transcript_161681
F01_transcript_49749	NA	F01_transcript_49749	NA	F01_transcript_49749
F01_transcript_160574	F01_transcript_160574	F01_transcript_160574	NA	F01_transcript_160574
F01_transcript_165795	NA	F01_transcript_165795	NA	NA
F01_transcript_163367	NA	F01_transcript_163367	NA	F01_transcript_163367
F01_transcript_4616	F01_transcript_4616	F01_transcript_4616	F01_transcript_4616	F01_transcript_4616
F01_transcript_72835	NA	NA	NA	F01_transcript_72835

F01_transcript_65149	NA	NA	F01_transcript_65149	F01_transcript_65149
F01_transcript_58147	NA	F01_transcript_58147	NA	NA
F01_transcript_86239	NA	F01_transcript_86239	F01_transcript_86239	F01_transcript_86239
F01_transcript_7569	NA	F01_transcript_7569	NA	F01_transcript_7569
F01_transcript_148429	F01_transcript_148429	F01_transcript_148429	F01_transcript_148429	F01_transcript_148429
F01_transcript_68214	NA	F01_transcript_68214	F01_transcript_68214	NA
F01_transcript_1468	NA	F01_transcript_1468	F01_transcript_1468	F01_transcript_1468
F01_transcript_162475	NA	NA	NA	F01_transcript_162475
F01_transcript_103110	NA	F01_transcript_103110	F01_transcript_103110	NA
F01_transcript_56744	NA	F01_transcript_56744	NA	NA
F01_transcript_158282	F01_transcript_158282	F01_transcript_158282	NA	NA
F01_transcript_94453	F01_transcript_94453	F01_transcript_94453	F01_transcript_94453	F01_transcript_94453
F01_transcript_87383	NA	F01_transcript_87383	NA	F01_transcript_87383
F01_transcript_47120	NA	NA	NA	F01_transcript_47120

F01_transcript_128845	NA	F01_transcript_128845	NA	F01_transcript_128845
F01_transcript_113637	NA	NA	F01_transcript_113637	F01_transcript_113637
F01_transcript_133210	NA	F01_transcript_133210	NA	F01_transcript_133210
F01_transcript_50041	NA	F01_transcript_50041	NA	F01_transcript_50041
F01_transcript_140433	NA	NA	NA	F01_transcript_140433
F01_transcript_126112	NA	NA	NA	F01_transcript_126112
F01_transcript_120649	NA	F01_transcript_120649	F01_transcript_120649	F01_transcript_120649
F01_transcript_95855	F01_transcript_95855	F01_transcript_95855	F01_transcript_95855	F01_transcript_95855
F01_transcript_57080	NA	NA	F01_transcript_57080	F01_transcript_57080
F01_transcript_86766	NA	NA	NA	F01_transcript_86766
F01_transcript_55625	F01_transcript_55625	F01_transcript_55625	F01_transcript_55625	F01_transcript_55625
F01_transcript_61731	NA	F01_transcript_61731	NA	NA
F01_transcript_136299	NA	F01_transcript_136299	F01_transcript_136299	NA
F01_transcript_100418	NA	F01_transcript_100418	NA	NA

F01_transcript_119046	NA	F01_transcript_119046	NA	NA
F01_transcript_156419	F01_transcript_156419	NA	NA	NA
F01_transcript_84235	NA	NA	NA	F01_transcript_84235
F01_transcript_85021	NA	NA	NA	F01_transcript_85021
F01_transcript_15264	NA	F01_transcript_15264	NA	NA
F01_transcript_49407	NA	F01_transcript_49407	NA	F01_transcript_49407
F01_transcript_99282	NA	F01_transcript_99282	NA	NA
F01_transcript_65203	NA	NA	F01_transcript_65203	NA
F01_transcript_127215	NA	NA	NA	F01_transcript_127215
F01_transcript_160230	NA	NA	NA	F01_transcript_160230
F01_transcript_154944	NA	F01_transcript_154944	NA	NA
F01_transcript_94652	NA	F01_transcript_94652	F01_transcript_94652	F01_transcript_94652
F01_transcript_88027	NA	NA	F01_transcript_88027	F01_transcript_88027
F01_transcript_139488	F01_transcript_139488	F01_transcript_139488	F01_transcript_139488	F01_transcript_139488

F01_transcript_164207	NA	F01_transcript_164207	F01_transcript_164207	F01_transcript_164207
F01_transcript_155566	NA	F01_transcript_155566	F01_transcript_155566	NA
F01_transcript_139462	NA	NA	NA	F01_transcript_139462
F01_transcript_125375	F01_transcript_125375	F01_transcript_125375	F01_transcript_125375	NA
F01_transcript_58134	F01_transcript_58134	F01_transcript_58134	F01_transcript_58134	F01_transcript_58134
F01_transcript_90304	NA	F01_transcript_90304	F01_transcript_90304	F01_transcript_90304
F01_transcript_86699	NA	F01_transcript_86699	NA	NA
F01_transcript_85730	NA	NA	NA	F01_transcript_85730
F01_transcript_112707	NA	F01_transcript_112707	NA	NA
F01_transcript_152673	NA	NA	NA	F01_transcript_152673
F01_transcript_112389	NA	NA	NA	F01_transcript_112389
F01_transcript_64715	NA	F01_transcript_64715	F01_transcript_64715	F01_transcript_64715
F01_transcript_115347	F01_transcript_115347	F01_transcript_115347	F01_transcript_115347	F01_transcript_115347
F01_transcript_123060	NA	F01_transcript_123060	NA	NA

F01_transcript_83228	F01_transcript_83228	F01_transcript_83228	F01_transcript_83228	F01_transcript_83228
F01_transcript_156871	NA	F01_transcript_156871	NA	F01_transcript_156871
F01_transcript_70048	NA	NA	NA	F01_transcript_70048
F01_transcript_78294	F01_transcript_78294	F01_transcript_78294	F01_transcript_78294	F01_transcript_78294
F01_transcript_162965	NA	F01_transcript_162965	NA	F01_transcript_162965
F01_transcript_157391	NA	F01_transcript_157391	NA	NA
F01_transcript_39450	NA	NA	NA	F01_transcript_39450
F01_transcript_106945	NA	NA	NA	F01_transcript_106945
F01_transcript_117016	NA	NA	NA	F01_transcript_117016
F01_transcript_163749	F01_transcript_163749	F01_transcript_163749	NA	F01_transcript_163749
F01_transcript_48966	F01_transcript_48966	F01_transcript_48966	F01_transcript_48966	F01_transcript_48966
F01_transcript_147392	NA	NA	NA	F01_transcript_147392
F01_transcript_69140	NA	NA	NA	F01_transcript_69140
F01_transcript_50173	F01_transcript_50173	F01_transcript_50173	NA	NA

F01_transcript_76339	NA	NA	NA	F01_transcript_76339
F01_transcript_49834	F01_transcript_49834	F01_transcript_49834	NA	NA
F01_transcript_112414	NA	F01_transcript_112414	NA	F01_transcript_112414
F01_transcript_64445	F01_transcript_64445	F01_transcript_64445	F01_transcript_64445	F01_transcript_64445
F01_transcript_153098	NA	F01_transcript_153098	NA	NA
F01_transcript_121430	NA	F01_transcript_121430	NA	F01_transcript_121430
F01_transcript_56459	NA	F01_transcript_56459	F01_transcript_56459	F01_transcript_56459
F01_transcript_125165	F01_transcript_125165	F01_transcript_125165	NA	F01_transcript_125165
F01_transcript_162666	NA	F01_transcript_162666	F01_transcript_162666	NA
F01_transcript_18023	NA	NA	NA	F01_transcript_18023
F01_transcript_16688	NA	NA	NA	F01_transcript_16688
F01_transcript_113178	NA	F01_transcript_113178	NA	NA
F01_transcript_88197	F01_transcript_88197	F01_transcript_88197	F01_transcript_88197	F01_transcript_88197
F01_transcript_157715	NA	NA	NA	F01_transcript_157715

F01_transcript_112782	NA	F01_transcript_112782	NA	F01_transcript_112782
F01_transcript_51766	NA	NA	NA	F01_transcript_51766
F01_transcript_119285	NA	F01_transcript_119285	NA	NA
F01_transcript_137723	NA	NA	NA	F01_transcript_137723
F01_transcript_74658	NA	F01_transcript_74658	NA	NA
F01_transcript_106191	NA	NA	NA	F01_transcript_106191
F01_transcript_59186	F01_transcript_59186	F01_transcript_59186	F01_transcript_59186	F01_transcript_59186
F01_transcript_149710	F01_transcript_149710	F01_transcript_149710	F01_transcript_149710	F01_transcript_149710
F01_transcript_160837	NA	NA	NA	F01_transcript_160837
F01_transcript_16357	NA	NA	NA	F01_transcript_16357
F01_transcript_163644	NA	F01_transcript_163644	NA	NA
F01_transcript_165378	NA	F01_transcript_165378	NA	NA
F01_transcript_2833	NA	NA	NA	F01_transcript_2833
F01_transcript_6133	NA	NA	NA	F01_transcript_6133

F01_transcript_155371	NA	NA	NA	F01_transcript_155371
F01_transcript_72694	F01_transcript_72694	F01_transcript_72694	F01_transcript_72694	F01_transcript_72694
F01_transcript_90180	F01_transcript_90180	F01_transcript_90180	F01_transcript_90180	F01_transcript_90180
F01_transcript_65957	NA	F01_transcript_65957	F01_transcript_65957	F01_transcript_65957
F01_transcript_107540	NA	F01_transcript_107540	NA	F01_transcript_107540
F01_transcript_152297	NA	NA	NA	F01_transcript_152297
F01_transcript_86994	NA	NA	NA	F01_transcript_86994
F01_transcript_77576	F01_transcript_77576	F01_transcript_77576	F01_transcript_77576	F01_transcript_77576
F01_transcript_69591	NA	F01_transcript_69591	F01_transcript_69591	F01_transcript_69591
F01_transcript_79551	NA	NA	NA	F01_transcript_79551
F01_transcript_25579	NA	NA	NA	F01_transcript_25579
F01_transcript_77060	NA	NA	NA	F01_transcript_77060
F01_transcript_156629	F01_transcript_156629	F01_transcript_156629	NA	NA
F01_transcript_45973	NA	F01_transcript_45973	NA	F01_transcript_45973

F01_transcript_108629	NA	NA	NA	F01_transcript_108629
F01_transcript_92662	F01_transcript_92662	F01_transcript_92662	NA	NA
F01_transcript_118665	F01_transcript_118665	F01_transcript_118665	F01_transcript_118665	F01_transcript_118665
F01_transcript_62166	NA	F01_transcript_62166	F01_transcript_62166	F01_transcript_62166
F01_transcript_26890	NA	NA	NA	F01_transcript_26890
F01_transcript_127243	F01_transcript_127243	F01_transcript_127243	F01_transcript_127243	F01_transcript_127243
F01_transcript_104446	F01_transcript_104446	F01_transcript_104446	F01_transcript_104446	F01_transcript_104446
F01_transcript_8183	NA	F01_transcript_8183	NA	F01_transcript_8183
F01_transcript_29673	NA	NA	NA	F01_transcript_29673
F01_transcript_115340	F01_transcript_115340	F01_transcript_115340	F01_transcript_115340	F01_transcript_115340
F01_transcript_104424	NA	F01_transcript_104424	NA	NA
F01_transcript_124775	NA	NA	NA	F01_transcript_124775
F01_transcript_48326	NA	NA	NA	F01_transcript_48326
F01_transcript_155906	NA	NA	NA	F01_transcript_155906

F01_transcript_127881	F01_transcript_127881	F01_transcript_127881	F01_transcript_127881	F01_transcript_127881
F01_transcript_83827	F01_transcript_83827	F01_transcript_83827	F01_transcript_83827	F01_transcript_83827
F01_transcript_46759	NA	F01_transcript_46759	F01_transcript_46759	NA
F01_transcript_140697	NA	NA	NA	F01_transcript_140697
F01_transcript_97325	NA	F01_transcript_97325	NA	F01_transcript_97325
F01_transcript_155503	F01_transcript_155503	F01_transcript_155503	F01_transcript_155503	F01_transcript_155503
F01_transcript_95865	NA	NA	NA	F01_transcript_95865
F01_transcript_126328	NA	NA	NA	F01_transcript_126328
F01_transcript_8523	F01_transcript_8523	F01_transcript_8523	F01_transcript_8523	F01_transcript_8523
F01_transcript_106111	F01_transcript_106111	F01_transcript_106111	F01_transcript_106111	F01_transcript_106111
F01_transcript_108425	F01_transcript_108425	F01_transcript_108425	NA	F01_transcript_108425
F01_transcript_28201	NA	NA	NA	F01_transcript_28201
F01_transcript_138488	NA	NA	NA	F01_transcript_138488
F01_transcript_87484	F01_transcript_87484	F01_transcript_87484	NA	F01_transcript_87484

F01_transcript_161503	NA	F01_transcript_161503	F01_transcript_161503	NA
F01_transcript_88035	NA	F01_transcript_88035	NA	NA
F01_transcript_156348	NA	F01_transcript_156348	F01_transcript_156348	F01_transcript_156348
F01_transcript_48975	F01_transcript_48975	F01_transcript_48975	NA	NA
F01_transcript_59501	NA	NA	F01_transcript_59501	NA
F01_transcript_43027	NA	NA	NA	F01_transcript_43027
F01_transcript_101765	NA	NA	NA	F01_transcript_101765
F01_transcript_17466	NA	F01_transcript_17466	NA	F01_transcript_17466
F01_transcript_129309	NA	F01_transcript_129309	NA	F01_transcript_129309
F01_transcript_66730	NA	NA	NA	F01_transcript_66730
F01_transcript_50798	NA	F01_transcript_50798	NA	NA
F01_transcript_42061	NA	NA	NA	F01_transcript_42061
F01_transcript_116845	NA	F01_transcript_116845	NA	NA
F01_transcript_79289	NA	F01_transcript_79289	F01_transcript_79289	F01_transcript_79289

F01_transcript_167356	NA	F01_transcript_167356	NA	NA
F01_transcript_104914	F01_transcript_104914	F01_transcript_104914	F01_transcript_104914	F01_transcript_104914
F01_transcript_97410	F01_transcript_97410	F01_transcript_97410	NA	NA
F01_transcript_79654	NA	NA	NA	F01_transcript_79654
F01_transcript_98377	NA	NA	NA	F01_transcript_98377
F01_transcript_132553	NA	NA	NA	F01_transcript_132553
F01_transcript_38921	NA	NA	NA	F01_transcript_38921
F01_transcript_166770	F01_transcript_166770	F01_transcript_166770	F01_transcript_166770	F01_transcript_166770
F01_transcript_157053	NA	F01_transcript_157053	NA	F01_transcript_157053
F01_transcript_57863	F01_transcript_57863	F01_transcript_57863	F01_transcript_57863	F01_transcript_57863
F01_transcript_157594	F01_transcript_157594	F01_transcript_157594	F01_transcript_157594	F01_transcript_157594
F01_transcript_112520	NA	F01_transcript_112520	NA	F01_transcript_112520
F01_transcript_110287	NA	F01_transcript_110287	F01_transcript_110287	NA
F01_transcript_134558	NA	NA	NA	F01_transcript_134558

F01_transcript_114050	NA	NA	F01_transcript_114050	F01_transcript_114050
F01_transcript_68152	NA	F01_transcript_68152	NA	NA
F01_transcript_146779	NA	NA	NA	F01_transcript_146779
F01_transcript_138987	NA	NA	NA	F01_transcript_138987
F01_transcript_97077	NA	NA	NA	F01_transcript_97077
F01_transcript_5691	NA	NA	F01_transcript_5691	NA
F01_transcript_142727	NA	F01_transcript_142727	NA	NA
F01_transcript_81456	NA	F01_transcript_81456	F01_transcript_81456	F01_transcript_81456
F01_transcript_107863	NA	F01_transcript_107863	NA	NA
F01_transcript_66293	F01_transcript_66293	F01_transcript_66293	F01_transcript_66293	F01_transcript_66293
F01_transcript_53471	NA	F01_transcript_53471	NA	NA
F01_transcript_30249	NA	F01_transcript_30249	F01_transcript_30249	F01_transcript_30249
F01_transcript_131933	F01_transcript_131933	F01_transcript_131933	F01_transcript_131933	F01_transcript_131933
F01_transcript_123833	F01_transcript_123833	F01_transcript_123833	NA	F01_transcript_123833

F01_transcript_4791	NA	NA	NA	F01_transcript_4791
F01_transcript_57287	NA	NA	NA	F01_transcript_57287
F01_transcript_95290	NA	F01_transcript_95290	NA	F01_transcript_95290
F01_transcript_87867	NA	NA	NA	F01_transcript_87867
F01_transcript_99146	NA	NA	NA	F01_transcript_99146
F01_transcript_87198	NA	F01_transcript_87198	F01_transcript_87198	F01_transcript_87198
F01_transcript_19103	NA	F01_transcript_19103	NA	NA
F01_transcript_94986	NA	F01_transcript_94986	NA	NA
F01_transcript_129246	F01_transcript_129246	F01_transcript_129246	F01_transcript_129246	F01_transcript_129246
F01_transcript_87690	NA	NA	NA	F01_transcript_87690
F01_transcript_36277	NA	F01_transcript_36277	NA	NA
F01_transcript_143156	F01_transcript_143156	F01_transcript_143156	F01_transcript_143156	F01_transcript_143156
F01_transcript_140597	F01_transcript_140597	F01_transcript_140597	NA	F01_transcript_140597
F01_transcript_103551	F01_transcript_103551	NA	NA	NA

F01_transcript_55406	NA	F01_transcript_55406	NA	NA
F01_transcript_52966	NA	F01_transcript_52966	F01_transcript_52966	NA
F01_transcript_151356	NA	F01_transcript_151356	NA	F01_transcript_151356
F01_transcript_166538	NA	F01_transcript_166538	NA	NA
F01_transcript_20943	NA	NA	NA	F01_transcript_20943
F01_transcript_105941	F01_transcript_105941	F01_transcript_105941	F01_transcript_105941	F01_transcript_105941
F01_transcript_75333	NA	F01_transcript_75333	NA	F01_transcript_75333
F01_transcript_127902	NA	F01_transcript_127902	NA	F01_transcript_127902
F01_transcript_117443	NA	NA	NA	F01_transcript_117443
F01_transcript_62513	NA	NA	NA	F01_transcript_62513
F01_transcript_156106	F01_transcript_156106	F01_transcript_156106	F01_transcript_156106	NA
F01_transcript_103778	NA	NA	NA	F01_transcript_103778
F01_transcript_59187	F01_transcript_59187	F01_transcript_59187	NA	F01_transcript_59187
F01_transcript_88495	NA	F01_transcript_88495	NA	F01_transcript_88495

F01_transcript_162565	F01_transcript_162565	F01_transcript_162565	F01_transcript_162565	F01_transcript_162565
F01_transcript_55979	NA	NA	NA	F01_transcript_55979
F01_transcript_16233	F01_transcript_16233	F01_transcript_16233	NA	F01_transcript_16233
F01_transcript_125784	NA	F01_transcript_125784	NA	NA
F01_transcript_158824	NA	F01_transcript_158824	F01_transcript_158824	F01_transcript_158824
F01_transcript_55165	NA	NA	NA	F01_transcript_55165
F01_transcript_108872	NA	NA	NA	F01_transcript_108872
F01_transcript_57876	NA	NA	NA	F01_transcript_57876
F01_transcript_83428	NA	NA	NA	F01_transcript_83428
F01_transcript_111636	NA	NA	NA	F01_transcript_111636
F01_transcript_121552	F01_transcript_121552	F01_transcript_121552	F01_transcript_121552	F01_transcript_121552
F01_transcript_149269	NA	NA	NA	F01_transcript_149269
F01_transcript_85033	F01_transcript_85033	F01_transcript_85033	F01_transcript_85033	F01_transcript_85033
F01_transcript_95304	NA	NA	NA	F01_transcript_95304

F01_transcript_71091	NA	NA	F01_transcript_71091	NA
F01_transcript_64860	NA	F01_transcript_64860	F01_transcript_64860	F01_transcript_64860
F01_transcript_141970	NA	NA	NA	F01_transcript_141970
F01_transcript_73013	NA	F01_transcript_73013	NA	F01_transcript_73013
F01_transcript_123505	NA	NA	NA	F01_transcript_123505
F01_transcript_128574	NA	NA	NA	F01_transcript_128574
F01_transcript_69694	NA	NA	NA	F01_transcript_69694
F01_transcript_148524	NA	F01_transcript_148524	NA	F01_transcript_148524
F01_transcript_158615	NA	NA	NA	F01_transcript_158615
F01_transcript_114342	NA	F01_transcript_114342	F01_transcript_114342	F01_transcript_114342
F01_transcript_116267	NA	F01_transcript_116267	NA	NA
F01_transcript_46642	NA	NA	NA	F01_transcript_46642
F01_transcript_59109	NA	NA	NA	F01_transcript_59109
F01_transcript_110757	F01_transcript_110757	F01_transcript_110757	NA	NA

F01_transcript_55570	F01_transcript_55570	F01_transcript_55570	F01_transcript_55570	F01_transcript_55570
F01_transcript_157240	NA	NA	NA	F01_transcript_157240
F01_transcript_94996	F01_transcript_94996	F01_transcript_94996	F01_transcript_94996	F01_transcript_94996
F01_transcript_81117	NA	NA	NA	F01_transcript_81117
F01_transcript_57072	NA	F01_transcript_57072	F01_transcript_57072	NA
F01_transcript_105730	NA	NA	NA	F01_transcript_105730
F01_transcript_72028	NA	F01_transcript_72028	F01_transcript_72028	F01_transcript_72028
F01_transcript_83818	NA	F01_transcript_83818	F01_transcript_83818	F01_transcript_83818
F01_transcript_142942	NA	NA	NA	F01_transcript_142942
F01_transcript_106902	NA	F01_transcript_106902	NA	NA
F01_transcript_158863	NA	NA	F01_transcript_158863	NA
F01_transcript_145195	NA	F01_transcript_145195	NA	NA
F01_transcript_103883	NA	F01_transcript_103883	NA	F01_transcript_103883
F01_transcript_133565	NA	F01_transcript_133565	NA	NA

F01_transcript_132515	NA	NA	NA	F01_transcript_132515
F01_transcript_106766	NA	F01_transcript_106766	NA	NA
F01_transcript_60474	NA	F01_transcript_60474	F01_transcript_60474	F01_transcript_60474
F01_transcript_12721	NA	F01_transcript_12721	F01_transcript_12721	F01_transcript_12721
F01_transcript_75036	NA	F01_transcript_75036	F01_transcript_75036	F01_transcript_75036
F01_transcript_106636	NA	F01_transcript_106636	NA	NA
F01_transcript_43426	NA	NA	NA	F01_transcript_43426
F01_transcript_72818	NA	F01_transcript_72818	NA	NA
F01_transcript_50927	NA	F01_transcript_50927	F01_transcript_50927	F01_transcript_50927
F01_transcript_61137	NA	NA	F01_transcript_61137	NA
F01_transcript_116686	NA	F01_transcript_116686	NA	NA
F01_transcript_58400	F01_transcript_58400	F01_transcript_58400	F01_transcript_58400	F01_transcript_58400
F01_transcript_116339	F01_transcript_116339	F01_transcript_116339	F01_transcript_116339	NA
F01_transcript_127773	F01_transcript_127773	F01_transcript_127773	F01_transcript_127773	F01_transcript_127773

F01_transcript_146326	NA	NA	NA	F01_transcript_146326
F01_transcript_141766	NA	F01_transcript_141766	NA	NA
F01_transcript_157571	F01_transcript_157571	F01_transcript_157571	F01_transcript_157571	F01_transcript_157571
F01_transcript_157560	NA	F01_transcript_157560	NA	NA
F01_transcript_13510	NA	NA	NA	F01_transcript_13510
F01_transcript_56383	NA	NA	NA	F01_transcript_56383
F01_transcript_69046	F01_transcript_69046	F01_transcript_69046	F01_transcript_69046	F01_transcript_69046
F01_transcript_166098	NA	NA	NA	F01_transcript_166098
F01_transcript_34850	F01_transcript_34850	F01_transcript_34850	NA	F01_transcript_34850
F01_transcript_86206	NA	F01_transcript_86206	F01_transcript_86206	F01_transcript_86206
F01_transcript_50713	F01_transcript_50713	F01_transcript_50713	NA	F01_transcript_50713
F01_transcript_28839	NA	NA	NA	F01_transcript_28839
F01_transcript_50902	F01_transcript_50902	F01_transcript_50902	NA	NA
F01_transcript_95095	NA	F01_transcript_95095	NA	F01_transcript_95095

F01_transcript_107163	NA	F01_transcript_107163	NA	NA
F01_transcript_63053	F01_transcript_63053	F01_transcript_63053	F01_transcript_63053	F01_transcript_63053
F01_transcript_60846	NA	F01_transcript_60846	F01_transcript_60846	F01_transcript_60846
F01_transcript_125371	NA	NA	NA	F01_transcript_125371
F01_transcript_89287	NA	F01_transcript_89287	NA	NA
F01_transcript_154466	NA	NA	NA	F01_transcript_154466
F01_transcript_7662	NA	NA	NA	F01_transcript_7662
F01_transcript_96783	NA	F01_transcript_96783	F01_transcript_96783	F01_transcript_96783
F01_transcript_61485	NA	NA	NA	F01_transcript_61485
F01_transcript_45687	NA	NA	NA	F01_transcript_45687
F01_transcript_31208	NA	NA	NA	F01_transcript_31208
F01_transcript_145767	F01_transcript_145767	F01_transcript_145767	F01_transcript_145767	F01_transcript_145767
F01_transcript_33281	NA	NA	NA	F01_transcript_33281
F01_transcript_158873	NA	NA	F01_transcript_158873	NA

F01_transcript_154657	NA	NA	NA	F01_transcript_154657
F01_transcript_84780	F01_transcript_84780	F01_transcript_84780	NA	NA
F01_transcript_155670	NA	NA	NA	F01_transcript_155670
F01_transcript_156515	F01_transcript_156515	F01_transcript_156515	F01_transcript_156515	F01_transcript_156515
F01_transcript_114055	NA	NA	NA	F01_transcript_114055
F01_transcript_119806	F01_transcript_119806	F01_transcript_119806	NA	F01_transcript_119806
F01_transcript_80010	NA	NA	NA	F01_transcript_80010
F01_transcript_165167	NA	F01_transcript_165167	NA	F01_transcript_165167
F01_transcript_110929	NA	NA	NA	F01_transcript_110929
F01_transcript_122302	NA	F01_transcript_122302	NA	NA
F01_transcript_81016	F01_transcript_81016	F01_transcript_81016	F01_transcript_81016	F01_transcript_81016
F01_transcript_51373	NA	F01_transcript_51373	NA	NA
F01_transcript_144132	NA	F01_transcript_144132	NA	NA
F01_transcript_157348	NA	F01_transcript_157348	NA	NA

F01_transcript_88284	NA	NA	NA	F01_transcript_88284
F01_transcript_103380	NA	F01_transcript_103380	NA	F01_transcript_103380
F01_transcript_70873	NA	F01_transcript_70873	F01_transcript_70873	F01_transcript_70873
F01_transcript_51131	F01_transcript_51131	F01_transcript_51131	F01_transcript_51131	F01_transcript_51131
F01_transcript_106900	NA	F01_transcript_106900	NA	F01_transcript_106900
F01_transcript_47577	NA	F01_transcript_47577	F01_transcript_47577	F01_transcript_47577
F01_transcript_127470	NA	NA	NA	F01_transcript_127470
F01_transcript_152931	NA	NA	NA	F01_transcript_152931
F01_transcript_157608	F01_transcript_157608	F01_transcript_157608	F01_transcript_157608	F01_transcript_157608
F01_transcript_133116	F01_transcript_133116	F01_transcript_133116	F01_transcript_133116	F01_transcript_133116
F01_transcript_33810	NA	NA	NA	F01_transcript_33810
F01_transcript_51648	NA	F01_transcript_51648	NA	F01_transcript_51648
F01_transcript_31472	F01_transcript_31472	F01_transcript_31472	F01_transcript_31472	F01_transcript_31472
F01_transcript_118550	NA	NA	NA	F01_transcript_118550

F01_transcript_72783	F01_transcript_72783	F01_transcript_72783	F01_transcript_72783	F01_transcript_72783
F01_transcript_14839	NA	NA	NA	F01_transcript_14839
F01_transcript_375	NA	NA	NA	F01_transcript_375
F01_transcript_20836	NA	NA	NA	F01_transcript_20836
F01_transcript_86603	NA	F01_transcript_86603	NA	NA
F01_transcript_75392	NA	F01_transcript_75392	NA	NA
F01_transcript_54466	NA	NA	NA	F01_transcript_54466
F01_transcript_83654	F01_transcript_83654	F01_transcript_83654	F01_transcript_83654	F01_transcript_83654
F01_transcript_79366	F01_transcript_79366	F01_transcript_79366	NA	NA
F01_transcript_119293	NA	NA	NA	F01_transcript_119293
F01_transcript_126276	NA	NA	NA	F01_transcript_126276
F01_transcript_154565	NA	F01_transcript_154565	NA	F01_transcript_154565
F01_transcript_99212	F01_transcript_99212	F01_transcript_99212	NA	F01_transcript_99212
F01_transcript_123115	NA	F01_transcript_123115	NA	NA

F01_transcript_25783	NA	NA	NA	F01_transcript_25783
F01_transcript_82555	F01_transcript_82555	F01_transcript_82555	NA	F01_transcript_82555
F01_transcript_1462	NA	NA	NA	F01_transcript_1462
F01_transcript_96029	NA	F01_transcript_96029	F01_transcript_96029	NA
F01_transcript_87532	NA	F01_transcript_87532	NA	NA
F01_transcript_159088	NA	NA	F01_transcript_159088	F01_transcript_159088
F01_transcript_73870	NA	NA	NA	F01_transcript_73870
F01_transcript_76243	NA	NA	NA	F01_transcript_76243
F01_transcript_57645	F01_transcript_57645	F01_transcript_57645	F01_transcript_57645	F01_transcript_57645
F01_transcript_5004	NA	NA	NA	F01_transcript_5004
F01_transcript_94348	NA	NA	NA	F01_transcript_94348
F01_transcript_69111	F01_transcript_69111	F01_transcript_69111	NA	NA
F01_transcript_45943	NA	NA	NA	F01_transcript_45943
F01_transcript_163250	F01_transcript_163250	F01_transcript_163250	NA	NA

F01_transcript_7919	F01_transcript_7919	F01_transcript_7919	F01_transcript_7919	F01_transcript_7919
F01_transcript_100445	F01_transcript_100445	F01_transcript_100445	NA	NA
F01_transcript_164399	F01_transcript_164399	F01_transcript_164399	NA	NA
F01_transcript_75684	F01_transcript_75684	F01_transcript_75684	F01_transcript_75684	F01_transcript_75684
F01_transcript_95359	NA	NA	NA	F01_transcript_95359
F01_transcript_50177	NA	F01_transcript_50177	NA	NA
F01_transcript_1365	F01_transcript_1365	F01_transcript_1365	F01_transcript_1365	F01_transcript_1365
F01_transcript_6250	F01_transcript_6250	F01_transcript_6250	NA	NA
F01_transcript_156428	NA	NA	NA	F01_transcript_156428
F01_transcript_65047	F01_transcript_65047	F01_transcript_65047	NA	F01_transcript_65047
F01_transcript_152160	NA	F01_transcript_152160	NA	NA
F01_transcript_101525	F01_transcript_101525	F01_transcript_101525	NA	F01_transcript_101525
F01_transcript_85169	F01_transcript_85169	F01_transcript_85169	NA	NA
F01_transcript_148271	NA	F01_transcript_148271	NA	F01_transcript_148271

F01_transcript_117841	F01_transcript_117841	F01_transcript_117841	F01_transcript_117841	F01_transcript_117841
F01_transcript_26397	F01_transcript_26397	F01_transcript_26397	F01_transcript_26397	F01_transcript_26397
F01_transcript_65992	NA	NA	NA	F01_transcript_65992
F01_transcript_96085	NA	F01_transcript_96085	F01_transcript_96085	F01_transcript_96085
F01_transcript_147083	NA	F01_transcript_147083	NA	NA
F01_transcript_83730	NA	F01_transcript_83730	NA	F01_transcript_83730
F01_transcript_135668	NA	F01_transcript_135668	NA	F01_transcript_135668
F01_transcript_114422	NA	NA	NA	F01_transcript_114422
F01_transcript_30539	NA	F01_transcript_30539	NA	NA
F01_transcript_128060	F01_transcript_128060	F01_transcript_128060	F01_transcript_128060	F01_transcript_128060
F01_transcript_57386	NA	F01_transcript_57386	NA	NA
F01_transcript_130720	NA	NA	NA	F01_transcript_130720
F01_transcript_50725	F01_transcript_50725	F01_transcript_50725	F01_transcript_50725	F01_transcript_50725
F01_transcript_69770	NA	NA	NA	F01_transcript_69770

F01_transcript_105467	NA	F01_transcript_105467	NA	NA
F01_transcript_69220	NA	F01_transcript_69220	NA	F01_transcript_69220
F01_transcript_63657	NA	NA	NA	F01_transcript_63657
F01_transcript_105740	NA	F01_transcript_105740	NA	NA
F01_transcript_151779	NA	F01_transcript_151779	NA	NA
F01_transcript_80872	F01_transcript_80872	F01_transcript_80872	NA	NA
F01_transcript_143392	NA	NA	NA	F01_transcript_143392
F01_transcript_23102	F01_transcript_23102	F01_transcript_23102	F01_transcript_23102	F01_transcript_23102
F01_transcript_154061	NA	F01_transcript_154061	NA	F01_transcript_154061
F01_transcript_105775	NA	F01_transcript_105775	F01_transcript_105775	NA
F01_transcript_165451	F01_transcript_165451	F01_transcript_165451	F01_transcript_165451	F01_transcript_165451
F01_transcript_110529	NA	NA	NA	F01_transcript_110529
F01_transcript_152753	NA	F01_transcript_152753	NA	F01_transcript_152753
F01_transcript_75380	F01_transcript_75380	F01_transcript_75380	F01_transcript_75380	F01_transcript_75380

F01_transcript_77432	F01_transcript_77432	F01_transcript_77432	NA	F01_transcript_77432
F01_transcript_145005	F01_transcript_145005	F01_transcript_145005	F01_transcript_145005	F01_transcript_145005
F01_transcript_152830	NA	NA	NA	F01_transcript_152830
F01_transcript_37080	NA	NA	NA	F01_transcript_37080
F01_transcript_78237	NA	NA	NA	F01_transcript_78237
F01_transcript_96835	NA	F01_transcript_96835	F01_transcript_96835	F01_transcript_96835
F01_transcript_98408	F01_transcript_98408	F01_transcript_98408	F01_transcript_98408	NA
F01_transcript_113079	NA	NA	NA	F01_transcript_113079
F01_transcript_154132	NA	F01_transcript_154132	NA	F01_transcript_154132
F01_transcript_56639	NA	NA	NA	F01_transcript_56639
F01_transcript_143135	NA	NA	NA	F01_transcript_143135
F01_transcript_66236	F01_transcript_66236	F01_transcript_66236	F01_transcript_66236	F01_transcript_66236
F01_transcript_67238	NA	NA	NA	F01_transcript_67238
F01_transcript_48585	NA	NA	NA	F01_transcript_48585

F01_transcript_156261	NA	F01_transcript_156261	NA	NA
F01_transcript_95844	NA	NA	NA	F01_transcript_95844
F01_transcript_60113	NA	NA	NA	F01_transcript_60113
F01_transcript_158826	NA	NA	NA	F01_transcript_158826
F01_transcript_147023	NA	NA	NA	F01_transcript_147023
F01_transcript_61903	NA	F01_transcript_61903	NA	NA
F01_transcript_93286	NA	F01_transcript_93286	NA	F01_transcript_93286
F01_transcript_80649	NA	NA	NA	F01_transcript_80649
F01_transcript_33948	NA	NA	NA	F01_transcript_33948
F01_transcript_121205	NA	NA	NA	F01_transcript_121205
F01_transcript_109776	F01_transcript_109776	F01_transcript_109776	F01_transcript_109776	F01_transcript_109776
F01_transcript_81718	NA	F01_transcript_81718	NA	NA
F01_transcript_136942	F01_transcript_136942	F01_transcript_136942	F01_transcript_136942	F01_transcript_136942
F01_transcript_103608	F01_transcript_103608	NA	F01_transcript_103608	NA

F01_transcript_113108	NA	NA	NA	F01_transcript_113108
F01_transcript_75025	NA	F01_transcript_75025	NA	F01_transcript_75025
F01_transcript_36920	NA	NA	NA	F01_transcript_36920
F01_transcript_122837	NA	NA	NA	F01_transcript_122837
F01_transcript_53331	NA	NA	NA	F01_transcript_53331
F01_transcript_69711	NA	F01_transcript_69711	NA	NA
F01_transcript_164252	NA	NA	NA	F01_transcript_164252
F01_transcript_134650	NA	F01_transcript_134650	NA	F01_transcript_134650
F01_transcript_97728	NA	F01_transcript_97728	NA	F01_transcript_97728
F01_transcript_131859	F01_transcript_131859	F01_transcript_131859	NA	NA
F01_transcript_75288	NA	F01_transcript_75288	NA	F01_transcript_75288
F01_transcript_48726	NA	NA	NA	F01_transcript_48726
F01_transcript_163097	NA	F01_transcript_163097	NA	NA
F01_transcript_49816	NA	NA	NA	F01_transcript_49816

F01_transcript_6451	NA	F01_transcript_6451	NA	NA
F01_transcript_66211	NA	NA	NA	F01_transcript_66211
F01_transcript_153146	NA	NA	NA	F01_transcript_153146
F01_transcript_135450	F01_transcript_135450	F01_transcript_135450	F01_transcript_135450	F01_transcript_135450
F01_transcript_120696	NA	F01_transcript_120696	NA	NA
F01_transcript_68291	NA	NA	NA	F01_transcript_68291
F01_transcript_78219	NA	F01_transcript_78219	F01_transcript_78219	F01_transcript_78219
F01_transcript_98037	NA	F01_transcript_98037	F01_transcript_98037	NA
F01_transcript_87628	NA	NA	NA	F01_transcript_87628
F01_transcript_100939	F01_transcript_100939	F01_transcript_100939	NA	F01_transcript_100939
F01_transcript_68292	NA	F01_transcript_68292	F01_transcript_68292	F01_transcript_68292
F01_transcript_136293	F01_transcript_136293	F01_transcript_136293	F01_transcript_136293	F01_transcript_136293
F01_transcript_3728	F01_transcript_3728	F01_transcript_3728	F01_transcript_3728	F01_transcript_3728
F01_transcript_164187	NA	NA	NA	F01_transcript_164187

F01_transcript_138890	F01_transcript_138890	NA	NA	F01_transcript_138890
F01_transcript_95092	NA	F01_transcript_95092	NA	NA
F01_transcript_93731	F01_transcript_93731	F01_transcript_93731	NA	NA
F01_transcript_55886	NA	F01_transcript_55886	NA	NA
F01_transcript_129778	F01_transcript_129778	F01_transcript_129778	NA	NA
F01_transcript_122694	NA	NA	NA	F01_transcript_122694
F01_transcript_132430	F01_transcript_132430	F01_transcript_132430	F01_transcript_132430	F01_transcript_132430
F01_transcript_110162	NA	F01_transcript_110162	F01_transcript_110162	F01_transcript_110162
F01_transcript_133054	NA	F01_transcript_133054	NA	F01_transcript_133054
F01_transcript_146668	NA	F01_transcript_146668	NA	NA
F01_transcript_98128	NA	F01_transcript_98128	NA	NA
F01_transcript_63922	NA	F01_transcript_63922	F01_transcript_63922	F01_transcript_63922
F01_transcript_55992	NA	NA	NA	F01_transcript_55992
F01_transcript_133397	NA	F01_transcript_133397	NA	NA

F01_transcript_120632	F01_transcript_120632	F01_transcript_120632	F01_transcript_120632	NA
F01_transcript_65573	NA	F01_transcript_65573	NA	F01_transcript_65573
F01_transcript_59341	NA	F01_transcript_59341	F01_transcript_59341	F01_transcript_59341
F01_transcript_107441	F01_transcript_107441	F01_transcript_107441	NA	F01_transcript_107441
F01_transcript_102777	NA	NA	NA	F01_transcript_102777
F01_transcript_93340	NA	F01_transcript_93340	NA	NA
F01_transcript_10594	F01_transcript_10594	F01_transcript_10594	F01_transcript_10594	F01_transcript_10594
F01_transcript_133597	F01_transcript_133597	F01_transcript_133597	NA	F01_transcript_133597
F01_transcript_164204	F01_transcript_164204	F01_transcript_164204	NA	NA
F01_transcript_99620	F01_transcript_99620	F01_transcript_99620	F01_transcript_99620	F01_transcript_99620
F01_transcript_137948	NA	NA	NA	F01_transcript_137948
F01_transcript_71103	F01_transcript_71103	F01_transcript_71103	F01_transcript_71103	NA
F01_transcript_67728	NA	NA	NA	F01_transcript_67728
F01_transcript_165636	F01_transcript_165636	F01_transcript_165636	NA	NA

F01_transcript_58761	NA	NA	F01_transcript_58761	F01_transcript_58761
F01_transcript_33755	NA	NA	NA	F01_transcript_33755
F01_transcript_123750	NA	F01_transcript_123750	NA	NA
F01_transcript_55094	NA	NA	NA	F01_transcript_55094
F01_transcript_97339	NA	F01_transcript_97339	NA	F01_transcript_97339
F01_transcript_96163	NA	F01_transcript_96163	NA	F01_transcript_96163
F01_transcript_120221	NA	NA	NA	F01_transcript_120221
F01_transcript_70454	NA	F01_transcript_70454	NA	NA
F01_transcript_58216	F01_transcript_58216	F01_transcript_58216	F01_transcript_58216	F01_transcript_58216
F01_transcript_64008	F01_transcript_64008	F01_transcript_64008	F01_transcript_64008	F01_transcript_64008
F01_transcript_75283	NA	F01_transcript_75283	NA	NA
F01_transcript_82464	NA	F01_transcript_82464	NA	NA
F01_transcript_112901	F01_transcript_112901	F01_transcript_112901	NA	NA
F01_transcript_153762	F01_transcript_153762	F01_transcript_153762	F01_transcript_153762	F01_transcript_153762

F01_transcript_166030	F01_transcript_166030	F01_transcript_166030	F01_transcript_166030	NA
F01_transcript_75157	NA	NA	NA	F01_transcript_75157
F01_transcript_65036	NA	NA	NA	F01_transcript_65036
F01_transcript_82732	F01_transcript_82732	F01_transcript_82732	F01_transcript_82732	F01_transcript_82732
F01_transcript_107256	F01_transcript_107256	F01_transcript_107256	F01_transcript_107256	F01_transcript_107256
F01_transcript_28329	NA	NA	NA	F01_transcript_28329
F01_transcript_110846	NA	NA	F01_transcript_110846	F01_transcript_110846
F01_transcript_76865	F01_transcript_76865	F01_transcript_76865	NA	F01_transcript_76865
F01_transcript_157920	NA	F01_transcript_157920	NA	NA
F01_transcript_103338	F01_transcript_103338	F01_transcript_103338	NA	F01_transcript_103338
F01_transcript_117776	F01_transcript_117776	F01_transcript_117776	NA	F01_transcript_117776
F01_transcript_163441	NA	F01_transcript_163441	NA	NA
F01_transcript_104955	F01_transcript_104955	F01_transcript_104955	F01_transcript_104955	F01_transcript_104955
F01_transcript_97996	F01_transcript_97996	F01_transcript_97996	F01_transcript_97996	F01_transcript_97996

F01_transcript_60668	F01_transcript_60668	F01_transcript_60668	NA	NA
F01_transcript_110205	F01_transcript_110205	F01_transcript_110205	NA	F01_transcript_110205
F01_transcript_51967	NA	F01_transcript_51967	NA	NA
F01_transcript_113169	F01_transcript_113169	F01_transcript_113169	NA	F01_transcript_113169
F01_transcript_123595	NA	F01_transcript_123595	NA	NA
F01_transcript_167371	F01_transcript_167371	F01_transcript_167371	NA	F01_transcript_167371
F01_transcript_38550	NA	NA	NA	F01_transcript_38550
F01_transcript_105284	NA	NA	NA	F01_transcript_105284
F01_transcript_109885	F01_transcript_109885	F01_transcript_109885	NA	F01_transcript_109885
F01_transcript_84334	F01_transcript_84334	F01_transcript_84334	NA	F01_transcript_84334
F01_transcript_95363	NA	NA	NA	F01_transcript_95363
F01_transcript_49385	NA	F01_transcript_49385	NA	F01_transcript_49385
F01_transcript_158010	NA	F01_transcript_158010	NA	F01_transcript_158010
F01_transcript_96649	NA	NA	NA	F01_transcript_96649

F01_transcript_121133	F01_transcript_121133	F01_transcript_121133	F01_transcript_121133	F01_transcript_121133
F01_transcript_91838	NA	F01_transcript_91838	F01_transcript_91838	F01_transcript_91838
F01_transcript_53259	NA	NA	NA	F01_transcript_53259
F01_transcript_155308	NA	NA	NA	F01_transcript_155308
F01_transcript_137970	NA	F01_transcript_137970	NA	F01_transcript_137970
F01_transcript_110405	NA	F01_transcript_110405	NA	NA
F01_transcript_107538	F01_transcript_107538	F01_transcript_107538	F01_transcript_107538	F01_transcript_107538
F01_transcript_82313	NA	F01_transcript_82313	NA	F01_transcript_82313
F01_transcript_114821	F01_transcript_114821	F01_transcript_114821	F01_transcript_114821	F01_transcript_114821
F01_transcript_48190	F01_transcript_48190	NA	F01_transcript_48190	NA
F01_transcript_65371	NA	NA	F01_transcript_65371	NA
F01_transcript_141566	F01_transcript_141566	F01_transcript_141566	F01_transcript_141566	F01_transcript_141566
F01_transcript_87907	NA	NA	NA	F01_transcript_87907
F01_transcript_114152	F01_transcript_114152	F01_transcript_114152	F01_transcript_114152	F01_transcript_114152

F01_transcript_122926	NA	NA	NA	F01_transcript_122926
F01_transcript_65784	NA	F01_transcript_65784	NA	NA
F01_transcript_144482	NA	F01_transcript_144482	NA	NA
F01_transcript_98170	F01_transcript_98170	F01_transcript_98170	F01_transcript_98170	F01_transcript_98170
F01_transcript_124524	NA	NA	NA	F01_transcript_124524
F01_transcript_67896	NA	F01_transcript_67896	NA	F01_transcript_67896
F01_transcript_123978	NA	F01_transcript_123978	NA	NA
F01_transcript_105414	F01_transcript_105414	F01_transcript_105414	F01_transcript_105414	F01_transcript_105414
F01_transcript_37216	NA	NA	NA	F01_transcript_37216
F01_transcript_159820	NA	F01_transcript_159820	F01_transcript_159820	F01_transcript_159820
F01_transcript_35648	NA	NA	NA	F01_transcript_35648
F01_transcript_135214	F01_transcript_135214	F01_transcript_135214	F01_transcript_135214	NA
F01_transcript_127410	F01_transcript_127410	F01_transcript_127410	F01_transcript_127410	F01_transcript_127410
F01_transcript_49204	NA	F01_transcript_49204	NA	NA

F01_transcript_155268	F01_transcript_155268	F01_transcript_155268	F01_transcript_155268	F01_transcript_155268
F01_transcript_142948	NA	F01_transcript_142948	NA	NA
F01_transcript_148125	F01_transcript_148125	F01_transcript_148125	NA	F01_transcript_148125
F01_transcript_61782	F01_transcript_61782	F01_transcript_61782	F01_transcript_61782	F01_transcript_61782
F01_transcript_93573	F01_transcript_93573	F01_transcript_93573	F01_transcript_93573	F01_transcript_93573
F01_transcript_123345	NA	F01_transcript_123345	NA	NA
F01_transcript_71864	NA	F01_transcript_71864	NA	NA
F01_transcript_136036	NA	NA	NA	F01_transcript_136036
F01_transcript_121876	NA	F01_transcript_121876	NA	NA
F01_transcript_118766	NA	F01_transcript_118766	NA	NA
F01_transcript_121085	NA	NA	NA	F01_transcript_121085
F01_transcript_145978	NA	F01_transcript_145978	NA	NA
F01_transcript_61647	F01_transcript_61647	F01_transcript_61647	F01_transcript_61647	F01_transcript_61647
F01_transcript_122744	NA	F01_transcript_122744	NA	NA

F01_transcript_90940	NA	F01_transcript_90940	NA	NA
F01_transcript_151973	NA	NA	NA	F01_transcript_151973
F01_transcript_148828	NA	NA	NA	F01_transcript_148828
F01_transcript_156950	NA	F01_transcript_156950	F01_transcript_156950	F01_transcript_156950
F01_transcript_66980	F01_transcript_66980	F01_transcript_66980	F01_transcript_66980	F01_transcript_66980
F01_transcript_131980	NA	F01_transcript_131980	NA	NA
F01_transcript_134291	NA	NA	NA	F01_transcript_134291
F01_transcript_103524	NA	F01_transcript_103524	NA	NA
F01_transcript_130632	NA	NA	NA	F01_transcript_130632
F01_transcript_128847	F01_transcript_128847	F01_transcript_128847	F01_transcript_128847	NA
F01_transcript_159613	NA	F01_transcript_159613	NA	NA
F01_transcript_96595	NA	NA	NA	F01_transcript_96595
F01_transcript_307	NA	NA	NA	F01_transcript_307
F01_transcript_10635	NA	F01_transcript_10635	NA	NA

F01_transcript_56131	NA	F01_transcript_56131	NA	F01_transcript_56131
F01_transcript_141861	NA	F01_transcript_141861	NA	F01_transcript_141861
F01_transcript_138872	NA	F01_transcript_138872	F01_transcript_138872	F01_transcript_138872
F01_transcript_6282	F01_transcript_6282	F01_transcript_6282	F01_transcript_6282	NA
F01_transcript_133832	F01_transcript_133832	F01_transcript_133832	F01_transcript_133832	F01_transcript_133832
F01_transcript_81300	F01_transcript_81300	F01_transcript_81300	NA	F01_transcript_81300
F01_transcript_93442	F01_transcript_93442	F01_transcript_93442	F01_transcript_93442	F01_transcript_93442
F01_transcript_6232	NA	F01_transcript_6232	NA	NA
F01_transcript_83950	F01_transcript_83950	F01_transcript_83950	F01_transcript_83950	F01_transcript_83950
F01_transcript_141380	NA	NA	NA	F01_transcript_141380
F01_transcript_72419	F01_transcript_72419	F01_transcript_72419	F01_transcript_72419	F01_transcript_72419
F01_transcript_14447	NA	NA	NA	F01_transcript_14447
F01_transcript_52573	NA	NA	NA	F01_transcript_52573
F01_transcript_91543	NA	F01_transcript_91543	NA	NA

F01_transcript_139882	NA	NA	NA	F01_transcript_139882
F01_transcript_25924	NA	NA	NA	F01_transcript_25924
F01_transcript_143269	NA	NA	F01_transcript_143269	F01_transcript_143269
F01_transcript_9841	F01_transcript_9841	F01_transcript_9841	F01_transcript_9841	F01_transcript_9841
F01_transcript_90455	NA	F01_transcript_90455	NA	NA
F01_transcript_45778	NA	NA	NA	F01_transcript_45778
F01_transcript_153755	NA	F01_transcript_153755	NA	NA
F01_transcript_59318	NA	NA	F01_transcript_59318	NA
F01_transcript_166647	NA	NA	NA	F01_transcript_166647
F01_transcript_54117	NA	NA	NA	F01_transcript_54117
F01_transcript_49417	F01_transcript_49417	F01_transcript_49417	F01_transcript_49417	F01_transcript_49417
F01_transcript_150516	NA	F01_transcript_150516	NA	NA
F01_transcript_87453	NA	F01_transcript_87453	NA	NA
F01_transcript_132424	NA	F01_transcript_132424	NA	NA

F01_transcript_158146	F01_transcript_158146	F01_transcript_158146	F01_transcript_158146	F01_transcript_158146
F01_transcript_60553	F01_transcript_60553	F01_transcript_60553	NA	NA
F01_transcript_115135	NA	F01_transcript_115135	NA	F01_transcript_115135
F01_transcript_165211	NA	F01_transcript_165211	F01_transcript_165211	F01_transcript_165211
F01_transcript_58620	NA	F01_transcript_58620	NA	F01_transcript_58620
F01_transcript_99600	NA	F01_transcript_99600	NA	NA
F01_transcript_151397	F01_transcript_151397	F01_transcript_151397	NA	F01_transcript_151397
F01_transcript_142376	F01_transcript_142376	F01_transcript_142376	F01_transcript_142376	NA
F01_transcript_67979	NA	F01_transcript_67979	NA	NA
F01_transcript_148365	NA	F01_transcript_148365	NA	NA
F01_transcript_60390	F01_transcript_60390	F01_transcript_60390	F01_transcript_60390	NA
F01_transcript_142329	F01_transcript_142329	F01_transcript_142329	F01_transcript_142329	F01_transcript_142329
F01_transcript_106814	F01_transcript_106814	F01_transcript_106814	F01_transcript_106814	F01_transcript_106814
F01_transcript_77478	NA	F01_transcript_77478	NA	NA

F01_transcript_41585	NA	NA	F01_transcript_41585	NA
F01_transcript_54131	NA	F01_transcript_54131	NA	NA
F01_transcript_26030	NA	NA	NA	F01_transcript_26030
F01_transcript_55001	NA	NA	NA	F01_transcript_55001
F01_transcript_49238	NA	F01_transcript_49238	NA	F01_transcript_49238
F01_transcript_150963	NA	NA	F01_transcript_150963	F01_transcript_150963
F01_transcript_86673	F01_transcript_86673	F01_transcript_86673	F01_transcript_86673	NA
F01_transcript_22983	NA	F01_transcript_22983	NA	NA
F01_transcript_129390	F01_transcript_129390	F01_transcript_129390	F01_transcript_129390	F01_transcript_129390
F01_transcript_103493	F01_transcript_103493	F01_transcript_103493	NA	NA
F01_transcript_111920	NA	F01_transcript_111920	NA	NA
F01_transcript_127494	F01_transcript_127494	F01_transcript_127494	NA	F01_transcript_127494
F01_transcript_85816	NA	NA	NA	F01_transcript_85816
F01_transcript_131699	F01_transcript_131699	F01_transcript_131699	F01_transcript_131699	F01_transcript_131699

F01_transcript_143793	F01_transcript_143793	F01_transcript_143793	NA	NA
F01_transcript_83913	F01_transcript_83913	F01_transcript_83913	F01_transcript_83913	F01_transcript_83913
F01_transcript_48414	NA	F01_transcript_48414	NA	NA
F01_transcript_116317	F01_transcript_116317	F01_transcript_116317	F01_transcript_116317	F01_transcript_116317
F01_transcript_126278	NA	F01_transcript_126278	NA	NA
F01_transcript_159554	NA	F01_transcript_159554	NA	NA
F01_transcript_58757	F01_transcript_58757	F01_transcript_58757	F01_transcript_58757	F01_transcript_58757
F01_transcript_126451	NA	NA	NA	F01_transcript_126451
F01_transcript_157043	NA	F01_transcript_157043	NA	NA
F01_transcript_140930	NA	F01_transcript_140930	NA	F01_transcript_140930
F01_transcript_109130	NA	F01_transcript_109130	NA	NA
F01_transcript_55890	NA	F01_transcript_55890	NA	NA
F01_transcript_78832	F01_transcript_78832	F01_transcript_78832	F01_transcript_78832	NA
F01_transcript_37472	NA	NA	NA	F01_transcript_37472

F01_transcript_125863	NA	NA	NA	F01_transcript_125863
F01_transcript_110087	NA	F01_transcript_110087	NA	NA
F01_transcript_21724	F01_transcript_21724	F01_transcript_21724	NA	F01_transcript_21724
F01_transcript_22862	NA	F01_transcript_22862	NA	NA
F01_transcript_157450	NA	F01_transcript_157450	F01_transcript_157450	F01_transcript_157450
F01_transcript_164547	NA	NA	NA	F01_transcript_164547
F01_transcript_121923	NA	F01_transcript_121923	NA	NA
F01_transcript_50897	NA	F01_transcript_50897	NA	F01_transcript_50897
F01_transcript_114738	NA	F01_transcript_114738	NA	F01_transcript_114738
F01_transcript_20586	F01_transcript_20586	F01_transcript_20586	F01_transcript_20586	F01_transcript_20586
F01_transcript_96156	F01_transcript_96156	F01_transcript_96156	F01_transcript_96156	NA
F01_transcript_146164	NA	F01_transcript_146164	F01_transcript_146164	F01_transcript_146164
F01_transcript_124023	F01_transcript_124023	F01_transcript_124023	F01_transcript_124023	F01_transcript_124023
F01_transcript_153805	F01_transcript_153805	F01_transcript_153805	NA	F01_transcript_153805

F01_transcript_150211	F01_transcript_150211	F01_transcript_150211	F01_transcript_150211	F01_transcript_150211
F01_transcript_70250	NA	NA	NA	F01_transcript_70250
F01_transcript_89136	NA	F01_transcript_89136	NA	F01_transcript_89136
F01_transcript_107336	NA	F01_transcript_107336	F01_transcript_107336	F01_transcript_107336
F01_transcript_73116	F01_transcript_73116	F01_transcript_73116	F01_transcript_73116	F01_transcript_73116
F01_transcript_41496	NA	NA	NA	F01_transcript_41496
F01_transcript_124162	NA	NA	NA	F01_transcript_124162
F01_transcript_47183	NA	F01_transcript_47183	NA	NA
F01_transcript_86130	NA	F01_transcript_86130	NA	NA
F01_transcript_154705	F01_transcript_154705	F01_transcript_154705	NA	F01_transcript_154705
F01_transcript_142219	NA	F01_transcript_142219	NA	NA
F01_transcript_69449	F01_transcript_69449	F01_transcript_69449	F01_transcript_69449	F01_transcript_69449
F01_transcript_154564	NA	NA	NA	F01_transcript_154564
F01_transcript_162221	NA	NA	NA	F01_transcript_162221

F01_transcript_157353	F01_transcript_157353	F01_transcript_157353	NA	NA
F01_transcript_91966	NA	NA	NA	F01_transcript_91966
F01_transcript_45249	NA	NA	NA	F01_transcript_45249
F01_transcript_163240	NA	F01_transcript_163240	F01_transcript_163240	NA
F01_transcript_97700	NA	F01_transcript_97700	NA	NA
F01_transcript_96534	NA	F01_transcript_96534	NA	NA
F01_transcript_152101	NA	F01_transcript_152101	NA	NA
F01_transcript_104665	NA	F01_transcript_104665	NA	F01_transcript_104665
F01_transcript_54836	F01_transcript_54836	F01_transcript_54836	NA	NA
F01_transcript_94961	NA	F01_transcript_94961	F01_transcript_94961	F01_transcript_94961
F01_transcript_103480	F01_transcript_103480	F01_transcript_103480	NA	NA
F01_transcript_148495	NA	F01_transcript_148495	NA	NA
F01_transcript_54837	NA	NA	NA	F01_transcript_54837
F01_transcript_124188	NA	F01_transcript_124188	NA	NA

F01_transcript_17873	NA	NA	NA	F01_transcript_17873
F01_transcript_161649	NA	NA	NA	F01_transcript_161649
F01_transcript_59483	NA	NA	NA	F01_transcript_59483
F01_transcript_53948	NA	F01_transcript_53948	NA	NA
F01_transcript_50920	F01_transcript_50920	F01_transcript_50920	F01_transcript_50920	F01_transcript_50920
F01_transcript_16892	F01_transcript_16892	F01_transcript_16892	F01_transcript_16892	NA
F01_transcript_110263	F01_transcript_110263	F01_transcript_110263	F01_transcript_110263	F01_transcript_110263
F01_transcript_73403	F01_transcript_73403	F01_transcript_73403	F01_transcript_73403	F01_transcript_73403
F01_transcript_139082	NA	F01_transcript_139082	NA	NA
F01_transcript_111217	F01_transcript_111217	F01_transcript_111217	F01_transcript_111217	F01_transcript_111217
F01_transcript_126125	NA	F01_transcript_126125	NA	F01_transcript_126125
F01_transcript_138616	F01_transcript_138616	F01_transcript_138616	NA	F01_transcript_138616
F01_transcript_49959	NA	NA	NA	F01_transcript_49959
F01_transcript_92628	NA	NA	NA	F01_transcript_92628

F01_transcript_127635	NA	NA	NA	F01_transcript_127635
F01_transcript_89044	NA	F01_transcript_89044	F01_transcript_89044	NA
F01_transcript_119736	NA	F01_transcript_119736	NA	NA
F01_transcript_13873	NA	F01_transcript_13873	NA	NA
F01_transcript_153380	NA	F01_transcript_153380	NA	NA
F01_transcript_49491	NA	NA	F01_transcript_49491	F01_transcript_49491
F01_transcript_146137	NA	NA	NA	F01_transcript_146137
F01_transcript_8039	NA	NA	NA	F01_transcript_8039
F01_transcript_71641	NA	NA	NA	F01_transcript_71641
F01_transcript_140578	NA	NA	NA	F01_transcript_140578
F01_transcript_119337	NA	F01_transcript_119337	F01_transcript_119337	F01_transcript_119337
F01_transcript_49873	NA	F01_transcript_49873	NA	NA
F01_transcript_156153	NA	F01_transcript_156153	F01_transcript_156153	F01_transcript_156153
F01_transcript_115761	NA	NA	NA	F01_transcript_115761

F01_transcript_103095	NA	F01_transcript_103095	F01_transcript_103095	F01_transcript_103095
F01_transcript_54287	NA	F01_transcript_54287	NA	NA
F01_transcript_144185	NA	NA	NA	F01_transcript_144185
F01_transcript_143230	NA	F01_transcript_143230	NA	F01_transcript_143230
F01_transcript_102322	NA	F01_transcript_102322	NA	F01_transcript_102322
F01_transcript_90212	NA	F01_transcript_90212	NA	F01_transcript_90212
F01_transcript_161803	NA	F01_transcript_161803	NA	F01_transcript_161803
F01_transcript_156199	F01_transcript_156199	F01_transcript_156199	F01_transcript_156199	F01_transcript_156199
F01_transcript_110067	NA	F01_transcript_110067	NA	NA
F01_transcript_6617	NA	NA	NA	F01_transcript_6617
F01_transcript_82869	F01_transcript_82869	F01_transcript_82869	NA	F01_transcript_82869
F01_transcript_57404	NA	F01_transcript_57404	NA	NA
F01_transcript_66302	NA	NA	NA	F01_transcript_66302
F01_transcript_116428	NA	NA	NA	F01_transcript_116428

F01_transcript_5	NA	F01_transcript_5	NA	NA
F01_transcript_63694	F01_transcript_63694	F01_transcript_63694	F01_transcript_63694	F01_transcript_63694
F01_transcript_92410	NA	NA	NA	F01_transcript_92410
F01_transcript_10795	NA	F01_transcript_10795	F01_transcript_10795	F01_transcript_10795
F01_transcript_139472	F01_transcript_139472	F01_transcript_139472	NA	NA
F01_transcript_150322	NA	NA	NA	F01_transcript_150322
F01_transcript_63591	NA	NA	NA	F01_transcript_63591
F01_transcript_117346	NA	F01_transcript_117346	NA	NA
F01_transcript_66129	F01_transcript_66129	F01_transcript_66129	NA	NA
F01_transcript_61493	NA	F01_transcript_61493	NA	F01_transcript_61493
F01_transcript_75427	NA	F01_transcript_75427	NA	NA
F01_transcript_131781	NA	F01_transcript_131781	NA	NA
F01_transcript_152492	F01_transcript_152492	F01_transcript_152492	F01_transcript_152492	F01_transcript_152492
F01_transcript_80140	F01_transcript_80140	F01_transcript_80140	F01_transcript_80140	F01_transcript_80140

F01_transcript_117545	NA	F01_transcript_117545	NA	NA
F01_transcript_56367	NA	NA	F01_transcript_56367	NA
F01_transcript_1812	NA	NA	NA	F01_transcript_1812
F01_transcript_26584	NA	F01_transcript_26584	NA	NA
F01_transcript_90481	F01_transcript_90481	F01_transcript_90481	NA	F01_transcript_90481
F01_transcript_152408	NA	F01_transcript_152408	F01_transcript_152408	F01_transcript_152408
F01_transcript_16661	NA	NA	NA	F01_transcript_16661
F01_transcript_90034	NA	F01_transcript_90034	F01_transcript_90034	NA
F01_transcript_2289	NA	NA	NA	F01_transcript_2289
F01_transcript_50943	F01_transcript_50943	F01_transcript_50943	F01_transcript_50943	F01_transcript_50943
F01_transcript_137000	NA	F01_transcript_137000	NA	F01_transcript_137000
F01_transcript_1310	NA	F01_transcript_1310	NA	F01_transcript_1310
F01_transcript_88600	NA	F01_transcript_88600	NA	NA
F01_transcript_5737	F01_transcript_5737	F01_transcript_5737	F01_transcript_5737	F01_transcript_5737

F01_transcript_87865	F01_transcript_87865	F01_transcript_87865	NA	F01_transcript_87865
F01_transcript_27763	NA	NA	NA	F01_transcript_27763
F01_transcript_121463	NA	F01_transcript_121463	NA	F01_transcript_121463
F01_transcript_112061	NA	F01_transcript_112061	F01_transcript_112061	F01_transcript_112061
F01_transcript_90319	F01_transcript_90319	F01_transcript_90319	F01_transcript_90319	F01_transcript_90319
F01_transcript_41268	NA	NA	NA	F01_transcript_41268
F01_transcript_165808	NA	F01_transcript_165808	F01_transcript_165808	F01_transcript_165808
F01_transcript_52133	NA	F01_transcript_52133	NA	NA
F01_transcript_162733	F01_transcript_162733	F01_transcript_162733	F01_transcript_162733	F01_transcript_162733
F01_transcript_162869	F01_transcript_162869	F01_transcript_162869	NA	F01_transcript_162869
F01_transcript_167242	NA	F01_transcript_167242	NA	NA
F01_transcript_133513	F01_transcript_133513	F01_transcript_133513	NA	NA
F01_transcript_136979	NA	F01_transcript_136979	NA	NA
F01_transcript_92218	NA	F01_transcript_92218	NA	F01_transcript_92218

F01_transcript_160328	F01_transcript_160328	F01_transcript_160328	NA	F01_transcript_160328
F01_transcript_113077	NA	NA	NA	F01_transcript_113077
F01_transcript_94795	NA	NA	NA	F01_transcript_94795
F01_transcript_86596	F01_transcript_86596	F01_transcript_86596	F01_transcript_86596	F01_transcript_86596
F01_transcript_92413	NA	NA	NA	F01_transcript_92413
F01_transcript_65442	F01_transcript_65442	F01_transcript_65442	F01_transcript_65442	F01_transcript_65442
F01_transcript_27815	NA	NA	NA	F01_transcript_27815
F01_transcript_164832	NA	NA	NA	F01_transcript_164832
F01_transcript_107544	NA	NA	NA	F01_transcript_107544
F01_transcript_126965	F01_transcript_126965	F01_transcript_126965	NA	NA
F01_transcript_35726	NA	F01_transcript_35726	NA	NA
F01_transcript_117485	F01_transcript_117485	F01_transcript_117485	F01_transcript_117485	F01_transcript_117485
F01_transcript_144777	F01_transcript_144777	F01_transcript_144777	NA	F01_transcript_144777
F01_transcript_161100	NA	F01_transcript_161100	NA	NA

F01_transcript_85477	NA	F01_transcript_85477	NA	NA
F01_transcript_156743	NA	NA	NA	F01_transcript_156743
F01_transcript_146311	NA	NA	NA	F01_transcript_146311
F01_transcript_87903	NA	F01_transcript_87903	NA	NA
F01_transcript_58755	NA	NA	NA	F01_transcript_58755
F01_transcript_124613	F01_transcript_124613	F01_transcript_124613	F01_transcript_124613	F01_transcript_124613
F01_transcript_64659	NA	NA	NA	F01_transcript_64659
F01_transcript_32248	NA	NA	NA	F01_transcript_32248
F01_transcript_88769	NA	F01_transcript_88769	NA	NA
F01_transcript_151095	NA	NA	NA	F01_transcript_151095
F01_transcript_17119	NA	F01_transcript_17119	NA	NA
F01_transcript_50448	F01_transcript_50448	F01_transcript_50448	NA	NA
F01_transcript_137308	NA	NA	NA	F01_transcript_137308
F01_transcript_132224	F01_transcript_132224	F01_transcript_132224	F01_transcript_132224	F01_transcript_132224

F01_transcript_34239	NA	NA	NA	F01_transcript_34239
F01_transcript_106252	NA	NA	NA	F01_transcript_106252
F01_transcript_151890	NA	NA	NA	F01_transcript_151890
F01_transcript_110306	NA	F01_transcript_110306	NA	F01_transcript_110306
F01_transcript_108325	F01_transcript_108325	F01_transcript_108325	F01_transcript_108325	F01_transcript_108325
F01_transcript_144468	NA	F01_transcript_144468	NA	NA
F01_transcript_40825	NA	NA	NA	F01_transcript_40825
F01_transcript_71633	NA	F01_transcript_71633	NA	NA
F01_transcript_90517	NA	F01_transcript_90517	NA	NA
F01_transcript_61230	F01_transcript_61230	F01_transcript_61230	F01_transcript_61230	F01_transcript_61230
F01_transcript_163604	NA	NA	NA	F01_transcript_163604
F01_transcript_57085	NA	F01_transcript_57085	F01_transcript_57085	NA
F01_transcript_104406	F01_transcript_104406	F01_transcript_104406	F01_transcript_104406	F01_transcript_104406
F01_transcript_157488	NA	NA	NA	F01_transcript_157488

F01_transcript_26565	NA	NA	NA	F01_transcript_26565
F01_transcript_62463	NA	F01_transcript_62463	F01_transcript_62463	F01_transcript_62463
F01_transcript_132373	NA	NA	NA	F01_transcript_132373
F01_transcript_128449	NA	F01_transcript_128449	NA	NA
F01_transcript_94788	NA	F01_transcript_94788	NA	NA
F01_transcript_146138	F01_transcript_146138	F01_transcript_146138	F01_transcript_146138	NA
F01_transcript_92538	NA	F01_transcript_92538	NA	NA
F01_transcript_166082	NA	F01_transcript_166082	NA	NA
F01_transcript_106188	NA	F01_transcript_106188	F01_transcript_106188	F01_transcript_106188
F01_transcript_158591	NA	F01_transcript_158591	NA	NA
F01_transcript_149333	NA	NA	NA	F01_transcript_149333
F01_transcript_57103	NA	NA	NA	F01_transcript_57103
F01_transcript_51996	NA	NA	NA	F01_transcript_51996
F01_transcript_128868	NA	NA	NA	F01_transcript_128868

F01_transcript_117820	NA	F01_transcript_117820	NA	NA
F01_transcript_101203	NA	NA	NA	F01_transcript_101203
F01_transcript_97210	NA	NA	NA	F01_transcript_97210
F01_transcript_75051	NA	F01_transcript_75051	NA	NA
F01_transcript_140571	F01_transcript_140571	F01_transcript_140571	F01_transcript_140571	NA
F01_transcript_80830	NA	F01_transcript_80830	NA	NA
F01_transcript_145580	NA	NA	NA	F01_transcript_145580
F01_transcript_70666	F01_transcript_70666	F01_transcript_70666	F01_transcript_70666	F01_transcript_70666
F01_transcript_150831	F01_transcript_150831	F01_transcript_150831	F01_transcript_150831	F01_transcript_150831
F01_transcript_142060	NA	NA	NA	F01_transcript_142060
F01_transcript_63716	NA	F01_transcript_63716	NA	NA
F01_transcript_142228	NA	F01_transcript_142228	F01_transcript_142228	F01_transcript_142228
F01_transcript_96833	NA	F01_transcript_96833	F01_transcript_96833	F01_transcript_96833
F01_transcript_118149	NA	NA	NA	F01_transcript_118149

F01_transcript_126071	NA	NA	NA	F01_transcript_126071
F01_transcript_37401	NA	NA	F01_transcript_37401	F01_transcript_37401
F01_transcript_93123	NA	F01_transcript_93123	NA	NA
F01_transcript_105726	NA	F01_transcript_105726	NA	NA
F01_transcript_56670	F01_transcript_56670	F01_transcript_56670	NA	F01_transcript_56670
F01_transcript_117629	NA	F01_transcript_117629	NA	F01_transcript_117629
F01_transcript_136680	NA	F01_transcript_136680	NA	NA
F01_transcript_123442	NA	F01_transcript_123442	NA	F01_transcript_123442
F01_transcript_68169	F01_transcript_68169	NA	NA	NA
F01_transcript_161732	NA	NA	NA	F01_transcript_161732
F01_transcript_167216	NA	NA	NA	F01_transcript_167216
F01_transcript_7804	NA	F01_transcript_7804	NA	NA
F01_transcript_159325	NA	F01_transcript_159325	F01_transcript_159325	F01_transcript_159325
F01_transcript_141606	F01_transcript_141606	F01_transcript_141606	NA	F01_transcript_141606

F01_transcript_80378	F01_transcript_80378	F01_transcript_80378	NA	NA
F01_transcript_129203	NA	F01_transcript_129203	F01_transcript_129203	F01_transcript_129203
F01_transcript_143398	F01_transcript_143398	F01_transcript_143398	F01_transcript_143398	F01_transcript_143398
F01_transcript_71526	NA	F01_transcript_71526	NA	F01_transcript_71526
F01_transcript_59955	NA	NA	NA	F01_transcript_59955
F01_transcript_80336	NA	NA	NA	F01_transcript_80336
F01_transcript_74915	NA	F01_transcript_74915	NA	NA
F01_transcript_134203	NA	F01_transcript_134203	NA	NA
F01_transcript_94213	NA	NA	NA	F01_transcript_94213
F01_transcript_6469	F01_transcript_6469	F01_transcript_6469	F01_transcript_6469	F01_transcript_6469
F01_transcript_79896	NA	F01_transcript_79896	NA	NA
F01_transcript_108110	NA	F01_transcript_108110	F01_transcript_108110	F01_transcript_108110
F01_transcript_72313	NA	F01_transcript_72313	NA	NA
F01_transcript_5396	NA	NA	NA	F01_transcript_5396

F01_transcript_45382	NA	NA	NA	F01_transcript_45382
F01_transcript_138610	NA	NA	NA	F01_transcript_138610
F01_transcript_89586	F01_transcript_89586	F01_transcript_89586	F01_transcript_89586	F01_transcript_89586
F01_transcript_7688	F01_transcript_7688	F01_transcript_7688	NA	NA
F01_transcript_140779	NA	F01_transcript_140779	NA	NA
F01_transcript_77974	NA	F01_transcript_77974	F01_transcript_77974	F01_transcript_77974
F01_transcript_128012	NA	NA	NA	F01_transcript_128012
F01_transcript_81667	NA	NA	NA	F01_transcript_81667
F01_transcript_132093	NA	F01_transcript_132093	NA	NA
F01_transcript_109659	F01_transcript_109659	F01_transcript_109659	F01_transcript_109659	F01_transcript_109659
F01_transcript_99852	NA	F01_transcript_99852	NA	NA
F01_transcript_158094	NA	NA	NA	F01_transcript_158094
F01_transcript_50950	F01_transcript_50950	F01_transcript_50950	NA	NA
F01_transcript_160631	NA	NA	NA	F01_transcript_160631

F01_transcript_125519	F01_transcript_125519	F01_transcript_125519	F01_transcript_125519	F01_transcript_125519
F01_transcript_53845	F01_transcript_53845	NA	NA	NA
F01_transcript_65074	NA	F01_transcript_65074	F01_transcript_65074	NA
F01_transcript_125245	NA	F01_transcript_125245	F01_transcript_125245	F01_transcript_125245
F01_transcript_123168	F01_transcript_123168	F01_transcript_123168	F01_transcript_123168	F01_transcript_123168
F01_transcript_138341	NA	F01_transcript_138341	NA	NA
F01_transcript_144686	NA	NA	NA	F01_transcript_144686
F01_transcript_105242	NA	F01_transcript_105242	NA	NA
F01_transcript_164116	F01_transcript_164116	F01_transcript_164116	F01_transcript_164116	F01_transcript_164116
F01_transcript_126687	F01_transcript_126687	F01_transcript_126687	NA	NA
F01_transcript_102492	NA	F01_transcript_102492	NA	NA
F01_transcript_64364	NA	NA	NA	F01_transcript_64364
F01_transcript_24931	NA	F01_transcript_24931	NA	F01_transcript_24931
F01_transcript_145315	NA	F01_transcript_145315	NA	F01_transcript_145315

F01_transcript_58857	NA	F01_transcript_58857	NA	F01_transcript_58857
F01_transcript_130505	NA	NA	NA	F01_transcript_130505
F01_transcript_158621	NA	F01_transcript_158621	NA	F01_transcript_158621
F01_transcript_126781	NA	F01_transcript_126781	NA	F01_transcript_126781
F01_transcript_120959	NA	NA	NA	F01_transcript_120959
F01_transcript_64978	NA	NA	NA	F01_transcript_64978
F01_transcript_53180	F01_transcript_53180	F01_transcript_53180	F01_transcript_53180	F01_transcript_53180
F01_transcript_17340	NA	F01_transcript_17340	NA	NA
F01_transcript_57173	NA	NA	F01_transcript_57173	F01_transcript_57173
F01_transcript_78707	NA	F01_transcript_78707	NA	NA
F01_transcript_64085	NA	NA	NA	F01_transcript_64085
F01_transcript_69276	NA	NA	NA	F01_transcript_69276
F01_transcript_115853	NA	NA	NA	F01_transcript_115853
F01_transcript_91678	F01_transcript_91678	F01_transcript_91678	F01_transcript_91678	F01_transcript_91678

F01_transcript_164593	F01_transcript_164593	F01_transcript_164593	F01_transcript_164593	NA
F01_transcript_58205	NA	NA	NA	F01_transcript_58205
F01_transcript_154511	NA	F01_transcript_154511	NA	NA
F01_transcript_160486	F01_transcript_160486	F01_transcript_160486	NA	NA
F01_transcript_126961	F01_transcript_126961	F01_transcript_126961	F01_transcript_126961	F01_transcript_126961
F01_transcript_116917	NA	F01_transcript_116917	NA	F01_transcript_116917
F01_transcript_132941	F01_transcript_132941	F01_transcript_132941	F01_transcript_132941	F01_transcript_132941
F01_transcript_144481	NA	F01_transcript_144481	F01_transcript_144481	F01_transcript_144481
F01_transcript_129331	NA	F01_transcript_129331	NA	NA
F01_transcript_148600	NA	NA	NA	F01_transcript_148600
F01_transcript_95599	F01_transcript_95599	NA	F01_transcript_95599	F01_transcript_95599
F01_transcript_166263	F01_transcript_166263	F01_transcript_166263	F01_transcript_166263	F01_transcript_166263
F01_transcript_133338	F01_transcript_133338	F01_transcript_133338	NA	F01_transcript_133338
F01_transcript_102993	NA	NA	NA	F01_transcript_102993

F01_transcript_59986	NA	NA	NA	F01_transcript_59986
F01_transcript_28445	NA	F01_transcript_28445	NA	NA
F01_transcript_4231	F01_transcript_4231	F01_transcript_4231	F01_transcript_4231	F01_transcript_4231
F01_transcript_66485	F01_transcript_66485	F01_transcript_66485	NA	F01_transcript_66485
F01_transcript_118432	F01_transcript_118432	F01_transcript_118432	F01_transcript_118432	F01_transcript_118432
F01_transcript_9429	F01_transcript_9429	F01_transcript_9429	F01_transcript_9429	F01_transcript_9429
F01_transcript_3909	NA	NA	NA	F01_transcript_3909
F01_transcript_104501	NA	NA	NA	F01_transcript_104501
F01_transcript_80118	F01_transcript_80118	F01_transcript_80118	NA	NA
F01_transcript_51415	F01_transcript_51415	NA	NA	NA
F01_transcript_20964	F01_transcript_20964	F01_transcript_20964	F01_transcript_20964	F01_transcript_20964
F01_transcript_104362	F01_transcript_104362	F01_transcript_104362	F01_transcript_104362	F01_transcript_104362
F01_transcript_119324	NA	F01_transcript_119324	NA	NA
F01_transcript_87938	F01_transcript_87938	F01_transcript_87938	NA	NA

F01_transcript_48910	F01_transcript_48910	F01_transcript_48910	F01_transcript_48910	F01_transcript_48910
F01_transcript_20618	NA	NA	NA	F01_transcript_20618
F01_transcript_164494	F01_transcript_164494	F01_transcript_164494	F01_transcript_164494	F01_transcript_164494
F01_transcript_70983	NA	F01_transcript_70983	F01_transcript_70983	F01_transcript_70983
F01_transcript_68780	F01_transcript_68780	NA	F01_transcript_68780	NA
F01_transcript_73915	NA	NA	NA	F01_transcript_73915
F01_transcript_60793	NA	NA	NA	F01_transcript_60793
F01_transcript_51923	NA	F01_transcript_51923	NA	F01_transcript_51923
F01_transcript_147024	F01_transcript_147024	F01_transcript_147024	NA	F01_transcript_147024
F01_transcript_68213	NA	NA	NA	F01_transcript_68213
F01_transcript_133754	NA	NA	NA	F01_transcript_133754
F01_transcript_4990	F01_transcript_4990	F01_transcript_4990	F01_transcript_4990	F01_transcript_4990
F01_transcript_66225	NA	F01_transcript_66225	NA	NA
F01_transcript_114525	F01_transcript_114525	F01_transcript_114525	NA	NA

F01_transcript_83198	F01_transcript_83198	F01_transcript_83198	F01_transcript_83198	F01_transcript_83198
F01_transcript_123033	NA	NA	NA	F01_transcript_123033
F01_transcript_134742	NA	NA	F01_transcript_134742	NA
F01_transcript_69616	F01_transcript_69616	F01_transcript_69616	F01_transcript_69616	F01_transcript_69616
F01_transcript_126678	NA	F01_transcript_126678	NA	NA
F01_transcript_125365	F01_transcript_125365	F01_transcript_125365	F01_transcript_125365	F01_transcript_125365
F01_transcript_107759	NA	F01_transcript_107759	F01_transcript_107759	NA
F01_transcript_159891	NA	F01_transcript_159891	NA	NA
F01_transcript_32483	NA	NA	NA	F01_transcript_32483
F01_transcript_107133	NA	F01_transcript_107133	NA	F01_transcript_107133
F01_transcript_97130	F01_transcript_97130	F01_transcript_97130	F01_transcript_97130	F01_transcript_97130
F01_transcript_126567	NA	F01_transcript_126567	NA	NA
F01_transcript_79608	NA	F01_transcript_79608	NA	NA
F01_transcript_97415	F01_transcript_97415	F01_transcript_97415	NA	F01_transcript_97415

F01_transcript_118222	NA	NA	NA	F01_transcript_118222
F01_transcript_110520	F01_transcript_110520	F01_transcript_110520	NA	NA
F01_transcript_47945	F01_transcript_47945	F01_transcript_47945	NA	NA
F01_transcript_57126	NA	NA	NA	F01_transcript_57126
F01_transcript_143744	NA	F01_transcript_143744	NA	NA
F01_transcript_74250	NA	NA	NA	F01_transcript_74250
F01_transcript_120901	NA	F01_transcript_120901	NA	NA
F01_transcript_49118	F01_transcript_49118	F01_transcript_49118	NA	F01_transcript_49118
F01_transcript_163100	NA	F01_transcript_163100	NA	NA
F01_transcript_51227	F01_transcript_51227	F01_transcript_51227	F01_transcript_51227	F01_transcript_51227
F01_transcript_2085	NA	NA	NA	F01_transcript_2085
F01_transcript_110805	F01_transcript_110805	F01_transcript_110805	F01_transcript_110805	F01_transcript_110805
F01_transcript_88406	F01_transcript_88406	F01_transcript_88406	F01_transcript_88406	F01_transcript_88406
F01_transcript_55509	NA	NA	NA	F01_transcript_55509

F01_transcript_88670	NA	NA	NA	F01_transcript_88670
F01_transcript_111985	F01_transcript_111985	F01_transcript_111985	F01_transcript_111985	F01_transcript_111985
F01_transcript_84217	NA	NA	F01_transcript_84217	F01_transcript_84217
F01_transcript_84234	F01_transcript_84234	F01_transcript_84234	F01_transcript_84234	F01_transcript_84234
F01_transcript_28800	NA	NA	NA	F01_transcript_28800
F01_transcript_99178	NA	F01_transcript_99178	F01_transcript_99178	NA
F01_transcript_3163	NA	NA	NA	F01_transcript_3163
F01_transcript_159366	NA	F01_transcript_159366	NA	NA
F01_transcript_64095	NA	F01_transcript_64095	NA	NA
F01_transcript_96392	NA	F01_transcript_96392	NA	F01_transcript_96392
F01_transcript_78240	NA	F01_transcript_78240	F01_transcript_78240	F01_transcript_78240
F01_transcript_141238	NA	NA	NA	F01_transcript_141238
F01_transcript_161210	F01_transcript_161210	F01_transcript_161210	F01_transcript_161210	F01_transcript_161210
F01_transcript_74000	NA	NA	F01_transcript_74000	F01_transcript_74000

F01_transcript_24499	NA	NA	NA	F01_transcript_24499
F01_transcript_90279	NA	F01_transcript_90279	NA	NA
F01_transcript_50040	F01_transcript_50040	NA	NA	F01_transcript_50040
F01_transcript_134942	NA	F01_transcript_134942	F01_transcript_134942	NA
F01_transcript_104873	NA	F01_transcript_104873	NA	NA
F01_transcript_96347	F01_transcript_96347	F01_transcript_96347	F01_transcript_96347	F01_transcript_96347
F01_transcript_133997	NA	NA	F01_transcript_133997	F01_transcript_133997
F01_transcript_99523	NA	F01_transcript_99523	NA	NA
F01_transcript_95085	NA	NA	NA	F01_transcript_95085
F01_transcript_110641	NA	NA	NA	F01_transcript_110641
F01_transcript_78557	NA	F01_transcript_78557	NA	F01_transcript_78557
F01_transcript_77062	NA	NA	F01_transcript_77062	NA
F01_transcript_105227	NA	NA	NA	F01_transcript_105227
F01_transcript_100061	NA	F01_transcript_100061	NA	NA

F01_transcript_141312	F01_transcript_141312	F01_transcript_141312	NA	NA
F01_transcript_128430	NA	F01_transcript_128430	NA	NA
F01_transcript_49801	NA	F01_transcript_49801	F01_transcript_49801	F01_transcript_49801
F01_transcript_81859	F01_transcript_81859	F01_transcript_81859	F01_transcript_81859	F01_transcript_81859
F01_transcript_112919	F01_transcript_112919	F01_transcript_112919	NA	NA
F01_transcript_133455	NA	NA	NA	F01_transcript_133455
F01_transcript_35527	NA	NA	NA	F01_transcript_35527
F01_transcript_158629	NA	NA	NA	F01_transcript_158629
F01_transcript_65843	NA	F01_transcript_65843	F01_transcript_65843	F01_transcript_65843
F01_transcript_136872	NA	F01_transcript_136872	NA	NA
F01_transcript_156929	NA	NA	NA	F01_transcript_156929
F01_transcript_98441	NA	NA	NA	F01_transcript_98441
F01_transcript_13019	NA	NA	NA	F01_transcript_13019
F01_transcript_27330	NA	NA	NA	F01_transcript_27330

F01_transcript_110003	NA	F01_transcript_110003	NA	NA
F01_transcript_141105	F01_transcript_141105	F01_transcript_141105	F01_transcript_141105	F01_transcript_141105
F01_transcript_53977	NA	F01_transcript_53977	NA	NA
F01_transcript_82446	NA	NA	NA	F01_transcript_82446
F01_transcript_98122	NA	NA	NA	F01_transcript_98122
F01_transcript_36524	NA	NA	NA	F01_transcript_36524
F01_transcript_165764	NA	F01_transcript_165764	NA	NA
F01_transcript_114164	F01_transcript_114164	F01_transcript_114164	NA	F01_transcript_114164
F01_transcript_40675	NA	NA	NA	F01_transcript_40675
F01_transcript_93326	NA	F01_transcript_93326	NA	F01_transcript_93326
F01_transcript_106934	NA	F01_transcript_106934	NA	NA
F01_transcript_157333	NA	NA	NA	F01_transcript_157333
F01_transcript_119685	NA	NA	NA	F01_transcript_119685
F01_transcript_99814	F01_transcript_99814	F01_transcript_99814	F01_transcript_99814	F01_transcript_99814

F01_transcript_7049	NA	NA	NA	F01_transcript_7049
F01_transcript_86659	NA	NA	NA	F01_transcript_86659
F01_transcript_116856	NA	F01_transcript_116856	NA	NA
F01_transcript_161092	F01_transcript_161092	F01_transcript_161092	NA	F01_transcript_161092
F01_transcript_13029	NA	NA	NA	F01_transcript_13029
F01_transcript_57396	F01_transcript_57396	F01_transcript_57396	NA	NA
F01_transcript_138992	NA	F01_transcript_138992	NA	NA
F01_transcript_78933	NA	F01_transcript_78933	NA	NA
F01_transcript_133270	NA	F01_transcript_133270	NA	F01_transcript_133270
F01_transcript_127274	NA	F01_transcript_127274	NA	NA
F01_transcript_152947	F01_transcript_152947	F01_transcript_152947	NA	F01_transcript_152947
F01_transcript_102200	F01_transcript_102200	F01_transcript_102200	F01_transcript_102200	F01_transcript_102200
F01_transcript_158612	NA	F01_transcript_158612	NA	F01_transcript_158612
F01_transcript_117983	NA	F01_transcript_117983	NA	NA

F01_transcript_108899	F01_transcript_108899	F01_transcript_108899	F01_transcript_108899	F01_transcript_108899
F01_transcript_116392	NA	NA	NA	F01_transcript_116392
F01_transcript_103362	F01_transcript_103362	F01_transcript_103362	NA	F01_transcript_103362
F01_transcript_107602	NA	NA	NA	F01_transcript_107602
F01_transcript_113641	NA	F01_transcript_113641	NA	F01_transcript_113641
F01_transcript_63864	F01_transcript_63864	F01_transcript_63864	F01_transcript_63864	F01_transcript_63864
F01_transcript_135464	F01_transcript_135464	F01_transcript_135464	F01_transcript_135464	F01_transcript_135464
F01_transcript_157178	NA	NA	NA	F01_transcript_157178
F01_transcript_44080	F01_transcript_44080	F01_transcript_44080	NA	NA
F01_transcript_150408	NA	F01_transcript_150408	F01_transcript_150408	NA
F01_transcript_28389	NA	NA	NA	F01_transcript_28389
F01_transcript_44432	NA	NA	NA	F01_transcript_44432
F01_transcript_150334	NA	NA	NA	F01_transcript_150334
F01_transcript_166792	NA	F01_transcript_166792	F01_transcript_166792	NA

F01_transcript_46751	NA	NA	NA	F01_transcript_46751
F01_transcript_80259	NA	F01_transcript_80259	NA	F01_transcript_80259
F01_transcript_132763	NA	NA	F01_transcript_132763	NA
F01_transcript_99700	NA	NA	NA	F01_transcript_99700
F01_transcript_166040	F01_transcript_166040	F01_transcript_166040	F01_transcript_166040	F01_transcript_166040
F01_transcript_120433	NA	NA	F01_transcript_120433	F01_transcript_120433
F01_transcript_104456	NA	NA	NA	F01_transcript_104456
F01_transcript_165358	F01_transcript_165358	F01_transcript_165358	F01_transcript_165358	F01_transcript_165358
F01_transcript_161425	NA	F01_transcript_161425	F01_transcript_161425	F01_transcript_161425
F01_transcript_65386	NA	NA	F01_transcript_65386	NA
F01_transcript_53037	NA	NA	NA	F01_transcript_53037
F01_transcript_105710	NA	NA	NA	F01_transcript_105710
F01_transcript_60850	F01_transcript_60850	F01_transcript_60850	F01_transcript_60850	F01_transcript_60850
F01_transcript_137847	F01_transcript_137847	F01_transcript_137847	F01_transcript_137847	F01_transcript_137847

F01_transcript_105332	F01_transcript_105332	F01_transcript_105332	F01_transcript_105332	F01_transcript_105332
F01_transcript_85470	NA	NA	NA	F01_transcript_85470
F01_transcript_89271	F01_transcript_89271	F01_transcript_89271	F01_transcript_89271	F01_transcript_89271
F01_transcript_92281	NA	F01_transcript_92281	NA	NA
F01_transcript_8473	NA	NA	NA	F01_transcript_8473
F01_transcript_77286	NA	F01_transcript_77286	NA	F01_transcript_77286
F01_transcript_55232	NA	F01_transcript_55232	NA	NA
F01_transcript_52001	NA	NA	NA	F01_transcript_52001
F01_transcript_10631	NA	NA	NA	F01_transcript_10631
F01_transcript_45579	NA	NA	NA	F01_transcript_45579
F01_transcript_111403	NA	F01_transcript_111403	F01_transcript_111403	F01_transcript_111403
F01_transcript_92905	NA	F01_transcript_92905	NA	NA
F01_transcript_49308	NA	F01_transcript_49308	NA	F01_transcript_49308
F01_transcript_91320	NA	NA	NA	F01_transcript_91320

F01_transcript_117210	NA	F01_transcript_117210	NA	NA
F01_transcript_150991	F01_transcript_150991	F01_transcript_150991	F01_transcript_150991	F01_transcript_150991
F01_transcript_141957	F01_transcript_141957	F01_transcript_141957	NA	F01_transcript_141957
F01_transcript_48874	NA	F01_transcript_48874	F01_transcript_48874	F01_transcript_48874
F01_transcript_85592	NA	F01_transcript_85592	NA	F01_transcript_85592
F01_transcript_108403	NA	F01_transcript_108403	NA	F01_transcript_108403
F01_transcript_24449	NA	NA	NA	F01_transcript_24449
F01_transcript_75216	NA	F01_transcript_75216	NA	NA
F01_transcript_75938	F01_transcript_75938	F01_transcript_75938	NA	F01_transcript_75938
F01_transcript_21433	NA	NA	NA	F01_transcript_21433
F01_transcript_155150	F01_transcript_155150	F01_transcript_155150	F01_transcript_155150	F01_transcript_155150
F01_transcript_160029	NA	F01_transcript_160029	NA	NA
F01_transcript_66476	F01_transcript_66476	F01_transcript_66476	NA	F01_transcript_66476
F01_transcript_46491	NA	NA	F01_transcript_46491	F01_transcript_46491

F01_transcript_138667	NA	F01_transcript_138667	NA	NA
F01_transcript_100439	NA	NA	F01_transcript_100439	NA
F01_transcript_60264	NA	NA	NA	F01_transcript_60264
F01_transcript_70910	NA	NA	NA	F01_transcript_70910
F01_transcript_56256	NA	NA	F01_transcript_56256	NA
F01_transcript_165073	F01_transcript_165073	F01_transcript_165073	F01_transcript_165073	F01_transcript_165073
F01_transcript_159127	NA	NA	NA	F01_transcript_159127
F01_transcript_138126	F01_transcript_138126	F01_transcript_138126	F01_transcript_138126	F01_transcript_138126
F01_transcript_73377	F01_transcript_73377	F01_transcript_73377	F01_transcript_73377	F01_transcript_73377
F01_transcript_115784	F01_transcript_115784	F01_transcript_115784	NA	NA
F01_transcript_154629	NA	NA	NA	F01_transcript_154629
F01_transcript_99753	NA	NA	NA	F01_transcript_99753
F01_transcript_166288	NA	NA	NA	F01_transcript_166288
F01_transcript_49538	F01_transcript_49538	F01_transcript_49538	NA	NA

F01_transcript_61888	NA	F01_transcript_61888	NA	F01_transcript_61888
F01_transcript_138158	NA	F01_transcript_138158	NA	F01_transcript_138158
F01_transcript_102003	NA	F01_transcript_102003	F01_transcript_102003	NA
F01_transcript_69123	F01_transcript_69123	F01_transcript_69123	NA	NA
F01_transcript_78054	F01_transcript_78054	F01_transcript_78054	F01_transcript_78054	F01_transcript_78054
F01_transcript_56841	NA	NA	F01_transcript_56841	NA
F01_transcript_56333	F01_transcript_56333	F01_transcript_56333	F01_transcript_56333	F01_transcript_56333
F01_transcript_125767	NA	NA	F01_transcript_125767	NA
F01_transcript_57154	NA	F01_transcript_57154	F01_transcript_57154	F01_transcript_57154
F01_transcript_102570	NA	NA	NA	F01_transcript_102570
F01_transcript_141303	NA	F01_transcript_141303	F01_transcript_141303	F01_transcript_141303
F01_transcript_72602	NA	F01_transcript_72602	F01_transcript_72602	F01_transcript_72602
F01_transcript_53124	NA	NA	NA	F01_transcript_53124
F01_transcript_109795	NA	NA	NA	F01_transcript_109795

F01_transcript_140199	NA	F01_transcript_140199	NA	F01_transcript_140199
F01_transcript_108407	NA	F01_transcript_108407	NA	F01_transcript_108407
F01_transcript_35801	NA	NA	NA	F01_transcript_35801
F01_transcript_48574	F01_transcript_48574	F01_transcript_48574	NA	NA
F01_transcript_37979	F01_transcript_37979	F01_transcript_37979	NA	F01_transcript_37979
F01_transcript_11856	NA	NA	NA	F01_transcript_11856
F01_transcript_75965	NA	F01_transcript_75965	NA	NA
F01_transcript_148348	NA	NA	NA	F01_transcript_148348
F01_transcript_112544	NA	F01_transcript_112544	NA	NA
F01_transcript_76326	NA	NA	NA	F01_transcript_76326
F01_transcript_144622	F01_transcript_144622	F01_transcript_144622	F01_transcript_144622	F01_transcript_144622
F01_transcript_119011	NA	NA	NA	F01_transcript_119011
F01_transcript_66034	F01_transcript_66034	F01_transcript_66034	NA	NA
F01_transcript_100818	NA	F01_transcript_100818	NA	F01_transcript_100818

F01_transcript_123136	NA	F01_transcript_123136	F01_transcript_123136	F01_transcript_123136
F01_transcript_107204	NA	F01_transcript_107204	NA	NA
F01_transcript_90681	NA	F01_transcript_90681	NA	F01_transcript_90681
F01_transcript_143584	NA	F01_transcript_143584	NA	NA
F01_transcript_149143	F01_transcript_149143	F01_transcript_149143	F01_transcript_149143	F01_transcript_149143
F01_transcript_38619	NA	F01_transcript_38619	NA	NA
F01_transcript_147296	F01_transcript_147296	F01_transcript_147296	F01_transcript_147296	F01_transcript_147296
F01_transcript_166754	NA	F01_transcript_166754	F01_transcript_166754	F01_transcript_166754
F01_transcript_98295	F01_transcript_98295	F01_transcript_98295	F01_transcript_98295	NA
F01_transcript_164335	F01_transcript_164335	F01_transcript_164335	F01_transcript_164335	F01_transcript_164335
F01_transcript_130054	F01_transcript_130054	F01_transcript_130054	F01_transcript_130054	F01_transcript_130054
F01_transcript_71890	NA	NA	NA	F01_transcript_71890
F01_transcript_121567	NA	F01_transcript_121567	NA	NA
F01_transcript_80812	F01_transcript_80812	F01_transcript_80812	F01_transcript_80812	F01_transcript_80812

F01_transcript_102048	F01_transcript_102048	F01_transcript_102048	NA	F01_transcript_102048
F01_transcript_93900	F01_transcript_93900	F01_transcript_93900	F01_transcript_93900	F01_transcript_93900
F01_transcript_75756	F01_transcript_75756	F01_transcript_75756	F01_transcript_75756	NA
F01_transcript_118791	F01_transcript_118791	F01_transcript_118791	F01_transcript_118791	F01_transcript_118791
F01_transcript_109806	NA	F01_transcript_109806	NA	NA
F01_transcript_116073	F01_transcript_116073	F01_transcript_116073	F01_transcript_116073	F01_transcript_116073
F01_transcript_148076	F01_transcript_148076	F01_transcript_148076	F01_transcript_148076	F01_transcript_148076
F01_transcript_55876	NA	F01_transcript_55876	NA	NA
F01_transcript_104889	NA	F01_transcript_104889	NA	F01_transcript_104889
F01_transcript_167636	NA	NA	NA	F01_transcript_167636
F01_transcript_143633	NA	F01_transcript_143633	NA	NA
F01_transcript_60380	F01_transcript_60380	F01_transcript_60380	F01_transcript_60380	F01_transcript_60380
F01_transcript_164815	NA	F01_transcript_164815	F01_transcript_164815	F01_transcript_164815
F01_transcript_155188	F01_transcript_155188	F01_transcript_155188	NA	NA

F01_transcript_105911	NA	NA	NA	F01_transcript_105911
F01_transcript_117380	F01_transcript_117380	F01_transcript_117380	NA	NA
F01_transcript_588	F01_transcript_588	F01_transcript_588	NA	F01_transcript_588
F01_transcript_46369	NA	NA	F01_transcript_46369	F01_transcript_46369
F01_transcript_96053	NA	NA	NA	F01_transcript_96053
F01_transcript_16659	NA	F01_transcript_16659	NA	NA
F01_transcript_146222	F01_transcript_146222	F01_transcript_146222	F01_transcript_146222	F01_transcript_146222
F01_transcript_86088	F01_transcript_86088	F01_transcript_86088	F01_transcript_86088	F01_transcript_86088
F01_transcript_56739	NA	F01_transcript_56739	NA	NA
F01_transcript_28561	NA	NA	NA	F01_transcript_28561
F01_transcript_74188	F01_transcript_74188	F01_transcript_74188	F01_transcript_74188	F01_transcript_74188
F01_transcript_56377	NA	NA	NA	F01_transcript_56377
F01_transcript_100779	NA	F01_transcript_100779	F01_transcript_100779	F01_transcript_100779
F01_transcript_62128	F01_transcript_62128	F01_transcript_62128	NA	NA

F01_transcript_51244	F01_transcript_51244	F01_transcript_51244	F01_transcript_51244	F01_transcript_51244
F01_transcript_43223	NA	NA	NA	F01_transcript_43223
F01_transcript_126311	F01_transcript_126311	F01_transcript_126311	NA	NA
F01_transcript_57981	NA	F01_transcript_57981	NA	F01_transcript_57981
F01_transcript_43779	NA	NA	NA	F01_transcript_43779
F01_transcript_76821	NA	NA	NA	F01_transcript_76821
F01_transcript_163302	NA	NA	NA	F01_transcript_163302
F01_transcript_112788	F01_transcript_112788	F01_transcript_112788	F01_transcript_112788	F01_transcript_112788
F01_transcript_39595	F01_transcript_39595	F01_transcript_39595	NA	F01_transcript_39595
F01_transcript_163960	NA	F01_transcript_163960	NA	NA
F01_transcript_114369	F01_transcript_114369	F01_transcript_114369	F01_transcript_114369	NA
F01_transcript_84468	NA	NA	NA	F01_transcript_84468
F01_transcript_37701	NA	NA	NA	F01_transcript_37701
F01_transcript_141918	F01_transcript_141918	F01_transcript_141918	F01_transcript_141918	F01_transcript_141918

F01_transcript_102410	NA	NA	NA	F01_transcript_102410
F01_transcript_105133	F01_transcript_105133	F01_transcript_105133	NA	NA
F01_transcript_103347	NA	F01_transcript_103347	NA	F01_transcript_103347
F01_transcript_167749	F01_transcript_167749	F01_transcript_167749	F01_transcript_167749	NA
F01_transcript_87594	F01_transcript_87594	F01_transcript_87594	F01_transcript_87594	F01_transcript_87594
F01_transcript_111782	F01_transcript_111782	F01_transcript_111782	NA	NA
F01_transcript_61292	NA	F01_transcript_61292	NA	NA
F01_transcript_95541	NA	NA	NA	F01_transcript_95541
F01_transcript_156449	F01_transcript_156449	F01_transcript_156449	F01_transcript_156449	F01_transcript_156449
F01_transcript_107227	NA	NA	NA	F01_transcript_107227
F01_transcript_147085	NA	F01_transcript_147085	NA	NA
F01_transcript_166745	NA	F01_transcript_166745	NA	NA
F01_transcript_156592	F01_transcript_156592	F01_transcript_156592	F01_transcript_156592	F01_transcript_156592
F01_transcript_53635	F01_transcript_53635	F01_transcript_53635	F01_transcript_53635	F01_transcript_53635

F01_transcript_112108	NA	NA	F01_transcript_112108	NA
F01_transcript_10939	NA	F01_transcript_10939	NA	NA
F01_transcript_146897	F01_transcript_146897	F01_transcript_146897	F01_transcript_146897	F01_transcript_146897
F01_transcript_104647	F01_transcript_104647	F01_transcript_104647	F01_transcript_104647	F01_transcript_104647
F01_transcript_430	NA	NA	NA	F01_transcript_430
F01_transcript_125891	NA	F01_transcript_125891	NA	NA
F01_transcript_72122	NA	F01_transcript_72122	F01_transcript_72122	F01_transcript_72122
F01_transcript_73523	NA	NA	NA	F01_transcript_73523
F01_transcript_38608	NA	NA	NA	F01_transcript_38608
F01_transcript_92075	F01_transcript_92075	F01_transcript_92075	F01_transcript_92075	F01_transcript_92075
F01_transcript_141460	NA	NA	NA	F01_transcript_141460
F01_transcript_74471	F01_transcript_74471	F01_transcript_74471	F01_transcript_74471	F01_transcript_74471
F01_transcript_58327	NA	F01_transcript_58327	NA	NA
F01_transcript_118139	NA	F01_transcript_118139	NA	F01_transcript_118139

F01_transcript_67273	F01_transcript_67273	F01_transcript_67273	NA	NA
F01_transcript_123981	NA	F01_transcript_123981	NA	F01_transcript_123981
F01_transcript_93753	NA	F01_transcript_93753	NA	NA
F01_transcript_98049	NA	F01_transcript_98049	NA	NA
F01_transcript_18307	NA	NA	NA	F01_transcript_18307
F01_transcript_116946	NA	NA	NA	F01_transcript_116946
F01_transcript_63903	NA	F01_transcript_63903	NA	NA
F01_transcript_56375	NA	NA	F01_transcript_56375	NA
F01_transcript_106146	F01_transcript_106146	F01_transcript_106146	NA	NA
F01_transcript_103803	F01_transcript_103803	F01_transcript_103803	F01_transcript_103803	F01_transcript_103803
F01_transcript_76583	NA	NA	NA	F01_transcript_76583
F01_transcript_154755	F01_transcript_154755	F01_transcript_154755	F01_transcript_154755	F01_transcript_154755
F01_transcript_10025	NA	F01_transcript_10025	NA	NA
F01_transcript_127306	NA	F01_transcript_127306	NA	NA

F01_transcript_142671	F01_transcript_142671	F01_transcript_142671	F01_transcript_142671	F01_transcript_142671
F01_transcript_163780	NA	F01_transcript_163780	NA	NA
F01_transcript_61491	NA	F01_transcript_61491	NA	NA
F01_transcript_43567	NA	NA	NA	F01_transcript_43567
F01_transcript_133329	NA	NA	F01_transcript_133329	F01_transcript_133329
F01_transcript_161140	NA	F01_transcript_161140	NA	NA
F01_transcript_161162	NA	F01_transcript_161162	NA	F01_transcript_161162
F01_transcript_152228	NA	NA	NA	F01_transcript_152228
F01_transcript_78531	NA	F01_transcript_78531	NA	NA
F01_transcript_102172	NA	F01_transcript_102172	NA	F01_transcript_102172
F01_transcript_150953	NA	NA	NA	F01_transcript_150953
F01_transcript_63928	F01_transcript_63928	F01_transcript_63928	F01_transcript_63928	F01_transcript_63928
F01_transcript_107871	F01_transcript_107871	F01_transcript_107871	F01_transcript_107871	F01_transcript_107871
F01_transcript_118306	NA	F01_transcript_118306	NA	F01_transcript_118306

F01_transcript_153433	F01_transcript_153433	F01_transcript_153433	F01_transcript_153433	F01_transcript_153433
F01_transcript_160557	NA	NA	NA	F01_transcript_160557
F01_transcript_139943	NA	NA	NA	F01_transcript_139943
F01_transcript_155779	NA	F01_transcript_155779	NA	NA
F01_transcript_91289	F01_transcript_91289	F01_transcript_91289	F01_transcript_91289	F01_transcript_91289
F01_transcript_13571	NA	NA	NA	F01_transcript_13571
F01_transcript_50090	F01_transcript_50090	F01_transcript_50090	F01_transcript_50090	F01_transcript_50090
F01_transcript_91744	NA	NA	NA	F01_transcript_91744
F01_transcript_58527	NA	NA	NA	F01_transcript_58527
F01_transcript_167004	F01_transcript_167004	F01_transcript_167004	NA	F01_transcript_167004
F01_transcript_247	NA	NA	NA	F01_transcript_247
F01_transcript_132207	NA	F01_transcript_132207	NA	NA
F01_transcript_89089	NA	NA	NA	F01_transcript_89089
F01_transcript_132459	NA	F01_transcript_132459	NA	F01_transcript_132459

F01_transcript_110845	NA	F01_transcript_110845	NA	NA
F01_transcript_20768	NA	NA	NA	F01_transcript_20768
F01_transcript_160827	NA	F01_transcript_160827	NA	F01_transcript_160827
F01_transcript_147699	F01_transcript_147699	F01_transcript_147699	F01_transcript_147699	F01_transcript_147699
F01_transcript_61132	NA	NA	NA	F01_transcript_61132
F01_transcript_166373	NA	NA	NA	F01_transcript_166373
F01_transcript_107409	F01_transcript_107409	F01_transcript_107409	F01_transcript_107409	F01_transcript_107409
F01_transcript_62763	F01_transcript_62763	F01_transcript_62763	F01_transcript_62763	F01_transcript_62763
F01_transcript_70159	NA	NA	NA	F01_transcript_70159
F01_transcript_50282	F01_transcript_50282	F01_transcript_50282	F01_transcript_50282	F01_transcript_50282
F01_transcript_113955	NA	F01_transcript_113955	F01_transcript_113955	F01_transcript_113955
F01_transcript_82949	NA	F01_transcript_82949	NA	NA
F01_transcript_10961	NA	F01_transcript_10961	F01_transcript_10961	F01_transcript_10961
F01_transcript_78551	NA	NA	NA	F01_transcript_78551

F01_transcript_145028	NA	F01_transcript_145028	NA	F01_transcript_145028
F01_transcript_136595	NA	NA	NA	F01_transcript_136595
F01_transcript_75107	NA	NA	NA	F01_transcript_75107
F01_transcript_133209	NA	NA	F01_transcript_133209	NA
F01_transcript_72280	NA	NA	NA	F01_transcript_72280
F01_transcript_122000	F01_transcript_122000	F01_transcript_122000	NA	F01_transcript_122000
F01_transcript_86105	NA	F01_transcript_86105	NA	NA
F01_transcript_115315	NA	F01_transcript_115315	NA	F01_transcript_115315
F01_transcript_90830	F01_transcript_90830	F01_transcript_90830	F01_transcript_90830	F01_transcript_90830
F01_transcript_48917	NA	F01_transcript_48917	NA	NA
F01_transcript_98881	NA	F01_transcript_98881	NA	NA
F01_transcript_92810	NA	F01_transcript_92810	NA	NA
F01_transcript_29194	F01_transcript_29194	F01_transcript_29194	F01_transcript_29194	F01_transcript_29194
F01_transcript_124232	NA	NA	NA	F01_transcript_124232

F01_transcript_160622	NA	F01_transcript_160622	NA	NA
F01_transcript_144527	F01_transcript_144527	NA	NA	NA
F01_transcript_31115	NA	NA	NA	F01_transcript_31115
F01_transcript_107567	NA	F01_transcript_107567	NA	F01_transcript_107567
F01_transcript_93370	NA	NA	NA	F01_transcript_93370
F01_transcript_85019	NA	NA	NA	F01_transcript_85019
F01_transcript_93401	NA	NA	NA	F01_transcript_93401
F01_transcript_162389	NA	F01_transcript_162389	NA	NA
F01_transcript_125608	NA	F01_transcript_125608	NA	F01_transcript_125608
F01_transcript_140140	NA	NA	NA	F01_transcript_140140
F01_transcript_124992	NA	F01_transcript_124992	NA	F01_transcript_124992
F01_transcript_15833	NA	NA	NA	F01_transcript_15833
F01_transcript_88613	F01_transcript_88613	F01_transcript_88613	F01_transcript_88613	F01_transcript_88613
F01_transcript_128300	NA	F01_transcript_128300	NA	NA

F01_transcript_69518	NA	NA	F01_transcript_69518	NA
F01_transcript_65804	NA	F01_transcript_65804	NA	NA
F01_transcript_90888	NA	NA	F01_transcript_90888	F01_transcript_90888
F01_transcript_166416	NA	F01_transcript_166416	NA	NA
F01_transcript_121107	NA	NA	NA	F01_transcript_121107
F01_transcript_135166	NA	F01_transcript_135166	NA	NA
F01_transcript_92934	F01_transcript_92934	F01_transcript_92934	NA	NA
F01_transcript_72277	NA	F01_transcript_72277	NA	F01_transcript_72277
F01_transcript_133876	F01_transcript_133876	F01_transcript_133876	NA	NA
F01_transcript_57110	NA	F01_transcript_57110	F01_transcript_57110	F01_transcript_57110
F01_transcript_146487	NA	NA	NA	F01_transcript_146487
F01_transcript_149392	NA	F01_transcript_149392	F01_transcript_149392	NA
F01_transcript_109035	F01_transcript_109035	F01_transcript_109035	NA	NA
F01_transcript_99277	F01_transcript_99277	F01_transcript_99277	F01_transcript_99277	F01_transcript_99277

F01_transcript_42983	F01_transcript_42983	NA	NA	NA
F01_transcript_115296	NA	F01_transcript_115296	NA	NA
F01_transcript_46417	NA	NA	NA	F01_transcript_46417
F01_transcript_56584	F01_transcript_56584	F01_transcript_56584	F01_transcript_56584	F01_transcript_56584
F01_transcript_138963	NA	F01_transcript_138963	NA	NA
F01_transcript_81355	NA	F01_transcript_81355	NA	F01_transcript_81355
F01_transcript_121699	NA	NA	NA	F01_transcript_121699
F01_transcript_159694	F01_transcript_159694	F01_transcript_159694	F01_transcript_159694	F01_transcript_159694
F01_transcript_108655	NA	NA	NA	F01_transcript_108655
F01_transcript_152615	NA	F01_transcript_152615	NA	F01_transcript_152615
F01_transcript_153784	NA	F01_transcript_153784	NA	F01_transcript_153784
F01_transcript_127016	NA	F01_transcript_127016	F01_transcript_127016	NA
F01_transcript_70893	F01_transcript_70893	F01_transcript_70893	F01_transcript_70893	NA
F01_transcript_89108	F01_transcript_89108	F01_transcript_89108	NA	NA

F01_transcript_63136	F01_transcript_63136	F01_transcript_63136	F01_transcript_63136	F01_transcript_63136
F01_transcript_72125	NA	F01_transcript_72125	NA	NA
F01_transcript_105147	F01_transcript_105147	F01_transcript_105147	F01_transcript_105147	F01_transcript_105147
F01_transcript_98757	NA	NA	NA	F01_transcript_98757
F01_transcript_166869	NA	NA	NA	F01_transcript_166869
F01_transcript_73898	NA	F01_transcript_73898	NA	NA
F01_transcript_41536	NA	NA	F01_transcript_41536	NA
F01_transcript_145249	NA	NA	NA	F01_transcript_145249
F01_transcript_155843	NA	F01_transcript_155843	NA	F01_transcript_155843
F01_transcript_163439	NA	F01_transcript_163439	NA	NA
F01_transcript_68671	NA	NA	F01_transcript_68671	NA
F01_transcript_155836	NA	F01_transcript_155836	NA	NA
F01_transcript_121005	NA	F01_transcript_121005	NA	NA
F01_transcript_96777	F01_transcript_96777	F01_transcript_96777	F01_transcript_96777	F01_transcript_96777

F01_transcript_68832	F01_transcript_68832	F01_transcript_68832	NA	NA
F01_transcript_107777	NA	F01_transcript_107777	NA	NA
F01_transcript_132116	F01_transcript_132116	F01_transcript_132116	F01_transcript_132116	NA
F01_transcript_145781	NA	NA	NA	F01_transcript_145781
F01_transcript_91419	NA	NA	NA	F01_transcript_91419
F01_transcript_8019	NA	NA	NA	F01_transcript_8019
F01_transcript_91565	NA	NA	NA	F01_transcript_91565
F01_transcript_127271	NA	F01_transcript_127271	NA	NA
F01_transcript_107611	F01_transcript_107611	F01_transcript_107611	F01_transcript_107611	F01_transcript_107611
F01_transcript_110254	NA	NA	NA	F01_transcript_110254
F01_transcript_77412	F01_transcript_77412	F01_transcript_77412	F01_transcript_77412	F01_transcript_77412
F01_transcript_37288	NA	NA	NA	F01_transcript_37288
F01_transcript_94700	F01_transcript_94700	F01_transcript_94700	F01_transcript_94700	F01_transcript_94700
F01_transcript_161678	NA	F01_transcript_161678	NA	NA

F01_transcript_150808	F01_transcript_150808	F01_transcript_150808	F01_transcript_150808	F01_transcript_150808
F01_transcript_130493	NA	F01_transcript_130493	NA	F01_transcript_130493
F01_transcript_119589	F01_transcript_119589	F01_transcript_119589	NA	NA
F01_transcript_140446	NA	F01_transcript_140446	F01_transcript_140446	F01_transcript_140446
F01_transcript_145770	NA	NA	NA	F01_transcript_145770
F01_transcript_92061	NA	F01_transcript_92061	NA	NA
F01_transcript_19950	F01_transcript_19950	F01_transcript_19950	F01_transcript_19950	NA
F01_transcript_84898	NA	NA	NA	F01_transcript_84898
F01_transcript_81500	NA	F01_transcript_81500	NA	NA
F01_transcript_101469	F01_transcript_101469	F01_transcript_101469	F01_transcript_101469	F01_transcript_101469
F01_transcript_137159	NA	NA	F01_transcript_137159	F01_transcript_137159
F01_transcript_94035	NA	F01_transcript_94035	NA	F01_transcript_94035
F01_transcript_139746	NA	NA	NA	F01_transcript_139746
F01_transcript_156597	NA	F01_transcript_156597	NA	NA

F01_transcript_56417	NA	NA	F01_transcript_56417	NA
F01_transcript_65233	NA	NA	NA	F01_transcript_65233
F01_transcript_60515	NA	NA	NA	F01_transcript_60515
F01_transcript_103226	NA	F01_transcript_103226	NA	NA
F01_transcript_79434	NA	F01_transcript_79434	NA	NA
F01_transcript_67453	NA	NA	F01_transcript_67453	F01_transcript_67453
F01_transcript_122658	F01_transcript_122658	F01_transcript_122658	F01_transcript_122658	F01_transcript_122658
F01_transcript_58277	F01_transcript_58277	F01_transcript_58277	F01_transcript_58277	F01_transcript_58277
F01_transcript_160749	F01_transcript_160749	F01_transcript_160749	NA	NA
F01_transcript_155713	NA	F01_transcript_155713	NA	NA
F01_transcript_135160	NA	NA	NA	F01_transcript_135160
F01_transcript_52786	NA	NA	NA	F01_transcript_52786
F01_transcript_73400	F01_transcript_73400	F01_transcript_73400	F01_transcript_73400	F01_transcript_73400
F01_transcript_79263	NA	F01_transcript_79263	NA	NA

F01_transcript_18392	F01_transcript_18392	F01_transcript_18392	F01_transcript_18392	F01_transcript_18392
F01_transcript_116921	NA	F01_transcript_116921	F01_transcript_116921	F01_transcript_116921
F01_transcript_133549	NA	F01_transcript_133549	NA	NA
F01_transcript_164077	NA	F01_transcript_164077	NA	NA
F01_transcript_119221	F01_transcript_119221	F01_transcript_119221	NA	F01_transcript_119221
F01_transcript_34532	F01_transcript_34532	F01_transcript_34532	F01_transcript_34532	F01_transcript_34532
F01_transcript_157330	NA	F01_transcript_157330	NA	F01_transcript_157330
F01_transcript_153921	NA	F01_transcript_153921	F01_transcript_153921	F01_transcript_153921
F01_transcript_111786	NA	F01_transcript_111786	NA	F01_transcript_111786
F01_transcript_61116	NA	F01_transcript_61116	NA	NA
F01_transcript_106970	NA	F01_transcript_106970	NA	NA
F01_transcript_154284	NA	NA	NA	F01_transcript_154284
F01_transcript_98437	F01_transcript_98437	F01_transcript_98437	NA	NA
F01_transcript_103070	F01_transcript_103070	F01_transcript_103070	F01_transcript_103070	F01_transcript_103070

F01_transcript_12475	F01_transcript_12475	F01_transcript_12475	F01_transcript_12475	F01_transcript_12475
F01_transcript_11995	NA	NA	NA	F01_transcript_11995
F01_transcript_90140	NA	F01_transcript_90140	NA	F01_transcript_90140
F01_transcript_144431	F01_transcript_144431	F01_transcript_144431	F01_transcript_144431	F01_transcript_144431
F01_transcript_105495	NA	NA	NA	F01_transcript_105495
F01_transcript_53383	NA	F01_transcript_53383	F01_transcript_53383	F01_transcript_53383
F01_transcript_85922	NA	F01_transcript_85922	NA	NA
F01_transcript_63659	NA	NA	NA	F01_transcript_63659
F01_transcript_166555	NA	F01_transcript_166555	NA	NA
F01_transcript_132502	F01_transcript_132502	F01_transcript_132502	F01_transcript_132502	F01_transcript_132502
F01_transcript_125560	NA	NA	NA	F01_transcript_125560
F01_transcript_32521	NA	F01_transcript_32521	F01_transcript_32521	NA
F01_transcript_167692	NA	F01_transcript_167692	NA	F01_transcript_167692
F01_transcript_118983	F01_transcript_118983	F01_transcript_118983	NA	F01_transcript_118983

F01_transcript_108202	F01_transcript_108202	F01_transcript_108202	F01_transcript_108202	F01_transcript_108202
F01_transcript_11939	NA	NA	NA	F01_transcript_11939
F01_transcript_93831	NA	F01_transcript_93831	NA	NA
F01_transcript_103636	NA	F01_transcript_103636	NA	F01_transcript_103636
F01_transcript_64105	NA	F01_transcript_64105	F01_transcript_64105	NA
F01_transcript_143921	NA	NA	NA	F01_transcript_143921
F01_transcript_129324	NA	NA	NA	F01_transcript_129324
F01_transcript_113720	NA	NA	NA	F01_transcript_113720
F01_transcript_129580	F01_transcript_129580	F01_transcript_129580	F01_transcript_129580	F01_transcript_129580
F01_transcript_94713	NA	NA	NA	F01_transcript_94713
F01_transcript_123215	NA	F01_transcript_123215	NA	NA
F01_transcript_64596	F01_transcript_64596	F01_transcript_64596	F01_transcript_64596	F01_transcript_64596
F01_transcript_13825	NA	NA	NA	F01_transcript_13825
F01_transcript_148588	F01_transcript_148588	F01_transcript_148588	F01_transcript_148588	F01_transcript_148588

F01_transcript_139293	NA	F01_transcript_139293	NA	NA
F01_transcript_147922	NA	F01_transcript_147922	NA	NA
F01_transcript_155084	F01_transcript_155084	F01_transcript_155084	NA	NA
F01_transcript_86340	NA	F01_transcript_86340	NA	F01_transcript_86340
F01_transcript_23128	F01_transcript_23128	F01_transcript_23128	F01_transcript_23128	F01_transcript_23128
F01_transcript_157263	NA	F01_transcript_157263	NA	F01_transcript_157263
F01_transcript_147897	NA	NA	NA	F01_transcript_147897
F01_transcript_61922	NA	F01_transcript_61922	NA	NA
F01_transcript_1909	NA	NA	NA	F01_transcript_1909
F01_transcript_126576	NA	NA	NA	F01_transcript_126576
F01_transcript_100173	NA	NA	NA	F01_transcript_100173
F01_transcript_160310	F01_transcript_160310	F01_transcript_160310	F01_transcript_160310	F01_transcript_160310
F01_transcript_144572	NA	F01_transcript_144572	NA	NA
F01_transcript_76613	F01_transcript_76613	NA	F01_transcript_76613	NA

F01_transcript_149009	F01_transcript_149009	F01_transcript_149009	F01_transcript_149009	F01_transcript_149009
F01_transcript_77544	NA	NA	NA	F01_transcript_77544
F01_transcript_149689	NA	F01_transcript_149689	NA	F01_transcript_149689
F01_transcript_102215	NA	NA	F01_transcript_102215	NA
F01_transcript_160682	F01_transcript_160682	F01_transcript_160682	F01_transcript_160682	F01_transcript_160682
F01_transcript_89262	NA	F01_transcript_89262	NA	F01_transcript_89262
F01_transcript_166761	NA	F01_transcript_166761	NA	NA
F01_transcript_83031	F01_transcript_83031	F01_transcript_83031	F01_transcript_83031	F01_transcript_83031
F01_transcript_119735	F01_transcript_119735	F01_transcript_119735	F01_transcript_119735	F01_transcript_119735
F01_transcript_78107	NA	NA	NA	F01_transcript_78107
F01_transcript_111551	NA	F01_transcript_111551	NA	F01_transcript_111551
F01_transcript_33577	F01_transcript_33577	F01_transcript_33577	NA	F01_transcript_33577
F01_transcript_121643	NA	F01_transcript_121643	NA	NA
F01_transcript_31842	F01_transcript_31842	F01_transcript_31842	F01_transcript_31842	NA

F01_transcript_63310	NA	NA	NA	F01_transcript_63310
F01_transcript_161629	F01_transcript_161629	F01_transcript_161629	F01_transcript_161629	F01_transcript_161629
F01_transcript_114388	NA	F01_transcript_114388	F01_transcript_114388	F01_transcript_114388
F01_transcript_152616	F01_transcript_152616	F01_transcript_152616	F01_transcript_152616	F01_transcript_152616
F01_transcript_15457	NA	NA	NA	F01_transcript_15457
F01_transcript_4178	NA	F01_transcript_4178	NA	F01_transcript_4178
F01_transcript_160429	F01_transcript_160429	F01_transcript_160429	F01_transcript_160429	NA
F01_transcript_23234	NA	NA	NA	F01_transcript_23234
F01_transcript_49035	F01_transcript_49035	F01_transcript_49035	NA	NA
F01_transcript_143153	NA	F01_transcript_143153	NA	NA
F01_transcript_99300	NA	F01_transcript_99300	NA	F01_transcript_99300
F01_transcript_144176	NA	F01_transcript_144176	NA	NA
F01_transcript_52136	NA	NA	NA	F01_transcript_52136
F01_transcript_70419	F01_transcript_70419	F01_transcript_70419	F01_transcript_70419	F01_transcript_70419

F01_transcript_50627	NA	F01_transcript_50627	NA	NA
F01_transcript_69497	NA	F01_transcript_69497	NA	NA
F01_transcript_63846	NA	NA	NA	F01_transcript_63846
F01_transcript_42805	NA	NA	NA	F01_transcript_42805
F01_transcript_133946	F01_transcript_133946	F01_transcript_133946	F01_transcript_133946	F01_transcript_133946
F01_transcript_128093	NA	F01_transcript_128093	NA	F01_transcript_128093
F01_transcript_34380	NA	NA	NA	F01_transcript_34380
F01_transcript_126336	NA	NA	NA	F01_transcript_126336
F01_transcript_51470	F01_transcript_51470	F01_transcript_51470	NA	NA
F01_transcript_26993	NA	F01_transcript_26993	NA	F01_transcript_26993
F01_transcript_13260	NA	NA	NA	F01_transcript_13260
F01_transcript_119588	NA	NA	NA	F01_transcript_119588
F01_transcript_49563	NA	F01_transcript_49563	F01_transcript_49563	NA
F01_transcript_147293	NA	F01_transcript_147293	NA	NA

F01_transcript_88472	NA	NA	NA	F01_transcript_88472
F01_transcript_88424	NA	F01_transcript_88424	NA	NA
F01_transcript_104867	F01_transcript_104867	F01_transcript_104867	F01_transcript_104867	F01_transcript_104867
F01_transcript_51596	F01_transcript_51596	F01_transcript_51596	F01_transcript_51596	F01_transcript_51596
F01_transcript_128387	NA	NA	NA	F01_transcript_128387
F01_transcript_99151	F01_transcript_99151	F01_transcript_99151	F01_transcript_99151	F01_transcript_99151
F01_transcript_138438	NA	NA	NA	F01_transcript_138438
F01_transcript_127365	NA	NA	NA	F01_transcript_127365
F01_transcript_125778	NA	NA	NA	F01_transcript_125778
F01_transcript_166820	NA	NA	NA	F01_transcript_166820
F01_transcript_86857	F01_transcript_86857	F01_transcript_86857	F01_transcript_86857	F01_transcript_86857
F01_transcript_107726	NA	NA	NA	F01_transcript_107726
F01_transcript_160662	NA	F01_transcript_160662	NA	NA
F01_transcript_51349	NA	F01_transcript_51349	NA	F01_transcript_51349

F01_transcript_96816	F01_transcript_96816	F01_transcript_96816	NA	NA
F01_transcript_30025	NA	NA	NA	F01_transcript_30025
F01_transcript_56068	F01_transcript_56068	F01_transcript_56068	NA	F01_transcript_56068
F01_transcript_68689	NA	NA	NA	F01_transcript_68689
F01_transcript_57011	F01_transcript_57011	F01_transcript_57011	F01_transcript_57011	F01_transcript_57011
F01_transcript_52401	F01_transcript_52401	F01_transcript_52401	F01_transcript_52401	F01_transcript_52401
F01_transcript_119211	NA	F01_transcript_119211	NA	NA
F01_transcript_109350	NA	NA	F01_transcript_109350	F01_transcript_109350
F01_transcript_94121	F01_transcript_94121	F01_transcript_94121	F01_transcript_94121	F01_transcript_94121
F01_transcript_83612	F01_transcript_83612	F01_transcript_83612	NA	F01_transcript_83612
F01_transcript_57146	NA	NA	F01_transcript_57146	NA
F01_transcript_51062	F01_transcript_51062	F01_transcript_51062	F01_transcript_51062	F01_transcript_51062
F01_transcript_136281	NA	F01_transcript_136281	F01_transcript_136281	NA
F01_transcript_6930	F01_transcript_6930	F01_transcript_6930	F01_transcript_6930	NA

F01_transcript_70614	F01_transcript_70614	F01_transcript_70614	F01_transcript_70614	F01_transcript_70614
F01_transcript_88326	F01_transcript_88326	F01_transcript_88326	NA	NA
F01_transcript_120515	NA	F01_transcript_120515	F01_transcript_120515	F01_transcript_120515
F01_transcript_110032	NA	F01_transcript_110032	F01_transcript_110032	F01_transcript_110032
F01_transcript_134386	NA	F01_transcript_134386	F01_transcript_134386	NA
F01_transcript_113896	NA	NA	NA	F01_transcript_113896
F01_transcript_70340	NA	F01_transcript_70340	NA	NA
F01_transcript_75135	NA	NA	NA	F01_transcript_75135
F01_transcript_8884	NA	NA	NA	F01_transcript_8884
F01_transcript_148740	NA	NA	NA	F01_transcript_148740
F01_transcript_108142	NA	F01_transcript_108142	NA	F01_transcript_108142
F01_transcript_133109	NA	F01_transcript_133109	NA	NA
F01_transcript_117004	NA	F01_transcript_117004	F01_transcript_117004	NA
F01_transcript_135240	F01_transcript_135240	F01_transcript_135240	F01_transcript_135240	F01_transcript_135240

F01_transcript_74450	NA	F01_transcript_74450	F01_transcript_74450	F01_transcript_74450
F01_transcript_24326	NA	F01_transcript_24326	NA	F01_transcript_24326
F01_transcript_92470	NA	F01_transcript_92470	NA	NA
F01_transcript_97153	NA	F01_transcript_97153	NA	NA
F01_transcript_18902	NA	NA	NA	F01_transcript_18902
F01_transcript_159830	F01_transcript_159830	F01_transcript_159830	NA	NA
F01_transcript_52360	F01_transcript_52360	F01_transcript_52360	F01_transcript_52360	F01_transcript_52360
F01_transcript_125615	NA	F01_transcript_125615	NA	NA
F01_transcript_91033	NA	F01_transcript_91033	NA	NA
F01_transcript_76823	NA	NA	NA	F01_transcript_76823
F01_transcript_156162	NA	NA	NA	F01_transcript_156162
F01_transcript_125678	F01_transcript_125678	F01_transcript_125678	F01_transcript_125678	F01_transcript_125678
F01_transcript_156774	NA	F01_transcript_156774	NA	NA
F01_transcript_90812	NA	F01_transcript_90812	NA	NA

F01_transcript_107722	NA	NA	NA	F01_transcript_107722
F01_transcript_165912	F01_transcript_165912	F01_transcript_165912	F01_transcript_165912	F01_transcript_165912
F01_transcript_58072	NA	F01_transcript_58072	NA	NA
F01_transcript_96067	NA	NA	NA	F01_transcript_96067
F01_transcript_55352	NA	F01_transcript_55352	F01_transcript_55352	F01_transcript_55352
F01_transcript_81876	F01_transcript_81876	F01_transcript_81876	F01_transcript_81876	NA
F01_transcript_70160	NA	NA	NA	F01_transcript_70160
F01_transcript_161608	F01_transcript_161608	F01_transcript_161608	NA	F01_transcript_161608
F01_transcript_166829	NA	NA	NA	F01_transcript_166829
F01_transcript_137182	F01_transcript_137182	F01_transcript_137182	F01_transcript_137182	F01_transcript_137182
F01_transcript_139585	F01_transcript_139585	F01_transcript_139585	F01_transcript_139585	F01_transcript_139585
F01_transcript_90662	NA	NA	NA	F01_transcript_90662
F01_transcript_11314	NA	NA	NA	F01_transcript_11314
F01_transcript_75676	NA	F01_transcript_75676	NA	NA

F01_transcript_163663	NA	NA	NA	F01_transcript_163663
F01_transcript_164913	NA	NA	NA	F01_transcript_164913
F01_transcript_132910	F01_transcript_132910	F01_transcript_132910	NA	F01_transcript_132910
F01_transcript_150711	NA	NA	NA	F01_transcript_150711
F01_transcript_149922	F01_transcript_149922	F01_transcript_149922	F01_transcript_149922	F01_transcript_149922
F01_transcript_138020	NA	NA	NA	F01_transcript_138020
F01_transcript_119824	F01_transcript_119824	F01_transcript_119824	NA	F01_transcript_119824
F01_transcript_133523	NA	NA	NA	F01_transcript_133523
F01_transcript_74409	NA	F01_transcript_74409	F01_transcript_74409	F01_transcript_74409
F01_transcript_88787	NA	NA	NA	F01_transcript_88787
F01_transcript_115048	F01_transcript_115048	F01_transcript_115048	F01_transcript_115048	NA
F01_transcript_145884	F01_transcript_145884	F01_transcript_145884	NA	F01_transcript_145884
F01_transcript_70843	NA	F01_transcript_70843	F01_transcript_70843	NA
F01_transcript_135420	NA	F01_transcript_135420	NA	F01_transcript_135420

F01_transcript_98995	F01_transcript_98995	F01_transcript_98995	F01_transcript_98995	F01_transcript_98995
F01_transcript_160376	F01_transcript_160376	F01_transcript_160376	F01_transcript_160376	NA
F01_transcript_92778	F01_transcript_92778	F01_transcript_92778	NA	NA
F01_transcript_145643	F01_transcript_145643	F01_transcript_145643	F01_transcript_145643	F01_transcript_145643
F01_transcript_16663	NA	F01_transcript_16663	NA	F01_transcript_16663
F01_transcript_23999	NA	NA	NA	F01_transcript_23999
F01_transcript_141269	NA	NA	NA	F01_transcript_141269
F01_transcript_158941	NA	NA	F01_transcript_158941	NA
F01_transcript_82823	F01_transcript_82823	F01_transcript_82823	F01_transcript_82823	F01_transcript_82823
F01_transcript_86471	NA	F01_transcript_86471	NA	F01_transcript_86471
F01_transcript_137480	F01_transcript_137480	F01_transcript_137480	NA	NA
F01_transcript_128153	F01_transcript_128153	F01_transcript_128153	F01_transcript_128153	F01_transcript_128153
F01_transcript_90469	NA	NA	NA	F01_transcript_90469
F01_transcript_48544	NA	NA	NA	F01_transcript_48544

F01_transcript_116177	NA	NA	NA	F01_transcript_116177
F01_transcript_11057	NA	NA	NA	F01_transcript_11057
F01_transcript_110348	NA	F01_transcript_110348	NA	F01_transcript_110348
F01_transcript_24489	NA	NA	NA	F01_transcript_24489
F01_transcript_106960	NA	NA	NA	F01_transcript_106960
F01_transcript_140918	NA	F01_transcript_140918	NA	NA
F01_transcript_49662	NA	F01_transcript_49662	F01_transcript_49662	F01_transcript_49662
F01_transcript_85484	NA	NA	F01_transcript_85484	F01_transcript_85484
F01_transcript_77055	NA	F01_transcript_77055	NA	NA
F01_transcript_51724	F01_transcript_51724	F01_transcript_51724	F01_transcript_51724	F01_transcript_51724
F01_transcript_61503	NA	NA	NA	F01_transcript_61503
F01_transcript_34335	NA	NA	NA	F01_transcript_34335
F01_transcript_9849	F01_transcript_9849	F01_transcript_9849	F01_transcript_9849	F01_transcript_9849
F01_transcript_123038	F01_transcript_123038	F01_transcript_123038	NA	F01_transcript_123038

F01_transcript_52997	NA	F01_transcript_52997	NA	NA
F01_transcript_156884	F01_transcript_156884	F01_transcript_156884	F01_transcript_156884	F01_transcript_156884
F01_transcript_54578	NA	F01_transcript_54578	NA	F01_transcript_54578
F01_transcript_62868	F01_transcript_62868	F01_transcript_62868	F01_transcript_62868	F01_transcript_62868
F01_transcript_111614	NA	NA	F01_transcript_111614	F01_transcript_111614
F01_transcript_115077	NA	F01_transcript_115077	NA	NA
F01_transcript_67471	F01_transcript_67471	F01_transcript_67471	F01_transcript_67471	F01_transcript_67471
F01_transcript_133749	NA	F01_transcript_133749	NA	NA
F01_transcript_66036	NA	NA	NA	F01_transcript_66036
F01_transcript_82384	NA	F01_transcript_82384	F01_transcript_82384	NA
F01_transcript_90870	NA	NA	NA	F01_transcript_90870
F01_transcript_43403	NA	NA	NA	F01_transcript_43403
F01_transcript_86246	NA	F01_transcript_86246	NA	NA
F01_transcript_66146	NA	F01_transcript_66146	NA	F01_transcript_66146

F01_transcript_2752	F01_transcript_2752	F01_transcript_2752	F01_transcript_2752	F01_transcript_2752
F01_transcript_137312	NA	F01_transcript_137312	NA	F01_transcript_137312
F01_transcript_88913	NA	F01_transcript_88913	F01_transcript_88913	NA
F01_transcript_121102	NA	F01_transcript_121102	NA	NA
F01_transcript_83698	NA	F01_transcript_83698	NA	F01_transcript_83698
F01_transcript_162645	NA	F01_transcript_162645	NA	NA
F01_transcript_49820	NA	F01_transcript_49820	NA	F01_transcript_49820
F01_transcript_156643	NA	NA	NA	F01_transcript_156643
F01_transcript_47230	NA	NA	NA	F01_transcript_47230
F01_transcript_77888	F01_transcript_77888	F01_transcript_77888	F01_transcript_77888	F01_transcript_77888
F01_transcript_93197	NA	F01_transcript_93197	NA	F01_transcript_93197
F01_transcript_52194	NA	NA	NA	F01_transcript_52194
F01_transcript_100987	NA	NA	NA	F01_transcript_100987
F01_transcript_59746	NA	F01_transcript_59746	F01_transcript_59746	NA

F01_transcript_115713	NA	F01_transcript_115713	F01_transcript_115713	F01_transcript_115713
F01_transcript_144128	NA	NA	NA	F01_transcript_144128
F01_transcript_155011	NA	NA	F01_transcript_155011	F01_transcript_155011
F01_transcript_129312	NA	F01_transcript_129312	F01_transcript_129312	NA
F01_transcript_132198	NA	F01_transcript_132198	NA	NA
F01_transcript_143076	NA	F01_transcript_143076	NA	F01_transcript_143076
F01_transcript_122020	F01_transcript_122020	F01_transcript_122020	NA	NA
F01_transcript_60460	F01_transcript_60460	F01_transcript_60460	F01_transcript_60460	F01_transcript_60460
F01_transcript_72064	NA	NA	NA	F01_transcript_72064
F01_transcript_72441	NA	F01_transcript_72441	NA	NA
F01_transcript_82275	NA	NA	NA	F01_transcript_82275
F01_transcript_142195	NA	F01_transcript_142195	NA	F01_transcript_142195
F01_transcript_106297	NA	NA	NA	F01_transcript_106297
F01_transcript_145291	F01_transcript_145291	F01_transcript_145291	NA	NA

F01_transcript_65344	NA	NA	F01_transcript_65344	NA
F01_transcript_138340	F01_transcript_138340	F01_transcript_138340	F01_transcript_138340	F01_transcript_138340
F01_transcript_152905	F01_transcript_152905	F01_transcript_152905	F01_transcript_152905	F01_transcript_152905
F01_transcript_95025	NA	F01_transcript_95025	NA	NA
F01_transcript_1871	NA	NA	NA	F01_transcript_1871
F01_transcript_134988	F01_transcript_134988	F01_transcript_134988	NA	F01_transcript_134988
F01_transcript_151666	NA	F01_transcript_151666	NA	NA
F01_transcript_79229	NA	F01_transcript_79229	NA	NA
F01_transcript_95964	NA	NA	NA	F01_transcript_95964
F01_transcript_93499	NA	F01_transcript_93499	NA	F01_transcript_93499
F01_transcript_4342	F01_transcript_4342	F01_transcript_4342	F01_transcript_4342	NA
F01_transcript_124354	F01_transcript_124354	F01_transcript_124354	NA	F01_transcript_124354
F01_transcript_76177	F01_transcript_76177	F01_transcript_76177	F01_transcript_76177	F01_transcript_76177
F01_transcript_160879	NA	NA	NA	F01_transcript_160879

F01_transcript_75082	NA	F01_transcript_75082	F01_transcript_75082	F01_transcript_75082
F01_transcript_152182	NA	F01_transcript_152182	F01_transcript_152182	F01_transcript_152182
F01_transcript_140299	NA	F01_transcript_140299	NA	F01_transcript_140299
F01_transcript_121293	NA	F01_transcript_121293	F01_transcript_121293	NA
F01_transcript_159511	F01_transcript_159511	F01_transcript_159511	NA	F01_transcript_159511
F01_transcript_94282	NA	NA	NA	F01_transcript_94282
F01_transcript_80906	F01_transcript_80906	F01_transcript_80906	NA	F01_transcript_80906
F01_transcript_67908	NA	NA	NA	F01_transcript_67908
F01_transcript_118017	NA	NA	NA	F01_transcript_118017
F01_transcript_72199	NA	NA	NA	F01_transcript_72199
F01_transcript_16774	NA	NA	NA	F01_transcript_16774
F01_transcript_151753	NA	NA	NA	F01_transcript_151753
F01_transcript_106539	NA	F01_transcript_106539	NA	F01_transcript_106539
F01_transcript_39816	NA	NA	NA	F01_transcript_39816

F01_transcript_87096	NA	NA	NA	F01_transcript_87096
F01_transcript_106695	NA	F01_transcript_106695	NA	NA
F01_transcript_46564	NA	F01_transcript_46564	NA	F01_transcript_46564
F01_transcript_161397	F01_transcript_161397	F01_transcript_161397	F01_transcript_161397	F01_transcript_161397
F01_transcript_139439	NA	F01_transcript_139439	NA	F01_transcript_139439
F01_transcript_82561	NA	NA	NA	F01_transcript_82561
F01_transcript_151240	F01_transcript_151240	NA	F01_transcript_151240	NA
F01_transcript_133773	F01_transcript_133773	F01_transcript_133773	F01_transcript_133773	F01_transcript_133773
F01_transcript_53624	F01_transcript_53624	F01_transcript_53624	F01_transcript_53624	F01_transcript_53624
F01_transcript_106841	F01_transcript_106841	F01_transcript_106841	NA	NA
F01_transcript_74165	F01_transcript_74165	F01_transcript_74165	F01_transcript_74165	NA
F01_transcript_127518	F01_transcript_127518	F01_transcript_127518	F01_transcript_127518	F01_transcript_127518
F01_transcript_130409	NA	F01_transcript_130409	NA	NA
F01_transcript_156689	NA	NA	NA	F01_transcript_156689

F01_transcript_149844	NA	NA	F01_transcript_149844	F01_transcript_149844
F01_transcript_137001	F01_transcript_137001	F01_transcript_137001	F01_transcript_137001	F01_transcript_137001
F01_transcript_147704	NA	F01_transcript_147704	NA	NA
F01_transcript_126297	F01_transcript_126297	F01_transcript_126297	NA	NA
F01_transcript_106172	NA	NA	NA	F01_transcript_106172
F01_transcript_75447	NA	NA	NA	F01_transcript_75447
F01_transcript_138050	NA	F01_transcript_138050	NA	NA
F01_transcript_65095	NA	NA	NA	F01_transcript_65095
F01_transcript_166128	NA	F01_transcript_166128	NA	NA
F01_transcript_132621	NA	F01_transcript_132621	NA	NA
F01_transcript_25446	NA	F01_transcript_25446	NA	NA
F01_transcript_73012	NA	F01_transcript_73012	NA	F01_transcript_73012
F01_transcript_68189	NA	F01_transcript_68189	F01_transcript_68189	F01_transcript_68189
F01_transcript_155413	F01_transcript_155413	F01_transcript_155413	NA	NA

F01_transcript_129922	NA	F01_transcript_129922	F01_transcript_129922	F01_transcript_129922
F01_transcript_104492	NA	NA	F01_transcript_104492	F01_transcript_104492
F01_transcript_43182	NA	NA	NA	F01_transcript_43182
F01_transcript_50059	NA	NA	F01_transcript_50059	F01_transcript_50059
F01_transcript_54199	NA	F01_transcript_54199	NA	NA
F01_transcript_84470	F01_transcript_84470	F01_transcript_84470	F01_transcript_84470	F01_transcript_84470
F01_transcript_4076	NA	NA	F01_transcript_4076	NA
F01_transcript_161168	F01_transcript_161168	F01_transcript_161168	NA	F01_transcript_161168
F01_transcript_118867	NA	F01_transcript_118867	NA	NA
F01_transcript_113207	NA	NA	NA	F01_transcript_113207
F01_transcript_118496	NA	F01_transcript_118496	NA	NA
F01_transcript_108440	NA	F01_transcript_108440	NA	F01_transcript_108440
F01_transcript_137669	NA	NA	NA	F01_transcript_137669
F01_transcript_89401	F01_transcript_89401	F01_transcript_89401	F01_transcript_89401	F01_transcript_89401

F01_transcript_106561	F01_transcript_106561	F01_transcript_106561	F01_transcript_106561	F01_transcript_106561
F01_transcript_575	NA	F01_transcript_575	NA	NA
F01_transcript_137968	NA	F01_transcript_137968	F01_transcript_137968	F01_transcript_137968
F01_transcript_90218	NA	F01_transcript_90218	NA	F01_transcript_90218
F01_transcript_110238	NA	F01_transcript_110238	NA	NA
F01_transcript_162068	F01_transcript_162068	F01_transcript_162068	NA	F01_transcript_162068
F01_transcript_152566	F01_transcript_152566	F01_transcript_152566	F01_transcript_152566	F01_transcript_152566
F01_transcript_13253	NA	NA	NA	F01_transcript_13253
F01_transcript_50384	F01_transcript_50384	F01_transcript_50384	NA	NA
F01_transcript_140736	NA	F01_transcript_140736	F01_transcript_140736	NA
F01_transcript_113264	NA	F01_transcript_113264	NA	F01_transcript_113264
F01_transcript_75374	NA	NA	NA	F01_transcript_75374
F01_transcript_134482	F01_transcript_134482	F01_transcript_134482	F01_transcript_134482	F01_transcript_134482
F01_transcript_71675	NA	NA	NA	F01_transcript_71675

F01_transcript_91626	NA	NA	NA	F01_transcript_91626
F01_transcript_142979	F01_transcript_142979	F01_transcript_142979	F01_transcript_142979	NA
F01_transcript_166147	NA	F01_transcript_166147	NA	F01_transcript_166147
F01_transcript_90984	NA	NA	F01_transcript_90984	F01_transcript_90984
F01_transcript_128623	F01_transcript_128623	F01_transcript_128623	NA	NA
F01_transcript_80440	NA	NA	NA	F01_transcript_80440
F01_transcript_90537	NA	NA	F01_transcript_90537	NA
F01_transcript_91170	F01_transcript_91170	F01_transcript_91170	NA	F01_transcript_91170
F01_transcript_129099	NA	F01_transcript_129099	NA	F01_transcript_129099
F01_transcript_17005	F01_transcript_17005	F01_transcript_17005	NA	F01_transcript_17005
F01_transcript_26465	NA	NA	NA	F01_transcript_26465
F01_transcript_123739	NA	NA	NA	F01_transcript_123739
F01_transcript_72954	F01_transcript_72954	F01_transcript_72954	F01_transcript_72954	F01_transcript_72954
F01_transcript_86663	NA	NA	NA	F01_transcript_86663

F01_transcript_134246	F01_transcript_134246	F01_transcript_134246	F01_transcript_134246	F01_transcript_134246
F01_transcript_48873	F01_transcript_48873	F01_transcript_48873	F01_transcript_48873	NA
F01_transcript_89779	NA	NA	NA	F01_transcript_89779
F01_transcript_14257	NA	NA	NA	F01_transcript_14257
F01_transcript_78319	NA	F01_transcript_78319	NA	F01_transcript_78319
F01_transcript_79198	NA	F01_transcript_79198	NA	NA
F01_transcript_155954	NA	NA	NA	F01_transcript_155954
F01_transcript_56963	NA	NA	F01_transcript_56963	F01_transcript_56963
F01_transcript_88581	NA	NA	NA	F01_transcript_88581
F01_transcript_46431	NA	NA	NA	F01_transcript_46431
F01_transcript_128267	F01_transcript_128267	F01_transcript_128267	F01_transcript_128267	F01_transcript_128267
F01_transcript_166483	F01_transcript_166483	F01_transcript_166483	F01_transcript_166483	F01_transcript_166483
F01_transcript_61568	NA	F01_transcript_61568	NA	NA
F01_transcript_135317	NA	NA	NA	F01_transcript_135317

F01_transcript_155168	F01_transcript_155168	F01_transcript_155168	F01_transcript_155168	F01_transcript_155168
F01_transcript_129634	NA	NA	NA	F01_transcript_129634
F01_transcript_81908	NA	NA	NA	F01_transcript_81908
F01_transcript_163619	NA	NA	NA	F01_transcript_163619
F01_transcript_158530	NA	F01_transcript_158530	NA	NA
F01_transcript_64135	NA	NA	NA	F01_transcript_64135
F01_transcript_162295	NA	NA	NA	F01_transcript_162295
F01_transcript_41510	NA	NA	F01_transcript_41510	F01_transcript_41510
F01_transcript_14093	NA	F01_transcript_14093	NA	NA
F01_transcript_138107	F01_transcript_138107	F01_transcript_138107	F01_transcript_138107	F01_transcript_138107
F01_transcript_113710	F01_transcript_113710	F01_transcript_113710	F01_transcript_113710	F01_transcript_113710
F01_transcript_122813	F01_transcript_122813	F01_transcript_122813	NA	F01_transcript_122813
F01_transcript_81929	NA	NA	NA	F01_transcript_81929
F01_transcript_88706	F01_transcript_88706	F01_transcript_88706	NA	F01_transcript_88706

F01_transcript_101795	NA	F01_transcript_101795	NA	F01_transcript_101795
F01_transcript_35465	F01_transcript_35465	F01_transcript_35465	NA	NA
F01_transcript_110401	F01_transcript_110401	F01_transcript_110401	F01_transcript_110401	F01_transcript_110401
F01_transcript_59435	NA	F01_transcript_59435	F01_transcript_59435	NA
F01_transcript_154386	NA	NA	NA	F01_transcript_154386
F01_transcript_99319	F01_transcript_99319	F01_transcript_99319	F01_transcript_99319	F01_transcript_99319
F01_transcript_77647	NA	F01_transcript_77647	NA	NA
F01_transcript_53192	F01_transcript_53192	F01_transcript_53192	NA	F01_transcript_53192
F01_transcript_87822	NA	F01_transcript_87822	NA	NA
F01_transcript_134759	NA	F01_transcript_134759	NA	NA
F01_transcript_85257	NA	NA	F01_transcript_85257	NA
F01_transcript_141142	NA	NA	NA	F01_transcript_141142
F01_transcript_79466	F01_transcript_79466	F01_transcript_79466	F01_transcript_79466	F01_transcript_79466
F01_transcript_166278	NA	F01_transcript_166278	NA	NA

F01_transcript_138699	NA	F01_transcript_138699	NA	NA
F01_transcript_69080	NA	F01_transcript_69080	NA	F01_transcript_69080
F01_transcript_140198	NA	F01_transcript_140198	F01_transcript_140198	F01_transcript_140198
F01_transcript_78019	F01_transcript_78019	F01_transcript_78019	NA	NA
F01_transcript_56174	NA	NA	NA	F01_transcript_56174
F01_transcript_151978	NA	F01_transcript_151978	NA	F01_transcript_151978
F01_transcript_39800	NA	NA	NA	F01_transcript_39800
F01_transcript_150678	F01_transcript_150678	F01_transcript_150678	F01_transcript_150678	F01_transcript_150678
F01_transcript_162370	F01_transcript_162370	F01_transcript_162370	F01_transcript_162370	F01_transcript_162370
F01_transcript_109992	NA	NA	NA	F01_transcript_109992
F01_transcript_91504	F01_transcript_91504	F01_transcript_91504	F01_transcript_91504	F01_transcript_91504
F01_transcript_4028	F01_transcript_4028	NA	F01_transcript_4028	NA
F01_transcript_73269	NA	NA	F01_transcript_73269	NA
F01_transcript_62931	F01_transcript_62931	F01_transcript_62931	NA	F01_transcript_62931

F01_transcript_149152	NA	F01_transcript_149152	NA	NA
F01_transcript_152304	NA	F01_transcript_152304	NA	F01_transcript_152304
F01_transcript_126493	NA	F01_transcript_126493	NA	NA
F01_transcript_3182	NA	F01_transcript_3182	NA	NA
F01_transcript_10885	NA	NA	NA	F01_transcript_10885
F01_transcript_131283	F01_transcript_131283	F01_transcript_131283	F01_transcript_131283	F01_transcript_131283
F01_transcript_78737	NA	NA	NA	F01_transcript_78737
F01_transcript_60443	NA	NA	F01_transcript_60443	NA
F01_transcript_158953	NA	NA	NA	F01_transcript_158953
F01_transcript_95782	NA	NA	NA	F01_transcript_95782
F01_transcript_54090	NA	F01_transcript_54090	NA	NA
F01_transcript_96431	NA	NA	NA	F01_transcript_96431
F01_transcript_67271	F01_transcript_67271	F01_transcript_67271	F01_transcript_67271	F01_transcript_67271
F01_transcript_154983	NA	F01_transcript_154983	F01_transcript_154983	F01_transcript_154983

F01_transcript_85621	NA	NA	NA	F01_transcript_85621
F01_transcript_80005	NA	NA	NA	F01_transcript_80005
F01_transcript_134065	F01_transcript_134065	F01_transcript_134065	NA	NA
F01_transcript_49817	NA	NA	NA	F01_transcript_49817
F01_transcript_117639	NA	F01_transcript_117639	NA	NA
F01_transcript_222	NA	NA	NA	F01_transcript_222
F01_transcript_1517	NA	NA	NA	F01_transcript_1517
F01_transcript_44682	NA	NA	NA	F01_transcript_44682
F01_transcript_79660	NA	F01_transcript_79660	NA	NA
F01_transcript_82204	F01_transcript_82204	F01_transcript_82204	F01_transcript_82204	F01_transcript_82204
F01_transcript_91982	NA	NA	NA	F01_transcript_91982
F01_transcript_67976	NA	NA	NA	F01_transcript_67976
F01_transcript_72661	NA	NA	NA	F01_transcript_72661
F01_transcript_51146	F01_transcript_51146	F01_transcript_51146	F01_transcript_51146	F01_transcript_51146

F01_transcript_76241	NA	NA	NA	F01_transcript_76241
F01_transcript_38333	NA	NA	NA	F01_transcript_38333
F01_transcript_9351	NA	F01_transcript_9351	NA	NA
F01_transcript_93714	NA	NA	NA	F01_transcript_93714
F01_transcript_66558	NA	F01_transcript_66558	NA	NA
F01_transcript_6066	F01_transcript_6066	F01_transcript_6066	F01_transcript_6066	F01_transcript_6066
F01_transcript_121989	NA	F01_transcript_121989	NA	NA
F01_transcript_139415	NA	NA	NA	F01_transcript_139415
F01_transcript_61364	NA	F01_transcript_61364	NA	F01_transcript_61364
F01_transcript_127829	NA	NA	NA	F01_transcript_127829
F01_transcript_120950	NA	F01_transcript_120950	NA	NA
F01_transcript_94604	F01_transcript_94604	F01_transcript_94604	F01_transcript_94604	F01_transcript_94604
F01_transcript_129392	NA	F01_transcript_129392	NA	F01_transcript_129392
F01_transcript_66990	NA	NA	NA	F01_transcript_66990

F01_transcript_62748	NA	F01_transcript_62748	F01_transcript_62748	F01_transcript_62748
F01_transcript_106268	NA	NA	NA	F01_transcript_106268
F01_transcript_91236	NA	NA	NA	F01_transcript_91236
F01_transcript_92720	F01_transcript_92720	F01_transcript_92720	F01_transcript_92720	F01_transcript_92720
F01_transcript_68611	F01_transcript_68611	F01_transcript_68611	NA	NA
F01_transcript_166876	F01_transcript_166876	F01_transcript_166876	F01_transcript_166876	F01_transcript_166876
F01_transcript_52746	F01_transcript_52746	F01_transcript_52746	F01_transcript_52746	NA
F01_transcript_137788	F01_transcript_137788	F01_transcript_137788	F01_transcript_137788	F01_transcript_137788
F01_transcript_153420	NA	NA	NA	F01_transcript_153420
F01_transcript_136728	NA	F01_transcript_136728	NA	NA
F01_transcript_154298	NA	F01_transcript_154298	NA	F01_transcript_154298
F01_transcript_131442	F01_transcript_131442	F01_transcript_131442	F01_transcript_131442	F01_transcript_131442
F01_transcript_116349	NA	F01_transcript_116349	NA	NA
F01_transcript_123058	NA	F01_transcript_123058	NA	NA

F01_transcript_51521	NA	F01_transcript_51521	NA	F01_transcript_51521
F01_transcript_140962	NA	NA	NA	F01_transcript_140962
F01_transcript_68159	NA	F01_transcript_68159	F01_transcript_68159	F01_transcript_68159
F01_transcript_78388	NA	F01_transcript_78388	F01_transcript_78388	F01_transcript_78388
F01_transcript_141514	NA	NA	NA	F01_transcript_141514
F01_transcript_93137	NA	NA	F01_transcript_93137	F01_transcript_93137
F01_transcript_63815	NA	NA	NA	F01_transcript_63815
F01_transcript_21587	NA	NA	NA	F01_transcript_21587
F01_transcript_68447	NA	F01_transcript_68447	NA	NA
F01_transcript_10340	F01_transcript_10340	F01_transcript_10340	F01_transcript_10340	F01_transcript_10340
F01_transcript_124890	NA	F01_transcript_124890	NA	F01_transcript_124890
F01_transcript_58847	NA	F01_transcript_58847	NA	F01_transcript_58847
F01_transcript_150393	F01_transcript_150393	F01_transcript_150393	F01_transcript_150393	F01_transcript_150393
F01_transcript_166204	NA	NA	NA	F01_transcript_166204

F01_transcript_152331	NA	F01_transcript_152331	NA	F01_transcript_152331
F01_transcript_146468	NA	F01_transcript_146468	NA	F01_transcript_146468
F01_transcript_126675	NA	NA	NA	F01_transcript_126675
F01_transcript_103066	NA	F01_transcript_103066	NA	NA
F01_transcript_56222	F01_transcript_56222	F01_transcript_56222	F01_transcript_56222	NA
F01_transcript_65744	NA	NA	NA	F01_transcript_65744
F01_transcript_144802	F01_transcript_144802	F01_transcript_144802	F01_transcript_144802	F01_transcript_144802
F01_transcript_82304	F01_transcript_82304	F01_transcript_82304	F01_transcript_82304	F01_transcript_82304
F01_transcript_142885	NA	F01_transcript_142885	NA	NA
F01_transcript_101034	F01_transcript_101034	F01_transcript_101034	NA	NA
F01_transcript_157452	NA	NA	NA	F01_transcript_157452
F01_transcript_45217	NA	NA	NA	F01_transcript_45217
F01_transcript_84265	NA	NA	NA	F01_transcript_84265
F01_transcript_113370	NA	F01_transcript_113370	NA	NA

F01_transcript_45893	NA	NA	NA	F01_transcript_45893
F01_transcript_3134	NA	NA	NA	F01_transcript_3134
F01_transcript_147397	F01_transcript_147397	F01_transcript_147397	F01_transcript_147397	F01_transcript_147397
F01_transcript_111703	F01_transcript_111703	F01_transcript_111703	F01_transcript_111703	F01_transcript_111703
F01_transcript_77050	NA	NA	NA	F01_transcript_77050
F01_transcript_100813	NA	F01_transcript_100813	NA	F01_transcript_100813
F01_transcript_64683	NA	F01_transcript_64683	NA	F01_transcript_64683
F01_transcript_36195	NA	NA	NA	F01_transcript_36195
F01_transcript_28909	NA	F01_transcript_28909	NA	NA
F01_transcript_111384	NA	NA	NA	F01_transcript_111384
F01_transcript_100739	NA	NA	F01_transcript_100739	F01_transcript_100739
F01_transcript_73900	F01_transcript_73900	F01_transcript_73900	F01_transcript_73900	NA
F01_transcript_40120	NA	F01_transcript_40120	F01_transcript_40120	NA
F01_transcript_44334	NA	NA	NA	F01_transcript_44334

F01_transcript_47264	F01_transcript_47264	F01_transcript_47264	NA	NA
F01_transcript_115341	NA	F01_transcript_115341	F01_transcript_115341	NA
F01_transcript_144722	F01_transcript_144722	F01_transcript_144722	NA	F01_transcript_144722
F01_transcript_62482	NA	NA	NA	F01_transcript_62482
F01_transcript_74207	NA	F01_transcript_74207	NA	NA
F01_transcript_156669	NA	F01_transcript_156669	NA	NA
F01_transcript_62289	NA	F01_transcript_62289	F01_transcript_62289	F01_transcript_62289
F01_transcript_58615	F01_transcript_58615	F01_transcript_58615	F01_transcript_58615	F01_transcript_58615
F01_transcript_166430	NA	F01_transcript_166430	NA	NA
F01_transcript_119679	NA	NA	NA	F01_transcript_119679
F01_transcript_76552	NA	NA	NA	F01_transcript_76552
F01_transcript_95641	NA	NA	NA	F01_transcript_95641
F01_transcript_152234	F01_transcript_152234	F01_transcript_152234	F01_transcript_152234	F01_transcript_152234
F01_transcript_142080	NA	F01_transcript_142080	NA	NA

F01_transcript_108652	F01_transcript_108652	F01_transcript_108652	F01_transcript_108652	F01_transcript_108652
F01_transcript_101078	NA	F01_transcript_101078	NA	NA
F01_transcript_82969	NA	NA	NA	F01_transcript_82969
F01_transcript_4486	NA	NA	F01_transcript_4486	F01_transcript_4486
F01_transcript_110235	NA	F01_transcript_110235	NA	NA
F01_transcript_112110	NA	NA	NA	F01_transcript_112110
F01_transcript_134535	NA	NA	NA	F01_transcript_134535
F01_transcript_167319	NA	NA	NA	F01_transcript_167319
F01_transcript_111554	NA	F01_transcript_111554	NA	F01_transcript_111554
F01_transcript_160060	F01_transcript_160060	F01_transcript_160060	F01_transcript_160060	F01_transcript_160060
F01_transcript_137470	NA	NA	NA	F01_transcript_137470
F01_transcript_22904	NA	NA	NA	F01_transcript_22904
F01_transcript_150290	F01_transcript_150290	F01_transcript_150290	F01_transcript_150290	F01_transcript_150290
F01_transcript_160253	NA	NA	NA	F01_transcript_160253

F01_transcript_154262	NA	NA	NA	F01_transcript_154262
F01_transcript_21079	NA	NA	NA	F01_transcript_21079
F01_transcript_20306	NA	F01_transcript_20306	NA	NA
F01_transcript_90855	NA	NA	NA	F01_transcript_90855
F01_transcript_155793	NA	NA	NA	F01_transcript_155793
F01_transcript_80007	NA	F01_transcript_80007	NA	NA
F01_transcript_144221	NA	NA	NA	F01_transcript_144221
F01_transcript_159735	NA	NA	NA	F01_transcript_159735
F01_transcript_45476	NA	NA	NA	F01_transcript_45476
F01_transcript_109836	F01_transcript_109836	F01_transcript_109836	NA	F01_transcript_109836
F01_transcript_50816	NA	F01_transcript_50816	F01_transcript_50816	F01_transcript_50816
F01_transcript_117060	NA	NA	NA	F01_transcript_117060
F01_transcript_82432	NA	F01_transcript_82432	NA	NA
F01_transcript_55816	NA	F01_transcript_55816	NA	NA

F01_transcript_133190	F01_transcript_133190	F01_transcript_133190	NA	NA
F01_transcript_158738	NA	F01_transcript_158738	NA	NA
F01_transcript_82686	NA	F01_transcript_82686	NA	NA
F01_transcript_131643	NA	NA	NA	F01_transcript_131643
F01_transcript_69529	NA	NA	NA	F01_transcript_69529
F01_transcript_74672	NA	NA	NA	F01_transcript_74672
F01_transcript_137295	NA	F01_transcript_137295	F01_transcript_137295	F01_transcript_137295
F01_transcript_166666	F01_transcript_166666	F01_transcript_166666	F01_transcript_166666	F01_transcript_166666
F01_transcript_116968	NA	NA	NA	F01_transcript_116968
F01_transcript_148963	NA	F01_transcript_148963	NA	NA
F01_transcript_51251	F01_transcript_51251	F01_transcript_51251	F01_transcript_51251	F01_transcript_51251
F01_transcript_140255	NA	NA	NA	F01_transcript_140255
F01_transcript_90648	F01_transcript_90648	F01_transcript_90648	F01_transcript_90648	F01_transcript_90648
F01_transcript_151004	F01_transcript_151004	F01_transcript_151004	F01_transcript_151004	F01_transcript_151004

F01_transcript_164701	NA	NA	NA	F01_transcript_164701
F01_transcript_46107	NA	NA	F01_transcript_46107	F01_transcript_46107
F01_transcript_60097	NA	NA	NA	F01_transcript_60097
F01_transcript_134878	NA	NA	F01_transcript_134878	F01_transcript_134878
F01_transcript_154108	NA	F01_transcript_154108	NA	NA
F01_transcript_72583	NA	F01_transcript_72583	F01_transcript_72583	F01_transcript_72583
F01_transcript_95469	NA	NA	NA	F01_transcript_95469
F01_transcript_106275	NA	NA	NA	F01_transcript_106275
F01_transcript_61356	NA	NA	NA	F01_transcript_61356
F01_transcript_159422	NA	NA	NA	F01_transcript_159422
F01_transcript_134996	F01_transcript_134996	F01_transcript_134996	F01_transcript_134996	F01_transcript_134996
F01_transcript_4441	NA	NA	NA	F01_transcript_4441
F01_transcript_119862	NA	F01_transcript_119862	NA	NA
F01_transcript_84163	F01_transcript_84163	F01_transcript_84163	F01_transcript_84163	F01_transcript_84163

F01_transcript_74908	F01_transcript_74908	F01_transcript_74908	F01_transcript_74908	F01_transcript_74908
F01_transcript_49383	NA	F01_transcript_49383	F01_transcript_49383	NA
F01_transcript_20958	NA	NA	NA	F01_transcript_20958
F01_transcript_65448	NA	F01_transcript_65448	F01_transcript_65448	F01_transcript_65448
F01_transcript_150271	F01_transcript_150271	F01_transcript_150271	F01_transcript_150271	F01_transcript_150271
F01_transcript_120015	F01_transcript_120015	F01_transcript_120015	F01_transcript_120015	NA
F01_transcript_146574	NA	NA	NA	F01_transcript_146574
F01_transcript_80955	NA	NA	NA	F01_transcript_80955
F01_transcript_119584	F01_transcript_119584	F01_transcript_119584	NA	NA
F01_transcript_43222	NA	NA	NA	F01_transcript_43222
F01_transcript_146785	NA	F01_transcript_146785	NA	NA
F01_transcript_147510	NA	F01_transcript_147510	F01_transcript_147510	F01_transcript_147510
F01_transcript_94841	NA	F01_transcript_94841	F01_transcript_94841	NA
F01_transcript_119227	F01_transcript_119227	F01_transcript_119227	F01_transcript_119227	F01_transcript_119227

F01_transcript_164369	NA	F01_transcript_164369	NA	NA
F01_transcript_160374	NA	F01_transcript_160374	NA	NA
F01_transcript_109384	F01_transcript_109384	F01_transcript_109384	NA	NA
F01_transcript_53804	F01_transcript_53804	F01_transcript_53804	NA	F01_transcript_53804
F01_transcript_63874	NA	NA	NA	F01_transcript_63874
F01_transcript_134686	NA	NA	NA	F01_transcript_134686
F01_transcript_123088	F01_transcript_123088	F01_transcript_123088	F01_transcript_123088	F01_transcript_123088
F01_transcript_147979	NA	F01_transcript_147979	NA	NA
F01_transcript_39272	NA	NA	NA	F01_transcript_39272
F01_transcript_165088	NA	F01_transcript_165088	F01_transcript_165088	F01_transcript_165088
F01_transcript_70882	NA	F01_transcript_70882	F01_transcript_70882	F01_transcript_70882
F01_transcript_102298	F01_transcript_102298	F01_transcript_102298	NA	F01_transcript_102298
F01_transcript_167219	NA	NA	NA	F01_transcript_167219
F01_transcript_114531	NA	F01_transcript_114531	NA	F01_transcript_114531

F01_transcript_86834	NA	NA	NA	F01_transcript_86834
F01_transcript_88828	F01_transcript_88828	F01_transcript_88828	NA	F01_transcript_88828
F01_transcript_119026	NA	F01_transcript_119026	NA	NA
F01_transcript_58201	NA	F01_transcript_58201	NA	NA
F01_transcript_148764	F01_transcript_148764	F01_transcript_148764	NA	NA
F01_transcript_75423	F01_transcript_75423	F01_transcript_75423	NA	F01_transcript_75423
F01_transcript_79897	NA	NA	NA	F01_transcript_79897
F01_transcript_117909	NA	NA	NA	F01_transcript_117909
F01_transcript_154608	F01_transcript_154608	F01_transcript_154608	F01_transcript_154608	F01_transcript_154608
F01_transcript_164068	F01_transcript_164068	F01_transcript_164068	F01_transcript_164068	F01_transcript_164068
F01_transcript_48970	F01_transcript_48970	F01_transcript_48970	F01_transcript_48970	F01_transcript_48970
F01_transcript_94684	F01_transcript_94684	F01_transcript_94684	F01_transcript_94684	F01_transcript_94684
F01_transcript_60575	NA	F01_transcript_60575	F01_transcript_60575	F01_transcript_60575
F01_transcript_109540	NA	NA	NA	F01_transcript_109540

F01_transcript_120288	NA	NA	NA	F01_transcript_120288
F01_transcript_138712	NA	NA	NA	F01_transcript_138712
F01_transcript_1721	F01_transcript_1721	F01_transcript_1721	F01_transcript_1721	F01_transcript_1721
F01_transcript_85715	NA	F01_transcript_85715	NA	F01_transcript_85715
F01_transcript_142559	NA	F01_transcript_142559	NA	NA
F01_transcript_59685	NA	F01_transcript_59685	NA	NA
F01_transcript_5592	NA	NA	NA	F01_transcript_5592
F01_transcript_10074	F01_transcript_10074	F01_transcript_10074	F01_transcript_10074	F01_transcript_10074
F01_transcript_104216	NA	NA	NA	F01_transcript_104216
F01_transcript_124417	NA	F01_transcript_124417	NA	F01_transcript_124417
F01_transcript_132375	F01_transcript_132375	F01_transcript_132375	F01_transcript_132375	F01_transcript_132375
F01_transcript_32748	F01_transcript_32748	F01_transcript_32748	F01_transcript_32748	F01_transcript_32748
F01_transcript_97675	NA	F01_transcript_97675	F01_transcript_97675	F01_transcript_97675
F01_transcript_127699	NA	F01_transcript_127699	NA	F01_transcript_127699

F01_transcript_113612	NA	F01_transcript_113612	NA	F01_transcript_113612
F01_transcript_38150	NA	NA	NA	F01_transcript_38150
F01_transcript_118129	F01_transcript_118129	F01_transcript_118129	F01_transcript_118129	NA
F01_transcript_139993	NA	F01_transcript_139993	NA	F01_transcript_139993
F01_transcript_128043	NA	NA	NA	F01_transcript_128043
F01_transcript_8084	NA	NA	NA	F01_transcript_8084
F01_transcript_64697	NA	F01_transcript_64697	NA	NA
F01_transcript_23433	NA	F01_transcript_23433	NA	NA
F01_transcript_124375	NA	NA	NA	F01_transcript_124375
F01_transcript_34097	NA	F01_transcript_34097	NA	NA
F01_transcript_126240	F01_transcript_126240	F01_transcript_126240	F01_transcript_126240	F01_transcript_126240
F01_transcript_43940	NA	F01_transcript_43940	NA	NA
F01_transcript_124839	NA	F01_transcript_124839	NA	NA
F01_transcript_126323	F01_transcript_126323	F01_transcript_126323	F01_transcript_126323	F01_transcript_126323

F01_transcript_146990	F01_transcript_146990	F01_transcript_146990	NA	F01_transcript_146990
F01_transcript_88544	NA	F01_transcript_88544	NA	NA
F01_transcript_60020	NA	F01_transcript_60020	NA	NA
F01_transcript_75744	F01_transcript_75744	F01_transcript_75744	NA	NA
F01_transcript_51121	NA	F01_transcript_51121	NA	F01_transcript_51121
F01_transcript_162625	F01_transcript_162625	F01_transcript_162625	F01_transcript_162625	F01_transcript_162625
F01_transcript_99566	NA	F01_transcript_99566	NA	NA
F01_transcript_149058	F01_transcript_149058	F01_transcript_149058	F01_transcript_149058	F01_transcript_149058
F01_transcript_91974	NA	F01_transcript_91974	NA	F01_transcript_91974
F01_transcript_61501	NA	F01_transcript_61501	NA	F01_transcript_61501
F01_transcript_149045	F01_transcript_149045	F01_transcript_149045	F01_transcript_149045	F01_transcript_149045
F01_transcript_120977	NA	NA	NA	F01_transcript_120977
F01_transcript_109604	NA	F01_transcript_109604	NA	NA
F01_transcript_164362	F01_transcript_164362	F01_transcript_164362	F01_transcript_164362	F01_transcript_164362

F01_transcript_49058	NA	F01_transcript_49058	NA	F01_transcript_49058
F01_transcript_105263	F01_transcript_105263	F01_transcript_105263	F01_transcript_105263	F01_transcript_105263
F01_transcript_100928	F01_transcript_100928	F01_transcript_100928	F01_transcript_100928	F01_transcript_100928
F01_transcript_134784	F01_transcript_134784	F01_transcript_134784	F01_transcript_134784	F01_transcript_134784
F01_transcript_153082	NA	NA	NA	F01_transcript_153082
F01_transcript_50903	NA	F01_transcript_50903	NA	NA
F01_transcript_125999	NA	NA	NA	F01_transcript_125999
F01_transcript_46696	NA	NA	NA	F01_transcript_46696
F01_transcript_69092	NA	F01_transcript_69092	NA	NA
F01_transcript_137713	NA	F01_transcript_137713	NA	NA
F01_transcript_116011	NA	F01_transcript_116011	NA	NA
F01_transcript_77700	NA	F01_transcript_77700	NA	NA
F01_transcript_101511	NA	NA	NA	F01_transcript_101511
F01_transcript_69872	NA	F01_transcript_69872	F01_transcript_69872	F01_transcript_69872

F01_transcript_69820	NA	NA	NA	F01_transcript_69820
F01_transcript_70834	NA	NA	F01_transcript_70834	NA
F01_transcript_110053	NA	NA	NA	F01_transcript_110053
F01_transcript_59500	NA	NA	F01_transcript_59500	NA
F01_transcript_127165	F01_transcript_127165	F01_transcript_127165	NA	F01_transcript_127165
F01_transcript_143038	F01_transcript_143038	F01_transcript_143038	NA	F01_transcript_143038
F01_transcript_102454	F01_transcript_102454	F01_transcript_102454	NA	F01_transcript_102454
F01_transcript_119620	NA	NA	NA	F01_transcript_119620
F01_transcript_4929	NA	F01_transcript_4929	NA	NA
F01_transcript_111028	NA	F01_transcript_111028	NA	NA
F01_transcript_61410	F01_transcript_61410	F01_transcript_61410	F01_transcript_61410	F01_transcript_61410
F01_transcript_65577	NA	F01_transcript_65577	NA	F01_transcript_65577
F01_transcript_130889	NA	NA	NA	F01_transcript_130889
F01_transcript_159662	NA	F01_transcript_159662	NA	F01_transcript_159662

F01_transcript_157186	NA	NA	NA	F01_transcript_157186
F01_transcript_149113	NA	NA	NA	F01_transcript_149113
F01_transcript_103699	NA	F01_transcript_103699	NA	F01_transcript_103699
F01_transcript_164807	F01_transcript_164807	F01_transcript_164807	F01_transcript_164807	F01_transcript_164807
F01_transcript_72283	F01_transcript_72283	F01_transcript_72283	F01_transcript_72283	F01_transcript_72283
F01_transcript_56328	NA	NA	NA	F01_transcript_56328
F01_transcript_46083	NA	NA	NA	F01_transcript_46083
F01_transcript_77029	NA	NA	NA	F01_transcript_77029
F01_transcript_136429	NA	NA	NA	F01_transcript_136429
F01_transcript_59417	F01_transcript_59417	NA	F01_transcript_59417	F01_transcript_59417
F01_transcript_167780	NA	NA	NA	F01_transcript_167780
F01_transcript_130706	NA	F01_transcript_130706	NA	F01_transcript_130706
F01_transcript_158203	NA	NA	NA	F01_transcript_158203
F01_transcript_56640	F01_transcript_56640	F01_transcript_56640	F01_transcript_56640	F01_transcript_56640

F01_transcript_98579	NA	NA	NA	F01_transcript_98579
F01_transcript_104411	NA	NA	NA	F01_transcript_104411
F01_transcript_60121	F01_transcript_60121	F01_transcript_60121	F01_transcript_60121	F01_transcript_60121
F01_transcript_161920	F01_transcript_161920	F01_transcript_161920	NA	NA
F01_transcript_162233	F01_transcript_162233	F01_transcript_162233	NA	F01_transcript_162233
F01_transcript_54344	NA	F01_transcript_54344	NA	NA
F01_transcript_69562	NA	F01_transcript_69562	NA	F01_transcript_69562
F01_transcript_64131	F01_transcript_64131	F01_transcript_64131	F01_transcript_64131	F01_transcript_64131
F01_transcript_53777	NA	NA	NA	F01_transcript_53777
F01_transcript_111958	NA	NA	NA	F01_transcript_111958
F01_transcript_160710	NA	F01_transcript_160710	NA	NA
F01_transcript_132725	NA	F01_transcript_132725	NA	F01_transcript_132725
F01_transcript_155987	NA	F01_transcript_155987	NA	NA
F01_transcript_78673	NA	F01_transcript_78673	F01_transcript_78673	F01_transcript_78673

F01_transcript_10699	F01_transcript_10699	F01_transcript_10699	F01_transcript_10699	F01_transcript_10699
F01_transcript_59946	NA	NA	NA	F01_transcript_59946
F01_transcript_163133	NA	F01_transcript_163133	NA	NA
F01_transcript_61252	NA	F01_transcript_61252	F01_transcript_61252	NA
F01_transcript_98403	F01_transcript_98403	F01_transcript_98403	F01_transcript_98403	F01_transcript_98403
F01_transcript_141966	F01_transcript_141966	F01_transcript_141966	F01_transcript_141966	NA
F01_transcript_54157	F01_transcript_54157	F01_transcript_54157	F01_transcript_54157	F01_transcript_54157
F01_transcript_18786	NA	NA	NA	F01_transcript_18786
F01_transcript_67243	NA	F01_transcript_67243	NA	F01_transcript_67243
F01_transcript_9748	NA	NA	NA	F01_transcript_9748
F01_transcript_126007	F01_transcript_126007	F01_transcript_126007	NA	NA
F01_transcript_75565	F01_transcript_75565	F01_transcript_75565	F01_transcript_75565	F01_transcript_75565
F01_transcript_82900	NA	F01_transcript_82900	NA	F01_transcript_82900
F01_transcript_1763	F01_transcript_1763	F01_transcript_1763	F01_transcript_1763	F01_transcript_1763

F01_transcript_29031	NA	NA	NA	F01_transcript_29031
F01_transcript_95563	F01_transcript_95563	F01_transcript_95563	F01_transcript_95563	F01_transcript_95563
F01_transcript_121041	F01_transcript_121041	F01_transcript_121041	F01_transcript_121041	NA
F01_transcript_43044	NA	F01_transcript_43044	NA	NA
F01_transcript_155633	F01_transcript_155633	NA	NA	NA
F01_transcript_120410	NA	NA	NA	F01_transcript_120410
F01_transcript_125556	NA	NA	NA	F01_transcript_125556
F01_transcript_157102	NA	F01_transcript_157102	NA	NA
F01_transcript_111552	NA	F01_transcript_111552	NA	NA
F01_transcript_59748	F01_transcript_59748	F01_transcript_59748	F01_transcript_59748	F01_transcript_59748
F01_transcript_82005	F01_transcript_82005	F01_transcript_82005	F01_transcript_82005	F01_transcript_82005
F01_transcript_74251	F01_transcript_74251	F01_transcript_74251	NA	F01_transcript_74251
F01_transcript_80892	NA	F01_transcript_80892	NA	F01_transcript_80892
F01_transcript_68394	NA	F01_transcript_68394	F01_transcript_68394	NA

F01_transcript_31680	NA	NA	NA	F01_transcript_31680
F01_transcript_64164	F01_transcript_64164	F01_transcript_64164	F01_transcript_64164	F01_transcript_64164
F01_transcript_24120	NA	NA	NA	F01_transcript_24120
F01_transcript_89267	NA	NA	NA	F01_transcript_89267
F01_transcript_79859	F01_transcript_79859	F01_transcript_79859	F01_transcript_79859	F01_transcript_79859
F01_transcript_105581	NA	F01_transcript_105581	NA	NA
F01_transcript_205	NA	F01_transcript_205	F01_transcript_205	NA
F01_transcript_104680	F01_transcript_104680	F01_transcript_104680	F01_transcript_104680	F01_transcript_104680
F01_transcript_12562	NA	F01_transcript_12562	NA	F01_transcript_12562
F01_transcript_130556	NA	F01_transcript_130556	NA	NA
F01_transcript_110128	NA	F01_transcript_110128	F01_transcript_110128	F01_transcript_110128
F01_transcript_49786	NA	F01_transcript_49786	NA	NA
F01_transcript_50522	F01_transcript_50522	F01_transcript_50522	NA	NA
F01_transcript_158886	NA	F01_transcript_158886	F01_transcript_158886	F01_transcript_158886

F01_transcript_117683	F01_transcript_117683	F01_transcript_117683	NA	F01_transcript_117683
F01_transcript_32106	NA	NA	NA	F01_transcript_32106
F01_transcript_106344	NA	F01_transcript_106344	NA	F01_transcript_106344
F01_transcript_86343	NA	F01_transcript_86343	F01_transcript_86343	F01_transcript_86343
F01_transcript_145839	F01_transcript_145839	F01_transcript_145839	NA	F01_transcript_145839
F01_transcript_140208	NA	NA	NA	F01_transcript_140208
F01_transcript_167226	NA	F01_transcript_167226	F01_transcript_167226	F01_transcript_167226
F01_transcript_98571	NA	NA	NA	F01_transcript_98571
F01_transcript_99692	NA	F01_transcript_99692	NA	F01_transcript_99692
F01_transcript_154152	F01_transcript_154152	F01_transcript_154152	NA	NA
F01_transcript_158356	NA	NA	NA	F01_transcript_158356
F01_transcript_124336	F01_transcript_124336	F01_transcript_124336	F01_transcript_124336	F01_transcript_124336
F01_transcript_134416	F01_transcript_134416	F01_transcript_134416	NA	F01_transcript_134416
F01_transcript_128145	F01_transcript_128145	F01_transcript_128145	F01_transcript_128145	F01_transcript_128145

F01_transcript_101483	F01_transcript_101483	F01_transcript_101483	F01_transcript_101483	F01_transcript_101483
F01_transcript_124331	NA	NA	NA	F01_transcript_124331
F01_transcript_78005	F01_transcript_78005	F01_transcript_78005	F01_transcript_78005	F01_transcript_78005
F01_transcript_132949	F01_transcript_132949	F01_transcript_132949	NA	F01_transcript_132949
F01_transcript_153551	NA	F01_transcript_153551	F01_transcript_153551	F01_transcript_153551
F01_transcript_119672	NA	F01_transcript_119672	F01_transcript_119672	F01_transcript_119672
F01_transcript_161217	NA	NA	NA	F01_transcript_161217
F01_transcript_153127	F01_transcript_153127	F01_transcript_153127	F01_transcript_153127	F01_transcript_153127
F01_transcript_57004	NA	NA	F01_transcript_57004	NA
F01_transcript_124029	F01_transcript_124029	F01_transcript_124029	NA	NA
F01_transcript_91583	F01_transcript_91583	F01_transcript_91583	F01_transcript_91583	F01_transcript_91583
F01_transcript_121308	NA	F01_transcript_121308	NA	F01_transcript_121308
F01_transcript_50954	NA	F01_transcript_50954	F01_transcript_50954	NA
F01_transcript_81509	NA	F01_transcript_81509	NA	NA

F01_transcript_69330	F01_transcript_69330	F01_transcript_69330	NA	F01_transcript_69330
F01_transcript_58264	NA	NA	NA	F01_transcript_58264
F01_transcript_7156	NA	NA	F01_transcript_7156	F01_transcript_7156
F01_transcript_62734	NA	F01_transcript_62734	NA	NA
F01_transcript_82542	NA	F01_transcript_82542	NA	NA
F01_transcript_67431	NA	NA	NA	F01_transcript_67431
F01_transcript_142150	NA	F01_transcript_142150	NA	NA
F01_transcript_67018	F01_transcript_67018	F01_transcript_67018	F01_transcript_67018	NA
F01_transcript_25685	NA	NA	NA	F01_transcript_25685
F01_transcript_166904	F01_transcript_166904	F01_transcript_166904	F01_transcript_166904	F01_transcript_166904
F01_transcript_146465	NA	NA	NA	F01_transcript_146465
F01_transcript_164224	NA	F01_transcript_164224	NA	NA
F01_transcript_104306	NA	NA	NA	F01_transcript_104306
F01_transcript_51469	NA	F01_transcript_51469	NA	NA

F01_transcript_112936	NA	NA	NA	F01_transcript_112936
F01_transcript_23874	NA	NA	NA	F01_transcript_23874
F01_transcript_162072	NA	NA	NA	F01_transcript_162072
F01_transcript_2021	F01_transcript_2021	F01_transcript_2021	F01_transcript_2021	F01_transcript_2021
F01_transcript_64518	NA	F01_transcript_64518	NA	NA
F01_transcript_133678	NA	F01_transcript_133678	F01_transcript_133678	NA
F01_transcript_140364	F01_transcript_140364	F01_transcript_140364	F01_transcript_140364	F01_transcript_140364
F01_transcript_134309	F01_transcript_134309	F01_transcript_134309	NA	F01_transcript_134309
F01_transcript_143340	F01_transcript_143340	F01_transcript_143340	NA	F01_transcript_143340
F01_transcript_158389	NA	F01_transcript_158389	NA	F01_transcript_158389
F01_transcript_152997	NA	F01_transcript_152997	NA	NA
F01_transcript_166817	NA	NA	NA	F01_transcript_166817
F01_transcript_60811	NA	F01_transcript_60811	NA	NA
F01_transcript_143142	NA	NA	NA	F01_transcript_143142

F01_transcript_154437	NA	NA	NA	F01_transcript_154437
F01_transcript_132665	NA	F01_transcript_132665	F01_transcript_132665	F01_transcript_132665
F01_transcript_163618	NA	F01_transcript_163618	NA	F01_transcript_163618
F01_transcript_148642	F01_transcript_148642	F01_transcript_148642	F01_transcript_148642	F01_transcript_148642
F01_transcript_67485	NA	F01_transcript_67485	NA	NA
F01_transcript_48604	F01_transcript_48604	NA	NA	NA
F01_transcript_151091	NA	F01_transcript_151091	NA	NA
F01_transcript_100986	NA	F01_transcript_100986	F01_transcript_100986	F01_transcript_100986
F01_transcript_114017	NA	NA	NA	F01_transcript_114017
F01_transcript_46650	NA	NA	NA	F01_transcript_46650
F01_transcript_12256	F01_transcript_12256	F01_transcript_12256	F01_transcript_12256	F01_transcript_12256
F01_transcript_81927	NA	F01_transcript_81927	NA	F01_transcript_81927
F01_transcript_75671	F01_transcript_75671	F01_transcript_75671	NA	F01_transcript_75671
F01_transcript_109438	F01_transcript_109438	F01_transcript_109438	F01_transcript_109438	F01_transcript_109438

F01_transcript_129478	F01_transcript_129478	F01_transcript_129478	F01_transcript_129478	F01_transcript_129478
F01_transcript_148245	NA	F01_transcript_148245	NA	F01_transcript_148245
F01_transcript_139747	F01_transcript_139747	F01_transcript_139747	F01_transcript_139747	F01_transcript_139747
F01_transcript_69168	NA	NA	NA	F01_transcript_69168
F01_transcript_165002	NA	F01_transcript_165002	NA	NA
F01_transcript_122709	F01_transcript_122709	F01_transcript_122709	NA	F01_transcript_122709
F01_transcript_93530	NA	NA	NA	F01_transcript_93530
F01_transcript_123836	NA	F01_transcript_123836	NA	F01_transcript_123836
F01_transcript_95718	F01_transcript_95718	F01_transcript_95718	F01_transcript_95718	F01_transcript_95718
F01_transcript_64082	NA	F01_transcript_64082	NA	NA
F01_transcript_62725	NA	F01_transcript_62725	NA	NA
F01_transcript_37290	NA	NA	NA	F01_transcript_37290
F01_transcript_150978	NA	F01_transcript_150978	NA	F01_transcript_150978
F01_transcript_119659	NA	F01_transcript_119659	F01_transcript_119659	F01_transcript_119659

F01_transcript_47170	NA	NA	NA	F01_transcript_47170
F01_transcript_139054	NA	F01_transcript_139054	NA	NA
F01_transcript_104686	F01_transcript_104686	F01_transcript_104686	F01_transcript_104686	F01_transcript_104686
F01_transcript_119241	NA	NA	NA	F01_transcript_119241
F01_transcript_67170	F01_transcript_67170	F01_transcript_67170	F01_transcript_67170	F01_transcript_67170
F01_transcript_77947	NA	NA	NA	F01_transcript_77947
F01_transcript_102758	NA	NA	NA	F01_transcript_102758
F01_transcript_125196	NA	F01_transcript_125196	NA	NA
F01_transcript_36918	NA	NA	NA	F01_transcript_36918
F01_transcript_26034	NA	NA	NA	F01_transcript_26034
F01_transcript_151330	F01_transcript_151330	F01_transcript_151330	F01_transcript_151330	F01_transcript_151330
F01_transcript_103676	NA	F01_transcript_103676	NA	F01_transcript_103676
F01_transcript_45401	NA	NA	NA	F01_transcript_45401
F01_transcript_138829	NA	F01_transcript_138829	NA	NA

F01_transcript_153143	NA	NA	NA	F01_transcript_153143
F01_transcript_140796	NA	NA	NA	F01_transcript_140796
F01_transcript_103851	F01_transcript_103851	F01_transcript_103851	NA	NA
F01_transcript_46621	NA	NA	NA	F01_transcript_46621
F01_transcript_46364	NA	NA	NA	F01_transcript_46364
F01_transcript_48368	NA	NA	NA	F01_transcript_48368
F01_transcript_102053	NA	NA	NA	F01_transcript_102053
F01_transcript_47246	NA	NA	F01_transcript_47246	F01_transcript_47246
F01_transcript_147227	NA	NA	NA	F01_transcript_147227
F01_transcript_48213	NA	F01_transcript_48213	F01_transcript_48213	F01_transcript_48213
F01_transcript_107146	NA	F01_transcript_107146	F01_transcript_107146	F01_transcript_107146
F01_transcript_83052	NA	F01_transcript_83052	NA	F01_transcript_83052
F01_transcript_149377	F01_transcript_149377	F01_transcript_149377	NA	F01_transcript_149377
F01_transcript_35558	NA	NA	NA	F01_transcript_35558

F01_transcript_164614	NA	F01_transcript_164614	NA	NA
F01_transcript_83105	NA	F01_transcript_83105	NA	NA
F01_transcript_44085	NA	NA	NA	F01_transcript_44085
F01_transcript_110980	NA	NA	NA	F01_transcript_110980
F01_transcript_57199	NA	NA	F01_transcript_57199	NA
F01_transcript_146081	F01_transcript_146081	F01_transcript_146081	F01_transcript_146081	F01_transcript_146081
F01_transcript_114629	F01_transcript_114629	F01_transcript_114629	NA	NA
F01_transcript_157863	NA	F01_transcript_157863	NA	F01_transcript_157863
F01_transcript_131481	NA	NA	NA	F01_transcript_131481
F01_transcript_64695	NA	F01_transcript_64695	F01_transcript_64695	F01_transcript_64695
F01_transcript_140867	F01_transcript_140867	F01_transcript_140867	F01_transcript_140867	F01_transcript_140867
F01_transcript_147842	F01_transcript_147842	F01_transcript_147842	F01_transcript_147842	F01_transcript_147842
F01_transcript_48984	NA	NA	F01_transcript_48984	NA
F01_transcript_122504	NA	NA	NA	F01_transcript_122504

F01_transcript_62893	NA	NA	NA	F01_transcript_62893
F01_transcript_65086	NA	F01_transcript_65086	F01_transcript_65086	NA
F01_transcript_2770	NA	NA	NA	F01_transcript_2770
F01_transcript_105134	NA	F01_transcript_105134	NA	NA
F01_transcript_103579	NA	NA	NA	F01_transcript_103579
F01_transcript_22665	F01_transcript_22665	F01_transcript_22665	F01_transcript_22665	F01_transcript_22665
F01_transcript_154315	NA	F01_transcript_154315	F01_transcript_154315	F01_transcript_154315
F01_transcript_125372	NA	F01_transcript_125372	NA	F01_transcript_125372
F01_transcript_154784	NA	F01_transcript_154784	NA	NA
F01_transcript_105363	F01_transcript_105363	F01_transcript_105363	NA	NA
F01_transcript_107879	NA	F01_transcript_107879	NA	NA
F01_transcript_85262	NA	NA	NA	F01_transcript_85262
F01_transcript_71133	NA	NA	F01_transcript_71133	NA
F01_transcript_60785	NA	F01_transcript_60785	NA	NA

F01_transcript_59206	NA	F01_transcript_59206	NA	NA
F01_transcript_22982	NA	NA	NA	F01_transcript_22982
F01_transcript_55046	NA	F01_transcript_55046	NA	NA
F01_transcript_72473	F01_transcript_72473	F01_transcript_72473	F01_transcript_72473	F01_transcript_72473
F01_transcript_65859	NA	F01_transcript_65859	NA	NA
F01_transcript_54242	NA	NA	NA	F01_transcript_54242
F01_transcript_49599	NA	F01_transcript_49599	NA	NA
F01_transcript_95753	NA	F01_transcript_95753	F01_transcript_95753	NA
F01_transcript_99506	NA	NA	NA	F01_transcript_99506
F01_transcript_9124	NA	F01_transcript_9124	F01_transcript_9124	NA
F01_transcript_162169	NA	NA	F01_transcript_162169	NA
F01_transcript_138247	NA	NA	NA	F01_transcript_138247
F01_transcript_94093	NA	NA	F01_transcript_94093	NA
F01_transcript_46557	NA	F01_transcript_46557	NA	F01_transcript_46557

F01_transcript_158864	F01_transcript_158864	F01_transcript_158864	F01_transcript_158864	F01_transcript_158864
F01_transcript_100776	NA	F01_transcript_100776	NA	F01_transcript_100776
F01_transcript_129803	F01_transcript_129803	F01_transcript_129803	NA	F01_transcript_129803
F01_transcript_49705	NA	F01_transcript_49705	F01_transcript_49705	NA
F01_transcript_105795	NA	NA	NA	F01_transcript_105795
F01_transcript_158971	NA	NA	NA	F01_transcript_158971
F01_transcript_164425	NA	F01_transcript_164425	NA	NA
F01_transcript_113331	NA	NA	NA	F01_transcript_113331
F01_transcript_125078	NA	F01_transcript_125078	NA	F01_transcript_125078
F01_transcript_118007	NA	NA	NA	F01_transcript_118007
F01_transcript_132196	NA	NA	F01_transcript_132196	F01_transcript_132196
F01_transcript_96786	NA	NA	NA	F01_transcript_96786
F01_transcript_128874	F01_transcript_128874	F01_transcript_128874	NA	NA
F01_transcript_97485	F01_transcript_97485	F01_transcript_97485	F01_transcript_97485	F01_transcript_97485

F01_transcript_87530	NA	NA	F01_transcript_87530	F01_transcript_87530
F01_transcript_64081	NA	NA	F01_transcript_64081	F01_transcript_64081
F01_transcript_109678	NA	F01_transcript_109678	F01_transcript_109678	NA
F01_transcript_159337	F01_transcript_159337	F01_transcript_159337	NA	NA
F01_transcript_29321	NA	NA	NA	F01_transcript_29321
F01_transcript_161882	NA	F01_transcript_161882	NA	F01_transcript_161882
F01_transcript_164251	F01_transcript_164251	F01_transcript_164251	F01_transcript_164251	F01_transcript_164251
F01_transcript_45570	NA	F01_transcript_45570	NA	NA
F01_transcript_74833	F01_transcript_74833	F01_transcript_74833	F01_transcript_74833	NA
F01_transcript_69274	F01_transcript_69274	F01_transcript_69274	F01_transcript_69274	F01_transcript_69274
F01_transcript_72921	F01_transcript_72921	F01_transcript_72921	NA	NA
F01_transcript_99204	NA	NA	NA	F01_transcript_99204
F01_transcript_149677	F01_transcript_149677	F01_transcript_149677	F01_transcript_149677	F01_transcript_149677
F01_transcript_124427	NA	NA	NA	F01_transcript_124427

F01_transcript_109112	F01_transcript_109112	F01_transcript_109112	NA	F01_transcript_109112
F01_transcript_72130	NA	F01_transcript_72130	F01_transcript_72130	NA
F01_transcript_70609	NA	F01_transcript_70609	NA	F01_transcript_70609
F01_transcript_150461	NA	F01_transcript_150461	NA	NA
F01_transcript_146209	NA	F01_transcript_146209	NA	F01_transcript_146209
F01_transcript_61968	NA	F01_transcript_61968	F01_transcript_61968	F01_transcript_61968
F01_transcript_140267	NA	F01_transcript_140267	NA	NA
F01_transcript_3941	NA	F01_transcript_3941	NA	F01_transcript_3941
F01_transcript_42636	NA	NA	NA	F01_transcript_42636
F01_transcript_119450	NA	NA	NA	F01_transcript_119450
F01_transcript_120775	NA	NA	F01_transcript_120775	F01_transcript_120775
F01_transcript_64059	NA	F01_transcript_64059	NA	NA
F01_transcript_158096	NA	F01_transcript_158096	F01_transcript_158096	F01_transcript_158096
F01_transcript_110029	NA	F01_transcript_110029	NA	NA

F01_transcript_43937	NA	NA	F01_transcript_43937	F01_transcript_43937
F01_transcript_80598	NA	NA	NA	F01_transcript_80598
F01_transcript_52032	NA	F01_transcript_52032	NA	NA
F01_transcript_68388	NA	NA	NA	F01_transcript_68388
F01_transcript_56806	NA	NA	F01_transcript_56806	NA
F01_transcript_152554	NA	F01_transcript_152554	NA	NA
F01_transcript_123183	NA	F01_transcript_123183	F01_transcript_123183	F01_transcript_123183
F01_transcript_69249	NA	F01_transcript_69249	F01_transcript_69249	NA
F01_transcript_166120	NA	F01_transcript_166120	NA	NA
F01_transcript_118130	NA	F01_transcript_118130	F01_transcript_118130	F01_transcript_118130
F01_transcript_54184	NA	F01_transcript_54184	NA	NA
F01_transcript_167255	F01_transcript_167255	F01_transcript_167255	NA	NA
F01_transcript_47631	NA	NA	NA	F01_transcript_47631
F01_transcript_22928	NA	NA	NA	F01_transcript_22928

F01_transcript_128581	NA	NA	NA	F01_transcript_128581
F01_transcript_6331	NA	F01_transcript_6331	F01_transcript_6331	F01_transcript_6331
F01_transcript_38917	NA	F01_transcript_38917	NA	NA
F01_transcript_139035	F01_transcript_139035	F01_transcript_139035	F01_transcript_139035	F01_transcript_139035
F01_transcript_15676	NA	F01_transcript_15676	NA	NA
F01_transcript_138363	NA	F01_transcript_138363	NA	NA
F01_transcript_140397	F01_transcript_140397	F01_transcript_140397	NA	F01_transcript_140397
F01_transcript_65778	NA	F01_transcript_65778	F01_transcript_65778	F01_transcript_65778
F01_transcript_123960	NA	NA	NA	F01_transcript_123960
F01_transcript_31941	NA	NA	NA	F01_transcript_31941
F01_transcript_108659	NA	F01_transcript_108659	NA	NA
F01_transcript_105254	NA	NA	NA	F01_transcript_105254
F01_transcript_95470	NA	NA	NA	F01_transcript_95470
F01_transcript_40562	F01_transcript_40562	F01_transcript_40562	F01_transcript_40562	F01_transcript_40562

F01_transcript_158620	F01_transcript_158620	F01_transcript_158620	F01_transcript_158620	F01_transcript_158620
F01_transcript_44985	NA	NA	NA	F01_transcript_44985
F01_transcript_99306	NA	NA	NA	F01_transcript_99306
F01_transcript_90290	NA	F01_transcript_90290	F01_transcript_90290	F01_transcript_90290
F01_transcript_76189	NA	NA	NA	F01_transcript_76189
F01_transcript_130571	NA	NA	NA	F01_transcript_130571
F01_transcript_154836	F01_transcript_154836	F01_transcript_154836	NA	NA
F01_transcript_75071	NA	F01_transcript_75071	F01_transcript_75071	F01_transcript_75071
F01_transcript_164272	NA	F01_transcript_164272	NA	F01_transcript_164272
F01_transcript_124385	NA	NA	NA	F01_transcript_124385
F01_transcript_109735	F01_transcript_109735	F01_transcript_109735	NA	NA
F01_transcript_121278	NA	F01_transcript_121278	NA	NA
F01_transcript_102699	NA	NA	NA	F01_transcript_102699
F01_transcript_122664	NA	F01_transcript_122664	NA	F01_transcript_122664

F01_transcript_102480	NA	NA	NA	F01_transcript_102480
F01_transcript_54306	NA	NA	NA	F01_transcript_54306
F01_transcript_136292	F01_transcript_136292	F01_transcript_136292	F01_transcript_136292	F01_transcript_136292
F01_transcript_81202	NA	F01_transcript_81202	NA	NA
F01_transcript_57122	NA	NA	F01_transcript_57122	NA
F01_transcript_114246	NA	NA	NA	F01_transcript_114246
F01_transcript_18918	NA	NA	NA	F01_transcript_18918
F01_transcript_79695	NA	NA	NA	F01_transcript_79695
F01_transcript_72520	NA	NA	NA	F01_transcript_72520
F01_transcript_79256	F01_transcript_79256	F01_transcript_79256	F01_transcript_79256	F01_transcript_79256
F01_transcript_142653	F01_transcript_142653	F01_transcript_142653	NA	NA
F01_transcript_106485	NA	F01_transcript_106485	NA	F01_transcript_106485
F01_transcript_101418	F01_transcript_101418	F01_transcript_101418	NA	NA
F01_transcript_130156	F01_transcript_130156	F01_transcript_130156	NA	F01_transcript_130156

F01_transcript_152976	NA	F01_transcript_152976	NA	NA
F01_transcript_71178	F01_transcript_71178	NA	F01_transcript_71178	F01_transcript_71178
F01_transcript_130811	NA	NA	NA	F01_transcript_130811
F01_transcript_99160	F01_transcript_99160	NA	NA	F01_transcript_99160
F01_transcript_141722	NA	F01_transcript_141722	F01_transcript_141722	F01_transcript_141722
F01_transcript_48464	F01_transcript_48464	F01_transcript_48464	F01_transcript_48464	NA
F01_transcript_57937	F01_transcript_57937	F01_transcript_57937	F01_transcript_57937	F01_transcript_57937
F01_transcript_70089	NA	NA	NA	F01_transcript_70089
F01_transcript_46061	NA	NA	NA	F01_transcript_46061
F01_transcript_6548	NA	NA	F01_transcript_6548	NA
F01_transcript_48036	NA	NA	F01_transcript_48036	F01_transcript_48036
F01_transcript_129280	NA	F01_transcript_129280	NA	F01_transcript_129280
F01_transcript_149475	F01_transcript_149475	F01_transcript_149475	F01_transcript_149475	F01_transcript_149475
F01_transcript_60578	NA	NA	NA	F01_transcript_60578

F01_transcript_50708	F01_transcript_50708	F01_transcript_50708	F01_transcript_50708	F01_transcript_50708
F01_transcript_119067	F01_transcript_119067	F01_transcript_119067	F01_transcript_119067	F01_transcript_119067
F01_transcript_114102	F01_transcript_114102	F01_transcript_114102	F01_transcript_114102	F01_transcript_114102
F01_transcript_155179	F01_transcript_155179	F01_transcript_155179	F01_transcript_155179	F01_transcript_155179
F01_transcript_58848	F01_transcript_58848	F01_transcript_58848	F01_transcript_58848	F01_transcript_58848
F01_transcript_97731	NA	NA	NA	F01_transcript_97731
F01_transcript_48383	NA	F01_transcript_48383	NA	F01_transcript_48383
F01_transcript_167083	NA	F01_transcript_167083	NA	NA
F01_transcript_47769	NA	F01_transcript_47769	NA	F01_transcript_47769
F01_transcript_81692	NA	F01_transcript_81692	F01_transcript_81692	NA
F01_transcript_103765	NA	F01_transcript_103765	F01_transcript_103765	NA
F01_transcript_158594	NA	F01_transcript_158594	NA	NA
F01_transcript_115444	NA	NA	NA	F01_transcript_115444
F01_transcript_148753	NA	F01_transcript_148753	NA	F01_transcript_148753

F01_transcript_50394	NA	F01_transcript_50394	NA	NA
F01_transcript_160977	NA	NA	NA	F01_transcript_160977
F01_transcript_49625	NA	F01_transcript_49625	F01_transcript_49625	F01_transcript_49625
F01_transcript_50256	NA	F01_transcript_50256	NA	F01_transcript_50256
F01_transcript_60086	F01_transcript_60086	F01_transcript_60086	F01_transcript_60086	F01_transcript_60086
F01_transcript_89105	NA	NA	NA	F01_transcript_89105
F01_transcript_124734	NA	F01_transcript_124734	NA	NA
F01_transcript_74213	F01_transcript_74213	F01_transcript_74213	F01_transcript_74213	F01_transcript_74213
F01_transcript_164636	F01_transcript_164636	F01_transcript_164636	F01_transcript_164636	F01_transcript_164636
F01_transcript_141521	F01_transcript_141521	F01_transcript_141521	F01_transcript_141521	F01_transcript_141521
F01_transcript_112006	NA	F01_transcript_112006	NA	NA
F01_transcript_85434	NA	F01_transcript_85434	NA	NA
F01_transcript_160320	F01_transcript_160320	F01_transcript_160320	F01_transcript_160320	F01_transcript_160320
F01_transcript_10780	F01_transcript_10780	F01_transcript_10780	F01_transcript_10780	F01_transcript_10780

F01_transcript_141816	NA	F01_transcript_141816	NA	NA
F01_transcript_149004	F01_transcript_149004	F01_transcript_149004	F01_transcript_149004	F01_transcript_149004
F01_transcript_140662	NA	NA	NA	F01_transcript_140662
F01_transcript_158514	NA	F01_transcript_158514	NA	F01_transcript_158514
F01_transcript_50251	NA	F01_transcript_50251	F01_transcript_50251	F01_transcript_50251
F01_transcript_127224	F01_transcript_127224	F01_transcript_127224	F01_transcript_127224	F01_transcript_127224
F01_transcript_110699	F01_transcript_110699	F01_transcript_110699	F01_transcript_110699	NA
F01_transcript_163783	F01_transcript_163783	F01_transcript_163783	NA	NA
F01_transcript_71266	NA	NA	F01_transcript_71266	NA
F01_transcript_17961	NA	NA	NA	F01_transcript_17961
F01_transcript_104829	F01_transcript_104829	F01_transcript_104829	F01_transcript_104829	F01_transcript_104829
F01_transcript_149764	NA	F01_transcript_149764	F01_transcript_149764	F01_transcript_149764
F01_transcript_4394	F01_transcript_4394	F01_transcript_4394	NA	NA
F01_transcript_66820	NA	NA	NA	F01_transcript_66820

F01_transcript_92320	NA	NA	NA	F01_transcript_92320
F01_transcript_90689	F01_transcript_90689	F01_transcript_90689	F01_transcript_90689	NA
F01_transcript_159564	NA	F01_transcript_159564	NA	NA
F01_transcript_58565	F01_transcript_58565	F01_transcript_58565	NA	NA
F01_transcript_91579	NA	NA	F01_transcript_91579	NA
F01_transcript_67641	NA	NA	NA	F01_transcript_67641
F01_transcript_148408	NA	NA	NA	F01_transcript_148408
F01_transcript_87152	NA	F01_transcript_87152	NA	NA
F01_transcript_143162	NA	F01_transcript_143162	NA	F01_transcript_143162
F01_transcript_67774	NA	F01_transcript_67774	NA	NA
F01_transcript_133480	NA	NA	NA	F01_transcript_133480
F01_transcript_51119	F01_transcript_51119	F01_transcript_51119	F01_transcript_51119	F01_transcript_51119
F01_transcript_57276	NA	F01_transcript_57276	F01_transcript_57276	NA
F01_transcript_135425	F01_transcript_135425	F01_transcript_135425	F01_transcript_135425	F01_transcript_135425

F01_transcript_97391	NA	NA	NA	F01_transcript_97391
F01_transcript_94318	NA	NA	NA	F01_transcript_94318
F01_transcript_16838	NA	NA	NA	F01_transcript_16838
F01_transcript_163737	NA	F01_transcript_163737	NA	F01_transcript_163737
F01_transcript_89542	F01_transcript_89542	F01_transcript_89542	F01_transcript_89542	F01_transcript_89542
F01_transcript_125610	NA	NA	NA	F01_transcript_125610
F01_transcript_166384	F01_transcript_166384	F01_transcript_166384	F01_transcript_166384	F01_transcript_166384
F01_transcript_69039	NA	F01_transcript_69039	NA	F01_transcript_69039
F01_transcript_96273	F01_transcript_96273	F01_transcript_96273	F01_transcript_96273	F01_transcript_96273
F01_transcript_959	NA	F01_transcript_959	NA	NA
F01_transcript_106732	NA	NA	NA	F01_transcript_106732
F01_transcript_81053	F01_transcript_81053	F01_transcript_81053	NA	F01_transcript_81053
F01_transcript_154128	NA	NA	NA	F01_transcript_154128
F01_transcript_157075	NA	F01_transcript_157075	NA	NA

F01_transcript_55130	NA	F01_transcript_55130	NA	F01_transcript_55130
F01_transcript_62462	NA	F01_transcript_62462	NA	NA
F01_transcript_110807	NA	F01_transcript_110807	F01_transcript_110807	F01_transcript_110807
F01_transcript_165543	NA	F01_transcript_165543	NA	F01_transcript_165543
F01_transcript_63437	NA	NA	NA	F01_transcript_63437
F01_transcript_123280	NA	NA	NA	F01_transcript_123280
F01_transcript_79615	NA	F01_transcript_79615	NA	NA
F01_transcript_148913	NA	NA	NA	F01_transcript_148913
F01_transcript_161067	F01_transcript_161067	F01_transcript_161067	NA	F01_transcript_161067
F01_transcript_111414	F01_transcript_111414	F01_transcript_111414	F01_transcript_111414	F01_transcript_111414
F01_transcript_167001	NA	NA	NA	F01_transcript_167001
F01_transcript_118223	NA	F01_transcript_118223	NA	F01_transcript_118223
F01_transcript_12607	NA	NA	NA	F01_transcript_12607
F01_transcript_123550	F01_transcript_123550	F01_transcript_123550	NA	F01_transcript_123550

F01_transcript_50587	NA	F01_transcript_50587	NA	F01_transcript_50587
F01_transcript_104789	NA	F01_transcript_104789	NA	NA
F01_transcript_160212	F01_transcript_160212	F01_transcript_160212	NA	NA
F01_transcript_107887	NA	NA	NA	F01_transcript_107887
F01_transcript_127569	NA	NA	NA	F01_transcript_127569
F01_transcript_162600	NA	F01_transcript_162600	F01_transcript_162600	NA
F01_transcript_61618	F01_transcript_61618	F01_transcript_61618	F01_transcript_61618	F01_transcript_61618
F01_transcript_49125	NA	F01_transcript_49125	NA	F01_transcript_49125
F01_transcript_111914	NA	F01_transcript_111914	NA	NA
F01_transcript_143137	NA	NA	F01_transcript_143137	F01_transcript_143137
F01_transcript_71950	F01_transcript_71950	F01_transcript_71950	F01_transcript_71950	F01_transcript_71950
F01_transcript_146483	NA	F01_transcript_146483	F01_transcript_146483	NA
F01_transcript_128474	NA	NA	NA	F01_transcript_128474
F01_transcript_138756	F01_transcript_138756	F01_transcript_138756	F01_transcript_138756	F01_transcript_138756

F01_transcript_83274	F01_transcript_83274	F01_transcript_83274	F01_transcript_83274	F01_transcript_83274
F01_transcript_9724	NA	F01_transcript_9724	NA	NA
F01_transcript_119271	NA	NA	NA	F01_transcript_119271
F01_transcript_138118	NA	F01_transcript_138118	NA	NA
F01_transcript_104416	F01_transcript_104416	F01_transcript_104416	F01_transcript_104416	F01_transcript_104416
F01_transcript_82751	NA	F01_transcript_82751	NA	NA
F01_transcript_111758	NA	NA	NA	F01_transcript_111758
F01_transcript_90023	NA	F01_transcript_90023	F01_transcript_90023	F01_transcript_90023
F01_transcript_35449	NA	F01_transcript_35449	F01_transcript_35449	F01_transcript_35449
F01_transcript_83750	NA	NA	F01_transcript_83750	NA
F01_transcript_27480	F01_transcript_27480	F01_transcript_27480	F01_transcript_27480	F01_transcript_27480
F01_transcript_101144	F01_transcript_101144	F01_transcript_101144	F01_transcript_101144	F01_transcript_101144
F01_transcript_115873	F01_transcript_115873	F01_transcript_115873	NA	NA
F01_transcript_106736	NA	F01_transcript_106736	NA	NA

F01_transcript_79271	NA	F01_transcript_79271	NA	NA
F01_transcript_110255	NA	NA	NA	F01_transcript_110255
F01_transcript_15515	NA	NA	NA	F01_transcript_15515
F01_transcript_164902	F01_transcript_164902	F01_transcript_164902	NA	F01_transcript_164902
F01_transcript_21122	NA	F01_transcript_21122	F01_transcript_21122	F01_transcript_21122
F01_transcript_161957	F01_transcript_161957	NA	F01_transcript_161957	NA
F01_transcript_126229	NA	F01_transcript_126229	NA	F01_transcript_126229
F01_transcript_59124	F01_transcript_59124	F01_transcript_59124	NA	NA
F01_transcript_149029	F01_transcript_149029	F01_transcript_149029	NA	F01_transcript_149029
F01_transcript_117068	NA	F01_transcript_117068	NA	NA
F01_transcript_166241	NA	F01_transcript_166241	NA	NA
F01_transcript_102761	F01_transcript_102761	F01_transcript_102761	NA	NA
F01_transcript_152318	F01_transcript_152318	F01_transcript_152318	F01_transcript_152318	F01_transcript_152318
F01_transcript_119796	F01_transcript_119796	F01_transcript_119796	NA	F01_transcript_119796

F01_transcript_131277	NA	F01_transcript_131277	F01_transcript_131277	F01_transcript_131277
F01_transcript_38131	NA	NA	NA	F01_transcript_38131
F01_transcript_92776	NA	NA	NA	F01_transcript_92776
F01_transcript_96688	NA	F01_transcript_96688	NA	NA
F01_transcript_4610	NA	NA	NA	F01_transcript_4610
F01_transcript_132750	NA	NA	F01_transcript_132750	F01_transcript_132750
F01_transcript_156824	NA	F01_transcript_156824	NA	NA
F01_transcript_57279	NA	NA	F01_transcript_57279	F01_transcript_57279
F01_transcript_104442	NA	F01_transcript_104442	F01_transcript_104442	NA
F01_transcript_138549	NA	F01_transcript_138549	NA	NA
F01_transcript_132654	F01_transcript_132654	F01_transcript_132654	F01_transcript_132654	F01_transcript_132654
F01_transcript_157328	F01_transcript_157328	F01_transcript_157328	NA	F01_transcript_157328
F01_transcript_76038	F01_transcript_76038	F01_transcript_76038	F01_transcript_76038	F01_transcript_76038
F01_transcript_54186	NA	NA	NA	F01_transcript_54186

F01_transcript_78725	NA	NA	NA	F01_transcript_78725
F01_transcript_119028	F01_transcript_119028	F01_transcript_119028	F01_transcript_119028	F01_transcript_119028
F01_transcript_100994	F01_transcript_100994	F01_transcript_100994	F01_transcript_100994	F01_transcript_100994
F01_transcript_161698	F01_transcript_161698	F01_transcript_161698	F01_transcript_161698	F01_transcript_161698
F01_transcript_117813	NA	NA	NA	F01_transcript_117813
F01_transcript_24360	F01_transcript_24360	F01_transcript_24360	F01_transcript_24360	F01_transcript_24360
F01_transcript_157259	NA	NA	NA	F01_transcript_157259
F01_transcript_153425	F01_transcript_153425	F01_transcript_153425	F01_transcript_153425	F01_transcript_153425
F01_transcript_13113	NA	F01_transcript_13113	NA	NA
F01_transcript_82681	NA	NA	NA	F01_transcript_82681
F01_transcript_108007	NA	F01_transcript_108007	NA	F01_transcript_108007
F01_transcript_114684	NA	F01_transcript_114684	NA	NA
F01_transcript_82767	NA	NA	NA	F01_transcript_82767
F01_transcript_153286	NA	NA	NA	F01_transcript_153286

F01_transcript_37294	F01_transcript_37294	F01_transcript_37294	NA	F01_transcript_37294
F01_transcript_49358	F01_transcript_49358	F01_transcript_49358	NA	F01_transcript_49358
F01_transcript_123477	NA	F01_transcript_123477	NA	NA
F01_transcript_101073	F01_transcript_101073	F01_transcript_101073	F01_transcript_101073	F01_transcript_101073
F01_transcript_55212	F01_transcript_55212	F01_transcript_55212	F01_transcript_55212	F01_transcript_55212
F01_transcript_131115	F01_transcript_131115	F01_transcript_131115	NA	NA
F01_transcript_154860	NA	NA	NA	F01_transcript_154860
F01_transcript_119115	NA	NA	F01_transcript_119115	NA
F01_transcript_83232	NA	F01_transcript_83232	NA	NA
F01_transcript_95672	NA	F01_transcript_95672	NA	F01_transcript_95672
F01_transcript_63492	F01_transcript_63492	F01_transcript_63492	F01_transcript_63492	F01_transcript_63492
F01_transcript_123628	NA	F01_transcript_123628	NA	NA
F01_transcript_63622	F01_transcript_63622	F01_transcript_63622	NA	NA
F01_transcript_98220	F01_transcript_98220	F01_transcript_98220	NA	F01_transcript_98220

F01_transcript_66019	NA	NA	NA	F01_transcript_66019
F01_transcript_54079	NA	F01_transcript_54079	NA	F01_transcript_54079
F01_transcript_66104	F01_transcript_66104	F01_transcript_66104	F01_transcript_66104	F01_transcript_66104
F01_transcript_5868	F01_transcript_5868	F01_transcript_5868	F01_transcript_5868	F01_transcript_5868
F01_transcript_86083	NA	F01_transcript_86083	NA	NA
F01_transcript_164380	NA	NA	NA	F01_transcript_164380
F01_transcript_69340	F01_transcript_69340	F01_transcript_69340	F01_transcript_69340	F01_transcript_69340
F01_transcript_99397	F01_transcript_99397	F01_transcript_99397	F01_transcript_99397	NA
F01_transcript_99507	F01_transcript_99507	F01_transcript_99507	F01_transcript_99507	F01_transcript_99507
F01_transcript_85793	NA	F01_transcript_85793	F01_transcript_85793	NA
F01_transcript_157836	NA	NA	NA	F01_transcript_157836
F01_transcript_135733	NA	F01_transcript_135733	NA	NA
F01_transcript_83174	NA	NA	NA	F01_transcript_83174
F01_transcript_148163	F01_transcript_148163	F01_transcript_148163	F01_transcript_148163	F01_transcript_148163

F01_transcript_130298	NA	NA	NA	F01_transcript_130298
F01_transcript_66283	NA	NA	NA	F01_transcript_66283
F01_transcript_9558	NA	NA	NA	F01_transcript_9558
F01_transcript_87390	NA	F01_transcript_87390	NA	F01_transcript_87390
F01_transcript_134826	NA	F01_transcript_134826	NA	NA
F01_transcript_104173	NA	F01_transcript_104173	F01_transcript_104173	F01_transcript_104173
F01_transcript_167472	NA	NA	NA	F01_transcript_167472
F01_transcript_164642	NA	F01_transcript_164642	NA	F01_transcript_164642
F01_transcript_129083	NA	F01_transcript_129083	F01_transcript_129083	NA
F01_transcript_69193	NA	F01_transcript_69193	NA	NA
F01_transcript_74016	NA	NA	NA	F01_transcript_74016
F01_transcript_84501	NA	F01_transcript_84501	F01_transcript_84501	F01_transcript_84501
F01_transcript_56563	NA	NA	F01_transcript_56563	F01_transcript_56563
F01_transcript_163565	NA	F01_transcript_163565	NA	NA

F01_transcript_8242	NA	NA	NA	F01_transcript_8242
F01_transcript_89006	NA	F01_transcript_89006	NA	F01_transcript_89006
F01_transcript_115502	NA	NA	NA	F01_transcript_115502
F01_transcript_18568	NA	NA	NA	F01_transcript_18568
F01_transcript_124460	F01_transcript_124460	F01_transcript_124460	F01_transcript_124460	F01_transcript_124460
F01_transcript_43570	NA	NA	NA	F01_transcript_43570
F01_transcript_43201	NA	NA	NA	F01_transcript_43201
F01_transcript_101586	NA	NA	NA	F01_transcript_101586
F01_transcript_141545	F01_transcript_141545	F01_transcript_141545	NA	F01_transcript_141545
F01_transcript_114493	F01_transcript_114493	F01_transcript_114493	F01_transcript_114493	F01_transcript_114493
F01_transcript_153941	NA	NA	NA	F01_transcript_153941
F01_transcript_147549	NA	F01_transcript_147549	F01_transcript_147549	NA
F01_transcript_114314	F01_transcript_114314	F01_transcript_114314	NA	NA
F01_transcript_68994	F01_transcript_68994	F01_transcript_68994	F01_transcript_68994	F01_transcript_68994

F01_transcript_79786	NA	F01_transcript_79786	NA	NA
F01_transcript_110406	NA	NA	NA	F01_transcript_110406
F01_transcript_121368	NA	F01_transcript_121368	NA	NA
F01_transcript_23455	F01_transcript_23455	F01_transcript_23455	F01_transcript_23455	F01_transcript_23455
F01_transcript_55778	NA	NA	NA	F01_transcript_55778
F01_transcript_120636	F01_transcript_120636	F01_transcript_120636	NA	F01_transcript_120636
F01_transcript_120027	NA	F01_transcript_120027	NA	NA
F01_transcript_86690	NA	F01_transcript_86690	NA	NA
F01_transcript_99522	F01_transcript_99522	F01_transcript_99522	NA	F01_transcript_99522
F01_transcript_90877	NA	F01_transcript_90877	NA	NA
F01_transcript_57237	F01_transcript_57237	F01_transcript_57237	F01_transcript_57237	F01_transcript_57237
F01_transcript_150143	NA	F01_transcript_150143	NA	NA
F01_transcript_85103	F01_transcript_85103	F01_transcript_85103	F01_transcript_85103	F01_transcript_85103
F01_transcript_34949	NA	NA	NA	F01_transcript_34949

F01_transcript_82326	NA	F01_transcript_82326	F01_transcript_82326	NA
F01_transcript_151606	NA	F01_transcript_151606	F01_transcript_151606	F01_transcript_151606
F01_transcript_83935	NA	NA	NA	F01_transcript_83935
F01_transcript_148723	NA	F01_transcript_148723	NA	NA
F01_transcript_5126	NA	NA	NA	F01_transcript_5126
F01_transcript_49776	NA	F01_transcript_49776	NA	F01_transcript_49776
F01_transcript_11608	NA	NA	NA	F01_transcript_11608
F01_transcript_33268	F01_transcript_33268	F01_transcript_33268	F01_transcript_33268	F01_transcript_33268
F01_transcript_78785	NA	NA	NA	F01_transcript_78785
F01_transcript_93100	F01_transcript_93100	NA	NA	NA
F01_transcript_153837	NA	F01_transcript_153837	NA	NA
F01_transcript_13057	NA	NA	NA	F01_transcript_13057
F01_transcript_136551	NA	NA	NA	F01_transcript_136551
F01_transcript_149738	F01_transcript_149738	F01_transcript_149738	F01_transcript_149738	F01_transcript_149738

F01_transcript_3747	NA	F01_transcript_3747	NA	F01_transcript_3747
F01_transcript_77612	NA	F01_transcript_77612	NA	NA
F01_transcript_115514	F01_transcript_115514	F01_transcript_115514	F01_transcript_115514	F01_transcript_115514
F01_transcript_25875	NA	NA	NA	F01_transcript_25875
F01_transcript_127671	NA	F01_transcript_127671	NA	NA
F01_transcript_138946	NA	F01_transcript_138946	NA	NA
F01_transcript_125350	F01_transcript_125350	F01_transcript_125350	F01_transcript_125350	F01_transcript_125350
F01_transcript_148904	NA	F01_transcript_148904	NA	NA
F01_transcript_81797	NA	NA	NA	F01_transcript_81797
F01_transcript_60549	F01_transcript_60549	F01_transcript_60549	F01_transcript_60549	F01_transcript_60549
F01_transcript_51422	F01_transcript_51422	F01_transcript_51422	F01_transcript_51422	NA
F01_transcript_86856	NA	F01_transcript_86856	NA	NA
F01_transcript_82925	NA	F01_transcript_82925	NA	NA
F01_transcript_134121	NA	F01_transcript_134121	NA	NA

F01_transcript_47450	NA	NA	NA	F01_transcript_47450
F01_transcript_141087	NA	NA	NA	F01_transcript_141087
F01_transcript_8866	NA	NA	NA	F01_transcript_8866
F01_transcript_63386	NA	F01_transcript_63386	NA	NA
F01_transcript_129604	F01_transcript_129604	F01_transcript_129604	F01_transcript_129604	F01_transcript_129604
F01_transcript_138566	NA	NA	NA	F01_transcript_138566
F01_transcript_103175	NA	F01_transcript_103175	NA	NA
F01_transcript_128526	NA	NA	NA	F01_transcript_128526
F01_transcript_121497	F01_transcript_121497	F01_transcript_121497	F01_transcript_121497	F01_transcript_121497
F01_transcript_118489	NA	NA	NA	F01_transcript_118489
F01_transcript_69637	F01_transcript_69637	F01_transcript_69637	F01_transcript_69637	F01_transcript_69637
F01_transcript_47201	NA	F01_transcript_47201	NA	F01_transcript_47201
F01_transcript_66978	F01_transcript_66978	F01_transcript_66978	F01_transcript_66978	F01_transcript_66978
F01_transcript_145724	NA	F01_transcript_145724	NA	F01_transcript_145724

F01_transcript_17021	F01_transcript_17021	NA	NA	NA
F01_transcript_147190	F01_transcript_147190	F01_transcript_147190	F01_transcript_147190	F01_transcript_147190
F01_transcript_18900	F01_transcript_18900	F01_transcript_18900	NA	NA
F01_transcript_57347	NA	NA	NA	F01_transcript_57347
F01_transcript_128853	NA	F01_transcript_128853	NA	NA
F01_transcript_119757	NA	F01_transcript_119757	NA	NA
F01_transcript_59145	NA	F01_transcript_59145	F01_transcript_59145	F01_transcript_59145
F01_transcript_57024	NA	F01_transcript_57024	F01_transcript_57024	NA
F01_transcript_79748	F01_transcript_79748	F01_transcript_79748	F01_transcript_79748	F01_transcript_79748
F01_transcript_90576	NA	F01_transcript_90576	F01_transcript_90576	F01_transcript_90576
F01_transcript_89829	NA	NA	NA	F01_transcript_89829
F01_transcript_66686	F01_transcript_66686	F01_transcript_66686	NA	F01_transcript_66686
F01_transcript_11281	NA	F01_transcript_11281	NA	NA
F01_transcript_106465	NA	NA	NA	F01_transcript_106465

F01_transcript_49739	F01_transcript_49739	F01_transcript_49739	NA	NA
F01_transcript_61120	NA	F01_transcript_61120	NA	F01_transcript_61120
F01_transcript_88618	NA	F01_transcript_88618	F01_transcript_88618	F01_transcript_88618
F01_transcript_159473	NA	F01_transcript_159473	NA	F01_transcript_159473
F01_transcript_134818	NA	F01_transcript_134818	NA	NA
F01_transcript_112457	F01_transcript_112457	F01_transcript_112457	NA	NA
F01_transcript_107965	NA	NA	NA	F01_transcript_107965
F01_transcript_148577	F01_transcript_148577	F01_transcript_148577	F01_transcript_148577	F01_transcript_148577
F01_transcript_120100	NA	NA	NA	F01_transcript_120100
F01_transcript_138859	F01_transcript_138859	F01_transcript_138859	NA	NA
F01_transcript_144639	NA	NA	F01_transcript_144639	F01_transcript_144639
F01_transcript_53909	NA	NA	NA	F01_transcript_53909
F01_transcript_51357	NA	F01_transcript_51357	NA	NA
F01_transcript_166687	F01_transcript_166687	F01_transcript_166687	F01_transcript_166687	F01_transcript_166687

F01_transcript_109987	NA	F01_transcript_109987	F01_transcript_109987	F01_transcript_109987
F01_transcript_102926	F01_transcript_102926	F01_transcript_102926	F01_transcript_102926	F01_transcript_102926
F01_transcript_100539	NA	NA	NA	F01_transcript_100539
F01_transcript_90498	F01_transcript_90498	F01_transcript_90498	F01_transcript_90498	F01_transcript_90498
F01_transcript_74041	NA	NA	NA	F01_transcript_74041
F01_transcript_51635	NA	F01_transcript_51635	F01_transcript_51635	NA
F01_transcript_119078	NA	F01_transcript_119078	F01_transcript_119078	F01_transcript_119078
F01_transcript_5883	NA	NA	NA	F01_transcript_5883
F01_transcript_50937	F01_transcript_50937	F01_transcript_50937	F01_transcript_50937	F01_transcript_50937
F01_transcript_144350	NA	NA	NA	F01_transcript_144350
F01_transcript_164130	F01_transcript_164130	F01_transcript_164130	NA	F01_transcript_164130
F01_transcript_107792	NA	F01_transcript_107792	NA	NA
F01_transcript_104871	F01_transcript_104871	F01_transcript_104871	F01_transcript_104871	F01_transcript_104871
F01_transcript_131203	F01_transcript_131203	F01_transcript_131203	F01_transcript_131203	F01_transcript_131203

F01_transcript_107171	NA	F01_transcript_107171	F01_transcript_107171	F01_transcript_107171
F01_transcript_126247	NA	F01_transcript_126247	F01_transcript_126247	F01_transcript_126247
F01_transcript_100975	F01_transcript_100975	F01_transcript_100975	F01_transcript_100975	F01_transcript_100975
F01_transcript_140782	NA	F01_transcript_140782	NA	F01_transcript_140782
F01_transcript_98697	NA	NA	NA	F01_transcript_98697
F01_transcript_2286	NA	NA	NA	F01_transcript_2286
F01_transcript_38438	NA	NA	NA	F01_transcript_38438
F01_transcript_113820	F01_transcript_113820	F01_transcript_113820	F01_transcript_113820	F01_transcript_113820
F01_transcript_156709	NA	F01_transcript_156709	NA	NA
F01_transcript_143871	NA	NA	NA	F01_transcript_143871
F01_transcript_99862	NA	F01_transcript_99862	NA	F01_transcript_99862
F01_transcript_35350	NA	NA	NA	F01_transcript_35350
F01_transcript_111432	F01_transcript_111432	F01_transcript_111432	F01_transcript_111432	F01_transcript_111432
F01_transcript_136124	NA	NA	NA	F01_transcript_136124

F01_transcript_105695	NA	F01_transcript_105695	NA	NA
F01_transcript_100747	NA	F01_transcript_100747	F01_transcript_100747	F01_transcript_100747
F01_transcript_27649	NA	NA	NA	F01_transcript_27649
F01_transcript_102325	F01_transcript_102325	F01_transcript_102325	F01_transcript_102325	F01_transcript_102325
F01_transcript_99013	NA	NA	NA	F01_transcript_99013
F01_transcript_136213	F01_transcript_136213	F01_transcript_136213	F01_transcript_136213	F01_transcript_136213
F01_transcript_87258	NA	F01_transcript_87258	NA	NA
F01_transcript_92653	NA	NA	NA	F01_transcript_92653
F01_transcript_45496	NA	NA	NA	F01_transcript_45496
F01_transcript_125480	NA	NA	NA	F01_transcript_125480
F01_transcript_102283	F01_transcript_102283	F01_transcript_102283	F01_transcript_102283	F01_transcript_102283
F01_transcript_110090	F01_transcript_110090	F01_transcript_110090	F01_transcript_110090	F01_transcript_110090
F01_transcript_5784	NA	NA	NA	F01_transcript_5784
F01_transcript_77657	F01_transcript_77657	F01_transcript_77657	NA	NA

F01_transcript_84394	NA	NA	NA	F01_transcript_84394
F01_transcript_131136	NA	F01_transcript_131136	F01_transcript_131136	NA
F01_transcript_112740	NA	F01_transcript_112740	NA	F01_transcript_112740
F01_transcript_84011	NA	F01_transcript_84011	NA	F01_transcript_84011
F01_transcript_62889	NA	F01_transcript_62889	NA	NA
F01_transcript_132155	NA	F01_transcript_132155	NA	NA
F01_transcript_57042	NA	NA	F01_transcript_57042	NA
F01_transcript_165983	NA	NA	NA	F01_transcript_165983
F01_transcript_53426	NA	F01_transcript_53426	NA	NA
F01_transcript_136722	NA	F01_transcript_136722	NA	NA
F01_transcript_5137	NA	NA	NA	F01_transcript_5137
F01_transcript_158440	NA	F01_transcript_158440	NA	F01_transcript_158440
F01_transcript_127969	NA	F01_transcript_127969	F01_transcript_127969	F01_transcript_127969
F01_transcript_49057	NA	F01_transcript_49057	NA	F01_transcript_49057

F01_transcript_67345	F01_transcript_67345	F01_transcript_67345	NA	NA
F01_transcript_44550	F01_transcript_44550	F01_transcript_44550	NA	F01_transcript_44550
F01_transcript_92375	NA	F01_transcript_92375	NA	NA
F01_transcript_96929	NA	F01_transcript_96929	NA	NA
F01_transcript_126771	F01_transcript_126771	F01_transcript_126771	F01_transcript_126771	F01_transcript_126771
F01_transcript_81062	F01_transcript_81062	F01_transcript_81062	F01_transcript_81062	NA
F01_transcript_105956	F01_transcript_105956	F01_transcript_105956	NA	F01_transcript_105956
F01_transcript_64101	NA	NA	NA	F01_transcript_64101
F01_transcript_82992	F01_transcript_82992	F01_transcript_82992	F01_transcript_82992	F01_transcript_82992
F01_transcript_61910	NA	F01_transcript_61910	NA	F01_transcript_61910
F01_transcript_54722	F01_transcript_54722	F01_transcript_54722	NA	NA
F01_transcript_94686	NA	F01_transcript_94686	F01_transcript_94686	F01_transcript_94686
F01_transcript_1583	NA	NA	NA	F01_transcript_1583
F01_transcript_134449	NA	F01_transcript_134449	NA	NA

F01_transcript_164535	NA	F01_transcript_164535	NA	F01_transcript_164535
F01_transcript_151453	NA	F01_transcript_151453	F01_transcript_151453	F01_transcript_151453
F01_transcript_125092	NA	NA	NA	F01_transcript_125092
F01_transcript_99916	F01_transcript_99916	F01_transcript_99916	NA	F01_transcript_99916
F01_transcript_56438	NA	F01_transcript_56438	F01_transcript_56438	F01_transcript_56438
F01_transcript_143721	F01_transcript_143721	F01_transcript_143721	NA	F01_transcript_143721
F01_transcript_88091	NA	NA	NA	F01_transcript_88091
F01_transcript_112607	NA	F01_transcript_112607	NA	F01_transcript_112607
F01_transcript_138090	NA	F01_transcript_138090	NA	NA
F01_transcript_134729	NA	F01_transcript_134729	NA	F01_transcript_134729
F01_transcript_165700	F01_transcript_165700	F01_transcript_165700	F01_transcript_165700	F01_transcript_165700
F01_transcript_99644	NA	F01_transcript_99644	NA	F01_transcript_99644
F01_transcript_6936	F01_transcript_6936	F01_transcript_6936	F01_transcript_6936	F01_transcript_6936
F01_transcript_107491	F01_transcript_107491	F01_transcript_107491	NA	F01_transcript_107491

F01_transcript_94914	NA	F01_transcript_94914	NA	NA
F01_transcript_71680	NA	NA	NA	F01_transcript_71680
F01_transcript_55714	NA	F01_transcript_55714	NA	F01_transcript_55714
F01_transcript_153662	NA	F01_transcript_153662	NA	NA
F01_transcript_27485	NA	F01_transcript_27485	NA	NA
F01_transcript_82827	NA	F01_transcript_82827	NA	NA
F01_transcript_148398	NA	F01_transcript_148398	F01_transcript_148398	F01_transcript_148398
F01_transcript_121363	NA	NA	NA	F01_transcript_121363
F01_transcript_131211	F01_transcript_131211	F01_transcript_131211	F01_transcript_131211	F01_transcript_131211
F01_transcript_53215	NA	NA	NA	F01_transcript_53215
F01_transcript_88961	NA	NA	NA	F01_transcript_88961
F01_transcript_115290	NA	F01_transcript_115290	NA	F01_transcript_115290
F01_transcript_144867	F01_transcript_144867	F01_transcript_144867	F01_transcript_144867	F01_transcript_144867
F01_transcript_157790	NA	NA	NA	F01_transcript_157790

F01_transcript_11089	NA	NA	NA	F01_transcript_11089
F01_transcript_123775	F01_transcript_123775	F01_transcript_123775	NA	F01_transcript_123775
F01_transcript_67417	NA	F01_transcript_67417	NA	F01_transcript_67417
F01_transcript_89601	NA	F01_transcript_89601	NA	NA
F01_transcript_501	NA	NA	NA	F01_transcript_501
F01_transcript_159706	NA	F01_transcript_159706	NA	NA
F01_transcript_104186	F01_transcript_104186	F01_transcript_104186	F01_transcript_104186	F01_transcript_104186
F01_transcript_102210	NA	F01_transcript_102210	NA	NA
F01_transcript_121925	F01_transcript_121925	F01_transcript_121925	F01_transcript_121925	F01_transcript_121925
F01_transcript_3618	NA	NA	NA	F01_transcript_3618
F01_transcript_4901	NA	F01_transcript_4901	NA	NA
F01_transcript_111537	NA	NA	NA	F01_transcript_111537
F01_transcript_150297	NA	F01_transcript_150297	NA	NA
F01_transcript_48654	NA	NA	NA	F01_transcript_48654

F01_transcript_154794	F01_transcript_154794	F01_transcript_154794	F01_transcript_154794	F01_transcript_154794
F01_transcript_107252	NA	F01_transcript_107252	NA	NA
F01_transcript_151048	NA	F01_transcript_151048	NA	NA
F01_transcript_48245	NA	NA	NA	F01_transcript_48245
F01_transcript_53064	F01_transcript_53064	F01_transcript_53064	NA	NA
F01_transcript_121855	NA	NA	NA	F01_transcript_121855
F01_transcript_69311	F01_transcript_69311	F01_transcript_69311	NA	F01_transcript_69311
F01_transcript_89993	NA	NA	NA	F01_transcript_89993
F01_transcript_130181	F01_transcript_130181	F01_transcript_130181	F01_transcript_130181	F01_transcript_130181
F01_transcript_56056	NA	F01_transcript_56056	NA	F01_transcript_56056
F01_transcript_108639	F01_transcript_108639	F01_transcript_108639	F01_transcript_108639	NA
F01_transcript_101952	NA	NA	NA	F01_transcript_101952
F01_transcript_162848	F01_transcript_162848	F01_transcript_162848	F01_transcript_162848	F01_transcript_162848
F01_transcript_142786	F01_transcript_142786	F01_transcript_142786	F01_transcript_142786	F01_transcript_142786

F01_transcript_146909	NA	NA	NA	F01_transcript_146909
F01_transcript_19757	NA	NA	NA	F01_transcript_19757
F01_transcript_99756	NA	F01_transcript_99756	NA	NA
F01_transcript_66765	NA	F01_transcript_66765	NA	NA
F01_transcript_87506	NA	F01_transcript_87506	NA	F01_transcript_87506
F01_transcript_72454	NA	NA	NA	F01_transcript_72454
F01_transcript_27640	NA	NA	NA	F01_transcript_27640
F01_transcript_21089	NA	F01_transcript_21089	NA	F01_transcript_21089
F01_transcript_141647	F01_transcript_141647	F01_transcript_141647	F01_transcript_141647	F01_transcript_141647
F01_transcript_50831	F01_transcript_50831	F01_transcript_50831	F01_transcript_50831	F01_transcript_50831
F01_transcript_39804	NA	NA	NA	F01_transcript_39804
F01_transcript_159227	F01_transcript_159227	F01_transcript_159227	F01_transcript_159227	F01_transcript_159227
F01_transcript_165345	NA	NA	NA	F01_transcript_165345
F01_transcript_18480	NA	NA	NA	F01_transcript_18480

F01_transcript_109681	NA	F01_transcript_109681	NA	F01_transcript_109681
F01_transcript_127961	F01_transcript_127961	F01_transcript_127961	NA	F01_transcript_127961
F01_transcript_132748	NA	F01_transcript_132748	F01_transcript_132748	F01_transcript_132748
F01_transcript_88723	NA	NA	F01_transcript_88723	F01_transcript_88723
F01_transcript_156124	NA	F01_transcript_156124	NA	NA
F01_transcript_94040	F01_transcript_94040	F01_transcript_94040	NA	F01_transcript_94040
F01_transcript_162058	F01_transcript_162058	F01_transcript_162058	F01_transcript_162058	NA
F01_transcript_113025	NA	NA	NA	F01_transcript_113025
F01_transcript_30736	F01_transcript_30736	F01_transcript_30736	NA	F01_transcript_30736
F01_transcript_154660	F01_transcript_154660	F01_transcript_154660	F01_transcript_154660	F01_transcript_154660
F01_transcript_47371	NA	NA	NA	F01_transcript_47371
F01_transcript_103437	NA	F01_transcript_103437	F01_transcript_103437	NA
F01_transcript_111368	NA	F01_transcript_111368	NA	F01_transcript_111368
F01_transcript_166861	NA	F01_transcript_166861	F01_transcript_166861	F01_transcript_166861

F01_transcript_74780	NA	F01_transcript_74780	NA	NA
F01_transcript_121140	NA	F01_transcript_121140	F01_transcript_121140	F01_transcript_121140
F01_transcript_160928	NA	NA	NA	F01_transcript_160928
F01_transcript_90794	F01_transcript_90794	F01_transcript_90794	NA	NA
F01_transcript_49415	F01_transcript_49415	F01_transcript_49415	NA	NA
F01_transcript_124030	NA	F01_transcript_124030	NA	F01_transcript_124030
F01_transcript_91637	F01_transcript_91637	F01_transcript_91637	NA	NA
F01_transcript_123139	NA	NA	NA	F01_transcript_123139
F01_transcript_65173	F01_transcript_65173	F01_transcript_65173	F01_transcript_65173	F01_transcript_65173
F01_transcript_80172	NA	F01_transcript_80172	F01_transcript_80172	F01_transcript_80172
F01_transcript_150060	NA	NA	NA	F01_transcript_150060
F01_transcript_52864	F01_transcript_52864	F01_transcript_52864	NA	NA
F01_transcript_83519	F01_transcript_83519	F01_transcript_83519	F01_transcript_83519	F01_transcript_83519
F01_transcript_3729	NA	NA	NA	F01_transcript_3729

F01_transcript_71197	NA	F01_transcript_71197	F01_transcript_71197	NA
F01_transcript_136240	NA	F01_transcript_136240	F01_transcript_136240	NA
F01_transcript_56465	NA	NA	F01_transcript_56465	NA
F01_transcript_75167	F01_transcript_75167	F01_transcript_75167	F01_transcript_75167	F01_transcript_75167
F01_transcript_67249	NA	F01_transcript_67249	NA	NA
F01_transcript_20162	NA	NA	NA	F01_transcript_20162
F01_transcript_145357	F01_transcript_145357	F01_transcript_145357	NA	F01_transcript_145357
F01_transcript_124855	NA	NA	NA	F01_transcript_124855
F01_transcript_130411	NA	NA	NA	F01_transcript_130411
F01_transcript_101810	F01_transcript_101810	F01_transcript_101810	F01_transcript_101810	NA
F01_transcript_144022	NA	F01_transcript_144022	NA	NA
F01_transcript_132715	F01_transcript_132715	F01_transcript_132715	F01_transcript_132715	NA
F01_transcript_29385	NA	F01_transcript_29385	NA	NA
F01_transcript_69171	NA	NA	NA	F01_transcript_69171

F01_transcript_87761	NA	F01_transcript_87761	NA	NA
F01_transcript_52452	F01_transcript_52452	F01_transcript_52452	F01_transcript_52452	F01_transcript_52452
F01_transcript_15612	F01_transcript_15612	F01_transcript_15612	F01_transcript_15612	F01_transcript_15612
F01_transcript_137348	NA	F01_transcript_137348	NA	NA
F01_transcript_49668	NA	NA	F01_transcript_49668	F01_transcript_49668
F01_transcript_109492	F01_transcript_109492	F01_transcript_109492	NA	NA
F01_transcript_137863	NA	NA	NA	F01_transcript_137863
F01_transcript_8308	F01_transcript_8308	F01_transcript_8308	NA	F01_transcript_8308
F01_transcript_78215	F01_transcript_78215	F01_transcript_78215	NA	F01_transcript_78215
F01_transcript_50616	F01_transcript_50616	F01_transcript_50616	NA	F01_transcript_50616
F01_transcript_112611	NA	F01_transcript_112611	NA	NA
F01_transcript_132928	F01_transcript_132928	F01_transcript_132928	F01_transcript_132928	F01_transcript_132928
F01_transcript_94907	NA	NA	F01_transcript_94907	F01_transcript_94907
F01_transcript_125487	F01_transcript_125487	F01_transcript_125487	NA	F01_transcript_125487

F01_transcript_81526	F01_transcript_81526	F01_transcript_81526	NA	F01_transcript_81526
F01_transcript_77705	NA	F01_transcript_77705	NA	NA
F01_transcript_130758	NA	NA	NA	F01_transcript_130758
F01_transcript_84133	NA	F01_transcript_84133	F01_transcript_84133	NA
F01_transcript_65403	NA	NA	F01_transcript_65403	F01_transcript_65403
F01_transcript_145758	NA	F01_transcript_145758	NA	F01_transcript_145758
F01_transcript_43515	NA	NA	NA	F01_transcript_43515
F01_transcript_17417	NA	NA	NA	F01_transcript_17417
F01_transcript_83430	NA	NA	NA	F01_transcript_83430
F01_transcript_132534	NA	NA	NA	F01_transcript_132534
F01_transcript_79333	F01_transcript_79333	F01_transcript_79333	F01_transcript_79333	F01_transcript_79333
F01_transcript_107176	F01_transcript_107176	F01_transcript_107176	F01_transcript_107176	NA
F01_transcript_96819	F01_transcript_96819	F01_transcript_96819	F01_transcript_96819	F01_transcript_96819
F01_transcript_81782	F01_transcript_81782	F01_transcript_81782	NA	NA

F01_transcript_105237	NA	NA	NA	F01_transcript_105237
F01_transcript_45678	NA	NA	NA	F01_transcript_45678
F01_transcript_13727	NA	F01_transcript_13727	NA	NA
F01_transcript_55929	NA	NA	F01_transcript_55929	F01_transcript_55929
F01_transcript_113942	NA	NA	NA	F01_transcript_113942
F01_transcript_115727	NA	F01_transcript_115727	NA	F01_transcript_115727
F01_transcript_64010	F01_transcript_64010	F01_transcript_64010	F01_transcript_64010	F01_transcript_64010
F01_transcript_101298	F01_transcript_101298	NA	F01_transcript_101298	F01_transcript_101298
F01_transcript_158502	NA	NA	F01_transcript_158502	F01_transcript_158502
F01_transcript_90028	F01_transcript_90028	F01_transcript_90028	F01_transcript_90028	F01_transcript_90028
F01_transcript_72341	NA	F01_transcript_72341	NA	NA
F01_transcript_77417	NA	NA	F01_transcript_77417	F01_transcript_77417
F01_transcript_146154	F01_transcript_146154	F01_transcript_146154	F01_transcript_146154	F01_transcript_146154
F01_transcript_156357	NA	NA	NA	F01_transcript_156357

F01_transcript_14589	NA	NA	NA	F01_transcript_14589
F01_transcript_151295	F01_transcript_151295	F01_transcript_151295	NA	NA
F01_transcript_116639	F01_transcript_116639	F01_transcript_116639	F01_transcript_116639	F01_transcript_116639
F01_transcript_149676	F01_transcript_149676	F01_transcript_149676	F01_transcript_149676	F01_transcript_149676
F01_transcript_162855	F01_transcript_162855	F01_transcript_162855	F01_transcript_162855	F01_transcript_162855
F01_transcript_78714	NA	F01_transcript_78714	NA	NA
F01_transcript_154949	NA	F01_transcript_154949	NA	F01_transcript_154949
F01_transcript_76730	NA	NA	NA	F01_transcript_76730
F01_transcript_114336	NA	NA	NA	F01_transcript_114336
F01_transcript_132632	NA	NA	NA	F01_transcript_132632
F01_transcript_72851	NA	F01_transcript_72851	NA	NA
F01_transcript_63823	NA	F01_transcript_63823	NA	F01_transcript_63823
F01_transcript_154868	NA	F01_transcript_154868	NA	F01_transcript_154868
F01_transcript_162610	NA	F01_transcript_162610	NA	NA

F01_transcript_138910	F01_transcript_138910	F01_transcript_138910	F01_transcript_138910	NA
F01_transcript_115792	NA	NA	NA	F01_transcript_115792
F01_transcript_103927	F01_transcript_103927	F01_transcript_103927	NA	NA
F01_transcript_142250	F01_transcript_142250	F01_transcript_142250	F01_transcript_142250	F01_transcript_142250
F01_transcript_114357	NA	NA	NA	F01_transcript_114357
F01_transcript_124632	NA	NA	NA	F01_transcript_124632
F01_transcript_121537	NA	F01_transcript_121537	NA	NA
F01_transcript_153196	F01_transcript_153196	F01_transcript_153196	F01_transcript_153196	NA
F01_transcript_72319	NA	NA	NA	F01_transcript_72319
F01_transcript_9244	NA	F01_transcript_9244	NA	NA
F01_transcript_62471	NA	F01_transcript_62471	F01_transcript_62471	F01_transcript_62471
F01_transcript_111906	NA	F01_transcript_111906	NA	NA
F01_transcript_61047	NA	F01_transcript_61047	NA	F01_transcript_61047
F01_transcript_157838	NA	NA	NA	F01_transcript_157838

F01_transcript_104761	NA	NA	NA	F01_transcript_104761
F01_transcript_117979	NA	F01_transcript_117979	NA	NA
F01_transcript_138871	NA	F01_transcript_138871	NA	F01_transcript_138871
F01_transcript_79396	F01_transcript_79396	F01_transcript_79396	F01_transcript_79396	F01_transcript_79396
F01_transcript_157641	F01_transcript_157641	F01_transcript_157641	F01_transcript_157641	F01_transcript_157641
F01_transcript_32080	F01_transcript_32080	NA	NA	F01_transcript_32080
F01_transcript_131902	NA	NA	NA	F01_transcript_131902
F01_transcript_111010	F01_transcript_111010	F01_transcript_111010	F01_transcript_111010	F01_transcript_111010
F01_transcript_51192	F01_transcript_51192	F01_transcript_51192	F01_transcript_51192	F01_transcript_51192
F01_transcript_155916	F01_transcript_155916	F01_transcript_155916	F01_transcript_155916	F01_transcript_155916
F01_transcript_118511	NA	NA	NA	F01_transcript_118511
F01_transcript_146892	NA	F01_transcript_146892	NA	NA
F01_transcript_41283	NA	NA	NA	F01_transcript_41283
F01_transcript_146083	NA	F01_transcript_146083	NA	NA

F01_transcript_140128	NA	NA	NA	F01_transcript_140128
F01_transcript_50343	F01_transcript_50343	NA	F01_transcript_50343	NA
F01_transcript_148286	F01_transcript_148286	F01_transcript_148286	NA	NA
F01_transcript_81196	F01_transcript_81196	F01_transcript_81196	F01_transcript_81196	F01_transcript_81196
F01_transcript_71222	NA	NA	F01_transcript_71222	NA
F01_transcript_103584	NA	F01_transcript_103584	NA	NA
F01_transcript_83546	F01_transcript_83546	F01_transcript_83546	NA	F01_transcript_83546
F01_transcript_153908	NA	F01_transcript_153908	F01_transcript_153908	NA
F01_transcript_156216	NA	F01_transcript_156216	F01_transcript_156216	F01_transcript_156216
F01_transcript_102134	NA	F01_transcript_102134	NA	NA
F01_transcript_120254	NA	F01_transcript_120254	F01_transcript_120254	F01_transcript_120254
F01_transcript_75819	NA	NA	NA	F01_transcript_75819
F01_transcript_106242	F01_transcript_106242	F01_transcript_106242	NA	F01_transcript_106242
F01_transcript_110968	NA	NA	NA	F01_transcript_110968

F01_transcript_50159	F01_transcript_50159	F01_transcript_50159	F01_transcript_50159	F01_transcript_50159
F01_transcript_139567	NA	F01_transcript_139567	NA	NA
F01_transcript_129956	F01_transcript_129956	F01_transcript_129956	NA	F01_transcript_129956
F01_transcript_138558	F01_transcript_138558	F01_transcript_138558	F01_transcript_138558	F01_transcript_138558
F01_transcript_142234	NA	F01_transcript_142234	NA	F01_transcript_142234
F01_transcript_79563	F01_transcript_79563	F01_transcript_79563	NA	NA
F01_transcript_110820	F01_transcript_110820	F01_transcript_110820	F01_transcript_110820	F01_transcript_110820
F01_transcript_118224	NA	NA	NA	F01_transcript_118224
F01_transcript_96546	NA	NA	NA	F01_transcript_96546
F01_transcript_112976	F01_transcript_112976	F01_transcript_112976	NA	F01_transcript_112976
F01_transcript_83041	NA	F01_transcript_83041	NA	F01_transcript_83041
F01_transcript_110220	NA	F01_transcript_110220	NA	NA
F01_transcript_49914	NA	NA	F01_transcript_49914	F01_transcript_49914
F01_transcript_71469	NA	F01_transcript_71469	NA	NA

F01_transcript_119634	NA	F01_transcript_119634	F01_transcript_119634	F01_transcript_119634
F01_transcript_49908	F01_transcript_49908	F01_transcript_49908	F01_transcript_49908	F01_transcript_49908
F01_transcript_121326	NA	F01_transcript_121326	NA	NA
F01_transcript_140421	F01_transcript_140421	F01_transcript_140421	F01_transcript_140421	F01_transcript_140421
F01_transcript_21446	NA	F01_transcript_21446	F01_transcript_21446	F01_transcript_21446
F01_transcript_85001	F01_transcript_85001	F01_transcript_85001	F01_transcript_85001	F01_transcript_85001
F01_transcript_164370	NA	NA	NA	F01_transcript_164370
F01_transcript_45966	NA	F01_transcript_45966	NA	NA
F01_transcript_104054	NA	NA	NA	F01_transcript_104054
F01_transcript_161283	NA	NA	NA	F01_transcript_161283
F01_transcript_44229	NA	NA	NA	F01_transcript_44229
F01_transcript_62685	NA	F01_transcript_62685	F01_transcript_62685	NA
F01_transcript_118846	NA	F01_transcript_118846	NA	F01_transcript_118846
F01_transcript_8994	NA	F01_transcript_8994	NA	F01_transcript_8994

F01_transcript_45352	NA	NA	NA	F01_transcript_45352
F01_transcript_84682	F01_transcript_84682	F01_transcript_84682	F01_transcript_84682	NA
F01_transcript_147483	NA	F01_transcript_147483	NA	NA
F01_transcript_105959	F01_transcript_105959	F01_transcript_105959	F01_transcript_105959	F01_transcript_105959
F01_transcript_79934	F01_transcript_79934	F01_transcript_79934	F01_transcript_79934	F01_transcript_79934
F01_transcript_52721	NA	F01_transcript_52721	NA	NA
F01_transcript_111489	F01_transcript_111489	F01_transcript_111489	F01_transcript_111489	NA
F01_transcript_37499	F01_transcript_37499	F01_transcript_37499	NA	NA
F01_transcript_109819	NA	F01_transcript_109819	F01_transcript_109819	NA
F01_transcript_59313	NA	NA	NA	F01_transcript_59313
F01_transcript_151512	F01_transcript_151512	F01_transcript_151512	F01_transcript_151512	F01_transcript_151512
F01_transcript_63162	NA	F01_transcript_63162	F01_transcript_63162	F01_transcript_63162
F01_transcript_120599	F01_transcript_120599	F01_transcript_120599	F01_transcript_120599	F01_transcript_120599
F01_transcript_98089	NA	NA	NA	F01_transcript_98089

F01_transcript_123478	NA	F01_transcript_123478	NA	NA
F01_transcript_75315	NA	F01_transcript_75315	NA	NA
F01_transcript_45840	NA	NA	NA	F01_transcript_45840
F01_transcript_126860	NA	NA	NA	F01_transcript_126860
F01_transcript_112218	NA	NA	NA	F01_transcript_112218
F01_transcript_162946	NA	F01_transcript_162946	NA	NA
F01_transcript_48006	NA	F01_transcript_48006	NA	NA
F01_transcript_167612	NA	NA	NA	F01_transcript_167612
F01_transcript_161875	F01_transcript_161875	F01_transcript_161875	F01_transcript_161875	F01_transcript_161875
F01_transcript_41928	NA	F01_transcript_41928	F01_transcript_41928	NA
F01_transcript_101873	F01_transcript_101873	F01_transcript_101873	F01_transcript_101873	F01_transcript_101873
F01_transcript_137954	NA	NA	NA	F01_transcript_137954
F01_transcript_142831	F01_transcript_142831	F01_transcript_142831	NA	NA
F01_transcript_2834	F01_transcript_2834	F01_transcript_2834	F01_transcript_2834	F01_transcript_2834

F01_transcript_148998	NA	F01_transcript_148998	F01_transcript_148998	F01_transcript_148998
F01_transcript_105381	NA	F01_transcript_105381	NA	NA
F01_transcript_49234	NA	NA	NA	F01_transcript_49234
F01_transcript_145314	F01_transcript_145314	F01_transcript_145314	NA	NA
F01_transcript_77923	NA	NA	NA	F01_transcript_77923
F01_transcript_157306	NA	F01_transcript_157306	NA	NA
F01_transcript_17344	NA	NA	NA	F01_transcript_17344
F01_transcript_103516	NA	NA	NA	F01_transcript_103516
F01_transcript_123298	NA	NA	NA	F01_transcript_123298
F01_transcript_45247	NA	NA	NA	F01_transcript_45247
F01_transcript_150314	NA	NA	NA	F01_transcript_150314
F01_transcript_73454	F01_transcript_73454	F01_transcript_73454	F01_transcript_73454	F01_transcript_73454
F01_transcript_154968	NA	NA	F01_transcript_154968	NA
F01_transcript_59428	NA	NA	F01_transcript_59428	NA

F01_transcript_157700	NA	F01_transcript_157700	F01_transcript_157700	NA
F01_transcript_85606	NA	NA	NA	F01_transcript_85606
F01_transcript_129019	NA	NA	NA	F01_transcript_129019
F01_transcript_84277	NA	NA	NA	F01_transcript_84277
F01_transcript_153204	NA	NA	NA	F01_transcript_153204
F01_transcript_65347	NA	NA	NA	F01_transcript_65347
F01_transcript_15887	NA	NA	F01_transcript_15887	NA
F01_transcript_110228	NA	NA	NA	F01_transcript_110228
F01_transcript_104270	NA	NA	NA	F01_transcript_104270
F01_transcript_39289	NA	NA	NA	F01_transcript_39289
F01_transcript_85283	NA	NA	NA	F01_transcript_85283
F01_transcript_135193	F01_transcript_135193	F01_transcript_135193	F01_transcript_135193	F01_transcript_135193
F01_transcript_120804	NA	NA	NA	F01_transcript_120804
F01_transcript_54000	F01_transcript_54000	F01_transcript_54000	F01_transcript_54000	F01_transcript_54000

F01_transcript_55861	NA	F01_transcript_55861	NA	NA
F01_transcript_103449	F01_transcript_103449	F01_transcript_103449	F01_transcript_103449	NA
F01_transcript_154493	NA	NA	NA	F01_transcript_154493
F01_transcript_66930	NA	NA	NA	F01_transcript_66930
F01_transcript_162788	NA	F01_transcript_162788	NA	NA
F01_transcript_102774	NA	F01_transcript_102774	NA	NA
F01_transcript_121659	NA	F01_transcript_121659	NA	F01_transcript_121659
F01_transcript_150482	F01_transcript_150482	F01_transcript_150482	F01_transcript_150482	F01_transcript_150482
F01_transcript_50231	NA	F01_transcript_50231	NA	F01_transcript_50231
F01_transcript_53391	F01_transcript_53391	F01_transcript_53391	F01_transcript_53391	F01_transcript_53391
F01_transcript_68417	NA	F01_transcript_68417	F01_transcript_68417	NA
F01_transcript_110990	NA	NA	NA	F01_transcript_110990
F01_transcript_114555	NA	F01_transcript_114555	NA	F01_transcript_114555
F01_transcript_145695	NA	F01_transcript_145695	NA	NA

F01_transcript_62426	NA	NA	NA	F01_transcript_62426
F01_transcript_89787	NA	NA	NA	F01_transcript_89787
F01_transcript_18281	NA	NA	NA	F01_transcript_18281
F01_transcript_63379	NA	NA	NA	F01_transcript_63379
F01_transcript_83672	NA	F01_transcript_83672	NA	F01_transcript_83672
F01_transcript_124263	NA	F01_transcript_124263	F01_transcript_124263	NA
F01_transcript_105865	NA	NA	NA	F01_transcript_105865
F01_transcript_19067	F01_transcript_19067	F01_transcript_19067	F01_transcript_19067	F01_transcript_19067
F01_transcript_6150	NA	NA	NA	F01_transcript_6150
F01_transcript_26304	F01_transcript_26304	F01_transcript_26304	F01_transcript_26304	F01_transcript_26304
F01_transcript_118637	NA	F01_transcript_118637	NA	NA
F01_transcript_81147	F01_transcript_81147	F01_transcript_81147	F01_transcript_81147	F01_transcript_81147
F01_transcript_6671	F01_transcript_6671	F01_transcript_6671	F01_transcript_6671	NA
F01_transcript_165365	NA	NA	NA	F01_transcript_165365

F01_transcript_81169	NA	F01_transcript_81169	NA	NA
F01_transcript_63370	F01_transcript_63370	F01_transcript_63370	NA	F01_transcript_63370
F01_transcript_96939	NA	NA	NA	F01_transcript_96939
F01_transcript_66405	NA	F01_transcript_66405	NA	NA
F01_transcript_153679	NA	F01_transcript_153679	NA	NA
F01_transcript_86971	NA	NA	NA	F01_transcript_86971
F01_transcript_48122	NA	NA	NA	F01_transcript_48122
F01_transcript_1689	NA	NA	NA	F01_transcript_1689
F01_transcript_106910	NA	NA	NA	F01_transcript_106910
F01_transcript_66371	F01_transcript_66371	F01_transcript_66371	F01_transcript_66371	F01_transcript_66371
F01_transcript_63956	F01_transcript_63956	F01_transcript_63956	F01_transcript_63956	NA
F01_transcript_131799	NA	F01_transcript_131799	NA	NA
F01_transcript_20373	NA	NA	NA	F01_transcript_20373
F01_transcript_155999	NA	NA	NA	F01_transcript_155999

F01_transcript_88743	NA	NA	NA	F01_transcript_88743
F01_transcript_127544	F01_transcript_127544	F01_transcript_127544	NA	NA
F01_transcript_86727	NA	F01_transcript_86727	NA	F01_transcript_86727
F01_transcript_56143	NA	F01_transcript_56143	NA	NA
F01_transcript_165326	NA	F01_transcript_165326	NA	F01_transcript_165326
F01_transcript_112612	NA	NA	NA	F01_transcript_112612
F01_transcript_142917	F01_transcript_142917	F01_transcript_142917	F01_transcript_142917	F01_transcript_142917
F01_transcript_112811	NA	F01_transcript_112811	NA	NA
F01_transcript_7737	NA	F01_transcript_7737	F01_transcript_7737	F01_transcript_7737
F01_transcript_153928	NA	F01_transcript_153928	NA	NA
F01_transcript_108360	F01_transcript_108360	F01_transcript_108360	F01_transcript_108360	F01_transcript_108360
F01_transcript_71695	F01_transcript_71695	F01_transcript_71695	F01_transcript_71695	F01_transcript_71695
F01_transcript_54481	NA	F01_transcript_54481	F01_transcript_54481	NA
F01_transcript_101149	NA	F01_transcript_101149	NA	NA

F01_transcript_136648	NA	NA	NA	F01_transcript_136648
F01_transcript_82110	NA	F01_transcript_82110	NA	NA
F01_transcript_116906	NA	F01_transcript_116906	F01_transcript_116906	NA
F01_transcript_140350	NA	NA	NA	F01_transcript_140350
F01_transcript_78151	NA	NA	NA	F01_transcript_78151
F01_transcript_63378	NA	NA	NA	F01_transcript_63378
F01_transcript_115934	NA	F01_transcript_115934	NA	NA
F01_transcript_59853	NA	F01_transcript_59853	NA	NA
F01_transcript_136468	NA	F01_transcript_136468	NA	F01_transcript_136468
F01_transcript_73439	NA	NA	NA	F01_transcript_73439
F01_transcript_85971	NA	NA	NA	F01_transcript_85971
F01_transcript_41604	NA	F01_transcript_41604	F01_transcript_41604	NA
F01_transcript_123682	F01_transcript_123682	F01_transcript_123682	F01_transcript_123682	F01_transcript_123682
F01_transcript_118436	F01_transcript_118436	F01_transcript_118436	NA	NA

F01_transcript_63867	NA	NA	NA	F01_transcript_63867
F01_transcript_140922	NA	F01_transcript_140922	NA	F01_transcript_140922
F01_transcript_120407	F01_transcript_120407	F01_transcript_120407	F01_transcript_120407	F01_transcript_120407
F01_transcript_5748	F01_transcript_5748	F01_transcript_5748	NA	F01_transcript_5748
F01_transcript_88084	NA	NA	NA	F01_transcript_88084
F01_transcript_107546	NA	NA	NA	F01_transcript_107546
F01_transcript_25405	NA	F01_transcript_25405	NA	F01_transcript_25405
F01_transcript_128782	F01_transcript_128782	F01_transcript_128782	F01_transcript_128782	F01_transcript_128782
F01_transcript_17350	NA	NA	NA	F01_transcript_17350
F01_transcript_55952	NA	F01_transcript_55952	NA	NA
F01_transcript_122498	NA	NA	NA	F01_transcript_122498
F01_transcript_107570	F01_transcript_107570	F01_transcript_107570	F01_transcript_107570	F01_transcript_107570
F01_transcript_111124	F01_transcript_111124	F01_transcript_111124	NA	NA
F01_transcript_48998	NA	F01_transcript_48998	F01_transcript_48998	F01_transcript_48998

F01_transcript_154966	F01_transcript_154966	F01_transcript_154966	F01_transcript_154966	F01_transcript_154966
F01_transcript_5107	NA	F01_transcript_5107	NA	NA
F01_transcript_56374	NA	NA	F01_transcript_56374	F01_transcript_56374
F01_transcript_128600	NA	F01_transcript_128600	NA	F01_transcript_128600
F01_transcript_31858	NA	NA	NA	F01_transcript_31858
F01_transcript_64772	NA	F01_transcript_64772	NA	NA
F01_transcript_77717	NA	F01_transcript_77717	NA	NA
F01_transcript_90914	NA	NA	NA	F01_transcript_90914
F01_transcript_159703	NA	NA	NA	F01_transcript_159703
F01_transcript_75124	F01_transcript_75124	F01_transcript_75124	F01_transcript_75124	F01_transcript_75124
F01_transcript_156853	F01_transcript_156853	F01_transcript_156853	NA	NA
F01_transcript_108321	NA	F01_transcript_108321	F01_transcript_108321	F01_transcript_108321
F01_transcript_115511	F01_transcript_115511	F01_transcript_115511	F01_transcript_115511	F01_transcript_115511
F01_transcript_131786	NA	F01_transcript_131786	NA	F01_transcript_131786

F01_transcript_59032	NA	NA	F01_transcript_59032	NA
F01_transcript_125950	NA	F01_transcript_125950	NA	NA
F01_transcript_55515	NA	F01_transcript_55515	NA	NA
F01_transcript_149024	NA	NA	NA	F01_transcript_149024
F01_transcript_105727	F01_transcript_105727	F01_transcript_105727	F01_transcript_105727	F01_transcript_105727
F01_transcript_108789	F01_transcript_108789	F01_transcript_108789	NA	NA
F01_transcript_95315	NA	NA	NA	F01_transcript_95315
F01_transcript_100698	F01_transcript_100698	F01_transcript_100698	F01_transcript_100698	F01_transcript_100698
F01_transcript_68420	NA	F01_transcript_68420	NA	NA
F01_transcript_108301	F01_transcript_108301	F01_transcript_108301	F01_transcript_108301	F01_transcript_108301
F01_transcript_124852	NA	NA	NA	F01_transcript_124852
F01_transcript_72683	F01_transcript_72683	F01_transcript_72683	F01_transcript_72683	F01_transcript_72683
F01_transcript_14802	NA	F01_transcript_14802	F01_transcript_14802	NA
F01_transcript_86393	F01_transcript_86393	F01_transcript_86393	F01_transcript_86393	NA

F01_transcript_15317	NA	NA	NA	F01_transcript_15317
F01_transcript_163798	NA	F01_transcript_163798	NA	NA
F01_transcript_58945	F01_transcript_58945	F01_transcript_58945	F01_transcript_58945	NA
F01_transcript_41291	NA	NA	NA	F01_transcript_41291
F01_transcript_116476	F01_transcript_116476	F01_transcript_116476	F01_transcript_116476	F01_transcript_116476
F01_transcript_68755	F01_transcript_68755	F01_transcript_68755	F01_transcript_68755	NA
F01_transcript_117797	NA	NA	NA	F01_transcript_117797
F01_transcript_95992	NA	NA	NA	F01_transcript_95992
F01_transcript_100088	NA	F01_transcript_100088	NA	NA
F01_transcript_76261	F01_transcript_76261	F01_transcript_76261	F01_transcript_76261	F01_transcript_76261
F01_transcript_89345	NA	NA	NA	F01_transcript_89345
F01_transcript_107121	NA	F01_transcript_107121	NA	NA
F01_transcript_63159	F01_transcript_63159	F01_transcript_63159	NA	NA
F01_transcript_108361	NA	F01_transcript_108361	NA	F01_transcript_108361

F01_transcript_77808	NA	NA	NA	F01_transcript_77808
F01_transcript_67150	F01_transcript_67150	F01_transcript_67150	F01_transcript_67150	F01_transcript_67150
F01_transcript_53139	NA	F01_transcript_53139	F01_transcript_53139	F01_transcript_53139
F01_transcript_111458	F01_transcript_111458	F01_transcript_111458	F01_transcript_111458	F01_transcript_111458
F01_transcript_123035	NA	NA	NA	F01_transcript_123035
F01_transcript_109566	F01_transcript_109566	F01_transcript_109566	NA	F01_transcript_109566
F01_transcript_10090	NA	NA	NA	F01_transcript_10090
F01_transcript_100771	NA	NA	NA	F01_transcript_100771
F01_transcript_105584	F01_transcript_105584	F01_transcript_105584	NA	F01_transcript_105584
F01_transcript_12665	F01_transcript_12665	F01_transcript_12665	F01_transcript_12665	F01_transcript_12665
F01_transcript_156148	F01_transcript_156148	NA	NA	F01_transcript_156148
F01_transcript_66263	NA	F01_transcript_66263	NA	NA
F01_transcript_97618	NA	F01_transcript_97618	NA	NA
F01_transcript_22699	NA	NA	NA	F01_transcript_22699

F01_transcript_119008	F01_transcript_119008	F01_transcript_119008	F01_transcript_119008	F01_transcript_119008
F01_transcript_104688	NA	NA	NA	F01_transcript_104688
F01_transcript_50017	NA	F01_transcript_50017	NA	NA
F01_transcript_123885	F01_transcript_123885	F01_transcript_123885	F01_transcript_123885	F01_transcript_123885
F01_transcript_120242	NA	NA	NA	F01_transcript_120242
F01_transcript_126498	NA	NA	NA	F01_transcript_126498
F01_transcript_94769	F01_transcript_94769	F01_transcript_94769	F01_transcript_94769	F01_transcript_94769
F01_transcript_79030	NA	NA	NA	F01_transcript_79030
F01_transcript_62437	NA	F01_transcript_62437	NA	F01_transcript_62437
F01_transcript_132089	NA	F01_transcript_132089	NA	NA
F01_transcript_121173	F01_transcript_121173	F01_transcript_121173	F01_transcript_121173	F01_transcript_121173
F01_transcript_117229	NA	F01_transcript_117229	F01_transcript_117229	F01_transcript_117229
F01_transcript_61621	NA	F01_transcript_61621	NA	NA
F01_transcript_8107	F01_transcript_8107	F01_transcript_8107	F01_transcript_8107	F01_transcript_8107

F01_transcript_82364	F01_transcript_82364	F01_transcript_82364	F01_transcript_82364	F01_transcript_82364
F01_transcript_54251	NA	F01_transcript_54251	NA	NA
F01_transcript_125983	NA	F01_transcript_125983	NA	NA
F01_transcript_104595	NA	F01_transcript_104595	NA	F01_transcript_104595
F01_transcript_79009	NA	NA	NA	F01_transcript_79009
F01_transcript_80401	NA	F01_transcript_80401	NA	F01_transcript_80401
F01_transcript_134815	F01_transcript_134815	F01_transcript_134815	NA	F01_transcript_134815
F01_transcript_82678	NA	NA	NA	F01_transcript_82678
F01_transcript_90804	F01_transcript_90804	F01_transcript_90804	NA	NA
F01_transcript_77798	NA	F01_transcript_77798	NA	NA
F01_transcript_120296	NA	F01_transcript_120296	NA	F01_transcript_120296
F01_transcript_62981	NA	NA	NA	F01_transcript_62981
F01_transcript_161777	F01_transcript_161777	F01_transcript_161777	NA	F01_transcript_161777
F01_transcript_92903	NA	F01_transcript_92903	NA	NA

F01_transcript_122015	NA	F01_transcript_122015	NA	F01_transcript_122015
F01_transcript_23586	F01_transcript_23586	F01_transcript_23586	F01_transcript_23586	F01_transcript_23586
F01_transcript_106363	NA	F01_transcript_106363	NA	F01_transcript_106363
F01_transcript_58981	NA	NA	F01_transcript_58981	NA
F01_transcript_163053	NA	F01_transcript_163053	F01_transcript_163053	F01_transcript_163053
F01_transcript_64886	NA	F01_transcript_64886	NA	NA
F01_transcript_92814	F01_transcript_92814	F01_transcript_92814	F01_transcript_92814	F01_transcript_92814
F01_transcript_102988	NA	NA	NA	F01_transcript_102988
F01_transcript_123729	NA	NA	NA	F01_transcript_123729
F01_transcript_39713	NA	NA	NA	F01_transcript_39713
F01_transcript_27080	NA	NA	F01_transcript_27080	F01_transcript_27080
F01_transcript_117767	NA	NA	NA	F01_transcript_117767
F01_transcript_58480	NA	NA	NA	F01_transcript_58480
F01_transcript_165413	F01_transcript_165413	NA	F01_transcript_165413	F01_transcript_165413

F01_transcript_160762	NA	F01_transcript_160762	F01_transcript_160762	F01_transcript_160762
F01_transcript_59118	F01_transcript_59118	F01_transcript_59118	NA	F01_transcript_59118
F01_transcript_81891	NA	F01_transcript_81891	NA	NA
F01_transcript_68695	F01_transcript_68695	F01_transcript_68695	NA	NA
F01_transcript_59377	NA	F01_transcript_59377	F01_transcript_59377	NA
F01_transcript_123829	F01_transcript_123829	F01_transcript_123829	NA	NA
F01_transcript_157597	NA	F01_transcript_157597	F01_transcript_157597	F01_transcript_157597
F01_transcript_116409	F01_transcript_116409	F01_transcript_116409	F01_transcript_116409	F01_transcript_116409
F01_transcript_72381	F01_transcript_72381	F01_transcript_72381	NA	NA
F01_transcript_127827	F01_transcript_127827	F01_transcript_127827	NA	F01_transcript_127827
F01_transcript_71583	NA	F01_transcript_71583	NA	F01_transcript_71583
F01_transcript_96371	F01_transcript_96371	F01_transcript_96371	F01_transcript_96371	F01_transcript_96371
F01_transcript_127517	F01_transcript_127517	F01_transcript_127517	NA	NA
F01_transcript_130638	NA	NA	NA	F01_transcript_130638

F01_transcript_93971	NA	F01_transcript_93971	NA	F01_transcript_93971
F01_transcript_81431	F01_transcript_81431	F01_transcript_81431	F01_transcript_81431	F01_transcript_81431
F01_transcript_72422	NA	F01_transcript_72422	NA	NA
F01_transcript_22507	F01_transcript_22507	F01_transcript_22507	NA	F01_transcript_22507
F01_transcript_149390	NA	NA	NA	F01_transcript_149390
F01_transcript_99234	NA	F01_transcript_99234	NA	NA
F01_transcript_488	NA	NA	NA	F01_transcript_488
F01_transcript_124876	NA	NA	NA	F01_transcript_124876
F01_transcript_131310	F01_transcript_131310	F01_transcript_131310	F01_transcript_131310	F01_transcript_131310
F01_transcript_139036	NA	F01_transcript_139036	NA	NA
F01_transcript_71828	NA	F01_transcript_71828	NA	NA
F01_transcript_75831	NA	F01_transcript_75831	NA	NA
F01_transcript_126181	F01_transcript_126181	F01_transcript_126181	NA	NA
F01_transcript_102481	NA	F01_transcript_102481	F01_transcript_102481	NA

F01_transcript_120365	NA	F01_transcript_120365	NA	F01_transcript_120365
F01_transcript_47479	NA	F01_transcript_47479	NA	F01_transcript_47479
F01_transcript_165875	NA	F01_transcript_165875	NA	NA
F01_transcript_157297	NA	NA	NA	F01_transcript_157297
F01_transcript_41605	NA	NA	F01_transcript_41605	F01_transcript_41605
F01_transcript_103878	NA	NA	NA	F01_transcript_103878
F01_transcript_79345	F01_transcript_79345	NA	F01_transcript_79345	F01_transcript_79345
F01_transcript_153387	NA	NA	NA	F01_transcript_153387
F01_transcript_82743	NA	F01_transcript_82743	F01_transcript_82743	NA
F01_transcript_120836	F01_transcript_120836	F01_transcript_120836	NA	F01_transcript_120836
F01_transcript_126628	NA	F01_transcript_126628	F01_transcript_126628	NA
F01_transcript_141476	NA	F01_transcript_141476	NA	F01_transcript_141476
F01_transcript_149567	F01_transcript_149567	F01_transcript_149567	F01_transcript_149567	F01_transcript_149567
F01_transcript_103544	F01_transcript_103544	F01_transcript_103544	NA	NA

F01_transcript_72604	NA	NA	NA	F01_transcript_72604
F01_transcript_130242	F01_transcript_130242	F01_transcript_130242	NA	NA
F01_transcript_86899	NA	F01_transcript_86899	F01_transcript_86899	F01_transcript_86899
F01_transcript_124085	NA	NA	NA	F01_transcript_124085
F01_transcript_96565	F01_transcript_96565	F01_transcript_96565	F01_transcript_96565	F01_transcript_96565
F01_transcript_112010	F01_transcript_112010	F01_transcript_112010	F01_transcript_112010	F01_transcript_112010
F01_transcript_95046	NA	F01_transcript_95046	NA	F01_transcript_95046
F01_transcript_150686	F01_transcript_150686	F01_transcript_150686	F01_transcript_150686	NA
F01_transcript_156697	NA	F01_transcript_156697	NA	NA
F01_transcript_153860	NA	F01_transcript_153860	NA	NA
F01_transcript_22539	NA	NA	NA	F01_transcript_22539
F01_transcript_68277	NA	NA	NA	F01_transcript_68277
F01_transcript_146314	NA	NA	NA	F01_transcript_146314
F01_transcript_84963	NA	F01_transcript_84963	NA	F01_transcript_84963

F01_transcript_154539	NA	F01_transcript_154539	NA	F01_transcript_154539
F01_transcript_9873	NA	F01_transcript_9873	NA	NA
F01_transcript_100393	NA	F01_transcript_100393	NA	NA
F01_transcript_167622	NA	F01_transcript_167622	NA	NA
F01_transcript_109491	NA	NA	NA	F01_transcript_109491
F01_transcript_93165	NA	NA	NA	F01_transcript_93165
F01_transcript_84525	NA	F01_transcript_84525	NA	NA
F01_transcript_140063	NA	NA	F01_transcript_140063	F01_transcript_140063
F01_transcript_147346	F01_transcript_147346	F01_transcript_147346	F01_transcript_147346	F01_transcript_147346
F01_transcript_75625	F01_transcript_75625	F01_transcript_75625	F01_transcript_75625	F01_transcript_75625
F01_transcript_62023	NA	NA	NA	F01_transcript_62023
F01_transcript_102130	NA	NA	NA	F01_transcript_102130
F01_transcript_9820	NA	NA	NA	F01_transcript_9820
F01_transcript_66183	F01_transcript_66183	F01_transcript_66183	F01_transcript_66183	F01_transcript_66183

F01_transcript_137455	NA	F01_transcript_137455	NA	F01_transcript_137455
F01_transcript_142196	NA	F01_transcript_142196	NA	F01_transcript_142196
F01_transcript_1800	NA	NA	NA	F01_transcript_1800
F01_transcript_24247	NA	NA	NA	F01_transcript_24247
F01_transcript_122400	NA	NA	NA	F01_transcript_122400
F01_transcript_60149	F01_transcript_60149	F01_transcript_60149	F01_transcript_60149	F01_transcript_60149
F01_transcript_162029	NA	F01_transcript_162029	NA	NA
F01_transcript_162326	NA	NA	NA	F01_transcript_162326
F01_transcript_145706	NA	F01_transcript_145706	F01_transcript_145706	NA
F01_transcript_126025	F01_transcript_126025	F01_transcript_126025	NA	NA
F01_transcript_149419	NA	NA	NA	F01_transcript_149419
F01_transcript_75799	NA	F01_transcript_75799	NA	NA
F01_transcript_144816	NA	NA	NA	F01_transcript_144816
F01_transcript_140083	NA	NA	NA	F01_transcript_140083

F01_transcript_77363	NA	NA	NA	F01_transcript_77363
F01_transcript_22869	NA	NA	NA	F01_transcript_22869
F01_transcript_131342	F01_transcript_131342	F01_transcript_131342	F01_transcript_131342	F01_transcript_131342
F01_transcript_75257	NA	NA	NA	F01_transcript_75257
F01_transcript_69755	NA	NA	NA	F01_transcript_69755
F01_transcript_57929	NA	NA	NA	F01_transcript_57929
F01_transcript_47392	NA	F01_transcript_47392	NA	NA
F01_transcript_88682	F01_transcript_88682	F01_transcript_88682	F01_transcript_88682	F01_transcript_88682
F01_transcript_50476	NA	F01_transcript_50476	NA	NA
F01_transcript_91403	NA	NA	NA	F01_transcript_91403
F01_transcript_90440	F01_transcript_90440	F01_transcript_90440	F01_transcript_90440	F01_transcript_90440
F01_transcript_115017	NA	NA	NA	F01_transcript_115017
F01_transcript_53190	F01_transcript_53190	F01_transcript_53190	F01_transcript_53190	F01_transcript_53190
F01_transcript_112559	NA	NA	NA	F01_transcript_112559

F01_transcript_41827	NA	NA	NA	F01_transcript_41827
F01_transcript_32393	NA	F01_transcript_32393	NA	NA
F01_transcript_114121	NA	NA	NA	F01_transcript_114121
F01_transcript_124169	NA	F01_transcript_124169	NA	NA
F01_transcript_142067	NA	NA	NA	F01_transcript_142067
F01_transcript_88203	NA	F01_transcript_88203	NA	NA
F01_transcript_117930	NA	F01_transcript_117930	NA	F01_transcript_117930
F01_transcript_157430	NA	NA	NA	F01_transcript_157430
F01_transcript_31431	NA	NA	NA	F01_transcript_31431
F01_transcript_77169	NA	F01_transcript_77169	NA	NA
F01_transcript_144996	NA	NA	NA	F01_transcript_144996
F01_transcript_84798	NA	NA	NA	F01_transcript_84798
F01_transcript_148082	NA	F01_transcript_148082	NA	NA
F01_transcript_129400	NA	F01_transcript_129400	F01_transcript_129400	F01_transcript_129400

F01_transcript_154998	NA	F01_transcript_154998	NA	NA
F01_transcript_77559	NA	F01_transcript_77559	NA	NA
F01_transcript_159381	NA	F01_transcript_159381	NA	F01_transcript_159381
F01_transcript_128271	NA	F01_transcript_128271	NA	NA
F01_transcript_88510	NA	F01_transcript_88510	NA	NA
F01_transcript_162719	F01_transcript_162719	F01_transcript_162719	NA	F01_transcript_162719
F01_transcript_132074	F01_transcript_132074	F01_transcript_132074	F01_transcript_132074	F01_transcript_132074
F01_transcript_127521	F01_transcript_127521	F01_transcript_127521	NA	NA
F01_transcript_160637	NA	F01_transcript_160637	NA	NA
F01_transcript_160471	NA	NA	NA	F01_transcript_160471
F01_transcript_154302	F01_transcript_154302	F01_transcript_154302	F01_transcript_154302	F01_transcript_154302
F01_transcript_49060	NA	NA	NA	F01_transcript_49060
F01_transcript_127781	NA	F01_transcript_127781	NA	NA
F01_transcript_57919	NA	F01_transcript_57919	F01_transcript_57919	F01_transcript_57919

F01_transcript_31758	NA	NA	NA	F01_transcript_31758
F01_transcript_108706	F01_transcript_108706	F01_transcript_108706	NA	F01_transcript_108706
F01_transcript_143977	NA	NA	NA	F01_transcript_143977
F01_transcript_148000	F01_transcript_148000	F01_transcript_148000	F01_transcript_148000	F01_transcript_148000
F01_transcript_139960	NA	NA	NA	F01_transcript_139960
F01_transcript_165313	NA	NA	NA	F01_transcript_165313
F01_transcript_82442	F01_transcript_82442	F01_transcript_82442	F01_transcript_82442	F01_transcript_82442
F01_transcript_116426	NA	NA	NA	F01_transcript_116426
F01_transcript_85208	NA	NA	F01_transcript_85208	NA
F01_transcript_43617	NA	NA	NA	F01_transcript_43617
F01_transcript_125612	NA	F01_transcript_125612	NA	NA
F01_transcript_71244	F01_transcript_71244	F01_transcript_71244	F01_transcript_71244	F01_transcript_71244
F01_transcript_100684	F01_transcript_100684	F01_transcript_100684	F01_transcript_100684	F01_transcript_100684
F01_transcript_72075	NA	F01_transcript_72075	NA	NA

F01_transcript_104240	F01_transcript_104240	F01_transcript_104240	F01_transcript_104240	F01_transcript_104240
F01_transcript_119626	NA	NA	NA	F01_transcript_119626
F01_transcript_104883	NA	NA	NA	F01_transcript_104883
F01_transcript_107217	NA	F01_transcript_107217	NA	F01_transcript_107217
F01_transcript_122186	F01_transcript_122186	F01_transcript_122186	NA	F01_transcript_122186
F01_transcript_126712	NA	NA	NA	F01_transcript_126712
F01_transcript_56235	NA	NA	F01_transcript_56235	F01_transcript_56235
F01_transcript_141227	NA	NA	NA	F01_transcript_141227
F01_transcript_141838	NA	F01_transcript_141838	NA	NA
F01_transcript_164241	NA	NA	NA	F01_transcript_164241
F01_transcript_71596	NA	F01_transcript_71596	NA	NA
F01_transcript_156444	NA	F01_transcript_156444	F01_transcript_156444	F01_transcript_156444
F01_transcript_142828	NA	F01_transcript_142828	NA	NA
F01_transcript_36301	NA	F01_transcript_36301	NA	NA

F01_transcript_118945	NA	F01_transcript_118945	NA	F01_transcript_118945
F01_transcript_67229	F01_transcript_67229	F01_transcript_67229	F01_transcript_67229	F01_transcript_67229
F01_transcript_37479	NA	NA	NA	F01_transcript_37479
F01_transcript_125875	F01_transcript_125875	F01_transcript_125875	F01_transcript_125875	F01_transcript_125875
F01_transcript_125894	F01_transcript_125894	F01_transcript_125894	NA	F01_transcript_125894
F01_transcript_117584	NA	F01_transcript_117584	F01_transcript_117584	F01_transcript_117584
F01_transcript_16833	NA	NA	NA	F01_transcript_16833
F01_transcript_49245	NA	F01_transcript_49245	F01_transcript_49245	F01_transcript_49245
F01_transcript_125957	NA	NA	NA	F01_transcript_125957
F01_transcript_134432	NA	NA	NA	F01_transcript_134432
F01_transcript_2844	NA	NA	NA	F01_transcript_2844
F01_transcript_81761	NA	F01_transcript_81761	NA	NA
F01_transcript_4845	NA	NA	NA	F01_transcript_4845
F01_transcript_50441	F01_transcript_50441	F01_transcript_50441	F01_transcript_50441	F01_transcript_50441

F01_transcript_143454	NA	NA	NA	F01_transcript_143454
F01_transcript_56474	F01_transcript_56474	F01_transcript_56474	F01_transcript_56474	NA
F01_transcript_140343	NA	F01_transcript_140343	NA	NA
F01_transcript_121825	NA	F01_transcript_121825	NA	NA
F01_transcript_56927	NA	NA	F01_transcript_56927	NA
F01_transcript_92879	NA	F01_transcript_92879	NA	NA
F01_transcript_70861	NA	F01_transcript_70861	F01_transcript_70861	F01_transcript_70861
F01_transcript_44258	NA	F01_transcript_44258	NA	NA
F01_transcript_5580	F01_transcript_5580	F01_transcript_5580	F01_transcript_5580	F01_transcript_5580
F01_transcript_44493	NA	NA	F01_transcript_44493	NA
F01_transcript_103791	NA	F01_transcript_103791	NA	NA
F01_transcript_105960	NA	F01_transcript_105960	NA	F01_transcript_105960
F01_transcript_88501	NA	NA	NA	F01_transcript_88501
F01_transcript_140996	NA	F01_transcript_140996	NA	F01_transcript_140996

F01_transcript_108444	NA	NA	NA	F01_transcript_108444
F01_transcript_57978	NA	NA	NA	F01_transcript_57978
F01_transcript_94555	F01_transcript_94555	F01_transcript_94555	F01_transcript_94555	F01_transcript_94555
F01_transcript_146533	NA	F01_transcript_146533	NA	F01_transcript_146533
F01_transcript_109247	F01_transcript_109247	F01_transcript_109247	NA	NA
F01_transcript_121315	NA	F01_transcript_121315	F01_transcript_121315	F01_transcript_121315
F01_transcript_53724	NA	F01_transcript_53724	NA	NA
F01_transcript_60692	F01_transcript_60692	F01_transcript_60692	F01_transcript_60692	F01_transcript_60692
F01_transcript_130584	F01_transcript_130584	F01_transcript_130584	NA	NA
F01_transcript_21346	NA	NA	NA	F01_transcript_21346
F01_transcript_147686	F01_transcript_147686	F01_transcript_147686	NA	NA
F01_transcript_69643	F01_transcript_69643	F01_transcript_69643	F01_transcript_69643	F01_transcript_69643
F01_transcript_118721	F01_transcript_118721	F01_transcript_118721	NA	NA
F01_transcript_106227	NA	NA	NA	F01_transcript_106227

F01_transcript_166337	NA	NA	NA	F01_transcript_166337
F01_transcript_43306	NA	NA	NA	F01_transcript_43306
F01_transcript_136803	F01_transcript_136803	F01_transcript_136803	F01_transcript_136803	F01_transcript_136803
F01_transcript_78068	NA	F01_transcript_78068	NA	NA
F01_transcript_158809	NA	F01_transcript_158809	NA	F01_transcript_158809
F01_transcript_135660	NA	NA	NA	F01_transcript_135660
F01_transcript_90741	NA	NA	NA	F01_transcript_90741
F01_transcript_126390	NA	NA	NA	F01_transcript_126390
F01_transcript_91649	F01_transcript_91649	F01_transcript_91649	F01_transcript_91649	NA
F01_transcript_103323	NA	NA	NA	F01_transcript_103323
F01_transcript_134187	F01_transcript_134187	F01_transcript_134187	F01_transcript_134187	F01_transcript_134187
F01_transcript_156432	NA	F01_transcript_156432	NA	F01_transcript_156432
F01_transcript_109049	NA	F01_transcript_109049	NA	NA
F01_transcript_91463	NA	NA	F01_transcript_91463	F01_transcript_91463

F01_transcript_54511	NA	F01_transcript_54511	NA	F01_transcript_54511
F01_transcript_46236	NA	NA	NA	F01_transcript_46236
F01_transcript_61601	NA	NA	NA	F01_transcript_61601
F01_transcript_129718	NA	F01_transcript_129718	F01_transcript_129718	F01_transcript_129718
F01_transcript_137852	F01_transcript_137852	F01_transcript_137852	NA	F01_transcript_137852
F01_transcript_79967	NA	F01_transcript_79967	NA	NA
F01_transcript_62381	F01_transcript_62381	F01_transcript_62381	F01_transcript_62381	F01_transcript_62381
F01_transcript_4593	NA	NA	NA	F01_transcript_4593
F01_transcript_164475	NA	NA	NA	F01_transcript_164475
F01_transcript_77225	F01_transcript_77225	F01_transcript_77225	NA	F01_transcript_77225
F01_transcript_80325	NA	F01_transcript_80325	NA	NA
F01_transcript_64247	NA	F01_transcript_64247	NA	NA
F01_transcript_111694	NA	F01_transcript_111694	F01_transcript_111694	F01_transcript_111694
F01_transcript_139680	F01_transcript_139680	F01_transcript_139680	NA	NA

F01_transcript_166751	NA	NA	NA	F01_transcript_166751
F01_transcript_73263	NA	F01_transcript_73263	NA	F01_transcript_73263
F01_transcript_1639	NA	NA	NA	F01_transcript_1639
F01_transcript_117139	NA	F01_transcript_117139	NA	NA
F01_transcript_161874	NA	F01_transcript_161874	NA	NA
F01_transcript_59981	NA	F01_transcript_59981	NA	F01_transcript_59981
F01_transcript_3107	NA	NA	NA	F01_transcript_3107
F01_transcript_159437	NA	NA	NA	F01_transcript_159437
F01_transcript_58895	NA	NA	NA	F01_transcript_58895
F01_transcript_97840	NA	F01_transcript_97840	F01_transcript_97840	F01_transcript_97840
F01_transcript_148181	F01_transcript_148181	F01_transcript_148181	F01_transcript_148181	NA
F01_transcript_27259	F01_transcript_27259	F01_transcript_27259	NA	NA
F01_transcript_79486	F01_transcript_79486	F01_transcript_79486	F01_transcript_79486	F01_transcript_79486
F01_transcript_113937	NA	NA	NA	F01_transcript_113937

F01_transcript_150136	NA	F01_transcript_150136	NA	NA
F01_transcript_87078	NA	F01_transcript_87078	NA	F01_transcript_87078
F01_transcript_46383	NA	NA	NA	F01_transcript_46383
F01_transcript_44314	NA	NA	NA	F01_transcript_44314
F01_transcript_165327	NA	F01_transcript_165327	NA	NA
F01_transcript_99002	F01_transcript_99002	F01_transcript_99002	F01_transcript_99002	F01_transcript_99002
F01_transcript_127932	NA	F01_transcript_127932	NA	NA
F01_transcript_71875	NA	F01_transcript_71875	NA	NA
F01_transcript_94253	NA	F01_transcript_94253	NA	NA
F01_transcript_49878	NA	F01_transcript_49878	NA	F01_transcript_49878
F01_transcript_85713	NA	NA	F01_transcript_85713	F01_transcript_85713
F01_transcript_31085	NA	F01_transcript_31085	NA	F01_transcript_31085
F01_transcript_83787	F01_transcript_83787	F01_transcript_83787	F01_transcript_83787	F01_transcript_83787
F01_transcript_162504	F01_transcript_162504	F01_transcript_162504	NA	F01_transcript_162504

F01_transcript_98576	NA	NA	NA	F01_transcript_98576
F01_transcript_53408	NA	NA	NA	F01_transcript_53408
F01_transcript_59499	F01_transcript_59499	F01_transcript_59499	F01_transcript_59499	F01_transcript_59499
F01_transcript_77924	F01_transcript_77924	F01_transcript_77924	F01_transcript_77924	F01_transcript_77924
F01_transcript_154924	NA	F01_transcript_154924	F01_transcript_154924	F01_transcript_154924
F01_transcript_109295	NA	NA	F01_transcript_109295	F01_transcript_109295
F01_transcript_135355	NA	F01_transcript_135355	F01_transcript_135355	F01_transcript_135355
F01_transcript_77170	NA	NA	NA	F01_transcript_77170
F01_transcript_104612	NA	F01_transcript_104612	F01_transcript_104612	F01_transcript_104612
F01_transcript_51595	NA	F01_transcript_51595	NA	NA
F01_transcript_90954	NA	NA	NA	F01_transcript_90954
F01_transcript_153485	NA	F01_transcript_153485	NA	NA
F01_transcript_105068	NA	F01_transcript_105068	F01_transcript_105068	F01_transcript_105068
F01_transcript_96391	NA	F01_transcript_96391	NA	NA

F01_transcript_130307	NA	NA	NA	F01_transcript_130307
F01_transcript_72124	NA	F01_transcript_72124	NA	NA
F01_transcript_167160	NA	NA	NA	F01_transcript_167160
F01_transcript_84591	F01_transcript_84591	F01_transcript_84591	NA	NA
F01_transcript_128784	NA	NA	NA	F01_transcript_128784
F01_transcript_125779	NA	F01_transcript_125779	F01_transcript_125779	F01_transcript_125779
F01_transcript_28482	NA	NA	NA	F01_transcript_28482
F01_transcript_103899	NA	NA	NA	F01_transcript_103899
F01_transcript_136025	NA	F01_transcript_136025	F01_transcript_136025	NA
F01_transcript_117194	NA	F01_transcript_117194	NA	F01_transcript_117194
F01_transcript_50123	F01_transcript_50123	F01_transcript_50123	NA	NA
F01_transcript_44179	F01_transcript_44179	F01_transcript_44179	NA	NA
F01_transcript_75305	NA	F01_transcript_75305	NA	NA
F01_transcript_135504	NA	F01_transcript_135504	NA	NA

F01_transcript_159413	F01_transcript_159413	F01_transcript_159413	F01_transcript_159413	F01_transcript_159413
F01_transcript_47311	NA	NA	NA	F01_transcript_47311
F01_transcript_92217	NA	NA	NA	F01_transcript_92217
F01_transcript_49168	NA	NA	NA	F01_transcript_49168
F01_transcript_84032	F01_transcript_84032	F01_transcript_84032	F01_transcript_84032	F01_transcript_84032
F01_transcript_167754	F01_transcript_167754	F01_transcript_167754	F01_transcript_167754	F01_transcript_167754
F01_transcript_69057	NA	F01_transcript_69057	NA	F01_transcript_69057
F01_transcript_109576	NA	NA	NA	F01_transcript_109576
F01_transcript_77108	NA	F01_transcript_77108	NA	NA
F01_transcript_24674	NA	NA	NA	F01_transcript_24674
F01_transcript_63186	NA	NA	NA	F01_transcript_63186
F01_transcript_164974	NA	NA	NA	F01_transcript_164974
F01_transcript_121823	NA	F01_transcript_121823	NA	NA
F01_transcript_49788	NA	F01_transcript_49788	F01_transcript_49788	NA

F01_transcript_57600	NA	NA	NA	F01_transcript_57600
F01_transcript_56922	F01_transcript_56922	F01_transcript_56922	F01_transcript_56922	NA
F01_transcript_70027	NA	NA	NA	F01_transcript_70027
F01_transcript_132526	NA	F01_transcript_132526	NA	NA
F01_transcript_140212	F01_transcript_140212	F01_transcript_140212	NA	NA
F01_transcript_61249	NA	NA	NA	F01_transcript_61249
F01_transcript_30075	NA	F01_transcript_30075	NA	F01_transcript_30075
F01_transcript_108574	F01_transcript_108574	F01_transcript_108574	F01_transcript_108574	F01_transcript_108574
F01_transcript_76130	NA	F01_transcript_76130	NA	NA
F01_transcript_92128	NA	NA	NA	F01_transcript_92128
F01_transcript_167330	NA	F01_transcript_167330	NA	F01_transcript_167330
F01_transcript_131253	NA	F01_transcript_131253	NA	NA
F01_transcript_116990	NA	F01_transcript_116990	F01_transcript_116990	F01_transcript_116990
F01_transcript_121142	NA	NA	NA	F01_transcript_121142

F01_transcript_54540	NA	F01_transcript_54540	NA	NA
F01_transcript_69244	NA	F01_transcript_69244	NA	F01_transcript_69244
F01_transcript_154774	NA	NA	NA	F01_transcript_154774
F01_transcript_60591	NA	F01_transcript_60591	F01_transcript_60591	F01_transcript_60591
F01_transcript_94251	NA	F01_transcript_94251	F01_transcript_94251	F01_transcript_94251
F01_transcript_103234	NA	NA	NA	F01_transcript_103234
F01_transcript_130062	NA	F01_transcript_130062	NA	NA
F01_transcript_163758	F01_transcript_163758	F01_transcript_163758	NA	F01_transcript_163758
F01_transcript_56715	NA	F01_transcript_56715	NA	NA
F01_transcript_152620	NA	NA	F01_transcript_152620	F01_transcript_152620
F01_transcript_66610	F01_transcript_66610	NA	F01_transcript_66610	F01_transcript_66610
F01_transcript_130580	F01_transcript_130580	F01_transcript_130580	F01_transcript_130580	F01_transcript_130580
F01_transcript_125282	NA	NA	NA	F01_transcript_125282
F01_transcript_125158	F01_transcript_125158	F01_transcript_125158	F01_transcript_125158	F01_transcript_125158

F01_transcript_67914	F01_transcript_67914	F01_transcript_67914	NA	F01_transcript_67914
F01_transcript_30214	NA	F01_transcript_30214	NA	NA
F01_transcript_101273	NA	F01_transcript_101273	NA	NA
F01_transcript_115468	NA	NA	NA	F01_transcript_115468
F01_transcript_75858	NA	F01_transcript_75858	NA	NA
F01_transcript_18450	F01_transcript_18450	F01_transcript_18450	NA	NA
F01_transcript_124903	NA	F01_transcript_124903	NA	NA
F01_transcript_126618	NA	F01_transcript_126618	NA	NA
F01_transcript_54485	F01_transcript_54485	F01_transcript_54485	F01_transcript_54485	F01_transcript_54485
F01_transcript_25840	NA	NA	NA	F01_transcript_25840
F01_transcript_162648	NA	F01_transcript_162648	NA	NA
F01_transcript_55043	NA	NA	NA	F01_transcript_55043
F01_transcript_98480	F01_transcript_98480	F01_transcript_98480	F01_transcript_98480	F01_transcript_98480
F01_transcript_160504	NA	NA	NA	F01_transcript_160504

F01_transcript_59861	NA	NA	NA	F01_transcript_59861
F01_transcript_61586	F01_transcript_61586	F01_transcript_61586	F01_transcript_61586	F01_transcript_61586
F01_transcript_78165	NA	NA	NA	F01_transcript_78165
F01_transcript_87220	NA	NA	NA	F01_transcript_87220
F01_transcript_57719	F01_transcript_57719	F01_transcript_57719	F01_transcript_57719	F01_transcript_57719
F01_transcript_133795	NA	NA	NA	F01_transcript_133795
F01_transcript_125457	F01_transcript_125457	F01_transcript_125457	F01_transcript_125457	F01_transcript_125457
F01_transcript_73863	F01_transcript_73863	F01_transcript_73863	F01_transcript_73863	F01_transcript_73863
F01_transcript_83922	NA	NA	NA	F01_transcript_83922
F01_transcript_14159	NA	F01_transcript_14159	NA	NA
F01_transcript_115954	NA	F01_transcript_115954	F01_transcript_115954	F01_transcript_115954
F01_transcript_91602	NA	NA	F01_transcript_91602	F01_transcript_91602
F01_transcript_58429	F01_transcript_58429	F01_transcript_58429	F01_transcript_58429	F01_transcript_58429
F01_transcript_161344	NA	F01_transcript_161344	NA	NA

F01_transcript_37135	F01_transcript_37135	F01_transcript_37135	NA	NA
F01_transcript_134194	NA	NA	NA	F01_transcript_134194
F01_transcript_87503	NA	NA	NA	F01_transcript_87503
F01_transcript_74790	NA	NA	F01_transcript_74790	F01_transcript_74790
F01_transcript_149953	F01_transcript_149953	NA	NA	NA
F01_transcript_53760	NA	NA	NA	F01_transcript_53760
F01_transcript_127672	F01_transcript_127672	F01_transcript_127672	F01_transcript_127672	F01_transcript_127672
F01_transcript_103988	F01_transcript_103988	F01_transcript_103988	F01_transcript_103988	F01_transcript_103988
F01_transcript_81993	NA	F01_transcript_81993	NA	NA
F01_transcript_73087	F01_transcript_73087	F01_transcript_73087	F01_transcript_73087	F01_transcript_73087
F01_transcript_145886	F01_transcript_145886	F01_transcript_145886	F01_transcript_145886	F01_transcript_145886
F01_transcript_48879	NA	F01_transcript_48879	F01_transcript_48879	F01_transcript_48879
F01_transcript_52917	NA	NA	NA	F01_transcript_52917
F01_transcript_113009	NA	F01_transcript_113009	NA	NA

F01_transcript_132588	F01_transcript_132588	F01_transcript_132588	NA	NA
F01_transcript_132882	NA	F01_transcript_132882	NA	NA
F01_transcript_26170	NA	NA	NA	F01_transcript_26170
F01_transcript_90372	NA	F01_transcript_90372	NA	F01_transcript_90372
F01_transcript_3841	NA	F01_transcript_3841	NA	F01_transcript_3841
F01_transcript_61068	NA	F01_transcript_61068	NA	F01_transcript_61068
F01_transcript_143272	NA	F01_transcript_143272	F01_transcript_143272	F01_transcript_143272
F01_transcript_151762	NA	F01_transcript_151762	NA	NA
F01_transcript_78285	NA	NA	NA	F01_transcript_78285
F01_transcript_48951	NA	NA	NA	F01_transcript_48951
F01_transcript_49294	NA	NA	NA	F01_transcript_49294
F01_transcript_64665	NA	F01_transcript_64665	NA	F01_transcript_64665
F01_transcript_24530	NA	NA	NA	F01_transcript_24530
F01_transcript_139500	NA	NA	NA	F01_transcript_139500

F01_transcript_155279	F01_transcript_155279	F01_transcript_155279	F01_transcript_155279	F01_transcript_155279
F01_transcript_88757	NA	F01_transcript_88757	NA	NA
F01_transcript_116718	NA	F01_transcript_116718	NA	NA
F01_transcript_50000	F01_transcript_50000	F01_transcript_50000	NA	F01_transcript_50000
F01_transcript_109904	F01_transcript_109904	F01_transcript_109904	NA	F01_transcript_109904
F01_transcript_19526	F01_transcript_19526	F01_transcript_19526	F01_transcript_19526	F01_transcript_19526
F01_transcript_96793	NA	F01_transcript_96793	NA	F01_transcript_96793
F01_transcript_6963	NA	F01_transcript_6963	F01_transcript_6963	NA
F01_transcript_65475	NA	NA	F01_transcript_65475	NA
F01_transcript_60505	F01_transcript_60505	F01_transcript_60505	NA	F01_transcript_60505
F01_transcript_148127	F01_transcript_148127	F01_transcript_148127	F01_transcript_148127	F01_transcript_148127
F01_transcript_153436	NA	NA	NA	F01_transcript_153436
F01_transcript_85988	NA	NA	NA	F01_transcript_85988
F01_transcript_71424	NA	F01_transcript_71424	NA	NA

F01_transcript_162927	F01_transcript_162927	F01_transcript_162927	F01_transcript_162927	F01_transcript_162927
F01_transcript_130699	NA	F01_transcript_130699	F01_transcript_130699	NA
F01_transcript_125860	NA	F01_transcript_125860	NA	NA
F01_transcript_8407	NA	NA	NA	F01_transcript_8407
F01_transcript_154576	F01_transcript_154576	F01_transcript_154576	NA	F01_transcript_154576
F01_transcript_138838	F01_transcript_138838	F01_transcript_138838	F01_transcript_138838	F01_transcript_138838
F01_transcript_164934	NA	F01_transcript_164934	NA	NA
F01_transcript_146750	NA	F01_transcript_146750	F01_transcript_146750	NA
F01_transcript_74602	NA	NA	NA	F01_transcript_74602
F01_transcript_126260	NA	NA	NA	F01_transcript_126260
F01_transcript_66251	NA	F01_transcript_66251	NA	NA
F01_transcript_86950	NA	NA	NA	F01_transcript_86950
F01_transcript_123245	NA	NA	NA	F01_transcript_123245
F01_transcript_50609	F01_transcript_50609	F01_transcript_50609	F01_transcript_50609	NA

F01_transcript_87996	NA	NA	NA	F01_transcript_87996
F01_transcript_120968	F01_transcript_120968	F01_transcript_120968	F01_transcript_120968	F01_transcript_120968
F01_transcript_88630	NA	F01_transcript_88630	NA	F01_transcript_88630
F01_transcript_1576	NA	NA	NA	F01_transcript_1576
F01_transcript_117091	F01_transcript_117091	F01_transcript_117091	NA	NA
F01_transcript_94462	NA	F01_transcript_94462	NA	NA
F01_transcript_151783	F01_transcript_151783	F01_transcript_151783	NA	NA
F01_transcript_28825	NA	NA	F01_transcript_28825	F01_transcript_28825
F01_transcript_57063	NA	NA	F01_transcript_57063	NA
F01_transcript_95418	F01_transcript_95418	F01_transcript_95418	F01_transcript_95418	F01_transcript_95418
F01_transcript_158047	NA	F01_transcript_158047	F01_transcript_158047	F01_transcript_158047
F01_transcript_141733	NA	F01_transcript_141733	F01_transcript_141733	F01_transcript_141733
F01_transcript_66949	NA	F01_transcript_66949	F01_transcript_66949	F01_transcript_66949
F01_transcript_97318	F01_transcript_97318	F01_transcript_97318	F01_transcript_97318	F01_transcript_97318

F01_transcript_872	F01_transcript_872	F01_transcript_872	F01_transcript_872	F01_transcript_872
F01_transcript_90011	NA	F01_transcript_90011	NA	NA
F01_transcript_81420	NA	F01_transcript_81420	NA	NA
F01_transcript_161933	NA	F01_transcript_161933	NA	NA
F01_transcript_65250	NA	NA	F01_transcript_65250	F01_transcript_65250
F01_transcript_841	NA	F01_transcript_841	NA	F01_transcript_841
F01_transcript_144059	NA	NA	NA	F01_transcript_144059
F01_transcript_131737	F01_transcript_131737	F01_transcript_131737	F01_transcript_131737	F01_transcript_131737
F01_transcript_92995	F01_transcript_92995	F01_transcript_92995	NA	F01_transcript_92995
F01_transcript_62530	F01_transcript_62530	F01_transcript_62530	F01_transcript_62530	F01_transcript_62530
F01_transcript_164921	NA	NA	NA	F01_transcript_164921
F01_transcript_53088	F01_transcript_53088	F01_transcript_53088	F01_transcript_53088	F01_transcript_53088
F01_transcript_117124	NA	NA	NA	F01_transcript_117124
F01_transcript_94010	NA	F01_transcript_94010	NA	NA

F01_transcript_90388	F01_transcript_90388	F01_transcript_90388	NA	F01_transcript_90388
F01_transcript_163005	NA	F01_transcript_163005	NA	NA
F01_transcript_148121	F01_transcript_148121	F01_transcript_148121	NA	F01_transcript_148121
F01_transcript_84415	NA	NA	NA	F01_transcript_84415
F01_transcript_71300	NA	F01_transcript_71300	F01_transcript_71300	F01_transcript_71300
F01_transcript_73184	NA	F01_transcript_73184	NA	F01_transcript_73184
F01_transcript_49348	F01_transcript_49348	NA	F01_transcript_49348	NA
F01_transcript_161196	NA	NA	NA	F01_transcript_161196
F01_transcript_20504	NA	NA	NA	F01_transcript_20504
F01_transcript_81611	NA	NA	NA	F01_transcript_81611
F01_transcript_94965	NA	F01_transcript_94965	F01_transcript_94965	NA
F01_transcript_119239	F01_transcript_119239	F01_transcript_119239	F01_transcript_119239	F01_transcript_119239
F01_transcript_124863	F01_transcript_124863	NA	NA	NA
F01_transcript_159622	NA	NA	NA	F01_transcript_159622

F01_transcript_3547	NA	F01_transcript_3547	F01_transcript_3547	F01_transcript_3547
F01_transcript_124356	F01_transcript_124356	F01_transcript_124356	NA	F01_transcript_124356
F01_transcript_145514	NA	NA	NA	F01_transcript_145514
F01_transcript_152299	F01_transcript_152299	F01_transcript_152299	F01_transcript_152299	F01_transcript_152299
F01_transcript_92301	F01_transcript_92301	F01_transcript_92301	F01_transcript_92301	F01_transcript_92301
F01_transcript_70072	NA	F01_transcript_70072	NA	NA
F01_transcript_50285	NA	F01_transcript_50285	NA	F01_transcript_50285
F01_transcript_48216	NA	F01_transcript_48216	NA	NA
F01_transcript_44161	NA	NA	NA	F01_transcript_44161
F01_transcript_59408	NA	NA	F01_transcript_59408	F01_transcript_59408
F01_transcript_52379	NA	F01_transcript_52379	NA	F01_transcript_52379
F01_transcript_136095	NA	NA	NA	F01_transcript_136095
F01_transcript_85504	NA	F01_transcript_85504	F01_transcript_85504	F01_transcript_85504
F01_transcript_165647	F01_transcript_165647	F01_transcript_165647	F01_transcript_165647	F01_transcript_165647

F01_transcript_50647	F01_transcript_50647	F01_transcript_50647	F01_transcript_50647	F01_transcript_50647
F01_transcript_21444	NA	NA	NA	F01_transcript_21444
F01_transcript_94073	NA	F01_transcript_94073	NA	NA
F01_transcript_65505	NA	NA	F01_transcript_65505	NA
F01_transcript_81913	NA	NA	NA	F01_transcript_81913
F01_transcript_56975	NA	NA	F01_transcript_56975	NA
F01_transcript_108168	NA	F01_transcript_108168	NA	F01_transcript_108168
F01_transcript_97094	F01_transcript_97094	F01_transcript_97094	F01_transcript_97094	F01_transcript_97094
F01_transcript_68442	NA	NA	NA	F01_transcript_68442
F01_transcript_157283	NA	NA	NA	F01_transcript_157283
F01_transcript_124555	NA	F01_transcript_124555	NA	F01_transcript_124555
F01_transcript_7493	F01_transcript_7493	F01_transcript_7493	F01_transcript_7493	F01_transcript_7493
F01_transcript_77800	NA	F01_transcript_77800	NA	F01_transcript_77800
F01_transcript_50347	F01_transcript_50347	F01_transcript_50347	NA	F01_transcript_50347

F01_transcript_88751	NA	F01_transcript_88751	NA	F01_transcript_88751
F01_transcript_113514	NA	F01_transcript_113514	F01_transcript_113514	F01_transcript_113514
F01_transcript_102949	NA	NA	F01_transcript_102949	F01_transcript_102949
F01_transcript_69213	NA	NA	NA	F01_transcript_69213
F01_transcript_101434	NA	F01_transcript_101434	NA	NA
F01_transcript_150331	F01_transcript_150331	F01_transcript_150331	NA	F01_transcript_150331
F01_transcript_49690	NA	NA	NA	F01_transcript_49690
F01_transcript_164323	F01_transcript_164323	F01_transcript_164323	F01_transcript_164323	F01_transcript_164323
F01_transcript_129945	NA	F01_transcript_129945	NA	NA
F01_transcript_127104	F01_transcript_127104	F01_transcript_127104	F01_transcript_127104	F01_transcript_127104
F01_transcript_97209	NA	NA	NA	F01_transcript_97209
F01_transcript_102249	NA	F01_transcript_102249	NA	F01_transcript_102249
F01_transcript_96304	NA	F01_transcript_96304	NA	F01_transcript_96304
F01_transcript_103958	NA	F01_transcript_103958	F01_transcript_103958	F01_transcript_103958

F01_transcript_65043	F01_transcript_65043	F01_transcript_65043	F01_transcript_65043	F01_transcript_65043
F01_transcript_114098	NA	F01_transcript_114098	NA	NA
F01_transcript_123923	F01_transcript_123923	F01_transcript_123923	F01_transcript_123923	F01_transcript_123923
F01_transcript_92406	NA	F01_transcript_92406	NA	NA
F01_transcript_64916	NA	F01_transcript_64916	NA	NA
F01_transcript_11432	NA	NA	NA	F01_transcript_11432
F01_transcript_129239	NA	NA	F01_transcript_129239	NA
F01_transcript_44992	NA	NA	NA	F01_transcript_44992
F01_transcript_28436	NA	NA	NA	F01_transcript_28436
F01_transcript_66691	NA	NA	NA	F01_transcript_66691
F01_transcript_76164	NA	F01_transcript_76164	F01_transcript_76164	NA
F01_transcript_135335	NA	NA	NA	F01_transcript_135335
F01_transcript_77607	NA	NA	NA	F01_transcript_77607
F01_transcript_156423	F01_transcript_156423	F01_transcript_156423	F01_transcript_156423	NA

F01_transcript_27677	NA	NA	NA	F01_transcript_27677
F01_transcript_62143	NA	NA	NA	F01_transcript_62143
F01_transcript_152051	NA	F01_transcript_152051	NA	F01_transcript_152051
F01_transcript_104799	F01_transcript_104799	F01_transcript_104799	F01_transcript_104799	NA
F01_transcript_62521	NA	NA	NA	F01_transcript_62521
F01_transcript_85320	NA	F01_transcript_85320	NA	F01_transcript_85320
F01_transcript_145614	NA	F01_transcript_145614	NA	F01_transcript_145614
F01_transcript_111479	NA	F01_transcript_111479	NA	F01_transcript_111479
F01_transcript_126750	F01_transcript_126750	F01_transcript_126750	F01_transcript_126750	F01_transcript_126750
F01_transcript_128871	F01_transcript_128871	F01_transcript_128871	NA	F01_transcript_128871
F01_transcript_85424	NA	F01_transcript_85424	NA	F01_transcript_85424
F01_transcript_145265	NA	NA	NA	F01_transcript_145265
F01_transcript_104755	F01_transcript_104755	F01_transcript_104755	F01_transcript_104755	F01_transcript_104755
F01_transcript_101487	F01_transcript_101487	F01_transcript_101487	NA	NA

F01_transcript_115352	NA	F01_transcript_115352	NA	NA
F01_transcript_156666	NA	F01_transcript_156666	NA	F01_transcript_156666
F01_transcript_68182	NA	NA	NA	F01_transcript_68182
F01_transcript_103513	NA	F01_transcript_103513	NA	NA
F01_transcript_162991	NA	NA	NA	F01_transcript_162991
F01_transcript_119911	NA	F01_transcript_119911	NA	NA
F01_transcript_152589	F01_transcript_152589	F01_transcript_152589	F01_transcript_152589	F01_transcript_152589
F01_transcript_142988	NA	F01_transcript_142988	F01_transcript_142988	NA
F01_transcript_102202	NA	NA	NA	F01_transcript_102202
F01_transcript_3315	NA	NA	NA	F01_transcript_3315
F01_transcript_31174	NA	NA	NA	F01_transcript_31174
F01_transcript_111989	NA	F01_transcript_111989	NA	NA
F01_transcript_6828	NA	NA	NA	F01_transcript_6828
F01_transcript_59328	F01_transcript_59328	F01_transcript_59328	F01_transcript_59328	F01_transcript_59328

F01_transcript_100453	F01_transcript_100453	F01_transcript_100453	NA	NA
F01_transcript_77374	F01_transcript_77374	F01_transcript_77374	NA	F01_transcript_77374
F01_transcript_47185	NA	NA	NA	F01_transcript_47185
F01_transcript_124792	NA	F01_transcript_124792	F01_transcript_124792	NA
F01_transcript_103784	NA	NA	NA	F01_transcript_103784
F01_transcript_121842	NA	F01_transcript_121842	NA	NA
F01_transcript_121690	NA	NA	NA	F01_transcript_121690
F01_transcript_2818	F01_transcript_2818	F01_transcript_2818	F01_transcript_2818	F01_transcript_2818
F01_transcript_911	NA	NA	NA	F01_transcript_911
F01_transcript_102022	NA	F01_transcript_102022	NA	NA
F01_transcript_113667	NA	NA	NA	F01_transcript_113667
F01_transcript_80730	NA	NA	NA	F01_transcript_80730
F01_transcript_20702	NA	NA	NA	F01_transcript_20702
F01_transcript_67090	NA	NA	NA	F01_transcript_67090

F01_transcript_121799	NA	NA	F01_transcript_121799	F01_transcript_121799
F01_transcript_50306	F01_transcript_50306	F01_transcript_50306	NA	F01_transcript_50306
F01_transcript_29123	NA	NA	NA	F01_transcript_29123
F01_transcript_77227	F01_transcript_77227	F01_transcript_77227	NA	NA
F01_transcript_79190	NA	NA	F01_transcript_79190	F01_transcript_79190
F01_transcript_54618	NA	NA	NA	F01_transcript_54618
F01_transcript_106755	F01_transcript_106755	F01_transcript_106755	F01_transcript_106755	F01_transcript_106755
F01_transcript_16128	NA	NA	NA	F01_transcript_16128
F01_transcript_166878	NA	F01_transcript_166878	NA	NA
F01_transcript_138809	F01_transcript_138809	F01_transcript_138809	NA	F01_transcript_138809
F01_transcript_56139	NA	F01_transcript_56139	NA	NA
F01_transcript_119048	NA	F01_transcript_119048	NA	NA
F01_transcript_59988	NA	F01_transcript_59988	NA	NA
F01_transcript_83555	NA	NA	F01_transcript_83555	F01_transcript_83555

F01_transcript_66642	NA	F01_transcript_66642	NA	F01_transcript_66642
F01_transcript_57131	NA	NA	F01_transcript_57131	NA
F01_transcript_139590	NA	F01_transcript_139590	NA	F01_transcript_139590
F01_transcript_56350	F01_transcript_56350	F01_transcript_56350	F01_transcript_56350	F01_transcript_56350
F01_transcript_115571	NA	F01_transcript_115571	NA	F01_transcript_115571
F01_transcript_41883	NA	NA	NA	F01_transcript_41883
F01_transcript_88000	F01_transcript_88000	F01_transcript_88000	F01_transcript_88000	F01_transcript_88000
F01_transcript_94074	NA	NA	NA	F01_transcript_94074
F01_transcript_38490	F01_transcript_38490	F01_transcript_38490	F01_transcript_38490	F01_transcript_38490
F01_transcript_92229	NA	NA	NA	F01_transcript_92229
F01_transcript_121089	NA	NA	NA	F01_transcript_121089
F01_transcript_61964	NA	F01_transcript_61964	NA	NA
F01_transcript_148909	F01_transcript_148909	F01_transcript_148909	F01_transcript_148909	F01_transcript_148909
F01_transcript_65247	NA	F01_transcript_65247	F01_transcript_65247	NA

F01_transcript_117622	NA	F01_transcript_117622	NA	F01_transcript_117622
F01_transcript_110164	F01_transcript_110164	F01_transcript_110164	F01_transcript_110164	F01_transcript_110164
F01_transcript_72526	F01_transcript_72526	F01_transcript_72526	F01_transcript_72526	F01_transcript_72526
F01_transcript_60648	NA	NA	NA	F01_transcript_60648
F01_transcript_104627	NA	F01_transcript_104627	NA	NA
F01_transcript_66068	NA	F01_transcript_66068	NA	NA
F01_transcript_143899	F01_transcript_143899	F01_transcript_143899	F01_transcript_143899	F01_transcript_143899
F01_transcript_58984	NA	F01_transcript_58984	NA	NA
F01_transcript_17328	F01_transcript_17328	F01_transcript_17328	F01_transcript_17328	F01_transcript_17328
F01_transcript_43649	NA	F01_transcript_43649	NA	F01_transcript_43649
F01_transcript_70354	NA	F01_transcript_70354	F01_transcript_70354	F01_transcript_70354
F01_transcript_96955	NA	NA	F01_transcript_96955	F01_transcript_96955
F01_transcript_150107	F01_transcript_150107	F01_transcript_150107	F01_transcript_150107	F01_transcript_150107
F01_transcript_120875	NA	NA	NA	F01_transcript_120875

F01_transcript_4942	NA	NA	NA	F01_transcript_4942
F01_transcript_59477	NA	F01_transcript_59477	F01_transcript_59477	NA
F01_transcript_117512	NA	F01_transcript_117512	NA	NA
F01_transcript_97637	NA	NA	NA	F01_transcript_97637
F01_transcript_74641	F01_transcript_74641	F01_transcript_74641	F01_transcript_74641	F01_transcript_74641
F01_transcript_160126	NA	NA	NA	F01_transcript_160126
F01_transcript_156981	NA	F01_transcript_156981	F01_transcript_156981	NA
F01_transcript_85767	NA	NA	NA	F01_transcript_85767
F01_transcript_106157	F01_transcript_106157	F01_transcript_106157	F01_transcript_106157	F01_transcript_106157
F01_transcript_72658	NA	F01_transcript_72658	F01_transcript_72658	F01_transcript_72658
F01_transcript_81472	NA	F01_transcript_81472	NA	NA
F01_transcript_128461	NA	NA	NA	F01_transcript_128461
F01_transcript_160035	F01_transcript_160035	F01_transcript_160035	NA	F01_transcript_160035
F01_transcript_108957	NA	NA	NA	F01_transcript_108957

F01_transcript_67027	F01_transcript_67027	F01_transcript_67027	F01_transcript_67027	F01_transcript_67027
F01_transcript_135494	NA	NA	NA	F01_transcript_135494
F01_transcript_68229	F01_transcript_68229	F01_transcript_68229	F01_transcript_68229	F01_transcript_68229
F01_transcript_27372	F01_transcript_27372	F01_transcript_27372	NA	NA
F01_transcript_81493	NA	F01_transcript_81493	NA	NA
F01_transcript_104370	NA	NA	NA	F01_transcript_104370
F01_transcript_147475	F01_transcript_147475	F01_transcript_147475	F01_transcript_147475	NA
F01_transcript_52130	NA	F01_transcript_52130	NA	F01_transcript_52130
F01_transcript_102463	F01_transcript_102463	F01_transcript_102463	NA	F01_transcript_102463
F01_transcript_70280	F01_transcript_70280	F01_transcript_70280	F01_transcript_70280	F01_transcript_70280
F01_transcript_111168	NA	F01_transcript_111168	NA	NA
F01_transcript_59323	F01_transcript_59323	F01_transcript_59323	F01_transcript_59323	F01_transcript_59323
F01_transcript_72069	NA	NA	NA	F01_transcript_72069
F01_transcript_93472	F01_transcript_93472	F01_transcript_93472	NA	NA

F01_transcript_92241	NA	F01_transcript_92241	NA	NA
F01_transcript_112842	NA	F01_transcript_112842	NA	F01_transcript_112842
F01_transcript_48925	NA	NA	NA	F01_transcript_48925
F01_transcript_117358	F01_transcript_117358	F01_transcript_117358	F01_transcript_117358	F01_transcript_117358
F01_transcript_59506	NA	NA	F01_transcript_59506	NA
F01_transcript_110764	NA	NA	NA	F01_transcript_110764
F01_transcript_142349	F01_transcript_142349	F01_transcript_142349	F01_transcript_142349	F01_transcript_142349
F01_transcript_119596	NA	F01_transcript_119596	NA	NA
F01_transcript_49852	NA	F01_transcript_49852	NA	NA
F01_transcript_144405	NA	F01_transcript_144405	NA	NA
F01_transcript_130274	NA	NA	NA	F01_transcript_130274
F01_transcript_61840	NA	F01_transcript_61840	NA	NA
F01_transcript_56882	NA	F01_transcript_56882	F01_transcript_56882	NA
F01_transcript_144382	F01_transcript_144382	F01_transcript_144382	NA	NA

F01_transcript_56023	F01_transcript_56023	F01_transcript_56023	F01_transcript_56023	F01_transcript_56023
F01_transcript_126444	NA	NA	NA	F01_transcript_126444
F01_transcript_83956	NA	F01_transcript_83956	NA	F01_transcript_83956
F01_transcript_99551	NA	NA	NA	F01_transcript_99551
F01_transcript_71224	NA	NA	F01_transcript_71224	F01_transcript_71224
F01_transcript_78968	NA	F01_transcript_78968	NA	F01_transcript_78968
F01_transcript_147055	F01_transcript_147055	F01_transcript_147055	F01_transcript_147055	F01_transcript_147055
F01_transcript_95862	F01_transcript_95862	F01_transcript_95862	F01_transcript_95862	F01_transcript_95862
F01_transcript_48694	F01_transcript_48694	NA	NA	F01_transcript_48694
F01_transcript_120161	NA	NA	NA	F01_transcript_120161
F01_transcript_139059	NA	NA	NA	F01_transcript_139059
F01_transcript_162528	NA	F01_transcript_162528	NA	F01_transcript_162528
F01_transcript_91576	NA	F01_transcript_91576	F01_transcript_91576	NA
F01_transcript_136504	NA	NA	NA	F01_transcript_136504

F01_transcript_54120	F01_transcript_54120	F01_transcript_54120	F01_transcript_54120	F01_transcript_54120
F01_transcript_55793	NA	F01_transcript_55793	NA	F01_transcript_55793
F01_transcript_98903	NA	F01_transcript_98903	NA	F01_transcript_98903
F01_transcript_161339	F01_transcript_161339	F01_transcript_161339	NA	NA
F01_transcript_127283	NA	F01_transcript_127283	NA	NA
F01_transcript_80759	NA	NA	NA	F01_transcript_80759
F01_transcript_166948	F01_transcript_166948	F01_transcript_166948	NA	NA
F01_transcript_166708	F01_transcript_166708	F01_transcript_166708	NA	NA
F01_transcript_62941	NA	F01_transcript_62941	NA	NA
F01_transcript_13269	NA	F01_transcript_13269	F01_transcript_13269	NA
F01_transcript_49130	NA	F01_transcript_49130	NA	F01_transcript_49130
F01_transcript_66364	NA	F01_transcript_66364	NA	F01_transcript_66364
F01_transcript_66398	NA	F01_transcript_66398	NA	F01_transcript_66398
F01_transcript_126919	NA	F01_transcript_126919	NA	NA

F01_transcript_96891	NA	F01_transcript_96891	NA	F01_transcript_96891
F01_transcript_3189	NA	NA	NA	F01_transcript_3189
F01_transcript_33132	NA	NA	NA	F01_transcript_33132
F01_transcript_124969	NA	F01_transcript_124969	NA	NA
F01_transcript_100910	NA	F01_transcript_100910	NA	F01_transcript_100910
F01_transcript_103533	NA	F01_transcript_103533	NA	F01_transcript_103533
F01_transcript_51545	F01_transcript_51545	F01_transcript_51545	NA	NA
F01_transcript_108850	NA	NA	NA	F01_transcript_108850
F01_transcript_79947	F01_transcript_79947	F01_transcript_79947	F01_transcript_79947	F01_transcript_79947
F01_transcript_70887	NA	NA	F01_transcript_70887	NA
F01_transcript_114610	NA	F01_transcript_114610	NA	NA
F01_transcript_100637	NA	F01_transcript_100637	NA	NA
F01_transcript_95874	NA	NA	NA	F01_transcript_95874
F01_transcript_53245	NA	F01_transcript_53245	NA	NA

F01_transcript_153748	NA	NA	NA	F01_transcript_153748
F01_transcript_72808	NA	F01_transcript_72808	NA	NA
F01_transcript_62154	NA	F01_transcript_62154	F01_transcript_62154	F01_transcript_62154
F01_transcript_88645	NA	F01_transcript_88645	F01_transcript_88645	F01_transcript_88645
F01_transcript_137048	NA	NA	NA	F01_transcript_137048
F01_transcript_167491	NA	NA	NA	F01_transcript_167491
F01_transcript_109796	NA	NA	NA	F01_transcript_109796
F01_transcript_56469	NA	NA	F01_transcript_56469	NA
F01_transcript_62971	NA	NA	NA	F01_transcript_62971
F01_transcript_85743	NA	NA	NA	F01_transcript_85743
F01_transcript_28113	NA	NA	NA	F01_transcript_28113
F01_transcript_95169	NA	NA	NA	F01_transcript_95169
F01_transcript_116480	NA	F01_transcript_116480	F01_transcript_116480	F01_transcript_116480
F01_transcript_85613	F01_transcript_85613	F01_transcript_85613	NA	NA

F01_transcript_127572	F01_transcript_127572	F01_transcript_127572	F01_transcript_127572	F01_transcript_127572
F01_transcript_161258	F01_transcript_161258	F01_transcript_161258	NA	NA
F01_transcript_117768	NA	F01_transcript_117768	NA	NA
F01_transcript_77991	NA	NA	NA	F01_transcript_77991
F01_transcript_133364	NA	F01_transcript_133364	F01_transcript_133364	F01_transcript_133364
F01_transcript_107177	NA	NA	NA	F01_transcript_107177
F01_transcript_97598	NA	F01_transcript_97598	NA	NA
F01_transcript_9885	NA	F01_transcript_9885	NA	NA
F01_transcript_56911	F01_transcript_56911	F01_transcript_56911	F01_transcript_56911	F01_transcript_56911
F01_transcript_114968	NA	NA	NA	F01_transcript_114968
F01_transcript_157141	NA	NA	F01_transcript_157141	NA
F01_transcript_112434	NA	NA	NA	F01_transcript_112434
F01_transcript_163698	F01_transcript_163698	F01_transcript_163698	F01_transcript_163698	F01_transcript_163698
F01_transcript_123321	F01_transcript_123321	F01_transcript_123321	NA	F01_transcript_123321

F01_transcript_88170	F01_transcript_88170	F01_transcript_88170	F01_transcript_88170	F01_transcript_88170
F01_transcript_94758	NA	F01_transcript_94758	NA	F01_transcript_94758
F01_transcript_100952	NA	F01_transcript_100952	NA	F01_transcript_100952
F01_transcript_166650	NA	F01_transcript_166650	F01_transcript_166650	F01_transcript_166650
F01_transcript_76213	NA	NA	NA	F01_transcript_76213
F01_transcript_21292	F01_transcript_21292	F01_transcript_21292	NA	NA
F01_transcript_77356	NA	NA	NA	F01_transcript_77356
F01_transcript_157638	NA	NA	NA	F01_transcript_157638
F01_transcript_64102	NA	NA	NA	F01_transcript_64102
F01_transcript_148872	NA	NA	NA	F01_transcript_148872
F01_transcript_155360	NA	F01_transcript_155360	F01_transcript_155360	F01_transcript_155360
F01_transcript_144080	F01_transcript_144080	F01_transcript_144080	NA	F01_transcript_144080
F01_transcript_40698	NA	NA	NA	F01_transcript_40698
F01_transcript_97787	NA	F01_transcript_97787	NA	NA

F01_transcript_96890	NA	NA	NA	F01_transcript_96890
F01_transcript_141149	NA	F01_transcript_141149	NA	F01_transcript_141149
F01_transcript_56436	NA	NA	NA	F01_transcript_56436
F01_transcript_99787	F01_transcript_99787	F01_transcript_99787	F01_transcript_99787	F01_transcript_99787
F01_transcript_99573	NA	NA	NA	F01_transcript_99573
F01_transcript_59347	NA	F01_transcript_59347	F01_transcript_59347	NA
F01_transcript_144906	F01_transcript_144906	F01_transcript_144906	F01_transcript_144906	F01_transcript_144906
F01_transcript_153141	NA	NA	NA	F01_transcript_153141
F01_transcript_117990	F01_transcript_117990	F01_transcript_117990	NA	NA
F01_transcript_95745	F01_transcript_95745	F01_transcript_95745	F01_transcript_95745	F01_transcript_95745
F01_transcript_25451	NA	NA	NA	F01_transcript_25451
F01_transcript_142761	NA	NA	NA	F01_transcript_142761
F01_transcript_129880	NA	F01_transcript_129880	NA	NA
F01_transcript_33196	NA	F01_transcript_33196	NA	F01_transcript_33196

F01_transcript_1159	NA	F01_transcript_1159	NA	NA
F01_transcript_39287	NA	NA	NA	F01_transcript_39287
F01_transcript_162327	NA	F01_transcript_162327	NA	NA
F01_transcript_106028	NA	NA	NA	F01_transcript_106028
F01_transcript_117031	NA	F01_transcript_117031	NA	NA
F01_transcript_135717	NA	F01_transcript_135717	NA	NA
F01_transcript_84103	NA	F01_transcript_84103	F01_transcript_84103	F01_transcript_84103
F01_transcript_5987	NA	NA	NA	F01_transcript_5987
F01_transcript_164597	NA	NA	NA	F01_transcript_164597
F01_transcript_60762	F01_transcript_60762	F01_transcript_60762	NA	F01_transcript_60762
F01_transcript_72217	F01_transcript_72217	F01_transcript_72217	NA	F01_transcript_72217
F01_transcript_38785	NA	NA	NA	F01_transcript_38785
F01_transcript_85171	NA	F01_transcript_85171	NA	F01_transcript_85171
F01_transcript_121496	NA	NA	NA	F01_transcript_121496

F01_transcript_167656	F01_transcript_167656	F01_transcript_167656	NA	NA
F01_transcript_64231	NA	NA	NA	F01_transcript_64231
F01_transcript_73515	NA	F01_transcript_73515	F01_transcript_73515	F01_transcript_73515
F01_transcript_7154	NA	NA	NA	F01_transcript_7154
F01_transcript_66923	F01_transcript_66923	F01_transcript_66923	NA	NA
F01_transcript_87192	NA	F01_transcript_87192	NA	F01_transcript_87192
F01_transcript_159073	NA	NA	F01_transcript_159073	NA
F01_transcript_164094	NA	F01_transcript_164094	NA	F01_transcript_164094
F01_transcript_165781	NA	F01_transcript_165781	NA	NA
F01_transcript_108335	NA	F01_transcript_108335	NA	NA
F01_transcript_70016	F01_transcript_70016	F01_transcript_70016	NA	NA
F01_transcript_100517	NA	NA	NA	F01_transcript_100517
F01_transcript_70457	NA	F01_transcript_70457	NA	NA
F01_transcript_105823	NA	F01_transcript_105823	NA	NA

F01_transcript_64218	NA	F01_transcript_64218	NA	NA
F01_transcript_130010	NA	NA	NA	F01_transcript_130010
F01_transcript_95669	F01_transcript_95669	F01_transcript_95669	NA	NA
F01_transcript_7004	NA	F01_transcript_7004	NA	F01_transcript_7004
F01_transcript_35382	NA	NA	F01_transcript_35382	NA
F01_transcript_118123	NA	F01_transcript_118123	NA	NA
F01_transcript_148104	F01_transcript_148104	F01_transcript_148104	NA	NA
F01_transcript_158585	NA	NA	NA	F01_transcript_158585
F01_transcript_83921	NA	NA	NA	F01_transcript_83921
F01_transcript_120514	NA	F01_transcript_120514	NA	NA
F01_transcript_122784	F01_transcript_122784	F01_transcript_122784	NA	F01_transcript_122784
F01_transcript_154591	NA	F01_transcript_154591	NA	NA
F01_transcript_136980	NA	F01_transcript_136980	NA	NA
F01_transcript_148138	F01_transcript_148138	F01_transcript_148138	F01_transcript_148138	F01_transcript_148138

F01_transcript_89302	NA	F01_transcript_89302	NA	F01_transcript_89302
F01_transcript_102706	NA	F01_transcript_102706	F01_transcript_102706	F01_transcript_102706
F01_transcript_120153	F01_transcript_120153	F01_transcript_120153	NA	NA
F01_transcript_117162	F01_transcript_117162	F01_transcript_117162	F01_transcript_117162	NA
F01_transcript_42191	NA	NA	NA	F01_transcript_42191
F01_transcript_94227	NA	F01_transcript_94227	F01_transcript_94227	F01_transcript_94227
F01_transcript_47053	NA	NA	NA	F01_transcript_47053
F01_transcript_89631	F01_transcript_89631	F01_transcript_89631	F01_transcript_89631	F01_transcript_89631
F01_transcript_146572	NA	F01_transcript_146572	NA	NA
F01_transcript_104818	NA	F01_transcript_104818	NA	F01_transcript_104818
F01_transcript_15693	NA	F01_transcript_15693	NA	F01_transcript_15693
F01_transcript_63020	NA	F01_transcript_63020	NA	NA
F01_transcript_75433	F01_transcript_75433	F01_transcript_75433	F01_transcript_75433	F01_transcript_75433
F01_transcript_126355	NA	NA	NA	F01_transcript_126355

F01_transcript_5409	F01_transcript_5409	F01_transcript_5409	NA	F01_transcript_5409
F01_transcript_74252	F01_transcript_74252	F01_transcript_74252	F01_transcript_74252	F01_transcript_74252
F01_transcript_153468	NA	NA	NA	F01_transcript_153468
F01_transcript_20097	NA	NA	NA	F01_transcript_20097
F01_transcript_165509	NA	NA	NA	F01_transcript_165509
F01_transcript_124145	F01_transcript_124145	F01_transcript_124145	F01_transcript_124145	NA
F01_transcript_156658	F01_transcript_156658	F01_transcript_156658	NA	F01_transcript_156658
F01_transcript_116005	F01_transcript_116005	F01_transcript_116005	NA	F01_transcript_116005
F01_transcript_101537	NA	F01_transcript_101537	F01_transcript_101537	NA
F01_transcript_165409	NA	F01_transcript_165409	F01_transcript_165409	NA
F01_transcript_87447	F01_transcript_87447	F01_transcript_87447	F01_transcript_87447	F01_transcript_87447
F01_transcript_65456	NA	NA	F01_transcript_65456	NA
F01_transcript_127420	NA	NA	F01_transcript_127420	NA
F01_transcript_74224	NA	F01_transcript_74224	NA	F01_transcript_74224

F01_transcript_99657	NA	NA	NA	F01_transcript_99657
F01_transcript_21141	NA	F01_transcript_21141	F01_transcript_21141	NA
F01_transcript_83871	F01_transcript_83871	F01_transcript_83871	NA	F01_transcript_83871
F01_transcript_154683	NA	NA	NA	F01_transcript_154683
F01_transcript_106547	NA	F01_transcript_106547	F01_transcript_106547	F01_transcript_106547
F01_transcript_37099	NA	NA	NA	F01_transcript_37099
F01_transcript_135700	NA	F01_transcript_135700	NA	NA
F01_transcript_49909	F01_transcript_49909	NA	NA	NA
F01_transcript_137976	NA	F01_transcript_137976	NA	NA
F01_transcript_137244	NA	NA	NA	F01_transcript_137244
F01_transcript_31300	F01_transcript_31300	F01_transcript_31300	F01_transcript_31300	F01_transcript_31300
F01_transcript_98274	NA	NA	NA	F01_transcript_98274
F01_transcript_123348	F01_transcript_123348	F01_transcript_123348	F01_transcript_123348	F01_transcript_123348
F01_transcript_98537	NA	F01_transcript_98537	NA	NA

F01_transcript_141882	F01_transcript_141882	F01_transcript_141882	F01_transcript_141882	NA
F01_transcript_127035	NA	F01_transcript_127035	NA	NA
F01_transcript_83538	NA	F01_transcript_83538	NA	NA
F01_transcript_116396	NA	NA	NA	F01_transcript_116396
F01_transcript_91895	NA	NA	NA	F01_transcript_91895
F01_transcript_16335	NA	F01_transcript_16335	NA	NA
F01_transcript_29129	NA	NA	NA	F01_transcript_29129
F01_transcript_72696	NA	NA	NA	F01_transcript_72696
F01_transcript_159886	NA	NA	NA	F01_transcript_159886
F01_transcript_138222	NA	F01_transcript_138222	NA	NA
F01_transcript_86495	NA	NA	NA	F01_transcript_86495
F01_transcript_146069	F01_transcript_146069	F01_transcript_146069	F01_transcript_146069	F01_transcript_146069
F01_transcript_104951	NA	F01_transcript_104951	NA	NA
F01_transcript_17429	NA	NA	NA	F01_transcript_17429

F01_transcript_85726	F01_transcript_85726	F01_transcript_85726	NA	NA
F01_transcript_143395	F01_transcript_143395	F01_transcript_143395	F01_transcript_143395	F01_transcript_143395
F01_transcript_31321	NA	NA	NA	F01_transcript_31321
F01_transcript_10414	NA	F01_transcript_10414	NA	NA
F01_transcript_131777	F01_transcript_131777	F01_transcript_131777	F01_transcript_131777	F01_transcript_131777
F01_transcript_150866	NA	F01_transcript_150866	NA	NA
F01_transcript_64163	NA	F01_transcript_64163	NA	F01_transcript_64163
F01_transcript_58843	NA	F01_transcript_58843	NA	F01_transcript_58843
F01_transcript_14074	NA	F01_transcript_14074	NA	F01_transcript_14074
F01_transcript_109478	F01_transcript_109478	F01_transcript_109478	NA	F01_transcript_109478
F01_transcript_77623	NA	F01_transcript_77623	NA	NA
F01_transcript_62901	NA	NA	NA	F01_transcript_62901
F01_transcript_90127	NA	NA	F01_transcript_90127	F01_transcript_90127
F01_transcript_69225	NA	F01_transcript_69225	NA	NA

F01_transcript_111709	NA	F01_transcript_111709	F01_transcript_111709	F01_transcript_111709
F01_transcript_12364	NA	F01_transcript_12364	NA	NA
F01_transcript_42016	NA	NA	NA	F01_transcript_42016
F01_transcript_57530	NA	NA	NA	F01_transcript_57530
F01_transcript_154238	F01_transcript_154238	F01_transcript_154238	NA	NA
F01_transcript_7755	NA	NA	NA	F01_transcript_7755
F01_transcript_146352	NA	NA	NA	F01_transcript_146352
F01_transcript_67613	NA	NA	NA	F01_transcript_67613
F01_transcript_118879	NA	F01_transcript_118879	NA	NA
F01_transcript_54074	NA	NA	NA	F01_transcript_54074
F01_transcript_99779	NA	F01_transcript_99779	F01_transcript_99779	F01_transcript_99779
F01_transcript_156995	NA	F01_transcript_156995	NA	F01_transcript_156995
F01_transcript_150932	NA	NA	NA	F01_transcript_150932
F01_transcript_124190	NA	F01_transcript_124190	NA	NA

F01_transcript_11446	F01_transcript_11446	F01_transcript_11446	F01_transcript_11446	F01_transcript_11446
F01_transcript_94322	NA	NA	NA	F01_transcript_94322
F01_transcript_108073	F01_transcript_108073	F01_transcript_108073	F01_transcript_108073	F01_transcript_108073
F01_transcript_145913	NA	F01_transcript_145913	NA	F01_transcript_145913
F01_transcript_56617	NA	F01_transcript_56617	F01_transcript_56617	NA
F01_transcript_129420	NA	F01_transcript_129420	NA	NA
F01_transcript_59914	NA	F01_transcript_59914	NA	F01_transcript_59914
F01_transcript_111225	NA	NA	F01_transcript_111225	NA
F01_transcript_44064	NA	F01_transcript_44064	NA	NA
F01_transcript_68607	F01_transcript_68607	F01_transcript_68607	NA	F01_transcript_68607
F01_transcript_164074	NA	NA	NA	F01_transcript_164074
F01_transcript_141601	NA	NA	NA	F01_transcript_141601
F01_transcript_11059	F01_transcript_11059	NA	NA	NA
F01_transcript_69928	NA	F01_transcript_69928	NA	NA

F01_transcript_132056	F01_transcript_132056	F01_transcript_132056	F01_transcript_132056	F01_transcript_132056
F01_transcript_115169	NA	F01_transcript_115169	NA	F01_transcript_115169
F01_transcript_142916	NA	F01_transcript_142916	NA	NA
F01_transcript_162355	F01_transcript_162355	F01_transcript_162355	NA	F01_transcript_162355
F01_transcript_52876	NA	F01_transcript_52876	NA	NA
F01_transcript_149216	F01_transcript_149216	F01_transcript_149216	F01_transcript_149216	F01_transcript_149216
F01_transcript_80472	F01_transcript_80472	F01_transcript_80472	F01_transcript_80472	F01_transcript_80472
F01_transcript_56308	NA	F01_transcript_56308	F01_transcript_56308	NA
F01_transcript_134762	F01_transcript_134762	F01_transcript_134762	F01_transcript_134762	F01_transcript_134762
F01_transcript_14064	NA	F01_transcript_14064	NA	F01_transcript_14064
F01_transcript_148977	NA	F01_transcript_148977	NA	NA
F01_transcript_127472	NA	F01_transcript_127472	NA	NA
F01_transcript_112187	NA	F01_transcript_112187	NA	F01_transcript_112187
F01_transcript_109488	F01_transcript_109488	F01_transcript_109488	F01_transcript_109488	F01_transcript_109488

F01_transcript_110039	F01_transcript_110039	F01_transcript_110039	F01_transcript_110039	F01_transcript_110039
F01_transcript_66187	NA	NA	NA	F01_transcript_66187
F01_transcript_163345	NA	F01_transcript_163345	NA	F01_transcript_163345
F01_transcript_74180	NA	F01_transcript_74180	F01_transcript_74180	F01_transcript_74180
F01_transcript_111622	F01_transcript_111622	F01_transcript_111622	NA	NA
F01_transcript_49279	NA	F01_transcript_49279	NA	NA
F01_transcript_114214	NA	NA	NA	F01_transcript_114214
F01_transcript_134895	NA	NA	NA	F01_transcript_134895
F01_transcript_99992	NA	NA	NA	F01_transcript_99992
F01_transcript_80595	NA	NA	NA	F01_transcript_80595
F01_transcript_100212	NA	F01_transcript_100212	NA	NA
F01_transcript_82608	NA	NA	NA	F01_transcript_82608
F01_transcript_27519	F01_transcript_27519	NA	F01_transcript_27519	F01_transcript_27519
F01_transcript_126145	NA	F01_transcript_126145	NA	NA

F01_transcript_27704	NA	NA	NA	F01_transcript_27704
F01_transcript_126768	F01_transcript_126768	F01_transcript_126768	NA	NA
F01_transcript_161208	F01_transcript_161208	F01_transcript_161208	F01_transcript_161208	F01_transcript_161208
F01_transcript_107119	NA	NA	NA	F01_transcript_107119
F01_transcript_101590	NA	NA	NA	F01_transcript_101590
F01_transcript_87775	F01_transcript_87775	F01_transcript_87775	F01_transcript_87775	F01_transcript_87775
F01_transcript_52563	F01_transcript_52563	F01_transcript_52563	F01_transcript_52563	F01_transcript_52563
F01_transcript_150712	F01_transcript_150712	F01_transcript_150712	F01_transcript_150712	F01_transcript_150712
F01_transcript_150071	NA	F01_transcript_150071	F01_transcript_150071	F01_transcript_150071
F01_transcript_120953	F01_transcript_120953	F01_transcript_120953	F01_transcript_120953	F01_transcript_120953
F01_transcript_61591	NA	F01_transcript_61591	F01_transcript_61591	F01_transcript_61591
F01_transcript_158349	NA	F01_transcript_158349	NA	NA
F01_transcript_51391	NA	F01_transcript_51391	F01_transcript_51391	NA
F01_transcript_57521	NA	NA	NA	F01_transcript_57521

F01_transcript_139335	NA	F01_transcript_139335	NA	F01_transcript_139335
F01_transcript_58618	F01_transcript_58618	F01_transcript_58618	F01_transcript_58618	F01_transcript_58618
F01_transcript_70862	NA	NA	F01_transcript_70862	F01_transcript_70862
F01_transcript_164245	NA	NA	NA	F01_transcript_164245
F01_transcript_140963	NA	NA	NA	F01_transcript_140963
F01_transcript_28497	F01_transcript_28497	F01_transcript_28497	F01_transcript_28497	F01_transcript_28497
F01_transcript_104699	NA	F01_transcript_104699	NA	NA
F01_transcript_132254	NA	F01_transcript_132254	NA	NA
F01_transcript_114019	F01_transcript_114019	F01_transcript_114019	NA	NA
F01_transcript_40212	NA	NA	NA	F01_transcript_40212
F01_transcript_88631	NA	F01_transcript_88631	NA	NA
F01_transcript_44949	NA	NA	NA	F01_transcript_44949
F01_transcript_73757	NA	F01_transcript_73757	F01_transcript_73757	F01_transcript_73757
F01_transcript_129286	NA	F01_transcript_129286	NA	NA

F01_transcript_49448	NA	NA	NA	F01_transcript_49448
F01_transcript_93779	NA	NA	NA	F01_transcript_93779
F01_transcript_65691	F01_transcript_65691	F01_transcript_65691	NA	NA
F01_transcript_68744	NA	NA	F01_transcript_68744	NA
F01_transcript_122329	NA	NA	NA	F01_transcript_122329
F01_transcript_128542	NA	NA	NA	F01_transcript_128542
F01_transcript_84463	NA	F01_transcript_84463	NA	NA
F01_transcript_162206	NA	F01_transcript_162206	NA	NA
F01_transcript_30151	NA	NA	NA	F01_transcript_30151
F01_transcript_74655	NA	NA	NA	F01_transcript_74655
F01_transcript_5191	NA	NA	NA	F01_transcript_5191
F01_transcript_72708	NA	NA	NA	F01_transcript_72708
F01_transcript_161277	NA	NA	NA	F01_transcript_161277
F01_transcript_167317	NA	NA	NA	F01_transcript_167317

F01_transcript_71652	NA	NA	NA	F01_transcript_71652
F01_transcript_78793	NA	F01_transcript_78793	NA	NA
F01_transcript_161925	NA	F01_transcript_161925	NA	NA
F01_transcript_89507	F01_transcript_89507	F01_transcript_89507	F01_transcript_89507	NA
F01_transcript_107705	NA	NA	NA	F01_transcript_107705
F01_transcript_146534	F01_transcript_146534	F01_transcript_146534	F01_transcript_146534	F01_transcript_146534
F01_transcript_9070	NA	F01_transcript_9070	NA	NA
F01_transcript_119447	NA	F01_transcript_119447	F01_transcript_119447	F01_transcript_119447
F01_transcript_56909	NA	F01_transcript_56909	F01_transcript_56909	NA
F01_transcript_90449	NA	F01_transcript_90449	NA	NA
F01_transcript_49927	NA	F01_transcript_49927	NA	NA
F01_transcript_81675	NA	F01_transcript_81675	NA	F01_transcript_81675
F01_transcript_76253	NA	NA	NA	F01_transcript_76253
F01_transcript_115700	F01_transcript_115700	F01_transcript_115700	F01_transcript_115700	F01_transcript_115700

F01_transcript_117279	NA	F01_transcript_117279	NA	NA
F01_transcript_98034	F01_transcript_98034	F01_transcript_98034	NA	F01_transcript_98034
F01_transcript_58961	F01_transcript_58961	F01_transcript_58961	F01_transcript_58961	F01_transcript_58961
F01_transcript_106821	NA	F01_transcript_106821	NA	NA
F01_transcript_150117	F01_transcript_150117	F01_transcript_150117	F01_transcript_150117	F01_transcript_150117
F01_transcript_135771	NA	F01_transcript_135771	NA	NA
F01_transcript_119470	NA	NA	NA	F01_transcript_119470
F01_transcript_155913	NA	F01_transcript_155913	NA	NA
F01_transcript_118972	F01_transcript_118972	F01_transcript_118972	F01_transcript_118972	F01_transcript_118972
F01_transcript_63252	NA	NA	NA	F01_transcript_63252
F01_transcript_108330	F01_transcript_108330	F01_transcript_108330	F01_transcript_108330	F01_transcript_108330
F01_transcript_162003	NA	NA	NA	F01_transcript_162003
F01_transcript_121536	NA	F01_transcript_121536	NA	NA
F01_transcript_149115	F01_transcript_149115	F01_transcript_149115	F01_transcript_149115	F01_transcript_149115

F01_transcript_83302	NA	NA	NA	F01_transcript_83302
F01_transcript_45225	NA	NA	NA	F01_transcript_45225
F01_transcript_95606	NA	F01_transcript_95606	NA	F01_transcript_95606
F01_transcript_162510	F01_transcript_162510	F01_transcript_162510	F01_transcript_162510	F01_transcript_162510
F01_transcript_98253	F01_transcript_98253	F01_transcript_98253	F01_transcript_98253	F01_transcript_98253
F01_transcript_89522	F01_transcript_89522	F01_transcript_89522	F01_transcript_89522	NA
F01_transcript_51490	F01_transcript_51490	F01_transcript_51490	F01_transcript_51490	F01_transcript_51490
F01_transcript_64403	NA	NA	NA	F01_transcript_64403
F01_transcript_17397	F01_transcript_17397	F01_transcript_17397	F01_transcript_17397	F01_transcript_17397
F01_transcript_55848	NA	F01_transcript_55848	NA	NA
F01_transcript_102592	NA	NA	NA	F01_transcript_102592
F01_transcript_91870	NA	F01_transcript_91870	NA	F01_transcript_91870
F01_transcript_112232	NA	F01_transcript_112232	NA	NA
F01_transcript_65782	F01_transcript_65782	F01_transcript_65782	NA	NA

F01_transcript_65772	NA	F01_transcript_65772	F01_transcript_65772	F01_transcript_65772
F01_transcript_133314	NA	F01_transcript_133314	NA	F01_transcript_133314
F01_transcript_108843	F01_transcript_108843	F01_transcript_108843	F01_transcript_108843	F01_transcript_108843
F01_transcript_71138	F01_transcript_71138	F01_transcript_71138	F01_transcript_71138	NA
F01_transcript_46661	NA	NA	NA	F01_transcript_46661
F01_transcript_68027	NA	F01_transcript_68027	F01_transcript_68027	F01_transcript_68027
F01_transcript_144302	NA	F01_transcript_144302	F01_transcript_144302	F01_transcript_144302
F01_transcript_143459	NA	F01_transcript_143459	NA	NA
F01_transcript_102494	NA	F01_transcript_102494	NA	NA
F01_transcript_46702	NA	NA	NA	F01_transcript_46702
F01_transcript_48137	NA	NA	F01_transcript_48137	F01_transcript_48137
F01_transcript_99283	NA	NA	NA	F01_transcript_99283
F01_transcript_103962	F01_transcript_103962	F01_transcript_103962	F01_transcript_103962	F01_transcript_103962
F01_transcript_88210	NA	F01_transcript_88210	F01_transcript_88210	F01_transcript_88210

F01_transcript_67478	NA	F01_transcript_67478	NA	NA
F01_transcript_34521	NA	NA	NA	F01_transcript_34521
F01_transcript_123034	NA	NA	NA	F01_transcript_123034
F01_transcript_160250	NA	NA	NA	F01_transcript_160250
F01_transcript_104107	NA	F01_transcript_104107	NA	F01_transcript_104107
F01_transcript_108213	NA	NA	NA	F01_transcript_108213
F01_transcript_54002	NA	NA	NA	F01_transcript_54002
F01_transcript_1915	NA	NA	NA	F01_transcript_1915
F01_transcript_71872	F01_transcript_71872	F01_transcript_71872	F01_transcript_71872	F01_transcript_71872
F01_transcript_92367	F01_transcript_92367	F01_transcript_92367	NA	NA
F01_transcript_144628	NA	F01_transcript_144628	NA	F01_transcript_144628
F01_transcript_54720	NA	NA	NA	F01_transcript_54720
F01_transcript_74003	NA	F01_transcript_74003	NA	NA
F01_transcript_141416	NA	NA	F01_transcript_141416	NA

F01_transcript_1082	NA	NA	NA	F01_transcript_1082
F01_transcript_98283	NA	NA	NA	F01_transcript_98283
F01_transcript_51804	F01_transcript_51804	F01_transcript_51804	NA	NA
F01_transcript_138527	F01_transcript_138527	F01_transcript_138527	F01_transcript_138527	F01_transcript_138527
F01_transcript_152291	NA	F01_transcript_152291	F01_transcript_152291	F01_transcript_152291
F01_transcript_117472	F01_transcript_117472	F01_transcript_117472	F01_transcript_117472	F01_transcript_117472
F01_transcript_63994	NA	F01_transcript_63994	NA	NA
F01_transcript_137073	F01_transcript_137073	NA	F01_transcript_137073	F01_transcript_137073
F01_transcript_31739	NA	NA	NA	F01_transcript_31739
F01_transcript_7785	NA	NA	F01_transcript_7785	NA
F01_transcript_44357	NA	F01_transcript_44357	NA	NA
F01_transcript_125840	F01_transcript_125840	F01_transcript_125840	F01_transcript_125840	F01_transcript_125840
F01_transcript_163394	NA	F01_transcript_163394	F01_transcript_163394	F01_transcript_163394
F01_transcript_129107	F01_transcript_129107	F01_transcript_129107	F01_transcript_129107	F01_transcript_129107

F01_transcript_94630	NA	NA	F01_transcript_94630	F01_transcript_94630
F01_transcript_66507	NA	NA	NA	F01_transcript_66507
F01_transcript_92079	NA	NA	NA	F01_transcript_92079
F01_transcript_59111	F01_transcript_59111	F01_transcript_59111	NA	NA
F01_transcript_141063	NA	NA	NA	F01_transcript_141063
F01_transcript_43666	NA	NA	NA	F01_transcript_43666
F01_transcript_90038	F01_transcript_90038	F01_transcript_90038	NA	NA
F01_transcript_106836	NA	NA	NA	F01_transcript_106836
F01_transcript_120622	NA	F01_transcript_120622	NA	F01_transcript_120622
F01_transcript_146234	NA	NA	NA	F01_transcript_146234
F01_transcript_163415	F01_transcript_163415	F01_transcript_163415	F01_transcript_163415	NA
F01_transcript_50513	F01_transcript_50513	F01_transcript_50513	F01_transcript_50513	F01_transcript_50513
F01_transcript_96512	F01_transcript_96512	F01_transcript_96512	F01_transcript_96512	F01_transcript_96512
F01_transcript_20535	F01_transcript_20535	NA	NA	F01_transcript_20535

F01_transcript_53890	NA	NA	NA	F01_transcript_53890
F01_transcript_73123	NA	NA	NA	F01_transcript_73123
F01_transcript_109648	NA	F01_transcript_109648	NA	NA
F01_transcript_104973	NA	F01_transcript_104973	NA	NA
F01_transcript_74663	F01_transcript_74663	F01_transcript_74663	F01_transcript_74663	F01_transcript_74663
F01_transcript_72349	NA	F01_transcript_72349	NA	NA
F01_transcript_50890	F01_transcript_50890	F01_transcript_50890	F01_transcript_50890	F01_transcript_50890
F01_transcript_107527	NA	NA	NA	F01_transcript_107527
F01_transcript_61956	NA	NA	NA	F01_transcript_61956
F01_transcript_150343	NA	NA	NA	F01_transcript_150343
F01_transcript_72363	NA	NA	NA	F01_transcript_72363
F01_transcript_161422	NA	F01_transcript_161422	NA	NA
F01_transcript_141074	NA	NA	NA	F01_transcript_141074
F01_transcript_58816	F01_transcript_58816	F01_transcript_58816	NA	F01_transcript_58816

F01_transcript_160688	F01_transcript_160688	F01_transcript_160688	F01_transcript_160688	F01_transcript_160688
F01_transcript_164929	NA	F01_transcript_164929	NA	F01_transcript_164929
F01_transcript_166884	F01_transcript_166884	F01_transcript_166884	F01_transcript_166884	NA
F01_transcript_62425	F01_transcript_62425	F01_transcript_62425	F01_transcript_62425	F01_transcript_62425
F01_transcript_151873	NA	NA	NA	F01_transcript_151873
F01_transcript_144258	NA	NA	NA	F01_transcript_144258
F01_transcript_155927	NA	F01_transcript_155927	NA	F01_transcript_155927
F01_transcript_73351	NA	F01_transcript_73351	F01_transcript_73351	F01_transcript_73351
F01_transcript_102788	NA	NA	NA	F01_transcript_102788
F01_transcript_140189	NA	NA	NA	F01_transcript_140189
F01_transcript_60477	F01_transcript_60477	F01_transcript_60477	F01_transcript_60477	F01_transcript_60477
F01_transcript_153765	NA	F01_transcript_153765	NA	NA
F01_transcript_132262	F01_transcript_132262	F01_transcript_132262	NA	F01_transcript_132262
F01_transcript_100538	NA	NA	NA	F01_transcript_100538

F01_transcript_11890	NA	NA	NA	F01_transcript_11890
F01_transcript_111725	NA	NA	NA	F01_transcript_111725
F01_transcript_43953	NA	NA	NA	F01_transcript_43953
F01_transcript_43058	NA	NA	NA	F01_transcript_43058
F01_transcript_163738	F01_transcript_163738	F01_transcript_163738	F01_transcript_163738	F01_transcript_163738
F01_transcript_143400	NA	NA	F01_transcript_143400	F01_transcript_143400
F01_transcript_63839	NA	F01_transcript_63839	NA	F01_transcript_63839
F01_transcript_141244	NA	NA	NA	F01_transcript_141244
F01_transcript_72622	NA	NA	NA	F01_transcript_72622
F01_transcript_117365	NA	NA	NA	F01_transcript_117365
F01_transcript_11052	NA	NA	NA	F01_transcript_11052
F01_transcript_73858	NA	F01_transcript_73858	NA	F01_transcript_73858
F01_transcript_71140	NA	F01_transcript_71140	F01_transcript_71140	F01_transcript_71140
F01_transcript_6647	F01_transcript_6647	F01_transcript_6647	NA	F01_transcript_6647

F01_transcript_5240	F01_transcript_5240	F01_transcript_5240	F01_transcript_5240	F01_transcript_5240
F01_transcript_151979	F01_transcript_151979	F01_transcript_151979	NA	F01_transcript_151979
F01_transcript_14980	NA	NA	NA	F01_transcript_14980
F01_transcript_49644	NA	F01_transcript_49644	F01_transcript_49644	F01_transcript_49644
F01_transcript_100047	F01_transcript_100047	F01_transcript_100047	F01_transcript_100047	F01_transcript_100047
F01_transcript_136144	NA	NA	NA	F01_transcript_136144
F01_transcript_142612	NA	F01_transcript_142612	NA	NA
F01_transcript_129256	NA	F01_transcript_129256	NA	NA
F01_transcript_128210	NA	NA	F01_transcript_128210	F01_transcript_128210
F01_transcript_106863	NA	NA	NA	F01_transcript_106863
F01_transcript_87663	NA	F01_transcript_87663	NA	NA
F01_transcript_127404	NA	NA	NA	F01_transcript_127404
F01_transcript_59862	NA	F01_transcript_59862	F01_transcript_59862	F01_transcript_59862
F01_transcript_42941	NA	NA	NA	F01_transcript_42941

F01_transcript_162429	NA	NA	NA	F01_transcript_162429
F01_transcript_105308	F01_transcript_105308	F01_transcript_105308	F01_transcript_105308	F01_transcript_105308
F01_transcript_106844	F01_transcript_106844	F01_transcript_106844	F01_transcript_106844	F01_transcript_106844
F01_transcript_121948	F01_transcript_121948	F01_transcript_121948	F01_transcript_121948	F01_transcript_121948
F01_transcript_86942	NA	NA	NA	F01_transcript_86942
F01_transcript_26504	NA	NA	F01_transcript_26504	NA
F01_transcript_6827	NA	NA	NA	F01_transcript_6827
F01_transcript_152913	NA	F01_transcript_152913	F01_transcript_152913	F01_transcript_152913
F01_transcript_72370	NA	F01_transcript_72370	NA	F01_transcript_72370
F01_transcript_149939	F01_transcript_149939	F01_transcript_149939	F01_transcript_149939	F01_transcript_149939
F01_transcript_53986	NA	F01_transcript_53986	NA	NA
F01_transcript_67061	NA	NA	NA	F01_transcript_67061
F01_transcript_5707	NA	NA	F01_transcript_5707	NA
F01_transcript_24359	NA	NA	NA	F01_transcript_24359

F01_transcript_127561	NA	F01_transcript_127561	F01_transcript_127561	F01_transcript_127561
F01_transcript_113038	NA	NA	NA	F01_transcript_113038
F01_transcript_125746	F01_transcript_125746	F01_transcript_125746	F01_transcript_125746	F01_transcript_125746
F01_transcript_158685	F01_transcript_158685	F01_transcript_158685	F01_transcript_158685	F01_transcript_158685
F01_transcript_158958	NA	NA	F01_transcript_158958	NA
F01_transcript_104964	F01_transcript_104964	F01_transcript_104964	F01_transcript_104964	F01_transcript_104964
F01_transcript_32964	NA	NA	NA	F01_transcript_32964
F01_transcript_30830	NA	F01_transcript_30830	NA	NA
F01_transcript_3344	NA	F01_transcript_3344	F01_transcript_3344	F01_transcript_3344
F01_transcript_69372	F01_transcript_69372	F01_transcript_69372	F01_transcript_69372	F01_transcript_69372
F01_transcript_63155	F01_transcript_63155	F01_transcript_63155	F01_transcript_63155	F01_transcript_63155
F01_transcript_80361	NA	F01_transcript_80361	NA	F01_transcript_80361
F01_transcript_101321	F01_transcript_101321	F01_transcript_101321	F01_transcript_101321	F01_transcript_101321
F01_transcript_116679	F01_transcript_116679	F01_transcript_116679	F01_transcript_116679	NA

F01_transcript_114474	NA	F01_transcript_114474	NA	F01_transcript_114474
F01_transcript_47951	NA	NA	NA	F01_transcript_47951
F01_transcript_124546	F01_transcript_124546	F01_transcript_124546	F01_transcript_124546	F01_transcript_124546
F01_transcript_86924	F01_transcript_86924	F01_transcript_86924	NA	F01_transcript_86924
F01_transcript_124595	F01_transcript_124595	F01_transcript_124595	F01_transcript_124595	F01_transcript_124595
F01_transcript_70213	NA	F01_transcript_70213	NA	NA
F01_transcript_124986	F01_transcript_124986	F01_transcript_124986	NA	NA
F01_transcript_80393	NA	F01_transcript_80393	NA	F01_transcript_80393
F01_transcript_149279	NA	NA	NA	F01_transcript_149279
F01_transcript_118178	NA	NA	F01_transcript_118178	F01_transcript_118178
F01_transcript_41415	NA	NA	NA	F01_transcript_41415
F01_transcript_97460	NA	F01_transcript_97460	NA	NA
F01_transcript_95712	NA	F01_transcript_95712	NA	F01_transcript_95712
F01_transcript_72047	F01_transcript_72047	F01_transcript_72047	NA	NA

F01_transcript_123956	F01_transcript_123956	F01_transcript_123956	NA	NA
F01_transcript_65907	F01_transcript_65907	F01_transcript_65907	F01_transcript_65907	F01_transcript_65907
F01_transcript_109268	F01_transcript_109268	NA	NA	F01_transcript_109268
F01_transcript_105992	NA	F01_transcript_105992	F01_transcript_105992	F01_transcript_105992
F01_transcript_84248	F01_transcript_84248	F01_transcript_84248	F01_transcript_84248	F01_transcript_84248
F01_transcript_77207	NA	F01_transcript_77207	NA	NA
F01_transcript_97440	F01_transcript_97440	F01_transcript_97440	F01_transcript_97440	F01_transcript_97440
F01_transcript_158185	F01_transcript_158185	F01_transcript_158185	NA	NA
F01_transcript_32469	NA	NA	NA	F01_transcript_32469
F01_transcript_17406	NA	F01_transcript_17406	NA	NA
F01_transcript_120477	F01_transcript_120477	F01_transcript_120477	F01_transcript_120477	NA
F01_transcript_120190	NA	NA	NA	F01_transcript_120190
F01_transcript_58853	F01_transcript_58853	F01_transcript_58853	F01_transcript_58853	F01_transcript_58853
F01_transcript_163920	NA	NA	NA	F01_transcript_163920

F01_transcript_66101	NA	F01_transcript_66101	NA	NA
F01_transcript_76481	F01_transcript_76481	F01_transcript_76481	F01_transcript_76481	F01_transcript_76481
F01_transcript_143590	NA	F01_transcript_143590	NA	NA
F01_transcript_140165	F01_transcript_140165	F01_transcript_140165	F01_transcript_140165	F01_transcript_140165
F01_transcript_156729	NA	F01_transcript_156729	F01_transcript_156729	F01_transcript_156729
F01_transcript_149757	NA	NA	NA	F01_transcript_149757
F01_transcript_149298	NA	F01_transcript_149298	NA	NA
F01_transcript_62064	NA	F01_transcript_62064	NA	NA
F01_transcript_116911	NA	F01_transcript_116911	NA	F01_transcript_116911
F01_transcript_149176	F01_transcript_149176	F01_transcript_149176	F01_transcript_149176	F01_transcript_149176
F01_transcript_60965	NA	F01_transcript_60965	F01_transcript_60965	NA
F01_transcript_144878	NA	F01_transcript_144878	F01_transcript_144878	NA
F01_transcript_15856	NA	NA	NA	F01_transcript_15856
F01_transcript_126362	NA	F01_transcript_126362	NA	NA

F01_transcript_144532	NA	F01_transcript_144532	NA	NA
F01_transcript_129882	F01_transcript_129882	F01_transcript_129882	NA	NA
F01_transcript_98601	NA	NA	NA	F01_transcript_98601
F01_transcript_165854	NA	NA	NA	F01_transcript_165854
F01_transcript_79664	NA	F01_transcript_79664	NA	F01_transcript_79664
F01_transcript_86353	NA	F01_transcript_86353	NA	NA
F01_transcript_138375	F01_transcript_138375	F01_transcript_138375	F01_transcript_138375	F01_transcript_138375
F01_transcript_149227	F01_transcript_149227	F01_transcript_149227	NA	NA
F01_transcript_18051	NA	NA	NA	F01_transcript_18051
F01_transcript_100636	F01_transcript_100636	F01_transcript_100636	F01_transcript_100636	F01_transcript_100636
F01_transcript_69525	F01_transcript_69525	F01_transcript_69525	NA	F01_transcript_69525
F01_transcript_49750	NA	NA	NA	F01_transcript_49750
F01_transcript_150526	NA	F01_transcript_150526	NA	NA
F01_transcript_107126	F01_transcript_107126	F01_transcript_107126	F01_transcript_107126	F01_transcript_107126

F01_transcript_158770	NA	F01_transcript_158770	NA	NA
F01_transcript_163208	NA	F01_transcript_163208	NA	F01_transcript_163208
F01_transcript_150354	NA	F01_transcript_150354	NA	F01_transcript_150354
F01_transcript_105519	F01_transcript_105519	F01_transcript_105519	F01_transcript_105519	F01_transcript_105519
F01_transcript_55930	NA	NA	NA	F01_transcript_55930
F01_transcript_147303	NA	F01_transcript_147303	NA	NA
F01_transcript_154463	NA	F01_transcript_154463	NA	NA
F01_transcript_69625	NA	NA	NA	F01_transcript_69625
F01_transcript_84791	NA	NA	NA	F01_transcript_84791
F01_transcript_75459	NA	NA	NA	F01_transcript_75459
F01_transcript_150472	F01_transcript_150472	F01_transcript_150472	F01_transcript_150472	F01_transcript_150472
F01_transcript_102942	NA	F01_transcript_102942	NA	F01_transcript_102942
F01_transcript_157229	NA	F01_transcript_157229	NA	F01_transcript_157229
F01_transcript_95980	NA	F01_transcript_95980	NA	NA

F01_transcript_153349	NA	F01_transcript_153349	NA	NA
F01_transcript_144679	NA	F01_transcript_144679	F01_transcript_144679	F01_transcript_144679
F01_transcript_59041	NA	F01_transcript_59041	NA	F01_transcript_59041
F01_transcript_79091	NA	NA	NA	F01_transcript_79091
F01_transcript_138958	NA	NA	NA	F01_transcript_138958
F01_transcript_47335	NA	F01_transcript_47335	NA	NA
F01_transcript_97855	NA	F01_transcript_97855	NA	NA
F01_transcript_147137	NA	NA	F01_transcript_147137	NA
F01_transcript_126816	NA	F01_transcript_126816	NA	NA
F01_transcript_62682	NA	NA	NA	F01_transcript_62682
F01_transcript_166673	F01_transcript_166673	F01_transcript_166673	F01_transcript_166673	F01_transcript_166673
F01_transcript_3337	NA	NA	NA	F01_transcript_3337
F01_transcript_79528	NA	NA	NA	F01_transcript_79528
F01_transcript_57194	NA	NA	F01_transcript_57194	NA

F01_transcript_108853	NA	NA	NA	F01_transcript_108853
F01_transcript_153419	F01_transcript_153419	F01_transcript_153419	NA	F01_transcript_153419
F01_transcript_141777	NA	F01_transcript_141777	NA	NA
F01_transcript_55674	F01_transcript_55674	F01_transcript_55674	F01_transcript_55674	NA
F01_transcript_92377	NA	NA	NA	F01_transcript_92377
F01_transcript_63371	NA	F01_transcript_63371	NA	NA
F01_transcript_88668	F01_transcript_88668	F01_transcript_88668	F01_transcript_88668	F01_transcript_88668
F01_transcript_44960	NA	F01_transcript_44960	NA	F01_transcript_44960
F01_transcript_142514	NA	NA	NA	F01_transcript_142514
F01_transcript_94480	NA	NA	NA	F01_transcript_94480
F01_transcript_59214	NA	NA	NA	F01_transcript_59214
F01_transcript_47787	NA	F01_transcript_47787	NA	NA
F01_transcript_83068	F01_transcript_83068	F01_transcript_83068	NA	NA
F01_transcript_24989	F01_transcript_24989	F01_transcript_24989	F01_transcript_24989	F01_transcript_24989

F01_transcript_126757	NA	F01_transcript_126757	NA	NA
F01_transcript_113048	NA	F01_transcript_113048	NA	F01_transcript_113048
F01_transcript_61777	F01_transcript_61777	F01_transcript_61777	NA	NA
F01_transcript_104563	NA	NA	NA	F01_transcript_104563
F01_transcript_49667	NA	F01_transcript_49667	NA	NA
F01_transcript_105968	NA	F01_transcript_105968	NA	F01_transcript_105968
F01_transcript_61836	NA	F01_transcript_61836	NA	NA
F01_transcript_101030	NA	NA	NA	F01_transcript_101030
F01_transcript_68047	NA	F01_transcript_68047	NA	NA
F01_transcript_64299	F01_transcript_64299	F01_transcript_64299	F01_transcript_64299	F01_transcript_64299
F01_transcript_70928	NA	NA	NA	F01_transcript_70928
F01_transcript_83463	NA	NA	NA	F01_transcript_83463
F01_transcript_79104	NA	NA	NA	F01_transcript_79104
F01_transcript_113840	NA	NA	NA	F01_transcript_113840

F01_transcript_59796	NA	F01_transcript_59796	NA	NA
F01_transcript_52559	NA	F01_transcript_52559	F01_transcript_52559	NA
F01_transcript_87494	NA	F01_transcript_87494	NA	F01_transcript_87494
F01_transcript_144307	NA	F01_transcript_144307	NA	NA
F01_transcript_71892	NA	F01_transcript_71892	NA	NA
F01_transcript_135795	NA	NA	F01_transcript_135795	F01_transcript_135795
F01_transcript_67723	NA	NA	NA	F01_transcript_67723
F01_transcript_61066	NA	NA	NA	F01_transcript_61066
F01_transcript_100915	NA	NA	NA	F01_transcript_100915
F01_transcript_79744	NA	F01_transcript_79744	F01_transcript_79744	F01_transcript_79744
F01_transcript_100292	NA	F01_transcript_100292	NA	F01_transcript_100292
F01_transcript_7840	NA	F01_transcript_7840	NA	F01_transcript_7840
F01_transcript_63983	NA	NA	NA	F01_transcript_63983
F01_transcript_84466	NA	NA	NA	F01_transcript_84466

F01_transcript_921	NA	NA	NA	F01_transcript_921
F01_transcript_49189	NA	F01_transcript_49189	NA	NA
F01_transcript_146548	NA	NA	NA	F01_transcript_146548
F01_transcript_107547	NA	NA	NA	F01_transcript_107547
F01_transcript_59294	F01_transcript_59294	F01_transcript_59294	F01_transcript_59294	F01_transcript_59294
F01_transcript_105885	NA	F01_transcript_105885	NA	F01_transcript_105885
F01_transcript_76685	NA	NA	NA	F01_transcript_76685
F01_transcript_55101	NA	F01_transcript_55101	NA	NA
F01_transcript_73243	NA	NA	NA	F01_transcript_73243
F01_transcript_82215	NA	F01_transcript_82215	NA	F01_transcript_82215
F01_transcript_160476	NA	F01_transcript_160476	NA	NA
F01_transcript_69185	F01_transcript_69185	F01_transcript_69185	NA	F01_transcript_69185
F01_transcript_118716	F01_transcript_118716	F01_transcript_118716	NA	F01_transcript_118716
F01_transcript_1641	NA	F01_transcript_1641	NA	NA

F01_transcript_53855	NA	NA	NA	F01_transcript_53855
F01_transcript_96924	NA	NA	NA	F01_transcript_96924
F01_transcript_32200	NA	NA	NA	F01_transcript_32200
F01_transcript_110649	NA	NA	NA	F01_transcript_110649
F01_transcript_111827	NA	NA	NA	F01_transcript_111827
F01_transcript_124317	NA	NA	NA	F01_transcript_124317
F01_transcript_156157	NA	NA	NA	F01_transcript_156157
F01_transcript_49103	NA	F01_transcript_49103	F01_transcript_49103	F01_transcript_49103
F01_transcript_127226	F01_transcript_127226	F01_transcript_127226	F01_transcript_127226	F01_transcript_127226
F01_transcript_125617	NA	NA	NA	F01_transcript_125617
F01_transcript_10018	NA	NA	NA	F01_transcript_10018
F01_transcript_89068	NA	NA	F01_transcript_89068	F01_transcript_89068
F01_transcript_71066	NA	F01_transcript_71066	F01_transcript_71066	NA
F01_transcript_48126	NA	NA	NA	F01_transcript_48126

F01_transcript_165767	NA	F01_transcript_165767	NA	F01_transcript_165767
F01_transcript_51844	NA	F01_transcript_51844	F01_transcript_51844	NA
F01_transcript_60184	NA	NA	NA	F01_transcript_60184
F01_transcript_38307	NA	NA	NA	F01_transcript_38307
F01_transcript_145385	NA	F01_transcript_145385	NA	F01_transcript_145385
F01_transcript_150942	NA	NA	NA	F01_transcript_150942
F01_transcript_106961	F01_transcript_106961	F01_transcript_106961	F01_transcript_106961	F01_transcript_106961
F01_transcript_161133	F01_transcript_161133	F01_transcript_161133	F01_transcript_161133	F01_transcript_161133
F01_transcript_60888	NA	NA	NA	F01_transcript_60888
F01_transcript_123548	F01_transcript_123548	F01_transcript_123548	F01_transcript_123548	F01_transcript_123548
F01_transcript_73237	NA	F01_transcript_73237	F01_transcript_73237	F01_transcript_73237
F01_transcript_75478	NA	F01_transcript_75478	NA	NA
F01_transcript_130394	F01_transcript_130394	F01_transcript_130394	F01_transcript_130394	F01_transcript_130394
F01_transcript_160803	F01_transcript_160803	F01_transcript_160803	F01_transcript_160803	F01_transcript_160803

F01_transcript_156577	NA	F01_transcript_156577	F01_transcript_156577	F01_transcript_156577
F01_transcript_151708	NA	NA	F01_transcript_151708	F01_transcript_151708
F01_transcript_31928	NA	NA	NA	F01_transcript_31928
F01_transcript_51397	NA	F01_transcript_51397	NA	NA
F01_transcript_118399	F01_transcript_118399	F01_transcript_118399	F01_transcript_118399	F01_transcript_118399
F01_transcript_144133	NA	F01_transcript_144133	NA	F01_transcript_144133
F01_transcript_94647	NA	NA	NA	F01_transcript_94647
F01_transcript_63091	NA	F01_transcript_63091	F01_transcript_63091	F01_transcript_63091
F01_transcript_87337	NA	NA	NA	F01_transcript_87337
F01_transcript_56900	NA	NA	F01_transcript_56900	NA
F01_transcript_95437	F01_transcript_95437	F01_transcript_95437	F01_transcript_95437	F01_transcript_95437
F01_transcript_38634	NA	F01_transcript_38634	NA	NA
F01_transcript_149185	F01_transcript_149185	F01_transcript_149185	F01_transcript_149185	F01_transcript_149185
F01_transcript_82859	NA	NA	NA	F01_transcript_82859

F01_transcript_139731	NA	F01_transcript_139731	NA	NA
F01_transcript_144259	NA	F01_transcript_144259	NA	F01_transcript_144259
F01_transcript_124939	F01_transcript_124939	F01_transcript_124939	F01_transcript_124939	F01_transcript_124939
F01_transcript_146469	NA	F01_transcript_146469	NA	NA
F01_transcript_56316	F01_transcript_56316	F01_transcript_56316	F01_transcript_56316	F01_transcript_56316
F01_transcript_70902	NA	F01_transcript_70902	F01_transcript_70902	NA
F01_transcript_56503	F01_transcript_56503	F01_transcript_56503	F01_transcript_56503	F01_transcript_56503
F01_transcript_52682	NA	F01_transcript_52682	NA	NA
F01_transcript_134951	NA	F01_transcript_134951	NA	NA
F01_transcript_121393	NA	F01_transcript_121393	NA	NA
F01_transcript_89192	F01_transcript_89192	F01_transcript_89192	F01_transcript_89192	F01_transcript_89192
F01_transcript_166743	NA	F01_transcript_166743	NA	F01_transcript_166743
F01_transcript_137786	F01_transcript_137786	F01_transcript_137786	F01_transcript_137786	F01_transcript_137786
F01_transcript_125938	F01_transcript_125938	F01_transcript_125938	F01_transcript_125938	F01_transcript_125938

F01_transcript_51168	F01_transcript_51168	F01_transcript_51168	F01_transcript_51168	F01_transcript_51168
F01_transcript_63369	NA	F01_transcript_63369	NA	F01_transcript_63369
F01_transcript_103781	NA	F01_transcript_103781	NA	F01_transcript_103781
F01_transcript_26355	NA	NA	NA	F01_transcript_26355
F01_transcript_50491	NA	NA	NA	F01_transcript_50491
F01_transcript_52976	F01_transcript_52976	F01_transcript_52976	F01_transcript_52976	F01_transcript_52976
F01_transcript_128140	NA	NA	NA	F01_transcript_128140
F01_transcript_139392	F01_transcript_139392	F01_transcript_139392	F01_transcript_139392	F01_transcript_139392
F01_transcript_51395	NA	F01_transcript_51395	NA	NA
F01_transcript_101096	F01_transcript_101096	F01_transcript_101096	F01_transcript_101096	NA
F01_transcript_87003	NA	NA	NA	F01_transcript_87003
F01_transcript_136758	NA	NA	NA	F01_transcript_136758
F01_transcript_163094	F01_transcript_163094	F01_transcript_163094	F01_transcript_163094	F01_transcript_163094
F01_transcript_143934	F01_transcript_143934	F01_transcript_143934	F01_transcript_143934	F01_transcript_143934

F01_transcript_54343	NA	F01_transcript_54343	F01_transcript_54343	NA
F01_transcript_102276	NA	NA	NA	F01_transcript_102276
F01_transcript_117910	NA	NA	NA	F01_transcript_117910
F01_transcript_62086	NA	F01_transcript_62086	NA	F01_transcript_62086
F01_transcript_72312	F01_transcript_72312	F01_transcript_72312	F01_transcript_72312	F01_transcript_72312
F01_transcript_15677	F01_transcript_15677	F01_transcript_15677	NA	NA
F01_transcript_42360	NA	F01_transcript_42360	NA	F01_transcript_42360
F01_transcript_93701	NA	NA	NA	F01_transcript_93701
F01_transcript_24340	NA	NA	NA	F01_transcript_24340
F01_transcript_67248	NA	NA	F01_transcript_67248	NA
F01_transcript_122996	NA	NA	NA	F01_transcript_122996
F01_transcript_68332	NA	NA	NA	F01_transcript_68332
F01_transcript_54949	NA	NA	NA	F01_transcript_54949
F01_transcript_48003	NA	NA	NA	F01_transcript_48003

F01_transcript_34300	F01_transcript_34300	F01_transcript_34300	F01_transcript_34300	F01_transcript_34300
F01_transcript_95194	NA	NA	NA	F01_transcript_95194
F01_transcript_107844	F01_transcript_107844	F01_transcript_107844	F01_transcript_107844	F01_transcript_107844
F01_transcript_116938	F01_transcript_116938	F01_transcript_116938	F01_transcript_116938	F01_transcript_116938
F01_transcript_24371	NA	NA	NA	F01_transcript_24371
F01_transcript_126541	NA	NA	NA	F01_transcript_126541
F01_transcript_6100	F01_transcript_6100	F01_transcript_6100	NA	NA
F01_transcript_53075	NA	F01_transcript_53075	NA	F01_transcript_53075
F01_transcript_111240	F01_transcript_111240	F01_transcript_111240	F01_transcript_111240	F01_transcript_111240
F01_transcript_130590	NA	F01_transcript_130590	F01_transcript_130590	F01_transcript_130590
F01_transcript_92711	F01_transcript_92711	F01_transcript_92711	NA	NA
F01_transcript_124920	NA	NA	NA	F01_transcript_124920
F01_transcript_154387	NA	NA	F01_transcript_154387	NA
F01_transcript_144327	NA	NA	NA	F01_transcript_144327

F01_transcript_115785	F01_transcript_115785	F01_transcript_115785	NA	NA
F01_transcript_108779	F01_transcript_108779	F01_transcript_108779	F01_transcript_108779	F01_transcript_108779
F01_transcript_110430	NA	NA	NA	F01_transcript_110430
F01_transcript_150681	NA	F01_transcript_150681	NA	F01_transcript_150681
F01_transcript_134425	NA	F01_transcript_134425	NA	F01_transcript_134425
F01_transcript_100258	NA	F01_transcript_100258	F01_transcript_100258	NA
F01_transcript_57183	NA	NA	F01_transcript_57183	NA
F01_transcript_110536	F01_transcript_110536	F01_transcript_110536	F01_transcript_110536	F01_transcript_110536
F01_transcript_64712	NA	F01_transcript_64712	NA	F01_transcript_64712
F01_transcript_56496	NA	NA	NA	F01_transcript_56496
F01_transcript_72476	NA	F01_transcript_72476	NA	F01_transcript_72476
F01_transcript_96450	F01_transcript_96450	F01_transcript_96450	F01_transcript_96450	F01_transcript_96450
F01_transcript_68309	NA	F01_transcript_68309	NA	F01_transcript_68309
F01_transcript_93545	F01_transcript_93545	F01_transcript_93545	NA	F01_transcript_93545

F01_transcript_32290	NA	NA	NA	F01_transcript_32290
F01_transcript_14148	NA	NA	NA	F01_transcript_14148
F01_transcript_85206	NA	NA	NA	F01_transcript_85206
F01_transcript_148039	NA	NA	NA	F01_transcript_148039
F01_transcript_97319	NA	NA	NA	F01_transcript_97319
F01_transcript_142932	NA	NA	NA	F01_transcript_142932
F01_transcript_90313	NA	NA	NA	F01_transcript_90313
F01_transcript_106602	NA	F01_transcript_106602	NA	NA
F01_transcript_91177	F01_transcript_91177	F01_transcript_91177	F01_transcript_91177	F01_transcript_91177
F01_transcript_107593	NA	F01_transcript_107593	NA	NA
F01_transcript_72747	NA	F01_transcript_72747	NA	NA
F01_transcript_164719	F01_transcript_164719	F01_transcript_164719	NA	NA
F01_transcript_55458	F01_transcript_55458	F01_transcript_55458	F01_transcript_55458	F01_transcript_55458
F01_transcript_65307	NA	F01_transcript_65307	F01_transcript_65307	F01_transcript_65307

F01_transcript_110304	NA	F01_transcript_110304	F01_transcript_110304	F01_transcript_110304
F01_transcript_46811	NA	F01_transcript_46811	NA	NA
F01_transcript_61212	F01_transcript_61212	F01_transcript_61212	F01_transcript_61212	F01_transcript_61212
F01_transcript_27745	NA	NA	NA	F01_transcript_27745
F01_transcript_107350	NA	NA	NA	F01_transcript_107350
F01_transcript_147580	NA	NA	NA	F01_transcript_147580
F01_transcript_58247	NA	F01_transcript_58247	NA	F01_transcript_58247
F01_transcript_93031	F01_transcript_93031	F01_transcript_93031	NA	F01_transcript_93031
F01_transcript_45945	NA	F01_transcript_45945	NA	NA
F01_transcript_145525	NA	NA	NA	F01_transcript_145525
F01_transcript_79139	NA	NA	NA	F01_transcript_79139
F01_transcript_5224	NA	NA	NA	F01_transcript_5224
F01_transcript_63292	F01_transcript_63292	F01_transcript_63292	F01_transcript_63292	F01_transcript_63292
F01_transcript_74044	NA	NA	NA	F01_transcript_74044

F01_transcript_72488	NA	NA	NA	F01_transcript_72488
F01_transcript_86650	F01_transcript_86650	F01_transcript_86650	NA	F01_transcript_86650
F01_transcript_115399	NA	NA	NA	F01_transcript_115399
F01_transcript_102704	NA	NA	NA	F01_transcript_102704
F01_transcript_155375	NA	F01_transcript_155375	NA	NA
F01_transcript_52749	F01_transcript_52749	F01_transcript_52749	F01_transcript_52749	F01_transcript_52749
F01_transcript_17494	F01_transcript_17494	F01_transcript_17494	F01_transcript_17494	F01_transcript_17494
F01_transcript_161872	NA	F01_transcript_161872	NA	NA
F01_transcript_95285	NA	NA	NA	F01_transcript_95285
F01_transcript_52898	NA	NA	NA	F01_transcript_52898
F01_transcript_108365	F01_transcript_108365	F01_transcript_108365	NA	F01_transcript_108365
F01_transcript_110632	NA	NA	F01_transcript_110632	F01_transcript_110632
F01_transcript_159050	F01_transcript_159050	F01_transcript_159050	F01_transcript_159050	F01_transcript_159050
F01_transcript_142343	NA	F01_transcript_142343	F01_transcript_142343	F01_transcript_142343

F01_transcript_65148	NA	NA	NA	F01_transcript_65148
F01_transcript_44430	NA	NA	NA	F01_transcript_44430
F01_transcript_50117	NA	F01_transcript_50117	NA	F01_transcript_50117
F01_transcript_101158	F01_transcript_101158	F01_transcript_101158	NA	F01_transcript_101158
F01_transcript_143608	F01_transcript_143608	F01_transcript_143608	F01_transcript_143608	F01_transcript_143608
F01_transcript_94101	NA	NA	NA	F01_transcript_94101
F01_transcript_76550	F01_transcript_76550	F01_transcript_76550	F01_transcript_76550	F01_transcript_76550
F01_transcript_158380	NA	F01_transcript_158380	NA	NA
F01_transcript_145899	NA	F01_transcript_145899	NA	NA
F01_transcript_134832	NA	F01_transcript_134832	F01_transcript_134832	F01_transcript_134832
F01_transcript_126199	F01_transcript_126199	F01_transcript_126199	F01_transcript_126199	F01_transcript_126199
F01_transcript_136640	F01_transcript_136640	F01_transcript_136640	NA	NA
F01_transcript_155346	NA	F01_transcript_155346	NA	NA
F01_transcript_98639	NA	F01_transcript_98639	NA	F01_transcript_98639

F01_transcript_85010	F01_transcript_85010	F01_transcript_85010	F01_transcript_85010	F01_transcript_85010
F01_transcript_117304	F01_transcript_117304	F01_transcript_117304	F01_transcript_117304	F01_transcript_117304
F01_transcript_153315	NA	NA	NA	F01_transcript_153315
F01_transcript_84121	F01_transcript_84121	F01_transcript_84121	F01_transcript_84121	F01_transcript_84121
F01_transcript_110368	NA	NA	NA	F01_transcript_110368
F01_transcript_133611	NA	F01_transcript_133611	NA	NA
F01_transcript_141664	F01_transcript_141664	F01_transcript_141664	F01_transcript_141664	F01_transcript_141664
F01_transcript_82017	NA	NA	NA	F01_transcript_82017
F01_transcript_134648	NA	NA	NA	F01_transcript_134648
F01_transcript_47661	NA	NA	NA	F01_transcript_47661
F01_transcript_118796	NA	F01_transcript_118796	NA	F01_transcript_118796
F01_transcript_142132	NA	F01_transcript_142132	F01_transcript_142132	F01_transcript_142132
F01_transcript_54890	NA	NA	NA	F01_transcript_54890
F01_transcript_65116	NA	NA	F01_transcript_65116	NA

F01_transcript_135855	F01_transcript_135855	F01_transcript_135855	F01_transcript_135855	NA
F01_transcript_118031	NA	F01_transcript_118031	NA	F01_transcript_118031
F01_transcript_66750	NA	NA	NA	F01_transcript_66750
F01_transcript_75762	NA	NA	NA	F01_transcript_75762
F01_transcript_136136	F01_transcript_136136	F01_transcript_136136	F01_transcript_136136	F01_transcript_136136
F01_transcript_12248	NA	F01_transcript_12248	F01_transcript_12248	NA
F01_transcript_97279	NA	F01_transcript_97279	NA	NA
F01_transcript_100609	NA	NA	NA	F01_transcript_100609
F01_transcript_148768	F01_transcript_148768	F01_transcript_148768	NA	NA
F01_transcript_69961	NA	NA	NA	F01_transcript_69961
F01_transcript_48850	F01_transcript_48850	NA	NA	F01_transcript_48850
F01_transcript_87409	NA	F01_transcript_87409	NA	NA
F01_transcript_51069	F01_transcript_51069	F01_transcript_51069	F01_transcript_51069	F01_transcript_51069
F01_transcript_69803	F01_transcript_69803	F01_transcript_69803	F01_transcript_69803	F01_transcript_69803

F01_transcript_52129	F01_transcript_52129	F01_transcript_52129	NA	F01_transcript_52129
F01_transcript_162676	F01_transcript_162676	F01_transcript_162676	F01_transcript_162676	F01_transcript_162676
F01_transcript_28412	NA	NA	NA	F01_transcript_28412
F01_transcript_139442	F01_transcript_139442	F01_transcript_139442	F01_transcript_139442	NA
F01_transcript_80164	NA	NA	NA	F01_transcript_80164
F01_transcript_113741	F01_transcript_113741	F01_transcript_113741	F01_transcript_113741	NA
F01_transcript_99785	F01_transcript_99785	F01_transcript_99785	F01_transcript_99785	F01_transcript_99785
F01_transcript_53477	NA	NA	NA	F01_transcript_53477
F01_transcript_119050	NA	NA	NA	F01_transcript_119050
F01_transcript_158470	NA	F01_transcript_158470	NA	F01_transcript_158470
F01_transcript_81139	F01_transcript_81139	F01_transcript_81139	F01_transcript_81139	NA
F01_transcript_144790	NA	NA	NA	F01_transcript_144790
F01_transcript_84027	NA	NA	NA	F01_transcript_84027
F01_transcript_64066	NA	NA	NA	F01_transcript_64066

F01_transcript_117528	F01_transcript_117528	F01_transcript_117528	NA	NA
F01_transcript_108540	F01_transcript_108540	F01_transcript_108540	NA	NA
F01_transcript_76386	NA	F01_transcript_76386	NA	NA
F01_transcript_130953	F01_transcript_130953	F01_transcript_130953	NA	F01_transcript_130953
F01_transcript_2799	NA	NA	NA	F01_transcript_2799
F01_transcript_86484	NA	F01_transcript_86484	NA	F01_transcript_86484
F01_transcript_62528	F01_transcript_62528	F01_transcript_62528	NA	F01_transcript_62528
F01_transcript_86221	F01_transcript_86221	F01_transcript_86221	NA	F01_transcript_86221
F01_transcript_93493	NA	F01_transcript_93493	NA	NA
F01_transcript_54084	NA	NA	NA	F01_transcript_54084
F01_transcript_70241	NA	NA	NA	F01_transcript_70241
F01_transcript_130937	F01_transcript_130937	F01_transcript_130937	F01_transcript_130937	F01_transcript_130937
F01_transcript_108857	NA	F01_transcript_108857	NA	F01_transcript_108857
F01_transcript_46218	F01_transcript_46218	F01_transcript_46218	NA	NA

F01_transcript_50576	NA	NA	NA	F01_transcript_50576
F01_transcript_68257	NA	F01_transcript_68257	F01_transcript_68257	F01_transcript_68257
F01_transcript_150253	NA	F01_transcript_150253	F01_transcript_150253	F01_transcript_150253
F01_transcript_127044	NA	F01_transcript_127044	NA	NA
F01_transcript_64882	F01_transcript_64882	F01_transcript_64882	F01_transcript_64882	F01_transcript_64882
F01_transcript_44045	F01_transcript_44045	F01_transcript_44045	F01_transcript_44045	F01_transcript_44045
F01_transcript_113143	NA	F01_transcript_113143	NA	NA
F01_transcript_99773	NA	F01_transcript_99773	NA	NA
F01_transcript_109872	NA	NA	NA	F01_transcript_109872
F01_transcript_72420	NA	F01_transcript_72420	NA	NA
F01_transcript_157599	F01_transcript_157599	F01_transcript_157599	NA	NA
F01_transcript_53806	NA	F01_transcript_53806	NA	NA
F01_transcript_62248	NA	NA	NA	F01_transcript_62248
F01_transcript_164221	F01_transcript_164221	F01_transcript_164221	F01_transcript_164221	F01_transcript_164221

F01_transcript_61795	NA	F01_transcript_61795	F01_transcript_61795	F01_transcript_61795
F01_transcript_167676	NA	NA	NA	F01_transcript_167676
F01_transcript_129371	F01_transcript_129371	F01_transcript_129371	F01_transcript_129371	F01_transcript_129371
F01_transcript_96710	F01_transcript_96710	F01_transcript_96710	F01_transcript_96710	F01_transcript_96710
F01_transcript_69822	NA	NA	NA	F01_transcript_69822
F01_transcript_105390	NA	NA	F01_transcript_105390	F01_transcript_105390
F01_transcript_132912	NA	F01_transcript_132912	NA	NA
F01_transcript_149778	NA	F01_transcript_149778	NA	NA
F01_transcript_141615	NA	NA	NA	F01_transcript_141615
F01_transcript_166343	NA	NA	NA	F01_transcript_166343
F01_transcript_101374	NA	F01_transcript_101374	NA	NA
F01_transcript_154479	NA	NA	NA	F01_transcript_154479
F01_transcript_109353	F01_transcript_109353	F01_transcript_109353	F01_transcript_109353	F01_transcript_109353
F01_transcript_145574	NA	F01_transcript_145574	NA	F01_transcript_145574

F01_transcript_128257	NA	NA	NA	F01_transcript_128257
F01_transcript_107738	NA	NA	NA	F01_transcript_107738
F01_transcript_127963	NA	F01_transcript_127963	NA	NA
F01_transcript_7498	NA	NA	NA	F01_transcript_7498
F01_transcript_163047	NA	F01_transcript_163047	NA	NA
F01_transcript_94319	NA	F01_transcript_94319	NA	NA
F01_transcript_66614	NA	F01_transcript_66614	NA	F01_transcript_66614
F01_transcript_52288	NA	F01_transcript_52288	NA	NA
F01_transcript_73673	F01_transcript_73673	F01_transcript_73673	F01_transcript_73673	NA
F01_transcript_156749	NA	F01_transcript_156749	NA	NA
F01_transcript_125190	F01_transcript_125190	F01_transcript_125190	NA	NA
F01_transcript_71166	NA	F01_transcript_71166	F01_transcript_71166	F01_transcript_71166
F01_transcript_156014	F01_transcript_156014	F01_transcript_156014	NA	F01_transcript_156014
F01_transcript_152278	F01_transcript_152278	F01_transcript_152278	F01_transcript_152278	F01_transcript_152278

F01_transcript_27800	NA	NA	NA	F01_transcript_27800
F01_transcript_86339	NA	NA	NA	F01_transcript_86339
F01_transcript_62545	F01_transcript_62545	F01_transcript_62545	F01_transcript_62545	F01_transcript_62545
F01_transcript_160016	F01_transcript_160016	F01_transcript_160016	F01_transcript_160016	F01_transcript_160016
F01_transcript_59389	NA	F01_transcript_59389	F01_transcript_59389	F01_transcript_59389
F01_transcript_46046	NA	NA	NA	F01_transcript_46046
F01_transcript_148914	NA	F01_transcript_148914	NA	NA
F01_transcript_56409	NA	F01_transcript_56409	F01_transcript_56409	F01_transcript_56409
F01_transcript_28685	NA	NA	NA	F01_transcript_28685
F01_transcript_153320	NA	F01_transcript_153320	NA	NA
F01_transcript_70802	NA	F01_transcript_70802	NA	NA
F01_transcript_122684	NA	NA	NA	F01_transcript_122684
F01_transcript_81032	NA	NA	NA	F01_transcript_81032
F01_transcript_54736	NA	NA	NA	F01_transcript_54736

F01_transcript_114039	NA	F01_transcript_114039	NA	F01_transcript_114039
F01_transcript_54834	F01_transcript_54834	F01_transcript_54834	F01_transcript_54834	F01_transcript_54834
F01_transcript_121453	NA	NA	NA	F01_transcript_121453
F01_transcript_151370	F01_transcript_151370	F01_transcript_151370	NA	NA
F01_transcript_94642	NA	NA	NA	F01_transcript_94642
F01_transcript_135673	F01_transcript_135673	F01_transcript_135673	F01_transcript_135673	F01_transcript_135673
F01_transcript_95477	NA	F01_transcript_95477	F01_transcript_95477	NA
F01_transcript_24072	NA	F01_transcript_24072	NA	F01_transcript_24072
F01_transcript_135698	NA	F01_transcript_135698	NA	NA
F01_transcript_163928	NA	NA	NA	F01_transcript_163928
F01_transcript_9324	NA	F01_transcript_9324	NA	F01_transcript_9324
F01_transcript_104225	F01_transcript_104225	F01_transcript_104225	NA	F01_transcript_104225
F01_transcript_144014	NA	NA	NA	F01_transcript_144014
F01_transcript_106178	F01_transcript_106178	F01_transcript_106178	F01_transcript_106178	F01_transcript_106178

F01_transcript_37444	NA	NA	NA	F01_transcript_37444
F01_transcript_59620	NA	NA	NA	F01_transcript_59620
F01_transcript_52861	NA	F01_transcript_52861	NA	NA
F01_transcript_115913	NA	NA	NA	F01_transcript_115913
F01_transcript_151743	F01_transcript_151743	F01_transcript_151743	F01_transcript_151743	F01_transcript_151743
F01_transcript_64103	NA	NA	NA	F01_transcript_64103
F01_transcript_161369	NA	NA	NA	F01_transcript_161369
F01_transcript_132589	NA	NA	NA	F01_transcript_132589
F01_transcript_150476	NA	F01_transcript_150476	NA	NA
F01_transcript_162152	F01_transcript_162152	F01_transcript_162152	NA	F01_transcript_162152
F01_transcript_79160	F01_transcript_79160	F01_transcript_79160	F01_transcript_79160	F01_transcript_79160
F01_transcript_3921	F01_transcript_3921	F01_transcript_3921	NA	F01_transcript_3921
F01_transcript_81414	NA	NA	NA	F01_transcript_81414
F01_transcript_100745	NA	NA	NA	F01_transcript_100745

F01_transcript_59360	NA	NA	NA	F01_transcript_59360
F01_transcript_82314	NA	NA	NA	F01_transcript_82314
F01_transcript_34198	NA	NA	NA	F01_transcript_34198
F01_transcript_63425	NA	F01_transcript_63425	NA	F01_transcript_63425
F01_transcript_140248	NA	NA	NA	F01_transcript_140248
F01_transcript_29362	NA	F01_transcript_29362	NA	F01_transcript_29362
F01_transcript_64639	NA	F01_transcript_64639	NA	NA
F01_transcript_143629	NA	F01_transcript_143629	NA	NA
F01_transcript_57119	NA	NA	F01_transcript_57119	NA
F01_transcript_5344	NA	NA	NA	F01_transcript_5344
F01_transcript_128005	F01_transcript_128005	F01_transcript_128005	F01_transcript_128005	F01_transcript_128005
F01_transcript_115243	NA	NA	NA	F01_transcript_115243
F01_transcript_70786	NA	NA	F01_transcript_70786	F01_transcript_70786
F01_transcript_58258	NA	F01_transcript_58258	NA	F01_transcript_58258

F01_transcript_32420	NA	F01_transcript_32420	NA	F01_transcript_32420
F01_transcript_95994	NA	F01_transcript_95994	NA	NA
F01_transcript_160294	F01_transcript_160294	F01_transcript_160294	NA	NA
F01_transcript_155564	NA	NA	F01_transcript_155564	F01_transcript_155564
F01_transcript_100642	NA	F01_transcript_100642	F01_transcript_100642	F01_transcript_100642
F01_transcript_79770	F01_transcript_79770	F01_transcript_79770	F01_transcript_79770	F01_transcript_79770
F01_transcript_81338	NA	NA	NA	F01_transcript_81338
F01_transcript_143709	NA	NA	NA	F01_transcript_143709
F01_transcript_167113	NA	F01_transcript_167113	F01_transcript_167113	F01_transcript_167113
F01_transcript_37829	NA	NA	NA	F01_transcript_37829
F01_transcript_25297	NA	F01_transcript_25297	NA	NA
F01_transcript_74983	NA	F01_transcript_74983	NA	NA
F01_transcript_24047	NA	F01_transcript_24047	NA	NA
F01_transcript_163562	F01_transcript_163562	F01_transcript_163562	F01_transcript_163562	F01_transcript_163562

F01_transcript_53034	NA	F01_transcript_53034	NA	NA
F01_transcript_70078	F01_transcript_70078	F01_transcript_70078	NA	NA
F01_transcript_92966	F01_transcript_92966	F01_transcript_92966	F01_transcript_92966	NA
F01_transcript_77330	NA	F01_transcript_77330	NA	NA
F01_transcript_165915	NA	F01_transcript_165915	NA	NA
F01_transcript_106622	NA	F01_transcript_106622	NA	NA
F01_transcript_1407	NA	NA	NA	F01_transcript_1407
F01_transcript_150142	F01_transcript_150142	F01_transcript_150142	F01_transcript_150142	F01_transcript_150142
F01_transcript_154588	NA	NA	NA	F01_transcript_154588
F01_transcript_72710	NA	F01_transcript_72710	NA	NA
F01_transcript_67386	NA	NA	NA	F01_transcript_67386
F01_transcript_28927	NA	NA	NA	F01_transcript_28927
F01_transcript_5625	NA	NA	NA	F01_transcript_5625
F01_transcript_108368	F01_transcript_108368	F01_transcript_108368	NA	F01_transcript_108368

F01_transcript_119146	NA	F01_transcript_119146	NA	NA
F01_transcript_120837	NA	F01_transcript_120837	NA	NA
F01_transcript_77871	NA	NA	NA	F01_transcript_77871
F01_transcript_276	NA	F01_transcript_276	NA	NA
F01_transcript_110435	F01_transcript_110435	F01_transcript_110435	F01_transcript_110435	F01_transcript_110435
F01_transcript_81701	F01_transcript_81701	F01_transcript_81701	NA	NA
F01_transcript_99802	NA	NA	NA	F01_transcript_99802
F01_transcript_143461	NA	NA	NA	F01_transcript_143461
F01_transcript_75734	F01_transcript_75734	F01_transcript_75734	F01_transcript_75734	F01_transcript_75734
F01_transcript_110759	NA	F01_transcript_110759	NA	F01_transcript_110759
F01_transcript_162732	NA	NA	NA	F01_transcript_162732
F01_transcript_58002	NA	NA	NA	F01_transcript_58002
F01_transcript_107302	F01_transcript_107302	F01_transcript_107302	NA	F01_transcript_107302
F01_transcript_143471	F01_transcript_143471	F01_transcript_143471	NA	F01_transcript_143471

F01_transcript_46570	NA	F01_transcript_46570	NA	NA
F01_transcript_46315	NA	F01_transcript_46315	NA	F01_transcript_46315
F01_transcript_165594	F01_transcript_165594	F01_transcript_165594	F01_transcript_165594	F01_transcript_165594
F01_transcript_135811	NA	F01_transcript_135811	NA	F01_transcript_135811
F01_transcript_134166	NA	F01_transcript_134166	NA	F01_transcript_134166
F01_transcript_49597	NA	F01_transcript_49597	F01_transcript_49597	NA
F01_transcript_164502	NA	F01_transcript_164502	NA	F01_transcript_164502
F01_transcript_60716	F01_transcript_60716	F01_transcript_60716	NA	F01_transcript_60716
F01_transcript_97423	NA	F01_transcript_97423	NA	F01_transcript_97423
F01_transcript_84481	NA	F01_transcript_84481	NA	NA
F01_transcript_113269	NA	NA	NA	F01_transcript_113269
F01_transcript_69714	NA	F01_transcript_69714	NA	NA
F01_transcript_118133	NA	F01_transcript_118133	NA	F01_transcript_118133
F01_transcript_149820	F01_transcript_149820	F01_transcript_149820	NA	NA

F01_transcript_134755	NA	F01_transcript_134755	NA	NA
F01_transcript_23795	NA	NA	NA	F01_transcript_23795
F01_transcript_83853	NA	NA	F01_transcript_83853	F01_transcript_83853
F01_transcript_86669	NA	F01_transcript_86669	NA	NA
F01_transcript_30271	NA	NA	NA	F01_transcript_30271
F01_transcript_99541	NA	F01_transcript_99541	NA	NA
F01_transcript_139363	NA	F01_transcript_139363	NA	NA
F01_transcript_157900	F01_transcript_157900	F01_transcript_157900	NA	NA
F01_transcript_68957	NA	NA	NA	F01_transcript_68957
F01_transcript_135110	NA	F01_transcript_135110	NA	NA
F01_transcript_57878	F01_transcript_57878	F01_transcript_57878	F01_transcript_57878	F01_transcript_57878
F01_transcript_167544	NA	NA	NA	F01_transcript_167544
F01_transcript_75908	NA	NA	NA	F01_transcript_75908
F01_transcript_68805	F01_transcript_68805	F01_transcript_68805	F01_transcript_68805	F01_transcript_68805

F01_transcript_107418	F01_transcript_107418	F01_transcript_107418	F01_transcript_107418	F01_transcript_107418
F01_transcript_102112	F01_transcript_102112	F01_transcript_102112	F01_transcript_102112	F01_transcript_102112
F01_transcript_147976	NA	NA	NA	F01_transcript_147976
F01_transcript_58937	F01_transcript_58937	F01_transcript_58937	F01_transcript_58937	F01_transcript_58937
F01_transcript_110693	NA	NA	NA	F01_transcript_110693
F01_transcript_77804	F01_transcript_77804	F01_transcript_77804	F01_transcript_77804	F01_transcript_77804
F01_transcript_82074	NA	F01_transcript_82074	NA	NA
F01_transcript_63303	NA	NA	NA	F01_transcript_63303
F01_transcript_58854	F01_transcript_58854	F01_transcript_58854	F01_transcript_58854	F01_transcript_58854
F01_transcript_76706	NA	NA	NA	F01_transcript_76706
F01_transcript_157110	F01_transcript_157110	F01_transcript_157110	NA	F01_transcript_157110
F01_transcript_101217	NA	NA	NA	F01_transcript_101217
F01_transcript_144155	F01_transcript_144155	F01_transcript_144155	NA	NA
F01_transcript_161881	NA	NA	NA	F01_transcript_161881

F01_transcript_45248	NA	NA	NA	F01_transcript_45248
F01_transcript_80175	F01_transcript_80175	F01_transcript_80175	F01_transcript_80175	F01_transcript_80175
F01_transcript_158579	F01_transcript_158579	F01_transcript_158579	F01_transcript_158579	F01_transcript_158579
F01_transcript_151305	F01_transcript_151305	F01_transcript_151305	F01_transcript_151305	NA
F01_transcript_74674	NA	F01_transcript_74674	NA	NA
F01_transcript_100417	NA	NA	NA	F01_transcript_100417
F01_transcript_88831	NA	F01_transcript_88831	NA	NA
F01_transcript_42830	NA	NA	NA	F01_transcript_42830
F01_transcript_56043	F01_transcript_56043	F01_transcript_56043	F01_transcript_56043	F01_transcript_56043
F01_transcript_96937	NA	F01_transcript_96937	NA	NA
F01_transcript_65314	F01_transcript_65314	F01_transcript_65314	F01_transcript_65314	F01_transcript_65314
F01_transcript_163658	F01_transcript_163658	F01_transcript_163658	F01_transcript_163658	F01_transcript_163658
F01_transcript_161895	F01_transcript_161895	F01_transcript_161895	NA	NA
F01_transcript_150433	NA	NA	NA	F01_transcript_150433

F01_transcript_125425	F01_transcript_125425	F01_transcript_125425	F01_transcript_125425	F01_transcript_125425
F01_transcript_69222	F01_transcript_69222	F01_transcript_69222	NA	F01_transcript_69222
F01_transcript_95441	NA	F01_transcript_95441	NA	NA
F01_transcript_41595	NA	NA	NA	F01_transcript_41595
F01_transcript_9121	F01_transcript_9121	F01_transcript_9121	F01_transcript_9121	F01_transcript_9121
F01_transcript_136324	NA	NA	NA	F01_transcript_136324
F01_transcript_117692	NA	F01_transcript_117692	NA	NA
F01_transcript_137589	F01_transcript_137589	F01_transcript_137589	NA	F01_transcript_137589
F01_transcript_63945	NA	F01_transcript_63945	NA	NA
F01_transcript_131907	NA	F01_transcript_131907	NA	NA
F01_transcript_57466	F01_transcript_57466	F01_transcript_57466	F01_transcript_57466	F01_transcript_57466
F01_transcript_155874	NA	NA	NA	F01_transcript_155874
F01_transcript_5752	NA	NA	F01_transcript_5752	F01_transcript_5752
F01_transcript_84541	NA	F01_transcript_84541	NA	F01_transcript_84541

F01_transcript_66395	NA	F01_transcript_66395	NA	NA
F01_transcript_49666	NA	NA	NA	F01_transcript_49666
F01_transcript_139865	NA	F01_transcript_139865	NA	NA
F01_transcript_97317	NA	F01_transcript_97317	F01_transcript_97317	F01_transcript_97317
F01_transcript_55455	NA	F01_transcript_55455	NA	NA
F01_transcript_156442	NA	NA	NA	F01_transcript_156442
F01_transcript_50650	NA	F01_transcript_50650	NA	NA
F01_transcript_159076	NA	NA	F01_transcript_159076	NA
F01_transcript_125251	NA	F01_transcript_125251	NA	NA
F01_transcript_108575	F01_transcript_108575	F01_transcript_108575	F01_transcript_108575	F01_transcript_108575
F01_transcript_8522	NA	NA	NA	F01_transcript_8522
F01_transcript_108241	NA	NA	NA	F01_transcript_108241
F01_transcript_131535	NA	F01_transcript_131535	NA	NA
F01_transcript_57200	NA	NA	F01_transcript_57200	F01_transcript_57200

F01_transcript_66518	NA	F01_transcript_66518	NA	NA
F01_transcript_149432	F01_transcript_149432	F01_transcript_149432	F01_transcript_149432	F01_transcript_149432
F01_transcript_130747	NA	F01_transcript_130747	NA	F01_transcript_130747
F01_transcript_77760	F01_transcript_77760	F01_transcript_77760	NA	NA
F01_transcript_143143	NA	F01_transcript_143143	F01_transcript_143143	F01_transcript_143143
F01_transcript_82773	NA	NA	NA	F01_transcript_82773
F01_transcript_113091	NA	NA	NA	F01_transcript_113091
F01_transcript_61456	NA	NA	NA	F01_transcript_61456
F01_transcript_108858	F01_transcript_108858	F01_transcript_108858	F01_transcript_108858	F01_transcript_108858
F01_transcript_57086	F01_transcript_57086	F01_transcript_57086	F01_transcript_57086	F01_transcript_57086
F01_transcript_11903	NA	NA	NA	F01_transcript_11903
F01_transcript_107714	F01_transcript_107714	F01_transcript_107714	F01_transcript_107714	F01_transcript_107714
F01_transcript_147716	NA	NA	NA	F01_transcript_147716
F01_transcript_53636	NA	NA	NA	F01_transcript_53636

F01_transcript_50199	F01_transcript_50199	F01_transcript_50199	F01_transcript_50199	F01_transcript_50199
F01_transcript_160939	NA	F01_transcript_160939	NA	NA
F01_transcript_59602	NA	NA	F01_transcript_59602	NA
F01_transcript_127106	F01_transcript_127106	F01_transcript_127106	NA	NA
F01_transcript_83700	NA	F01_transcript_83700	NA	NA
F01_transcript_93102	F01_transcript_93102	F01_transcript_93102	F01_transcript_93102	F01_transcript_93102
F01_transcript_158439	F01_transcript_158439	NA	NA	NA
F01_transcript_135485	NA	F01_transcript_135485	F01_transcript_135485	F01_transcript_135485
F01_transcript_68131	NA	F01_transcript_68131	NA	F01_transcript_68131
F01_transcript_156343	F01_transcript_156343	F01_transcript_156343	F01_transcript_156343	F01_transcript_156343
F01_transcript_96680	NA	F01_transcript_96680	NA	NA
F01_transcript_108859	NA	F01_transcript_108859	F01_transcript_108859	NA
F01_transcript_88076	NA	NA	NA	F01_transcript_88076
F01_transcript_79614	NA	F01_transcript_79614	NA	NA

F01_transcript_103733	NA	NA	NA	F01_transcript_103733
F01_transcript_137431	NA	NA	NA	F01_transcript_137431
F01_transcript_65786	NA	F01_transcript_65786	NA	NA
F01_transcript_56576	NA	NA	F01_transcript_56576	NA
F01_transcript_134348	NA	NA	NA	F01_transcript_134348
F01_transcript_105520	NA	F01_transcript_105520	NA	NA
F01_transcript_39083	NA	NA	NA	F01_transcript_39083
F01_transcript_108943	NA	NA	NA	F01_transcript_108943
F01_transcript_95189	NA	F01_transcript_95189	F01_transcript_95189	F01_transcript_95189
F01_transcript_32998	NA	NA	NA	F01_transcript_32998
F01_transcript_87939	NA	F01_transcript_87939	F01_transcript_87939	F01_transcript_87939
F01_transcript_115363	NA	NA	NA	F01_transcript_115363
F01_transcript_96526	NA	F01_transcript_96526	NA	NA
F01_transcript_88167	NA	F01_transcript_88167	F01_transcript_88167	F01_transcript_88167

F01_transcript_92008	NA	NA	NA	F01_transcript_92008
F01_transcript_81934	F01_transcript_81934	F01_transcript_81934	F01_transcript_81934	F01_transcript_81934
F01_transcript_158869	NA	NA	F01_transcript_158869	NA
F01_transcript_71975	F01_transcript_71975	F01_transcript_71975	NA	F01_transcript_71975
F01_transcript_97146	NA	NA	F01_transcript_97146	NA
F01_transcript_60563	NA	NA	NA	F01_transcript_60563
F01_transcript_119527	NA	F01_transcript_119527	F01_transcript_119527	NA
F01_transcript_115208	NA	F01_transcript_115208	NA	NA
F01_transcript_165334	NA	NA	NA	F01_transcript_165334
F01_transcript_81669	F01_transcript_81669	F01_transcript_81669	NA	NA
F01_transcript_29444	NA	NA	NA	F01_transcript_29444
F01_transcript_113933	F01_transcript_113933	F01_transcript_113933	F01_transcript_113933	NA
F01_transcript_91292	NA	NA	NA	F01_transcript_91292
F01_transcript_151575	F01_transcript_151575	F01_transcript_151575	F01_transcript_151575	F01_transcript_151575

F01_transcript_82628	NA	F01_transcript_82628	F01_transcript_82628	NA
F01_transcript_148235	NA	F01_transcript_148235	NA	F01_transcript_148235
F01_transcript_146742	NA	NA	NA	F01_transcript_146742
F01_transcript_34422	NA	F01_transcript_34422	NA	NA
F01_transcript_21549	NA	F01_transcript_21549	F01_transcript_21549	F01_transcript_21549
F01_transcript_47797	NA	NA	NA	F01_transcript_47797
F01_transcript_145170	NA	F01_transcript_145170	NA	NA
F01_transcript_114564	F01_transcript_114564	F01_transcript_114564	NA	NA
F01_transcript_73173	NA	F01_transcript_73173	NA	NA
F01_transcript_113874	NA	NA	NA	F01_transcript_113874
F01_transcript_41242	NA	F01_transcript_41242	NA	F01_transcript_41242
F01_transcript_118871	NA	NA	NA	F01_transcript_118871
F01_transcript_104884	NA	F01_transcript_104884	NA	NA
F01_transcript_153668	NA	NA	NA	F01_transcript_153668

F01_transcript_7395	NA	F01_transcript_7395	NA	NA
F01_transcript_79136	NA	F01_transcript_79136	NA	NA
F01_transcript_116522	NA	NA	NA	F01_transcript_116522
F01_transcript_143141	NA	F01_transcript_143141	NA	NA
F01_transcript_138265	NA	NA	NA	F01_transcript_138265
F01_transcript_116524	NA	NA	NA	F01_transcript_116524
F01_transcript_40467	NA	F01_transcript_40467	NA	NA
F01_transcript_149414	NA	NA	NA	F01_transcript_149414
F01_transcript_96987	F01_transcript_96987	F01_transcript_96987	F01_transcript_96987	F01_transcript_96987
F01_transcript_103245	NA	F01_transcript_103245	NA	NA
F01_transcript_53077	F01_transcript_53077	F01_transcript_53077	NA	F01_transcript_53077
F01_transcript_10430	NA	F01_transcript_10430	NA	NA
F01_transcript_566	NA	NA	NA	F01_transcript_566
F01_transcript_142779	NA	NA	NA	F01_transcript_142779

F01_transcript_115977	NA	NA	NA	F01_transcript_115977
F01_transcript_71461	NA	F01_transcript_71461	NA	F01_transcript_71461
F01_transcript_60424	F01_transcript_60424	F01_transcript_60424	NA	NA
F01_transcript_23132	NA	NA	NA	F01_transcript_23132
F01_transcript_121086	NA	F01_transcript_121086	NA	NA
F01_transcript_70684	NA	F01_transcript_70684	NA	F01_transcript_70684
F01_transcript_111131	NA	NA	NA	F01_transcript_111131
F01_transcript_148146	NA	NA	NA	F01_transcript_148146
F01_transcript_68307	NA	F01_transcript_68307	NA	NA
F01_transcript_12450	NA	NA	NA	F01_transcript_12450
F01_transcript_119106	NA	F01_transcript_119106	NA	F01_transcript_119106
F01_transcript_146937	NA	F01_transcript_146937	NA	NA
F01_transcript_109868	NA	F01_transcript_109868	NA	NA
F01_transcript_116588	NA	F01_transcript_116588	NA	NA

F01_transcript_40521	NA	NA	NA	F01_transcript_40521
F01_transcript_114287	F01_transcript_114287	F01_transcript_114287	NA	F01_transcript_114287
F01_transcript_75056	F01_transcript_75056	F01_transcript_75056	NA	F01_transcript_75056
F01_transcript_137860	NA	F01_transcript_137860	F01_transcript_137860	NA
F01_transcript_97466	NA	F01_transcript_97466	F01_transcript_97466	F01_transcript_97466
F01_transcript_73416	F01_transcript_73416	F01_transcript_73416	F01_transcript_73416	F01_transcript_73416
F01_transcript_147454	NA	F01_transcript_147454	NA	NA
F01_transcript_22683	NA	NA	NA	F01_transcript_22683
F01_transcript_101510	F01_transcript_101510	F01_transcript_101510	NA	F01_transcript_101510
F01_transcript_151544	F01_transcript_151544	F01_transcript_151544	F01_transcript_151544	F01_transcript_151544
F01_transcript_150969	F01_transcript_150969	F01_transcript_150969	F01_transcript_150969	F01_transcript_150969
F01_transcript_53505	NA	NA	NA	F01_transcript_53505
F01_transcript_93431	F01_transcript_93431	F01_transcript_93431	F01_transcript_93431	NA
F01_transcript_101272	NA	F01_transcript_101272	NA	NA

F01_transcript_125736	NA	F01_transcript_125736	NA	F01_transcript_125736
F01_transcript_134515	NA	NA	NA	F01_transcript_134515
F01_transcript_143755	NA	F01_transcript_143755	NA	NA
F01_transcript_140028	NA	NA	NA	F01_transcript_140028
F01_transcript_16907	NA	NA	NA	F01_transcript_16907
F01_transcript_14211	NA	NA	NA	F01_transcript_14211
F01_transcript_54200	NA	F01_transcript_54200	NA	NA
F01_transcript_72727	NA	NA	NA	F01_transcript_72727
F01_transcript_89511	F01_transcript_89511	F01_transcript_89511	NA	NA
F01_transcript_42305	F01_transcript_42305	F01_transcript_42305	F01_transcript_42305	F01_transcript_42305
F01_transcript_146616	F01_transcript_146616	F01_transcript_146616	F01_transcript_146616	F01_transcript_146616
F01_transcript_141365	NA	F01_transcript_141365	NA	F01_transcript_141365
F01_transcript_122027	NA	NA	F01_transcript_122027	NA
F01_transcript_59203	NA	NA	NA	F01_transcript_59203

F01_transcript_145540	NA	NA	NA	F01_transcript_145540
F01_transcript_106746	NA	F01_transcript_106746	NA	NA
F01_transcript_108541	NA	F01_transcript_108541	NA	NA
F01_transcript_96841	NA	NA	NA	F01_transcript_96841
F01_transcript_81122	NA	NA	NA	F01_transcript_81122
F01_transcript_132423	NA	NA	NA	F01_transcript_132423
F01_transcript_125257	NA	F01_transcript_125257	NA	F01_transcript_125257
F01_transcript_152839	NA	F01_transcript_152839	NA	F01_transcript_152839
F01_transcript_102830	F01_transcript_102830	F01_transcript_102830	F01_transcript_102830	F01_transcript_102830
F01_transcript_15875	NA	NA	NA	F01_transcript_15875
F01_transcript_101852	F01_transcript_101852	F01_transcript_101852	F01_transcript_101852	F01_transcript_101852
F01_transcript_84477	NA	NA	NA	F01_transcript_84477
F01_transcript_141346	NA	F01_transcript_141346	NA	F01_transcript_141346
F01_transcript_107627	F01_transcript_107627	F01_transcript_107627	F01_transcript_107627	F01_transcript_107627

F01_transcript_44856	NA	NA	NA	F01_transcript_44856
F01_transcript_110230	F01_transcript_110230	F01_transcript_110230	F01_transcript_110230	NA
F01_transcript_104120	NA	F01_transcript_104120	F01_transcript_104120	F01_transcript_104120
F01_transcript_158485	F01_transcript_158485	F01_transcript_158485	NA	NA
F01_transcript_165026	F01_transcript_165026	F01_transcript_165026	F01_transcript_165026	F01_transcript_165026
F01_transcript_13240	F01_transcript_13240	F01_transcript_13240	F01_transcript_13240	NA
F01_transcript_154277	NA	F01_transcript_154277	NA	NA
F01_transcript_134433	NA	NA	NA	F01_transcript_134433
F01_transcript_66828	NA	F01_transcript_66828	NA	NA
F01_transcript_150701	NA	F01_transcript_150701	F01_transcript_150701	F01_transcript_150701
F01_transcript_102508	NA	F01_transcript_102508	NA	NA
F01_transcript_133183	NA	F01_transcript_133183	NA	NA
F01_transcript_38255	F01_transcript_38255	F01_transcript_38255	F01_transcript_38255	F01_transcript_38255
F01_transcript_47009	NA	NA	NA	F01_transcript_47009

F01_transcript_159234	NA	NA	NA	F01_transcript_159234
F01_transcript_123218	NA	F01_transcript_123218	F01_transcript_123218	NA
F01_transcript_90743	NA	NA	NA	F01_transcript_90743
F01_transcript_103751	F01_transcript_103751	F01_transcript_103751	NA	NA
F01_transcript_92189	NA	NA	NA	F01_transcript_92189
F01_transcript_109426	F01_transcript_109426	F01_transcript_109426	F01_transcript_109426	F01_transcript_109426
F01_transcript_153846	NA	NA	NA	F01_transcript_153846
F01_transcript_63967	NA	NA	NA	F01_transcript_63967
F01_transcript_105496	F01_transcript_105496	F01_transcript_105496	F01_transcript_105496	F01_transcript_105496
F01_transcript_148798	NA	NA	NA	F01_transcript_148798
F01_transcript_140437	NA	F01_transcript_140437	NA	NA
F01_transcript_163794	NA	F01_transcript_163794	F01_transcript_163794	F01_transcript_163794
F01_transcript_70639	F01_transcript_70639	F01_transcript_70639	F01_transcript_70639	NA
F01_transcript_123579	F01_transcript_123579	F01_transcript_123579	F01_transcript_123579	F01_transcript_123579

F01_transcript_78318	F01_transcript_78318	F01_transcript_78318	F01_transcript_78318	F01_transcript_78318
F01_transcript_118272	F01_transcript_118272	F01_transcript_118272	NA	F01_transcript_118272
F01_transcript_160729	F01_transcript_160729	F01_transcript_160729	F01_transcript_160729	F01_transcript_160729
F01_transcript_51713	F01_transcript_51713	F01_transcript_51713	F01_transcript_51713	F01_transcript_51713
F01_transcript_61360	NA	NA	NA	F01_transcript_61360
F01_transcript_80338	NA	NA	NA	F01_transcript_80338
F01_transcript_146923	NA	F01_transcript_146923	NA	NA
F01_transcript_116441	F01_transcript_116441	F01_transcript_116441	NA	NA
F01_transcript_143788	NA	F01_transcript_143788	NA	NA
F01_transcript_95738	F01_transcript_95738	F01_transcript_95738	F01_transcript_95738	F01_transcript_95738
F01_transcript_80132	NA	F01_transcript_80132	NA	NA
F01_transcript_94559	NA	F01_transcript_94559	NA	NA
F01_transcript_25304	NA	F01_transcript_25304	NA	F01_transcript_25304
F01_transcript_166807	F01_transcript_166807	F01_transcript_166807	F01_transcript_166807	F01_transcript_166807

F01_transcript_79960	NA	F01_transcript_79960	NA	F01_transcript_79960
F01_transcript_106026	NA	F01_transcript_106026	NA	F01_transcript_106026
F01_transcript_33272	NA	NA	NA	F01_transcript_33272
F01_transcript_58773	NA	F01_transcript_58773	NA	NA
F01_transcript_159040	NA	NA	F01_transcript_159040	NA
F01_transcript_120831	NA	F01_transcript_120831	NA	NA
F01_transcript_131227	NA	F01_transcript_131227	NA	NA
F01_transcript_158239	NA	F01_transcript_158239	NA	NA
F01_transcript_152723	NA	F01_transcript_152723	NA	NA
F01_transcript_98563	F01_transcript_98563	F01_transcript_98563	NA	F01_transcript_98563
F01_transcript_75892	NA	NA	NA	F01_transcript_75892
F01_transcript_154067	NA	NA	NA	F01_transcript_154067
F01_transcript_103993	NA	F01_transcript_103993	NA	NA
F01_transcript_56457	NA	NA	F01_transcript_56457	NA

F01_transcript_68573	NA	F01_transcript_68573	NA	NA
F01_transcript_83952	F01_transcript_83952	F01_transcript_83952	F01_transcript_83952	F01_transcript_83952
F01_transcript_104084	F01_transcript_104084	F01_transcript_104084	NA	F01_transcript_104084
F01_transcript_157837	NA	NA	NA	F01_transcript_157837
F01_transcript_158883	F01_transcript_158883	F01_transcript_158883	F01_transcript_158883	NA
F01_transcript_150655	NA	F01_transcript_150655	NA	NA
F01_transcript_152146	F01_transcript_152146	F01_transcript_152146	F01_transcript_152146	F01_transcript_152146
F01_transcript_79922	NA	NA	NA	F01_transcript_79922
F01_transcript_95707	NA	NA	NA	F01_transcript_95707
F01_transcript_155742	F01_transcript_155742	F01_transcript_155742	F01_transcript_155742	F01_transcript_155742
F01_transcript_102800	NA	F01_transcript_102800	NA	NA
F01_transcript_148615	NA	F01_transcript_148615	NA	NA
F01_transcript_78097	NA	NA	NA	F01_transcript_78097
F01_transcript_54282	NA	NA	NA	F01_transcript_54282

F01_transcript_88249	NA	NA	NA	F01_transcript_88249
F01_transcript_152707	NA	F01_transcript_152707	NA	NA
F01_transcript_118789	F01_transcript_118789	F01_transcript_118789	F01_transcript_118789	F01_transcript_118789
F01_transcript_166095	F01_transcript_166095	F01_transcript_166095	NA	F01_transcript_166095
F01_transcript_100851	NA	NA	NA	F01_transcript_100851
F01_transcript_128015	F01_transcript_128015	F01_transcript_128015	NA	NA
F01_transcript_86531	NA	F01_transcript_86531	NA	NA
F01_transcript_71877	NA	F01_transcript_71877	F01_transcript_71877	NA
F01_transcript_110539	NA	NA	NA	F01_transcript_110539
F01_transcript_127370	F01_transcript_127370	F01_transcript_127370	F01_transcript_127370	F01_transcript_127370
F01_transcript_143957	NA	F01_transcript_143957	NA	NA
F01_transcript_138702	NA	F01_transcript_138702	NA	NA
F01_transcript_75329	F01_transcript_75329	F01_transcript_75329	F01_transcript_75329	NA
F01_transcript_119183	NA	F01_transcript_119183	NA	NA

F01_transcript_104855	F01_transcript_104855	F01_transcript_104855	NA	F01_transcript_104855
F01_transcript_102196	F01_transcript_102196	F01_transcript_102196	NA	NA
F01_transcript_86921	NA	F01_transcript_86921	NA	NA
F01_transcript_136122	F01_transcript_136122	F01_transcript_136122	F01_transcript_136122	F01_transcript_136122
F01_transcript_107938	NA	NA	NA	F01_transcript_107938
F01_transcript_145398	NA	NA	NA	F01_transcript_145398
F01_transcript_15069	NA	F01_transcript_15069	NA	NA
F01_transcript_71575	NA	NA	NA	F01_transcript_71575
F01_transcript_164019	F01_transcript_164019	F01_transcript_164019	NA	NA
F01_transcript_66058	NA	NA	F01_transcript_66058	NA
F01_transcript_21969	NA	NA	NA	F01_transcript_21969
F01_transcript_6172	NA	F01_transcript_6172	NA	NA
F01_transcript_95819	F01_transcript_95819	F01_transcript_95819	F01_transcript_95819	F01_transcript_95819
F01_transcript_148367	F01_transcript_148367	F01_transcript_148367	F01_transcript_148367	F01_transcript_148367

F01_transcript_143689	NA	F01_transcript_143689	NA	F01_transcript_143689
F01_transcript_127583	NA	NA	NA	F01_transcript_127583
F01_transcript_149421	F01_transcript_149421	F01_transcript_149421	F01_transcript_149421	F01_transcript_149421
F01_transcript_10186	NA	F01_transcript_10186	F01_transcript_10186	F01_transcript_10186
F01_transcript_70672	F01_transcript_70672	F01_transcript_70672	F01_transcript_70672	F01_transcript_70672
F01_transcript_1691	NA	NA	NA	F01_transcript_1691
F01_transcript_77687	F01_transcript_77687	F01_transcript_77687	F01_transcript_77687	F01_transcript_77687
F01_transcript_93514	F01_transcript_93514	F01_transcript_93514	NA	F01_transcript_93514
F01_transcript_165337	F01_transcript_165337	F01_transcript_165337	NA	NA
F01_transcript_156876	NA	F01_transcript_156876	NA	NA
F01_transcript_11244	NA	NA	NA	F01_transcript_11244
F01_transcript_120182	NA	NA	NA	F01_transcript_120182
F01_transcript_21399	NA	F01_transcript_21399	NA	NA
F01_transcript_62895	F01_transcript_62895	F01_transcript_62895	F01_transcript_62895	F01_transcript_62895

F01_transcript_22987	NA	NA	NA	F01_transcript_22987
F01_transcript_127144	NA	F01_transcript_127144	NA	F01_transcript_127144
F01_transcript_58561	NA	F01_transcript_58561	F01_transcript_58561	F01_transcript_58561
F01_transcript_149571	NA	NA	NA	F01_transcript_149571
F01_transcript_93046	NA	F01_transcript_93046	NA	NA
F01_transcript_156673	NA	F01_transcript_156673	NA	F01_transcript_156673
F01_transcript_81881	NA	NA	NA	F01_transcript_81881
F01_transcript_85924	NA	F01_transcript_85924	F01_transcript_85924	NA
F01_transcript_71271	NA	F01_transcript_71271	F01_transcript_71271	NA
F01_transcript_144466	F01_transcript_144466	F01_transcript_144466	NA	F01_transcript_144466
F01_transcript_139404	NA	F01_transcript_139404	NA	NA
F01_transcript_62525	NA	NA	NA	F01_transcript_62525
F01_transcript_155062	F01_transcript_155062	F01_transcript_155062	F01_transcript_155062	F01_transcript_155062
F01_transcript_13589	NA	NA	NA	F01_transcript_13589

F01_transcript_87536	NA	NA	NA	F01_transcript_87536
F01_transcript_68663	F01_transcript_68663	F01_transcript_68663	NA	NA
F01_transcript_136374	NA	NA	F01_transcript_136374	NA
F01_transcript_56248	NA	NA	F01_transcript_56248	NA
F01_transcript_161282	F01_transcript_161282	F01_transcript_161282	NA	F01_transcript_161282
F01_transcript_34768	NA	NA	F01_transcript_34768	NA
F01_transcript_127338	NA	F01_transcript_127338	F01_transcript_127338	F01_transcript_127338
F01_transcript_50319	F01_transcript_50319	F01_transcript_50319	NA	NA
F01_transcript_110184	NA	F01_transcript_110184	NA	NA
F01_transcript_104860	NA	F01_transcript_104860	NA	NA
F01_transcript_67230	NA	F01_transcript_67230	F01_transcript_67230	F01_transcript_67230
F01_transcript_57697	NA	F01_transcript_57697	NA	NA
F01_transcript_54277	NA	NA	NA	F01_transcript_54277
F01_transcript_45625	NA	NA	NA	F01_transcript_45625

F01_transcript_129137	NA	NA	NA	F01_transcript_129137
F01_transcript_151289	NA	NA	NA	F01_transcript_151289
F01_transcript_116113	NA	NA	NA	F01_transcript_116113
F01_transcript_150844	NA	F01_transcript_150844	F01_transcript_150844	NA
F01_transcript_49088	NA	F01_transcript_49088	NA	NA
F01_transcript_59998	NA	NA	NA	F01_transcript_59998
F01_transcript_115554	NA	F01_transcript_115554	NA	NA
F01_transcript_73581	F01_transcript_73581	F01_transcript_73581	F01_transcript_73581	F01_transcript_73581
F01_transcript_54085	NA	NA	NA	F01_transcript_54085
F01_transcript_73642	NA	F01_transcript_73642	NA	NA
F01_transcript_137567	NA	F01_transcript_137567	NA	NA
F01_transcript_159746	F01_transcript_159746	NA	NA	F01_transcript_159746
F01_transcript_64058	NA	F01_transcript_64058	NA	NA
F01_transcript_53590	NA	NA	NA	F01_transcript_53590

F01_transcript_101951	NA	NA	NA	F01_transcript_101951
F01_transcript_56319	NA	NA	F01_transcript_56319	NA
F01_transcript_134932	NA	F01_transcript_134932	F01_transcript_134932	F01_transcript_134932
F01_transcript_92417	F01_transcript_92417	F01_transcript_92417	F01_transcript_92417	F01_transcript_92417
F01_transcript_108151	F01_transcript_108151	F01_transcript_108151	F01_transcript_108151	F01_transcript_108151
F01_transcript_164797	NA	F01_transcript_164797	NA	NA
F01_transcript_37321	NA	NA	NA	F01_transcript_37321
F01_transcript_74804	F01_transcript_74804	F01_transcript_74804	F01_transcript_74804	F01_transcript_74804
F01_transcript_102071	NA	F01_transcript_102071	NA	NA
F01_transcript_96944	NA	F01_transcript_96944	F01_transcript_96944	F01_transcript_96944
F01_transcript_56391	NA	NA	F01_transcript_56391	NA
F01_transcript_63918	F01_transcript_63918	F01_transcript_63918	F01_transcript_63918	F01_transcript_63918
F01_transcript_3895	F01_transcript_3895	F01_transcript_3895	NA	NA
F01_transcript_112727	NA	F01_transcript_112727	NA	F01_transcript_112727

F01_transcript_68242	F01_transcript_68242	F01_transcript_68242	F01_transcript_68242	F01_transcript_68242
F01_transcript_16840	NA	NA	NA	F01_transcript_16840
F01_transcript_83404	NA	F01_transcript_83404	NA	F01_transcript_83404
F01_transcript_77217	NA	F01_transcript_77217	NA	NA
F01_transcript_11897	F01_transcript_11897	F01_transcript_11897	NA	F01_transcript_11897
F01_transcript_48314	F01_transcript_48314	F01_transcript_48314	NA	F01_transcript_48314
F01_transcript_157892	NA	F01_transcript_157892	NA	F01_transcript_157892
F01_transcript_71554	NA	NA	NA	F01_transcript_71554
F01_transcript_166418	NA	F01_transcript_166418	NA	NA
F01_transcript_92992	NA	NA	NA	F01_transcript_92992
F01_transcript_96384	F01_transcript_96384	NA	NA	NA
F01_transcript_59030	NA	F01_transcript_59030	NA	NA
F01_transcript_103574	NA	F01_transcript_103574	NA	F01_transcript_103574
F01_transcript_27297	NA	NA	NA	F01_transcript_27297

F01_transcript_20446	NA	NA	NA	F01_transcript_20446
F01_transcript_79917	NA	NA	NA	F01_transcript_79917
F01_transcript_40282	NA	NA	NA	F01_transcript_40282
F01_transcript_21649	NA	NA	NA	F01_transcript_21649
F01_transcript_163944	NA	NA	NA	F01_transcript_163944
F01_transcript_113497	F01_transcript_113497	F01_transcript_113497	NA	NA
F01_transcript_135031	NA	F01_transcript_135031	NA	F01_transcript_135031
F01_transcript_109509	NA	F01_transcript_109509	F01_transcript_109509	F01_transcript_109509
F01_transcript_57083	NA	NA	F01_transcript_57083	NA
F01_transcript_59054	NA	NA	NA	F01_transcript_59054
F01_transcript_129524	F01_transcript_129524	F01_transcript_129524	F01_transcript_129524	F01_transcript_129524
F01_transcript_124149	NA	F01_transcript_124149	NA	NA
F01_transcript_33080	NA	F01_transcript_33080	NA	NA
F01_transcript_165018	NA	F01_transcript_165018	NA	F01_transcript_165018

F01_transcript_129927	F01_transcript_129927	F01_transcript_129927	F01_transcript_129927	F01_transcript_129927
F01_transcript_136373	F01_transcript_136373	F01_transcript_136373	F01_transcript_136373	F01_transcript_136373
F01_transcript_18353	F01_transcript_18353	F01_transcript_18353	F01_transcript_18353	F01_transcript_18353
F01_transcript_49141	F01_transcript_49141	F01_transcript_49141	F01_transcript_49141	F01_transcript_49141
F01_transcript_77277	NA	NA	NA	F01_transcript_77277
F01_transcript_109198	F01_transcript_109198	F01_transcript_109198	F01_transcript_109198	F01_transcript_109198
F01_transcript_90227	NA	F01_transcript_90227	NA	NA
F01_transcript_6008	F01_transcript_6008	F01_transcript_6008	F01_transcript_6008	F01_transcript_6008
F01_transcript_59276	F01_transcript_59276	F01_transcript_59276	F01_transcript_59276	F01_transcript_59276
F01_transcript_153328	NA	NA	NA	F01_transcript_153328
F01_transcript_126730	NA	NA	NA	F01_transcript_126730
F01_transcript_165098	NA	F01_transcript_165098	F01_transcript_165098	F01_transcript_165098
F01_transcript_102622	NA	NA	NA	F01_transcript_102622
F01_transcript_48577	NA	NA	NA	F01_transcript_48577

F01_transcript_71150	NA	F01_transcript_71150	F01_transcript_71150	F01_transcript_71150
F01_transcript_147101	NA	F01_transcript_147101	F01_transcript_147101	F01_transcript_147101
F01_transcript_49595	NA	F01_transcript_49595	F01_transcript_49595	F01_transcript_49595
F01_transcript_78728	NA	NA	NA	F01_transcript_78728
F01_transcript_141665	NA	F01_transcript_141665	NA	NA
F01_transcript_90947	NA	F01_transcript_90947	NA	NA
F01_transcript_159739	NA	NA	NA	F01_transcript_159739
F01_transcript_94909	NA	NA	NA	F01_transcript_94909
F01_transcript_29291	NA	NA	NA	F01_transcript_29291
F01_transcript_52264	F01_transcript_52264	F01_transcript_52264	F01_transcript_52264	F01_transcript_52264
F01_transcript_77828	NA	F01_transcript_77828	NA	NA
F01_transcript_56430	NA	NA	F01_transcript_56430	NA
F01_transcript_133792	F01_transcript_133792	F01_transcript_133792	F01_transcript_133792	F01_transcript_133792
F01_transcript_156044	NA	F01_transcript_156044	NA	F01_transcript_156044

F01_transcript_120628	NA	NA	NA	F01_transcript_120628
F01_transcript_67133	NA	F01_transcript_67133	NA	F01_transcript_67133
F01_transcript_148073	NA	F01_transcript_148073	NA	NA
F01_transcript_139269	NA	F01_transcript_139269	NA	NA
F01_transcript_26860	F01_transcript_26860	F01_transcript_26860	F01_transcript_26860	F01_transcript_26860
F01_transcript_137827	NA	NA	NA	F01_transcript_137827
F01_transcript_150698	NA	NA	NA	F01_transcript_150698
F01_transcript_73063	F01_transcript_73063	F01_transcript_73063	NA	NA
F01_transcript_109282	NA	F01_transcript_109282	NA	NA
F01_transcript_120472	NA	F01_transcript_120472	F01_transcript_120472	F01_transcript_120472
F01_transcript_8248	NA	F01_transcript_8248	NA	NA
F01_transcript_139988	F01_transcript_139988	F01_transcript_139988	F01_transcript_139988	NA
F01_transcript_57403	NA	NA	NA	F01_transcript_57403
F01_transcript_141185	NA	NA	NA	F01_transcript_141185

F01_transcript_48972	NA	F01_transcript_48972	NA	F01_transcript_48972
F01_transcript_102343	NA	NA	NA	F01_transcript_102343
F01_transcript_136575	NA	NA	NA	F01_transcript_136575
F01_transcript_154665	NA	F01_transcript_154665	NA	NA
F01_transcript_126996	F01_transcript_126996	F01_transcript_126996	NA	F01_transcript_126996
F01_transcript_109317	NA	NA	NA	F01_transcript_109317
F01_transcript_136598	NA	F01_transcript_136598	NA	NA
F01_transcript_110897	F01_transcript_110897	F01_transcript_110897	F01_transcript_110897	F01_transcript_110897
F01_transcript_124858	NA	F01_transcript_124858	NA	NA
F01_transcript_113478	NA	F01_transcript_113478	NA	NA
F01_transcript_50298	NA	F01_transcript_50298	F01_transcript_50298	F01_transcript_50298
F01_transcript_124335	NA	F01_transcript_124335	NA	NA
F01_transcript_107096	NA	F01_transcript_107096	NA	NA
F01_transcript_48635	F01_transcript_48635	F01_transcript_48635	NA	NA

F01_transcript_106463	NA	F01_transcript_106463	NA	NA
F01_transcript_125225	NA	F01_transcript_125225	NA	NA
F01_transcript_54654	NA	NA	NA	F01_transcript_54654
F01_transcript_135903	NA	F01_transcript_135903	NA	F01_transcript_135903
F01_transcript_47081	NA	F01_transcript_47081	F01_transcript_47081	F01_transcript_47081
F01_transcript_85444	NA	F01_transcript_85444	NA	NA
F01_transcript_139709	F01_transcript_139709	F01_transcript_139709	F01_transcript_139709	F01_transcript_139709
F01_transcript_40001	NA	NA	NA	F01_transcript_40001
F01_transcript_124535	NA	F01_transcript_124535	NA	NA
F01_transcript_52798	F01_transcript_52798	F01_transcript_52798	NA	NA
F01_transcript_49565	NA	NA	NA	F01_transcript_49565
F01_transcript_99410	NA	F01_transcript_99410	NA	F01_transcript_99410
F01_transcript_54663	NA	NA	NA	F01_transcript_54663
F01_transcript_25990	NA	NA	NA	F01_transcript_25990

F01_transcript_147164	NA	F01_transcript_147164	NA	NA
F01_transcript_125298	NA	F01_transcript_125298	NA	F01_transcript_125298
F01_transcript_151992	NA	F01_transcript_151992	F01_transcript_151992	NA
F01_transcript_71313	NA	F01_transcript_71313	F01_transcript_71313	F01_transcript_71313
F01_transcript_30477	NA	NA	NA	F01_transcript_30477
F01_transcript_55668	NA	NA	NA	F01_transcript_55668
F01_transcript_119012	NA	F01_transcript_119012	NA	NA
F01_transcript_93911	NA	F01_transcript_93911	F01_transcript_93911	NA
F01_transcript_43987	NA	NA	NA	F01_transcript_43987
F01_transcript_46181	NA	NA	NA	F01_transcript_46181
F01_transcript_69634	F01_transcript_69634	F01_transcript_69634	NA	NA
F01_transcript_97538	NA	NA	NA	F01_transcript_97538
F01_transcript_86999	NA	NA	NA	F01_transcript_86999
F01_transcript_26233	NA	F01_transcript_26233	F01_transcript_26233	F01_transcript_26233

F01_transcript_83481	NA	F01_transcript_83481	F01_transcript_83481	NA
F01_transcript_71397	NA	F01_transcript_71397	F01_transcript_71397	F01_transcript_71397
F01_transcript_52070	F01_transcript_52070	F01_transcript_52070	NA	NA
F01_transcript_86368	NA	NA	F01_transcript_86368	F01_transcript_86368
F01_transcript_143952	F01_transcript_143952	F01_transcript_143952	NA	NA
F01_transcript_97191	NA	F01_transcript_97191	NA	F01_transcript_97191
F01_transcript_125845	NA	F01_transcript_125845	F01_transcript_125845	F01_transcript_125845
F01_transcript_161614	NA	F01_transcript_161614	NA	F01_transcript_161614
F01_transcript_138883	F01_transcript_138883	F01_transcript_138883	F01_transcript_138883	F01_transcript_138883
F01_transcript_76086	F01_transcript_76086	F01_transcript_76086	NA	NA
F01_transcript_88709	F01_transcript_88709	F01_transcript_88709	NA	NA
F01_transcript_126701	NA	NA	F01_transcript_126701	NA
F01_transcript_123697	NA	NA	NA	F01_transcript_123697
F01_transcript_72983	NA	NA	NA	F01_transcript_72983

F01_transcript_140428	NA	F01_transcript_140428	F01_transcript_140428	F01_transcript_140428
F01_transcript_101145	NA	NA	NA	F01_transcript_101145
F01_transcript_35887	NA	F01_transcript_35887	NA	NA
F01_transcript_122997	F01_transcript_122997	F01_transcript_122997	NA	NA
F01_transcript_94353	NA	F01_transcript_94353	NA	F01_transcript_94353
F01_transcript_118987	NA	NA	NA	F01_transcript_118987
F01_transcript_44495	NA	F01_transcript_44495	NA	NA
F01_transcript_12902	NA	NA	NA	F01_transcript_12902
F01_transcript_125622	NA	NA	NA	F01_transcript_125622
F01_transcript_85392	NA	F01_transcript_85392	NA	NA
F01_transcript_72877	NA	NA	NA	F01_transcript_72877
F01_transcript_39675	NA	NA	NA	F01_transcript_39675
F01_transcript_124059	NA	F01_transcript_124059	NA	NA
F01_transcript_50377	F01_transcript_50377	NA	NA	NA

F01_transcript_149042	NA	F01_transcript_149042	NA	NA
F01_transcript_108891	NA	F01_transcript_108891	NA	NA
F01_transcript_52619	F01_transcript_52619	F01_transcript_52619	F01_transcript_52619	F01_transcript_52619
F01_transcript_110222	NA	NA	NA	F01_transcript_110222
F01_transcript_50631	F01_transcript_50631	F01_transcript_50631	NA	NA
F01_transcript_127839	NA	NA	NA	F01_transcript_127839
F01_transcript_156706	F01_transcript_156706	F01_transcript_156706	F01_transcript_156706	NA
F01_transcript_67907	NA	NA	NA	F01_transcript_67907
F01_transcript_136405	F01_transcript_136405	F01_transcript_136405	F01_transcript_136405	F01_transcript_136405
F01_transcript_87254	NA	NA	NA	F01_transcript_87254
F01_transcript_143956	NA	NA	NA	F01_transcript_143956
F01_transcript_122933	NA	F01_transcript_122933	F01_transcript_122933	F01_transcript_122933
F01_transcript_112729	NA	F01_transcript_112729	NA	F01_transcript_112729
F01_transcript_86859	NA	F01_transcript_86859	F01_transcript_86859	NA

F01_transcript_111267	NA	F01_transcript_111267	NA	NA
F01_transcript_128990	NA	NA	NA	F01_transcript_128990
F01_transcript_83311	F01_transcript_83311	F01_transcript_83311	F01_transcript_83311	F01_transcript_83311
F01_transcript_166366	NA	F01_transcript_166366	NA	NA
F01_transcript_120644	NA	F01_transcript_120644	NA	NA
F01_transcript_47344	NA	NA	NA	F01_transcript_47344
F01_transcript_62388	NA	NA	NA	F01_transcript_62388
F01_transcript_165456	NA	F01_transcript_165456	NA	NA
F01_transcript_47621	NA	F01_transcript_47621	NA	NA
F01_transcript_158952	NA	F01_transcript_158952	F01_transcript_158952	F01_transcript_158952
F01_transcript_57100	F01_transcript_57100	F01_transcript_57100	F01_transcript_57100	NA
F01_transcript_75946	NA	NA	NA	F01_transcript_75946
F01_transcript_83211	NA	NA	NA	F01_transcript_83211
F01_transcript_50588	F01_transcript_50588	F01_transcript_50588	NA	F01_transcript_50588

F01_transcript_93926	F01_transcript_93926	F01_transcript_93926	NA	F01_transcript_93926
F01_transcript_55792	NA	NA	NA	F01_transcript_55792
F01_transcript_50502	NA	F01_transcript_50502	F01_transcript_50502	F01_transcript_50502
F01_transcript_124389	NA	F01_transcript_124389	F01_transcript_124389	F01_transcript_124389
F01_transcript_149266	NA	F01_transcript_149266	NA	F01_transcript_149266
F01_transcript_150030	F01_transcript_150030	F01_transcript_150030	NA	NA
F01_transcript_9815	NA	NA	NA	F01_transcript_9815
F01_transcript_35077	NA	NA	NA	F01_transcript_35077
F01_transcript_87487	NA	NA	F01_transcript_87487	F01_transcript_87487
F01_transcript_127046	NA	F01_transcript_127046	F01_transcript_127046	NA
F01_transcript_96872	NA	NA	NA	F01_transcript_96872
F01_transcript_99603	NA	NA	NA	F01_transcript_99603
F01_transcript_109709	NA	NA	NA	F01_transcript_109709
F01_transcript_33050	NA	NA	NA	F01_transcript_33050

F01_transcript_75212	NA	F01_transcript_75212	NA	NA
F01_transcript_149811	F01_transcript_149811	F01_transcript_149811	NA	NA
F01_transcript_164271	NA	NA	NA	F01_transcript_164271
F01_transcript_148315	F01_transcript_148315	F01_transcript_148315	F01_transcript_148315	F01_transcript_148315
F01_transcript_79539	NA	NA	NA	F01_transcript_79539
F01_transcript_95064	F01_transcript_95064	F01_transcript_95064	NA	F01_transcript_95064
F01_transcript_95496	NA	F01_transcript_95496	NA	F01_transcript_95496
F01_transcript_57792	NA	NA	NA	F01_transcript_57792
F01_transcript_46938	NA	NA	NA	F01_transcript_46938
F01_transcript_156091	NA	F01_transcript_156091	NA	NA
F01_transcript_101768	NA	F01_transcript_101768	NA	NA
F01_transcript_59351	NA	NA	F01_transcript_59351	NA
F01_transcript_151626	NA	F01_transcript_151626	NA	NA
F01_transcript_47909	NA	NA	NA	F01_transcript_47909

F01_transcript_150181	F01_transcript_150181	F01_transcript_150181	NA	F01_transcript_150181
F01_transcript_85160	NA	NA	NA	F01_transcript_85160
F01_transcript_129565	NA	NA	NA	F01_transcript_129565
F01_transcript_114680	NA	F01_transcript_114680	NA	F01_transcript_114680
F01_transcript_59781	F01_transcript_59781	F01_transcript_59781	F01_transcript_59781	NA
F01_transcript_128767	NA	NA	NA	F01_transcript_128767
F01_transcript_128082	NA	NA	NA	F01_transcript_128082
F01_transcript_159117	NA	NA	F01_transcript_159117	NA
F01_transcript_152420	NA	NA	NA	F01_transcript_152420
F01_transcript_154123	F01_transcript_154123	F01_transcript_154123	F01_transcript_154123	F01_transcript_154123
F01_transcript_39517	F01_transcript_39517	F01_transcript_39517	NA	F01_transcript_39517
F01_transcript_23366	NA	NA	NA	F01_transcript_23366
F01_transcript_49209	NA	NA	F01_transcript_49209	F01_transcript_49209
F01_transcript_114754	NA	F01_transcript_114754	NA	NA

F01_transcript_147126	F01_transcript_147126	F01_transcript_147126	F01_transcript_147126	F01_transcript_147126
F01_transcript_50103	NA	F01_transcript_50103	NA	F01_transcript_50103
F01_transcript_54112	NA	NA	NA	F01_transcript_54112
F01_transcript_110629	NA	NA	NA	F01_transcript_110629
F01_transcript_135891	NA	F01_transcript_135891	NA	NA
F01_transcript_167132	F01_transcript_167132	F01_transcript_167132	F01_transcript_167132	F01_transcript_167132
F01_transcript_96576	NA	NA	NA	F01_transcript_96576
F01_transcript_167439	NA	F01_transcript_167439	NA	NA
F01_transcript_128259	F01_transcript_128259	F01_transcript_128259	F01_transcript_128259	F01_transcript_128259
F01_transcript_47693	NA	F01_transcript_47693	F01_transcript_47693	F01_transcript_47693
F01_transcript_88796	NA	NA	NA	F01_transcript_88796
F01_transcript_54865	NA	F01_transcript_54865	F01_transcript_54865	F01_transcript_54865
F01_transcript_100575	NA	F01_transcript_100575	F01_transcript_100575	F01_transcript_100575
F01_transcript_154227	NA	NA	NA	F01_transcript_154227

F01_transcript_59571	NA	NA	F01_transcript_59571	F01_transcript_59571
F01_transcript_71976	NA	F01_transcript_71976	NA	NA
F01_transcript_135531	F01_transcript_135531	F01_transcript_135531	F01_transcript_135531	F01_transcript_135531
F01_transcript_148087	F01_transcript_148087	F01_transcript_148087	F01_transcript_148087	F01_transcript_148087
F01_transcript_167349	F01_transcript_167349	F01_transcript_167349	F01_transcript_167349	F01_transcript_167349
F01_transcript_77491	NA	NA	NA	F01_transcript_77491
F01_transcript_97007	NA	NA	NA	F01_transcript_97007
F01_transcript_64526	F01_transcript_64526	F01_transcript_64526	F01_transcript_64526	F01_transcript_64526
F01_transcript_107326	NA	NA	F01_transcript_107326	NA
F01_transcript_44879	F01_transcript_44879	F01_transcript_44879	NA	F01_transcript_44879
F01_transcript_124773	NA	F01_transcript_124773	NA	F01_transcript_124773
F01_transcript_5686	NA	NA	NA	F01_transcript_5686
F01_transcript_129869	NA	F01_transcript_129869	NA	F01_transcript_129869
F01_transcript_112079	NA	F01_transcript_112079	F01_transcript_112079	NA

F01_transcript_141166	F01_transcript_141166	F01_transcript_141166	F01_transcript_141166	F01_transcript_141166
F01_transcript_35870	NA	NA	NA	F01_transcript_35870
F01_transcript_80284	NA	F01_transcript_80284	NA	F01_transcript_80284
F01_transcript_131918	F01_transcript_131918	F01_transcript_131918	F01_transcript_131918	F01_transcript_131918
F01_transcript_117292	F01_transcript_117292	F01_transcript_117292	F01_transcript_117292	NA
F01_transcript_165069	NA	F01_transcript_165069	NA	F01_transcript_165069
F01_transcript_17463	NA	F01_transcript_17463	NA	NA
F01_transcript_152176	F01_transcript_152176	F01_transcript_152176	NA	NA
F01_transcript_11559	NA	F01_transcript_11559	F01_transcript_11559	F01_transcript_11559
F01_transcript_126255	NA	F01_transcript_126255	NA	NA
F01_transcript_152618	F01_transcript_152618	F01_transcript_152618	NA	NA
F01_transcript_116446	NA	NA	NA	F01_transcript_116446
F01_transcript_67657	NA	F01_transcript_67657	NA	NA
F01_transcript_139155	NA	NA	NA	F01_transcript_139155

F01_transcript_60864	F01_transcript_60864	F01_transcript_60864	F01_transcript_60864	F01_transcript_60864
F01_transcript_127507	F01_transcript_127507	F01_transcript_127507	F01_transcript_127507	F01_transcript_127507
F01_transcript_106782	F01_transcript_106782	F01_transcript_106782	NA	NA
F01_transcript_108025	F01_transcript_108025	F01_transcript_108025	F01_transcript_108025	F01_transcript_108025
F01_transcript_159841	F01_transcript_159841	F01_transcript_159841	F01_transcript_159841	F01_transcript_159841
F01_transcript_98797	NA	NA	NA	F01_transcript_98797
F01_transcript_161070	F01_transcript_161070	F01_transcript_161070	NA	F01_transcript_161070
F01_transcript_21261	NA	NA	NA	F01_transcript_21261
F01_transcript_161327	F01_transcript_161327	F01_transcript_161327	NA	NA
F01_transcript_98038	NA	NA	NA	F01_transcript_98038
F01_transcript_50417	NA	NA	F01_transcript_50417	F01_transcript_50417
F01_transcript_1056	NA	NA	NA	F01_transcript_1056
F01_transcript_93785	F01_transcript_93785	F01_transcript_93785	NA	NA
F01_transcript_133471	NA	F01_transcript_133471	NA	NA

F01_transcript_58026	NA	F01_transcript_58026	NA	F01_transcript_58026
F01_transcript_66117	NA	NA	NA	F01_transcript_66117
F01_transcript_167695	NA	NA	NA	F01_transcript_167695
F01_transcript_159674	F01_transcript_159674	F01_transcript_159674	F01_transcript_159674	F01_transcript_159674
F01_transcript_72334	NA	F01_transcript_72334	NA	F01_transcript_72334
F01_transcript_78894	NA	F01_transcript_78894	F01_transcript_78894	F01_transcript_78894
F01_transcript_130886	NA	F01_transcript_130886	NA	NA
F01_transcript_54746	NA	NA	NA	F01_transcript_54746
F01_transcript_166843	F01_transcript_166843	F01_transcript_166843	NA	NA
F01_transcript_36136	NA	NA	NA	F01_transcript_36136
F01_transcript_29727	F01_transcript_29727	F01_transcript_29727	NA	F01_transcript_29727
F01_transcript_71386	NA	NA	NA	F01_transcript_71386
F01_transcript_145213	NA	F01_transcript_145213	NA	F01_transcript_145213
F01_transcript_146225	F01_transcript_146225	F01_transcript_146225	F01_transcript_146225	F01_transcript_146225

F01_transcript_97204	F01_transcript_97204	F01_transcript_97204	NA	F01_transcript_97204
F01_transcript_57179	NA	NA	F01_transcript_57179	NA
F01_transcript_131511	F01_transcript_131511	F01_transcript_131511	NA	NA
F01_transcript_105601	NA	F01_transcript_105601	NA	NA
F01_transcript_78	NA	NA	NA	F01_transcript_78
F01_transcript_148711	NA	F01_transcript_148711	NA	NA
F01_transcript_2128	F01_transcript_2128	F01_transcript_2128	F01_transcript_2128	F01_transcript_2128
F01_transcript_23492	F01_transcript_23492	F01_transcript_23492	NA	F01_transcript_23492
F01_transcript_67798	NA	NA	NA	F01_transcript_67798
F01_transcript_104833	NA	NA	NA	F01_transcript_104833
F01_transcript_20191	NA	NA	NA	F01_transcript_20191
F01_transcript_60014	NA	F01_transcript_60014	NA	F01_transcript_60014
F01_transcript_8973	NA	F01_transcript_8973	NA	NA
F01_transcript_134588	NA	NA	NA	F01_transcript_134588

F01_transcript_116690	NA	NA	NA	F01_transcript_116690
F01_transcript_106575	NA	F01_transcript_106575	NA	F01_transcript_106575
F01_transcript_64531	F01_transcript_64531	F01_transcript_64531	F01_transcript_64531	F01_transcript_64531
F01_transcript_71230	NA	NA	F01_transcript_71230	NA
F01_transcript_361	NA	NA	NA	F01_transcript_361
F01_transcript_49075	NA	F01_transcript_49075	F01_transcript_49075	F01_transcript_49075
F01_transcript_116732	NA	NA	NA	F01_transcript_116732
F01_transcript_67118	NA	NA	NA	F01_transcript_67118
F01_transcript_162120	F01_transcript_162120	F01_transcript_162120	F01_transcript_162120	F01_transcript_162120
F01_transcript_144436	NA	NA	NA	F01_transcript_144436
F01_transcript_69107	F01_transcript_69107	F01_transcript_69107	NA	F01_transcript_69107
F01_transcript_24900	NA	NA	NA	F01_transcript_24900
F01_transcript_138622	NA	NA	NA	F01_transcript_138622
F01_transcript_146294	NA	F01_transcript_146294	NA	NA

F01_transcript_100505	NA	NA	NA	F01_transcript_100505
F01_transcript_36286	F01_transcript_36286	F01_transcript_36286	F01_transcript_36286	F01_transcript_36286
F01_transcript_165882	NA	F01_transcript_165882	NA	NA
F01_transcript_135790	NA	F01_transcript_135790	NA	NA
F01_transcript_91285	NA	F01_transcript_91285	NA	F01_transcript_91285
F01_transcript_62733	NA	F01_transcript_62733	NA	NA
F01_transcript_70648	F01_transcript_70648	F01_transcript_70648	F01_transcript_70648	NA
F01_transcript_115562	NA	F01_transcript_115562	F01_transcript_115562	F01_transcript_115562
F01_transcript_149959	NA	NA	NA	F01_transcript_149959
F01_transcript_106548	NA	NA	NA	F01_transcript_106548
F01_transcript_116249	NA	NA	NA	F01_transcript_116249
F01_transcript_57261	F01_transcript_57261	F01_transcript_57261	NA	F01_transcript_57261
F01_transcript_72824	F01_transcript_72824	F01_transcript_72824	F01_transcript_72824	F01_transcript_72824
F01_transcript_5495	NA	NA	NA	F01_transcript_5495

F01_transcript_81389	NA	F01_transcript_81389	NA	F01_transcript_81389
F01_transcript_88507	NA	NA	NA	F01_transcript_88507
F01_transcript_57732	NA	F01_transcript_57732	NA	NA
F01_transcript_84913	F01_transcript_84913	F01_transcript_84913	F01_transcript_84913	F01_transcript_84913
F01_transcript_50028	F01_transcript_50028	F01_transcript_50028	F01_transcript_50028	F01_transcript_50028
F01_transcript_93502	NA	F01_transcript_93502	NA	NA
F01_transcript_153182	NA	NA	NA	F01_transcript_153182
F01_transcript_100054	NA	NA	NA	F01_transcript_100054
F01_transcript_151671	NA	NA	NA	F01_transcript_151671
F01_transcript_129838	NA	NA	NA	F01_transcript_129838
F01_transcript_72777	NA	F01_transcript_72777	NA	F01_transcript_72777
F01_transcript_154559	F01_transcript_154559	F01_transcript_154559	F01_transcript_154559	F01_transcript_154559
F01_transcript_124709	NA	NA	NA	F01_transcript_124709
F01_transcript_156474	NA	NA	NA	F01_transcript_156474

F01_transcript_80541	NA	NA	NA	F01_transcript_80541
F01_transcript_123601	NA	F01_transcript_123601	NA	NA
F01_transcript_151897	F01_transcript_151897	F01_transcript_151897	F01_transcript_151897	F01_transcript_151897
F01_transcript_155280	F01_transcript_155280	F01_transcript_155280	F01_transcript_155280	NA
F01_transcript_74923	NA	NA	NA	F01_transcript_74923
F01_transcript_41334	NA	NA	NA	F01_transcript_41334
F01_transcript_71824	NA	NA	NA	F01_transcript_71824
F01_transcript_47759	NA	F01_transcript_47759	NA	F01_transcript_47759
F01_transcript_147138	F01_transcript_147138	F01_transcript_147138	NA	F01_transcript_147138
F01_transcript_62335	NA	NA	NA	F01_transcript_62335
F01_transcript_79435	NA	F01_transcript_79435	NA	F01_transcript_79435
F01_transcript_120396	NA	F01_transcript_120396	NA	NA
F01_transcript_97616	NA	NA	NA	F01_transcript_97616
F01_transcript_49568	NA	F01_transcript_49568	NA	NA

F01_transcript_163827	F01_transcript_163827	F01_transcript_163827	F01_transcript_163827	NA
F01_transcript_71018	NA	F01_transcript_71018	F01_transcript_71018	NA
F01_transcript_133788	NA	F01_transcript_133788	NA	F01_transcript_133788
F01_transcript_154856	NA	NA	NA	F01_transcript_154856
F01_transcript_162226	NA	NA	NA	F01_transcript_162226
F01_transcript_55116	NA	NA	NA	F01_transcript_55116
F01_transcript_153960	NA	NA	NA	F01_transcript_153960
F01_transcript_59503	NA	F01_transcript_59503	F01_transcript_59503	F01_transcript_59503
F01_transcript_71176	NA	F01_transcript_71176	F01_transcript_71176	NA
F01_transcript_109816	F01_transcript_109816	F01_transcript_109816	F01_transcript_109816	NA
F01_transcript_42545	NA	NA	NA	F01_transcript_42545
F01_transcript_71709	NA	F01_transcript_71709	F01_transcript_71709	F01_transcript_71709
F01_transcript_43307	NA	NA	NA	F01_transcript_43307
F01_transcript_155621	NA	F01_transcript_155621	F01_transcript_155621	NA

F01_transcript_18567	NA	NA	F01_transcript_18567	F01_transcript_18567
F01_transcript_26177	NA	F01_transcript_26177	NA	NA
F01_transcript_133509	F01_transcript_133509	F01_transcript_133509	F01_transcript_133509	F01_transcript_133509
F01_transcript_74907	NA	F01_transcript_74907	NA	F01_transcript_74907
F01_transcript_146522	F01_transcript_146522	F01_transcript_146522	F01_transcript_146522	F01_transcript_146522
F01_transcript_100704	F01_transcript_100704	F01_transcript_100704	F01_transcript_100704	NA
F01_transcript_12497	NA	NA	F01_transcript_12497	F01_transcript_12497
F01_transcript_25403	F01_transcript_25403	F01_transcript_25403	NA	NA
F01_transcript_158415	F01_transcript_158415	F01_transcript_158415	F01_transcript_158415	F01_transcript_158415
F01_transcript_104297	NA	NA	NA	F01_transcript_104297
F01_transcript_162506	F01_transcript_162506	F01_transcript_162506	F01_transcript_162506	F01_transcript_162506
F01_transcript_99848	NA	F01_transcript_99848	NA	NA
F01_transcript_54943	NA	F01_transcript_54943	NA	NA
F01_transcript_166171	NA	NA	NA	F01_transcript_166171

F01_transcript_148353	F01_transcript_148353	NA	NA	F01_transcript_148353
F01_transcript_134421	NA	F01_transcript_134421	F01_transcript_134421	NA
F01_transcript_70434	NA	F01_transcript_70434	NA	F01_transcript_70434
F01_transcript_61315	F01_transcript_61315	F01_transcript_61315	NA	NA
F01_transcript_9055	NA	NA	NA	F01_transcript_9055
F01_transcript_61821	NA	F01_transcript_61821	NA	NA
F01_transcript_127899	NA	NA	NA	F01_transcript_127899
F01_transcript_120768	NA	NA	NA	F01_transcript_120768
F01_transcript_4870	F01_transcript_4870	F01_transcript_4870	NA	F01_transcript_4870
F01_transcript_155724	F01_transcript_155724	F01_transcript_155724	F01_transcript_155724	F01_transcript_155724
F01_transcript_113249	NA	F01_transcript_113249	NA	NA
F01_transcript_66059	NA	NA	NA	F01_transcript_66059
F01_transcript_66575	NA	F01_transcript_66575	NA	F01_transcript_66575
F01_transcript_127594	NA	F01_transcript_127594	F01_transcript_127594	F01_transcript_127594

F01_transcript_20855	NA	NA	NA	F01_transcript_20855
F01_transcript_62029	NA	F01_transcript_62029	NA	NA
F01_transcript_50484	NA	NA	NA	F01_transcript_50484
F01_transcript_74371	NA	F01_transcript_74371	NA	NA
F01_transcript_12858	NA	NA	NA	F01_transcript_12858
F01_transcript_106446	F01_transcript_106446	F01_transcript_106446	F01_transcript_106446	F01_transcript_106446
F01_transcript_99643	F01_transcript_99643	F01_transcript_99643	F01_transcript_99643	F01_transcript_99643
F01_transcript_93603	F01_transcript_93603	F01_transcript_93603	NA	NA
F01_transcript_81277	NA	NA	NA	F01_transcript_81277
F01_transcript_152705	NA	F01_transcript_152705	NA	NA
F01_transcript_147633	NA	NA	NA	F01_transcript_147633
F01_transcript_110813	NA	NA	NA	F01_transcript_110813
F01_transcript_90912	F01_transcript_90912	F01_transcript_90912	NA	NA
F01_transcript_110995	F01_transcript_110995	F01_transcript_110995	F01_transcript_110995	NA

F01_transcript_7651	NA	F01_transcript_7651	NA	NA
F01_transcript_127509	NA	F01_transcript_127509	F01_transcript_127509	F01_transcript_127509
F01_transcript_136697	NA	NA	NA	F01_transcript_136697
F01_transcript_135066	NA	NA	F01_transcript_135066	F01_transcript_135066
F01_transcript_142523	F01_transcript_142523	F01_transcript_142523	NA	F01_transcript_142523
F01_transcript_21735	NA	NA	NA	F01_transcript_21735
F01_transcript_157721	NA	NA	NA	F01_transcript_157721
F01_transcript_145573	NA	F01_transcript_145573	F01_transcript_145573	F01_transcript_145573
F01_transcript_164403	NA	NA	NA	F01_transcript_164403
F01_transcript_60936	NA	F01_transcript_60936	NA	NA
F01_transcript_112511	F01_transcript_112511	F01_transcript_112511	NA	F01_transcript_112511
F01_transcript_56298	NA	NA	NA	F01_transcript_56298
F01_transcript_58845	NA	F01_transcript_58845	NA	F01_transcript_58845
F01_transcript_163710	NA	F01_transcript_163710	NA	NA

F01_transcript_160530	NA	F01_transcript_160530	NA	NA
F01_transcript_132038	NA	F01_transcript_132038	NA	F01_transcript_132038
F01_transcript_39121	NA	NA	NA	F01_transcript_39121
F01_transcript_111196	NA	F01_transcript_111196	NA	NA
F01_transcript_51066	F01_transcript_51066	F01_transcript_51066	F01_transcript_51066	F01_transcript_51066
F01_transcript_109477	F01_transcript_109477	F01_transcript_109477	F01_transcript_109477	F01_transcript_109477
F01_transcript_71441	NA	F01_transcript_71441	NA	NA
F01_transcript_167660	NA	F01_transcript_167660	NA	NA
F01_transcript_84376	NA	F01_transcript_84376	F01_transcript_84376	NA
F01_transcript_129182	NA	F01_transcript_129182	NA	F01_transcript_129182
F01_transcript_102560	F01_transcript_102560	F01_transcript_102560	F01_transcript_102560	F01_transcript_102560
F01_transcript_115064	F01_transcript_115064	F01_transcript_115064	F01_transcript_115064	F01_transcript_115064
F01_transcript_76928	NA	NA	NA	F01_transcript_76928
F01_transcript_18616	NA	NA	NA	F01_transcript_18616

F01_transcript_34983	NA	NA	NA	F01_transcript_34983
F01_transcript_159370	NA	F01_transcript_159370	NA	NA
F01_transcript_153310	F01_transcript_153310	F01_transcript_153310	NA	F01_transcript_153310
F01_transcript_157184	F01_transcript_157184	F01_transcript_157184	F01_transcript_157184	F01_transcript_157184
F01_transcript_56001	NA	NA	NA	F01_transcript_56001
F01_transcript_113799	NA	F01_transcript_113799	NA	NA
F01_transcript_105419	NA	NA	NA	F01_transcript_105419
F01_transcript_56405	NA	F01_transcript_56405	F01_transcript_56405	NA
F01_transcript_50062	NA	F01_transcript_50062	F01_transcript_50062	F01_transcript_50062
F01_transcript_81027	F01_transcript_81027	F01_transcript_81027	F01_transcript_81027	F01_transcript_81027
F01_transcript_125997	NA	NA	F01_transcript_125997	NA
F01_transcript_64133	NA	NA	NA	F01_transcript_64133
F01_transcript_155012	F01_transcript_155012	F01_transcript_155012	F01_transcript_155012	F01_transcript_155012
F01_transcript_7294	F01_transcript_7294	NA	NA	NA

F01_transcript_76167	NA	F01_transcript_76167	NA	F01_transcript_76167
F01_transcript_50375	NA	F01_transcript_50375	F01_transcript_50375	NA
F01_transcript_112002	NA	NA	NA	F01_transcript_112002
F01_transcript_157124	NA	F01_transcript_157124	NA	NA
F01_transcript_137481	NA	NA	F01_transcript_137481	F01_transcript_137481
F01_transcript_139982	F01_transcript_139982	F01_transcript_139982	F01_transcript_139982	F01_transcript_139982
F01_transcript_152911	F01_transcript_152911	F01_transcript_152911	F01_transcript_152911	NA
F01_transcript_66214	NA	NA	NA	F01_transcript_66214
F01_transcript_161631	NA	NA	NA	F01_transcript_161631
F01_transcript_58795	NA	F01_transcript_58795	NA	NA
F01_transcript_109687	F01_transcript_109687	F01_transcript_109687	F01_transcript_109687	F01_transcript_109687
F01_transcript_167401	NA	F01_transcript_167401	F01_transcript_167401	NA
F01_transcript_129916	NA	NA	NA	F01_transcript_129916
F01_transcript_57	NA	F01_transcript_57	F01_transcript_57	F01_transcript_57

F01_transcript_114958	F01_transcript_114958	F01_transcript_114958	NA	F01_transcript_114958
F01_transcript_51808	NA	NA	NA	F01_transcript_51808
F01_transcript_45738	NA	NA	NA	F01_transcript_45738
F01_transcript_122592	F01_transcript_122592	F01_transcript_122592	NA	NA
F01_transcript_66528	NA	F01_transcript_66528	F01_transcript_66528	F01_transcript_66528
F01_transcript_125231	F01_transcript_125231	F01_transcript_125231	NA	NA
F01_transcript_105498	F01_transcript_105498	F01_transcript_105498	F01_transcript_105498	NA
F01_transcript_51063	F01_transcript_51063	F01_transcript_51063	F01_transcript_51063	F01_transcript_51063
F01_transcript_102056	NA	F01_transcript_102056	NA	NA
F01_transcript_158581	NA	F01_transcript_158581	NA	F01_transcript_158581
F01_transcript_70763	NA	F01_transcript_70763	NA	NA
F01_transcript_29033	NA	NA	NA	F01_transcript_29033
F01_transcript_148789	NA	NA	NA	F01_transcript_148789
F01_transcript_153242	NA	NA	NA	F01_transcript_153242

F01_transcript_91334	NA	NA	F01_transcript_91334	F01_transcript_91334
F01_transcript_155645	F01_transcript_155645	F01_transcript_155645	NA	NA
F01_transcript_146029	F01_transcript_146029	F01_transcript_146029	F01_transcript_146029	F01_transcript_146029
F01_transcript_91609	NA	NA	NA	F01_transcript_91609
F01_transcript_144567	NA	NA	NA	F01_transcript_144567
F01_transcript_58038	NA	F01_transcript_58038	F01_transcript_58038	F01_transcript_58038
F01_transcript_125314	NA	F01_transcript_125314	NA	NA
F01_transcript_25720	NA	F01_transcript_25720	NA	F01_transcript_25720
F01_transcript_127019	NA	F01_transcript_127019	NA	NA
F01_transcript_76662	F01_transcript_76662	F01_transcript_76662	F01_transcript_76662	F01_transcript_76662
F01_transcript_64395	NA	F01_transcript_64395	NA	F01_transcript_64395
F01_transcript_107005	F01_transcript_107005	F01_transcript_107005	F01_transcript_107005	F01_transcript_107005
F01_transcript_133150	NA	F01_transcript_133150	NA	NA
F01_transcript_109134	NA	NA	NA	F01_transcript_109134

F01_transcript_49849	NA	F01_transcript_49849	NA	F01_transcript_49849
F01_transcript_92080	NA	NA	NA	F01_transcript_92080
F01_transcript_52959	F01_transcript_52959	F01_transcript_52959	F01_transcript_52959	F01_transcript_52959
F01_transcript_45162	NA	NA	NA	F01_transcript_45162
F01_transcript_84680	NA	NA	NA	F01_transcript_84680
F01_transcript_6891	NA	F01_transcript_6891	NA	NA
F01_transcript_5567	NA	F01_transcript_5567	NA	NA
F01_transcript_140067	NA	NA	NA	F01_transcript_140067
F01_transcript_76957	NA	NA	NA	F01_transcript_76957
F01_transcript_36934	NA	NA	NA	F01_transcript_36934
F01_transcript_142014	NA	F01_transcript_142014	F01_transcript_142014	NA
F01_transcript_36441	NA	NA	NA	F01_transcript_36441
F01_transcript_158604	F01_transcript_158604	F01_transcript_158604	NA	NA
F01_transcript_109572	NA	NA	NA	F01_transcript_109572

F01_transcript_133349	F01_transcript_133349	F01_transcript_133349	F01_transcript_133349	NA
F01_transcript_153064	F01_transcript_153064	F01_transcript_153064	F01_transcript_153064	F01_transcript_153064
F01_transcript_101656	F01_transcript_101656	F01_transcript_101656	NA	F01_transcript_101656
F01_transcript_73606	NA	F01_transcript_73606	NA	NA
F01_transcript_118373	NA	F01_transcript_118373	NA	NA
F01_transcript_157606	F01_transcript_157606	F01_transcript_157606	F01_transcript_157606	F01_transcript_157606
F01_transcript_90783	NA	F01_transcript_90783	NA	NA
F01_transcript_74447	NA	NA	NA	F01_transcript_74447
F01_transcript_107869	NA	NA	NA	F01_transcript_107869
F01_transcript_155076	NA	NA	NA	F01_transcript_155076
F01_transcript_60684	F01_transcript_60684	F01_transcript_60684	F01_transcript_60684	F01_transcript_60684
F01_transcript_91734	NA	NA	NA	F01_transcript_91734
F01_transcript_130257	F01_transcript_130257	F01_transcript_130257	F01_transcript_130257	F01_transcript_130257
F01_transcript_162988	NA	NA	NA	F01_transcript_162988

F01_transcript_131846	NA	F01_transcript_131846	NA	F01_transcript_131846
F01_transcript_5054	NA	F01_transcript_5054	NA	NA
F01_transcript_155600	NA	F01_transcript_155600	NA	NA
F01_transcript_100314	NA	F01_transcript_100314	NA	NA
F01_transcript_54437	F01_transcript_54437	F01_transcript_54437	F01_transcript_54437	F01_transcript_54437
F01_transcript_107155	NA	F01_transcript_107155	NA	NA
F01_transcript_59306	NA	NA	F01_transcript_59306	NA
F01_transcript_117772	F01_transcript_117772	F01_transcript_117772	F01_transcript_117772	F01_transcript_117772
F01_transcript_153572	NA	F01_transcript_153572	NA	NA
F01_transcript_164950	F01_transcript_164950	F01_transcript_164950	NA	NA
F01_transcript_57188	NA	NA	NA	F01_transcript_57188
F01_transcript_86753	NA	F01_transcript_86753	NA	NA
F01_transcript_106946	F01_transcript_106946	F01_transcript_106946	F01_transcript_106946	F01_transcript_106946
F01_transcript_144008	NA	F01_transcript_144008	NA	F01_transcript_144008

F01_transcript_26521	NA	F01_transcript_26521	NA	NA
F01_transcript_59337	NA	NA	NA	F01_transcript_59337
F01_transcript_107728	NA	F01_transcript_107728	NA	F01_transcript_107728
F01_transcript_57038	NA	NA	NA	F01_transcript_57038
F01_transcript_109619	F01_transcript_109619	F01_transcript_109619	F01_transcript_109619	F01_transcript_109619
F01_transcript_66346	NA	F01_transcript_66346	NA	F01_transcript_66346
F01_transcript_129997	NA	F01_transcript_129997	NA	NA
F01_transcript_46189	NA	F01_transcript_46189	F01_transcript_46189	F01_transcript_46189
F01_transcript_48336	NA	F01_transcript_48336	NA	F01_transcript_48336
F01_transcript_51298	F01_transcript_51298	F01_transcript_51298	F01_transcript_51298	F01_transcript_51298
F01_transcript_104488	NA	F01_transcript_104488	NA	NA
F01_transcript_75422	F01_transcript_75422	F01_transcript_75422	F01_transcript_75422	F01_transcript_75422
F01_transcript_132688	NA	NA	NA	F01_transcript_132688
F01_transcript_167065	NA	F01_transcript_167065	F01_transcript_167065	NA

F01_transcript_153756	NA	F01_transcript_153756	NA	F01_transcript_153756
F01_transcript_156418	NA	F01_transcript_156418	NA	NA
F01_transcript_60140	NA	NA	NA	F01_transcript_60140
F01_transcript_88559	NA	NA	NA	F01_transcript_88559
F01_transcript_112961	F01_transcript_112961	F01_transcript_112961	NA	NA
F01_transcript_100024	NA	F01_transcript_100024	NA	NA
F01_transcript_149465	NA	F01_transcript_149465	NA	NA
F01_transcript_56795	NA	F01_transcript_56795	F01_transcript_56795	F01_transcript_56795
F01_transcript_130187	F01_transcript_130187	F01_transcript_130187	NA	NA
F01_transcript_52981	NA	F01_transcript_52981	NA	NA
F01_transcript_164894	NA	F01_transcript_164894	NA	NA
F01_transcript_100297	F01_transcript_100297	F01_transcript_100297	F01_transcript_100297	F01_transcript_100297
F01_transcript_76053	NA	F01_transcript_76053	NA	NA
F01_transcript_155584	F01_transcript_155584	F01_transcript_155584	F01_transcript_155584	NA

F01_transcript_136238	F01_transcript_136238	F01_transcript_136238	NA	NA
F01_transcript_54408	F01_transcript_54408	F01_transcript_54408	NA	NA
F01_transcript_50585	F01_transcript_50585	F01_transcript_50585	F01_transcript_50585	F01_transcript_50585
F01_transcript_49187	NA	F01_transcript_49187	NA	F01_transcript_49187
F01_transcript_70492	NA	NA	NA	F01_transcript_70492
F01_transcript_11021	NA	NA	NA	F01_transcript_11021
F01_transcript_55965	NA	F01_transcript_55965	NA	F01_transcript_55965
F01_transcript_87393	NA	F01_transcript_87393	NA	NA
F01_transcript_72581	NA	F01_transcript_72581	NA	F01_transcript_72581
F01_transcript_135562	NA	NA	NA	F01_transcript_135562
F01_transcript_167173	F01_transcript_167173	F01_transcript_167173	NA	NA
F01_transcript_107179	F01_transcript_107179	F01_transcript_107179	NA	NA
F01_transcript_100099	F01_transcript_100099	F01_transcript_100099	F01_transcript_100099	F01_transcript_100099
F01_transcript_36898	NA	NA	NA	F01_transcript_36898

F01_transcript_76487	NA	F01_transcript_76487	NA	NA
F01_transcript_86120	F01_transcript_86120	F01_transcript_86120	F01_transcript_86120	F01_transcript_86120
F01_transcript_26902	NA	F01_transcript_26902	F01_transcript_26902	NA
F01_transcript_130495	F01_transcript_130495	F01_transcript_130495	F01_transcript_130495	F01_transcript_130495
F01_transcript_58153	NA	F01_transcript_58153	NA	F01_transcript_58153
F01_transcript_98642	NA	NA	F01_transcript_98642	F01_transcript_98642
F01_transcript_45634	NA	F01_transcript_45634	NA	NA
F01_transcript_89724	F01_transcript_89724	F01_transcript_89724	F01_transcript_89724	F01_transcript_89724
F01_transcript_47608	NA	NA	F01_transcript_47608	F01_transcript_47608
F01_transcript_46378	NA	NA	NA	F01_transcript_46378
F01_transcript_155590	NA	NA	NA	F01_transcript_155590
F01_transcript_150098	NA	F01_transcript_150098	NA	F01_transcript_150098
F01_transcript_60720	F01_transcript_60720	F01_transcript_60720	F01_transcript_60720	F01_transcript_60720
F01_transcript_148139	NA	NA	NA	F01_transcript_148139

F01_transcript_78493	NA	F01_transcript_78493	NA	F01_transcript_78493
F01_transcript_85469	NA	NA	NA	F01_transcript_85469
F01_transcript_74990	NA	F01_transcript_74990	NA	NA
F01_transcript_93309	F01_transcript_93309	F01_transcript_93309	NA	NA
F01_transcript_49797	NA	NA	F01_transcript_49797	F01_transcript_49797
F01_transcript_126534	NA	F01_transcript_126534	F01_transcript_126534	NA
F01_transcript_143397	NA	NA	NA	F01_transcript_143397
F01_transcript_61961	NA	F01_transcript_61961	NA	F01_transcript_61961
F01_transcript_154838	F01_transcript_154838	F01_transcript_154838	F01_transcript_154838	F01_transcript_154838
F01_transcript_72460	NA	F01_transcript_72460	NA	NA
F01_transcript_114069	NA	NA	NA	F01_transcript_114069
F01_transcript_152765	F01_transcript_152765	F01_transcript_152765	F01_transcript_152765	F01_transcript_152765
F01_transcript_103336	NA	NA	NA	F01_transcript_103336
F01_transcript_53753	NA	F01_transcript_53753	NA	NA

F01_transcript_110037	NA	F01_transcript_110037	NA	NA
F01_transcript_65305	NA	NA	NA	F01_transcript_65305
F01_transcript_143138	F01_transcript_143138	F01_transcript_143138	NA	NA
F01_transcript_165549	NA	NA	NA	F01_transcript_165549
F01_transcript_156025	NA	NA	NA	F01_transcript_156025
F01_transcript_159828	F01_transcript_159828	F01_transcript_159828	NA	F01_transcript_159828
F01_transcript_80861	NA	NA	NA	F01_transcript_80861
F01_transcript_116087	NA	F01_transcript_116087	NA	NA
F01_transcript_69362	NA	F01_transcript_69362	F01_transcript_69362	F01_transcript_69362
F01_transcript_78605	F01_transcript_78605	F01_transcript_78605	NA	NA
F01_transcript_73851	NA	NA	F01_transcript_73851	F01_transcript_73851
F01_transcript_105906	NA	NA	F01_transcript_105906	F01_transcript_105906
F01_transcript_129974	NA	F01_transcript_129974	NA	NA
F01_transcript_92723	NA	F01_transcript_92723	NA	NA

F01_transcript_96953	F01_transcript_96953	F01_transcript_96953	F01_transcript_96953	F01_transcript_96953
F01_transcript_119083	NA	F01_transcript_119083	F01_transcript_119083	F01_transcript_119083
F01_transcript_156843	F01_transcript_156843	F01_transcript_156843	F01_transcript_156843	F01_transcript_156843
F01_transcript_95452	NA	F01_transcript_95452	F01_transcript_95452	NA
F01_transcript_114126	NA	F01_transcript_114126	F01_transcript_114126	NA
F01_transcript_100312	NA	NA	NA	F01_transcript_100312
F01_transcript_154870	NA	NA	NA	F01_transcript_154870
F01_transcript_111326	NA	NA	NA	F01_transcript_111326
F01_transcript_18911	NA	NA	NA	F01_transcript_18911
F01_transcript_102021	NA	NA	NA	F01_transcript_102021
F01_transcript_131290	F01_transcript_131290	F01_transcript_131290	F01_transcript_131290	F01_transcript_131290
F01_transcript_70986	NA	F01_transcript_70986	F01_transcript_70986	F01_transcript_70986
F01_transcript_84856	NA	F01_transcript_84856	NA	NA
F01_transcript_62592	F01_transcript_62592	NA	NA	F01_transcript_62592

F01_transcript_94281	F01_transcript_94281	F01_transcript_94281	F01_transcript_94281	F01_transcript_94281
F01_transcript_65361	NA	NA	NA	F01_transcript_65361
F01_transcript_15710	NA	NA	NA	F01_transcript_15710
F01_transcript_111281	NA	NA	NA	F01_transcript_111281
F01_transcript_78090	F01_transcript_78090	F01_transcript_78090	F01_transcript_78090	F01_transcript_78090
F01_transcript_117176	NA	F01_transcript_117176	NA	NA
F01_transcript_13639	F01_transcript_13639	F01_transcript_13639	F01_transcript_13639	F01_transcript_13639
F01_transcript_22558	NA	NA	NA	F01_transcript_22558
F01_transcript_24226	NA	F01_transcript_24226	NA	NA
F01_transcript_139379	NA	NA	NA	F01_transcript_139379
F01_transcript_139095	NA	NA	NA	F01_transcript_139095
F01_transcript_115724	F01_transcript_115724	F01_transcript_115724	F01_transcript_115724	F01_transcript_115724
F01_transcript_156711	F01_transcript_156711	F01_transcript_156711	F01_transcript_156711	F01_transcript_156711
F01_transcript_158806	NA	F01_transcript_158806	NA	NA

F01_transcript_22877	NA	F01_transcript_22877	NA	F01_transcript_22877
F01_transcript_69191	F01_transcript_69191	F01_transcript_69191	F01_transcript_69191	F01_transcript_69191
F01_transcript_98086	F01_transcript_98086	F01_transcript_98086	F01_transcript_98086	F01_transcript_98086
F01_transcript_157077	F01_transcript_157077	F01_transcript_157077	NA	F01_transcript_157077
F01_transcript_142895	F01_transcript_142895	F01_transcript_142895	F01_transcript_142895	F01_transcript_142895
F01_transcript_50264	F01_transcript_50264	F01_transcript_50264	F01_transcript_50264	F01_transcript_50264
F01_transcript_6908	NA	F01_transcript_6908	NA	F01_transcript_6908
F01_transcript_119586	NA	NA	NA	F01_transcript_119586
F01_transcript_90335	NA	F01_transcript_90335	F01_transcript_90335	NA
F01_transcript_114820	F01_transcript_114820	F01_transcript_114820	F01_transcript_114820	F01_transcript_114820
F01_transcript_72426	NA	F01_transcript_72426	F01_transcript_72426	NA
F01_transcript_62892	F01_transcript_62892	F01_transcript_62892	F01_transcript_62892	NA
F01_transcript_89712	F01_transcript_89712	F01_transcript_89712	F01_transcript_89712	F01_transcript_89712
F01_transcript_70899	NA	F01_transcript_70899	F01_transcript_70899	F01_transcript_70899

F01_transcript_123036	NA	F01_transcript_123036	NA	NA
F01_transcript_76555	F01_transcript_76555	F01_transcript_76555	F01_transcript_76555	F01_transcript_76555
F01_transcript_75230	F01_transcript_75230	F01_transcript_75230	NA	NA
F01_transcript_130992	NA	F01_transcript_130992	NA	NA
F01_transcript_4661	NA	NA	NA	F01_transcript_4661
F01_transcript_155589	NA	F01_transcript_155589	NA	NA
F01_transcript_112603	NA	NA	NA	F01_transcript_112603
F01_transcript_127805	F01_transcript_127805	F01_transcript_127805	NA	F01_transcript_127805
F01_transcript_93783	NA	NA	NA	F01_transcript_93783
F01_transcript_8883	NA	NA	NA	F01_transcript_8883
F01_transcript_62318	NA	F01_transcript_62318	F01_transcript_62318	F01_transcript_62318
F01_transcript_134441	NA	NA	F01_transcript_134441	F01_transcript_134441
F01_transcript_131881	NA	F01_transcript_131881	F01_transcript_131881	F01_transcript_131881
F01_transcript_51346	NA	F01_transcript_51346	NA	NA

F01_transcript_153261	NA	NA	NA	F01_transcript_153261
F01_transcript_158906	NA	F01_transcript_158906	F01_transcript_158906	NA
F01_transcript_158908	F01_transcript_158908	F01_transcript_158908	F01_transcript_158908	F01_transcript_158908
F01_transcript_68150	NA	NA	NA	F01_transcript_68150
F01_transcript_78490	F01_transcript_78490	F01_transcript_78490	NA	F01_transcript_78490
F01_transcript_3306	NA	NA	NA	F01_transcript_3306
F01_transcript_33966	NA	NA	NA	F01_transcript_33966
F01_transcript_118617	NA	NA	NA	F01_transcript_118617
F01_transcript_13119	NA	NA	NA	F01_transcript_13119
F01_transcript_49214	NA	NA	NA	F01_transcript_49214
F01_transcript_112126	NA	F01_transcript_112126	F01_transcript_112126	NA
F01_transcript_96096	NA	NA	NA	F01_transcript_96096
F01_transcript_51932	F01_transcript_51932	F01_transcript_51932	F01_transcript_51932	F01_transcript_51932
F01_transcript_101275	NA	NA	F01_transcript_101275	F01_transcript_101275

F01_transcript_63650	NA	F01_transcript_63650	NA	NA
F01_transcript_128977	F01_transcript_128977	F01_transcript_128977	F01_transcript_128977	F01_transcript_128977
F01_transcript_109008	NA	F01_transcript_109008	F01_transcript_109008	NA
F01_transcript_126270	NA	F01_transcript_126270	F01_transcript_126270	F01_transcript_126270
F01_transcript_45735	NA	NA	NA	F01_transcript_45735
F01_transcript_52808	F01_transcript_52808	F01_transcript_52808	F01_transcript_52808	F01_transcript_52808
F01_transcript_82123	NA	F01_transcript_82123	F01_transcript_82123	NA
F01_transcript_151164	NA	F01_transcript_151164	NA	F01_transcript_151164
F01_transcript_45087	NA	NA	F01_transcript_45087	NA
F01_transcript_31235	NA	NA	NA	F01_transcript_31235
F01_transcript_161843	NA	F01_transcript_161843	NA	NA
F01_transcript_156338	F01_transcript_156338	F01_transcript_156338	NA	NA
F01_transcript_150627	NA	F01_transcript_150627	F01_transcript_150627	F01_transcript_150627
F01_transcript_81336	F01_transcript_81336	F01_transcript_81336	F01_transcript_81336	F01_transcript_81336

F01_transcript_121452	NA	NA	NA	F01_transcript_121452
F01_transcript_115952	NA	NA	NA	F01_transcript_115952
F01_transcript_158867	NA	NA	NA	F01_transcript_158867
F01_transcript_164069	NA	NA	NA	F01_transcript_164069
F01_transcript_166028	F01_transcript_166028	F01_transcript_166028	F01_transcript_166028	F01_transcript_166028
F01_transcript_146623	NA	NA	NA	F01_transcript_146623
F01_transcript_74285	NA	F01_transcript_74285	NA	NA
F01_transcript_162384	F01_transcript_162384	F01_transcript_162384	NA	F01_transcript_162384
F01_transcript_142508	NA	F01_transcript_142508	NA	NA
F01_transcript_43087	NA	F01_transcript_43087	NA	NA
F01_transcript_93745	F01_transcript_93745	F01_transcript_93745	F01_transcript_93745	F01_transcript_93745
F01_transcript_134789	F01_transcript_134789	F01_transcript_134789	F01_transcript_134789	F01_transcript_134789
F01_transcript_94729	NA	NA	NA	F01_transcript_94729
F01_transcript_131813	NA	NA	NA	F01_transcript_131813

F01_transcript_76415	NA	NA	NA	F01_transcript_76415
F01_transcript_70385	NA	F01_transcript_70385	F01_transcript_70385	F01_transcript_70385
F01_transcript_146301	NA	NA	NA	F01_transcript_146301
F01_transcript_154560	NA	NA	NA	F01_transcript_154560
F01_transcript_71401	NA	NA	NA	F01_transcript_71401
F01_transcript_45030	NA	NA	NA	F01_transcript_45030
F01_transcript_162266	NA	F01_transcript_162266	F01_transcript_162266	F01_transcript_162266
F01_transcript_114423	F01_transcript_114423	F01_transcript_114423	F01_transcript_114423	F01_transcript_114423
F01_transcript_112430	F01_transcript_112430	F01_transcript_112430	NA	F01_transcript_112430
F01_transcript_54965	F01_transcript_54965	F01_transcript_54965	F01_transcript_54965	NA
F01_transcript_92548	F01_transcript_92548	F01_transcript_92548	F01_transcript_92548	F01_transcript_92548
F01_transcript_147436	F01_transcript_147436	F01_transcript_147436	F01_transcript_147436	F01_transcript_147436
F01_transcript_116187	F01_transcript_116187	F01_transcript_116187	F01_transcript_116187	F01_transcript_116187
F01_transcript_110291	NA	F01_transcript_110291	NA	NA

F01_transcript_135377	NA	F01_transcript_135377	NA	NA
F01_transcript_138523	NA	F01_transcript_138523	F01_transcript_138523	F01_transcript_138523
F01_transcript_114822	NA	F01_transcript_114822	NA	F01_transcript_114822
F01_transcript_108744	NA	NA	NA	F01_transcript_108744
F01_transcript_137072	NA	F01_transcript_137072	F01_transcript_137072	F01_transcript_137072
F01_transcript_158722	NA	F01_transcript_158722	NA	NA
F01_transcript_132795	NA	F01_transcript_132795	F01_transcript_132795	F01_transcript_132795
F01_transcript_167182	NA	NA	F01_transcript_167182	NA
F01_transcript_74011	F01_transcript_74011	F01_transcript_74011	F01_transcript_74011	F01_transcript_74011
F01_transcript_42577	NA	NA	F01_transcript_42577	NA
F01_transcript_97110	F01_transcript_97110	F01_transcript_97110	F01_transcript_97110	F01_transcript_97110
F01_transcript_62196	NA	F01_transcript_62196	NA	NA
F01_transcript_88265	NA	NA	NA	F01_transcript_88265
F01_transcript_941	F01_transcript_941	F01_transcript_941	F01_transcript_941	NA

F01_transcript_113950	NA	F01_transcript_113950	F01_transcript_113950	NA
F01_transcript_105140	F01_transcript_105140	F01_transcript_105140	F01_transcript_105140	F01_transcript_105140
F01_transcript_80019	NA	NA	NA	F01_transcript_80019
F01_transcript_148195	F01_transcript_148195	F01_transcript_148195	F01_transcript_148195	F01_transcript_148195
F01_transcript_135671	F01_transcript_135671	F01_transcript_135671	F01_transcript_135671	F01_transcript_135671
F01_transcript_56451	NA	NA	NA	F01_transcript_56451
F01_transcript_137493	NA	F01_transcript_137493	NA	NA
F01_transcript_158065	NA	F01_transcript_158065	NA	NA
F01_transcript_54181	F01_transcript_54181	F01_transcript_54181	F01_transcript_54181	F01_transcript_54181
F01_transcript_62560	NA	NA	NA	F01_transcript_62560
F01_transcript_92597	F01_transcript_92597	F01_transcript_92597	F01_transcript_92597	F01_transcript_92597
F01_transcript_140074	NA	F01_transcript_140074	NA	NA
F01_transcript_105178	F01_transcript_105178	F01_transcript_105178	F01_transcript_105178	F01_transcript_105178
F01_transcript_3515	NA	F01_transcript_3515	F01_transcript_3515	NA

F01_transcript_65054	F01_transcript_65054	F01_transcript_65054	F01_transcript_65054	F01_transcript_65054
F01_transcript_125357	NA	F01_transcript_125357	NA	F01_transcript_125357
F01_transcript_94379	NA	NA	NA	F01_transcript_94379
F01_transcript_110292	NA	F01_transcript_110292	NA	NA
F01_transcript_136604	NA	NA	NA	F01_transcript_136604
F01_transcript_135224	F01_transcript_135224	F01_transcript_135224	NA	NA
F01_transcript_58438	NA	NA	NA	F01_transcript_58438
F01_transcript_48634	NA	F01_transcript_48634	NA	NA
F01_transcript_58985	NA	F01_transcript_58985	NA	NA
F01_transcript_134955	NA	F01_transcript_134955	NA	NA
F01_transcript_52685	NA	NA	NA	F01_transcript_52685
F01_transcript_153459	F01_transcript_153459	F01_transcript_153459	NA	F01_transcript_153459
F01_transcript_131857	NA	NA	NA	F01_transcript_131857
F01_transcript_156227	NA	F01_transcript_156227	F01_transcript_156227	F01_transcript_156227

F01_transcript_47785	NA	NA	NA	F01_transcript_47785
F01_transcript_89185	F01_transcript_89185	F01_transcript_89185	F01_transcript_89185	F01_transcript_89185
F01_transcript_157046	NA	F01_transcript_157046	NA	NA
F01_transcript_57025	NA	NA	F01_transcript_57025	NA
F01_transcript_1926	F01_transcript_1926	F01_transcript_1926	F01_transcript_1926	F01_transcript_1926
F01_transcript_160782	F01_transcript_160782	F01_transcript_160782	F01_transcript_160782	F01_transcript_160782
F01_transcript_121602	NA	F01_transcript_121602	NA	NA
F01_transcript_80389	NA	NA	NA	F01_transcript_80389
F01_transcript_117233	NA	F01_transcript_117233	NA	NA
F01_transcript_56105	F01_transcript_56105	F01_transcript_56105	F01_transcript_56105	F01_transcript_56105
F01_transcript_132786	NA	F01_transcript_132786	NA	F01_transcript_132786
F01_transcript_67272	NA	NA	NA	F01_transcript_67272
F01_transcript_4498	NA	NA	F01_transcript_4498	F01_transcript_4498
F01_transcript_135204	NA	NA	NA	F01_transcript_135204

F01_transcript_160089	NA	NA	NA	F01_transcript_160089
F01_transcript_70779	NA	F01_transcript_70779	F01_transcript_70779	NA
F01_transcript_154781	NA	F01_transcript_154781	NA	NA
F01_transcript_163864	NA	F01_transcript_163864	NA	NA
F01_transcript_67028	NA	NA	NA	F01_transcript_67028
F01_transcript_142520	NA	NA	NA	F01_transcript_142520
F01_transcript_93523	NA	NA	NA	F01_transcript_93523
F01_transcript_47378	NA	NA	NA	F01_transcript_47378
F01_transcript_100010	NA	F01_transcript_100010	NA	NA
F01_transcript_124882	NA	F01_transcript_124882	NA	NA
F01_transcript_4467	NA	NA	NA	F01_transcript_4467
F01_transcript_143204	NA	F01_transcript_143204	NA	NA
F01_transcript_112449	F01_transcript_112449	F01_transcript_112449	F01_transcript_112449	F01_transcript_112449
F01_transcript_162738	NA	F01_transcript_162738	NA	NA

F01_transcript_55118	NA	NA	NA	F01_transcript_55118
F01_transcript_75344	F01_transcript_75344	F01_transcript_75344	NA	F01_transcript_75344
F01_transcript_163382	NA	NA	NA	F01_transcript_163382
F01_transcript_21818	NA	NA	NA	F01_transcript_21818
F01_transcript_117759	NA	F01_transcript_117759	NA	NA
F01_transcript_127490	NA	NA	NA	F01_transcript_127490
F01_transcript_157932	NA	F01_transcript_157932	NA	NA
F01_transcript_79324	NA	F01_transcript_79324	F01_transcript_79324	NA
F01_transcript_101664	F01_transcript_101664	F01_transcript_101664	NA	NA
F01_transcript_129433	NA	F01_transcript_129433	NA	NA
F01_transcript_97260	F01_transcript_97260	F01_transcript_97260	F01_transcript_97260	F01_transcript_97260
F01_transcript_96765	F01_transcript_96765	F01_transcript_96765	F01_transcript_96765	F01_transcript_96765
F01_transcript_153449	NA	F01_transcript_153449	NA	NA
F01_transcript_106600	NA	F01_transcript_106600	F01_transcript_106600	F01_transcript_106600

F01_transcript_9089	NA	NA	NA	F01_transcript_9089
F01_transcript_82620	NA	NA	NA	F01_transcript_82620
F01_transcript_82695	NA	NA	NA	F01_transcript_82695
F01_transcript_103534	NA	F01_transcript_103534	NA	NA
F01_transcript_26964	NA	F01_transcript_26964	NA	NA
F01_transcript_138814	NA	F01_transcript_138814	F01_transcript_138814	F01_transcript_138814
F01_transcript_56714	NA	F01_transcript_56714	NA	NA
F01_transcript_117532	F01_transcript_117532	F01_transcript_117532	NA	NA
F01_transcript_94623	NA	F01_transcript_94623	NA	NA
F01_transcript_64955	NA	NA	F01_transcript_64955	NA
F01_transcript_130841	NA	F01_transcript_130841	NA	NA
F01_transcript_79205	NA	F01_transcript_79205	NA	F01_transcript_79205
F01_transcript_48037	NA	NA	NA	F01_transcript_48037
F01_transcript_138608	NA	F01_transcript_138608	NA	NA

F01_transcript_135561	NA	NA	NA	F01_transcript_135561
F01_transcript_111182	F01_transcript_111182	F01_transcript_111182	F01_transcript_111182	F01_transcript_111182
F01_transcript_12789	NA	NA	NA	F01_transcript_12789
F01_transcript_115154	F01_transcript_115154	F01_transcript_115154	F01_transcript_115154	F01_transcript_115154
F01_transcript_130824	NA	F01_transcript_130824	F01_transcript_130824	NA
F01_transcript_74466	NA	NA	NA	F01_transcript_74466
F01_transcript_124454	F01_transcript_124454	F01_transcript_124454	F01_transcript_124454	F01_transcript_124454
F01_transcript_48598	NA	F01_transcript_48598	NA	NA
F01_transcript_95108	NA	F01_transcript_95108	NA	NA
F01_transcript_92867	NA	NA	NA	F01_transcript_92867
F01_transcript_60392	NA	NA	NA	F01_transcript_60392
F01_transcript_113096	NA	NA	NA	F01_transcript_113096
F01_transcript_11648	NA	NA	NA	F01_transcript_11648
F01_transcript_92931	NA	F01_transcript_92931	NA	F01_transcript_92931

F01_transcript_80198	F01_transcript_80198	F01_transcript_80198	F01_transcript_80198	F01_transcript_80198
F01_transcript_157650	NA	F01_transcript_157650	NA	NA
F01_transcript_149940	NA	NA	NA	F01_transcript_149940
F01_transcript_90007	NA	NA	F01_transcript_90007	F01_transcript_90007
F01_transcript_26992	NA	NA	NA	F01_transcript_26992
F01_transcript_9586	NA	NA	F01_transcript_9586	NA
F01_transcript_138918	F01_transcript_138918	F01_transcript_138918	NA	NA
F01_transcript_77551	NA	NA	NA	F01_transcript_77551
F01_transcript_39773	NA	NA	NA	F01_transcript_39773
F01_transcript_10982	NA	NA	F01_transcript_10982	F01_transcript_10982
F01_transcript_83124	NA	NA	F01_transcript_83124	F01_transcript_83124
F01_transcript_57672	NA	NA	NA	F01_transcript_57672
F01_transcript_43849	NA	NA	NA	F01_transcript_43849
F01_transcript_15745	NA	NA	NA	F01_transcript_15745

F01_transcript_91695	NA	F01_transcript_91695	NA	NA
F01_transcript_97145	NA	NA	NA	F01_transcript_97145
F01_transcript_51229	F01_transcript_51229	F01_transcript_51229	F01_transcript_51229	F01_transcript_51229
F01_transcript_133596	NA	NA	NA	F01_transcript_133596
F01_transcript_65330	F01_transcript_65330	F01_transcript_65330	F01_transcript_65330	F01_transcript_65330
F01_transcript_151311	F01_transcript_151311	F01_transcript_151311	F01_transcript_151311	F01_transcript_151311
F01_transcript_67637	NA	NA	NA	F01_transcript_67637
F01_transcript_64404	NA	NA	NA	F01_transcript_64404
F01_transcript_147988	NA	F01_transcript_147988	F01_transcript_147988	F01_transcript_147988
F01_transcript_70756	NA	NA	NA	F01_transcript_70756
F01_transcript_64107	F01_transcript_64107	F01_transcript_64107	NA	F01_transcript_64107
F01_transcript_77505	NA	NA	F01_transcript_77505	NA
F01_transcript_74026	NA	F01_transcript_74026	F01_transcript_74026	F01_transcript_74026
F01_transcript_127609	NA	F01_transcript_127609	NA	NA

F01_transcript_41257	NA	NA	NA	F01_transcript_41257
F01_transcript_146347	NA	F01_transcript_146347	NA	NA
F01_transcript_29929	NA	NA	NA	F01_transcript_29929
F01_transcript_71006	F01_transcript_71006	F01_transcript_71006	F01_transcript_71006	F01_transcript_71006
F01_transcript_164144	NA	NA	NA	F01_transcript_164144
F01_transcript_100743	NA	NA	NA	F01_transcript_100743
F01_transcript_125511	F01_transcript_125511	NA	F01_transcript_125511	F01_transcript_125511
F01_transcript_166376	F01_transcript_166376	F01_transcript_166376	F01_transcript_166376	F01_transcript_166376
F01_transcript_71683	NA	NA	NA	F01_transcript_71683
F01_transcript_121600	NA	F01_transcript_121600	F01_transcript_121600	F01_transcript_121600
F01_transcript_34848	NA	NA	F01_transcript_34848	F01_transcript_34848
F01_transcript_52191	F01_transcript_52191	F01_transcript_52191	F01_transcript_52191	F01_transcript_52191
F01_transcript_146007	F01_transcript_146007	F01_transcript_146007	F01_transcript_146007	F01_transcript_146007
F01_transcript_69432	NA	F01_transcript_69432	NA	NA

F01_transcript_66498	NA	NA	NA	F01_transcript_66498
F01_transcript_132962	NA	F01_transcript_132962	NA	NA
F01_transcript_70402	NA	NA	F01_transcript_70402	NA
F01_transcript_105050	NA	F01_transcript_105050	NA	NA
F01_transcript_131944	F01_transcript_131944	F01_transcript_131944	NA	F01_transcript_131944
F01_transcript_108739	F01_transcript_108739	F01_transcript_108739	F01_transcript_108739	NA
F01_transcript_56668	NA	F01_transcript_56668	NA	F01_transcript_56668
F01_transcript_85148	NA	F01_transcript_85148	F01_transcript_85148	F01_transcript_85148
F01_transcript_128268	NA	F01_transcript_128268	F01_transcript_128268	F01_transcript_128268
F01_transcript_110674	F01_transcript_110674	F01_transcript_110674	NA	NA
F01_transcript_83821	F01_transcript_83821	F01_transcript_83821	F01_transcript_83821	F01_transcript_83821
F01_transcript_116233	F01_transcript_116233	F01_transcript_116233	F01_transcript_116233	F01_transcript_116233
F01_transcript_163811	F01_transcript_163811	F01_transcript_163811	F01_transcript_163811	F01_transcript_163811
F01_transcript_148405	NA	F01_transcript_148405	NA	NA

F01_transcript_97290	F01_transcript_97290	F01_transcript_97290	F01_transcript_97290	F01_transcript_97290
F01_transcript_60319	F01_transcript_60319	F01_transcript_60319	F01_transcript_60319	F01_transcript_60319
F01_transcript_114612	NA	F01_transcript_114612	F01_transcript_114612	F01_transcript_114612
F01_transcript_149643	NA	F01_transcript_149643	NA	NA
F01_transcript_581	F01_transcript_581	F01_transcript_581	F01_transcript_581	NA
F01_transcript_45313	NA	NA	NA	F01_transcript_45313
F01_transcript_123534	NA	F01_transcript_123534	NA	NA
F01_transcript_86549	NA	NA	F01_transcript_86549	F01_transcript_86549
F01_transcript_21830	F01_transcript_21830	F01_transcript_21830	NA	F01_transcript_21830
F01_transcript_140275	F01_transcript_140275	F01_transcript_140275	F01_transcript_140275	F01_transcript_140275
F01_transcript_146927	F01_transcript_146927	F01_transcript_146927	F01_transcript_146927	F01_transcript_146927
F01_transcript_68160	F01_transcript_68160	F01_transcript_68160	NA	NA
F01_transcript_90450	NA	F01_transcript_90450	F01_transcript_90450	F01_transcript_90450
F01_transcript_620	NA	F01_transcript_620	NA	NA

F01_transcript_50439	NA	F01_transcript_50439	NA	NA
F01_transcript_65395	F01_transcript_65395	F01_transcript_65395	F01_transcript_65395	F01_transcript_65395
F01_transcript_77956	NA	NA	NA	F01_transcript_77956
F01_transcript_86262	NA	NA	NA	F01_transcript_86262
F01_transcript_1702	F01_transcript_1702	F01_transcript_1702	NA	NA
F01_transcript_131220	NA	F01_transcript_131220	F01_transcript_131220	F01_transcript_131220
F01_transcript_167384	NA	F01_transcript_167384	NA	NA
F01_transcript_31145	NA	NA	NA	F01_transcript_31145
F01_transcript_141808	NA	F01_transcript_141808	NA	NA
F01_transcript_67156	NA	F01_transcript_67156	F01_transcript_67156	F01_transcript_67156
F01_transcript_96008	NA	F01_transcript_96008	NA	NA
F01_transcript_148304	F01_transcript_148304	F01_transcript_148304	F01_transcript_148304	F01_transcript_148304
F01_transcript_93460	NA	NA	NA	F01_transcript_93460
F01_transcript_139357	NA	F01_transcript_139357	NA	NA

F01_transcript_16178	NA	NA	NA	F01_transcript_16178
F01_transcript_47824	NA	F01_transcript_47824	NA	NA
F01_transcript_138955	NA	NA	F01_transcript_138955	F01_transcript_138955
F01_transcript_116165	F01_transcript_116165	F01_transcript_116165	F01_transcript_116165	F01_transcript_116165
F01_transcript_31176	F01_transcript_31176	F01_transcript_31176	F01_transcript_31176	F01_transcript_31176
F01_transcript_112378	F01_transcript_112378	F01_transcript_112378	F01_transcript_112378	F01_transcript_112378
F01_transcript_148923	NA	F01_transcript_148923	NA	F01_transcript_148923
F01_transcript_127112	NA	F01_transcript_127112	NA	NA
F01_transcript_108090	F01_transcript_108090	F01_transcript_108090	F01_transcript_108090	F01_transcript_108090
F01_transcript_128949	F01_transcript_128949	F01_transcript_128949	F01_transcript_128949	F01_transcript_128949
F01_transcript_57988	NA	F01_transcript_57988	NA	NA
F01_transcript_126692	NA	NA	NA	F01_transcript_126692
F01_transcript_159722	NA	F01_transcript_159722	NA	NA
F01_transcript_18519	NA	F01_transcript_18519	NA	F01_transcript_18519

F01_transcript_51701	NA	F01_transcript_51701	NA	F01_transcript_51701
F01_transcript_107221	F01_transcript_107221	F01_transcript_107221	F01_transcript_107221	NA
F01_transcript_150753	F01_transcript_150753	F01_transcript_150753	F01_transcript_150753	F01_transcript_150753
F01_transcript_8906	NA	NA	NA	F01_transcript_8906
F01_transcript_163366	NA	F01_transcript_163366	F01_transcript_163366	F01_transcript_163366
F01_transcript_113001	NA	NA	NA	F01_transcript_113001
F01_transcript_64177	NA	F01_transcript_64177	F01_transcript_64177	F01_transcript_64177
F01_transcript_156723	F01_transcript_156723	F01_transcript_156723	F01_transcript_156723	F01_transcript_156723
F01_transcript_151910	NA	NA	NA	F01_transcript_151910
F01_transcript_26510	NA	NA	NA	F01_transcript_26510
F01_transcript_163647	NA	NA	NA	F01_transcript_163647
F01_transcript_24850	NA	NA	NA	F01_transcript_24850
F01_transcript_48766	NA	NA	F01_transcript_48766	F01_transcript_48766
F01_transcript_69064	NA	NA	NA	F01_transcript_69064

F01_transcript_11576	NA	F01_transcript_11576	NA	NA
F01_transcript_82101	F01_transcript_82101	F01_transcript_82101	F01_transcript_82101	F01_transcript_82101
F01_transcript_124251	NA	F01_transcript_124251	NA	F01_transcript_124251
F01_transcript_135836	NA	NA	NA	F01_transcript_135836
F01_transcript_95298	F01_transcript_95298	F01_transcript_95298	NA	NA
F01_transcript_15346	NA	F01_transcript_15346	NA	NA
F01_transcript_140483	NA	NA	F01_transcript_140483	NA
F01_transcript_135893	NA	F01_transcript_135893	F01_transcript_135893	F01_transcript_135893
F01_transcript_50490	NA	F01_transcript_50490	NA	NA
F01_transcript_165422	NA	NA	NA	F01_transcript_165422
F01_transcript_149984	F01_transcript_149984	F01_transcript_149984	F01_transcript_149984	F01_transcript_149984
F01_transcript_115249	NA	NA	NA	F01_transcript_115249
F01_transcript_111155	NA	NA	NA	F01_transcript_111155
F01_transcript_68183	NA	F01_transcript_68183	NA	F01_transcript_68183

F01_transcript_58332	F01_transcript_58332	F01_transcript_58332	F01_transcript_58332	F01_transcript_58332
F01_transcript_127444	NA	NA	NA	F01_transcript_127444
F01_transcript_150423	NA	F01_transcript_150423	NA	NA
F01_transcript_73951	NA	F01_transcript_73951	NA	NA
F01_transcript_144247	NA	F01_transcript_144247	F01_transcript_144247	F01_transcript_144247
F01_transcript_128672	NA	F01_transcript_128672	F01_transcript_128672	F01_transcript_128672
F01_transcript_87718	NA	F01_transcript_87718	F01_transcript_87718	F01_transcript_87718
F01_transcript_151768	NA	NA	NA	F01_transcript_151768
F01_transcript_157465	F01_transcript_157465	F01_transcript_157465	F01_transcript_157465	F01_transcript_157465
F01_transcript_79646	NA	NA	NA	F01_transcript_79646
F01_transcript_89127	NA	F01_transcript_89127	NA	NA
F01_transcript_68859	NA	F01_transcript_68859	NA	NA
F01_transcript_147521	NA	F01_transcript_147521	NA	NA
F01_transcript_21319	NA	F01_transcript_21319	NA	NA

F01_transcript_4554	NA	F01_transcript_4554	NA	NA
F01_transcript_93463	F01_transcript_93463	F01_transcript_93463	F01_transcript_93463	F01_transcript_93463
F01_transcript_164577	NA	F01_transcript_164577	NA	F01_transcript_164577
F01_transcript_109232	NA	NA	NA	F01_transcript_109232
F01_transcript_73626	NA	NA	F01_transcript_73626	NA
F01_transcript_68312	NA	F01_transcript_68312	NA	NA
F01_transcript_123342	NA	F01_transcript_123342	NA	NA
F01_transcript_78245	NA	F01_transcript_78245	NA	F01_transcript_78245
F01_transcript_117518	F01_transcript_117518	F01_transcript_117518	F01_transcript_117518	F01_transcript_117518
F01_transcript_141539	F01_transcript_141539	F01_transcript_141539	F01_transcript_141539	F01_transcript_141539
F01_transcript_119623	NA	NA	NA	F01_transcript_119623
F01_transcript_2588	F01_transcript_2588	F01_transcript_2588	F01_transcript_2588	F01_transcript_2588
F01_transcript_112432	NA	F01_transcript_112432	NA	NA
F01_transcript_56816	NA	NA	F01_transcript_56816	NA

F01_transcript_153209	NA	F01_transcript_153209	F01_transcript_153209	F01_transcript_153209
F01_transcript_166788	F01_transcript_166788	F01_transcript_166788	F01_transcript_166788	F01_transcript_166788
F01_transcript_136765	NA	F01_transcript_136765	F01_transcript_136765	F01_transcript_136765
F01_transcript_75194	NA	NA	NA	F01_transcript_75194
F01_transcript_84827	NA	F01_transcript_84827	NA	F01_transcript_84827
F01_transcript_98551	NA	NA	NA	F01_transcript_98551
F01_transcript_106484	NA	F01_transcript_106484	NA	F01_transcript_106484
F01_transcript_166801	NA	NA	NA	F01_transcript_166801
F01_transcript_91010	F01_transcript_91010	F01_transcript_91010	F01_transcript_91010	F01_transcript_91010
F01_transcript_152649	NA	F01_transcript_152649	NA	NA
F01_transcript_62015	NA	F01_transcript_62015	NA	F01_transcript_62015
F01_transcript_152779	NA	NA	NA	F01_transcript_152779
F01_transcript_68628	F01_transcript_68628	F01_transcript_68628	F01_transcript_68628	F01_transcript_68628
F01_transcript_53928	NA	NA	NA	F01_transcript_53928

F01_transcript_61679	F01_transcript_61679	F01_transcript_61679	F01_transcript_61679	F01_transcript_61679
F01_transcript_149888	NA	NA	NA	F01_transcript_149888
F01_transcript_65537	NA	NA	NA	F01_transcript_65537
F01_transcript_13765	NA	F01_transcript_13765	NA	NA
F01_transcript_120360	NA	NA	NA	F01_transcript_120360
F01_transcript_99176	NA	F01_transcript_99176	NA	NA
F01_transcript_146304	NA	F01_transcript_146304	NA	NA
F01_transcript_83156	NA	NA	NA	F01_transcript_83156
F01_transcript_73211	NA	F01_transcript_73211	F01_transcript_73211	F01_transcript_73211
F01_transcript_147382	NA	NA	NA	F01_transcript_147382
F01_transcript_84876	F01_transcript_84876	F01_transcript_84876	F01_transcript_84876	F01_transcript_84876
F01_transcript_155757	NA	F01_transcript_155757	NA	NA
F01_transcript_43267	NA	NA	NA	F01_transcript_43267
F01_transcript_91564	F01_transcript_91564	F01_transcript_91564	F01_transcript_91564	F01_transcript_91564

F01_transcript_2364	NA	NA	NA	F01_transcript_2364
F01_transcript_113034	NA	NA	F01_transcript_113034	NA
F01_transcript_121646	NA	NA	NA	F01_transcript_121646
F01_transcript_73294	NA	F01_transcript_73294	NA	NA
F01_transcript_151703	F01_transcript_151703	F01_transcript_151703	F01_transcript_151703	F01_transcript_151703
F01_transcript_58143	NA	F01_transcript_58143	NA	NA
F01_transcript_51677	F01_transcript_51677	F01_transcript_51677	F01_transcript_51677	F01_transcript_51677
F01_transcript_51160	F01_transcript_51160	F01_transcript_51160	F01_transcript_51160	F01_transcript_51160
F01_transcript_92327	F01_transcript_92327	F01_transcript_92327	NA	NA
F01_transcript_77471	F01_transcript_77471	F01_transcript_77471	F01_transcript_77471	F01_transcript_77471
F01_transcript_154459	NA	F01_transcript_154459	NA	NA
F01_transcript_114886	F01_transcript_114886	F01_transcript_114886	F01_transcript_114886	F01_transcript_114886
F01_transcript_163557	NA	F01_transcript_163557	F01_transcript_163557	F01_transcript_163557
F01_transcript_133503	NA	NA	NA	F01_transcript_133503

F01_transcript_100984	NA	NA	NA	F01_transcript_100984
F01_transcript_97900	NA	F01_transcript_97900	NA	NA
F01_transcript_95530	NA	F01_transcript_95530	NA	F01_transcript_95530
F01_transcript_95039	F01_transcript_95039	F01_transcript_95039	NA	NA
F01_transcript_141236	NA	F01_transcript_141236	NA	NA
F01_transcript_2473	NA	NA	NA	F01_transcript_2473
F01_transcript_56472	NA	NA	F01_transcript_56472	F01_transcript_56472
F01_transcript_4248	NA	NA	NA	F01_transcript_4248
F01_transcript_120760	NA	F01_transcript_120760	NA	NA
F01_transcript_82192	F01_transcript_82192	F01_transcript_82192	F01_transcript_82192	F01_transcript_82192
F01_transcript_124198	F01_transcript_124198	F01_transcript_124198	NA	F01_transcript_124198
F01_transcript_54303	NA	F01_transcript_54303	NA	NA
F01_transcript_82207	NA	F01_transcript_82207	NA	NA
F01_transcript_5773	NA	NA	F01_transcript_5773	F01_transcript_5773

F01_transcript_96407	NA	NA	NA	F01_transcript_96407
F01_transcript_159002	NA	NA	F01_transcript_159002	NA
F01_transcript_108572	F01_transcript_108572	F01_transcript_108572	F01_transcript_108572	F01_transcript_108572
F01_transcript_81332	NA	NA	NA	F01_transcript_81332
F01_transcript_157175	NA	NA	NA	F01_transcript_157175
F01_transcript_38546	NA	F01_transcript_38546	NA	NA
F01_transcript_41272	NA	NA	NA	F01_transcript_41272
F01_transcript_113464	NA	NA	NA	F01_transcript_113464
F01_transcript_3339	F01_transcript_3339	F01_transcript_3339	F01_transcript_3339	F01_transcript_3339
F01_transcript_126446	NA	F01_transcript_126446	NA	NA
F01_transcript_67597	NA	F01_transcript_67597	NA	NA
F01_transcript_159433	NA	F01_transcript_159433	NA	F01_transcript_159433
F01_transcript_152195	NA	F01_transcript_152195	NA	NA
F01_transcript_116908	NA	F01_transcript_116908	NA	NA

F01_transcript_165894	NA	NA	NA	F01_transcript_165894
F01_transcript_95876	NA	F01_transcript_95876	NA	F01_transcript_95876
F01_transcript_52276	F01_transcript_52276	F01_transcript_52276	F01_transcript_52276	F01_transcript_52276
F01_transcript_62683	NA	F01_transcript_62683	NA	NA
F01_transcript_54107	NA	NA	NA	F01_transcript_54107
F01_transcript_119193	NA	NA	NA	F01_transcript_119193
F01_transcript_84102	NA	NA	NA	F01_transcript_84102
F01_transcript_156783	NA	F01_transcript_156783	NA	NA
F01_transcript_50825	F01_transcript_50825	F01_transcript_50825	F01_transcript_50825	F01_transcript_50825
F01_transcript_103453	NA	NA	NA	F01_transcript_103453
F01_transcript_157991	F01_transcript_157991	NA	NA	F01_transcript_157991
F01_transcript_18389	F01_transcript_18389	F01_transcript_18389	F01_transcript_18389	F01_transcript_18389
F01_transcript_42598	NA	NA	NA	F01_transcript_42598
F01_transcript_16459	NA	NA	NA	F01_transcript_16459

F01_transcript_88827	NA	F01_transcript_88827	NA	NA
F01_transcript_46821	NA	F01_transcript_46821	NA	NA
F01_transcript_108127	NA	F01_transcript_108127	NA	NA
F01_transcript_120122	F01_transcript_120122	NA	NA	F01_transcript_120122
F01_transcript_80002	NA	F01_transcript_80002	NA	NA
F01_transcript_66140	F01_transcript_66140	F01_transcript_66140	F01_transcript_66140	F01_transcript_66140
F01_transcript_158182	NA	NA	NA	F01_transcript_158182
F01_transcript_71105	F01_transcript_71105	F01_transcript_71105	F01_transcript_71105	F01_transcript_71105
F01_transcript_56905	NA	F01_transcript_56905	F01_transcript_56905	NA
F01_transcript_36785	NA	F01_transcript_36785	NA	NA
F01_transcript_51662	NA	NA	NA	F01_transcript_51662
F01_transcript_128864	NA	F01_transcript_128864	NA	NA
F01_transcript_55028	NA	F01_transcript_55028	NA	NA
F01_transcript_54900	NA	NA	NA	F01_transcript_54900

F01_transcript_139401	NA	NA	NA	F01_transcript_139401
F01_transcript_99804	NA	F01_transcript_99804	NA	NA
F01_transcript_85204	NA	NA	NA	F01_transcript_85204
F01_transcript_50561	NA	F01_transcript_50561	NA	NA
F01_transcript_151277	NA	NA	NA	F01_transcript_151277
F01_transcript_69685	NA	NA	NA	F01_transcript_69685
F01_transcript_145268	F01_transcript_145268	F01_transcript_145268	F01_transcript_145268	F01_transcript_145268
F01_transcript_135235	NA	NA	NA	F01_transcript_135235
F01_transcript_95815	NA	NA	NA	F01_transcript_95815
F01_transcript_83788	NA	F01_transcript_83788	F01_transcript_83788	F01_transcript_83788
F01_transcript_91575	NA	F01_transcript_91575	NA	F01_transcript_91575
F01_transcript_60889	F01_transcript_60889	F01_transcript_60889	F01_transcript_60889	F01_transcript_60889
F01_transcript_78865	F01_transcript_78865	F01_transcript_78865	NA	NA
F01_transcript_72077	NA	F01_transcript_72077	NA	NA

F01_transcript_155691	F01_transcript_155691	F01_transcript_155691	F01_transcript_155691	F01_transcript_155691
F01_transcript_106159	NA	NA	NA	F01_transcript_106159
F01_transcript_39690	NA	NA	NA	F01_transcript_39690
F01_transcript_75720	NA	NA	NA	F01_transcript_75720
F01_transcript_95043	NA	F01_transcript_95043	NA	NA
F01_transcript_111177	F01_transcript_111177	F01_transcript_111177	F01_transcript_111177	F01_transcript_111177
F01_transcript_83099	NA	F01_transcript_83099	NA	NA
F01_transcript_53621	F01_transcript_53621	F01_transcript_53621	F01_transcript_53621	F01_transcript_53621
F01_transcript_114359	F01_transcript_114359	F01_transcript_114359	NA	F01_transcript_114359
F01_transcript_55799	NA	F01_transcript_55799	NA	NA
F01_transcript_68121	F01_transcript_68121	F01_transcript_68121	F01_transcript_68121	F01_transcript_68121
F01_transcript_78792	NA	F01_transcript_78792	NA	F01_transcript_78792
F01_transcript_54229	NA	F01_transcript_54229	NA	NA
F01_transcript_71201	NA	NA	F01_transcript_71201	NA

F01_transcript_52020	F01_transcript_52020	F01_transcript_52020	F01_transcript_52020	F01_transcript_52020
F01_transcript_65273	NA	F01_transcript_65273	F01_transcript_65273	F01_transcript_65273
F01_transcript_66997	NA	F01_transcript_66997	F01_transcript_66997	NA
F01_transcript_134299	NA	NA	NA	F01_transcript_134299
F01_transcript_64774	NA	F01_transcript_64774	NA	NA
F01_transcript_158127	NA	F01_transcript_158127	NA	F01_transcript_158127
F01_transcript_88396	F01_transcript_88396	F01_transcript_88396	F01_transcript_88396	F01_transcript_88396
F01_transcript_152534	F01_transcript_152534	F01_transcript_152534	F01_transcript_152534	F01_transcript_152534
F01_transcript_63200	NA	NA	NA	F01_transcript_63200
F01_transcript_140746	F01_transcript_140746	F01_transcript_140746	F01_transcript_140746	F01_transcript_140746
F01_transcript_38201	NA	NA	NA	F01_transcript_38201
F01_transcript_37809	NA	F01_transcript_37809	NA	NA
F01_transcript_50424	F01_transcript_50424	F01_transcript_50424	F01_transcript_50424	F01_transcript_50424
F01_transcript_75246	NA	NA	NA	F01_transcript_75246

F01_transcript_115705	F01_transcript_115705	F01_transcript_115705	F01_transcript_115705	NA
F01_transcript_137530	NA	NA	NA	F01_transcript_137530
F01_transcript_81455	NA	F01_transcript_81455	NA	NA
F01_transcript_75742	NA	NA	NA	F01_transcript_75742
F01_transcript_42092	NA	NA	NA	F01_transcript_42092
F01_transcript_154171	NA	F01_transcript_154171	NA	NA
F01_transcript_61044	NA	F01_transcript_61044	NA	F01_transcript_61044
F01_transcript_84037	NA	NA	F01_transcript_84037	F01_transcript_84037
F01_transcript_59181	F01_transcript_59181	F01_transcript_59181	F01_transcript_59181	F01_transcript_59181
F01_transcript_72752	F01_transcript_72752	F01_transcript_72752	F01_transcript_72752	F01_transcript_72752
F01_transcript_124888	NA	NA	NA	F01_transcript_124888
F01_transcript_91723	NA	F01_transcript_91723	NA	F01_transcript_91723
F01_transcript_164871	F01_transcript_164871	NA	NA	NA
F01_transcript_128722	NA	F01_transcript_128722	NA	NA

F01_transcript_45406	NA	NA	F01_transcript_45406	F01_transcript_45406
F01_transcript_160094	F01_transcript_160094	F01_transcript_160094	F01_transcript_160094	F01_transcript_160094
F01_transcript_139857	NA	F01_transcript_139857	NA	NA
F01_transcript_11424	F01_transcript_11424	F01_transcript_11424	F01_transcript_11424	F01_transcript_11424
F01_transcript_130316	F01_transcript_130316	F01_transcript_130316	F01_transcript_130316	F01_transcript_130316
F01_transcript_92921	NA	NA	NA	F01_transcript_92921
F01_transcript_66084	F01_transcript_66084	F01_transcript_66084	F01_transcript_66084	F01_transcript_66084
F01_transcript_123798	NA	NA	NA	F01_transcript_123798
F01_transcript_125015	NA	F01_transcript_125015	NA	F01_transcript_125015
F01_transcript_6067	NA	NA	NA	F01_transcript_6067
F01_transcript_90057	NA	NA	NA	F01_transcript_90057
F01_transcript_4485	NA	NA	NA	F01_transcript_4485
F01_transcript_113940	F01_transcript_113940	F01_transcript_113940	F01_transcript_113940	F01_transcript_113940
F01_transcript_149178	NA	NA	NA	F01_transcript_149178

F01_transcript_73946	NA	F01_transcript_73946	NA	F01_transcript_73946
F01_transcript_64386	NA	NA	NA	F01_transcript_64386
F01_transcript_161105	F01_transcript_161105	F01_transcript_161105	F01_transcript_161105	F01_transcript_161105
F01_transcript_106943	NA	NA	NA	F01_transcript_106943
F01_transcript_29746	F01_transcript_29746	F01_transcript_29746	F01_transcript_29746	F01_transcript_29746
F01_transcript_112800	NA	NA	NA	F01_transcript_112800
F01_transcript_134746	NA	NA	NA	F01_transcript_134746
F01_transcript_166814	NA	F01_transcript_166814	NA	NA
F01_transcript_139992	NA	F01_transcript_139992	NA	NA
F01_transcript_114552	NA	NA	NA	F01_transcript_114552
F01_transcript_10641	NA	NA	NA	F01_transcript_10641
F01_transcript_124576	NA	F01_transcript_124576	F01_transcript_124576	F01_transcript_124576
F01_transcript_120911	F01_transcript_120911	F01_transcript_120911	F01_transcript_120911	NA
F01_transcript_93739	NA	F01_transcript_93739	NA	NA

F01_transcript_152546	NA	NA	NA	F01_transcript_152546
F01_transcript_37616	NA	F01_transcript_37616	NA	F01_transcript_37616
F01_transcript_32168	NA	NA	NA	F01_transcript_32168
F01_transcript_76673	F01_transcript_76673	F01_transcript_76673	F01_transcript_76673	F01_transcript_76673
F01_transcript_13555	NA	NA	NA	F01_transcript_13555
F01_transcript_15559	NA	NA	F01_transcript_15559	F01_transcript_15559
F01_transcript_70532	F01_transcript_70532	F01_transcript_70532	F01_transcript_70532	F01_transcript_70532
F01_transcript_55120	F01_transcript_55120	F01_transcript_55120	F01_transcript_55120	F01_transcript_55120
F01_transcript_113521	NA	F01_transcript_113521	F01_transcript_113521	NA
F01_transcript_44217	F01_transcript_44217	F01_transcript_44217	F01_transcript_44217	F01_transcript_44217
F01_transcript_74505	NA	NA	NA	F01_transcript_74505
F01_transcript_118775	NA	F01_transcript_118775	NA	F01_transcript_118775
F01_transcript_47393	NA	NA	NA	F01_transcript_47393
F01_transcript_50679	F01_transcript_50679	F01_transcript_50679	F01_transcript_50679	F01_transcript_50679

F01_transcript_57675	NA	NA	NA	F01_transcript_57675
F01_transcript_86303	NA	NA	NA	F01_transcript_86303
F01_transcript_94470	F01_transcript_94470	F01_transcript_94470	F01_transcript_94470	F01_transcript_94470
F01_transcript_86237	F01_transcript_86237	F01_transcript_86237	NA	F01_transcript_86237
F01_transcript_88136	NA	NA	NA	F01_transcript_88136
F01_transcript_112197	F01_transcript_112197	F01_transcript_112197	NA	NA
F01_transcript_58918	NA	F01_transcript_58918	F01_transcript_58918	NA
F01_transcript_154213	NA	F01_transcript_154213	NA	F01_transcript_154213
F01_transcript_146710	F01_transcript_146710	F01_transcript_146710	F01_transcript_146710	NA
F01_transcript_51340	NA	NA	NA	F01_transcript_51340
F01_transcript_62817	NA	F01_transcript_62817	NA	NA
F01_transcript_146313	NA	F01_transcript_146313	NA	F01_transcript_146313
F01_transcript_92790	NA	F01_transcript_92790	NA	NA
F01_transcript_90548	F01_transcript_90548	F01_transcript_90548	F01_transcript_90548	F01_transcript_90548

F01_transcript_101812	NA	F01_transcript_101812	NA	F01_transcript_101812
F01_transcript_74103	F01_transcript_74103	F01_transcript_74103	NA	NA
F01_transcript_130862	F01_transcript_130862	F01_transcript_130862	F01_transcript_130862	F01_transcript_130862
F01_transcript_148381	F01_transcript_148381	F01_transcript_148381	NA	NA
F01_transcript_23105	NA	F01_transcript_23105	NA	NA
F01_transcript_84536	NA	F01_transcript_84536	NA	NA
F01_transcript_114670	F01_transcript_114670	F01_transcript_114670	F01_transcript_114670	F01_transcript_114670
F01_transcript_88795	NA	F01_transcript_88795	NA	NA
F01_transcript_136667	NA	F01_transcript_136667	NA	NA
F01_transcript_55567	NA	F01_transcript_55567	NA	F01_transcript_55567
F01_transcript_2621	NA	F01_transcript_2621	NA	F01_transcript_2621
F01_transcript_103658	NA	NA	NA	F01_transcript_103658
F01_transcript_158431	F01_transcript_158431	F01_transcript_158431	F01_transcript_158431	F01_transcript_158431
F01_transcript_35389	F01_transcript_35389	F01_transcript_35389	F01_transcript_35389	F01_transcript_35389

F01_transcript_61539	NA	NA	NA	F01_transcript_61539
F01_transcript_72996	NA	F01_transcript_72996	NA	NA
F01_transcript_122928	NA	F01_transcript_122928	F01_transcript_122928	F01_transcript_122928
F01_transcript_128244	F01_transcript_128244	F01_transcript_128244	F01_transcript_128244	F01_transcript_128244
F01_transcript_78362	NA	F01_transcript_78362	NA	NA
F01_transcript_165320	F01_transcript_165320	F01_transcript_165320	F01_transcript_165320	F01_transcript_165320
F01_transcript_112463	NA	NA	NA	F01_transcript_112463
F01_transcript_150871	NA	F01_transcript_150871	F01_transcript_150871	F01_transcript_150871
F01_transcript_105645	NA	F01_transcript_105645	NA	NA
F01_transcript_10192	F01_transcript_10192	F01_transcript_10192	NA	F01_transcript_10192
F01_transcript_26982	NA	NA	NA	F01_transcript_26982
F01_transcript_157761	F01_transcript_157761	F01_transcript_157761	F01_transcript_157761	F01_transcript_157761
F01_transcript_148742	F01_transcript_148742	F01_transcript_148742	F01_transcript_148742	F01_transcript_148742
F01_transcript_141413	NA	F01_transcript_141413	NA	NA

F01_transcript_158658	F01_transcript_158658	F01_transcript_158658	NA	F01_transcript_158658
F01_transcript_111140	NA	F01_transcript_111140	NA	F01_transcript_111140
F01_transcript_92887	NA	F01_transcript_92887	F01_transcript_92887	F01_transcript_92887
F01_transcript_32285	NA	NA	NA	F01_transcript_32285
F01_transcript_81031	NA	NA	NA	F01_transcript_81031
F01_transcript_44051	F01_transcript_44051	F01_transcript_44051	F01_transcript_44051	F01_transcript_44051
F01_transcript_144166	NA	F01_transcript_144166	NA	NA
F01_transcript_153650	NA	F01_transcript_153650	NA	NA
F01_transcript_56455	NA	F01_transcript_56455	F01_transcript_56455	NA
F01_transcript_70924	F01_transcript_70924	NA	F01_transcript_70924	F01_transcript_70924
F01_transcript_86184	NA	NA	NA	F01_transcript_86184
F01_transcript_112962	NA	F01_transcript_112962	NA	NA
F01_transcript_48830	F01_transcript_48830	NA	F01_transcript_48830	F01_transcript_48830
F01_transcript_138314	NA	F01_transcript_138314	NA	NA

F01_transcript_76181	NA	NA	NA	F01_transcript_76181
F01_transcript_114097	NA	NA	NA	F01_transcript_114097
F01_transcript_59018	F01_transcript_59018	F01_transcript_59018	F01_transcript_59018	NA
F01_transcript_9001	NA	F01_transcript_9001	F01_transcript_9001	NA
F01_transcript_112473	NA	F01_transcript_112473	NA	NA
F01_transcript_110705	NA	F01_transcript_110705	NA	F01_transcript_110705
F01_transcript_66811	F01_transcript_66811	F01_transcript_66811	F01_transcript_66811	F01_transcript_66811
F01_transcript_164732	NA	F01_transcript_164732	NA	NA
F01_transcript_48991	NA	NA	NA	F01_transcript_48991
F01_transcript_151020	NA	F01_transcript_151020	NA	NA
F01_transcript_78226	F01_transcript_78226	F01_transcript_78226	F01_transcript_78226	F01_transcript_78226
F01_transcript_165659	NA	F01_transcript_165659	NA	F01_transcript_165659
F01_transcript_110856	F01_transcript_110856	F01_transcript_110856	F01_transcript_110856	F01_transcript_110856
F01_transcript_65296	NA	NA	F01_transcript_65296	NA

F01_transcript_155388	F01_transcript_155388	F01_transcript_155388	F01_transcript_155388	F01_transcript_155388
F01_transcript_80136	F01_transcript_80136	F01_transcript_80136	NA	NA
F01_transcript_144020	NA	NA	NA	F01_transcript_144020
F01_transcript_149758	NA	F01_transcript_149758	NA	F01_transcript_149758
F01_transcript_148350	NA	F01_transcript_148350	F01_transcript_148350	F01_transcript_148350
F01_transcript_110609	NA	NA	NA	F01_transcript_110609
F01_transcript_37380	NA	NA	NA	F01_transcript_37380
F01_transcript_80091	F01_transcript_80091	F01_transcript_80091	NA	NA
F01_transcript_108618	NA	F01_transcript_108618	NA	NA
F01_transcript_18913	NA	F01_transcript_18913	NA	F01_transcript_18913
F01_transcript_156066	NA	NA	NA	F01_transcript_156066
F01_transcript_166987	F01_transcript_166987	F01_transcript_166987	F01_transcript_166987	F01_transcript_166987
F01_transcript_76240	NA	NA	NA	F01_transcript_76240
F01_transcript_39316	NA	NA	NA	F01_transcript_39316

F01_transcript_119869	NA	F01_transcript_119869	NA	NA
F01_transcript_106826	NA	F01_transcript_106826	NA	NA
F01_transcript_149260	NA	NA	NA	F01_transcript_149260
F01_transcript_40153	F01_transcript_40153	F01_transcript_40153	F01_transcript_40153	F01_transcript_40153
F01_transcript_68762	NA	NA	NA	F01_transcript_68762
F01_transcript_145746	NA	NA	NA	F01_transcript_145746
F01_transcript_86116	NA	F01_transcript_86116	NA	NA
F01_transcript_61368	NA	NA	NA	F01_transcript_61368
F01_transcript_123188	NA	F01_transcript_123188	NA	NA
F01_transcript_77002	NA	F01_transcript_77002	NA	NA
F01_transcript_70424	NA	F01_transcript_70424	NA	NA
F01_transcript_39638	NA	NA	NA	F01_transcript_39638
F01_transcript_73071	NA	NA	NA	F01_transcript_73071
F01_transcript_162426	F01_transcript_162426	F01_transcript_162426	F01_transcript_162426	F01_transcript_162426

F01_transcript_116728	F01_transcript_116728	F01_transcript_116728	NA	NA
F01_transcript_18338	F01_transcript_18338	F01_transcript_18338	F01_transcript_18338	F01_transcript_18338
F01_transcript_63604	NA	F01_transcript_63604	NA	NA
F01_transcript_59564	NA	NA	F01_transcript_59564	NA
F01_transcript_111158	NA	F01_transcript_111158	NA	F01_transcript_111158
F01_transcript_82651	NA	NA	NA	F01_transcript_82651
F01_transcript_75431	NA	NA	NA	F01_transcript_75431
F01_transcript_71043	NA	NA	NA	F01_transcript_71043
F01_transcript_86974	F01_transcript_86974	F01_transcript_86974	F01_transcript_86974	F01_transcript_86974
F01_transcript_17268	NA	F01_transcript_17268	NA	F01_transcript_17268
F01_transcript_59441	NA	F01_transcript_59441	F01_transcript_59441	NA
F01_transcript_81434	NA	NA	NA	F01_transcript_81434
F01_transcript_78691	NA	NA	NA	F01_transcript_78691
F01_transcript_115054	F01_transcript_115054	F01_transcript_115054	NA	F01_transcript_115054

F01_transcript_114953	NA	NA	NA	F01_transcript_114953
F01_transcript_140471	NA	F01_transcript_140471	NA	NA
F01_transcript_80434	NA	F01_transcript_80434	NA	NA
F01_transcript_50811	F01_transcript_50811	F01_transcript_50811	F01_transcript_50811	F01_transcript_50811
F01_transcript_54115	NA	F01_transcript_54115	F01_transcript_54115	F01_transcript_54115
F01_transcript_90723	NA	F01_transcript_90723	F01_transcript_90723	F01_transcript_90723
F01_transcript_116776	NA	F01_transcript_116776	NA	NA
F01_transcript_144144	NA	F01_transcript_144144	NA	NA
F01_transcript_153977	NA	F01_transcript_153977	NA	F01_transcript_153977
F01_transcript_41568	NA	NA	NA	F01_transcript_41568
F01_transcript_56731	F01_transcript_56731	F01_transcript_56731	F01_transcript_56731	F01_transcript_56731
F01_transcript_158507	F01_transcript_158507	F01_transcript_158507	NA	F01_transcript_158507
F01_transcript_162090	F01_transcript_162090	F01_transcript_162090	F01_transcript_162090	F01_transcript_162090
F01_transcript_166567	NA	NA	NA	F01_transcript_166567

F01_transcript_110127	NA	NA	NA	F01_transcript_110127
F01_transcript_148244	NA	NA	NA	F01_transcript_148244
F01_transcript_54216	NA	NA	NA	F01_transcript_54216
F01_transcript_109147	NA	F01_transcript_109147	NA	NA
F01_transcript_85423	NA	F01_transcript_85423	NA	F01_transcript_85423
F01_transcript_5819	NA	F01_transcript_5819	NA	NA
F01_transcript_106182	NA	F01_transcript_106182	NA	F01_transcript_106182
F01_transcript_132094	NA	F01_transcript_132094	NA	F01_transcript_132094
F01_transcript_126135	NA	F01_transcript_126135	NA	NA
F01_transcript_51283	F01_transcript_51283	F01_transcript_51283	F01_transcript_51283	F01_transcript_51283
F01_transcript_824	NA	NA	NA	F01_transcript_824
F01_transcript_149747	NA	F01_transcript_149747	NA	F01_transcript_149747
F01_transcript_104747	NA	F01_transcript_104747	NA	F01_transcript_104747
F01_transcript_139621	F01_transcript_139621	F01_transcript_139621	F01_transcript_139621	F01_transcript_139621

F01_transcript_113150	NA	NA	NA	F01_transcript_113150
F01_transcript_60382	F01_transcript_60382	F01_transcript_60382	F01_transcript_60382	F01_transcript_60382
F01_transcript_39373	NA	NA	NA	F01_transcript_39373
F01_transcript_76183	NA	NA	NA	F01_transcript_76183
F01_transcript_107467	NA	NA	NA	F01_transcript_107467
F01_transcript_61304	NA	NA	NA	F01_transcript_61304
F01_transcript_48679	NA	F01_transcript_48679	NA	NA
F01_transcript_89740	F01_transcript_89740	F01_transcript_89740	NA	F01_transcript_89740
F01_transcript_143338	F01_transcript_143338	F01_transcript_143338	NA	NA
F01_transcript_61059	F01_transcript_61059	F01_transcript_61059	F01_transcript_61059	F01_transcript_61059
F01_transcript_59859	NA	NA	NA	F01_transcript_59859
F01_transcript_142870	NA	F01_transcript_142870	NA	F01_transcript_142870
F01_transcript_51663	F01_transcript_51663	F01_transcript_51663	F01_transcript_51663	NA
F01_transcript_85272	NA	F01_transcript_85272	NA	NA

F01_transcript_90264	NA	NA	NA	F01_transcript_90264
F01_transcript_135217	F01_transcript_135217	F01_transcript_135217	F01_transcript_135217	F01_transcript_135217
F01_transcript_85749	NA	F01_transcript_85749	NA	NA
F01_transcript_85804	NA	NA	F01_transcript_85804	F01_transcript_85804
F01_transcript_28838	NA	F01_transcript_28838	NA	NA
F01_transcript_106876	NA	F01_transcript_106876	NA	NA
F01_transcript_164134	NA	F01_transcript_164134	NA	F01_transcript_164134
F01_transcript_116731	NA	F01_transcript_116731	NA	NA
F01_transcript_6534	NA	NA	F01_transcript_6534	NA
F01_transcript_162470	NA	NA	NA	F01_transcript_162470
F01_transcript_78935	NA	NA	NA	F01_transcript_78935
F01_transcript_64187	F01_transcript_64187	F01_transcript_64187	F01_transcript_64187	F01_transcript_64187
F01_transcript_159105	NA	F01_transcript_159105	NA	NA
F01_transcript_121586	NA	NA	NA	F01_transcript_121586

F01_transcript_145502	NA	NA	NA	F01_transcript_145502
F01_transcript_162085	NA	NA	NA	F01_transcript_162085
F01_transcript_102156	F01_transcript_102156	F01_transcript_102156	F01_transcript_102156	F01_transcript_102156
F01_transcript_64696	NA	NA	NA	F01_transcript_64696
F01_transcript_78042	F01_transcript_78042	F01_transcript_78042	NA	NA
F01_transcript_110617	NA	F01_transcript_110617	NA	F01_transcript_110617
F01_transcript_105387	F01_transcript_105387	F01_transcript_105387	NA	F01_transcript_105387
F01_transcript_62404	F01_transcript_62404	F01_transcript_62404	F01_transcript_62404	F01_transcript_62404
F01_transcript_89219	NA	F01_transcript_89219	NA	NA
F01_transcript_106845	NA	NA	NA	F01_transcript_106845
F01_transcript_143822	NA	NA	NA	F01_transcript_143822
F01_transcript_70059	F01_transcript_70059	F01_transcript_70059	F01_transcript_70059	F01_transcript_70059
F01_transcript_38558	F01_transcript_38558	F01_transcript_38558	F01_transcript_38558	F01_transcript_38558
F01_transcript_166077	NA	NA	F01_transcript_166077	F01_transcript_166077

F01_transcript_130963	NA	NA	NA	F01_transcript_130963
F01_transcript_47051	NA	NA	NA	F01_transcript_47051
F01_transcript_50720	F01_transcript_50720	F01_transcript_50720	F01_transcript_50720	F01_transcript_50720
F01_transcript_22797	NA	NA	NA	F01_transcript_22797
F01_transcript_97744	NA	NA	NA	F01_transcript_97744
F01_transcript_138223	NA	F01_transcript_138223	NA	F01_transcript_138223
F01_transcript_167418	NA	F01_transcript_167418	NA	F01_transcript_167418
F01_transcript_108657	NA	NA	F01_transcript_108657	F01_transcript_108657
F01_transcript_148884	F01_transcript_148884	F01_transcript_148884	F01_transcript_148884	F01_transcript_148884
F01_transcript_128791	NA	NA	F01_transcript_128791	F01_transcript_128791
F01_transcript_55530	NA	F01_transcript_55530	NA	NA
F01_transcript_91356	NA	F01_transcript_91356	F01_transcript_91356	F01_transcript_91356
F01_transcript_165518	NA	F01_transcript_165518	NA	NA
F01_transcript_131617	NA	NA	NA	F01_transcript_131617

F01_transcript_78672	F01_transcript_78672	F01_transcript_78672	F01_transcript_78672	F01_transcript_78672
F01_transcript_166986	NA	F01_transcript_166986	F01_transcript_166986	F01_transcript_166986
F01_transcript_109311	NA	NA	NA	F01_transcript_109311
F01_transcript_6003	NA	NA	NA	F01_transcript_6003
F01_transcript_101833	F01_transcript_101833	F01_transcript_101833	F01_transcript_101833	F01_transcript_101833
F01_transcript_153443	F01_transcript_153443	F01_transcript_153443	F01_transcript_153443	NA
F01_transcript_64441	NA	F01_transcript_64441	NA	NA
F01_transcript_115388	F01_transcript_115388	F01_transcript_115388	F01_transcript_115388	F01_transcript_115388
F01_transcript_68509	NA	F01_transcript_68509	F01_transcript_68509	F01_transcript_68509
F01_transcript_100856	NA	NA	NA	F01_transcript_100856
F01_transcript_86576	F01_transcript_86576	F01_transcript_86576	F01_transcript_86576	F01_transcript_86576
F01_transcript_61823	NA	F01_transcript_61823	F01_transcript_61823	F01_transcript_61823
F01_transcript_109683	NA	NA	NA	F01_transcript_109683
F01_transcript_117265	NA	F01_transcript_117265	NA	NA

F01_transcript_64302	F01_transcript_64302	F01_transcript_64302	F01_transcript_64302	F01_transcript_64302
F01_transcript_151759	F01_transcript_151759	F01_transcript_151759	F01_transcript_151759	NA
F01_transcript_88064	NA	NA	NA	F01_transcript_88064
F01_transcript_50363	F01_transcript_50363	F01_transcript_50363	F01_transcript_50363	NA
F01_transcript_109649	NA	NA	NA	F01_transcript_109649
F01_transcript_124142	NA	NA	NA	F01_transcript_124142
F01_transcript_56473	NA	NA	F01_transcript_56473	NA
F01_transcript_18086	NA	NA	NA	F01_transcript_18086
F01_transcript_95083	F01_transcript_95083	F01_transcript_95083	F01_transcript_95083	F01_transcript_95083
F01_transcript_50438	F01_transcript_50438	F01_transcript_50438	F01_transcript_50438	F01_transcript_50438
F01_transcript_96362	NA	F01_transcript_96362	F01_transcript_96362	F01_transcript_96362
F01_transcript_87712	NA	NA	NA	F01_transcript_87712
F01_transcript_117041	NA	F01_transcript_117041	NA	F01_transcript_117041
F01_transcript_154455	NA	NA	NA	F01_transcript_154455

F01_transcript_159532	NA	NA	NA	F01_transcript_159532
F01_transcript_50965	F01_transcript_50965	F01_transcript_50965	F01_transcript_50965	F01_transcript_50965
F01_transcript_147629	F01_transcript_147629	F01_transcript_147629	NA	NA
F01_transcript_86061	F01_transcript_86061	F01_transcript_86061	NA	F01_transcript_86061
F01_transcript_75068	NA	F01_transcript_75068	NA	NA
F01_transcript_50271	F01_transcript_50271	F01_transcript_50271	F01_transcript_50271	F01_transcript_50271
F01_transcript_127547	F01_transcript_127547	F01_transcript_127547	NA	NA
F01_transcript_119576	F01_transcript_119576	F01_transcript_119576	F01_transcript_119576	F01_transcript_119576
F01_transcript_133627	NA	F01_transcript_133627	NA	NA
F01_transcript_112888	F01_transcript_112888	F01_transcript_112888	F01_transcript_112888	F01_transcript_112888
F01_transcript_88468	NA	F01_transcript_88468	NA	F01_transcript_88468
F01_transcript_92010	NA	F01_transcript_92010	NA	NA
F01_transcript_138345	F01_transcript_138345	F01_transcript_138345	NA	NA
F01_transcript_100691	F01_transcript_100691	F01_transcript_100691	NA	NA

F01_transcript_149486	F01_transcript_149486	F01_transcript_149486	NA	F01_transcript_149486
F01_transcript_104593	F01_transcript_104593	F01_transcript_104593	F01_transcript_104593	F01_transcript_104593
F01_transcript_67452	F01_transcript_67452	F01_transcript_67452	F01_transcript_67452	F01_transcript_67452
F01_transcript_136295	NA	F01_transcript_136295	NA	F01_transcript_136295
F01_transcript_52399	F01_transcript_52399	F01_transcript_52399	F01_transcript_52399	F01_transcript_52399
F01_transcript_23357	NA	F01_transcript_23357	NA	NA
F01_transcript_39269	F01_transcript_39269	F01_transcript_39269	NA	F01_transcript_39269
F01_transcript_117454	NA	F01_transcript_117454	F01_transcript_117454	F01_transcript_117454
F01_transcript_53611	NA	NA	NA	F01_transcript_53611
F01_transcript_17045	F01_transcript_17045	F01_transcript_17045	F01_transcript_17045	F01_transcript_17045
F01_transcript_140029	NA	F01_transcript_140029	NA	NA
F01_transcript_43387	NA	NA	NA	F01_transcript_43387
F01_transcript_64134	F01_transcript_64134	F01_transcript_64134	NA	F01_transcript_64134
F01_transcript_137680	F01_transcript_137680	F01_transcript_137680	F01_transcript_137680	F01_transcript_137680

F01_transcript_77583	F01_transcript_77583	F01_transcript_77583	NA	F01_transcript_77583
F01_transcript_52612	NA	NA	NA	F01_transcript_52612
F01_transcript_146532	F01_transcript_146532	F01_transcript_146532	NA	NA
F01_transcript_152958	NA	F01_transcript_152958	NA	NA
F01_transcript_113716	NA	F01_transcript_113716	NA	NA
F01_transcript_157224	NA	NA	NA	F01_transcript_157224
F01_transcript_158076	NA	NA	NA	F01_transcript_158076
F01_transcript_110098	NA	NA	NA	F01_transcript_110098
F01_transcript_96064	NA	F01_transcript_96064	NA	NA
F01_transcript_97147	NA	F01_transcript_97147	NA	NA
F01_transcript_77071	NA	F01_transcript_77071	NA	NA
F01_transcript_118534	NA	F01_transcript_118534	NA	F01_transcript_118534
F01_transcript_111630	NA	F01_transcript_111630	NA	F01_transcript_111630
F01_transcript_137327	NA	F01_transcript_137327	F01_transcript_137327	F01_transcript_137327

F01_transcript_134123	NA	F01_transcript_134123	F01_transcript_134123	F01_transcript_134123
F01_transcript_63904	NA	NA	NA	F01_transcript_63904
F01_transcript_152310	NA	NA	NA	F01_transcript_152310
F01_transcript_84177	NA	NA	F01_transcript_84177	NA
F01_transcript_129542	NA	F01_transcript_129542	NA	F01_transcript_129542
F01_transcript_69441	F01_transcript_69441	F01_transcript_69441	F01_transcript_69441	F01_transcript_69441
F01_transcript_74507	NA	F01_transcript_74507	NA	NA
F01_transcript_92636	NA	F01_transcript_92636	NA	NA
F01_transcript_65670	NA	F01_transcript_65670	NA	F01_transcript_65670
F01_transcript_84379	F01_transcript_84379	F01_transcript_84379	F01_transcript_84379	F01_transcript_84379
F01_transcript_80751	NA	F01_transcript_80751	F01_transcript_80751	F01_transcript_80751
F01_transcript_67340	NA	F01_transcript_67340	F01_transcript_67340	NA
F01_transcript_85391	NA	NA	NA	F01_transcript_85391
F01_transcript_134381	NA	F01_transcript_134381	NA	NA

F01_transcript_5980	NA	NA	NA	F01_transcript_5980
F01_transcript_126496	NA	F01_transcript_126496	NA	NA
F01_transcript_104151	NA	NA	NA	F01_transcript_104151
F01_transcript_104323	NA	F01_transcript_104323	NA	NA
F01_transcript_166618	NA	F01_transcript_166618	NA	NA
F01_transcript_106723	NA	F01_transcript_106723	NA	F01_transcript_106723
F01_transcript_131567	NA	F01_transcript_131567	NA	F01_transcript_131567
F01_transcript_7425	NA	F01_transcript_7425	NA	F01_transcript_7425
F01_transcript_87632	NA	NA	NA	F01_transcript_87632
F01_transcript_112862	NA	F01_transcript_112862	NA	F01_transcript_112862
F01_transcript_60175	NA	F01_transcript_60175	NA	F01_transcript_60175
F01_transcript_162250	NA	NA	NA	F01_transcript_162250
F01_transcript_147926	NA	F01_transcript_147926	NA	F01_transcript_147926
F01_transcript_2505	NA	NA	NA	F01_transcript_2505

F01_transcript_49045	F01_transcript_49045	F01_transcript_49045	F01_transcript_49045	F01_transcript_49045
F01_transcript_116993	F01_transcript_116993	F01_transcript_116993	F01_transcript_116993	F01_transcript_116993
F01_transcript_160647	F01_transcript_160647	F01_transcript_160647	F01_transcript_160647	F01_transcript_160647
F01_transcript_57548	NA	F01_transcript_57548	NA	NA
F01_transcript_24268	NA	NA	NA	F01_transcript_24268
F01_transcript_136407	NA	NA	NA	F01_transcript_136407
F01_transcript_104128	NA	NA	NA	F01_transcript_104128
F01_transcript_157436	NA	F01_transcript_157436	NA	NA
F01_transcript_107621	F01_transcript_107621	F01_transcript_107621	NA	NA
F01_transcript_123958	NA	F01_transcript_123958	NA	NA
F01_transcript_136693	F01_transcript_136693	F01_transcript_136693	F01_transcript_136693	F01_transcript_136693
F01_transcript_51246	F01_transcript_51246	F01_transcript_51246	F01_transcript_51246	F01_transcript_51246
F01_transcript_132460	NA	F01_transcript_132460	NA	NA
F01_transcript_55534	F01_transcript_55534	F01_transcript_55534	F01_transcript_55534	F01_transcript_55534

F01_transcript_118962	F01_transcript_118962	F01_transcript_118962	NA	F01_transcript_118962
F01_transcript_47513	NA	F01_transcript_47513	NA	NA
F01_transcript_15533	NA	NA	NA	F01_transcript_15533
F01_transcript_2816	NA	NA	NA	F01_transcript_2816
F01_transcript_143891	F01_transcript_143891	F01_transcript_143891	F01_transcript_143891	F01_transcript_143891
F01_transcript_144297	NA	F01_transcript_144297	NA	NA
F01_transcript_153739	F01_transcript_153739	F01_transcript_153739	NA	F01_transcript_153739
F01_transcript_106132	F01_transcript_106132	F01_transcript_106132	NA	F01_transcript_106132
F01_transcript_151398	NA	NA	NA	F01_transcript_151398
F01_transcript_166213	NA	NA	NA	F01_transcript_166213
F01_transcript_79311	NA	F01_transcript_79311	NA	NA
F01_transcript_139310	NA	F01_transcript_139310	NA	NA
F01_transcript_76313	NA	NA	NA	F01_transcript_76313
F01_transcript_77372	NA	NA	NA	F01_transcript_77372

F01_transcript_167567	F01_transcript_167567	F01_transcript_167567	F01_transcript_167567	F01_transcript_167567
F01_transcript_130730	NA	F01_transcript_130730	F01_transcript_130730	F01_transcript_130730
F01_transcript_56844	NA	NA	F01_transcript_56844	F01_transcript_56844
F01_transcript_160756	NA	F01_transcript_160756	F01_transcript_160756	NA
F01_transcript_30865	NA	NA	NA	F01_transcript_30865
F01_transcript_86325	F01_transcript_86325	F01_transcript_86325	F01_transcript_86325	F01_transcript_86325
F01_transcript_118780	NA	F01_transcript_118780	NA	NA
F01_transcript_100096	F01_transcript_100096	F01_transcript_100096	NA	F01_transcript_100096
F01_transcript_81079	F01_transcript_81079	F01_transcript_81079	F01_transcript_81079	F01_transcript_81079
F01_transcript_81710	NA	NA	NA	F01_transcript_81710
F01_transcript_89715	F01_transcript_89715	F01_transcript_89715	NA	NA
F01_transcript_164757	NA	NA	NA	F01_transcript_164757
F01_transcript_55900	NA	F01_transcript_55900	NA	NA
F01_transcript_117496	NA	F01_transcript_117496	NA	F01_transcript_117496

F01_transcript_68355	NA	F01_transcript_68355	NA	F01_transcript_68355
F01_transcript_153368	F01_transcript_153368	F01_transcript_153368	NA	NA
F01_transcript_121520	NA	NA	NA	F01_transcript_121520
F01_transcript_160154	F01_transcript_160154	F01_transcript_160154	F01_transcript_160154	NA
F01_transcript_81646	NA	F01_transcript_81646	F01_transcript_81646	F01_transcript_81646
F01_transcript_119199	NA	F01_transcript_119199	NA	NA
F01_transcript_86222	NA	NA	NA	F01_transcript_86222
F01_transcript_61555	NA	F01_transcript_61555	NA	NA
F01_transcript_113935	F01_transcript_113935	F01_transcript_113935	F01_transcript_113935	F01_transcript_113935
F01_transcript_45905	NA	NA	NA	F01_transcript_45905
F01_transcript_120393	F01_transcript_120393	F01_transcript_120393	NA	F01_transcript_120393
F01_transcript_64591	F01_transcript_64591	F01_transcript_64591	NA	NA
F01_transcript_30707	NA	NA	F01_transcript_30707	F01_transcript_30707
F01_transcript_78163	F01_transcript_78163	F01_transcript_78163	F01_transcript_78163	F01_transcript_78163

F01_transcript_106944	NA	F01_transcript_106944	NA	F01_transcript_106944
F01_transcript_3796	NA	NA	NA	F01_transcript_3796
F01_transcript_148361	F01_transcript_148361	F01_transcript_148361	F01_transcript_148361	F01_transcript_148361
F01_transcript_1994	NA	F01_transcript_1994	NA	NA
F01_transcript_138080	NA	F01_transcript_138080	NA	F01_transcript_138080
F01_transcript_117663	NA	NA	NA	F01_transcript_117663
F01_transcript_47962	NA	NA	NA	F01_transcript_47962
F01_transcript_83243	NA	F01_transcript_83243	NA	NA
F01_transcript_136367	NA	F01_transcript_136367	NA	F01_transcript_136367
F01_transcript_90503	F01_transcript_90503	F01_transcript_90503	NA	NA
F01_transcript_83807	NA	F01_transcript_83807	NA	NA
F01_transcript_49453	NA	NA	F01_transcript_49453	F01_transcript_49453
F01_transcript_83194	NA	F01_transcript_83194	F01_transcript_83194	F01_transcript_83194
F01_transcript_115894	NA	F01_transcript_115894	NA	NA

F01_transcript_151175	NA	F01_transcript_151175	NA	F01_transcript_151175
F01_transcript_60056	F01_transcript_60056	F01_transcript_60056	F01_transcript_60056	F01_transcript_60056
F01_transcript_133474	F01_transcript_133474	F01_transcript_133474	NA	NA
F01_transcript_100023	NA	F01_transcript_100023	NA	NA
F01_transcript_138192	NA	F01_transcript_138192	NA	F01_transcript_138192
F01_transcript_117634	F01_transcript_117634	F01_transcript_117634	F01_transcript_117634	NA
F01_transcript_149729	NA	F01_transcript_149729	NA	NA
F01_transcript_137791	F01_transcript_137791	F01_transcript_137791	NA	F01_transcript_137791
F01_transcript_90418	F01_transcript_90418	F01_transcript_90418	F01_transcript_90418	F01_transcript_90418
F01_transcript_61614	NA	F01_transcript_61614	NA	F01_transcript_61614
F01_transcript_71260	NA	F01_transcript_71260	F01_transcript_71260	NA
F01_transcript_125025	NA	F01_transcript_125025	NA	F01_transcript_125025
F01_transcript_55916	NA	NA	NA	F01_transcript_55916
F01_transcript_123935	F01_transcript_123935	F01_transcript_123935	F01_transcript_123935	F01_transcript_123935

F01_transcript_65592	NA	NA	NA	F01_transcript_65592
F01_transcript_158147	NA	F01_transcript_158147	NA	NA
F01_transcript_143824	NA	F01_transcript_143824	NA	NA
F01_transcript_107496	NA	F01_transcript_107496	NA	NA
F01_transcript_122601	NA	F01_transcript_122601	NA	NA
F01_transcript_12626	NA	F01_transcript_12626	NA	NA
F01_transcript_80303	NA	NA	NA	F01_transcript_80303
F01_transcript_160592	NA	F01_transcript_160592	NA	NA
F01_transcript_119645	NA	F01_transcript_119645	NA	NA
F01_transcript_130340	NA	F01_transcript_130340	NA	F01_transcript_130340
F01_transcript_114697	NA	F01_transcript_114697	NA	F01_transcript_114697
F01_transcript_111882	NA	F01_transcript_111882	NA	F01_transcript_111882
F01_transcript_54228	F01_transcript_54228	F01_transcript_54228	F01_transcript_54228	F01_transcript_54228
F01_transcript_58744	NA	F01_transcript_58744	NA	F01_transcript_58744

F01_transcript_49025	F01_transcript_49025	F01_transcript_49025	F01_transcript_49025	F01_transcript_49025
F01_transcript_71570	NA	F01_transcript_71570	NA	F01_transcript_71570
F01_transcript_87764	NA	F01_transcript_87764	NA	F01_transcript_87764
F01_transcript_75158	F01_transcript_75158	F01_transcript_75158	F01_transcript_75158	F01_transcript_75158
F01_transcript_85491	NA	NA	NA	F01_transcript_85491
F01_transcript_12825	NA	NA	NA	F01_transcript_12825
F01_transcript_123430	F01_transcript_123430	F01_transcript_123430	NA	NA
F01_transcript_120424	NA	NA	F01_transcript_120424	F01_transcript_120424
F01_transcript_57157	NA	NA	F01_transcript_57157	NA
F01_transcript_116416	NA	F01_transcript_116416	NA	F01_transcript_116416
F01_transcript_29855	NA	F01_transcript_29855	NA	NA
F01_transcript_154091	F01_transcript_154091	F01_transcript_154091	F01_transcript_154091	F01_transcript_154091
F01_transcript_119519	NA	F01_transcript_119519	NA	NA
F01_transcript_51997	F01_transcript_51997	F01_transcript_51997	F01_transcript_51997	F01_transcript_51997

F01_transcript_124318	NA	F01_transcript_124318	F01_transcript_124318	F01_transcript_124318
F01_transcript_126677	NA	F01_transcript_126677	NA	NA
F01_transcript_102285	NA	F01_transcript_102285	F01_transcript_102285	NA
F01_transcript_64589	NA	F01_transcript_64589	NA	F01_transcript_64589
F01_transcript_96923	NA	NA	NA	F01_transcript_96923
F01_transcript_81382	F01_transcript_81382	F01_transcript_81382	F01_transcript_81382	F01_transcript_81382
F01_transcript_87629	NA	F01_transcript_87629	NA	NA
F01_transcript_74076	NA	NA	NA	F01_transcript_74076
F01_transcript_153446	F01_transcript_153446	F01_transcript_153446	F01_transcript_153446	F01_transcript_153446
F01_transcript_81231	F01_transcript_81231	F01_transcript_81231	F01_transcript_81231	F01_transcript_81231
F01_transcript_68969	NA	F01_transcript_68969	NA	NA
F01_transcript_148311	F01_transcript_148311	F01_transcript_148311	F01_transcript_148311	F01_transcript_148311
F01_transcript_155848	NA	F01_transcript_155848	F01_transcript_155848	F01_transcript_155848
F01_transcript_162687	NA	NA	NA	F01_transcript_162687

F01_transcript_103209	NA	NA	NA	F01_transcript_103209
F01_transcript_70480	F01_transcript_70480	F01_transcript_70480	F01_transcript_70480	F01_transcript_70480
F01_transcript_112643	F01_transcript_112643	F01_transcript_112643	NA	NA
F01_transcript_93095	NA	NA	NA	F01_transcript_93095
F01_transcript_36702	NA	F01_transcript_36702	NA	NA
F01_transcript_62940	F01_transcript_62940	F01_transcript_62940	F01_transcript_62940	NA
F01_transcript_116111	F01_transcript_116111	F01_transcript_116111	F01_transcript_116111	F01_transcript_116111
F01_transcript_41456	NA	NA	NA	F01_transcript_41456
F01_transcript_121053	F01_transcript_121053	F01_transcript_121053	NA	NA
F01_transcript_85509	NA	F01_transcript_85509	NA	F01_transcript_85509
F01_transcript_46523	NA	F01_transcript_46523	F01_transcript_46523	F01_transcript_46523
F01_transcript_165991	NA	NA	F01_transcript_165991	F01_transcript_165991
F01_transcript_38755	NA	NA	NA	F01_transcript_38755
F01_transcript_63408	F01_transcript_63408	F01_transcript_63408	F01_transcript_63408	F01_transcript_63408

F01_transcript_82178	NA	F01_transcript_82178	NA	F01_transcript_82178
F01_transcript_72758	F01_transcript_72758	F01_transcript_72758	F01_transcript_72758	F01_transcript_72758
F01_transcript_13585	NA	NA	NA	F01_transcript_13585
F01_transcript_139014	NA	F01_transcript_139014	NA	NA
F01_transcript_53504	NA	F01_transcript_53504	NA	F01_transcript_53504
F01_transcript_19225	NA	F01_transcript_19225	NA	F01_transcript_19225
F01_transcript_65646	NA	NA	NA	F01_transcript_65646
F01_transcript_56634	NA	F01_transcript_56634	NA	NA
F01_transcript_48969	NA	NA	F01_transcript_48969	F01_transcript_48969
F01_transcript_67875	F01_transcript_67875	F01_transcript_67875	F01_transcript_67875	F01_transcript_67875
F01_transcript_153519	NA	F01_transcript_153519	F01_transcript_153519	NA
F01_transcript_31807	NA	NA	NA	F01_transcript_31807
F01_transcript_160583	NA	F01_transcript_160583	F01_transcript_160583	F01_transcript_160583
F01_transcript_96464	NA	NA	NA	F01_transcript_96464

F01_transcript_41070	NA	NA	NA	F01_transcript_41070
F01_transcript_127688	NA	F01_transcript_127688	NA	NA
F01_transcript_49824	NA	F01_transcript_49824	NA	NA
F01_transcript_130510	NA	F01_transcript_130510	NA	F01_transcript_130510
F01_transcript_99597	F01_transcript_99597	F01_transcript_99597	F01_transcript_99597	F01_transcript_99597
F01_transcript_144074	F01_transcript_144074	F01_transcript_144074	NA	NA
F01_transcript_129074	NA	F01_transcript_129074	NA	NA
F01_transcript_64728	NA	NA	NA	F01_transcript_64728
F01_transcript_141027	F01_transcript_141027	F01_transcript_141027	F01_transcript_141027	F01_transcript_141027
F01_transcript_59704	F01_transcript_59704	F01_transcript_59704	F01_transcript_59704	F01_transcript_59704
F01_transcript_3085	F01_transcript_3085	F01_transcript_3085	NA	NA
F01_transcript_125721	NA	F01_transcript_125721	NA	F01_transcript_125721
F01_transcript_49340	NA	F01_transcript_49340	NA	NA
F01_transcript_58368	NA	F01_transcript_58368	NA	NA

F01_transcript_110081	F01_transcript_110081	F01_transcript_110081	NA	NA
F01_transcript_12078	F01_transcript_12078	F01_transcript_12078	F01_transcript_12078	F01_transcript_12078
F01_transcript_141550	NA	NA	NA	F01_transcript_141550
F01_transcript_161513	NA	F01_transcript_161513	NA	F01_transcript_161513
F01_transcript_81792	NA	NA	NA	F01_transcript_81792
F01_transcript_80525	NA	F01_transcript_80525	NA	NA
F01_transcript_119693	NA	F01_transcript_119693	NA	F01_transcript_119693
F01_transcript_129972	NA	NA	F01_transcript_129972	NA
F01_transcript_79277	NA	F01_transcript_79277	NA	NA
F01_transcript_111318	NA	NA	F01_transcript_111318	F01_transcript_111318
F01_transcript_124028	F01_transcript_124028	F01_transcript_124028	F01_transcript_124028	F01_transcript_124028
F01_transcript_19570	NA	NA	NA	F01_transcript_19570
F01_transcript_98655	NA	F01_transcript_98655	NA	NA
F01_transcript_108072	NA	NA	F01_transcript_108072	NA

F01_transcript_26932	F01_transcript_26932	F01_transcript_26932	F01_transcript_26932	F01_transcript_26932
F01_transcript_126823	F01_transcript_126823	F01_transcript_126823	F01_transcript_126823	NA
F01_transcript_6911	NA	F01_transcript_6911	F01_transcript_6911	F01_transcript_6911
F01_transcript_115863	NA	F01_transcript_115863	NA	NA
F01_transcript_42900	NA	F01_transcript_42900	NA	NA
F01_transcript_118551	NA	NA	NA	F01_transcript_118551
F01_transcript_51489	NA	F01_transcript_51489	NA	NA
F01_transcript_149852	F01_transcript_149852	F01_transcript_149852	F01_transcript_149852	F01_transcript_149852
F01_transcript_84843	F01_transcript_84843	F01_transcript_84843	F01_transcript_84843	F01_transcript_84843
F01_transcript_108593	NA	F01_transcript_108593	NA	NA
F01_transcript_45921	NA	NA	NA	F01_transcript_45921
F01_transcript_10673	NA	NA	NA	F01_transcript_10673
F01_transcript_72547	NA	F01_transcript_72547	NA	NA
F01_transcript_64570	F01_transcript_64570	NA	F01_transcript_64570	NA

F01_transcript_93334	NA	F01_transcript_93334	F01_transcript_93334	F01_transcript_93334
F01_transcript_57891	NA	NA	NA	F01_transcript_57891
F01_transcript_22107	NA	F01_transcript_22107	NA	F01_transcript_22107
F01_transcript_166476	NA	F01_transcript_166476	NA	NA
F01_transcript_150187	F01_transcript_150187	F01_transcript_150187	NA	F01_transcript_150187
F01_transcript_165093	NA	NA	NA	F01_transcript_165093
F01_transcript_74284	NA	NA	NA	F01_transcript_74284
F01_transcript_148458	F01_transcript_148458	F01_transcript_148458	NA	F01_transcript_148458
F01_transcript_3930	NA	NA	NA	F01_transcript_3930
F01_transcript_88362	NA	NA	F01_transcript_88362	F01_transcript_88362
F01_transcript_48967	NA	NA	NA	F01_transcript_48967
F01_transcript_95633	NA	F01_transcript_95633	NA	NA
F01_transcript_125924	NA	NA	NA	F01_transcript_125924
F01_transcript_89794	NA	F01_transcript_89794	NA	F01_transcript_89794

F01_transcript_50455	F01_transcript_50455	NA	F01_transcript_50455	F01_transcript_50455
F01_transcript_144109	F01_transcript_144109	F01_transcript_144109	F01_transcript_144109	NA
F01_transcript_50809	F01_transcript_50809	F01_transcript_50809	F01_transcript_50809	F01_transcript_50809
F01_transcript_82434	NA	NA	NA	F01_transcript_82434
F01_transcript_142224	NA	NA	NA	F01_transcript_142224
F01_transcript_71736	F01_transcript_71736	F01_transcript_71736	NA	NA
F01_transcript_44823	NA	NA	NA	F01_transcript_44823
F01_transcript_5981	NA	NA	NA	F01_transcript_5981
F01_transcript_56572	F01_transcript_56572	F01_transcript_56572	F01_transcript_56572	NA
F01_transcript_167003	NA	F01_transcript_167003	NA	NA
F01_transcript_134423	NA	NA	F01_transcript_134423	F01_transcript_134423
F01_transcript_46928	NA	NA	F01_transcript_46928	F01_transcript_46928
F01_transcript_77082	NA	F01_transcript_77082	NA	NA
F01_transcript_60963	NA	NA	NA	F01_transcript_60963

F01_transcript_48920	NA	NA	F01_transcript_48920	F01_transcript_48920
F01_transcript_86467	NA	NA	NA	F01_transcript_86467
F01_transcript_106663	F01_transcript_106663	F01_transcript_106663	NA	NA
F01_transcript_134075	NA	F01_transcript_134075	NA	NA
F01_transcript_13514	NA	NA	NA	F01_transcript_13514
F01_transcript_41039	NA	NA	NA	F01_transcript_41039
F01_transcript_101946	F01_transcript_101946	F01_transcript_101946	F01_transcript_101946	F01_transcript_101946
F01_transcript_107614	NA	F01_transcript_107614	NA	NA
F01_transcript_161539	NA	NA	NA	F01_transcript_161539
F01_transcript_166629	NA	NA	NA	F01_transcript_166629
F01_transcript_144968	NA	NA	F01_transcript_144968	F01_transcript_144968
F01_transcript_57205	NA	F01_transcript_57205	F01_transcript_57205	NA
F01_transcript_156075	NA	NA	NA	F01_transcript_156075
F01_transcript_160177	NA	F01_transcript_160177	NA	F01_transcript_160177

F01_transcript_89934	NA	NA	NA	F01_transcript_89934
F01_transcript_149215	NA	NA	NA	F01_transcript_149215
F01_transcript_127425	F01_transcript_127425	F01_transcript_127425	F01_transcript_127425	F01_transcript_127425
F01_transcript_157226	NA	F01_transcript_157226	NA	F01_transcript_157226
F01_transcript_137838	F01_transcript_137838	F01_transcript_137838	F01_transcript_137838	F01_transcript_137838
F01_transcript_114520	F01_transcript_114520	F01_transcript_114520	NA	F01_transcript_114520
F01_transcript_49616	NA	F01_transcript_49616	NA	NA
F01_transcript_86212	F01_transcript_86212	F01_transcript_86212	F01_transcript_86212	F01_transcript_86212
F01_transcript_66881	F01_transcript_66881	F01_transcript_66881	NA	NA
F01_transcript_164376	NA	NA	NA	F01_transcript_164376
F01_transcript_151661	F01_transcript_151661	F01_transcript_151661	NA	NA
F01_transcript_14172	NA	F01_transcript_14172	NA	NA
F01_transcript_82874	NA	NA	F01_transcript_82874	NA
F01_transcript_3973	NA	NA	F01_transcript_3973	NA

F01_transcript_160206	F01_transcript_160206	F01_transcript_160206	NA	F01_transcript_160206
F01_transcript_69341	NA	NA	NA	F01_transcript_69341
F01_transcript_137885	NA	F01_transcript_137885	F01_transcript_137885	F01_transcript_137885
F01_transcript_123631	NA	NA	NA	F01_transcript_123631
F01_transcript_159106	NA	F01_transcript_159106	NA	NA
F01_transcript_133654	NA	NA	NA	F01_transcript_133654
F01_transcript_116422	NA	NA	NA	F01_transcript_116422
F01_transcript_12674	NA	NA	NA	F01_transcript_12674
F01_transcript_90230	NA	F01_transcript_90230	NA	F01_transcript_90230
F01_transcript_96383	NA	NA	NA	F01_transcript_96383
F01_transcript_154017	NA	F01_transcript_154017	NA	F01_transcript_154017
F01_transcript_92498	F01_transcript_92498	F01_transcript_92498	F01_transcript_92498	F01_transcript_92498
F01_transcript_130656	NA	NA	NA	F01_transcript_130656
F01_transcript_154271	NA	NA	NA	F01_transcript_154271

F01_transcript_76991	NA	F01_transcript_76991	F01_transcript_76991	NA
F01_transcript_138677	NA	F01_transcript_138677	NA	NA
F01_transcript_156691	F01_transcript_156691	F01_transcript_156691	F01_transcript_156691	F01_transcript_156691
F01_transcript_134567	F01_transcript_134567	F01_transcript_134567	F01_transcript_134567	F01_transcript_134567
F01_transcript_124268	NA	F01_transcript_124268	NA	NA
F01_transcript_120658	NA	F01_transcript_120658	NA	NA
F01_transcript_110142	NA	NA	NA	F01_transcript_110142
F01_transcript_118891	F01_transcript_118891	F01_transcript_118891	F01_transcript_118891	F01_transcript_118891
F01_transcript_141833	NA	F01_transcript_141833	NA	NA
F01_transcript_2497	NA	NA	NA	F01_transcript_2497
F01_transcript_82162	NA	F01_transcript_82162	F01_transcript_82162	F01_transcript_82162
F01_transcript_124764	NA	F01_transcript_124764	NA	NA
F01_transcript_54559	NA	NA	NA	F01_transcript_54559
F01_transcript_132031	F01_transcript_132031	F01_transcript_132031	F01_transcript_132031	F01_transcript_132031

F01_transcript_120483	F01_transcript_120483	F01_transcript_120483	F01_transcript_120483	F01_transcript_120483
F01_transcript_56699	NA	F01_transcript_56699	NA	NA
F01_transcript_111426	NA	NA	NA	F01_transcript_111426
F01_transcript_67268	NA	NA	NA	F01_transcript_67268
F01_transcript_5442	NA	NA	NA	F01_transcript_5442
F01_transcript_72258	NA	F01_transcript_72258	NA	NA
F01_transcript_134903	NA	NA	NA	F01_transcript_134903
F01_transcript_52691	NA	NA	NA	F01_transcript_52691
F01_transcript_100954	NA	F01_transcript_100954	NA	F01_transcript_100954
F01_transcript_153952	NA	F01_transcript_153952	NA	NA
F01_transcript_111061	NA	NA	NA	F01_transcript_111061
F01_transcript_33713	NA	F01_transcript_33713	NA	NA
F01_transcript_25195	NA	NA	NA	F01_transcript_25195
F01_transcript_162216	F01_transcript_162216	F01_transcript_162216	F01_transcript_162216	F01_transcript_162216

F01_transcript_53629	NA	NA	NA	F01_transcript_53629
F01_transcript_112959	NA	F01_transcript_112959	NA	NA
F01_transcript_118709	NA	F01_transcript_118709	NA	F01_transcript_118709
F01_transcript_84328	NA	F01_transcript_84328	NA	F01_transcript_84328
F01_transcript_148397	NA	F01_transcript_148397	F01_transcript_148397	F01_transcript_148397
F01_transcript_151058	NA	F01_transcript_151058	NA	NA
F01_transcript_135396	F01_transcript_135396	F01_transcript_135396	F01_transcript_135396	F01_transcript_135396
F01_transcript_116489	NA	F01_transcript_116489	NA	NA
F01_transcript_93885	NA	NA	NA	F01_transcript_93885
F01_transcript_56990	NA	NA	F01_transcript_56990	F01_transcript_56990
F01_transcript_925	NA	F01_transcript_925	NA	NA
F01_transcript_62364	NA	NA	NA	F01_transcript_62364
F01_transcript_87854	NA	F01_transcript_87854	F01_transcript_87854	F01_transcript_87854
F01_transcript_149863	F01_transcript_149863	F01_transcript_149863	F01_transcript_149863	NA

F01_transcript_131614	F01_transcript_131614	F01_transcript_131614	NA	NA
F01_transcript_102074	NA	F01_transcript_102074	F01_transcript_102074	F01_transcript_102074
F01_transcript_90322	NA	F01_transcript_90322	NA	F01_transcript_90322
F01_transcript_50760	F01_transcript_50760	F01_transcript_50760	NA	F01_transcript_50760
F01_transcript_119615	NA	F01_transcript_119615	NA	NA
F01_transcript_49762	F01_transcript_49762	F01_transcript_49762	NA	NA
F01_transcript_135966	NA	F01_transcript_135966	F01_transcript_135966	F01_transcript_135966
F01_transcript_74319	NA	F01_transcript_74319	NA	NA
F01_transcript_108983	NA	F01_transcript_108983	NA	NA
F01_transcript_29897	NA	NA	NA	F01_transcript_29897
F01_transcript_59664	NA	F01_transcript_59664	NA	F01_transcript_59664
F01_transcript_160788	NA	NA	NA	F01_transcript_160788
F01_transcript_52954	F01_transcript_52954	F01_transcript_52954	NA	NA
F01_transcript_106556	NA	F01_transcript_106556	NA	NA

F01_transcript_142176	NA	NA	F01_transcript_142176	NA
F01_transcript_61437	F01_transcript_61437	F01_transcript_61437	NA	F01_transcript_61437
F01_transcript_161979	NA	F01_transcript_161979	F01_transcript_161979	NA
F01_transcript_157008	NA	NA	F01_transcript_157008	F01_transcript_157008
F01_transcript_166311	NA	NA	NA	F01_transcript_166311
F01_transcript_153052	NA	NA	NA	F01_transcript_153052
F01_transcript_133484	NA	F01_transcript_133484	NA	NA
F01_transcript_113873	F01_transcript_113873	F01_transcript_113873	NA	F01_transcript_113873
F01_transcript_61936	NA	NA	NA	F01_transcript_61936
F01_transcript_134725	NA	NA	NA	F01_transcript_134725
F01_transcript_131841	NA	F01_transcript_131841	NA	NA
F01_transcript_165796	NA	F01_transcript_165796	NA	NA
F01_transcript_79077	NA	F01_transcript_79077	NA	NA
F01_transcript_125760	F01_transcript_125760	F01_transcript_125760	F01_transcript_125760	F01_transcript_125760

F01_transcript_103795	NA	F01_transcript_103795	NA	NA
F01_transcript_113989	NA	F01_transcript_113989	NA	NA
F01_transcript_100760	NA	F01_transcript_100760	NA	NA
F01_transcript_145221	NA	NA	NA	F01_transcript_145221
F01_transcript_11091	NA	F01_transcript_11091	NA	F01_transcript_11091
F01_transcript_108982	F01_transcript_108982	F01_transcript_108982	NA	F01_transcript_108982
F01_transcript_55842	NA	F01_transcript_55842	NA	F01_transcript_55842
F01_transcript_156787	F01_transcript_156787	F01_transcript_156787	F01_transcript_156787	F01_transcript_156787
F01_transcript_145891	NA	F01_transcript_145891	NA	NA
F01_transcript_97255	NA	F01_transcript_97255	NA	F01_transcript_97255
F01_transcript_59494	NA	F01_transcript_59494	F01_transcript_59494	NA
F01_transcript_107395	F01_transcript_107395	F01_transcript_107395	F01_transcript_107395	F01_transcript_107395
F01_transcript_160010	NA	F01_transcript_160010	NA	NA
F01_transcript_91998	F01_transcript_91998	F01_transcript_91998	F01_transcript_91998	F01_transcript_91998

F01_transcript_6364	NA	NA	NA	F01_transcript_6364
F01_transcript_33870	NA	NA	NA	F01_transcript_33870
F01_transcript_158405	NA	NA	NA	F01_transcript_158405
F01_transcript_17614	NA	F01_transcript_17614	NA	F01_transcript_17614
F01_transcript_107838	NA	NA	NA	F01_transcript_107838
F01_transcript_119611	F01_transcript_119611	F01_transcript_119611	NA	NA
F01_transcript_77323	NA	NA	NA	F01_transcript_77323
F01_transcript_156226	NA	F01_transcript_156226	NA	F01_transcript_156226
F01_transcript_164460	NA	F01_transcript_164460	NA	NA
F01_transcript_73445	F01_transcript_73445	F01_transcript_73445	NA	NA
F01_transcript_28866	NA	NA	NA	F01_transcript_28866
F01_transcript_87994	F01_transcript_87994	F01_transcript_87994	F01_transcript_87994	F01_transcript_87994
F01_transcript_122998	NA	NA	NA	F01_transcript_122998
F01_transcript_121019	F01_transcript_121019	F01_transcript_121019	F01_transcript_121019	F01_transcript_121019

F01_transcript_166138	NA	F01_transcript_166138	NA	NA
F01_transcript_69750	F01_transcript_69750	F01_transcript_69750	F01_transcript_69750	F01_transcript_69750
F01_transcript_104197	F01_transcript_104197	F01_transcript_104197	F01_transcript_104197	F01_transcript_104197
F01_transcript_86632	NA	NA	NA	F01_transcript_86632
F01_transcript_69025	NA	NA	NA	F01_transcript_69025
F01_transcript_80886	F01_transcript_80886	F01_transcript_80886	F01_transcript_80886	F01_transcript_80886
F01_transcript_95409	F01_transcript_95409	F01_transcript_95409	NA	F01_transcript_95409
F01_transcript_129031	NA	NA	NA	F01_transcript_129031
F01_transcript_75536	NA	NA	NA	F01_transcript_75536
F01_transcript_127198	F01_transcript_127198	F01_transcript_127198	F01_transcript_127198	F01_transcript_127198
F01_transcript_112109	NA	F01_transcript_112109	NA	NA
F01_transcript_84126	NA	F01_transcript_84126	F01_transcript_84126	NA
F01_transcript_3604	NA	NA	NA	F01_transcript_3604
F01_transcript_85595	NA	NA	NA	F01_transcript_85595

F01_transcript_65406	F01_transcript_65406	F01_transcript_65406	F01_transcript_65406	F01_transcript_65406
F01_transcript_68964	NA	NA	NA	F01_transcript_68964
F01_transcript_111203	F01_transcript_111203	F01_transcript_111203	F01_transcript_111203	F01_transcript_111203
F01_transcript_135077	F01_transcript_135077	F01_transcript_135077	F01_transcript_135077	NA
F01_transcript_20082	NA	NA	NA	F01_transcript_20082
F01_transcript_94866	F01_transcript_94866	F01_transcript_94866	NA	F01_transcript_94866
F01_transcript_144961	NA	NA	NA	F01_transcript_144961
F01_transcript_122212	F01_transcript_122212	F01_transcript_122212	F01_transcript_122212	F01_transcript_122212
F01_transcript_90059	NA	NA	NA	F01_transcript_90059
F01_transcript_113595	NA	F01_transcript_113595	NA	F01_transcript_113595
F01_transcript_83016	F01_transcript_83016	F01_transcript_83016	NA	F01_transcript_83016
F01_transcript_63715	NA	NA	NA	F01_transcript_63715
F01_transcript_100490	F01_transcript_100490	F01_transcript_100490	NA	NA
F01_transcript_130818	F01_transcript_130818	F01_transcript_130818	F01_transcript_130818	F01_transcript_130818

F01_transcript_118369	NA	NA	NA	F01_transcript_118369
F01_transcript_131678	NA	F01_transcript_131678	F01_transcript_131678	F01_transcript_131678
F01_transcript_103848	F01_transcript_103848	F01_transcript_103848	NA	NA
F01_transcript_149551	F01_transcript_149551	F01_transcript_149551	F01_transcript_149551	F01_transcript_149551
F01_transcript_164230	F01_transcript_164230	F01_transcript_164230	F01_transcript_164230	F01_transcript_164230
F01_transcript_89686	NA	NA	NA	F01_transcript_89686
F01_transcript_85930	F01_transcript_85930	F01_transcript_85930	F01_transcript_85930	NA
F01_transcript_51735	NA	F01_transcript_51735	NA	F01_transcript_51735
F01_transcript_86269	F01_transcript_86269	F01_transcript_86269	NA	NA
F01_transcript_40222	NA	NA	NA	F01_transcript_40222
F01_transcript_135897	F01_transcript_135897	F01_transcript_135897	F01_transcript_135897	F01_transcript_135897
F01_transcript_140344	F01_transcript_140344	F01_transcript_140344	F01_transcript_140344	F01_transcript_140344
F01_transcript_159068	NA	NA	F01_transcript_159068	F01_transcript_159068
F01_transcript_103698	NA	NA	NA	F01_transcript_103698

F01_transcript_147400	F01_transcript_147400	F01_transcript_147400	NA	F01_transcript_147400
F01_transcript_109652	F01_transcript_109652	F01_transcript_109652	F01_transcript_109652	F01_transcript_109652
F01_transcript_161835	NA	F01_transcript_161835	NA	F01_transcript_161835
F01_transcript_86777	NA	NA	F01_transcript_86777	NA
F01_transcript_23437	NA	NA	NA	F01_transcript_23437
F01_transcript_109006	F01_transcript_109006	F01_transcript_109006	F01_transcript_109006	NA
F01_transcript_85551	NA	F01_transcript_85551	NA	NA
F01_transcript_130898	F01_transcript_130898	F01_transcript_130898	NA	F01_transcript_130898
F01_transcript_88163	F01_transcript_88163	F01_transcript_88163	NA	F01_transcript_88163
F01_transcript_67917	NA	F01_transcript_67917	NA	F01_transcript_67917
F01_transcript_76605	F01_transcript_76605	F01_transcript_76605	F01_transcript_76605	F01_transcript_76605
F01_transcript_102028	NA	NA	NA	F01_transcript_102028
F01_transcript_13007	NA	NA	NA	F01_transcript_13007
F01_transcript_79376	NA	F01_transcript_79376	NA	NA

F01_transcript_58831	F01_transcript_58831	F01_transcript_58831	NA	NA
F01_transcript_158870	NA	NA	F01_transcript_158870	F01_transcript_158870
F01_transcript_53278	F01_transcript_53278	F01_transcript_53278	F01_transcript_53278	F01_transcript_53278
F01_transcript_159977	NA	NA	NA	F01_transcript_159977
F01_transcript_58308	NA	F01_transcript_58308	NA	NA
F01_transcript_46217	NA	NA	NA	F01_transcript_46217
F01_transcript_116136	NA	NA	NA	F01_transcript_116136
F01_transcript_62858	NA	F01_transcript_62858	NA	NA
F01_transcript_121969	NA	NA	NA	F01_transcript_121969
F01_transcript_147786	F01_transcript_147786	F01_transcript_147786	F01_transcript_147786	F01_transcript_147786
F01_transcript_58707	NA	NA	NA	F01_transcript_58707
F01_transcript_138639	NA	NA	NA	F01_transcript_138639
F01_transcript_144755	NA	NA	F01_transcript_144755	F01_transcript_144755
F01_transcript_48372	F01_transcript_48372	NA	F01_transcript_48372	F01_transcript_48372

F01_transcript_86166	NA	NA	NA	F01_transcript_86166
F01_transcript_51052	F01_transcript_51052	NA	F01_transcript_51052	F01_transcript_51052
F01_transcript_82939	NA	F01_transcript_82939	F01_transcript_82939	F01_transcript_82939
F01_transcript_49609	NA	F01_transcript_49609	NA	NA
F01_transcript_94114	NA	NA	NA	F01_transcript_94114
F01_transcript_10565	NA	F01_transcript_10565	NA	NA
F01_transcript_59606	NA	F01_transcript_59606	F01_transcript_59606	NA
F01_transcript_138257	F01_transcript_138257	F01_transcript_138257	F01_transcript_138257	NA
F01_transcript_120697	NA	NA	NA	F01_transcript_120697
F01_transcript_57902	F01_transcript_57902	F01_transcript_57902	F01_transcript_57902	F01_transcript_57902
F01_transcript_24740	F01_transcript_24740	F01_transcript_24740	NA	F01_transcript_24740
F01_transcript_70180	F01_transcript_70180	F01_transcript_70180	NA	NA
F01_transcript_73055	NA	F01_transcript_73055	NA	F01_transcript_73055
F01_transcript_73254	F01_transcript_73254	F01_transcript_73254	NA	F01_transcript_73254

F01_transcript_112116	NA	F01_transcript_112116	NA	NA
F01_transcript_50362	NA	F01_transcript_50362	NA	F01_transcript_50362
F01_transcript_71730	NA	F01_transcript_71730	F01_transcript_71730	F01_transcript_71730
F01_transcript_70536	NA	NA	NA	F01_transcript_70536
F01_transcript_150303	NA	F01_transcript_150303	NA	NA
F01_transcript_60979	NA	NA	NA	F01_transcript_60979
F01_transcript_78641	NA	F01_transcript_78641	NA	F01_transcript_78641
F01_transcript_145136	F01_transcript_145136	F01_transcript_145136	NA	F01_transcript_145136
F01_transcript_138861	F01_transcript_138861	F01_transcript_138861	NA	F01_transcript_138861
F01_transcript_57081	NA	NA	F01_transcript_57081	NA
F01_transcript_120137	F01_transcript_120137	F01_transcript_120137	F01_transcript_120137	F01_transcript_120137
F01_transcript_129807	NA	NA	NA	F01_transcript_129807
F01_transcript_65780	NA	F01_transcript_65780	NA	NA
F01_transcript_107380	NA	NA	F01_transcript_107380	F01_transcript_107380

F01_transcript_102004	NA	F01_transcript_102004	NA	F01_transcript_102004
F01_transcript_108830	NA	NA	NA	F01_transcript_108830
F01_transcript_26691	NA	F01_transcript_26691	NA	NA
F01_transcript_95410	F01_transcript_95410	F01_transcript_95410	F01_transcript_95410	F01_transcript_95410
F01_transcript_130515	NA	F01_transcript_130515	F01_transcript_130515	F01_transcript_130515
F01_transcript_135346	F01_transcript_135346	F01_transcript_135346	F01_transcript_135346	NA
F01_transcript_133875	NA	F01_transcript_133875	NA	NA
F01_transcript_83255	NA	F01_transcript_83255	NA	F01_transcript_83255
F01_transcript_166659	F01_transcript_166659	F01_transcript_166659	F01_transcript_166659	F01_transcript_166659
F01_transcript_117484	NA	F01_transcript_117484	NA	NA
F01_transcript_101403	NA	NA	NA	F01_transcript_101403
F01_transcript_86505	NA	NA	NA	F01_transcript_86505
F01_transcript_122028	NA	NA	NA	F01_transcript_122028
F01_transcript_135002	NA	NA	NA	F01_transcript_135002

F01_transcript_86759	F01_transcript_86759	F01_transcript_86759	NA	NA
F01_transcript_121223	NA	F01_transcript_121223	NA	NA
F01_transcript_51658	NA	F01_transcript_51658	NA	NA
F01_transcript_148746	F01_transcript_148746	F01_transcript_148746	F01_transcript_148746	F01_transcript_148746
F01_transcript_138502	F01_transcript_138502	F01_transcript_138502	F01_transcript_138502	F01_transcript_138502
F01_transcript_86265	F01_transcript_86265	F01_transcript_86265	F01_transcript_86265	F01_transcript_86265
F01_transcript_49440	F01_transcript_49440	F01_transcript_49440	F01_transcript_49440	F01_transcript_49440
F01_transcript_164643	NA	F01_transcript_164643	F01_transcript_164643	NA
F01_transcript_131645	F01_transcript_131645	F01_transcript_131645	NA	NA
F01_transcript_68935	NA	NA	NA	F01_transcript_68935
F01_transcript_119399	NA	NA	NA	F01_transcript_119399
F01_transcript_153445	NA	NA	NA	F01_transcript_153445
F01_transcript_118099	NA	F01_transcript_118099	F01_transcript_118099	F01_transcript_118099
F01_transcript_137526	NA	NA	NA	F01_transcript_137526

F01_transcript_67280	F01_transcript_67280	F01_transcript_67280	NA	NA
F01_transcript_83210	NA	NA	NA	F01_transcript_83210
F01_transcript_137875	NA	F01_transcript_137875	NA	NA
F01_transcript_68567	NA	NA	NA	F01_transcript_68567
F01_transcript_155563	NA	NA	NA	F01_transcript_155563
F01_transcript_75206	NA	F01_transcript_75206	NA	NA
F01_transcript_109411	NA	NA	NA	F01_transcript_109411
F01_transcript_136585	NA	F01_transcript_136585	NA	F01_transcript_136585
F01_transcript_65258	NA	F01_transcript_65258	F01_transcript_65258	NA
F01_transcript_81400	NA	NA	NA	F01_transcript_81400
F01_transcript_131666	NA	F01_transcript_131666	NA	NA
F01_transcript_99680	NA	F01_transcript_99680	NA	F01_transcript_99680
F01_transcript_101846	NA	F01_transcript_101846	NA	NA
F01_transcript_163877	NA	F01_transcript_163877	F01_transcript_163877	F01_transcript_163877

F01_transcript_53309	NA	F01_transcript_53309	NA	NA
F01_transcript_80424	NA	F01_transcript_80424	F01_transcript_80424	F01_transcript_80424
F01_transcript_115779	NA	NA	NA	F01_transcript_115779
F01_transcript_118810	NA	F01_transcript_118810	NA	NA
F01_transcript_786	F01_transcript_786	F01_transcript_786	F01_transcript_786	NA
F01_transcript_85666	NA	F01_transcript_85666	NA	NA
F01_transcript_154159	NA	F01_transcript_154159	F01_transcript_154159	NA
F01_transcript_92177	NA	F01_transcript_92177	NA	F01_transcript_92177
F01_transcript_164385	NA	F01_transcript_164385	F01_transcript_164385	F01_transcript_164385
F01_transcript_84540	NA	F01_transcript_84540	F01_transcript_84540	NA
F01_transcript_102163	NA	NA	NA	F01_transcript_102163
F01_transcript_89718	NA	F01_transcript_89718	NA	F01_transcript_89718
F01_transcript_63917	NA	NA	NA	F01_transcript_63917
F01_transcript_85993	NA	NA	F01_transcript_85993	F01_transcript_85993

F01_transcript_139046	NA	NA	NA	F01_transcript_139046
F01_transcript_7723	NA	NA	NA	F01_transcript_7723
F01_transcript_110750	NA	F01_transcript_110750	F01_transcript_110750	F01_transcript_110750
F01_transcript_105557	NA	F01_transcript_105557	NA	NA
F01_transcript_143215	NA	NA	NA	F01_transcript_143215
F01_transcript_167449	NA	NA	NA	F01_transcript_167449
F01_transcript_8562	NA	NA	NA	F01_transcript_8562
F01_transcript_142716	NA	NA	NA	F01_transcript_142716
F01_transcript_132335	F01_transcript_132335	F01_transcript_132335	NA	NA
F01_transcript_130136	NA	NA	NA	F01_transcript_130136
F01_transcript_71453	NA	F01_transcript_71453	NA	NA
F01_transcript_57196	NA	NA	F01_transcript_57196	NA
F01_transcript_53538	NA	F01_transcript_53538	NA	F01_transcript_53538
F01_transcript_96488	F01_transcript_96488	F01_transcript_96488	F01_transcript_96488	F01_transcript_96488

F01_transcript_7539	NA	NA	NA	F01_transcript_7539
F01_transcript_79177	NA	F01_transcript_79177	NA	NA
F01_transcript_49012	F01_transcript_49012	NA	NA	NA
F01_transcript_108180	F01_transcript_108180	F01_transcript_108180	F01_transcript_108180	F01_transcript_108180
F01_transcript_111704	F01_transcript_111704	F01_transcript_111704	F01_transcript_111704	F01_transcript_111704
F01_transcript_23919	F01_transcript_23919	F01_transcript_23919	NA	F01_transcript_23919
F01_transcript_76883	NA	F01_transcript_76883	NA	NA
F01_transcript_101864	F01_transcript_101864	F01_transcript_101864	NA	NA
F01_transcript_151225	F01_transcript_151225	F01_transcript_151225	F01_transcript_151225	F01_transcript_151225
F01_transcript_137986	F01_transcript_137986	F01_transcript_137986	F01_transcript_137986	F01_transcript_137986
F01_transcript_157019	NA	F01_transcript_157019	F01_transcript_157019	F01_transcript_157019
F01_transcript_132425	NA	NA	NA	F01_transcript_132425
F01_transcript_84337	F01_transcript_84337	F01_transcript_84337	F01_transcript_84337	NA
F01_transcript_146166	NA	F01_transcript_146166	NA	F01_transcript_146166

F01_transcript_61422	NA	NA	NA	F01_transcript_61422
F01_transcript_157623	F01_transcript_157623	F01_transcript_157623	NA	F01_transcript_157623
F01_transcript_136569	NA	F01_transcript_136569	NA	NA
F01_transcript_134408	NA	NA	NA	F01_transcript_134408
F01_transcript_160610	NA	NA	NA	F01_transcript_160610
F01_transcript_166594	NA	F01_transcript_166594	F01_transcript_166594	F01_transcript_166594
F01_transcript_111949	F01_transcript_111949	F01_transcript_111949	F01_transcript_111949	F01_transcript_111949
F01_transcript_71183	F01_transcript_71183	F01_transcript_71183	F01_transcript_71183	F01_transcript_71183
F01_transcript_58834	F01_transcript_58834	F01_transcript_58834	F01_transcript_58834	NA
F01_transcript_103852	F01_transcript_103852	F01_transcript_103852	NA	NA
F01_transcript_97194	NA	NA	NA	F01_transcript_97194
F01_transcript_70353	NA	F01_transcript_70353	F01_transcript_70353	F01_transcript_70353
F01_transcript_17958	NA	F01_transcript_17958	NA	NA
F01_transcript_133432	NA	NA	NA	F01_transcript_133432

F01_transcript_107354	NA	NA	NA	F01_transcript_107354
F01_transcript_82302	NA	F01_transcript_82302	NA	NA
F01_transcript_124840	NA	F01_transcript_124840	NA	F01_transcript_124840
F01_transcript_48408	F01_transcript_48408	F01_transcript_48408	F01_transcript_48408	F01_transcript_48408
F01_transcript_66934	NA	F01_transcript_66934	F01_transcript_66934	F01_transcript_66934
F01_transcript_66583	NA	F01_transcript_66583	NA	F01_transcript_66583
F01_transcript_67278	NA	NA	NA	F01_transcript_67278
F01_transcript_158043	F01_transcript_158043	F01_transcript_158043	F01_transcript_158043	F01_transcript_158043
F01_transcript_50232	F01_transcript_50232	F01_transcript_50232	NA	F01_transcript_50232
F01_transcript_77925	NA	NA	NA	F01_transcript_77925
F01_transcript_32706	NA	NA	NA	F01_transcript_32706
F01_transcript_47321	NA	F01_transcript_47321	NA	NA
F01_transcript_126935	F01_transcript_126935	F01_transcript_126935	F01_transcript_126935	F01_transcript_126935
F01_transcript_63165	NA	F01_transcript_63165	NA	F01_transcript_63165

F01_transcript_71895	NA	NA	F01_transcript_71895	NA
F01_transcript_3365	NA	NA	NA	F01_transcript_3365
F01_transcript_99285	NA	F01_transcript_99285	NA	NA
F01_transcript_164185	F01_transcript_164185	F01_transcript_164185	F01_transcript_164185	F01_transcript_164185
F01_transcript_64265	F01_transcript_64265	F01_transcript_64265	F01_transcript_64265	NA
F01_transcript_97027	NA	F01_transcript_97027	NA	NA
F01_transcript_112952	F01_transcript_112952	F01_transcript_112952	F01_transcript_112952	F01_transcript_112952
F01_transcript_85624	NA	F01_transcript_85624	NA	F01_transcript_85624
F01_transcript_53445	NA	NA	NA	F01_transcript_53445
F01_transcript_68656	F01_transcript_68656	F01_transcript_68656	F01_transcript_68656	F01_transcript_68656
F01_transcript_55350	F01_transcript_55350	F01_transcript_55350	F01_transcript_55350	F01_transcript_55350
F01_transcript_13280	NA	F01_transcript_13280	NA	NA
F01_transcript_12948	F01_transcript_12948	F01_transcript_12948	F01_transcript_12948	F01_transcript_12948
F01_transcript_150315	F01_transcript_150315	F01_transcript_150315	NA	NA

F01_transcript_130785	NA	NA	NA	F01_transcript_130785
F01_transcript_63560	NA	NA	NA	F01_transcript_63560
F01_transcript_56818	NA	NA	F01_transcript_56818	NA
F01_transcript_90072	NA	NA	NA	F01_transcript_90072
F01_transcript_127780	F01_transcript_127780	F01_transcript_127780	F01_transcript_127780	NA
F01_transcript_101299	F01_transcript_101299	F01_transcript_101299	F01_transcript_101299	F01_transcript_101299
F01_transcript_68361	NA	F01_transcript_68361	NA	NA
F01_transcript_56915	F01_transcript_56915	F01_transcript_56915	F01_transcript_56915	F01_transcript_56915
F01_transcript_86358	NA	NA	NA	F01_transcript_86358
F01_transcript_137107	F01_transcript_137107	F01_transcript_137107	F01_transcript_137107	F01_transcript_137107
F01_transcript_53912	NA	NA	NA	F01_transcript_53912
F01_transcript_49172	NA	F01_transcript_49172	NA	NA
F01_transcript_133487	NA	F01_transcript_133487	NA	NA
F01_transcript_114302	NA	F01_transcript_114302	NA	NA

F01_transcript_260	NA	NA	NA	F01_transcript_260
F01_transcript_156071	F01_transcript_156071	F01_transcript_156071	F01_transcript_156071	F01_transcript_156071
F01_transcript_58142	NA	F01_transcript_58142	NA	F01_transcript_58142
F01_transcript_48771	NA	F01_transcript_48771	NA	NA
F01_transcript_154054	NA	F01_transcript_154054	NA	F01_transcript_154054
F01_transcript_103833	F01_transcript_103833	NA	NA	NA
F01_transcript_56370	NA	NA	F01_transcript_56370	NA
F01_transcript_70237	F01_transcript_70237	F01_transcript_70237	F01_transcript_70237	F01_transcript_70237
F01_transcript_146224	F01_transcript_146224	F01_transcript_146224	F01_transcript_146224	F01_transcript_146224
F01_transcript_70833	NA	NA	F01_transcript_70833	NA
F01_transcript_121612	NA	F01_transcript_121612	F01_transcript_121612	F01_transcript_121612
F01_transcript_69519	F01_transcript_69519	F01_transcript_69519	NA	NA
F01_transcript_154929	F01_transcript_154929	F01_transcript_154929	F01_transcript_154929	F01_transcript_154929
F01_transcript_133499	NA	F01_transcript_133499	NA	NA

F01_transcript_167624	NA	F01_transcript_167624	F01_transcript_167624	F01_transcript_167624
F01_transcript_61534	NA	NA	NA	F01_transcript_61534
F01_transcript_155861	F01_transcript_155861	F01_transcript_155861	F01_transcript_155861	F01_transcript_155861
F01_transcript_72204	NA	F01_transcript_72204	NA	NA
F01_transcript_151405	NA	F01_transcript_151405	NA	NA
F01_transcript_28208	NA	NA	NA	F01_transcript_28208
F01_transcript_94476	NA	F01_transcript_94476	F01_transcript_94476	F01_transcript_94476
F01_transcript_113063	NA	F01_transcript_113063	NA	F01_transcript_113063
F01_transcript_154124	NA	NA	F01_transcript_154124	NA
F01_transcript_84312	NA	F01_transcript_84312	NA	NA
F01_transcript_2467	NA	NA	NA	F01_transcript_2467
F01_transcript_122710	NA	F01_transcript_122710	F01_transcript_122710	F01_transcript_122710
F01_transcript_33152	NA	F01_transcript_33152	NA	NA
F01_transcript_89200	NA	F01_transcript_89200	NA	NA

F01_transcript_66116	NA	NA	NA	F01_transcript_66116
F01_transcript_114070	NA	F01_transcript_114070	NA	NA
F01_transcript_107800	NA	F01_transcript_107800	NA	NA
F01_transcript_162751	NA	F01_transcript_162751	NA	NA
F01_transcript_152626	NA	NA	NA	F01_transcript_152626
F01_transcript_121912	F01_transcript_121912	F01_transcript_121912	F01_transcript_121912	F01_transcript_121912
F01_transcript_41266	F01_transcript_41266	F01_transcript_41266	NA	NA
F01_transcript_106531	F01_transcript_106531	F01_transcript_106531	F01_transcript_106531	F01_transcript_106531
F01_transcript_41590	NA	NA	NA	F01_transcript_41590
F01_transcript_103583	NA	F01_transcript_103583	NA	NA
F01_transcript_92848	F01_transcript_92848	F01_transcript_92848	NA	F01_transcript_92848
F01_transcript_131334	NA	F01_transcript_131334	NA	F01_transcript_131334
F01_transcript_57191	F01_transcript_57191	F01_transcript_57191	F01_transcript_57191	F01_transcript_57191
F01_transcript_146553	F01_transcript_146553	F01_transcript_146553	F01_transcript_146553	F01_transcript_146553

F01_transcript_77882	F01_transcript_77882	F01_transcript_77882	NA	NA
F01_transcript_776	NA	NA	NA	F01_transcript_776
F01_transcript_58359	F01_transcript_58359	F01_transcript_58359	F01_transcript_58359	F01_transcript_58359
F01_transcript_92637	NA	F01_transcript_92637	NA	NA
F01_transcript_71024	NA	NA	F01_transcript_71024	NA
F01_transcript_65885	NA	F01_transcript_65885	NA	F01_transcript_65885
F01_transcript_149346	NA	NA	NA	F01_transcript_149346
F01_transcript_88165	NA	NA	NA	F01_transcript_88165
F01_transcript_132302	NA	F01_transcript_132302	NA	NA
F01_transcript_62075	NA	NA	NA	F01_transcript_62075
F01_transcript_147106	NA	NA	NA	F01_transcript_147106
F01_transcript_158719	NA	NA	NA	F01_transcript_158719
F01_transcript_108138	NA	NA	NA	F01_transcript_108138
F01_transcript_137668	NA	F01_transcript_137668	F01_transcript_137668	F01_transcript_137668

F01_transcript_144907	F01_transcript_144907	F01_transcript_144907	F01_transcript_144907	F01_transcript_144907
F01_transcript_32583	NA	NA	NA	F01_transcript_32583
F01_transcript_96800	NA	F01_transcript_96800	NA	F01_transcript_96800
F01_transcript_151319	F01_transcript_151319	F01_transcript_151319	F01_transcript_151319	F01_transcript_151319
F01_transcript_71873	F01_transcript_71873	F01_transcript_71873	F01_transcript_71873	F01_transcript_71873
F01_transcript_116869	NA	NA	NA	F01_transcript_116869
F01_transcript_22757	NA	F01_transcript_22757	NA	NA
F01_transcript_118221	NA	F01_transcript_118221	NA	NA
F01_transcript_66715	F01_transcript_66715	F01_transcript_66715	NA	F01_transcript_66715
F01_transcript_105958	F01_transcript_105958	F01_transcript_105958	NA	NA
F01_transcript_123554	NA	NA	NA	F01_transcript_123554
F01_transcript_122705	F01_transcript_122705	F01_transcript_122705	NA	NA
F01_transcript_82003	F01_transcript_82003	F01_transcript_82003	F01_transcript_82003	F01_transcript_82003
F01_transcript_69040	NA	NA	NA	F01_transcript_69040

F01_transcript_54132	F01_transcript_54132	F01_transcript_54132	F01_transcript_54132	NA
F01_transcript_157725	F01_transcript_157725	F01_transcript_157725	NA	NA
F01_transcript_167685	F01_transcript_167685	F01_transcript_167685	F01_transcript_167685	F01_transcript_167685
F01_transcript_162721	NA	NA	NA	F01_transcript_162721
F01_transcript_132088	F01_transcript_132088	F01_transcript_132088	NA	NA
F01_transcript_17422	NA	NA	F01_transcript_17422	F01_transcript_17422
F01_transcript_96870	F01_transcript_96870	F01_transcript_96870	F01_transcript_96870	F01_transcript_96870
F01_transcript_155186	F01_transcript_155186	F01_transcript_155186	F01_transcript_155186	F01_transcript_155186
F01_transcript_62505	NA	F01_transcript_62505	NA	NA
F01_transcript_167086	F01_transcript_167086	F01_transcript_167086	NA	F01_transcript_167086
F01_transcript_135222	NA	F01_transcript_135222	NA	F01_transcript_135222
F01_transcript_135935	NA	NA	NA	F01_transcript_135935
F01_transcript_120554	NA	F01_transcript_120554	NA	NA
F01_transcript_6114	NA	NA	F01_transcript_6114	F01_transcript_6114

F01_transcript_58914	F01_transcript_58914	F01_transcript_58914	F01_transcript_58914	F01_transcript_58914
F01_transcript_160246	F01_transcript_160246	F01_transcript_160246	NA	F01_transcript_160246
F01_transcript_109626	F01_transcript_109626	F01_transcript_109626	NA	F01_transcript_109626
F01_transcript_111450	NA	F01_transcript_111450	NA	NA
F01_transcript_89941	NA	F01_transcript_89941	NA	NA
F01_transcript_166627	F01_transcript_166627	F01_transcript_166627	NA	F01_transcript_166627
F01_transcript_130596	NA	F01_transcript_130596	F01_transcript_130596	NA
F01_transcript_141985	F01_transcript_141985	F01_transcript_141985	F01_transcript_141985	F01_transcript_141985
F01_transcript_164551	NA	F01_transcript_164551	NA	NA
F01_transcript_4176	NA	NA	NA	F01_transcript_4176
F01_transcript_93301	F01_transcript_93301	F01_transcript_93301	F01_transcript_93301	F01_transcript_93301
F01_transcript_53480	NA	NA	NA	F01_transcript_53480
F01_transcript_88950	NA	NA	F01_transcript_88950	NA
F01_transcript_134829	NA	F01_transcript_134829	NA	NA

F01_transcript_113093	NA	NA	NA	F01_transcript_113093
F01_transcript_33767	NA	F01_transcript_33767	NA	NA
F01_transcript_123256	F01_transcript_123256	F01_transcript_123256	F01_transcript_123256	F01_transcript_123256
F01_transcript_39719	NA	NA	NA	F01_transcript_39719
F01_transcript_131789	NA	NA	NA	F01_transcript_131789
F01_transcript_25442	NA	F01_transcript_25442	NA	NA
F01_transcript_70686	NA	NA	NA	F01_transcript_70686
F01_transcript_106154	F01_transcript_106154	F01_transcript_106154	F01_transcript_106154	F01_transcript_106154
F01_transcript_108323	NA	F01_transcript_108323	NA	NA
F01_transcript_116539	NA	F01_transcript_116539	NA	NA
F01_transcript_4327	NA	F01_transcript_4327	NA	F01_transcript_4327
F01_transcript_120007	NA	F01_transcript_120007	NA	NA
F01_transcript_5767	NA	F01_transcript_5767	F01_transcript_5767	NA
F01_transcript_81867	NA	NA	NA	F01_transcript_81867

F01_transcript_60928	NA	F01_transcript_60928	NA	NA
F01_transcript_99581	F01_transcript_99581	F01_transcript_99581	F01_transcript_99581	F01_transcript_99581
F01_transcript_45985	NA	NA	NA	F01_transcript_45985
F01_transcript_166190	F01_transcript_166190	F01_transcript_166190	F01_transcript_166190	F01_transcript_166190
F01_transcript_108056	NA	NA	NA	F01_transcript_108056
F01_transcript_75371	NA	NA	NA	F01_transcript_75371
F01_transcript_104298	NA	NA	NA	F01_transcript_104298
F01_transcript_116453	NA	F01_transcript_116453	NA	NA
F01_transcript_54258	NA	F01_transcript_54258	NA	NA
F01_transcript_52929	NA	F01_transcript_52929	NA	NA
F01_transcript_56567	NA	NA	NA	F01_transcript_56567
F01_transcript_33636	F01_transcript_33636	F01_transcript_33636	NA	F01_transcript_33636
F01_transcript_54325	NA	NA	NA	F01_transcript_54325
F01_transcript_151452	NA	F01_transcript_151452	F01_transcript_151452	F01_transcript_151452

F01_transcript_91446	NA	NA	NA	F01_transcript_91446
F01_transcript_116332	NA	F01_transcript_116332	NA	NA
F01_transcript_140780	F01_transcript_140780	F01_transcript_140780	F01_transcript_140780	F01_transcript_140780
F01_transcript_131105	NA	NA	NA	F01_transcript_131105
F01_transcript_49503	NA	F01_transcript_49503	F01_transcript_49503	NA
F01_transcript_2760	F01_transcript_2760	F01_transcript_2760	F01_transcript_2760	F01_transcript_2760
F01_transcript_165603	NA	NA	F01_transcript_165603	NA
F01_transcript_69891	NA	F01_transcript_69891	NA	F01_transcript_69891
F01_transcript_59592	F01_transcript_59592	F01_transcript_59592	F01_transcript_59592	F01_transcript_59592
F01_transcript_13520	NA	NA	NA	F01_transcript_13520
F01_transcript_61714	NA	F01_transcript_61714	F01_transcript_61714	F01_transcript_61714
F01_transcript_156323	NA	NA	NA	F01_transcript_156323
F01_transcript_65397	F01_transcript_65397	F01_transcript_65397	F01_transcript_65397	F01_transcript_65397
F01_transcript_99774	F01_transcript_99774	F01_transcript_99774	NA	F01_transcript_99774

F01_transcript_150565	F01_transcript_150565	F01_transcript_150565	F01_transcript_150565	F01_transcript_150565
F01_transcript_68138	F01_transcript_68138	F01_transcript_68138	F01_transcript_68138	F01_transcript_68138
F01_transcript_140912	NA	F01_transcript_140912	NA	NA
F01_transcript_134949	NA	NA	NA	F01_transcript_134949
F01_transcript_59559	NA	NA	F01_transcript_59559	NA
F01_transcript_124037	F01_transcript_124037	F01_transcript_124037	NA	NA
F01_transcript_123609	NA	NA	NA	F01_transcript_123609
F01_transcript_167322	NA	F01_transcript_167322	NA	NA
F01_transcript_130554	NA	NA	NA	F01_transcript_130554
F01_transcript_13337	NA	NA	NA	F01_transcript_13337
F01_transcript_59216	NA	NA	NA	F01_transcript_59216
F01_transcript_88116	NA	F01_transcript_88116	NA	NA
F01_transcript_45613	F01_transcript_45613	F01_transcript_45613	F01_transcript_45613	F01_transcript_45613
F01_transcript_150025	NA	NA	NA	F01_transcript_150025

F01_transcript_98465	NA	NA	NA	F01_transcript_98465
F01_transcript_154048	NA	F01_transcript_154048	NA	NA
F01_transcript_153171	NA	NA	NA	F01_transcript_153171
F01_transcript_61808	NA	NA	NA	F01_transcript_61808
F01_transcript_9564	F01_transcript_9564	F01_transcript_9564	F01_transcript_9564	NA
F01_transcript_138416	F01_transcript_138416	F01_transcript_138416	F01_transcript_138416	F01_transcript_138416
F01_transcript_42406	NA	NA	NA	F01_transcript_42406
F01_transcript_140377	NA	NA	NA	F01_transcript_140377
F01_transcript_1887	NA	NA	NA	F01_transcript_1887
F01_transcript_131204	NA	F01_transcript_131204	NA	NA
F01_transcript_97959	NA	F01_transcript_97959	NA	F01_transcript_97959
F01_transcript_100306	F01_transcript_100306	F01_transcript_100306	F01_transcript_100306	F01_transcript_100306
F01_transcript_148302	NA	F01_transcript_148302	NA	F01_transcript_148302
F01_transcript_108990	NA	F01_transcript_108990	NA	NA

F01_transcript_127668	NA	NA	F01_transcript_127668	F01_transcript_127668
F01_transcript_131403	NA	NA	NA	F01_transcript_131403
F01_transcript_33439	NA	NA	NA	F01_transcript_33439
F01_transcript_88467	NA	NA	NA	F01_transcript_88467
F01_transcript_60388	NA	NA	NA	F01_transcript_60388
F01_transcript_132651	NA	F01_transcript_132651	F01_transcript_132651	F01_transcript_132651
F01_transcript_152645	F01_transcript_152645	F01_transcript_152645	F01_transcript_152645	F01_transcript_152645
F01_transcript_95856	NA	F01_transcript_95856	NA	NA
F01_transcript_65004	NA	F01_transcript_65004	NA	F01_transcript_65004
F01_transcript_99296	NA	NA	NA	F01_transcript_99296
F01_transcript_67023	F01_transcript_67023	F01_transcript_67023	F01_transcript_67023	F01_transcript_67023
F01_transcript_72176	NA	F01_transcript_72176	NA	NA
F01_transcript_132291	NA	NA	NA	F01_transcript_132291
F01_transcript_42641	NA	NA	NA	F01_transcript_42641

F01_transcript_62139	F01_transcript_62139	F01_transcript_62139	F01_transcript_62139	F01_transcript_62139
F01_transcript_159756	F01_transcript_159756	F01_transcript_159756	F01_transcript_159756	F01_transcript_159756
F01_transcript_59736	F01_transcript_59736	F01_transcript_59736	NA	F01_transcript_59736
F01_transcript_160808	NA	F01_transcript_160808	F01_transcript_160808	NA
F01_transcript_40699	NA	NA	NA	F01_transcript_40699
F01_transcript_128717	NA	F01_transcript_128717	NA	NA
F01_transcript_2472	NA	NA	F01_transcript_2472	NA
F01_transcript_49709	F01_transcript_49709	NA	NA	NA
F01_transcript_52224	NA	NA	NA	F01_transcript_52224
F01_transcript_80867	NA	F01_transcript_80867	F01_transcript_80867	F01_transcript_80867
F01_transcript_117671	NA	NA	NA	F01_transcript_117671
F01_transcript_80169	F01_transcript_80169	F01_transcript_80169	NA	NA
F01_transcript_148415	F01_transcript_148415	F01_transcript_148415	F01_transcript_148415	F01_transcript_148415
F01_transcript_95960	F01_transcript_95960	F01_transcript_95960	F01_transcript_95960	F01_transcript_95960

F01_transcript_46876	NA	NA	NA	F01_transcript_46876
F01_transcript_79344	NA	F01_transcript_79344	NA	F01_transcript_79344
F01_transcript_87084	NA	F01_transcript_87084	NA	F01_transcript_87084
F01_transcript_94412	NA	NA	NA	F01_transcript_94412
F01_transcript_75618	F01_transcript_75618	F01_transcript_75618	F01_transcript_75618	F01_transcript_75618
F01_transcript_136706	NA	F01_transcript_136706	F01_transcript_136706	NA
F01_transcript_164680	NA	F01_transcript_164680	NA	NA
F01_transcript_60045	NA	F01_transcript_60045	NA	NA
F01_transcript_137920	F01_transcript_137920	F01_transcript_137920	F01_transcript_137920	F01_transcript_137920
F01_transcript_156993	NA	NA	NA	F01_transcript_156993
F01_transcript_35040	NA	NA	NA	F01_transcript_35040
F01_transcript_65308	NA	NA	F01_transcript_65308	NA
F01_transcript_57886	F01_transcript_57886	F01_transcript_57886	F01_transcript_57886	F01_transcript_57886
F01_transcript_152694	NA	NA	NA	F01_transcript_152694

F01_transcript_102125	F01_transcript_102125	F01_transcript_102125	NA	F01_transcript_102125
F01_transcript_116481	NA	NA	NA	F01_transcript_116481
F01_transcript_145320	NA	F01_transcript_145320	NA	F01_transcript_145320
F01_transcript_150373	NA	NA	NA	F01_transcript_150373
F01_transcript_31879	NA	NA	NA	F01_transcript_31879
F01_transcript_58432	NA	NA	NA	F01_transcript_58432
F01_transcript_9650	NA	NA	F01_transcript_9650	F01_transcript_9650
F01_transcript_149318	NA	F01_transcript_149318	NA	F01_transcript_149318
F01_transcript_122615	NA	NA	NA	F01_transcript_122615
F01_transcript_125143	F01_transcript_125143	F01_transcript_125143	F01_transcript_125143	F01_transcript_125143
F01_transcript_58676	NA	NA	NA	F01_transcript_58676
F01_transcript_90758	NA	F01_transcript_90758	F01_transcript_90758	F01_transcript_90758
F01_transcript_52262	F01_transcript_52262	F01_transcript_52262	NA	NA
F01_transcript_11733	F01_transcript_11733	F01_transcript_11733	NA	NA

F01_transcript_123287	NA	NA	NA	F01_transcript_123287
F01_transcript_68701	NA	NA	F01_transcript_68701	NA
F01_transcript_85358	NA	F01_transcript_85358	NA	NA
F01_transcript_78644	NA	F01_transcript_78644	NA	NA
F01_transcript_115572	F01_transcript_115572	F01_transcript_115572	F01_transcript_115572	F01_transcript_115572
F01_transcript_118516	NA	NA	NA	F01_transcript_118516
F01_transcript_151265	NA	NA	NA	F01_transcript_151265
F01_transcript_136313	NA	NA	NA	F01_transcript_136313
F01_transcript_30686	F01_transcript_30686	F01_transcript_30686	F01_transcript_30686	F01_transcript_30686
F01_transcript_59446	NA	F01_transcript_59446	F01_transcript_59446	F01_transcript_59446
F01_transcript_126644	NA	NA	NA	F01_transcript_126644
F01_transcript_54288	F01_transcript_54288	F01_transcript_54288	F01_transcript_54288	F01_transcript_54288
F01_transcript_49911	NA	NA	NA	F01_transcript_49911
F01_transcript_67456	NA	F01_transcript_67456	NA	NA

F01_transcript_60395	NA	NA	NA	F01_transcript_60395
F01_transcript_65729	NA	NA	NA	F01_transcript_65729
F01_transcript_139723	NA	NA	NA	F01_transcript_139723
F01_transcript_95328	NA	F01_transcript_95328	NA	NA
F01_transcript_248	NA	NA	NA	F01_transcript_248
F01_transcript_164100	NA	NA	NA	F01_transcript_164100
F01_transcript_71981	NA	NA	NA	F01_transcript_71981
F01_transcript_9956	NA	NA	NA	F01_transcript_9956
F01_transcript_79648	F01_transcript_79648	F01_transcript_79648	F01_transcript_79648	F01_transcript_79648
F01_transcript_129150	F01_transcript_129150	F01_transcript_129150	F01_transcript_129150	F01_transcript_129150
F01_transcript_20606	NA	F01_transcript_20606	NA	NA
F01_transcript_147771	NA	F01_transcript_147771	NA	F01_transcript_147771
F01_transcript_134967	NA	F01_transcript_134967	NA	NA
F01_transcript_125024	F01_transcript_125024	F01_transcript_125024	F01_transcript_125024	F01_transcript_125024

F01_transcript_134397	NA	NA	NA	F01_transcript_134397
F01_transcript_31738	NA	NA	NA	F01_transcript_31738
F01_transcript_56243	F01_transcript_56243	F01_transcript_56243	F01_transcript_56243	F01_transcript_56243
F01_transcript_8885	NA	NA	NA	F01_transcript_8885
F01_transcript_132345	NA	NA	NA	F01_transcript_132345
F01_transcript_75341	NA	NA	NA	F01_transcript_75341
F01_transcript_15142	NA	NA	NA	F01_transcript_15142
F01_transcript_150806	F01_transcript_150806	F01_transcript_150806	F01_transcript_150806	F01_transcript_150806
F01_transcript_139492	NA	NA	NA	F01_transcript_139492
F01_transcript_167414	NA	F01_transcript_167414	NA	F01_transcript_167414
F01_transcript_76477	F01_transcript_76477	F01_transcript_76477	F01_transcript_76477	F01_transcript_76477
F01_transcript_86848	F01_transcript_86848	F01_transcript_86848	F01_transcript_86848	F01_transcript_86848
F01_transcript_117858	F01_transcript_117858	F01_transcript_117858	F01_transcript_117858	F01_transcript_117858
F01_transcript_23542	NA	NA	NA	F01_transcript_23542

F01_transcript_67480	NA	F01_transcript_67480	F01_transcript_67480	F01_transcript_67480
F01_transcript_64513	F01_transcript_64513	F01_transcript_64513	NA	F01_transcript_64513
F01_transcript_57798	NA	F01_transcript_57798	NA	NA
F01_transcript_75875	NA	NA	NA	F01_transcript_75875
F01_transcript_152583	NA	NA	NA	F01_transcript_152583
F01_transcript_157017	NA	F01_transcript_157017	F01_transcript_157017	NA
F01_transcript_64962	F01_transcript_64962	F01_transcript_64962	NA	F01_transcript_64962
F01_transcript_157902	NA	NA	NA	F01_transcript_157902
F01_transcript_65429	NA	NA	F01_transcript_65429	NA
F01_transcript_140598	NA	NA	NA	F01_transcript_140598
F01_transcript_93051	NA	NA	NA	F01_transcript_93051
F01_transcript_88808	F01_transcript_88808	F01_transcript_88808	F01_transcript_88808	F01_transcript_88808
F01_transcript_103716	NA	NA	NA	F01_transcript_103716
F01_transcript_128929	NA	F01_transcript_128929	NA	F01_transcript_128929

F01_transcript_87375	NA	NA	NA	F01_transcript_87375
F01_transcript_50078	NA	F01_transcript_50078	NA	NA
F01_transcript_87742	F01_transcript_87742	F01_transcript_87742	F01_transcript_87742	F01_transcript_87742
F01_transcript_134628	NA	NA	NA	F01_transcript_134628
F01_transcript_8418	NA	NA	NA	F01_transcript_8418
F01_transcript_37243	NA	NA	NA	F01_transcript_37243
F01_transcript_88748	NA	F01_transcript_88748	NA	NA
F01_transcript_41121	NA	NA	NA	F01_transcript_41121
F01_transcript_159979	NA	F01_transcript_159979	NA	NA
F01_transcript_163534	NA	NA	NA	F01_transcript_163534
F01_transcript_133301	NA	F01_transcript_133301	NA	F01_transcript_133301
F01_transcript_145345	NA	F01_transcript_145345	NA	NA
F01_transcript_118515	F01_transcript_118515	F01_transcript_118515	F01_transcript_118515	F01_transcript_118515
F01_transcript_31039	NA	NA	NA	F01_transcript_31039

F01_transcript_74083	NA	F01_transcript_74083	F01_transcript_74083	F01_transcript_74083
F01_transcript_105316	NA	F01_transcript_105316	NA	F01_transcript_105316
F01_transcript_111153	NA	F01_transcript_111153	F01_transcript_111153	NA
F01_transcript_149082	F01_transcript_149082	F01_transcript_149082	F01_transcript_149082	F01_transcript_149082
F01_transcript_132014	NA	F01_transcript_132014	NA	NA
F01_transcript_89536	NA	F01_transcript_89536	NA	NA
F01_transcript_160292	NA	NA	NA	F01_transcript_160292
F01_transcript_72815	F01_transcript_72815	F01_transcript_72815	NA	F01_transcript_72815
F01_transcript_104336	NA	NA	NA	F01_transcript_104336
F01_transcript_89339	NA	F01_transcript_89339	NA	F01_transcript_89339
F01_transcript_73907	NA	NA	NA	F01_transcript_73907
F01_transcript_52698	NA	NA	NA	F01_transcript_52698
F01_transcript_70003	F01_transcript_70003	NA	NA	NA
F01_transcript_118878	NA	NA	NA	F01_transcript_118878

F01_transcript_70701	NA	F01_transcript_70701	F01_transcript_70701	F01_transcript_70701
F01_transcript_121949	F01_transcript_121949	F01_transcript_121949	F01_transcript_121949	F01_transcript_121949
F01_transcript_76380	NA	F01_transcript_76380	NA	NA
F01_transcript_18434	NA	F01_transcript_18434	NA	NA
F01_transcript_80006	NA	F01_transcript_80006	NA	NA
F01_transcript_142994	F01_transcript_142994	F01_transcript_142994	F01_transcript_142994	F01_transcript_142994
F01_transcript_117018	NA	F01_transcript_117018	NA	F01_transcript_117018
F01_transcript_71776	F01_transcript_71776	F01_transcript_71776	F01_transcript_71776	F01_transcript_71776
F01_transcript_57507	NA	F01_transcript_57507	F01_transcript_57507	F01_transcript_57507
F01_transcript_105099	NA	F01_transcript_105099	F01_transcript_105099	NA
F01_transcript_67148	NA	NA	NA	F01_transcript_67148
F01_transcript_93297	NA	NA	NA	F01_transcript_93297
F01_transcript_81766	F01_transcript_81766	F01_transcript_81766	F01_transcript_81766	F01_transcript_81766
F01_transcript_110316	NA	F01_transcript_110316	NA	NA

F01_transcript_42549	NA	NA	NA	F01_transcript_42549
F01_transcript_84207	F01_transcript_84207	F01_transcript_84207	F01_transcript_84207	F01_transcript_84207
F01_transcript_75541	NA	F01_transcript_75541	F01_transcript_75541	F01_transcript_75541
F01_transcript_163853	F01_transcript_163853	F01_transcript_163853	F01_transcript_163853	F01_transcript_163853
F01_transcript_64540	F01_transcript_64540	F01_transcript_64540	F01_transcript_64540	F01_transcript_64540
F01_transcript_54048	NA	F01_transcript_54048	NA	F01_transcript_54048
F01_transcript_51303	NA	F01_transcript_51303	F01_transcript_51303	F01_transcript_51303
F01_transcript_151190	NA	F01_transcript_151190	NA	NA
F01_transcript_155956	NA	F01_transcript_155956	NA	NA
F01_transcript_107995	NA	F01_transcript_107995	NA	NA
F01_transcript_153361	NA	NA	NA	F01_transcript_153361
F01_transcript_119756	NA	NA	NA	F01_transcript_119756
F01_transcript_105396	F01_transcript_105396	F01_transcript_105396	F01_transcript_105396	F01_transcript_105396
F01_transcript_73383	NA	NA	NA	F01_transcript_73383

F01_transcript_26493	NA	NA	NA	F01_transcript_26493
F01_transcript_72229	F01_transcript_72229	F01_transcript_72229	NA	F01_transcript_72229
F01_transcript_109840	NA	F01_transcript_109840	NA	F01_transcript_109840
F01_transcript_102985	NA	NA	F01_transcript_102985	F01_transcript_102985
F01_transcript_75574	NA	NA	NA	F01_transcript_75574
F01_transcript_50844	F01_transcript_50844	F01_transcript_50844	F01_transcript_50844	F01_transcript_50844
F01_transcript_73950	NA	F01_transcript_73950	NA	NA
F01_transcript_164821	NA	NA	NA	F01_transcript_164821
F01_transcript_119118	NA	NA	NA	F01_transcript_119118
F01_transcript_65110	NA	NA	F01_transcript_65110	NA
F01_transcript_79624	NA	F01_transcript_79624	F01_transcript_79624	F01_transcript_79624
F01_transcript_58912	F01_transcript_58912	F01_transcript_58912	F01_transcript_58912	F01_transcript_58912
F01_transcript_127201	F01_transcript_127201	F01_transcript_127201	F01_transcript_127201	F01_transcript_127201
F01_transcript_119308	NA	NA	NA	F01_transcript_119308

F01_transcript_65963	NA	NA	NA	F01_transcript_65963
F01_transcript_73699	NA	F01_transcript_73699	NA	NA
F01_transcript_34051	NA	NA	NA	F01_transcript_34051
F01_transcript_103133	NA	F01_transcript_103133	F01_transcript_103133	NA
F01_transcript_69930	NA	F01_transcript_69930	NA	NA
F01_transcript_99904	NA	F01_transcript_99904	NA	F01_transcript_99904
F01_transcript_144625	NA	F01_transcript_144625	NA	NA
F01_transcript_72436	NA	NA	F01_transcript_72436	NA
F01_transcript_161323	F01_transcript_161323	F01_transcript_161323	F01_transcript_161323	F01_transcript_161323
F01_transcript_147312	NA	F01_transcript_147312	NA	F01_transcript_147312
F01_transcript_82225	F01_transcript_82225	F01_transcript_82225	F01_transcript_82225	F01_transcript_82225
F01_transcript_82289	NA	NA	NA	F01_transcript_82289
F01_transcript_151903	F01_transcript_151903	F01_transcript_151903	F01_transcript_151903	F01_transcript_151903
F01_transcript_153645	F01_transcript_153645	F01_transcript_153645	F01_transcript_153645	F01_transcript_153645

F01_transcript_59295	NA	NA	F01_transcript_59295	F01_transcript_59295
F01_transcript_45035	NA	NA	NA	F01_transcript_45035
F01_transcript_63228	NA	NA	NA	F01_transcript_63228
F01_transcript_53934	NA	F01_transcript_53934	NA	F01_transcript_53934
F01_transcript_144120	NA	NA	NA	F01_transcript_144120
F01_transcript_63960	NA	F01_transcript_63960	F01_transcript_63960	F01_transcript_63960
F01_transcript_119516	NA	F01_transcript_119516	NA	NA
F01_transcript_97453	NA	NA	NA	F01_transcript_97453
F01_transcript_157501	NA	F01_transcript_157501	NA	NA
F01_transcript_125070	F01_transcript_125070	F01_transcript_125070	F01_transcript_125070	F01_transcript_125070
F01_transcript_92065	NA	F01_transcript_92065	NA	NA
F01_transcript_122269	F01_transcript_122269	F01_transcript_122269	F01_transcript_122269	F01_transcript_122269
F01_transcript_94931	NA	F01_transcript_94931	F01_transcript_94931	F01_transcript_94931
F01_transcript_81443	NA	F01_transcript_81443	F01_transcript_81443	F01_transcript_81443

F01_transcript_163199	F01_transcript_163199	F01_transcript_163199	NA	F01_transcript_163199
F01_transcript_106383	NA	F01_transcript_106383	F01_transcript_106383	F01_transcript_106383
F01_transcript_68997	NA	F01_transcript_68997	NA	F01_transcript_68997
F01_transcript_50389	F01_transcript_50389	F01_transcript_50389	F01_transcript_50389	F01_transcript_50389
F01_transcript_50469	F01_transcript_50469	F01_transcript_50469	NA	NA
F01_transcript_142237	NA	F01_transcript_142237	F01_transcript_142237	F01_transcript_142237
F01_transcript_93136	NA	F01_transcript_93136	F01_transcript_93136	F01_transcript_93136
F01_transcript_57255	NA	F01_transcript_57255	F01_transcript_57255	F01_transcript_57255
F01_transcript_43572	NA	NA	NA	F01_transcript_43572
F01_transcript_48281	NA	NA	NA	F01_transcript_48281
F01_transcript_83303	NA	NA	NA	F01_transcript_83303
F01_transcript_74933	NA	NA	NA	F01_transcript_74933
F01_transcript_134438	NA	NA	NA	F01_transcript_134438
F01_transcript_139165	F01_transcript_139165	F01_transcript_139165	F01_transcript_139165	F01_transcript_139165

F01_transcript_60164	NA	NA	NA	F01_transcript_60164
F01_transcript_102243	F01_transcript_102243	F01_transcript_102243	F01_transcript_102243	F01_transcript_102243
F01_transcript_24851	NA	NA	NA	F01_transcript_24851
F01_transcript_147336	NA	F01_transcript_147336	NA	NA
F01_transcript_75940	F01_transcript_75940	F01_transcript_75940	F01_transcript_75940	F01_transcript_75940
F01_transcript_65360	NA	NA	F01_transcript_65360	NA
F01_transcript_119548	NA	F01_transcript_119548	NA	NA
F01_transcript_69926	NA	F01_transcript_69926	NA	NA
F01_transcript_95580	NA	NA	NA	F01_transcript_95580
F01_transcript_141662	NA	F01_transcript_141662	NA	NA
F01_transcript_57557	F01_transcript_57557	F01_transcript_57557	F01_transcript_57557	NA
F01_transcript_73448	NA	NA	NA	F01_transcript_73448
F01_transcript_105893	NA	F01_transcript_105893	NA	F01_transcript_105893
F01_transcript_144548	NA	F01_transcript_144548	F01_transcript_144548	F01_transcript_144548

F01_transcript_156456	F01_transcript_156456	F01_transcript_156456	NA	NA
F01_transcript_90430	NA	NA	NA	F01_transcript_90430
F01_transcript_147998	NA	F01_transcript_147998	F01_transcript_147998	F01_transcript_147998
F01_transcript_135046	NA	F01_transcript_135046	NA	F01_transcript_135046
F01_transcript_8314	NA	F01_transcript_8314	NA	F01_transcript_8314
F01_transcript_75929	NA	NA	NA	F01_transcript_75929
F01_transcript_87420	NA	F01_transcript_87420	NA	NA
F01_transcript_41525	NA	NA	F01_transcript_41525	NA
F01_transcript_59195	F01_transcript_59195	F01_transcript_59195	F01_transcript_59195	F01_transcript_59195
F01_transcript_144555	F01_transcript_144555	F01_transcript_144555	NA	F01_transcript_144555
F01_transcript_84555	NA	F01_transcript_84555	NA	NA
F01_transcript_25918	NA	NA	NA	F01_transcript_25918
F01_transcript_113046	NA	F01_transcript_113046	NA	NA
F01_transcript_101037	NA	F01_transcript_101037	F01_transcript_101037	F01_transcript_101037

F01_transcript_112498	NA	F01_transcript_112498	NA	NA
F01_transcript_109901	F01_transcript_109901	F01_transcript_109901	NA	NA
F01_transcript_14865	NA	NA	NA	F01_transcript_14865
F01_transcript_143219	NA	F01_transcript_143219	F01_transcript_143219	F01_transcript_143219
F01_transcript_109561	F01_transcript_109561	F01_transcript_109561	F01_transcript_109561	F01_transcript_109561
F01_transcript_104681	F01_transcript_104681	F01_transcript_104681	F01_transcript_104681	F01_transcript_104681
F01_transcript_36082	NA	NA	NA	F01_transcript_36082
F01_transcript_14699	NA	NA	NA	F01_transcript_14699
F01_transcript_140301	NA	NA	F01_transcript_140301	NA
F01_transcript_161045	NA	F01_transcript_161045	F01_transcript_161045	NA
F01_transcript_73773	NA	F01_transcript_73773	NA	NA
F01_transcript_122353	NA	F01_transcript_122353	NA	NA
F01_transcript_153288	NA	F01_transcript_153288	NA	NA
F01_transcript_139695	NA	F01_transcript_139695	F01_transcript_139695	F01_transcript_139695

F01_transcript_62292	NA	NA	F01_transcript_62292	F01_transcript_62292
F01_transcript_133545	NA	F01_transcript_133545	NA	NA
F01_transcript_115007	NA	NA	NA	F01_transcript_115007
F01_transcript_141169	NA	NA	NA	F01_transcript_141169
F01_transcript_68068	NA	NA	NA	F01_transcript_68068
F01_transcript_87206	F01_transcript_87206	F01_transcript_87206	F01_transcript_87206	F01_transcript_87206
F01_transcript_62838	F01_transcript_62838	F01_transcript_62838	F01_transcript_62838	F01_transcript_62838
F01_transcript_103052	NA	NA	NA	F01_transcript_103052
F01_transcript_20185	NA	F01_transcript_20185	F01_transcript_20185	F01_transcript_20185
F01_transcript_154741	NA	F01_transcript_154741	NA	F01_transcript_154741
F01_transcript_128162	F01_transcript_128162	F01_transcript_128162	F01_transcript_128162	F01_transcript_128162
F01_transcript_112445	NA	F01_transcript_112445	NA	NA
F01_transcript_42284	F01_transcript_42284	F01_transcript_42284	NA	NA
F01_transcript_86572	NA	F01_transcript_86572	F01_transcript_86572	F01_transcript_86572

F01_transcript_111898	F01_transcript_111898	F01_transcript_111898	F01_transcript_111898	NA
F01_transcript_147823	F01_transcript_147823	F01_transcript_147823	NA	NA
F01_transcript_137513	NA	NA	NA	F01_transcript_137513
F01_transcript_59297	NA	NA	F01_transcript_59297	NA
F01_transcript_150265	NA	NA	NA	F01_transcript_150265
F01_transcript_42350	NA	NA	NA	F01_transcript_42350
F01_transcript_103289	NA	NA	NA	F01_transcript_103289
F01_transcript_152946	F01_transcript_152946	F01_transcript_152946	F01_transcript_152946	F01_transcript_152946
F01_transcript_93826	NA	NA	NA	F01_transcript_93826
F01_transcript_54256	NA	NA	NA	F01_transcript_54256
F01_transcript_152714	F01_transcript_152714	F01_transcript_152714	F01_transcript_152714	F01_transcript_152714
F01_transcript_48318	F01_transcript_48318	F01_transcript_48318	NA	NA
F01_transcript_71223	NA	F01_transcript_71223	F01_transcript_71223	NA
F01_transcript_53349	NA	F01_transcript_53349	NA	NA

F01_transcript_137314	NA	NA	NA	F01_transcript_137314
F01_transcript_52488	NA	NA	NA	F01_transcript_52488
F01_transcript_145383	NA	F01_transcript_145383	NA	NA
F01_transcript_128099	F01_transcript_128099	F01_transcript_128099	F01_transcript_128099	F01_transcript_128099
F01_transcript_125742	F01_transcript_125742	F01_transcript_125742	F01_transcript_125742	F01_transcript_125742
F01_transcript_156708	NA	F01_transcript_156708	NA	NA
F01_transcript_92749	NA	F01_transcript_92749	NA	NA
F01_transcript_118376	F01_transcript_118376	F01_transcript_118376	F01_transcript_118376	F01_transcript_118376
F01_transcript_164978	NA	NA	NA	F01_transcript_164978
F01_transcript_163516	NA	NA	NA	F01_transcript_163516
F01_transcript_108615	F01_transcript_108615	F01_transcript_108615	F01_transcript_108615	F01_transcript_108615
F01_transcript_100990	NA	NA	NA	F01_transcript_100990
F01_transcript_87601	NA	NA	NA	F01_transcript_87601
F01_transcript_540	NA	NA	NA	F01_transcript_540

F01_transcript_90863	F01_transcript_90863	F01_transcript_90863	F01_transcript_90863	F01_transcript_90863
F01_transcript_11082	F01_transcript_11082	F01_transcript_11082	NA	NA
F01_transcript_26349	NA	NA	NA	F01_transcript_26349
F01_transcript_47526	F01_transcript_47526	F01_transcript_47526	F01_transcript_47526	F01_transcript_47526
F01_transcript_23144	NA	NA	NA	F01_transcript_23144
F01_transcript_126216	F01_transcript_126216	F01_transcript_126216	F01_transcript_126216	F01_transcript_126216
F01_transcript_95401	NA	NA	NA	F01_transcript_95401
F01_transcript_11862	NA	NA	NA	F01_transcript_11862
F01_transcript_106022	NA	F01_transcript_106022	NA	NA
F01_transcript_62580	F01_transcript_62580	F01_transcript_62580	F01_transcript_62580	F01_transcript_62580
F01_transcript_56792	F01_transcript_56792	F01_transcript_56792	NA	NA
F01_transcript_157655	F01_transcript_157655	F01_transcript_157655	F01_transcript_157655	F01_transcript_157655
F01_transcript_155029	NA	F01_transcript_155029	NA	F01_transcript_155029
F01_transcript_119094	F01_transcript_119094	NA	NA	F01_transcript_119094

F01_transcript_128727	NA	F01_transcript_128727	NA	F01_transcript_128727
F01_transcript_24330	NA	F01_transcript_24330	NA	F01_transcript_24330
F01_transcript_119213	NA	F01_transcript_119213	NA	F01_transcript_119213
F01_transcript_158884	NA	NA	F01_transcript_158884	F01_transcript_158884
F01_transcript_54655	NA	F01_transcript_54655	NA	F01_transcript_54655
F01_transcript_86142	NA	NA	NA	F01_transcript_86142
F01_transcript_40940	NA	NA	NA	F01_transcript_40940
F01_transcript_128543	NA	NA	NA	F01_transcript_128543
F01_transcript_145233	NA	F01_transcript_145233	NA	NA
F01_transcript_56607	NA	NA	F01_transcript_56607	F01_transcript_56607
F01_transcript_81763	NA	F01_transcript_81763	NA	NA
F01_transcript_110930	NA	NA	NA	F01_transcript_110930
F01_transcript_6851	NA	F01_transcript_6851	NA	NA
F01_transcript_148731	NA	F01_transcript_148731	F01_transcript_148731	NA

F01_transcript_142477	F01_transcript_142477	F01_transcript_142477	F01_transcript_142477	F01_transcript_142477
F01_transcript_8993	NA	NA	NA	F01_transcript_8993
F01_transcript_117471	F01_transcript_117471	F01_transcript_117471	NA	F01_transcript_117471
F01_transcript_52603	NA	F01_transcript_52603	F01_transcript_52603	F01_transcript_52603
F01_transcript_62399	NA	F01_transcript_62399	F01_transcript_62399	NA
F01_transcript_50047	NA	F01_transcript_50047	NA	F01_transcript_50047
F01_transcript_77677	NA	F01_transcript_77677	NA	NA
F01_transcript_68245	NA	F01_transcript_68245	NA	NA
F01_transcript_50089	NA	NA	NA	F01_transcript_50089
F01_transcript_151171	NA	F01_transcript_151171	F01_transcript_151171	NA
F01_transcript_4172	F01_transcript_4172	F01_transcript_4172	F01_transcript_4172	NA
F01_transcript_61604	NA	NA	NA	F01_transcript_61604
F01_transcript_133673	NA	F01_transcript_133673	F01_transcript_133673	NA
F01_transcript_89595	NA	NA	NA	F01_transcript_89595

F01_transcript_24553	F01_transcript_24553	F01_transcript_24553	F01_transcript_24553	F01_transcript_24553
F01_transcript_106212	NA	F01_transcript_106212	NA	NA
F01_transcript_45122	NA	F01_transcript_45122	NA	NA
F01_transcript_81453	NA	NA	NA	F01_transcript_81453
F01_transcript_137692	NA	NA	NA	F01_transcript_137692
F01_transcript_155553	NA	F01_transcript_155553	NA	NA
F01_transcript_52591	F01_transcript_52591	F01_transcript_52591	F01_transcript_52591	F01_transcript_52591
F01_transcript_118460	NA	F01_transcript_118460	NA	NA
F01_transcript_98305	F01_transcript_98305	F01_transcript_98305	NA	NA
F01_transcript_122161	F01_transcript_122161	F01_transcript_122161	F01_transcript_122161	F01_transcript_122161
F01_transcript_81021	NA	NA	NA	F01_transcript_81021
F01_transcript_26514	NA	F01_transcript_26514	NA	NA
F01_transcript_50411	NA	NA	F01_transcript_50411	F01_transcript_50411
F01_transcript_117343	F01_transcript_117343	F01_transcript_117343	F01_transcript_117343	F01_transcript_117343

F01_transcript_84066	NA	F01_transcript_84066	NA	NA
F01_transcript_53712	NA	F01_transcript_53712	NA	F01_transcript_53712
F01_transcript_136496	F01_transcript_136496	F01_transcript_136496	F01_transcript_136496	F01_transcript_136496
F01_transcript_145784	NA	F01_transcript_145784	F01_transcript_145784	F01_transcript_145784
F01_transcript_128798	NA	F01_transcript_128798	NA	NA
F01_transcript_52974	NA	F01_transcript_52974	F01_transcript_52974	NA
F01_transcript_135782	NA	F01_transcript_135782	F01_transcript_135782	F01_transcript_135782
F01_transcript_80717	NA	NA	F01_transcript_80717	F01_transcript_80717
F01_transcript_93999	NA	NA	NA	F01_transcript_93999
F01_transcript_166048	NA	F01_transcript_166048	NA	NA
F01_transcript_128062	F01_transcript_128062	F01_transcript_128062	NA	NA
F01_transcript_73482	F01_transcript_73482	F01_transcript_73482	F01_transcript_73482	F01_transcript_73482
F01_transcript_34041	NA	NA	NA	F01_transcript_34041
F01_transcript_13455	NA	F01_transcript_13455	NA	NA

F01_transcript_134698	F01_transcript_134698	NA	NA	NA
F01_transcript_40375	NA	NA	NA	F01_transcript_40375
F01_transcript_84545	NA	F01_transcript_84545	NA	F01_transcript_84545
F01_transcript_135878	NA	F01_transcript_135878	NA	NA
F01_transcript_163140	F01_transcript_163140	F01_transcript_163140	F01_transcript_163140	F01_transcript_163140
F01_transcript_116474	NA	F01_transcript_116474	F01_transcript_116474	F01_transcript_116474
F01_transcript_99929	NA	F01_transcript_99929	NA	NA
F01_transcript_46591	NA	NA	NA	F01_transcript_46591
F01_transcript_78045	NA	F01_transcript_78045	NA	F01_transcript_78045
F01_transcript_53220	NA	NA	NA	F01_transcript_53220
F01_transcript_87985	NA	NA	NA	F01_transcript_87985
F01_transcript_152627	NA	F01_transcript_152627	NA	NA
F01_transcript_102006	NA	F01_transcript_102006	NA	NA
F01_transcript_97617	NA	NA	F01_transcript_97617	F01_transcript_97617

F01_transcript_143987	F01_transcript_143987	F01_transcript_143987	F01_transcript_143987	F01_transcript_143987
F01_transcript_79169	NA	NA	NA	F01_transcript_79169
F01_transcript_166847	NA	NA	NA	F01_transcript_166847
F01_transcript_130540	NA	NA	NA	F01_transcript_130540
F01_transcript_95340	NA	NA	NA	F01_transcript_95340
F01_transcript_132905	NA	NA	NA	F01_transcript_132905
F01_transcript_9560	NA	NA	NA	F01_transcript_9560
F01_transcript_456	NA	NA	NA	F01_transcript_456
F01_transcript_148752	F01_transcript_148752	F01_transcript_148752	NA	F01_transcript_148752
F01_transcript_78448	F01_transcript_78448	F01_transcript_78448	F01_transcript_78448	F01_transcript_78448
F01_transcript_159614	NA	NA	NA	F01_transcript_159614
F01_transcript_114091	NA	F01_transcript_114091	NA	F01_transcript_114091
F01_transcript_42581	NA	NA	NA	F01_transcript_42581
F01_transcript_103484	NA	F01_transcript_103484	NA	F01_transcript_103484

F01_transcript_147664	F01_transcript_147664	F01_transcript_147664	F01_transcript_147664	F01_transcript_147664
F01_transcript_141237	F01_transcript_141237	F01_transcript_141237	NA	NA
F01_transcript_86129	F01_transcript_86129	F01_transcript_86129	F01_transcript_86129	F01_transcript_86129
F01_transcript_164784	NA	NA	NA	F01_transcript_164784
F01_transcript_70136	F01_transcript_70136	F01_transcript_70136	F01_transcript_70136	NA
F01_transcript_864	F01_transcript_864	F01_transcript_864	F01_transcript_864	F01_transcript_864
F01_transcript_3514	NA	F01_transcript_3514	F01_transcript_3514	F01_transcript_3514
F01_transcript_100327	NA	NA	NA	F01_transcript_100327
F01_transcript_72929	NA	F01_transcript_72929	NA	F01_transcript_72929
F01_transcript_31605	NA	NA	NA	F01_transcript_31605
F01_transcript_95791	NA	NA	NA	F01_transcript_95791
F01_transcript_92326	F01_transcript_92326	F01_transcript_92326	F01_transcript_92326	F01_transcript_92326
F01_transcript_103829	NA	NA	NA	F01_transcript_103829
F01_transcript_69575	NA	F01_transcript_69575	NA	F01_transcript_69575

F01_transcript_119416	F01_transcript_119416	F01_transcript_119416	F01_transcript_119416	F01_transcript_119416
F01_transcript_49093	NA	NA	NA	F01_transcript_49093
F01_transcript_158901	NA	F01_transcript_158901	F01_transcript_158901	F01_transcript_158901
F01_transcript_63027	NA	NA	NA	F01_transcript_63027
F01_transcript_14509	F01_transcript_14509	F01_transcript_14509	NA	NA
F01_transcript_96905	NA	NA	NA	F01_transcript_96905
F01_transcript_151118	F01_transcript_151118	F01_transcript_151118	F01_transcript_151118	F01_transcript_151118
F01_transcript_143989	NA	F01_transcript_143989	NA	NA
F01_transcript_48913	NA	NA	NA	F01_transcript_48913
F01_transcript_136175	NA	NA	NA	F01_transcript_136175
F01_transcript_120394	NA	NA	NA	F01_transcript_120394
F01_transcript_61594	NA	F01_transcript_61594	F01_transcript_61594	F01_transcript_61594
F01_transcript_94265	NA	NA	NA	F01_transcript_94265
F01_transcript_114536	NA	F01_transcript_114536	NA	F01_transcript_114536

F01_transcript_3341	NA	NA	NA	F01_transcript_3341
F01_transcript_77436	NA	NA	F01_transcript_77436	NA
F01_transcript_124285	F01_transcript_124285	F01_transcript_124285	NA	NA
F01_transcript_70171	F01_transcript_70171	F01_transcript_70171	F01_transcript_70171	F01_transcript_70171
F01_transcript_57650	NA	NA	NA	F01_transcript_57650
F01_transcript_67378	NA	NA	NA	F01_transcript_67378
F01_transcript_31954	NA	NA	NA	F01_transcript_31954
F01_transcript_9313	F01_transcript_9313	F01_transcript_9313	NA	F01_transcript_9313
F01_transcript_63195	NA	NA	NA	F01_transcript_63195
F01_transcript_109671	F01_transcript_109671	F01_transcript_109671	F01_transcript_109671	F01_transcript_109671
F01_transcript_87086	NA	F01_transcript_87086	NA	F01_transcript_87086
F01_transcript_62615	NA	F01_transcript_62615	NA	NA
F01_transcript_63011	NA	NA	NA	F01_transcript_63011
F01_transcript_73657	NA	NA	NA	F01_transcript_73657

F01_transcript_115956	NA	NA	NA	F01_transcript_115956
F01_transcript_23259	F01_transcript_23259	F01_transcript_23259	F01_transcript_23259	F01_transcript_23259
F01_transcript_154833	NA	NA	NA	F01_transcript_154833
F01_transcript_166479	F01_transcript_166479	F01_transcript_166479	NA	F01_transcript_166479
F01_transcript_144070	F01_transcript_144070	F01_transcript_144070	F01_transcript_144070	F01_transcript_144070
F01_transcript_128713	F01_transcript_128713	F01_transcript_128713	F01_transcript_128713	NA
F01_transcript_9788	NA	F01_transcript_9788	NA	F01_transcript_9788
F01_transcript_48229	NA	F01_transcript_48229	NA	F01_transcript_48229
F01_transcript_43236	NA	NA	NA	F01_transcript_43236
F01_transcript_157367	NA	NA	NA	F01_transcript_157367
F01_transcript_80706	NA	NA	NA	F01_transcript_80706
F01_transcript_94921	F01_transcript_94921	F01_transcript_94921	F01_transcript_94921	F01_transcript_94921
F01_transcript_90392	NA	NA	NA	F01_transcript_90392
F01_transcript_71817	NA	NA	NA	F01_transcript_71817

F01_transcript_108741	F01_transcript_108741	F01_transcript_108741	F01_transcript_108741	F01_transcript_108741
F01_transcript_77135	F01_transcript_77135	F01_transcript_77135	NA	F01_transcript_77135
F01_transcript_120339	NA	NA	NA	F01_transcript_120339
F01_transcript_106	NA	NA	NA	F01_transcript_106
F01_transcript_155086	F01_transcript_155086	F01_transcript_155086	F01_transcript_155086	NA
F01_transcript_141549	F01_transcript_141549	F01_transcript_141549	F01_transcript_141549	F01_transcript_141549
F01_transcript_110437	NA	F01_transcript_110437	NA	F01_transcript_110437
F01_transcript_14786	NA	NA	NA	F01_transcript_14786
F01_transcript_49155	F01_transcript_49155	NA	F01_transcript_49155	F01_transcript_49155
F01_transcript_118467	F01_transcript_118467	F01_transcript_118467	F01_transcript_118467	F01_transcript_118467
F01_transcript_95843	NA	F01_transcript_95843	NA	NA
F01_transcript_109918	NA	NA	NA	F01_transcript_109918
F01_transcript_72050	NA	F01_transcript_72050	F01_transcript_72050	F01_transcript_72050
F01_transcript_158368	F01_transcript_158368	F01_transcript_158368	F01_transcript_158368	F01_transcript_158368

F01_transcript_142122	NA	F01_transcript_142122	NA	NA
F01_transcript_138948	F01_transcript_138948	F01_transcript_138948	NA	NA
F01_transcript_69485	NA	F01_transcript_69485	F01_transcript_69485	NA
F01_transcript_131055	F01_transcript_131055	F01_transcript_131055	NA	F01_transcript_131055
F01_transcript_31741	NA	NA	NA	F01_transcript_31741
F01_transcript_41131	NA	F01_transcript_41131	NA	NA
F01_transcript_123053	F01_transcript_123053	F01_transcript_123053	F01_transcript_123053	F01_transcript_123053
F01_transcript_82684	NA	NA	NA	F01_transcript_82684
F01_transcript_133010	NA	NA	NA	F01_transcript_133010
F01_transcript_75914	NA	F01_transcript_75914	NA	F01_transcript_75914
F01_transcript_149211	NA	NA	NA	F01_transcript_149211
F01_transcript_113008	F01_transcript_113008	F01_transcript_113008	F01_transcript_113008	F01_transcript_113008
F01_transcript_113135	NA	NA	NA	F01_transcript_113135
F01_transcript_19474	NA	NA	NA	F01_transcript_19474

F01_transcript_148625	NA	NA	F01_transcript_148625	F01_transcript_148625
F01_transcript_108610	F01_transcript_108610	F01_transcript_108610	NA	NA
F01_transcript_76330	F01_transcript_76330	F01_transcript_76330	F01_transcript_76330	F01_transcript_76330
F01_transcript_102526	NA	NA	NA	F01_transcript_102526
F01_transcript_79322	F01_transcript_79322	F01_transcript_79322	F01_transcript_79322	F01_transcript_79322
F01_transcript_156951	NA	NA	NA	F01_transcript_156951
F01_transcript_162912	F01_transcript_162912	F01_transcript_162912	NA	NA
F01_transcript_113983	NA	F01_transcript_113983	NA	NA
F01_transcript_80528	NA	NA	NA	F01_transcript_80528
F01_transcript_154480	NA	F01_transcript_154480	F01_transcript_154480	F01_transcript_154480
F01_transcript_53496	NA	F01_transcript_53496	NA	F01_transcript_53496
F01_transcript_97296	NA	NA	F01_transcript_97296	F01_transcript_97296
F01_transcript_110275	NA	NA	NA	F01_transcript_110275
F01_transcript_130333	NA	F01_transcript_130333	NA	NA

F01_transcript_86123	NA	F01_transcript_86123	NA	F01_transcript_86123
F01_transcript_75299	NA	F01_transcript_75299	F01_transcript_75299	NA
F01_transcript_105522	NA	F01_transcript_105522	NA	NA
F01_transcript_137013	NA	NA	NA	F01_transcript_137013
F01_transcript_33202	NA	NA	NA	F01_transcript_33202
F01_transcript_32431	NA	NA	NA	F01_transcript_32431
F01_transcript_165486	NA	F01_transcript_165486	NA	NA
F01_transcript_129856	NA	NA	NA	F01_transcript_129856
F01_transcript_90092	F01_transcript_90092	F01_transcript_90092	F01_transcript_90092	NA
F01_transcript_108105	F01_transcript_108105	F01_transcript_108105	F01_transcript_108105	F01_transcript_108105
F01_transcript_120833	NA	NA	NA	F01_transcript_120833
F01_transcript_53161	NA	F01_transcript_53161	NA	NA
F01_transcript_102561	NA	NA	NA	F01_transcript_102561
F01_transcript_105707	NA	NA	NA	F01_transcript_105707

F01_transcript_51775	NA	NA	NA	F01_transcript_51775
F01_transcript_55366	NA	NA	NA	F01_transcript_55366
F01_transcript_150809	F01_transcript_150809	F01_transcript_150809	F01_transcript_150809	F01_transcript_150809
F01_transcript_102255	NA	F01_transcript_102255	NA	F01_transcript_102255
F01_transcript_5698	NA	NA	F01_transcript_5698	NA
F01_transcript_12096	NA	NA	NA	F01_transcript_12096
F01_transcript_155248	NA	F01_transcript_155248	NA	NA
F01_transcript_156202	F01_transcript_156202	F01_transcript_156202	F01_transcript_156202	F01_transcript_156202
F01_transcript_150233	F01_transcript_150233	F01_transcript_150233	F01_transcript_150233	F01_transcript_150233
F01_transcript_4145	NA	NA	NA	F01_transcript_4145
F01_transcript_146569	NA	NA	NA	F01_transcript_146569
F01_transcript_144662	NA	NA	NA	F01_transcript_144662
F01_transcript_114546	F01_transcript_114546	F01_transcript_114546	F01_transcript_114546	F01_transcript_114546
F01_transcript_127873	F01_transcript_127873	F01_transcript_127873	NA	NA

F01_transcript_154295	NA	F01_transcript_154295	NA	F01_transcript_154295
F01_transcript_57427	NA	F01_transcript_57427	F01_transcript_57427	F01_transcript_57427
F01_transcript_115898	NA	NA	NA	F01_transcript_115898
F01_transcript_104592	NA	F01_transcript_104592	NA	NA
F01_transcript_106108	F01_transcript_106108	F01_transcript_106108	F01_transcript_106108	F01_transcript_106108
F01_transcript_118071	NA	F01_transcript_118071	F01_transcript_118071	NA
F01_transcript_115982	NA	NA	NA	F01_transcript_115982
F01_transcript_49303	NA	F01_transcript_49303	F01_transcript_49303	NA
F01_transcript_21046	F01_transcript_21046	F01_transcript_21046	F01_transcript_21046	F01_transcript_21046
F01_transcript_55285	NA	F01_transcript_55285	NA	NA
F01_transcript_165072	NA	NA	NA	F01_transcript_165072
F01_transcript_2586	NA	NA	NA	F01_transcript_2586
F01_transcript_73103	F01_transcript_73103	F01_transcript_73103	NA	F01_transcript_73103
F01_transcript_147546	NA	F01_transcript_147546	NA	NA

F01_transcript_37352	NA	NA	NA	F01_transcript_37352
F01_transcript_79247	NA	NA	NA	F01_transcript_79247
F01_transcript_125804	NA	F01_transcript_125804	NA	NA
F01_transcript_48445	NA	NA	F01_transcript_48445	F01_transcript_48445
F01_transcript_62000	NA	F01_transcript_62000	NA	F01_transcript_62000
F01_transcript_67059	NA	NA	NA	F01_transcript_67059
F01_transcript_139540	F01_transcript_139540	F01_transcript_139540	F01_transcript_139540	F01_transcript_139540
F01_transcript_40947	NA	NA	NA	F01_transcript_40947
F01_transcript_65977	NA	F01_transcript_65977	NA	NA
F01_transcript_49230	NA	NA	NA	F01_transcript_49230
F01_transcript_88541	NA	F01_transcript_88541	NA	NA
F01_transcript_165750	NA	NA	NA	F01_transcript_165750
F01_transcript_157277	F01_transcript_157277	F01_transcript_157277	F01_transcript_157277	F01_transcript_157277
F01_transcript_43761	NA	NA	NA	F01_transcript_43761

F01_transcript_136013	NA	NA	F01_transcript_136013	F01_transcript_136013
F01_transcript_158633	NA	F01_transcript_158633	NA	F01_transcript_158633
F01_transcript_9936	NA	F01_transcript_9936	NA	F01_transcript_9936
F01_transcript_77296	NA	NA	NA	F01_transcript_77296
F01_transcript_39011	NA	NA	NA	F01_transcript_39011
F01_transcript_137176	NA	F01_transcript_137176	NA	NA
F01_transcript_130395	NA	F01_transcript_130395	NA	NA
F01_transcript_129285	NA	NA	NA	F01_transcript_129285
F01_transcript_107344	F01_transcript_107344	F01_transcript_107344	F01_transcript_107344	F01_transcript_107344
F01_transcript_162674	NA	F01_transcript_162674	F01_transcript_162674	NA
F01_transcript_4973	NA	NA	NA	F01_transcript_4973
F01_transcript_16991	NA	F01_transcript_16991	F01_transcript_16991	F01_transcript_16991
F01_transcript_141632	NA	NA	NA	F01_transcript_141632
F01_transcript_148958	F01_transcript_148958	F01_transcript_148958	F01_transcript_148958	NA

F01_transcript_126036	NA	NA	NA	F01_transcript_126036
F01_transcript_53679	F01_transcript_53679	F01_transcript_53679	F01_transcript_53679	NA
F01_transcript_89600	NA	F01_transcript_89600	NA	NA
F01_transcript_84392	NA	F01_transcript_84392	F01_transcript_84392	NA
F01_transcript_48308	NA	F01_transcript_48308	NA	NA
F01_transcript_149039	F01_transcript_149039	F01_transcript_149039	F01_transcript_149039	F01_transcript_149039
F01_transcript_130707	F01_transcript_130707	F01_transcript_130707	NA	F01_transcript_130707
F01_transcript_132944	NA	F01_transcript_132944	F01_transcript_132944	F01_transcript_132944
F01_transcript_66770	F01_transcript_66770	F01_transcript_66770	F01_transcript_66770	F01_transcript_66770
F01_transcript_126727	F01_transcript_126727	F01_transcript_126727	F01_transcript_126727	F01_transcript_126727
F01_transcript_104578	NA	F01_transcript_104578	NA	F01_transcript_104578
F01_transcript_72986	NA	F01_transcript_72986	F01_transcript_72986	F01_transcript_72986
F01_transcript_111	NA	NA	NA	F01_transcript_111
F01_transcript_75933	NA	NA	NA	F01_transcript_75933

F01_transcript_166117	NA	NA	NA	F01_transcript_166117
F01_transcript_96333	NA	F01_transcript_96333	F01_transcript_96333	F01_transcript_96333
F01_transcript_150581	NA	F01_transcript_150581	NA	F01_transcript_150581
F01_transcript_122198	NA	F01_transcript_122198	NA	F01_transcript_122198
F01_transcript_58237	NA	F01_transcript_58237	NA	F01_transcript_58237
F01_transcript_85652	NA	NA	NA	F01_transcript_85652
F01_transcript_14281	F01_transcript_14281	F01_transcript_14281	F01_transcript_14281	F01_transcript_14281
F01_transcript_116662	F01_transcript_116662	F01_transcript_116662	F01_transcript_116662	F01_transcript_116662
F01_transcript_155256	NA	F01_transcript_155256	NA	NA
F01_transcript_93079	NA	F01_transcript_93079	NA	NA
F01_transcript_8287	NA	F01_transcript_8287	NA	NA
F01_transcript_83214	NA	F01_transcript_83214	F01_transcript_83214	F01_transcript_83214
F01_transcript_145834	NA	F01_transcript_145834	NA	NA
F01_transcript_52437	NA	NA	NA	F01_transcript_52437

F01_transcript_75295	NA	F01_transcript_75295	NA	NA
F01_transcript_118625	NA	F01_transcript_118625	F01_transcript_118625	F01_transcript_118625
F01_transcript_39237	NA	NA	NA	F01_transcript_39237
F01_transcript_150149	F01_transcript_150149	F01_transcript_150149	F01_transcript_150149	F01_transcript_150149
F01_transcript_18442	NA	NA	NA	F01_transcript_18442
F01_transcript_1210	NA	NA	NA	F01_transcript_1210
F01_transcript_79245	NA	NA	NA	F01_transcript_79245
F01_transcript_18302	NA	F01_transcript_18302	NA	NA
F01_transcript_164027	NA	F01_transcript_164027	NA	F01_transcript_164027
F01_transcript_129481	NA	F01_transcript_129481	NA	F01_transcript_129481
F01_transcript_124810	NA	NA	F01_transcript_124810	F01_transcript_124810
F01_transcript_144165	NA	F01_transcript_144165	NA	NA
F01_transcript_93659	NA	NA	NA	F01_transcript_93659
F01_transcript_112052	NA	NA	F01_transcript_112052	F01_transcript_112052

F01_transcript_147001	NA	F01_transcript_147001	NA	F01_transcript_147001
F01_transcript_82238	NA	NA	F01_transcript_82238	NA
F01_transcript_105137	NA	F01_transcript_105137	NA	NA
F01_transcript_44158	NA	F01_transcript_44158	NA	NA
F01_transcript_85280	NA	NA	NA	F01_transcript_85280
F01_transcript_11702	NA	NA	NA	F01_transcript_11702
F01_transcript_126165	NA	NA	NA	F01_transcript_126165
F01_transcript_108699	NA	NA	NA	F01_transcript_108699
F01_transcript_135054	NA	F01_transcript_135054	NA	F01_transcript_135054
F01_transcript_57143	NA	NA	F01_transcript_57143	NA
F01_transcript_91397	NA	F01_transcript_91397	F01_transcript_91397	F01_transcript_91397
F01_transcript_113015	NA	NA	NA	F01_transcript_113015
F01_transcript_114733	F01_transcript_114733	F01_transcript_114733	F01_transcript_114733	NA
F01_transcript_135047	NA	F01_transcript_135047	NA	NA

F01_transcript_131855	NA	NA	NA	F01_transcript_131855
F01_transcript_94571	F01_transcript_94571	F01_transcript_94571	NA	NA
F01_transcript_94751	F01_transcript_94751	F01_transcript_94751	F01_transcript_94751	F01_transcript_94751
F01_transcript_144034	NA	NA	NA	F01_transcript_144034
F01_transcript_145292	NA	NA	NA	F01_transcript_145292
F01_transcript_165978	NA	NA	NA	F01_transcript_165978
F01_transcript_123655	NA	NA	NA	F01_transcript_123655
F01_transcript_131622	NA	NA	NA	F01_transcript_131622
F01_transcript_102131	NA	F01_transcript_102131	NA	F01_transcript_102131
F01_transcript_93931	F01_transcript_93931	F01_transcript_93931	F01_transcript_93931	F01_transcript_93931
F01_transcript_139386	NA	F01_transcript_139386	NA	NA
F01_transcript_106044	NA	F01_transcript_106044	NA	NA
F01_transcript_115685	NA	F01_transcript_115685	NA	F01_transcript_115685
F01_transcript_97879	F01_transcript_97879	NA	NA	F01_transcript_97879

F01_transcript_107999	NA	NA	NA	F01_transcript_107999
F01_transcript_158307	NA	NA	NA	F01_transcript_158307
F01_transcript_134473	F01_transcript_134473	F01_transcript_134473	NA	NA
F01_transcript_122262	NA	F01_transcript_122262	NA	NA
F01_transcript_163272	NA	NA	F01_transcript_163272	F01_transcript_163272
F01_transcript_166940	F01_transcript_166940	F01_transcript_166940	F01_transcript_166940	F01_transcript_166940
F01_transcript_73553	F01_transcript_73553	F01_transcript_73553	F01_transcript_73553	F01_transcript_73553
F01_transcript_133853	NA	F01_transcript_133853	NA	F01_transcript_133853
F01_transcript_81217	F01_transcript_81217	F01_transcript_81217	NA	NA
F01_transcript_113324	NA	F01_transcript_113324	NA	NA
F01_transcript_82710	NA	F01_transcript_82710	F01_transcript_82710	NA
F01_transcript_155234	NA	NA	NA	F01_transcript_155234
F01_transcript_93708	NA	NA	NA	F01_transcript_93708
F01_transcript_16678	NA	NA	NA	F01_transcript_16678

F01_transcript_108528	F01_transcript_108528	F01_transcript_108528	F01_transcript_108528	F01_transcript_108528
F01_transcript_51274	F01_transcript_51274	F01_transcript_51274	F01_transcript_51274	F01_transcript_51274
F01_transcript_101441	NA	F01_transcript_101441	NA	NA
F01_transcript_68685	NA	F01_transcript_68685	F01_transcript_68685	F01_transcript_68685
F01_transcript_87226	NA	NA	F01_transcript_87226	F01_transcript_87226
F01_transcript_54225	F01_transcript_54225	F01_transcript_54225	F01_transcript_54225	F01_transcript_54225
F01_transcript_121911	NA	NA	NA	F01_transcript_121911
F01_transcript_117786	F01_transcript_117786	F01_transcript_117786	F01_transcript_117786	F01_transcript_117786
F01_transcript_77926	NA	NA	NA	F01_transcript_77926
F01_transcript_93036	NA	F01_transcript_93036	NA	F01_transcript_93036
F01_transcript_67825	NA	F01_transcript_67825	NA	NA
F01_transcript_156165	F01_transcript_156165	F01_transcript_156165	NA	F01_transcript_156165
F01_transcript_50385	NA	F01_transcript_50385	NA	NA
F01_transcript_64235	NA	F01_transcript_64235	NA	NA

F01_transcript_19467	NA	NA	NA	F01_transcript_19467
F01_transcript_128957	NA	F01_transcript_128957	NA	F01_transcript_128957
F01_transcript_59027	NA	F01_transcript_59027	F01_transcript_59027	NA
F01_transcript_125908	F01_transcript_125908	F01_transcript_125908	NA	F01_transcript_125908
F01_transcript_73545	F01_transcript_73545	F01_transcript_73545	F01_transcript_73545	F01_transcript_73545
F01_transcript_133090	NA	F01_transcript_133090	NA	NA
F01_transcript_89284	NA	F01_transcript_89284	NA	F01_transcript_89284
F01_transcript_94728	NA	NA	NA	F01_transcript_94728
F01_transcript_155945	NA	F01_transcript_155945	NA	NA
F01_transcript_99062	F01_transcript_99062	F01_transcript_99062	F01_transcript_99062	NA
F01_transcript_65833	NA	NA	NA	F01_transcript_65833
F01_transcript_151194	F01_transcript_151194	F01_transcript_151194	F01_transcript_151194	F01_transcript_151194
F01_transcript_152988	F01_transcript_152988	F01_transcript_152988	NA	NA
F01_transcript_101295	NA	F01_transcript_101295	NA	F01_transcript_101295

F01_transcript_61397	NA	NA	NA	F01_transcript_61397
F01_transcript_166795	NA	NA	NA	F01_transcript_166795
F01_transcript_50907	F01_transcript_50907	F01_transcript_50907	F01_transcript_50907	F01_transcript_50907
F01_transcript_57424	F01_transcript_57424	F01_transcript_57424	F01_transcript_57424	F01_transcript_57424
F01_transcript_56268	NA	NA	F01_transcript_56268	NA
F01_transcript_108255	NA	F01_transcript_108255	NA	NA
F01_transcript_86348	NA	F01_transcript_86348	NA	NA
F01_transcript_149287	NA	F01_transcript_149287	NA	NA
F01_transcript_101489	NA	F01_transcript_101489	NA	F01_transcript_101489
F01_transcript_146136	NA	F01_transcript_146136	NA	NA
F01_transcript_102879	F01_transcript_102879	F01_transcript_102879	F01_transcript_102879	F01_transcript_102879
F01_transcript_155300	F01_transcript_155300	F01_transcript_155300	F01_transcript_155300	F01_transcript_155300
F01_transcript_68553	F01_transcript_68553	F01_transcript_68553	F01_transcript_68553	F01_transcript_68553
F01_transcript_139105	NA	F01_transcript_139105	NA	NA

F01_transcript_86395	NA	NA	NA	F01_transcript_86395
F01_transcript_73782	NA	NA	NA	F01_transcript_73782
F01_transcript_161088	F01_transcript_161088	F01_transcript_161088	NA	F01_transcript_161088
F01_transcript_151727	F01_transcript_151727	F01_transcript_151727	F01_transcript_151727	F01_transcript_151727
F01_transcript_16772	NA	NA	NA	F01_transcript_16772
F01_transcript_78409	F01_transcript_78409	F01_transcript_78409	F01_transcript_78409	F01_transcript_78409
F01_transcript_124453	NA	F01_transcript_124453	NA	NA
F01_transcript_111344	NA	F01_transcript_111344	NA	NA
F01_transcript_62285	NA	NA	NA	F01_transcript_62285
F01_transcript_110439	NA	NA	NA	F01_transcript_110439
F01_transcript_166790	NA	NA	NA	F01_transcript_166790
F01_transcript_143373	NA	NA	NA	F01_transcript_143373
F01_transcript_58426	NA	F01_transcript_58426	F01_transcript_58426	NA
F01_transcript_152644	NA	F01_transcript_152644	NA	NA

F01_transcript_82019	F01_transcript_82019	F01_transcript_82019	F01_transcript_82019	F01_transcript_82019
F01_transcript_149887	F01_transcript_149887	F01_transcript_149887	F01_transcript_149887	F01_transcript_149887
F01_transcript_113954	NA	F01_transcript_113954	NA	F01_transcript_113954
F01_transcript_47690	NA	NA	NA	F01_transcript_47690
F01_transcript_147017	F01_transcript_147017	F01_transcript_147017	F01_transcript_147017	NA
F01_transcript_158370	NA	NA	F01_transcript_158370	NA
F01_transcript_91158	F01_transcript_91158	F01_transcript_91158	NA	F01_transcript_91158
F01_transcript_29364	NA	NA	NA	F01_transcript_29364
F01_transcript_140497	F01_transcript_140497	F01_transcript_140497	F01_transcript_140497	F01_transcript_140497
F01_transcript_109381	NA	NA	NA	F01_transcript_109381
F01_transcript_143905	NA	F01_transcript_143905	F01_transcript_143905	F01_transcript_143905
F01_transcript_143048	NA	F01_transcript_143048	NA	NA
F01_transcript_128759	NA	F01_transcript_128759	NA	NA
F01_transcript_70629	F01_transcript_70629	F01_transcript_70629	NA	F01_transcript_70629

F01_transcript_117745	NA	F01_transcript_117745	NA	F01_transcript_117745
F01_transcript_110386	NA	F01_transcript_110386	NA	F01_transcript_110386
F01_transcript_82037	NA	NA	F01_transcript_82037	F01_transcript_82037
F01_transcript_87031	NA	F01_transcript_87031	NA	NA
F01_transcript_137679	NA	F01_transcript_137679	NA	NA
F01_transcript_122566	F01_transcript_122566	F01_transcript_122566	F01_transcript_122566	F01_transcript_122566
F01_transcript_56024	NA	NA	NA	F01_transcript_56024
F01_transcript_102625	NA	NA	NA	F01_transcript_102625
F01_transcript_23608	NA	NA	NA	F01_transcript_23608
F01_transcript_60067	NA	NA	NA	F01_transcript_60067
F01_transcript_121304	NA	F01_transcript_121304	NA	F01_transcript_121304
F01_transcript_162747	F01_transcript_162747	NA	NA	NA
F01_transcript_76570	F01_transcript_76570	F01_transcript_76570	F01_transcript_76570	F01_transcript_76570
F01_transcript_109394	NA	F01_transcript_109394	NA	F01_transcript_109394

F01_transcript_9259	NA	NA	NA	F01_transcript_9259
F01_transcript_128962	NA	NA	NA	F01_transcript_128962
F01_transcript_70258	NA	F01_transcript_70258	F01_transcript_70258	F01_transcript_70258
F01_transcript_151135	F01_transcript_151135	F01_transcript_151135	F01_transcript_151135	F01_transcript_151135
F01_transcript_137583	NA	NA	NA	F01_transcript_137583
F01_transcript_85990	NA	F01_transcript_85990	NA	NA
F01_transcript_83843	NA	F01_transcript_83843	F01_transcript_83843	F01_transcript_83843
F01_transcript_112320	F01_transcript_112320	F01_transcript_112320	NA	F01_transcript_112320
F01_transcript_141567	NA	NA	NA	F01_transcript_141567
F01_transcript_101097	NA	F01_transcript_101097	NA	NA
F01_transcript_113770	NA	NA	NA	F01_transcript_113770
F01_transcript_64653	F01_transcript_64653	F01_transcript_64653	F01_transcript_64653	F01_transcript_64653
F01_transcript_97222	F01_transcript_97222	F01_transcript_97222	NA	F01_transcript_97222
F01_transcript_10331	NA	NA	NA	F01_transcript_10331

F01_transcript_66232	F01_transcript_66232	F01_transcript_66232	F01_transcript_66232	F01_transcript_66232
F01_transcript_67009	NA	NA	NA	F01_transcript_67009
F01_transcript_56904	F01_transcript_56904	F01_transcript_56904	F01_transcript_56904	F01_transcript_56904
F01_transcript_90631	NA	F01_transcript_90631	F01_transcript_90631	F01_transcript_90631
F01_transcript_117324	NA	NA	NA	F01_transcript_117324
F01_transcript_7424	NA	NA	NA	F01_transcript_7424
F01_transcript_137817	NA	F01_transcript_137817	NA	F01_transcript_137817
F01_transcript_113078	NA	F01_transcript_113078	F01_transcript_113078	F01_transcript_113078
F01_transcript_66655	NA	NA	F01_transcript_66655	NA
F01_transcript_68284	F01_transcript_68284	NA	NA	NA
F01_transcript_117344	NA	NA	NA	F01_transcript_117344
F01_transcript_148325	NA	NA	NA	F01_transcript_148325
F01_transcript_7725	F01_transcript_7725	F01_transcript_7725	NA	NA
F01_transcript_19471	NA	F01_transcript_19471	NA	F01_transcript_19471

F01_transcript_51697	NA	NA	NA	F01_transcript_51697
F01_transcript_134920	NA	F01_transcript_134920	NA	F01_transcript_134920
F01_transcript_90666	F01_transcript_90666	F01_transcript_90666	F01_transcript_90666	F01_transcript_90666
F01_transcript_50290	NA	F01_transcript_50290	F01_transcript_50290	F01_transcript_50290
F01_transcript_51182	F01_transcript_51182	F01_transcript_51182	F01_transcript_51182	F01_transcript_51182
F01_transcript_47326	NA	NA	NA	F01_transcript_47326
F01_transcript_56804	F01_transcript_56804	F01_transcript_56804	F01_transcript_56804	F01_transcript_56804
F01_transcript_70788	F01_transcript_70788	F01_transcript_70788	F01_transcript_70788	F01_transcript_70788
F01_transcript_101018	NA	F01_transcript_101018	NA	NA
F01_transcript_64600	NA	F01_transcript_64600	NA	NA
F01_transcript_157586	NA	NA	NA	F01_transcript_157586
F01_transcript_49267	NA	F01_transcript_49267	NA	NA
F01_transcript_153335	NA	F01_transcript_153335	NA	F01_transcript_153335
F01_transcript_131826	NA	NA	NA	F01_transcript_131826

F01_transcript_64906	NA	F01_transcript_64906	NA	NA
F01_transcript_162996	NA	NA	NA	F01_transcript_162996
F01_transcript_130901	NA	NA	NA	F01_transcript_130901
F01_transcript_22976	NA	NA	NA	F01_transcript_22976
F01_transcript_71328	NA	NA	NA	F01_transcript_71328
F01_transcript_91116	NA	NA	NA	F01_transcript_91116
F01_transcript_64607	NA	F01_transcript_64607	F01_transcript_64607	F01_transcript_64607
F01_transcript_159007	NA	NA	F01_transcript_159007	F01_transcript_159007
F01_transcript_85077	NA	NA	NA	F01_transcript_85077
F01_transcript_98047	F01_transcript_98047	F01_transcript_98047	NA	F01_transcript_98047
F01_transcript_46723	NA	NA	F01_transcript_46723	F01_transcript_46723
F01_transcript_73533	F01_transcript_73533	F01_transcript_73533	F01_transcript_73533	F01_transcript_73533
F01_transcript_54734	NA	NA	F01_transcript_54734	NA
F01_transcript_7533	NA	NA	NA	F01_transcript_7533

F01_transcript_109601	NA	NA	NA	F01_transcript_109601
F01_transcript_105339	F01_transcript_105339	F01_transcript_105339	F01_transcript_105339	F01_transcript_105339
F01_transcript_27155	NA	F01_transcript_27155	NA	F01_transcript_27155
F01_transcript_83045	NA	NA	NA	F01_transcript_83045
F01_transcript_149728	F01_transcript_149728	F01_transcript_149728	F01_transcript_149728	F01_transcript_149728
F01_transcript_153070	NA	NA	NA	F01_transcript_153070
F01_transcript_133464	F01_transcript_133464	F01_transcript_133464	NA	NA
F01_transcript_69911	NA	F01_transcript_69911	NA	NA
F01_transcript_48317	NA	F01_transcript_48317	NA	F01_transcript_48317
F01_transcript_60120	NA	F01_transcript_60120	F01_transcript_60120	F01_transcript_60120
F01_transcript_161390	F01_transcript_161390	F01_transcript_161390	F01_transcript_161390	F01_transcript_161390
F01_transcript_61544	NA	NA	NA	F01_transcript_61544
F01_transcript_118291	NA	NA	NA	F01_transcript_118291
F01_transcript_47196	NA	NA	NA	F01_transcript_47196

F01_transcript_15653	NA	NA	NA	F01_transcript_15653
F01_transcript_97249	F01_transcript_97249	F01_transcript_97249	NA	NA
F01_transcript_163228	NA	F01_transcript_163228	NA	F01_transcript_163228
F01_transcript_65301	NA	NA	NA	F01_transcript_65301
F01_transcript_160192	NA	F01_transcript_160192	NA	F01_transcript_160192
F01_transcript_61111	NA	F01_transcript_61111	F01_transcript_61111	F01_transcript_61111
F01_transcript_145866	NA	F01_transcript_145866	F01_transcript_145866	F01_transcript_145866
F01_transcript_29355	F01_transcript_29355	F01_transcript_29355	F01_transcript_29355	NA
F01_transcript_164525	F01_transcript_164525	F01_transcript_164525	NA	NA
F01_transcript_91768	NA	NA	NA	F01_transcript_91768
F01_transcript_138783	NA	F01_transcript_138783	NA	NA
F01_transcript_76217	NA	F01_transcript_76217	NA	NA
F01_transcript_121390	NA	NA	NA	F01_transcript_121390
F01_transcript_21243	NA	NA	NA	F01_transcript_21243

F01_transcript_158328	F01_transcript_158328	F01_transcript_158328	F01_transcript_158328	NA
F01_transcript_121343	NA	NA	NA	F01_transcript_121343
F01_transcript_89774	NA	F01_transcript_89774	NA	F01_transcript_89774
F01_transcript_38566	NA	NA	NA	F01_transcript_38566
F01_transcript_154116	NA	F01_transcript_154116	NA	NA
F01_transcript_134647	NA	NA	NA	F01_transcript_134647
F01_transcript_134769	NA	NA	F01_transcript_134769	F01_transcript_134769
F01_transcript_134487	NA	NA	NA	F01_transcript_134487
F01_transcript_17043	NA	NA	NA	F01_transcript_17043
F01_transcript_47854	F01_transcript_47854	F01_transcript_47854	F01_transcript_47854	F01_transcript_47854
F01_transcript_59456	NA	F01_transcript_59456	F01_transcript_59456	F01_transcript_59456
F01_transcript_103300	F01_transcript_103300	F01_transcript_103300	F01_transcript_103300	F01_transcript_103300
F01_transcript_109600	NA	NA	NA	F01_transcript_109600
F01_transcript_139901	NA	NA	NA	F01_transcript_139901

F01_transcript_55335	NA	F01_transcript_55335	NA	NA
F01_transcript_155712	NA	NA	NA	F01_transcript_155712
F01_transcript_63992	NA	F01_transcript_63992	NA	NA
F01_transcript_72679	F01_transcript_72679	F01_transcript_72679	NA	NA
F01_transcript_38071	NA	NA	NA	F01_transcript_38071
F01_transcript_60416	NA	F01_transcript_60416	NA	F01_transcript_60416
F01_transcript_147898	NA	F01_transcript_147898	NA	NA
F01_transcript_128952	NA	NA	NA	F01_transcript_128952
F01_transcript_98657	NA	NA	NA	F01_transcript_98657
F01_transcript_58140	F01_transcript_58140	F01_transcript_58140	F01_transcript_58140	F01_transcript_58140
F01_transcript_56826	NA	NA	F01_transcript_56826	NA
F01_transcript_151984	F01_transcript_151984	F01_transcript_151984	F01_transcript_151984	F01_transcript_151984
F01_transcript_35089	NA	NA	NA	F01_transcript_35089
F01_transcript_40559	NA	NA	NA	F01_transcript_40559

F01_transcript_10650	NA	NA	NA	F01_transcript_10650
F01_transcript_99800	NA	F01_transcript_99800	NA	F01_transcript_99800
F01_transcript_157548	NA	F01_transcript_157548	F01_transcript_157548	NA
F01_transcript_74931	NA	F01_transcript_74931	NA	NA
F01_transcript_68759	NA	F01_transcript_68759	F01_transcript_68759	F01_transcript_68759
F01_transcript_71032	NA	NA	NA	F01_transcript_71032
F01_transcript_134682	F01_transcript_134682	F01_transcript_134682	F01_transcript_134682	F01_transcript_134682
F01_transcript_90006	NA	NA	NA	F01_transcript_90006
F01_transcript_51221	F01_transcript_51221	F01_transcript_51221	F01_transcript_51221	F01_transcript_51221
F01_transcript_120099	NA	NA	NA	F01_transcript_120099
F01_transcript_85957	NA	NA	NA	F01_transcript_85957
F01_transcript_117065	NA	F01_transcript_117065	NA	NA
F01_transcript_113233	NA	F01_transcript_113233	NA	NA
F01_transcript_49107	NA	NA	NA	F01_transcript_49107

F01_transcript_115226	NA	F01_transcript_115226	F01_transcript_115226	F01_transcript_115226
F01_transcript_119877	F01_transcript_119877	F01_transcript_119877	F01_transcript_119877	F01_transcript_119877
F01_transcript_50575	F01_transcript_50575	NA	F01_transcript_50575	F01_transcript_50575
F01_transcript_102617	NA	F01_transcript_102617	NA	F01_transcript_102617
F01_transcript_80243	NA	F01_transcript_80243	NA	NA
F01_transcript_77279	NA	F01_transcript_77279	NA	NA
F01_transcript_158304	NA	NA	NA	F01_transcript_158304
F01_transcript_57314	F01_transcript_57314	F01_transcript_57314	F01_transcript_57314	F01_transcript_57314
F01_transcript_53873	NA	NA	NA	F01_transcript_53873
F01_transcript_165895	NA	NA	NA	F01_transcript_165895
F01_transcript_70780	NA	F01_transcript_70780	NA	F01_transcript_70780
F01_transcript_154053	NA	NA	NA	F01_transcript_154053
F01_transcript_147768	NA	F01_transcript_147768	NA	NA
F01_transcript_153263	NA	NA	NA	F01_transcript_153263

F01_transcript_10056	NA	F01_transcript_10056	NA	NA
F01_transcript_82158	F01_transcript_82158	F01_transcript_82158	F01_transcript_82158	F01_transcript_82158
F01_transcript_50203	F01_transcript_50203	F01_transcript_50203	F01_transcript_50203	F01_transcript_50203
F01_transcript_133682	NA	F01_transcript_133682	NA	F01_transcript_133682
F01_transcript_65321	F01_transcript_65321	F01_transcript_65321	F01_transcript_65321	F01_transcript_65321
F01_transcript_66448	NA	F01_transcript_66448	NA	NA
F01_transcript_142119	NA	F01_transcript_142119	NA	NA
F01_transcript_57773	NA	NA	NA	F01_transcript_57773
F01_transcript_52422	NA	F01_transcript_52422	F01_transcript_52422	F01_transcript_52422
F01_transcript_90901	NA	F01_transcript_90901	NA	NA
F01_transcript_104394	F01_transcript_104394	F01_transcript_104394	NA	F01_transcript_104394
F01_transcript_88173	NA	F01_transcript_88173	NA	NA
F01_transcript_144888	F01_transcript_144888	F01_transcript_144888	F01_transcript_144888	F01_transcript_144888
F01_transcript_48477	NA	F01_transcript_48477	NA	NA

F01_transcript_56034	NA	F01_transcript_56034	NA	NA
F01_transcript_95102	F01_transcript_95102	F01_transcript_95102	F01_transcript_95102	F01_transcript_95102
F01_transcript_67088	NA	NA	NA	F01_transcript_67088
F01_transcript_72833	F01_transcript_72833	F01_transcript_72833	F01_transcript_72833	F01_transcript_72833
F01_transcript_64227	NA	F01_transcript_64227	NA	NA
F01_transcript_44306	NA	NA	NA	F01_transcript_44306
F01_transcript_38449	NA	NA	NA	F01_transcript_38449
F01_transcript_135756	NA	NA	NA	F01_transcript_135756
F01_transcript_106834	F01_transcript_106834	F01_transcript_106834	F01_transcript_106834	F01_transcript_106834
F01_transcript_166390	NA	F01_transcript_166390	NA	NA
F01_transcript_124797	NA	F01_transcript_124797	NA	NA
F01_transcript_71351	F01_transcript_71351	F01_transcript_71351	F01_transcript_71351	F01_transcript_71351
F01_transcript_134659	NA	F01_transcript_134659	NA	NA
F01_transcript_61141	NA	NA	NA	F01_transcript_61141

F01_transcript_120322	NA	NA	NA	F01_transcript_120322
F01_transcript_92642	F01_transcript_92642	F01_transcript_92642	F01_transcript_92642	F01_transcript_92642
F01_transcript_141022	F01_transcript_141022	F01_transcript_141022	NA	NA
F01_transcript_57649	NA	F01_transcript_57649	NA	F01_transcript_57649
F01_transcript_130384	F01_transcript_130384	F01_transcript_130384	NA	F01_transcript_130384
F01_transcript_122720	NA	NA	NA	F01_transcript_122720
F01_transcript_121075	NA	NA	NA	F01_transcript_121075
F01_transcript_83246	NA	F01_transcript_83246	NA	F01_transcript_83246
F01_transcript_138379	NA	F01_transcript_138379	NA	NA
F01_transcript_88851	NA	F01_transcript_88851	NA	NA
F01_transcript_151557	NA	F01_transcript_151557	NA	NA
F01_transcript_98502	NA	F01_transcript_98502	NA	NA
F01_transcript_129593	F01_transcript_129593	F01_transcript_129593	F01_transcript_129593	F01_transcript_129593
F01_transcript_49330	NA	NA	F01_transcript_49330	F01_transcript_49330

F01_transcript_71578	NA	NA	NA	F01_transcript_71578
F01_transcript_113275	F01_transcript_113275	F01_transcript_113275	F01_transcript_113275	F01_transcript_113275
F01_transcript_94764	NA	F01_transcript_94764	NA	NA
F01_transcript_79241	NA	NA	NA	F01_transcript_79241
F01_transcript_126593	NA	NA	NA	F01_transcript_126593
F01_transcript_162382	F01_transcript_162382	F01_transcript_162382	NA	F01_transcript_162382
F01_transcript_67225	F01_transcript_67225	F01_transcript_67225	NA	NA
F01_transcript_68662	NA	F01_transcript_68662	NA	NA
F01_transcript_155101	F01_transcript_155101	F01_transcript_155101	F01_transcript_155101	F01_transcript_155101
F01_transcript_59309	NA	NA	F01_transcript_59309	F01_transcript_59309
F01_transcript_66979	NA	NA	NA	F01_transcript_66979
F01_transcript_129425	NA	F01_transcript_129425	NA	NA
F01_transcript_70737	NA	F01_transcript_70737	NA	NA
F01_transcript_112252	NA	NA	NA	F01_transcript_112252

F01_transcript_52886	NA	NA	NA	F01_transcript_52886
F01_transcript_81767	NA	F01_transcript_81767	NA	F01_transcript_81767
F01_transcript_121029	NA	NA	NA	F01_transcript_121029
F01_transcript_51764	NA	F01_transcript_51764	F01_transcript_51764	F01_transcript_51764
F01_transcript_59448	NA	NA	F01_transcript_59448	NA
F01_transcript_91094	NA	NA	NA	F01_transcript_91094
F01_transcript_56233	NA	NA	F01_transcript_56233	NA
F01_transcript_85261	F01_transcript_85261	F01_transcript_85261	F01_transcript_85261	F01_transcript_85261
F01_transcript_94085	NA	NA	NA	F01_transcript_94085
F01_transcript_121950	F01_transcript_121950	F01_transcript_121950	NA	NA
F01_transcript_14230	NA	F01_transcript_14230	NA	F01_transcript_14230
F01_transcript_127725	NA	NA	NA	F01_transcript_127725
F01_transcript_87581	NA	F01_transcript_87581	NA	F01_transcript_87581
F01_transcript_166321	NA	F01_transcript_166321	NA	NA

F01_transcript_152578	F01_transcript_152578	F01_transcript_152578	F01_transcript_152578	F01_transcript_152578
F01_transcript_140006	NA	NA	F01_transcript_140006	F01_transcript_140006
F01_transcript_94378	NA	F01_transcript_94378	NA	NA
F01_transcript_94831	NA	NA	NA	F01_transcript_94831
F01_transcript_50108	F01_transcript_50108	F01_transcript_50108	F01_transcript_50108	F01_transcript_50108
F01_transcript_158684	F01_transcript_158684	F01_transcript_158684	F01_transcript_158684	F01_transcript_158684
F01_transcript_88371	NA	NA	NA	F01_transcript_88371
F01_transcript_80844	NA	NA	NA	F01_transcript_80844
F01_transcript_18138	NA	F01_transcript_18138	NA	F01_transcript_18138
F01_transcript_143298	NA	NA	NA	F01_transcript_143298
F01_transcript_54605	NA	NA	NA	F01_transcript_54605
F01_transcript_119295	NA	F01_transcript_119295	NA	NA
F01_transcript_97261	F01_transcript_97261	F01_transcript_97261	F01_transcript_97261	F01_transcript_97261
F01_transcript_28164	NA	NA	NA	F01_transcript_28164

F01_transcript_83391	NA	NA	NA	F01_transcript_83391
F01_transcript_165829	NA	NA	NA	F01_transcript_165829
F01_transcript_70772	NA	NA	F01_transcript_70772	F01_transcript_70772
F01_transcript_90266	F01_transcript_90266	F01_transcript_90266	F01_transcript_90266	F01_transcript_90266
F01_transcript_99348	F01_transcript_99348	F01_transcript_99348	NA	NA
F01_transcript_65381	NA	F01_transcript_65381	F01_transcript_65381	F01_transcript_65381
F01_transcript_118080	NA	NA	NA	F01_transcript_118080
F01_transcript_111189	NA	F01_transcript_111189	F01_transcript_111189	F01_transcript_111189
F01_transcript_134834	NA	F01_transcript_134834	NA	F01_transcript_134834
F01_transcript_45803	NA	NA	NA	F01_transcript_45803
F01_transcript_112363	NA	F01_transcript_112363	NA	NA
F01_transcript_97148	NA	NA	NA	F01_transcript_97148
F01_transcript_145629	NA	F01_transcript_145629	NA	NA
F01_transcript_86819	F01_transcript_86819	F01_transcript_86819	F01_transcript_86819	F01_transcript_86819

F01_transcript_156207	NA	F01_transcript_156207	F01_transcript_156207	F01_transcript_156207
F01_transcript_10511	NA	NA	NA	F01_transcript_10511
F01_transcript_111650	F01_transcript_111650	F01_transcript_111650	NA	NA
F01_transcript_115848	NA	NA	NA	F01_transcript_115848
F01_transcript_125901	NA	NA	NA	F01_transcript_125901
F01_transcript_77515	F01_transcript_77515	F01_transcript_77515	NA	NA
F01_transcript_98842	NA	NA	NA	F01_transcript_98842
F01_transcript_125562	NA	NA	NA	F01_transcript_125562
F01_transcript_164164	F01_transcript_164164	F01_transcript_164164	NA	NA
F01_transcript_125979	NA	F01_transcript_125979	NA	F01_transcript_125979
F01_transcript_274	F01_transcript_274	F01_transcript_274	NA	F01_transcript_274
F01_transcript_11044	NA	NA	NA	F01_transcript_11044
F01_transcript_119739	NA	NA	NA	F01_transcript_119739
F01_transcript_104179	NA	NA	NA	F01_transcript_104179

F01_transcript_115662	NA	NA	NA	F01_transcript_115662
F01_transcript_92267	NA	NA	F01_transcript_92267	F01_transcript_92267
F01_transcript_109246	F01_transcript_109246	F01_transcript_109246	F01_transcript_109246	F01_transcript_109246
F01_transcript_69683	F01_transcript_69683	F01_transcript_69683	NA	NA
F01_transcript_98085	F01_transcript_98085	F01_transcript_98085	NA	F01_transcript_98085
F01_transcript_54162	F01_transcript_54162	F01_transcript_54162	F01_transcript_54162	NA
F01_transcript_122573	NA	NA	NA	F01_transcript_122573
F01_transcript_156574	NA	NA	NA	F01_transcript_156574
F01_transcript_36559	NA	NA	NA	F01_transcript_36559
F01_transcript_19387	NA	NA	NA	F01_transcript_19387
F01_transcript_50948	NA	F01_transcript_50948	NA	NA
F01_transcript_93503	NA	NA	NA	F01_transcript_93503
F01_transcript_157933	NA	F01_transcript_157933	NA	NA
F01_transcript_96168	NA	NA	NA	F01_transcript_96168

F01_transcript_113312	NA	F01_transcript_113312	NA	NA
F01_transcript_63360	F01_transcript_63360	F01_transcript_63360	F01_transcript_63360	F01_transcript_63360
F01_transcript_45093	NA	F01_transcript_45093	NA	NA
F01_transcript_58328	NA	NA	NA	F01_transcript_58328
F01_transcript_133547	NA	NA	NA	F01_transcript_133547
F01_transcript_72843	F01_transcript_72843	F01_transcript_72843	F01_transcript_72843	F01_transcript_72843
F01_transcript_100631	NA	NA	NA	F01_transcript_100631
F01_transcript_151814	NA	F01_transcript_151814	NA	NA
F01_transcript_79510	NA	NA	NA	F01_transcript_79510
F01_transcript_81166	F01_transcript_81166	F01_transcript_81166	F01_transcript_81166	F01_transcript_81166
F01_transcript_126642	NA	NA	NA	F01_transcript_126642
F01_transcript_135124	NA	NA	NA	F01_transcript_135124
F01_transcript_41254	NA	NA	NA	F01_transcript_41254
F01_transcript_135411	NA	NA	NA	F01_transcript_135411

F01_transcript_79672	NA	NA	NA	F01_transcript_79672
F01_transcript_114583	F01_transcript_114583	F01_transcript_114583	F01_transcript_114583	F01_transcript_114583
F01_transcript_156252	NA	F01_transcript_156252	NA	NA
F01_transcript_122527	NA	NA	NA	F01_transcript_122527
F01_transcript_80599	NA	NA	NA	F01_transcript_80599
F01_transcript_138312	F01_transcript_138312	F01_transcript_138312	F01_transcript_138312	F01_transcript_138312
F01_transcript_101847	F01_transcript_101847	F01_transcript_101847	F01_transcript_101847	NA
F01_transcript_85580	NA	F01_transcript_85580	NA	NA
F01_transcript_118144	NA	F01_transcript_118144	NA	F01_transcript_118144
F01_transcript_136642	NA	NA	NA	F01_transcript_136642
F01_transcript_49907	F01_transcript_49907	NA	NA	NA
F01_transcript_106870	NA	F01_transcript_106870	NA	NA
F01_transcript_3121	NA	NA	NA	F01_transcript_3121
F01_transcript_53135	NA	NA	NA	F01_transcript_53135

F01_transcript_125524	NA	NA	NA	F01_transcript_125524
F01_transcript_72126	NA	F01_transcript_72126	NA	NA
F01_transcript_146882	F01_transcript_146882	F01_transcript_146882	NA	F01_transcript_146882
F01_transcript_134265	NA	F01_transcript_134265	NA	NA
F01_transcript_90921	F01_transcript_90921	F01_transcript_90921	F01_transcript_90921	F01_transcript_90921
F01_transcript_73544	NA	F01_transcript_73544	NA	F01_transcript_73544
F01_transcript_117402	NA	F01_transcript_117402	NA	F01_transcript_117402
F01_transcript_149340	NA	F01_transcript_149340	NA	NA
F01_transcript_61165	NA	F01_transcript_61165	NA	NA
F01_transcript_7714	NA	NA	NA	F01_transcript_7714
F01_transcript_152400	NA	F01_transcript_152400	NA	NA
F01_transcript_14714	F01_transcript_14714	F01_transcript_14714	F01_transcript_14714	F01_transcript_14714
F01_transcript_71377	NA	F01_transcript_71377	NA	NA
F01_transcript_149990	NA	F01_transcript_149990	F01_transcript_149990	NA

F01_transcript_54954	NA	F01_transcript_54954	NA	NA
F01_transcript_122805	F01_transcript_122805	F01_transcript_122805	F01_transcript_122805	F01_transcript_122805
F01_transcript_111379	NA	F01_transcript_111379	NA	NA
F01_transcript_58809	F01_transcript_58809	F01_transcript_58809	F01_transcript_58809	F01_transcript_58809
F01_transcript_98500	NA	F01_transcript_98500	NA	NA
F01_transcript_58551	NA	NA	NA	F01_transcript_58551
F01_transcript_85095	NA	F01_transcript_85095	NA	F01_transcript_85095
F01_transcript_60090	F01_transcript_60090	F01_transcript_60090	F01_transcript_60090	F01_transcript_60090
F01_transcript_52558	NA	NA	NA	F01_transcript_52558
F01_transcript_139097	F01_transcript_139097	F01_transcript_139097	F01_transcript_139097	F01_transcript_139097
F01_transcript_134824	F01_transcript_134824	F01_transcript_134824	NA	NA
F01_transcript_95998	NA	F01_transcript_95998	NA	NA
F01_transcript_69761	NA	F01_transcript_69761	NA	NA
F01_transcript_70855	NA	NA	F01_transcript_70855	NA

F01_transcript_88391	NA	NA	F01_transcript_88391	NA
F01_transcript_2678	NA	NA	NA	F01_transcript_2678
F01_transcript_25141	F01_transcript_25141	F01_transcript_25141	F01_transcript_25141	F01_transcript_25141
F01_transcript_135103	F01_transcript_135103	F01_transcript_135103	NA	F01_transcript_135103
F01_transcript_166149	NA	F01_transcript_166149	NA	NA
F01_transcript_117677	F01_transcript_117677	F01_transcript_117677	F01_transcript_117677	F01_transcript_117677
F01_transcript_20410	NA	NA	NA	F01_transcript_20410
F01_transcript_24956	NA	NA	NA	F01_transcript_24956
F01_transcript_105107	NA	NA	NA	F01_transcript_105107
F01_transcript_94244	F01_transcript_94244	F01_transcript_94244	NA	F01_transcript_94244
F01_transcript_57481	NA	F01_transcript_57481	NA	NA
F01_transcript_162240	NA	NA	NA	F01_transcript_162240
F01_transcript_75179	F01_transcript_75179	F01_transcript_75179	NA	F01_transcript_75179
F01_transcript_6313	NA	NA	NA	F01_transcript_6313

F01_transcript_51329	NA	F01_transcript_51329	NA	NA
F01_transcript_58078	NA	NA	NA	F01_transcript_58078
F01_transcript_159356	NA	F01_transcript_159356	F01_transcript_159356	F01_transcript_159356
F01_transcript_71761	F01_transcript_71761	F01_transcript_71761	F01_transcript_71761	F01_transcript_71761
F01_transcript_148576	NA	NA	NA	F01_transcript_148576
F01_transcript_46788	NA	NA	NA	F01_transcript_46788
F01_transcript_54007	NA	F01_transcript_54007	NA	NA
F01_transcript_101058	F01_transcript_101058	F01_transcript_101058	NA	NA
F01_transcript_162089	NA	F01_transcript_162089	NA	F01_transcript_162089
F01_transcript_97168	F01_transcript_97168	F01_transcript_97168	F01_transcript_97168	F01_transcript_97168
F01_transcript_150017	NA	NA	NA	F01_transcript_150017
F01_transcript_89789	NA	F01_transcript_89789	NA	F01_transcript_89789
F01_transcript_67508	NA	F01_transcript_67508	NA	F01_transcript_67508
F01_transcript_156745	NA	NA	NA	F01_transcript_156745

F01_transcript_47674	NA	F01_transcript_47674	NA	NA
F01_transcript_51917	F01_transcript_51917	F01_transcript_51917	F01_transcript_51917	F01_transcript_51917
F01_transcript_165124	NA	NA	NA	F01_transcript_165124
F01_transcript_76495	NA	F01_transcript_76495	NA	F01_transcript_76495
F01_transcript_99317	F01_transcript_99317	F01_transcript_99317	F01_transcript_99317	F01_transcript_99317
F01_transcript_150257	NA	F01_transcript_150257	F01_transcript_150257	F01_transcript_150257
F01_transcript_130147	NA	F01_transcript_130147	NA	NA
F01_transcript_116902	F01_transcript_116902	F01_transcript_116902	F01_transcript_116902	F01_transcript_116902
F01_transcript_119326	NA	F01_transcript_119326	NA	NA
F01_transcript_29350	NA	NA	NA	F01_transcript_29350
F01_transcript_103865	NA	NA	NA	F01_transcript_103865
F01_transcript_145955	NA	F01_transcript_145955	NA	NA
F01_transcript_163083	F01_transcript_163083	F01_transcript_163083	F01_transcript_163083	F01_transcript_163083
F01_transcript_152337	NA	F01_transcript_152337	NA	NA

F01_transcript_59962	NA	NA	NA	F01_transcript_59962
F01_transcript_120243	F01_transcript_120243	F01_transcript_120243	NA	NA
F01_transcript_52873	F01_transcript_52873	F01_transcript_52873	F01_transcript_52873	F01_transcript_52873
F01_transcript_50758	F01_transcript_50758	F01_transcript_50758	F01_transcript_50758	F01_transcript_50758
F01_transcript_110402	F01_transcript_110402	F01_transcript_110402	F01_transcript_110402	F01_transcript_110402
F01_transcript_18471	NA	F01_transcript_18471	F01_transcript_18471	F01_transcript_18471
F01_transcript_97566	NA	F01_transcript_97566	NA	NA
F01_transcript_129284	F01_transcript_129284	F01_transcript_129284	F01_transcript_129284	F01_transcript_129284
F01_transcript_49713	F01_transcript_49713	F01_transcript_49713	F01_transcript_49713	F01_transcript_49713
F01_transcript_115757	NA	F01_transcript_115757	NA	NA
F01_transcript_66626	NA	F01_transcript_66626	NA	NA
F01_transcript_103896	NA	F01_transcript_103896	NA	NA
F01_transcript_106756	F01_transcript_106756	F01_transcript_106756	F01_transcript_106756	F01_transcript_106756
F01_transcript_104754	NA	F01_transcript_104754	NA	F01_transcript_104754

F01_transcript_89807	NA	F01_transcript_89807	NA	NA
F01_transcript_65440	F01_transcript_65440	F01_transcript_65440	F01_transcript_65440	F01_transcript_65440
F01_transcript_128530	F01_transcript_128530	F01_transcript_128530	NA	F01_transcript_128530
F01_transcript_165775	F01_transcript_165775	F01_transcript_165775	F01_transcript_165775	F01_transcript_165775
F01_transcript_68099	NA	F01_transcript_68099	NA	F01_transcript_68099
F01_transcript_54195	NA	F01_transcript_54195	F01_transcript_54195	NA
F01_transcript_144003	NA	NA	NA	F01_transcript_144003
F01_transcript_89240	NA	NA	NA	F01_transcript_89240
F01_transcript_63336	F01_transcript_63336	F01_transcript_63336	F01_transcript_63336	F01_transcript_63336
F01_transcript_155318	NA	F01_transcript_155318	NA	NA
F01_transcript_119545	F01_transcript_119545	F01_transcript_119545	F01_transcript_119545	F01_transcript_119545
F01_transcript_79040	NA	NA	NA	F01_transcript_79040
F01_transcript_160946	NA	NA	NA	F01_transcript_160946
F01_transcript_151239	NA	NA	NA	F01_transcript_151239

F01_transcript_21705	F01_transcript_21705	F01_transcript_21705	NA	NA
F01_transcript_68490	F01_transcript_68490	F01_transcript_68490	F01_transcript_68490	F01_transcript_68490
F01_transcript_76203	NA	F01_transcript_76203	NA	NA
F01_transcript_65852	F01_transcript_65852	NA	NA	F01_transcript_65852
F01_transcript_110546	NA	F01_transcript_110546	NA	NA
F01_transcript_117670	F01_transcript_117670	F01_transcript_117670	F01_transcript_117670	F01_transcript_117670
F01_transcript_149398	NA	F01_transcript_149398	NA	NA
F01_transcript_88272	NA	F01_transcript_88272	NA	F01_transcript_88272
F01_transcript_12880	NA	NA	NA	F01_transcript_12880
F01_transcript_66489	NA	F01_transcript_66489	NA	NA
F01_transcript_103089	F01_transcript_103089	F01_transcript_103089	F01_transcript_103089	F01_transcript_103089
F01_transcript_127981	NA	NA	NA	F01_transcript_127981
F01_transcript_133912	NA	NA	NA	F01_transcript_133912
F01_transcript_167050	NA	F01_transcript_167050	NA	NA

F01_transcript_150120	F01_transcript_150120	F01_transcript_150120	F01_transcript_150120	F01_transcript_150120
F01_transcript_146297	NA	F01_transcript_146297	F01_transcript_146297	F01_transcript_146297
F01_transcript_95674	NA	F01_transcript_95674	NA	NA
F01_transcript_122743	F01_transcript_122743	F01_transcript_122743	NA	F01_transcript_122743
F01_transcript_144831	NA	NA	NA	F01_transcript_144831
F01_transcript_59951	F01_transcript_59951	F01_transcript_59951	NA	F01_transcript_59951
F01_transcript_68552	NA	F01_transcript_68552	NA	F01_transcript_68552
F01_transcript_140895	NA	NA	NA	F01_transcript_140895
F01_transcript_5454	NA	NA	NA	F01_transcript_5454
F01_transcript_157135	NA	NA	NA	F01_transcript_157135
F01_transcript_115605	NA	F01_transcript_115605	NA	NA
F01_transcript_71231	NA	NA	F01_transcript_71231	F01_transcript_71231
F01_transcript_123634	NA	F01_transcript_123634	NA	F01_transcript_123634
F01_transcript_113760	NA	F01_transcript_113760	NA	NA

F01_transcript_134841	F01_transcript_134841	F01_transcript_134841	F01_transcript_134841	NA
F01_transcript_55015	NA	NA	NA	F01_transcript_55015
F01_transcript_146260	NA	F01_transcript_146260	NA	NA
F01_transcript_37814	NA	F01_transcript_37814	NA	NA
F01_transcript_133212	NA	F01_transcript_133212	NA	NA
F01_transcript_158895	F01_transcript_158895	F01_transcript_158895	F01_transcript_158895	F01_transcript_158895
F01_transcript_114844	NA	NA	NA	F01_transcript_114844
F01_transcript_78624	NA	NA	NA	F01_transcript_78624
F01_transcript_107682	NA	F01_transcript_107682	F01_transcript_107682	F01_transcript_107682
F01_transcript_83544	NA	F01_transcript_83544	NA	F01_transcript_83544
F01_transcript_161184	NA	F01_transcript_161184	NA	NA
F01_transcript_138697	F01_transcript_138697	F01_transcript_138697	F01_transcript_138697	F01_transcript_138697
F01_transcript_48845	F01_transcript_48845	F01_transcript_48845	NA	F01_transcript_48845
F01_transcript_109319	F01_transcript_109319	F01_transcript_109319	NA	NA

F01_transcript_82263	F01_transcript_82263	F01_transcript_82263	F01_transcript_82263	F01_transcript_82263
F01_transcript_134238	NA	F01_transcript_134238	F01_transcript_134238	F01_transcript_134238
F01_transcript_123797	F01_transcript_123797	F01_transcript_123797	NA	NA
F01_transcript_165593	NA	F01_transcript_165593	NA	F01_transcript_165593
F01_transcript_89546	NA	NA	NA	F01_transcript_89546
F01_transcript_50916	F01_transcript_50916	F01_transcript_50916	F01_transcript_50916	F01_transcript_50916
F01_transcript_125601	NA	F01_transcript_125601	NA	F01_transcript_125601
F01_transcript_106161	NA	F01_transcript_106161	NA	NA
F01_transcript_12333	NA	NA	NA	F01_transcript_12333
F01_transcript_91898	NA	NA	NA	F01_transcript_91898
F01_transcript_134406	NA	NA	NA	F01_transcript_134406
F01_transcript_15023	F01_transcript_15023	F01_transcript_15023	NA	NA
F01_transcript_96810	NA	F01_transcript_96810	NA	F01_transcript_96810
F01_transcript_54782	F01_transcript_54782	F01_transcript_54782	F01_transcript_54782	F01_transcript_54782

F01_transcript_81483	NA	NA	NA	F01_transcript_81483
F01_transcript_158925	NA	NA	F01_transcript_158925	NA
F01_transcript_164858	F01_transcript_164858	F01_transcript_164858	NA	F01_transcript_164858
F01_transcript_5889	NA	NA	NA	F01_transcript_5889
F01_transcript_129382	NA	NA	NA	F01_transcript_129382
F01_transcript_70179	NA	NA	NA	F01_transcript_70179
F01_transcript_5309	NA	NA	NA	F01_transcript_5309
F01_transcript_22376	NA	NA	NA	F01_transcript_22376
F01_transcript_136858	NA	F01_transcript_136858	NA	NA
F01_transcript_58639	NA	F01_transcript_58639	NA	NA
F01_transcript_54585	F01_transcript_54585	F01_transcript_54585	F01_transcript_54585	F01_transcript_54585
F01_transcript_30965	NA	NA	NA	F01_transcript_30965
F01_transcript_124560	NA	F01_transcript_124560	NA	NA
F01_transcript_163130	F01_transcript_163130	F01_transcript_163130	NA	NA

F01_transcript_27164	NA	NA	NA	F01_transcript_27164
F01_transcript_113087	NA	NA	NA	F01_transcript_113087
F01_transcript_67416	NA	NA	NA	F01_transcript_67416
F01_transcript_79373	NA	NA	NA	F01_transcript_79373
F01_transcript_11109	NA	NA	NA	F01_transcript_11109
F01_transcript_52141	NA	F01_transcript_52141	NA	F01_transcript_52141
F01_transcript_106651	NA	F01_transcript_106651	NA	NA
F01_transcript_172	NA	NA	NA	F01_transcript_172
F01_transcript_110104	F01_transcript_110104	F01_transcript_110104	NA	NA
F01_transcript_129001	NA	NA	NA	F01_transcript_129001
F01_transcript_122909	NA	NA	NA	F01_transcript_122909
F01_transcript_54770	NA	F01_transcript_54770	NA	F01_transcript_54770
F01_transcript_147147	NA	NA	NA	F01_transcript_147147
F01_transcript_137996	F01_transcript_137996	F01_transcript_137996	F01_transcript_137996	F01_transcript_137996

F01_transcript_140161	NA	F01_transcript_140161	NA	NA
F01_transcript_66889	NA	NA	NA	F01_transcript_66889
F01_transcript_82846	NA	NA	NA	F01_transcript_82846
F01_transcript_64885	NA	NA	NA	F01_transcript_64885
F01_transcript_133107	NA	F01_transcript_133107	NA	NA
F01_transcript_52842	NA	F01_transcript_52842	F01_transcript_52842	NA
F01_transcript_110662	F01_transcript_110662	NA	F01_transcript_110662	F01_transcript_110662
F01_transcript_68876	NA	F01_transcript_68876	NA	F01_transcript_68876
F01_transcript_150100	NA	NA	NA	F01_transcript_150100
F01_transcript_113215	NA	F01_transcript_113215	NA	NA
F01_transcript_90406	NA	NA	NA	F01_transcript_90406
F01_transcript_2229	NA	F01_transcript_2229	NA	NA
F01_transcript_95318	NA	NA	NA	F01_transcript_95318
F01_transcript_90036	NA	F01_transcript_90036	NA	F01_transcript_90036

F01_transcript_88847	NA	NA	NA	F01_transcript_88847
F01_transcript_161452	NA	NA	NA	F01_transcript_161452
F01_transcript_88338	NA	F01_transcript_88338	NA	NA
F01_transcript_148085	F01_transcript_148085	F01_transcript_148085	NA	F01_transcript_148085
F01_transcript_78875	NA	NA	NA	F01_transcript_78875
F01_transcript_106940	NA	F01_transcript_106940	NA	NA
F01_transcript_158210	NA	F01_transcript_158210	NA	NA
F01_transcript_155068	F01_transcript_155068	F01_transcript_155068	F01_transcript_155068	F01_transcript_155068
F01_transcript_34290	NA	F01_transcript_34290	NA	NA
F01_transcript_112933	NA	F01_transcript_112933	NA	F01_transcript_112933
F01_transcript_102311	NA	NA	NA	F01_transcript_102311
F01_transcript_67775	NA	NA	NA	F01_transcript_67775
F01_transcript_105030	F01_transcript_105030	F01_transcript_105030	F01_transcript_105030	F01_transcript_105030
F01_transcript_92865	NA	NA	NA	F01_transcript_92865

F01_transcript_55987	F01_transcript_55987	F01_transcript_55987	F01_transcript_55987	F01_transcript_55987
F01_transcript_161009	F01_transcript_161009	F01_transcript_161009	F01_transcript_161009	F01_transcript_161009
F01_transcript_146087	NA	NA	NA	F01_transcript_146087
F01_transcript_84980	NA	F01_transcript_84980	NA	NA
F01_transcript_115145	NA	F01_transcript_115145	NA	NA
F01_transcript_89031	NA	NA	NA	F01_transcript_89031
F01_transcript_4043	NA	NA	NA	F01_transcript_4043
F01_transcript_150741	NA	F01_transcript_150741	NA	NA
F01_transcript_100279	NA	NA	NA	F01_transcript_100279
F01_transcript_166000	NA	F01_transcript_166000	NA	NA
F01_transcript_86614	NA	NA	NA	F01_transcript_86614
F01_transcript_85871	F01_transcript_85871	F01_transcript_85871	F01_transcript_85871	F01_transcript_85871
F01_transcript_128744	NA	NA	F01_transcript_128744	F01_transcript_128744
F01_transcript_80318	F01_transcript_80318	F01_transcript_80318	F01_transcript_80318	F01_transcript_80318

F01_transcript_132746	NA	NA	NA	F01_transcript_132746
F01_transcript_63224	NA	F01_transcript_63224	NA	NA
F01_transcript_138092	NA	NA	NA	F01_transcript_138092
F01_transcript_133793	F01_transcript_133793	F01_transcript_133793	NA	F01_transcript_133793
F01_transcript_123806	NA	NA	NA	F01_transcript_123806
F01_transcript_75269	NA	F01_transcript_75269	F01_transcript_75269	F01_transcript_75269
F01_transcript_159555	F01_transcript_159555	F01_transcript_159555	F01_transcript_159555	F01_transcript_159555
F01_transcript_12586	F01_transcript_12586	F01_transcript_12586	NA	F01_transcript_12586
F01_transcript_106007	F01_transcript_106007	F01_transcript_106007	F01_transcript_106007	F01_transcript_106007
F01_transcript_56863	NA	NA	NA	F01_transcript_56863
F01_transcript_152929	NA	F01_transcript_152929	NA	NA
F01_transcript_144457	NA	F01_transcript_144457	NA	F01_transcript_144457
F01_transcript_143795	F01_transcript_143795	F01_transcript_143795	NA	F01_transcript_143795
F01_transcript_149646	NA	NA	F01_transcript_149646	F01_transcript_149646

F01_transcript_89778	F01_transcript_89778	F01_transcript_89778	F01_transcript_89778	F01_transcript_89778
F01_transcript_144274	NA	F01_transcript_144274	NA	NA
F01_transcript_74294	NA	NA	NA	F01_transcript_74294
F01_transcript_93864	NA	F01_transcript_93864	NA	NA
F01_transcript_23616	NA	NA	NA	F01_transcript_23616
F01_transcript_107396	NA	NA	NA	F01_transcript_107396
F01_transcript_107921	F01_transcript_107921	F01_transcript_107921	F01_transcript_107921	F01_transcript_107921
F01_transcript_81606	F01_transcript_81606	F01_transcript_81606	F01_transcript_81606	F01_transcript_81606
F01_transcript_39651	NA	NA	NA	F01_transcript_39651
F01_transcript_61905	F01_transcript_61905	F01_transcript_61905	F01_transcript_61905	F01_transcript_61905
F01_transcript_22439	NA	F01_transcript_22439	NA	NA
F01_transcript_12983	NA	NA	NA	F01_transcript_12983
F01_transcript_105250	F01_transcript_105250	F01_transcript_105250	F01_transcript_105250	F01_transcript_105250
F01_transcript_157237	NA	F01_transcript_157237	NA	NA

F01_transcript_135777	NA	F01_transcript_135777	NA	F01_transcript_135777
F01_transcript_57109	F01_transcript_57109	F01_transcript_57109	F01_transcript_57109	F01_transcript_57109
F01_transcript_112958	NA	F01_transcript_112958	NA	F01_transcript_112958
F01_transcript_123162	NA	F01_transcript_123162	NA	F01_transcript_123162
F01_transcript_36044	NA	NA	NA	F01_transcript_36044
F01_transcript_91299	NA	F01_transcript_91299	NA	NA
F01_transcript_30261	NA	NA	NA	F01_transcript_30261
F01_transcript_131914	NA	NA	NA	F01_transcript_131914
F01_transcript_145413	F01_transcript_145413	F01_transcript_145413	F01_transcript_145413	F01_transcript_145413
F01_transcript_67013	NA	NA	NA	F01_transcript_67013
F01_transcript_91711	NA	F01_transcript_91711	NA	NA
F01_transcript_166541	NA	NA	NA	F01_transcript_166541
F01_transcript_122098	F01_transcript_122098	F01_transcript_122098	F01_transcript_122098	F01_transcript_122098
F01_transcript_141754	NA	F01_transcript_141754	NA	NA

F01_transcript_125564	F01_transcript_125564	F01_transcript_125564	F01_transcript_125564	F01_transcript_125564
F01_transcript_67250	F01_transcript_67250	F01_transcript_67250	F01_transcript_67250	F01_transcript_67250
F01_transcript_40757	NA	F01_transcript_40757	F01_transcript_40757	F01_transcript_40757
F01_transcript_88835	NA	NA	NA	F01_transcript_88835
F01_transcript_141124	NA	NA	F01_transcript_141124	F01_transcript_141124
F01_transcript_109122	F01_transcript_109122	F01_transcript_109122	F01_transcript_109122	F01_transcript_109122
F01_transcript_155955	F01_transcript_155955	F01_transcript_155955	NA	F01_transcript_155955
F01_transcript_8044	F01_transcript_8044	F01_transcript_8044	F01_transcript_8044	F01_transcript_8044
F01_transcript_147220	NA	NA	NA	F01_transcript_147220
F01_transcript_116830	F01_transcript_116830	F01_transcript_116830	F01_transcript_116830	F01_transcript_116830
F01_transcript_92432	NA	NA	NA	F01_transcript_92432
F01_transcript_130575	NA	F01_transcript_130575	F01_transcript_130575	F01_transcript_130575
F01_transcript_45626	NA	NA	NA	F01_transcript_45626
F01_transcript_87750	NA	NA	NA	F01_transcript_87750

F01_transcript_146055	NA	F01_transcript_146055	F01_transcript_146055	F01_transcript_146055
F01_transcript_112383	NA	NA	NA	F01_transcript_112383
F01_transcript_112613	NA	NA	NA	F01_transcript_112613
F01_transcript_25320	F01_transcript_25320	F01_transcript_25320	NA	NA
F01_transcript_126045	NA	F01_transcript_126045	NA	NA
F01_transcript_63613	NA	NA	NA	F01_transcript_63613
F01_transcript_164753	NA	F01_transcript_164753	NA	NA
F01_transcript_16526	NA	F01_transcript_16526	NA	NA
F01_transcript_50525	F01_transcript_50525	NA	NA	F01_transcript_50525
F01_transcript_28516	NA	F01_transcript_28516	NA	NA
F01_transcript_138065	NA	F01_transcript_138065	NA	NA
F01_transcript_162724	F01_transcript_162724	F01_transcript_162724	F01_transcript_162724	NA
F01_transcript_95926	F01_transcript_95926	F01_transcript_95926	NA	NA
F01_transcript_119650	NA	F01_transcript_119650	NA	F01_transcript_119650

F01_transcript_47602	NA	F01_transcript_47602	NA	F01_transcript_47602
F01_transcript_60427	NA	F01_transcript_60427	NA	NA
F01_transcript_152018	NA	F01_transcript_152018	NA	F01_transcript_152018
F01_transcript_127753	NA	F01_transcript_127753	NA	NA
F01_transcript_74656	NA	F01_transcript_74656	NA	NA
F01_transcript_142091	NA	NA	NA	F01_transcript_142091
F01_transcript_92282	NA	F01_transcript_92282	NA	NA
F01_transcript_55414	F01_transcript_55414	F01_transcript_55414	F01_transcript_55414	NA
F01_transcript_153002	NA	NA	NA	F01_transcript_153002
F01_transcript_88020	NA	NA	NA	F01_transcript_88020
F01_transcript_158407	NA	F01_transcript_158407	NA	NA
F01_transcript_162378	NA	F01_transcript_162378	NA	NA
F01_transcript_133909	NA	NA	F01_transcript_133909	NA
F01_transcript_49977	NA	NA	F01_transcript_49977	NA

F01_transcript_90185	NA	F01_transcript_90185	NA	NA
F01_transcript_76638	NA	NA	NA	F01_transcript_76638
F01_transcript_62702	NA	F01_transcript_62702	F01_transcript_62702	NA
F01_transcript_156635	F01_transcript_156635	F01_transcript_156635	F01_transcript_156635	F01_transcript_156635
F01_transcript_124980	NA	F01_transcript_124980	NA	NA
F01_transcript_134133	NA	F01_transcript_134133	NA	NA
F01_transcript_14738	NA	NA	NA	F01_transcript_14738
F01_transcript_123456	NA	NA	NA	F01_transcript_123456
F01_transcript_154597	NA	F01_transcript_154597	NA	F01_transcript_154597
F01_transcript_100563	F01_transcript_100563	F01_transcript_100563	F01_transcript_100563	F01_transcript_100563
F01_transcript_66158	NA	NA	NA	F01_transcript_66158
F01_transcript_109136	F01_transcript_109136	F01_transcript_109136	NA	NA
F01_transcript_103097	NA	NA	NA	F01_transcript_103097
F01_transcript_93426	NA	F01_transcript_93426	NA	NA

F01_transcript_64562	NA	F01_transcript_64562	F01_transcript_64562	F01_transcript_64562
F01_transcript_144198	NA	F01_transcript_144198	NA	F01_transcript_144198
F01_transcript_163261	F01_transcript_163261	F01_transcript_163261	F01_transcript_163261	NA
F01_transcript_100507	NA	NA	NA	F01_transcript_100507
F01_transcript_115199	F01_transcript_115199	F01_transcript_115199	F01_transcript_115199	NA
F01_transcript_163034	F01_transcript_163034	F01_transcript_163034	F01_transcript_163034	F01_transcript_163034
F01_transcript_97937	NA	F01_transcript_97937	NA	NA
F01_transcript_87301	F01_transcript_87301	F01_transcript_87301	F01_transcript_87301	F01_transcript_87301
F01_transcript_137778	NA	NA	NA	F01_transcript_137778
F01_transcript_142358	NA	NA	NA	F01_transcript_142358
F01_transcript_125842	NA	F01_transcript_125842	F01_transcript_125842	NA
F01_transcript_81462	NA	F01_transcript_81462	NA	NA
F01_transcript_107049	NA	NA	NA	F01_transcript_107049
F01_transcript_81774	NA	F01_transcript_81774	NA	F01_transcript_81774

F01_transcript_59945	F01_transcript_59945	F01_transcript_59945	F01_transcript_59945	F01_transcript_59945
F01_transcript_7928	F01_transcript_7928	F01_transcript_7928	NA	NA
F01_transcript_75897	F01_transcript_75897	F01_transcript_75897	NA	NA
F01_transcript_91495	F01_transcript_91495	F01_transcript_91495	NA	F01_transcript_91495
F01_transcript_81878	NA	F01_transcript_81878	F01_transcript_81878	F01_transcript_81878
F01_transcript_41503	NA	F01_transcript_41503	NA	NA
F01_transcript_62235	F01_transcript_62235	F01_transcript_62235	NA	F01_transcript_62235
F01_transcript_123872	NA	F01_transcript_123872	NA	NA
F01_transcript_122141	NA	F01_transcript_122141	NA	NA
F01_transcript_73989	NA	NA	NA	F01_transcript_73989
F01_transcript_50418	F01_transcript_50418	F01_transcript_50418	F01_transcript_50418	F01_transcript_50418
F01_transcript_141942	F01_transcript_141942	F01_transcript_141942	F01_transcript_141942	F01_transcript_141942
F01_transcript_150252	NA	NA	NA	F01_transcript_150252
F01_transcript_100338	F01_transcript_100338	F01_transcript_100338	F01_transcript_100338	F01_transcript_100338

F01_transcript_116837	F01_transcript_116837	F01_transcript_116837	F01_transcript_116837	F01_transcript_116837
F01_transcript_81094	NA	NA	NA	F01_transcript_81094
F01_transcript_64044	NA	F01_transcript_64044	F01_transcript_64044	F01_transcript_64044
F01_transcript_157514	F01_transcript_157514	F01_transcript_157514	F01_transcript_157514	F01_transcript_157514
F01_transcript_121137	NA	F01_transcript_121137	NA	NA
F01_transcript_132605	NA	F01_transcript_132605	NA	NA
F01_transcript_105839	NA	F01_transcript_105839	F01_transcript_105839	F01_transcript_105839
F01_transcript_135438	NA	F01_transcript_135438	NA	F01_transcript_135438
F01_transcript_71157	NA	NA	F01_transcript_71157	NA
F01_transcript_156685	NA	NA	NA	F01_transcript_156685
F01_transcript_76064	NA	F01_transcript_76064	NA	NA
F01_transcript_90249	F01_transcript_90249	F01_transcript_90249	F01_transcript_90249	F01_transcript_90249
F01_transcript_161169	NA	F01_transcript_161169	NA	F01_transcript_161169
F01_transcript_104290	NA	F01_transcript_104290	NA	F01_transcript_104290

F01_transcript_130935	NA	NA	NA	F01_transcript_130935
F01_transcript_95595	F01_transcript_95595	F01_transcript_95595	F01_transcript_95595	F01_transcript_95595
F01_transcript_155558	NA	F01_transcript_155558	NA	NA
F01_transcript_117015	NA	NA	NA	F01_transcript_117015
F01_transcript_55005	F01_transcript_55005	F01_transcript_55005	F01_transcript_55005	F01_transcript_55005
F01_transcript_104983	NA	F01_transcript_104983	NA	F01_transcript_104983
F01_transcript_140009	F01_transcript_140009	F01_transcript_140009	F01_transcript_140009	F01_transcript_140009
F01_transcript_56346	NA	NA	F01_transcript_56346	F01_transcript_56346
F01_transcript_18106	NA	NA	NA	F01_transcript_18106
F01_transcript_61161	F01_transcript_61161	F01_transcript_61161	F01_transcript_61161	F01_transcript_61161
F01_transcript_75951	NA	NA	NA	F01_transcript_75951
F01_transcript_102740	NA	NA	NA	F01_transcript_102740
F01_transcript_56979	NA	F01_transcript_56979	F01_transcript_56979	NA
F01_transcript_139868	NA	NA	NA	F01_transcript_139868

F01_transcript_167257	NA	F01_transcript_167257	NA	NA
F01_transcript_54928	F01_transcript_54928	F01_transcript_54928	F01_transcript_54928	F01_transcript_54928
F01_transcript_85036	F01_transcript_85036	F01_transcript_85036	F01_transcript_85036	F01_transcript_85036
F01_transcript_60829	NA	F01_transcript_60829	F01_transcript_60829	F01_transcript_60829
F01_transcript_130212	F01_transcript_130212	F01_transcript_130212	F01_transcript_130212	F01_transcript_130212
F01_transcript_106487	NA	F01_transcript_106487	F01_transcript_106487	F01_transcript_106487
F01_transcript_136396	F01_transcript_136396	F01_transcript_136396	F01_transcript_136396	F01_transcript_136396
F01_transcript_12756	NA	F01_transcript_12756	NA	NA
F01_transcript_120794	NA	NA	NA	F01_transcript_120794
F01_transcript_124397	NA	F01_transcript_124397	NA	NA
F01_transcript_139900	NA	F01_transcript_139900	NA	F01_transcript_139900
F01_transcript_93492	NA	NA	NA	F01_transcript_93492
F01_transcript_130206	NA	F01_transcript_130206	NA	NA
F01_transcript_50226	F01_transcript_50226	F01_transcript_50226	F01_transcript_50226	F01_transcript_50226

F01_transcript_74941	NA	NA	NA	F01_transcript_74941
F01_transcript_51336	NA	F01_transcript_51336	NA	NA
F01_transcript_150714	NA	F01_transcript_150714	NA	NA
F01_transcript_125221	NA	NA	NA	F01_transcript_125221
F01_transcript_138559	NA	F01_transcript_138559	F01_transcript_138559	F01_transcript_138559
F01_transcript_139029	F01_transcript_139029	F01_transcript_139029	F01_transcript_139029	F01_transcript_139029
F01_transcript_125655	NA	F01_transcript_125655	NA	F01_transcript_125655
F01_transcript_82640	F01_transcript_82640	F01_transcript_82640	NA	F01_transcript_82640
F01_transcript_153981	F01_transcript_153981	F01_transcript_153981	F01_transcript_153981	F01_transcript_153981
F01_transcript_166578	NA	F01_transcript_166578	NA	NA
F01_transcript_96040	NA	F01_transcript_96040	NA	F01_transcript_96040
F01_transcript_84274	NA	NA	NA	F01_transcript_84274
F01_transcript_90274	NA	F01_transcript_90274	NA	NA
F01_transcript_120525	F01_transcript_120525	F01_transcript_120525	F01_transcript_120525	F01_transcript_120525

F01_transcript_61815	F01_transcript_61815	F01_transcript_61815	F01_transcript_61815	F01_transcript_61815
F01_transcript_59364	NA	NA	NA	F01_transcript_59364
F01_transcript_108063	NA	F01_transcript_108063	NA	NA
F01_transcript_131603	NA	F01_transcript_131603	NA	NA
F01_transcript_53833	NA	F01_transcript_53833	NA	NA
F01_transcript_141993	NA	NA	NA	F01_transcript_141993
F01_transcript_32983	NA	NA	NA	F01_transcript_32983
F01_transcript_81679	NA	F01_transcript_81679	F01_transcript_81679	F01_transcript_81679
F01_transcript_50959	F01_transcript_50959	F01_transcript_50959	F01_transcript_50959	F01_transcript_50959
F01_transcript_82374	NA	F01_transcript_82374	NA	NA
F01_transcript_75666	NA	NA	NA	F01_transcript_75666
F01_transcript_24974	NA	NA	NA	F01_transcript_24974
F01_transcript_129010	NA	NA	NA	F01_transcript_129010
F01_transcript_56655	F01_transcript_56655	F01_transcript_56655	F01_transcript_56655	F01_transcript_56655

F01_transcript_64122	NA	NA	NA	F01_transcript_64122
F01_transcript_41688	NA	NA	NA	F01_transcript_41688
F01_transcript_65490	F01_transcript_65490	F01_transcript_65490	F01_transcript_65490	F01_transcript_65490
F01_transcript_156480	NA	F01_transcript_156480	NA	NA
F01_transcript_65264	NA	NA	F01_transcript_65264	NA
F01_transcript_131378	NA	F01_transcript_131378	NA	NA
F01_transcript_151084	NA	F01_transcript_151084	NA	NA
F01_transcript_119653	F01_transcript_119653	F01_transcript_119653	F01_transcript_119653	F01_transcript_119653
F01_transcript_128222	F01_transcript_128222	F01_transcript_128222	NA	NA
F01_transcript_53705	NA	F01_transcript_53705	NA	F01_transcript_53705
F01_transcript_95446	NA	F01_transcript_95446	NA	NA
F01_transcript_53388	NA	NA	NA	F01_transcript_53388
F01_transcript_51961	NA	F01_transcript_51961	NA	NA
F01_transcript_88262	F01_transcript_88262	F01_transcript_88262	F01_transcript_88262	F01_transcript_88262

F01_transcript_72009	NA	F01_transcript_72009	NA	NA
F01_transcript_128559	F01_transcript_128559	F01_transcript_128559	NA	NA
F01_transcript_141186	NA	F01_transcript_141186	F01_transcript_141186	NA
F01_transcript_149763	NA	NA	NA	F01_transcript_149763
F01_transcript_123097	NA	F01_transcript_123097	NA	NA
F01_transcript_99333	NA	F01_transcript_99333	NA	F01_transcript_99333
F01_transcript_167066	NA	NA	F01_transcript_167066	NA
F01_transcript_70134	NA	NA	NA	F01_transcript_70134
F01_transcript_143034	NA	NA	NA	F01_transcript_143034
F01_transcript_56204	F01_transcript_56204	F01_transcript_56204	F01_transcript_56204	F01_transcript_56204
F01_transcript_140769	NA	F01_transcript_140769	NA	F01_transcript_140769
F01_transcript_76779	F01_transcript_76779	F01_transcript_76779	F01_transcript_76779	F01_transcript_76779
F01_transcript_139484	NA	F01_transcript_139484	NA	NA
F01_transcript_98214	F01_transcript_98214	F01_transcript_98214	NA	NA

F01_transcript_158088	F01_transcript_158088	F01_transcript_158088	F01_transcript_158088	F01_transcript_158088
F01_transcript_126662	NA	NA	NA	F01_transcript_126662
F01_transcript_162505	NA	NA	NA	F01_transcript_162505
F01_transcript_153203	F01_transcript_153203	F01_transcript_153203	F01_transcript_153203	F01_transcript_153203
F01_transcript_138489	F01_transcript_138489	NA	NA	NA
F01_transcript_70944	NA	F01_transcript_70944	F01_transcript_70944	NA
F01_transcript_53633	NA	NA	NA	F01_transcript_53633
F01_transcript_149162	NA	NA	NA	F01_transcript_149162
F01_transcript_45341	NA	NA	F01_transcript_45341	F01_transcript_45341
F01_transcript_6481	NA	NA	NA	F01_transcript_6481
F01_transcript_74938	F01_transcript_74938	F01_transcript_74938	F01_transcript_74938	F01_transcript_74938
F01_transcript_64641	F01_transcript_64641	F01_transcript_64641	F01_transcript_64641	F01_transcript_64641
F01_transcript_89528	NA	NA	NA	F01_transcript_89528
F01_transcript_65221	F01_transcript_65221	F01_transcript_65221	F01_transcript_65221	F01_transcript_65221

F01_transcript_123479	NA	NA	NA	F01_transcript_123479
F01_transcript_96676	F01_transcript_96676	F01_transcript_96676	F01_transcript_96676	F01_transcript_96676
F01_transcript_90594	NA	NA	NA	F01_transcript_90594
F01_transcript_47435	NA	NA	NA	F01_transcript_47435
F01_transcript_99280	NA	NA	NA	F01_transcript_99280
F01_transcript_40220	NA	NA	NA	F01_transcript_40220
F01_transcript_40073	NA	NA	NA	F01_transcript_40073
F01_transcript_77011	NA	NA	NA	F01_transcript_77011
F01_transcript_94255	NA	F01_transcript_94255	NA	NA
F01_transcript_58844	NA	NA	NA	F01_transcript_58844
F01_transcript_60892	NA	F01_transcript_60892	NA	F01_transcript_60892
F01_transcript_118179	NA	NA	NA	F01_transcript_118179
F01_transcript_63551	F01_transcript_63551	F01_transcript_63551	NA	F01_transcript_63551
F01_transcript_125342	NA	NA	NA	F01_transcript_125342

F01_transcript_68040	NA	NA	NA	F01_transcript_68040
F01_transcript_93418	NA	NA	NA	F01_transcript_93418
F01_transcript_132377	NA	F01_transcript_132377	NA	NA
F01_transcript_130090	NA	NA	NA	F01_transcript_130090
F01_transcript_118640	NA	NA	NA	F01_transcript_118640
F01_transcript_58284	NA	NA	F01_transcript_58284	F01_transcript_58284
F01_transcript_116843	NA	F01_transcript_116843	NA	NA
F01_transcript_121295	NA	F01_transcript_121295	NA	NA
F01_transcript_8330	NA	NA	NA	F01_transcript_8330
F01_transcript_81218	NA	NA	NA	F01_transcript_81218
F01_transcript_128009	NA	F01_transcript_128009	NA	NA
F01_transcript_147402	NA	F01_transcript_147402	F01_transcript_147402	F01_transcript_147402
F01_transcript_50249	NA	F01_transcript_50249	NA	NA
F01_transcript_112009	F01_transcript_112009	F01_transcript_112009	NA	F01_transcript_112009

F01_transcript_53984	NA	NA	NA	F01_transcript_53984
F01_transcript_75702	NA	F01_transcript_75702	NA	NA
F01_transcript_149444	NA	F01_transcript_149444	NA	NA
F01_transcript_48784	NA	F01_transcript_48784	NA	F01_transcript_48784
F01_transcript_109111	NA	NA	NA	F01_transcript_109111
F01_transcript_68459	F01_transcript_68459	F01_transcript_68459	F01_transcript_68459	F01_transcript_68459
F01_transcript_103604	F01_transcript_103604	F01_transcript_103604	F01_transcript_103604	F01_transcript_103604
F01_transcript_91114	NA	NA	F01_transcript_91114	NA
F01_transcript_117830	NA	F01_transcript_117830	NA	NA
F01_transcript_105574	NA	F01_transcript_105574	NA	F01_transcript_105574
F01_transcript_133200	NA	F01_transcript_133200	F01_transcript_133200	NA
F01_transcript_74164	F01_transcript_74164	F01_transcript_74164	F01_transcript_74164	F01_transcript_74164
F01_transcript_135739	NA	F01_transcript_135739	NA	NA
F01_transcript_49074	NA	NA	NA	F01_transcript_49074

F01_transcript_140333	NA	NA	NA	F01_transcript_140333
F01_transcript_61541	F01_transcript_61541	F01_transcript_61541	F01_transcript_61541	F01_transcript_61541
F01_transcript_77880	F01_transcript_77880	F01_transcript_77880	NA	NA
F01_transcript_94507	NA	NA	NA	F01_transcript_94507
F01_transcript_8992	NA	NA	NA	F01_transcript_8992
F01_transcript_138474	NA	F01_transcript_138474	NA	NA
F01_transcript_146833	NA	F01_transcript_146833	NA	F01_transcript_146833
F01_transcript_58769	NA	F01_transcript_58769	NA	F01_transcript_58769
F01_transcript_76838	NA	NA	NA	F01_transcript_76838
F01_transcript_104310	NA	F01_transcript_104310	NA	F01_transcript_104310
F01_transcript_160170	NA	NA	NA	F01_transcript_160170
F01_transcript_49509	NA	F01_transcript_49509	NA	NA
F01_transcript_132694	NA	NA	NA	F01_transcript_132694
F01_transcript_70513	NA	F01_transcript_70513	F01_transcript_70513	F01_transcript_70513

F01_transcript_41588	NA	NA	F01_transcript_41588	NA
F01_transcript_147225	NA	NA	NA	F01_transcript_147225
F01_transcript_126894	F01_transcript_126894	F01_transcript_126894	F01_transcript_126894	F01_transcript_126894
F01_transcript_24396	NA	NA	NA	F01_transcript_24396
F01_transcript_40183	NA	NA	NA	F01_transcript_40183
F01_transcript_79090	NA	NA	NA	F01_transcript_79090
F01_transcript_115597	NA	F01_transcript_115597	NA	NA
F01_transcript_61997	NA	NA	NA	F01_transcript_61997
F01_transcript_40053	F01_transcript_40053	F01_transcript_40053	NA	NA
F01_transcript_165044	NA	NA	F01_transcript_165044	NA
F01_transcript_143016	F01_transcript_143016	F01_transcript_143016	NA	NA
F01_transcript_23120	F01_transcript_23120	F01_transcript_23120	F01_transcript_23120	F01_transcript_23120
F01_transcript_67094	F01_transcript_67094	F01_transcript_67094	NA	NA
F01_transcript_27714	NA	F01_transcript_27714	NA	F01_transcript_27714

F01_transcript_49746	NA	F01_transcript_49746	NA	NA
F01_transcript_49962	NA	NA	NA	F01_transcript_49962
F01_transcript_150822	NA	NA	NA	F01_transcript_150822
F01_transcript_163014	NA	F01_transcript_163014	NA	NA
F01_transcript_110997	NA	NA	NA	F01_transcript_110997
F01_transcript_93509	NA	F01_transcript_93509	NA	NA
F01_transcript_65286	NA	NA	F01_transcript_65286	F01_transcript_65286
F01_transcript_77226	F01_transcript_77226	NA	NA	F01_transcript_77226
F01_transcript_150215	F01_transcript_150215	F01_transcript_150215	NA	F01_transcript_150215
F01_transcript_160658	NA	F01_transcript_160658	F01_transcript_160658	NA
F01_transcript_165769	NA	NA	NA	F01_transcript_165769
F01_transcript_51925	NA	NA	NA	F01_transcript_51925
F01_transcript_151825	F01_transcript_151825	F01_transcript_151825	F01_transcript_151825	F01_transcript_151825
F01_transcript_145735	NA	NA	NA	F01_transcript_145735

F01_transcript_142057	NA	F01_transcript_142057	F01_transcript_142057	F01_transcript_142057
F01_transcript_166864	NA	NA	F01_transcript_166864	F01_transcript_166864
F01_transcript_165697	F01_transcript_165697	F01_transcript_165697	NA	F01_transcript_165697
F01_transcript_162597	NA	NA	NA	F01_transcript_162597
F01_transcript_110403	NA	F01_transcript_110403	F01_transcript_110403	F01_transcript_110403
F01_transcript_149035	NA	NA	NA	F01_transcript_149035
F01_transcript_146223	NA	F01_transcript_146223	NA	NA
F01_transcript_117117	F01_transcript_117117	F01_transcript_117117	F01_transcript_117117	F01_transcript_117117
F01_transcript_72486	NA	F01_transcript_72486	NA	NA
F01_transcript_91217	NA	F01_transcript_91217	NA	NA
F01_transcript_19530	NA	NA	NA	F01_transcript_19530
F01_transcript_71433	NA	NA	NA	F01_transcript_71433
F01_transcript_76713	NA	F01_transcript_76713	NA	NA
F01_transcript_11203	F01_transcript_11203	NA	F01_transcript_11203	NA

F01_transcript_45464	NA	NA	NA	F01_transcript_45464
F01_transcript_91493	NA	F01_transcript_91493	NA	NA
F01_transcript_124717	NA	NA	NA	F01_transcript_124717
F01_transcript_56152	NA	F01_transcript_56152	NA	F01_transcript_56152
F01_transcript_117469	NA	NA	NA	F01_transcript_117469
F01_transcript_114554	NA	NA	NA	F01_transcript_114554
F01_transcript_60141	NA	F01_transcript_60141	NA	NA
F01_transcript_91202	F01_transcript_91202	F01_transcript_91202	NA	NA
F01_transcript_92918	NA	F01_transcript_92918	NA	NA
F01_transcript_54432	NA	F01_transcript_54432	NA	NA
F01_transcript_153587	NA	F01_transcript_153587	F01_transcript_153587	NA
F01_transcript_189	NA	F01_transcript_189	NA	NA
F01_transcript_121778	NA	F01_transcript_121778	F01_transcript_121778	F01_transcript_121778
F01_transcript_141913	NA	F01_transcript_141913	NA	NA

F01_transcript_77245	F01_transcript_77245	F01_transcript_77245	F01_transcript_77245	F01_transcript_77245
F01_transcript_142603	NA	F01_transcript_142603	NA	F01_transcript_142603
F01_transcript_69048	F01_transcript_69048	F01_transcript_69048	F01_transcript_69048	F01_transcript_69048
F01_transcript_160969	NA	F01_transcript_160969	NA	F01_transcript_160969
F01_transcript_110842	NA	F01_transcript_110842	NA	F01_transcript_110842
F01_transcript_102435	F01_transcript_102435	F01_transcript_102435	F01_transcript_102435	F01_transcript_102435
F01_transcript_101337	NA	NA	NA	F01_transcript_101337
F01_transcript_138190	NA	NA	NA	F01_transcript_138190
F01_transcript_74254	NA	F01_transcript_74254	NA	NA
F01_transcript_131438	F01_transcript_131438	F01_transcript_131438	F01_transcript_131438	NA
F01_transcript_133175	NA	NA	NA	F01_transcript_133175
F01_transcript_132961	NA	F01_transcript_132961	NA	NA
F01_transcript_136524	NA	NA	NA	F01_transcript_136524
F01_transcript_76764	F01_transcript_76764	F01_transcript_76764	NA	F01_transcript_76764

F01_transcript_120751	F01_transcript_120751	F01_transcript_120751	F01_transcript_120751	F01_transcript_120751
F01_transcript_139240	NA	NA	F01_transcript_139240	F01_transcript_139240
F01_transcript_65058	NA	NA	F01_transcript_65058	NA
F01_transcript_160454	NA	F01_transcript_160454	F01_transcript_160454	F01_transcript_160454
F01_transcript_86860	NA	F01_transcript_86860	NA	NA
F01_transcript_97172	NA	F01_transcript_97172	F01_transcript_97172	F01_transcript_97172
F01_transcript_145102	NA	F01_transcript_145102	F01_transcript_145102	F01_transcript_145102
F01_transcript_110071	NA	F01_transcript_110071	NA	NA
F01_transcript_54129	F01_transcript_54129	F01_transcript_54129	NA	F01_transcript_54129
F01_transcript_126689	NA	F01_transcript_126689	NA	NA
F01_transcript_64657	NA	NA	NA	F01_transcript_64657
F01_transcript_65414	NA	NA	F01_transcript_65414	NA
F01_transcript_124432	NA	F01_transcript_124432	NA	F01_transcript_124432
F01_transcript_55537	F01_transcript_55537	F01_transcript_55537	NA	NA

F01_transcript_46653	NA	NA	NA	F01_transcript_46653
F01_transcript_154806	F01_transcript_154806	F01_transcript_154806	F01_transcript_154806	F01_transcript_154806
F01_transcript_72971	NA	F01_transcript_72971	NA	NA
F01_transcript_132673	F01_transcript_132673	F01_transcript_132673	NA	NA
F01_transcript_88766	NA	NA	NA	F01_transcript_88766
F01_transcript_158086	NA	NA	NA	F01_transcript_158086
F01_transcript_132975	NA	F01_transcript_132975	NA	NA
F01_transcript_33393	NA	F01_transcript_33393	NA	NA
F01_transcript_87806	F01_transcript_87806	F01_transcript_87806	NA	NA
F01_transcript_70502	F01_transcript_70502	NA	NA	F01_transcript_70502
F01_transcript_164092	NA	F01_transcript_164092	NA	F01_transcript_164092
F01_transcript_2858	NA	NA	NA	F01_transcript_2858
F01_transcript_104885	NA	F01_transcript_104885	NA	F01_transcript_104885
F01_transcript_65476	NA	NA	F01_transcript_65476	F01_transcript_65476

F01_transcript_51903	NA	F01_transcript_51903	NA	NA
F01_transcript_53288	NA	F01_transcript_53288	NA	NA
F01_transcript_93306	NA	NA	NA	F01_transcript_93306
F01_transcript_134415	NA	NA	NA	F01_transcript_134415
F01_transcript_151619	NA	NA	NA	F01_transcript_151619
F01_transcript_135237	F01_transcript_135237	F01_transcript_135237	F01_transcript_135237	F01_transcript_135237
F01_transcript_44655	NA	NA	NA	F01_transcript_44655
F01_transcript_86228	F01_transcript_86228	NA	F01_transcript_86228	F01_transcript_86228
F01_transcript_72390	NA	NA	NA	F01_transcript_72390
F01_transcript_65242	NA	NA	F01_transcript_65242	NA
F01_transcript_110991	F01_transcript_110991	F01_transcript_110991	NA	NA
F01_transcript_97431	NA	NA	F01_transcript_97431	F01_transcript_97431
F01_transcript_119443	F01_transcript_119443	F01_transcript_119443	NA	F01_transcript_119443
F01_transcript_138707	NA	NA	F01_transcript_138707	NA

F01_transcript_77850	F01_transcript_77850	F01_transcript_77850	NA	F01_transcript_77850
F01_transcript_67699	F01_transcript_67699	F01_transcript_67699	F01_transcript_67699	F01_transcript_67699
F01_transcript_42427	F01_transcript_42427	F01_transcript_42427	F01_transcript_42427	F01_transcript_42427
F01_transcript_89636	F01_transcript_89636	F01_transcript_89636	NA	NA
F01_transcript_104119	NA	F01_transcript_104119	NA	F01_transcript_104119
F01_transcript_70092	NA	F01_transcript_70092	NA	F01_transcript_70092
F01_transcript_48791	NA	NA	NA	F01_transcript_48791
F01_transcript_83467	NA	F01_transcript_83467	F01_transcript_83467	F01_transcript_83467
F01_transcript_138200	NA	F01_transcript_138200	NA	NA
F01_transcript_62557	NA	NA	NA	F01_transcript_62557
F01_transcript_146383	NA	F01_transcript_146383	NA	NA
F01_transcript_102440	F01_transcript_102440	F01_transcript_102440	F01_transcript_102440	F01_transcript_102440
F01_transcript_117216	NA	F01_transcript_117216	F01_transcript_117216	F01_transcript_117216
F01_transcript_5600	F01_transcript_5600	F01_transcript_5600	F01_transcript_5600	F01_transcript_5600

F01_transcript_87328	NA	NA	NA	F01_transcript_87328
F01_transcript_159664	NA	NA	NA	F01_transcript_159664
F01_transcript_103359	NA	F01_transcript_103359	F01_transcript_103359	F01_transcript_103359
F01_transcript_42125	NA	F01_transcript_42125	NA	NA
F01_transcript_117598	NA	NA	NA	F01_transcript_117598
F01_transcript_142498	F01_transcript_142498	F01_transcript_142498	F01_transcript_142498	F01_transcript_142498
F01_transcript_120659	F01_transcript_120659	F01_transcript_120659	F01_transcript_120659	F01_transcript_120659
F01_transcript_60348	F01_transcript_60348	F01_transcript_60348	F01_transcript_60348	F01_transcript_60348
F01_transcript_46781	F01_transcript_46781	F01_transcript_46781	F01_transcript_46781	F01_transcript_46781
F01_transcript_114500	F01_transcript_114500	F01_transcript_114500	F01_transcript_114500	F01_transcript_114500
F01_transcript_79425	NA	NA	NA	F01_transcript_79425
F01_transcript_58459	NA	NA	NA	F01_transcript_58459
F01_transcript_164693	NA	NA	NA	F01_transcript_164693
F01_transcript_34739	NA	NA	NA	F01_transcript_34739

F01_transcript_149450	F01_transcript_149450	F01_transcript_149450	NA	F01_transcript_149450
F01_transcript_132579	NA	F01_transcript_132579	NA	NA
F01_transcript_94165	NA	F01_transcript_94165	F01_transcript_94165	F01_transcript_94165
F01_transcript_133709	F01_transcript_133709	F01_transcript_133709	F01_transcript_133709	F01_transcript_133709
F01_transcript_165492	NA	F01_transcript_165492	NA	F01_transcript_165492
F01_transcript_119561	F01_transcript_119561	F01_transcript_119561	F01_transcript_119561	NA
F01_transcript_109013	F01_transcript_109013	F01_transcript_109013	F01_transcript_109013	F01_transcript_109013
F01_transcript_89758	NA	F01_transcript_89758	NA	NA
F01_transcript_116074	NA	F01_transcript_116074	NA	F01_transcript_116074
F01_transcript_110112	NA	F01_transcript_110112	NA	NA
F01_transcript_101181	NA	F01_transcript_101181	NA	NA
F01_transcript_58280	NA	NA	NA	F01_transcript_58280
F01_transcript_118226	NA	F01_transcript_118226	F01_transcript_118226	F01_transcript_118226
F01_transcript_151124	F01_transcript_151124	F01_transcript_151124	NA	F01_transcript_151124

F01_transcript_65201	F01_transcript_65201	F01_transcript_65201	F01_transcript_65201	F01_transcript_65201
F01_transcript_60531	F01_transcript_60531	F01_transcript_60531	F01_transcript_60531	NA
F01_transcript_84802	NA	F01_transcript_84802	NA	NA
F01_transcript_54701	NA	F01_transcript_54701	NA	F01_transcript_54701
F01_transcript_116137	NA	F01_transcript_116137	NA	NA
F01_transcript_60885	NA	NA	NA	F01_transcript_60885
F01_transcript_149831	NA	NA	NA	F01_transcript_149831
F01_transcript_90504	F01_transcript_90504	F01_transcript_90504	F01_transcript_90504	F01_transcript_90504
F01_transcript_160026	NA	NA	NA	F01_transcript_160026
F01_transcript_47273	NA	F01_transcript_47273	NA	NA
F01_transcript_12520	NA	NA	NA	F01_transcript_12520
F01_transcript_72969	NA	F01_transcript_72969	NA	NA
F01_transcript_90908	NA	NA	NA	F01_transcript_90908
F01_transcript_48790	NA	NA	NA	F01_transcript_48790

F01_transcript_114829	NA	NA	NA	F01_transcript_114829
F01_transcript_132412	NA	F01_transcript_132412	NA	F01_transcript_132412
F01_transcript_1968	NA	F01_transcript_1968	NA	NA
F01_transcript_135736	NA	F01_transcript_135736	NA	NA
F01_transcript_105853	F01_transcript_105853	F01_transcript_105853	NA	NA
F01_transcript_133655	NA	F01_transcript_133655	NA	NA
F01_transcript_116678	NA	NA	NA	F01_transcript_116678
F01_transcript_119079	NA	NA	NA	F01_transcript_119079
F01_transcript_167458	NA	F01_transcript_167458	NA	NA
F01_transcript_70931	NA	NA	F01_transcript_70931	F01_transcript_70931
F01_transcript_153454	F01_transcript_153454	F01_transcript_153454	F01_transcript_153454	F01_transcript_153454
F01_transcript_121714	NA	F01_transcript_121714	NA	NA
F01_transcript_127913	NA	NA	NA	F01_transcript_127913
F01_transcript_101918	NA	NA	NA	F01_transcript_101918

F01_transcript_100969	NA	F01_transcript_100969	F01_transcript_100969	F01_transcript_100969
F01_transcript_125294	F01_transcript_125294	F01_transcript_125294	F01_transcript_125294	NA
F01_transcript_156896	NA	F01_transcript_156896	NA	NA
F01_transcript_143546	NA	F01_transcript_143546	NA	F01_transcript_143546
F01_transcript_8370	F01_transcript_8370	F01_transcript_8370	NA	NA
F01_transcript_100974	F01_transcript_100974	F01_transcript_100974	F01_transcript_100974	F01_transcript_100974
F01_transcript_54133	F01_transcript_54133	F01_transcript_54133	F01_transcript_54133	NA
F01_transcript_48981	NA	NA	NA	F01_transcript_48981
F01_transcript_74666	NA	F01_transcript_74666	NA	F01_transcript_74666
F01_transcript_108140	NA	NA	NA	F01_transcript_108140
F01_transcript_48638	F01_transcript_48638	F01_transcript_48638	F01_transcript_48638	F01_transcript_48638
F01_transcript_57391	F01_transcript_57391	F01_transcript_57391	F01_transcript_57391	F01_transcript_57391
F01_transcript_158253	NA	NA	NA	F01_transcript_158253
F01_transcript_155134	F01_transcript_155134	F01_transcript_155134	F01_transcript_155134	F01_transcript_155134

F01_transcript_119658	NA	F01_transcript_119658	F01_transcript_119658	NA
F01_transcript_91532	NA	NA	NA	F01_transcript_91532
F01_transcript_36392	NA	NA	NA	F01_transcript_36392
F01_transcript_122553	NA	NA	NA	F01_transcript_122553
F01_transcript_127953	F01_transcript_127953	F01_transcript_127953	F01_transcript_127953	F01_transcript_127953
F01_transcript_16636	NA	F01_transcript_16636	NA	F01_transcript_16636
F01_transcript_51199	F01_transcript_51199	F01_transcript_51199	F01_transcript_51199	F01_transcript_51199
F01_transcript_84566	NA	NA	NA	F01_transcript_84566
F01_transcript_76075	NA	F01_transcript_76075	NA	NA
F01_transcript_59789	NA	NA	F01_transcript_59789	F01_transcript_59789
F01_transcript_63772	NA	F01_transcript_63772	NA	NA
F01_transcript_123901	F01_transcript_123901	F01_transcript_123901	F01_transcript_123901	F01_transcript_123901
F01_transcript_147238	NA	F01_transcript_147238	NA	NA
F01_transcript_50834	F01_transcript_50834	F01_transcript_50834	NA	NA

F01_transcript_42689	NA	NA	NA	F01_transcript_42689
F01_transcript_100001	F01_transcript_100001	F01_transcript_100001	NA	NA
F01_transcript_28437	NA	F01_transcript_28437	NA	F01_transcript_28437
F01_transcript_143344	NA	NA	NA	F01_transcript_143344
F01_transcript_109565	NA	F01_transcript_109565	F01_transcript_109565	F01_transcript_109565
F01_transcript_135993	F01_transcript_135993	F01_transcript_135993	NA	NA
F01_transcript_116969	NA	F01_transcript_116969	NA	NA
F01_transcript_153255	F01_transcript_153255	F01_transcript_153255	F01_transcript_153255	F01_transcript_153255
F01_transcript_70769	NA	F01_transcript_70769	NA	NA
F01_transcript_99687	NA	F01_transcript_99687	NA	F01_transcript_99687
F01_transcript_627	NA	NA	NA	F01_transcript_627
F01_transcript_106474	NA	F01_transcript_106474	NA	F01_transcript_106474
F01_transcript_112791	NA	F01_transcript_112791	NA	NA
F01_transcript_159521	F01_transcript_159521	F01_transcript_159521	F01_transcript_159521	F01_transcript_159521

F01_transcript_59850	NA	NA	NA	F01_transcript_59850
F01_transcript_99633	NA	F01_transcript_99633	NA	NA
F01_transcript_82853	F01_transcript_82853	F01_transcript_82853	F01_transcript_82853	F01_transcript_82853
F01_transcript_74958	F01_transcript_74958	F01_transcript_74958	NA	F01_transcript_74958
F01_transcript_10587	NA	NA	NA	F01_transcript_10587
F01_transcript_53379	NA	NA	NA	F01_transcript_53379
F01_transcript_105493	NA	NA	NA	F01_transcript_105493
F01_transcript_159271	F01_transcript_159271	F01_transcript_159271	F01_transcript_159271	F01_transcript_159271
F01_transcript_145183	NA	F01_transcript_145183	NA	F01_transcript_145183
F01_transcript_131148	NA	F01_transcript_131148	NA	NA
F01_transcript_58428	F01_transcript_58428	F01_transcript_58428	F01_transcript_58428	F01_transcript_58428
F01_transcript_135049	NA	F01_transcript_135049	NA	NA
F01_transcript_156019	NA	NA	NA	F01_transcript_156019
F01_transcript_134046	F01_transcript_134046	F01_transcript_134046	NA	NA

F01_transcript_80806	NA	F01_transcript_80806	NA	NA
F01_transcript_102868	F01_transcript_102868	F01_transcript_102868	NA	NA
F01_transcript_81939	F01_transcript_81939	F01_transcript_81939	NA	NA
F01_transcript_69924	NA	NA	NA	F01_transcript_69924
F01_transcript_108115	F01_transcript_108115	F01_transcript_108115	F01_transcript_108115	F01_transcript_108115
F01_transcript_68930	NA	F01_transcript_68930	NA	NA
F01_transcript_51345	F01_transcript_51345	F01_transcript_51345	F01_transcript_51345	F01_transcript_51345
F01_transcript_81088	F01_transcript_81088	F01_transcript_81088	F01_transcript_81088	F01_transcript_81088
F01_transcript_71003	NA	NA	F01_transcript_71003	F01_transcript_71003
F01_transcript_134952	NA	NA	NA	F01_transcript_134952
F01_transcript_71114	NA	NA	F01_transcript_71114	F01_transcript_71114
F01_transcript_39627	NA	NA	NA	F01_transcript_39627
F01_transcript_112569	NA	F01_transcript_112569	NA	NA
F01_transcript_46945	NA	NA	NA	F01_transcript_46945

F01_transcript_39913	F01_transcript_39913	F01_transcript_39913	NA	F01_transcript_39913
F01_transcript_66986	NA	NA	NA	F01_transcript_66986
F01_transcript_123841	NA	NA	F01_transcript_123841	F01_transcript_123841
F01_transcript_88737	NA	F01_transcript_88737	NA	NA
F01_transcript_111823	F01_transcript_111823	F01_transcript_111823	NA	F01_transcript_111823
F01_transcript_27085	F01_transcript_27085	F01_transcript_27085	F01_transcript_27085	F01_transcript_27085
F01_transcript_114828	F01_transcript_114828	F01_transcript_114828	F01_transcript_114828	F01_transcript_114828
F01_transcript_145069	NA	F01_transcript_145069	NA	NA
F01_transcript_50004	NA	NA	NA	F01_transcript_50004
F01_transcript_47853	NA	NA	NA	F01_transcript_47853
F01_transcript_73153	NA	NA	F01_transcript_73153	F01_transcript_73153
F01_transcript_69160	F01_transcript_69160	F01_transcript_69160	F01_transcript_69160	F01_transcript_69160
F01_transcript_90032	NA	NA	NA	F01_transcript_90032
F01_transcript_81759	NA	F01_transcript_81759	NA	NA

F01_transcript_106342	F01_transcript_106342	F01_transcript_106342	F01_transcript_106342	F01_transcript_106342
F01_transcript_92907	NA	NA	NA	F01_transcript_92907
F01_transcript_85853	NA	NA	NA	F01_transcript_85853
F01_transcript_87634	F01_transcript_87634	F01_transcript_87634	F01_transcript_87634	F01_transcript_87634
F01_transcript_37752	NA	NA	NA	F01_transcript_37752
F01_transcript_97732	NA	F01_transcript_97732	NA	NA
F01_transcript_105955	NA	NA	NA	F01_transcript_105955
F01_transcript_29229	NA	F01_transcript_29229	NA	NA
F01_transcript_162287	NA	NA	NA	F01_transcript_162287
F01_transcript_135730	NA	NA	F01_transcript_135730	F01_transcript_135730
F01_transcript_167163	F01_transcript_167163	F01_transcript_167163	F01_transcript_167163	F01_transcript_167163
F01_transcript_65916	NA	F01_transcript_65916	NA	F01_transcript_65916
F01_transcript_91207	F01_transcript_91207	F01_transcript_91207	F01_transcript_91207	F01_transcript_91207
F01_transcript_127596	F01_transcript_127596	F01_transcript_127596	NA	F01_transcript_127596

F01_transcript_81283	F01_transcript_81283	F01_transcript_81283	NA	NA
F01_transcript_137842	NA	F01_transcript_137842	NA	NA
F01_transcript_137972	NA	F01_transcript_137972	F01_transcript_137972	F01_transcript_137972
F01_transcript_46917	NA	F01_transcript_46917	NA	NA
F01_transcript_131488	NA	NA	NA	F01_transcript_131488
F01_transcript_135732	F01_transcript_135732	F01_transcript_135732	F01_transcript_135732	NA
F01_transcript_156399	NA	NA	NA	F01_transcript_156399
F01_transcript_84972	NA	F01_transcript_84972	NA	NA
F01_transcript_45151	NA	NA	NA	F01_transcript_45151
F01_transcript_109507	NA	F01_transcript_109507	NA	F01_transcript_109507
F01_transcript_5117	F01_transcript_5117	F01_transcript_5117	NA	NA
F01_transcript_128789	F01_transcript_128789	F01_transcript_128789	F01_transcript_128789	F01_transcript_128789
F01_transcript_31556	NA	NA	NA	F01_transcript_31556
F01_transcript_122106	NA	F01_transcript_122106	NA	NA

F01_transcript_160992	F01_transcript_160992	F01_transcript_160992	F01_transcript_160992	F01_transcript_160992
F01_transcript_85418	NA	F01_transcript_85418	NA	NA
F01_transcript_50210	NA	F01_transcript_50210	NA	NA
F01_transcript_586	NA	NA	NA	F01_transcript_586
F01_transcript_146998	F01_transcript_146998	F01_transcript_146998	F01_transcript_146998	F01_transcript_146998
F01_transcript_68912	F01_transcript_68912	F01_transcript_68912	NA	F01_transcript_68912
F01_transcript_135248	NA	NA	NA	F01_transcript_135248
F01_transcript_58001	NA	NA	NA	F01_transcript_58001
F01_transcript_19217	NA	F01_transcript_19217	NA	F01_transcript_19217
F01_transcript_152252	NA	NA	NA	F01_transcript_152252
F01_transcript_161749	NA	NA	NA	F01_transcript_161749
F01_transcript_114579	F01_transcript_114579	NA	NA	F01_transcript_114579
F01_transcript_151375	F01_transcript_151375	F01_transcript_151375	F01_transcript_151375	NA
F01_transcript_60278	NA	NA	NA	F01_transcript_60278

F01_transcript_157375	F01_transcript_157375	F01_transcript_157375	F01_transcript_157375	F01_transcript_157375
F01_transcript_12639	NA	F01_transcript_12639	F01_transcript_12639	F01_transcript_12639
F01_transcript_106489	NA	F01_transcript_106489	NA	NA
F01_transcript_129624	F01_transcript_129624	F01_transcript_129624	F01_transcript_129624	F01_transcript_129624
F01_transcript_75790	NA	NA	NA	F01_transcript_75790
F01_transcript_147445	NA	F01_transcript_147445	NA	NA
F01_transcript_114841	F01_transcript_114841	F01_transcript_114841	F01_transcript_114841	NA
F01_transcript_162374	F01_transcript_162374	F01_transcript_162374	F01_transcript_162374	F01_transcript_162374
F01_transcript_56044	F01_transcript_56044	F01_transcript_56044	NA	F01_transcript_56044
F01_transcript_102264	NA	F01_transcript_102264	F01_transcript_102264	NA
F01_transcript_82141	NA	F01_transcript_82141	NA	NA
F01_transcript_50787	F01_transcript_50787	F01_transcript_50787	NA	NA
F01_transcript_27200	NA	NA	NA	F01_transcript_27200
F01_transcript_117104	NA	F01_transcript_117104	NA	F01_transcript_117104

F01_transcript_113411	NA	F01_transcript_113411	NA	F01_transcript_113411
F01_transcript_110264	F01_transcript_110264	F01_transcript_110264	NA	F01_transcript_110264
F01_transcript_125503	F01_transcript_125503	F01_transcript_125503	F01_transcript_125503	F01_transcript_125503
F01_transcript_112656	F01_transcript_112656	F01_transcript_112656	F01_transcript_112656	F01_transcript_112656
F01_transcript_45971	NA	NA	NA	F01_transcript_45971
F01_transcript_140551	NA	F01_transcript_140551	F01_transcript_140551	F01_transcript_140551
F01_transcript_125848	NA	NA	NA	F01_transcript_125848
F01_transcript_85822	F01_transcript_85822	F01_transcript_85822	NA	F01_transcript_85822
F01_transcript_128008	F01_transcript_128008	F01_transcript_128008	F01_transcript_128008	F01_transcript_128008
F01_transcript_86355	F01_transcript_86355	F01_transcript_86355	F01_transcript_86355	F01_transcript_86355
F01_transcript_107698	NA	NA	NA	F01_transcript_107698
F01_transcript_107016	NA	F01_transcript_107016	NA	F01_transcript_107016
F01_transcript_150986	NA	NA	NA	F01_transcript_150986
F01_transcript_33318	NA	NA	NA	F01_transcript_33318

F01_transcript_131179	NA	NA	NA	F01_transcript_131179
F01_transcript_44519	NA	NA	NA	F01_transcript_44519
F01_transcript_138755	NA	NA	F01_transcript_138755	NA
F01_transcript_147948	F01_transcript_147948	F01_transcript_147948	NA	F01_transcript_147948
F01_transcript_152603	F01_transcript_152603	F01_transcript_152603	F01_transcript_152603	F01_transcript_152603
F01_transcript_115019	NA	F01_transcript_115019	NA	NA
F01_transcript_78321	NA	F01_transcript_78321	F01_transcript_78321	NA
F01_transcript_79839	NA	NA	NA	F01_transcript_79839
F01_transcript_147433	F01_transcript_147433	F01_transcript_147433	F01_transcript_147433	F01_transcript_147433
F01_transcript_106083	NA	NA	NA	F01_transcript_106083
F01_transcript_46338	NA	NA	F01_transcript_46338	F01_transcript_46338
F01_transcript_71592	NA	NA	NA	F01_transcript_71592
F01_transcript_123353	NA	F01_transcript_123353	NA	F01_transcript_123353
F01_transcript_58919	F01_transcript_58919	F01_transcript_58919	F01_transcript_58919	F01_transcript_58919

F01_transcript_59512	NA	NA	F01_transcript_59512	NA
F01_transcript_52962	F01_transcript_52962	F01_transcript_52962	F01_transcript_52962	F01_transcript_52962
F01_transcript_148841	NA	NA	NA	F01_transcript_148841
F01_transcript_93599	F01_transcript_93599	F01_transcript_93599	F01_transcript_93599	F01_transcript_93599
F01_transcript_146809	F01_transcript_146809	F01_transcript_146809	F01_transcript_146809	NA
F01_transcript_69268	F01_transcript_69268	F01_transcript_69268	F01_transcript_69268	F01_transcript_69268
F01_transcript_113020	NA	F01_transcript_113020	NA	NA
F01_transcript_58446	NA	F01_transcript_58446	NA	F01_transcript_58446
F01_transcript_157873	F01_transcript_157873	F01_transcript_157873	NA	F01_transcript_157873
F01_transcript_89491	NA	F01_transcript_89491	NA	NA
F01_transcript_103135	NA	NA	NA	F01_transcript_103135
F01_transcript_167283	NA	F01_transcript_167283	NA	NA
F01_transcript_73608	F01_transcript_73608	F01_transcript_73608	F01_transcript_73608	F01_transcript_73608
F01_transcript_164417	NA	NA	NA	F01_transcript_164417

F01_transcript_51130	F01_transcript_51130	F01_transcript_51130	F01_transcript_51130	F01_transcript_51130
F01_transcript_48426	F01_transcript_48426	F01_transcript_48426	F01_transcript_48426	F01_transcript_48426
F01_transcript_64331	NA	F01_transcript_64331	NA	NA
F01_transcript_105326	NA	F01_transcript_105326	NA	NA
F01_transcript_81107	F01_transcript_81107	F01_transcript_81107	F01_transcript_81107	F01_transcript_81107
F01_transcript_151183	NA	NA	NA	F01_transcript_151183
F01_transcript_143110	NA	NA	NA	F01_transcript_143110
F01_transcript_122827	NA	NA	NA	F01_transcript_122827
F01_transcript_129328	NA	F01_transcript_129328	NA	NA
F01_transcript_55499	NA	NA	NA	F01_transcript_55499
F01_transcript_150858	F01_transcript_150858	F01_transcript_150858	F01_transcript_150858	F01_transcript_150858
F01_transcript_150956	NA	F01_transcript_150956	F01_transcript_150956	F01_transcript_150956
F01_transcript_112483	NA	NA	NA	F01_transcript_112483
F01_transcript_112381	F01_transcript_112381	F01_transcript_112381	F01_transcript_112381	F01_transcript_112381

F01_transcript_133950	F01_transcript_133950	F01_transcript_133950	NA	NA
F01_transcript_77022	NA	NA	F01_transcript_77022	F01_transcript_77022
F01_transcript_46161	NA	F01_transcript_46161	NA	F01_transcript_46161
F01_transcript_74600	F01_transcript_74600	F01_transcript_74600	F01_transcript_74600	F01_transcript_74600
F01_transcript_53529	NA	NA	NA	F01_transcript_53529
F01_transcript_10792	F01_transcript_10792	F01_transcript_10792	NA	F01_transcript_10792
F01_transcript_57916	F01_transcript_57916	F01_transcript_57916	F01_transcript_57916	F01_transcript_57916
F01_transcript_119669	NA	NA	NA	F01_transcript_119669
F01_transcript_143170	F01_transcript_143170	F01_transcript_143170	F01_transcript_143170	F01_transcript_143170
F01_transcript_75787	NA	NA	NA	F01_transcript_75787
F01_transcript_111260	F01_transcript_111260	F01_transcript_111260	F01_transcript_111260	F01_transcript_111260
F01_transcript_135473	NA	NA	F01_transcript_135473	NA
F01_transcript_70940	NA	NA	F01_transcript_70940	NA
F01_transcript_139849	NA	NA	F01_transcript_139849	F01_transcript_139849

F01_transcript_101592	NA	NA	NA	F01_transcript_101592
F01_transcript_77236	NA	F01_transcript_77236	NA	NA
F01_transcript_79841	F01_transcript_79841	F01_transcript_79841	F01_transcript_79841	F01_transcript_79841
F01_transcript_139324	F01_transcript_139324	F01_transcript_139324	NA	F01_transcript_139324
F01_transcript_76626	NA	NA	NA	F01_transcript_76626
F01_transcript_3452	NA	F01_transcript_3452	F01_transcript_3452	F01_transcript_3452
F01_transcript_78834	F01_transcript_78834	F01_transcript_78834	NA	NA
F01_transcript_158949	NA	F01_transcript_158949	F01_transcript_158949	F01_transcript_158949
F01_transcript_50574	NA	NA	F01_transcript_50574	NA
F01_transcript_157826	NA	NA	NA	F01_transcript_157826
F01_transcript_151921	F01_transcript_151921	F01_transcript_151921	F01_transcript_151921	F01_transcript_151921
F01_transcript_75471	NA	F01_transcript_75471	NA	NA
F01_transcript_94975	F01_transcript_94975	F01_transcript_94975	F01_transcript_94975	F01_transcript_94975
F01_transcript_50369	F01_transcript_50369	F01_transcript_50369	NA	F01_transcript_50369

F01_transcript_2088	NA	NA	NA	F01_transcript_2088
F01_transcript_81903	NA	F01_transcript_81903	F01_transcript_81903	F01_transcript_81903
F01_transcript_89057	F01_transcript_89057	F01_transcript_89057	F01_transcript_89057	NA
F01_transcript_163838	NA	NA	NA	F01_transcript_163838
F01_transcript_131891	NA	F01_transcript_131891	NA	NA
F01_transcript_99273	F01_transcript_99273	F01_transcript_99273	F01_transcript_99273	F01_transcript_99273
F01_transcript_138381	F01_transcript_138381	F01_transcript_138381	F01_transcript_138381	F01_transcript_138381
F01_transcript_99763	NA	F01_transcript_99763	NA	NA
F01_transcript_142109	F01_transcript_142109	F01_transcript_142109	F01_transcript_142109	F01_transcript_142109
F01_transcript_113997	NA	NA	NA	F01_transcript_113997
F01_transcript_61853	F01_transcript_61853	F01_transcript_61853	F01_transcript_61853	F01_transcript_61853
F01_transcript_152076	NA	NA	NA	F01_transcript_152076
F01_transcript_3015	NA	F01_transcript_3015	F01_transcript_3015	F01_transcript_3015
F01_transcript_42317	NA	NA	NA	F01_transcript_42317

F01_transcript_43129	NA	NA	NA	F01_transcript_43129
F01_transcript_96144	NA	F01_transcript_96144	NA	F01_transcript_96144
F01_transcript_153683	F01_transcript_153683	F01_transcript_153683	F01_transcript_153683	F01_transcript_153683
F01_transcript_97342	NA	F01_transcript_97342	NA	NA
F01_transcript_153072	NA	NA	NA	F01_transcript_153072
F01_transcript_66884	NA	NA	NA	F01_transcript_66884
F01_transcript_93949	NA	F01_transcript_93949	NA	F01_transcript_93949
F01_transcript_64672	NA	NA	NA	F01_transcript_64672
F01_transcript_131164	NA	NA	NA	F01_transcript_131164
F01_transcript_3478	NA	NA	NA	F01_transcript_3478
F01_transcript_2619	NA	F01_transcript_2619	NA	F01_transcript_2619
F01_transcript_37489	NA	NA	NA	F01_transcript_37489
F01_transcript_99019	NA	F01_transcript_99019	NA	NA
F01_transcript_36592	NA	NA	NA	F01_transcript_36592

F01_transcript_138597	NA	F01_transcript_138597	NA	F01_transcript_138597
F01_transcript_450	NA	NA	NA	F01_transcript_450
F01_transcript_159446	NA	F01_transcript_159446	NA	NA
F01_transcript_109553	NA	F01_transcript_109553	NA	NA
F01_transcript_87926	F01_transcript_87926	F01_transcript_87926	F01_transcript_87926	F01_transcript_87926
F01_transcript_60713	F01_transcript_60713	F01_transcript_60713	F01_transcript_60713	F01_transcript_60713
F01_transcript_96206	F01_transcript_96206	F01_transcript_96206	F01_transcript_96206	F01_transcript_96206
F01_transcript_79584	F01_transcript_79584	F01_transcript_79584	F01_transcript_79584	F01_transcript_79584
F01_transcript_163848	NA	NA	NA	F01_transcript_163848
F01_transcript_75409	NA	F01_transcript_75409	NA	NA
F01_transcript_157271	NA	NA	NA	F01_transcript_157271
F01_transcript_147829	NA	NA	NA	F01_transcript_147829
F01_transcript_147405	F01_transcript_147405	F01_transcript_147405	F01_transcript_147405	F01_transcript_147405
F01_transcript_7453	F01_transcript_7453	F01_transcript_7453	F01_transcript_7453	F01_transcript_7453

F01_transcript_123859	F01_transcript_123859	NA	NA	NA
F01_transcript_60967	F01_transcript_60967	F01_transcript_60967	F01_transcript_60967	F01_transcript_60967
F01_transcript_63469	NA	NA	F01_transcript_63469	F01_transcript_63469
F01_transcript_73711	F01_transcript_73711	F01_transcript_73711	F01_transcript_73711	F01_transcript_73711
F01_transcript_72628	NA	NA	NA	F01_transcript_72628
F01_transcript_103951	NA	F01_transcript_103951	NA	NA
F01_transcript_60180	F01_transcript_60180	F01_transcript_60180	F01_transcript_60180	F01_transcript_60180
F01_transcript_144791	NA	NA	NA	F01_transcript_144791
F01_transcript_49764	NA	NA	F01_transcript_49764	NA
F01_transcript_150810	F01_transcript_150810	F01_transcript_150810	F01_transcript_150810	F01_transcript_150810
F01_transcript_54318	F01_transcript_54318	F01_transcript_54318	F01_transcript_54318	F01_transcript_54318
F01_transcript_14487	F01_transcript_14487	F01_transcript_14487	NA	NA
F01_transcript_4150	F01_transcript_4150	F01_transcript_4150	F01_transcript_4150	F01_transcript_4150
F01_transcript_121573	F01_transcript_121573	F01_transcript_121573	NA	NA

F01_transcript_121979	NA	F01_transcript_121979	NA	NA
F01_transcript_127802	F01_transcript_127802	F01_transcript_127802	F01_transcript_127802	F01_transcript_127802
F01_transcript_96282	F01_transcript_96282	F01_transcript_96282	F01_transcript_96282	F01_transcript_96282
F01_transcript_98615	NA	F01_transcript_98615	NA	NA
F01_transcript_66769	F01_transcript_66769	F01_transcript_66769	F01_transcript_66769	F01_transcript_66769
F01_transcript_140826	NA	F01_transcript_140826	NA	F01_transcript_140826
F01_transcript_101226	NA	NA	NA	F01_transcript_101226
F01_transcript_142350	NA	NA	NA	F01_transcript_142350
F01_transcript_62310	NA	NA	NA	F01_transcript_62310
F01_transcript_139192	NA	F01_transcript_139192	NA	NA
F01_transcript_149822	NA	F01_transcript_149822	NA	NA
F01_transcript_90916	NA	NA	NA	F01_transcript_90916
F01_transcript_32911	NA	NA	NA	F01_transcript_32911
F01_transcript_148522	NA	F01_transcript_148522	F01_transcript_148522	F01_transcript_148522

F01_transcript_147518	F01_transcript_147518	F01_transcript_147518	F01_transcript_147518	NA
F01_transcript_69594	NA	NA	NA	F01_transcript_69594
F01_transcript_140937	NA	NA	NA	F01_transcript_140937
F01_transcript_98060	NA	NA	NA	F01_transcript_98060
F01_transcript_142838	NA	F01_transcript_142838	NA	F01_transcript_142838
F01_transcript_7673	NA	NA	NA	F01_transcript_7673
F01_transcript_115696	NA	NA	NA	F01_transcript_115696
F01_transcript_126978	F01_transcript_126978	F01_transcript_126978	F01_transcript_126978	F01_transcript_126978
F01_transcript_96812	NA	NA	NA	F01_transcript_96812
F01_transcript_76488	NA	F01_transcript_76488	F01_transcript_76488	F01_transcript_76488
F01_transcript_69409	NA	F01_transcript_69409	NA	NA
F01_transcript_80206	NA	F01_transcript_80206	NA	NA
F01_transcript_127700	NA	F01_transcript_127700	NA	F01_transcript_127700
F01_transcript_142217	NA	NA	NA	F01_transcript_142217

F01_transcript_116953	F01_transcript_116953	F01_transcript_116953	NA	NA
F01_transcript_147894	F01_transcript_147894	F01_transcript_147894	F01_transcript_147894	F01_transcript_147894
F01_transcript_65902	NA	F01_transcript_65902	NA	NA
F01_transcript_137497	NA	F01_transcript_137497	NA	NA
F01_transcript_120979	F01_transcript_120979	F01_transcript_120979	F01_transcript_120979	F01_transcript_120979
F01_transcript_102640	NA	F01_transcript_102640	NA	NA
F01_transcript_46167	NA	NA	F01_transcript_46167	F01_transcript_46167
F01_transcript_126673	NA	NA	NA	F01_transcript_126673
F01_transcript_16172	NA	NA	NA	F01_transcript_16172
F01_transcript_6511	NA	NA	NA	F01_transcript_6511
F01_transcript_113326	F01_transcript_113326	F01_transcript_113326	F01_transcript_113326	NA
F01_transcript_123482	NA	NA	NA	F01_transcript_123482
F01_transcript_137960	NA	F01_transcript_137960	NA	F01_transcript_137960
F01_transcript_155148	F01_transcript_155148	F01_transcript_155148	NA	F01_transcript_155148

F01_transcript_165024	F01_transcript_165024	F01_transcript_165024	F01_transcript_165024	F01_transcript_165024
F01_transcript_58060	F01_transcript_58060	F01_transcript_58060	F01_transcript_58060	F01_transcript_58060
F01_transcript_153627	NA	F01_transcript_153627	NA	NA
F01_transcript_50547	NA	F01_transcript_50547	NA	F01_transcript_50547
F01_transcript_110508	F01_transcript_110508	F01_transcript_110508	F01_transcript_110508	F01_transcript_110508
F01_transcript_90513	NA	NA	NA	F01_transcript_90513
F01_transcript_54830	NA	F01_transcript_54830	NA	NA
F01_transcript_89790	NA	F01_transcript_89790	NA	NA
F01_transcript_50925	F01_transcript_50925	F01_transcript_50925	F01_transcript_50925	F01_transcript_50925
F01_transcript_146525	NA	NA	NA	F01_transcript_146525
F01_transcript_56850	NA	NA	F01_transcript_56850	F01_transcript_56850
F01_transcript_127608	NA	NA	F01_transcript_127608	F01_transcript_127608
F01_transcript_102610	F01_transcript_102610	F01_transcript_102610	F01_transcript_102610	F01_transcript_102610
F01_transcript_129948	NA	NA	NA	F01_transcript_129948

F01_transcript_134010	NA	F01_transcript_134010	F01_transcript_134010	NA
F01_transcript_99794	F01_transcript_99794	F01_transcript_99794	F01_transcript_99794	F01_transcript_99794
F01_transcript_85277	NA	NA	NA	F01_transcript_85277
F01_transcript_58766	NA	F01_transcript_58766	NA	F01_transcript_58766
F01_transcript_99384	NA	NA	NA	F01_transcript_99384
F01_transcript_44843	NA	F01_transcript_44843	NA	NA
F01_transcript_142675	F01_transcript_142675	F01_transcript_142675	NA	F01_transcript_142675
F01_transcript_91117	NA	F01_transcript_91117	NA	F01_transcript_91117
F01_transcript_50034	F01_transcript_50034	F01_transcript_50034	F01_transcript_50034	F01_transcript_50034
F01_transcript_17107	NA	NA	NA	F01_transcript_17107
F01_transcript_111940	F01_transcript_111940	F01_transcript_111940	F01_transcript_111940	F01_transcript_111940
F01_transcript_14697	F01_transcript_14697	F01_transcript_14697	F01_transcript_14697	F01_transcript_14697
F01_transcript_141338	NA	NA	NA	F01_transcript_141338
F01_transcript_119431	F01_transcript_119431	F01_transcript_119431	NA	F01_transcript_119431

F01_transcript_114490	F01_transcript_114490	F01_transcript_114490	F01_transcript_114490	F01_transcript_114490
F01_transcript_64119	NA	NA	NA	F01_transcript_64119
F01_transcript_35748	NA	NA	NA	F01_transcript_35748
F01_transcript_129598	NA	NA	NA	F01_transcript_129598
F01_transcript_111076	NA	NA	NA	F01_transcript_111076
F01_transcript_118121	NA	NA	NA	F01_transcript_118121
F01_transcript_138155	NA	NA	NA	F01_transcript_138155
F01_transcript_73794	NA	F01_transcript_73794	NA	F01_transcript_73794
F01_transcript_101364	F01_transcript_101364	F01_transcript_101364	NA	F01_transcript_101364
F01_transcript_115706	NA	F01_transcript_115706	NA	NA
F01_transcript_95492	NA	F01_transcript_95492	NA	F01_transcript_95492
F01_transcript_135316	NA	NA	NA	F01_transcript_135316
F01_transcript_50698	F01_transcript_50698	F01_transcript_50698	NA	NA
F01_transcript_76074	NA	F01_transcript_76074	F01_transcript_76074	F01_transcript_76074

F01_transcript_123547	F01_transcript_123547	F01_transcript_123547	NA	NA
F01_transcript_126699	NA	F01_transcript_126699	NA	NA
F01_transcript_74808	NA	F01_transcript_74808	NA	F01_transcript_74808
F01_transcript_104511	NA	F01_transcript_104511	NA	NA
F01_transcript_129513	NA	NA	NA	F01_transcript_129513
F01_transcript_154841	F01_transcript_154841	F01_transcript_154841	F01_transcript_154841	F01_transcript_154841
F01_transcript_94716	NA	NA	NA	F01_transcript_94716
F01_transcript_149324	NA	NA	NA	F01_transcript_149324
F01_transcript_107712	F01_transcript_107712	F01_transcript_107712	NA	NA
F01_transcript_92474	NA	F01_transcript_92474	NA	NA
F01_transcript_95162	NA	F01_transcript_95162	NA	NA
F01_transcript_106957	NA	F01_transcript_106957	NA	F01_transcript_106957
F01_transcript_125229	NA	F01_transcript_125229	NA	F01_transcript_125229
F01_transcript_122986	NA	NA	NA	F01_transcript_122986

F01_transcript_8428	NA	F01_transcript_8428	NA	F01_transcript_8428
F01_transcript_120582	F01_transcript_120582	F01_transcript_120582	F01_transcript_120582	F01_transcript_120582
F01_transcript_21696	F01_transcript_21696	F01_transcript_21696	NA	F01_transcript_21696
F01_transcript_59901	NA	F01_transcript_59901	NA	NA
F01_transcript_98529	NA	NA	NA	F01_transcript_98529
F01_transcript_128434	NA	F01_transcript_128434	NA	F01_transcript_128434
F01_transcript_85986	NA	NA	NA	F01_transcript_85986
F01_transcript_78686	F01_transcript_78686	F01_transcript_78686	NA	F01_transcript_78686
F01_transcript_47331	F01_transcript_47331	NA	F01_transcript_47331	F01_transcript_47331
F01_transcript_49889	NA	F01_transcript_49889	NA	NA
F01_transcript_128166	NA	F01_transcript_128166	NA	NA
F01_transcript_76544	F01_transcript_76544	F01_transcript_76544	NA	F01_transcript_76544
F01_transcript_145162	F01_transcript_145162	F01_transcript_145162	F01_transcript_145162	F01_transcript_145162
F01_transcript_43291	NA	NA	NA	F01_transcript_43291

F01_transcript_59708	NA	NA	NA	F01_transcript_59708
F01_transcript_40686	NA	NA	NA	F01_transcript_40686
F01_transcript_112529	F01_transcript_112529	F01_transcript_112529	F01_transcript_112529	F01_transcript_112529
F01_transcript_100191	NA	NA	F01_transcript_100191	NA
F01_transcript_97816	NA	F01_transcript_97816	NA	NA
F01_transcript_83206	NA	F01_transcript_83206	NA	NA
F01_transcript_156760	NA	F01_transcript_156760	NA	NA
F01_transcript_95592	NA	NA	NA	F01_transcript_95592
F01_transcript_43248	NA	F01_transcript_43248	NA	F01_transcript_43248
F01_transcript_147844	NA	F01_transcript_147844	NA	F01_transcript_147844
F01_transcript_109960	NA	F01_transcript_109960	F01_transcript_109960	F01_transcript_109960
F01_transcript_150152	F01_transcript_150152	F01_transcript_150152	F01_transcript_150152	F01_transcript_150152
F01_transcript_135943	F01_transcript_135943	F01_transcript_135943	F01_transcript_135943	F01_transcript_135943
F01_transcript_77817	NA	F01_transcript_77817	F01_transcript_77817	F01_transcript_77817

F01_transcript_45611	NA	F01_transcript_45611	NA	NA
F01_transcript_93740	NA	F01_transcript_93740	F01_transcript_93740	F01_transcript_93740
F01_transcript_100612	NA	F01_transcript_100612	NA	NA
F01_transcript_21078	F01_transcript_21078	F01_transcript_21078	F01_transcript_21078	F01_transcript_21078
F01_transcript_159591	NA	F01_transcript_159591	NA	NA
F01_transcript_77806	NA	NA	NA	F01_transcript_77806
F01_transcript_107557	NA	NA	NA	F01_transcript_107557
F01_transcript_18090	NA	F01_transcript_18090	F01_transcript_18090	NA
F01_transcript_17436	NA	NA	NA	F01_transcript_17436
F01_transcript_106259	NA	F01_transcript_106259	NA	F01_transcript_106259
F01_transcript_129064	NA	F01_transcript_129064	F01_transcript_129064	NA
F01_transcript_150527	NA	F01_transcript_150527	NA	NA
F01_transcript_52899	F01_transcript_52899	F01_transcript_52899	F01_transcript_52899	F01_transcript_52899
F01_transcript_55907	NA	NA	NA	F01_transcript_55907

F01_transcript_76265	F01_transcript_76265	F01_transcript_76265	F01_transcript_76265	F01_transcript_76265
F01_transcript_52040	NA	F01_transcript_52040	NA	NA
F01_transcript_15877	NA	NA	NA	F01_transcript_15877
F01_transcript_116120	NA	NA	NA	F01_transcript_116120
F01_transcript_155674	NA	F01_transcript_155674	NA	NA
F01_transcript_131671	NA	NA	NA	F01_transcript_131671
F01_transcript_111262	F01_transcript_111262	F01_transcript_111262	F01_transcript_111262	F01_transcript_111262
F01_transcript_66639	NA	NA	NA	F01_transcript_66639
F01_transcript_151969	NA	NA	NA	F01_transcript_151969
F01_transcript_101636	NA	NA	NA	F01_transcript_101636
F01_transcript_86896	NA	NA	NA	F01_transcript_86896
F01_transcript_106299	F01_transcript_106299	F01_transcript_106299	F01_transcript_106299	F01_transcript_106299
F01_transcript_82876	NA	NA	NA	F01_transcript_82876
F01_transcript_66895	NA	F01_transcript_66895	NA	NA

F01_transcript_135163	F01_transcript_135163	F01_transcript_135163	F01_transcript_135163	F01_transcript_135163
F01_transcript_44822	NA	NA	NA	F01_transcript_44822
F01_transcript_51921	F01_transcript_51921	F01_transcript_51921	NA	NA
F01_transcript_110727	NA	NA	NA	F01_transcript_110727
F01_transcript_145787	F01_transcript_145787	F01_transcript_145787	F01_transcript_145787	F01_transcript_145787
F01_transcript_118296	F01_transcript_118296	F01_transcript_118296	F01_transcript_118296	NA
F01_transcript_93798	NA	F01_transcript_93798	NA	NA
F01_transcript_134344	NA	NA	NA	F01_transcript_134344
F01_transcript_83613	NA	NA	NA	F01_transcript_83613
F01_transcript_133220	F01_transcript_133220	F01_transcript_133220	NA	F01_transcript_133220
F01_transcript_135182	NA	F01_transcript_135182	NA	NA
F01_transcript_162074	NA	NA	NA	F01_transcript_162074
F01_transcript_76557	NA	F01_transcript_76557	NA	F01_transcript_76557
F01_transcript_129176	NA	NA	NA	F01_transcript_129176

F01_transcript_147931	NA	F01_transcript_147931	F01_transcript_147931	F01_transcript_147931
F01_transcript_40224	NA	NA	NA	F01_transcript_40224
F01_transcript_54699	NA	F01_transcript_54699	NA	NA
F01_transcript_53020	NA	F01_transcript_53020	F01_transcript_53020	NA
F01_transcript_132237	F01_transcript_132237	F01_transcript_132237	NA	NA
F01_transcript_2172	NA	F01_transcript_2172	NA	F01_transcript_2172
F01_transcript_50883	F01_transcript_50883	F01_transcript_50883	NA	NA
F01_transcript_84132	NA	F01_transcript_84132	NA	NA
F01_transcript_13738	F01_transcript_13738	F01_transcript_13738	NA	NA
F01_transcript_116707	NA	NA	NA	F01_transcript_116707
F01_transcript_70605	NA	NA	NA	F01_transcript_70605
F01_transcript_108890	F01_transcript_108890	F01_transcript_108890	F01_transcript_108890	F01_transcript_108890
F01_transcript_121955	NA	F01_transcript_121955	NA	NA
F01_transcript_79144	NA	F01_transcript_79144	NA	NA

F01_transcript_82379	F01_transcript_82379	F01_transcript_82379	NA	NA
F01_transcript_75687	NA	NA	NA	F01_transcript_75687
F01_transcript_71664	F01_transcript_71664	F01_transcript_71664	F01_transcript_71664	F01_transcript_71664
F01_transcript_122270	F01_transcript_122270	F01_transcript_122270	F01_transcript_122270	F01_transcript_122270
F01_transcript_123684	NA	F01_transcript_123684	NA	NA
F01_transcript_80665	NA	NA	NA	F01_transcript_80665
F01_transcript_166070	F01_transcript_166070	F01_transcript_166070	F01_transcript_166070	F01_transcript_166070
F01_transcript_149011	NA	F01_transcript_149011	NA	F01_transcript_149011
F01_transcript_28020	F01_transcript_28020	F01_transcript_28020	NA	NA
F01_transcript_10923	NA	NA	NA	F01_transcript_10923
F01_transcript_74036	NA	F01_transcript_74036	NA	NA
F01_transcript_148396	F01_transcript_148396	F01_transcript_148396	F01_transcript_148396	F01_transcript_148396
F01_transcript_97586	NA	F01_transcript_97586	NA	F01_transcript_97586
F01_transcript_79444	NA	NA	F01_transcript_79444	F01_transcript_79444

F01_transcript_48301	NA	F01_transcript_48301	NA	NA
F01_transcript_62948	NA	NA	NA	F01_transcript_62948
F01_transcript_106328	NA	F01_transcript_106328	NA	F01_transcript_106328
F01_transcript_125910	NA	NA	NA	F01_transcript_125910
F01_transcript_76520	NA	NA	NA	F01_transcript_76520
F01_transcript_163209	NA	NA	NA	F01_transcript_163209
F01_transcript_147014	NA	F01_transcript_147014	F01_transcript_147014	F01_transcript_147014
F01_transcript_93867	NA	NA	F01_transcript_93867	F01_transcript_93867
F01_transcript_104773	F01_transcript_104773	F01_transcript_104773	F01_transcript_104773	NA
F01_transcript_56676	NA	NA	NA	F01_transcript_56676
F01_transcript_3392	NA	F01_transcript_3392	NA	NA
F01_transcript_93578	NA	NA	NA	F01_transcript_93578
F01_transcript_61100	NA	NA	NA	F01_transcript_61100
F01_transcript_132040	NA	F01_transcript_132040	NA	NA

F01_transcript_70432	NA	F01_transcript_70432	NA	F01_transcript_70432
F01_transcript_115865	F01_transcript_115865	F01_transcript_115865	F01_transcript_115865	F01_transcript_115865
F01_transcript_157443	NA	F01_transcript_157443	NA	NA
F01_transcript_54385	NA	F01_transcript_54385	F01_transcript_54385	F01_transcript_54385
F01_transcript_122541	F01_transcript_122541	F01_transcript_122541	F01_transcript_122541	F01_transcript_122541
F01_transcript_144525	F01_transcript_144525	NA	F01_transcript_144525	F01_transcript_144525
F01_transcript_37454	NA	NA	NA	F01_transcript_37454
F01_transcript_24714	NA	NA	NA	F01_transcript_24714
F01_transcript_70903	NA	NA	F01_transcript_70903	NA
F01_transcript_111369	F01_transcript_111369	F01_transcript_111369	F01_transcript_111369	F01_transcript_111369
F01_transcript_162930	NA	F01_transcript_162930	F01_transcript_162930	F01_transcript_162930
F01_transcript_114207	NA	NA	NA	F01_transcript_114207
F01_transcript_84100	F01_transcript_84100	F01_transcript_84100	F01_transcript_84100	F01_transcript_84100
F01_transcript_61589	F01_transcript_61589	F01_transcript_61589	F01_transcript_61589	F01_transcript_61589

F01_transcript_94933	NA	NA	NA	F01_transcript_94933
F01_transcript_86458	F01_transcript_86458	F01_transcript_86458	NA	NA
F01_transcript_53882	NA	F01_transcript_53882	NA	NA
F01_transcript_127407	NA	NA	NA	F01_transcript_127407
F01_transcript_156259	F01_transcript_156259	F01_transcript_156259	F01_transcript_156259	F01_transcript_156259
F01_transcript_116031	F01_transcript_116031	F01_transcript_116031	F01_transcript_116031	F01_transcript_116031
F01_transcript_42484	NA	NA	NA	F01_transcript_42484
F01_transcript_132709	F01_transcript_132709	F01_transcript_132709	F01_transcript_132709	F01_transcript_132709
F01_transcript_111767	F01_transcript_111767	F01_transcript_111767	F01_transcript_111767	F01_transcript_111767
F01_transcript_59126	NA	NA	NA	F01_transcript_59126
F01_transcript_150304	NA	F01_transcript_150304	NA	NA
F01_transcript_86982	F01_transcript_86982	F01_transcript_86982	F01_transcript_86982	F01_transcript_86982
F01_transcript_77772	NA	NA	NA	F01_transcript_77772
F01_transcript_47453	NA	F01_transcript_47453	NA	NA

F01_transcript_58039	NA	F01_transcript_58039	NA	F01_transcript_58039
F01_transcript_58306	NA	NA	NA	F01_transcript_58306
F01_transcript_34443	NA	NA	NA	F01_transcript_34443
F01_transcript_46671	NA	NA	NA	F01_transcript_46671
F01_transcript_50973	F01_transcript_50973	F01_transcript_50973	F01_transcript_50973	F01_transcript_50973
F01_transcript_141537	NA	NA	NA	F01_transcript_141537
F01_transcript_93383	NA	F01_transcript_93383	F01_transcript_93383	F01_transcript_93383
F01_transcript_108590	F01_transcript_108590	F01_transcript_108590	F01_transcript_108590	F01_transcript_108590
F01_transcript_104614	F01_transcript_104614	F01_transcript_104614	F01_transcript_104614	F01_transcript_104614
F01_transcript_78631	NA	F01_transcript_78631	F01_transcript_78631	F01_transcript_78631
F01_transcript_85228	NA	F01_transcript_85228	NA	F01_transcript_85228
F01_transcript_80737	NA	F01_transcript_80737	NA	F01_transcript_80737
F01_transcript_161028	NA	F01_transcript_161028	NA	NA
F01_transcript_60706	NA	F01_transcript_60706	NA	NA

F01_transcript_143988	NA	NA	NA	F01_transcript_143988
F01_transcript_101970	NA	NA	NA	F01_transcript_101970
F01_transcript_150445	NA	F01_transcript_150445	NA	NA
F01_transcript_144450	F01_transcript_144450	F01_transcript_144450	F01_transcript_144450	NA
F01_transcript_14456	NA	F01_transcript_14456	NA	NA
F01_transcript_129878	F01_transcript_129878	F01_transcript_129878	F01_transcript_129878	F01_transcript_129878
F01_transcript_5688	NA	NA	NA	F01_transcript_5688
F01_transcript_90789	F01_transcript_90789	F01_transcript_90789	F01_transcript_90789	F01_transcript_90789
F01_transcript_149697	F01_transcript_149697	F01_transcript_149697	F01_transcript_149697	F01_transcript_149697
F01_transcript_158432	NA	NA	NA	F01_transcript_158432
F01_transcript_143675	NA	F01_transcript_143675	F01_transcript_143675	NA
F01_transcript_9415	NA	NA	NA	F01_transcript_9415
F01_transcript_165818	F01_transcript_165818	F01_transcript_165818	F01_transcript_165818	NA
F01_transcript_69382	F01_transcript_69382	F01_transcript_69382	NA	NA

F01_transcript_57304	F01_transcript_57304	F01_transcript_57304	NA	F01_transcript_57304
F01_transcript_163220	NA	NA	NA	F01_transcript_163220
F01_transcript_25259	NA	NA	NA	F01_transcript_25259
F01_transcript_54096	F01_transcript_54096	F01_transcript_54096	F01_transcript_54096	F01_transcript_54096
F01_transcript_124520	NA	NA	NA	F01_transcript_124520
F01_transcript_144953	NA	NA	NA	F01_transcript_144953
F01_transcript_125098	NA	F01_transcript_125098	F01_transcript_125098	F01_transcript_125098
F01_transcript_97258	NA	NA	NA	F01_transcript_97258
F01_transcript_96224	NA	NA	NA	F01_transcript_96224
F01_transcript_93191	NA	F01_transcript_93191	F01_transcript_93191	F01_transcript_93191
F01_transcript_65176	NA	NA	F01_transcript_65176	NA
F01_transcript_8706	NA	NA	F01_transcript_8706	F01_transcript_8706
F01_transcript_56774	NA	F01_transcript_56774	F01_transcript_56774	F01_transcript_56774
F01_transcript_29285	NA	NA	NA	F01_transcript_29285

F01_transcript_62634	F01_transcript_62634	F01_transcript_62634	NA	F01_transcript_62634
F01_transcript_151617	NA	NA	F01_transcript_151617	NA
F01_transcript_153978	NA	F01_transcript_153978	NA	F01_transcript_153978
F01_transcript_106767	NA	NA	NA	F01_transcript_106767
F01_transcript_28533	NA	NA	NA	F01_transcript_28533
F01_transcript_51742	NA	F01_transcript_51742	NA	F01_transcript_51742
F01_transcript_88639	NA	F01_transcript_88639	NA	F01_transcript_88639
F01_transcript_31863	NA	NA	NA	F01_transcript_31863
F01_transcript_122474	F01_transcript_122474	F01_transcript_122474	NA	F01_transcript_122474
F01_transcript_71188	F01_transcript_71188	F01_transcript_71188	F01_transcript_71188	F01_transcript_71188
F01_transcript_131449	NA	NA	NA	F01_transcript_131449
F01_transcript_153942	F01_transcript_153942	F01_transcript_153942	F01_transcript_153942	F01_transcript_153942
F01_transcript_164818	F01_transcript_164818	NA	NA	NA
F01_transcript_54495	F01_transcript_54495	F01_transcript_54495	F01_transcript_54495	F01_transcript_54495

F01_transcript_68563	NA	NA	NA	F01_transcript_68563
F01_transcript_70152	F01_transcript_70152	F01_transcript_70152	F01_transcript_70152	F01_transcript_70152
F01_transcript_95360	NA	NA	NA	F01_transcript_95360
F01_transcript_92820	F01_transcript_92820	F01_transcript_92820	NA	NA
F01_transcript_57517	F01_transcript_57517	F01_transcript_57517	F01_transcript_57517	F01_transcript_57517
F01_transcript_111660	NA	F01_transcript_111660	NA	F01_transcript_111660
F01_transcript_125509	NA	F01_transcript_125509	NA	F01_transcript_125509
F01_transcript_86782	NA	NA	NA	F01_transcript_86782
F01_transcript_166942	NA	F01_transcript_166942	NA	F01_transcript_166942
F01_transcript_39419	NA	NA	NA	F01_transcript_39419
F01_transcript_87847	NA	NA	NA	F01_transcript_87847
F01_transcript_162252	F01_transcript_162252	F01_transcript_162252	F01_transcript_162252	F01_transcript_162252
F01_transcript_108608	NA	NA	NA	F01_transcript_108608
F01_transcript_126762	NA	F01_transcript_126762	NA	F01_transcript_126762

F01_transcript_80500	NA	F01_transcript_80500	NA	F01_transcript_80500
F01_transcript_107408	NA	NA	NA	F01_transcript_107408
F01_transcript_145693	NA	F01_transcript_145693	NA	NA
F01_transcript_96544	NA	NA	NA	F01_transcript_96544
F01_transcript_125296	F01_transcript_125296	F01_transcript_125296	NA	F01_transcript_125296
F01_transcript_153241	NA	F01_transcript_153241	F01_transcript_153241	F01_transcript_153241
F01_transcript_138820	NA	NA	NA	F01_transcript_138820
F01_transcript_128063	NA	NA	NA	F01_transcript_128063
F01_transcript_129276	NA	NA	F01_transcript_129276	F01_transcript_129276
F01_transcript_9550	NA	NA	NA	F01_transcript_9550
F01_transcript_159667	NA	F01_transcript_159667	NA	NA
F01_transcript_106246	NA	F01_transcript_106246	NA	NA
F01_transcript_362	NA	NA	NA	F01_transcript_362
F01_transcript_129621	F01_transcript_129621	F01_transcript_129621	F01_transcript_129621	F01_transcript_129621

F01_transcript_122583	F01_transcript_122583	F01_transcript_122583	F01_transcript_122583	F01_transcript_122583
F01_transcript_30402	F01_transcript_30402	F01_transcript_30402	F01_transcript_30402	F01_transcript_30402
F01_transcript_110247	NA	F01_transcript_110247	NA	NA
F01_transcript_100181	NA	F01_transcript_100181	NA	NA
F01_transcript_114151	NA	NA	NA	F01_transcript_114151
F01_transcript_49811	NA	F01_transcript_49811	NA	NA
F01_transcript_86115	NA	NA	NA	F01_transcript_86115
F01_transcript_230	NA	NA	NA	F01_transcript_230
F01_transcript_142348	NA	F01_transcript_142348	NA	NA
F01_transcript_15089	NA	NA	NA	F01_transcript_15089
F01_transcript_124870	NA	F01_transcript_124870	F01_transcript_124870	F01_transcript_124870
F01_transcript_115087	NA	F01_transcript_115087	NA	NA
F01_transcript_91200	NA	NA	NA	F01_transcript_91200
F01_transcript_59480	F01_transcript_59480	F01_transcript_59480	F01_transcript_59480	F01_transcript_59480

F01_transcript_152008	NA	NA	NA	F01_transcript_152008
F01_transcript_61969	NA	F01_transcript_61969	NA	NA
F01_transcript_69752	NA	NA	NA	F01_transcript_69752
F01_transcript_147807	NA	F01_transcript_147807	F01_transcript_147807	NA
F01_transcript_97829	NA	F01_transcript_97829	NA	NA
F01_transcript_33807	NA	NA	NA	F01_transcript_33807
F01_transcript_140197	NA	NA	NA	F01_transcript_140197
F01_transcript_100071	NA	F01_transcript_100071	F01_transcript_100071	F01_transcript_100071
F01_transcript_79567	NA	NA	F01_transcript_79567	F01_transcript_79567
F01_transcript_143997	F01_transcript_143997	F01_transcript_143997	F01_transcript_143997	F01_transcript_143997
F01_transcript_130839	NA	F01_transcript_130839	NA	NA
F01_transcript_163423	NA	F01_transcript_163423	NA	NA
F01_transcript_167231	NA	NA	NA	F01_transcript_167231
F01_transcript_148650	NA	NA	NA	F01_transcript_148650

F01_transcript_120185	NA	NA	NA	F01_transcript_120185
F01_transcript_61408	NA	NA	NA	F01_transcript_61408
F01_transcript_117267	NA	F01_transcript_117267	F01_transcript_117267	F01_transcript_117267
F01_transcript_65293	NA	NA	F01_transcript_65293	NA
F01_transcript_164907	NA	NA	NA	F01_transcript_164907
F01_transcript_59628	F01_transcript_59628	F01_transcript_59628	NA	F01_transcript_59628
F01_transcript_73393	F01_transcript_73393	F01_transcript_73393	NA	NA
F01_transcript_55261	NA	NA	NA	F01_transcript_55261
F01_transcript_136421	F01_transcript_136421	F01_transcript_136421	F01_transcript_136421	F01_transcript_136421
F01_transcript_147841	NA	F01_transcript_147841	NA	NA
F01_transcript_8991	NA	F01_transcript_8991	NA	F01_transcript_8991
F01_transcript_29211	NA	NA	NA	F01_transcript_29211
F01_transcript_8372	NA	NA	NA	F01_transcript_8372
F01_transcript_98303	NA	F01_transcript_98303	F01_transcript_98303	NA

F01_transcript_152613	NA	F01_transcript_152613	NA	F01_transcript_152613
F01_transcript_88412	NA	NA	NA	F01_transcript_88412
F01_transcript_50292	F01_transcript_50292	F01_transcript_50292	F01_transcript_50292	F01_transcript_50292
F01_transcript_71149	NA	F01_transcript_71149	F01_transcript_71149	NA
F01_transcript_131365	NA	F01_transcript_131365	NA	NA
F01_transcript_96129	NA	NA	NA	F01_transcript_96129
F01_transcript_60353	F01_transcript_60353	NA	F01_transcript_60353	NA
F01_transcript_83799	NA	F01_transcript_83799	F01_transcript_83799	NA
F01_transcript_13517	NA	NA	NA	F01_transcript_13517
F01_transcript_51666	F01_transcript_51666	F01_transcript_51666	F01_transcript_51666	F01_transcript_51666
F01_transcript_97161	NA	NA	NA	F01_transcript_97161
F01_transcript_123178	F01_transcript_123178	F01_transcript_123178	F01_transcript_123178	F01_transcript_123178
F01_transcript_87979	F01_transcript_87979	F01_transcript_87979	F01_transcript_87979	F01_transcript_87979
F01_transcript_100255	NA	F01_transcript_100255	NA	NA

F01_transcript_76977	NA	F01_transcript_76977	NA	NA
F01_transcript_123820	F01_transcript_123820	F01_transcript_123820	NA	F01_transcript_123820
F01_transcript_139538	NA	F01_transcript_139538	F01_transcript_139538	F01_transcript_139538
F01_transcript_138089	F01_transcript_138089	F01_transcript_138089	F01_transcript_138089	NA
F01_transcript_55786	NA	F01_transcript_55786	F01_transcript_55786	F01_transcript_55786
F01_transcript_126718	F01_transcript_126718	F01_transcript_126718	F01_transcript_126718	F01_transcript_126718
F01_transcript_57503	NA	F01_transcript_57503	NA	NA
F01_transcript_86985	NA	F01_transcript_86985	NA	F01_transcript_86985
F01_transcript_63888	NA	NA	NA	F01_transcript_63888
F01_transcript_88444	F01_transcript_88444	F01_transcript_88444	NA	F01_transcript_88444
F01_transcript_134378	NA	NA	NA	F01_transcript_134378
F01_transcript_159018	NA	F01_transcript_159018	F01_transcript_159018	NA
F01_transcript_63853	F01_transcript_63853	F01_transcript_63853	NA	NA
F01_transcript_149580	NA	NA	NA	F01_transcript_149580

F01_transcript_56182	NA	NA	F01_transcript_56182	NA
F01_transcript_83072	F01_transcript_83072	F01_transcript_83072	NA	F01_transcript_83072
F01_transcript_75142	NA	F01_transcript_75142	NA	NA
F01_transcript_148823	NA	F01_transcript_148823	NA	F01_transcript_148823
F01_transcript_79469	F01_transcript_79469	NA	F01_transcript_79469	F01_transcript_79469
F01_transcript_85545	F01_transcript_85545	F01_transcript_85545	F01_transcript_85545	F01_transcript_85545
F01_transcript_66327	NA	NA	NA	F01_transcript_66327
F01_transcript_51638	F01_transcript_51638	F01_transcript_51638	F01_transcript_51638	F01_transcript_51638
F01_transcript_124550	NA	F01_transcript_124550	NA	NA
F01_transcript_162146	NA	NA	NA	F01_transcript_162146
F01_transcript_88911	NA	NA	NA	F01_transcript_88911
F01_transcript_56991	NA	F01_transcript_56991	F01_transcript_56991	NA
F01_transcript_131959	NA	NA	F01_transcript_131959	NA
F01_transcript_97864	F01_transcript_97864	F01_transcript_97864	NA	F01_transcript_97864

F01_transcript_130220	NA	NA	NA	F01_transcript_130220
F01_transcript_57765	NA	NA	NA	F01_transcript_57765
F01_transcript_20891	NA	F01_transcript_20891	NA	F01_transcript_20891
F01_transcript_112424	F01_transcript_112424	F01_transcript_112424	F01_transcript_112424	F01_transcript_112424
F01_transcript_86569	NA	F01_transcript_86569	NA	NA
F01_transcript_55045	NA	F01_transcript_55045	F01_transcript_55045	F01_transcript_55045
F01_transcript_103000	NA	NA	NA	F01_transcript_103000
F01_transcript_105682	NA	F01_transcript_105682	NA	NA
F01_transcript_152851	F01_transcript_152851	F01_transcript_152851	F01_transcript_152851	F01_transcript_152851
F01_transcript_54087	NA	F01_transcript_54087	NA	NA
F01_transcript_125750	NA	NA	NA	F01_transcript_125750
F01_transcript_164249	F01_transcript_164249	F01_transcript_164249	F01_transcript_164249	F01_transcript_164249
F01_transcript_141544	F01_transcript_141544	NA	NA	F01_transcript_141544
F01_transcript_142367	NA	F01_transcript_142367	NA	F01_transcript_142367

F01_transcript_14126	NA	NA	NA	F01_transcript_14126
F01_transcript_86714	NA	NA	NA	F01_transcript_86714
F01_transcript_113587	NA	F01_transcript_113587	NA	NA
F01_transcript_56865	F01_transcript_56865	F01_transcript_56865	F01_transcript_56865	F01_transcript_56865
F01_transcript_45571	NA	F01_transcript_45571	NA	F01_transcript_45571
F01_transcript_112340	NA	F01_transcript_112340	NA	NA
F01_transcript_98696	NA	F01_transcript_98696	NA	F01_transcript_98696
F01_transcript_99636	NA	F01_transcript_99636	NA	F01_transcript_99636
F01_transcript_157683	NA	F01_transcript_157683	F01_transcript_157683	NA
F01_transcript_159331	NA	NA	NA	F01_transcript_159331
F01_transcript_163303	F01_transcript_163303	F01_transcript_163303	F01_transcript_163303	F01_transcript_163303
F01_transcript_132189	F01_transcript_132189	F01_transcript_132189	F01_transcript_132189	F01_transcript_132189
F01_transcript_133563	NA	NA	NA	F01_transcript_133563
F01_transcript_48220	NA	F01_transcript_48220	NA	NA

F01_transcript_149893	F01_transcript_149893	F01_transcript_149893	F01_transcript_149893	NA
F01_transcript_77884	NA	NA	NA	F01_transcript_77884
F01_transcript_63545	F01_transcript_63545	F01_transcript_63545	F01_transcript_63545	F01_transcript_63545
F01_transcript_87131	NA	NA	NA	F01_transcript_87131
F01_transcript_88157	NA	NA	NA	F01_transcript_88157
F01_transcript_92209	NA	F01_transcript_92209	NA	NA
F01_transcript_69896	F01_transcript_69896	NA	NA	F01_transcript_69896
F01_transcript_159500	NA	NA	NA	F01_transcript_159500
F01_transcript_19753	NA	NA	NA	F01_transcript_19753
F01_transcript_165678	F01_transcript_165678	F01_transcript_165678	F01_transcript_165678	NA
F01_transcript_88155	NA	NA	NA	F01_transcript_88155
F01_transcript_135496	F01_transcript_135496	F01_transcript_135496	F01_transcript_135496	F01_transcript_135496
F01_transcript_132969	NA	F01_transcript_132969	NA	NA
F01_transcript_10296	NA	NA	NA	F01_transcript_10296

F01_transcript_65192	NA	NA	NA	F01_transcript_65192
F01_transcript_64031	NA	NA	NA	F01_transcript_64031
F01_transcript_49212	NA	F01_transcript_49212	NA	NA
F01_transcript_82050	F01_transcript_82050	F01_transcript_82050	F01_transcript_82050	NA
F01_transcript_91549	NA	F01_transcript_91549	NA	NA
F01_transcript_167168	NA	F01_transcript_167168	NA	NA
F01_transcript_63480	NA	F01_transcript_63480	NA	NA
F01_transcript_70054	NA	NA	NA	F01_transcript_70054
F01_transcript_78236	F01_transcript_78236	F01_transcript_78236	F01_transcript_78236	F01_transcript_78236
F01_transcript_88509	NA	F01_transcript_88509	NA	NA
F01_transcript_90091	NA	NA	NA	F01_transcript_90091
F01_transcript_88580	NA	NA	NA	F01_transcript_88580
F01_transcript_164922	NA	NA	F01_transcript_164922	NA
F01_transcript_112652	NA	F01_transcript_112652	F01_transcript_112652	NA

F01_transcript_116063	F01_transcript_116063	F01_transcript_116063	NA	NA
F01_transcript_126528	NA	F01_transcript_126528	NA	NA
F01_transcript_96703	NA	F01_transcript_96703	F01_transcript_96703	F01_transcript_96703
F01_transcript_150605	NA	NA	NA	F01_transcript_150605
F01_transcript_120370	F01_transcript_120370	F01_transcript_120370	F01_transcript_120370	F01_transcript_120370
F01_transcript_41578	NA	NA	NA	F01_transcript_41578
F01_transcript_114957	F01_transcript_114957	F01_transcript_114957	F01_transcript_114957	F01_transcript_114957
F01_transcript_137265	NA	F01_transcript_137265	NA	F01_transcript_137265
F01_transcript_122140	NA	F01_transcript_122140	NA	F01_transcript_122140
F01_transcript_82682	NA	F01_transcript_82682	NA	F01_transcript_82682
F01_transcript_29390	NA	NA	NA	F01_transcript_29390
F01_transcript_49712	F01_transcript_49712	F01_transcript_49712	NA	NA
F01_transcript_159023	NA	NA	F01_transcript_159023	F01_transcript_159023
F01_transcript_158452	NA	F01_transcript_158452	NA	F01_transcript_158452

F01_transcript_154314	F01_transcript_154314	F01_transcript_154314	NA	F01_transcript_154314
F01_transcript_135825	NA	F01_transcript_135825	NA	F01_transcript_135825
F01_transcript_63785	F01_transcript_63785	F01_transcript_63785	NA	NA
F01_transcript_102019	NA	NA	NA	F01_transcript_102019
F01_transcript_92705	NA	F01_transcript_92705	NA	NA
F01_transcript_91867	NA	F01_transcript_91867	NA	F01_transcript_91867
F01_transcript_164297	F01_transcript_164297	F01_transcript_164297	F01_transcript_164297	F01_transcript_164297
F01_transcript_135657	NA	F01_transcript_135657	NA	NA
F01_transcript_93780	F01_transcript_93780	F01_transcript_93780	F01_transcript_93780	F01_transcript_93780
F01_transcript_82573	NA	F01_transcript_82573	NA	NA
F01_transcript_34613	NA	NA	NA	F01_transcript_34613
F01_transcript_109512	F01_transcript_109512	F01_transcript_109512	NA	F01_transcript_109512
F01_transcript_121792	F01_transcript_121792	F01_transcript_121792	NA	NA
F01_transcript_47642	NA	NA	NA	F01_transcript_47642

F01_transcript_109645	F01_transcript_109645	NA	NA	NA
F01_transcript_1884	F01_transcript_1884	F01_transcript_1884	F01_transcript_1884	NA
F01_transcript_155319	F01_transcript_155319	F01_transcript_155319	F01_transcript_155319	F01_transcript_155319
F01_transcript_38724	NA	F01_transcript_38724	NA	NA
F01_transcript_82698	NA	F01_transcript_82698	NA	F01_transcript_82698
F01_transcript_104701	F01_transcript_104701	F01_transcript_104701	F01_transcript_104701	F01_transcript_104701
F01_transcript_163909	NA	F01_transcript_163909	NA	NA
F01_transcript_116676	NA	F01_transcript_116676	F01_transcript_116676	NA
F01_transcript_82191	F01_transcript_82191	F01_transcript_82191	F01_transcript_82191	F01_transcript_82191
F01_transcript_68622	NA	F01_transcript_68622	F01_transcript_68622	F01_transcript_68622
F01_transcript_53626	NA	F01_transcript_53626	NA	NA
F01_transcript_6433	NA	NA	NA	F01_transcript_6433
F01_transcript_111528	NA	NA	F01_transcript_111528	F01_transcript_111528
F01_transcript_158693	NA	NA	NA	F01_transcript_158693

F01_transcript_109930	NA	F01_transcript_109930	NA	NA
F01_transcript_130327	NA	F01_transcript_130327	NA	NA
F01_transcript_76659	F01_transcript_76659	F01_transcript_76659	NA	F01_transcript_76659
F01_transcript_166910	NA	NA	NA	F01_transcript_166910
F01_transcript_99016	F01_transcript_99016	F01_transcript_99016	F01_transcript_99016	F01_transcript_99016
F01_transcript_60364	NA	F01_transcript_60364	NA	NA
F01_transcript_85620	F01_transcript_85620	F01_transcript_85620	NA	F01_transcript_85620
F01_transcript_49089	NA	F01_transcript_49089	NA	NA
F01_transcript_89347	NA	F01_transcript_89347	NA	NA
F01_transcript_121697	NA	NA	NA	F01_transcript_121697
F01_transcript_23387	NA	NA	NA	F01_transcript_23387
F01_transcript_93670	NA	F01_transcript_93670	NA	NA
F01_transcript_76011	NA	F01_transcript_76011	NA	NA
F01_transcript_68532	NA	F01_transcript_68532	F01_transcript_68532	F01_transcript_68532

F01_transcript_76987	F01_transcript_76987	F01_transcript_76987	F01_transcript_76987	F01_transcript_76987
F01_transcript_55405	NA	F01_transcript_55405	NA	NA
F01_transcript_52460	NA	F01_transcript_52460	F01_transcript_52460	F01_transcript_52460
F01_transcript_64278	F01_transcript_64278	F01_transcript_64278	F01_transcript_64278	F01_transcript_64278
F01_transcript_50838	F01_transcript_50838	F01_transcript_50838	NA	F01_transcript_50838
F01_transcript_134223	NA	NA	NA	F01_transcript_134223
F01_transcript_141410	NA	F01_transcript_141410	F01_transcript_141410	F01_transcript_141410
F01_transcript_122359	NA	NA	NA	F01_transcript_122359
F01_transcript_119845	NA	NA	NA	F01_transcript_119845
F01_transcript_52290	NA	F01_transcript_52290	NA	NA
F01_transcript_74328	NA	F01_transcript_74328	NA	NA
F01_transcript_86776	NA	F01_transcript_86776	F01_transcript_86776	F01_transcript_86776
F01_transcript_124175	NA	NA	NA	F01_transcript_124175
F01_transcript_133604	NA	F01_transcript_133604	NA	NA

F01_transcript_36370	NA	NA	F01_transcript_36370	F01_transcript_36370
F01_transcript_77522	NA	NA	NA	F01_transcript_77522
F01_transcript_123665	F01_transcript_123665	NA	NA	NA
F01_transcript_97666	NA	F01_transcript_97666	NA	NA
F01_transcript_163739	NA	F01_transcript_163739	NA	F01_transcript_163739
F01_transcript_144027	NA	F01_transcript_144027	NA	NA
F01_transcript_142703	NA	F01_transcript_142703	F01_transcript_142703	F01_transcript_142703
F01_transcript_111238	NA	F01_transcript_111238	NA	NA
F01_transcript_167091	F01_transcript_167091	F01_transcript_167091	F01_transcript_167091	F01_transcript_167091
F01_transcript_73443	NA	F01_transcript_73443	NA	NA
F01_transcript_119768	NA	NA	NA	F01_transcript_119768
F01_transcript_48893	NA	F01_transcript_48893	NA	NA
F01_transcript_49958	NA	F01_transcript_49958	NA	NA
F01_transcript_157508	F01_transcript_157508	F01_transcript_157508	NA	F01_transcript_157508

F01_transcript_56424	NA	F01_transcript_56424	F01_transcript_56424	NA
F01_transcript_49955	NA	F01_transcript_49955	NA	NA
F01_transcript_60856	NA	F01_transcript_60856	NA	NA
F01_transcript_34989	NA	F01_transcript_34989	NA	NA
F01_transcript_138051	NA	F01_transcript_138051	NA	NA
F01_transcript_95899	NA	F01_transcript_95899	F01_transcript_95899	F01_transcript_95899
F01_transcript_75289	NA	NA	NA	F01_transcript_75289
F01_transcript_87125	F01_transcript_87125	F01_transcript_87125	F01_transcript_87125	F01_transcript_87125
F01_transcript_38390	NA	F01_transcript_38390	NA	F01_transcript_38390
F01_transcript_164459	F01_transcript_164459	F01_transcript_164459	F01_transcript_164459	F01_transcript_164459
F01_transcript_100433	NA	F01_transcript_100433	NA	NA
F01_transcript_9234	F01_transcript_9234	F01_transcript_9234	F01_transcript_9234	F01_transcript_9234
F01_transcript_60877	NA	NA	NA	F01_transcript_60877
F01_transcript_135293	F01_transcript_135293	F01_transcript_135293	F01_transcript_135293	F01_transcript_135293

F01_transcript_139134	NA	F01_transcript_139134	NA	NA
F01_transcript_100349	NA	F01_transcript_100349	F01_transcript_100349	F01_transcript_100349
F01_transcript_74984	NA	F01_transcript_74984	NA	F01_transcript_74984
F01_transcript_144403	F01_transcript_144403	F01_transcript_144403	F01_transcript_144403	F01_transcript_144403
F01_transcript_163393	NA	F01_transcript_163393	NA	NA
F01_transcript_56464	NA	F01_transcript_56464	F01_transcript_56464	NA
F01_transcript_156858	NA	F01_transcript_156858	NA	F01_transcript_156858
F01_transcript_51307	NA	F01_transcript_51307	F01_transcript_51307	F01_transcript_51307
F01_transcript_124748	NA	NA	NA	F01_transcript_124748
F01_transcript_117334	F01_transcript_117334	F01_transcript_117334	NA	F01_transcript_117334
F01_transcript_149771	NA	F01_transcript_149771	NA	NA
F01_transcript_75979	F01_transcript_75979	F01_transcript_75979	F01_transcript_75979	NA
F01_transcript_49615	NA	F01_transcript_49615	F01_transcript_49615	F01_transcript_49615
F01_transcript_114219	NA	F01_transcript_114219	NA	F01_transcript_114219

F01_transcript_48792	NA	F01_transcript_48792	NA	F01_transcript_48792
F01_transcript_138263	NA	NA	NA	F01_transcript_138263
F01_transcript_129396	NA	NA	NA	F01_transcript_129396
F01_transcript_79719	NA	F01_transcript_79719	NA	NA
F01_transcript_143906	NA	F01_transcript_143906	NA	F01_transcript_143906
F01_transcript_56357	F01_transcript_56357	F01_transcript_56357	F01_transcript_56357	NA
F01_transcript_70442	F01_transcript_70442	F01_transcript_70442	F01_transcript_70442	F01_transcript_70442
F01_transcript_99632	NA	NA	NA	F01_transcript_99632
F01_transcript_127847	NA	NA	NA	F01_transcript_127847
F01_transcript_20583	NA	NA	NA	F01_transcript_20583
F01_transcript_105577	NA	F01_transcript_105577	NA	NA
F01_transcript_148419	NA	NA	NA	F01_transcript_148419
F01_transcript_66515	F01_transcript_66515	F01_transcript_66515	NA	F01_transcript_66515
F01_transcript_143057	NA	NA	NA	F01_transcript_143057

F01_transcript_133393	NA	F01_transcript_133393	F01_transcript_133393	NA
F01_transcript_142756	F01_transcript_142756	F01_transcript_142756	NA	NA
F01_transcript_109283	NA	F01_transcript_109283	NA	F01_transcript_109283
F01_transcript_51018	F01_transcript_51018	F01_transcript_51018	F01_transcript_51018	F01_transcript_51018
F01_transcript_29484	NA	NA	NA	F01_transcript_29484
F01_transcript_130980	F01_transcript_130980	F01_transcript_130980	NA	F01_transcript_130980
F01_transcript_152972	F01_transcript_152972	F01_transcript_152972	NA	NA
F01_transcript_76453	F01_transcript_76453	F01_transcript_76453	F01_transcript_76453	F01_transcript_76453
F01_transcript_61160	NA	NA	NA	F01_transcript_61160
F01_transcript_95711	NA	F01_transcript_95711	F01_transcript_95711	F01_transcript_95711
F01_transcript_104076	NA	F01_transcript_104076	NA	F01_transcript_104076
F01_transcript_114683	NA	F01_transcript_114683	NA	F01_transcript_114683
F01_transcript_17640	NA	NA	NA	F01_transcript_17640
F01_transcript_51424	NA	NA	NA	F01_transcript_51424

F01_transcript_96063	NA	NA	NA	F01_transcript_96063
F01_transcript_55102	NA	F01_transcript_55102	NA	NA
F01_transcript_128912	F01_transcript_128912	F01_transcript_128912	F01_transcript_128912	F01_transcript_128912
F01_transcript_12478	F01_transcript_12478	F01_transcript_12478	F01_transcript_12478	F01_transcript_12478
F01_transcript_142587	F01_transcript_142587	F01_transcript_142587	F01_transcript_142587	F01_transcript_142587
F01_transcript_70201	NA	NA	NA	F01_transcript_70201
F01_transcript_52316	NA	F01_transcript_52316	F01_transcript_52316	F01_transcript_52316
F01_transcript_159816	NA	NA	NA	F01_transcript_159816
F01_transcript_163028	NA	F01_transcript_163028	NA	NA
F01_transcript_111720	F01_transcript_111720	F01_transcript_111720	F01_transcript_111720	F01_transcript_111720
F01_transcript_132720	NA	NA	NA	F01_transcript_132720
F01_transcript_67282	NA	F01_transcript_67282	F01_transcript_67282	NA
F01_transcript_133283	NA	F01_transcript_133283	F01_transcript_133283	F01_transcript_133283
F01_transcript_166010	F01_transcript_166010	F01_transcript_166010	F01_transcript_166010	F01_transcript_166010

F01_transcript_90726	NA	F01_transcript_90726	F01_transcript_90726	F01_transcript_90726
F01_transcript_149846	F01_transcript_149846	F01_transcript_149846	F01_transcript_149846	NA
F01_transcript_86056	NA	F01_transcript_86056	NA	NA
F01_transcript_124967	NA	NA	F01_transcript_124967	NA
F01_transcript_167584	NA	F01_transcript_167584	NA	F01_transcript_167584
F01_transcript_113800	NA	NA	NA	F01_transcript_113800
F01_transcript_96767	NA	F01_transcript_96767	NA	NA
F01_transcript_143670	F01_transcript_143670	F01_transcript_143670	NA	F01_transcript_143670
F01_transcript_134589	NA	NA	NA	F01_transcript_134589
F01_transcript_93782	F01_transcript_93782	F01_transcript_93782	F01_transcript_93782	F01_transcript_93782
F01_transcript_153539	F01_transcript_153539	F01_transcript_153539	F01_transcript_153539	F01_transcript_153539
F01_transcript_45667	NA	NA	NA	F01_transcript_45667
F01_transcript_107169	F01_transcript_107169	F01_transcript_107169	F01_transcript_107169	F01_transcript_107169
F01_transcript_92878	F01_transcript_92878	F01_transcript_92878	NA	NA

F01_transcript_14325	NA	NA	NA	F01_transcript_14325
F01_transcript_50581	NA	F01_transcript_50581	NA	NA
F01_transcript_92919	NA	NA	NA	F01_transcript_92919
F01_transcript_114145	NA	F01_transcript_114145	NA	NA
F01_transcript_41558	NA	NA	F01_transcript_41558	NA
F01_transcript_155964	NA	NA	NA	F01_transcript_155964
F01_transcript_155239	NA	F01_transcript_155239	NA	NA
F01_transcript_49780	NA	NA	NA	F01_transcript_49780
F01_transcript_16733	F01_transcript_16733	F01_transcript_16733	NA	F01_transcript_16733
F01_transcript_145485	F01_transcript_145485	F01_transcript_145485	NA	NA
F01_transcript_135321	F01_transcript_135321	F01_transcript_135321	NA	F01_transcript_135321
F01_transcript_122388	NA	NA	F01_transcript_122388	F01_transcript_122388
F01_transcript_136898	NA	F01_transcript_136898	F01_transcript_136898	F01_transcript_136898
F01_transcript_79628	F01_transcript_79628	F01_transcript_79628	NA	F01_transcript_79628

F01_transcript_88302	NA	F01_transcript_88302	NA	NA
F01_transcript_129860	NA	NA	NA	F01_transcript_129860
F01_transcript_83697	NA	F01_transcript_83697	NA	F01_transcript_83697
F01_transcript_28630	NA	F01_transcript_28630	NA	F01_transcript_28630
F01_transcript_66976	F01_transcript_66976	F01_transcript_66976	F01_transcript_66976	F01_transcript_66976
F01_transcript_47872	NA	F01_transcript_47872	NA	NA
F01_transcript_153657	F01_transcript_153657	F01_transcript_153657	F01_transcript_153657	F01_transcript_153657
F01_transcript_42362	NA	F01_transcript_42362	NA	F01_transcript_42362
F01_transcript_122915	NA	NA	NA	F01_transcript_122915
F01_transcript_133016	NA	NA	NA	F01_transcript_133016
F01_transcript_121024	NA	F01_transcript_121024	NA	F01_transcript_121024
F01_transcript_146435	F01_transcript_146435	F01_transcript_146435	NA	F01_transcript_146435
F01_transcript_117476	NA	NA	NA	F01_transcript_117476
F01_transcript_111514	NA	NA	NA	F01_transcript_111514

F01_transcript_125335	NA	NA	NA	F01_transcript_125335
F01_transcript_132561	NA	NA	NA	F01_transcript_132561
F01_transcript_52609	NA	F01_transcript_52609	NA	F01_transcript_52609
F01_transcript_150567	NA	NA	F01_transcript_150567	F01_transcript_150567
F01_transcript_34399	F01_transcript_34399	F01_transcript_34399	F01_transcript_34399	F01_transcript_34399
F01_transcript_147963	NA	NA	NA	F01_transcript_147963
F01_transcript_148951	F01_transcript_148951	F01_transcript_148951	NA	NA
F01_transcript_71142	NA	NA	NA	F01_transcript_71142
F01_transcript_158564	NA	F01_transcript_158564	NA	F01_transcript_158564
F01_transcript_137720	NA	NA	NA	F01_transcript_137720
F01_transcript_167201	NA	NA	NA	F01_transcript_167201
F01_transcript_26756	NA	NA	NA	F01_transcript_26756
F01_transcript_129961	NA	F01_transcript_129961	NA	NA
F01_transcript_1904	NA	NA	NA	F01_transcript_1904

F01_transcript_79163	F01_transcript_79163	F01_transcript_79163	F01_transcript_79163	F01_transcript_79163
F01_transcript_61117	NA	F01_transcript_61117	NA	F01_transcript_61117
F01_transcript_68562	NA	NA	NA	F01_transcript_68562
F01_transcript_134073	NA	F01_transcript_134073	NA	NA
F01_transcript_112034	F01_transcript_112034	F01_transcript_112034	F01_transcript_112034	F01_transcript_112034
F01_transcript_141313	F01_transcript_141313	F01_transcript_141313	F01_transcript_141313	F01_transcript_141313
F01_transcript_165733	F01_transcript_165733	F01_transcript_165733	F01_transcript_165733	F01_transcript_165733
F01_transcript_72906	F01_transcript_72906	F01_transcript_72906	F01_transcript_72906	F01_transcript_72906
F01_transcript_122445	F01_transcript_122445	F01_transcript_122445	F01_transcript_122445	F01_transcript_122445
F01_transcript_110357	NA	F01_transcript_110357	F01_transcript_110357	NA
F01_transcript_165502	F01_transcript_165502	F01_transcript_165502	F01_transcript_165502	NA
F01_transcript_132223	NA	NA	NA	F01_transcript_132223
F01_transcript_163372	NA	NA	NA	F01_transcript_163372
F01_transcript_76483	NA	F01_transcript_76483	NA	NA

F01_transcript_58120	NA	F01_transcript_58120	NA	F01_transcript_58120
F01_transcript_165390	NA	F01_transcript_165390	NA	NA
F01_transcript_80982	NA	NA	NA	F01_transcript_80982
F01_transcript_97652	NA	NA	NA	F01_transcript_97652
F01_transcript_74918	NA	NA	NA	F01_transcript_74918
F01_transcript_117223	F01_transcript_117223	F01_transcript_117223	F01_transcript_117223	F01_transcript_117223
F01_transcript_79737	F01_transcript_79737	F01_transcript_79737	F01_transcript_79737	NA
F01_transcript_163102	NA	F01_transcript_163102	NA	F01_transcript_163102
F01_transcript_113533	F01_transcript_113533	F01_transcript_113533	F01_transcript_113533	F01_transcript_113533
F01_transcript_80437	NA	F01_transcript_80437	NA	F01_transcript_80437
F01_transcript_2187	NA	NA	NA	F01_transcript_2187
F01_transcript_22056	NA	F01_transcript_22056	NA	F01_transcript_22056
F01_transcript_77561	NA	NA	NA	F01_transcript_77561
F01_transcript_165927	NA	F01_transcript_165927	NA	NA

F01_transcript_124177	NA	F01_transcript_124177	NA	NA
F01_transcript_93316	NA	F01_transcript_93316	NA	NA
F01_transcript_83715	NA	F01_transcript_83715	NA	F01_transcript_83715
F01_transcript_87689	F01_transcript_87689	F01_transcript_87689	F01_transcript_87689	F01_transcript_87689
F01_transcript_88108	NA	F01_transcript_88108	NA	F01_transcript_88108
F01_transcript_82091	F01_transcript_82091	F01_transcript_82091	NA	F01_transcript_82091
F01_transcript_154598	NA	F01_transcript_154598	NA	F01_transcript_154598
F01_transcript_49240	NA	F01_transcript_49240	NA	NA
F01_transcript_166101	NA	F01_transcript_166101	NA	F01_transcript_166101
F01_transcript_99248	F01_transcript_99248	F01_transcript_99248	F01_transcript_99248	F01_transcript_99248
F01_transcript_83621	F01_transcript_83621	F01_transcript_83621	F01_transcript_83621	F01_transcript_83621
F01_transcript_55661	F01_transcript_55661	F01_transcript_55661	F01_transcript_55661	F01_transcript_55661
F01_transcript_109445	NA	NA	NA	F01_transcript_109445
F01_transcript_98233	F01_transcript_98233	F01_transcript_98233	F01_transcript_98233	NA

F01_transcript_69178	F01_transcript_69178	F01_transcript_69178	NA	NA
F01_transcript_20	NA	NA	NA	F01_transcript_20
F01_transcript_15675	F01_transcript_15675	F01_transcript_15675	F01_transcript_15675	F01_transcript_15675
F01_transcript_143081	NA	NA	NA	F01_transcript_143081
F01_transcript_58442	F01_transcript_58442	F01_transcript_58442	F01_transcript_58442	F01_transcript_58442
F01_transcript_104690	NA	F01_transcript_104690	F01_transcript_104690	F01_transcript_104690
F01_transcript_161483	NA	F01_transcript_161483	NA	F01_transcript_161483
F01_transcript_80943	F01_transcript_80943	F01_transcript_80943	NA	F01_transcript_80943
F01_transcript_34052	NA	NA	NA	F01_transcript_34052
F01_transcript_48591	NA	NA	F01_transcript_48591	F01_transcript_48591
F01_transcript_120055	NA	F01_transcript_120055	NA	F01_transcript_120055
F01_transcript_32274	NA	NA	NA	F01_transcript_32274
F01_transcript_66745	NA	NA	NA	F01_transcript_66745
F01_transcript_91546	NA	F01_transcript_91546	NA	NA

F01_transcript_54606	NA	F01_transcript_54606	NA	NA
F01_transcript_123128	NA	NA	NA	F01_transcript_123128
F01_transcript_166245	NA	NA	NA	F01_transcript_166245
F01_transcript_92013	NA	NA	NA	F01_transcript_92013
F01_transcript_65655	F01_transcript_65655	F01_transcript_65655	F01_transcript_65655	F01_transcript_65655
F01_transcript_60902	F01_transcript_60902	F01_transcript_60902	F01_transcript_60902	F01_transcript_60902
F01_transcript_141120	F01_transcript_141120	F01_transcript_141120	F01_transcript_141120	F01_transcript_141120
F01_transcript_156872	NA	F01_transcript_156872	NA	F01_transcript_156872
F01_transcript_12826	NA	F01_transcript_12826	NA	F01_transcript_12826
F01_transcript_131951	NA	NA	NA	F01_transcript_131951
F01_transcript_103843	NA	NA	NA	F01_transcript_103843
F01_transcript_7799	NA	NA	NA	F01_transcript_7799
F01_transcript_167348	NA	NA	F01_transcript_167348	F01_transcript_167348
F01_transcript_38129	NA	NA	NA	F01_transcript_38129

F01_transcript_11807	NA	NA	NA	F01_transcript_11807
F01_transcript_138562	F01_transcript_138562	F01_transcript_138562	F01_transcript_138562	F01_transcript_138562
F01_transcript_87074	NA	NA	NA	F01_transcript_87074
F01_transcript_124886	NA	F01_transcript_124886	NA	F01_transcript_124886
F01_transcript_48166	NA	NA	NA	F01_transcript_48166
F01_transcript_107842	NA	F01_transcript_107842	NA	NA
F01_transcript_96257	NA	NA	NA	F01_transcript_96257
F01_transcript_72281	F01_transcript_72281	F01_transcript_72281	NA	NA
F01_transcript_80834	NA	F01_transcript_80834	NA	NA
F01_transcript_149151	NA	NA	NA	F01_transcript_149151
F01_transcript_89630	NA	NA	NA	F01_transcript_89630
F01_transcript_115990	NA	F01_transcript_115990	NA	NA
F01_transcript_104429	NA	NA	F01_transcript_104429	F01_transcript_104429
F01_transcript_127948	NA	NA	F01_transcript_127948	NA

F01_transcript_160407	NA	NA	NA	F01_transcript_160407
F01_transcript_111011	NA	F01_transcript_111011	NA	NA
F01_transcript_161980	NA	F01_transcript_161980	NA	NA
F01_transcript_33671	NA	NA	F01_transcript_33671	NA
F01_transcript_133922	NA	NA	NA	F01_transcript_133922
F01_transcript_75828	NA	NA	NA	F01_transcript_75828
F01_transcript_10376	NA	NA	NA	F01_transcript_10376
F01_transcript_127888	NA	F01_transcript_127888	NA	F01_transcript_127888
F01_transcript_42871	NA	NA	NA	F01_transcript_42871
F01_transcript_101365	NA	F01_transcript_101365	NA	NA
F01_transcript_159688	NA	NA	NA	F01_transcript_159688
F01_transcript_118770	F01_transcript_118770	F01_transcript_118770	F01_transcript_118770	F01_transcript_118770
F01_transcript_67744	NA	F01_transcript_67744	NA	F01_transcript_67744
F01_transcript_73292	NA	F01_transcript_73292	NA	F01_transcript_73292

F01_transcript_166314	NA	NA	NA	F01_transcript_166314
F01_transcript_152771	F01_transcript_152771	F01_transcript_152771	NA	F01_transcript_152771
F01_transcript_69129	NA	F01_transcript_69129	NA	NA
F01_transcript_155145	NA	F01_transcript_155145	NA	NA
F01_transcript_110328	F01_transcript_110328	F01_transcript_110328	NA	F01_transcript_110328
F01_transcript_135288	F01_transcript_135288	F01_transcript_135288	F01_transcript_135288	F01_transcript_135288
F01_transcript_114679	NA	NA	NA	F01_transcript_114679
F01_transcript_161399	F01_transcript_161399	F01_transcript_161399	NA	NA
F01_transcript_131977	NA	F01_transcript_131977	NA	NA
F01_transcript_73734	F01_transcript_73734	F01_transcript_73734	F01_transcript_73734	F01_transcript_73734
F01_transcript_474	NA	NA	NA	F01_transcript_474
F01_transcript_43560	NA	NA	NA	F01_transcript_43560
F01_transcript_94404	F01_transcript_94404	F01_transcript_94404	F01_transcript_94404	F01_transcript_94404
F01_transcript_57510	NA	F01_transcript_57510	NA	NA

F01_transcript_63575	F01_transcript_63575	F01_transcript_63575	NA	NA
F01_transcript_74681	NA	F01_transcript_74681	F01_transcript_74681	F01_transcript_74681
F01_transcript_75749	NA	F01_transcript_75749	NA	NA
F01_transcript_91404	F01_transcript_91404	F01_transcript_91404	NA	NA
F01_transcript_129261	F01_transcript_129261	F01_transcript_129261	NA	F01_transcript_129261
F01_transcript_139093	F01_transcript_139093	F01_transcript_139093	NA	F01_transcript_139093
F01_transcript_163079	NA	NA	NA	F01_transcript_163079
F01_transcript_124348	NA	F01_transcript_124348	NA	F01_transcript_124348
F01_transcript_60642	NA	NA	NA	F01_transcript_60642
F01_transcript_138849	NA	F01_transcript_138849	NA	NA
F01_transcript_151341	NA	F01_transcript_151341	NA	F01_transcript_151341
F01_transcript_119763	NA	F01_transcript_119763	NA	NA
F01_transcript_98971	NA	F01_transcript_98971	NA	F01_transcript_98971
F01_transcript_65858	NA	NA	NA	F01_transcript_65858

F01_transcript_41888	NA	NA	NA	F01_transcript_41888
F01_transcript_148322	F01_transcript_148322	F01_transcript_148322	F01_transcript_148322	F01_transcript_148322
F01_transcript_90278	NA	NA	NA	F01_transcript_90278
F01_transcript_51218	F01_transcript_51218	F01_transcript_51218	F01_transcript_51218	F01_transcript_51218
F01_transcript_64341	NA	NA	NA	F01_transcript_64341
F01_transcript_166435	NA	NA	NA	F01_transcript_166435
F01_transcript_157073	NA	NA	NA	F01_transcript_157073
F01_transcript_104937	F01_transcript_104937	F01_transcript_104937	NA	NA
F01_transcript_113806	NA	F01_transcript_113806	NA	NA
F01_transcript_108069	NA	F01_transcript_108069	NA	NA
F01_transcript_50733	F01_transcript_50733	F01_transcript_50733	NA	F01_transcript_50733
F01_transcript_82604	NA	NA	NA	F01_transcript_82604
F01_transcript_161493	NA	F01_transcript_161493	NA	NA
F01_transcript_20666	F01_transcript_20666	F01_transcript_20666	NA	NA

F01_transcript_121134	NA	NA	NA	F01_transcript_121134
F01_transcript_148550	F01_transcript_148550	F01_transcript_148550	F01_transcript_148550	F01_transcript_148550
F01_transcript_58730	F01_transcript_58730	F01_transcript_58730	F01_transcript_58730	F01_transcript_58730
F01_transcript_55771	NA	NA	NA	F01_transcript_55771
F01_transcript_136248	NA	NA	NA	F01_transcript_136248
F01_transcript_94246	NA	NA	NA	F01_transcript_94246
F01_transcript_103139	NA	NA	NA	F01_transcript_103139
F01_transcript_51734	F01_transcript_51734	F01_transcript_51734	NA	F01_transcript_51734
F01_transcript_40024	NA	NA	NA	F01_transcript_40024
F01_transcript_142709	NA	NA	NA	F01_transcript_142709
F01_transcript_118319	F01_transcript_118319	F01_transcript_118319	NA	F01_transcript_118319
F01_transcript_136929	NA	F01_transcript_136929	NA	NA
F01_transcript_134443	NA	NA	NA	F01_transcript_134443
F01_transcript_48135	NA	F01_transcript_48135	NA	NA

F01_transcript_103760	NA	F01_transcript_103760	F01_transcript_103760	F01_transcript_103760
F01_transcript_7734	NA	NA	NA	F01_transcript_7734
F01_transcript_80894	NA	F01_transcript_80894	NA	NA
F01_transcript_51285	F01_transcript_51285	F01_transcript_51285	F01_transcript_51285	F01_transcript_51285
F01_transcript_8963	NA	F01_transcript_8963	NA	NA
F01_transcript_29086	NA	NA	NA	F01_transcript_29086
F01_transcript_127557	F01_transcript_127557	F01_transcript_127557	F01_transcript_127557	F01_transcript_127557
F01_transcript_107543	NA	NA	NA	F01_transcript_107543
F01_transcript_122870	NA	F01_transcript_122870	NA	F01_transcript_122870
F01_transcript_108937	NA	F01_transcript_108937	NA	NA
F01_transcript_138745	NA	NA	NA	F01_transcript_138745
F01_transcript_56448	NA	NA	F01_transcript_56448	NA
F01_transcript_115214	NA	F01_transcript_115214	NA	NA
F01_transcript_5453	NA	NA	NA	F01_transcript_5453

F01_transcript_167490	NA	NA	NA	F01_transcript_167490
F01_transcript_155733	NA	F01_transcript_155733	NA	F01_transcript_155733
F01_transcript_108085	F01_transcript_108085	F01_transcript_108085	NA	F01_transcript_108085
F01_transcript_126807	NA	NA	NA	F01_transcript_126807
F01_transcript_115378	NA	F01_transcript_115378	NA	NA
F01_transcript_145648	F01_transcript_145648	F01_transcript_145648	NA	F01_transcript_145648
F01_transcript_71246	NA	F01_transcript_71246	F01_transcript_71246	NA
F01_transcript_121938	NA	F01_transcript_121938	F01_transcript_121938	F01_transcript_121938
F01_transcript_152717	NA	F01_transcript_152717	NA	F01_transcript_152717
F01_transcript_114803	NA	F01_transcript_114803	NA	NA
F01_transcript_60598	F01_transcript_60598	F01_transcript_60598	F01_transcript_60598	F01_transcript_60598
F01_transcript_164725	F01_transcript_164725	F01_transcript_164725	NA	F01_transcript_164725
F01_transcript_114527	NA	NA	F01_transcript_114527	F01_transcript_114527
F01_transcript_99087	NA	NA	F01_transcript_99087	F01_transcript_99087

F01_transcript_106137	NA	NA	NA	F01_transcript_106137
F01_transcript_48469	F01_transcript_48469	F01_transcript_48469	NA	F01_transcript_48469
F01_transcript_139963	NA	NA	NA	F01_transcript_139963
F01_transcript_141710	F01_transcript_141710	F01_transcript_141710	NA	F01_transcript_141710
F01_transcript_97310	NA	F01_transcript_97310	NA	NA
F01_transcript_141499	NA	F01_transcript_141499	NA	F01_transcript_141499
F01_transcript_24918	NA	NA	F01_transcript_24918	F01_transcript_24918
F01_transcript_54887	NA	NA	NA	F01_transcript_54887
F01_transcript_82169	NA	F01_transcript_82169	F01_transcript_82169	NA
F01_transcript_153936	F01_transcript_153936	F01_transcript_153936	F01_transcript_153936	F01_transcript_153936
F01_transcript_103121	F01_transcript_103121	F01_transcript_103121	NA	F01_transcript_103121
F01_transcript_119555	NA	F01_transcript_119555	NA	NA
F01_transcript_72214	F01_transcript_72214	F01_transcript_72214	NA	NA
F01_transcript_49014	NA	NA	F01_transcript_49014	F01_transcript_49014

F01_transcript_50144	F01_transcript_50144	NA	NA	NA
F01_transcript_142782	NA	F01_transcript_142782	NA	NA
F01_transcript_109701	F01_transcript_109701	F01_transcript_109701	NA	NA
F01_transcript_108549	NA	F01_transcript_108549	NA	NA
F01_transcript_156482	NA	F01_transcript_156482	F01_transcript_156482	NA
F01_transcript_23036	NA	NA	NA	F01_transcript_23036
F01_transcript_97033	NA	F01_transcript_97033	NA	NA
F01_transcript_121355	NA	NA	NA	F01_transcript_121355
F01_transcript_10343	NA	F01_transcript_10343	NA	F01_transcript_10343
F01_transcript_46779	NA	NA	NA	F01_transcript_46779
F01_transcript_112060	F01_transcript_112060	F01_transcript_112060	F01_transcript_112060	F01_transcript_112060
F01_transcript_100026	F01_transcript_100026	F01_transcript_100026	NA	NA
F01_transcript_49457	NA	NA	F01_transcript_49457	F01_transcript_49457
F01_transcript_160828	NA	F01_transcript_160828	NA	NA

F01_transcript_143828	NA	F01_transcript_143828	NA	NA
F01_transcript_138428	F01_transcript_138428	F01_transcript_138428	NA	NA
F01_transcript_89664	NA	NA	NA	F01_transcript_89664
F01_transcript_5475	NA	NA	NA	F01_transcript_5475
F01_transcript_34804	F01_transcript_34804	F01_transcript_34804	F01_transcript_34804	F01_transcript_34804
F01_transcript_136358	NA	NA	NA	F01_transcript_136358
F01_transcript_58502	NA	F01_transcript_58502	NA	NA
F01_transcript_24194	NA	F01_transcript_24194	NA	NA
F01_transcript_112466	F01_transcript_112466	F01_transcript_112466	NA	F01_transcript_112466
F01_transcript_60813	F01_transcript_60813	F01_transcript_60813	F01_transcript_60813	F01_transcript_60813
F01_transcript_25500	NA	NA	NA	F01_transcript_25500
F01_transcript_80296	NA	F01_transcript_80296	NA	NA
F01_transcript_27379	NA	NA	NA	F01_transcript_27379
F01_transcript_59739	NA	F01_transcript_59739	NA	NA

F01_transcript_77229	NA	F01_transcript_77229	NA	F01_transcript_77229
F01_transcript_48116	NA	NA	NA	F01_transcript_48116
F01_transcript_74909	F01_transcript_74909	F01_transcript_74909	NA	NA
F01_transcript_68196	NA	NA	NA	F01_transcript_68196
F01_transcript_137225	NA	NA	NA	F01_transcript_137225
F01_transcript_152395	NA	NA	NA	F01_transcript_152395
F01_transcript_68254	NA	F01_transcript_68254	NA	F01_transcript_68254
F01_transcript_65191	NA	NA	F01_transcript_65191	NA
F01_transcript_120152	F01_transcript_120152	F01_transcript_120152	F01_transcript_120152	F01_transcript_120152
F01_transcript_73429	F01_transcript_73429	F01_transcript_73429	F01_transcript_73429	F01_transcript_73429
F01_transcript_92114	F01_transcript_92114	F01_transcript_92114	NA	F01_transcript_92114
F01_transcript_52311	NA	NA	NA	F01_transcript_52311
F01_transcript_118150	NA	F01_transcript_118150	F01_transcript_118150	F01_transcript_118150
F01_transcript_101936	NA	NA	NA	F01_transcript_101936

F01_transcript_99305	NA	F01_transcript_99305	NA	NA
F01_transcript_156591	NA	F01_transcript_156591	F01_transcript_156591	F01_transcript_156591
F01_transcript_33018	NA	NA	NA	F01_transcript_33018
F01_transcript_153010	NA	NA	NA	F01_transcript_153010
F01_transcript_71468	NA	NA	NA	F01_transcript_71468
F01_transcript_118338	NA	F01_transcript_118338	NA	F01_transcript_118338
F01_transcript_52086	NA	F01_transcript_52086	NA	F01_transcript_52086
F01_transcript_128072	F01_transcript_128072	F01_transcript_128072	F01_transcript_128072	F01_transcript_128072
F01_transcript_117743	NA	NA	NA	F01_transcript_117743
F01_transcript_48359	F01_transcript_48359	F01_transcript_48359	NA	NA
F01_transcript_144787	NA	NA	NA	F01_transcript_144787
F01_transcript_99917	NA	NA	NA	F01_transcript_99917
F01_transcript_67515	NA	NA	NA	F01_transcript_67515
F01_transcript_81311	NA	NA	NA	F01_transcript_81311

F01_transcript_137254	NA	NA	NA	F01_transcript_137254
F01_transcript_150583	NA	F01_transcript_150583	NA	F01_transcript_150583
F01_transcript_120164	NA	NA	NA	F01_transcript_120164
F01_transcript_94324	F01_transcript_94324	F01_transcript_94324	F01_transcript_94324	F01_transcript_94324
F01_transcript_166325	NA	F01_transcript_166325	NA	NA
F01_transcript_145814	NA	NA	NA	F01_transcript_145814
F01_transcript_103681	NA	F01_transcript_103681	F01_transcript_103681	NA
F01_transcript_54650	NA	NA	NA	F01_transcript_54650
F01_transcript_165911	NA	NA	F01_transcript_165911	NA
F01_transcript_6388	NA	F01_transcript_6388	NA	NA
F01_transcript_109208	F01_transcript_109208	F01_transcript_109208	NA	F01_transcript_109208
F01_transcript_7335	NA	F01_transcript_7335	F01_transcript_7335	F01_transcript_7335
F01_transcript_160584	NA	F01_transcript_160584	NA	NA
F01_transcript_113205	F01_transcript_113205	F01_transcript_113205	NA	NA

F01_transcript_63056	F01_transcript_63056	F01_transcript_63056	F01_transcript_63056	F01_transcript_63056
F01_transcript_101644	NA	F01_transcript_101644	NA	NA
F01_transcript_134733	NA	NA	NA	F01_transcript_134733
F01_transcript_118308	NA	NA	NA	F01_transcript_118308
F01_transcript_146559	F01_transcript_146559	F01_transcript_146559	F01_transcript_146559	F01_transcript_146559
F01_transcript_78037	NA	F01_transcript_78037	NA	F01_transcript_78037
F01_transcript_107609	NA	NA	NA	F01_transcript_107609
F01_transcript_105213	F01_transcript_105213	F01_transcript_105213	F01_transcript_105213	F01_transcript_105213
F01_transcript_60116	NA	F01_transcript_60116	NA	NA
F01_transcript_101332	F01_transcript_101332	F01_transcript_101332	NA	NA
F01_transcript_51495	F01_transcript_51495	F01_transcript_51495	F01_transcript_51495	F01_transcript_51495
F01_transcript_101071	NA	F01_transcript_101071	NA	NA
F01_transcript_50742	F01_transcript_50742	F01_transcript_50742	NA	NA
F01_transcript_78906	NA	F01_transcript_78906	NA	NA

F01_transcript_72864	NA	NA	NA	F01_transcript_72864
F01_transcript_132348	NA	F01_transcript_132348	NA	F01_transcript_132348
F01_transcript_137264	NA	F01_transcript_137264	NA	NA
F01_transcript_89933	F01_transcript_89933	F01_transcript_89933	NA	NA
F01_transcript_150316	NA	F01_transcript_150316	NA	F01_transcript_150316
F01_transcript_4890	NA	NA	NA	F01_transcript_4890
F01_transcript_75424	NA	F01_transcript_75424	NA	NA
F01_transcript_33832	NA	NA	NA	F01_transcript_33832
F01_transcript_108218	F01_transcript_108218	F01_transcript_108218	F01_transcript_108218	F01_transcript_108218
F01_transcript_107492	NA	F01_transcript_107492	F01_transcript_107492	NA
F01_transcript_142208	F01_transcript_142208	F01_transcript_142208	F01_transcript_142208	F01_transcript_142208
F01_transcript_35842	NA	NA	NA	F01_transcript_35842
F01_transcript_135	NA	NA	NA	F01_transcript_135
F01_transcript_101751	NA	F01_transcript_101751	NA	NA

F01_transcript_167651	NA	F01_transcript_167651	NA	NA
F01_transcript_98394	NA	NA	NA	F01_transcript_98394
F01_transcript_94948	F01_transcript_94948	F01_transcript_94948	F01_transcript_94948	NA
F01_transcript_48812	NA	NA	F01_transcript_48812	F01_transcript_48812
F01_transcript_84792	NA	NA	F01_transcript_84792	F01_transcript_84792
F01_transcript_113859	NA	F01_transcript_113859	NA	F01_transcript_113859
F01_transcript_98226	NA	NA	NA	F01_transcript_98226
F01_transcript_103637	NA	NA	NA	F01_transcript_103637
F01_transcript_167407	NA	F01_transcript_167407	F01_transcript_167407	NA
F01_transcript_141456	NA	NA	NA	F01_transcript_141456
F01_transcript_62915	NA	F01_transcript_62915	NA	NA
F01_transcript_92712	F01_transcript_92712	F01_transcript_92712	NA	F01_transcript_92712
F01_transcript_67329	NA	NA	NA	F01_transcript_67329
F01_transcript_107165	NA	F01_transcript_107165	NA	NA

F01_transcript_44902	NA	NA	NA	F01_transcript_44902
F01_transcript_132797	F01_transcript_132797	F01_transcript_132797	NA	F01_transcript_132797
F01_transcript_163038	F01_transcript_163038	F01_transcript_163038	NA	F01_transcript_163038
F01_transcript_145185	NA	F01_transcript_145185	NA	NA
F01_transcript_28333	NA	NA	NA	F01_transcript_28333
F01_transcript_92702	NA	F01_transcript_92702	NA	NA
F01_transcript_149080	NA	F01_transcript_149080	F01_transcript_149080	F01_transcript_149080
F01_transcript_58191	NA	NA	NA	F01_transcript_58191
F01_transcript_77281	NA	NA	F01_transcript_77281	F01_transcript_77281
F01_transcript_83305	NA	NA	NA	F01_transcript_83305
F01_transcript_116885	F01_transcript_116885	F01_transcript_116885	F01_transcript_116885	F01_transcript_116885
F01_transcript_140740	NA	NA	NA	F01_transcript_140740
F01_transcript_167162	NA	NA	NA	F01_transcript_167162
F01_transcript_137575	NA	F01_transcript_137575	NA	NA

F01_transcript_154954	NA	F01_transcript_154954	NA	NA
F01_transcript_151207	NA	NA	F01_transcript_151207	F01_transcript_151207
F01_transcript_53578	F01_transcript_53578	F01_transcript_53578	NA	F01_transcript_53578
F01_transcript_138249	F01_transcript_138249	F01_transcript_138249	NA	NA
F01_transcript_8560	NA	NA	NA	F01_transcript_8560
F01_transcript_148321	NA	NA	NA	F01_transcript_148321
F01_transcript_123350	NA	F01_transcript_123350	NA	F01_transcript_123350
F01_transcript_11460	F01_transcript_11460	F01_transcript_11460	NA	NA
F01_transcript_78873	F01_transcript_78873	F01_transcript_78873	F01_transcript_78873	NA
F01_transcript_40193	NA	NA	NA	F01_transcript_40193
F01_transcript_27751	NA	NA	NA	F01_transcript_27751
F01_transcript_93400	NA	NA	NA	F01_transcript_93400
F01_transcript_131556	NA	NA	NA	F01_transcript_131556
F01_transcript_116183	F01_transcript_116183	F01_transcript_116183	NA	F01_transcript_116183

F01_transcript_60701	F01_transcript_60701	F01_transcript_60701	F01_transcript_60701	F01_transcript_60701
F01_transcript_48032	NA	NA	NA	F01_transcript_48032
F01_transcript_69078	NA	F01_transcript_69078	NA	F01_transcript_69078
F01_transcript_53552	F01_transcript_53552	F01_transcript_53552	F01_transcript_53552	NA
F01_transcript_411	NA	F01_transcript_411	NA	NA
F01_transcript_52301	F01_transcript_52301	F01_transcript_52301	NA	F01_transcript_52301
F01_transcript_108123	F01_transcript_108123	F01_transcript_108123	NA	F01_transcript_108123
F01_transcript_100198	NA	NA	NA	F01_transcript_100198
F01_transcript_126404	F01_transcript_126404	F01_transcript_126404	F01_transcript_126404	F01_transcript_126404
F01_transcript_157938	NA	NA	NA	F01_transcript_157938
F01_transcript_153803	F01_transcript_153803	F01_transcript_153803	NA	F01_transcript_153803
F01_transcript_98255	F01_transcript_98255	F01_transcript_98255	F01_transcript_98255	F01_transcript_98255
F01_transcript_4024	NA	NA	NA	F01_transcript_4024
F01_transcript_63019	NA	F01_transcript_63019	NA	F01_transcript_63019

F01_transcript_115069	NA	NA	NA	F01_transcript_115069
F01_transcript_112543	NA	NA	NA	F01_transcript_112543
F01_transcript_3424	F01_transcript_3424	F01_transcript_3424	F01_transcript_3424	F01_transcript_3424
F01_transcript_56136	NA	F01_transcript_56136	NA	NA
F01_transcript_166293	F01_transcript_166293	F01_transcript_166293	F01_transcript_166293	F01_transcript_166293
F01_transcript_73339	NA	F01_transcript_73339	NA	NA
F01_transcript_90281	F01_transcript_90281	F01_transcript_90281	F01_transcript_90281	F01_transcript_90281
F01_transcript_94048	NA	NA	NA	F01_transcript_94048
F01_transcript_55607	F01_transcript_55607	F01_transcript_55607	NA	F01_transcript_55607
F01_transcript_1366	NA	NA	NA	F01_transcript_1366
F01_transcript_147215	NA	F01_transcript_147215	NA	NA
F01_transcript_5657	NA	F01_transcript_5657	F01_transcript_5657	F01_transcript_5657
F01_transcript_134155	NA	NA	NA	F01_transcript_134155
F01_transcript_131827	NA	NA	F01_transcript_131827	NA

F01_transcript_111242	NA	F01_transcript_111242	NA	NA
F01_transcript_161928	F01_transcript_161928	F01_transcript_161928	NA	F01_transcript_161928
F01_transcript_150094	NA	F01_transcript_150094	F01_transcript_150094	NA
F01_transcript_28264	NA	F01_transcript_28264	F01_transcript_28264	F01_transcript_28264
F01_transcript_139529	NA	NA	NA	F01_transcript_139529
F01_transcript_160556	NA	NA	NA	F01_transcript_160556
F01_transcript_75162	F01_transcript_75162	F01_transcript_75162	F01_transcript_75162	NA
F01_transcript_8845	NA	F01_transcript_8845	F01_transcript_8845	F01_transcript_8845
F01_transcript_80287	NA	F01_transcript_80287	NA	NA
F01_transcript_115515	F01_transcript_115515	F01_transcript_115515	NA	F01_transcript_115515
F01_transcript_164138	NA	NA	NA	F01_transcript_164138
F01_transcript_118030	F01_transcript_118030	F01_transcript_118030	F01_transcript_118030	F01_transcript_118030
F01_transcript_96827	NA	F01_transcript_96827	F01_transcript_96827	NA
F01_transcript_54970	NA	F01_transcript_54970	NA	F01_transcript_54970

F01_transcript_119563	NA	NA	NA	F01_transcript_119563
F01_transcript_312	NA	NA	NA	F01_transcript_312
F01_transcript_105506	NA	NA	NA	F01_transcript_105506
F01_transcript_42355	NA	NA	NA	F01_transcript_42355
F01_transcript_44511	NA	NA	NA	F01_transcript_44511
F01_transcript_101634	NA	F01_transcript_101634	F01_transcript_101634	NA
F01_transcript_91407	NA	NA	NA	F01_transcript_91407
F01_transcript_52803	NA	NA	NA	F01_transcript_52803
F01_transcript_68933	NA	NA	NA	F01_transcript_68933
F01_transcript_112327	NA	F01_transcript_112327	NA	NA
F01_transcript_86420	NA	NA	NA	F01_transcript_86420
F01_transcript_89697	NA	F01_transcript_89697	NA	F01_transcript_89697
F01_transcript_110049	NA	NA	NA	F01_transcript_110049
F01_transcript_130364	NA	F01_transcript_130364	NA	F01_transcript_130364

F01_transcript_16239	NA	NA	NA	F01_transcript_16239
F01_transcript_58434	NA	NA	NA	F01_transcript_58434
F01_transcript_36525	NA	NA	NA	F01_transcript_36525
F01_transcript_102458	NA	NA	NA	F01_transcript_102458
F01_transcript_91927	NA	NA	NA	F01_transcript_91927
F01_transcript_79932	NA	F01_transcript_79932	NA	NA
F01_transcript_59927	NA	F01_transcript_59927	NA	F01_transcript_59927
F01_transcript_90597	F01_transcript_90597	F01_transcript_90597	F01_transcript_90597	F01_transcript_90597
F01_transcript_133972	NA	NA	NA	F01_transcript_133972
F01_transcript_82106	NA	NA	NA	F01_transcript_82106
F01_transcript_152469	F01_transcript_152469	F01_transcript_152469	F01_transcript_152469	F01_transcript_152469
F01_transcript_74633	F01_transcript_74633	F01_transcript_74633	F01_transcript_74633	F01_transcript_74633
F01_transcript_106429	F01_transcript_106429	F01_transcript_106429	F01_transcript_106429	F01_transcript_106429
F01_transcript_99586	F01_transcript_99586	F01_transcript_99586	F01_transcript_99586	F01_transcript_99586

F01_transcript_134130	NA	F01_transcript_134130	NA	NA
F01_transcript_48798	NA	NA	NA	F01_transcript_48798
F01_transcript_95124	NA	F01_transcript_95124	NA	NA
F01_transcript_95375	F01_transcript_95375	F01_transcript_95375	F01_transcript_95375	F01_transcript_95375
F01_transcript_61820	NA	NA	NA	F01_transcript_61820
F01_transcript_137799	F01_transcript_137799	F01_transcript_137799	F01_transcript_137799	F01_transcript_137799
F01_transcript_98552	NA	F01_transcript_98552	NA	F01_transcript_98552
F01_transcript_132831	NA	NA	NA	F01_transcript_132831
F01_transcript_116043	F01_transcript_116043	F01_transcript_116043	F01_transcript_116043	F01_transcript_116043
F01_transcript_78994	NA	F01_transcript_78994	F01_transcript_78994	F01_transcript_78994
F01_transcript_12268	NA	NA	F01_transcript_12268	F01_transcript_12268
F01_transcript_90328	NA	F01_transcript_90328	NA	F01_transcript_90328
F01_transcript_87291	F01_transcript_87291	F01_transcript_87291	NA	NA
F01_transcript_123754	F01_transcript_123754	F01_transcript_123754	NA	F01_transcript_123754

F01_transcript_121159	NA	NA	NA	F01_transcript_121159
F01_transcript_163037	NA	NA	NA	F01_transcript_163037
F01_transcript_55059	F01_transcript_55059	F01_transcript_55059	F01_transcript_55059	F01_transcript_55059
F01_transcript_67015	NA	F01_transcript_67015	NA	NA
F01_transcript_163395	F01_transcript_163395	F01_transcript_163395	F01_transcript_163395	F01_transcript_163395
F01_transcript_90529	F01_transcript_90529	F01_transcript_90529	NA	F01_transcript_90529
F01_transcript_9493	NA	NA	NA	F01_transcript_9493
F01_transcript_166722	NA	F01_transcript_166722	NA	NA
F01_transcript_100614	NA	NA	F01_transcript_100614	F01_transcript_100614
F01_transcript_92572	NA	F01_transcript_92572	NA	NA
F01_transcript_146008	NA	NA	NA	F01_transcript_146008
F01_transcript_151755	NA	F01_transcript_151755	NA	NA
F01_transcript_140515	NA	NA	NA	F01_transcript_140515
F01_transcript_52940	F01_transcript_52940	NA	NA	F01_transcript_52940

F01_transcript_71007	NA	F01_transcript_71007	F01_transcript_71007	F01_transcript_71007
F01_transcript_151149	NA	F01_transcript_151149	NA	NA
F01_transcript_57187	NA	NA	NA	F01_transcript_57187
F01_transcript_44256	NA	NA	NA	F01_transcript_44256
F01_transcript_110518	F01_transcript_110518	F01_transcript_110518	F01_transcript_110518	F01_transcript_110518
F01_transcript_87857	NA	F01_transcript_87857	NA	NA
F01_transcript_18306	NA	NA	NA	F01_transcript_18306
F01_transcript_28162	NA	NA	NA	F01_transcript_28162
F01_transcript_143030	NA	F01_transcript_143030	NA	F01_transcript_143030
F01_transcript_165682	NA	F01_transcript_165682	NA	NA
F01_transcript_32172	NA	F01_transcript_32172	NA	NA
F01_transcript_128616	F01_transcript_128616	F01_transcript_128616	F01_transcript_128616	F01_transcript_128616
F01_transcript_167539	NA	F01_transcript_167539	NA	NA
F01_transcript_81112	NA	F01_transcript_81112	NA	NA

F01_transcript_144256	NA	NA	NA	F01_transcript_144256
F01_transcript_64847	F01_transcript_64847	F01_transcript_64847	NA	F01_transcript_64847
F01_transcript_162463	NA	F01_transcript_162463	NA	F01_transcript_162463
F01_transcript_33562	F01_transcript_33562	F01_transcript_33562	F01_transcript_33562	F01_transcript_33562
F01_transcript_89504	NA	F01_transcript_89504	NA	F01_transcript_89504
F01_transcript_104603	NA	F01_transcript_104603	NA	F01_transcript_104603
F01_transcript_116842	F01_transcript_116842	F01_transcript_116842	NA	F01_transcript_116842
F01_transcript_86074	F01_transcript_86074	F01_transcript_86074	F01_transcript_86074	F01_transcript_86074
F01_transcript_164304	NA	F01_transcript_164304	NA	NA
F01_transcript_93519	NA	NA	NA	F01_transcript_93519
F01_transcript_71276	NA	NA	F01_transcript_71276	NA
F01_transcript_166881	F01_transcript_166881	F01_transcript_166881	NA	F01_transcript_166881
F01_transcript_120681	NA	F01_transcript_120681	NA	NA
F01_transcript_80619	NA	F01_transcript_80619	F01_transcript_80619	F01_transcript_80619

F01_transcript_99451	F01_transcript_99451	F01_transcript_99451	NA	NA
F01_transcript_74783	F01_transcript_74783	F01_transcript_74783	F01_transcript_74783	F01_transcript_74783
F01_transcript_140369	F01_transcript_140369	F01_transcript_140369	F01_transcript_140369	F01_transcript_140369
F01_transcript_103268	NA	F01_transcript_103268	NA	F01_transcript_103268
F01_transcript_141015	F01_transcript_141015	F01_transcript_141015	F01_transcript_141015	F01_transcript_141015
F01_transcript_161149	NA	NA	NA	F01_transcript_161149
F01_transcript_108949	NA	F01_transcript_108949	F01_transcript_108949	F01_transcript_108949
F01_transcript_2885	NA	NA	NA	F01_transcript_2885
F01_transcript_36905	NA	NA	NA	F01_transcript_36905
F01_transcript_62593	NA	F01_transcript_62593	NA	NA
F01_transcript_160587	NA	F01_transcript_160587	NA	NA
F01_transcript_126930	NA	F01_transcript_126930	F01_transcript_126930	NA
F01_transcript_54614	F01_transcript_54614	F01_transcript_54614	F01_transcript_54614	F01_transcript_54614
F01_transcript_96315	F01_transcript_96315	F01_transcript_96315	F01_transcript_96315	F01_transcript_96315

F01_transcript_125248	NA	NA	NA	F01_transcript_125248
F01_transcript_82083	F01_transcript_82083	F01_transcript_82083	F01_transcript_82083	F01_transcript_82083
F01_transcript_48606	NA	NA	NA	F01_transcript_48606
F01_transcript_117867	F01_transcript_117867	F01_transcript_117867	NA	F01_transcript_117867
F01_transcript_120160	NA	NA	NA	F01_transcript_120160
F01_transcript_107142	NA	F01_transcript_107142	NA	NA
F01_transcript_89466	F01_transcript_89466	F01_transcript_89466	F01_transcript_89466	F01_transcript_89466
F01_transcript_109483	F01_transcript_109483	F01_transcript_109483	F01_transcript_109483	NA
F01_transcript_108545	F01_transcript_108545	F01_transcript_108545	F01_transcript_108545	F01_transcript_108545
F01_transcript_131293	NA	F01_transcript_131293	NA	NA
F01_transcript_78137	NA	NA	NA	F01_transcript_78137
F01_transcript_115654	NA	F01_transcript_115654	NA	NA
F01_transcript_84842	NA	F01_transcript_84842	NA	NA
F01_transcript_143519	NA	F01_transcript_143519	NA	NA

F01_transcript_133923	NA	NA	NA	F01_transcript_133923
F01_transcript_161248	F01_transcript_161248	F01_transcript_161248	F01_transcript_161248	F01_transcript_161248
F01_transcript_77885	NA	NA	NA	F01_transcript_77885
F01_transcript_78699	NA	NA	NA	F01_transcript_78699
F01_transcript_102007	NA	F01_transcript_102007	NA	NA
F01_transcript_103611	F01_transcript_103611	F01_transcript_103611	NA	NA
F01_transcript_64453	NA	NA	NA	F01_transcript_64453
F01_transcript_138767	F01_transcript_138767	F01_transcript_138767	F01_transcript_138767	F01_transcript_138767
F01_transcript_93510	NA	F01_transcript_93510	NA	NA
F01_transcript_81274	NA	NA	NA	F01_transcript_81274
F01_transcript_84131	F01_transcript_84131	F01_transcript_84131	F01_transcript_84131	NA
F01_transcript_140254	NA	F01_transcript_140254	NA	NA
F01_transcript_132708	NA	F01_transcript_132708	NA	F01_transcript_132708
F01_transcript_71480	NA	NA	F01_transcript_71480	NA

F01_transcript_167473	NA	NA	NA	F01_transcript_167473
F01_transcript_115241	NA	F01_transcript_115241	NA	NA
F01_transcript_89055	NA	NA	NA	F01_transcript_89055
F01_transcript_78639	NA	NA	NA	F01_transcript_78639
F01_transcript_29618	NA	NA	NA	F01_transcript_29618
F01_transcript_87153	F01_transcript_87153	F01_transcript_87153	F01_transcript_87153	F01_transcript_87153
F01_transcript_23005	NA	NA	NA	F01_transcript_23005
F01_transcript_99227	NA	F01_transcript_99227	NA	F01_transcript_99227
F01_transcript_14833	NA	NA	NA	F01_transcript_14833
F01_transcript_113579	F01_transcript_113579	F01_transcript_113579	F01_transcript_113579	NA
F01_transcript_61489	F01_transcript_61489	F01_transcript_61489	F01_transcript_61489	F01_transcript_61489
F01_transcript_73494	NA	F01_transcript_73494	NA	F01_transcript_73494
F01_transcript_166034	F01_transcript_166034	NA	NA	NA
F01_transcript_164033	NA	NA	NA	F01_transcript_164033

F01_transcript_111334	NA	F01_transcript_111334	NA	NA
F01_transcript_86949	NA	NA	NA	F01_transcript_86949
F01_transcript_91670	NA	F01_transcript_91670	NA	NA
F01_transcript_141651	NA	NA	NA	F01_transcript_141651
F01_transcript_101787	F01_transcript_101787	NA	NA	F01_transcript_101787
F01_transcript_66760	NA	NA	NA	F01_transcript_66760
F01_transcript_148594	NA	NA	NA	F01_transcript_148594
F01_transcript_86490	NA	NA	NA	F01_transcript_86490
F01_transcript_69324	F01_transcript_69324	F01_transcript_69324	F01_transcript_69324	F01_transcript_69324
F01_transcript_146636	NA	F01_transcript_146636	F01_transcript_146636	F01_transcript_146636
F01_transcript_156363	NA	NA	NA	F01_transcript_156363
F01_transcript_92692	NA	F01_transcript_92692	NA	F01_transcript_92692
F01_transcript_71731	NA	F01_transcript_71731	F01_transcript_71731	F01_transcript_71731
F01_transcript_47816	NA	F01_transcript_47816	NA	F01_transcript_47816

F01_transcript_117009	NA	F01_transcript_117009	NA	NA
F01_transcript_119575	NA	F01_transcript_119575	NA	NA
F01_transcript_103021	NA	NA	NA	F01_transcript_103021
F01_transcript_50589	NA	F01_transcript_50589	NA	NA
F01_transcript_135625	NA	NA	NA	F01_transcript_135625
F01_transcript_146438	F01_transcript_146438	F01_transcript_146438	F01_transcript_146438	F01_transcript_146438
F01_transcript_53006	NA	NA	NA	F01_transcript_53006
F01_transcript_52510	NA	F01_transcript_52510	NA	NA
F01_transcript_141938	F01_transcript_141938	F01_transcript_141938	F01_transcript_141938	F01_transcript_141938
F01_transcript_24027	NA	F01_transcript_24027	F01_transcript_24027	F01_transcript_24027
F01_transcript_109314	NA	F01_transcript_109314	NA	F01_transcript_109314
F01_transcript_77521	NA	NA	NA	F01_transcript_77521
F01_transcript_57747	NA	NA	NA	F01_transcript_57747
F01_transcript_126473	F01_transcript_126473	F01_transcript_126473	F01_transcript_126473	F01_transcript_126473

F01_transcript_76873	F01_transcript_76873	F01_transcript_76873	F01_transcript_76873	F01_transcript_76873
F01_transcript_85429	F01_transcript_85429	F01_transcript_85429	F01_transcript_85429	F01_transcript_85429
F01_transcript_48457	NA	F01_transcript_48457	NA	NA
F01_transcript_156555	NA	F01_transcript_156555	NA	F01_transcript_156555
F01_transcript_100549	NA	F01_transcript_100549	F01_transcript_100549	F01_transcript_100549
F01_transcript_98660	NA	F01_transcript_98660	F01_transcript_98660	F01_transcript_98660
F01_transcript_93015	NA	F01_transcript_93015	NA	F01_transcript_93015
F01_transcript_62579	NA	NA	NA	F01_transcript_62579
F01_transcript_158921	NA	NA	F01_transcript_158921	NA
F01_transcript_163576	NA	F01_transcript_163576	NA	NA
F01_transcript_114791	F01_transcript_114791	F01_transcript_114791	NA	F01_transcript_114791
F01_transcript_86170	NA	NA	NA	F01_transcript_86170
F01_transcript_96477	F01_transcript_96477	F01_transcript_96477	F01_transcript_96477	F01_transcript_96477
F01_transcript_67576	F01_transcript_67576	F01_transcript_67576	F01_transcript_67576	F01_transcript_67576

F01_transcript_89178	NA	F01_transcript_89178	NA	F01_transcript_89178
F01_transcript_53619	F01_transcript_53619	F01_transcript_53619	NA	F01_transcript_53619
F01_transcript_149019	F01_transcript_149019	F01_transcript_149019	F01_transcript_149019	F01_transcript_149019
F01_transcript_147332	NA	F01_transcript_147332	NA	NA
F01_transcript_100947	F01_transcript_100947	F01_transcript_100947	F01_transcript_100947	F01_transcript_100947
F01_transcript_146190	NA	NA	NA	F01_transcript_146190
F01_transcript_137482	NA	NA	NA	F01_transcript_137482
F01_transcript_26774	F01_transcript_26774	F01_transcript_26774	F01_transcript_26774	F01_transcript_26774
F01_transcript_140969	NA	NA	NA	F01_transcript_140969
F01_transcript_63980	F01_transcript_63980	F01_transcript_63980	F01_transcript_63980	F01_transcript_63980
F01_transcript_128422	NA	F01_transcript_128422	NA	F01_transcript_128422
F01_transcript_13339	NA	F01_transcript_13339	NA	NA
F01_transcript_58754	NA	NA	NA	F01_transcript_58754
F01_transcript_136566	NA	NA	NA	F01_transcript_136566

F01_transcript_69564	NA	F01_transcript_69564	NA	NA
F01_transcript_50112	F01_transcript_50112	F01_transcript_50112	NA	NA
F01_transcript_166612	F01_transcript_166612	F01_transcript_166612	NA	NA
F01_transcript_37680	NA	NA	NA	F01_transcript_37680
F01_transcript_135841	NA	F01_transcript_135841	F01_transcript_135841	F01_transcript_135841
F01_transcript_104704	NA	F01_transcript_104704	NA	F01_transcript_104704
F01_transcript_69780	NA	F01_transcript_69780	NA	F01_transcript_69780
F01_transcript_160958	NA	F01_transcript_160958	NA	NA
F01_transcript_109605	NA	F01_transcript_109605	NA	NA
F01_transcript_91295	NA	NA	NA	F01_transcript_91295
F01_transcript_61309	F01_transcript_61309	F01_transcript_61309	F01_transcript_61309	F01_transcript_61309
F01_transcript_125154	NA	NA	NA	F01_transcript_125154
F01_transcript_166150	F01_transcript_166150	F01_transcript_166150	F01_transcript_166150	NA
F01_transcript_124424	F01_transcript_124424	F01_transcript_124424	F01_transcript_124424	F01_transcript_124424

F01_transcript_80036	F01_transcript_80036	F01_transcript_80036	F01_transcript_80036	F01_transcript_80036
F01_transcript_48186	NA	F01_transcript_48186	NA	NA
F01_transcript_62740	NA	NA	NA	F01_transcript_62740
F01_transcript_94301	F01_transcript_94301	F01_transcript_94301	F01_transcript_94301	F01_transcript_94301
F01_transcript_156802	NA	F01_transcript_156802	F01_transcript_156802	F01_transcript_156802
F01_transcript_83985	NA	NA	NA	F01_transcript_83985
F01_transcript_167597	NA	F01_transcript_167597	NA	NA
F01_transcript_74621	NA	F01_transcript_74621	NA	NA
F01_transcript_68686	NA	NA	NA	F01_transcript_68686
F01_transcript_140196	NA	F01_transcript_140196	NA	NA
F01_transcript_162037	NA	NA	NA	F01_transcript_162037
F01_transcript_105778	NA	F01_transcript_105778	F01_transcript_105778	F01_transcript_105778
F01_transcript_117191	NA	NA	NA	F01_transcript_117191
F01_transcript_131368	F01_transcript_131368	F01_transcript_131368	NA	F01_transcript_131368

F01_transcript_92101	NA	F01_transcript_92101	NA	NA
F01_transcript_41342	NA	NA	NA	F01_transcript_41342
F01_transcript_100069	NA	F01_transcript_100069	NA	NA
F01_transcript_95587	NA	NA	NA	F01_transcript_95587
F01_transcript_98197	NA	F01_transcript_98197	NA	F01_transcript_98197
F01_transcript_84524	F01_transcript_84524	F01_transcript_84524	NA	NA
F01_transcript_104742	F01_transcript_104742	F01_transcript_104742	NA	F01_transcript_104742
F01_transcript_130163	NA	F01_transcript_130163	NA	NA
F01_transcript_127332	F01_transcript_127332	F01_transcript_127332	F01_transcript_127332	F01_transcript_127332
F01_transcript_151476	F01_transcript_151476	F01_transcript_151476	F01_transcript_151476	F01_transcript_151476
F01_transcript_53905	F01_transcript_53905	F01_transcript_53905	F01_transcript_53905	F01_transcript_53905
F01_transcript_165635	NA	NA	NA	F01_transcript_165635
F01_transcript_102892	NA	NA	NA	F01_transcript_102892
F01_transcript_89382	F01_transcript_89382	F01_transcript_89382	F01_transcript_89382	F01_transcript_89382

F01_transcript_157646	NA	F01_transcript_157646	F01_transcript_157646	F01_transcript_157646
F01_transcript_143452	F01_transcript_143452	F01_transcript_143452	F01_transcript_143452	F01_transcript_143452
F01_transcript_70477	F01_transcript_70477	F01_transcript_70477	F01_transcript_70477	F01_transcript_70477
F01_transcript_98231	NA	F01_transcript_98231	NA	NA
F01_transcript_55940	NA	F01_transcript_55940	NA	NA
F01_transcript_103241	NA	F01_transcript_103241	NA	NA
F01_transcript_167475	F01_transcript_167475	F01_transcript_167475	F01_transcript_167475	F01_transcript_167475
F01_transcript_89175	F01_transcript_89175	F01_transcript_89175	F01_transcript_89175	F01_transcript_89175
F01_transcript_64807	NA	F01_transcript_64807	F01_transcript_64807	F01_transcript_64807
F01_transcript_124108	NA	NA	NA	F01_transcript_124108
F01_transcript_134863	NA	F01_transcript_134863	NA	F01_transcript_134863
F01_transcript_58405	NA	F01_transcript_58405	NA	NA
F01_transcript_62543	NA	F01_transcript_62543	NA	NA
F01_transcript_53770	NA	NA	NA	F01_transcript_53770

F01_transcript_70313	F01_transcript_70313	F01_transcript_70313	NA	NA
F01_transcript_91156	NA	NA	NA	F01_transcript_91156
F01_transcript_8082	NA	F01_transcript_8082	NA	F01_transcript_8082
F01_transcript_137742	F01_transcript_137742	F01_transcript_137742	F01_transcript_137742	F01_transcript_137742
F01_transcript_91143	F01_transcript_91143	F01_transcript_91143	F01_transcript_91143	F01_transcript_91143
F01_transcript_37950	NA	NA	NA	F01_transcript_37950
F01_transcript_135169	NA	NA	NA	F01_transcript_135169
F01_transcript_124365	F01_transcript_124365	F01_transcript_124365	F01_transcript_124365	NA
F01_transcript_166879	F01_transcript_166879	F01_transcript_166879	NA	F01_transcript_166879
F01_transcript_137665	NA	F01_transcript_137665	NA	NA
F01_transcript_117116	NA	F01_transcript_117116	NA	F01_transcript_117116
F01_transcript_109706	F01_transcript_109706	F01_transcript_109706	F01_transcript_109706	F01_transcript_109706
F01_transcript_166184	NA	F01_transcript_166184	NA	NA
F01_transcript_85512	NA	NA	NA	F01_transcript_85512

F01_transcript_97323	NA	F01_transcript_97323	F01_transcript_97323	F01_transcript_97323
F01_transcript_49331	NA	F01_transcript_49331	NA	NA
F01_transcript_160333	NA	NA	NA	F01_transcript_160333
F01_transcript_93245	NA	F01_transcript_93245	F01_transcript_93245	F01_transcript_93245
F01_transcript_101473	NA	F01_transcript_101473	NA	F01_transcript_101473
F01_transcript_159365	NA	F01_transcript_159365	NA	F01_transcript_159365
F01_transcript_132305	NA	NA	NA	F01_transcript_132305
F01_transcript_141414	F01_transcript_141414	F01_transcript_141414	NA	F01_transcript_141414
F01_transcript_74940	F01_transcript_74940	F01_transcript_74940	NA	F01_transcript_74940
F01_transcript_146399	NA	F01_transcript_146399	F01_transcript_146399	F01_transcript_146399
F01_transcript_61196	NA	F01_transcript_61196	NA	NA
F01_transcript_92862	NA	F01_transcript_92862	NA	NA
F01_transcript_50401	F01_transcript_50401	F01_transcript_50401	NA	NA
F01_transcript_75678	NA	F01_transcript_75678	NA	NA

F01_transcript_105452	NA	F01_transcript_105452	NA	NA
F01_transcript_69482	NA	F01_transcript_69482	NA	F01_transcript_69482
F01_transcript_83111	NA	NA	NA	F01_transcript_83111
F01_transcript_167433	NA	NA	NA	F01_transcript_167433
F01_transcript_132339	NA	F01_transcript_132339	NA	NA
F01_transcript_161321	NA	NA	F01_transcript_161321	NA
F01_transcript_48646	NA	NA	NA	F01_transcript_48646
F01_transcript_63751	NA	F01_transcript_63751	F01_transcript_63751	F01_transcript_63751
F01_transcript_66164	F01_transcript_66164	F01_transcript_66164	NA	NA
F01_transcript_91288	NA	NA	NA	F01_transcript_91288
F01_transcript_152959	NA	F01_transcript_152959	NA	NA
F01_transcript_154289	F01_transcript_154289	F01_transcript_154289	F01_transcript_154289	F01_transcript_154289
F01_transcript_49700	NA	NA	NA	F01_transcript_49700
F01_transcript_53625	NA	F01_transcript_53625	NA	NA

F01_transcript_112981	NA	NA	NA	F01_transcript_112981
F01_transcript_113371	F01_transcript_113371	F01_transcript_113371	F01_transcript_113371	F01_transcript_113371
F01_transcript_158996	F01_transcript_158996	F01_transcript_158996	F01_transcript_158996	NA
F01_transcript_48403	NA	F01_transcript_48403	NA	NA
F01_transcript_90747	F01_transcript_90747	F01_transcript_90747	F01_transcript_90747	F01_transcript_90747
F01_transcript_118393	F01_transcript_118393	F01_transcript_118393	F01_transcript_118393	F01_transcript_118393
F01_transcript_127784	NA	F01_transcript_127784	NA	F01_transcript_127784
F01_transcript_148607	NA	F01_transcript_148607	F01_transcript_148607	F01_transcript_148607
F01_transcript_126464	NA	NA	NA	F01_transcript_126464
F01_transcript_110300	NA	NA	NA	F01_transcript_110300
F01_transcript_154197	NA	F01_transcript_154197	NA	NA
F01_transcript_69073	F01_transcript_69073	F01_transcript_69073	NA	F01_transcript_69073
F01_transcript_94070	F01_transcript_94070	F01_transcript_94070	F01_transcript_94070	F01_transcript_94070
F01_transcript_153258	NA	F01_transcript_153258	NA	F01_transcript_153258

F01_transcript_62752	F01_transcript_62752	F01_transcript_62752	NA	F01_transcript_62752
F01_transcript_68035	NA	F01_transcript_68035	NA	F01_transcript_68035
F01_transcript_6021	F01_transcript_6021	F01_transcript_6021	F01_transcript_6021	F01_transcript_6021
F01_transcript_55832	NA	F01_transcript_55832	NA	NA
F01_transcript_166391	F01_transcript_166391	F01_transcript_166391	F01_transcript_166391	NA
F01_transcript_58163	NA	F01_transcript_58163	NA	F01_transcript_58163
F01_transcript_144087	NA	NA	NA	F01_transcript_144087
F01_transcript_159420	F01_transcript_159420	F01_transcript_159420	F01_transcript_159420	F01_transcript_159420
F01_transcript_128026	F01_transcript_128026	F01_transcript_128026	F01_transcript_128026	F01_transcript_128026
F01_transcript_116684	NA	F01_transcript_116684	NA	NA
F01_transcript_153533	NA	NA	NA	F01_transcript_153533
F01_transcript_123607	F01_transcript_123607	F01_transcript_123607	NA	NA
F01_transcript_78573	NA	F01_transcript_78573	NA	F01_transcript_78573
F01_transcript_70199	NA	NA	NA	F01_transcript_70199

F01_transcript_63935	NA	F01_transcript_63935	NA	F01_transcript_63935
F01_transcript_63347	NA	F01_transcript_63347	NA	F01_transcript_63347
F01_transcript_112176	NA	F01_transcript_112176	NA	NA
F01_transcript_76353	NA	NA	NA	F01_transcript_76353
F01_transcript_99163	NA	NA	F01_transcript_99163	NA
F01_transcript_163879	NA	F01_transcript_163879	F01_transcript_163879	F01_transcript_163879
F01_transcript_125773	NA	NA	NA	F01_transcript_125773
F01_transcript_130655	NA	F01_transcript_130655	NA	F01_transcript_130655
F01_transcript_60238	NA	NA	F01_transcript_60238	F01_transcript_60238
F01_transcript_110165	F01_transcript_110165	F01_transcript_110165	F01_transcript_110165	F01_transcript_110165
F01_transcript_154923	NA	F01_transcript_154923	NA	F01_transcript_154923
F01_transcript_61040	NA	NA	NA	F01_transcript_61040
F01_transcript_53600	NA	NA	NA	F01_transcript_53600
F01_transcript_6977	NA	NA	NA	F01_transcript_6977

F01_transcript_88161	NA	F01_transcript_88161	NA	NA
F01_transcript_102815	NA	F01_transcript_102815	F01_transcript_102815	F01_transcript_102815
F01_transcript_92365	NA	NA	NA	F01_transcript_92365
F01_transcript_43015	NA	NA	NA	F01_transcript_43015
F01_transcript_26682	NA	NA	NA	F01_transcript_26682
F01_transcript_95424	NA	F01_transcript_95424	NA	NA
F01_transcript_74209	F01_transcript_74209	F01_transcript_74209	NA	NA
F01_transcript_56113	NA	NA	NA	F01_transcript_56113
F01_transcript_146714	NA	F01_transcript_146714	NA	F01_transcript_146714
F01_transcript_112781	NA	F01_transcript_112781	NA	NA
F01_transcript_134007	F01_transcript_134007	F01_transcript_134007	NA	F01_transcript_134007
F01_transcript_58926	F01_transcript_58926	F01_transcript_58926	F01_transcript_58926	F01_transcript_58926
F01_transcript_55702	NA	NA	F01_transcript_55702	F01_transcript_55702
F01_transcript_53843	NA	F01_transcript_53843	F01_transcript_53843	F01_transcript_53843

F01_transcript_126564	F01_transcript_126564	F01_transcript_126564	F01_transcript_126564	F01_transcript_126564
F01_transcript_86267	F01_transcript_86267	F01_transcript_86267	F01_transcript_86267	F01_transcript_86267
F01_transcript_96122	F01_transcript_96122	F01_transcript_96122	F01_transcript_96122	F01_transcript_96122
F01_transcript_51266	F01_transcript_51266	F01_transcript_51266	F01_transcript_51266	F01_transcript_51266
F01_transcript_55217	F01_transcript_55217	F01_transcript_55217	NA	NA
F01_transcript_41895	NA	NA	NA	F01_transcript_41895
F01_transcript_61611	F01_transcript_61611	F01_transcript_61611	NA	NA
F01_transcript_78525	NA	F01_transcript_78525	NA	NA
F01_transcript_56277	F01_transcript_56277	F01_transcript_56277	F01_transcript_56277	F01_transcript_56277
F01_transcript_58975	NA	NA	NA	F01_transcript_58975
F01_transcript_143599	NA	NA	NA	F01_transcript_143599
F01_transcript_162632	NA	F01_transcript_162632	F01_transcript_162632	F01_transcript_162632
F01_transcript_113613	NA	F01_transcript_113613	NA	NA
F01_transcript_91815	F01_transcript_91815	F01_transcript_91815	F01_transcript_91815	F01_transcript_91815

F01_transcript_19924	NA	F01_transcript_19924	NA	NA
F01_transcript_90357	NA	NA	NA	F01_transcript_90357
F01_transcript_79384	NA	F01_transcript_79384	F01_transcript_79384	NA
F01_transcript_166049	NA	F01_transcript_166049	NA	NA
F01_transcript_152155	NA	F01_transcript_152155	F01_transcript_152155	F01_transcript_152155
F01_transcript_86407	NA	F01_transcript_86407	NA	NA
F01_transcript_55384	NA	NA	NA	F01_transcript_55384
F01_transcript_38839	NA	NA	NA	F01_transcript_38839
F01_transcript_135999	NA	NA	NA	F01_transcript_135999
F01_transcript_109371	F01_transcript_109371	F01_transcript_109371	F01_transcript_109371	F01_transcript_109371
F01_transcript_114132	NA	F01_transcript_114132	F01_transcript_114132	NA
F01_transcript_152640	NA	NA	NA	F01_transcript_152640
F01_transcript_71672	F01_transcript_71672	F01_transcript_71672	NA	NA
F01_transcript_124110	NA	NA	NA	F01_transcript_124110

F01_transcript_126715	NA	F01_transcript_126715	NA	NA
F01_transcript_89004	F01_transcript_89004	F01_transcript_89004	F01_transcript_89004	F01_transcript_89004
F01_transcript_133847	F01_transcript_133847	F01_transcript_133847	NA	NA
F01_transcript_165250	F01_transcript_165250	F01_transcript_165250	F01_transcript_165250	F01_transcript_165250
F01_transcript_55186	F01_transcript_55186	F01_transcript_55186	F01_transcript_55186	F01_transcript_55186
F01_transcript_142854	NA	NA	NA	F01_transcript_142854
F01_transcript_25576	NA	F01_transcript_25576	NA	F01_transcript_25576
F01_transcript_122438	F01_transcript_122438	F01_transcript_122438	NA	F01_transcript_122438
F01_transcript_75772	NA	NA	NA	F01_transcript_75772
F01_transcript_90295	F01_transcript_90295	F01_transcript_90295	NA	NA
F01_transcript_125446	NA	NA	NA	F01_transcript_125446
F01_transcript_144698	NA	F01_transcript_144698	F01_transcript_144698	F01_transcript_144698
F01_transcript_5937	NA	NA	NA	F01_transcript_5937
F01_transcript_164539	F01_transcript_164539	F01_transcript_164539	F01_transcript_164539	F01_transcript_164539

F01_transcript_53304	NA	NA	NA	F01_transcript_53304
F01_transcript_61776	F01_transcript_61776	F01_transcript_61776	F01_transcript_61776	F01_transcript_61776
F01_transcript_83506	NA	F01_transcript_83506	NA	NA
F01_transcript_50739	F01_transcript_50739	F01_transcript_50739	F01_transcript_50739	NA
F01_transcript_63010	NA	F01_transcript_63010	NA	NA
F01_transcript_62375	NA	F01_transcript_62375	NA	NA
F01_transcript_112167	NA	F01_transcript_112167	NA	NA
F01_transcript_95546	NA	F01_transcript_95546	NA	NA
F01_transcript_148152	F01_transcript_148152	F01_transcript_148152	F01_transcript_148152	F01_transcript_148152
F01_transcript_161161	NA	F01_transcript_161161	NA	NA
F01_transcript_163428	NA	NA	NA	F01_transcript_163428
F01_transcript_96485	NA	F01_transcript_96485	NA	F01_transcript_96485
F01_transcript_73746	NA	NA	NA	F01_transcript_73746
F01_transcript_53574	NA	F01_transcript_53574	NA	NA

F01_transcript_56072	NA	NA	NA	F01_transcript_56072
F01_transcript_165828	NA	NA	NA	F01_transcript_165828
F01_transcript_98930	F01_transcript_98930	NA	NA	NA
F01_transcript_151964	NA	F01_transcript_151964	NA	NA
F01_transcript_49351	NA	F01_transcript_49351	NA	NA
F01_transcript_103592	F01_transcript_103592	F01_transcript_103592	NA	F01_transcript_103592
F01_transcript_125571	F01_transcript_125571	F01_transcript_125571	F01_transcript_125571	F01_transcript_125571
F01_transcript_48657	NA	F01_transcript_48657	F01_transcript_48657	F01_transcript_48657
F01_transcript_84396	NA	F01_transcript_84396	NA	NA
F01_transcript_97560	F01_transcript_97560	F01_transcript_97560	F01_transcript_97560	F01_transcript_97560
F01_transcript_76146	NA	F01_transcript_76146	NA	F01_transcript_76146
F01_transcript_100266	NA	F01_transcript_100266	F01_transcript_100266	F01_transcript_100266
F01_transcript_151499	F01_transcript_151499	F01_transcript_151499	F01_transcript_151499	F01_transcript_151499
F01_transcript_61746	F01_transcript_61746	F01_transcript_61746	F01_transcript_61746	F01_transcript_61746

F01_transcript_130701	NA	F01_transcript_130701	NA	F01_transcript_130701
F01_transcript_69028	NA	F01_transcript_69028	F01_transcript_69028	F01_transcript_69028
F01_transcript_157036	NA	NA	NA	F01_transcript_157036
F01_transcript_122532	NA	F01_transcript_122532	NA	NA
F01_transcript_118735	F01_transcript_118735	F01_transcript_118735	F01_transcript_118735	F01_transcript_118735
F01_transcript_106389	NA	F01_transcript_106389	NA	NA
F01_transcript_80112	NA	F01_transcript_80112	NA	NA
F01_transcript_98667	NA	NA	F01_transcript_98667	F01_transcript_98667
F01_transcript_124955	F01_transcript_124955	F01_transcript_124955	F01_transcript_124955	F01_transcript_124955
F01_transcript_94766	NA	NA	F01_transcript_94766	F01_transcript_94766
F01_transcript_75768	NA	NA	NA	F01_transcript_75768
F01_transcript_85397	F01_transcript_85397	F01_transcript_85397	F01_transcript_85397	F01_transcript_85397
F01_transcript_153786	F01_transcript_153786	F01_transcript_153786	NA	NA
F01_transcript_78174	NA	NA	NA	F01_transcript_78174

F01_transcript_61779	F01_transcript_61779	F01_transcript_61779	F01_transcript_61779	F01_transcript_61779
F01_transcript_128469	F01_transcript_128469	F01_transcript_128469	F01_transcript_128469	F01_transcript_128469
F01_transcript_79565	NA	NA	NA	F01_transcript_79565
F01_transcript_47802	NA	F01_transcript_47802	NA	NA
F01_transcript_126352	F01_transcript_126352	F01_transcript_126352	NA	NA
F01_transcript_15645	NA	NA	NA	F01_transcript_15645
F01_transcript_24815	NA	NA	NA	F01_transcript_24815
F01_transcript_154299	NA	F01_transcript_154299	NA	NA
F01_transcript_68847	NA	NA	NA	F01_transcript_68847
F01_transcript_13486	NA	F01_transcript_13486	F01_transcript_13486	F01_transcript_13486
F01_transcript_49548	NA	NA	NA	F01_transcript_49548
F01_transcript_49154	NA	NA	NA	F01_transcript_49154
F01_transcript_101621	NA	NA	NA	F01_transcript_101621
F01_transcript_116027	NA	F01_transcript_116027	NA	NA

F01_transcript_159477	NA	NA	NA	F01_transcript_159477
F01_transcript_92738	NA	F01_transcript_92738	F01_transcript_92738	F01_transcript_92738
F01_transcript_94871	NA	NA	F01_transcript_94871	F01_transcript_94871
F01_transcript_129124	NA	NA	NA	F01_transcript_129124
F01_transcript_43432	NA	F01_transcript_43432	NA	NA
F01_transcript_115766	F01_transcript_115766	F01_transcript_115766	NA	NA
F01_transcript_66722	NA	F01_transcript_66722	NA	NA
F01_transcript_76952	NA	NA	NA	F01_transcript_76952
F01_transcript_88316	F01_transcript_88316	F01_transcript_88316	F01_transcript_88316	NA
F01_transcript_92414	NA	F01_transcript_92414	F01_transcript_92414	F01_transcript_92414
F01_transcript_127357	NA	NA	NA	F01_transcript_127357
F01_transcript_50450	NA	F01_transcript_50450	NA	NA
F01_transcript_70352	NA	F01_transcript_70352	NA	F01_transcript_70352
F01_transcript_70141	F01_transcript_70141	F01_transcript_70141	F01_transcript_70141	F01_transcript_70141

F01_transcript_105116	F01_transcript_105116	F01_transcript_105116	NA	NA
F01_transcript_80219	NA	NA	NA	F01_transcript_80219
F01_transcript_104930	NA	F01_transcript_104930	NA	NA
F01_transcript_4517	NA	NA	NA	F01_transcript_4517
F01_transcript_98277	NA	NA	NA	F01_transcript_98277
F01_transcript_48707	NA	NA	NA	F01_transcript_48707
F01_transcript_159257	NA	F01_transcript_159257	NA	NA
F01_transcript_151148	NA	F01_transcript_151148	F01_transcript_151148	F01_transcript_151148
F01_transcript_133288	NA	F01_transcript_133288	F01_transcript_133288	F01_transcript_133288
F01_transcript_81281	F01_transcript_81281	F01_transcript_81281	F01_transcript_81281	F01_transcript_81281
F01_transcript_75346	F01_transcript_75346	F01_transcript_75346	NA	F01_transcript_75346
F01_transcript_101086	NA	F01_transcript_101086	NA	NA
F01_transcript_126462	NA	F01_transcript_126462	NA	NA
F01_transcript_137460	F01_transcript_137460	F01_transcript_137460	F01_transcript_137460	F01_transcript_137460

F01_transcript_55359	NA	NA	NA	F01_transcript_55359
F01_transcript_96385	F01_transcript_96385	F01_transcript_96385	NA	F01_transcript_96385
F01_transcript_128983	NA	NA	NA	F01_transcript_128983
F01_transcript_94098	F01_transcript_94098	F01_transcript_94098	F01_transcript_94098	F01_transcript_94098
F01_transcript_56312	NA	NA	F01_transcript_56312	NA
F01_transcript_133496	NA	F01_transcript_133496	NA	NA
F01_transcript_49161	F01_transcript_49161	F01_transcript_49161	F01_transcript_49161	F01_transcript_49161
F01_transcript_153871	NA	F01_transcript_153871	NA	F01_transcript_153871
F01_transcript_72429	NA	NA	NA	F01_transcript_72429
F01_transcript_110094	NA	NA	NA	F01_transcript_110094
F01_transcript_89714	F01_transcript_89714	F01_transcript_89714	NA	NA
F01_transcript_36804	NA	NA	NA	F01_transcript_36804
F01_transcript_74001	NA	NA	NA	F01_transcript_74001
F01_transcript_69561	NA	F01_transcript_69561	NA	F01_transcript_69561

F01_transcript_107299	NA	NA	NA	F01_transcript_107299
F01_transcript_108837	F01_transcript_108837	F01_transcript_108837	F01_transcript_108837	F01_transcript_108837
F01_transcript_87091	NA	F01_transcript_87091	NA	NA
F01_transcript_146694	F01_transcript_146694	F01_transcript_146694	F01_transcript_146694	F01_transcript_146694
F01_transcript_127871	F01_transcript_127871	F01_transcript_127871	F01_transcript_127871	F01_transcript_127871
F01_transcript_55560	NA	F01_transcript_55560	NA	NA
F01_transcript_97413	F01_transcript_97413	F01_transcript_97413	F01_transcript_97413	NA
F01_transcript_112317	F01_transcript_112317	F01_transcript_112317	F01_transcript_112317	F01_transcript_112317
F01_transcript_84354	F01_transcript_84354	F01_transcript_84354	F01_transcript_84354	F01_transcript_84354
F01_transcript_149341	NA	F01_transcript_149341	NA	F01_transcript_149341
F01_transcript_160444	F01_transcript_160444	F01_transcript_160444	F01_transcript_160444	F01_transcript_160444
F01_transcript_85365	NA	NA	NA	F01_transcript_85365
F01_transcript_42257	NA	NA	NA	F01_transcript_42257
F01_transcript_113421	NA	NA	NA	F01_transcript_113421

F01_transcript_159379	NA	NA	NA	F01_transcript_159379
F01_transcript_61363	NA	F01_transcript_61363	NA	NA
F01_transcript_69085	NA	F01_transcript_69085	NA	NA
F01_transcript_137219	NA	F01_transcript_137219	NA	NA
F01_transcript_51970	NA	F01_transcript_51970	NA	NA
F01_transcript_23066	NA	NA	NA	F01_transcript_23066
F01_transcript_74504	NA	NA	NA	F01_transcript_74504
F01_transcript_134795	NA	NA	NA	F01_transcript_134795
F01_transcript_52797	F01_transcript_52797	F01_transcript_52797	F01_transcript_52797	F01_transcript_52797
F01_transcript_112701	NA	F01_transcript_112701	NA	NA
F01_transcript_15619	NA	NA	NA	F01_transcript_15619
F01_transcript_84991	NA	F01_transcript_84991	F01_transcript_84991	F01_transcript_84991
F01_transcript_71625	NA	F01_transcript_71625	NA	NA
F01_transcript_59819	F01_transcript_59819	F01_transcript_59819	F01_transcript_59819	F01_transcript_59819

F01_transcript_149203	F01_transcript_149203	F01_transcript_149203	NA	NA
F01_transcript_158376	F01_transcript_158376	F01_transcript_158376	F01_transcript_158376	F01_transcript_158376
F01_transcript_138272	NA	F01_transcript_138272	NA	F01_transcript_138272
F01_transcript_149876	F01_transcript_149876	F01_transcript_149876	NA	NA
F01_transcript_106270	NA	NA	NA	F01_transcript_106270
F01_transcript_167230	NA	F01_transcript_167230	NA	F01_transcript_167230
F01_transcript_68557	NA	F01_transcript_68557	NA	NA
F01_transcript_158968	NA	NA	F01_transcript_158968	NA
F01_transcript_60732	NA	F01_transcript_60732	NA	NA
F01_transcript_45373	NA	NA	NA	F01_transcript_45373
F01_transcript_121540	NA	NA	F01_transcript_121540	F01_transcript_121540
F01_transcript_92635	NA	NA	NA	F01_transcript_92635
F01_transcript_4129	F01_transcript_4129	F01_transcript_4129	NA	NA
F01_transcript_99465	F01_transcript_99465	F01_transcript_99465	F01_transcript_99465	F01_transcript_99465

F01_transcript_100592	F01_transcript_100592	F01_transcript_100592	F01_transcript_100592	F01_transcript_100592
F01_transcript_160197	NA	NA	NA	F01_transcript_160197
F01_transcript_116542	NA	NA	NA	F01_transcript_116542
F01_transcript_81458	NA	NA	NA	F01_transcript_81458
F01_transcript_99844	F01_transcript_99844	F01_transcript_99844	NA	NA
F01_transcript_141122	F01_transcript_141122	F01_transcript_141122	NA	NA
F01_transcript_23865	NA	NA	NA	F01_transcript_23865
F01_transcript_80390	NA	F01_transcript_80390	NA	NA
F01_transcript_46742	NA	NA	NA	F01_transcript_46742
F01_transcript_165010	NA	F01_transcript_165010	F01_transcript_165010	F01_transcript_165010
F01_transcript_144161	NA	NA	NA	F01_transcript_144161
F01_transcript_72467	F01_transcript_72467	F01_transcript_72467	F01_transcript_72467	F01_transcript_72467
F01_transcript_128149	NA	F01_transcript_128149	NA	F01_transcript_128149
F01_transcript_67146	NA	F01_transcript_67146	NA	NA

F01_transcript_97953	NA	NA	NA	F01_transcript_97953
F01_transcript_120640	NA	F01_transcript_120640	NA	NA
F01_transcript_86789	NA	F01_transcript_86789	F01_transcript_86789	F01_transcript_86789
F01_transcript_54972	NA	NA	NA	F01_transcript_54972
F01_transcript_62737	F01_transcript_62737	F01_transcript_62737	F01_transcript_62737	F01_transcript_62737
F01_transcript_49270	NA	F01_transcript_49270	NA	NA
F01_transcript_167495	NA	NA	NA	F01_transcript_167495
F01_transcript_116124	NA	F01_transcript_116124	F01_transcript_116124	F01_transcript_116124
F01_transcript_69963	NA	NA	NA	F01_transcript_69963
F01_transcript_58404	NA	F01_transcript_58404	NA	NA
F01_transcript_161458	NA	F01_transcript_161458	F01_transcript_161458	F01_transcript_161458
F01_transcript_155591	F01_transcript_155591	F01_transcript_155591	F01_transcript_155591	F01_transcript_155591
F01_transcript_125108	F01_transcript_125108	F01_transcript_125108	NA	NA
F01_transcript_116343	NA	F01_transcript_116343	NA	NA

F01_transcript_33943	NA	NA	NA	F01_transcript_33943
F01_transcript_22860	F01_transcript_22860	F01_transcript_22860	NA	NA
F01_transcript_103341	F01_transcript_103341	F01_transcript_103341	F01_transcript_103341	F01_transcript_103341
F01_transcript_134547	NA	F01_transcript_134547	NA	F01_transcript_134547
F01_transcript_147297	NA	F01_transcript_147297	NA	NA
F01_transcript_111063	F01_transcript_111063	F01_transcript_111063	NA	F01_transcript_111063
F01_transcript_91674	F01_transcript_91674	F01_transcript_91674	F01_transcript_91674	F01_transcript_91674
F01_transcript_91518	NA	NA	NA	F01_transcript_91518
F01_transcript_89166	F01_transcript_89166	F01_transcript_89166	F01_transcript_89166	F01_transcript_89166
F01_transcript_69925	F01_transcript_69925	F01_transcript_69925	F01_transcript_69925	F01_transcript_69925
F01_transcript_118240	NA	NA	NA	F01_transcript_118240
F01_transcript_143248	NA	NA	NA	F01_transcript_143248
F01_transcript_112335	F01_transcript_112335	F01_transcript_112335	F01_transcript_112335	F01_transcript_112335
F01_transcript_14411	F01_transcript_14411	F01_transcript_14411	F01_transcript_14411	F01_transcript_14411

F01_transcript_70053	NA	NA	NA	F01_transcript_70053
F01_transcript_135746	F01_transcript_135746	F01_transcript_135746	NA	NA
F01_transcript_116994	F01_transcript_116994	F01_transcript_116994	F01_transcript_116994	F01_transcript_116994
F01_transcript_94198	NA	NA	NA	F01_transcript_94198
F01_transcript_95002	NA	F01_transcript_95002	F01_transcript_95002	F01_transcript_95002
F01_transcript_153902	F01_transcript_153902	F01_transcript_153902	F01_transcript_153902	F01_transcript_153902
F01_transcript_73956	NA	F01_transcript_73956	NA	NA
F01_transcript_83390	F01_transcript_83390	F01_transcript_83390	F01_transcript_83390	F01_transcript_83390
F01_transcript_88393	NA	NA	NA	F01_transcript_88393
F01_transcript_142331	F01_transcript_142331	NA	F01_transcript_142331	F01_transcript_142331
F01_transcript_158490	NA	F01_transcript_158490	NA	NA
F01_transcript_90776	NA	NA	NA	F01_transcript_90776
F01_transcript_159894	NA	F01_transcript_159894	NA	NA
F01_transcript_102520	F01_transcript_102520	F01_transcript_102520	F01_transcript_102520	F01_transcript_102520

F01_transcript_48535	NA	F01_transcript_48535	NA	NA
F01_transcript_53973	F01_transcript_53973	F01_transcript_53973	F01_transcript_53973	F01_transcript_53973
F01_transcript_164781	F01_transcript_164781	F01_transcript_164781	F01_transcript_164781	F01_transcript_164781
F01_transcript_124371	F01_transcript_124371	F01_transcript_124371	F01_transcript_124371	F01_transcript_124371
F01_transcript_157964	NA	F01_transcript_157964	F01_transcript_157964	NA
F01_transcript_62377	F01_transcript_62377	F01_transcript_62377	NA	NA
F01_transcript_49720	NA	F01_transcript_49720	F01_transcript_49720	F01_transcript_49720
F01_transcript_100060	NA	NA	NA	F01_transcript_100060
F01_transcript_107019	NA	F01_transcript_107019	NA	NA
F01_transcript_4353	F01_transcript_4353	F01_transcript_4353	F01_transcript_4353	F01_transcript_4353
F01_transcript_109581	NA	NA	NA	F01_transcript_109581
F01_transcript_73075	NA	NA	NA	F01_transcript_73075
F01_transcript_72637	NA	NA	NA	F01_transcript_72637
F01_transcript_117819	NA	NA	NA	F01_transcript_117819

F01_transcript_154182	F01_transcript_154182	F01_transcript_154182	NA	F01_transcript_154182
F01_transcript_64198	F01_transcript_64198	F01_transcript_64198	F01_transcript_64198	F01_transcript_64198
F01_transcript_148227	NA	F01_transcript_148227	NA	NA
F01_transcript_111970	NA	NA	NA	F01_transcript_111970
F01_transcript_93594	F01_transcript_93594	F01_transcript_93594	NA	NA
F01_transcript_143345	F01_transcript_143345	F01_transcript_143345	F01_transcript_143345	NA
F01_transcript_116582	NA	NA	NA	F01_transcript_116582
F01_transcript_102106	NA	F01_transcript_102106	F01_transcript_102106	F01_transcript_102106
F01_transcript_157958	NA	F01_transcript_157958	F01_transcript_157958	F01_transcript_157958
F01_transcript_105321	NA	F01_transcript_105321	NA	NA
F01_transcript_67772	NA	F01_transcript_67772	NA	F01_transcript_67772
F01_transcript_66540	NA	NA	NA	F01_transcript_66540
F01_transcript_154780	F01_transcript_154780	F01_transcript_154780	NA	F01_transcript_154780
F01_transcript_99927	NA	NA	F01_transcript_99927	F01_transcript_99927

F01_transcript_44123	F01_transcript_44123	NA	NA	F01_transcript_44123
F01_transcript_68102	NA	NA	F01_transcript_68102	NA
F01_transcript_88677	NA	F01_transcript_88677	F01_transcript_88677	F01_transcript_88677
F01_transcript_68436	F01_transcript_68436	F01_transcript_68436	F01_transcript_68436	NA
F01_transcript_78752	F01_transcript_78752	F01_transcript_78752	F01_transcript_78752	F01_transcript_78752
F01_transcript_134791	NA	NA	NA	F01_transcript_134791
F01_transcript_159344	NA	F01_transcript_159344	F01_transcript_159344	NA
F01_transcript_67101	NA	F01_transcript_67101	NA	F01_transcript_67101
F01_transcript_67732	NA	NA	NA	F01_transcript_67732
F01_transcript_128834	F01_transcript_128834	F01_transcript_128834	NA	NA
F01_transcript_7554	NA	NA	NA	F01_transcript_7554
F01_transcript_147192	F01_transcript_147192	F01_transcript_147192	F01_transcript_147192	F01_transcript_147192
F01_transcript_101048	F01_transcript_101048	F01_transcript_101048	F01_transcript_101048	F01_transcript_101048
F01_transcript_154935	F01_transcript_154935	F01_transcript_154935	NA	F01_transcript_154935

F01_transcript_75588	F01_transcript_75588	F01_transcript_75588	F01_transcript_75588	F01_transcript_75588
F01_transcript_81129	NA	NA	NA	F01_transcript_81129
F01_transcript_80750	F01_transcript_80750	F01_transcript_80750	F01_transcript_80750	F01_transcript_80750
F01_transcript_53514	F01_transcript_53514	F01_transcript_53514	F01_transcript_53514	F01_transcript_53514
F01_transcript_126691	NA	F01_transcript_126691	NA	F01_transcript_126691
F01_transcript_97614	NA	NA	NA	F01_transcript_97614
F01_transcript_153531	NA	NA	NA	F01_transcript_153531
F01_transcript_82671	F01_transcript_82671	F01_transcript_82671	F01_transcript_82671	F01_transcript_82671
F01_transcript_147748	NA	F01_transcript_147748	NA	NA
F01_transcript_46392	NA	NA	NA	F01_transcript_46392
F01_transcript_109287	F01_transcript_109287	F01_transcript_109287	F01_transcript_109287	F01_transcript_109287
F01_transcript_102189	F01_transcript_102189	F01_transcript_102189	NA	NA
F01_transcript_152609	F01_transcript_152609	F01_transcript_152609	F01_transcript_152609	F01_transcript_152609
F01_transcript_1121	NA	NA	NA	F01_transcript_1121

F01_transcript_158166	F01_transcript_158166	NA	NA	NA
F01_transcript_48211	NA	NA	NA	F01_transcript_48211
F01_transcript_143239	NA	F01_transcript_143239	F01_transcript_143239	NA
F01_transcript_62283	F01_transcript_62283	F01_transcript_62283	NA	NA
F01_transcript_153779	NA	NA	NA	F01_transcript_153779
F01_transcript_87794	NA	F01_transcript_87794	NA	NA
F01_transcript_143569	NA	F01_transcript_143569	F01_transcript_143569	F01_transcript_143569
F01_transcript_52119	NA	NA	NA	F01_transcript_52119
F01_transcript_82909	NA	F01_transcript_82909	NA	NA
F01_transcript_40780	NA	F01_transcript_40780	NA	NA
F01_transcript_21694	NA	NA	NA	F01_transcript_21694
F01_transcript_141176	F01_transcript_141176	F01_transcript_141176	NA	F01_transcript_141176
F01_transcript_149785	NA	F01_transcript_149785	NA	NA
F01_transcript_59092	F01_transcript_59092	F01_transcript_59092	F01_transcript_59092	F01_transcript_59092

F01_transcript_166341	NA	NA	NA	F01_transcript_166341
F01_transcript_133352	F01_transcript_133352	F01_transcript_133352	NA	F01_transcript_133352
F01_transcript_133152	F01_transcript_133152	F01_transcript_133152	F01_transcript_133152	F01_transcript_133152
F01_transcript_65245	NA	NA	F01_transcript_65245	NA
F01_transcript_67823	NA	F01_transcript_67823	F01_transcript_67823	F01_transcript_67823
F01_transcript_37735	NA	F01_transcript_37735	NA	NA
F01_transcript_141579	F01_transcript_141579	F01_transcript_141579	F01_transcript_141579	F01_transcript_141579
F01_transcript_149958	NA	NA	NA	F01_transcript_149958
F01_transcript_95464	NA	F01_transcript_95464	F01_transcript_95464	F01_transcript_95464
F01_transcript_70190	NA	NA	NA	F01_transcript_70190
F01_transcript_119133	F01_transcript_119133	F01_transcript_119133	NA	F01_transcript_119133
F01_transcript_163403	F01_transcript_163403	F01_transcript_163403	NA	NA
F01_transcript_49623	F01_transcript_49623	F01_transcript_49623	F01_transcript_49623	F01_transcript_49623
F01_transcript_119350	F01_transcript_119350	F01_transcript_119350	NA	F01_transcript_119350

F01_transcript_121190	NA	F01_transcript_121190	NA	F01_transcript_121190
F01_transcript_13781	F01_transcript_13781	F01_transcript_13781	NA	NA
F01_transcript_84428	NA	F01_transcript_84428	NA	F01_transcript_84428
F01_transcript_49423	NA	NA	NA	F01_transcript_49423
F01_transcript_85377	F01_transcript_85377	F01_transcript_85377	F01_transcript_85377	F01_transcript_85377
F01_transcript_123095	NA	F01_transcript_123095	NA	NA
F01_transcript_52762	F01_transcript_52762	F01_transcript_52762	NA	NA
F01_transcript_88899	NA	F01_transcript_88899	NA	NA
F01_transcript_162715	F01_transcript_162715	F01_transcript_162715	NA	F01_transcript_162715
F01_transcript_80028	F01_transcript_80028	F01_transcript_80028	F01_transcript_80028	F01_transcript_80028
F01_transcript_162014	NA	F01_transcript_162014	NA	F01_transcript_162014
F01_transcript_81802	F01_transcript_81802	F01_transcript_81802	F01_transcript_81802	F01_transcript_81802
F01_transcript_61402	NA	F01_transcript_61402	NA	F01_transcript_61402
F01_transcript_124346	NA	F01_transcript_124346	NA	F01_transcript_124346

F01_transcript_157239	NA	F01_transcript_157239	NA	NA
F01_transcript_126111	F01_transcript_126111	F01_transcript_126111	F01_transcript_126111	NA
F01_transcript_57615	NA	F01_transcript_57615	F01_transcript_57615	F01_transcript_57615
F01_transcript_85650	F01_transcript_85650	F01_transcript_85650	F01_transcript_85650	NA
F01_transcript_45716	NA	F01_transcript_45716	NA	NA
F01_transcript_85761	NA	F01_transcript_85761	NA	NA
F01_transcript_165126	NA	F01_transcript_165126	F01_transcript_165126	F01_transcript_165126
F01_transcript_106359	F01_transcript_106359	F01_transcript_106359	F01_transcript_106359	NA
F01_transcript_86209	NA	NA	NA	F01_transcript_86209
F01_transcript_23149	NA	NA	NA	F01_transcript_23149
F01_transcript_49450	NA	F01_transcript_49450	NA	NA
F01_transcript_146718	NA	F01_transcript_146718	NA	NA
F01_transcript_76114	NA	F01_transcript_76114	NA	F01_transcript_76114
F01_transcript_167345	NA	F01_transcript_167345	NA	F01_transcript_167345

F01_transcript_94305	NA	F01_transcript_94305	F01_transcript_94305	F01_transcript_94305
F01_transcript_145530	NA	NA	NA	F01_transcript_145530
F01_transcript_29093	NA	NA	NA	F01_transcript_29093
F01_transcript_118812	F01_transcript_118812	F01_transcript_118812	NA	F01_transcript_118812
F01_transcript_79597	F01_transcript_79597	F01_transcript_79597	F01_transcript_79597	F01_transcript_79597
F01_transcript_48210	NA	F01_transcript_48210	NA	NA
F01_transcript_139090	F01_transcript_139090	F01_transcript_139090	F01_transcript_139090	F01_transcript_139090
F01_transcript_115425	F01_transcript_115425	F01_transcript_115425	F01_transcript_115425	F01_transcript_115425
F01_transcript_11797	NA	NA	NA	F01_transcript_11797
F01_transcript_82065	F01_transcript_82065	F01_transcript_82065	F01_transcript_82065	F01_transcript_82065
F01_transcript_64022	NA	F01_transcript_64022	NA	NA
F01_transcript_95428	F01_transcript_95428	F01_transcript_95428	F01_transcript_95428	NA
F01_transcript_133098	NA	F01_transcript_133098	F01_transcript_133098	F01_transcript_133098
F01_transcript_148525	NA	NA	NA	F01_transcript_148525

F01_transcript_155942	NA	NA	NA	F01_transcript_155942
F01_transcript_123289	NA	NA	NA	F01_transcript_123289
F01_transcript_158569	F01_transcript_158569	F01_transcript_158569	NA	NA
F01_transcript_52727	NA	NA	NA	F01_transcript_52727
F01_transcript_44092	NA	NA	NA	F01_transcript_44092
F01_transcript_21994	NA	NA	NA	F01_transcript_21994
F01_transcript_113969	NA	F01_transcript_113969	NA	NA
F01_transcript_85991	F01_transcript_85991	F01_transcript_85991	NA	F01_transcript_85991
F01_transcript_140298	NA	NA	NA	F01_transcript_140298
F01_transcript_8609	NA	NA	NA	F01_transcript_8609
F01_transcript_151097	NA	F01_transcript_151097	NA	NA
F01_transcript_110311	F01_transcript_110311	F01_transcript_110311	F01_transcript_110311	F01_transcript_110311
F01_transcript_111333	NA	F01_transcript_111333	NA	F01_transcript_111333
F01_transcript_122102	NA	F01_transcript_122102	NA	NA

F01_transcript_127463	NA	NA	NA	F01_transcript_127463
F01_transcript_150842	NA	NA	NA	F01_transcript_150842
F01_transcript_91742	NA	NA	NA	F01_transcript_91742
F01_transcript_147308	NA	NA	NA	F01_transcript_147308
F01_transcript_107	NA	F01_transcript_107	NA	NA
F01_transcript_121743	F01_transcript_121743	F01_transcript_121743	NA	F01_transcript_121743
F01_transcript_152867	NA	NA	NA	F01_transcript_152867
F01_transcript_64621	NA	F01_transcript_64621	NA	NA
F01_transcript_132580	F01_transcript_132580	F01_transcript_132580	F01_transcript_132580	F01_transcript_132580
F01_transcript_155801	F01_transcript_155801	F01_transcript_155801	F01_transcript_155801	NA
F01_transcript_49085	NA	F01_transcript_49085	F01_transcript_49085	F01_transcript_49085
F01_transcript_17507	NA	NA	NA	F01_transcript_17507
F01_transcript_165610	NA	F01_transcript_165610	NA	F01_transcript_165610
F01_transcript_106321	NA	NA	NA	F01_transcript_106321

F01_transcript_136810	NA	NA	NA	F01_transcript_136810
F01_transcript_13074	F01_transcript_13074	F01_transcript_13074	F01_transcript_13074	NA
F01_transcript_105500	NA	NA	F01_transcript_105500	F01_transcript_105500
F01_transcript_99127	NA	F01_transcript_99127	NA	NA
F01_transcript_123596	NA	NA	NA	F01_transcript_123596
F01_transcript_134703	NA	F01_transcript_134703	NA	NA
F01_transcript_2122	NA	NA	NA	F01_transcript_2122
F01_transcript_78394	F01_transcript_78394	F01_transcript_78394	NA	NA
F01_transcript_154055	F01_transcript_154055	F01_transcript_154055	F01_transcript_154055	F01_transcript_154055
F01_transcript_89086	F01_transcript_89086	F01_transcript_89086	NA	F01_transcript_89086
F01_transcript_46622	NA	NA	NA	F01_transcript_46622
F01_transcript_40047	NA	NA	NA	F01_transcript_40047
F01_transcript_127722	F01_transcript_127722	F01_transcript_127722	NA	NA
F01_transcript_134469	F01_transcript_134469	F01_transcript_134469	F01_transcript_134469	NA

F01_transcript_62760	NA	F01_transcript_62760	NA	F01_transcript_62760
F01_transcript_116028	NA	NA	NA	F01_transcript_116028
F01_transcript_118659	NA	NA	NA	F01_transcript_118659
F01_transcript_74141	NA	NA	NA	F01_transcript_74141
F01_transcript_148274	NA	F01_transcript_148274	NA	NA
F01_transcript_120742	F01_transcript_120742	F01_transcript_120742	F01_transcript_120742	F01_transcript_120742
F01_transcript_163388	NA	F01_transcript_163388	NA	NA
F01_transcript_101648	NA	NA	NA	F01_transcript_101648
F01_transcript_97942	NA	NA	NA	F01_transcript_97942
F01_transcript_153287	NA	NA	NA	F01_transcript_153287
F01_transcript_108161	F01_transcript_108161	F01_transcript_108161	NA	F01_transcript_108161
F01_transcript_52394	NA	F01_transcript_52394	NA	NA
F01_transcript_110922	NA	NA	NA	F01_transcript_110922
F01_transcript_156452	NA	F01_transcript_156452	NA	NA

F01_transcript_100912	NA	F01_transcript_100912	NA	NA
F01_transcript_58091	NA	F01_transcript_58091	NA	F01_transcript_58091
F01_transcript_76697	F01_transcript_76697	F01_transcript_76697	F01_transcript_76697	F01_transcript_76697
F01_transcript_109538	NA	F01_transcript_109538	F01_transcript_109538	F01_transcript_109538
F01_transcript_155751	NA	F01_transcript_155751	NA	F01_transcript_155751
F01_transcript_160329	NA	F01_transcript_160329	NA	F01_transcript_160329
F01_transcript_126594	F01_transcript_126594	F01_transcript_126594	F01_transcript_126594	F01_transcript_126594
F01_transcript_63885	NA	NA	F01_transcript_63885	NA
F01_transcript_31344	NA	NA	NA	F01_transcript_31344
F01_transcript_110305	NA	NA	NA	F01_transcript_110305
F01_transcript_138055	NA	F01_transcript_138055	NA	F01_transcript_138055
F01_transcript_85147	F01_transcript_85147	F01_transcript_85147	F01_transcript_85147	F01_transcript_85147
F01_transcript_79230	F01_transcript_79230	F01_transcript_79230	F01_transcript_79230	NA
F01_transcript_102546	NA	NA	NA	F01_transcript_102546

F01_transcript_155292	NA	F01_transcript_155292	NA	F01_transcript_155292
F01_transcript_103628	NA	NA	NA	F01_transcript_103628
F01_transcript_92857	F01_transcript_92857	F01_transcript_92857	F01_transcript_92857	F01_transcript_92857
F01_transcript_408	F01_transcript_408	F01_transcript_408	NA	F01_transcript_408
F01_transcript_106974	F01_transcript_106974	F01_transcript_106974	NA	F01_transcript_106974
F01_transcript_58964	F01_transcript_58964	F01_transcript_58964	F01_transcript_58964	F01_transcript_58964
F01_transcript_4472	F01_transcript_4472	F01_transcript_4472	NA	F01_transcript_4472
F01_transcript_62366	NA	F01_transcript_62366	NA	NA
F01_transcript_97284	NA	NA	NA	F01_transcript_97284
F01_transcript_48926	NA	F01_transcript_48926	NA	NA
F01_transcript_155785	NA	NA	NA	F01_transcript_155785
F01_transcript_105692	NA	F01_transcript_105692	NA	NA
F01_transcript_56790	NA	NA	NA	F01_transcript_56790
F01_transcript_163419	NA	NA	NA	F01_transcript_163419

F01_transcript_9925	F01_transcript_9925	F01_transcript_9925	F01_transcript_9925	F01_transcript_9925
F01_transcript_64553	NA	NA	NA	F01_transcript_64553
F01_transcript_107613	NA	F01_transcript_107613	NA	NA
F01_transcript_160559	NA	NA	NA	F01_transcript_160559
F01_transcript_77789	NA	F01_transcript_77789	NA	F01_transcript_77789
F01_transcript_63962	F01_transcript_63962	F01_transcript_63962	NA	F01_transcript_63962
F01_transcript_67258	NA	F01_transcript_67258	F01_transcript_67258	F01_transcript_67258
F01_transcript_30028	NA	NA	NA	F01_transcript_30028
F01_transcript_88939	NA	NA	NA	F01_transcript_88939
F01_transcript_63466	NA	NA	NA	F01_transcript_63466
F01_transcript_128345	NA	F01_transcript_128345	NA	F01_transcript_128345
F01_transcript_102460	NA	NA	NA	F01_transcript_102460
F01_transcript_76302	F01_transcript_76302	F01_transcript_76302	F01_transcript_76302	F01_transcript_76302
F01_transcript_91413	NA	F01_transcript_91413	NA	F01_transcript_91413

F01_transcript_91253	F01_transcript_91253	F01_transcript_91253	F01_transcript_91253	F01_transcript_91253
F01_transcript_107284	NA	F01_transcript_107284	NA	F01_transcript_107284
F01_transcript_119749	NA	F01_transcript_119749	NA	NA
F01_transcript_96209	NA	NA	NA	F01_transcript_96209
F01_transcript_94697	NA	NA	NA	F01_transcript_94697
F01_transcript_153646	F01_transcript_153646	F01_transcript_153646	F01_transcript_153646	F01_transcript_153646
F01_transcript_32756	NA	NA	NA	F01_transcript_32756
F01_transcript_95785	F01_transcript_95785	F01_transcript_95785	F01_transcript_95785	F01_transcript_95785
F01_transcript_147218	F01_transcript_147218	F01_transcript_147218	F01_transcript_147218	F01_transcript_147218
F01_transcript_7075	F01_transcript_7075	F01_transcript_7075	NA	NA
F01_transcript_86058	NA	F01_transcript_86058	NA	NA
F01_transcript_54496	NA	F01_transcript_54496	F01_transcript_54496	NA
F01_transcript_125770	NA	NA	F01_transcript_125770	F01_transcript_125770
F01_transcript_124470	NA	NA	NA	F01_transcript_124470

F01_transcript_134660	NA	F01_transcript_134660	NA	NA
F01_transcript_35338	NA	NA	NA	F01_transcript_35338
F01_transcript_87109	F01_transcript_87109	F01_transcript_87109	F01_transcript_87109	F01_transcript_87109
F01_transcript_101698	NA	NA	NA	F01_transcript_101698
F01_transcript_144265	F01_transcript_144265	F01_transcript_144265	F01_transcript_144265	F01_transcript_144265
F01_transcript_115536	F01_transcript_115536	F01_transcript_115536	NA	F01_transcript_115536
F01_transcript_83776	F01_transcript_83776	F01_transcript_83776	NA	F01_transcript_83776
F01_transcript_151073	NA	F01_transcript_151073	NA	NA
F01_transcript_67226	F01_transcript_67226	F01_transcript_67226	F01_transcript_67226	NA
F01_transcript_81839	F01_transcript_81839	F01_transcript_81839	F01_transcript_81839	NA
F01_transcript_136346	F01_transcript_136346	F01_transcript_136346	F01_transcript_136346	F01_transcript_136346
F01_transcript_93616	NA	F01_transcript_93616	NA	NA
F01_transcript_125077	NA	F01_transcript_125077	NA	NA
F01_transcript_1575	NA	NA	NA	F01_transcript_1575

F01_transcript_98243	NA	F01_transcript_98243	NA	NA
F01_transcript_40976	NA	F01_transcript_40976	NA	F01_transcript_40976
F01_transcript_86106	NA	F01_transcript_86106	NA	F01_transcript_86106
F01_transcript_79785	F01_transcript_79785	F01_transcript_79785	F01_transcript_79785	F01_transcript_79785
F01_transcript_144240	F01_transcript_144240	F01_transcript_144240	F01_transcript_144240	F01_transcript_144240
F01_transcript_84720	F01_transcript_84720	F01_transcript_84720	F01_transcript_84720	F01_transcript_84720
F01_transcript_81097	NA	NA	NA	F01_transcript_81097
F01_transcript_88266	F01_transcript_88266	F01_transcript_88266	F01_transcript_88266	F01_transcript_88266
F01_transcript_147136	NA	NA	NA	F01_transcript_147136
F01_transcript_122097	NA	F01_transcript_122097	NA	NA
F01_transcript_116090	NA	F01_transcript_116090	F01_transcript_116090	F01_transcript_116090
F01_transcript_44838	NA	NA	NA	F01_transcript_44838
F01_transcript_9938	NA	F01_transcript_9938	NA	NA
F01_transcript_154194	NA	F01_transcript_154194	NA	F01_transcript_154194

F01_transcript_80621	NA	F01_transcript_80621	NA	F01_transcript_80621
F01_transcript_73897	F01_transcript_73897	F01_transcript_73897	F01_transcript_73897	F01_transcript_73897
F01_transcript_57996	NA	F01_transcript_57996	NA	F01_transcript_57996
F01_transcript_35474	NA	F01_transcript_35474	NA	NA
F01_transcript_58011	NA	F01_transcript_58011	NA	NA
F01_transcript_52481	NA	NA	NA	F01_transcript_52481
F01_transcript_32671	NA	NA	NA	F01_transcript_32671
F01_transcript_90632	NA	NA	NA	F01_transcript_90632
F01_transcript_133574	NA	NA	NA	F01_transcript_133574
F01_transcript_54967	NA	NA	NA	F01_transcript_54967
F01_transcript_113267	NA	F01_transcript_113267	NA	F01_transcript_113267
F01_transcript_58413	NA	NA	NA	F01_transcript_58413
F01_transcript_73258	NA	F01_transcript_73258	NA	F01_transcript_73258
F01_transcript_147761	NA	NA	NA	F01_transcript_147761

F01_transcript_103734	NA	NA	NA	F01_transcript_103734
F01_transcript_106272	F01_transcript_106272	F01_transcript_106272	F01_transcript_106272	F01_transcript_106272
F01_transcript_53123	F01_transcript_53123	F01_transcript_53123	F01_transcript_53123	F01_transcript_53123
F01_transcript_57218	F01_transcript_57218	NA	F01_transcript_57218	F01_transcript_57218
F01_transcript_113361	NA	NA	NA	F01_transcript_113361
F01_transcript_106604	F01_transcript_106604	F01_transcript_106604	NA	F01_transcript_106604
F01_transcript_159469	NA	F01_transcript_159469	NA	F01_transcript_159469
F01_transcript_157851	NA	F01_transcript_157851	NA	NA
F01_transcript_127799	F01_transcript_127799	F01_transcript_127799	F01_transcript_127799	F01_transcript_127799
F01_transcript_131760	F01_transcript_131760	F01_transcript_131760	F01_transcript_131760	F01_transcript_131760
F01_transcript_67311	NA	F01_transcript_67311	NA	NA
F01_transcript_377	NA	NA	NA	F01_transcript_377
F01_transcript_121371	NA	F01_transcript_121371	NA	NA
F01_transcript_97962	NA	NA	NA	F01_transcript_97962

F01_transcript_60156	NA	NA	NA	F01_transcript_60156
F01_transcript_114643	F01_transcript_114643	F01_transcript_114643	F01_transcript_114643	F01_transcript_114643
F01_transcript_66044	F01_transcript_66044	F01_transcript_66044	NA	F01_transcript_66044
F01_transcript_16	NA	NA	NA	F01_transcript_16
F01_transcript_65572	F01_transcript_65572	NA	F01_transcript_65572	NA
F01_transcript_126982	F01_transcript_126982	F01_transcript_126982	NA	NA
F01_transcript_51802	NA	NA	NA	F01_transcript_51802
F01_transcript_51637	NA	F01_transcript_51637	F01_transcript_51637	F01_transcript_51637
F01_transcript_69787	NA	NA	NA	F01_transcript_69787
F01_transcript_49434	NA	F01_transcript_49434	F01_transcript_49434	F01_transcript_49434
F01_transcript_26640	NA	NA	NA	F01_transcript_26640
F01_transcript_41166	NA	NA	NA	F01_transcript_41166
F01_transcript_9110	NA	F01_transcript_9110	NA	F01_transcript_9110
F01_transcript_71571	F01_transcript_71571	F01_transcript_71571	NA	F01_transcript_71571

F01_transcript_151857	NA	F01_transcript_151857	NA	F01_transcript_151857
F01_transcript_108950	F01_transcript_108950	F01_transcript_108950	NA	NA
F01_transcript_140417	F01_transcript_140417	F01_transcript_140417	NA	F01_transcript_140417
F01_transcript_59311	NA	NA	F01_transcript_59311	NA
F01_transcript_164319	NA	F01_transcript_164319	NA	NA
F01_transcript_130711	NA	NA	NA	F01_transcript_130711
F01_transcript_135315	NA	F01_transcript_135315	NA	NA
F01_transcript_66792	NA	F01_transcript_66792	NA	F01_transcript_66792
F01_transcript_53160	NA	NA	NA	F01_transcript_53160
F01_transcript_44163	NA	NA	NA	F01_transcript_44163
F01_transcript_36148	NA	NA	NA	F01_transcript_36148
F01_transcript_128092	NA	F01_transcript_128092	NA	NA
F01_transcript_130500	NA	NA	NA	F01_transcript_130500
F01_transcript_63931	NA	F01_transcript_63931	NA	NA

F01_transcript_79750	NA	NA	NA	F01_transcript_79750
F01_transcript_129750	NA	NA	NA	F01_transcript_129750
F01_transcript_101878	NA	F01_transcript_101878	F01_transcript_101878	F01_transcript_101878
F01_transcript_133050	NA	NA	NA	F01_transcript_133050
F01_transcript_113410	F01_transcript_113410	F01_transcript_113410	F01_transcript_113410	F01_transcript_113410
F01_transcript_2133	F01_transcript_2133	F01_transcript_2133	NA	NA
F01_transcript_142919	NA	NA	NA	F01_transcript_142919
F01_transcript_123081	NA	F01_transcript_123081	NA	NA
F01_transcript_167716	NA	F01_transcript_167716	NA	F01_transcript_167716
F01_transcript_114209	F01_transcript_114209	F01_transcript_114209	F01_transcript_114209	F01_transcript_114209
F01_transcript_108631	NA	F01_transcript_108631	NA	F01_transcript_108631
F01_transcript_119428	F01_transcript_119428	F01_transcript_119428	NA	NA
F01_transcript_48712	F01_transcript_48712	F01_transcript_48712	NA	NA
F01_transcript_71013	NA	NA	F01_transcript_71013	F01_transcript_71013

F01_transcript_149987	NA	F01_transcript_149987	F01_transcript_149987	F01_transcript_149987
F01_transcript_81413	NA	F01_transcript_81413	NA	F01_transcript_81413
F01_transcript_51289	F01_transcript_51289	F01_transcript_51289	F01_transcript_51289	F01_transcript_51289
F01_transcript_62611	NA	F01_transcript_62611	NA	NA
F01_transcript_104218	F01_transcript_104218	F01_transcript_104218	F01_transcript_104218	F01_transcript_104218
F01_transcript_94953	NA	F01_transcript_94953	NA	F01_transcript_94953
F01_transcript_111084	NA	F01_transcript_111084	NA	NA
F01_transcript_48971	NA	NA	NA	F01_transcript_48971
F01_transcript_142488	NA	NA	NA	F01_transcript_142488
F01_transcript_29483	NA	NA	NA	F01_transcript_29483
F01_transcript_122519	NA	NA	NA	F01_transcript_122519
F01_transcript_144204	NA	NA	NA	F01_transcript_144204
F01_transcript_71696	NA	NA	NA	F01_transcript_71696
F01_transcript_96552	NA	NA	NA	F01_transcript_96552

F01_transcript_36300	NA	NA	NA	F01_transcript_36300
F01_transcript_151985	NA	F01_transcript_151985	NA	NA
F01_transcript_120937	NA	F01_transcript_120937	NA	NA
F01_transcript_143797	F01_transcript_143797	F01_transcript_143797	NA	F01_transcript_143797
F01_transcript_154756	F01_transcript_154756	F01_transcript_154756	F01_transcript_154756	F01_transcript_154756
F01_transcript_115890	NA	NA	NA	F01_transcript_115890
F01_transcript_83370	NA	NA	NA	F01_transcript_83370
F01_transcript_156244	F01_transcript_156244	F01_transcript_156244	F01_transcript_156244	F01_transcript_156244
F01_transcript_744	NA	F01_transcript_744	NA	NA
F01_transcript_122568	F01_transcript_122568	F01_transcript_122568	NA	NA
F01_transcript_82586	NA	F01_transcript_82586	NA	NA
F01_transcript_68045	NA	NA	NA	F01_transcript_68045
F01_transcript_37123	F01_transcript_37123	F01_transcript_37123	F01_transcript_37123	NA
F01_transcript_124325	NA	NA	NA	F01_transcript_124325

F01_transcript_79307	NA	F01_transcript_79307	F01_transcript_79307	NA
F01_transcript_24521	F01_transcript_24521	F01_transcript_24521	NA	F01_transcript_24521
F01_transcript_151357	F01_transcript_151357	F01_transcript_151357	F01_transcript_151357	F01_transcript_151357
F01_transcript_165611	NA	NA	NA	F01_transcript_165611
F01_transcript_104830	NA	F01_transcript_104830	NA	F01_transcript_104830
F01_transcript_99932	NA	NA	NA	F01_transcript_99932
F01_transcript_117069	NA	F01_transcript_117069	NA	NA
F01_transcript_109417	NA	F01_transcript_109417	NA	NA
F01_transcript_50481	F01_transcript_50481	F01_transcript_50481	F01_transcript_50481	F01_transcript_50481
F01_transcript_48740	NA	NA	NA	F01_transcript_48740
F01_transcript_87770	F01_transcript_87770	F01_transcript_87770	F01_transcript_87770	F01_transcript_87770
F01_transcript_57890	NA	NA	NA	F01_transcript_57890
F01_transcript_123821	F01_transcript_123821	F01_transcript_123821	F01_transcript_123821	F01_transcript_123821
F01_transcript_128570	F01_transcript_128570	F01_transcript_128570	NA	NA

F01_transcript_52944	NA	NA	NA	F01_transcript_52944
F01_transcript_151324	F01_transcript_151324	F01_transcript_151324	F01_transcript_151324	F01_transcript_151324
F01_transcript_83424	NA	NA	NA	F01_transcript_83424
F01_transcript_85722	NA	NA	NA	F01_transcript_85722
F01_transcript_3164	NA	F01_transcript_3164	F01_transcript_3164	F01_transcript_3164
F01_transcript_162914	NA	NA	NA	F01_transcript_162914
F01_transcript_106380	NA	NA	NA	F01_transcript_106380
F01_transcript_110627	NA	NA	NA	F01_transcript_110627
F01_transcript_56917	NA	F01_transcript_56917	F01_transcript_56917	F01_transcript_56917
F01_transcript_51124	F01_transcript_51124	F01_transcript_51124	F01_transcript_51124	F01_transcript_51124
F01_transcript_124818	NA	F01_transcript_124818	NA	NA
F01_transcript_88868	F01_transcript_88868	F01_transcript_88868	F01_transcript_88868	NA
F01_transcript_29436	F01_transcript_29436	F01_transcript_29436	F01_transcript_29436	F01_transcript_29436
F01_transcript_91453	NA	F01_transcript_91453	NA	NA

F01_transcript_157099	NA	NA	F01_transcript_157099	F01_transcript_157099
F01_transcript_148339	NA	F01_transcript_148339	F01_transcript_148339	F01_transcript_148339
F01_transcript_97975	NA	NA	NA	F01_transcript_97975
F01_transcript_122692	NA	F01_transcript_122692	NA	F01_transcript_122692
F01_transcript_105335	NA	F01_transcript_105335	NA	NA
F01_transcript_106133	NA	F01_transcript_106133	NA	NA
F01_transcript_81986	NA	NA	NA	F01_transcript_81986
F01_transcript_92459	NA	F01_transcript_92459	NA	NA
F01_transcript_127563	NA	F01_transcript_127563	NA	NA
F01_transcript_51102	F01_transcript_51102	F01_transcript_51102	F01_transcript_51102	NA
F01_transcript_157275	NA	NA	NA	F01_transcript_157275
F01_transcript_68022	F01_transcript_68022	F01_transcript_68022	F01_transcript_68022	F01_transcript_68022
F01_transcript_113699	NA	F01_transcript_113699	NA	NA
F01_transcript_51542	NA	F01_transcript_51542	NA	F01_transcript_51542

F01_transcript_125802	NA	NA	NA	F01_transcript_125802
F01_transcript_81115	NA	F01_transcript_81115	NA	NA
F01_transcript_8732	NA	NA	NA	F01_transcript_8732
F01_transcript_111452	NA	F01_transcript_111452	NA	F01_transcript_111452
F01_transcript_166885	NA	NA	NA	F01_transcript_166885
F01_transcript_118244	NA	F01_transcript_118244	NA	F01_transcript_118244
F01_transcript_63699	NA	NA	NA	F01_transcript_63699
F01_transcript_1748	NA	NA	F01_transcript_1748	NA
F01_transcript_139263	F01_transcript_139263	NA	NA	F01_transcript_139263
F01_transcript_113728	NA	F01_transcript_113728	NA	NA
F01_transcript_32377	NA	NA	NA	F01_transcript_32377
F01_transcript_83899	NA	NA	NA	F01_transcript_83899
F01_transcript_132215	NA	F01_transcript_132215	NA	NA
F01_transcript_98485	NA	NA	NA	F01_transcript_98485

F01_transcript_4505	NA	NA	NA	F01_transcript_4505
F01_transcript_2186	NA	F01_transcript_2186	NA	F01_transcript_2186
F01_transcript_87340	NA	F01_transcript_87340	NA	NA
F01_transcript_163035	NA	F01_transcript_163035	F01_transcript_163035	NA
F01_transcript_102042	NA	F01_transcript_102042	NA	F01_transcript_102042
F01_transcript_97933	NA	NA	F01_transcript_97933	F01_transcript_97933
F01_transcript_46752	NA	NA	NA	F01_transcript_46752
F01_transcript_73869	NA	NA	NA	F01_transcript_73869
F01_transcript_60916	NA	NA	NA	F01_transcript_60916
F01_transcript_90021	NA	F01_transcript_90021	NA	F01_transcript_90021
F01_transcript_69398	NA	F01_transcript_69398	NA	F01_transcript_69398
F01_transcript_138187	NA	NA	NA	F01_transcript_138187
F01_transcript_53646	NA	NA	NA	F01_transcript_53646
F01_transcript_68996	F01_transcript_68996	F01_transcript_68996	F01_transcript_68996	F01_transcript_68996

F01_transcript_54982	F01_transcript_54982	F01_transcript_54982	NA	F01_transcript_54982
F01_transcript_167229	F01_transcript_167229	F01_transcript_167229	NA	F01_transcript_167229
F01_transcript_58627	F01_transcript_58627	F01_transcript_58627	F01_transcript_58627	F01_transcript_58627
F01_transcript_93153	F01_transcript_93153	F01_transcript_93153	F01_transcript_93153	F01_transcript_93153
F01_transcript_52726	NA	F01_transcript_52726	NA	NA
F01_transcript_42813	NA	NA	NA	F01_transcript_42813
F01_transcript_136644	NA	F01_transcript_136644	NA	NA
F01_transcript_114917	NA	NA	NA	F01_transcript_114917
F01_transcript_144646	NA	NA	NA	F01_transcript_144646
F01_transcript_43545	NA	NA	NA	F01_transcript_43545
F01_transcript_164013	NA	NA	NA	F01_transcript_164013
F01_transcript_158922	NA	NA	F01_transcript_158922	NA
F01_transcript_92932	NA	NA	NA	F01_transcript_92932
F01_transcript_123132	NA	F01_transcript_123132	NA	NA

F01_transcript_111408	NA	F01_transcript_111408	NA	F01_transcript_111408
F01_transcript_30267	NA	NA	NA	F01_transcript_30267
F01_transcript_64051	NA	F01_transcript_64051	NA	F01_transcript_64051
F01_transcript_112766	F01_transcript_112766	F01_transcript_112766	F01_transcript_112766	F01_transcript_112766
F01_transcript_121890	NA	NA	NA	F01_transcript_121890
F01_transcript_148253	F01_transcript_148253	F01_transcript_148253	F01_transcript_148253	F01_transcript_148253
F01_transcript_33278	NA	F01_transcript_33278	NA	F01_transcript_33278
F01_transcript_63060	NA	NA	NA	F01_transcript_63060
F01_transcript_18390	NA	NA	NA	F01_transcript_18390
F01_transcript_43905	NA	F01_transcript_43905	NA	NA
F01_transcript_73092	NA	F01_transcript_73092	NA	NA
F01_transcript_50514	NA	F01_transcript_50514	F01_transcript_50514	F01_transcript_50514
F01_transcript_161929	NA	F01_transcript_161929	NA	F01_transcript_161929
F01_transcript_120869	NA	NA	NA	F01_transcript_120869

F01_transcript_150513	NA	F01_transcript_150513	NA	NA
F01_transcript_164455	F01_transcript_164455	F01_transcript_164455	F01_transcript_164455	F01_transcript_164455
F01_transcript_105873	F01_transcript_105873	F01_transcript_105873	F01_transcript_105873	F01_transcript_105873
F01_transcript_75468	NA	F01_transcript_75468	NA	NA
F01_transcript_69419	NA	F01_transcript_69419	NA	NA
F01_transcript_139828	NA	F01_transcript_139828	F01_transcript_139828	F01_transcript_139828
F01_transcript_114563	NA	NA	NA	F01_transcript_114563
F01_transcript_14755	F01_transcript_14755	F01_transcript_14755	F01_transcript_14755	F01_transcript_14755
F01_transcript_156280	NA	F01_transcript_156280	NA	NA
F01_transcript_14251	NA	NA	F01_transcript_14251	NA
F01_transcript_107153	F01_transcript_107153	NA	NA	NA
F01_transcript_98588	NA	F01_transcript_98588	F01_transcript_98588	F01_transcript_98588
F01_transcript_33120	F01_transcript_33120	F01_transcript_33120	F01_transcript_33120	F01_transcript_33120
F01_transcript_107046	NA	NA	NA	F01_transcript_107046

F01_transcript_65777	F01_transcript_65777	F01_transcript_65777	F01_transcript_65777	NA
F01_transcript_135123	NA	F01_transcript_135123	NA	NA
F01_transcript_106688	F01_transcript_106688	F01_transcript_106688	F01_transcript_106688	F01_transcript_106688
F01_transcript_114310	NA	NA	NA	F01_transcript_114310
F01_transcript_132479	NA	NA	NA	F01_transcript_132479
F01_transcript_47396	NA	NA	F01_transcript_47396	F01_transcript_47396
F01_transcript_66817	NA	NA	NA	F01_transcript_66817
F01_transcript_53560	NA	F01_transcript_53560	NA	F01_transcript_53560
F01_transcript_110073	NA	F01_transcript_110073	F01_transcript_110073	F01_transcript_110073
F01_transcript_41651	F01_transcript_41651	F01_transcript_41651	F01_transcript_41651	F01_transcript_41651
F01_transcript_92741	NA	F01_transcript_92741	NA	NA
F01_transcript_86605	NA	F01_transcript_86605	F01_transcript_86605	F01_transcript_86605
F01_transcript_63157	F01_transcript_63157	F01_transcript_63157	F01_transcript_63157	F01_transcript_63157
F01_transcript_85798	NA	NA	NA	F01_transcript_85798

F01_transcript_125459	NA	NA	NA	F01_transcript_125459
F01_transcript_27012	NA	NA	NA	F01_transcript_27012
F01_transcript_65239	NA	NA	F01_transcript_65239	NA
F01_transcript_21732	NA	F01_transcript_21732	NA	NA
F01_transcript_149883	NA	F01_transcript_149883	NA	F01_transcript_149883
F01_transcript_91539	NA	NA	NA	F01_transcript_91539
F01_transcript_132988	NA	NA	NA	F01_transcript_132988
F01_transcript_91736	F01_transcript_91736	F01_transcript_91736	F01_transcript_91736	F01_transcript_91736
F01_transcript_136993	NA	F01_transcript_136993	NA	F01_transcript_136993
F01_transcript_126998	NA	F01_transcript_126998	NA	NA
F01_transcript_163773	F01_transcript_163773	F01_transcript_163773	F01_transcript_163773	F01_transcript_163773
F01_transcript_82362	NA	NA	NA	F01_transcript_82362
F01_transcript_30114	NA	F01_transcript_30114	NA	NA
F01_transcript_126144	NA	NA	NA	F01_transcript_126144

F01_transcript_106291	F01_transcript_106291	F01_transcript_106291	F01_transcript_106291	F01_transcript_106291
F01_transcript_66025	NA	F01_transcript_66025	NA	NA
F01_transcript_54866	NA	NA	NA	F01_transcript_54866
F01_transcript_134665	F01_transcript_134665	F01_transcript_134665	F01_transcript_134665	NA
F01_transcript_113698	F01_transcript_113698	F01_transcript_113698	NA	NA
F01_transcript_77782	NA	NA	NA	F01_transcript_77782
F01_transcript_106025	NA	F01_transcript_106025	NA	NA
F01_transcript_75814	F01_transcript_75814	F01_transcript_75814	F01_transcript_75814	F01_transcript_75814
F01_transcript_133740	NA	F01_transcript_133740	NA	NA
F01_transcript_102001	NA	F01_transcript_102001	F01_transcript_102001	F01_transcript_102001
F01_transcript_47539	F01_transcript_47539	F01_transcript_47539	F01_transcript_47539	F01_transcript_47539
F01_transcript_56749	NA	F01_transcript_56749	F01_transcript_56749	F01_transcript_56749
F01_transcript_147757	NA	F01_transcript_147757	NA	NA
F01_transcript_101128	NA	NA	NA	F01_transcript_101128

F01_transcript_9247	NA	NA	F01_transcript_9247	F01_transcript_9247
F01_transcript_104668	NA	NA	NA	F01_transcript_104668
F01_transcript_138439	NA	NA	F01_transcript_138439	F01_transcript_138439
F01_transcript_145030	F01_transcript_145030	F01_transcript_145030	NA	F01_transcript_145030
F01_transcript_50466	F01_transcript_50466	F01_transcript_50466	F01_transcript_50466	NA
F01_transcript_65236	NA	F01_transcript_65236	F01_transcript_65236	NA
F01_transcript_155699	F01_transcript_155699	F01_transcript_155699	F01_transcript_155699	NA
F01_transcript_93732	F01_transcript_93732	F01_transcript_93732	F01_transcript_93732	F01_transcript_93732
F01_transcript_135014	F01_transcript_135014	F01_transcript_135014	F01_transcript_135014	F01_transcript_135014
F01_transcript_50120	NA	F01_transcript_50120	NA	NA
F01_transcript_59450	NA	NA	NA	F01_transcript_59450
F01_transcript_131496	F01_transcript_131496	F01_transcript_131496	F01_transcript_131496	F01_transcript_131496
F01_transcript_107301	F01_transcript_107301	F01_transcript_107301	F01_transcript_107301	F01_transcript_107301
F01_transcript_128410	F01_transcript_128410	F01_transcript_128410	NA	F01_transcript_128410

F01_transcript_30921	NA	NA	NA	F01_transcript_30921
F01_transcript_157507	NA	F01_transcript_157507	NA	NA
F01_transcript_110985	NA	NA	NA	F01_transcript_110985
F01_transcript_62224	F01_transcript_62224	F01_transcript_62224	F01_transcript_62224	F01_transcript_62224
F01_transcript_147211	F01_transcript_147211	F01_transcript_147211	F01_transcript_147211	F01_transcript_147211
F01_transcript_91769	NA	F01_transcript_91769	NA	NA
F01_transcript_85745	NA	NA	F01_transcript_85745	NA
F01_transcript_52442	NA	F01_transcript_52442	NA	NA
F01_transcript_102898	F01_transcript_102898	F01_transcript_102898	NA	NA
F01_transcript_75204	NA	NA	NA	F01_transcript_75204
F01_transcript_77143	NA	F01_transcript_77143	NA	NA
F01_transcript_24664	NA	NA	NA	F01_transcript_24664
F01_transcript_45062	NA	NA	NA	F01_transcript_45062
F01_transcript_83760	F01_transcript_83760	F01_transcript_83760	F01_transcript_83760	F01_transcript_83760

F01_transcript_68982	F01_transcript_68982	F01_transcript_68982	F01_transcript_68982	NA
F01_transcript_49610	NA	F01_transcript_49610	NA	F01_transcript_49610
F01_transcript_125965	F01_transcript_125965	F01_transcript_125965	F01_transcript_125965	F01_transcript_125965
F01_transcript_148810	NA	NA	NA	F01_transcript_148810
F01_transcript_124155	NA	F01_transcript_124155	NA	NA
F01_transcript_128376	NA	F01_transcript_128376	NA	NA
F01_transcript_164110	NA	F01_transcript_164110	NA	NA
F01_transcript_68434	NA	NA	NA	F01_transcript_68434
F01_transcript_110906	NA	F01_transcript_110906	NA	NA
F01_transcript_70006	F01_transcript_70006	F01_transcript_70006	F01_transcript_70006	NA
F01_transcript_164166	NA	F01_transcript_164166	NA	NA
F01_transcript_101931	NA	NA	NA	F01_transcript_101931
F01_transcript_102175	F01_transcript_102175	F01_transcript_102175	F01_transcript_102175	F01_transcript_102175
F01_transcript_119688	NA	NA	NA	F01_transcript_119688

F01_transcript_152442	F01_transcript_152442	F01_transcript_152442	F01_transcript_152442	F01_transcript_152442
F01_transcript_57176	NA	NA	F01_transcript_57176	NA
F01_transcript_151019	F01_transcript_151019	F01_transcript_151019	F01_transcript_151019	NA
F01_transcript_102059	F01_transcript_102059	F01_transcript_102059	F01_transcript_102059	F01_transcript_102059
F01_transcript_60235	NA	F01_transcript_60235	NA	F01_transcript_60235
F01_transcript_71111	NA	F01_transcript_71111	F01_transcript_71111	F01_transcript_71111
F01_transcript_55936	NA	F01_transcript_55936	NA	F01_transcript_55936
F01_transcript_4074	NA	F01_transcript_4074	NA	NA
F01_transcript_148507	NA	NA	F01_transcript_148507	NA
F01_transcript_151112	F01_transcript_151112	F01_transcript_151112	F01_transcript_151112	NA
F01_transcript_63859	NA	F01_transcript_63859	NA	NA
F01_transcript_48333	NA	F01_transcript_48333	NA	F01_transcript_48333
F01_transcript_18679	NA	NA	F01_transcript_18679	F01_transcript_18679
F01_transcript_80899	NA	F01_transcript_80899	NA	NA

F01_transcript_101891	F01_transcript_101891	F01_transcript_101891	F01_transcript_101891	F01_transcript_101891
F01_transcript_157095	NA	NA	NA	F01_transcript_157095
F01_transcript_87675	NA	F01_transcript_87675	NA	F01_transcript_87675
F01_transcript_35296	NA	NA	NA	F01_transcript_35296
F01_transcript_104958	NA	F01_transcript_104958	NA	F01_transcript_104958
F01_transcript_73718	NA	F01_transcript_73718	F01_transcript_73718	NA
F01_transcript_104330	NA	F01_transcript_104330	NA	NA
F01_transcript_110580	F01_transcript_110580	F01_transcript_110580	NA	F01_transcript_110580
F01_transcript_54290	F01_transcript_54290	F01_transcript_54290	F01_transcript_54290	F01_transcript_54290
F01_transcript_103859	F01_transcript_103859	F01_transcript_103859	F01_transcript_103859	F01_transcript_103859
F01_transcript_93501	NA	NA	NA	F01_transcript_93501
F01_transcript_48854	NA	NA	F01_transcript_48854	F01_transcript_48854
F01_transcript_72446	NA	F01_transcript_72446	NA	F01_transcript_72446
F01_transcript_76878	NA	F01_transcript_76878	NA	F01_transcript_76878

F01_transcript_101982	F01_transcript_101982	F01_transcript_101982	F01_transcript_101982	F01_transcript_101982
F01_transcript_105347	F01_transcript_105347	F01_transcript_105347	F01_transcript_105347	F01_transcript_105347
F01_transcript_97270	NA	NA	NA	F01_transcript_97270
F01_transcript_157441	NA	NA	NA	F01_transcript_157441
F01_transcript_155502	NA	F01_transcript_155502	F01_transcript_155502	F01_transcript_155502
F01_transcript_145930	NA	F01_transcript_145930	NA	F01_transcript_145930
F01_transcript_59235	F01_transcript_59235	F01_transcript_59235	F01_transcript_59235	F01_transcript_59235
F01_transcript_158966	F01_transcript_158966	F01_transcript_158966	F01_transcript_158966	F01_transcript_158966
F01_transcript_123046	NA	NA	NA	F01_transcript_123046
F01_transcript_88431	NA	NA	NA	F01_transcript_88431
F01_transcript_47887	NA	NA	NA	F01_transcript_47887
F01_transcript_35793	NA	F01_transcript_35793	NA	NA
F01_transcript_106816	NA	NA	NA	F01_transcript_106816
F01_transcript_86966	NA	NA	NA	F01_transcript_86966

F01_transcript_164996	NA	NA	NA	F01_transcript_164996
F01_transcript_128557	F01_transcript_128557	F01_transcript_128557	F01_transcript_128557	F01_transcript_128557
F01_transcript_84942	NA	F01_transcript_84942	F01_transcript_84942	F01_transcript_84942
F01_transcript_114749	NA	NA	NA	F01_transcript_114749
F01_transcript_113956	NA	NA	F01_transcript_113956	F01_transcript_113956
F01_transcript_66169	NA	NA	NA	F01_transcript_66169
F01_transcript_155871	F01_transcript_155871	F01_transcript_155871	F01_transcript_155871	F01_transcript_155871
F01_transcript_32761	NA	NA	NA	F01_transcript_32761
F01_transcript_104607	NA	F01_transcript_104607	NA	F01_transcript_104607
F01_transcript_164968	NA	NA	NA	F01_transcript_164968
F01_transcript_73842	NA	NA	NA	F01_transcript_73842
F01_transcript_82410	F01_transcript_82410	F01_transcript_82410	F01_transcript_82410	F01_transcript_82410
F01_transcript_138750	NA	F01_transcript_138750	NA	NA
F01_transcript_125964	F01_transcript_125964	F01_transcript_125964	F01_transcript_125964	F01_transcript_125964

F01_transcript_127032	NA	NA	NA	F01_transcript_127032
F01_transcript_88535	NA	F01_transcript_88535	NA	F01_transcript_88535
F01_transcript_115813	NA	F01_transcript_115813	NA	F01_transcript_115813
F01_transcript_82403	NA	NA	NA	F01_transcript_82403
F01_transcript_117719	NA	NA	NA	F01_transcript_117719
F01_transcript_102882	F01_transcript_102882	F01_transcript_102882	NA	F01_transcript_102882
F01_transcript_144220	NA	F01_transcript_144220	F01_transcript_144220	F01_transcript_144220
F01_transcript_47180	NA	NA	NA	F01_transcript_47180
F01_transcript_60226	F01_transcript_60226	F01_transcript_60226	F01_transcript_60226	F01_transcript_60226
F01_transcript_36903	NA	NA	NA	F01_transcript_36903
F01_transcript_152646	F01_transcript_152646	F01_transcript_152646	F01_transcript_152646	F01_transcript_152646
F01_transcript_53271	NA	F01_transcript_53271	NA	NA
F01_transcript_54159	NA	F01_transcript_54159	NA	F01_transcript_54159
F01_transcript_88729	F01_transcript_88729	F01_transcript_88729	F01_transcript_88729	F01_transcript_88729

F01_transcript_144992	NA	F01_transcript_144992	NA	F01_transcript_144992
F01_transcript_65092	NA	NA	F01_transcript_65092	NA
F01_transcript_19914	NA	NA	F01_transcript_19914	NA
F01_transcript_11429	NA	NA	NA	F01_transcript_11429
F01_transcript_147070	NA	F01_transcript_147070	NA	F01_transcript_147070
F01_transcript_35640	NA	F01_transcript_35640	NA	NA
F01_transcript_90813	NA	NA	NA	F01_transcript_90813
F01_transcript_105330	NA	F01_transcript_105330	F01_transcript_105330	F01_transcript_105330
F01_transcript_78589	F01_transcript_78589	F01_transcript_78589	F01_transcript_78589	F01_transcript_78589
F01_transcript_120449	NA	NA	NA	F01_transcript_120449
F01_transcript_80780	NA	F01_transcript_80780	NA	NA
F01_transcript_55561	NA	F01_transcript_55561	NA	NA
F01_transcript_92619	NA	NA	NA	F01_transcript_92619
F01_transcript_84046	NA	F01_transcript_84046	NA	NA

F01_transcript_75502	F01_transcript_75502	F01_transcript_75502	F01_transcript_75502	F01_transcript_75502
F01_transcript_145815	NA	F01_transcript_145815	F01_transcript_145815	F01_transcript_145815
F01_transcript_142486	NA	NA	NA	F01_transcript_142486
F01_transcript_108184	NA	F01_transcript_108184	NA	NA
F01_transcript_130426	NA	F01_transcript_130426	NA	F01_transcript_130426
F01_transcript_107268	NA	NA	NA	F01_transcript_107268
F01_transcript_112648	F01_transcript_112648	F01_transcript_112648	F01_transcript_112648	F01_transcript_112648
F01_transcript_140758	NA	F01_transcript_140758	NA	NA
F01_transcript_9217	NA	NA	NA	F01_transcript_9217
F01_transcript_127640	NA	F01_transcript_127640	NA	NA
F01_transcript_15680	NA	F01_transcript_15680	F01_transcript_15680	F01_transcript_15680
F01_transcript_91197	NA	NA	NA	F01_transcript_91197
F01_transcript_151314	F01_transcript_151314	F01_transcript_151314	NA	F01_transcript_151314
F01_transcript_112991	NA	F01_transcript_112991	F01_transcript_112991	F01_transcript_112991

F01_transcript_127998	F01_transcript_127998	F01_transcript_127998	NA	NA
F01_transcript_119122	NA	F01_transcript_119122	NA	NA
F01_transcript_82505	NA	NA	NA	F01_transcript_82505
F01_transcript_147603	NA	F01_transcript_147603	NA	NA
F01_transcript_54638	NA	F01_transcript_54638	NA	NA
F01_transcript_139077	NA	F01_transcript_139077	NA	F01_transcript_139077
F01_transcript_85342	NA	NA	NA	F01_transcript_85342
F01_transcript_49443	NA	NA	NA	F01_transcript_49443
F01_transcript_75021	NA	NA	NA	F01_transcript_75021
F01_transcript_160548	NA	NA	NA	F01_transcript_160548
F01_transcript_151076	NA	NA	NA	F01_transcript_151076
F01_transcript_61892	F01_transcript_61892	F01_transcript_61892	NA	F01_transcript_61892
F01_transcript_64481	NA	NA	NA	F01_transcript_64481
F01_transcript_96678	NA	F01_transcript_96678	NA	NA

F01_transcript_130567	NA	NA	NA	F01_transcript_130567
F01_transcript_118325	F01_transcript_118325	F01_transcript_118325	NA	F01_transcript_118325
F01_transcript_124287	F01_transcript_124287	F01_transcript_124287	F01_transcript_124287	F01_transcript_124287
F01_transcript_138568	NA	NA	NA	F01_transcript_138568
F01_transcript_82025	NA	NA	NA	F01_transcript_82025
F01_transcript_164375	NA	NA	NA	F01_transcript_164375
F01_transcript_63866	F01_transcript_63866	F01_transcript_63866	F01_transcript_63866	F01_transcript_63866
F01_transcript_156035	NA	NA	NA	F01_transcript_156035
F01_transcript_30045	NA	NA	NA	F01_transcript_30045
F01_transcript_111752	NA	NA	NA	F01_transcript_111752
F01_transcript_158002	F01_transcript_158002	F01_transcript_158002	NA	F01_transcript_158002
F01_transcript_159516	NA	F01_transcript_159516	NA	F01_transcript_159516
F01_transcript_89812	NA	NA	NA	F01_transcript_89812
F01_transcript_107533	NA	F01_transcript_107533	NA	F01_transcript_107533

F01_transcript_166547	F01_transcript_166547	F01_transcript_166547	NA	NA
F01_transcript_110613	F01_transcript_110613	F01_transcript_110613	F01_transcript_110613	NA
F01_transcript_87685	F01_transcript_87685	F01_transcript_87685	NA	NA
F01_transcript_97397	NA	F01_transcript_97397	F01_transcript_97397	F01_transcript_97397
F01_transcript_45198	NA	NA	F01_transcript_45198	NA
F01_transcript_123235	F01_transcript_123235	F01_transcript_123235	F01_transcript_123235	F01_transcript_123235
F01_transcript_162537	F01_transcript_162537	F01_transcript_162537	F01_transcript_162537	F01_transcript_162537
F01_transcript_67986	F01_transcript_67986	F01_transcript_67986	F01_transcript_67986	F01_transcript_67986
F01_transcript_67797	NA	F01_transcript_67797	F01_transcript_67797	F01_transcript_67797
F01_transcript_148384	F01_transcript_148384	F01_transcript_148384	F01_transcript_148384	F01_transcript_148384
F01_transcript_115141	NA	F01_transcript_115141	NA	F01_transcript_115141
F01_transcript_8697	F01_transcript_8697	F01_transcript_8697	NA	F01_transcript_8697
F01_transcript_113738	NA	F01_transcript_113738	NA	F01_transcript_113738
F01_transcript_153183	NA	F01_transcript_153183	NA	NA

F01_transcript_64706	NA	F01_transcript_64706	NA	F01_transcript_64706
F01_transcript_140576	NA	NA	NA	F01_transcript_140576
F01_transcript_73008	NA	F01_transcript_73008	NA	NA
F01_transcript_99789	NA	F01_transcript_99789	NA	NA
F01_transcript_61379	NA	NA	NA	F01_transcript_61379
F01_transcript_16712	NA	NA	NA	F01_transcript_16712
F01_transcript_72785	F01_transcript_72785	F01_transcript_72785	F01_transcript_72785	F01_transcript_72785
F01_transcript_140125	NA	NA	NA	F01_transcript_140125
F01_transcript_167215	F01_transcript_167215	F01_transcript_167215	NA	F01_transcript_167215
F01_transcript_107813	F01_transcript_107813	F01_transcript_107813	F01_transcript_107813	F01_transcript_107813
F01_transcript_165612	NA	NA	NA	F01_transcript_165612
F01_transcript_72862	NA	NA	NA	F01_transcript_72862
F01_transcript_129787	NA	F01_transcript_129787	F01_transcript_129787	F01_transcript_129787
F01_transcript_144494	NA	NA	NA	F01_transcript_144494

F01_transcript_105919	F01_transcript_105919	F01_transcript_105919	NA	NA
F01_transcript_59329	NA	NA	NA	F01_transcript_59329
F01_transcript_1129	NA	F01_transcript_1129	NA	F01_transcript_1129
F01_transcript_67467	NA	F01_transcript_67467	F01_transcript_67467	F01_transcript_67467
F01_transcript_71267	NA	F01_transcript_71267	F01_transcript_71267	F01_transcript_71267
F01_transcript_119988	NA	NA	NA	F01_transcript_119988
F01_transcript_164698	NA	NA	NA	F01_transcript_164698
F01_transcript_49138	F01_transcript_49138	F01_transcript_49138	NA	F01_transcript_49138
F01_transcript_151273	NA	F01_transcript_151273	NA	NA
F01_transcript_85166	NA	F01_transcript_85166	F01_transcript_85166	F01_transcript_85166
F01_transcript_45210	NA	NA	NA	F01_transcript_45210
F01_transcript_96740	NA	F01_transcript_96740	F01_transcript_96740	F01_transcript_96740
F01_transcript_110215	NA	F01_transcript_110215	NA	F01_transcript_110215
F01_transcript_96332	NA	F01_transcript_96332	NA	NA

F01_transcript_98319	NA	F01_transcript_98319	NA	NA
F01_transcript_145513	F01_transcript_145513	F01_transcript_145513	F01_transcript_145513	F01_transcript_145513
F01_transcript_37432	NA	F01_transcript_37432	NA	NA
F01_transcript_147832	F01_transcript_147832	F01_transcript_147832	F01_transcript_147832	NA
F01_transcript_162767	NA	F01_transcript_162767	NA	NA
F01_transcript_50432	NA	NA	NA	F01_transcript_50432
F01_transcript_102411	NA	F01_transcript_102411	F01_transcript_102411	F01_transcript_102411
F01_transcript_74947	F01_transcript_74947	F01_transcript_74947	F01_transcript_74947	F01_transcript_74947
F01_transcript_3563	F01_transcript_3563	F01_transcript_3563	F01_transcript_3563	F01_transcript_3563
F01_transcript_98599	F01_transcript_98599	F01_transcript_98599	NA	NA
F01_transcript_97727	NA	NA	NA	F01_transcript_97727
F01_transcript_73832	NA	F01_transcript_73832	NA	NA
F01_transcript_144754	NA	F01_transcript_144754	NA	NA
F01_transcript_82426	F01_transcript_82426	F01_transcript_82426	F01_transcript_82426	F01_transcript_82426

F01_transcript_72256	NA	NA	NA	F01_transcript_72256
F01_transcript_87708	F01_transcript_87708	F01_transcript_87708	NA	F01_transcript_87708
F01_transcript_164520	F01_transcript_164520	F01_transcript_164520	F01_transcript_164520	F01_transcript_164520
F01_transcript_40528	NA	F01_transcript_40528	NA	F01_transcript_40528
F01_transcript_116619	NA	NA	NA	F01_transcript_116619
F01_transcript_113451	F01_transcript_113451	F01_transcript_113451	F01_transcript_113451	F01_transcript_113451
F01_transcript_113265	NA	NA	NA	F01_transcript_113265
F01_transcript_67463	NA	NA	NA	F01_transcript_67463
F01_transcript_129264	NA	NA	F01_transcript_129264	F01_transcript_129264
F01_transcript_91766	NA	NA	NA	F01_transcript_91766
F01_transcript_46246	NA	NA	NA	F01_transcript_46246
F01_transcript_136767	NA	F01_transcript_136767	NA	NA
F01_transcript_97661	NA	F01_transcript_97661	NA	NA
F01_transcript_46866	NA	NA	NA	F01_transcript_46866

F01_transcript_108595	F01_transcript_108595	F01_transcript_108595	F01_transcript_108595	F01_transcript_108595
F01_transcript_68411	NA	F01_transcript_68411	NA	NA
F01_transcript_70050	NA	F01_transcript_70050	F01_transcript_70050	F01_transcript_70050
F01_transcript_137117	F01_transcript_137117	F01_transcript_137117	F01_transcript_137117	F01_transcript_137117
F01_transcript_71499	F01_transcript_71499	F01_transcript_71499	NA	NA
F01_transcript_32641	NA	NA	NA	F01_transcript_32641
F01_transcript_5148	F01_transcript_5148	F01_transcript_5148	NA	NA
F01_transcript_142339	NA	F01_transcript_142339	NA	NA
F01_transcript_64691	NA	F01_transcript_64691	NA	F01_transcript_64691
F01_transcript_154797	F01_transcript_154797	F01_transcript_154797	NA	F01_transcript_154797
F01_transcript_157580	F01_transcript_157580	F01_transcript_157580	F01_transcript_157580	F01_transcript_157580
F01_transcript_141374	NA	F01_transcript_141374	F01_transcript_141374	F01_transcript_141374
F01_transcript_163458	NA	NA	NA	F01_transcript_163458
F01_transcript_57621	NA	F01_transcript_57621	F01_transcript_57621	F01_transcript_57621

F01_transcript_73346	NA	F01_transcript_73346	NA	F01_transcript_73346
F01_transcript_16565	NA	F01_transcript_16565	F01_transcript_16565	NA
F01_transcript_113374	NA	F01_transcript_113374	F01_transcript_113374	NA
F01_transcript_56958	F01_transcript_56958	F01_transcript_56958	F01_transcript_56958	NA
F01_transcript_108861	NA	F01_transcript_108861	NA	F01_transcript_108861
F01_transcript_106042	F01_transcript_106042	F01_transcript_106042	NA	F01_transcript_106042
F01_transcript_60467	F01_transcript_60467	F01_transcript_60467	F01_transcript_60467	F01_transcript_60467
F01_transcript_115834	F01_transcript_115834	F01_transcript_115834	NA	NA
F01_transcript_110958	NA	NA	NA	F01_transcript_110958
F01_transcript_134586	NA	F01_transcript_134586	NA	F01_transcript_134586
F01_transcript_31457	NA	F01_transcript_31457	F01_transcript_31457	F01_transcript_31457
F01_transcript_65949	NA	NA	NA	F01_transcript_65949
F01_transcript_92664	NA	F01_transcript_92664	F01_transcript_92664	NA
F01_transcript_52744	F01_transcript_52744	F01_transcript_52744	F01_transcript_52744	F01_transcript_52744

F01_transcript_121599	NA	NA	NA	F01_transcript_121599
F01_transcript_52971	NA	F01_transcript_52971	NA	F01_transcript_52971
F01_transcript_109849	F01_transcript_109849	F01_transcript_109849	F01_transcript_109849	F01_transcript_109849
F01_transcript_117966	NA	F01_transcript_117966	NA	NA
F01_transcript_67277	NA	F01_transcript_67277	NA	NA
F01_transcript_111686	F01_transcript_111686	F01_transcript_111686	F01_transcript_111686	F01_transcript_111686
F01_transcript_10471	F01_transcript_10471	F01_transcript_10471	F01_transcript_10471	NA
F01_transcript_132188	F01_transcript_132188	F01_transcript_132188	NA	NA
F01_transcript_143456	NA	NA	NA	F01_transcript_143456
F01_transcript_99675	F01_transcript_99675	F01_transcript_99675	F01_transcript_99675	F01_transcript_99675
F01_transcript_160983	F01_transcript_160983	F01_transcript_160983	NA	F01_transcript_160983
F01_transcript_61070	NA	NA	NA	F01_transcript_61070
F01_transcript_58624	NA	NA	NA	F01_transcript_58624
F01_transcript_144368	NA	NA	NA	F01_transcript_144368

F01_transcript_48129	NA	NA	F01_transcript_48129	F01_transcript_48129
F01_transcript_141224	NA	F01_transcript_141224	F01_transcript_141224	NA
F01_transcript_76	NA	NA	NA	F01_transcript_76
F01_transcript_126972	F01_transcript_126972	F01_transcript_126972	F01_transcript_126972	NA
F01_transcript_68449	NA	NA	NA	F01_transcript_68449
F01_transcript_44835	NA	NA	NA	F01_transcript_44835
F01_transcript_90760	NA	F01_transcript_90760	NA	NA
F01_transcript_86441	NA	F01_transcript_86441	NA	NA
F01_transcript_130381	NA	NA	NA	F01_transcript_130381
F01_transcript_1381	NA	NA	NA	F01_transcript_1381
F01_transcript_159854	NA	F01_transcript_159854	NA	NA
F01_transcript_96684	NA	NA	F01_transcript_96684	NA
F01_transcript_51010	F01_transcript_51010	F01_transcript_51010	F01_transcript_51010	F01_transcript_51010
F01_transcript_13315	NA	NA	NA	F01_transcript_13315

F01_transcript_101653	F01_transcript_101653	F01_transcript_101653	F01_transcript_101653	F01_transcript_101653
F01_transcript_72641	F01_transcript_72641	F01_transcript_72641	F01_transcript_72641	F01_transcript_72641
F01_transcript_58248	NA	F01_transcript_58248	F01_transcript_58248	F01_transcript_58248
F01_transcript_92216	NA	F01_transcript_92216	F01_transcript_92216	NA
F01_transcript_136793	NA	NA	NA	F01_transcript_136793
F01_transcript_52963	F01_transcript_52963	F01_transcript_52963	F01_transcript_52963	NA
F01_transcript_108578	F01_transcript_108578	F01_transcript_108578	F01_transcript_108578	F01_transcript_108578
F01_transcript_133656	NA	NA	NA	F01_transcript_133656
F01_transcript_64250	NA	F01_transcript_64250	NA	NA
F01_transcript_60385	F01_transcript_60385	F01_transcript_60385	F01_transcript_60385	F01_transcript_60385
F01_transcript_113642	NA	F01_transcript_113642	NA	NA
F01_transcript_120758	NA	NA	NA	F01_transcript_120758
F01_transcript_154700	F01_transcript_154700	F01_transcript_154700	F01_transcript_154700	F01_transcript_154700
F01_transcript_117940	NA	F01_transcript_117940	NA	NA

F01_transcript_115963	F01_transcript_115963	F01_transcript_115963	F01_transcript_115963	F01_transcript_115963
F01_transcript_98322	NA	F01_transcript_98322	NA	NA
F01_transcript_66837	NA	F01_transcript_66837	NA	NA
F01_transcript_137767	NA	NA	NA	F01_transcript_137767
F01_transcript_78721	NA	NA	NA	F01_transcript_78721
F01_transcript_105994	NA	F01_transcript_105994	NA	F01_transcript_105994
F01_transcript_152248	F01_transcript_152248	F01_transcript_152248	F01_transcript_152248	F01_transcript_152248
F01_transcript_93416	NA	NA	NA	F01_transcript_93416
F01_transcript_59404	NA	NA	F01_transcript_59404	F01_transcript_59404
F01_transcript_48306	NA	F01_transcript_48306	NA	NA
F01_transcript_158371	NA	NA	NA	F01_transcript_158371
F01_transcript_2388	NA	F01_transcript_2388	F01_transcript_2388	F01_transcript_2388
F01_transcript_6385	NA	F01_transcript_6385	NA	F01_transcript_6385
F01_transcript_84013	F01_transcript_84013	F01_transcript_84013	F01_transcript_84013	F01_transcript_84013

F01_transcript_128373	F01_transcript_128373	F01_transcript_128373	NA	NA
F01_transcript_85447	NA	F01_transcript_85447	F01_transcript_85447	F01_transcript_85447
F01_transcript_159162	NA	F01_transcript_159162	NA	NA
F01_transcript_72811	F01_transcript_72811	F01_transcript_72811	F01_transcript_72811	F01_transcript_72811
F01_transcript_153501	NA	F01_transcript_153501	F01_transcript_153501	F01_transcript_153501
F01_transcript_98837	NA	NA	NA	F01_transcript_98837
F01_transcript_71440	NA	F01_transcript_71440	NA	NA
F01_transcript_107536	NA	NA	NA	F01_transcript_107536
F01_transcript_57799	NA	NA	NA	F01_transcript_57799
F01_transcript_147575	NA	F01_transcript_147575	NA	NA
F01_transcript_146843	F01_transcript_146843	F01_transcript_146843	F01_transcript_146843	F01_transcript_146843
F01_transcript_48954	NA	NA	F01_transcript_48954	F01_transcript_48954
F01_transcript_89094	F01_transcript_89094	F01_transcript_89094	F01_transcript_89094	F01_transcript_89094
F01_transcript_139485	NA	NA	NA	F01_transcript_139485

F01_transcript_106017	F01_transcript_106017	F01_transcript_106017	NA	F01_transcript_106017
F01_transcript_32531	NA	NA	NA	F01_transcript_32531
F01_transcript_125433	F01_transcript_125433	F01_transcript_125433	F01_transcript_125433	F01_transcript_125433
F01_transcript_65868	NA	F01_transcript_65868	NA	NA
F01_transcript_17887	NA	F01_transcript_17887	NA	NA
F01_transcript_74524	NA	F01_transcript_74524	NA	NA
F01_transcript_106318	NA	NA	NA	F01_transcript_106318
F01_transcript_4846	NA	NA	F01_transcript_4846	F01_transcript_4846
F01_transcript_161026	NA	NA	NA	F01_transcript_161026
F01_transcript_86530	NA	NA	NA	F01_transcript_86530
F01_transcript_91809	F01_transcript_91809	F01_transcript_91809	F01_transcript_91809	F01_transcript_91809
F01_transcript_151331	F01_transcript_151331	F01_transcript_151331	F01_transcript_151331	F01_transcript_151331
F01_transcript_40412	NA	NA	NA	F01_transcript_40412
F01_transcript_141471	F01_transcript_141471	F01_transcript_141471	F01_transcript_141471	F01_transcript_141471

F01_transcript_103626	F01_transcript_103626	F01_transcript_103626	F01_transcript_103626	F01_transcript_103626
F01_transcript_121078	F01_transcript_121078	NA	F01_transcript_121078	NA
F01_transcript_123733	NA	F01_transcript_123733	NA	NA
F01_transcript_129121	NA	NA	NA	F01_transcript_129121
F01_transcript_69507	F01_transcript_69507	F01_transcript_69507	F01_transcript_69507	F01_transcript_69507
F01_transcript_125971	NA	F01_transcript_125971	F01_transcript_125971	F01_transcript_125971
F01_transcript_162872	NA	NA	NA	F01_transcript_162872
F01_transcript_118579	NA	NA	NA	F01_transcript_118579
F01_transcript_84427	F01_transcript_84427	F01_transcript_84427	F01_transcript_84427	F01_transcript_84427
F01_transcript_100791	NA	F01_transcript_100791	NA	NA
F01_transcript_157994	NA	NA	NA	F01_transcript_157994
F01_transcript_150281	F01_transcript_150281	F01_transcript_150281	F01_transcript_150281	F01_transcript_150281
F01_transcript_58029	NA	F01_transcript_58029	NA	NA
F01_transcript_44795	NA	NA	NA	F01_transcript_44795

F01_transcript_80710	NA	F01_transcript_80710	F01_transcript_80710	F01_transcript_80710
F01_transcript_89039	NA	F01_transcript_89039	NA	NA
F01_transcript_70052	NA	F01_transcript_70052	F01_transcript_70052	F01_transcript_70052
F01_transcript_56662	NA	F01_transcript_56662	F01_transcript_56662	NA
F01_transcript_19494	NA	F01_transcript_19494	F01_transcript_19494	F01_transcript_19494
F01_transcript_92598	F01_transcript_92598	F01_transcript_92598	F01_transcript_92598	F01_transcript_92598
F01_transcript_59258	F01_transcript_59258	F01_transcript_59258	F01_transcript_59258	F01_transcript_59258
F01_transcript_50342	F01_transcript_50342	F01_transcript_50342	F01_transcript_50342	F01_transcript_50342
F01_transcript_99728	NA	NA	NA	F01_transcript_99728
F01_transcript_392	NA	F01_transcript_392	NA	F01_transcript_392
F01_transcript_155580	NA	NA	NA	F01_transcript_155580
F01_transcript_57014	NA	NA	F01_transcript_57014	NA
F01_transcript_757	NA	NA	NA	F01_transcript_757
F01_transcript_132143	NA	F01_transcript_132143	NA	F01_transcript_132143

F01_transcript_69181	NA	F01_transcript_69181	NA	NA
F01_transcript_163046	F01_transcript_163046	F01_transcript_163046	NA	F01_transcript_163046
F01_transcript_72798	NA	F01_transcript_72798	NA	NA
F01_transcript_87664	NA	F01_transcript_87664	NA	F01_transcript_87664
F01_transcript_114237	NA	NA	NA	F01_transcript_114237
F01_transcript_63202	NA	F01_transcript_63202	NA	NA
F01_transcript_132077	NA	NA	F01_transcript_132077	F01_transcript_132077
F01_transcript_120141	NA	F01_transcript_120141	NA	NA
F01_transcript_147601	NA	NA	NA	F01_transcript_147601
F01_transcript_85679	NA	NA	NA	F01_transcript_85679
F01_transcript_104808	NA	F01_transcript_104808	NA	NA
F01_transcript_149492	NA	F01_transcript_149492	F01_transcript_149492	F01_transcript_149492
F01_transcript_167282	F01_transcript_167282	F01_transcript_167282	F01_transcript_167282	F01_transcript_167282
F01_transcript_53923	NA	NA	NA	F01_transcript_53923

F01_transcript_59640	NA	F01_transcript_59640	NA	NA
F01_transcript_159434	NA	NA	NA	F01_transcript_159434
F01_transcript_24301	NA	NA	NA	F01_transcript_24301
F01_transcript_122539	F01_transcript_122539	F01_transcript_122539	F01_transcript_122539	F01_transcript_122539
F01_transcript_107292	NA	F01_transcript_107292	NA	NA
F01_transcript_75084	F01_transcript_75084	F01_transcript_75084	F01_transcript_75084	F01_transcript_75084
F01_transcript_33554	F01_transcript_33554	F01_transcript_33554	F01_transcript_33554	F01_transcript_33554
F01_transcript_18040	NA	NA	NA	F01_transcript_18040
F01_transcript_46691	NA	NA	NA	F01_transcript_46691
F01_transcript_134501	NA	NA	NA	F01_transcript_134501
F01_transcript_87592	NA	F01_transcript_87592	NA	NA
F01_transcript_134243	F01_transcript_134243	F01_transcript_134243	NA	NA
F01_transcript_113019	NA	NA	NA	F01_transcript_113019
F01_transcript_49156	NA	F01_transcript_49156	NA	NA

F01_transcript_56602	NA	NA	NA	F01_transcript_56602
F01_transcript_76616	NA	NA	NA	F01_transcript_76616
F01_transcript_165561	NA	F01_transcript_165561	NA	NA
F01_transcript_166002	NA	NA	NA	F01_transcript_166002
F01_transcript_105628	NA	NA	NA	F01_transcript_105628
F01_transcript_150076	F01_transcript_150076	F01_transcript_150076	F01_transcript_150076	F01_transcript_150076
F01_transcript_159012	NA	NA	F01_transcript_159012	NA
F01_transcript_103953	NA	NA	NA	F01_transcript_103953
F01_transcript_124673	NA	F01_transcript_124673	NA	F01_transcript_124673
F01_transcript_111779	F01_transcript_111779	F01_transcript_111779	NA	NA
F01_transcript_150972	NA	F01_transcript_150972	NA	F01_transcript_150972
F01_transcript_3585	NA	NA	NA	F01_transcript_3585
F01_transcript_82706	NA	NA	F01_transcript_82706	F01_transcript_82706
F01_transcript_140329	F01_transcript_140329	F01_transcript_140329	NA	NA

F01_transcript_126457	NA	F01_transcript_126457	NA	NA
F01_transcript_162497	NA	F01_transcript_162497	NA	NA
F01_transcript_100298	F01_transcript_100298	F01_transcript_100298	F01_transcript_100298	NA
F01_transcript_104364	F01_transcript_104364	F01_transcript_104364	F01_transcript_104364	F01_transcript_104364
F01_transcript_113092	NA	NA	NA	F01_transcript_113092
F01_transcript_100811	NA	F01_transcript_100811	NA	NA
F01_transcript_44880	NA	NA	NA	F01_transcript_44880
F01_transcript_48560	F01_transcript_48560	F01_transcript_48560	F01_transcript_48560	F01_transcript_48560
F01_transcript_127533	F01_transcript_127533	F01_transcript_127533	F01_transcript_127533	F01_transcript_127533
F01_transcript_79461	F01_transcript_79461	F01_transcript_79461	NA	NA
F01_transcript_55152	NA	NA	NA	F01_transcript_55152
F01_transcript_127228	NA	NA	NA	F01_transcript_127228
F01_transcript_110879	NA	F01_transcript_110879	NA	NA
F01_transcript_98036	NA	NA	NA	F01_transcript_98036

F01_transcript_78549	NA	NA	NA	F01_transcript_78549
F01_transcript_140898	NA	NA	NA	F01_transcript_140898
F01_transcript_95312	F01_transcript_95312	F01_transcript_95312	NA	F01_transcript_95312
F01_transcript_66773	F01_transcript_66773	F01_transcript_66773	F01_transcript_66773	F01_transcript_66773
F01_transcript_58790	F01_transcript_58790	F01_transcript_58790	NA	F01_transcript_58790
F01_transcript_116289	NA	F01_transcript_116289	NA	NA
F01_transcript_76818	NA	F01_transcript_76818	F01_transcript_76818	F01_transcript_76818
F01_transcript_23176	NA	NA	NA	F01_transcript_23176
F01_transcript_113362	NA	F01_transcript_113362	NA	NA
F01_transcript_42367	NA	F01_transcript_42367	NA	NA
F01_transcript_89128	F01_transcript_89128	F01_transcript_89128	NA	NA
F01_transcript_160289	F01_transcript_160289	F01_transcript_160289	F01_transcript_160289	F01_transcript_160289
F01_transcript_47938	NA	NA	NA	F01_transcript_47938
F01_transcript_56650	NA	NA	NA	F01_transcript_56650

F01_transcript_93850	NA	NA	NA	F01_transcript_93850
F01_transcript_56029	NA	F01_transcript_56029	NA	F01_transcript_56029
F01_transcript_54193	NA	F01_transcript_54193	F01_transcript_54193	F01_transcript_54193
F01_transcript_166094	NA	NA	NA	F01_transcript_166094
F01_transcript_96586	NA	F01_transcript_96586	F01_transcript_96586	NA
F01_transcript_73681	NA	NA	NA	F01_transcript_73681
F01_transcript_93597	NA	NA	NA	F01_transcript_93597
F01_transcript_51688	F01_transcript_51688	F01_transcript_51688	F01_transcript_51688	F01_transcript_51688
F01_transcript_122920	F01_transcript_122920	F01_transcript_122920	F01_transcript_122920	F01_transcript_122920
F01_transcript_114004	NA	F01_transcript_114004	NA	F01_transcript_114004
F01_transcript_133268	NA	F01_transcript_133268	NA	F01_transcript_133268
F01_transcript_67188	NA	NA	NA	F01_transcript_67188
F01_transcript_91646	NA	NA	NA	F01_transcript_91646
F01_transcript_103937	F01_transcript_103937	F01_transcript_103937	NA	F01_transcript_103937

F01_transcript_95	NA	NA	NA	F01_transcript_95
F01_transcript_61472	NA	F01_transcript_61472	NA	NA
F01_transcript_109297	NA	F01_transcript_109297	NA	NA
F01_transcript_62781	NA	F01_transcript_62781	NA	NA
F01_transcript_105402	F01_transcript_105402	F01_transcript_105402	F01_transcript_105402	F01_transcript_105402
F01_transcript_70255	NA	F01_transcript_70255	NA	NA
F01_transcript_149060	NA	NA	NA	F01_transcript_149060
F01_transcript_128396	NA	F01_transcript_128396	NA	NA
F01_transcript_54764	NA	F01_transcript_54764	NA	NA
F01_transcript_132954	NA	NA	NA	F01_transcript_132954
F01_transcript_61251	F01_transcript_61251	F01_transcript_61251	NA	NA
F01_transcript_107224	NA	F01_transcript_107224	NA	NA
F01_transcript_105041	F01_transcript_105041	F01_transcript_105041	F01_transcript_105041	F01_transcript_105041
F01_transcript_136546	NA	NA	F01_transcript_136546	NA

F01_transcript_94825	NA	NA	NA	F01_transcript_94825
F01_transcript_131460	NA	NA	NA	F01_transcript_131460
F01_transcript_93477	NA	NA	NA	F01_transcript_93477
F01_transcript_93441	F01_transcript_93441	F01_transcript_93441	F01_transcript_93441	NA
F01_transcript_3786	F01_transcript_3786	F01_transcript_3786	NA	NA
F01_transcript_128783	F01_transcript_128783	F01_transcript_128783	F01_transcript_128783	F01_transcript_128783
F01_transcript_90252	NA	NA	F01_transcript_90252	NA
F01_transcript_15224	NA	F01_transcript_15224	NA	F01_transcript_15224
F01_transcript_155274	F01_transcript_155274	F01_transcript_155274	F01_transcript_155274	F01_transcript_155274
F01_transcript_71330	NA	F01_transcript_71330	F01_transcript_71330	F01_transcript_71330
F01_transcript_25978	F01_transcript_25978	F01_transcript_25978	F01_transcript_25978	F01_transcript_25978
F01_transcript_72098	NA	F01_transcript_72098	NA	F01_transcript_72098
F01_transcript_15296	NA	NA	NA	F01_transcript_15296
F01_transcript_80197	NA	F01_transcript_80197	NA	NA

F01_transcript_127878	NA	NA	NA	F01_transcript_127878
F01_transcript_74484	F01_transcript_74484	F01_transcript_74484	F01_transcript_74484	F01_transcript_74484
F01_transcript_128058	NA	NA	NA	F01_transcript_128058
F01_transcript_73520	NA	NA	NA	F01_transcript_73520
F01_transcript_43865	NA	F01_transcript_43865	NA	NA
F01_transcript_164398	F01_transcript_164398	F01_transcript_164398	NA	F01_transcript_164398
F01_transcript_127788	F01_transcript_127788	F01_transcript_127788	NA	NA
F01_transcript_49760	NA	F01_transcript_49760	NA	NA
F01_transcript_152602	F01_transcript_152602	F01_transcript_152602	NA	F01_transcript_152602
F01_transcript_135292	NA	NA	NA	F01_transcript_135292
F01_transcript_150377	F01_transcript_150377	F01_transcript_150377	F01_transcript_150377	F01_transcript_150377
F01_transcript_75680	F01_transcript_75680	F01_transcript_75680	F01_transcript_75680	F01_transcript_75680
F01_transcript_129957	NA	NA	NA	F01_transcript_129957
F01_transcript_17759	NA	NA	NA	F01_transcript_17759

F01_transcript_49607	NA	F01_transcript_49607	NA	F01_transcript_49607
F01_transcript_118519	NA	F01_transcript_118519	F01_transcript_118519	F01_transcript_118519
F01_transcript_166215	NA	NA	NA	F01_transcript_166215
F01_transcript_162279	NA	NA	NA	F01_transcript_162279
F01_transcript_144827	NA	NA	NA	F01_transcript_144827
F01_transcript_73185	NA	F01_transcript_73185	F01_transcript_73185	NA
F01_transcript_107822	F01_transcript_107822	F01_transcript_107822	F01_transcript_107822	F01_transcript_107822
F01_transcript_15814	NA	F01_transcript_15814	F01_transcript_15814	NA
F01_transcript_38425	NA	F01_transcript_38425	NA	F01_transcript_38425
F01_transcript_167550	NA	NA	NA	F01_transcript_167550
F01_transcript_121364	NA	F01_transcript_121364	NA	NA
F01_transcript_22744	NA	NA	NA	F01_transcript_22744
F01_transcript_131026	NA	F01_transcript_131026	F01_transcript_131026	NA
F01_transcript_81528	F01_transcript_81528	F01_transcript_81528	NA	F01_transcript_81528

F01_transcript_24245	NA	F01_transcript_24245	NA	NA
F01_transcript_65645	NA	NA	NA	F01_transcript_65645
F01_transcript_17890	NA	F01_transcript_17890	NA	F01_transcript_17890
F01_transcript_153076	NA	F01_transcript_153076	NA	NA
F01_transcript_83975	NA	F01_transcript_83975	NA	F01_transcript_83975
F01_transcript_155798	F01_transcript_155798	F01_transcript_155798	F01_transcript_155798	F01_transcript_155798
F01_transcript_50869	F01_transcript_50869	F01_transcript_50869	F01_transcript_50869	F01_transcript_50869
F01_transcript_120148	NA	NA	NA	F01_transcript_120148
F01_transcript_92437	NA	NA	NA	F01_transcript_92437
F01_transcript_85902	F01_transcript_85902	F01_transcript_85902	NA	NA
F01_transcript_75511	NA	NA	NA	F01_transcript_75511
F01_transcript_146800	F01_transcript_146800	F01_transcript_146800	F01_transcript_146800	F01_transcript_146800
F01_transcript_98616	NA	NA	NA	F01_transcript_98616
F01_transcript_163491	NA	F01_transcript_163491	NA	NA

F01_transcript_21759	NA	NA	NA	F01_transcript_21759
F01_transcript_132796	F01_transcript_132796	F01_transcript_132796	NA	F01_transcript_132796
F01_transcript_155734	F01_transcript_155734	F01_transcript_155734	F01_transcript_155734	F01_transcript_155734
F01_transcript_161380	NA	NA	NA	F01_transcript_161380
F01_transcript_89059	NA	NA	NA	F01_transcript_89059
F01_transcript_127230	NA	NA	NA	F01_transcript_127230
F01_transcript_120585	F01_transcript_120585	F01_transcript_120585	NA	F01_transcript_120585
F01_transcript_96446	F01_transcript_96446	F01_transcript_96446	F01_transcript_96446	F01_transcript_96446
F01_transcript_76755	F01_transcript_76755	F01_transcript_76755	F01_transcript_76755	F01_transcript_76755
F01_transcript_65094	NA	F01_transcript_65094	F01_transcript_65094	F01_transcript_65094
F01_transcript_82568	F01_transcript_82568	F01_transcript_82568	F01_transcript_82568	F01_transcript_82568
F01_transcript_87242	NA	NA	NA	F01_transcript_87242
F01_transcript_47402	NA	NA	NA	F01_transcript_47402
F01_transcript_157504	F01_transcript_157504	F01_transcript_157504	F01_transcript_157504	F01_transcript_157504

F01_transcript_110303	F01_transcript_110303	F01_transcript_110303	F01_transcript_110303	F01_transcript_110303
F01_transcript_101363	NA	NA	NA	F01_transcript_101363
F01_transcript_86740	NA	NA	NA	F01_transcript_86740
F01_transcript_146376	F01_transcript_146376	F01_transcript_146376	NA	NA
F01_transcript_94279	F01_transcript_94279	F01_transcript_94279	F01_transcript_94279	F01_transcript_94279
F01_transcript_31776	NA	F01_transcript_31776	F01_transcript_31776	F01_transcript_31776
F01_transcript_135090	NA	F01_transcript_135090	NA	NA
F01_transcript_77393	F01_transcript_77393	F01_transcript_77393	F01_transcript_77393	F01_transcript_77393
F01_transcript_109284	F01_transcript_109284	F01_transcript_109284	NA	F01_transcript_109284
F01_transcript_108274	F01_transcript_108274	F01_transcript_108274	F01_transcript_108274	F01_transcript_108274
F01_transcript_90902	NA	F01_transcript_90902	NA	NA
F01_transcript_156902	F01_transcript_156902	F01_transcript_156902	F01_transcript_156902	F01_transcript_156902
F01_transcript_112600	NA	F01_transcript_112600	NA	NA
F01_transcript_93696	NA	F01_transcript_93696	NA	NA

F01_transcript_113330	NA	F01_transcript_113330	NA	NA
F01_transcript_89745	NA	F01_transcript_89745	NA	F01_transcript_89745
F01_transcript_139620	NA	NA	NA	F01_transcript_139620
F01_transcript_86272	NA	F01_transcript_86272	NA	NA
F01_transcript_74521	NA	F01_transcript_74521	NA	NA
F01_transcript_105781	NA	NA	NA	F01_transcript_105781
F01_transcript_71095	NA	NA	F01_transcript_71095	NA
F01_transcript_119786	NA	NA	NA	F01_transcript_119786
F01_transcript_18942	NA	NA	NA	F01_transcript_18942
F01_transcript_65959	NA	F01_transcript_65959	NA	F01_transcript_65959
F01_transcript_87997	NA	NA	NA	F01_transcript_87997
F01_transcript_128471	NA	F01_transcript_128471	NA	NA
F01_transcript_54639	NA	F01_transcript_54639	F01_transcript_54639	F01_transcript_54639
F01_transcript_100604	F01_transcript_100604	NA	NA	NA

F01_transcript_98602	NA	F01_transcript_98602	NA	NA
F01_transcript_105400	NA	F01_transcript_105400	NA	NA
F01_transcript_124862	NA	NA	NA	F01_transcript_124862
F01_transcript_157957	NA	F01_transcript_157957	NA	NA
F01_transcript_156991	F01_transcript_156991	F01_transcript_156991	F01_transcript_156991	F01_transcript_156991
F01_transcript_147182	NA	NA	NA	F01_transcript_147182
F01_transcript_145698	NA	F01_transcript_145698	F01_transcript_145698	F01_transcript_145698
F01_transcript_51501	F01_transcript_51501	F01_transcript_51501	F01_transcript_51501	F01_transcript_51501
F01_transcript_120213	NA	F01_transcript_120213	NA	NA
F01_transcript_61762	F01_transcript_61762	F01_transcript_61762	NA	NA
F01_transcript_57602	NA	F01_transcript_57602	NA	NA
F01_transcript_43699	NA	NA	NA	F01_transcript_43699
F01_transcript_85495	NA	F01_transcript_85495	NA	NA
F01_transcript_154114	F01_transcript_154114	F01_transcript_154114	NA	NA

F01_transcript_125465	NA	F01_transcript_125465	NA	NA
F01_transcript_63413	F01_transcript_63413	F01_transcript_63413	NA	NA
F01_transcript_109731	F01_transcript_109731	F01_transcript_109731	F01_transcript_109731	F01_transcript_109731
F01_transcript_160975	F01_transcript_160975	F01_transcript_160975	NA	F01_transcript_160975
F01_transcript_152203	NA	F01_transcript_152203	F01_transcript_152203	F01_transcript_152203
F01_transcript_139558	NA	NA	NA	F01_transcript_139558
F01_transcript_24376	NA	F01_transcript_24376	NA	F01_transcript_24376
F01_transcript_128611	NA	F01_transcript_128611	NA	NA
F01_transcript_65274	NA	F01_transcript_65274	F01_transcript_65274	F01_transcript_65274
F01_transcript_74084	NA	NA	NA	F01_transcript_74084
F01_transcript_153705	NA	NA	NA	F01_transcript_153705
F01_transcript_156342	NA	NA	NA	F01_transcript_156342
F01_transcript_35711	NA	NA	NA	F01_transcript_35711
F01_transcript_86574	NA	NA	NA	F01_transcript_86574

F01_transcript_117866	F01_transcript_117866	F01_transcript_117866	NA	NA
F01_transcript_63366	NA	F01_transcript_63366	NA	NA
F01_transcript_48552	NA	F01_transcript_48552	F01_transcript_48552	F01_transcript_48552
F01_transcript_61807	NA	NA	NA	F01_transcript_61807
F01_transcript_144251	F01_transcript_144251	NA	NA	NA
F01_transcript_89272	NA	F01_transcript_89272	NA	F01_transcript_89272
F01_transcript_82700	F01_transcript_82700	F01_transcript_82700	NA	NA
F01_transcript_166144	NA	F01_transcript_166144	NA	NA
F01_transcript_131735	F01_transcript_131735	F01_transcript_131735	F01_transcript_131735	F01_transcript_131735
F01_transcript_118667	F01_transcript_118667	NA	NA	NA
F01_transcript_26709	NA	NA	NA	F01_transcript_26709
F01_transcript_103492	F01_transcript_103492	F01_transcript_103492	F01_transcript_103492	F01_transcript_103492
F01_transcript_120273	F01_transcript_120273	F01_transcript_120273	F01_transcript_120273	F01_transcript_120273
F01_transcript_75491	NA	F01_transcript_75491	NA	NA

F01_transcript_86522	NA	NA	NA	F01_transcript_86522
F01_transcript_90042	F01_transcript_90042	F01_transcript_90042	F01_transcript_90042	F01_transcript_90042
F01_transcript_104011	NA	NA	NA	F01_transcript_104011
F01_transcript_99823	F01_transcript_99823	F01_transcript_99823	F01_transcript_99823	F01_transcript_99823
F01_transcript_111221	F01_transcript_111221	F01_transcript_111221	F01_transcript_111221	F01_transcript_111221
F01_transcript_74433	NA	NA	NA	F01_transcript_74433
F01_transcript_109042	NA	F01_transcript_109042	NA	NA
F01_transcript_96314	F01_transcript_96314	F01_transcript_96314	NA	NA
F01_transcript_108147	NA	F01_transcript_108147	F01_transcript_108147	F01_transcript_108147
F01_transcript_78114	F01_transcript_78114	F01_transcript_78114	F01_transcript_78114	F01_transcript_78114
F01_transcript_85582	NA	F01_transcript_85582	NA	NA
F01_transcript_121081	NA	NA	NA	F01_transcript_121081
F01_transcript_82806	NA	NA	NA	F01_transcript_82806
F01_transcript_83001	F01_transcript_83001	F01_transcript_83001	F01_transcript_83001	F01_transcript_83001

F01_transcript_77489	NA	F01_transcript_77489	NA	NA
F01_transcript_53756	NA	F01_transcript_53756	NA	F01_transcript_53756
F01_transcript_134477	NA	F01_transcript_134477	NA	NA
F01_transcript_146946	F01_transcript_146946	F01_transcript_146946	F01_transcript_146946	F01_transcript_146946
F01_transcript_83671	F01_transcript_83671	F01_transcript_83671	F01_transcript_83671	F01_transcript_83671
F01_transcript_147020	NA	NA	NA	F01_transcript_147020
F01_transcript_87277	NA	F01_transcript_87277	NA	NA
F01_transcript_99339	NA	F01_transcript_99339	NA	NA
F01_transcript_164448	NA	F01_transcript_164448	NA	NA
F01_transcript_149755	F01_transcript_149755	F01_transcript_149755	F01_transcript_149755	F01_transcript_149755
F01_transcript_33234	NA	NA	NA	F01_transcript_33234
F01_transcript_159374	NA	F01_transcript_159374	F01_transcript_159374	F01_transcript_159374
F01_transcript_110168	NA	NA	NA	F01_transcript_110168
F01_transcript_115599	NA	NA	NA	F01_transcript_115599

F01_transcript_56941	NA	NA	NA	F01_transcript_56941
F01_transcript_118830	NA	NA	NA	F01_transcript_118830
F01_transcript_105833	NA	NA	NA	F01_transcript_105833
F01_transcript_78130	F01_transcript_78130	F01_transcript_78130	F01_transcript_78130	F01_transcript_78130
F01_transcript_44538	NA	F01_transcript_44538	NA	NA
F01_transcript_130252	F01_transcript_130252	F01_transcript_130252	F01_transcript_130252	F01_transcript_130252
F01_transcript_111231	NA	NA	NA	F01_transcript_111231
F01_transcript_65603	NA	NA	NA	F01_transcript_65603
F01_transcript_48827	NA	F01_transcript_48827	F01_transcript_48827	NA
F01_transcript_128901	F01_transcript_128901	F01_transcript_128901	F01_transcript_128901	F01_transcript_128901
F01_transcript_78587	NA	F01_transcript_78587	F01_transcript_78587	F01_transcript_78587
F01_transcript_122299	F01_transcript_122299	F01_transcript_122299	NA	F01_transcript_122299
F01_transcript_103057	NA	NA	NA	F01_transcript_103057
F01_transcript_163235	NA	NA	NA	F01_transcript_163235

F01_transcript_73993	NA	NA	NA	F01_transcript_73993
F01_transcript_93106	F01_transcript_93106	F01_transcript_93106	F01_transcript_93106	F01_transcript_93106
F01_transcript_108622	F01_transcript_108622	NA	NA	NA
F01_transcript_133001	NA	NA	NA	F01_transcript_133001
F01_transcript_142901	NA	NA	NA	F01_transcript_142901
F01_transcript_151196	NA	F01_transcript_151196	F01_transcript_151196	NA
F01_transcript_37968	NA	NA	NA	F01_transcript_37968
F01_transcript_96467	F01_transcript_96467	F01_transcript_96467	NA	F01_transcript_96467
F01_transcript_74576	NA	NA	NA	F01_transcript_74576
F01_transcript_131367	NA	F01_transcript_131367	NA	NA
F01_transcript_104355	F01_transcript_104355	F01_transcript_104355	F01_transcript_104355	NA
F01_transcript_89436	F01_transcript_89436	F01_transcript_89436	F01_transcript_89436	F01_transcript_89436
F01_transcript_104376	NA	NA	NA	F01_transcript_104376
F01_transcript_73352	NA	NA	NA	F01_transcript_73352

F01_transcript_137284	F01_transcript_137284	F01_transcript_137284	F01_transcript_137284	F01_transcript_137284
F01_transcript_76934	NA	NA	NA	F01_transcript_76934
F01_transcript_126258	NA	NA	NA	F01_transcript_126258
F01_transcript_638	NA	NA	NA	F01_transcript_638
F01_transcript_100522	F01_transcript_100522	F01_transcript_100522	F01_transcript_100522	F01_transcript_100522
F01_transcript_136565	NA	F01_transcript_136565	NA	NA
F01_transcript_151778	F01_transcript_151778	F01_transcript_151778	F01_transcript_151778	F01_transcript_151778
F01_transcript_6187	NA	F01_transcript_6187	NA	F01_transcript_6187
F01_transcript_79993	NA	NA	NA	F01_transcript_79993
F01_transcript_161300	NA	F01_transcript_161300	NA	NA
F01_transcript_63312	NA	NA	NA	F01_transcript_63312
F01_transcript_135509	F01_transcript_135509	F01_transcript_135509	F01_transcript_135509	F01_transcript_135509
F01_transcript_54465	NA	F01_transcript_54465	NA	NA
F01_transcript_88068	NA	NA	NA	F01_transcript_88068

F01_transcript_3891	NA	F01_transcript_3891	NA	F01_transcript_3891
F01_transcript_130203	F01_transcript_130203	F01_transcript_130203	F01_transcript_130203	F01_transcript_130203
F01_transcript_59296	NA	NA	F01_transcript_59296	NA
F01_transcript_119628	NA	NA	F01_transcript_119628	F01_transcript_119628
F01_transcript_161468	NA	NA	NA	F01_transcript_161468
F01_transcript_119866	NA	F01_transcript_119866	NA	NA
F01_transcript_127831	F01_transcript_127831	F01_transcript_127831	NA	NA
F01_transcript_64089	F01_transcript_64089	F01_transcript_64089	NA	F01_transcript_64089
F01_transcript_16796	F01_transcript_16796	F01_transcript_16796	F01_transcript_16796	F01_transcript_16796
F01_transcript_160257	NA	NA	NA	F01_transcript_160257
F01_transcript_20975	NA	NA	NA	F01_transcript_20975
F01_transcript_48947	F01_transcript_48947	F01_transcript_48947	F01_transcript_48947	F01_transcript_48947
F01_transcript_46290	NA	NA	NA	F01_transcript_46290
F01_transcript_149856	NA	F01_transcript_149856	NA	NA

F01_transcript_114545	F01_transcript_114545	F01_transcript_114545	F01_transcript_114545	F01_transcript_114545
F01_transcript_107984	NA	NA	NA	F01_transcript_107984
F01_transcript_95054	NA	F01_transcript_95054	NA	NA
F01_transcript_50716	F01_transcript_50716	F01_transcript_50716	F01_transcript_50716	NA
F01_transcript_141339	NA	F01_transcript_141339	NA	NA
F01_transcript_103154	F01_transcript_103154	F01_transcript_103154	NA	F01_transcript_103154
F01_transcript_160145	F01_transcript_160145	F01_transcript_160145	F01_transcript_160145	F01_transcript_160145
F01_transcript_156316	NA	F01_transcript_156316	NA	NA
F01_transcript_107986	NA	F01_transcript_107986	NA	F01_transcript_107986
F01_transcript_8792	NA	F01_transcript_8792	F01_transcript_8792	F01_transcript_8792
F01_transcript_58041	NA	F01_transcript_58041	NA	F01_transcript_58041
F01_transcript_99411	NA	F01_transcript_99411	F01_transcript_99411	NA
F01_transcript_53640	NA	F01_transcript_53640	NA	NA
F01_transcript_128618	F01_transcript_128618	F01_transcript_128618	F01_transcript_128618	F01_transcript_128618

F01_transcript_52125	NA	F01_transcript_52125	F01_transcript_52125	F01_transcript_52125
F01_transcript_81137	NA	F01_transcript_81137	F01_transcript_81137	F01_transcript_81137
F01_transcript_69400	F01_transcript_69400	F01_transcript_69400	F01_transcript_69400	F01_transcript_69400
F01_transcript_120267	F01_transcript_120267	F01_transcript_120267	NA	F01_transcript_120267
F01_transcript_58595	NA	NA	NA	F01_transcript_58595
F01_transcript_59445	F01_transcript_59445	F01_transcript_59445	F01_transcript_59445	F01_transcript_59445
F01_transcript_49229	F01_transcript_49229	F01_transcript_49229	F01_transcript_49229	F01_transcript_49229
F01_transcript_118849	NA	NA	NA	F01_transcript_118849
F01_transcript_153376	NA	F01_transcript_153376	F01_transcript_153376	F01_transcript_153376
F01_transcript_75164	NA	NA	NA	F01_transcript_75164
F01_transcript_54295	NA	NA	NA	F01_transcript_54295
F01_transcript_121328	F01_transcript_121328	F01_transcript_121328	NA	NA
F01_transcript_74856	NA	F01_transcript_74856	F01_transcript_74856	F01_transcript_74856
F01_transcript_63245	NA	F01_transcript_63245	NA	NA

F01_transcript_22749	NA	F01_transcript_22749	NA	NA
F01_transcript_73861	F01_transcript_73861	F01_transcript_73861	F01_transcript_73861	F01_transcript_73861
F01_transcript_138753	NA	NA	NA	F01_transcript_138753
F01_transcript_89563	NA	F01_transcript_89563	NA	F01_transcript_89563
F01_transcript_14579	NA	NA	NA	F01_transcript_14579
F01_transcript_112807	NA	NA	NA	F01_transcript_112807
F01_transcript_105110	NA	F01_transcript_105110	NA	NA
F01_transcript_63204	NA	F01_transcript_63204	NA	NA
F01_transcript_161284	NA	F01_transcript_161284	NA	F01_transcript_161284
F01_transcript_125445	NA	NA	NA	F01_transcript_125445
F01_transcript_144994	NA	F01_transcript_144994	F01_transcript_144994	F01_transcript_144994
F01_transcript_79894	NA	F01_transcript_79894	NA	NA
F01_transcript_152892	NA	NA	NA	F01_transcript_152892
F01_transcript_106432	NA	F01_transcript_106432	NA	NA

F01_transcript_152079	NA	NA	NA	F01_transcript_152079
F01_transcript_122643	NA	NA	NA	F01_transcript_122643
F01_transcript_79246	NA	NA	NA	F01_transcript_79246
F01_transcript_139755	NA	F01_transcript_139755	NA	NA
F01_transcript_126967	F01_transcript_126967	F01_transcript_126967	F01_transcript_126967	F01_transcript_126967
F01_transcript_70298	NA	NA	F01_transcript_70298	F01_transcript_70298
F01_transcript_151784	F01_transcript_151784	F01_transcript_151784	F01_transcript_151784	F01_transcript_151784
F01_transcript_158468	NA	F01_transcript_158468	NA	F01_transcript_158468
F01_transcript_53786	F01_transcript_53786	F01_transcript_53786	NA	NA
F01_transcript_122626	NA	F01_transcript_122626	NA	NA
F01_transcript_152084	NA	F01_transcript_152084	NA	F01_transcript_152084
F01_transcript_151982	F01_transcript_151982	F01_transcript_151982	F01_transcript_151982	F01_transcript_151982
F01_transcript_5360	NA	F01_transcript_5360	NA	F01_transcript_5360
F01_transcript_69844	NA	F01_transcript_69844	NA	F01_transcript_69844

F01_transcript_73584	NA	F01_transcript_73584	NA	NA
F01_transcript_79596	F01_transcript_79596	F01_transcript_79596	F01_transcript_79596	F01_transcript_79596
F01_transcript_143409	F01_transcript_143409	F01_transcript_143409	NA	NA
F01_transcript_63703	F01_transcript_63703	F01_transcript_63703	NA	F01_transcript_63703
F01_transcript_53587	NA	NA	F01_transcript_53587	F01_transcript_53587
F01_transcript_111173	F01_transcript_111173	F01_transcript_111173	F01_transcript_111173	F01_transcript_111173
F01_transcript_158999	NA	NA	NA	F01_transcript_158999
F01_transcript_150242	NA	F01_transcript_150242	NA	NA
F01_transcript_53601	NA	F01_transcript_53601	NA	F01_transcript_53601
F01_transcript_147525	NA	F01_transcript_147525	NA	NA
F01_transcript_123855	NA	NA	NA	F01_transcript_123855
F01_transcript_108395	NA	NA	NA	F01_transcript_108395
F01_transcript_75260	F01_transcript_75260	F01_transcript_75260	F01_transcript_75260	F01_transcript_75260
F01_transcript_162472	F01_transcript_162472	NA	F01_transcript_162472	NA

F01_transcript_123219	NA	NA	F01_transcript_123219	F01_transcript_123219
F01_transcript_125949	F01_transcript_125949	F01_transcript_125949	F01_transcript_125949	F01_transcript_125949
F01_transcript_55245	NA	NA	NA	F01_transcript_55245
F01_transcript_40369	NA	NA	NA	F01_transcript_40369
F01_transcript_87477	NA	F01_transcript_87477	NA	NA
F01_transcript_98447	F01_transcript_98447	F01_transcript_98447	F01_transcript_98447	F01_transcript_98447
F01_transcript_166449	NA	NA	NA	F01_transcript_166449
F01_transcript_150855	NA	NA	NA	F01_transcript_150855
F01_transcript_125877	F01_transcript_125877	NA	NA	NA
F01_transcript_96253	NA	NA	NA	F01_transcript_96253
F01_transcript_27773	NA	NA	NA	F01_transcript_27773
F01_transcript_157968	F01_transcript_157968	F01_transcript_157968	F01_transcript_157968	F01_transcript_157968
F01_transcript_115185	NA	NA	NA	F01_transcript_115185
F01_transcript_122702	NA	F01_transcript_122702	NA	F01_transcript_122702

F01_transcript_36767	NA	NA	NA	F01_transcript_36767
F01_transcript_60953	NA	F01_transcript_60953	NA	NA
F01_transcript_72482	NA	NA	NA	F01_transcript_72482
F01_transcript_62946	F01_transcript_62946	F01_transcript_62946	F01_transcript_62946	F01_transcript_62946
F01_transcript_71961	NA	NA	NA	F01_transcript_71961
F01_transcript_136325	NA	F01_transcript_136325	NA	NA
F01_transcript_140887	NA	NA	NA	F01_transcript_140887
F01_transcript_159913	NA	F01_transcript_159913	NA	NA
F01_transcript_67591	NA	F01_transcript_67591	F01_transcript_67591	F01_transcript_67591
F01_transcript_113325	F01_transcript_113325	F01_transcript_113325	F01_transcript_113325	F01_transcript_113325
F01_transcript_86046	NA	F01_transcript_86046	NA	F01_transcript_86046
F01_transcript_109598	NA	F01_transcript_109598	NA	NA
F01_transcript_118476	NA	F01_transcript_118476	F01_transcript_118476	F01_transcript_118476
F01_transcript_138328	NA	F01_transcript_138328	NA	NA

F01_transcript_33649	NA	NA	NA	F01_transcript_33649
F01_transcript_119828	F01_transcript_119828	F01_transcript_119828	NA	NA
F01_transcript_46228	NA	F01_transcript_46228	NA	NA
F01_transcript_73862	F01_transcript_73862	F01_transcript_73862	F01_transcript_73862	F01_transcript_73862
F01_transcript_45531	NA	F01_transcript_45531	NA	F01_transcript_45531
F01_transcript_129670	F01_transcript_129670	F01_transcript_129670	F01_transcript_129670	F01_transcript_129670
F01_transcript_133963	F01_transcript_133963	F01_transcript_133963	F01_transcript_133963	F01_transcript_133963
F01_transcript_62226	NA	F01_transcript_62226	NA	NA
F01_transcript_64741	NA	F01_transcript_64741	NA	NA
F01_transcript_150440	NA	F01_transcript_150440	NA	NA
F01_transcript_23938	NA	F01_transcript_23938	NA	F01_transcript_23938
F01_transcript_109758	NA	F01_transcript_109758	NA	NA
F01_transcript_50958	NA	F01_transcript_50958	F01_transcript_50958	F01_transcript_50958
F01_transcript_107333	NA	F01_transcript_107333	NA	F01_transcript_107333

F01_transcript_49101	NA	F01_transcript_49101	NA	F01_transcript_49101
F01_transcript_153268	NA	F01_transcript_153268	NA	F01_transcript_153268
F01_transcript_150011	NA	F01_transcript_150011	NA	F01_transcript_150011
F01_transcript_66987	NA	NA	NA	F01_transcript_66987
F01_transcript_152852	NA	F01_transcript_152852	NA	F01_transcript_152852
F01_transcript_76452	F01_transcript_76452	F01_transcript_76452	F01_transcript_76452	F01_transcript_76452
F01_transcript_97315	NA	NA	NA	F01_transcript_97315
F01_transcript_102498	NA	F01_transcript_102498	NA	F01_transcript_102498
F01_transcript_125559	NA	NA	NA	F01_transcript_125559
F01_transcript_166100	NA	F01_transcript_166100	F01_transcript_166100	F01_transcript_166100
F01_transcript_137731	F01_transcript_137731	F01_transcript_137731	F01_transcript_137731	F01_transcript_137731
F01_transcript_1690	NA	F01_transcript_1690	NA	NA
F01_transcript_122557	NA	F01_transcript_122557	NA	F01_transcript_122557
F01_transcript_150306	NA	NA	NA	F01_transcript_150306

F01_transcript_17915	NA	NA	NA	F01_transcript_17915
F01_transcript_19236	NA	F01_transcript_19236	F01_transcript_19236	F01_transcript_19236
F01_transcript_90024	NA	NA	NA	F01_transcript_90024
F01_transcript_155500	F01_transcript_155500	F01_transcript_155500	F01_transcript_155500	F01_transcript_155500
F01_transcript_93818	F01_transcript_93818	F01_transcript_93818	NA	F01_transcript_93818
F01_transcript_143457	F01_transcript_143457	F01_transcript_143457	F01_transcript_143457	F01_transcript_143457
F01_transcript_63124	NA	NA	F01_transcript_63124	F01_transcript_63124
F01_transcript_54562	NA	NA	NA	F01_transcript_54562
F01_transcript_1654	NA	NA	NA	F01_transcript_1654
F01_transcript_52813	NA	F01_transcript_52813	NA	NA
F01_transcript_162836	NA	NA	NA	F01_transcript_162836
F01_transcript_27189	NA	F01_transcript_27189	F01_transcript_27189	F01_transcript_27189
F01_transcript_85081	NA	NA	NA	F01_transcript_85081
F01_transcript_165970	NA	F01_transcript_165970	NA	NA

F01_transcript_59801	NA	F01_transcript_59801	NA	NA
F01_transcript_153557	F01_transcript_153557	F01_transcript_153557	F01_transcript_153557	F01_transcript_153557
F01_transcript_153423	F01_transcript_153423	F01_transcript_153423	F01_transcript_153423	NA
F01_transcript_130828	NA	F01_transcript_130828	NA	NA
F01_transcript_151163	F01_transcript_151163	F01_transcript_151163	F01_transcript_151163	F01_transcript_151163
F01_transcript_145284	F01_transcript_145284	F01_transcript_145284	F01_transcript_145284	F01_transcript_145284
F01_transcript_54453	NA	NA	NA	F01_transcript_54453
F01_transcript_113844	NA	F01_transcript_113844	NA	NA
F01_transcript_157552	F01_transcript_157552	F01_transcript_157552	F01_transcript_157552	F01_transcript_157552
F01_transcript_150140	F01_transcript_150140	F01_transcript_150140	F01_transcript_150140	NA
F01_transcript_126874	F01_transcript_126874	F01_transcript_126874	NA	F01_transcript_126874
F01_transcript_78692	NA	F01_transcript_78692	NA	NA
F01_transcript_50077	F01_transcript_50077	F01_transcript_50077	F01_transcript_50077	F01_transcript_50077
F01_transcript_71147	NA	F01_transcript_71147	F01_transcript_71147	NA

F01_transcript_76247	NA	F01_transcript_76247	NA	F01_transcript_76247
F01_transcript_117780	F01_transcript_117780	F01_transcript_117780	F01_transcript_117780	F01_transcript_117780
F01_transcript_95033	NA	NA	NA	F01_transcript_95033
F01_transcript_164433	NA	NA	NA	F01_transcript_164433
F01_transcript_50035	F01_transcript_50035	F01_transcript_50035	NA	NA
F01_transcript_80376	NA	F01_transcript_80376	NA	NA
F01_transcript_122503	F01_transcript_122503	F01_transcript_122503	F01_transcript_122503	F01_transcript_122503
F01_transcript_112668	NA	F01_transcript_112668	NA	NA
F01_transcript_104514	F01_transcript_104514	F01_transcript_104514	NA	NA
F01_transcript_59943	NA	F01_transcript_59943	F01_transcript_59943	F01_transcript_59943
F01_transcript_52956	F01_transcript_52956	F01_transcript_52956	F01_transcript_52956	F01_transcript_52956
F01_transcript_160156	NA	NA	NA	F01_transcript_160156
F01_transcript_130600	NA	F01_transcript_130600	NA	NA
F01_transcript_154920	F01_transcript_154920	F01_transcript_154920	NA	F01_transcript_154920

F01_transcript_61313	NA	F01_transcript_61313	F01_transcript_61313	F01_transcript_61313
F01_transcript_12433	NA	NA	NA	F01_transcript_12433
F01_transcript_165122	NA	F01_transcript_165122	NA	NA
F01_transcript_151294	F01_transcript_151294	F01_transcript_151294	F01_transcript_151294	F01_transcript_151294
F01_transcript_53360	F01_transcript_53360	F01_transcript_53360	F01_transcript_53360	F01_transcript_53360
F01_transcript_58085	NA	NA	NA	F01_transcript_58085
F01_transcript_28607	NA	NA	NA	F01_transcript_28607
F01_transcript_119536	F01_transcript_119536	F01_transcript_119536	NA	NA
F01_transcript_143172	F01_transcript_143172	F01_transcript_143172	F01_transcript_143172	F01_transcript_143172
F01_transcript_63738	NA	F01_transcript_63738	NA	NA
F01_transcript_111539	NA	F01_transcript_111539	NA	F01_transcript_111539
F01_transcript_125152	F01_transcript_125152	F01_transcript_125152	NA	NA
F01_transcript_92808	NA	F01_transcript_92808	NA	NA
F01_transcript_151770	NA	F01_transcript_151770	F01_transcript_151770	F01_transcript_151770

F01_transcript_79513	NA	F01_transcript_79513	NA	F01_transcript_79513
F01_transcript_142667	NA	F01_transcript_142667	NA	NA
F01_transcript_155074	NA	F01_transcript_155074	NA	NA
F01_transcript_147162	NA	F01_transcript_147162	F01_transcript_147162	F01_transcript_147162
F01_transcript_69563	NA	F01_transcript_69563	F01_transcript_69563	F01_transcript_69563
F01_transcript_49313	NA	F01_transcript_49313	NA	NA
F01_transcript_74982	NA	NA	NA	F01_transcript_74982
F01_transcript_123653	NA	NA	NA	F01_transcript_123653
F01_transcript_84533	NA	NA	NA	F01_transcript_84533
F01_transcript_53564	NA	NA	NA	F01_transcript_53564
F01_transcript_130793	NA	NA	NA	F01_transcript_130793
F01_transcript_127905	F01_transcript_127905	F01_transcript_127905	F01_transcript_127905	F01_transcript_127905
F01_transcript_50217	NA	F01_transcript_50217	NA	F01_transcript_50217
F01_transcript_164374	F01_transcript_164374	F01_transcript_164374	F01_transcript_164374	F01_transcript_164374

F01_transcript_46174	NA	NA	F01_transcript_46174	F01_transcript_46174
F01_transcript_161215	NA	NA	NA	F01_transcript_161215
F01_transcript_108449	F01_transcript_108449	F01_transcript_108449	F01_transcript_108449	NA
F01_transcript_27665	NA	NA	NA	F01_transcript_27665
F01_transcript_156999	F01_transcript_156999	F01_transcript_156999	NA	F01_transcript_156999
F01_transcript_142963	NA	F01_transcript_142963	NA	NA
F01_transcript_29578	NA	NA	NA	F01_transcript_29578
F01_transcript_128844	NA	NA	NA	F01_transcript_128844
F01_transcript_154508	NA	NA	NA	F01_transcript_154508
F01_transcript_93893	NA	F01_transcript_93893	NA	F01_transcript_93893
F01_transcript_79218	NA	NA	NA	F01_transcript_79218
F01_transcript_119651	NA	F01_transcript_119651	NA	NA
F01_transcript_154113	NA	F01_transcript_154113	NA	NA
F01_transcript_35624	NA	NA	NA	F01_transcript_35624

F01_transcript_28548	NA	NA	NA	F01_transcript_28548
F01_transcript_90009	NA	F01_transcript_90009	F01_transcript_90009	F01_transcript_90009
F01_transcript_70842	F01_transcript_70842	F01_transcript_70842	F01_transcript_70842	F01_transcript_70842
F01_transcript_57441	F01_transcript_57441	F01_transcript_57441	F01_transcript_57441	F01_transcript_57441
F01_transcript_148052	F01_transcript_148052	F01_transcript_148052	F01_transcript_148052	F01_transcript_148052
F01_transcript_60401	NA	F01_transcript_60401	NA	F01_transcript_60401
F01_transcript_112923	NA	F01_transcript_112923	NA	NA
F01_transcript_145167	NA	F01_transcript_145167	NA	NA
F01_transcript_77496	NA	NA	NA	F01_transcript_77496
F01_transcript_152535	NA	F01_transcript_152535	NA	NA
F01_transcript_120910	NA	F01_transcript_120910	F01_transcript_120910	F01_transcript_120910
F01_transcript_100162	NA	NA	NA	F01_transcript_100162
F01_transcript_68635	NA	NA	NA	F01_transcript_68635
F01_transcript_74490	NA	NA	F01_transcript_74490	F01_transcript_74490

F01_transcript_150540	NA	NA	NA	F01_transcript_150540
F01_transcript_27308	NA	NA	NA	F01_transcript_27308
F01_transcript_49071	NA	F01_transcript_49071	NA	F01_transcript_49071
F01_transcript_109216	NA	NA	NA	F01_transcript_109216
F01_transcript_160866	NA	F01_transcript_160866	NA	NA
F01_transcript_136736	NA	NA	NA	F01_transcript_136736
F01_transcript_107331	F01_transcript_107331	F01_transcript_107331	F01_transcript_107331	F01_transcript_107331
F01_transcript_8323	F01_transcript_8323	F01_transcript_8323	NA	NA
F01_transcript_20264	NA	NA	NA	F01_transcript_20264
F01_transcript_2976	NA	NA	NA	F01_transcript_2976
F01_transcript_96440	F01_transcript_96440	F01_transcript_96440	F01_transcript_96440	F01_transcript_96440
F01_transcript_112869	F01_transcript_112869	F01_transcript_112869	F01_transcript_112869	F01_transcript_112869
F01_transcript_39694	NA	NA	NA	F01_transcript_39694
F01_transcript_157870	NA	F01_transcript_157870	NA	F01_transcript_157870

F01_transcript_27878	NA	NA	NA	F01_transcript_27878
F01_transcript_39206	F01_transcript_39206	F01_transcript_39206	NA	F01_transcript_39206
F01_transcript_160440	NA	NA	NA	F01_transcript_160440
F01_transcript_134687	F01_transcript_134687	F01_transcript_134687	F01_transcript_134687	NA
F01_transcript_163854	NA	F01_transcript_163854	NA	F01_transcript_163854
F01_transcript_62199	F01_transcript_62199	F01_transcript_62199	F01_transcript_62199	F01_transcript_62199
F01_transcript_55154	F01_transcript_55154	F01_transcript_55154	F01_transcript_55154	F01_transcript_55154
F01_transcript_117539	NA	F01_transcript_117539	NA	F01_transcript_117539
F01_transcript_99167	NA	F01_transcript_99167	NA	NA
F01_transcript_21914	NA	NA	NA	F01_transcript_21914
F01_transcript_71467	NA	NA	NA	F01_transcript_71467
F01_transcript_129915	NA	F01_transcript_129915	F01_transcript_129915	F01_transcript_129915
F01_transcript_61146	NA	NA	NA	F01_transcript_61146
F01_transcript_125974	NA	NA	NA	F01_transcript_125974

F01_transcript_69236	NA	F01_transcript_69236	F01_transcript_69236	F01_transcript_69236
F01_transcript_59566	NA	NA	F01_transcript_59566	NA
F01_transcript_62536	NA	NA	NA	F01_transcript_62536
F01_transcript_59548	NA	NA	F01_transcript_59548	NA
F01_transcript_116286	NA	NA	NA	F01_transcript_116286
F01_transcript_162582	F01_transcript_162582	F01_transcript_162582	F01_transcript_162582	F01_transcript_162582
F01_transcript_45986	NA	NA	NA	F01_transcript_45986
F01_transcript_30161	NA	NA	NA	F01_transcript_30161
F01_transcript_62376	NA	NA	NA	F01_transcript_62376
F01_transcript_76783	F01_transcript_76783	F01_transcript_76783	F01_transcript_76783	F01_transcript_76783
F01_transcript_119888	NA	F01_transcript_119888	F01_transcript_119888	F01_transcript_119888
F01_transcript_57888	NA	F01_transcript_57888	NA	NA
F01_transcript_36692	NA	NA	NA	F01_transcript_36692
F01_transcript_70754	NA	F01_transcript_70754	NA	NA

F01_transcript_49236	NA	NA	NA	F01_transcript_49236
F01_transcript_139141	F01_transcript_139141	F01_transcript_139141	F01_transcript_139141	F01_transcript_139141
F01_transcript_135174	NA	F01_transcript_135174	F01_transcript_135174	F01_transcript_135174
F01_transcript_129552	NA	NA	NA	F01_transcript_129552
F01_transcript_13386	F01_transcript_13386	F01_transcript_13386	NA	NA
F01_transcript_90168	NA	F01_transcript_90168	F01_transcript_90168	F01_transcript_90168
F01_transcript_45857	NA	NA	NA	F01_transcript_45857
F01_transcript_49148	F01_transcript_49148	F01_transcript_49148	NA	NA
F01_transcript_60714	NA	F01_transcript_60714	NA	NA
F01_transcript_136415	NA	NA	F01_transcript_136415	F01_transcript_136415
F01_transcript_106117	NA	F01_transcript_106117	NA	NA
F01_transcript_153093	F01_transcript_153093	F01_transcript_153093	F01_transcript_153093	F01_transcript_153093
F01_transcript_50571	NA	F01_transcript_50571	NA	NA
F01_transcript_100257	F01_transcript_100257	F01_transcript_100257	F01_transcript_100257	F01_transcript_100257

F01_transcript_53926	NA	NA	NA	F01_transcript_53926
F01_transcript_130968	F01_transcript_130968	F01_transcript_130968	F01_transcript_130968	F01_transcript_130968
F01_transcript_132630	NA	NA	NA	F01_transcript_132630
F01_transcript_5531	F01_transcript_5531	F01_transcript_5531	NA	NA
F01_transcript_162492	NA	F01_transcript_162492	NA	NA
F01_transcript_148223	F01_transcript_148223	F01_transcript_148223	NA	F01_transcript_148223
F01_transcript_96376	NA	F01_transcript_96376	NA	F01_transcript_96376
F01_transcript_131762	NA	NA	NA	F01_transcript_131762
F01_transcript_68582	NA	NA	NA	F01_transcript_68582
F01_transcript_90350	NA	NA	NA	F01_transcript_90350
F01_transcript_114878	NA	F01_transcript_114878	NA	NA
F01_transcript_42023	F01_transcript_42023	NA	NA	NA
F01_transcript_65970	NA	NA	NA	F01_transcript_65970
F01_transcript_29405	NA	NA	NA	F01_transcript_29405

F01_transcript_46264	NA	NA	NA	F01_transcript_46264
F01_transcript_83954	NA	F01_transcript_83954	NA	NA
F01_transcript_96986	NA	F01_transcript_96986	NA	F01_transcript_96986
F01_transcript_50146	NA	NA	NA	F01_transcript_50146
F01_transcript_72999	NA	F01_transcript_72999	NA	NA
F01_transcript_42984	NA	NA	NA	F01_transcript_42984
F01_transcript_126028	F01_transcript_126028	F01_transcript_126028	F01_transcript_126028	F01_transcript_126028
F01_transcript_61432	F01_transcript_61432	F01_transcript_61432	F01_transcript_61432	F01_transcript_61432
F01_transcript_146605	NA	NA	NA	F01_transcript_146605
F01_transcript_86360	NA	F01_transcript_86360	NA	F01_transcript_86360
F01_transcript_79634	NA	NA	NA	F01_transcript_79634
F01_transcript_27075	NA	F01_transcript_27075	NA	NA
F01_transcript_106218	F01_transcript_106218	F01_transcript_106218	F01_transcript_106218	F01_transcript_106218
F01_transcript_132740	NA	F01_transcript_132740	NA	NA

F01_transcript_129067	F01_transcript_129067	F01_transcript_129067	F01_transcript_129067	F01_transcript_129067
F01_transcript_144862	NA	NA	NA	F01_transcript_144862
F01_transcript_124494	NA	NA	F01_transcript_124494	F01_transcript_124494
F01_transcript_110572	NA	NA	NA	F01_transcript_110572
F01_transcript_11786	F01_transcript_11786	F01_transcript_11786	F01_transcript_11786	F01_transcript_11786
F01_transcript_19903	NA	NA	NA	F01_transcript_19903
F01_transcript_138925	NA	F01_transcript_138925	NA	F01_transcript_138925
F01_transcript_80053	NA	NA	NA	F01_transcript_80053
F01_transcript_130948	NA	F01_transcript_130948	NA	F01_transcript_130948
F01_transcript_117506	NA	F01_transcript_117506	NA	F01_transcript_117506
F01_transcript_78229	F01_transcript_78229	F01_transcript_78229	F01_transcript_78229	F01_transcript_78229
F01_transcript_138333	NA	NA	NA	F01_transcript_138333
F01_transcript_88931	F01_transcript_88931	F01_transcript_88931	F01_transcript_88931	F01_transcript_88931
F01_transcript_109547	NA	NA	NA	F01_transcript_109547

F01_transcript_94151	F01_transcript_94151	F01_transcript_94151	NA	NA
F01_transcript_98761	NA	F01_transcript_98761	NA	NA
F01_transcript_135483	F01_transcript_135483	F01_transcript_135483	F01_transcript_135483	F01_transcript_135483
F01_transcript_65437	NA	NA	F01_transcript_65437	NA
F01_transcript_107949	F01_transcript_107949	F01_transcript_107949	NA	F01_transcript_107949
F01_transcript_7431	F01_transcript_7431	F01_transcript_7431	F01_transcript_7431	F01_transcript_7431
F01_transcript_138511	F01_transcript_138511	F01_transcript_138511	F01_transcript_138511	F01_transcript_138511
F01_transcript_134891	NA	NA	NA	F01_transcript_134891
F01_transcript_158164	F01_transcript_158164	F01_transcript_158164	F01_transcript_158164	F01_transcript_158164
F01_transcript_138164	NA	NA	NA	F01_transcript_138164
F01_transcript_64896	F01_transcript_64896	F01_transcript_64896	F01_transcript_64896	F01_transcript_64896
F01_transcript_41042	NA	NA	NA	F01_transcript_41042
F01_transcript_147266	NA	NA	NA	F01_transcript_147266
F01_transcript_81308	F01_transcript_81308	F01_transcript_81308	F01_transcript_81308	F01_transcript_81308

F01_transcript_108906	F01_transcript_108906	F01_transcript_108906	F01_transcript_108906	F01_transcript_108906
F01_transcript_166914	F01_transcript_166914	F01_transcript_166914	F01_transcript_166914	F01_transcript_166914
F01_transcript_30712	NA	NA	NA	F01_transcript_30712
F01_transcript_126945	NA	NA	NA	F01_transcript_126945
F01_transcript_120967	NA	F01_transcript_120967	NA	NA
F01_transcript_153934	NA	F01_transcript_153934	F01_transcript_153934	F01_transcript_153934
F01_transcript_75924	NA	F01_transcript_75924	F01_transcript_75924	F01_transcript_75924
F01_transcript_32680	F01_transcript_32680	F01_transcript_32680	F01_transcript_32680	F01_transcript_32680
F01_transcript_100477	F01_transcript_100477	F01_transcript_100477	F01_transcript_100477	F01_transcript_100477
F01_transcript_57682	NA	F01_transcript_57682	NA	NA
F01_transcript_49365	F01_transcript_49365	F01_transcript_49365	NA	NA
F01_transcript_80776	NA	F01_transcript_80776	NA	NA
F01_transcript_42779	NA	NA	NA	F01_transcript_42779
F01_transcript_96050	NA	F01_transcript_96050	NA	NA

F01_transcript_54688	F01_transcript_54688	F01_transcript_54688	F01_transcript_54688	F01_transcript_54688
F01_transcript_135785	NA	NA	NA	F01_transcript_135785
F01_transcript_72459	NA	F01_transcript_72459	NA	F01_transcript_72459
F01_transcript_158881	NA	F01_transcript_158881	F01_transcript_158881	F01_transcript_158881
F01_transcript_165648	NA	F01_transcript_165648	NA	NA
F01_transcript_143190	NA	F01_transcript_143190	NA	NA
F01_transcript_86779	NA	NA	F01_transcript_86779	F01_transcript_86779
F01_transcript_39397	NA	F01_transcript_39397	NA	NA
F01_transcript_1687	NA	F01_transcript_1687	NA	F01_transcript_1687
F01_transcript_135523	NA	NA	NA	F01_transcript_135523
F01_transcript_132886	NA	F01_transcript_132886	NA	F01_transcript_132886
F01_transcript_98625	F01_transcript_98625	F01_transcript_98625	F01_transcript_98625	F01_transcript_98625
F01_transcript_124258	NA	F01_transcript_124258	F01_transcript_124258	F01_transcript_124258
F01_transcript_54670	NA	F01_transcript_54670	NA	NA

F01_transcript_92358	NA	F01_transcript_92358	F01_transcript_92358	NA
F01_transcript_164774	F01_transcript_164774	F01_transcript_164774	F01_transcript_164774	F01_transcript_164774
F01_transcript_102927	NA	F01_transcript_102927	F01_transcript_102927	F01_transcript_102927
F01_transcript_122958	F01_transcript_122958	F01_transcript_122958	NA	NA
F01_transcript_87991	F01_transcript_87991	F01_transcript_87991	NA	F01_transcript_87991
F01_transcript_152614	F01_transcript_152614	F01_transcript_152614	NA	F01_transcript_152614
F01_transcript_102635	F01_transcript_102635	F01_transcript_102635	F01_transcript_102635	NA
F01_transcript_162620	NA	NA	NA	F01_transcript_162620
F01_transcript_27197	NA	F01_transcript_27197	NA	NA
F01_transcript_99264	NA	NA	NA	F01_transcript_99264
F01_transcript_139765	NA	NA	F01_transcript_139765	F01_transcript_139765
F01_transcript_57791	NA	F01_transcript_57791	NA	F01_transcript_57791
F01_transcript_107138	NA	NA	NA	F01_transcript_107138
F01_transcript_40789	NA	NA	NA	F01_transcript_40789

F01_transcript_132879	NA	NA	NA	F01_transcript_132879
F01_transcript_54154	F01_transcript_54154	F01_transcript_54154	NA	F01_transcript_54154
F01_transcript_148501	NA	F01_transcript_148501	NA	NA
F01_transcript_124105	NA	F01_transcript_124105	NA	NA
F01_transcript_86036	NA	NA	NA	F01_transcript_86036
F01_transcript_151635	NA	F01_transcript_151635	F01_transcript_151635	F01_transcript_151635
F01_transcript_126388	NA	F01_transcript_126388	NA	NA
F01_transcript_22001	NA	NA	NA	F01_transcript_22001
F01_transcript_161010	F01_transcript_161010	F01_transcript_161010	F01_transcript_161010	F01_transcript_161010
F01_transcript_51241	F01_transcript_51241	F01_transcript_51241	F01_transcript_51241	F01_transcript_51241
F01_transcript_58081	F01_transcript_58081	F01_transcript_58081	F01_transcript_58081	F01_transcript_58081
F01_transcript_103555	NA	F01_transcript_103555	NA	NA
F01_transcript_164140	NA	NA	NA	F01_transcript_164140
F01_transcript_166165	NA	F01_transcript_166165	NA	NA

F01_transcript_105424	NA	NA	NA	F01_transcript_105424
F01_transcript_37646	NA	NA	NA	F01_transcript_37646
F01_transcript_104897	F01_transcript_104897	F01_transcript_104897	F01_transcript_104897	F01_transcript_104897
F01_transcript_85252	NA	F01_transcript_85252	F01_transcript_85252	F01_transcript_85252
F01_transcript_24441	NA	NA	NA	F01_transcript_24441
F01_transcript_44971	NA	NA	NA	F01_transcript_44971
F01_transcript_98125	NA	F01_transcript_98125	NA	NA
F01_transcript_90936	F01_transcript_90936	F01_transcript_90936	F01_transcript_90936	F01_transcript_90936
F01_transcript_82476	F01_transcript_82476	F01_transcript_82476	F01_transcript_82476	F01_transcript_82476
F01_transcript_134035	NA	F01_transcript_134035	NA	NA
F01_transcript_57928	NA	F01_transcript_57928	NA	NA
F01_transcript_3762	NA	NA	NA	F01_transcript_3762
F01_transcript_166569	F01_transcript_166569	F01_transcript_166569	NA	NA
F01_transcript_86464	NA	F01_transcript_86464	NA	NA

F01_transcript_52614	NA	NA	NA	F01_transcript_52614
F01_transcript_68385	F01_transcript_68385	F01_transcript_68385	NA	NA
F01_transcript_159773	NA	F01_transcript_159773	NA	F01_transcript_159773
F01_transcript_86506	NA	NA	NA	F01_transcript_86506
F01_transcript_96275	NA	F01_transcript_96275	NA	NA
F01_transcript_101044	NA	NA	NA	F01_transcript_101044
F01_transcript_124234	NA	F01_transcript_124234	F01_transcript_124234	F01_transcript_124234
F01_transcript_103522	NA	F01_transcript_103522	NA	NA
F01_transcript_37503	F01_transcript_37503	F01_transcript_37503	NA	F01_transcript_37503
F01_transcript_164127	F01_transcript_164127	F01_transcript_164127	F01_transcript_164127	F01_transcript_164127
F01_transcript_98284	NA	F01_transcript_98284	NA	NA
F01_transcript_139771	F01_transcript_139771	F01_transcript_139771	F01_transcript_139771	F01_transcript_139771
F01_transcript_139464	NA	F01_transcript_139464	NA	NA
F01_transcript_88288	F01_transcript_88288	F01_transcript_88288	F01_transcript_88288	F01_transcript_88288

F01_transcript_78749	F01_transcript_78749	F01_transcript_78749	F01_transcript_78749	F01_transcript_78749
F01_transcript_151054	NA	NA	NA	F01_transcript_151054
F01_transcript_124525	F01_transcript_124525	F01_transcript_124525	F01_transcript_124525	F01_transcript_124525
F01_transcript_163154	NA	F01_transcript_163154	NA	NA
F01_transcript_157633	NA	F01_transcript_157633	NA	NA
F01_transcript_118462	F01_transcript_118462	F01_transcript_118462	NA	NA
F01_transcript_21070	NA	NA	NA	F01_transcript_21070
F01_transcript_116954	NA	F01_transcript_116954	NA	F01_transcript_116954
F01_transcript_85498	NA	NA	NA	F01_transcript_85498
F01_transcript_125236	F01_transcript_125236	F01_transcript_125236	NA	F01_transcript_125236
F01_transcript_16967	NA	NA	NA	F01_transcript_16967
F01_transcript_60087	F01_transcript_60087	F01_transcript_60087	NA	NA
F01_transcript_167007	NA	F01_transcript_167007	NA	F01_transcript_167007
F01_transcript_151448	NA	F01_transcript_151448	F01_transcript_151448	F01_transcript_151448

F01_transcript_87880	NA	F01_transcript_87880	NA	NA
F01_transcript_136174	F01_transcript_136174	F01_transcript_136174	NA	NA
F01_transcript_76771	F01_transcript_76771	F01_transcript_76771	F01_transcript_76771	F01_transcript_76771
F01_transcript_136792	NA	NA	NA	F01_transcript_136792
F01_transcript_114051	NA	F01_transcript_114051	NA	NA
F01_transcript_137689	F01_transcript_137689	F01_transcript_137689	F01_transcript_137689	F01_transcript_137689
F01_transcript_24681	NA	F01_transcript_24681	NA	F01_transcript_24681
F01_transcript_122083	NA	NA	NA	F01_transcript_122083
F01_transcript_134412	NA	NA	NA	F01_transcript_134412
F01_transcript_97502	NA	NA	NA	F01_transcript_97502
F01_transcript_149753	F01_transcript_149753	F01_transcript_149753	F01_transcript_149753	F01_transcript_149753
F01_transcript_123265	NA	NA	F01_transcript_123265	NA
F01_transcript_111742	NA	NA	NA	F01_transcript_111742
F01_transcript_134466	NA	F01_transcript_134466	F01_transcript_134466	F01_transcript_134466

F01_transcript_62098	F01_transcript_62098	F01_transcript_62098	F01_transcript_62098	F01_transcript_62098
F01_transcript_156182	NA	NA	NA	F01_transcript_156182
F01_transcript_14181	NA	NA	NA	F01_transcript_14181
F01_transcript_149913	NA	F01_transcript_149913	NA	F01_transcript_149913
F01_transcript_6651	NA	F01_transcript_6651	F01_transcript_6651	F01_transcript_6651
F01_transcript_74089	NA	F01_transcript_74089	NA	NA
F01_transcript_142209	NA	F01_transcript_142209	NA	NA
F01_transcript_150243	NA	F01_transcript_150243	NA	NA
F01_transcript_50968	NA	F01_transcript_50968	NA	NA
F01_transcript_127259	F01_transcript_127259	F01_transcript_127259	F01_transcript_127259	F01_transcript_127259
F01_transcript_93078	NA	F01_transcript_93078	NA	NA
F01_transcript_39550	NA	NA	NA	F01_transcript_39550
F01_transcript_105639	NA	F01_transcript_105639	NA	F01_transcript_105639
F01_transcript_121264	F01_transcript_121264	F01_transcript_121264	F01_transcript_121264	F01_transcript_121264

F01_transcript_62861	NA	NA	NA	F01_transcript_62861
F01_transcript_107768	NA	NA	NA	F01_transcript_107768
F01_transcript_31467	NA	NA	NA	F01_transcript_31467
F01_transcript_160641	NA	F01_transcript_160641	NA	F01_transcript_160641
F01_transcript_111721	NA	NA	NA	F01_transcript_111721
F01_transcript_120129	F01_transcript_120129	F01_transcript_120129	F01_transcript_120129	F01_transcript_120129
F01_transcript_56931	F01_transcript_56931	F01_transcript_56931	F01_transcript_56931	F01_transcript_56931
F01_transcript_87151	NA	F01_transcript_87151	F01_transcript_87151	F01_transcript_87151
F01_transcript_49038	NA	NA	F01_transcript_49038	F01_transcript_49038
F01_transcript_59797	F01_transcript_59797	F01_transcript_59797	NA	NA
F01_transcript_96193	NA	F01_transcript_96193	F01_transcript_96193	NA
F01_transcript_78349	NA	F01_transcript_78349	NA	F01_transcript_78349
F01_transcript_114572	NA	F01_transcript_114572	NA	NA
F01_transcript_29271	NA	NA	NA	F01_transcript_29271

F01_transcript_94123	NA	NA	NA	F01_transcript_94123
F01_transcript_155365	NA	F01_transcript_155365	NA	NA
F01_transcript_101945	NA	NA	NA	F01_transcript_101945
F01_transcript_68493	NA	NA	NA	F01_transcript_68493
F01_transcript_71403	NA	NA	NA	F01_transcript_71403
F01_transcript_147900	NA	F01_transcript_147900	NA	NA
F01_transcript_78821	F01_transcript_78821	F01_transcript_78821	F01_transcript_78821	NA
F01_transcript_71847	NA	NA	NA	F01_transcript_71847
F01_transcript_159593	NA	F01_transcript_159593	NA	NA
F01_transcript_38007	NA	NA	NA	F01_transcript_38007
F01_transcript_142985	NA	F01_transcript_142985	NA	NA
F01_transcript_116961	NA	F01_transcript_116961	NA	NA
F01_transcript_57026	NA	F01_transcript_57026	F01_transcript_57026	NA
F01_transcript_114591	F01_transcript_114591	F01_transcript_114591	F01_transcript_114591	F01_transcript_114591

F01_transcript_68132	NA	NA	NA	F01_transcript_68132
F01_transcript_139654	F01_transcript_139654	F01_transcript_139654	NA	F01_transcript_139654
F01_transcript_111463	NA	F01_transcript_111463	F01_transcript_111463	NA
F01_transcript_95367	F01_transcript_95367	F01_transcript_95367	F01_transcript_95367	F01_transcript_95367
F01_transcript_106908	F01_transcript_106908	F01_transcript_106908	F01_transcript_106908	F01_transcript_106908
F01_transcript_13931	NA	NA	NA	F01_transcript_13931
F01_transcript_94478	F01_transcript_94478	F01_transcript_94478	F01_transcript_94478	NA
F01_transcript_77713	NA	F01_transcript_77713	NA	NA
F01_transcript_148861	F01_transcript_148861	F01_transcript_148861	F01_transcript_148861	F01_transcript_148861
F01_transcript_146936	NA	F01_transcript_146936	NA	F01_transcript_146936
F01_transcript_52793	NA	NA	F01_transcript_52793	NA
F01_transcript_164729	F01_transcript_164729	F01_transcript_164729	F01_transcript_164729	F01_transcript_164729
F01_transcript_102779	NA	NA	NA	F01_transcript_102779
F01_transcript_105417	F01_transcript_105417	F01_transcript_105417	F01_transcript_105417	F01_transcript_105417

F01_transcript_90369	NA	NA	F01_transcript_90369	F01_transcript_90369
F01_transcript_73229	NA	NA	NA	F01_transcript_73229
F01_transcript_64705	F01_transcript_64705	F01_transcript_64705	F01_transcript_64705	NA
F01_transcript_166660	NA	F01_transcript_166660	F01_transcript_166660	F01_transcript_166660
F01_transcript_55319	NA	F01_transcript_55319	NA	NA
F01_transcript_118601	NA	NA	NA	F01_transcript_118601
F01_transcript_165119	NA	NA	NA	F01_transcript_165119
F01_transcript_141473	NA	F01_transcript_141473	NA	NA
F01_transcript_28405	NA	F01_transcript_28405	NA	F01_transcript_28405
F01_transcript_56974	NA	NA	F01_transcript_56974	NA
F01_transcript_139247	NA	NA	NA	F01_transcript_139247
F01_transcript_26376	F01_transcript_26376	F01_transcript_26376	F01_transcript_26376	F01_transcript_26376
F01_transcript_166769	NA	NA	NA	F01_transcript_166769
F01_transcript_152530	F01_transcript_152530	F01_transcript_152530	F01_transcript_152530	F01_transcript_152530

F01_transcript_56854	F01_transcript_56854	F01_transcript_56854	F01_transcript_56854	F01_transcript_56854
F01_transcript_120906	NA	F01_transcript_120906	NA	NA
F01_transcript_50462	F01_transcript_50462	F01_transcript_50462	F01_transcript_50462	F01_transcript_50462
F01_transcript_11149	NA	F01_transcript_11149	NA	NA
F01_transcript_78567	NA	F01_transcript_78567	NA	NA
F01_transcript_65378	NA	NA	F01_transcript_65378	F01_transcript_65378
F01_transcript_157363	NA	F01_transcript_157363	NA	NA
F01_transcript_71213	NA	F01_transcript_71213	F01_transcript_71213	F01_transcript_71213
F01_transcript_90050	NA	NA	F01_transcript_90050	F01_transcript_90050
F01_transcript_115872	F01_transcript_115872	F01_transcript_115872	F01_transcript_115872	F01_transcript_115872
F01_transcript_161916	NA	F01_transcript_161916	NA	NA
F01_transcript_74321	NA	NA	NA	F01_transcript_74321
F01_transcript_28969	NA	NA	NA	F01_transcript_28969
F01_transcript_152910	F01_transcript_152910	F01_transcript_152910	NA	F01_transcript_152910

F01_transcript_88736	NA	NA	NA	F01_transcript_88736
F01_transcript_136571	NA	NA	NA	F01_transcript_136571
F01_transcript_82186	F01_transcript_82186	F01_transcript_82186	F01_transcript_82186	NA
F01_transcript_129671	NA	F01_transcript_129671	NA	NA
F01_transcript_96977	F01_transcript_96977	F01_transcript_96977	NA	NA
F01_transcript_47510	NA	NA	NA	F01_transcript_47510
F01_transcript_139114	NA	F01_transcript_139114	NA	NA
F01_transcript_157705	F01_transcript_157705	NA	F01_transcript_157705	F01_transcript_157705
F01_transcript_131796	NA	F01_transcript_131796	NA	NA
F01_transcript_58819	F01_transcript_58819	F01_transcript_58819	F01_transcript_58819	F01_transcript_58819
F01_transcript_95621	NA	F01_transcript_95621	F01_transcript_95621	F01_transcript_95621
F01_transcript_21537	NA	NA	NA	F01_transcript_21537
F01_transcript_78809	NA	NA	NA	F01_transcript_78809
F01_transcript_32599	NA	NA	NA	F01_transcript_32599

F01_transcript_153073	NA	NA	NA	F01_transcript_153073
F01_transcript_139393	NA	NA	NA	F01_transcript_139393
F01_transcript_128897	NA	NA	NA	F01_transcript_128897
F01_transcript_69401	F01_transcript_69401	F01_transcript_69401	F01_transcript_69401	NA
F01_transcript_141281	NA	NA	NA	F01_transcript_141281
F01_transcript_88536	F01_transcript_88536	F01_transcript_88536	F01_transcript_88536	F01_transcript_88536
F01_transcript_153666	NA	F01_transcript_153666	NA	NA
F01_transcript_129422	F01_transcript_129422	F01_transcript_129422	F01_transcript_129422	F01_transcript_129422
F01_transcript_111254	F01_transcript_111254	F01_transcript_111254	F01_transcript_111254	F01_transcript_111254
F01_transcript_115598	F01_transcript_115598	F01_transcript_115598	F01_transcript_115598	F01_transcript_115598
F01_transcript_108364	F01_transcript_108364	F01_transcript_108364	F01_transcript_108364	F01_transcript_108364
F01_transcript_75740	F01_transcript_75740	F01_transcript_75740	F01_transcript_75740	NA
F01_transcript_99304	NA	NA	NA	F01_transcript_99304
F01_transcript_162741	NA	F01_transcript_162741	NA	NA

F01_transcript_150893	NA	F01_transcript_150893	NA	NA
F01_transcript_67365	F01_transcript_67365	F01_transcript_67365	F01_transcript_67365	F01_transcript_67365
F01_transcript_125406	NA	NA	NA	F01_transcript_125406
F01_transcript_141761	F01_transcript_141761	F01_transcript_141761	F01_transcript_141761	F01_transcript_141761
F01_transcript_56564	F01_transcript_56564	F01_transcript_56564	F01_transcript_56564	F01_transcript_56564
F01_transcript_111438	NA	NA	F01_transcript_111438	F01_transcript_111438
F01_transcript_113396	F01_transcript_113396	F01_transcript_113396	F01_transcript_113396	F01_transcript_113396
F01_transcript_71962	NA	F01_transcript_71962	NA	NA
F01_transcript_88539	NA	NA	NA	F01_transcript_88539
F01_transcript_134325	NA	NA	F01_transcript_134325	F01_transcript_134325
F01_transcript_143094	NA	NA	NA	F01_transcript_143094
F01_transcript_147886	F01_transcript_147886	F01_transcript_147886	F01_transcript_147886	F01_transcript_147886
F01_transcript_118395	NA	NA	NA	F01_transcript_118395
F01_transcript_97899	F01_transcript_97899	F01_transcript_97899	F01_transcript_97899	F01_transcript_97899

F01_transcript_66252	F01_transcript_66252	F01_transcript_66252	F01_transcript_66252	F01_transcript_66252
F01_transcript_114088	NA	F01_transcript_114088	NA	NA
F01_transcript_102332	F01_transcript_102332	F01_transcript_102332	F01_transcript_102332	F01_transcript_102332
F01_transcript_148986	NA	F01_transcript_148986	NA	F01_transcript_148986
F01_transcript_160432	NA	F01_transcript_160432	NA	NA
F01_transcript_132314	NA	NA	NA	F01_transcript_132314
F01_transcript_139476	NA	F01_transcript_139476	NA	F01_transcript_139476
F01_transcript_154416	NA	F01_transcript_154416	NA	NA
F01_transcript_78682	NA	F01_transcript_78682	NA	NA
F01_transcript_70183	NA	F01_transcript_70183	NA	NA
F01_transcript_75695	F01_transcript_75695	F01_transcript_75695	F01_transcript_75695	F01_transcript_75695
F01_transcript_62842	NA	F01_transcript_62842	F01_transcript_62842	NA
F01_transcript_156255	NA	NA	NA	F01_transcript_156255
F01_transcript_65281	NA	F01_transcript_65281	F01_transcript_65281	NA

F01_transcript_125041	NA	F01_transcript_125041	F01_transcript_125041	NA
F01_transcript_150151	F01_transcript_150151	F01_transcript_150151	NA	NA
F01_transcript_39542	NA	F01_transcript_39542	NA	NA
F01_transcript_97718	F01_transcript_97718	F01_transcript_97718	NA	F01_transcript_97718
F01_transcript_2185	NA	NA	NA	F01_transcript_2185
F01_transcript_112217	F01_transcript_112217	F01_transcript_112217	NA	NA
F01_transcript_148150	NA	F01_transcript_148150	NA	NA
F01_transcript_104172	F01_transcript_104172	F01_transcript_104172	NA	NA
F01_transcript_57816	NA	F01_transcript_57816	NA	F01_transcript_57816
F01_transcript_144460	NA	F01_transcript_144460	F01_transcript_144460	F01_transcript_144460
F01_transcript_137086	NA	F01_transcript_137086	NA	NA
F01_transcript_56359	NA	F01_transcript_56359	F01_transcript_56359	NA
F01_transcript_114843	F01_transcript_114843	F01_transcript_114843	F01_transcript_114843	F01_transcript_114843
F01_transcript_144163	NA	NA	NA	F01_transcript_144163

F01_transcript_117054	NA	F01_transcript_117054	NA	NA
F01_transcript_45674	NA	NA	NA	F01_transcript_45674
F01_transcript_116374	F01_transcript_116374	F01_transcript_116374	F01_transcript_116374	NA
F01_transcript_162449	NA	F01_transcript_162449	NA	F01_transcript_162449
F01_transcript_87716	F01_transcript_87716	F01_transcript_87716	F01_transcript_87716	F01_transcript_87716
F01_transcript_88244	NA	NA	NA	F01_transcript_88244
F01_transcript_56347	F01_transcript_56347	F01_transcript_56347	F01_transcript_56347	F01_transcript_56347
F01_transcript_56275	NA	NA	NA	F01_transcript_56275
F01_transcript_93703	F01_transcript_93703	F01_transcript_93703	F01_transcript_93703	F01_transcript_93703
F01_transcript_138607	F01_transcript_138607	F01_transcript_138607	NA	F01_transcript_138607
F01_transcript_63232	NA	NA	NA	F01_transcript_63232
F01_transcript_141064	F01_transcript_141064	F01_transcript_141064	NA	NA
F01_transcript_41227	NA	NA	NA	F01_transcript_41227
F01_transcript_146518	NA	F01_transcript_146518	NA	NA

F01_transcript_6725	F01_transcript_6725	F01_transcript_6725	F01_transcript_6725	F01_transcript_6725
F01_transcript_163494	NA	NA	NA	F01_transcript_163494
F01_transcript_31110	NA	NA	NA	F01_transcript_31110
F01_transcript_125246	NA	F01_transcript_125246	NA	NA
F01_transcript_166533	NA	F01_transcript_166533	NA	NA
F01_transcript_21438	NA	F01_transcript_21438	NA	NA
F01_transcript_56521	NA	F01_transcript_56521	F01_transcript_56521	F01_transcript_56521
F01_transcript_1220	NA	NA	NA	F01_transcript_1220
F01_transcript_130288	NA	F01_transcript_130288	NA	NA
F01_transcript_145143	NA	NA	NA	F01_transcript_145143
F01_transcript_147813	NA	NA	NA	F01_transcript_147813
F01_transcript_49469	NA	F01_transcript_49469	NA	F01_transcript_49469
F01_transcript_129374	NA	F01_transcript_129374	NA	F01_transcript_129374
F01_transcript_109001	NA	NA	F01_transcript_109001	F01_transcript_109001

F01_transcript_64727	F01_transcript_64727	F01_transcript_64727	F01_transcript_64727	F01_transcript_64727
F01_transcript_109589	F01_transcript_109589	F01_transcript_109589	F01_transcript_109589	F01_transcript_109589
F01_transcript_122315	NA	F01_transcript_122315	NA	NA
F01_transcript_140148	NA	F01_transcript_140148	NA	F01_transcript_140148
F01_transcript_55611	NA	NA	NA	F01_transcript_55611
F01_transcript_110687	NA	NA	NA	F01_transcript_110687
F01_transcript_105963	NA	F01_transcript_105963	NA	F01_transcript_105963
F01_transcript_145942	F01_transcript_145942	F01_transcript_145942	F01_transcript_145942	F01_transcript_145942
F01_transcript_51040	F01_transcript_51040	F01_transcript_51040	F01_transcript_51040	F01_transcript_51040
F01_transcript_123239	NA	F01_transcript_123239	NA	NA
F01_transcript_156079	F01_transcript_156079	F01_transcript_156079	F01_transcript_156079	F01_transcript_156079
F01_transcript_51480	F01_transcript_51480	F01_transcript_51480	F01_transcript_51480	NA
F01_transcript_73777	F01_transcript_73777	F01_transcript_73777	F01_transcript_73777	F01_transcript_73777
F01_transcript_115773	F01_transcript_115773	F01_transcript_115773	F01_transcript_115773	F01_transcript_115773

F01_transcript_145402	NA	NA	NA	F01_transcript_145402
F01_transcript_156493	NA	F01_transcript_156493	NA	NA
F01_transcript_77313	NA	F01_transcript_77313	NA	NA
F01_transcript_98587	NA	NA	NA	F01_transcript_98587
F01_transcript_152251	NA	NA	NA	F01_transcript_152251
F01_transcript_62443	NA	F01_transcript_62443	F01_transcript_62443	F01_transcript_62443
F01_transcript_56306	NA	NA	F01_transcript_56306	F01_transcript_56306
F01_transcript_148042	NA	F01_transcript_148042	NA	NA
F01_transcript_74822	NA	NA	NA	F01_transcript_74822
F01_transcript_90766	NA	F01_transcript_90766	NA	F01_transcript_90766
F01_transcript_152945	F01_transcript_152945	F01_transcript_152945	F01_transcript_152945	F01_transcript_152945
F01_transcript_147203	NA	F01_transcript_147203	F01_transcript_147203	F01_transcript_147203
F01_transcript_62138	F01_transcript_62138	F01_transcript_62138	NA	F01_transcript_62138
F01_transcript_102213	NA	NA	NA	F01_transcript_102213

F01_transcript_142506	NA	F01_transcript_142506	NA	NA
F01_transcript_73240	F01_transcript_73240	F01_transcript_73240	F01_transcript_73240	F01_transcript_73240
F01_transcript_68670	NA	F01_transcript_68670	F01_transcript_68670	F01_transcript_68670
F01_transcript_123369	NA	F01_transcript_123369	NA	NA
F01_transcript_54778	NA	NA	NA	F01_transcript_54778
F01_transcript_55870	F01_transcript_55870	F01_transcript_55870	F01_transcript_55870	F01_transcript_55870
F01_transcript_98420	F01_transcript_98420	F01_transcript_98420	F01_transcript_98420	F01_transcript_98420
F01_transcript_116744	NA	NA	NA	F01_transcript_116744
F01_transcript_26922	NA	NA	F01_transcript_26922	F01_transcript_26922
F01_transcript_116644	NA	NA	NA	F01_transcript_116644
F01_transcript_150327	F01_transcript_150327	F01_transcript_150327	F01_transcript_150327	F01_transcript_150327
F01_transcript_24160	NA	F01_transcript_24160	NA	F01_transcript_24160
F01_transcript_153476	F01_transcript_153476	F01_transcript_153476	F01_transcript_153476	F01_transcript_153476
F01_transcript_94500	NA	NA	NA	F01_transcript_94500

F01_transcript_147437	NA	F01_transcript_147437	NA	NA
F01_transcript_155524	NA	NA	NA	F01_transcript_155524
F01_transcript_56885	F01_transcript_56885	F01_transcript_56885	F01_transcript_56885	F01_transcript_56885
F01_transcript_78799	NA	NA	NA	F01_transcript_78799
F01_transcript_77591	F01_transcript_77591	F01_transcript_77591	NA	F01_transcript_77591
F01_transcript_26561	NA	F01_transcript_26561	NA	NA
F01_transcript_104461	NA	NA	NA	F01_transcript_104461
F01_transcript_65497	NA	NA	F01_transcript_65497	F01_transcript_65497
F01_transcript_85475	F01_transcript_85475	F01_transcript_85475	F01_transcript_85475	F01_transcript_85475
F01_transcript_90025	F01_transcript_90025	F01_transcript_90025	NA	F01_transcript_90025
F01_transcript_75429	NA	NA	F01_transcript_75429	NA
F01_transcript_131523	NA	F01_transcript_131523	NA	NA
F01_transcript_86887	NA	F01_transcript_86887	NA	NA
F01_transcript_136201	NA	F01_transcript_136201	F01_transcript_136201	F01_transcript_136201

F01_transcript_166394	NA	NA	NA	F01_transcript_166394
F01_transcript_122263	F01_transcript_122263	F01_transcript_122263	F01_transcript_122263	NA
F01_transcript_91241	F01_transcript_91241	F01_transcript_91241	F01_transcript_91241	F01_transcript_91241
F01_transcript_125614	NA	F01_transcript_125614	NA	NA
F01_transcript_572	NA	NA	NA	F01_transcript_572
F01_transcript_112324	NA	NA	NA	F01_transcript_112324
F01_transcript_132251	NA	F01_transcript_132251	NA	F01_transcript_132251
F01_transcript_24849	NA	NA	NA	F01_transcript_24849
F01_transcript_8699	NA	NA	NA	F01_transcript_8699
F01_transcript_52361	NA	NA	NA	F01_transcript_52361
F01_transcript_117689	NA	F01_transcript_117689	NA	NA
F01_transcript_50242	F01_transcript_50242	F01_transcript_50242	F01_transcript_50242	F01_transcript_50242
F01_transcript_150818	NA	NA	NA	F01_transcript_150818
F01_transcript_129859	NA	F01_transcript_129859	F01_transcript_129859	F01_transcript_129859

F01_transcript_108999	F01_transcript_108999	F01_transcript_108999	F01_transcript_108999	F01_transcript_108999
F01_transcript_45130	NA	NA	NA	F01_transcript_45130
F01_transcript_115768	NA	F01_transcript_115768	NA	NA
F01_transcript_47581	NA	NA	NA	F01_transcript_47581
F01_transcript_162905	F01_transcript_162905	F01_transcript_162905	NA	F01_transcript_162905
F01_transcript_90462	NA	NA	NA	F01_transcript_90462
F01_transcript_56537	NA	F01_transcript_56537	F01_transcript_56537	F01_transcript_56537
F01_transcript_134297	NA	F01_transcript_134297	NA	F01_transcript_134297
F01_transcript_49803	F01_transcript_49803	F01_transcript_49803	NA	NA
F01_transcript_72620	F01_transcript_72620	F01_transcript_72620	F01_transcript_72620	F01_transcript_72620
F01_transcript_121557	NA	F01_transcript_121557	NA	NA
F01_transcript_147978	F01_transcript_147978	F01_transcript_147978	F01_transcript_147978	F01_transcript_147978
F01_transcript_147962	F01_transcript_147962	F01_transcript_147962	F01_transcript_147962	F01_transcript_147962
F01_transcript_142690	NA	F01_transcript_142690	F01_transcript_142690	F01_transcript_142690

F01_transcript_127212	NA	F01_transcript_127212	F01_transcript_127212	F01_transcript_127212
F01_transcript_150116	NA	F01_transcript_150116	F01_transcript_150116	F01_transcript_150116
F01_transcript_104075	NA	NA	NA	F01_transcript_104075
F01_transcript_92935	NA	F01_transcript_92935	NA	F01_transcript_92935
F01_transcript_78864	NA	F01_transcript_78864	NA	F01_transcript_78864
F01_transcript_70922	F01_transcript_70922	F01_transcript_70922	F01_transcript_70922	F01_transcript_70922
F01_transcript_112322	NA	NA	NA	F01_transcript_112322
F01_transcript_161029	NA	NA	NA	F01_transcript_161029
F01_transcript_27198	NA	NA	NA	F01_transcript_27198
F01_transcript_130620	NA	NA	NA	F01_transcript_130620
F01_transcript_78565	NA	NA	NA	F01_transcript_78565
F01_transcript_131958	NA	F01_transcript_131958	NA	NA
F01_transcript_118479	F01_transcript_118479	F01_transcript_118479	NA	F01_transcript_118479
F01_transcript_139031	NA	NA	NA	F01_transcript_139031

F01_transcript_58970	NA	NA	NA	F01_transcript_58970
F01_transcript_165266	NA	F01_transcript_165266	F01_transcript_165266	NA
F01_transcript_30844	NA	NA	NA	F01_transcript_30844
F01_transcript_139595	NA	F01_transcript_139595	NA	NA
F01_transcript_50911	F01_transcript_50911	NA	NA	NA
F01_transcript_52404	NA	F01_transcript_52404	F01_transcript_52404	F01_transcript_52404
F01_transcript_114895	NA	NA	NA	F01_transcript_114895
F01_transcript_103587	NA	NA	F01_transcript_103587	F01_transcript_103587
F01_transcript_88327	NA	F01_transcript_88327	NA	F01_transcript_88327
F01_transcript_166136	NA	NA	NA	F01_transcript_166136
F01_transcript_87262	NA	F01_transcript_87262	NA	NA
F01_transcript_50467	NA	F01_transcript_50467	NA	F01_transcript_50467
F01_transcript_13374	NA	F01_transcript_13374	NA	NA
F01_transcript_124394	NA	F01_transcript_124394	NA	F01_transcript_124394

F01_transcript_130073	NA	NA	NA	F01_transcript_130073
F01_transcript_86962	NA	NA	NA	F01_transcript_86962
F01_transcript_14880	NA	NA	NA	F01_transcript_14880
F01_transcript_68296	NA	F01_transcript_68296	NA	F01_transcript_68296
F01_transcript_87597	NA	F01_transcript_87597	NA	NA
F01_transcript_149981	NA	NA	NA	F01_transcript_149981
F01_transcript_85720	F01_transcript_85720	F01_transcript_85720	F01_transcript_85720	F01_transcript_85720
F01_transcript_127520	NA	F01_transcript_127520	NA	NA
F01_transcript_142023	NA	F01_transcript_142023	NA	NA
F01_transcript_81177	NA	F01_transcript_81177	NA	NA
F01_transcript_111676	F01_transcript_111676	F01_transcript_111676	F01_transcript_111676	F01_transcript_111676
F01_transcript_71122	NA	F01_transcript_71122	F01_transcript_71122	NA
F01_transcript_100412	F01_transcript_100412	F01_transcript_100412	NA	F01_transcript_100412
F01_transcript_167198	F01_transcript_167198	F01_transcript_167198	F01_transcript_167198	F01_transcript_167198

F01_transcript_158584	NA	NA	NA	F01_transcript_158584
F01_transcript_65222	NA	NA	F01_transcript_65222	NA
F01_transcript_70660	NA	NA	NA	F01_transcript_70660
F01_transcript_102614	F01_transcript_102614	F01_transcript_102614	F01_transcript_102614	F01_transcript_102614
F01_transcript_148792	NA	F01_transcript_148792	NA	NA
F01_transcript_63518	F01_transcript_63518	F01_transcript_63518	F01_transcript_63518	F01_transcript_63518
F01_transcript_88140	NA	F01_transcript_88140	NA	NA
F01_transcript_131289	NA	F01_transcript_131289	NA	NA
F01_transcript_146955	NA	F01_transcript_146955	NA	F01_transcript_146955
F01_transcript_130996	NA	F01_transcript_130996	NA	NA
F01_transcript_95234	F01_transcript_95234	F01_transcript_95234	F01_transcript_95234	F01_transcript_95234
F01_transcript_51859	NA	NA	NA	F01_transcript_51859
F01_transcript_143232	NA	F01_transcript_143232	NA	NA
F01_transcript_157054	F01_transcript_157054	F01_transcript_157054	F01_transcript_157054	F01_transcript_157054

F01_transcript_10524	NA	NA	NA	F01_transcript_10524
F01_transcript_133056	NA	NA	NA	F01_transcript_133056
F01_transcript_97181	NA	F01_transcript_97181	NA	F01_transcript_97181
F01_transcript_59741	NA	NA	NA	F01_transcript_59741
F01_transcript_157781	F01_transcript_157781	F01_transcript_157781	F01_transcript_157781	F01_transcript_157781
F01_transcript_158575	NA	F01_transcript_158575	NA	NA
F01_transcript_109075	NA	F01_transcript_109075	NA	F01_transcript_109075
F01_transcript_94327	NA	F01_transcript_94327	NA	F01_transcript_94327
F01_transcript_84247	NA	F01_transcript_84247	NA	NA
F01_transcript_166633	F01_transcript_166633	F01_transcript_166633	F01_transcript_166633	F01_transcript_166633
F01_transcript_122216	NA	F01_transcript_122216	NA	NA
F01_transcript_131713	NA	F01_transcript_131713	NA	NA
F01_transcript_130079	NA	NA	NA	F01_transcript_130079
F01_transcript_48815	NA	F01_transcript_48815	NA	NA

F01_transcript_86956	NA	NA	NA	F01_transcript_86956
F01_transcript_148188	F01_transcript_148188	F01_transcript_148188	F01_transcript_148188	F01_transcript_148188
F01_transcript_55255	F01_transcript_55255	F01_transcript_55255	F01_transcript_55255	F01_transcript_55255
F01_transcript_59690	NA	NA	NA	F01_transcript_59690
F01_transcript_119867	NA	NA	NA	F01_transcript_119867
F01_transcript_91988	F01_transcript_91988	F01_transcript_91988	F01_transcript_91988	F01_transcript_91988
F01_transcript_126160	NA	F01_transcript_126160	F01_transcript_126160	NA
F01_transcript_124710	NA	NA	NA	F01_transcript_124710
F01_transcript_106645	NA	F01_transcript_106645	NA	F01_transcript_106645
F01_transcript_104651	F01_transcript_104651	F01_transcript_104651	F01_transcript_104651	F01_transcript_104651
F01_transcript_63877	F01_transcript_63877	F01_transcript_63877	F01_transcript_63877	F01_transcript_63877
F01_transcript_132759	NA	NA	NA	F01_transcript_132759
F01_transcript_107628	NA	F01_transcript_107628	NA	NA
F01_transcript_149122	F01_transcript_149122	F01_transcript_149122	F01_transcript_149122	F01_transcript_149122

F01_transcript_158250	NA	F01_transcript_158250	F01_transcript_158250	NA
F01_transcript_74873	NA	F01_transcript_74873	NA	NA
F01_transcript_111276	NA	F01_transcript_111276	F01_transcript_111276	NA
F01_transcript_91045	F01_transcript_91045	F01_transcript_91045	F01_transcript_91045	F01_transcript_91045
F01_transcript_127415	F01_transcript_127415	F01_transcript_127415	NA	NA
F01_transcript_145900	NA	F01_transcript_145900	NA	NA
F01_transcript_129813	F01_transcript_129813	F01_transcript_129813	F01_transcript_129813	F01_transcript_129813
F01_transcript_109971	NA	NA	NA	F01_transcript_109971
F01_transcript_82	NA	NA	NA	F01_transcript_82
F01_transcript_50710	NA	F01_transcript_50710	F01_transcript_50710	F01_transcript_50710
F01_transcript_62997	NA	NA	NA	F01_transcript_62997
F01_transcript_63040	NA	NA	NA	F01_transcript_63040
F01_transcript_101039	F01_transcript_101039	F01_transcript_101039	F01_transcript_101039	F01_transcript_101039
F01_transcript_162453	F01_transcript_162453	F01_transcript_162453	F01_transcript_162453	F01_transcript_162453

F01_transcript_151221	NA	NA	NA	F01_transcript_151221
F01_transcript_50372	NA	F01_transcript_50372	F01_transcript_50372	NA
F01_transcript_150255	F01_transcript_150255	F01_transcript_150255	NA	NA
F01_transcript_91091	NA	F01_transcript_91091	NA	NA
F01_transcript_137472	NA	NA	NA	F01_transcript_137472
F01_transcript_146969	F01_transcript_146969	F01_transcript_146969	NA	F01_transcript_146969
F01_transcript_52800	NA	F01_transcript_52800	NA	NA
F01_transcript_60207	NA	F01_transcript_60207	NA	F01_transcript_60207
F01_transcript_59248	F01_transcript_59248	F01_transcript_59248	NA	F01_transcript_59248
F01_transcript_56587	NA	F01_transcript_56587	F01_transcript_56587	NA
F01_transcript_59647	F01_transcript_59647	F01_transcript_59647	F01_transcript_59647	F01_transcript_59647
F01_transcript_90378	NA	NA	F01_transcript_90378	F01_transcript_90378
F01_transcript_91682	NA	F01_transcript_91682	NA	F01_transcript_91682
F01_transcript_158491	F01_transcript_158491	F01_transcript_158491	F01_transcript_158491	F01_transcript_158491

F01_transcript_26593	NA	NA	NA	F01_transcript_26593
F01_transcript_81285	NA	NA	NA	F01_transcript_81285
F01_transcript_90831	F01_transcript_90831	F01_transcript_90831	F01_transcript_90831	F01_transcript_90831
F01_transcript_75306	F01_transcript_75306	F01_transcript_75306	NA	F01_transcript_75306
F01_transcript_91423	NA	NA	NA	F01_transcript_91423
F01_transcript_69573	F01_transcript_69573	F01_transcript_69573	NA	F01_transcript_69573
F01_transcript_138811	NA	NA	NA	F01_transcript_138811
F01_transcript_143741	NA	NA	NA	F01_transcript_143741
F01_transcript_66822	F01_transcript_66822	F01_transcript_66822	F01_transcript_66822	F01_transcript_66822
F01_transcript_102260	NA	NA	NA	F01_transcript_102260
F01_transcript_124643	NA	F01_transcript_124643	NA	NA
F01_transcript_144335	NA	F01_transcript_144335	NA	NA
F01_transcript_41693	NA	F01_transcript_41693	NA	NA
F01_transcript_45535	NA	NA	NA	F01_transcript_45535

F01_transcript_126887	F01_transcript_126887	F01_transcript_126887	NA	F01_transcript_126887
F01_transcript_145532	NA	NA	NA	F01_transcript_145532
F01_transcript_131769	NA	NA	NA	F01_transcript_131769
F01_transcript_56404	NA	NA	NA	F01_transcript_56404
F01_transcript_77390	F01_transcript_77390	F01_transcript_77390	F01_transcript_77390	F01_transcript_77390
F01_transcript_55412	NA	NA	NA	F01_transcript_55412
F01_transcript_120750	F01_transcript_120750	F01_transcript_120750	F01_transcript_120750	NA
F01_transcript_75201	F01_transcript_75201	F01_transcript_75201	F01_transcript_75201	F01_transcript_75201
F01_transcript_156300	F01_transcript_156300	F01_transcript_156300	F01_transcript_156300	F01_transcript_156300
F01_transcript_107721	NA	NA	NA	F01_transcript_107721
F01_transcript_130598	NA	NA	NA	F01_transcript_130598
F01_transcript_145091	NA	F01_transcript_145091	NA	F01_transcript_145091
F01_transcript_105413	F01_transcript_105413	NA	F01_transcript_105413	F01_transcript_105413
F01_transcript_130012	NA	NA	NA	F01_transcript_130012

F01_transcript_49851	F01_transcript_49851	F01_transcript_49851	NA	F01_transcript_49851
F01_transcript_142622	NA	NA	NA	F01_transcript_142622
F01_transcript_98182	NA	F01_transcript_98182	NA	F01_transcript_98182
F01_transcript_156920	NA	F01_transcript_156920	NA	NA
F01_transcript_133081	NA	NA	NA	F01_transcript_133081
F01_transcript_145045	NA	NA	NA	F01_transcript_145045
F01_transcript_86001	NA	F01_transcript_86001	NA	F01_transcript_86001
F01_transcript_88599	NA	NA	NA	F01_transcript_88599
F01_transcript_134646	F01_transcript_134646	F01_transcript_134646	F01_transcript_134646	F01_transcript_134646
F01_transcript_167064	NA	F01_transcript_167064	F01_transcript_167064	F01_transcript_167064
F01_transcript_125129	NA	F01_transcript_125129	NA	F01_transcript_125129
F01_transcript_54362	NA	NA	NA	F01_transcript_54362
F01_transcript_166415	NA	F01_transcript_166415	NA	F01_transcript_166415
F01_transcript_106149	NA	F01_transcript_106149	NA	NA

F01_transcript_161204	NA	F01_transcript_161204	F01_transcript_161204	NA
F01_transcript_51268	F01_transcript_51268	F01_transcript_51268	F01_transcript_51268	F01_transcript_51268
F01_transcript_48884	NA	F01_transcript_48884	F01_transcript_48884	F01_transcript_48884
F01_transcript_112043	NA	NA	NA	F01_transcript_112043
F01_transcript_124468	NA	F01_transcript_124468	NA	NA
F01_transcript_64410	NA	F01_transcript_64410	NA	F01_transcript_64410
F01_transcript_71099	NA	F01_transcript_71099	F01_transcript_71099	F01_transcript_71099
F01_transcript_119172	F01_transcript_119172	F01_transcript_119172	F01_transcript_119172	F01_transcript_119172
F01_transcript_152437	NA	F01_transcript_152437	NA	NA
F01_transcript_57002	NA	NA	F01_transcript_57002	NA
F01_transcript_110685	F01_transcript_110685	F01_transcript_110685	F01_transcript_110685	F01_transcript_110685
F01_transcript_93775	NA	NA	NA	F01_transcript_93775
F01_transcript_53911	F01_transcript_53911	F01_transcript_53911	F01_transcript_53911	F01_transcript_53911
F01_transcript_71960	F01_transcript_71960	F01_transcript_71960	NA	F01_transcript_71960

F01_transcript_66028	NA	NA	NA	F01_transcript_66028
F01_transcript_5283	NA	NA	NA	F01_transcript_5283
F01_transcript_111850	NA	NA	NA	F01_transcript_111850
F01_transcript_81165	F01_transcript_81165	F01_transcript_81165	F01_transcript_81165	F01_transcript_81165
F01_transcript_135287	NA	NA	NA	F01_transcript_135287
F01_transcript_123982	NA	F01_transcript_123982	F01_transcript_123982	F01_transcript_123982
F01_transcript_78640	NA	F01_transcript_78640	F01_transcript_78640	F01_transcript_78640
F01_transcript_137257	NA	NA	NA	F01_transcript_137257
F01_transcript_125205	F01_transcript_125205	NA	NA	NA
F01_transcript_75614	F01_transcript_75614	F01_transcript_75614	F01_transcript_75614	F01_transcript_75614
F01_transcript_29950	F01_transcript_29950	F01_transcript_29950	F01_transcript_29950	F01_transcript_29950
F01_transcript_91624	F01_transcript_91624	F01_transcript_91624	F01_transcript_91624	F01_transcript_91624
F01_transcript_96278	NA	NA	NA	F01_transcript_96278
F01_transcript_136363	NA	F01_transcript_136363	F01_transcript_136363	F01_transcript_136363

F01_transcript_157693	F01_transcript_157693	F01_transcript_157693	F01_transcript_157693	F01_transcript_157693
F01_transcript_65020	NA	F01_transcript_65020	NA	NA
F01_transcript_103091	NA	NA	NA	F01_transcript_103091
F01_transcript_96624	F01_transcript_96624	F01_transcript_96624	NA	F01_transcript_96624
F01_transcript_82201	NA	NA	NA	F01_transcript_82201
F01_transcript_114862	NA	NA	NA	F01_transcript_114862
F01_transcript_77732	NA	NA	NA	F01_transcript_77732
F01_transcript_83636	NA	F01_transcript_83636	F01_transcript_83636	F01_transcript_83636
F01_transcript_118727	NA	NA	NA	F01_transcript_118727
F01_transcript_91945	F01_transcript_91945	F01_transcript_91945	F01_transcript_91945	F01_transcript_91945
F01_transcript_100231	NA	F01_transcript_100231	F01_transcript_100231	NA
F01_transcript_148601	F01_transcript_148601	F01_transcript_148601	F01_transcript_148601	F01_transcript_148601
F01_transcript_137552	NA	F01_transcript_137552	NA	NA
F01_transcript_97295	F01_transcript_97295	F01_transcript_97295	F01_transcript_97295	F01_transcript_97295

F01_transcript_72953	F01_transcript_72953	F01_transcript_72953	F01_transcript_72953	F01_transcript_72953
F01_transcript_55722	NA	F01_transcript_55722	NA	F01_transcript_55722
F01_transcript_24312	F01_transcript_24312	F01_transcript_24312	NA	F01_transcript_24312
F01_transcript_121346	NA	F01_transcript_121346	NA	NA
F01_transcript_76336	F01_transcript_76336	F01_transcript_76336	NA	NA
F01_transcript_95502	NA	F01_transcript_95502	NA	F01_transcript_95502
F01_transcript_118599	NA	NA	NA	F01_transcript_118599
F01_transcript_98063	NA	NA	NA	F01_transcript_98063
F01_transcript_135328	NA	NA	NA	F01_transcript_135328
F01_transcript_56524	NA	NA	F01_transcript_56524	NA
F01_transcript_89329	F01_transcript_89329	F01_transcript_89329	F01_transcript_89329	F01_transcript_89329
F01_transcript_124102	NA	F01_transcript_124102	NA	NA
F01_transcript_150402	NA	NA	NA	F01_transcript_150402
F01_transcript_85071	NA	NA	NA	F01_transcript_85071

F01_transcript_73251	F01_transcript_73251	F01_transcript_73251	F01_transcript_73251	F01_transcript_73251
F01_transcript_157386	F01_transcript_157386	F01_transcript_157386	F01_transcript_157386	F01_transcript_157386
F01_transcript_34139	NA	NA	NA	F01_transcript_34139
F01_transcript_32777	F01_transcript_32777	F01_transcript_32777	F01_transcript_32777	F01_transcript_32777
F01_transcript_139619	NA	NA	NA	F01_transcript_139619
F01_transcript_101528	NA	F01_transcript_101528	NA	NA
F01_transcript_48924	NA	NA	F01_transcript_48924	F01_transcript_48924
F01_transcript_145002	NA	NA	NA	F01_transcript_145002
F01_transcript_163061	F01_transcript_163061	F01_transcript_163061	F01_transcript_163061	F01_transcript_163061
F01_transcript_148484	NA	F01_transcript_148484	F01_transcript_148484	F01_transcript_148484
F01_transcript_67130	F01_transcript_67130	F01_transcript_67130	F01_transcript_67130	F01_transcript_67130
F01_transcript_47543	NA	F01_transcript_47543	NA	NA
F01_transcript_50913	F01_transcript_50913	F01_transcript_50913	F01_transcript_50913	F01_transcript_50913
F01_transcript_159958	NA	NA	NA	F01_transcript_159958

F01_transcript_136125	F01_transcript_136125	F01_transcript_136125	F01_transcript_136125	F01_transcript_136125
F01_transcript_13011	NA	F01_transcript_13011	NA	NA
F01_transcript_89569	NA	F01_transcript_89569	NA	NA
F01_transcript_153935	F01_transcript_153935	F01_transcript_153935	NA	NA
F01_transcript_20359	NA	NA	NA	F01_transcript_20359
F01_transcript_63568	NA	NA	F01_transcript_63568	NA
F01_transcript_37191	NA	F01_transcript_37191	NA	NA
F01_transcript_38185	NA	NA	NA	F01_transcript_38185
F01_transcript_59540	NA	NA	F01_transcript_59540	F01_transcript_59540
F01_transcript_152217	NA	NA	F01_transcript_152217	NA
F01_transcript_147213	NA	F01_transcript_147213	F01_transcript_147213	NA
F01_transcript_52771	NA	F01_transcript_52771	NA	NA
F01_transcript_100769	NA	F01_transcript_100769	NA	NA
F01_transcript_109293	NA	NA	NA	F01_transcript_109293

F01_transcript_70036	F01_transcript_70036	F01_transcript_70036	NA	NA
F01_transcript_116107	F01_transcript_116107	F01_transcript_116107	NA	F01_transcript_116107
F01_transcript_144095	F01_transcript_144095	F01_transcript_144095	NA	NA
F01_transcript_131908	NA	F01_transcript_131908	NA	F01_transcript_131908
F01_transcript_149540	F01_transcript_149540	F01_transcript_149540	F01_transcript_149540	NA
F01_transcript_58357	NA	NA	NA	F01_transcript_58357
F01_transcript_71841	NA	F01_transcript_71841	NA	NA
F01_transcript_15510	F01_transcript_15510	F01_transcript_15510	F01_transcript_15510	F01_transcript_15510
F01_transcript_120607	F01_transcript_120607	F01_transcript_120607	NA	NA
F01_transcript_131751	NA	F01_transcript_131751	NA	F01_transcript_131751
F01_transcript_72879	NA	NA	F01_transcript_72879	NA
F01_transcript_156224	NA	F01_transcript_156224	NA	NA
F01_transcript_89656	F01_transcript_89656	F01_transcript_89656	NA	F01_transcript_89656
F01_transcript_46955	NA	F01_transcript_46955	F01_transcript_46955	NA

F01_transcript_115426	NA	NA	NA	F01_transcript_115426
F01_transcript_71839	NA	F01_transcript_71839	NA	F01_transcript_71839
F01_transcript_104676	F01_transcript_104676	F01_transcript_104676	NA	F01_transcript_104676
F01_transcript_68698	NA	F01_transcript_68698	F01_transcript_68698	F01_transcript_68698
F01_transcript_8105	NA	NA	NA	F01_transcript_8105
F01_transcript_164214	NA	NA	F01_transcript_164214	NA
F01_transcript_69571	NA	NA	NA	F01_transcript_69571
F01_transcript_150910	NA	NA	NA	F01_transcript_150910
F01_transcript_109113	NA	F01_transcript_109113	NA	NA
F01_transcript_163467	NA	F01_transcript_163467	NA	NA
F01_transcript_7523	NA	NA	NA	F01_transcript_7523
F01_transcript_91077	NA	NA	NA	F01_transcript_91077
F01_transcript_47597	NA	NA	NA	F01_transcript_47597
F01_transcript_100346	F01_transcript_100346	F01_transcript_100346	F01_transcript_100346	F01_transcript_100346

F01_transcript_149025	NA	F01_transcript_149025	NA	NA
F01_transcript_87888	NA	NA	F01_transcript_87888	F01_transcript_87888
F01_transcript_160503	F01_transcript_160503	NA	NA	NA
F01_transcript_76205	NA	NA	NA	F01_transcript_76205
F01_transcript_128913	NA	NA	NA	F01_transcript_128913
F01_transcript_50127	NA	F01_transcript_50127	NA	NA
F01_transcript_156406	F01_transcript_156406	F01_transcript_156406	F01_transcript_156406	F01_transcript_156406
F01_transcript_119633	F01_transcript_119633	F01_transcript_119633	F01_transcript_119633	F01_transcript_119633
F01_transcript_117196	F01_transcript_117196	F01_transcript_117196	NA	NA
F01_transcript_117709	NA	F01_transcript_117709	NA	F01_transcript_117709
F01_transcript_146084	NA	NA	NA	F01_transcript_146084
F01_transcript_87407	NA	F01_transcript_87407	NA	NA
F01_transcript_102663	NA	NA	NA	F01_transcript_102663
F01_transcript_123795	NA	NA	NA	F01_transcript_123795

F01_transcript_76014	NA	F01_transcript_76014	NA	F01_transcript_76014
F01_transcript_106253	F01_transcript_106253	F01_transcript_106253	F01_transcript_106253	F01_transcript_106253
F01_transcript_70947	NA	F01_transcript_70947	F01_transcript_70947	NA
F01_transcript_37539	NA	NA	NA	F01_transcript_37539
F01_transcript_1098	F01_transcript_1098	F01_transcript_1098	NA	F01_transcript_1098
F01_transcript_132601	F01_transcript_132601	F01_transcript_132601	F01_transcript_132601	F01_transcript_132601
F01_transcript_123800	NA	F01_transcript_123800	NA	NA
F01_transcript_146653	NA	F01_transcript_146653	NA	F01_transcript_146653
F01_transcript_122674	NA	NA	NA	F01_transcript_122674
F01_transcript_150145	F01_transcript_150145	F01_transcript_150145	F01_transcript_150145	F01_transcript_150145
F01_transcript_80962	NA	NA	NA	F01_transcript_80962
F01_transcript_156211	NA	NA	NA	F01_transcript_156211
F01_transcript_123783	F01_transcript_123783	F01_transcript_123783	F01_transcript_123783	F01_transcript_123783
F01_transcript_121756	NA	NA	NA	F01_transcript_121756

F01_transcript_89305	NA	NA	NA	F01_transcript_89305
F01_transcript_150948	NA	F01_transcript_150948	NA	NA
F01_transcript_55972	F01_transcript_55972	F01_transcript_55972	NA	NA
F01_transcript_64	NA	NA	NA	F01_transcript_64
F01_transcript_87769	F01_transcript_87769	F01_transcript_87769	F01_transcript_87769	F01_transcript_87769
F01_transcript_110823	NA	NA	NA	F01_transcript_110823
F01_transcript_72834	NA	NA	NA	F01_transcript_72834
F01_transcript_75738	NA	NA	NA	F01_transcript_75738
F01_transcript_109916	F01_transcript_109916	F01_transcript_109916	NA	F01_transcript_109916
F01_transcript_139297	NA	NA	NA	F01_transcript_139297
F01_transcript_157062	F01_transcript_157062	F01_transcript_157062	F01_transcript_157062	F01_transcript_157062
F01_transcript_71513	F01_transcript_71513	F01_transcript_71513	F01_transcript_71513	F01_transcript_71513
F01_transcript_54708	F01_transcript_54708	F01_transcript_54708	NA	F01_transcript_54708
F01_transcript_61259	NA	NA	F01_transcript_61259	F01_transcript_61259

F01_transcript_160419	NA	NA	NA	F01_transcript_160419
F01_transcript_109636	F01_transcript_109636	F01_transcript_109636	F01_transcript_109636	F01_transcript_109636
F01_transcript_91049	NA	F01_transcript_91049	NA	NA
F01_transcript_89153	F01_transcript_89153	F01_transcript_89153	NA	NA
F01_transcript_69024	NA	NA	NA	F01_transcript_69024
F01_transcript_166704	NA	F01_transcript_166704	NA	F01_transcript_166704
F01_transcript_9331	NA	F01_transcript_9331	NA	NA
F01_transcript_146293	NA	NA	NA	F01_transcript_146293
F01_transcript_137564	F01_transcript_137564	F01_transcript_137564	F01_transcript_137564	F01_transcript_137564
F01_transcript_115722	NA	F01_transcript_115722	NA	F01_transcript_115722
F01_transcript_93362	F01_transcript_93362	F01_transcript_93362	NA	NA
F01_transcript_149191	F01_transcript_149191	F01_transcript_149191	F01_transcript_149191	F01_transcript_149191
F01_transcript_124784	NA	F01_transcript_124784	F01_transcript_124784	F01_transcript_124784
F01_transcript_124423	NA	NA	NA	F01_transcript_124423

F01_transcript_21392	NA	F01_transcript_21392	F01_transcript_21392	NA
F01_transcript_54591	F01_transcript_54591	F01_transcript_54591	F01_transcript_54591	F01_transcript_54591
F01_transcript_155980	F01_transcript_155980	F01_transcript_155980	F01_transcript_155980	F01_transcript_155980
F01_transcript_68882	NA	NA	NA	F01_transcript_68882
F01_transcript_125899	NA	NA	NA	F01_transcript_125899
F01_transcript_15852	NA	F01_transcript_15852	NA	NA
F01_transcript_70415	NA	NA	NA	F01_transcript_70415
F01_transcript_50807	NA	F01_transcript_50807	NA	NA
F01_transcript_147135	NA	F01_transcript_147135	NA	NA
F01_transcript_57458	NA	NA	NA	F01_transcript_57458
F01_transcript_142650	NA	F01_transcript_142650	NA	NA
F01_transcript_118539	F01_transcript_118539	F01_transcript_118539	F01_transcript_118539	NA
F01_transcript_20726	F01_transcript_20726	F01_transcript_20726	F01_transcript_20726	F01_transcript_20726
F01_transcript_30030	F01_transcript_30030	F01_transcript_30030	F01_transcript_30030	F01_transcript_30030

F01_transcript_91779	NA	F01_transcript_91779	NA	F01_transcript_91779
F01_transcript_118323	NA	F01_transcript_118323	NA	NA
F01_transcript_50562	F01_transcript_50562	F01_transcript_50562	F01_transcript_50562	NA
F01_transcript_54176	NA	F01_transcript_54176	NA	NA
F01_transcript_48844	NA	F01_transcript_48844	NA	NA
F01_transcript_5856	F01_transcript_5856	F01_transcript_5856	F01_transcript_5856	F01_transcript_5856
F01_transcript_99021	NA	F01_transcript_99021	F01_transcript_99021	F01_transcript_99021
F01_transcript_80691	NA	NA	NA	F01_transcript_80691
F01_transcript_27016	NA	NA	NA	F01_transcript_27016
F01_transcript_142563	F01_transcript_142563	F01_transcript_142563	F01_transcript_142563	F01_transcript_142563
F01_transcript_135009	NA	F01_transcript_135009	NA	F01_transcript_135009
F01_transcript_139250	NA	F01_transcript_139250	NA	F01_transcript_139250
F01_transcript_112692	F01_transcript_112692	F01_transcript_112692	NA	NA
F01_transcript_98596	NA	F01_transcript_98596	NA	F01_transcript_98596

F01_transcript_66677	NA	F01_transcript_66677	NA	NA
F01_transcript_67136	NA	F01_transcript_67136	NA	NA
F01_transcript_50915	F01_transcript_50915	F01_transcript_50915	F01_transcript_50915	F01_transcript_50915
F01_transcript_18861	NA	NA	NA	F01_transcript_18861
F01_transcript_63253	NA	F01_transcript_63253	NA	F01_transcript_63253
F01_transcript_120771	NA	NA	NA	F01_transcript_120771
F01_transcript_113727	F01_transcript_113727	F01_transcript_113727	NA	NA
F01_transcript_48485	NA	F01_transcript_48485	NA	NA
F01_transcript_48609	NA	F01_transcript_48609	NA	F01_transcript_48609
F01_transcript_73656	NA	NA	NA	F01_transcript_73656
F01_transcript_69083	NA	NA	NA	F01_transcript_69083
F01_transcript_119250	NA	F01_transcript_119250	NA	F01_transcript_119250
F01_transcript_48355	NA	F01_transcript_48355	NA	F01_transcript_48355
F01_transcript_65889	NA	F01_transcript_65889	F01_transcript_65889	F01_transcript_65889

F01_transcript_70668	NA	F01_transcript_70668	F01_transcript_70668	NA
F01_transcript_118598	NA	F01_transcript_118598	F01_transcript_118598	F01_transcript_118598
F01_transcript_82270	NA	NA	NA	F01_transcript_82270
F01_transcript_65719	F01_transcript_65719	F01_transcript_65719	F01_transcript_65719	F01_transcript_65719
F01_transcript_102138	NA	F01_transcript_102138	NA	NA
F01_transcript_24383	NA	NA	NA	F01_transcript_24383
F01_transcript_107048	NA	NA	NA	F01_transcript_107048
F01_transcript_32000	NA	F01_transcript_32000	NA	NA
F01_transcript_80392	F01_transcript_80392	F01_transcript_80392	F01_transcript_80392	F01_transcript_80392
F01_transcript_88419	NA	F01_transcript_88419	NA	F01_transcript_88419
F01_transcript_122760	F01_transcript_122760	F01_transcript_122760	F01_transcript_122760	F01_transcript_122760
F01_transcript_60919	NA	F01_transcript_60919	NA	NA
F01_transcript_71463	NA	F01_transcript_71463	NA	F01_transcript_71463
F01_transcript_130777	NA	F01_transcript_130777	NA	F01_transcript_130777

F01_transcript_111688	NA	NA	NA	F01_transcript_111688
F01_transcript_82011	F01_transcript_82011	F01_transcript_82011	NA	NA
F01_transcript_104162	NA	F01_transcript_104162	NA	NA
F01_transcript_144589	NA	NA	NA	F01_transcript_144589
F01_transcript_48407	NA	NA	NA	F01_transcript_48407
F01_transcript_84912	NA	NA	NA	F01_transcript_84912
F01_transcript_29867	NA	F01_transcript_29867	NA	F01_transcript_29867
F01_transcript_120261	F01_transcript_120261	F01_transcript_120261	NA	F01_transcript_120261
F01_transcript_44600	NA	NA	NA	F01_transcript_44600
F01_transcript_90429	F01_transcript_90429	F01_transcript_90429	NA	NA
F01_transcript_67046	F01_transcript_67046	F01_transcript_67046	NA	NA
F01_transcript_66202	F01_transcript_66202	F01_transcript_66202	F01_transcript_66202	F01_transcript_66202
F01_transcript_126285	NA	NA	NA	F01_transcript_126285
F01_transcript_85608	NA	NA	NA	F01_transcript_85608

F01_transcript_115396	NA	NA	NA	F01_transcript_115396
F01_transcript_134678	F01_transcript_134678	F01_transcript_134678	NA	NA
F01_transcript_123529	NA	F01_transcript_123529	NA	NA
F01_transcript_116587	NA	F01_transcript_116587	NA	F01_transcript_116587
F01_transcript_52407	F01_transcript_52407	F01_transcript_52407	F01_transcript_52407	F01_transcript_52407
F01_transcript_99005	NA	NA	F01_transcript_99005	F01_transcript_99005
F01_transcript_125427	NA	NA	NA	F01_transcript_125427
F01_transcript_66030	NA	NA	NA	F01_transcript_66030
F01_transcript_63273	NA	F01_transcript_63273	NA	F01_transcript_63273
F01_transcript_167463	NA	F01_transcript_167463	NA	NA
F01_transcript_84825	NA	NA	NA	F01_transcript_84825
F01_transcript_10783	NA	NA	NA	F01_transcript_10783
F01_transcript_99574	F01_transcript_99574	NA	F01_transcript_99574	F01_transcript_99574
F01_transcript_98433	F01_transcript_98433	F01_transcript_98433	F01_transcript_98433	F01_transcript_98433

F01_transcript_23618	NA	F01_transcript_23618	NA	NA
F01_transcript_37442	NA	NA	NA	F01_transcript_37442
F01_transcript_126073	F01_transcript_126073	F01_transcript_126073	F01_transcript_126073	F01_transcript_126073
F01_transcript_93259	NA	NA	NA	F01_transcript_93259
F01_transcript_91275	NA	NA	NA	F01_transcript_91275
F01_transcript_159107	NA	F01_transcript_159107	NA	F01_transcript_159107
F01_transcript_24596	NA	NA	NA	F01_transcript_24596
F01_transcript_94495	NA	F01_transcript_94495	NA	NA
F01_transcript_130984	NA	NA	NA	F01_transcript_130984
F01_transcript_129377	NA	F01_transcript_129377	F01_transcript_129377	F01_transcript_129377
F01_transcript_125785	NA	NA	NA	F01_transcript_125785
F01_transcript_9154	NA	NA	NA	F01_transcript_9154
F01_transcript_114254	NA	F01_transcript_114254	NA	F01_transcript_114254
F01_transcript_93412	NA	F01_transcript_93412	NA	NA

F01_transcript_68675	F01_transcript_68675	F01_transcript_68675	NA	F01_transcript_68675
F01_transcript_86653	NA	NA	NA	F01_transcript_86653
F01_transcript_54859	F01_transcript_54859	F01_transcript_54859	F01_transcript_54859	F01_transcript_54859
F01_transcript_145539	F01_transcript_145539	F01_transcript_145539	NA	F01_transcript_145539
F01_transcript_165142	F01_transcript_165142	F01_transcript_165142	NA	NA
F01_transcript_146353	NA	F01_transcript_146353	NA	NA
F01_transcript_139249	NA	F01_transcript_139249	NA	F01_transcript_139249
F01_transcript_71661	F01_transcript_71661	F01_transcript_71661	F01_transcript_71661	F01_transcript_71661
F01_transcript_89221	NA	F01_transcript_89221	NA	NA
F01_transcript_28383	NA	F01_transcript_28383	NA	NA
F01_transcript_96373	F01_transcript_96373	F01_transcript_96373	F01_transcript_96373	F01_transcript_96373
F01_transcript_158437	NA	F01_transcript_158437	NA	F01_transcript_158437
F01_transcript_55298	NA	F01_transcript_55298	F01_transcript_55298	F01_transcript_55298
F01_transcript_58891	NA	NA	NA	F01_transcript_58891

F01_transcript_25638	NA	NA	NA	F01_transcript_25638
F01_transcript_89399	NA	NA	NA	F01_transcript_89399
F01_transcript_65710	F01_transcript_65710	F01_transcript_65710	NA	F01_transcript_65710
F01_transcript_6581	NA	NA	F01_transcript_6581	F01_transcript_6581
F01_transcript_98152	NA	NA	NA	F01_transcript_98152
F01_transcript_36474	NA	NA	NA	F01_transcript_36474
F01_transcript_119474	NA	NA	F01_transcript_119474	F01_transcript_119474
F01_transcript_125156	F01_transcript_125156	F01_transcript_125156	F01_transcript_125156	F01_transcript_125156
F01_transcript_130467	NA	NA	NA	F01_transcript_130467
F01_transcript_122992	NA	NA	NA	F01_transcript_122992
F01_transcript_20848	NA	F01_transcript_20848	NA	F01_transcript_20848
F01_transcript_83639	F01_transcript_83639	F01_transcript_83639	F01_transcript_83639	F01_transcript_83639
F01_transcript_31590	NA	NA	NA	F01_transcript_31590
F01_transcript_125322	F01_transcript_125322	F01_transcript_125322	NA	F01_transcript_125322

F01_transcript_51141	F01_transcript_51141	F01_transcript_51141	F01_transcript_51141	F01_transcript_51141
F01_transcript_27719	F01_transcript_27719	NA	NA	F01_transcript_27719
F01_transcript_157845	F01_transcript_157845	F01_transcript_157845	F01_transcript_157845	F01_transcript_157845
F01_transcript_101707	NA	NA	NA	F01_transcript_101707
F01_transcript_166140	F01_transcript_166140	F01_transcript_166140	NA	F01_transcript_166140
F01_transcript_101208	NA	F01_transcript_101208	F01_transcript_101208	NA
F01_transcript_147552	NA	F01_transcript_147552	NA	NA
F01_transcript_67284	F01_transcript_67284	F01_transcript_67284	F01_transcript_67284	F01_transcript_67284
F01_transcript_93039	NA	F01_transcript_93039	NA	F01_transcript_93039
F01_transcript_110388	F01_transcript_110388	F01_transcript_110388	F01_transcript_110388	F01_transcript_110388
F01_transcript_83130	F01_transcript_83130	F01_transcript_83130	F01_transcript_83130	F01_transcript_83130
F01_transcript_98258	F01_transcript_98258	F01_transcript_98258	NA	F01_transcript_98258
F01_transcript_135443	F01_transcript_135443	F01_transcript_135443	NA	F01_transcript_135443
F01_transcript_73990	NA	NA	NA	F01_transcript_73990

F01_transcript_69917	NA	NA	NA	F01_transcript_69917
F01_transcript_152451	NA	NA	NA	F01_transcript_152451
F01_transcript_64593	F01_transcript_64593	F01_transcript_64593	NA	NA
F01_transcript_26196	F01_transcript_26196	F01_transcript_26196	F01_transcript_26196	F01_transcript_26196
F01_transcript_79979	NA	F01_transcript_79979	NA	NA
F01_transcript_46279	NA	NA	NA	F01_transcript_46279
F01_transcript_139273	F01_transcript_139273	F01_transcript_139273	NA	F01_transcript_139273
F01_transcript_120484	NA	NA	NA	F01_transcript_120484
F01_transcript_140555	F01_transcript_140555	F01_transcript_140555	NA	F01_transcript_140555
F01_transcript_120399	NA	F01_transcript_120399	F01_transcript_120399	F01_transcript_120399
F01_transcript_120233	NA	F01_transcript_120233	NA	NA
F01_transcript_75806	NA	F01_transcript_75806	NA	F01_transcript_75806
F01_transcript_160198	NA	NA	NA	F01_transcript_160198
F01_transcript_121030	NA	F01_transcript_121030	NA	F01_transcript_121030

F01_transcript_80594	F01_transcript_80594	F01_transcript_80594	F01_transcript_80594	F01_transcript_80594
F01_transcript_104465	NA	F01_transcript_104465	NA	NA
F01_transcript_128634	NA	NA	F01_transcript_128634	NA
F01_transcript_166268	NA	NA	NA	F01_transcript_166268
F01_transcript_59716	NA	NA	NA	F01_transcript_59716
F01_transcript_147364	NA	F01_transcript_147364	NA	F01_transcript_147364
F01_transcript_108864	F01_transcript_108864	F01_transcript_108864	F01_transcript_108864	F01_transcript_108864
F01_transcript_111738	NA	NA	NA	F01_transcript_111738
F01_transcript_1217	NA	F01_transcript_1217	F01_transcript_1217	F01_transcript_1217
F01_transcript_144582	F01_transcript_144582	F01_transcript_144582	F01_transcript_144582	NA
F01_transcript_162019	NA	F01_transcript_162019	F01_transcript_162019	NA
F01_transcript_69904	NA	F01_transcript_69904	NA	F01_transcript_69904
F01_transcript_124844	NA	NA	NA	F01_transcript_124844
F01_transcript_126449	NA	F01_transcript_126449	NA	NA

F01_transcript_88236	F01_transcript_88236	F01_transcript_88236	F01_transcript_88236	F01_transcript_88236
F01_transcript_93357	F01_transcript_93357	F01_transcript_93357	NA	NA
F01_transcript_36688	NA	F01_transcript_36688	NA	NA
F01_transcript_51750	NA	F01_transcript_51750	NA	F01_transcript_51750
F01_transcript_121189	NA	F01_transcript_121189	F01_transcript_121189	F01_transcript_121189
F01_transcript_64966	NA	NA	NA	F01_transcript_64966
F01_transcript_59253	NA	F01_transcript_59253	F01_transcript_59253	F01_transcript_59253
F01_transcript_109820	NA	F01_transcript_109820	NA	NA
F01_transcript_62379	NA	F01_transcript_62379	NA	NA
F01_transcript_141187	NA	NA	NA	F01_transcript_141187
F01_transcript_157286	NA	F01_transcript_157286	NA	NA
F01_transcript_75294	F01_transcript_75294	F01_transcript_75294	NA	NA
F01_transcript_153389	NA	F01_transcript_153389	NA	F01_transcript_153389
F01_transcript_141685	NA	NA	NA	F01_transcript_141685

F01_transcript_121903	NA	F01_transcript_121903	NA	NA
F01_transcript_152438	F01_transcript_152438	F01_transcript_152438	F01_transcript_152438	F01_transcript_152438
F01_transcript_112566	F01_transcript_112566	F01_transcript_112566	NA	F01_transcript_112566
F01_transcript_56444	NA	F01_transcript_56444	F01_transcript_56444	NA
F01_transcript_150437	NA	NA	NA	F01_transcript_150437
F01_transcript_147289	NA	F01_transcript_147289	F01_transcript_147289	F01_transcript_147289
F01_transcript_41138	NA	F01_transcript_41138	NA	NA
F01_transcript_15326	NA	NA	NA	F01_transcript_15326
F01_transcript_137737	F01_transcript_137737	F01_transcript_137737	F01_transcript_137737	NA
F01_transcript_118924	NA	F01_transcript_118924	NA	NA
F01_transcript_44728	NA	NA	NA	F01_transcript_44728
F01_transcript_77849	NA	NA	NA	F01_transcript_77849
F01_transcript_87610	NA	F01_transcript_87610	NA	F01_transcript_87610
F01_transcript_120902	NA	F01_transcript_120902	NA	F01_transcript_120902

F01_transcript_115755	NA	F01_transcript_115755	NA	NA
F01_transcript_102430	NA	F01_transcript_102430	NA	F01_transcript_102430
F01_transcript_79769	F01_transcript_79769	F01_transcript_79769	F01_transcript_79769	NA
F01_transcript_148566	NA	NA	NA	F01_transcript_148566
F01_transcript_60132	NA	F01_transcript_60132	NA	F01_transcript_60132
F01_transcript_122411	NA	F01_transcript_122411	F01_transcript_122411	F01_transcript_122411
F01_transcript_103598	NA	F01_transcript_103598	NA	NA
F01_transcript_65712	NA	F01_transcript_65712	NA	NA
F01_transcript_167332	NA	F01_transcript_167332	NA	F01_transcript_167332
F01_transcript_95852	NA	F01_transcript_95852	F01_transcript_95852	F01_transcript_95852
F01_transcript_89019	NA	F01_transcript_89019	F01_transcript_89019	F01_transcript_89019
F01_transcript_135613	NA	F01_transcript_135613	NA	NA
F01_transcript_103084	F01_transcript_103084	F01_transcript_103084	F01_transcript_103084	F01_transcript_103084
F01_transcript_134410	NA	F01_transcript_134410	F01_transcript_134410	NA

F01_transcript_120285	NA	F01_transcript_120285	NA	NA
F01_transcript_158420	F01_transcript_158420	F01_transcript_158420	NA	NA
F01_transcript_135221	NA	F01_transcript_135221	NA	F01_transcript_135221
F01_transcript_132036	NA	NA	NA	F01_transcript_132036
F01_transcript_146307	NA	NA	NA	F01_transcript_146307
F01_transcript_121478	NA	F01_transcript_121478	NA	NA
F01_transcript_69368	NA	NA	NA	F01_transcript_69368
F01_transcript_65266	NA	NA	F01_transcript_65266	NA
F01_transcript_131141	F01_transcript_131141	F01_transcript_131141	F01_transcript_131141	F01_transcript_131141
F01_transcript_63611	NA	NA	NA	F01_transcript_63611
F01_transcript_157990	F01_transcript_157990	F01_transcript_157990	F01_transcript_157990	F01_transcript_157990
F01_transcript_67157	F01_transcript_67157	F01_transcript_67157	NA	F01_transcript_67157
F01_transcript_93168	F01_transcript_93168	F01_transcript_93168	NA	NA
F01_transcript_159777	NA	F01_transcript_159777	NA	F01_transcript_159777

F01_transcript_66147	NA	NA	NA	F01_transcript_66147
F01_transcript_131560	NA	F01_transcript_131560	NA	F01_transcript_131560
F01_transcript_70012	NA	F01_transcript_70012	F01_transcript_70012	F01_transcript_70012
F01_transcript_139850	NA	NA	NA	F01_transcript_139850
F01_transcript_46730	NA	NA	NA	F01_transcript_46730
F01_transcript_141984	NA	F01_transcript_141984	NA	NA
F01_transcript_41083	NA	NA	NA	F01_transcript_41083
F01_transcript_120118	NA	F01_transcript_120118	F01_transcript_120118	F01_transcript_120118
F01_transcript_78602	F01_transcript_78602	F01_transcript_78602	F01_transcript_78602	F01_transcript_78602
F01_transcript_148968	NA	NA	F01_transcript_148968	F01_transcript_148968
F01_transcript_16358	NA	NA	NA	F01_transcript_16358
F01_transcript_72627	NA	NA	NA	F01_transcript_72627
F01_transcript_58987	F01_transcript_58987	F01_transcript_58987	NA	F01_transcript_58987
F01_transcript_100224	F01_transcript_100224	F01_transcript_100224	F01_transcript_100224	F01_transcript_100224

F01_transcript_114324	NA	F01_transcript_114324	NA	NA
F01_transcript_97892	NA	NA	NA	F01_transcript_97892
F01_transcript_55685	F01_transcript_55685	F01_transcript_55685	NA	NA
F01_transcript_122650	NA	NA	F01_transcript_122650	F01_transcript_122650
F01_transcript_61329	F01_transcript_61329	F01_transcript_61329	NA	NA
F01_transcript_84677	NA	NA	NA	F01_transcript_84677
F01_transcript_126614	NA	NA	NA	F01_transcript_126614
F01_transcript_53649	NA	F01_transcript_53649	NA	NA
F01_transcript_53782	NA	NA	NA	F01_transcript_53782
F01_transcript_106408	F01_transcript_106408	F01_transcript_106408	F01_transcript_106408	F01_transcript_106408
F01_transcript_155050	NA	F01_transcript_155050	F01_transcript_155050	NA
F01_transcript_70417	NA	NA	NA	F01_transcript_70417
F01_transcript_137404	F01_transcript_137404	F01_transcript_137404	F01_transcript_137404	F01_transcript_137404
F01_transcript_101822	F01_transcript_101822	F01_transcript_101822	F01_transcript_101822	F01_transcript_101822

F01_transcript_119125	NA	F01_transcript_119125	NA	F01_transcript_119125
F01_transcript_99679	NA	NA	F01_transcript_99679	F01_transcript_99679
F01_transcript_47128	F01_transcript_47128	F01_transcript_47128	NA	NA
F01_transcript_78495	NA	NA	NA	F01_transcript_78495
F01_transcript_142573	NA	NA	NA	F01_transcript_142573
F01_transcript_77468	F01_transcript_77468	F01_transcript_77468	F01_transcript_77468	F01_transcript_77468
F01_transcript_135710	F01_transcript_135710	F01_transcript_135710	NA	F01_transcript_135710
F01_transcript_82733	F01_transcript_82733	F01_transcript_82733	F01_transcript_82733	F01_transcript_82733
F01_transcript_17616	NA	NA	NA	F01_transcript_17616
F01_transcript_127821	NA	NA	NA	F01_transcript_127821
F01_transcript_64315	NA	NA	NA	F01_transcript_64315
F01_transcript_158625	NA	F01_transcript_158625	NA	F01_transcript_158625
F01_transcript_138508	NA	F01_transcript_138508	F01_transcript_138508	F01_transcript_138508
F01_transcript_73676	F01_transcript_73676	F01_transcript_73676	F01_transcript_73676	F01_transcript_73676

F01_transcript_126911	NA	F01_transcript_126911	NA	NA
F01_transcript_75884	NA	NA	NA	F01_transcript_75884
F01_transcript_122714	F01_transcript_122714	F01_transcript_122714	NA	NA
F01_transcript_16740	NA	NA	NA	F01_transcript_16740
F01_transcript_112184	NA	NA	NA	F01_transcript_112184
F01_transcript_96876	NA	F01_transcript_96876	NA	NA
F01_transcript_30524	NA	F01_transcript_30524	NA	F01_transcript_30524
F01_transcript_50731	F01_transcript_50731	F01_transcript_50731	NA	F01_transcript_50731
F01_transcript_16912	NA	NA	NA	F01_transcript_16912
F01_transcript_154967	NA	NA	NA	F01_transcript_154967
F01_transcript_106928	NA	F01_transcript_106928	NA	F01_transcript_106928
F01_transcript_51745	NA	NA	NA	F01_transcript_51745
F01_transcript_4061	NA	F01_transcript_4061	NA	F01_transcript_4061
F01_transcript_143732	F01_transcript_143732	F01_transcript_143732	F01_transcript_143732	F01_transcript_143732

F01_transcript_75713	F01_transcript_75713	F01_transcript_75713	F01_transcript_75713	NA
F01_transcript_7222	NA	NA	NA	F01_transcript_7222
F01_transcript_146514	NA	NA	NA	F01_transcript_146514
F01_transcript_147160	F01_transcript_147160	F01_transcript_147160	F01_transcript_147160	F01_transcript_147160
F01_transcript_10877	NA	F01_transcript_10877	NA	F01_transcript_10877
F01_transcript_100204	F01_transcript_100204	F01_transcript_100204	F01_transcript_100204	F01_transcript_100204
F01_transcript_134876	NA	NA	NA	F01_transcript_134876
F01_transcript_119811	NA	NA	NA	F01_transcript_119811
F01_transcript_109338	NA	NA	NA	F01_transcript_109338
F01_transcript_47719	F01_transcript_47719	F01_transcript_47719	F01_transcript_47719	F01_transcript_47719
F01_transcript_114673	F01_transcript_114673	F01_transcript_114673	F01_transcript_114673	F01_transcript_114673
F01_transcript_69856	F01_transcript_69856	F01_transcript_69856	F01_transcript_69856	F01_transcript_69856
F01_transcript_29872	NA	NA	NA	F01_transcript_29872
F01_transcript_146959	NA	F01_transcript_146959	NA	NA

F01_transcript_50101	NA	NA	F01_transcript_50101	NA
F01_transcript_81656	F01_transcript_81656	F01_transcript_81656	NA	NA
F01_transcript_62017	NA	NA	NA	F01_transcript_62017
F01_transcript_127655	NA	NA	NA	F01_transcript_127655
F01_transcript_59921	F01_transcript_59921	F01_transcript_59921	NA	F01_transcript_59921
F01_transcript_135076	NA	F01_transcript_135076	F01_transcript_135076	NA
F01_transcript_100647	NA	NA	NA	F01_transcript_100647
F01_transcript_17085	NA	NA	NA	F01_transcript_17085
F01_transcript_114600	NA	F01_transcript_114600	F01_transcript_114600	F01_transcript_114600
F01_transcript_50037	NA	NA	NA	F01_transcript_50037
F01_transcript_161319	NA	F01_transcript_161319	NA	NA
F01_transcript_65443	F01_transcript_65443	F01_transcript_65443	F01_transcript_65443	F01_transcript_65443
F01_transcript_100654	F01_transcript_100654	F01_transcript_100654	NA	NA
F01_transcript_128733	NA	NA	NA	F01_transcript_128733

F01_transcript_79623	NA	F01_transcript_79623	NA	F01_transcript_79623
F01_transcript_50549	NA	F01_transcript_50549	F01_transcript_50549	F01_transcript_50549
F01_transcript_114905	NA	F01_transcript_114905	NA	NA
F01_transcript_97134	NA	F01_transcript_97134	NA	NA
F01_transcript_103409	NA	F01_transcript_103409	NA	NA
F01_transcript_102451	F01_transcript_102451	F01_transcript_102451	F01_transcript_102451	NA
F01_transcript_59599	NA	NA	F01_transcript_59599	F01_transcript_59599
F01_transcript_22840	NA	NA	NA	F01_transcript_22840
F01_transcript_108518	NA	NA	NA	F01_transcript_108518
F01_transcript_131861	NA	NA	NA	F01_transcript_131861
F01_transcript_99090	NA	F01_transcript_99090	NA	F01_transcript_99090
F01_transcript_142601	NA	F01_transcript_142601	NA	NA
F01_transcript_39408	NA	NA	NA	F01_transcript_39408
F01_transcript_46931	NA	NA	NA	F01_transcript_46931

F01_transcript_164905	NA	F01_transcript_164905	NA	NA
F01_transcript_6476	NA	NA	NA	F01_transcript_6476
F01_transcript_68390	NA	NA	NA	F01_transcript_68390
F01_transcript_52107	F01_transcript_52107	F01_transcript_52107	F01_transcript_52107	F01_transcript_52107
F01_transcript_88649	NA	NA	NA	F01_transcript_88649
F01_transcript_142552	NA	NA	F01_transcript_142552	NA
F01_transcript_113887	F01_transcript_113887	F01_transcript_113887	F01_transcript_113887	F01_transcript_113887
F01_transcript_43423	NA	NA	F01_transcript_43423	F01_transcript_43423
F01_transcript_114378	NA	F01_transcript_114378	NA	F01_transcript_114378
F01_transcript_115576	NA	F01_transcript_115576	NA	NA
F01_transcript_83207	F01_transcript_83207	F01_transcript_83207	F01_transcript_83207	F01_transcript_83207
F01_transcript_4335	F01_transcript_4335	F01_transcript_4335	NA	F01_transcript_4335
F01_transcript_125369	NA	NA	NA	F01_transcript_125369
F01_transcript_30803	NA	NA	NA	F01_transcript_30803

F01_transcript_112245	F01_transcript_112245	F01_transcript_112245	F01_transcript_112245	F01_transcript_112245
F01_transcript_143418	NA	F01_transcript_143418	NA	NA
F01_transcript_12462	NA	NA	NA	F01_transcript_12462
F01_transcript_140300	NA	F01_transcript_140300	F01_transcript_140300	NA
F01_transcript_142572	F01_transcript_142572	F01_transcript_142572	F01_transcript_142572	NA
F01_transcript_115901	NA	NA	NA	F01_transcript_115901
F01_transcript_124515	NA	NA	NA	F01_transcript_124515
F01_transcript_139565	NA	NA	NA	F01_transcript_139565
F01_transcript_83082	NA	F01_transcript_83082	F01_transcript_83082	F01_transcript_83082
F01_transcript_45184	NA	NA	NA	F01_transcript_45184
F01_transcript_66962	F01_transcript_66962	F01_transcript_66962	F01_transcript_66962	F01_transcript_66962
F01_transcript_83218	NA	F01_transcript_83218	NA	NA
F01_transcript_106293	NA	F01_transcript_106293	NA	NA
F01_transcript_90454	F01_transcript_90454	F01_transcript_90454	F01_transcript_90454	F01_transcript_90454

F01_transcript_87677	NA	F01_transcript_87677	NA	F01_transcript_87677
F01_transcript_99246	NA	NA	NA	F01_transcript_99246
F01_transcript_112995	NA	F01_transcript_112995	NA	NA
F01_transcript_101242	NA	NA	NA	F01_transcript_101242
F01_transcript_122671	F01_transcript_122671	F01_transcript_122671	NA	F01_transcript_122671
F01_transcript_135781	NA	NA	NA	F01_transcript_135781
F01_transcript_96143	NA	NA	NA	F01_transcript_96143
F01_transcript_139899	F01_transcript_139899	F01_transcript_139899	F01_transcript_139899	F01_transcript_139899
F01_transcript_147698	NA	NA	F01_transcript_147698	NA
F01_transcript_140678	F01_transcript_140678	F01_transcript_140678	F01_transcript_140678	F01_transcript_140678
F01_transcript_84198	NA	NA	NA	F01_transcript_84198
F01_transcript_87085	F01_transcript_87085	F01_transcript_87085	NA	F01_transcript_87085
F01_transcript_58440	NA	NA	NA	F01_transcript_58440
F01_transcript_164408	NA	NA	NA	F01_transcript_164408

F01_transcript_158193	NA	NA	F01_transcript_158193	F01_transcript_158193
F01_transcript_16873	F01_transcript_16873	F01_transcript_16873	F01_transcript_16873	F01_transcript_16873
F01_transcript_125310	NA	NA	NA	F01_transcript_125310
F01_transcript_145993	NA	F01_transcript_145993	F01_transcript_145993	F01_transcript_145993
F01_transcript_33071	NA	NA	NA	F01_transcript_33071
F01_transcript_35513	NA	NA	NA	F01_transcript_35513
F01_transcript_63778	NA	F01_transcript_63778	F01_transcript_63778	F01_transcript_63778
F01_transcript_63761	F01_transcript_63761	F01_transcript_63761	F01_transcript_63761	F01_transcript_63761
F01_transcript_55115	F01_transcript_55115	F01_transcript_55115	F01_transcript_55115	NA
F01_transcript_158756	NA	NA	NA	F01_transcript_158756
F01_transcript_118653	NA	NA	NA	F01_transcript_118653
F01_transcript_14130	F01_transcript_14130	NA	NA	F01_transcript_14130
F01_transcript_71167	NA	NA	F01_transcript_71167	NA
F01_transcript_150082	NA	F01_transcript_150082	NA	NA

F01_transcript_149353	F01_transcript_149353	F01_transcript_149353	F01_transcript_149353	NA
F01_transcript_157281	NA	F01_transcript_157281	NA	F01_transcript_157281
F01_transcript_79971	NA	NA	F01_transcript_79971	NA
F01_transcript_102069	NA	NA	NA	F01_transcript_102069
F01_transcript_66689	F01_transcript_66689	F01_transcript_66689	F01_transcript_66689	F01_transcript_66689
F01_transcript_108201	NA	F01_transcript_108201	F01_transcript_108201	F01_transcript_108201
F01_transcript_46646	NA	NA	NA	F01_transcript_46646
F01_transcript_156748	NA	F01_transcript_156748	F01_transcript_156748	F01_transcript_156748
F01_transcript_115304	F01_transcript_115304	F01_transcript_115304	F01_transcript_115304	F01_transcript_115304
F01_transcript_72202	F01_transcript_72202	F01_transcript_72202	NA	F01_transcript_72202
F01_transcript_48665	NA	F01_transcript_48665	NA	NA
F01_transcript_45431	NA	NA	NA	F01_transcript_45431
F01_transcript_60574	NA	F01_transcript_60574	NA	NA
F01_transcript_165443	NA	F01_transcript_165443	F01_transcript_165443	F01_transcript_165443

F01_transcript_85159	NA	NA	NA	F01_transcript_85159
F01_transcript_156233	NA	F01_transcript_156233	NA	NA
F01_transcript_24131	NA	NA	NA	F01_transcript_24131
F01_transcript_69655	NA	F01_transcript_69655	NA	NA
F01_transcript_94484	F01_transcript_94484	F01_transcript_94484	NA	F01_transcript_94484
F01_transcript_133297	NA	NA	NA	F01_transcript_133297
F01_transcript_44923	NA	NA	NA	F01_transcript_44923
F01_transcript_2152	NA	NA	NA	F01_transcript_2152
F01_transcript_131947	F01_transcript_131947	F01_transcript_131947	F01_transcript_131947	F01_transcript_131947
F01_transcript_93673	NA	NA	NA	F01_transcript_93673
F01_transcript_129568	NA	NA	NA	F01_transcript_129568
F01_transcript_53678	NA	NA	F01_transcript_53678	NA
F01_transcript_110844	NA	F01_transcript_110844	F01_transcript_110844	NA
F01_transcript_101559	NA	NA	NA	F01_transcript_101559

F01_transcript_72900	F01_transcript_72900	F01_transcript_72900	NA	F01_transcript_72900
F01_transcript_78038	NA	F01_transcript_78038	F01_transcript_78038	F01_transcript_78038
F01_transcript_98139	F01_transcript_98139	F01_transcript_98139	NA	NA
F01_transcript_153825	F01_transcript_153825	F01_transcript_153825	F01_transcript_153825	F01_transcript_153825
F01_transcript_113529	F01_transcript_113529	F01_transcript_113529	F01_transcript_113529	F01_transcript_113529
F01_transcript_98898	NA	NA	NA	F01_transcript_98898
F01_transcript_73479	NA	F01_transcript_73479	NA	NA
F01_transcript_161279	NA	F01_transcript_161279	NA	NA
F01_transcript_65359	NA	F01_transcript_65359	F01_transcript_65359	NA
F01_transcript_147321	NA	F01_transcript_147321	NA	NA
F01_transcript_133712	NA	F01_transcript_133712	NA	NA
F01_transcript_143004	NA	NA	NA	F01_transcript_143004
F01_transcript_50118	NA	F01_transcript_50118	NA	F01_transcript_50118
F01_transcript_113290	NA	NA	NA	F01_transcript_113290

F01_transcript_71315	NA	NA	F01_transcript_71315	NA
F01_transcript_120180	NA	NA	NA	F01_transcript_120180
F01_transcript_105082	F01_transcript_105082	F01_transcript_105082	F01_transcript_105082	F01_transcript_105082
F01_transcript_33518	NA	NA	NA	F01_transcript_33518
F01_transcript_50367	NA	NA	NA	F01_transcript_50367
F01_transcript_90149	NA	F01_transcript_90149	NA	NA
F01_transcript_69325	F01_transcript_69325	F01_transcript_69325	NA	NA
F01_transcript_68303	NA	F01_transcript_68303	NA	NA
F01_transcript_52849	F01_transcript_52849	F01_transcript_52849	NA	NA
F01_transcript_49793	NA	F01_transcript_49793	NA	F01_transcript_49793
F01_transcript_1513	F01_transcript_1513	F01_transcript_1513	NA	F01_transcript_1513
F01_transcript_68403	NA	NA	NA	F01_transcript_68403
F01_transcript_143194	NA	NA	NA	F01_transcript_143194
F01_transcript_57770	NA	NA	NA	F01_transcript_57770

F01_transcript_82404	F01_transcript_82404	F01_transcript_82404	F01_transcript_82404	F01_transcript_82404
F01_transcript_21779	NA	F01_transcript_21779	NA	F01_transcript_21779
F01_transcript_166201	F01_transcript_166201	F01_transcript_166201	F01_transcript_166201	F01_transcript_166201
F01_transcript_156213	F01_transcript_156213	F01_transcript_156213	F01_transcript_156213	NA
F01_transcript_93602	F01_transcript_93602	F01_transcript_93602	F01_transcript_93602	F01_transcript_93602
F01_transcript_71871	NA	F01_transcript_71871	NA	F01_transcript_71871
F01_transcript_135550	NA	F01_transcript_135550	NA	NA
F01_transcript_153859	NA	F01_transcript_153859	NA	NA
F01_transcript_154534	F01_transcript_154534	NA	NA	NA
F01_transcript_101609	NA	F01_transcript_101609	NA	NA
F01_transcript_27211	NA	NA	NA	F01_transcript_27211
F01_transcript_48786	NA	NA	NA	F01_transcript_48786
F01_transcript_67501	NA	NA	NA	F01_transcript_67501
F01_transcript_119498	NA	F01_transcript_119498	NA	NA

F01_transcript_150220	F01_transcript_150220	F01_transcript_150220	F01_transcript_150220	F01_transcript_150220
F01_transcript_126925	F01_transcript_126925	F01_transcript_126925	NA	NA
F01_transcript_86111	NA	NA	NA	F01_transcript_86111
F01_transcript_47342	NA	NA	NA	F01_transcript_47342
F01_transcript_86666	F01_transcript_86666	NA	F01_transcript_86666	F01_transcript_86666
F01_transcript_158063	F01_transcript_158063	F01_transcript_158063	F01_transcript_158063	F01_transcript_158063
F01_transcript_25724	NA	NA	NA	F01_transcript_25724
F01_transcript_83199	NA	NA	NA	F01_transcript_83199
F01_transcript_133473	NA	NA	NA	F01_transcript_133473
F01_transcript_72886	F01_transcript_72886	F01_transcript_72886	F01_transcript_72886	F01_transcript_72886
F01_transcript_145911	F01_transcript_145911	F01_transcript_145911	F01_transcript_145911	F01_transcript_145911
F01_transcript_119781	NA	F01_transcript_119781	NA	NA
F01_transcript_65263	NA	F01_transcript_65263	F01_transcript_65263	NA
F01_transcript_58192	F01_transcript_58192	NA	F01_transcript_58192	F01_transcript_58192

F01_transcript_145302	NA	F01_transcript_145302	NA	NA
F01_transcript_96588	NA	F01_transcript_96588	NA	F01_transcript_96588
F01_transcript_128435	NA	F01_transcript_128435	F01_transcript_128435	F01_transcript_128435
F01_transcript_93762	NA	F01_transcript_93762	NA	NA
F01_transcript_121649	NA	F01_transcript_121649	NA	NA
F01_transcript_73376	F01_transcript_73376	F01_transcript_73376	F01_transcript_73376	F01_transcript_73376
F01_transcript_140008	NA	F01_transcript_140008	NA	NA
F01_transcript_107231	NA	F01_transcript_107231	NA	F01_transcript_107231
F01_transcript_166874	NA	F01_transcript_166874	NA	F01_transcript_166874
F01_transcript_125340	NA	NA	NA	F01_transcript_125340
F01_transcript_59583	NA	NA	F01_transcript_59583	NA
F01_transcript_87607	NA	F01_transcript_87607	NA	F01_transcript_87607
F01_transcript_99604	F01_transcript_99604	F01_transcript_99604	NA	NA
F01_transcript_42676	F01_transcript_42676	F01_transcript_42676	F01_transcript_42676	F01_transcript_42676

F01_transcript_128134	NA	F01_transcript_128134	NA	NA
F01_transcript_87725	NA	F01_transcript_87725	NA	NA
F01_transcript_84502	NA	F01_transcript_84502	F01_transcript_84502	F01_transcript_84502
F01_transcript_52259	F01_transcript_52259	F01_transcript_52259	F01_transcript_52259	F01_transcript_52259
F01_transcript_115096	F01_transcript_115096	F01_transcript_115096	NA	NA
F01_transcript_58454	F01_transcript_58454	F01_transcript_58454	F01_transcript_58454	F01_transcript_58454
F01_transcript_58075	NA	NA	NA	F01_transcript_58075
F01_transcript_62546	NA	NA	NA	F01_transcript_62546
F01_transcript_101718	NA	F01_transcript_101718	NA	NA
F01_transcript_136200	NA	F01_transcript_136200	NA	F01_transcript_136200
F01_transcript_139575	F01_transcript_139575	F01_transcript_139575	NA	F01_transcript_139575
F01_transcript_134068	F01_transcript_134068	F01_transcript_134068	F01_transcript_134068	F01_transcript_134068
F01_transcript_51169	F01_transcript_51169	F01_transcript_51169	F01_transcript_51169	F01_transcript_51169
F01_transcript_58345	NA	NA	NA	F01_transcript_58345

F01_transcript_50458	F01_transcript_50458	NA	NA	NA
F01_transcript_70546	F01_transcript_70546	F01_transcript_70546	F01_transcript_70546	F01_transcript_70546
F01_transcript_54255	NA	NA	NA	F01_transcript_54255
F01_transcript_123708	NA	F01_transcript_123708	NA	NA
F01_transcript_48454	NA	F01_transcript_48454	NA	NA
F01_transcript_22223	NA	F01_transcript_22223	NA	NA
F01_transcript_4350	NA	NA	NA	F01_transcript_4350
F01_transcript_139239	F01_transcript_139239	F01_transcript_139239	F01_transcript_139239	F01_transcript_139239
F01_transcript_150110	F01_transcript_150110	F01_transcript_150110	F01_transcript_150110	F01_transcript_150110
F01_transcript_155419	F01_transcript_155419	F01_transcript_155419	NA	NA
F01_transcript_137944	NA	NA	NA	F01_transcript_137944
F01_transcript_128677	NA	NA	NA	F01_transcript_128677
F01_transcript_64319	NA	F01_transcript_64319	NA	F01_transcript_64319
F01_transcript_156205	F01_transcript_156205	F01_transcript_156205	F01_transcript_156205	F01_transcript_156205

F01_transcript_59609	F01_transcript_59609	F01_transcript_59609	F01_transcript_59609	F01_transcript_59609
F01_transcript_165449	F01_transcript_165449	F01_transcript_165449	F01_transcript_165449	F01_transcript_165449
F01_transcript_116344	NA	NA	NA	F01_transcript_116344
F01_transcript_31502	NA	F01_transcript_31502	NA	F01_transcript_31502
F01_transcript_1138	F01_transcript_1138	F01_transcript_1138	NA	NA
F01_transcript_44017	NA	NA	NA	F01_transcript_44017
F01_transcript_7399	NA	NA	NA	F01_transcript_7399
F01_transcript_75960	NA	F01_transcript_75960	NA	F01_transcript_75960
F01_transcript_108841	NA	F01_transcript_108841	F01_transcript_108841	F01_transcript_108841
F01_transcript_121864	F01_transcript_121864	F01_transcript_121864	F01_transcript_121864	F01_transcript_121864
F01_transcript_164567	NA	F01_transcript_164567	NA	F01_transcript_164567
F01_transcript_41107	NA	NA	NA	F01_transcript_41107
F01_transcript_5159	NA	F01_transcript_5159	NA	F01_transcript_5159
F01_transcript_43461	NA	F01_transcript_43461	NA	NA

F01_transcript_91786	NA	NA	NA	F01_transcript_91786
F01_transcript_108103	NA	NA	NA	F01_transcript_108103
F01_transcript_28761	NA	NA	NA	F01_transcript_28761
F01_transcript_147856	F01_transcript_147856	F01_transcript_147856	F01_transcript_147856	F01_transcript_147856
F01_transcript_127108	NA	NA	NA	F01_transcript_127108
F01_transcript_105235	NA	NA	NA	F01_transcript_105235
F01_transcript_60792	NA	F01_transcript_60792	NA	F01_transcript_60792
F01_transcript_135260	F01_transcript_135260	F01_transcript_135260	F01_transcript_135260	F01_transcript_135260
F01_transcript_128832	F01_transcript_128832	F01_transcript_128832	F01_transcript_128832	F01_transcript_128832
F01_transcript_145931	NA	NA	NA	F01_transcript_145931
F01_transcript_164867	F01_transcript_164867	F01_transcript_164867	F01_transcript_164867	F01_transcript_164867
F01_transcript_107504	NA	NA	NA	F01_transcript_107504
F01_transcript_111244	NA	F01_transcript_111244	NA	NA
F01_transcript_109091	F01_transcript_109091	F01_transcript_109091	NA	F01_transcript_109091

F01_transcript_55474	NA	F01_transcript_55474	NA	NA
F01_transcript_73415	NA	F01_transcript_73415	NA	NA
F01_transcript_10420	NA	NA	NA	F01_transcript_10420
F01_transcript_1161	NA	NA	NA	F01_transcript_1161
F01_transcript_166690	F01_transcript_166690	F01_transcript_166690	NA	F01_transcript_166690
F01_transcript_64981	NA	F01_transcript_64981	NA	NA
F01_transcript_124013	NA	NA	NA	F01_transcript_124013
F01_transcript_157158	NA	NA	NA	F01_transcript_157158
F01_transcript_1376	NA	NA	NA	F01_transcript_1376
F01_transcript_98478	NA	F01_transcript_98478	NA	NA
F01_transcript_110761	F01_transcript_110761	F01_transcript_110761	F01_transcript_110761	F01_transcript_110761
F01_transcript_114882	F01_transcript_114882	F01_transcript_114882	F01_transcript_114882	F01_transcript_114882
F01_transcript_117756	NA	F01_transcript_117756	NA	F01_transcript_117756
F01_transcript_94416	F01_transcript_94416	F01_transcript_94416	NA	NA

F01_transcript_163552	NA	F01_transcript_163552	NA	NA
F01_transcript_127482	NA	NA	NA	F01_transcript_127482
F01_transcript_142721	NA	NA	NA	F01_transcript_142721
F01_transcript_71506	NA	NA	F01_transcript_71506	F01_transcript_71506
F01_transcript_159034	NA	F01_transcript_159034	F01_transcript_159034	NA
F01_transcript_9524	NA	NA	F01_transcript_9524	F01_transcript_9524
F01_transcript_150047	NA	F01_transcript_150047	F01_transcript_150047	F01_transcript_150047
F01_transcript_150965	NA	NA	NA	F01_transcript_150965
F01_transcript_118366	F01_transcript_118366	F01_transcript_118366	F01_transcript_118366	F01_transcript_118366
F01_transcript_124823	NA	F01_transcript_124823	NA	NA
F01_transcript_59980	NA	F01_transcript_59980	NA	NA
F01_transcript_28141	NA	NA	NA	F01_transcript_28141
F01_transcript_126548	F01_transcript_126548	F01_transcript_126548	F01_transcript_126548	F01_transcript_126548
F01_transcript_67092	NA	NA	NA	F01_transcript_67092

F01_transcript_55797	F01_transcript_55797	F01_transcript_55797	NA	F01_transcript_55797
F01_transcript_114480	NA	F01_transcript_114480	NA	NA
F01_transcript_126033	NA	NA	NA	F01_transcript_126033
F01_transcript_21891	F01_transcript_21891	F01_transcript_21891	NA	NA
F01_transcript_58821	NA	NA	NA	F01_transcript_58821
F01_transcript_159865	F01_transcript_159865	F01_transcript_159865	F01_transcript_159865	F01_transcript_159865
F01_transcript_115278	NA	NA	NA	F01_transcript_115278
F01_transcript_79535	NA	F01_transcript_79535	NA	F01_transcript_79535
F01_transcript_93743	F01_transcript_93743	NA	NA	NA
F01_transcript_61826	NA	F01_transcript_61826	NA	NA
F01_transcript_116704	F01_transcript_116704	F01_transcript_116704	F01_transcript_116704	F01_transcript_116704
F01_transcript_154019	NA	F01_transcript_154019	NA	NA
F01_transcript_111604	F01_transcript_111604	F01_transcript_111604	NA	NA
F01_transcript_160904	NA	NA	NA	F01_transcript_160904

F01_transcript_122330	F01_transcript_122330	F01_transcript_122330	F01_transcript_122330	F01_transcript_122330
F01_transcript_116464	NA	NA	NA	F01_transcript_116464
F01_transcript_161173	NA	NA	NA	F01_transcript_161173
F01_transcript_135197	NA	F01_transcript_135197	NA	F01_transcript_135197
F01_transcript_80870	NA	F01_transcript_80870	NA	NA
F01_transcript_106499	NA	F01_transcript_106499	NA	F01_transcript_106499
F01_transcript_9689	NA	F01_transcript_9689	NA	F01_transcript_9689
F01_transcript_19572	NA	NA	NA	F01_transcript_19572
F01_transcript_145976	F01_transcript_145976	F01_transcript_145976	NA	F01_transcript_145976
F01_transcript_15284	F01_transcript_15284	F01_transcript_15284	NA	NA
F01_transcript_152342	NA	F01_transcript_152342	NA	NA
F01_transcript_83793	NA	NA	NA	F01_transcript_83793
F01_transcript_135133	NA	NA	NA	F01_transcript_135133
F01_transcript_162441	NA	F01_transcript_162441	NA	NA

F01_transcript_137346	NA	NA	NA	F01_transcript_137346
F01_transcript_97565	F01_transcript_97565	F01_transcript_97565	F01_transcript_97565	F01_transcript_97565
F01_transcript_90501	F01_transcript_90501	F01_transcript_90501	F01_transcript_90501	F01_transcript_90501
F01_transcript_83193	NA	NA	NA	F01_transcript_83193
F01_transcript_157354	F01_transcript_157354	F01_transcript_157354	F01_transcript_157354	F01_transcript_157354
F01_transcript_71069	NA	F01_transcript_71069	F01_transcript_71069	NA
F01_transcript_131499	F01_transcript_131499	F01_transcript_131499	NA	F01_transcript_131499
F01_transcript_81412	NA	F01_transcript_81412	F01_transcript_81412	NA
F01_transcript_148059	NA	NA	NA	F01_transcript_148059
F01_transcript_52839	NA	NA	NA	F01_transcript_52839
F01_transcript_104669	NA	NA	NA	F01_transcript_104669
F01_transcript_167092	F01_transcript_167092	F01_transcript_167092	F01_transcript_167092	F01_transcript_167092
F01_transcript_86879	NA	NA	NA	F01_transcript_86879
F01_transcript_104156	NA	F01_transcript_104156	F01_transcript_104156	F01_transcript_104156

F01_transcript_129032	F01_transcript_129032	F01_transcript_129032	F01_transcript_129032	F01_transcript_129032
F01_transcript_12805	F01_transcript_12805	F01_transcript_12805	F01_transcript_12805	F01_transcript_12805
F01_transcript_121597	NA	NA	NA	F01_transcript_121597
F01_transcript_134127	NA	NA	NA	F01_transcript_134127
F01_transcript_144135	NA	NA	NA	F01_transcript_144135
F01_transcript_75537	NA	F01_transcript_75537	NA	NA
F01_transcript_101733	F01_transcript_101733	F01_transcript_101733	F01_transcript_101733	F01_transcript_101733
F01_transcript_76657	NA	F01_transcript_76657	F01_transcript_76657	F01_transcript_76657
F01_transcript_59598	NA	NA	F01_transcript_59598	NA
F01_transcript_152179	NA	F01_transcript_152179	NA	NA
F01_transcript_102606	NA	F01_transcript_102606	NA	NA
F01_transcript_54816	NA	NA	NA	F01_transcript_54816
F01_transcript_59386	NA	NA	F01_transcript_59386	F01_transcript_59386
F01_transcript_79572	NA	F01_transcript_79572	NA	NA

F01_transcript_161831	F01_transcript_161831	F01_transcript_161831	NA	F01_transcript_161831
F01_transcript_111340	F01_transcript_111340	F01_transcript_111340	NA	NA
F01_transcript_70373	F01_transcript_70373	F01_transcript_70373	NA	NA
F01_transcript_48068	F01_transcript_48068	F01_transcript_48068	F01_transcript_48068	F01_transcript_48068
F01_transcript_154880	F01_transcript_154880	F01_transcript_154880	NA	F01_transcript_154880
F01_transcript_103538	NA	NA	NA	F01_transcript_103538
F01_transcript_134001	NA	NA	NA	F01_transcript_134001
F01_transcript_57219	F01_transcript_57219	F01_transcript_57219	F01_transcript_57219	F01_transcript_57219
F01_transcript_72096	NA	F01_transcript_72096	NA	NA
F01_transcript_68992	NA	F01_transcript_68992	NA	NA
F01_transcript_5404	NA	NA	NA	F01_transcript_5404
F01_transcript_60690	NA	NA	NA	F01_transcript_60690
F01_transcript_165400	NA	F01_transcript_165400	NA	NA
F01_transcript_57114	F01_transcript_57114	NA	F01_transcript_57114	F01_transcript_57114

F01_transcript_110622	NA	NA	NA	F01_transcript_110622
F01_transcript_131234	F01_transcript_131234	F01_transcript_131234	NA	NA
F01_transcript_163934	F01_transcript_163934	F01_transcript_163934	F01_transcript_163934	F01_transcript_163934
F01_transcript_132594	NA	NA	NA	F01_transcript_132594
F01_transcript_81838	NA	NA	NA	F01_transcript_81838
F01_transcript_122169	NA	NA	NA	F01_transcript_122169
F01_transcript_166265	NA	NA	F01_transcript_166265	F01_transcript_166265
F01_transcript_97486	NA	F01_transcript_97486	NA	F01_transcript_97486
F01_transcript_15651	NA	NA	NA	F01_transcript_15651
F01_transcript_56353	NA	F01_transcript_56353	F01_transcript_56353	NA
F01_transcript_23781	NA	NA	NA	F01_transcript_23781
F01_transcript_48152	NA	F01_transcript_48152	F01_transcript_48152	F01_transcript_48152
F01_transcript_132925	F01_transcript_132925	F01_transcript_132925	NA	F01_transcript_132925
F01_transcript_148481	NA	NA	F01_transcript_148481	F01_transcript_148481

F01_transcript_60649	NA	F01_transcript_60649	NA	NA
F01_transcript_118825	F01_transcript_118825	F01_transcript_118825	F01_transcript_118825	F01_transcript_118825
F01_transcript_67695	NA	NA	NA	F01_transcript_67695
F01_transcript_58005	F01_transcript_58005	F01_transcript_58005	NA	NA
F01_transcript_55209	NA	NA	NA	F01_transcript_55209
F01_transcript_149577	NA	F01_transcript_149577	NA	NA
F01_transcript_66539	NA	F01_transcript_66539	NA	NA
F01_transcript_32958	NA	NA	NA	F01_transcript_32958
F01_transcript_78757	NA	NA	NA	F01_transcript_78757
F01_transcript_151547	NA	F01_transcript_151547	NA	F01_transcript_151547
F01_transcript_112721	F01_transcript_112721	F01_transcript_112721	NA	F01_transcript_112721
F01_transcript_82788	F01_transcript_82788	F01_transcript_82788	F01_transcript_82788	F01_transcript_82788
F01_transcript_104275	F01_transcript_104275	F01_transcript_104275	NA	NA
F01_transcript_84572	NA	F01_transcript_84572	NA	NA

F01_transcript_96062	NA	NA	NA	F01_transcript_96062
F01_transcript_132920	NA	F01_transcript_132920	NA	F01_transcript_132920
F01_transcript_47678	F01_transcript_47678	F01_transcript_47678	F01_transcript_47678	F01_transcript_47678
F01_transcript_61007	F01_transcript_61007	F01_transcript_61007	F01_transcript_61007	F01_transcript_61007
F01_transcript_72191	NA	F01_transcript_72191	NA	NA
F01_transcript_50977	F01_transcript_50977	F01_transcript_50977	F01_transcript_50977	F01_transcript_50977
F01_transcript_158760	NA	NA	F01_transcript_158760	F01_transcript_158760
F01_transcript_101794	NA	NA	NA	F01_transcript_101794
F01_transcript_109465	F01_transcript_109465	F01_transcript_109465	F01_transcript_109465	F01_transcript_109465
F01_transcript_156380	NA	F01_transcript_156380	NA	NA
F01_transcript_72689	NA	NA	NA	F01_transcript_72689
F01_transcript_43141	NA	F01_transcript_43141	NA	NA
F01_transcript_160777	NA	NA	NA	F01_transcript_160777
F01_transcript_110472	NA	F01_transcript_110472	NA	NA

F01_transcript_122946	F01_transcript_122946	F01_transcript_122946	F01_transcript_122946	F01_transcript_122946
F01_transcript_25934	NA	F01_transcript_25934	NA	F01_transcript_25934
F01_transcript_102262	NA	F01_transcript_102262	NA	F01_transcript_102262
F01_transcript_101979	NA	F01_transcript_101979	NA	F01_transcript_101979
F01_transcript_143674	F01_transcript_143674	F01_transcript_143674	F01_transcript_143674	F01_transcript_143674
F01_transcript_100687	F01_transcript_100687	F01_transcript_100687	F01_transcript_100687	F01_transcript_100687
F01_transcript_90475	NA	F01_transcript_90475	F01_transcript_90475	F01_transcript_90475
F01_transcript_108089	NA	NA	NA	F01_transcript_108089
F01_transcript_12319	NA	NA	NA	F01_transcript_12319
F01_transcript_101666	NA	F01_transcript_101666	NA	NA
F01_transcript_139069	F01_transcript_139069	F01_transcript_139069	F01_transcript_139069	F01_transcript_139069
F01_transcript_40491	NA	NA	NA	F01_transcript_40491
F01_transcript_53598	NA	F01_transcript_53598	NA	NA
F01_transcript_76661	NA	F01_transcript_76661	NA	NA

F01_transcript_61143	NA	F01_transcript_61143	F01_transcript_61143	F01_transcript_61143
F01_transcript_78262	NA	F01_transcript_78262	NA	NA
F01_transcript_60411	F01_transcript_60411	F01_transcript_60411	F01_transcript_60411	F01_transcript_60411
F01_transcript_26119	NA	F01_transcript_26119	F01_transcript_26119	F01_transcript_26119
F01_transcript_80066	NA	NA	NA	F01_transcript_80066
F01_transcript_82351	F01_transcript_82351	F01_transcript_82351	NA	NA
F01_transcript_86606	F01_transcript_86606	NA	NA	NA
F01_transcript_129783	F01_transcript_129783	F01_transcript_129783	F01_transcript_129783	F01_transcript_129783
F01_transcript_115055	NA	F01_transcript_115055	F01_transcript_115055	F01_transcript_115055
F01_transcript_85823	NA	F01_transcript_85823	NA	NA
F01_transcript_77887	NA	F01_transcript_77887	NA	NA
F01_transcript_87882	NA	NA	NA	F01_transcript_87882
F01_transcript_155064	NA	F01_transcript_155064	NA	NA
F01_transcript_115327	NA	NA	NA	F01_transcript_115327

F01_transcript_91402	NA	NA	NA	F01_transcript_91402
F01_transcript_109446	F01_transcript_109446	F01_transcript_109446	F01_transcript_109446	NA
F01_transcript_134281	NA	NA	NA	F01_transcript_134281
F01_transcript_160601	NA	F01_transcript_160601	NA	NA
F01_transcript_108852	F01_transcript_108852	F01_transcript_108852	F01_transcript_108852	NA
F01_transcript_30828	NA	NA	NA	F01_transcript_30828
F01_transcript_66265	F01_transcript_66265	F01_transcript_66265	F01_transcript_66265	F01_transcript_66265
F01_transcript_103849	NA	NA	NA	F01_transcript_103849
F01_transcript_100474	F01_transcript_100474	F01_transcript_100474	NA	NA
F01_transcript_118078	NA	F01_transcript_118078	NA	F01_transcript_118078
F01_transcript_80477	NA	F01_transcript_80477	F01_transcript_80477	NA
F01_transcript_152233	NA	F01_transcript_152233	NA	NA
F01_transcript_105789	NA	F01_transcript_105789	NA	NA
F01_transcript_87260	NA	F01_transcript_87260	NA	F01_transcript_87260

F01_transcript_89113	NA	NA	NA	F01_transcript_89113
F01_transcript_98836	F01_transcript_98836	F01_transcript_98836	NA	NA
F01_transcript_146686	F01_transcript_146686	F01_transcript_146686	F01_transcript_146686	F01_transcript_146686
F01_transcript_119678	F01_transcript_119678	F01_transcript_119678	F01_transcript_119678	F01_transcript_119678
F01_transcript_68814	F01_transcript_68814	F01_transcript_68814	F01_transcript_68814	F01_transcript_68814
F01_transcript_148975	NA	NA	NA	F01_transcript_148975
F01_transcript_22678	NA	NA	NA	F01_transcript_22678
F01_transcript_65216	NA	F01_transcript_65216	F01_transcript_65216	F01_transcript_65216
F01_transcript_72551	NA	F01_transcript_72551	NA	F01_transcript_72551
F01_transcript_119029	NA	NA	F01_transcript_119029	NA
F01_transcript_75905	NA	F01_transcript_75905	NA	NA
F01_transcript_107648	NA	F01_transcript_107648	NA	NA
F01_transcript_138387	NA	NA	NA	F01_transcript_138387
F01_transcript_158940	NA	NA	F01_transcript_158940	NA

F01_transcript_76420	F01_transcript_76420	F01_transcript_76420	F01_transcript_76420	F01_transcript_76420
F01_transcript_66369	NA	F01_transcript_66369	NA	NA
F01_transcript_18711	NA	F01_transcript_18711	F01_transcript_18711	NA
F01_transcript_91902	F01_transcript_91902	F01_transcript_91902	F01_transcript_91902	F01_transcript_91902
F01_transcript_154039	NA	F01_transcript_154039	NA	F01_transcript_154039
F01_transcript_104159	NA	F01_transcript_104159	NA	NA
F01_transcript_68222	NA	F01_transcript_68222	NA	NA
F01_transcript_102290	F01_transcript_102290	F01_transcript_102290	F01_transcript_102290	F01_transcript_102290
F01_transcript_72511	F01_transcript_72511	F01_transcript_72511	NA	F01_transcript_72511
F01_transcript_87936	NA	NA	NA	F01_transcript_87936
F01_transcript_140454	F01_transcript_140454	F01_transcript_140454	F01_transcript_140454	F01_transcript_140454
F01_transcript_50301	F01_transcript_50301	F01_transcript_50301	F01_transcript_50301	NA
F01_transcript_145367	NA	F01_transcript_145367	NA	NA
F01_transcript_127116	F01_transcript_127116	F01_transcript_127116	NA	NA

F01_transcript_140666	NA	NA	NA	F01_transcript_140666
F01_transcript_114748	F01_transcript_114748	F01_transcript_114748	NA	NA
F01_transcript_53336	NA	F01_transcript_53336	NA	NA
F01_transcript_114182	NA	F01_transcript_114182	NA	NA
F01_transcript_123205	F01_transcript_123205	F01_transcript_123205	F01_transcript_123205	F01_transcript_123205
F01_transcript_145542	NA	F01_transcript_145542	NA	NA
F01_transcript_98979	F01_transcript_98979	F01_transcript_98979	F01_transcript_98979	NA
F01_transcript_114100	NA	F01_transcript_114100	NA	F01_transcript_114100
F01_transcript_49007	F01_transcript_49007	F01_transcript_49007	NA	F01_transcript_49007
F01_transcript_144721	F01_transcript_144721	F01_transcript_144721	NA	F01_transcript_144721
F01_transcript_111557	NA	F01_transcript_111557	NA	F01_transcript_111557
F01_transcript_102835	F01_transcript_102835	F01_transcript_102835	F01_transcript_102835	F01_transcript_102835
F01_transcript_18888	NA	F01_transcript_18888	F01_transcript_18888	F01_transcript_18888
F01_transcript_12246	NA	NA	NA	F01_transcript_12246

F01_transcript_115335	NA	F01_transcript_115335	F01_transcript_115335	F01_transcript_115335
F01_transcript_110768	F01_transcript_110768	F01_transcript_110768	F01_transcript_110768	F01_transcript_110768
F01_transcript_155167	NA	NA	NA	F01_transcript_155167
F01_transcript_143039	NA	NA	NA	F01_transcript_143039
F01_transcript_97232	NA	F01_transcript_97232	F01_transcript_97232	F01_transcript_97232
F01_transcript_131971	F01_transcript_131971	F01_transcript_131971	F01_transcript_131971	F01_transcript_131971
F01_transcript_80180	NA	NA	NA	F01_transcript_80180
F01_transcript_57778	F01_transcript_57778	F01_transcript_57778	F01_transcript_57778	F01_transcript_57778
F01_transcript_130666	F01_transcript_130666	F01_transcript_130666	F01_transcript_130666	F01_transcript_130666
F01_transcript_136841	NA	F01_transcript_136841	NA	NA
F01_transcript_154370	F01_transcript_154370	F01_transcript_154370	F01_transcript_154370	F01_transcript_154370
F01_transcript_138965	NA	F01_transcript_138965	F01_transcript_138965	F01_transcript_138965
F01_transcript_54062	NA	F01_transcript_54062	NA	NA
F01_transcript_33756	NA	F01_transcript_33756	NA	F01_transcript_33756

F01_transcript_87591	NA	F01_transcript_87591	NA	NA
F01_transcript_79385	F01_transcript_79385	F01_transcript_79385	F01_transcript_79385	NA
F01_transcript_89474	NA	NA	NA	F01_transcript_89474
F01_transcript_40399	F01_transcript_40399	F01_transcript_40399	F01_transcript_40399	F01_transcript_40399
F01_transcript_139124	F01_transcript_139124	F01_transcript_139124	F01_transcript_139124	F01_transcript_139124
F01_transcript_50079	F01_transcript_50079	NA	NA	F01_transcript_50079
F01_transcript_4522	NA	NA	NA	F01_transcript_4522
F01_transcript_80926	NA	F01_transcript_80926	NA	NA
F01_transcript_42964	NA	NA	NA	F01_transcript_42964
F01_transcript_92882	F01_transcript_92882	F01_transcript_92882	F01_transcript_92882	F01_transcript_92882
F01_transcript_150199	F01_transcript_150199	F01_transcript_150199	F01_transcript_150199	NA
F01_transcript_151261	F01_transcript_151261	F01_transcript_151261	NA	NA
F01_transcript_153622	NA	NA	NA	F01_transcript_153622
F01_transcript_82408	NA	NA	NA	F01_transcript_82408

F01_transcript_73056	NA	NA	NA	F01_transcript_73056
F01_transcript_77997	NA	NA	NA	F01_transcript_77997
F01_transcript_74174	NA	F01_transcript_74174	NA	F01_transcript_74174
F01_transcript_118057	NA	F01_transcript_118057	NA	NA
F01_transcript_59394	NA	F01_transcript_59394	F01_transcript_59394	F01_transcript_59394
F01_transcript_162973	NA	NA	NA	F01_transcript_162973
F01_transcript_115313	NA	NA	NA	F01_transcript_115313
F01_transcript_42250	NA	NA	NA	F01_transcript_42250
F01_transcript_137839	F01_transcript_137839	F01_transcript_137839	F01_transcript_137839	F01_transcript_137839
F01_transcript_116893	F01_transcript_116893	F01_transcript_116893	NA	F01_transcript_116893
F01_transcript_89173	NA	NA	NA	F01_transcript_89173
F01_transcript_103390	NA	F01_transcript_103390	NA	F01_transcript_103390
F01_transcript_89695	NA	NA	NA	F01_transcript_89695
F01_transcript_75981	NA	F01_transcript_75981	F01_transcript_75981	F01_transcript_75981

F01_transcript_92373	NA	NA	NA	F01_transcript_92373
F01_transcript_129638	NA	F01_transcript_129638	NA	NA
F01_transcript_143484	NA	F01_transcript_143484	F01_transcript_143484	F01_transcript_143484
F01_transcript_45213	NA	NA	NA	F01_transcript_45213
F01_transcript_80875	F01_transcript_80875	F01_transcript_80875	F01_transcript_80875	F01_transcript_80875
F01_transcript_139242	NA	F01_transcript_139242	NA	NA
F01_transcript_10965	NA	NA	NA	F01_transcript_10965
F01_transcript_86761	F01_transcript_86761	F01_transcript_86761	F01_transcript_86761	F01_transcript_86761
F01_transcript_104103	NA	F01_transcript_104103	NA	F01_transcript_104103
F01_transcript_10272	NA	NA	NA	F01_transcript_10272
F01_transcript_152113	F01_transcript_152113	F01_transcript_152113	F01_transcript_152113	F01_transcript_152113
F01_transcript_164155	NA	F01_transcript_164155	NA	NA
F01_transcript_8390	NA	NA	NA	F01_transcript_8390
F01_transcript_142252	NA	F01_transcript_142252	NA	F01_transcript_142252

F01_transcript_151748	F01_transcript_151748	F01_transcript_151748	NA	NA
F01_transcript_116643	NA	NA	NA	F01_transcript_116643
F01_transcript_1928	NA	F01_transcript_1928	NA	NA
F01_transcript_142783	F01_transcript_142783	F01_transcript_142783	NA	NA
F01_transcript_94459	NA	F01_transcript_94459	NA	NA
F01_transcript_140089	NA	F01_transcript_140089	NA	NA
F01_transcript_60167	NA	NA	NA	F01_transcript_60167
F01_transcript_58110	NA	F01_transcript_58110	NA	NA
F01_transcript_144580	NA	F01_transcript_144580	NA	NA
F01_transcript_1289	NA	NA	NA	F01_transcript_1289
F01_transcript_156641	NA	NA	F01_transcript_156641	NA
F01_transcript_132504	NA	F01_transcript_132504	NA	NA
F01_transcript_32173	F01_transcript_32173	F01_transcript_32173	F01_transcript_32173	F01_transcript_32173
F01_transcript_92783	NA	F01_transcript_92783	F01_transcript_92783	F01_transcript_92783

F01_transcript_124879	NA	NA	NA	F01_transcript_124879
F01_transcript_87842	NA	F01_transcript_87842	F01_transcript_87842	F01_transcript_87842
F01_transcript_38545	NA	NA	NA	F01_transcript_38545
F01_transcript_112157	F01_transcript_112157	F01_transcript_112157	F01_transcript_112157	F01_transcript_112157
F01_transcript_90785	NA	F01_transcript_90785	NA	NA
F01_transcript_81521	NA	F01_transcript_81521	NA	F01_transcript_81521
F01_transcript_103707	F01_transcript_103707	F01_transcript_103707	NA	F01_transcript_103707
F01_transcript_134484	NA	NA	NA	F01_transcript_134484
F01_transcript_60240	NA	F01_transcript_60240	F01_transcript_60240	F01_transcript_60240
F01_transcript_134160	F01_transcript_134160	F01_transcript_134160	NA	NA
F01_transcript_61720	NA	NA	NA	F01_transcript_61720
F01_transcript_135451	NA	NA	NA	F01_transcript_135451
F01_transcript_66961	NA	F01_transcript_66961	NA	F01_transcript_66961
F01_transcript_149859	F01_transcript_149859	F01_transcript_149859	NA	NA

F01_transcript_104846	NA	NA	NA	F01_transcript_104846
F01_transcript_66470	NA	F01_transcript_66470	F01_transcript_66470	F01_transcript_66470
F01_transcript_130259	F01_transcript_130259	F01_transcript_130259	F01_transcript_130259	NA
F01_transcript_3489	NA	NA	NA	F01_transcript_3489
F01_transcript_125019	F01_transcript_125019	F01_transcript_125019	F01_transcript_125019	F01_transcript_125019
F01_transcript_147503	NA	F01_transcript_147503	NA	F01_transcript_147503
F01_transcript_118623	NA	NA	NA	F01_transcript_118623
F01_transcript_49787	NA	F01_transcript_49787	NA	F01_transcript_49787
F01_transcript_128691	NA	F01_transcript_128691	F01_transcript_128691	F01_transcript_128691
F01_transcript_164026	F01_transcript_164026	F01_transcript_164026	F01_transcript_164026	F01_transcript_164026
F01_transcript_153691	F01_transcript_153691	F01_transcript_153691	NA	F01_transcript_153691
F01_transcript_108828	F01_transcript_108828	F01_transcript_108828	F01_transcript_108828	F01_transcript_108828
F01_transcript_67436	NA	NA	NA	F01_transcript_67436
F01_transcript_152478	F01_transcript_152478	F01_transcript_152478	NA	NA

F01_transcript_64384	NA	NA	NA	F01_transcript_64384
F01_transcript_118733	NA	NA	NA	F01_transcript_118733
F01_transcript_52	NA	NA	NA	F01_transcript_52
F01_transcript_97762	NA	NA	NA	F01_transcript_97762
F01_transcript_147466	F01_transcript_147466	F01_transcript_147466	F01_transcript_147466	F01_transcript_147466
F01_transcript_100752	NA	NA	NA	F01_transcript_100752
F01_transcript_129359	F01_transcript_129359	F01_transcript_129359	F01_transcript_129359	F01_transcript_129359
F01_transcript_119052	F01_transcript_119052	F01_transcript_119052	F01_transcript_119052	F01_transcript_119052
F01_transcript_137771	NA	NA	NA	F01_transcript_137771
F01_transcript_158267	NA	F01_transcript_158267	NA	F01_transcript_158267
F01_transcript_71022	F01_transcript_71022	F01_transcript_71022	F01_transcript_71022	F01_transcript_71022
F01_transcript_118669	NA	F01_transcript_118669	NA	F01_transcript_118669
F01_transcript_146237	NA	F01_transcript_146237	NA	NA
F01_transcript_102794	F01_transcript_102794	NA	NA	NA

F01_transcript_117327	NA	F01_transcript_117327	NA	NA
F01_transcript_133968	NA	F01_transcript_133968	NA	F01_transcript_133968
F01_transcript_140626	NA	F01_transcript_140626	NA	NA
F01_transcript_165881	F01_transcript_165881	NA	F01_transcript_165881	F01_transcript_165881
F01_transcript_69842	NA	NA	F01_transcript_69842	F01_transcript_69842
F01_transcript_124646	NA	F01_transcript_124646	NA	F01_transcript_124646
F01_transcript_40137	NA	NA	NA	F01_transcript_40137
F01_transcript_133738	F01_transcript_133738	F01_transcript_133738	NA	F01_transcript_133738
F01_transcript_10750	NA	F01_transcript_10750	NA	NA
F01_transcript_78013	F01_transcript_78013	NA	NA	NA
F01_transcript_150413	NA	F01_transcript_150413	F01_transcript_150413	F01_transcript_150413
F01_transcript_136302	NA	F01_transcript_136302	NA	NA
F01_transcript_93315	NA	NA	NA	F01_transcript_93315
F01_transcript_99859	NA	F01_transcript_99859	NA	F01_transcript_99859

F01_transcript_82882	F01_transcript_82882	F01_transcript_82882	NA	F01_transcript_82882
F01_transcript_120414	F01_transcript_120414	F01_transcript_120414	NA	NA
F01_transcript_117660	F01_transcript_117660	F01_transcript_117660	F01_transcript_117660	F01_transcript_117660
F01_transcript_131537	NA	NA	NA	F01_transcript_131537
F01_transcript_80885	NA	NA	NA	F01_transcript_80885
F01_transcript_136806	NA	F01_transcript_136806	F01_transcript_136806	F01_transcript_136806
F01_transcript_164303	F01_transcript_164303	F01_transcript_164303	F01_transcript_164303	F01_transcript_164303
F01_transcript_98693	NA	NA	NA	F01_transcript_98693
F01_transcript_60738	F01_transcript_60738	F01_transcript_60738	F01_transcript_60738	F01_transcript_60738
F01_transcript_126916	NA	F01_transcript_126916	NA	NA
F01_transcript_30894	NA	NA	NA	F01_transcript_30894
F01_transcript_152684	NA	F01_transcript_152684	NA	NA
F01_transcript_67519	NA	F01_transcript_67519	NA	NA
F01_transcript_110611	F01_transcript_110611	F01_transcript_110611	F01_transcript_110611	F01_transcript_110611

F01_transcript_113318	NA	F01_transcript_113318	NA	NA
F01_transcript_30066	F01_transcript_30066	NA	F01_transcript_30066	NA
F01_transcript_48500	F01_transcript_48500	F01_transcript_48500	F01_transcript_48500	F01_transcript_48500
F01_transcript_63134	F01_transcript_63134	F01_transcript_63134	F01_transcript_63134	F01_transcript_63134
F01_transcript_111172	NA	NA	NA	F01_transcript_111172
F01_transcript_128828	NA	NA	NA	F01_transcript_128828
F01_transcript_85235	NA	F01_transcript_85235	NA	NA
F01_transcript_84519	F01_transcript_84519	F01_transcript_84519	F01_transcript_84519	F01_transcript_84519
F01_transcript_137573	F01_transcript_137573	F01_transcript_137573	F01_transcript_137573	F01_transcript_137573
F01_transcript_122815	NA	NA	NA	F01_transcript_122815
F01_transcript_79421	NA	NA	NA	F01_transcript_79421
F01_transcript_54604	NA	F01_transcript_54604	NA	NA
F01_transcript_148985	F01_transcript_148985	F01_transcript_148985	F01_transcript_148985	F01_transcript_148985
F01_transcript_96356	NA	NA	NA	F01_transcript_96356

F01_transcript_83204	NA	NA	NA	F01_transcript_83204
F01_transcript_84571	F01_transcript_84571	F01_transcript_84571	F01_transcript_84571	F01_transcript_84571
F01_transcript_95070	NA	F01_transcript_95070	F01_transcript_95070	F01_transcript_95070
F01_transcript_558	NA	NA	NA	F01_transcript_558
F01_transcript_89530	F01_transcript_89530	F01_transcript_89530	F01_transcript_89530	F01_transcript_89530
F01_transcript_79285	F01_transcript_79285	F01_transcript_79285	NA	F01_transcript_79285
F01_transcript_87124	F01_transcript_87124	F01_transcript_87124	F01_transcript_87124	F01_transcript_87124
F01_transcript_91553	NA	NA	NA	F01_transcript_91553
F01_transcript_85693	NA	F01_transcript_85693	NA	F01_transcript_85693
F01_transcript_121880	NA	NA	NA	F01_transcript_121880
F01_transcript_59814	NA	F01_transcript_59814	NA	NA
F01_transcript_5689	NA	NA	F01_transcript_5689	NA
F01_transcript_60130	F01_transcript_60130	F01_transcript_60130	F01_transcript_60130	F01_transcript_60130
F01_transcript_154623	NA	NA	NA	F01_transcript_154623

F01_transcript_166765	NA	NA	NA	F01_transcript_166765
F01_transcript_5576	NA	NA	NA	F01_transcript_5576
F01_transcript_160345	NA	F01_transcript_160345	NA	F01_transcript_160345
F01_transcript_106378	F01_transcript_106378	F01_transcript_106378	F01_transcript_106378	F01_transcript_106378
F01_transcript_48575	NA	F01_transcript_48575	NA	NA
F01_transcript_167193	NA	NA	F01_transcript_167193	NA
F01_transcript_115548	F01_transcript_115548	F01_transcript_115548	F01_transcript_115548	F01_transcript_115548
F01_transcript_62321	NA	F01_transcript_62321	F01_transcript_62321	NA
F01_transcript_58983	NA	NA	NA	F01_transcript_58983
F01_transcript_110004	NA	NA	NA	F01_transcript_110004
F01_transcript_155928	NA	NA	NA	F01_transcript_155928
F01_transcript_12452	F01_transcript_12452	F01_transcript_12452	NA	NA
F01_transcript_43437	NA	NA	NA	F01_transcript_43437
F01_transcript_69805	NA	F01_transcript_69805	F01_transcript_69805	F01_transcript_69805

F01_transcript_110089	F01_transcript_110089	F01_transcript_110089	F01_transcript_110089	F01_transcript_110089
F01_transcript_126591	F01_transcript_126591	F01_transcript_126591	F01_transcript_126591	F01_transcript_126591
F01_transcript_137880	F01_transcript_137880	F01_transcript_137880	F01_transcript_137880	F01_transcript_137880
F01_transcript_76628	NA	NA	NA	F01_transcript_76628
F01_transcript_10966	NA	F01_transcript_10966	NA	NA
F01_transcript_77041	NA	F01_transcript_77041	NA	NA
F01_transcript_156305	NA	F01_transcript_156305	NA	F01_transcript_156305
F01_transcript_48311	NA	F01_transcript_48311	NA	NA
F01_transcript_106468	NA	NA	NA	F01_transcript_106468
F01_transcript_75822	F01_transcript_75822	F01_transcript_75822	NA	F01_transcript_75822
F01_transcript_100274	NA	F01_transcript_100274	NA	NA
F01_transcript_132989	NA	NA	NA	F01_transcript_132989
F01_transcript_23087	NA	F01_transcript_23087	F01_transcript_23087	F01_transcript_23087
F01_transcript_72837	F01_transcript_72837	F01_transcript_72837	NA	NA

F01_transcript_76357	NA	F01_transcript_76357	NA	F01_transcript_76357
F01_transcript_112858	NA	NA	NA	F01_transcript_112858
F01_transcript_81734	NA	F01_transcript_81734	NA	NA
F01_transcript_163077	F01_transcript_163077	F01_transcript_163077	F01_transcript_163077	F01_transcript_163077
F01_transcript_127724	NA	F01_transcript_127724	NA	NA
F01_transcript_60302	F01_transcript_60302	F01_transcript_60302	NA	F01_transcript_60302
F01_transcript_154875	NA	F01_transcript_154875	NA	NA
F01_transcript_119051	NA	F01_transcript_119051	NA	NA
F01_transcript_5832	NA	NA	NA	F01_transcript_5832
F01_transcript_49487	NA	F01_transcript_49487	NA	NA
F01_transcript_136717	F01_transcript_136717	F01_transcript_136717	F01_transcript_136717	F01_transcript_136717
F01_transcript_50655	F01_transcript_50655	F01_transcript_50655	F01_transcript_50655	F01_transcript_50655
F01_transcript_97522	NA	NA	NA	F01_transcript_97522
F01_transcript_152030	NA	NA	NA	F01_transcript_152030

F01_transcript_133486	F01_transcript_133486	NA	NA	NA
F01_transcript_4219	NA	F01_transcript_4219	NA	NA
F01_transcript_44565	F01_transcript_44565	F01_transcript_44565	F01_transcript_44565	F01_transcript_44565
F01_transcript_95807	NA	NA	NA	F01_transcript_95807
F01_transcript_142354	NA	NA	NA	F01_transcript_142354
F01_transcript_133695	F01_transcript_133695	F01_transcript_133695	F01_transcript_133695	F01_transcript_133695
F01_transcript_131512	F01_transcript_131512	F01_transcript_131512	F01_transcript_131512	F01_transcript_131512
F01_transcript_124615	NA	NA	NA	F01_transcript_124615
F01_transcript_109467	NA	NA	NA	F01_transcript_109467
F01_transcript_7989	NA	F01_transcript_7989	NA	NA
F01_transcript_104538	F01_transcript_104538	F01_transcript_104538	F01_transcript_104538	F01_transcript_104538
F01_transcript_116311	NA	F01_transcript_116311	NA	NA
F01_transcript_70273	NA	F01_transcript_70273	NA	F01_transcript_70273
F01_transcript_96287	NA	NA	NA	F01_transcript_96287

F01_transcript_102129	NA	F01_transcript_102129	NA	F01_transcript_102129
F01_transcript_95514	NA	NA	NA	F01_transcript_95514
F01_transcript_66295	NA	F01_transcript_66295	NA	NA
F01_transcript_159652	F01_transcript_159652	F01_transcript_159652	NA	F01_transcript_159652
F01_transcript_69522	F01_transcript_69522	F01_transcript_69522	NA	NA
F01_transcript_94717	F01_transcript_94717	F01_transcript_94717	F01_transcript_94717	F01_transcript_94717
F01_transcript_155878	NA	NA	NA	F01_transcript_155878
F01_transcript_143706	F01_transcript_143706	F01_transcript_143706	NA	NA
F01_transcript_112486	NA	F01_transcript_112486	NA	NA
F01_transcript_154963	F01_transcript_154963	F01_transcript_154963	NA	NA
F01_transcript_158893	NA	NA	F01_transcript_158893	NA
F01_transcript_126186	F01_transcript_126186	F01_transcript_126186	F01_transcript_126186	NA
F01_transcript_97281	NA	NA	NA	F01_transcript_97281
F01_transcript_95740	NA	F01_transcript_95740	NA	NA

F01_transcript_28101	NA	NA	NA	F01_transcript_28101
F01_transcript_102034	NA	NA	NA	F01_transcript_102034
F01_transcript_3651	F01_transcript_3651	F01_transcript_3651	F01_transcript_3651	F01_transcript_3651
F01_transcript_160877	NA	F01_transcript_160877	NA	NA
F01_transcript_113967	NA	F01_transcript_113967	NA	NA
F01_transcript_79927	NA	F01_transcript_79927	NA	NA
F01_transcript_162249	NA	NA	NA	F01_transcript_162249
F01_transcript_86868	NA	NA	NA	F01_transcript_86868
F01_transcript_129364	NA	NA	NA	F01_transcript_129364
F01_transcript_141593	NA	NA	NA	F01_transcript_141593
F01_transcript_68377	NA	F01_transcript_68377	NA	NA
F01_transcript_22595	NA	NA	NA	F01_transcript_22595
F01_transcript_77907	F01_transcript_77907	F01_transcript_77907	NA	NA
F01_transcript_4099	NA	NA	NA	F01_transcript_4099

F01_transcript_45207	NA	NA	NA	F01_transcript_45207
F01_transcript_145186	NA	NA	NA	F01_transcript_145186
F01_transcript_100300	F01_transcript_100300	F01_transcript_100300	F01_transcript_100300	F01_transcript_100300
F01_transcript_158089	F01_transcript_158089	F01_transcript_158089	F01_transcript_158089	F01_transcript_158089
F01_transcript_78392	NA	NA	NA	F01_transcript_78392
F01_transcript_149827	F01_transcript_149827	F01_transcript_149827	F01_transcript_149827	F01_transcript_149827
F01_transcript_132421	NA	F01_transcript_132421	NA	NA
F01_transcript_19488	NA	NA	NA	F01_transcript_19488
F01_transcript_162106	F01_transcript_162106	F01_transcript_162106	F01_transcript_162106	F01_transcript_162106
F01_transcript_156064	NA	NA	NA	F01_transcript_156064
F01_transcript_135904	NA	NA	NA	F01_transcript_135904
F01_transcript_88914	NA	F01_transcript_88914	NA	F01_transcript_88914
F01_transcript_119920	F01_transcript_119920	F01_transcript_119920	NA	F01_transcript_119920
F01_transcript_82675	NA	F01_transcript_82675	NA	NA

F01_transcript_50770	F01_transcript_50770	F01_transcript_50770	F01_transcript_50770	F01_transcript_50770
F01_transcript_153236	NA	NA	NA	F01_transcript_153236
F01_transcript_112439	NA	F01_transcript_112439	NA	F01_transcript_112439
F01_transcript_59625	NA	F01_transcript_59625	F01_transcript_59625	NA
F01_transcript_126358	F01_transcript_126358	F01_transcript_126358	F01_transcript_126358	F01_transcript_126358
F01_transcript_142799	F01_transcript_142799	F01_transcript_142799	NA	NA
F01_transcript_96080	NA	NA	NA	F01_transcript_96080
F01_transcript_147152	F01_transcript_147152	F01_transcript_147152	F01_transcript_147152	F01_transcript_147152
F01_transcript_128795	NA	NA	NA	F01_transcript_128795
F01_transcript_111486	NA	NA	NA	F01_transcript_111486
F01_transcript_88584	NA	NA	NA	F01_transcript_88584
F01_transcript_129796	NA	NA	NA	F01_transcript_129796
F01_transcript_57549	NA	NA	NA	F01_transcript_57549
F01_transcript_49683	NA	F01_transcript_49683	NA	NA

F01_transcript_16920	NA	NA	NA	F01_transcript_16920
F01_transcript_85610	NA	F01_transcript_85610	NA	NA
F01_transcript_110640	F01_transcript_110640	F01_transcript_110640	F01_transcript_110640	F01_transcript_110640
F01_transcript_116909	NA	NA	NA	F01_transcript_116909
F01_transcript_145154	NA	F01_transcript_145154	F01_transcript_145154	NA
F01_transcript_120276	NA	F01_transcript_120276	F01_transcript_120276	F01_transcript_120276
F01_transcript_132625	NA	NA	NA	F01_transcript_132625
F01_transcript_100761	F01_transcript_100761	NA	NA	NA
F01_transcript_143531	NA	NA	NA	F01_transcript_143531
F01_transcript_122563	NA	F01_transcript_122563	F01_transcript_122563	F01_transcript_122563
F01_transcript_93787	NA	NA	NA	F01_transcript_93787
F01_transcript_82834	NA	F01_transcript_82834	F01_transcript_82834	F01_transcript_82834
F01_transcript_140625	NA	F01_transcript_140625	NA	F01_transcript_140625
F01_transcript_12531	F01_transcript_12531	F01_transcript_12531	F01_transcript_12531	F01_transcript_12531

F01_transcript_62982	F01_transcript_62982	F01_transcript_62982	F01_transcript_62982	F01_transcript_62982
F01_transcript_32472	NA	NA	F01_transcript_32472	F01_transcript_32472
F01_transcript_137170	NA	F01_transcript_137170	NA	NA
F01_transcript_47645	NA	NA	NA	F01_transcript_47645
F01_transcript_120456	NA	F01_transcript_120456	NA	NA
F01_transcript_70541	NA	NA	NA	F01_transcript_70541
F01_transcript_50223	NA	F01_transcript_50223	NA	NA
F01_transcript_157444	F01_transcript_157444	F01_transcript_157444	NA	NA
F01_transcript_135780	NA	F01_transcript_135780	F01_transcript_135780	F01_transcript_135780
F01_transcript_138979	NA	NA	NA	F01_transcript_138979
F01_transcript_63512	NA	F01_transcript_63512	NA	NA
F01_transcript_94133	NA	F01_transcript_94133	F01_transcript_94133	F01_transcript_94133
F01_transcript_86364	F01_transcript_86364	F01_transcript_86364	F01_transcript_86364	NA
F01_transcript_79347	F01_transcript_79347	F01_transcript_79347	F01_transcript_79347	F01_transcript_79347

F01_transcript_118973	NA	F01_transcript_118973	NA	F01_transcript_118973
F01_transcript_127249	NA	F01_transcript_127249	NA	NA
F01_transcript_92686	F01_transcript_92686	F01_transcript_92686	F01_transcript_92686	F01_transcript_92686
F01_transcript_136463	NA	F01_transcript_136463	F01_transcript_136463	F01_transcript_136463
F01_transcript_37957	NA	NA	NA	F01_transcript_37957
F01_transcript_21158	NA	NA	NA	F01_transcript_21158
F01_transcript_167492	F01_transcript_167492	F01_transcript_167492	NA	F01_transcript_167492
F01_transcript_49122	NA	F01_transcript_49122	NA	F01_transcript_49122
F01_transcript_61359	NA	NA	NA	F01_transcript_61359
F01_transcript_146813	NA	F01_transcript_146813	F01_transcript_146813	NA
F01_transcript_107802	F01_transcript_107802	F01_transcript_107802	F01_transcript_107802	NA
F01_transcript_20093	NA	NA	NA	F01_transcript_20093
F01_transcript_106613	F01_transcript_106613	F01_transcript_106613	NA	F01_transcript_106613
F01_transcript_126424	NA	F01_transcript_126424	NA	F01_transcript_126424

F01_transcript_64137	NA	NA	NA	F01_transcript_64137
F01_transcript_72227	NA	NA	NA	F01_transcript_72227
F01_transcript_46144	F01_transcript_46144	F01_transcript_46144	F01_transcript_46144	F01_transcript_46144
F01_transcript_129166	NA	F01_transcript_129166	NA	F01_transcript_129166
F01_transcript_78661	NA	NA	NA	F01_transcript_78661
F01_transcript_149657	F01_transcript_149657	F01_transcript_149657	F01_transcript_149657	F01_transcript_149657
F01_transcript_167361	NA	NA	F01_transcript_167361	NA
F01_transcript_38018	NA	NA	NA	F01_transcript_38018
F01_transcript_61864	NA	NA	NA	F01_transcript_61864
F01_transcript_39574	NA	NA	NA	F01_transcript_39574
F01_transcript_44353	NA	NA	NA	F01_transcript_44353
F01_transcript_35246	NA	NA	NA	F01_transcript_35246
F01_transcript_53016	F01_transcript_53016	F01_transcript_53016	F01_transcript_53016	F01_transcript_53016
F01_transcript_69658	F01_transcript_69658	F01_transcript_69658	F01_transcript_69658	F01_transcript_69658

F01_transcript_42891	NA	F01_transcript_42891	NA	F01_transcript_42891
F01_transcript_13158	NA	F01_transcript_13158	NA	NA
F01_transcript_71158	NA	F01_transcript_71158	F01_transcript_71158	NA
F01_transcript_158435	F01_transcript_158435	F01_transcript_158435	F01_transcript_158435	F01_transcript_158435
F01_transcript_56051	NA	F01_transcript_56051	NA	NA
F01_transcript_129743	NA	F01_transcript_129743	F01_transcript_129743	NA
F01_transcript_105118	NA	NA	NA	F01_transcript_105118
F01_transcript_78824	NA	F01_transcript_78824	NA	NA
F01_transcript_31674	NA	F01_transcript_31674	NA	F01_transcript_31674
F01_transcript_79821	NA	F01_transcript_79821	NA	F01_transcript_79821
F01_transcript_122634	NA	NA	NA	F01_transcript_122634
F01_transcript_4993	F01_transcript_4993	F01_transcript_4993	F01_transcript_4993	F01_transcript_4993
F01_transcript_101020	NA	F01_transcript_101020	NA	NA
F01_transcript_103402	NA	F01_transcript_103402	NA	F01_transcript_103402

F01_transcript_47288	NA	F01_transcript_47288	NA	NA
F01_transcript_154786	NA	F01_transcript_154786	NA	NA
F01_transcript_131118	NA	F01_transcript_131118	NA	F01_transcript_131118
F01_transcript_41917	NA	F01_transcript_41917	NA	NA
F01_transcript_35487	NA	F01_transcript_35487	NA	NA
F01_transcript_130867	F01_transcript_130867	F01_transcript_130867	F01_transcript_130867	F01_transcript_130867
F01_transcript_152376	F01_transcript_152376	F01_transcript_152376	F01_transcript_152376	F01_transcript_152376
F01_transcript_73954	NA	NA	NA	F01_transcript_73954
F01_transcript_114165	NA	F01_transcript_114165	NA	NA
F01_transcript_6792	NA	NA	NA	F01_transcript_6792
F01_transcript_148687	NA	F01_transcript_148687	NA	NA
F01_transcript_99827	F01_transcript_99827	F01_transcript_99827	F01_transcript_99827	F01_transcript_99827
F01_transcript_151473	NA	NA	NA	F01_transcript_151473
F01_transcript_116886	NA	NA	NA	F01_transcript_116886

F01_transcript_91352	NA	NA	NA	F01_transcript_91352
F01_transcript_106238	NA	NA	NA	F01_transcript_106238
F01_transcript_26549	NA	NA	NA	F01_transcript_26549
F01_transcript_88129	NA	NA	NA	F01_transcript_88129
F01_transcript_79516	NA	NA	NA	F01_transcript_79516
F01_transcript_165066	F01_transcript_165066	F01_transcript_165066	F01_transcript_165066	F01_transcript_165066
F01_transcript_154970	F01_transcript_154970	F01_transcript_154970	NA	F01_transcript_154970
F01_transcript_156961	NA	NA	NA	F01_transcript_156961
F01_transcript_71185	NA	NA	F01_transcript_71185	NA
F01_transcript_99262	F01_transcript_99262	F01_transcript_99262	NA	F01_transcript_99262
F01_transcript_67422	F01_transcript_67422	F01_transcript_67422	F01_transcript_67422	F01_transcript_67422
F01_transcript_134546	NA	F01_transcript_134546	F01_transcript_134546	NA
F01_transcript_59515	NA	F01_transcript_59515	F01_transcript_59515	F01_transcript_59515
F01_transcript_93465	F01_transcript_93465	F01_transcript_93465	F01_transcript_93465	NA

F01_transcript_83644	NA	F01_transcript_83644	NA	F01_transcript_83644
F01_transcript_165676	NA	F01_transcript_165676	NA	NA
F01_transcript_83725	NA	NA	F01_transcript_83725	NA
F01_transcript_84917	NA	NA	NA	F01_transcript_84917
F01_transcript_129984	NA	NA	NA	F01_transcript_129984
F01_transcript_143862	NA	F01_transcript_143862	NA	F01_transcript_143862
F01_transcript_48943	NA	NA	F01_transcript_48943	F01_transcript_48943
F01_transcript_75995	NA	NA	NA	F01_transcript_75995
F01_transcript_156503	NA	NA	NA	F01_transcript_156503
F01_transcript_85506	NA	NA	NA	F01_transcript_85506
F01_transcript_75937	NA	NA	NA	F01_transcript_75937
F01_transcript_117103	NA	F01_transcript_117103	NA	NA
F01_transcript_85187	F01_transcript_85187	F01_transcript_85187	F01_transcript_85187	F01_transcript_85187
F01_transcript_111167	F01_transcript_111167	F01_transcript_111167	F01_transcript_111167	F01_transcript_111167

F01_transcript_151170	F01_transcript_151170	F01_transcript_151170	F01_transcript_151170	F01_transcript_151170
F01_transcript_61593	NA	NA	NA	F01_transcript_61593
F01_transcript_141171	F01_transcript_141171	F01_transcript_141171	F01_transcript_141171	F01_transcript_141171
F01_transcript_145472	NA	F01_transcript_145472	NA	NA
F01_transcript_162702	NA	F01_transcript_162702	NA	F01_transcript_162702
F01_transcript_77844	NA	NA	NA	F01_transcript_77844
F01_transcript_141934	NA	NA	F01_transcript_141934	NA
F01_transcript_60869	NA	NA	NA	F01_transcript_60869
F01_transcript_92504	NA	NA	NA	F01_transcript_92504
F01_transcript_129486	NA	F01_transcript_129486	NA	NA
F01_transcript_138172	NA	F01_transcript_138172	NA	NA
F01_transcript_131969	NA	NA	NA	F01_transcript_131969
F01_transcript_66483	NA	NA	NA	F01_transcript_66483
F01_transcript_25971	NA	NA	NA	F01_transcript_25971

F01_transcript_92852	F01_transcript_92852	F01_transcript_92852	F01_transcript_92852	F01_transcript_92852
F01_transcript_58025	NA	F01_transcript_58025	NA	NA
F01_transcript_111812	NA	NA	NA	F01_transcript_111812
F01_transcript_57278	NA	NA	NA	F01_transcript_57278
F01_transcript_71800	NA	F01_transcript_71800	NA	NA
F01_transcript_43418	NA	F01_transcript_43418	NA	NA
F01_transcript_166780	F01_transcript_166780	F01_transcript_166780	NA	F01_transcript_166780
F01_transcript_157664	F01_transcript_157664	F01_transcript_157664	F01_transcript_157664	F01_transcript_157664
F01_transcript_121692	NA	F01_transcript_121692	NA	NA
F01_transcript_41657	NA	F01_transcript_41657	NA	NA
F01_transcript_146708	F01_transcript_146708	F01_transcript_146708	NA	F01_transcript_146708
F01_transcript_150618	NA	F01_transcript_150618	NA	NA
F01_transcript_111683	NA	F01_transcript_111683	NA	NA
F01_transcript_90505	NA	NA	NA	F01_transcript_90505

F01_transcript_12130	NA	F01_transcript_12130	NA	NA
F01_transcript_104700	NA	NA	NA	F01_transcript_104700
F01_transcript_13975	NA	NA	NA	F01_transcript_13975
F01_transcript_70080	NA	F01_transcript_70080	NA	NA
F01_transcript_147339	F01_transcript_147339	F01_transcript_147339	F01_transcript_147339	F01_transcript_147339
F01_transcript_71168	F01_transcript_71168	F01_transcript_71168	F01_transcript_71168	F01_transcript_71168
F01_transcript_149614	NA	NA	NA	F01_transcript_149614
F01_transcript_111757	NA	F01_transcript_111757	F01_transcript_111757	F01_transcript_111757
F01_transcript_116049	NA	F01_transcript_116049	NA	NA
F01_transcript_134157	NA	NA	NA	F01_transcript_134157
F01_transcript_87860	NA	NA	NA	F01_transcript_87860
F01_transcript_97289	NA	NA	NA	F01_transcript_97289
F01_transcript_49207	NA	NA	NA	F01_transcript_49207
F01_transcript_3115	NA	F01_transcript_3115	NA	NA

F01_transcript_125522	F01_transcript_125522	F01_transcript_125522	NA	F01_transcript_125522
F01_transcript_113047	NA	NA	NA	F01_transcript_113047
F01_transcript_162334	F01_transcript_162334	F01_transcript_162334	F01_transcript_162334	F01_transcript_162334
F01_transcript_100504	NA	F01_transcript_100504	NA	F01_transcript_100504
F01_transcript_130040	NA	F01_transcript_130040	NA	F01_transcript_130040
F01_transcript_104632	NA	F01_transcript_104632	NA	NA
F01_transcript_49000	NA	NA	NA	F01_transcript_49000
F01_transcript_108612	F01_transcript_108612	F01_transcript_108612	NA	NA
F01_transcript_114476	NA	NA	NA	F01_transcript_114476
F01_transcript_81035	F01_transcript_81035	F01_transcript_81035	NA	F01_transcript_81035
F01_transcript_164545	NA	F01_transcript_164545	NA	NA
F01_transcript_31841	NA	F01_transcript_31841	F01_transcript_31841	NA
F01_transcript_58286	NA	NA	NA	F01_transcript_58286
F01_transcript_137878	F01_transcript_137878	F01_transcript_137878	F01_transcript_137878	F01_transcript_137878

F01_transcript_101186	NA	NA	NA	F01_transcript_101186
F01_transcript_111087	NA	NA	NA	F01_transcript_111087
F01_transcript_56999	NA	NA	F01_transcript_56999	NA
F01_transcript_137469	NA	F01_transcript_137469	NA	NA
F01_transcript_61476	NA	NA	NA	F01_transcript_61476
F01_transcript_129446	NA	F01_transcript_129446	NA	NA
F01_transcript_22135	F01_transcript_22135	F01_transcript_22135	NA	NA
F01_transcript_47835	NA	F01_transcript_47835	F01_transcript_47835	NA
F01_transcript_86199	NA	F01_transcript_86199	NA	NA
F01_transcript_130566	NA	F01_transcript_130566	NA	NA
F01_transcript_54387	NA	F01_transcript_54387	F01_transcript_54387	F01_transcript_54387
F01_transcript_138580	F01_transcript_138580	F01_transcript_138580	F01_transcript_138580	F01_transcript_138580
F01_transcript_61237	NA	F01_transcript_61237	NA	NA
F01_transcript_119893	NA	NA	NA	F01_transcript_119893

F01_transcript_128200	F01_transcript_128200	F01_transcript_128200	F01_transcript_128200	F01_transcript_128200
F01_transcript_138929	F01_transcript_138929	F01_transcript_138929	F01_transcript_138929	F01_transcript_138929
F01_transcript_97822	NA	NA	NA	F01_transcript_97822
F01_transcript_107211	NA	F01_transcript_107211	NA	NA
F01_transcript_163511	NA	F01_transcript_163511	NA	NA
F01_transcript_76587	F01_transcript_76587	F01_transcript_76587	F01_transcript_76587	F01_transcript_76587
F01_transcript_104502	F01_transcript_104502	F01_transcript_104502	F01_transcript_104502	F01_transcript_104502
F01_transcript_122350	F01_transcript_122350	F01_transcript_122350	F01_transcript_122350	F01_transcript_122350
F01_transcript_100111	F01_transcript_100111	F01_transcript_100111	F01_transcript_100111	F01_transcript_100111
F01_transcript_89029	NA	NA	NA	F01_transcript_89029
F01_transcript_4094	F01_transcript_4094	F01_transcript_4094	F01_transcript_4094	F01_transcript_4094
F01_transcript_109628	NA	F01_transcript_109628	NA	F01_transcript_109628
F01_transcript_127471	F01_transcript_127471	F01_transcript_127471	F01_transcript_127471	F01_transcript_127471
F01_transcript_83690	NA	F01_transcript_83690	NA	F01_transcript_83690

F01_transcript_97350	NA	F01_transcript_97350	NA	NA
F01_transcript_73052	NA	NA	NA	F01_transcript_73052
F01_transcript_65230	NA	NA	NA	F01_transcript_65230
F01_transcript_159358	F01_transcript_159358	F01_transcript_159358	NA	NA
F01_transcript_78976	NA	NA	NA	F01_transcript_78976
F01_transcript_53167	NA	F01_transcript_53167	NA	NA
F01_transcript_167436	NA	NA	NA	F01_transcript_167436
F01_transcript_102420	F01_transcript_102420	F01_transcript_102420	NA	F01_transcript_102420
F01_transcript_36654	NA	NA	NA	F01_transcript_36654
F01_transcript_156593	F01_transcript_156593	F01_transcript_156593	NA	F01_transcript_156593
F01_transcript_159110	NA	NA	NA	F01_transcript_159110
F01_transcript_136561	NA	NA	NA	F01_transcript_136561
F01_transcript_117413	F01_transcript_117413	F01_transcript_117413	F01_transcript_117413	F01_transcript_117413
F01_transcript_40401	F01_transcript_40401	F01_transcript_40401	F01_transcript_40401	F01_transcript_40401

F01_transcript_101164	NA	NA	NA	F01_transcript_101164
F01_transcript_89982	NA	NA	NA	F01_transcript_89982
F01_transcript_111872	NA	F01_transcript_111872	NA	NA
F01_transcript_165444	NA	F01_transcript_165444	NA	NA
F01_transcript_2890	NA	NA	NA	F01_transcript_2890
F01_transcript_145805	NA	F01_transcript_145805	NA	NA
F01_transcript_30870	NA	NA	NA	F01_transcript_30870
F01_transcript_3826	NA	F01_transcript_3826	NA	NA
F01_transcript_37963	NA	NA	NA	F01_transcript_37963
F01_transcript_95253	NA	NA	NA	F01_transcript_95253
F01_transcript_6242	NA	NA	NA	F01_transcript_6242
F01_transcript_145081	NA	NA	NA	F01_transcript_145081
F01_transcript_49090	F01_transcript_49090	F01_transcript_49090	F01_transcript_49090	F01_transcript_49090
F01_transcript_81195	F01_transcript_81195	F01_transcript_81195	F01_transcript_81195	F01_transcript_81195

F01_transcript_101579	NA	F01_transcript_101579	NA	F01_transcript_101579
F01_transcript_4078	F01_transcript_4078	F01_transcript_4078	NA	F01_transcript_4078
F01_transcript_22957	NA	NA	NA	F01_transcript_22957
F01_transcript_113435	F01_transcript_113435	F01_transcript_113435	F01_transcript_113435	F01_transcript_113435
F01_transcript_103379	F01_transcript_103379	F01_transcript_103379	F01_transcript_103379	NA
F01_transcript_71686	F01_transcript_71686	F01_transcript_71686	NA	F01_transcript_71686
F01_transcript_85531	NA	NA	NA	F01_transcript_85531
F01_transcript_138996	NA	NA	NA	F01_transcript_138996
F01_transcript_93257	NA	F01_transcript_93257	NA	NA
F01_transcript_162005	F01_transcript_162005	F01_transcript_162005	F01_transcript_162005	F01_transcript_162005
F01_transcript_160176	NA	NA	NA	F01_transcript_160176
F01_transcript_66201	NA	NA	NA	F01_transcript_66201
F01_transcript_141099	NA	F01_transcript_141099	F01_transcript_141099	F01_transcript_141099
F01_transcript_113775	NA	NA	F01_transcript_113775	F01_transcript_113775

F01_transcript_56726	NA	NA	NA	F01_transcript_56726
F01_transcript_162662	NA	F01_transcript_162662	NA	F01_transcript_162662
F01_transcript_122232	NA	F01_transcript_122232	F01_transcript_122232	F01_transcript_122232
F01_transcript_85958	NA	NA	F01_transcript_85958	F01_transcript_85958
F01_transcript_87391	F01_transcript_87391	F01_transcript_87391	NA	NA
F01_transcript_45417	NA	F01_transcript_45417	F01_transcript_45417	F01_transcript_45417
F01_transcript_3985	F01_transcript_3985	F01_transcript_3985	NA	NA
F01_transcript_144546	NA	NA	NA	F01_transcript_144546
F01_transcript_102461	NA	F01_transcript_102461	NA	F01_transcript_102461
F01_transcript_10226	NA	NA	NA	F01_transcript_10226
F01_transcript_17971	NA	NA	NA	F01_transcript_17971
F01_transcript_59363	F01_transcript_59363	F01_transcript_59363	F01_transcript_59363	F01_transcript_59363
F01_transcript_112270	NA	NA	NA	F01_transcript_112270
F01_transcript_28108	F01_transcript_28108	F01_transcript_28108	NA	NA

F01_transcript_161787	NA	NA	NA	F01_transcript_161787
F01_transcript_93272	NA	NA	NA	F01_transcript_93272
F01_transcript_71990	NA	NA	NA	F01_transcript_71990
F01_transcript_96492	F01_transcript_96492	F01_transcript_96492	F01_transcript_96492	F01_transcript_96492
F01_transcript_23179	NA	NA	NA	F01_transcript_23179
F01_transcript_93600	NA	NA	NA	F01_transcript_93600
F01_transcript_146966	F01_transcript_146966	F01_transcript_146966	F01_transcript_146966	F01_transcript_146966
F01_transcript_103990	NA	NA	NA	F01_transcript_103990
F01_transcript_156160	NA	F01_transcript_156160	NA	NA
F01_transcript_162340	NA	F01_transcript_162340	NA	NA
F01_transcript_154291	NA	F01_transcript_154291	NA	F01_transcript_154291
F01_transcript_50707	F01_transcript_50707	F01_transcript_50707	F01_transcript_50707	F01_transcript_50707
F01_transcript_44381	NA	NA	NA	F01_transcript_44381
F01_transcript_104404	F01_transcript_104404	F01_transcript_104404	NA	NA

F01_transcript_137979	NA	NA	F01_transcript_137979	NA
F01_transcript_138398	NA	F01_transcript_138398	NA	F01_transcript_138398
F01_transcript_104134	NA	NA	NA	F01_transcript_104134
F01_transcript_105014	NA	NA	NA	F01_transcript_105014
F01_transcript_67428	NA	F01_transcript_67428	NA	NA
F01_transcript_96484	NA	NA	NA	F01_transcript_96484
F01_transcript_56550	F01_transcript_56550	F01_transcript_56550	F01_transcript_56550	F01_transcript_56550
F01_transcript_63834	NA	F01_transcript_63834	NA	NA
F01_transcript_49653	F01_transcript_49653	F01_transcript_49653	NA	F01_transcript_49653
F01_transcript_49664	NA	F01_transcript_49664	NA	NA
F01_transcript_140173	NA	NA	NA	F01_transcript_140173
F01_transcript_54019	NA	NA	NA	F01_transcript_54019
F01_transcript_64179	NA	F01_transcript_64179	NA	NA
F01_transcript_85979	F01_transcript_85979	F01_transcript_85979	F01_transcript_85979	F01_transcript_85979

F01_transcript_129254	NA	F01_transcript_129254	F01_transcript_129254	F01_transcript_129254
F01_transcript_140888	NA	NA	NA	F01_transcript_140888
F01_transcript_70735	NA	NA	NA	F01_transcript_70735
F01_transcript_103671	NA	NA	NA	F01_transcript_103671
F01_transcript_130326	F01_transcript_130326	F01_transcript_130326	F01_transcript_130326	F01_transcript_130326
F01_transcript_56953	NA	NA	F01_transcript_56953	NA
F01_transcript_130285	NA	F01_transcript_130285	NA	F01_transcript_130285
F01_transcript_121817	NA	NA	NA	F01_transcript_121817
F01_transcript_6302	NA	NA	NA	F01_transcript_6302
F01_transcript_95107	NA	NA	NA	F01_transcript_95107
F01_transcript_88500	NA	NA	F01_transcript_88500	NA
F01_transcript_160807	F01_transcript_160807	F01_transcript_160807	F01_transcript_160807	F01_transcript_160807
F01_transcript_50174	F01_transcript_50174	F01_transcript_50174	F01_transcript_50174	NA
F01_transcript_142933	NA	NA	NA	F01_transcript_142933

F01_transcript_151997	NA	F01_transcript_151997	NA	NA
F01_transcript_67528	NA	NA	NA	F01_transcript_67528
F01_transcript_30520	NA	F01_transcript_30520	F01_transcript_30520	NA
F01_transcript_65200	F01_transcript_65200	F01_transcript_65200	F01_transcript_65200	F01_transcript_65200
F01_transcript_149456	NA	NA	NA	F01_transcript_149456
F01_transcript_158663	NA	NA	NA	F01_transcript_158663
F01_transcript_150725	NA	NA	NA	F01_transcript_150725
F01_transcript_54671	NA	NA	NA	F01_transcript_54671
F01_transcript_13357	NA	NA	NA	F01_transcript_13357
F01_transcript_67540	F01_transcript_67540	F01_transcript_67540	NA	NA
F01_transcript_40434	NA	F01_transcript_40434	NA	NA
F01_transcript_2346	NA	F01_transcript_2346	NA	NA
F01_transcript_154343	NA	F01_transcript_154343	NA	NA
F01_transcript_117681	NA	NA	NA	F01_transcript_117681

F01_transcript_43013	NA	F01_transcript_43013	NA	NA
F01_transcript_77210	NA	F01_transcript_77210	NA	NA
F01_transcript_122008	F01_transcript_122008	F01_transcript_122008	F01_transcript_122008	NA
F01_transcript_127587	NA	F01_transcript_127587	NA	NA
F01_transcript_156404	NA	NA	F01_transcript_156404	NA
F01_transcript_162796	F01_transcript_162796	F01_transcript_162796	NA	NA
F01_transcript_10356	NA	NA	NA	F01_transcript_10356
F01_transcript_67308	NA	NA	NA	F01_transcript_67308
F01_transcript_27812	NA	NA	NA	F01_transcript_27812
F01_transcript_103240	NA	NA	NA	F01_transcript_103240
F01_transcript_157244	F01_transcript_157244	F01_transcript_157244	F01_transcript_157244	F01_transcript_157244
F01_transcript_66658	F01_transcript_66658	F01_transcript_66658	F01_transcript_66658	F01_transcript_66658
F01_transcript_62130	NA	F01_transcript_62130	F01_transcript_62130	F01_transcript_62130
F01_transcript_157136	NA	F01_transcript_157136	NA	NA

F01_transcript_167536	NA	F01_transcript_167536	NA	NA
F01_transcript_117976	F01_transcript_117976	F01_transcript_117976	NA	F01_transcript_117976
F01_transcript_101309	F01_transcript_101309	F01_transcript_101309	NA	F01_transcript_101309
F01_transcript_65465	NA	NA	F01_transcript_65465	NA
F01_transcript_133799	F01_transcript_133799	F01_transcript_133799	NA	NA
F01_transcript_145600	NA	NA	NA	F01_transcript_145600
F01_transcript_102107	F01_transcript_102107	NA	F01_transcript_102107	NA
F01_transcript_122884	NA	NA	NA	F01_transcript_122884
F01_transcript_163833	NA	F01_transcript_163833	NA	NA
F01_transcript_2520	NA	NA	NA	F01_transcript_2520
F01_transcript_29006	NA	F01_transcript_29006	NA	F01_transcript_29006
F01_transcript_134564	NA	F01_transcript_134564	NA	F01_transcript_134564
F01_transcript_46805	NA	NA	NA	F01_transcript_46805
F01_transcript_62844	NA	F01_transcript_62844	NA	F01_transcript_62844

F01_transcript_121033	NA	NA	NA	F01_transcript_121033
F01_transcript_113793	NA	F01_transcript_113793	NA	NA
F01_transcript_166713	NA	F01_transcript_166713	NA	NA
F01_transcript_6710	NA	NA	NA	F01_transcript_6710
F01_transcript_122881	F01_transcript_122881	F01_transcript_122881	NA	NA
F01_transcript_60841	NA	NA	NA	F01_transcript_60841
F01_transcript_60473	NA	NA	F01_transcript_60473	F01_transcript_60473
F01_transcript_166178	NA	F01_transcript_166178	NA	NA
F01_transcript_114264	F01_transcript_114264	F01_transcript_114264	F01_transcript_114264	F01_transcript_114264
F01_transcript_73558	NA	F01_transcript_73558	F01_transcript_73558	F01_transcript_73558
F01_transcript_94776	NA	NA	NA	F01_transcript_94776
F01_transcript_46118	NA	F01_transcript_46118	NA	NA
F01_transcript_148783	F01_transcript_148783	F01_transcript_148783	F01_transcript_148783	F01_transcript_148783
F01_transcript_151957	F01_transcript_151957	F01_transcript_151957	F01_transcript_151957	F01_transcript_151957

F01_transcript_92420	NA	NA	NA	F01_transcript_92420
F01_transcript_135312	NA	NA	NA	F01_transcript_135312
F01_transcript_69322	F01_transcript_69322	F01_transcript_69322	NA	NA
F01_transcript_122298	NA	NA	NA	F01_transcript_122298
F01_transcript_92603	NA	NA	NA	F01_transcript_92603
F01_transcript_133622	F01_transcript_133622	F01_transcript_133622	NA	NA
F01_transcript_96581	NA	F01_transcript_96581	NA	NA
F01_transcript_9893	NA	F01_transcript_9893	NA	F01_transcript_9893
F01_transcript_56017	NA	NA	NA	F01_transcript_56017
F01_transcript_122433	NA	NA	NA	F01_transcript_122433
F01_transcript_66992	NA	F01_transcript_66992	NA	F01_transcript_66992
F01_transcript_124815	NA	F01_transcript_124815	NA	NA
F01_transcript_27441	F01_transcript_27441	F01_transcript_27441	F01_transcript_27441	NA
F01_transcript_150157	NA	F01_transcript_150157	F01_transcript_150157	F01_transcript_150157

F01_transcript_133231	F01_transcript_133231	F01_transcript_133231	F01_transcript_133231	F01_transcript_133231
F01_transcript_103040	NA	F01_transcript_103040	NA	F01_transcript_103040
F01_transcript_116517	NA	NA	NA	F01_transcript_116517
F01_transcript_129946	NA	F01_transcript_129946	NA	NA
F01_transcript_147657	NA	NA	NA	F01_transcript_147657
F01_transcript_144947	NA	NA	NA	F01_transcript_144947
F01_transcript_129865	NA	F01_transcript_129865	NA	NA
F01_transcript_153688	F01_transcript_153688	F01_transcript_153688	F01_transcript_153688	F01_transcript_153688
F01_transcript_53798	NA	NA	NA	F01_transcript_53798
F01_transcript_64279	F01_transcript_64279	F01_transcript_64279	F01_transcript_64279	NA
F01_transcript_52666	F01_transcript_52666	F01_transcript_52666	F01_transcript_52666	F01_transcript_52666
F01_transcript_163004	NA	NA	NA	F01_transcript_163004
F01_transcript_48897	NA	F01_transcript_48897	NA	F01_transcript_48897
F01_transcript_95322	NA	NA	NA	F01_transcript_95322

F01_transcript_83930	NA	F01_transcript_83930	NA	NA
F01_transcript_134509	NA	F01_transcript_134509	NA	NA
F01_transcript_135347	NA	NA	F01_transcript_135347	NA
F01_transcript_75161	NA	NA	NA	F01_transcript_75161
F01_transcript_28844	NA	F01_transcript_28844	F01_transcript_28844	F01_transcript_28844
F01_transcript_103530	F01_transcript_103530	F01_transcript_103530	F01_transcript_103530	F01_transcript_103530
F01_transcript_89678	NA	F01_transcript_89678	NA	NA
F01_transcript_12085	F01_transcript_12085	F01_transcript_12085	F01_transcript_12085	F01_transcript_12085
F01_transcript_161376	NA	NA	NA	F01_transcript_161376
F01_transcript_51622	NA	F01_transcript_51622	NA	F01_transcript_51622
F01_transcript_72529	NA	F01_transcript_72529	NA	NA
F01_transcript_98954	F01_transcript_98954	F01_transcript_98954	F01_transcript_98954	F01_transcript_98954
F01_transcript_152796	NA	NA	NA	F01_transcript_152796
F01_transcript_129460	NA	NA	NA	F01_transcript_129460

F01_transcript_64340	NA	F01_transcript_64340	F01_transcript_64340	F01_transcript_64340
F01_transcript_55835	NA	F01_transcript_55835	NA	NA
F01_transcript_147095	NA	F01_transcript_147095	NA	NA
F01_transcript_139675	F01_transcript_139675	F01_transcript_139675	F01_transcript_139675	F01_transcript_139675
F01_transcript_108880	NA	NA	NA	F01_transcript_108880
F01_transcript_114628	NA	NA	NA	F01_transcript_114628
F01_transcript_50705	NA	F01_transcript_50705	F01_transcript_50705	NA
F01_transcript_113592	F01_transcript_113592	F01_transcript_113592	F01_transcript_113592	F01_transcript_113592
F01_transcript_50361	NA	NA	NA	F01_transcript_50361
F01_transcript_142160	F01_transcript_142160	F01_transcript_142160	F01_transcript_142160	F01_transcript_142160
F01_transcript_129506	NA	F01_transcript_129506	NA	NA
F01_transcript_141257	NA	NA	NA	F01_transcript_141257
F01_transcript_162372	NA	NA	NA	F01_transcript_162372
F01_transcript_59865	NA	NA	NA	F01_transcript_59865

F01_transcript_20849	NA	NA	NA	F01_transcript_20849
F01_transcript_167500	NA	NA	NA	F01_transcript_167500
F01_transcript_113484	F01_transcript_113484	F01_transcript_113484	NA	F01_transcript_113484
F01_transcript_108556	NA	F01_transcript_108556	F01_transcript_108556	F01_transcript_108556
F01_transcript_159899	F01_transcript_159899	F01_transcript_159899	F01_transcript_159899	F01_transcript_159899
F01_transcript_45031	F01_transcript_45031	F01_transcript_45031	F01_transcript_45031	F01_transcript_45031
F01_transcript_23572	NA	NA	NA	F01_transcript_23572
F01_transcript_94503	NA	F01_transcript_94503	NA	NA
F01_transcript_64867	F01_transcript_64867	F01_transcript_64867	NA	NA
F01_transcript_32238	NA	NA	NA	F01_transcript_32238
F01_transcript_68794	F01_transcript_68794	F01_transcript_68794	NA	F01_transcript_68794
F01_transcript_131107	NA	NA	NA	F01_transcript_131107
F01_transcript_167573	F01_transcript_167573	F01_transcript_167573	F01_transcript_167573	F01_transcript_167573
F01_transcript_127227	F01_transcript_127227	F01_transcript_127227	F01_transcript_127227	F01_transcript_127227

F01_transcript_96664	NA	NA	NA	F01_transcript_96664
F01_transcript_145895	NA	NA	NA	F01_transcript_145895
F01_transcript_44910	F01_transcript_44910	F01_transcript_44910	F01_transcript_44910	NA
F01_transcript_29860	NA	NA	NA	F01_transcript_29860
F01_transcript_116363	NA	F01_transcript_116363	NA	NA
F01_transcript_50421	NA	F01_transcript_50421	NA	NA
F01_transcript_148337	F01_transcript_148337	F01_transcript_148337	NA	NA
F01_transcript_122062	NA	NA	NA	F01_transcript_122062
F01_transcript_123948	NA	F01_transcript_123948	F01_transcript_123948	NA
F01_transcript_138870	NA	F01_transcript_138870	NA	F01_transcript_138870
F01_transcript_48295	NA	F01_transcript_48295	NA	NA
F01_transcript_60726	F01_transcript_60726	F01_transcript_60726	NA	NA
F01_transcript_129560	NA	F01_transcript_129560	NA	NA
F01_transcript_87896	F01_transcript_87896	F01_transcript_87896	F01_transcript_87896	F01_transcript_87896

F01_transcript_77213	NA	F01_transcript_77213	NA	NA
F01_transcript_28337	NA	NA	F01_transcript_28337	NA
F01_transcript_8667	NA	NA	NA	F01_transcript_8667
F01_transcript_50180	F01_transcript_50180	F01_transcript_50180	NA	NA
F01_transcript_110041	NA	F01_transcript_110041	NA	NA
F01_transcript_135666	F01_transcript_135666	F01_transcript_135666	NA	NA
F01_transcript_119722	F01_transcript_119722	F01_transcript_119722	F01_transcript_119722	NA
F01_transcript_52294	F01_transcript_52294	F01_transcript_52294	NA	F01_transcript_52294
F01_transcript_108264	NA	F01_transcript_108264	NA	NA
F01_transcript_84753	NA	NA	NA	F01_transcript_84753
F01_transcript_110725	NA	NA	NA	F01_transcript_110725
F01_transcript_80210	NA	F01_transcript_80210	NA	F01_transcript_80210
F01_transcript_73542	NA	F01_transcript_73542	NA	NA
F01_transcript_86536	F01_transcript_86536	F01_transcript_86536	F01_transcript_86536	F01_transcript_86536

F01_transcript_167380	F01_transcript_167380	F01_transcript_167380	NA	F01_transcript_167380
F01_transcript_118560	NA	NA	NA	F01_transcript_118560
F01_transcript_95049	F01_transcript_95049	F01_transcript_95049	NA	NA
F01_transcript_125220	NA	NA	NA	F01_transcript_125220
F01_transcript_117953	NA	F01_transcript_117953	NA	F01_transcript_117953
F01_transcript_36887	F01_transcript_36887	F01_transcript_36887	F01_transcript_36887	F01_transcript_36887
F01_transcript_117415	NA	F01_transcript_117415	NA	NA
F01_transcript_58219	F01_transcript_58219	F01_transcript_58219	F01_transcript_58219	F01_transcript_58219
F01_transcript_150054	NA	F01_transcript_150054	NA	F01_transcript_150054
F01_transcript_164070	F01_transcript_164070	F01_transcript_164070	F01_transcript_164070	F01_transcript_164070
F01_transcript_123145	NA	NA	NA	F01_transcript_123145
F01_transcript_97908	NA	NA	NA	F01_transcript_97908
F01_transcript_136556	NA	F01_transcript_136556	F01_transcript_136556	F01_transcript_136556
F01_transcript_107157	NA	F01_transcript_107157	NA	F01_transcript_107157

F01_transcript_124866	NA	NA	NA	F01_transcript_124866
F01_transcript_132793	NA	F01_transcript_132793	NA	NA
F01_transcript_114390	NA	F01_transcript_114390	NA	F01_transcript_114390
F01_transcript_117466	NA	F01_transcript_117466	NA	F01_transcript_117466
F01_transcript_164603	NA	F01_transcript_164603	F01_transcript_164603	F01_transcript_164603
F01_transcript_60632	NA	NA	NA	F01_transcript_60632
F01_transcript_94741	NA	NA	NA	F01_transcript_94741
F01_transcript_59038	F01_transcript_59038	F01_transcript_59038	NA	NA
F01_transcript_81955	NA	NA	NA	F01_transcript_81955
F01_transcript_111122	F01_transcript_111122	F01_transcript_111122	NA	NA
F01_transcript_124650	F01_transcript_124650	F01_transcript_124650	F01_transcript_124650	F01_transcript_124650
F01_transcript_159451	F01_transcript_159451	F01_transcript_159451	F01_transcript_159451	F01_transcript_159451
F01_transcript_161860	F01_transcript_161860	F01_transcript_161860	F01_transcript_161860	NA
F01_transcript_6223	F01_transcript_6223	F01_transcript_6223	NA	F01_transcript_6223

F01_transcript_167291	NA	NA	NA	F01_transcript_167291
F01_transcript_973	NA	NA	NA	F01_transcript_973
F01_transcript_56724	NA	F01_transcript_56724	NA	NA
F01_transcript_145535	NA	NA	NA	F01_transcript_145535
F01_transcript_124475	NA	NA	NA	F01_transcript_124475
F01_transcript_122144	NA	NA	NA	F01_transcript_122144
F01_transcript_84226	F01_transcript_84226	F01_transcript_84226	F01_transcript_84226	F01_transcript_84226
F01_transcript_74543	NA	NA	NA	F01_transcript_74543
F01_transcript_83328	NA	F01_transcript_83328	NA	F01_transcript_83328
F01_transcript_153838	NA	NA	NA	F01_transcript_153838
F01_transcript_50540	F01_transcript_50540	F01_transcript_50540	F01_transcript_50540	NA
F01_transcript_53565	NA	F01_transcript_53565	F01_transcript_53565	F01_transcript_53565
F01_transcript_118153	NA	NA	NA	F01_transcript_118153
F01_transcript_60940	NA	F01_transcript_60940	F01_transcript_60940	F01_transcript_60940

F01_transcript_165926	F01_transcript_165926	F01_transcript_165926	F01_transcript_165926	F01_transcript_165926
F01_transcript_118465	NA	F01_transcript_118465	NA	F01_transcript_118465
F01_transcript_26865	NA	NA	NA	F01_transcript_26865
F01_transcript_123255	NA	F01_transcript_123255	NA	F01_transcript_123255
F01_transcript_135840	NA	F01_transcript_135840	NA	NA
F01_transcript_71062	F01_transcript_71062	F01_transcript_71062	F01_transcript_71062	F01_transcript_71062
F01_transcript_90981	F01_transcript_90981	F01_transcript_90981	F01_transcript_90981	F01_transcript_90981
F01_transcript_102600	NA	F01_transcript_102600	NA	NA
F01_transcript_31444	NA	NA	NA	F01_transcript_31444
F01_transcript_115619	NA	NA	NA	F01_transcript_115619
F01_transcript_15221	NA	NA	NA	F01_transcript_15221
F01_transcript_151665	F01_transcript_151665	F01_transcript_151665	NA	F01_transcript_151665
F01_transcript_161555	F01_transcript_161555	F01_transcript_161555	NA	NA
F01_transcript_101512	NA	F01_transcript_101512	NA	NA

F01_transcript_106924	F01_transcript_106924	F01_transcript_106924	F01_transcript_106924	F01_transcript_106924
F01_transcript_110704	NA	F01_transcript_110704	NA	F01_transcript_110704
F01_transcript_69657	NA	NA	NA	F01_transcript_69657
F01_transcript_142745	NA	F01_transcript_142745	NA	NA
F01_transcript_100611	NA	F01_transcript_100611	NA	F01_transcript_100611
F01_transcript_85268	NA	F01_transcript_85268	NA	NA
F01_transcript_154203	NA	F01_transcript_154203	NA	NA
F01_transcript_55802	F01_transcript_55802	F01_transcript_55802	F01_transcript_55802	F01_transcript_55802
F01_transcript_36438	F01_transcript_36438	F01_transcript_36438	NA	F01_transcript_36438
F01_transcript_32283	NA	NA	NA	F01_transcript_32283
F01_transcript_165742	NA	NA	NA	F01_transcript_165742
F01_transcript_137328	NA	NA	NA	F01_transcript_137328
F01_transcript_48307	NA	NA	F01_transcript_48307	F01_transcript_48307
F01_transcript_148220	F01_transcript_148220	NA	NA	NA

F01_transcript_160752	NA	F01_transcript_160752	NA	NA
F01_transcript_83356	F01_transcript_83356	F01_transcript_83356	F01_transcript_83356	F01_transcript_83356
F01_transcript_127618	NA	NA	NA	F01_transcript_127618
F01_transcript_71907	NA	NA	NA	F01_transcript_71907
F01_transcript_16081	NA	NA	NA	F01_transcript_16081
F01_transcript_112972	F01_transcript_112972	F01_transcript_112972	F01_transcript_112972	F01_transcript_112972
F01_transcript_61180	NA	NA	NA	F01_transcript_61180
F01_transcript_13058	F01_transcript_13058	F01_transcript_13058	F01_transcript_13058	F01_transcript_13058
F01_transcript_137395	NA	F01_transcript_137395	NA	F01_transcript_137395
F01_transcript_108065	NA	F01_transcript_108065	NA	NA
F01_transcript_13227	NA	F01_transcript_13227	NA	NA
F01_transcript_83981	NA	F01_transcript_83981	NA	NA
F01_transcript_71775	NA	F01_transcript_71775	NA	NA
F01_transcript_58321	NA	F01_transcript_58321	NA	NA

F01_transcript_21659	NA	NA	NA	F01_transcript_21659
F01_transcript_134796	NA	NA	NA	F01_transcript_134796
F01_transcript_60636	NA	F01_transcript_60636	F01_transcript_60636	F01_transcript_60636
F01_transcript_120402	NA	F01_transcript_120402	NA	NA
F01_transcript_5575	NA	NA	NA	F01_transcript_5575
F01_transcript_120899	NA	NA	NA	F01_transcript_120899
F01_transcript_140039	F01_transcript_140039	F01_transcript_140039	F01_transcript_140039	F01_transcript_140039
F01_transcript_128477	NA	F01_transcript_128477	NA	NA
F01_transcript_113242	NA	F01_transcript_113242	NA	F01_transcript_113242
F01_transcript_158765	NA	NA	NA	F01_transcript_158765
F01_transcript_52569	NA	F01_transcript_52569	NA	F01_transcript_52569
F01_transcript_70810	NA	F01_transcript_70810	NA	NA
F01_transcript_45402	NA	F01_transcript_45402	NA	NA
F01_transcript_60853	F01_transcript_60853	F01_transcript_60853	F01_transcript_60853	NA

F01_transcript_120921	NA	F01_transcript_120921	NA	NA
F01_transcript_73219	NA	NA	NA	F01_transcript_73219
F01_transcript_88962	NA	NA	NA	F01_transcript_88962
F01_transcript_100431	NA	NA	NA	F01_transcript_100431
F01_transcript_115476	F01_transcript_115476	F01_transcript_115476	F01_transcript_115476	F01_transcript_115476
F01_transcript_144879	NA	NA	NA	F01_transcript_144879
F01_transcript_54043	NA	F01_transcript_54043	NA	F01_transcript_54043
F01_transcript_103762	F01_transcript_103762	F01_transcript_103762	NA	NA
F01_transcript_156462	NA	F01_transcript_156462	NA	NA
F01_transcript_99624	NA	F01_transcript_99624	NA	NA
F01_transcript_139586	NA	F01_transcript_139586	NA	NA
F01_transcript_48235	NA	NA	NA	F01_transcript_48235
F01_transcript_160820	F01_transcript_160820	F01_transcript_160820	NA	F01_transcript_160820
F01_transcript_143883	F01_transcript_143883	F01_transcript_143883	F01_transcript_143883	F01_transcript_143883

F01_transcript_167114	F01_transcript_167114	F01_transcript_167114	NA	NA
F01_transcript_118861	NA	F01_transcript_118861	NA	NA
F01_transcript_74133	F01_transcript_74133	F01_transcript_74133	F01_transcript_74133	F01_transcript_74133
F01_transcript_164477	NA	NA	F01_transcript_164477	NA
F01_transcript_165065	F01_transcript_165065	F01_transcript_165065	F01_transcript_165065	F01_transcript_165065
F01_transcript_135628	NA	NA	NA	F01_transcript_135628
F01_transcript_143904	F01_transcript_143904	F01_transcript_143904	F01_transcript_143904	F01_transcript_143904
F01_transcript_90818	NA	NA	NA	F01_transcript_90818
F01_transcript_65135	NA	NA	F01_transcript_65135	F01_transcript_65135
F01_transcript_145075	F01_transcript_145075	NA	NA	F01_transcript_145075
F01_transcript_42847	F01_transcript_42847	F01_transcript_42847	F01_transcript_42847	F01_transcript_42847
F01_transcript_30558	NA	NA	NA	F01_transcript_30558
F01_transcript_52828	F01_transcript_52828	F01_transcript_52828	F01_transcript_52828	F01_transcript_52828
F01_transcript_147774	NA	F01_transcript_147774	F01_transcript_147774	NA

F01_transcript_166306	NA	NA	NA	F01_transcript_166306
F01_transcript_81405	F01_transcript_81405	F01_transcript_81405	F01_transcript_81405	F01_transcript_81405
F01_transcript_111981	F01_transcript_111981	F01_transcript_111981	F01_transcript_111981	F01_transcript_111981
F01_transcript_85821	NA	NA	NA	F01_transcript_85821
F01_transcript_64203	F01_transcript_64203	F01_transcript_64203	F01_transcript_64203	F01_transcript_64203
F01_transcript_157005	F01_transcript_157005	F01_transcript_157005	F01_transcript_157005	F01_transcript_157005
F01_transcript_68415	NA	F01_transcript_68415	F01_transcript_68415	F01_transcript_68415
F01_transcript_18982	NA	NA	NA	F01_transcript_18982
F01_transcript_164512	NA	F01_transcript_164512	NA	NA
F01_transcript_167363	NA	F01_transcript_167363	NA	NA
F01_transcript_162299	NA	F01_transcript_162299	NA	F01_transcript_162299
F01_transcript_71115	NA	NA	NA	F01_transcript_71115
F01_transcript_131687	F01_transcript_131687	F01_transcript_131687	F01_transcript_131687	F01_transcript_131687
F01_transcript_77634	NA	F01_transcript_77634	NA	F01_transcript_77634

F01_transcript_114760	NA	F01_transcript_114760	F01_transcript_114760	F01_transcript_114760
F01_transcript_162232	NA	F01_transcript_162232	NA	F01_transcript_162232
F01_transcript_152230	NA	NA	NA	F01_transcript_152230
F01_transcript_115198	NA	F01_transcript_115198	NA	NA
F01_transcript_95969	NA	NA	NA	F01_transcript_95969
F01_transcript_155428	NA	NA	NA	F01_transcript_155428
F01_transcript_50944	F01_transcript_50944	F01_transcript_50944	F01_transcript_50944	F01_transcript_50944
F01_transcript_124125	NA	NA	NA	F01_transcript_124125
F01_transcript_106358	NA	F01_transcript_106358	NA	NA
F01_transcript_113467	NA	NA	NA	F01_transcript_113467
F01_transcript_119169	NA	F01_transcript_119169	NA	NA
F01_transcript_54468	NA	NA	NA	F01_transcript_54468
F01_transcript_49366	NA	F01_transcript_49366	F01_transcript_49366	F01_transcript_49366
F01_transcript_72687	NA	F01_transcript_72687	NA	NA

F01_transcript_140518	NA	NA	NA	F01_transcript_140518
F01_transcript_111284	NA	F01_transcript_111284	F01_transcript_111284	F01_transcript_111284
F01_transcript_85412	NA	F01_transcript_85412	NA	NA
F01_transcript_161764	NA	F01_transcript_161764	NA	F01_transcript_161764
F01_transcript_21651	NA	NA	NA	F01_transcript_21651
F01_transcript_72087	NA	NA	NA	F01_transcript_72087
F01_transcript_147316	NA	F01_transcript_147316	NA	F01_transcript_147316
F01_transcript_62816	NA	F01_transcript_62816	F01_transcript_62816	NA
F01_transcript_73749	NA	F01_transcript_73749	NA	NA
F01_transcript_162623	F01_transcript_162623	F01_transcript_162623	F01_transcript_162623	F01_transcript_162623
F01_transcript_123277	NA	F01_transcript_123277	NA	NA
F01_transcript_156349	NA	NA	NA	F01_transcript_156349
F01_transcript_57153	NA	NA	F01_transcript_57153	NA
F01_transcript_150351	NA	F01_transcript_150351	NA	NA

F01_transcript_93814	F01_transcript_93814	F01_transcript_93814	NA	NA
F01_transcript_150616	F01_transcript_150616	F01_transcript_150616	F01_transcript_150616	F01_transcript_150616
F01_transcript_4428	F01_transcript_4428	F01_transcript_4428	F01_transcript_4428	F01_transcript_4428
F01_transcript_90512	NA	F01_transcript_90512	NA	NA
F01_transcript_45037	NA	F01_transcript_45037	NA	NA
F01_transcript_59388	NA	F01_transcript_59388	F01_transcript_59388	NA
F01_transcript_88894	NA	NA	NA	F01_transcript_88894
F01_transcript_28898	NA	NA	NA	F01_transcript_28898
F01_transcript_123867	NA	F01_transcript_123867	NA	NA
F01_transcript_48811	NA	NA	NA	F01_transcript_48811
F01_transcript_145799	NA	NA	NA	F01_transcript_145799
F01_transcript_113307	F01_transcript_113307	F01_transcript_113307	F01_transcript_113307	F01_transcript_113307
F01_transcript_159387	F01_transcript_159387	F01_transcript_159387	F01_transcript_159387	F01_transcript_159387
F01_transcript_116688	F01_transcript_116688	F01_transcript_116688	NA	F01_transcript_116688

F01_transcript_165579	F01_transcript_165579	F01_transcript_165579	F01_transcript_165579	F01_transcript_165579
F01_transcript_59443	NA	F01_transcript_59443	F01_transcript_59443	NA
F01_transcript_119891	NA	NA	NA	F01_transcript_119891
F01_transcript_56510	F01_transcript_56510	F01_transcript_56510	F01_transcript_56510	F01_transcript_56510
F01_transcript_68931	NA	F01_transcript_68931	NA	NA
F01_transcript_149276	NA	F01_transcript_149276	NA	NA
F01_transcript_89628	NA	F01_transcript_89628	NA	NA
F01_transcript_110420	NA	NA	NA	F01_transcript_110420
F01_transcript_66110	NA	F01_transcript_66110	NA	NA
F01_transcript_106349	F01_transcript_106349	F01_transcript_106349	NA	F01_transcript_106349
F01_transcript_89797	NA	NA	NA	F01_transcript_89797
F01_transcript_160057	NA	F01_transcript_160057	NA	NA
F01_transcript_70366	NA	F01_transcript_70366	F01_transcript_70366	F01_transcript_70366
F01_transcript_15705	F01_transcript_15705	F01_transcript_15705	F01_transcript_15705	F01_transcript_15705

F01_transcript_106635	F01_transcript_106635	F01_transcript_106635	F01_transcript_106635	F01_transcript_106635
F01_transcript_99943	NA	NA	NA	F01_transcript_99943
F01_transcript_624	NA	NA	NA	F01_transcript_624
F01_transcript_101420	NA	NA	NA	F01_transcript_101420
F01_transcript_87137	F01_transcript_87137	F01_transcript_87137	F01_transcript_87137	F01_transcript_87137
F01_transcript_137516	NA	F01_transcript_137516	NA	NA
F01_transcript_80330	NA	F01_transcript_80330	NA	NA
F01_transcript_58539	NA	NA	F01_transcript_58539	F01_transcript_58539
F01_transcript_2590	F01_transcript_2590	NA	NA	NA
F01_transcript_100478	NA	NA	NA	F01_transcript_100478
F01_transcript_82293	F01_transcript_82293	F01_transcript_82293	NA	NA
F01_transcript_89141	F01_transcript_89141	NA	F01_transcript_89141	F01_transcript_89141
F01_transcript_65292	NA	NA	F01_transcript_65292	NA
F01_transcript_53355	NA	NA	NA	F01_transcript_53355

F01_transcript_133829	NA	F01_transcript_133829	NA	NA
F01_transcript_61407	NA	F01_transcript_61407	NA	NA
F01_transcript_138026	NA	NA	NA	F01_transcript_138026
F01_transcript_140032	NA	NA	NA	F01_transcript_140032
F01_transcript_140415	NA	F01_transcript_140415	NA	F01_transcript_140415
F01_transcript_100246	NA	NA	NA	F01_transcript_100246
F01_transcript_94426	NA	F01_transcript_94426	F01_transcript_94426	F01_transcript_94426
F01_transcript_64002	F01_transcript_64002	F01_transcript_64002	F01_transcript_64002	F01_transcript_64002
F01_transcript_104892	NA	NA	NA	F01_transcript_104892
F01_transcript_137667	NA	F01_transcript_137667	NA	NA
F01_transcript_65662	NA	NA	NA	F01_transcript_65662
F01_transcript_155220	NA	F01_transcript_155220	NA	F01_transcript_155220
F01_transcript_161064	NA	NA	NA	F01_transcript_161064
F01_transcript_97010	F01_transcript_97010	F01_transcript_97010	F01_transcript_97010	NA

F01_transcript_6398	NA	NA	NA	F01_transcript_6398
F01_transcript_137694	NA	NA	NA	F01_transcript_137694
F01_transcript_76781	NA	NA	NA	F01_transcript_76781
F01_transcript_119145	F01_transcript_119145	F01_transcript_119145	F01_transcript_119145	F01_transcript_119145
F01_transcript_105675	NA	F01_transcript_105675	NA	F01_transcript_105675
F01_transcript_126698	F01_transcript_126698	F01_transcript_126698	NA	NA
F01_transcript_89138	NA	F01_transcript_89138	NA	F01_transcript_89138
F01_transcript_151733	F01_transcript_151733	F01_transcript_151733	NA	F01_transcript_151733
F01_transcript_160947	NA	F01_transcript_160947	NA	F01_transcript_160947
F01_transcript_52988	NA	F01_transcript_52988	NA	NA
F01_transcript_102558	NA	NA	NA	F01_transcript_102558
F01_transcript_26885	NA	NA	NA	F01_transcript_26885
F01_transcript_166909	F01_transcript_166909	F01_transcript_166909	F01_transcript_166909	F01_transcript_166909
F01_transcript_91255	F01_transcript_91255	F01_transcript_91255	NA	F01_transcript_91255

F01_transcript_114032	NA	F01_transcript_114032	NA	NA
F01_transcript_62638	F01_transcript_62638	F01_transcript_62638	F01_transcript_62638	F01_transcript_62638
F01_transcript_5922	F01_transcript_5922	F01_transcript_5922	NA	NA
F01_transcript_74311	NA	NA	NA	F01_transcript_74311
F01_transcript_136195	NA	NA	NA	F01_transcript_136195
F01_transcript_30101	NA	NA	NA	F01_transcript_30101
F01_transcript_134286	NA	F01_transcript_134286	NA	NA
F01_transcript_55794	F01_transcript_55794	F01_transcript_55794	NA	F01_transcript_55794
F01_transcript_160076	NA	F01_transcript_160076	NA	NA
F01_transcript_112166	F01_transcript_112166	F01_transcript_112166	F01_transcript_112166	NA
F01_transcript_140874	F01_transcript_140874	F01_transcript_140874	F01_transcript_140874	F01_transcript_140874
F01_transcript_77025	F01_transcript_77025	F01_transcript_77025	NA	NA
F01_transcript_66544	F01_transcript_66544	F01_transcript_66544	NA	F01_transcript_66544
F01_transcript_9024	F01_transcript_9024	F01_transcript_9024	NA	NA

F01_transcript_66740	F01_transcript_66740	F01_transcript_66740	F01_transcript_66740	F01_transcript_66740
F01_transcript_156740	NA	NA	NA	F01_transcript_156740
F01_transcript_82913	F01_transcript_82913	F01_transcript_82913	NA	NA
F01_transcript_52154	F01_transcript_52154	F01_transcript_52154	F01_transcript_52154	F01_transcript_52154
F01_transcript_165257	F01_transcript_165257	F01_transcript_165257	F01_transcript_165257	NA
F01_transcript_103577	F01_transcript_103577	F01_transcript_103577	F01_transcript_103577	F01_transcript_103577
F01_transcript_119035	F01_transcript_119035	F01_transcript_119035	F01_transcript_119035	F01_transcript_119035
F01_transcript_84505	NA	F01_transcript_84505	NA	NA
F01_transcript_69675	F01_transcript_69675	F01_transcript_69675	F01_transcript_69675	F01_transcript_69675
F01_transcript_70801	NA	NA	NA	F01_transcript_70801
F01_transcript_113302	NA	F01_transcript_113302	NA	F01_transcript_113302
F01_transcript_109183	NA	F01_transcript_109183	NA	NA
F01_transcript_49779	NA	NA	NA	F01_transcript_49779
F01_transcript_8762	NA	NA	NA	F01_transcript_8762

F01_transcript_135528	NA	F01_transcript_135528	NA	NA
F01_transcript_134538	NA	F01_transcript_134538	F01_transcript_134538	NA
F01_transcript_9909	NA	F01_transcript_9909	NA	NA
F01_transcript_111309	F01_transcript_111309	F01_transcript_111309	F01_transcript_111309	F01_transcript_111309
F01_transcript_109059	NA	F01_transcript_109059	NA	NA
F01_transcript_93522	NA	NA	NA	F01_transcript_93522
F01_transcript_81980	NA	NA	NA	F01_transcript_81980
F01_transcript_87278	NA	F01_transcript_87278	NA	NA
F01_transcript_152878	NA	F01_transcript_152878	NA	NA
F01_transcript_52845	NA	F01_transcript_52845	NA	NA
F01_transcript_96926	NA	NA	F01_transcript_96926	F01_transcript_96926
F01_transcript_153611	NA	F01_transcript_153611	NA	F01_transcript_153611
F01_transcript_166515	F01_transcript_166515	F01_transcript_166515	F01_transcript_166515	F01_transcript_166515
F01_transcript_163277	F01_transcript_163277	F01_transcript_163277	NA	F01_transcript_163277

F01_transcript_117826	F01_transcript_117826	F01_transcript_117826	NA	F01_transcript_117826
F01_transcript_135573	NA	F01_transcript_135573	NA	F01_transcript_135573
F01_transcript_153114	F01_transcript_153114	F01_transcript_153114	F01_transcript_153114	F01_transcript_153114
F01_transcript_96294	NA	NA	NA	F01_transcript_96294
F01_transcript_9403	NA	NA	NA	F01_transcript_9403
F01_transcript_96622	F01_transcript_96622	F01_transcript_96622	F01_transcript_96622	F01_transcript_96622
F01_transcript_112517	NA	NA	NA	F01_transcript_112517
F01_transcript_164956	F01_transcript_164956	F01_transcript_164956	NA	F01_transcript_164956
F01_transcript_96471	F01_transcript_96471	F01_transcript_96471	F01_transcript_96471	F01_transcript_96471
F01_transcript_92605	F01_transcript_92605	F01_transcript_92605	F01_transcript_92605	F01_transcript_92605
F01_transcript_118863	NA	F01_transcript_118863	NA	NA
F01_transcript_95426	NA	NA	NA	F01_transcript_95426
F01_transcript_117307	NA	NA	NA	F01_transcript_117307
F01_transcript_105925	F01_transcript_105925	F01_transcript_105925	F01_transcript_105925	F01_transcript_105925

F01_transcript_8448	NA	F01_transcript_8448	NA	NA
F01_transcript_108933	NA	NA	NA	F01_transcript_108933
F01_transcript_114562	F01_transcript_114562	F01_transcript_114562	NA	NA
F01_transcript_89378	NA	NA	NA	F01_transcript_89378
F01_transcript_74733	NA	F01_transcript_74733	NA	F01_transcript_74733
F01_transcript_164091	F01_transcript_164091	F01_transcript_164091	F01_transcript_164091	F01_transcript_164091
F01_transcript_91476	NA	F01_transcript_91476	NA	F01_transcript_91476
F01_transcript_47641	F01_transcript_47641	F01_transcript_47641	NA	F01_transcript_47641
F01_transcript_96847	NA	F01_transcript_96847	NA	NA
F01_transcript_18246	NA	NA	NA	F01_transcript_18246
F01_transcript_120861	F01_transcript_120861	F01_transcript_120861	F01_transcript_120861	NA
F01_transcript_82704	F01_transcript_82704	F01_transcript_82704	F01_transcript_82704	F01_transcript_82704
F01_transcript_102733	F01_transcript_102733	F01_transcript_102733	NA	NA
F01_transcript_31964	NA	NA	NA	F01_transcript_31964

F01_transcript_69721	NA	F01_transcript_69721	NA	NA
F01_transcript_147009	NA	F01_transcript_147009	NA	NA
F01_transcript_162578	NA	NA	NA	F01_transcript_162578
F01_transcript_104659	F01_transcript_104659	F01_transcript_104659	NA	F01_transcript_104659
F01_transcript_60211	F01_transcript_60211	F01_transcript_60211	NA	NA
F01_transcript_9354	NA	NA	NA	F01_transcript_9354
F01_transcript_78730	F01_transcript_78730	F01_transcript_78730	NA	NA
F01_transcript_159741	NA	NA	F01_transcript_159741	F01_transcript_159741
F01_transcript_130211	NA	F01_transcript_130211	F01_transcript_130211	F01_transcript_130211
F01_transcript_129701	F01_transcript_129701	F01_transcript_129701	NA	F01_transcript_129701
F01_transcript_53957	NA	NA	NA	F01_transcript_53957
F01_transcript_145262	NA	F01_transcript_145262	NA	F01_transcript_145262
F01_transcript_54440	F01_transcript_54440	F01_transcript_54440	NA	NA
F01_transcript_72542	F01_transcript_72542	F01_transcript_72542	NA	F01_transcript_72542

F01_transcript_128277	NA	F01_transcript_128277	NA	NA
F01_transcript_89746	NA	NA	F01_transcript_89746	F01_transcript_89746
F01_transcript_21087	NA	NA	NA	F01_transcript_21087
F01_transcript_156564	F01_transcript_156564	F01_transcript_156564	NA	F01_transcript_156564
F01_transcript_49926	NA	F01_transcript_49926	F01_transcript_49926	F01_transcript_49926
F01_transcript_130151	NA	NA	NA	F01_transcript_130151
F01_transcript_153492	NA	NA	NA	F01_transcript_153492
F01_transcript_131504	NA	NA	NA	F01_transcript_131504
F01_transcript_101447	NA	NA	NA	F01_transcript_101447
F01_transcript_85648	F01_transcript_85648	F01_transcript_85648	F01_transcript_85648	F01_transcript_85648
F01_transcript_140170	NA	NA	NA	F01_transcript_140170
F01_transcript_104249	NA	F01_transcript_104249	NA	NA
F01_transcript_99707	F01_transcript_99707	F01_transcript_99707	F01_transcript_99707	F01_transcript_99707
F01_transcript_115721	NA	NA	NA	F01_transcript_115721

F01_transcript_116429	F01_transcript_116429	F01_transcript_116429	NA	NA
F01_transcript_92317	NA	F01_transcript_92317	NA	F01_transcript_92317
F01_transcript_78958	NA	F01_transcript_78958	F01_transcript_78958	F01_transcript_78958
F01_transcript_73441	NA	NA	NA	F01_transcript_73441
F01_transcript_138605	NA	F01_transcript_138605	NA	NA
F01_transcript_165384	F01_transcript_165384	F01_transcript_165384	F01_transcript_165384	F01_transcript_165384
F01_transcript_109131	NA	NA	NA	F01_transcript_109131
F01_transcript_67931	F01_transcript_67931	F01_transcript_67931	F01_transcript_67931	F01_transcript_67931
F01_transcript_149086	F01_transcript_149086	F01_transcript_149086	F01_transcript_149086	F01_transcript_149086
F01_transcript_104490	NA	NA	NA	F01_transcript_104490
F01_transcript_55885	F01_transcript_55885	F01_transcript_55885	F01_transcript_55885	F01_transcript_55885
F01_transcript_147810	NA	NA	F01_transcript_147810	F01_transcript_147810
F01_transcript_49250	NA	NA	NA	F01_transcript_49250
F01_transcript_122340	F01_transcript_122340	F01_transcript_122340	F01_transcript_122340	F01_transcript_122340

F01_transcript_50314	NA	F01_transcript_50314	NA	F01_transcript_50314
F01_transcript_83138	F01_transcript_83138	F01_transcript_83138	NA	F01_transcript_83138
F01_transcript_167559	NA	F01_transcript_167559	NA	NA
F01_transcript_92245	NA	F01_transcript_92245	NA	F01_transcript_92245
F01_transcript_11040	F01_transcript_11040	F01_transcript_11040	NA	F01_transcript_11040
F01_transcript_138733	F01_transcript_138733	F01_transcript_138733	NA	NA
F01_transcript_127014	F01_transcript_127014	F01_transcript_127014	F01_transcript_127014	F01_transcript_127014
F01_transcript_10990	F01_transcript_10990	F01_transcript_10990	F01_transcript_10990	F01_transcript_10990
F01_transcript_117166	NA	F01_transcript_117166	NA	NA
F01_transcript_84959	F01_transcript_84959	F01_transcript_84959	F01_transcript_84959	F01_transcript_84959
F01_transcript_104361	NA	NA	NA	F01_transcript_104361
F01_transcript_54543	NA	NA	NA	F01_transcript_54543
F01_transcript_109437	NA	F01_transcript_109437	NA	NA
F01_transcript_90053	NA	F01_transcript_90053	F01_transcript_90053	F01_transcript_90053

F01_transcript_83608	F01_transcript_83608	F01_transcript_83608	NA	F01_transcript_83608
F01_transcript_100046	F01_transcript_100046	F01_transcript_100046	NA	F01_transcript_100046
F01_transcript_23417	NA	NA	NA	F01_transcript_23417
F01_transcript_143186	NA	NA	NA	F01_transcript_143186
F01_transcript_71331	F01_transcript_71331	F01_transcript_71331	F01_transcript_71331	NA
F01_transcript_61834	NA	NA	NA	F01_transcript_61834
F01_transcript_131702	NA	F01_transcript_131702	F01_transcript_131702	F01_transcript_131702
F01_transcript_158157	NA	NA	NA	F01_transcript_158157
F01_transcript_147572	NA	F01_transcript_147572	NA	F01_transcript_147572
F01_transcript_83145	NA	F01_transcript_83145	NA	NA
F01_transcript_36962	NA	F01_transcript_36962	NA	F01_transcript_36962
F01_transcript_77958	F01_transcript_77958	F01_transcript_77958	F01_transcript_77958	F01_transcript_77958
F01_transcript_115818	F01_transcript_115818	F01_transcript_115818	F01_transcript_115818	F01_transcript_115818
F01_transcript_23264	NA	NA	NA	F01_transcript_23264

F01_transcript_92064	NA	NA	NA	F01_transcript_92064
F01_transcript_163974	F01_transcript_163974	F01_transcript_163974	F01_transcript_163974	F01_transcript_163974
F01_transcript_86556	NA	F01_transcript_86556	F01_transcript_86556	NA
F01_transcript_51161	F01_transcript_51161	F01_transcript_51161	F01_transcript_51161	F01_transcript_51161
F01_transcript_103216	NA	F01_transcript_103216	NA	NA
F01_transcript_148208	NA	F01_transcript_148208	NA	NA
F01_transcript_131937	F01_transcript_131937	F01_transcript_131937	F01_transcript_131937	F01_transcript_131937
F01_transcript_138476	NA	NA	NA	F01_transcript_138476
F01_transcript_17910	NA	NA	NA	F01_transcript_17910
F01_transcript_141565	NA	F01_transcript_141565	NA	F01_transcript_141565
F01_transcript_55083	NA	F01_transcript_55083	NA	F01_transcript_55083
F01_transcript_149429	NA	F01_transcript_149429	NA	F01_transcript_149429
F01_transcript_163248	NA	NA	NA	F01_transcript_163248
F01_transcript_105733	F01_transcript_105733	F01_transcript_105733	F01_transcript_105733	NA

F01_transcript_69540	NA	NA	F01_transcript_69540	F01_transcript_69540
F01_transcript_28007	NA	NA	NA	F01_transcript_28007
F01_transcript_126295	F01_transcript_126295	F01_transcript_126295	F01_transcript_126295	F01_transcript_126295
F01_transcript_166205	F01_transcript_166205	F01_transcript_166205	F01_transcript_166205	F01_transcript_166205
F01_transcript_96515	NA	NA	NA	F01_transcript_96515
F01_transcript_162862	NA	NA	NA	F01_transcript_162862
F01_transcript_90884	NA	F01_transcript_90884	NA	NA
F01_transcript_80925	NA	F01_transcript_80925	F01_transcript_80925	NA
F01_transcript_113872	NA	NA	NA	F01_transcript_113872
F01_transcript_84136	NA	NA	NA	F01_transcript_84136
F01_transcript_120186	NA	F01_transcript_120186	NA	NA
F01_transcript_103456	NA	F01_transcript_103456	NA	NA
F01_transcript_15593	F01_transcript_15593	F01_transcript_15593	NA	NA
F01_transcript_68904	NA	NA	NA	F01_transcript_68904

F01_transcript_28717	NA	NA	NA	F01_transcript_28717
F01_transcript_42228	NA	NA	NA	F01_transcript_42228
F01_transcript_119921	F01_transcript_119921	F01_transcript_119921	F01_transcript_119921	NA
F01_transcript_141910	NA	NA	NA	F01_transcript_141910
F01_transcript_128040	F01_transcript_128040	F01_transcript_128040	F01_transcript_128040	F01_transcript_128040
F01_transcript_99530	F01_transcript_99530	F01_transcript_99530	NA	NA
F01_transcript_58880	NA	NA	NA	F01_transcript_58880
F01_transcript_132937	NA	NA	NA	F01_transcript_132937
F01_transcript_115718	NA	NA	NA	F01_transcript_115718
F01_transcript_115600	NA	F01_transcript_115600	NA	NA
F01_transcript_108924	F01_transcript_108924	F01_transcript_108924	F01_transcript_108924	F01_transcript_108924
F01_transcript_63733	F01_transcript_63733	F01_transcript_63733	F01_transcript_63733	F01_transcript_63733
F01_transcript_158686	F01_transcript_158686	F01_transcript_158686	NA	F01_transcript_158686
F01_transcript_153989	NA	NA	NA	F01_transcript_153989

F01_transcript_106271	F01_transcript_106271	F01_transcript_106271	F01_transcript_106271	F01_transcript_106271
F01_transcript_120737	NA	F01_transcript_120737	NA	NA
F01_transcript_108311	NA	F01_transcript_108311	NA	NA
F01_transcript_153728	NA	NA	NA	F01_transcript_153728
F01_transcript_65325	F01_transcript_65325	F01_transcript_65325	F01_transcript_65325	F01_transcript_65325
F01_transcript_50328	NA	NA	NA	F01_transcript_50328
F01_transcript_149363	F01_transcript_149363	F01_transcript_149363	F01_transcript_149363	F01_transcript_149363
F01_transcript_138463	NA	NA	NA	F01_transcript_138463
F01_transcript_109074	NA	F01_transcript_109074	F01_transcript_109074	NA
F01_transcript_105699	NA	F01_transcript_105699	F01_transcript_105699	F01_transcript_105699
F01_transcript_46235	NA	F01_transcript_46235	NA	NA
F01_transcript_153506	F01_transcript_153506	F01_transcript_153506	F01_transcript_153506	F01_transcript_153506
F01_transcript_149202	NA	F01_transcript_149202	NA	F01_transcript_149202
F01_transcript_82658	NA	F01_transcript_82658	NA	NA

F01_transcript_98719	NA	NA	NA	F01_transcript_98719
F01_transcript_86183	NA	NA	F01_transcript_86183	NA
F01_transcript_82928	NA	NA	NA	F01_transcript_82928
F01_transcript_28518	NA	NA	NA	F01_transcript_28518
F01_transcript_55601	NA	F01_transcript_55601	NA	NA
F01_transcript_95358	NA	NA	NA	F01_transcript_95358
F01_transcript_157596	NA	NA	NA	F01_transcript_157596
F01_transcript_53818	NA	F01_transcript_53818	NA	NA
F01_transcript_147	NA	NA	NA	F01_transcript_147
F01_transcript_113902	F01_transcript_113902	F01_transcript_113902	NA	F01_transcript_113902
F01_transcript_71859	F01_transcript_71859	F01_transcript_71859	F01_transcript_71859	F01_transcript_71859
F01_transcript_138321	NA	F01_transcript_138321	NA	NA
F01_transcript_46561	NA	NA	NA	F01_transcript_46561
F01_transcript_84098	NA	F01_transcript_84098	NA	NA

F01_transcript_43312	NA	NA	NA	F01_transcript_43312
F01_transcript_71268	NA	NA	F01_transcript_71268	NA
F01_transcript_86388	NA	F01_transcript_86388	F01_transcript_86388	F01_transcript_86388
F01_transcript_139443	F01_transcript_139443	F01_transcript_139443	F01_transcript_139443	F01_transcript_139443
F01_transcript_128438	NA	F01_transcript_128438	NA	F01_transcript_128438
F01_transcript_135409	NA	NA	NA	F01_transcript_135409
F01_transcript_97519	NA	F01_transcript_97519	NA	NA
F01_transcript_103599	NA	F01_transcript_103599	NA	NA
F01_transcript_73910	NA	F01_transcript_73910	NA	F01_transcript_73910
F01_transcript_92169	F01_transcript_92169	F01_transcript_92169	F01_transcript_92169	F01_transcript_92169
F01_transcript_165476	NA	NA	NA	F01_transcript_165476
F01_transcript_64365	NA	F01_transcript_64365	NA	NA
F01_transcript_48626	NA	NA	NA	F01_transcript_48626
F01_transcript_146715	NA	NA	NA	F01_transcript_146715

F01_transcript_119564	F01_transcript_119564	F01_transcript_119564	F01_transcript_119564	F01_transcript_119564
F01_transcript_167765	NA	NA	NA	F01_transcript_167765
F01_transcript_126758	NA	F01_transcript_126758	NA	F01_transcript_126758
F01_transcript_131068	F01_transcript_131068	F01_transcript_131068	NA	F01_transcript_131068
F01_transcript_22682	NA	NA	NA	F01_transcript_22682
F01_transcript_141070	F01_transcript_141070	F01_transcript_141070	F01_transcript_141070	F01_transcript_141070
F01_transcript_128898	F01_transcript_128898	F01_transcript_128898	F01_transcript_128898	F01_transcript_128898
F01_transcript_108059	NA	NA	NA	F01_transcript_108059
F01_transcript_96640	NA	F01_transcript_96640	NA	NA
F01_transcript_77178	F01_transcript_77178	F01_transcript_77178	F01_transcript_77178	NA
F01_transcript_49865	NA	F01_transcript_49865	F01_transcript_49865	NA
F01_transcript_76761	F01_transcript_76761	F01_transcript_76761	F01_transcript_76761	F01_transcript_76761
F01_transcript_159842	F01_transcript_159842	F01_transcript_159842	NA	F01_transcript_159842
F01_transcript_123906	NA	F01_transcript_123906	NA	NA

F01_transcript_116784	NA	NA	NA	F01_transcript_116784
F01_transcript_138109	NA	NA	NA	F01_transcript_138109
F01_transcript_46078	NA	NA	F01_transcript_46078	F01_transcript_46078
F01_transcript_125374	F01_transcript_125374	F01_transcript_125374	F01_transcript_125374	F01_transcript_125374
F01_transcript_92091	F01_transcript_92091	F01_transcript_92091	F01_transcript_92091	F01_transcript_92091
F01_transcript_62697	F01_transcript_62697	F01_transcript_62697	F01_transcript_62697	F01_transcript_62697
F01_transcript_130201	F01_transcript_130201	F01_transcript_130201	F01_transcript_130201	F01_transcript_130201
F01_transcript_163435	NA	F01_transcript_163435	NA	F01_transcript_163435
F01_transcript_161355	NA	F01_transcript_161355	NA	NA
F01_transcript_65235	NA	NA	F01_transcript_65235	F01_transcript_65235
F01_transcript_143140	NA	NA	F01_transcript_143140	F01_transcript_143140
F01_transcript_151772	NA	F01_transcript_151772	F01_transcript_151772	NA
F01_transcript_77805	NA	NA	NA	F01_transcript_77805
F01_transcript_71226	NA	NA	F01_transcript_71226	NA

F01_transcript_100284	NA	NA	F01_transcript_100284	NA
F01_transcript_122809	NA	F01_transcript_122809	F01_transcript_122809	F01_transcript_122809
F01_transcript_471	NA	NA	NA	F01_transcript_471
F01_transcript_153893	NA	F01_transcript_153893	NA	F01_transcript_153893
F01_transcript_26417	F01_transcript_26417	F01_transcript_26417	F01_transcript_26417	F01_transcript_26417
F01_transcript_163776	F01_transcript_163776	F01_transcript_163776	F01_transcript_163776	F01_transcript_163776
F01_transcript_62644	NA	NA	NA	F01_transcript_62644
F01_transcript_47014	NA	NA	NA	F01_transcript_47014
F01_transcript_162396	NA	F01_transcript_162396	NA	NA
F01_transcript_103934	NA	NA	NA	F01_transcript_103934
F01_transcript_68223	NA	NA	NA	F01_transcript_68223
F01_transcript_61135	NA	F01_transcript_61135	F01_transcript_61135	F01_transcript_61135
F01_transcript_98772	F01_transcript_98772	F01_transcript_98772	F01_transcript_98772	F01_transcript_98772
F01_transcript_46554	NA	NA	NA	F01_transcript_46554

F01_transcript_88428	F01_transcript_88428	F01_transcript_88428	F01_transcript_88428	F01_transcript_88428
F01_transcript_135336	F01_transcript_135336	NA	NA	NA
F01_transcript_107966	NA	F01_transcript_107966	NA	NA
F01_transcript_119546	F01_transcript_119546	F01_transcript_119546	F01_transcript_119546	F01_transcript_119546
F01_transcript_163911	F01_transcript_163911	F01_transcript_163911	NA	NA
F01_transcript_28673	NA	NA	NA	F01_transcript_28673
F01_transcript_7783	NA	F01_transcript_7783	NA	F01_transcript_7783
F01_transcript_87297	NA	NA	NA	F01_transcript_87297
F01_transcript_19175	NA	F01_transcript_19175	NA	NA
F01_transcript_7285	F01_transcript_7285	F01_transcript_7285	NA	NA
F01_transcript_136856	NA	F01_transcript_136856	NA	NA
F01_transcript_139714	NA	F01_transcript_139714	NA	NA
F01_transcript_131396	F01_transcript_131396	F01_transcript_131396	F01_transcript_131396	F01_transcript_131396
F01_transcript_167277	F01_transcript_167277	F01_transcript_167277	F01_transcript_167277	NA

F01_transcript_167515	NA	NA	NA	F01_transcript_167515
F01_transcript_157582	F01_transcript_157582	F01_transcript_157582	F01_transcript_157582	F01_transcript_157582
F01_transcript_143264	NA	F01_transcript_143264	NA	F01_transcript_143264
F01_transcript_49743	NA	F01_transcript_49743	NA	NA
F01_transcript_29516	NA	NA	NA	F01_transcript_29516
F01_transcript_145430	NA	F01_transcript_145430	NA	NA
F01_transcript_117302	NA	F01_transcript_117302	NA	NA
F01_transcript_101119	NA	F01_transcript_101119	NA	NA
F01_transcript_62101	F01_transcript_62101	F01_transcript_62101	NA	NA
F01_transcript_153226	NA	F01_transcript_153226	F01_transcript_153226	F01_transcript_153226
F01_transcript_93576	NA	NA	NA	F01_transcript_93576
F01_transcript_130520	NA	F01_transcript_130520	F01_transcript_130520	NA
F01_transcript_100500	F01_transcript_100500	F01_transcript_100500	F01_transcript_100500	F01_transcript_100500
F01_transcript_28976	NA	NA	NA	F01_transcript_28976

F01_transcript_148534	NA	NA	NA	F01_transcript_148534
F01_transcript_148079	NA	F01_transcript_148079	F01_transcript_148079	F01_transcript_148079
F01_transcript_77786	NA	NA	NA	F01_transcript_77786
F01_transcript_107404	F01_transcript_107404	F01_transcript_107404	F01_transcript_107404	F01_transcript_107404
F01_transcript_122125	NA	F01_transcript_122125	NA	NA
F01_transcript_101619	NA	F01_transcript_101619	F01_transcript_101619	F01_transcript_101619
F01_transcript_79575	NA	NA	NA	F01_transcript_79575
F01_transcript_66185	NA	F01_transcript_66185	F01_transcript_66185	NA
F01_transcript_41606	NA	NA	F01_transcript_41606	NA
F01_transcript_64301	F01_transcript_64301	F01_transcript_64301	NA	F01_transcript_64301
F01_transcript_73737	NA	NA	NA	F01_transcript_73737
F01_transcript_65424	NA	NA	F01_transcript_65424	NA
F01_transcript_53374	NA	NA	NA	F01_transcript_53374
F01_transcript_68771	NA	NA	NA	F01_transcript_68771

F01_transcript_100511	NA	F01_transcript_100511	NA	NA
F01_transcript_130506	NA	NA	NA	F01_transcript_130506
F01_transcript_66964	NA	F01_transcript_66964	NA	NA
F01_transcript_47531	NA	NA	NA	F01_transcript_47531
F01_transcript_103116	NA	NA	NA	F01_transcript_103116
F01_transcript_152815	NA	F01_transcript_152815	NA	NA
F01_transcript_78329	F01_transcript_78329	F01_transcript_78329	NA	NA
F01_transcript_7275	NA	F01_transcript_7275	NA	F01_transcript_7275
F01_transcript_19826	NA	NA	NA	F01_transcript_19826
F01_transcript_139750	NA	NA	NA	F01_transcript_139750
F01_transcript_82190	NA	F01_transcript_82190	F01_transcript_82190	NA
F01_transcript_65441	F01_transcript_65441	F01_transcript_65441	F01_transcript_65441	F01_transcript_65441
F01_transcript_141250	F01_transcript_141250	F01_transcript_141250	F01_transcript_141250	F01_transcript_141250
F01_transcript_48042	NA	F01_transcript_48042	F01_transcript_48042	F01_transcript_48042

F01_transcript_82457	NA	F01_transcript_82457	NA	NA
F01_transcript_155738	NA	F01_transcript_155738	NA	F01_transcript_155738
F01_transcript_118960	NA	F01_transcript_118960	F01_transcript_118960	F01_transcript_118960
F01_transcript_100732	NA	F01_transcript_100732	NA	F01_transcript_100732
F01_transcript_43593	NA	NA	NA	F01_transcript_43593
F01_transcript_94573	NA	NA	NA	F01_transcript_94573
F01_transcript_143443	F01_transcript_143443	F01_transcript_143443	F01_transcript_143443	F01_transcript_143443
F01_transcript_113010	F01_transcript_113010	F01_transcript_113010	F01_transcript_113010	F01_transcript_113010
F01_transcript_93397	F01_transcript_93397	F01_transcript_93397	F01_transcript_93397	F01_transcript_93397
F01_transcript_56142	F01_transcript_56142	F01_transcript_56142	F01_transcript_56142	F01_transcript_56142
F01_transcript_94517	NA	F01_transcript_94517	F01_transcript_94517	F01_transcript_94517
F01_transcript_48059	NA	F01_transcript_48059	NA	NA
F01_transcript_138931	NA	NA	NA	F01_transcript_138931
F01_transcript_117359	F01_transcript_117359	F01_transcript_117359	NA	NA

F01_transcript_123349	NA	F01_transcript_123349	NA	NA
F01_transcript_60737	NA	F01_transcript_60737	NA	F01_transcript_60737
F01_transcript_118694	NA	F01_transcript_118694	NA	NA
F01_transcript_121644	F01_transcript_121644	F01_transcript_121644	F01_transcript_121644	F01_transcript_121644
F01_transcript_55736	NA	F01_transcript_55736	NA	F01_transcript_55736
F01_transcript_95975	NA	F01_transcript_95975	NA	F01_transcript_95975
F01_transcript_45242	NA	NA	NA	F01_transcript_45242
F01_transcript_11204	NA	NA	NA	F01_transcript_11204
F01_transcript_146792	F01_transcript_146792	F01_transcript_146792	F01_transcript_146792	F01_transcript_146792
F01_transcript_22361	F01_transcript_22361	F01_transcript_22361	NA	F01_transcript_22361
F01_transcript_117086	F01_transcript_117086	F01_transcript_117086	F01_transcript_117086	F01_transcript_117086
F01_transcript_54945	NA	F01_transcript_54945	NA	NA
F01_transcript_51459	NA	NA	NA	F01_transcript_51459
F01_transcript_131208	F01_transcript_131208	F01_transcript_131208	NA	NA

F01_transcript_469	NA	NA	NA	F01_transcript_469
F01_transcript_165083	NA	F01_transcript_165083	F01_transcript_165083	F01_transcript_165083
F01_transcript_101684	F01_transcript_101684	F01_transcript_101684	F01_transcript_101684	F01_transcript_101684
F01_transcript_52468	NA	F01_transcript_52468	NA	F01_transcript_52468
F01_transcript_29202	NA	NA	NA	F01_transcript_29202
F01_transcript_134475	NA	NA	NA	F01_transcript_134475
F01_transcript_48390	NA	NA	NA	F01_transcript_48390
F01_transcript_93034	NA	F01_transcript_93034	NA	NA
F01_transcript_151035	NA	F01_transcript_151035	NA	NA
F01_transcript_55373	F01_transcript_55373	F01_transcript_55373	NA	F01_transcript_55373
F01_transcript_104170	F01_transcript_104170	NA	NA	NA
F01_transcript_38829	NA	F01_transcript_38829	NA	F01_transcript_38829
F01_transcript_103826	NA	NA	NA	F01_transcript_103826
F01_transcript_74458	F01_transcript_74458	F01_transcript_74458	F01_transcript_74458	F01_transcript_74458

F01_transcript_99340	NA	F01_transcript_99340	NA	NA
F01_transcript_131108	NA	NA	F01_transcript_131108	NA
F01_transcript_76531	F01_transcript_76531	F01_transcript_76531	NA	F01_transcript_76531
F01_transcript_3835	F01_transcript_3835	F01_transcript_3835	NA	F01_transcript_3835
F01_transcript_93188	NA	NA	F01_transcript_93188	F01_transcript_93188
F01_transcript_101721	F01_transcript_101721	F01_transcript_101721	F01_transcript_101721	F01_transcript_101721
F01_transcript_124786	NA	NA	NA	F01_transcript_124786
F01_transcript_105492	F01_transcript_105492	F01_transcript_105492	NA	NA
F01_transcript_55691	F01_transcript_55691	F01_transcript_55691	F01_transcript_55691	F01_transcript_55691
F01_transcript_121351	NA	F01_transcript_121351	F01_transcript_121351	F01_transcript_121351
F01_transcript_83667	NA	NA	NA	F01_transcript_83667
F01_transcript_88550	NA	F01_transcript_88550	NA	NA
F01_transcript_84093	NA	F01_transcript_84093	NA	NA
F01_transcript_157376	F01_transcript_157376	F01_transcript_157376	F01_transcript_157376	F01_transcript_157376

F01_transcript_145106	NA	NA	NA	F01_transcript_145106
F01_transcript_111951	NA	F01_transcript_111951	NA	NA
F01_transcript_154440	NA	F01_transcript_154440	NA	NA
F01_transcript_94939	NA	NA	F01_transcript_94939	F01_transcript_94939
F01_transcript_88057	NA	F01_transcript_88057	NA	NA
F01_transcript_128266	F01_transcript_128266	F01_transcript_128266	NA	F01_transcript_128266
F01_transcript_118912	NA	NA	NA	F01_transcript_118912
F01_transcript_70368	NA	NA	NA	F01_transcript_70368
F01_transcript_71778	NA	F01_transcript_71778	F01_transcript_71778	F01_transcript_71778
F01_transcript_124630	F01_transcript_124630	F01_transcript_124630	F01_transcript_124630	F01_transcript_124630
F01_transcript_108336	F01_transcript_108336	F01_transcript_108336	F01_transcript_108336	F01_transcript_108336
F01_transcript_121713	NA	F01_transcript_121713	F01_transcript_121713	NA
F01_transcript_163662	NA	NA	NA	F01_transcript_163662
F01_transcript_140559	F01_transcript_140559	F01_transcript_140559	F01_transcript_140559	F01_transcript_140559

F01_transcript_157941	F01_transcript_157941	F01_transcript_157941	F01_transcript_157941	F01_transcript_157941
F01_transcript_53808	F01_transcript_53808	F01_transcript_53808	NA	F01_transcript_53808
F01_transcript_92016	F01_transcript_92016	F01_transcript_92016	F01_transcript_92016	F01_transcript_92016
F01_transcript_131119	F01_transcript_131119	F01_transcript_131119	F01_transcript_131119	F01_transcript_131119
F01_transcript_130534	F01_transcript_130534	F01_transcript_130534	F01_transcript_130534	F01_transcript_130534
F01_transcript_54480	NA	F01_transcript_54480	NA	NA
F01_transcript_5053	NA	F01_transcript_5053	F01_transcript_5053	NA
F01_transcript_132055	NA	NA	NA	F01_transcript_132055
F01_transcript_71550	NA	F01_transcript_71550	NA	NA
F01_transcript_92730	F01_transcript_92730	F01_transcript_92730	NA	NA
F01_transcript_102821	NA	NA	NA	F01_transcript_102821
F01_transcript_144019	NA	F01_transcript_144019	NA	F01_transcript_144019
F01_transcript_150357	F01_transcript_150357	F01_transcript_150357	NA	F01_transcript_150357
F01_transcript_6283	NA	F01_transcript_6283	NA	NA

F01_transcript_131482	NA	F01_transcript_131482	F01_transcript_131482	NA
F01_transcript_132104	F01_transcript_132104	F01_transcript_132104	F01_transcript_132104	F01_transcript_132104
F01_transcript_56505	NA	F01_transcript_56505	F01_transcript_56505	NA
F01_transcript_70757	NA	NA	NA	F01_transcript_70757
F01_transcript_97044	NA	F01_transcript_97044	NA	NA
F01_transcript_123214	NA	F01_transcript_123214	F01_transcript_123214	NA
F01_transcript_84734	F01_transcript_84734	F01_transcript_84734	F01_transcript_84734	F01_transcript_84734
F01_transcript_121421	F01_transcript_121421	F01_transcript_121421	F01_transcript_121421	F01_transcript_121421
F01_transcript_96802	NA	NA	NA	F01_transcript_96802
F01_transcript_55365	NA	F01_transcript_55365	NA	NA
F01_transcript_55466	NA	NA	NA	F01_transcript_55466
F01_transcript_138840	F01_transcript_138840	F01_transcript_138840	F01_transcript_138840	NA
F01_transcript_158212	F01_transcript_158212	F01_transcript_158212	F01_transcript_158212	F01_transcript_158212
F01_transcript_104131	NA	F01_transcript_104131	NA	F01_transcript_104131

F01_transcript_127419	NA	NA	NA	F01_transcript_127419
F01_transcript_163918	F01_transcript_163918	F01_transcript_163918	F01_transcript_163918	NA
F01_transcript_86314	NA	F01_transcript_86314	NA	NA
F01_transcript_165499	NA	F01_transcript_165499	NA	NA
F01_transcript_93962	NA	NA	NA	F01_transcript_93962
F01_transcript_125661	NA	F01_transcript_125661	F01_transcript_125661	F01_transcript_125661
F01_transcript_147846	NA	F01_transcript_147846	NA	NA
F01_transcript_50857	F01_transcript_50857	F01_transcript_50857	F01_transcript_50857	F01_transcript_50857
F01_transcript_167521	NA	F01_transcript_167521	F01_transcript_167521	F01_transcript_167521
F01_transcript_160116	NA	F01_transcript_160116	NA	NA
F01_transcript_151382	F01_transcript_151382	F01_transcript_151382	F01_transcript_151382	F01_transcript_151382
F01_transcript_45991	NA	NA	F01_transcript_45991	F01_transcript_45991
F01_transcript_113159	NA	NA	NA	F01_transcript_113159
F01_transcript_94501	NA	NA	NA	F01_transcript_94501

F01_transcript_47279	NA	NA	F01_transcript_47279	F01_transcript_47279
F01_transcript_66714	F01_transcript_66714	F01_transcript_66714	F01_transcript_66714	F01_transcript_66714
F01_transcript_137141	NA	F01_transcript_137141	F01_transcript_137141	NA
F01_transcript_141819	F01_transcript_141819	F01_transcript_141819	F01_transcript_141819	F01_transcript_141819
F01_transcript_82414	NA	NA	NA	F01_transcript_82414
F01_transcript_123249	NA	NA	NA	F01_transcript_123249
F01_transcript_102588	NA	NA	NA	F01_transcript_102588
F01_transcript_160458	NA	NA	NA	F01_transcript_160458
F01_transcript_64775	F01_transcript_64775	F01_transcript_64775	F01_transcript_64775	F01_transcript_64775
F01_transcript_26753	NA	F01_transcript_26753	NA	F01_transcript_26753
F01_transcript_57382	F01_transcript_57382	F01_transcript_57382	F01_transcript_57382	F01_transcript_57382
F01_transcript_117865	NA	F01_transcript_117865	NA	NA
F01_transcript_143019	NA	F01_transcript_143019	NA	NA
F01_transcript_133796	F01_transcript_133796	F01_transcript_133796	F01_transcript_133796	F01_transcript_133796

F01_transcript_105416	NA	F01_transcript_105416	F01_transcript_105416	F01_transcript_105416
F01_transcript_91743	NA	F01_transcript_91743	NA	NA
F01_transcript_120165	NA	NA	NA	F01_transcript_120165
F01_transcript_112690	NA	NA	NA	F01_transcript_112690
F01_transcript_338	NA	F01_transcript_338	NA	NA
F01_transcript_108091	F01_transcript_108091	F01_transcript_108091	F01_transcript_108091	F01_transcript_108091
F01_transcript_161416	NA	F01_transcript_161416	NA	F01_transcript_161416
F01_transcript_74256	NA	F01_transcript_74256	NA	NA
F01_transcript_157871	NA	F01_transcript_157871	F01_transcript_157871	F01_transcript_157871
F01_transcript_21559	NA	NA	NA	F01_transcript_21559
F01_transcript_72673	NA	F01_transcript_72673	NA	NA
F01_transcript_100370	NA	NA	NA	F01_transcript_100370
F01_transcript_88529	NA	NA	NA	F01_transcript_88529
F01_transcript_147416	F01_transcript_147416	F01_transcript_147416	F01_transcript_147416	F01_transcript_147416

F01_transcript_108886	NA	F01_transcript_108886	NA	NA
F01_transcript_69741	NA	NA	NA	F01_transcript_69741
F01_transcript_133145	NA	F01_transcript_133145	NA	NA
F01_transcript_113033	NA	NA	NA	F01_transcript_113033
F01_transcript_166406	NA	NA	NA	F01_transcript_166406
F01_transcript_145656	NA	NA	NA	F01_transcript_145656
F01_transcript_71393	NA	NA	NA	F01_transcript_71393
F01_transcript_159130	NA	F01_transcript_159130	F01_transcript_159130	F01_transcript_159130
F01_transcript_67946	F01_transcript_67946	F01_transcript_67946	F01_transcript_67946	F01_transcript_67946
F01_transcript_67638	NA	F01_transcript_67638	F01_transcript_67638	F01_transcript_67638
F01_transcript_56718	NA	NA	NA	F01_transcript_56718
F01_transcript_141239	NA	F01_transcript_141239	NA	F01_transcript_141239
F01_transcript_138347	NA	F01_transcript_138347	NA	F01_transcript_138347
F01_transcript_77196	NA	NA	NA	F01_transcript_77196

F01_transcript_65073	NA	F01_transcript_65073	F01_transcript_65073	NA
F01_transcript_52356	NA	NA	NA	F01_transcript_52356
F01_transcript_59964	NA	F01_transcript_59964	NA	NA
F01_transcript_166917	NA	NA	NA	F01_transcript_166917
F01_transcript_143687	NA	NA	F01_transcript_143687	F01_transcript_143687
F01_transcript_57608	NA	NA	NA	F01_transcript_57608
F01_transcript_136185	NA	NA	NA	F01_transcript_136185
F01_transcript_126056	NA	NA	NA	F01_transcript_126056
F01_transcript_123113	NA	F01_transcript_123113	NA	NA
F01_transcript_151042	NA	F01_transcript_151042	NA	NA
F01_transcript_48130	NA	NA	F01_transcript_48130	F01_transcript_48130
F01_transcript_156972	NA	NA	NA	F01_transcript_156972
F01_transcript_118127	NA	F01_transcript_118127	NA	NA
F01_transcript_135827	F01_transcript_135827	F01_transcript_135827	F01_transcript_135827	F01_transcript_135827

F01_transcript_42834	NA	NA	NA	F01_transcript_42834
F01_transcript_107342	NA	NA	NA	F01_transcript_107342
F01_transcript_110229	NA	NA	NA	F01_transcript_110229
F01_transcript_45724	NA	NA	NA	F01_transcript_45724
F01_transcript_114191	NA	F01_transcript_114191	NA	NA
F01_transcript_149409	NA	NA	NA	F01_transcript_149409
F01_transcript_130700	F01_transcript_130700	F01_transcript_130700	NA	NA
F01_transcript_97940	NA	F01_transcript_97940	NA	NA
F01_transcript_124996	NA	NA	NA	F01_transcript_124996
F01_transcript_49488	NA	F01_transcript_49488	F01_transcript_49488	F01_transcript_49488
F01_transcript_156151	F01_transcript_156151	F01_transcript_156151	F01_transcript_156151	F01_transcript_156151
F01_transcript_1550	NA	NA	F01_transcript_1550	F01_transcript_1550
F01_transcript_134872	F01_transcript_134872	F01_transcript_134872	F01_transcript_134872	F01_transcript_134872
F01_transcript_79014	F01_transcript_79014	F01_transcript_79014	NA	F01_transcript_79014

F01_transcript_152332	NA	F01_transcript_152332	F01_transcript_152332	NA
F01_transcript_79158	F01_transcript_79158	F01_transcript_79158	F01_transcript_79158	F01_transcript_79158
F01_transcript_116825	F01_transcript_116825	F01_transcript_116825	NA	F01_transcript_116825
F01_transcript_50596	F01_transcript_50596	F01_transcript_50596	NA	F01_transcript_50596
F01_transcript_85411	NA	F01_transcript_85411	NA	NA
F01_transcript_86520	NA	NA	NA	F01_transcript_86520
F01_transcript_74455	NA	NA	NA	F01_transcript_74455
F01_transcript_54252	F01_transcript_54252	F01_transcript_54252	F01_transcript_54252	F01_transcript_54252
F01_transcript_4354	NA	F01_transcript_4354	NA	NA
F01_transcript_150728	NA	F01_transcript_150728	NA	F01_transcript_150728
F01_transcript_162151	F01_transcript_162151	F01_transcript_162151	F01_transcript_162151	F01_transcript_162151
F01_transcript_2163	F01_transcript_2163	F01_transcript_2163	F01_transcript_2163	F01_transcript_2163
F01_transcript_111053	NA	NA	NA	F01_transcript_111053
F01_transcript_52708	NA	F01_transcript_52708	NA	F01_transcript_52708

F01_transcript_66338	NA	F01_transcript_66338	F01_transcript_66338	F01_transcript_66338
F01_transcript_157038	NA	NA	NA	F01_transcript_157038
F01_transcript_56244	NA	NA	F01_transcript_56244	F01_transcript_56244
F01_transcript_70847	NA	NA	NA	F01_transcript_70847
F01_transcript_79224	NA	F01_transcript_79224	NA	NA
F01_transcript_62202	NA	F01_transcript_62202	NA	F01_transcript_62202
F01_transcript_51953	NA	NA	NA	F01_transcript_51953
F01_transcript_9100	NA	NA	NA	F01_transcript_9100
F01_transcript_145680	NA	F01_transcript_145680	NA	F01_transcript_145680
F01_transcript_61667	NA	F01_transcript_61667	NA	F01_transcript_61667
F01_transcript_147974	NA	F01_transcript_147974	NA	NA
F01_transcript_60260	NA	NA	NA	F01_transcript_60260
F01_transcript_56028	F01_transcript_56028	F01_transcript_56028	NA	NA
F01_transcript_105786	NA	NA	NA	F01_transcript_105786

F01_transcript_55471	NA	F01_transcript_55471	NA	F01_transcript_55471
F01_transcript_156463	NA	NA	NA	F01_transcript_156463
F01_transcript_114451	F01_transcript_114451	F01_transcript_114451	F01_transcript_114451	F01_transcript_114451
F01_transcript_121769	F01_transcript_121769	F01_transcript_121769	F01_transcript_121769	F01_transcript_121769
F01_transcript_158754	NA	F01_transcript_158754	NA	F01_transcript_158754
F01_transcript_105176	F01_transcript_105176	F01_transcript_105176	F01_transcript_105176	F01_transcript_105176
F01_transcript_85000	NA	NA	NA	F01_transcript_85000
F01_transcript_59895	NA	F01_transcript_59895	F01_transcript_59895	F01_transcript_59895
F01_transcript_1601	NA	F01_transcript_1601	NA	NA
F01_transcript_161269	NA	NA	NA	F01_transcript_161269
F01_transcript_110734	NA	F01_transcript_110734	NA	NA
F01_transcript_101041	NA	NA	NA	F01_transcript_101041
F01_transcript_114839	NA	NA	NA	F01_transcript_114839
F01_transcript_103674	NA	NA	NA	F01_transcript_103674

F01_transcript_136532	NA	NA	NA	F01_transcript_136532
F01_transcript_67214	NA	NA	NA	F01_transcript_67214
F01_transcript_8507	NA	NA	NA	F01_transcript_8507
F01_transcript_84841	NA	F01_transcript_84841	F01_transcript_84841	NA
F01_transcript_46231	NA	NA	NA	F01_transcript_46231
F01_transcript_52928	NA	NA	NA	F01_transcript_52928
F01_transcript_113824	NA	NA	NA	F01_transcript_113824
F01_transcript_98819	NA	F01_transcript_98819	NA	NA
F01_transcript_90891	NA	F01_transcript_90891	NA	NA
F01_transcript_157160	F01_transcript_157160	F01_transcript_157160	F01_transcript_157160	NA
F01_transcript_142066	NA	F01_transcript_142066	NA	NA
F01_transcript_62910	F01_transcript_62910	F01_transcript_62910	F01_transcript_62910	F01_transcript_62910
F01_transcript_64936	NA	F01_transcript_64936	NA	NA
F01_transcript_146058	NA	NA	NA	F01_transcript_146058

F01_transcript_7507	NA	F01_transcript_7507	NA	NA
F01_transcript_100429	NA	F01_transcript_100429	NA	NA
F01_transcript_64291	NA	F01_transcript_64291	NA	NA
F01_transcript_91237	NA	F01_transcript_91237	NA	NA
F01_transcript_167668	NA	NA	NA	F01_transcript_167668
F01_transcript_160101	NA	F01_transcript_160101	NA	F01_transcript_160101
F01_transcript_144355	NA	NA	NA	F01_transcript_144355
F01_transcript_135675	NA	NA	NA	F01_transcript_135675
F01_transcript_348	NA	NA	NA	F01_transcript_348
F01_transcript_113136	NA	F01_transcript_113136	NA	NA
F01_transcript_78403	NA	NA	NA	F01_transcript_78403
F01_transcript_120689	NA	F01_transcript_120689	NA	NA
F01_transcript_55391	F01_transcript_55391	F01_transcript_55391	NA	NA
F01_transcript_50360	F01_transcript_50360	F01_transcript_50360	F01_transcript_50360	F01_transcript_50360

F01_transcript_98027	NA	NA	NA	F01_transcript_98027
F01_transcript_60430	F01_transcript_60430	F01_transcript_60430	F01_transcript_60430	F01_transcript_60430
F01_transcript_85049	NA	F01_transcript_85049	NA	NA
F01_transcript_66380	NA	F01_transcript_66380	NA	NA
F01_transcript_117202	NA	NA	NA	F01_transcript_117202
F01_transcript_106907	NA	NA	NA	F01_transcript_106907
F01_transcript_133170	F01_transcript_133170	F01_transcript_133170	F01_transcript_133170	F01_transcript_133170
F01_transcript_94276	NA	F01_transcript_94276	NA	NA
F01_transcript_164135	F01_transcript_164135	F01_transcript_164135	F01_transcript_164135	F01_transcript_164135
F01_transcript_62860	NA	F01_transcript_62860	NA	F01_transcript_62860
F01_transcript_97046	NA	F01_transcript_97046	F01_transcript_97046	NA
F01_transcript_61893	NA	NA	NA	F01_transcript_61893
F01_transcript_150608	F01_transcript_150608	F01_transcript_150608	NA	F01_transcript_150608
F01_transcript_26965	NA	NA	NA	F01_transcript_26965

F01_transcript_139468	F01_transcript_139468	F01_transcript_139468	NA	NA
F01_transcript_163633	NA	F01_transcript_163633	NA	NA
F01_transcript_114228	NA	NA	NA	F01_transcript_114228
F01_transcript_83158	NA	NA	NA	F01_transcript_83158
F01_transcript_103002	NA	F01_transcript_103002	NA	NA
F01_transcript_162188	F01_transcript_162188	F01_transcript_162188	F01_transcript_162188	F01_transcript_162188
F01_transcript_158332	F01_transcript_158332	F01_transcript_158332	NA	F01_transcript_158332
F01_transcript_72040	NA	NA	NA	F01_transcript_72040
F01_transcript_60254	NA	F01_transcript_60254	F01_transcript_60254	F01_transcript_60254
F01_transcript_72156	NA	F01_transcript_72156	NA	NA
F01_transcript_94465	NA	F01_transcript_94465	NA	NA
F01_transcript_143291	NA	F01_transcript_143291	NA	NA
F01_transcript_157974	NA	F01_transcript_157974	NA	NA
F01_transcript_156036	F01_transcript_156036	F01_transcript_156036	F01_transcript_156036	F01_transcript_156036

F01_transcript_117896	NA	NA	NA	F01_transcript_117896
F01_transcript_105148	NA	NA	NA	F01_transcript_105148
F01_transcript_84096	NA	NA	F01_transcript_84096	NA
F01_transcript_63529	NA	NA	NA	F01_transcript_63529
F01_transcript_79147	NA	NA	NA	F01_transcript_79147
F01_transcript_96310	NA	NA	NA	F01_transcript_96310
F01_transcript_131529	NA	NA	NA	F01_transcript_131529
F01_transcript_133099	NA	F01_transcript_133099	NA	F01_transcript_133099
F01_transcript_83027	NA	NA	NA	F01_transcript_83027
F01_transcript_165504	F01_transcript_165504	F01_transcript_165504	F01_transcript_165504	F01_transcript_165504
F01_transcript_105671	NA	F01_transcript_105671	NA	NA
F01_transcript_135604	F01_transcript_135604	F01_transcript_135604	F01_transcript_135604	F01_transcript_135604
F01_transcript_49991	NA	F01_transcript_49991	NA	NA
F01_transcript_159011	NA	NA	F01_transcript_159011	NA

F01_transcript_18532	NA	NA	NA	F01_transcript_18532
F01_transcript_135112	NA	F01_transcript_135112	NA	NA
F01_transcript_33035	NA	NA	NA	F01_transcript_33035
F01_transcript_84029	NA	NA	NA	F01_transcript_84029
F01_transcript_58898	NA	NA	NA	F01_transcript_58898
F01_transcript_95075	F01_transcript_95075	F01_transcript_95075	F01_transcript_95075	NA
F01_transcript_68565	NA	NA	NA	F01_transcript_68565
F01_transcript_11885	NA	NA	NA	F01_transcript_11885
F01_transcript_127187	NA	NA	NA	F01_transcript_127187
F01_transcript_124820	NA	F01_transcript_124820	NA	NA
F01_transcript_94757	NA	F01_transcript_94757	F01_transcript_94757	F01_transcript_94757
F01_transcript_114649	NA	F01_transcript_114649	NA	F01_transcript_114649
F01_transcript_150683	NA	NA	NA	F01_transcript_150683
F01_transcript_68232	NA	NA	NA	F01_transcript_68232

F01_transcript_113648	F01_transcript_113648	F01_transcript_113648	F01_transcript_113648	F01_transcript_113648
F01_transcript_154415	NA	F01_transcript_154415	F01_transcript_154415	F01_transcript_154415
F01_transcript_48801	NA	NA	NA	F01_transcript_48801
F01_transcript_115707	NA	F01_transcript_115707	F01_transcript_115707	F01_transcript_115707
F01_transcript_5971	NA	F01_transcript_5971	NA	F01_transcript_5971
F01_transcript_80356	F01_transcript_80356	F01_transcript_80356	F01_transcript_80356	F01_transcript_80356
F01_transcript_160959	F01_transcript_160959	F01_transcript_160959	NA	NA
F01_transcript_29157	NA	NA	F01_transcript_29157	F01_transcript_29157
F01_transcript_135835	NA	F01_transcript_135835	NA	F01_transcript_135835
F01_transcript_110522	NA	NA	NA	F01_transcript_110522
F01_transcript_67629	NA	NA	NA	F01_transcript_67629
F01_transcript_148941	NA	F01_transcript_148941	NA	NA
F01_transcript_34304	NA	NA	F01_transcript_34304	NA
F01_transcript_73850	F01_transcript_73850	F01_transcript_73850	NA	F01_transcript_73850

F01_transcript_156230	NA	NA	NA	F01_transcript_156230
F01_transcript_108626	NA	NA	NA	F01_transcript_108626
F01_transcript_153040	NA	NA	NA	F01_transcript_153040
F01_transcript_60010	NA	F01_transcript_60010	NA	NA
F01_transcript_90132	NA	F01_transcript_90132	NA	NA
F01_transcript_124430	F01_transcript_124430	F01_transcript_124430	F01_transcript_124430	F01_transcript_124430
F01_transcript_86349	NA	F01_transcript_86349	NA	NA
F01_transcript_125570	F01_transcript_125570	F01_transcript_125570	F01_transcript_125570	F01_transcript_125570
F01_transcript_124533	NA	F01_transcript_124533	NA	NA
F01_transcript_159693	NA	F01_transcript_159693	NA	NA
F01_transcript_11521	NA	F01_transcript_11521	NA	NA
F01_transcript_82371	NA	F01_transcript_82371	NA	NA
F01_transcript_128317	NA	F01_transcript_128317	NA	F01_transcript_128317
F01_transcript_69834	NA	NA	NA	F01_transcript_69834

F01_transcript_151120	F01_transcript_151120	F01_transcript_151120	F01_transcript_151120	F01_transcript_151120
F01_transcript_123184	NA	F01_transcript_123184	F01_transcript_123184	NA
F01_transcript_163166	NA	NA	NA	F01_transcript_163166
F01_transcript_109219	F01_transcript_109219	F01_transcript_109219	F01_transcript_109219	F01_transcript_109219
F01_transcript_87819	NA	F01_transcript_87819	NA	F01_transcript_87819
F01_transcript_86647	NA	F01_transcript_86647	NA	F01_transcript_86647
F01_transcript_45369	NA	NA	NA	F01_transcript_45369
F01_transcript_69003	NA	NA	NA	F01_transcript_69003
F01_transcript_136088	NA	F01_transcript_136088	NA	NA
F01_transcript_131206	NA	F01_transcript_131206	NA	NA
F01_transcript_163686	F01_transcript_163686	F01_transcript_163686	F01_transcript_163686	F01_transcript_163686
F01_transcript_141892	NA	NA	NA	F01_transcript_141892
F01_transcript_114225	NA	F01_transcript_114225	NA	NA
F01_transcript_107809	NA	F01_transcript_107809	F01_transcript_107809	F01_transcript_107809

F01_transcript_50095	NA	NA	F01_transcript_50095	F01_transcript_50095
F01_transcript_109357	NA	F01_transcript_109357	NA	NA
F01_transcript_133026	NA	NA	F01_transcript_133026	NA
F01_transcript_159654	NA	NA	NA	F01_transcript_159654
F01_transcript_134049	F01_transcript_134049	F01_transcript_134049	F01_transcript_134049	F01_transcript_134049
F01_transcript_31566	NA	NA	NA	F01_transcript_31566
F01_transcript_129334	F01_transcript_129334	F01_transcript_129334	NA	NA
F01_transcript_165047	NA	NA	NA	F01_transcript_165047
F01_transcript_29151	NA	NA	NA	F01_transcript_29151
F01_transcript_30128	NA	F01_transcript_30128	NA	NA
F01_transcript_48402	NA	NA	NA	F01_transcript_48402
F01_transcript_107086	NA	F01_transcript_107086	NA	NA
F01_transcript_158972	F01_transcript_158972	F01_transcript_158972	F01_transcript_158972	F01_transcript_158972
F01_transcript_49536	NA	NA	NA	F01_transcript_49536

F01_transcript_91100	NA	F01_transcript_91100	NA	F01_transcript_91100
F01_transcript_55050	NA	NA	NA	F01_transcript_55050
F01_transcript_66602	NA	F01_transcript_66602	NA	NA
F01_transcript_55123	NA	F01_transcript_55123	NA	NA
F01_transcript_158691	NA	F01_transcript_158691	NA	NA
F01_transcript_65332	F01_transcript_65332	F01_transcript_65332	F01_transcript_65332	F01_transcript_65332
F01_transcript_157493	NA	NA	NA	F01_transcript_157493
F01_transcript_103790	NA	NA	NA	F01_transcript_103790
F01_transcript_66970	NA	NA	NA	F01_transcript_66970
F01_transcript_127126	F01_transcript_127126	F01_transcript_127126	F01_transcript_127126	F01_transcript_127126
F01_transcript_115111	NA	NA	NA	F01_transcript_115111
F01_transcript_69555	NA	NA	NA	F01_transcript_69555
F01_transcript_70514	NA	F01_transcript_70514	NA	NA
F01_transcript_90560	F01_transcript_90560	F01_transcript_90560	F01_transcript_90560	F01_transcript_90560

F01_transcript_51312	F01_transcript_51312	F01_transcript_51312	F01_transcript_51312	F01_transcript_51312
F01_transcript_86708	NA	NA	F01_transcript_86708	F01_transcript_86708
F01_transcript_72880	F01_transcript_72880	F01_transcript_72880	F01_transcript_72880	F01_transcript_72880
F01_transcript_49980	F01_transcript_49980	F01_transcript_49980	F01_transcript_49980	F01_transcript_49980
F01_transcript_103497	NA	NA	NA	F01_transcript_103497
F01_transcript_85174	F01_transcript_85174	F01_transcript_85174	F01_transcript_85174	F01_transcript_85174
F01_transcript_98952	F01_transcript_98952	F01_transcript_98952	F01_transcript_98952	F01_transcript_98952
F01_transcript_31730	NA	F01_transcript_31730	NA	NA
F01_transcript_88689	F01_transcript_88689	F01_transcript_88689	F01_transcript_88689	NA
F01_transcript_122846	NA	NA	NA	F01_transcript_122846
F01_transcript_53283	NA	F01_transcript_53283	NA	F01_transcript_53283
F01_transcript_134009	NA	NA	NA	F01_transcript_134009
F01_transcript_157769	NA	F01_transcript_157769	NA	F01_transcript_157769
F01_transcript_59644	NA	F01_transcript_59644	F01_transcript_59644	F01_transcript_59644

F01_transcript_145169	NA	F01_transcript_145169	F01_transcript_145169	F01_transcript_145169
F01_transcript_137088	NA	F01_transcript_137088	NA	NA
F01_transcript_131956	F01_transcript_131956	F01_transcript_131956	F01_transcript_131956	F01_transcript_131956
F01_transcript_136726	F01_transcript_136726	NA	F01_transcript_136726	F01_transcript_136726
F01_transcript_46182	F01_transcript_46182	F01_transcript_46182	F01_transcript_46182	F01_transcript_46182
F01_transcript_131882	NA	F01_transcript_131882	F01_transcript_131882	F01_transcript_131882
F01_transcript_76089	NA	NA	NA	F01_transcript_76089
F01_transcript_102997	NA	F01_transcript_102997	NA	NA
F01_transcript_145414	F01_transcript_145414	F01_transcript_145414	F01_transcript_145414	F01_transcript_145414
F01_transcript_59293	NA	F01_transcript_59293	F01_transcript_59293	NA
F01_transcript_100710	NA	F01_transcript_100710	NA	NA
F01_transcript_118895	F01_transcript_118895	F01_transcript_118895	F01_transcript_118895	F01_transcript_118895
F01_transcript_148606	NA	F01_transcript_148606	NA	NA
F01_transcript_4907	NA	F01_transcript_4907	F01_transcript_4907	F01_transcript_4907

F01_transcript_22945	NA	F01_transcript_22945	NA	NA
F01_transcript_121238	NA	F01_transcript_121238	NA	NA
F01_transcript_155922	F01_transcript_155922	F01_transcript_155922	NA	NA
F01_transcript_124597	NA	NA	NA	F01_transcript_124597
F01_transcript_80602	NA	NA	NA	F01_transcript_80602
F01_transcript_125542	NA	NA	NA	F01_transcript_125542
F01_transcript_85425	NA	F01_transcript_85425	NA	F01_transcript_85425
F01_transcript_109266	NA	F01_transcript_109266	NA	F01_transcript_109266
F01_transcript_78815	NA	NA	NA	F01_transcript_78815
F01_transcript_149934	F01_transcript_149934	F01_transcript_149934	F01_transcript_149934	F01_transcript_149934
F01_transcript_155436	NA	F01_transcript_155436	NA	NA
F01_transcript_77195	NA	NA	NA	F01_transcript_77195
F01_transcript_127623	NA	NA	NA	F01_transcript_127623
F01_transcript_133243	NA	F01_transcript_133243	NA	F01_transcript_133243

F01_transcript_59484	NA	NA	F01_transcript_59484	NA
F01_transcript_64272	F01_transcript_64272	F01_transcript_64272	F01_transcript_64272	F01_transcript_64272
F01_transcript_145590	F01_transcript_145590	F01_transcript_145590	F01_transcript_145590	F01_transcript_145590
F01_transcript_102279	NA	NA	NA	F01_transcript_102279
F01_transcript_56511	NA	NA	F01_transcript_56511	NA
F01_transcript_40469	NA	NA	NA	F01_transcript_40469
F01_transcript_139200	F01_transcript_139200	F01_transcript_139200	F01_transcript_139200	F01_transcript_139200
F01_transcript_164942	NA	NA	NA	F01_transcript_164942
F01_transcript_90769	F01_transcript_90769	F01_transcript_90769	NA	F01_transcript_90769
F01_transcript_167465	NA	F01_transcript_167465	NA	NA
F01_transcript_167777	NA	NA	NA	F01_transcript_167777
F01_transcript_90213	NA	F01_transcript_90213	NA	NA
F01_transcript_38748	NA	NA	NA	F01_transcript_38748
F01_transcript_109536	NA	NA	NA	F01_transcript_109536

F01_transcript_143769	F01_transcript_143769	F01_transcript_143769	F01_transcript_143769	F01_transcript_143769
F01_transcript_148211	NA	NA	NA	F01_transcript_148211
F01_transcript_149991	NA	F01_transcript_149991	F01_transcript_149991	F01_transcript_149991
F01_transcript_167725	NA	F01_transcript_167725	NA	NA
F01_transcript_58162	NA	F01_transcript_58162	NA	NA
F01_transcript_157539	NA	F01_transcript_157539	NA	NA
F01_transcript_60746	F01_transcript_60746	F01_transcript_60746	F01_transcript_60746	F01_transcript_60746
F01_transcript_48411	NA	NA	NA	F01_transcript_48411
F01_transcript_59705	NA	F01_transcript_59705	NA	NA
F01_transcript_79192	NA	F01_transcript_79192	NA	F01_transcript_79192
F01_transcript_86302	F01_transcript_86302	F01_transcript_86302	NA	NA
F01_transcript_122455	NA	F01_transcript_122455	NA	NA
F01_transcript_115902	NA	F01_transcript_115902	NA	NA
F01_transcript_153401	F01_transcript_153401	F01_transcript_153401	F01_transcript_153401	F01_transcript_153401

F01_transcript_143240	F01_transcript_143240	F01_transcript_143240	F01_transcript_143240	F01_transcript_143240
F01_transcript_157405	F01_transcript_157405	F01_transcript_157405	NA	F01_transcript_157405
F01_transcript_98330	NA	F01_transcript_98330	NA	NA
F01_transcript_92985	F01_transcript_92985	F01_transcript_92985	NA	F01_transcript_92985
F01_transcript_72032	NA	F01_transcript_72032	NA	F01_transcript_72032
F01_transcript_149967	F01_transcript_149967	F01_transcript_149967	NA	F01_transcript_149967
F01_transcript_29003	NA	NA	NA	F01_transcript_29003
F01_transcript_52314	NA	F01_transcript_52314	NA	NA
F01_transcript_84851	NA	F01_transcript_84851	NA	NA
F01_transcript_57592	NA	NA	F01_transcript_57592	NA
F01_transcript_126313	NA	NA	NA	F01_transcript_126313
F01_transcript_54558	NA	NA	NA	F01_transcript_54558
F01_transcript_35063	NA	NA	NA	F01_transcript_35063
F01_transcript_80757	NA	NA	NA	F01_transcript_80757

F01_transcript_58549	NA	NA	NA	F01_transcript_58549
F01_transcript_65151	NA	NA	F01_transcript_65151	NA
F01_transcript_14027	NA	F01_transcript_14027	NA	NA
F01_transcript_48063	NA	NA	NA	F01_transcript_48063
F01_transcript_143238	NA	NA	NA	F01_transcript_143238
F01_transcript_49964	NA	F01_transcript_49964	NA	NA
F01_transcript_128137	NA	F01_transcript_128137	NA	NA
F01_transcript_129912	NA	NA	NA	F01_transcript_129912
F01_transcript_137495	F01_transcript_137495	F01_transcript_137495	NA	F01_transcript_137495
F01_transcript_54553	NA	F01_transcript_54553	NA	NA
F01_transcript_130521	F01_transcript_130521	F01_transcript_130521	NA	F01_transcript_130521
F01_transcript_54820	NA	F01_transcript_54820	F01_transcript_54820	F01_transcript_54820
F01_transcript_69205	F01_transcript_69205	F01_transcript_69205	F01_transcript_69205	F01_transcript_69205
F01_transcript_144550	NA	NA	NA	F01_transcript_144550

F01_transcript_15419	NA	NA	NA	F01_transcript_15419
F01_transcript_103261	F01_transcript_103261	F01_transcript_103261	NA	NA
F01_transcript_152390	NA	F01_transcript_152390	F01_transcript_152390	NA
F01_transcript_145721	F01_transcript_145721	F01_transcript_145721	NA	F01_transcript_145721
F01_transcript_129343	NA	F01_transcript_129343	NA	NA
F01_transcript_20335	NA	NA	NA	F01_transcript_20335
F01_transcript_145972	NA	F01_transcript_145972	NA	F01_transcript_145972
F01_transcript_49812	NA	F01_transcript_49812	NA	F01_transcript_49812
F01_transcript_25447	F01_transcript_25447	F01_transcript_25447	F01_transcript_25447	F01_transcript_25447
F01_transcript_113130	NA	NA	NA	F01_transcript_113130
F01_transcript_119188	NA	NA	F01_transcript_119188	F01_transcript_119188
F01_transcript_88784	NA	F01_transcript_88784	NA	NA
F01_transcript_37520	NA	NA	NA	F01_transcript_37520
F01_transcript_160765	NA	NA	NA	F01_transcript_160765

F01_transcript_144211	NA	F01_transcript_144211	NA	NA
F01_transcript_72624	F01_transcript_72624	F01_transcript_72624	F01_transcript_72624	F01_transcript_72624
F01_transcript_114894	NA	NA	NA	F01_transcript_114894
F01_transcript_137230	NA	NA	NA	F01_transcript_137230
F01_transcript_73208	F01_transcript_73208	F01_transcript_73208	NA	NA
F01_transcript_76636	NA	F01_transcript_76636	F01_transcript_76636	F01_transcript_76636
F01_transcript_54407	NA	NA	NA	F01_transcript_54407
F01_transcript_96569	F01_transcript_96569	F01_transcript_96569	NA	F01_transcript_96569
F01_transcript_39880	NA	NA	NA	F01_transcript_39880
F01_transcript_56305	NA	NA	F01_transcript_56305	NA
F01_transcript_91715	NA	F01_transcript_91715	NA	NA
F01_transcript_4166	NA	NA	NA	F01_transcript_4166
F01_transcript_93500	F01_transcript_93500	F01_transcript_93500	F01_transcript_93500	F01_transcript_93500
F01_transcript_54004	NA	NA	NA	F01_transcript_54004

F01_transcript_147044	F01_transcript_147044	NA	NA	F01_transcript_147044
F01_transcript_31783	NA	NA	NA	F01_transcript_31783
F01_transcript_91221	F01_transcript_91221	F01_transcript_91221	NA	F01_transcript_91221
F01_transcript_139775	F01_transcript_139775	F01_transcript_139775	NA	NA
F01_transcript_136173	NA	F01_transcript_136173	NA	F01_transcript_136173
F01_transcript_54724	NA	NA	NA	F01_transcript_54724
F01_transcript_59546	NA	NA	F01_transcript_59546	NA
F01_transcript_41303	F01_transcript_41303	F01_transcript_41303	NA	F01_transcript_41303
F01_transcript_158528	NA	NA	NA	F01_transcript_158528
F01_transcript_80379	NA	F01_transcript_80379	NA	NA
F01_transcript_43026	NA	NA	NA	F01_transcript_43026
F01_transcript_150450	NA	F01_transcript_150450	NA	F01_transcript_150450
F01_transcript_61537	NA	F01_transcript_61537	F01_transcript_61537	F01_transcript_61537
F01_transcript_130998	NA	F01_transcript_130998	NA	F01_transcript_130998

F01_transcript_109878	NA	F01_transcript_109878	NA	NA
F01_transcript_153522	F01_transcript_153522	F01_transcript_153522	F01_transcript_153522	NA
F01_transcript_151359	NA	NA	NA	F01_transcript_151359
F01_transcript_154765	NA	F01_transcript_154765	NA	F01_transcript_154765
F01_transcript_132195	NA	NA	NA	F01_transcript_132195
F01_transcript_26291	NA	F01_transcript_26291	NA	NA
F01_transcript_62904	NA	F01_transcript_62904	NA	NA
F01_transcript_107070	NA	F01_transcript_107070	NA	NA
F01_transcript_80332	NA	NA	NA	F01_transcript_80332
F01_transcript_155056	NA	NA	NA	F01_transcript_155056
F01_transcript_53205	NA	NA	NA	F01_transcript_53205
F01_transcript_163504	NA	F01_transcript_163504	NA	F01_transcript_163504
F01_transcript_149466	NA	F01_transcript_149466	F01_transcript_149466	F01_transcript_149466
F01_transcript_122059	NA	NA	NA	F01_transcript_122059

F01_transcript_79659	NA	NA	NA	F01_transcript_79659
F01_transcript_72184	NA	NA	NA	F01_transcript_72184
F01_transcript_52486	NA	NA	NA	F01_transcript_52486
F01_transcript_144789	NA	NA	NA	F01_transcript_144789
F01_transcript_146424	NA	NA	NA	F01_transcript_146424
F01_transcript_135427	NA	F01_transcript_135427	NA	NA
F01_transcript_1108	NA	NA	NA	F01_transcript_1108
F01_transcript_150992	F01_transcript_150992	F01_transcript_150992	F01_transcript_150992	F01_transcript_150992
F01_transcript_51005	NA	F01_transcript_51005	F01_transcript_51005	F01_transcript_51005
F01_transcript_59576	F01_transcript_59576	F01_transcript_59576	F01_transcript_59576	F01_transcript_59576
F01_transcript_33534	NA	F01_transcript_33534	NA	F01_transcript_33534
F01_transcript_20546	NA	F01_transcript_20546	NA	NA
F01_transcript_122602	NA	F01_transcript_122602	NA	F01_transcript_122602
F01_transcript_104007	NA	F01_transcript_104007	F01_transcript_104007	F01_transcript_104007

F01_transcript_62043	F01_transcript_62043	F01_transcript_62043	NA	F01_transcript_62043
F01_transcript_14048	NA	NA	NA	F01_transcript_14048
F01_transcript_155982	NA	F01_transcript_155982	NA	F01_transcript_155982
F01_transcript_164002	NA	F01_transcript_164002	NA	F01_transcript_164002
F01_transcript_139161	NA	NA	NA	F01_transcript_139161
F01_transcript_165597	F01_transcript_165597	F01_transcript_165597	F01_transcript_165597	NA
F01_transcript_140871	F01_transcript_140871	F01_transcript_140871	NA	NA
F01_transcript_148385	NA	F01_transcript_148385	NA	F01_transcript_148385
F01_transcript_160413	NA	F01_transcript_160413	NA	F01_transcript_160413
F01_transcript_142857	NA	F01_transcript_142857	NA	NA
F01_transcript_148897	NA	F01_transcript_148897	NA	F01_transcript_148897
F01_transcript_47050	NA	NA	NA	F01_transcript_47050
F01_transcript_38374	NA	F01_transcript_38374	NA	NA
F01_transcript_62126	NA	NA	NA	F01_transcript_62126

F01_transcript_124725	F01_transcript_124725	F01_transcript_124725	F01_transcript_124725	F01_transcript_124725
F01_transcript_31359	NA	NA	NA	F01_transcript_31359
F01_transcript_29421	NA	NA	NA	F01_transcript_29421
F01_transcript_132929	F01_transcript_132929	F01_transcript_132929	F01_transcript_132929	F01_transcript_132929
F01_transcript_63462	NA	NA	NA	F01_transcript_63462
F01_transcript_73867	NA	NA	NA	F01_transcript_73867
F01_transcript_76910	NA	F01_transcript_76910	NA	F01_transcript_76910
F01_transcript_104924	NA	F01_transcript_104924	F01_transcript_104924	F01_transcript_104924
F01_transcript_119849	F01_transcript_119849	F01_transcript_119849	NA	NA
F01_transcript_146473	NA	NA	NA	F01_transcript_146473
F01_transcript_91710	F01_transcript_91710	F01_transcript_91710	F01_transcript_91710	F01_transcript_91710
F01_transcript_154316	NA	F01_transcript_154316	NA	F01_transcript_154316
F01_transcript_56664	NA	F01_transcript_56664	F01_transcript_56664	F01_transcript_56664
F01_transcript_71565	F01_transcript_71565	F01_transcript_71565	F01_transcript_71565	F01_transcript_71565

F01_transcript_131593	F01_transcript_131593	F01_transcript_131593	F01_transcript_131593	F01_transcript_131593
F01_transcript_65421	F01_transcript_65421	F01_transcript_65421	F01_transcript_65421	F01_transcript_65421
F01_transcript_142876	NA	NA	NA	F01_transcript_142876
F01_transcript_57838	NA	F01_transcript_57838	NA	NA
F01_transcript_133559	NA	F01_transcript_133559	F01_transcript_133559	NA
F01_transcript_157522	F01_transcript_157522	F01_transcript_157522	NA	NA
F01_transcript_95709	F01_transcript_95709	F01_transcript_95709	F01_transcript_95709	F01_transcript_95709
F01_transcript_128995	NA	F01_transcript_128995	NA	F01_transcript_128995
F01_transcript_120245	F01_transcript_120245	F01_transcript_120245	F01_transcript_120245	F01_transcript_120245
F01_transcript_125402	NA	NA	NA	F01_transcript_125402
F01_transcript_156696	NA	F01_transcript_156696	NA	NA
F01_transcript_61126	F01_transcript_61126	F01_transcript_61126	F01_transcript_61126	F01_transcript_61126
F01_transcript_161742	NA	NA	NA	F01_transcript_161742
F01_transcript_122686	NA	NA	NA	F01_transcript_122686

F01_transcript_132363	NA	NA	NA	F01_transcript_132363
F01_transcript_124580	F01_transcript_124580	F01_transcript_124580	NA	F01_transcript_124580
F01_transcript_68084	F01_transcript_68084	F01_transcript_68084	F01_transcript_68084	F01_transcript_68084
F01_transcript_99754	NA	NA	NA	F01_transcript_99754
F01_transcript_99879	NA	F01_transcript_99879	NA	F01_transcript_99879
F01_transcript_45045	NA	NA	NA	F01_transcript_45045
F01_transcript_129724	F01_transcript_129724	F01_transcript_129724	F01_transcript_129724	F01_transcript_129724
F01_transcript_1	NA	F01_transcript_1	F01_transcript_1	NA
F01_transcript_66421	NA	NA	NA	F01_transcript_66421
F01_transcript_138271	NA	NA	NA	F01_transcript_138271
F01_transcript_52272	F01_transcript_52272	F01_transcript_52272	F01_transcript_52272	F01_transcript_52272
F01_transcript_144775	NA	F01_transcript_144775	NA	NA
F01_transcript_87647	NA	F01_transcript_87647	NA	NA
F01_transcript_67399	NA	F01_transcript_67399	NA	NA

F01_transcript_158792	F01_transcript_158792	F01_transcript_158792	F01_transcript_158792	F01_transcript_158792
F01_transcript_42703	NA	F01_transcript_42703	NA	NA
F01_transcript_80366	F01_transcript_80366	F01_transcript_80366	F01_transcript_80366	F01_transcript_80366
F01_transcript_117947	F01_transcript_117947	F01_transcript_117947	NA	NA
F01_transcript_96660	NA	F01_transcript_96660	NA	NA
F01_transcript_149536	NA	NA	NA	F01_transcript_149536
F01_transcript_91411	NA	NA	NA	F01_transcript_91411
F01_transcript_106630	NA	F01_transcript_106630	F01_transcript_106630	F01_transcript_106630
F01_transcript_151333	NA	F01_transcript_151333	NA	NA
F01_transcript_116802	NA	F01_transcript_116802	NA	NA
F01_transcript_58951	NA	NA	NA	F01_transcript_58951
F01_transcript_123355	F01_transcript_123355	F01_transcript_123355	F01_transcript_123355	F01_transcript_123355
F01_transcript_136893	NA	NA	NA	F01_transcript_136893
F01_transcript_140495	F01_transcript_140495	F01_transcript_140495	F01_transcript_140495	F01_transcript_140495

F01_transcript_113708	F01_transcript_113708	F01_transcript_113708	F01_transcript_113708	F01_transcript_113708
F01_transcript_156109	F01_transcript_156109	F01_transcript_156109	NA	F01_transcript_156109
F01_transcript_152091	NA	F01_transcript_152091	NA	NA
F01_transcript_52374	NA	NA	NA	F01_transcript_52374
F01_transcript_103630	NA	F01_transcript_103630	F01_transcript_103630	F01_transcript_103630
F01_transcript_55752	NA	F01_transcript_55752	NA	NA
F01_transcript_113387	NA	F01_transcript_113387	NA	F01_transcript_113387
F01_transcript_85072	F01_transcript_85072	F01_transcript_85072	F01_transcript_85072	NA
F01_transcript_115759	NA	NA	NA	F01_transcript_115759
F01_transcript_80616	NA	NA	NA	F01_transcript_80616
F01_transcript_136732	NA	NA	F01_transcript_136732	F01_transcript_136732
F01_transcript_146875	NA	NA	NA	F01_transcript_146875
F01_transcript_33578	NA	F01_transcript_33578	F01_transcript_33578	F01_transcript_33578
F01_transcript_135295	F01_transcript_135295	F01_transcript_135295	NA	NA

F01_transcript_115102	F01_transcript_115102	F01_transcript_115102	F01_transcript_115102	F01_transcript_115102
F01_transcript_129782	NA	F01_transcript_129782	F01_transcript_129782	NA
F01_transcript_67813	NA	NA	NA	F01_transcript_67813
F01_transcript_84859	NA	NA	NA	F01_transcript_84859
F01_transcript_35512	NA	NA	NA	F01_transcript_35512
F01_transcript_152412	NA	NA	NA	F01_transcript_152412
F01_transcript_54649	NA	F01_transcript_54649	NA	F01_transcript_54649
F01_transcript_77757	NA	F01_transcript_77757	NA	NA
F01_transcript_79499	F01_transcript_79499	F01_transcript_79499	NA	F01_transcript_79499
F01_transcript_28327	NA	NA	NA	F01_transcript_28327
F01_transcript_62387	F01_transcript_62387	F01_transcript_62387	F01_transcript_62387	F01_transcript_62387
F01_transcript_71744	NA	NA	NA	F01_transcript_71744
F01_transcript_91287	NA	F01_transcript_91287	NA	F01_transcript_91287
F01_transcript_50316	F01_transcript_50316	F01_transcript_50316	NA	F01_transcript_50316

F01_transcript_101805	F01_transcript_101805	F01_transcript_101805	F01_transcript_101805	F01_transcript_101805
F01_transcript_114044	NA	NA	NA	F01_transcript_114044
F01_transcript_137419	NA	F01_transcript_137419	NA	NA
F01_transcript_133635	NA	NA	NA	F01_transcript_133635
F01_transcript_125171	NA	F01_transcript_125171	NA	F01_transcript_125171
F01_transcript_52668	F01_transcript_52668	F01_transcript_52668	NA	NA
F01_transcript_90658	NA	F01_transcript_90658	NA	F01_transcript_90658
F01_transcript_150420	NA	F01_transcript_150420	NA	F01_transcript_150420
F01_transcript_93737	NA	F01_transcript_93737	NA	NA
F01_transcript_60181	F01_transcript_60181	F01_transcript_60181	F01_transcript_60181	F01_transcript_60181
F01_transcript_105848	NA	NA	NA	F01_transcript_105848
F01_transcript_124093	F01_transcript_124093	F01_transcript_124093	NA	F01_transcript_124093
F01_transcript_54713	NA	NA	NA	F01_transcript_54713
F01_transcript_127094	NA	F01_transcript_127094	F01_transcript_127094	F01_transcript_127094

F01_transcript_55990	NA	NA	NA	F01_transcript_55990
F01_transcript_21355	NA	F01_transcript_21355	NA	NA
F01_transcript_119690	NA	F01_transcript_119690	NA	NA
F01_transcript_104390	NA	F01_transcript_104390	F01_transcript_104390	F01_transcript_104390
F01_transcript_12929	NA	F01_transcript_12929	NA	NA
F01_transcript_9643	NA	NA	NA	F01_transcript_9643
F01_transcript_60169	F01_transcript_60169	F01_transcript_60169	F01_transcript_60169	F01_transcript_60169
F01_transcript_98247	NA	NA	NA	F01_transcript_98247
F01_transcript_164415	F01_transcript_164415	F01_transcript_164415	NA	F01_transcript_164415
F01_transcript_79844	NA	F01_transcript_79844	NA	NA
F01_transcript_148663	F01_transcript_148663	F01_transcript_148663	F01_transcript_148663	F01_transcript_148663
F01_transcript_122071	F01_transcript_122071	F01_transcript_122071	F01_transcript_122071	F01_transcript_122071
F01_transcript_166232	F01_transcript_166232	F01_transcript_166232	NA	NA
F01_transcript_135775	NA	F01_transcript_135775	F01_transcript_135775	F01_transcript_135775

F01_transcript_6979	NA	NA	NA	F01_transcript_6979
F01_transcript_139266	NA	NA	NA	F01_transcript_139266
F01_transcript_55630	NA	F01_transcript_55630	NA	NA
F01_transcript_62773	NA	NA	NA	F01_transcript_62773
F01_transcript_34580	NA	NA	NA	F01_transcript_34580
F01_transcript_63878	F01_transcript_63878	F01_transcript_63878	F01_transcript_63878	F01_transcript_63878
F01_transcript_130830	NA	NA	NA	F01_transcript_130830
F01_transcript_86677	F01_transcript_86677	F01_transcript_86677	F01_transcript_86677	F01_transcript_86677
F01_transcript_132719	NA	F01_transcript_132719	NA	NA
F01_transcript_92929	NA	NA	NA	F01_transcript_92929
F01_transcript_60183	NA	NA	NA	F01_transcript_60183
F01_transcript_93557	NA	NA	NA	F01_transcript_93557
F01_transcript_159345	F01_transcript_159345	F01_transcript_159345	F01_transcript_159345	NA
F01_transcript_92784	NA	F01_transcript_92784	NA	NA

F01_transcript_137690	F01_transcript_137690	F01_transcript_137690	F01_transcript_137690	F01_transcript_137690
F01_transcript_66973	F01_transcript_66973	F01_transcript_66973	F01_transcript_66973	F01_transcript_66973
F01_transcript_79485	NA	NA	NA	F01_transcript_79485
F01_transcript_103979	NA	F01_transcript_103979	NA	NA
F01_transcript_38838	NA	NA	NA	F01_transcript_38838
F01_transcript_106773	NA	F01_transcript_106773	F01_transcript_106773	F01_transcript_106773
F01_transcript_8111	NA	F01_transcript_8111	NA	F01_transcript_8111
F01_transcript_101422	NA	F01_transcript_101422	NA	NA
F01_transcript_113547	NA	F01_transcript_113547	F01_transcript_113547	F01_transcript_113547
F01_transcript_47217	NA	NA	NA	F01_transcript_47217
F01_transcript_85344	NA	NA	NA	F01_transcript_85344
F01_transcript_74397	NA	NA	NA	F01_transcript_74397
F01_transcript_54448	F01_transcript_54448	F01_transcript_54448	F01_transcript_54448	F01_transcript_54448
F01_transcript_114040	F01_transcript_114040	F01_transcript_114040	NA	NA

F01_transcript_136194	NA	F01_transcript_136194	NA	F01_transcript_136194
F01_transcript_50747	F01_transcript_50747	F01_transcript_50747	F01_transcript_50747	F01_transcript_50747
F01_transcript_45773	NA	F01_transcript_45773	F01_transcript_45773	F01_transcript_45773
F01_transcript_82188	NA	NA	NA	F01_transcript_82188
F01_transcript_167770	NA	NA	NA	F01_transcript_167770
F01_transcript_12267	NA	F01_transcript_12267	NA	NA
F01_transcript_160088	NA	NA	NA	F01_transcript_160088
F01_transcript_45167	NA	F01_transcript_45167	NA	F01_transcript_45167
F01_transcript_56502	NA	NA	F01_transcript_56502	NA
F01_transcript_152937	NA	F01_transcript_152937	NA	NA
F01_transcript_114307	NA	NA	F01_transcript_114307	NA
F01_transcript_7267	NA	NA	NA	F01_transcript_7267
F01_transcript_110216	NA	NA	NA	F01_transcript_110216
F01_transcript_128578	NA	F01_transcript_128578	NA	F01_transcript_128578

F01_transcript_76327	NA	F01_transcript_76327	NA	NA
F01_transcript_159732	F01_transcript_159732	F01_transcript_159732	F01_transcript_159732	F01_transcript_159732
F01_transcript_49215	NA	NA	NA	F01_transcript_49215
F01_transcript_96950	NA	F01_transcript_96950	NA	NA
F01_transcript_9792	NA	NA	F01_transcript_9792	F01_transcript_9792
F01_transcript_39445	NA	NA	NA	F01_transcript_39445
F01_transcript_66253	NA	F01_transcript_66253	F01_transcript_66253	F01_transcript_66253
F01_transcript_118111	NA	F01_transcript_118111	NA	NA
F01_transcript_100189	NA	F01_transcript_100189	NA	NA
F01_transcript_53933	NA	F01_transcript_53933	NA	NA
F01_transcript_30915	NA	NA	NA	F01_transcript_30915
F01_transcript_131137	NA	NA	F01_transcript_131137	F01_transcript_131137
F01_transcript_59549	NA	F01_transcript_59549	F01_transcript_59549	NA
F01_transcript_65160	F01_transcript_65160	F01_transcript_65160	F01_transcript_65160	F01_transcript_65160

F01_transcript_78417	NA	NA	NA	F01_transcript_78417
F01_transcript_155847	F01_transcript_155847	F01_transcript_155847	NA	F01_transcript_155847
F01_transcript_32381	NA	NA	NA	F01_transcript_32381
F01_transcript_131590	F01_transcript_131590	F01_transcript_131590	F01_transcript_131590	F01_transcript_131590
F01_transcript_81778	F01_transcript_81778	F01_transcript_81778	F01_transcript_81778	F01_transcript_81778
F01_transcript_8064	NA	F01_transcript_8064	NA	F01_transcript_8064
F01_transcript_3050	F01_transcript_3050	F01_transcript_3050	F01_transcript_3050	F01_transcript_3050
F01_transcript_166920	F01_transcript_166920	F01_transcript_166920	NA	F01_transcript_166920
F01_transcript_111100	NA	F01_transcript_111100	NA	F01_transcript_111100
F01_transcript_36019	NA	NA	NA	F01_transcript_36019
F01_transcript_86583	F01_transcript_86583	F01_transcript_86583	F01_transcript_86583	NA
F01_transcript_143526	NA	NA	NA	F01_transcript_143526
F01_transcript_90795	F01_transcript_90795	F01_transcript_90795	F01_transcript_90795	F01_transcript_90795
F01_transcript_73430	NA	NA	F01_transcript_73430	F01_transcript_73430

F01_transcript_114408	NA	NA	NA	F01_transcript_114408
F01_transcript_113186	F01_transcript_113186	F01_transcript_113186	NA	NA
F01_transcript_743	NA	F01_transcript_743	NA	F01_transcript_743
F01_transcript_86760	F01_transcript_86760	F01_transcript_86760	NA	NA
F01_transcript_2642	NA	NA	NA	F01_transcript_2642
F01_transcript_104569	F01_transcript_104569	F01_transcript_104569	F01_transcript_104569	F01_transcript_104569
F01_transcript_97087	NA	F01_transcript_97087	F01_transcript_97087	F01_transcript_97087
F01_transcript_46344	NA	NA	NA	F01_transcript_46344
F01_transcript_127426	NA	F01_transcript_127426	NA	F01_transcript_127426
F01_transcript_105741	NA	NA	NA	F01_transcript_105741
F01_transcript_120121	NA	F01_transcript_120121	NA	F01_transcript_120121
F01_transcript_94116	NA	F01_transcript_94116	NA	NA
F01_transcript_55898	NA	F01_transcript_55898	NA	NA
F01_transcript_67423	NA	NA	NA	F01_transcript_67423

F01_transcript_91807	NA	F01_transcript_91807	F01_transcript_91807	NA
F01_transcript_74802	NA	NA	NA	F01_transcript_74802
F01_transcript_46010	NA	NA	NA	F01_transcript_46010
F01_transcript_88924	NA	NA	NA	F01_transcript_88924
F01_transcript_146517	NA	NA	NA	F01_transcript_146517
F01_transcript_107148	NA	NA	NA	F01_transcript_107148
F01_transcript_65717	NA	F01_transcript_65717	NA	F01_transcript_65717
F01_transcript_58159	F01_transcript_58159	F01_transcript_58159	NA	F01_transcript_58159
F01_transcript_96213	F01_transcript_96213	F01_transcript_96213	F01_transcript_96213	F01_transcript_96213
F01_transcript_147124	NA	F01_transcript_147124	F01_transcript_147124	F01_transcript_147124
F01_transcript_132087	NA	F01_transcript_132087	F01_transcript_132087	F01_transcript_132087
F01_transcript_116509	NA	F01_transcript_116509	NA	NA
F01_transcript_87247	F01_transcript_87247	F01_transcript_87247	F01_transcript_87247	F01_transcript_87247
F01_transcript_166810	F01_transcript_166810	F01_transcript_166810	F01_transcript_166810	F01_transcript_166810

F01_transcript_58939	NA	F01_transcript_58939	NA	NA
F01_transcript_132828	F01_transcript_132828	F01_transcript_132828	NA	F01_transcript_132828
F01_transcript_33849	NA	F01_transcript_33849	NA	NA
F01_transcript_38167	NA	NA	NA	F01_transcript_38167
F01_transcript_89752	F01_transcript_89752	F01_transcript_89752	F01_transcript_89752	F01_transcript_89752
F01_transcript_64401	NA	NA	NA	F01_transcript_64401
F01_transcript_79665	F01_transcript_79665	F01_transcript_79665	NA	F01_transcript_79665
F01_transcript_126171	F01_transcript_126171	F01_transcript_126171	NA	NA
F01_transcript_57587	NA	NA	NA	F01_transcript_57587
F01_transcript_37710	NA	NA	NA	F01_transcript_37710
F01_transcript_161415	NA	F01_transcript_161415	NA	NA
F01_transcript_59858	NA	F01_transcript_59858	NA	NA
F01_transcript_89654	F01_transcript_89654	F01_transcript_89654	F01_transcript_89654	F01_transcript_89654
F01_transcript_130224	NA	F01_transcript_130224	NA	NA

F01_transcript_113791	NA	F01_transcript_113791	NA	NA
F01_transcript_71623	NA	F01_transcript_71623	NA	NA
F01_transcript_109476	NA	F01_transcript_109476	F01_transcript_109476	F01_transcript_109476
F01_transcript_150950	F01_transcript_150950	F01_transcript_150950	NA	F01_transcript_150950
F01_transcript_66816	NA	F01_transcript_66816	NA	NA
F01_transcript_155363	F01_transcript_155363	F01_transcript_155363	F01_transcript_155363	F01_transcript_155363
F01_transcript_92228	NA	NA	NA	F01_transcript_92228
F01_transcript_143926	NA	F01_transcript_143926	F01_transcript_143926	NA
F01_transcript_115774	F01_transcript_115774	F01_transcript_115774	F01_transcript_115774	F01_transcript_115774
F01_transcript_64994	NA	F01_transcript_64994	F01_transcript_64994	NA
F01_transcript_94434	NA	NA	F01_transcript_94434	NA
F01_transcript_83043	NA	NA	NA	F01_transcript_83043
F01_transcript_116762	NA	F01_transcript_116762	NA	NA
F01_transcript_112909	NA	F01_transcript_112909	NA	NA

F01_transcript_83341	NA	NA	NA	F01_transcript_83341
F01_transcript_76891	F01_transcript_76891	F01_transcript_76891	F01_transcript_76891	F01_transcript_76891
F01_transcript_96884	F01_transcript_96884	F01_transcript_96884	NA	NA
F01_transcript_66245	NA	F01_transcript_66245	F01_transcript_66245	F01_transcript_66245
F01_transcript_64671	F01_transcript_64671	F01_transcript_64671	NA	F01_transcript_64671
F01_transcript_65931	F01_transcript_65931	F01_transcript_65931	NA	F01_transcript_65931
F01_transcript_96302	F01_transcript_96302	F01_transcript_96302	F01_transcript_96302	F01_transcript_96302
F01_transcript_39052	NA	F01_transcript_39052	NA	F01_transcript_39052
F01_transcript_71217	NA	F01_transcript_71217	F01_transcript_71217	NA
F01_transcript_83771	NA	NA	NA	F01_transcript_83771
F01_transcript_46260	NA	NA	NA	F01_transcript_46260
F01_transcript_55211	NA	F01_transcript_55211	NA	NA
F01_transcript_32908	F01_transcript_32908	NA	NA	NA
F01_transcript_70889	NA	NA	F01_transcript_70889	NA

F01_transcript_128016	NA	NA	NA	F01_transcript_128016
F01_transcript_17495	NA	NA	NA	F01_transcript_17495
F01_transcript_60866	F01_transcript_60866	F01_transcript_60866	F01_transcript_60866	F01_transcript_60866
F01_transcript_118904	NA	NA	NA	F01_transcript_118904
F01_transcript_156982	F01_transcript_156982	F01_transcript_156982	F01_transcript_156982	F01_transcript_156982
F01_transcript_102743	F01_transcript_102743	F01_transcript_102743	NA	F01_transcript_102743
F01_transcript_156159	NA	F01_transcript_156159	NA	NA
F01_transcript_69329	NA	NA	NA	F01_transcript_69329
F01_transcript_156505	NA	F01_transcript_156505	NA	NA
F01_transcript_155461	NA	NA	NA	F01_transcript_155461
F01_transcript_146564	NA	NA	NA	F01_transcript_146564
F01_transcript_124103	NA	F01_transcript_124103	NA	NA
F01_transcript_150964	NA	F01_transcript_150964	NA	NA
F01_transcript_165884	NA	F01_transcript_165884	NA	F01_transcript_165884

F01_transcript_96045	F01_transcript_96045	F01_transcript_96045	F01_transcript_96045	F01_transcript_96045
F01_transcript_105403	NA	F01_transcript_105403	NA	NA
F01_transcript_144195	NA	F01_transcript_144195	NA	F01_transcript_144195
F01_transcript_86320	F01_transcript_86320	F01_transcript_86320	NA	NA
F01_transcript_133489	F01_transcript_133489	F01_transcript_133489	F01_transcript_133489	F01_transcript_133489
F01_transcript_72575	NA	F01_transcript_72575	NA	NA
F01_transcript_90777	NA	F01_transcript_90777	NA	F01_transcript_90777
F01_transcript_71055	NA	F01_transcript_71055	F01_transcript_71055	NA
F01_transcript_142630	NA	NA	NA	F01_transcript_142630
F01_transcript_136704	NA	NA	NA	F01_transcript_136704
F01_transcript_165102	F01_transcript_165102	F01_transcript_165102	F01_transcript_165102	F01_transcript_165102
F01_transcript_59432	F01_transcript_59432	F01_transcript_59432	F01_transcript_59432	NA
F01_transcript_115671	NA	F01_transcript_115671	F01_transcript_115671	F01_transcript_115671
F01_transcript_15191	F01_transcript_15191	F01_transcript_15191	F01_transcript_15191	NA

F01_transcript_108774	NA	NA	NA	F01_transcript_108774
F01_transcript_39350	NA	NA	NA	F01_transcript_39350
F01_transcript_54573	NA	F01_transcript_54573	F01_transcript_54573	NA
F01_transcript_108096	NA	F01_transcript_108096	NA	NA
F01_transcript_100821	NA	F01_transcript_100821	NA	F01_transcript_100821
F01_transcript_62876	NA	NA	NA	F01_transcript_62876
F01_transcript_153675	NA	F01_transcript_153675	NA	F01_transcript_153675
F01_transcript_112033	NA	NA	NA	F01_transcript_112033
F01_transcript_144990	NA	F01_transcript_144990	NA	NA
F01_transcript_95974	NA	F01_transcript_95974	NA	NA
F01_transcript_113721	NA	NA	NA	F01_transcript_113721
F01_transcript_140538	NA	NA	F01_transcript_140538	F01_transcript_140538
F01_transcript_120293	NA	NA	NA	F01_transcript_120293
F01_transcript_107249	NA	F01_transcript_107249	NA	NA

F01_transcript_127489	NA	NA	NA	F01_transcript_127489
F01_transcript_46178	NA	NA	F01_transcript_46178	F01_transcript_46178
F01_transcript_71152	NA	F01_transcript_71152	F01_transcript_71152	NA
F01_transcript_50020	NA	F01_transcript_50020	NA	NA
F01_transcript_92991	NA	F01_transcript_92991	NA	NA
F01_transcript_138529	NA	F01_transcript_138529	NA	NA
F01_transcript_146665	NA	NA	NA	F01_transcript_146665
F01_transcript_98443	NA	F01_transcript_98443	NA	NA
F01_transcript_61780	F01_transcript_61780	F01_transcript_61780	NA	F01_transcript_61780
F01_transcript_32409	NA	F01_transcript_32409	NA	NA
F01_transcript_117385	NA	NA	NA	F01_transcript_117385
F01_transcript_60000	NA	NA	NA	F01_transcript_60000
F01_transcript_34164	NA	NA	NA	F01_transcript_34164
F01_transcript_70468	F01_transcript_70468	F01_transcript_70468	F01_transcript_70468	F01_transcript_70468

F01_transcript_154412	F01_transcript_154412	F01_transcript_154412	NA	NA
F01_transcript_62709	NA	NA	NA	F01_transcript_62709
F01_transcript_128851	NA	NA	NA	F01_transcript_128851
F01_transcript_76325	NA	F01_transcript_76325	NA	NA
F01_transcript_87051	F01_transcript_87051	F01_transcript_87051	F01_transcript_87051	F01_transcript_87051
F01_transcript_75918	F01_transcript_75918	F01_transcript_75918	F01_transcript_75918	F01_transcript_75918
F01_transcript_74308	NA	F01_transcript_74308	NA	F01_transcript_74308
F01_transcript_556	NA	F01_transcript_556	NA	NA
F01_transcript_165993	NA	F01_transcript_165993	NA	NA
F01_transcript_125635	NA	NA	NA	F01_transcript_125635
F01_transcript_156663	F01_transcript_156663	F01_transcript_156663	F01_transcript_156663	F01_transcript_156663
F01_transcript_32841	F01_transcript_32841	F01_transcript_32841	NA	NA
F01_transcript_69965	F01_transcript_69965	F01_transcript_69965	NA	NA
F01_transcript_166352	NA	F01_transcript_166352	NA	NA

F01_transcript_64866	NA	NA	NA	F01_transcript_64866
F01_transcript_163902	NA	F01_transcript_163902	NA	F01_transcript_163902
F01_transcript_54311	NA	NA	NA	F01_transcript_54311
F01_transcript_65012	NA	NA	NA	F01_transcript_65012
F01_transcript_157684	NA	NA	NA	F01_transcript_157684
F01_transcript_47883	NA	F01_transcript_47883	NA	NA
F01_transcript_150940	F01_transcript_150940	F01_transcript_150940	NA	NA
F01_transcript_82030	F01_transcript_82030	F01_transcript_82030	F01_transcript_82030	NA
F01_transcript_69135	NA	F01_transcript_69135	NA	NA
F01_transcript_157985	NA	F01_transcript_157985	NA	NA
F01_transcript_130080	NA	F01_transcript_130080	NA	NA
F01_transcript_105538	NA	NA	NA	F01_transcript_105538
F01_transcript_136378	NA	F01_transcript_136378	F01_transcript_136378	NA
F01_transcript_157130	NA	NA	NA	F01_transcript_157130

F01_transcript_71232	F01_transcript_71232	F01_transcript_71232	NA	F01_transcript_71232
F01_transcript_115119	NA	F01_transcript_115119	NA	NA
F01_transcript_56495	NA	NA	F01_transcript_56495	NA
F01_transcript_60628	F01_transcript_60628	F01_transcript_60628	F01_transcript_60628	NA
F01_transcript_99890	NA	NA	NA	F01_transcript_99890
F01_transcript_101894	F01_transcript_101894	F01_transcript_101894	F01_transcript_101894	F01_transcript_101894
F01_transcript_156615	NA	NA	NA	F01_transcript_156615
F01_transcript_161587	NA	NA	NA	F01_transcript_161587
F01_transcript_116508	NA	F01_transcript_116508	NA	NA
F01_transcript_69378	NA	F01_transcript_69378	F01_transcript_69378	F01_transcript_69378
F01_transcript_17986	NA	NA	NA	F01_transcript_17986
F01_transcript_45414	NA	NA	NA	F01_transcript_45414
F01_transcript_76147	NA	NA	NA	F01_transcript_76147
F01_transcript_163683	NA	F01_transcript_163683	NA	F01_transcript_163683

F01_transcript_102954	NA	F01_transcript_102954	NA	NA
F01_transcript_159447	NA	NA	NA	F01_transcript_159447
F01_transcript_91999	F01_transcript_91999	NA	NA	NA
F01_transcript_54005	NA	NA	NA	F01_transcript_54005
F01_transcript_160191	NA	NA	NA	F01_transcript_160191
F01_transcript_147994	F01_transcript_147994	F01_transcript_147994	NA	F01_transcript_147994
F01_transcript_95515	NA	NA	NA	F01_transcript_95515
F01_transcript_106405	F01_transcript_106405	F01_transcript_106405	F01_transcript_106405	F01_transcript_106405
F01_transcript_102567	NA	F01_transcript_102567	NA	NA
F01_transcript_43484	NA	NA	NA	F01_transcript_43484
F01_transcript_119091	F01_transcript_119091	F01_transcript_119091	F01_transcript_119091	F01_transcript_119091
F01_transcript_96269	F01_transcript_96269	F01_transcript_96269	NA	NA
F01_transcript_82507	NA	NA	NA	F01_transcript_82507
F01_transcript_25131	NA	NA	NA	F01_transcript_25131

F01_transcript_154650	NA	NA	NA	F01_transcript_154650
F01_transcript_139610	NA	F01_transcript_139610	NA	NA
F01_transcript_86881	NA	NA	NA	F01_transcript_86881
F01_transcript_101556	NA	NA	NA	F01_transcript_101556
F01_transcript_161956	NA	NA	NA	F01_transcript_161956
F01_transcript_162153	NA	NA	NA	F01_transcript_162153
F01_transcript_108981	F01_transcript_108981	F01_transcript_108981	F01_transcript_108981	F01_transcript_108981
F01_transcript_55716	F01_transcript_55716	F01_transcript_55716	F01_transcript_55716	F01_transcript_55716
F01_transcript_72985	NA	F01_transcript_72985	NA	F01_transcript_72985
F01_transcript_13135	NA	F01_transcript_13135	NA	NA
F01_transcript_83172	F01_transcript_83172	F01_transcript_83172	F01_transcript_83172	F01_transcript_83172
F01_transcript_162343	NA	F01_transcript_162343	NA	NA
F01_transcript_78079	F01_transcript_78079	F01_transcript_78079	F01_transcript_78079	F01_transcript_78079
F01_transcript_104207	NA	F01_transcript_104207	F01_transcript_104207	F01_transcript_104207

F01_transcript_39523	NA	NA	NA	F01_transcript_39523
F01_transcript_2986	NA	NA	NA	F01_transcript_2986
F01_transcript_60320	NA	NA	NA	F01_transcript_60320
F01_transcript_11621	NA	F01_transcript_11621	NA	F01_transcript_11621
F01_transcript_54391	F01_transcript_54391	F01_transcript_54391	F01_transcript_54391	NA
F01_transcript_81490	F01_transcript_81490	F01_transcript_81490	NA	NA
F01_transcript_133491	NA	NA	NA	F01_transcript_133491
F01_transcript_77635	NA	F01_transcript_77635	NA	F01_transcript_77635
F01_transcript_87264	NA	F01_transcript_87264	NA	NA
F01_transcript_43475	F01_transcript_43475	NA	NA	NA
F01_transcript_157357	NA	NA	NA	F01_transcript_157357
F01_transcript_66074	NA	NA	NA	F01_transcript_66074
F01_transcript_154775	F01_transcript_154775	F01_transcript_154775	F01_transcript_154775	F01_transcript_154775
F01_transcript_66848	F01_transcript_66848	F01_transcript_66848	NA	NA

F01_transcript_158162	NA	NA	NA	F01_transcript_158162
F01_transcript_152695	NA	NA	NA	F01_transcript_152695
F01_transcript_72700	NA	NA	NA	F01_transcript_72700
F01_transcript_90394	F01_transcript_90394	F01_transcript_90394	F01_transcript_90394	F01_transcript_90394
F01_transcript_138983	F01_transcript_138983	F01_transcript_138983	F01_transcript_138983	F01_transcript_138983
F01_transcript_162587	F01_transcript_162587	F01_transcript_162587	NA	NA
F01_transcript_117712	F01_transcript_117712	F01_transcript_117712	F01_transcript_117712	F01_transcript_117712
F01_transcript_48809	NA	NA	NA	F01_transcript_48809
F01_transcript_14242	NA	NA	NA	F01_transcript_14242
F01_transcript_94381	NA	NA	NA	F01_transcript_94381
F01_transcript_155933	NA	NA	NA	F01_transcript_155933
F01_transcript_167626	NA	NA	NA	F01_transcript_167626
F01_transcript_101738	F01_transcript_101738	F01_transcript_101738	F01_transcript_101738	NA
F01_transcript_102612	NA	F01_transcript_102612	F01_transcript_102612	NA

F01_transcript_35815	NA	NA	NA	F01_transcript_35815
F01_transcript_86792	NA	F01_transcript_86792	NA	NA
F01_transcript_57766	F01_transcript_57766	F01_transcript_57766	F01_transcript_57766	F01_transcript_57766
F01_transcript_99426	NA	F01_transcript_99426	NA	NA
F01_transcript_129744	NA	F01_transcript_129744	NA	NA
F01_transcript_33739	NA	F01_transcript_33739	NA	F01_transcript_33739
F01_transcript_14536	F01_transcript_14536	F01_transcript_14536	F01_transcript_14536	F01_transcript_14536
F01_transcript_63850	F01_transcript_63850	F01_transcript_63850	F01_transcript_63850	F01_transcript_63850
F01_transcript_145591	NA	F01_transcript_145591	F01_transcript_145591	F01_transcript_145591
F01_transcript_86456	F01_transcript_86456	F01_transcript_86456	NA	F01_transcript_86456
F01_transcript_46697	F01_transcript_46697	F01_transcript_46697	NA	NA
F01_transcript_65145	F01_transcript_65145	F01_transcript_65145	F01_transcript_65145	NA
F01_transcript_154663	NA	NA	NA	F01_transcript_154663
F01_transcript_103711	NA	F01_transcript_103711	NA	NA

F01_transcript_8297	NA	NA	NA	F01_transcript_8297
F01_transcript_103207	NA	F01_transcript_103207	NA	F01_transcript_103207
F01_transcript_19870	NA	F01_transcript_19870	NA	NA
F01_transcript_10218	F01_transcript_10218	F01_transcript_10218	F01_transcript_10218	F01_transcript_10218
F01_transcript_108026	NA	NA	NA	F01_transcript_108026
F01_transcript_137401	F01_transcript_137401	F01_transcript_137401	NA	F01_transcript_137401
F01_transcript_91250	NA	F01_transcript_91250	NA	NA
F01_transcript_65435	NA	NA	F01_transcript_65435	NA
F01_transcript_148693	NA	F01_transcript_148693	NA	NA
F01_transcript_158001	F01_transcript_158001	F01_transcript_158001	NA	NA
F01_transcript_46512	NA	NA	NA	F01_transcript_46512
F01_transcript_159245	NA	F01_transcript_159245	NA	NA
F01_transcript_49671	F01_transcript_49671	F01_transcript_49671	F01_transcript_49671	F01_transcript_49671
F01_transcript_78212	F01_transcript_78212	F01_transcript_78212	NA	F01_transcript_78212

F01_transcript_100527	F01_transcript_100527	F01_transcript_100527	F01_transcript_100527	F01_transcript_100527
F01_transcript_1534	NA	NA	NA	F01_transcript_1534
F01_transcript_123566	NA	F01_transcript_123566	NA	NA
F01_transcript_45557	NA	NA	NA	F01_transcript_45557
F01_transcript_124043	NA	NA	NA	F01_transcript_124043
F01_transcript_133516	NA	NA	NA	F01_transcript_133516
F01_transcript_141107	F01_transcript_141107	F01_transcript_141107	F01_transcript_141107	F01_transcript_141107
F01_transcript_83568	NA	F01_transcript_83568	NA	F01_transcript_83568
F01_transcript_54294	NA	F01_transcript_54294	NA	F01_transcript_54294
F01_transcript_142662	F01_transcript_142662	F01_transcript_142662	NA	F01_transcript_142662
F01_transcript_56924	NA	NA	F01_transcript_56924	NA
F01_transcript_84357	NA	NA	NA	F01_transcript_84357
F01_transcript_162306	NA	F01_transcript_162306	NA	F01_transcript_162306
F01_transcript_127085	F01_transcript_127085	F01_transcript_127085	NA	F01_transcript_127085

F01_transcript_162235	NA	F01_transcript_162235	F01_transcript_162235	F01_transcript_162235
F01_transcript_54753	NA	NA	NA	F01_transcript_54753
F01_transcript_14368	NA	NA	NA	F01_transcript_14368
F01_transcript_85940	F01_transcript_85940	F01_transcript_85940	F01_transcript_85940	F01_transcript_85940
F01_transcript_54599	NA	NA	NA	F01_transcript_54599
F01_transcript_75267	NA	F01_transcript_75267	NA	NA
F01_transcript_117447	NA	F01_transcript_117447	NA	F01_transcript_117447
F01_transcript_162637	F01_transcript_162637	F01_transcript_162637	F01_transcript_162637	F01_transcript_162637
F01_transcript_33560	NA	NA	NA	F01_transcript_33560
F01_transcript_74436	NA	F01_transcript_74436	NA	NA
F01_transcript_101166	NA	F01_transcript_101166	NA	NA
F01_transcript_54623	NA	F01_transcript_54623	NA	F01_transcript_54623
F01_transcript_162956	NA	F01_transcript_162956	NA	NA
F01_transcript_148651	NA	NA	NA	F01_transcript_148651

F01_transcript_121946	F01_transcript_121946	F01_transcript_121946	NA	F01_transcript_121946
F01_transcript_47280	NA	NA	NA	F01_transcript_47280
F01_transcript_107806	NA	F01_transcript_107806	NA	F01_transcript_107806
F01_transcript_51339	NA	F01_transcript_51339	F01_transcript_51339	NA
F01_transcript_88048	NA	NA	NA	F01_transcript_88048
F01_transcript_127880	NA	NA	NA	F01_transcript_127880
F01_transcript_108106	NA	F01_transcript_108106	NA	F01_transcript_108106
F01_transcript_75141	NA	F01_transcript_75141	NA	NA
F01_transcript_58667	NA	F01_transcript_58667	NA	NA
F01_transcript_101271	NA	NA	NA	F01_transcript_101271
F01_transcript_151217	NA	NA	NA	F01_transcript_151217
F01_transcript_124223	F01_transcript_124223	F01_transcript_124223	NA	F01_transcript_124223
F01_transcript_95042	NA	F01_transcript_95042	NA	F01_transcript_95042
F01_transcript_89784	NA	NA	NA	F01_transcript_89784

F01_transcript_8646	NA	F01_transcript_8646	NA	NA
F01_transcript_81057	NA	F01_transcript_81057	NA	NA
F01_transcript_151061	NA	F01_transcript_151061	F01_transcript_151061	F01_transcript_151061
F01_transcript_109807	NA	NA	NA	F01_transcript_109807
F01_transcript_155311	F01_transcript_155311	F01_transcript_155311	F01_transcript_155311	F01_transcript_155311
F01_transcript_59911	NA	F01_transcript_59911	NA	NA
F01_transcript_159940	NA	NA	NA	F01_transcript_159940
F01_transcript_159967	NA	NA	NA	F01_transcript_159967
F01_transcript_53924	NA	F01_transcript_53924	NA	NA
F01_transcript_153982	NA	NA	NA	F01_transcript_153982
F01_transcript_158587	NA	NA	NA	F01_transcript_158587
F01_transcript_151344	NA	F01_transcript_151344	NA	F01_transcript_151344
F01_transcript_55968	F01_transcript_55968	F01_transcript_55968	F01_transcript_55968	F01_transcript_55968
F01_transcript_69358	F01_transcript_69358	F01_transcript_69358	NA	F01_transcript_69358

F01_transcript_87130	NA	F01_transcript_87130	NA	NA
F01_transcript_17274	NA	NA	NA	F01_transcript_17274
F01_transcript_112879	F01_transcript_112879	F01_transcript_112879	NA	NA
F01_transcript_107523	NA	NA	F01_transcript_107523	F01_transcript_107523
F01_transcript_161461	F01_transcript_161461	F01_transcript_161461	F01_transcript_161461	F01_transcript_161461
F01_transcript_166386	NA	F01_transcript_166386	NA	F01_transcript_166386
F01_transcript_158056	NA	F01_transcript_158056	F01_transcript_158056	NA
F01_transcript_79075	F01_transcript_79075	F01_transcript_79075	F01_transcript_79075	F01_transcript_79075
F01_transcript_133518	NA	F01_transcript_133518	F01_transcript_133518	NA
F01_transcript_52557	F01_transcript_52557	F01_transcript_52557	NA	F01_transcript_52557
F01_transcript_40758	NA	NA	NA	F01_transcript_40758
F01_transcript_44433	NA	NA	NA	F01_transcript_44433
F01_transcript_119223	F01_transcript_119223	F01_transcript_119223	NA	F01_transcript_119223
F01_transcript_99441	F01_transcript_99441	F01_transcript_99441	F01_transcript_99441	F01_transcript_99441

F01_transcript_57473	NA	F01_transcript_57473	NA	NA
F01_transcript_149665	F01_transcript_149665	F01_transcript_149665	F01_transcript_149665	NA
F01_transcript_141594	NA	NA	NA	F01_transcript_141594
F01_transcript_61868	NA	NA	NA	F01_transcript_61868
F01_transcript_122306	NA	F01_transcript_122306	F01_transcript_122306	F01_transcript_122306
F01_transcript_64258	NA	NA	NA	F01_transcript_64258
F01_transcript_116210	NA	F01_transcript_116210	NA	NA
F01_transcript_149848	F01_transcript_149848	F01_transcript_149848	F01_transcript_149848	F01_transcript_149848
F01_transcript_165905	NA	F01_transcript_165905	NA	NA
F01_transcript_64225	NA	NA	NA	F01_transcript_64225
F01_transcript_134488	NA	F01_transcript_134488	NA	NA
F01_transcript_108617	NA	F01_transcript_108617	NA	F01_transcript_108617
F01_transcript_101068	F01_transcript_101068	F01_transcript_101068	F01_transcript_101068	F01_transcript_101068
F01_transcript_78384	NA	NA	NA	F01_transcript_78384

F01_transcript_67303	NA	F01_transcript_67303	NA	NA
F01_transcript_86658	NA	F01_transcript_86658	NA	F01_transcript_86658
F01_transcript_123002	NA	NA	NA	F01_transcript_123002
F01_transcript_2941	NA	NA	NA	F01_transcript_2941
F01_transcript_112502	F01_transcript_112502	F01_transcript_112502	NA	F01_transcript_112502
F01_transcript_86513	NA	F01_transcript_86513	NA	NA
F01_transcript_167143	NA	F01_transcript_167143	NA	F01_transcript_167143
F01_transcript_90307	NA	F01_transcript_90307	NA	F01_transcript_90307
F01_transcript_58764	NA	NA	NA	F01_transcript_58764
F01_transcript_122490	NA	NA	NA	F01_transcript_122490
F01_transcript_51232	F01_transcript_51232	F01_transcript_51232	F01_transcript_51232	F01_transcript_51232
F01_transcript_93279	NA	NA	NA	F01_transcript_93279
F01_transcript_167557	NA	F01_transcript_167557	NA	NA
F01_transcript_19875	NA	NA	NA	F01_transcript_19875

F01_transcript_13034	NA	NA	NA	F01_transcript_13034
F01_transcript_84078	NA	NA	NA	F01_transcript_84078
F01_transcript_113534	NA	NA	NA	F01_transcript_113534
F01_transcript_47464	NA	NA	NA	F01_transcript_47464
F01_transcript_12573	NA	NA	NA	F01_transcript_12573
F01_transcript_91910	NA	NA	NA	F01_transcript_91910
F01_transcript_100938	F01_transcript_100938	F01_transcript_100938	NA	NA
F01_transcript_123070	F01_transcript_123070	F01_transcript_123070	F01_transcript_123070	F01_transcript_123070
F01_transcript_137793	NA	NA	NA	F01_transcript_137793
F01_transcript_120196	NA	NA	NA	F01_transcript_120196
F01_transcript_24481	NA	NA	NA	F01_transcript_24481
F01_transcript_140775	NA	F01_transcript_140775	NA	NA
F01_transcript_164981	NA	F01_transcript_164981	F01_transcript_164981	F01_transcript_164981
F01_transcript_143610	F01_transcript_143610	NA	NA	NA

F01_transcript_56601	NA	F01_transcript_56601	F01_transcript_56601	F01_transcript_56601
F01_transcript_93115	F01_transcript_93115	F01_transcript_93115	F01_transcript_93115	F01_transcript_93115
F01_transcript_63439	F01_transcript_63439	F01_transcript_63439	F01_transcript_63439	F01_transcript_63439
F01_transcript_163459	NA	F01_transcript_163459	NA	F01_transcript_163459
F01_transcript_130379	NA	NA	NA	F01_transcript_130379
F01_transcript_151247	F01_transcript_151247	F01_transcript_151247	F01_transcript_151247	F01_transcript_151247
F01_transcript_133924	F01_transcript_133924	F01_transcript_133924	NA	F01_transcript_133924
F01_transcript_72975	F01_transcript_72975	F01_transcript_72975	F01_transcript_72975	F01_transcript_72975
F01_transcript_51035	NA	NA	F01_transcript_51035	F01_transcript_51035
F01_transcript_96219	F01_transcript_96219	F01_transcript_96219	F01_transcript_96219	NA
F01_transcript_91685	NA	F01_transcript_91685	NA	NA
F01_transcript_132757	NA	F01_transcript_132757	F01_transcript_132757	F01_transcript_132757
F01_transcript_86328	NA	F01_transcript_86328	NA	NA
F01_transcript_115305	NA	F01_transcript_115305	NA	F01_transcript_115305

F01_transcript_125624	NA	F01_transcript_125624	NA	NA
F01_transcript_52632	NA	NA	F01_transcript_52632	F01_transcript_52632
F01_transcript_147758	F01_transcript_147758	F01_transcript_147758	NA	F01_transcript_147758
F01_transcript_128292	NA	NA	F01_transcript_128292	F01_transcript_128292
F01_transcript_131052	NA	F01_transcript_131052	NA	F01_transcript_131052
F01_transcript_55224	NA	NA	NA	F01_transcript_55224
F01_transcript_113037	NA	F01_transcript_113037	F01_transcript_113037	F01_transcript_113037
F01_transcript_53663	F01_transcript_53663	F01_transcript_53663	NA	NA
F01_transcript_106757	NA	NA	NA	F01_transcript_106757
F01_transcript_76138	F01_transcript_76138	F01_transcript_76138	NA	NA
F01_transcript_161182	NA	F01_transcript_161182	NA	NA
F01_transcript_150044	NA	NA	NA	F01_transcript_150044
F01_transcript_98430	NA	NA	NA	F01_transcript_98430
F01_transcript_159529	NA	F01_transcript_159529	NA	NA

F01_transcript_121401	NA	F01_transcript_121401	NA	NA
F01_transcript_85055	NA	F01_transcript_85055	NA	F01_transcript_85055
F01_transcript_122549	NA	NA	NA	F01_transcript_122549
F01_transcript_141644	F01_transcript_141644	F01_transcript_141644	F01_transcript_141644	F01_transcript_141644
F01_transcript_133277	NA	NA	NA	F01_transcript_133277
F01_transcript_50337	F01_transcript_50337	F01_transcript_50337	F01_transcript_50337	F01_transcript_50337
F01_transcript_95773	F01_transcript_95773	F01_transcript_95773	NA	NA
F01_transcript_90201	NA	NA	NA	F01_transcript_90201
F01_transcript_93763	NA	NA	NA	F01_transcript_93763
F01_transcript_98793	F01_transcript_98793	F01_transcript_98793	F01_transcript_98793	F01_transcript_98793
F01_transcript_90056	NA	NA	NA	F01_transcript_90056
F01_transcript_77263	NA	NA	NA	F01_transcript_77263
F01_transcript_131184	NA	F01_transcript_131184	NA	NA
F01_transcript_80417	NA	F01_transcript_80417	NA	F01_transcript_80417

F01_transcript_32576	NA	NA	NA	F01_transcript_32576
F01_transcript_40778	NA	F01_transcript_40778	F01_transcript_40778	F01_transcript_40778
F01_transcript_116264	F01_transcript_116264	F01_transcript_116264	F01_transcript_116264	F01_transcript_116264
F01_transcript_154083	F01_transcript_154083	F01_transcript_154083	NA	NA
F01_transcript_39337	NA	NA	NA	F01_transcript_39337
F01_transcript_164694	F01_transcript_164694	F01_transcript_164694	F01_transcript_164694	F01_transcript_164694
F01_transcript_50110	NA	F01_transcript_50110	NA	NA
F01_transcript_110767	NA	NA	NA	F01_transcript_110767
F01_transcript_118958	NA	F01_transcript_118958	NA	NA
F01_transcript_157965	NA	NA	NA	F01_transcript_157965
F01_transcript_47741	NA	F01_transcript_47741	NA	F01_transcript_47741
F01_transcript_51585	NA	NA	NA	F01_transcript_51585
F01_transcript_96885	F01_transcript_96885	F01_transcript_96885	F01_transcript_96885	F01_transcript_96885
F01_transcript_149842	NA	F01_transcript_149842	NA	NA

F01_transcript_123347	NA	NA	NA	F01_transcript_123347
F01_transcript_3497	F01_transcript_3497	F01_transcript_3497	F01_transcript_3497	F01_transcript_3497
F01_transcript_106320	F01_transcript_106320	F01_transcript_106320	F01_transcript_106320	F01_transcript_106320
F01_transcript_62825	F01_transcript_62825	F01_transcript_62825	F01_transcript_62825	F01_transcript_62825
F01_transcript_138178	NA	NA	F01_transcript_138178	F01_transcript_138178
F01_transcript_83773	NA	NA	NA	F01_transcript_83773
F01_transcript_92926	F01_transcript_92926	F01_transcript_92926	F01_transcript_92926	F01_transcript_92926
F01_transcript_125181	NA	NA	NA	F01_transcript_125181
F01_transcript_123013	NA	F01_transcript_123013	NA	NA
F01_transcript_160994	NA	F01_transcript_160994	NA	F01_transcript_160994
F01_transcript_19685	NA	F01_transcript_19685	F01_transcript_19685	F01_transcript_19685
F01_transcript_133335	NA	F01_transcript_133335	NA	F01_transcript_133335
F01_transcript_107053	F01_transcript_107053	F01_transcript_107053	NA	F01_transcript_107053
F01_transcript_93227	NA	NA	NA	F01_transcript_93227

F01_transcript_77273	F01_transcript_77273	F01_transcript_77273	F01_transcript_77273	F01_transcript_77273
F01_transcript_71772	NA	NA	NA	F01_transcript_71772
F01_transcript_123907	F01_transcript_123907	F01_transcript_123907	F01_transcript_123907	F01_transcript_123907
F01_transcript_112382	NA	NA	NA	F01_transcript_112382
F01_transcript_150237	NA	NA	NA	F01_transcript_150237
F01_transcript_82044	NA	NA	NA	F01_transcript_82044
F01_transcript_153460	F01_transcript_153460	F01_transcript_153460	NA	F01_transcript_153460
F01_transcript_151734	NA	F01_transcript_151734	NA	NA
F01_transcript_84918	F01_transcript_84918	F01_transcript_84918	NA	NA
F01_transcript_161199	NA	NA	NA	F01_transcript_161199
F01_transcript_48412	NA	NA	NA	F01_transcript_48412
F01_transcript_98235	F01_transcript_98235	F01_transcript_98235	F01_transcript_98235	NA
F01_transcript_55839	NA	NA	NA	F01_transcript_55839
F01_transcript_56258	NA	NA	F01_transcript_56258	NA

F01_transcript_158156	NA	NA	NA	F01_transcript_158156
F01_transcript_144222	NA	NA	NA	F01_transcript_144222
F01_transcript_105451	F01_transcript_105451	F01_transcript_105451	NA	F01_transcript_105451
F01_transcript_81586	NA	F01_transcript_81586	NA	NA
F01_transcript_146563	F01_transcript_146563	F01_transcript_146563	F01_transcript_146563	NA
F01_transcript_136739	NA	F01_transcript_136739	NA	NA
F01_transcript_105384	NA	F01_transcript_105384	NA	NA
F01_transcript_60897	F01_transcript_60897	F01_transcript_60897	NA	F01_transcript_60897
F01_transcript_56635	NA	NA	NA	F01_transcript_56635
F01_transcript_81573	NA	NA	F01_transcript_81573	NA
F01_transcript_156910	F01_transcript_156910	F01_transcript_156910	F01_transcript_156910	F01_transcript_156910
F01_transcript_72152	NA	NA	NA	F01_transcript_72152
F01_transcript_48108	NA	F01_transcript_48108	NA	NA
F01_transcript_70838	NA	NA	NA	F01_transcript_70838

F01_transcript_66239	NA	NA	NA	F01_transcript_66239
F01_transcript_9456	NA	NA	NA	F01_transcript_9456
F01_transcript_128053	NA	NA	NA	F01_transcript_128053
F01_transcript_83484	NA	F01_transcript_83484	F01_transcript_83484	F01_transcript_83484
F01_transcript_63267	NA	F01_transcript_63267	F01_transcript_63267	F01_transcript_63267
F01_transcript_152444	NA	F01_transcript_152444	F01_transcript_152444	NA
F01_transcript_161122	F01_transcript_161122	F01_transcript_161122	F01_transcript_161122	F01_transcript_161122
F01_transcript_55906	NA	NA	NA	F01_transcript_55906
F01_transcript_19840	NA	NA	NA	F01_transcript_19840
F01_transcript_123393	NA	F01_transcript_123393	NA	NA
F01_transcript_55986	NA	F01_transcript_55986	NA	F01_transcript_55986
F01_transcript_105485	NA	F01_transcript_105485	NA	NA
F01_transcript_73798	NA	NA	NA	F01_transcript_73798
F01_transcript_77754	NA	NA	NA	F01_transcript_77754

F01_transcript_128915	NA	NA	NA	F01_transcript_128915
F01_transcript_37704	F01_transcript_37704	F01_transcript_37704	NA	NA
F01_transcript_35216	NA	F01_transcript_35216	NA	NA
F01_transcript_65630	NA	NA	NA	F01_transcript_65630
F01_transcript_74364	F01_transcript_74364	F01_transcript_74364	F01_transcript_74364	F01_transcript_74364
F01_transcript_76803	NA	F01_transcript_76803	NA	F01_transcript_76803
F01_transcript_159060	NA	NA	F01_transcript_159060	NA
F01_transcript_48944	F01_transcript_48944	F01_transcript_48944	F01_transcript_48944	F01_transcript_48944
F01_transcript_162185	F01_transcript_162185	F01_transcript_162185	F01_transcript_162185	F01_transcript_162185
F01_transcript_148056	NA	F01_transcript_148056	NA	NA
F01_transcript_150230	NA	F01_transcript_150230	NA	NA
F01_transcript_51087	F01_transcript_51087	F01_transcript_51087	F01_transcript_51087	F01_transcript_51087
F01_transcript_43420	F01_transcript_43420	F01_transcript_43420	F01_transcript_43420	F01_transcript_43420
F01_transcript_147564	NA	F01_transcript_147564	NA	NA

F01_transcript_9398	NA	NA	NA	F01_transcript_9398
F01_transcript_160181	NA	F01_transcript_160181	F01_transcript_160181	F01_transcript_160181
F01_transcript_89264	NA	NA	NA	F01_transcript_89264
F01_transcript_63119	F01_transcript_63119	F01_transcript_63119	F01_transcript_63119	F01_transcript_63119
F01_transcript_18561	NA	NA	NA	F01_transcript_18561
F01_transcript_128130	F01_transcript_128130	F01_transcript_128130	F01_transcript_128130	F01_transcript_128130
F01_transcript_158501	F01_transcript_158501	F01_transcript_158501	NA	NA
F01_transcript_121972	F01_transcript_121972	F01_transcript_121972	NA	NA
F01_transcript_52043	NA	NA	NA	F01_transcript_52043
F01_transcript_92407	NA	NA	NA	F01_transcript_92407
F01_transcript_92502	NA	F01_transcript_92502	F01_transcript_92502	F01_transcript_92502
F01_transcript_138456	NA	NA	NA	F01_transcript_138456
F01_transcript_100411	F01_transcript_100411	F01_transcript_100411	F01_transcript_100411	F01_transcript_100411
F01_transcript_137438	NA	F01_transcript_137438	NA	NA

F01_transcript_37810	F01_transcript_37810	F01_transcript_37810	NA	NA
F01_transcript_57089	NA	NA	F01_transcript_57089	NA
F01_transcript_4067	NA	F01_transcript_4067	NA	NA
F01_transcript_103429	F01_transcript_103429	F01_transcript_103429	NA	NA
F01_transcript_64700	NA	NA	NA	F01_transcript_64700
F01_transcript_113769	F01_transcript_113769	F01_transcript_113769	F01_transcript_113769	F01_transcript_113769
F01_transcript_78249	F01_transcript_78249	F01_transcript_78249	F01_transcript_78249	F01_transcript_78249
F01_transcript_1962	NA	F01_transcript_1962	NA	NA
F01_transcript_70674	NA	F01_transcript_70674	NA	F01_transcript_70674
F01_transcript_156720	NA	NA	NA	F01_transcript_156720
F01_transcript_53313	NA	NA	NA	F01_transcript_53313
F01_transcript_167146	NA	F01_transcript_167146	NA	F01_transcript_167146
F01_transcript_141146	NA	F01_transcript_141146	NA	F01_transcript_141146
F01_transcript_109299	NA	NA	NA	F01_transcript_109299

F01_transcript_134737	NA	F01_transcript_134737	F01_transcript_134737	NA
F01_transcript_51537	NA	F01_transcript_51537	NA	NA
F01_transcript_67503	F01_transcript_67503	F01_transcript_67503	F01_transcript_67503	F01_transcript_67503
F01_transcript_14431	NA	F01_transcript_14431	NA	NA
F01_transcript_129000	F01_transcript_129000	F01_transcript_129000	F01_transcript_129000	F01_transcript_129000
F01_transcript_68353	F01_transcript_68353	F01_transcript_68353	F01_transcript_68353	F01_transcript_68353
F01_transcript_74268	NA	F01_transcript_74268	NA	NA
F01_transcript_77861	F01_transcript_77861	F01_transcript_77861	F01_transcript_77861	F01_transcript_77861
F01_transcript_88083	NA	NA	NA	F01_transcript_88083
F01_transcript_136649	NA	F01_transcript_136649	NA	NA
F01_transcript_159488	NA	F01_transcript_159488	NA	NA
F01_transcript_60570	F01_transcript_60570	F01_transcript_60570	F01_transcript_60570	F01_transcript_60570
F01_transcript_144351	NA	F01_transcript_144351	F01_transcript_144351	F01_transcript_144351
F01_transcript_121932	NA	NA	NA	F01_transcript_121932

F01_transcript_87222	F01_transcript_87222	F01_transcript_87222	NA	NA
F01_transcript_54595	NA	F01_transcript_54595	NA	F01_transcript_54595
F01_transcript_123501	NA	NA	NA	F01_transcript_123501
F01_transcript_81491	NA	NA	NA	F01_transcript_81491
F01_transcript_135643	NA	F01_transcript_135643	NA	F01_transcript_135643
F01_transcript_156425	NA	NA	NA	F01_transcript_156425
F01_transcript_92489	F01_transcript_92489	F01_transcript_92489	F01_transcript_92489	NA
F01_transcript_72819	NA	NA	NA	F01_transcript_72819
F01_transcript_155918	NA	NA	NA	F01_transcript_155918
F01_transcript_136641	F01_transcript_136641	F01_transcript_136641	F01_transcript_136641	F01_transcript_136641
F01_transcript_80733	NA	NA	F01_transcript_80733	F01_transcript_80733
F01_transcript_93896	NA	F01_transcript_93896	F01_transcript_93896	F01_transcript_93896
F01_transcript_116247	NA	NA	NA	F01_transcript_116247
F01_transcript_166199	NA	F01_transcript_166199	F01_transcript_166199	F01_transcript_166199

F01_transcript_67447	NA	NA	NA	F01_transcript_67447
F01_transcript_137522	F01_transcript_137522	F01_transcript_137522	F01_transcript_137522	F01_transcript_137522
F01_transcript_106391	NA	F01_transcript_106391	NA	NA
F01_transcript_148417	NA	NA	NA	F01_transcript_148417
F01_transcript_18836	NA	NA	NA	F01_transcript_18836
F01_transcript_132016	NA	F01_transcript_132016	NA	F01_transcript_132016
F01_transcript_15074	NA	NA	NA	F01_transcript_15074
F01_transcript_37639	NA	NA	NA	F01_transcript_37639
F01_transcript_65187	NA	NA	F01_transcript_65187	NA
F01_transcript_144732	NA	F01_transcript_144732	F01_transcript_144732	NA
F01_transcript_44113	NA	NA	NA	F01_transcript_44113
F01_transcript_61564	F01_transcript_61564	F01_transcript_61564	F01_transcript_61564	F01_transcript_61564
F01_transcript_160003	NA	NA	NA	F01_transcript_160003
F01_transcript_163418	NA	NA	NA	F01_transcript_163418

F01_transcript_143965	F01_transcript_143965	F01_transcript_143965	NA	F01_transcript_143965
F01_transcript_37143	NA	NA	F01_transcript_37143	F01_transcript_37143
F01_transcript_52640	F01_transcript_52640	F01_transcript_52640	F01_transcript_52640	F01_transcript_52640
F01_transcript_126110	NA	F01_transcript_126110	NA	NA
F01_transcript_50039	F01_transcript_50039	NA	NA	F01_transcript_50039
F01_transcript_76813	NA	F01_transcript_76813	NA	NA
F01_transcript_9395	NA	NA	NA	F01_transcript_9395
F01_transcript_61452	NA	NA	NA	F01_transcript_61452
F01_transcript_90417	NA	NA	NA	F01_transcript_90417
F01_transcript_115450	NA	F01_transcript_115450	F01_transcript_115450	NA
F01_transcript_39485	NA	NA	NA	F01_transcript_39485
F01_transcript_22999	NA	NA	NA	F01_transcript_22999
F01_transcript_158688	NA	F01_transcript_158688	F01_transcript_158688	F01_transcript_158688
F01_transcript_49414	NA	F01_transcript_49414	NA	NA

F01_transcript_83006	NA	F01_transcript_83006	F01_transcript_83006	NA
F01_transcript_59487	NA	NA	F01_transcript_59487	NA
F01_transcript_50373	NA	F01_transcript_50373	NA	NA
F01_transcript_80475	NA	F01_transcript_80475	NA	NA
F01_transcript_63	NA	NA	NA	F01_transcript_63
F01_transcript_85502	F01_transcript_85502	F01_transcript_85502	F01_transcript_85502	F01_transcript_85502
F01_transcript_31189	NA	NA	NA	F01_transcript_31189
F01_transcript_15448	NA	NA	F01_transcript_15448	F01_transcript_15448
F01_transcript_49770	NA	F01_transcript_49770	NA	NA
F01_transcript_66259	NA	NA	NA	F01_transcript_66259
F01_transcript_97659	NA	NA	NA	F01_transcript_97659
F01_transcript_150234	NA	NA	F01_transcript_150234	F01_transcript_150234
F01_transcript_134044	NA	F01_transcript_134044	NA	NA
F01_transcript_59359	NA	NA	F01_transcript_59359	NA

F01_transcript_144158	NA	F01_transcript_144158	NA	F01_transcript_144158
F01_transcript_60951	F01_transcript_60951	F01_transcript_60951	F01_transcript_60951	F01_transcript_60951
F01_transcript_154503	NA	NA	NA	F01_transcript_154503
F01_transcript_18808	NA	NA	NA	F01_transcript_18808
F01_transcript_82246	NA	NA	NA	F01_transcript_82246
F01_transcript_109889	F01_transcript_109889	F01_transcript_109889	F01_transcript_109889	F01_transcript_109889
F01_transcript_79287	F01_transcript_79287	F01_transcript_79287	F01_transcript_79287	F01_transcript_79287
F01_transcript_68524	NA	NA	NA	F01_transcript_68524
F01_transcript_112492	F01_transcript_112492	F01_transcript_112492	NA	F01_transcript_112492
F01_transcript_166582	NA	NA	NA	F01_transcript_166582
F01_transcript_99286	NA	NA	NA	F01_transcript_99286
F01_transcript_23994	NA	NA	NA	F01_transcript_23994
F01_transcript_80340	NA	F01_transcript_80340	F01_transcript_80340	F01_transcript_80340
F01_transcript_155136	NA	F01_transcript_155136	F01_transcript_155136	F01_transcript_155136

F01_transcript_23768	NA	NA	NA	F01_transcript_23768
F01_transcript_148876	NA	F01_transcript_148876	NA	NA
F01_transcript_53356	NA	F01_transcript_53356	NA	NA
F01_transcript_71742	NA	F01_transcript_71742	NA	NA
F01_transcript_127895	NA	F01_transcript_127895	NA	NA
F01_transcript_145422	NA	F01_transcript_145422	F01_transcript_145422	NA
F01_transcript_109712	NA	NA	NA	F01_transcript_109712
F01_transcript_23723	NA	NA	NA	F01_transcript_23723
F01_transcript_116629	NA	NA	NA	F01_transcript_116629
F01_transcript_103322	F01_transcript_103322	F01_transcript_103322	F01_transcript_103322	F01_transcript_103322
F01_transcript_702	NA	NA	NA	F01_transcript_702
F01_transcript_58720	F01_transcript_58720	F01_transcript_58720	F01_transcript_58720	F01_transcript_58720
F01_transcript_150538	F01_transcript_150538	F01_transcript_150538	F01_transcript_150538	F01_transcript_150538
F01_transcript_158068	F01_transcript_158068	F01_transcript_158068	F01_transcript_158068	F01_transcript_158068

F01_transcript_113255	NA	F01_transcript_113255	NA	NA
F01_transcript_79496	F01_transcript_79496	F01_transcript_79496	F01_transcript_79496	F01_transcript_79496
F01_transcript_94834	NA	F01_transcript_94834	NA	NA
F01_transcript_94682	F01_transcript_94682	F01_transcript_94682	F01_transcript_94682	F01_transcript_94682
F01_transcript_69282	NA	F01_transcript_69282	NA	NA
F01_transcript_142012	F01_transcript_142012	F01_transcript_142012	F01_transcript_142012	F01_transcript_142012
F01_transcript_166452	NA	NA	NA	F01_transcript_166452
F01_transcript_152286	NA	NA	F01_transcript_152286	F01_transcript_152286
F01_transcript_110673	F01_transcript_110673	F01_transcript_110673	F01_transcript_110673	F01_transcript_110673
F01_transcript_73585	F01_transcript_73585	F01_transcript_73585	F01_transcript_73585	F01_transcript_73585
F01_transcript_70380	NA	F01_transcript_70380	NA	F01_transcript_70380
F01_transcript_149407	NA	NA	NA	F01_transcript_149407
F01_transcript_52039	NA	NA	NA	F01_transcript_52039
F01_transcript_106616	NA	NA	NA	F01_transcript_106616

F01_transcript_151847	NA	F01_transcript_151847	NA	F01_transcript_151847
F01_transcript_139684	NA	F01_transcript_139684	NA	F01_transcript_139684
F01_transcript_101394	NA	NA	NA	F01_transcript_101394
F01_transcript_155393	NA	NA	F01_transcript_155393	NA
F01_transcript_74973	NA	NA	NA	F01_transcript_74973
F01_transcript_74160	NA	NA	NA	F01_transcript_74160
F01_transcript_46954	NA	NA	NA	F01_transcript_46954
F01_transcript_46386	NA	NA	F01_transcript_46386	NA
F01_transcript_127551	NA	F01_transcript_127551	NA	NA
F01_transcript_100569	F01_transcript_100569	F01_transcript_100569	F01_transcript_100569	F01_transcript_100569
F01_transcript_36710	NA	NA	NA	F01_transcript_36710
F01_transcript_48629	NA	F01_transcript_48629	NA	NA
F01_transcript_79740	NA	NA	NA	F01_transcript_79740
F01_transcript_72076	F01_transcript_72076	F01_transcript_72076	F01_transcript_72076	F01_transcript_72076

F01_transcript_18883	NA	NA	NA	F01_transcript_18883
F01_transcript_288	NA	NA	NA	F01_transcript_288
F01_transcript_80305	NA	NA	NA	F01_transcript_80305
F01_transcript_128739	F01_transcript_128739	F01_transcript_128739	NA	NA
F01_transcript_694	NA	F01_transcript_694	F01_transcript_694	F01_transcript_694
F01_transcript_74479	NA	NA	NA	F01_transcript_74479
F01_transcript_100058	NA	NA	NA	F01_transcript_100058
F01_transcript_139562	F01_transcript_139562	F01_transcript_139562	F01_transcript_139562	F01_transcript_139562
F01_transcript_125525	NA	F01_transcript_125525	NA	NA
F01_transcript_79777	NA	F01_transcript_79777	F01_transcript_79777	NA
F01_transcript_40074	NA	NA	NA	F01_transcript_40074
F01_transcript_162435	NA	NA	NA	F01_transcript_162435
F01_transcript_133996	NA	F01_transcript_133996	NA	F01_transcript_133996
F01_transcript_83133	NA	F01_transcript_83133	NA	F01_transcript_83133

F01_transcript_132635	F01_transcript_132635	F01_transcript_132635	F01_transcript_132635	F01_transcript_132635
F01_transcript_111885	NA	F01_transcript_111885	F01_transcript_111885	F01_transcript_111885
F01_transcript_34717	NA	NA	NA	F01_transcript_34717
F01_transcript_367	NA	NA	NA	F01_transcript_367
F01_transcript_135252	NA	F01_transcript_135252	F01_transcript_135252	F01_transcript_135252
F01_transcript_49471	NA	NA	NA	F01_transcript_49471
F01_transcript_85487	NA	NA	NA	F01_transcript_85487
F01_transcript_49574	NA	F01_transcript_49574	NA	NA
F01_transcript_155370	F01_transcript_155370	F01_transcript_155370	F01_transcript_155370	F01_transcript_155370
F01_transcript_97988	F01_transcript_97988	F01_transcript_97988	F01_transcript_97988	F01_transcript_97988
F01_transcript_95950	NA	F01_transcript_95950	NA	NA
F01_transcript_90852	F01_transcript_90852	F01_transcript_90852	F01_transcript_90852	F01_transcript_90852
F01_transcript_102238	NA	F01_transcript_102238	NA	NA
F01_transcript_61932	F01_transcript_61932	F01_transcript_61932	F01_transcript_61932	F01_transcript_61932

F01_transcript_61590	NA	F01_transcript_61590	NA	NA
F01_transcript_128711	F01_transcript_128711	F01_transcript_128711	F01_transcript_128711	F01_transcript_128711
F01_transcript_24957	F01_transcript_24957	F01_transcript_24957	NA	F01_transcript_24957
F01_transcript_163424	F01_transcript_163424	F01_transcript_163424	F01_transcript_163424	F01_transcript_163424
F01_transcript_50500	F01_transcript_50500	F01_transcript_50500	NA	NA
F01_transcript_15905	NA	NA	F01_transcript_15905	NA
F01_transcript_135007	NA	F01_transcript_135007	NA	NA
F01_transcript_98009	NA	F01_transcript_98009	NA	NA
F01_transcript_84167	NA	F01_transcript_84167	F01_transcript_84167	F01_transcript_84167
F01_transcript_74225	NA	NA	NA	F01_transcript_74225
F01_transcript_115384	NA	F01_transcript_115384	NA	F01_transcript_115384
F01_transcript_92116	NA	NA	NA	F01_transcript_92116
F01_transcript_101411	NA	F01_transcript_101411	NA	NA
F01_transcript_162642	F01_transcript_162642	F01_transcript_162642	F01_transcript_162642	NA

F01_transcript_59922	NA	F01_transcript_59922	NA	NA
F01_transcript_54602	NA	NA	NA	F01_transcript_54602
F01_transcript_71299	NA	NA	F01_transcript_71299	NA
F01_transcript_54793	NA	F01_transcript_54793	NA	NA
F01_transcript_62177	NA	NA	NA	F01_transcript_62177
F01_transcript_30721	NA	F01_transcript_30721	F01_transcript_30721	F01_transcript_30721
F01_transcript_125892	NA	F01_transcript_125892	NA	F01_transcript_125892
F01_transcript_54236	NA	NA	NA	F01_transcript_54236
F01_transcript_67387	NA	F01_transcript_67387	NA	F01_transcript_67387
F01_transcript_148706	F01_transcript_148706	F01_transcript_148706	F01_transcript_148706	F01_transcript_148706
F01_transcript_134683	NA	F01_transcript_134683	NA	NA
F01_transcript_9538	NA	F01_transcript_9538	NA	NA
F01_transcript_86601	NA	F01_transcript_86601	NA	F01_transcript_86601
F01_transcript_158725	NA	F01_transcript_158725	NA	F01_transcript_158725

F01_transcript_122719	F01_transcript_122719	F01_transcript_122719	F01_transcript_122719	F01_transcript_122719
F01_transcript_138975	NA	NA	NA	F01_transcript_138975
F01_transcript_133333	F01_transcript_133333	NA	NA	F01_transcript_133333
F01_transcript_5578	F01_transcript_5578	F01_transcript_5578	NA	F01_transcript_5578
F01_transcript_50893	NA	F01_transcript_50893	F01_transcript_50893	F01_transcript_50893
F01_transcript_162328	NA	NA	NA	F01_transcript_162328
F01_transcript_19370	NA	NA	NA	F01_transcript_19370
F01_transcript_160952	F01_transcript_160952	F01_transcript_160952	F01_transcript_160952	F01_transcript_160952
F01_transcript_126391	NA	NA	NA	F01_transcript_126391
F01_transcript_85433	F01_transcript_85433	F01_transcript_85433	F01_transcript_85433	F01_transcript_85433
F01_transcript_44065	NA	NA	NA	F01_transcript_44065
F01_transcript_81724	NA	F01_transcript_81724	NA	F01_transcript_81724
F01_transcript_9758	F01_transcript_9758	F01_transcript_9758	NA	NA
F01_transcript_112979	NA	NA	NA	F01_transcript_112979

F01_transcript_125566	NA	F01_transcript_125566	NA	NA
F01_transcript_125063	NA	NA	NA	F01_transcript_125063
F01_transcript_71332	F01_transcript_71332	F01_transcript_71332	F01_transcript_71332	F01_transcript_71332
F01_transcript_145022	F01_transcript_145022	F01_transcript_145022	NA	NA
F01_transcript_100358	NA	F01_transcript_100358	NA	NA
F01_transcript_157274	F01_transcript_157274	F01_transcript_157274	F01_transcript_157274	F01_transcript_157274
F01_transcript_81065	NA	NA	NA	F01_transcript_81065
F01_transcript_165375	NA	NA	NA	F01_transcript_165375
F01_transcript_125428	NA	F01_transcript_125428	NA	NA
F01_transcript_92988	NA	F01_transcript_92988	NA	NA
F01_transcript_109045	F01_transcript_109045	F01_transcript_109045	F01_transcript_109045	F01_transcript_109045
F01_transcript_60783	NA	NA	NA	F01_transcript_60783
F01_transcript_76559	NA	F01_transcript_76559	NA	NA
F01_transcript_54111	NA	NA	NA	F01_transcript_54111

F01_transcript_90608	NA	F01_transcript_90608	F01_transcript_90608	F01_transcript_90608
F01_transcript_98573	NA	F01_transcript_98573	F01_transcript_98573	NA
F01_transcript_83349	NA	F01_transcript_83349	NA	NA
F01_transcript_86843	NA	F01_transcript_86843	NA	F01_transcript_86843
F01_transcript_160865	F01_transcript_160865	F01_transcript_160865	F01_transcript_160865	F01_transcript_160865
F01_transcript_135525	NA	NA	NA	F01_transcript_135525
F01_transcript_142929	NA	NA	F01_transcript_142929	F01_transcript_142929
F01_transcript_133931	NA	F01_transcript_133931	NA	NA
F01_transcript_50006	F01_transcript_50006	NA	NA	F01_transcript_50006
F01_transcript_152871	NA	NA	NA	F01_transcript_152871
F01_transcript_134032	NA	F01_transcript_134032	F01_transcript_134032	NA
F01_transcript_142260	F01_transcript_142260	F01_transcript_142260	F01_transcript_142260	F01_transcript_142260
F01_transcript_13221	NA	F01_transcript_13221	NA	NA
F01_transcript_147186	NA	F01_transcript_147186	F01_transcript_147186	F01_transcript_147186

F01_transcript_129610	NA	NA	NA	F01_transcript_129610
F01_transcript_146933	NA	NA	NA	F01_transcript_146933
F01_transcript_109555	F01_transcript_109555	F01_transcript_109555	F01_transcript_109555	F01_transcript_109555
F01_transcript_11633	NA	NA	NA	F01_transcript_11633
F01_transcript_80476	NA	F01_transcript_80476	NA	NA
F01_transcript_43459	NA	NA	NA	F01_transcript_43459
F01_transcript_69197	NA	F01_transcript_69197	NA	NA
F01_transcript_142022	F01_transcript_142022	F01_transcript_142022	NA	NA
F01_transcript_82160	NA	NA	NA	F01_transcript_82160
F01_transcript_132114	NA	NA	NA	F01_transcript_132114
F01_transcript_2965	NA	F01_transcript_2965	NA	NA
F01_transcript_123591	NA	F01_transcript_123591	F01_transcript_123591	F01_transcript_123591
F01_transcript_82649	F01_transcript_82649	F01_transcript_82649	F01_transcript_82649	F01_transcript_82649
F01_transcript_97782	F01_transcript_97782	F01_transcript_97782	F01_transcript_97782	F01_transcript_97782

F01_transcript_101116	F01_transcript_101116	F01_transcript_101116	F01_transcript_101116	F01_transcript_101116
F01_transcript_30961	NA	NA	NA	F01_transcript_30961
F01_transcript_49886	NA	NA	NA	F01_transcript_49886
F01_transcript_109766	NA	NA	NA	F01_transcript_109766
F01_transcript_105225	NA	NA	NA	F01_transcript_105225
F01_transcript_106388	NA	F01_transcript_106388	NA	NA
F01_transcript_146962	NA	F01_transcript_146962	NA	NA
F01_transcript_136366	NA	F01_transcript_136366	NA	NA
F01_transcript_106947	NA	NA	F01_transcript_106947	NA
F01_transcript_64588	F01_transcript_64588	F01_transcript_64588	F01_transcript_64588	F01_transcript_64588
F01_transcript_115430	F01_transcript_115430	F01_transcript_115430	F01_transcript_115430	F01_transcript_115430
F01_transcript_145451	F01_transcript_145451	F01_transcript_145451	F01_transcript_145451	F01_transcript_145451
F01_transcript_138834	F01_transcript_138834	F01_transcript_138834	F01_transcript_138834	F01_transcript_138834
F01_transcript_61079	NA	F01_transcript_61079	NA	NA

F01_transcript_99772	NA	F01_transcript_99772	NA	NA
F01_transcript_115771	F01_transcript_115771	F01_transcript_115771	NA	F01_transcript_115771
F01_transcript_97979	NA	F01_transcript_97979	F01_transcript_97979	NA
F01_transcript_140928	NA	NA	NA	F01_transcript_140928
F01_transcript_66511	NA	F01_transcript_66511	NA	F01_transcript_66511
F01_transcript_25440	NA	NA	NA	F01_transcript_25440
F01_transcript_28960	NA	NA	NA	F01_transcript_28960
F01_transcript_77562	NA	F01_transcript_77562	NA	F01_transcript_77562
F01_transcript_57547	F01_transcript_57547	F01_transcript_57547	F01_transcript_57547	F01_transcript_57547
F01_transcript_52495	NA	F01_transcript_52495	NA	F01_transcript_52495
F01_transcript_116884	F01_transcript_116884	F01_transcript_116884	F01_transcript_116884	F01_transcript_116884
F01_transcript_77106	NA	F01_transcript_77106	F01_transcript_77106	F01_transcript_77106
F01_transcript_66113	NA	NA	NA	F01_transcript_66113
F01_transcript_27988	F01_transcript_27988	F01_transcript_27988	NA	NA

F01_transcript_150598	F01_transcript_150598	F01_transcript_150598	F01_transcript_150598	F01_transcript_150598
F01_transcript_107617	NA	F01_transcript_107617	F01_transcript_107617	F01_transcript_107617
F01_transcript_93482	F01_transcript_93482	F01_transcript_93482	F01_transcript_93482	F01_transcript_93482
F01_transcript_131950	F01_transcript_131950	F01_transcript_131950	NA	NA
F01_transcript_167640	F01_transcript_167640	F01_transcript_167640	NA	NA
F01_transcript_128059	NA	NA	NA	F01_transcript_128059
F01_transcript_145240	NA	F01_transcript_145240	NA	F01_transcript_145240
F01_transcript_68135	F01_transcript_68135	F01_transcript_68135	F01_transcript_68135	F01_transcript_68135
F01_transcript_120145	F01_transcript_120145	F01_transcript_120145	F01_transcript_120145	F01_transcript_120145
F01_transcript_147932	F01_transcript_147932	F01_transcript_147932	F01_transcript_147932	F01_transcript_147932
F01_transcript_96679	NA	F01_transcript_96679	NA	NA
F01_transcript_164508	NA	F01_transcript_164508	NA	NA
F01_transcript_128139	NA	F01_transcript_128139	NA	NA
F01_transcript_130609	F01_transcript_130609	F01_transcript_130609	F01_transcript_130609	F01_transcript_130609

F01_transcript_118059	NA	F01_transcript_118059	NA	F01_transcript_118059
F01_transcript_51908	NA	F01_transcript_51908	NA	NA
F01_transcript_108551	NA	NA	NA	F01_transcript_108551
F01_transcript_18788	NA	NA	NA	F01_transcript_18788
F01_transcript_52474	NA	NA	NA	F01_transcript_52474
F01_transcript_101189	NA	F01_transcript_101189	NA	F01_transcript_101189
F01_transcript_151915	F01_transcript_151915	F01_transcript_151915	F01_transcript_151915	F01_transcript_151915
F01_transcript_30739	F01_transcript_30739	F01_transcript_30739	F01_transcript_30739	NA
F01_transcript_131387	NA	F01_transcript_131387	NA	NA
F01_transcript_99806	NA	F01_transcript_99806	NA	NA
F01_transcript_114974	NA	NA	NA	F01_transcript_114974
F01_transcript_53185	NA	NA	NA	F01_transcript_53185
F01_transcript_65367	NA	NA	F01_transcript_65367	NA
F01_transcript_148263	F01_transcript_148263	F01_transcript_148263	F01_transcript_148263	F01_transcript_148263

F01_transcript_78480	F01_transcript_78480	F01_transcript_78480	F01_transcript_78480	NA
F01_transcript_159467	NA	NA	NA	F01_transcript_159467
F01_transcript_13324	NA	F01_transcript_13324	F01_transcript_13324	F01_transcript_13324
F01_transcript_60833	NA	F01_transcript_60833	NA	NA
F01_transcript_61175	NA	NA	NA	F01_transcript_61175
F01_transcript_108380	F01_transcript_108380	NA	NA	NA
F01_transcript_133653	NA	F01_transcript_133653	F01_transcript_133653	NA
F01_transcript_5804	NA	NA	NA	F01_transcript_5804
F01_transcript_60292	NA	F01_transcript_60292	NA	NA
F01_transcript_167122	F01_transcript_167122	F01_transcript_167122	F01_transcript_167122	NA
F01_transcript_152193	NA	F01_transcript_152193	F01_transcript_152193	F01_transcript_152193
F01_transcript_94567	F01_transcript_94567	F01_transcript_94567	F01_transcript_94567	F01_transcript_94567
F01_transcript_88652	NA	NA	NA	F01_transcript_88652
F01_transcript_137251	NA	F01_transcript_137251	NA	NA

F01_transcript_153212	NA	NA	NA	F01_transcript_153212
F01_transcript_51738	NA	F01_transcript_51738	NA	NA
F01_transcript_57368	F01_transcript_57368	F01_transcript_57368	F01_transcript_57368	F01_transcript_57368
F01_transcript_127927	NA	NA	NA	F01_transcript_127927
F01_transcript_165402	NA	NA	NA	F01_transcript_165402
F01_transcript_127438	F01_transcript_127438	F01_transcript_127438	F01_transcript_127438	F01_transcript_127438
F01_transcript_85468	F01_transcript_85468	F01_transcript_85468	F01_transcript_85468	F01_transcript_85468
F01_transcript_23041	NA	F01_transcript_23041	NA	NA
F01_transcript_152597	NA	NA	NA	F01_transcript_152597
F01_transcript_55788	NA	NA	NA	F01_transcript_55788
F01_transcript_108465	NA	F01_transcript_108465	NA	NA
F01_transcript_57472	F01_transcript_57472	F01_transcript_57472	F01_transcript_57472	F01_transcript_57472
F01_transcript_59002	NA	F01_transcript_59002	NA	NA
F01_transcript_89730	NA	F01_transcript_89730	F01_transcript_89730	NA

F01_transcript_137292	NA	F01_transcript_137292	NA	NA
F01_transcript_47309	NA	F01_transcript_47309	F01_transcript_47309	F01_transcript_47309
F01_transcript_95935	NA	F01_transcript_95935	F01_transcript_95935	NA
F01_transcript_108001	NA	F01_transcript_108001	NA	F01_transcript_108001
F01_transcript_149014	NA	NA	NA	F01_transcript_149014
F01_transcript_145425	NA	NA	NA	F01_transcript_145425
F01_transcript_65981	NA	F01_transcript_65981	NA	F01_transcript_65981
F01_transcript_63668	NA	F01_transcript_63668	NA	NA
F01_transcript_85674	NA	NA	NA	F01_transcript_85674
F01_transcript_80953	F01_transcript_80953	F01_transcript_80953	F01_transcript_80953	F01_transcript_80953
F01_transcript_55635	NA	F01_transcript_55635	NA	F01_transcript_55635
F01_transcript_49551	NA	F01_transcript_49551	NA	F01_transcript_49551
F01_transcript_139678	NA	NA	F01_transcript_139678	NA
F01_transcript_125744	NA	F01_transcript_125744	NA	F01_transcript_125744

F01_transcript_14458	NA	F01_transcript_14458	F01_transcript_14458	F01_transcript_14458
F01_transcript_20084	F01_transcript_20084	F01_transcript_20084	F01_transcript_20084	F01_transcript_20084
F01_transcript_51559	NA	NA	NA	F01_transcript_51559
F01_transcript_77618	F01_transcript_77618	F01_transcript_77618	F01_transcript_77618	F01_transcript_77618
F01_transcript_155862	F01_transcript_155862	F01_transcript_155862	F01_transcript_155862	NA
F01_transcript_160404	NA	NA	NA	F01_transcript_160404
F01_transcript_167400	NA	F01_transcript_167400	NA	F01_transcript_167400
F01_transcript_135115	NA	F01_transcript_135115	NA	NA
F01_transcript_110044	NA	F01_transcript_110044	NA	NA
F01_transcript_103051	NA	F01_transcript_103051	NA	NA
F01_transcript_87068	F01_transcript_87068	F01_transcript_87068	F01_transcript_87068	F01_transcript_87068
F01_transcript_49422	F01_transcript_49422	NA	NA	NA
F01_transcript_139145	NA	NA	NA	F01_transcript_139145
F01_transcript_133714	F01_transcript_133714	F01_transcript_133714	F01_transcript_133714	F01_transcript_133714

F01_transcript_160653	NA	NA	NA	F01_transcript_160653
F01_transcript_65064	NA	NA	F01_transcript_65064	NA
F01_transcript_110983	NA	F01_transcript_110983	NA	NA
F01_transcript_50364	NA	F01_transcript_50364	NA	NA
F01_transcript_96820	NA	NA	NA	F01_transcript_96820
F01_transcript_106266	NA	NA	NA	F01_transcript_106266
F01_transcript_157065	F01_transcript_157065	F01_transcript_157065	F01_transcript_157065	F01_transcript_157065
F01_transcript_113909	NA	NA	NA	F01_transcript_113909
F01_transcript_116625	NA	NA	NA	F01_transcript_116625
F01_transcript_144076	F01_transcript_144076	F01_transcript_144076	NA	NA
F01_transcript_51128	F01_transcript_51128	F01_transcript_51128	F01_transcript_51128	F01_transcript_51128
F01_transcript_32824	NA	NA	NA	F01_transcript_32824
F01_transcript_58703	NA	F01_transcript_58703	NA	NA
F01_transcript_162067	NA	F01_transcript_162067	NA	NA

F01_transcript_123683	NA	F01_transcript_123683	NA	F01_transcript_123683
F01_transcript_148621	F01_transcript_148621	F01_transcript_148621	F01_transcript_148621	F01_transcript_148621
F01_transcript_65641	NA	F01_transcript_65641	F01_transcript_65641	F01_transcript_65641
F01_transcript_51854	NA	F01_transcript_51854	F01_transcript_51854	F01_transcript_51854
F01_transcript_133585	NA	NA	NA	F01_transcript_133585
F01_transcript_155039	NA	F01_transcript_155039	NA	NA
F01_transcript_85326	NA	F01_transcript_85326	NA	NA
F01_transcript_125003	NA	NA	NA	F01_transcript_125003
F01_transcript_39017	F01_transcript_39017	F01_transcript_39017	NA	F01_transcript_39017
F01_transcript_70010	NA	F01_transcript_70010	NA	NA
F01_transcript_157779	NA	NA	NA	F01_transcript_157779
F01_transcript_111455	NA	F01_transcript_111455	NA	NA
F01_transcript_50600	F01_transcript_50600	F01_transcript_50600	NA	F01_transcript_50600
F01_transcript_40930	NA	NA	NA	F01_transcript_40930

F01_transcript_128555	F01_transcript_128555	F01_transcript_128555	F01_transcript_128555	F01_transcript_128555
F01_transcript_71106	NA	F01_transcript_71106	F01_transcript_71106	NA
F01_transcript_119947	NA	NA	NA	F01_transcript_119947
F01_transcript_139961	F01_transcript_139961	F01_transcript_139961	F01_transcript_139961	F01_transcript_139961
F01_transcript_79714	F01_transcript_79714	F01_transcript_79714	F01_transcript_79714	F01_transcript_79714
F01_transcript_49113	NA	F01_transcript_49113	F01_transcript_49113	F01_transcript_49113
F01_transcript_126596	F01_transcript_126596	F01_transcript_126596	F01_transcript_126596	NA
F01_transcript_145476	NA	NA	F01_transcript_145476	F01_transcript_145476
F01_transcript_126132	F01_transcript_126132	F01_transcript_126132	F01_transcript_126132	F01_transcript_126132
F01_transcript_161171	NA	NA	NA	F01_transcript_161171
F01_transcript_143539	NA	F01_transcript_143539	NA	F01_transcript_143539
F01_transcript_33232	NA	NA	NA	F01_transcript_33232
F01_transcript_151707	NA	NA	NA	F01_transcript_151707
F01_transcript_101291	F01_transcript_101291	F01_transcript_101291	F01_transcript_101291	F01_transcript_101291

F01_transcript_127176	NA	NA	NA	F01_transcript_127176
F01_transcript_50395	F01_transcript_50395	F01_transcript_50395	F01_transcript_50395	F01_transcript_50395
F01_transcript_141206	NA	F01_transcript_141206	NA	F01_transcript_141206
F01_transcript_164853	NA	NA	NA	F01_transcript_164853
F01_transcript_58338	NA	NA	NA	F01_transcript_58338
F01_transcript_150493	NA	NA	NA	F01_transcript_150493
F01_transcript_133936	NA	NA	NA	F01_transcript_133936
F01_transcript_56079	NA	F01_transcript_56079	NA	F01_transcript_56079
F01_transcript_154965	NA	F01_transcript_154965	F01_transcript_154965	F01_transcript_154965
F01_transcript_135106	NA	NA	NA	F01_transcript_135106
F01_transcript_165041	NA	NA	NA	F01_transcript_165041
F01_transcript_99824	F01_transcript_99824	F01_transcript_99824	F01_transcript_99824	F01_transcript_99824
F01_transcript_148580	NA	NA	NA	F01_transcript_148580
F01_transcript_100967	NA	F01_transcript_100967	NA	NA

F01_transcript_162557	F01_transcript_162557	F01_transcript_162557	F01_transcript_162557	F01_transcript_162557
F01_transcript_97116	NA	NA	NA	F01_transcript_97116
F01_transcript_22460	NA	F01_transcript_22460	NA	NA
F01_transcript_134278	NA	F01_transcript_134278	NA	NA
F01_transcript_140589	F01_transcript_140589	F01_transcript_140589	F01_transcript_140589	F01_transcript_140589
F01_transcript_76361	NA	NA	NA	F01_transcript_76361
F01_transcript_144937	NA	F01_transcript_144937	NA	NA
F01_transcript_59472	NA	NA	F01_transcript_59472	F01_transcript_59472
F01_transcript_24387	F01_transcript_24387	F01_transcript_24387	F01_transcript_24387	F01_transcript_24387
F01_transcript_163712	NA	NA	NA	F01_transcript_163712
F01_transcript_75195	NA	F01_transcript_75195	NA	NA
F01_transcript_92501	NA	F01_transcript_92501	NA	NA
F01_transcript_93086	F01_transcript_93086	F01_transcript_93086	NA	NA
F01_transcript_50922	NA	F01_transcript_50922	NA	F01_transcript_50922

F01_transcript_115668	F01_transcript_115668	F01_transcript_115668	NA	NA
F01_transcript_101744	NA	NA	NA	F01_transcript_101744
F01_transcript_164227	NA	F01_transcript_164227	NA	F01_transcript_164227
F01_transcript_51963	F01_transcript_51963	F01_transcript_51963	F01_transcript_51963	F01_transcript_51963
F01_transcript_155883	NA	NA	F01_transcript_155883	F01_transcript_155883
F01_transcript_126108	NA	F01_transcript_126108	NA	NA
F01_transcript_46394	NA	NA	NA	F01_transcript_46394
F01_transcript_56050	F01_transcript_56050	F01_transcript_56050	F01_transcript_56050	F01_transcript_56050
F01_transcript_114034	NA	F01_transcript_114034	NA	NA
F01_transcript_98774	NA	NA	NA	F01_transcript_98774
F01_transcript_127007	NA	F01_transcript_127007	F01_transcript_127007	F01_transcript_127007
F01_transcript_85847	F01_transcript_85847	F01_transcript_85847	NA	NA
F01_transcript_14090	NA	NA	NA	F01_transcript_14090
F01_transcript_108734	F01_transcript_108734	F01_transcript_108734	F01_transcript_108734	F01_transcript_108734

F01_transcript_66672	NA	F01_transcript_66672	NA	NA
F01_transcript_84865	NA	F01_transcript_84865	NA	NA
F01_transcript_86694	F01_transcript_86694	F01_transcript_86694	F01_transcript_86694	NA
F01_transcript_146450	F01_transcript_146450	F01_transcript_146450	F01_transcript_146450	F01_transcript_146450
F01_transcript_159687	NA	F01_transcript_159687	NA	NA
F01_transcript_62242	NA	F01_transcript_62242	NA	NA
F01_transcript_135159	F01_transcript_135159	F01_transcript_135159	F01_transcript_135159	F01_transcript_135159
F01_transcript_64189	NA	NA	NA	F01_transcript_64189
F01_transcript_82495	NA	F01_transcript_82495	NA	NA
F01_transcript_90161	NA	NA	NA	F01_transcript_90161
F01_transcript_32592	NA	NA	NA	F01_transcript_32592
F01_transcript_66891	NA	NA	NA	F01_transcript_66891
F01_transcript_80790	NA	F01_transcript_80790	NA	NA
F01_transcript_6675	NA	NA	NA	F01_transcript_6675

F01_transcript_146221	NA	F01_transcript_146221	NA	NA
F01_transcript_109472	NA	F01_transcript_109472	F01_transcript_109472	F01_transcript_109472
F01_transcript_100407	NA	F01_transcript_100407	NA	NA
F01_transcript_94360	F01_transcript_94360	F01_transcript_94360	NA	F01_transcript_94360
F01_transcript_51610	NA	NA	NA	F01_transcript_51610
F01_transcript_154141	NA	F01_transcript_154141	NA	NA
F01_transcript_129895	F01_transcript_129895	F01_transcript_129895	NA	NA
F01_transcript_77674	NA	F01_transcript_77674	NA	F01_transcript_77674
F01_transcript_8031	NA	NA	NA	F01_transcript_8031
F01_transcript_52904	F01_transcript_52904	F01_transcript_52904	NA	F01_transcript_52904
F01_transcript_30205	F01_transcript_30205	F01_transcript_30205	NA	F01_transcript_30205
F01_transcript_45796	NA	NA	NA	F01_transcript_45796
F01_transcript_141909	NA	F01_transcript_141909	NA	NA
F01_transcript_76711	NA	NA	NA	F01_transcript_76711

F01_transcript_61285	NA	NA	NA	F01_transcript_61285
F01_transcript_47681	NA	NA	NA	F01_transcript_47681
F01_transcript_152693	NA	NA	NA	F01_transcript_152693
F01_transcript_133634	NA	F01_transcript_133634	F01_transcript_133634	F01_transcript_133634
F01_transcript_118055	F01_transcript_118055	F01_transcript_118055	F01_transcript_118055	F01_transcript_118055
F01_transcript_112773	NA	F01_transcript_112773	NA	NA
F01_transcript_124309	F01_transcript_124309	F01_transcript_124309	F01_transcript_124309	F01_transcript_124309
F01_transcript_16735	NA	F01_transcript_16735	NA	F01_transcript_16735
F01_transcript_81239	NA	F01_transcript_81239	F01_transcript_81239	F01_transcript_81239
F01_transcript_92446	F01_transcript_92446	F01_transcript_92446	F01_transcript_92446	F01_transcript_92446
F01_transcript_162561	F01_transcript_162561	F01_transcript_162561	F01_transcript_162561	F01_transcript_162561
F01_transcript_127964	F01_transcript_127964	F01_transcript_127964	F01_transcript_127964	F01_transcript_127964
F01_transcript_137537	NA	NA	NA	F01_transcript_137537
F01_transcript_46322	NA	NA	NA	F01_transcript_46322

F01_transcript_116174	NA	NA	NA	F01_transcript_116174
F01_transcript_63982	NA	NA	NA	F01_transcript_63982
F01_transcript_166309	NA	NA	NA	F01_transcript_166309
F01_transcript_98688	NA	NA	NA	F01_transcript_98688
F01_transcript_55935	F01_transcript_55935	F01_transcript_55935	F01_transcript_55935	F01_transcript_55935
F01_transcript_134969	NA	NA	NA	F01_transcript_134969
F01_transcript_81844	NA	NA	NA	F01_transcript_81844
F01_transcript_51954	NA	NA	NA	F01_transcript_51954
F01_transcript_5302	NA	F01_transcript_5302	NA	NA
F01_transcript_110749	F01_transcript_110749	F01_transcript_110749	F01_transcript_110749	F01_transcript_110749
F01_transcript_95177	NA	NA	F01_transcript_95177	F01_transcript_95177
F01_transcript_54598	NA	F01_transcript_54598	NA	F01_transcript_54598
F01_transcript_109	NA	NA	NA	F01_transcript_109
F01_transcript_166695	NA	NA	F01_transcript_166695	NA

F01_transcript_101426	F01_transcript_101426	F01_transcript_101426	F01_transcript_101426	F01_transcript_101426
F01_transcript_164234	F01_transcript_164234	NA	NA	F01_transcript_164234
F01_transcript_116554	NA	F01_transcript_116554	NA	F01_transcript_116554
F01_transcript_35345	F01_transcript_35345	F01_transcript_35345	F01_transcript_35345	F01_transcript_35345
F01_transcript_3012	NA	F01_transcript_3012	NA	NA
F01_transcript_135058	NA	NA	NA	F01_transcript_135058
F01_transcript_12940	NA	NA	NA	F01_transcript_12940
F01_transcript_62356	NA	NA	NA	F01_transcript_62356
F01_transcript_118957	NA	NA	NA	F01_transcript_118957
F01_transcript_92771	F01_transcript_92771	F01_transcript_92771	NA	NA
F01_transcript_140716	NA	F01_transcript_140716	NA	NA
F01_transcript_120705	F01_transcript_120705	F01_transcript_120705	F01_transcript_120705	F01_transcript_120705
F01_transcript_56255	NA	NA	NA	F01_transcript_56255
F01_transcript_104783	NA	NA	NA	F01_transcript_104783

F01_transcript_118245	NA	F01_transcript_118245	F01_transcript_118245	F01_transcript_118245
F01_transcript_41621	F01_transcript_41621	F01_transcript_41621	NA	NA
F01_transcript_122941	NA	NA	NA	F01_transcript_122941
F01_transcript_153081	NA	F01_transcript_153081	NA	F01_transcript_153081
F01_transcript_54784	NA	NA	NA	F01_transcript_54784
F01_transcript_71204	NA	NA	NA	F01_transcript_71204
F01_transcript_31940	NA	NA	NA	F01_transcript_31940
F01_transcript_163792	NA	F01_transcript_163792	NA	NA
F01_transcript_64249	NA	NA	NA	F01_transcript_64249
F01_transcript_147129	NA	F01_transcript_147129	NA	F01_transcript_147129
F01_transcript_76154	F01_transcript_76154	F01_transcript_76154	F01_transcript_76154	F01_transcript_76154
F01_transcript_143316	NA	NA	NA	F01_transcript_143316
F01_transcript_133918	NA	F01_transcript_133918	NA	F01_transcript_133918
F01_transcript_118644	NA	NA	NA	F01_transcript_118644

F01_transcript_92359	F01_transcript_92359	F01_transcript_92359	F01_transcript_92359	F01_transcript_92359
F01_transcript_156251	F01_transcript_156251	F01_transcript_156251	F01_transcript_156251	F01_transcript_156251
F01_transcript_149308	F01_transcript_149308	F01_transcript_149308	NA	NA
F01_transcript_11009	NA	NA	NA	F01_transcript_11009
F01_transcript_6871	NA	NA	NA	F01_transcript_6871
F01_transcript_112304	NA	F01_transcript_112304	NA	NA
F01_transcript_111024	F01_transcript_111024	F01_transcript_111024	F01_transcript_111024	F01_transcript_111024
F01_transcript_58045	F01_transcript_58045	F01_transcript_58045	NA	NA
F01_transcript_136245	F01_transcript_136245	F01_transcript_136245	F01_transcript_136245	F01_transcript_136245
F01_transcript_55637	F01_transcript_55637	F01_transcript_55637	F01_transcript_55637	F01_transcript_55637
F01_transcript_136603	NA	F01_transcript_136603	NA	NA
F01_transcript_42879	NA	NA	NA	F01_transcript_42879
F01_transcript_81648	NA	NA	NA	F01_transcript_81648
F01_transcript_124845	NA	F01_transcript_124845	F01_transcript_124845	F01_transcript_124845

F01_transcript_111954	NA	F01_transcript_111954	NA	NA
F01_transcript_93790	F01_transcript_93790	F01_transcript_93790	F01_transcript_93790	F01_transcript_93790
F01_transcript_89927	NA	F01_transcript_89927	NA	NA
F01_transcript_44000	NA	NA	NA	F01_transcript_44000
F01_transcript_50402	F01_transcript_50402	F01_transcript_50402	F01_transcript_50402	NA
F01_transcript_71037	NA	NA	F01_transcript_71037	NA
F01_transcript_51350	NA	NA	NA	F01_transcript_51350
F01_transcript_138803	F01_transcript_138803	F01_transcript_138803	F01_transcript_138803	F01_transcript_138803
F01_transcript_93552	NA	NA	NA	F01_transcript_93552
F01_transcript_84366	NA	NA	NA	F01_transcript_84366
F01_transcript_72607	F01_transcript_72607	F01_transcript_72607	NA	F01_transcript_72607
F01_transcript_30579	NA	NA	NA	F01_transcript_30579
F01_transcript_154112	NA	NA	F01_transcript_154112	F01_transcript_154112
F01_transcript_131654	F01_transcript_131654	F01_transcript_131654	F01_transcript_131654	F01_transcript_131654

F01_transcript_101772	NA	F01_transcript_101772	NA	NA
F01_transcript_65218	F01_transcript_65218	F01_transcript_65218	F01_transcript_65218	F01_transcript_65218
F01_transcript_81264	NA	NA	NA	F01_transcript_81264
F01_transcript_10867	NA	NA	NA	F01_transcript_10867
F01_transcript_158975	NA	NA	NA	F01_transcript_158975
F01_transcript_49239	NA	F01_transcript_49239	NA	NA
F01_transcript_60941	F01_transcript_60941	F01_transcript_60941	NA	NA
F01_transcript_119502	NA	NA	NA	F01_transcript_119502
F01_transcript_11600	NA	NA	NA	F01_transcript_11600
F01_transcript_60412	NA	F01_transcript_60412	NA	F01_transcript_60412
F01_transcript_117340	NA	NA	NA	F01_transcript_117340
F01_transcript_48527	F01_transcript_48527	F01_transcript_48527	NA	NA
F01_transcript_89372	NA	F01_transcript_89372	NA	NA
F01_transcript_145898	NA	NA	NA	F01_transcript_145898

F01_transcript_2942	NA	NA	NA	F01_transcript_2942
F01_transcript_85050	NA	F01_transcript_85050	F01_transcript_85050	F01_transcript_85050
F01_transcript_146993	NA	F01_transcript_146993	NA	F01_transcript_146993
F01_transcript_101987	NA	NA	NA	F01_transcript_101987
F01_transcript_52000	NA	F01_transcript_52000	NA	NA
F01_transcript_68569	F01_transcript_68569	F01_transcript_68569	F01_transcript_68569	F01_transcript_68569
F01_transcript_19802	NA	NA	NA	F01_transcript_19802
F01_transcript_71498	F01_transcript_71498	F01_transcript_71498	F01_transcript_71498	F01_transcript_71498
F01_transcript_20293	F01_transcript_20293	F01_transcript_20293	NA	F01_transcript_20293
F01_transcript_124529	NA	F01_transcript_124529	F01_transcript_124529	NA
F01_transcript_160697	F01_transcript_160697	NA	NA	NA
F01_transcript_129440	NA	NA	NA	F01_transcript_129440
F01_transcript_128985	F01_transcript_128985	F01_transcript_128985	F01_transcript_128985	F01_transcript_128985
F01_transcript_38955	NA	F01_transcript_38955	NA	NA

F01_transcript_66455	F01_transcript_66455	F01_transcript_66455	F01_transcript_66455	F01_transcript_66455
F01_transcript_119601	NA	F01_transcript_119601	NA	NA
F01_transcript_56432	NA	NA	F01_transcript_56432	F01_transcript_56432
F01_transcript_113203	F01_transcript_113203	F01_transcript_113203	NA	NA
F01_transcript_48386	NA	NA	F01_transcript_48386	F01_transcript_48386
F01_transcript_112824	NA	NA	NA	F01_transcript_112824
F01_transcript_93074	NA	F01_transcript_93074	NA	F01_transcript_93074
F01_transcript_99792	NA	F01_transcript_99792	NA	F01_transcript_99792
F01_transcript_109410	NA	F01_transcript_109410	NA	NA
F01_transcript_81655	NA	NA	NA	F01_transcript_81655
F01_transcript_112528	NA	NA	F01_transcript_112528	F01_transcript_112528
F01_transcript_120548	F01_transcript_120548	F01_transcript_120548	NA	F01_transcript_120548
F01_transcript_149717	F01_transcript_149717	F01_transcript_149717	F01_transcript_149717	F01_transcript_149717
F01_transcript_125212	F01_transcript_125212	F01_transcript_125212	F01_transcript_125212	F01_transcript_125212

F01_transcript_73440	NA	NA	NA	F01_transcript_73440
F01_transcript_158981	NA	NA	F01_transcript_158981	NA
F01_transcript_102688	NA	F01_transcript_102688	NA	F01_transcript_102688
F01_transcript_46321	NA	F01_transcript_46321	F01_transcript_46321	NA
F01_transcript_52449	NA	F01_transcript_52449	F01_transcript_52449	F01_transcript_52449
F01_transcript_138499	NA	F01_transcript_138499	NA	NA
F01_transcript_150979	NA	NA	NA	F01_transcript_150979
F01_transcript_50851	F01_transcript_50851	F01_transcript_50851	F01_transcript_50851	F01_transcript_50851
F01_transcript_36271	NA	NA	NA	F01_transcript_36271
F01_transcript_78430	NA	NA	NA	F01_transcript_78430
F01_transcript_80731	NA	NA	NA	F01_transcript_80731
F01_transcript_69149	NA	F01_transcript_69149	F01_transcript_69149	F01_transcript_69149
F01_transcript_80510	NA	F01_transcript_80510	NA	NA
F01_transcript_136639	F01_transcript_136639	F01_transcript_136639	F01_transcript_136639	NA

F01_transcript_124541	F01_transcript_124541	F01_transcript_124541	F01_transcript_124541	NA
F01_transcript_116620	F01_transcript_116620	F01_transcript_116620	F01_transcript_116620	F01_transcript_116620
F01_transcript_155089	NA	NA	NA	F01_transcript_155089
F01_transcript_161050	F01_transcript_161050	F01_transcript_161050	F01_transcript_161050	F01_transcript_161050
F01_transcript_7583	F01_transcript_7583	F01_transcript_7583	F01_transcript_7583	NA
F01_transcript_138358	NA	NA	NA	F01_transcript_138358
F01_transcript_157089	F01_transcript_157089	F01_transcript_157089	F01_transcript_157089	F01_transcript_157089
F01_transcript_152292	F01_transcript_152292	F01_transcript_152292	NA	F01_transcript_152292
F01_transcript_110969	F01_transcript_110969	F01_transcript_110969	NA	NA
F01_transcript_160008	NA	F01_transcript_160008	NA	NA
F01_transcript_100225	NA	NA	NA	F01_transcript_100225
F01_transcript_130097	F01_transcript_130097	F01_transcript_130097	F01_transcript_130097	F01_transcript_130097
F01_transcript_84445	NA	F01_transcript_84445	NA	F01_transcript_84445
F01_transcript_92613	NA	F01_transcript_92613	NA	NA

F01_transcript_83499	NA	NA	NA	F01_transcript_83499
F01_transcript_292	NA	F01_transcript_292	NA	F01_transcript_292
F01_transcript_80494	NA	NA	NA	F01_transcript_80494
F01_transcript_70203	F01_transcript_70203	F01_transcript_70203	NA	F01_transcript_70203
F01_transcript_4887	NA	NA	NA	F01_transcript_4887
F01_transcript_152514	F01_transcript_152514	F01_transcript_152514	F01_transcript_152514	F01_transcript_152514
F01_transcript_16176	NA	NA	F01_transcript_16176	F01_transcript_16176
F01_transcript_46864	NA	NA	NA	F01_transcript_46864
F01_transcript_147949	NA	NA	NA	F01_transcript_147949
F01_transcript_48207	NA	F01_transcript_48207	NA	F01_transcript_48207
F01_transcript_98493	NA	NA	NA	F01_transcript_98493
F01_transcript_48923	F01_transcript_48923	NA	NA	NA
F01_transcript_128437	F01_transcript_128437	F01_transcript_128437	F01_transcript_128437	F01_transcript_128437
F01_transcript_95635	NA	NA	NA	F01_transcript_95635

F01_transcript_79255	NA	F01_transcript_79255	NA	NA
F01_transcript_139958	NA	F01_transcript_139958	NA	NA
F01_transcript_53754	NA	F01_transcript_53754	NA	NA
F01_transcript_78333	NA	F01_transcript_78333	NA	NA
F01_transcript_89235	NA	F01_transcript_89235	NA	NA
F01_transcript_53087	F01_transcript_53087	F01_transcript_53087	NA	F01_transcript_53087
F01_transcript_161237	NA	NA	NA	F01_transcript_161237
F01_transcript_60299	NA	NA	NA	F01_transcript_60299
F01_transcript_107701	NA	F01_transcript_107701	NA	F01_transcript_107701
F01_transcript_65899	NA	NA	NA	F01_transcript_65899
F01_transcript_112047	NA	NA	NA	F01_transcript_112047
F01_transcript_83101	F01_transcript_83101	F01_transcript_83101	F01_transcript_83101	F01_transcript_83101
F01_transcript_80455	F01_transcript_80455	F01_transcript_80455	F01_transcript_80455	F01_transcript_80455
F01_transcript_152339	NA	F01_transcript_152339	NA	NA

F01_transcript_7308	NA	NA	NA	F01_transcript_7308
F01_transcript_163637	F01_transcript_163637	F01_transcript_163637	F01_transcript_163637	F01_transcript_163637
F01_transcript_89985	F01_transcript_89985	F01_transcript_89985	F01_transcript_89985	NA
F01_transcript_89592	NA	NA	NA	F01_transcript_89592
F01_transcript_74176	NA	NA	NA	F01_transcript_74176
F01_transcript_62835	NA	F01_transcript_62835	F01_transcript_62835	NA
F01_transcript_164961	F01_transcript_164961	F01_transcript_164961	F01_transcript_164961	F01_transcript_164961
F01_transcript_88993	F01_transcript_88993	F01_transcript_88993	NA	F01_transcript_88993
F01_transcript_111019	F01_transcript_111019	F01_transcript_111019	F01_transcript_111019	F01_transcript_111019
F01_transcript_115467	NA	F01_transcript_115467	NA	NA
F01_transcript_30781	NA	NA	NA	F01_transcript_30781
F01_transcript_47911	NA	NA	NA	F01_transcript_47911
F01_transcript_86326	NA	NA	NA	F01_transcript_86326
F01_transcript_50979	NA	F01_transcript_50979	NA	NA

F01_transcript_108376	NA	F01_transcript_108376	F01_transcript_108376	F01_transcript_108376
F01_transcript_2996	NA	NA	NA	F01_transcript_2996
F01_transcript_101290	NA	F01_transcript_101290	NA	NA
F01_transcript_77355	NA	F01_transcript_77355	NA	NA
F01_transcript_2178	NA	NA	NA	F01_transcript_2178
F01_transcript_110574	NA	F01_transcript_110574	NA	NA
F01_transcript_162659	NA	F01_transcript_162659	NA	F01_transcript_162659
F01_transcript_51353	NA	NA	NA	F01_transcript_51353
F01_transcript_49649	F01_transcript_49649	F01_transcript_49649	F01_transcript_49649	F01_transcript_49649
F01_transcript_67194	NA	F01_transcript_67194	F01_transcript_67194	F01_transcript_67194
F01_transcript_114663	F01_transcript_114663	F01_transcript_114663	F01_transcript_114663	F01_transcript_114663
F01_transcript_91969	NA	NA	NA	F01_transcript_91969
F01_transcript_86363	F01_transcript_86363	F01_transcript_86363	F01_transcript_86363	F01_transcript_86363
F01_transcript_106100	NA	NA	NA	F01_transcript_106100

F01_transcript_149469	NA	NA	NA	F01_transcript_149469
F01_transcript_81270	F01_transcript_81270	F01_transcript_81270	F01_transcript_81270	F01_transcript_81270
F01_transcript_650	NA	NA	NA	F01_transcript_650
F01_transcript_19097	NA	NA	NA	F01_transcript_19097
F01_transcript_31649	F01_transcript_31649	F01_transcript_31649	NA	F01_transcript_31649
F01_transcript_77046	NA	NA	NA	F01_transcript_77046
F01_transcript_112100	F01_transcript_112100	F01_transcript_112100	F01_transcript_112100	F01_transcript_112100
F01_transcript_58329	NA	NA	NA	F01_transcript_58329
F01_transcript_97056	F01_transcript_97056	F01_transcript_97056	F01_transcript_97056	F01_transcript_97056
F01_transcript_138875	NA	F01_transcript_138875	NA	F01_transcript_138875
F01_transcript_164612	NA	NA	NA	F01_transcript_164612
F01_transcript_106168	NA	F01_transcript_106168	NA	F01_transcript_106168
F01_transcript_144600	NA	F01_transcript_144600	NA	F01_transcript_144600
F01_transcript_161446	NA	NA	NA	F01_transcript_161446

F01_transcript_139126	NA	F01_transcript_139126	NA	NA
F01_transcript_11323	NA	NA	NA	F01_transcript_11323
F01_transcript_72976	NA	NA	NA	F01_transcript_72976
F01_transcript_136936	F01_transcript_136936	F01_transcript_136936	F01_transcript_136936	NA
F01_transcript_109583	NA	F01_transcript_109583	NA	F01_transcript_109583
F01_transcript_147944	NA	NA	NA	F01_transcript_147944
F01_transcript_134107	NA	NA	NA	F01_transcript_134107
F01_transcript_154361	F01_transcript_154361	F01_transcript_154361	F01_transcript_154361	F01_transcript_154361
F01_transcript_104118	NA	F01_transcript_104118	NA	NA
F01_transcript_143711	NA	NA	NA	F01_transcript_143711
F01_transcript_56984	NA	NA	NA	F01_transcript_56984
F01_transcript_123730	F01_transcript_123730	F01_transcript_123730	F01_transcript_123730	F01_transcript_123730
F01_transcript_122917	F01_transcript_122917	F01_transcript_122917	NA	NA
F01_transcript_57078	F01_transcript_57078	F01_transcript_57078	F01_transcript_57078	F01_transcript_57078

F01_transcript_74834	NA	NA	NA	F01_transcript_74834
F01_transcript_68974	NA	F01_transcript_68974	NA	F01_transcript_68974
F01_transcript_144616	NA	F01_transcript_144616	NA	NA
F01_transcript_153034	NA	NA	F01_transcript_153034	F01_transcript_153034
F01_transcript_144181	NA	F01_transcript_144181	NA	NA
F01_transcript_30008	F01_transcript_30008	F01_transcript_30008	F01_transcript_30008	F01_transcript_30008
F01_transcript_68693	F01_transcript_68693	F01_transcript_68693	F01_transcript_68693	F01_transcript_68693
F01_transcript_80648	NA	NA	NA	F01_transcript_80648
F01_transcript_148796	NA	NA	NA	F01_transcript_148796
F01_transcript_74616	NA	F01_transcript_74616	NA	NA
F01_transcript_136444	NA	F01_transcript_136444	NA	F01_transcript_136444
F01_transcript_61125	NA	NA	NA	F01_transcript_61125
F01_transcript_79912	F01_transcript_79912	F01_transcript_79912	F01_transcript_79912	F01_transcript_79912
F01_transcript_77487	F01_transcript_77487	F01_transcript_77487	F01_transcript_77487	F01_transcript_77487

F01_transcript_90843	NA	F01_transcript_90843	NA	NA
F01_transcript_113550	F01_transcript_113550	F01_transcript_113550	F01_transcript_113550	F01_transcript_113550
F01_transcript_162816	F01_transcript_162816	F01_transcript_162816	F01_transcript_162816	F01_transcript_162816
F01_transcript_26755	NA	NA	NA	F01_transcript_26755
F01_transcript_100996	F01_transcript_100996	F01_transcript_100996	F01_transcript_100996	F01_transcript_100996
F01_transcript_146870	NA	NA	NA	F01_transcript_146870
F01_transcript_5678	F01_transcript_5678	F01_transcript_5678	F01_transcript_5678	F01_transcript_5678
F01_transcript_121624	F01_transcript_121624	F01_transcript_121624	NA	NA
F01_transcript_83129	NA	NA	NA	F01_transcript_83129
F01_transcript_139101	NA	NA	NA	F01_transcript_139101
F01_transcript_154075	NA	NA	NA	F01_transcript_154075
F01_transcript_109511	NA	NA	NA	F01_transcript_109511
F01_transcript_66535	NA	NA	NA	F01_transcript_66535
F01_transcript_107892	F01_transcript_107892	F01_transcript_107892	F01_transcript_107892	F01_transcript_107892

F01_transcript_75487	NA	F01_transcript_75487	NA	NA
F01_transcript_50031	NA	F01_transcript_50031	NA	NA
F01_transcript_148850	F01_transcript_148850	F01_transcript_148850	NA	NA
F01_transcript_75419	F01_transcript_75419	F01_transcript_75419	F01_transcript_75419	F01_transcript_75419
F01_transcript_97802	F01_transcript_97802	F01_transcript_97802	F01_transcript_97802	F01_transcript_97802
F01_transcript_79877	NA	NA	NA	F01_transcript_79877
F01_transcript_43404	NA	NA	NA	F01_transcript_43404
F01_transcript_96148	NA	F01_transcript_96148	NA	NA
F01_transcript_64388	NA	NA	NA	F01_transcript_64388
F01_transcript_84116	F01_transcript_84116	F01_transcript_84116	F01_transcript_84116	F01_transcript_84116
F01_transcript_132756	F01_transcript_132756	F01_transcript_132756	F01_transcript_132756	F01_transcript_132756
F01_transcript_132480	NA	F01_transcript_132480	NA	NA
F01_transcript_111730	NA	F01_transcript_111730	NA	NA
F01_transcript_133700	F01_transcript_133700	F01_transcript_133700	F01_transcript_133700	F01_transcript_133700

F01_transcript_148072	NA	F01_transcript_148072	NA	NA
F01_transcript_636	F01_transcript_636	F01_transcript_636	F01_transcript_636	F01_transcript_636
F01_transcript_77609	NA	NA	NA	F01_transcript_77609
F01_transcript_74495	NA	F01_transcript_74495	NA	NA
F01_transcript_163602	NA	F01_transcript_163602	NA	NA
F01_transcript_91849	NA	NA	NA	F01_transcript_91849
F01_transcript_57463	F01_transcript_57463	F01_transcript_57463	F01_transcript_57463	F01_transcript_57463
F01_transcript_54383	F01_transcript_54383	F01_transcript_54383	F01_transcript_54383	F01_transcript_54383
F01_transcript_138278	NA	NA	F01_transcript_138278	NA
F01_transcript_93627	F01_transcript_93627	F01_transcript_93627	NA	NA
F01_transcript_141704	F01_transcript_141704	F01_transcript_141704	F01_transcript_141704	F01_transcript_141704
F01_transcript_105927	NA	NA	NA	F01_transcript_105927
F01_transcript_127851	NA	NA	NA	F01_transcript_127851
F01_transcript_119617	F01_transcript_119617	F01_transcript_119617	F01_transcript_119617	F01_transcript_119617

F01_transcript_67596	NA	F01_transcript_67596	NA	NA
F01_transcript_165860	F01_transcript_165860	F01_transcript_165860	F01_transcript_165860	F01_transcript_165860
F01_transcript_109508	NA	F01_transcript_109508	F01_transcript_109508	NA
F01_transcript_94334	NA	F01_transcript_94334	NA	NA
F01_transcript_152326	NA	NA	NA	F01_transcript_152326
F01_transcript_109163	NA	F01_transcript_109163	NA	NA
F01_transcript_149869	NA	F01_transcript_149869	NA	F01_transcript_149869
F01_transcript_158321	NA	NA	NA	F01_transcript_158321
F01_transcript_65433	NA	F01_transcript_65433	F01_transcript_65433	NA
F01_transcript_80062	NA	F01_transcript_80062	NA	NA
F01_transcript_57325	NA	NA	NA	F01_transcript_57325
F01_transcript_50138	NA	F01_transcript_50138	NA	NA
F01_transcript_130535	NA	F01_transcript_130535	NA	NA
F01_transcript_109653	NA	NA	NA	F01_transcript_109653

F01_transcript_71200	NA	F01_transcript_71200	F01_transcript_71200	F01_transcript_71200
F01_transcript_163288	NA	NA	NA	F01_transcript_163288
F01_transcript_162704	NA	NA	NA	F01_transcript_162704
F01_transcript_151463	NA	NA	NA	F01_transcript_151463
F01_transcript_91384	NA	NA	NA	F01_transcript_91384
F01_transcript_6255	NA	NA	NA	F01_transcript_6255
F01_transcript_122596	NA	NA	NA	F01_transcript_122596
F01_transcript_128534	NA	F01_transcript_128534	NA	F01_transcript_128534
F01_transcript_35720	NA	NA	NA	F01_transcript_35720
F01_transcript_63042	NA	NA	NA	F01_transcript_63042
F01_transcript_123201	F01_transcript_123201	F01_transcript_123201	F01_transcript_123201	F01_transcript_123201
F01_transcript_124384	F01_transcript_124384	F01_transcript_124384	F01_transcript_124384	F01_transcript_124384
F01_transcript_111331	NA	F01_transcript_111331	NA	NA
F01_transcript_129834	NA	F01_transcript_129834	F01_transcript_129834	NA

F01_transcript_125170	NA	F01_transcript_125170	F01_transcript_125170	NA
F01_transcript_92145	NA	F01_transcript_92145	NA	F01_transcript_92145
F01_transcript_137489	F01_transcript_137489	F01_transcript_137489	F01_transcript_137489	F01_transcript_137489
F01_transcript_75321	NA	NA	NA	F01_transcript_75321
F01_transcript_107200	NA	F01_transcript_107200	NA	F01_transcript_107200
F01_transcript_99238	F01_transcript_99238	F01_transcript_99238	F01_transcript_99238	F01_transcript_99238
F01_transcript_77863	NA	NA	NA	F01_transcript_77863
F01_transcript_155817	NA	F01_transcript_155817	NA	NA
F01_transcript_95413	F01_transcript_95413	F01_transcript_95413	F01_transcript_95413	F01_transcript_95413
F01_transcript_91963	NA	NA	NA	F01_transcript_91963
F01_transcript_66615	NA	NA	F01_transcript_66615	NA
F01_transcript_163406	NA	F01_transcript_163406	NA	F01_transcript_163406
F01_transcript_55012	F01_transcript_55012	F01_transcript_55012	F01_transcript_55012	F01_transcript_55012
F01_transcript_162194	NA	F01_transcript_162194	F01_transcript_162194	F01_transcript_162194

F01_transcript_57005	NA	NA	F01_transcript_57005	NA
F01_transcript_134601	F01_transcript_134601	F01_transcript_134601	F01_transcript_134601	NA
F01_transcript_120284	F01_transcript_120284	F01_transcript_120284	NA	F01_transcript_120284
F01_transcript_104713	NA	F01_transcript_104713	NA	F01_transcript_104713
F01_transcript_29721	NA	F01_transcript_29721	NA	NA
F01_transcript_115641	NA	F01_transcript_115641	NA	NA
F01_transcript_46518	NA	NA	NA	F01_transcript_46518
F01_transcript_148036	F01_transcript_148036	F01_transcript_148036	F01_transcript_148036	F01_transcript_148036
F01_transcript_29636	NA	NA	F01_transcript_29636	NA
F01_transcript_98229	NA	F01_transcript_98229	NA	F01_transcript_98229
F01_transcript_75174	F01_transcript_75174	F01_transcript_75174	F01_transcript_75174	F01_transcript_75174
F01_transcript_153638	NA	NA	F01_transcript_153638	NA
F01_transcript_122107	NA	F01_transcript_122107	F01_transcript_122107	NA
F01_transcript_23131	F01_transcript_23131	F01_transcript_23131	F01_transcript_23131	F01_transcript_23131

F01_transcript_49704	F01_transcript_49704	F01_transcript_49704	F01_transcript_49704	F01_transcript_49704
F01_transcript_150258	NA	F01_transcript_150258	NA	NA
F01_transcript_70670	NA	F01_transcript_70670	NA	NA
F01_transcript_70215	NA	F01_transcript_70215	NA	F01_transcript_70215
F01_transcript_16305	NA	NA	NA	F01_transcript_16305
F01_transcript_150805	F01_transcript_150805	F01_transcript_150805	F01_transcript_150805	F01_transcript_150805
F01_transcript_85841	NA	F01_transcript_85841	NA	NA
F01_transcript_136381	NA	NA	NA	F01_transcript_136381
F01_transcript_78866	NA	F01_transcript_78866	NA	NA
F01_transcript_148626	NA	NA	NA	F01_transcript_148626
F01_transcript_101749	F01_transcript_101749	F01_transcript_101749	F01_transcript_101749	F01_transcript_101749
F01_transcript_117653	NA	F01_transcript_117653	NA	NA
F01_transcript_90494	F01_transcript_90494	F01_transcript_90494	F01_transcript_90494	F01_transcript_90494
F01_transcript_86190	F01_transcript_86190	F01_transcript_86190	F01_transcript_86190	NA

F01_transcript_140970	NA	F01_transcript_140970	NA	NA
F01_transcript_82784	NA	NA	F01_transcript_82784	NA
F01_transcript_132331	F01_transcript_132331	F01_transcript_132331	F01_transcript_132331	F01_transcript_132331
F01_transcript_104235	NA	NA	NA	F01_transcript_104235
F01_transcript_150996	NA	F01_transcript_150996	NA	NA
F01_transcript_123888	NA	F01_transcript_123888	F01_transcript_123888	F01_transcript_123888
F01_transcript_161959	F01_transcript_161959	F01_transcript_161959	F01_transcript_161959	F01_transcript_161959
F01_transcript_93991	NA	F01_transcript_93991	NA	F01_transcript_93991
F01_transcript_82611	F01_transcript_82611	F01_transcript_82611	F01_transcript_82611	F01_transcript_82611
F01_transcript_74434	NA	NA	NA	F01_transcript_74434
F01_transcript_107978	F01_transcript_107978	F01_transcript_107978	F01_transcript_107978	F01_transcript_107978
F01_transcript_154373	NA	F01_transcript_154373	F01_transcript_154373	NA
F01_transcript_91764	NA	F01_transcript_91764	NA	F01_transcript_91764
F01_transcript_109502	NA	F01_transcript_109502	NA	NA

F01_transcript_67987	NA	F01_transcript_67987	NA	NA
F01_transcript_89525	NA	F01_transcript_89525	NA	NA
F01_transcript_75389	NA	NA	NA	F01_transcript_75389
F01_transcript_80980	NA	F01_transcript_80980	F01_transcript_80980	F01_transcript_80980
F01_transcript_99547	NA	NA	NA	F01_transcript_99547
F01_transcript_56603	NA	F01_transcript_56603	F01_transcript_56603	NA
F01_transcript_167313	NA	F01_transcript_167313	NA	F01_transcript_167313
F01_transcript_98120	NA	NA	NA	F01_transcript_98120
F01_transcript_139501	F01_transcript_139501	F01_transcript_139501	NA	NA
F01_transcript_81565	NA	NA	F01_transcript_81565	NA
F01_transcript_116002	F01_transcript_116002	F01_transcript_116002	NA	F01_transcript_116002
F01_transcript_92990	NA	NA	NA	F01_transcript_92990
F01_transcript_80072	NA	F01_transcript_80072	NA	NA
F01_transcript_111293	NA	F01_transcript_111293	F01_transcript_111293	F01_transcript_111293

F01_transcript_119981	NA	F01_transcript_119981	NA	NA
F01_transcript_85224	NA	F01_transcript_85224	NA	F01_transcript_85224
F01_transcript_148624	F01_transcript_148624	F01_transcript_148624	F01_transcript_148624	F01_transcript_148624
F01_transcript_148912	NA	F01_transcript_148912	NA	NA
F01_transcript_93280	NA	F01_transcript_93280	F01_transcript_93280	F01_transcript_93280
F01_transcript_150791	F01_transcript_150791	F01_transcript_150791	F01_transcript_150791	F01_transcript_150791
F01_transcript_90111	NA	F01_transcript_90111	NA	NA
F01_transcript_25615	NA	NA	NA	F01_transcript_25615
F01_transcript_12597	NA	NA	NA	F01_transcript_12597
F01_transcript_147806	F01_transcript_147806	F01_transcript_147806	F01_transcript_147806	NA
F01_transcript_66479	NA	F01_transcript_66479	NA	F01_transcript_66479
F01_transcript_120873	F01_transcript_120873	F01_transcript_120873	F01_transcript_120873	F01_transcript_120873
F01_transcript_130737	NA	NA	NA	F01_transcript_130737
F01_transcript_32347	NA	NA	NA	F01_transcript_32347

F01_transcript_129061	NA	F01_transcript_129061	NA	NA
F01_transcript_158556	NA	NA	F01_transcript_158556	F01_transcript_158556
F01_transcript_60194	F01_transcript_60194	F01_transcript_60194	NA	F01_transcript_60194
F01_transcript_109865	F01_transcript_109865	F01_transcript_109865	NA	NA
F01_transcript_87957	NA	NA	NA	F01_transcript_87957
F01_transcript_150173	NA	F01_transcript_150173	NA	F01_transcript_150173
F01_transcript_54317	NA	F01_transcript_54317	NA	F01_transcript_54317
F01_transcript_129613	F01_transcript_129613	F01_transcript_129613	NA	NA
F01_transcript_71698	NA	F01_transcript_71698	NA	F01_transcript_71698
F01_transcript_86734	NA	NA	NA	F01_transcript_86734
F01_transcript_46989	NA	F01_transcript_46989	NA	NA
F01_transcript_74883	NA	F01_transcript_74883	NA	NA
F01_transcript_121480	NA	NA	NA	F01_transcript_121480
F01_transcript_131990	NA	NA	F01_transcript_131990	NA

F01_transcript_437	NA	F01_transcript_437	NA	F01_transcript_437
F01_transcript_15424	NA	F01_transcript_15424	NA	NA
F01_transcript_69464	NA	F01_transcript_69464	NA	NA
F01_transcript_59200	F01_transcript_59200	F01_transcript_59200	F01_transcript_59200	F01_transcript_59200
F01_transcript_56211	NA	NA	NA	F01_transcript_56211
F01_transcript_99819	NA	F01_transcript_99819	NA	NA
F01_transcript_109548	NA	F01_transcript_109548	NA	NA
F01_transcript_153861	NA	F01_transcript_153861	F01_transcript_153861	F01_transcript_153861
F01_transcript_96874	F01_transcript_96874	F01_transcript_96874	F01_transcript_96874	F01_transcript_96874
F01_transcript_104079	NA	NA	NA	F01_transcript_104079
F01_transcript_75420	NA	NA	NA	F01_transcript_75420
F01_transcript_18168	F01_transcript_18168	F01_transcript_18168	F01_transcript_18168	F01_transcript_18168
F01_transcript_127493	NA	F01_transcript_127493	NA	NA
F01_transcript_37785	NA	NA	NA	F01_transcript_37785

F01_transcript_111263	NA	F01_transcript_111263	NA	F01_transcript_111263
F01_transcript_22083	NA	F01_transcript_22083	NA	NA
F01_transcript_131682	F01_transcript_131682	F01_transcript_131682	F01_transcript_131682	F01_transcript_131682
F01_transcript_107257	NA	F01_transcript_107257	NA	NA
F01_transcript_83955	NA	NA	NA	F01_transcript_83955
F01_transcript_127516	F01_transcript_127516	F01_transcript_127516	F01_transcript_127516	F01_transcript_127516
F01_transcript_123262	F01_transcript_123262	F01_transcript_123262	NA	NA
F01_transcript_53296	NA	NA	NA	F01_transcript_53296
F01_transcript_113633	F01_transcript_113633	F01_transcript_113633	F01_transcript_113633	F01_transcript_113633
F01_transcript_107472	F01_transcript_107472	F01_transcript_107472	F01_transcript_107472	F01_transcript_107472
F01_transcript_14810	NA	NA	NA	F01_transcript_14810
F01_transcript_92334	NA	F01_transcript_92334	F01_transcript_92334	F01_transcript_92334
F01_transcript_123332	NA	NA	NA	F01_transcript_123332
F01_transcript_161950	F01_transcript_161950	F01_transcript_161950	NA	NA

F01_transcript_141191	F01_transcript_141191	F01_transcript_141191	F01_transcript_141191	F01_transcript_141191
F01_transcript_92746	NA	F01_transcript_92746	NA	F01_transcript_92746
F01_transcript_88046	NA	NA	NA	F01_transcript_88046
F01_transcript_78450	NA	NA	NA	F01_transcript_78450
F01_transcript_31599	NA	NA	NA	F01_transcript_31599
F01_transcript_71540	NA	F01_transcript_71540	NA	NA
F01_transcript_100362	NA	F01_transcript_100362	NA	F01_transcript_100362
F01_transcript_106634	NA	NA	NA	F01_transcript_106634
F01_transcript_91134	NA	F01_transcript_91134	NA	NA
F01_transcript_112065	NA	NA	NA	F01_transcript_112065
F01_transcript_142243	NA	F01_transcript_142243	F01_transcript_142243	NA
F01_transcript_162483	NA	NA	NA	F01_transcript_162483
F01_transcript_70110	NA	F01_transcript_70110	NA	NA
F01_transcript_76501	NA	F01_transcript_76501	NA	NA

F01_transcript_112783	NA	F01_transcript_112783	NA	NA
F01_transcript_64334	F01_transcript_64334	F01_transcript_64334	F01_transcript_64334	F01_transcript_64334
F01_transcript_98750	NA	F01_transcript_98750	NA	NA
F01_transcript_114736	F01_transcript_114736	F01_transcript_114736	F01_transcript_114736	F01_transcript_114736
F01_transcript_142445	NA	F01_transcript_142445	NA	NA
F01_transcript_79166	NA	F01_transcript_79166	F01_transcript_79166	NA
F01_transcript_67539	NA	NA	NA	F01_transcript_67539
F01_transcript_127348	F01_transcript_127348	F01_transcript_127348	NA	F01_transcript_127348
F01_transcript_63766	NA	F01_transcript_63766	NA	F01_transcript_63766
F01_transcript_91935	F01_transcript_91935	F01_transcript_91935	NA	NA
F01_transcript_127531	F01_transcript_127531	F01_transcript_127531	F01_transcript_127531	F01_transcript_127531
F01_transcript_59522	NA	F01_transcript_59522	F01_transcript_59522	NA
F01_transcript_17796	NA	NA	NA	F01_transcript_17796
F01_transcript_36066	NA	F01_transcript_36066	NA	NA

F01_transcript_54109	NA	NA	NA	F01_transcript_54109
F01_transcript_72360	NA	NA	NA	F01_transcript_72360
F01_transcript_52287	F01_transcript_52287	F01_transcript_52287	F01_transcript_52287	F01_transcript_52287
F01_transcript_155990	F01_transcript_155990	F01_transcript_155990	NA	F01_transcript_155990
F01_transcript_137726	NA	F01_transcript_137726	NA	NA
F01_transcript_51751	NA	NA	NA	F01_transcript_51751
F01_transcript_16554	NA	F01_transcript_16554	NA	NA
F01_transcript_110077	NA	F01_transcript_110077	NA	F01_transcript_110077
F01_transcript_95622	NA	F01_transcript_95622	NA	F01_transcript_95622
F01_transcript_116330	NA	F01_transcript_116330	NA	F01_transcript_116330
F01_transcript_85942	NA	NA	NA	F01_transcript_85942
F01_transcript_51112	F01_transcript_51112	F01_transcript_51112	NA	NA
F01_transcript_99834	NA	NA	NA	F01_transcript_99834
F01_transcript_65159	NA	NA	F01_transcript_65159	NA

F01_transcript_64449	F01_transcript_64449	F01_transcript_64449	F01_transcript_64449	F01_transcript_64449
F01_transcript_73723	NA	F01_transcript_73723	NA	F01_transcript_73723
F01_transcript_103897	NA	F01_transcript_103897	NA	NA
F01_transcript_84197	NA	F01_transcript_84197	F01_transcript_84197	F01_transcript_84197
F01_transcript_138221	NA	NA	NA	F01_transcript_138221
F01_transcript_142414	NA	F01_transcript_142414	NA	NA
F01_transcript_115252	NA	F01_transcript_115252	NA	F01_transcript_115252
F01_transcript_3700	NA	NA	NA	F01_transcript_3700
F01_transcript_16074	NA	F01_transcript_16074	NA	NA
F01_transcript_150679	NA	NA	NA	F01_transcript_150679
F01_transcript_61053	NA	F01_transcript_61053	NA	F01_transcript_61053
F01_transcript_101960	NA	NA	NA	F01_transcript_101960
F01_transcript_34677	NA	F01_transcript_34677	NA	F01_transcript_34677
F01_transcript_163599	F01_transcript_163599	F01_transcript_163599	F01_transcript_163599	F01_transcript_163599

F01_transcript_103313	NA	F01_transcript_103313	NA	F01_transcript_103313
F01_transcript_76037	NA	NA	NA	F01_transcript_76037
F01_transcript_152576	F01_transcript_152576	F01_transcript_152576	F01_transcript_152576	F01_transcript_152576
F01_transcript_125590	NA	NA	NA	F01_transcript_125590
F01_transcript_142685	NA	F01_transcript_142685	NA	F01_transcript_142685
F01_transcript_72796	NA	F01_transcript_72796	NA	F01_transcript_72796
F01_transcript_114782	F01_transcript_114782	F01_transcript_114782	F01_transcript_114782	NA
F01_transcript_114823	NA	F01_transcript_114823	NA	NA
F01_transcript_80171	NA	NA	NA	F01_transcript_80171
F01_transcript_77373	NA	F01_transcript_77373	NA	NA
F01_transcript_105200	NA	F01_transcript_105200	NA	NA
F01_transcript_85641	F01_transcript_85641	F01_transcript_85641	F01_transcript_85641	F01_transcript_85641
F01_transcript_108750	F01_transcript_108750	F01_transcript_108750	F01_transcript_108750	F01_transcript_108750
F01_transcript_71116	NA	NA	F01_transcript_71116	F01_transcript_71116

F01_transcript_114553	NA	F01_transcript_114553	NA	NA
F01_transcript_54725	F01_transcript_54725	F01_transcript_54725	NA	NA
F01_transcript_83567	NA	NA	NA	F01_transcript_83567
F01_transcript_48865	NA	F01_transcript_48865	NA	NA
F01_transcript_57851	NA	F01_transcript_57851	NA	NA
F01_transcript_163426	NA	NA	NA	F01_transcript_163426
F01_transcript_80079	NA	F01_transcript_80079	NA	F01_transcript_80079
F01_transcript_21492	F01_transcript_21492	NA	NA	F01_transcript_21492
F01_transcript_165165	NA	NA	NA	F01_transcript_165165
F01_transcript_128887	NA	F01_transcript_128887	NA	NA
F01_transcript_54160	NA	F01_transcript_54160	NA	NA
F01_transcript_18721	NA	NA	F01_transcript_18721	F01_transcript_18721
F01_transcript_64330	NA	F01_transcript_64330	NA	NA
F01_transcript_51106	F01_transcript_51106	F01_transcript_51106	F01_transcript_51106	F01_transcript_51106

F01_transcript_119942	NA	F01_transcript_119942	F01_transcript_119942	F01_transcript_119942
F01_transcript_46341	NA	NA	NA	F01_transcript_46341
F01_transcript_92977	F01_transcript_92977	F01_transcript_92977	F01_transcript_92977	F01_transcript_92977
F01_transcript_154518	NA	F01_transcript_154518	F01_transcript_154518	F01_transcript_154518
F01_transcript_135655	NA	NA	F01_transcript_135655	F01_transcript_135655
F01_transcript_122560	F01_transcript_122560	F01_transcript_122560	F01_transcript_122560	F01_transcript_122560
F01_transcript_126695	NA	NA	NA	F01_transcript_126695
F01_transcript_131351	F01_transcript_131351	NA	F01_transcript_131351	F01_transcript_131351
F01_transcript_79733	NA	NA	NA	F01_transcript_79733
F01_transcript_99419	NA	NA	NA	F01_transcript_99419
F01_transcript_108385	NA	F01_transcript_108385	NA	NA
F01_transcript_44205	NA	F01_transcript_44205	NA	NA
F01_transcript_75585	F01_transcript_75585	F01_transcript_75585	NA	NA
F01_transcript_69814	NA	F01_transcript_69814	F01_transcript_69814	F01_transcript_69814

F01_transcript_106833	NA	F01_transcript_106833	NA	F01_transcript_106833
F01_transcript_48899	NA	NA	NA	F01_transcript_48899
F01_transcript_153939	NA	F01_transcript_153939	NA	F01_transcript_153939
F01_transcript_100310	NA	NA	NA	F01_transcript_100310
F01_transcript_151044	NA	NA	F01_transcript_151044	NA
F01_transcript_84645	NA	F01_transcript_84645	NA	NA
F01_transcript_96318	NA	NA	NA	F01_transcript_96318
F01_transcript_12670	F01_transcript_12670	F01_transcript_12670	NA	NA
F01_transcript_57019	F01_transcript_57019	F01_transcript_57019	F01_transcript_57019	NA
F01_transcript_133439	NA	F01_transcript_133439	NA	NA
F01_transcript_150579	F01_transcript_150579	F01_transcript_150579	F01_transcript_150579	F01_transcript_150579
F01_transcript_75117	NA	F01_transcript_75117	NA	NA
F01_transcript_149563	F01_transcript_149563	F01_transcript_149563	F01_transcript_149563	F01_transcript_149563
F01_transcript_36871	NA	NA	NA	F01_transcript_36871

F01_transcript_68913	NA	F01_transcript_68913	NA	NA
F01_transcript_165097	NA	F01_transcript_165097	NA	NA
F01_transcript_78805	NA	NA	NA	F01_transcript_78805
F01_transcript_98310	NA	NA	NA	F01_transcript_98310
F01_transcript_100350	NA	NA	NA	F01_transcript_100350
F01_transcript_151686	F01_transcript_151686	F01_transcript_151686	NA	NA
F01_transcript_24625	NA	NA	NA	F01_transcript_24625
F01_transcript_120994	NA	NA	NA	F01_transcript_120994
F01_transcript_159124	NA	F01_transcript_159124	NA	NA
F01_transcript_79937	F01_transcript_79937	F01_transcript_79937	F01_transcript_79937	F01_transcript_79937
F01_transcript_53655	NA	F01_transcript_53655	F01_transcript_53655	F01_transcript_53655
F01_transcript_145375	NA	NA	NA	F01_transcript_145375
F01_transcript_75249	NA	NA	NA	F01_transcript_75249
F01_transcript_47694	NA	NA	NA	F01_transcript_47694

F01_transcript_54188	NA	F01_transcript_54188	F01_transcript_54188	NA
F01_transcript_78303	NA	NA	NA	F01_transcript_78303
F01_transcript_11718	F01_transcript_11718	F01_transcript_11718	F01_transcript_11718	NA
F01_transcript_69021	F01_transcript_69021	F01_transcript_69021	NA	F01_transcript_69021
F01_transcript_53354	F01_transcript_53354	F01_transcript_53354	F01_transcript_53354	F01_transcript_53354
F01_transcript_16302	NA	NA	NA	F01_transcript_16302
F01_transcript_148186	F01_transcript_148186	F01_transcript_148186	NA	F01_transcript_148186
F01_transcript_72790	F01_transcript_72790	F01_transcript_72790	F01_transcript_72790	F01_transcript_72790
F01_transcript_160739	NA	F01_transcript_160739	NA	NA
F01_transcript_131692	NA	F01_transcript_131692	NA	F01_transcript_131692
F01_transcript_139377	NA	F01_transcript_139377	F01_transcript_139377	F01_transcript_139377
F01_transcript_106891	F01_transcript_106891	F01_transcript_106891	F01_transcript_106891	F01_transcript_106891
F01_transcript_156590	NA	F01_transcript_156590	NA	NA
F01_transcript_29141	NA	NA	NA	F01_transcript_29141

F01_transcript_111269	NA	F01_transcript_111269	F01_transcript_111269	NA
F01_transcript_94132	NA	F01_transcript_94132	NA	NA
F01_transcript_18489	NA	NA	NA	F01_transcript_18489
F01_transcript_125388	F01_transcript_125388	F01_transcript_125388	NA	NA
F01_transcript_75072	F01_transcript_75072	F01_transcript_75072	NA	NA
F01_transcript_54315	F01_transcript_54315	F01_transcript_54315	F01_transcript_54315	F01_transcript_54315
F01_transcript_115126	NA	NA	NA	F01_transcript_115126
F01_transcript_141729	NA	NA	NA	F01_transcript_141729
F01_transcript_1681	NA	NA	NA	F01_transcript_1681
F01_transcript_3030	F01_transcript_3030	F01_transcript_3030	NA	F01_transcript_3030
F01_transcript_60970	F01_transcript_60970	F01_transcript_60970	F01_transcript_60970	F01_transcript_60970
F01_transcript_119202	NA	F01_transcript_119202	NA	F01_transcript_119202
F01_transcript_78791	F01_transcript_78791	F01_transcript_78791	NA	F01_transcript_78791
F01_transcript_142878	F01_transcript_142878	F01_transcript_142878	F01_transcript_142878	F01_transcript_142878

F01_transcript_81791	NA	F01_transcript_81791	NA	F01_transcript_81791
F01_transcript_66620	NA	NA	NA	F01_transcript_66620
F01_transcript_42875	NA	F01_transcript_42875	NA	F01_transcript_42875
F01_transcript_131120	NA	F01_transcript_131120	NA	NA
F01_transcript_93413	NA	NA	NA	F01_transcript_93413
F01_transcript_108525	NA	F01_transcript_108525	NA	F01_transcript_108525
F01_transcript_117167	F01_transcript_117167	F01_transcript_117167	NA	F01_transcript_117167
F01_transcript_166050	NA	F01_transcript_166050	NA	F01_transcript_166050
F01_transcript_51026	F01_transcript_51026	F01_transcript_51026	F01_transcript_51026	F01_transcript_51026
F01_transcript_136840	NA	NA	NA	F01_transcript_136840
F01_transcript_125981	NA	F01_transcript_125981	F01_transcript_125981	NA
F01_transcript_165711	NA	F01_transcript_165711	NA	NA
F01_transcript_134463	NA	F01_transcript_134463	NA	NA
F01_transcript_119925	NA	NA	F01_transcript_119925	NA

F01_transcript_56833	NA	NA	F01_transcript_56833	NA
F01_transcript_90012	NA	F01_transcript_90012	NA	F01_transcript_90012
F01_transcript_72797	F01_transcript_72797	F01_transcript_72797	NA	F01_transcript_72797
F01_transcript_76065	NA	NA	NA	F01_transcript_76065
F01_transcript_128721	F01_transcript_128721	F01_transcript_128721	NA	F01_transcript_128721
F01_transcript_32316	NA	NA	NA	F01_transcript_32316
F01_transcript_60316	F01_transcript_60316	F01_transcript_60316	F01_transcript_60316	F01_transcript_60316
F01_transcript_58786	NA	F01_transcript_58786	NA	NA
F01_transcript_161427	NA	NA	NA	F01_transcript_161427
F01_transcript_100553	NA	NA	NA	F01_transcript_100553
F01_transcript_31047	F01_transcript_31047	F01_transcript_31047	F01_transcript_31047	F01_transcript_31047
F01_transcript_105831	NA	F01_transcript_105831	F01_transcript_105831	F01_transcript_105831
F01_transcript_148802	NA	NA	NA	F01_transcript_148802
F01_transcript_112586	NA	NA	F01_transcript_112586	F01_transcript_112586

F01_transcript_86229	F01_transcript_86229	F01_transcript_86229	F01_transcript_86229	F01_transcript_86229
F01_transcript_54567	F01_transcript_54567	F01_transcript_54567	NA	F01_transcript_54567
F01_transcript_61942	F01_transcript_61942	F01_transcript_61942	F01_transcript_61942	F01_transcript_61942
F01_transcript_65412	NA	NA	F01_transcript_65412	NA
F01_transcript_21093	NA	NA	NA	F01_transcript_21093
F01_transcript_157496	F01_transcript_157496	F01_transcript_157496	F01_transcript_157496	F01_transcript_157496
F01_transcript_45959	NA	NA	NA	F01_transcript_45959
F01_transcript_82127	NA	NA	NA	F01_transcript_82127
F01_transcript_86639	NA	F01_transcript_86639	NA	NA
F01_transcript_51847	NA	NA	NA	F01_transcript_51847
F01_transcript_144842	NA	NA	NA	F01_transcript_144842
F01_transcript_60488	NA	NA	F01_transcript_60488	NA
F01_transcript_85961	NA	F01_transcript_85961	NA	F01_transcript_85961
F01_transcript_56785	F01_transcript_56785	F01_transcript_56785	NA	F01_transcript_56785

F01_transcript_77503	F01_transcript_77503	F01_transcript_77503	F01_transcript_77503	F01_transcript_77503
F01_transcript_112938	NA	F01_transcript_112938	NA	NA
F01_transcript_151373	NA	NA	NA	F01_transcript_151373
F01_transcript_116613	F01_transcript_116613	F01_transcript_116613	F01_transcript_116613	F01_transcript_116613
F01_transcript_141839	NA	F01_transcript_141839	NA	NA
F01_transcript_47298	NA	NA	NA	F01_transcript_47298
F01_transcript_113688	NA	NA	NA	F01_transcript_113688
F01_transcript_5857	F01_transcript_5857	F01_transcript_5857	NA	F01_transcript_5857
F01_transcript_124200	NA	NA	NA	F01_transcript_124200
F01_transcript_121534	F01_transcript_121534	F01_transcript_121534	F01_transcript_121534	F01_transcript_121534
F01_transcript_50910	F01_transcript_50910	F01_transcript_50910	NA	NA
F01_transcript_84990	F01_transcript_84990	F01_transcript_84990	F01_transcript_84990	NA
F01_transcript_103980	F01_transcript_103980	F01_transcript_103980	NA	F01_transcript_103980
F01_transcript_164864	F01_transcript_164864	F01_transcript_164864	F01_transcript_164864	F01_transcript_164864

F01_transcript_146900	NA	NA	NA	F01_transcript_146900
F01_transcript_155497	NA	NA	NA	F01_transcript_155497
F01_transcript_162268	NA	F01_transcript_162268	F01_transcript_162268	F01_transcript_162268
F01_transcript_82609	NA	F01_transcript_82609	NA	NA
F01_transcript_81253	NA	F01_transcript_81253	NA	NA
F01_transcript_97684	NA	F01_transcript_97684	NA	NA
F01_transcript_73395	NA	F01_transcript_73395	NA	F01_transcript_73395
F01_transcript_91643	F01_transcript_91643	F01_transcript_91643	F01_transcript_91643	F01_transcript_91643
F01_transcript_112581	F01_transcript_112581	F01_transcript_112581	NA	NA
F01_transcript_78900	NA	NA	NA	F01_transcript_78900
F01_transcript_98410	NA	NA	NA	F01_transcript_98410
F01_transcript_103366	F01_transcript_103366	F01_transcript_103366	F01_transcript_103366	F01_transcript_103366
F01_transcript_57827	F01_transcript_57827	NA	NA	NA
F01_transcript_58097	F01_transcript_58097	F01_transcript_58097	F01_transcript_58097	F01_transcript_58097

F01_transcript_76785	NA	F01_transcript_76785	NA	NA
F01_transcript_5661	NA	F01_transcript_5661	F01_transcript_5661	F01_transcript_5661
F01_transcript_152754	NA	NA	NA	F01_transcript_152754
F01_transcript_106051	F01_transcript_106051	F01_transcript_106051	F01_transcript_106051	F01_transcript_106051
F01_transcript_92302	F01_transcript_92302	F01_transcript_92302	NA	F01_transcript_92302
F01_transcript_126212	NA	F01_transcript_126212	NA	F01_transcript_126212
F01_transcript_32214	NA	NA	NA	F01_transcript_32214
F01_transcript_167551	NA	F01_transcript_167551	NA	NA
F01_transcript_52641	NA	F01_transcript_52641	NA	NA
F01_transcript_87116	NA	F01_transcript_87116	NA	NA
F01_transcript_125456	NA	NA	NA	F01_transcript_125456
F01_transcript_89703	F01_transcript_89703	F01_transcript_89703	F01_transcript_89703	NA
F01_transcript_72561	NA	F01_transcript_72561	NA	F01_transcript_72561
F01_transcript_91079	F01_transcript_91079	F01_transcript_91079	F01_transcript_91079	F01_transcript_91079

F01_transcript_165607	NA	NA	NA	F01_transcript_165607
F01_transcript_49940	NA	NA	F01_transcript_49940	NA
F01_transcript_63105	NA	F01_transcript_63105	F01_transcript_63105	NA
F01_transcript_78458	F01_transcript_78458	F01_transcript_78458	NA	F01_transcript_78458
F01_transcript_49114	NA	F01_transcript_49114	NA	NA
F01_transcript_85997	NA	F01_transcript_85997	NA	NA
F01_transcript_166228	NA	F01_transcript_166228	NA	F01_transcript_166228
F01_transcript_153732	NA	NA	NA	F01_transcript_153732
F01_transcript_159885	NA	NA	NA	F01_transcript_159885
F01_transcript_94062	F01_transcript_94062	F01_transcript_94062	F01_transcript_94062	F01_transcript_94062
F01_transcript_119842	NA	F01_transcript_119842	F01_transcript_119842	F01_transcript_119842
F01_transcript_91993	NA	F01_transcript_91993	NA	NA
F01_transcript_94144	NA	NA	NA	F01_transcript_94144
F01_transcript_11875	NA	F01_transcript_11875	NA	NA

F01_transcript_104334	NA	F01_transcript_104334	NA	F01_transcript_104334
F01_transcript_89966	NA	F01_transcript_89966	NA	F01_transcript_89966
F01_transcript_2710	NA	F01_transcript_2710	NA	NA
F01_transcript_70196	F01_transcript_70196	F01_transcript_70196	F01_transcript_70196	F01_transcript_70196
F01_transcript_100566	F01_transcript_100566	F01_transcript_100566	F01_transcript_100566	NA
F01_transcript_148045	NA	NA	NA	F01_transcript_148045
F01_transcript_51355	NA	NA	F01_transcript_51355	NA
F01_transcript_63845	NA	NA	NA	F01_transcript_63845
F01_transcript_65508	NA	F01_transcript_65508	NA	NA
F01_transcript_162753	NA	NA	NA	F01_transcript_162753
F01_transcript_75714	F01_transcript_75714	F01_transcript_75714	F01_transcript_75714	F01_transcript_75714
F01_transcript_117695	NA	NA	NA	F01_transcript_117695
F01_transcript_75669	F01_transcript_75669	F01_transcript_75669	F01_transcript_75669	NA
F01_transcript_19197	NA	F01_transcript_19197	F01_transcript_19197	NA

F01_transcript_162606	NA	F01_transcript_162606	NA	NA
F01_transcript_121245	NA	F01_transcript_121245	NA	F01_transcript_121245
F01_transcript_89741	NA	F01_transcript_89741	NA	NA
F01_transcript_104074	NA	F01_transcript_104074	NA	F01_transcript_104074
F01_transcript_48912	NA	NA	F01_transcript_48912	F01_transcript_48912
F01_transcript_166510	NA	F01_transcript_166510	F01_transcript_166510	NA
F01_transcript_87643	NA	NA	NA	F01_transcript_87643
F01_transcript_79980	F01_transcript_79980	NA	NA	F01_transcript_79980
F01_transcript_118574	NA	F01_transcript_118574	NA	NA
F01_transcript_102838	F01_transcript_102838	F01_transcript_102838	F01_transcript_102838	NA
F01_transcript_142138	NA	NA	NA	F01_transcript_142138
F01_transcript_13285	NA	F01_transcript_13285	NA	NA
F01_transcript_109419	F01_transcript_109419	F01_transcript_109419	NA	F01_transcript_109419
F01_transcript_113706	NA	F01_transcript_113706	NA	NA

F01_transcript_101480	NA	NA	NA	F01_transcript_101480
F01_transcript_84229	NA	F01_transcript_84229	F01_transcript_84229	NA
F01_transcript_61387	NA	NA	NA	F01_transcript_61387
F01_transcript_85701	F01_transcript_85701	F01_transcript_85701	F01_transcript_85701	F01_transcript_85701
F01_transcript_60159	NA	NA	NA	F01_transcript_60159
F01_transcript_83081	NA	NA	NA	F01_transcript_83081
F01_transcript_93402	NA	NA	F01_transcript_93402	NA
F01_transcript_145033	F01_transcript_145033	F01_transcript_145033	NA	NA
F01_transcript_141292	NA	NA	F01_transcript_141292	F01_transcript_141292
F01_transcript_115410	NA	F01_transcript_115410	NA	NA
F01_transcript_89050	NA	NA	NA	F01_transcript_89050
F01_transcript_85041	NA	NA	NA	F01_transcript_85041
F01_transcript_62680	NA	F01_transcript_62680	NA	NA
F01_transcript_98364	NA	F01_transcript_98364	NA	NA

F01_transcript_125054	NA	NA	NA	F01_transcript_125054
F01_transcript_103232	NA	NA	F01_transcript_103232	F01_transcript_103232
F01_transcript_116100	F01_transcript_116100	F01_transcript_116100	NA	F01_transcript_116100
F01_transcript_47595	NA	NA	F01_transcript_47595	F01_transcript_47595
F01_transcript_160778	F01_transcript_160778	F01_transcript_160778	F01_transcript_160778	NA
F01_transcript_159604	NA	NA	NA	F01_transcript_159604
F01_transcript_71915	NA	F01_transcript_71915	NA	F01_transcript_71915
F01_transcript_95474	NA	F01_transcript_95474	NA	NA
F01_transcript_93948	F01_transcript_93948	F01_transcript_93948	F01_transcript_93948	F01_transcript_93948
F01_transcript_36299	F01_transcript_36299	F01_transcript_36299	F01_transcript_36299	F01_transcript_36299
F01_transcript_93209	NA	F01_transcript_93209	NA	NA
F01_transcript_127980	NA	F01_transcript_127980	NA	NA
F01_transcript_58838	NA	F01_transcript_58838	NA	F01_transcript_58838
F01_transcript_150311	F01_transcript_150311	F01_transcript_150311	F01_transcript_150311	F01_transcript_150311

F01_transcript_119464	F01_transcript_119464	F01_transcript_119464	F01_transcript_119464	F01_transcript_119464
F01_transcript_95486	NA	NA	NA	F01_transcript_95486
F01_transcript_128421	NA	NA	NA	F01_transcript_128421
F01_transcript_135874	NA	F01_transcript_135874	F01_transcript_135874	F01_transcript_135874
F01_transcript_90673	F01_transcript_90673	F01_transcript_90673	F01_transcript_90673	F01_transcript_90673
F01_transcript_66344	NA	F01_transcript_66344	NA	NA
F01_transcript_49825	NA	F01_transcript_49825	NA	F01_transcript_49825
F01_transcript_132973	NA	F01_transcript_132973	F01_transcript_132973	F01_transcript_132973
F01_transcript_90531	NA	F01_transcript_90531	NA	NA
F01_transcript_106829	NA	F01_transcript_106829	NA	NA
F01_transcript_74097	F01_transcript_74097	F01_transcript_74097	F01_transcript_74097	F01_transcript_74097
F01_transcript_158479	NA	NA	NA	F01_transcript_158479
F01_transcript_107147	F01_transcript_107147	F01_transcript_107147	F01_transcript_107147	F01_transcript_107147
F01_transcript_127759	NA	NA	NA	F01_transcript_127759

F01_transcript_123089	NA	F01_transcript_123089	F01_transcript_123089	F01_transcript_123089
F01_transcript_150188	NA	NA	NA	F01_transcript_150188
F01_transcript_20802	NA	NA	NA	F01_transcript_20802
F01_transcript_166919	F01_transcript_166919	F01_transcript_166919	F01_transcript_166919	F01_transcript_166919
F01_transcript_92762	NA	F01_transcript_92762	NA	NA
F01_transcript_76864	NA	F01_transcript_76864	NA	NA
F01_transcript_99211	F01_transcript_99211	F01_transcript_99211	F01_transcript_99211	F01_transcript_99211
F01_transcript_103840	F01_transcript_103840	F01_transcript_103840	F01_transcript_103840	F01_transcript_103840
F01_transcript_95990	F01_transcript_95990	F01_transcript_95990	F01_transcript_95990	F01_transcript_95990
F01_transcript_121905	NA	F01_transcript_121905	NA	F01_transcript_121905
F01_transcript_20382	NA	NA	NA	F01_transcript_20382
F01_transcript_154930	NA	NA	NA	F01_transcript_154930
F01_transcript_140563	F01_transcript_140563	F01_transcript_140563	F01_transcript_140563	F01_transcript_140563
F01_transcript_130683	NA	NA	NA	F01_transcript_130683

F01_transcript_79424	NA	F01_transcript_79424	NA	F01_transcript_79424
F01_transcript_131060	NA	NA	NA	F01_transcript_131060
F01_transcript_138189	NA	F01_transcript_138189	NA	NA
F01_transcript_127882	NA	F01_transcript_127882	NA	F01_transcript_127882
F01_transcript_122517	NA	F01_transcript_122517	NA	NA
F01_transcript_160892	NA	F01_transcript_160892	F01_transcript_160892	NA
F01_transcript_97306	NA	F01_transcript_97306	NA	NA
F01_transcript_103559	F01_transcript_103559	F01_transcript_103559	F01_transcript_103559	F01_transcript_103559
F01_transcript_7951	NA	NA	NA	F01_transcript_7951
F01_transcript_117815	NA	F01_transcript_117815	NA	NA
F01_transcript_146245	F01_transcript_146245	F01_transcript_146245	NA	F01_transcript_146245
F01_transcript_101179	F01_transcript_101179	F01_transcript_101179	F01_transcript_101179	F01_transcript_101179
F01_transcript_108432	NA	NA	NA	F01_transcript_108432
F01_transcript_61774	NA	F01_transcript_61774	F01_transcript_61774	F01_transcript_61774

F01_transcript_125765	NA	NA	NA	F01_transcript_125765
F01_transcript_100757	F01_transcript_100757	F01_transcript_100757	NA	F01_transcript_100757
F01_transcript_154874	NA	F01_transcript_154874	NA	NA
F01_transcript_114351	NA	NA	NA	F01_transcript_114351
F01_transcript_110776	F01_transcript_110776	F01_transcript_110776	F01_transcript_110776	F01_transcript_110776
F01_transcript_124147	F01_transcript_124147	F01_transcript_124147	NA	NA
F01_transcript_133933	NA	F01_transcript_133933	NA	NA
F01_transcript_150282	NA	NA	NA	F01_transcript_150282
F01_transcript_106417	NA	NA	NA	F01_transcript_106417
F01_transcript_131517	NA	NA	NA	F01_transcript_131517
F01_transcript_17500	NA	F01_transcript_17500	NA	NA
F01_transcript_98466	NA	F01_transcript_98466	NA	NA
F01_transcript_106979	F01_transcript_106979	F01_transcript_106979	F01_transcript_106979	F01_transcript_106979
F01_transcript_3624	NA	NA	NA	F01_transcript_3624

F01_transcript_46664	NA	NA	F01_transcript_46664	F01_transcript_46664
F01_transcript_78087	NA	NA	NA	F01_transcript_78087
F01_transcript_83841	NA	NA	NA	F01_transcript_83841
F01_transcript_23954	NA	NA	NA	F01_transcript_23954
F01_transcript_7015	F01_transcript_7015	F01_transcript_7015	F01_transcript_7015	F01_transcript_7015
F01_transcript_93160	F01_transcript_93160	F01_transcript_93160	F01_transcript_93160	F01_transcript_93160
F01_transcript_56336	NA	NA	F01_transcript_56336	F01_transcript_56336
F01_transcript_152188	F01_transcript_152188	NA	NA	NA
F01_transcript_47229	NA	NA	NA	F01_transcript_47229
F01_transcript_76088	NA	NA	F01_transcript_76088	NA
F01_transcript_87023	F01_transcript_87023	F01_transcript_87023	NA	NA
F01_transcript_161426	NA	F01_transcript_161426	NA	NA
F01_transcript_142713	NA	NA	NA	F01_transcript_142713
F01_transcript_51731	NA	F01_transcript_51731	NA	NA

F01_transcript_83157	NA	F01_transcript_83157	NA	NA
F01_transcript_43894	NA	NA	NA	F01_transcript_43894
F01_transcript_104903	NA	NA	NA	F01_transcript_104903
F01_transcript_60249	NA	F01_transcript_60249	F01_transcript_60249	NA
F01_transcript_115308	NA	NA	F01_transcript_115308	NA
F01_transcript_49783	NA	F01_transcript_49783	NA	NA
F01_transcript_129776	NA	NA	F01_transcript_129776	F01_transcript_129776
F01_transcript_112951	NA	F01_transcript_112951	NA	F01_transcript_112951
F01_transcript_147775	NA	F01_transcript_147775	NA	NA
F01_transcript_25311	NA	NA	NA	F01_transcript_25311
F01_transcript_144281	F01_transcript_144281	F01_transcript_144281	F01_transcript_144281	F01_transcript_144281
F01_transcript_151646	NA	F01_transcript_151646	NA	F01_transcript_151646
F01_transcript_74636	NA	NA	NA	F01_transcript_74636
F01_transcript_147819	NA	F01_transcript_147819	NA	NA

F01_transcript_128707	NA	NA	NA	F01_transcript_128707
F01_transcript_20212	F01_transcript_20212	F01_transcript_20212	F01_transcript_20212	F01_transcript_20212
F01_transcript_151355	F01_transcript_151355	F01_transcript_151355	F01_transcript_151355	F01_transcript_151355
F01_transcript_104952	F01_transcript_104952	F01_transcript_104952	F01_transcript_104952	F01_transcript_104952
F01_transcript_50268	NA	NA	NA	F01_transcript_50268
F01_transcript_81272	NA	NA	NA	F01_transcript_81272
F01_transcript_153482	NA	NA	NA	F01_transcript_153482
F01_transcript_84462	NA	F01_transcript_84462	NA	NA
F01_transcript_128478	NA	F01_transcript_128478	NA	F01_transcript_128478
F01_transcript_142144	NA	NA	F01_transcript_142144	F01_transcript_142144
F01_transcript_161270	NA	F01_transcript_161270	NA	NA
F01_transcript_131133	NA	F01_transcript_131133	F01_transcript_131133	F01_transcript_131133
F01_transcript_110018	F01_transcript_110018	F01_transcript_110018	F01_transcript_110018	F01_transcript_110018
F01_transcript_57738	NA	F01_transcript_57738	NA	NA

F01_transcript_63137	NA	F01_transcript_63137	NA	NA
F01_transcript_44218	NA	F01_transcript_44218	F01_transcript_44218	F01_transcript_44218
F01_transcript_119386	NA	F01_transcript_119386	NA	NA
F01_transcript_101781	NA	F01_transcript_101781	NA	F01_transcript_101781
F01_transcript_80769	NA	F01_transcript_80769	NA	NA
F01_transcript_95898	NA	NA	NA	F01_transcript_95898
F01_transcript_132118	NA	F01_transcript_132118	NA	NA
F01_transcript_151491	NA	F01_transcript_151491	NA	F01_transcript_151491
F01_transcript_110361	F01_transcript_110361	F01_transcript_110361	NA	NA
F01_transcript_83584	F01_transcript_83584	F01_transcript_83584	NA	NA
F01_transcript_116521	NA	F01_transcript_116521	NA	NA
F01_transcript_157313	F01_transcript_157313	F01_transcript_157313	F01_transcript_157313	F01_transcript_157313
F01_transcript_162373	F01_transcript_162373	F01_transcript_162373	F01_transcript_162373	F01_transcript_162373
F01_transcript_68680	F01_transcript_68680	F01_transcript_68680	F01_transcript_68680	F01_transcript_68680

F01_transcript_73367	NA	F01_transcript_73367	NA	F01_transcript_73367
F01_transcript_57414	NA	NA	NA	F01_transcript_57414
F01_transcript_82763	NA	F01_transcript_82763	NA	NA
F01_transcript_14652	F01_transcript_14652	F01_transcript_14652	F01_transcript_14652	F01_transcript_14652
F01_transcript_113147	F01_transcript_113147	F01_transcript_113147	F01_transcript_113147	F01_transcript_113147
F01_transcript_99094	NA	F01_transcript_99094	NA	F01_transcript_99094
F01_transcript_31746	NA	NA	NA	F01_transcript_31746
F01_transcript_117507	NA	F01_transcript_117507	NA	NA
F01_transcript_156314	NA	F01_transcript_156314	NA	F01_transcript_156314
F01_transcript_104184	NA	NA	F01_transcript_104184	NA
F01_transcript_73947	F01_transcript_73947	F01_transcript_73947	F01_transcript_73947	F01_transcript_73947
F01_transcript_48688	F01_transcript_48688	F01_transcript_48688	NA	F01_transcript_48688
F01_transcript_97503	NA	NA	NA	F01_transcript_97503
F01_transcript_143501	NA	NA	NA	F01_transcript_143501

F01_transcript_165728	F01_transcript_165728	F01_transcript_165728	F01_transcript_165728	F01_transcript_165728
F01_transcript_22158	NA	NA	NA	F01_transcript_22158
F01_transcript_156725	NA	F01_transcript_156725	NA	NA
F01_transcript_97902	F01_transcript_97902	F01_transcript_97902	NA	NA
F01_transcript_55813	F01_transcript_55813	F01_transcript_55813	F01_transcript_55813	F01_transcript_55813
F01_transcript_129361	F01_transcript_129361	F01_transcript_129361	F01_transcript_129361	F01_transcript_129361
F01_transcript_84051	NA	NA	NA	F01_transcript_84051
F01_transcript_59536	NA	NA	F01_transcript_59536	NA
F01_transcript_132744	F01_transcript_132744	F01_transcript_132744	NA	NA
F01_transcript_157789	F01_transcript_157789	F01_transcript_157789	F01_transcript_157789	NA
F01_transcript_160433	F01_transcript_160433	F01_transcript_160433	F01_transcript_160433	F01_transcript_160433
F01_transcript_90489	F01_transcript_90489	F01_transcript_90489	F01_transcript_90489	F01_transcript_90489
F01_transcript_101509	F01_transcript_101509	F01_transcript_101509	NA	F01_transcript_101509
F01_transcript_154401	F01_transcript_154401	F01_transcript_154401	F01_transcript_154401	NA

F01_transcript_154857	NA	F01_transcript_154857	F01_transcript_154857	F01_transcript_154857
F01_transcript_149549	NA	F01_transcript_149549	F01_transcript_149549	NA
F01_transcript_145464	F01_transcript_145464	F01_transcript_145464	F01_transcript_145464	F01_transcript_145464
F01_transcript_143682	NA	F01_transcript_143682	NA	NA
F01_transcript_154706	NA	F01_transcript_154706	NA	NA
F01_transcript_75187	F01_transcript_75187	F01_transcript_75187	F01_transcript_75187	F01_transcript_75187
F01_transcript_121869	F01_transcript_121869	F01_transcript_121869	F01_transcript_121869	F01_transcript_121869
F01_transcript_3084	NA	NA	NA	F01_transcript_3084
F01_transcript_135318	NA	F01_transcript_135318	NA	NA
F01_transcript_74800	NA	F01_transcript_74800	NA	F01_transcript_74800
F01_transcript_132144	NA	NA	NA	F01_transcript_132144
F01_transcript_134905	NA	F01_transcript_134905	NA	NA
F01_transcript_68122	F01_transcript_68122	F01_transcript_68122	F01_transcript_68122	F01_transcript_68122
F01_transcript_84282	F01_transcript_84282	F01_transcript_84282	F01_transcript_84282	F01_transcript_84282

F01_transcript_67003	NA	NA	NA	F01_transcript_67003
F01_transcript_116209	NA	NA	NA	F01_transcript_116209
F01_transcript_70675	NA	NA	NA	F01_transcript_70675
F01_transcript_88152	NA	NA	NA	F01_transcript_88152
F01_transcript_61274	NA	NA	NA	F01_transcript_61274
F01_transcript_141383	F01_transcript_141383	F01_transcript_141383	F01_transcript_141383	F01_transcript_141383
F01_transcript_94261	NA	NA	NA	F01_transcript_94261
F01_transcript_47961	NA	F01_transcript_47961	F01_transcript_47961	F01_transcript_47961
F01_transcript_37207	NA	F01_transcript_37207	NA	NA
F01_transcript_140486	F01_transcript_140486	F01_transcript_140486	F01_transcript_140486	F01_transcript_140486
F01_transcript_49019	NA	F01_transcript_49019	NA	NA
F01_transcript_30955	NA	NA	NA	F01_transcript_30955
F01_transcript_106864	NA	F01_transcript_106864	F01_transcript_106864	F01_transcript_106864
F01_transcript_144363	NA	F01_transcript_144363	F01_transcript_144363	F01_transcript_144363

F01_transcript_53498	NA	NA	NA	F01_transcript_53498
F01_transcript_82020	F01_transcript_82020	F01_transcript_82020	NA	F01_transcript_82020
F01_transcript_81418	NA	F01_transcript_81418	NA	NA
F01_transcript_151693	NA	F01_transcript_151693	NA	NA
F01_transcript_69611	F01_transcript_69611	F01_transcript_69611	F01_transcript_69611	F01_transcript_69611
F01_transcript_151106	NA	F01_transcript_151106	NA	NA
F01_transcript_77115	NA	NA	NA	F01_transcript_77115
F01_transcript_54488	NA	NA	NA	F01_transcript_54488
F01_transcript_43089	NA	NA	NA	F01_transcript_43089
F01_transcript_135262	NA	NA	NA	F01_transcript_135262
F01_transcript_131965	NA	F01_transcript_131965	NA	NA
F01_transcript_93328	NA	F01_transcript_93328	NA	F01_transcript_93328
F01_transcript_143445	NA	F01_transcript_143445	F01_transcript_143445	F01_transcript_143445
F01_transcript_81999	F01_transcript_81999	F01_transcript_81999	F01_transcript_81999	F01_transcript_81999

F01_transcript_77140	NA	NA	NA	F01_transcript_77140
F01_transcript_81830	NA	NA	NA	F01_transcript_81830
F01_transcript_148873	NA	NA	NA	F01_transcript_148873
F01_transcript_119098	NA	NA	NA	F01_transcript_119098
F01_transcript_155403	NA	F01_transcript_155403	F01_transcript_155403	F01_transcript_155403
F01_transcript_138604	NA	F01_transcript_138604	F01_transcript_138604	F01_transcript_138604
F01_transcript_124708	F01_transcript_124708	F01_transcript_124708	F01_transcript_124708	F01_transcript_124708
F01_transcript_48258	NA	NA	F01_transcript_48258	F01_transcript_48258
F01_transcript_69300	NA	NA	NA	F01_transcript_69300
F01_transcript_85894	NA	F01_transcript_85894	NA	NA
F01_transcript_147582	NA	NA	NA	F01_transcript_147582
F01_transcript_97534	NA	F01_transcript_97534	NA	NA
F01_transcript_150048	NA	F01_transcript_150048	NA	F01_transcript_150048
F01_transcript_39042	NA	NA	NA	F01_transcript_39042

F01_transcript_82295	F01_transcript_82295	F01_transcript_82295	NA	NA
F01_transcript_91286	NA	NA	NA	F01_transcript_91286
F01_transcript_115377	NA	F01_transcript_115377	NA	F01_transcript_115377
F01_transcript_161573	F01_transcript_161573	F01_transcript_161573	F01_transcript_161573	F01_transcript_161573
F01_transcript_145971	NA	F01_transcript_145971	NA	NA
F01_transcript_117664	NA	NA	NA	F01_transcript_117664
F01_transcript_50322	NA	F01_transcript_50322	NA	NA
F01_transcript_146346	F01_transcript_146346	F01_transcript_146346	NA	F01_transcript_146346
F01_transcript_161148	F01_transcript_161148	F01_transcript_161148	NA	NA
F01_transcript_79611	NA	NA	F01_transcript_79611	F01_transcript_79611
F01_transcript_138701	NA	NA	NA	F01_transcript_138701
F01_transcript_54320	NA	NA	NA	F01_transcript_54320
F01_transcript_51634	F01_transcript_51634	NA	NA	NA
F01_transcript_159772	F01_transcript_159772	F01_transcript_159772	F01_transcript_159772	F01_transcript_159772

F01_transcript_103434	NA	NA	NA	F01_transcript_103434
F01_transcript_156938	F01_transcript_156938	F01_transcript_156938	F01_transcript_156938	F01_transcript_156938
F01_transcript_56700	NA	NA	NA	F01_transcript_56700
F01_transcript_102407	F01_transcript_102407	F01_transcript_102407	F01_transcript_102407	F01_transcript_102407
F01_transcript_53008	F01_transcript_53008	F01_transcript_53008	F01_transcript_53008	F01_transcript_53008
F01_transcript_32088	NA	NA	NA	F01_transcript_32088
F01_transcript_146888	F01_transcript_146888	F01_transcript_146888	F01_transcript_146888	F01_transcript_146888
F01_transcript_62158	F01_transcript_62158	F01_transcript_62158	F01_transcript_62158	F01_transcript_62158
F01_transcript_98983	NA	NA	NA	F01_transcript_98983
F01_transcript_49686	NA	F01_transcript_49686	NA	NA
F01_transcript_136154	NA	NA	NA	F01_transcript_136154
F01_transcript_10290	NA	F01_transcript_10290	NA	F01_transcript_10290
F01_transcript_129599	F01_transcript_129599	F01_transcript_129599	F01_transcript_129599	F01_transcript_129599
F01_transcript_23082	NA	NA	NA	F01_transcript_23082

F01_transcript_148720	F01_transcript_148720	F01_transcript_148720	F01_transcript_148720	F01_transcript_148720
F01_transcript_113282	F01_transcript_113282	F01_transcript_113282	F01_transcript_113282	F01_transcript_113282
F01_transcript_22585	NA	NA	NA	F01_transcript_22585
F01_transcript_58139	F01_transcript_58139	F01_transcript_58139	F01_transcript_58139	F01_transcript_58139
F01_transcript_123760	NA	F01_transcript_123760	NA	F01_transcript_123760
F01_transcript_6654	NA	NA	NA	F01_transcript_6654
F01_transcript_122681	NA	NA	NA	F01_transcript_122681
F01_transcript_115728	F01_transcript_115728	F01_transcript_115728	F01_transcript_115728	F01_transcript_115728
F01_transcript_161643	F01_transcript_161643	F01_transcript_161643	NA	NA
F01_transcript_100547	NA	NA	F01_transcript_100547	F01_transcript_100547
F01_transcript_82768	NA	NA	NA	F01_transcript_82768
F01_transcript_46151	NA	F01_transcript_46151	NA	F01_transcript_46151
F01_transcript_79865	NA	F01_transcript_79865	NA	F01_transcript_79865
F01_transcript_104999	NA	NA	F01_transcript_104999	F01_transcript_104999

F01_transcript_51187	F01_transcript_51187	F01_transcript_51187	F01_transcript_51187	F01_transcript_51187
F01_transcript_53231	NA	NA	NA	F01_transcript_53231
F01_transcript_151212	F01_transcript_151212	F01_transcript_151212	F01_transcript_151212	F01_transcript_151212
F01_transcript_128709	NA	NA	NA	F01_transcript_128709
F01_transcript_106792	F01_transcript_106792	F01_transcript_106792	F01_transcript_106792	F01_transcript_106792
F01_transcript_131747	NA	F01_transcript_131747	F01_transcript_131747	F01_transcript_131747
F01_transcript_138245	F01_transcript_138245	F01_transcript_138245	NA	F01_transcript_138245
F01_transcript_87807	F01_transcript_87807	F01_transcript_87807	F01_transcript_87807	F01_transcript_87807
F01_transcript_24507	NA	F01_transcript_24507	NA	NA
F01_transcript_112918	NA	NA	NA	F01_transcript_112918
F01_transcript_110714	F01_transcript_110714	F01_transcript_110714	F01_transcript_110714	F01_transcript_110714
F01_transcript_103008	NA	F01_transcript_103008	NA	NA
F01_transcript_109021	NA	NA	F01_transcript_109021	NA
F01_transcript_127645	F01_transcript_127645	F01_transcript_127645	F01_transcript_127645	F01_transcript_127645

F01_transcript_39302	NA	NA	NA	F01_transcript_39302
F01_transcript_72164	NA	NA	NA	F01_transcript_72164
F01_transcript_99188	NA	F01_transcript_99188	NA	NA
F01_transcript_15168	F01_transcript_15168	F01_transcript_15168	F01_transcript_15168	F01_transcript_15168
F01_transcript_57418	NA	F01_transcript_57418	NA	NA
F01_transcript_152741	NA	NA	NA	F01_transcript_152741
F01_transcript_83249	NA	F01_transcript_83249	F01_transcript_83249	F01_transcript_83249
F01_transcript_66269	F01_transcript_66269	F01_transcript_66269	F01_transcript_66269	F01_transcript_66269
F01_transcript_61014	NA	NA	NA	F01_transcript_61014
F01_transcript_154950	NA	F01_transcript_154950	NA	NA
F01_transcript_42686	NA	NA	NA	F01_transcript_42686
F01_transcript_74288	F01_transcript_74288	F01_transcript_74288	NA	F01_transcript_74288
F01_transcript_137277	NA	NA	NA	F01_transcript_137277
F01_transcript_143517	NA	NA	NA	F01_transcript_143517

F01_transcript_99502	NA	F01_transcript_99502	NA	NA
F01_transcript_107816	NA	NA	F01_transcript_107816	NA
F01_transcript_51196	F01_transcript_51196	F01_transcript_51196	F01_transcript_51196	F01_transcript_51196
F01_transcript_10053	NA	F01_transcript_10053	F01_transcript_10053	NA
F01_transcript_50335	F01_transcript_50335	F01_transcript_50335	F01_transcript_50335	F01_transcript_50335
F01_transcript_53519	NA	F01_transcript_53519	NA	NA
F01_transcript_137823	F01_transcript_137823	F01_transcript_137823	F01_transcript_137823	NA
F01_transcript_69183	F01_transcript_69183	F01_transcript_69183	F01_transcript_69183	F01_transcript_69183
F01_transcript_133148	F01_transcript_133148	F01_transcript_133148	F01_transcript_133148	F01_transcript_133148
F01_transcript_115331	NA	F01_transcript_115331	NA	NA
F01_transcript_34059	NA	NA	F01_transcript_34059	F01_transcript_34059
F01_transcript_127524	F01_transcript_127524	F01_transcript_127524	F01_transcript_127524	F01_transcript_127524
F01_transcript_108912	F01_transcript_108912	F01_transcript_108912	F01_transcript_108912	F01_transcript_108912
F01_transcript_38379	NA	NA	NA	F01_transcript_38379

F01_transcript_109166	NA	F01_transcript_109166	NA	F01_transcript_109166
F01_transcript_4921	NA	NA	F01_transcript_4921	F01_transcript_4921
F01_transcript_166565	F01_transcript_166565	F01_transcript_166565	NA	NA
F01_transcript_62212	NA	NA	NA	F01_transcript_62212
F01_transcript_148290	NA	F01_transcript_148290	NA	NA
F01_transcript_64035	F01_transcript_64035	F01_transcript_64035	NA	NA
F01_transcript_121608	NA	F01_transcript_121608	NA	NA
F01_transcript_151786	NA	F01_transcript_151786	F01_transcript_151786	F01_transcript_151786
F01_transcript_133719	NA	NA	NA	F01_transcript_133719
F01_transcript_94338	F01_transcript_94338	F01_transcript_94338	NA	NA
F01_transcript_101783	NA	NA	NA	F01_transcript_101783
F01_transcript_6015	NA	F01_transcript_6015	NA	NA
F01_transcript_113395	F01_transcript_113395	F01_transcript_113395	F01_transcript_113395	F01_transcript_113395
F01_transcript_81860	F01_transcript_81860	F01_transcript_81860	F01_transcript_81860	F01_transcript_81860

F01_transcript_154446	F01_transcript_154446	F01_transcript_154446	F01_transcript_154446	F01_transcript_154446
F01_transcript_157055	NA	F01_transcript_157055	NA	NA
F01_transcript_81176	NA	NA	NA	F01_transcript_81176
F01_transcript_138896	NA	F01_transcript_138896	F01_transcript_138896	F01_transcript_138896
F01_transcript_27624	NA	F01_transcript_27624	NA	F01_transcript_27624
F01_transcript_129140	NA	F01_transcript_129140	NA	NA
F01_transcript_36303	NA	NA	NA	F01_transcript_36303
F01_transcript_134112	F01_transcript_134112	F01_transcript_134112	NA	NA
F01_transcript_11714	NA	NA	NA	F01_transcript_11714
F01_transcript_115484	NA	F01_transcript_115484	NA	NA
F01_transcript_115491	NA	F01_transcript_115491	NA	F01_transcript_115491
F01_transcript_90215	NA	F01_transcript_90215	NA	NA
F01_transcript_89896	NA	F01_transcript_89896	NA	NA
F01_transcript_60079	NA	F01_transcript_60079	NA	NA

F01_transcript_3428	F01_transcript_3428	F01_transcript_3428	F01_transcript_3428	F01_transcript_3428
F01_transcript_109474	F01_transcript_109474	F01_transcript_109474	F01_transcript_109474	F01_transcript_109474
F01_transcript_85501	F01_transcript_85501	F01_transcript_85501	F01_transcript_85501	F01_transcript_85501
F01_transcript_164875	F01_transcript_164875	F01_transcript_164875	NA	NA
F01_transcript_105218	NA	F01_transcript_105218	NA	NA
F01_transcript_47466	NA	F01_transcript_47466	NA	NA
F01_transcript_78040	NA	NA	NA	F01_transcript_78040
F01_transcript_97324	NA	F01_transcript_97324	NA	F01_transcript_97324
F01_transcript_49748	NA	F01_transcript_49748	NA	NA
F01_transcript_4004	F01_transcript_4004	F01_transcript_4004	F01_transcript_4004	F01_transcript_4004
F01_transcript_104335	F01_transcript_104335	F01_transcript_104335	F01_transcript_104335	F01_transcript_104335
F01_transcript_104224	NA	NA	NA	F01_transcript_104224
F01_transcript_18102	NA	F01_transcript_18102	NA	F01_transcript_18102
F01_transcript_122964	F01_transcript_122964	F01_transcript_122964	F01_transcript_122964	F01_transcript_122964

F01_transcript_148794	NA	F01_transcript_148794	NA	NA
F01_transcript_141533	NA	F01_transcript_141533	NA	NA
F01_transcript_64415	NA	F01_transcript_64415	F01_transcript_64415	F01_transcript_64415
F01_transcript_145245	NA	NA	NA	F01_transcript_145245
F01_transcript_75113	NA	F01_transcript_75113	NA	NA
F01_transcript_115646	NA	F01_transcript_115646	NA	F01_transcript_115646
F01_transcript_70881	NA	NA	F01_transcript_70881	NA
F01_transcript_88417	F01_transcript_88417	F01_transcript_88417	F01_transcript_88417	F01_transcript_88417
F01_transcript_166767	NA	F01_transcript_166767	NA	F01_transcript_166767
F01_transcript_59552	NA	NA	F01_transcript_59552	NA
F01_transcript_55442	NA	F01_transcript_55442	NA	NA
F01_transcript_149243	F01_transcript_149243	F01_transcript_149243	F01_transcript_149243	F01_transcript_149243
F01_transcript_162519	F01_transcript_162519	F01_transcript_162519	F01_transcript_162519	F01_transcript_162519
F01_transcript_142872	F01_transcript_142872	F01_transcript_142872	F01_transcript_142872	F01_transcript_142872

F01_transcript_164952	NA	F01_transcript_164952	F01_transcript_164952	NA
F01_transcript_123892	NA	F01_transcript_123892	NA	NA
F01_transcript_71595	NA	NA	NA	F01_transcript_71595
F01_transcript_79350	NA	NA	NA	F01_transcript_79350
F01_transcript_149500	NA	F01_transcript_149500	F01_transcript_149500	F01_transcript_149500
F01_transcript_100988	F01_transcript_100988	F01_transcript_100988	NA	NA
F01_transcript_149424	F01_transcript_149424	F01_transcript_149424	F01_transcript_149424	F01_transcript_149424
F01_transcript_70584	NA	F01_transcript_70584	NA	NA
F01_transcript_38640	NA	NA	NA	F01_transcript_38640
F01_transcript_39410	NA	NA	NA	F01_transcript_39410
F01_transcript_89154	F01_transcript_89154	F01_transcript_89154	F01_transcript_89154	F01_transcript_89154
F01_transcript_150178	F01_transcript_150178	F01_transcript_150178	F01_transcript_150178	F01_transcript_150178
F01_transcript_148578	F01_transcript_148578	F01_transcript_148578	NA	NA
F01_transcript_78509	F01_transcript_78509	NA	F01_transcript_78509	F01_transcript_78509

F01_transcript_50124	NA	F01_transcript_50124	NA	F01_transcript_50124
F01_transcript_161391	NA	F01_transcript_161391	F01_transcript_161391	F01_transcript_161391
F01_transcript_104389	NA	NA	NA	F01_transcript_104389
F01_transcript_121492	NA	NA	NA	F01_transcript_121492
F01_transcript_109500	NA	F01_transcript_109500	NA	NA
F01_transcript_131849	NA	NA	NA	F01_transcript_131849
F01_transcript_145554	NA	F01_transcript_145554	F01_transcript_145554	F01_transcript_145554
F01_transcript_62091	NA	F01_transcript_62091	NA	NA
F01_transcript_113252	NA	NA	NA	F01_transcript_113252
F01_transcript_38063	NA	NA	NA	F01_transcript_38063
F01_transcript_167285	NA	F01_transcript_167285	NA	F01_transcript_167285
F01_transcript_113305	NA	NA	NA	F01_transcript_113305
F01_transcript_107939	NA	F01_transcript_107939	F01_transcript_107939	F01_transcript_107939
F01_transcript_61897	F01_transcript_61897	F01_transcript_61897	F01_transcript_61897	F01_transcript_61897

F01_transcript_156953	F01_transcript_156953	F01_transcript_156953	F01_transcript_156953	F01_transcript_156953
F01_transcript_137510	NA	NA	NA	F01_transcript_137510
F01_transcript_105747	F01_transcript_105747	F01_transcript_105747	F01_transcript_105747	F01_transcript_105747
F01_transcript_61688	NA	NA	NA	F01_transcript_61688
F01_transcript_31670	F01_transcript_31670	F01_transcript_31670	F01_transcript_31670	F01_transcript_31670
F01_transcript_153958	NA	F01_transcript_153958	NA	F01_transcript_153958
F01_transcript_139704	F01_transcript_139704	F01_transcript_139704	F01_transcript_139704	F01_transcript_139704
F01_transcript_47977	NA	NA	F01_transcript_47977	NA
F01_transcript_162046	F01_transcript_162046	F01_transcript_162046	F01_transcript_162046	F01_transcript_162046
F01_transcript_50133	NA	F01_transcript_50133	NA	F01_transcript_50133
F01_transcript_132777	F01_transcript_132777	F01_transcript_132777	F01_transcript_132777	F01_transcript_132777
F01_transcript_150286	NA	F01_transcript_150286	NA	F01_transcript_150286
F01_transcript_47379	NA	F01_transcript_47379	NA	NA
F01_transcript_145248	NA	NA	F01_transcript_145248	F01_transcript_145248

F01_transcript_72385	NA	F01_transcript_72385	NA	NA
F01_transcript_104187	NA	NA	NA	F01_transcript_104187
F01_transcript_142611	NA	F01_transcript_142611	NA	F01_transcript_142611
F01_transcript_55502	NA	F01_transcript_55502	NA	NA
F01_transcript_100911	F01_transcript_100911	F01_transcript_100911	NA	NA
F01_transcript_131032	NA	NA	NA	F01_transcript_131032
F01_transcript_84316	NA	F01_transcript_84316	NA	NA
F01_transcript_112631	F01_transcript_112631	F01_transcript_112631	F01_transcript_112631	NA
F01_transcript_50179	NA	F01_transcript_50179	NA	F01_transcript_50179
F01_transcript_59451	NA	F01_transcript_59451	F01_transcript_59451	F01_transcript_59451
F01_transcript_55529	NA	F01_transcript_55529	NA	F01_transcript_55529
F01_transcript_124568	NA	F01_transcript_124568	F01_transcript_124568	F01_transcript_124568
F01_transcript_86280	NA	NA	NA	F01_transcript_86280
F01_transcript_68657	NA	F01_transcript_68657	NA	NA

F01_transcript_132029	F01_transcript_132029	F01_transcript_132029	NA	NA
F01_transcript_59588	NA	NA	F01_transcript_59588	F01_transcript_59588
F01_transcript_124587	NA	F01_transcript_124587	NA	NA
F01_transcript_68610	NA	F01_transcript_68610	NA	NA
F01_transcript_116862	NA	F01_transcript_116862	NA	NA
F01_transcript_90538	NA	F01_transcript_90538	NA	NA
F01_transcript_87240	NA	F01_transcript_87240	NA	F01_transcript_87240
F01_transcript_71567	NA	NA	NA	F01_transcript_71567
F01_transcript_53729	NA	NA	NA	F01_transcript_53729
F01_transcript_94460	F01_transcript_94460	F01_transcript_94460	F01_transcript_94460	F01_transcript_94460
F01_transcript_161806	NA	F01_transcript_161806	NA	NA
F01_transcript_63340	NA	NA	NA	F01_transcript_63340
F01_transcript_90472	F01_transcript_90472	F01_transcript_90472	NA	NA
F01_transcript_112658	NA	NA	NA	F01_transcript_112658

F01_transcript_158748	F01_transcript_158748	F01_transcript_158748	NA	F01_transcript_158748
F01_transcript_923	NA	NA	NA	F01_transcript_923
F01_transcript_64713	NA	NA	NA	F01_transcript_64713
F01_transcript_113634	NA	F01_transcript_113634	NA	NA
F01_transcript_132253	NA	NA	NA	F01_transcript_132253
F01_transcript_68899	NA	F01_transcript_68899	NA	F01_transcript_68899
F01_transcript_68168	NA	F01_transcript_68168	NA	F01_transcript_68168
F01_transcript_119823	NA	NA	NA	F01_transcript_119823
F01_transcript_167189	NA	NA	NA	F01_transcript_167189
F01_transcript_51009	F01_transcript_51009	F01_transcript_51009	F01_transcript_51009	NA
F01_transcript_49937	F01_transcript_49937	F01_transcript_49937	F01_transcript_49937	F01_transcript_49937
F01_transcript_8549	NA	F01_transcript_8549	F01_transcript_8549	F01_transcript_8549
F01_transcript_51768	NA	F01_transcript_51768	NA	NA
F01_transcript_85488	NA	NA	NA	F01_transcript_85488

F01_transcript_79790	NA	F01_transcript_79790	F01_transcript_79790	F01_transcript_79790
F01_transcript_141415	NA	F01_transcript_141415	NA	NA
F01_transcript_119908	NA	F01_transcript_119908	NA	NA
F01_transcript_144416	NA	F01_transcript_144416	NA	F01_transcript_144416
F01_transcript_71627	NA	F01_transcript_71627	F01_transcript_71627	NA
F01_transcript_84239	NA	F01_transcript_84239	F01_transcript_84239	NA
F01_transcript_86403	NA	NA	NA	F01_transcript_86403
F01_transcript_68082	F01_transcript_68082	F01_transcript_68082	F01_transcript_68082	F01_transcript_68082
F01_transcript_84769	NA	F01_transcript_84769	F01_transcript_84769	NA
F01_transcript_114338	NA	NA	NA	F01_transcript_114338
F01_transcript_40797	NA	F01_transcript_40797	F01_transcript_40797	NA
F01_transcript_83960	F01_transcript_83960	F01_transcript_83960	F01_transcript_83960	F01_transcript_83960
F01_transcript_16089	NA	NA	NA	F01_transcript_16089
F01_transcript_112234	F01_transcript_112234	F01_transcript_112234	F01_transcript_112234	F01_transcript_112234

F01_transcript_93553	NA	F01_transcript_93553	NA	NA
F01_transcript_77366	NA	F01_transcript_77366	NA	F01_transcript_77366
F01_transcript_75699	F01_transcript_75699	F01_transcript_75699	NA	F01_transcript_75699
F01_transcript_159764	F01_transcript_159764	F01_transcript_159764	F01_transcript_159764	F01_transcript_159764
F01_transcript_154189	NA	NA	NA	F01_transcript_154189
F01_transcript_47850	NA	F01_transcript_47850	NA	F01_transcript_47850
F01_transcript_67685	NA	F01_transcript_67685	NA	NA
F01_transcript_134142	NA	F01_transcript_134142	NA	NA
F01_transcript_136479	NA	F01_transcript_136479	NA	F01_transcript_136479
F01_transcript_118969	NA	NA	NA	F01_transcript_118969
F01_transcript_151808	F01_transcript_151808	F01_transcript_151808	F01_transcript_151808	F01_transcript_151808
F01_transcript_32416	NA	F01_transcript_32416	F01_transcript_32416	F01_transcript_32416
F01_transcript_76749	NA	F01_transcript_76749	F01_transcript_76749	F01_transcript_76749
F01_transcript_60499	F01_transcript_60499	F01_transcript_60499	F01_transcript_60499	F01_transcript_60499

F01_transcript_117113	NA	F01_transcript_117113	NA	NA
F01_transcript_97644	NA	NA	NA	F01_transcript_97644
F01_transcript_145596	NA	F01_transcript_145596	NA	F01_transcript_145596
F01_transcript_90089	NA	NA	NA	F01_transcript_90089
F01_transcript_36225	NA	F01_transcript_36225	NA	F01_transcript_36225
F01_transcript_159287	NA	NA	NA	F01_transcript_159287
F01_transcript_34115	NA	F01_transcript_34115	NA	NA
F01_transcript_101314	F01_transcript_101314	F01_transcript_101314	F01_transcript_101314	F01_transcript_101314
F01_transcript_53418	F01_transcript_53418	F01_transcript_53418	F01_transcript_53418	F01_transcript_53418
F01_transcript_110144	NA	NA	NA	F01_transcript_110144
F01_transcript_84342	NA	F01_transcript_84342	NA	NA
F01_transcript_58839	NA	F01_transcript_58839	NA	NA
F01_transcript_98406	NA	F01_transcript_98406	NA	NA
F01_transcript_136209	NA	F01_transcript_136209	NA	NA

F01_transcript_72568	NA	F01_transcript_72568	F01_transcript_72568	F01_transcript_72568
F01_transcript_124058	F01_transcript_124058	F01_transcript_124058	F01_transcript_124058	F01_transcript_124058
F01_transcript_153152	F01_transcript_153152	F01_transcript_153152	F01_transcript_153152	F01_transcript_153152
F01_transcript_121383	NA	NA	NA	F01_transcript_121383
F01_transcript_78870	F01_transcript_78870	F01_transcript_78870	NA	F01_transcript_78870
F01_transcript_53671	NA	NA	NA	F01_transcript_53671
F01_transcript_96428	NA	F01_transcript_96428	NA	NA
F01_transcript_63684	NA	F01_transcript_63684	NA	NA
F01_transcript_167455	NA	F01_transcript_167455	NA	F01_transcript_167455
F01_transcript_16577	NA	F01_transcript_16577	NA	NA
F01_transcript_63724	NA	F01_transcript_63724	NA	F01_transcript_63724
F01_transcript_122555	NA	NA	NA	F01_transcript_122555
F01_transcript_130741	NA	NA	NA	F01_transcript_130741
F01_transcript_90325	NA	NA	NA	F01_transcript_90325

F01_transcript_76133	NA	NA	NA	F01_transcript_76133
F01_transcript_7365	NA	NA	NA	F01_transcript_7365
F01_transcript_113155	F01_transcript_113155	F01_transcript_113155	F01_transcript_113155	F01_transcript_113155
F01_transcript_121508	NA	NA	NA	F01_transcript_121508
F01_transcript_158622	NA	F01_transcript_158622	NA	NA
F01_transcript_104300	F01_transcript_104300	F01_transcript_104300	NA	NA
F01_transcript_112147	NA	F01_transcript_112147	NA	F01_transcript_112147
F01_transcript_37545	F01_transcript_37545	NA	NA	NA
F01_transcript_144915	F01_transcript_144915	F01_transcript_144915	NA	NA
F01_transcript_36906	NA	NA	NA	F01_transcript_36906
F01_transcript_148697	F01_transcript_148697	F01_transcript_148697	NA	NA
F01_transcript_94139	NA	F01_transcript_94139	F01_transcript_94139	F01_transcript_94139
F01_transcript_144797	NA	F01_transcript_144797	F01_transcript_144797	NA
F01_transcript_64953	NA	F01_transcript_64953	NA	NA

F01_transcript_91409	NA	F01_transcript_91409	NA	NA
F01_transcript_53232	NA	F01_transcript_53232	NA	NA
F01_transcript_62994	F01_transcript_62994	F01_transcript_62994	F01_transcript_62994	F01_transcript_62994
F01_transcript_143103	NA	F01_transcript_143103	NA	F01_transcript_143103
F01_transcript_48862	NA	F01_transcript_48862	NA	NA
F01_transcript_143025	NA	F01_transcript_143025	NA	NA
F01_transcript_130442	NA	F01_transcript_130442	NA	NA
F01_transcript_149386	F01_transcript_149386	F01_transcript_149386	NA	NA
F01_transcript_4826	NA	NA	F01_transcript_4826	F01_transcript_4826
F01_transcript_145862	NA	F01_transcript_145862	NA	F01_transcript_145862
F01_transcript_84578	F01_transcript_84578	F01_transcript_84578	F01_transcript_84578	F01_transcript_84578
F01_transcript_63237	NA	F01_transcript_63237	NA	NA
F01_transcript_159037	NA	F01_transcript_159037	F01_transcript_159037	NA
F01_transcript_61519	F01_transcript_61519	F01_transcript_61519	F01_transcript_61519	F01_transcript_61519

F01_transcript_13841	NA	NA	F01_transcript_13841	F01_transcript_13841
F01_transcript_115745	NA	F01_transcript_115745	NA	NA
F01_transcript_90881	NA	F01_transcript_90881	F01_transcript_90881	F01_transcript_90881
F01_transcript_62780	NA	F01_transcript_62780	NA	NA
F01_transcript_4766	NA	NA	NA	F01_transcript_4766
F01_transcript_37042	NA	NA	NA	F01_transcript_37042
F01_transcript_116901	NA	NA	NA	F01_transcript_116901
F01_transcript_154762	F01_transcript_154762	F01_transcript_154762	F01_transcript_154762	F01_transcript_154762
F01_transcript_78339	NA	NA	F01_transcript_78339	NA
F01_transcript_103120	NA	NA	NA	F01_transcript_103120
F01_transcript_27244	F01_transcript_27244	F01_transcript_27244	F01_transcript_27244	F01_transcript_27244
F01_transcript_12289	NA	F01_transcript_12289	NA	NA
F01_transcript_85549	NA	F01_transcript_85549	NA	F01_transcript_85549
F01_transcript_90177	F01_transcript_90177	F01_transcript_90177	F01_transcript_90177	F01_transcript_90177

F01_transcript_94215	NA	NA	NA	F01_transcript_94215
F01_transcript_127001	F01_transcript_127001	F01_transcript_127001	F01_transcript_127001	F01_transcript_127001
F01_transcript_74047	NA	NA	NA	F01_transcript_74047
F01_transcript_53403	NA	F01_transcript_53403	F01_transcript_53403	F01_transcript_53403
F01_transcript_50873	F01_transcript_50873	F01_transcript_50873	F01_transcript_50873	F01_transcript_50873
F01_transcript_53507	F01_transcript_53507	F01_transcript_53507	F01_transcript_53507	F01_transcript_53507
F01_transcript_108300	F01_transcript_108300	F01_transcript_108300	NA	F01_transcript_108300
F01_transcript_113126	NA	F01_transcript_113126	NA	NA
F01_transcript_150913	NA	F01_transcript_150913	NA	F01_transcript_150913
F01_transcript_81809	NA	F01_transcript_81809	NA	NA
F01_transcript_82118	NA	NA	NA	F01_transcript_82118
F01_transcript_145954	NA	F01_transcript_145954	F01_transcript_145954	F01_transcript_145954
F01_transcript_153502	F01_transcript_153502	F01_transcript_153502	F01_transcript_153502	F01_transcript_153502
F01_transcript_55737	NA	NA	NA	F01_transcript_55737

F01_transcript_114303	NA	NA	NA	F01_transcript_114303
F01_transcript_149607	NA	F01_transcript_149607	F01_transcript_149607	F01_transcript_149607
F01_transcript_104158	NA	NA	NA	F01_transcript_104158
F01_transcript_25544	NA	F01_transcript_25544	NA	NA
F01_transcript_121635	NA	F01_transcript_121635	NA	NA
F01_transcript_130890	NA	NA	NA	F01_transcript_130890
F01_transcript_128424	NA	F01_transcript_128424	F01_transcript_128424	NA
F01_transcript_164933	NA	F01_transcript_164933	NA	NA
F01_transcript_136065	NA	F01_transcript_136065	NA	NA
F01_transcript_10458	NA	F01_transcript_10458	NA	NA
F01_transcript_77818	NA	NA	NA	F01_transcript_77818
F01_transcript_154212	NA	NA	NA	F01_transcript_154212
F01_transcript_111280	NA	F01_transcript_111280	NA	F01_transcript_111280
F01_transcript_63139	NA	F01_transcript_63139	NA	NA

F01_transcript_51284	F01_transcript_51284	F01_transcript_51284	F01_transcript_51284	F01_transcript_51284
F01_transcript_40703	NA	NA	NA	F01_transcript_40703
F01_transcript_58536	NA	NA	NA	F01_transcript_58536
F01_transcript_163650	NA	NA	NA	F01_transcript_163650
F01_transcript_53319	NA	NA	NA	F01_transcript_53319
F01_transcript_166672	NA	NA	NA	F01_transcript_166672
F01_transcript_48915	F01_transcript_48915	F01_transcript_48915	F01_transcript_48915	F01_transcript_48915
F01_transcript_121995	NA	F01_transcript_121995	NA	NA
F01_transcript_157677	F01_transcript_157677	F01_transcript_157677	NA	F01_transcript_157677
F01_transcript_154380	F01_transcript_154380	F01_transcript_154380	F01_transcript_154380	F01_transcript_154380
F01_transcript_100325	F01_transcript_100325	F01_transcript_100325	F01_transcript_100325	F01_transcript_100325
F01_transcript_63552	F01_transcript_63552	F01_transcript_63552	NA	F01_transcript_63552
F01_transcript_156299	NA	F01_transcript_156299	NA	NA
F01_transcript_72477	NA	NA	F01_transcript_72477	F01_transcript_72477

F01_transcript_123402	F01_transcript_123402	F01_transcript_123402	F01_transcript_123402	F01_transcript_123402
F01_transcript_47006	NA	F01_transcript_47006	NA	NA
F01_transcript_64310	NA	F01_transcript_64310	NA	NA
F01_transcript_71293	NA	NA	F01_transcript_71293	NA
F01_transcript_111713	F01_transcript_111713	F01_transcript_111713	NA	NA
F01_transcript_115459	NA	NA	NA	F01_transcript_115459
F01_transcript_166680	F01_transcript_166680	F01_transcript_166680	F01_transcript_166680	F01_transcript_166680
F01_transcript_52631	F01_transcript_52631	F01_transcript_52631	F01_transcript_52631	F01_transcript_52631
F01_transcript_29227	NA	NA	NA	F01_transcript_29227
F01_transcript_132667	F01_transcript_132667	F01_transcript_132667	F01_transcript_132667	NA
F01_transcript_104445	F01_transcript_104445	F01_transcript_104445	NA	NA
F01_transcript_156499	NA	NA	NA	F01_transcript_156499
F01_transcript_159449	NA	F01_transcript_159449	NA	NA
F01_transcript_79806	NA	NA	NA	F01_transcript_79806

F01_transcript_158935	NA	NA	F01_transcript_158935	F01_transcript_158935
F01_transcript_53311	NA	F01_transcript_53311	F01_transcript_53311	F01_transcript_53311
F01_transcript_110417	F01_transcript_110417	F01_transcript_110417	F01_transcript_110417	F01_transcript_110417
F01_transcript_105115	F01_transcript_105115	F01_transcript_105115	F01_transcript_105115	F01_transcript_105115
F01_transcript_82739	NA	F01_transcript_82739	NA	NA
F01_transcript_98327	NA	F01_transcript_98327	NA	NA
F01_transcript_122120	F01_transcript_122120	F01_transcript_122120	NA	NA
F01_transcript_161261	NA	NA	NA	F01_transcript_161261
F01_transcript_108908	NA	F01_transcript_108908	F01_transcript_108908	F01_transcript_108908
F01_transcript_64336	NA	NA	NA	F01_transcript_64336
F01_transcript_76622	NA	F01_transcript_76622	NA	NA
F01_transcript_109858	NA	F01_transcript_109858	NA	F01_transcript_109858
F01_transcript_121784	F01_transcript_121784	F01_transcript_121784	F01_transcript_121784	F01_transcript_121784
F01_transcript_96856	F01_transcript_96856	F01_transcript_96856	F01_transcript_96856	F01_transcript_96856

F01_transcript_105264	F01_transcript_105264	F01_transcript_105264	F01_transcript_105264	F01_transcript_105264
F01_transcript_59229	F01_transcript_59229	F01_transcript_59229	F01_transcript_59229	NA
F01_transcript_153896	NA	F01_transcript_153896	NA	NA
F01_transcript_106914	F01_transcript_106914	F01_transcript_106914	F01_transcript_106914	F01_transcript_106914
F01_transcript_71825	F01_transcript_71825	F01_transcript_71825	F01_transcript_71825	F01_transcript_71825
F01_transcript_148831	NA	F01_transcript_148831	F01_transcript_148831	F01_transcript_148831
F01_transcript_108995	NA	NA	NA	F01_transcript_108995
F01_transcript_117059	NA	NA	NA	F01_transcript_117059
F01_transcript_57181	NA	NA	F01_transcript_57181	F01_transcript_57181
F01_transcript_23070	NA	NA	NA	F01_transcript_23070
F01_transcript_149705	NA	NA	NA	F01_transcript_149705
F01_transcript_88166	NA	NA	NA	F01_transcript_88166
F01_transcript_145837	F01_transcript_145837	F01_transcript_145837	F01_transcript_145837	F01_transcript_145837
F01_transcript_142491	F01_transcript_142491	F01_transcript_142491	F01_transcript_142491	F01_transcript_142491

F01_transcript_132772	NA	NA	NA	F01_transcript_132772
F01_transcript_105105	NA	F01_transcript_105105	NA	F01_transcript_105105
F01_transcript_27860	NA	NA	NA	F01_transcript_27860
F01_transcript_23706	NA	NA	NA	F01_transcript_23706
F01_transcript_136541	NA	F01_transcript_136541	NA	F01_transcript_136541
F01_transcript_133557	NA	F01_transcript_133557	F01_transcript_133557	NA
F01_transcript_148391	F01_transcript_148391	NA	NA	F01_transcript_148391
F01_transcript_138879	F01_transcript_138879	F01_transcript_138879	F01_transcript_138879	F01_transcript_138879
F01_transcript_101288	F01_transcript_101288	F01_transcript_101288	F01_transcript_101288	F01_transcript_101288
F01_transcript_9785	NA	F01_transcript_9785	NA	F01_transcript_9785
F01_transcript_126230	NA	NA	NA	F01_transcript_126230
F01_transcript_52359	NA	NA	NA	F01_transcript_52359
F01_transcript_136509	NA	F01_transcript_136509	NA	NA
F01_transcript_100749	F01_transcript_100749	F01_transcript_100749	F01_transcript_100749	F01_transcript_100749

F01_transcript_35150	NA	NA	NA	F01_transcript_35150
F01_transcript_156624	NA	F01_transcript_156624	NA	NA
F01_transcript_97119	NA	F01_transcript_97119	NA	F01_transcript_97119
F01_transcript_131053	NA	NA	NA	F01_transcript_131053
F01_transcript_80157	F01_transcript_80157	F01_transcript_80157	F01_transcript_80157	F01_transcript_80157
F01_transcript_135145	F01_transcript_135145	F01_transcript_135145	F01_transcript_135145	F01_transcript_135145
F01_transcript_136262	NA	NA	NA	F01_transcript_136262
F01_transcript_89283	F01_transcript_89283	F01_transcript_89283	F01_transcript_89283	NA
F01_transcript_139833	F01_transcript_139833	F01_transcript_139833	NA	F01_transcript_139833
F01_transcript_20473	NA	NA	NA	F01_transcript_20473
F01_transcript_13879	F01_transcript_13879	F01_transcript_13879	F01_transcript_13879	F01_transcript_13879
F01_transcript_4295	NA	NA	NA	F01_transcript_4295
F01_transcript_132866	F01_transcript_132866	F01_transcript_132866	F01_transcript_132866	F01_transcript_132866
F01_transcript_78363	NA	F01_transcript_78363	F01_transcript_78363	F01_transcript_78363

F01_transcript_101021	F01_transcript_101021	NA	F01_transcript_101021	NA
F01_transcript_81816	NA	F01_transcript_81816	NA	NA
F01_transcript_108587	NA	NA	F01_transcript_108587	F01_transcript_108587
F01_transcript_134434	NA	NA	NA	F01_transcript_134434
F01_transcript_4994	NA	NA	NA	F01_transcript_4994
F01_transcript_65680	NA	F01_transcript_65680	NA	F01_transcript_65680
F01_transcript_127292	F01_transcript_127292	F01_transcript_127292	NA	NA
F01_transcript_79089	F01_transcript_79089	F01_transcript_79089	F01_transcript_79089	F01_transcript_79089
F01_transcript_100065	NA	F01_transcript_100065	NA	NA
F01_transcript_159763	NA	F01_transcript_159763	F01_transcript_159763	F01_transcript_159763
F01_transcript_156833	NA	NA	NA	F01_transcript_156833
F01_transcript_62213	F01_transcript_62213	F01_transcript_62213	F01_transcript_62213	F01_transcript_62213
F01_transcript_125799	F01_transcript_125799	F01_transcript_125799	F01_transcript_125799	NA
F01_transcript_25045	NA	NA	NA	F01_transcript_25045

F01_transcript_46016	NA	F01_transcript_46016	NA	NA
F01_transcript_131437	NA	NA	NA	F01_transcript_131437
F01_transcript_86861	NA	F01_transcript_86861	NA	F01_transcript_86861
F01_transcript_35841	NA	NA	NA	F01_transcript_35841
F01_transcript_52343	NA	NA	NA	F01_transcript_52343
F01_transcript_134668	NA	NA	NA	F01_transcript_134668
F01_transcript_137850	NA	F01_transcript_137850	NA	NA
F01_transcript_104724	F01_transcript_104724	F01_transcript_104724	F01_transcript_104724	F01_transcript_104724
F01_transcript_49437	F01_transcript_49437	F01_transcript_49437	F01_transcript_49437	F01_transcript_49437
F01_transcript_134486	NA	NA	NA	F01_transcript_134486
F01_transcript_83385	NA	F01_transcript_83385	NA	NA
F01_transcript_114407	F01_transcript_114407	F01_transcript_114407	NA	F01_transcript_114407
F01_transcript_88032	F01_transcript_88032	F01_transcript_88032	NA	F01_transcript_88032
F01_transcript_74444	F01_transcript_74444	F01_transcript_74444	F01_transcript_74444	F01_transcript_74444

F01_transcript_17337	NA	NA	NA	F01_transcript_17337
F01_transcript_16169	NA	NA	NA	F01_transcript_16169
F01_transcript_123564	NA	F01_transcript_123564	NA	F01_transcript_123564
F01_transcript_111446	NA	NA	F01_transcript_111446	NA
F01_transcript_44606	NA	NA	NA	F01_transcript_44606
F01_transcript_116175	NA	NA	NA	F01_transcript_116175
F01_transcript_118258	F01_transcript_118258	NA	F01_transcript_118258	NA
F01_transcript_89067	NA	NA	NA	F01_transcript_89067
F01_transcript_38541	NA	NA	NA	F01_transcript_38541
F01_transcript_98239	NA	NA	NA	F01_transcript_98239
F01_transcript_141052	NA	F01_transcript_141052	F01_transcript_141052	NA
F01_transcript_109213	F01_transcript_109213	F01_transcript_109213	F01_transcript_109213	F01_transcript_109213
F01_transcript_164932	F01_transcript_164932	F01_transcript_164932	F01_transcript_164932	F01_transcript_164932
F01_transcript_52150	F01_transcript_52150	NA	NA	F01_transcript_52150

F01_transcript_129171	NA	NA	NA	F01_transcript_129171
F01_transcript_21048	F01_transcript_21048	F01_transcript_21048	F01_transcript_21048	F01_transcript_21048
F01_transcript_126208	NA	F01_transcript_126208	NA	NA
F01_transcript_153056	F01_transcript_153056	F01_transcript_153056	F01_transcript_153056	F01_transcript_153056
F01_transcript_147360	NA	NA	NA	F01_transcript_147360
F01_transcript_134619	NA	F01_transcript_134619	F01_transcript_134619	F01_transcript_134619
F01_transcript_132703	NA	F01_transcript_132703	NA	F01_transcript_132703
F01_transcript_64190	NA	F01_transcript_64190	NA	F01_transcript_64190
F01_transcript_142656	NA	NA	NA	F01_transcript_142656
F01_transcript_83849	NA	NA	NA	F01_transcript_83849
F01_transcript_85807	NA	F01_transcript_85807	NA	F01_transcript_85807
F01_transcript_150600	F01_transcript_150600	F01_transcript_150600	F01_transcript_150600	F01_transcript_150600
F01_transcript_140167	NA	F01_transcript_140167	F01_transcript_140167	F01_transcript_140167
F01_transcript_110513	NA	NA	NA	F01_transcript_110513

F01_transcript_78543	NA	F01_transcript_78543	NA	NA
F01_transcript_149111	NA	NA	NA	F01_transcript_149111
F01_transcript_129757	NA	F01_transcript_129757	NA	NA
F01_transcript_72386	NA	F01_transcript_72386	NA	NA
F01_transcript_100516	NA	F01_transcript_100516	NA	NA
F01_transcript_60909	F01_transcript_60909	F01_transcript_60909	F01_transcript_60909	F01_transcript_60909
F01_transcript_82735	NA	NA	NA	F01_transcript_82735
F01_transcript_59650	F01_transcript_59650	F01_transcript_59650	NA	F01_transcript_59650
F01_transcript_108736	NA	NA	F01_transcript_108736	F01_transcript_108736
F01_transcript_86845	NA	F01_transcript_86845	NA	NA
F01_transcript_86496	NA	NA	NA	F01_transcript_86496
F01_transcript_131595	NA	F01_transcript_131595	NA	NA
F01_transcript_132998	F01_transcript_132998	F01_transcript_132998	F01_transcript_132998	F01_transcript_132998
F01_transcript_15743	NA	F01_transcript_15743	NA	NA

F01_transcript_62507	F01_transcript_62507	F01_transcript_62507	F01_transcript_62507	F01_transcript_62507
F01_transcript_80816	NA	F01_transcript_80816	NA	NA
F01_transcript_85012	NA	NA	NA	F01_transcript_85012
F01_transcript_102204	NA	F01_transcript_102204	NA	NA
F01_transcript_11193	NA	NA	NA	F01_transcript_11193
F01_transcript_97896	NA	NA	F01_transcript_97896	F01_transcript_97896
F01_transcript_131146	F01_transcript_131146	F01_transcript_131146	F01_transcript_131146	F01_transcript_131146
F01_transcript_81478	NA	F01_transcript_81478	NA	NA
F01_transcript_58905	NA	F01_transcript_58905	NA	F01_transcript_58905
F01_transcript_93308	F01_transcript_93308	F01_transcript_93308	F01_transcript_93308	F01_transcript_93308
F01_transcript_114593	NA	F01_transcript_114593	NA	F01_transcript_114593
F01_transcript_105869	NA	F01_transcript_105869	NA	NA
F01_transcript_63321	NA	F01_transcript_63321	NA	F01_transcript_63321
F01_transcript_81247	NA	F01_transcript_81247	NA	NA

F01_transcript_80084	F01_transcript_80084	F01_transcript_80084	NA	F01_transcript_80084
F01_transcript_66590	NA	F01_transcript_66590	NA	NA
F01_transcript_71701	F01_transcript_71701	F01_transcript_71701	F01_transcript_71701	F01_transcript_71701
F01_transcript_77922	NA	F01_transcript_77922	NA	F01_transcript_77922
F01_transcript_166946	NA	NA	NA	F01_transcript_166946
F01_transcript_149670	NA	F01_transcript_149670	NA	NA
F01_transcript_165802	F01_transcript_165802	F01_transcript_165802	NA	F01_transcript_165802
F01_transcript_164421	NA	NA	NA	F01_transcript_164421
F01_transcript_152152	NA	NA	NA	F01_transcript_152152
F01_transcript_49660	F01_transcript_49660	F01_transcript_49660	NA	NA
F01_transcript_117840	NA	NA	NA	F01_transcript_117840
F01_transcript_98509	NA	F01_transcript_98509	NA	NA
F01_transcript_97316	NA	F01_transcript_97316	NA	NA
F01_transcript_113921	NA	F01_transcript_113921	NA	NA

F01_transcript_50392	F01_transcript_50392	F01_transcript_50392	F01_transcript_50392	F01_transcript_50392
F01_transcript_83179	F01_transcript_83179	F01_transcript_83179	NA	F01_transcript_83179
F01_transcript_7066	NA	F01_transcript_7066	NA	F01_transcript_7066
F01_transcript_126155	F01_transcript_126155	F01_transcript_126155	NA	F01_transcript_126155
F01_transcript_151639	NA	NA	NA	F01_transcript_151639
F01_transcript_19804	NA	F01_transcript_19804	NA	F01_transcript_19804
F01_transcript_46618	NA	NA	NA	F01_transcript_46618
F01_transcript_17335	F01_transcript_17335	F01_transcript_17335	F01_transcript_17335	F01_transcript_17335
F01_transcript_55579	NA	F01_transcript_55579	NA	F01_transcript_55579
F01_transcript_108126	NA	F01_transcript_108126	NA	NA
F01_transcript_165487	NA	NA	NA	F01_transcript_165487
F01_transcript_74234	F01_transcript_74234	F01_transcript_74234	NA	NA
F01_transcript_52745	NA	NA	NA	F01_transcript_52745
F01_transcript_152633	NA	NA	NA	F01_transcript_152633

F01_transcript_54301	NA	F01_transcript_54301	NA	NA
F01_transcript_121084	NA	F01_transcript_121084	NA	NA
F01_transcript_117112	F01_transcript_117112	F01_transcript_117112	F01_transcript_117112	F01_transcript_117112
F01_transcript_108412	F01_transcript_108412	F01_transcript_108412	F01_transcript_108412	F01_transcript_108412
F01_transcript_108022	NA	F01_transcript_108022	NA	F01_transcript_108022
F01_transcript_86165	NA	NA	NA	F01_transcript_86165
F01_transcript_59909	F01_transcript_59909	F01_transcript_59909	F01_transcript_59909	F01_transcript_59909
F01_transcript_36698	NA	NA	NA	F01_transcript_36698
F01_transcript_107956	NA	F01_transcript_107956	F01_transcript_107956	F01_transcript_107956
F01_transcript_149459	NA	NA	NA	F01_transcript_149459
F01_transcript_67107	NA	NA	F01_transcript_67107	NA
F01_transcript_129799	NA	F01_transcript_129799	NA	F01_transcript_129799
F01_transcript_20269	F01_transcript_20269	F01_transcript_20269	F01_transcript_20269	F01_transcript_20269
F01_transcript_77650	F01_transcript_77650	F01_transcript_77650	F01_transcript_77650	F01_transcript_77650

F01_transcript_93541	F01_transcript_93541	F01_transcript_93541	NA	F01_transcript_93541
F01_transcript_13969	NA	NA	NA	F01_transcript_13969
F01_transcript_121650	NA	F01_transcript_121650	NA	NA
F01_transcript_143867	NA	NA	NA	F01_transcript_143867
F01_transcript_79041	F01_transcript_79041	F01_transcript_79041	F01_transcript_79041	F01_transcript_79041
F01_transcript_77091	F01_transcript_77091	F01_transcript_77091	F01_transcript_77091	F01_transcript_77091
F01_transcript_100562	NA	NA	NA	F01_transcript_100562
F01_transcript_148549	NA	F01_transcript_148549	NA	NA
F01_transcript_81699	F01_transcript_81699	F01_transcript_81699	NA	NA
F01_transcript_74516	NA	F01_transcript_74516	NA	NA
F01_transcript_46635	NA	F01_transcript_46635	NA	NA
F01_transcript_83990	F01_transcript_83990	F01_transcript_83990	NA	F01_transcript_83990
F01_transcript_130355	NA	F01_transcript_130355	NA	NA
F01_transcript_96654	NA	F01_transcript_96654	NA	NA

F01_transcript_137809	NA	NA	NA	F01_transcript_137809
F01_transcript_52018	NA	NA	NA	F01_transcript_52018
F01_transcript_82422	NA	F01_transcript_82422	NA	F01_transcript_82422
F01_transcript_77092	NA	NA	NA	F01_transcript_77092
F01_transcript_139969	F01_transcript_139969	F01_transcript_139969	F01_transcript_139969	F01_transcript_139969
F01_transcript_1009	NA	F01_transcript_1009	F01_transcript_1009	F01_transcript_1009
F01_transcript_63538	F01_transcript_63538	F01_transcript_63538	F01_transcript_63538	F01_transcript_63538
F01_transcript_73721	NA	NA	NA	F01_transcript_73721
F01_transcript_4817	NA	F01_transcript_4817	NA	NA
F01_transcript_102564	NA	NA	NA	F01_transcript_102564
F01_transcript_145907	F01_transcript_145907	F01_transcript_145907	F01_transcript_145907	F01_transcript_145907
F01_transcript_67211	NA	F01_transcript_67211	NA	NA
F01_transcript_58309	F01_transcript_58309	F01_transcript_58309	F01_transcript_58309	NA
F01_transcript_102581	NA	F01_transcript_102581	F01_transcript_102581	NA

F01_transcript_60420	NA	F01_transcript_60420	F01_transcript_60420	F01_transcript_60420
F01_transcript_144899	NA	NA	NA	F01_transcript_144899
F01_transcript_114440	NA	F01_transcript_114440	NA	F01_transcript_114440
F01_transcript_137938	F01_transcript_137938	F01_transcript_137938	F01_transcript_137938	F01_transcript_137938
F01_transcript_36295	NA	NA	NA	F01_transcript_36295
F01_transcript_155778	NA	NA	NA	F01_transcript_155778
F01_transcript_142545	NA	NA	NA	F01_transcript_142545
F01_transcript_74473	F01_transcript_74473	F01_transcript_74473	F01_transcript_74473	F01_transcript_74473
F01_transcript_159812	F01_transcript_159812	F01_transcript_159812	F01_transcript_159812	F01_transcript_159812
F01_transcript_92181	F01_transcript_92181	F01_transcript_92181	F01_transcript_92181	F01_transcript_92181
F01_transcript_65129	NA	NA	F01_transcript_65129	NA
F01_transcript_129905	F01_transcript_129905	F01_transcript_129905	F01_transcript_129905	F01_transcript_129905
F01_transcript_82627	NA	F01_transcript_82627	NA	NA
F01_transcript_129762	F01_transcript_129762	F01_transcript_129762	F01_transcript_129762	F01_transcript_129762

F01_transcript_106302	NA	F01_transcript_106302	F01_transcript_106302	NA
F01_transcript_135548	NA	NA	F01_transcript_135548	NA
F01_transcript_97987	NA	F01_transcript_97987	NA	NA
F01_transcript_106768	NA	NA	NA	F01_transcript_106768
F01_transcript_57165	NA	NA	F01_transcript_57165	NA
F01_transcript_85471	NA	NA	NA	F01_transcript_85471
F01_transcript_50349	F01_transcript_50349	F01_transcript_50349	F01_transcript_50349	F01_transcript_50349
F01_transcript_48547	NA	F01_transcript_48547	NA	NA
F01_transcript_132895	F01_transcript_132895	F01_transcript_132895	F01_transcript_132895	F01_transcript_132895
F01_transcript_104705	F01_transcript_104705	F01_transcript_104705	F01_transcript_104705	F01_transcript_104705
F01_transcript_122308	NA	F01_transcript_122308	NA	NA
F01_transcript_166345	F01_transcript_166345	F01_transcript_166345	F01_transcript_166345	F01_transcript_166345
F01_transcript_48842	NA	F01_transcript_48842	F01_transcript_48842	F01_transcript_48842
F01_transcript_78322	F01_transcript_78322	F01_transcript_78322	F01_transcript_78322	F01_transcript_78322

F01_transcript_136083	NA	F01_transcript_136083	NA	NA
F01_transcript_141508	NA	NA	NA	F01_transcript_141508
F01_transcript_105888	NA	NA	NA	F01_transcript_105888
F01_transcript_152205	NA	F01_transcript_152205	F01_transcript_152205	F01_transcript_152205
F01_transcript_38743	F01_transcript_38743	F01_transcript_38743	F01_transcript_38743	NA
F01_transcript_5306	NA	F01_transcript_5306	NA	F01_transcript_5306
F01_transcript_78432	NA	F01_transcript_78432	NA	NA
F01_transcript_132851	NA	NA	NA	F01_transcript_132851
F01_transcript_139824	NA	F01_transcript_139824	NA	NA
F01_transcript_84945	F01_transcript_84945	F01_transcript_84945	NA	F01_transcript_84945
F01_transcript_125002	F01_transcript_125002	F01_transcript_125002	F01_transcript_125002	F01_transcript_125002
F01_transcript_80746	NA	F01_transcript_80746	NA	F01_transcript_80746
F01_transcript_53900	NA	NA	NA	F01_transcript_53900
F01_transcript_78305	NA	F01_transcript_78305	NA	NA

F01_transcript_11455	NA	NA	NA	F01_transcript_11455
F01_transcript_110484	F01_transcript_110484	F01_transcript_110484	F01_transcript_110484	F01_transcript_110484
F01_transcript_61021	NA	NA	NA	F01_transcript_61021
F01_transcript_137108	NA	NA	NA	F01_transcript_137108
F01_transcript_65051	NA	NA	NA	F01_transcript_65051
F01_transcript_60287	F01_transcript_60287	F01_transcript_60287	NA	NA
F01_transcript_152808	NA	F01_transcript_152808	F01_transcript_152808	NA
F01_transcript_73532	F01_transcript_73532	F01_transcript_73532	F01_transcript_73532	F01_transcript_73532
F01_transcript_63489	F01_transcript_63489	F01_transcript_63489	NA	NA
F01_transcript_3875	NA	F01_transcript_3875	F01_transcript_3875	F01_transcript_3875
F01_transcript_48256	NA	NA	NA	F01_transcript_48256
F01_transcript_51314	F01_transcript_51314	F01_transcript_51314	F01_transcript_51314	F01_transcript_51314
F01_transcript_57345	F01_transcript_57345	F01_transcript_57345	NA	NA
F01_transcript_50391	NA	F01_transcript_50391	NA	NA

F01_transcript_164747	NA	F01_transcript_164747	NA	NA
F01_transcript_40079	NA	F01_transcript_40079	NA	NA
F01_transcript_122548	NA	F01_transcript_122548	NA	NA
F01_transcript_55596	NA	F01_transcript_55596	NA	NA
F01_transcript_50195	NA	F01_transcript_50195	NA	NA
F01_transcript_50681	F01_transcript_50681	F01_transcript_50681	NA	F01_transcript_50681
F01_transcript_117477	NA	F01_transcript_117477	NA	F01_transcript_117477
F01_transcript_134126	NA	F01_transcript_134126	NA	NA
F01_transcript_56388	F01_transcript_56388	F01_transcript_56388	F01_transcript_56388	F01_transcript_56388
F01_transcript_125913	NA	F01_transcript_125913	F01_transcript_125913	F01_transcript_125913
F01_transcript_152180	NA	NA	NA	F01_transcript_152180
F01_transcript_120842	NA	F01_transcript_120842	NA	NA
F01_transcript_165154	NA	F01_transcript_165154	NA	NA
F01_transcript_81206	F01_transcript_81206	F01_transcript_81206	NA	NA

F01_transcript_100685	NA	F01_transcript_100685	NA	NA
F01_transcript_82945	F01_transcript_82945	F01_transcript_82945	F01_transcript_82945	F01_transcript_82945
F01_transcript_144563	NA	F01_transcript_144563	NA	NA
F01_transcript_145822	F01_transcript_145822	F01_transcript_145822	F01_transcript_145822	F01_transcript_145822
F01_transcript_137105	NA	F01_transcript_137105	NA	NA
F01_transcript_18617	NA	F01_transcript_18617	NA	NA
F01_transcript_154529	F01_transcript_154529	F01_transcript_154529	F01_transcript_154529	F01_transcript_154529
F01_transcript_127446	NA	NA	NA	F01_transcript_127446
F01_transcript_33420	NA	F01_transcript_33420	NA	F01_transcript_33420
F01_transcript_53500	NA	NA	NA	F01_transcript_53500
F01_transcript_57958	NA	F01_transcript_57958	NA	F01_transcript_57958
F01_transcript_101075	NA	F01_transcript_101075	NA	NA
F01_transcript_4200	F01_transcript_4200	F01_transcript_4200	NA	F01_transcript_4200
F01_transcript_89722	NA	NA	NA	F01_transcript_89722

F01_transcript_87027	NA	NA	NA	F01_transcript_87027
F01_transcript_131715	NA	F01_transcript_131715	NA	NA
F01_transcript_90153	NA	F01_transcript_90153	NA	F01_transcript_90153
F01_transcript_7575	NA	F01_transcript_7575	NA	NA
F01_transcript_4212	NA	F01_transcript_4212	F01_transcript_4212	NA
F01_transcript_139734	NA	NA	NA	F01_transcript_139734
F01_transcript_165224	NA	NA	NA	F01_transcript_165224
F01_transcript_56858	F01_transcript_56858	F01_transcript_56858	F01_transcript_56858	F01_transcript_56858
F01_transcript_135111	F01_transcript_135111	F01_transcript_135111	F01_transcript_135111	F01_transcript_135111
F01_transcript_47476	NA	F01_transcript_47476	NA	NA
F01_transcript_53792	F01_transcript_53792	F01_transcript_53792	F01_transcript_53792	F01_transcript_53792
F01_transcript_152047	NA	NA	NA	F01_transcript_152047
F01_transcript_135653	NA	NA	NA	F01_transcript_135653
F01_transcript_120904	F01_transcript_120904	F01_transcript_120904	F01_transcript_120904	F01_transcript_120904

F01_transcript_150033	F01_transcript_150033	F01_transcript_150033	F01_transcript_150033	F01_transcript_150033
F01_transcript_154226	F01_transcript_154226	F01_transcript_154226	F01_transcript_154226	F01_transcript_154226
F01_transcript_158222	F01_transcript_158222	NA	F01_transcript_158222	F01_transcript_158222
F01_transcript_147120	NA	NA	NA	F01_transcript_147120
F01_transcript_124971	NA	F01_transcript_124971	NA	F01_transcript_124971
F01_transcript_11931	NA	F01_transcript_11931	NA	F01_transcript_11931
F01_transcript_150049	NA	F01_transcript_150049	NA	NA
F01_transcript_132984	F01_transcript_132984	F01_transcript_132984	NA	NA
F01_transcript_82922	NA	F01_transcript_82922	NA	NA
F01_transcript_57048	NA	NA	F01_transcript_57048	NA
F01_transcript_128495	NA	NA	NA	F01_transcript_128495
F01_transcript_154913	NA	F01_transcript_154913	NA	NA
F01_transcript_158007	NA	NA	NA	F01_transcript_158007
F01_transcript_115777	F01_transcript_115777	F01_transcript_115777	F01_transcript_115777	F01_transcript_115777

F01_transcript_118259	NA	NA	NA	F01_transcript_118259
F01_transcript_35779	NA	NA	NA	F01_transcript_35779
F01_transcript_48939	NA	F01_transcript_48939	NA	NA
F01_transcript_137715	NA	F01_transcript_137715	NA	NA
F01_transcript_73210	F01_transcript_73210	F01_transcript_73210	NA	NA
F01_transcript_148545	NA	F01_transcript_148545	NA	NA
F01_transcript_114372	F01_transcript_114372	F01_transcript_114372	F01_transcript_114372	F01_transcript_114372
F01_transcript_97632	NA	NA	NA	F01_transcript_97632
F01_transcript_81904	F01_transcript_81904	F01_transcript_81904	F01_transcript_81904	F01_transcript_81904
F01_transcript_89095	NA	F01_transcript_89095	NA	NA
F01_transcript_56199	NA	F01_transcript_56199	NA	NA
F01_transcript_63883	F01_transcript_63883	F01_transcript_63883	F01_transcript_63883	F01_transcript_63883
F01_transcript_86322	F01_transcript_86322	F01_transcript_86322	NA	NA
F01_transcript_13925	F01_transcript_13925	F01_transcript_13925	F01_transcript_13925	F01_transcript_13925

F01_transcript_163001	NA	F01_transcript_163001	NA	NA
F01_transcript_105000	NA	NA	NA	F01_transcript_105000
F01_transcript_84866	NA	F01_transcript_84866	NA	NA
F01_transcript_73658	F01_transcript_73658	F01_transcript_73658	F01_transcript_73658	F01_transcript_73658
F01_transcript_91136	F01_transcript_91136	F01_transcript_91136	F01_transcript_91136	F01_transcript_91136
F01_transcript_151868	F01_transcript_151868	F01_transcript_151868	F01_transcript_151868	F01_transcript_151868
F01_transcript_29334	NA	NA	NA	F01_transcript_29334
F01_transcript_95585	NA	NA	NA	F01_transcript_95585
F01_transcript_83996	NA	NA	NA	F01_transcript_83996
F01_transcript_143852	NA	F01_transcript_143852	NA	F01_transcript_143852
F01_transcript_152572	NA	F01_transcript_152572	NA	NA
F01_transcript_105318	NA	F01_transcript_105318	NA	NA
F01_transcript_120228	NA	F01_transcript_120228	NA	NA
F01_transcript_68207	NA	F01_transcript_68207	NA	F01_transcript_68207

F01_transcript_40849	NA	NA	NA	F01_transcript_40849
F01_transcript_144942	F01_transcript_144942	F01_transcript_144942	F01_transcript_144942	F01_transcript_144942
F01_transcript_104400	NA	NA	NA	F01_transcript_104400
F01_transcript_127866	NA	F01_transcript_127866	NA	F01_transcript_127866
F01_transcript_126472	NA	NA	NA	F01_transcript_126472
F01_transcript_167610	NA	F01_transcript_167610	NA	NA
F01_transcript_115076	NA	NA	NA	F01_transcript_115076
F01_transcript_167342	F01_transcript_167342	F01_transcript_167342	NA	NA
F01_transcript_73171	NA	NA	NA	F01_transcript_73171
F01_transcript_50996	F01_transcript_50996	F01_transcript_50996	F01_transcript_50996	F01_transcript_50996
F01_transcript_8894	NA	NA	NA	F01_transcript_8894
F01_transcript_54224	NA	NA	NA	F01_transcript_54224
F01_transcript_57035	NA	NA	F01_transcript_57035	NA
F01_transcript_62662	NA	F01_transcript_62662	NA	NA

F01_transcript_89655	NA	NA	NA	F01_transcript_89655
F01_transcript_74244	NA	NA	NA	F01_transcript_74244
F01_transcript_86862	NA	NA	NA	F01_transcript_86862
F01_transcript_27328	NA	NA	NA	F01_transcript_27328
F01_transcript_162223	NA	F01_transcript_162223	NA	NA
F01_transcript_142515	NA	NA	NA	F01_transcript_142515
F01_transcript_96583	F01_transcript_96583	F01_transcript_96583	NA	F01_transcript_96583
F01_transcript_96780	NA	F01_transcript_96780	NA	NA
F01_transcript_74569	F01_transcript_74569	F01_transcript_74569	F01_transcript_74569	F01_transcript_74569
F01_transcript_69587	NA	NA	NA	F01_transcript_69587
F01_transcript_161519	F01_transcript_161519	F01_transcript_161519	F01_transcript_161519	F01_transcript_161519
F01_transcript_106817	NA	NA	NA	F01_transcript_106817
F01_transcript_90250	NA	F01_transcript_90250	NA	NA
F01_transcript_133187	NA	F01_transcript_133187	NA	NA

F01_transcript_119001	NA	NA	F01_transcript_119001	F01_transcript_119001
F01_transcript_28877	F01_transcript_28877	F01_transcript_28877	F01_transcript_28877	F01_transcript_28877
F01_transcript_41155	NA	NA	NA	F01_transcript_41155
F01_transcript_156988	NA	F01_transcript_156988	F01_transcript_156988	F01_transcript_156988
F01_transcript_154671	NA	NA	NA	F01_transcript_154671
F01_transcript_108647	NA	NA	NA	F01_transcript_108647
F01_transcript_140179	F01_transcript_140179	F01_transcript_140179	NA	NA
F01_transcript_81290	NA	F01_transcript_81290	F01_transcript_81290	F01_transcript_81290
F01_transcript_113801	NA	NA	NA	F01_transcript_113801
F01_transcript_4416	F01_transcript_4416	F01_transcript_4416	F01_transcript_4416	F01_transcript_4416
F01_transcript_146865	F01_transcript_146865	F01_transcript_146865	F01_transcript_146865	F01_transcript_146865
F01_transcript_113057	NA	F01_transcript_113057	NA	F01_transcript_113057
F01_transcript_99157	NA	F01_transcript_99157	NA	NA
F01_transcript_104876	NA	F01_transcript_104876	NA	F01_transcript_104876

F01_transcript_131822	NA	F01_transcript_131822	NA	NA
F01_transcript_126794	F01_transcript_126794	F01_transcript_126794	F01_transcript_126794	F01_transcript_126794
F01_transcript_82711	NA	NA	NA	F01_transcript_82711
F01_transcript_62382	F01_transcript_62382	F01_transcript_62382	F01_transcript_62382	F01_transcript_62382
F01_transcript_127221	NA	F01_transcript_127221	NA	NA
F01_transcript_142370	NA	NA	NA	F01_transcript_142370
F01_transcript_48224	F01_transcript_48224	NA	NA	NA
F01_transcript_134831	NA	NA	NA	F01_transcript_134831
F01_transcript_4539	NA	NA	NA	F01_transcript_4539
F01_transcript_74260	NA	NA	NA	F01_transcript_74260
F01_transcript_99531	NA	NA	NA	F01_transcript_99531
F01_transcript_108242	NA	F01_transcript_108242	F01_transcript_108242	F01_transcript_108242
F01_transcript_15727	NA	NA	NA	F01_transcript_15727
F01_transcript_150169	F01_transcript_150169	F01_transcript_150169	F01_transcript_150169	F01_transcript_150169

F01_transcript_149696	F01_transcript_149696	F01_transcript_149696	F01_transcript_149696	F01_transcript_149696
F01_transcript_20927	NA	NA	NA	F01_transcript_20927
F01_transcript_136596	NA	F01_transcript_136596	NA	F01_transcript_136596
F01_transcript_85952	NA	NA	NA	F01_transcript_85952
F01_transcript_82793	NA	NA	NA	F01_transcript_82793
F01_transcript_85106	NA	F01_transcript_85106	NA	NA
F01_transcript_129714	F01_transcript_129714	F01_transcript_129714	F01_transcript_129714	NA
F01_transcript_131371	NA	F01_transcript_131371	NA	NA
F01_transcript_152577	NA	NA	NA	F01_transcript_152577
F01_transcript_82084	NA	F01_transcript_82084	NA	NA
F01_transcript_89483	NA	F01_transcript_89483	NA	NA
F01_transcript_75050	NA	F01_transcript_75050	NA	NA
F01_transcript_136202	F01_transcript_136202	F01_transcript_136202	NA	F01_transcript_136202
F01_transcript_82342	F01_transcript_82342	F01_transcript_82342	F01_transcript_82342	F01_transcript_82342

F01_transcript_43298	NA	NA	NA	F01_transcript_43298
F01_transcript_85671	NA	NA	NA	F01_transcript_85671
F01_transcript_59230	NA	NA	NA	F01_transcript_59230
F01_transcript_163500	F01_transcript_163500	NA	NA	NA
F01_transcript_125604	F01_transcript_125604	F01_transcript_125604	F01_transcript_125604	F01_transcript_125604
F01_transcript_43873	NA	NA	NA	F01_transcript_43873
F01_transcript_101730	NA	F01_transcript_101730	NA	NA
F01_transcript_132884	NA	F01_transcript_132884	NA	NA
F01_transcript_95329	F01_transcript_95329	F01_transcript_95329	F01_transcript_95329	F01_transcript_95329
F01_transcript_124	NA	NA	NA	F01_transcript_124
F01_transcript_98833	F01_transcript_98833	F01_transcript_98833	F01_transcript_98833	F01_transcript_98833
F01_transcript_56074	NA	F01_transcript_56074	NA	F01_transcript_56074
F01_transcript_80404	NA	NA	NA	F01_transcript_80404
F01_transcript_154205	NA	NA	NA	F01_transcript_154205

F01_transcript_130589	F01_transcript_130589	F01_transcript_130589	NA	F01_transcript_130589
F01_transcript_109349	F01_transcript_109349	F01_transcript_109349	F01_transcript_109349	F01_transcript_109349
F01_transcript_61931	NA	F01_transcript_61931	NA	F01_transcript_61931
F01_transcript_133027	NA	NA	NA	F01_transcript_133027
F01_transcript_92829	NA	F01_transcript_92829	NA	F01_transcript_92829
F01_transcript_80548	NA	F01_transcript_80548	NA	NA
F01_transcript_44952	F01_transcript_44952	F01_transcript_44952	F01_transcript_44952	F01_transcript_44952
F01_transcript_148936	NA	F01_transcript_148936	NA	NA
F01_transcript_55135	NA	F01_transcript_55135	NA	F01_transcript_55135
F01_transcript_131425	NA	F01_transcript_131425	NA	NA
F01_transcript_60148	F01_transcript_60148	F01_transcript_60148	NA	F01_transcript_60148
F01_transcript_126213	F01_transcript_126213	F01_transcript_126213	F01_transcript_126213	F01_transcript_126213
F01_transcript_68081	NA	F01_transcript_68081	NA	NA
F01_transcript_85105	F01_transcript_85105	F01_transcript_85105	F01_transcript_85105	F01_transcript_85105

F01_transcript_88891	F01_transcript_88891	F01_transcript_88891	F01_transcript_88891	F01_transcript_88891
F01_transcript_141454	NA	F01_transcript_141454	F01_transcript_141454	F01_transcript_141454
F01_transcript_72140	F01_transcript_72140	F01_transcript_72140	NA	F01_transcript_72140
F01_transcript_156616	F01_transcript_156616	F01_transcript_156616	NA	F01_transcript_156616
F01_transcript_113024	NA	F01_transcript_113024	NA	F01_transcript_113024
F01_transcript_107616	NA	F01_transcript_107616	NA	NA
F01_transcript_48160	NA	NA	NA	F01_transcript_48160
F01_transcript_104342	NA	NA	NA	F01_transcript_104342
F01_transcript_165094	NA	NA	NA	F01_transcript_165094
F01_transcript_120035	F01_transcript_120035	F01_transcript_120035	NA	NA
F01_transcript_32839	NA	NA	NA	F01_transcript_32839
F01_transcript_91522	NA	NA	NA	F01_transcript_91522
F01_transcript_48841	NA	NA	NA	F01_transcript_48841
F01_transcript_15862	NA	NA	NA	F01_transcript_15862

F01_transcript_66830	NA	NA	NA	F01_transcript_66830
F01_transcript_52979	NA	F01_transcript_52979	NA	NA
F01_transcript_6893	NA	F01_transcript_6893	NA	F01_transcript_6893
F01_transcript_127302	NA	F01_transcript_127302	NA	NA
F01_transcript_79243	NA	F01_transcript_79243	NA	NA
F01_transcript_44073	NA	NA	NA	F01_transcript_44073
F01_transcript_7030	NA	NA	NA	F01_transcript_7030
F01_transcript_105251	NA	F01_transcript_105251	NA	F01_transcript_105251
F01_transcript_62510	NA	F01_transcript_62510	NA	NA
F01_transcript_154788	F01_transcript_154788	F01_transcript_154788	F01_transcript_154788	F01_transcript_154788
F01_transcript_119161	NA	NA	NA	F01_transcript_119161
F01_transcript_65372	F01_transcript_65372	F01_transcript_65372	F01_transcript_65372	NA
F01_transcript_115801	NA	NA	NA	F01_transcript_115801
F01_transcript_51051	F01_transcript_51051	F01_transcript_51051	F01_transcript_51051	F01_transcript_51051

F01_transcript_19308	NA	F01_transcript_19308	NA	NA
F01_transcript_328	NA	F01_transcript_328	NA	NA
F01_transcript_143492	NA	F01_transcript_143492	NA	F01_transcript_143492
F01_transcript_54748	NA	F01_transcript_54748	NA	NA
F01_transcript_128580	NA	NA	NA	F01_transcript_128580
F01_transcript_65368	F01_transcript_65368	F01_transcript_65368	F01_transcript_65368	F01_transcript_65368
F01_transcript_83329	NA	F01_transcript_83329	NA	F01_transcript_83329
F01_transcript_81475	NA	F01_transcript_81475	F01_transcript_81475	F01_transcript_81475
F01_transcript_67426	NA	NA	NA	F01_transcript_67426
F01_transcript_107832	NA	NA	F01_transcript_107832	NA
F01_transcript_121184	NA	F01_transcript_121184	F01_transcript_121184	NA
F01_transcript_133221	NA	NA	NA	F01_transcript_133221
F01_transcript_2904	NA	F01_transcript_2904	NA	F01_transcript_2904
F01_transcript_98269	F01_transcript_98269	F01_transcript_98269	F01_transcript_98269	F01_transcript_98269

F01_transcript_58592	F01_transcript_58592	F01_transcript_58592	NA	F01_transcript_58592
F01_transcript_102140	NA	NA	NA	F01_transcript_102140
F01_transcript_109153	F01_transcript_109153	F01_transcript_109153	F01_transcript_109153	F01_transcript_109153
F01_transcript_105356	NA	F01_transcript_105356	F01_transcript_105356	F01_transcript_105356
F01_transcript_15997	NA	NA	NA	F01_transcript_15997
F01_transcript_91231	NA	NA	NA	F01_transcript_91231
F01_transcript_144668	F01_transcript_144668	F01_transcript_144668	NA	F01_transcript_144668
F01_transcript_98753	NA	F01_transcript_98753	NA	NA
F01_transcript_49152	NA	NA	NA	F01_transcript_49152
F01_transcript_97009	NA	F01_transcript_97009	NA	NA
F01_transcript_134101	NA	NA	NA	F01_transcript_134101
F01_transcript_86104	NA	NA	NA	F01_transcript_86104
F01_transcript_95690	F01_transcript_95690	F01_transcript_95690	F01_transcript_95690	F01_transcript_95690
F01_transcript_71693	NA	NA	F01_transcript_71693	F01_transcript_71693

F01_transcript_142634	NA	F01_transcript_142634	NA	NA
F01_transcript_57757	NA	NA	NA	F01_transcript_57757
F01_transcript_149891	NA	F01_transcript_149891	NA	NA
F01_transcript_80864	NA	NA	NA	F01_transcript_80864
F01_transcript_76304	NA	NA	NA	F01_transcript_76304
F01_transcript_22199	NA	NA	NA	F01_transcript_22199
F01_transcript_161754	F01_transcript_161754	F01_transcript_161754	NA	NA
F01_transcript_66189	F01_transcript_66189	F01_transcript_66189	F01_transcript_66189	F01_transcript_66189
F01_transcript_112846	NA	NA	NA	F01_transcript_112846
F01_transcript_157471	NA	F01_transcript_157471	NA	NA
F01_transcript_82615	NA	F01_transcript_82615	NA	NA
F01_transcript_76867	NA	NA	NA	F01_transcript_76867
F01_transcript_138116	F01_transcript_138116	F01_transcript_138116	NA	F01_transcript_138116
F01_transcript_57023	NA	NA	F01_transcript_57023	NA

F01_transcript_128177	NA	F01_transcript_128177	F01_transcript_128177	F01_transcript_128177
F01_transcript_49528	F01_transcript_49528	F01_transcript_49528	F01_transcript_49528	F01_transcript_49528
F01_transcript_140781	NA	F01_transcript_140781	F01_transcript_140781	F01_transcript_140781
F01_transcript_141207	F01_transcript_141207	F01_transcript_141207	NA	NA
F01_transcript_4286	NA	F01_transcript_4286	NA	NA
F01_transcript_67753	NA	NA	F01_transcript_67753	F01_transcript_67753
F01_transcript_92033	F01_transcript_92033	F01_transcript_92033	NA	NA
F01_transcript_84724	NA	F01_transcript_84724	NA	NA
F01_transcript_105358	NA	F01_transcript_105358	F01_transcript_105358	F01_transcript_105358
F01_transcript_119062	F01_transcript_119062	F01_transcript_119062	F01_transcript_119062	F01_transcript_119062
F01_transcript_82934	NA	F01_transcript_82934	F01_transcript_82934	F01_transcript_82934
F01_transcript_164677	NA	F01_transcript_164677	NA	NA
F01_transcript_53794	F01_transcript_53794	F01_transcript_53794	NA	F01_transcript_53794
F01_transcript_57022	F01_transcript_57022	F01_transcript_57022	F01_transcript_57022	NA

F01_transcript_124272	NA	F01_transcript_124272	NA	F01_transcript_124272
F01_transcript_55067	NA	F01_transcript_55067	NA	F01_transcript_55067
F01_transcript_99795	NA	F01_transcript_99795	NA	F01_transcript_99795
F01_transcript_52157	F01_transcript_52157	F01_transcript_52157	F01_transcript_52157	F01_transcript_52157
F01_transcript_49902	NA	NA	NA	F01_transcript_49902
F01_transcript_121997	F01_transcript_121997	F01_transcript_121997	F01_transcript_121997	F01_transcript_121997
F01_transcript_160588	NA	F01_transcript_160588	NA	NA
F01_transcript_63689	NA	F01_transcript_63689	NA	NA
F01_transcript_103294	F01_transcript_103294	F01_transcript_103294	F01_transcript_103294	F01_transcript_103294
F01_transcript_151018	NA	F01_transcript_151018	NA	NA
F01_transcript_81471	F01_transcript_81471	F01_transcript_81471	F01_transcript_81471	F01_transcript_81471
F01_transcript_123252	F01_transcript_123252	F01_transcript_123252	F01_transcript_123252	F01_transcript_123252
F01_transcript_85810	NA	F01_transcript_85810	NA	F01_transcript_85810
F01_transcript_48345	NA	NA	NA	F01_transcript_48345

F01_transcript_122745	NA	NA	NA	F01_transcript_122745
F01_transcript_157741	NA	NA	F01_transcript_157741	NA
F01_transcript_91301	NA	NA	NA	F01_transcript_91301
F01_transcript_163925	NA	NA	NA	F01_transcript_163925
F01_transcript_142623	NA	NA	NA	F01_transcript_142623
F01_transcript_166079	NA	NA	NA	F01_transcript_166079
F01_transcript_26785	NA	NA	NA	F01_transcript_26785
F01_transcript_38	NA	NA	NA	F01_transcript_38
F01_transcript_72200	F01_transcript_72200	F01_transcript_72200	F01_transcript_72200	F01_transcript_72200
F01_transcript_72822	NA	NA	NA	F01_transcript_72822
F01_transcript_162205	NA	NA	NA	F01_transcript_162205
F01_transcript_41706	NA	NA	NA	F01_transcript_41706
F01_transcript_116176	NA	F01_transcript_116176	F01_transcript_116176	F01_transcript_116176
F01_transcript_102958	NA	F01_transcript_102958	NA	F01_transcript_102958

F01_transcript_55109	NA	F01_transcript_55109	NA	NA
F01_transcript_108553	NA	F01_transcript_108553	NA	F01_transcript_108553
F01_transcript_149777	NA	NA	NA	F01_transcript_149777
F01_transcript_91881	F01_transcript_91881	F01_transcript_91881	F01_transcript_91881	F01_transcript_91881
F01_transcript_144125	NA	F01_transcript_144125	F01_transcript_144125	F01_transcript_144125
F01_transcript_3652	NA	F01_transcript_3652	NA	NA
F01_transcript_97570	NA	NA	NA	F01_transcript_97570
F01_transcript_154086	F01_transcript_154086	F01_transcript_154086	F01_transcript_154086	F01_transcript_154086
F01_transcript_42538	NA	NA	NA	F01_transcript_42538
F01_transcript_153892	NA	F01_transcript_153892	F01_transcript_153892	F01_transcript_153892
F01_transcript_116284	F01_transcript_116284	F01_transcript_116284	NA	NA
F01_transcript_58556	NA	F01_transcript_58556	NA	NA
F01_transcript_144761	NA	F01_transcript_144761	NA	NA
F01_transcript_36407	NA	NA	NA	F01_transcript_36407

F01_transcript_85394	NA	F01_transcript_85394	NA	NA
F01_transcript_103382	NA	F01_transcript_103382	F01_transcript_103382	NA
F01_transcript_71488	NA	F01_transcript_71488	NA	NA
F01_transcript_155575	NA	NA	NA	F01_transcript_155575
F01_transcript_95256	F01_transcript_95256	F01_transcript_95256	F01_transcript_95256	F01_transcript_95256
F01_transcript_112005	NA	NA	NA	F01_transcript_112005
F01_transcript_100143	NA	NA	NA	F01_transcript_100143
F01_transcript_83738	NA	F01_transcript_83738	F01_transcript_83738	F01_transcript_83738
F01_transcript_163455	NA	NA	NA	F01_transcript_163455
F01_transcript_135211	NA	F01_transcript_135211	NA	NA
F01_transcript_60623	F01_transcript_60623	F01_transcript_60623	F01_transcript_60623	F01_transcript_60623
F01_transcript_83587	NA	F01_transcript_83587	NA	NA
F01_transcript_136982	NA	NA	NA	F01_transcript_136982
F01_transcript_71773	NA	F01_transcript_71773	F01_transcript_71773	F01_transcript_71773

F01_transcript_143185	NA	NA	NA	F01_transcript_143185
F01_transcript_97050	NA	F01_transcript_97050	NA	NA
F01_transcript_2262	NA	F01_transcript_2262	NA	NA
F01_transcript_68668	NA	F01_transcript_68668	F01_transcript_68668	F01_transcript_68668
F01_transcript_109696	NA	F01_transcript_109696	NA	NA
F01_transcript_96460	F01_transcript_96460	F01_transcript_96460	NA	NA
F01_transcript_88264	F01_transcript_88264	F01_transcript_88264	F01_transcript_88264	F01_transcript_88264
F01_transcript_91414	NA	NA	NA	F01_transcript_91414
F01_transcript_22570	NA	F01_transcript_22570	F01_transcript_22570	F01_transcript_22570
F01_transcript_31166	NA	NA	NA	F01_transcript_31166
F01_transcript_134797	F01_transcript_134797	F01_transcript_134797	F01_transcript_134797	F01_transcript_134797
F01_transcript_93672	F01_transcript_93672	F01_transcript_93672	NA	NA
F01_transcript_110109	NA	F01_transcript_110109	NA	F01_transcript_110109
F01_transcript_58293	F01_transcript_58293	F01_transcript_58293	F01_transcript_58293	F01_transcript_58293

F01_transcript_45305	F01_transcript_45305	F01_transcript_45305	F01_transcript_45305	F01_transcript_45305
F01_transcript_114063	NA	F01_transcript_114063	NA	F01_transcript_114063
F01_transcript_52478	NA	F01_transcript_52478	F01_transcript_52478	F01_transcript_52478
F01_transcript_49121	F01_transcript_49121	NA	NA	F01_transcript_49121
F01_transcript_46406	NA	NA	NA	F01_transcript_46406
F01_transcript_160543	NA	F01_transcript_160543	NA	F01_transcript_160543
F01_transcript_130106	NA	NA	NA	F01_transcript_130106
F01_transcript_24276	NA	NA	NA	F01_transcript_24276
F01_transcript_155552	F01_transcript_155552	F01_transcript_155552	F01_transcript_155552	F01_transcript_155552
F01_transcript_110938	NA	F01_transcript_110938	NA	NA
F01_transcript_100576	F01_transcript_100576	F01_transcript_100576	NA	F01_transcript_100576
F01_transcript_6980	F01_transcript_6980	F01_transcript_6980	NA	F01_transcript_6980
F01_transcript_10015	NA	NA	NA	F01_transcript_10015
F01_transcript_81856	NA	NA	NA	F01_transcript_81856

F01_transcript_119322	NA	F01_transcript_119322	NA	F01_transcript_119322
F01_transcript_125955	NA	F01_transcript_125955	NA	NA
F01_transcript_111074	NA	F01_transcript_111074	NA	F01_transcript_111074
F01_transcript_11876	NA	F01_transcript_11876	NA	F01_transcript_11876
F01_transcript_117159	NA	NA	NA	F01_transcript_117159
F01_transcript_155642	NA	F01_transcript_155642	NA	NA
F01_transcript_51269	F01_transcript_51269	F01_transcript_51269	F01_transcript_51269	F01_transcript_51269
F01_transcript_141231	NA	F01_transcript_141231	NA	F01_transcript_141231
F01_transcript_66536	NA	F01_transcript_66536	NA	NA
F01_transcript_18420	NA	NA	F01_transcript_18420	F01_transcript_18420
F01_transcript_67091	NA	NA	NA	F01_transcript_67091
F01_transcript_147314	NA	NA	NA	F01_transcript_147314
F01_transcript_17975	NA	NA	NA	F01_transcript_17975
F01_transcript_71164	NA	NA	F01_transcript_71164	NA

F01_transcript_158109	NA	F01_transcript_158109	NA	F01_transcript_158109
F01_transcript_131140	F01_transcript_131140	F01_transcript_131140	NA	F01_transcript_131140
F01_transcript_91022	NA	F01_transcript_91022	F01_transcript_91022	F01_transcript_91022
F01_transcript_70822	NA	NA	F01_transcript_70822	NA
F01_transcript_105653	NA	F01_transcript_105653	NA	NA
F01_transcript_81345	NA	NA	NA	F01_transcript_81345
F01_transcript_55721	NA	NA	NA	F01_transcript_55721
F01_transcript_79919	NA	F01_transcript_79919	F01_transcript_79919	F01_transcript_79919
F01_transcript_71611	NA	NA	NA	F01_transcript_71611
F01_transcript_49412	NA	F01_transcript_49412	F01_transcript_49412	F01_transcript_49412
F01_transcript_154306	F01_transcript_154306	F01_transcript_154306	F01_transcript_154306	F01_transcript_154306
F01_transcript_153817	F01_transcript_153817	F01_transcript_153817	F01_transcript_153817	F01_transcript_153817
F01_transcript_134640	F01_transcript_134640	F01_transcript_134640	NA	NA
F01_transcript_86757	NA	F01_transcript_86757	NA	NA

F01_transcript_131771	F01_transcript_131771	F01_transcript_131771	F01_transcript_131771	F01_transcript_131771
F01_transcript_164365	NA	NA	NA	F01_transcript_164365
F01_transcript_98241	NA	F01_transcript_98241	NA	F01_transcript_98241
F01_transcript_134941	NA	NA	NA	F01_transcript_134941
F01_transcript_130796	F01_transcript_130796	F01_transcript_130796	F01_transcript_130796	F01_transcript_130796
F01_transcript_51047	F01_transcript_51047	F01_transcript_51047	NA	F01_transcript_51047
F01_transcript_63039	NA	NA	NA	F01_transcript_63039
F01_transcript_154077	NA	F01_transcript_154077	NA	F01_transcript_154077
F01_transcript_85921	NA	F01_transcript_85921	NA	NA
F01_transcript_53918	NA	F01_transcript_53918	NA	F01_transcript_53918
F01_transcript_96327	NA	NA	F01_transcript_96327	NA
F01_transcript_109399	NA	F01_transcript_109399	NA	NA
F01_transcript_131382	F01_transcript_131382	F01_transcript_131382	F01_transcript_131382	F01_transcript_131382
F01_transcript_122457	F01_transcript_122457	F01_transcript_122457	F01_transcript_122457	F01_transcript_122457

F01_transcript_95242	NA	NA	NA	F01_transcript_95242
F01_transcript_56940	F01_transcript_56940	F01_transcript_56940	F01_transcript_56940	F01_transcript_56940
F01_transcript_62625	NA	F01_transcript_62625	NA	NA
F01_transcript_83125	NA	NA	NA	F01_transcript_83125
F01_transcript_70962	NA	NA	F01_transcript_70962	NA
F01_transcript_113530	NA	F01_transcript_113530	NA	NA
F01_transcript_153684	NA	NA	NA	F01_transcript_153684
F01_transcript_69098	F01_transcript_69098	F01_transcript_69098	NA	F01_transcript_69098
F01_transcript_78180	NA	F01_transcript_78180	NA	NA
F01_transcript_138550	F01_transcript_138550	F01_transcript_138550	F01_transcript_138550	F01_transcript_138550
F01_transcript_61396	NA	F01_transcript_61396	F01_transcript_61396	F01_transcript_61396
F01_transcript_161216	NA	F01_transcript_161216	NA	NA
F01_transcript_20572	NA	NA	NA	F01_transcript_20572
F01_transcript_111353	NA	F01_transcript_111353	NA	NA

F01_transcript_160903	NA	F01_transcript_160903	NA	NA
F01_transcript_69440	NA	NA	NA	F01_transcript_69440
F01_transcript_142775	NA	NA	NA	F01_transcript_142775
F01_transcript_14800	NA	NA	NA	F01_transcript_14800
F01_transcript_76739	NA	NA	NA	F01_transcript_76739
F01_transcript_96443	F01_transcript_96443	F01_transcript_96443	NA	F01_transcript_96443
F01_transcript_141609	NA	F01_transcript_141609	NA	NA
F01_transcript_147163	NA	F01_transcript_147163	F01_transcript_147163	F01_transcript_147163
F01_transcript_109228	F01_transcript_109228	F01_transcript_109228	NA	NA
F01_transcript_93546	NA	F01_transcript_93546	F01_transcript_93546	F01_transcript_93546
F01_transcript_40144	NA	NA	NA	F01_transcript_40144
F01_transcript_144551	NA	NA	NA	F01_transcript_144551
F01_transcript_99601	NA	F01_transcript_99601	NA	NA
F01_transcript_110021	NA	F01_transcript_110021	NA	F01_transcript_110021

F01_transcript_45161	NA	NA	NA	F01_transcript_45161
F01_transcript_149173	NA	F01_transcript_149173	NA	NA
F01_transcript_125029	F01_transcript_125029	F01_transcript_125029	NA	F01_transcript_125029
F01_transcript_57056	NA	NA	F01_transcript_57056	F01_transcript_57056
F01_transcript_165129	F01_transcript_165129	F01_transcript_165129	NA	F01_transcript_165129
F01_transcript_48016	NA	NA	NA	F01_transcript_48016
F01_transcript_99606	NA	NA	NA	F01_transcript_99606
F01_transcript_79013	F01_transcript_79013	F01_transcript_79013	F01_transcript_79013	F01_transcript_79013
F01_transcript_70126	F01_transcript_70126	F01_transcript_70126	F01_transcript_70126	F01_transcript_70126
F01_transcript_136300	NA	NA	NA	F01_transcript_136300
F01_transcript_135680	NA	F01_transcript_135680	F01_transcript_135680	F01_transcript_135680
F01_transcript_155491	NA	F01_transcript_155491	NA	F01_transcript_155491
F01_transcript_38705	F01_transcript_38705	F01_transcript_38705	NA	F01_transcript_38705
F01_transcript_138587	NA	F01_transcript_138587	NA	NA

F01_transcript_108363	NA	F01_transcript_108363	NA	F01_transcript_108363
F01_transcript_54882	NA	F01_transcript_54882	NA	F01_transcript_54882
F01_transcript_60070	NA	F01_transcript_60070	F01_transcript_60070	NA
F01_transcript_156334	NA	NA	NA	F01_transcript_156334
F01_transcript_29091	NA	NA	NA	F01_transcript_29091
F01_transcript_84330	F01_transcript_84330	F01_transcript_84330	F01_transcript_84330	F01_transcript_84330
F01_transcript_166943	NA	F01_transcript_166943	F01_transcript_166943	F01_transcript_166943
F01_transcript_122848	NA	NA	F01_transcript_122848	F01_transcript_122848
F01_transcript_128640	NA	F01_transcript_128640	NA	F01_transcript_128640
F01_transcript_121188	NA	NA	NA	F01_transcript_121188
F01_transcript_127301	F01_transcript_127301	F01_transcript_127301	F01_transcript_127301	NA
F01_transcript_119356	NA	NA	NA	F01_transcript_119356
F01_transcript_87891	F01_transcript_87891	F01_transcript_87891	F01_transcript_87891	F01_transcript_87891
F01_transcript_54049	NA	F01_transcript_54049	F01_transcript_54049	F01_transcript_54049

F01_transcript_75009	NA	NA	NA	F01_transcript_75009
F01_transcript_116757	F01_transcript_116757	F01_transcript_116757	F01_transcript_116757	F01_transcript_116757
F01_transcript_151614	NA	NA	NA	F01_transcript_151614
F01_transcript_50519	F01_transcript_50519	F01_transcript_50519	F01_transcript_50519	F01_transcript_50519
F01_transcript_59864	NA	F01_transcript_59864	NA	F01_transcript_59864
F01_transcript_114428	NA	F01_transcript_114428	NA	F01_transcript_114428
F01_transcript_105202	NA	NA	NA	F01_transcript_105202
F01_transcript_123861	NA	NA	NA	F01_transcript_123861
F01_transcript_114574	NA	NA	NA	F01_transcript_114574
F01_transcript_122644	NA	NA	F01_transcript_122644	F01_transcript_122644
F01_transcript_167395	NA	NA	NA	F01_transcript_167395
F01_transcript_87620	F01_transcript_87620	F01_transcript_87620	F01_transcript_87620	F01_transcript_87620
F01_transcript_129399	F01_transcript_129399	NA	NA	NA
F01_transcript_48906	NA	F01_transcript_48906	NA	NA

F01_transcript_69567	NA	F01_transcript_69567	NA	NA
F01_transcript_59234	F01_transcript_59234	F01_transcript_59234	F01_transcript_59234	F01_transcript_59234
F01_transcript_121013	NA	NA	NA	F01_transcript_121013
F01_transcript_79585	NA	NA	NA	F01_transcript_79585
F01_transcript_155709	NA	NA	F01_transcript_155709	NA
F01_transcript_64394	NA	NA	NA	F01_transcript_64394
F01_transcript_164538	NA	F01_transcript_164538	NA	NA
F01_transcript_103822	F01_transcript_103822	F01_transcript_103822	F01_transcript_103822	NA
F01_transcript_113422	NA	NA	NA	F01_transcript_113422
F01_transcript_143191	NA	F01_transcript_143191	NA	NA
F01_transcript_80105	NA	F01_transcript_80105	F01_transcript_80105	F01_transcript_80105
F01_transcript_72220	F01_transcript_72220	F01_transcript_72220	NA	NA
F01_transcript_28246	NA	NA	NA	F01_transcript_28246
F01_transcript_20150	NA	NA	NA	F01_transcript_20150

F01_transcript_122133	NA	NA	NA	F01_transcript_122133
F01_transcript_136733	NA	NA	NA	F01_transcript_136733
F01_transcript_58628	F01_transcript_58628	F01_transcript_58628	F01_transcript_58628	F01_transcript_58628
F01_transcript_36635	NA	NA	NA	F01_transcript_36635
F01_transcript_122489	NA	NA	NA	F01_transcript_122489
F01_transcript_75693	F01_transcript_75693	F01_transcript_75693	NA	F01_transcript_75693
F01_transcript_18140	NA	NA	NA	F01_transcript_18140
F01_transcript_76525	NA	NA	NA	F01_transcript_76525
F01_transcript_92109	NA	NA	NA	F01_transcript_92109
F01_transcript_75065	NA	F01_transcript_75065	NA	NA
F01_transcript_49291	F01_transcript_49291	F01_transcript_49291	NA	NA
F01_transcript_52199	NA	F01_transcript_52199	NA	NA
F01_transcript_119542	NA	NA	NA	F01_transcript_119542
F01_transcript_130180	NA	NA	NA	F01_transcript_130180

F01_transcript_113018	NA	NA	NA	F01_transcript_113018
F01_transcript_60708	F01_transcript_60708	F01_transcript_60708	NA	F01_transcript_60708
F01_transcript_140642	NA	NA	NA	F01_transcript_140642
F01_transcript_111999	NA	NA	NA	F01_transcript_111999
F01_transcript_138873	F01_transcript_138873	F01_transcript_138873	NA	NA
F01_transcript_80405	NA	NA	NA	F01_transcript_80405
F01_transcript_59509	NA	NA	F01_transcript_59509	NA
F01_transcript_64812	NA	NA	NA	F01_transcript_64812
F01_transcript_62181	F01_transcript_62181	F01_transcript_62181	F01_transcript_62181	NA
F01_transcript_128914	F01_transcript_128914	F01_transcript_128914	F01_transcript_128914	F01_transcript_128914
F01_transcript_130663	NA	NA	NA	F01_transcript_130663
F01_transcript_140863	NA	NA	NA	F01_transcript_140863
F01_transcript_64998	NA	F01_transcript_64998	NA	NA
F01_transcript_54635	NA	F01_transcript_54635	NA	NA

F01_transcript_25759	F01_transcript_25759	F01_transcript_25759	F01_transcript_25759	F01_transcript_25759
F01_transcript_68371	NA	NA	NA	F01_transcript_68371
F01_transcript_6554	NA	F01_transcript_6554	NA	NA
F01_transcript_100673	F01_transcript_100673	F01_transcript_100673	F01_transcript_100673	F01_transcript_100673
F01_transcript_77159	F01_transcript_77159	F01_transcript_77159	F01_transcript_77159	F01_transcript_77159
F01_transcript_71997	NA	NA	NA	F01_transcript_71997
F01_transcript_15557	NA	NA	NA	F01_transcript_15557
F01_transcript_145263	NA	F01_transcript_145263	NA	NA
F01_transcript_78466	NA	F01_transcript_78466	F01_transcript_78466	F01_transcript_78466
F01_transcript_125348	F01_transcript_125348	F01_transcript_125348	F01_transcript_125348	F01_transcript_125348
F01_transcript_53294	NA	F01_transcript_53294	NA	NA
F01_transcript_131588	NA	F01_transcript_131588	NA	F01_transcript_131588
F01_transcript_116603	NA	F01_transcript_116603	NA	F01_transcript_116603
F01_transcript_109922	NA	F01_transcript_109922	NA	F01_transcript_109922

F01_transcript_71541	NA	NA	NA	F01_transcript_71541
F01_transcript_55250	NA	NA	NA	F01_transcript_55250
F01_transcript_166590	NA	F01_transcript_166590	NA	NA
F01_transcript_109756	NA	NA	NA	F01_transcript_109756
F01_transcript_46606	NA	NA	NA	F01_transcript_46606
F01_transcript_32946	NA	NA	NA	F01_transcript_32946
F01_transcript_83590	NA	F01_transcript_83590	F01_transcript_83590	NA
F01_transcript_150368	NA	F01_transcript_150368	NA	NA
F01_transcript_106020	NA	NA	NA	F01_transcript_106020
F01_transcript_81203	NA	NA	NA	F01_transcript_81203
F01_transcript_97248	NA	F01_transcript_97248	NA	NA
F01_transcript_139785	NA	NA	NA	F01_transcript_139785
F01_transcript_49814	F01_transcript_49814	F01_transcript_49814	F01_transcript_49814	NA
F01_transcript_57259	NA	F01_transcript_57259	NA	NA

F01_transcript_26820	NA	NA	NA	F01_transcript_26820
F01_transcript_20140	NA	NA	NA	F01_transcript_20140
F01_transcript_114672	F01_transcript_114672	F01_transcript_114672	F01_transcript_114672	F01_transcript_114672
F01_transcript_140390	NA	NA	NA	F01_transcript_140390
F01_transcript_121892	NA	F01_transcript_121892	NA	NA
F01_transcript_138600	NA	F01_transcript_138600	NA	NA
F01_transcript_51032	F01_transcript_51032	F01_transcript_51032	F01_transcript_51032	F01_transcript_51032
F01_transcript_79970	NA	NA	NA	F01_transcript_79970
F01_transcript_74762	NA	NA	NA	F01_transcript_74762
F01_transcript_145728	NA	NA	NA	F01_transcript_145728
F01_transcript_128284	NA	NA	NA	F01_transcript_128284
[truncated: 475,974 more chars]
